# A New Method for Predicting the Subcellular Localization of Eukaryotic Proteins with Both Single and Multiple Sites: Euk-mPLoc 2.0

**DOI:** 10.1371/journal.pone.0009931

**Published:** 2010-04-01

**Authors:** Kuo-Chen Chou, Hong-Bin Shen

**Affiliations:** 1 Gordon Life Science Institute, San Diego, California, United States of America; 2 Institute of Image Processing & Pattern Recognition, Shanghai Jiaotong University, Shanghai, China; Institute of Infectious Disease and Molecular Medicine, South Africa

## Abstract

Information of subcellular locations of proteins is important for in-depth studies of cell biology. It is very useful for proteomics, system biology and drug development as well. However, most existing methods for predicting protein subcellular location can only cover 5 to 12 location sites. Also, they are limited to deal with single-location proteins and hence failed to work for multiplex proteins, which can simultaneously exist at, or move between, two or more location sites. Actually, multiplex proteins of this kind usually posses some important biological functions worthy of our special notice. A new predictor called “**Euk-mPLoc 2.0**” is developed by hybridizing the gene ontology information, functional domain information, and sequential evolutionary information through three different modes of pseudo amino acid composition. It can be used to identify eukaryotic proteins among the following 22 locations: (1) acrosome, (2) cell wall, (3) centriole, (4) chloroplast, (5) cyanelle, (6) cytoplasm, (7) cytoskeleton, (8) endoplasmic reticulum, (9) endosome, (10) extracell, (11) Golgi apparatus, (12) hydrogenosome, (13) lysosome, (14) melanosome, (15) microsome (16) mitochondria, (17) nucleus, (18) peroxisome, (19) plasma membrane, (20) plastid, (21) spindle pole body, and (22) vacuole. Compared with the existing methods for predicting eukaryotic protein subcellular localization, the new predictor is much more powerful and flexible, particularly in dealing with proteins with multiple locations and proteins without available accession numbers. For a newly-constructed stringent benchmark dataset which contains both single- and multiple-location proteins and in which none of proteins has 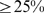 pairwise sequence identity to any other in a same location, the overall jackknife success rate achieved by **Euk-mPLoc 2.0** is more than 24% higher than those by any of the existing predictors. As a user-friendly web-server, Euk-mPLoc 2.0 is freely accessible at http://www.csbio.sjtu.edu.cn/bioinf/euk-multi-2/. For a query protein sequence of 400 amino acids, it will take about 15 seconds for the web-server to yield the predicted result; the longer the sequence is, the more time it may usually need. It is anticipated that the novel approach and the powerful predictor as presented in this paper will have a significant impact to Molecular Cell Biology, System Biology, Proteomics, Bioinformatics, and Drug Development.

## Introduction

With the avalanche of protein sequences generated in the post-genomic era, numerous efforts have been made to develop various methods for predicting protein subcellular localization based on the sequence information (see, e.g., [1], [2], [3], [4], [5], [6], [7], [8] as well as a long list of references cited in two comprehensive review articles [9], [10]). However, relatively much less efforts have been made to address those proteins which may simultaneously exist at, or move between, two or more different subcellular locations. Actually, proteins with multiple locations or dynamic feature of this kind are particularly interesting because they may have some very special biological functions worthy of our notice [11], [12]. Particularly, as pointed out by Millar et al. [13], recent evidences indicate that an increasing number of proteins have multiple locations in the cell.

About two years ago, a web-server predictor [14] was developed for dealing with the eukaryotic systems that contain both single-location and multiple-location proteins. The predictor is called **Euk-mPLoc**, where “m” stands for “multiple” meaning it can be used to deal with multiplex proteins as well. The **Euk-mPLoc** predictor was established by hybridizing the “higher-level” GO (gene ontology [15]) approach and PseAAC (pseudo amino acid composition [16], [17]) approach. Its power mainly came from the GO approach because proteins formulated in the GO database space would be clustered in a manner much better reflecting the distribution of their subcellular locations, as elucidated in [18].

However, the existing version of **Euk-mPLoc** has the following shortcomings. **(1)** In order to make the prediction engine able to use the advantage of the GO approach, the accession number for a query protein is required as a part of input; many proteins, such as synthetic and hypothetical proteins, or newly-discovered sequences without being deposited into databanks yet, do not have accession numbers, and hence cannot be treated with the GO approach. **(2)** Even though their accession numbers are available, it is not always certain for them to be meaningfully formulated in a GO space because the current GO database is far from complete yet. **(3)** Although the PseAAC approach, a complement to the GO approach in **Euk-mPLoc**, can take into account some partial sequence order effects, the original PseAAC [16], [19] missed the functional domain and sequential evolution information that may considerably affect the prediction quality.

The present study was devoted to develop a new and more powerful predictor for predicting eukaryotic protein subcellular localization by addressing the above three problems.

## Materials and Methods

**Figure 1:**
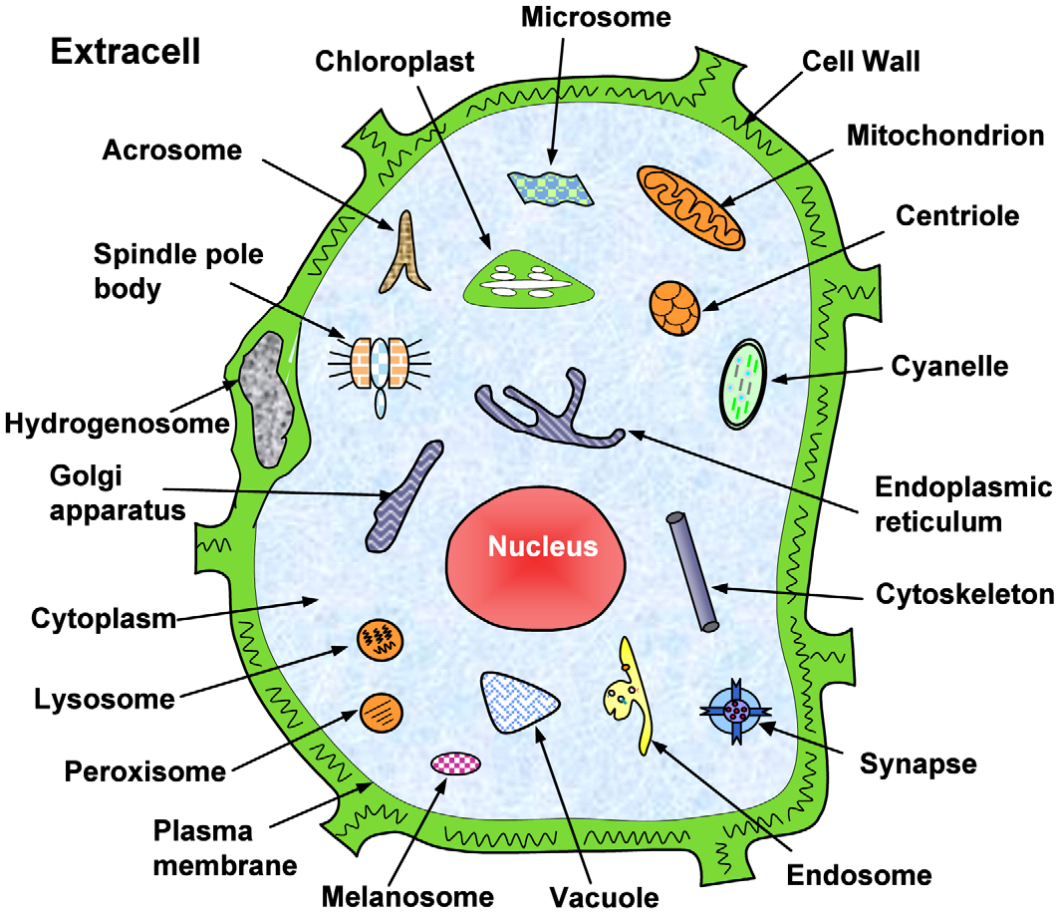
Illustration to show the 22 subcellular locations of eukaryotic proteins. The 22 location sites are: (1) acrosome, (2) cell wall, (3) centriole, (4) chloroplast, (5) cyanelle, (6) cytoplasm, (7) cytoskeleton, (8) endoplasmic reticulum, (9) endosome, (10) extracell, (11) Golgi apparatus, (12) hydrogenosome, (13) lysosome, (14) melanosome, (15) microsome (16) mitochondria, (17) nucleus, (18) peroxisome, (19) plasma membrane, (20) plastid, (21) spindle pole body, and (22) vacuole. Reprinted from [14] with permission.

Protein sequences were collected from the Swiss-Prot database at http://www.ebi.ac.uk/swissprot/. The detailed procedures are basically the same as described in [14]; the only difference is: in order to establish a more updated benchmark dataset, instead of version 50.7 of the Swiss-Prot database released on 9-Sept-2006, the version 55.3 released on 29-Apr-2008 was adopted. After strictly following the procedures as described in [14], we finally obtained a benchmark dataset 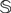 containing 7,766 different protein sequences that are distributed among 22 subcellular locations (**Fig. 1**); i.e.,
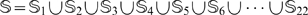
(1)where 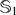 represents the subset for the subcellular location of “acrosome”, 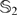 for “cell membrane”, 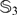 for “cell wall”, and so forth; while 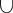 represents the symbol for “union” in the set theory. A breakdown of the 7,766 eukaryotic proteins in the benchmark dataset 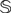 according to their 22 location sites is given in **Table 1**. To avoid redundancy and homology bias, none of the proteins in 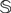 has 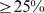 pairwise sequence identity to any other in a same subset. The corresponding accession numbers and protein sequences are given in Online Supporting Information S1.

**Table 1:** Breakdown of the eukaryotic protein benchmark dataset 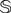 derived from Swiss-Prot database (release 55.3) according to the procedures described in the Materials section.

Subset^a^	Subcellular location	Number of proteins
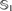	Acrosome	14
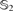	Cell membrane	697
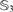	Cell wall	49
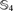	Centrosome	96
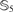	Chloroplast	385
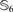	Cyanelle	79
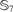	Cytoplasm	2186
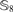	Cytoskeleton	139
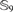	Endoplasmic reticulum	457
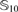	Endosome	41
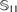	Extracell	1048
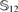	Golgi apparatus	254
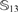	Hydrogenosome	10
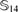	Lysosome	57
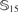	Melanosome	47
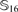	Microsome	13
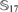	Mitochondrion	610
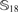	Nucleus	2320
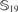	Peroxisome	110
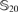	Spindle pole body	68
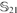	Synapse	47
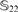	Vacuole	170
Number of total virtual proteins 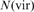		8,897^b^
Number of total different proteins 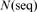		7,766^c^

None of the proteins included here has 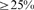 sequence identity to any other in a same subcellular location.

a See Fig. 1 and Eq.1 as well as the relevant text for the definitions of the subsets listed in this table.

b See Eqs.2–3 for the definition about the number of virtual proteins, and its relation with the number of different proteins.

c Of the 7,766 different proteins, 6,687 belong to one subcellular location, 1,029 to two locations, 48 to three locations, and 2 to four locations. See Online Supporting Information S1 for the protein sequences.

Because the system investigated now contains both the single-location and the multiple-location proteins, some of the proteins in 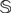 may occur in two or more location sites. Therefore, it is instructive to introduce the concept of “virtual sample”, as illustrated as follows. A protein sample coexisting at two different location sites will be counted as 2 virtual samples even though they have an identical sequence; if coexisting at three different sites, 3 virtual samples; and so forth. Accordingly, the total number of the different virtual protein samples is generally greater than that of the total different sequence samples. Their relationship can be formulated as follows
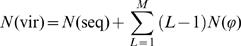
(2)where 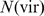 is the number of total different virtual protein samples in 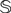, 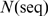 the number of total different protein sequences, 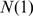 the number of proteins with one location, 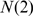 the number of proteins with two locations, and so forth; while 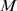 is the number of total subcellular location sites (for the current case, 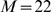 as shown in **Fig. 1** and **Table 1**).

For the current 7,766 different protein sequences, 6,687 occur in one subcellular location, 1,029 in two locations, 48 in three locations, 2 in four locations, and none in five or more locations. Substituting these data into **Eq.2**, we have
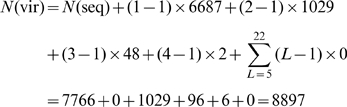
(3)which is fully consistent with the figures in **Table 1** and the data in Online Supporting Information S1.

As stated in a recent comprehensive review [20], to develop a powerful method for statistically predicting protein subcellular localization, one of the most important things is to formulate the sample of a protein with the core features that have intrinsic correlation with its localization in a cell. Since the concept of pseudo amino acid composition (PseAAC) was proposed [16], it has provided a very flexible mathematical frame for investigators to incorporate their desired information into the representation of protein samples. According to its original definition, the PseAAC is actually formulated by a set of discrete numbers [16] as long as it is different from the classical amino acid composition (AAC) and that it is derived from a protein sequence that is able to harbor some sort of its sequence order and pattern information, or able to reflect some physicochemical and biochemical properties of the constituent amino acids. Since the concept of PseAAC was proposed, it has been widely used to deal with many protein-related problems and sequence-related systems (see, e.g., [21], [22], [23], [24], [25], [26], [27], [28], [29], [30], [31], [32], [33], [34], [35], [36], [37], [38], [39], [40], [41], [42] and a long list of PseAAC-related references cited in a recent review [20]). As summarized in [20], until now 16 different PseAAC modes have been used to represent the samples of proteins for predicting their attributes. Each of these modes has its own advantage and disadvantage. In this study, we are to formulate the protein samples by hybridizing the following three different modes of PseAAC.

### 1. GO (Gene Ontology) Representation Mode

GO database [15] was established according to the molecular function, biological process, and cellular component. Accordingly, protein samples defined in a GO database space would be clustered in a way better reflecting their subcellular locations [10], [18]. However, the way of using GO mode to represent a protein sample in the original **Euk-mPLoc** predictor [14] was derived through its accession number from the GO database [43]. Thus, when using **Euk-mPLoc** to perform prediction, the accession number of a query protein would be indispensable. To avoid such a requirement, the following different procedures are proposed to derive the GO representation mode.

#### Step 1

Use BLAST [44] to search the homologous proteins of the query protein 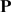 from the Swiss-Prot database (version 55.3), with the expect value 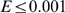 for the BLAST parameter.

#### Step 2

Those proteins which have 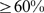 pairwise sequence identity with the query protein 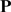 are collected into a set, 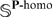, called the “homology set” of 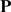. All the elements in 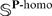 can be deemed as the “representative proteins” of 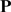. Because they were retrieved from the Swiss-Prot database, these representative proteins must each have their own accession numbers.

#### Step 3

Search each of these accession numbers collected in Step 2 against the GO database at http://www.ebi.ac.uk/GOA/ to find the corresponding GO numbers [43].

#### Step 4

The current GO database (version 70.0 released 10 March 2008) contains 60,020 GO numbers, thus the query protein 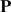 can be expressed via its representative proteins in 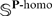 by the following formulation
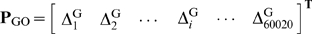
(4)where 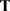 is the transposing operator, and
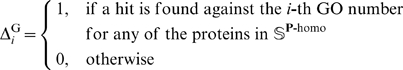
(5)


Through the above steps, we can use the GO information derived from its representative proteins in 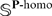 to formulate the query protein 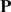. The rationale of so doing is based on the fact that homology proteins generally share similar attributes, such as structural conformations and biological functions [45], [46], [47]. Thus, the accession number is no longer indispensable for the input of the query protein even if using the high-level GO approach to predict its subcellular localization as required in **Euk-mPLoc** [14].

The above homology-based GO extraction method is particularly useful for studying those proteins which do not have UniProt accession numbers. However, it would still fail to work under any one of the following situations: **(1)** the query protein does not have significant homology to any protein in the Swiss-Prot database, i.e., 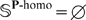 meaning the homology set is an empty one; **(2)** its representative proteins do not contain any useful GO information for statistical prediction based on a given training dataset.

Therefore, it is necessary to consider the following representation modes for those proteins which fail to be meaningfully defined in the GO space.

### 2. FunD (Functional Domain) Representation Mode

FunD is the core of a protein that plays the major role for its function. That is why in determining the 3-D (dimensional) structure of a protein by experiments (see, e.g., [48], [49]) or by computational modeling (see, e.g., [47], [50]) the first priority was always focused on its FunD. Actually, using the FunD information to formulate protein samples for statistical predictions was originally proposed in [51], [52], and quite encouraged results were achieved. In that time, the 2005 FunDs in the SBASE-A database [53] were used as bases to formulate the protein samples. Since then, a series of follow-up protein FunD databases were established, such as COG [54], KOG [54], SMART [55], Pfam [56], and CDD [57]. Of these databases, CDD contains the domains imported from COG, Pfam and SMART, and hence is relatively much more complete [57]. The version 2.11 of CDD contains 17,402 characteristic domains. Using each of these domains as a base vector, we can define a FunD space with 17,402 dimensions. Thus, by following the similar procedures in [51], a protein sample can be uniquely defined through the steps described below:

#### Step 1

Use RPS-BLAST (Reverse PSI-BLAST) program [44] to conduct sequence alignment of the protein sequence with each of the 17,402 domain sequences in the CDD database.

#### Step 2

If the significance threshold value (expect value) is 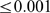 for the 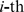 domain meaning that a “hit” is found, then the 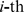 component of the protein in the 17402-D space is assigned 1; otherwise, 0.

#### Step 3

The protein sample 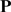 in the FunD space can thus be formulated as
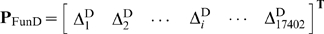
(6)where 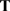 is the transpose operator, and
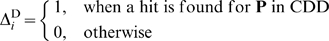
(7)


Defined this way, the protein sample becomes corresponding to a 17402-D vector 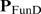 with each of the 17402 functional domain sequences as a base for the vector space. By using such a representation, not only some sequence-order effects but also some functional information is included. Since the function of a protein is closely related to its subcellular location, the FunD formulation of Eq.6 would naturally incorporate those factors that might be directly correlated with the protein subcellular location.

### 3. SeqEvo (Sequential Evolution) Representation Mode

Since biology is a natural science with historic dimension, all biological species have actually developed continuously starting out from a very limited number of ancestral species. It is quite typical for protein sequences [47]. Their evolution involves changes of single residues, insertions and deletions of several residues, gene doubling, and gene fusion. With such changes accumulated for a long period of time, many similarities between initial and resultant amino acid sequences are eliminated, but the corresponding proteins may still share many common attributes, such as their location site in a cell. Therefore, to catch the core feature and intrinsic relationship from a huge number of complicated protein sequences, it is particularly important to take into account the evolution effects. To realize this, here we are to incorporate the evolution information through the “Position-Specific Scoring Matrix” or “PSSM” [44], i.e., to express the protein 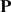 by a 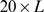 matrix as formulated by
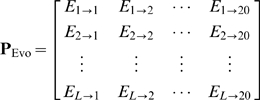
(8)where 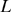 is the length of 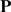 (counted in the total number of its constituent amino acids), 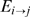 represents the score of the amino acid residue in the 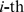 position of the protein sequence being changed to amino acid type 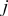 during the evolutionary process. Here, the numerical codes 1, 2, …, 20 are used to denote the 20 native amino acid types according to the alphabetical order of their single character codes. The 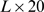 scores in Eq.8 were generated by using PSI-BLAST [44] to search the Swiss-Prot database (version 55.3 released on 29-Apr-2007) through three iterations with 0.001 as the 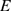-value cutoff for multiple sequence alignment against the sequence of the protein 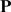, followed by a standard conversion given below:
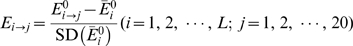
(9)where 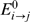 represent the original scores directly created by PSI-BLAST [44] that are generally shown as positive or negative integers (the positive score means that the corresponding mutation occurs more frequently than expected by chance, while the negative means just the opposite); the symbol 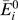 means taking the average of 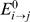 over 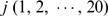, and 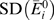 means the corresponding standard deviation. The converted values obtained by Eq.9 will have a zero mean value over the 20 amino acids and will remain unchanged if going through the same conversion procedure again. However, according Eq.8, a protein with 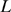 length is corresponding to a matrix of 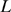 rows. Hence, proteins with different lengths will correspond to matrices of different dimensions. This will become a hurdle for us to develop a predictor able to unanimously cover proteins of any length. To overcome such a hurdle, one possible avenue is to represent a protein sample 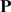 by
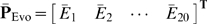
(10)where
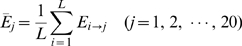
(11)where 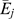 represents the average score of the amino acid residues in the protein 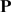 being changed to amino acid type 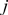 during the evolutionary process. However, if 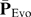 of Eq.10 was used to represent the protein 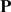, all the sequence-order information during the evolutionary process would be erased. To avoid completely erasing the sequence-order information, the concept of PseAAC as originally proposed in [16] was utilized; i.e., instead of Eq.10, let us use the pseudo position-specific scoring matrix as given by
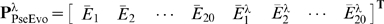
(12)to represent the protein 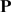, where
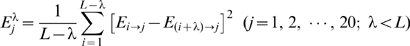
(13)meaning that 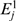 is the correlation factor by coupling the most contiguous position-specific scoring matrix scores along the protein chain for the amino acid type 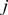; 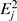 that by coupling the second-most contiguous position-specific scoring matrix scores; and so forth. Note that, as mentioned in the Material section of [14], the length of the shortest protein sequence in the benchmark dataset is 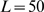, and hence the value allowed for 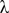 in Eq.13 must be smaller than 50. When 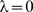, 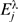 becomes a naught element and Eq.12 is degenerated to Eq.10.

A hybridization of the above three different PseAAC modes, i.e., Eq.4, Eq.6, and Eq.12, will be used to represent protein samples for establishing a new classifier for predicting eukaryotic protein subcellular localization, as described below.

### 4. Prediction Engine 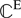 and Computing Procedures

The prediction engine used in this study is the ensemble classifier 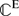 formed by fusing many individual OET-KNN (Optimized Evidence-Theoretic K-Nearest Neighbor) classifiers [58], [59]. According to the underlying rule of the OET-KNN classifier, a query protein should be assigned to the class the majority of its *K* nearest neighbors belongs to. However, for most benchmark datasets, when 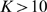 the success rate thus obtained would decrease markedly. Therefore, our consideration for *K* can be confined within the range from 1 to 10. Accordingly, the ensemble classifier 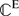 can be formulated as
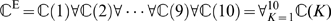
(14)where the symbol 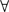 denotes the fusing operator, 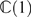 is the individual OET-KNN classifier based on 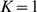 nearest neighbor, 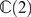 that based on 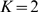 nearest neighbors, and so forth. The detailed mathematical formulations for OET-KNN and 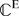 have been given in Eqs.22–29 in [10], where it has also been clearly elaborated how the ensemble classifier 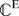 worked during the process of prediction. To avoid redundancy, we are not to repeat the details here.

The prediction is processed according to the following order.

#### Step 1

If the query protein 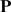 can be expressed as a meaningful or productive descriptor in the GO database via its representative proteins in its homology set 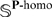, then 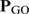 of Eq.4 should be input into the prediction engine for identifying its subcellular location site(s); i.e.
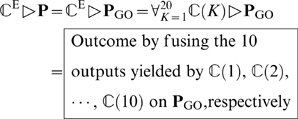
(15)where 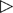 represents the identification operator, and the fusion is made via a voting operation as formulated by Eqs.32–35 in [10].

#### Step 2

If the query protein 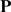 does not have significant homology to any protein in the Swiss-Prot database, i.e., 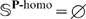 (empty set), or its representative proteins in 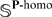 do not contain any useful GO information, then both the FunD representation 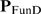 of Eq.6 and the pseudo position-specific scoring matrix representation 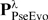 of Eq.12 should be inputted into the prediction engine 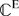. The output will be determined by fusing many preliminary outcomes associated with different *K* of 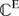 (cf. Eq.14) and different possible 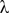 of the pseudo sequential evolution descriptor (cf. Eq.12); i.e.,
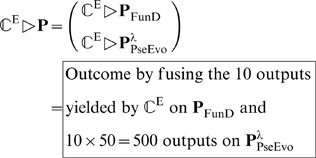
(16)where the factor 10 is because 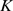 in 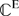 can be 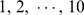 and the factor 50 is because 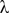 in 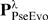 can be 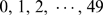 (cf. Eqs.12–13).

#### Step 3

To make Eqs.15–16 capable to handle proteins with multiple locations as well, the ensemble classifier 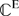 needed to be modified to 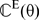, where 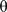 is a threshold parameter for controlling the count of multiple location sites and optimizing the predicted results, as formulated by Eqs.39–48 in [10] where it was also elaborated how to evaluate the overall success rate when using 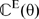 on a benchmark dataset containing both single and multiple location proteins.

**Figure 2:**
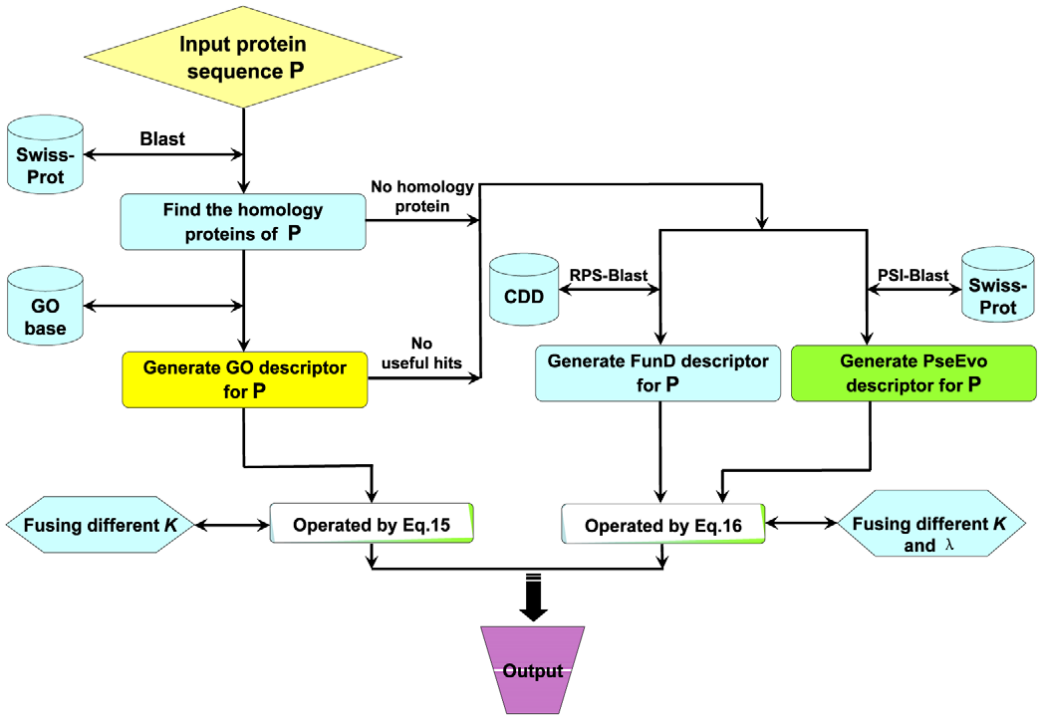
A flowchart to show the prediction process of Euk-mPLoc 2.0.

The entire ensemble classifier thus established is called “**Euk-mPLoc 2.0**”, where “2.0” refers to an updated version evolved from Euk-mPLoc [14]. To provide an intuitive picture, a flowchart is given in **Fig. 2** to illustrate the prediction process of **Euk-mPLoc 2.0**.

### Protocol Guide

For the convenience of experimental scientists, a user-friendly web-server was established for **Euk-mPLoc 2.0**. Below, let us give a step-by-step guide on how to use it to get the desired results.

#### Step 1

**Figure 3:**
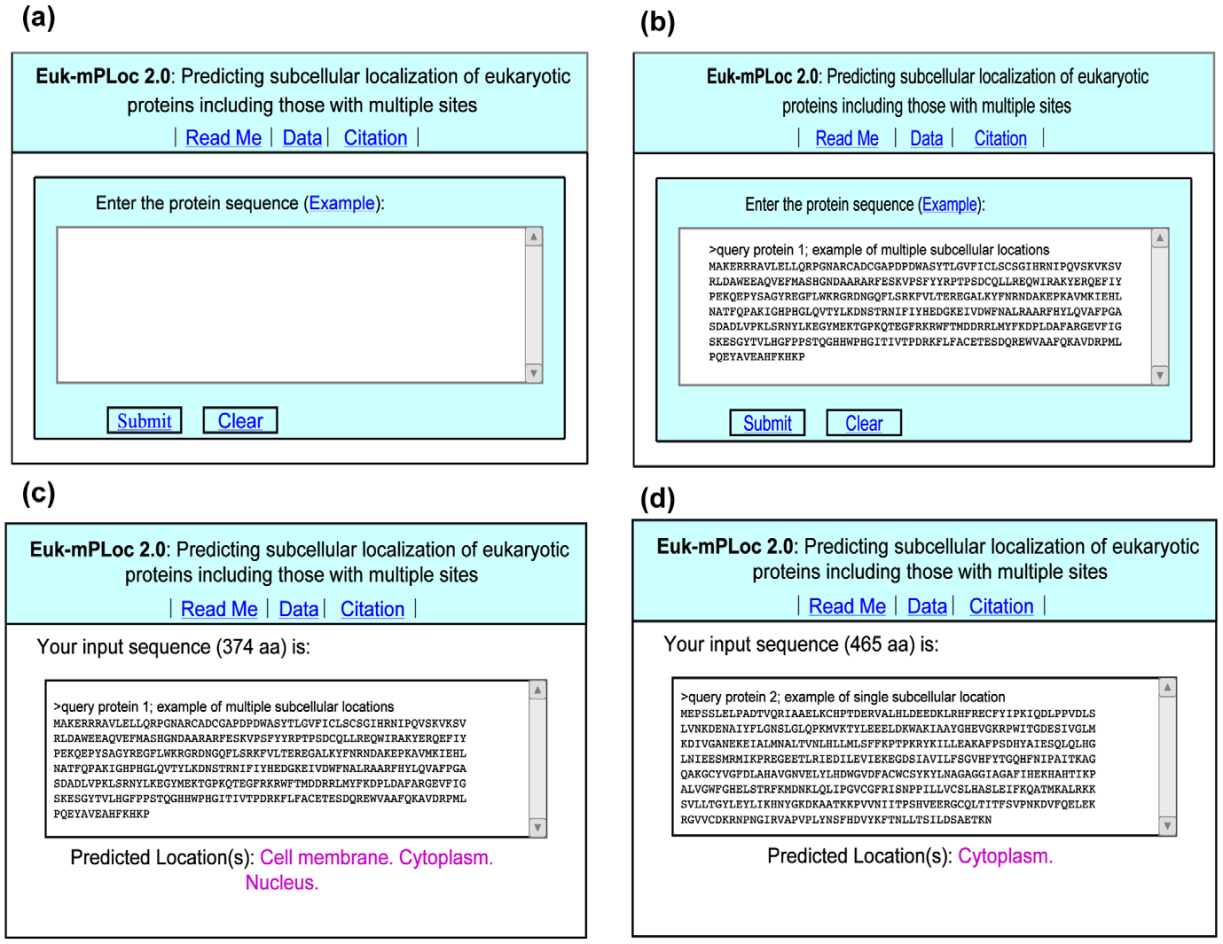
Semi-screenshot to show the prediction steps. (**a**) The top page of the **Euk-mPLoc 2.0** web server at http://www.csbio.sjtu.edu.cn/bioinf/euk-multi-2/. (**b**) The input of a query protein in FASTA format. (**c**) The output predicted by **Euk-mPLoc 2.0** for the query protein 1 in the Example window. (**d**) The output for the query protein 2 in the Example window.

Open the web server at http://www.csbio.sjtu.edu.cn/bioinf/euk-multi-2/ and you will see the top page of the predictor on your computer screen, as shown in **Fig. 3a**. Click on the Read Me button to see a brief introduction about **Euk-mPLoc 2.0** predictor and the caveat when using it.

#### Step 2

Either type or copy and paste the query protein sequence into the input box at the center of **Fig. 3a**. The input sequence should be in the FASTA format. A sequence in FASTA format consists of a single initial line beginning with a greater-than symbol (“>”) in the first column, followed by lines of sequence data. The words right after the “>” symbol in the single initial line are optional and only used for the purpose of identification and description. All lines should be no longer than 120 characters and usually do not exceed 80 characters. The sequence ends if another line starting with a “>” appears; this indicates the start of another sequence. Example sequences in FASTA format can be seen by clicking on the Example button right above the input box. For more information about FASTA format, visit http://en.wikipedia.org/wiki/Fasta_format.

#### Step 3

Click on the Submit button to see the predicted result. For example, if you use the sequence of query protein 1 in the Example window, the input screen should look like the illustration in **Fig. 3b**; after clicking the Submit button, you will see “**Cell membrane;** Cytoplasm; Nucleus” shown on the predicted result window (**Fig. 3c**), meaning that the protein is a multiplex one, which can simultaneously occur in “cell membrane”, “cytoplasm”, and “nucleus” organelles, fully consistent with experimental observations. However, if using the sequence of query protein 2 in the Example window as an input, you will instead see “**Cytoplasm**” shown on the predicted result window (**Fig. 3d**), meaning that the protein is a single-location one residing in “cytoplasm” compartment only, also fully consistent with experimental observations. It takes about 15 seconds for a protein sequence of 400 amino acids before the predicted result appears on your computer screen; the longer the sequence is, the more time it is usually needed.

#### Step 4

Click on the Citation button to find the relevant papers that document the detailed development and algorithm of **Euk-mPLoc 2.0**.

#### Step 5

Click on the Data button to download the benchmark datasets used to train and test the **Euk-mPLoc 2.0** predictor.

#### Caveat

To obtain the predicted result with the expected success rate, the entire sequence of the query protein rather than its fragment should be used as an input. A sequence with less than 50 amino acid residues is generally deemed as a fragment. Also, if the query protein is known not one of the 22 locations as shown in **Fig. 1**, stop the prediction because the result thus obtained will not make any sense.

## Results and Discussion

In statistical prediction, it would be meaningless to simply say a success rate of a predictor without specifying what method and benchmark dataset were used to test its accuracy. The following three cross-validation methods are often used to evaluate the accuracy of a statistical predictor: independent dataset test, sub-sampling (K-fold) test, and jackknife test [60]. Of these three, the jackknife test is deemed the most objective because the independent dataset test and sub-sampling test cannot avoid arbitrariness, as elaborated in a comprehensive review [10]. Therefore, the jackknife test has been increasingly and widely adopted to examine the power of various predictors (see, e.g., [23], [24], [25], [27], [29], [31], [34], [37], [61], [62], [63], [64], [65], [66], [67]). However, even if tested by the jackknife cross-validation, a same predictor can still yield different success rates for different benchmark datasets. This is because the more stringent of a benchmark dataset in excluding homologous sequences, or the more subcellular locations it covers, the more difficult for a predictor to yield a high overall success rate. For instance, ProtLock [2] and HSLPred [68] are two predictors developed for identifying protein subcellular localization. Both were reported with the success rates over 70–80% [2], [68] when tested by the benchmark datasets that allow inclusion of homologous proteins with up to 90% pairwise sequence identity and cover only 4 or 5 subcellular location sites. However, when the two predictors were tested by the stringent dataset covering 16 different subcellular locations in which none of proteins included has 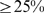 pairwise sequence identity to any other in a same subset, the overall jackknife success rate achieved by ProtLock [2] would drop down to 28.7% and that by HSLPred [68] down to 33.1%, as reported in [58].

Now the current benchmark dataset is even more stringent because, in addition to the same threshold to rigorously exclude the homologous sequences, it covers even more, i.e., 22 location sites. Besides, to the best of our knowledge, except **Euk-mPLoc** [14], so far there is no other web-server predictor whatsoever that can be used to predict a system with both single- and multiple-location proteins distributed among 22 different location sites. Accordingly, to demonstrate the advantage of **Euk-mPLoc 2.0**, it would be sufficient to simply compare the success rates achieved by the new predictor with those by **Euk-mPLoc** [14].

Listed in **Table 2** are the results obtained with **Euk-mPLoc** [14] and **Euk-mPLoc 2.0** on the benchmark dataset 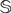 (cf. **Table 1**) by the jackknife cross-validation test. During the testing process, only the sequences of proteins in Online Supporting Information S1 but not their accession numbers were used as inputs in order to make the comparison between the two predictors under exactly the same condition. During the course of the jackknife cross-validation by **Euk-mPLoc 2.0** and **Euk-mPLoc**, the false positives (over-predictions) and false negatives (under-predictions) were also taken into account to reduce the scores for calculating the success rate. Note that it is more complicated to count the over-predictions and under-predictions for a system containing both single-location and multiple-location proteins. For the detailed calculation process, refer to Eqs.43–48 as well as Fig. 4 in a comprehensive review [10]. As we can see from **Table 2**, for such a stringent and multiplex benchmark dataset, the overall success rate achieved by **Euk-mPLoc 2.0** is over 64%, which is about 25% higher than that by **Euk-mPLoc**.

**Table 2:** A comparison of Euk-mPLoc 2.0 with Euk-PLoc in the jackknife cross-validation test on the benchmark dataset covering 22 location sites where none of the eukaryotic proteins included has 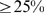 pairwise sequence identity to any other in a same location.

Subcellular location site	Success rate by jackknife cross-validation^a^	
	Euk-mPLoc	Euk-mPLoc 2.0
Acrosome	0/14 = 0.00%	1/14 = 7.14%
Cell membrane	262/697 = 37.58%	452/697 = 64.85%
Cell wall	4/49 = 8.16%	6/49 = 12.24%
Centrosome	9/96 = 9.38%	22/96 = 22.92%
Chloroplast	117/385 = 30.39%	318/385 = 82.60%
Cyanelle	12/79 = 15.19%	47/79 = 59.49%
Cytoplasm	918/2186 = 41.99%	1418/2186 = 64.87%
Cytoskeleton	4/139 = 2.88%	44/139 = 31.65%
Endoplasmic reticulum	115/457 = 25.16%	348/457 = 76.15%
Endosome	1/41 = 2.44%	2/41 = 4.88%
Extracell	678/1048 = 64.69%	858/1048 = 81.87%
Golgi apparatus	5/254 = 1.97%	56/254 = 22.05%
Hydrogenosome	0/10 = 0.00%	2/10 = 20.00%
Lysosome	5/57 = 8.77%	26/57 = 45.61%
Melanosome	0/47 = 0.00%	0/47 = 0.00%
Microsome	0/13 = 0.00%	1/13 = 7.69%
Mitochondrion	143/610 = 23.44%	427/610 = 70.00%
Nucleus	1212/2320 = 52.24%	1501/2320 = 64.70%
Peroxisome	1/110 = 0.91%	56/110 = 50.91%
Spindle pole body	0/68 = 0.00%	23/68 = 0.3382
Synapse	0/47 = 0.00%	0/47 = 0.00%
Vacuole	7/170 = 4.12%	101/170 = 59.41%
**Total**	**3493/8897 = 39.26%**	**5709/8897 = 64.17%**

a Note that in order to make the comparison under exactly the same condition, only the sequences of proteins in the Online Supporting Information S1 but not their accession numbers were used as inputs during the prediction.

Finally, it should be pointed out that although **Euk-mPLoc 2.0** is more powerful than the existing predictors in identifying the subcellular locations of eukaryotic proteins, there is much room for further improvement in future studies. As shown in **Table 2**, the success rates by **Euk-mPLoc 2.0** for proteins belonging to “melanosome” and “synapse” locations are very low. This is because of that, compared with the most of the other 20 location sites, the numbers of proteins in the two sites are not sufficiently large (cf. **Table 1** and Online Supporting Information S1) to train the prediction engine in a more effective way. It is anticipated that with more experimental data available for the two sites in the future, the situation will be improved and **Euk-mPLoc 2.0** will become even more powerful.

## Supporting Information

Supporting Information S1 (4.45 MB PDF)
