# Supplementary material for: A New Method for Predicting the Subcellular Localization of Eukaryotic Proteins with Both Single and Multiple Sites: Euk-mPLoc 2.0
Source: PLoS One. 2010 Apr 1;5(4):e9931. doi: 10.1371/journal.pone.0009931 (PMC2848569; doi:10.1371/journal.pone.0009931)
Supplement: Supporting Information S1 — (4.45 MB PDF) [file pone.0009931.s001.pdf]

Online Supporting Information S1. The benchmark dataset $\mathbb{S}$ used in this study contains 7,766 different eukaryotic protein sequences classified into 22 subsets according to their subcellular locations. Of the 7,766 different proteins, 6,687 belong to one subcellular location, 1,029 to two locations, 48 to three locations, and 2 to four locations. Both the accession numbers and sequences are given. None of the proteins included has $\geq 25\%$ pairwise sequence identity to any other in the same subset. See **Table 1** and the relevant text of the paper for further explanation.

---

(1) 14 acrosome proteins ($\mathbb{S}_1$)

```
>Q80WG7
MSESEEISEFGYIMELLAKGKVTIKNIEKELICPACKELFTHPLILPCQHSVCHKCVKEL
LLSLDDSFNDVASDSSNQSSPRLRLTSPSMDKIDKINRPGWKRNSLTPRPTTFPCPGCEH
DVDLGERGVSGLFRNFTLETIVERYRQAARAATAIMCDLCKPPPQESTKSCMDCSASYCN
ECFKIYHPWGTVKAQHEYVGPTTNFRPKVLMCPEHETERINMYCELCRRPVCHLCKLGGN
HSNHRVTTMSSAYKTLKEKLSKDIDFLIGKESQVKSQISELNLLMKETECNGERAKEEAL
AHFEKLFEILEDRKSSVLKAIDASKKLRLDKFHTQMEEYQGLLENNGLVGYAQEVLKETD
QSCFVQTAKQLHLRIQKATESLKSFRPAAQASFEDYVVNISKQTEVLGELSFFSSGIDIP
EINEEQSKVYNNALIDWHHPEKDKADSYVLEYRKINRDEEMISWNEIEVHGTSKVVSNLE
SNSPYAFRVRAYRGSICSPCSRELILHTPPAPVFSFLFDEKCGYNTEHLLLNLKRDRVES
RAGFNVLLAAERIQVGHYTSLDYIIGDVGVTKGKHFWACRVEPYSYLVKVGVASSDKLQE
WLRSPRDAASPRYEQDSGHDSGSEDACFDSSQPFTLVTIGMKKFFIPKSPTSSNEPENRV
LPMPTSIGIFLDCDKGKVSFYDMDHMKCLYERQVDCSHTMYPAFALMGSGGIQLEEAITA
KYLEYEEDV
>Q9R0N6
MQADRSMKMGHALNPFSTSAPLDATAGPSLIPDLITRIPWPRWTLFIAILAAGVLLVSCL
LCVICCYCHRHRHRKQPKDKETVGLGSARNSTTTHLVQPDVDCLEPCSGGDQQWGRLLLS
LEYDFGSQEIRVGLRQAGNLKAEGTADPYAWVSVSTQSGRRHETKVHRGTLSPMFEETCC
FLVPPAELPKATLKVQLWDFKRFSEHEPLGELQLPLGTVDLQHVLESWYQLGPPGTTEPE
QMGELCFSLRYVPSSGSLTVVVLEARGLNPGLAEAYVKIQLMLNQRKWKKSKTSSKKGTT
TPYFNEAFVFLVPVSQLQSVDLVLAVWARGLQLRTEPVGKVLLGSRASGQPLQHWADMLA
HARRPIAQWHHLRSPREVDRVLALQPRLPLLRPRS
>P23118
MGFHQISVIIVVLALASARAADEFPSHTDTPTDCPEADHGCWCHGSFAQCWRTYEDSRMT
EEIGNRITQLELLYQPSEEVVTYIRRISALRELRISEDGMSLDCSCDVIYALDDKQVTLV
NQAELTFGNCRERGWPRERMAARPFVHRCHVLRMQDGETRKRRGADDNDGDDVSKRASPR
KGDEPAGHKLKDLAPQNTHHLVNIHDADKHPASEFVNFISGHRRSRRSTDDDAAVSDDSE
RGARKKRYGNQGNYPQAMNPQSRGVNYGQPAQQGYGAQGMGGAFGGGQGMGGAVRGGQGM
GGAVGGGQFGAFSPGEAEADNADYDEYSDSLDEGDTTISAAVMDDIKAVLGATKIDLPVD
INDPYDLGLLLRHLRHHSNLLANIGDPAVREQVLSAMQEEEEEEEEDAANGVRQNVLNNI
NANAPGNAGYGGQGGMGAFGGGGGGMGAIGGGGGAMMGQQGMGGVPQRMGGQPQGNAYNQ
GYRQG
>Q91XV4
MDLGLAGRRALVTGAGKGIGRSTVLALQAAGAHVVAVSRTQADLDSLVSECPGVETVCVD
LADWEATEQALSSVGPVDLLVNNAAVALLQPFLEVTKEAFDMSFNVNLRAVIQVSQIVAR
```

GMIARGAPGAIVNVSSQASQRALANHSVYCSTKGALDMLTKMMALELGPHKIRVNAVNPT
VVMTSMGRTNWSDPHKAKVMLDRIPLGKFAEVENVVDAILFLLSHRSNMTTGSTLPVDGG
FLVT
>Q99932
METNESTEGSRSRSRSLDIQPSSEGLGPTSEPFPSSDDSPRSALAAATAAAAAAASAAAA
TAAFTTAKAAALSTKTPAPCSEFMEPSSDPSLLGEPCAGPGFTHNIAHGSLGFEPVYVSC
IAQDTCTTTDHSSNPGPVPGSSSGPVLGSSSGAGHGSGSGSGPGCGSVPGSGSGPGPGSG
PGSGPGHGSGSHPGPASGPGPDTGPDSELSPCIPPGFRNLVADRVPNYTSWSQHCPWEPQ
KQPPWEFLQVLEPGARGLWKPPDIKGKLMVCYETLPRGQCLLYNWEEERATNHLDQVPSM
QDGSESFFFRHGHRGLLTMQLKSPMPSSTTQKDSYQPPGNVYWPLRGKREAMLEMLLQHQ
ICKEVQAEQEPTRKLFEVESVTHHDYRMELAQAGTPAPTKPHDYRQEQPETFWIQRAPQL
PVCEGD
>Q8C636
MDSGKSRSLETTVKRVYRDLLLPKINTSKKMSTLTNSPSSLEGTPGIKKNSGEMQAEASA
ERVKLTKSIKEKQNNELEKVALKRKADSEEKLVGKKEAKIMELDNQLVTTVPLPHIPLKN
IMDVEMKLVYVDEEDVSYEFAQPNMCLGLQATGQTATTMPPASPRDLTTLPQIDKWLQVA
LKDASSCYRQKKYAVAAGQFRTALELCSKGAALGKPFEAHAEDIASIASFIETKLVTCYL
RMRKPDLALNHAHRSIVLNPAYFRNHLRQAAVFRCLERYSEAARSAMIADYMFWLCGGSE
HSVSKLIKLYWQAMIEEAITRAEAFSVMYTPFATRIKPENIEKVKEVFMRTHPTYVDCIY
TDTQGLHVLPQTADWSCFPPQQYLLTLGFKNKEDGKFLEKVSSRKLPTYTEHKTPFSPLT
REDTVRHMETVGKRILPILDFIRSTQLNGNFHACSGVMEKLHYASLLSRLQRVKEQAQVI
NQAMAELATVPYLQDISQQEAELLQSLMADAMDTLEGKKSDKERVWNTIQKVGRIEDFLY
QLEDSFLKTKKLRTARRQKTKMKRLQTVQQN
>P47970
MLALLAAGVAFAVVVLAQDKPLPGSHFVCSAIPPEALFAGCPLPATPMQGVSLSPEEELR
AAVLQLRETVVMQKETLGAQREAIRELTSKLARCEGLMAGKAESSKDTMGDLPRDPSRVV
EQLSRSLQVLKDRLESLELQLRTNASNTGLPSDFREVLQRRLGELERQLLRKVAELEDEK
SLLHNETSAHQQKTENTLNALLQRVTELERGNSAFKSPDAFKVSLPFRTNYLYGKIKKTL
PELYSFTICLWLRSSASPGIGTPFSYAVPGQANEIVLIEWGNNPIELLINDKVAQLPLFV
SDGKWHHICITWTTRDGLWEAFQDGEKLGTGENLAPWHPIKSGGVLILGQEQDTVGGRFD
ATQAFVGELSQFNIWDRVLRPQEISNIANCSLNMAGNIIPWVDNNVDVFGGASKWPVETC
EERLLDL
>Q6UW49
MKPLVLLVALLLWPSSVPAYPSITVTPDEEQNLNHYIQVLENLVRSVPSGEPGREKKSNS
PKHVYSIASKGSKFKELVTHGDASTENDVLTNPISEETTTFPTGGFTPEIGKKKHTESTP
FWSIKPNNVSIVLHAEEPYIENEEPEPEPEPAAKQTEAPRMLPVVTESSTSPYVTSYKSP
VTTLDKSTGIEISTESEDVPQLSGETAIEKPEEFGKHPESWNNDDILKKILDINSQVQQA
LLSDTSNPAYREDIEASKDHLKRSLALAAAAEHKLKTMYKSQLLPVGRTSNKIDDIETVI
NMLCNSRSKLYEYLDIKCVPPEMREKAATVFNTLKNMCRSRRVTALLKVY
>O88987
MADRVDWLQSQSGVCKVGVYSPGDNQHQDWKMDTSTDPVRVLSWLRKDLEKSTAGFQDSR
FKPGESSFVEEVAYPVDQRKGFCVDYYNTTNKGSPGRLHFEMSHKENPSQGLISHVGNGG
SIDEVSFYANRLTNLVIAMARKEINEKIHGAENKCVHQSLYMGDEPTPHKSLSTVASELV
NETVTACSKNISSDKAPGSGDRASGSSQAPGLRYMSTLKIKESTKEGKCPDDKPGTKKSF

FYKEVFESRNAGDAKEGGRSLPGDQKLFRTSPDNRPDDFSNSISQGIMTYANSVVSDMMV
SIMKTLKIQVKDTTIATILLKKVLMKHAKEVVSDLIDSFMKNLHGVTGSLMTDTDFVSAV
KRSFFSHGSQKATDIMDAMLGKLYNVMFAKKFPENIRRARDKSESYSLISTKSRAGDPKL
SNLNFAMKSESKLKENLFSTCKLEKEKTCAETLGEHIIKEGLHMWHKSQQKSPGLERAAK
LGNAPQEVSFECPDPCEANPPHQPQPPENFANFMCDSDSWAKDLIVSALLLIQYHLAQGG
KMDAQSFLEAAASTNFPTNKPPPPSPVVQDECKLKSPPHKICDQEQTEKKDLMSVIFNFI
RNLLSETIFKSSRNCESNVHEQNTQEEEIHPCERPKTPCERPITPPAPKFCEDEEATGGA
LSGLTKMVANQLDNCMNGQMVEHLMDSVMKLCLIIAKSCDSPLSELGEEKCGDASRPNSA
FPDNLYECLPVKGTGTAEALLQNAYLTIHNELRGLSGQPPEGCEIPKVIVSNHNLADTVQ
NKQLQAVLQWVAASELNVPILYFAGDDEGIQEKLLQLSATAVEKGRSVGEVLQSVLRYEK
ERQLDEAVGNVTRLQLLDWLMANL
>Q8TDM5
MVLCWLLLLVMALPPGTTGVKDCVFCELTDSMQCPGTYMHCGDDEDCFTGHGVAPGTGPV
INKGCLRATSCGLEEPVSYRGVTYSLTTNCCTGRLCNRAPSSQTVGATTSLALGLGMLLP
PRLL
>Q60736
MITWSFIDLWRTSHSTLFQMTLATVLMAPVLGDCGPPPLLPFASPTNQLYESTTFPSGTV
LKYTCHHGFKRVNSSHLSCDENGSWVYSTFCARKRCKNPGELVNGKVEIPSDLLVGSIIE
FSCSKGYLLIGSATSRCEVQGKGVDWSDSLPECVIATCEPPPPISNGKHSGRDDDLYTFG
SVVIYNCDPTFTLLGNASIVCTVVNRTVGVWRPHPPACQKIVCHRPQIPKGYLAPGFRQF
YAYRDALEIRCKKGFILRGSSVIHCEANGEWFPSIPTCEPNGCTNIPDISYASWEGYKFP
LRNFEVFEIGAKLKYQCKPGYRASLNDPQTVTCQENLTWSSTNGCERICCPTPDMEKIKI
VSERRDFTGTCIYAYGDYVFYICNEGSYPMSTDGRSSCQADGKWDPAIPSCQADSGLQNR
LALFTFPNISETNVTNKTYLFGHEENSTEHAMKGVCLKPMVINGNLSVERVIYAELENIT
IQCDPGYTIVGSPNIICSNRTWYPEVPSCQMEVLEDCRIVSRGAQLLHCLSSPEDVHRAL
KVYKLFLEIERLEHQKEKWIQLHRKPQSMKINRSFRLCN
>Q63564
MDDYRYRDNYEGYAPNDGYYRGNEQNPEEDAQSDVTEGHDEEDEIYEGEYQGIPHPDDVK
SKQTKMAPSRADGLRGQADLMAERMEDEEQLAHQYETIIDECGHGRFQWTLFFVLVLALM
ADGVEVFVVSFALPSAEKDMCLSSSKKGMLGLIVYLGMMAGAFILGGLADKLGRKKVLSM
SLAINASFASLSSFVQGYGAFLFCRLISGIGIGGSLPIVFAYFSEFLSREKRGEHLSWLG
IFWMTGGIYASAMAWSIIPHYGWGFSMGTNYHFHSWRVFVIVCALPATVSMVALKFMPES
PRFLLEMGKHDEAWMILKQVHDTNMRAKGTPEKVFTVSHIKTPKQMDEFIEIQSSTGTWY
QRWLVRFMTIFKQVWDNALYCVMGPYRMNTLILAVVWFTMALSYYGLTVWFPDMIRYFQD
EEYKSKMKVFFGEHVHGATINFTMENQIHQHGKLVNDKFIKMYFKHVLFEDTFFDKCYFE
DVTSTDTYFKNCTIESTTFYNTDLYKHKFIDCRFINSTFLEQKEGCHMDFEEDNDFLIYL
VSFLGSLSVLPGNIISALLMDRIGRLKMIGGSMLISAVCCFFLFFGNSESAMIGWQCLFC
GTSIAAWNALDVITVELYPTNQRATAFGILNGLCKLGAILGNTIFASFVGITKVVPILLA
AASLVGGGLVALRLPETREQVLM
>Q9H0H5
MDTMMLNVRNLFEQLVRRVEILSEGNEVQFIQLAKDFEDFRKKWQRTDHELGKYKDLLMK
AETERSALDVKLKHARNQVDVEIKRRQRAEADCEKLERQIQLIREMLMCDTSGSIQLSEE
QKSALAFLNRGQPSSSNAGNKRLSTIDESGSILSDISFDKTDESLDWDSSLVKTFKLKKR
EKRRSTSRQFVDGPPGPVKKTRSIGSAVDQGNESIVAKTTVTVPNDGGPIEAVSTIETVP

YWTRSRRKTGTLQPWNSDSTLNSRQLEPRTETDSVGTPQSNGGMRLHDFVSKTVIKPESC
VPCGKRIKFGKLSLKCRDCRVVSHPECRDRCPLPCIPTLIGTPVKIGEGMLADFVSQTSP
MIPSIVVHCVNEIEQRGLTETGLYRISGCDRTVKELKEKFLRVKTVPLLSKVDDIHAICS
LLKDFLRNLKEPLLTFRLNRAFMEAAEITDEDNSIAAMYQAVGELPQANRDTLAFLMIHL
QRVAQSPHTKMDVANLAKVFGPTIVAHAVPNPDPVTMLQDIKRQPKVVERLLSLPLEYWS
QFMMVEQENIDPLHVIENSNAFSTPQTPDIKVSLLGPVTTPEHQLLKTPSSSSLSQRVRS
TLTKNTPRFGSKSKSATNLGRQGNFFASPMLK
>Q06990
MNMFLLLMSLYLLGSARGTSGQSDESSGSIDHQTSVQQLSGEFFSLENPSDAEALYETAS
GLNTLSEHGSSEHGSREHTVAEHTPGEHAESEHASGEPAATGHAEGEHTVGEQPSGEQPS
GEHLSGEQSLGEHASGEQPSDEQLSGEHASGEQPSGEHASGEQPSGEQPSGEHASGEQSL
GEHALSEKPSGEQPSGAPISSISTGTILNCYTCAYMNDQGRCLRGEGTCITQNSQQCMLK
KIFEGGKLQFMVQGCENMCPSMNLFSHGTRMQIICCRNQSFCNKI

(2) 697 cell membrane proteins ($\mathbb{S}_2$)
>Q9SV84
MAPPEAEVGAVMVMAPPTPGTPGTPGGPLITGMRVDSMSFDHRKPTPRCKCLPVMGSTWG
QHDTCFTDFPSPDVSLTRKLGAEFVGTFILIFTATAGPIVNQKYDGAETLIGNAACAGLA
VMIIILSTGHISGAHLNPSLTIAFAALRHFPWAHVPAYIAAQVSASICASFALKGVFHPF
MSGGVTIPSVSLGQAFALEFIITFILLFVVTAVATDTRAVGELAGIAVGATVMLNILVAG
PSTGGSMNPVRTLGPAVASGNYRSLWVYLVAPTLGAISGAAVYTGVKLNDSVTDPPRPVR
SFRR
>Q99NF2
MGAAASRRRALRSEAMSSVAAKVRAARAFGEYLSQSHPENRNGADHLLADAYSGHDGSPE
MQPAPQNKRRLSLVSNGRYEGSISDEAVSGKPAIEGPQPHVYTISREPALLPGSEAEAIE
LAVVKGRRQRERHPHHHSQPLRASPGSSREDISRPCQSWAGSRQGSKECPGCAQLVPGPS
SRAFGLEQPPLPEAPGRHKKLERMYSVDGVSDDVPIRTWFPKENLFSFQTATTTMQAISV
FRGYAERKRRKRENDSASVIQRNFRKHLRMVGSRRVKAQTFAERRERSFSRSWSDPTPMK
ADTSHDSRDSSDLQSSHCTLDEACEDLDWDTEKGLEAMACNTEGFLPPKVMLISSKVPKA
EYIPTIIRRDDPSIIPILYDHEHATFEDILEEIEKKLNIYHKGAKIWKMLIFCQGGPGHL
YLLKNKVATFAKVEKEEDMIHFWKRLSRLMSKVNPEPNVIHIMGCYILGNPNGEKLFQNL
RTLMTPYKVTFESPLELSAQGKQMIETYFDFRLYRLWKSRQHSKLLDFDDVL
>Q9ES57
MFCFWRTSALAVLLIWGVFVAGSSCTDKNQTTQNNSSSPLTQVNTTVSVQIGTKALLCCF
SIPLTKAVLITWIIKLRGLPSCTIAYKVDTKTNETSCLGRNITWASTPDHSPELQISAVT
LQHEGTYTCETVTPEGNFEKNYDLQVLVPPEVTYFPEKNRSAVCEAMAGKPAAQISWSPD
GDCVTTSESHSNGTVTVRSTCHWEQNNVSDVSCIVSHLTGNQSLSIELSRGGNQSLRPYI
PYIIPSIIILIIIGCICLLKISGFRKCKLPKLEATSAIEEDEMQPYASYTEKSNPLYDTV
TKVEAFPVSQGEVNGTDCLTLSAIGI
>Q0VDU3
MEPATALLIVDYYDYTSPDPPILETPSHLSYTSVFLPIFYTVVFLTGVVGNFILMIALHF
KRGNRRLIDIFIINLAASDFIFLVTVPLWMDKEASLGLWRTGSFLCKGSSYVISVNMHCS
VFLLTCMSMDRYLAIMHPALAKRLRRRSSAYAVCAVVWIISCVLGLPTLLSRELTHIEGK
PYCAEKKPTSLKLMWGLVALITTFFVPLLSIVTCYCCITRRLCAHYQQSGKHNKKLKKSI
KIVIIAVAAFTVSWVPFNTFKLLAIVSGFQPEGLFHSEALQLAMNVTGPLAFASSCVNPL

IYYVFDSYIRRAIVRCLCPCLKTHNFGSSTETSDSHLTKALSNFIHAEDFIRRRKRSVSL

>P36408
MSSDISEKIQQARRDAESMKEQIRANRDVMNDTTLKTFTRDLPGLPKMEGKIKVRRNLKG
HLAKIYAMHWAEDNVHLVSASQDGKLLVWDGLTTNKVHAIPLRSSWVMTCAYSPTANFVA
CGGLDNICSIYNLRSREQPIRVCRELNSHTGYLSCCRFLNDRQIVTSSGDMTCILWDVEN
GTKITEFSDHNGDVMSVSVSPDKNYFISGACDATAKLWDLRSGKCVQTFTGHEADINAVQ
YFPNGLSFGTGSDDASCRLFDIRADRELMQYTHDNILCGITSVGFSFSGRFLFAGYDDFT
CNVWDTLKGERVLSLTGHGNRVSCLGVPTDGMALCTGSWDSLLKIWA
>Q8JG54
MGRGAGSTALGLFQILPVFLCIFPPVTSPCKILKCNSEFWAATSGSHHLGAEETPEFCTA
LRAYAHCTRRTARTCRGDLAYHSAVHGIDDLMVQHNCSKDGPTSQPRLRTLPPGDSQERS
DSPEICHYEKSFHKHSAAPNYTHCGLFGDPHLRTFTDTFQTCKVQGAWPLIDNNYLNVQV
TNTPVLPGSSATATSKLTIIFKSFQECVEQKVYQAEMDELPAAFADGSKNGGDKHGANSL
KITEKVSGQHIEIQAKYIGTTIVVRQVGRYLTFAVRMPEEVVNAVEDRDSQGLYLCLRGC
PLNQQIDFQTFRLAQAAEGRARRKGPSLPAPPEAFTYESATAKCREKLPVEDLYFQSCVF
DLLTTGDVNFMLAAYYAFEDVKMLHSNKDKLHLYERTRALAPGNAAPSEHPWALPALWVA
LLSLSQCWLGLL
>Q8WVF1
MSVRTLPLLFLNLGGEMLYILDQRLRAQNIPGDKARKVLNDIISTMFNRKFMEELFKPQE
LYSKKALRTVYERLAHASIMKLNQASMDKLYDLMTMAFKYQVLLCPRPKDVLLVTFNHLD
TIKGFIRDSPTILQQVDETLRQLTEIYGGLSAGEFQLIRQTLLIFFQDLHIRVSMFLKDK
VQNNNGRFVLPVSGPVPWGTEVPGLIRMFNNKGEEVKRIEFKHGGNYVPAPKEGSFELYG
DRVLKLGTNMYSVNQPVETHVSGSSKNLASWTQESIAPNPLAKEELNFLARLMGGMEIKK
PSGPEPGFRLNLFTTDEEEEQAALTRPEELSYEVINIQATQDQQRSEELARIMGEFEITE
QPRLSTSKGDDLLAMMDEL
>P43322
MSERKEGRGKGKGKKKDRGSRGKPGPAEGDPSPALPPRLKEMKSQESAAGSKLVLRCETS
SEYSSLRFKWFKNGNELNRKNKPENIKIQKKPGKSELRINKASLADSGEYMCKVISKLGN
DSASANITIVESNEFITGMPASTETAYVSSESPIRISVSTEGANTSSSTSTSTTGTSHLI
KCAEKEKTFCVNGGECFTVKDLSNPSRYLCKCPNEFTGDRCQNYVMASFYMTSRRKRQET
EKPLERKLDHSLVKESKAEELYQKRVLTITGICIALLVVGIMCVVAYCKTKKQRQKLHDR
LRQSLRSERSNLVNIANGPHHPNPPPENVQLVNQYVSKNVISSEHIVEREVETSFSTSHY
TSTAHHSTTVTQTPSHSWSNGHTESVISESNSVIMMSSVENSRHSSPAGGPRGRLHGLGG
PRDNSFLRHARETPDSYRDSPHSERYVSAMTTPARMSPVDFHTPSSPKSPPSEMSPPVSS
MTVSMPSVAVSPFVEEERPLLLVTPPRLREKKYDHHPQQLNSFHHNPAHQSTSLPPSPLR
IVEDEEYETTQEYESVQEPVKKVTNSRRAKRTKPNGHIANRLEMDSNTSSVSSNSESETE
DERVGEDTPFLGIQNPLAASLEVAPAFRLAESRTNPAGRFSTQEELQARLSSVIANQDPI
AV
>P07666
MEQLLAQLHTCSELIAEGYSSTGNIGWLNEFCATFLDFASDLKARLPEVAPSGANLDVET
IFLCLTQVVTCITHLERTISMEAPHMTRQHFLDRLDWCLRRLLVSLTQLEGNVTPVKNLE
DHSFVELMDLALDHLDDYMEKLAQQRNNSLHILEESFTEDTYQLASIVNHIVRHALAFAN
VAIHSDKKALTALCETLLAECATFHEEAGEPNSGHRKLEALSLERALYALESFLNEALLH

LLFVSLIDLENASVEKLKDALQRDPAGAQELISAFDTNMDRIQQIGVLAIAFSQDIKTKT
IVRSCLASLESLDACIVPALQLPESTSSAHHAEVLQEHFNQELLIFRNVIHEIIDSCSLI
NNYLDMLGERIHVQDKSHLKLIVQRGGVVVDHFRLPVNYSGLSEDGKRVHKDLILILREC
QAVVNLDVPVDPKRIVKRLKILYSVLAKLRDLICRDNLEPDSSVASEAQVPSSATRTFVR
SSRSFGKRHRSFVKQTGNCSVFGPQDSLAESGHSESDLISFQITEILRLD
>O65493
MAFSAPSLSKFSLLVAISASALLCCAFARDFSIVGYTPEHLTNTDKLLELFESWMSEHSK
AYKSVEEKVHRFEVFRENLMHIDQRNNEINSYWLGLNEFADLTHEEFKGRYLGLAKPQFS
RKRQPSANFRYRDITDLPKSVDWRKKGAVAPVKDQGQCGSCWAFSTVAAVEGINQITTGN
LSSLSEQELIDCDTTFNSGCNGGLMDYAFQYIISTGGLHKEDDYPYLMEEGICQEQKEDV
ERVTISGYEDVPENDDESLVKALAHQPVSVAIEASGRDFQFYKGGVFNGKCGTDLDHGVA
AVGYGSSKGSDYVIVKNSWGPRWGEKGFIRMKRNTGKPEGLCGINKMASYPTKTK
>Q8BI36
MAVDIQPACLGLYCGKTLLFKNGSSEIYGECGVCPRGQRTNAQKYCQPCTESPELYDWLY
LGFMAMLPLVLHWFFIEWYSGKKSSSALFQHITALFECTMAAIITLLVSDPVGVLYIRSC
RVLMLSDWYTMLYNPSPDYVTTVHCTHEAVYPLYTIVFVYYAFCLVLMMLLRPLLVKKIA
CGLGKSDRFKSIYAALYFFPILTVLQAVGGGLLYYAFPYIILVLSLVTLAVYMSASEIEN
CYDLLVRKKRLIVLFSHWLLHAYGIVSISRVDRLEHDLPLLALVPTPALFYLFTAKFTEP
SRILSEGANGH
>Q969Z4
MKPSLLCRPLSCFLMLLPWPLATLTSTTLWQCPPGEEPDLDPGQGTLCRPCPPGTFSAAW
GSSPCQPHARCSLWRRLEAQVGMATRDTLCGDCWPGWFGPWGVPRVPCQPCSWAPLGTHG
CDEWGRRARRGVEVAAGASSGGETRQPGNGTRAGGPEETAAQYAVIAIVPVFCLMGLLGI
LVCNLLKRKGYHCTAHKEVGPGPGGGGSGINPAYRTEDANEDTIGVLVRLITEKKENAAA
LEELLKEYHSKQLVQTSHRPVSKLPPAPPNVPHICPHRHHLHTVQGLASLSGPCCSRCSQ
KKWPEVLLSPEAVAATTPVPSLLPNPTRVPKAGAKAGRQGEITILSVGRFRVARIPEQRT
SSMVSEVKTITEAGPSWGDLPDSPQPGLPPEQQALLGSGGSRTKWLKPPAENKAEENRYV
VRLSESNLVI
>O00443
MAQISSNSGFKECPSSHPEPTRAKDVDKEEALQMEAEALAKLQKDRQVTDNQRGFELSSS
TRKKAQVYNKQDYDLMVFPESDSQKRALDIDVEKLTQAELEKLLLDDSFETKKTPVLPVT
PILSPSFSAQLYFRPTIQRGQWPPGLPGPSTYALPSIYPSTYSKQAAFQNGFNPRMPTFP
STEPIYLSLPGQSPYFSYPLTPATPFHPQGSLPIYRPVVSTDMAKLFDKIASTSEFLKNG
KARTDLEITDSKVSNLQVSPKSEDISKFDWLDLDPLSKPKVDNVEVLDHEEEKNVSSLLA
KDPWDAVLLEERSTANCHLERKVNGKSLSVATVTRSQSLNIRTTQLAKAQGHISQKDPNG
TSSLPTGSSLLQEVEVQNEEMAAFCRSITKLKTKFPYTNHRTNPGYLLSPVTAQRNICGE
NASVKVSIDIEGFQLPVTFTCDVSSTVEIIIMQALCWVHDDLNQVDVGSYVLKVCGQEEV
LQNNHCLGSHEHIQNCRKWDTEIRLQLLTFSAMCQNLARTAEDDETPVDLNKHLYQIEKP
CKEAMTRHPVEELLDSYHNQVELALQIENQHRAVDQVIKAVRKICSALDGVETLAITESV
KKLKRAVNLPRSKTADVTSLFGGEDTSRSSTRGSLNPENPVQVSINQLTAAIYDLLRLHA
NSGRSPTDCAQSSKSVKEAWTTTEQLQFTIFAAHGISSNWVSNYEKYYLICSLSHNGKDL
FKPIQSKKVGTYKNFFYLIKWDELIIFPIQISQLPLESVLHLTLFGILNQSSGSSPDSNK
QRKGPEALGKVSLPLFDFKRFLTCGTKLLYLWTSSHTNSVPGTVTKKGYVMERIVLQVDF
PSPAFDIIYTTPQVDRSIIQQHNLETLENDIKGKLLDILHKDSSLGLSKEDKAFLWEKRY

YCFKHPNCLPKILASAPNWKWVNLAKTYSLLHQWPALYPLIALELLDSKFADQEVRSLAV
TWIEAISDDELTDLLPQFVQALKYEIYLNSSLVQFLLSRALGNIQIAHNLYWLLKDALHD
VQFSTRYEHVLGALLSVGGKRLREELLKQTKLVQLLGGVAEKVRQASGSARQVVLQRSME
RVQSFFQKNKCRLPLKPSLVAKELNIKSCSFFSSNAVPLKVTMVNADPMGEEINVMFKVG
EDLRQDMLALQMIKIMDKIWLKEGLDLRMVIFKCLSTGRDRGMVELVPASDTLRKIQVEY
GVTGSFKDKPLAEWLRKYNPSEEEYEKASENFIYSCAGCCVATYVLGICDRHNDNIMLRS
TGHMFHIDFGKFLGHAQMFGSFKRDRAPFVLTSDMAYVINGGEKPTIRFQLFVDLCCQAY
NLIRKQTNLFLNLLSLMIPSGLPELTSIQDLKYVRDALQPQTTDAEATIFFTRLIESSLG
SIATKFNFFIHNLAQLRFSGLPSNDEPILSFSPKTYSFRQDGRIKEVSVFTYHKKYNPDK
HYIYVVRILREGQIEPSFVFRTFDEFQELHNKLSIIFPLWKLPGFPNRMVLGRTHIKDVA
AKRKIELNSYLQSLMNASTDVAECDLVCTFFHPLLRDEKAEGIARSADAGSFSPTPGQIG
GAVKLSISYRNGTLFIMVMHIKDLVTEDGADPNPYVKTYLLPDNHKTSKRKTKISRKTRN
PTFNEMLVYSGYSKETLRQRELQLSVLSAESLRENFFLGGVTLPLKDFNLSKETVKWYQL
TAATYL
>Q24372
MWRPSISNCVWSTLLLAIFVQQTLAQRTPTISYITQEQIKDIGGTVEFDCSVQYAKEYNV
LFLKTDSDPVFLSTGSTLVIKDSRFSLRYDPNSSTYKLQIKDIQETDAGTYTCQVVISTV
HKVSAEVKLSVRRPPVISDNSTQSVVASEGSEVQMECYASGYPTPTITWRRENNAILPTD
SATYVGNTLRIKSVKKEDRGTYYCVADNGVSKGDRRNINVEVEFAPVITVPRPRLGQALQ
YDMDLECHIEAYPPPAIVWTKDDIQLANNQHYSISHFATADEYTDSTLRVITVEKRQYGD
YVCKATNRFGEAEARVNLFETIIPVCPPACGQAYIAGAEDVSATSFALVGILAALLFAR
>P13265
MAGTVRTACLLVAMLLGLGCLGQAQPPPPPDATCHQVRSFFQRLQPGLKWVPETPVPGSD
LQVCLPKGPTCCSRKMEEKYQLTARLNMEQLLQSASMELKFLIIQNAAVFQEAFEIVVRH
AKNYTNAMFKNNYPSLTPQAFEFVGEFFTDVSLYILGSDINVDDMVNELFDSLFPVIYTQ
MMNPGLPESVLDINECLRGARRDLKVFGSFPKLIMTQVSKSLQVTRIFLQALNLGIEVIN
TTDHLKFSKDCGRMLTRMWYCSYCQGLMMVKPCGGYCNVVMQGCMAGVVEIDKYWREYIL
SLEELVNGMYRIYDMENVLLGLFSTIHDSIQYVQKNGGKLTTTIGKLCAHSQQRQYRSAY
YPEDLFIDKKVLKVARVEHEETLSSRRRELIQKLKSFISFYSALPGYICSHSPVAENDTL
CWNGQELVERYSQKAARNGMKNQFNLHELKMKGPEPVVSQIIDKLKHINQLLRTMSVPKG
KVVDKSLDEEGLESGDCGDDEDECIGSSGDGMMKVKNQLRFLAELAYDLDVDDAPGNKQH
GNQKDNEITTSHSVGNMPSPLKILISVAIYVACFFSWCTDLPCPCLCCPAAPCGPPT
>Q9Y3P8
MNQADPRLRAVCLWTLTSAAMSRGDNCTDLLALGIPSITQAWGLWVLLGAVTLLFLISLA
AHLSQWTRGRSRSHPGQGRSGESVEEVPLYGNLHYLQTGRLSQDPEPDQQDPTLGGPARA
AEEVMCYTSLQLRPPQGRIPGPGTPVKYSEVVLDSEPKSQASGPEPELYASVCAQTRRAR
ASFPDQAYANSQPAAS
>Q05812
MVFGFTKRDRRVPDLSRYDYYYQNHEDYNKSPQLSAAAASAASAASPDRTNYSRSHSLVS
HAPSIPRQRSSVKSPGRRLSTSSAAPPTSRAAAKQYSQKTYSLRSQRSGEYHLHPPGYTT
NGSRMNSMTSGANVRRNYGKNKSTAGNNNDSRANSITVKTTQVTDPSGRTQSITKKTIRK
INGYEYVETTTTTKNLVPLGDSQRHFDEFSENYMLQDDDILEEQASDNIHDIIEENETDN
EKPYSPVSESHLQDDSELNVEKPDFPLGSYFHHKYSTDVMPLEEESSLSNFSDALDYIPP
THQTSSKYIHNKRKQASTTRRKKRPPAVKNAEAEAKKPLTEAEMYLKALEVAKRNVYHTD

AASDNASAPLGSNKSRKSRMGQKMTLRSSSDSPTATANLVKSNVEVQPKRFTSSFFSRNT
KSAPHEVHNHSVSTHFKSNKAVDPVPEPKSANTGLTDKEMYDQALKIAQARYYNSHGIQP
EAVDNSTTAAKPRQVGVSHLGSTGSIPPNEQHYLGDSEIPVQSEVHEYEPIPLQKTKTTG
SSKNKFKTMFDKVLQFSQENYGYQHKKEQGEQTPVTRNAEESFPAASISEGVTTAKPSSN
EGVMTNPVVTDSPSPLQQQIDSTTASSNGQSQGNVPTSAVASTTRTRSPELQDNLKSSSS
LLQDQTPQRQEDATDPTTSSTNELSAAEPTMVTSTHATKTIQAQTQDPPTKHKKSSFFTK
LFKKKSSR
>P26952
MAANLWLILGLLASHSSDLAAVREAPPTAVTTPIQNLHIDPAHYTLSWDPAPGADITTGA
FCRKGRDIFVWADPGLARCSFQSLSLCHVTNFTVFLGKDRAVAGSIQFPPDDDGDHEAAA
QDLRCWVHEGQLSCQWERGPKATGDVHYRMFWRDVRLGPAHNRECPHYHSLDVNTAGPAP
HGGHEGCTLDLDTVLGSTPNSPDLVPQVTITVNGSGRAGPVPCMDNTVDLQRAEVLAPPT
LTVECNGSEAHARWVARNRFHHGLLGYTLQVNQSSRSEPQEYNVSIPHFWVPNAGAISFR
VKSRSEVYPRKLSSWSEAWGLVCPPEVMPVKTALVTSVATVLGAGLVAAGLLLWWRKSLL
YRLCPPIPRLRLPLAGEMVVWEPALEDCEVTPVTDA
>P21849
MFGRFLLAIVILQLARTACTQEADDGKCKTCGVTIGQDTWCSECNGANYAPVNGQCVDVN
AEGPSKTLCPQHSAGKCTQCGGNSFMYKDGCYSSGEGLPGHSLCLSSDGDGVCTEAAPGY
FAPVGAANTEQSVIACGDTTGVTIAAGGNTYKGIADCAECSAPDATAGAEAGKVATCTKC
GVSKYLKDNVCVDKAQCNSGSTNKFVAVDDSENGNKCVSCSDNLNGGVANCDTCSYDEQS
KKIKCTKCTDNNYLKTTSEGTSCVQKDQCKDGFFPKDDSSAGNKCLPCNDSTDGIANCAT
CALVSGRSGAALVTCSACTDGYKPSADKTTCEAVSNCKTPGCKACSNEGKENEVCTDCDG
STYLTPTSQCIDSCAKIGNYYGATEGAKKLCKECTAANCKTCDDQGQCQACNDGFYKNGD
ACSPCHESCKTCSAGTASDCTECPTGKALRYGDDGTKGTCGEGCTTGTGAGACKTCGLTI
DGASYCSECATTTEYPQNGVCAPKASRATPTCNDSPIQNGVCGTCADNYFKMNGGCYETV
KYPGKTVCISAPNGGTCQKAADGYKLDSGTLTVCSEGCKECASSTDCTTCLDGYVKSASA
CTKCDASCETCNGAATTCKACATGYYKTASGEGACTSCESDSNGVTGIKGCLNCAPPPNN
KGSVLCYLIKDSGSTNKSGLSTGAIAGISVAVIVVVGGLIGFLCWWFLCRGKA
>Q6T3U4
MAAAWQGWLLWALLLNSAQGELYTPTHKAGFCTFYEECGKNPELSGGLTSLSNISCLSNT
PARHVTGDHLALLQRVCPRLYNGPNDTYACCSTKQLVSLDSSLSITKALLTRCPACSENF
VSIHCHNTCSPDQSLFINVTRVVQRDPGQLPAVVAYEAFYQRSFAEKAYESCSRVRIPAA
ASLAVGSMCGVYGSALCNAQRWLNFQGDTGNGLAPLDITFHLLEPGQALADGMKPLDGKI
TPCNESQGEDSAACSCQDCAASCPVIPPPPALRPSFYMGRMPGWLALIIIFTAVFVLLSV
VLVYLRVASNRNKNKTAGSQEAPNLPRKRRFSPHTVLGRFFESWGTRVASWPLTVLALSF
IVVIALSVGLTFIELTTDPVELWSAPKSQARKEKAFHDEHFGPFFRTNQIFVTAKNRSSY
KYDSLLLGPKNFSGILSLDLLQELLELQERLRHLQVWSHEAQRNISLQDICYAPLNPHNT
SLTDCCVNSLLQYFQNNHTLLLLTANQTLNGQTSLVDWKDHFLYCANAPLTYKDGTALAL
SCIADYGAPVFPFLAVGGYQGTDYSEAEALIITFSINNYPADDPRMAHAKLWEEAFLKEM
QSFQRSTADKFQIAFSAERSLEDEINRTTIQDLPVFAISYLIVFLYISLALGSYSRWSRV
AVDSKATLGLGGVAVVLGAVVAAMGFYSYLGVPSSLVIIQVVPFLVLAVGADNIFIFVLE
YQRLPRMPGEQREAHIGRTLGSVAPSMLLCSLSEAICFFLGALTSMPAVRTFALTSGLAI
IFDFLLQMTAFVALLSLDSKRQEASRPDVVCCFSSRNLPPPKQKEGLLLCFFRKIYTPFL
LHRFIRPVVLLLFLVLFGANLYLMCNISVGLDQDLALPKDSYLIDYFLFLNRYLEVGPPV

YFDTTSGYNFSTEAGMNAICSSAGCESFSLTQKIQYASEFPNQSYVAIAASSWVDDFIDW
LTPSSSCCRIYTRGPHKDEFCPSTDTSFNCLKNCMNRTLGPVRPTTEQFHKYLPWFLNDT
PNIRCPKGGLAAYRTSVNLSSDGQIIASQFMAYHKPLRNSQDFTEALRASRLLAANITAE
LRKVPGTDPNFEVFPYTISNVFYQQYLTVLPEGIFTLALCFVPTFVVCYLLLGLDIRSGI
LNLLSIIMILVDTIGLMAVWGISYNAVSLINLVTAVGMSVEFVSHITRSFAVSTKPTRLE
RAKDATIFMGSAVFAGVAMTNFPGILILGFAQAQLIQIFFFRLNLLITLLGLLHGLVFLP
VVLSYLGPDVNQALVLEEKLATEAAMVSEPSCPQYPFPADANTSDYVNYGFNPEFIPEIN
AASSSLPKSDQKF
>P54244
MTLKIRTIIILFCVISVTTTSQSLNATLKTFDPRLLNSTADRDIAMKNVPLVRLTRHLLS
PERYDVRVRPILDHKKSLKVHISISLYQIIEVDEPSQNIKLNVWMIQKWRDEYLDWNPNE
YGMINSTIIPFHHLWIPDTYLYNSVKMSRDETERYMNIQATSNYWKGEKGAELSFLYPAI
YTITCRLNIRFFPYDRQNCTLTISSWTNSKSALDYYADTEVSMQSFIPNEEWQVKSFKIH
RHEYKYACCAEPWVILQASLVIQRKPLYYLVNLIIPTSIITLVAITGFFTPASTDDDRTE
KINLGITTLLAMSILMLMVSDQMPTTSEFVPLIAWFYLSIIIIISIGTFLTSVVLSVQGR
RQYGRNPPQFIRYIFFVLLPQVLLLNVPPPLQTLWGELDDDPLNVRRRKKSHYLSRNVNN
GSTKMASPMSTLRVPQSAGSVSEKRQSFQMIDVTSPNSPNTARSRAPSLAPSTAKATMWE
GTMSALAGTNTQLRRTSNVFNKEVDEMRRKRQCSLEWEFLATVLDRFLLIVFVGAVVIVT
AGLILVGRMAQYSYDHPDDRFFNV
>O14654
MASCSFTRDQATRRLRGAAAAAAAALAAVVTTPLLSSGTPTALIGTGSSCPGAMWLSTAT
GSRSDSESEEEDLPVGEEVCKRGYLRKQKHGHRRYFVLKLETADAPARLEYYENARKFRH
SVRAAAAAAAAAASGAAIPPLIPPRRVITLYQCFSVSQRADARYRHLIALFTQDEYFAMV
AENESEQESWYLLLSRLILESKRRRCGTLGAQPDGEPAALAAAAAAEPPFYKDVWQVIVK
PRGLGHRKELSGVFRLCLTDEEVVFVRLNTEVASVVVQLLSIRRCGHSEQYFFLEVGRST
VIGPGELWMQVDDCVVAQNMHELFLEKMRALCADEYRARCRSYSISIGAHLLTLLSARRH
LGLVPLEPGGWLRRSRFEQFCHLRAIGDGEDEMLFTRRFVTPSEPVAHSRRGRLHLPRGR
RSRRAVSVPASFFRRLAPSPARPRHPAEAPNNGARLSSEVSGSGSGNFGEEGNPQGKEDQ
EGSGGDYMPMNNWGSGNGRGSGGGQGSNGQGSSSHSSGGNQCSGEGQGSRGGQGSNGQGS
GGNQCSRDGQGTAGGHGSGGGQRPGGGHGSGGGQGPGDGHGSGGGKNSGGGKGSGSGKGS
DGDGERGKSLKKRSYFGKLTQSKQQQMPPPPPPPPPPPPAGGTGGKGKSGGRFRLYFCVD
RGATKECKEAKEVKDAEIPEGAARGPHRARAFDEDEDDPYVPMRPGVATPLVSSSDYMPM
APQNVSASKKRHSRSPFEDSRGYMMMFPRVSPPPAPSPPKAPDTNKEDDSKDNDSESDYM
FMAPGAGAIPKNPRNPQGGSSSKSWSSYFSLPNPFRSSPLGQNDNSEYVPMLPGKFLGRG
LDKEVSYNWDPKDAASKPSGEGSFSKPGDGGSPSKPSDHEPPKNKAKRPNRLSFITKGYK
IKPKPQKPTHEQREADSSSDYVNMDFTKRESNTPAPSTQGLPDSWGIIAEPRQSAFSNYV
NVEFGVPFPNPANDLSDLLRAIPRANPLSLDSARWPLPPLPLSATGSNAIEEEGDYIEVI
FNSAMTPAMALADSAIRYDAETGRIYVVDPFSECCMDISLSPSRCSEPPPVARLLQEEEQ
ERRRPQSRSQSFFAAARAAVSAFPTDSLERDLSPSSAPAVASAAEPTLALSQVVAAASAL
AAAPGIGAAAAAAGFDSASARWFQPVANAADAEAVRGAQDVAGGSNPGAHNPSANLARGD
NQAGGAAAAAAAPEPPPRSRRVPRPPEREDSDNDDDTHVRMDFARRDNQFDSPKRGR
>Q9NS86
MGETMSKRLKLHLGGEAEMEERAFVNPFPDYEAAAGALLASGAAEETGCVRPPATTDEPG
LPFHQDGKIIHNFIRRIQTKIKDLLQQMEEGLKTADPHDCSAYTGWTGIALLYLQLYRVT

CDQTYLLRSLDYVKRTLRNLNGRRVTFLCGDAGPLAVGAVIYHKLRSDCESQECVTKLLQ
LQRSVVCQESDLPDELLYGRAGYLYALLYLNTEIGPGTVCESAIKEVVNAIIESGKTLSR
EERKTERCPLLYQWHRKQYVGAAHGMAGIYYMLMQPAAKVDQETLTEMVKPSIDYVRHKK
FRSGNYPSSLSNETDRLVHWCHGAPGVIHMLMQAYKVFKEEKYLKEAMECSDVIWQRGLL
RKGYGICHGTAGNGYSFLSLYRLTQDKKYLYRACKFAEWCLDYGAHGCRIPDRPYSLFEG
MAGAIHFLSDVLGPETSRFPAFELDSSKRD
>Q08902
MSTSSSVTQKNLDTNAEALKKEDKVLSEFDIQDERPKSLLWESAFVGVLCSAQLMTQAGL
GQSLAPLHIIGNSFGTTNAGQLSWFASAYSLTVGTFILIAGRLGDIFGHKKFFVLGFFWY
ALWSLLAGFSVYSNQIFFDCCRAFQGMGPAFLLPNAIAILGRTYKPGRRKNMVFSLFGAS
APGGFFLGAVFSSMLGQLAWWPWAYWIMGIACFVLAVAGYFVIPHTPMPSRDASSFKLLE
RIDFAGSVTGVVGLILFNFAWNQGPVVGWQTPYTYALLIVGTFFLVIFAYIESRAAFPLL
PFAALSSDTAFVLSCIAAGWASFGIWIFYTWQFMEDSRGQTPLLSSAQFSPVAISGFCAA
VTTGFLLSHTPPSTVMLFAMTAFTVGTILIATAPVHQTYWAQTFVSIIVMPWGMDMSFPA
ATIMLSDSMPHEHQGLAASLVNTVVNYSISIGLGIAGTIESRVNDGGAKPLKGYRCSWYM
GIGLSGLGIFVAATYAWSTFMKSKKRISEKQHFIE
>Q12489
MSAQDYYGNSASKQSYSRPSAPPPGYETASRGYAPSQSQQNYYPPQQQQQQYQQQPQYYQ
QQQPQYYQQHPQQPIYVQQQPASSGNEDCLAGCLAGLCLCCTLDMLF
>Q3U9N9
MVPSQEEPAAERETNEAQPPGPAPSDDAPLPGPGPSDVSDVAAEKVEVELTRSAGSEPPV
PPEGGWGWLVMLAAMWCNGSVFGIQNAYGVLFVSMLDTFKAKDDDNMAFKTAWVGSLSMG
MIFFCCPIVSVFTDMFGCRRTAVVGAAVGFIGLMSSSFVSSIEPLYLTYGIIFACGCSFA
YQPSLVILGHYFKKRLGLVNGIVTAGSSVFTILLPLLLGNLISSVKLFNTLRILCIFMFV
LFLAGFTYRPLVPSTKEKESGGSRSSFFSRRKLSPPKKVFNFALFKETTYAVWAAGIPLA
LFGYFVPYVHLMNHVKERFQDVNNKEVLFMCIGITSGVGRLLFGRIADYLPGVKKVYLQV
LSFFFIGLMSMMIPLCSAFGALIAVCLAMGLFDGCFISIMAPIAFELVGPQDASQAIGFL
LGFMSIPMTVGPPIAGLLHDKLGTYDVAFYLAGIPPFVGGVVLCLIPWIHSKKQRKISKN
AGGEKMEKMLENQSSLLSGSSGIFKKDSASII
>O44783
MDRRNGGDPLAPPRPPKLLPRVHRPRAPEPTLSGVDHNAGATASALASGASSAAPVAIHN
NNSQQQLSISAAASNNNTISIIPASPDFDDYQIHHLTFLPQRPSSLSRNSSTASSTTATG
ISVSGSGSVSGSSSSFTRRRPPAPVPLNNSISNNNNNSINNNFLSHFQSAEPASNALGQP
PASPVTLAQPRPESERLTNEYVDTPLQHATRSQHPAGQQDNGQTTTHHLLLLPQRNQHLH
LQQHQQHLQQQQQQQQQQQQQQHLQHQQNQQHARLATTTQATSVGSDHTDGLLHSHLQNS
TTKPPASKQPAPPRLGMGLGLGLGLGLNQPIITKQPTPATQKERMHALEELLQPGGAGGN
GGPLVMAGDPSLLNPIVCPRCGRCRCEQCQSPRPLPQTWVCNKTCLCSAESVIDYASCLC
CAKALFYHCARDNDLDCDDGNGTPCVDNPCSCGPYKRTQRWGWLGALSIFLPCLWFYWPM
RGCMKLCEKCYGRFAGRGCRCQGIGGGGAGSGGGVGSIGSTSSMLPIVPLGVNGSGLGGG
VSLSGGVTDGGLNQANGKAMDHGCSAARSILRKGDLTPEKRLLDSSPDY
>Q90632
MGRADREEGQLPAPVKPPDGGWGWIVLFGCFVITGFSYAFPKAVSVYFKELMKDFHVGYS
DTAWISSIMLAMLYGTGPVCSIMVNQFGCRPVMLIGGLLASSGMILASFTTNIIELYLTA
GVLTGLGMALNFQPSLIMLGTYFDKRRPLANGLAAAGSPVFLSSLSPLGQVLLEKFGWRG

GFLIMGGLLLNCCTCGAVMRPLDAGMKRKTEKAQDKYEAKEMLPIGGKSEEGISTTDGTK
KTKKAKKKPKKGKKLLDFSIFSNRGFIIYTISKFILVLGLFVPPILLVNYPKDTGVPDTE
AAFLLSIIGFIDIFARPACGMVAGLKWVRPHVAYLFSFAMLFNGSTDICSARASNYTGLV
IFCVFFGISYGMVGALQFEVLMAIVGSQKFSSAIGLVLLIEAFAVLIGPPSAGRLVDALK
NYEVIFYLAGSEVVLSALFLPWATYCCLNRGKKTPPPEKNPSAGGGSDTEEAESDVQEAE
EHSSDNHQPAHGTDKATVAANEEANHVEDEQSGEGGRCPEADGEASSRAGCNADQTVERD
SF
>P40061
MSIPHSAKQSSPLSSRRRSVTNTTPLLTPRHSRDNSSTQISSAKNITSSSPSTITNESSK
RNKQNLVLSTSFISTKRLENSAPSPTSPLMARRTRSTMTKALLNLKAEINNQYQELARLR
KKKDDIEHLRDSTISDIYSGSYSTNHLQKHSMRIRANTQLREIDNSIKRVEKHIFDLKQQ
FDKKRQRSLTTSSSIKADVGSIRNDDGQNNDSEELGDHDSLTDQVTLDDEYLTTPTSGTE
RNSQQNLNRNSTVNSRNNENHSTLSIPDLDGSNKVNLTGDTEKDLGDLENENQIFTSTTT
EAATWLVSDYMQSFQEKNVNPDFIAQKANGLVTLLKEHSEIRKDLVLTSFMSSIQNLLLN
GNKLIAASAYRVCRYLINSSIFIDELLELRLDAFIIISLAKDNSFQIEREQALKMVRRFI
EYNNGVTQGIMQAIISCVEKPEDSLRHMALETLLELCFVAPEMVKECRGMRVIEGFLQDY
TSFSLASVILDTILQLMATHKTRQHFLEDFNVSVLTTVFSDTNTKSNVNVEKMQNASTLI
SITLNSYNGFMLFSNNNFKPLKQLVSFFQIPICAQYLIDIFLDVLKIKPLPYKPRGRHSH
SFKPIPSQYYKECMSVNQRLALIVLILENSEFVPHLLELLNEEDRDDHLVAKGRYLLTEY
FNLRMNLVDKKYTSVSKPIYKENFTYVNETFQFKKIAYKMNRNRNTIGMSGIDYAQNIKS
FSKNIKENTLLREVDDFRFRRMVYDSKVLQTKDFTRWNWNIINELLEGPLLNKKQLEELV
KSTKFIRRLLVFYRPLRLRFSNVNKGAKLSQKYVQVGCQFFKTLTATPEGMKILMDDTKI
IPQLASLMFRAMEGNISGNIFNKNKLREKIIFGYFKFIGILTQSKNGVHILTRWNFFTVI
YKMFQFESKLGLEFLLLTIPELDLKYSSHCRVIIGKALVVANEKVRIEATKHIGDKLKEL
LSTKESDLKLKANKVKLQQFKMEMLTRQLYDLSPSVVAVADQALYECIVAGNGSEELGTS
FRMFLNQMVFIRSPILFELLSRPYGFQLLNEINFVKEERDSWLSKKNIEYVHIVEEFLKK
NESINAKSLTFQQKSRLPLHFYESLTKTEDGILLLSQTGDLVTFMNVIKKYVNGNNMATV
ENAKEILDLKAALWCVGFIGSTELGIGLLDNYSLVEDIIEVAYNASVTSVRFTAFYVLGL
ISMTREGCEILDEMGWNCCVSVQDEPIGIALPNRLDRFLSYNEHKWSAFGEYSDEMIVFN
KSDGDLIEKCLPIEFDLDKLLKEKDTAENPLNEKIITNKYDNDITSQTITVSGENSSLFA
NEGLSSPYVTQYRNDDDSIESKVLHIVSQLGNHILSNHAVKEITEINNKYGPRLFENEKM
FFKVFNMMSKYRFKPHVRKFLCGLFINNRALENVIRHDNKRDKRPANFTR
>Q86UT5
MVTPSPPGNHSLSLEAPRLHTASDLLGNHSLGLPLITALVGSRDRRGRVFSPVPVPLPTN
PTTQHPTRQKLPSTLSGHRVCQAHGEPVLGLCPLLPLFCCPPHPPDPWSLERPRFCLLSK
EEGKSFGFHLQQELGRAGHVVCRVDPGTSAQRQGLQEGDRILAVNNDVVEHEDYAVVVRR
IRASSPRVLLTVLARHAHDVARAQLGEDAHLCPTLGPGVRPRLCHIVKDEGGFGFSVTHG
NQGPFWLVLSTGGAAERAGVPPGARLLEVNGVSVEKFTHNQLTRKLWQSGQQVTLLVAGP
EVEEQCRQLGLPLAAPLAEGWALPTKPRCLHLEKGPQGFGFLLREEKGLDGRPGQFLWEV
DPGLPAKKAGMQAGDRLVAVAGESVEGLGHEETVSRIQGQGSCVSLTVVDPEADRFFSMV
RLSPLLFLENTEAPASPQGSSSASLVETEDPSLEDTSVPSVPLGSRQCFLYPGPGGSYGF
RLSCVASGPRLFISQVTPGGSAARAGLQVGDVILEVNGYPVGGQNDLERLQQLPEAEPPL
CLKLAARSLRGLEAWIPPGAAEDWALASDLL
>Q62976

MANGGGGGGGGGSSGSSGGGGGGGGGGETALRMSSNIHANHLSLDASSSSSSSSSSSSSSSS
SVHEPKMDALIIPVTMEVPCDSRGQRMWWAFLASSMVTFFGGLFIILLWRTLKYLWTVCC
HCGGKTKEAQKINNGSSQADGTLKPVDEKEEVVAAEVGWMTSVKDWAGVMISAQTLTGRV
LVVLVFALSIGALVIYFIDSSNPIESCQNFYKDFTLQIDMAFNVFFLLYFGLRFIAANDK
LWFWLEVNSVVDFFTVPPVFVSVYLNRSWLGLRFLRALRLIQFSEILQFLNILKTSNSIK
LVNLLSIFISTWLTAAGFIHLVENSGDPWENFQNNQALTYWECVYLLMVTMSTVGYGDVY
AKTTLGRLFMVFFILGGLAMFASYVPEIIELIGNRKKYGGSYSAVSGRKHIVVCGHITLE
SVSNFLKDFLHKDRDDVNVEIVFLHNISPNLELEALFKRHFTQVEFYQGSVLNPHDLARV
KIESADACLILANKYCADPDAEDASNIMRVISIKNYHPKIRIITQMLQYHNKAHLLNIPS
WNWKEGDDAICLAELKLGFIAQSCLAQGLSTMLANLFSMRSFIKIEEDTWQKYYLEGVSN
EMYTEYLSSAFVGLSFPTVCELCFVKLKLLMIAIEYKSANRESRSRKRILINPGNHLKIQ
EGTLGFFIASDAKEVKRAFFYCKACHDDVTDPKRIKKCGCRRLEDEQPPTLSPKKKQRNG
GMRNSPNTSPKLMRHDPLLIPGNDQIDNMDSNVKKYDSTGMFHWCAPKEIEKVILTRSEA
AMTVLSGHVVVCIFGDVSSALIGLRNLVMPLRASNFHYHELKHIVFVGSIEYLKREWETL
HNFPKVSILPGTPLSRADLRAVNINLCDMCVILSANQNNIDDTSLQDKECILASLNIKSM
QFDDSIGVLQANSQGFTPPGMDRSSPDNSPVHGMLRQPSITTGVNIPIITELAKPGKLPL
VSVNQEKNSGTHILMITELVNDTNVQFLDQDDDDDPDTELYLTQPFACGTAFAVSVLDSL
MSATYFNDNILTLIRTLVTGGATPELEALIAEENALRGGYSTPQTLANRDRCRVAQLALL
DGPFADLGDGGCYGDLFCKALKTYNMLCFGIYRLRDAHLSTPSQCTKRYVITNPPYEFEL
VPTDLIFCLMQFDHNAGQSRASLSHSSHSSQSSSKKSSSVHSIPSTANRPNRPKSRESRD
KQKKEMVYR
>Q5GH77
METVFEEMDEESTGGVSSSKEEIVLGQRLHLSFPFSIIFSTVLYCGEVAFGLYMFEIYRK
ANDTFWMSFTISFIIVGAILDQIILMFFNKDLRRNKAALLFWHILLLGPIVRCLHTIRNY
HKWLKNLKQEKEETQVSITKRNTMLEREIAFSIRDNFMQQKAFKYMSVIQAFLGSVPQLI
LQMYISLTIREWPLNRALLMTFSLLSVTYGAIRCNILAIQISNDDTTIKLPPIEFFCVVM
WRFLEVISRVVTLAFFIASLKLKSLPVLLIIYFVSLLAPWLEFWKSGAHLPGNKENNSNM
VGTVLMLFLITLLYAAINFSCWSAVKLQLSDDKIIDGRQRWGHRILHYSFQFLENVIMIL
VFRFFGGKTLLNCCDSLIAVQLIISYLLATGFMLLFYQYLYPWQSGKVLPGRTENQPEAP
YYYVNIEKTEKNKNKQLRNYCHSCNRVGYFSIRKSMTCS
>Q96K78
MASCRAWNLRVLVAVVCGLLTGIILGLGIWRIVIRIQRGKSTSSSSTPTEFCRNGGTWEN
GRCICTEEWKGLRCTIANFCENSTYMGFTFARIPVGRYGPSLQTCGKDTPNAGNPMAVRL
CSLSLYGEIELQKVTIGNCNENLETLEKQVEDVTAPLNNISSEVQILTSDANKLTAENIT
SATRVVGQIFNTSRNASPEAKKVAIVTVSQLLDASEDAFQRVAATANDDALTTLIEQMET
YSLSLGNQSVVEPNIAIQSANFSSENAVGPSNVRFSVQKGASSSLVSSSTFIHTNVDGLN
PDAQTELQVLLNMTKNYTKTCGFVVYQNDKLFQSKTFTAKSDFSQKIISSKTDENEQDQS
ASVDMVFSPKYNQKEFQLYSYACVYWNLSAKDWDTYGCQKDKGTDGFLRCRCNHTTNFAV
LMTFKKDYQYPKSLDILSNVGCALSVTGLALTVIFQIVTRKVRKTSVTWVLVNLCISMLI
FNLLFVFGIENSNKNLQTSDGDINNIDFDNNDIPRTDTINIPNPMCTAIAALLHYFLLVT
FTWNALSAAQLYYLLIRTMKPLPRHFILFISLIGWGVPAIVVAITVGVIYSQNGNNPQWE
LDYRQEKICWLAIPEPNGVIKSPLLWSFIVPVTIILISNVVMFITISIKVLWKNNQNLTS
TKKVSSMKKIVSTLSVAVVFGITWILAYLMLVNDDSIRIVFSYIFCLFNTTQGLQIFILY
TVRTKVFQSEASKVLMLLSSIGRRKSLPSVTRPRLRVKMYNFLRSLPTLHERFRLLETSP

STEEITLSESDNAKESI
>Q9Y5X5
MNSFFGTPAASWCLLESDVSSAPDKEAGRERRALSVQQRGGPAWSGSLEWSRQSAGDRRR
LGLSRQTAKSSWSRSRDRTCCCRRAWWILVPAADRARRERFIMNEKWDTNSSENWHPIWN
VNDTKHHLYSDINITYVNYYLHQPQVAAIFIISYFLIFFLCMMGNTVVCFIVMRNKHMHT
VTNLFILNLAISDLLVGIFCMPITLLDNIIAGWPFGNTMCKISGLVQGISVAASVFTLVA
IAVDRFQCVVYPFKPKLTIKTAFVIIMIIWVLAITIMSPSAVMLHVQEEKYYRVRLNSQN
KTSPVYWCREDWPNQEMRKIYTTVLFANIYLAPLSLIVIMYGRIGISLFRAAVPHTGRKN
QEQWHVVSRKKQKIIKMLLIVALLFILSWLPLWTLMMLSDYADLSPNELQIINIYIYPFA
HWLAFGNSSVNPIIYGFFNENFRRGFQEAFQLQLCQKRAKPMEAYALKAKSHVLINTSNQ
LVQESTFQNPHGETLLYRKSAEKPQQELVMEELKETTNSSEI
>O95278
MRFRFGVVVPPAVAGARPELLVVGSRPELGRWEPRGAVRLRPAGTAAGDGALALQEPGLW
LGEVELAAEEAAQDGAEPGRVDTFWYKFLKREPGGELSWEGNGPHHDRCCTYNENNLVDG
VYCLPIGHWIEATGHTNEMKHTTDFYFNIAGHQAMHYSRILPNIWLGSCPRQVEHVTIKL
KHELGITAVMNFQTEWDIVQNSSGCNRYPEPMTPDTMIKLYREEGLAYIWMPTPDMSTEG
RVQMLPQAVCLLHALLEKGHIVYVHCNAGVGRSTAAVCGWLQYVMGWNLRKVQYFLMAKR
PAVYIDEEALARAQEDFFQKFGKVRSSVCSL
>Q91X85
MVNESLNQEESNDRPAPESEFQMDTSYSTQPSGSIHPSVSGHPSVSGHPSVSGHPSVSIH
PSVSIDPSVSVRPSSSALPSTLAQPSGLTHHSSLVREDSVIKVSKRRWVVVLVFSCYSLC
NAFQWIQYGSINNIFMNFYGVSAFAIDWLSMCYMLTYIPLLLPVAWMLEKFGLRTIAITG
SALNCLGAWVKLGSLEPHLFPVTMVGQVICSVAQVFILGMPSRIASVWFGADEVSTACSV
AVFGNQLGIAIGFLVPPVLVPNIKDPEKLAYHISIMFYIIGGVATFLFILVIIVFKEKPK
HPPSRAQSLSYALATTDASYLSSIVRLFKNLNFVLLVITYGLNAGAFYALSTLLNRMVIL
HFPGEEVNAGRIGLTIVIAGMFGAMISGIWLDKSKTYKETTLVVYIMTLVGMVVYTFTLN
LNHLWIVFITAGTLGFFMTGYLPLGFEFAVEFTYPESEGVSSGLLNVSAQVFGIVFTISQ
GQIIDNHGTMFGNIFLCVFLALGSALTAFIKSDLRRQRANKDAPETKVQEEEEEEEGSNT
SKVPVVSEAHL
>Q9WTY2
MPVPYKHQLRRAQAVDELDWPPQASSSGSSDSLGSGEAALAQKDGVFKVMLLGESGVGKS
TLAGTFGGLQGDNAHEMENSEDTYERRIMVDKEEVTLIVYDIWEQGDAGGWLQDHCLQTG
DAFLIVFSVTDRRSFSKVPETLLRLRAGRPHHDLPVILVGNKSDLARSREVSLEEGRHLA
GTLSCKHIETSAALHHNTRELFEGAVRQIRLRRGRGHAGGQRPEPSSPDGPAPPTRRESL
TKKAKRFLANLVPRNAKFFKQRSRSCHDLSVL
>P59822
MTLLWCVVSLYFYGILQSDASERCDDWGLDTMRQIQVFEDEPARIKCPLFEHFLKFNYST
AHSAGLTLIWYWTRQDRDLEEPINFRLPENRISKEKDVLWFRPTLLNDTGNYTCMLRNTT
YCSKVAFPLEVVQKDSCFNSPMKLPVHKLYIEYGIQRITCPNVDGYFPSSVKPTITWYMG
CYKIQNFNNVIPEGMNLSFLIAFISNNGNYTCVVTYPENGRTFHLTRTLTVKVVGSPKNA
VPPVIHSPNDHVVYEKEPGEELLIPCTVYFSFLMDSRNEVWWTIDGKKPDDIPIDVTINE
SISHSRTEDETRTQILSIKKVTSEDLKRSYVCHARSAKGEVAKAATVKQKVPAPRYTVEL
ACGFGATVLLVVILIVVYHVYWLEMVLFYRAHFGTDETILDGKEYDIYVSYARNAEEEEF
VLLTLRGVLENEFGYKLCIFDRDSLPGGIVTDETLSFIQKSRRLLVVLSPNYVLQGTQAL

LELKAGLENMASQGNINVILVQYKAVKETKVKELKRAKTVLTVIKWKGEKSKYPQGRFWK
QLQVAMPVKKSPRRSSSDEQGLSYSSLKNV
>Q3E756
MSFIPIVCGMKSFDSSYDTVPGHQNLYCPNCHNYSVGPIKRKEFFTIWFIPLVPVFWGKQ
LHCPICNWRQDFKNDEQLNKVIQEQQNLRQKQPN
>Q9Z1Y0
MPPRELSEAEPPPLPASTPPPRRRSAPPELGIKCVLVGDGAVGKSSLIVSYTCNGYPSRY
RPTALDTFSVQVLVDGAPVRIELWDTAGQEDFDRLRSLCYPDTDVFLACFSVVQPSSFQN
ITEKWLPEIRTHNPQAPVLLVGTQADLRDDVNVLIQLDQGGREGPVPEPQAQGLAEKIRA
CCYLECSALTQKNLKEVFDSAILSAIEHKARLEKKLNAKGVRTLSRCRWKKFFCFV
>Q94BT2
MASSSSSLLILAVACFVSLISPAISQQACKSQNLNSAGPFDSCEDLPVLNSYLHYTYNSS
NSSLSVAFVATPSQANGGWVAWAINPTGTKMAGSQAFLAYRSGGGAAPVVKTYNISSYSS
LVEGKLAFDFWNLRAESLSGGRIAIFTTVKVPAGADSVNQVWQIGGNVTNGRPGVHPFGP
DNLGSHRVLSFTEDAAPGSAPSPGSAPAPGTSGSTTPGTAAGGPGNAGSLTRNVNFGVNL
GILVLLGSIFIF
>Q86SU0
MAWPKLPAPWLLLCTWLPAGCLSLLVTVQHTERYVTLFASIILKCDYTTSAQLQDVVVTW
RFKSFCKDPIFDYYSASYQAALSLGQDPSNDCNDNQREVRIVAQRRGQNEPVLGVDYRQR
KITIQNRADLVINEVMWWDHGVYYCTIEAPGDTSGDPDKEVKLIVLHWLTVIFIILGALL
LLLLIGVCWCQCCPQYCCCYIRCPCCPAHCCCPEEALARHRYMKQAQALGPQMMGKPLYW
GADRSSQVSSYPMHPLLQRDLSLPSSLPQMPMTQTTNQPPIANGVLEYLEKELRNLNLAQ
PLPPDLKGRFGHPCSMLSSLGSEVVERRIIHLPPLIRDLSSSRRTSDSLHQQWLTPIPSR
PWDLREGRSHHHYPDFHQELQDRGPKSWALERRELDPSWSGRHRSSRLNGSPIHWSDRDS
LSDVPSSSEARWRPSHPPFRSRCQERPRRPSPRESTQRHGRRRRHRSYSPPLPSGLSSWS
SEEDKERQPQSWRAHRRGSHSPHWPEEKPPSYRSLDITPGKNSRKKGSVERRSEKDSSHS
GRSVVI
>Q12361
MITEGFPPNLNALKGSSLLEKRVDSLRQLNTTTVNQLLGLPGMTSTFTAPQLLQLRIIAI
TASAVSLIAGCLGMFFLSKMDKRRKVFRHDLIAFLIICDFLKAFILMIYPMIILINNSVY
ATPAFFNTLGWFTAFAIEGADMAIMIFAIHFAILIFKPNWKWRNKRSGNMEGGLYKKRSY
IWPITALVPAILASLAFINYNKLNDDSDTTIILDNNNYNFPDSPRQGGYKPWSAWCYLPP
KPYWYKIVLSWGPRYFIIIFIFAVYLSIYIFITSESKRIKAQIGDFNHNVLEEEKEKKKL
FGLGHWGKAKWYFRSYFKLPLLHLLRNLKNFFTISFIDPNEETDDSGSSNGTFNFGESSN
EIPTLFRKTNTGSDENVSASGGVRLLDYNSAKPLDMSKYAMSEQPDLERNNPFDCENDIT
LNPSELVSKQKEHKVTFSVENEGLDTRKSSMLGHQTFSCQNSLESPLAMYDNKNDNSDIT
SNIKEKGGIINNNSNNDDDDNNNNNDNDNDNNNSNNNNNNNNNNNNNNNNNNNNNNNNNN
NNNNSNNIKNNVDNNNTNPADNIPTLSNEAFTPSQQFSQERVNNNADRCENSSFTNVQQH
FQAQTYKQMKKRRAQIQKNLRAIFIYPLSYIGIWLFPIIADALQYNHEIKHGPTMWVTYI
DTCVRPLSCLVDVIVYLFKEKPWNYSWAKTESKYLIEKYILKGELGEKEILKFCHSNWGK
RGWYYRGKWKKRKCWKYSTNPLKRILWFVERFFKQLFELKLHFSFYDNCDDFEYWENYYS
AKDSNDNKRTESDETKTNSSDRSLPSNSLELQAMLNNITAEEVEVPLFWRIIHHIPMLGG
IDLDELNRLLKIRYNNDHFSLPGLKFALNQNKSHDKHQDVSTNSMVKSSFFSSNIVTNDD
ENSIEEDKNLRYSDASASENYLVKPTIPGTTPDPIIEAQNDNDSSDSSGIDLIAFLRNGP

L
>Q8BG67
MPTRVCCCCSALRPRYKRLVDNIFPEDPKDGLVKADMEKLTFYAVSAPEKLDRIGAYLAE
RLSRDVVRHRSGYVLIAMEALDQLLMACHSQSIKPFVESFLHMVAKLLESGEPKLQVLGT
NSFVKFANIEEDTPSYHRRYDFFVSRFSAMCHSCHSDPEIRTEIRIAGIRGIQGVVRKTV
NDELRATIWEPQHMDKIVPSLLFNMQKIEEVDSRLGPPSSPSAADKEENPAVLAESCFRE
LLGRATFGNMNNAVRPVFAHLDHHKLWDPNEFAVHCFKIIMYSIQAQYSHHVIQEILGHL
DARRKDSPRVRAGIIQVLLEAVAIAAKGSIGPTVLEVFNTLLKHLRLSVELEANDSQKGS
VGSVTVSSKDNDEKIVQNAVIQTIGFFGSNLPDYQRSEIMMFIMGKVPVFGTSTHTLDIS
QLGDLGTRRIQIMLLRSLLMVTSGYKAKTIVTALPGSFLDPLLSPSLMEDYELRQLVLEV
MHNLMDRHDNRAKLRGIRIIPDVADLKIKREKICRQDTSFMKKNGQQLYRHIYLGCKEED
NVQKNYELLYTSLALITIELANEEVVIDLIRLAIALQDSAIINEDNLSMFHRCGIMALVA
AYLNFVSQMIAVPAFCQHVSKVIETRTMEAPYFLPEHIFRDKCMLPKSLEKHDKNLYFLT
NKIAESLGGSGYSVERLTVPYVPQVTDEDRLSRRKSIVDTVSIQVDILSNSVPSDDVVSN
TEEITFEALKKAIDTNGMEEQEKEKRRLVIEKFQKAPFEEIAAQCESKANLLHDRLAQIL
ELTIRPPPSPSGTLTVTSGHTQYQSVPVYEMKFPDLCVY
>Q28175
MSSQVEHPAGGYKKLFETVEELSSPLTAHVTGRIPLWLTGSLLRCGPGLFEVGSEPFYHL
FDGQALLHKFDFKEGHVTYHRRFIRTDAYVRAMTEKRIVITEFGTCAFPDPCKNIFSRFF
SYFRGVEVTDNALVNIYPVGEDYYACTETNFITKVNPETLETIKQVDLCNYVSVNGATAH
PHIENDGTVYNIGNCFGKNFSIAYNIVKIPPLQADKEDPISKSEIVVQFPCSDRFKPSYV
HSFGLTPNYIVFVETPVKINLFKFLSSWSLWGANYMDCFESNETMGVWLHIADKKRKKYI
NNKYRTSPFNLFHHINTYEDHEFLIVDLCCWKGFEFVYNYSYLANLRENWEEVKKNARKA
PQPEVRRYVLPLNIDKADTGKNLVTLPNTTATAILCSDETIWLEPEVLFSGPRQAFEFPQ
INYQKYGGKPYTYAYGLGLNHFVPDRLCKLNVKTKETWVWQEPDSYPSEPIFVSHPDALE
EDDGVVLSVVVSPGAGQKPAYLLILNAKDLSEVARAEVEINIPVTFHGLFKKS
>Q9NRM6
MSLVLLSLAALCRSAVPREPTVQCGSETGPSPEWMLQHDLIPGDLRDLRVEPVTTSVATG
DYSILMNVSWVLRADASIRLLKATKICVTGKSNFQSYSCVRCNYTEAFQTQTRPSGGKWT
FSYIGFPVELNTVYFIGAHNIPNANMNEDGPSMSVNFTSPGCLDHIMKYKKKCVKAGSLW
DPNITACKKNEETVEVNFTTTPLGNRYMALIQHSTIIGFSQVFEPHQKKQTRASVVIPVT
GDSEGATVQLTPYFPTCGSDCIRHKGTVVLCPQTGVPFPLDNNKSKPGGWLPLLLLSLLV
ATWVLVAGIYLMWRHERIKKTSFSTTTLLPPIKVLVVYPSEICFHHTICYFTEFLQNHCR
SEVILEKWQKKKIAEMGPVQWLATQKKAADKVVFLLSNDVNSVCDGTCGKSEGSPSENSQ
DLFPLAFNLFCSDLRSQIHLHKYVVVYFREIDTKDDYNALSVCPKYHLMKDATAFCAELL
HVKQQVSAGKRSQACHDGCCSL
>Q9ES73
MAQKPDGGAGLRGFQAEASVEDSALLVQTLMEAIQISEAPPTSQATAAASGPNASPQSSQ
PPTANEKADTEVSAAAARPKTGFKAQNTTTKGPNDYSQARNAKEMPKNQPKVAFKSQNAT
SKGPHAASDFSHAASTGKSAAKKSEMAFKGQNTTTKAGPSATYNFTQSPSANEMTNNQPK
TAKAWNDTTKIPGADAQTQNVNQAKMADVGTSAGISETDGAAAQTSADGSQAQNVESRTI
IRGKRTRKINNLNVEENSNGDQRRASLASGNWRSAPVPVTTQNPPGAPPNVLWQTPLAWQ
NPSGWQNQTARQTPPARQSPPARQTPSAWQNPVAWQNPVIWPNPVIWQNPVIWPNPIVWP
GPIVWPNPMAWQSTPGWQSPPSWQAPPSWQSPQDWQGPPDWQLPPDWSMPPDWSFPSDWP

FPPDWIPADWPIPPDWQNLRPSPNLRSSPNSRASQNQGPPQPRDVALLQERANKLVKYLM
LKDYTKVPIKRSEMLRDIIREYTDVYPEIIERACFVLEKKFGIQLKEIDKEEHLYILIST
PESLAGILGTTKDTPKLGLLLVILGIIFMNGNRATEAVLWEALRKMGLRPGVRHPLLGDL
RKLLTYEFVKQKYLDYRRVPNSNPPEYEFLWGLRSYHETSKMKVLRFIAEVQKRDPRDWT
AQFMEAADEALDALDAAAAEAEARAEARNRMGIGDEAVSGPWSWDDIEFELLTWDEEGDF
GDPWSRIPFTFWARYHQNARSRFPQAFTGPIIGPSGTATANFAANFGAIGFFWVE
>Q06220
MKKTQTWIITCIYLQLLLFNPLVKTKGICGKRVTDDVKDVTKLVANLPKDYKIALKYVPG
MDVLPSHCWISVMVEQLSVSLTDLLDKFSNISEGLSNYSIIDKLVKIVDDLVECTEGYSF
ENVKKAPKSPELRLFTPEEFFRIFNRSIDAFKDLETVASKSSECVVSSTLSPDKDSRVSV
TKPFMLPPVAASSLRNDSSSSNRKASNSIGDSNLQWAAMALPAFFSLVIGFAFGALYWKK
KQPNLTRTVENIQINEEDNEISMLQEKEREFQEV
>O35433
MEQRASLDSEESESPPQENSCLDPPDRDPNCKPPPVKPHIFTTRSRTRLFGKGDSEEASP
LDCPYEEGGLASCPIITVSSVLTIQRPGDGPASVRPSSQDSVSAGEKPPRLYDRRSIFDA
VAQSNCQELESLLPFLQRSKKRLTDSEFKDPETGKTCLLKAMLNLHNGQNDTIALLLDVA
RKTDSLKQFVNASYTDSYYKGQTALHIAIERRNMTLVTLLVENGADVQAAANGDFFKKTK
GRPGFYFGELPLSLAACTNQLAIVKFLLQNSWQPADISARDSVGNTVLHALVEVADNTVD
NTKFVTSMYNEILILGAKLHPTLKLEEITNRKGLTPLALAASSGKIGVLAYILQREIHEP
ECRHLSRKFTEWAYGPVHSSLYDLSCIDTCEKNSVLEVIAYSSSETPNRHDMLLVEPLNR
LLQDKWDRFVKRIFYFNFFVYCLYMIIFTAAAYYRPVEGLPPYKLKNTVGDYFRVTGEIL
SVSGGVYFFFRGIQYFLQRRPSLKSLFVDSYSEILFFVQSLFMLVSVVLYFSQRKEYVAS
MVFSLAMGWTNMLYYTRGFQQMGIYAVMIEKMILRDLCRFMFVYLVFLFGFSTAVVTLIE
DGKNNSLPMESTPHKCRGSACKPGNSYNSLYSTCLELFKFTIGMGDLEFTENYDFKAVFI
ILLLAYVILTYILLLNMLIALMGETVNKIAQESKNIWKLQRAITILDTEKSFLKCMRKAF
RSGKLLQVGFTPDGKDDYRWCFRVDEVNWTTWNTNVGIINEDPGNCEGVKRTLSFSLRSG
RVSGRNWKNFALVPLLRDASTRDRHATQQEEVQLKHYTGSLKPEDAEVFKDSMVPGEK
>Q8NH09
MALGNHSTITEFLLLGLSADPNIRALLFVLFLGIYLLTIMENLMLLLVIRADSCLHKPMY
FFLSHLSFVDLCFSSVIVPKMLENLLSQRKTISVEGCLAQVFFVFVTAGTEACLLSGMAY
DRHAAIRRPLLYGQIMGKQLYMHLVWGSWGLGFLDALINVLLAVNMVFCEAKIIHHYSYE
MPSLLPLSCSDISRSLIVLLCSTLLHGLGNFLLVFLSYTRIISTILSISSTSGRSKAFST
CSAHLTAVTLYYGSGLLRHLMPNSGSPIELIFSVQYTVVTPMLNSLIYSLKNKEVKGERS
LRDSSHLPQLHKGQARWKRPAFTEGRREPGHPELSIPVTPQPQGACACSALRAAPTALP
>O55225
MGARMPRRCLLLLSCFCLLRVESTAEVQHQASALTWKISAELQQEPAPEPSHTYQEMSLA
VEDVTTVMEGQEAEALAASAMSSWERRLHRAKCAPSYLFSCFNGGECVHPALCDCRRFNA
TGPRCQLVYNVGPERDSICRTWGQHHVETFDGLYYYFSGKGSYTLVGHHEPEGQSFSIQV
HNDPQCGSAHYTCPRSVSLFLSGEREICLAKEVTHGGVRVQLPQVVGGVQLQQLAGYVIA
RHPSAFTLAWDGISAIYIKMSPEFLGWTHGLCGNNNADPQDDLVTSYGKVTDDVGEFVHS
WQEQVPNSPPGPVTTSLPRPPCLQQSPASMQGVYERCEVLLRPPFDTCHAYVSPLPFAAS
CTSDLCQSGGDEATWCRALMEYARACAQAGRPLQGWRTQLSQCTVHCKEKAFIYNECIAC
CPASCQSRASCVDSEITCVDGCYCPNGLIFEDGGCMSPAECPCEFHGTLHPPGSVVTEDC
NACTCTAGKWVCSSSVCPAECSVTGDIHFTTFDGRRYTFPATCQYILAKSRSSGTFTVTL

QNAPCGPNQDGACVQSVSVILHQDPRRQVTLTQAGDVLLFDQYKITPPYSDDAFEIRRLS
SVFLRVRTNVGVRILYDREGLRLYLQVDQRWVEDTVGLCGTFNGNTQDDFLSPVGVPEST
PQLFGNSWKTLSACVPLVPGSLLDPCDVHLQAASYALQSCSVLTGELFAPCSAYLSPIPY
FEQCRRDACRCGQPCLCATLAHYARLCRRHGLPVDFRAHLPACALSCEATKEYSPCVTLC
APTCQDLASPDVCGANGGGNFSREECVEGCTCPPDTFLDTQADLCVPRNQCSCHFQGVDY
PPGDSDIPSLGHCHCKDGVMSCDSRAPAAACPAGQVFVNCSELHPDPELSRERTCEQQLL
NLSMPARGPCLSGCACPQGLLRHGDACFPPEECPCTWKGKEYFPGDQVVSSCHTCVCQRG
SFQCTLHPCASTCTAYGDRHYRTFDGLPYDFVGACKVHLVKSTSELSFSVMVEDVNCYGS
GVICRKSISINVGSSLIIFDDDSGDPSPESFLDEKQAVHIWRAGFFTLVHFPREHITLLW
DQRTTVHVQAGPQWQGQLVGLCGNFDLKTINEMRTPENLELTNPQEFGSSWAAVECPDTP
DPGDTCVLNPLREPFARKECGILLSEVFETCHPVVDVTWFYSNCLTDTCGCSRGGDCECF
CASVAAYAHQCCQHGVVIDWRTPRICPYDCDFFNKALGKGPYQLSSVAAGGTLVATKAVD
SDIALVRAEDLAPGDISSFLLTAALYKAKAHDPDVVSLEAADRPNFFLHTTANGSIGLAK
WQRDEAFHQHASFSLHRGTWQAGLVALESLAKPGSFLHSSGLELALRAYEHTEVFRGGAL
FRLLDAKPLGAAYPTCEWRYDACASPCFQTCRDPRATSCQDVPGRSCVPVCPTPKVLDEV
TQRCVYLEDCVEPAPRGPTETLGNETLVPGQVPPTTSAEQQLPQGLPGASAYSPAPVPVA
PPTSAPNPPMAATEGQAPSPGSTQPTLQTPLGLTTSNFPAGHTEATAREEGAASLLTTSH
PPGFSSSLPSSLQMPTSGIVSGATETTKVTITFTGSPNTTVASRSPPIPRFPLMTKAVTV
PSHDSFPVKTTPLQPSWLWSLSSRPMTSLGATSWPPTSPGSHLSTAVTKVANKTMTSLSV
LAQSTSSSSQPLAAVTTAHRAPASPLVTKGLEVVSATEKGEAGHSQLTELPVSPPPSPAP
IDLPHPAQHTTTAPGPSALSPGILAAGSPSTGAHRPGATALASLEPTRPPHLLSGLPLDT
SLPLAKVGTSAPVATPGSKGYIPTPPPQHQATTLATAMTVSPLTQSLSLTVPLMSAVEEQ
AHSPSPKPPQGTGMAPDQMLGATLPSFGASSVIAGVPPTVSAAPRKSTTQRAAILSKKVS
PPTLISDSVQGGFTELTPIVSHTVTPLATEAEGPRAGTVPLVPTTYSLSRVSARTASREG
PLVLLPQLAEAYGTPAGLQPQEDLMRQATTEQSGRSAPAQSIAEESMEAEVNTSATCVPI
AEQDCVHHICLEGQLIRVNQTQHCPQGAVRPRCGVLGLAVRVGGDRCCPQWECACRCSIF
PDLSFVTFDGSHAALFKEAIYVLSQSPDETISVHVLDCKSANLGHLNWPPFCLVILNVTH
LAHHVSIDRFNRKVTVDSQVVWPPMSRYGFRIEDTGHMYMVRTPSHIQIQWLHSSGLMIL
EASKVSKTQGHGLCGICDGDAANDLTLKDGSVLGEAEDPAPFLDSWQVPSSLTSEGQTRF
RPDSCATADCSPCLRMVSNRTFSACHSFVSPESFCELWIRDTKYVQQPCVALTVYVAMCH
KFHVCIEWRGSDYCPFLCSSDSTYQACVAACEPPDTCQDGVLGPLDPEQCQVLGEGCVCT
EGTILHRRHSALCIPEDKCACTDSTGVPRALGETWNSSLSGCCQQQCQAPDTIIPVDLDC
PGPRPESCPRFGEVILLQPTEDPCCLGSVCVCNQTLCEGLAPTCRPGHSLITHFQEDSCC
PSYSCECDPGLCEAEQVPTCREDQILIEGRLGDSCCTSYFCGCGECSDPMPECQEGEALT
VHRNTTELCCPLYQCVCENFRCPQVQCGMGTSLVEVWSPDRCCPYKSCECDCDTIPVPRC
HLWEKSQLDEEFMHSVENVCGCAKYECVKAPVCLSRELGVMQPGQTVVELSADGVCHTSR
CTDVLDPLTNFYQINITSVLCDMHCEANQEYDHPRDLAACCGSCRNVSCLFTFPNGTTSL
FLPGASWIADCARHHCGSTPLGAVLVRSPISCPPFNETECAKVGGSVVPSLEGCCRACKE
DGRSCKKVTIRMTIRKNDCRSNTPVNLVSCDGRCPSASIYNHNINTYARFCKCCREVGLQ
RRSVQLFCATNATWVPYTVQEPTDCACQWS
>Q03677
MVKVYIHDNKVDSDYRAPHNSGTELSLDELAKLGVIYKYCANEEEVNEIARQREYKNRDV
VNICEGSFKSEAEFNEKLATFYQEHLHEDEEIRYCLEGAGYFDVRDASTPENWIRCLVES
GDLLILPPGIYHRFTLTTSNHIKALRLFKDEPKWQAINRSNQADSLPVRKDYIALINQY

>Q8N1M1
MTVTYSSKVANATFFGFHRLLLKWRGSIYKLLYREFIVFAVLYTAISLVYRLLLTGVQKR
YFEKLSIYCDRYAEQIPVTFVLGFYVTLVVNRWWNQFVNLPWPDRLMFLISSSVHGSDEH
GRLLRRTLMRYVNLTSLLIFRSVSTAVYKRFPTMDHVVEAGFMTTDERKLFNHLKSPHLK
YWVPFIWFGNLATKARNEGRIRDSVDLQSLMTEMNRYRSWCSLLFGYDWVGIPLVYTQVV
TLAVYTFFFACLIGRQFLDPTKGYAGHDLDLYIPIFTLLQFFFYAGWLKVAEQLINPFGE
DDDDFETNWCIDRNLQVSLLAVDEMHMSLPKMKKDIYWDDSAARPPYTLAAADYCIPSFL
GSTVQMGLSGSDFPDEEWLWDYEKHGHRHSMIRRVKRFLSAHEHPSSPRRRSYRRQTSDS
SMFLPRDDLSPARDLLDVPSRNPPRASPTWKKSCFPEGSPTLHFSMGELSTIRETSQTST
LQSLTPQSSVRTSPIKMPLVPEVLITAAEAPVPTSGGYHHDSATSILSSEFTGVQPSKTE
QQQGPMGSILSPSEKETPPGGPSPQTVSASAEENIFNCEEDPGDTFLKRWSLPGFLGSSH
TSLGNLSPDPMSSQPALLIDTETSSEISGINIVAGSRVSSDMLYLMENLDTKETDIIELN
KETEESPK
>Q9NHB0
MPGLCIGILLLIGFGCTTAYKIPTPTVELLETGFSVSIPDEEGVKVVAFNVNRNRNFTSF
INEGQYNVRLTEPQNGRWTTNFSSVPLRSQDVLYLWTSVQHQKAVYQDLAQPLPVCNLGG
EYRPRGCSPGDDDFTDDNQLSTEDSALEPTAPSVCEPSESQVSPQIGVSICKGQLLFEET
FDQLNESLWIHDVRLPLDSKDAEFVLYDGKAKVHDGNLVIEPLLWSSYRPDLSIANSRLD
LSERCTGTHNRIKECILHSTGSGPSGIMPPIVTPRISTKETFAFQYGRIEIRAKLPKGDW
IVPLLLLEPLTEWYGQSGYESGQLRVALARGNSVLRMPRGKLVDGRSLYGGPVLSTDAHQ
REDLWLSKRKISHFGDDFHTYSLDWSSNRLLFSVDGQVYGEMLNGFTELDENPRWKQGGP
MAPFDKMFYISLGVSVGGFGDFVDHLRTATYEKPWANYHPQAKLQFHQAQDQWLPTWKQP
ALKIDYVRVFATDN
>P28285
MAHETSFNDALDYIYIANSMNDRAFLIAEPHPEQPNVDGQDQDDAELEELDDMAVTDDGQ
LEDTNNNNNSKRYYSSGKRRADFIGSLALKPPPTDVNTTTTTAGSPLATAALAAAAASAS
VAAAAARITAKAAHRALTTKQDATSSPASSPALQLIDMDNNYTNVAVGLGAMLLNDTLLL
EGNDSSLFGEMLANRSGQLDLINGTGGLNVTTSKVAEDDFTQLLRMAVTSVLLGLMILVT
IIGNVFVIAAIILERNLQNVANYLVASLAVADLFVACLVMPLGAVYEISQGWILGPELCD
IWTSCDVLCCTASILHLVAIAVDRYWAVTNIDYIHSRTSNRVFMMIFCVWTAAVIVSLAP
QFGWKDPDYLQRIEQQKCMVSQDVSYQVFATCCTFYVPLLVILALYWKIYQTARKRIHRR
RPRPVDAAVNNNQPDGGAATDTKLHRLRLRLGRFSTAKSKTGSAVGVSGPASGGRALGLV
DGNSTNTVNTVEDTEFSSSNVDSKSRAGVEAPSTSGNQIATVSHLVALAKQQGKSTAKSS
AAVNGMAPSGRQEDDGQRPEHGEQEDREELEDQDEQVGPQPTTATSATTAAGTNESEDQC
KANGVEVLEDPQLQQQLEQVQQLQKSVKSGGGGGASTSNATTITSISALSPQTPTSQGVG
IAAAAAGPMTAKTSTLTSCNQSHPLCGTANESPSTPEPRSRQPTTPQQQPHQQAHQQQQQ
QQQLSSIANPMQKVNKRKETLEAKRERKAAKTLAIITGAFVVCWLPFFVMALTMPLCAAC
QISDSVASLFLWLGYFNSTLNPVIYTIFSPEFRQAFKRILFGGHRPVHYRSGKL
>P97837
MGTAQVLPGILQKHCCILPDRNTESQCTLCGEPEEEEGGDLAQPGLSFPGPAEEDIDQQY
SWSPTQHFSEERYSPAPRNMKGLTGSRNQPQLCVGHTCGLSPTDECEHPHDHVRHGPDVR
QPYLLSPAESCPMDHHRCSPRSSVHSECMMMPVMLGDHVSSSTFPRMHYSSHYDTRDDCA
TSHASTKVNRIPANLLDQFEKQLPLHRDGFHTLQYHRASAATEQRNESPGRIRHLVHSVQ
KLFTKSHSLEGSSKSNINGTKSEGRMDDHHQSHLSKHSKRSKSKERKPESKHKSGMSSWW

SSDDNLDSDSTYRTPSVAHRHHMDHIPHCYPEALQSPFGDLSLKTSKSNSDVKCSACEGL
ALTPDTRYMKRSSWSTLTVSQAKEAYRKSSLNLDKPLVHPEIKPSLQPCHYLQVPQDDWG
AYPTGGKEEEIPCRRMRSGSYIKAMGDEESGESDSSPKTSPTVALRPEPLLKSIIQRPLG
DHQTQSYLQAATEVPVGHSLDPSVNYNSPKFRSRNQSYMRAVSTLSQASCVSQMSEAEVN
GQFESVCESVFSEVESQAMDALDLPGCFRTRSHSYLRAIQAGYSQDDECIPVMTPSNMTS
TIRSTAAVSYTNYKKTPPPVPPRTTSKPLISVTAQSSTESTQDAYQDSRAQRMSPWPQDS
RGGLYNSMDSLDSNKAMNLALESAAAQRHAADTQSSSTRSIDKAVLVSKAEELLKSRCSS
IGVQDSEFPDHQPYPRSDVETATDSDTESRGLREYHSVGVQVEDEKRHGRFKRSNSVTAA
VQADLELEGFPGHVSMEDKGLQFGSSFQRHSEPSTPTQYGALRTVRTQGLFSYREDYRTQ
VDTSTLPPPDPWLEPSLDTVETGRMSPCRRDGSWFLKLLHTETKKMEGWCKEMEREAEEN
DLSEEILGKIRSAVGSAQLLMSQKFQQFYWLCQQNMDPSAMPRPTSQDLAGYWDMLQLSV
EDVSMKFDELHQLKLNDWKIMESPERKEERKIPPPIPKKPPKGKFPITREKSLDLPDRQR
QEARRRLMAAKRAASFRQNSATERADSIEIYIPEAQTRL
>O75882
MVAAAAATEARLRRRTAATAALAGRSGGPHWDWDVTRAGRPGLGAGLRLPRLLSPPLRPR
LLLLLLLLSPPLLLLLLPCEAEAAAAAAAVSGSAAAEAKECDRPCVNGGRCNPGTGQCVC
PAGWVGEQCQHCGGRFRLTGSSGFVTDGPGNYKYKTKCTWLIEGQPNRIMRLRFNHFATE
CSWDHLYVYDGDSIYAPLVAAFSGLIVPERDGNETVPEVVATSGYALLHFFSDAAYNLTG
FNITYSFDMCPNNCSGRGECKISNSSDTVECECSENWKGEACDIPHCTDNCGFPHRGICN
SSDVRGCSCFSDWQGPGCSVPVPANQSFWTREEYSNLKLPRASHKAVVNGNIMWVVGGYM
FNHSDYNMVLAYDLASREWLPLNRSVNNVVVRYGHSLALYKDKIYMYGGKIDSTGNVTNE
LRVFHIHNESWVLLTPKAKEQYAVVGHSAHIVTLKNGRVVMLVIFGHCPLYGYISNVQEY
DLDKNTWSILHTQGALVQGGYGHSSVYDHRTRALYVHGGYKAFSANKYRLADDLYRYDVD
TQMWTILKDSRFFRYLHTAVIVSGTMLVFGGNTHNDTSMSHGAKCFSSDFMAYDIACDRW
SVLPRPDLHHDVNRFGHSAVLHNSTMYVFGGFNSLLLSDILVFTSEQCDAHRSEAACLAA
GPGIRCVWNTGSSQCISWALATDEQEEKLKSECFSKRTLDHDRCDQHTDCYSCTANTNDC
HWCNDHCVPRNHSCSEGQISIFRYENCPKDNPMYYCNKKTSCRSCALDQNCQWEPRNQEC
IALPENICGIGWHLVGNSCLKITTAKENYDNAKLFCRNHNALLASLTTQKKVEFVLKQLR
IMQSSQSMSKLTLTPWVGLRKINVSYWCWEDMSPFTNSLLQWMPSEPSDAGFCGILSEPS
TRGLKAATCINPLNGSVCERPANHSAKQCRTPCALRTACGDCTSGSSECMWCSNMKQCVD
SNAYVASFPFGQCMEWYTMSTCPPENCSGYCTCSHCLEQPGCGWCTDPSNTGKGKCIEGS
YKGPVKMPSQAPTGNFYPQPLLNSSMCLEDSRYNWSFIHCPACQCNGHSKCINQSICEKC
ENLTTGKHCETCISGFYGDPTNGGKCQPCKCNGHASLCNTNTGKCFCTTKGVKGDECQLC
EVENRYQGNPLRGTCYYTLLIDYQFTFSLSQEDDRYYTAINFVATPDEQNRDLDMFINAS
KNFNLNITWAASFSAGTQAGEEMPVVSKTNIKEYKDSFSNEKFDFRNHPNITFFVYVSNF
TWPIKIQIAFSQHSNFMDLVQFFVTFFSCFLSLLLVAAVVWKIKQSCWASRRREQLLREM
QQMASRPFASVNVALETDEEPPDLIGGSIKTVPKPIALEPCFGNKAAVLSVFVRLPRGLG
GIPPPGQSGLAVASALVDISQQMPIVYKEKSGAVRNRKQQPPAQPGTCI
>Q64096
MSDCWCFIFCKEHVRSNPLSPQHDGASREEADHQVDVSDGIRLVPDKAEATAATASDEIM
HQDIVPLCAADIQEQLKKRFAYLSGGRGQDGSPVITFPDYPAFSEIPDKEFQNVMTYLTS
IPSLQDAGIGFILVIDRRQDKWTSVKASVLRIAASFPANLQLVLVLRPTGFFQRTLSDIA
FKFNRDEFKMKVPVMMLSSVPELHGYIDKSQLTEDLGGTLDYCHSRWLCHRTAIESFALM
VKQTAQMLQAFGTELAETELPNDVQSTSLVLSAHTEKKAKVKEDLQLALKEGNSILESLR

EPLAESAAHSVNQDQLDNQATVQRLLAQLNETEAAFDEFWAKHQQKLEQCLQLRHFEQGF
REVKTTLDSMSQKIAAFTDVGNSLAHVQHLLKDLTAFEEKSSVAVDKARALSLEGQQLIE
NRHYAVDSIHPKCEELQHLCDHFASEVTRRRGLLSKSLELHSLLETSMKWSDEGIFLLAS
QPVDKCQSQDGAEASFQEIEKFLETGAENKIQELNEIYKEYECILNQDLLEHVQKVFQKQ
ESTEEMFHRRQASLKKLAAKQTRPVQPVAPRPEALTKSPSPSPGSWRSSENSSSEGNALR
RGPYRRAKSEMSEPRQGRTSSTGEEEESLAILRRHVMNELLDTERAYVEELLCVLEGYAA
EMDNPLMAHLISTGLQNKKNILFGNMEEIYHFHNRNIPAGVESCIDCPELVGRCFLERME
EFQIYEKYCQNKPRSESLWRQCSDCPFFQECQKKLDHKLSLDSYLLKPVQRITKYQLLLK
EMLKYSKHCEGAEDLQEALSSILGILKAVNDSMHLIAITGYDGNLGDLGKLLMQGSFSVW
TDHKKGHTKVKELARFKPMQRHLFLHEKAVLFCKKREENGEGYEKAPSYSYKQSLNMTAV
GITENVKGDTKKFEIWYNAREEVYIIQAPTPEIKAAWVNEIRKVLTSQLQACREASQHRA
LEQSHSLPLPTPSSTSPTKGNTRNVKKLEDRKTDPLSLEGYVSSSLPKPPEKGKGWSKTS
HSLEAPEEDGGWSSAEELINSSDAEEDGGVGPKKLVPGKYTVVMDDEKGGPDTLAMRSGD
MVEVVEEGAEGLWYVRDLTSSKEGWVPASSLSTLLGKSSSAQCLSSSGKIHCARQLCPEP
AEILSPEPV
>Q9C0H2
MAGVSYAAPWWVSLLHRLPHFDLSWEATSSQFRPEDTDYQQALLLLGAAALACLALDLLF
LLFYSFWLCCRRRKSEEHLDADCCCTAWCVIIATLVCSAGIAVGFYGNGETSDGIHRATY
SLRHANRTVAGVQDRVWDTAVGLNHTAEPSLQTLERQLAGRPEPLRAVQRLQGLLETLLG
YTAAIPFWRNTAVSLEVLAEQVDLYDWYRWLGYLGLLLLDVIICLLVLVGLIRSSKGILV
GVCLLGVLALVISWGALGLELAVSVGSSDFCVDPDAYVTKMVEEYSVLSGDILQYYLACS
PRAANPFQQKLSGSHKALVEMQDVVAELLRTVPWEQPATKDPLLRVQEVLNGTEVNLQHL
TALVDCRSLHLDYVQALTGFCYDGVEGLIYLALFSFVTALMFSSIVCSVPHTWQQKRGPD
EDGEEEAAPGPRQAHDSLYRVHMPSLYSCGSSYGSETSIPAAAHTVSNAPVTEYMSQNAN
FQNPRCENTPLIGRESPPPSYTSSMRAKYLATSQPRPDSSGSH
>P91926
MAPVRGDGMRGLAVFISDIRNCKSKEAEVKRINKELANIRSKFKGDKTLDGYQKKKYVCK
LLFIFLLGHDIDFGHMEAVNLLSSNKYSEKQIGYLFISVLVNTNSDLIRLIIQSIKNDLQ
SRNPVHVNLALQCIANIGSRDMAESFSNEIPKLLVSGDTMDVVKQSAALCLLRLFRSSPD
IIPGGEWTSRIIHLLNDQHMGVVTAATSLIDALVKRNPDEYKGCVNLAVSRLSRIVTASY
TDLQDYTYYFVPAPWLSVKLLRLLQNYNPVTEEAGVRARLNETLETILNKAQEPPKSKKV
QHSNAKNAVLFEAINLIIHSDSEPNLLVRACNQLGQFLSNRETNLRYLALESMCHLATSE
FSHEEVKKHQEVVILSMKMEKDVSVRQMAVDLLYAMCDRGNAEEIVQEMLNYLETADYSI
REEMVLKVAILAEKYATDYTWYVDVILNLIRIAGDYVSEEVWYRVIQIVINREEVQGYAA
KTVFEALQAPACHENMVKVGGYILGEFGNLIAGDSRSAPLVQFKLLHSKYHLCSPMTRAL
LLSTYIKFINLFPEIRTNIQDVFRQHSNLRSADAELQQRASEYLQLSIVASTDVLATVLE
EMPSFPERESSILAVLKKKKPGRVPENEIRESKSPAPLTSAAQNNALVNNSHSKLNNSNA
NTDLLGLSTPPSNNIGSGSNSNSTLIDVLGDMYGSNSNNNSSAVYNTKKFLFKNNGVLFE
NEMLQIGVKSEFRQNLGRLGLFYGNKTQVPLTNFNPVLQWSAEDALKLNVQMKVVEPTLE
AGAQIQQLLTAECIEDYADAPTIEISFRYNGTQQKFSIKLPLSVNKFFEPTEMNAESFFA
RWKNLSGEQQRSQKVFKAAQPLDLPGARNKLMGFGMQLLDQVDPNPDNMVCAGIIHTQSQ
QVGCLMRLEPNKQAQMFRLTVRASKETVTREICDLLTDQF
>P30273
MIPAVVLLLLLLVEQAAALGEPQLCYILDAILFLYGIVLTLLYCRLKIQVRKAAITSYEK

SDGVYTGLSTRNQETYETLKHEKPPQ
>Q6H2Y3
MAQRQPHSPNQTLISITNDTESSSVVSNDNTNKGRSGDNSPGIEALCAIYITYAVIISVG
ILGNAILIKVFFKTKSMQTVPNIFITSLAFGDLLLLLTCVPVDATHYLAEGWLFGRIGCK
VLSFIRLTSVGVSVFTLTILSADRYKAVVKPLERQPSNAILKTCIKAGCVWIVSMIFALP
EAIFSNVYSFRDPNKNVTFESCTSYPVSKKLLQEIHSLLCFLVFYIIPLSIISVYYSLIA
RTLYKSTLNIPTEEQGHARKQIESRKRIARTVLVLVALFALCWLPNHLLYLYHSFTSQTY
VDPSAMHFIFTIFSRVLAFSNSCVNPFALYWLSKTFQKHFKAQLFCCKAEQPEPPVADTS
LTTLAVMGRVPGTGNMQMSEISVTSFPGCSVKQAEDRV
>Q93052
MSHPSWLPPKSTGEPLGHVPARMETTHSFGNPSISVSTQQPPKKFAPVVAPKPKYNPYKQ
PGGEGDFLPPPPPPLDDSSALPSISGNFPPPPPLDEEAFKVQGNPGGKTLEERRSSLDAE
IDSLTSILADLECSSPYKPRPPQSSTGSTASPPVSTPVTGHKRMVIPNQPPLTATKKSTL
KPQPAPQAGPIPVAPIGTLKPQPQPVPASYTTASTSSRPTFNVQVKSAQPSPHYMAAPSS
GQIYGSGPQGYNTQPVPVSGQCPPPSTRGGMDYAYIPPPGLQPEPGYGYAPNQGRYYEGY
YAAGPGYGGRNDSDPTYGQQGHPNTWKREPGYTPPGAGNQNPPGMYPVTGPKKTYITDPV
SAPCAPPLQPKGGHSGQLGPSSVAPSFRPEDELEHLTKKMLYDMENPPADEYFGRCARCG
ENVVGEGTGCTAMDQVFHVDCFTCIICNNKLRGQPFYAVEKKAYCEPCYINTLEQCNVCS
KPIMERILRATGKAYHPHCFTCVMCHRSLDGIPFTVDAGGLIHCIEDFHKKFAPRCSVCK
EPIMPAPGQEETVRIVALDRDFHVHCYRCEDCGGLLSEGDNQGCYPLDGHILCKTCNSAR
IRVLTAKASTDL
>Q8IYJ3
MPQRGHPSQEGLWALPSLPMAHGPKPETEGLLDLSFLTEEEQEAIAGVLQRDARLRQLEE
GRVSKLRASVADPGQLKILTGDWFQEARSQRHHNAHFGSDLVRASMRRKKSTRGDQAPGH
DREAEAAVKEKEEGPEPRLTIDEAPQERLRETEGPDFPSPSVPLKASDPEEASQAQEDPG
QGDQQVCAEEADPELEPASGGEQEPRPQQAQTKAASQILENGEEAPGPDPSLDRMLSSSS
SVSSLNSSTLSGSQMSLSGDAEAVQVRGSVHFALHYEPGAAELRVHVIQCQGLAAARRRR
SDPYVKSYLLPDKQSKRKTAVKKRNLNPVFNETLRYSVPQAELQGRVLSLSVWHRESLGR
NIFLGEVEVPLDTWDWGSEPTWLPLQPRVPPSPDDLPSRGLLALSLKYVPAGSEGAGLPP
SGELHFWVKEARDLLPLRAGSLDTYVQCFVLPDDSQASRQRTRVVRRSLSPVFNHTMVYD
GFGPADLRQACAELSLWDHGALANRQLGGTRLSLGTGSSYGLQVPWMDSTPEEKQLWQAL
LEQPCEWVDGLLPLRTNLAPRT
>Q9UIB8
MAQHHLWILLLCLQTWPEAAGKDSEIFTVNGILGESVTFPVNIQEPRQVKIIAWTSKTSV
AYVTPGDSETAPVVTVTHRNYYERIHALGPNYNLVISDLRMEDAGDYKADINTQADPYTT
TKRYNLQIYRRLGKPKITQSLMASVNSTCNVTLTCSVEKEEKNVTYNWSPLGEEGNVLQI
FQTPEDQELTYTCTAQNPVSNNSDSISARQLCADIAMGFRTHHTGLLSVLAMFFLLVLIL
SSVFLFRLFKRRQGRIFPEGSCLNTFTKNPYAASKKTIYTYIMASRNTQPAESRIYDEIL
QSKVLPSKEEPVNTVYSEVQFADKMGKASTQDSKPPGTSSYEIVI
>O14512
MVFRNVGRPPEEEDVEAAPEPGPSELLCPRHRCALDPKALPPGLALERTWGPAAGLEAQL
AALGLGQPAGPGVKTVGGGCCPCPCPPQPPPPQPQPPAAAPQAGEDPTETSDALLVLEGL
ESEAESLETNSCSEEELSSPGRGGGGGGRLLLQPPGPELPPVPFPLQDLVPLGRLSRGEQ
QQQQQQQPPPPPPPPGPLRPLAGPSRKGSFKIRLSRLFRTKSCNGGSGGGDGTGKRPSGE

LAASAASLTDMGGSAGRELDAGRKPKLTRTQSAFSPVSFSPLFTGETVSLVDVDISQRGL
TSPHPPTPPPPPRRSLSLLDDISGTLPTSVLVAPMGSSLQSFPLPPPPPPHAPDAFPRIA
PIRAAESLHSQPPQHLQCPLYRPDSSSFAASLRELEKCGWYWGPMNWEDAEMKLKGKPDG
SFLVRDSSDPRYILSLSFRSQGITHHTRMEHYRGTFSLWCHPKFEDRCQSVVEFIKRAIM
HSKNGKFLYFLRSRVPGLPPTPVQLLYPVSRFSNVKSLQHLCRFRIRQLVRIDHIPDLPL
PKPLISYIRKFYYYDPQEEVYLSLKEAQLISKQKQEVEPST
>P34082
MGELPPNSVGVFLALLLCSCSLIELTRAQSPILEIYPKQEVQRKPVGKPLILTCRPTVPE
PSLVADLQWKDNRNNTILPKPNGRNQPPMYTETLPGESLALMITSLSVEMGGKYYCTASY
ANTEILEKGVTIKTYVAITWTNAPENQYPTLGQDYVVMCEVKADPNPTIDWLRNGDPIRT
TNDKYVVQTNGLLIRNVQESDEGIYTCRAAVIETGELLERTIRVEVFIQPEIISLPTNLE
AVEGKPFAANCTARGKPVPEISWIRDATQLNVATADRFQVNPQTGLVTISSVSQDDYGTY
TCLAKNRAGVVDQKTKLNVLVRPQIYELYNVTGARTKEIAITCRAKGRPAPAITFRRWGT
QEEYTNGQQDDDPRIILEPNFDEERGESTGTLRISNAERSDDGLYQCIARNKGADAYKTG
HITVEFAPDFSHMKELPPVFSWEQRKANLSCLAMGIPNATIEWHWNGRKIKDLYDTNLKI
VGTGPRSDLIVHPVTRQYYSGYKCIATNIHGTAEHDMQLKEARVPDFVSEAKPSQLTATT
MTFDIRGPSTELGLPILAYSVQYKEALNPDWSTAYNRSWSPDSPYIVEGLRPQTEYSFRF
AARNQVGLGNWGVNQQQSTPRRSAPEEPKPLHNPVQHDKEEPVVVSPYSDHFELRWGVPA
DNGEPIDRYQIKYCPGVKISGTWTELENSCNTVEVMETTSFEMTQLVGNTYYRIELKAHN
AIGYSSPASIIMKTTRGIDVIQVAERQVFSSAAIVGIAIGGVLLLLFVVDLLCCITVHMG
VMATMCRKAKRSPSEIDDEAKLGSGQLVKEPPPSPLPLPPPVKLGGSPMSTPLDEKEPLR
TPTGSIKQNSTIEFDGRFVHSRSGEIIGKNSAV
>Q8K400
MRKFNIRKVLDGLTAGSSSASQQQQQQQHPPGNREPEIQETLQSEHFQLCKTVRHGFPYQ
PSALAFDPVQKILAVGTQTGALRLFGRPGAECYCQHDSGAAVIQLQFLINEGALVSALAD
DTLHLWNLRQKRPAVLHSLKFCRERVTFCHLPFQSKWLYVGTERGNIHIVNVESFTLSGY
VIMWNKAIELSSKSHPGPVVHISDNPMDEGKLPIGFESGTVVLWDLKSKKADYRYTYDEA
IHSVAWHHEGKQFICSHSDGTLTIWNVRSPAKPVQTITPHGKQLKDGKKPEPCKPILKVE
FKTTRSGEPFIILSGGLSYDTVGRRPCLTVMHGKSTAVLEMDYSIVDFLTLCETPYPNDF
QEPYAVVVLLEKDLVLIDLAQNGYPIFENPYPLSIHESPVTCCEYFADCPVDLIPALYSV
GARQKRQGYSKKEWPINGGNWGLGAQSYPEIIITGHADGSVKFWDASAITLQVLYKLKTS
KVFEKSRNKDDRQNTDIVDEDPYAIQIISWCPESRMLCIAGVSAHVIIYRFSKQEVLTEV
IPMLEVRLLYEINDVDTPEGEQPPPLSTPVGSSNPQPIPPQSHPSTSSSSSDGLRDNVPC
LKVKNSPLKQSPGYQTELVIQLVWVGGEPPQQITSLALNSSYGLVVFGNCNGIAMVDYLQ
KAVLLNLSTIELYGSNDPYRREPRSPRKSRQPSGAGLCDITEGTVVPEDRCKSPTSGSSS
PHNSDDEQKVNNFIEKVKTQSRKFSKMVANDLAKMSRKLSLPTDLKPDLDVKDNSFSRSR
SSSVTSIDKESRETISALHFCETLTRKADSSPSPCLWVGTTVGTAFVITLNLPPGPEQRL
LQPVIVSPSGTILRLKGAILRMAFLDATGCLMSPAYEPWKEHNVAEEKDEKEKLKKRRPV
SVSPSSSQEISENQYAVICSEKQAKVMSLPTQSCAYKQNITETSFVLRGDIVALSNSVCL
ACFCANGHIMTFSLPSLRPLLDVYYLPLTNMRIARTFCFANNGQALYLVSPTEIQRLTYS
QETCENLQEMLGELFTPVETPEAPNRGFFKGLFGGGAQSLDREELFGESSSGKASRSLAQ
HIPGPGGIEGVKGAASGVVGELARARLALDERGQKLSDLEERTAAMMSSADSFSKHAHEM
MLKYKDKKWYQF
>P09119

MSAIPITPTKRIRRNLFDDAPATPPRPLKRKKLQFTDVTPESSPEKLQFGSQSIFLRTKA
LLQKSSELVNLNSSDGALPARTAEYEQVMNFLAKAISEHRSDSLYITGPPGTGKTAQLDM
IIRQKFQSLPLSLSTPRSKDVLRHTNPNLQNLSWFELPDGRLESVAVTSINCISLGEPSS
IFQKIFDSFQDLNGPTLQIKNMQHLQKFLEPYHKKTTFVVVLDEMDRLLHANTSETQSVR
TILELFLLAKLPTVSFVLIGMANSLDMKDRFLSRLNLDRGLLPQTIVFQPYTAEQMYEIV
IQKMSSLPTIIFQPMAIKFAAKKCAGNTGDLRKLFDVLRGSIEIYELEKRFLLSPTRGSL
NSAQVPLTPTTSPVKKSYPEPQGKIGLNYIAKVFSKFVNNNSTRTRIAKLNIQQKLILCT
IIQSLKLNSDATIDESFDHYIKAITKTDTLAPLQRNEFLEICTILETCGLVSIKKTKCKG
KTKRFVDKIDVDLDMREFYDEMTKISILKPFLH
>Q8TDV2
MGDELAPCPVGTTAWPALIQLISKTPCMPQAASNTSLGLGDLRVPSSMLYWLFLPSSLLA
AATLAVSPLLLVTILRNQRLRQEPHYLLPANILLSDLAYILLHMLISSSSLGGWELGRMA
CGILTDAVFAACTSTILSFTAIVLHTYLAVIHPLRYLSFMSHGAAWKAVALIWLVACCFP
TFLIWLSKWQDAQLEEQGASYILPPSMGTQPGCGLLVIVTYTSILCVLFLCTALIANCFW
RIYAEAKTSGIWGQGYSRARGTLLIHSVLITLYVSTGVVFSLDMVLTRYHHIDSGTHTWL
LAANSEVLMMLPRAMLPYLYLLRYRQLLGMVRGHLPSRRHQAIFTIS
>Q9EPF2
MGLPRLVCAFLFAACCCCRSATGVPGEEKQPTPTPDPVEVEVGNTALLKCGPAHPSGNFS
QVEWFLIHKERQIPIFRVHQGKGQSEPGEYEHRLSLHGPGATLALSQVTPHDDRMFLCKS
KQPRPQDHYVQLQVYKAPEEPTIQANVLGIHVDIQELKEVATCVGRNGYPIPQVIWYKNG
RPLQEEENRVHIQSSQTVESSGLYTLKSVLSARLVKEDKDAQFYCELSYRLPSGNRMKES
KEVTVPVLYPAEKVWVEVEPVGLLKEGDHVKIRCLTDGNPQPHFTINKKNPSTEEMEEES
TDENGLLSLEPAQKHHSGVYQCQSLDLETTVMLSSDPLELLVNYVSDVQVDPTAPEVQEG
DSLTLTCKAESNQDLEFEWLRDKTGQLLGKGPILQLNNVKREAGGRYLCVASVPSVPGLN
RTRRVSVGIFGSPWMAAKERKVWAQENAMLNLSCEASGHPQPTISWNINGSATEWNPDPQ
TVVSTLNVLVTPELLETGAECTASNSLGSNTTVIILKLVTLTTLTPDSSQTTGLSTPTVS
PHSRANSTSTEKKLPQQESKGVVIVAVIVCTLVLAVLGATLYYFYKKGKLPCGRSGKQEI
TLPPTRKSEFVVEVKSDKLPEEMALLQGSNGDKRAPGDQGEKYIDLRH
>Q96RD9
MLLWVILLVLAPVSGQFARTPRPIIFLQPPWTTVFQGERVTLTCKGFRFYSPQKTKWYHR
YLGKEILRETPDNILEVQESGEYRCQAQGSPLSSPVHLDFSSASLILQAPLSVFEGDSVV
LRCRAKAEVTLNNTIYKNDNVLAFLNKRTDFHIPHACLKDNGAYRCTGYKESCCPVSSNT
VKIQVQEPFTRPVLRASSFQPISGNPVTLTCETQLSLERSDVPLRFRFFRDDQTLGLGWS
LSPNFQITAMWSKDSGFYWCKAATMPHSVISDSPRSWIQVQIPASHPVLTLSPEKALNFE
GTKVTLHCETQEDSLRTLYRFYHEGVPLRHKSVRCERGASISFSLTTENSGNYYCTADNG
LGAKPSKAVSLSVTVPVSHPVLNLSSPEDLIFEGAKVTLHCEAQRGSLPILYQFHHEDAA
LERRSANSAGGVAISFSLTAEHSGNYYCTADNGFGPQRSKAVSLSITVPVSHPVLTLSSA
EALTFEGATVTLHCEVQRGSPQILYQFYHEDMPLWSSSTPSVGRVSFSFSLTEGHSGNYY
CTADNGFGPQRSEVVSLFVTVPVSRPILTLRVPRAQAVVGDLLELHCEAPRGSPPILYWF
YHEDVTLGSSSAPSGGEASFNLSLTAEHSGNYSCEANNGLVAQHSDTISLSVIVPVSRPI
LTFRAPRAQAVVGDLLELHCEALRGSSPILYWFYHEDVTLGKISAPSGGGASFNLSLTTE
HSGIYSCEADNGLEAQRSEMVTLKVAVPVSRPVLTLRAPGTHAAVGDLLELHCEALRGSP
LILYRFFHEDVTLGNRSSPSGGASLNLSLTAEHSGNYSCEADNGLGAQRSETVTLYITGL
TANRSGPFATGVAGGLLSIAGLAAGALLLYCWLSRKAGRKPASDPARSPSDSDSQEPTYH

NVPAWEELQPVYTNANPRGENVVYSEVRIIQEKKKHAVASDPRHLRNKGSPIIYSEVKVA
STPVSGSLFLASSAPHR
>Q9R1M7
MRRLSLWWLLSRVCLLLPPPCALVLAGVPSSSSHPQPCQILKRIGHAVRVGAVHLQPWTT
APRAASRAQEGGRAGAQRDDPESGTWRPPAPSQGARWLGSALHGRGPPGSRKLGEGAGAE
TLWPRDALLFAVENLNRVEGLLPYNLSLEVVMAIEAGLGDLPLMPFSSPSSPWSSDPFSF
LQSVCHTVVVQGVSALLAFPQSQGEMMELDLVSSVLHIPVLSIVRHEFPRESQNPLHLQL
SLENSLSSDADVTVSILTMNNWYNFSLLLCQEDWNITDFLLLTENNSKFHLESVINITAN
LSSTKDLLSFLQVQMDNIRNSTPTMVMFGCDMDSIRQIFEMSTQFGLSPPELHWVLGDSQ
NVEELRTEGLPLGLIAHGKTTQSVFEYYVQDAMELVARAVATATMIQPELALLPSTMNCM
DVKTTNLTSGQYLSRFLANTTFRGLSGSIKVKGSTIISSENNFFIWNLQHDPMGKPMWTR
LGSWQGGRIVMDSGIWPEQAQRHKTHFQHPNKLHLRVVTLIEHPFVFTREVDDEGLCPAG
QLCLDPMTNDSSMLDRLFSSLHSSNDTVPIKFKKCCYGYCIDLLEQLAEDMNFDFDLYIV
GDGKYGAWKNGHWTGLVGDLLSGTANMAVTSFSINTARSQVIDFTSPFFSTSLGILVRTR
DTAAPIGAFMWPLHWTMWLGIFVALHITAIFLTLYEWKSPFGMTPKGRNRNKVFSFSSAL
NVCYALLFGRTAAIKPPKCWTGRFLMNLWAIFCMFCLSTYTANLAAVMVGEKIYEELSGI
HDPKLHHPSQGFRFGTVRESSAEDYVRQSFPEMHEYMRRYNVPATPDGVQYLKNDPEKLD
AFIMDKALLDYEVSIDADCKLLTVGKPFAIEGYGIGLPPNSPLTSNISELISQYKSHGFM
DVLHDKWYKVVPCGKRSFAVTETLQMGIKHFSGLFVLLCIGFGLSILTTIGEHIVHRLLL
PRIKNKSKLQYWLHTSQRFHRALNTSFVEEKQPRSKTKRVEKSRWRRWTCKTEGDSELSL
FPRSNLGPQQLMVWNTSNLSHDNQRKYIFNDEEGQNQLGTQAHQDIPLPQRRRELPASLT
TNGKADSLNVTRSSVIQELSELEKQIQVIRQELQLAVSRKTELEEYQKTNRTCES
>P38928
MTQLQISLLLTATISLLHLVVATPYEAYPIGKQYPPVARVNESFTFQISNDTYKSSVDKT
AQITYNCFDLPSWLSFDSSSRTFSGEPSSDLLSDANTTLYFNVILEGTDSADSTSLNNTY
QFVVTNRPSISLSSDFNLLALLKNYGYTNGKNALKLDPNEVFNVTFDRSMFTNEESIVSY
YGRSQLYNAPLPNWLFFDSGELKFTGTAPVINSAIAPETSYSFVIIATDIEGFSAVEVEF
ELVIGAHQLTTSIQNSLIINVTDTGNVSYDLPLNYVYLDDDPISSDKLGSINLLDAPDWV
ALDNATISGSVPDELLGKNSNPANFSVSIYDTYGDVIYFNFEVVSTTDLFAISSLPNINA
TRGEWFSYYFLPSQFTDYVNTNVSLEFTNSSQDHDWVKFQSSNLTLAGEVPKNFDKLSLG
LKANQGSQSQELYFNIIGMDSKITHSNHSANATSTRSSHHSTSTSSYTSSTYTAKISSTS
AAATSSAPAALPAANKTSSHNKKAVAIACGVAIPLGVILVALICFLIFWRRRRENPDDEN
LPHAISGPDLNNPANKPNQENATPLNNPFDDDASSYDDTSIARRLAALNTLKLDNHSATE
SDISSVDEKRDSLSGMNTYNDQFQSQSKEELLAKPPVQPPESPFFDPQNRSSSVYMDSEP
AVNKSWRYTGNLSPVSDIVRDSYGSQKTVDTEKLFDLEAPEKEKRTSRDVTMSSLDPWNS
NISPSPVRKSVTPSPYNVTKHRNRHLQNIQDSQSGKNGITPTTMSTSSSDDFVPVKDGEN
FCWVHSMEPDRRPSKKRLVDFSNKSNVNVGQVKDIHGRIPEML
>Q9Z0U4
MLLLLLVPLFLRPLGAGGAQTPNATSEGCQIIHPPWEGGIRYRGLTRDQVKAINFLPVDY
EIEYVCRGEREVVGPKVRKCLANGSWTDMDTPSRCVRICSKSYLTLENGKVFLTGGDLPA
LDGARVEFRCDPDFHLVGSSRSVCSQGQWSTPKPHCQVNRTPHSERRAVYIGALFPMSGG
WPGGQACQPAVEMALEDVNSRRDILPDYELKLIHHDSKCDPGQATKYLYELLYNDPIKII
LMPGCSSVSTLVAEAARMWNLIVLSYGSSSPALSNRQRFPTFFRTHPSATLHNPTRVKLF
EKWGWKKIATIQQTTEVFTSTLDDLEERVKEAGIEITFRQSFFSDPAVPVKNLKRQDARI

IVGLFYETEARKVFCEVYKERLFGKKYVWFLIGWYADNWFKTYDPSINCTVEEMTEAVEG
HITTEIVMLNPANTRSISNMTSQEFVEKLTKRLKRHPEETGGFQEAPLAYDAIWALALAL
NKTSGGGGRSGVRLEDFNYNNQTITDQIYRAMNSSSFEGVSGHVVFDASGSRMAWTLIEQ
LQGGSYKKIGYYDSTKDDLSWSKTDKWIGGSPPADQTLVIKTFRFLSQKLFISVSVLSSL
GIVLAVVCLSFNIYNSHVRYIQNSQPNLNNLTAVGCSLALAAVFPLGLDGYHIGRSQFPF
VCQARLWLLGLGFSLGYGSMFTKIWWVHTVFTKKEEKKEWRKTLEPWKLYATVGLLVGMD
VLTLAIWQIVDPLHRTIETFAKEEPKEDIDVSILPQLEHCSSKKMNTWLGELWSFAVSSD
VQRRATVGGDSPICVWPAPESIFYGYKGLLLLLGIFLAYETKSVSTEKINDHRAVGMAIY
NVAVLCLITAPVTMILSSQQDAAFAFASLAIVFSSYITLVVLFVPKMRRLITRGEWQSET
QDTMKTGSSTNNNEEEKSRLLEKENRELEKIIAEKEERVSELRHQLQSRQQLRSRRHPPT
PPDPSGGLPRGPSEPPDRLSCDGSRVHLLYK
>Q99680
MCFSPILEINMQSESNITVRDDIDDINTNMYQPLSYPLSFQVSLTGFLMLEIVLGLGSNL
TVLVLYCMKSNLINSVSNIITMNLHVLDVIICVGCIPLTIVILLLSLESNTALICCFHEA
CVSFASVSTAINVFAITLDRYDISVKPANRILTMGRAVMLMISIWIFSFFSFLIPFIEVN
FFSLQSGNTWENKTLLCVSTNEYYTELGMYYHLLVQIPIFFFTVVVMLITYTKILQALNI
RIGTRFSTGQKKKARKKKTISLTTQHEATDMSQSSGGRNVVFGVRTSVSVIIALRRAVKR
HRERRERQKRVFRMSLLIISTFLLCWTPISVLNTTILCLGPSDLLVKLRLCFLVMAYGTT
IFHPLLYAFTRQKFQKVLKSKMKKRVVSIVEADPLPNNAVIHNSWIDPKRNKKITFEDSE
IREKCLVPQVVTD
>O42918
MKLSLKDLALSIILVLGVAPPVQALSAEEWKKQSVYNVMTDRFATSKVVPHCDVKAEKYC
GGNWQGIVDHLDYIRDLGFTAISISPVVEQLEGPEYHGEAYHGHRPKNYYRLNPHFGNEE
DLKELSDALHGKGMYLMVDVAINHTISEYFEDDYFKNTYFDKTHIDHKCKEHCSCHHDKF
PRPVPHNGTKPDHKPWKHEEHCHHGKFPRPVPHNGTKPDHKPWKHEEHCSCHHDKFPRPV
PHNGTKPDHKPWKHEEHCSCHHDKFPRPVPHNGTKPDHKPWKHEEHCHHGKFPRPIPHNG
TKPDHKPWKHEEHCHHGRFPRPVPHNGTKPDHKPWKHEEHCSCHHDKFSRPVPHNGTKPD
HKPWKHEEHCHHGKFLRPVPHNVTKPDHKPWKHEEHCHHGKFPRPVPHNGTKPDHKPWKH
EEHCSCHEDHSVHERPSAKGALEDYIQTFVETFQIDGIRFDAMGDKYRKYWPDFCKAAGV
FCMGDLKSSDSLKVCDWQNDLEGLSNFPVRESAVKAFLMNNPEGIVDLADKMTDMRGLCV
NPLLLGNFVENKDLPRMASISSDPATLKNAIAFVLMGDGIPMMYNGQELAMRGEEVPYNR
PAIWEKGFPKRNPYYKFTSTINRFRKAMIESKDGEAFLNMQSDISIVDKRILLLRRGKVI
TVLNNLGSYYIHFHYTVSAEEHKWMDVLSCKSVPVQDNRQVFMVRNGEPMILYPEESAYE
MGLCPSPIQLTEDYNSEGSLSINVEEAQTSKAPEQNRGFTNVLAALLLSLLMIL
>Q14210
MRTALLLLAALAVATGPALTLRCHVCTSSSNCKHSVVCPASSRFCKTTNTVEPLRGNLVK
KDCAESCTPSYTLQGQVSSGTSSTQCCQEDLCNEKLHNAAPTRTALAHSALSLGLALSLL
AVILAPSL
>O95866
MAVFLQLLPLLLSRAQGNPGASLDGRPGDRVNLSCGGVSHPIRWVWAPSFPACKGLSKGR
RPILWASSSGTPTVPPLQPFVGRLRSLDSGIRRLELLLSAGDSGTFFCKGRHEDESRTVL
HVLGDRTYCKAPGPTHGSVYPQLLIPLLGAGLVLGLGALGLVWWLHRRLPPQPIRPLPRF
APLVKTEPQRPVKEEEPKIPGDLDQEPSLLYADLDHLALSRPRRLSTADPADASTIYAVV
V

>Q8BWU1
MNWTAATCWALLLAAAFLCDSCSAKGGRGGARGSARGVRGGARGASRVRVRPAPRYGSSL
RVAAAGAAAGAAAGVAAGLATGSGWRRTSGPGELGLEDDENGAMGGNGTDRGVYSYWAWT
SGSGSVHSPRICLLLGGTLGALELLRP
>Q8NFM7
MAPWLQLCSVFFTVNACLNGSQLAVAAGGSGRARGADTCGWRGVGPASRNSGLYNITFKY
DNCTTYLNPVGKHVIADAQNITISQYACHDQVAVTILWSPGALGIEFLKGFRVILEELKS
EGRQCQQLILKDPKQLNSSFKRTGMESQPFLNMKFETDYFVKVVPFPSIKNESNYHPFFF
RTRACDLLLQPDNLACKPFWKPRNLNISQHGSDMQVSFDHAPHNFGFRFFYLHYKLKHEG
PFKRKTCKQEQTTETTSCLLQNVSPGDYIIELVDDTNTTRKVMHYALKPVHSPWAGPIRA
VAITVPLVVISAFATLFTVMCRKKQQENIYSHLDEESSESSTYTAALPRERLRPRPKVFL
CYSSKDGQNHMNVVQCFAYFLQDFCGCEVALDLWEDFSLCREGQREWVIQKIHESQFIIV
VCSKGMKYFVDKKNYKHKGGGRGSGKGELFLVAVSAIAEKLRQAKQSSSAALSKFIAVYF
DYSCEGDVPGILDLSTKYRLMDNLPQLCSHLHSRDHGLQEPGQHTRQGSRRNYFRSKSGR
SLYVAICNMHQFIDEEPDWFEKQFVPFHPPPLRYREPVLEKFDSGLVLNDVMCKPGPESD
FCLKVEAAVLGATGPADSQHESQHGGLDQDGEARPALDGSAALQPLLHTVKAGSPSDMPR
DSGIYDSSVPSSELSLPLMEGLSTDQTETSSLTESVSSSSGLGEEEPPALPSKLLSSGSC
KADLGCRSYTDELHAVAPL
>Q8BR86
MRPFQLDLLFLCFFLFSQELGLQKRGCCLVLGYMAKDKFRRMNEGQVYSFSQQPQDQVVV
SGQPVTLLCAIPEYDGFVLWIKDGLALGVGRDLSSYPQYLVVGNHLSGEHHLKILRAELQ
DDAVYECQAIQAAIRSRPARLTVLVPPDDPIILGGPVISLRAGDPLNLTCHADNAKPAAS
IIWLRKGEVINGATYSKTLLRDGKRESIVSTLFISPGDVENGQSIVCRATNKAIPGGKET
SVTIDIQHPPLVNLSVEPQPVLEDNIVTFHCSAKANPAVTQYRWAKRGHIIKEASGELYR
TTVDYTYFSEPVSCEVTNALGSTNLSRTVDVYFGPRMTSEPQSLLVDLGSDAVFSCAWIG
NPSLTIVWMKRGSGVVLSNEKTLTLKSVRQEDAGKYVCRAVVPRVGAGEREVTLTVNGPP
IISSTQTQHALHGEKGQIKCFIRSTPPPDRIAWSWKENVLESGTSGRYTVETVNTEEGVI
STLTISNIVRADFQTIYNCTAWNSFGSDTEIIRLKEQGSEMKSGAGLEAESVPMAVIIGV
AVGAGVAFLVLMATIVAFCCARSQRNLKGVVSAKNDIRVEIVHKEPSSGREAEDHTTIKQ
LMMDRGEFQQDSVLKQLEVLKEEEKEFQNLKDPTNGYYSVNTFKEHHSTPTISLSSCQPD
LRPTGKQRVPTGMSFTNIYSTLSGQGRLYDYGQRFVLGMGSSSIELCEREFQRGSLSDSS
SFLDTQCDSSVSSSGKQDGYVQFDKASKASASSSHHSQSSSQNSDPSRPLQRRMQTHV
>P58019
MRAQRGLILLLLLLLAVFCSTAVSLKCYNCFQFVSSCKINTTCSPNLDSCLYAVAGRQVYQ
QCWKLSDCNSNYIMSRLDVAGIQSKCCQWGLCNKNLDGLEEPNNAETSSLRKTALLGTSV
LVAILKFCF
>Q6AXS0
MAGRWLDPLWAPGFLCVALILETASGAGDLSTKAHGHIQFSARGVNQTAMADCRAVCSLN
TSDRCDFVKRNPDCHSEGGYLDYLKGIFCYFPPNLLPLAITLYVFWLLYLFLILGVTAAK
FFCPNLSAISTSLKLSHNVAGVTFLAFGNGAPDIFSALVAFSDPRTAGLAIGALFGAGVL
VTTVVAGGITILRPFMAASRPFLRDITFYMVAVFLTFTALYLGRITLVWALGYLGLYVFY
VVTVIICTWVYQRQRSRSLVHSISETPELLTDSEEDQMSSNTNSYDYGEEYRPLLLGEET
TGQILLQALNPLDYRKWRTQSISCKLLKVAKLPVEFLLLLTVPVVDPDKDDRNWKRPLNC
LQLVISPLVLVLTLQSGVYGIYEIGGLLPVWAVVVIVGTALASVTFFATSNSEPPRLHWL

FAFLGFLTSALWINAAATEVVNILRSLGVVFRLSNTVLGLTLLAWGNSIGDAFSDFTLAR
QGYPRMAFSACFGGIIFNILVGVGLGCLLQIVRSHASEVKLEPDGLLVWVLASALGLSLV
FSLVSVPLQCFQLSKAYGLCLLLFYICFIVVVLLTEFGVIHLKAD
>Q12675
MSSPSKPTSPFVDDIEHESGSASNGLSSMSPFDDSFQFEKPSSAHGNIEVAKTGGSVLKR
QSKPMKDISTPDLSKVTFDGIDDYSNDNDINDDDELNGKKTEIHEHENEVDDDLHSFQAT
PMPNTGGFEDVELDNNEGSNNDSQADHKLKRVRFGTRRNKSGRIDINRSKTLKWAKKNFH
NAIDEFSTKEDSLENSALQNRSDELRTVYYNLPLPEDMLDEDGLPLAVYPRNKIRTTKYT
PLTFFPKNILFQFHNFANIYFLILLILGAFQIFGVTNPGFASVPLIVIVIITAIKDGIED
SRRTVLDLEVNNTRTHILSGVKNENVAVDNVSLWRRFKKANTRALIKIFEYFSENLTAAG
REKKLQKKREELRRKRNSRSFGPRGSLDSIGSYRMSADFGRPSLDYENLNQTMSQANRYN
DGENLVDRTLQPNPECRFAKDYWKNVKVGDIVRVHNNDEIPADMILLSTSDVDGACYVET
KNLDGETNLKVRQSLKCSKIIKSSRDITRTKFWVESEGPHANLYSYQGNFKWQDTQNGNI
RNEPVNINNLLLRGCTLRNTKWAMGMVIFTGDDTKIMINAGVTPTKKSRISRELNFSVIL
NFVLLFILCFTAGIVNGVYYKQKPRSRDYFEFGTIGGSASTNGFVSFWVAVILYQSLVPI
SLYISVEIIKTAQAIFIYTDVLLYNAKLDYPCTPKSWNISDDLGQIEYIFSDKTGTLTQN
VMEFKKCTINGVSYGRAYTEALAGLRKRQGVDVESEGRREKEEIAKDRETMIDELRSMSD
NTQFCPEDLTFVSKEIVEDLKGSSGDHQQKCCEHFLLALALCHSVLVEPNKDDPKKLDIK
AQSPDESALVSTARQLGYSFVGSSKSGLIVEIQGVQKEFQVLNVLEFNSSRKRMSCIIKI
PGSTPKDEPKALLICKGADSVIYSRLDRTQNDATLLEKTALHLEEYATEGLRTLCLAQRE
LTWSEYERWVKTYDVAAASVTNREEELDKVTDVIERELILLGGTAIEDRLQDGVPDSIAL
LAEAGIKLWVLTGDKVETAINIGFSCNVLNNDMELLVVKASGEDVEEFGSDPIQVVNNLV
TKYLREKFGMSGSEEELKEAKREHGLPQGNFAVIIDGDALKVALNGEEMRRKFLLLCKNC
KAVLCCRVSPAQKAAVVKLVKKTLDVMTLAIGDGSNDVAMIQSADVGVGIAGEEGRQAVM
CSDYAIGQFRYVTRLVLVHGKWCYKRLAEMIPQFFYKNVIFTLSLFWYGIYNNFDGSYLF
EYTYLTFYNLAFTSVPVILLAVLDQDVSDTVSMLVPQLYRVGILRKEWNQTKFLWYMLDG
VYQSVICFFFPYLAYHKNMVVTENGLGLDHRYFVGVFVTAIAVTSCNFYVFMEQYRWDWF
CGLFICLSLAVFYGWTGIWTSSSSSNEFYKGAARVFAQPAYWAVLFVGVLFCLLPRFTID
CIRKIFYPKDIEIVREMWLRGDFDLYPQGYDPTDPSRPRINEIRPLTDFKEPISLDTHFD
GVSHSQETIVTEEIPMSILNGEQGSRKGYRVSTTLERRDQLSPVTTTNNLPRRSMASARG
NKLRTSLDRTREEMLANHQLDTRYSVERARASLDLPGINHAETLLSQRSRDR
>O55162
MDAARRGDTQPVMWTTRWLLLLPLLLCEGAQALECYSCVQKADDGCSPHKMKTVKCGPGV
DVCTEAVGAVESIHGQFSVAVRGCGSGIPGKNDRGLDLHGLLAFIQLQQCTEDRCNAKLN
LTLRGLNPAGNESAYEHNGAECYSCMGLSREKCQGAMPPVVNCYNASGRVYKGCFDGNVT
LTAANVTVSLPVRGCVQDEACTRDGVTGPGFTLSGSCCQGPRCNSDLRNKTYFSPRIPPL
VLLPPPTTPAPSTRTQNSSSTTSTTAPTTATTTIKPTTVQASHTSSTHETEHEVIQEEGS
HLSGGATGHQDRSNMGKFPEKGGAQIPSKGGSDALGSWLSAILLTVVAGAML
>P41698
MIQSDEDNLDSSETTASTSYSGTSSVSSRLQLRTSLFFENLNGAHGNPDAETEMATVAYE
TTSRGQGFAVYINNERFSQIMGASTSSSSSSNSSSITQFHDTQDNNIPSNTTVRPTSLRR
DNEDTVPLRNVTPSQNAAVRPERAVNSPSSQRLSCALTISTSVLMGEDVEGSPIEQEHSR
VVSSLYSSLANRGNDESKNGTPPRPTSIEPNETTEHSFFSYHYDDTLEPDVEEAVRLTKN
KTSNVNFISSTGSKGEGETEDEVIDQYEPVNESKFIPHKLKIPEKAGSIKSSTSDDSHSP

GAPGTSARKIKIPQSPSLIGNILIPSHNSDSSNESSPKDHIGHNNEEKFSSKSTRKPSTS
LEEEGPPIGLPSIPVLRSVSGPSKWTKTPLRLESGNSTKSDPFSRYEGHKTPSPLTKMNK
KKNKTLPEHGQPLVLAPIKSQSSESDTGQNSIIEKPARSIRRKQQEKTDNRKEDRHDAEN
IDLEARMPIQHIDTASIHSFDSGQNGFRDVYSIENIIVILLCCSIVPPLFFIIGCSSRRK
LVSDYRLMRLLMNKEHRAALLQGFIWDVDLRWFRMFCLILGAAETVIVMAGIAIGFGVGI
TRE
>Q08245
MSEIQNKAETAAQDVQQKLEETKESLQNKGQEVKEQAEASIDNLKNEATPEAEQVKKEEQ
NIADGVEQKKTEAANKVEETKKQASAAVSEKKETKKEGGFLKKLNRKIASIFN
>Q03770
MSSVNQIYDLFPNKHNIQFTDSHSQEHDTSSSLAKNDTDGTISIPGSIDTGILKSIIEEQ
GWNDAELYRSSIQNQRFFLTDKYTKKKHLTMEDMLSPEEEQIYQEPIQDFQTYNKRVQRE
YELRERMEEFFRQNTKNDLHILNEDSLNQQYSPLGPADYVLPLDRYSRMKHIASNFFRKK
LGIPRKLKRRSHYNPNAEGHTKGNSSILSSTTDVIDNASYRNIAIDENVDITHKEHAIDE
INEQGASGSESVVEGGSLLHDIEKVFNRSRATRKYHIQRKLKVRHIQMLSIGACFSVGLF
LTSGKAFSIAGPFGTLLGFGLTGSIILATMLSFTELSTLIPVSSGFSGLASRFVEDAFGF
ALGWTYWISCMLALPAQVSSSTFYLSYYNNVNISKGVTAGFITLFSAFSIVVNLLDVSIM
GEIVYVAGISKVIIAILMVFTMIILNAGHGNDIHEGVGFRYWDSSKSVRNLTYGLYRPTF
DLADAGEGSKKGISGPKGRFLATASVMLISTFAFSGVEMTFLASGEAINPRKTIPSATKR
TFSIVLISYVFLIFSVGINIYSGDPRLLSYFPGISEKRYEAIIKGTGMDWRLRTNCRGGI
DYRQISVGTGYSSPWVVALQNFGLCTFASAFNAILIFFTATAGISSLFSCSRTLYAMSVQ
RKAPPVFEICSKRGVPYVSVIFSSLFSVIAYIAVDQTAIENFDVLANVSSASTSIIWMGL
NLSFLRFYYALKQRKDIISRNDSSYPYKSPFQPYLAIYGLVGCSLFVIFMGYPNFIHHFW
STKAFFSAYGGLMFFFISYTAYKVLGTSKIQRLDQLDMDSGRREMDRTDWTEHSQYLGTY
RERAKKLVTWLI
>Q03405
MGHPPLLPLLLLLHTCVPASWGLRCMQCKTNGDCRVEECALGQDLCRTTIVRLWEEGEEL
ELVEKSCTHSEKTNRTLSYRTGLKITSLTEVVCGLDLCNQGNSGRAVTYSRSRYLECISC
GSSDMSCERGRHQSLQCRSPEEQCLDVVTHWIQEGEEGRPKDDRHLRGCGYLPGCPGSNG
FHNNDTFHFLKCCNTTKCNEGPILELENLPQNGRQCYSCKGNSTHGCSSEETFLIDCRGP
MNQCLVATGTHEPKNQSYMVRGCATASMCQHAHLGDAFSMNHIDVSCCTKSGCNHPDLDV
QYRSGAAPQPGPAHLSLTITLLMTARLWGGTLLWT
>Q14542
MARGDAPRDSYHLVGISFFILGLGTLLPWNFFITAIPYFQARLAGAGNSTARILSTNHTG
PEDAFNFNNWVTLLSQLPLLLFTLLNSFLYQCVPETVRILGSLLAILLLFALTAALVKVD
MSPGPFFSITMASVCFINSFSAVLQGSLFGQLGTMPSTYSTLFLSGQGLAGIFAALAMLL
SMASGVDAETSALGYFITPCVGILMSIVCYLSLPHLKFARYYLANKSSQAQAQELETKAE
LLQSDENGIPSSPQKVALTLDLDLEKEPESEPDEPQKPGKPSVFTVFQKIWLTALCLVLV
FTVTLSVFPAITAMVTSSTSPGKWSQFFNPICCFLLFNIMDWLGRSLTSYFLWPDEDSRL
LPLLVCLRFLFVPLFMLCHVPQRSRLPILFPQDAYFITFMLLFAVSNGYLVSLTMCLAPR
QVLPHEREVAGALMTFFLALGLSCGASLSFLFKALL
>Q9NY59
MVLYTTPFPNSCLSALHCVSWALIFPCYWLVDRLAASFIPTTYEKRQRADDPCCLQLLCT
ALFTPIYLALLVASLPFAFLGFLFWSPLQSARRPYIYSRLEDKGLAGGAALLSEWKGTGP

GKSFCFATANVCLLPDSLARVNNLFNTQARAKEIGQRIRNGAARPQIKIYIDSPTNTSIS
AASFSSLVSPQGGDGVARAVPGSIKRTASVEYKGDGGRHPGDEAANGPASGDPVDSSSPE
DACIVRIGGEEGGRPPEADDPVPGGQARNGAGGGPRGQTPNHNQQDGDSGSLGSPSASRE
SLVKGRAGPDTSASGEPGANSKLLYKASVVKKAAARRRRHPDEAFDHEVSAFFPANLDFL
CLQEVFDKRAATKLKEQLHGYFEYILYDVGVYGCQGCCSFKCLNSGLLFASRYPIMDVAY
HCYPNKCNDDALASKGALFLKVQVGSTPQDQRIVGYIACTHLHAPQEDSAIRCGQLDLLQ
DWLADFRKSTSSSSAANPEELVAFDVVCGDFNFDNCSSDDKLEQQHSLFTHYRDPCRLGP
GEEKPWAIGTLLDTNGLYDEDVCTPDNLQKVLESEEGRREYLAFPTSKSSGQKGRKELLK
GNGRRIDYMLHAEEGLCPDWKAEVEEFSFITQLSGLTDHLPVAMRLMVSSGEEEA
>P35409
MEDRGICPRVLQVLFLVVLILISPVYAAKNDACTKCSCNPKGMVSCFELQEFPPLATFPR
NTTTLHVSFSGEISIPSDILQHLEKLKYLTLNNNKIKNIAKFRVKNGYSSLITLSYTHNI
IETIENGAFDDLQQLTQLDLSNNRLKEFPIFNKTSSVTKLYLRGNPGITKLPRQSLGNLP
SLENLFMERTGIQEIPAGIFRQNTRLINLYFNKTKALERINEDAFDEDSSLKTLVLDETS
VTSLPSRGLKNLHFLSLKDVPNFWQLPELDSIREVYLSPYNGFLCCEFESGEKYGKDCTM
QKPSTEENNGQTTASSPTKEPATSGLGGGTHLSTQPHTTSGFGGGGFPGGGGGFPGGGGF
PAGGSKTSTQPHTTSGFGGGGFPGGGGGFPGGGGFPAGGSKTSTQPHTTSGFGGGGFPGG
GGGFPGGGGFPGGSNTSTQPHTTSNSGGGGFPGGGGFPGGGTPFTNQFTIPHIPNVHQST
ADPPTLIPHSNHTPNGTQFHQCSKIPVQCVPKSDAFHPCEDIMGYVWLTVVSFMVGAVAL
VANLVVALVLLTSQRRLNVTRFLMCNLAFADFILGLYIFILTSVSAVTRGDYHNYVQQWQ
NGAGCKILGFLAVFSSELSLFTLVMMTIERFYAIVHAMHMNARLSFRKTVRFMIGGWIFA
LVMAVVPLTGVSGYSKVAICLPFDVSDATSTAYVAFLLLVNGASFISVMYLYSRMLYVVV
SGGDMEGAPKRNDSKVAKRMAILVFTDMLCWAPIAFFGLLAAFGQTLLTVTQSKILLVFF
FPINSICNPFLYAFFTKAFKRELFTALSRIGFCKFRALKYNGTLSSFLYSRSRRHHSTVN
AEHSTPKSKHASTMSLRQSHQDLYRKESKTAESLNGICNAGFNAHEETRTSPGSVRYVRS
LRGVTKSSSPPHLKLQKQKILQSPS
>O76689
MSVSSNDPEQRNGRGMASGNNVDMSLYPPFIKQLDAKLPDYTREGDIEYPFEEITGVGDE
NRIRGNWSNKSDYLLAVIGFTAGVGSFWKFPFLVFQHGGAAFLVPYLCMLCLASLPMFFM
EMVLGQFSSSAAISVWKVVPLFKGIGFAQVTISGFFAVFFNIISAWTLFYLINSFSFSIP
WSNCANSWSGENCTLGTRIQCKEMNGTLLVNGSCIVEHASSNETTVIPLHDLGSIPSLKY
FQNDVLMLSKGVDDFGTLNWYLGLCVLACWIAVFLCLFQGVKSSGKVVYVAVIVPFIILT
VLLTRLLTLDGSLAAVFYFLTPKWEILMDLHVWGEAAVQAFYSVSCCSGGLFTIASYSRF
HNNIYKDIWLVLIVDVIVSLVGCLLTFSAIGFTCYEFAISLDKFHIRDGFHLVFVFLAEA
LAGVSVAPLYAGLFFIMILLVVHATQMFVVETIVSSICDEYPERLRRNRRHVLTTVCALF
ILLSIPFCLSSGLFWMELLTQFVLTWPLVVIAFLECMAINWVYGVDNMLDNAKWIVGYWP
PCYIFWKILFKFICPMVYLAILCFLWLDWNSIQYESYQFPYWSILTAWCIASFPLILIPI
VGIWQFCIAKGTITQKWWRVLYPDDAWGPAMAIHRAEKFPLQIPEARRLLLPPEVEIASS
RGVLQEEMPMSYDYNTSSAADVRSNRSTGHGATDVRSVAATNNTIPKFERETAI
>O54728
MESWPGVSLVGLLLLLLLGQGPSQIHGSSGENTSQPQQVFRTLKNFSFPCKPKKLELSVL
SKSVHSLRPSDIKLVAAIGNLETPPAPGSGVVNMEKPQSLESELQNVCIGIMTALSDIIR
HFNPSVLMPTCSPGKGTAGHTTIAEDLWIQAKELVRHLKDNPELDFEKDWKLITVLFSNT
SQCHLCSSDQQKRHLMKHMEMLSGVLDYLHREVPRAFVNLVDLSEVLTMAQQHQETGFSP

APEICKCSEEITKLSKAVMQWSYQEAWEDLLASSKFNKHETFAVVFQSFFSEVELPLERP
SPQDSTTLALRIWNSMMEPVGRKDGTLNEAERKTMKCPSQESPYLFTYRNSNYQARQLKP
IGKFQMKEGTKFTCPDKDPSDSIPTTVHRLRPADIKVIGAMGDSLTAGNGAGSSPGNVLD
VLTQYRGLSWSVGGDETIETVTTLANILREFNPSLKGFSVGTGKENTPRASFNQAVAGAK
SDGLAAQAKKLVSLMKDDKTINFQEDWKIITVFIGGNDLCGSCNNLARFSPQTFTDNIKT
ALDILHAEVPRAFVNMVSVIEITPLRELFNEPKVSCPRMILRSLCPCVLNLGENSAELAQ
LVERNRQYQEETGKLIESGRYDTRDDFTVVLQPMFENVVMPRTLEGLPDSSFFAPDCFHF
NVKTHARSAIALWKNMLEPVGRKTRHQNFEIKVPIMCPNQTSPFLSTTKNSNLGHGTSMS
CEEKAPSASPPTSVHTLRPADIQVVAALGDSVTAGNGISSQEGDLADVTTQYRGLSYSAG
GDKFLENVTTLPNILREFNGNLTGYSVGTGDVNSASAFLNQAVPGAKAENLASQVQTLIQ
KMKNDTRVNFHQDWKVITVMIGASDLCDFCKDSNRYSAANFSDHLRNALDILHKEVPRAL
VNLVDFMNPSIIRQVFLKNPDKCPVNQTSVLCNCVLTPGEDSHELARLEAFTKSYQSSML
QLVESGRYDTREDFSVVLQPFLFNIRLPILENGNPDTSFFAPDCILLSQKFHTQLARALW
ANMLEPLGKKMDTLDPKELIALACPTKDKPFLRTFRNSNYTYPIKPAIENWGSDFLCTEQ
SPSSKVPTSVHELRPSDIKVVAAMGDFLTTATGARPSESSSLDTPWRGLSWSIGGDGTLE
THTTLPNILKKFNPSILGFSTGTLENTAGLNVAEEGARAQDMPAQAQALVKKMKSTPTIN
IQEDWKLITLLIGNNDLCLYCEDPENYSTREYVKYIQHALDIFYEELPRVFINVVEVMEL
SGLLHDQGGKCAMPLAVQKNCSCLKRSQNLMAMQELKKVNGNLQSALSELSYWHRYMQRE
DFAVTVQPFFRNTFVPLDERGGLDLTFFSEDCFHFSVRGHAEMAIALWNNMLEPVGKKTT
SNNFTYNRTKLKCPSPENPFLYTVRNSQILLDKAKENSNTLYWAVPVAAVGGLVVGILGM
MLWRTVRLVQ
>Q9NJ15
MPPAIVILALFTAALCAVNLRTVAADGPRIYRNEWALHVEGGTAAADRLASKHGFINKGQ
IGSLEDHYLFVHRRTWKRSLRSSSHRHALLQREPEVRWLQQQVVKRRVKRRVKRVYSMYP
WEQRVQHSSPQVNNPAQQDNLWDPHFNDEKWDKMWYLHCDRPEFACQWSDMNVEAAWKKG
YTGKGVVVSILDDGSETDHPDLAGNYDPDASSDINGGTLDPTPRYEYTNENRHGTRCAGE
VAAMGNNSFCSVGVAYKASIGGVRMLDGDVTDSVEAASLGLNPQHIMIYSASWGPDDDGK
TVDGPANLAQKTFQAGAENGRDKLGSIFVWASGNGGRTHDSCGCDGYTNSIYTISVSSAS
EQGKVPWYLEPCASTLATTYSSGAPHERKVITTDLRKGCTESHTGTSASAPMAAGILALA
LEANPMLTWRDMQYIVVMAANPSPLDRDTESAYPRDPRKESDFVTNGAGLRVSHNFGFGL
MDAGKMVELAESWRRVPEQHVCEEDPNAQQRAITKGETIVDTKTTGGCNGTDHHVKYLEH
VVVEISLDHPCRGHLSIHITSPSGTRSTLLPERQFDSSSDGLKDWAFMTTHCWGEQPEGD
WILEVKDLGQQNCQRYGLRTVLPVLRKWKLILYGTAEHPLYKRDEESRPHTPQTREEPTD
EEECEDGDYYDRSKQRCRHCHDSCATCHGRHSGQCLSCHEGNYFVEDEGTCSEECGQGYY
KDEEERKCLDCSADCLTCQVSADHCTSCDDEDGLKLFENTCVAQCSEGRYMDENDVCQDC
DDSCDTCTGPDATDCVTCADEDLLQESQCVESCSSGYFQQEYECLKCHATCASCSGSRDD
QCLTCSGHLELDEDTHRCITSCEDGEYGTEEGKCEDCNIICKKCNGSQADQCLECHHDTN
LYDTTCVQYCGNRRYPENGECHPCHPSCLGCIGGEINQCNQCITDYEGEDHFLYQGTCHV
TCPPGLYGDTTDQVCKACAPGCIACDGPADNQCTLCEEERAPTDGRCQSEGSQTDEAECA
EGCHSCEEGPDICDSCDEDYYLTEDTCVRRTNCPSFTYPDDQDRECRPCHDNCEACDGPN
NQNCNSCKEGFYKTPDGCSTGCPNRYYKDDTNKECKPCDSSCFTCSGPASFHCLSCADGD
FLHESSCRSTCPAGFIGNAESHECVESSCEQDQYYSSETGRCEDCPYNCRACDNDGDCAE
CAPTYIVVDGRCRPEETCEDGEYQDRDRDTAELSCRPCHQSCKTCSGPSDTDCDSCKGDD
TILDRGECITSCGPGEYMDRREKKCKACHPTCKECSDEYDDTCTACNDGFLLTDASSCEA

GCPPGQFLHHGDCDSCHRECKTCDGPHHDNCLSCQPGSYLNDQQCSTHCPEGTFEETYED
DSGETVLQCRLCHVNCKTCHGEGEEDCMECANDIKYKQDGRCVTECQEGHYPDLTNECQQ
CWSDCETCDGPRNDQCVTCPYNYYLVLGKCLEDCPEGYYDTMRQEKECGECHPSCATCNE
GGNYNCLSCPYGSKLGEGVCYPMCEEHEYYVEKTQICEECDNSCKTCRGSTAHDCLSCEA
PYGYHAMKHLCTACCEEGSPENEYCCICHESTRLCITDREAEGVQFSSADSIPTNVAYIA
VATFICVVIVVLFFVVFGMLQARSNGRLCWAHKYQQVPTTRYEKMNDHVNILSQEDFYNE
DSLSEDEIHSIDSTRH
>O70491
MESQASENGSQTSSGVTDDYSSWYIEEPLGAEEVQPEGVIPLCQLTAPPALLHACLASLS
FLVLLLLALLVRRRRLWPRCGHRGLGLPSPVDFLAGDLSWTVPAAVFVVLFSNLCLLLPD
ENPLPFLNLTAASSPDGEMETSRGPWKLLALLYYPALYYPLAACASAGHQAAFLLGTVLS
WAHFGVQVWQKAECPQDPKIYKHYSLLASLPLLLGLGFLSLWYPVQLVQSLRHRTGAGSQ
GLQTSYSEKYLRTLLCPKKLDSCSHPASKRSLLSRAWAFSHHSIYTPQPGFRLPLKLVIS
ATLTGTATYQVALLLLVSVVPTVQKVRAGINTDVSYLLAGFGIVLSEDRQEVVELVKHHL
WTVEACYISALVLSCASTFLLLIRSLRTHRANLQALHRGAALDLDPPLQSIHPSRQAIVS
WMSFCAYQTAFSCLGLLVQQVIFFLGTTSLAFLVFVPLLHGRNLLLLRSLESTWPFWLTV
ALAVILQNIAANWIFLRTHHGYPELTNRRMLCVATFLLFPINMLVGAIMAVWRVLISSLY
NTVHLGQMDLSLLPQRAASLDPGYHTYQNFLRIEASQSHPGVIAFCALLLHAPSPQPRPP
LAPQDSLRPAEEEEGMQLLQTKDLMAKGAGHKGSQSRARWGLAYTLLHNPSLQAFRKAAL
TSAKANGTQP
>Q6PEM8
MEGRVSSVGSPHSFLNAPVLFRGPVEPLVFLANFALVLQGPLTTQYLWHRFSTELGYNGT
RHRENCGNQSADPLMKEVETLTSHWTLYMNVGGFLVGLFWSTLLGAWSDRVGRRPLLVLA
SLGLLLQAVVSIFVVQLELHVGFFVLGRALCALLGDFNGLLAASFASVADVSSNHSRTFR
MALLEACIGVAGTLASLLGGHWLRAQGYANPFWLALALLIVMALYAAFCFGETVKEPKST
RLFTLRHHRSIARLYVVPAPEKSRMHLALYSLAIFVVVTVHFGAQDILTLYELSAPLCWD
SKLIGYGSAAQHLPYLTSLLGLRLLQFCLADTWVAEIGLAFNILGMVVFAFATITPLMFT
GYGLLFLSLVTTPVIRAKLSKLVSESEQGALFSAVACVNSLAMLMASGIFNSIYPATLNF
MKGFPFLLGAGLLFIPAILIGVLEKVNPHPEFQQFPQSP
>P11279
MAPRSARRPLLLLLPVAAARPHALSSAAMFMVKNGNGTACIMANFSAAFSVNYDTKSGPK
NMTFDLPSDATVVLNRSSCGKENTSDPSLVIAFGRGHTLTLNFTRNATRYSVQLMSFVYN
LSDTHLFPNASSKEIKTVESITDIRADIDKKYRCVSGTQVHMNNVTVTLHDATIQAYLSN
SSFSRGETRCEQDRPSPTTAPPAPPSPSPSPVPKSPSVDKYNVSGTNGTCLLASMGLQLN
LTYERKDNTTVTRLLNINPNKTSASGSCGAHLVTLELHSEGTTVLLFQFGMNASSSRFFL
QGIQLNTILPDARDPAFKAANGSLRALQATVGNSYKCNAEEHVRVTKAFSVNIFKVWVQA
FKVEGGQFGSVEECLLDENSTLIPIAVGGALAGLVLIVLIAYLVGRKRSHAGYQTI
>Q13224
MKPRAECCSPKFWLVLAVLAVSGSRARSQKSPPSIGIAVILVGTSDEVAIKDAHEKDDFH
HLSVVPRVELVAMNETDPKSIITRICDLMSDRKIQGVVFADDTDQEAIAQILDFISAQTL
TPILGIHGGSSMIMADKDESSMFFQFGPSIEQQASVMLNIMEEYDWYIFSIVTTYFPGYQ
DFVNKIRSTIENSFVGWELEEVLLLDMSLDDGDSKIQNQLKKLQSPIILLYCTKEEATYI
FEVANSVGLTGYGYTWIVPSLVAGDTDTVPAEFPTGLISVSYDEWDYGLPARVRDGIAII
TTAASDMLSEHSFIPEPKSSCYNTHEKRIYQSNMLNRYLINVTFEGRNLSFSEDGYQMHP

KLVIILLNKERKWERVGKWKDKSLQMKYYVWPRMCPETEEQEDDHLSIVTLEEAPFVIVE
SVDPLSGTCMRNTVPCQKRIVTENKTDEEPGYIKKCCKGFCIDILKKISKSVKFTYDLYL
VTNGKHGKKINGTWNGMIGEVVMKRAYMAVGSLTINEERSEVVDFSVPFIETGISVMVSR
SNGTVSPSAFLEPFSADVWVMMFVMLLIVSAVAVFVFEYFSPVGYNRCLADGREPGGPSF
TIGKAIWLLWGLVFNNSVPVQNPKGTTSKIMVSVWAFFAVIFLASYTANLAAFMIQEEYV
DQVSGLSDKKFQRPNDFSPPFRFGTVPNGSTERNIRNNYAEMHAYMGKFNQRGVDDALLS
LKTGKLDAFIYDAAVLNYMAGRDEGCKLVTIGSGKVFASTGYGIAIQKDSGWKRQVDLAI
LQLFGDGEMEELEALWLTGICHNEKNEVMSSQLDIDNMAGVFYMLGAAMALSLITFICEH
LFYWQFRHCFMGVCSGKPGMVFSISRGIYSCIHGVAIEERQSVMNSPTATMNNTHSNILR
LLRTAKNMANLSGVNGSPQSALDFIRRESSVYDISEHRRSFTHSDCKSYNNPPCEENLFS
DYISEVERTFGNLQLKDSNVYQDHYHHHHRPHSIGSASSIDGLYDCDNPPFTTQSRSISK
KPLDIGLPSSKHSQLSDLYGKFSFKSDRYSGHDDLIRSDVSDISTHTVTYGNIEGNAAKR
RKQQYKDSLKKRPASAKSRREFDEIELAYRRRPPRSPDHKRYFRDKEGLRDFYLDQFRTK
ENSPHWEHVDLTDIYKERSDDFKRDSVSGGGPCTNRSHIKHGTGDKHGVVSGVPAPWEKN
LTNVEWEDRSGGNFCRSCPSKLHNYSTTVTGQNSGRQACIRCEACKKAGNLYDISEDNSL
QELDQPAAPVAVTSNASTTKYPQSPTNSKAQKKNRNKLRRQHSYDTFVDLQKEEAALAPR
SVSLKDKGRFMDGSPYAHMFEMSAGESTFANNKSSVPTAGHHHHNNPGGGYMLSKSLYPD
RVTQNPFIPTFGDDQCLLHGSKSYFFRQPTVAGASKARPDFRALVTNKPVVSALHGAVPA
RFQKDICIGNQSNPCVPNNKNPRAFNGSSNGHVYEKLSSIESDV
>Q06315
MKRIFSGVKSPKLSAPPKVFKNDESPSTPSSPKFDQGLRSLSASASRLFSNSISTPGSPT
LDLPQEHSINGDISPELVPIVTLLSAQAHRRYHYGIFLILHDLKTDGTPAARQWEECYGV
LLGTQLALWDAKELSDSKNNKNTSTMKKAASRPSFINFTDASVRSLDANDQVIIASENEK
TKKDLDNVLVVSTTLKNRYFLKFKNSKSFKTWNAAIRLSLFEFTALQEAYTGSFLSSRGV
KLGDIKVVMADTKFTYEDWVSVRFGTGMPWKRCYAVISPQSGKKKKNSKGSICFYENNKK
TKKSNIMTTVVDARALYAVYPSSPILIDTSTIIKLEGFVSFDKSEEPQETNLFIMPEKHQ
GVPGYDTIIRFLIPAMNAFYLYGRPKGLIANRTDPDSLLFALPTLPHIYYLQVDDVLSLT
KDKNYIHWSAADWRNNIVQVLQKKLSKGYKGCGNKTVSVSSGMMKSPAISSAELFEGYDS
LPERQMESPQKSKMKSPTLASTDDINSASASVNSHATSVKQTELFVTDNSSKINDSVSAQ
SSVTTNFKDTFTTPMTSGMLNHENSERSFGSGLKLKITDSNLENMEDVEAKSANEFSTTP
EDKHIHLANAAELSALYDKYSTSPFGKSEANSSPKPQKLEVKDRSKNENRSPYERYVGTS
AESKTFEIGNVRESKSTINTSLSSPLRVEDSRRSKNEDLGSLKEFEELSQKISNMGMANI
SSEALSDTAENSSFVTDLNLNINNSSSVNLNEEQRVPDFGEENVFDPDYMEQNQMLETES
RYTTDEFDFSDNQDAASSNYSNGQTNRTVTETLSASDRNDKIPHSSLFTNLNQLTSNGGN
YQDREDFSGDQINKPQQSQPLHVKGPQTSSFGYRNSSANSSQPQAPYPVGRPLGKIRTGP
LTVQPMQQGGNSSMYSFQSSQHRFHSSQQRQNQSLSFRNNTYGSGNNQNTFHPSPQLQQQ
PQNMRYLNNKLPINDRSPIPQTQHHVPDGRPSLHINTTNRTNPLTAQSGFSQFMPPNSTS
TNPYSS
>P06734
MEEGQYSEIEELPRRRCCRRGTQIVLLGLVTAALWAGLLTLLLLWHWDTTQSLKQLEERA
ARNVSQVSKNLESHHGDQMAQKSQSTQISQELEELRAEQQRLKSQDLELSWNLNGLQADL
SSFKSQELNERNEASDLLERLREEVTKLRMELQVSSGFVCNTCPEKWINFQRKCYYFGKG
TKQWVHARYACDDMEGQLVSIHSPEEQDFLTKHASHTGSWIGLRNLDLKGEFIWVDGSHV
DYSNWAPGEPTSRSQGEDCVMMRGSGRWNDAFCDRKLGAWVCDRLATCTPPASEGSAESM

GPDSRPDPDGRLPTPSAPLHS
>Q5U431
MASSSGSNHICSRVIDHSHVPEFEVATWIKITLILVYLIIFVVGILGNSVTIRVTQVLQK
KGYLQKEVTDHMVSLACSDILVFLIGMPMEFYSIIWNPLTTPSYALSCKLHTFLFETCSY
ATLLHVLTLSFERYIAICHPFKYKAVSGPRQVKLLIGFVWVTSALVALPLLFAMGIEYPL
VNVPTHKGLNCNLSRTRHHDEPGNSNMSICTNLSNRWEVFQSSIFGAFAVYLVVLASVAF
MCWNMMKVLMKSKQGTLAGTGPQLQLRKSESEESRTARRQTIIFLRLIVVTLAVCWMPNQ
IRRIMAAAKPKHDWTRTYFRAYMILLPFSDTFFYLSSVVNPLLYNVSSQQFRKVFWQVLC
CRLTLQHANQEKRQRARFISTKDSTSSARSPLIFLASRRSNSSSRRTNKVFLSTFQTEAK
PGEAKPQPLSPESPQTGSETKPAGSTTENSLQEQEV
>P35735
MIQFKSPGNWLFIVPWIAFIPWYGMLIAMLICWASQGHPIYWFMHSEQFPVYISDIGATN
LRPLFISCAGWQGLGYVITVALEFFQRSGYLPFQLKKKDPSISDSTSYAEKLHSGKYLMP
PYYTKDERNLIFAAFVLGSIGELALLFSSIFSTALYHRVHIAMVSVFVVFMFLSTCCLIA
EYFLMGRHYASVHPLASPHFNPQSSEKSFNQDYNTVDELPWYKWKGHVWNKFTISATLKV
IWLTLAVVWAICFGAINDRSKSACFEWLLAFWFGIIFMILSADFYLGGRYRQSRYFNHVE
SFSGYYKYDKALGLYHSEDVLPSDDNAGVIATETASSNIYNNSSSNESIQVVV
>Q9NP59
MTRAGDHNRQRGCCGSLADYLTSAKFLLYLGHSLSTWGDRMWHFAVSVFLVELYGNSLLL
TAVYGLVVAGSVLVLGAIIGDWVDKNARLKVAQTSLVVQNVSVILCGIILMMVFLHKHEL
LTMYHGWVLTSCYILIITIANIANLASTATAITIQRDWIVVVAGEDRSKLANMNATIRRI
DQLTNILAPMAVGQIMTFGSPVIGCGFISGWNLVSMCVEYVLLWKVYQKTPALAVKAGLK
EEETELKQLNLHKDTEPKPLEGTHLMGVKDSNIHELEHEQEPTCASQMAEPFRTFRDGWV
SYYNQPVFLAGMGLAFLYMTVLGFDCITTGYAYTQGLSGSILSILMGASAITGIMGTVAF
TWLRRKCGLVRTGLISGLAQLSCLILCVISVFMPGSPLDLSVSPFEDIRSRFIQGESITP
TKIPEITTEIYMSNGSNSANIVPETSPESVPIISVSLLFAGVIAARIGLWSFDLTVTQLL
QENVIESERGIINGVQNSMNYLLDLLHFIMVILAPNPEAFGLLVLISVSFVAMGHIMYFR
FAQNTLGNKLFACGPDAKEVRKENQANTSVV
>Q8R1S9
MDPMELNNVSIEPDGDSCSGDSIQDSYTGMENSDKDAMNSQFANEDAESQKFLTNGFLGK
KKLADYADEHHPGMTSFGMSSFNLSNAIMGSGILGLSYAMANTGIILFIIMLLTVAILSL
YSVHLLLKTAKEGGSLIYEKLGEKAFGWPGKIGAFISITMQNIGAMSSYLFIIKYELPEV
IRAFMGLEENTGEWYLNGNYLVLFVSVGIILPLSLLKNLGYLGYTSGFSLSCMVFFVSVV
IYKKFQIPCPLPALDHNNGNLTFNNTLPIHMISLPNDSESSGVNFMMDYAHHNPAGLDEK
QVAGPLHSNGVEYEAQGAEKCQPKYFVFNSRTAYAIPILAFAFVCHPEVLPIYSELKDRS
RRKMQTVSNISISGMLVMYLLAALFGYLSFYGDVEDELLHAYSKVYTFDTALLMVRLAVL
VAVTLTVPIVLFPIRTSVITLLFPRKPFSWLKHFGIAAIIIALNNILVILVPTIKYIFGF
IGASSATMLIFILPAAFYLKLVKKEPLRSPQKIGALVFLVTGIIFMMGSMALIILDWIYN
PPNPNHH
>Q05518
MENRNSSTSSRPFSVNNPFRNATVDSSINQYKNDSQFQEWAKNQSRTNSFDMPQLNTRTS
SQLSFPNIPEDEPQRNADQQGAFYSGLESFSSGSLSPPSRPLSSKNPFLDDVSSATDFRR
SPPPVSRNKNHPTAKEEKEQLRQRYLEESDVSTVGNTRENTDLPPSYEEITSTNGSRRAY
PKEKVSRPSSHREHSNSGTYISRRSSSHHHREASSSSTPSKKGKRKSKVIVPKNVDTIDK

LDVTGLFGGSFHHDGPFDAVTPHRNKNNKAAPVLAFPVDGPNSTIGGASTKKSALDEVFG
RDDTDDSDIYQYSSQTLRRGGDTQDAIKANVGNVQQMDAKNKTELVHGPVTAGLGSSTFL
DGAPASSAAIRNDIKAHSYHNRNGGLQRNKSLSQRLGLGGSGDSNAPMTGVRRNLSLSRD
NYDVGHSNEGVRRSKTVNSPNRTHKSNYTTDFDGQDDHNEDEEDVYLGVRYNEPNMKKKS
TGSKLLSRVKSLKVGRKSQ
>Q9Z0E8
MRDYDEVTAFLGEWGPFQRLIFFLLSASIIPNGFNGMSIVFLAGTPEHRCLVPHTVNLSS
AWRNHSIPLETKDGRQVPQKCRRYRLATIANFSELGLEPGRDVDLEQLEQESCLDGWEYD
KDVFLSTIVTEWDLVCKDDWKAPLTTSLFFVGVLMGSFISGQLSDRFGRKNVLFLTMGMQ
TGFSFLQVFSVNFEMFTVLFVLVGMGQISNYVAAFVLGTEILSKSIRIIFATLGVCIFYA
FGFMVLPLFAYFIRDWRMLLLALTVPGVLCGALWWFIPESPRWLISQGRIKEAEVIIRKA
AKINGIVAPSTIFDPSELQDLNSTKPQLHHIYDLIRTRNIRVITIMSIILWLTISVGYFG
LSLDTPNLHGDIYVNCFLLAAVEVPAYVLAWLLLQYLPRRYSISAALFLGGSVLLFMQLV
PSELFYLSTALVMVGKFGITSAYSMVYVYTAELYPTVVRNMGVGVSSTASRLGSILSPYF
VYLGAYDRFLPYILMGSLTILTAILTLFFPESFGVPLPDTIDQMLRVKGIKQWQIQSQTR
MQKDGEESPTVLKSTAF
>P79391
MTVDDPKGMKDQLDQKPNGKTAKGFVSSWRWYPAAVTLGVLCLGLLVTVILLILQLSQVS
DLIKKQQANITHQEDILEGQILAQRRSEKSAQESQKELKEMIETLAHKLDEKSKKLMELH
RQNLNLQEVLKEAANYSGPCPQDWLWHEENCYQFSSGSFNWEKSQENCLSLDAHLLKINS
TDELEFIQQMIAHSSFPFWMGLSMRKPNYSWLWEDGTPLTPHLFRIQGAVSRMYPSGTCA
YIQRGTVFAENCILTAFSICQKKANLLRAQ
>P38566
MGVLKFKHIFFGSAVELSGVFQIVFIFLLIPCCLTANFRAPPVIPNVPFLWAWNAPTEFC
LGKSGEPLDMSLFSLFGSPRKNKTGQGITIFYVDRLGYYPYIDPHTGAIVHGRIPQLGPL
QQHLTKLRQEILYYMPKDNVGLAVIDWEEWLPTWLRNWKPKDIYRIKSIELVKSQHPQYN
HSYATEKAKRDFEKAGKDFMEETLKLGRLLRPNHLWGYYLFPDCYNHHYDKPNLYKGSCF
DIEKKRNDDLSWLWKESTALFPSVYLTSRARSATALSKLYVVRNRVHEAIRVSKIPDDKS
PLPNFVYTRLVFTDQIFQFLSHHDLVYTIGEIVALGASGIVVWGSQSLARSMKSCLHLDN
YMKTILNPYLINVTLAAKMCNQVLCQEQGVCTRKNWNPNDYLHLNPGNFAIQLGSNGTYK
VDGKPTLTDLEQFSKNFQCSCYTNLNCKERTDMNNVRTVNVCAVENVCIDTNVGPQAVTY
APKEKKDVAHILSNTTSINSSTTMSLPFPRKHVSGCLLVLCMYSQYLNICYRLVAIGIQH
GYYLK
>Q84TI7
MDRVVAKIAKIRSQLTKLRSLFFLYFIYFLFFSFLGFLALKITKPRTTSRPHDFDLFFTS
VSAITVSSMSTVDMEVFSNTQLIFLTILMFLGGEIFTSFLNLYVSYFTKFVFPHNKIRHI
LGSYNSDSSIEDRCDVETVTDYREGLIKIDERASKCLYSVVLSYHLVTNLVGSVLLLVYV
NFVKTARDVLSSKEISPLTFSVFTTVSTFANCGFVPTNENMIIFRKNSGLIWLLIPQVLM
GNTLFPCFLVLLIWGLYKITKRDEYGYILKNHNKMGYSHLLSVRLCVLLGVTVLGFLIIQ
LLFFCAFEWTSESLEGMSSYEKLVGSLFQVVNSRHTGETIVDLSTLSPAILVLFILMMYL
PPYTLFMPLTEQKTIEKEGGDDDSENGKKVKKSGLIVSQLSFLTICIFLISITERQNLQR
DPINFNVLNITLEVISAYGNVGFTTGYSCERRVDISDGGCKDASYGFAGRWSPMGKFVLI
IVMFYGRFKQFTAKSGRAWILYPSSS
>Q9UJ42

MTALSSENCSFQYQLRQTNQPLDVNYLLFLIILGKILLNILTLGMRRKNTCQNFMEYFCI
SLAFVDLLLLVNISIILYFRDFVLLSIRFTKYHICLFTQIISFTYGFLHYPVFLTACIDY
CLNFSKTTKLSFKCQKLFYFFTVILIWISVLAYVLGDPAIYQSLKAQNAYSRHCPFYVSI
QSYWLSFFMVMILFVAFITCWEEVTTLVQAIRITSYMNETILYFPFSSHSSYTVRSKKIF
LSKLIVCFLSTWLPFVLLQVIIVLLKVQIPAYIEMNIPWLYFVNSFLIATVYWFNCHKLN
LKDIGLPLDPFVNWKCCFIPLTIPNLEQIEKPISIMIC
>Q08417
MSNATNNTLGSLLPQLEAAANRNSLYGGMVPNLRFNITMIVIWGILLTIHVVQLLMRQYW
FSIAFICTGILEVLGFIGRTWSHSNVADMDAFLLNMICLTIAPVFTMGGIYYQLAKLIEV
YGHRFSLLPSPMAYSFIFICSDIVSLVVQAVGGGLCGVAVTDGTSTTTGNHVFIAGLAIQ
VASMAIFLMLWFHFLFRIYISVRWEHINSRPISLSLLKISQTEVDYLYREKFHFLRLEPK
RWVFHYFNLAITVAVLTIFTRCCYRLAELVVGWDGYLITHEWYFIILDALMMAIATVTLT
IFHPGFAFKGRSTSIPITPGHVDPETLPHTDDVEDILDTSDSKQFDIEKEEFQASMKYPI
STFKQFMSKIANLFSSKKKAKL
>Q5XXA6
MRVNEKYSTLPAEDRSVHIINICAIEDIGYLPSEGTLLNSLSVDPDAECKYGLYFRDGRR
KVDYILVYHHKRPSGNRTLVRRVQHSDTPSGARSVKQDHPLPGKGASLDAGSGEPPMDYH
EDDKRFRREEYEGNLLEAGLELERDEDTKIHGVGFVKIHAPWNVLCREAEFLKLKMPTKK
MYHINETRGLLKKINSVLQKITDPIQPKVAEHRPQTMKRLSYPFSREKQHLFDLSDKDSF
FDSKTRSTIVYEILKRTTCTKAKYSMGITSLLANGVYAAAYPLHDGDYNGENVEFNDRKL
LYEEWARYGVFYKYQPIDLVRKYFGEKIGLYFAWLGVYTQMLIPASIVGIIVFLYGCATM
DENIPSMEMCDQRHNITMCPLCDKTCSYWKMSSACATARASHLFDNPATVFFSVFMALWA
ATFMEHWKRKQMRLNYRWDLTGFEEEEEAVKDHPRAEYEARVLEKSLKKESRNKEKRRHI
PEESTNKWKQRVKTAMAGVKLTDKVKLTWRDRFPAYLTNLVSIIFMIAVTFAIVLGVIIY
RISMAAALAMNSSPSVRSNIRVTVTATAVIINLVVIILLDEVYGCIARWLTKIEVPKTEK
SFEERLIFKAFLLKFVNSYTPIFYVAFFKGRFVGRPGDYVYIFRSFRMEECAPGGCLMEL
CIQLSIIMLGKQLIQNNLFEIGIPKMKKLIRYLKLKQQSPPDHEECVKRKQRYEVDYNLE
PFAGLTPEYMEMIIQFGFVTLFVASFPLAPLFALLNNIIEIRLDAKKFVTELRRPVAVRA
KDIGIWYNILRGIGKLAVIINAFVISFTSDFIPRLVYLYMYSKNGTMHGFVNHTLSSFNV
SDFQNGTAPNDPLDLGYEVQICRYKDYREPPWSENKYDISKDFWAVLAARLAFVIVFQNL
VMFMSDFVDWVIPDIPKDISQQIHKEKVLMVELFMREEQDKQQLLETWMEKERQKDEPPC
NHHNTKACPDSLGSPAPSHAYHGGVL
>O08602
MAKAAVTKRHHFMIQKLLILLSYGYTNGLDDAHSLRCNLTIKDPTPADPLWYEAKCLVDE
ILILHLSNINKTMTSGDPGETANATEVGECLTQPLKDLCQKLRNKVSNTKVDTHKTNGYP
HLQVTMIYLQSQGQIPSATWEFNISDSYFFTFYTENMSWRSANDESGVIMNKWKDDGEFV
KRLKFLIPECRQEVDEFLKQPKEKPRSTSRSPSITQLTSTSPLPPPSHSTSKKGFISVGL
IFISLLFAFAFAM
>Q8NFP4
MEVTCLLLLALIPFHCRGQGVYAPAQAQIVHAGQACVVKEDNISERVYTIREGDTLMLQC
LVTGHPRPQVRWTKTAGSASDKFQETSVFNETLRIERIARTQGGRYYCKAENGVGVPAIK
SIRVDVQYLDEPMLTVHQTVSDVRGNFYQEKTVFLRCTVNSNPPARFIWKRGSDTLSHSQ
DNGVDIYEPLYTQGETKVLKLKNLRPQDYASYTCQVSVRNVCGIPDKAITFRLTNTTAPP
ALKLSVNETLVVNPGENVTVQCLLTGGDPLPQLQWSHGPGPLPLGALAQGGTLSIPSVQA

RDSGYYNCTATNNVGNPAKKTVNLLVRSMKNATFQITPDVIKESENIQLGQDLKLSCHVD
AVPQEKVTYQWFKNGKPARMSKRLLVTRNDPELPAVTSSLELIDLHFSDYGTYLCMASFP
GAPVPDLSVEVNISSETVPPTISVPKGRAVVTVREGSPAELQCEVRGKPRPPVLWSRVDK
EAALLPSGLPLEETPDGKLRLERVSRDMSGTYRCQTARYNGFNVRPREAQVQLNVQFPPE
VEPSSQDVRQALGRPVLLRCSLLRGSPQRIASAVWRFKGQLLPPPPVVPAAAEAPDHAEL
RLDAVTRDSSGSYECSVSNDVGSAACLFQVSAKAYSPEFYFDTPNPTRSHKLSKNYSYVL
QWTQREPDAVDPVLNYRLSIRQLNQHNAVVKAIPVRRVEKGQLLEYILTDLRVPHSYEVR
LTPYTTFGAGDMASRIIHYTEPINSPNLSDNTCHFEDEKICGYTQDLTDNFDWTRQNALT
QNPKRSPNTGPPTDISGTPEGYYMFIETSRPRELGDRARLVSPLYNASAKFYCVSFFYHM
YGKHIGSLNLLVRSRNKGALDTHAWSLSGNKGNVWQQAHVPISPSGPFQIIFEGVRGPGY
LGDIAIDDVTLKKGECPRKQTDPNKVVVMPGSGAPCQSSPQLWGPMAIFLLALQR
>P22224
MDQEGQPLLSKDFQQVLLATASGNNSSWTERAVLNNESTDAVKHEPALGQNDVFDLDPLS
FDKWVPFLRRALDKNQLDPVIDELENSIEDNFQGLELQLLQDSQMNDKLETSIDEIANIQ
GMVQDTLSSEISKFQIRLSESANELIVKKQMYVNNKKISLKISEATILITKVVRILELSS
KCQELITERKFFKVLQNLDSLEKLYLQEFKNYNFQFLIEIYNSIPFLQKVTKDECINLIR
NSLNLNLGKNLIKVGQEFVAIYENELLPQWLETRSKMKLTNFKFNSPIEISMRDESFLAK
LNLGEFFQLDDFHDSIMIFQNLNELSVLSGEFNKEYELRKTKLMYPLIWKKNKTAAYQMD
SLLRGTGTTPGSTAHDVSTDDPFTQSLSLHFLQDYFLKILGFLLYDINLNKATEFILVDN
NYNSTNEFWDGLMDRLSPYLSYFIDEKLKTEEDMIKLKDFLCIYVAILENFKLNIEPLYK
ILVSIFEKFCSVSLRAFDDEFQILLNDDDFMPLSINDKTLYEKVLKICWMKEGEHLSLPD
PTNGEPFAVTLPFSPLYPMTCTLAKKTYSKITAFLSIFYRHELHTLNNILVKTMDDIFND
IVNKKIRSKLESTSREEIAQILVNLDYFIIAAKEFSNFMTRENILQNPDMEIRLSSIKYL
AESRKLAETKLIELIDSKISDILETIEIDWQITEVRQDPDISIIDLAQFLEMMFASTLQN
LPYSVQTLLIFREFDSLTRQFMGLLLHDTPSTITHESIMNFEVDVNYLESIIPRIFPSTP
GTIDSNGYQSPMTPSTPTFPNANGVDAPTLFENNIKSLEATFMELKQCIELLKTQGKDYN
EPEIRLRKYSRIRQEDAALLLSKIQHFVSSVEGANGDDTSVMDSSSIFNSESASVIDSNT
SRIAKFFNRR
>O15374
MLKREGKVQPYTKTLDGGWGWMIVIHFFLVNVFVMGMTKTFAIFFVVFQEEFEGTSEQIG
WIGSIMSSLRFCAGPLVAIICDILGEKTTSILGAFVVTGGYLISSWATSIPFLCVTMGLL
PGLGSAFLYQVAAVVTTKYFKKRLALSTAIARSGMGLTFLLAPFTKFLIDLYDWTGALIL
FGAIALNLVPSSMLLRPIHIKSENNSGIKDKGSSLSAHGPEAHATETHCHETEESTIKDS
TTQKAGLPSKNLTVSQNQSEEFYNGPNRNRLLLKSDEESDKVISWSCKQLFDISLFRNPF
FYIFTWSFLLSQLAYFIPTFHLVARAKTLGIDIMDASYLVSVAGILETVSQIISGWVADQ
NWIKKYHYHKSYLILCGITNLLAPLATTFPLLMTYTICFAIFAGGYLALILPVLVDLCRN
STVNRFLGLASFFAGMAVLSGPPIAGWLYDYTQTYNGSFYFSGICYLLSSVSFFFVPLAE
RWKNSLT
>P35603
MIGGLFVYNHKGEVLISRIYRDDVTRNAVDAFRVNVIHARQQVRSPVTNMARTSFFHVKR
GNVWICAVTRQNVNAAMVFEFLKRFADTMQSYFGKLNEENVKNNFVLIYELLDEILDFGY
PQNTDPGVLKTFITQQGVRTADAPVPVTKEEQSQITSQVTGQIGWRREGIKYRRNELFLD
VIEYVNLLMNQQGQVLSAHVAGKVAMKSYLSGMPECKFGINDKITIEGKSKPGSDDPNKA
SRAAVAIDDCQFHQCVKLTKFETEHAISFIPPDGEYELMRYRTTKDIQLPFRVIPLVREV

SRNKMEVKVVVKSNFKPSLLAQKLEVRIPTPPNTSGVQLICMKGKAKYKAGENAIVWKIK
RMAGMKESQISAEIDLLSTGNVEKKKWNRPPVSMNFEVPFAPSGLKVRYLKVFEPKLNYS
DHDVIKWVRYIGRSGLYETRC
>Q00381
MAVQFILCFNKQGVVRLVRWFDVHSSDPQRSQDAIAQIYRLISSRDHKHQSNFVEFSDST
KLIYRRYAGLYFVMGVDLLDDEPIYLCHIHLFVEVLDAFFGNVCELDIVFNFYKVYMIMD
EMFIGGEIQEISKDMLLERLSILDRLD
>P49578
MLTHYQLCFQKRSSQNYTVPDPTSCFDVPPWTILCQRATMIMHNNSSALPLFSNVSSFWK
RDSHGPGLLDEGASLIGSYDSPETTESFPFSTVETTNSSLNATIKDPLGGHAVWQVVLIA
FLTGIIALVTIIGNILVIVSFKVNKQLKTVNNYFLLSLACADLIIGVISMNLFTTYIIMG
HWALGNLACDLWLSIDYVASNASVMNLLVISFDRYFSITRPLTYRAKRTTKRAGVMIGLA
WIISFVLWAPAILFWQYFVGKRTVPLDECFIQFLSEPIITFGTAIAAFYLPVTIMSILYW
RIYKETEKRTKELAGLQASGSEAETARFVHQTGSSRSLSSYELQRQSTKRSSRRKYRRCH
FWLTMKSWEPNTDQGDQEHSSSDSWNNNDAAASLENSASSDEEDITAETRAIYSIVLKLP
GHSAILNSTKLPSSEDLNESADELQKSDTDSQEKKPKKLQPPKSIQDGGSFQKSFSKLPI
QPGSAETATASDGISSVTKTSAALPLSFKEATLAKKFALKTRSQITKRKRMSLIKEKKAA
QTLSAILFAFIITWTPYNIMVLVNTFCDCVPKTVWNLGYWLCYINSTVNPVCYALCNKMF
RNTFKMLLLCQCDKRKRRKQQYQQRQSVIFHKRIPREAS
>Q04656
MDPSMGVNSVTISVEGMTCNSCVWTIEQQIGKVNGVHHIKVSLEEKNATIIYDPKLQTPK
TLQEAIDDMGFDAVIHNPDPLPVLTDTLFLTVTASLTLPWDHIQSTLLKTKGVTDIKIYP
QKRTVAVTIIPSIVNANQIKELVPELSLDTGTLEKKSGACEDHSMAQAGEVVLKMKVEGM
TCHSCTSTIEGKIGKLQGVQRIKVSLDNQEATIVYQPHLISVEEMKKQIEAMGFPAFVKK
QPKYLKLGAIDVERLKNTPVKSSEGSQQRSPSYTNDSTATFIIDGMHCKSCVSNIESTLS
ALQYVSSIVVSLENRSAIVKYNASSVTPESLRKAIEAVSPGLYRVSITSEVESTSNSPSS
SSLQKIPLNVVSQPLTQETVINIDGMTCNSCVQSIEGVISKKPGVKSIRVSLANSNGTVE
YDPLLTSPETLRGAIEDMGFDATLSDTNEPLVVIAQPSSEMPLLTSTNEFYTKGMTPVQD
KEEGKNSSKCYIQVTGMTCASCVANIERNLRREEGIYSILVALMAGKAEVRYNPAVIQPP
MIAEFIRELGFGATVIENADEGDGVLELVVRGMTCASCVHKIESSLTKHRGILYCSVALA
TNKAHIKYDPEIIGPRDIIHTIESLGFEASLVKKDRSASHLDHKREIRQWRRSFLVSLFF
CIPVMGLMTYMMVMDHHFATLHHNQNMSKEEMINLHSSMFLERQILPGLSVMNLLSFLLC
VPVQFFGGWYFYIQAYKALKHKTANMDVLIVLATTIAFAYSLIILLVAMYERAKVNPITF
FDTPPMLFVFIALGRWLEHIAKGKTSEALAKLISLQATEATIVTLDSDNILLSEEQVDVE
LVQRGDIIKVVPGGKFPVDGRVIEGHSMVDESLITGEAMPVAKKPGSTVIAGSINQNGSL
LICATHVGADTTLSQIVKLVEEAQTSKAPIQQFADKLSGYFVPFIVFVSIATLLVWIVIG
FLNFEIVETYFPGYNRSISRTETIIRFAFQASITVLCIACPCSLGLATPTAVMVGTGVGA
QNGILIKGGEPLEMAHKVKVVVFDKTGTITHGTPVVNQVKVLTESNRISHHKILAIVGTA
ESNSEHPLGTAITKYCKQELDTETLGTCIDFQVVPGCGISCKVTNIEGLLHKNNWNIEDN
NIKNASLVQIDASNEQSSTSSSMIIDAQISNALNAQQHKVLIGNREWMIRNGLVINNDVN
DFMTEHERKGRTAVLVAVDDELCGLIAIADTVKPEAELAIHILKSMGLEVVLMTGDNSKT
ARSIASQVGITKVFAEVLPSHKVAKVKQLQEEGKRVAMVGDGINDSPALAMANVGIAIGT
GTDVAIEAADVVLIRNDLLDVVASIDLSRKTVKRIRINFVFALIYNLVGIPIAAGVFMPI
GLVLQPWMGSAAMAASSVSVVLSSLFLKLYRKPTYESYELPARSQIGQKSPSEISVHGI

DDTSRNSPKLGLLDRIVNYSRASINSLLSDKRSLNSVVTSEPDKHSLLVGDFREDDDTAL

>P28660
MSRSVLQPSQQKLAEKLTILNDRGVGMLTRLYNIKKACGDPKAKPSYLIDKNLESAVKFI
VRKFPAVETRNNNQQLAQLQKEKSEILKNLALYYFTFVDVMEFKDHVCDLLNTIDVCQVF
FDITVNFDLTKNYLDLTVTYTTLMILLSRIEERKAIIGLYNYAHEMTHGASDREYPRLGQ
MIVDYENPLKKMMEEFVPHSKSLSDALISLQMVYPRRNLSADQWRNAQLLSLISAPSTML
NPAQSDTMPCEYLSLDAMEKWIIFGFILCHGMLNTEATALNLWKLALQSSSCLSLFRDEV
FHIHKAAEDLFVNIRGYNKRINDIRECKEAAVSHAGSMHRERRKFLRSALKELATVLSDQ
PGLLGPKALFVFMALSFARDEIIWLLRHADNMPKKSADDFIDKHIAELIFYMEELRAHVR
KYGPVMQRYYVQYLSGFDAVVLNELVQNLSVCPEDESIIMSSFVNTMTSLSVKQVEDGEV
FDFRGMRLDWFRLQAYTSVSKASLSLADHRELGKMMNTIIFHTKMVDSLVEMLVETSDLS
IFCFYSRAFEKMFQQCLELPSQSRYSIAFPLLCTHFMSCTHELCPEERHHIGDRSLSLCN
MFLDEMAKQARNLITDICTEQCTLSDQLLPKHCAKTISQAVNKKSKKQTGKKGEPEREKP
GVESMRKNRLVVTNLDKLHTALSELCFSINYVPNMAVWEHTFTPREYLTSHLEIRFTKSI
VGMTMYNQATQEIAKPSELLTSVRAYMTVLQSIENYVQIDITRVFNNVLLQQTQHLDSHG
EPTITSLYTNWYLETLLRQVSNGHIAYFPAMKAFVNLPTENELTFNAEEYSDISEMRSLS
ELLGPYGMKFLSESLMWHISSQVAELKKLVVENVDVLTQMRTSFDKPDQMAALFKRLSSV
DSVLKRMTIIGVILSFRSLAQEALRDVLSYHIPFLVSSIEDFKDHIPRETDMKVAMNVYE
LSSAAGLPCEIDPALVVALSSQKSENISPEEEYKIACLLMVFVAVSLPTLASNVMSQYSP
AIEGHCNNIHCLAKAINQIAAALFTIHKGSIEDRLKEFLALASSSLLKIGQETDKTTTRN
RESVYLLLDMIVQESPFLTMDLLESCFPYVLLRNAYHAVYKQSVTSSA
>Q96P67
MNNNTTCIQPSMISSMALPIIYILLCIVGVFGNTLSQWIFLTKIGKKTSTHIYLSHLVTA
NLLVCSAMPFMSIYFLKGFQWEYQSAQCRVVNFLGTLSMHASMFVSLLILSWIAISRYAT
LMQKDSSQETTSCYEKIFYGHLLKKFRQPNFARKLCIYIWGVVLGIIIPVTVYYSVIEAT
EGEESLCYNRQMELGAMISQIAGLIGTTFIGFSFLVVLTSYYSFVSHLRKIRTCTSIMEK
DLTYSSVKRHLLVIQILLIVCFLPYSIFKPIFYVLHQRDNCQQLNYLIETKNILTCLASA
RSSTDPIIFLLLDKTFKKTLYNLFTKSNSAHMQSYG
>Q6U820
MKTTTVACAVAGLLFSCVSGAPDPVHVEIQQRALPNAPDGYTPSTVGCPASRPTIRSAAS
LSPNETSWLETRRGKTTSAMKDFFNHVKIQDFDAAGYIDRHSSNSSDLPNIGIAVSGGGY
RALMNGAGAIKAFDSRTPNSTSAGQLGGLLQSATYLSGLSGGSWLVGSIYINNFTTISAL
QTHQKGTVWQFQNSIFEGPDGGSIQILDSATYYRDISNAVSGKSDAGYPTSITDYWGRAL
SYQMINATNGGPSYTWSSIALTDAFQKAEMPMPLVVADGRYPGELLISSNATVYEFNPWE
FGTFDPTVFGFAPLEYLGTKFNGGSVPSNESCVRGFDNVGFVMGTSSTLFNQFLLQINST
ALPDWLKSVFTDILKDIGENDEDIAQYAPNPFYHFSNTTNPSAAELELDLVDGGEDLQNI
PLHPLIQPERHVDVIFAVDSSADTTYSWPNGTALVATYERSLNSSGIANGTSFPAIPDQN
TFVNKGLNTRPTFFGCNSSNTTGPSPLIVYLPNYPYTAYSNFSTFQPDYTEQERDSTILN
GYDVVTMGNSTRDGNWSTCVGCAILSRSLERTNTNVPEICKQCFQRYCWDGSLNSTTPAG
YEPVTILDSAASGIIPSISTVAMAVVFAAWTIF
>Q9FHD4
METTKKLFALLCLFVTMNQAISVSDPDDMETFCMKSSRNTTSNTTYNKNLNTLLSTLSNQ
SSFANYYNLTTGLASDTVHGMFLCTGDVNRTTCNACVKNATIEIAKNCTNHREAIIYNVD

CMVRYSDKFFLTTLETNPSYWWSSNDLIPKSFGKFGQRLSDKMGEVIVRSSLLSSSFTPY
YLMDTTRFDNLYDLESIVQCTPDLDPRNCTTCLKLALQELTECCGNQVWAFIYTPNCMVS
FDTYNSSLPPLPPPSRSGSFSHRGNNKLLGGMVLAVSVSVFAFLSLV
>P42519
MSQQVFSAHPALAVDQLQQAEQEEHNTSSNHHQQRSATQSRRHTKAAPKKFTLSRSCAGG
GSGTLSGVHQQVKPSPSNPAISPECRKTLPVRTNYAAVDDDDDIECEDVDEVNFGQQEKE
RERETRQPTKDCGTDETDHVQQRHKNTMTTSATAASRHHHQDGGGGDQSDLSSVISSPSV
STVSSPLSTPTRLPQALQQQLHCCQKSTGMESRARTSPQQIQHPHRQHHQQQHHHHHHHH
HLTAAGCTGGGGGGGGSGGSGSCKAKKLDPRLNPSPYRQLLPIALCLLSFAAVFATLIVY
MDTTEIRHQQFRLNMSRDYELNGVAQDDPALIDFLRQIHMGKYLGKASPKVAAAASVGVG
PPPNSPRLAAAGSTFGSGNSSGSGADQLAHYVADLVGGKMNGAVIQSLSGPLAHLITAPW
LSEQLNWMGVLVEPEPRWYFTLRKQNAQRARMQVVHACVSPNTYPKEITIHNEDVRINSL
HDEETSWFNSRVKCFPLYTIMLACERTEYDLLSLGVQGHELEILQTLPFDKVKIDVISIH
LLEDHEDVADYVLDITRFLAGKSYKLQRKIGRNYFYQRLNASASRTRKKDILLLKTP
>P33241
MAEASSDPGAEEREELLGPTAQWSVEDEEEAVHEQCQHERDRQLQAQDEEGGGHVPERPK
QEMLLSLKPSEAPELDEDEGFGDWSQRPEQRQQHEGAQGALDSGEPPQCRSPEGEQEDRP
GLHAYEKEDSDEVHLEELSLSKEGPGPEDTVQDNLGAAGAEEEQEEHQKCQQPRTPSPLV
LEGTIEQSSPPLSPTTKLIDRTESLNRSIEKSNSVKKSQPDLPISKIDQWLEQYTQAIET
AGRTPKLARQASIELPSMAVASTKSRWETGEVQAQSAAKTPSCKDIVAGDMSKKSLWEQK
GGSKTSSTIKSTPSGKRYKFVATGHGKYEKVLVEGGPAP
>Q6UX01
MEAPDYEVLSVREQLFHERIRECIISTLLFATLYILCHIFLTRFKKPAEFTTVDDEDATV
NKIALELCTFTLAIALGAVLLLPFSIISNEVLLSLPRNYYIQWLNGSLIHGLWNLVFLFS
NLSLIFLMPFAYFFTESEGFAGSRKGVLGRVYETVVMLMLLTLLVLGMVWVASAIVDKNK
ANRESLYDFWEYYLPYLYSCISFLGVLLLLVCTPLGLARMFSVTGKLLVKPRLLEDLEEQ
LYCSAFEEAALTRRICNPTSCWLPLDMELLHRQVLALQTQRVLLEKRRKASAWQRNLGYP
LAMLCLLVLTGLSVLIVAIHILELLIDEAAMPRGMQGTSLGQVSFSKLGSFGAVIQVVLI
FYLMVSSVVGFYSSPLFRSLRPRWHDTAMTQIIGNCVCLLVLSSALPVFSRTLGLTRFDL
LGDFGRFNWLGNFYIVFLYNAAFAGLTTLCLVKTFTAAVRAELIRAFGLDRLPLPVSGFP
QASRKTQHQ
>P42951
MSNEAYQHDHTVNPHQKIVVNSYDWLQFRDEQDHCKSKNPITHASPGVGSNAQNSDIAEA
PQVFHPSYQSLVNVPSESPRPDQTSGSNPAVGLLHNAEDKASGQEEEGSQYEIQYSVFRP
LHAYPTKGLAYEQLRRKEEQEQRENFNHLVSDCIEAVETFGRELERIQTGSSGSYFVYGT
RADESVPVGVFKPKDEEPYGPFSPKWTKWAHRTFFPCLFGRSCLIPNLGYICESAASLLD
RRLETHLVPYTDTASIESFNFYDNRKKWVLGYNLQKKKQKKLGSFQLFLKEYINADEFFH
KYPLPGMYSDVKHSFHRKSSGEDINHKPETTRNLTDETEPSKQINSSPISTESEENSKFE
WTESSLSQFRLELEKLIILDYIMRNTDRGLDNWMVKLIKLSNNKWRLKLAAIDNGLSFPW
KHPDEWRLYPYGWLYLPLQLLAKPFSEQMRSHFLPILTSTNWWEESYQEFLALFSRDQDF
NVRMWKKQWAVLKGQAFNVVETLKDPRQGPLELVRRTRCQVIDEKMQVPCCPPPVSIFKN
AIDEPIGSYSTSPMVLPSTPSTIPFHAHNQSNSNPVYYDSTLHPFANKTVIAERLQIVNS
TPVFTWC
>Q5DRK1

MRLSWKERVFMVLLGVAAASGLTMLILILVKATNVLLPADTKFGILFDAGSSHTSLFVYQ
WPANKEKDTGVVSQALACQVEGPGISSYTSDPTQAGESLKSCLQEALALIPQTQHPVTPA
FLGATAGMRLLSQKNSSQAQDILAAVSQTLSRAPVDFWGARILAGQDEGAFGWITVNYVL
GMLLKYSSGQWILPEDGTLVGALDLGGASTQISFVPQGPILDQSTQVTFRLYGANYSVYT
HSYLCFGRDQILRRLLAELVQSSQVARVRHPCYHSGYQATLSLASLYDSPCVHTPDSLNY
TQNLTVEGIGNPGNCVAALRGLFNFSSCKGQEDCAFNGVYQPPVHGQFYAFSNFYYTFQF
LNLTSRQPLNIVNDTIWKFCQKPWRLVEDSYPGQERWLRDYCASGLYILVLLLEGYKFSE
ETWPNIQFQKQAGGTDIGWTLGFMLNLTGMIPAEALTQWRAQSYSIWIAGVVFAVLTLVA
ILGAAAVQLFWTQD
>O43895
MARAHWGCCPWLVLLCACAWGHTKPVDLGGQDVRNCSTNPPYLPVTVVNTTMSLTALRQQ
MQTQNLSAYIIPGTDAHMNEYIGQHDERRAWITGFTGSAGTAVVTMKKAAVWTDSRYWTQ
AERQMDCNWELHKEVGTTPIVTWLLTEIPAGGRVGFDPFLLSIDTWESYDLALQGSNRQL
VSITTNLVDLVWGSERPPVPNQPIYALQEAFTGSTWQEKVSGVRSQMQKHQKVPTAVLLS
ALEETAWLFNLRASDIPYNPFFYSYTLLTDSSIRLFANKSRFSSETLSYLNSSCTGPMCV
QIEDYSQVRDSIQAYSLGDVRIWIGTSYTMYGIYEMIPKEKLVTDTYSPVMMTKAVKNSK
EQALLKASHVRDAVAVIRYLVWLEKNVPKGTVDEFSGAEIVDKFRGEEQFSSGPSFETIS
ASGLNAALAHYSPTKELNRKLSSDEMYLLDSGGQYWDGTTDITRTVHWGTPSAFQKEAYT
RVLIGNIDLSRLIFPAATSGRMVEAFARRALWDAGLNYGHGTGHGIGNFLCVHEWPVGFQ
SNNIAMAKGMFTSIEPGYYKDGEFGIRLEDVALVVEAKTKYPGSYLTFEVVSFVPYDRNL
IDVSLLSPEHLQYLNRYYQTIREKVGPELQRRQLLEEFEWLQQHTEPLAARAPDTASWAS
VLVVSTLAILGWSV
>P46951
MPNSNVRIPPTVPSKIIDVVDQALRARLLGGSTFNSGFDSLDSVLNLQFRLHYHVIGSNG
PAKPVCDVLLKESQNLEKNMSMMEELNDYPEITKLVEKILFNCLGILFFHRGQFQESQRC
LLHSLKIHNNTASQKTALMEQYDRYLIVENLYYRGLVSQDINIMQNVFYKELLAHVDTIP
PESNGLLFEYISLIVAKLRFNQIQDLAENFKTTVENPFILFLYMIKKFQSPLKKHIDNDD
LYLKFGQNVLLKAKFPTASETNDEALEHFNVFLQYYFKFTHIKKIKVNPSWYNFIISSME
KTFQSIEVSKTAMFLFQNLSDNSNDEIKKKTFKRESILNFVNFVKYNDKYYQLHDNSHRD
IISFIDAYSFILQNSSKTDSIENVFDYDNTVSTFATSLNSFYKEYNLPLMSQSESLDWLE
NSTRCVYPGNISKVLTNAWSTLYEIRKYQLDFLVSNNLTSYLCNAMMLSTKEKDNADVEE
QEEGEEEKALRELQFKYSYTLAQQRHIETAIKTLESLILSKNPNYYKAWHLLALCRSVQE
DKEMSYKIVCSVLEAMNESLQNNTLLLNDRWQFIHLKLTQLALIEEIFGTLEALETLPEV
FELYATLFPDSQPELNSMGPKYSQTKEYLLQMVWIFAANMYMRTKDNDEDAKAAIKEASN
VESKFKNLNCNIANGYLSIIKDEPGVALKEFETVLYYDENNLDALVGFAELIFPEELGVE
ETNLERYYTLSLDKKPGKRAKLTFVNDTDRSAAYARLKFLLECAILESIEAYYSPEVWWY
LSLIYEKYQDDEYKNSLLKCIKYQELNPIRSLRYCNY
>P32600
MNKYINKYTTPPNLLSLRQRAEGKHRTRKKLTHKSHSHDDEMSTTSNTDSNHNGPNDSGR
VITGSAGHIGKISFVDSELDTTFSTLNLIFDKLKSDVPQERASGANELSTTLTSLAREVS
AEQFQRFSNSLNNKIFELIHGFTSSEKIGGILAVDTLISFYLSTEELPNQTSRLANYLRV
LIPSSDIEVMRLAANTLGRLTVPGGTLTSDFVEFEVRTCIDWLTLTADNNSSSSKLEYRR
HAALLIIKALADNSPYLLYPYVNSILDNIWVPLRDAKLIIRLDAAVALGKCLTIIQDRDP
ALGKQWFQRLFQGCTHGLSLNTNDSVHATLLVFRELLSLKAPYLRDKYDDIYKSTMKYKE

YKFDVIRREVYAILPLLAAFDPAIFTKKYLDRIMVHYLRYLKNIDMNAANNSDKPFILVS
IGDIAFEVGSSISPYMTLILDNIREGLRTKFKVRKQFEKDLFYCIGKLACALGPAFAKHL
NKDLLNLMLNCPMSDHMQETLMILNEKIPSLESTVNSRILNLLSISLSGEKFIQSNQYDF
NNQFSIEKARKSRNQSFMKKTGESNDDITDAQILIQCFKMLQLIHHQYSLTEFVRLITIS
YIEHEDSSVRKLAALTSCDLFIKDDICKQTSVHALHSVSEVLSKLLMIAITDPVAEIRLE
ILQHLGSNFDPQLAQPDNLRLLFMALNDEIFGIQLEAIKIIGRLSSVNPAYVVPSLRKTL
LELLTQLKFSNMPKKKEESATLLCTLINSSDEVAKPYIDPILDVILPKCQDASSAVASTA
LKVLGELSVVGGKEMTRYLKELMPLIINTFQDQSNSFKRDAALTTLGQLAASSGYVVGPL
LDYPELLGILINILKTENNPHIRRGTVRLIGILGALDPYKHREIEVTSNSKSSVEQNAPS
IDIALLMQGVSPSNDEYYPTVVIHNLMKILNDPSLSIHHTAAIQAIMHIFQNLGLRCVSF
LDQIIPGIILVMRSCPPSQLDFYFQQLGSLISIVKQHIRPHVEKIYGVIREFFPIIKLQI
TIISVIESISKALEGEFKRFVPETLTFFLDILENDQSNKRIVPIRILKSLVTFGPNLEDY
SHLIMPIVVRMTEYSAGSLKKISIITLGRLAKNINLSEMSSRIVQALVRILNNGDRELTK
ATMNTLSLLLLQLGTDFVVFVPVINKALLRNRIQHSVYDQLVNKLLNNECLPTNIIFDKE
NEVPERKNYEDEMQVTKLPVNQNILKNAWYCSQQKTKEDWQEWIRRLSIQLLKESPSACL
RSCSSLVSVYYPLARELFNASFSSCWVELQTSYQEDLIQALCKALSSSENPPEIYQMLLN
LVEFMEHDDKPLPIPIHTLGKYAQKCHAFAKALHYKEVEFLEEPKNSTIEALISINNQLH
QTDSAIGILKHAQQHNELQLKETWYEKLQRWEDALAAYNEKEAAGEDSVEVMMGKLRSLY
ALGEWEELSKLASEKWGTAKPEVKKAMAPLAAGAAWGLEQWDEIAQYTSVMKSQSPDKEF
YDAILCLHRNNFKKAEVHIFNARDLLVTELSALVNESYNRAYNVVVRAQIIAELEEIIKY
KKLPQNSDKRLTMRETWNTRLLGCQKNIDVWQRILRVRSLVIKPKEDAQVRIKFANLCRK
SGRMALAKKVLNTLLEETDDPDHPNTAKASPPVVYAQLKYLWATGLQDEALKQLINFTSR
MAHDLGLDPNNMIAQSVPQQSKRVPRHVEDYTKLLARCFLKQGEWRVCLQPKWRLSNPDS
ILGSYLLATHFDNTWYKAWHNWALANFEVISMLTSVSKKKQEGSDASSVTDINEFDNGMI
GVNTFDAKEVHYSSNLIHRHVIPAIKGFFHSISLSESSSLQDALRLLTLWFTFGGIPEAT
QAMHEGFNLIQIGTWLEVLPQLISRIHQPNQIVSRSLLSLLSDLGKAHPQALVYPLMVAI
KSESLSRQKAALSIIEKMRIHSPVLVDQAELVSHELIRMAVLWHEQWYEGLDDASRQFFG
EHNTEKMFAALEPLYEMLKRGPETLREISFQNSFGRDLNDAYEWLMNYKKSKDVSNLNQA
WDIYYNVFRKIGKQLPQLQTLELQHVSPKLLSAHDLELAVPGTRASGGKPIVKISKFEPV
FSVISSKQRPRKFCIKGSDGKDYKYVLKGHEDIRQDSLVMQLFGLVNTLLQNDAECFRRH
LDIQQYPAIPLSPKSGLLGWVPNSDTFHVLIREHREAKKIPLNIEHWVMLQMAPDYDNLT
LLQKVEVFTYALNNTEGQDLYKVLWLKSRSSETWLERRTTYTRSLAVMSMTGYILGLGDR
HPSNLMLDRITGKVIHIDFGDCFEAAILREKFPEKVPFRLTRMLTYAMEVSGIEGSFRIT
CENVMKVLRDNKGSLMAILEAFAFDPLINWGFDLPTKKIEEETGIQLPVMNANELLSNGA
ITEEEVQRVENEHKNAIRNARAMLVLKRITDKLTGNDIRRFNDLDVPEQVDKLIQQATSV
ENLCQHYIGWCPFW
>Q8VYI9
MATHSSFTATTPLFLIVLLSLSSVSVLGASHHHATAPAPSVDCSTLILNMADCLSFVSSG
GTVAKPEGTCCSGLKTVLKADSQCLCEAFKSSASLGVTLNITKASTLPAACKLHAPSIAT
CGLSVAPSTAPGLAPGVAAAGPETAGFLAPNPSSGNDGSSLIPTSFTTVLSAVLFVLFFS
SA
>P21796
MAVPPTYADLGKSARDVFTKGYGFGLIKLDLKTKSENGLEFTSSGSANTETTKVTGSLET
KYRWTEYGLTFTEKWNTDNTLGTEITVEDQLARGLKLTFDSSFSPNTGKKNAKIKTGYKR

EHINLGCDMDFDIAGPSIRGALVLGYEGWLAGYQMNFETAKSRVTQSNFAVGYKTDEFQL
HTNVNDGTEFGGSIYQKVNKKLETAVNLAWTAGNSNTRFGIAAKYQIDPDACFSAKVNNS
SLIGLGYTQTLKPGIKLTLSALLDGKNVNAGGHKLGLGLEFQA
>O13547
MRATTLLSSVVSLALLSKEVLATPPACLLACVAQVGKSSSTCDSLNQVTCYCEHENSAVK
KCLDSICPNNDADAAYSAFKSSCSEQNASLGDSSSSASSSASSSSKASSSTKASSSSASS
STKASSSSASSSTKASSSSAAPSSSKASSTESSSSSSSSTKAPSSEESSSTYVSSSKQAS
STSEAHSSSAASSTVSQETVSSALPTSTAVISTFSEGSGNVLEAGKSVFIAAVAAMLI
>Q04749
MLKEPSVRLREAIIEGNLLIVKRLLRRNPDLLTNIDSENGWSSLHYASYHGRYLICVYLI
QLGHDKHELIKTFKGNTCVHLALMKGHEQTLHLLLQQFPRFINHRGENGRAPIHIACMND
YYQCLSLLIGVGADLWVMDTNGDTPLHVCLEYGSISCMKMLLNEGEVSLDDNVRDKGNWK
PIDVAQTFEVGNIYSKVLKEVKKKGPPLGAGKKPSSFRTPILNAKATFEDGPSPVLSMNS
PYSLYSNNSPLPVLPRRISTHTTSGNGGNRRSSITNPVFNPRKPTLSTDSFSSSSNSSSR
LRVNSINVKTPVGVSPKKELVSESVRHSATPTSPHNNIALINRYLLPNKSNDNVRGDSQT
ATINDDGGGGNGGDATIGMGLRKDPDDENENKYKIKVNNGEPRRRVSLLNIPISKLRNSN
NTRAED
>P30546
MTCPNSSCVFEDKMCQGNKTAPANDAQLTPLVVVLSTISLVTVGLNLLVLYAVRSERKLH
TVGNLYIVSLSVADLIVGVVVMPMNILYLLMSRWSLGRPLCLFWLSMDYVASTASIFSVF
ILCIDRYRSVQQPLKYLRYRTKTRASITILAAWFLSFLWIIPILGWRHFQPKTPEPREDK
CETDFYNVTWFKVMTAIINFYLPTLLMLWFYAKIYKAVRQHCQHRELINGSFPSFSDMKM
KPENLQVGAKKPGKESPWEVLKRKPKDTGGGPVLKPPSQEPKEVTSPGVFSQEKEEKDGE
LGKFYCFPLDTVQAQPEAEGSGRGYATINQSQNQLEMGEQGLSMPGAKEALEDQILGDSQ
SFSRTDSDTPAEPAPAKGKSRSESSTGLEYIKFTWKRLRSHSRQYVSGLHMNRERKAAKQ
LGFIMAAFIICWIPYFIFFMVIAFCESCCNQHVHMFTIWLGYINSTLNPLIYPLCNENFK
KTFKKILHIRS
>P23469
MEPLCPLLLVGFSLPLARALRGNETTADSNETTTTSGPPDPGASQPLLAWLLLPLLLLLL
VLLLAAYFFRFRKQRKAVVSTSDKKMPNGILEEQEQQRVMLLSRSPSGPKKYFPIPVEHL
EEEIRIRSADDCKQFREEFNSLPSGHIQGTFELANKEENREKNRYPNILPNDHSRVILSQ
LDGIPCSDYINASYIDGYKEKNKFIAAQGPKQETVNDFWRMVWEQKSATIVMLTNLKERK
EEKCHQYWPDQGCWTYGNIRVCVEDCVVLVDYTIRKFCIQPQLPDGCKAPRLVSQLHFTS
WPDFGVPFTPIGMLKFLKKVKTLNPVHAGPIVVHCSAGVGRTGTFIVIDAMMAMMHAEQK
VDVFEFVSRIRNQRPQMVQTDMQYTFIYQALLEYYLYGDTELDVSSLEKHLQTMHGTTTH
FDKIGLEEEFRKLTNVRIMKENMRTGNLPANMKKARVIQIIPYDFNRVILSMKRGQEYTD
YINASFIDGYRQKDYFIATQGPLAHTVEDFWRMIWEWKSHTIVMLTEVQEREQDKCYQYW
PTEGSVTHGEITIEIKNDTLSEAISIRDFLVTLNQPQARQEEQVRVVRQFHFHGWPEIGI
PAEGKGMIDLIAAVQKQQQQTGNHPITVHCSAGAGRTGTFIALSNILERVKAEGLLDVFQ
AVKSLRLQRPHMVQTLEQYEFCYKVVQDFIDIFSDYANFK
>Q8WXI7
MLKPSGLPGSSSPTRSLMTGSRSTKATPEMDSGLTGATLSPKTSTGAIVVTEHTLPFTSP
DKTLASPTSSVVGRTTQSLGVMSSALPESTSRGMTHSEQRTSPSLSPQVNGTPSRNYPAT
SMVSGLSSPRTRTSSTEGNFTKEASTYTLTVETTSGPVTEKYTVPTETSTTEGDSTETPW

DTRYIPVKITSPMKTFADSTASKENAPVSMTPAETTVTDSHTPGRTNPSFGTLYSSFLDL
SPKGTPNSRGETSLELILSTTGYPFSSPEPGSAGHSRISTSAPLSSSASVLDNKISETSI
FSGQSLTSPLSPGVPEARASTMPNSAIPFSMTLSNAETSAERVRSTISSLGTPSISTKQT
AETILTFHAFAETMDIPSTHIAKTLASEWLGSPGTLGGTSTSALTTTSPSTTLVSEETNT
HHSTSGKETEGTLNTSMTPLETSAPGEESEMTATLVPTLGFTTLDSKIRSPSQVSSSHPT
RELRTTGSTSGRQSSSTAAHGSSDILRATTSSTSKASSWTSESTAQQFSEPQHTQWVETS
PSMKTERPPASTSVAAPITTSVPSVVSGFTTLKTSSTKGIWLEETSADTLIGESTAGPTT
HQFAVPTGISMTGGSSTRGSQGTTHLLTRATASSETSADLTLATNGVPVSVSPAVSKTAA
GSSPPGGTKPSYTMVSSVIPETSSLQSSAFREGTSLGLTPLNTRHPFSSPEPDSAGHTKI
STSIPLLSSASVLEDKVSATSTFSHHKATSSITTGTPEISTKTKPSSAVLSSMTLSNAAT
SPERVRNATSPLTHPSPSGEETAGSVLTLSTSAETTDSPNIHPTGTLTSESSESPSTLSL
PSVSGVKTTFSSSTPSTHLFTSGEETEETSNPSVSQPETSVSRVRTTLASTSVPTPVFPT
MDTWPTRSAQFSSSHLVSELRATSSTSVTNSTGSALPKISHLTGTATMSQTNRDTFNDSA
APQSTTWPETSPRFKTGLPSATTTVSTSATSLSATVMVSKFTSPATSSMEATSIREPSTT
ILTTETTNGPGSMAVASTNIPIGKGYITEGRLDTSHLPIGTTASSETSMDFTMAKESVSM
SVSPSQSMDAAGSSTPGRTSQFVDTFSDDVYHLTSREITIPRDGTSSALTPQMTATHPPS
PDPGSARSTWLGILSSSPSSPTPKVTMSSTFSTQRVTTSMIMDTVETSRWNMPNLPSTTS
LTPSNIPTSGAIGKSTLVPLDTPSPATSLEASEGGLPTLSTYPESTNTPSIHLGAHASSE
SPSTIKLTMASVVKPGSYTPLTFPSIETHIHVSTARMAYSSGSSPEMTAPGETNTGSTWD
PTTYITTTDPKDTSSAQVSTPHSVRTLRTTENHPKTESATPAAYSGSPKISSSPNLTSPA
TKAWTITDTTEHSTQLHYTKLAEKSSGFETQSAPGPVSVVIPTSPTIGSSTLELTSDVPG
EPLVLAPSEQTTITLPMATWLSTSLTEEMASTDLDISSPSSPMSTFAIFPPMSTPSHELS
KSEADTSAIRNTDSTTLDQHLGIRSLGRTGDLTTVPITPLTTTWTSVIEHSTQAQDTLSA
TMSPTHVTQSLKDQTSIPASASPSHLTEVYPELGTQGRSSSEATTFWKPSTDTLSREIET
GPTNIQSTPPMDNTTTGSSSSGVTLGIAHLPIGTSSPAETSTNMALERRSSTATVSMAGT
MGLLVTSAPGRSISQSLGRVSSVLSESTTEGVTDSSKGSSPRLNTQGNTALSSSLEPSYA
EGSQMSTSIPLTSSPTTPDVEFIGGSTFWTKEVTTVMTSDISKSSARTESSSATLMSTAL
GSTENTGKEKLRTASMDLPSPTPSMEVTPWISLTLSNAPNTTDSLDLSHGVHTSSAGTLA
TDRSLNTGVTRASRLENGSDTSSKSLSMGNSTHTSMTDTEKSEVSSSIHPRPETSAPGAE
TTLTSTPGNRAISLTLPFSSIPVEEVISTGITSGPDINSAPMTHSPITPPTIVWTSTGTI
EQSTQPLHAVSSEKVSVQTQSTPYVNSVAVSASPTHENSVSSGSSTSSPYSSASLESLDS
TISRRNAITSWLWDLTTSLPTTTWPSTSLSEALSSGHSGVSNPSSTTTEFPLFSAASTSA
AKQRNPETETHGPQNTAASTLNTDASSVTGLSETPVGASISSEVPLPMAITSRSDVSGLT
SESTANPSLGTASSAGTKLTRTISLPTSESLVSFRMNKDPWTVSIPLGSHPTTNTETSIP
VNSAGPPGLSTVASDVIDTPSDGAESIPTVSFSPSPDTEVTTISHFPEKTTHSFRTISSL
THELTSRVTPIPGDWMSSAMSTKPTGASPSITLGERRTITSAAPTTSPIVLTASFTETST
VSLDNETTVKTSDILDARKTNELPSDSSSSSDLINTSIASSTMDVTKTASISPTSISGMT
ASSSPSLFSSDRPQVPTSTTETNTATSPSVSSNTYSLDGGSNVGGTPSTLPPFTITHPVE
TSSALLAWSRPVRTFSTMVSTDTASGENPTSSNSVVTSVPAPGTWASVGSTTDLPAMGFL
KTSPAGEAHSLLASTIEPATAFTPHLSAAVVTGSSATSEASLLTTSESKAIHSSPQTPTT
PTSGANWETSATPESLLVVTETSDTTLTSKILVTDTILFSTVSTPPSKFPSTGTLSGASF
PTLLPDTPAIPLTATEPTSSLATSFDSTPLVTIASDSLGTVPETTLTMSETSNGDALVLK
TVSNPDRSIPGITIQGVTESPLHPSSTSPSKIVAPRNTTYEGSITVALSTLPAGTTGSLV
FSQSSENSETTALVDSSAGLERASVMPLTTGSQGMASSGGIRSGSTHSTGTKTFSSLPLT

MNPGEVTAMSEITTNRLTATQSTAPKGIPVKPTSAESGLLTPVSASSSPSKAFASLTTAP
PSTWGIPQSTLTFEFSEVPSLDTKSASLPTPGQSLNTIPDSDASTASSSLSKSPEKNPRA
RMMTSTKAISASSFQSTGFTETPEGSASPSMAGHEPRVPTSGTGDPRYASESMSYPDPSK
ASSAMTSTSLASKLTTLFSTGQAARSGSSSSPISLSTEKETSFLSPTASTSRKTSLFLGP
SMARQPNILVHLQTSALTLSPTSTLNMSQEEPPELTSSQTIAEEEGTTAETQTLTFTPSE
TPTSLLPVSSPTEPTARRKSSPETWASSISVPAKTSLVETTDGTLVTTIKMSSQAAQGNS
TWPAPAEETGTSPAGTSPGSPEVSTTLKIMSSKEPSISPEIRSTVRNSPWKTPETTVPME
TTVEPVTLQSTALGSGSTSISHLPTGTTSPTKSPTENMLATERVSLSPSPPEAWTNLYSG
TPGGTRQSLATMSSVSLESPTARSITGTGQQSSPELVSKTTGMEFSMWHGSTGGTTGDTH
VSLSTSSNILEDPVTSPNSVSSLTDKSKHKTETWVSTTAIPSTVLNNKIMAAEQQTSRSV
DEAYSSTSSWSDQTSGSDITLGASPDVTNTLYITSTAQTTSLVSLPSGDQGITSLTNPSG
GKTSSASSVTSPSIGLETLRANVSAVKSDIAPTAGHLSQTSSPAEVSILDVTTAPTPGIS
TTITTMGTNSISTTTPNPEVGMSTMDSTPATERRTTSTEHPSTWSSTAASDSWTVTDMTS
NLKVARSPGTISTMHTTSFLASSTELDSMSTPHGRITVIGTSLVTPSSDASAVKTETSTS
ERTLSPSDTTASTPISTFSRVQRMSISVPDILSTSWTPSSTEAEDVPVSMVSTDHASTKT
DPNTPLSTFLFDSLSTLDWDTGRSLSSATATTSAPQGATTPQELTLETMISPATSQLPFS
IGHITSAVTPAAMARSSGVTFSRPDPTSKKAEQTSTQLPTTTSAHPGQVPRSAATTLDVI
PHTAKTPDATFQRQGQTALTTEARATSDSWNEKEKSTPSAPWITEMMNSVSEDTIKEVTS
SSSVLKDPEYAGHKLGIWDDFIPKFGKAAHMRELPLLSPPQDKEAIHPSTNTVETTGWVT
SSEHASHSTIPAHSASSKLTSPVVTTSTREQAIVSMSTTTWPESTRARTEPNSFLTIELR
DVSPYMDTSSTTQTSIISSPGSTAITKGPRTEITSSKRISSSFLAQSMRSSDSPSEAITR
LSNFPAMTESGGMILAMQTSPPGATSLSAPTLDTSATASWTGTPLATTQRFTYSEKTTLF
SKGPEDTSQPSPPSVEETSSSSSLVPIHATTSPSNILLTSQGHSPSSTPPVTSVFLSETS
GLGKTTDMSRISLEPGTSLPPNLSSTAGEALSTYEASRDTKAIHHSADTAVTNMEATSSE
YSPIPGHTKPSKATSPLVTSHIMGDITSSTSVFGSSETTEIETVSSVNQGLQERSTSQVA
SSATETSTVITHVSSGDATTHVTKTQATFSSGTSISSPHQFITSTNTFTDVSTNPSTSLI
MTESSGVTITTQTGPTGAATQGPYLLDTSTMPYLTETPLAVTPDFMQSEKTTLISKGPKD
VTWTSPPSVAETSYPSSLTPFLVTTIPPATSTLQGQHTSSPVSATSVLTSGLVKTTDMLN
TSMEPVTNSPQNLNNPSNEILATLAATTDIETIHPSINKAVTNMGTASSAHVLHSTLPVS
SEPSTATSPMVPASSMGDALASISIPGSETTDIEGEPTSSLTAGRKENSTLQEMNSTTES
NIILSNVSVGAITEATKMEVPSFDATFIPTPAQSTKFPDIFSVASSRLSNSPPMTISTHM
TTTQTGSSGATSKIPLALDTSTLETSAGTPSVVTEGFAHSKITTAMNNDVKDVSQTNPPF
QDEASSPSSQAPVLVTTLPSSVAFTPQWHSTSSPVSMSSVLTSSLVKTAGKVDTSLETVT
SSPQSMSNTLDDISVTSAATTDIETTHPSINTVVTNVGTTGSAFESHSTVSAYPEPSKVT
SPNVTTSTMEDTTISRSIPKSSKTTRTETETTSSLTPKLRETSISQEITSSTETSTVPYK
ELTGATTEVSRTDVTSSSSTSFPGPDQSTVSLDISTETNTRLSTSPIMTESAEITITTQT
GPHGATSQDTFTMDPSNTTP
>Q03193
MISEKAATALATIATVCWCVQLIPQIIYNWKKKDCTGLPPLMMFLWVVSGIPFAIYFCVS
KGNVILQVQPHLFMFFCSISFVQSCYYPPISMARSKIVMIVAAIIAADVGMEVGFILWLR
PLYEKGVKWPDLIFGISASVLLAVGLLPPYFELAKRKGRVIGINFAFLFIDSLGAWLSII
SVILGNMDIMGIILYSIVAGMELGIFASHFIWWCRFRFLAKGNTFDEESGQAQKEEPDEK
IEQDISKSDRNVTNYNLDNCSIPDDASSFADDFNIYDSTDGGTLSRAQTLHAVHGVVVRT
DPDRYSRLSV

>Q14512
MKICSLTLLSFLLLAAQVLLVEGKKKVKNGLHSKVVSEQKDTLGNTQIKQKSRPGNKGKF
VTKDQANCRWAATEQEEGISLKVECTQLDHEFSCVFAGNPTSCLKLKDERVYWKQVARNL
RSQKDICRYSKTAVKTRVCRKDFPESSLKLVSSTLFGNTKPRKEKTEMSPREHIKGKETT
PSSLAVTQTMATKAPECVEDPDMANQRKTALEFCGETWSSLCTFFLSIVQDTSC
>Q8BHK3
MSVTKSARSPQVATPLNLDLPESAKKLQSQDPSPANGSSSESSKKTKGITGFQTLVHLVK
GNMGTGILGLPLAVKNAGILMGPLSLLVMGLIACHCMHILVRCAQRFCHRLNKPFMDYGD
TVMHGLAFSPNAWLQNHAHWGRRVVSFFLIVTQLGFCCVYIVFLADNLKQVVEAVNSTTI
SCHKNETVVLTPTMDSRLYMLSFLPVLGLLVFVRNLRVLTIFSLLANISMLVSLVIIAQY
IIQEIPDASQLPLVASWKTYPLFFGTAIFSFESIGVVLPLENKMKDARGFPTILSLGMSI
ITTLYIAIGALGYLRFGDDIKASITLNLPNCWLYQSVKLLYVVGILCTYALQFYVPAEII
IPLAVSQVSKRWALPVDLSIRLALVCLTCMLAILIPRLDLVLSLVGSVSSSALALIIPPL
LEVVTYYGEGISPLTVTKDALISILGFMGFVVGTYQALDELIKSGNSPALSNSTMFIQ
>Q12355
MQLHSLIASTALLITSALAATSSSSSIPSSCTISSHATATAQSDLDKYSRCDTLVGNLTI
GGGLKTGALANVKEINGSLTIFNATNLTSFAADSLESITDSLNLQSLTILTSASFGSLQS
VDSIKLITLPAISSFTSNIKSANNIYISDTSLQSVDGFSALKKVNVFNVNNNKKLTSIKS
PVETVSDSLQFSFNGNQTKITFDDLVWANNISLTDVHSVSFANLQKINSSLGFINNSISS
LNFTKLNTIGQTFSIVSNDYLKNLSFSNLSTIGGALVVANNTGLQKIGGLDNLTTIGGTL
EVVGNFTSLNLDSLKSVKGGADVESKSSNFSCNALKALQKKGGIKGESFVCKNGASSTSV
KLSSTSKSQSSQTTAKVSKSSSKAEEKKFTSGDIKAAASASSVSSSGASSSSSKSSKGNA
AIMAPIGQTTPLVGLLTAIIMSIM
>P54211
MADIKEGVEEGSVKVDMIKEPLTQGDTGVDEVDFAKITLDDAFKYLNCNKHGLSSAEAAA
RLQQHGPNKLPDSSRNPVLVFLGYMWNPLAWAMEAAAIISIALLDVADFVLIVGLLLINA
IISFYEESNADKAIKALTAALAPKAMVVRDGAIVTIDAVNLVPGDVILIRLGNIVPADVK
LLEEEGADEGEQEAPMQIDQAALTGESLPAKKFTGDVAFSGSSIKQGERHAVVYATGVNT
FFGRAAALISGTNNVSNLQTVMNKMSAICIVTILLWVVVELAVQFGHYSHECVGGREGCP
TLLNMLVVLVGGIPIAMPTVLSVTLALGAYKLAREGAIVTRMSAVEEMAGMDVLCSDKTG
TLTLNKLSIDKSMVVPVGNMGVDEIMRMGALSANTVTEEPIDMVLWESYPDRETIKRDYK
HTKYFPFNPNDKITIATCLEIATGRVFRVLKGSPQVVLAKAWNAAELDATVNQKMVEFAN
RGFRALGLAMADGDGKDGTKWEMLALLPLFDPPRHDTKETIEHCQNQGIQVKMITGDHLL
IGKETAKMLGMGTEMFPSEVMIKARNGDASQLHGYKNFVEMVETCNGFAQVFPEHKFEIV
KILQDSNHVVGMTGDGVNDAPALKKADVGVAVADATDAARGAADIVLTEPGLSTIVTAVI
GARKIFQRMTTYSKYTIAMTFRICFTFGLITVIYDWYFPTILIVIMAVFNDGAMIALSKD
RVVASKTPNSWNITNIFIMGMVYGLYLTLSTWALYQTATKTTFFEDKTPLHSLNDQYSVL
QPWCEDEVRAKLGQTIDPYASLCESNSYAKQFDECEGYQKGSGVQVEDVPTLHAQCVTEQ
RYLRGAMTRSLIYTQVSISGQALVFVVRTAGYSLMERAGTSTYLAFFFAQVGATLFGIFG
LGGFEKPRHQLEDCQFCDYSFHEPVDWFDSGIVPESGTESDFTASVIGCGGYVIVAWIWS
AIWYVLLDPIKWILFWILNEEGFRDTMSWRESTKRSLDRRSKDDIGDKEFTGPSGMVPAN
YSNPLGRASMSKPVSAVLDRKSASLVAINRNSMTVSQDPNRALNIGRRSMIGRPSGPVGR
TSMPLGRISRTSNTLSTGSKDGQIGRGSKPLNSSSAEIKPDKYDFASTIRE
>Q96E35

MTDFKLGIVRLGRVAGKTKYTLIDEQDIPLVESYSFEARMEVDADGNGAKIFAYAFDKNR
GRGSGRLLHELLWERHRGGVAPGFQVVHLNAVTVDNRLDNLQLVPWGWRPKAEETSSKQR
EQSLYWLAIQQLPTDPIEEQFPVLNVTRYYNANGDVVEEEENSCTYYECHYPPCTVIEKQ
LREFNICGRCQVARYCGSQCQQKDWPAHKKHCRERKRPFQHELEPER
>Q62765
MALPRCMWPNYVWRAMMACVVHRGSGAPLTLCLLGCLLQTFHVLSQKLDDVDPLVTTNFG
KIRGIKKELNNEILGPVIQFLGVPYAAPPTGEHRFQPPEPPSPWSDIRNATQFAPVCPQN
IIDGRLPEVMLPVWFTNNLDVVSSYVQDQSEDCLYLNIYVPTEDVKRISKECARKPGKKI
CRKGDIRDSGGPKPVMVYIHGGSYMEGTGNLYDGSVLASYGNVIVITVNYRLGVLGFLST
GDQAAKGNYGLLDLIQALRWTSENIGFFGGDPLRITVFGSGAGGSCVNLLTLSHYSEGNR
WSNSTKGLFQRAIAQSGTALSSWAVSFQPAKYARILATKVGCNVSDTVELVECLQKKPYK
ELVDQDVQPARYHIAFGPVIDGDVIPDDPQILMEQGEFLNYDIMLGVNQGEGLKFVENIV
DSDDGVSASDFDFAVSNFVDNLYGYPEGKDVLRETIKFMYTDWADRHNPETRRKTLLALF
TDHQWVAPAVATADLHSNFGSPTYFYAFYHHCQTDQVPAWADAAHGDEVPYVLGIPMIGP
TELFPCNFSKNDVMLSAVVMTYWTNFAKTGDPNQPVPQDTKFIHTKPNRFEEVAWTRYSQ
KDQLYLHIGLKPRVKEHYRANKVNLWLELVPHLHNLNDISQYTSTTTKVPSTDITLRPTR
KNSTPVTSAFPTAKQDDPKQQPSPFSVDQRDYSTELSVTIAVGASLLFLNILAFAALYYK
KDKRRHDVHRRCSPQRTTTNDLTHAPEEEIMSLQMKHTDLDHECESIHPHEVVLRTACPP
DYTLAMRRSPDDVPLMTPNTITMIPNTIPGIQPLHTFNTFTGGQNNTLPHPHPHPHSHST
TRV
>Q96LC7
MLLPLLLSSLLGGSQAMDGRFWIRVQESVMVPEGLCISVPCSFSYPRQDWTGSTPAYGYW
FKAVTETTKGAPVATNHQSREVEMSTRGRFQLTGDPAKGNCSLVIRDAQMQDESQYFFRV
ERGSYVRYNFMNDGFFLKVTALTQKPDVYIPETLEPGQPVTVICVFNWAFEECPPPSFSW
TGAALSSQGTKPTTSHFSVLSFTPRPQDHNTDLTCHVDFSRKGVSAQRTVRLRVAYAPRD
LVISISRDNTPALEPQPQGNVPYLEAQKGQFLRLLCAADSQPPATLSWVLQNRVLSSSHP
WGPRPLGLELPGVKAGDSGRYTCRAENRLGSQQRALDLSVQYPPENLRVMVSQANRTVLE
NLGNGTSLPVLEGQSLCLVCVTHSSPPARLSWTQRGQVLSPSQPSDPGVLELPRVQVEHE
GEFTCHARHPLGSQHVSLSLSVHYSPKLLGPSCSWEAEGLHCSCSSQASPAPSLRWWLGE
ELLEGNSSQDSFEVTPSSAGPWANSSLSLHGGLSSGLRLRCEAWNVHGAQSGSILQLPDK
KGLISTAFSNGAFLGIGITALLFLCLALIIMKILPKRRTQTETPRPRFSRHSTILDYINV
VPTAGPLAQKRNQKATPNSPRTPLPPGAPSPESKKNQKKQYQLPSFPEPKSSTQAPESQE
SQEELHYATLNFPGVRPRPEARMPKGTQADYAEVKFQ
>P09483
MANSGTGAPPPLLLLPLLLLLGTGLLPASSHIETRAHAEERLLKRLFSGYNKWSRPVANI
SDVVLVRFGLSIAQLIDVDEKNQMMTTNVWVKQEWHDYKLRWDPGDYENVTSIRIPSELI
WRPDIVLYNNADGDFAVTHLTKAHLFYDGRVQWTPPAIYKSSCSIDVTFFPFDQQNCTMK
FGSWTYDKAKIDLVSMHSRVDQLDFWESGEWVIVDAVGTYNTRKYECCAEIYPDITYAFI
IRRLPLFYTINLIIPCLLISCLTVLVFYLPSECGEKVTLCISVLLSLTVFLLLITEIIPS
PTSLVIPLIGEYLLFTMIFVTLSIVITVFVLNVHHRSPRTHTMPAWVRRVFLDIVPRLLF
MKRPSVVKDNCRRLIESMHKMANAPRFWPEPVGEPGILSDICNQGLSPAPTFCNPTDTAV
ETQPTCRSPPLEVPDLKTSEVEKASPCPSPGSCPPPKSSSGAPMLIKARSLSVQHVPSSQ
EAAEDGIRCRSRSIQYCVSQDGAASLADSKPTSSPTSLKARPSQLPVSDQASPCKCTCKE
PSPVSPVTVLKAGGTKAPPQHLPLSPALTRAVEGVQYIADHLKAEDTDFSVKEDWKYVAM

VIDRIFLWMFIIVCLLGTVGLFLPPWLAAC
>P23276
MEGGDQSEEEPRERSQAGGMGTLWSQESTPEERLPVEGSRPWAVARRVLTAILILGLLLC
FSVLLFYNFQNCGPRPCETSVCLDLRDHYLASGNTSVAPCTDFFSFACGRAKETNNSFQE
LATKNKNRLRRILEVQNSWHPGSGEEKAFQFYNSCMDTLAIEAAGTGPLRQVIEELGGWR
ISGKWTSLNFNRTLRLLMSQYGHFPFFRAYLGPHPASPHTPVIQIDQPEFDVPLKQDQEQ
KIYAQIFREYLTYLNQLGTLLGGDPSKVQEHSSLSISITSRLFQFLRPLEQRRAQGKLFQ
MVTIDQLKEMAPAIDWLSCLQATFTPMSLSPSQSLVVHDVEYLKNMSQLVEEMLLKQRDF
LQSHMILGLVVTLSPALDSQFQEARRKLSQKLRELTEQPPMPARPRWMKCVEETGTFFEP
TLAALFVREAFGPSTRSAAMKLFTAIRDALITRLRNLPWMNEETQNMAQDKVAQLQVEMG
ASEWALKPELARQEYNDIQLGSSFLQSVLSCVRSLRARIVQSFLQPHPQHRWKVSPWDVN
AYYSVSDHVVVFPAGLLQPPFFHPGYPRAVNFGAAGSIMAHELLHIFYQLLLPGGCLACD
NHALQEAHLCLKRHYAAFPLPSRTSFNDSLTFLENAADVGGLAIALQAYSKRLLRHHGET
VLPSLDLSPQQIFFRSYAQVMCRKPSPQDSHDTHSPPHLRVHGPLSSTPAFARYFRCARG
ALLNPSSRCQLW
>Q13564
MAQLGKLLKEQKYDRQLRLWGDHGQEALESAHVCLINATATGTEILKNLVLPGIGSFTII
DGNQVSGEDAGNNFFLQRSSIGKNRAEAAMEFLQELNSDVSGSFVEESPENLLDNDPSFF
CRFTVVVATQLPESTSLRLADVLWNSQIPLLICRTYGLVGYMRIIIKEHPVIESHPDNAL
EDLRLDKPFPELREHFQSYDLDHMEKKDHSHTPWIVIIAKYLAQWYSETNGRIPKTYKEK
EDFRDLIRQGILKNENGAPEDEENFEEAIKNVNTALNTTQIPSSIEDIFNDDRCINITKQ
TPSFWILARALKEFVAKEGQGNLPVRGTIPDMIADSGKYIKLQNVYREKAKKDAAAVGNH
VAKLLQSIGQAPESISEKELKLLCSNSAFLRVVRCRSLAEEYGLDTINKDEIISSMDNPD
NEIVLYLMLRAVDRFHKQQGRYPGVSNYQVEEDIGKLKSCLTGFLQEYGLSVMVKDDYVH
EFCRYGAAEPHTIAAFLGGAAAQEVIKIITKQFVIFNNTYIYSGMSQTSATFQL
>O88735
MAEQGAGGDGHRGGDGATHSDPASDGYKVQEKRTAPSRPTSTVSGQTSNHSGNKPDPPPV
LRVDDRQRLARERREEREKQLAARETVWLEREERARQHYERHLEARKKKLEDQRLKEERR
RAAVEEKRRQRLEEDKERHEAVVRRTMERSQKPRQKSNRWSWGSPLHGSSSIHSGDPDRR
SVSTMNLSKHVDPVISKRLSSSSATLLNSPDRARRLQLSPWESSVVSRLLTPTHSFLARS
KSTAALSGDTASCSPIIMPFKAAHSRNPVDRPKLFVTPPEGSARRRTIHGLASHKRERER
EHVPFHVSPGARRTLSPSNLKARSPAPARLWLPSKSMPHLPGTPRPASSLPPGSVRAASA
QAPSSSPGNIRPFKREVKVEPEKKDPLPAVKSRVPLVKVEEVTVEEGTPVKPPEPAAPAS
APIATPAPAPATDPAPVPAPSSTVTVGVVPKTSAGTTDPEEATRLLAEKRRLAREQREKE
ERERKEKEELERQKIEELARRVAEERSRREEEARRLEEEQAREKEELALRLAEEERERWE
REEVERVQKQKEEEARAREEAERARQEREKHFQKEEQERLERKKRLEEIMRRTRRTETAD
KKTTEQRNGDIAKGVLTGEPEVPALPCMASSGNGESAESPHGVALQQSEVTTESSPDLEK
QPNENGMSIQNENFEEVINLPVGSKASRLDVTNENPEIPLKPILAFNDEGTLGPLPQVDG
VQTQQTAEVI
>P59833
MAISKLHLLFLLSVFLSLHPLVLSDTADEEDVLLTGINSYRASLNLTTLIHNHNAECLAD
EIADQFKNQPCTNTTGSASVPGTTPGFPNLPNLLSKCRLNPTVTRDGAILPACVPNLDPS
LVLTNFTMSQYSKDLNDSKFTGIGIGSDDNWIVVVLTTSTPEGSYSPASNSGAFAFGVNG
LVSSSLMFLLFCFFMF

>Q9QZI9
MGAVLGVFSLASWVPCLCSGASCLLCSCCPISKNSTVTRLIYAFILFLGTIVSCIMMTEG
IQTQLKKIPGFCEGGFQIKMVDTKAEKDCDVLVGFKAVYRINFAVAIFFFAFFLLMLKVK
TSKDPRAAVHNGFWFFKIAAIIGIMIGSFYIPGGSFTEVWFVAGMLGASFFIIIQLVLLV
DMAHSWNELWVNRMEEGNPRLWYAALLSFTSLFYILSIVFAALLYVFYTKPDDCTENKVF
ISLNLIFCVAVSIVSILPKVQEHQPRSGLLQSSIITLYTLYLTWSAMTNEPERSCNPSLM
SIITHLTSPTVSPANSTTLAPAYAPPSQSGHFMNLDDIWGLIIFVFCLIYSSFRTSSNSQ
VNKLTLSGSDSVILGDTTNGANDEEDGQPRRAVDNEKEGVQYSYSFFHLMLCCASLYIMM
TITSWYSPDAKFQKVSSKWLAVWFKMGSSWLCLLLYLWTLVAPLVLTGRDFS
>P41832
MLKNSGSKHSNSKESHSNSSSGIFQNLKRLANSNATNSNTGSPTYASQQQHSPVGNEVST
SPASSSSFRKLNAPSRSTSTEARPLNKKSTLNTQNLSQYMNGKLSGDVPVSSQHARSHSM
QSKYSYSKRNSSQASNKLTRQHTGQSHSASSLLSQGSLTNLSKFTTPDGKIYLEMPSDPY
EVEVLFEDIMYKRNIFQSLSEDKQEALMGYSIEKKWLIVKQDLQNELKKMRANTTSSSTA
SRTSMASDHHPILTANSSLSSPKSVLMTSASSPTSTVYSNSLNHSTTLSSVGTSTSKGKK
LVSGSLKKQPSLNNIYRGGAENNTSASTLPGDRTNRPPIHYVQRILADKLTSDEMKDLWV
TLRTEQLDWVDAFIDHQGHIAMANVLMNSIYKTAPRENLTKELLEKENSFFKCFRVLSML
SQGLYEFSTHRLMTDTVAEGLFSTKLATRKMATEIFVCMLEKKNKSRFEAVLTSLDKKFR
IGQNLHMIQNFKKMPQYFSHLTLESHLKIIQAWLFAVEQTLDGRGKMGSLVGASDEFKNG
GGENAILEYCQWTMVFINHLCSCSDNINQRMLLRTKLENCGILRIMNKIKLLDYDKVIDQ
IELYDNNKLDDFNVKLEANNKAFNVDLHDPLSLLKNLWDICKGTENEKLLVSLVQHLFLS
SSKLIEENQNSSKLTKQLKLMDSLVTNVSVASTSDEETNMNMAIQRLYDAMQTDEVARRA
ILESRALTKKLEEIQAERDSLSEKLSKAEHGLVGQLEDELHERDRILAKNQRVMQQLEAE
LEELKKKHLLEKHQQEVELRKMLTILNSRPEESFNKNEGTRGMNSSLNSSEKANIQKVLQ
DGLSRAKKDYKDDSKKFGMTLQPNKRLKMLRMQMENIENEARQLEMTNFAEFEKDRLEPP
IHIKKPKVKKMKNKDRKPLVKPQEADVNKLNDLRRALTEIQMESNDISKFNVEERVNELF
NEKKSLALKRLKELETKYKGFGIDFNVDEIMDSPKKNTGDVETEEDANYASLDPKTYQKK
LDEINRITDQLLDIQTQTEHEIQVEEDGESDLSSSSSDDESEEIYQDASPTQELRSEHSE
LSSGSGPGSFLDALSQKYGTGQNVTASAAFGENNNGSGIGPLHSKVEKTFMNRLRKSTVS
SAPYLEELTQKVNKVEPYEQNEDEGLDKKSLPENSTASAASAFDKAEKDMRQHVENGKQG
RVVNHEEDKTADFSAVSKLNNTDGAEDLSTQSSVLSSQPPPPPPPPPPVPAKLFGESLEK
EKKSEDDTVKQETTGDSPAPPPPPPPPPPPPMALFGKPKGETPPPPPLPSVLSSSTDGVI
PPAPPMMPASQIKSAVTSPLLPQSPSLFEKYPRPHKKLKQLHWEKLDCTDNSIWGTGKAE
KFADDLYEKGVLADLEKAFAAREIKSLASKRKEDLQKITFLSRDISQQFGINLHMYSSLS
VADLVKKILNCDRDFLQTPSVVEFLSKSEIIEVSVNLARNYAPYSTDWEGVRNLEDAKPP
EKDPNDLQRADQIYLQLMVNLESYWGSRMRALTVVTSYEREYNELLAKLRKVDKAVSALQ
ESDNLRNVFNVILAVGNFMNDTSKQAQGFKLSTLQRLTFIKDTTNSMTFLNYVEKIVRLN
YPSFNDFLSELEPVLDVVKVSIEQLVNDCKDFSQSIVNVERSVEIGNLSDSSKFHPLDKV
LIKTLPVLPEARKKGDLLEDEVKLTIMEFESLMHTYGEDSGDKFAKISFFKKFADFINEY
KKAQAQNLAAEEEERLYIKHKKIVEEQQKRAQEKEKQKENSNSPSSEGNEEDEAEDRRAV
MDKLLEQLKNAGPAKSDPSSARKRALVRKKYLSEKDNAPQLLNDLDTEEGSILYSPEAMD
PTADTVIHAESPTPLATRGVMNTSEDLPSPSKTSALEDQEEISDRARMLLKELRGSDTPV
KQNSILDEHLEKLRARKERSIGEASTGNRLSFK
>P01375

MSTESMIRDVELAEEALPKKTGGPQGSRRCLFLSLFSFLIVAGATTLFCLLHFGVIGPQR
EEFPRDLSLISPLAQAVRSSSRTPSDKPVAHVVANPQAEGQLQWLNRRANALLANGVELR
DNQLVVPSEGLYLIYSQVLFKGQGCPSTHVLLTHTISRIAVSYQTKVNLLSAIKSPCQRE
TPEGAEAKPWYEPIYLGGVFQLEKGDRLSAEINRPDYLDFAESGQVYFGIIAL
>Q12465
MFVHRLWTLAFPFLVEISKASQLENIKSLLDIEDNVLPNLNISQNNSNAVQILGGVDALS
FYEYTGQQNFTKEIGPETSSHGLVYYSNNTYIQLEDASDDTRIDKITPFGVDSFILSGSG
TINNISVGNQILYNLSTLSMTPIFNQSLGAVQAVLADNSSIYFGGNFSYNNGSMTGYSAL
IWDSISNTTQLLPFGGFGENSSVNSIVKLNNDNILFAGQFYTLDDPSALISSSNNGTNST
SSLNATTLELGQRIPLRYASWDSQGSTTFASDSLVCPNTNEDAWLYPDTSGSLVCNLPYE
VSPTKIRLYNSQRSDSEISVFQILTDPSSSIMNLTYLDPLSGELKNCGEFCPLYSRATLL
SASQNVSSSMDMITFIDNNKTDVKWTSDFQDFAFVNELPVSSLKFVALNSYGGSVGLSGL
ELYQDTFSTYANDSLNEYGCSALTNDSSSSTLSSNDWYNGLTGESYIAAKYVPDQNEPIP
RVKFYPNIIHPGHYTINMYTPGCLQDNTCSARGIVNVTMWNQQNNTIMKTYLIYQNNDNL
KYDQIYSGYLDFSPEIVLEYVSGIYTTNTATVVVADQVNVITVSLDAFNTLSDSSNAKKE
TLLNGILQYQKSNFTSTRLNETKVGNTTLNLFPVKNYPKNSSLYADIYDNKLVIGGVSNR
ISIVDLNDDFEVTSSKNQTIQGDVHGITKTNQGLLIFGDILSSNNQSAVFLFNGSFENVF
NQSRTVNSALNISLANNDFIVLDNDYVVNASSNALIRNSSSFSLSLWAAGNNGDGDVLFS
GAVSHMQYGNLNGSVRFLNENEIEPLNLEGGIVPYLGAYLNESATAYAYEVDSLNKIYFS
NEVYPSWNWSSGITQMLYADNQTLLAVSAGSSTTAELSIFDLRNLTMIANETLGSNARIN
ALVNFEKNCSMLVGGDFQMTEPNCTGLCLYNYESKTWSTFLNNTIFGEITQLSFTNSSEL
IISGLFETKEYQSIRLGSFNLTNSTMIPLLSGSEGKLNSFTVTEDSIVAWNDTSLFIYRN
QEWNITSLPGNASSISSVSAIYTDIESNTLNKRGINNVNNGSILLLNGNFNISQYGYLQS
LLFDFQKWTPYFISETTNTSNYNPIIFINRDVSTEFNSQSPLANVNITVTSPQSTSSQPP
SSSASSESKSKSKKKKIGRGFVVLIGLALALGTVSVLGIAGVILAYVFKDPEGDYKPIKP
RIDENEMLDTVPPEKLMKFV
>Q8MJ88
MNPFHSSCWNTSAELSNKSWNKEFAYQTASVVDTVILPSMIGIICSTGLVGNILIVFTII
RSRKKTVPDIYICNLAVADLVHIIGMPFLIHQWARGGEWVFGGPLCTIITSLDTCNQFAC
SAIMTVMSVDRYFALVQPFRLTSWRTRYKTIRINLGLWAASFVLALPVWIYSKVIKFKDG
VESCAFDLTSPDDVLWYTLYLTITTFFFPLPLILVCYILILCYTWEMYQQNKDARCCNPS
VPKQRVMKLTKMVLVLVAVFILSAAPYHVIQLVNLQMEQPTLAFYVGYYLSICLSYASSS
INPFLYILLSGNFQKRLPQIQRRVTDKEIKNMGNTLKSHF
>Q5ZHX6
MTNCKGRSTITKTNSKVHDREGFVNWTISLSTVQSDKFLNLLLSMVPVVYQINQDERHKK
VNGVWQDGLQPAGHNYNVKSDQHVEYHHFSEQTFHGSNGHSPSSCSPKYDDFSSYNYCDG
MDASETDAMLQEDNISSDSNEDIIVEGIRKQPKESSSIMALQILVPFLLAGFGTVTAGMV
LDIVQHWDVFKNVTEVFILVPALLGLKGNLEMTLASRLSTAVNIGKMDSPIEKWNLIIGN
LALKQVQATVVGFLAAVAAVILGWIPEGKYSFSHSILLCSSSVATAFIASLLQGIIMVGV
IVGSKKTGINPDNVATPIAASFGDLITLAILAWISQGLYTCLETYYYVSPLVGAFFLALT
PMGIVIAAKHPATRTVLHSGWEPVITAMIISSIGGLILDTTVSDPNLVGIVVYTPVINGI
GGNLVAIQASRISTYLHLHSIPGELPEEAKGCYYPCRTYYGTGVNNKSAQVLLLLVIPGH
LIFLYTIHLMKSGHTSLTPIFIAVYLFAALLQVFTLLWIADWMVHHFWKKGKDPDSFSIP
YLTALGDLLGTALLAVGFHFLWLIGDRDGDVGD

>Q8BUE1
MGPAMFMAFRLWNWLLLLAVLTRSEATSYVNESSNPTAQQAPDARFAASSSDPDEGISVF
ELDYDYVQIPYEVTLWILLASLAKIGFHLYHRLPHLMPESCLLIIVGALVGGIIFGTHHK
SPPVMDSSIYFLYLLPPIVLESGYFMPTRPFFENIGSILWWAGLGALINAFGIGLSLYFI
CQIKAFGLGDINLLHNLLFGSLISAVDPVAVLAVFEEARVNEQLYMMIFGEALLNDGISV
VLYNILIAFTKMHKFEDIEAVDILAGCARFVIVGCGGVFFGIIFGFISAFITRFTQNISA
IEPLIVFMFSYLSYLAAETLYLSGILAITACAVTMKKYVEENVSQTSYTTIKYFMKMLSS
VSETLIFIFMGVSTIGKNHEWNWAFICFTLLFCQIWRAISVFTLFYVSNQFRTFPFSIKD
QFIIFYSGVRGAGSFSLAFLLPLSLFPRKKLFVTATLVVTYFTVFFQGITIGPLVRYLDV
RKTNKKESINEELHSRLMDHLKAGIEDVCGQWSHYQVRDKFKKFDHRYLRKILIRRNLPK
SSIVSLYKKLEMKQAIEMVETGILSSVASPTPYQSERIQGIKRLSPEDVESMRDILTRSM
YQVRQRTLSYNKYNLKPQTSEKQAKEILIRRQNTLRESMRKGQSLPWGKPAGTKNFRYLS
FPYSNPQAARREARAAEPTDDDGTDSGFQPLMFSIHSRAGSLQERRQTQAVIPMKRLQRG
EKALSFSYRSNTSWEDQAGWRRMDVLRPKPLFYAVAEEYDSGEQTEEETSAILSRWTAEH
RHSTEHHKSHSPLLHRK
>Q9VVV9
MAGNIVKWWKHKILGGYKQFSVQECTTDSEELMYHQVRASSSCSAPPDLLLVSERDNNIQ
LRSPVVNIITTPPGNASGAGSKQQSHHQTNHHSSGRSHPGHTAHPQDVSSGGSHSKHLRI
SSTSNGKHGKYSNMQQQLPQDEDVVDAAATMQQQQHTGHAHSRHLHHHKEERIRLEEFTC
DVSVEGGKSSQPLQFSFTFYDLDGHHGKITKDDIVGIVYTIYESIGKSVVVPHCGSKTIN
VRLTVSPEGKSKSQPVVPVPVAAGFSSSHASKLKKLPTGLAAMSKPLAGGGVGSGGASAL
TTSAGNRRQHRYRPRKLIKSDDEDDDSNSEKEKDAAHAPAADQPSGSGTKATGKSHHHQS
QSARYHQKNNSRAEQCCTEQNTPDNGHNTYENMLNLKCCKPEVDQVDCPSHRQHHQSHPN
HQMRQQDIYMKQATQRVKMLRRARKQKYQDHCLETRQRSLSVGNDSACPNRHLQLQQPPV
GHPQPQSLNHKSASGSPPLGVGGGGDMMLDGVQLRQPRPHSLTPHQHQQQNQQQQQQQRK
SAECWKSALNRNDLISIIRESMEKNRLCFQLNGKPQANVSPIRQPAAQQQPQQQQRQRCN
TGSKIPTLITNHSPVAQQSPLSCSPPTAEPTTPSIPAAPPAIEVNGQQHHPTHPTHPSHH
NHHEHPQPHIPIYHQQLAINPAVLAAQQTHNTAHNKLNLCGYDSFLHATICGGGAAAHSP
PATPSNVATVQPIPKKSQKNLLQGYQRLEQSQQQQQQQRSSKDYKNYGNLIYAKLSEQLQ
QKDREQRRQRHKQQQHQMLQDQPKDASRSEQRPPTSNSSSAGSKIYGDAVECAHLLASEE
EDLPPSPQLTSTPSKVVSTDTLIDLNDDVGEAVAEAVTEGGKQSLEAEESGQQVEVELDT
SASSSMIHRYVHEHIHHHYHHFKEQQDV
>Q9EPQ1
MTKPNSLIFYCIIVLGLTLMKIQLSEECELIIKRPNANLTRVPKDLPLQTTTLDLSQNNI
SELQTSDILSLSKLRVLIMSYNRLQYLNISVFKFNTELEYLDLSHNELKVILCHPTVSLK
HLDLSFNAFDALPICKEFGNMSQLQFLGLSGSRVQSSSVQLIAHLNISKVLLVLGDAYGE
KEDPESLRHVSTETLHIVFPSKREFRFLLDVSVSTTIGLELSNIKCVLEDQGCSYFLRAL
SKLGKNLKLSNLTLNNVETTWNSFINILQIVWHTPVKYFSISNVKLQGQLAFRMFNYSDT
SLKALSIHQVVTDVFSFPQSYIYSIFANMNIQNFTMSGTHMVHMLCPSQVSPFLHVDFTD
NLLTDMVFKDCRNLVRLKTLSLQKNQLKNLENIILTSAKMTSLQKLDISQNSLRYSDGGI
PCAWTQSLLVLNLSSNMLTGSVFRCLPPKVKVLDLHNNRIMSIPKDVTHLQALQELNVAS
NSLTDLPGCGAFSSLSVLVIDHNSVSHPSEDFFQSCQNIRSLTAGNNPFQCTCELRDFVK
NIGWVAREVVEGWPDSYRCDYPESSRGTALRDFHMSPLSCDTVLLTVTIGATMLVLAVTG
AFLCLYFDLPWYVRMLCQWTQTRHRARHIPLEELQRNLQFHAFVSYSGHDSAWVKNELLP

NLEKDDIQICLHERNFVPGKSIVENIINFIEKSYKSIFVLSPHFIQSEWCHYELYFAHHN
LFHEGSDNLILILLAPIPQYSIPTNYHKLKTLMSRRTYLEWPTEKNKHGLFWANLRASIN
VKLVNQAEGTCYTQQ
>Q9XFM6
MALELWQTLKEAIHAYTGLSPVVFFTALALAFAIYQVISGWFASPFDDVNRHQRARSLAQ
EEEPPIPQPVQVGEITEEELKQYDGSDPQKPLLMAIKHQIYDVTQSRMFYGPGGPYALFA
GKDASRALAKMSFEEKDLTWDVSGLGPFELDALQDWEYKFMSKYAKVGTVKVAGSEPETA
SVSEPTENVEQDAHVTTTPGKTVVDKSDDAPAETVLKKEE
>Q01889
MVSLIILFRLTFAIANRVRTLMKVLVIVSFFVLTGSASADSGALSLSGAAEALCNASKRL
KSVYAFVQAKTKYATEKVREFEDMVELVRLKIVRVRGNEGGNGNWTSCTGIAKFLKRVGS
KVNRVKRRELKRLRYLGYSAVGAAGIAAGRLDEMINVWRRAYSSGKGDFCVGNSEHHATR
NQLLNCYLSDSEKDEFISLGDMHRMERVEEMNMTRESMNELLRLTGGGGNPLERYVHSAN
CHLTNTRPGGGYLSENSTSRRILWGDGIISLKRSGRGFVGGTGKTRAEVLIHDVTWEEDP
VNNVPVLRNAYRELRKFVNLYAYIEELAHETGAHWNWEYAQAIVNTGTHGNKSTVVVDED
SGKSFVVLGNRETVQEEKLLEEMAICGVGRADSLRRTLALLFLLF
>Q5MD62
MDLGKPMKNVLVVALLVIFQVCLCQDEVTDNYIGDNTTVDYTLYESVCFKKDVRNFKAWF
LPIMYSIICFVGLLGNGLVMLTYIYFKRLKTMTDTYLLNLALADILFLLTLPFWAYSAAK
SWVFGVHVCKLIFGIYKISFFSGMLLLLCISIDRYVAIVQAVSAHRHRARVLLISKLSCL
GIWMLAIVLSTPEVMYSGIQKSSSEQALRCSLVTEHVEALITIQVAQMVVGFLIPLMAMS
FCYLVIIRTLLQARNFERNKAIKVIIAVVVVFVAFQLPYNGVVLAHTVANFNITSGTSCE
LSKQLNIAYDVTYSLACVRCCVNPFLYAFIGVKFRSDLFKLFKDLGCLSQEQLRQWSFCR
HTRRSSMSVEAETTTTFSP
>Q90343
MPGSLKEETALLLEDYFQHRAGGAALPPSATAAELRRAAAELERRERPFFRSCAPLARAE
PREAAALLRKVAAQLETDGGLNWGRLLALVVFAGTLAAALAESACEEGPSRLAAALTAYL
AEEQGEWMEEHGGWDGFCRFFGRHGSQPADQNSTLSNAIMAAAGFGIAGLAFLLVVR
>P23292
MSQVQSPLTATNSGLAVNNNTMNSQMPNRSNVRLVNGTLPPSLHVSSNLNHNTGNSSASY
SGSQSRDDSTIVGLHYKIGKKIGEGSFGVLFEGTNMINGLPVAIKFEPRKTEAPQLKDEY
RTYKILAGTPGIPQEYYFGQEGLHNILVIDLLGPSLEDLFDWCGRRFSVKTVVQVAVQMI
TLIEDLHAHDLIYRDIKPDNFLIGRPGQPDANKVHLIDFGMAKQYRDPKTKQHIPYREKK
SLSGTARYMSINTHLGREQSRRDDMEAMGHVFFYFLRGQLPWQGLKAPNNKQKYEKIGEK
KRLTNVYDLAQGLPIQFGRYLEIVRNLSFEETPDYEGYRMLLLSVLDDLGETADGQYDWM
KLNGGRGWDLSINKKPNLHGYGHPNPPNEKSKRHRSKNHQYSSPDHHHHYNQQQQQQQAQ
AQAQAQAQAKVQQQQLQQAQAQQQANRYQLQPDDSHYDEEREASKLDPTSYEAYQQQTQQ
KYAQQQQKQMQQKSKQFANTGANGQTNKYPYNAQPTANDEQNAKNAAQDRNSNKSSKGFF
SKLGCC
>Q9LW07
MKKKTWFLNFSLFFLQIFTSSNALDVTQFGAVGDGVTDDSQAFLKAWEAVCSGTGDGQFV
VPAGMTFMLQPLKFQGSCKSTPVFVQMLGKLVAPSKGNWKGDKDQWILFTDIEGLVIEGD
GEINGQGSSWWEHKGSRPTALKFRSCNNLRLSGLTHLDSPMAHIHISECNYVTISSLRIN
APESSPNTDGIDVGASSNVVIQDCIIATGDDCIAINSGTSNIHISGIDCGPGHGISIGSL

GKDGETATVENVCVQNCNFRGTMNGARIKTWQGGSGYARMITFNGITLDNVENPIIIDQF
YNGGDSDNAKDRKSSAVEVSKVVFSNFIGTSKSEYGVDFRCSERVPCTEIFLRDMKIETA
SSGSGQVAQGQCLNVRGASTIAVPGLECLELSTDMFSSAQLLEQTCMSAQSVQPRTTTQP
MQDPIWVFQSRGKQLRVYNIAILVSFISLVTYILAR
>O00161
MDNLSSEEIQQRAHQITDESLESTRRILGLAIESQDAGIKTITMLDEQKEQLNRIEEGLD
QINKDMRETEKTLTELNKCCGLCVCPCNRTKNFESGKAYKTTWGDGGENSPCNVVSKQPG
PVTNGQLQQPTTGAASGGYIKRITNDAREDEMEENLTQVGSILGNLKDMALNIGNEIDAQ
NPQIKRITDKADTNRDRIDIANARAKKLIDS
>P97789
MGVPKFYRWISERYPCLSEVVKEHQIPEFDNLYLDMNGIIHQCSHPNDDDVHFRISDDKI
FTDIFHYLEVLFRIIKPRKVFFMAVDGVAPRAKMNQQRGRRFRSAKEAEDKIKKAIEKGE
TLPTEARFDSNCITPGTEFMARLHEHLKYFVNMKISTDKSWQGVTIYFSGHETPGEGEHK
IMEFIRSEKAKPDHDPNTRHCLYGLDADLIMLGLTSHEAHFSLLREEVRFGGKKTQRVCA
PEETTFHLLHLSLMREYIDYEFSALKEKITFKYDIEKIIDDWILMGFLVGNDFIPHLPHL
HINHDALPLLYGTYIAILPELGGYINESGHLNLPRFERYLVKLSDFDREHFSEVFVDLKW
FESKVGNKYLNEAAGAAAEEAKNCKEKRKPKGQENSLSWAALDKSEGEGVASRDNFEDET
EDDDLFETEFRQYKRTYYMTKMGVDVVSDEFLANQAACYVQAIQWILHYYYHGVQSWSWY
YPYHYAPFLSDIRSISTLKIHFELGKPFKPFEQLLAVLPSASKNLLPTCYQHLMTSEDSP
IIEYYPPDFKTDLNGKQQEWEAVVLIPFIDETRLLEAMETCNHSLKKEERKRNQHSECLM
CWYDRDTEFTYSSPWPEKFPAIERCCTRYKMISLDAWRVDINKNKITRVDQKALYFCGFP
TLKHIKHKFFLKKSGVQVFQQSSRGENLMLEISVNAEPDELRIENIASAVLGKAVFVNWP
HLEEARVVAVSDGETKFYIEEPPGTQKVYLGKTAPPSKVIQLTDKEQSNWTKEIQGISEQ
YLRRKGIIINETSAVVYAQLLTGRKYQISQNGEVRLEKQWSKQILPFVYQTIVKDIRAFD
SRFSNIKTLDDLFPPRTMVFMLGTPYYGCTGEVQDSGDLITEGRIRVVFSIPCEPNLDAL
IQNQHKYSIKYNPGYVLAGRLGVSGYLVSRFTGSIFIGRGSRRNPHGDHKANVGLNLKFN
KKNEEVPGYTKKVGNEWMYSSAAEQLLAEYIERAPELFSYIAKNSQEDVFYEDDIWPGEN
ENGAEKVQEIITWLKGHPVSTLSRSSCDLHILDAAIVEKIEEEVEKCKQRKSNKKVRVTV
KPHLMYRPLEQQHGVIPDRDAEFRLFDRVVNVRESFSVPVGLRGTVIGIKGASREADVLF
EVLFDEEFPGGLTIRCSPGRGYRLPTSALVNLSHGSRCETGNQKLTAIVKPQPSVSHCSA
APSGHLGGLNHSPQSPFLPTQVPTKGDDEFCNIWQSLQGAGKTQHLQPTVQEKGAVLPQE
ISQVTEGHKSGFTDHSVRHQQRKHDSQRKFKEEYKSPKAECQSQKLSSKQTSGGSARCSI
KLLKRNESPGTSEAQKVVTSYPNAVHKPPSGIENFLASLNLSKENEAQLPHHGEPPDEAD
LSPQSFAMKGTRMLKEILKIDSPDTRDSKNDMKKSDNEATVSSRRDERGVSAHPKPTCHM
NKPHGTNEFQNVASVDSVCWPGQMPPVSTPVTELSRICSLVGMPQPDFSFLRTTQTMTVC
QVKLSNGLLVHGPQCHSESEAKERAALFALQQLGSLGVSFPLPPPIFTNYPPAVPPGAVP
PVFTQPTANIMPSSSHLFGSVSWRPPVPVAGNAFHYPSYPGTMPLAGGVPGGVHSQFIPL
QVTKKRVANRKNFENKEAQSSQATPLQTNKPGSSEATKMTPQESPPASSSSSQAAQPVSS
HVETASQGHVGSQPRSAPSSSKRKSRKLAVNFSVSKPSE
>P15291
MRLREPLLSGSAAMPGASLQRACRLLVAVCALHLGVTLVYYLAGRDLSRLPQLVGVSTPL
QGGSNSAAAIGQSSGELRTGGARPPPPLGASSQPRPGGDSSPVVDSGPGPASNLTSVPVP
HTTALSLPACPEESPLLVGPMLIEFNMPVDLELVAKQNPNVKMGGRYAPRDCVSPHKVAI
IIPFRNRQEHLKYWLYYLHPVLQRQQLDYGIYVINQAGDTIFNRAKLLNVGFQEALKDYD

YTCFVFSDVDLIPMNDHNAYRCFSQPRHISVAMDKFGFSLPYVQYFGGVSALSKQQFLTI
NGFPNNYWGWGGEDDDIFNRLVFRGMSISRPNAVVGRCRMIRHSRDKKNEPNPQRFDRIA
HTKETMLSDGLNSLTYQVLDVQRYPLYTQITVDIGTPS
>Q6NZI2
MEDPTLYIVERPLPGYPDAEAPEPSSAGAQAAEEPSGAGSEELIKSDQVNGVLVLSLLDK
IIGAVDQIQLTQAQLEERQAEMEGAVQSIQGELSKLGKAHATTSNTVSKLLEKVRKVSVN
VKTVRGSLERQAGQIKKLEVNEAELLRRRNFKVMIYQDEVKLPAKLSISKSLKESEALPE
KEGEELGEGERPEEDAAALELSSDEAVEVEEVIEESRAERIKRSGLRRVDDFKKAFSKEK
MEKTKVRTRENLEKTRLKTKENLEKTRHTLEKRMNKLGTRLVPAERREKLKTSRDKLRKS
FTPDHVVYARSKTAVYKVPPFTFHVKKIREGQVEVLKATEMVEVGADDDEGGAERGEAGD
LRRGSSPDVHALLEITEESDAVLVDKSDSD
>P38993
MTNALLSIAVLLFSMLSLAQAETHTFNWTTGWDYRNVDGLKSRPVITCNGQFPWPDITVN
KGDRVQIYLTNGMNNTNTSMHFHGLFQNGTASMDGVPFLTQCPIAPGSTMLYNFTVDYNV
GTYWYHSHTDGQYEDGMKGLFIIKDDSFPYDYDEELSLSLSEWYHDLVTDLTKSFMSVYN
PTGAEPIPQNLIVNNTMNLTWEVQPDTTYLLRIVNVGGFVSQYFWIEDHEMTVVEIDGIT
TEKNVTDMLYITVAQRYTVLVHTKNDTDKNFAIMQKFDDTMLDVIPSDLQLNATSYMVYN
KTAALPTQNYVDSIDNFLDDFYLQPYEKEAIYGEPDHVITVDVVMDNLKNGVNYAFFNNI
TYTAPKVPTLMTVLSSGDQANNSEIYGSNTHTFILEKDEIVEIVLNNQDTGTHPFHLHGH
AFQTIQRDRTYDDALGEVPHSFDPDNHPAFPEYPMRRDTLYVRPQSNFVIRFKADNPGVW
FFHCHIEWHLLQGLGLVLVEDPFGIQDAHSQQLSENHLEVCQSCSVATEGNAAANTLDLT
DLTGENVQHAFIPTGFTKKGIIAMTFSCFAGILGIITIAIYGMMDMEDATEKVIRDLHVD
PEVLLNEVDENEERQVNEDRHSTEKHQFLTKAKRFF
>P25063
MGRAMVARLGLGLLLLALLLPTQIYSSETTTGTSSNSSQSTSNTGLAPNPTNATTKAAGG
ALQSTASLFVVSLSLLHLYS
>P38065
MDRKKTLINSSVSNNNSTIKGLQLFIADLRSAQQAQEQEKRIQSEIVKIKQHFDAAKKKQ
GNHDRLGGYQRKKYVAKLAYIYITSNTTKLNEILFGLEQTVELLKSSIFSEKFIGYMTLE
LLYERSEVVAKVNDEVNYQLMKDLSSSDDNFVMLALNFVGVVGELTNRLAYNDDITTGVF
KILRSPTSSIYLKKKSALSFLALLKSNHSILTEDLQRKQLWIQRILSLLDDTENYRLTLA
TIPLIEFIAKYIDPSYCTRLLPQLTEILYNCVVVGTSRSSDNQFPLEYTFANMPNPWLIT
KVVSLLSILIASPTERDSGSLLQTNNIDNELLNKLRKCVSVAIELGTRQAQDPMERIVQN
TVLFSLINFASKLDPSDEAISNSVTALCSLLTSKEINIRYLTLDSLVKLCSSSGKPAIDA
VRYKNLDMIFHLLNTERDSSIVRKVVDLLYTFTDVENVKIIVDGLLQYILSPKNLAEPQI
KSDIAVKIAILTEKYATDINWFVIISLQLLSLTSNTTINDDEIWQRLCQIVVNNPSLHRI
TCERLVDYLCKKQASEAIIKAAAFLLGEYSSLITDRISSANLFTLFAEKYFSAPNVAKAM
ILTTMIKLYKTSPEIGSNVIKFFQLELNSLDIELQTRSFEYLNIIQLAKVNGNTDILQIL
FEPMPPFNSKSNPLLKRLGSLPASAGSTTLINTPSEASSSTPDLLSKRANSSRSIMVPMP
PPSRRNTIDDVNSKISSSEDFSGKDSYYSRQILAPNWREGFTRMISHKQGVLFTSSLMKV
FYRITTPDAQQPYVFHISLAFINLTEWEITGLSTQIIPSKTQGNPEYLIMNINTPSTATI
GPHKRAEQSYEVSIRKPFDVEDSPILAIHFKCGGSTNTINLKTAIGMTTTLISSDVNPSM
HLNLAQFISRWKTLSDALGKEGEYQKSGIKLNKDFRKVETISLEDGLLLLTQTVKRLGFD
IVDQTSVRSTLFVSGIIHTKSEGNFGCLMKIQYQVNGTVNVTCKTTTAGPLAKYIVECIK

NVLTK
>O43561
MEEAILVPCVLGLLLLPILAMLMALCVHCHRLPGSYDSTSSDSLYPRGIQFKRPHTVAPW
PPAYPPVTSYPPLSQPDLLPIPRSPQPLGGSHRTPSSRRDSDGANSVASYENEGASGIRG
AQAGWGVWGPSWTRLTPVSLPPEPACEDADEDEDDYHNPGYLVVLPDSTPATSTAAPSAP
ALSTPGIRDSAFSMESIDDYVNVPESGESAEASLDGSREYVNVSQELHPGAAKTEPAALS
SQEAEEVEEEGAPDYENLQELN
>Q15391
MINSTSTQPPDESCSQNLLITQQIIPVLYCMVFIAGILLNGVSGWIFFYVPSSKSFIIYL
KNIVIADFVMSLTFPFKILGDSGLGPWQLNVFVCRVSAVLFYVNMYVSIVFFGLISFDRY
YKIVKPLWTSFIQSVSYSKLLSVIVWMLMLLLAVPNIILTNQSVREVTQIKCIELKSELG
RKWHKASNYIFVAIFWIVFLLLIVFYTAITKKIFKSHLKSSRNSTSVKKKSSRNIFSIVF
VFFVCFVPYHIARIPYTKSQTEAHYSCQSKEILRYMKEFTLLLSAANVCLDPIIYFFLCQ
PFREILCKKLHIPLKAQNDLDISRIKRGNTTLESTDTL
>Q03646
MKVIKTLSIINFFIFVTFNIKNESKYSNTFINNAYNMSIRRSMAESKPPTGDGAVASAGN
GAVASAGNGAVASAGNGAVASAGNGAGNGAGNGAGNGAGNGAGNGAGNGAGNGAGNGAGN
GAGNGAGNGAGNGAGNGAGNGAGNGAVASAGNGAGNGAVASAGNGAVAERSSSTPATTTT
TTTTNDAEASTSTSSENSNHNNAETNPKGNGEVQPNQANKETQNNSNVQQDSQTKSNVPR
TQDADTKSPTAQPEQAENSAPTAEQTESPELQSAPENKGTGQHGHMHGSRNNHPQNTSDS
QKECTDGNKENCGAATSLLNNSSNIASINKFVVLISATLVLSFAIFI
>P33302
MPEAKLNNNVNDVTSYSSASSSTENAADLHNYNGFDEHTEARIQKLARTLTAQSMQNSTQ
SAPNKSDAQSIFSSGVEGVNPIFSDPEAPGYDPKLDPNSENFSSAAWVKNMAHLSAADPD
FYKPYSLGCAWKNLSASGASADVAYQSTVVNIPYKILKSGLRKFQRSKETNTFQILKPMD
GCLNPGELLVVLGRPGSGCTTLLKSISSNTHGFDLGADTKISYSGYSGDDIKKHFRGEVV
YNAEADVHLPHLTVFETLVTVARLKTPQNRIKGVDRESYANHLAEVAMATYGLSHTRNTK
VGNDIVRGVSGGERKRVSIAEVSICGSKFQCWDNATRGLDSATALEFIRALKTQADISNT
SATVAIYQCSQDAYDLFNKVCVLDDGYQIYYGPADKAKKYFEDMGYVCPSRQTTADFLTS
VTSPSERTLNKDMLKKGIHIPQTPKEMNDYWVKSPNYKELMKEVDQRLLNDDEASREAIK
EAHIAKQSKRARPSSPYTVSYMMQVKYLLIRNMWRLRNNIGFTLFMILGNCSMALILGSM
FFKIMKKGDTSTFYFRGSAMFFAILFNAFSSLLEIFSLYEARPITEKHRTYSLYHPSADA
FASVLSEIPSKLIIAVCFNIIFYFLVDFRRNGGVFFFYLLINIVAVFSMSHLFRCVGSLT
KTLSEAMVPASMLLLALSMYTGFAIPKKKILRWSKWIWYINPLAYLFESLLINEFHGIKF
PCAEYVPRGPAYANISSTESVCTVVGAVPGQDYVLGDDFIRGTYQYYHKDKWRGFGIGMA
YVVFFFFVYLFLCEYNEGAKQKGEILVFPRSIVKRMKKRGVLTEKNANDPENVGERSDLS
SDRKMLQESSEEESDTYGEIGLSKSEAIFHWRNLCYEVQIKAETRRILNNVDGWVKPGTL
TALMGASGAGKTTLLDCLAERVTMGVITGDILVNGIPRDKSFPRSIGYCQQQDLHLKTAT
VRESLRFSAYLRQPAEVSIEEKNRYVEEVIKILEMEKYADAVVGVAGEGLNVEQRKRLTI
GVELTAKPKLLVFLDEPTSGLDSQTAWSICQLMKKLANHGQAILCTIHQPSAILMQEFDR
LLFMQRGGKTVYFGDLGEGCKTMIDYFESHGAHKCPADANPAEWMLEVVGAAPGSHANQD
YYEVWRNSEEYRAVQSELDWMERELPKKGSITAAEDKHEFSQSIIYQTKLVSIRLFQQYW
RSPDYLWSKFILTIFNQLFIGFTFFKAGTSLQGLQNQMLAVFMFTVIFNPILQQYLPSFV
QQRDLYEARERPSRTFSWISFIFAQIFVEVPWNILAGTIAYFIYYYPIGFYSNASAAGQL

HERGALFWLFSCAFYVYVGSMGLLVISFNQVAESAANLASLLFTMSLSFCGVMTTPSAMP
RFWIFMYRVSPLTYFIQALLAVGVANVDVKCADYELLEFTPPSGMTCGQYMEPYLQLAKT
GYLTDENATDTCSFCQISTTNDYLANVNSFYSERWRNYGIFICYIAFNYIAGVFFYWLAR
VPKKNGKLSKK
>P51790
MTNGGSINSSTHLLDLLDEPIPGVGTYDDFHTIDWVREKCKDRERHRRINSKKKESAWEM
TKSLYDAWSGWLVVTLTGLASGALAGLIDIAADWMTDLKEGICLSALWYNHEQCCWGSNE
TTFEERDKCPQWKTWAELIIGQAEGPGSYIMNYIMYIFWALSFAFLAVSLVKVFAPYACG
SGIPEIKTILSGFIIRGYLGKWTLMIKTITLVLAVASGLSLGKEGPLVHVACCCGNIFSY
LFPKYSTNEAKKREVLSAASAAGVSVAFGAPIGGVLFSLEEVSYYFPLKTLWRSFFAALV
AAFVLRSINPFGNSRLVLFYVEYHTPWYLFELFPFILLGVFGGLWGAFFIRANIAWCRRR
KSTKFGKYPVLEVIIVAAITAVIAFPNPYTRLNTSELIKELFTDCGPLESSSLCDYRNDM
NASKIVDDIPDRPAGIGVYSAIWQLCLALIFKIIMTVFTFGIKVPSGLFIPSMAIGAIAG
RIVGIAVEQLAYYHHDWFIFKEWCEVGADCITPGLYAMVGAAACLGGVTRMTVSLVVIVF
ELTGGLEYIVPLMAAVMTSKWVGDAFGREGIYEAHIRLNGYPFLDAKEEFEFTHTTLAAD
VMRPRRNDPPLAVLTQDNMTVDDIENMINETSYNGFPVIMSKESQRLVGFALRRDLTIAI
ESARKKQEGIVGSSRVCFAQHTPSLPAESPRPLKLRSILDMSPFTVTDHTPMEIVVDIFR
KLGLRQCLVTHNGRLLGIITKKDILRHMAQTANQDPASIMFN
>Q9NZU7
MGNCVKYPLRNLSRKMCQEEQTSYMVVQTSEEGLAADAELPGPLLMLAQNCAVMHNLLGP
ACIFLRKGFAENRQPDRSLRPEEIEELREAFREFDKDKDGYINCRDLGNCMRTMGYMPTE
MELIELSQQINMNLGGHVDFDDFVELMGPKLLAETADMIGVKELRDAFREFDTNGDGEIS
TSELREAMRKLLGHQVGHRDIEEIIRDVDLNGDGRVDFEEFVRMMSR
>P30999
MDDSEVESTASILASVKEQEAQFEKLTRALEEERRHVSAQLERVRVSPQDANSLMANGTL
TRRHQNGRFVGDADLERQKFSDLKLNGPQDHNHLLYSTIPRMQEPGQIVETYTEEDPEGA
MSVVSVETTDDGTTRRTETTVKKVVKTMTTRTVQPVPMGPDGLPVDASAVSNNYIQTLGR
DFRKNGNGGPGPYVGQAGTATLPRNFHYPPDGYGRHYEDGYPGGSDNYGSLSRVTRIEER
YRPSMEGYRAPSRQDVYGPQPQVRVGGSSVDLHRFHPEPYGLEDDQRSMGYDDLDYGMMS
DYGTARRTGTPSDPRRRLRSYEDMIGEEVPPDQYYWAPLAQHERGSLASLDSLRKGMPPP
SNWRQPELPEVIAMLGFRLDAVKSNAAAYLQHLCYRNDKVKTDVRKLKGIPILVGLLDHP
KKEVHLGACGALKNISFGRDQDNKIAIKNCDGVPALVRLLRKARDMDLTEVITGTLWNLS
SHDSIKMEIVDHALHALTDEVIIPHSGWEREPNEDCKPRHIEWESVLTNTAGCLRNVSSE
RSEARRKLRECDGLVDALIFIVQAEIGQKDSDSKLVENCVCLLRNLSYQVHREIPQAERY
QEALPTVANSTGPHAASCFGAKKGKDEWFSRGKKPTEDPANDTVDFPKRTSPARGYELLF
QPEVVRIYISLLKESKTPAILEASAGAIQNLCAGRWTYGRYIRSALRQEKALSAIAELLT
SEHERVVKAASGALRNLAVDARNKELIGKHAIPNLVKNLPGGQQNSSWNFSEDTVVSILN
TINEVIAENLEAAKKLRETQGIEKLVLINKSGNRSEKEVRAAALVLQTIWGYKELRKPLE
KEGWKKSDFQVNLNNASRSQSSHSYDDSTLPLIDRNQKSDKKPDREEIPMSNMGSNTKSL
DNNYSTLNERGDHNRTLDRSGDLGDMEPLKGAPLMQKI
>A6QLE7
MNDSRWTEWRILNTSSGILNVSERHSCPLGFGHYSAVDVCIFETIVIVLLTFLIIAGNLT
VIFVFHCAPLLHHYTTSYFIQTMAYADLFVGVSCLVPTLSLLHYSTGIHESLTCQVFGYI
ISVLKSVSMACLACISVDRYLAITKPLSYNQLVTPCRLRICIILIWIYSCLIFLPSFFGW

GKPGYHGDIFEWCATSWLTSAYFTGFIVCLLYAPAALVVCFTYFHIFKICRQHTKEINDR
RARFPSHEAAASRDAGHSPDRRYAMVLFRITSVFYMLWLPYIIYFLLESSRVLDNPTLSF
LTTWLAISNSFCNCVIYSLSNSVFRLGLRRLSETMCTSCMCVKDKEARDPKPRKRANSCS
I
>Q60902
MAAPLVPLSQQIPSGNPLYESYYKQVDPAYTGRVGASEAALFLKKSGLSDIILGKIWDLA
DPEGKGFLDKQGFYVALRLVACAQSGHEVTLSSLSLTMPPPKFHDTSSPLMATQSSAETH
WAVRVEEKAKFDGIFESLLPVNGLLSGDKVKPVLMNSKLPLDVLGRVWDLSDIDKDGHLD
RDEFAVAMHLVYRALEKEPVPSILPPPLIPPSKRKKTVFAGAVPVLPASPPPKDSLRSTP
SHGSVSSLNSTGSLSPKHSVKQPPVAWVVPVADKMRFDEIFLKTDLDLDGYVSGQEVKEI
FMHSGLTQNLLAHIWALADTRQTGKLSKEQFALAMYFIQQKVSKGIDPPQVLSPDMVPPS
ERGTPIPDSSSTLASGEFTGVKELDDISQEIAQLQREKYSLEQDIREKEEAIKQKTSEVQ
ELQNDLDRETSSLQELEAQKQDAQDRLDEMDQQKAKLRDMLSDVRQKCQDETQTISSLKT
QIQSQESDLKSQEDDLNRAKSELNRLQQEETQLEQSIQAGRAQLETILRSLKCTQDDINQ
ARSKLSQLQESHLEAHRSLEQYDQVPDGVSGTSLPDLATLNEGILLAERGGFGAMDDPFK
NKALLFSNNSQELHPDPFQAEDPFKSDPFKGADPFKGDPFQSDPFSEQQTAATDPFGGDP
FKESDPFHSSSSDDFFKKQTKNDPFTSDPFTKNPSLPSKLDPFESSDPFSSSSISSKGSD
PFGTLDPFGSSSFSSAEGFADFSQMSKPPPSGPFSSSLGGTGFSDDPFKSKQDTPALPPK
KPAPPRPKPPSGQSTPVSQLGSSDFPESPDPFQPLGADSGEPFQNKKGFGDPFSGKDPFA
PSSSAKPPKASSSGFADFTSFGNEEQQLAWAKRESEKAEQERLARLRRQEQEDLELAIAL
SKADMPA
>Q09917
MRLWRSPLLLLVVVVELFSWADALTRFISADSLSTCMTDSQLSASKLYASYFPDNQSVAF
DISIQSLVSTNVSIDVDISAYGIEIKKVIDPCDMEISGFCPMQTGNIALSGSHTLTGEAL
SILDSIPSIAYTVPDLDAVVTINIYESDTNTQLACVRTTVQNGRSVYHRAVYWVMCMVIG
IPLLIFLLISPVLQTPALWEIVETMITLFQFAQIQALYSMMATSLPAIIYSWGRNFMWSM
GIIRIGFMQDVFTWYVKSTGGTPSTLVDLGIHANVALAKRGIDLGSLAKRATTTVTTSTS
DSITLRGIKRISYMMGIETTNFFATGFSFFIILLFFSLLVAMASRFIVEMVLLASRNQAL
KKQRIRLYWKSISKGFFYRVIFVGFTQMSVLSMWEIYTRDSSALAFLSMYVIVDMAVLLC
YAFVRTIQIIRKTGPYSHPDVLYNLYSDTQHLMRWGFMYVQLDVRFFYFTFPLLLITLVR
SMFIGFGQGSPKVQGCAMFGISVVVFALMVILRPYATKHMNTLHIGVALMNLISGSFILV
MCQAFYVEELARQVIGIIFFALNAITMLLLILGIFIRTLIVLFRKSGHGTYYRILDDQSE
KATSYNKSIKDMSSSDMAFSDPAYSGTTLRSSVDLNTPEYPFSNRNDSDSTFTNNKYVSP
WDAIEEASYANLRGNTDVEQPFMESDYTRISENNNNAERRRKPLPNNAFR
>P97849
MRTPGAGTASVASLGLLWLLGLPWTWSAAAAFGVYVGSGGWRFLRIVCKTARRDLFGLSV
LIRVRLELRRHRRAGDTIPRIFQAVAQRQPERLALVDASSGICWTFAQLDTYSNAVANLF
LQLGFAPGDVVAVFLEGRPEFVGLWLGLAKAGVVAALLNVNLRREPLAFCLGTSAAKALI
YGGEMAAAVAEVSEQLGKSLLKFCSGDLGPESVLPDTQLLDPMLAEAPTTPLAQAPGKGM
DDRLFYIYTSGTTGLPKAAIVVHSRYYRIAAFGHHSYSMRANDVLYDCLPLYHSAGNIMG
VGQCIIYGLTVVLRKKFSASRFWDDCVKYNCTVVQYIGEICRYLLRQPVRDVERRHRVRL
AVGNGLRPAIWEEFTQGFGVRQIGEFYGATECNCSIANMDGKVGSCGFNSRILTHVYPIR
LVKVNEDTMEPLRDSQGLCIPCQPGEPGLLVGQINQQDPLRRFDGYVSDSATNKKIAHSV
FRKGDSAYLSGDVLVMDELGYMYFRDRSGDTFRWRGENVSTTEVEAVLSRLLGQTDVAVY

GVAVPGVEGKSGMAAIADPHNQLDPNSMYQELQKVLASYAQPIFLRLLPQVDTTGTFKIQ
KTRLQREGFDPRQTSDRLFFLDLKQGRYLPLDERVHARICAGDFSL
>Q18685
MAHLVEGTSIIDGKWQLPILVTDLNIQRSISVLGNLNVGGLMLELVSECDVERDWSDHAL
WWPEKRRWLQHTRSTLDQNGITAETQLEFTPMHKEARIQLPDMQMIDARVDFSVNSFKAT
KKLCRDLGIRYSEELSLKRYIPPEDLRRGTSDADNMNGPLSMRPGEESVGPMTLRKAAPI
FASQSNLDMRRRGQSPALSQSGHIFNAHEMGTLPRHGTLPRGVSPSPGAYNDTMRRTPIM
PSISFSEGLENEQFDDALIHSPRLAPSRDTPVFRPQNYVEKAAINRGWLDSSRSLMEQGI
FEGDIILLRFKFMNFFDLNPKYDPVRINQLYEQAKWSILLDEFDHTEEEATLFAALQLQA
TLQRDSPEPEENNKDDVDILLDELEQNLDAAALNRRSDLTQVPELADYLKYMKPKKLAAF
KGFKRAFFSFRDLYLSYHQSSSDVNSAPLGHFSLKGCEVSQDVSVGQQKYHIKLLLPTAE
GMIDFILKCDSEHQYARWMAACRLASRGKSMADSSYQQEVESIKNLLKMQSGNGNENGNS
NTASRKAAAVKLPNDFNVDEYISSKYVRRARSKQQIQQRVSDAHGNVRQLTATEAKLQYI
RAWQALPEHGIHYFIVRFRNARKAELVAVAVNRLAKLNMDNGESLKTWRFANMKKWHVNW
EIRHLKIQFEDEDIEFKPLSADCKVVHEFIGGYIFLSMRSKEHSQNLDEELFHKLTGGWA

>O35394
MAAQKDQQKDAEVEGLSATTLLPKLIPSGAGREWLERRRATIRPWGTFVDQQRFSRPRNV
GELCQRLVRNVEYYQSNYVFVFLGLILYCVVTSPMLLVALAVFFGACYILYLRTLQSKLV
LFGREVSPAHQYALAGGVSFPFFWLAGAGSAVFWVLGATLVLIGSHAAFHQIEPADGEEL
QMEPV
>Q9ES19
MTEMSFLNSEVLAGDLMSPFDQSGLGAEESLGLLDDYLEVAKHFKPHGFSSDKAGSSEWL
AMDGLVSASDTGKEDAFSGTDWMLEKMDLKEFDFDALFRMDDLETMPDELLATLDDTCDL
FAPLVQETNKEPPQTVNPIGHLPESVIKVDQAAPFTFLQPLPCSPGFLSSTPDHSFSLEL
GSEVDISEGDRKPDSAAYITLTPQCVKEEDTPSDSDSGICMSPESYLGSPQHSPSTSRAP
PDSLPSPGVPRGSRPKPYDPPGVSVTAKVKTEKLDKKLKKMEQNKTAATRYRQKKRAEQE
ALTGECKELEKKNEALKEKADSLAKEIQYLKDLIEEVRKARGKKRVP
>Q18179
MGSVNESCDNYVEIFNKINYFFRDDQVINGTEYSPKEFGYFITFAYMLIIILFGAIGNFLT
IIVVILNPAMRTTRNFFILNLALSDFFVCIVTAPTTLYTVLYMFWPFSRTLCKIAGSLQG
FNIFLSTFSIASIAVDRYVLIIFPTKRERQQNLSFCFFIMIWVISLILAVPLLQASDLTP
VFVEPSCDLALYICHEQNEIWEKMIISKGTYTLAVLITQYAFPLFSLVFAYSRIAHRMKL
RFANRNQNVTTNTNTSQRRRSVVERQRRTHLLLVCVVAVFAVAWLPLNVFHIFNTFELVN
SFSVTTFSICHCLAMCSACLNPLIYAFFNHNFRIEFMHLFDRVGLRSLRVVIFGEQESLK
KSMRTEFRSRGGCKTVTTAEPATFQRMNESMILSAMEQDEQLSSGGKLFVLKKYILKMFQ
KGGHKQSTPASPRLGFGYNSIMTSELFSIVEGVLS
>Q98895
MKLALHRIAGATMAALTTEVQYLGSNDASYEDPQADAALMKSRFNFEKPYSASSSLHRLI
PGNKELIYGGLSTILPTNASDFPLSNGSGEATQCGEDIVDNMECFMILTPAQQLVIVILA
ITLGTFTVLENFVVLCVILHSHTLRSRPSYHFIGSLAVADLIGSIIFVYSFLDFHVLHRK
DSPSIFLFKLAGVIASFTASVGSLFLTAIDRYVSIHRPMAYKRIITKTKAVIAFSVMWAI
SIEFSLLPLLGWNCKRLHSVCSDIFPLIDEKYLMFWIGMTTVLLLFIIYAYMFILWKSHH
HAVRMLSRSSQRSIIVYTSEGTKVQTVRPEQARMDLRLAKTLVLILVALIICWGPLLAIM

VYDLFGRVNDFIKTVFAFCSMLCLLNSTINPVIYAMRSKDLRRAFVNICHMCRGTTQSLD
SSAESDWNSRSVRSTGGRAGKDRSVGGKPQVKVAQVTVSGVTASSPAEAV
>Q62768
MSLLCVGVKKAKFDGAQEKFNTYVTLKVQNVKSTTIAVRGSQPSWEQDFMFEINRLDLGL
TVEVWNKGLIWDTMVGTVWIPLRTIRQSNEEGPGEWLTLDSQAIMADSEICGTKDPTFHR
ILLDAHFELPLDIPEEEARYWAKKLEQLNAMRDQDEYSFQDQQDKPLPVPSSQCCNWNYF
GWGEQNDDPDSAVDDRDSDYRSETSNSIPPPYYTTSQPNASVHQYSVRPPPLGSRESYSD
SMHSYEEFSEPRALSPTGSSRYASSGELSQGSSQLSEDFDPDEHSLQGSELDDERDRDSY
HSCHSSVSYHKDSPRWDQDEEDLEDLEDLEDEELPEEEELEEEELEEEEELEEEELELEE
EEEVPDDLASYTQQEDTTVAEPKEFKRISFPTAAPQKEDKVSAVPIEAPDVSKGIPKAAT
PEEKAAAECAQEAEPPKSEESFRSREAEEGQEGQDAMSRAKANWLRAFNKVRMQLQEARG
EGEMSKSLWFKGGPGGGLIIIDSMPDIRKRKPIPLVSDLAMSLVQSRKAGITSALASSTL
NNEELKNHVYKKTLQALIYPISCTTPHNFEVWTATTPTYCYECEGLLWGIARQGMRCTEC
GVKCHEKCQDLLNADCLQRAAEKSSKHGAEDRTQNIIMVLKDRMKIRERNKPEIFELIQE
VFAVTKSAHTQQMKAVKQSVLDGTSKWSAKISITVVCAQGLQAKDKTGSSDPYVTVQVGK
TKKRTKTIYGNLNPVWEENFHFECHNSSDRIKVRVLDEDDDIKSRVKQRFKRESDDFLGQ
TIIEVRTLSGEMDVWYNLDKRTDKSAVSGAIRLHISVEIKGEEKVAPYHVQYTCLHENLF
HFVTDVQNNGVVKIPDAKGDDAWKVYYDETAQEIVDEFAMRYGVESIYQAMTHFACLSSK
YMCPGVPAVMSTLLANINAYYAHTTASTNVSASDRFAASNFGKERFVKLLDQLHNSLRID
LSMYRNNFPASSPERLQDLKSTVDLLTSITFFRMKVQELQSPPRASQVVKDCVKACLNST
YEYIFNNCHELYGREYQTDPAKKGEVPPEEQGPSIKNLDFWSKLITLIVSIIEEDKNSYT
PCLNQFPQELNVGKISAEVMWSLFAQDMKYAMEEHDKHRLCKSADYMNLHFKVKWLYNEY
VAELPTFKDRVPEYPAWFEPFVIQWLDENEEVSRDFLHGALERDKKDGFQQTSEHALFSC
SVVDVFSQLNQSFEIIKKLECPDPQIVGHYMRRFAKTISNVLLQYADIVSKDFASYCSKE
KEKVPCILMNNTQQLRVQLEKMFEAMGGKELDAEASGTLKELQVKLNNVLDELSHVFATS
FQPHIEECVRQMGDILSQVKGTGNVPASACSSVAQDADNVLQPIMDLLDSNLTLFAKICE
KTVLKRVLKELWKLVMNTMERTIVLPPLTDQTMIGTLLRKHGKGLEKGRVKLPSHSDGTQ
MIFNAAKELGQLSKLKDHMVREEAKSLTPKQCAVVELALDTIKQYFHAGGVGLKKTFLEK
SPDLQSLRYALSLYTQATDLLIKTFVQTQSAQVHGGKGTRFTLSEDVCPEMGSGVEDPVG
EVSVHVELFTHPGTGEQKVTVKVVAANDLKWQTSGIFRPFIEVNIVGPQLSDKKRKFATK
SKNNSWAPKYNESFQFSLSADAGPECYELQVCVKDYCFAREDRTVELAVLQLRELAQRGS
AACWLPLGRRIHMDDTGLTVLRILSQRSNDEVAKEFVKLKSDTRSAEEGGAAPAP
>P16444
MWSGWWLWPLVAVCTADFFRDEAERIMRDSPVIDGHNDLPWQLLDMFNNRLQDERANLTT
LAGTHTNIPKLRAGFVGGQFWSVYTPCDTQNKDAVRRTLEQMDVVHRMCRMYPETFLYVT
SSAGIRQAFREGKVASLIGVEGGHSIDSSLGVLRALYQLGMRYLTLTHSCNTPWADNWLV
DTGDSEPQSQGLSPFGQRVVKELNRLGVLIDLAHVSVATMKATLQLSRAPVIFSHSSAYS
VCASRRNVPDDVLRLVKQTDSLVMVNFYNNYISCTNKANLSQVADHLDHIKEVAGARAVG
FGGDFDGVPRVPEGLEDVSKYPDLIAELLRRNWTEAEVKGALADNLLRVFEAVEQASNLT
QAPEEEPIPLDQLGGSCRTHYGYSSGASSLHRHWGLLLASLAPLVLCLSLL
>P39690
MPRFAIIFALLIAYSLFLSTLFTGSIPDRANTVTSNAPCQVVIWDWIRTRRICNCCSRLC
YSLLGRSNLSRTAKRGVCTIAGAVLATAAVIVAAVLVGKSSGSATKRGLTKTISVLNHTI
PFTDHILNGQTLSNGTGSNFVTIGFSGYAVHATIKRASTTDIISWVIPESMEPTLARVAS

YVSSSSINLAAVPDTGGNASALSFQNAVQEFATSWVSMTYDQSYGDLRNVANDEGGEEIL
ILMRKRSYRISFQVIETGSTALLLRTRRVVSQLITMTYLVTVQARVGIQIGDIFQHYGGI
DNYVMTSISVLRTLEDKAFHENKLLIVREPPNKSNQDANQSYRLRPFSANDLIQNLKSVD
IGFLAFCSFFDKYAHYPEIIMMKITIFISKGNLWSIIYVIQARYVRKRVMKVRGQMPGGL
LTNMESLLNIVSTPNLNISEFHIQTHSMSQSKPMYFQKQCYSSQNNIIYIYNSIHITCGA
VYVIVHDVRTPSVFVLIELRNCKPLKNSWCETTKTSPRDTKIKKNEYNETVCRRAGALLD
GRVRTIRFLMMRTHWSRVKGVSCNTANRLSRFCNHVVSYYPSQNATIHLLPTSLRAESLE
QQYTTRPLSSSNNRFCCLKSIFINNCKKACESPSLVSCNLQQTAELLMVYYLYICEACYV
SRNHDLLSKQCMSTVRAVYVARMRLPKFRSTFPCMPRLCWLVNGVVVV
>Q02092
MDLRHLLFTLALVCANDSLSASDDLLQWPQISKCRSPELETFSCYWTDGKVTTSGTIQLL
YMKRSDEDWKECPDYITAGENSCYFNTSYTSIWIPYCVKLANKDEVFDEKCFSVDEIVLP
DPPVHLNWTLLNTSQTGIHGDIQVRWDPPPTADVQKGWITLEYELQYKEVNETKWKELEP
RLSTVVPLYSLKMGRDYEIRVRSRQRTSEKFGEFSEILYVSFTQAGIEFVHCAEEIEFPW
FLVVVFGVCGLAVTAILILLSKQPRLKMLIFPPVPVPKIKGIDPDLLKKGKLDEVNSILA
SHDNYKTQLYNDDLWVEFIELDIDDSDEKNRVSDTDRLLSDDHLKSHSCLGAKDDDSGRA
SCYEPDIPETDFSASDTCDAISDIDQFKKVTEKEEDLLCLHRKDDVEALQSLANTDTQQP
HTSTQSESRESWPPFADSTDSANPSVQTQLSNQNSLTNTDFYAQVSDITPAGSVVLSPGQ
KSKVGRAQCESCTEQNFTMDNAYFCEADVKKCIAVISQEEDEPRVQEQSCNEDTYFTTES
LTTTGINLGASMAETPSMEMPVPDYTSIHIVHSPQGLVLNATALPVPEKEFNMSCGYVST
DQLNKIMP
>P46011
MSMQQETSHMTAAPQTNGHQIFPEIDMSAGDSSSIVRATVVQASTVFYDTPATLDKAERL
LSEAAENGSQLVVFPEAFIGGYPRGSTFELAIGSRTAKGRDDFRKYHASAIDVPGPEVER
LALMAKKYKVYLVMGVIEREGYTLYCTVLFFDSQGLFLGKHRKLMPTALERCIWGFGDGS
TIPVFDTPIGKIGAAICWENRMPSLRTAMYAKGIEIYCAPTADSRETWLASMTHIALEGG
CFVLSANQFCRRKDYPSPPEYMFSGSEESLTPDSVVCAGGSSIISPLGIVLAGPNYRGEA
LITADLDLGDIARAKFDFDVVGHYSRPEVFSLNIREHPRKAVSFKTSKVMEDESV
>P01732
MALPVTALLLPLALLLHAARPSQFRVSPLDRTWNLGETVELKCQVLLSNPTSGCSWLFQP
RGAAASPTFLLYLSQNKPKAAEGLDTQRFSGKRLGDTFVLTLSDFRRENEGYYFCSALSN
SIMYFSHFVPVFLPAKPTTTPAPRPPTPAPTIASQPLSLRPEACRPAAGGAVHTRGLDFA
CDIYIWAPLAGTCGVLLLSLVITLYCNHRNRRRVCKCPRPVVKSGDKPSLSARYV
>P34576
MQAGISIFFLFLHIPIFFVNCSNSTSCVAREEFQCKMDDSCISMKKWQDGVDDCYDGSDE
VCLPWQFDCQFGSPRCISKNKLHDKKIDCYSGFDEGCPAHYFVCRDRSACIEPSKYLNGV
ADCKDKSDEPCAQNQFQCSDGTKCIPKAQFQDGKEDCDDGSDEECTTSQFACQCGTIKCV
SDTFIMDGNWDCEDGSDEFINKTLTANCTRNNKVNIATNSLSLGKLKFCSGKNPCKPELG
QVCVIIGGTWRCVCKLGTFRPLGSDKCIPIELLTRYRNAPSSKCSNSHEEQFGLLQGFQK
SLSSRKELERWSNIRRAPPRTLSGTTPSGSDGFLKSGAEQVPFMFQEDKNYVSVIPDEII
DSYGTIMTPPEFHFNRDDIRCGNKTCGLHESCQKNSESKYECICREGFTIFEGTCRELID
ECAQGKHDCHPEARCVDALIGYECLCREGYLDTSIDPKARPGRKCRKLINECTNALMNDC
SQNARCLDKPIGYTCRCQDDYVDVSREGARKPGRNCTQAINECASNLHNCDTHAICQDQP
VGYSCRCPFGFIDSSPTALEPGRKCVQANNEAATTSTTTSQCIKEKNGETVCKCLLGYKN

VGTKTHLNCQMEKRANPCQDYSLHDCDPVAECFSEQPGYFQCQCPKGFTDSSADKRFPGR
KCVRAVDECALGRHTCDPHADCIDTHQGYTCKCRSGWSDTSLDPLRSPGRSCKKADMCSN
IDCAAEAECRETPIGPMCQCVSGYVDVSRQHGRPAGRVCRAVVNECAEGRHDCSSHATCI
DTADGFTCRCKDSYRDESSDTLKHPGKNCVRTVQPDPPECDVSDPMSCDPAKREVCIFVE
NTYKCRCANGYSRLPDGRCVVINECAEPRLNTCGKNAECIDLAEGYTCQCRSGYADISPV
SQPGRICRARVNECSNKEKYNVDCSENAICADTEHSYSCRCRPGFADVSAAFNKLPGRRC
IEAVNECASPSLNDCSKNAFCEDAKEGYICTCRPGYVDNSPNAARHPGRICTKPVEKIKT
DLKDTSFSTDDGCDPKNPKCGANEACVQRHGQHNCECVETAFRYTDGSCRVYSACSKRNT
CDKNAICLNRFDSYTCQCRPGYIDLSADLTNAPGRICKELINECASSDNECSPYARCIDA
TNGYACQCLDGFIDVSSRYNKPPGRQCTNSNNECSEKSLNTCDENADCVDTPDGYTCQCY
GGFVDVSSNANLPPGRVCTVQTTCPKQKTDLVFLIDGSGSIGSYVFKNEVLRFVREFVEL
FEIGRSKTRVGLIQYSDQIRHEFDLDQYGDRDSLLKGISETQYLTGLTRTGAAIQHMVQE
GFSERRGARPQQSDIARVAIILTDGRSQDNVTGPADSARKLSINTFAIGVTDHVLASELE
SIAGSPNRWFYVDKFKDLDTRLRSMIQKAACPSPTKQETPSEDVCNPRTQTGCDRSLNEH
CAVENGRPRCVCPEGFTRHPFTRVCGGDLCNPQLITSCIFPEECQITPYKNFRCSCPEGY
NRDYRSGFCVSVKEVQISPQHDANCHNGGVRCSENERCTNDGSDWFCECLPGFERIRNGQ
CAYPGSCNPNDPMSCDVRKRQQCLPRGNIYTCQCGRNEKRHPITDICLKNECLTGEHDCD
RSARCIDTDESYICACQSGFIDHSPNPSERPGRVCVALQNECLDGSNRCSPNALCTDTEE
GYVCRCKSGFVDYSPNPQTFPGMVCKELVNECTNPRLNQCDRNAHCIDTIEGYSCICKPG
FVDMDEFGNPGRRCEQIKTNDKCSPGKNDCDRNARCIQIGDDDYSCACPPGFKDKSPSSS
RPGRLCIPVIPECDNPTLNDCDSPDRAVCTDTDDGYMCRCRQGFLDISPSISVKPGRLCK
PLQNECALGIDDCARDGGICEDNPDSFTCRCAMNYLDVSFDRVTRPGRKCKRLINECQTG
QNDCSEEATCTDTEDSYICACPQSHIDLSPDTVNRPGRRCLMRINECTSNRHDCSPNADC
IDTPESYKCRCRDDFVDESPDSSRRPGRICRPALVDECRTGKHDCHVNAICQDLPQGYTC
QCSADFVDVSPHRASHPGRLCQPRPTPPPPECRLDGGNQCKVHLNEVCRLMGGEPKCSCP
VNYQRDSSGSCSIINECLFTQLNDCHTAADCIDQVQGYTCQCRDGFKDIGDRRRPGRMCK
PMVNECQYPHLNDCHQNAACIDLEEGYECKCNQGFMDHSHGRPGRICKQLTNECLRPSLN
SCDRNARCIDKEEGYECECRDGFIDVSPSPTLKGRACRELVNECANSRLNDCDKNARCKD
TMDSYECDCPVNSKDISPSPSFPGRVCLMFINECESGVHDCDPSATCRDNEQSFTCECPS
GFVDRSPNKHARPGRVCVKLVDECREGRHTCSSHADCRDLEEGYTCECRDGYVDRSPNLA
SQPGRVCSAPEVCPPNHDCSSAAVCEPLGGMKYQCVCIQGYVDQSPGSQKGRVCVRNNAC
HDPRLNTCSRNAICYDEPRGYRCECKRGFMDRSPDSSQRGRVCEPPPPPSPPPRHPCQDP
ERNDCHPAGTCRATGAQSYTCECLSGYADRSPDPRNKPGRLCVLTEPVCLDPEQNDCHAA
AICSEVNGPEKYTCKCRDGYTDESPDPLRRPGRICKGLINECLDRSLNDCHSLAVCKDLP
NGYTCQCPINAKDQSPDPRKPGRICSLSVNECANPSLNSCSAFADCFDEENGYRCRCRNG
YHDDDPAHPGHRCSFMINECDSSNLNDCDRNANCIDTAGGYDCACKAPYRDEGPPQSPGR
ICRLNECLNPNRNTCDRNADCRDLDYGYTCTCRHGFYDQSPNPQEPGRICIEFQQEEHIE
RVKVTTVQSEPRREFPCGRDDCIKARGEVCISGEYCGCKPGEGRSASTGKCQEVQETPFE
LRVVTRDQRPLMYSTEFGSQKSPSYVEIVELFEKNMARTFGGTSLAPRYVNTKVDYITHP
KTKNSSWDQGLLFKYEVQTTKSQSQPIDECELWKQMQASLDRTNGAIGGGSLRVASDTDL
LNPCKQQEEWGNCGGMSCKEHLKEVCIAGHICGCPDGMKRRDANSECRVVESWNVPLWVV
RDKEKPIVFSESFDNPQTPVYKDYSKRLEKGIEGCYPHTELKNAFVTAEVNDIVNPVLMN
ASYDTGLLFNTTVHFRKGMVHVPSDAYYQLIKYVTKENNNEVGDSELYLNPTQPDPFNPC
FKNDCDPHGKCIEISKYAYKCECGVGYRDINPQSPGKKCLPVHGFNECERKEDNECSENA

RCIDLEHLYKCECLPSYYDTSPVGSVPGSLCVLDYCSDVNFCPTNTTCKNMEQQAECKCD
AGFMDIRKSEKRTALMLGDDTLCMHVRDVDECALGLNNCSGVAHCIDRAVGYTCKCPDGY
IDGNPDEPGRVCGALLCDLCNAHGDCVHNTATNNITCVCTDGWTGPQCQVAPSNASLVLL
ILLALLFLLLTLCCLLYFCTKCHCFKGRRFAGAGANGFGYRRGGAWPWSTLEGSASSESG
AEFSAMSAAGNDYYPDIGIPRAKLKSGMMASGNTAEVRNMEVARLDQYLDENAVRIPRAH
LVDAHGDTSFDSLSEASSEYTIKEEIERKVITDVTTKEIKTTTTTDEQGNTIVTTTEAVH
PRDTTIVHGGGYTQNESSSSSFSGGERAYQSQSQQQQSMSQGMSQSMSQHATSAGYSSSG
MESSAHNSGYASIRHTGERERGGSEEEFSIGRARGMAAASSGYQHSSSENREVEEYCSEE
EDVEHSVGDKRTIVTKNHSYEPFVNGESERFKTEVVTSQTSTHVTKK
>P01120
MPLNKSNIREYKLVVVGGGGVGKSALTIQLTQSHFVDEYDPTIEDSYRKQVVIDDEVSIL
DILDTAGQEEYSAMREQYMRNGEGFLLVYSITSKSSLDELMTYYQQILRVKDTDYVPIVV
VGNKSDLENEKQVSYQDGLNMAKQMNAPFLETSAKQAINVEEAFYTLARLVRDEGGKYNK
TLTENDNSKQTSQDTKGSGANSVPRNSGGHRKMSNAANGKNVNSSTTVVNARNASIESKT
GLAGNQATNGKTQTDRTNIDNSTGQAGQANAQSANTVNNRVNNNSKAGQVSNAKQARKQQ
AAPGGNTSEASKSGSGGCCIIS
>P52797
MAAAPLLLLLLLVPVPLLPLLAQGPGGALGNRHAVYWNSSNQHLRREGYTVQVNVNDYLD
IYCPHYNSSGVGPGAGPGPGGGAEQYVLYMVSRNGYRTCNASQGFKRWECNRPHAPHSPI
KFSEKFQRYSAFSLGYEFHAGHEYYYISTPTHNLHWKCLRMKVFVCCASTSHSGEKPVPT
LPQFTMGPNVKINVLEDFEGENPQVPKLEKSISGTSPKREHLPLAVGIAFFLMTFLAS
>Q94F08
MAKTNQAITICSLLLLLLLSETTSHLLCSDSKTPVNNNETLQFCDSYKERSCCNSKDDLQ
LQNRFNSMNISDSNCSSLLKSILCSKCDEFSGQLFGDDDSSLVPILCNSTSQDLCSKLWD
SCQNISIVSSPFSPTLLGGATSPSTSSNSSTLTDLWKSQTEFCTAFGGPSQTNNNKTKCF
NGEPVNRDTSDDDEDDVKTPKGICLEKIGTGSYLNMVAHPDGSNRAFFSNQPGKIWLGTI
PDQDSGKPMEIDESTPFVDITDQVSFDTQFGMMGMAFHPKFAENGRFFASFNCDKVKSPG
CSGRCACNSDVNCDPSKLPKDDGTTPCRYQTVVSEYTANGTSSSPSTAKIGKASEVRRIF
TMGLPYSSSHGGQILFGPDGYLYLMTGDGGGVSDTHNFAQNKKSLLGKILRLDVDVMPSV
SEISKLGLWGNYSIPKNNPFQGNENEQPEIWALGLRNPWRCSFDSERPDYFLCADVGKDT
YEEVDIITMGGNYGWRTYEGPYVFSPLSPFGENVSSDSNLTFPILGYNHSEVNKHEGSAS
IIGGYFYRSNTDPCSYGTYLYADLYANAMWAAIESPEDSGNFTDSLIPFSCSKDSPMKCT
AAPGGASSGPALGYIYSFGQDNNKDIHLLTSSGVYRIVRPSRCNLACSKENTTASAGKQN
PAGSAPPQPLPSSARKLCFSVFLLLSLLMMFLTLLD
>O94898
MAPAPLGVPEEQLLGCRSRVLSRLLFIAQTALLLLPAAGAGLCPAPCSCRIPLLDCSRRK
LPAPSWRALSGLLPPDTAILDFSHNRLSNWNISLESQTLQEVKMNYNELTEIPYFGEPTS
NITLLSLVHNIIPEINAQALQFYPALESLDLSSNIISEIKTSSFPRMQLKYLNLSNNRIT
TLEAGCFDNLSSSLLVVKLNRNRMSMIPPKIFKLPHLQFLELKRNRIKIVEGLTFQGLDS
LRSLKMQRNGISKLKDGAFFGLNNMEELELEHNNLTRVNKGWLYGLRMLQQLYVSQNAIE
RISPDAWEFCQRLSELDLSYNQLTRLDESAFVGLSLLERLNLGDNRVTHIADGVFRFLSN
LQTLDLRNNEISWAIEDASEAFAGLTSLTKLILQGNQIKSITKKAFIGLESLEHLDLNNN
AIMSIQENAFSQTHLKELILNTSSLLCDCHLKWLLQWLVDNNFQHSVNVSCAHPEWLAGQ
SILNVDLKDFVCDDFLKPQIRTHPETIIALRGMNVTLTCTAVSSSDSPMSTVWRKDSEIL

YDVDTENFVRYWQQAGEALEYTSILHLFNVNFTDEGKYQCIVTNHFGSNYSQKAKLTVNE
MPSFLKTPMDLTIRTGAMARLECAAEGHPAPQISWQKDGGTDFPAARERRMHVMPEDDVF
FIANVKIEDMGIYSCMAQNTAGGLSANASLTVLETPSFIRPLEDKTVTRGETAVLQCIAG
GSPAPRLNWTKDDGPLLVTERHFFAAANQLLIIVDAGLEDAGKYTCIMSNTLGTERGHIY
LNVISSPNCDSSQSSIGHEDDGWTTVGIVIIVVVCCVVGTSLIWVIVIYHMRRKNEDYSI
TNTEELNLPADIPSYLSSQGTLSEPQEGYSNSEAGSHQQLMPPANGYIHKGTDGGTGTRV
ICSDCYDNANIYSRTREYCPYTYIAEEDVLDQTLSSLMVQMPKETYLVHPPQDTTALESL
IPSANREPSAFPTNHERISEKKLPSTQMSGETLQRPVWNINRELGLPHPPFSQQPVHESP
QLHQNEGLAGREPDCSASSMSCHRLQDHAFDFSRTRNIQDGSEGT
>P42838
MVNFDLGQVGEVFRRKDKGAIVSGDNPEEEEDVDASEFEEDEVKPVRTKNRRPKEDAFTQ
QRLAAINPVLTPRTVLPLYLLIAVVFVIVGGCILAQNSKVDEVTIYYQDCMTNATSSWSD
IPSEHWQFVFHKYKTYNTAPQWRFVDDESDDFTKQRGTCQIRFTTPSDMKNNVYLNYVLE
KFAANHRRYVLSFSEDQIRGEDASYETVHDATGINCKPLSKNADGKIYYPCGLIANSMFN
DTFPLQLTNVGDTSNNYSLTNKGINWESDKKRYKKTKYNYTQIAPPPYWEKMYPDGYNET
NIPDIQDWEEFQNWMRPGAFDKITKLIRINKNDTLPAGEYQLDIGLHWPVLEFNGKKGIY
LTHGSHLGGRNPFLGIVYLIGGCICAAMALILLTFWLFGGRKIADASSLSWNMK
>O88339
MSTSSLRRQMKNIVHNYSEAEIKVREATSNDPWGPSSSLMSEIADLTYNVVAFSEIMSMI
WKRLNDHGKNWRHVYKAMTLMEYLIKTGSERVSQQCKENMYAVQTLKDFQYVDRDGKDQG
VNVREKAKQLVALLRDEDRLREERAHALKTKEKLAQTATASSAAVGSGPPPEAEQAWPQS
SGEEELQLQLALAMSKEEADQPPSCGPEDDVQLQLALSLSREEHDKEERIRRGDDLRLQM
AIEESKRETGGKEESSLMDLADVFTTPAPPQASDPWGGPASVPTAVPVAAAASDPWGAPA
VPPAADPWGGAAPTPASGDPWRPAAPTGPSVDPWGGTPAPAAGEGPTSDPWGSADGGAPV
SGPPSSDPWAPAPAFSDPWGGSPAKPSSNGTAVGGFDTEPDEFSDFDRLRTALPTSGSST
GELELLAGEVPARSPGAFDMSGVGGSLAESVGSPPPAATPTPTPPTRKTPESFLGPNAAL
VDLDSLVSRPGPTPPGAKASNPFLPSGAPATGPSVTNPFQPAPPATLTLNQLRLSPVPPV
PGAPPTYISPLGGGPGLPPMMPPGPPAPNTNPFLL
>Q9Z0F8
MRRRLLILTTLVPFVLAPRPPEEAGSGAHPRLEKLDSLLSDYDILSLANIQQHSIRKRDL
QSATHLETLLTFSALKRHFKLYLTSSTERFSQNLRVVVVDGKEESEYSVKWQDFFSGHVV
GEPDSRVLAHIGDDDVTVRINTDGAEYNIEPLWRFVNDTKDKRMLVYKSEDIKDFSRLQS
PKVCGYLNADSEELLPKGLIDREPSEEFVRRVKRRAEPNPLKNTCKLLVVADHRFYKYMG
RGEESTTTNYLIELIDRVDDIYRNTSWDNAGFKGYGVQIEQIRILKSPQEVKPGERHFNM
AKSFPNEEKDAWDVKMLLEQFSFDIAEEASKVCLAHLFTYQDFDMGTLGLAYVGSPRANS
HGGVCPKAYYNPTVKKNIYLNSGLTSTKNYGKTILTKEADLVTTHELGHNFGAEHDPDGL
AECAPNEDQGGKYVMYPIAVSGDHENNKMFSNCSKQSIYKTIESKAQECFQERSNKVCGN
SRVDEGEECDPGIMYLNNDTCCNSDCTLKPGVQCSDRNSPCCKNCQFETAQKKCQEAINA
TCKGVSYCTGNSSECPPPGDAEDDTVCLDLGKCKAGKCIPFCKREQELESCACVDTDNSC
KVCCRNLSGPCVPYVDAEQKNLFLRKGKPCTVGFCDMNGKCEKRVQDVIERFWDFIDQLS
INTFGKFLADNIVGSVLVFSLIFWIPFSILVHCVDKKLDKQYESLSLFHHSNIEMLSSMD
SASVRIIKPFPAPQTPGRLQALQPAAMMPPVSAAPKLDHQRMDTIQEDPSTDSHVDDDGF
EKDPFPNSSTAAKSFEDLTDHPVTRSEKAASFKLQRQSRVDSKETEC
>Q5SDL7

MVAMEAMAAMEVMVAAMAATADTVASSAASATATEATVAMDTASLSLPLQLSPRSLPQSS
LSATAATVATDTVVSSADTEVSDTEDSAATVSATASLSMLPQSSPRSLPQSSLSATAATV
DSVTDMADTAMDTKQFISKGNEHFFAASYLCAWADQSAAGS
>Q9QUM4
MDPKGSLSWRILLFLSLAFELSYGTGGGVMDCPVILQKLGQDTWLPLTNEHQINKSVNKS
VRILVTMATSPGSKSNKKIVSFDLSKGSYPDHLEDGYHFQSKNLSLKILGNRRESEGWYL
VSVEENVSVQQFCKQLKLYEQVSPPEIKVLNKTQENENGTCSLLLACTVKKGDHVTYSWS
DEAGTHLLSRANRSHLLHITLSNQHQDSIYNCTASNPVSSISRTFNLSSQACKQESSSES
SPWMQYTLVPLGVVIIFILVFTAIIMMKRQGKSNHCQPPVEEKSLTIYAQVQKSGPQEKK
LHDALTDQDPCTTIYVAATEPAPESVQEPNPTTVYASVTLPES
>Q01113
MGLGRCIWEGWTLESEALRRDMGTWLLACICICTCVCLGVSVTGEGQGPRSRTFTCLTNN
ILRIDCHWSAPELGQGSSPWLLFTSNQAPGGTHKCILRGSECTVVLPPEAVLVPSDNFTI
TFHHCMSGREQVSLVDPEYLPRRHVKLDPPSDLQSNISSGHCILTWSISPALEPMTTLLS
YELAFKKQEEAWEQAQHRDHIVGVTWLILEAFELDPGFIHEARLRVQMATLEDDVVEERR
YTGQWSEWSQPVCFQAPQRQGPLIPPWGWPGNTLVAVSIFLLLTGPTYLLFKLSPRVKRI
FYQNVPSPAMFFQPLYSVHNGNFQTWMGAHGAGVLLSQDCAGTPQGALEPCVQEATALLT
CGPARPWKSVALEEEQEGPGTRLPGNLSSEDVLPAGCTEWRVQTLAYLPQEDWAPTSLTR
PAPPDSEGSRSSSSSSSSNNNNYCALGCYGGWHLSALPGNTQSSGPIPALACGLSCDHQG
LETQQGVAWVLAGHCQRPGLHEDLQGMLLPSVLSKARSWTF
>O88788
MAQLEGYYFSAALSCTFLVSCLLFSAFSRALREPYMDEIFHLPQAQRYCEGRFSLSQWDP
MITTLPGLYLVSVGVVKPASWILGWSEHVVCSIGMLRFVNLLFSVGNFYLLYLLFRKIQP
RNKASSSIQRILSTLTLAVFPTLYFFNFLYYTEAGSVFFTLFAYLMCLYGNHRTSALLGF
CGFMFRQTNIIWAAFCAGHIIAQKCSEAWKTELQKKKEERLPPAKGPLSELRRVLQFLLM
YSMSLKNLSMLFLLTWPYMLLLLAFFVFVVVNGGIVVGDRSSHEACLHFPQLFYFFSFTA
FFSFPHLLSPTKVKTFLSLVWKRRVQFSVITLVSVFLVWKFTYVHKYLLADNRHYTFYVW
KRVFQRHEIVKYLLVPAYMFAGWAVADSLKSKSIFWNLMFFVCLVASTVPQKLLEFRYFI
LPYIIYRLNMPLPPISRLVCELGCYAVVNFLTFYIFLNKTFQWSDSHDIQRFMW
>P0C5H6
MIWCLRLTILSLILSQGADGRRKPEVVSVVGRAGESAVLGCDLLPPAGRPPLHVIEWLRF
GFLLPIFIQFGLYSPRIDPDYVGRVRLQTGASLQIEGLRVEDQGWYECRVLFLDQHSPEQ
DFANGSWVHLTVNSPPQFQETPPLVLEVKELEAVTLRCVALGSPQPYVTWKFRGQDLGKG
QGQVQVRNGTLWIRRVERGSAGDYTCQASSTEGSVTHTTQLLVLGPPVIVVPPNNNTVNA
SQDVSLACRAEAYPANLTYSWFQDRINVFHISRLQSRVRILVDGSLWLQATQPDDAGHYT
CVPSNGFPHPPSASAYLTVLYPAQVTVMPPETPLPIGMRGVIRCPVRANPPLLFVTWTKD
GQALQLDKFPGWSLGPEGSLVIALGNEDALGEYSCTPYNSLGTAGSSPVTRVLLKAPPAF
IDQPKEEYFQEVGRDLLIPCSARGDPPPIVSWAKVGRGLQGQAQVDSNNSLILRPLTKEA
HGRWECSARNAVAHVTISTNVYVLGTSPHVVTNVSVVPLPKGANVSWEPGFDGGYLQRFS
VWYTPLAKRPDRAHHDWVSLAVPMGATHLLVPGLQAYTQYQFSVLAQNKLGSGPFSEIVL
SIPEGLPTTPAVPRLPPTEMPPPLSPPRGLVAVRTPRGVLLHWDPPELIPERLDGYILEG
RQGSQGWEILDQGVAGTEIQLLVPGLIKDVLYEFRLVAFADSYVSDPSNIANISTSGLEV
YPSRTQLPGLLPQPVLAGVVGGVCFLGVAVLVSILAACLMNRRRAARRHRKRLRQDPPLI
FSPRGRSGPHSAPGSDSPDSVTKFKLQGSPVPSLRQSLLWGEPARPPSPHPDSPLGRGPL

PLEPICRGPDGRFVMGPTVAPPQEKLCLERSEPRTSAKRLAQSLDCSSSSPSGVPQPLCI
TDISPVGQPPAAMPSPLPGPGPLLQYLSLPFFREMNVDGDWPPLEEPTPASPPDFMGSHP
CPTSSFLPPPDSPPTNLRAVLPGTLMGVGVSSEPPYTALADWTLRERVLPGLLSAAPRGS
LTSQSSGRGSASFLRPPSTAPSAGGSYLSPAPGDTSSWASGPERWPRREHVVTVSKRRNT
SVDENYEWDSEFPGDMELLETWHPGLASSRAHPELEPELGVKTPEKSCLLNTTHAPGPEA
RCAALREEFLAFRRRRDATRARLPVCQQSISYPEQATLL
>Q8CJ52
MTRTLDLMVPLLGLSLGLALSLPRAVAADCGSLGRVEHLAFAPVPGTEPTAPRVRAPWPL
DSLYGTVRRFLSVVQLNPFPAELIKTLLNDPSSVKTDEVVRYEAGYVVCAVIAGLYLLLV
PITGLCFCCCRCRRRCGGRVKTEHKAMACERGTLMTFLLLTTLMLLIGMVCAFATNQFTH
SQTGPSVEAVPETLLSLRGLVSDVPKKLQAVADQFSSPQKQVSKDLDGVGENLGNIIHNQ
LKSTVYPVLASVHGLGQALQVSIDHLQSVNATSVELREGQQHLGPPVQAHRERLLALLQE
SWCHENCKGALSQASALQLGADFSQMPPVDDVLHRLQGVPEANFSSMVQEENATFNNLPI
LVHMQMVSVVKDLKKALAEQPEGVRMLAQAFPGSEATSRWSQALEGLEQRSRPYLQDVQR
YETYRWILGCVLCSAILLVVICNLLGLSLGIWGLFAREDPSHSETKGEAGARFLMAGVAF
SFLFAAPLILLVFATFLVGGNVQTLVCRSWESGELYEFVDTPGNLPPSMNLSYLLGLRKN
ISVFQAYRQCKAGTALWKVLQLNDSYDLDKHLDIKQYTHELQQELQSFKVDLKDLDLLNP
AARQNLEALQSSGLEKIHYRDFLVQIQKPVVKTDMGQLAKELEGLAQAQNESLRRQQLQE
EARELRSLYQEKVVPQENLVAKLNPSIRVLESSAPKLQVNTSDLLDIVARLKGELPAQLN
HILRNATECFLTREMGYFSQYVTWVREEVTQHIATCQPFSKALDDGHMILCDMMANPWNA
FWFCLGWCTFFLIPSIIFAVKTSKYFRPIRKRLSSTSSEETQLFHIPRVTSLKL
>P22146
MLFKSLSKLATAAAFFAGVATADDVPAIEVVGNKFFYSNNGSQFYIRGVAYQADTANETS
GSTVNDPLANYESCSRDIPYLKKLNTNVIRVYAINTTLDHSECMKALNDADIYVIADLAA
PATSINRDDPTWTVDLFNSYKTVVDTFANYTNVLGFFAGNEVTNNYTNTDASAFVKAAIR
DVRQYISDKNYRKIPVGYSSNDDEDTRVKMTDYFACGDDDVKADFYGINMYEWCGKSDFK
TSGYADRTAEFKNLSIPVFFSEYGCNEVTPRLFTEVEALYGSNMTDVWSGGIVYMYFEET
NKYGLVSIDGNDVKTLDDFNNYSSEINKISPTSANTKSYSATTSDVACPATGKYWSAATE
LPPTPNGGLCSCMNAANSCVVSDDVDSDDYETLFNWICNEVDCSGISANGTAGKYGAYSF
CTPKEQLSFVMNLYYEKSGGSKSDCSFSGSATLQTATTQASCSSALKEIGSMGTNSASGS
VDLGSGTESSTASSNASGSSSKSNSGSSGSSSSSSSSASSSSSSKKNAATNVKANLAQV
VFTSIISLSIAAGVGFALV
>O15354
MRAPGALLARMSRLLLLLLLKVSASSALGVAPASRNETCLGESCAPTVIQRRGRDAWGPG
NSARDVLRARAPREEQGAAFLAGPSWDLPAAPGRDPAAGRGAEASAAGPPGPPTRPPGPW
RWKGARGQEPSETLGRGNPTALQLFLQISEEEEKGPRGAGISGRSQEQSVKTVPGASDLF
YWPRRAGKLQGSHHKPLSKTANGLAGHEGWTIALPGRALAQNGSLGEGIHEPGGPRRGNS
TNRRVRLKNPFYPLTQESYGAYAVMCLSVVIFGTGIIGNLAVMCIVCHNYYMRSISNSLL
ANLAFWDFLIIFFCLPLVIFHELTKKWLLEDFSCKIVPYIEVASLGVTTFTLCALCIDRF
RAATNVQMYYEMIENCSSTTAKLAVIWVGALLLALPEVVLRQLSKEDLGFSGRAPAERCI
IKISPDLPDTIYVLALTYDSARLWWYFGCYFCLPTLFTITCSLVTARKIRKAEKACTRGN
KRQIQLESQMNCTVVALTILYGFCIIPENICNIVTAYMATGVSQQTMDLLNIISQFLLFF
KSCVTPVLLFCLCKPFSRAFMECCCCCCEECIQKSSTVTSDDNDNEYTTELELSPFSTIR
REMSTFASVGTHC

>Q02401
MELPWTALFLSTVLLGLSCQGSDWESDRNFISAAGPLTNDLVLNLNYPPGKQGSDVVSGN
TDHLLCQQPLPSFLSQYFSSLRASQVTHYKVLLSWAQLLPTGSSKNPDQEAVQCYRQLLQ
SLKDAQLEPMVVLCHQTPPTSSAIQREGAFADLFADYATLAFQSFGDLVEIWFTFSDLEK
VIMDLPHKDLKASALQTLSNAHRRAFEIYHRKFSSQGGKLSVVLKAEDIPELLPDPALAA
LVQGSVDFLSLDLSYECQSVATLPQKLSELQNLEPKVKVFIYTLKLEDCPATGTSPSSLL
ISLLEAINKDQIQTVGFDVNAFLSCTSNSEESPSCSLTDSLALQTEQQQETAVPSSPGSA
YQRVWAAFANQSREERDAFLQDVFPEGFLWGISTGAFNVEGGWAEGGRGPSIWDHYGNLN
AAEGQATAKVASDSYHKPASDVALLRGIRAQVYKFSISWSGLFPLGQKSTPNRQGVAYYN
KLIDRLLDSHIEPMATLFHWDLPQALQEQGGWQNESVVEAFLDYAAFCFSTFGDRVKLWV
TFHEPWVMSYAGYGTGQHAPAISDPGMASFKVAHLILKAHARTWHLYDLHHRLQQQGRVG
IVLNSDLAEPLDRKSPQDLAAAERFLHFMLGWFAHPIFVDGDYPTTSAQIQHINQQCGHP
LAQLPEFTEAEKRLLKGSADFLGLSHYTSRLISKAGRQTCTSSYDNIGGFSQHVDPEWPQ
TASPWIRVVPWGIRRLLRFASMEYTKGKLPIFLAGNGMPVGEEADLFDDSVRVNYFNWYI
NEVLKAVKEDLVDVRSYIVRSLIDGYEGPLGFSQRFGLYHVNFNDSSRPRTPRKSAYLFT
SIIEKNGFSAKKVKRNPLPVRADFTSRARVTDSLPSEVPSKAKISVEKFSKQPRFERDLF
YDGRFRDDFLWGVSSSPYQIEGGWNADGKGPSIWDNFTHTPGNGVKDNATGDVACDSYHQ
LDADLNILRTLKVKSYRFSISWSRIFPTGRNSTINKQGVDYYNRLIDSLVDNNIFPMVTL
FHWDLPQALQDIGGWENPSLIELFDSYADYCFKTFGDRVKFWMTFNEPWCHVVLGYSSGI
FPPSVQEPGWLPYKVSHIVIKAHARVYHTYDEKYRSEQKGVISLSLNTHWAEPKDPGLQR
DVEAADRMLQFTMGWFAHPIFKNGDYPDVMKWTVGNRSELQHLASSRLPTFTEEEKNYVR
GTADVFCHNTYTSVFVQHSTPRLNPPSYDDDMELKLIEMNSSTGVMHQDVPWGTRRLLNW
IKEEYGNIPIYITENGQGLENPTLDDTERIFYHKTYINEALKAYKLDGVDLRGYSAWTLM
DDFEWLLGYTMRFGLYYVDFNHVSRPRTARASARYYPDLIANNGMPLAREDEFLYGEFPK
GFIWSAASASYQVEGAWRADGKGLSIWDTFSHTPLRIGNDDNGDVACDSYHKIAEDVVAL
QNLGVSHYRFSIAWSRILPDGTTKFINEAGLSYYVRFIDALLAAGITPQVTIYHWDLPQA
LQDVGGWENETIVQRFKEYADVLFQRLGDRVKFWITLNEPFVIAAQGYGTGVSAPGISFR
PGTAPYIAGHNLIKAHAEAWHLYNDVYRARQGGTISITISSDWGEPRDPTNREHVEAARS
YVQFMGGWFAHPIFKNGDYPEVMKTRIRDRSLGAGLNKSRLPEFTESEKSRIKGTFDFFG
FNHNTTVLAYNLDYPAAFSSFDADRGVASIADSSWPVSGSFWLKVTPFGFRRILNWLKEE
YNNPPIYVTENGVSRRGEPELNDTDRIYYLRSYINEALKAVHDKVDLRGYTVWSIMDNFE
WATGFAERFGVHFVNRSDPSLPRIPRASAKFYATIVRCNGFPDPAQGPHPCLQQPEDAAP
TASPVQSEVPFLGLMLGIAEAQTALYVLFALLLLGACSLAFLTYNTGRRSKQGNAQPSQH
QLSPISSF
>Q969F8
MHTVATSGPNASWGAPANASGCPGCGANASDGPVPSPRAVDAWLVPLFFAALMLLGLVGN
SLVIYVICRHKPMRTVTNFYIANLAATDVTFLLCCVPFTALLYPLPGWVLGDFMCKFVNY
IQQVSVQATCATLTAMSVDRWYVTVFPLRALHRRTPRLALAVSLSIWVGSAAVSAPVLAL
HRLSPGPRAYCSEAFPSRALERAFALYNLLALYLLPLLATCACYAAMLRHLGRVAVRPAP
ADSALQGQVLAERAGAVRAKVSRLVAAVVLLFAACWGPIQLFLVLQALGPAGSWHPRSYA
AYALKTWAHCMSYSNSALNPLLYAFLGSHFRQAFRRVCPCAPRRPRRPRRPGPSDPAAPH
AELLRLGSHPAPARAQKPGSSGLAARGLCVLGEDNAPL
>Q91X56
MEPGLLRPAPVSEVIVLHYNYTGKLRGARYQPGAGLRADAAVCLAVCAFIVLENLAVLLV

LVRHPRFHAPMFLLLGSLTLSDLLAGAAYATNILLSGPLTLRLSPALWFAREGGVFVALA
ASVLSLLAIALERHLTMARRGPAPAASRARTLAMAVAAWGASLLLGLLPALGWNCLGRLE
TCSTVLPLYAKAYVLFCVLAFLGILAAICALYARIYCQVRANARRLRAGPGSRRATSSSR
SRHTPRSLALLRTLSVVLLAFVACWGPLFLLLLLDVACPARACPVLLQADPFLGLAMANS
LLNPIIYTFTNRDLRHALLRLLCCGRGPCNQDSSNSLQRSPSAAGPSGGGLRRCLPPTLD
RSSSPSEHLSPQQDGVDTSCSTGSPGVATANRSLVPTATD
>Q9GV77
MASALLCFLAAILPGMIAAQNTWVLGTSDVRTVEPVNPVGGGVSLGGIDIDSTENRIIVQ
NTGIAVPFGREKAIDPNSELVINVQAGDSCSIKVLPRQSDPLSQIPGRLVPPSFPCDFSP
GEVKYVHFGSRKPQTDKVKLQLRYDTATDVYIIPFTIDVRVESKQLEIVTRNVPLVVQDL
MGTSDALDADKLEFEFDSNTEVCKVTVLSSTSGLPRYGEVMNHDEQGQMIDCNDFLELGI
QYRHTAATSSPREDYIPLVVELQNQQGQVIKQEYFQSMVRIIDGDDNTPPSLVLSSDMMM
EVDQFVMTAITPSILAAEDVETPADMLIFNITSQTLGPDDGMIVSTDDRNQPITSFTQKD
LRDLKIAYKPPPRDTDVQTIYQIELEIVDSELATSETHSLLIVVKPKNTLAPVVTTNTGL
VLFEGQSRPLLGGQNLGISDEDNLQDVIIAPINGSRYGELRIGNQRIKQFTIADLIEGAV
TYHHYGTDTYSDNIIFRMTDGQHEVEFLFPITIAPIDDEAPIVDVNTGVTVNENEVVAIT
NFVLSATDIDSDDSEIRFVLEQPLSDMGNLFLRQVNIPEDPQNWISQDNFYEREVTEFTL
EDIQNGHLFYQHGGSHNADPVFDRILFRVVDSADPQPNESPVQELLVKVMPQDLQPPEMF
GGTTLQLSVDEFQITPILKKNLRFTDMDSNDRELKYTIVSPLTDSDSNNNLPVGDIVLTD
EPNTPINMFTQAQINHMKVSYKPPSTELGIAPRAITFQFVVQDTQGNMGSPHNFIILLRP
VDNQPPTITNTGVQVFERGTVIIDQTMLDATDPDTDRNSIRVVLVQPPVFGTMNLNDIAL
EKGDEFTLGDIENSRVKYVSGDAEEQSDEIHLEITDGVHVVPIVIHINVAPIDDEAPTLD
LPPGTIGSFLEVQENSFSLITSNILSASDPDTEDLLLTFIVDRQPNEGRIESNGVVADVF
TQQDIVNGLVRYVHTGGEIGPSKRDDSFNLTLSDMSPDWILGGNEITQVEVYVTVLPVDN
LAPNVTMGVQFYVDEAGKGNINMTHLQAPDVDTEDDDILCTIVVAPSVGYLENISPAPGS
EKSRGGMPISAFSIKDLRLNHINYVQSIHQGMEPEEDQFTFRCTDGVNESPNFLFPINII
PVNDEEPQVYAREIIVDEGGQRIIDEPLLRAEDGDVPADELHFFIVTPPQHGTITYTRLE
GDIPILNFTMDQIANGNDIKYIHDDSETTEDSFTVLLTDGKYEITKEITITILEVDDETP
RLTINDGIDIEIGESRIISNRILKATDLDSADSNLTYTVRYAPEKGLLQRLSKFDGSVVE
NITLGMNFTQWEVDNQRIRYVHTDGDGGRDLIKFDITDGTNPLIDRYFYVTVDHIDNVHP
SIINAGVTMQEGSRVTLTTSIISTSDLNSPDEDLLFTITTAPTKGHLESTDNPGMPINSF
TQLDLAGSKIYYVHTADDEVKMDSFQFQVTDGFNTVVRTFRISFTDVDNKEPVVRYDTIR
LQEGDNKLITPFELGIDDRDTPANELRFTITQLPIHGNILRNNTALVTEFTMHDINENLI
SYQHDGSEQTADSFSFIVTDGTHNEFYVLPDITTLTRQPQQVPIEIVPVDNGAPQIVVNR
GAPTLDLLGTGELGFMITNKYLMSEDRDSVDNSLLYVITTQPQHGYIMNIALGNISITNF
TQSDVNNMYIQYIVYPNVDATSDTFFVEVRDAGGNTLPNQPFRLNWSWISLEKEYYEVNE
TERYLNIKLVRRGYLGETSFVGIQTADGTAIADEDFRGKSARQVQFNPGQTEGFWRVRIL
NDRLYEQAEVFEIILHDPVMGALEYPDRAVVTIFDAEDESGVFIDLPDNYVIEEDIGEFL
VPIRRTGDLSQELMASCSTMPGSATGSDPSPVLSFSDYISRMEEDPDNMVAFDKGEDLAY
CRILIIDDSLYEEDETFQVKLSNPMGGRIGNPSAINVIIAGDTDDVPSFYFGEPEYKVDE
NAPFVEVTVFRTGTDVSKMASVTVRSRASNPVSAVAGEDYAGISRNLDFAPGVNQQTVKV
YIIDDRGQPRLEGPETFELVLNMPMNGVLGAPSKTVITINDTISDLPKVEFRHPTYEVNE
NDIRITAEVVRSGDLSIESSVRCYTRQGSAQVMMDYDERPNTEASIITFLPGERSKTCTV
LLMDDNVFEPDEAFRLVLGSPRTASGVPAVVGEQNVTVVTVHDVGDAPIIKFPETKFSID

EPTDLDSVVTVSIPVIRMGDNTQTSIVRVFTKDGSARSGIDYNPLSQVLEFGFNVTERVV
EIEILPDEDRNEMREAFTLHITNDQMMIADVQMNHAIIYIEQEGQASGVTFPSQPVVVSL
LDYDDIPNARTNPPRGYPLICVSPCNPKYPDFATTGPICDSEGLNDTVTQFRWMVSAPTS
ESGVTSPLRQTDSDTFFSSTKSITLDSVYFGPGSRVQCVARAVGSEGDAGREHPSNSIVI
STTDGMCMPRVANAIGAEPFTARMRYTGPADPDYPNKVRLTVTMPHVDGMLPVISTRQLS
NFELALSKDGYRVGTHRCSNLLDYNEIPTDFGFITEETKNPNVVGDTYAYQYSPELRGEE
TLRFYRNLNLEACLWEFNAYYDMSELLDECGGLVGTDGQVLDLVQSYVSMRIPLFVSFVF
HSPVATGGWKHFDQQSTLQLTFVYDTSILWQNGIGSQVTTGTQSLQGNLYPTSMRIDEDG
RLVVNFRTEALFNGLFVQSHQSTDVVSTVNSIDHPGITYSLSLLRTEPTYAQPEQLWQFV
SDLSVSDYSGTYTIQLVPCTTLPNTVYSQPPVCNPEDIITFELPIRFQQVSDPVPEEYSL
NTEFVLVGKESIYLSDGSMGFGEGSDVAYNPGDTIFGRIHVDPVQNLGAGFNLDIQKVFL
CTGRDGYIPKYNPAANEYGCVADTPNLLYAFKILDRGAPDTIVREFNGLPFNATLAIDNA
ADLELVQQPGADGFRLASDALFEVDYGRTWYLHSIYSMRSSESSGIGKRETEHHAISSRQ
RRQANSEALVDPAQGQGTNMKRVALQGPQDVDNNLGGTYELAPKGTNVVMIAVVIGVILI
ILLVALVIGVVVRRRQAKQQPVVVVNGSAKVVSNVHFDDNTEV
>O88492
MSASGDGTRVPPKSKGKTLSSFFGSLPGFSSARNLVSHTHSSTSTKDLQTATDPSGTPAP
SSKVSTNSQMAGDAAGLLQPSEQTAGDKDMGSFSVTSSEDAFSGVFGIMDAAKGMVQGGL
GATQSALVGTKEAVSGGVMGAVGVAKGLVKGGLDTSKNVLTNTKDTVTTGVMGAANMAKG
TVQTGLDTTKSVVMGTKDTVATGLAGAVNVAKGTIQGGLDTTKSVVMGTKDTVTTGLTGA
ANVAKGVVQGGLDTTKSVVMGTKDTVTTGLTGAMNVAKGTAQMGIDTSKTVLTGTKDTVC
AGATGAINVAKGAAQGGLDTTKSVLIGTKDTVTTGLTGAVNVAKGAVQGGLDTTKSVVMG
TKDTVTTGLTGAMNVAKGTAQMGLGTSKTVLTGTKDTVCAGLTGAINVAKGAAQGGLDTT
KSVLMGTKDTVTTGLTGAVNVAKGTIQGGLDTTKSVVMGTKDTVTTGLTGAVNVAKGTIQ
GGLDTTKSVVMGTKDTVTTGLTGAVNVAKGAAQGGLDTTKSVVMGTKDTVTTGLTGAMNV
AKGTAQMGLGTSKTVLTGTKDTVCAGLTGAINVAKGAAQGGLDTTKSVLMGTKDTVTTGL
TGAVNVAKGTIQGGLDTTKSVVMGTKDTVTTGLTGAVNVAKGAVQGGLDTTKSVVMGTKD
TVTTGLTGALNVAKGTAQMGIDTSKTVLIGTKDTVCAGATGAINMAKGAAQGGLDTTKSV
LMGTKDTVTTGLTGAINVAKGSAQGGLDTTKSVLIGTKDTVTTGLTGALNVAKGTVQTGL
DTSQRVLTGTKDNVYAGVTGAVNVAKGTIQGGLDTTKSVVMGTKDTVTTGLTGAVNVAKG
AVQGGLDTTKSVVMGTKDTVTTGLTGAMNVAKGTAQMGIDTSKTVLTGTKDTVCAGLTGA
INVAKGATQGGLDTTKSVLMGTKDTVTTGLTGAINVAKGAAQGGLDTTKSVLLGTKDTVT
TGLTGAANVAKETVQMGLDTSKNILMDTKDSICAGATGAITVVKGAAQGGLDTSNAALTG
TMDTAKGTVQTSLDTSKHMLIGMKDTVCAGVTSAMNMAKGIHKNTDTTRDTQSSVLAHSG
NVATNAIHTGVHTVPSSLSGSHSIICHEPSIYRATNHGVGHAILTSTESLCCETSSFSDK
YGLGHVTEPRADTKTLVSGMASSACAATRSVEECGQLAATGFAALPDELKGLGDIFQPMT
TEEQAQLAVSESGPRVLSADRGSYYIRLGDLAPSFRQRAFEHALSHIQHNQFQARAAVAQ
LQEAFQMTDMTMEAACGKLCSDQSLNTMVEAVGSHEMRASVAQDRLCTLAHQLHAAYSSL
VTSLQGLPEVQQQAGQARHSLCKLYGLVSSEAGSELQTEQLAQSSAGVVEAWQGLEVLLE
KLQQNPPLSWLVGPFTSMPCGQL
>P25023
MDTRSSLCPKTQAVVAVFWGPGCHLSTCIEMFNITTQALGSAHNGTFSEVNCPDTEWWSW
LNAIQAPFLWVLFLLAALENIFVLSVFCLHKTNCTVAEIYLGNLAAADLILACGLPFWAI
TIANNFDWLFGEVLCRVVNTMIYMNLYSSICFLMLVSIDRYLALVKTMSMGRMRGVRWAK

LYSLVIWSCTLLLSSPMLVFRTMKDYREEGHNVTACVIVYPSRSWEVFTNMLLNLVGFLL
PLSIITFCTVRIMQVLRNNEMKKFKEVQTEKKATVLVLAVLGLFVLCWFPFQISTFLDTL
LRLGVLSGCWNERAVDIVTQISSYVAYSNSCLNPLVYVIVGKRFRKKSREVYQAICRKGG
CMGESVQMENSMGTLRTSISVDRQIHKLQDWAGNKQ
>Q868T3
MLHLRLFDSSLYYTLASASESSGLASSTSTERSFNGTQGAGGVAAGGESLTPTDVAAVNL
TYFTPAISHVMLAPTTIATTTASATMVQIQTTAAPSHDLETGGNSTSSDPGEFDNLNSFY
FYETEQFAVLWILFTVIVLGNSAVLFVMFINKNRKSRMNYFIKQLALADLCVGLLNVLTD
IIWRITISWRAGNLACKAIRFSQVCVTYSSTYVLVAMSIDRYDAITHPMNFSKSWKRARH
LVAGAWLISALFSLPILVLYEEKLIQGHPQCWIELGSPIAWQVYMSLVSATLFAIPALII
SACYAIIVKTIWAKGSIFVPTERAGFGAAPARRASSRGIIPRAKVKTVKMTLTIVFVFII
CWSPYIIFDLLQVFGQIPHSQTNIAIATFIQSLAPLNSAANPLIYCLFSSQVFRTLSRFP
PFKWFTCCCKSYRNNSQQNRCHTVGRRLHNSCDSMRTLTTSLTVSRRSTNKTNARVVICE
RPTKVVTVPAMSERRGVSLKGNTDIL
>P19256
MVAGSDAGRALGVLSVVCLLHCFGFISCFSQQIYGVVYGNVTFHVPSNVPLKEVLWKKQK
DKVAELENSEFRAFSSFKNRVYLDTVSGSLTIYNLTSSDEDEYEMESPNITDTMKFFLYV
LESLPSPTLTCALTNGSIEVQCMIPEHYNSHRGLIMYSWDCPMEQCKRNSTSIYFKMEND
LPQKIQCTLSNPLFNTTSSIILTTCIPSSGHSRHRYALIPIPLAVITTCIVLYMNGILKC
DRKPDRTNSN
>P08953
MSRLKAASELALLVIILQLLQWPGSEASFGRDACSEMSIDGLCQCAPIMSEYEIICPANA
ENPTFRLTIQPKDYVQIMCNLTDTTDYQQLPKKLRIGEVDRVQMRRCMLPGHTPIASILD
YLGIVSPTTLIFESDNLGMNITRQHLDRLHGLKRFRFTTRRLTHIPANLLTDMRNLSHLE
LRANIEEMPSHLFDDLENLESIEFGSNKLRQMPRGIFGKMPKLKQLNLWSNQLHNLTKHD
FEGATSVLGIDIHDNGIEQLPHDVFAHLTNVTDINLSANLFRSLPQGLFDHNKHLNEVRL
MNNRVPLATLPSRLFANQPELQILRLRAELQSLPGDLFEHSTQITNISLGDNLLKTLPAT
LLEHQVNLLSLDLSNNRLTHLPDSLFAHTTNLTDLRLEDNLLTGISGDIFSNLGNLVTLV
MSRNRLRTIDSRAFVSTNGLRHLHLDHNDIDLQQPLLDIMLQTQINSPFGYMHGLLTLNL
RNNSIIFVYNDWKNTMLQLRELDLSYNNISSLGYEDLAFLSQNRLHVNMTHNKIRRIALP
EDVHLGEGYNNNLVHVDLNDNPLVCDCTILWFIQLVRGVHKPQYSRQFKLRTDRLVCSQP
NVLEGTPVRQIEPQTLICPLDFSDDPRERKCPRGCNCHVRTYDKALVINCHSGNLTHVPR
LPNLHKNMQLMELHLENNTLLRLPSANTPGYESVTSLHLAGNNLTSIDVDQLPTNLTHLD
ISWNHLQMLNATVLGFLNRTMKWRSVKLSGNPWMCDCTAKPLLLFTQDNFERIGDRNEMM
CVNAEMPTRMVELSTNDICPAEKGVFIALAVVIALTGLLAGFTAALYYKFQTEIKIWLYA
HNLLLWFVTEEDLDKDKKFDAFISYSHKDQSFIEDYLVPQLEHGPQKFQLCVHERDWLVG
GHIPENIMRSVADSRRTIIVLSQNFIKSEWARLEFRAAHRSALNEGRSRIIVIIYSDIGD
VEKLDEELKAYLKMNTYLKWGDPWFWDKLRFALPHRRPVGNIGNGALIKTALKGSTDDKL
ELIKPSPVTPPLTTPPAEATKNPLVAQLNGVTPHQAIMIANGKNGLTNLYTPNGKSHGNG
HINGAFIINTNAKQSDV
>Q9FHN6
MAATDFFFAFVFSFALIFGFSFAGDPYVSYDFTLSYITASPLGVPQQVIAVNGKFPGPVI
NATTNYNVHVNVLNHLDEPLLLTWPGVQMRRNSWQDGVLGTNCPIPPNWNFTYDFQLKDQ
IGSYFYSPSLNFQRASGGFGALIINNRDLVPIPFTEPDGEIIFIIGDWYTQNHTALRRIL

DSGKELGMPDGVLINGKGPFKYNSSVPDGIEHETVNVDPGKTYRIRVHNVGISTSLNFRI
QNHKLLLIETEGRYTSQMNFTDFDVHVGQSYSFLVTMDQNATSDYYIVASARFVNETVWQ
RVTGVGILHYSNSKGPASGPLPVSATDVNHPWSAMNQPRAIKQNTSASGARPNPQGSFHY
GQINITRTYILRSLPPTKINGKLRATLNGISFVNPSTPMRLADDHKVKGDYMLDFPDRPL
DEKLPRLSSSIINATYKGFIQVIFQNNDTKIQSFHIDGYAFYVVAMDFGIWSEDRNSSYN
NWDAVARSTVEVYPGAWTAVLISLDNVGVWNIRVENLDRWYLGQETYMRIINPEENGSTE
MDPPENVMYCGALQAMQKEQHHSSATKSMTNGQLILIFSMMMVLLSSFSSFC
>Q24352
MCTMPATRDASGSGDASTDLIAARSLSSHQGQRSNLRIFKLLISCCLLMLCIYPNAWPWS
IGPGSGPFSVSADSIKGRGDTHRLGEMGTSLSSSLPSSWLTQSNNHANISELLDNLLRGY
DNSIRPDFGGPPATIEVDIMVRSMGPISEVDMTYSMDCYFRQSWVDKRLAFEGAQDTLAL
SVSMLARIWKPDTYFYNGKQSYLHTITTPNKFVRIYQNGRVLYSSRLTIKAGCPMNLADF
PMDIQKCPLKFGSFGYTTSDVIYRWNKERPPVAIAEDMKLSQFDLVDCPAGNLTDIVYKA
AAPRPQRRPFNNKDPPRPTSKVMTTFAGPAAKNQHVRGTGLKLDKGAFGTGRDATGGSGS
TTGLSGTITLETNHPSEYSMLMVNFHLQRHMGNFLIQVYGPCCLLVVLSWVSFWLNREAT
ADRVSLGITTVLTMTFLGLEARTDLPKVSYPTALDFFVFLSFGFIFATILQFAVVHYYTK
YGSGECYFIIEELDSESGESETEPLTSDFRGSTESKIYEVIPLSMCAISMPPPPTRLGML
TSRNRRPRNRRHGLWSMKLLGLFDWRRRRKPPRADSDEDEDDEQTQLRANEAPTTSAAAA
AAQAAAQAARISPPTGGRRRMSYYRREEMEARRKGKRTPQYNSVSKIDRASRIVFPLLFI
LINVFYWYGYLSRSSRILANTPDAST
>Q8BQP9
MSSAPNGRKKRPSRSTRSSIFQISKPPLQSGDWERRGSGSESAHKTQRALDDCKMLVQEF
NTQVALYRELVISIGDVSVSCPSLRAEMHKTRTKGCEMARQAHQKLAAISGPEDGEIHPE
ICRLYIQLQCCLEMYTTEMLKSICLLGSLQFHRKGKEASGGAKNLDSKIEENAETPALED
SLSSPLESQQQCWQVATDIENTERDMREMKNLLSKLRETMPLPLKNQDDSSLLNLTPYPM
VRRRKRRFFGLCCLVSS
>Q16623
MKDRTQELRTAKDSDDDDDVAVTVDRDRFMDEFFEQVEEIRGFIDKIAENVEEVKRKHSA
ILASPNPDEKTKEELEELMSDIKKTANKVRSKLKSIEQSIEQEEGLNRSSADLRIRKTQH
STLSRKFVEVMSEYNATQSDYRERCKGRIQRQLEITGRTTTSEELEDMLESGNPAIFASG
IIMDSSISKQALSEIETRHSEIIKLENSIRELHDMFMDMAMLVESQGEMIDRIEYNVEHA
VDYVERAVSDTKKAVKYQSKARRKKIMIIICCVILGIVIASTVGGIFA
>P53386
MSSNDSNDTDKQHTRLDPTGVDDAYIPPEQPETKHHRFKISRDTLRNHFIAAVGEFCGTF
MFLWCAYVICNVANHDVALVAAPDGSHPGQLIMIAIGFGFSVMFSIWCFAGVSGGALNPA
VSLSLCLARAVSPTRCVVMWVSQIVAGMAAGGAASAMTPGEVLFANSLGLGCSRTRGLFL
EMFGTAILCLTVLMTAVEKRETNFMAALPIGISLFIAHVALTAYTGTGVNPARSLGAAVA
ARYFPHYHWIYWIGPLLGSILAWSVWQLLQILDYTTYVTAEKAASTKEKAQKKVKPAVPL
LWLKSNFPLLFFISRSLALNVIIFGKN
>P78746
MIVLFTLLRWAPISPVFSMRTMHANLAHRGIFLPVMIVTLPLPVHLRRRFPAQMVLMLQW
FAFGMFSVLLIIPWLLCVYRLVTHSPGRTKRIKQVLDDRTAPKTVVVMPVYKEAPETLIR
AIDSVVDCDYPANCIHVFLSYDGCLIDESYLRLIEHLGIPITLESYPQSIDVTYKDARIT
VSRFKHGGKRHCQKQTFRLIDMVYADYLERHDNLFVLFIDSDCILDRVCLQNFMYDMELK

PGSKHDMLAMTGVITSTTDRGSLLTLLQDMEYVHGQLFERSVESSCGAVTCLPGALTMLR
FSAFRKMAKYYFADKAEQCEDFFDYGKCHLGEDRWLTHLFMVGARKRYQIQMCAGAFCKT
EAVQTFSSLLKQRRRWFLGFITNEVCMLTDVRLWKRYPLLCLVRFMQNTIRTTALLFFII
ALSLITTSSSINDLPVGFIAISLGLNYVLMFYLGAKLKRYKAWLFPLMFILNPFFNWLYM
VYGILTAGQRTWGGPRADAATADEHTSPEEAVELAKAQGDELNVDLTTFRSRGDEKSVPI
HPSEKIDGRFSAPELPDGYDSNLNDSNAALTELMTPLPSVPRIGIHTYPSSDSILTSDSL
SSIHLPLKVEELTGDNDNMKPYPDRQPRDTSSLHQMQRTCSNGIVASDSCSSQDDASEMV
NKPEILSPSAHILPHPSQATESSSGEDIYPLHLPSPHQHEAHFAPLNASTRGSMEGNTPE
VQRPRRKLPGIPRPIRAQKDPESMV
>Q25532
MASQTHGIQQLLAAEKRAAEKVSEARKRKAKRLKQAKEEAQDEVEKYRQERERQFKEFEA
KHMGTREGVAAKIDAETRIKIDEMNKMVQTQKEAVIKDVLNLVYDIKPELHINYRVV
>Q5NUL3
MSPECARAAGDAPLRSLEQANRTRFPFFSDVKGDHRLVLAAVETTVLVLIFAVSLLGNVC
ALVLVARRRRRGATACLVLNLFCADLLFISAIPLVLAVRWTEAWLLGPVACHLLFYVMTL
SGSVTILTLAAVSLERMVCIVHLQRGVRGPGRRARAVLLALIWGYSAVAALPLCVFFRVV
PQRLPGADQEISICTLIWPTIPGEISWDVSFVTLNFLVPGLVIVISYSKILQTSEHLLDA
RAVVTHSEITKASRKRLTVSLAYSESHQIRVSQQDFRLFRTLFLLMVSFFIMWSPIIITI
LLILIQNFKQDLVIWPSLFFWVVAFTFANSALNPILYNMTLCRNEWKKIFCCFWFPEKGA
ILTDTSVKRNDLSIISG
>Q03877
MSRRVFTSAALHLLVVMWMCCGPCEAAAASTGENSVNARQPQRFDLFVPQKTVLLPRGGG
NSEKKWDSFASPSLVSAGGVIAAFAEGHLSSKNKDNKSTEPSSDAVAWYIDSAWEWSTLV
GEVNKSTWQAHTVLGKVDGKERFDVVLRPTTTTKDNKVFLLAGSSVASNVNGSWSHGGLK
LKLVVGDVRKPTDSEQSGRINWGEVQSPLNENSGAVQERKLTAFVASGGAGVLMEDGTIV
FSLMARNEEEDVYSMIIYSKDDGSTWALSNSVSSAKCVNPRITEWEGSLLMIVDCEDEQK
VYVSRDMGTTWTEAVGKLLGVWVNSGSGASQDSSLHVDALITATIEGRRVMLYTQRGNSL
GENANPLYLWVTDNNRSFHVGPVGMDNAEKEELESALLYSDGKLHLLQRRVSGEGSVISL
SRLTEELKEIESVLSTWAQKDIFFSSLSIPTAGLVAVLSDAAGDGRWNDEYLCLNATVKN
AVKVKDGFQLTESNSRVLWSVNTRDNNLRHVFLSHDFTVVATVIIQNVPSGKTSLLTATL
ANTESNYTMGLSYTADNKWETIFKGDKKPTTESRPWEPKKEYQVALMLQGKKASVYIDGR
SLGEGEALLTDEKSLEFVHFCFGACVQESSPTAAQKTKVTVTNVFLYNRPLNSTEMRAIK
DRIPIPKRGPGSQVEGGTERRHIPRIEGVRANAPVGSGLLPLLLLLGLWVFAAL
>Q9LX20
MVSRSAFLLFCVLFLATEETLASLFSSRLIHRFSDEGRASIKTPSSSDSLPNKQSLEYYR
LLAESDFRRQRMNLGAKVQSLVPSEGSKTISSGNDFGWLHYTWIDIGTPSVSFLVALDTG
SNLLWIPCNCVQCAPLTSTYYSSLATKDLNEYNPSSSSTSKVFLCSHKLCDSASDCESPK
EQCPYTVNYLSGNTSSSGLLVEDILHLTYNTNNRLMNGSSSVKARVVIGCGKKQSGDYLD
GVAPDGLMGLGPAEISVPSFLSKAGLMRNSFSLCFDEEDSGRIYFGDMGPSIQQSTPFLQ
LDNNKYSGYIVGVEACCIGNSCLKQTSFTTFIDSGQSFTYLPEEIYRKVALEIDRHINAT
SKNFEGVSWEYCYESSAEPKVPAIKLKFSHNNTFVIHKPLFVFQQSQGLVQFCLPISPSG
QEGIGSIGQNYMRGYRMVFDRENMKLGWSPSKCQEDKIEPPQASPGSTSSPNPLPTDEQQ
SRGGHAVSPAIAGKTPSKTPSSSSSYSFSSIMRLFNSLLLLHWLASLM
>P87889

MGQTKSKIKSKYASYLSFIKILLKRGGVKVSTKNLIKLFQIIEQFCPWFPEQGTSDLKDW
KRIGKELKQAGRKGNIIPLTVWNDWAIIKAALEPFQTEEDSISVSDAPGSCLIDCNENTR
KKSQKETESLHCEYVAEPVMAQSTQNVDYNQLQEVIYPETLKLEGKGPELMGPSESKPRG
TSPLPAGQVLVRLQPQKQVKENKTQPQVAYQYWPLAELQYRPPPESQYGYPGMPPAPQGR
APYHQPPTRRLNPMAPPSRQGSELHEIIDKSRKEGDTEAWQFPVTLEPMPPGEGAQEGEP
PTVEARYKSFSIKMLKDMKEGVKQYGPNSPYMRTLLDSIAYGHRLIPYDWEILAKSSLSP
SQFLQFKTWWIDGVQEQVRRNRAANPPVNIDADQLLGIGQNWSTISQQALMQNEAIEQVR
AICLRAWEKIQDPGSTCPSFNTVRQGSKEPYPDFVARLQDVAQKSIADEKAGKVIVELMA
YENANPECQSAIKPLKGKVPAGSDVISEYVKACDGIGGAMHKAMLMAQAITGVVLGGQVR
TFGGKCYNCGQIGHLKKNCPVLNKQNITIQATTGREPPDLCPRCKKGKHWASQCRSKFD
KNGQPLSGNEQRGQPQAPQQTGAFPIQPFVPQGFQGQQPPLSQVFQGISQLPQYNNCPSP
QAAVQQ
>Q03445
MHSRLKFLAYLHFICASSIFWPEFSSAQQQQQTVSLTEKIPLGAIFEQGTDDVQSAFKYA
MLNHNLNVSSRRFELQAYVDVINTADAFKLSRLICNQFSRGVYSMLGAVSPDSFDTLHSY
SNTFQMPFVTPWFPEKVLAPSSGLLDFAISMRPDYHQAIIDTIQYYGWQSIIYLYDSHDG
LLRLQQIYQELKPGNETFRVQMVKRIANVTMAIEFLHTLEDLGRFSKKRIVLDCPAEMAK
EIIVQHVRDIKLGRRTYHYLLSGLVMDNHWPSDVVEFGAINITGFRIVDSNRRAVRDFHD
SRKRLEPSGQSQSQNAGGPNSLPAISAQAALMYDAVFVLVEAFNRILRKKPDQFRSNHLQ
RRSHGGSSSSSATGTNESSALLDCNTSKGWVTPWEQGEKISRVLRKVEIDGLSGEIRFDE
DGRRINYTLHVVEMSVNSTLQQVAEWRDDAGLLPLHSHNYASSSRSASASTGDYDRNHTY
IVSSLLEEPYLSLKQYTYGESLVGNDRFEGYCKDLADMLAAQLGIKYEIRLVQDGNYGAE
NQYAPGGWDGMVGELIRKEADIAISAMTITAERERVIDFSKPFMTLGISIMIKKPVKQTP
GVFSFLNPLSQEIWISVILSYVGVSFVLYFVTRFPPYEWRIVRRPQADSTAQQPPGIIGG
ATLSEPQAHVPPVPPNEFTMLNSFWYSLAAFMQQGCDITPPSIAGRIAAAVWWFFTIILI
SSYTANLAAFLTVERMVAPIKTPEDLTMQTDVNYGTLLYGSTWEFFRRSQIGLHNKMWEY
MNANQHHSVHTYDEGIRRVRQSKGKYALLVESPKNEYVNARPPCDTMKVGRNIDTKGFGV
ATPIGSPLRKRLNEAVLTLKENGELLRIRNKWWFDKTECNLDQETSTPNELSLSNVAGIY
YILIGGLLLAVIVAIMEFFCRNKTPQLKSPGSNGSAGGVPGMLASSTYQRDSLSDAIMHS
QAKLAMQASSEYDERLVGVELASNVRYQYSM
>O88628
MSSCNFTHATFMLIGIPGLEEAHFWFGFPLLSMYAVALFGNCIVVFIVRTERSLHAPMYL
FLCMLAAIDLALSTSTMPKILALFWFDSREITFDACLAQMFFIHALSAIESTILLAMAFD
RYVAICHPLRHAAVLNNTVTVQIGMVALVRGSLFFFPLPLLIKRLAFCHSNVLSHSYCVH
QDVMKLAYTDTLPNVVYGLTAILLVMGVDVMFISLSYFLIIRAVLQLPSKSERAKAFGTC
VSHIGVVLAFYVPLIGLSVVHRFGNSLDPIVHVLMGDVYLLLPPVINPIIYGAKTKQIRT
RVLAMFKISCDKDIEAGGNT
>P30975
MENRSDFEADDYGDISWSNWSNWSTPAGVLFSAMSSVLSASNHTPLPDFGQELALSTSSF
NHSQTLSTDLPAVGDVEDAAEDAAASMETGSFAFVVPWWRQVLWSILFGGMVIVATGGNL
IVVWIVMTTKRMRTVTNYFIVNLSIADAMVSSLNVTFNYYYMLDSDWPFGEFYCKLSQFI
AMLSICASVFTLMAISIDRYVAIIRPLQPRMSKRCNLAIAAVIWLASTLISCPMMIIYRT
EEVPVRGLSNRTVCYPEWPDGPTNHSTMESLYNILIIILTYFLPIVSMTVTYSRVGIELW
GSKTIGECTPRQVENVRSKRRVVKMMIVVVLIFAICWLPFHSYFIITSCYPAITEAPFIQ

ELYLAIYWLAMSNSMYNPIIYCWMNSRFRYGFKMVFRWCLFVRVGTEPFSRRENLTSRYS
CSGSPDHNRIKRNDTQKSILYTCPSSPKSHRISHSGTGRSATLRNSLPAESLSSGGSGGG
GHRKRLSYQQEMQQRWSGPNSATAVTNSSSTANTTQLLS
>Q9ET77
MSSGGRFNFDDGGSYCGGWEDGKAHGHGVCTGPKGQGEYTGSWSHGFEVLGVYTWPSGNT
YQGTWAQGKRHGIGLESKGKWVYKGEWTHGFKGRYGVRECTGNGAKYEGTWSNGLQDGYG
TETYSDGGTYQGQWVGGMRQGYGVRQSVPYGMAAVIRSPLRTSINSLRSEHTNGAALHPD
ASPAVAGSPAVSRGGFVLVAHSDSEILKSKKKGLFRRSLLSGLKLRKSESKSSLASQRSK
QSSFRSEAGMSTVSSTASDIHSTISLGEAEAELAVIEDDIDATTTETYVGEWKNDKRSGF
GVSQRSDGLKYEGEWVSNRRHGYGCMTFPDGTKEEGKYKQNVLVSGKRKNLIPLRASKIR
EKVDRAVEAAERAATIAKQKAEIAASRTSHSRAKAEAALTAAQKAQEEARIARITAKEFS
PSFQHRENGLEYQRPKHQMSCDDIEVLSTGTPLQQESPELYRKGTTPSDLTPDDSPLQSF
PASPTSTPPPAPASRTKMAHFSRQVSVDEERSGDIQMLLEGRGGDYARNSWGEEKAGASR
GIRSGALRSGQPTEDFRTRGSGHKQPGNPKPRERRTESPTTFSWTSHHRAGNPCSGGPKL
LEPDEEQLSNYKLEMKPLLRMDACPQDTHPQRRRHSRGAGGDRGFGLQRLRSKSQNKENL
RPASSAEPTVQKLESLRLGDRPEPRLLRWDLTFSPPQKSLPVALESDEETGDELKSSTGS
APILVVMVILLNIGVAILFINFFI
>Q9VN14
MLAKIGLLASILVLNLVGQITPQFSENLPDPDPQSGQQPQNYQPSYNKDYSPRYNPLYTG
QQSADPNQFDNTLVDGQSPNTYKGYYDGRAGGGGLGGNVVGPGNNLGGLGPQYDPFNRNS
IGSAGVSYRDAYTDEDNFCPEHWVSFRQTCYRFIRSPKRNWAEAKKICKAHNADLINVDN
VEKHSFILKNLILQNQRQNRFFISARQTGPLNWVNDDNTQLVQIEDSFSMDEQVPLENED
LHDNRFLVQNDLNNQNINNPNQFYNSLPGTVNQRNQNNLRGFIGPNQPYGDNRYVRDRVV
YAFSKKRDRWMFMPAYEIELNLFICESKVLYSSDNVNIKLDDKRPYHYGLDINDMERIPR
GPYFVKQPNDTTFDVNKNRLINDVTLSCLANGYPTPSYTWYREVYVDDRLEYQKIDPLAQ
DRYTISGGNLIIYEPKQALDQGAYHCVAENKFGRIRSESAHLNFGFIMEFNLKRSAETSE
MNWGKSIFCDPPQHYPDVRYYWARDYFPNFVEEDQRVFVSRDGALYFSFIETVDRANYSC
TVQTLVSDTGRNGPFFPLRVTPNSNYQALIFANTFPKVFPEAPVAGDEIRLECMAFGYPI
PSYNWTRQGLPLQRNAYTINYGRVLIIQNATTNDNGEYSCTITNPRKTLMKSIYINIQMR
PQFTIPLKDMIKDYNSDVTFICEAFAIPDANYTWYKNAERLDPANINRDRYIIQDNVLTI
KFLEKDKDDAMYQCGAQNQLKTSFSSAQLRVLSMKPSFKKHPLESEVYAVYNGNTTIVCD
PEAAPRPKFQWKKDGQVIGSGGHRRILPSGTLTISPTSRDDEGIYTCIASNQAGTDESHA
RVIVLQEIRFIETPPQRIVSKEHDLIFLHCEAAFDELLDIAYVWKHNGEVLKNNHDGTGR
IIVDWNRLTVHNTSMRDAGDYECVVKSAVNEISSKTSVSIEGAPGAPGGVQVIQISKTKA
IIEWVDGSHNGRAIRYYNILGRTNWNRTWVNVSTHVQAREVDRYTSRQQAEVVNLTPWSA
YEFSVTAVNDLGIGTPSAPSPIYSTYEDKPYIAPRNVGGGGGKIGDLTITWDPLLPQEQH
SHGIHYKVFWKLKGAIEWASDEIKKQDHMGVAVVNIPLNNYYTEYEVKVQAINSVGKGPE
SEIAVIHSAEDMPQVAPQKPIALAYNSTCFNVTWQPIDMSRENIRGKLIGHRLKYWKTTH
QEEDSVYYLSRTTRNWALIVGLQPDTYYFVKVMAYNAAGEGPESERFEERTYRKAPQKPP
SSVHVYGINPSTVRVVWRYVSPSQDEEPVEGYKVRIWESDQNMITANNTIVPIGQKLESY
INNLTPGKSYNMRVLAYSNGGDGRMSSPTLHFQMGKTTRNGANTRHGHNINTALILSTLL
LISTFLYTSQ
>P49291
MIRRGLLSVTAALVLLSVSCSAQETMGCADRTAINDFNATLYMGKWYEYAKMGSMPYEEG

GVCVTAEYSMSSNNITVVNSMKDNTTHEVNTTTGWAEFASELHTDGKLSVHFPNSPSVGN
YWILSTDYDNYSIVWSCVKRPDSAASTEISWILLRSRNSSNMTLERVEDELKNLQLDLNK
YTKTEQSAKYCAGAEHVVGAMLSVAIASLFALLH
>Q13421
MALPTARPLLGSCGTPALGSLLFLLFSLGWVQPSRTLAGETGQEAAPLDGVLANPPNISS
LSPRQLLGFPCAEVSGLSTERVRELAVALAQKNVKLSTEQLRCLAHRLSEPPEDLDALPL
DLLLFLNPDAFSGPQACTRFFSRITKANVDLLPRGAPERQRLLPAALACWGVRGSLLSEA
DVRALGGLACDLPGRFVAESAEVLLPRLVSCPGPLDQDQQEAARAALQGGGPPYGPPSTW
SVSTMDALRGLLPVLGQPIIRSIPQGIVAAWRQRSSRDPSWRQPERTILRPRFRREVEKT
ACPSGKKAREIDESLIFYKKWELEACVDAALLATQMDRVNAIPFTYEQLDVLKHKLDELY
PQGYPESVIQHLGYLFLKMSPEDIRKWNVTSLETLKALLEVNKGHEMSPQAPRRPLPQVA
TLIDRFVKGRGQLDKDTLDTLTAFYPGYLCSLSPEELSSVPPSSIWAVRPQDLDTCDPRQ
LDVLYPKARLAFQNMNGSEYFVKIQSFLGGAPTEDLKALSQQNVSMDLATFMKLRTDAVL
PLTVAEVQKLLGPHVEGLKAEERHRPVRDWILRQRQDDLDTLGLGLQGGIPNGYLVLDLS
MQEALSGTPCLLGPGPVLTVLALLLASTLA
>P35611
MNGDSRAAVVTSPPPTTAPHKERYFDRVDENNPEYLRERNMAPDLRQDFNMMEQKKRVSM
ILQSPAFCEELESMIQEQFKKGKNPTGLLALQQIADFMTTNVPNVYPAAPQGGMAALNMS
LGMVTPVNDLRGSDSIAYDKGEKLLRCKLAAFYRLADLFGWSQLIYNHITTRVNSEQEHF
LIVPFGLLYSEVTASSLVKINLQGDIVDRGSTNLGVNQAGFTLHSAIYAARPDVKCVVHI
HTPAGAAVSAMKCGLLPISPEALSLGEVAYHDYHGILVDEEEKVLIQKNLGPKSKVLILR
NHGLVSVGESVEEAFYYIHNLVVACEIQVRTLASAGGPDNLVLLNPEKYKAKSRSPGSPV
GEGTGSPPKWQIGEQEFEALMRMLDNLGYRTGYPYRYPALREKSKKYSDVEVPASVTGYS
FASDGDSGTCSPLRHSFQKQQREKTRWLNSGRGDEASEEGQNGSSPKSKTKWTKEDGHRT
STSAVPNLFVPLNTNPKEVQEMRNKIREQNLQDIKTAGPQSQVLCGVVMDRSLVQGELVT
ASKAIIEKEYQPHVIVSTTGPNPFTTLTDRELEEYRREVERKQKGSEENLDEAREQKEKS
PPDQPAVPHPPPSTPIKLEEDLVPEPTTGDDSDAATFKPTLPDLSPDEPSEALGFPMLEK
EEEAHRPPSPTEAPTEASPEPAPDPAPVAEEAAPSAVEEGAAADPGSDGSPGKSPSKKKK
KFRTPSFLKKSKKKSDS
>O88799
MALPVWTLMLLVGAAWGQEQVPAWRPNSPDLGPMVHTSREDSILSKCDFEDNSRPFCDWS
QMSADDGDWIRTTGPSLTGTSGPPGGYPNGEGYYLHMDPKTFPQGGVARLRSPDIWEQGP
LCVHFAFHMFGLSWGAQLRLLLLRGRKHLRPYVLWKHVNTQSPSWMPTTVTVPADHDIPS
WLMFEGMRGNTAYLDISLDGLSIQRGTCNQVCMSQMCTFDTLNDLCGWSWVPTATGAKWT
QKKGPTGKQGVGPAEDFSNPGNGYYMLLDSTNARPGQKAVLLSPLSHSRGCMTLSFHYIM
HGQGHEEGLFVYATFLGNIRKYTLFSGHPGPDWQAVSVNYTGQGQIQFMVVGMFGNIPEP
AIAVDAISIAPCGESFPQCDFEDRVHPFCDWNQVYGDMGHWSWGSKSVPTLIAGSPREFP
YGGEHYIFFDSVKLSQEGQSARLVSPPFCAPGGICVEFAYHMYGLGKGTTLKLLLGSPAG
SSPIPLWNRVGSQSSGWMNSSVTIPKGYQQPMQLFIEATRGTSTAFVVALNFILISHGPC
RVLLQTEIPSSPLLPPTGPSESTVPTLPMEQPTSPTKATTVTIEIPTTPTEEATIPTETT
TVPTEVINVSPKETSIPPEVTIPTEVITVSPEEIISPTEVTPVPTDVTAAYVEATNASPE
ETSVPPEVTILTEVTTVSPEETTVPTEVPIVLIEATAFPTGETTLYTEVPTVPTEVTGVH
TEVTNVSPEETSVPTEETISTEVTTVSPEETTVPTEVPIVLIEATASPTGEITLYTEVPT
VPTEVTGVHTEVTNVSPEETSVPTEETISTEVTTVSPEETTLPTEVPTVSTEVTNVSPEE

TSVPPEETILTTLYTEVPTVPTEVTGVHTEVTNVSPEETSVPTEETISTEVTTVSPEETT
LPTEVPTVSTEVTNVSPEETSVPPEETILTEITTVSPEETVFPIEGTTLPTEVLTVPIEV
TTFPTGETTVPTEVPTVSTEMTGVHTEVTTVFPEETSIPTEVATVLPASIPPEETTTPTE
VTTTPPEETTIPAEVTTVPPASIPPEETASLTEVTTTPPEETTTPTEVTTVPPEKTTIPT
EVTTVPPASIFPEETTVPPEETTIASEETTVSTQETTLLTEQSAVTQTSIACRPPCPSPP
LMPIGPLLSKPPGVSMFSLAPTTGVSTTESCPPNAHIELCACPASCESPKPSCQPPCIPG
CVCNPGFLFSNNQCINESSCNCPYNNKHYKPGEEWFTPNCTERCRCLPGSLMECQISQCG
THTVCQLKSDQYQCEPYGKATCLVYGDLHFVTFDERHIGFTGTCTYILTQTCSNSTDHFF
RITANTEERGVEGVSCLDKVVISLPETTVTMISGRHTLIGDQEVTLPAILSDDTYVGLSG
RFVELRTTFGLRVRWDGDQQLFVTVSSTFSGKLCGFCGNYDGDSSNDNLKSDGMMTHDEE
ELRLSWQVEEDEDKDWVSSRCQKKKNPPSCDAALGSTMSGPKLCGQLVNPSGPFEACLLH
LKASSFLDNCVTDMCSFQGLQQKLCARMSAMTATCQDAGYPVKPWREPQFCPLVCPKNSR
YSLCAKPCPETCHPISTTQHCSDKCVEGCECDPGFILSGSECVPSSQCGCTSFQGRYFKL
QEQWFNPDCKEICTCESHNHILCKPWKCKAQEACSYKNGVLGCHAQGAATCMVSGDPHYL
TFDGALHHFMGTCTYVLTQPCWSKSQENNFVVSATNEIHDGNLEVSYVKAVHVQVFDLKI
SMFKGQKVVLNNQRVVLPVWPSQGRVTIRLSGIFVLLYTNFGLQVRYDGRHLVEVTVPSS
YTGSLCGLCGNYNNNSMDDNLRADMKPAGNSLLLGAAWKILEASDPGCFLAGGKPSRCAD
SDMDDVWTKKCAILMNPLGPFSNCHEAVPPQASFSSCVYGQCETNGDNLTFCHSLQAYAS
LCAQAGQVTTWRNSTFCPMRCPPRSSYNPCANSCPATCLTLSTPRDCPTLPCVEGCECQS
GHILSGTTCVPLRQCGCSDQDGSYHLLGESWYTEKTCTTLCTCSAHSNITCSPTACKANH
VCLRQEGLLRCAAEMGECRISEDSQIVSFDDHSHPIQDTCTYILVKVCHPNTNMPFFMIS
AKTDINTNGKNKTFGVYQLYIDIFNFHITLQKDHLVLISLINDSIVTLPTTTHIPGVSVM
TEDVYTIVTIKDEIQVKFESNNFLDVKIPASSNGKVCGVCGNFNGEEEDELMTPSGELAE
DEQEFMNSWKDKSMDPNCQKIEGQNLQVEQQEIMNGKCRPIDFEKAQANCQTALQGPAWA
HCSSRVPIKPFLLKCMNSFCEFRELFRALCDSLQSFEDACQNQGLKPPIWRNSSFCPLEC
PAHSHYTNCLPSCPPSCLDPDSRCEGSGHKVPATCREGCICQPDYVLLNDKCVLRSHCGC
KDAQGVFIPAGKTWISEDCTQSCTCMKGSMRCWDFQCPPGTYCKNSNDGSSNCVKISLQC
PAHSKFTDCLPPCHPSCSDPDGHCEGISTNAHSNCKEGCVCQPGYVLRNDKCVLRIECGC
QHTQGGFIPAGKNWTSRGCSQSCDCMEGVIRCQNFQCPSGTYCQDIEDGTSNCANITLQC
PAHSSFTNCLPPCQPSCSDPEGHCGGSTTKAPSACQEGCVCEPDYVVLNNKCVPRIECGC
KDAQGVLIPADKIWINKGCTQTCACVTGTIHCRDFQCPSGTYCKDIKDDASNCTEIILQC
PDHSLYTHCLPSCLLSCSDPDGLCRGTSPEAPSTCKEGCVCDPDYVLSNDKCVLRIECGC
KDAQGVLIPAGKTWINRGCTQSCSCMGGAIQCQNFKCPSEAYCQDMEDGNSNCTSIPLQC
PAHSHYTNCLPTCQPSCSDPDGHCEGSSTKAPSACKEGCVCEPDYVMLNNKCVPRIECGC
KDTQGVLIPADKTWINRGCTQSCTCRGGAIQCQKYHCSSGTYCKDMEDDSSSCATITLQC
PAHSHFTNCLPPCQPSCLDSEGHCEGSTTKAPSACQEGCVCEPDYVVLNNKCVPRIECGC
KDAQGVLIPADKTWINRGCTQSCTCKGGAIQCQKFQCPSETYCKDIEDGNSNCTRISLQC
PANSNFTSCLPSCQPSCSNTDVHCEGSSPNTLSSCREGCVCQSGYVLHNDKCILRNQCGC
KDAQGALIPEGKTWITSGCTQSCNCTGGAIQCQNFQCPLKTYCKDLKDGSSNCTNIPLQC
PAHSRYTNCLPSCPPLCLDPEGLCEGTSPKVPSTCREGCICQPGYLMHKNKCVLRIFCGC
KNTQGAFISADKTWISRGCTQSCTCPAGAIHCRNFKCPSGTYCKNGDNGSSNCTEITLQC
PTNSQFTDCLPSCVPSCSNRCEVTSPSVPSSCREGCLCNHGFVFSEDKCVPRTQCGCKDA
RGAIIPAGKTWTSKGCTQSCACVEGNIQCQNFQCPPETYCKDNSEGSSTCTKITLQCPAH
TQYTSCLPSCLPSCLDPEGLCKDISPKVPSTCKEGCVCQSGYVLNSDKCVLRAECDCKDA

QGALIPAGKTWTSPGCTQSCACMGGAVQCQSSQCPPGTYCKDNEDGNSNCAKITLQCPAH
SLFTNCLPPCLPSCLDPDGLCKGASPKVPSTCKEGCICQSGYVLSNNKCLLRNRCGCKDA
HGALIPEDKTWVSRGCTQSCVCTGGSIQCLSSQCPPGAYCKDNEDGSSNCARIPPQCPAN
SHYTDCFPPCPPSCSDPEGHCEASGPRVLSTCREGCLCNPGFVLDRDKCVPRVECGCKDA
QGALIPSGKTWTSPGCTQSCACMGGVVQCQSSQCPPGTYCKDNEDGNSNCAKITLQCPTH
SNYTDCLPFCLPSCLDPSALCGGTSPKGPSTCKEGCVCQPGYVLDKDKCILKIECGCRDT
QGAVIPAGKTWLSTGCIQSCACVEGTIQCQNFQCPPGTYCNHNNNCAKIPLQCPAHSHFT
SCLPSCPPSCANLDGSCEQTSPKVPSTCKEGCLCQPGYFLNNGKCVLQTHCDCKDAEGGL
VPAGKTWTSKDCTQSCACTGGAVQCQNFQCPLGTYCKDSGDGSSNCTKIHKGAMGDGVLM
AGGIRALQCPAHSHFTSCLPSCPPSCSNLDGSCVESNFKAPSVCKKGCICQPGYLLNNDK
CVLRIQCGCKDTQGGLIPAGRTWISSDCTKSCSCMGGIIQCRDFQCPPGTYCKESNDSSR
TCAKIPLQCPAHSHYTNCLPACSRSCTDLDGHCEGTSPKVPSPCKEGCLCQPGYVVHNHK
CVLQIHCGCKDAQGGFVPAGKTWISRGCTQSCACVGGAVQCHNFTCPTGTQCQNSSCSKI
TVQCPAHSQYTTCLPSCLPSCFDPEGLCGGASPRAPSTCREGCVCEADYVLREDKCVLRT
QCGCKDAQGDLIPANKTWLTRGCAQKCTCKGGNIHCWNFKCPLGTECKDSVDGGSNCTKI
ALQCPAHSHHTYCLPSCIPSCSNVNDRCESTSLQRPSTCIEGCLCHSGFVFSKDKCVPRT
QCGCKDSQGTLIPAGKNWITTGCSQRCTCTGGLVQCHDFQCPSGAECQDIEDGNSNCVEI
TVQCPAHSHYSKCLPPCQPSCSDPDGHCEGTSPEAPSTCEEGCVCEPDYVLSNDKCVPSS
ECGCKDAHGVLIPESKTWVSRGCTKNCTCKGGTVQCHDFSCPTGSRCLDNNEGNSNCVTY
ALKCPAHSLYTNCLPSCLPSCSDPEGLCGGTSPEVPSTCKEGCICQSGYVLHKNKCMLRI
HCDCKDFQGSLIKTGQTWISSGCSKICTCKGGFFQCQSYKCPSGTQCEESEDGSSNCVSS
TMKCPANSLYTHCLPTCLPSCSNPDGRCEGTSHKAPSTCREGCVCQPGYLLNKDTCVHKN
QCGCKDIRGNIIPAGNTWISSDCTQSCACTDGVIQCQNFVCPSGSHCQYNEDGSSDCAAN
KLERCTIFGDPYYLTFDGFTYHFLGRMNYYLIKTVDKLPRGIEPLIMEGRNKISPKGSST
LHEVTTIVYGYKIQLQEELVVLVNDEKVAVPYNPNEHLRVMLRAQRLLLVTDFEMVLDFD
GKHSAVISLPTTYRGLTRGL
>Q8NAC3
MPVPWFLLSLALGRSPVVLSLERLVGPQDATHCSPVSLEPWGDEERLRVQFLAQQSLSLA
PVTAATARTALSGLSGADGRREERGRGKSWVCLSLGGSGNTEPQKKGLSCRLWDSDILCL
PGDIVPAPGPVLAPTHLQTELVLRCQKETDCDLCLRVAVHLAVHGHWEEPEDEEKFGGAA
DLGVEEPRNASLQAQVVLSFQAYPTARCVLLEVQVPAALVQFGQSVGSVVYDCFEAALGS
EVRIWSYTQPRYEKELNHTQQLPDCRGLEVWNSIPSCWALPWLNVSADGDNVHLVLNVSE
EQHFGLSLYWNQVQGPPKPRWHKNLTGPQIITLNHTDLVPCLCIQVWPLEPDSVRTNICP
FREDPRAHQNLWQAARLRLLTLQSWLLDAPCSLPAEAALCWRAPGGDPCQPLVPPLSWEN
VTVDKVLEFPLLKGHPNLCVQVNSSEKLQLQECLWADSLGPLKDDVLLLETRGPQDNRSL
CALEPSGCTSLPSKASTRAARLGEYLLQDLQSGQCLQLWDDDLGALWACPMDKYIHKRWA
LVWLACLLFAAALSLILLLKKDHAKGWLRLLKQDVRSGAAARGRAALLLYSADDSGFERL
VGALASALCQLPLRVAVDLWSRRELSAQGPVAWFHAQRRQTLQEGGVVVLLFSPGAVALC
SEWLQDGVSGPGAHGPHDAFRASLSCVLPDFLQGRAPGSYVGACFDRLLHPDAVPALFRT
VPVFTLPSQLPDFLGALQQPRAPRSGRLQERAEQVSRALQPALDSYFHPPGTPAPGRGVG
PGAGPGAGDGT
>P13231
MGNRAFKSHHGHFLSAEGEAVKTHHGHHDHHTHFHVENHGGKVALKTHCGKYLSIGDHKQ
VYLSHHLHGDHSLFHLEHHGGKVSIKGHHHHYISADHHGHVSTKEHHDHDTTFEEIII

>Q8TDU6
MTPNSTGEVPSPIPKGALGLSLALASLIITANLLLALGIAWDRRLRSPPAGCFFLSLLLA
GLLTGLALPTLPGLWNQSRRGYWSCLLVYLAPNFSFLSLLANLLLVHGERYMAVLRPLQP
PGSIRLALLLTWAGPLLFASLPALGWNHWTPGANCSSQAIFPAPYLYLEVYGLLLPAVGA
AAFLSVRVLATAHRQLQDICRLERAVCRDEPSALARALTWRQARAQAGAMLLFGLCWGPY
VATLLLSVLAYEQRPPLGPGTLLSLLSLGSASAAAVPVAMGLGDQRYTAPWRAAAQRCLQ
GLWGRASRDSPGPSIAYHPSSQSSVDLDLN
>P54660
MLVLRNLLALVTLALLFTLSSAQYTLSVSNSASGSKCTTAVSAKLNACNTGCLNSFNIVE
SSNGKGLVFKTFINAACSGEYESLSQFTCAANQKIPTTSYIVSCNSTPSSNSTTDSDSSS
GSTVMIGLASSLLFAFATLLALF
>P51653
MSAVRPLLLLLLPLCPGPGPGHGSEAKVVRSCAETRQVLGARGYSLNLIPPSLISGEHLQ
ICPQEYTCCSSETEQKLIRDAEVTFRGLVEDSGSFLIHTLAARHRKFNEFFREMLSISQH
SLAQLFSHSYGRLYSQHAVIFNSLFSGLRDYYEKSGEGLDDTLADFWAQLLERAFPLLHP
QYSFPPDFLLCLTRLTSTADGSLQPFGDSPRRLRLQITRALVAARALVQGLETGRNVVSE
ALKVPMLEGCRQALMRLIGCPLCRGVPSLMPCRGFCLNVAHGCLSSRGLEPEWGGYLDGL
LLLAEKLQGPFSFELAAESIGVKISEGLMHLQENSVKVSAKVFQECGTPHPVQSRNRRAP
APREETSRSWRSSAEEERPTTAAGTNLHRLVWELRERLSRVRGFWAGLPVTVCGDSRMAA
DLSQEAAPCWTGVGRGRYMSPVVVGSLNEQLHNPELDTSSPDVPTRRRRLHLRAATARMK
AAALGQDLDMHDADEDASGSGGGQQYADDWKAGAAPVVPPARPPRPPRPPRRDGLGVRGG
SGSARYNQGRSRNLGSSVGLHAPRVFILLPSALTLLGLR
>P07207
MQSQRSRRRSRAPNTWICFWINKMHAVASLPASLPLLLLTLAFANLPNTVRGTDTALVAA
SCTSVGCQNGGTCVTQLNGKTYCACDSHYVGDYCEHRNPCNSMRCQNGGTCQVTFRNGRP
GISCKCPLGFDESLCEIAVPNACDHVTCLNGGTCQLKTLEEYTCACANGYTGERCETKNL
CASSPCRNGATCTALAGSSSFTCSCPPGFTGDTCSYDIEECQSNPCKYGGTCVNTHGSYQ
CMCPTGYTGKDCDTKYKPCSPSPCQNGGICRSNGLSYECKCPKGFEGKNCEQNYDDCLGH
LCQNGGTCIDGISDYTCRCPPNFTGRFCQDDVDECAQRDHPVCQNGATCTNTHGSYSCIC
VNGWAGLDCSNNTDDCKQAACFYGATCIDGVGSFYCQCTKGKTGLLCHLDDACTSNPCHA
DAICDTSPINGSYACSCATGYKGVDCSEDIDECDQGSPCEHNGICVNTPGSYRCNCSQGF
TGPRCETNINECESHPCQNEGSCLDDPGTFRCVCMPGFTGTQCEIDIDECQSNPCLNDGT
CHDKINGFKCSCALGFTGARCQINIDDCQSQPCRNRGICHDSIAGYSCECPPGYTGTSCE
ININDCDSNPCHRGKCIDDVNSFKCLCDPGYTGYICQKQINECESNPCQFDGHCQDRVGS
YYCQCQAGTSGKNCEVNVNECHSNPCNNGATCIDGINSYKCQCVPGFTGQHCEKNVDECI
SSPCANNGVCIDQVNGYKCECPRGFYDAHCLSDVDECASNPCVNEGRCEDGINEFICHCP
PGYTGKRCELDIDECSSNPCQHGGTCYDKLNAFSCQCMPGYTGQKCETNIDDCVTNPCGN
GGTCIDKVNGYKCVCKVPFTGRDCESKMDPCASNRCKNEAKCTPSSNFLDFSCTCKLGYT
GRYCDEDIDECSLSSPCRNGASCLNVPGSYRCLCTKGYEGRDCAINTDDCASFPCQNGGT
CLDGIGDYSCLCVDGFDGKHCETDINECLSQPCQNGATCSQYVNSYTCTCPLGFSGINCQ
TNDEDCTESSCLNGGSCIDGINGYNCSCLAGYSGANCQYKLNKCDSNPCLNGATCHEQNN
EYTCHCPSGFTGKQCSEYVDWCGQSPCENGATCSQMKHQFSCKCSAGWTGKLCDVQTISC
QDAADRKGLSLRQLCNNGTCKDYGNSHVCYCSQGYAGSYCQKEIDECQSQPCQNGGTCRD
LIGAYECQCRQGFQGQNCELNIDDCAPNPCQNGGTCHDRVMNFSCSCPPGTMGIICEINK

DDCKPGACHNNGSCIDRVGGFECVCQPGFVGARCEGDINECLSNPCSNAGTLDCVQLVNN
YHCNCRPGHMGRHCEHKVDFCAQSPCQNGGNCNIRQSGHHCICNNGFYGKNCELSGQDCD
SNPCRVGNCVVADEGFGYRCECPRGTLGEHCEIDTLDECSPNPCAQGAACEDLLGDYECL
CPSKWKGKRCDIYDANYPGWNGGSGSGNDRYAADLEQQRAMCDKRGCTEKQGNGICDSDC
NTYACNFDGNDCSLGINPWANCTANECWNKFKNGKCNEECNNAACHYDGHDCERKLKSCD
SLFDAYCQKHYGDGFCDYGCNNAECSWDGLDCENKTQSPVLAEGAMSVVMLMNVEAFREI
QAQFLRNMSHMLRTTVRLKKDALGHDIIINWKDNVRVPEIEDTDFARKNKILYTQQVHQT
GIQIYLEIDNRKCTECFTHAVEAAEFLAATAAKHQLRNDFQIHSVRGIKNPGDEDNGEPP
ANVKYVITGIILVIIALAFFGMVLSTQRKRAHGVTWFPEGFRAPAAVMSRRRRDPHGQEM
RNLNKQVAMQSQGVGQPGAHWSDDESDMPLPKRQRSDPVSGVGLGNNGGYASDHTMVSEY
EEADQRVWSQAHLDVVDVRAIMTPPAHQDGGKHDVDARGPCGLTPLMIAAVRGGGLDTGE
DIENNEDSTAQVISDLLAQGAELNATMDKTGETSLHLAARFARADAAKRLLDAGADANCQ
DNTGRTPLHAAVAADAMGVFQILLRNRATNLNARMHDGTTPLILAARLAIEGMVEDLITA
DADINAADNSGKTALHWAAAVNNTEAVNILLMHHANRDAQDDKDETPLFLAAREGSYEAC
KALLDNFANREITDHMDRLPRDVASERLHHDIVRLLDEHVPRSPQMLSMTPQAMIGSPPP
GQQQPQLITQPTVISAGNGGNNGNGNASGKQSNQTAKQKAAKKAKLIEGSPDNGLDATGS
LRRKASSKKTSAASKKAANLNGLNPGQLTGGVSGVPGVPPTNSAAQAAAAAAAAVAAMSH
ELEGSPVGVGMGGNLPSPYDTSSMYSNAMAAPLANGNPNTGAKQPPSYEDCIKNAQSMQS
LQGNGLDMIKLDNYAYSMGSPFQQELLNGQGLGMNGNGQRNGVGPGVLPGGLCGMGGLSG
AGNGNSHEQGLSPPYSNQSPPHSVQSSLALSPHAYLGSPSPAKSRPSLPTSPTHIQAMRH
ATQQKQFGGSNLNSLLGGANGGGVVGGGGGGGGGVGQGPQNSPVSLGIISPTGSDMGIML
APPQSSKNSAIMQTISPQQQQQQQQQQQQQHQQQQQQQQQQQQQQQQQLGGLEFGSAGLD
LNGFCGSPDSFHSGQMNPPSIQSSMSGSSPSTNMLSPSSQHNQQAFYQYLTPSSQHSGGH
TPQHLVQTLDSYPTPSPESPGHWSSSSPRSNSDWSEGVQSPAANNLYISGGHQANKGSEA
IYI
>P40189
MLTLQTWVVQALFIFLTTESTGELLDPCGYISPESPVVQLHSNFTAVCVLKEKCMDYFHV
NANYIVWKTNHFTIPKEQYTIINRTASSVTFTDIASLNIQLTCNILTFGQLEQNVYGITI
ISGLPPEKPKNLSCIVNEGKKMRCEWDGGRETHLETNFTLKSEWATHKFADCKAKRDTPT
SCTVDYSTVYFVNIEVWVEAENALGKVTSDHINFDPVYKVKPNPPHNLSVINSEELSSIL
KLTWTNPSIKSVIILKYNIQYRTKDASTWSQIPPEDTASTRSSFTVQDLKPFTEYVFRIR
CMKEDGKGYWSDWSEEASGITYEDRPSKAPSFWYKIDPSHTQGYRTVQLVWKTLPPFEAN
GKILDYEVTLTRWKSHLQNYTVNATKLTVNLTNDRYLATLTVRNLVGKSDAAVLTIPACD
FQATHPVMDLKAFPKDNMLWVEWTTPRESVKKYILEWCVLSDKAPCITDWQQEDGTVHRT
YLRGNLAESKCYLITVTPVYADGPGSPESIKAYLKQAPPSKGPTVRTKKVGKNEAVLEWD
QLPVDVQNGFIRNYTIFYRTIIGNETAVNVDSSHTEYTLSSLTSDTLYMVRMAAYTDEGG
KDGPEFTFTTPKFAQGEIEAIVVPVCLAFLLTTLLGVLFCFNKRDLIKKHIWPNVPDPSK
SHIAQWSPHTPPRHNFNSKDQMYSDGNFTDVSVVEIEANDKKPFPEDLKSLDLFKKEKIN
TEGHSSGIGGSSCMSSSRPSISSSDENESSQNTSSTVQYSTVVHSGYRHQVPSVQVFSRS
ESTQPLLDSEERPEDLQLVDHVDGGDGILPRQQYFKQNCSQHESSPDISHFERSKQVSSV
NEEDFVRLKQQISDHISQSCGSGQMKMFQEVSAADAFGPGTEGQVERFETVGMEAATDEG
MPKSYLPQTVRQGGYMPQ
>O43493
MRFVVALVLLNVAAAGAVPLLATESVKQEEAGVRPSAGNVSTHPSLSQRPGGSTKSHPEP

QTPKDSPSKSSAEAQTPEDTPNKSGAEAKTQKDSSNKSGAEAKTQKGSTSKSGSEAQTTK
DSTSKSHPELQTPKDSTGKSGAEAQTPEDSPNRSGAEAKTQKDSPSKSGSEAQTTKDVPN
KSGADGQTPKDGSSKSGAEDQTPKDVPNKSGAEKQTPKDGSNKSGAEEQGPIDGPSKSGA
EEQTSKDSPNKVVPEQPSRKDHSKPISNPSDNKELPKADTNQLADKGKLSPHAFKTESGE
ETDLISPPQEEVKSSEPTEDVEPKEAEDDDTGPEEGSPPKEEKEKMSGSASSENREGTLS
DSTGSEKDDLYPNGSGNGSAESSHFFAYLVTAAILVAVLYIAHHNKRKIIAFVLEGKRSK
VTRRPKASDYQRLDQKYVLILNVFPAPPKRSFLPQVLTEWYIPLEKDERHQWIVLLSFQL

>Q8NFY4
MRVFLLCAYILLLMVSQLRAVSFPEDDEPLNTVDYHYSRQYPVFRGRPSGNESQHRLDFQ
LMLKIRDTLYIAGRDQVYTVNLNEMPKTEVIPNKKLTWRSRQQDRENCAMKGKHKDECHN
FIKVFVPRNDEMVFVCGTNAFNPMCRYYRLSTLEYDGEEISGLARCPFDARQTNVALFAD
GKLYSATVADFLASDAVIYRSMGDGSALRTIKYDSKWIKEPHFLHAIEYGNYVYFFFREI
AVEHNNLGKAVYSRVARICKNDMGGSQRVLEKHWTSFLKARLNCSVPGDSFFYFDVLQSI
TDIIQINGIPTVVGVFTTQLNSIPGSAVCAFSMDDIEKVFKGRFKEQKTPDSVWTAVPED
KVPKPRPGCCAKHGLAEAYKTSIDFPDETLSFIKSHPLMDSAVPPIADEPWFTKTRVRYR
LTAISVDHSAGPYQNYTVIFVGSEAGMVLKVLAKTSPFSLNDSVLLEEIEAYNHAKCSAE
NEEDKKVISLQLDKDHHALYVAFSSCIIRIPLSRCERYGSCKKSCIASRDPYCGWLSQGS
CGRVTPGMLAEGYEQDTEFGNTAHLGDCHEILPTSTTPDYKIFGGPTSDMEVSSSSVTTM
ASIPEITPKVIDTWRPKLTSSRKFVVQDDPNTSDFTDPLSGIPKGVRWEVQSGESNQMVH
MNVLITCVFAAFVLGAFIAGVAVYCYRDMFVRKNRKIHKDAESAQSCTDSSGSFAKLNGL
FDSPVKEYQQNIDSPKLYSNLLTSRKELPPNGDTKSMVMDHRGQPPELAALPTPESTPVL
HQKTLQAMKSHSEKAHGHGASRKETPQFFPSSPPPHSPLSHGHIPSAIVLPNATHDYNTS
FSNSNAHKAEKKLQNIDHPLTKSSSKRDHRRSVDSRNTLNDLLKHLNDPNSNPKAIMGDI
QMAHQNLMLDPMGSMSEVPPKVPNREASLYSPPSTLPRNSPTKRVDVPTTPGVPMTSLER
QRGYHKNSSQRHSISAMPKNLNSPNGVLLSRQPSMNRGGYMPTPTGAKVDYIQGTPVSVH
LQPSLSRQSSYTSNGTLPRTGLKRTPSLKPDVPPKPSFVPQTPSVRPLNKYTY
>Q9LQU4
MEAQHLHAKPHAEGEWSTGFCDCFSDCKNCCITFWCPCITFGQVAEIVDRGSTSCGTAGA
LYALIAVVTGCACIYSCFYRGKMRAQYNIKGDDCTDCLKHFCCELCSLTQQYRELKHRGY
DMSLGWAGNVERQQNQGGVAMGAPVFQGGMTR
>Q9SD53
MADKTDIISSSSDKASPPPPSAFRNYLSSGSKEPVLLLESAGKESCCIFRVPESFVALNP
KAYKPKVVSIGPYHYGEKHLQMIQQHKPRLLQLFLDEAKKKDVEENVLVKAVVDLEDKIR
KSYSEELKTGHDLMFMMVLDGCFILMVFLIMSGNIELSEDPIFSIPWLLSSIQSDLLLLE
NQVPFFVLQTLYVGSKIGVSSDLNRIAFHFFKNPIDKEGSYWEKHRNYKAKHLLDLIRET
FLPNTSESDKASSPHVQVQLHEGKSGNVPSVDSKAVPLILSAKRLRLQGIKFRLRRSKED
SILNVRLKKNKLQIPQLRFDGFISSFFLNCVAFEQFYTDSSNEITTYIVFMGCLLNNEED
VTFLRNDKLIIENHFGSNNEVSEFFKTISKDVVFEVDTSYLNNVFKGVNEYTKKWYNGLW
AGFRHTHFESPWTFLSSCAVLFVILLTMLQSTVAILSYLNDKKGNGNAAPPPLGLP
>Q92597
MSREMQDVDLAEVKPLVEKGETITGLLQEFDVQEQDIETLHGSVHVTLCGTPKGNRPVIL
TYHDIGMNHKTCYNPLFNYEDMQEITQHFAVCHVDAPGQQDGAASFPAGYMYPSMDQLAE
MLPGVLQQFGLKSIIGMGTGAGAYILTRFALNNPEMVEGLVLINVNPCAEGWMDWAASKI

SGWTQALPDMVVSHLFGKEEMQSNVEVVHTYRQHIVNDMNPGNLHLFINAYNSRRDLEIE
RPMPGTHTVTLQCPALLVVGDSSPAVDAVVECNSKLDPTKTTLLKMADCGGLPQISQPAK
LAEAFKYFVQGMGYMPSASMTRLMRSRTASGSSVTSLDGTRSRSHTSEGTRSRSHTSEGT
RSRSHTSEGAHLDITPNSGAAGNSAGPKSMEVSC
>Q9QZE9
MTAWILLPVSLSAFSITGIWTVYAMAVMNRHVCPVENWSYNDSCSPDPAEQGGPKTCCTL
DDVPLISKCGTYPPESCLFSLIGNMGAFMVALICLLRYGQLLEQNRHSWINTTALITGCT
NAAGLVVVGNFQVDHAKSLHYIGAGVAFPAGLLFVCLHCVLFYHGATTPLDMAMAYLRSV
LAVIAFVTLVLSGVFFLHESSELQHGAALCEWVFVLDILIFYGTFSYEFGAVSSDTLVAA
LQPAPGRACKSSGSSSTSTHLNCAPESIAMI
>Q8NGU9
MEDLFSPSILPPAPNISVPILLGWGLNLTLGQGAPASGPPSRRVRLVFLGVILVVAVAGN
TTVLCRLCGGGGPWAGPKRRKMDFLLVQLALADLYACGGTALSQLAWELLGEPRAATGDL
ACRFLQLLQASGRGASAHLVVLIALERRRAVRLPHGRPLPARALAALGWLLALLLALPPA
FVVRGDSPSPLPPPPPPTSLQPGAPPAARAWPGERRCHGIFAPLPRWHLQVYAFYEAVAG
FVAPVTVLGVACGHLLSVWWRHRPQAPAAAAPWSASPGRAPAPSALPRAKVQSLKMSLLL
ALLFVGCELPYFAARLAAAWSSGPAGDWEGEGLSAALRVVAMANSALNPFVYLFFQAGDC
RLRRQLRKRLGSLCCAPQGGAEDEEGPRGHQALYRQRWPHPHYHHARREPLDEGGLRPPP
PRPRPLPCSCESAF
>Q9N2G5
MAETAEPEGGAPSPQGPPEGSALLEERPGEPDPAGPEASEGAAKAPSGEGAGAAAKAGAT
EEASGGRDGEGAGEQAPDAGTESGGETPDAKGAQIEAEGAPEGTKAPQLGEEGSGGKQVE
ESGPDCELRGEAAREAEGQAAAPAAPGAQEEAVPGDSVDAEGSIDAGGSVDAAGSVDAGG
SIDAGGSMDAGGSVDAGGSIDTGGSVDAAGSVDAGGSIDTGRNVDAGGSIDAGGSVDAGG
SMDAEGPAGGAHGAGGEPQDLGAGSPQPRSEAVEVAAAENEGHSPGESVEDAAAEEAAGT
REPEGSEDAAGEDGDQGRPQEETEQQAERQEPGPETQSEEEERPPDRSPDGEAAASTRAA
QPEAELSNHLAAEEGGQRGEGPANGRGEDGEASEEGDPGQEHDITLFVKAGYDGESIGNC
PFSQRLFMILWLKGVIFNVTTVDLKRKPADLQNLAPGTNPPFMTFDGDVKTDVNKIEEFL
EEKLAPPRYPKLATQHPESNSAGNDVFAKFSAFIKNTKKDANEIYEKSLLKALKKLDAYL
NSPLPDEVDAYSTEDVAVSGRKFLDGDDLTLADCNLLPKLHIIKIVAKKYRDFEFPPEMT
GIWRYLNNAYARDEFINTCPADQEIEHAYSDVAKRMK
>Q8IZF6
MDDNSRYWMAFSYITNNALLGREDIDLGLAGDHQQLILYRLGKTFSIRHHLASFQWHTIC
LIWDGVKGKLELFLNKERILEVTDQPHNLTPHGTLFLGHFLKNESSEVKSMMRSFPGSLY
YFQLWDHILENEEFMKCLDGNIVSWEEDVWLVNKIIPTVDRTLRCVPENMTIQEKSTTVS
QQIDMTTPSQITGVKPQNTAHSSTLLSQSIPIFATDYTTISYSNTTSPPLETMTAQKILK
TLVDETATFAVDVLSTSSAISLPTQSISIDNTTNSMKKTKSPSSESTKTTKMVEAMATEI
FQPPTPSNFLSTSRFTKNSVVSTTSAIKSQSAVTKTTSLFSTIESTSMSTTPCLKQKSTN
TGALPISTAGQEFIESTAAGTVPWFTVEKTSPASTHVGTASSFPPEPVLISTAAPVDSVF
PRNQTAFPLATTDMKIAFTVHSLTLPTRLIETTPAPRTAETELTSTNFQDVSLPRVEDAM
STSMSKETSSKTFSFLTSFSFTGTESVQTVIDAEATRTALTPEITLASTVAETMLSSTIT
GRVYTQNTPTADGHLLTLMSTRSASTSKAPESGPTSTTDEAAHLFSSNETIWTSRPDQAL
LASMNTTTILTFVPNENFTSAFHENTTYTEYLSATTNITPLKASPEGKGTTANDATTARY
TTAVSKLTSPWFANFSIVSGTTSITNMPEFKLTTLLLKTIPMSTKPANELPLTPRETVVP

SVDIISTLACIQPNFSTEESASETTQTEINGAIVFGGTTTPVPKSATTQRLNATVTRKEA
TSHYLMRKSTIAAVAEVSPFSTMLEVTDESAQRVTASVTVSSFPDIEKLSTPLDNKTATT
EVRESWLLTKLVKTTPRSSYNEMTEMFNFNHTYVAHWTSETSEGISAGSPTSGSTHIFGE
PLGASTTRISETSFSTTPTDRTATSLSDGILPPQPTAAHSSATPVPVTHMFSLPVNGSSV
VAEETEVTMSEPSTLARAFSTSVLSDVSNLSSTTMTTALVPPLDQTASTTIVIVPTHGDL
IRTTSEATVISVRKTSMAVPSLTETPFHSLRLSTPVTAKAETTLFSTSVDTVTPSTHTLV
CSKPPPDNIPPASSTHVISTTSTPEATQPISQVEETSTYALSFPYTFSGGGVVASLATGT
TETSVVDETTPSHISANKLTTSVNSHISSSATYRVHTPVSIQLVTSTSVLSSDKDQMTIS
LGKTPRTMEVTEMSPSKNSFISYSRGTPSLEMTDTGFPETTKISSHQTHSPSEIPLGTPS
DGNLASSPTSGSTQITPTLTSSNTVGVHIPEMSTSLGKTALPSQALTITTFLCPEKESTS
ALPAYTPRTVEMIVNSTYVTHSVSYGQDTSFVDTTTSSSTRISNPMDINTTFSHLHSLRT
QPEVTSVASFISESTQTFPESLSLSTAGLYNDGFTVLSDRITTAFSVPNVPTMLPRESSM
ATSTPIYQMSSLPVNVTAFTSKKVSDTPPIVITKSSKTMHPGCLKSPCTATSGPMSEMSS
IPVNNSAFTPATVSSDTSTRVGLFSTLLSSVTPRTTMTMQTSTLDVTPVIYAGATSKNKM
VSSAFTTEMIEAPSRITPTTFLSPTEPTLPFVKTVPTTIMAGIVTPFVGTTAFSPLSSKS
TGAISSIPKTTFSPFLSATQQSSQADEATTLGILSGITNRSLSTVNSGTGVALTDTYSRI
TVPENMLSPTHADSLHTSFNIQVSPSLTSFKSASGPTKNVKTTTNCFSSNTRKMTSLLEK
TSLTNYATSLNTPVSYPPWTPSSATLPSLTSFVYSPHSTEAEISTPKTSPPPTSQMVEFP
VLGTRMTSSNTQPLLMTSWNIPTAEGSQFPISTTINVPTSNEMETETLHLVPGPLSTFTA
SQTGLVSKDVMAMSSIPMSGILPNHGLSENPSLSTSLRAITSTLADVKHTFEKMTTSVTP
GTTLPSILSGATSGSVISKSPILTWLLSSLPSGSPPATVSNAPHVMTSSTVEVSKSTFLT
SDMISAHPFTNLTTLPSATMSTILTRTIPTPTLGGITTGFPTSLPMSINVTDDIVYISTH
PEASSRTTITANPRTVSHPSSFSRKTMSPSTTDHTLSVGAMPLPSSTITSSWNRIPTASS
PSTLIIPKPTLDSLLNIMTTTSTVPGASFPLISTGVTYPFTATVSSPISSFFETTWLDST
PSFLSTEASTSPTATKSTEPGNCKADETASKYKGTYKWLLTNPTETAQTRCIKNEDGNAT
RFSISINTGKSQWEKPKFKQCKLLQELPDKIVDLANITISDENAEDVAEHILNLINESPA
LGKEETKIIVSKISDISQCDEISMNLTHVMLQIINVVLEKQNNSASDLHEISNEILRIIE
RTGHKMEFSGQIANLTVAGLALAVLRGDHTFDGMAFSIHSYEEGTDPETKNVTKALTTYV
VSASISDDMFIQNLADPVVITLQHIGGNQNYGQVHCAFWDFENNGLGGWNSSGCKVKETN
VNYTICQCDHLTHFGVLMDLSRSTVDSVNEQILALITYTGCGISSIFLGVAVVTYIAFKL
RKDYPAKILINLCTALLMLNLVFLINSWLSSFQKVGVCITAAVALHYFLLVSFTWMGLEA
VHMYLALVKVFNIYIPNYILKFCLVGWGIPAIMVAITVSVKKDLYGTLSPTTPCWIKDDS
IFYISVVAYFCLIFLMNLSMFCTVLVQLNSVKSQIQKTRRKMILHDLKGTMSLTFLLGLT
WGFAFFAWGPMRNFFLYLFAIFNTLQDGSSRCQIKVGYKQEGLKKIFEHKLLTPSLKSTA
TSSTFKSLGSAQGTPSEISFPNAPELSALHVVASEPTTG
>P31414
MVAPALLPELWTEILVPICAVIGIAFSLFQWYVVSRVKLTSDLGASSSGGANNGKNGYGD
YLIEEEEGVNDQSVVAKCAEIQTAISEGATSFLFTEYKYVGVFMIFFAAVIFVFLGSVEG
FSTDNKPCTYDTTRTCKPALATAAFSTIAFVLGAVTSVLSGFLGMKIATYANARTTLEAR
KGVGKAFIVAFRSGAVMGFLLAASGLLVLYITINVFKIYYGDDWEGLFEAITGYGLGGSS
MALFGRVGGGIYTKAADVGADLVGKIERNIPEDDPRNPAVIADNVGDNVGDIAGMGSDLF
GSYAEASCAALVVASISSFGINHDFTAMCYPLLISSMGILVCLITTLFATDFFEIKLVKE
IEPALKNQLIISTVIMTVGIAIVSWVGLPTSFTIFNFGTQKVVKNWQLFLCVCVGLWAGL
IIGFVTEYYTSNAYSPVQDVADSCRTGAATNVIFGLALGYKSVIIPIFAIAISIFVSFSF

AAMYGVAVAALGMLSTIATGLAIDAYGPISDNAGGIAEMAGMSHRIRERTDALDAAGNTT
AAIGKGFAIGSAALVSLALFGAFVSRAGIHTVDVLTPKVIIGLLVGAMLPYWFSAMTMKS
VGSAALKMVEEVRRQFNTIPGLMEGTAKPDYATCVKISTDASIKEMIPPGCLVMLTPLIV
GFFFGVETLSGVLAGSLVSGVQIAISASNTGGAWDNAKKYIEAGVSEHAKSLGPKGSEPH
KAAVIGDTIGDPLKDTSGPSLNILIKLMAVESLVFAPFFATHGGILFKYF
>P43630
MSLTVVSMACVGFFLLQGAWPLMGGQDKPFLSARPSTVVPRGGHVALQCHYRRGFNNFML
YKEDRSHVPIFHGRIFQESFIMGPVTPAHAGTYRCRGSRPHSLTGWSAPSNPLVIMVTGN
HRKPSLLAHPGPLLKSGETVILQCWSDVMFEHFFLHREGISEDPSRLVGQIHDGVSKANF
SIGPLMPVLAGTYRCYGSVPHSPYQLSAPSDPLDIVITGLYEKPSLSAQPGPTVQAGENV
TLSCSSWSSYDIYHLSREGEAHERRLRAVPKVNRTFQADFPLGPATHGGTYRCFGSFRAL
PCVWSNSSDPLLVSVTGNPSSSWPSPTEPSSKSGICRHLHVLIGTSVVIFLFILLLFFLL
YRWCSNKKNAAVMDQEPAGDRTVNRQDSDEQDPQEVTYAQLDHCVFIQRKISRPSQRPKT
PLTDTSVYTELPNAEPRSKVVSCPRAPQSGLEGVF
>Q9JM59
MRGQGRKESLSESRDLDGSYDQLTGHPPGPSKKALKQRFLKLLPCCGPQALPSVSETLAA
PASLRPHRPRPLDPDSVEDEFELSTVCHRPEGLEQLQEQTKFTRRELQVLYRGFKNECPS
GIVNEENFKQIYSQFFPQGDSSNYATFLFNAFDTNHDGSVSFEDFVAGLSVILRGTIDDR
LSWAFNLYDLNKDGCITKEEMLDIMKSIYDMMGKYTYPALREEAPREHVESFFQKMDRNK
DGVVTIEEFIESCQQDENIMRSMQLFDNVI
>Q9VZW5
MSGTAVARLLLRLELPSPGVMPPPPTDYDYGGPISDDEFLASAMATEGPTVRYDLFPQNN
SQPTLQIVLNHTEVQTDLQYPHYEDLGLDPDPNWTRICEDVYNPLLENNRIEFWVCGVLI
NIVGVLGILGNIISMIILSRPQMRSSINYLLTGLARCDTVLIITSILLFGIPSIYPYTGH
FFGYYNYVYPFISPAVFPIGMIAQTASIYMTFTVTLERYVAVCHPLKARALCTYGRAKIY
FIVCVCFSLAYNMPRFWEVLTVTYPEPGKDVILHCVRPSRLRRSETYINIYIHWCYLIVN
YIIPFLTLAILNCLIYRQVKRANRERQRLSRSEKREIGLATMLLCVVIVFFMLNFLPLVL
NISEAFYSTIDHKITKISNLLITINSSVNFLIYIIFGEKFKRIFLLIFFKRRLSRDQPDL
IHYESSISNNGDGTLNHRSSGRFSRHGTQRSTTTTYLVATGGPGGGGCGGGGGNNSLNNV
RLTQVSGSPGLVKIKRNRAPSPGPVVYFPAREMQRSASTTNSTTNNNTSIGYDWTLPDSK
KLGHVSSGF
>P38856
MTTYFKLVKGATKIKSAPPKQKYLDPILLGTSNEEDFYEIVKGLDSRINDTAWTIVYKSL
LVVHLMIREGSKDVALRYYSRNLEFFDIENIRGSNGSASGDMRALDRYDNYLKVRCREFG
KIKKDYVRDGYRTLKLNSGNYGSSRNKQHSINIALDHVESLEVQIQALIKNKYTQYDLSN
ELIIFGFKLLIQDLLALYNALNEGIITLLESFFELSHHNAERTLDLYKTFVDLTEHVVRY
LKSGKTAGLKIPVIKHITTKLVRSLEEHLIEDDKTHNTFVPVDSSQGSAGAVVAKSTAQE
RLEQIREQKRILEAQLKNEQVAISPALTTVTAAQSYNPFGTDSSMHTNIPMAVANQTQQI
ANNPFVSQTQPQVMNTPTAHTEPANLNVPEYAAVQHTVNFNPVQDAGVSAQQTGYYSINN
HLTPTFTGAGFGGYSVSQDTTAASNQQVSHSQTGSNNPFALHNAATIATGNPAHENVLNN
PFSRPNFDEQNTNMPLQQQIISNPFQNQTYNQQQFQQQKMPLSSINSVMTTPTSMQGSMN
IPQRFDKMEFQAHYTQNHLQQQQQQQQQQQQQQQQPQQGYYVPATAGANPVTNITGTVQ
PQNFPFYPQQQPQPEQSQTQQPVLGNQYANNLNLIDM
>O43653

MKAVLLALLMAGLALQPGTALLCYSCKAQVSNEDCLQVENCTQLGEQCWTARIRAVGLLT
VISKGCSLNCVDDSQDYYVGKKNITCCDTDLCNASGAHALQPAAAILALLPALGLLLWGP
GQL
>Q19705
MGFLKILRKQRAREREMRILILGLDNAGKTTLMKKFLDEPTDTIEPTLGFDIKTVHFKDF
QLNLWDVGGQKSLRSYWKNYFESTDALIWVVDSSDRERLLQCSEELKKLLGEERLAGASL
LVLANKSDLPGAIDVNSIAQVLDLHSIKSHHWKIFSCCALSGDRLVQAMTWLCDDVGSRL
FILD
>Q6R3K9
MENERVEREQSQFQEDEFIDSRKPPPWRKQITVRAIVASLLIGIVYSVICLKLNLTTGLV
PNLNISSALLAFVFLKSWTKVLQKAGIATTPFTRQENTIAQTCAVACYSISLAGGFASYL
LGLNRRTYEETGVNTEGNNPRGIKEPGVGWMTSFLFVTSFIGLVVLVPLRKVMIIDYKLT
YPSGTATAVLINGFHTSKGDKTAKKQIRGFIKSFGLSFFWAFFGWFYSGGEKCGFSQFPT
FGLQALDKTFYFDFSMTYVGAGMICSHLVNLSLLFGAILSWGIMWPLIARLKGEWFPATL
KDNSMQGLNGYKVFICIALILGDGLYNFVKILFFTGRSFHSRLSKTNSISTLVEVPEDST
KESDNLKRENEVFVRESIPLWMACVGYLFFSLVSIIAIPLMFPQLKWYFVLVAYLLAPSL
SFCNAYGAGLTDMNMAYNYGKAALFVMAALAGKNDGVVAGMVACGLIKSIVSVSADLMHD
FKTGHLTQTSPRSMLVAQAIGTAIGCVVAPLTFFLFYKAFDVGNQNGEYKAPYAMIYRNM
AIIGVQGPSALPKHCLELCYGFFAFAVAANLARDLLPDKPGKWIPLPMAMAVPFLVGGSF
AIDMCIGSLVVYVWKKVNRKKADVMVPAVASGLICGDGLWILPSSLLALAKVRPPICMNF
TAAH
>Q9S9K4
MELRRKLCIVVAVFVIVIEFASANFVFKAQHKFAGKKKNLEHFKSHDTRRHSRMLASIDL
PLGGDSRVDSVGLYFTKIKLGSPPKEYHVQVDTGSDILWINCKPCPKCPTKTNLNFRLSL
FDMNASSTSKKVGCDDDFCSFISQSDSCQPALGCSYHIVYADESTSDGKFIRDMLTLEQV
TGDLKTGPLGQEVVFGCGSDQSGQLGNGDSAVDGVMGFGQSNTSVLSQLAATGDAKRVFS
HCLDNVKGGGIFAVGVVDSPKVKTTPMVPNQMHYNVMLMGMDVDGTSLDLPRSIVRNGGT
IVDSGTTLAYFPKVLYDSLIETILARQPVKLHIVEETFQCFSFSTNVDEAFPPVSFEFED
SVKLTVYPHDYLFTLEEELYCFGWQAGGLTTDERSEVILLGDLVLSNKLVVYDLDNEVIG
WADHNCSSSIKIKDGSGGVYSVGADNLSSAPRLLMITKLLTILSPLIVMAFTSLA
>Q12451
MSREDLSIAEDLNQVSKPLLKVKLLEVLGQGDFKHLKALVDNEFQPKDDPSVQQVLNLIL
HYAVQVAPILLIKEIVAHWVDQVGDEKSSSKSDDGIHLDLNYQDENGNTPLHLAAAQSRS
DVISFLLSQKSINDCVKNKAHQQPLDMCKDLNVAQMIQLKRDDYFLETVHSLRAAMNKRD
FSKLDSIWKNPRNLNLLDINGIDPETGTTLLYEYSQKKDIEMCQWLLKHGAEATVKDGKG
RSPLDLVKNIKLPAKPSNNVTPEIKLKNLLEKNLREQAIVHEDVASSKPPTYKGFLKKWT
NFAHGYKLRWFILSGDGNLSYYKDQSHVDRPRGTLKVSTCRLHIDSSEKLNFELLGGITG
TTRWRLKGNHPIETTRWVNAIQSAIRFAKDKEILNKKKAVPPSLALKNKSPALISHSKTQ
GSLPEASQYYQHTLHKEVIQPSSVSLYRRPSNNLSVVSSEIQLNDNLTESGKRFVSKMIE
NRLDGSKTPVGVHTGSALQRVRSSNTLKSNRSMQSGSGVASPIDKVPNGANLSQSNTTTG
STASLSDNNYIDNFEGDEANSDDEEEDLGINFDRDEEYIKAQYGPYKEKLDMYEQAISIE
LSSLIELIEQEEPSPEVWLTIKKSLINTSTIFGKLKDLTYKRDKRLVDMVSKQGDVNNVW
VQSVKELEMELSNKTERLASIDKERRGLKKILHKKLLESHATAGNKESLENDKEQESDTT
ASTLGQIAKFISATKEEDEASDADEFYDAAELVDEVTELTEAHPEISTAAAPKHAPPPVP

NETDNDSQYVQDEKSKIESNVEKTSQKFEKQNNLVTEDEPKTDQSLKNFKAEDKESQVKE
KTKEIASSVIGEKTIVAVTTVQKRKEEYLLKEGSYLGYEDGIRKRLSMDKDDRPKISLWA
VLKSMVGKDMTRMTLPVTFNEPTSLLQRVAEDLEYSELLDQAATFEDSTLRTLYVAAFTA
SSYASTTKRVAKPFNPLLGETFEYSRPDKQYRFFTEQVSHHPPISATWTESPRWDFWGES
FVDTKFNGRSFNVKHLGLWHIKLRPNDNEKEELYTWKKPNNTVIGILIGNPQVDNHGEVN
VVNHTTGDHCKLYFKARGWRSSGAYEITGEVYNKKKQKVWILGGHWNEAIFAKKVVKDGD
LSLEKTRTAASAGNGPTDDGTKFLIWKANDRPEEPFNLTPFAITLNAPQPHLLPWLPPTD
TRLRPDQRAMEDGRYDEAGDEKFRVEEKQRAARRKREENNLEYHPQWFVRDTHPITKAKY
WRYTGKYWVKRRDHDLKDCGDIF
>P12624
MGAQFSKTAAKGEATAERPGEAAVASSPSKANGQENGHVKVNGDASPAAAEPGAKEELQA
NGSAPAADKEEPAAAGSGAASPAAAEKDEPAAAAPDAGASPVEKEAPVEGEAAEPGSPTA
AEGEAASAASSTSSPKAEDGATPSPSNETPKKKKKRFSFKKSFKLSGFSFKKNKKEAGEG
GEAEGAAGASAEGGKDEASGGAAAAAGEAGAAPGEPTAAPGEEAAAGEEGAAGGDPQEAK
PEEAAVAPEKPPASEEAKAVEEPSKAEEKAEEAGVSAAGCEAPSAAGPGVPPEQEAAPAE
EAAAAPASSACAAPSQEAQPECSPEAPPAEAAE
>Q8TEZ7
MTTAILERLSTLSVSGQQLRRLPKILEDGLPKMPCTVPETDVPQLFREPYIRTGYRPTGH
EWRYYFFSLFQKHNEVVNVWTHLLAALAVLLRFWAFAEAEALPWASTHSLPLLLFILSSI
TYLTCSLLAHLLQSKSELSHYTFYFVDYVGVSVYQYGSALAHFFYSSDQAWYDRFWLFFL
PAAAFCGWLSCAGCCYAKYRYRRPYPVMRKICQVVPAGLAFILDISPVAHRVALCHLAGC
QEQAAWYHTLQILFFLVSAYFFSCPVPEKYFPGSCDIVGHGHQIFHAFLSICTLSQLEAI
LLDYQGRQEIFLQRHGPLSVHMACLSFFFLAACSAATAALLRHKVKARLTKKDS
>Q9VS24
MHELFNKVLAKRDLSRAGDLFSVPDADIVDDITEVLSEISPIISHADYVKNNNDQSVVEI
CVTRVLSCIRETKTAERYCAALVDLLRTCLLWNLQPSGTTKEEPPHAKIAADIISSIFLN
YDKKELMKIALPVAVQFLPKGNRELSRNLASYLSLAAIDHAGLLSPHTESVMESILSGNY
GLLRVLSQVYEVAPEAVTPHAPLLMPLLPQCDPQERMAVFQLYLLIVQKSPEVLESCVPQ
LCGSLLDSDTSSITMQILLKLSQHSPSLLVDHFEQLRLAAKTNPGTIPLCAQIMTTAGIG
SKETAQQALDFVLEHLPTQALLQQEATRLCSAYPVLFTDKVLACVRQKNAALSSQQDVGN
GLANKTSGGVTIVSLNSSSPSPPLKPVGAPAINTTIVQPASVVSATPTPPPSSASAPIAS
STPVSAATPTPQHAGYTRRVKLGDSRSTGRLHPASNTHRSVTRLNVASGSVGGLHKSMTR
LSNSQINQQPGGSSNGNVVVQSGATPKTPTGLSNPPVTPVPPLSNNVVITGHNRHGIPVT
SGGVTVTTSPSKVRPHSQGPSTLLNSSTVLMKYSTDALNQSVGSISIPQSAAVATLPSTQ
TQNAVSVHHASPALPQSASNGHAKSVMKLPVNGNSEVIVSGPTTNVAPRRSDNTSRTLLN
ANSVMDQRMSTFEPYQIMRDPVQQFCEKNFNSIKSYMDEVSQHLPPPTRCSIEVSNEFAE
RRTKKVAKLHFACQIRGPHCLYSKTCFTMRTRNPKTWIHMMFLDFQVRHFVKEKCVLSTR
ESGISNLKNIWQILKCENRSFTELVTSQFPQVKDREILVNELRHSGFLDVFEVSKTDKSN
PNCNELEYQWGCFLCNHPDKAVGFLNGSNQPMIEGQLKEKKGKWRLFRRWRTRYFTLSGA
HLSCKGSSGGESIDVNQIRSVKVSRGARNIPKAFEIFTADQTLILKPKDGKNAEEWVQCL
SIVVAHSQARDNPTAKTNSLPARSIGSSKPSF
>P32940
MATVILFVAWMACLMVGVCYQEFQTQQNFPDISNPSQELNQEPAHRIVQLDSIQNNGALN
MSTGNVLNMSPPPPSPCLSRAKIRHAFKYVTTILSCVIFLVGIVGNSTLLRIIYKNKCMR

NGPNVLIASLALGDLFYILIAIPIISISFWLSTGHSEYIYQLVHLYRARVYSLSLCALSI
DRYRAVASWNRIRSIGIPVRKAIELTLIWAVAIIVAVPEAIAFNLVELDFRGQTILVCML
PMEQTSDFMRFYQEVKVWWLFGFYFCLPLACTGVFYTLMSCEMLSIKNGMRIALNDHMKQ
RREVAKTVFCLVVIFALCWLPLHVSSIFVRLSATVKRACILKNKRSCIMAEIQTGVNYQL
LMVMNYTGINMASLNSCIGPVALYFVSRKFKNCFQSCLCCWCHRPTLTITPMDEKGSGGK
WKANGHDLDLDRSSSRLSNKYSSS
>Q9BWV1
MLRGTMTAWRGMRPEVTLACLLLATAGCFADLNEVPQVTVQPASTVQKPGGTVILGCVVE
PPRMNVTWRLNGKELNGSDDALGVLITHGTLVITALNNHTVGRYQCVARMPAGAVASVPA
TVTLANLQDFKLDVQHVIEVDEGNTAVIACHLPESHPKAQVRYSVKQEWLEASRGNYLIM
PSGNLQIVNASQEDEGMYKCAAYNPVTQEVKTSGSSDRLRVRRSTAEAARIIYPPEAQTI
IVTKGQSLILECVASGIPPPRVTWAKDGSSVTGYNKTRFLLSNLLIDTTSEEDSGTYRCM
ADNGVGQPGAAVILYNVQVFEPPEVTMELSQLVIPWGQSAKLTCEVRGNPPPSVLWLRNA
VPLISSQRLRLSRRALRVLSMGPEDEGVYQCMAENEVGSAHAVVQLRTSRPSITPRLWQD
AELATGTPPVSPSKLGNPEQMLRGQPALPRPPTSVGPASPQCPGEKGQGAPAEAPIILSS
PRTSKTDSYELVWRPRHEGSGRAPILYYVVKHRKVTNSSDDWTISGIPANQHRLTLTRLD
PGSLYEVEMAAYNCAGEGQTAMVTFRTGRRPKPEIMASKEQQIQRDDPGASPQSSSQPDH
GRLSPPEAPDRPTISTASETSVYVTWIPRGNGGFPIQSFRVEYKKLKKVGDWILATSAIP
PSRLSVEITGLEKGTSYKFRVRALNMLGESEPSAPSRPYVVSGYSGRVYERPVAGPYITF
TDAVNETTIMLKWMYIPASNNNTPIHGFYIYYRPTDSDNDSDYKKDMVEGDKYWHSISHL
QPETSYDIKMQCFNEGGESEFSNVMICETKARKSSGQPGRLPPPTLAPPQPPLPETIERP
VGTGAMVARSSDLPYLIVGVVLGSIVLIIVTFIPFCLWRAWSKQKHTTDLGFPRSALPPS
CPYTMVPLGGLPGHQASGQPYLSGISGRACANGIHMNRGCPSAAVGYPGMKPQQHCPGEL
QQQSDTSSLLRQTHLGNGYDPQSHQITRGPKSSPDEGSFLYTLPDDSTHQLLQPHHDCCQ
RQEQPAAVGQSGVRRAPDSPVLEAVWDPPFHSGPPCCLGLVPVEEVDSPDSCQVSGGDWC
PQHPVGAYVGQEPGMQLSPGPLVRVSFETPPLTI
>P40897
MSTIYRESDSLESEPSPTPTTIPIQINMEEEKKDAFVKNIDEDVNNLTATTDEEDRDPES
QKFDRHSIQEEGLVWKGDPTYLPNSPYPEVRSAVSIEDDPTIRLNHWRTWFLTTVFVVVF
AGVNQFFSLRYPSLEINFLVAQVVCYPIGRILALLPDWKCSKVPFFDLNPGPFTKKEHAV
VTIAVALTSSTAYAMYILNAQGSFYNMKLNVGYQFLLVWTSQMIGYGAAGLTRRWVVNPA
SSIWPQTLISVSLFDSLHSRKVEKTVANGWTMPRYRFFLIVLIGSFIWYWVPGFLFTGLS
YFNVILWGSKTRHNFIANTIFGTQSGLGALPITFDYTQVSQAMSGSVFATPFYVSANTYA
SVLIFFVIVLPCLYFTNTWYAKYMPVISGSTYDNTQNKYNVTKILNEDYSINLEKYKEYS
PVFVPFSYLLSYALNFAAVIAVFVHCILYHGKDIVAKFKDRKNGGTDIHMRIYSKNYKDC
PDWWYLLLQIVMIGLGFVAVCCFDTKFPAWAFVIAILISLVNFIPQGILEAMTNQHVGLN
IITELICGYMLPLRPMANLLFKLYGFIVMRQGLNLSRDLKLAMYMKVSPRLIFAVQIYAT
IISGMVNVGVQEWMMHNIDGLCTTDQPNGFTCANGRTVFNASIIWSLPKYLFSSGRIYNP
LMWFFLIGLLFPLAVYAVQWKFPKFKFAKHIHTPVFFTGPGNIPPSTPYNYSLFFAMSFC
LNLIRKRWRAWFNKYNFVMGAGVEAGVAISVVIIFLCVQYPGGKLSWWGNNVWKRTYDND
YKKFYTLKKGETFGYDKWW
>Q03555
MATEGMILTNHDHQIRVGVLTVSDSCFRNLAEDRSGINLKDLVQDPSLLGGTISAYKIVP
DEIEEIKETLIDWCDEKELNLILTTGGTGFAPRDVTPEKFPTFPFCGLQKGATKEVIERE

APGMALAMLMGSLNVTPLGMLSRPVCGIRGKTLIINLPGSKKGSQECFQFILPALPHAID
LLRDAIVKVKEVHDELEDLPSPPPPLSPPPTTSPHKQTEDKGVQCEEEEEEKKDSGVAST
EDSSSSHITAAALAAKIPDSIISRGVQVLPRDTASLSTTPSESPRAQATSRLSTASCPTP
KQIRRPDESKGVASRVGSLKVQSRCSSKENILRASHSAVDITKVARRHRMSPFPLTSMDK
AFITVLEMTPVLGTEIINYRDGMGRVLAQDVYAKDNLPPFPASVKDGYAVRAADGPGDRF
IIGESQAGEQPTQTVMPGQVMRVTTGAPIPCGADAVVQVEDTELIRESDDGTEELEVRIL
VQARPGQDIRPIGHDIKRGECVLAKGTHMGPSEIGLLATVGVTEVEVNKFPVVAVMSTGN
ELLNPEDDLLPGKIRDSNRSTLLATIQEHGYPTINLGIVGDNPDDLLNALNEGISRADVI
ITSGGVSMGEKDYLKQVLDIDLHAQIHFGRVFMKPGLPTTFATLDIDGVRKIIFALPGNP
VSAVVTCNLFVVPALRKMQGILDPRPTIIKARLSCDVKLDPRPEYHRCILTWHHQEPLPW
AQSTGNQMSSRLMSMRSANGLLMLPPKTEQYVELHKGEVVDVMVIGRL
>P32240
MAEVGGTIPRSNRELQRCVLLTTTIMSIPGVNASFSSTPERLNSPVTIPAVMFIFGVVGN
LVAIVVLCKSRKEQKETTFYTLVCGLAVTDLLGTLLVSPVTIATYMKGQWPGDQALCDYS
TFILLFFGLSGLSIICAMSIERYLAINHAYFYSHYVDKRLAGLTLFAIYASNVLFCALPN
MGLGRSERQYPGTWCFIDWTTNVTAYAAFSYMYAGFSSFLILATVLCNVLVCGALLRMHR
QFMRRTSLGTEQHHAAAAAAVASVACRGHAGASPALQRLSDFRRRRSFRRIAGAEIQMVI
LLIATSLVVLICSIPLVVRVFINQLYQPNVVKDISRNPDLQAIRIASVNPILDPWIYILL
RKTVLSKAIEKIKCLFCRIGGSGRDSSAQHCSESRRTSSAMSGHSRSFLARELKEISSTS
QTLLYLPDLTESSLGGRNLLPGSHGMGLTQADTTSLRTLRISETSDSSQGQDSESVLLVD
EVSGSHREEPASKGNSLQVTFPSETLKLSEKCI
>P41594
MVLLLILSVLLLKEDVRGSAQSSERRVVAHMPGDIIIGALFSVHHQPTVDKVHERKCGAV
REQYGIQRVEAMLHTLERINSDPTLLPNITLGCEIRDSCWHSAVALEQSIEFIRDSLISS
EEEEGLVRCVDGSSSSFRSKKPIVGVIGPGSSSVAIQVQNLLQLFNIPQIAYSATSMDLS
DKTLFKYFMRVVPSDAQQARAMVDIVKRYNWTYVSAVHTEGNYGESGMEAFKDMSAKEGI
CIAHSYKIYSNAGEQSFDKLLKKLTSHLPKARVVACFCEGMTVRGLLMAMRRLGLAGEFL
LLGSDGWADRYDVTDGYQREAVGGITIKLQSPDVKWFDDYYLKLRPETNHRNPWFQEFWQ
HRFQCRLEGFPQENSKYNKTCNSSLTLKTHHVQDSKMGFVINAIYSMAYGLHNMQMSLCP
GYAGLCDAMKPIDGRKLLESLMKTNFTGVSGDTILFDENGDSPGRYEIMNFKEMGKDYFD
YINVGSWDNGELKMDDDEVWSKKSNIIRSVCSEPCEKGQIKVIRKGEVSCCWTCTPCKEN
EYVFDEYTCKACQLGSWPTDDLTGCDLIPVQYLRWGDPEPIAAVVFACLGLLATLFVTVV
FIIYRDTPVVKSSSRELCYIILAGICLGYLCTFCLIAKPKQIYCYLQRIGIGLSPAMSYS
ALVTKTNRIARILAGSKKKICTKKPRFMSACAQLVIAFILICIQLGIIVALFIMEPPDIM
HDYPSIREVYLICNTTNLGVVTPLGYNGLLILSCTFYAFKTRNVPANFNEAKYIAFTMYT
TCIIWLAFVPIYFGSNYKIITMCFSVSLSATVALGCMFVPKVYIILAKPERNVRSAFTTS
TVVRMHVGDGKSSSAASRSSSLVNLWKRRGSSGETLRYKDRRLAQHKSEIECFTPKGSMG
NGGRATMSSSNGKSVTWAQNEKSSRGQHLWQRLSIHINKKENPNQTAVIKPFPKSTESRG
LGAGAGAGGSAGGVGATGGAGCAGAGPGGPESPDAGPKALYDVAEAEEHFPAPARPRSPS
PISTLSHRAGSASRTDDDVPSLHSEPVARSSSSQGSLMEQISSVVTRFTANISELNSMML
STAAPSPGVGAPLCSSYLIPKEIQLPTTMTTFAEIQPLPAIEVTGGAQPAAGAQAAGDAA
RESPAAGPEAAAAKPDLEELVALTPPSPFRDSVDSGSTTPNSPVSESALCIPSSPKYDTL
IIRDYTQSSSSL
>P14719

MIDRQRMGLWALAILTLPMYLTVTEGSKSSWGLENEALIVRCPQRGRSTYPVEWYYSDTN
ESIPTQKRNRIFVSRDRLKFLPARVEDSGIYACVIRSPNLNKTGYLNVTIHKKPPSCNIP
DYLMYSTVRGSDKNFKITCPTIDLYNWTAPVQWFKNCKALQEPRFRAHRSYLFIDNVTHD
DEGDYTCQFTHAENGTNYIVTATRSFTVEEKGFSMFPVITNPPYNHTMEVEIGKPASIAC
SACFGKGSHFLADVLWQINKTVVGNFGEARIQEEEGRNESSSNDMDCLTSVLRITGVTEK
DLSLEYDCLALNLHGMIRHTIRLRRKQPIDHRSIYYIVAGCSLLLMFINVLVIVLKVFWI
EVALFWRDIVTPYKTRNDGKLYDAYIIYPRVFRGSAAGTHSVEYFVHHTLPDVLENKCGY
KLCIYGRDLLPGQDAATVVESSIQNSRRQVFVLAPHMMHSKEFAYEQEIALHSALIQNNS
KVILIEMEPLGEASRLQVGDLQDSLQHLVKIQGTIKWREDHVADKQSLSSKFWKHVRYQM
PVPERASKTASVAAPLSGKACLDLKHF
>Q8R416
METTVGTLGENTTNTFTDFFSARDGSGAETSPLPFTFSYGDYDMPSDEEEDVTNSRTFFA
AKIVIGMALVGIMLVCGIGNFIFITALARYKKLRNLTNLLIANLAISDFLVAIVCCPFEM
DYYVVRQLSWEHGHVLCASVNYLRTVSLYVSTNALLAIAIDRYLAIVHPLRPRMKCQTAA
GLIFLVWSVSILIAIPAAYFTTETVLVIVESQEKIFCGQIWPVDQQFYYRSYFLLVFGLE
FVGPVIAMTLCYARVSRELWFKAVPGFQTEQIRRRLRCRRRTVLGLVCVLSAYVLCWAPF
YGFTIVRDFFPSVFVKEKHYLTAFYVVECIAMSNSMINTLCFVTVRNNTSKYLKRILRLQ
WRASPSGSKASADLDLRTTGIPATEEVDCIRLK
>Q9JJZ1
MSPEDPQETQPLLRSPGARAPGGRRVFLATFAAALGPLSFGFALGYSSPAIPSLRRTAPP
ALRLGDTAASWFGAVVTLGAAAGGVLGGWLLDRAGRKLSLLLCTVPFVTGFAVITAARDV
WMLLGGRLLTGLACGVASLVAPVYISEIAYPAVRGLLGSCVQLMVVTGILLAYVAGWVLE
WRWLAVLGCVPPTLMLLLMCYMPETPRFLLTQHQYQEAMAALRFLWGSEEGWEEPPVGAE
HQGFQLAMLRRPGVHKPLIIGICLMVFQQLSGVNAIMFYANTIFEEAKFKDSSLASVTVG
IIQVLFTAVAALIMDRAGRKLLLALSGVIMVFSMSAFGTYFKLTQSGPSNSSHVGLLVPI
SAEPADVHLGLAWLAVGSMCLFIAGFAVGWGPIPWLLMSEIFPLHIKGVATGVCVLTNWF
MAFLVTKEFNSIMEILRPYGAFWLTAAFCILSVLFTLTFVPETKGRTLEQITAHFEGR
>P40528
MKFADHLTESAIPEWRDKYIDYKVGKKKLRRYKEKLDAEEEQSSSYRSWMPSVSVYQTAF
QQREPGKSRSDGDYRSGPAFKKDYSALQREFVADFIEDWLISFQLSKCNEFYLWLLKECD
KKFEVLQSQLHYYSLQKNYERDNLNRSSSNVDMSTSLYAAGLAGRSDSRVNSIDSDSRSV
MYGSMPCTKEAKKPRLSLLAYCQKVLKDNRLLPSWPKRGFSLLQDLRQDASSRGRETFAF
GASFLETMTTTQARNLLSNAIIEYYLYLQLVKSFRDINVTGFRKMVKKFDKTCHTRELTT
FMSYARTHYTLFKHADANVQLVAQKMQQITSSQPTPTSELSSAQRDKEPITWLETQITEW
FTTALTNSPKDRKHNTHKLKKLTIQYSISEQMVHRNNRSIVQMLVVGLGIGVSMTLITYT
LYLGISSEETSFTHKILFPLWGGWYMVLLIAFLFLVNCFIWHRTGINYRFIMLGEIQSKN
GTQFFNNDFATSKIPLKLYFLTFFIVPCAVCSMLSFALEKLTPLGFLYIGIVSFLFLCPS
GLIPYWDKVVHTRKWLVVTLIRLMMSGFFPVEFGDFFLGDIICSLTYSIADIAMFFCVYS
HTPNNLCGSSHSRAMGVLSCLPSYWRFMQCLRRFADSGDWFPHLLNAAKYTLGIAYNATL
CAYRLSDRSEQRRTPFIVCATLNSILTSAWDLVMDWSFAHNTTSYNWLLRDDLYLAGKKN
WENGSYSFSRKLVYYFAMIWDILIRFEWIVYAIAPQTIQQSAVTSFILALLEVLRRFVWI
IFRVENEHVANVHLFRVTGDAPLPYPIAQVGDDSMDSSDLGSKAFSSLNDIPITPSHDNN
PHSFAEPMPAYRGTFRRRSSVFENISRSIPWAHATDFQRPTVNTVDDRSPETDSESEVES
IM

>P20695
MPKNKKRNAPHRGGGGGGGSGAATSAATTGGPHRTVQPFSDEDASIETMSHCSGYSDPSS
FAEDGPEVLDEEGTQEDLEYKLKGLIDLTLDKSAKTRQAALEGVKNALSSKVLYEFVLER
RMTLTDSIERCLKKGKSDGQRAAAALASVLCIQLGPGLESEEILKTLGPILKKIICDGTA
SIQARQTCATCFGVCCFIATDDITELYSTLECLEGIFTKSYLKEKDTNVPCSTPNTVLHI
SSLLAWTLLLTICPISEVKKKLELHFHKLPSLLSCDDVNMRIAAGESLALLFELARGMES
DFFYEDMDSLTQMLRALATDGNKHRAKVDKRKQRSVFRDVLRAVEERDFPTETVKFGPER
MYIDSWVKKHTYDTFKEALGSGMQYHLQTNEFLRNVFELGPPVMLDAATLKTMKIPRFER
HLYNSAAFKARTKARSKCRDKRADVGEFF
>Q9C7F7
MKGLHLHLVLVTMTIVASIAAAAPAAPGGALADECNQDFQKVTLCLDFATGKATIPSKKC
CDAVEDIKERDPKCLCFVIQQAKTGGQALKDLGVQEDKLIQLPTSCQLHNASITNCPKLL
GISPSSPDAAVFTNNATTTPVAPAGKSPATPATSTDKGGSASAKDGHAVVALAVALMAVS
FVLTLPRHVTLGM
>Q9BPZ7
MAFLDNPTIILAHIRQSHVTSDDTGMCEMVLIDHDVDLEKIHPPSMPGDSGSEIQGSNGE
TQGYVYAQSVDITSSWDFGIRRRSNTAQRLERLRKERQNQIKCKNIQWKERNSKQSAQEL
KSLFEKKSLKEKPPISGKQSILSVRLEQCPLQLNNPFNEYSKFDGKGHVGTTATKKIDVY
LPLHSSQDRLLPMTVVTMASARVQDLIGLICWQYTSEGREPKLNDNVSAYCLHIAEDDGE
VDTDFPPLDSNEPIHKFGFSTLALVEKYSSPGLTSKESLFVRINAAHGFSLIQVDNTKVT
MKEILLKAVKRRKGSQKVSGPQYRLEKQSEPNVAVDLDSTLESQSAWEFCLVRENSSRAD
GVFEEDSQIDIATVQDMLSSHHYKSFKVSMIHRLRFTTDVQLGISGDKVEIDPVTNQKAS
TKFWIKQKPISIDSDLLCACDLAEEKSPSHAIFKLTYLSNHDYKHLYFESDAATVNEIVL
KVNYILESRASTARADYFAQKQRKLNRRTSFSFQKEKKSGQQ
>P10040
MAKIANASLSQQQKQRQAETATTTTTTVAASVETATTTARSRDRTKSAAQITSHLLKRAI
SVYSSPQWIPLFILIYLATDVASVAVPTKEAYFNGSTYLRLTTPMPIWDHSAISFRSCRG
GEILAQQYNKNSIVISVLNDFLQISLAGPAVHGPNNRLDVKLPYQLLDNRWHTLQFKYEY
GNLYLHVDRAASIFANSTYNSQFLTNQDIGYKDAILILGNSFSGCLLDGPGLQFVNNSTV
QNVVFGHCPLTPGPCSDHDLFTRLPDNFCLNDPCMGHGTCSSSPEGYECRCTARYSGKNC
QKDNGSPCAKNPCENGGSCLENSRGDYQCFCDPNHSGQHCETEVNIHPLCQTNPCLNNGA
CVVIGGSGALTCECPKGYAGARCEVDTDECASQPCQNNGSCIDRINGFSCDCSGTGYTGA
FCQTNVDECDKNPCLNGGRCFDTYGWYTCQCLDGWGGEICDRPMTCQTQQCLNGGTCLDK
PIGFQCLCPPEYTGELCQIAPSCAQQCPIDSECVGGKCVCKPGSSGYNCQTSTGDGASAL
ALTPINCNATNGKCLNGGTCSMNGTHCYCAVGYSGDRCEKAENCSPLNCQEPMVCVQNQC
LCPENKVCNQCATQPCQNGGECVDLPNGDYECKCTRGWTGRTCGNDVDECTLHPKICGNG
ICKNEKGSYKCYCTPGFTGVHCDSDVDECLSFPCLNGATCHNKINAYECVCQPGYEGENC
EVDIDECGSNPCSNGSTCIDRINNFTCNCIPGMTGRICDIDIDDCVGDPCLNGGQCIDQL
GGFRCDCSGTGYEGENCELNIDECLSNPCTNGAKCLDRVKDYFCDCHNGYKGKNCEQDIN
ECESNPCQYNGNCLERSNITLYQMSRITDLPKVFSQPFSFENASGYECVCVPGIIGKNCE
ININECDSNPCSKHGNCNDGIGTYTCECEPGFEGTHCEINIDECDRYNPCQRGTCYDQID
DYDCDCDANYGGKNCSVLLKGCDQNPCLNGGACLPYLINEVTHLYNCTCENGFQGDKCEK
TTTLSMVATSLISVTTEREEGYDINLQFRTTLPNGVLAFGTTGEKNEPVSYILELINGRL
NLHSSLLNKWEGVFIGSKLNDSNWHKVFVAINTSHLVLSANDEQAIFPVGSYETANNSQP

SFPRTYLGGTIPNLKSYLRHLTHQPSAFVGCMQDIMVNGKWIFPDEQDANISYTKLENVQ
SGCPRTEQCKPNPCHSNGECTDLWHTFACHCPRPFFGHTCQHNMTAATFGHENTTHSAVI
VETTDVARRAIRSILDISMFIRTREPTGQVFYLGTDPRKAPTKNIGDSYVAAKLHGGELL
VKMQFSGTPEAYTVGGQKLDNGYNHLIEVVRNQTLVQVKLNGTEYFRKTLSTTGLLDAQV
LYLGGPAPTRESLLGATTEPGIIPVPGAGIPIEDTTVPKEADDSRDYFKGIIQDVKVSNG
SLNLIVEMYSLNVTDVQVNAKPLGAVTIDRASVLPGEVSDDLCRKNPCLHNAECRNTWND
YTCKCPNGYKGKNCQEIEFCQHVTCPGQSLCQNLDDGYECVTNTTFTGQERSPLAFFYFQ
EQQSDDIVSEASPKQTLKPVIDIAFRTRAGGTLLYIDNVDGFFEIGVNGGRVTITWKLSA
LHFGESARFEKENTDGEWSRIYLRAHNSKLEGGWKGWESMVDPTPAFSTDIDQAAFQSLI
ATSTQVYLGGMPESRQARGSTLSAQQGSQFKGCVGEARVGDLLLPYFSMAELYSRTNVSV
QQKAQFRLNATRPEEGCILCFQSDCKNDGFCQSPSDEYACTCQPGFEGDDCGTDIDECLN
TECLNNGTCINQVAAFFCQCQPGFEGQHCEQNIDECADQPCHNGGNCTDLIASYVCDCPE
DYMGPQCDVLKQMTCENEPCRNGSTCQNGFNASTGNNFTCTCVPGFEGPLCDIPFCEITP
CDNGGLCLTTGAVPMCKCSLGYTGRLCEQDINECESNPCQNGGQCKDLVGRYECDCQGTG
FEGIRCENDIDECNMEGDYCGGLGRCFNKPGSFQCICQKPYCGAYCNFTDPCNATDLCSN
GGRCVESCGAKPDYYCECPEGFAGKNCTAPITAKEDGPSTTDIAIIVIPVVVVLLLIAGA
LLGTFLVMARNKRATRGTYSPSAQEYCNPRLEMDNVLKPPPEERLI
>Q9JLN5
MERPSPCGSWLVGCLFTIAVFQPPVQVLGDAGKVYIAPLRDTANLPCPLFLWPNMVLSEM
RWYRPGHLPRTQAVHVFRDGQDRDEDLMPEYKGRTALVRDAHKESYILQISNVRLEDRGL
YQCQVWVGNSSREDNVTLQVAVLGSDPYIHVKGYDAGWIELLCQSVGWFPKPWTEWRDTT
GRALLSLSEVHSLDENGLFRTAVSSRIRDNALGNVSCTIHNEALGQEKTTAMIIGAPERG
SLSSPAVALSVVLPVLGLLILLGIWLICKQKKSKEKLLYEQAMEVENLLEDHAKEKGRLH
KALKKLRSELKLKRAAANAGWRRARLHFVAVTLDPDTAHPKLILSEDRRCVRLGDRKRPV
PDNPERFDFVVSVLGSEYFTTGCHYWEVYVGEKTKWILGVCSESVSRKGKVTASPANGHW
LVRQSRGNEYEALTSPQTSFRLKESPKCVGIFLDYEAGIISFYNVTDKSHIFTFTHSFSS
PLRPFFEPCLHDEGKNTAPLIICTELQKSEESIVPKQEGKDRANGDVSLKMNPSLLSPQG
SELFLLNDTWPSNLGPALKGLKVPSL
>P58406
MERAPPDGLMNASGALAGEAAAAGGARGFSAAWTAVLAALMALLIVATVLGNALVMLAFV
ADSSLRTQNNFFLLNLAISDFLVGAFCIPLYVPYVLTGRWTFGRGLCKLWLVVDYLLCAS
SVFNIVLISYDRFLSVTRAVSYRAQQGDTRRAVRKMALVWVLAFLLYGPAILSWEYLSGG
SSIPEGHCYAEFFYNWYFLITASTLEFFTPFLSVTFFNLSIYLNIQRRTRLRLDGGREAG
PEPPPDAQPSPPPAPPSCWGCWPKGHGEAMPLHRYGVGEAGPGVETGEAGLGGGSGGGAA
ASPTSSSGSSSRGTERPRSLKRGSKPSASSASLEKRMKMVSQSITQRFRLSRDKKVAKSL
AIIVSIFGLCWAPYTLLMIIRAACHGHCVPDYWYETSFWLLWANSAVNPVLYPLCHYSFR
RAFTKLLCPQKLKVQPHGSLEQCWK
>Q08157
MKKNSSVVFFLVGLSQFVTMAFLIIGSITAPIFKQIGYSKYDEITYGTFGYCKEGSCSKA
SYNYHPDELSDSDSNWKLNSNARSILGKIIFITPIAAGLNFLGFLCTIMSVLLINVLSSD
RVGSASAIMFFVNLTFSTLGFLSASLICIVVFLLFYPHVTWCSWVLIPGAALSLLVIPLI
FSAYSRSSGSRDDDETEELEEKGMLLNDPYLSSKSGRFDIDADSEANLRGDSRTNLLGDN
FKNGTNITVVPDIISHNQDPKLSNITTSTTSDISTFDKEAKDMENSNGSGLNEEEDDGMA
YDKRRSTSTYSVIESESGLKNGSVSNNYVRNNGSNTSNNINYKVPLGKTEISSSASLASS

DYSQREVIPHRNPSRLLNDIMETSFNEPNDSHINSMSSYNDKDSTLTSISQRGVNPEVYN
QMPRETAAGPANIRPYAGQPHPAPLVYPQQRLQPQQQQPQQQYHQYNLYQRTTPAGPDPS
NVILQSNPYFNVAPNQVPQHRNPVPGVGFAPNPLPNQSPITQGYKPAYKRRMQNKNLPRA
TTSLNNPYGFR
>Q8R5G7
MAAPQDLDIAVWLALVHLEQYADTFRRHGLATAGAAQHLGHEELRHLGISATGHRKRILR
LLRAGSAEGFLDSHLDNTMEPTPSPAPDAQPPKPVPKPRTVFGLSNPATAQRPGLSPIFW
DPEVSRNSECTQRSSPLLPSSSEHPSVPNTMEMMPNAIYFGLDLRGRAQAAQDVTPDSSQ
ATVPTPAFRPTTGTVHIMDPGCLYYGVQPVGIPGASDRRDGRGVCQERAEHRQDLETRED
AGYASLELPGDSILSLPTQDAETSDDLISPYASFSSTADRPVPLLSGWLDKLSPQGNYVF
QRRFVQFNGRSLMYFGSDKDPFPKGVIPLTAIEMTRSSKDNKFQVITGQRVFVFRTESEA
QRDLWCSTLQSCLKEQRLLGHPRPPHPPRPLRTGTLELRGHKAKVFAALIPGELALYKSE
QAFSLGIGICFIELQGCSVRETKSRSFDLLTPHRCFSFTAESGGARQSWAAALQEAVTET
LSDYEVAEKVWSNPANRHCADCRASRPDWAAVNLGVVICKQCAGQHRALGSGISKVQSLK
LDTSVWSNEIVQLFIVLGNDRANCFWAGALPPGEGLHPDSAPGPRGEFISRKYKLGLFRK
PHPRHPDHSQLLQALCAAMAGPNLLKNMAQLLCVETSEGEEPLSPSALNGSLLSLLPSDS
PGVYNEVVVPATYRGFLYCGSISNKAGAPPLRRGRDAPPRLWCVLGAALEMFASESSPEP
LSLLQPQDIVCLGVSPPPADPGDLDRFPFSFELILTGGRIQHFATDGADSLEAWISAVGK
WFSPLSCHQLLGPGLLRMGRLWLRSPSHAGLAPGLWLSGFGLLRGDHLFLCPAPGPGPPA
PEDMVHLRRLQEISVVSAADTPDKKEHLVLVETGRTLYLQGEGRLDFAAWNTAIGGAAGG
GGTGLQEQQMSRGDIPIIVDACISFVTQHGLRLEGVYRKGGARARSLRLLAEFRRDARSV
KLRPREHFVEDVTDTLKRFFRELDDPVTSARLLPRWREAAELSQKNQRLEKYKEVISCLP
RVNRRTLATLIGHLYRVQKCASLNQMCTRNLALLFAPSVFQTDGRGEHEVRVLQELIDGY
ISVFDIDSDQAAQIDLEVSLITTWKDVQLSQAGDLIMEVYIEQQLPDNCVTLKVSPTLTA
EELTNQVLEMRGAASGTDLWVTFEILEHGELERPLHPKEKVLEQALQWCQLPEPCSASLL
LRKVSMAHAGCLFTGVRRESPRVGLLRCREEPPRLLGNRFQERFFLVRGRCLLLLKEKKS
SKPEREWSLEGAKVYLGIRKKLKPPTLWGFTLILEKMHLCLSCMDEEEMWDWTTSILKAQ
HDDQQSVVLRRRSSSDLARQKFGTMPLLPIRGDDSGATLLSANQTLRRLHNRRTLSMFFP
MKSPQGSVEEQDELEEPVYEEPVYEEVGAFPELTKDTTFSSTWEWSAKSDPSLTSQRSFD
QPPLSKASMLGHEERIPDPPPGPPSKSSSQARGSLEEQLLQELNNLILRKGEPASCPESS
SQPTSPQAPSPTSLPTPTPSLPTQPPCTSNPPSSQPLT
>Q9P0L0
MASASGAMAKHEQILVLDPPTDLKFKGPFTDVVTTNLKLRNPSDRKVCFKVKTTAPRRYC
VRPNSGIIDPGSTVTVSVMLQPFDYDPNEKSKHKFMVQTIFAPPNTSDMEAVWKEAKPDE
LMDSKLRCVFEMPNENDKLNDMEPSKAVPLNASKQDGPMPKPHSVSLNDTETRKLMEECK
RLQGEMMKLSEENRHLRDEGLRLRKVAHSDKPGSTSTASFRDNVTSPLPSLLVVIAAIFI
GFFLGKFIL
>P84903
MDVCARLALWLLWGLLLHQGQSLSHSHSEKNTGASSGATSEESTEAEFCRIDKPLCHSED
EKLSFEAVRNIHKLMDDDANGDVDVEESDEFLREDLNYHDPTVKHSTFHGEDKLISVEDL
WKAWKASEVYNWTVDEVIQWLITYVELPQYEETFRKLQLTGHAMPRLAVTNTTMTGTVLK
MTDRSHRQKLQLKALDTVLFGPPLLTRHNHLKDFMLVVSIVIGVGGCWFAYIQNRYSKEH
MKKMMKDLEGLHRAEQSLHDLQERLHKAQEEHRTVEVEKVHLEKKLRDEINLAKQEAQRL
KELREGTENERSRQKYAEEELEQVREALRKAEKELESHSSWYAPEALQKWLQLTHEVEVQ

YYNIKKQNAERQLLVAKEGAEKIKKKRNTLFGTFHVAHSSSLDDVDHKILTAKQALSEVT
AALRERLHRWQQIEILCGFQIVNNPGIHSLVAALNIDPSWMGSTRPNPAHFIMTDDVDDM
DEEIVSPLSMQSPSLQSSVRQRLTEPQHGLGSQRDLTHSDSESSLHTSDRQRVAPKPPQM
GRAADEALNATSSNGSHRLIEGVHPGSLVEKLPDSPALAKKTILALNHGLDKAHSLMELN
PSVPPGGSPLLDSSHSHSPSSPDPDTPSPVGDSRALQGSRNTRIPHLAGKKAMAEEDNGS
IGEETDSSPGRKKFPLKIFKKPLKK
>Q9UBT7
MAASPGPAGVGGAGAVYGSGSSGFALDSGLEIKTRSVEQTLLPLVSQITTLINHKDNTKK
SDKTLQAIQRVGQAVNLAVGRFVKVGEAIANENWDLKEEINIACIEAKQAGETIAALTDI
TNLNHLESDGQITIFTDKTGVIKAARLLLSSVTKVLLLADRVVIKQIITSRNKVLATMER
LEKVNSFQEFVQIFSQFGNEMVEFAHLSGDRQNDLKDEKKKAKMAAARAVLEKCTMMLLT
ASKTCLRHPNCESAHKNKEGVFDRMKVALDKVIEIVTDCKPNGETDISSISIFTGIKEFK
MNIEALRENLYFQSKENLSVTLEVILERMEDFTDSAYTSHEHRERILELSTQARMELQQL
ISVWIQAQSKKTKSIAEELELSILKISHSLNELKKELHSTATQLAADLLKYHADHVVLKA
LKLTGVEGNLEALAEYACKLSEQKEQLVETCRLLRHISGTEPLEITCIHAEETFQVTGQQ
IISAAETLTLHPSSKIAKENLDVFCEAWESQISDMSTLLREINDVFEGRRGEKYGYLSLP
KPMKNNANLKSLKPDKPDSEEQAKIAKLGLKLGLLTSDADCEIEKWEDQENEIVQYGRNM
SSMAYSLYLFTRGEGPLKTSQDLIHQLEVFAAEGLKLTSSVQAFSKQLKDDDKLMLLLEI
NKLIPLCHQLQTVTKTSLQNKVFLKVDKCITKTRSMMALLVQLLSLCYKLLKKLQMENNG
WVSVTNKDTMDSKT
>Q5QD08
MSIADETVSWNQDSILSRDLFSATSAELCYENLNRSCVRSPYSPGPRLILYAVFGFGAVL
AVCGNLLVMTSILHFRQLHSPANFLVASLACADFLVGVMVMPFSMVRSVEGCWYFGDSYC
KLHTCFDVSFCYCSLFHLCFISVDRYIAVSDPLAYPTRFTASVSGKCITFSWLLSISYGF
SLIYTGASEAGLEDLVSSLTCVGGCQIAVNQTWVFINFSVFLIPTLVMITVYSKIFLIAK
QQAQNIEKMSKQTARASDSYKDRVAKRERKAAKTLGIAVAAFLLSWLPYFIDSFIDAFLG
FITPTYVYEILVWIVYYNSAMNPLIYAFFYPWFRKAIKLTVTGKILRENSSTTNLFSE
>P10870
MDPNSNSSSETLRQEKQGFLDKALQRVKGIALRRNNSNKDHTTDDTTGSIRTPTSLQRQN
SDRQSNMTSVFTDDISTIDDNSILFSEPPQKQSMMMSICVGVFVAVGGFLFGYDTGLINS
ITSMNYVKSHVAPNHDSFTAQQMSILVSFLSLGTFFGALTAPFISDSYGRKPTIIFSTIF
IFSIGNSLQVGAGGITLLIVGRVISGIGIGAISAVVPLYQAEATHKSLRGAIISTYQWAI
TWGLLVSSAVSQGTHARNDASSYRIPIGLQYVWSSFLAIGMFFLPESPRYYVLKDKLDEA
AKSLSFLRGVPVHDSGLLEELVEIKATYDYEASFGSSNFIDCFISSKSRPKQTLRMFTGI
ALQAFQQFSGINFIFYYGVNFFNKTGVSNSYLVSFITYAVNVVFNVPGLFFVEFFGRRKV
LVVGGVIMTIANFIVAIVGCSLKTVAAAKVMIAFICLFIAAFSATWGGVVWVISAELYPL
GVRSKCTAICAAANWLVNFICALITPYIVDTGSHTSSLGAKIFFIWGSLNAMGVIVVYLT
VYETKGLTLEEIDELYIKSSTGVVSPKFNKDIRERALKFQYDPLQRLEDGKNTFVAKRNN
FDDETPRNDFRNTISGEIDHSPNQKEVHSIPERVDIPTSTEILESPNKSSGMTVPVSPSL
QDVPIPQTTEPAEIRTKYVDLGNGLGLNTYNRGPPSLSSDSSEDYTEDEIGGPSSQGDQS
NRSTMNDINDYMARLIHSTSTASNTTDKFSGNQSTLRYHTASSHSDTTEEDSNLMDLGNG
LALNAYNRGPPSILMNSSDEEANGGETSDNLNTAQDLAGMKERMAQFAQSYIDKRGGLEP
ETQSNILSTSLSVMADTNEHNNEILHSSEENATNQPVNENNDLK
>O35235

MRRASRDYGKYLRSSEEMGSGPGVPHEGPLHPAPSAPAPAPPPAASRSMFLALLGLGLGQ
VVCSIALFLYFRAQMDPNRISEDSTHCFYRILRLHENAGLQDSTLESEDTLPDSCRRMKQ
AFQGAVQKELQHIVGPQRFSGAPAMMEGSWLDVAQRGKPEAQPFAHLTINAASIPSGSHK
VTLSSWYHDRGWAKISNMTLSNGKLRVNQDGFYYLYANICFRHHETSGSVPTDYLQLMVY
VVKTSIKIPSSHNLMKGGSTKNWSGNSEFHFYSINVGGFFKLRAGEEISIQVSNPSLLDP
DQDATYFGAFKVQDID
>P37088
MEGNKLEEQDSSPPQSTPGLMKGNKREEQGLGPEPAAPQQPTAEEEALIEFHRSYRELFE
FFCNNTTIHGAIRLVCSQHNRMKTAFWAVLWLCTFGMMYWQFGLLFGEYFSYPVSLNINL
NSDKLVFPAVTICTLNPYRYPEIKEELEELDRITEQTLFDLYKYSSFTTLVAGSRSRRDL
RGTLPHPLQRLRVPPPPHGARRARSVASSLRDNNPQVDWKDWKIGFQLCNQNKSDCFYQT
YSSGVDAVREWYRFHYINILSRLPETLPSLEEDTLGNFIFACRFNQVSCNQANYSHFHHP
MYGNCYTFNDKNNSNLWMSSMPGINNGLSLMLRAEQNDFIPLLSTVTGARVMVHGQDEPA
FMDDGGFNLRPGVETSISMRKETLDRLGGDYGDCTKNGSDVPVENLYPSKYTQQVCIHSC
FQESMIKECGCAYIFYPRPQNVEYCDYRKHSSWGYCYYKLQVDFSSDHLGCFTKCRKPCS
VTSYQLSAGYSRWPSVTSQEWVFQMLSRQNNYTVNNKRNGVAKVNIFFKELNYKTNSESP
SVTMVTLLSNLGSQWSLWFGSSVLSVVEMAELVFDLLVIMFLMLLRRFRSRYWSPGRGGR
GAQEVASTLASSPPSHFCPHPMSLSLSQPGPAPSPALTAPPPAYATLGPRPSPGGSAGAS
SSTCPLGGP
>Q6P7G9
MWSCEDLNYTNSGEEQYLCNEFHLFLFIFSVLYLIICFPVGLCYNVQLVLVNLYNKATMT
MPDVYFVNMAIAGLIINAVAPVYLFGPAYTKWSLWSFGNEVYITLLILFNVSSLVIMYST
TLLSLDYYIECALPRTYMSSVYNTKHVCGFIWGGAVLTSFSSLLFYICNHVSTKIIECSK
MQNREAADAIMVLIGYVVPIIAVIYALVLILQIRKEATPLDQESGRLDPSVHRLLIATVC
TQFILWTPYYVTLLVNTFMDARVKSSNTFYIRIFQFTEGLSNFLAFSSSFVLPLIHRHIN
KNFSGKLQRLLKRLHCGSQGCTHEHTVVQQVMT
>Q9NR71
MAKRTFSNLETFLIFLLVMMSAITVALLSLLFITSGTIENHKDLGGHFFSTTQSPPATQG
STAAQRSTATQHSTATQSSTATQTSPVPLTPESPLFQNFSGYHIGVGRADCTGQVADINL
MGYGKSGQNAQGILTRLYSRAFIMAEPDGSNRTVFVSIDIGMVSQRLRLEVLNRLQSKYG
SLYRRDNVILSGTHTHSGPAGYFQYTVFVIASEGFSNQTFQHMVTGILKSIDIAHTNMKP
GKIFINKGNVDGVQINRSPYSYLQNPQSERARYSSNTDKEMIVLKMVDLNGDDLGLISWF
AIHPVSMNNSNHLVNSDNVGYASYLLEQEKNKGYLPGQGPFVAAFASSNLGDVSPNILGP
RCINTGESCDNANSTCPIGGPSMCIAKGPGQDMFDSTQIIGRAMYQRAKELYASASQEVT
GPLASAHQWVDMTDVTVWLNSTHASKTCKPALGYSFAAGTIDGVGGLNFTQGKTEGDPFW
DTIRDQILGKPSEEIKECHKPKPILLHTGELSKPHPWHPDIVDVQIITLGSLAITAIPGE
FTTMSGRRLREAVQAEFASHGMQNMTVVISGLCNVYTHYITTYEEYQAQRYEAASTIYGP
HTLSAYIQLFRNLAKAIATDTVANLSRGPEPPFFKQLIVPLIPSIVDRAPKGRTFGDVLQ
PAKPEYRVGEVAEVIFVGANPKNSVQNQTHQTFLTVEKYEATSTSWQIVCNDASWETRFY
WHKGLLGLSNATVEWHIPDTAQPGIYRIRYFGHNRKQDILKPAVILSFEGTSPAFEVVTI

>P35410
MVWGKICWFSQRAGWTVFAESQISLSCSLCLHSGDQEAQNPNLVSQLCGVFLQNETNETI
HMQMSMAVGQQALPLNIIAPKAVLVSLCGVLLNGTVFWLLCCGATNPYMVYILHLVAADV

IYLCCSAVGFLQVTLLTYHGVVFFIPDFLAILSPFSFEVCLCLLVAISTERCVCVLFPIW
YRCHRPKYTSNVVCTLIWGLPFCINIVKSLFLTYWKHVKACVIFLKLSGLFHAILSLVMC
VSSLTLLIRFLCCSQQQKATRVYAVVQISAPMFLLWALPLSVAPLITDFKMFVTTSYLIS
LFLIINSSANPIIYFFVGSLRKKRLKESLRVILQRALADKPEVGRNKKAAGIDPMEQPHS
TQHVENLLPREHRVDVET
>O75695
MGCFFSKRRKADKESRPENEEERPKQYSWDQREKVDPKDYMFSGLKDETVGRLPGTVAGQ
QFLIQDCENCNIYIFDHSATVTIDDCTNCIIFLGPVKGSVFFRNCRDCKCTLACQQFRVR
DCRKLEVFLCCATQPIIESSSNIKFGCFQWYYPELAFQFKDAGLSIFNNTWSNIHDFTPV
SGELNWSLLPEDAVVQDYVPIPTTEELKAVRVSTEANRSIVPISRGQRQKSSDESCLVVL
FAGDYTIANARKLIDEMVGKGFFLVQTKEVSMKAEDAQRVFREKAPDFLPLLNKGPVIAL
EFNGDGAVEVCQLIVNEIFNGTKMFVSESKETASGDVDSFYNFADIQMGI
>Q9EPH0
MDHAEENEIPAETQKYLVERPIFSHPVLQERLHVKDKVTDSIGDKLKQAFTCTPKKVRNI
IYMFLPITKWLPAYKFKEYVLGDLVSGISTGVLQLPQGLAFAMLAAVPPVFGLYSSFYPV
IMYCFFGTSRHISIGPFAVISLMIGGVAVRLVPDDIVIPGGVNATNGTEARDALRVKVAM
SVTLLSGIIQFCLGVCRFGFVAIYLTEPLVRGFTTAAAVHVFTSMLKYLFGVKTKRYSGI
FSVVYSTVAVLQNVKNLNVCSLGVGLMVFGLLLGGKEFNERFKEKLPAPIPLEFFAVVMG
TGISAGFNLHESYSVDVVGTLPLGLLPPANPDTSLFHLVYVDAIAIAIVGFSVTISMAKT
LANKHGYQVDGNQELIALGICNSIGSLFQTFSISCSLSRSLVQEGTGGKTQLAGCLASLM
ILLVILATGFLFESLPQAVLSAIVIVNLKGMFMQFSDLPFFWRTSKIELTIWLTTFVSSL
FLGLDYGLITAVIIALLTVIYRTQSPSYTVLGQLPDTDVYIDIDAYEEVKEIPGIKIFQI
NAPIYYANSDLYSSALKRKTGVNPAIIMGARRKAMRKYAKEVGNANIANATVVKVDAEVD
GENATKPEEEDDEVKFPPIVIKTTFPEELQRFLPQGENIHTVILDFTQVNFMDSVGVKTL
AGIVKEYGDVGIYVYLAGCSAQVVNDLTSNRFFENPALKELLFHSIHDAVLGSQVREAMA
EQETTVLPPQEDMEPNATPTTPEA
>Q08229
MSEEREENGISRATLNTQRLSAMIDSLNNEKDDRLFPSPTTTRTMITEEKADQSDVFKPP
SRLLRSPAGDVSLPPGDNRSSMISNYSGIIQEGVEVSYVVKNRQQTQERRTSKDSNSLYS
LKEPVSKNELPSLPMLPSEATLTKHLSDNQSTKSNTNADEIVIKPVTNAKPVGRFNSNTS
KKVEGRGSLKLLSSPLRQEKVMRSSIGSGNLASESGSSTYNTKFHQSIQEQLEEEEEGNV
SDKLSIVSSVIPELYTTTNEAPKAINPIRSETNDYNPTIPPRSKDRPRSRLFIEEGDGEG
DLLTEEILPTPVQPGGHYKNSSQISTVSEQKSESYYSAATSMPPEEETYLTRPLPSTPNE
DSRVTSNLKRDDTLKAIHDRANHTSTSTNKQDDDMYEDIIEETPKKTKLKKDTKKKLNKK
KSVKELRSFDIDTLNQLLSVTKGTLIGSEFAQLGMKIEEKRALERLVDSLSRLTADMVLD
PDRYEEGLKRLDKATKALEGF
>O70342
MEVKLEEHFNKTFVTENNTAASQNTASPAWEDYRGTENNTSAARNTAFPVWEDYRGSVDD
LQYFLIGLYTFVSLLGFMGNLLILMAVMKKRNQKTTVNFLIGNLAFSDILVVLFCSPFTL
TSVLLDQWMFGKAMCHIMPFLQCVSVLVSTLILISIAIVRYHMIKHPISNNLTANHGYFL
IATVWTLGFAICSPLPVFHSLVELKETFGSALLSSKYLCVESWPSDSYRIAFTISLLLVQ
YILPLVCLTVSHTSVCRSISCGLSHKENRLEENEMINLTLHPSKKSRDQAKPPSTQKWSY
SFIRKHRRRYSKKTACVLPAPAGPSQEKHLTVPENPGSVRSQLSPSSKVIPGVPICFEVK
PEESSDAQEMRVKRSLTRIKKRSRSVFYRLTILILVFAVSWMPLHVFHVVTDFNDNLISN

RHFKLVYCICHLLGMMSCCLNPILYGFLNNGIKADLRALIHCLHMS

>Q14439

MGHNGSWISPNASEPHNASGAEAAGVNRSALGEFGEAQLYRQFTTTVQVVIFIGSLLGNF
MVLWSTCRTTVFKSVTNRFIKNLACSGICASLVCVPFDIILSTSPHCCWWIYTMLFCKVV
KFLHKVFCSVTILSFPAIALDRYYSVLYPLERKISDAKSRELVMYIWAHAVVASVPVFAV
TNVADIYATSTCTEVWSNSLGHLVYVLVYNITTVIVPVVVVFLFLILIRRALSASQKKKV
IIAALRTPQNTISIPYASQREAELHATLLSMVMVFILCSVPYATLVVYQTVLNVPDTSVF
LLLTAVWLPKVSLLANPVLFLTVNKSVRKCLIGTLVQLHHRYSRRNVVSTGSGMAEASLE
PSIRSGSQLLEMFHIGQQQIFKPTEDEEESEAKYIGSADFQAKEIFSTCLEGEQGPQFAP
SAPPLSTVDSVSQVAPAAPVEPETFPDKYSLQFGFGPFELPPQWLSETRNSKKRLLPPLG
NTPEELIQTKVPKVGRVERKMSRNNKVSIFPKVDS

>Q4W815

MGAPSSLLFSTAHWRTVPFLLAFWVLLSTGTAEDPTMTPEFLRHISEKIPEEYQNPHCFT
RDLNDFICFWEGERRKNASFSYSEDDQIKWCQLRTEVASNNTWWYICEFPVTDVVLFAGI
TISAYPCHKCQTAREIYINELVLLNPPLNVTVKEKQDPRGLLISWKPPHFQKNHDINNEI
KYQVNYSTPGADMQTVEVEAGNTEIFLTDIVPAAYTVTVRCKADGVSYNGYWSDWTAPIT
IATIIDLRLLLLLSIAIFVALIAGVGVYIFMRHGMYLKHKVWPQVPTPENNFQGLFTTHK
GNFKLWLGQADAYLLWISRHVFQEDPSSTLEVLSELPPAALPQSFNPNPLKDSYVVLDEN
RMPCSLEWLEAQRHKTVIVGAESMDSRLQTVNKDVVLEDTSKGQIAVKANNRVHSLEGDG
SQGEAFREDEYVEAPRMEHERHRVSRENSVSSDGKQSIPSSFEYTELQTCEGLLSPKPRP
VPPRMPLKYAYLDMSSSGEHSPPPSPNFYQNSPITNFLAPIYSQS

>O42393

MGQDQTKQQIEKGLHLYQSNQTEKALQVWMRVLEKSADPAGRFRVLGCLITAHAEMGRYK
DMLKFAVVQIDTARELEDPNYLTEGYLNLARSNEKLCEFQKTISYCKTCLNMQGTTVSLQ
LNGQVSLSMGNAFLGLSIFQKALECFEKALRYAHNNDDKMLECRVCCSLGNFYAQIKDYE
KALFFPCKAAELVNDYGAGWSLKYRAMSQYHMAVAYRKLGRLADAMDCCEESMKIALQHG
DRPLQALCLLCFADIHLSRRDVQTAFPRYDSAMSIMTEIGNRLGQIQVLLGVAKCWMIQK
ELDKALESIEKAQELAEGLGNKLGLLKLHCLCERIYRTKGLQQELRDHVVKFHECVEEME
LYCGMCGESIGEKNNQLQALPCSHFFHLKCLQTNGTRGCPNCRRLSVKPGYV

>Q38856

MKTIFLVLIFVSFAISPATSTAPEECGSESANPCVNKAKALPLKVIAIFVILIASMIGVG
APLFSRNVSFLQPDGNIFTIIKCFASGIILGTGFMHVLPDSFEMLSSICLEENPWHKFPF
SGFLAMLSGLITLAIDSMATSLYTSKNAVGIMPHGHGHGHGPANDVTLPIKEDDSSNAQL
LRYRVIAMVLELGIIVHSVVIGLSLGATSDTCTIKGLIAALCFHQMFEGMGLGGCILQAE
YTNMKKFVMAFFFAVTTPFGIALGIALSTVYQDNSPKALITVGLLNACSAGLLIYMALVD
LLAAEFMGPKLQGSIKMQFKCLIAALLGCGGMSIIAKWA

>Q5I6B8

MELEVPDEAESAEAGAVTAEAAWSAESGAAAGMTQKKAILAEAPLVTGQPGPGHGKKLGH
RGVDASGETTYKKTTSSTLKGAIQLGIGYTVGNLSSKPERDVLMQDFYVVESIFFPSEGS
NLTPAHHFQDFRFKTYAPVAFRYFRELFGIRPDDYLYSLCNEPLIELSNPGASGSVFYVT
SDDEFIIKTVMHKEAEFLQKLLPGYYMNLNQNPRTLLPKFYGLYCVQSGGKNIRVVVMNN
VLPRVVKMHLKFDLKGSTYKRRASKKEKEKSLPTYKDLDFMQDMPEGLLLDSDTFGALVK
TLQRDCLVLESFKIMDYSLLLGVHNIDQQERERQAEGAQSKADEKRPVAQKALYSTARES
IQGGAARGEAIETDDTMGGIPAVNGRGERLLLHIGIIDILQSYRFIKKLEHTWKALVHDG

DTVSVHRPSFYAERFFKFMSSTVFRKSSSLKSSPSKKGRGALLAVKPLGPTAAFSASQIP
SEREDVQYDLRGARSYPTLEDEGRPDLLPCTPPSFEEATTASIATTLSSTSLSIPERSPS
DTSEQPRYRRRTQSSGQDGRPQEELHAEDLQKITVQVEPVCGVGVVVPKEQGAGVEVPPS
GASAAATVEVDAASQASEPASQASDEEDAPSTDIYFFAHGRYWLFSPRRRRLRAVTPSHT
GAPTDGRSWVYSPLHYSARPASDGESDT
>Q9BX66
MSSECDGGSKAVMNGLAPGSNGQDKATADPLRARSISAVKIIPVKTVKNASGLVLPTDMD
PTKICTGKGAVTLRASSSYRETPSSSPASPQETRQHESKPGLEPEPSSADEWRLSSSADA
NGNAQPSSLAAKGYRSVHPNLPSDKSQDATSSSAAQPEVIVVPLYLVNTDRGQEGTARPP
TPLGPLGCVPTIPATASAASPLTFPTLDDFIPPHLQRWPHHSQPARASGSFAPISQTPPS
FSPPPPLVPPAPEDLRRVSEPDLTGAVSSTDSSPLLNEVSSSLIGTDSQAFPSVSKPSSA
YPSTTIVNPTIVLLQHNREQQKRLSSLSDPVSERRVGEQDSAPTQEKPTSPGKAIEKRAK
DDSRRVVKSTQDLSDVSMDEVGIPLRNTERSKDWYKTMFKQIHKLNRDTPEENPYFPTYK
FPELPEIQQTSEEDNPYTPTYQFPASTPSPKSEDDDSDLYSPRYSFSEDTKSPLSVPRSK
SEMSYIDGEKVVKRSATLPLPARSSSLKSSSERNDWEPPDKKVDTRKYRAEPKSIYEYQP
GKSSVLTNEKMSRDISPEEIDLKNEPWYKFFSELEFGKPPPKKIWDYTPGDCSILPREDR
KTNLDKDLSLCQTELEADLEKMETLNKAPSANVPQSSAISPTPEISSETPGYIYSSNFHA
VKRESDGAPGDLTSLENERQIYKSVLEGGDIPLQGLSGLKRPSSSASTKDSESPRHFIPA
DYLESTEEFIRRRHDDKEKLLADQRRLKREQEEADIAARRHTGVIPTHHQFITNERFGDL
LNIDDTAKRKSGSEMRPARAKFDFKAQTLKELPLQKGDIVYIYKQIDQNWYEGEHHGRVG
IFPRTYIELLPPAEKAQPKKLTPVQVLEYGEAIAKFNFNGDTQVEMSFRKGERITLLRQV
DENWYEGRIPGTSRQGIFPITYVDVIKRPLVKNPVDYMDLPFSSSPSRSATASPQFSSHS
KLITPAPSSLPHSRRALSPEMHAVTSEWISLTVGVPGRRSLALTPPLPPLPEASIYNTDH
LALSPRASPSLSLSLPHLSWSDRPTPRSVASPLALPSPHKTYSLAPTSQASLHMNGDGGV
HTPSSGIHQDSFLQLPLGSSDSVISQLSDAFSSQSKRQPWREESGQYERKAERGAGERGP
GGPKISKKSCLKPSDGVRCLSTEQRLSDLNTPEESRPGKPLGSAFPGSEAEQTERHRGGE
QAGRKAARRGGSQQPQAQQRRVTPDRSQTSQDLFSYQALYSYIPQNDDELELRDGDIVDV
MEKCDDGWFVGTSRRTKQFGTFPGNYVKPLYL
>P08571
MERASCLLLLLLPLVHVSATTPEPCELDDEDFRCVCNFSEPQPDWSEAFQCVSAVEVEIH
AGGLNLEPFLKRVDADADPRQYADTVKALRVRRLTVGAAQVPAQLLVGALRVLAYSRLKE
LTLEDLKITGTMPPLPLEATGLALSSLRLRNVSWATGRSWLAELQQWLKPGLKVLSIAQA
HSPAFSCEQVRAFPALTSLDLSDNPGLGERGLMAALCPHKFPAIQNLALRNTGMETPTGV
CAALAAAGVQPHSLDLSHNSLRATVNPSAPRCMWSSALNSLNLSFAGLEQVPKGLPAKLR
VLDLSCNRLNRAPQPDELPEVDNLTLDGNPFLVPGTALPHEGSMNSGVVPACARSTLSVG
VSGTLVLLQGARGFA
>P21978
MAQCVRRLVLAAPLAAVVALLLCTSSAPVARAAGTSDFTGAQQKNTLTVLQAFARAIPAL
GDTWTGSDFCSWEHIICYSSGVGVWMHNVDYTGTLPEMPASVDYKDVMILALDFGAMGQG
LSGTLPPSWSSMKHLIVLDLEGTKVSGTLPPEWSEMTSAEALQLENCGLSGSLPTSWSSM
PKLRIVSLSGNHFCGCVPDSWREKDRLDVTIEEWHMGEDCKLANACRPTAAPGTTTTNPP
TTTGTPAASSTPSPGSGCEVDGCEVCEGDSAARCARCREGYSLTDEKTCLGEPRWRRGGG
VERTAGCRCCVGGCAVERGAGGVRVRRAPLLCGAPGACPRPRHGVVAALSPPPADGETDS
HTRTRTRRRASRVLSAVVAPARMHGHAEACMRVRVPALVCLSVWPAVGTRRRSNVRAAAV

CRLGQRRCGARPSPCASVCVSWPRERRTECACPALFDGARLRCCALVVCAGAAPAG
>Q3V6T2
MENEIFTPLLEQFMTSPLVTWVKTFGPLAAGNGTNLDEYVALVDGVFLNQVMLQINPKLE
SQRVNKKVNNDASLRMHNLSILVRQIKFYYQETLQQLIMMSLPNVLIIGKNPFSEQGTEE
VKKLLLLLLLGCAVQCQKKEEFIERIQGLDFDTKAAVAAHIQEVTHNQENVFDLQWMEVTD
MSQEDIEPLLKNMALHLKRLIDERDEHSETIIELSEERDGLHFLPHASSSAQSPCGSPGM
KRTESRQHLSVELADAKAKIRRLRQELEEKTEQLLDCKQELEQMEIELKRLQQENMNLLS
DARSARMYRDELDALREKAVRVDKLESEVSRYKERLHDIEFYKARVEELKEDNQVLLETK
TMLEDQLEGTRARSDKLHELEKENLQLKAKLHDMEMERDMDRKKIEELMEENMTLEMAQK
QSMDESLHLGWELEQISRTSELSEAPQKSLGHEVNELTSSRLLKLEMENQSLTKTVEELR
TTVDSVEGNASKILKMEKENQRLSKKVEILENEIVQEKQSLQNCQNLSKDLMKEKAQLEK
TIETLRENSERQIKILEQENEHLNQTVSSLRQRSQISAEARVKDIEKENKILHESIKETS
SKLSKIEFEKRQIKKELEHYKEKGERAEELENELHHLEKENELLQKKITNLKITCEKIEA
LEQENSELERENRKLKKTLDSFKNLTFQLESLEKENSQLDEENLELRRNVESLKCASMKM
AQLQLENKELESEKEQLKKGLELLKASFKKTERLEVSYQGLDIENQRLQKTLENSNKKIQ
QLESELQDLEMENQTLQKNLEELKISSKRLEQLEKENKSLEQETSQLEKDKKQLEKENKR
LRQQAEIKDTTLEENNVKIGNLEKENKTLSKEIGIYKESCVRLKELEKENKELVKRATID
IKTLVTLREDLVSEKLKTQQMNNDLEKLTHELEKIGLNKERLLHDEQSTDDSRYKLLESK
LESTLKKSLEIKEEKIAALEARLEESTNYNQQLRQELKTVKKNYEALKQRQDEERMVQSS
PPISGEDNKWERESQETTRELLKVKDRLIEVERNNATLQAEKQALKTQLKQLETQNNNLQ
AQILALQRQTVSLQEQNTTLQTQNAKLQVENSTLNSQSTSLMNQNAQLLIQQSSLENENE
SVIKEREDLKSLYDSLIKDHEKLELLHERQASEYESLISKHGTLKSAHKNLEVEHRDLED
RYNQLLKQKGQLEDLEKMLKVEQEKMLLENKNHETVAAEYKKLCGENDRLNHTYSQLLKE
TEVLQTDHKNLKSLLNNSKLEQTRLEAEFSKLKEQYQQLDITSTKLNNQCELLSQLKGNL
EEENRHLLDQIQTLMLQNRTLLEQNMESKDLFHVEQRQYIDKLNELRRQKEKLEEKIMDQ
YKFYDPSPPRRRGNWITLKMRKLIKSKKDINRERQKSLTLTPTRSDSSEGFLQLPHQDSQ
DSSSVGSNSLEDGQTLGTKKSSMVALKRLPFLRNRPKDKDKMKACYRRSMSMNDLVQSMV
LAGQWTGSTENLEVPDDISTGKRRKELGAMAFSTTAINFSTVNSSAGFRSKQLVNNKDTT
SFEDISPQGVSDDSSTGSRVHASRPASLDSGRTSTSNSNNNASLHEVKAGAVNNQSRPQS
HSSGEFSLLHDHEAWSSSGSSPIQYLKRQTRSSPVLQHKISETLESRHHKIKTGSPGSEV
VTLQQFLEESNKLTSVQIKSSSQENLLDEVMKSLSVSSDFLGKDKPVSCGLARSVSGKTP
GDFYDRRTTKPEFLRPGPRKTEDTYFISSAGKPTPGTQGKIKLVKESSLSRQSKDSNPYA
TLPRASSVISTAEGTTRRTSIHDFLTKDSRLPISVDSPPAAADSNTTAASNVDKVQESRN
SKSRSREQQSS
>Q9FJ62
MINMRDNPTMHVLQASKFLFLALILIQLLSTQLFAQRSKSPWQTLTGDAPLVIARGGFSG
LLPDSSLDAYSFVSQTSVPGAVLWCDVQLTKDAIGLCFPDVKMMNASNIQDVYPKRKTSY
LLNGVPTQDWFTIDFNFKDLTKVILKQGILSRSAAFDGNSYGISTVKDISTQLKPEGFWL
NVQHDAFYAQHNLSMSSFLLSISKTVIIDYLSSPEVNFFRNIGRRFGRNGPKFVFRFLEK
DDVEVSTNQTYGSLAGNLTFLKTFASGVLVPKSYIWPIESQYLLPRTSFVQDAHKAGLEV
YASGFGNDFDLAYNYSFDPLAEYLSFMDNGDFSVDGLLSDFPLTASSAVDCFSHLGSNAS
SQVDFLVISKNGASGDYPGCTDLAYTKAIKDGADVIDCSLQMSSDGIPFCLSSINLGEST
NVVQSPFRNRSTTVPEIGSLPGIYSFSLAWSEIQTLRPAIENPYSREFTMFRNPRERSSG
KFVSLSDFLNLAKNSSSLTGVLISVENATYLREKQGLDAVKAVLDTLTEAGYSNKTTTTR

VMIQSTNSSVLIDFKKQSRYETVYKVEETIRDILDTAIEDIKKFADAVVISKKSVFPTSE
SFTTGQTKLVERLQKFQLPVYVEVFRNEFVSQPWDFFADATVEINSHVTGAGINGTITEF
PLTAARYKRNSCLTRKDVPPYMIPVQPAGLLTIVSPASLPPAEAPSPVFTDADVTEPPLP
PVSARAPTTTPGPQSTGEKSPNGQTRVALSLLLSAFATVFASLLLL
>Q96247
MSEGVEAIVANDNGTDQVNGNRTGKDNEEHDGSTGSNLSNFLWHGGSVWDAWFSCASNQV
AQVLLTLPYSFSQLGMLSGIVLQIFYGLLGSWTAYLISVLYVEYRARKEKEGKSFKNHVI
QWFEVLDGLLGSYWKALGLAFNCTFLLFGSVIQLIACASNIYYINDHLDKRTWTYIFGAC
CATTVFIPSFHNYRIWSFLGLGMTTYTAWYLAIASIIHGQAEGVKHSGPTKLVLYFTGAT
NILYTFGGHAVTVEIMHAMWKPQKFKYIYLMATLYVFTLTIPSAAAVYWAFGDALLDHSN
AFSLMPKNAWRDAAVILMLIHQFITFGFACTPLYFVWEKVIGMHDTKSICLRALARLPVV
IPIWFLAIIFPFFGPINSAVGALLVSFTVYIIPSLAHMLTYRSASARQNAAEKPPFFMPS
WTAMYVLNAFVVVWVLIVGFGFGGWASVTNFVRQVDTFGLFAKCYQCKPAAAAAHAPVSA
LHHRL
>A5X5Y0
MEGSWFHRKRFSFYLLLGFLLQGRGVTFTINCSGFGQHGADPTALNSVFNRKPFRPVTNI
SVPTQVNISFAMSAILDVNEQLHLLSSFLWLEMVWDNPFISWNPEECEGITKMSMAAKNL
WLPDIFIIELMDVDKTPKGLTAYVSNEGRIRYKKPMKVDSICNLDIFYFPFDQQNCTLTF
SSFLYTVDSMLLDMEKEVWEITDASRNILQTHGEWELLGLSKATAKLSRGGNLYDQIVFY
VAIRRRPSLYVINLLVPSGFLVAIDALSFYLPVKSGNRVPFKITLLLGYNVFLLMMSDLL
PTSGTPLIGVYFALCLSLMVGSLLETIFITHLLHVATTQPPPLPRWLHSLLLHCNSPGRC
CPTAPQKENKGPGLTPTHLPGVKEPEVSAGQMPGPAEAELTGGSEWTRAQREHEAQKQHS
VELWLQFSHAMDAMLFRLYLLFMASSIITVICLWNT
>Q63072
MAVQACALSLRLGLWMSLLLPVLPGAGARAAGARWSGEGTTPHLQSIFLGRCAEYTTLLS
LEPGNKNCTAIWEAFKVVLDKDPCSVLPSDYDLFINLSRHAIPRDKSLFWENNHLLVMSY
AENTRRLMPLCDVLYGKVGDFLSWCRQENASGLDYQSCPTAEDCENNAVDAYWKSASMQY
SRDSSGVINVMLNGSEPKGAYPTKGFFADFEIPYLQKDKITRIEIWVMHEVGGPHVESCG
EGSVKILEDRLEALGFQHSCINDYPPVKFLMCVDHSTHPDCAMNSASASMWRESPALHAI
GDISLIISLLVALASSSQA
>Q8TEW0
MKVTVCFGRTRVVVPCGDGHMKVFSLIQQAVTRYRKAIAKDPNYWIQVHRLEHGDGGILD
LDDILCDVADDKDRLVAVFDEQDPHHGGDGTSASSTGTQSPEIFGSELGTNNVSAFQPYQ
ATSEIEVTPSVLRANMPLHVRRSSDPALIGLSTSVSDSNFSSEEPSRKNPTRWSTTAGFL
KQNTAGSPKTCDRKKDENYRSLPRDTSNWSNQFQRDNARSSLSASHPMVGKWLEKQEQDE
DGTEEDNSRVEPVGHADTGLEHIPNFSLDDMVKLVEVPNDGGPLGIHVVPFSARGGRTLG
LLVKRLEKGGKAEHENLFRENDCIVRINDGDLRNRRFEQAQHMFRQAMRTPIIWFHVVPA
ANKEQYEQLSQSEKNNYYSSRFSPDSQYIDNRSVNSAGLHTVQRAPRLNHPPEQIDSHSR
LPHSAHPSGKPPSAPASAPQNVFSTTVSSGYNTKKIGKRLNIQLKKGTEGLGFSITSRDV
TIGGSAPIYVKNILPRGAAIQDGRLKAGDRLIEVNGVDLVGKSQEEVVSLLRSTKMEGTV
SLLVFRQEDAFHPRELNAEPSQMQIPKETKAEDEDIVLTPDGTREFLTFEVPLNDSGSAG
LGVSVKGNRSKENHADLGIFVKSIINGGAASKDGRLRVNDQLIAVNGESLLGKTNQDAME
TLRRSMSTEGNKRGMIQLIVARRISKCNELKSPGSPPGPELPIETALDDRERRISHSLYS
GIEGLDESPSRNAALSRIMGESGKYQLSPTVNMPQDDTVIIEDDRLPVLPPHLSDQSSSS

SHDDVGFVTADAGTWAKAAISDSADCSLSPDVDPVLAFQREGFGRQSMSEKRTKQFSDAS
QLDFVKTRKSKSMDLGIADETKLNTVDDQKAGSPSRDVGPSLGLKKSSSLESLQTAVAEV
TLNGDIPFHRPRPRIIRGRGCNESFRAAIDKSYDKPAVDDDDEGMETLEEDTEESSRSGR
ESVSTASDQPSHSLERQMNGNQEKGDKTDRKKDKTGKEKKKDRDKEKDKMKAKKGMLKGL
GDMFRFGKHRKDDKIEKTGKIKIQESFTSEEERIRMKQEQERIQAKTREFRERQARERDY
AEIQDFHRTFGCDDELMYGGVSSYEGSMALNARPQSPREGHMMDALYAQVKKPRNSKPSP
VDSNRSTPSNHDRIQRLRQEFQQAKQDEDVEDRRRTYSFEQPWPNARPATQSGRHSVSVE
VQMQRQRQEERESSQQAQRQYSSLPRQSRKNASSVSQDSWEQNYSPGEGFQSAKENPRYS
SYQGSRNGYLGGHGFNARVMLETQELLRQEQRRKEQQMKKQPPSEGPSNYDSYKKVQDPS
YAPPKGPFRQDVPPSPSQVARLNRLQTPEKGRPFYS
>Q9H2K8
MRKGVLKDPEIADLFYKDDPEELFIGLHEIGHGSFGAVYFATNAHTSEVVAIKKMSYSGK
QTHEKWQDILKEVKFLRQLKHPNTIEYKGCYLKEHTAWLVMEYCLGSASDLLEVHKKPLQ
EVEIAAITHGALHGLAYLHSHALIHRDIKAGNILLTEPGQVKLADFGSASMASPANSFVG
TPYWMAPEVILAMDEGQYDGKVDIWSLGITCIELAERKPPLFNMNAMSALYHIAQNDSPT
LQSNEWTDSFRRFVDYCLQKIPQERPTSAELLRHDFVRRDRPLRVLIDLIQRTKDAVREL
DNLQYRKMKKILFQETRNGPLNESQEDEEDSEHGTSLNREMDSLGSNHSIPSMSVSTGSQ
SSSVNSMQEVMDESSSELVMMHDDESTINSSSSVVHKKDHVFIRDEAGHGDPRPEPRPTQ
SVQSQALHYRNRERFATIKSASLVTRQIHEHEQENELREQMSGYKRMRRQHQKQLIALEN
KLKAEMDEHRLKLQKEVETHANNSSIELEKLAKKQVAIIEKEAKVAAADEKKFQQQILAQ
QKKDLTTFLESQKKQYKICKEKIKEEMNEDHSTPKKEKQERISKHKENLQHTQAEEEAHL
LTQQRLYYDKNCRFFKRKIMIKRHEVEQQNIREELNKKRTQKEMEHAMLIRHDESTRELE
YRQLHTLQKLRMDLIRLQHQTELENQLEYNKRRERELHRKHVMELRQQPKNLKAMEMQIK
KQFQDTCKVQTKQYKALKNHQLEVTPKNEHKTILKTLKDEQTRKLAILAEQYEQSINEMM
ASQALRLDEAQEAECQALRLQLQQEMELLNAYQSKIKMQTEAQHERELQKLEQRVSLRRA
HLEQKIEEELAALQKERSERIKNLLERQEREIETFDMESLRMGFGNLVTLDFPKEDYR
>Q86UW1
MEPGRTQIKLDPRYTADLLEVLKTNYGIPSACFSQPPTAAQLLRALGPVELALTSILTLL
ALGSIAIFLEDAVYLYKNTLCPIKRRTLLWKSSAPTVVSVLCCFGLWIPRSLVLVEMTIT
SFYAVCFYLLMLVMVEGFGGKEAVLRTLRDTPMMVHTGPCCCCCPCCPRLLLTRKKLQLL
MLGPFQYAFLKITLTLVGLFLVPDGIYDPADISEGSTALWINTFLGVSTLLALWTLGIIS
RQARLHLGEQNMGAKFALFQVLLILTALQPSIFSVLANGGQIACSPPYSSKTRSQVMNCH
LLILETFLMTVLTRMYYRRKDHKVGYETFSSPDLDLNLKA
>Q6GTX8
MSPHPTALLGLVLCLAQTIHTQEEDLPRPSISAEPGTVIPLGSHVTFVCRGPVGVQTFRL
ERESRSTYNDTEDVSQASPSESEARFRIDSVSEGNAGPYRCIYYKPPKWSEQSDYLELLV
KETSGGPDSPDTEPGSSAGPTQRPSDNSHNEHAPASQGLKAEHLYILIGVSVVFLFCLLL
LVLFCLHRQNQIKQGPPRSKDEEQKPQQRPDLAVDVLERTADKATVNGLPEKDRETDTSA
LAAGSSQEVTYAQLDHWALTQRTARAVSPQSTKPMAESITYAAVARH
>Q12117
MSTFETLIKRGGNEAIKINPPTGADFHITSRGSDWFWTCFCCYLLFGLILTFLMFRKPVN
DRFFYLTGIAPNFFMCIAYFTMASNLGWIPVKAKYNHVQTSTQKEHPGYRQIFYSRFVGW
FLALPWPIIQICMLAGTPFWQMAFNVCITEFFTVCWLIAACVHSTYKWGYYTIGLGAAIV
VSISVMTTSYNLVKQRDNDIRLTFLVFFSIIMFLWIIAYPTCFGITDGGNVLQPDSAGIF

YGIIDLILMCFIPTLLVPIANHFGADKLGYHFGPSDAEAVMAPKAPVASPRPAATPNLSK
DKKKKSKKSKKSKKSKKSEE

>Q9JL55

MWLWEDQGGLLGPFSFVLVLLLVVTRSPFNACVLTGSLYLLLRFFSFEPVPSRRALQVLK
PRDRVSAIAHRGGSHDAPENTLAAIRQAAKNGATGVELDIEFTSDGVPVLMHDNTVDRTT
DGSGRLCDLTFEQVRKLNPAANHRLRNEFPDERIPTLREAVTECLCHNLTIFFDVKGHAD
MASAALKNIYMEFPQLYNNSMVCSFLPEVIYKMRQTDQKVITALTHRPWSLSHTGDGKPR
YSVFWKQSVFVVLDILLDWSMHNVLWYLCGISAFLMQKDFVSPDYLKKWSAKGIQVVSWT
VNTFDEKNYYESHLGSSYITDSMLEDCAPHF

>Q9R269

MHSLFRKRNKGKYSPTVQTRSISNKELSDLIEQLQKNADQVERNIVDTEAKMQSDLARMQ
EGQLPEHRDAALQNVSDSEKLLYVLEADSAIAKHMKHPQGDMIAEDIRQLKERVTNLRGK
HKQMYSLAVKEADPRVNWDTLVDEKLDKLSSQSFGTDLPLVDSQVEQHNIFHNEVKAIGP
HLAKDKEQNSELQAKYQKLLTASQARQQHLSSLQDYMQRCTNELYWLDQQAKGRMQYDWS
DRNLDYPSRRRQYENFINRNLEAKEERINKLHTEGDQLLTAEHPGRNSIEAHMEAVHAEW
KEYLNLLICEESHLKYMEDYHQFHKDMKDAQELLRKVDSDLNQKYSPDFKDRYQIELLLR
ELDDQEKALDKYEDVVRGLQRRGQQVVPLKYRRETPLKPIPVEALCDFESDQGLISRGYS
YTLQKNNGESWELTDSTGKKLAAPAVCFIIPPTDPEALALADSLGSQYRSVRQKATGSKH
ALQQRHEVLRTENPGDASDLQGRQLLAGLDKVASDLDRQEKAITGILRPPLEQGRAIEDS
AERAKGLKNITNELLQIEPEKTQCTAECEAFVQALPASGTAPLLKTRVEDTNQKYERLVW
LLEAAQEKVDVANRLENSLQRGRELLASYENRLIQDDTMPESGHVLDRKRQELEAMASEL
QAHKSLLGEVGKNLQVAKQCSSSLASRFQEHCPDLERQEAEVHKLNQRFNNLSQQVERRA
QSLQSARAAYDEYCSGYNRVLQFLAKTPSYEPQETDSLGQMETKLKNQKNLLDELASREQ
EVQKVYADSQQYQQAVKDYELEAEKLRSLLDLENGRNSHVNKRARLQSPAAKVKEEEAAL
AAKFTEVNAINRQRLQNLEFALNLLRQQPEAGVTHETLQGGKLSSGMEETWKIKKELEEE
IERRQQLENEVKSAQEEIQTLKDQGPQESLVRKEVLKKVPDPALEESFQQLQQTLAEEQH
KNQLLQEELGALQLRLQALEQETRDGGQEYVVKEVLRIEPDRAQEDEVLQLREELEALRR
QKGAREAEVLLLQQRVAALAAEKSRVQEKVTEREVVKLQNDPQLEAEYRRLQEEHQREGT
LREKQEEELSFLQAKLRRLEKERAMAEGKITVKEVLKVEKDAAVEREVNDLTRQYEDEAA
KARSGQREKTELLRKIWALEEENAKVVVQEKVREIVRPDPKAESEVANLRLELVEQERKF
RGAEEQLKSYQSELEALRNRGPQVEVKEVTKEVIKYTTDPETEQELQRLREEIMDKTRLI
ERCDLEIYQLKQEIQALKDTKPQVQTREVVQEILQFQEDPQTKKEVESLRIQLSEEQKKQ
VDLEGERASQEEKIKRKEEELAQQRKERVVRQEVVQYEDEPDLRAEVTAFTNSIDAELRQ
IDKLHVELRRLQHRRAELERQLEELERERQARRAAELEVQRLQQRLAALEQEEAKTGEKV
THTQKVVLQQDPQQTREHALLRAQLEEERHRRQLLEGELEPLRRKLAALEKAEIKEKVVF
SESVQVEKGDTEQEIQRLKKSLEEESQSKRELDSEVTRLEAKLSELEFYNSKSSKELDFL
REENHKLQLERQNLQLETRRLQSEIEMAATETRDLKNITTIDSGTHLNSRLWSLEKELDD
LKKMSKDKDLEIDELQRRLGSVAVKREQRENHLRRSIVVIDPDTGRELSPEEAHRAGLID
WKMFVKLRSQECDWEEISVKGPNGESSVIHDRKSGKKFSIEDALQSGRLTAAQYDRYVNK
DMSIQELAVLVSGQK

>Q7TNJ2

MAFCTQLMLLLWKNYTYRRRQPIQLVVELLWPLFLFFILVAVRHSHPPLEHHECHFPNKP
LPSAGTVPWLQGLVCNVNNSCFQHPTPGEKPGVLSNFKDSLISRLLADAHTVLGGHSTQD
MLAALGKLIPVLRAVGSGAWPQESNQPAKQGSVTELLEKILQRASLETVLGQAQDSMRKF

SDATRTVAQELLTLPSLVELRALLRRPRGSAGSLELISEALCSTKGPSSPGGLSLNWYEA
NQINEFMGPELAPTLPDSSLSPACSEFVGALDDHPVSRLLWRRLKPLILGKILFAPDTNF
TRKLMAQVNQTFEELALLRDLHELWGVLGPQIFNFMNDSTNVAMLQKLLDVEGTGWQQQT
PKGQKQLEAIRDFLDPSRGRYNWQEAHADMGRLAEILGQILECVSLDKLEAVPSEEALVS
RALELLGERRLWAGIVFLSPEHPLDSSEPPSPTTTGPGHLRVKIRMDIDDVTRTNKIRDK
FWDPGPSADPLMDLRYVWGGFVYLQDLLEQAAVRVLSGRDSRAGLYLQQMPHPCYVDDVF
LRVLSRSLPLFLTLAWIYSVALTVKAVVREKETRLRETMRAMGLSRAVLWLGWFLSCLGP
FLVSAALLVLVLKLGNILPYSHPVVVFLFLAAFAVATVAQSFLLSAFFSRANLAAACGGL
AYFALYLPYVLCVAWRERLPLGGLLAVSLLSPVAFGFGCESLALLEEQGDGAQWHNLGTG
PAEDVFSLAQVSAFLLLDAVIYGLALWYLEAVCPGQYGIPEPWNFPFRRSYWCGPGPPKS
SVLAPAPQDPKVLVEEPPPGLVPGVSIRGLKKHFRGSPQPALRGLNLDFYEGHITAFLGH
NGAGKTTTLSILSGLFPPSSGSASILGHDVQTNMAAIRPHLGICPQYNVLFDMLTVEEHV
WFYGRLKGVSAAAIDSEQEHLIRDVGLIPKRDTQTRHLSGGMQRKLSVAIAFVGGSRVVI
MDEPTAGVDPASRRGIWELLLKYREGRTLILSTHHLDEAELLGDRVAMVASGSLCCCGSP
LFLRRHLGCGYYLTLVKSSQSLVTHDLKGDTEDPRREKKSGSEGKTADTVLTRDGPHRSS
QVPAPDAVPVTPSAALILELVQRHVPGAQLVEELPHELVLALPYAGALDGSFATVFQELD
QQLERLGLTGYGISDTNLEEIFLKVVEEAHAHGEGGDPRQQQHLLTATPQPHTGPEASVL
ENGELAKLVLDPQAPKGSAPTTAQVQGWTLTCQQLRALLHKRFLLARRSRRGLFAQIVLP
ALFVGLALFFTLIVPPFGQYPPLQLSPAMYGPQVSFFSEDAPADPNRMKLLEALLGEAGL
QDPSVQGKGSRGSECTHSLACYFTVPEVPPDVASILASGNWTPDSPSPACQCSQPGARRL
LPDCPAGAGGPPPPQAMAGFGEVVQNLTGRNVSDFLVKTYPSLVRRGLKTKKWVDEVRYG
GFSLGGRDPDLPSGREVVRTVAEMRALLSPQPGNTLDRILNNLTQWALGLDARNSLKIWF
NNKGWHAMVAFVNRANNGLLRAFLPSGSVRHAHSITTLNHPLNLTKEQLSEATLIASSVD
VLVSICVVFAMSFVPASFTLVLIEERITRAKHLQLVSGLPQTLYWLGNFLWDMCNYLVAV
CIVVLIFLAFQQKAYVAPENLPALLLLLLLYGWSITPLMYPASFFFSVPSTAYVVLTCIN
LFIGINSSMATFVLELLSDQNLQEVSRILKQVFLIFPHFCLGRGLIDMVRNQAMADAFER
LGDKQFQSPLRWDIIGKNLLAMVAQGPLFLLITLLLQHRNRLLPQPKSRLPPPLGEEDED
VVRERERVTKGATQGDVLVLRDLTKVYRGQRSPAVDHLCLGIPPGECFGLLGVNGAGKTS
TFRMVTGDTLPSSGEAVLAGHNVAQEPSAAHRSMGYCPQSDAIFDLLTGREHLELFARLR
GVPEAQVAQTALSGLVRLGLPSYADRPAGTYSGGNKRKLATALALVGDPAVVFLDEPTTG
MDPSARRFLWNNLLSVVREGRSVVLTSHSMEECEALCTRLAIMVNGRFRCLGSAQHLKSR
FGAGHTLTLRVPPDQPEPAIAFIVTTFPDAELREVHGSRLRFQLPPGGGCTLARVFRELA
AQGKAHGVEDFSVSQTTLEEVFLYFSKDQGEEEEGSGQETETREVSTPGLQHPKRVSRFL
EDPSSVETVI
>Q9H1K0
MASLDDPGEVREGFLCPLCLKDLQSFYQLHSHYEEEHSGEDRDVKGQIKSLVQKAKKAKD
RLLKREGDDRAESGTQGYESFSYGGVDPYMWEPQELGAVRSHLSDFKKHRAARIDHYVVE
VNKLIIRLEKLTAFDRTNTESAKIRAIEKSVVPWVNDQDVPFCPDCGNKFSIRNRRHHCR
LCGSIMCKKCMELISLPLANKLTSASKESLSTHTSPSQSPNSVHGSRRGSISSMSSVSSV
LDEKDDDRIRCCTHCKDTLLKREQQIDEKEHTPDIVKLYEKLRLCMEKVDQKAPEYIRMA
ASLNAGETTYSLEHASDLRVEVQKVYELIDALSKKILTLGLNQDPPPHPSNLRLQRMIRY
SATLFVQEKLLGLMSLPTKEQFEELKKKRKEEMERKRAVERQAALESQRRLEERQSGLAS
RAANGEVASLRRGPAPLRKAEGWLPLSGGQGQSEDSDPLLQQIHNITSFIRQAKAAGRMD
EVRTLQENLRQLQDEYDQQQTEKAIELSRRQAEEEDLQREQLQMLRERELEREREQFRVA

SLHTRTRSLDFREIGPFQLEPSREPRTHLAYALDLGSSPVPSSTAPKTPSLSSTQPTRVW
SGPPAVGQERLPQSSMPQQHEGPSLNPFDEEDLSSPMEEATTGPPAAGVSLDPSARILKE
YNPFEEEDEEEEAVAGNPFIQPDSPAPNPFSEEDEHPQQRLSSPLVPGNPFEEPTCINPF
EMDSDSGPEAEEPIEEELLLQQIDNIKAYIFDAKQCGRLDEVEVLTENLRELKHTLAKQK
GGTD
>Q7TSN5
MGKATLAVFADSDSSNMNESFAHLHFAGGYLPSDSKGWRTIIPSLLAAVCLVGFVGNLCV
IGLLLHGVWKRKPSMIHSLILNLSLADISLLLFSAPVRATAYVKGVWDLGWFVCKSSDWF
THMCMAAKSLTFVVVAKVCFMYASDPAKPVGTHNCTIWSLLGAIWVVASLLPLPEWFFST
TRHHAGVEMCLVDVPAVAAEFMSLFGKLYPLLVFCLPLLLAGFYFWRAYNQCKIRCAKTQ
NLRNQMRSKQLTVMLLSTAVTSALLWLPEWIAWLWVWHLKAGGPMPPQGFIALSQVLMFS
ISTVNPLIFLMMSEEFKAGLKGIWKWMITRKPVVTSEVQEVPAGNIETLPGKAPSPETQT
CIPDTDRCGSPDSSKETTDKVMVPILPDVEQFWHERDVGPSAQDNDPIPWEHEGQETKGC
N
>Q8QFX1
MREAAERRQQLELEHEQALAVLNAKQQEIELLQKAQVEAKKEHEGAVQLLEAKVRELEEK
CRTQSEQFNLLSRELEKFRQQAGKIDLLSSNPLTSSDISGSPSKSLSQLMNGIATSLGKG
HESPSGSRCVISEFIRPLQISGDKPEQLSVKPTFLSKSRSDTPRCRFDSDMDNDQNSNTS
KQRYSGKVHLCIARYSYNPFDGPNENPEAELPLTAGKYLYVYGDMDEDGFYEGELLDGQR
GLVPSNFVDFVQDNETRLSSTLSSEQDQNFINHSGSTLEGDILEISPPSHIDSSVISNGA
GTLDVNIDEIGEDIVPYPRKITLIKQLAKSVIVGWEPPVVPPGWGTINSYNVLVDKEVRM
NIALGSRTKALIEKLNISTCTYRISIQSITNKGNSDELQCTLLVGKDVVVAPSNLKVDNI
TQISAELSWLPTNSNYSHVIFLNEEEFDIVKAASYKYHFFNLKPNMAYKVKVMAKPHQMP
WQLPLEQREKKEAFVEFSTLPAGPPAPPQDVTVRAGSTQATIQVSWKPPALTATGTSHGA
NVTGYGVYAKGQRVAEVIFPTAENTLVELMRLRNLEAKEVTVRTLSAQGESVDSSVAAIP
SDLLVPPSPHPRTAPKSKPLASAGAPETKEEHLGPHLKIDESWEQTHSASPVHGHTLEPP
VPNFPSSLQGRRSPSPNRILPQPQGTPVPNTVAKAMAREAAQRVAESNRMERRSVFSERS
NAAQYANSDDEEDGYDSPNVKRRGASVDDFLKGSELGKQPHYCHGEDYHTESSRGSDLSD
IMEEDEEELYSEMQLEDGGRRRVSLTSHNALKECNKNKTTDATFLEQPDFSQQIHHSKKL
FSIPEVAEEDGEYSELLYKQGLGMPYKKKSTIARDSRPPRPYNQDQQHNFWYPAKHRISG
MEDFTADDKGCKYSRSLSRSPDSGLDCGSEEEESRFTFRYTCDSVSANVASSCCADTADC
SCRKSMRPLLARRKTLTRQTSIEEDFGDLGSSFVEPRSEQVKSSYEKKYETQKCNRTDNL
SNEDIQGGWKNDLKMADSRAAGPLAKSSHRDAEDSLLLGNPSSAGRPERVEHAGRRSSHG
SAVPQRSRPMLVPSIDGYGGHDHLSPDIYEESETDPGTEDISTRIFVALFDYDPLTMSPN
PDAAEEELPFKEGQIIKVYGDKDADGFYRGETCTRIGLIPCNMVSEIQADDEEMMDQLLK
QGFLPLNTPVEKIERNRRSGRQHSVSTRRMVALYDYDPRESSPNVDVEAELTFCTGDIIT
VFGEIDEDGFYYGELNGQKGLVPSNFLEEVPDDVEVYLSDAPSRYLHDTPMRTKAKRKKS
VHFTP
>Q7M720
MGSNVYGILTMVMIAEFVFGNMSNGFIVLINCIDWVRKGTLSSIGWILLFLAISRMVLIW
EMLITWIKYMKYSFSFVTGTELRGIMFTWVISNHFSLWLATILSIFYLLKIASFSKPVFL
YLKWREKKVLLIVLLGNLIFLMLNILQINKHIEHWMYQYERNITWSSRVSDFAGFSNLVL
LEMIVFSVTPFTVALVSFILLIFSLWKHLQKMHLNSRGERDPSTKAHVNALRIMVSFLLL
YATYFISFFLSLIPMAHKTRLGLMFSITVGLFYPSSHSFILILGHSNLRQASLWVMTYLK

CGQKH
>Q12256
MPSSLTKTESNSDPRTNIQQVPKALDKNVTNSGNLDSTSSSTGSITEDEKRSEPNADSNN
MTGGEPIDPRDLDWDGPDDPDNPHNWSSLKKWYTTMTSAFLCLVVTMGSSLYVSSVPELV
ERYHVSQTLALAGLTFYLLGLSTVIGAPLSEVFGRKPVYLFSLPVSMLFTMGVGLSNGHM
RIILPLRFLSGVFASPALSVGSGTILDIFDVDQVSVAMTYFVLSPFLGPVLSPIMAGFAT
EAKGWRWSEWIQLIAGGLILPFIALMPETHKGIILRKRAKKRNIALKKFSREAQKEFLKT
TVTITILRPLKMLVVEPIVFVFSVYVAFIFAILFGFFEAYAVIYRGVYHMSMGISGLPFI
GIGVGLWIGAFFYLYIDRKYLFPKPPAGTQPLTEKERTSKRTTPYRGARDAETGELLPVV
PEKFLIACKFGSVALPIGLFWQAWTARSDVHWMAPVAAGVPFGFGLILIFFSVLMYFSTC
YPPLTVASCLAANNLLRYVMSSVFPLFTIQMYTKMKIKWASTLFALVCVVMIPIPWVFEK
WGSKLRHKSQFGYAAMEKEAETEGGIDDVNAVDGELNLTRMTTLRTMETDPSTREKPGER
LSLRRTHTQPVPASFDREDGQHAQNRNEPISNSLYSAIKDNEDGYSYTEMATDASARMV
>P41968
MSIQKTYLEGDFVFPVSSSSFLRTLLEPQLGSALLTAMNASCCLPSVQPTLPNGSEHLQA
PFFSNQSSSAFCEQVFIKPEVFLSLGIVSLLENILVILAVVRNGNLHSPMYFFLCSLAVA
DMLVSVSNALETIMIAIVHSDYLTFEDQFIQHMDNIFDSMICISLVASICNLLAIAVDRY
VTIFYALRYHSIMTVRKALTLIVAIWVCCGVCGVVFIVYSESKMVIVCLITMFFAMMLLM
GTLYVHMFLFARLHVKRIAALPPADGVAPQQHSCMKGAVTITILLGVFIFCWAPFFLHLV
LIITCPTNPYCICYTAHFNTYLVLIMCNSVIDPLIYAFRSLELRNTFREILCGCNGMNLG

>P40485
MSKNNTMTSAVSDMLSQQQLNLQHLHNLQQHTRSMTSADHANVLQQQQQQQQQQQQQQQQ
QQQSASFQNGSLTSDINQQSYLNGQPVPSTSNSTFQNNRTLTMNSGGLQGIISNGSPNID
SNTNVTIAVPDPNNNNGKQLQGKNSLTNTSILSRARSSLQRQRLAQQQQQQQDPRSPLVI
LVPTAAQPTDILAARFSAWRNVIKSVIVYLTEIASIQDEIVRQQLRLSHAVQFPFFSIEN
QYQPSSQEDKSVQKFFLPLGNGSIQDLPTILNQYHESLASSASKASRELTNDVIPRLEDL
RRDLIVKIKEIKSLQSDFKNSCSKELQQTKQAMKQFQESLKDARYSVPKQDPFLTKLALD
RQIKKQLQEENFLHEAFDNLETSGAELEKIVVMEIQNSLTIYARLLGQEAQLVFDILISK
LDSGFFNVDPQFEWDNFISRDPNFLLPNLPMRTFKEIVYKYQFDPLTYEIKSGFLERRSK
FLKSYSKGYYVLTPNFLHEFKTADRKKDLVPVMSLALSECTVTEHSRKNSTSSPNSTGSD
AKFVLHAKQNGIIRRGHNWVFKADSYESMMSWFDNLKILTSTSNIQDKYKFITQKLNLNS
DGKPKLTNNHTSINKYQLSNANSTMVENDENDDINSNYVGSTVTPKLDNQTNTNTSMSSL
PDTNDSELQDQVPNIYIQTPINDFKS
>P83861
MQALNITAEQFSRLLSAHNLTREQFIHRYGLRPLVYTPELPARAKLAFALAGALIFALAL
FGNSLVIYVVTRSKAMRTVTNIFICSLALSDLLIAFFCIPVTMLQNISDKWLGGAFICKM
VPFVQSTAVVTEILTMTCIAVERHQGLIHPFKMKWQYTTRRAFTILGVVWLAAIIVGSPM
WHVQRLEIKYDFLYEKEHVCCLEEWASPMHQRIYTTFILVILFLLPLVVMLVLYSKIGYE
LWIKKRVGDSSALQTIHGKEMSKIARKKKRAVVMMVTVVALFAACWAPFHVVHMMVEYSN
FEKEYDDVTIKMVFAVAQTIGFFNSICNPFVYAFMNENFKKNFLSAVCYCIVRETFSPGQ
KPGNSGISMMQKRAKLSRSQRPVAEAKGDLFSDANVDVKLCEQPGEKRQLKRQLAFFSSE
LSENSTFGSGHEL
>P0C673

MTRRRSAPASWLLVSLLGVATSLEVSESPGSVQVARGQTAVLPCAFSTSAALLNLNVIWM
VIPLSNANQPEQVILYQGGQMFDGALRFHGRVGFTGTMPATNVSIFINNTQLSDTGTYQC
LVNNLPDRGGRNIGVTGLTVLVPPSAPQCQIQGSQDLGSDVILLCSSEEGIPRPTYLWEK
LDNTLKLPPTATQDQVQGTVTIRNISALSSGLYQCVASNAIGTSTCLLDLQVISPQPRSV
GVIAGAVGTGAVLIVICLALISGAFFYWRSKNKEEEEEEIPNEIREDDLPPKCSSAKAFH
TEISSSENNTLTSSNTYNSRYWNNNPKPHRNTESFNHFSDLRQSFSGNAVIPSIYANGNH
LVLGPHKTLVVTANRGSSPQVLPRNNGSVSRKPWPQHTHSYTVSQMTLERIGAVPVMVPA
QSRAGSLV
>P70585
MGFDHRMETDQPPVVTATLLVPLQNGSCVEAAEALLPHGLMELHEEHSWMSNRTDLQYEL
NPGEVATASIFFGALWLFSIFGNSLVCLVIHRSRRTQSTTNYFVVSMACADLLISVASTP
FVVLQFTTGRWTLGSAMCKVVRYFQYLTPGVQIYVLLSICIDRFYTIVYPLSFKVSREKA
KRMIAASWILDAAFVTPVFFFYGSNWDSHCNYFLPPSWEGTAYTVIHFLVGFVIPSVLII
LFYQKVIKYIWRIGTDGRTLRRTMNIVPRTKVKTVKMFLLLNLVFLFSWLPFHVAQLWHP
HEQDYRKSSLVFTAVTWVSFSSSASKPTLYSIYNANFRRGMKETFCMSSMKCYRSNAYTI
TTSSRMAKRNYVGISEIPPVSRTITKDSIYDSFDREAREKKLAWPINSNPPNTFV
>P70310
METSSLWPPRPSPSAGLSLEARLGVDTRLWAKVLFTALYSLIFALGTAGNALSVHVVLKA
RTGRPGRLRYHVLSLALSALLLLLISVPMELYNFVWSHYPWVFGDLGCRGYYFVRELCAY
ATVLSVASLSAERCLAVCQPLRARRLLTPRRTCRLLSLVWVASLGLALPMAVIMGQKHEM
ERADGEPEPASRVCTVLVSRASSRSTFQVKRAGLLRSPLWELTAILNGITVNHLVALYSQ
VPSASAQVNSIPSRLELLSEEGLLGFITWRKTLSLGVQASLVRHKDASQIRSLQHSAQVL
RAIVAVYVICWLPYHARRLMYCYIPDDGWTDELYDFYHYFYMVTNTLFYVSSAVTPVLYN
AVSSSFRKLFLESLSSLCGEQRSVVPLPQEAPESTTSTYSFRLWGSPRNPSLGEIQV
>P79928
MTEDIMATSYPTFLTTPYLPMKLLMNLTNDTEDICVFDEGFKFLLLPVSYSAVFMVGLPL
NIAAMWIFIAKMRPWNPTTVYMFNLALSDTLYVLSLPTLVYYYADKNNWPFGEVLCKLVR
FLFYANLYSSILFLTCISVHRYRGVCHPITSLRRMNAKHAYVICALVWLSVTLCLVPNLI
FVTVSPKVKNTICHDTTRPEDFARYVEYSTAIMCLLFGIPCLIIAGCYGLMTRELMKPIV
SGNQQTLPSYKKRSIKTIIFVMIAFAICFMPFHITRTLYYYARLLGIKCYALNVINVTYK
VTRPLASANSCIDPILYFLANDRYRRRLIRTVRRRSSVPNRRCMHTNHPQTEPHMTAGPL
PVISAEEIPSNGSMVRDENGEGSREHRVEWTDTKEINQMMNRRSTIKRNSTDKNDMKENR
HGENYLPYVEVVEKEDYETKRENRKTTEQSSKTNAEQDELQTQIDSRLKRGKWQLSSKKG
AAQENEKGHMEPSFEGEGTSTWNLLTPKMYGKKDRLAKNVEEVGYGKEKELQNFPKA
>Q9P904
MEDDGRLAPRQIWARPTSTTTRSYVPGCTPFLLPSDGYVYLNRTYSISLGENAIYDPACT
STPTTTEHAGAALDIHDPFYASVTPLLYAMGCATVVSYLLVIILLITPRTFYVGGPGGGA
NFLGRHGMVSGSYSNNSSVVGVGGRPWLQKVAALLVAISLTIATADSFRVAEKQYDHGYS
DAEALTSEVIDGTEIKVVRIISSTFLWLAQVQTLIRLFPRHKEKVMIKWAGFALIVLDTI
FAILDKFLVKTNTTRPRLYEDAIPALSYLFELALNLLYAAWVIFYSLSKHRFAFFHPKMR
NICLVALLSLCAVLIPVVFFVLDIAKPEIAGWGTYIRWVGSAAASVVVWEWVERIEALER
DERKDGILGREVFDGDEMIEVTPSEEVDWPRQQFHGHDRGGGTGMSSAWGGVMGLAHRPL
RPRGGRITQTQREAEGATAAKSRKRRSARPTPPPAAVTPVSRADTTSAASTVYNVHYYPV
SSPTPPVAMPFMEEEDEGSDGDGEKELAVVQNQQSSLPTQDTYTEPRRQNSPQIVNVDNR

WRFILNPFKNRHAALPREVASAQAEEEGFLSPDDQAPRQGDEENPHYTPRHRGLFSFHPM
SDGASRRQSGADQPLPVTVIPARRRGQDTWSPQWFTNSNLVDRSSSRRTASRPRDQRMKV
IQPQVQSAAPWTAADMEASFSSMDYELRYDPEAAALVSEDIPDHHSQPTQADSPAHPTMS
GGNGPSGGETQSALDTGPGIEGSVNEGSVIEGPEESHSQR
>P27590
MGQLLSLTWLLLVMVVTPWFTVAGANDSPEARRCSECHDNATCVLDGVVTTCSCQAGFTG
DGLVCEDIDECATPWTHNCSNSICMNTLGSYECSCQDGFRLTPGLGCIDVNECTEQGLSN
CHSLATCVNTEGSYSCVCPKGYRGDGWYCECSPGFCEPGLDCLPQGPSGKLVCQDPCNVY
ETLTEYWRSTDYGAGYSCDSDMHGWYRFTGQGGVRMAETCVPVLRCNTAAPMWLNGSHPS
SREGIVSRTACAHWSDHCCLWSTEIQVKACPGGFYVYNLTEPPECNLAYCTDPSSVEGTC
EECGVDEDCVSDNGRWRCQCKQDFNVTDVSLLEHRLECEANEIKISLSKCQLQSLGFMKV
FMYLNDRQCSGFSERGERDWMSIVTPARDGPCGTVLRRNETHATYSNTLYLASEIIIRDI
NIRINFECSYPLDMKVSLKTSLQPMVSALNISLGGTGKFTVQMALFQNPTYTQPYQGPSV
MLSTEAFLYVGTMLDGGDLSRFVLLMTNCYATPSSNSTDPVKYFIIQDRCPHTEDTTIQV
TENGESSQARFSIQMFRFAGNSDLVYLHCEVYLCDTMSEQCKPTCSGTRYRSGNFIDQTR
VLNLGPITRQGVQASVSKAASSNLGFLSIWLLLFLSATLTLMVH
>P69682
MAAELEYESVLCVKPDVSVYRIPPRASNRGYRASDWKLDQPDWTGRLRITSKGKIAYIKL
EDKVSGELFAQAPVEQYPGIAVETVADSSRYFVIRIQDGTGRSAFIGIGFTDRGDAFDFN
VSLQDHFKWVKQETEISKESQEMDSRPKLDLGFKEGQTIKLSIGNITAKKGGTSKPRASG
TGGLSLLPPPPGGKVTIPPPSSSVAISNHVTPPPIPKSNHGSNDSDILLDLDSPAPVPTS
APAPAPASTSNDLWGDFSTASSSVPNQAPQPSNWVQF
>O55242
MPWAAGRRWAWITLILTIIAVLIQAAWLWLGTQNFVFSREEIAQLARQYAGLDHELAFSR
LIVELRRLHPGHVLPDEELQWVFVNAGGWMGAMCILHASLSEYVLLFGTALGSHGHSGRY
WAEISDTIISGTFHQWKEGTTKSEVFYPGETVVHGPGEATALEWGPNTWMVEYGRGVIPS
TLFFALADTFFSTQDYLTLFYTLRAYARGLRLELTTYLFGQDS
>Q6PRD1
MGTRGAVMPPPMWGLLGCCFVCAWALGGPRPIRSLPPLSSQVKPGSVPMQVPLEGAEAAL
AYLYSGDAQQLSQVNCSERYEARGAGAMPGLPPSLQGAAGTLAQAANFLNMLLQANDIRE
SSVEEDVEWYQALVRSVAEGDPRVYRALLTFNPPPGASHLQLALQATRTGEETILQDLSG
NWVQEENPPGDLDTPALKKRVLTNDLGSLGSPKWPQADGYVGDTQQVRLSPPFLECQEGR
LRPGWLITLSATFYGLKPDLSPEVRGQVQMDVDLQSVDINQCASGPGWYSNTHLCDLNST
QCVPLESQGFVLGRYLCRCRPGFYGASPSGGLEESDFQTTGQFGFPEGRSGRLLQCLPCP
EGCTSCMDATPCLVEEAAVLRAAVLACQACCMLAIFLSMLVSYRCRRNKRIWASGVVLLE
TVLFGFLLLYFPVFILYFKPSVFRCIALRWVRLLGFAIVYGTIILKLYRVLQLFLSRTAQ
RSALLSSGRLLRRLGLLLLPVLGFLAVWTVGALERGIQHAPLVIRGHTPSGRHFYLCHHD
RWDYIMVVAELLLLCWGSFLCYATRAVLSAFHEPRYMGIALHNELLLSAAFHTARFVLVP
SLHPDWTLLLFFFHTHSTVTTTLALIFIPKFWKLGAPPREEMVDEVCEDELDLQHSGSYL
GSSIASAWSEHSLDPGDIRDELKKLYAQLEVHKTKEMAANNPHLPKKRGSSCQGLGRSFM
RYLAEFPEALARQHSRDSGSPGHGSLPGSSRRRLLSSSLQEPEGTPALHKSRSTYDQRRE
QDPPLLDSLLRRKLAKKASRTESRESVEGPPALGFRSASAHNLTVGERLPRARPASLQKS
LSVASSREKALLMASQAYLEETYRQAKEREERKKAKAAMASLVRRPSARRLERPRGAPLS
APPSPAKSSSVDSSHTSGRLHEEARRRLPHPPIRHQVSTPILALSGGLGEPRMLSPTSTL

APALLPALAPTPAPALAPVPVSPQSPNLLTYICPWENAELPAKQENVPQEGPSGPERGHH
SPAPARARLWRALSVAVEKSRAGENEMDAEDAHHQREANDVDEDRPKIFPKSHSLKAPVQ
QGSMRSLGLAIKALTRSRSTYREKESVEESPEGQNSGTAGESMGAPSRSPRLGRPKAVSK
QAALIPSDDKESLQNQQNAHTSRMLQVCQREGSREQEDRGRRMTQGLGERKAERAGKTGL
AMLRQVSRDKNIKQSKETPVGWQELPKAGLQSLGSADHRVAEVCPWEVTESETRQPDSGN
KAEICPWETSEGAPESRALRQDPGDSQKKRGEARGKSEPIDVVPMMRKKPERLVREQEAV
CPWESADRGGLSPGSAPQDPGRIRDKSEAGDSVEARKVEKPGWEAAGPEAHTPDITKAEP
CPWEASEGGEDGKPAQEAVKDLPQEKQKTRKATFWKEQKPGGDLESLCPWESTDFRGPSA
VSIQAPGSSECSGSLGSGIAEVCLWEAGDAPAIQKAEICPWELDDNVMGQEMLSLGTGRE
SLQEKEKASRKGSFGEMGEQTVKAVQKLSQQQESVCPRESTVPGHSSPCLDNSSSKAGSQ
FLCNGGSRATQVCPQEDLRPEAQEATPAKTEICPWEVNERTREEWTSAQVPRGGESQKDK
EKMPGKSEIEDVTAWEKPEGQIQKQEAVGPWESVDPGSFSPQPRPQDTERPQTLLQMSGS
VGSKAADICPLDVEENLTAGKAEICPWEVGAGAGEERALGAEAIRKSPNDTGKVSADLGP
RERAVTAPEKPQKPTPEWEVACPWGSVGPGACSQHPGTLDADGPKAGFQELDHMGCRPGE
VCPWEAQEAATSEKAKICPWEVSEGTTGKGLDQKAGSESAEQREKALEKGRLTSLGEDVS
KGMAKLCQQQETICIWENKDLRESPAQAPKISDLPSSMSSEVAEGHSLEATEKGDLRQDP
KTGSFPEHITQEKAPAADTEEFTTEDGEKTSHELQSVCPWETTAPADSVSHLDRQRPDQP
KASSQRLVSTGGRAADVCPWDVPDAGVYKSDSSAKAETCPWEVTERIPVKGVSRQDGKGD
SQEEKGRAPEKSEPKGVPVQKKPEMADFRQQEAVCPWESQDGKGLSPQPAPDASDRSRGS
SEAAGSVETRVAEVCLWEVVEAPSAKKAEICPWEAGGGAAEEGEQERESQGQGEMFLQKA
GPGGTEEHFSKAAAKPREQEAVCPGEGTGSGGLLPQSGALDPELKVSPKEAGSMGSRMAE
LCQWEITDPEGNKIKGTMADICPGEETGVPSEESGLLALTATRREFFPTAPEKPLCLLVH
GPLDHFFPESKIPCPKVSRPASTFTLEGVRELQGPSGLEPRTSLAPEPSLQEAESQSSSL
TEDSGQVAFEAQYEEFTPPTVYPWDWE
>Q96P69
MGPGEALLAGLLVMVLAVALLSNALVLLCCAYSAELRTRASGVLLVNLSLGHLLLAALDM
PFTLLGVMRGRTPSAPGACQVIGFLDTFLASNAALSVAALSADQWLAVGFPLRYAGRLRP
RYAGLLLGCAWGQSLAFSGAALGCSWLGYSSAFASCSLRLPPEPERPRFAAFTATLHAVG
FVLPLAVLCLTSLQVHRVARRHCQRMDTVTMKALALLADLHPSVRQRCLIQQKRRRHRAT
RKIGIAIATFLICFAPYVMTRLAELVPFVTVNAQWGILSKCLTYSKAVADPFTYSLLRRP
FRQVLAGMVHRLLKRTPRPASTHDSSLDVAGMVHQLLKRTPRPASTHNGSVDTENDSCLQ
QTH
>O43687
MGQLCCFPFSRDEGKISELESSSSAVLQRYSKDIPSWSSGEKNGGEPDDAELVRLSKRLV
ENAVLKAVQQYLEETQNKNKPGEGSSVKTEAADQNGNDNENNRK
>P40169
MSYKKFVYFINLFFLLGATLLTFFLILAGGRTTGVLKNFYWFQASTSGFNSAPSVTRWYN
YNWCGWESRGIAVNCSSKMAAQPFSPRDNFGSSPLMPSTFLNNRNAYYYLSRVGWAMLLI
GLFFLLITLVSVIASLIRYNRRTAALATAMSWITLFFITLSACLYTGCYAKAVKAFHHEN
RDARLGPKNFGLIWTTVFLLIVNAICCTIMVATHKRNEYIYDRSFASTKTVDSQTPTPVP
TNGGIPSSVPVTEVQQSQSHQNHRFFKKLRTKKRTVTSAGDEPDRVQEERVYTEQNVPVV
S
>Q7Z7M1
MDAPWGAGERWLHGAAVDRSGVSLGPPPTPQVNQGTLGPQVAPVAAGEVVKTAGGVCKFS

GQRLSWWQAQESCEQQFGHLALQPPDGVLASRLRDPVWVGQREAPLRRPPQRRARTTAVL
VFDERTADRAARLRSPLPELAALTACTHVQWDCASPDPAALFSVAAPALPNALQLRAFAE
PGGVVRAALVVRGQHAPFLAAFRADGRWHHVCATWEQRGGRWALFSDGRRRAGARGLGAG
HPVPSGGILVLGQDQDSLGGGFSVRHALSGNLTDFHLWARALSPAQLHRARACAPPSEGL
LFRWDPGALDVTPSLLPTVWVRLLCPVPSEECPTWNPGPRSEGSELCLEPQPFLCCYRTE
PYRRLQDAQSWPGQDVISRVNALANDIVLLPDPLSEVHGALSPAEASSFLGLLEHVLAME
MAPLGPAALLAVVRFLKRVVALGAGDPELLLTGPWEQLSQGVVSVASLVLEEQVADTWLS
LREVIGGPMALVASVQRLAPLLSTSMTSERPRMRIQHRHAGLSGVTVIHSWFTSRVFQHT
LEGPDLEPQAPASSEEANRVQRFLSTQVGSAIISSEVWDVTGEVNVAMTFHLQHRAQSPL
FPPHPPSPYTGGAWATTGCSVAALYLDSTACFCNHSTSFAILLQIYEVQRGPEEESLLRT
LSFVGCGVSFCALTTTFLLFLVAGVPKSERTTVHKNLTFSLASAEGFLMTSEWAKANEVA
CVAVTVAMHFLFLVAFSWMLVEGLLLWRKVVAVSMHPGPGMRLYHATGWGVPVGIVAVTL
AMLPHDYVAPGHCWLNVHTNAIWAFVGPVLFVLTANTCILARVVMITVSSARRRARMLSP
QPCLQQQIWTQIWATVKPVLVLLPVLGLTWLAGILVHLSPAWAYAAVGLNSIQGLYIFLV
YAACNEEVRSALQRMAEKKVAEVLRALGVWGGAAKEHSLPFSVLPLFLPPKPSTPRHPLK
APA
>Q60766
MKPSHSSCEAAPLLPNMAETHYAPLSSAFPFVTSYQTGSSRLPEVSRSTERALREGKLLE
LVYGIKETVATLSQIPVSIFVTGDSGNGMSSFINALRVIGHDEDASAPTGVVRTTKTRTE
YSSSHFPNVVLWDLPGLGATAQTVEDYVEEMKFSTCDLFIIIASEQFSSNHVKLSKIIQS
MGKRFYIVWTKLDRDLSTSVLSEVRLLQNIQENIRENLQKEKVKYPPVFLVSSLDPLLYD
FPKLRDTLHKDLSNIRCCEPLKTLYGTYEKIVGDKVAVWKQRIANESLKNSLGVRDDDNM
GECLKVYRLIFGVDDESVQQVAQSMGTVVMEYKDNMKSQNFYTLRREDWKLRLMTCAIVN
AFFRLLRFLPCVCCCLRRLRHKRMLFLVAQDTKNILEKILRDSIFPPQI
>Q9H400
MGLPVSWAPPALWVLGCCALLLSLWALCTACRRPEDAVAPRKRARRQRARLQGSATAAEA
SLLRRTHLCSLSKSDTRLHELHRGPRSSRALRPASMDLLRPHWLEVSRDITGPQAAPSAF
PHQELPRALPAAAATAGCAGLEATYSNVGLAALPGVSLAASPVVAEYARVQKRKGTHRSP
QEPQQGKTEVTPAAQVDVLYSRVCKPKRRDPGPTTDPLDPKGQGAILALAGDLAYQTLPL
RALDVDSGPLENVYESIRELGDPAGRSSTCGAGTPPASSCPSLGRGWRPLPASLP
>P00507
MALLHSGRVLSGMAAAFHPGLAAAASARASSWWTHVEMGPPDPILGVTEAFKRDTNSKKM
NLGVGAYRDDNGKPYVLPSVRKAEAQIAGKNLDKEYLPIGGLADFCKASAELALGENSEV
LKSGRFVTVQTISGTGALRVGASFLQRFFKFSRDVFLPKPSWGNHTPIFRDAGMQLQGYR
YYDPKTCGFDFSGALEDISKIPEQSVLLLHACAHNPTGVDPRPEQWKEMAAVVKKKNLFA
FFDMAYQGFASGDGDKDAWAVRHFIEQGINVCLCQSYAKNMGLYGERVGAFTVVCKDAEE
AKRVESQLKILIRPLYSNPPLNGARIAATILTSPDLRKQWLQEVKGMADRIISMRTQLVS
NLKKEGSSHNWQHITDQIGMFCFTGLKPEQVERLTKEFSVYMTKDGRISVAGVTSGNVGY
LAHAIHQVTK
>Q28634
MVPAWLWLLCFSVPQALVEVSPTTLHVEVPENYGGNFPLYLTKLPWPHKEAGGRVVLSGD
SGVAAEGPFSVEAESGFLLVTRALDREEQAEYQIQVTLEAEDGHVLWGPQSVTVHVKDEN
DQVPQFSQALYSARLSQGTRPGVPFLFLEASDGDEPGTANSDLRFHILSQTPAQPSPDVF
RLEPRLGALALSPEGSAGFDHALEGPYQLLVQVKDMGDQASGHQATATVEISIVESTWTP

LEPVHLAENLKVPYPHHLAQVHWSGGDVHYRLESQPPGPFDVDTEGKLYVTGELDREAQE
QYVLQVQAQNSRGEDYAEPLELHVVVTDENDHAPVCPPRGPPVSVPELSPPGTAVTTLSA
EDADAPGSPNSHVVYRLLSPEPQEGPEGGAFQLDPTSGSVSLGAAPLEAGQNMLLQVLAV
DLAGAEAGLSSTCEVAVTVTDVNDHAPEFTSSQVGPVSLPEDTEPGTLVATLTATDADLE
PAFRLMDFTIEAGDGEGTFGLDWEPDSGHVQLYLLKNLSYEAAPSHTVVVVVRNVVETVG
PGPGPGATATVTVLVEKVMPPPRLEQKSYEADIPVNAPAGSFLLTIQPAEPWNGALRFSL
VNDSEGWFCIQKVSGEVHTARPLQGARPGDSYTVLVEAQDADAPRLSTSAALVIHFLRAP
PAPALPLAPMPSRHLCTPRQDHGVLIPAPSEDPDMATGHGPYSFALGPNPTVQRDWRLQP
LNDSHAFLTLALHWVEPREHIVPVVVSQDARVWQLPVRVVVCRCNTEGECMRKVGRMKGM
PTKLSAVGILVGTLAAIGFFLILIFTHLALARKKDLDAPADNVPLKAAA
>Q62668
MSDHPLKEMSDNNRSPPLPEPLSSRYKLYESELSSPTWPSSSQDTHPALPLLEMPEEKDL
RSSDEDSHIVKIEKPNERSKRRESELPRRASAGRGAFSLFQAVSYLTGDMKECKNWLKDK
PLVLQFLDWVLRGAAQVMFVNNPLSGLIIFIGLLIQNPWWTIAGALGTVVSTLAALALSQ
DRSAIASGLHGYNGMLVGLLVAVFSEKLDYYWWLLFPVTFASMACPVISSALSTVFAKWD
LPVFTLPFNIALTLYLAATGHYNLFFPTTLVKPASSAPNITWSEIEMPLLLQTIPVGVGQ
VYGCDNPWTGGVILVALFISSPLICLHAAIGSIVGLLAALTVATPFETIYTGLWSYNCVL
SCVAIGGMFYVLTWQTHLLALVCALFCAYTGAALSNMMAVVGVPPGTWAFCLSTLTFLLL
TSNNPGIHKLPLSKVTYPEANRIYFLTAKRSDEQKPPNGGGGEQSHGGGQRKAEEGSETV
FPRRKSVFHIEWSSIRRRSKVFGKSEHQERQTKEPLPYLYRKPTVELLDLNTMEESSEIK
VETNTTRTTWIQSSMIAGGKRVSKALSYITGEMKECGEGLKDKSPVFQFLDWVLRGTSQV
MFVNNPLSGILIVLGLFVQNPWWAISGCLGTIMSTLTALILSQDKSAIAAGLHGYNGVLV
GLLMAVFSDKGNYYWWLLLPVIVMSMTCPILSSALSTVFSKWDLPVFTLPFNIAVTLYLA
ATGHYNLFFPTKLLQPAVTTPNITWSDVQVPLLLRAIPVGIGQVYGCDNPWTGGIFLVAL
FVSSPLICLHAAIGSTIGMLAALSIATPFDSIYFGLCGFNSTLACIAIGGMFYVITWQTH
LLAIACALFAAYLGAALANMLSVFGLPPCTWPFCLSALTFLLLTTNNPGIYKLPLSKVTY
PEANRIYFLSQEKNRRASMITKYQAYDVS
>Q16790
MAPLCPSPWLPLLIPAPAPGLTVQLLLSLLLLVPVHPQRLPRMQEDSPLGGGSSGEDDPL
GEEDLPSEEDSPREEDPPGEEDLPGEEDLPGEEDLPEVKPKSEEEGSLKLEDLPTVEAPG
DPQEPQNNAHRDKEGDDQSHWRYGGDPPWPRVSPACAGRFQSPVDIRPQLAAFCPALRPL
ELLGFQLPPLPELRLRNNGHSVQLTLPPGLEMALGPGREYRALQLHLHWGAAGRPGSEHT
VEGHRFPAEIHVVHLSTAFARVDEALGRPGGLAVLAAFLEEGPEENSAYEQLLSRLEEIA
EEGSETQVPGLDISALLPSDFSRYFQYEGSLTTPPCAQGVIWTVFNQTVMLSAKQLHTLS
DTLWGPGDSRLQLNFRATQPLNGRVIEASFPAGVDSSPRAAEPVQLNSCLAAGDILALVF
GLLFAVTSVAFLVQMRRQHRRGTKGGVSYRPAEVAETGA
>P0C0A9
MGLCFPCPAESAPPSPSPEEKRAKLAEAAERRQKEAASRGILDIQSVEAKKKKKEQLEKQ
METSGPPAGGLRWTVS
>P27177
MARLLTTCCLLALLLAACTDVALSKKGKGKPSGGGWGAGSHRQPSYPRQPGYPHNPGYPH
NPGYPHNPGYPHNPGYPHNPGYPQNPGYPHNPGYPGWGQGYNPSSGGSYHNQKPWKPPKT
NFKHVAGAAAAGAVVGGLGGYAMGRVMSGMNYHFDSPDEYRWWSENSARYPNRVYYRDYS
SPVPQDVFVADCFNITVTEYSIGPAAKKNTSEAVAAANQTEVEMENKVVTKVIREMCVQQ

YREYRLASGIQLHPADTWLAVLLLLLTTLFAMH
>P29240
MPRVPSASATGSSALLSLLCAFSLGRAAPFQLTILHTNDVHARVEETNQDSGKCFTQSFA
GVARRWTKIEELRARDKNVLLLDAGDQYQGTIWFNYYKGAEAAHFIEAVGYNAMALGNHE
FDNGAEGLLDPFLLNVSFPVLSANLEQGEDQVPSLIGYYKPSTVLDVNGEKIGVVGYTSK
ETPTLSSPGPHLIFKDEIQAVQHEVDILVSQGIDKIIALGHSGFETDKLIAQKVRGVDVV
VGGHSNTFLYTGKAPSNDVPVGPYPFLVNSDDQRTIPVVQAYAYGKYLGYLKLTFDKGEV
IKREGNPILLNSSIIQDPVLLAEVNKWKESLANFGKEVIGRTVVYLNGTTEECRNRECNM
GNLICDAMIQQNIRNPDEKFWNHVSICIFQGGGIRAPINEQNNGTIQVDSLLAVLPFGST
IDLLEVYGSTLRAAFDHSVRRYGQNTGEFLQVSGIQVQFNLKRPPGSRVVKIDVLCADCR
VPHYQPLLDNKIYKIVTNSYIAEGGDGFTMLKNERLRYDTGSTDISVVSSYIKQMKVVYP
AVEGRILFVENSATLPIINLKIGLSLFAFLTWFLHCS
>P26342
MAVTSHHMIPVMVVLMSACLATAGPEPSTRCELSPINASHPVQALMESFTVLSGCASRGT
TGLPREVHVLNLRSTDQGPGQRQREVTLHLNPIASVHTHHKPIVFLLNSPQPLVWHLKTE
RLAAGVPRLFLVSEGSVVQFPSGNFSLTAETEERNFPQENEHLLRWAQKEYGAVTSFTEL
KIARNIYIKVGEDQVFPPTCNIGKNFLSLNYLAEYLQPKAAEGCVLPSQPHEKEVHIIEL
ITPSSNPYSAFQVDIIVDIRPAQEDPEVVKNLVLILKCKKSVNWVIKSFDVKGNLKVIAP
NSIGFGKESERSMTMTKLVRDDIPSTQENLMKWALDNGYRPVTSYTMAPVANRFHLRLEN
NEEMRDEEVHTIPPELRILLDPDHPPALDNPLFPGEGSPNGGLPFPFPDIPRRGWKEGED
RIPRPKQPIVPSVQLLPDHREPEEVQGGVDIALSVKCDHEKMVVAVDKDSFQTNGYSGME
LTLLDPSCKAKMNGTHFVLESPLNGCGTRHRRSTPDGVVYYNSIVVQAPSPGDSSGWPDG
YEDLESGDNGFPGDGDEGETAPLSRAGVVVFNCSLRQLRNPSGFQGQLDGNATFNMELYN
TDLFLVPSPGVFSVAENEHVYVEVSVTKADQDLGFAIQTCFLSPYSNPDRMSDYTIIENI
CPKDDSVKFYSSKRVHFPIPHAEVDKKRFSFLFKSVFNTSLLFLHCELTLCSRKKGSLKL
PRCVTPDDACTSLDATMIWTMMQNKKTFTKPLAVVLQVDYKENVPSTKDSSPIPPPPPQI
FHGLDTLTVMGIAFAAFVIGALLTGALWYIYSHTGETARRQQVPTSPPASENSSAAHSIG
STQSTPCSSSSTA
>P51740
MQGAWVLLLLGFRLQLSLSVIPVEEENPAFWTQKAADALNVAKKLQPIQTSAKNLIIFLG
DGMGVATVTATRILKGQLEGNLGPETPLAMDHFPYMALSKTYSVDRQVPDSASTATAYLC
GVKTNYKTIGVSAAARFDQCNTTFGNEVLSVMYRAKKAGKSVGVGDHTRVQHASPAGTYV
HTVTSNWYGDADMPALPLQEGCKDIATQLISNMDINVILGGGRKYMFPAGTPDPEYPNDV
NETGTRLDGKNLVQEWLSKHQGSQYVWNRQELIQKSLDPSVTYLMGLFEPVDTKFEIQRD
PLMDPSLKDMTEAALHVLSRNPKGFYLFVEGGRIDRGHHLGTAYLALTEAVMFDSAIERA
SLQASEQDTLTIVTADHSHVFSFGGYTLRGTSIFGLAPLNALDGKPYTSILYGNGPGYVG
TGERPNVTDAESHDPSYQQQAAVPVKSETTVGKDVAIFARGPQAHLLHGVQEQNYIAHVM
AFAGCLEPYTDCGLAPPADENRPTTPVQNSTTTTTTTTTTTTTTTTRVQNSASSLGPAT
APLAWHYWPRR
>Q8TC92
MVDAGGVENITQLPQELPQMMAAAADGLGSIAIDTTQLNMSVTDPTAWATAMNNLGMVPV
GLPGQQLVSDSICVPGFDPSLNMMTGITPINPMIPGLGLVPPPPPTEVAVVKEIIHCKSC
TLFPQNPNLPPPSTRERPPGCKTVFVGGLPENATEEIIQEVFEQCGDITAIRKSKKNFCH
IRFAEEFMVDKAIYLSGYRMRLGSSTDKKDSGRLHVDFAQARDDFYEWECKQRMRAREER

HRRKLEEDRLRPPSPPAIMHYSEHEAALLAEKLKDDSKFSEAITVLLSWIERGEVNRRSA
NQFYSMVQSANSHVRRLMNEKATHEQEMEEAKENFKNALTGILTQFEQIVAVFNASTRQK
AWDHFSKAQRKNIDIWRKHSEELRNAQSEQLMGIRREEEMEMSDDENCDSPTKKMRVDES
ALAAQAYALKEENDSLRWQLDAYRNEVELLKQEKEQLFRTEENLTKDQQLQFLQQTMQGM
QQQLLTIQEELNNKKSELEQAKEEQSHTQALLKVLQEQLKGTKELVETNGHSHEDSNEIN
VLTVALVNQDRENNIEKRSQGLKSEKEALLIGIISTFLHVHPFGANIEYLWSYMQQLDSK
ISANEIEMLLMRLPRMFKQEFTGVGATLEKRWKLCAFEGIKTT
>P54657
MSVDANKVKFFFGKNCTGESFFEYNKGETVRFNNGDKWNDKFMSCLVGSNVRCNIWEHNEI
DTPTPGKFQELAQGSTNNDLTSINGLSKFQVLPGAFQWAVDVKIVNKVNSTAGSYEMTIT
PYQVDKVACKDGDDFVQLPIPKLTPPDSEIVSHLTVRQTHTPYDYVVNGSVYFKYSPTTG
QVTVIKKDETFPKNMTVTQDDNTSFIFNLNSEK
>Q969L2
MSAGGASVPPPPNPAVSFPPPRVTLPAGPDILRTYSGAFVCLEILFGGLVWILVASSNVP
LPLLQGWVMFVSVTAFFFSLLFLGMFLSGMVAQIDANWNFLDFAYHFTVFVFYFGAFLLE
AAATSLHDLHCNTTITGQPLLSDNQYNINVAASIFAFMTTACYGCSLGLALRRWRP
>Q9JK97
MAEAPPRRLGLGPPPGDAPRAELVALTAVQSEQGEAGGGGSPRRLGLLGSPLPPGAPLPG
PGSGSGSACGGQRSSAAQKRYRRLQNWVYNVLERPRGWAFVYHVFIFLLVFSCLVLSVLS
TIQEHQELANECLLILEFVMIVVFGLEYIIRVWSAGCCCRYRGWQGRFRFARKPFCVIDF
IVFVASVAVIAAGTQGNIFATSALRSMRFLQILRMVRMDRRGGTWKLLGSVVYAHSKELI
TAWYIGFLVLIFASFLVYLAEKDANSDFSSYADSLWWGTITLTTIGYGDKTPHTWLGRVL
AAGFALLGISFFALPAGILGSGFALKVQEQHRQKHFEKRRMPAANLIQAAWRLYSTDTSR
AYLTATWYYYDSILPSFRELALLFEHIQRARNGGLRPLEVRRAPVPDGAPSRYPPVATCH
RPGSASFCPGESSRMGIKDRIRISSSQKRTGPSKQHLAPPPIPTSPSSEQVGEASSPSKV
QKSWSFNDRTRFRASLRLKPRCSAEEGPSEEVAEEKSYQCELTVDDVMPAVKTVIRSVRI
LKFLVAKRKFKETLRPYDVKDVIEQYSAGHLDMLGRIKSLQARVDQIVGRGPGDRKTREK
GDKGPSDTEAVDEISMMGRVVKVEKQVQSIEHKLDLLLGFYSRCLRSGTSASLGTVQVPL
FDPDITSDYHSPVDHEDISVSAQTLSISRSVSTNMD
>Q05329
MASPGSGFWSFGSEDGSGDSENPGTARAWCQVAQKFTGGIGNKLCALLYGDAEKPAESGG
SQPPRAAARKAACACDQKPCSCSKVDVNYAFLHATDLLPACDGERPTLAFLQDVMNILLQ
YVVKSFDRSTKVIDFHYPNELLQEYNWELADQPQNLEEILMHCQTTLKYAIKTGHPRYFN
QLSTGLDMVGLAADWLTSTANTNMFTYEIAPVFVLLEYVTLKKMREIIGWPGGSGDGIFS
PGGAISNMYAMMIARFKMFPEVKEKGMAALPRLIAFTSEHSHFSLKKGAAALGIGTDSVI
LIKCDERGKMIPSDLERRILEAKQKGFVPFLVSATAGTTVYGAFDPLLAVADICKKYKIW
MHVDAAWGGGLLMSRKHKWKLSGVERANSVTWNPHKMMGVPLQCSALLVREEGLMQNCNQ
MHASYLFQQDKHYDLSYDTGDKALQCGRHVDVFKLWLMWRAKGTTGFEAHVDKCLELAEY
LYNIIKNREGYEMVFDGKPQHTNVCFWYIPPSLRTLEDNEERMSRLSKVAPVIKARMMEY
GTTMVSYQPLGDKVNFFRMVISNPAATHQDIDFLIEEIERLGQDL
>Q08470
MLKFIRGKGQQPTAERQRLQKDLFAYRKTAQHGFPHKPSALAYDPVAKLMAIGTQTGAIK
VFGQPGVELYAQHTLVNNSAAELNVQLLEWVYGTGRILSLTAANQLILWEPVGTTLVPIK
TLPFDGKLKKVSSLCCSLNKDLLWIGTEGGNIYQLDLKSFTIREPVIYHDVVLEQVPPTY

KLNPGAIESIRQLPNSLNKLLIAYNRGLCVLWDMQTSAVERAYIAPGHGQSVGLSVNSTG
TEFSWYHADGSYATWSIDNGEPPQNVNYVPYGPDPCKSINRLYKGQRGTNDVIVFSGGMP
RSAYGDHNCVSVHVSDGHKVCLDFTSKVIDFFVTYKEGSDDVEVLIVLLEEELCAYDLTD
PSILSIKAPYLHSVHASAVTCNYLASQVTQGVYERILRAGDEQDIDYSSIDWPITGGVLT
DDSVESDEDEVVKDYEILLTGHEDGSVKFWDCTGVLLKPIYNFKTASIFGNEHEYREDGA
ADISAEQLDEGEPPFRKAGLFDPYSDDPRLAVKKIAFCPKTGQLVVGGTAGQIVIADFDT
AAEDQSSLKYNSMNLVSDRDGFVWKGHDQLNVRANLLEDNAVPLTENGVNITGVLQVLPP
ASITCMALEANWGLVSGGTAHGLVLFDFKNFVPVFHRCTLNPNDLTGAGEQLSRRKSFKK
SLRESFRKLRKGRSTRNNPTNQVPTTATRPVERQIEARCTDDGLGSMVRCLLFAKTYVTN
VNITSPTLWSATNASTVSVFLLHLPPAQTAATTVPPASGNVPPQASRRISAQLAKEIQLK
HRAPVVGISIFDQTGSPVDQFNAGENGSPPHRLLIASEEQFKVFSLPQLKPINKYKLTAN
EGARIRRIHFGSFSCRISPELLQSLHSGSPTKSVRSHGEGDGAGNVSATSAAGRGDLYHE
MALICLTNMGDIMVLSVPELKRQLNAAAVRREDINGISSLCFTNDGEALYMMSSSELQRI
ALSTSKVVQPTGIVEVEPLESEDNETASLVNDGNESDKDGEANKELANTASDTQPPVATL
RAKPLELNADRSSLHLTNGISNSNSPNRANETITSSIGDITVDSVRDHLNTTTTTLCSTT
TEETVGRLSVLSTQTNQATTTVNMKDIPDINIPNLMDLASKSNTTETSTSSVVIKSVITS
ISHEKTNGESENVTTKTTAHEESQF
>P97433
MELSCSEVPLYGQKTVYAKFGKNVYLPEDAEFYFVYGGSHQRHVVIADRVQDNVLQSSIP
GHWLQETVTVSVCLCSEGYSPVTMGSGSVTYVDNMACRLARLLVTQADRLTACSHQTLLT
PFALTVEALPALDEELVLALTQLELPLGWTVLGNSSLEVSLHRESLLHLAVRWALPKLFH
FLLCLPGGVKALKLPNEEATTPLDLALQGGHSTLVEDITNFQGSHSPGFSRLRLNEEATL
QFVHSSETLTLTVNHTAEHLLEADIKLFRKYFWDRAFLVKALEQEAKTEKATMPSGAAET
EEEVRNLESGRSPSEEEEDAKSIKSQVDGPSEHEDQDRLALDRSFDGLKKSKHVPASLAA
GQLSDVLNGGDEVYANCMVIDQVGDLDINYINLEGLSTHTSPESGRSMLGPQACMHTLPP
DTSPCGRPLIENSEGTLDAAASQSFVTPSSSRTSNLNLSFGLHGFEKEQSHLKKRSSSLD
ALVADSEGEGGSEPPICYAVGSQSSPRTGLPSGDELDSFETNTEPDCNISRTESLSLSST
LHSKESLLSGIRSRSYSCSSPKISSGKSRLVRDFTVCSTSEEQRSYSFQEPPGEKRIQEE
EWDEYVIPAKSESEKYKVSRTFSFLMNRMTSPRNKSKMKNKDTKEKEKMNRHQFVPGTFS
GVLQCSGCDKTLLGKESLQCANCKANTHKGCKDAVPPCTKKFQEKYNKNKPQSILGSSSV
RDVPAPGLSLHPSSSMPIGLPAGRKEFAAQVHPLSRSVPGTTLESFRRAVTSLESEGDSW
RSRSHSDELFQSMGSSPSTESFMMEDVVDSSLWIDLSSDAQEFEAESWSLVVDPSFCSRQ
EKDVIKRQDVIFELMQTEVHHIQTLLIMSEVFRKGMKEELQLDHSTVDKIFPCLDELLET
HRHFFFSMKERRQESCAGSDRNFVINQIGDILVQQFSEENASKMKRIYGEFCSHHKEAMS
LFKELQQNKKFQNFIKIRNSNLLARRRGIPECILLVTQRITKYPVLVERILQYTKERTEE
HRDLCKALGLIKDMIAAVDLKVSEYEKNQKWLEILNKIENKTYTKLKNGHVFRKQALLSQ
ERALLHDGLVYWKTATGRFKDILALLLTDVLLFLQEKDQKYIFAAVDQKPSVISLQKLIA
REVANEERGMFLISASSAGPEMYEIHTNSKEERNNWMRRIQQAVESCPEEEGGRTSESDE
ERRKAEARVAKIQQCQEILSNQDQQICTYLEEKLHIYAELGELSGFEDVHLEPHLLIKPD
PGEPPQAASLLAAALREAESLQVAVKASKMGDVSQSSEESPGGTVLMDTPSTQDVPASPT
ASLVTEGTEGRGCWDVDPGLQGVVTDLAVSDAGEKVEYRSFSGSSQSEIIQAIQNLTRLL
YSLQAALTIQDSHIEIHKLVLQQRESLAPSHSFRGGPLQDQEKSRYLEKQREELANIHKL
QHQFQQEQRRWHRTCDQQQREQEAQESWLQARERECQSQEELLLRHRSELDHQLQEYQQS
LERLREGQRMVERERQKMRVQQGLLGHCKHSRQRSLPAVFSPGSKEVTELNRAESLCHEN

SFFINEAFGHMSLNTSNKPNPSGVPWDAHPLEGSHFDLARTSESPTELKIDISQPPDVNS
ELWTTGPGHQRPALQENSKESYKNVADLDSFQSESSSPQDSNQRWPPATDTHNRSKTKSS
DGGWTRWRCWRRG
>P36027
MLSFTTKNSFRLLLLILSCISTIRAQFFVQSSSSNSSAVSTARSSVSRVSSSSSILSSSM
VSSSSADSSSLTSSTSSRSLVSHTSSSTSIASISFTSFSFSSDSSTSSSSSASSDSSSSS
SFSISSTSATSESSTSSTQTSTSSSSSLSSTPSSSSSPSTITSAPSTSSTPSTTAYNQGS
TITSIINGKTILSNHYTTVTYTPSATADSSNKSKSSGLSKKNRNIVIGCVVGIGVPLILV
ILALIYMFCIQSSRTDFIDSDGKVVTAYRANKFTKWWYMLLGKKVSDEYHSDSPLGGSAS
SAGGLDLDEADDVMEQSSLFDVRIRDSDSVLPNANTADHNNTNSGGEPINSSVASNDIIE
EKFYDEQGNELSPRNY
>Q8VYM2
MAEQQLGVLKALDVAKTQLYHFTAIVIAGMGFFTDAYDLFCVSLVTKLLGRIYYFNPESA
KPGSLPPHVAAAVNGVALCGTLSGQLFFGWLGDKLGRKKVYGLTLVMMILCSVASGLSFG
HEAKGVMTTLCFFRFWLGFGIGGDYPLSATIMSEYANKKTRGAFIAAVFAMQGVGILAGG
FVALAVSSIFDKKFPAPTYAVNRALSTPPQVDYIWRIIVMFGALPAALTYYWRMKMPETA
RYTALVAKNIKQATADMSKVLQTDIELEERVEDDVKDPKQNYGLFSKEFLRRHGLHLLGT
TSTWFLLDIAFYSQNLFQKDIFSAIGWIPKAATMNATHEVFRIARAQTLIALCSTVPGYW
FTVAFIDTIGRFKIQLNGFFMMTVFMFAIAFPYNHWIKPENRIGFVVMYSLTFFFANFGP
NATTFIVPAEIFPARLRSTCHGISAAAGKAGAIVGAFGFLYAAQSQDKAKVDAGYPPGIG
VKNSLIMLGVLNFIGMLFTFLVPEPKGKSLEELSGEAEVSHDEK
>Q9UKB5
MWIQQLLGLSSMSIRWPGRPLGSHAWILIAMFQLAVDLPACEALGPGPEFWLLPRSPPRP
PRLWSFRSGQPARVPAPVWSPRPPRVERIHGQMQMPRARRAHRPRDQAAALVPKAGLAKP
PAAAKSSPSLASSSSSSSSAVAGGAPEQQALLRRGKRHLQGDGLSSFDSRGSRPTTETEF
IAWGPTGDEEALESNTFPGVYGPTTVSILQTRKTTVAATTTTTTTATPMTLQTKGFTESL
DPRRRIPGGVSTTEPSTSPSNNGEVTQPPRILGEASGLAVHQIITITVSLIMVIAALITT
LVLKNCCAQSGNTRRNSHQRKTNQQEESCQNLTDFPSARVPSSLDIFTAYNETLQCSHEC
VRASVPVYTDETLHSTTGEYKSTFNGNRPSSSDRHLIPVAFVSEKWFEISC
>O97827
MWPSQLLVFMMLLAPIIHGGKHSERHPALASPLRHAERGPGGALPPRHLLQQPAAERATA
HRGPGPRGATRGVRGPGAHGAQISAQAFSRAPIPMAVVRRELSCESYPIELRCPGTDVIM
IESANYGRTDDKICDSDPAQMENIRCYLPDAYKIMSQRCNNRTQCAVVAGPDVFPDPCPG
TYKYLEVQYECVPYKVEQKVFLCPGLLKGVYQSEHLFESDHQSGAWCKDPLQASDKIYYM
PWTPYRTDTLTEYSSKDDFIAGRPTTTYKLPHRVDGTGFVVYDGALFFNKERTRNIVKFD
LRTRIKSGEAIIANANYHDTSPYRWGGKSDIDLAVDENGLWVIYATEQNNGKIVISQLNP
YTLRIEGTWDTAYDKRSASNAFMICGILYVVKSVYEDDDNEATGNKIDYIYNTDQSKDSL
VDVPFPNSYQYIAAVDYNPRDNLLYVWNNYHVVKYSLDFGPLDSRSGQAHHGQVSYISPP
IHLDSDLERPPVREISTTGPLGTGSTTTSTTLRTTTWSPGRSTTPSVSGRRNRSTSTPSP
AIEVLNDITTHVPSASPQIPALEESCEAVEAREIMWFKTRQGQMAKQPCPAGTIGVSTYL
CLAPDGIWDPQGPDLSNCSSPWVNHITQKLKSGETAANIARELAEQTRNHLNAGDITYSV
RAMDQLVGLLDVQLRNLTPGGKDSAARSLNKLQKRERSCRAYVQAMVETVNNLLQPQALN
AWRDLTTSDQLRAATMLLDTVEESAFVLADNLLKTDIVRENTDNIQLEVARLSTEGNLED
LKFPENTGHGSTIQLSANTLKQNGRNGEIRVAFVLYNNLGPYLSTENASMKLGTEAMSTN

HSVIVNSPVITAAINKEFSNKVYLADPVVFTVKHIKQSEENFNPNCSFWSYSKRTMTGYW
STQGCRLLTTNKTHTTCSCNHLTNFAVLMAHVEVKHSDAVHDLLLDVITWVGILLSLVCL
LICIFTFCFFRGLQSDRNTIHKNLCISLFVAELLFLIGINRTDQPIACAVFAALLHFFFL
AAFTWMFLEGVQLYIMLVEVFESEHSRRKYFYLVGYGMPALIVAVSAAVDYRSYGTDKVC
WLRLDTYFIWSFIGPATLIIMLNVIFLGIALYKMFHHTAILKPESGCLDNINYEDNRPFI
KSWVIGAIALLCLLGLTWAFGLMYINESTVIMAYLFTIFNSLQGMFIFIFHCVLQKKVRK
EYGKCLRTHCCSGRSTESSIGSGKTSGSRTPGRYSTGSQSRIRRMWNDTVRKQSESSFIT
GDINSSASLNRGAMANHLISNALLRPHGTNNPYNTLLGEPAVCNNPSVSMYNAQEGLLNN
ARDTSVMDTLPLNGNHGNSYSIASGEYLSNCVQIIDRGYNHNETALEKKILKELTSNYIP
SYLNNHERSSEQNRNLMNKLVNNLGSGSEDDAIVLDDATSFNHEESLGLELIHEESDAPL
LPPRVYSTENHQLHHYTRRRIPQDHSESFFPLLTNEHTEDLQSPHRDSLYTSMPALAGVP
TAESVTTSTQTEPPPAKCGDAEDVYYKSMPNLGSRNHVHQLHTYYQLGRGSSDGFIVPPN
KDGTPPEGSSKGPAHLVTSL
>Q9SVG4
MLTTPPRTFVSVPFFFFFLLFLSLPLSSFSQSNSVYNSFLKCFSDKTKSPQSQITDNVFS
QTNPAFSSVLRAYIRNARFNTSSTLKPTIIITPRSESHVSAAVTCSKTLNFLLKIRSGGH
DYDGLSYISDKPFFILDMSNIRDVSVDIASNSAWISAGATLGEVYYRIWEKSRVHGFPAG
VCPTVGVGGHLSGGGYGNMVRKFGLSVDYVEDAKIVDVNGRVLDRKAMGEDLFWAITGGG
GGSYGVVLGYKVKLVPVPSVVTVFRVEQYMDSGAVDMVHKWQSVGPKTDPNLFMRMLIQP
VTRKKVKTVRASVVALFLGRADEVVALLSKEFPELGLKKENCSEMTWFQSALWWDNRLNA
TQVDPKVFLDRNLDTSSFGKRKSDYVATAIPKKGIESLFKKMIELGKIGLVFNPYGGKMA
EVAVNAKPFPHRNKLFKIQYSVNWKENSAEIEKGYLNQAKVLYSFMTGFVSKNPRSSYFN
YRDVDIGVNDHGANSYKEGEVYGRKYFGENFDRLVKIKTAVDPGNFFRNEQSIPTLKNEK
GMLLPEPGKARRWSRVGGATVVATVVLHVF
>P40087
MDLTISNELTGEIYGPIEVSEDMALTDLIALLQADCGFDKTKHDLYYNMDILDSNRTQSL
KELGLKTDDLLLIRGKISNSIQTDAATLSDEAFIEQFRQELLNNQMLRSQLILQIPGLND
LVNDPLLFRERLGPLILQRRYGGYNTAMNPFGIPQDEYTRLMANPDDPDNKKRIAELLDQ
QAIDEQLRNAIEYTPEMFTQVPMLYINIEINNYPVKAFVDTGAQTTIMSTRLAKKTGLSR
MIDKRFIGEARGVGTGKIIGRIHQAQVKIETQYIPCSFTVLDTDIDVLIGLDMLKRHLAC
VDLKENVLRIAEVETSFLSEAEIPKSFQEGLPAPTSVTTSSDKPLTPTKTSSTLPPQPGA
VPALAPRTGMGPTPTGRSTAGATTATGRTFPEQTIKQLMDLGFPRDAVVKALKQTNGNAE
FAASLLFQ
>Q07436
MRAFCTVSAPLEVCASSAEQLSPGSRFLALRLLGQQQPKTLYFLVDAKSRVREVYTQTCL
HFATQGMLDTELFGLAVLIDGEYMFADPESKLSKYGPKSWRSSHTHGLDANGRPLLELHF
RVQFYIESPFMLKDETSRHNYYLQLRHNILQRDLPREQAEQALVFLAGLALQADLGDAPP
GTSNSKDDSGEETSASPSNGGRGLSATTTLPKISKRANERMLRLSTYVASTSKRETIPLP
PSLPPNGADYYRIEDYLPSGLHTPWARSAMRACHREHLGMATAEAELLYIQQACSLHETI
NAHTYRMRLAKSEQGSGSAWFVVYAKGIKILGGESTNSSSNPETTTFLWPNITKLSFERK
KFEIRSGESRITLYAASDEKNKLLLTLCKDTHQWSMKLAARLKEVSKREEEEAAESQRLH
ASYACSRSLLLPYKSKNEQRISVISSTSSNTTSGIVSDRVHSEDELEIMINTPPAPLAAP
STESLALAHLLDRPSVSRQTSSVGQMSLKDLEEQLAALSVRPQDASSNGATIVTNSSVQR
NSMGTTANDSSTATDSPSSQHNIGSQCSSTCSTVVVTSPVNGAGASSSGAPIPVHSTSSS

LELGFSHTAQNSALSETSPDDFLSTSAREETESVSGASGVYTLAHGAPPTETSGVYTMHS
SELTGQSSEIAESEKSSHYGMFQPQKLEETHVQHSDSVDGKKKEDFRPRSDSNVSTGSSF
RGDGSDPTDNKHSLLSAEELTNLIVGRGTYPSRKTVSSSLHSDCDYVTLPLGDQGEEEVD
QPPAPPPPYSARHEKTGLCGPPIAKPIPKPIAVVAPKPDSPPCSPPVPPAPIPAPPPAIR
RRDPPPYSISSKPRPTSLISVSSSAHPAPSAAGSMSSLKSEEVTARFITTRPQISILKAH
TSLIPDGAKPSYAAPHHCSSVASSNGSVCSHQLSQQSLHNSNYAGGSQASLHHHHVPSHH
RHSGSAAIGIVPYGLHKSTASLHHQQSCVLLPVIKPRQFLAPPPPSLPRQPPPPPPPNHP
HLASHLYEREMARKQLELYQQQLYSDVDYVIYPIQDPAVSQQEYLDAKQGSLLAAMAQAA
PPPPHHPYLAMQVSPAIYRSTPYLPLTLSTHSRYASTQNLSDTYVQLPGPGYSPLYSPSM
ASLCSSYEPPPPPPLHPAALAAAAAAGAGSSSSSMFARSRSDDNILNSLDLLPKGKRLPP
PPPPPYVNRRLKKPPMPAPSEKPPPIPSKPIPSRMSPIPPRKPPTLNPHHANSPLTKTSS
GAQWAGERPRPDLGLGLGLNRGNNSILAQLQASMVAQSHAQAQAQALDIALLREKSKHLD
LPLISALCNDRSLLKQTKVVINPKTGQEMPTSSAQPSGATTNGVANSSAGAGTLSKARKG
STVSHRHPQDKLPPLPVQQLAEANNYVIDPAVMMKQQQQQQQHNKTS
>Q9QUG3
MKNRLGTWWVAILCMLLASHLSTVKARGIKHRFKWNRKVLPSSGGQITEARVAENRPGAF
IKQGRKLDIDFGAEGNRYYAANYWQFPDGIYYEGCSEANVTKEMLVTSCVNATQAANQAE
FSREKQDSKLHQRVLWRLIKEICSAKHCDFWLERGAALRVAVDQPAMVCLLGFVWFIVK
>P11602
MERGRGMGKTALLAVLCLCLRGAAGSDPEAEMNFEGIESKFSLRTPAEPDEDVCYLVPGQ
MDSLAQCNFNHTSKTFVVIHGWTVTGMYESWVPKLVDALYKREPDSNVIVVDWLVRAQQH
YPVSAAYTKLVGKDVAMFIDWMEEKFNYPLNNVHLLGYSLGAHAAGIAGSLTKKKVNRIT
GLDPAGPTFEYADAPIRLSPDDADFVDVLHTYTRGSPDRSIGIQKPVGHIDIYPNGGGFQ
PGCNLGEALRLIAEKGFSDVDQLVKCSHERSIHLFIDSLLYEEKPSMAYRCNTKEAFEKG
LCLSCRKNRCNNLGYKVNRVRTKRNTKMYLKTRAQMPYKVFHYQVKIHFFGKTNVTKVDQ
PFLISLYGTLDESENIPFTLPEVSSNKTFSFLIYTEVDIGDLLMLKLQWEKDTFFSWSDW
WTPFAFTIQRVRVKSGETQKKVVFCSRDGSSRLGKGEEAAIFVKCLEQPVSRKRGGAKKA
SKENSAHESA
>Q8WTV0
MGCSAKARWAAGALGVAGLLCAVLGAVMIVMVPSLIKQQVLKNVRIDPSSLSFNMWKEIP
IPFYLSVYFFDVMNPSEILKGEKPQVRERGPYVYREFRHKSNITFNNNDTVSFLEYRTFQ
FQPSKSHGSESDYIVMPNILVLGAAVMMENKPMTLKLIMTLAFTTLGERAFMNRTVGEIM
WGYKDPLVNLINKYFPGMFPFKDKFGLFAELNNSDSGLFTVFTGVQNISRIHLVDKWNGL
SKVDFWHSDQCNMINGTSGQMWPPFMTPESSLEFYSPEACRSMKLMYKESGVFEGIPTYR
FVAPKTLFANGSIYPPNEGFCPCLESGIQNVSTCRFSAPLFLSHPHFLNADPVLAEAVTG
LHPNQEAHSLFLDIHPVTGIPMNCSVKLQLSLYMKSVAGIGQTGKIEPVVLPLLWFAESG
AMEGETLHTFYTQLVLMPKVMHYAQYVLLALGCVLLLVPVICQIRSQVGAGQRAARADSH
SLACWGKGASDRTLWPTAAWSPPPAAVLRLCRSGSGHCWGLRSTLASFACRVATTLPVLE
GLGPSLGGGTGS
>Q8HYC3
MALTPESPSSFPGLAAIGSSVPEPPGSPNATLNSSWASPTEPSSLEDLVATGAIGTLLSA
MGVVGVVGNAYTLVVTCRSLRAVASMYIYVVNLALADLLYLLSIPFIVATYITKEWHFGD
VGCRVLFSLDFLTMHASIFTLTVMSSERYAAVLRPLDTVQRPKGYRKLLALGTWLLALLL
TLPVMLAMRLVRRGPKSLCLPAWGPRAHRAYLTLLFATSIAGPGLLIGLLYARLARAYRR

SQRASFKRARRPGARALRLVLGIVLLFWACFLPFWLWQLLAQYREAPLAPRTARIVNYLT
TCLTYGNSCANPFLYTLLTRNYRDHLRGRVRSPGSGGVRGPVPSLQPRARFQRGSGRSLS
SCSPQPTESLVLAAAAPAGPALESPGDPA
>Q13433
MARKLSVILILTFALSVTNPLHELKAAAFPQTTEKISPNWESGINVDLAISTRQYHLQQL
FYRYGENNSLSVEGFRKLLQNIGIDKIKRIHIHHDHDHHSDHEHHSDHERHSDHEHHSDH
EHHSDHNHAASGKNKRKALCPDHDSDSSGKDPRNSQGKGAHRPEHASGRRNVKDSVSASE
VTSTVYNTVSEGTHFLETIETPRPGKLFPKDVSSSTPPSVTSKSRVSRLAGRKTNESVSE
PRKGFMYSRNTNENPQECFNASKLLTSHGMGIQVPLNATEFNYLCPAIINQIDARSCLIH
TSEKKAEIPPKTYSLQIAWVGGFIAISIISFLSLLGVILVPLMNRVFFKFLLSFLVALAV
GTLSGDAFLHLLPHSHASHHHSHSHEEPAMEMKRGPLFSHLSSQNIEESAYFDSTWKGLT
ALGGLYFMFLVEHVLTLIKQFKDKKKKNQKKPENDDDVEIKKQLSKYESQLSTNEEKVDT
DDRTEGYLRADSQEPSHFDSQQPAVLEEEEVMIAHAHPQEVYNEYVPRGCKNKCHSHFHD
TLGQSDDLIHHHHDYHHILHHHHHQNHHPHSHSQRYSREELKDAGVATLAWMVIMGDGLH
NFSDGLAIGAAFTEGLSSGLSTSVAVFCHELPHELGDFAVLLKAGMTVKQAVLYNALSAM
LAYLGMATGIFIGHYAENVSMWIFALTAGLFMYVALVDMVPEMLHNDASDHGCSRWGYFF
LQNAGMLLGFGIMLLISIFEHKIVFRINF
>Q06810
MSSSSKASASSSLSSTATSSTSATRGSDGCVVCDSTASCPVCASGEYCVMTSLTCDKCPS
TYCAKQSDSQLSSLSSSSSSSSSNSNEKTSLIVGFTVGIVGGAMLIALVALYFINKRYW
KPKRQKNKALKLEEASQSYGNEEEYFDDEDDDDEDDEDDGGMRKDESHTLFNTSLVPPTL
NVPGNRSSASTTRTRASNILPIAYIPGVTSGLSTDKLQSKLRSSSKRQNAAGDIRSHITL
GSSILDGLDDEDDEHNQVLNKDADDNLITAIRAKPKLVQIAEEESDKEIQDLDVIEEQTE
ADDLSHMAKSEASHGNNDEDDDEEGSFILDLEIPESIRESTQGSRTESPFEDKFEIHDER

>Q8VIM4
MADEKTFRIGFIVLGLFLLSLGTFLMSHDRPQVYGTFYAMGSVMVIGGVIWSMCQCYPKI
TFVPADSDFQGILSPKALSLLETGLSEVKSPQPPYVRLWEEAAYDQSLPDFTHIQMKVMG
YSEDPRPLLAPELKTGASSVREGEPRTAQAWMEAPVVVHRGSDENEGEKSHSQSSPSVGP
QGSAPLASFHDDLDVGSSEGSSLQPSPNRDEPHRQVPWASRGPLDRFSDFALIDDTPTSE
DTVLDGQAREAALPRKQQWSLRMKGETVQARAEEPEQEEEDLYYGLPDSPGDPLPDKELG
FEPDIQG
>Q9HCG7
MGTQDPGNMGTGVPASEQISCAKEDPQVYCPEETGGTKDVQVTDCKSPEDSRPPKETDCC
NPEDSGQLMVSYEGKAMGYQVPPFGWRICLAHEFTEKRKPFQANNVSLSNMIKHIGMGLR
YLQWWYRKTHVEKKTPFIDMINSVPLRQIYGCPLGGIGGGTITRGWRGQFCRWQLNPGMY
QHRTVIADQFTVCLRREGQTVYQQVLSLERPSVLRSWNWGLCGYFAFYHALYPRAWTVYQ
LPGQNVTLTCRQITPILPHDYQDSSLPVGVFVWDVENEGDEALDVSIMFSMRNGLGGGDD
APGGLWNEPFCLERSGETVRGLLLHHPTLPNPYTMAVAARVTAATTVTHITAFDPDSTGQ
QVWQDLLQDGQLDSPTGQSTPTQKGVGIAGAVCVSSKLRPRGQCRLEFSLAWDMPRIMFG
AKGQVHYRRYTRFFGQDGDAAPALSHYALCRYAEWEERISAWQSPVLDDRSLPAWYKSAL
FNELYFLADGGTVWLEVLEDSLPEELGRNMCHLRPTLRDYGRFGYLEGQEYRMYNTYDVH
FYASFALIMLWPKLELSLQYDMALATLREDLTRRRYLMSGVMAPVKRRNVIPHDIGDPDD
EPWLRVNAYLIHDTADWKDLNLKFVLQVYRDYYLTGDQNFLKDMWPVCLAVMESEMKFDK

DHDGLIENGGYADQTYDGWVTTGPSAYCGGLWLAAVAVMVQMAALCGAQDIQDKFSSILS
RGQEAYERLLWNGRYYNYDSSSRPQSRSVMSDQCAGQWFLKACGLGEGDTEVFPTQHVVR
ALQTIFELNVQAFAGGAMGAVNGMQPHGVPDKSSVQSDEVWVGVVYGLAATMIQEGLTWE
GFQTAEGCYRTVWERLGLAFQTPEAYCQQRVFRSLAYMRPLSIWAMQLALQQQQHKKASW
PKVKQGTGLRTGPMFGPKEAMANLSPE
>Q8NFZ6
MTHTLYPTPFALYPINISAAWHLGPLPVSCFVSNKYQCSLAFGATTGLRVLVVVVPQTQL
SFLSSLCLVSLFLHSLVSAHGEKPTKPVGLDPTLFQVVVGILGNFSLLYYYMFLYFRGYK
PRSTDLILRHLTVADSLVILSKRIPETMATFGLKHFDNYFGCKFLLYAHRVGRGVSIGST
CLLSVFQVITINPRNSRWAEMKVKAPTYIGLSNILCWAFHMLVNAIFPIYTTGKWSNNNI
TKKGDLGYCSAPLSDEVTKSVYAALTSFHDVLCLGLMLWASSSIVLVLYRHKQQVQHICR
NNLYPNSSPGNRAIQSILALVSTFALCYALSFITYVYLALFDNSSWWLVNTAALIIACFP
TISPFVLMCRDPSRSRLCSICCRRNRRFFHDFRKM
>Q9NZQ7
MRIFAVFIFMTYWHLLNAFTVTVPKDLYVVEYGSNMTIECKFPVEKQLDLAALIVYWEME
DKNIIQFVHGEEDLKVQHSSYRQRARLLKDQLSLGNAALQITDVKLQDAGVYRCMISYGG
ADYKRITVKVNAPYNKINQRILVVDPVTSEHELTCQAEGYPKAEVIWTSSDHQVLSGKTT
TTNSKREEKLFNVTSTLRINTTTNEIFYCTFRRLDPEENHTAELVIPELPLAHPPNERTH
LVILGAILLCLGVALTFIFRLRKGRMMDVKKCGIQDTNSKKQSDTHLEET
>P29533
MPVKMVAVLGASTVLWILFAVSQAFKIEISPEYKTIAQIGDSMALTCSTTGCESPLFSWR
TQIDSPLNAKVRTEGSKSVLTMEPVSFENEHSYLCTATCGSGKLERSIHVDIYSFPKDPE
IQFSGPLEVGKPVTVKCLAPDIYPVYRLEIDLFKGDQLMNRQEFSSEEMTKSLETKSLEV
TFTPVIEDIGKALVCRAKLHIDQIDSTLKERETVKELQVYISPRNTTISVHPSTRLQEGG
AVTMTCSSEGLPAPEIFWGRKLDNEVLQLLSGNATLTLIAMRMEDSGVYVCEGVNLIGRD
KAEVELVVQEKPFIVDISPGSQVAAQVGDSVVLTCAAIGCDSPSFSWRTQTDSPLNGVVR
NEGAKSTLVLSSVGFEDEHSYLCAVTCLQRTLEKRTQVEVYSFPEDPVIKMSGPLVHGRP
VTVNCTVPNVYPFDHLEIELLKGETTLMKKYFLEEMGIKSLETKILETTFIPTIEDTGKS
LVCLARLHSGEMESEPKQRQSVQPLYVNVAPKETTIWVSPSPILEEGSPVNLTCSSDGIP
APKILWSRQLNNGELQPLSENTTLTFMSTKRDDSGIYVCEGINEAGISRKSVELIIQVSP
KDIQLTVFPSKSVKEGDTVIISCTCGNVPETWIILKKKAKTGDMVLKSVDGSYTIRQAQL
QDAGIYECESKTEVGSQLRSLTLDVKGKEHNKDYFSPELLALYCASSLVIPAIGMIVYFA
RKANMKGSYSLVEAQKSKV
>Q8K4Z6
MALLITVVTCFMIILDTSQSCHTPDDFVAITSPGHIMIGGLFAIHEKMLSSDDHPRRPQI
QKCAGFEISVFLQTLAMIHSIEMINNSTLLSGVKLGYEIYDTCTEVTAAMAATLRFLSKF
NCSRETVVFQCDYSSYMPRVKAVIGAGYSETSIAVSRMLNLQLMPQVSYESTAEILSDKI
RFPSFLRTVPSDFYQTKAMAHLIRQSGWNWIGAITTDDDYGRLALNTFAIQAAENNVCIA
FKEVLPAFLSDNTIEVRINQTLEKIIAEAQVNVIVVFLRKFHVFNLFTKAIERKISKIWI
ASDNWSTATKIITIPNVKKLGKVVGFAFRRGNTSSFHSFLQTLHMYPNDNNKPLHEFAML
VSACKYIKDGDLSQCISNYSQATLTYDTTKTIENHLFKRNDFLWHYTEPGLIYSIQLAVF
ALGHAIRDLCQARDCKKPNAFQPWELLAVLKNVTFTDGRNSFHFDAHGDLNTGYDVVLWK
ETNGLMTVTKMAEYDLQRDVFITTNQETKHEFRKLKQILSKCSKECSPGQMKKATGSQHS
CCYECVSCPENHYSNETDMDHCLLCNNETHWAPVRSTTCFEKEVEYLDWDDSLALLLIAL

SLLGIAFVLAIGIIFTRNLKTPVVKSSGGLVVCYVMLICHALNFASTGFFIGEPQDFACK
TRQTLFGVSFTLCVSCILTKSLKILLAFSFDPKLTMFLKCLYRPVPIVLTCTGIQVVICT
LWLVLAAPSVEENISLPRVIILECEEGSALAFGTMLGYITVLAFICFVFAFKGRKLPENY
NEAKFLTFGMLIYFIAWITFIPVYTTTFGKYLPAVEIIVILISNYGILCCIFFPKCYIIL
CKQKTNTKSAFLQMVYNYSAHSVDSLALSHVSLDSTSYDTATTNQSPGNKMTACQNDNHL
PAQVLPHTGTAKTIKASKTLRQKRSSSI
>P38925
MVNVGPSHAAVAVDASEARKRNISEEVFELRDKKDSTVVIEGEAPVRTFTSSSSNHERED
TYVSKRQVMRDIFAKYLKFIGPGLMVSVAYIDPGNYSTAVDAGASNQFSLLCIILLSNFI
AIFLQCLCIKLGSVTGLDLSRACREYLPRWLNWTLYFFAECAVIATDIAEVIGTAIALNI
LIKVPLPAGVAITVVDVFLIMFTYKPGASSIRFIRIFECFVAVLVVGVCICFAIELAYIP
KSTSVKQVFRGFVPSAQMFDHNGIYTAISILGATVMPHSLFLGSALVQPRLLDYDVKHGN
YTVSEEQDKVKKSKSTEEIMEEKYFNYRPTNAAIKYCMKYSMVELSITLFTLALFVNCAI
LVVAGSTLYNSPEADGADLFTIHELLSRNLAPAAGTIFMLALLLSGQSAGVVCTMSGQIV
SEGHINWKLQPWQRRLATRCISIIPCLVISICIGREALSKALNASQVVLSIVLPFLVAPL
IFFTCKKSIMKTEITVDHTEEDSHNHQNNNDRSAGSVIEQDGSSGMEIENGKDVKIVYMA
NNWIITVIAIIVWLFLSLLNVYAIVQLGMSHGDIS
>P12024
MGLEFFFKFGYAFLTITLMIMIWMSLARASMFDREMEETHYPPCTYNVMCTCSKSSTDLG
IVHCKNVPFPALPRMVNQSKVFMLHMENTGLREIEPYFLQSTGMYRLKISGNHLTEIPDD
AFTGLERSLWELILPQNDLVEIPSKSLRHLQKLRHLDLGYNHITHIQHDSFRGLEDSLQT
LILRENCISQLMSHSFSGLLILETLDLSGNNLFEIDPNVFVDGMPRLTRLLLTDNILSEI
PYDALGPLKSLRTLDISHNVIWSLSGNETYEIKASTKLNLDNLHLEYNHIEVLPPNSFKY
FDTVNRTFFDGNPIHTLREDAFKPARIREIYMRYCGLTNISPVAFDSLVNSLQILDLSGN
NLTKLHHKLFNNFDVLRVISMRDNKIKIQKPTETFNAVHYTLLKLDLSGDRNDPTNLQTL
RNMTRMRNMRSLSISRLGSSSVGPEDFKDFGVELEDLQITRASLSGIQSHAFKHVRGLKR
LDFSENGISSIENDAFHEIGHSLISLKMSHGYSGSALPAEPLRHLTSLQELDFSNNHISS
MSDTSFHFLKNLRLLELHDNRIEQVLKGTFQGDIHSKLEEISLRFNHLTSISQHTFFDLE
ALRKLHLDDNKIDKIERRAFMNLDELEYLSLRGNKINNLADESFQNLPKLEILDMAFNQL
PNFNFDYFDQVGTLSNLNVNVSHNQIRQLMYNSSWSGRNEHGGMYHSNIKILDLSHNNIS
IIHPGYFRPAEISLTHLHLGYNSLMNTTRDVFGNMPHLQWLDLSYNWIHELDFDAFKNTK
QLQLVFFGHNYLSDIPQDIFKPVQGLRIVDFSHNHLRGLPDNLFYNGGMEKLDVSHNMML
KIPSSSLSSLAALTLCELHLSNNFISTIHSMDLSNKFRSLRYLDISYNYLLRIDDAVFAT
MPKLAVLDLSHNRDLKVMDKSFMGLENSLIKLGLENISLSTVPEIRLKYLREFRLGYNEL
PSIPQELAHNMSNLRMLDLSNNDLTNVPLMTQALPHLRRLMLSGNPITSLNNNSFDGVNE
DLEMLDISNFRLHYFEYGCLDSLPHLRSLKLTAYSHLEHFNIPHLLRHHYNIRQLWIEAP
QPFTRIVKKGSGPTQEMQTLQLGNPTDLQREMEGHLPSKLTNITFSGPQFTNLNERILRG
MRSPYLYMQLFNTSLQALPPNFFKYMGRVRNISLDIRYHNRNLKKIPNPNTGAVPYLPNS
VFLTDLKMSHTDLNCDCDLGWVEFWQRKRRQYICSSQTWTDTVFRTFMNSPCQVYGRHNC
DEHDDDLRETRCENKGGQQLMEALKFDLECGWDNANCREAAFVVVMVCVAMVFWM
>Q62997
MFLATLYFALPLLDLLMSAEVSGGDRLDCVKASDQCLKEQSCSTKYRTLRQCVAGKETNF
SLTSGLEAKDECRSAMEALKQKSLYNCRCKRGMKKEKNCLRIYWSMYQSLQGNDLLEDSP
YEPVNSRLSDIFRAVPFISDVFQQVEHISKGNNCLDAAKACNLDDTCKKYRSAYITPCTT

SMSNEVCNRRKCHKALRQFFDKVPAKHSYGMLFCSCRDIACTERRRQTIVPVCSYEERER
PNCLSLQDSCKTNYICRSRLADFFTNCQPESRSVSNCLKENYADCLLAYSGLIGTVMTPN
YVDSSSLSVAPWCDCSNSGNDLEDCLKFLNFFKDNTCLKNAIQAFGNGSDVTMWQPAPPV
QTTTATTTTAFRVKNKPLGPAGSENEIPTHVLPPCANLQAQKLKSNVSGSTHLCLSDSDF
GKDGLAGASSHITTKSMAAPPSCSLSSLPVLMLTALAALLSVSLAETS
>Q9BZJ8
MESSPIPQSSGNSSTLGRVPQTPGPSTASGVPEVGLRDVASESVALFFMLLLDLTAVAGN
AAVMAVIAKTPALRKFVFVFHLCLVDLLAALTLMPLAMLSSSALFDHALFGEVACRLYLF
LSVCFVSLAILSVSAINVERYYYVVHPMRYEVRMTLGLVASVLVGVWVKALAMASVPVLG
RVSWEEGAPSVPPGCSLQWSHSAYCQLFVVVFAVLYFLLPLLLILVVYCSMFRVARVAAM
QHGPLPTWMETPRQRSESLSSRSTMVTSSGAPQTTPHRTFGGGKAAVVLLAVGGQFLLCW
LPYFSFHLYVALSAQPISTGQVESVVTWIGYFCFTSNPFFYGCLNRQIRGELSKQFVCFF
KPAPEEELRLPSREGSIEENFLQFLQGTGCPSESWVSRPLPSPKQEPPAVDFRIPGQIAE
ETSEFLEQQLTSDIIMSDSYLRPAASPRLES
>O15552
MLPDWKSSLILMAYIIIFLTGLPANLLALRAFVGRIRQPQPAPVHILLLSLTLADLLLLL
LLPFKIIEAASNFRWYLPKVVCALTSFGFYSSIYCSTWLLAGISIERYLGVAFPVQYKLS
RRPLYGVIAALVAWVMSFGHCTIVIIVQYLNTTEQVRSGNEITCYENFTDNQLDVVLPVR
LELCLVLFFIPMAVTIFCYWRFVWIMLSQPLVGAQRRRRAVGLAVVTLLNFLVCFGPYNV
SHLVGYHQRKSPWWRSIAVVFSSLNASLDPLLFYFSSSVVRRAFGRGLQVLRNQGSSLLG
RRGKDTAEGTNEDRGVGQGEGMPSSDFTTE
>Q90380
MAANCERTFIAIKPDGVQRGLVGEIIKRFEQKGFRLVGMKFVHASEELLKQHYIDLKDRP
FYPGLVKYMNSGPIVAMVWEGLNVVKTGRVMLGETNPADSKPGTIRGDFCIQVGRNIIHG
SDSVESAQKEINLWFKPAELIDFKSCAHDWIYE
>P38873
MPEIYGPQPLKPLNTVMRHGFEEQYQSDQLLQSLANDFIFYFDDKRHKTNGNPIPEEDKQ
RDVNRYYQPITDWKIMKDRQKTVSAALLLCLNLGVDPPDVMKTHPCARVEAWVDPLNFQD
SKKAIEQIGKNLQAQYETLSLRTRYKQSLDPCVEDVKRFCNSLRRTSKEDRILFHYNGHG
VPKPTKSGEIWVFNRGYTQYIPVSLYDLQTWLGAPCIFVYDCNSAENILINFQKFVQKRI
KDDEEGNHDVAAPSPTSAYQDCFQLASCTSDELLLMSPELPADLFSCCLTCPIEISIRIF
LMQSPLKDSKYKIFFENSTSNQPFGDSKNSFKSKIPNVNIPGMLSDRRTPLGELNWIFTA
ITDTIAWTSLPRPLFKKLFRHDLMIAALFRNFLLAKRIMPWYNCHPVSDPELPDSITTHP
MWKSWDLAMDEVLTKIVIDLKNAPPATALESQMILQQQETLQNGGSSKSNAQDTKAGSIQ
TQSRFAVANLSTMSLVNNPALQSRKSISLQSSQQQLQQQQQQQQFTGFFEQNLTAFELW
LKYASNVRHPPEQLPIVLQVLLSQVHRIRALVLLSRFLDLGPWAVYLSLSIGIFPYVLKL
LQSPAPELKPILVFIWARIMSIDYKNTQSELIKEKGYMYFVTVLVPDWGVNGMSATNGSA
MINSGNPLTMTASQNINGPSSRYYERQQGNRTSNLGHNNLPFYHSNDTTDEQKAMAVFVL
ASFVRNFPLGQKNCFSLELVNKLCFYIDNSEIPLLRQWCVILLGLLFADNPLNRFVCMNT
GAVEILLKSLKDPVPEVRTASIFALKHFISGFQDAEVILRLQQEFEEQYQQLHSQLQHLQ
NQSHLQQQQSQQQQQHLEQQQMKIEKQIRHCQVMQNQLEVIDLRKLKRQEIGNLISILPL
INDGSSLVRKELVVYFSHIVSRYSNFFIVVVFNDLLEEIKLLEKSDINTRNTSDKYSVSQ
GSIFYTVWKSLLILAEDPFLENKELSKQVIDYILLELSAHKELGGPFAVMEKFLLKRSSK
AHQTGKFGFNSSQVQFVKSSLRSFSPNERVDNNAFKKEQQQHDPKISHPMRTSLAKLFQS

LGFSESNSDSDTQSSNTSMKSHTSKKGPSGLYLLNGNNNIYPTAETPRFRKHTEPLQLPL
NSSFLDYSREYFQEPQMKKQEADEPGSVEYNARLWRRNRNETIIQETQGEKKLSIYGNWS
KKLISLNNKSQPKLMKFAQFEDQLITADDRSTITVFDWEKGKTLSKFSNGTPFGTKVTDL
KLINEDDSALLLTGSSDGVIKIYRDYQDVDTFKIVSAWRGLTDMLLTPRSTGLLTEWLQI
RGSLLTTGDVKVIRVWDAHTETVEVDIPAKTSSLITSLTADQLAGNIFVAGFADGSLRVY
DRRLDPRDSMIRRWRAGNDKQGVWINNVHLQRGGYRELVSGATNGVVELWDIRSEDPVES
FVDQNVTSQYGSQQKPTTMTCMQVHEHAPIIATGTKQIKIWTTSGDLLNSFKNSHNNGVT
STLAATGIPKSLSYSSTSDAFLSSMAFHPHRMMIAATNSHDSIVNIYKCEDERIDYF
>O43451
MARKKLKKFTTLEIVLSVLLLVLFIISIVLIVLLAKESLKSTAPDPGTTGTPDPGTTGTP
DPGTTGTTHARTTGPPDPGTTGTTPVSAECPVVNELERINCIPDQPPTKATCDQRGCCWN
PQGAVSVPWCYYSKNHSYHVEGNLVNTNAGFTARLKNLPSSPVFGSNVDNVLLTAEYQTS
NRFHFKLTDQTNNRFEVPHEHVQSFSGNAAASLTYQVEISRQPFSIKVTRRSNNRVLFDS
SIGPLLFADQFLQLSTRLPSTNVYGLGEHVHQQYRHDMNWKTWPIFNRDTTPNGNGTNLY
GAQTFFLCLEDASGLSFGVFLMNSNAMEVVLQPAPAITYRTIGGILDFYVFLGNTPEQVV
QEYLELIGRPALPSYWALGFHLSRYEYGTLDNMREVVERNRAAQLPYDVQHADIDYMDER
RDFTYDSVDFKGFPEFVNELHNNGQKLVIIVDPAISNNSSSSKPYGPYDRGSDMKIWVNS
SDGVTPLIGEVWPGQTVFPDYTNPNCAVWWTKEFELFHNQVEFDGIWIDMNEVSNFVDGS
VSGCSTNNLNNPPFTPRILDGYLFCKTLCMDAVQHWGKQYDIHNLYGYSMAVATAEAAKT
VFPNKRSFILTRSTFAGSGKFAAHWLGDNTATWDDLRWSIPGVLEFNLFGIPMVGPDICG
FALDTPEELCRRWMQLGAFYPFSRNHNGQGYKDQDPASFGADSLLLNSSRHYLNIRYTLL
PYLYTLFFRAHSRGDTVARPLLHEFYEDNSTWDVHQQFLWGPGLLITPVLDEGAEKVMAY
VPDAVWYDYETGSQVRWRKQKVEMELPGDKIGLHLRGGYIFPTQQPNTTTLASRKNPLGL
IIALDENKEAKGELFWDDGETKDTVANKVYLLCEFSVTQNRLEVNISQSTYKDPNNLAFN
EIKILGTEEPSNVTVKHNGVPSQTSPTVTYDSNLKVAIITDIDLLLGEAYTVEWSIKIRD
EEKIDCYPDENGASAENCTARGCIWEASNSSGVPFCYFVNDLYSVSDVQYNSHGATADIS
LKSSVYANAFPSTPVNPLRLDVTYHKNEMLQFKIYDPNKNRYEVPVPLNIPSMPSSTPEG
QLYDVLIKKNPFGIEIRRKSTGTIIWDSQLLGFTFSDMFIRISTRLPSKYLYGFGETEHR
SYRRDLEWHTWGMFSRDQPPGYKKNSYGVHPYYMGLEEDGSAHGVLLLNSNAMDVTFQPL
PALTYRTTGGVLDFYVFLGPTPELVTQQYTELIGRPVMVPYWSLGFQLCRYGYQNDSEIA
SLYDEMVAAQIPYDVQYSDIDYMERQLDFTLSPKFAGFPALINRMKADGMRVILILDPAI
SGNETQPYPAFTRGVEDDVFIKYPNDGDIVWGKVWPDFPDVVVNGSLDWDSQVELYRAYV
AFPDFFRNSTAKWWKREIEELYNNPQNPERSLKFDGMWIDMNEPSSFVNGAVSPGCRDAS
LNHPPYMPHLESRDRGLSSKTLCMESQQILPDGSLVQHYNVHNLYGWSQTRPTYEAVQEV
TGQRGVVITRSTFPSSGRWAGHWLGDNTAAWDQLKKSIIGMMEFSLFGISYTGADICGFF
QDAEYEMCVRWMQLGAFYPFSRNHNTIGTRRQDPVSWDVAFVNISRTVLQTRYTLLPYLY
TLMHKAHTEGVTVVRPLLHEFVSDQVTWDIDSQFLLGPAFLVSPVLERNARNVTAYFPRA
RWYDYYTGVDINARGEWKTLPAPLDHINLHVRGGYILPWQEPALNTHLSRQKFMGFKIAL
DDEGTAGGWLFWDDGQSIDTYGKGLYYLASFSASQNTMQSHIIFNNYITGTNPLKLGYIE
IWGVGSVPVTSVSISVSGMVITPSFNNDPTTQVLSIDVTDRNISLHNFTSLTWISTL
>Q08921
MVHRGRTLKSDTDVTSLNASTVSHQSKPFRQFSTRSRAKSNASFKGLRRVLTHDGTLDND
YFNKHNVSQKCKSSDALFRKRTISGLNMTALTRVKSNQGKRSASFHSPVHNTLLSPKNSS
HSNTGTAGFGLKPRRSKSTQSVLSLRDAQESKKSESTTDEEVECFSEDNIEDGKVNNDKV

IAEHVMPEEKKNVQQLNQNELQSPDSIDEQEEDKSGTDGKENHRAVSLPLPHLSSNNYFG
ESSHSIEHQKDGETSPSSIETKLNATSVINEEGQSKVTKEADIDDLSSHSQNLRASLVKA
GDNISEAPYDKEKKILDVGNTLAAHKSNQKPSHSDEQFDQEDHIDAPRSNSSRKSDSSFM
SLRRQSSKQHKLLNEEEDLIKPDDISSAGTKDIEGHSLLENYAPNMILSQSTGVERRFEN
SSSIQNSLGNEIHDSGEHMASGDTFNELDDGKLRKSKKNGGRSQLGQNIPNSQSTFPTIA
NIGSKDNNVPQHNFSTSISSLTNNLRRAAPESFHGSRMNNIFHKKGNQNLLLRSNDLNKN
SAAPASPLSNEHITSSTNSGSDANRQSNSGAKFNSFAQFLKSDGIDAESRTQRKLWLQRE
NSIMDLSSQNDGSDSIFMAGNIDAKREFERISHEYSNVKRFYNPLDEALLRVQPIITGNA
NNIRKKSHNDAQSIAHSSSDTDHKDEDDLLFTNYDKKFDDLYPHLASAKIQAVLSGIWKS
ESYLFNKDVNPINKNRTTSTNHSVGHTASQNARNLLRGPMGSSTTLHHQRVINSLQPTTR
AVNRRMENVGYMHTQPQQR
>O14492
MNGAGPGPAAAAPVPVPVPVPDWRQFCELHAQAAAVDFAHKFCRFLRDNPAYDTPDAGAS
FSRHFAANFLDVFGEEVRRVLVAGPTTRGAAVSAEAMEPELADTSALKAASYGHSRSSED
VSTHAATKARVRKGFSLRNMSLCVVDGVRDMWHRRASPEPDAAAAPRTAEPRDKWTRRLR
LSRTLAAKVELVDIQREGALRFMVADDAAAGSGGSAQWQKCRLLLRRAVAEERFRLEFFV
PPKASRPKVSIPLSAIIEVRTTMPLEMPEKDNTFVLKVENGAEYILETIDSLQKHSWVAD
IQGCVDPGDSEEDTELSCTRGGCLASRVASCSCELLTDAVDLPRPPETTAVGAVVTAPHS
RGRDAVRESLIHVPLETFLQTLESPGGSGSDSNNTGEQGAETDPEAEPELELSDYPWFHG
TLSRVKAAQLVLAGGPRNHGLFVIRQSETRPGEYVLTFNFQGKAKHLRLSLNGHGQCHVQ
HLWFQSVLDMLRHFHTHPIPLESGGSADITLRSYVRAQDPPPEPGPTPPAAPASPACWSD
SPGQHYFSSLAAAACPPASPSDAAGASSSSASSSSAASGPAPPRPVEGQLSARSRSNSAE
RLLEAVAATAAEEPPEAAPGRARAVENQYSFY
>P12729
MKFQHTFIALLSLLTYANAYDYFTTTLANQNPVCASVDVIQNVCTEVCGRFVRYIPDATN
TNQFTFAEYTTNQCTVQVTPAVTNTFTCADQTSSHALGSDWSGVCKITATPAPTVTPTVT
PTVTPTVTPTPTNTPNPTPSQTSTTTGSASTVVASLSLIIFSMILSLC
>Q96PE1
MRGAPARLLLPLLPWLLLLLAPEARGAPGCPLSIRSCKCSGERPKGLSGGVPGPARRRVV
CSGGDLPEPPEPGLLPNGTVTLLLSNNKITGLRNGSFLGLSLLEKLDLRNNIISTVQPGA
FLGLGELKRLDLSNNRIGCLTSETFQGLPRLLRLNISGNIFSSLQPGVFDELPALKVVDL
GTEFLTCDCHLRWLLPWAQNRSLQLSEHTLCAYPSALHAQALGSLQEAQLCCEGALELHT
HHLIPSLRQVVFQGDRLPFQCSASYLGNDTRIRWYHNRAPVEGDEQAGILLAESLIHDCT
FITSELTLSHIGVWASGEWECTVSMAQGNASKKVEIVVLETSASYCPAERVANNRGDFRW
PRTLAGITAYQSCLQYPFTSVPLGGGAPGTRASRRCDRAGRWEPGDYSHCLYTNDITRVL
YTFVLMPINASNALTLAHQLRVYTAEAASFSDMMDVVYVAQMIQKFLGYVDQIKELVEVM
VDMASNLMLVDEHLLWLAQREDKACSRIVGALERIGGAALSPHAQHISVNARNVALEAYL
IKPHSYVGLTCTAFQRREGGVPGTRPGSPGQNPPPEPEPPADQQLRFRCTTGRPNVSLSS
FHIKNSVALASIQLPPSLFSSLPAALAPPVPPDCTLQLLVFRNGRLFHSHSNTSRPGAAG
PGKRRGVATPVIFAGTSGCGVGNLTEPVAVSLRHWAEGAEPVAAWWSQEGPGEAGGWTSE
GCQLRSSQPNVSALHCQHLGNVAVLMELSAFPREVGGAGAGLHPVVYPCTALLLLCLFAT
IITYILNHSSIRVSRKGWHMLLNLCFHIAMTSAVFAGGITLTNYQMVCQAVGITLHYSSL
STLLWMGVKARVLHKELTWRAPPPQEGDPALPTPSPMLRFYLIAGGIPLIICGITAAVNI
HNYRDHSPYCWLVWRPSLGAFYIPVALILLITWIYFLCAGLRLRGPLAQNPKAGNSRASL

EAGEELRGSTRLRGSGPLLSDSGSLLATGSARVGTPGPPEDGDSLYSPGVQLGALVTTHF
LYLAMWACGALAVSQRWLPRVVCSCLYGVAASALGLFVFTHHCARRRDVRASWRACCPPA
SPAAPHAPPRALPAAAEDGSPVFGEGPPSLKSSPSGSSGHPLALGPCKLTNLQLAQSQVC
EAGAAAGGEGEPEPAGTRGNLAHRHPNNVHHGRRAHKSRAKGHRAGEACGKNRLKALRGG
AAGALELLSSESGSLHNSPTDSYLGSSRNSPGAGLQLEGEPMLTPSEGSDTSAAPLSEAG
RAGQRRSASRDSLKGGGALEKESHRRSYPLNAASLNGAPKGGKYDDVTLMGAEVASGGCM
KTGLWKSETTV
>O95425
MKRKERIARRLEGIENDTQPILLQSCTGLVTHRLLEEDTPRYMRASDPASPHIGRSNEEE
ETSDSSLEKQTRSKYCTETSGVHGDSPYGSGTMDTHSLESKAERIARYKAERRRQLAEKY
GLTLDPEADSEYLSRYTKSRKEPDAVEKRGGKSDKQEESSRDASSLYPGTETMGLRTCAG
ESKDYALHAGDGSSDPEVLLNIENQRRGQELSATRQAHDLSPAAESSSTFSFSGRDSSFT
EVPRSPKHAHSSSLQQAASRSPSFGDPQLSPEARPSTGKPKHEWFLQKDSEGDTPSLINW
PSRVKVREKLVKEESARNSPELASESVTQRRHQPAPVHYVSFQSEHSAFDRVPSKAAGST
RQPIRGYVQPADTGHTAKLVTPETPENASECSWVASATQNVPKPPSLTVLEGDGRDSPVL
HICESKAEEEEGEGEGEEKEEDVCFTEALEQSKKTLLALEGDGLVRSPEDPSRNEDFGKP
AVSTVTLEHQKELENVAQPPQAPHQPTERTGRSEMVLYIQSEPVSQDAKPTGHNREASKK
RKVRTRSLSDFTGPPQLQALKYKDPASRRELELPSSKTEGPYGEISMLDTKVSVAQLRSA
FLASANACRRPELKSRVERSAEGPGLPTGVERERGSRKPRRYFSPGESRKTSERFRTQPI
TSAERKESDRCTSHSETPTVDDEEKVDERAKLSVAAKRLLFREMEKSFDEQNVPKRRSRN
TAVEQRLRRLQDRSLTQPITTEEVVIAATEPIPASCSGGTHPVMARLPSPTVARSAVQPA
RLQASAHQKALAKDQTNEGKELAEQGEPDSSTLSLAEKLALFNKLSQPVSKAISTRNRID
TRQRRMNARYQTQPVTLGEVEQVQSGKLIPFSPAVNTSVSTVASTVAPMYAGDLRTKPPL
DHNASATDYKFSSSIENSDSPVRSILKSQAWQPLVEGSENKGMLREYGETESKRALTGRD
SGMEKYGSFEEAEASYPILNRAREGDSHKESKYAVPRRGSLERANPPITHLGDEPKEFSM
AKMNAQGNLDLRDRLPFEEKVEVENVMKRKFSLRAAEFGEPTSEQTGTAAGKTIAQTTAP
VSWKPQDSSEQPQEKLCKNPCAMFAAGEIKTPTGEGLLDSPSKTMSIKERLALLKKSGEE
DWRNRLSRRQEGGKAPASSLHTQEAGRSLIKKRVTESRESQMTIEERKQLITVREEAWKT
RGRGAANDSTQFTVAGRMVKKGLASPTAITPVASAICGKTRGTTPVSKPLEDIEARPDMQ
LESDLKLDRLETFLRRLNNKVGGMHETVLTVTGKSVKEVMKPDDDETFAKFYRSVDYNMP
RSPVEMDEDFDVIFDPYAPKLTSSVAEHKRAVRPKRRVQASKNPLKMLAAREDLLQEYTE
QRLNVAFMESKRMKVEKMSSNSNFSEVTLAGLASKENFSNVSLRSVNLTEQNSNNSAVPY
KRLMLLQIKGRRHVQTRLVEPRASALNSGDCFLLLSPHCCFLWVGEFANVIEKAKASELA
TLIQTKRELGCRATYIQTIEEGINTHTHAAKDFWKLLGGQTSYQSAGDPKEDELYEAAII
ETNCIYRLMDDKLVPDDDYWGKIPKCSLLQPKEVLVFDFGSEVYVWHGKEVTLAQRKIAF
QLAKHLWNGTFDYENCDINPLDPGECNPLIPRKGQGRPDWAIFGRLTEHNETILFKEKFL
DWTELKRSNEKNPGELAQHKEDPRTDVKAYDVTRMVSMPQTTAGTILDGVNVGRGYGLVE
GHDRRQFEITSVSVDVWHILEFDYSRLPKQSIGQFHEGDAYVVKWKFMVSTAVGSRQKGE
HSVRAAGKEKCVYFFWQGRHSTVSEKGTSALMTVELDEERGAQVQVLQGKEPPCFLQCFQ
GGMVVHSGRREEEEENVQSEWRLYCVRGEVPVEGNLLEVACHCSSLRSRTSMVVLNVNKA
LIYLWHGCKAQAHTKEVGRTAANKIKEQCPLEAGLHSSSKVTIHECDEGSEPLGFWDALG
RRDRKAYDCMLQDPGSFNFAPRLFILSSSSGDFAATEFVYPARAPSVVSSMPFLQEDLYS
APQPALFLVDNHHEVYLWQGWWPIENKITGSARIRWASDRKSAMETVLQYCKGKNLKKPA
PKSYLIHAGLEPLTFTNMFPSWEHREDIAEITEMDTEVSNQITLVEDVLAKLCKTIYPLA

DLLARPLPEGVDPLKLEIYLTDEDFEFALDMTRDEYNALPAWKQVNLKKAKGLF
>Q8IZ08
MEEPQPPRPPASMALLGSQHSGAPSAAGPPGGTSSAATAAVLSFSTVATAALGNLSDASG
GGTAAAPGGGGLGGSGAAREAGAAVRRPLGPEAAPLLSHGAAVAAQALVLLLIFLLSSLG
NCAVMGVIVKHRQLRTVTNAFILSLSLSDLLTALLCLPAAFLDLFTPPGGSAPAAAAGPW
RGFCAASRFFSSCFGIVSTLSVALISLDRYCAIVRPPREKIGRRRALQLLAGAWLTALGF
SLPWELLGAPRELAAAQSFHGCLYRTSPDPAQLGAAFSVGLVVACYLLPFLLMCFCHYHI
CKTVRLSDVRVRPVNTYARVLRFFSEVRTATTVLIMIVFVICCWGPYCFLVLLAAARQAQ
TMQAPSLLSVVAVWLTWANGAINPVIYAIRNPNISMLLGRNREEGYRTRNVDAFLPSQGP
GLQARSRSRLRNRYANRLGACNRMSSSNPASGVAGDVAMWARKNPVVLFCREGPPEPVTA
VTKQPKSEAGDTSL
>Q9BYE9
MAQLWLSCFLLPALVVSVAANVAPKFLANMTSVILPEDLPVGAQAFWLVAEDQDNDPLTY
GMSSPNAYFFAVTPKTGEVKLASALDYETLYTFKVTISVSDPYIQVQREMLVIVEDRNDN
APVFQNTAFSTSINETLPVGSVVFSVLAVDKDMGSAGMVVYSIEKVIPSTGDSEHLFRIL
ANGSIVLNGSLSYNNKSAFYQLELKACDLGGMYHNTFTIQCSLPVFLSISVVDQPDLDPQ
FVREFYSASVAEDAAKGTSVLTVEAVDGDKGINDPVIYSISYSTRPGWFDIGADGVIRVN
GSLDREQLLEADEEVQLQVTATETHLNIYGQEAKVSIWVTVRVMDVNDHKPEFYNCSLPA
CTFTPEEAQVNFTGYVDEHASPRIPIDDLTMVVYDPDKGSNGTFLLSLGGPDAEAFSVSP
ERAAGSASVQVLVRVSALVDYERQTAMAVQVVATDSVSQNFSVAMVTIHLRDINDHRPTF
PQSLYVLTVPEHSATGSVVTDSIHATDPDTGAWGQITYSLLPGNGADLFQVDPVSGTVTV
RNGELLDRESQAVYYLTLQATDGGNLSSSTTLQIHLLDINDNAPVVSGSYNIFVQEEEGN
VSVTIQAHDNDEPGTNNSRLLFNLLPGPYSHNFSLDPDTGLLRNLGPLDREAIDPALEGR
IVLTVLVSDCGEPVLGTKVNVTITVEDINDNLPIFNQSSYNFTVKEEDPGVLVGVVKAWD
ADQTEANNRISFSLSGSGANYFMIRGLVLGAGWAEGYLRLPPDVSLDYETQPVFNLTVSA
ENPDPQGGETIVDVCVNVKDVNDNPPTLDVASLRGIRVAENGSQHGQVAVVVASDVDTSA
QLEIQLVNILCTKAGVDVGSLCWGWFSVAANGSVYINQSKAIDYEACDLVTLVVRACDLA
TDPGFQAYSNNGSLLITIEDVNDNAPYFLPENKTFVIIPELVLPNREVASVRARDDDSGN
NGVILFSILRVDFISKDGATIPFQGVFSIFTSSEADVFAGSIQPVTSLDSTLQGTYQVTV
QARDRPSLGPFLEATTTLNLFTVDQSYRSRLQFSTPKEEVGANRQAINAALTQATRTTVY
IVDIQDIDSAARARPHSYLDAYFVFPNGSALTLDELSVMIRNDQDSLMQLLQLGLVVLGS
QESQESDLSKQLISVIIGLGVALLLVLVIMTMAFVCVRKSYNRKLQAMKAAKEARKTAAG
VMPSAPAIPGTNMYNTERANPMLNLPNKDLGLEYLSPSNDLDSVSVNSLDDNSVDVDKNS
QEIKEHRPPHTPPEPDPEPLSVVLLGRQAGASGQLEGPSYTNAGLDTTDL
>P25618
MLIINGKIIPIAHTICAFSAFFAALVTGYSLHFHKIVTNAHYTYPDEWFPSVSATIGDRY
PERSIFQILIALTAFPRFLLLLGHYYLNQSKVCFLVGVLRTVSCGGWVYITSTDDHDIHD
IFMITYIVLTLPWDIMITRYSSPLTSKNKGLTATIFFGTLFPMIYWYIQHSVQQRAGAYS
IYAYFEWSLILLDIAFDAFAYADFKKIDIVLAFNEKPGNTSFFQIRDSSPINYGEEKSSE
LQKSGEKKVEKEKPVARSATGSYFRFDSFFYLLTNIFNGFLFWSNVTSLLCSIWHFPLWY
MGISGYEAAILGYLGPIFLYLPFVSEAFTQYGVLLGGIIAIGAYIVQMPELRLISVAVGT
SITVATFVQNLRYITNAETSFSFALTWLLGLVASVILKMGFYTNNPTWVILDERNGGYNK
TALVLTVLFGMLSPYVNSINFEGKRNAQAKSASLIGKLFLAVGFGSLLFGIHQLLTDSST
TIYWAWEGYNESHGPLPWPWGALTCTVMLFASLSSVKFMGKPLVPCLLLLISTAVLSARS

ITQWPKYIFGGLLYAIAMLWLVPSYFSALGQVQNIWVYVLSFSVYIIFVLAHVWVVAYAF
VPMGWVLREKIETVLAFSSTFIIIGALTCKNLNIQLVTMGKKFFIYVFFFAVALLSLTAR
FVYDIRPTGIPQPYHPDSQLITAGIWTIHFGLDNDMWASEDRMINLIKDMELDVVGLLET
DTQRITMGNRDLTSKLAHDLNMYADFGPGPNKHTWGCVLLSKFPIVNSTHHLLPSPVGEL
APAIHATLQTYNDTLVDVFVFHSGQEEDEEDRRLQSNYMAKLMGNTTRPAILLSYLVVDP
GEGNYNTYVSETSGMHDIDPSDDDRWCEYILYRGLRRTGYARVARGTITDTELQVGKFQV
LSEQALVEHSDSMYEYGHMSEPEYEDMKFPDKFLGEGERGHFYHVFDEPRYYL
>Q9Z0J8
MVLLAQGACCSNQWLAAVLLSLCSCLPAGQSVDFPWAAVDNMLVRKGDTAVLRCYLEDGA
SKGAWLNRSSIIFAGGDKWSVDPRVSISTLNKRDYSLQIQNVDVTDDGPYTCSVQTQHTP
RTMQVHLTVQVPPKIYDISNDMTINEGTNVTLTCLATGKPEPAISWRHISPSAKPFENGQ
YLDIYGITRDQAGEYECSAENDVSFPDVKKVRVVVNFAPTIQEIKSGTVTPGRSGLIRCE
GAGVPPPAFEWYKGEKRLFNGQQGIIIQNFSTRSILTVTNVTQEHFGNYTCVAANKLGTT
NASLPLNPPSTAQYGITGSACDLFSCWSLALTLSSVISIFYLKNAILQ
>P97798
MAAEREAGRLLCTSSSRRCCPPPPLLLLLPLLLLLGRPASGAAATKSGPRRQSQGASVRT
FTPFYFLVEPVDTLSVRGSSVILNCSAYSEPSPNIEWKKDGTFLNLESDDRRQLLPDGSL
FISNVVHSKHNKPDEGFYQCVATVDNLGTIVSRTAKLTVAGLPRFTSQPEPSSVYVGNSA
ILNCEVNADLVPFVRWEQNRQPLLLDDRIVKLPSGTLVISNATEGDGGLYRCIVESGGPP
KFSDEAELKVLQDPEEIVDLVFLMRPSSMMKVTGQSAVLPCVVSGLPAPVVRWMKNEEVL
DTESSGRLVLLAGGCLEISDVTEDDAGTYFCIADNGNKTVEAQAELTVQVPPGFLKQPAN
IYAHESMDIVFECEVTGKPTPTVKWVKNGDVVIPSDNFKIVKEHNLQVLGLVKSDEGFYQ
CIAENDVGNAQAGAQLIILEHDVAIPTLPPTSLTSATTDHLAPATTGPLPSAPRDVVASL
VSTRFIKLTWRTPASDPHGDNLTYSVFYTKEGVDRERVENTSQPGEMQVTIQNLMPATVY
IFKVMAQNKHGSGESSAPLRVETQPEVQLPGPAPNIRAYATSPTSITVTWETPLSGNGEI
QNYKLYYMEKGTDKEQDIDVSSHSYTINGLKKYTEYSFRVVAYNKHGPGVSTQDVAVRTL
SDVPSAAPQNLSLEVRNSKSIVIHWQPPSSTTQNGQITGYKIRYRKASRKSDVTETLVTG
TQLSQLIEGLDRGTEYNFRVAALTVNGTGPATDWLSAETFESDLDETRVPEVPSSLHVRP
LVTSIVVSWTPPENQNIVVRGYAIGYGIGSPHAQTIKVDYKQRYYTIENLDPSSHYVITL
KAFNNVGEGIPLYESAVTRPHTDTSEVDLFVINAPYTPVPDPTPMMPPVGVQASILSHDT
IRITWADNSLPKHQKITDSRYYTVRWKTNIPANTKYKNANATTLSYLVTGLKPNTLYEFS
VMVTKGRRSSTWSMTAHGATFELVPTSPPKDVTVVSKEGKPRTIIVNWQPPSEANGKITG
YIIYYSTDVNAEIHDWVIEPVVGNRLTHQIQELTLDTPYYFKIQARNSKGMGPMSEAVQF
RTPKADSSDKMPNDQALGSAGKGSRLPDLGSDYKPPMSGSNSPHGSPTSPLDSNMLLVII
VSVGVITIVVVVVIAVFCTRRTTSHQKKKRAACKSVNGSHKYKGNCKDVKPPDLWIHHER
LELKPIDKSPDPNPVMTDTPIPRNSQDITPVDNSMDSNIHQRRNSYRGHESEDSMSTLAG
RRGMRPKMMMPFDSQPPQPVISAHPIHSLDNPHHHFHSSSLASPARSHLYHPSSPWPIGT
SMSLSDRANSTESVRNTPSTDTMPASSSQTCCTDHQDPEGATSSSYLASSQEEDSGQSLP
TAHVRPSHPLKSFAVPAIPPPGPPLYDPALPSTPLLSQQALEPSTFHSVKTASIGTLGRS
RPPMPVVVPSAPEVQETTRMLEDSESSYEPDELTKEMAHLEGLMKDLNAITTA
>P26043
MPKPINVRVTTMDAELEFAIQPNTTGKQLFDQVVKTVGLREVWFFGLQYVDSKGYSTWLK
LNKKVTQQDVKKENPLQFKFRAKFFPEDVSEELIQEITQRLFFLQVKEAILNDEIYCPPE
TAVLLASYAVQAKYGDYNKEIHKPGYLANDRLLPQRVLEQHKLTKEQWEERIQNWHEEHR

GMLREDSMMEYLKIAQDLEMYGVNYFEIKNKKGTELWLGVDALGLNIYEHDDKLTPKIGF
PWSEIRNISFNDKKFVIKPIDKKAPDFVFYAPRLRINKRILALCMGNHELYMRRRKPDTI
EVQQMKAQAREEKHQKQLERAQLENEKKKREIAEKEKERIEREKEELMERLRQIEEQTVK
AQKELEEQTRKALELEQERQRAKEEAERLDRERRAAEEAKSAIAKQAADQMKNQEQLAEE
LAEFTAKIALLEEAKKKKEEEATEWQHKAFAAQEDLEKTKEELKTVMSAPPPPPPPPVIP
PTENEHDEQDENSAEASAELSSEGVMNHRSEEERVTETQKNERVKKQLQALSSELAQARD
ETKKTQNDVLHAENVKAGRDKYKTLRQIRQGNTKQRIDEFEAM
>P34116
MKIILTLSIFLICFLQLGQSVIDPSQNEVMSDLLFNLYGYDKSLDPCNSNSVECDDINST
STIKTVISLNLPTPLQEYVITQDLTPLQNLTYMELYEKIYLTLSFFKNINKLTQLETIVT
LSFNVTIPDDTIFPASLETFSIYKPSVPLSIAIFGSNIKNLYVNSPLTGYSIPTLINVNP
YLENLQLPVTYYSGFPSNISLAFPNLQYLTIYVNNDMDQNNYHNFSISNIGVFKNLKGLD
IEFTDSYNPQEFSINSFLSNVPVIDSLYIYGQGVTIDPSVGIIDLSYVKSKKFLSINIQE
SSLLNNCKGKSFKSPKKAYFRSNYNTFSYACIDFSNLAYFYDYYNEYEQYLPNIDNAPLL
NEIYISESVVVGDIPESYCRINYLGLNYNQLNGTAPSCILCLGGNRGGDIVLPNPLLNFN
KTSEPYCPTFKIDQNYTNLVATDGIGKLIITGTNLGWYGNDITPITANSKLAITIPKGVG
TNKSITVTFQNGEQRTFNYSYVPPFIKSYGFLELDSNKYLTINGTGFDFENPNIITINGQ
QITFSIALGGGDNDGLIALPIDELPNFDSETKFTVSTLVGGQSSNEVTFYYFNSINITEE
KLVLNNTGGSVDINGSFGTNNISLVSISINGTNCLVTSYTNSKLTIQYPSKQVGDNYVLT
LNVGGYAVNLVVEYIEGGETPTPSTTPSTTPSTTPSTTPSSTPTQSPGDDGSTSSTLSIS
FYLITLLLLTQQFI
>P01831
MNPAISVALLLSVLQVSRGQKVTSLTACLVNQNLRLDCRHENNTKDNSIQHEFSLTREKR
KHVLSGTLGIPEHTYRSRVTLSNQPYIKVLTLANFTTKDEGDYFCELQVSGANPMSSNKS
ISVYRDKLVKCGGISLLVQNTSWMLLLLLSLSLLQALDFISL
>Q7Z410
MEPTVADVHLVPRTTKEVPALDAACCRAASIGVVATSLVVLTLGVLLAFLSTQGFHVDHT
AELRGIRWTSSLRRETSDYHRTLTPTLEALLHFLLRPLQTLSLGLEEELLQRGIRARLRE
HGISLAAYGTIVSAELTGRHKGPLAERDFKSGRCPGNSFSCGNSQCVTKVNPECDDQEDC
SDGSDEAHCECGLQPAWRMAGRIVGGMEASPGEFPWQASLRENKEHFCGAAIINARWLVS
AAHCFNEFQDPTKWVAYVGATYLSGSEASTVRAQVVQIVKHPLYNADTADFDVAVLELTS
PLPFGRHIQPVCLPAATHIFPPSKKCLISGWGYLKEDFLVKPEVLQKATVELLDQALCAS
LYGHSLTDRMVCAGYLDGKVDSCQGDSGGPLVCEEPSGRFFLAGIVSWGIGCAEARRPGV
YARVTRLRDWILEATTKASMPLAPTMAPAPAAPSTAWPTSPESPVVSTPTKSMQALSTVP
LDWVTVPKLQECGARPAMEKPTRVVGGFGAASGEVPWQVSLKEGSRHFCGATVVGDRWLL
SAAHCFNHTKVEQVRAHLGTASLLGLGGSPVKIGLRRVVLHPLYNPGILDFDLAVLELAS
PLAFNKYIQPVCLPLAIQKFPVGRKCMISGWGNTQEGNATKPELLQKASVGIIDQKTCSV
LYNFSLTDRMICAGFLEGKVDSCQGDSGGPLACEEAPGVFYLAGIVSWGIGCAQVKKPGV
YTRITRLKGWILEIMSSQPLPMSPPSTTRMLATTSPRTTAGLTVPGATPSRPTPGAASRV
TGQPANSTLSAVSTTARGQTPFPDAPEATTHTQLPDCGLAPAALTRIVGGSAAGRGEWPW
QVSLWLRRREHRCGAVLVAERWLLSAAHCFDVYGDPKQWAAFLGTPFLSGAEGQLERVAR
IYKHPFYNLYTLDYDVALLELAGPVRRSRLVRPICLPEPAPRPPDGTRCVITGWGSVREG
GSMARQLQKAAVRLLSEQTCRRFYPVQISSRMLCAGFPQGGVDSCSGDAGGPLACREPSG
RWVLTGVTSWGYGCGRPHFPGVYTRVAAVRGWIGQHIQE

>Q08722
MWPLVAALLLGSACCGSAQLLFNKTKSVEFTFCNDTVVIPCFVTNMEAQNTTEVYVKWKF
KGRDIYTFDGALNKSTVPTDFSSAKIEVSQLLKGDASLKMDKSDAVSHTGNYTCEVTELT
REGETIIELKYRVVSWFSPNENILIVIFPIFAILLFWGQFGIKTLKYRSGGMDEKTIALL
VAGLVITVIVIVGAILFVPGEYSLKNATGLGLIVTSTGILILLHYYVFSTAIGLTSFVIA
ILVIQVIAYILAVVGLSLCIAACIPMHGPLLISGLSILALAQLLGLVYMKFVASNQKTIQ
PPRKAVEEPLNAFKESKGMMNDE
>P53241
MTISNKSWRSYFPHLRKLPEDDQYLYSDDTNSSIIAEEELHHSVDKSSKTDVTAETTAVE
PHPHNLRHDLPYEVRDEAGRKWWKYFDEFEYRVNKEYKKSRKWYEFLYPNHTTQTKAERR
LLYKLDIIIALYFFMLCWSKSVDLNNYTNAYVSNMKEDLNMKGNDYVYTSTIANVGAIVF
QLPFMYLLPRFPSHIILPVMDLGWTWFTFACYRANSLAELRAYRFILSAFGAAYYPVSQY
ILGCWYAPDEINSRVCLFFCGQQLGSVTSGLLQSRIFKSLNGVHGLAGWRWMFLIDAIAI
SLPTAIIGFFVIPGVPSKCYSLFLTDEEIRIARARNKRNQIKDGVDKSKLAPLWSRKLWK
KVFCTPAFWVLVVFDTCSWNNMTAYSGSYTLWLKSNTKYSIAQVNNLSVIPACLGFAYVI
FCAFGADLFRCKWIFMVFAAIMNTVSCALLIKWDIPSKAKWYAFFTTYFSVAASPCLWSF
INDFLRFDPQVKAITWIAIYSFSQSTYAWIPTLAWPTVESPRFKTGYTVSLIFGAIYGLW
TFVVLFFYKRNEKKHALGNGIILYDSNKGEELPEFVKKNMEERDGYYYLKRSS
>Q7TNN9
MAWNTNLRGRLPITCLILQVTMVVLFGVFVRYDIQADAHWWLEKKRKNISSDVENEFYYR
YPSFEDVHAMVFVGFGFLMTYLQRYGFSAVGFNFLLAAFGIQWALLMQGWFHFFEEGHIL
LSVENLIQADFCVASTCVAFGAVLGKISPMQLLIMTFFQVTLFTVNEFILLNLIEAKDAG
GSMTIHTFGAYFGLTVTWILYRKNLEQSKQRQSSVYHSDLFAMIGTLFLWIYWPSFNSAS
SFHGDTQHRAALNTYLSLAASVLTTVAVSSVIHKKGKLDMVHIQNATLAGGVGVGTAAEM
MLTPYGALIVGFFCGILSTLGFAYLSPFLESRLRIQDTCGIHNLHGIPGIIGGIVGAVTA
AYSSPDVYGEPGIVHSFGFGGYKADWTKRMQGRSQIFGLLLSLAMALVGGIIVGFILKLP
FWGQASDENCFEDAIYWEVPEEVNTVYIPEDLAHKHSTSLVPAIPLVLSTPSASIVPPVP
PTPPASLATVTSSSLVH
>Q9GZU1
MTAPAGPRGSETERLLTPNPGYGTQAGPSPAPPTPPEEEDLRRRLKYFFMSPCDKFRAKG
RKPCKLMLQVVKILVVTVQLILFGLSNQLAVTFREENTIAFRHLFLLGYSDGADDTFAAY
TREQLYQAIFHAVDQYLALPDVSLGRYAYVRGGGDPWTNGSGLALCQRYYHRGHVDPAND
TFDIDPMVVTDCIQVDPPERPPPPPSDDLTLLESSSSYKNLTLKFHKLVNVTIHFRLKTI
NLQSLINNEIPDCYTFSVLITFDNKAHSGRIPISLETQAHIQECKHPSVFQHGDNSFRLL
FDVVVILTCSLSFLLCARSLLRGFLLQNEFVGFMWRQRGRVISLWERLEFVNGWYILLVT
SDVLTISGTIMKIGIEAKNLASYDVCSILLGTSTLLVWVGVIRYLTFFHNYNILIATLRV
ALPSVMRFCCCVAVIYLGYCFCGWIVLGPYHVKFRSLSMVSECLFSLINGDDMFVTFAAM
QAQQGRSSLVWLFSQLYLYSFISLFIYMVLSLFIALITGAYDTIKHPGGAGAEESELQAY
IAQCQDSPTSGKFRRGSGSACSLLCCCGRDPSEEHSLLVN
>Q8L940
MEGTGVVAVYGNGAITEAKKSPFSVKVGLAQMLRGGVIMDVVNAEQARIAEEAGACAVMA
LERVPADIRAQGGVARMSDPQMIKEIKQAVTIPVMAKARIGHFVEAQILEAIGIDYIDES
EVLTLADEDHHINKHNFRIPFVCGCRNLGEALRRIREGAAMIRTKGEAGTGNIIEAVRHV
RSVNGDIRVLRNMDDDEVFTFAKKLAAPYDLVMQTKQLGRLPVVQFAAGGVATPADAALM

MQLGCDGVFVGSGIFKSGDPARRARAIVQAVTHYSDPEMLVEVSCGLGEAMVGINLNDEK
VERFANRSE
>Q9JHW1
MASGWDERPPWRLESLRLLPPPPLLLLLLLLRSSAQAAHIKKAEATTTTVGGSEAAEGQF
DHYYHEAALGEALEAAAAAGPPGLARLFSIGNSVEGRPLWVLRLTAGLGPPPTPAAVGLD
AAGPLLPGRPQVKLVGNMHGDETVSRQVLVYLARELASGYRRGDPRLVRLLNTTDVYLLP
SLNPDGFERAREGDCGLGDSGPPGTSGRDNSRGRDLNRSFPDQFSTGEPPSLDEVPEVRA
LIDWIRRNKFVLSGNLHGGSVVASYPFDDSPEHKTTGIYSKTSDDEVFRYLAKAYASNHP
IMRTGEPHCPGDEEETFKDGITNGAHWYDVEGGMQDYNYVWANCFEITLELSCCKYPPAS
QLRQEWENNRESLITLIEKVHIGIKGFVKDSVTGSGLENATISVAGINHNITTGRFGDFH
RLLIPGSYNLTAVSPGYMPLTINNIVVKEGPATEIDFSLQPTVMSVVPDSTEAVTTPGTV
AVPNIPPGTPSSHQPIQPKDFHHHHFPDMEIFLRRFANEYPNITRLYSLGKSVESRELYV
MEISDNPGVHEPGEPEFKYIGNMHGNEVVGRELLLNLIEYLCKNFGTDPEVTDLVRSTRI
HLMPSMNPDGYEKSQEGDSISVVGRNNSNNFDLNRNFPDQFVPITDPTQPETIAVMSWVK
AYPFVLSANLHGGSLVVNYPYDDNEQGVATYSKSPDDAVFQQIALSYSKENSQMFQGRPC
KDMYLNEYFPHGITNGASWYNVPGGMQDWNYLQTNCFEVTIELGCVKYPFEKELPKYWEQ
NRRSLIQFMKQVHQGVKGFVLDATDGRGILNATLSVAEINHPVTTYKAGDYWRLLVPGTY
KITASARGYNPVTKNVTVRSEGAIQVNFTLVRSSTDANNESKKGKGHSTSTDDTSDPTSK
EFEALIKHLSAENGLEGFMLSSSSDLALYRYHSYKDLSEFLRGLVMNYPHITNLTTLGQS
VEYRHIWSLEISNKPNISEPEEPKIRFVAGIHGNAPVGTELLLALAEFLCLNYKKNPVVT
QLVDRTRIVIVPSLNPDGRERAQEKDCTSKTGHTNARGRDLDTDFTSNASQPETKAIIEN
LIQKQDFSLSIALDGGSVLVTYPYDKPVQTVENKETLKHLASLYANNHPSMHMGQPSCPN
NSDENIPGGVMRGAEWHSHLGSMKDYSVTYGHCPEITVYTSCCYFPSAAQLPALWAENKK
SLLSMLVEVHKGVHGLVKDKTGKPISKAVIVLNEGIRVHTKEGGYFHVLLAPGVHNINAI
ADGYQQQHSQVFVHHDAASSVVIVFDTDNRIFGLPRELVVTVSGATMSALILTACIIWCI
CSIKSNRHKDGFHRLRQHHDEYEDEIRMMSTGSKKSLLSHEFQDETDTEEETLYSSKH
>P91885
MYSLIFLALIGAAFGVPLSTNEDSTRNQNLAALYVLPQTSYPTFYDVRLFIDPGYTEAFH
GNVSIRIIPNINIDQITIHAMAMRIDSIRVVSDVNPNEDLFSDFTLATDDTHLLTIRLTR
NITALQPHVIHIDYVAQYADDMFGVYVSTYEENGRTVNLVTSQLQPTFARRAFPCYDEPA
LKAVFRTTIYAPAAYATVRSNTPERRDSLKPNEPGYVKHEFEDTLVMSTYLIAYLVSNFN
YIENSQNPIYPIPFRVYSRPGTQNTAEFALEFGQQNMIALEEYTEFPYAFPKIDKAAVPD
FAAGAMENWGLVIYREVALLVREGVTTTSVKQNIGRIICHENTHMWFGNEVGPMSWTYTW
LNEGFANFFENYATDFVRPQWRMMDQFVIAMQNVFQSDAVLSVNPMTHPVYTPSQIIGTF
NAVAYQKSGSVIRMLQHFMTPEIFRRGLVIYIKANSRAAAAPSDLYVALQQALDESSHRI
PKPISTIMTEWSTQGGFPVLTVRRTAPNADSVFVAQERYLTDRSLTSTDRWHVPVNWVIS
SNVNFSDTSPQAWILPTFPATAVDVPGLSNADWYIFNKQQTGYYRVNYDVENWVALARVL
NNSHEIIHVLNRAQIVDDAFNLARNGRLHYKNAFEISRYLEMEKDYIPWAAANPAFNYLD
IVLSGANSYNLYRYYLLNLTAPMFEDLGFDVKSGEEFVTPYHRNIILDINCRFGNQRCIS
RAQEILQAFKNNPNQRPNPDIQTLVYCSSLRAGNVENFNFLWNMYLGTSDSSEQSILLSA
LGCTSNAERRNFYLNQIIDDNSAVREQDRHSIAVSVINSSPEGMNVALDFVVENFHRIQP
RVQALTGTTNILNTFARRLTTSAHNEKIDELVRRHESIFSAGERASIAAIRENIAASIAW
SNSNAGIVENWLKENYGPPSGAKSLTAGLLVLISLFVAIFNH
>Q94CD8

MLLPRWFAEALLLLLSILACSNAAFIGVNIGTDLTNMPPPSDIVTLLKSQQITHVRLYDA
NSHMLKAFANTSIEVMVGVTNEEILKIGRFPSAAAAWVNKNVAAYIPSTNITAIAVGSEV
LTTIPHVAPILASALNNIHKALVASNLNFKVKVSSPMSMDIMPKPFPPSTSTFSPSWNTT
VYQLLQFLKNTGSFFMLNAYPYYGYTTANGIFPLDYALFKQLSPVKQIVDPNTLLHYNSM
FDAMVDAAYYSMEALNFSKIPVVVTETGWPSSGGSDEAAATVANAETFNTNLIKRVLNNS
GPPSQPDIPINTYIYELYNEDKRSGPVSERNWGILFPNGTSVYPLSLSGGSSSAALNGSS
MFCVAKADADDDKLVDGLNWACGQGRANCAAIQPGQPCYLPNDVKSHASFAFNDYYQKMK
SAGGTCDFDGTAITTTRDPSYRTCAYTGSLNANATNGNFPPDALGPASPLGGNANARIIF
SYHLPILAPLALTLLQLLLQHDRLL
>P06731
MESPSAPPHRWCIPWQRLLLTASLLTFWNPPTTAKLTIESTPFNVAEGKEVLLLVHNLPQ
HLFGYSWYKGERVDGNRQIIGYVIGTQQATPGPAYSGREIIYPNASLLIQNIIQNDTGFY
TLHVIKSDLVNEEATGQFRVYPELPKPSISSNNSKPVEDKDAVAFTCEPETQDATYLWWV
NNQSLPVSPRLQLSNGNRTLTLFNVTRNDTASYKCETQNPVSARRSDSVILNVLYGPDAP
TISPLNTSYRSGENLNLSCHAASNPPAQYSWFVNGTFQQSTQELFIPNITVNNSGSYTCQ
AHNSDTGLNRTTVTTITVYAEPPKPFITSNNSNPVEDEDAVALTCEPEIQNTTYLWWVNN
QSLPVSPRLQLSNDNRTLTLLSVTRNDVGPYECGIQNELSVDHSDPVILNVLYGPDDPTI
SPSYTYYRPGVNLSLSCHAASNPPAQYSWLIDGNIQQHTQELFISNITEKNSGLYTCQAN
NSASGHSRTTVKTITVSAELPKPSISSNNSKPVEDKDAVAFTCEPEAQNTTYLWWVNGQS
LPVSPRLQLSNGNRTLTLFNVTRNDARAYVCGIQNSVSANRSDPVTLDVLYGPDTPIISP
PDSSYLSGANLNLSCHSASNPSPQYSWRINGIPQQHTQVLFIAKITPNNNGTYACFVSNL
ATGRNNSIVKSITVSASGTSPGLSAGATVGIMIGVLVGVALI
>P57773
MGDWNLLGDTLEEVHIHSTMIGKIWLTILFIFRMLVLGVAAEDVWNDEQSGFICNTEQPG
CRNVCYDQAFPISLIRYWVLQVIFVSSPSLVYMGHALYRLRVLEEERQRMKAQLRVELEE
VEFEMPRDRRRLEQELCQLEKRKLNKAPLRGTLLCTYVIHIFTRSVVEVGFMIGQYLLYG
FHLEPLFKCHGHPCPNIIDCFVSRPTEKTIFLLFMQSIATISLFLNILEIFHLGFKKIKR
GLWGKYKLKKEHNEFHANKAKQNVAKYQSTSANSLKRLPSAPDYNLLVEKQTHTAVYPSL
NSSSVFQPNPDNHSVNDEKCILDEQETVLSNEISTLSTSCSHFQHISSNNNKDTHKIFGK
ELNGNQLMEKRETEGKDSKRNYYSRGHRSIPGVAIDGENNMRQSPQTVFSLPANCDWKPR
WLRATWGSSTEHENRGSPPKGNLKGQFRKGTVRTLPPSQGDSQSLDIPNTADSLGGLSFE
PGLVRTCNNPVCPPNHVVSLTNNLIGRRVPTDLQI
>Q9VWE0
MVAQEQLVLLLMLLAGCRGGANAILDPGWVIPSKVEQLIGGDFNLSCTLNEDYFNGKSAE
DCPVEKLYFTGGGRVYRDSKHIRILNNTTILFSDTNAVEQENDYHCMCDEYVINKSKVYV
GTRPLLVRDFNCLDYDFQFMVCNFTQPPNTVITKYNISYNTNNDWRYSNTLDCNFDSAPV
VTCNLTDDNYKRFSETFYFRLSISNALGHETQPITINHFERLVPARPGQNLTLLNRTESS
VCLSWEMPRRSNYNRGLVWQVRVTPQNFEPITRPSWRNHTLTIKDTLCLTELPFAGYNYT
LRVRVRANQNNTLWSEPMIYAFATAPAPPRRPPRVTYGSFYVYSSEKAMRFYWEPLEEHE
LNGPDFRYSISEYRINGTAVDPGLIKVESNSAMIDHWSMSAVHHFLIRSSNSQGLSVNAT
PMTIGPISNRDFKVREPRNIRSVYHPTNKSYTLSWDPPSDQRELQNYTVFWCVPKPGLQS
ECEGSIRFAEVASGLHHFTTSPDQLLTLHMAVSANYQSHNTGLHWAICSSDKKDDLAKME
PSIDVATSTSLTVSWSERVCAVILAGYNLTYCQRSAGRPDNCTTVTIDRYTNKHVIQNLV
PYTDYSVKMLMYSDSRVSKYSDELVNRTGEAAPSQPRELQLIRVTSDSVELAWKPPLLAN

GVVRAYEGTFRSLHDNVTDTFRVSASADELVNNEKPITYRLGNLTAFTKYEISVRARTVY
PSEPSNVILFSTAIGVPSPPQLYVINNPDQSSRLDWEPPRTPAGRIDFYEISLRDNNASC
LTSTILPGRNLSYVMATPRCTSHNPFQLAVRAINVEQHPQLNGADAAEGAVLLMSTNGKG
CEARTDALGEEERLQFEAYAANMTAYRLYRSDWGIYGFICTPDTHSVKAMYQTIEVTVAI
LVLGVIFYLVYKKYRKMSDIGLVLPQGIMETMKKPIDMGGLGLGLGPDSSVSGGIVCTRV
DDSPPYTPQDLPHDFSSCGSESSKLLLRTASSSGGGGCVDRDGYDDNHETGPISAVGPPT
SYLAMRHGLLVQNDRERERERDREQEREREQQQQQRESEMDREQSCTNGYIKPTQMKSWG
GNGPSDNDHTFSVPSTAMTAPMSQPLSQIPLSGYVPVPIPQSRFNPAPVQPFGSPAVPSA
ATAAAASTFFPPAHLLNMDNYVQASDLHKLKPLVAAPLSQTGGPAFAGSSPATSPPLQLP
PVHAASPAAATPKMADIGYTTMEQLQLTGLIKPPLAATVGSPTHAAGGAPGGGNQHSRLQ
PQINGYVTPQDLNAMAHNRHVL
>Q7Z614
MASPEHPGSPGCMGPITQCTARTQQEAPATGPDLPHPGPDGHLDTHSGLSSNSSMTTREL
QQYWQNQKCRWKHVKLLFEIASARIEERKVSKFVVYQIIVIQTGSFDNNKAVLERRYSDF
AKLQKALLKTFREEIEDVEFPRKHLTGNFAEEMICERRRALQEYLGLLYAIRCVRRSREF
LDFLTRPELREAFGCLRAGQYPRALELLLRVLPLQEKLTAHCPAAAVPALCAVLLCHRDL
DRPAEAFAAGERALQRLQAREGHRYYAPLLDAMVRLAYALGKDFVTLQERLEESQLRRPT
PRGITLKELTVREYLH
>Q9VHR8
MFWLRGLQRSRQQLNRLCHFRSRLNYGTSPSIRSVQPSISRSLGFCSSLVRYSCSTNLPQ
PAEMASKTAHFVLPNTQPIADLDCKSAFENLTEKEKLYAHHFSQASWDGGLIALIQSSPE
APLIFSLLHRIFLAEKIPVLRAKALEAGVSADDFTAFLVYACGVFANAGNYKGMGDSKIV
PNLSEKQFETIVKASAAYSDEPRVGKIFEKVKNLIYALESRNEILGFAPDGITTYWSDNC
TKEDSEIVNAWLTSKRIEPYMCRTFKIVENGQTVYDVKLGSVAESTQDGITLPLEEYNGN
KFRVTRGDYQKLLQRVNQHLLQAQKYAANENESKMIEHYVRSFEQGSLDEHKNGSRWWIK
DKGPVIETYIGFIETYRDPAGGRAEFEGFVAMVNKESSAKFSELVNRAEKLIEYLPWTEP
YEKDSYLKPDFTSLDVLTFAGSGVPAGINIPNYDEIRQDEGFKNVSLGNVLANINRKDPI
PFLTEEDQTLMKEYKVKAFEVQVGLHELLGHGSGKLFRIDENGVYNFDKENTKNLVTGEP
ITKWYLPGETYDTKFGAIGSSYEECRAEAVGLYLSLQRDILEIFGFKDKAEQDNIIYVNW
LSLIWNGMGVALEMFNPKSKLWLQAHSRARFVIMKVLLEAGEGLVKVEETEKGKNLLLTV
DRSKIDTVGRKALGDFLTKLQVYKSTADIEAASKMYEHYSKVDESGSHPWAKWRDICLAH
KKPRMILVQANTAIGQDQKVQLKTYEPTHEGYIQSWVERYPNTDIDDLLESIVEKDKKYF
PTAFNN
>Q14699
MGCGLNKLEKRDEKRPGNIYSTLKRPQVETKIDVSYEYRFLEFTTLSAAELPGSSAVRLA
SLRDLPAQLLELYQQGFSLAALHPFVQPTHEREKTPLEHIFRAILIKKTDRSQKTDLHNE
GYILELDCCSSLDHPTDQKLIPEFIKKIQEAASQGLKFVGVIPQYHSSVNSAGSSAPVST
ANSTEDARDAKNARGDHASLENEKPGTGDVCSAPAGRNQSPEPSSGPRGEVPLAKQPSSP
SGEGDGGELSPQGVSKTLDGPESNPLEVHEEPLSGKMEIFTLFNKPKSHQKCRQYYPVTI
PLHVSKNGQTVSGLDANWLEHMSDHFRKGGMLVNAVFYLGIVNDSLHGLTDGVFIFEAVS
TEDSKTIQGYDAIVVEQWTVLEGVEVQTDYVPLLNSLAAYGWQLTCVLPTPVVKTTSEGS
VSTKQIVFLQRPCLPQKIKKKESKFQWRFSREEMHNRQMRKSKGKLSARDKQQAEENEKN
LEDQSSKAGDMGNCVSGQQQEGGVSEEMKGPVQEDKGEQLSPGGLLCGVGVEGEAVQNGP
ASHSRALVGICTGHSNPGEDARDGDAEEVRELGTVEEN

>P46940
MSAADEVDGLGVARPHYGSVLDNERLTAEEMDERRRQNVAYEYLCHLEEAKRWMEACLGE
DLPPTTELEEGLRNGVYLAKLGNFFSPKVVSLKKIYDREQTRYKATGLHFRHTDNVIQWL
NAMDEIGLPKIFYPETTDIYDRKNMPRCIYCIHALSLYLFKLGLAPQIQDLYGKVDFTEE
EINNMKTELEKYGIQMPAFSKIGGILANELSVDEAALHAAVIAINEAIDRRIPADTFAAL
KNPNAMLVNLEEPLASTYQDILYQAKQDKMTNAKNRTENSERERDVYEELLTQAEIQGNI
NKVNTFSALANIDLALEQGDALALFRALQSPALGLRGLQQQNSDWYLKQLLSDKQQKRQS
GQTDPLQKEELQSGVDAANSAAQQYQRRLAAVALINAAIQKGVAEKTVLELMNPEAQLPQ
VYPFAADLYQKELATLQRQSPEHNLTHPELSVAVEMLSSVALINRALESGDVNTVWKQLS
SSVTGLTNIEEENCQRYLDELMKLKAQAHAENNEFITWNDIQACVDHVNLVVQEEHERIL
AIGLINEALDEGDAQKTLQALQIPAAKLEGVLAEVAQHYQDTLIRAKREKAQEIQDESAV
LWLDEIQGGIWQSNKDTQEAQKFALGIFAINEAVESGDVGKTLSALRSPDVGLYGVIPEC
GETYHSDLAEAKKKKLAVGDNNSKWVKHWVKGGYYYYHNLETQEGGWDEPPNFVQNSMQL
SREEIQSSISGVTAAYNREQLWLANEGLITRLQARCRGYLVRQEFRSRMNFLKKQIPAIT
CIQSQWRGYKQKKAYQDRLAYLRSHKDEVVKIQSLARMHQARKRYRDRLQYFRDHINDII
KIQAFIRANKARDDYKTLINAEDPPMVVVRKFVHLLDQSDQDFQEELDLMKMREEVITLI
RSNQQLENDLNLMDIKIGLLVKNKITLQDVVSHSKKLTKKNKEQLSDMMMINKQKGGLKA
LSKEKREKLEAYQHLFYLLQTNPTYLAKLIFQMPQNKSTKFMDSVIFTLYNYASNQREEY
LLLRLFKTALQEEIKSKVDQIQEIVTGNPTVIKMVVSFNRGARGQNALRQILAPVVKEIM
DDKSLNIKTDPVDIYKSWVNQMESQTGEASKLPYDVTPEQALAHEEVKTRLDSSIRNMRA
VTDKFLSAIVSSVDKIPYGMRFIAKVLKDSLHEKFPDAGEDELLKIIGNLLYYRYMNPAI
VAPDAFDIIDLSAGGQLTTDQRRNLGSIAKMLQHAASNKMFLGDNAHLSIINEYLSQSYQ
KFRRFFQTACDVPELQDKFNVDEYSDLVTLTKPVIYISIGEIINTHTLLLDHQDAIAPEH
NDPIHELLDDLGEVPTIESLIGESSGNLNDPNKEALAKTEVSLTLTNKFDVPGDENAEMD
ARTILLNTKRLIVDVIRFQPGETLTEILETPATSEQEAEHQRAMQRRAIRDAKTPDKMKK
SKSVKEDSNLTLQEKKEKIQTGLKKLTELGTVDPKNKYQELINDIARDIRNQRRYRQRRK
AELVKLQQTYAALNSKATFYGEQVDYYKSYIKTCLDNLASKGKVSKKPREMKGKKSKKIS
LKYTAARLHEKGVLLEIEDLQVNQFKNVIFEISPTEEVGDFEVKAKFMGVQMETFMLHYQ
DLLQLQYEGVAVMKLFDRAKVNVNLLIFLLNKKFYGK
>Q8NFA2
MAGPRYPVSVQGAALVQIKRLQTFAFSVRWSDGSDTFVRRSWDEFRQLKKTLKETFPVEA
GLLRRSDRVLPKLLGQASLDAPLLGRVGRTSRGLARLQLLETYSRRLLATAERVARSPTI
TGFFAPQPLDLEPALPPGSRVILPTPEEQPLSRAAGRLSIHSLEAQSLRCLQPFCTQDTR
DRPFQAQAQESLDVLLRHPSGWWLVENEDRQTAWFPAPYLEEAAPGQGREGGPSLGSSGP
QFCASRAYESSRADELSVPAGARVRVLETSDRGWWLCRYGDRAGLLPAVLLRPEGLGALL
SGTGFRGGDDPAGEARGFPEPSQATAPPPTVPTRPSPGAIQSRCCTVTRRALERRPRRQG
RPRGCVDSVPHPTTEQ
>Q03482
MSQQQGYYQQGPPQQGYYQQGPPQQGYYQQGPPQQGYPQQQPVYVQQGQPKEESCLDSCL
KCLCCCFLLELVCDN
>P52183
MGNCCSTFRAVFKPNEGSGGGIPLMPVRGGSTRRPDSLPKPPAAVVPSPPSPGDVPDAGV
APEVASVKEVDVLLAENGDAHRTRHYELMDREKEPRLVVRRGQPFAVSVTLSRPYNPDID
AISFVFTVEDAEKPSYGQGTLVAVPLLAKGAESGAAWNAVLDSSADDILRIQITPAADAI

VGKWKMDIDTKLKNDGAVSYSYKDPFYILYNPWCRQDQVFLEGEELLQEYVLNDTGLIWR
GSYNRLRPCVWKYAQFEKEILDCALYLVSKIGGVRPSECGDPVRVCRAISAAVNSPDDNG
AVMGNWSNDYGGGTPPTKWIGSMKILQQFYKNKKPVKYGQCWVFAGVLTTVCRALGLPAR
TVTTYSAAHDTQNSLTVDYFVDDKGEIMEEMNSDSIWNFHVWTEVWMERPDLMPGDGAHY
GGWQAVDSTPQELSDNMYRCGPAPVVAVKQGEVLRPYDSAYVFAEVNADKVFWRYSGPTQ
PLKLIRKDMLGIGQNISTKAVGRFQREDITNTYKYPEKSVEERAAMLKALRQSESLFSRY
YLNEDFNDIHFNFELRDDIVIGSPFSVVVVMKNRSNQQDYTVTVLLRVDTVLYTGHVKDG
VKKEKVERLIKAGAVEEVRIDVSYEDYYKHLVDQCAFNIACLATVHDTNYEYFAQDDFRV
RKPDIKIKLEGEPVQGQEMSAVATLKNPLPIPVKKGQFLIEGPGIAKTQKIKLSQNIAPG
EEASVNFKFTPKYDGRATIAAKFSSKELDDVDGFLNFMVEPKKEVNGTGNAA
>P17655
MAGIAAKLAKDREAAEGLGSHERAIKYLNQDYEALRNECLEAGTLFQDPSFPAIPSALGF
KELGPYSSKTRGIEWKRPTEICADPQFIIGGATRTDICQGALGDCWLLAAIASLTLNEEI
LARVVPLNQSFQENYAGIFHFQFWQYGEWVEVVVDDRLPTKDGELLFVHSAEGSEFWSAL
LEKAYAKINGCYEALSGGATTEGFEDFTGGIAEWYELKKPPPNLFKIIQKALQKGSLLGC
SIDITSAADSEAITFQKLVKGHAYSVTGAEEVESNGSLQKLIRIRNPWGEVEWTGRWNDN
CPSWNTIDPEERERLTRRHEDGEFWMSFSDFLRHYSRLEICNLTPDTLTSDTYKKWKLTK
MDGNWRRGSTAGGCRNYPNTFWMNPQYLIKLEEEDEDEEDGESGCTFLVGLIQKHRRRQR
KMGEDMHTIGFGIYEVPEELSGQTNIHLSKNFFLTNRARERSDTFINLREVLNRFKLPPG
EYILVPSTFEPNKDGDFCIRVFSEKKADYQAVDDEIEANLEEFDISEDDIDDGFRRLFAQ
LAGEDAEISAFELQTILRRVLAKRQDIKSDGFSIETCKIMVDMLDSDGSGKLGLKEFYIL
WTKIQKYQKIYREIDVDRSGTMNSYEMRKALEEAGFKMPCQLHQVIVARFADDQLIIDFD
NFVRCLVRLETLFKIFKQLDPENTGTIELDLISWLCFSVL
>Q9YH92
MAHKGLQLLGFTLSLLGLIGLIIGTIMPQWKMSAYVGDNIITAIAMYQGLWMSCAYQSTG
QQQCKVYDSVLQLDSALQATRALMVVAILLTVAGLGVASMGMKCTNCGGDDKVKKSRIAM
TGGIILSVGALCSIVACGWFTSQIIRDFYNPFTPVNTKYEFGAAIFIAWAGAFLDIMGGG
MLASSCSKGQSSPNYPKSSRPVKSSRPPSSSKEYV
>P09326
MCSRGWDSCLALELLLLPLSLLVTSIQGHLVHMTVVSGSNVTLNISESLPENYKQLTWFY
TFDQKIVEWDSRKSKYFESKFKGRVRLDPQSGALYISKVQKEDNSTYIMRVLKKTGNEQE
WKIKLQVLDPVPKPVIKIEKIEDMDDNCYLKLSCVIPGESVNYTWYGDKRPFPKELQNSV
LETTLMPHNYSRCYTCQVSNSVSSKNGTVCLSPPCTLARSFGVEWIASWLVVTVPTILGL
LLT
>Q5MFV8
MIGKVVVSVASILLIVGVAIGVVAYINKNGDANLSPQMKAVRGICEATSDKASCVKTLEP
VKSDDPNKLIKAFMLATRDAITQSSNFTGKTEGNLGSGISPNNKAVLDYCKKVFMYALED
LSTIVEEMGEDLNQIGSKIDQLKQWLTGVYNYQTDCLDDIEEDDLRKTIGEGIASSKILT
SNAIDIFHTVVSAMAKLNLKVEDFKNMTGGIFAPSDKGAAPVNKGTPPVADDSPVADPDG
PARRLLEDIDETGIPTWVSGADRKLMTKAGRGSNDGGARIRATFVVAKDGSGQFKTVQQA
VNACPEKNPGRCIIHIKAGIYREQVIIPKKKNNIFMFGDGARKTVISYNRSVKLSPGTTT
SLSGTVQVESEGFMAKWIGFKNTAGPMGHQAVAIRVNGDRAVIFNCRFDGYQDTLYVNNG
RQFYRNIVVSGTVDFIFGKSATVIQNSLIVVRKGNKGQFNTVTADGNEKGLAMKIGIVLQ
NCRIVPDKKLAAERLIVESYLGRPWKKFSTTVIINSEIGDVIRPEGWKIWDGESFHKSCR

YVEYNNRGPGAITNRRVNWVKIARSAAEVNDFTVANWLGPINWIQEANVPVTLGL
>Q5QGZ9
MWIDFFTYSSMSEEVTYADLQFQNSSEMEKIPEIGKFGEKAPPAPSHVWRPAALFLTLLC
LLLLIGLGVLASMFHVTLKIEMKKMNKLQNISEELQRNISLQLMSNMNISNKIRNLSTTL
QTIATKLCRELYSKEQEHKCKPCPRRWIWHKDSCYFLSDDVQTWQESKMACAAQNASLLK
INNKNALEFIKSQSRSYDYWLGLSPEEDSTRGMRVDNIINSSAWVIRNAPDLNNMYCGYI
NRLYVQYYHCTYKQRMICEKMANPVQLGSTYFREA
>Q86WB7
MDRSLRNVLVVSFGFLLLFTAYGGLQSLQSSLYSEEGLGVTALSTLYGGMLLSSMFLPPL
LIERLGCKGTIILSMCGYVAFSVGNFFASWYTLIPTSILLGLGAAPLWSAQCTYLTITGN
THAEKAGKRGKDMVNQYFGIFFLIFQSSGVWGNLISSLVFGQTPSQETLPEEQLTSCGAS
DCLMATTTTNSTQRPSQQLVYTLLGIYTGSGVLAVLMIAAFLQPIRDVQRESEGEKKSVP
FWSTLLSTFKLYRDKRLCLLILLPLYSGLQQGFLSSEYTRSYVTCTLGIQFVGYVMICFS
ATDALCSVLYGKVSQYTGRAVLYVLGAVTHVSCMIALLLWRPRADHLAVFFVFSGLWGVA
DAVWQTQNNALYGVLFEKSKEAAFANYRLWEALGFVIAFGYSMFLCVHVKLYILLGVLSL
TMVAYGLVECVESKNPIRPHAPGQVNQAEDEEIQTKM
>Q7Z601
MSIMMLPMEQKIQWVPTSLQDITAVLGTEAYTEEDKSMVSHAQKSQHSCLSHSRWLRSPQ
VTGGSWDLRIRPSKDSSSFRQAQCLRKDPGANNHLESQGVRGTAGDADRELRGPSEKATA
GQPRVTLLPTPHVSGLSQEFESHWPEIAERSPCVAGVIPVIYYSVLLGLGLPVSLLTAVA
LARLATRTRRPSYYYLLALTASDIIIQVVIVFAGFLLQGAVLARQVPQAVVRTANILEFA
ANHASVWIAILLTVDRYTALCHPLHHRAASSPGRTRRAIAAVLSAALLTGIPFYWWLDMW
RDTDSPRTLDEVLKWAHCLTVYFIPCGVFLVTNSAIIHRLRRRGRSGLQPRVGKSTAILL
GITTLFTLLWAPRVFVMLYHMYVAPVHRDWRVHLALDVANMVAMLHTAANFGLYCFVSKT
FRATVRQVIHDAYLPCTLASQPEGMAAKPVMEPPGLPTGAEV
>O80725
MASESGLNGDPNILEEVSETKRDKEEEEEVKKTEKKDEEHEKTKTVPFYKLFAFADSFDF
LLMILGTLGSIGNGLGFPLMTLLFGDLIDAFGENQTNTTDKVSKVALKFVWLGIGTFAAA
FLQLSGWMISGERQAARIRSLYLKTILRQDIAFFDIDTNTGEVVGRMSGDTVLIQDAMGE
KVGKAIQLLATFVGGFVIAFVRGWLLTLVMLSSIPLLVMAGALLAIVIAKTASRGQTAYA
KAATVVEQTIGSIRTVASFTGEKQAISNYNKHLVTAYKAGVIEGGSTGLGLGTLFLVVFC
SYALAVWYGGKLILDKGYTGGQVLNIIIAVLTGSMSLGQTSPCLSAFAAGQAAAYKMFET
IERRPNIDSYSTNGKVLDDIKGDIELKDVYFTYPARPDEQIFRGFSLFISSGTTVALVGQ
SGSGKSTVVSLIERFYDPQAGDVLIDGINLKEFQLKWIRSKIGLVSQEPVLFTASIKDNI
AYGKEDATTEEIKAAAELANASKFVDKLPQGLDTMVGEHGTQLSGGQKQRIAVARAILKD
PRILLLDEATSALDAESERVVQEALDRIMVNRTTVVVAHRLSTVRNADMIAVIHQGKIVE
KGSHTELLKDPEGAYSQLIRLQEEKKSDENAAEEQKMSSIESFKQSSLRKSSLGRSLSKG
GSSRGNSSRHSFNMFGFPAGIDGNVVQDQEEDDTTQPKTEPKKVSIFRIAALNKPEIPVL
ILGSISAAANGVILPIFGILISSVIKAFFQPPKKLKEDTSFWAIIFMVLGFASIIAYPAQ
TFFFAIAGCKLVQRIRSMCFEKVVHMEVGWFDEPENSSGTIGARLSADAATIRGLVGDSL
AQTVQNLSSILAGLIIAFLACWQLAFVVLAMLPLIALNGFLYMKFMKGFSADAKKMYGEA
SQVANDAVGSIRTVASFCAEDKVMNMYSKKCEGPMKNGIRQGIVSGIGFGFSFFVLFSSY
AASFYVGARLVDDGKTTFDSVFRVFFALTMAAMAISQSSSLSPDSSKADVAAASIFAIMD
RESKIDPSVESGRVLDNVKGDIELRHVSFKYPARPDVQIFQDLCLSIRAGKTVALVGESG

SGKSTVIALLQRFYDPDSGEITLDGVEIKSLRLKWLRQQTGLVSQEPILFNETIRANIAY
GKGGDASESEIVSSAELSNAHGFISGLQQGYDTMVGERGIQLSGGQKQRVAIARAIVKDP
KVLLLDEATSALDAESERVVQDALDRVMVNRTTIVVAHRLSTIKNADVIAVVKNGVIVEK
GKHDTLINIKDGVYASLVQLHLTAAS
>O60245
MLRMRTAGWARGWCLGCCLLLPLSFSLAAAKQLLRYRLAEEGPADVRIGNVASDLGIVTG
SGEVTFSLESGSEYLKIDNLTGELSTSERRIDREKLPQCQMIFDENECFLDFEVSVIGPS
QSWVDLFEGQVIVLDINDNTPTFPSPVLTLTVEENRPVGTLYLLPTATDRDFGRNGIERY
ELLQEPGGGGSGGESRRAGAADSAPYPGGGGNGASGGGSGGSKRRLDASEGGGGTNPGGR
SSVFELQVADTPDGEKQPQLIVKGALDREQRDSYELTLRVRDGGDPPRSSQAILRVLITD
VNDNSPRFEKSVYEADLAENSAPGTPILQLRAADLDVGVNGQIEYVFGAATESVRRLLRL
DETSGWLSVLHRIDREEVNQLRFTVMARDRGQPPKTDKATVVLNIKDENDNVPSIEIRKI
GRIPLKDGVANVAEDVLVDTPIALVQVSDRDQGENGVVTCTVVGDVPFQLKPASDTEGDQ
NKKKYFLHTSTPLDYEATREFNVVIVAVDSGSPSLSSKNSLIVKVGDTNDNPPMFGQSVV
EVYFPENNIPGERVATVLATDADSGKNAEIAYSLDSSVMGIFAIDPDSGDILVNTVLDRE
QTDRYEFKVNAKDKGIPVLQGSTTVIVQVADKNDNDPKFMQDVFTFYVKENLQPNSPVGM
VTVMDADKGRNAEMSLYIEENNNIFSIENDTGTIYSTMSFDREHQTTYTFRVKAVDGGDP
PRSATATVSLFVMDENDNAPTVTLPKNISYTLLPPSSNVRTVVATVLATDSDDGINADLN
YSIVGGNPFKLFEIDPTSGVVSLVGKLTQKHYGLHRLVVQVNDSGQPSQSTTTVVHVFVN
ESVSNATAIDSQIARSLHIPLTQDIAGDPSYEISKQRLSIVIGVVAGIMTVILIIILIVVM
ARYCRSKNKNGYEAGKKDHEDFFTPQQHDKSKKPKKDKKNKKSKQPLYSSIVTVEASKPN
GQRYDSVNEKLSDSPSMGRYRSVNGGPGSPDLARHYKSSSPLPTVQLHPQSPTAGKKHQA
VQDLPPANTFVGAGDNISIGSDHCSEYSCQTNNKYSKQMRLHPYITVFG
>P14043
MAPRSLYLLAVLLFSANLFAGVGFAAAAEGPEDKGLTKGGKGKGEKGTKVGADDTNGTDP
DPEPEPEPEPEPEPEPEPEPEPEPEPEPEPEPEPGAATLKSVALPFAIAAAALVAAF
>Q810W7
MSDSLWTALSNFSMPSFPGGSMFRRTKSCRTSNRKSLILTSTSPTLPRPHSPLPGHLGSS
PLDSPRNFSPNTPAHFSFASSRRADGRRWSLASLPSSGYGTNTPSSTVSSSCSSQERLHQ
LPYQPTVDELHFLSKHFGSTESITDEDGGRRSPAVRPRSRSLSPGRSPSSYDNEIVMMNH
VYKERFPKATAQMEEKLRDFARAYEPDSVLPLADGVLSFIHHQIIELARDCLTKSRDGLI
TTVYFYELQENLEKLLQDAYERSESLEVAFVTQLVKKLLIIISRPARLLECLEFNPEEFY
HLLEAAEGHAKEGHLVKTDIPRYIIRQLGLTRDPFPDVVRLEEQDSGGSNTPEQDDTSEG
RSSTSKAKKPPGESDFDTIKLISNGAYGAVYLVRHRDTRQRFAMKKINKQNLILRNQIQQ
AFVERDILTFAENPFVVGMFCSFETRRHLCMVMEYVEGGDCATLLKNIGALPVEMARMYF
AETVLALEYLHNYGIVHRDLKPDNLLITSMGHIKLTDFGLSKMGLMSLTTNLYEGHIEKD
AREFLDKQVCGTPEYIAPEVILRQGYGKPVDWWAMGIILYEFLVGCVPFFGDTPEELFGQ
VISDDILWPEGDEALPTDAQLLISSLLQTNPLVRLGAGGAFEVKQHSFFRDLDWTGLLRQ
KAEFIPHLESEDDTSYFDTRSDRYHHVNSYDEDDTTEEEPVEIRQFSSCSPRFSKVYSSM
EQLSQHEPKTPVSASGASKRDPNAKGPEEKVAGKREGLGGLTLREKTWRGGSPEIKRFSA
SEASFLEGEASPPLGARRRFSALLEPSRFTAPQEDEDEARLRRPPRPSSDPPSSLDTRVP
KEAAQGEGTSTPGEPEATERSHPGDFCPPSKDGDPSGPRATNDLVLRRARHQQLSGDLSV
EKRPSRTGGKVIKSASATALSVMIPAVDPHGGSPLASPMSPRSLSSNPSSRDSSPSRDYS
PAVSGLRSPITIQRSGKKYGFTLRAIRVYMGDSDVYSVHHIVWHVEEGGPAQEAGLCAGD

LITHVNGEPVHGMVHPEVVELILKSGNKVAVTTTPFENTSIRIGPARRSSYKAKMARRNK
RPSAKDGQESKKRSSLFRKITKQSNLLHTSRSLSSLNRSLSSSDSLPGSPTHGLPARSPT
HSYRSTPDSAYLGASSQSSSPASSTPNSPASSASHHIRPSTLHGLSPKLHRQYRSARCKS
AGNIPLSPLAHTPSPTQASPPPLPGHTVGSSHTTQSFPAKLHSSPPIVRPRPKSAEPPRS
PLLKRVQSAEKLGASLGADKKGALRKHSLEVGHPDFRKDFHGELALHSLAESDGETPPIE
GPGATRQVAMRRLGRQESPLSLGADPLLPDGVQRPMASGKEDSAGGTEACTPPRATTPGS
RTLERDLGCTRHQSVQTEDGPGGVARALAKAALSPVQEHETGRRSSSGEAGTPPVPIVVE
PARPGVKTQTPQPLGTDSKGLKEPVAQIPLVPDAPRVRERWVLEEVEERTTLSGPRSKPA
SPKLSPDPQTPTVAPTKNVPRSAAPPVPPASLMVPGTKPEAGSNSRCPAEGVASAGLTKT
GAPSPASLGP
>Q63064
MNTFLLLLTISLLVVVQIQTGDLGQNSTAVTTPANKAATTAAATTKAAATTATKTTTAVR
KTPGKPPKAGASSITDVGACTFLFFANTLMCLFYLS
>Q1ERP8
MRPLVLLWGCLVLPGYEALKGPKEISGFEGDTVSLRCTYVEKMKEHRKYWCRQGGILVSR
CGDIVYANQDQEVTRGRMSIRDSPQELSMTVIMRDLTLKDSGKYWCGIDRLGRDESFEVT
LIVFPGKRDNAVPAGTCCPSSPTPSFQPLTPTRSLQPKAKAWQTQLPEPRSSRPVVWLPL
TTPQDSRAVASSVSKPSVSIPMVRMMAPVLILLSLLLAAGLIAFGSHMLRWRKKAWLATE
TQKNEKVYLETSLPGNGWTTEDSTIDLAVTPECLRNLNPSAVPSPETQNLSQSTEEEEAA
RSLDDDKEDVMAPPPLQMSAEELAFSEFISV
>Q6PIZ9
MSGISGCPFFLWGLLALLGLALVISLIFNISHYVEKQRQDKMYSYSSDHTRVDEYYIEDT
PIYGNLDDMISEPMDENCYEQMKARPEKSVNKMQEATPSAQATNETQMCYASLDHSVKGK
RRKPRKQNTHFSDKDGDEQLHAIDASVSKTTLVDSFSPESQAVEENIHDDPIRLFGLIRA
KREPIN
>Q86YD1
MVRPRRAPYRSGAGGPLGGRGRPPRPLVVRAVRSRSWPASPRGPQPPRIRARSAPPMEGA
RVFGALGPIGPSSPGLTLGGLAVSEHRLSNKLLAWSGVLEWQEKRRPYSDSTAKLKRTLP
CQAYVNQGENLETDQWPQKLIMQLIPQQLLTTLGPLFRNSQLAQFHFTNRDCDSLKGLCR
IMGNGFAGCMLFPHISPCEVRVLMLLYSSKKKIFMGLIPYDQSGFVSAIRQVITTRKQAV
GPGGVNSGPVQIVNNKFLAWSGVMEWQEPRPEPNSRSKRWLPSHVYVNQGEILRTEQWPR
KLYMQLIPQQLLTTLVPLFRNSRLVQFHFTKDLETLKSLCRIMDNGFAGCVHFSYKASCE
IRVLMLLYSSEKKIFIGLIPHDQGNFVNGIRRVIANQQQVLQRNLEQEQQQRGMGG
>Q00689
MSRDRSSTHRRRSVAARLIGFAAAGLVMAVGAAAVWAQAAGHHCIHDRLQARVLQSVAQQ
RSVPAAFSALGLPYVSTGTISSAHTVDWALADSTSPSVARAADWGTLRIAVSTADLTDPG
YHCTRVGQRVNNHNGEIVTCTAEDVLTEEKRDILVSYLIPQALQLHAERLKVRQVQGSWK
VTGMTGSICGDFSVPTAHLTAGVTNADFVLYVASVPSEPGVLAWATTCQVFSDDHPAVGV
INIPAANIVSRYDQGATRVVTHEVAHALGFSSTFFKSAGIVKSVTNLRGKPFAAPVINSS
TVVAKAREQYGCPTLEYLEVEDQGGSGSAGSHIKMRNAKDELMAPASAAGYYTALTMAVF
EDLGFYKADFTKAEVMPWGRNASCDFLTKKCMENNITQWPEMFCNTTERRYRCPTDRLRL
GTCGIRTYSTPMPPYFEYFNDTFLAGYSAFLDYCPFTLGYSNGACNQDPSTAPALLKEFS
VFSDASRCLDGAFQPTTAREVLMYNALCANVMCDTAARTYSVQVRGSSGYVACTPGQRVE
LATLSAAFVNGSYITCPPYVEVCQANIKGVIDFEGDAADTAAMRRWRERMTALATVTAAL

LGIVLAAMAILVVWLLLITIP
>Q8IWY4
MGAAAVRWHLCVLLALGTRGRLAGGSGLPGSVDVDECSEGTDDCHIDAICQNTPKSYKCL
CKPGYKGEGKQCEDIDECENDYYNGGCVHECINIPGNYRCTCFDGFMLAHDGHNCLDVDE
CQDNNGGCQQICVNAMGSYECQCHSGFFLSDNQHTCIHRSNEGMNCMNKDHGCAHICRET
PKGGVACDCRPGFDLAQNQKDCTLTCNYGNGGCQHSCEDTDTGPTCGCHQKYALHSDGRT
CIETCAVNNGGCDRTCKDTATGVRCSCPVGFTLQPDGKTCKDINECLVNNGGCDHFCRNT
VGSFECGCRKGYKLLTDERTCQDIDECSFERTCDHICINSPGSFQCLCHRGYILYGTTHC
GDVDECSMSNGSCDQGCVNTKGSYECVCPPGRRLHWNGKDCVETGKCLSRAKTSPRAQLS
CSKAGGVESCFLSCPAHTLFVPDSENSYVLSCGVPGPQGKALQKRNGTSSGLGPSCSDAP
TTPIKQKARFKIRDAKCHLRPHSQARAKETARQPLLDHCHVTFVTLKCDSSKKRRRGRKS
PSKEVSHITAEFEIETKMEEASDTCEADCLRKRAEQSLQAAIKTLRKSIGRQQFYVQVSG
TEYEVAQRPAKALEGQGACGAGQVLQDSKCVACGPGTHFGGELGQCVSCMPGTYQDMEGQ
LSCTPCPSSDGLGLPGARNVSECGGQCSPGFFSADGFKPCQACPVGTYQPEPGRTGCFPC
GGGLLTKHEGTTSFQDCEAKVHCSPGHHYNTTTHRCIRCPVGTYQPEFGQNHCITCPGNT
STDFDGSTNVTHCKNQHCGGELGDYTGYIESPNYPGDYPANAECVWHIAPPPKRRILIVV
PEIFLPIEDECGDVLVMRKSASPTSITTYETCQTYERPIAFTSRSRKLWIQFKSNEGNSG
KGFQVPYVTYDEDYQQLIEDIVRDGRLYASENHQEILKDKKLIKALFDVLAHPQNYFKYT
AQESKEMFPRSFIKLLRSKVSRFLRPYK
>Q96AD5
MFPREKTWNISFAGCGFLGVYYVGVASCLREHAPFLVANATHIYGASAGALTATALVTGV
CLGEAGAKFIEVSKEARKRFLGPLHPSFNLVKIIRSFLLKVLPADSHEHASGRLGISLTR
VSDGENVIISHFNSKDELIQANVCSGFIPVYCGLIPPSLQGVRYVDGGISDNLPLYELKN
TITVSPFSGESDICPQDSSTNIHELRVTNTSIQFNLRNLYRLSKALFPPEPLVLREMCKQ
GYRDGLRFLQRNGLLNRPNPLLALPPARPHGPEDKDQAVESAQAEDYSQLPGEDHILEHL
PARLNEALLEACVEPTDLLTTLSNMLPVRLATAMMVPYTLPLESALSFTIRLLEWLPDVP
EDIRWMKEQTGSICQYLVMRAKRKLGRHLPSRLPEQVELRRVQSLPSVPLSCAAYREALP
GWMRNNLSLGDALAKWEECQRQLLLGLFCTNVAFPPEALRMRAPADPAPAPADPASPQHQ
LAGPAPLLSTPAPEARPVIGALGL
>Q9R0S2
MPRSRGGRAAPGQASRWSGWRAPGRLLPLLPALCCLAAAAGAGKPTGADAPFAGQNWLKS
YGYLLPYESRASALHSGKALQSAVSTMQQFYGIPVTGVLDQTTIEWMKKPRCGVPDHPHL
SRRRRNKRYALTGQKWRQKHITYSIHNYTPKVGELDTRKAIRQAFDVWQKVTPLTFEEVP
YHEIKSDRKEADIMIFFASGFHGDSSPFDGEGGFLAHAYFPGPGIGGDTHFDSDEPWTLG
NANHDGNDLFLVAVHELGHALGLEHSNDPSAIMAPFYQYMETHNFKLPQDDLQGIQKIYG
PPAEPLEPTRPLPTLPVRRIHSPSERKHERHPRPPRPPLGDRPSTPGAKPNICDGNFNTV
ALFRGEMFVFKDRWFWRLRNNRVQEGYPMQIEQFWKGLPARIDAAYERADGRFVFFKGDK
YWVFKEVTVEPGYPHSLGELGSCLPREGIDTALRWEPVGKTYFFKGERYWRYSEERRATD
PGYPKPITVWKGIPQAPQGAFISKEGYYTYFYKGRDYWKFDNQKLSVEPGYPRNILRDWM
GCKQKEVERRKERRLPQDDVDIMVTIDDVPGSVNAVAVVVPCTLSLCLLVLLYTIFQFKN
KAGPQPVTYYKRPVQEWV
>P11505
MGDMANNSVAYSGVKNSLKEANHDGDFGITLAELRALMELRSTDALRKIQESYGDVYGIC
TKLKTSPNEGLSGNPADLERREAVFGKNFIPPKKPKTFLQLVWEALQDVTLIILEIAAIV

SLGLSFYQPPEGDNALCGEVSVGEEEGEGETGWIEGAAILLSVVCVVLVTAFNDWSKEKQ
FRGLQSRIEQEQKFTVIRGGQVIQIPVADITVGDIAQVKYGDLLPADGILIQGNDLKIDE
SSLTGESDHVKKSLDKDPLLLSGTHVMEGSGRMVVTAVGVNSQTGIIFTLLGAGGEEEEK
KDEKKKEKKNKKQDGAIENRNKAKAQDGAAMEMQPLKSEEGGDGDEKDKKKANLPKKEKS
VLQGKLTKLAVQIGKAGLLMSAITVIILVLYFVIDTFWVQKRPWLAECTPIYIQYFVKFF
IIGVTVLVVAVPEGLPLAVTISLAYSVKKMMKDNNLVRHLDACETMGNATAICSDKTGTL
TMNRMTVVQAYINEKHYKKVPEPEAIPPNILSYLVTGISVNCAYTSKILPPEKEGGLPRH
VGNKTECALLGFLLDLKRDYQDVRNEIPEEALYKVYTFNSVRKSMSTVLKNSDGSFRIFS
KGASEIILKKCFKILSANGEAKVFRPRDRDDIVKTVIEPMASEGLRTICLAFRDFPAGEP
EPEWDNENDVVTGLTCIAVVGIEDPVRPEVPEAIKKCQRAGITVRMVTGDNINTARAIAT
KCGILHPGEDFLCLEGKDFNRRIRNEKGEIEQERIDKIWPKLRVLARSSPTDKHTLVKGI
IDSTVSEQRQVVAVTGDGTNDGPALKKADVGFAMGIAGTDVAKEASDIILTDDNFTSIVK
AVMWGRNVYDSISKFLQFQLTVNVVAVIVAFTGACITQDSPLKAVQMLWVNLIMDTLASL
ALATEPPTESLLLRKPYGRNKPLISRTMMKNILGHAFYQLVVVFTLLFAGEKFFDIDSGR
NAPLHAPPSEHYTIVFNTFVLMQLFNEINARKIHGERNVFEGIFNNAIFCTIVLGTFVVQ
IIIVQFGGKPFSCSELSIEQWLWSIFLGMGTLLWGQLISTIPTSRLKFLKEAGHGTQKEE
IPEEELAEDVEEIDHAERELRRGQILWFRGLNRIQTQMDVVNAFQSGGSIQGALRRQPSI
ASQHHDVTNVSTPTHIRVVNAFRSSLYEGLEKPESRSSIHNFMTHPEFRIEDSEPLIPLI
DDTDAEDDAPTKRNSSPPPSPNKNNNAVDSGIHLTIEMNKSATSSSPGSPLHSLETSL
>Q8R4F1
MLRLLALFLHCLPLVSGDYDICKSWVTTDEGPTWEFYACQSKVMRLKDYVKVKVEPSGIT
CGDPPERFCSHENPYLCSNECDASNPDLAHPPRLMFDREDEGLATYWQSVTWSRYPSPLE
ANITLSWNKSVELTDDVVVTFEYGRPTVMVLEKSLDNGRTWQPYQFYAEDCMEAFGMSAR
RARDMSPSSAHRVLCTEEYSRWAGSKKEKHVRFEVRDRFAIFAGPDLRNMDNLYTRMESA
KGLKEFFTFTDLRMRLLRPALGGTYVQRENLYKYFYAISNIEVIGRCKCNLHANLCTVRE
GSLQCECEHNTTGPDCGRCKKNFRTRAWRAGSYLPLPHGSPNACAAAGSAFGSQTKPPTM
APLGDSSFWPQVSSSAEAVAISVAVPSQAKDFTLFELKPRSPQVIPIEEFQDCECYGHSN
RCSYIDFLNVVTCVSCKHNTRGQHCQHCRLGYYRNGSAELDDENVCIECNCNQIGSVHDR
CNETGFCECREGAVGPKCDDCLPTHYWRQGCYPNVCDDDQLLCQNGGTCQQNQRCACPPG
YTGIRCEQPRCDLADDAGPDCDRAPGIVPRPDTLLGCLLLLGLAARLAC
>P55073
MLRSLLLHSLRLCAQTASCLVLFPRFLGTAFMLWLLDFLCIRKHFLGRRRRGQPEPEVEL
NSEGEEVPPDDPPICVSDDNRLCTLASLKAVWHGQKLDFFKQAHEGGPAPNSEVVLPDGF
QSQHILDYAQGNRPLVLNFGSCTUPPFMARMSAFQRLVTKYQRDVDFLIIYIEEAHPSDG
WVTTDSPYIIPQHRSLEDRVSAARVLQQGAPGCALVLDTMANSSSSAYGAYFERLYVIQS
GTIMYQGGRGPDGYQVSELRTWLERYDEQLHGARPRRV
>Q6NVG5
MGLRRWLRSACCCCPCRCLEEPARPEKEPLVSGNNPYSSFGATLERDDEKNLWSMPHDVS
HTEADDDRILYNLIVIRNQQTKDSEEWQRLNYDIYTLRQIRREVRNRWRRILEDLGFQRE
ADSLLSVTKLSTMSDSKNTRKAREMLLKLAEETSIFPASWELSERYLLVVDRLIALDAAE
DFFKIASQMYPKKPGVPCLVDGQRKLHCLPFPSP
>Q9H8T0
MNPFWSMSTSSVRKRSEGEEKTLTGDVKTSPPRTAPKKQLPSIPKNALPITKPTSPAPAA
QSTNGTHASYGPFYLEYSLLAEFTLVVKQKLPGVYVQPSYRSALMWFGVIFIRHGLYQDG

VFKFTVYIPDNYPDGDCPRLVFDIPVFHPLVDPTSGELDVKRAFAKWRRNHNHIWQVLMY
ARRVFYKIDTASPLNPEAAVLYEKDIQLFKSKVVDSVKVCTARLFDQPKIEDPYAISFSP
WNPSVHDEAREKMLTQKKPEEQHNKSVHVAGLSWVKPGSVQPFSKEEKTVAT
>Q70E73
MEQLSDEEIDHGAEEDSDKEDQDLDKMFGAWLGELDKLTQSLDSDKPMEPVKRSPLRQET
NMANFSYRFSIYNLNEALNQGETVDLDALMADLCSIEQELSSIGSGNSKRQITETKATQK
LPVSRHTLKHGTLKGLSSSSNRIAKPSHASYSLDDVTAQLEQASLSMDEAAQQSVLEDTK
PLVTNQHRRTASAGTVSDAEVHSISNSSHSSITSAASSMDSLDIDKVTRPQELDLTHQGQ
PITEHAISLRCSSKQAKRHIDFTEEQAELTPHSYLDRETSLLLRNIAGKPSHLLTKEEQA
AKLKAEKIRVALEKIKEAQVKKLVIRVHMSDDSSKTMMVDERQTVRQVLDNLMDKSHCGY
SLDWSLVETVSELQMERIFEDHENLVENLLNWTRDSQNKLIFMERIEKYALFKNPQNYLL
GKKETAEMADRNKEVLLEECFCGSSVTVPEIEGVLWLKDDGKKSWKKRYFLLRASGIYYV
PKGKAKVSRDLVCFLQLDHVNVYYGQDYRNKYKAPTDYCLVLKHPQIQKKSQYIKYLCCD
DVRTLHQWVNGIRIAKYGKQLYMNYQEALKRTESAYDWTSLSSSSIKSGSSSSSIPESQS
NHSNQSDSGVSDTQPAGHVRSQSIVSSVFSEAWKRGTQLEESSKARMESMNRPYTSLVPP
LSPQPKIVTPYTASQPSPPLPPPPPPPPPPPPPPPPPPPPLPSQSAPSAGSAAPMFVKYS
TITRLQNASQHSGALFKPPTPPVMQSQSVKPQILVPPNGVVPPPPPPPPPPTPGSAMAQL
KPAPCAPSLPQFSAPPPPLKIHQVQHITQVAPPTPPPPPPIPAPLPPQAPPKPLVTIPAP
TSTKTVAPVVTQAAPPTPTPPVPPAKKQPAFPASYIPPSPPTPPVPVPPPTLPKQQSFCA
KPPPSPLSPVPSVVKQIASQFPPPPTPPAMESQPLKPVPANVAPQSPPAVKAKPKWQPSS
IPVPSPDFPPPPPESSLVFPPPPPSPVPAPPPPPPPTASPTPDKSGSPGKKTSKTSSPGG
KKPPPTPQRNSSIKSSSGAEHPEPKRPSVDSLVSKFTPPAESGSPSKETLPPPAAPPKPG
KLNLSGVNLPGVLQQGCVSAKAPVLSGRGKDSVVEFPSPPSDSDFPPPPPETDLPLPPIE
IPAVFSGNTSPKVAVVNPQPQQWSKMSVKKAPPPTRPKRNDSTRLTQAEISEQPTMATVV
PQVPTSPKSSLSVQPGFLADLNRTLQRKSITRHGSLSSRMSRAEPTATMDDMALPPPPPE
LLSDQQKAGYGGSHISGYATLRRGPPPAPPKRDQNTKLSRDW
>P13837
MNNKFIIFSLLLALVASQTYSLTSCTCAQLLSEGDCIKNVSLGCSWDTTKKTCGVSTTPV
TPTVTYAAYCDTFAETDCPKAKPCTDCGNYAACAWVESKCTFFTGCTPFAKTLDSECQAI
SNRCITDGTHCVEVDACSTYKKQLPCAKNAAGSLCYWDTTNNTCVDANTCDKLPATFATD
KDCRDVISTCTTKTGGGCVDSGNNCSDQTLEIQCVWNKLKTTSCYWDGAACKDRICDNAP
TSLTTDDACKTFRTDGTCTTKANGGCVTRTTCAAATIQASCIKNSSGGDCYWTGTACVDK
ACANTPTTIATNSACAGFVTGCITKSGGGCVVNGACSVANVQAACVKNPSNFDCIWDTTC
KEKTCANASTTNNTHDLCTSYLSTCTVKSGGGCQNRTCANAPTTMTTNDACEAYFTGNNC
ITKSGGGCVTNTTCAAITLEAACVKNSSGSTCFWDTASSSCKDKTCVNAPATNTTHDLCQ
PFLNTCTVNSTSAGCVEKTCENSLVLAICDKDTSSRACIWKGKCYKKQCVLASSATTTHA
DCQTYHSTCTLSNSGTGCVPLPLKCEAITIEAACNLKANGQPCGWNGSQCIDKACSTASK
TFTTTSQCTGHISTCVANNPVTVNGSLTIQGCQDLPTTCARRKSSENCEITRVGFPTCLW
VSSSTSCVEKSCATASTVGTTGALSAGGFTFSGCQTYLNTCISNNTADGCIAKPSSCSSL
VSSNCRDGSKASGDCYWNGSSCVDKTCANIIQTTHNSCNTTFNQCTVNNGGTACQTLATA
CTSYSTQENCKFTSTNKNCVWTGLACRNATCADAPDTTAYDSDTECLAYPTPSETCTVVY
KVGAQGCVSKSANCSDYMTSAQCHKTLTNLTANDDCKWIVDRCYALSSFATGACTTFKGT
KTMCEGYRAGCTNTVGAASSASCTLDCTLKTGSGLTFADCQALDSTCSVKKDGTGCIAIQ
STCAGYGSTAANCFRSSASGTAGYCAMNTNCQSVTSAAECAFVTGLTGLDHSKCQLYHSS

CTSLKDGTGCQEYKTTCSGYAATNNCATSGQGKCFFDVECLRFSNCASITGTGLTTAICG
TYDAGCVANVNGTACQEKLATCDLYLTQNSCSTSAAAATADKCAWSGTACLAVTTVGTHC
PYVTGTGLTDLICAAYNANCTANKAGTACQEKKATCNLYTTEATCSTSAAAATADKCAWS
GAACLAVTTVATECAYVTGTGLTDLICAAYNANCTANKAGTACQEKKATCNLYTTEATCS
TSAAAATADKCAWSGAACLAVTTVATECAYVTGTGLTNAICAAYNANCTANKAGTACQEK
KATCNLYTTEATCSTSAAAATADKCAWSGAACLAVTTVATECAYVTGTGLTNAICAAYNA
NCTANKAGTACQEKKATCNLYTTEATCSTSAAAATADKCAWSGAACLAVTTVATECAYVT
GTGLTKAICATYNAGCINLKDGTGCQEAKANCKDYTTSNKCTAQTTSTLSCLWIDNSCYP
VTDLNCSVITGLGFVHAQCQAYSTGCTSVSDGSKCQDFKSTCEQYPGTTLGCTKTASTKC
YLQGSACITISNVATDCAKITGSAGTITFEICQSYNTGCSVNRARSACVQQQAQCSGYTS
AMTSCYKSGAGLCIASTNTDTACVAATAATCDAVYLGAGNYSSANCNEMKAGCTNNGTTA
CVAKTCANAAGITFNHTNCNSYLNTCTVNSGNSACQTMASKCADQTQASCLYSVEGECVV
VGTSCVRKTCDTAATDATRDDDTECSTYQQSCTVARLGACQARAACATYKSSLQCKFNTS
GGKCFWNPTNKTCVDLNCGNIEATTLYDTHNECVAVDATLACTVRATNGAAAQGCMARGA
CASYTIEEQCKTNASNGVCVWNTNANLPAPACQDKSCTSAPTSTTTHNDCYAYYNTATVK
CTVVATPSNSGGNPTLGGCQQTAACSSYIDKEQCQINANGDPCGWNGTQCADKSCATASA
TADYDDDTKCRAYITNKCTVSDSGQGCVEIPATCETMTQKQCYYNKAGDPCYWTGTACIT
KSCDNAPDATATADECNTYLAGCTLNNVKCKTKVCEDFAFATDALCKQAISTCTTNGTNC
VTRGTCFQALSQAGCVTSSTNQQCEWIPAVLNASNVITSPAYCTIKNCSTAPITLTSEAA
CAGYFTNCTTKNGGGCVTKSTCSAVTIDVACTTALNGTVCAWDSAQNKCRDKDCQDFSGT
THAACQAQRAGCTAGAGGKCARVQNCEQTSVRAACIEGTNGPCLWIDKYQNTDGTKGACF
RYTSCKSLNWNNDSSCKWISNKCTTNGSNCVGITLCSETNTDGGCVTGYDGACIQSVPDL
NSSDPKVCKPYTSCADAFYTTHSDCQIASSKCTTNGTTGCIALGSCSSYTVQAGCYFNDK
GTLYTSGVITSTGICTWDTTSSSCRDQSCADLTGTTHATCSSQLSTCTSDGTTCLLKGAC
TSYTTQTACTTAVGSDGACYWELASATNNNTAKCRLLTCADIQNGTATNVCSVALSTCVS
NGTACIPKANCSTYTSKVACNSGGLDGICVFTQSTATGAAAGTGTCALMTACTVANNDQT
ACQAARDRCSWTAASGTRATAVASKCATHTCATNQATNGACTRFLNWDKKTQQVCTLVSG
ACTATDPSSFSSNDCFLVSGYTYTCNASTSKCGVCTAVVVQPNTTDNNTNTTDNNTTTDS
GYILGLSIVLGYLMF
>O95971
MLLEPGRGCCALAILLAIVDIQSGGCINITSSASQEGTRLNLICTVWHKKEEAEGFVVFL
CKDRSGDCSPETSLKQLRLKRDPGIDGVGEISSQLMFTISQVTPLHSGTYQCCARSQKSG
IRLQGHFFSILFTETGNYTVTGLKQRQHLEFSHNEGTLSSGFLQEKVWVMLVTSLVALQA
L
>Q8R5M8
MASAVLPSGSQCAAAAAVAAAAAPPGLRLRLLLLLLSAAALIPTGDGQNLFTKDVTVIEG
EVATISCQVNKSDDSVIQLLNPNRQTIYFRDFRPLKDSRFQLLNFSSSELKVSLTNVSIS
DEGRYFCQLYTDPPQESYTTITVLVPPRNLMIDIQKDTAVEGEEIEVNCTAMASKPATTI
RWFKGNKELKGKSEVEEWSDMYTVTSQLMLKVHKEDDGVPVICQVEHPAVTGNLQTQRYL
EVQYKPQVHIQMTYPLQGLTREGDAFELTCEAIGKPQPVMVTWVRVDDEMPQHAVLSGPN
LFINNLNKTDNGTYRCEASNIVGKAHSDYMLYVYDPPTTIPPPTTTTTTTTTTTTILTI
ITDTTATTEPAVHDSRAGEEGTIGAVDHAVIGGVVAVVVFAMLCLLIILGRYFARHKGTY
FTHEAKGADDAADADTAIINAEGGQNNSEEKKEYFI
>Q02223

MLQMAGQCSQNEYFDSLLHACIPCQLRCSSNTPPLTCQRYCNASVTNSVKGTNAILWTCL
GLSLIISLAVFVLMFLLRKISSEPLKDEFKNTGSGLLGMANIDLEKSRTGDEIILPRGLE
YTVEECTCEDCIKSKPKVDSDHCFPLPAMEEGATILVTTKTNDYCKSLPAALSATEIEKS
ISAR
>O35250
MIPPQEASARRREIEDKLKQEEETLSFIRDSLEKSDQLTRNMVSILSSFESRLMKLENSI
IPVHKQTENLQRLQENVEKTLSCLDHVISYYHVASDTEKIIREGPTGRLEEYLGSMAKIQ
KAVEYFQDNSPDSPELNKVKLLFERGKESLESEFRSLMTRHSKVVSPVLLLDLISADDEL
EVQEDVVLEHLPESVLRDVVRISRWLVEYGRNQDFMNVYYQIRSSQLDRSIKGLKEHFRK
SSSSSGVPYSPAIPNKRKDTPTKKPIKRPGTIRKAQNLLKQYSQHGLDGKKGGSNLIPLE
GRDDMLDVETDAYIHCVSAFVKLAQSEYRLLMEIIPEHHQKKTFDSLIQDALDGLMLEGE
NIVSAARKAIIRHDFSTVLTVFPILRHLKQTKPEFDQVLQGTAASTKNKLPGLITSMETI
GAKALEDFADNIKNDPDKEYNMPKDGTVHELTSNAILFLQQLLDFQETAGAMLASQVLGD
TYNIPLDPRETSSSATSYSSEFSKRLLSTYICKVLGNLQLNLLSKSKVYEDPALSAIFLH
NNYNYILKSLEKSELIQLVAVTQKTAERSYREHIEQQIQTYQRSWLKVTDYIAEKNLPVF
QPGVKLRDKERQMIKERFKGFNDGLEELCKIQKVWAIPDTEQRDKIRQAQKDIVKETYGA
FLHRYGSVPFTKNPEKYIKYRVEQVGDMIDRLFDTSA
>O14239
MAIYSSFWIRLYFTFRFFCYFLTSVVASDVSFLGDFSGFNVLSNSSANTDLSSQFFEQTN
NGSLSSLIDTSYSNSQICYSSWQGDLYVIVLDSEQQTVLLQSNFTSNPTSLEIFSSQNTS
FFGNISAIFCDDKFPYAYATFTFSDKPSFTSIYRWNVTNSNVTIEHFYNVKGNVDSLFFL
NNDSVAISGNFTEISPFSSSNGPQIAKLAFRSNNSFSQNSLNRNLSSVSCDLTDSYSLWD
PSSSGLVSIYAWAPYLIDFNRIRFYNYESSENSVAFFSAINPADGTVLPLTHYDLETGLS
STCDVNCSLQNFANYQDFYFSKGYNSYQIEIQMFGNGEATENSAFGLSSLQFFETNQNSY
FDDSYNQESCGFPGLNSLSSYEGNFEASFSNASMPYWIQTIAGEQASVSFFPNITVPTNG
TVQLLIPGCTYDNTCSQRGSVIANVYFAKNKQPATKLVQQASDFDQYVSLYSGYLQGFSD
NFRPYVELLPYKNSRMVTHSIRFLEQSYTNVSNGLVFVNTTTDVNKLPSIIEFPAASKLR
GTAISQIKSLSNGNFSLYMTGNFSDNYGNNVVYMDSLNHLHSFPNNGLNGWVSYIYVSGD
SSYFGGNFTHTGDGSIKLNYIAMYSETSRNWSSLGLGTNGPVTHIGSTSLFIDGKIESFI
SFQGDFNEVYTSEGYAISTSGFSLWNPSSKSWVSMEKLGFYMSGYLFDIPGFNSTQRIYS
GNLSAIASYSTRNIAHFSSDSLNDTFIPCYVNAFPSYIRLEDIAYPFANNSMIAILGSEE
MEDKCTAAVYFANSTEPIYPKRILSANCSSKFIVLEDCLIIYSNDTDESDIVKNTFVSFN
TTSNSLGNTTALSQLKGHINSVIVDDSYNNIFFGGNLSEQSSGCVGFCIFEYNSSSWRNI
SHNLISAEVQSILWVNETYSSMYLAGKFVWDTSDVDYLLMYNFDNNTIMSCKGSSSIPGP
VLLASLKSQSKDEYSVLLYGTEVSSSDTYLNVLNSEGAINSYSLDIHLNQSTINSIDFFE
SNQISQIPINDSIIVLSGLIVLDDSSKASAVYCVNKSCLPLLTAFKDNGEAGIVRKVVQQ
KSFSSSASKMIPVTTKYDHIGQPRYVVIISLGISIGVMFLIMSGSIVVEIIHWFFSEHVE
TLHDYSNFLKELKTQ
>Q07157
MSARAAAAKSTAMEETAIWEQHTVTLHRAPGFGFGIAISGGRDNPHFQSGETSIVISDVL
KGGPAEGQLQENDRVAMVNGVSMDNVEHAFAVQQLRKSGKNAKITIRRKKKVQIPVSRPD
PEPVSDNEEDSYDEEIHDPRSGRSGVVNRRSEKIWPRDRSASRERSLSPRSDRRSVASSQ
PAKPTKVTLVKSRKNEEYGLRLASHIFVKEISQDSLAARDGNIQEGDVVLKINGTVTENM
SLTDAKTLIERSKGKLKMVVQRDERATLLNVPDLSDSIHSANASERDDISEIQSLASDHS

GRSHDRPPRRSRSRSPDQRSEPSDHSRHSPQQPSNGSLRSRDEERISKPGAVSTPVKHAD
DHTPKTVEEVTVERNEKQTPSLPEPKPVYAQVGQPDVDLPVSPSDGVLPNSTHEDGILRP
SMKLVKFRKGDSVGLRLAGGNDVGIFVAGVLEDSPAAKEGLEEGDQILRVNNVDFTNIIR
EEAVLFLLDLPKGEEVTILAQKKKDVYRRIVESDVGDSFYIRTHFEYEKESPYGLSFNKG
EVFRVVDTLYNGKLGSWLAIRIGKNHKEVERGIIPNKNRAEQLASVQYTLPKTAGGDRAD
FWRFRGLRSSKRNLRKSREDLSAQPVQTKFPAYERVVLREAGFLRPVTIFGPIADVAREK
LAREEPDIYQIAKSEPRDAGTDQRSSGIIRLHTIKQIIDQDKHALLDVTPNAVDRLNYAQ
WYPIVVFLNPDSKQGVKTMRMRLCPESRKSARKLYERSHKLRKNNHHLFTTTINLNSMND
GWYGALKEAIQQQQNQLVWVSEGKADGATSDDLDLHDDRLSYLSAPGSEYSMYSTDSRHT
SDYEDTDTEGGAYTDQELDETLNDEVGTPPESAITRSSEPVREDSSGMHHENQTYPPYSP
QAQPQPIHRIDSPGFKPASQQKAEASSPVPYLSPETNPASSTSAVNHNVNLTNVRLEEPT
PAPSTSYSPQADSLRTPSTEAAHIMLRDQEPSLSSHVDPTKVYRKDPYPEEMMRQNHVLK
QPAVSHPGHRPDKEPNLTYEPQLPYVEKQASRDLEQPTYRYESSSYTDQFSRNYEHRLRY
EDRVPMYEEQWSYYDDKQPYPSRPPFDNQHSQDLDSRQHPEESSERGYFPRFEEPAPLSY
DSRPRYEQAPRASALRHEEQPAPGYDTHGRLRPEAQPHPSAGPKPAESKQYFEQYSRSYE
QVPPQGFTSRAGHFEPLHGAAAVPPLIPSSQHKPEALPSNTKPLPPPPTQTEEEEDPAMK
PQSVLTRVKMFENKRSASLETKKDVNDTGSFKPPEVASKPSGAPIIGPKPTSQNQFSEHD
KTLYRIPEPQKPQLKPPEDIVRSNHYDPEEDEEYYRKQLSYFDRRSFENKPPAHIAASHL
SEPAKPAHSQNQSNFSSYSSKGKPPEADGVDRSFGEKRYEPIQATPPPPPLPSQYAQPSQ
PVTSASLHIHSKGAHGEGNSVSLDFQNSLVSKPDPPPSQNKPATFRPPNREDTAQAAFYP
QKSFPDKAPVNGTEQTQKTVTPAYNRFTPKPYTSSARPFERKFESPKFNHNLLPSETAHK
PDLSSKTPTSPKTLVKSHSLAQPPEFDSGVETFSIHAEKPKYQINNISTVPKAIPVSPSA
VEEDEDEDGHTVVATARGIFNSNGGVLSSIETGVSIIIPQGAIPEGVEQEIYFKVCRDNS
ILPPLDKEKGETLLSPLVMCGPHGLKFLKPVELRLPHCDPKTWQNKCLPGDPNYLVGANC
VSVLIDHF
>Q2YDG0
MAIHRTVLMCLGLPLFLLPGARAQEQAPPGCSPDLNPLYYNLCDRSEAWGIILEAVAGAG
VVTTFVLTIILVASLPFVQDTKKRSLLGTQVFFLLGTLGLFCLVFACVVKPSFSTCASRR
FLFGVLFAICFSCLVAHVLALHFLVRKNHGPRGWVIFLVALLLSLVEVIINTEWLIITLV
RGAGTEGDALGNGSAGWVAVSPCAIANADFVMALIYVMLLLLCAFSGAWSALCGRFKRWR
KHGVFILLTTTASIAVWVVWIVMYTYGNRQHNSPTWDDPTLAIALATNAWAFVLFYVIPE
VSQVTRSSPEQSYQGDLYPTRGVGYETILKEQKGQSMFVENKAFSMDEPASAKRPVSPYS
GYNGQLLTSMYQPTEMTLMHKAPSDGAYDVILPRATANSQVTGSANSTLRAEDIYAAQGR
QEATLPKEGKNSQVFRNPYVWD
>Q8TDM5
MVLCWLLLLVMALPPGTTGVKDCVFCELTDSMQCPGTYMHCGDDEDCFTGHGVAPGTGPV
INKGCLRATSCGLEEPVSYRGVTYSLTTNCCTGRLCNRAPSSQTVGATTSLALGLGMLLP
PRLL
>Q9HB89
MTPLCLNCSVLPGDLYPGGARNPMACNGSAARGHFDPEDLNLTDEALRLKYLGPQQTELF
MPICATYLLIFVVGAVGNGLTCLVILRHKAMRTPTNYYLFSLAVSDLLVLLVGLPLELYE
MWHNYPFLLGVGGCYFRTLLFEMVCLASVLNVTALSVERYVAVVHPLQARSMVTRAHVRR
VLGAVWGLAMLCSLPNTSLHGIRQLHVPCRGPVPDSAVCMLVRPRALYNMVVQTTALLFF
CLPMAIMSVLYLLIGLRLRRERLLLMQEAKGRGSAAARSRYTCRLQQHDRGRRQVTKMLF

VLVVVFGICWAPFHADRVMWSVVSQWTDGLHLAFQHVHVISGIFFYLGSAANPVLYSLMS
SRFRETFQEALCLGACCHRLRPRHSSHSLSRMTTGSTLCDVGSLGSWVHPLAGNDGPEAQ
QETDPS
>Q9NZ20
MGVQAGLFGMLGFLGVALGGSPALRWYRTSCHLTKAVPGNPLGYLSFLAKDAQGLALIHA
RWDAHRRLQACSWEDEPELTAAYGALCAHETAWGSFIHTPGPELQRALATLQSQWEACRA
LEESPAGARKKRAAGQSGVPGGGHQREKRGWTMPGTLWCGVGDSAGNSSELGVFQGPDLC
CREHDRCPQNISPLQYNYGIRNYRFHTISHCDCDTRFQQCLQNQHDSISDIVGVAFFNVL
EIPCFVLEEQEACVAWYWWGGCRMYGTVPLARLQPRTFYNASWSSRATSPTPSSRSPAPP
KPRQKQHLRKGPPHQKGSKRPSKANTTALQDPMVSPRLDVAPTGLQGPQGGLKPQGARWV
CRSFRRHLDQCEHQIGPREIEFQLLNSAQEPLFHCNCTRRLARFLRLHSPPEVTNMLWEL
LGTTCFKLAPPLDCVEGKNCSRDPRAIRVSARHLRRLQQRRHQLQDKGTDERQPWPSEPL
RGPMSFYNQCLQLTQAARRPDRQQKSWSQ
>Q9ULC0
MELLQVTILFLLPSICSSNSTGVLEAANNSLVVTTTKPSITTPNTESLQKNVVTPTTGTT
PKGTITNELLKMSLMSTATFLTSKDEGLKATTTDVRKNDSIISNVTVTSVTLPNAVSTLQ
SSKPKTETQSSIKTTEIPGSVLQPDASPSKTGTLTSIPVTIPENTSQSQVIGTEGGKNAS
TSATSRSYSSIILPVVIALIVITLSVFVLVGLYRMCWKADPGTPENGNDQPQSDKESVKL
LTVKTISHESGEHSAQGKTKN
>Q92482
MGRQKELVSRCGEMLHIRYRLLRQALAECLGTLILVMFGCGSVAQVVLSRGTHGGFLTIN
LAFGFAVTLGILIAGQVSGAHLNPAVTFAMCFLAREPWIKLPIYTLAQTLGAFLGAGIVF
GLYYDAIWHFADNQLFVSGPNGTAGIFATYPSGHLDMINGFFDQFIGTASLIVCVLAIVD
PYNNPVPRGLEAFTVGLVVLVIGTSMGFNSGYAVNPARDFGPRLFTALAGWGSAVFTTGQ
HWWWVPIVSPLLGSIAGVFVYQLMIGCHLEQPPPSNEEENVKLAHVKHKEQI
>P62944
MTDSKYFTTNKKGEIFELKAELNNEKKEKRKEAVKKVIAAMTVGKDVSSLFPDVVNCMQT
DNLELKKLVYLYLMNYAKSQPDMAIMAVNSFVKDCEDPNPLIRALAVRTMGCIRVDKITE
YLCEPLRKCLKDEDPYVRKTAAVCVAKLHDINAQMVEDQGFLDSLRDLIADSNPMVVANA
VAALSEISESHPNSNLLDLNPQNINKLLTALNECTEWGQIFILDCLSNYNPKDDREAQSI
CERVTPRLSHANSAVVLSAVKVLMKFLELLPKDSDYYNMLLKKLAPPLVTLLSGEPEVQY
VALRNINLIVQKRPEILKQEIKVFFVKYNDPIYVKLEKLDIMIRLASQANIAQVLAELKE
YATEVDVDFVRKAVRAIGRCAIKVEQSAERCVSTLLDLIQTKVNYVVQEAIVVIRDIFRK
YPNKYESIIATLCENLDSLDEPDARAAMIWIVGEYAERIDNADELLESFLEGFHDESTQV
QLTLLTAIVKLFLKKPSETQELVQQVLSLATQDSDNPDLRDRGYIYWRLLSTDPVTAKEV
VLSEKPLISEETDLIEPTLLDELICHIGSLASVYHKPPNAFVEGSHGIHRKHLPIHHGST
DAGDSPVGTTTATNLEQPQVIPSQGDLLGDLLNLDLGPPVNVPQVSSMQMGAVDLLGGGL
DSLVGQSFIPSSVPATFAPSPTPAVVSSGLNDLFELSTGIGMAPGGYVAPKAVWLPAVKA
KGLEISGTFTHRQGHIYMEMNFTNKALQHMTDFAIQFNKNSFGVIPSTPLAIHTPLMPNQ
SIDVSLPLNTLGPVMKMEPLNNLQVAVKNNIDVFYFSCLIPLNVLFVEDGKMERQVFLAT
WKDIPNENELQFQIKECHLNADTVSSKLQNNNVYTIAKRNVEGQDMLYQSLKLTNGIWIL
AELRIQPGNPNYTLSLKCRAPEVSQYIYQVYDSILKN
>P97629
METFTNDRLQLPRNMIENSMFEEEPDVVDLAKEPCLHPLEPDEVEYEPRGSRLLVRGLGE

HEMDEDEEDYESSAKLLGMSFMNRSSGLRNSATGYRQSPDGTCSVPSARTLVICVFVIVV
AVSVIMVIYLLPRCTFTKEGCHKTNQSAELIQPIATNGKVFPWAQIRLPTAIIPQRYELS
LHPNLTSMTFRGSVTISLQALQDTRDIILHSTGHNISSVTFMSAVSSQEKQVEILEYPYH
EQIAVVAPESLLTGHNYTLKIEYSANISNSYYGFYGITYTDKSNEKKNFAATQFEPLAAR
SAFPCFDEPAFKATFIIKITRDEHHTALSNMPKKSSVPTEEGLIQDEFSESVKMSTYLVA
FIVGEMRNLSQDVNGTLVSVYAVPEKIDQVYHALDTTVKLLEFYQNYFEIQYPLKKLDLV
AIPDFEAGAMENWGLLTFREETLLYDNATSSVADRKLVTKIIAHELAHQWFGNLVTMQWW
NDLWLNEGFATFMEYFSVEKIFKELNSYEDFLDARFKTMRKDSLNSSHPISSSVQSSEQI
EEMFDSLSYFKGASLLLMLKSYLSEDVFQHAIILYLHNHSYAAIQSDDLWDSFNEVTGKT
LDVKKMMKTWTLQKGFPLVTVQRKGTELLLQQERFFPSMQPEIQDSDTSHLWHIPISYVT
DGRNYSEYRSVSLLDKKSDVINLTEQVQWVKVNTNMTGYYIVHYAHDGWAALINQLKRNP
YVLSDKDRANLINNIFELAGLGKVPLQMAFDLIDYLRNETHTAPITEALFQTDLIYNLLE
KLGHMDLSSRLVTRVHKLLQNQIQQQTWTDEGTPSMRELRSALLEFACAHSLENCTTMAT
KLFDGWMASNGTQSLPTDVMTTVFKVGARTEKGWLFLFSMYSSMGSEAEKDKILEALASS
ADAHKLYWLMKSSLDGDIIRTQKLSLIIRTVGRQFPGHLLAWDFVKENWNKLVHKFHLGS
YTIQSIVAGSTHLFSTKTHLSEVQEFFENQSEATLQLRCVQEAFEVIELNIQWMARNLKT
LTLWL
>A1Z6W3
MSSLSTGGGAGGSSGGPGGADAAAAPAAGQATVTATGNMEPAMVPRTANLLACKQWWRVC
FLYGDQQKYYRQLYSKAAAQRLADANQEPDNARDREYDTVDCDLIAGQLDAVEDADDGID
LGDHSSTPKGGATTAGRPLFPHSSSPRRSKKLLRSLRAHVRGEKLPKNDTTTANESSEVT
QRNARVTVLDDPFLFGIDADHLGDLVVRGKRYSTLDATENMARFYAEQEATAQVLEIIEQ
EEESPEQEAPKPALPPKQKQQRPVPPLPPPPANRVTQDQGTQPAAPQVPLQPLTAGDLQF
LNLSLRQRSLPRSMKPFKDAHDISFTFNELDTSAEPEVATGAAQQESNEPISRTPLTQIS
YLQKIPTLPRHFSPSGQGLATPPALGSGGMGLPSSSSASALYAAQAAAGILPTSPLPLQR
HQQYLPPHHQQHPGAGMGPGPGSGAAAGPPLGPQYSPGCSANPKYSNAQLPPPPHHHHQL
SPALSTPSPPSLLHHPAGGTSSASAHAPFLGGPHMDMQRQSHSDDDSGCALEEYTWVPPG
LRPDQVRLYFSQIPDDKVPYVNSPGEQYRVRQLLHQLPPHDNEVRYCHSLTDEERKELRL
FSTQRKRDALGRGNVRQLMSARPCDGCDDLISTGDIAVFATRLGPNASWHPACFACSVCR
ELLVDLIYFHRDGRMYCGRHHAETLKPRCSACDEIILADECTEAEGRAWHMNHFACHECD
KQLGGQRYIMREGKPYCLHCFDAMFAEYCDYCGEAIGVDQGQMSHDGQHWHATDECFSCN
TCRCSLLGRAFLPRRGAIYCSIACSKGEPPTPSDSSGTGMYTTPTPPTQRVRPHPQAPLP
ARIPSSHASSSPPMSPQQQQQHQATFNQAMYQMQSQQMEAAGGLVDQSKSYAASDSDAGV
VKDLEHGGHMGGGDLTDFSGGRASSTSQNLSPLNSPGDFQPHFLPKPMELQRDGVYNFNE
MSSNLDAAWSAKPTNSYHLQRQLLENPHTASMPELAGKLVAPPAHMQHLSQLHAVSSHQF
QQHEYADILHPPPPPPGEIPELPTPNLSVASTALPPELMGSPTHSAGDRSLNTPMSTQSA
SHAPPHPVSILSGASSSSPMSGEPAKKKGVRFEGIPDTLPRSRSYSGNGAGTSGGGERER
DRDKDKEGGGRHGHGHSSRRRRRRKSSSSSSHHRSGSGHRSHSTTRADTYAPAQPLSSSY
QGPPSVLQAANLVHESPSRQQRERERERERERESSEESDVCSTCSSSSSSSEDYMMMYQLPQ
RRHYGGVRVSYVPNDALAYDRKRKPSELGGDKDKNCIIS
>P35329
MRVHYLWLLLILGHAASAQYSSANDWTVDHPQTLFAWEGACIRIPCKYKTPLPKARLDNI
LLFQNYEFDKATKKFKGTVLYNKAEPELYPPKQRRVTFLGNSIDNCTLKIHPIRANDSGN
LGLRMTAGTERWMEPIHLNVSEKPFQPYIQMPSEIRESQSVTLTCGLNFSCFEYDILLQW

FLEDSKITSVTPSVTSITSSVTSSIKNVYTESKLTFQPKWTDHGKSVKCQVQHSSEVLSE
RTVRLDVKYTPKLEIKVNPTEVEKNNSVTMTCRVNSSNPKLRTVAVSWFKDGRPLEDQEL
EQEQQMSKLILHSVTKDMRGKYRCQASNDIGPGESEEVELTVHYAPEPSRVHIYPSPAEE
GQSVELICESLASPSATNYTWYHNRKPIPGDTQEKLRIPKVSPWHAGNYSCLAENRLGHG
KIDQEAKLDVHYAPKAVTTVIQSFTPILEGDSVTLVCRYNSSNPDVTSYRWNPQGSGSVL
KPGVLRIQKVTWDSMPVSCAACNHKCSWALPVILNVHYAPRDVKVLKVSPASEIRAGQRV
LLQCDFAESNPAEVRFFWKKNGSLVQEGRYLSFGSVSPEDSGNYNCMVNNSIGETLSQAW
NLQVLYAPRRLRVSISPGDHVMEGKKATLSCESDANPPISQYTWFDSSGQDLHSSGQKLR
LEPLEVQHTGSYRCKGTNGIGTGESPPSTLTVYYSPETIGKRVALGLGFCLTICILAIWG
MKIQKKWKQNRSQQGLQENSSGQSFFVRNKKARRTPLSEGPQSQGCYNPAMDDTVSYAIL
RFPESDMHNAGDAGTPATQAPPPNNSDSVTYSVIQKRPMGDYENVNPSCPEDESIHYSEL
VQFGAGKRPQAKEDVDYVTLKH
>O01670
MMSFPIALFADVNQSFRANLVVSSYHHAITFPTVRGANFSTFFPRNFSVSADVWLCGANF
SQEWQLMQPVCSTKYDSITIFITVAVVLTLITLWTILGNFFVLMALYRYGTLRTMSNCLI
GNLAISDLLLAVTVLPISTVHDLLGYWVFGEFTCTLWLCMDVLYCTASIWGLCTVAFDRY
LATVYPVWYHDQRSVRKAVGCIVFVWIFSIVISFAPFIGWQHMIPSFFSFNASIQRYQCI
LFTSSSYVLYSSMGSFVIPAILMAFMYVRIFVVLHNQSRGVKFKSGLKISSSKYNGCPVI
NEPSREGINGLGRDVTNTTLLSDAVGSSADLTSNGKDDPRVLATAPIELTEDVPPLNGHH
HRTVHETPYVSGLHTKRSNSFALPTELEKKCKPLTNNILHMMDFDRRNSHNAVIQRSASE
MVNLDVSKHELLISNVCHRSKSATALTSETGDPLGSLAGPRRSLQCNVGGLVRNKHMTLS
MKRRFELREQRATKRMLLIMACFCVCWMPFLFMYILRSVCDTCHMNQHFVAAIIWLGYVN
SSLNPVLYTLFNDDFKVAFKRLIGARSPSAYRSPGPRR
>Q9V5N8
MQTREFPQRPLGLLLVLLVVLLQSSLIKSYLIIVHEDTPPGTVIFNASVYKLGSERHYKI
NAHKSANFVHHLVSVNHKDGQIQLRKALKCDGIYYPNLFTFYVDSTSNRLRSIDYYSLPV
RIFVSGHSCNEDRRIEQELHHHHYEEEDNTGYSKRRRRRSTQEMIQLNGNQLEEVFRQNS
TEFRAGDLIFGDSFDNEMRHRILSRKRRAVGSPDPLHLQPALHRRISDAKQWISETYASY
AIHTTDKWNQICLRRSQFINSLNAFLPRSVCQHCKVSFLDVNDERFAIEHQSRDLVASRD
VCIAESMWKVSITFNIRCDRRDIVDSDHRLKIVYHHQEFNDTDIARRVRRELRNQSPYFE
QALYVASVLEEQPAGAAVTTVRARDPEDSPVVYSMVSLLDSRSQSLFKVDSRTGVVTTSA
SLDRELMDVHYFRVVATDDSFPPRSGTTTLQVNVLDCNDHSPTFEAEQFEASIREGATVG
STVITLRATDQDIGKNAEIEYGIEAVTDGAGLAQDQEMPIFRIDSRSGVISTRSSLDRET
SDSYHLLVTAADLASAQSERRTATASVQVKVLDDNDNYPQFSERTYTVQVPEDQWGGTED
NTVAHIRATDADQGNNAAIRYAIIGGNTQSQFSIDSMSGDVSLVKPLDYESVRSYRLVIR
AQDGGSPSRSNTTQLLVNVIDANDNAPRFYTSQFQESVLENVPVGYNIIRVQAYDSDEGA
NAEITYSISERDDNFPLAVDPRTGWVQTIKPLDREEQGRFAFQVVAKDGGVPPKSASSSV
VITVQDVNDNDPAFNPKYYEANVGEDQPPGTPVTTVTATDPDEDSRLHYEITTGNTRGRF
AITSQNGRGLITIAQSLDYKQEKRFLLTVAATDSGGRSDTATVHINITDANNFAPIFENA
PYSASVFEDAPVGTTVLVVSATDSDVGVNAQITYSLNEESINGLGSPDPFSINPQTGAIV
TNAPLDRETTSGYLLTVTAKDGGNPSLSDTTDVEIGVTDVNDNAPAFKSPLYQASILEDA
LVGTSVIQVAASDPDVGLNGRIKYLLSDRDIEDGSFVIDPTSGTIRTNKGLDRESVAVFH
LTAIAVDKGSPPLSSTVEVQIRLEDVNDSPPTFASDKITLYVPENSPVGSVVGEIHAHDP
DEGVNAVVHYSIIGGDDSNAFSLVTRPGSERAQLLTMTELDYESTRKRFELVVRAASPPL

RNDAHIEILVTDVNDNAPVLRDFQVIFNNFRDHFPSGEIGRIPAFDADVSDKLHYRILSG
NNANLLRLNSSSGGLVLSPQLNTNVPKFATMEVSVSDGINEAKAIMQLSVRLITEDMLFN
SVTVRLNEMTEEAFLSPLLNFFLDGLAAIIPCPKEHIFVFSIQDDTDVSSRILNVSFSAR
RPDVSHEEFYTPQYLQERVYLNRAILARLATVEVLPFDDNLCVREPCLNFEECLTVLKFG
NASEFIHSDTVLFRPIYPVNTFACSCPEGFTGSKEHYLCDTEVDLCYSDPCQNGGTCVRR
EGGYTCVCPSTHTGQNCETGVGHLRPCPSETCEGGLSCLSNYPSSQPPPYTATCELRARA
FGRNSFLTFESLKQRHRFNLKLRFATVQENGLLLYNGRYNELHDFIALEIHEGHVSFSFS
LGDHSERISVIQEAKVSDGKWHQVEVVYLNRSVTLVLDNCDTAIALSGQLGDRWSCANRT
TLKLDKRCSLLTETCHRFLDLTGPLQVGGLPRIPAHFPVTNRDFVGCISDLRIDDRFVDL
NSYVADNGTLAGCPQKAPLCQSEPCFNGGTCREGWGTYSCECPEGYAGNSCQDNIPAPWR
FSGDGSLSFNPLLRPIQLPWTTSFSLRTRQKEAFLLQIQIGQNSSAAVCLRQGVLYYIFD
GEPMYLAGAFLSDGEWHRVEIRWQQGSEIHFSVDYGQRSGSVPMSQKVQGLYVGKIVMGS
PDGSIGAVPEASPFEGCIQDVRIGAGQSVLSRPTIRENVEDGCESRAQCPDHCPNHSSCQ
SSWDLSTCECDSGYVGTDCAPICTVRPCASGVCRANTSLPRGYDCECNSSSRHGDYCEKE
LQQPCPGGWWGERVCGPCRCDLAQGYHPDCNKTTGQCYCKTNHYQPPNETACLSCDCYSI
GSFSGACNPLTGQCECREGVIGRRCDSCSNPYAEVTLSGCEVVYDACPRSFAGGVWWPRT
PLGGVAIEGCPPPARGKGQRSCDVQSGSWNTPDMYNCTSEPFVELRRQLSQLEKLELELN
SFVAIKMAEQLRKACEAVDRRGASKDQKISGNGRPNRRYKMESSFLLSNGGNVWSHELEM
DYLSDELKFTHDRLYGADLLVTEGLLQELINYELMQSGLNLSHSQDKYFIKNLVDAASVI
LDRKYEAEWRRATELIQRGPDDLVDAFNKYLVVLARSQHDTYTSPFEIVQPNMALGLDIV
TTESLFGYEPEQLSEYHRSKYLKPNAFTTESVVLPDTSGFLQHSARQRPVISFPKYNNYI
LDRRKFDQHTKVLVPLEMLGITPPESDEISQSGRRGSSHDHRAIVAYAQYKDVGQLLPDL
YDETITRRWGVDVELATPILSLQILVPSMEREQETQRLEIPSRKIFSSSSPSSSSSSGST
EQQFVEVFDVPKAPTSSSEQQIEDIRITAHEIPPPVSSVEQQEASSDEDGEEREPHIRLN
LDDIEFHGNSGEEVISPDSPEMLNPNYEGVSSTGSDEQPKGENEAVYRDRRLVKRQVEIT
YPSEQMQQTEQVVYRSLGSPHLAQPIKLQMWLDVDSARFGPRSNPQCVRWNSFTNQWTRL
GCQTEIPDFDGDFNPAAQQAILVNCSCTHISSYAVIVDVIDPEDIPEPSLLVQITSYSAF
LVSLPLLLGVLLALALLRGQQTNSNTIHQNIVLCVFCAELLFFVGMQSRRQLLESEFPCK
LTAICLHYFWLAAFAWTTVDCVHLYRMLTEMRDINHGPMGFYFAMGYGAPAIVVGLSVGV
RAHEYGNSLFCWLSVYEPVVWWLVGPIAGMSVVNLLILFVSVKAAFTLKDHVLGFGNLRT
LLWLSVVSLPLMGVMWVLAVLAASEHSQLLSLLLSGVVLLHALFCLIGYCIINKRVRENL
QRTCLRCMGRKVPLLDSSMVVSNSSHNVNAAARPSNFLASGYDTTTRRNIGISASSTTSR
STAKTSSSPYSDGQLRQTSTSTSNYNSASDAPSFLRGFESSTTGRSRGGEEKPSRRQRKD
SDSGSETDGRSLELASSHSSDDDESRTARSSGTHRSTAVSSTPAYLPNITEHVQATTPPE
LNVVQSPQLFPSVNKPVYAPRWSSQLPDAYLQSPPNIGRWSQDTGSDNEHVHGQAKMTIS
PNPLPNPDLTDTSYLQQHHNKINMPPSILENIRDAREGYEDSLYGRRGEYPDKYGSYKPP
SHYGSEKDYPGGGSGSQTIGHMRSFHPDAAYLSDNIYDKQRTLGSGYLGAKSESPYLSKD
RITPDIYGSRDGHYSLKRQPAYATDSLHSVHSLLKNDYHQQQQQQQHHLQDRLSEGSDK
NGYHFPYTAEEDHLPARKLSHTQPPSLHGSQLMQPPGVGLVNDVNNPGLMGRHTLNGGSR
HSSRASSPPSTMVAPMQPLGPLTSITDTDSEAEY
>Q05934
MRAMDTQVQSAERGLVLPPMNSTVSSATAATTATNTDTDTDGDRDEERESLAEDGSEWVP
AYMLTRDRSRYLGHFLGVDKMLEAVKCKYCGVIIRRQGNSISMAEASQTHLWSTHKIDPN
ANYYSGWTGVEAGSTFMVRPPLKNHQGGSATTNSIANLLEIDEDFLKRTREKEMALPLVQ

SLAIIIASENLPLSFVDNTAVRLLINQNANSLSFIDHDLILNAIRSIAYNLDRIIQRTAL
RNNSDLSLIIDKNYLLMDPTDRSNQLSNRLKNQLFEMQKINFFSLSHSVWNNTISILSIQ
YYDDFHSQVKTLPLIIQNLHEYNNDPKLSIPAQLLKISQELPGLQNTVISITLPRSQIVD
LLNVMDSQPFFPNTYTNAKNYYHNCIISIINSAILPLFGTPKSADITHPRQSSFSKEPLT
LLDSLIDLSNIDISNSIFSRINSFLDDLQSNSWQLDKFRSLCEKFGFEFVCSKFDLSRYS
TATVSLQTFLNLRPIIEEYQSSIQIEKFNEIDFQIIDYLLITLNSINRILKFFTSSKSLN
FTYVLFAIMSIEKHLLSTLGSLQFQRLIAPFETFLSKIQEFKTILFSDDMNLLAMFLCPA
ILFEREVLEYSFHTISLSEIVDKLSTSIFSLLKRFLNLHTIGNVNNSHNTSNHSNMNIHT
DNQTNNINNRSGNNSDNNDNEHDNDNDNHSNSNTPASRIDIDPTGGENSVLPEQQPQNSN
NNLSFGSLSDTHHLSDSTISKEIDSIFLQIIQEDLYDYLSTVNSIVPISYRSYCEQSNFI
RDSGRFKKRIITEDSIIGELEQPMNFIEELLDIHVPVCNAFWSQYLDNDAGPIIRILFKI
MQCQSSSSIRGEYSFLNDFIPRVHPDLTQEIIKIKLFNDQFVASKVDYDLDTLQTASQYL
P
>Q0QWG9
MPATNQGWPEDFGFQLGGSGPCFVIEVAEGSSAHAGGLRPGDQILEVEGLAVGGLSRERI
VRLARRCPRVPPSLGVLPGPEGGPTALTAAWLTRRFGRSLPLSRELLRLAGGPRPDAVHR
ERRRKAQEFSCQVDDILGDRLTAKEQVFTALKQFAAEQRVDELVWTLTLVLPSEAQGPVL
DNLRIFIPKKHRARFDEVVSQGLLGKLCRARRAQGAQRLRRSRSEERPERLLVSTRASAA
PRRPDEPPPRKATSLLGGRTGPGGPRRTVRVYKGNKSFGFTLRGHGPVWIESVLPGSPAE
NASLKSGDRILFLNGLDMRNCSHDKVVSMLQGSGAMPTLVVEEGPVPFASDSDSLDSPTR
ASALTSLQWVADILPSSIRVQGRTFSQQLDHLLTPPERYGVCRALERFFQHRNIDTLIVD
VYPVLDTPAKQVLWQFLYQLLTYEEQELCQEKIACFLGYTAMTEPESSLDLEPESTPEPT
PEPQPRSSLRASSMCRRSLRSQGLETSLSCGPGDCPEMPLPLIPGERQAGDGTSLPETPN
PKMMSAVYAELESRLNSSFKGKIGTMSKSRASPPVPSLVGTSGPRTLSGVSWPSDRLLPS
PCYDPLCSGGLASPSSSESHPYASLDSSRAPSPQPGLGSIHADSPPSPDPIRPPSRRKLF
AFSRPVRSRDTDRFLDALSEQLGPRLSIVDDFLTPENDYEEMSFHDDQGSFVTNERSSAS
ECVSSSEEGSSLTYSSISDHIPPPPLSPPPPPPLPFHDPKPSSRTSDGPRGPPQSLTKPL
TQINHPVPPPPPPPLPPPVPCAPPMLSRGVGHRRSETSHMSVKRLRWEQVENSEGTIWGQ
LGEDSDYDKLSDMVKYLDLELHFGTQKPPKPVPGPEPFRKKEVVEILSHKKAYNTSILLA
HLKLTPGELRQVLMSMEPRRLEPAHLAQLLLFAPDADEEQRYQAFREAPGRLSEPDQFVL
QMLSVPEYKTRLRSLHFQATLQEKTEEIRGSLECLRQASLELKNSRKLAKILEFVLAMGN
YLNDGQPKTNKTTGFKINFLTELNSTKTVDGKSTFLHILAKSLSQHFPELLGFAQDLPTV
PLAAKVNQRALTGDLADLHDTVSEIQVACQSMAPSSEDRFAVVMASFLETAQPALRALDG
LQREAMEELGKALAFFGEDSKATTSEAFFGIFSEFMSKFERALSDLQAGDGPRSSGMVSP
LAW
>Q9XYF6
MAAKPEQDCKTAAALIRAGSLIEGVESAGKDFLVWTTQGPAVKKDPDLLFSLCRVLPGST
QQTLKLQQVEPTADSQELTVQAKEVWQANPGIDPLTYGDIGGLPHTNEPCVLDFLARRYQ
SKVIYTTAEPLIVAVNPFQDLKNAGPDTIALYRDAPDVDKLPPHVFYASRRAMTNMHQLK
KPQTIIVSGESGAGKTETTKMLMKYLATSAGGNLDLKIQTAIMAANPVLEAFGNAKTVRN
NNSSRFGRFMLLDVAREGGIQHGKVVAFLLEKSRIVCQDKDERNYHIFYQFLKGAPGHMR
QRYMLQPLEAYTFINPHCLDAPGIVDTEDFEQTVKSLESMNMTETETCTIWSIVSGVLLM
GNAKPTGKTEAGVENAACFVGESEAALRNACSLLFLDYPSILHELTVKTTYAGSNKIESR
WTVPDSEMLRASLAKGMFEQLFLWIIRKLNADIEPKGGSFDVFMGLLDIFGFEVFQNNSL

EQLFINITNEVLQRNFTDIVFEKELQLYSKEGISSKKIEYTTNEKLIETLLGKGTSVLAA
LEDQCISPSGTDEKFVSSLASKLAGNKCFIPSKNTKSLEFTVVHTIGKVIYNADGFAFKN
KDVLRPEIIEITRASTNDVVRGLFEGVKVEKGKMAKGMLIGSQFMTQLKGLMEVIQKTES
HFIRCIKPNDDKVPLKWVNSKVLIQLHALSILEALHLRQLAFSYRRTFEEFAAQFRFINL
GVSNKPGADAKTICVELLKSTSISADEYALGKTMVFLKPQAAKMLVRLQREALSAWEPLV
GVFEGMTVLKRAKQLSTGRAVPATRICANVRRKLVQAGIKVC
>Q14644
MAVEDEGLRVFQSVKIKIGEAKNLPSYPGPSKMRDCYCTVNLDQEEVFRTKIVEKSLCPF
YGEDFYCEIPRSFRHLSFYIFDRDVFRRDSIIGKVAIQKEDLQKYHNRDTWFQLQHVDAD
SEVQGKVHLELRLSEVITDTGVVCHKLATRIVECQGLPIVNGQCDPYATVTLAGPFRSEA
KKTKVKRKTNNPQFDEVFYFEVTRPCSYSKKSHFDFEEEDVDKLEIRVDLWNASNLKFGD
EFLGELRIPLKVLRQSSSYEAWYFLQPRDNGSKSLKPDDLGSLRLNVVYTEDHVFSSDYY
SPLRDLLLKSADVEPVSASAAHILGEVCREKQEAAVPLVRLFLHYGRVVPFISAIASAEV
KRTQDPNTIFRGNSLASKCIDETMKLAGMHYLHVTLKPAIEEICQSHKPCEIDPVKLKDG
ENLENNMENLRQYVDRVFHAITESGVSCPTVMCDIFFSLREAAAKRFQDDPDVRYTAVSS
FIFLRFFAPAILSPNLFQLTPHHTDPQTSRTLTLISKTVQTLGSLSKSKSASFKESYMAT
FYEFFNEQKYADAVKNFLDLISSSGRRDPKSVEQPIVLKEGFMIKRAQGRKRFGMKNFKK
RWFRLTNHEFTYHKSKGDQPLYSIPIENILAVEKLEEESFKMKNMFQVIQPERALYIQAN
NCVEAKDWIDILTKVSQCNQKRLTVYHPSAYLSGHWLCCRAPSDSAPGCSPCTGGLPANI
QLDIDGDRETERIYSLFNLYMSKLEKMQEACGSKSVYDGPEQEEYSTFVIDDPQETYKTL
KQVIRWVGALEQEHAQYKRDKFKKTKYGSQEHPIGDKSFQNYIRQQSETSTHSI
>O77408
MSRDIFMKRLRLHLLFDEVAMVTHIVGDVLSSVLLCAVVLLVLVGNTLVVAAVATSRKLR
TVTNVFIVNLACADLLLGVLVLPFSAVNEIKDVWIFGHVWCQVWLAVDVWLCTASILNLC
CISLDRYLAITRPIRYPGLMSAKRAKTLVAGVWLFSFVICCPPLIGWNDGGDGIMDYNGT
TATPIPVTTTQTPVTGRDDVLCDNGFNYSTNSNMNTTCTYSGDSSLSTTCELTNSRGYRI
YAALGSFFIPMLVMVFFYLQIYRAAVKTISAYAKGELKTKYSVRENGSKTNSVTLRIHRG
GRGPSTGSSVYRHGSTYGGSAAGAATREGCGDKDAAGGRRFGRQEMDSHLPVRKCRSSDA
SLVTLTGLKCEIIDNGNAKHGPISELIKGRGKSFFWRKEKKRSVGGERESFENSTRNGRS
TRAKLCGGRCLAIETDICSSGECSPRTKRIKEHARATQHNSLPVTPSLSSQNEETDAVFV
RGTSNSEYKPRRSRLSAHKPGHAMRLHMQKFNREKKAAKTLAIIVGAFIMCWMPFFTIYL
VGAFCENCISPIVFSVAFWLGYCNSAMNPCVYALFSRDFRFAFRKLLTCSCKAWSKNRSF
RPQTSDVPAIQLHCATQDDAKSSSDIGPTASGGNGGYT
>Q4WCX9
MKFSPAYLLAFAPIVAAHFRLQYPTSRGFNADTMANFPCGDLAQSSNRTQVSLSSGSFPV
ALRMGHDETAVEVLLALGNDPGTNFNITLHPTFRIEGLGEFCLPNVVFDESVLGTKITDG
MNATLQVQSNGDPSGGLYACADIQFSTTAQDEKPSSCTNNTGISAIAFTGAAAERNANES
TADGQAQSGSSGSSSDSGSHSGHSSATQTSSTTASSTAGAVALETAAWGILGAAVVGGLA
VL
>Q9LKW9
MTTVIDATMAYRFLEEATDSSSSSSSSKLESSPVDAVLFVGMSLVLGIASRHLLRGTRVP
YTVALLVIGIALGSLEYGAKHNLGKIGHGIRIWNEIDPELLLAVFLPALLFESSFSMEVH
QIKRCLGQMVLLAVPGVLISTACLGSLVKVTFPYEWDWKTSLLLGGLLSATDPVAVVALL
KELGASKKLSTIIEGESLMNDGTAIVVFQLFLKMAMGQNSDWSSIIKFLLKVALGAVGIG

LAFGIASVIWLKFIFNDTVIEITLTIAVSYFAYYTAQEWAGASGVLTVMTLGMFYAAFAR
TAFKGDSQKSLHHFWEMVAYIANTLIFILSGVVIAEGILDSDKIAYQGNSWRFLFLLYVY
IQLSRVVVVGVLYPLLCRFGYGLDWKESIILVWSGLRGAVALALSLSVKQSSGNSHISKE
TGTLFLFFTGGIVFLTLIVNGSTTQFVLRLLRMDILPAPKKRILEYTKYEMLNKALRAFQ
DLGDDEELGPADWPTVESYISSLKGSEGELVHHPHNGSKIGSLDPKSLKDIRMRFLNGVQ
ATYWEMLDEGRISEVTANILMQSVDEALDQVSTTLCDWRGLKPHVNFPNYYNFLHSKVVP
RKLVTYFAVERLESACYISAAFLRAHTIARQQLYDFLGESNIGSIVINESEKEGEEAKKF
LEKVRSSFPQVLRVVKTKQVTYSVLNHLLGYIENLEKVGLLEEKEIAHLHDAVQTGLKKL
LRNPPIVKLPKLSDMITSHPLSVALPPAFCEPLKHSKKEPMKLRGVTLYKEGSKPTGVWL
IFDGIVKWKSKILSNNHSLHPTFSHGSTLGLYEVLTGKPYLCDLITDSMVLCFFIDSEKI
LSLQSDSTIDDFLWQESALVLLKLLRPQIFESVAMQELRALVSTESSKLTTYVTGESIEI
DCNSIGLLLEGFVKPVGIKEELISSPAALSPSNGNQSFHNSSEASGIMRVSFSQQATQYI
VETRARAIIFNIGAFGADRTLHRRPSSLTPPRSSSSDQLQRSFRKEHRGLMSWPENIYAK
QQQEINKTTLSLSERAMQLSIFGSMVNVYRRSVSFGGIYNNKLQDNLLYKKLPLNPAQGL
VSAKSESSIVTKKQLETRKHACQLPLKGESSTRQNTMVESSDEEDEDEGIVVRIDSPSKI
VFRNDL
>Q8GUI4
MTRRAEFEMGLFVILQSMFLISLCSSQKPEEFLPEISPDTSPQPFLPFIAPSPMVPYINS
TMPKLSGLCSLNFSASESLIQTTSHNCWTVFAPLLANVMCCPQLDATLTIILGKASKETG
LLALNRTQSKHCLSDLEQILVGKGASGQLNKICSIHSSNLTSSSCPVINVDEFESTVDTA
KLLLACEKIDPVKECCEEACQNAILDAATNISLKASETLTDNSDRINDCKNVVNRWLATK
LDPSRVKETLRGLANCKINRVCPLVFPHMKHIGGNCSNELSNQTGCCRAMESYVSHLQKQ
TLITNLQALDCATSLGTKLQKLNITKNIFSVCHISLKDFSLQVGNQESGCLLPSLPSDAI
FDKDTGISFTCDLNDNIPAPWPSSSLSSASTCKKPVRIPALPAAASSQPRLHDEGVTRLV
IFVLSMLLVMLLS
>Q673G8
MLGRKIPGSGVLGAGAGMDRGRTGERLHAALAGLQELHFLRDKQSAMVHWALTLNRDQPD
PSKQENVSKEELRLEATLSLLKQQLTRLRRQDVGLKTHLQQLDQQITELKLDVCKASTEH
LESDSRPSSGFYELSDGGSGSLSNSCTSVYSESLSSSSQTSLLPLLSTSYASHGRSSCGQ
TGVSRRCSADESTAQSDAPRSGVKLGSSLIRTATARADRARQRPVSTGDLDRMIGPGFGA
FKSTDVKSSTPCSSPQNPSVDPKYQSNLVSSNGTEVYRYPSPLHAVALQSPIFSCTSDQG
SSVALDEMPEEETQNLNEESTISSSVGYINKLLQRSSSRVNLLSSIKRIETVSGTHEQIP
RSQEMIYGSLNGPQLLGSLQQLSMPLENALETGKTSALNNNQKQMDPPCLGSNFKEVEVQ
MLHHGKHPESSLDLQKNNFPINNTAGKVGAESDNGASEKRSGHFPKDLSVVQKPVERKSS
FTSGREGSRASCHDKSSIPQSEFVHAQFVPAGSQRVKVRQADKKTKSVKLRKKSSEKPSA
KKQHQKPLSREFCTKNRTDLKQSGSCRGKVTYLEESQAQSCSDCSCNGLINSHCIQNNHQ
QIPSSKSTKSRKAPEPVYHPLDHAKKKPSSRKWPSSSEIPLPPTLHTQRSKEMLNSRKVA
MVRSVSARPRSGHWGCPPPRALPHSLSTSSYFSYLESRYPAAPVSSRHPPRCESEFSEYS
AECASLFHSTIAASSDGEMSDYTTNRFGDSESSQGSQTASESDSSLSLDEEDLLEEEEED
EGGLVWAQAAMGTTAAGFSLQQHHRSESAACRIKASRALKKKIRRFQPASLKVMTLV
>P32855
MDYLKPAQKGRRRGLSINSLSETQQSAMNSSLDHLQNDLNRINLQWNRILSDNTNPLELA
LAFLDDTSVGLGHRYEEFNQLKSQIGSHLQDVVNEHSQVFNTNVASYGKAVSSIMQAQEQ
TLNLKNCLKEANEKITTDKGSLQELNDNNLKYTKMIDVLVNIEELLQIPEKIEENIRKEN

FHQVQILLERGFILMNNKSLKTVEILKPINQQLELQEHLLFNNLIEEIHDIMYSKSNKTN
FTRVTNNDIFKIISISHNGFTSLENYLYNIVNIDIMEHSKTINKNLEQFIHDQSLNKGNI
MLQENAATQAPLAPSRNQENEGFNRIGFLLKTINNINKLPVAFNIITERAKEEIHNIIVK
STESIRSKHPSLLKMATSLKNDNHFGLPVQDILSIILRECFWEIFLKLLYAIQCHRAIFE
MSNILQPTSSAKPAFKFNKIWGKLLDEIELLLVRYINDPELISSNNGSIKPINGATNNAP
TLPKRKNPKIFSLEYNIEDNSSVKDQAFELKALLKDIFPGFSVSSNMDLDSIYVKDESFE
QDEPLVPPSVFNMKVILDPFLLFTQSTSTIVPSVLTQNTISSLTFFDDYMNKSFLPKIQM
TMDYLFTVEVESNNPYALELSDENHNIFKTALDFQRLFYNLLNVFNTANTFREKISYCIL
DLLNHFYNYYLGLFNSLIGTSDRHLTRKIITAWLQNGILMDQEQKILNGDETLFHEESIE
LFKEIPHFYQAGKGLSKSDLFNNLTLDTILQFSASVLWILNWLPGLKKAINIDEVSQEPM
LDADRLRSSWTFSESMDLNYSNPSSSPNSLGNLKILLDDKASKKFDETIDGFKTLKFKLI
TILRFNIRALCIYDIGSFFQNTKIWNMDVGSIELDQNIASLISELRRTESKLKQQLPEKE
KNSIFIGLDIVNNYALIKGAKSIKVLNHNGIKKMLRNVNVLQHAYRNLSSEPSKINMNVT
MNFYSLCGSSEAELFEYIKDNELPHCSVEDLKTILRLQFSEEMHRQLKRQSTSSTKGSIK
PSNKRYTEALEKLSNLEKEQSKEGARTKIGKLKSKLNAVHTANEK
>Q7TSN7
MEGSWRDVLAVLVILAQLTASGSSYQIIEGPQNVTVLKDSEAHFNCTVTHGWKLLMWTLN
QMVVLSLTTQGPIITNNRFTYASYNSTDSFISELIIHDVQPSDSGSVQCSLQNSHGFGSA
FLSVQVMGTLNIPSNNLIVTEGEPCNVTCYAVGWTSLPDISWELEVPVSHSSYNSFLESG
NFMRVLSVLDLTPLGNGTLTCVAELKDLQASKSLTVNLTVVQPPPDSIGEEGPALPTWAI
ILLAVAFSLLLILIIVLIIIFCCCCASRREKEESTYQNEIRKSANMRTNKADPETKLKGG
KENYGYSSDEAKAAQTASLPPKSAEVSLPEKRSSSLPYQELNKHQPGPATHPRVSFDIAS
PQKVRNVTLV
>P46939
MAKYGEHEASPDNGQNEFSDIIKSRSDEHNDVQKKTFTKWINARFSKSGKPPINDMFTDL
KDGRKLLDLLEGLTGTSLPKERGSTRVHALNNVNRVLQVLHQNNVELVNIGGTDIVDGNH
KLTLGLLWSIILHWQVKDVMKDVMSDLQQTNSEKILLSWVRQTTRPYSQVNVLNFTTSWT
DGLAFNAVLHRHKPDLFSWDKVVKMSPIERLEHAFSKAQTYLGIEKLLDPEDVAVRLPDK
KSIIMYLTSLFEVLPQQVTIDAIREVETLPRKYKKECEEEAINIQSTAPEEEHESPRAET
PSTVTEVDMDLDSYQIALEEVLTWLLSAEDTFQEQDDISDDVEEVKDQFATHEAFMMELT
AHQSSVGSVLQAGNQLITQGTLSDEEEFEIQEQMTLLNARWEALRVESMDRQSRLHDVLM
ELQKKQLQQLSAWLTLTEERIQKMETCPLDDDVKSLQKLLEEHKSLQSDLEAEQVKVNSL
THMVVIVDENSGESATAILEDQLQKLGERWTAVCRWTEERWNRLQEINILWQELLEEQCL
LKAWLTEKEEALNKVQTSNFKDQKELSVSVRRLAILKEDMEMKRQTLDQLSEIGQDVGQL
LDNSKASKKINSDSEELTQRWDSLVQRLEDSSNQVTQAVAKLGMSQIPQKDLLETVRVRE
QAITKKSKQELPPPPPPKKRQIHVDIEAKKKFDAISAELLNWILKWKTAIQTTEIKEYMK
MQDTSEMKKKLKALEKEQRERIPRADELNQTGQILVEQMGKEGLPTEEIKNVLEKVSSEW
KNVSQHLEDLERKIQLQEDINAYFKQLDELEKVIKTKEEWVKHTSISESSRQSLPSLKDS
CQRELTNLLGLHPKIEMARASCSALMSQPSAPDFVQRGFDSFLGRYQAVQEAVEDRQQHL
ENELKGQPGHAYLETLKTLKDVLNDSENKAQVSLNVLNDLAKVEKALQEKKTLDEILENQ
KPALHKLAEETKALEKNVHPDVEKLYKQEFDDVQGKWNKLKVLVSKDLHLLEEIALTLRA
FEADSTVIEKWMDGVKDFLMKQQAAQGDDAGLQRQLDQCSAFVNEIETIESSLKNMKEIE
TNLRSGPVAGIKTWVQTRLGDYQTQLEKLSKEIATQKSRLSESQEKAANLKKDLAEMQEW
MTQAEEEYLERDFEYKSPEELESAVEEMKRAKEDVLQKEVRVKILKDNIKLLAAKVPSGG

QELTSELNVVLENYQLLCNRIRGKCHTLEEVWSCWIELLHYLDLETTWLNTLEERMKSTE
VLPEKTDAVNEALESLESVLRHPADNRTQIRELGQTLIDGGILDDIISEKLEAFNSRYED
LSHLAESKQISLEKQLQVLRETDQMLQVLQESLGELDKQLTTYLTDRIDAFQVPQEAQKI
QAEISAHELTLEELRRNMRSQPLTSPESRTARGGSQMDVLQRKLREVSTKFQLFQKPANF
EQRMLDCKRVLDGVKAELHVLDVKDVDPDVIQTHLDKCMKLYKTLSEVKLEVETVIKTGR
HIVQKQQTDNPKGMDEQLTSLKVLYNDLGAQVTEGKQDLERASQLARKMKKEAASLSEWL
SATETELVQKSTSEGLLGDLDTEISWAKNVLKDLEKRKADLNTITESSAALQNLIEGSEP
ILEERLCVLNAGWSRVRTWTEDWCNTLMNHQNQLEIFDGNVAHISTWLYQAEALLDEIEK
KPTSKQEEIVKRLVSELDDANLQVENVRDQALILMNARGSSSRELVEPKLAELNRNFEKV
SQHIKSAKLLIAQEPLYQCLVTTETFETGVPFSDLEKLENDIENMLKFVEKHLESSDEDE
KMDEESAQIEEVLQRGEEMLHQPMEDNKKEKIRLQLLLLHTRYNKIKAIPIQQRKMGQLA
SGIRSSLLPTDYLVEINKILLCMDDVELSLNVPELNTAIYEDFSFQEDSLKNIKDQLDKL
GEQIAVIHEKQPDVILEASGPEAIQIRDTLTQLNAKWDRINRMYSDRKGCFDRAMEEWRQ
FHCDLNDLTQWITEAEELLVDTCAPGGSLDLEKARIHQQELEVGISSHQPSFAALNRTGD
GIVQKLSQADGSFLKEKLAGLNQRWDAIVAEVKDRQPRLKGESKQVMKYRHQLDEIICWL
TKAEHAMQKRSTTELGENLQELRDLTQEMEVHAEKLKWLNRTELEMLSDKSLSLPERDKI
SESLRTVNMTWNKICREVPTTLKECIQEPSSVSQTRIAAHPNVQKVVLVSSASDIPVQSH
RTSEISIPADLDKTITELADWLVLIDQMLKSNIVTVGDVEEINKTVSRMKITKADLEQRH
PQLDYVFTLAQNLKNKASSSDMRTAITEKLERVKNQWDGTQHGVELRQQQLEDMIIDSLQ
WDDHREETEELMRKYEARLYILQQARRDPLTKQISDNQILLQELGPGDGIVMAFDNVLQK
LLEEYGSDDTRNVKETTEYLKTSWINLKQSIADRQNALEAEWRTVQASRRDLENFLKWIQ
EAETTVNVLVDASHRENALQDSILARELKQQMQDIQAEIDAHNDIFKSIDGNRQKMVKAL
GNSEEATMLQHRLDDMNQRWNDLKAKSASIRAHLEASAEKWNRLLMSLEELIKWLNMKDE
ELKKQMPIGGDVPALQLQYDHCKALRRELKEKEYSVLNAVDQARVFLADQPIEAPEEPRR
NLQSKTELTPEERAQKIAKAMRKQSSEVKEKWESLNAVTSNWQKQVDKALEKLRDLQGAM
DDLDADMKEAESVRNGWKPVGDLLIDSLQDHIEKIMAFREEIAPINFKVKTVNDLSSQLS
PLDLHPSLKMSRQLDDLNMRWKLLQVSVDDRLKQLQEAHRDFGPSSQHFLSTSVQLPWQR
SISHNKVPYYINHQTQTTCWDHPKMTELFQSLADLNNVRFSAYRTAIKIRRLQKALCLDL
LELSTTNEIFKQHKLNQNDQLLSVPDVINCLTTTYDGLEQMHKDLVNVPLCVDMCLNWLL
NVYDTGRTGKIRVQSLKIGLMSLSKGLLEEKYRYLFKEVAGPTEMCDQRQLGLLLHDAIQ
IPRQLGEVAAFGGSNIEPSVRSCFQQNNNKPEISVKEFIDWMHLEPQSMVWLPVLHRVAA
AETAKHQAKCNICKECPIVGFRYRSLKHFNYDVCQSCFFSGRTAKGHKLHYPMVEYCIPT
TSGEDVRDFTKVLKNKFRSKKYFAKHPRLGYLPVQTVLEGDNLETPITLISMWPEHYDPS
QSPQLFHDDTHSRIEQYATRLAQMERTNGSFLTDSSSTTGSVEDEHALIQQYCQTLGGES
PVSQPQSPAQILKSVEREERGELERIIADLEEEQRNLQVEYEQLKDQHLRRGLPVGSPPE
SIISPHHTSEDSELIAEAKLLRQHKGRLEARMQILEDHNKQLESQLHRLRQLLEQPESDS
RINGVSPWASPQHSALSYSLDPDASGPQFHQAAGEDLLAPPHDTSTDLTEVMEQIHSTFP
SCCPNVPSRPQAM
>P13830
MRPFHAYSWIFSQQYMGTKNVKEKNPTIYSFDDEEKRNENKSFLKVLCSKRGVLPIIGIL
YIILNGNLGYNGSSSSGVQFTDRCSRNLYGETLPVNPYADSENPIVVSQVFGLPFEKPTF
TLESPPDIDHTNILGFNEKFMTDVNRYRYSNNYEAIPHISEFNPLIVDKVLFDYNEKVDN
LGRSGGDIIKKMQTLWDEIMDINKRKYDSLKEKLQKTYSQYKVQYDMPKEAYESKWTQCI
KLIDQGGENLEERLNSQFKNWYRQKYLNLEEYRRLTVLNQIAWKALSNQIQYSCRKIMNS

DISSFKHINELKSLEHRAAKAAEAEMKKRAQKPKKKKSRRGWLCCGGGDIETVEPQQEEP
VQTVQEQQVNEYGDILPSLRASITNSAINYYDTVKDGVYLDHETSDALYTDEDLLFDLEK
QKYMDMLDTSEEESVEENEEEHTVDDEHVEEHTADDEHVEEPTVADDEHVEEPTVADEHV
EEPTVAEEHVEEPTVAEEHVEEPASDVQQTSEAAPTIEIPDTLYYDILGVGVNADMNEIT
ERYFKLAENYYPYQRSGSTVFHNFRKVNEAYQVLGDIDKKRWYNKYGYDGIKQVNFMNPS
IFYLLSSLEKFKDFTGTPQIVTLLRFFFEKRLSMNDLENKSEHLLKFMEQYQKEREAHVS
EYLLNILQPCIAGDSKWNVPIITKLEGLKGSRFDIPILESLRWIFKHVAKTHLKKSSKSA
KKLQQRTQANKQELANINNNLMSTLKEYLGSSEQMNSITYNFENINSNVDNGNQSKNISD
LSYTDQKEILEKIVSYIVDISLYDIENTALNAAEQLLSDNSVDEKTLKKRAQSLKKLSSI
MERYAGGKRNDKKSKNFDTKDIVGYIMHGISTINTEMKNQNENVPEHVQHNAEENVEHDA
EENVEHDAEENVEHDAEENVEHDAEENVEHDAEENVEENVEEVEENVEENVEENVEENVE
EVEENVEENVEENVEENVEENVEENVEENVEENVEENVEEYDEENVEEVEENVEENVEEN
VEENVEENVEEVEENVEENVEENVEENVEENVEENVEEYDEENVEEHNEEYDE
>Q14126
MARSPGRAYALLLLLICFNVGSGLHLQVLSTRNENKLLPKHPHLVRQKRAWITAPVALRE
GEDLSKKNPIAKIHSDLAEERGLKITYKYTGKGITEPPFGIFVFNKDTGELNVTSILDRE
ETPFFLLTGYALDARGNNVEKPLELRIKVLDINDNEPVFTQDVFVGSVEELSAAHTLVMK
INATDADEPNTLNSKISYRIVSLEPAYPPVFYLNKDTGEIYTTSVTLDREEHSSYTLTVE
ARDGNGEVTDKPVKQAQVQIRILDVNDNIPVVENKVLEGMVEENQVNVEVTRIKVFDADE
IGSDNWLANFTFASGNEGGYFHIETDAQTNEGIVTLIKEVDYEEMKNLDFSVIVANKAAF
HKSIRSKYKPTPIPIKVKVKNVKEGIHFKSSVISIYVSESMDRSSKGQIIGNFQAFDEDT
GLPAHARYVKLEDRDNWISVDSVTSEIKLAKLPDFESRYVQNGTYTVKIVAISEDYPRKT
ITGTVLINVEDINDNCPTLIEPVQTICHDAEYVNVTAEDLDGHPNSGPFSFSVIDKPPGM
AEKWKIARQESTSVLLQQSEKKLGRSEIQFLISDNQGFSCPEKQVLTLTVCECLHGSGCR
EAQHDSYVGLGPAAIALMILAFLLLLLVPLLLLMCHCGKGAKGFTPIPGTIEMLHPWNNE
GAPPEDKVVPSFLPVDQGGSLVGRNGVGGMAKEATMKGSSSASIVKGQHEMSEMDGRWEE
HRSLLSGRATQFTGATGAIMTTETTKTARATGASRDMAGAQAAAVALNEEFLRNYFTDKA
ASYTEEDENHTAKDCLLVYSQEETESLNASIGCCSFIEGELDDRFLDDLGLKFKTLAEVC
LGQKIDINKEIEQRQKPATETSMNTASHSLCEQTMVNSENTYSSGSSFPVPKSLQEANAE
KVTQEIVTERSVSSRQAQKVATPLPDPMASRNVIATETSYVTGSTMPPTTVILGPSQPQS
LIVTERVYAPASTLVDQPYANEGTVVVTERVIQPHGGGSNPLEGTQHLQDVPYVMVRERE
SFLAPSSGVQPTLAMPNIAVGQNVTVTERVLAPASTLQSSYQIPTENSMTARNTTVSGAG
VPGPLPDFGLEESGHSNSTITTSSTRVTKHSTVQHSYS
>P38731
MESVHSRDPVKEEKKHVFMGMEHELNPETHNDSNSDSYGLPQLSEKYNALRQNRSLIIQQ
TEIIGSAYNKWYLQAILLLSAFICGYGYGLDGNIRYIYTGYATSSYSEHSLLSTINVINA
VVSAASQIIYARLSDVFGRLYLFISAVILYVVGTIIQSQAYDVQRYAAGAIFYNAGYVGV
ILILLIILSDFSSLKWRLLYQFVPTWPFIINTWIAGNITSRANPVVNWSWDVGMWAFIFP
LSCVPIVLCMLHMQWRARKTPEWHALKGQKSYYQEHGFIKILKQLFWMLDVVGVLLMGCS
LGCILVPLTLAGGVKTTWNDSRLIGPFVLGFVLIPILWIWEYRFARDPILPYRLVKDRAV
WSSMGISFLIDFIYYMAADYLYTVMIVAVNESVKSATRIATLSSFVSTVASPFFALLVTR
CTRLKPFIMFGCALWMVAMGLLYHFRGGSQSHSGIIGALCVWGVGTTLFTYPVTVSVQSA
VSHENMATVTALNYTLYRIGSAVGSAVSGAIWTQTLYKQILKRMGDVALATTAYESPYTF
IETYTWGTPQRNALMNAYKYVQRLETIVALVFCVPLIAFSLCLRDPKLTDTVAVEYIEDG

EYVDTKDNDPILDWFEKLPSKFTFKRE

>O88495

MGPTKAVPTPFGCIGCKLPKPDYPPALIIFMFCAMVITVVVDLIGNSMVILAVTKNKKLR
NSGNIFVASLSVADMLVAIYPYPLMLYAMSVGGWDLSQLQCQMVGLVTGLSVVGSIFNIT
AIAINRYCYICHSLQYKRIFSLRNTCIYLVVTWVMTVLAVLPNMYIGTIEYDPRTYTCIF
NYVNNPAFTVTIVCIHFVLPLIIVGYCYTKIWIKVLAARDPAGQNPDNQFAEVRNFLTMF
VIFLLFAVCWCPVNVLTVLVAVIPKEMAGKIPNWLYLAAYCIAYFNSCLNAIIYGILNES
FRREYWTIFHAMRHPILFISHLISDIRETWETRALTRARVRARDQVREQERARACVAVEG
TPRNVRNVLLPGDASAPHSDRASVRPKPQTRSTSVYRKPASIHHKSISGHPKSASVYPKP
ASSVHCKPASVHFKPASVHFKGDSVYFKGDTVHYRAASKLVTSHRISAGPSTSHPTSMAG
YIKSGTSHPATTTVDYLEPATTSHSVLTAVDLPEVSASHCLEMTSTGHLRADISASVLPS
VPFELAATPPDTTAIPIASGDYRKVVLIDDDSDDSDCSDEMAV

>Q8N6Q3

MSAVLLLALLGFILPLPGVQALLCQFGTVQHVWKVSDLPRQWTPKNTSCDSGLGCQDTLM
LIESGPQVSLVLSKGCTEAKDQEPRVTEHRMGPGLSLISYTFVCRQEDFCNNLVNSLPLW
APQPPADPGSLRCPVCLSMEGCLEGTTEEICPKGTTHCYDGLLRLRGGGIFSNLRVQGCM
PQPGCNLLNGTQEIGPVGMTENCNRKDFLTCHRGTTIMTHGNLAQEPTDWTTSNTEMCEV
GQVCQETLLLLDVGLTSTLVGTKGCSTVGAQNSQKTTIHSAPPGVLVASYTHFCSSDLCN
SASSSSVLLNSLPPQAAPVPGDRQCPTCVQPLGTCSSGSPRMTCPRGATHCYDGYIHLSG
GGLSTKMSIQGCVAQPSSFLLNHTRQIGIFSAREKRDVQPPASQHEGGGAEGLESLTWGV
GLALAPALWWGVVCPSC

>Q9Y653

MTPQSLLQTTLFLLSLLFLVQGAHGRGHREDFRFCSQRNQTHRSSLHYKPTPDLRISIEN
SEEALTVHAPFPAAHPASRSFPDPRGLYHFCLYWNRHAGRLHLLYGKRDFLLSDKASSLL
CFQHQEESLAQGPPLLATSVTSWWSPQNISLPSAASFTFSFHSPPHTAAHNASVDMCELK
RDLQLLSQFLKHPQKASRRPSAAPASQQLQSLESKLTSVRFMGDMVSFEEDRINATVWKL
QPTAGLQDLHIHSRQEEEQSEIMEYSVLLPRTLFQRTKGRSGEAEKRLLLVDFSSQALFQ
DKNSSQVLGEKVLGIVVQNTKVANLTEPVVLTFQHQLQPKNVTLQCVFWVEDPTLSSPGH
WSSAGCETVRRETQTSCFCNHLTYFAVLMVSSVEVDAVHKHYLSLLSYVGCVVSALACLV
TIAAYLCSRVPLPCRRKPRDYTIKVHMNLLLAVFLLDTSFLLSEPVALTGSEAGCRASAI
FLHFSLLTCLSWMGLEGYNLYRLVVEVFGTYVPGYLLKLSAMGWGFPIFLVTLVALVDVD
NYGPIILAVHRTPEGVIYPSMCWIRDSLVSYITNLGLFSLVFLFNMAMLATMVVQILRLR
PHTQKWSHVLTLLGLSLVLGLPWALIFFSFASGTFQLVVLYLFSIITSFQGFLIFIWYWS
MRLQARGGPSPLKSNSDSARLPISSGSTSSSRI

>Q8IS44

MLLLENFNDYFPNYNGSTVSGTSTIAPGVAITGSRGSGLLLEQNLTGLYLDGYRLNCTNE
TLNLTDSCGELRVVDHNYWALILILFPILTLFGNILVILSVCRERSLQTVTNYFIVSLAI
ADLLVAVVVMPFAVYFLVNGAWALPDVVCDFYIAMDVICSTSSIFNLVAISIDRYIAVTQ
PIKYAKHKNSRRVCLTILLVWAISAAIGSPIVLGLNNTPNREPDVCAFYNADFILYSSLS
SFYIPCIIMVFLYWNIFKALRSRARKQRAARKPHLSELTGGSVIENIAQTRRLAETALDS
SRHASRILPDEAATNTASGSNEEEDENAISPDIDDCHVIVNDKSTEFMLATVVEETGNVA
MKPLSFVRYGVQEAMTLARNDSTLSTTSKTSSRKDKKNSQASRFTIYKVHKASKKKREKS
SAKKERKATKTLAIVLGVFLFCWLPFFSCNIMDAMCAKFKKDCRPGLTAYMMTTWLGYIN
SFVNPVIYTIFNPEFRKAFKKIMHMG

>Q9UKQ2
MLQGLLPVSLLLSVAVSAIKELPGVKKYEVVYPIRLHPLHKREAKEPEQQEQFETELKYK
MTINGKIAVLYLKKNKNLLAPGYTETYYNSTGKEITTSPQIMDDCYYQGHILNEKVSDAS
ISTCRGLRGYFSQGDQRYFIEPLSPIHRDGQEHALFKYNPDEKNYDSTCGMDGVLWAHDL
QQNIALPATKLVKLKDRKVQEHEKYIEYYLVLDNGEFKRYNENQDEIRKRVFEMANYVNM
LYKKLNTHVALVGMEIWTDKDKIKITPNASFTLENFSKWRGSVLSRRKRHDIAQLITATE
LAGTTVGLAFMSTMCSPYSVGVVQDHSDNLLRVAGTMAHEMGHNFGMFHDDYSCKCPSTI
CVMDKALSFYIPTDFSSCSRLSYDKFFEDKLSNCLFNAPLPTDIISTPICGNQLVEMGED
CDCGTSEECTNICCDAKTCKIKATFQCALGECCEKCQFKKAGMVCRPAKDECDLPEMCNG
KSGNCPDDRFQVNGFPCHHGKGHCLMGTCPTLQEQCTELWGPGTEVADKSCYNRNEGGSK
YGYCRRVDDTLIPCKANDTMCGKLFCQGGSDNLPWKGRIVTFLTCKTFDPEDTSQEIGMV
ANGTKCGDNKVCINAECVDIEKAYKSTNCSSKCKGHAVCDHELQCQCEEGWIPPDCDDSS
VVFHFSIVVGVLFPMAVIFVVVAMVIRHQSSREKQKKDQRPLSTTGTRPHKQKRKPQMVK
AVQPQEMSQMKPHVYDLPVEGNEPPASFHKDTNALPPTVFKDNPMSTPKDSNPEA
>Q00731
MNFLLSWVHWTLALLLYLHHAKWSQAAPTTEGEQKSHEVIKFMDVYQRSYCRPIETLVDI
FQEYPDEIEYIFKPSCVPLMRCAGCCNDEALECVPTSESNITMQIMRIKPHQSQHIGEMS
FLQHSRCECRPKKDRTKPEKKSVRGKGKGQKRKRKKSRFKSWSVHCEPCSERRKHLFVQD
PQTCKCSCKNTDSRCKARQLELNERTCRCDKPRR
>Q86XR7
MGIGKSKINSCPLSLSWGKRHSVDTSPGYHESDSKKSEDLSLCNVAEHSNTTEGPTGKQE
GAQSVEEMFEEEAEEEVFLKFVILHAEDDTDEALRVQNLLQDDFGIKPGIIFAEMPCGRQ
HLQNLDDAVNGSAWTILLLTENFLRDTWCNFQFYTSLMNSVNRQHKYNSVIPMRPLNNPL
PRERTPFALQTINALEEESRGFPTQVERIFQESVYKTQQTIWKETRNMVQRQFIA
>P53217
MAKTIKVIRKKDPKKKNLSDPLAKQKLVWKIGHVLTLVFGLLFSITYFYHVLIFFKYRSW
KWLFLRVNKNYSFIQSKRWYMKLLSWSPQVMYRLSLIGVFMSESVTMQQNWVGLNPTWND
LLSSENFHTLLIACLWFFGGGKSFYKILPYMILSYLHLTKMNYELNANKEEKIPLTPKDR
KMLHLLAYSELLVILALTLDTILFKTGTSGFMLVIYVGIYWLRLNFSPYAQVAVLELLVK
FEKYVPKKYRDKWQVIKNFIYMKMKEHEKRTEEVARYA
>Q99519
MTGERPSTALPDRRWGPRILGFWGGCRVWVFAAIFLLLSLAASWSKAENDFGLVQPLVTM
EQLLWVSGRQIGSVDTFRIPLITATPRGTLLAFAEARKMSSSDEGAKFIALRRSMDQGST
WSPTAFIVNDGDVPDGLNLGAVVSDVETGVVFLFYSLCAHKAGCQVASTMLVWSKDDGVS
WSTPRNLSLDIGTEVFAPGPGSGIQKQREPRKGRLIVCGHGTLERDGVFCLLSDDHGASW
RYGSGVSGIPYGQPKQENDFNPDECQPYELPDGSVVINARNQNNYHCHCRIVLRSYDACD
TLRPRDVTFDPELVDPVVAAGAVVTSSGIVFFSNPAHPEFRVNLTLRWSFSNGTSWRKET
VQLWPGPSGYSSLATLEGSMDGEEQAPQLYVLYEKGRNHYTESISVAKISVYGTL
>O14495
MQNYKYDKAIVPESKNGGSPALNNNPRRSGSKRVLLICLDLFCLFMAGLPFLIIETSTIK
PYHRGFYCNDESIKYPLKTGETINDAVLCAVGIVIAILAIITGEFYRIYYLKKSRSTIQN
PYVAALYKQVGCFLFGCAISQSFTDIAKVSIGRLRPHFLSVCNPDFSQINCSEGYIQNYR
CRGDDSKVQEARKSFFSGHASFSMYTMLYLVLYLQARFTWRGARLLRPLLQFTLIMMAFY
TGLSRVSDHKHHPSDVLAGFAQGALVACCIVFFVSDLFKTKTTLSLPAPAIRKEILSPVD

IIDRNNHHNMM

>Q8BFU7

MGSNSTSSAESNCNATYLPFQYSLYATTYIFIFIPGLLANSAALWVLCRFISKKNKAIIF
MINLSVADLAHILSLPLRIYYYINRHWPFQRALCLLCFYLKYLNMYASIFFLTCISLQRC
LFLLKPFRARNWKRRYDVGISAVIWIVVGTACLPFPILRNAGLANSTDSCFADLGYKQMD
AVVLVTMVVIAELAGFVIPVITIACCTWKTTVSLKHPPIAFQGISERKKALRMVFMCAAV
FVICFTPYHINFIFYTMVKESIITSCPTVKSTLYFHPFSLCLASLCCLLDPILYYFMASE
FRDQLSRHGSSVTRSRLMSRESGSSMVN

>Q9WVT0

MKSSRTVTLYFVLIVICSSEATWSRPAEPIVHPLILQEHELAGEELLRPKRAVAVGGPVA
EEYTVDVEISFENVSFLESIRAHLNSLRFPVQGNGTDILSMAMTTVCTPTGNDLLCFCEK
GYQWPEERCLSSLTCQEHDSALPGRYCNCLKGLPPQGPFCQLPETYITLKIKVRLNIGFQ
EDLENTSSALYRSYKTDLERAFRAGYRTLPGFRSVTVTQFTKGSVVVDYIVEVASAPLPG
SIHKANEQVIQNLNQTYKMDYNSFQGTPSNETKFTVTPEFIFEGDNVTLECESEFVSSNT
SWFYGEKRSDIQNSDKFSIHTSIINNISLVTRLTIFNFTQHDAGLYGCNVTLDIFEYGTV
RKLDVTPIRILAKEERKVVCDNNPISLNCCSENIANWSRIEWKQEGKINIEGTPETDLES
SCSTYTLKADGTQCPSGSSGTTVIYTCEFVSVYGAKGSKNIAVTFTSVANLTITPDPISV
SEGQSFSITCLSDVSSFDEVYWNTSAGIKIHPRFYTMRRYRDGAESVLTVKTSTREWNGT
YHCIFRYKNSYSIATKDVTVHPLPLESDIMMDPLEASGLCTSSHQFKCCIEENDGEEYIV
TFHVDSSSFPAEREVIGKQACYTYSLPGKLPSRCPKDIDVFCHFTNAANSSVRSPSMKLT
LVPGKNITCQDPIIGIGEPGKVIQKLCQFAGVSRSPGQTIGGTVTYKCVGSQWKEETRAC
ISAPINGLLQLAKALIKSPSQDQKLPKYLRDLSVSTGKEEQDIRSSPGSLGAIISILDLL
STVPTQVNSEMMRDILATINVILDKSTLNSWEKLLQQQSNQSSQFLQSVERFSKALELGD
STPPFLFHPNVQMKSMVIKRGHAQMYQQKFVFTDSDLWGDVAIDECQLGSLQPDSSIVTV
AFPTLKAILAQDGQRKTPSNSLVMTTTVSHNIVKPFRISMTFKNNHRSGGKPQCVFWNFS
LANNTGGWDSSGCTVEDDGRDNRDRVFCKCNHLTSFSILMSPDSPDPGSLLKILLDIISY
IGLGFSIVSLAACLVVEAMVWKSVTKNRTSYMRHICIVNIALCLLIADIWFIVAGAIHDG
HYPLNETACVAATFFIHFFYLSVFFWMLTLGLMLFYRLIFILHDASKSTQKAIAFSLGYG
CPLIISSITVGVTQPQEVYMRKNACWLNWEDTRALLAFAIPALIIVVVNVSITVVVITKI
LRPSVGDKPGKQEKSSLFQISKSIGVLTPLLGLTWGFGLATVIQGSNAVFHIIFTLLNAF
QGLFILLFGCLWDQKVQEALLHKFSLSRWSSQHSKSTSLGSSTPVFSMSSPISRRFNNLF
GKTGTYNVSTPETTSSSVENSSSAYSLLN

>Q08760

MKEELSKVSSMQNFEMIQRERLPTLYEVLIQRTSQPVDLWTFYTFLSQFPYAINYLDFWV
DLMTHTRLCKNYIELVRKSLINFPQEQQQNGSTSTATFDLLNALIEEGHLDPEAPDKLLE
NSGPDVPFSPKLNELLGDWKHQSGIGQEALRNEDVALIVDEIMKRRSQQDGKPQITTKQL
LHSAVGLCNTYLVSPEQSERYLSNIPMETRNRIIESVQIERKYDIEIFDDLKNLTYQFLE
MDCFPKFLSRVALHNIHDEISDWRFHSVGVTNEKSNRSRGQTHISRSPFSNHTSISRIGF
GLLWLGIGFWIGYVLIFLAYSRAIRVVTVVPFTLGCYCIVCGMYQVDIVYSWFGVTQRLL
HRHKNAGNDEGDASSDTDHVPMILAVFGGRRRLTRIEHPFTRQLLRKRGLWCLLLVVGAT
AAFTVIFSCVPGRRV

>Q63803

MGMLNCLHGNNMSGQHDIPPEVGDQPEQEPLEAQGAAAPGAGVGPAEEMETEPSNNEPIP
DETDSEVCGPPEDSKSDIQSPSQAFEEVQVGGDYSPPPEEAMPFEIQQPSLGDFWPTLEQ

PGPSGTPSGIKAFNPAILEPGTPTGAHPGLGAYSPPPEEAMPFEFNEPAQEDRCQPPLQV
PDLAPGGPEAWVSRALPAEPGNLGFENTGFREDYSPPPEESVPFQLDGEEFGGDSPPPGL
PRVTPQIGIGGEFPTVAVPSTLCLAPAANAPPLWVQGAIGRPFREAVRSPNFAYDISPME
ITRPLLEIGRASTGVDDDTAVNMDSPPIASDGPPIEVSGAPVKSEHAKRPPLERQAAETG
NSPISSTTAEEAKVPSLERGEGSPTQPETVHIKPAPVAESGTDSSKADPDSATHAVLQIG
PEEVGGVPTMPTDLPPASEDAGPDVRAEPDGGTAPATPAESEDNREPAAAAAAEPAAEPA
AEPAAEPAAEPAAEPAAEAVPDTEAESASGAVPDTQEEPAAAAASATPAEPAARAAPVTP
TEPATRAVPSARAHPAAGAVPGASAMSAAARAAAARAAYAGPLVWGARSLSATPAARASL
PARAAAAARAASAARAVAAGRSASAAPSRAHLRPPSPEIQVADPPTPRPAPRPSAWPDKY
ERGRSCCRYEAASGICEIESSSDESEEGATGCFQWLLRRNRRPGQPRSHTVGSNPVRNFF
ARAFGSCFGLSECTRSRSLSPGKAKDPMEERRKQMRKEAMEMREQKRADKKRSKLIDKQL
EEEKMDYMCTHRLLLLGAGESGKSTIVKQMRILHVNGFNGEGGEEDPQAARSNSDGEKAT
KVQDIKNNLKEAIETIVAAMSNLVPPVELANPENQFRVDYILSVMNVPNFDFPPEFYEHA
KALWEDEGVRACYERSNEYQLIDCAQYFLDKIDVIKQADYVPSDQDLPRCRVLTSGIFET
KFQVDKVNFHMFDVGGQRDERRKWIQCFNDVTAIIFVVASSSYNMVIREDNQTNRLQEAL
NLFKSIWNNRWLRTISVILFLNKQDLLAEKVLAGKSKIEDYFPEFARYTTPEDATPEPGE
DPRVTRAKYFIRDEFLRISTASGDGRHYCYPHFTCAVDTENIRRVFNDCRDIIQRMHLRQ
YELL
>Q7L804
MMLSEQAQKWFPTHVQVTVLQAKDLKPKGKSGTNDTYTIIQLGKEKYSTSVAEKTLEPVW
KEEASFELPGLLIQGSPEKYILFLIVMHRSLVGLDKFLGQVAINLNDIFEDKQRRKTEWF
RLESKQGKRIKNRGEIKVNIQFMRNNMTASMFDLSMKDKTRSPFAKLKDKMKGRKNDGTF
SDTSSAIIPSTHMPDANSEFSSGEIQMKSKPKKPFLLGPQRLSSAHSMSDLSGSHMSSEK
LKAGTIGQTHLLGHQLDSFGTVPESGSLKSPHRRTLSFDTSKMNQPDSIVDEGELCFGRQ
NDPFTNVTASLPQKFATLPRKKNPFEESSETWDSSMNLFSKPIEIRKENKREKREKVSLF
ERVTGKKDSRRSDKLNNGGSDSPCDLKSPNAFSENRQDYFDYESTNPFTAKFRASNIMPS
SSFHMSPTSNEDLRKIPDSNPFDATAGYRSLTYEEVLQELVKHKELLRRKDTHIRELEDY
IDNLLVRVMEETPSILRVPYEPSRKAGKFSNS
>Q38890
MYGRDPWGGPLEINTADSATDDDRSRNLNDLDRAALSRPLDETQQSWLLGPTEQKKKKYV
DLGCIIVSRKIFVWTVGTLVAAALLAGFITLIVKTVPRHHPKTPPPDNYTIALHKALKFF
NAQKSGKLPKHNNVSWRGNSGLQDGKGETGSFYKDLVGGYYDAGDAIKFNFPMAYAMTML
SWSVIEYSAKYEAAGELTHVKELIKWGTDYFLKTFNSTADSIDDLVSQVGSGNTDDGNTD
PNDHYCWMRPEDMDYKRPVTTCNGGCSDLAAEMAAALASASIVFKDNKEYSKKLVHGAKV
VYQFGRTRRGRYSAGTAESSKFYNSSMYWDEFIWGGAWMYYATGNVTYLNLITQPTMAKH
AGAFWGGPYYGVFSWDNKLAGAQLLLSRLRLFLSPGYPYEEILRTFHNQTSIVMCSYLPI
FNKFNRTNGGLIELNHGAPQPLQYSVNAAFLATLYSDYLDAADTPGWYCGPNFYSTSVLR
DFARSQIDYILGKNPRKMSYVVGFGTKYPRHVHHRGASIPKNKVKYNCKGGWKWRDSKKP
NPNTIEGAMVAGPDKRDGYRDVRMNYNYTEPTLAGNAGLVAALVALSGEEEATGKIDKNT
IFSAVPPLFPTPPPPPAPWKP
>Q61543
MAVCGRVRGMFRLSAALPLLLLAAAGAQNGHGQGQGPGTNFGPFPGQGGGGSPAGQQPPQ
QPQLSQQQQQPPPQQQQQQQQQSLFAAGGLPARRGGAGPGGTGGGWKLAEEESCREDVTR
VCPKHTWSNNLAVLECLQDVREPENEISSDCNHLLWNYKLNLTTDPKFESVAREVCKSTI

SEIKECAEEPVGKGYMVSCLVDHRGNITEYQCHQYITKMTAIIFSDYRLICGFMDDCKND
INLLKCGSIRLGEKDAHSQGEVVSCLEKGLVKEAEEKEPKIQVSELCKKAILRVAELSSD
DFHLDRHLYFACRDDRERFCENTQAGEGRVYKCLFNHKFEESMSEKCREALTTRQKLIAQ
DYKVSYSLAKSCKSDLKKYRCNVENLPRSREARLSYLLMCLESAVHRGRQVSSECQGEML
DYRRMLMEDFSLSPEIILSCRGEIEHHCSGLHRKGRTLHCLMKVVRGEKGNLGMNCQQAL
QTLIQETDPGADYRIDRALNEACESVIQTACKHIRSGDPMILSCLMEHLYTEKMVEDCEH
RLLELQYFISRDWKLDPVLYRKCQGDASRLCHTHGWNETSELMPPGAVFSCLYRHAYRTE
EQGRRLSRECRAEVQRILHQRAMDVKLDPALQDKCLIDLGKWCSEKTETGQELECLQDHL
DDLAVECRDIVGNLTELESEDIQIEALLMRACEPIIQNFCHDVADNQIDSGDLMECLIQN
KHQKDMNEKCAIGVTHFQLVQMKDFRFSYKFKMACKEDVLKLCPNIKKKVDVVICLSTTV
RNDTLQEAKEHRVSLKCRKQLRVEELEMTEDIRLEPDLYEACKSDIKNYCSTVQYGNAQI
IECLKENKKQLSTRCHQKVFKLQETEMMDPELDYTLMRVCKQMIKRFCPEADSKTMLQCL
KQNKNSELMDPKCKQMITKRQITQNTDYRLNPVLRKACKADIPKFCHGILTKAKDDSELE
GQVISCLKLRYADQRLSSDCEDQIRIIIQESALDYRLDPQLQLHCSDEIANLCAEEAAAQ
EQTGQVEECLKVNLLKIKTELCKKEVLNMLKESKADIFVDPVLHTACALDIKHHCAAITP
GRGRQMSCLMEALEDKRVRLQPECKKRLNDRIEMWSYAAKVAPADGFSDLAMQVMTSPSK
NYILSVISGSICILFLIGLMCGRITKRVTRELKDR
>Q9NPD5
MDQHQHLNKTAESASSEKKKTRRCNGFKMFLAALSFSYIAKALGGIIMKISITQIERRFD
ISSSLAGLIDGSFEIGNLLVIVFVSYFGSKLHRPKLIGIGCLLMGTGSILTSLPHFFMGY
YRYSKETHINPSENSTSSLSTCLINQTLSFNGTSPEIVEKDCVKESGSHMWIYVFMGNML
RGIGETPIVPLGISYIDDFAKEGHSSLYLGSLNAIGMIGPVIGFALGSLFAKMYVDIGYV
DLSTIRITPKDSRWVGAWWLGFLVSGLFSIISSIPFFFLPKNPNKPQKERKISLSLHVLK
TNDDRNQTANLTNQGKNVTKNVTGFFQSLKSILTNPLYVIFLLLTLLQVSSFIGSFTYVF
KYMEQQYGQSASHANFLLGIITIPTVATGMFLGGFIIKKFKLSLVGIAKFSFLTSMISFL
FQLLYFPLICESKSVAGLTLTYDGNNSVASHVDVPLSYCNSECNCDESQWEPVCGNNGIT
YLSPCLAGCKSSSGIKKHTVFYNCSCVEVTGLQNRNYSAHLGECPRDNTCTRKFFIYVAI
QVINSLFSATGGTTFILLTVKIVQPELKALAMGFQSMVIRTLGGILAPIYFGALIDKTCM
KWSTNSCGAQGACRIYNSVFFGRVYLGLSIALRFPALVLYIVFIFAMKKKFQGKDTKASD
NERKVMDEANLEFLNNGEHFVPSAGTDSKTCNLDMQDNAAAN
>P51802
MEEIVGLREGSPRKPVPLQELWRPCPRIRRNIQGSLEWLKERLFRVGEDWYFLVALGVLM
ALISYAMNFAIGRVVRAHKWLYREIGDGHLLRYLSWTVYPVALLSFSSGFSQSITPSSGG
SGIPEVKTILTGVILEDYLDIKNFGAKVVGLSCTLATGSTIFLGKLGPFVHLSVMIAAYL
GRVRTKTVGEPENKTKEMELLAAGAAVGVATVFAAPISGVLFSIEVMSSHFSVWDYWRGF
FAATCGAFMFHLLAVFNSEQETITSIYKTSFPVDIPFDLPEIFFFVALGAICGILSCGYN
YCQRTSLFFLKSNGFTSKLLATSKPLYSALAAVVLASITYPPGVGRFMASRLSMSEYLET
LFDNNSWALMTKNSSPPWSAEPDPQNLWLEWCHPQMTVFGTLVFFLVMKFWMLILATTIP
IPAGYFLPIFVYGAAIGRLFGEVLSLAFPEGIVAGGKVSPIMPGAYALAGAAAFSGAVTH
TLSTALLAFEVSGQIVHALPVLMAVLAANAICQSYQPSFYDGTIIVKKLPYLPWIRGRKI
GSHLVTVGHFMNCTLTTLAKDMPLEEVIKVVISTDVTQYPLVETTESQVLVGIVKRTHLV
QSLHTDSASWAPGQQPCLQDILANGCPTQPVTLQLSTETSLHETHNLFELLNLQLLFVTS
RGRAVGSVSWVELKKAISTLTNPPAPK
>Q9NS93

MGFLQLLVVAVLASEHRVAGAAEVFGNSSEGLIEFSVGKFRYFELNRPFPEEAILHDISS
NVTFLIFQIHSQYQNTTVSFSPTLLSNSSETGTASGLVFILRPEQSTCTWYLGTSGIQPV
QNMAILLSYSERDPVPGGCNLEFDLDIDPNIYLEYNFFETTIKFAPANLGYARGVDPPPC
DAGTDQDSRWRLQYDVYQYFLPENDLTEEMLLKHLQRMVSVPQVKASALKVVTLTANDKT
SVSFSSLPGQGVIYNVIVWDPFLNTSAAYIPAHTYACSFEAGEGSCASLGRVSSKVFFTL
FALLGFFICFFGHRFWKTELFFIGFIIMGFFFYILITRLTPIKYDVNLILTAVTGSVGGM
FLVAVWWRFGILSICMLCVGLVLGFLISSVTFFTPLGNLKIFHDDGVFWVTFSCIAILIP
VVFMGCLRILNILTCGVIGSYSVVLAIDSYWSTSLSYITLNVLKRALNKDFHRAFTNVPF
QTNDFIILAVWGMLAVSGITLQIRRERGRPFFPPHPYKLWKQERERRVTNILDPSYHIPP
LRERLYGRLTQIKGLFQKEQPAGERTPLLL
>P54774
MSQQGESSDPKSGKKDFSTAILERKKSPNRLVVDEAVNDDNSVVTMHPQTMEKLQLFRGD
TILIKGKKRKDTICIALADENCEEPKIRMNKVVRSNLRVRLGDVVSVHQCPDVKYGKRVH
ILPIDDTIEGVTGNLFDAFLKPYFLEAYRPVRKGDLFLVRGGMRSVEFKVVETDPGEYCV
VAPDTEIFCEGEPLKREDEERLDEVGYDDVGGVRKQMAQIRELVELPLRHPQLFKSIGVK
PPKGILLYGPPGSGKTLIARAVANETGAFFFCINGPEIMSKLAGESESNLRKAFEEAEKN
APSIIFIDEIDSIAPKREKTHGEVERRIVSQLLTLMDGLKSRAHVIVIGATNRPNSIDPA
LRRFGRFDREIDIGVPDEVGRLEVLRIHTKNMKLSDDVDLERIAKDTHGYVGADLAALCT
EAALQCIREKMDVIDLEDETIDAEVLNSMAVTNEHFQTALGTSNPSALRETVVEVPNVSW
EDIGGLENVKRELQETVQYPVEHPEKFEKFGMSPSKGVLFYGPPGCGKTLLAKAIANECQ
ANFISVKGPELLTMWFGESEANVREIFDKARQSAPCVLFFDELDSIATQRGSSVGDAGGA
ADRVLNQLLTEMDGMSAKKTVFIIGATNRPDIIDPALLRPGRLDQLIYIPLPDEDSRHQI
FKACLRKSPIAKNVDLRALARHTQGFSGADITEICQRACKYAIRENIEKDIERERKSREN
PEAMDEDTVDDEVAEIKAAHFEESMKFARRSVSDADIRKYQAFAQTLQQSRGFGSEFRFP
ESGDRTTTGSDPFAASAGGADEDDLYS
>Q401N2
MMALWSLLHLTFLGFSITLLLVHGQGFQGTAAIWPSLFNVNLSKKVQESIQIPNNGSAPL
LVDVRVFVSNVFNVDILRYTMSSMLLLRLSWLDTRLAWNTSAHPRHAITLPWESLWTPRL
TILEALWVDWRDQSPQARVDQDGHVKLNLALATETNCNFELLHFPRDHSNCSLSFYALSN
TAMELEFQAHVVNEIVSVKREYVVYDLKTQVPPQQLVPCFQVTLRLKNTALKSIIALLVP
AEALLLADVCGGLLPLRAIERIGYKVTLLLSYLVLHSSLVQALPSSSSCNPLLIYYFTIL
LLLLFLSTIETVLLAGLLARGNLGAKSGPSPAPRGEQREHGNPGPHPAEEPSRGVKGSQR
SWPETADRIFFLVYVVGVLCTQFVFAGIWMWAACKSDAAPGEAAPHGRRPRL
>Q9R1N9
MVAERTRKAAASGSRGPGELGAPGPGTVALAEQCARLPSPGCCGLLALALCSLALSLLAH
FRTAELQARVLRLEAERGEQQMEKAILGRVNQLLDEKWKFYSRRRREAPKMSPGCNCPPG
PPGPTGRPGLPGDKGAIGMPGRVGIKGQPGEKGAPGDAGMSIVGPRGPPGQPGTRGFPGF
PGPIGLDGRPGHPGPKGEMGLVGPRGQPGPQGQKGEKGQCGEYPHREYPGGMLAALRSNP
IMSLKLLPLLNSVRLAPPPVIKRRTFQGEQSQTGIQGPPGPPGPPGPSGPLGHPGLPGPI
GPPGLPGPPGPKGDPGIQGYHGRKGERGMPGMPGKHGAKGVPGIAVAGMKGEPGTPGTKG
EKGAAGSPGLLGQKGEKGDAGNAIGGGRGEPGPPGLPGPPGPKGEAGVDGQAGPPGQQGD
KGQPGAAGEQGPSGPKGAKGEPGKGEMVDYNGSINEALQEIRTLALMGPPGLPGQTGPPG
PPGTPGQRGEIGLPGPPGHDGDKGPRGKPGDMGPAGPQGPPGKDGPPGMKGEVGPPGSPG
EKGETGQAGPQGLDGPTGEKGEPGDEGRPGATGLPGPIGLPGFTGEKGEAGEKGDPGAEV

PGPPGPEGPPGPPGLQGFPGPKGEAGLEGSKGEKGSQGEKGDRGPLGLPGASGLDGRPGP
PGTPGPIGVPGPAGPKGERGSKGDPGMTGPTGAAGLPGLHGPPGDKGNRGERGKKGSRGP
KGDKGDQGAPGLDAPCPLGEDGLPVQGCWNK
>Q9Z0M6
MRGVRCPGLLVVCILLSLSGAGTQKAESKNCAKWCPINSKCVSNRSCVCKPGFSSEKELI
TNPAESCEDINECLLPGFSCGDFAMCKNSEGSYTCVCNLGYKLLSGAESFVNESENTCQA
SVNTGTTPVPSRIHTVTTAPGNLPEQTTTVHQTQMGDSEERTPKDVNECISGQNHCHQST
HCINKLGGYSCICRQGWKPVPGSPNGPVSTVCEDVDECSSGQHQCHNSTVCKNTVGSYKC
HCRPGWKPTSGSLRGPDTICQEPPFPTWTLLPTAHSQTLLRFSVEVQNLLRDFNPATVNY
TIQKLIEAVDKLLEDPMETQTQQVAAQLLSNLEQSLRTLAQFLPKGPFTYTSPSNTELSL
MVKEQDNKDVTTVHHGQTWMELDWAVTAGAKISENGSSVAGILSSPNMEKLLGNTPLNLE
QRRASLEDFYGSPIPSVSLKLLSNINSVFLTNTNTEKLASNVTFKFDFTSVESIEPRHEL
ICAFWKAHNGNGYWDTDGCSMNGTGFCHCNHLTSFAILMAQYHVQDPRLELITKVGLLLS
LICLLLCILTFLLVKPIQSSRTMVHLHLCICLFLGSIIFLVGVENEGGEVGLRCRLVAMM
LHFCFLAAFCWMALEGVELYFLVVRVFQGQGLSTWQRCLIGYGVPLLIVAISMAVVKMDG
YGHATYCWLDFRKQGFLWSFSGPVAFIIFCNAAIFVITVWKLTKKFSEINPNMKKLRKAR
VLTITAIAQLLVLGCTWGFGLFLFNPHSTWLSYIFTLLNCLQGLFLYVMLCLLNKKVREE
YWKWACMVTGSKYTEFNSSTTGTGTSQTRALRSSESGM
>Q9FMF5
MWESESDGGVGVGGGGGREYGDGVLSSNKHGGVKTDGFELRGQSWFVATDIPSDLLVKIG
DMNFHLHKYPLLSRSGKMNRLIYESRDPDPTILILDDLPGGPEAFELASKFCYGVPVDLT
ATNISGLRCAAEYLEMTEDLEEGNLIFKTEAFLSYVVLSSWRDSILVLKSCEKLSPWAEN
LQIVRRCSESIAWKACSNPKGIRWAYTGKAPSPSTTNFAGSSPRWNESKDSSFYCSPSRN
TNSQPVPPDWWFEDVSILRIDHFVRVITAIKVKGMRFELLGAVIMHYAGKWLPGLIKEGG
VAIAPAMSSAIGGGLGLGGDEMSISCGSNSSGGSSGPDWKGGLHMVLSAGKTNGHQDSVA
CLAGLGISPKDQRMIVESLISIIPPQKDSVTCSFLLRLLRAANMLKVAPALITELEKRVG
MQFEQATLQDLLIPGYNNKGETMYDVDLVQRLLEHFLVQEQTEGSSPSRMSPSPSQSMYA
DIPRGNNNNGGGGGGNNQNAKMRVARLVDSYLTEVARDRNLPLTKFQVLAEALPESARTC
DDGLYRAIDSYLKAHPTLSEHERKRLCRVMDCQKLSMDACMHAAQNERLPLRVVVQVLFS
EQVKISNALANTSLKESTTLGEAMGTYQPMIPNRKTLIEATPQSFQARWAAAKKDINTLK
FELETVKTKYVELQNEMEVMQRQFEKTGKVKNTPSSSAWTSGWKKLSKLTKMSGQESHDI
SSGGEQAGVDHPPPRKPRRWRNSIS
>Q54QQ1
MDDQQPLQTYNQPPPPLPKHIQLQIQQRQLQLQQQEEQERLQQQQQQQQQQDQQQHKLE
EGSRRRSLSDVVGGVNISNKETPKRAFRIYWANDNHMYWTLLLDSTNTVGDVSEGLKDKV
PSSGTQCYLYKRTKANFGRWKDRRLSNEDSVWDIVDRRRKNHKSDPIFVLKRKKSTFSIS
QKKSPQNESDTSPLSSSGEFLNPYTNPTGSNNIVPSNAGHSRNTSSNSNNSANTSSNNLL
AHHQFNNNNNNNNNNNNNNNNNNNNNNNNNNNNNNNNNNNNNNSNNSSNSNSNNSSNTT
TTTTTTTTTTTSTSTNSINTSNNNNNNNSGSNINPSSFISPMSSLVSLSLSSSPASSAP
HSPTLSSQPSSPHTITPITTSPPPPQRNNSSKSLKSTMEMEPPAEYISLLSNNPPPTNGS
GSRPPSGSFIPGSIVNGSRSSASDLIGFKPPVCQQQQQQQQQQQQQQQQVQQQQQQPQQ
VQQLQQLQQLQQQYQQQQPQQQIQPLSPQQQSQQQQQSPVKQSESIPQSPNLQSITQSSS
SNKPLSGSTGMNRSSSPSPFAKKNIGGGGGGGSLADRKNRLSVQPKDIMRLMRFYFGDSL
DGTFFTIAVQNDTSAKDVCASVEQKLLLPSGSTFVSLVLPIQPNGSKIERVLGEDEIIIE

VKDTWEDPSQTFFQVTHKQQAPNNKRLSRQIQAEPHVIPSEWRRSTDAISLRASSDDMWK
RKSIPIFLQVTGPPNTHSHSHSPQRQLPNMSGSPHSHHHHQNNNNNNNNNNNNNNNNDLD
DDDSDDESDFDVSTLRGWVHWIPSADLEYIKRIGSGTYSKVYKGKYRDKFVAIKTMRGSN
MTTEQIESFKKECDILSTIQSPLLISFYGSCIEESQLSMVVEYCSKGTLHKVLNNPLLDF
DWDKWFKWMTEVVEGVRYLHSMNPPMVHRDLKTLNILISSDWTAKLCDFGLTRTMTMTNV
STLGMLRGTMAYTAPEIYDGLLFNTKSDVYSLGVIMWEAIQRCISGVYLRPFHEHPISMD
IQIIILTSKNKVRPKISENCPEQLKTLITRCWDENPDKRPNCDEILEILLTMKQAHDENK
DEWESIRTKLPKQTIKAVSSSSSNNNNNNNNNNNNNNNNNNNNNNNNNNNNNGTNTNSTVN
NVVVPSSSNATIVVAPSVSSNSSNINTSMDNKSMSSLSDSPVLPSVGIVSTSSLTGNSNI
NNNNNNIHDNNNPNNNNSNNSNNTSLDVPPVGVILTRDRRNSIIAVGEVPVIVKPPETPD
GTTMVTEVFDFEVPQDSPSLEPLSSNNIISTANILNIGYVNNENNSIIIEGNKSTLNEIG
NNNNNNNNNNNNNNNNNNSSNNNSINNNSSNNDENNCINNCINIESNESCIKSNTPEQIT
LKCVDER
>Q10951
MTEVECWDDDPDFASDVDNASLFTQSIATTSASTSVDDGFSFDDSISRLNSLSISDVPSA
HSRVEQWNQQVHNLNHELSSNKNRISSNVEGYEDDFSFDEEGEEGNNEFNTLRPNKYQDA
DVDEFNTIRASETASQRPPIPFSSDTLKKTYLSSENDARYPSVSDSPYCESEGFSSFEDD
FEIDPDTDLNSILHRKQNRMDPKASFSSVEQSSLRTPSSAHNDDGFWDDFDIDFNNETES
IFRKKIRSPNTINQKHPYISSTISYQPNVHQDAKYYPLCKDIFPSLANENPHSDNPNLKY
SSKTLSKRDTSSHYPETLKASSKHSSPVKGNSSISSTWTPSNLKIYHSKNSMGLMDLDAL
KTVASNSKYRTKPKNCKTYGDGTELDTLDELPVDYEFELKLRKKQTTKVSGTPKSKHAGS
TQEWHSHTTPRSTSKHENNLNNITNSAKNEHIRSQRQHKTHAAPSKELTKESLSNDQLSV
KEKRRHHKKAPTLIQNLNSPRTPKIVGKMRYNPTKHCWEGNDYAIRDFDTPISPSRPALI
SNISTKKGIQVVGNMVYDPTRLRWIDNSISGQEAEDPFSGFDDLEDTDSTSQYLNENSGS
FNGSINSIINFPDMSEIYDVGPEFEKKQFSEDIQWRKRIDGWFFSFKNDDRSRLWELYNI
LNAEQ
>P53379
MKLSVLTFVVDALLVCSSIVDAGVTDFPSLPSNEVYVKMNFQKKYGSSFENALDDTKGRT
RLMTRDDDYELVELTNQNSFYSVELDIGTPPQKVTVLVDTGSSDLWVTGSDNPYCSTKKK
DTTGSSFKQVNKDALASVVESVFTEISYDTTIVTSEATATFDSTASTSQLIDCATYGTFN
TSKSSTFNSNNTEFSIAYGDTTFASGTWGHDQLSLNDLNITGLSFAVANETNSTVGVLGI
GLPGLESTYSGVSLSSVQKSYTYNNFPMVLKNSGVIKSTAYSLFANDSDSKHGTILFGAV
DHGKYAGDLYTIPIINTLQHRGYKDPIQFQVTLQGLGTSKGDKEDNLTTLTTTKIPVLLD
SGTTISYMPTELVKMLADQVGATYSSAYGYYIMDCIKEMEEESSIIFDFGGFYLSNWLSD
FQLVTDSRSNICILGIAPQSDPTIILGDNFLANTYVVYDLDNMEISMAQANFSDDGEYIE
IIESAVPSALKAPGYSSTWSTYESIVSGGNMFSTAANSSISYFASTSHSATSSSSSKGQK
TQTSTTALSISKSTSSTSSTGMLSPTSSSSTRKENGGHNLNPPFFARFITAIFHHI
>P48974
MNSEPSWTATPSPGGTLPVPNATTPWLGRDEELAKVEIGILATVLVLATGGNLAVLLTLG
RHGHKRSRMHLFVLHLALTDLGVALFQVLPQLLWDITYRFQGSDLLCRAVKYLQVLSMFA
STYMLLAMTLDRYLAVCHPLRSLRQPSQSTYPLIAAPWLLAAILSLPQVFIFSLREVIQG
SGVLDCWADFYFSWGPRAYITWTTMAIFVLPVAVLSACYGLICHEIYKNLKVKTQAGREE
RRGWRTWDKSSSSAVATAATRGLPSRVSSISTISRAKIRTVKMTFVIVLAYIACWAPFFS
VQMWSVWDENAPNEDSTNVAFTISMLLGNLSSCCNPWIYMGFNSRLLPRSLSHHACCTGS

KPQVHRQLSTSSLTSRRTTLLTHACGSPTLRLSLNLSLRAKPRPAGSLKDLEQVDGEATM
ETSIF
>Q9BZZ2
MGFLPKLLLLASFFPAGQASWGVSSPQDVQGVKGSCLLIPCIFSFPADVEVPDGITAIWY
YDYSGQRQVVSHSADPKLVEARFRGRTEFMGNPEHRVCNLLLKDLQPEDSGSYNFRFEIS
EVNRWSDVKGTLVTVTEEPRVPTIASPVELLEGTEVDFNCSTPYVCLQEQVRLQWQGQDP
ARSVTFNSQKFEPTGVGHLETLHMAMSWQDHGRILRCQLSVANHRAQSEIHLQVKYAPKG
VKILLSPSGRNILPGELVTLTCQVNSSYPAVSSIKWLKDGVRLQTKTGVLHLPQAAWSDA
GVYTCQAENGVGSLVSPPISLHIFMAEVQVSPAGPILENQTVTLVCNTPNEAPSDLRYSW
YKNHVLLEDAHSHTLRLHLATRADTGFYFCEVQNVHGSERSGPVSVVVNHPPLTPVLTAF
LETQAGLVGILHCSVVSEPLATLVLSHGGHILASTSGDSDHSPRFSGTSGPNSLRLEIRD
LEETDSGEYKCSATNSLGNATSTLDFHANAARLLISPAAEVVEGQAVTLSCRSGLSPTPD
ARFSWYLNGALLHEGPGSSLLLPAASSTDAGSYHCRARDGHSASGPSSPAVLTVLYPPRQ
PTFTTRLDLDAAGAGAGRRGLLLCRVDSDPPARLQLLHKDRVVATSLPSGGGCSTCGGCS
PRMKVTKAPNLLRVEIHNPLLEEEGLYLCEASNALGNASTSATFNGQATVLAIAPSHTLQ
EGTEANLTCNVSREAAGSPANFSWFRNGVLWAQGPLETVTLLPVARTDAALYACRILTEA
GAQLSTPVLLSVLYPPDRPKLSALLDMGQGHMALFICTVDSRPLALLALFHGEHLLATSL
GPQVPSHGRFQAKAEANSLKLEVRELGLGDSGSYRCEATNVLGSSNTSLFFQVRGAWVQV
SPSPELQEGQAVVLSCQVHTGVPEGTSYRWYRDGQPLQESTSATLRFAAITLTQAGAYHC
QAQAPGSATTSLAAPISLHVSYAPRHVTLTTLMDTGPGRLGLLLCRVDSDPPAQLRLLHG
DRLVASTLQGVGGPEGSSPRLHVAVAPNTLRLEIHGAMLEDEGVYICEASNTLGQASASA
DFDAQAVNVQVWPGATVREGQLVNLTCLVWTTHPAQLTYTWYQDGQQRLDAHSIPLPNVT
VRDATSYRCGVGPPGRAPRLSRPITLDVLYAPRNLRLTYLLESHGGQLALVLCTVDSRPP
AQLALSHAGRLLASSTAASVPNTLRLELRGPQPRDEGFYSCSARSPLGQANTSLELRLEG
VRVILAPEAAVPEGAPITVTCADPAAHAPTLYTWYHNGRWLQEGPAASLSFLVATRAHAG
AYSCQAQDAQGTRSSRPAALQVLYAPQDAVLSSFRDSRARSMAVIQCTVDSEPPAELALS
HDGKVLATSSGVHSLASGTGHVQVARNALRLQVQDVPAGDDTYVCTAQNLLGSISTIGRL
QVEGARVVAEPGLDVPEGAALNLSCRLLGGPGPVGNSTFAWFWNDRRLHAEPVPTLAFTH
VARAQAGMYHCLAELPTGAAASAPVMLRVLYPPKTPTMMVFVEPEGGLRGILDCRVDSEP
LASLTLHLGSRLVASSQPQGAPAEPHIHVLASPNALRVDIEALRPSDQGEYICSASNVLG
SASTSTYFGVRALHRLHQFQQLLWVLGLLVGLLLLLLGLGACYTWRRRRVCKQSMGENSV
EMAFQKETTQLIDPDAATCETSTCAPPLG
>Q9US08
MGILSVIRIFIYCLIRLFSFNSRKRNSSIDELERGEINAFACDKQNTPSPNSECRPLTPL
NSPFRRTVEGDTANLQAPSPTACPSFDSASSIKSSKEDIACRKLSTERFFYSPNGIDEMK
KQQQFKKIPTVSITEVEPAFIPSNMEQALFSEEPFVIQDDLSHQINLDDNRKFSRIQKNK
LDPLITVNLVPIVNGENCFNFYETQMETNFLSPEFIKKPSMVDHKRDSEINAVTAVSADG
SFSPLTETLSSTISTLSNDSLDGLSSQKNEFTGFNSNSEWIPNDTVDYICNSDASSVVSN
LSDFEDCFDQFGVYDKEYEKKIEKTKRNFPKFQSSATYPPFSAHHQARRNNPQGFRNVKK
RFQLFKDDCTALTDSDFLDALPFFNARVIIALYVEWRLRVYETMIIKGEESLKNLQRARD
GPHNKLWLGVFGNKDTIKKLSNYDRRAISNPYVSNHLNFNVRRVKSDTVYIPTILQLLRS
STQLMTEQAFVASSNLKSTKGLRETRLIQKYKVKGDFEYVYAALYYAAFTQESTLKAVTI
DDILSEDELEDWWYLNLRYTSFITPQLCFEFLDKEADSCRDRLTEVKSEPPKAVIYEEPL
NKLSRFSSDRLFTSHELVAIAELISLNEPPLESGKKFYYEEFQKACQKKRDDYRYSPEIE

FKAAFREKFLQSNEKVPFPRPGEIYEKRCDYFSKYACQNIFISKPLKRSGSIYEVFSDGI
GRVIYGSVLDYEATRNNILLHFGFEIHCAPSEEELIEREEAFKDFHNMLSFKYSANEIYE
FCGTHSRAEVHKNEVLKRMAYYLIDENKEISILRRILSLRIVEPTTSFTRYEYDLWRTFY
PDVLYLNYSKSGRIPTGSPYIMNGQWRKDLCVSTPPMVDINSALFPHWFKLSASNLFAGA
EHAGLNRQKPDKIDLDLYEPLPENSYLVAAELRVLRALRHKNPENIPLLEKWEVRALHQL
MAKIRKYYTVPTDYLSLRMDGLQAMLRKREYMSCYTFNYFDYACLMDHAYRLYEGFNNQV
VNLPPRIM
>Q08236
MDTVTVLNELRAQFLRVCPEKDQMKRIIKPYIPVDEFNTEQCLDSSIRELYMNSDGVSLL
PELESPPVSKDFMENYASLGKMRIMRENEGQKGKANQNLIGAEKTERDEEETRNLQDKSA
KNTLIVEENGTLRYNPLNSSASNSLLNDDDHTSGKHHKTSSKEDSYLNSSMEMQKKSSKR
SSLPFVRIFKSRRDHSNTSGNKNVMNTTNTRAKSSTLHPPGARHNKKGSKFDMNFDFDEN
LEEEDDDDDDDEEGDDIHSQFFQLDDDFDAKGSGASPAHKGINGMSNNKNNTYTNNRNSI
SILDDRESSNGNIGSASRLKSHFPTSQKGKIFLTDNKNDGQKSDSLNANKGIHGDGSSAS
GNGSVSRDGLTETESNNISDMESYINEKDLDDLNFDTVTSNINKTVSDLGGHESTNDGTA
VMNRDSKDSRSNSNEFNAQNRDRITPGSSYGKSLLGSEYSEERYSNNDSSTMESGEMSLD
SDMQTNTIPSHSIPMSMQKYGIYHGDDDSTLNNVFDKAVLTMNSSRHPKERRDTVISGKE
PTSLTSSNRKFSVSSNLTSTRSPLLRGHGRTSSTASSEHMKAPKVSDSVLHRARKSTLTL
KQDHSQPSVPSSVHKSSKEGNILIEKTTDYLVSKPKASQLSNMFNKKKKRTNTNSVDVLE
YFSFVCGDKVPNYESMGLEIYIQASKKYKRNSFTTKVRKSSTIFEVIGFALFLYSTEKKP
DNFEEDGLTVEDISNPNNFSLKIVDEDGEPFEDNFGKLDRKSTIQSISDSEVVLCKVDDA
EKSQNEIETPLPFETGGGLMDASTLDANSSHDTTDGTINQLSFYKPIIGNEDDIDKTNGS
KIIDVTVYLYPNVNPKFNYTTISVLVTSHINDILVKYCKMKNMDPNEYALKVLGKNYILD
LNDTVLRLDGINKVELISKKDARELHLEKMKPDLKKPVLPTIQSNDLTPLTLEPLNSYLK
ADAGGAVAAIPENTKVTSKAKKISTKYKLGLAKQHSSSSVASGSVSTAGGLANGNGFFKN
KNSSKSSLHGTLQFHNINRSQSTMEHTPDTPNGVGDNFQDLFTGAYHKYKVWRRQQMSFI
NKHERTLAIDGDYIYIVPPEGRIHWHDNVKTKSLHISQVVLVKKSKRVPEHFKIFVRREG
QDDIKRYYFEAVSGQECTEIVTRLQNLLSAYRMNHK
>P31407
MAAEVDSRPRGLPGGGASLGAAREHVQAVTRNYITHPRITYRTVCSVNGPLVVLDQVKFA
QYAEIVNFTLPNGTQRSGQVLEVSGTKAIVQVFEGTSGIDAQKTTCEFTGDILRTPVSED
MLGRVFNGSGKPIDKGPVVMAEDFLDINGQPINPHDRIYPEEMIETGISPIDVMNSIARG
QKIPIFSAAGLPHNEIAAQICRQAGLVKKSKAVLDYHDDNFAIVFAAMGVNMETARFFKS
DFEQNGTMGNVCLFLNLANDPTIERIITPRLALTTAEFLAYQCEKHVLVILTDMSSYAEA
LREVSAAREEVPGRRGFPGYMYTDLATIYERAGRVEGRGGSITQIPILTMPNDDITHPIP
DLTGFITEGQIYVDRQLHNRQIYPPINVLPSLSRLMKSAIGEGMTRKDHGDVSNQLYACY
AIGKDVQAMKAVVGEEALTSEDLLYLEFLQKFEKKFINQGPYEKRSVFESLDLGWKLLRT
FPKEMLKRIPQNIIDEFFSREGAPQDTEADTAL
>Q9GZY6
MSSGTELLWPGAALLVLLGVAASLCVRCSRPGAKRSEKIYQQRSLREDQQSFTGSRTYSL
VGQAWPGPLADMAPTRKDKLLQFYPSLEDPASSRYQNFSKGSRHGSEEAYIDPIAMEYYN
WGRFSKPPEDDDANSYENVLICKQKTTETGAQQEGIGGLCRGDLSLSLALKTGPTSGLCP
SASPEEDEESEDYQNSASIHQWRESRKVMGQLQREASPGPVGSPDEEDGEPDYVNGEVAA
TEA

>Q96FL8
MEAPEEPAPVRGGPEATLEVRGSRCLRLSAFREELRALLVLAGPAFLVQLMVFLISFISS
VFCGHLGKLELDAVTLAIAVINVTGVSVGFGLSSACDTLISQTYGSQNLKHVGVILQRSA
LVLLLCCFPCWALFLNTQHILLLFRQDPDVSRLTQTYVTIFIPALPATFLYMLQVKYLLN
QGIVLPQIVTGVAANLVNALANYLFLHQLHLGVIGSALANLISQYTLALLLFLYILGKKL
HQATWGGWSLECLQDWASFLRLAIPSMLMLCMEWWAYEVGSFLSGILGMVELGAQSIVYE
LAIIVYMVPAGFSVAASVRVGNALGAGDMEQARKSSTVSLLITVLFAVAFSVLLLSCKDH
VGYIFTTDRDIINLVAQVVPIYAVSHLFEALACTSGGVLRGSGNQKVGAIVNTIGYYVVG
LPIGIALMFATTLGVMGLWSGIIICTVFQAVCFLGFIIQLNWKKACQQAQVHANLKVNNV
PRSGNSALPQDPLHPGCPENLEGILTNDVGKTGEPQSDQQMRQEEPLPEHPQDGAKLSRK
QLVLRRGLLLLGVFLILLVGILVRFYVRIQ
>P32249
MANNFTPPSATPQGNDCDLYAHHSTARIVMPLHYSLVFIIGLVGNLLALVVIVQNRKKIN
STTLYSTNLVISDILFTTALPTRIAYYAMGFDWRIGDALCRITALVFYINTYAGVNFMTC
LSIDRFIAVVHPLRYNKIKRIEHAKGVCIFVWILVFAQTLPLLINPMSKQEAERITCMEY
PNFEETKSLPWILLGACFIGYVLPLIIILICYSQICCKLFRTAKQNPLTEKSGVNKKALN
TIILIIVVFVLCFTPYHVAIIQHMIKKLRFSNFLECSQRHSFQISLHFTVCLMNFNCCMD
PFIYFFACKGYKRKVMRMLKRQVSVSISSAVKSAPEENSREMTETQMMIHSKSSNGK
>Q8BGE9
MQVASATPAATVRKAAAGDELSEFFALTPDLLEVANASGNASLQLQDLWWELGLELPDGA
APGHPPGGGGAESTDTEARVRILISAVYWVVCALGLAGNLLVLYLMKSKQGWRKSSINLF
VTNLALTDFQFVLTLPFWAVENALDFKWPFGKAMCKIVSMVTSMNMYASVFFLTAMSVAR
YHSVASALKSHRTRGRGRGDCCGQSLRESCCFSAKVLCGLIWASAALASLPNAIFSTTIR
VLGEELCLMHFPDKLLGWDRQFWLGLYHLQKVLLGFLLPLSIISLCYLLLVRFISDRRVV
GTTDAVGAAAAPGGGLSTASARRRSKVTKSVTIVVLSFFLCWLPNQALTTWSILIKFNAV
PFSQEYFQCQVYAFPVSVCLAHSNSCLNPILYCLVRREFRKALKNLLWRIASPSLTNMRP
FTATTKPEPEDHGLQALAPLNAAAEPDLIYYPPGVVVYSGGRYDLLPSSSAY
>P10966
MRPRLWLLLAAQLTVLHGNSVLQQTPAYIKVQTNKMVMLSCEAKISLSNMRIYWLRQRQA
PSSDSHHEFLALWDSAKGTIHGEEVEQEKIAVFRDASRFILNLTSVKPEDSGIYFCMIVG
SPELTFGKGTQLSVVDFLPTTAQPTKKSTLKKRVCRLPRPETQKGPLCSPITLGLLVAGV
LVLLVSLGVAIHLCCRRRRARLRFMKQFYK
>Q9JK83
MNRGHRHGASSGCLGTMEVKSKFGAEFRRFSLERSKPGKFEEFYGLLQHVHKIPNVDVLV
GYADIHGDLPPINNDDNYHKAVSTANPLLRIFIQKKEEADYSAFGTDTLIRKKNMLSNVL
RPDNHRKKPHIVISMPQDFRPVSSIIDVDILPETHRRVRLCKYGTEKPLGFYIRDGSSVR
VTPHGLEKVPGIFISRLVPGGLAQSTGLLAVNDEVLEVNGIEVSGKSLDQVTDMMIANSR
NLIITVRPANQRNNVVRNSRTSGSSSQSTDNSLLGFPQQVEASFEPEDQDSDEDDIIIED
SGEPQQIPKATPAQSLESLTQIELSFESGQNGFSPPQDTSLVPVPGSLDTELESRAPDQK
LLEEDGTIITL
>Q9VVT2
MATETTKMIYTPPPLDIKMEIEIGEQPQPPVKCSNFFANHWKGLVVFLVPLLCLPVMLLN
EGAEFRCMYLLLVMAIFWVTEALPLYVTSMIPIVAFPIMGIMSSDQTCRLYFKDTLVMFM
GGIMVALAVEYCNLHKRLALRVIQIVGCSPRRLHFGLIMVTMFLSMWISNAACTAMMCPI

IQAVLEELQAQGVCKINHEPQYQIVGGNKKNNEDEPPYPTKITLCYYLGIAYASSLGGCG
TIIGTATNLTFKGIYEARFKNSTEQMDFPTFMFYSVPSMLVYTLLTFVFLQWHFMGLWRP
KSKEAQEVQRGREGADVAKKVIDQRYKDLGPMSIHEIQVMILFIFMVVMYFTRKPGIFLG
WADLLNSKDIRNSMPTIFVVVMCFMLPANYAFLRYCTRRGGPVPTGPTPSLITWKFIQTK
VPWGLVFLLGGGFALAEGSKQSGMAKLIGNALIGLKVLPNSVLLLVVILVAVFLTAFSSN
VAIANIIIPVLAEMSLAIEIHPLYLILPAGLACSMAFHLPVSTPPNALVAGYANIRTKDM
AIAGIGPTIITIITLFVFCQTWGLVVYPNLNSFPEWAQIYAAAALGNKTH
>P53885
MQSSLPQFTFKWPKGPEAIILTGTFDDWKGTLPMVKDPSGAFEITLPVTFDSPSSKFYFK
FIVDGQWLPSKDYKVNIDEGVENNFITEEDVIKQRENGSSTLVPESAGLAVSKNAPLIEP
EAEKRAKKLRKFKIKRVIKTNKQTGERSIFSQEVVELPDSEDETQQVNKTGKNADGLSGT
TTIIENNVGVNEEKAIKPYEENHPKVNLVKSEGYVTDGLGKTQSSESRLYELSAEDLEKE
EEEEDEDKGGGKDTSTSADAEASEDQNKEPLSKSAKFEKPEEKVPVSSITSHAKETSVKP
TGKVATETQTYETKQGAPTAAAKKIEAKKATRPSKPKGTKETPNKGVQKNPAKNGGFFKK
LAQLLK
>P38727
MSSELLISNSKPRPEGLRKLCEGETVILPRDITPSKCAYFLKQNIVFISYIFIHIIITII
LNRLALSAHGNTLIIILAALLITISLFLLLLLPYLSCSRYKLRCLDDDCKFKLLAEVITH
KPNMDLSTWDRIAYDMNQFVYDRRICADRSFFYDGSYCYQVFKKLVATPYLVNSNMNSIY
ADLEMRSNGATNINDSGNSSLHIELGTYIFKALAVFRNSVDKYWEDKYPEMGVTV
>Q8C8C1
MRIFRPWRLRCPALHLPSFPTFSIKCSLPPLPTDEDMCKSVTTGEWKKVFYEKMEEVKPA
DSWDFIIDPNLKHNVLAPGWKQYLELHASGRFHCSWCWHTWQSPHVVILFHMYLDKAQRA
GSVRMRVFKQLCYECGTARLDESSMLEENIESLVDNLITSLREQCYGERGGHYRIHVASR
QDNRRHRGEFCEACQEGIVHWKPSEKLLEEEATTYTFSRAPSPTKPQAETGSGCNFCSIP
WCLFWATVLMLIIYLQFSFRTSV
>P21860
MRANDALQVLGLLFSLARGSEVGNSQAVCPGTLNGLSVTGDAENQYQTLYKLYERCEVVM
GNLEIVLTGHNADLSFLQWIREVTGYVLVAMNEFSTLPLPNLRVVRGTQVYDGKFAIFVM
LNYNTNSSHALRQLRLTQLTEILSGGVYIEKNDKLCHMDTIDWRDIVRDRDAEIVVKDNG
RSCPPCHEVCKGRCWGPGSEDCQTLTKTICAPQCNGHCFGPNPNQCCHDECAGGCSGPQD
TDCFACRHFNDSGACVPRCPQPLVYNKLTFQLEPNPHTKYQYGGVCVASCPHNFVVDQTS
CVRACPPDKMEVDKNGLKMCEPCGGLCPKACEGTGSGSRFQTVDSSNIDGFVNCTKILGN
LDFLITGLNGDPWHKIPALDPEKLNVFRTVREITGYLNIQSWPPHMHNFSVFSNLTTIGG
RSLYNRGFSLLIMKNLNVTSLGFRSLKEISAGRIYISANRQLCYHHSLNWTKVLRGPTEE
RLDIKHNRPRRDCVAEGKVCDPLCSSGGCWGPGPGQCLSCRNYSRGGVCVTHCNFLNGEP
REFAHEAECFSCHPECQPMEGTATCNGSGSDTCAQCAHFRDGPHCVSSCPHGVLGAKGPI
YKYPDVQNECRPCHENCTQGCKGPELQDCLGQTLVLIGKTHLTMALTVIAGLVVIFMMLG
GTFLYWRGRRIQNKRAMRRYLERGESIEPLDPSEKANKVLARIFKETELRKLKVLGSGVF
GTVHKGVWIPEGESIKIPVCIKVIEDKSGRQSFQAVTDHMLAIGSLDHAHIVRLLGLCPG
SSLQLVTQYLPLGSLLDHVRQHRGALGPQLLLNWGVQIAKGMYYLEEHGMVHRNLAARNV
LLKSPSQVQVADFGVADLLPPDDKQLLYSEAKTPIKWMALESIHFGKYTHQSDVWSYGVT
VWELMTFGAEPYAGLRLAEVPDLLEKGERLAQPQICTIDVYMVMVKCWMIDENIRPTFKE
LANEFTRMARDPPRYLVIKRESGPGIAPGPEPHGLTNKKLEEVELEPELDLDLDLEAEED

NLATTTLGSALSLPVGTLNRPRGSQSLLSPSSGYMPMNQGNLGESCQESAVSGSSERCPR
PVSLHPMPRGCLASESSEGHVTGSEAELQEKVSMCRSRSRSRSPRPRGDSAYHSQRHSLL
TPVTPLSPPGLEEEDVNGYVMPDTHLKGTPSSREGTLSSVGLSSVLGTEEEDEDEEYEYM
NRRRRHSPPHPPRPSSLEELGYEYMDVGSDLSASLGSTQSCPLHPVPIMPTAGTTPDEDY
EYMNRQRDGGGPGGDYAAMGACPASEQGYEEMRAFQGPGHQAPHVHYARLKTLRSLEATD
SAFDNPDYWHSRLFPKANAQRT
>Q92692
MARAAALLPSRSPPTPLLWPLLLLLLLETGAQDVRVQVLPEVRGQLGGTVELPCHLLPPV
PGLYISLVTWQRPDAPANHQNVAAFHPKMGPSFPSPKPGSERLSFVSAKQSTGQDTEAEL
QDATLALHGLTVEDEGNYTCEFATFPKGSVRGMTWLRVIAKPKNQAEAQKVTFSQDPTTV
ALCISKEGRPPARISWLSSLDWEAKETQVSGTLAGTVTVTSRFTLVPSGRADGVTVTCKV
EHESFEEPALIPVTLSVRYPPEVSISGYDDNWYLGRTDATLSCDVRSNPEPTGYDWSTTS
GTFPTSAVAQGSQLVIHAVDSLFNTTFVCTVTNAVGMGRAEQVIFVRETPNTAGAGATGG
IIGGIIAAIIATAVAATGILICRQQRKEQTLQGAEEDEDLEGPPSYKPPTPKAKLEAQEM
PSQLFTLGASEHSPLKTPYFDAGASCTEQEMPRYHELPTLEERSGPLHPGATSLGSPIPV
PPGPPAVEDVSLDLEDEEGEEEEEYLDKINPIYDALSYSSPSDSYQGKGFVMSRAMYV
>Q9NWQ8
MGPAGSLLGSGQMQITLWGSLAAVAIFFVITFLIFLCSSCDREKKPRQHSGDHENLMNVP
SDKEMFSRSVTSLATDAPASSEQNGALTNGDILSEDSTLTCMQHYEEVQTSASDLLDSQD
STGKPKCHQSRELPRIPPESAVDTMLTARSVDGDQGLGMEGPYEVLKDSSSQENMVEDCL
YETVKEIKEVAAAAHLEKGHSGKAKSTSASKELPGPQTEGKAEFAEYASVDRNKKCRQSV
NVESILGNSCDPEEEAPPPVPVKLLDENENLQEKEGGEAEESATDTTSETNKRFSSLSYK
SREEDPTLTEEEISAMYSSVNKPGQLVNKSGQSLTVPESTYTSIQGDPQRSPSSCNDLYA
TVKDFEKTPNSTLPPAGRPSEEPEPDYEAIQTLNREEEKATLGTNGHHGLVPKENDYESI
SDLQQGRDITRL
>Q7TQP0
MDGYNTSENSSCDPILAHHLTSIYFIVLIGGLVGLISILFLLVKMNSRSVTTMAVINLVV
VHGVFLLTVPFRLAYLIKGTWTFGLPFCKFVSAMLHIHMYLTFLFYVVILVIRYLIFFKR
RDKVEFYRKLHAVAASSAMWLLVIVIVVPLVVSQYGNSEEYNEQQCFRFHKELGHDSVRV
INYMIVIVVIAVALILLGFQVFITLSMVRKFRHSLLSHQEFWAQLKNLFFIGIIIICFLP
YQFFRIYYLYVVAHSKSCKNKVAFYNEILLSTTAISCCDLLLFVFGGSHWVKQKIVDMWN
CLLCH
>Q62897
MAAGVAAWLPFARAAAIGWMPVANCPMPLAPADKNKRQDELIVLNVSGRRFQTWRTTLER
YPDTLLGSTEKEFFFNEDTKEYFFDRDPEVFRCVLNFYRTGKLHYPRYECISAYDDELAF
YGILPEIIGDCCYEEYKDRKRENAERLMDDNESENNQESMPSLSFRQTMWRAFENPHTST
LALVFYYVTGFFIAVSVITNVVETVPCGTVPGSKELPCGERYSVAFFCLDTACVMIFTVE
YLLRLFAAPSRYRFIRSVMSIIDVVAIMPYYIGLVMTNNEDVSGAFVTLRVFRVFRIFKF
SRHSQGLRILGYTLKSCASELGFLLFSLTMAIIIFATVMFYAEKGSSASKFTSIPASFWY
TIVTMTTLGYGDMVPKTIAGKIFGSICSLSGVLVIALPVPVIVSNFSRIYHQNQRADKRR
AQKKARLARIRVAKTGSSNAYLHSKRNGLLNEALELTGTPEEEHMGKTTSLIESQHHHLL
HCLEKTTGLSYLVDDPLLSVRTSTIKNHEFIDEQMFEQNCMESSMQNYPSTRSPSLSSHS
GLTTTCCSRRSKKTTHLPNSNLPATRLRSMQELSTIHIQGSEQPSLTTSRSSLNLKADDG
LRPNCKTSQITTAIISIPTPPALTPEGESRPPPASPGPNTNIPSITSNVVKVSVL

>Q9PVZ4
MGQGVLRGEGHPNNNPNSKVGWKSLVGIITIFMLILCDQSDGKICYSMDIRNNISQFSML
EDCTVIEGHLQILLMFTSKPENFRGLRFPKLTTITDYLLLFRVYGLESLKDLFPNLTVIR
GTRLFFNYALVIFEMVHXKEIGLYNLMNITRGSVRIEKNNELCYLSTIDWSIILDSVEDN
YIELNRDNKEECGDVCPGTVKGKSKCKHTLVNGALVERCWTQDHCQKVCPSDCKGSGCLP
DGQCCHPECLGSCRKPNDPSECTACRHFQNEGVCVTACPKGSYQFQGWRCIDFNTCQELN
SRCQNSRDNSCPPYVIHKGECMPDCPSGYIANSTTRTCTPCAGPCPKVCTIFQNVKTIDS
VTSAQELRGCTVINGSLIINLRGGNNIATELEANLGLIEEISGYLKIRRSYALVSLSFFR
KLRLIRGEVLEAGNYSFYALDNPSLRQLWDWHKHNLTIIHGKLFFHHNPRLCLSQIHQME
EVTGTKGRQDKNDIATKTNGDQASCEDNLLTFNFIKTSHDMVLLRWDAYWPPDYRDLLGF
MVHYKEAPFQNVTEFDGQDACGSNSWTVVDMDAPERSADGKTQSPGCLLRSLKPWTQYAV
FVKTLVSGSDEGRTYGAKSKIIYIRTNETIPSVPLDPFSVSNSTSQIILKWKPPSEPNGN
VTHYLVYWQEQPEDSDLYEVDYCNKGLKLPSRTWTPPTEIDENGNENQTEHTSVNKCCPC
PKTEFQIQKEQDESAFRKTFENYLHNEVFIPRPVRKRRDLFGVANGTLPDPVTAPPLFNV
SSTRAPDEPEPKIYSQKVWFKESVLISGLKHFTGYRIEIHACNHELSMGCSVAAYVNART
MPEATADKVVGPITYEYVEPNIIHLKWQEPKDPNGLIVLYEVHYSRVGGIEEVITCVSQK
QYNTDKGGKLRVLTPGNYSVKIRATSLAGNGSWTEQAYFQVPDHPHSNIVKIITGPIIAV
FLLLIVLVYCVVQKKKDAEGPAGPLYTSSNPEYLSASEVYIPDEWEVPRDKINLLRELGQ
GSFGMVYEGIAKDIIKGEPEVRVAVKTVNESASLRERIEFLNEASVMKAFNCHHVVRLLG
VVSKGQPTLVIMELMAHGDLKSYLRSLRPDAENNPGRLAPTLKEIIQMAAEISDGMAYLN
AKKFVHRDLAARNCMVADDYAVKIGDFGMTRDIYETDYYRKGGKGLLPVRWMSPESLKDG
VFTAFSDVWSFGVVLWEITSLAEQPYQGLSNEQVLKFVMDGGSLDHPENCPPRLHSLMQM
CWQYNPKMRPTFLEIIDMLKDDLRPSFQDVSFYYSDENKPPETDDLEIDFENMESTPLDP
SSCSLRDQSSRTNIYEEHIPYTHMNGGRKNGRILSLPRSSPS
>P83326
MAFSYCSSSLFVSLLLVILFISPLSQRPSVKAENHLISEICPKTRNPSLCLQALESDPRS
ASKDLKGLGQFSIDIAQASAKQTSKIIASLTNQATDPKLKGRYETCSENYADAIDSLGQA
KQFLTSGDYNSLNIYASAAFDGAGTCEDSFEGPPNIPTQLHQADLKLEDLCDIVLVISNL
LPGSK
>Q9ERM2
MESALLLPSLLLVAAYPRGGSPQQEWMQSPPAPSVTSAPFWVRLNPELEAVPPGGSAWLN
CSHNCPLPVHSSLRTQLRQGKIVNGSGWVSYQLLDVRAWNSKVRCVVTCAGETREATARI
TAYKRPRSVILEPPVLVGHKYTLRCYVTHVFPVGFLVVSLRRGGRVIYHESLERFTGSDL
ANVTLTYVMRAGLNDLWQPLTCHARLNLDGLVVRSSSAPVMLTVLALSPASIALASTSIA
TLVGILLAVGAVYVRKYLAVQT
>Q07800
MGFISSILCCSSETTQSNSNSAYRQQQSSSLNKNRSVKHSNTKSRTRGVHQTNSPPSKTN
SAATFSSTERSTGKSGISTNDNEKKKPSSPTAAVTATTTNNMTKVEKRISKDDLYEEKYE
VDEDEEIDDEDNRRSRGIVQEKGDAVKDTSRQKKQQQQQQQQSQPQPQPQSQSQSQSQSQ
SQQRGPTVQVSSDHLIQDMNLSRVSSSSQASETSNDADDEDDEDEEYIDLTLLQQGQYHA
PGYNTLLPPQDESTKGKKCLILDLDETLVHSSFKYLRSADFVLSVEIDDQVHNVYVIKRP
GVEEFLERVGKLFEVVVFTASVSRYGDPLLDILDTDKVIHHRLFREACYNYEGNYIKNLS
QIGRPLSDIIILDNSPASYIFHPQHAIPISSWFSDTHDNELLDIIPLLEDLSVKTSLDVG
KILDVTI

>Q06991
MRNFFTLFFAAIFSLGALILAIVACAGSTKNYSPINKIYCAELDLSQMKVSTVLPSLSSA
TLSSLGLPSYINIGLWSYCTVDSSHNIQSCSSPHGIQNFNLSSLVYDNINNNEALELMDS
VASVVLPEKLKSKMTYYNNLVKCMFITILIGIVLTFVNLVFNVLRWIIHIRPLTWFGAFF
SFFAFAALLVSIGSCLGTYSYIKYILKHNYSDYGISMSIGRNYQGLMWGAVVGALLNFIL
WCSVRSRPTVIYANAPIEEKPLI
>Q9NY26
MGPWGEPELLVWRPEAVASEPPVPVGLEVKLGALVLLLVLTLLCSLVPICVLRRPGANHE
GSASRQKALSLVSCFAGGVFLATCLLDLLPDYLAAIDEALAALHVTLQFPLQEFILAMGF
FLVLVMEQITLAYKEQSGPSPLEETRALLGTVNGGPQHWHDGPGVPQASGAPATPSALRA
CVLVFSLALHSVFEGLAVGLQRDRARAMELCLALLLHKGILAVSLSLRLLQSHLRAQVVA
GCGILFSCMTPLGIGLGAALAESAGPLHQLAQSVLEGMAAGTFLYITFLEILPQELASSE
QRILKVILLLAGFALLTGLLFIQI
>Q7TN39
MLSIFNIWGTFNRVLFFLSLTVSLAGLAGNTLLLWHLGLRIKKGPFNTYLLHLAAADFLF
LSCQVGFSIAKIASGYEDTLYFPVTFLWFAVGLWLLAAFIVDCCLSYMFPSFCGPNCRPR
YTSFVLCLVIWALTMLAVLLPANACGLLYNRMSLLVCLKYHWVSVVWLGVLASTACGASM
FLLVFGNCCSSQPPSKFCKLAQCSGILLFFCRLPLVFYWCLRPVIKFLLPFFFPLATLLA
CIDSSAKPLLYYLKGRQLRKEPLQVALNRALGEESQSSSGGISLPMSRV
>O43508
MAARRSQRRRGRRGEPGTALLVPLALGLGLALACLGLLLAVVSLGSRASLSAQEPAQEEL
VAEEDQDPSELNPQTEESQDPAPFLNRLVRPRRSAPKGRKTRARRAIAAHYEVHPRPGQD
GAQAGVDGTVSGWEEARINSSSPLRYNRQIGEFIVTRAGLYYLYCQVHFDEGKAVYLKLD
LLVDGVLALRCLEEFSATAASSLGPQLRLCQVSGLLALRPGSSLRIRTLPWAHLKAAPFL
TYFGLFQVH
>Q7YRP1
MLKLVIIENMAEIMLFSLDLLLFSTDILCFNFPSKMIKLPGFITIQIFFYPQASFGISAN
TILFLFHIFTFVFSHRSKSIDMIISHLSLIHILLLFTQAILVSLDFFGSQNTQDDLRCKV
IVFLNKVMRGLSICTPCLLNVLQAIISPSIFSLAKLKHPSASHILGFFLFSWVLNMFIGV
IFCCTLWLPPVKWGQSSVCHTALFLFAHELHPQETVFHTNDFEGCHLYRVHGPLKRLHGD
YFIQTIRGYLSAFTQPACPPVSPVKRASQTILLLVSFVFIYWVDFMFSFSRGVTWINDSL
LVWFQVIVANSYATISPLMLIYADNQIFKTLQMLWFKYLSPPKLMLKFNRQCGSTKK
>Q8WY07
MPWQAFRRFGQKLVRRRTLESGMAETRLARCLSTLDLVALGVGSTLGAGVYVLAGEVAKD
KAGPSIVICFLVAALSSVLAGLCYAEFGARVPRSGSAYLYSYVTVGELWAFTTGWNLILS
YVIGTASVARAWSSAFDNLIGNHISKTLQGSIALHVPHVLAEYPDFFALGLVLLLTGLLA
LGASESALVTKVFTGVNLLVLGFVMISGFVKGDVHNWKLTEEDYELAMAELNDTYSLGPL
GSGGFVPFGFEGILRGAATCFYAFVGFDCIATTGEEAQNPQRSIPMGIVISLSVCFLAYF
AVSSALTLMMPYYQLQPESPLPEAFLYIGWAPARYVVAVGSLCALSTSLLGSMFPMPRVI
YAMAEDGLLFRVLARIHTGTRTPIIATVVSGIIAAFMAFLFKLTDLVDLMSIGTLLAYSL
VSICVLILRYQPDQETKTGEEVELQEEAITTESEKLTLWGLFFPLNSIPTPLSGQIVYVC
SSLLAVLLTALCLVLAQWSVPLLSGDLLWTAVVVLLLLLIIGIIVVIWRQPQSSTPLHFK
VPALPLLPLMSIFVNIYLMMQMTAGTWARFGVWMLIGFAIYFGYGIQHSLEEIKSNQPSR
KSRAKTVDLDPGTLYVHSV

>Q9LVM5
MAMEIGEDEWKVCCGSSEFAKQMSTSGPLTSQEAIYTARDIWFNQVNVTDWLEAFSAHPQ
IGNTPSPSINSDFARRSVSEQSTAFATTSASALQELAEWNVLYKKKFGFIFIICASGRTH
AEMLHALKERYENRPIVELEIAAMEQMKITELRMAKLFSDKAKVISETDSSSSPVSTKPQ
DRLRIIGGHLNVAAEAKAPKRSRPPITTHVLDVSRGAPAAGVEVHLEVWSGTTGPSFVHG
GGGVWSSVGTSATDRDGRSGPLMDLVDALNPGTYRISFDTAKYSPGCFFPYVSIVFQVTE
SQKWEHFHVPLLLAPFSFSTYRGS
>Q07950
MVNKVVDEVQRLVSAIILTSFMTGLFILSLWKNYVTVHFQHKNDPRDTRSSRTKIQPNDK
KKKRPARHSRPLSISSTTPLDLQRDQENNIEYDRTVTSKLSMTSNASLSENGDGNANIKM
ETNVNQAPYAAENPFQNIALAEDTKLVPDLKYYYKEYGIDIEEFEVETDDGFIIDLWHFK
SRLNDGVEEVKREPILLLHGLLQSCGAFASSGRKSLAYFLYESGFDVWLGNNRCGLNAKW
NMKKLGNDHSKKWDWDMHQMVQYDLKALINYVLDSTGYAKLSLVAHSQGTTQGFMGLVNG
EKLYASDFKLVDKLENFVALAPAVYPGPLLDEKAFVRLMAKGIDSPWYFGRRSFIPLMMT
MRKLMVGTKIFSFLSYIMFNYLFDWNDVLWDRVLRDRNFLFSPVHISVKLMQWWLSPLPN
KLSFKKGAEKIFPDKKTWFPIAKNDDDSGNNLDNNKLHLNPKRQNSEEFPHIIMFIPKQD
RLVDGERLINHFINHEANAVYKIWYIDEYSHLDVLWAHDVIDRIGKPMIENLRFPNAR
>Q9UKJ5
MADFDEIYEEEEDEERALEEQLLKYSPDPVVVRGSGHVTVFGLSNKFESEFPSSLTGKVA
PEEFKASINRVNSCLKKNLPVNVRWLLCGCLCCCCTLGCSMWPVICLSKRTRRSIEKLLE
WENNRLYHKLCLHWRLSKRKCETNNMMEYVILIEFLPKTPIFRPD
>Q9WU72
MDESAKTLPPPCLCFCSEKGEDMKVGYDPITPQKEEGAWFGICRDGRLLAATLLLALLSS
SFTAMSLYQLAALQADLMNLRMELQSYRGSATPAAAGAPELTAGVKLLTPAAPRPHNSSR
GHRNRRAFQGPEETEQDVDLSAPPAPCLPGCRHSQHDDNGMNLRNIIQDCLQLIADSDTP
TIRKGTYTFVPWLLSFKRGNALEEKENKIVVRQTGYFFIYSQVLYTDPIFAMGHVIQRKK
VHVFGDELSLVTLFRCIQNMPKTLPNNSCYSAGIARLEEGDEIQLAIPRENAQISRNGDD
TFFGALKLL
>Q9GZM5
MATTAAPAGGARNGAGPEWGGFEENIQGGGSAVIDMENMDDTSGSSFEDMGELHQRLREE
EVDADAADAAAAEEEDGEFLGMKGFKGQLSRQVADQMWQAGKRQASRAFSLYANIDILRP
YFDVEPAQVRSRLLESMIPIKMVNFPQKIAGELYGPLMLVFTLVAILLHGMKTSDTIIRE
GTLMGTAIGTCFGYWLGVSSFIYFLAYLCNAQITMLQMLALLGYGLFGHCIVLFITYNIH
LHALFYLFWLLVGGLSTLRMVAVLVSRTVGPTQRLLLCGTLAALHMLFLLYLHFAYHKVV
EGILDTLEGPNIPPIQRVPRDIPAMLPAARLPTTVLNATAKAVAVTLQSH
>Q8N6U8
MSLNSSLSCRKELSNLTEEEGGEGGVIITQFIAIIVITIFVCLGNLVIVVTLYKKSYLLT
LSNKFVFSLTLSNFLLSVLVLPFVVTSSIRREWIFGVVWCNFSALLYLLISSASMLTLGV
IAIDRYYAVLYPMVYPMKITGNRAVMALVYIWLHSLIGCLPPLFGWSSVEFDEFKWMCVA
AWHREPGYTAFWQIWCALFPFLVMLVCYGFIFRVARVKARKVHCGTVVIVEEDAQRTGRK
NSSTSTSSSGSRRNAFQGVVYSANQCKALITILVVLGAFMVTWGPYMVVIASEALWGKSS
VSPSLETWATWLSFASAVCHPLIYGLWNKTVRKELLGMCFGDRYYREPFVQRQRTSRLFS
ISNRITDLGLSPHLTALMAGGQPLGHSSSTGDTGFSCSQDSGTDMMLLEDYTSDDNPPSH
CTCPPKRRSSVTFEDEVEQIKEAAKNSILHVKAEVHKSLDSYAASLAKAIEAEAKINLFG

EEALPGVLVTARTVPGGGFGGRRGSRTLVSQRLQLQSIEEGDVLAAEQR
>Q8K1Z6
MATLSNHNQLDLSNGSHPEEYKIAALVFYSCIFLIGLFVNVTALWVFSCTTKKRTTVTIY
MMNVALLDLVFILSLPFRMFYYAKGEWPFGEYFCHILGALVVFYPSLALWLLAFISADRY
MAIVQPKYAKELKNTGKAVLACGGVWVMTLTTTVPLLLLYEDPDKASSPATCLKISDITH
LKAVNVLNFTRLIFFFLIPLFIMIGCYVVIIHSLLRGQTSKLKPKVKEKSIRIIMTLLLQ
VLVCFVPFHICFAVLMLQGQENSYSPWGAFTTFLMNLSTCLDVVLYYIVSKQFQARVISV
MLYRNYLRSVRRKSVRSGSLRSLSNMNSEML
>Q8TD43
MVVPEKEQSWIPKIFKKKTCTTFIVDSTDPGGTLCQCGRPRTAHPAVAMEDAFGAAVVTV
WDSDAHTTEKPTDAYGELDFTGAGRKHSNFLRLSDRTDPAAVYSLVTRTWGFRAPNLVVS
VLGGSGGPVLQTWLQDLLRRGLVRAAQSTGAWIVTGGLHTGIGRHVGVAVRDHQMASTGG
TKVVAMGVAPWGVVRNRDTLINPKGSFPARYRWRGDPEDGVQFPLDYNYSAFFLVDDGTH
GCLGGENRFRLRLESYISQQKTGVGGTGIDIPVLLLLIDGDEKMLTRIENATQAQLPCLL
VAGSGGAADCLAETLEDTLAPGSGGARQGEARDRIRRFFPKGDLEVLQAQVERIMTRKEL
LTVYSSEDGSEEFETIVLKALVKACGSSEASAYLDELRLAVAWNRVDIAQSELFRGDIQW
RSFHLEASLMDALLNDRPEFVRLLISHGLSLGHFLTPMRLAQLYSAAPSNSLIRNLLDQA
SHSAGTKAPALKGGAAELRPPDVGHVLRMLLGKMCAPRYPSGGAWDPHPGQGFGESMYLL
SDKATSPLSLDAGLGQAPWSDLLLWALLLNRAQMAMYFWEMGSNAVSSALGACLLLRVMA
RLEPDAEEAARRKDLAFKFEGMGVDLFGECYRSSEVRAARLLLRRCPLWGDATCLQLAMQ
ADARAFFAQDGVQSLLTQKWWGDMASTTPIWALVLAFFCPPLIYTRLITFRKSEEEPTRE
ELEFDMDSVINGEGPVGTADPAEKTPLGVPRQSGRPGCCGGRCGGRRCLRRWFHFWGAPV
TIFMGNVVSYLLFLLLFSRVLLVDFQPAPPGSLELLLYFWAFTLLCEELRQGLSGGGGSL
ASGGPGPGHASLSQRLRLYLADSWNQCDLVALTCFLLGVGCRLTPGLYHLGRTVLCIDFM
VFTVRLLHIFTVNKQLGPKIVIVSKMMKDVFFFLFFLGVWLVAYGVATEGLLRPRDSDFP
SILRRVFYRPYLQIFGQIPQEDMDVALMEHSNCSSEPGFWAHPPGAQAGTCVSQYANWLV
VLLLVIFLLVANILLVNLLIAMFSYTFGKVQGNSDLYWKAQRYRLIREFHSRPALAPPFI
VISHLRLLLRQLCRRPRSPQPSSPALEHFRVYLSKEAERKLLTWESVHKENFLLARARDK
RESDSERLKRTSQKVDLALKQLGHIREYEQRLKVLEREVQQCSRVLGWVAEALSRSALLP
PGGPPPPDLPGSKD
>Q8VZ80
MTGATPENRTAPSPPPVKHVPESVLPAKPPKRNNYAFACAILASMTSILLGYDIGVMSGA
MIYIKRDLKINDLQIGILAGSLNIYSLIGSCAAGRTSDWIGRRYTIVLAGAIFFAGAILM
GLSPNYAFLMFGRFIAGIGVGYALMIAPVYTAEVSPASSRGFLNSFPEVFINAGIMLGYV
SNLAFSNLPLKVGWRLMLGIGAVPSVILAIGVLAMPESPRWLVMQGRLGDAKRVLDKTSD
SPTEATLRLEDIKHAAGIPADCHDDVVQVSRRNSHGEGVWRELLIRPTPAVRRVMIAAIG
IHFFQQASGIDAVVLFSPRIFKTAGLKTDHQQLLATVAVGVVKTSFILVATFLLDRIGRR
PLLLTSVGGMVLSLAALGTSLTIIDQSEKKVMWAVVVAIATVMTYVATFSIGAGPITWVY
SSEIFPLRLRSQGSSMGVVVNRVTSGVISISFLPMSKAMTTGGAFYLFGGIATVAWVFFY
TFLPETQGRMLEDMDELFSGFRWRDSKSKPKGNPEKTVPNPEVEIGSNKQWKEGDTQSS
>Q8WWQ8
MMLQHLVIFCLGLVVQNFCSPAETTGQARRCDRKSLLTIRTECRSCALNLGVKCPDGYTM
ITSGSVGVRDCRYTFEVRTYSLSLPGCRHICRKDYLQPRCCPGRWGPDCIECPGGAGSPC
NGRGSCAEGMEGNGTCSCQEGFGGTACETCADDNLFGPSCSSVCNCVHGVCNSGLDGDGT

CECYSAYTGPKCDKPIPECAALLCPENSRCSPSTEDENKLECKCLPNYRGDGKYCDPINP
CLRKICHPHAHCTYLGPNRHSCTCQEGYRGDGQVCLPVDPCQINFGNCPTKSTVCKYDGP
GQSHCECKEHYQNFVPGVGCSMTDICKSDNPCHRNANCTTVAPGRTECICQKGYVGDGLT
CYGNIMERLRELNTEPRGKWQGRLTSFISLLDKAYAWPLSKLGPFTVLLPTDKGLKGFNV
NELLVDNKAAQYFVKLHIIAGQMNIEYMNNTDMFYTLTGKSGEIFNSDKDNQIKLKLHGG
KKKVKIIQGDIIASNGLLHILDRAMDKLEPTFESNNEQTIMTMLQPRYSKFRSLLEETNL
GHALDEDGVGGPYTIFVPNNEALNNMKDGTLDYLLSPEGSRKLLELVRYHIVPFTQLEVA
TLISTPHIRSMANQLIQFNTTDNGQILANDVAMEEIEITAKNGRIYTLTGVLIPPSIVPI
LPHRCDETKREMKLGTCVSCSLVYWSRCPANSEPTALFTHRCVYSGRFGSLKSGCARYCN
ATVKIPKCCKGFYGPDCNQCPGGFSNPCSGNGQCADSLGGNGTCICEEGFQGSQCQFCSD
PNKYGPRCNKKCLCVHGTCNNRIDSDGACLTGTCRDGSAGRLCDKQTSACGPYVQFCHIH
ATCEYSNGTASCICKAGYEGDGTLCSEMDPCTGLTPGGCSRNAECIKTGTGTHTCVCQQG
WTGNGRDCSEINNCLLPSAGGCHDNASCLYVGPGQNECECKKGFRGNGIDCEPITSCLEQ
TGKCHPLASCQSTSSGVWSCVCQEGYEGDGFLCYGNAAVELSFLSEAAIFNRWINNASLQ
PTLSATSNLTVLVPSQQATEDMDQDEKSFWLSQSNIPALIKYHMLLGTYRVADLQTLSSS
DMLATSLQGNFLHLAKVDGNITIEGASIVDGDNAATNGVIHIINKVLVPQRRLTGSLPNL
LMRLEQMPDYSIFRGYIIQYNLANAIEAADAYTVFAPNNNAIENYIREKKVLSLEEDVLR
YHVVLEEKLLKNDLHNGMHRETMLGFSYFLSFFLHNDQLYVNEAPINYTNVATDKGVIHG
LGKVLEIQKNRCDNNDTTIIRGRCRTCSSELTCPFGTKSLGNEKRRCIYTSYFMGRRTLF
IGCQPKCVRTVITRECCAGFFGPQCQPCPGNAQNVCFGNGICLDGVNGTGVCECGEGFSG
TACETCTEGKYGIHCDQACSCVHGRCNQGPLGDGSCDCDVGWRGVHCDNATTEDNCNGTC
HTSANCLTNSDGTASCKCAAGFQGNGTICTAINACEISNGGCSAKADCKRTTPGRRVCTC
KAGYTGDGIVCLEINPCLENHGGCDKNAECTQTGPNQAACNCLPAYTGDGKVCTLINVCL
TKNGGCSEFAICNHTGQVERTCTCKPNYIGDGFTCRGSIYQELPKNPKTSQYFFQLQEHF
VKDLVGPGPFTVFAPLSAAFDEEARVKDWDKYGLMPQVLRYHVVACHQLLLENLKLISNA
TSLQGEPIVISVSQSTVYINNKAKIISSDIISTNGIVHIIDKLLSPKNLLITPKDNSGRI
LQNLTTLATNNGYIKFSNLIQDSGLLSVITDPIHTPVTLFWPTDQALHALPAEQQDFLFN
QDNKDKLKEYLKFHVIRDAKVLAVDLPTSTAWKTLQGSELSVKCGAGRDIGDLFLNGQTC
RIVQRELLFDLGVAYGIDCLLIDPTLGGRCDTFTTFDASGECGSCVNTPSCPRWSKPKGV
KQKCLYNLPFKRNLEGCRERCSLVIQIPRCCKGYFGRDCQACPGGPDAPCNNRGVCLDQY
SATGECKCNTGFNGTACEMCWPGRFGPDCLPCGCSDHGQCDDGITGSGQCLCETGWTGPS
CDTQAVLPAVCTPPCSAHATCKENNTCECNLDYEGDGITCTVVDFCKQDNGGCAKVARCS
QKGTKVSCSCQKGYKGDGHSCTEIDPCADGLNGGCHEHATCKMTGPGKHKCECKSHYVGD
GLNCEPEQLPIDRCLQDNGQCHADAKCVDLHFQDTTVGVFHLRSPLGQYKLTFDKAREAC
ANEAATMATYNQLSYAQKAKYHLCSAGWLETGRVAYPTAFASQNCGSGVVGIVDYGPRPN
KSEMWDVFCYRMKDVNCTCKVGYVGDGFSCSGNLLQVLMSFPSLTNFLTEVLAYSNSSAR
GRAFLEHLTDLSIRGTLFVPQNSGLGENETLSGRDIEHHLANVSMFFYNDLVNGTTLQTR
LGSKLLITASQDPLQPTETRFVDGRAILQWDIFASNGIIHVISRPLKAPPAPVTLTHTGL
GAGIFFAIILVTGAVALAAYSYFRINRRTIGFQHFESEEDINVAALGKQQPENISNPLYE
STTSAPPEPSYDPFTDSEERQLEGNDPLRTL
>Q03157
MGPTSPAARGQGRRWRPPLPLLLPLSLLLLRAQLAVGNLAVGSPSAAEAPGSAQVAGLCG
RLTLHRDLRTGRWEPDPQRSRRCLLDPQRVLEYCRQMYPELHIARVEQAAQAIPMERWCG
GTRSGRCAHPHHEVVPFHCLPGEFVSEALLVPEGCRFLHQERMDQCESSTRRHQEAQEAC

SSQGLILHGSGMLLPCGSDRFRGVEYVCCPPPATPNPSGMAAGDPSTRSWPLGGRAEGGE
DEEEVESFPQPVDDYFVEPPQAEEEEEEEEEERAPPPSSHTPVMVSRVTPTPRPTDGVDVY
FGMPGEIGEHEGFLRAKMDLEERRMRQINEVMREWAMADSQSKNLPKADRQALNEHFQSI
LQTLEEQVSGERQRLVETHATRVIALINDQRRAALEGFLAALQGDPPQAERVLMALRRYL
RAEQKEQRHTLRHYQHVAAVDPEKAQQMRFQVQTHLQVIEERMNQSLGLLDQNPHLAQEL
RPQIQELLLAEHLGPSELDASVPGSSSEDKGSLQPPESKDDPPVTLPKGSTDQESSSSGR
EKLTPLEQYEQKVNASAPRGFPFHSSDIQRDELAPSGTGVSREALSGLLIMGAGGGSLIV
LSLLLLRKKKPYGTISHGVVEVDPMLTLEEQQLRELQRHGYENPTYRFLEERP
>Q967F4
MSWNILLILLISNLDEVLAKTLLKLPSNAPPGWLISDLQFQNLIGDSEIATLQPSIFSTN
FEVEDGYRIITNTTVTQFHGELFELFLNVKEQNFQRLVTLHVYVDPRGTSQQPATFLSTV
YHATVYTSQQPGSTVVFSKPITVRNRKNFVISPISKIDKISKYSSPFSVMTRGKSVDIVM
MKQKLEEDDITRHVIFLGAFTEKTGEMIAQTKVIIDVIDSGDVHFLLKSKKSIAKFASAI
PANSTVFDVEKRNLSEPLLFHLEEPSRFFKIDQFSGRVSTVLPVGYGTYHIHVVARNQKK
QRSDAWLEISVKKEQKLEPMTSSRSRRHLDDIVFRIPENTTMEDIEKKDMKIPLFAGETI
GEINVAKEWLKIDDDGKIHLLKPLNYEKTSSIIATVPINGLQSTRTQTIRIHVADIDEPP
SFVNSPLPMLAVVPLNPTIGRIVYQFVARDEHGDGDSNVLYKTIDVIPAGSFIVDPKSGV
VRTGWSKYERGDTYRISAQAMDLSPSDNTTSQLSEVAILEILADERPPQFAKQEYEVTVS
EDNLVDYSVVDVKAQSFRSFEDGRSKGPITYSLEGDTPEDETKWFRIDPSTGIIHLTRLL
DFDDPALPKLHKLKVTAREDNRESHVDLTIRIDDVNDNVPTFTRPLYTAQVREDIPLNQT
ILKVTAVDKDTGDNSRITYSVDNHNFSINSNGEISAKVRLDADQLNERHFVYRFNVTARD
HGEPVSLSSSAMIHIRTENTNDESAVFLPTSQYTAFVAEDAQGGTPVIQIQARDADRDEV
TYSFMDKNGRSTQKMNLFSIDEHTGLVKLRHGVSAADLAEAENPINLTVIVQDDGSCCVY
PSKTHTSYATLLIGIEDVNNNKPEFPDCAKYSDIAKIMEGTYKTDPPTIVKVEATDDDSS
ANGDIVYSLYYTQSESRKAFVIDRQTGVLTPSPHVVFDRETRPREDVTVKATDRGDRPLI
GFCQFSVEVVDINDNSPQFERPSYETSVSRFEAVGTSVITVFAFDNDAAHNAEITYSLEI
DTTAGEEHQNDLDFFELVNRRSGEITLIKPIPMKTQKFIFNVIADDNGIPEALQSSAQVT
LNVLDKQQKAPKWQTSPDCKPGITVDENVELNKVILRCRAVSSGDSRNSDVIYKLTASGG
PGNKAESKFRQFNKFENGNEWVEVVIMEGLDYEQVNNYTLTLTATDMTSRVASTKTFVVE
VRDVNDVVPQFTVDLFTGTIDEEMTPNEHLEKTNGKPIVTVKAIDTDSDGPQNEVHYRIV
GEANGEETKHFRIDELTGEIFPNEKFDREKIDMYILTVEASDRSVSALPGANGPNKDNVK
VQIVINDVNDNAPSFEEQKYIGRVKESEGEGHDVITIKAHDLDKHSNLRYHLIGAGGGRI
PFGVRTDSGTIFVKEPLDFEASDQYHLVLIASDGRHNATTNVYIHIEDVNDNAPQFEQQK
YATTVIEEDVDIPKVLFNVHATDADQDEKSSRIVYRLEGQGADEVFRIGKYSGTIELVKA
LDRDPPAGVPSWNFVVQAIDDDGNGLVGYADVQVNVRDINDNSPIFPERLFGYIEENREP
IHSDGVYFMDVQARDFDDPTTENANIEYGIVRNKLINGESVFRIDQNTGKIFAMRSLDRE
ISSEREFIIEVRANDRGVPSREGFANVTIKVTDMNDNAPFFEKTRYEGSVEETAPIGAAV
MSFSAFDADEEAKDNVFTYQLSEESDYFYVTTDKDSKQSSVGVLRVKQPLDYEDVTQRDG
FHLGIRVSDGRHDAEAAVHVALVDRNDHAPHIHGATEHRVREDVPRGTSIGRYTATDRDA
GDTARFRINRQSDPKRQFTIDQDGTLRVAHTLDREDIAVYNLIIEAYDNSNNIGRQMVAV
YLQDVNDNGPEPYTVPRPCIFRENTPVNQLGTCEIRATDRDTAEFGPPFTMEVSPSFKYS
QYLNVIFNANGDGGNGSMTITPLQEFDREAPVPGKILEIPLILADRAGRRNEASVHVIIG
DLNDNTMHDGRMTIHVNSYLGRLKETVIGRVYVDDADDWDLGDKTFSWKDSRPGFELSDK
GSITMAGEMAAGTYTMSANVHDNARDEDAVGYVTVIVNAVPQIAFDNQGSVQLLIAEETP

LQLPDDFIRADSNGQSLMDTFKQEMTAYMGGDVTVDVFSVQVGIATLQTRDVPVLNVRFN
ARGSTYRDTAQLNGLIAAHRADLQRKLNVEIVGVGIDMCKFTQCDAGCQTLNSADYDGIV
VSANSTVIVGVNATSRDDCTCPVWRAPPACQHSLCHNDGVCHNTNPGFFCECRNDGLKGA
RCQGTTRSFGGNGFAWYKPMPACTSLNISFSFMTTQSDALLFYNGPLETLRNDTHIEYSD
YIFIQLRGGRISLEVSMNGQSRSSLEVASTALNDGTWHDISVNQEGKRVELVVDNCRFLG
AGADDSSCRAELYTPDDDERLNIVTPVQIGGLAPLSGQDYPQTIPRAGLNGCVRNLNVNG
DQYDLATPAFEQNSEKGCRLWGATCDSNSVDSLNHCIHGDCFADVQGSGAMVAKCVCDPG
WGGARCERRMEWIQFAQGAFIEYSPRIAFPEQVSDIELLFISGKVNGAPAELSFGTDSQQ
SYVSTNLESGQNGVTAAGKFDIGTGGRRARQELRVSEVLLKENASYWLQFTRNPTRASLS
IDNAYTVSTQLDKGEPFSLQVNQITLGTQGQNKGFQGCIGTYRWSKQNLPLKRGGAMDEN
EESIVSISNMAGVQDGCDLRITCADLPAGYCGGSFVCVDFWKGPFCTCNDGANAILGDDG
QVVGCGETLAVSKLGISSPAIILILVSLALLILLVMMMVVYTRRSPGAFENVRPEEMNRD
NLRQYGVEGGGEADNDQYSMAGLRKPVMPLDTGMGPAIGGHPPHYPPRGMAPPKDDHELN
SKIKDLETDQNAAPYDELRIYDDERDNISVVTLESIESAQ
>P48357
MICQKFCVVLLHWEFIYVITAFNLSYPITPWRFKLSCMPPNSTYDYFLLPAGLSKNTSNS
NGHYETAVEPKFNSSGTHFSNLSKTTFHCCFRSEQDRNCSLCADNIEGKTFVSTVNSLVF
QQIDANWNIQCWLKGDLKLFICYVESLFKNLFRNYNYKVHLLYVLPEVLEDSPLVPQKGS
FQMVHCNCSVHECCECLVPVPTAKLNDTLLMCLKITSGGVIFQSPLMSVQPINMVKPDPP
LGLHMEITDDGNLKISWSSPPLVPFPLQYQVKYSENSTTVIREADKIVSATSLLVDSILP
GSSYEVQVRGKRLDGPGIWSDWSTPRVFTTQDVIYFPPKILTSVGSNVSFHCIYKKENKI
VPSKEIVWWMNLAEKIPQSQYDVVSDHVSKVTFFNLNETKPRGKFTYDAVYCCNEHECHH
RYAELYVIDVNINISCETDGYLTKMTCRWSTSTIQSLAESTLQLRYHRSSLYCSDIPSIH
PISEPKDCYLQSDGFYECIFQPIFLLSGYTMWIRINHSLGSLDSPPTCVLPDSVVKPLPP
SSVKAEITINIGLLKISWEKPVFPENNLQFQIRYGLSGKEVQWKMYEVYDAKSKSVSLPV
PDLCAVYAVQVRCKRLDGLGYWSNWSNPAYTVVMDIKVPMRGPEFWRIINGDTMKKEKNV
TLLWKPLMKNDSLCSVQRYVINHHTSCNGTWSEDVGNHTKFTFLWTEQAHTVTVLAINSI
GASVANFNLTFSWPMSKVNIVQSLSAYPLNSSCVIVSWILSPSDYKLMYFIIEWKNLNED
GEIKWLRISSSVKKYYIHDHFIPIEKYQFSLYPIFMEGVGKPKIINSFTQDDIEKHQSDA
GLYVIVPVIISSSILLLGTLLISHQRMKKLFWEDVPNPKNCSWAQGLNFQKPETFEHLFI
KHTASVTCGPLLLEPETISEDISVDTSWKNKDEMMPTTVVSLLSTTDLEKGSVCISDQFN
SVNFSEAEGTEVTYEDESQRQPFVKYATLISNSKPSETGEEQGLINSSVTKCFSSKNSPL
KDSFSNSSWEIEAQAFFILSDQHPNIISPHLTFSEGLDELLKLEGNFPEENNDKKSIYYL
GVTSIKKRESGVLLTDKSRVSCPFPAPCLFTDIRVLQDSCSHFVENNINLGTSSKKTFAS
YMPQFQTCSTQTHKIMENKMCDLTV
>P97577
MEAPLVSLDEEFEDIRPCCTEDPEEKPQSLYGTSPHHLEDPSLSELENFSSEIISFKSME
DLVNEFDEKLNVCFRNYNAKTENLAPVKNQLQIQEEEETLRDEEVWDALTDNYIPSLSED
WRDPNIEALNGNSSDTEIHEKEEEDEFIEKSENDSGINEEPLLTADQVIEEIEEMMQNSP
DPEEEVEVLEEEDGGEISSQADSVLLQEMQALTQTFNNNWSYEGLRHMSGSELTELLDQV
EGAIRDFSEELVHQLARRDELEFEKEVKNSFITVLIEVQNKQKEQRELMKKRRKEKGLSL
QSSRIEKGNQMPLKRFSMEGISNILQSGIRQTFGSSGADRQYLNTVIPYEKKSSPPSVED
LQMLTNILFAMKEDNEKVPTLLTDYILKVLCPT
>Q13508

MKTGHFEIVTMLLATMILVDIFQVKAEVLDMADNAFDDEYLKCTDRMEIKYVPQLLKEEK
ASHQQLDTVWENAKAKWAARKTQIFLPMNFKDNHGIALMAYISEAQEQTPFYHLFSEAVK
MAGQSREDYIYGFQFKAFHFYLTRALQLLRKPCEASSKTVVYRTSQGTSFTFGGLNQARF
GHFTLAYSAKPQAANDQLTVLSIYTCLGVDIENFLDKESERITLIPLNEVFQVSQEGAGN
NLILQSINKTCSHYECAFLGGLKTENCIENLEYFQPIYVYNPGEKNQKLEDHSEKNWKLE
DHGEKNQKLEDHGVKILEPTQIPGMKIPEPFPLPEDKSQGNINNPTPGPVPVPGPKSHPS
ASSGKLLLPQFGMVIILISVSAINLFVAL
>Q99467
MAFDVSCFFWVVLFSAGCKVITSWDQMCIEKEANKTYNCENLGLSEIPDTLPNTTEFLEF
SFNFLPTIHNRTFSRLMNLTFLDLTRCQINWIHEDTFQSHHQLSTLVLTGNPLIFMAETS
LNGPKSLKHLFLIQTGISNLEFIPVHNLENLESLYLGSNHISSIKFPKDFPARNLKVLDF
QNNAIHYISREDMRSLEQAINLSLNFNGNNVKGIELGAFDSTVFQSLNFGGTPNLSVIFN
GLQNSTTQSLWLGTFEDIDDEDISSAMLKGLCEMSVESLNLQEHRFSDISSTTFQCFTQL
QELDLTATHLKGLPSGMKGLNLLKKLVLSVNHFDQLCQISAANFPSLTHLYIRGNVKKLH
LGVGCLEKLGNLQTLDLSHNDIEASDCCSLQLKNLSHLQTLNLSHNEPLGLQSQAFKECP
QLELLDLAFTRLHINAPQSPFQNLHFLQVLNLTYCFLDTSNQHLLAGLPVLRHLNLKGNH
FQDGTITKTNLLQTVGSLEVLILSSCGLLSIDQQAFHSLGKMSHVDLSHNSLTCDSIDSL
SHLKGIYLNLAANSINIISPRLLPILSQQSTINLSHNPLDCTCSNIHFLTWYKENLHKLE
GSEETTCANPPSLRGVKLSDVKLSCGITAIGIFFLIVFLLLLAILLFFAVKYLLRWKYQH
I
>Q12246
MVVQKKLRAILTDEGVLIKSQSHHMFNKHGQLRSGDSLSLLSCLSCLDDGTLSSDGGSFD
EDDSLELLPLNTTIPFNRILNAKYVNVGQKGFNNGKISSNPFQTENLSSSSENDDVENHS
LSNDKAPVSESQSFPKKDKWDTKTNTVKVSPDDSQDNSPSLGIKDNQQLIELTFAVPKGH
DVIPQKLTLLIDHVSRKSRANTGEENISSGTVEEILEKSYENSKRNRSILVIINPHGGKG
TAKNLFLTKARPILVESGCKIEIAYTKYARHAIDIAKDLDISKYDTIACASGDGIPYEVI
NGLYRRPDRVDAFNKLAVTQLPCGSGNAMSISCHWTNNPSYAALCLVKSIETRIDLMCCS
QPSYMNEWPRLSFLSQTYGVIAESDINTEFIRWMGPVRFNLGVAFNIIQGKKYPCEVFVK
YAAKSKKELKVHFLENKDKNKGCLTFEPNPSPNSSPDLLSKNNINNSTKDELSPNFLNED
NFKLKYPMTEPVPRDWEKMDSELTDNLTIFYTGKMPYIAKDTKFFPAALPADGTIDLVIT
DARIPVTRMTPILLSLDKGSHVLEPEVIHSKILAYKIIPKVESGLFSVDGEKFPLEPLQV
EIMPMLCKTLLRNGRYIDTEFESM
>Q4WI46
MVRIGSSLLLATLAATTVSAASDPPKCSQDSHCPEEWPCCSLYGQCGTGAYCLGGCDPLM
SFSLDSCTPEPICQGKTYKDWSNLDNLASNTKYLGDASKSDWVYSGYPKVEDGNLLLTMP
KNSVGTLIANNHYIWYGKITAKIKSSRGAGVVTGFILLSDTKDEIDYEFVGADLTNVQTN
YYFQGVLDYNHGGNASVSGGNTFGDWHEYTIDWKPDAITWSVDGEVKRTLKKESTYNETS
KQYMYPQTPSRMQLSLWPAGQASNAPGTIAWAGGEIDWDSEDIKDPGYYYATFGEITVEC
YDPPSGADIKGTKAYIFKDKAGLESSVQITNNKTVLASFGATGLDMDVGASSSASGSANK
TSSSANTVPSGNGGSGNEPGNSHSGSSGSGTSTSDGSGSSTGFSQGSETSASSNKNAAPS
QNERVLNGSFFAVLVAVVALVTL
>P28908
MRVLLAALGLLFLGALRAFPQDRPFEDTCHGNPSHYYDKAVRRCCYRCPMGLFPTQQCPQ
RPTDCRKQCEPDYYLDEADRCTACVTCSRDDLVEKTPCAWNSSRVCECRPGMFCSTSAVN

SCARCFFHSVCPAGMIVKFPGTAQKNTVCEPASPGVSPACASPENCKEPSSGTIPQAKPT
PVSPATSSASTMPVRGGTRLAQEAASKLTRAPDSPSSVGRPSSDPGLSPTQPCPEGSGDC
RKQCEPDYYLDEAGRCTACVSCSRDDLVEKTPCAWNSSRTCECRPGMICATSATNSCARC
VPYPICAAETVTKPQDMAEKDTTFEAPPLGTQPDCNPTPENGEAPASTSPTQSLLVDSQA
SKTLPIPTSAPVALSSTGKPVLDAGPVLFWVILVLVVVVGSSAFLLCHRRACRKRIRQKL
HLCYPVQTSQPKLELVDSRPRRSSTQLRSGASVTEPVAEERGLMSQPLMETCHSVGAAYL
ESLPLQDASPAGGPSSPRDLPEPRVSTEHTNNKIEKIYIMKADTVIVGTVKAELPEGRGL
AGPAEPELEEELEADHTPHYPEQETEPPLGSCSDVMLSVEEEGKEDPLPTAASGK
>Q3UVY1
MSFFLSNLTNDSNLWKASHNSTETDLMNSPATLTLSLFCLICIMTLAALVGSIFSLVSLL
TMQYRTVLSILVTSWSVDDLLSVLSVAIFMVLQWPKEAQGYFQSLCTTSALLYMCQGLSS
NLKATLIVCYNFYTMNRTVESQSSSWRLGQVLGVTLTVWAVSLLLASLPLCGWGVFVRTP
WGCLTDCSSPYVLLLFAVYASAFGLLAVLSVPLTHQLLCSEEPLRLHANYQEISRGASTP
GTPAAAGRVLCLPPEDVEIPALRCTGGCSPSSDVVFAPGQPAASGAGAGRRENPGTPQGT
NSFPLSLAQKRFSLILALTKVILWLPMMIHMVVKHVVGFQSLPVDMLSFLLSLLASSVTP
VFVLSKRWAHLPCGCIINCQPDTYSVAFDGKKSKRKGFEFNLSFQQSYGLYKIPHADCYD
DDENSISYHNPKNYECEATKEPWGDNRSVFNTITVEISTTPPLDSATLTGVHKCTNTDIP
ESKQAMKEEKGAFSVKTESDINYGETTSFEGPERRLSHEENQKPDLSDWEWCRSKSERTP
RQRSGGGLAIPICAFQGTVSLQAPTGKTLSLSTYEVSAEGQKITPASKKIEVYRSKSVGH
EPNSEESPSTFADTSVKIHLEVLEICDNDEALDTVSIISNISQSSTKVRSPSLRYSRKEN
RFVSCDLGETASYSLFLPTSDPDGDINISIPDTVEAHRQNSRRQHEERDGYQEEIQLLNK
AYRKREAESKGD
>Q90X64
MDNDSQYSGYSYKSGHSRSSRKHRDRRERHRSKSREGSRGDKSVTIQAPGEPLLDNESTR
GEDRDDNWGETTTVVTGTSEHSISHDDITRITKDMEDSAKLDCSRHLGVVIGGALALLSF
LTPIAFMLLPQILWREDLEQCGTACEGLFISVAFKLLILLLGSWALFFRRPKAFFPRVFV
FRALLMVLVFLLVVSYWLFYGVRILESRDKNYQGIVQYAVSLVDALLFVHYLAVVLLELR
QLQPQFTIKVVRSTDGASRFYNIGHLSIQRVAVWILENYYHDFPVYNPALLNLPKSILSK
KMSGFKVYSLGEENTTNNSTGQSRAVIAAAARRRDNSHNEYYYEEAEHERRVRKRKARLV
VAVEEAFTHIKRLQDEDQKNPREIMDPREAAQAIFASMARAMQKYLRTTKQQPYHTMESI
LHHLEFCITHDMTPKAFLERYLGPGPTIQYHKDRWLAKQWTLVSEEPVTNGLKDGVVFVL
KRQDFSLVVSTKKIPFFKLSEEFVDPKSHKFVMRLQSETSV
>Q9C8K2
MELESTSNGRRPPPPAEIGRGAYLAWEDLTVVIPNFSGGPTRRLLDGLNGHAEPGRIMAI
MGPSGSGKSTLLDSLAGRLARNVIMTGNLLLNGKKARLDYGLVAYVTQEDILMGTLTVRE
TITYSAHLRLSSDLTKEEVNDIVEGTIIELGLQDCADRVIGNWHSRGVSGGERKRVSVAL
EILTRPQILFLDEPTSGLDSASAFFVIQALRNIARDGGRTVVSSIHQPSSEVFALFDDLF
LLSSGETVYFGESKFAVEFFAEAGFPCPKKRNPSDHFLRCINSDFDTVTATLKGSQRIRE
TPATSDPLMNLATSEIKARLVENYRRSVYAKSAKSRIRELASIEGHHGMEVRKGSEATWF
KQLRTLTKRSFVNMCRDIGYYWSRIVIYIVVSFCVGTIFYDVGHSYTSILARVSCGGFIT
GFMTFMSIGGFPSFIEEMKVFYKERLSGYYGVSVYIISNYVSSFPFLVAIALITGSITYN
MVKFRPGVSHWAFFCLNIFFSVSVIESLMMVVASLVPNFLMGLITGAGIIGIIMMTSGFF
RLLPDLPKVFWRYPISFMSYGSWAIQGAYKNDFLGLEFDPMFAGEPKMTGEQVINKIFGV
QVTHSKWWDLSAIVLILVCYRILFFIVLKLKERAEPALKAIQAKRTMKSLKKRPSFKKVP

SLSSLSSRRHQPLHSLSSQEGLTSPIN
>Q0GA42
MAAAAAAAAALGVRLRDCCSRGAVLLLFFSLSPRPPAAAAWLLGLRPEDTAGGRVSLEGG
TLRAAEGTSFLLRVYFQPGPPRCREQSDWASDVEVLGPLRPGGVAGSALVQLFSLCAWDG
RAWHHHGAAGGFLLRVRPRLYGPGGDLLPPAWLRALGALLLLALSALFSGLRLSLLSLDP
VELRVLRNSGSAAEQEQARRVQAVRGRGTHLLCTLLLGQAGANAALAGWLYASLPPGVGD
PGEDSGEAGVHFPWLPALVCTGAVFLGAEICPYSVCSRHGLAIASHSVCLTRLLMAAAFP
VCYPLGRLLDWALPQEISTFYTREKLLETLRAADPYSDLVKEELNIIQGALELRTKVVEE
VLTPLGDCFMLRSDAVLDFATVSEILRSGYTRIPVYEGDQRHNIVDILFVKDLAFVDPDD
CTPLLTVTRFYNRPLHCVFNDTRLDTVLEEFKKGKSHLAIVQRVNNEGEGDPFYEVMGIV
TLEDIIEEIIKSEILDETDLYTDNRKKQRVPHRERRRHDFSLFKLSDSEIRVKISPQLLL
ATHRFMATEVEPFKSLYLSEKILLRLLKHPNVIQELKFDERNKKAPEHYLYQRNRPVDYF
VLLLQGKVEVEVGKEGLRFENGAFTYYGVPAIMTSACSDNDVRKVGSLAGSSVFLNRSPS
RCSGLNRSESPNRERSDFGGSNTQLYSSSNNLYTPDYSVHILSDVQFVKITRQQYQNALT
ACHMDSSPQSPDMEAFTDGDSTKAPTTRGTPQTPKDDPVLTLLSNRTSLPCSRSDGLRSP
GEVVYLRMEEMAFPQEEMPNFEEHRSQQVSLSPVAVPTTAASDPECCNIHLDPEASPCSS
DSEENMGKKLLRTLSGRKRKKSADGERASEENSNLTPLIT
>P34979
MKATRDHASAPFCTRFNHSDPGIWAAERAVEAPNNLTLPPEPSEDCGSVSVAFSMTMMIT
GFVGNALAITLVSKSYRRREGKRKKSFLLCIGWLALTDMVGQLLTSPVVIVLYLSHQRWE
QLDPSGRLCTFFGLTMTVFGLSSLFIASAMAVERALATRAPHWYSSHMKTSVTRAVLLGV
WLAVLAFALLPVLGVGQYTIQWPGTWCFISTGPGGNGTNSRQNWGNVFFASAFAILGLSA
LVVTFACNLATIKALVSRCRAKATASQSSAQWGRITTETAIQLMGIMCVLSVCWSPLLIM
MLKMIFNHTSVEHCKTYTENQDECNFFLIAVRLASLNQILDPWVYLLLRKILLQKFCQLL
KGHSYGLDTEGGTENKDKEMKENLYISNLSRFFILLGHFTEARRGRGHIYLHTLEHQ
>P08101
MESNWTVHVFSRTLCHMLLWTAVLNLAAGTHDLPKAVVKLEPPWIQVLKEDTVTLTCEGT
HNPGNSSTQWFHNGRSIRSQVQASYTFKATVNDSGEYRCQMEQTRLSDPVDLGVISDWLL
LQTPQLVFLEGETITLRCHSWRNKLLNRISFFHNEKSVRYHHYSSNFSIPKANHSHSGDY
YCKGSLGRTLHQSKPVTITVQGPKSSRSLPVLTIVAAVTGIAVAAIVIILVSLVYLKKKQ
VPALPGNPDHREMGETLPEEVGEYRQPSGGSVPVSPGPPSGLEPTSSSPYNPPDLEEAAK
TEAENTITYSLLKHPEALDEETEHDYQNHI
>P13595
MLRTKDLIWTLFFLGTAVSLQVDIVPSQGEISVGESKFFLCQVAGDAKDKDISWFSPNGE
KLSPNQQRISVVWNDDDSSTLTIYNANIDDAGIYKCVVTAEDGTQSEATVNVKIFQKLMF
KNAPTPQEFKEGEDAVIVCDVVSSLPPTIIWKHKGRDVILKKDVRFIVLSNNYLQIRGIK
KTDEGTYRCEGRILARGEINFKDIQVIVNVPPTVQARQSIVNATANLGQSVTLVCDADGF
PEPTMSWTKDGEPIENEEEDERSRSSVSDSSEVTIRNVDKNDEAEYVCIAENKAGEQDAS
IHLKVFAKPKITYVENQTAMELEEQVTLTCEASGDPIPSITWRTSTRNISSEEQDLDGHM
VVRSHARVSSLTLKSIQYRDAGEYMCTASNTIGQDSQSIDLEFQYAPKLQGPVAVYTWEG
NQVNITCEVFAYPSATISWFRDGQLLPSSNYSNIKIYNTPSASYLEVTPDSENDFGNYNC
TAVNRIGQESLEFILVQADTPSSPSIDRVEPYSSTAQVQFDEPEATGGVPILKYKAEWKS
LGEESWHFKWYDAKEANMEGIVTIMGLKPETRYSVRLAALNGKGLGEISAATEFKTQPVR
EPSAPKLEGQMGEDGNSIKVNLIKQDDGGSPIRHYLVKYRALASEWKPEIRLPSGSDHVM

LKSLDWNAEYEVYVVAENQQGKSKAAHFVFRTSAQPTAIPANGSPTAGLSTGAIVGILIV
IFVLLLVVMDITCYFLNKCGLLMCIAVNLCGKAGPGAKGKDMEEGKAAFSKDESKEPIVE
VRTEEERTPNHDGGKHTEPNETTPLTEPELPADTTATVEDMLPSVTTVTTNSDTITETFA
TAQNSPTSETTTLTSSIAPPATTVPDSNSVPAGQATPSKGVTASSSSPASAPKVAPLVDL
SDTPTSAPSASNLSSTVLANQGAVLSPSTPASAGETSKAPPASKASPAPTPTPAGAASPL
AAVAAPATDAPQAKQEAPSTKGPDPEPTQPGTVKNPPEAATAPASPKSKAATTNPSQGED
LKMDEGNFKTPDIDLAKDVFAALGSPRPATGASGQASELAPSPADSAVPPAPAKTEKGPV
ETKSEPPESEAKPAPTEVKTVPNDATQTKENESKA
>Q8BXS7
MDTAVEANLGAAGHGPRTELSDEDYYPQGSWDTVFLVALLLLGLPANGLMAWLAGSQARH
GAGTRLALLLLSLALSDFLFLAAATFQILEIQHGGHWPLGTAACRFYYFLWGVSYSSGLF
LLTALSLDRCLLALCPRWYPGHRPARLPLWVCAGVWVLATLFSVPWLVFPEAAVWWYDLV
ICLDFWDTEELPLRMLEILGGFLPFLLLLVCHVLTQATACRTCCGHQPRRMACHGFARVA
KTILSAYVVLRLPYQLAQLLYLAFLWDVYPGYLLWEALVYSDYLILLNSCLSPFLCLAAS
ADLRALLRTVLSSFAAAVCEERPGSFIPAEPQTLPGPTSEGQSRLDSVVQPQVNPSVQLQ
SDSVVQPEVSPSAQPQSDSVAQPTVGSLIQPPLDTVVQLEVNPLTQPQLDPMAQPQVNPS
AQPQSKSVVQPQVDPLTQPQLDPVAQPQSNTETPIPAFGDESASNPGEENSSGPCPDPTP
GTPENLDRPAVPQEKSPSNVPPEEAPSAGPT
>Q96D31
MHPEPAPPPSRSSPELPPSGGSTTSGSRRSRRRSGDGEPPGAPPPPPSAVTYPDWIGQSY
SEVMSLNEHSMQALSWRKLYLSRAKLKASSRTSALLSGFAMVAMVEVQLDADHDYPPGLL
IAFSACTTVLVAVHLFALMISTCILPNIEAVSNVHNLNSVKESPHERMHRHIELAWAFST
VIGTLLFLAEVVLLCWVKFLPLKKQPGQPRPTSKPPASGAAANVSTSGITPGQAAAIAST
TIMVPFGLIFIVFAVHFYRSLVSHKTDRQFQELNELAEFARLQDQLDHRGDHPLTPGSHY
A
>Q9T076
MTFLKMKSLSFFFTILLSLSTLFTISNARKFNVGGSGAWVTNPPENYESWSGKNRFLVHD
TLYFSYAKGADSVLEVNKADYDACNTKNPIKRVDDGDSEISLDRYGPFYFISGNEDNCKK
GQKLNVVVISARIPSTAQSPHAAAPGSSTPGSMTPPGGAHSPKSSSPVSPTTSPPGSTTP
PGGAHSPKSSSAVSPATSPPGSMAPKSGSPVSPTTSPPAPPKSTSPVSPSSAPMTSPPAP
MAPKSSSTIPPSSAPMTSPPGSMAPKSSSPVSNSPTVSPSLAPGGSTSSSPSDSPSGSAM
GPSGDGPSAAGDISTPAGAPGQKKSSANGMTVMSITTVLSLVLTIFLSA
>P34886
MNRNPRMSKFQPNPRSRSRFQDETDLRSLRSFKTDFSNYLASDTNFLNVAEIMTSYAYGE
SNNAHEKEIQCDLLTENGGIEIDPTRLSYRERIRWHLQQFCYKTSSHGIPMLGQAPNSLY
RAAWVFLLLICAIQFINQAVAVIQKYQKMDKITDIQLKFDTAPFPAITLCNLNPYKDSVI
RSHDSISKILGVFKSVMKKAGDSSSEALEEEEETEYDMNGITIQAKRKKRGAGEKGTFEP
ANSACECDEEDGSNECEERSTEKPSGDNDMCICAFDRQTNDAWPCHRKEQWTNTTCQTCD
EHYLCSKKAKKGTKRSELKKEPCICESKGLFCIKHEHAAMVLNLWEYFGDSEDFSEISTE
EREALGFGNMTDEVAIVTKAKENIIFAMSALSEEQRILMSQAKHNLIHKCSFNGKPCDID
QDFELVADPTFGNCFVFNHDREIFKSSVRAGPQYGLRVMLFVNASDYLPTSEAVGIRLTI
HDKDDFPFPDTFGYSAPTGYISSFGMRMKKMSRLPAPYGDCVEDGATSNYIYKGYAYSTE
GCYRTCFQELIIDRCGCSDPRFPSIGGVQPCQVFNKNHRECLEKHTHQIGEIHGSFKCRC
QQPCNQTIYTTSYSEAIWPSQALNISLGQCEKEAEECNEEYKENAAMLEVFYEALNFEVL

SESEAYGIVKMMADFGGHLGLWSGVSVMTCCEFVCLAFELIYMAIAHHINQQRIRRRENA
ANEY
>P14740
MKTPWKVLLGLLGVAALVTIITVPVVLLNKDEAAADSRRTYTLADYLKNTFRVKSYSLRW
VSDSEYLYKQENNILLFNAEHGNSSIFLENSTFEIFGDSISDYSVSPDRLFVLLEYNYVK
QWRHSYTASYSIYDLNKRQLITEEKIPNNTQWITWSQEGHKLAYVWKNDIYVKIEPHLPS
HRITSTGKENVIFNGINDWVYEEEIFGAYSALWWSPNGTFLAYAQFNDTGVPLIEYSFYS
DESLQYPKTVWIPYPKAGAVNPTVKFFIVNTDSLSSTTTTIPMQITAPASVTTGDHYLCD
VAWVSEDRISLQWLRRIQNYSVMAICDYDKTTLVWNCPTTQEHIETSATGWCGRFRPAEP
HFTSDGSSFYKIVSDKDGYKHICQFQKDRKPEQVCTFITKGAWEVISIEALTSDYLYYIS
NEYKEMPGGRNLYKIQLTDHTNKKCLSCDLNPERCQYYSVSLSKEAKYYQLGCRGPGLPL
YTLHRSTDQKELRVLEDNSALDKMLQDVQMPSKKLDFIVLNETRFWYQMILPPHFDKSKK
YPLLIDVYAGPCSQKADAAFRLNWATYLASTENIIVASFDGRGSGYQGDKIMHAINKRLG
TLEVEDQIEAARQFLKMGFVDSKRVAIWGWSYGGYVTSMVLGSGSGVFKCGIAVAPVSRW
EYYDSVYTERYMGLPTPEDNLDHYRNSTVMSRAENFKQVEYLLIHGTADDNVHFQQSAQI
SKALVDAGVDFQAMWYTDEDHGIASSTAHQHIYSHMSHFLQQCFSLR
>P41318
MSVILVSAGYDHTIRFWEALTGVCSRTIQHSDSQVNRLEITNDKKLLATAGHQNVRLYDI
RTTNPNPVASFEGHRGNVTSVSFQQDNRWMVTSSEDGTIKVWDVRSPSIPRNYKHNAPVN
EVVIHPNQGELISCDRDGNIRIWDLGENQCTHQLTPEDDTSLQSLSMASDGSMLAAANTK
GNCYVWEMPNHTDASHLKPVTKFRAHSTYITRILLSSDVKHLATCSADHTARVWSIDDDF
KLETTLDGHQRWVWDCAFSADSAYLVTASSDHYVRLWDLSTREIVRQYGGHHKGAVCVAL
NDV
>Q8NFN8
MEPEINCSELCDSFPGQELDRRPLHDLCKTTITSSHHSSKTISSLSPVLLGIVWTFLSCG
LLLILFFLAFTIHCRKNRIVKMSSPNLNIVTLLGSCLTYSSAYLFGIQDVLVGSSMETLI
QTRLSMLCIGTSLVFGPILGKSWRLYKVFTQRVPDKRVIIKDLQLLGLVAALLMADVILL
MTWVLTDPIQCLQILSVSMTVTGKDVSCTSTSTHFCASRYSDVWIALIWGCKGLLLLYGA
YLAGLTGHVSSPPVNQSLTIMVGVNLLVLAAGLLFVVTRYLHSWPNLVFGLTSGGIFVCT
TTINCFIFIPQLKQWKAFEEENQTIRRMAKYFSTPNKSFHTQYGEEENCHPRGEKSSMER
LLTEKNAVIESLQEQVNNAKEKIVRLMSAECTYDLPEGAAPPASSPNKDVQAVASVHTLA
AAQGPSGHLSDFQNDPGMAARDSQCTSGPSSYAQSLEGPGKDSSFSPGKEEKISDSKDFS
DHLDSGCSQKPWTEQSLGPERGDQVPMNPSQSLLPDRGGSDPQRQRHLENSEEPPERRSR
VSSVIREKLQEVLQDLGLGPEASLSTAPSCHQQTWKNSAAFSPQKMPLSKELGFSPYMVR
RRRAAQRARSHFPGSAPSSVGHRANRTVPGAHSRLHVQNGDSPSLAPQTTDSRVRRPSSR
KPSLPSDPQDRPGTLEGSKQSQTEPEGARGSKAAFLRQPSGSGRAPSPAAPCLSKASPDL
PEQWQLWPPVPSGCASLSSQHSYFDTESSSSDEFFCRCHRPYCEICFQSSSDSSDSGTSD
TDPEPTGGLASWEKLWARSKPIVNFKDDLKPTLV
>Q28896
MKGFLFLLLTISLLVMIQIQTGVLGNSTTPRMTTKKVKSATPALSSLGGGSVLLFLANTL
IQLFYLS
>Q9Y5U5
MAQHGAMGAFRALCGLALLCALSLGQRPTGGPGCGPGRLLLGTGTDARCCRVHTTRCCRD
YPGEECCSEWDCMCVQPEFHCGDPCCTTCRHHPCPPGQGVQSQGKFSFGFQCIDCASGTF

SGGHEGHCKPWTDCTQFGFLTVFPGNKTHNAVCVPGSPPAEPLGWLTVVLLAVAACVLLL
TSAQLGLHIWQLRSQCMWPRETQLLLEVPPSTEDARSCQFPEEERGERSAEEKGRLGDLW
V
>P10636
MAEPRQEFEVMEDHAGTYGLGDRKDQGGYTMHQDQEGDTDAGLKESPLQTPTEDGSEEPG
SETSDAKSTPTAEDVTAPLVDEGAPGKQAAAQPHTEIPEGTTAEEAGIGDTPSLEDEAAG
HVTQEPESGKVVQEGFLREPGPPGLSHQLMSGMPGAPLLPEGPREATRQPSGTGPEDTEG
GRHAPELLKHQLLGDLHQEGPPLKGAGGKERPGSKEEVDEDRDVDESSPQDSPPSKASPA
QDGRPPQTAAREATSIPGFPAEGAIPLPVDFLSKVSTEIPASEPDGPSVGRAKGQDAPLE
FTFHVEITPNVQKEQAHSEEHLGRAAFPGAPGEGPEARGPSLGEDTKEADLPEPSEKQPA
AAPRGKPVSRVPQLKARMVSKSKDGTGSDDKKAKTSTRSSAKTLKNRPCLSPKLPTPGSS
DPLIQPSSPAVCPEPPSSPKHVSSVTSRTGSSGAKEMKLKGADGKTKIATPRGAAPPGQK
GQANATRIPAKTPPAPKTPPSSGEPPKSGDRSGYSSPGSPGTPGSRSRTPSLPTPPTREP
KKVAVVRTPPKSPSSAKSRLQTAPVPMPDLKNVKSKIGSTENLKHQPGGGKVQIINKKLD
LSNVQSKCGSKDNIKHVPGGGSVQIVYKPVDLSKVTSKCGSLGNIHHKPGGGQVEVKSEK
LDFKDRVQSKIGSLDNITHVPGGGNKKIETHKLTFRENAKAKTDHGAEIVYKSPVVSGDT
SPRHLSNVSSTGSIDMVDSPQLATLADEVSASLAKQGL
>Q9Z258
MARAKLPRSPSEGKAGPGDTPAGSAAPEEPHGLSPLLPTRGGGSVGSDVGQRLHVEDFSL
DSSLSQVQVEFYVNENTFKERLKLFFIKNQRSSLRIRLFNFSLKLLTCLLYIVRVLLDNP
DQGIGCWGCTKYNYTFNGSSSEFHWAPILWVERKMALWVIQVIVATISFLETMLLIYLSY
KGNIWEQIFHVSFVLEMINTLPFIITVFWPPLRNLFIPVFLNCWLAKHALENMINDFHRA
ILRTQSAMFNQVLILFCTLLCLVFTGTCGIQHLERAGGNLNLLTSFYFCIVTFSTVGFGD
VTPKIWPSQLLVVILICVTLVVLPLQFEELVYLWMERQKSGGNYSRHRARTEKHVVLCVS
SLKIDLLMDFLNEFYAHPRLQDYYVVILCPSEMDVQVRRVLQIPLWSQRVIYLQGSALKD
QDLMRAKMDNGEACFILSSRNEVDRTAADHQTILRAWAVKDFAPNCPLYVQILKPENKFH
VKFADHVVCEEECKYAMLALNCICPATSTLITLLVHTSRGQEGQESPEQWQRMYGRCSGN
EVYHIRMGDSKFFREYEGKSFTYAAFHAHKKYGVCLIGLKREENKSILLNPGPRHILAAS
DTCFYINITKEENSAFIFKQEEKQNRRGLAGQALYEGPSRLPVHSIIASMVAMDLQNTDC
RPSQGGSGGGGGKLTLPTENGSGSRRPSIAPVLELADSSALLPCDLLSDQSEDEVTPSDD
EGLSVVEYVKGYPPNSPYIGSSPTLCHLLPVKAPFCCLRLDKGCKHNSYEDAKAYGFKNK
LIIVSAETAGNGLYNFIVPLRAYYRSRRELNPIVLLLDNKPDHHFLEAICCFPMVYYMEG
SVDNLDSLLQCGIIYADNLVVVDKESTMSAEEDYMADAKTIVNVQTMFRLFPSLSITTEL
THPSNMRFMQFRAKDSYSLALSKLEKQERENGSNLAFMFRLPFAAGRVFSISMLDTLLYQ
SFVKDYMITITRLLLGLDTTPGSGYLCAMKVTEDDLWIRTYGRLFQKLCSSSAEIPIGIY
RTECHVFSSEPHDLRAQSQISVNMEDCEDTREAKGPWGTRAASGGGSTHGRHGGSADPVE
HPLLRRKSLQWARKLSRKSSKQAGKAPMTTDWITQQRLSLYRRSERQELSELVKNRMKHL
GLPTTGYEDVANLTASDVMNRVNLGYLQDEMNDHHQNTLSYVLINPPPDTRLEPNDIVYL
IRSDPLAHVTSSSQSRKSSCSNKLSSCNPETRDETQL
>O14514
MRGQAAAPGPVWILAPLLLLLLLLGRRARAAAGADAGPGPEPCATLVQGKFFGYFSAAAV
FPANASRCSWTLRNPDPRRYTLYMKVAKAPVPCSGPGRVRTYQFDSFLESTRTYLGVESF
DEVLRLCDPSAPLAFLQASKQFLQMRRQQPPQHDGLRPRAGPPGPTDDFSVEYLVVGNRN
PSRAACQMLCRWLDACLAGSRSSHPCGIMQTPCACLGGEAGGPAAGPLAPRGDVCLRDAV

AGGPENCLTSLTQDRGGHGATGGWKLWSLWGECTRDCGGGLQTRTRTCLPAPGVEGGGCE
GVLEEGRQCNREACGPAGRTSSRSQSLRSTDARRREELGDELQQFGFPAPQTGDPAAEEW
SPWSVCSSTCGEGWQTRTRFCVSSSYSTQCSGPLREQRLCNNSAVCPVHGAWDEWSPWSL
CSSTCGRGFRDRTRTCRPPQFGGNPCEGPEKQTKFCNIALCPGRAVDGNWNEWSSWSACS
ASCSQGRQQRTRECNGPSYGGAECQGHWVETRDCFLQQCPVDGKWQAWASWGSCSVTCGA
GSQRRERVCSGPFFGGAACQGPQDEYRQCGTQRCPEPHEICDEDNFGAVIWKETPAGEVA
AVRCPRNATGLILRRCELDEEGIAYWEPPTYIRCVSIDYRNIQMMTREHLAKAQRGLPGE
GVSEVIQTLVEISQDGTSYSGDLLSTIDVLRNMTEIFRRAYYSPTPGDVQNFVQILSNLL
AEENRDKWEEAQLAGPNAKELFRLVEDFVDVIGFRMKDLRDAYQVTDNLVLSIHKLPASG
ATDISFPMKGWRATGDWAKVPEDRVTVSKSVFSTGLTEADEASVFVVGTVLYRNLGSFLA
LQRNTTVLNSKVISVTVKPPPRSLRTPLEIEFAHMYNGTTNQTCILWDETDVPSSSAPPQ
LGPWSWRGCRTVPLDALRTRCLCDRLSTFAILAQLSADANMEKATLPSVTLIVGCGVSSL
TLLMLVIIYVSVWRYIRSERSVILINFCLSIISSNALILIGQTQTRNKVMCTLVAAFLHF
FFLSSFCWVLTEAWQSYMAVTGHLRNRLIRKRFLCLGWGLPALVVAISVGFTKAKGYSTM
NYCWLSLEGGLLYAFVGPAAAVVLVNMVIGILVFNKLVSKDGITDKKLKERAGASLWSSC
VVLPLLALTWMSAVLAVTDRRSALFQILFAVFDSLEGFVIVMVHCILRREVQDAVKCRVV
DRQEEGNGDSGGSFQNGHAQLMTDFEKDVDLACRSVLNKDIAACRTATITGTLKRPSLPE
EEKLKLAHAKGPPTNFNSLPANVSKLHLHGSPRYPGGPLPDFPNHSLTLKRDKAPKSSFV
GDGDIFKKLDSELSRAQEKALDTSYVILPTATATLRPKPKEEPKYSIHIDQMPQTRLIHL
STAPEASLPARSPPSRQPPSGGPPEAPPAQPPPPPPPPPPPPQQPLPPPPNLEPAPPSLG
DPGEPAAHPGPSTGPSTKNENVATLSVSSLERRKSRYAELDFEKIMHTRKRHQDMFQDLN
RKLQHAAEKDKEVLGPDSKPEKQQTPNKRPWESLRKAHGTPTWVKKELEPLQPSPLELRS
VEWERSGATIPLVGQDIIDLQTEV

>P25117

MRFTLTRWCLTLFIFLNRPLPVLPDSADGAHTPTLEPEPFLYILGKQRMLEAQHRCYDRM
QKLPPYQGEGLYCNRTWDGWSCWDDTPAGVLAEQYCPDYFPDFDAAEKVTKYCGEDGDWY
RHPESNISWSNYTMCNAFTPDKLQNAYILYYLAIVGHSLSILTLLISLGIFMFLRYFNLL
APFNALLYPTRSISCQRVTLHKNMFLTYVLNSIIIIVHLVVIVPNGELVKRDPPICKVLH
FFHQYMMSCNYFWMLCEGVYLHTLIVVSVFAEGQRLWWYHVLGWGFPLIPTTAHAITRAV
LFNDNCWLSVDTNLLYIIHGPVMAALVVNFFFLLNILRVLVKKLKESQEAESHMYLKAVR
ATLILVPLLGVQFVVLPWRPSTPLLGKIYDYVVHSLIHFQGFFVAIIYCFCNHEVQGALK
RQWNQYQAQRWAGRRSTRAANAAAATAAAAAALAETVEIPVYICHQEPREEPAGEEPVVE
VEGVEVIAMEVLEQETSA

>Q80VL0

MSRRRISCKDLGHADCQGWLYKKKEKGTFLSNKWKKFWVVLKGSSLYWYGNQLAEKADGF
VNLPDFTVERASECKKKNAFKINHPQIKTFYFAAENLQEMNMWLNKLGFAVTHKESTTKD
EECYSESEQEDPETAVEAPPPPSASATSSPVAARRASSSSPKRRETSCSFSSLENTVKAP
SRFSSSGSKERQSWLDIVNSSPATEDVGHPLSFAVQVHTLASSEASSCRVSENSSTTPES
GCLNSLSSDDTSSLNNSQDHLTVPDRASGSRMTDRDEIKSSEDDEMEKLYKSLEQASLSP
LGDRRPSTKKELRKSFVKRCKNPSINEKLHKIRTLNSTLKCKEHDLAMINQLLDDPKLTA
RKYREWKVMNTLLIQDIYQQQVPQDPEVTPQEIMNPTSSDCVENSL

>Q4WLB9

MKASVSLLLLSAASMASAAMSVSQCAQMCLSNMKAKAGELGCSAGDDKCLCSQANYGYGI
RDCTTEACPDDDAIAVLSSALSSCPSDSAAVTATGAGGSSSGSGSGSDSGSGSGSGSGSG

SGSGSGSGSSSGSGSGSGSGSGSGSNSGSGSASSTATGTATGTATGTATGTATGTATG
SENSTTGGAGAGAGAGASSTGTNASGTGATTSGANPSNTGATTTDTTLTTTTTSSENGSS
TGNSSSETGAGSGSSTATGSGSGSGAGSASTTAPNSSSTGNVAPRGAVVGSGAVGALALA
ALIIL
>Q9Z1Q3
MNSQLVGILLSALLGVALGHRTRCYDCGGGPSNSCKQTVITCGEGERCGFLDRKPQPSSE
QAKQPSATLSHHYPACVATHHCNQVAIESVGDVTFTTQKNCCFGDLCNSAVASSVTPLCI
LAAAVTTLAWLLPGL
>Q9SUC9
MKKSLTLLILLLCSLLFSTVLSNLLVEPVQPNTVPAFPVETQAQSCRLDLSNELFGGVNE
ACGRNLDRSRCCPVLAAWLFAAHARSALQLPAPAPTPESSDPDEPMKPDDSQKCVNTLQS
ALLTKQIKIPQPNSSCDAILCFCGIRLHQISSLSCPAAFNVSSGFKNATPTAAVKNLEKE
CRNSSYSGCTRCLGALQKLKVRGGNKKTTTERGTKMMSKDCQLMGLTWLLARNKTAYIPT
VSAVLRAIMYSPHPPHLNKCSPDQENMPLAVDSLQFQKSFSSSSHLFGVLPFLPLVLCIF
LFLL
>Q29960
MRVMAPRTLILLLSGALALTETWACSHSMRYFYTAVSRPGRGEPRFIAVGYVDDTQFVRF
DSDAASPRGEPRAPWVEQEGPEYWDRETQKYKRQAQTDRVSLRNLRGYYNQSEAGSHTLQ
WMYGCDLGPDGRLLRGYDQSAYDGKDYIALNEHLRSCTAADTAAQITQRKWEAARAAEQQ
RAYLEGTCVEWLRRYLENGKETLQRAEHPKTHVTHHLVSDHEATLRCWALGFYPAEITLT
WQRDGEDQTQDTELVETRPAGDGTFQKWAAVVVPSGEEQRYTCHVQHEGLPEPLTLRWEP
SSQPTIPIVGIVAGLAVLAVLAVLGAVVAVVMCRRKSSGGKGGSCSQAASSNSAQGSDES
LIACKA
>Q9Y4D2
MPGIVVFRRRWSVGSDDLVLPAIFLFLLHTTWFVILSVVLFGLVYNPHEACSLNLVDHGR
GYLGILLSCMIAEMAIIWLSMRGGILYTEPRDSMQYVLYVRLAILVIEFIYAIVGIVWLT
QYYTSCNDLTAKNVTLGMVVCNWVVILSVCITVLCVFDPTGRTFVKLRATKRRQRNLRTY
NLRHRLEEGQATSWSRRLKVFLCCTRTKDSQSDAYSEIAYLFAEFFRDLDIVPSDIIAGL
VLLRQRQRAKRNAVLDEANNDILAFLSGMPVTRNTKYLDLKNSQEMLRYKEVCYYMLFAL
AAYGWPMYLMRKPACGLCQLARSCSCCLCPARPRFAPGVTIEEDNCCGCNAIAIRRHFLD
ENMTAVDIVYTSCHDAVYETPFYVAVDHDKKKVVISIRGTLSPKDALTDLTGDAERLPVE
GHHGTWLGHKGMVLSAEYIKKKLEQEMVLSQAFGRDLGRGTKHYGLIVVGHSLGAGTAAI
LSFLLRPQYPTLKCFAYSPPGGLLSEDAMEYSKEFVTAVVLGKDLVPRIGLSQLEGFRRQ
LLDVLQRSTKPKWRIIVGATKCIPKSELPEEVEVTTLASTRLWTHPSDLTIALSASTPLY
PPGRIIHVVHNHPAEQCCCCEQEEPTYFAIWGDNKAFNEVIISPAMLHEHLPYVVMEGLN
KVLENYNKGKTALLSAAKVMVSPTEVDLTPELIFQQQPLPTGPPMPTGLALELPTADHRN
SSVRSKSQSEMSLEGFSEGRLLSPVVAAAARQDPVELLLLSTQERLAAELQARRAPLATM
ESLSDTESLYSFDSRRSSGFRSIRGSPSLHAVLERDEGHLFYIDPAIPEENPSLSSRTEL
LAADSLSKHSQDTQPLEAALGSGGVTPERPPSAAANDEEEEVGGGGGGPASRGELALHNG
RLGDSPSPQVLEFAEFIDSLFNLDSKSSSFQDLYCMVVPESPTSDYAEGPKSPSQQEILL
RAQFEPNLVPKPPRLFAGSADPSSGISLSPSFPLSSSGELMDLTPTGLSSQECLAADKIR
TSTPTGHGASPAKQDELVISAR
>P20398
MSLQQLKRKSLRDGWLMDGVVPSPGAEIDSPLFQTESKIQQLEKELESLQMQQLRLENPA

AVQPEAKAIQTPFLNGEKIQQGGGQAGDAKEVTAGQANNTHGIIHEEQPTKEDQDMGTVL
PIPAPRGKTVPKEDENQANPELKVDMEHQKVELVDLIQECPVENQTVEHVEKNKPHLDQE
HTEKNQDGQHGILEFLTQDQQLGNPNLQHLDRYLITEVTVKHFEKNQAHPGQEKNQDEQH
GILKYLTQDQQNENPNLGHLDQYLITEITLQSNPLENISVHDQTQSTSDQNMETKLPTDI
PQQKESQSEGKIQTKDQGPEFELLYKDHSHEKGTTDQTQHQELLPSSIEPKEENPKAQDE
KLDHHNESVSTVHEQKEVHDMDPRQLSTHQKSLSISEDQNQGSVSLSDPQNQDQSLALPE
KGLEETPQLNLSHEVQCEEPSLMEQISISLLQSMEQNQEGADQTPKSVALEELSELLSSD
MIKQSVLLSKNLEESPSTASTEETQETMCQAVIIPDLKKENSDPEVVQESSSHEPMSSTI
AQSSSAEGNSSPESRPLLQKSQGTDSQQGGNTATQQEERRKKKTCQCCVVM
>P23515
MEYQILKMSLCLFILLFLTPGILCICPLQCICTERHRHVDCSGRNLSTLPSGLQENIIHL
NLSYNHFTDLHNQLTQYTNLRTLDISNNRLESLPAHLPRSLWNMSAANNNIKLLDKSDTA
YQWNLKYLDVSKNMLEKVVLIKNTLRSLEVLNLSSNKLWTVPTNMPSKLHIVDLSNNSLT
QILPGTLINLTNLTHLYLHNNKFTFIPDQSFDQLFQLQEITLYNNRWSCDHKQNITYLLK
WMMETKAHVIGTPCSTQISSLKEHNMYPTPSGFTSSLFTVSGMQTVDTINSLSVVTQPKV
TKIPKQYRTKETTFGATLSKDTTFTSTDKAFVPYPEDTSTETINSHEAAAATLTIHLQDG
MVTNTSLTSSTKSSPTPMTLSITSGMPNNFSEMPQQSTTLNLWREETTTNVKTPLPSVAN
AWKVNASFLLLLNVVVMLAV
>Q9GP84
MRFISIFLIIVALCVSSSWAFNFTDQPNSFRISGTGCGSGTTTVYFSTDGRCNSACGGSI
RIKGEGNNVPNQQFTLNDYSKNVTNCSGTSNVASFRCPALVNTTSPTFTVNVGNSAYHVT
CQYAQVTETPAGNSADKVAVGIAIIFGALISLLAL
>A2ALU4
MEGAEPRARPERLAEAEAPATDGVRLVEVQLSGGAPWGFTLKGGREHGEPLVITKIEEGS
KAAAVDKLLAGDEIVAINDVSLSGFRQEAICLVKGSHKTLKLVVKRKSDPSWRPHSWHAT
KYFDVHPEPAASLFLNTSGSPSWKSQHQASSSSHDLSGSWEHTSLQRTSDHFSSMGSIDS
LDHSSQLYPSGHLSSAKSNSSIDHLGGHSKRDSAYGSFSTCSSTPDHTLPKADASSTENI
LYKVGLWEASRPGSSRQSQSTGDPQGLQDRPSCFIPRVPGNSSKSPRPEDNVEPKIATHG
RSNFGPVWYVPDKKKAPSPPPLGLPLRSDSFSVAARGHEKARGPPFSDLASMQHFITLPH
VQPRGDHRMETTDRQWKLTHLSSGKEIGNVGYQSEGHLDCRWLCSDDRAGRPSGPPGRLQ
FSDVHFLKSYHGSQHQQQCSDESPRAPSSPRELLHITPGGGLQEPPEPSQDDNPTQVRWP
GSAHQKLDDRGRSHYFPGSLRQPVQGSAQVVIPRGDYWHSDTTPVDLEYPLLRPVGQRTY
LQQHEETPASHEKEGYHQLNAGIEGCCSGIQEPPRASRTVRTGLQCPSNDFKLVDGESGR
ISRQRTPMLHSLTQDGTWRPGNSKDCGNDKPPLFDAQVGKPTRRSDRFATTLRNEIQMRR
AKLQKSKSTVTLAGDSEAEDCAGDWRADVGAVPEGSFPSTYKEHLKEAQTRVLKATSFQR
RDLDPTPADQYSGPSEHRTFDHSASSSLSSFPGEPDSAPRFCETGLAKAPSSGVGVPHVL
RIGGRKRFTAEQKLKSYSEPEKINEVGLSGDHRPHPTVRTPEDTVGTFADRWKFFEETSK
SLLQKAGHRQVHCGLPREKAERPQTGHHECESTEPWFQKRSLATSCGEILSDRKVEKASE
KLNPPRRLGTFAEYQASWKEQKKPLEARSSGRYHSADDILDAGLDQQQRPQYIHERSRSS
PSTDHYSQEVPVEPNRQAEDSGDHKEAILCTLQAEEGCSAPSAQPQDSQHVNEDTTFPQP
ETQLSSKCQHLQTSAMETSRSPSPQFAPQKLTDKPPLLIHEDNSARIERVMDNNTTVKMV
PIKIVHSESQPEKESRQSLACPAELPPLPSGLERDQIKTLSTSEQCYSRFCVYTRQEVEA
PHRARPPEPRPPSTPAPPVRDSCSSPPSLNYGKAKEKTMDDLKSEELAREIVGKDKSLAD
ILDPSVKIKTTMDLMEGIFPKDEYLLEEAQQRRKLLPKVPLPRVTEDKKQDPGMPGVVSL

ATNSTYYSTSAPKAELLIKMKDLQEPEEYSAGDLDHDLSVKKQELIDSISRKLQVLREAR
ESLLEDIQANNALGDEVEAIVKDVCKPNEFDKFRMFIGDLDKVVNLLLSLSGRLARVENA
LNNLDDNPSPGDRQSLLEKQRVLTQQHEDAKELKENLDRRERIVFDILATYLSEENLADY
EHFVKMKSALIIEQRELEDKIHLGEEQLKCLFDSLQPERSK
>O95800
MNSTGHLQDAPNATSLHVPHSQEGNSTSLQEGLQDLIHTATLVTCTFLLAVIFCLGSYGN
FIVFLSFFDPAFRKFRTNFDFMILNLSFCDLFICGVTAPMFTFVLFFSSASSIPDAFCFT
FHLTSSGFIIMSLKTVAVIALHRLRMVLGKQPNRTASFPCTVLLTLLLWATSFTLATLAT
LKTSKSHLCLPMSSLIAGKGKAILSLYVVDFTFCVAVVSVSYIMIAQTLRKNAQVRKCPP
VITVDASRPQPFMGVPVQGGGDPIQCAMPALYRNQNYNKLQHVQTRGYTKSPNQLVTPAA
SRLQLVSAINLSTAKDSKAVVTCVIIVLSVLVCCLPLGISLVQVVLSSNGSFILYQFELF
GFTLIFFKSGLNPFIYSRNSAGLRRKVLWCLQYIGLGFFCCKQKTRLRAMGKGNLEVNRN
KSSHHETNSAYMLSPKPQKKFVDQACGPSHSKESMVSPKISAGHQHCGQSSSTPINTRIE
PYYSIYNSSPSQEESSPCNLQPVNSFGFANSYIAMHYHTTNDLVQEYDSTSAKQIPVPSV

>P15920
MGSLFRSESMCLAQLFLQSGTAYECLSALGEKGLVQFRDLNQNVSSFQRKFVGEVKRCEE
LERILVYLVQEITRADIPLPEGEASPPAPPLKHVLEMQEQLQKLEVELREVTKNKEKLRK
NLLELVEYTHMLRVTKTFLKRNVEFEPTYEEFPALENDSLLDYSCMQRLGAKLGFVSGLI
QQGRVEAFERMLWRACKGYTIVTYAELDECLEDPETGEVIKWYVFLISFWGEQIGHKVKK
ICDCYHCHIYPYPNTAEERREIQEGLNTRIQDLYTVLHKTEDYLRQVLCKAAESVCSRVV
QVRKMKAIYHMLNMCSFDVTNKCLIAEVWCPEVDLPGLRRALEEGSRESGATIPSFMNTI
PTKETPPTLIRTNKFTEGFQNIVDAYGVGSYREVNPALFTIITFPFLFAVMFGDFGHGFV
MFLFALLLVLNENHPRLSQSQEILRMFFDGRYILLLMGLFSVYTGLIYNDCFSKSVNLFG
SGWNVSAMYSSSHSPEEQRKMVLWNDSTIRHSRTLQLDPNIPGVFRGPYPFGIDPIWNLA
TNRLTFLNSFKMKMSVILGIFHMTFGVVLGIFNHLHFRKKFNVYLVSVPEILFMLCIFGY
LIFMIIYKWLAYSAETSREAPSILIEFINMFLFPTSKTHGLYPGQAHVQRVLVALTVLAV
PVLFLGKPLFLLWLHNGRNCFGMSRSGYTLVRKDSEEEVSLLGNQDIEEGNSRMEEGCRE
VTCEEFNFGEILMTQAIHSIEYCLGCISNTASYLRLWALSLAHAQLSDVLWAMLMRVGLR
VDTTYGVLLLLPVMAFFAVLTIFILLVMEGLSAFLHAIRLHWVEFQNKFYVGAGTKFVPF
SFSLLSSKFSNDDSIA
>Q8X176
MKPSVATLLATVSLVYAQTATEKEPSLSAIESAAASIQPYSPVSNVEGVAFNRFFQVWLE
NIDYEDAAADENMKWLASQGILLTNFYAVTHPSEPNYCAAVGGDTFGMDNDNFNQIPANV
STVADLLDTKNIAWGEYQEHLPYPGFQGFNYSNQETYVNDYVRKHNPLVLYDSVTKNSTR
LRQIKNFTSFEDDLANKKLPQWAFITPNMTNDAHDTNITFGAKWERSWIAPLLNNSYFMN
DTLILLTFDEDGTYSKSNKIFSVLLGGAIPDELKGTQDDTFYTHYSVIASVSANWGLPSL
GRWDCGANILEIVANKTGYVNYDVDTTNLRLNETYPGPMSAGEYSKYSPVWPNALTRGDC
SAGHGILDIVKETYANTEPTYNYSSPFPYDTASNYNTKVTATKKNVTGTHRSSSSSSPSA
SSNAAVSAVAPAAGVSGLLLGLALNLL
>Q9UP52
MERLWGLFQRAQQLSPRSSQTVYQRVEGPRKGHLEEEEEDGEEGAETLAHFCPMELRGPE
PLGSRPRQPNLIPWAAAGRRAAPYLVLTALLIFTGAFLLGYVAFRGSCQACGDSVLVVSE
DVNYEPDLDFHQGRLYWSDLQAMFLQFLGEGRLEDTIRQTSLRERVAGSAGMAALTQDIR

AALSRQKLDHVWTDTHYVGLQFPDPAHPNTLHWVDEAGKVGEQLPLEDPDVYCPYSAIGN
VTGELVYAHYGRPEDLQDLRARGVDPVGRLLLVRVGVISFAQKVTNAQDFGAQGVLIYPE
PADFSQDPPKPSLSSQQAVYGHVHLGTGDPYTPGFPSFNQTQFPPVASSGLPSIPAQPIS
ADIASRLLRKLKGPVAPQEWQGSLLGSPYHLGPGPRLRLVVNNHRTSTPINNIFGCIEGR
SEPDHYVVIGAQRDAWGPGAAKSAVGTAILLELVRTFSSMVSNGFRPRRSLLFISWDGGD
FGSVGSTEWLEGYLSVLHLKAVVYVSLDNAVLGDDKFHAKTSPLLTSLIESVLKQVDSPN
HSGQTLYEQVVFTNPSWDAEVIRPLPMDSSAYSFTAFVGVPAVEFSFMEDDQAYPFLHTK
EDTYENLHKVLQGRLPAVAQAVAQLAGQLLIRLSHDRLLPLDFGRYGDVVLRHIGNLNEF
SGDLKARGLTLQWVYSARGDYIRAAEKLRQEIYSSEERDERLTRMYNVRIMRVEFYFLSQ
YVSPADSPFRHIFMGRGDHTLGALLDHLRLLRSNSSGTPGATSSTGFQESRFRRQLALLT
WTLQGAANALSGDVWNIDNNF
>Q12466
MAKEDTGVTAPKKPETAQVANINGIDKLEPPKTKEETESSKSVSSEKAAHASDESFKRSI
HEASYVGWKQIGGWEDKDELTLDDELMDMTRETFLDNIIPDSLYGDWYHSVAIFFIGGVA
SFALGHYKFSMGSAFFVIVITSLLYRTSAKKYRGSIRELVQKEFTVQKVENDYESLEWLN
AFLDKYWPILEPSVSQLIVQQANEQMATNEAIPKFITQLWIDELTLGVKPPRVDLVKTFQ
NTASDVVVMDWGISFTPHDLCDMSAKQVRNYVNELAVVKAKIFGITIPVSVSDIAFKAHA
RVKFKLMTPFPHVETVNIQLLKVPDFDFVATLFGRSIFNWEILAIPGLMTLIQKMAKKYM
GPILLPPFSLQLNIPQLLSGSNLSIGILEITVKNAKGLKRTSSILNESIDPYLSFEFNDI
SIAKTRTVRDTLNPVWDETLYVLLNSFTDPLTISVYDKRAKLKDKVLGRIQYNLNTLHDK
TTQRNLKAQFLRNSKPVGELTFDLRFFPTLEEKKLPDGSVEELPDLNTGIAKVVVEEGSR
FAEEEQKVTAYVEVYLNAKLVLTTGKATDTGTLKWNSDYEAVIADRRKTRYKFVVKDGKG
EEIGSTIQTLNDLIDRSQVNKNLIPLKNQKGDIKITTYWRPVRLEIGSNSVAYTPPIGAI
RVFIEKANDLRNLEKFGTIDPYCKVLVNGLSKGRTDFKSQTLNPVWNQVIYVAVTSPNQR
ITLQCMDVETVNKDRSLGEFNVNVQDLFKKDENDKYEETIDEKAKVGRLVMPKKKPKGTI
TYYTSFYPALPVLTLEEIQDLDKVNKKKKALELRKSAIDEKKISKEDKAKFDQEWNEVKE
LEDMYSNRQKLDLPELLQYNQGVLAVTVLNGELPDSGLYVQAFFDDNGHPRFVSPRIPSR
IVKNGWSGDVIIKELDKSITTFRVAKNKNYNRVEKCVCEVELPTQELVKNCYYKPSILHL
SGEGSAKLMLQISWFPIDTKQLPANDLITNSGDLTIMSRSAENLIASDLNGYSDPYLKYY
INNEEDCAYKTKVVKKTLNPKWNDEGTIQINNRLNDVLRIKVMDWDSTSADDTIGTAEIP
LNKVKVEGTTELDVPVEGLENAGQDGGMLHLAFSFKPRYTISVSKREKKVGDIASKGLGT
GLKAGTTVIGGGVGAIGKIKKGVFGGLGSLTNHKKNHEMGEEETKF
>P39551
MSPQYHFYFVSFRNLVLNEKCLRSKKQVMKSFNWYKTDRYFDPHNILQHHSRAIEKTRYK
LGMQTSSESTDAKSDFLDEPSAYLIEKNVALPKDIFGSYLSYWIYEVTRHKAAVILLVLI
VTSILLLVFFYNTEFCVAFEILLFSFCFPGTCMVVIAFSEPIGDREFKVKLLMEIITRKP
AVKGKEWRTITYKMNQYLFDHGLWDTPYYFYRDEDCHRYFLSLIKGRTFKKQKESSASNV
KDAQSNDETAGTPNEAAESSSFSAGPNFIKLLTKAAEIEQQFQKEYWRQEYPGVDEFF
>Q80T22
MEFRGSGATAVEQHLLQSETPGKNGLQATSSDQVGRTLRWFTTVVLNAAFLGMGVSAAVL
GPTFPDLARNVNRNISSLSEIFVGRALGYLGGSVVGGVLFDCMNHFLLLGLSHLLTAAGL
YLTPFCKTAALLTAMMSITGVSFGVLDTGGNVLILDLWGDKGAPHIQALHFSFALGAFLA
PLLAKLAWGTTASAQNHTEPQLDRSALNRSFEAASDSVLAVPDDMNLLWAYASIGTYVLV
LSVFLFAPFFKKRSKQKKSAASAQGARRAKYHRALLCLLFLFFFFYVGAEVTYGSYVFSF

ATTHVGMEESEAAGLNSIFWGTFAACRGLAIFFATLLQPGTMMVLCNIGSLASSFFLVLF
DKSPLCLWIASSVYGASMAATFPSGISWIEQYTTLTGKSAAFILVGAALGLMATPALSGI
LQGHYPDLPVILYMCLGSAVLTTVLFPVMYKVATLPLDRKQEKSINSEGQKILLSSSRLI
KEAK
>P08582
MRGPSGALWLLLALRTVLGGMEVRWCATSDPEQHKCGNMSEAFREAGIQPSLLCVRGTSA
DHCVQLIAAQEADAITLDGGAIYEAGKEHGLKPVVGEVYDQEVGTSYYAVAVVRRSSHVT
IDTLKGVKSCHTGINRTVGWNVPVGYLVESGRLSVMGCDVLKAVSDYFGGSCVPGAGETS
YSESLCRLCRGDSSGEGVCDKSPLERYYDYSGAFRCLAEGAGDVAFVKHSTVLENTDGKT
LPSWGQALLSQDFELLCRDGSRADVTEWRQCHLARVPAHAVVVRADTDGGLIFRLLNEGQ
RLFSHEGSSFQMFSSEAYGQKDLLFKDSTSELVPIATQTYEAWLGHEYLHAMKGLLCDPN
RLPPYLRWCVLSTPEIQKCGDMAVAFRRQRLKPEIQCVSAKSPQHCMERIQAEQVDAVTL
SGEDIYTAGKKYGLVPAAGEHYAPEDSSNSYYVVAVVRRDSSHAFTLDELRGKRSCHAGF
GSPAGWDVPVGALIQRGFIRPKDCDVLTAVSEFFNASCVPVNNPKNYPSSLCALCVGDEQ
GRNKCVGNSQERYYGYRGAFRCLVENAGDVAFVRHTTVFDNTNGHNSEPWAAELRSEDYE
LLCPNGARAEVSQFAACNLAQIPPHAVMVRPDTNIFTVYGLLDKAQDLFGDDHNKNGFKM
FDSSNYHGQDLLFKDATVRAVPVGEKTTYRGWLGLDYVAALEGMSSQQCSGAAAPAPGAP
LLPLLLPALAARLLPPAL
>P08962
MAVEGGMKCVKFLLYVLLLAFCACAVGLIAVGVGAQLVLSQTIIQGATPGSLLPVVIIAV
GVFLFLVAFVGCCGACKENYCLMITFAIFLSLIMLVEVAAAIAGYVFRDKVMSEFNNNFR
QQMENYPKNNHTASILDRMQADFKCCGAANYTDWEKIPSMSKNRVPDSCCINVTVGCGIN
FNEKAIHKEGCVEKIGGWLRKNVLVVAAAALGIAFVEVLGIVFACCLVKSIRSGYEVM
>P78423
MAPISLSWLLRLATFCHLTVLLAGQHHGVTKCNITCSKMTSKIPVALLIHYQQNQASCGK
RAIILETRQHRLFCADPKEQWVKDAMQHLDRQAAALTRNGGTFEKQIGEVKPRTTPAAGG
MDESVVLEPEATGESSSLEPTPSSQEAQRALGTSPELPTGVTGSSGTRLPPTPKAQDGGP
VGTELFRVPPVSTAATWQSSAPHQPGPSLWAEAKTSEAPSTQDPSTQASTASSPAPEENA
PSEGQRVWGQGQSPRPENSLEREEMGPVPAHTDAFQDWGPGSMAHVSVVPVSSEGTPSRE
PVASGSWTPKAEEPIHATMDPQRLGVLITPVPDAQAATRRQAVGLLAFLGLLFCLGVAMF
TYQSLQGCPRKMAGEMAEGLRYIPRSCGSNSYVLVPV
>Q7DM58
MWLLSSSPWLSELSCSYSAVVEHTSSVPVPIQWLRFVLLSPCPQRALFSAVDFIFLLCFA
LHKLFSSPSSSSEINGHAEIRKPLIGIRGRTPTRTTAWFKTTVAVTVLLSFCSVVLCVLA
FTGKRRTQRPWNLIDPLFWLIHAVTHLVIAVLVLHQKRFAALNHPLSLRIYWISSFVLTS
LFAVTGIFHFLSDAATSLRAEDVASFFSFPLTAFLLIASVRGITGLVTAETNSPTKPSDA
VSVEKSDNVSLYASASVFSKTFWLWMNPLLSKGYKSPLTLEQVPTLSPEHKAERLALLFE
SSWPKPSENSSHPIRTTLLRCFWKEILFTAILAIVRLGVMYVGPVLIQSFVDFTSGKRSS
PWQGYYLVLILLVAKFVEVLTTHQFNFDSQKLGMLIRSTLITALYKKGLKLTGSARQNHG
VGQIVNYMAVDAQQLSDMMLQLHAIWLMPLQVTVALVLLYGSLGASVITAVIGLTGVFVF
ILLGTQRNNGYQFSLMGNRDSRMKATNEMLNYMRVIKFQAWENHFNKRILKFRDMEFGWL
SKFLYSIAGNIIVLWSTPVLISALTFATALALGVKLDAGTVFTTTTIFKILQEPIRTFPQ
SMISLSQAMISLGRLDSYMMSKELSEDAVERALGCDGNTAVEVRDGSFSWDDEDNEPALS
DINFKVKKGELTAIVGTVGSGKSSLLASVLGEMHRISGQVRVCGSTGYVAQTSWIENGTV

QDNILFGLPMVREKYNKVLNVCSLEKDLQMMEFGDKTEIGERGINLSGGQKQRIQLARAV
YQECDVYLLDDVFSAVDAHTGSDIFKKCVRGALKGKTVLLVTHQVDFLHNVDCILVMRDG
KIVESGKYDELVSSGLDFGELVAAHETSMELVEAGADSAAVATSPRTPTSPHASSPRTSM
ESPHLSDLNDEHIKSFLGSHIVEDGSKLIKEEERETGQVSLGVYKQYCTEAYGWWGIVLV
LFFSLTWQGSLMASDYWLAYETSAKNAISFDASVFILGYVIIALVSIVLVSIRSYYVTHL
GLKTAQIFFRQILNSILHAPMSFFDTTPSGRILSRASTDQTNVDILIPFMLGLVVSMYTT
LLSIFIVTCQYAWPTAFFVIPLGWLNIWYRNYYLASSRELTRMDSITKAPIIHHFSESIA
GVMTIRSFRKQELFRQENVKRVNDNLRMDFHNNGSNEWLGFRLELVGSWVLCISALFMVL
LPSNVIRPENVGLSLSYGLSLNSVLFFAIYMSCFVENKMVSVERIKQFTDIPSESEWERK
ETLPPSNWPFHGNVHLEDLKVRYRPNTPLVLKGITLDIKGGEKVGVVGRTGSGKSTLIQV
LFRLVEPSGGKIIIDGIDISTLGLHDLRSRFGIIPQEPVLFEGTVRSNIDPTEQYSDEEI
WKSLERCQLKDVVATKPEKLDSLVVDNGENWSVGQRQLLCLGRVMLKRSRLLFLDEATAS
VDSQTDAVIQKIIREDFASCTIISIAHRIPTVMDGDRVLVIDAGKAKEFDSPARLLERPS
LFAALVQEYALRSAGI
>P54654
MSEATIVELLKRLDQATTRLEAVEKSIASGVASSSSSSPSSGAAGPSSASVKEFQNLVD
QHITPFVALSKKLAPEVGNQVEQLVKAIDAEKALINTASQSKKPSQETLLELIKPLNNFA
AEVGKIRDSNRSSKFFNNLSAISESIGFLSWVVVEPTPGPHVAEMRGSAEFYTNRILKEF
KGVNQDQVDWVSNYVNFLKDLEKYIKQYHTTGLTWNPKGGDAKSATPAPASSAPAAPVAP
AVSSTPVESKKGPGLGAVFGELSKGDGVTSGLKKVTNDMKSKNFTDKSSVVKAADTKVAK
VDAPSRPAVFALQGNKWSIEYQVNNKEIVIAEPDSRQTVYIFQCVNSLVQIKGKVNAITL
DGCKKTSIVFENAISSCEVVNCNGVEIQVTGRVPSIAIDKTSGCQIYLSKDSLETEIVSS
KSSEMNVLIPGATENDDLVELAIPEQYKTSVKGNKLHTESTSHI
>O42414
MVLHSHQLTYAGIAFALCLHHLISAIEVPLDSNIQSELPQPPTITKQSVKDYIVDPRDNI
FIECEAKGNPVPTFSWTRNGKFFNVAKDPKVSMRRRSGTLVIDFHGGGRPDDYEGEYQCF
ARNDYGTALSSKIHLQVSRSPLWPKEKVDVIEVDEGAPLSLQCNPPPGLPPPVIFWMSSS
MEPIHQDKRVSQGQNGDLYFSNVMLQDAQTDYSCNARFHFTHTIQQKNPYTLKVKTKKPH
NETSLRNHTDMYSARGVTETTPSFMYPYGTSSSQMVLRGVDLLLECIASGVPAPDIMWYK
KGGELPAGKTKLENFNKALRISNVSEEDSGEYFCLASNKMGSIRHTISVRVKAAPYWLDE
PQNLILAPGEDGRLVCRANGNPKPSIQWLVNGEPIEGSPPNPSREVAGDTIVFRDTQIGS
SAVYQCNASNEHGYLLANAFVSVLDVPPRILAPRNQLIKVIQYNRTRLDCPFFGSPIPTL
RWFKNGQGNMLDGGNYKAHENGSLEMSMARKEDQGIYTCVATNILGKVEAQVRLEVKDPT
RIVRGPEDQVVKRGSMPRLHCRVKHDPTLKLTVTWLKDDAPLYIGNRMKKEDDGLTIYGV
AEKDQGDYTCVASTELDKDSAKAYLTVLAIPANRLRDLPKERPDRPRDLELSDLAERSVK
LTWIPGDDNNSPITDYIVQFEEDRFQPGTWHNHSRYPGNVNSALLSLSPYVNYQFRVIAV
NDVGSSLPSMPSERYQTSGARPEINPTGVQGAGTQKNNMEITWTPLNATQAYGPNLRYIV
RWRRRDPRGSWYNETVKAPRHVVWNTPIYVPYEIKVQAENDFGRAPEPETYIGYSGEDYP
KAAPTDVRIRVLNSTAIALTWTRVHLDTIQGQLKEYRAYFWRDSSLLKNLWVSKKRQYVS
FPGDRNRGIVSRLFPYSNYKLEMVVTNGRGDGPRSEVKEFPTPEGVPSSPRYLRIRQPNL
ESINLEWDHPEHPNGVLTGYNLRYQASCLSSPVNGSKTGRTLVENFSPNQTRFTVQRTDP
ISRYRFFLRARTQVGDGEVIVEESPALLNEATPTPASTWLPPPTTELTPAATIATTTTTA
TPTTETPPTEIPTTAIPTTTTTTTTTAASTVASTTTTAERAAAATTKQELATNGSSIWDI
RAWANSNWANITWSHNYSAGTDFVVKYITSNKTEKSIPVKAQTPSSVQLANLTPGMVYKL

WVFPIWSSPSEHSYITFTTSSAYTKNHVDIATQGWFIGLMCAIALLVLILLIVCFIKRSR
GGKYPVRDNKDEHLNPEDKNVEDGSFDYRSLESDEDNKPLPNSQTSLDGTIKQQESDDSL
VDYGEGGEGQFNEDGSFIGQYTVKKDKEETEGNESSEATSPVNAIYSLA
>P04764
MSILKIHAREIFDSRGNPTVEVDLYTAKGLFRAAVPSGASTGIYEALELRDNDKTRFMGK
GVSKAVEHINKTIAPALVSKKLNVVEQEKIDQLMIEMDGTENKSKFGANAILGVSLAVCK
AGAVEKGVPLYRHIADLAGNPEVILPVPAFNVINGGSHAGNKLAMQEFMILPVGASSFRE
AMRIGAEVYHNLKNVIKEKYGKDATNVGDEGGFAPNILENKEALELLKSAIAKAGYTDQV
VIGMDVAASEFYRAGKYDLDFKSPDDASRYITPDQLADLYKSFIKDYPVVSIEDPFDQDD
WDAWQKFTATAGIQVVGDDLTVTNPKRIAKAAGEKSCNCLLLKVNQIGSVTESLQACKLA
QSNGWGVMVSHRSGETEDTFIADLVVGLCTGQIKTGAPCRSERLAKYNQILRIEEELGSK
AKFAGRSFRNPLAK
>Q95MG6
MSAVNGTPWGSSAREEVWAGSGVEVEGSELPTFSTAAKVRVGVTIVLFVSSAGGNLAVLW
SVTRPQPSQLRPSPVRRLFAHLAAADLLVTFVVMPLDATWNITVQWLAGDIACRTLMFLK
LMAMYAAAFLPVVIGLDRQAAVLNPLGSRSGVRKLLGAAWGLSFLLALPQLFLFHTVHRA
GPVPFTQCATKGSFKARWQETTYNLFTFCCLFLLPLTAMAICYSRIVLGVSSPRTRKGSH
APAGEFALRRSFDNRPRVRLRALRLALLVLLTFILCWTPYYLLGLWYWFSPSMLSEVPPS
LSHILFLFGLLNAPLDPLLYGAFTLGCRRGHQELSMDSSREEGSRRMFQQDIQALRQTEV
QKTVTSRKAGETKDIPITSI
>Q01717
MENETVSELNQTELPPQVAVALEYQVVTILLVVVICGLGIVGNIMVVLVVMRTKHMRTAT
NCYLVSLAVADLMVLVAAGLPNITDSIYGSWVYGYVGCLCITYLQYLGINASSCSITAFT
IERYIAICHPIKAQFLCTFSRAKKIIIFVWAFTSIYCMLWFFLLDLNISTYKDAIVISCG
YKISRNYYSPIYLMDFGVFYVMPMILATVLYGFIARILFLNPIPSDPKENSKTWKNDSTH
QNKNMNLNTTNRCFNSTVSSRKQVTKMLAVVVILFALLWMPYRTLVVVNSFLSSPFQENW
FLLFCRICIYLNSAINPVIYNLMSQKFRAAFRKLCNCKQKPTEKAANYSVALNYSVIKES
DRFSTELDDITVTDTYVSTTKVSFDDTCLASEKNGPSSCTYGYSLTAKQEKI
>Q29102
MSFSLNFTLPANTTSSPVVTSGKGADCGPSLGLAAGIPSLVATALLVALLLILIHRRRRS
SESTEEIERPCEISEIYDNPRVAENPRRSPTHEKNIMGAEEAHIYVKTVSGSQEPMRDTY
RPAVEMERRRGLWWLIPRLSLE
>Q9Y5Y4
MSANATLKPLCPILEQMSRLQSHSNTSIRYIDHAAVLLHGLASLLGLVENGVILFVVGCR
MRQTVVTTWVLHLALSDLLASASLPFFTYFLAVGHSWELGTTFCKLHSSIFFLNMFASGF
LLSAISLDRCLQVVRPVWAQNHRTVAAAHKVCLVLWALAVLNTVPYFVFRDTISRLDGRI
MCYYNVLLLNPGPDRDATCNSRQAALAVSKFLLAFLVPLAIIASSHAAVSLRLQHRGRRR
PGRFVRLVAAVVAAFALCWGPYHVFSLLEARAHANPGLRPLVWRGLPFVTSLAFFNSVAN
PVLYVLTCPDMLRKLRRSLRTVLESVLVDDSELGGAGSSRRRRTSSTARSASPLALCSRP
EEPRGPARLLGWLLGSCAASPQTGPLNRALSSTSS
>Q8VHN7
MSVTSEPGMISSFLLVYLSTLFISFVFGEAEIRFTGQTEFFVNETSTTVIRLVIERIGEP
ANVTAIVSLSGEDTGDFFDTYAAAFIPARGTNRTVYIAVCDDDLPEPDETFTFHLTLQKP
SANVKLGWPRAASVTILSNDNAFGIISFSTPSSISVIEPRSRNASVPLTLIREKGTYGMV

TVTFDVSGGPNPPEEDLNPVRGNITFPPGRATVIYNVTVLDDEVPENDELFLIQLRSVEG
GAEINASRSSVEIIVKKNDSPVNFMQSVYVVPEDDHVLTIPVLRGKDSDGNLIGSDETQV
SIRYKVMTWDSTAHAQQNVDFIDLQPDTTLVFPPFVHESHLKFQIIDDLIPEIAESFHIM
LLKNTLQGDAVLMGPSTVQVTIKPNDKPYGVLSFNSILFERPVIIDEDTASSSRFEEIAV
VRNGGTHGNVSVSWVLTRNSSDPSPVTADITPASGTLQFAQGQMLAPISLVVFDDDLPEE
AEAYLLTILPHTIQGGAEVSEPAQLLFYIQDSDNVYGEIAFFPGESQKIESSPSERSLSL
SLARRGGSKGDVRVIYSALYIPAGAMDPLRAKDGILNTSRRSSLLFPEQNQQVSIKLPIR
NDAFLQNGAHFLVQLEAVVLVNIFPPIPPVSPRFGEIRNISLLVTPAIANGEIGFLSNLP
IILHEPKDSSAEVVSIPLHRDGTDGQATVYWSLRPSGFNSKAVTLDDAGPFNGSVVFLSG
QNETSINITVKGDDIPELNETVTLSLDRVSVDSDVLKSGYTSRDLIILENDDPGGIFEFS
YDSRGPYVIKEGDAVELRITRSRGSLVKQFLRFHVEPRESNEFYGNMGVLEFTPGEREVV
ITLLTRLDGTPELDEHFWVILSSHGERESKLGRATLVNITILKNDYPHGIIEFVSDGLSA
SIKESKGEDIYHAVYGVIRTRGNFGAVNVSWMVSPDFTQDVFPVQGTVCFGDQEFFKNIT
VYSLVDEIPEEMEEFTIILLNATGGAQTGIRTTASLRILRNDDPVYFAEPCVLRVQEGET
ANFTVLRNGSVDGACTVQYATVDGKASGEEGDFAPVEKGETLVFEVGSREQSISVHVKDD
GIPETDEPFYIVLFNSTGDTVVYEYGVATVIIEANDDPNGVFSLEPIDKAVEEGKTNAFW
ILRHRGHFGNVSVAWQLFQNASLQPGQEFYETSGTVNFTDGEETKPVILRAFPDRIPEFN
EFYILRLVNISGPGGQLAETNFQVTVMIPFNDDPFGIFILDPECLEREVAEDVLSEDDMS
YITSFTILRQQGVFGDVRVGWEVLSREFTAGLPPMIDFILLGSFPSTVPLQPHMRRHHSG
TDVLYFSGLEGAFGTVDPKYQPFRNNTIANFTFSAWVMPNANTNGFLIAKDDSHGSIYYG
VKIQTNETHVTLSLHYKTFGSNVTYIAKSTVMKYLEEGVWLHVLIILDDGIIEFYLDGKA
MPRGIKSLKGEAITDGPGILRIGAGMDGGARFTGWMQDVRTYERKLTPEEIYELHAVPAR
TDLHPISGYLEFRQGESNKSFIVAARDDSEEEGEELFLLKLVSVDGGAQISKENTTARLR
IQKSDNANGLFGFTGACIPEMTEEGSTVSCVVERTRGALGYVHVFYTISQIESEGINYLV
DDFANASGTITFLPWQRSEVLNLYVLDEDMPELNEYFRVTLVSAVPGDGKLGSTPISGAS
IDPEKETTGITVKASDHPYGLMQFSTGLPPQPEDSMSLPASSVPHITVQEEDGEIRLLVI
RAQGLLGRVTVGFRTVSLTAFSPEDYQSTAGTLEFQSGERYKYIFVNITDNSIPELEKSF
KVELLNLDGGVSDLFRVDGSGSGEADTDFFLPPVLPHASLGVASQILVTIAASDHAHGVF
EFSPESLFVSGTEPEDGYSTVVLNVTRTRGALSAVTLQWKVDSDLDGDLAITSGNITFET
GQRIASITVEILSDEEPELDKALTVSILNVSSGSLGVLTNATLTILASDDPYGVFIFPNK
TRPLSVEEATQNVALSIIRLKGLMGEVAVSYATIDDMEKPPYFPPNLARATQGGDYISAS
GLALFRVNQTEATITISILDDAEPERSESVFIELFNSSLVDKVQNRPIPHSPRLGPKVET
VAHLVIVANDDAFGTVQLSATSVHVAENHVGPIINVTRTGGTFADVSVKFKAVPITAAAG
EDYSIASSDVVLLEGETTKAVPIYIINDIYPELEETFLVQLLNETTGGATLGPLREAVIT
IEASDDPYGLFGFQNTKFIVEEPEFNSVRVNVPIIRNSGTLGNVTVQWVAIINGQFATGD
LRVVSGNVTFAPGETIQTLLLEVLADDVPEIEEVVQVQLAAASGGGTIGLDRVANIVIPA
NDNPYGSVAFVQSVFRVQEPLERSSYANITVRRSGGHFGRLLLCYGTSDIDVVARAVEEG
EDVLSYYESPTQGVPDPLWRTWVNVSAVEETQYTCATLCLKERACSAFSVVSGAEGPRCF
WMTSWVSGTVNSSDFQTYKKNMTRVASLFSGQAVAGSDYEPVTRQWAVILEGDEFANLTV
SVLPDDAPEMDESFLISLLEVHLMNISDSFKNQPTIGHPNTSAVVIGLNGDAFGVFIIYS
VSPNTSEDGLCVEVQEQPQTSVELVIYRTGGSLGQVMVEWRVVGGTATEGLDFMGAGDIL
TFAEGETKKMAILTILDDSEPEDNESILVRLGATEGGSRILPSSDTVTVNILANDNVAGI
VSFQTASRSVIGHEGEMLQFHVVRTPPGRGNVTVNWKVVGQNLEVNFANFTGQLFFSEGT
LNKTIFVHLLDDNIPEEKEVYQVVLYDVKTQGVSPAGVALLDAQGYAAVLTVEASDEPHG

VLNFALSSRFVVLQEANVTIQLFVNREFGSLGAINVTYATVPGIVSLKNNTEGNLAEPES
DFIPVVGSLVLEEGETTAAISITVLEDDIPELKEYFLVNLTHVDLIMAPLTSSPPRLDSE
GLTAQIVIDANDGAQGMIEWQRNRFEVNETDGVVTLVAQRSRAALGQVSLFMYAQNLEAQ
AGLDYMRTPQILHFTDGERFKHVDVMILDDDMPEGDERFQLLLTNPSPGLELGKNTIALI
TVLANDDGPGVLSFNNSGHIFLREPTSLYVQESVAVLVIVREPAQGLFGTVAVQFVVTEV
NSSTESKDLSPSKGFIVLEEGVRSKTLRISAILDTEPEMDEHFVCTLFNPTGGARLGAHV
QTLITIFQNQAPLGLFSISAVENSATSIDVEESNRSVYLNVSRTNGLDLTASVQWETVSE
TAFGMRGMDVVFSIFQSFFDKTALDWCFFTVEGSVYGVMLRKSSLVVYRWQGTFVPVEDL
KVESPKTCEAFNIGVSPYLVITHGERSGEKPSINSVYMLTAGFRLVLIQTIIISGSCQVR
HFTSDSQDYFIIASRRNDSELTQVFRWNGNNFAWHQTLPVRGVLGMALFSRGGSVFLAIS
QANIRQTSLLFTWSGTQFINFQELPISGITQVEALSSGDDVYLCFAKNTFLGNQNAIDIF
VWEMGHSSLRYFQSLDFAAVKRIRSFTPASGIVHILLTAQDGSALYCWNSELNAFSFVLE
APAAHDAAFVTVKSLNSSKTLIALVGATDSHLYELTYVSSQSDFIPSLGELIFEPGDKEA
IIAVNVLDDTVPEKEESFRVQLKSPRGGAEIGINSSVRVTVLANDGAYGVVAFAQNSLHK
QLEELERDSLVTLNVERLRGTHGRITVAWEAAGSVSDVFPTSGVISFTEDQAMSMITLTV
LADDLPELSEAVVVTLTQIVTEGVEDPLKGATIDQSRSRSVLTILPSDSPYGAVRWHTES
LFNRVPEPTENITVVQLHIVRDKGLFGDISIHLIAKPNFLLHINNQATEDEDFVLQDSVI
IMKENIKETHAEVAILPDEVPELDEGLIVTIAAVNLVNPNFPAEQPRVQRPRMESAEILI
EENDDPRGIFNFHVVRDVGGVIIAHEGPPPLNVLQVPVVRMAGTFETVNVYWKATPDSAG
LEDFQPSHGMLQFADGQVIAPILVTIIDDSEFELLETFTISLVSVTGGGRLGDDVSVNVV
IAPNDSPFGIFGFEKKTVMVDGPLLSDDPDSYVTLTVVRSPGGKGAVRLHWAIEEKAKDD
LSPLNGTLYFDETESQKSVILHTLKDGMVGEDRRFIIELTAADEVEISPVKGSASVIIRG
DKSISEVGIASSSRHIIIGEPSATYNGTAIIDLVRGPGVSGEITVNWKILPPSRGEFVET
SGQLTMLDGQTAATVVIQVLNDDIPEEKCHYEFQLTEISEGRMLHEASVSARITMVASDA
PYGRFSFSHEQLHVSKAAQRVNVTVVRSGGSFGRARVLYETGSRTAEAGWDFVPASGELL
FEAREKMKSLYIDILDDDLPEGPEEFVLAITRVDLQGRGYDFTIQENGLQIDQPPEIGNI
SIVRIIIMKNDNAEGIIEFDPKYTDISVEEDAGVITLPVLRLHGTYGHVSADFSSRGFSA
VPGGYVLRGSSVTFQHGQNLSFINVSIIDDNGSEFEKQFEILLIGATGGAILGRHLVSKI
TIAKSDSPFGIIRFLNQSKISLPNPSSTMALHLVLERTGGLLGEIQVSWEVVGPDAEEPL
PPHNGDFADPVSGTVSFGDGEGGVRSIILRVCPHEETEAEETFIVQLKPLREAKLDPRAK
AVTLTIQKFGDPNGVIHFAPESLSKRRFSEPPPSDGPLLVSFLVTRSKGTSGDIKVHWEL
SSEFDITRDFLSTRGFFTIADGESDANFDVHLLPDDVPEIEEEYAVQLVSVEGGAELDLG
KCTARFSVSANDDPHGVFALYSDRQSVLIGQNLDRSIQINITRLAGAFGAVAVRVQILSD
NKEDPVATENEERQLVITDGARYKVGLVPLKNQVFLSLGSNFTLQLVSVRLLSGPFYGIP
TILQEAKNAILSVPEEAANSQVGFESAAFQLMDIKAGTSQVMVSRKGTYGRLSVAWTTGY
APGSEIPEPIVIGNMTPTLGSLSFVHGEERKGVLLWTFPSPGRPEAFVLHLSGLRSSAAG
GAQLRSGFTTAEIEPMGVFQ
>Q8GZ17
MDSAPNFIPRLLLLSLLIVSIPLTSSQSDANTTNPSPSPPSDSDLCNGVFVSYTHTKGSK
IPPNDTANQPYRFESVITVLNHGRDELKSWRVFVKFAHREILVSASNAVLSDGSSLPVSV
ENGTVFAGYPSSDLKSAIQTAGDVTQMQARVELVGTQFGVAPPNVPLPKNITLATDGWKC
PKATQKGTNVLQVCCIPDPDYDNREIIDNEFLPRKDGDLTIMYDVVRSYSSNYMAQVTME
NHNPLGRLDNWKLSFDWMRDEFIYTMKGAYPSIVDSSDCVDGPQAKHYQDLDFSNVLSCA
RRPTVIDLPPTKYNDSTFGLIPFCCRNGTILPRSMDPSKSSSVFQMQVYKMPPDLNISAL

SPPQNWRINGTLNPDYKCGPPVRVSPSQFVDPSGLPSNRTAFASWQVVCNITQPKDASPR
CCVSFSAYFNDSIVPCKTCACGCSSNKAARACSATAPSLLLPQQALLVPFENRTELTVAW
AYLKQRPVPNPMPCGDNCGVSINWHLATDYRGGWTARVTVFNWGETDFVDWFTAVQMKNA
APGFEKAYSFNASTIGINGKNNTIFMEGLPGLNYLVAERDGENPLKNPRIPGKQQSVMSF
TKKLTPGINVPGGDGFPSKVFFNGEECSLPTILPMRSSQHRKHISVFLLALPVLALLILR
A
>Q18007
MPNYTVPPDPADTSWDSPYSIPVQIVVWIIIIVLSLETIIGNAMVVMAYRIERNISKQVS
NRYIVSLAISDLIIGIEGFPFFTVYVLNGDRWPLGWVACQTWLFLDYTLCLVSILTVLLI
TADRYLSVCHTAKYLKWQSPTKTQLLIVMSWLLPAIIFGIMIYGWQAMTGQSTSMSGAEC
SAPFLSNPYVNMGMYVAYYWTTLVAMLILYKGIHQAAKNLEKKAKAKERRHIALILSQRL
GTQVGVSLMLQSKAEKEKAEEAQKDSGYTSNQAGDANNLRRFGFSEPETSQFRVDPNSNN
NLNVEGSLNTENDQNLGVIEEERSGFLSRRESNESYYPGPHPTAANSRRCSEMEKVSLLS
ESDGVPSTRPAKSYGRLSLRSRYSASESITTTHENDEKEVEKADSLQKLFADDELGSVLN
FKEEKLKNTDSNNDSDTTSVILQRSRKYKKNKRPRSSRRSEHSTPRQIAKVKQAEGTAAQ
LIEESVPDDDQTETIEVKRTDRWVVSMKKRIARALIRRRSTTRPERGSSSNSDDSSSEVE
GEEKPEVRNNGLKIPQLTVNNENRGETSSQPGRDRLAPPNKTDTFLSASGVSRKISTIST
VITREKVISSIFAPIAVFNRGRKQTKAEKRAHKAFRTITFIVGFFAILWSPYYIMATVYG
FCKGECIPSFLYTLSYYMCYLNSSGNPFAYALANRQFRSAFMRMFRGNFNKVA
>O15218
MSVKPSWGPGPSEGVTAVPTSDLGEIHNWTELLDLFNHTLSECHVELSQSTKRVVLFALY
LAMFVVGLVENLLVICVNWRGSGRAGLMNLYILNMAIADLGIVLSLPVWMLEVTLDYTWL
WGSFSCRFTHYFYFVNMYSSIFFLVCLSVDRYVTLTSASPSWQRYQHRVRRAMCAGIWVL
SAIIPLPEVVHIQLVEGPEPMCLFMAPFETYSTWALAVALSTTILGFLLPFPLITVFNVL
TACRLRQPGQPKSRRHCLLLCAYVAVFVMCWLPYHVTLLLLTLHGTHISLHCHLVHLLYF
FYDVIDCFSMLHCVINPILYNFLSPHFRGRLLNAVVHYLPKDQTKAGTCASSSSCSTQHS
IIITKGDSQPAAAAPHPEPSLSFQAHHLLPNTSPISPTQPLTPS
>Q6H3X3
MAAAASPAFLLRLPLLLLLSSWCRTGLADPHSLCYDITVIPKFRPGPRWCAVQGQVDEKT
FLHYDCGSKTVTPVSPLGKKLNVTTAWKAQNPVLREVVDILTEQLLDIQLENYIPKEPLT
LQARMSCEQKAEGHGSGSWQLSFDGQIFLLFDSENRMWTTVHPGARKMKEKWENDKDMTM
SFHYISMGDCTGWLEDFLMGMDSTLEPSAGAPPTMSSGTAQPRATATTLILCCLLIMCLL
ICSRHSLTQSHGHHPQSLQPPPHPPLLHPTWLLRRVLWSDSYQIAKRPLSGGHVTRVTLP
IIGDDSHSLPCPLALYTINNGAARYSEPLQVSIS
>O95867
MKALMLLTLSVLLCWVSADIRCHSCYKVPVLGCVDRQSCRLEPGGQQCLTTHAYLGKMWVF
SNLRCGTPEEPCQEAFNQTNRKLGLTYNTTCCNKDNCNSAGPRPTPALGLVFLTSLAGLG
LWLLH
>P32358
MRLFLLLVGLTTVIADDLPPTCIRPVYCNSTLLHYVQMARLYPDSKTFVDFQMRKDENAT
LSAFQELLDRTNHNPTKEDLQEFVVDFFDETSELEEWKPDDHKENPPFLAKIRDQGFREF
AKALNDIWPTLARRVKPSVLEKPEQSSLVPMTHGFIVPGGRFKEIYYWDAYWIIEGLLIT
DMTETAKGMIENLIELLYKFGHIPNGSRWYYQERSQPPLLAAMIKLYYEKTKDIEFIRKY
ISALEKELEYWLDTHLIAFNKDDRVYTLLRYYIPSAGPRPESYYEDYELAQKLDKNTDPN

DIYADLKSAAESGWDFSTRWFISESGDNSGNLTNLNTKNVIPVDLNAIFAGALQITANFQ
AILKNPRRAAHWGYMAEQWRSSIEQALWDEEDGVWHDYDILNNKPRRYFYTSNLAPLWMN
AVEKPFLAKHGARVLEYLHESQALEYPGGVPVSLVNSGEQWDFPNAWPPEVSIVVTAIQN
IGSEESSKLAKELAQVWVRACKSGFTEKKQMFEKYDALNAGKYGGGGEYTVQDGFGWSNG
VVLEFLDRYGAVLTSVDSVDASANNGQSNEESETDSKEK
>Q8IVI9
MRDPLTDCPYNKVYKNLKEFSQNGENFCKQVTSVLQQRANLEISYAKGLQKLASKLSKAL
QNTRKSCVSSAWAWASEGMKSTADLHQKLGKAIELEAIKPTYQVLNVQEKKRKSLDNEVE
KTANLVISNWNQQIKAKKKLMVSTKKHEALFQLVESSKQSMTEKEKRKLLNKLTKSTEKL
EKEDENYYQKNMAGYSTRLKWENTLENCYQSILELEKERIQLLCNNLNQYSQHISLFGQT
LTTCHTQIHCAISKIDIEKDIQAVMEETAILSTENKSEFLLTDYFEEDPNSAMDKERRKS
LLKPKLLRLQRDIEKASKDKEGLERMLKTYSSTSSFSDAKSQKDTAALMDENNLKLDLLE
ANSYKLSSMLAELEQRPQPSHPCSNSIFRWREKEHTHSYVKISRPFLMKRLENIVSKASS
GGQSNPGSSTPAPGAAQLSSRLCKALYSFQARQDDELNLEKGDIVIIHEKKEEGWWFGSL
NGKKGHFPAAYVEELPSNAGNTATKA
>Q93ZH0
MKIPEKPIFLIFVSLILASSLTFTATAKSTIEPCSSNDTCNALLGYTLYTDLKVSEVASL
FQVDPISILLANAIDISYPDVENHILPSKLFLKIPITCSCVDGIRKSVSTHYKTRPSDNL
GSIADSVYGGLVSAEQIQEANSVNDPSLLDVGTSLVIPLPCACFNGTDNSLPAVYLSYVV
KEIDTLVGIARRYSTTITDLMNVNAMGAPDVSSGDILAVPLSACASKFPRYASDFGLIVP
NGSYALAAGHCVQCSCALGSRNLYCEPASLAVSCSSMQCRNSNLMLGNITVQQTSAGCNV
TTCDYNGIANGTILTMLTRSLQPRCPGPQQFAPLLAPPDTVPRDVMYAPAPSPDFDGPGS
IASSPRSSMLPGGGILPGNPANGPAGSISTASASSVSYFFITFLISIASFSLALSS
>Q92556
MPPPADIVKVAIEWPGAYPKLMEIDQKKPLSAIIKEVCDGWSLANHEYFALQHADSSNFY
ITEKNRNEIKNGTILRLTTSPAQNAQQLHERIQSSSMDAKLEALKDLASLSRDVTFAQEF
INLDGISLLTQMVESGTERYQKLQKIMKPCFGDMLSFTLTAFVELMDHGIVSWDTFSVAF
IKKIASFVNKSAIDISILQRSLAILESMVLNSHDLYQKVAQEITIGQLIPHLQGSDQEIQ
TYTIAVINALFLKAPDERRQEMANILAQKQLRSIILTHVIRAQRAINNEMAHQLYVLQVL
TFNLLEDRMMTKMDPQDQAQRDIIFELRRIAFDAESEPNNSSGSMEKRKSMYTRDYKKLG
FINHVNPAMDFTQTPPGMLALDNMLYFAKHHQDAYIRIVLENSSREDKHECPFGRSSIEL
TKMLCEILKVGELPSETCNDFHPMFFTHDRSFEEFFCICIQLLNKTWKEMRATSEDFNKV
MQVVKEQVMRALTTKPSSLDQFKSKLQNLSYTEILKIRQSERMNQEDFQSRPILELKEKI
QPEILELIKQQRLNRLVEGTCFRKLNARRRQDKFWYCRLSPNHKVLHYGDLEESPQGEVP
HDSLQDKLPVADIKAVVTGKDCPHMKEKGALKQNKEVLELAFSILYDSNCQLNFIAPDKH
EYCIWTDGLNALLGKDMMSDLTRNDLDTLLSMEIKLRLLDLENIQIPDAPPPIPKEPSNY
DFVYDCN
>Q9NRD9
MGFCLALAWTLLVGAWTPLGAQNPISWEVQRFDGWYNNLMEHRWGSKGSRLQRLVPASYA
DGVYQPLGEPHLPNPRDLSNTISRGPAGLASLRNRTVLGVFFGYHVLSDLVSVETPGCPA
EFLNIRIPPGDPMFDPDQRGDVVLPFQRSRWDPETGRSPSNPRDPANQVTGWLDGSAIYG
SSHSWSDALRSFSRGQLASGPDPAFPRDSQNPLLMWAAPDPATGQNGPRGLYAFGAERGN
REPFLQALGLLWFRYHNLWAQRLARQHPDWEDEELFQHARKRVIATYQNIAVYEWLPSFL
QKTLPEYTGYRPFLDPSISSEFVAASEQFLSTMVPPGVYMRNASCHFQGVINRNSSVSRA

LRVCNSYWSREHPSLQSAEDVDALLLGMASQIAEREDHVLVEDVRDFWPGPLKFSRTDHL
ASCLQRGRDLGLPSYTKARAALGLSPITRWQDINPALSRSNDTVLEATAALYNQDLSWLE
LLPGGLLESHRDPGPLFSTIVLEQFVRLRDGDRYWFENTRNGLFSKKEIEEIRNTTLQDV
LVAVINIDPSALQPNVFVWHKGDPCPQPRQLSTEGLPACAPSVVRDYFEGSGFGFGVTIG
TLCCFPLVSLLSAWIVARLRMRNFKRLQGQDRQSIVSEKLVGGMEALEWQGHKEPCRPVL
VYLQPGQIRVVDGRLTVLRTIQLQPPQKVNFVLSSNRGRRTLLLKIPKEYDLVLLFNLEE
ERQALVENLRGALKESGLSIQEWELREQELMRAAVTREQRRHLLETFFRHLFSQVLDINQ
ADAGTLPLDSSQKVREALTCELSRAEFAESLGLKPQDMFVESMFSLADKDGNGYLSFREF
LDILVVFMKGSPEEKSRLMFRMYDFDGNGLISKDEFIRMLRSFIEISNNCLSKAQLAEVV
ESMFRESGFQDKEELTWEDFHFMLRDHNSELRFTQLCVKGVEVPEVIKDLCRRASYISQD
MICPSPRVSARCSRSDIETELTPQRLQCPMDTDPPQEIRRRFGKKVTSFQPLLFTEAHRE
KFQRSCLHQTVQQFKRFIENYRRHIGCVAVFYAIAGGLFLERAYYYAFAAHHTGITDTTR
VGIILSRGTAASISFMFSYILLTMCRNLITFLRETFLNRYVPFDAAVDFHRLIASTAIVL
TVLHSVGHVVNVYLFSISPLSVLSCLFPGLFHDDGSELPQKYYWWFFQTVPGLTGVVLLL
ILAIMYVFASHHFRRRSFRGFWLTHHLYILLYVLLIIHGSFALIQLPRFHIFFLVPAIIY
GGDKLVSLSRKKVEISVVKAELLPSGVTHLRFQRPQGFEYKSGQWVRIACLALGTTEYHP
FTLTSAPHEDTLSLHIRAAGPWTTRLREIYSAPTGDRCARYPKLYLDGPFGEGHQEWHKF
EVSVLVGGGIGVTPFASILKDLVFKSSVSCQVFCKKIYFIWVTRTQRQFEWLADIIREVE
ENDHQDLVSVHIYITQLAEKFDLRTTMLYICERHFQKVLNRSLFTGLRSITHFGRPPFEP
FFNSLQEVHPQVRKIGVFSCGPPGMTKNVEKACQLINRQDRTHFSHHYENF
>Q9R216
MAVPPLLRGALLLWQLLATGGAALEIGRFDPERGRGPAPCQAMEIPMCRGIGYNLTRMPN
LLGHTSQGEAAAQLAEFSPLVQYGCHSHLRFFLCSLYAPMCTDQVSTPIPACRPMCEQAR
LRCAPIMEQFNFGWPDSLDCARLPTRNDPHALCMEAPENATAGPTEPHKGLGMLPVAPRP
ARPPGDSAPGPGSGGTCDNPEKFQYVEKSRSCAPRCGPGVEVFWSRRDKDFALVWMAVWS
ALCFFSTAFTVFTFLLEPHRFQYPERPIIFLSMCYNVYSLAFLIRAVAGAQSVACDQEAG
ALYVIQEGLENTGCTLVFLLLYYFGMASSLWWVVLTLTWFLAAGKKWGHEAIEAHGSYFH
MAAWGLPALKTIVVLTLRKVAGDELTGLCYVASMDPAALTGFVLVPLSCYLVLGTSFLLT
GFVALFHIRKIMKTGGTNTEKLEKLMVKIGVFSILYTVPATCVIVCYVYERLNMDFWRLR
ATEQPCTAATVPGGRRDCSLPGGSVPTVAVFMLKIFMSLVVGITSGVWVWSSKTFQTWQS
LCYRKMAAGRARAKACRTPGGYGRGTHCHYKAPTVVLHMTKTDPSLENPTHL
>O54901
MGSLVFRRPFCHLSTYSLIWGMAAVALSTAQVEVVTQDERKALHTTASLRCSLKTSQEPL
IVTWQKKKAVSPENMVTYSKTHGVVIQPAYKDRINVTELGLWNSSITFWNTTLEDEGCYM
CLFNTFGSQKVSGTACLTLYVQPIVHLHYNYFEDHLNITCSATARPAPAISWKGTGTGIE
NSTESHFHSNGTTSVTSILRVKDPKTQVGKEVICQVLYLGNVIDYKQSLDKGFWFSVPLL
LSIVSLVILLVLISILLYWKRHRNQERGESSQGMQRMK
>Q9TU31
MGAVRIAPGLALLLCCPVLSSAYALVDADDVMTKEEQIFLLHRAQAQCQKRLKEVLQRPA
DIMESDKGWASASTSGKPKKEKASGKLYPESEEDKEVPTGSRHRGRPCLPEWDHILCWPL
GAPGEVVAVPCPDYIYDFNHKGHAYRRCDRNGSWELVPGHNRTWANYSECVKFLTNETRE
REVFDRLGMIYTVGYSVSLASLTVAVLILAYFRRLHCTRNYIHMHLFLSFMLRAVSIFVK
DAVLYSGATLDEAERLTEEELRAIAQAPPPPTAAAGYAGCRVAVTFFLYFLATNYYWILV
EGLYLHSLIFMAFFSEKKYLWGFTVFGWGLPAVFVAVWVSVRATLANTGCWDLSSGNKKW

IIQVPILASIVLNFILFINIVRVLATKLRETNAGRCDTRQQYRKLLKSTLVLMPLFGVHY
IVFMATPYTEVSGTLWQVQMHYEMLFNSFQGFFVAIIYCFCNGEVQAEIKKSWSRWTLAL
DFKRKARSGSSSYSYGPMVSHTSVTNVGPRAGLGLPLSPRLLPAAAATTTATTNGHPPIP
GHTKPGAPTLPATPPATAAPKDDGFLNGSCSGLDEEASAPERPPALLQEEWETVM
>Q9DDA9
MSGTYDDSVGVEVSSDSFWEVGNYKRTVKRIDDGHRLCNDLMNCIHERARIEKVYAQQLT
EWAKRWKQLVERGPQYGTVEKAWHNLMTEAEKVSELHLEVKNALMNEDFEKIKNWQKEAF
HKQMMGGFKETKEADDGFRKAQKPWAKKLKEVEAAKKSYHAACKEEKLATSRETNSKADP
AMNPEQLKKLQDKVEKSKQDSQKTKEKYEKSLKDLDGTTPQYMENMEQVFEQCQQFEDKR
LSFFREVLLEVEKHLDLSNVESYASIYRELEYAIKSADAMEDLKWFRNNHGPGMSMNWPQ
FEDWSADLNRTLSRREKKKPTDGVTLTGISQSGEQSSIQNQHSSHLSVQSAQSTNNPFED
EEETVSINETENKKIENVGSYEKTHPAEWSDDESNNPFNPSDTNGDNNPFDEDALTTLEV
RVRALYDYDGQELDELSFKAGEELTKIEDEDEQGWCKGRLEGGQVGLYPANYVESVQ
>P05533
MDTSHTTKSCLLILLVALLCAERAQGLECYQCYGVPFETSCPSITCPYPDGVCVTQEAAV
IVDSQTRKVKNNLCLPICPPNIESMEILGTKVNVKTSCCQEDLCNVAVPNGGSTWTMAGV
LLFSLSSVLLQTLL
>P70500
MPEENIFLFVPNLIGYARIVFAIISFYFMPCCPFTASSFYLLSGLLDAFDGHAARALNQG
TRFGAMLDMLTDRCATMCLLVNLALLYPRATLLFQLSMSLDVASHWLHLHSSVVRGSESH
KMIDLSGNPVLRIYYTSRPALFTLCAGNELFYCLLYLFNFSEGPLVGSVGLFRMGLWITA
PIALLKSIISVIHLVTAARNMAALDAADRAKKK
>Q62915
MADDDVLFEDVYELCEVIGKGPFSVVRRCINRETGQQFAVKIVDVAKFTSSPGLSTEDLK
REASICHMLKHPHIVELLETYSSDGMLYMVFEFMDGADLCFEIVKRADAGFVYSEAVASH
YMRQILEALRYCHDNNIIHRDVKPHCVLLASKENSAPVKLGGFGVAIQLGESGLVAGGRV
GTPHFMAPEVVKREPYGKPVDVWGCGVILFILLSGCLPFYGTKERLFEGIIKGKYKMNPR
QWSHISESAKDLVRRMLMLDPAERITVYEALNHPWLKERDRYAYKIHLPETVEQLRKFNA
RRKLKGAVLAAVSSHKFNSFYGDPPEELPDFSEDPTSSGLLAAERAVSQVLDSLEEIHAL
TDCSEKDLDFLHSVFQDQHLHTLLDLYDKINTKSSPQIRNPPSDAVQRAKEVLEEISCYP
ENNDAKELKRILTQPHFMALLQTHHVVAHEVYSDEALRVTPPPTSPYLNGDSPESANGDM
DMENVTRVRLVQFQKNTDEPMGITLKMNELNHCIVARIMHGGMIHRQGTLHVGDEIREIN
GISVANQTVEQLQKMLREMRGSITFKIVPSYRTQSSSCERDSPSTSRQSPANGHSSTNNS
VSIYVRAQFEYDPAKDDLIPCKEAGIRFRVGDIIQIISKDDHNWWQGKLENSKNGTAGLI
PSPELQEWRVACIAMEKTKQEQQASCTWFGKKKKQYKDKYLAKHNADLVTYEEVVKLPAF
KRKTLVLLGAHGVGRRHIKNTLITKHPERFAYPIPHTTRPPKKDEENGKNYYFVSHDQMM
QDISNNEYLEYGSHEDAMYGTKLETIRKIHEQGLIAILDVEPQALKVLRTAEFPPFVVFI
AAPTITPGLNEDESLQRLQKESDVLQRTYAHYFDLTIINNEIDETIRHLEEAVELVCTAP
QWVPVSWVY
>Q9BZJ6
MVFSAVLTAFHTGTSNTTFVVYENTYMNITLPPPFQHPDLSPLLRYSFETMAPTGLSSLT
VNSTAVPTTPAAFKSLNLPLQITLSAIMIFILFVSFLGNLVVCLMVYQKAAMRSAINILL
ASLAFADMLLAVLNMPFALVTILTTRWIFGKFFCRVSAMFFWLFVIEGVAILLIISIDRF
LIIVQRQDKLNPYRAKVLIAVSWATSFCVAFPLAVGNPDLQIPSRAPQCVFGYTTNPGYQ

AYVILISLISFFIPFLVILYSFMGILNTLRHNALRIHSYPEGICLSQASKLGLMSLQRPF
QMSIDMGFKTRAFTTILILFAVFIVCWAPFTTYSLVATFSKHFYYQHNFFEISTWLLWLC
YLKSALNPLIYYWRIKKFHDACLDMMPKSFKFLPQLPGHTKRRIRPSAVYVCGEHRTVV
>Q9FM65
MAKKMSSLIIIFNILLLLTTQTHAHNVTRLLANHPSFSSFSHFLTQTHLADEINRRRTIT
VCAVDNAAMSALTSKGYTLSTLKNILSLHVLLDYFGTKKLHQIRDGSALAATLFQATGAA
PGTSGFVNITDLRGGKVGFGPDGGDLSSFFVKSIEEVPYNISIIQISRVLPSETAAAPTP
APAEMNLTGIMSAHGCKVFAETLLTNPGASKTYQESLEGGMTVFCPGDDAMKGFLPKYKN
LTAPKKEAFLDFLAVPTYYSMAMLKSNNGPMNTLATDGANKFELTVQNDGEKVTLKTRIN
TVKIVDTLIDEQPLAIYATDKVLLPKELFKASAVEAPAPAPAPEDGDVADSPKAAKGKAK
GKKKKAAPSPDNDPFGDSDSPAEGPDGEADDATADDAGAVRIIGGAKAGLVVSLLCLFAS
SWLL
>P15941
MTPGTQSPFFLLLLLTVLTVVTGSGHASSTPGGEKETSATQRSSVPSSTEKNAVSMTSSV
LSSHSPGSGSSTTQGQDVTLAPATEPASGSAATWGQDVTSVPVTRPALGSTTPPAHDVTS
APDNKPAPGSTAPPAHGVTSAPDTRPAPGSTAPPAHGVTSAPDTRPAPGSTAPPAHGVTS
APDTRPAPGSTAPPAHGVTSAPDTRPAPGSTAPPAHGVTSAPDTRPAPGSTAPPAHGVTS
APDTRPAPGSTAPPAHGVTSAPDTRPAPGSTAPPAHGVTSAPDTRPAPGSTAPPAHGVTS
APDTRPAPGSTAPPAHGVTSAPDTRPAPGSTAPPAHGVTSAPDTRPAPGSTAPPAHGVTS
APDTRPAPGSTAPPAHGVTSAPDTRPAPGSTAPPAHGVTSAPDTRPAPGSTAPPAHGVTS
APDTRPAPGSTAPPAHGVTSAPDTRPAPGSTAPPAHGVTSAPDTRPAPGSTAPPAHGVTS
APDTRPAPGSTAPPAHGVTSAPDTRPAPGSTAPPAHGVTSAPDTRPAPGSTAPPAHGVTS
APDTRPAPGSTAPPAHGVTSAPDTRPAPGSTAPPAHGVTSAPDTRPAPGSTAPPAHGVTS
APDTRPAPGSTAPPAHGVTSAPDTRPAPGSTAPPAHGVTSAPDTRPAPGSTAPPAHGVTS
APDTRPAPGSTAPPAHGVTSAPDTRPAPGSTAPPAHGVTSAPDTRPAPGSTAPPAHGVTS
APDTRPAPGSTAPPAHGVTSAPDTRPAPGSTAPPAHGVTSAPDTRPAPGSTAPPAHGVTS
APDTRPAPGSTAPPAHGVTSAPDTRPAPGSTAPPAHGVTSAPDTRPAPGSTAPPAHGVTS
APDTRPAPGSTAPPAHGVTSAPDTRPAPGSTAPPAHGVTSAPDTRPAPGSTAPPAHGVTS
APDTRPAPGSTAPPAHGVTSAPDTRPAPGSTAPPAHGVTSAPDNRPALGSTAPPVHNVTS
ASGSASGSASTLVHNGTSARATTTPASKSTPFSIPSHHSDTPTTLASHSTKTDASSTHHS
SVPPLTSSNHSTSPQLSTGVSFFFLSFHISNLQFNSSLEDPSTDYYQELQRDISEMFLQI
YKQGGFLGLSNIKFRPGSVVVQLTLAFREGTINVHDVETQFNQYKTEAASRYNLTISDVS
VSDVPFPFSAQSGAGVPGWGIALLVLVCVLVALAIVYLIALAVCQCRRKNYGQLDIFPAR
DTYHPMSEYPTYHTHGRYVPPSSTDRSPYEKVSAGNGGSSLSYTNPAVAAASANL
>Q7TQN8
MESSFSFGVILAVLTILIIAVNALVVVAMLLSIYKNDGVGLCFTLNLAVADTLIGVAISG
LVTDQLSSSAQHTQKTLCSLRMAFVTSSAAASVLTVMLIAFDRYLAIKQPLRYFQIMNGL
VAGGCIAGLWLISYLIGFLPLGVSIFQQTTYHGPCTFFAVFHPRFVLTLSCAGFFPAVLL
FVFFYCDMLKIASVHSQHIRKMEHAGAMVGACRPPRPVNDFKAVRTVSVLIGSFTLSWSP
FLITSIVQVACHKCCLYQVLEKYLWLLGVGNSLLNPLIYAYWQREVRQQLCHMALGLLAD
GSTQPQIETLKGKEERKKVGRKTLYTCDAQTLYTCDAQTLYTCDAQTLYTCDACDTQTLY
TCDAQTLYTCDAQTLYTCDAQTLYTCDAQTLYTCDAQTLYTCDTQTLYTCDAQTLYTCDA
QTLYTCDAQTLYTCDAQTLYTSSLVTGQTEQTPLKRANMSDPLRTCRG
>Q86YW5

MGLTLLLLLLLGLEGQGIVGSLPEVLQAPVGSSILVQCHYRLQDVKAQKVWCRFLPEGCQ
PLVSSAVDRRAPAGRRTFLTDLGGGLLQVEMVTLQEEDAGEYGCMVDGARGPQILHRVSL
NILPPEEEEETHKIGSLAENAFSDPAGSANPLEPSQDEKSIPLIWGAVLLVGLLVAAVVL
FAVMAKRKQGNRLGVCGRFLSSRVSGMNPSSVVHHVSDSGPAAELPLDVPHIRLDSPPSF
DNTTYTSLPLDSPSGKPSLPAPSSLPPLPPKVLVCSKPVTYATVIFPGGNKGGGTSCGPA
QNPPNNQTPSS
>Q9QZR6
MERDRITALKRSFEVEEIEPPNSTPPRRVQTPLLRATVASSSQKFQDLGVKNSEPAARLV
DTLSQRSPKPSLRRVDLAGAKAPEPMSRRTELSIDISSKQVESTASTPGPSRFGLKRAEV
LGHKTPEPVPRRTEITIVKPQESGLRRVETPASKAPEGSAMPVTDAAPKRVEIQVPKPAE
APNCPLPPQTLENSEAPMSQLQSRLEPRPPVTEVPYRNQEDSEVAPSCVVDMADNPRDAM
LKQAPVSRNEKAPVDFGYVGIDSILEQMRRKAMKQGFEFNIMVVGQSGLGKSTLINTLFK
SKISRKSVQPISEERIPKTIEIKSITHDIEEKGVRMKLTVIDTPGFGDHINNENCWQPIM
KFINDQYEKYLQEEVNINRKKRIPDTRVHCCLYFIPATGHSLRPLDIEFMKRLSKVVNIV
PVIAKADTLTLEERVYFKQRITSDLLSNGIDVYPQKEFDEAEDRLVNEKFREMIPFAVVG
SDHEYQVNGKRILGRKTKWGTIEVENTTHCEFAYLRDLLIRTHMQNIKDITSNIHFEAYR
VKRLNEGNSAMANGIEKEPETQEM
>Q7KRY7
MFKCIPIFKGCNRQVEFVDKRHCSLPQVPEEILRYSRTLEELFLDANHIRDLPKNFFRLH
RLRKLGLSDNEIGRLPPDIQNFENLVELDVSRNDIPDIPDDIKHLQSLQVADFSSNPIPK
LPSGFSQLKNLTVLGLNDMSLTTLPADFGSLTQLESLELRENLLKHLPETISQLTKLKRL
DLGDNEIEDLPPYLGYLPGLHELWLDHNQLQRLPPELGLLTKLTYLDVSENRLEELPNEI
SGLVSLTDLDLAQNLLEALPDGIAKLSRLTILKLDQNRLQRLNDTLGNCENMQELILTEN
FLSELPASIGQMTKLNNLNVDRNALEYLPLEIGQCANLGVLSLRDNKLKKLPPELGNCTV
LHVLDVSGNQLLYLPYSLVNLQLKAVWLSENQSQPLLTFQPDTDAETGEQVLSCYLLPQQ
EYQPITPARDLESDSEPFEEREPSRTVVKFSEEATQEKETPFVRQNTPHPKDLKAKAQKL
KVERSRNEEHANLVTLPEENGTKLAETPTETRTIANNHQQQPHPVQQPIVGVNSKQPVVV
GVVTPTTTTVAPTGVQGGSEGASSTANNVKAATAAVVAELAATVGGSDEVQDDDEQEDEF
ESDRRVGFQVEGEDDDFYKRPPKLHRRDTPHHLKNKRVQHLTDKQASEILANALASQERN
DTTPQHSLSGKVTSPIEEEEQLEVEQEQQQQQQHPFDSSLSPISAGKTAEASTDPDNLD
GVTELRLEQYEIHIERTAAGLGLSIAGGKGSTPFKGDDDGIFISRVTEAGPADLAGLKVG
DKVIKVNGIVVVDADHYQAVQVLKACGAVLVLVVQREVTRLIGHPVFSEDGSVSQISVET
RPLVADAPPAASISHERYIPAPIEIVPQQQHLQQQQQQPIQQVAPTHSYSGNVFATPTAA
QTVQPAVSAAPNGLLLNGREAPLSYIQLHTTLIRDQIGQGLGFSIAGGKGSPPFKDDCDG
IFISRITEGGLAYRDGKIMVGDRVMAINGNDMTEAHHDAAVACLTEPQRFVRLVLQREYR
GPLEPPTSPRSPAVLNSLSPSGYLANRPANFSRSVVEVEQPYKYNTLATTTPTPKPTVPA
SISNNNNTLPSSKTNGFATAAAATIDSSTGQPVPAPRRTNSVPMGDGDIGAGSTTSGDSG
EAQPSSLRPLTSDDFQAMIPAHFLSGGSQHQVHVARPNEVGVSAVTVNVNKPQPDLPMFP
AAPTELGRVTETITKSTFTETVMTRITDNQLAEPLISEEVVLPKNQGSLGFSIIGGTDHS
CVPFGTREPGIFISHIVPGGIASKCGKLRMGDRILKVNEADVSKATHQDAVLELLKPGDE
IKLTIQHDPLPPGFQEVLLSKAEGERLGMHIKGGLNGQRGNPADPSDEGVFVSKINSVGA
ARRDGRLKVGMRLLEVNGHSLLGASHQDAVNVLRNAGNEIQLVVCKGYDKSNLIHSIGQA
GGMSTGFNSSASCSGGSRQGSRASETGSELSQSQSVSSLDHEEDERLRQDFDVFASQKPD
AQQPTGPSVLAAAAAMVHGASSPTPPAATSNITPLPTAAAVASADLTAPDTPATQTVALI

HAEQQAHQQQQQTQLAPLGQEKSTQEKVLEIVRAADAFTTVPPKSPSEHHEQDKIQKTTT
VVISKHTLDTNPTTPTTPAAPLSIAGAESANSAGAPSPAVPASTPGSAPVLPAVAVQTQT
QTTSTEKDEEEESQLQSTPASRDGAEEQQEEVRAKPTPTKVPKSVSDKKRFFESAMEDQH
KPTQKTDKVFSFLSKDEVEKLRQEEERKIATLRRDKNSRLLDAANDNIDKDAAQQRTKSN
SNSSSGDDNDDSDQEEGIARGDSVDNAALGHFDDAEDMRNPLDEIEAVFRS
>O08786
MDVVDSLLMNGSNITPPCELGLENETLFCLDQPQPSKEWQSAVQILLYSFIFLLSVLGNT
LVITVLIRNKRMRTVTNIFLLSLAVSDLMLCLFCMPFNLIPNLLKDFIFGSAVCKTTTYF
MGTSVSVSTFNLVAISLERYGAICRPLQSRVWQTKSHALKVIAATWCLSFTIMTPYPIYS
NLVPFTKNNNQTANMCRFLLPSDAMQQSWQTFLLLILFLIPGVVMVVAYGLISLELYQGI
KFDASQKKSAKEKRLSSGGGGGGGSSSSRYEDSDGCYLQKSRPPRKLELQQLSTSSSGGR
INRIRSSGSAANLIAKKRVIRMLIVIVVLFFLCWMPIFSANAWRAYDTVSAEKHLSGTPI
SFILLLSYTSSCVNPIIYCFMNKRFRLGFMATFPCCPNPGPTGVRGEVGEEEDGRTIRAS
LSRYSYSHMSTSAPPH
>P13587
MGEGTTKENNNAEFNAYHTLTAEEAAEFIGTSLTEGLTQDEFVHRLKTVGENTLGDDTKI
DYKAMVLHQVCNAMIMVLLISMIISFAMHDWITGGVISFVIAVNVLIGLVQEYKATKTMN
SLKNLSSPNAHVIRNGKSETINSKDVVPGDICLVKVGDTIPADLRLIETKNFDTDESLLT
GESLPVSKDANLVFGKEEETSVGDRLNLAFSSSAVVKGRAKGIVIKTALNSEIGKIAKSL
QGDSGLISRDPSKSWLQNTWISTKKVTGAFLGTNVGTPLHRKLSKLAVLLFWIAVLFAII
VMASQKFDVDKRVAIYAICVALSMIPSSLVVVLTITMSVGAAVMVSRNVIVRKLDSLEAL
GAVNDICSDKTGTLTQGKMLARQIWIPRFGTITISNSDDPFNPNEGNVSLIPRFSPYEYS
HNEDGDVGILQNFKDRLYEKDLPEDIDMDLFQKWLETATLANIATVFKDDATDCWKAHGD
PTEIAIQVFATKMDLPHNALTGEKSTNQSNENDQSSLSQHNEKPGSAQFEHIAEFPFDST
VKRMSSVYYNNHNETYNIYGKGAFESIISCCSSWYGKDGVKITPLTDCDVETIRKNVYSL
SNEGLRVLGFASKSFTKDQVNDDQLKNITSNRATAESDLVFLGLIGIYDPPRNETAGAVK
KFHQAGINVHMLTGDFVGTAKAIAQEVGILPTNLYHYSQEIVDSMVMTGSQFDGLSEEEV
DDLPVLPLVIARCSPQTKVRMIEALHRRKKFCTMTGDGVNDSPSLKMANVGIAMGINGSD
VSKEASDIVLSDDNFASILNAVEEGRRMTDNIQKFVLQLLAENVAQALYLIIGLVFRDEN
GKSVFPLSPVEVLWIIVVTSCFPAMGLGLEKAAPDLMDRPPHDSEVGIFTWEVIIDTFAY
GIIMTGSCMASFTGSLYGINSGRLGHDCDGTYNSSCRDVYRSRSAAFATMTWCALILAWE
VVDMRRSFFRMHPDTDSPVKEFFRSIWGNQFLFWSIIFGFVSAFPVVYIPVINDKVFLHK
PIGAEWGLAIAFTIAFWIGAELYKCGKRRYFKTQRAHNPENDLESNNKRDPFEAYSTSTT
IHTEVNIGIKQ
>Q62962
MDLKESPSEGSLQPSSIQIFANTSTLHGIRHIFVYGPLTIRRVLWAVAFVGSLGLLLVES
SERVSYYFSYQHVTKVDEVVAQSLVFPAVTLCNLNGFRFSRLTTNDLYHAGELLALLDVN
LQIPDPHLADPTVLEALRQKANFKHYKPKQFSMLEFLHRVGHDLKDMMLYCKFKGQECGH
QDFTTVFTKYGKCYMFNSGEDGKPLLTTVKGGTGNGLEIMLDIQQDEYLPIWGETEETTF
EAGVKVQIHSQSEPPFIQELGFGVAPGFQTFVATQEQRLTYLPPPWGECRSSEMGLDFFP
VYSITACRIDCETRYIVENCNCRMVHMPGDAPFCTPEQHKECAEPALGLLAEKDSNYCLC
RTPCNLTRYNKELSMVKIPSKTSAKYLEKKFNKSEKYISENILVLDIFFEALNYETIEQK
KAYEVAALLGDIGGQMGLFIGASLLTILELFDYIYELIKEKLLDLLGKEEEEGSHDENMS
TCDTMPNHSETISHTVNVPLQTALGTLEEIAC

>Q8TBR7
MLLTLAGGALFFPGLFALCTWALRRSQPGWSRTDCVMISTRLVSSVHAVLATGSGIVIIR
SCDDVITGRHWLAREYVWFLIPYMIYDSYAMYLCEWCRTRDQNRAPSLTLRNFLSRNRLM
ITHHAVILFVLVPVAQRLRGDLGDFFVGCIFTAELSTPFVSLGRVLIQLKQQHTLLYKVN
GILTLATFLSCRILLFPFMYWSYGRQQGLSLLQVPFSIPFYCNVANAFLVAPQIYWFCLL
CRKAVRLFDTPQAKKDG
>P38250
MSQTITSLDPNCVIVFNKTSSANEKSLNVEFKRLNIHSIIEPGHDLQTSYAFIRIHQDNA
KPLFSFLQNLDFIESIIPYHDTELSDDLHKLISISKSKILEAPKQYELYNLSNLTNNPKQ
SLYFAFLQNYIKWLIPFSFFGLSIRFLSNFTYEFNSTYSLFAILWTLSFTAFWLYKYEPF
WSDRLSKYSSFSTIEFLQDKQKAQKKASSVIMLKKCCFIPVALLFGAILLSFQLYCFALE
IFYKQIYNGPMISILSFLPTILICTFTPVLTVIYNKYFVEPMTKWENHSSVVNAKKSKEA
KNFVIIFLSSYVPLLITLFLYLPMGHLLTAEIRTKVFNAFSILARLPTHDSDFIIDTKRY
EDQFFYFIVINQLIQFSMENFVPSLVSIAQQKINGPNPNFVKAESEIGKAQLSSSDMKIW
SKVKSYQTDPWGATFDLDANFKKLLLQFGYLVMFSTIWPLAPFICLIVNLIVYQVDLRKA
VLYSKPEYFPFPIYDKPSSVSNTQKLTVGLWNSVLVMFSILGCVITATLTYMYQSCNIPG
VGAHTSIHTNKAWYLANPINHSWINIVLYAVFIEHVSVAIFFLFSSILKSSHDDVANGIV
PKHVVNVQNPPKQEVFEKIPSPEFNSNNEKELVQRKGSANEKLHQELGEKQPASSANGYE
AHAATHANNDPSSLSSASSPSLSSSSSSSKTGVVKAVDNDTAGSAGKKPLATESTEKRNS
LVKVPTVGSYGVAGATLPETIPTSKNYYLRFDEDGKSIRDAKSSAESSNATNNNTLGTES
KLLPDGDAVDALSRKIDQIPKIAVTGGENNENTQAKDDAATKTPLIKDANIKPVVNAAVN
DNQSKVSVATEQTKKTEVSTKNGPSRSISTKETKDSARPSNNNTTTTTTTDATQPHHHHH
HHRHRDAGVKNVTNNSKTTESSSSSSAAKEKPKHKKGLLHKLKKKL
>Q12252
MADSSSTSAFISTLIIYGLTAVVFVWLFLLLRPKNRRVYEPRSLKDIQTIPEEERTEPVP
EGYFGWVEYLLSKPHSFLIQHTSVDGYFLLRYIGIVGSLSFVGCLLLLPILLPVNATNGN
NLQGFELLSFSNVTNKNRFYAHVFLSWIFFGLFTYVIYKELYYYVVFRHAMQTTPLYDGL
LSSRTVIVTELHKSIAQEGEMQMRFPKASNVAFAYDLSDLQELCKERAKNAAKYEAALNK
VLNKCVKMTRNKTQKQLDKLYNNGTKPKDDLETYVPHKKRPKHRLGKLPLCLGGKKVNTL
SYSSKRIGELNEEIHEKQADWASNDRQPACFIQFETQLEAQRCYQSVEAILGKKNFGKRL
IGYSPEDVNWGSMRLSSKERHSRRAVANTIMVLLIIFWAFPVAVVGIISNVNFLTDKVPF
LRFINNMPTFLMGVITGLLPTIALVVLMSLVPPFIVMLGKLSGCVTRQETDLYSQAWYYA
FAVIQIFLVVTATSSASSTVDSIIDRPRSAMTLLANNLPKASNFYIMYFILKGLTGPTWT
ILQAVNLLLSKVLGRVLDSTPRQKWNRYNTLATPRMGIVYPGIEILVCIYICYSIIAPIL
LFFSTVMLTLLYVAYLYNLNYVFGFSFDLKGRNYPRALFQIFVGIYLSEVCLLGLFIMAK
TWGPLVLEVFWIVVTALAHIYMKRKFIPLFDAVPLSAIRHARGEPGYSYPTSDLGLQEIK
DIADEMKGKYEQDNTHGILTPVTKDDLKKANLIPDNDGSSENGTPSNPFESGSERASLSG
SNAESDSIKKLNDTVIKKSSTLSSSTKDNNESTFVPEGEKFRKFHYSDVEALRNKRPYDE
DDHSKHGPEGAVPVNADAGVIYSDPAAVMKEPQAFPPDVLETNTWTRRILQFFNPRRSYP
FDSVRMRFPLVFNTSIEYDEEYLSSAYTDPCVREKDPIVWCCKDPLGVSKQQIQEARSNG
LDVRDDFTRYDEKGKVIFTYNPPDYEPEAKK
>O94532
MASKMPEGSPPTSRSIQSRNSSYSTSSNERIGTPSTISLSENSDLSKLQSTNDFESREDL
SLTSDDNNDPEYVMCYNTVYHQKTKINDKLLSETEQLRKIYPLESRVFPKPTIVKEITNE

RTKRYTFYDDDAPLTNQHTVLDEATYNRILKRIDFFIEKVVEVPPTYHFLSLLNVQLRIQ
PIWWMDEFAERNGIESLLSALRNLGHYPERASKTPLESQIIQSLFHCLNSENCRRRYQSS
AKCSVPGFNALGTIAETVLSKSLNSARMATFLLKFLCNKKGLSYFKAVIRAFEWLVEQKL
SKTRFSAWMHSFNDVITGVRVCADSSPQAIVHMDEFEDTDCLIDYLVATLALIRDLCAAP
PDLQLRCALRHELLSAGLQKAIDSLLKWRNRHVRDALQLLIKEHNADARRFRSGSDVNNV
DRKCVKKQMNYREESHTPHGNTTRKTSTPVSNNRPTTPEQQAVWDVFQRIYTRFTGSEGS
KESFIKLLEYFVTEPDNGKIQKSMQLLTHTLEALEGFKTAKADTNVGLTILSQRLLDKLG
TAEEIAEYKTKYNGAMLENKHLKEQVESMLSQLNVGPRDPMQFLKKQLDELKAELNLRDN
LLASMQREFETRYRAQIQAYNKLQSQMEHVQNSNEQHLQPGLLNKVSKSFDSVHRRNLSQ
DSLDAMTEQFSYHVEPNILSGSGIPVRVHTPSKTEDLDESFSGSEISSSPSPLLPDVSDT
VEEQQKLLLKSPPPPPPAVIVPTPAPAPIPVPPPAPIMGGPPPPPPPPGVAGAGPPPPPP
PPPAVSAGGSRYYAPAPQAEPEPKIDETSLTEEQKIQLEEARKQRKAADDAARAAIEKYT
SIPSLRDLHKPTRPLKRVHWQRVDPLPGPNVFTKFCLNFDITAKVFIDNGLLDFLDEKFD
NTPREDFVAVEISDQRSSLLPDTVEQNMAIILRSVSNMPVEDLVQKFLVEPDFLPASILY
FDRASLASTNAYTDPFIPYSTDYTKKNPKEPTADVNSLSYFEKFFVLFVVNLRHYFQERM
KALKFRSTLFGDLEILEVRMKEVIDTSDSIMEDKNFAEFFQVLLIIGNYFNEPYDRASAF
SLYMIYRLETLRDSSSALTLMHYFDEIIRTRFPELLQAESTFKKIQSVSGYNIDAMVAGV
DGAYDEFCDFQTSLKDGALSKCDQHHPDDKAYDILSEWLPEAKERIRNIKKLKTDMLTKL
ENTVKYLCEYDSIDKVRNSFFKNLNSFYEMYSIAKAENEERFEKEKRRIMSEDRDKLIRG
RQKTSIVAKYRNKRELPEDSDDKQDTASKDKNSLETIDEKMEDASKIEGDAKTGDDNEME
DLDKMEDLEKPDYAEEKDPYITVMSELRSRIQNVPKRTVTVYSDEGVATLEPGAQGDDVV
DKAKMILEKMEGHSQLLTSSANPDEEVLRAKLKAAERLQKPAIPRTRRKGHTEPKSAKSL
LAELTNGSNASNLVENDRQKQ
>Q9R1N3
MEADGAGEQMRPLLTRGPDEEAVVDLGKTSSTVNTKFEKEELESHRAVYVGVHVPFSKES
RRRHRHRGHKHHHRRRKDKDSDKEDGRESPSYDTPSQRVQFILGTEDDDEEHIPHDLFTE
MDELCYRDGEEYEWKETARWLKFEEDVEDGGDRWSKPYVATLSLHSLFELRSCILNGTVM
LDMRASTLDEIADMVLDNMIASGQLDDSIRENVREALLKRHHHQNEKRFTSRIPLVRSFA
DIGKKHSDPHLLERNGEGLSASRHSLRTGLSASNLSLRGESPLSLLLSHLLPSSRAGTPA
GSRCTTPVPTPQNSPPSSPSLSRLTSRSSQQTQPQAPEVLVSPDRDDIPRVVIHPPEEDI
EALKGQEQKNEENTDFTPGILASPQSAPGNLDSSKSGEVKGNGSGGSRENSTVDFSKVDM
NFMRKIPTGAEASNVLVGEVDFLERPIIAFVRLAPAVLLSGLTEVPVPTRFLFLLLGPAG
KAPQYHEIGRSIATLMTDEIFHDVAYKAKDRNDLLSGIDEFLDQVTVLPPGEWDPSIRIE
PPKSVPSQEKRKIPAFPNGSAPVSADPPKEADHHAGPELQRTGRLFGGLILDIKRKAPFF
LSDFKDALSLQCLASILFLYCACMSPVITFGGLLGEATEGRISAIESLFGASLTGIAYSL
FAGQPLTILGSTGPVLVFEKILFKFCRDYHLSYLSLRTSIGLWTSFLCIVLVATDASSLV
CYITRFTEEAFAALICIIFIYEALEKLFHLGEIYAFNMHNNLDALTSYTCVCAEPSNPSN
ETVELWERKNVTAASISWANLTVSECKTFHGMFVGSACGPHGPYVPDVLFWCVVLFFTTF
FLSSFLKQFKTKRYFPTKVRSTISDFAVFLTIVIMVAIDYLVGIPSPKLHVPEKFEPTDP
SRGWIISPLGDNPWWTLLIAAVPALLCTILIFMDQQITAVIINRKEHKLKKGAGYHLDLL
MVAVMLGVCSIMGLPWFVAATVLSISHVNSLKVESECSAPGEQPKFLGIREQRVTGLMIF
ILMGLSVFMTSVLKFIPMPVLYGVFLYMGVSSLKGIQFFDRIKLFGMPAKHQPDLIYLRY
VPLWKVHVFTVVQLTCLVLLWVIKASAAAVVFPMMVLALVFVRKLMDLCFTKRELSWLDD
LMPESKKKKEDDKKKKEKEEAERMLQGDGDTVHLPFERGSLLQIPVKTLKYSPEKPVSVT

INFEDEPSKKYMDAETSL
>P49771
MTVLAPAWSPTTYLLLLLLLSSGLSGTQDCSFQHSPISSDFAVKIRELSDYLLQDYPVTV
ASNLQDEELCGGLWRLVLAQRWMERLKTVAGSKMQGLLERVNTEIHFVTKCAFQPPPSCL
RFVQTNISRLLQETSEQLVALKPWITRQNFSRCLELQCQPDSSTLPPPWSPRPLEATAPT
APQPPLLLLLLLPVGLLLLAAAWCLHWQRTRRRTPRPGEQVPPVPSPQDLLLVEH
>Q9ERD6
MDLMNGQASSVTIAATVSEKSSSSESLSEKGSELKKSFDAVVFDVLKVTPEEYAGQITLM
DVPVFKAIQPDELSSCGWNKKEKYSSAPNAVAFTRRFNHVSFWVVREILHAQTLKIRAEV
LSHYIKTAKKLYELNNLHALMAVVSGLQSAPIFRLTKTWALLSRKDKTTFEKLEYVMSKE
DNYKRLRDYISSLKMTPCIPYLGIYLSDLTYIDSAYPSTGSILENEQRSNLMNNILRIIS
DLQQSCEYDIPILPHVQKYLNSVQYIEELQKFVEDDNYKLSLKIEPGASTPRSAASREDL
AGPDIGASPQGGRKSSAAAAAAAAAEGALLPQTPPSPRNLIPHGHRKCHSLGYNFIHKMN
TAEFKSATFPNAGPRHLLDDSVMEPHAPSRGQAESSTLSSGISIGSSDGSELSEETSWPA
FERNRLYHSLGPVTRVPRNGYRSHTKASSSAESEDLAVHLYPGAVTIQGVLRRKTLLKEG
KKPTVASWTKYWAALCGTQLFYYAAKSLKATERKHFKSTSNKNVSVVGWMVMMADDPEHP
DLFLLTDSEKGNSYKFQAGSRMNAMLWFKHLSAACQSNKQQVPTNLMTFE
>P05556
MNLQPIFWIGLISSVCCVFAQTDENRCLKANAKSCGECIQAGPNCGWCTNSTFLQEGMPT
SARCDDLEALKKKGCPPDDIENPRGSKDIKKNKNVTNRSKGTAEKLKPEDIHQIQPQQLV
LRLRSGEPQTFTLKFKRAEDYPIDLYYLMDLSYSMKDDLENVKSLGTDLMNEMRRITSDF
RIGFGSFVEKTVMPYISTTPAKLRNPCTSEQNCTTPFSYKNVLSLTNKGEVFNELVGKQR
ISGNLDSPEGGFDAIMQVAVCGSLIGWRNVTRLLVFSTDAGFHFAGDGKLGGIVLPNDGQ
CHLENNMYTMSHYYDYPSIAHLVQKLSENNIQTIFAVTEEFQPVYKELKNLIPKSAVGTL
SANSSNVIQLIIDAYNSLSSEVILENGKLSEGVTISYKSYCKNGVNGTGENGRKCSNISI
GDEVQFEISITSNKCPKKDSDSFKIRPLGFTEEVEVILQYICECECQSEGIPESPKCHEG
NGTFECGACRCNEGRVGRHCECSTDEVNSEDMDAYCRKENSSEICSNNGECVCGQCVCRK
RDNTNEIYSGKFCECDNFNCDRSNGLICGGNGVCKCRVCECNPNYTGSACDCSLDTSTCE
ASNGQICNGRGICECGVCKCTDPKFQGQTCEMCQTCLGVCAEHKECVQCRAFNKGEKKDT
CTQECSYFNITKVESRDKLPQPVQPDPVSHCKEKDVDDCWFYFTYSVNGNNEVMVHVVEN
PECPTGPDIIPIVAGVVAGIVLIGLALLLIWKLLMIIHDRREFAKFEKEKMNAKWDTGEN
PIYKSAVTTVVNPKYEGK
>Q6YHK3
MQGPPLLTAAHLLCVCTAALAVAPGPRFLVTAPGIIRPGGNVTIGVELLEHCPSQVTVKA
ELLKTASNLTVSVLEAEGVFEKGSFKTLTLPSLPLNSADEIYELRVTGRTQDEILFSNST
RLSFETKRISVFIQTDKALYKPKQEVKFRIVTLFSDFKPYKTSLNILIKDPKSNLIQQWL
SQQSDLGVISKTFQLSSHPILGDWSIQVQVNDQTYYQSFQVSEYVLPKFEVTLQTPLYCS
MNSKHLNGTITAKYTYGKPVKGDVTLTFLPLSFWGKKKNITKTFKINGSANFSFNDEEMK
NVMDSSNGLSEYLDLSSPGPVEILTTVTESVTGISRNVSTNVFFKQHDYIIEFFDYTTVL
KPSLNFTATVKVTRADGNQLTLEERRNNVVITVTQRNYTEYWSGSNSGNQKMEAVQKINY
TVPQSGTFKIEFPILEDSSELQLKAYFLGSKSSMAVHSLFKSPSKTYIQLKTRDENIKVG
SPFELVVSGNKRLKELSYMVVSRGQLVAVGKQNSTMFSLTPENSWTPKACVIVYYIEDDG
EIISDVLKIPVQLVFKNKIKLYWSKVKAEPSEKVSLRISVTQPDSIVGIVAVDKSVNLMN
ASNDITMENVVHELELYNTGYYLGMFMNSFAVFQECGLWVLTDANLTKDYIDGVYDNAEY

AERFMEENEGHIVDIHDFSLGSSPHVRKHFPETWIWLDTNMGYRIYQEFEVTVPDSITSW
VATGFVISEDLGLGLTTTPVELQAFQPFFIFLNLPYSVIRGEEFALEITIFNYLKDATEV
KVIIEKSDKFDILMTSNEINATGHQQTLLVPSEDGATVLFPIRPTHLGEIPITVTALSPT
ASDAVTQMILVKAEGIEKSYSQSILLDLTDNRLQSTLKTLSFSFPPNTVTGSERVQITAI
GDVLGPSINGLASLIRMPYGCGEQNMINFAPNIYILDYLTKKKQLTDNLKEKALSFMRQG
YQRELLYQREDGSFSAFGNYDPSGSTWLSAFVLRCFLEADPYIDIDQNVLHRTYTWLKGH
QKSNGEFWDPGRVIHSELQGGNKSPVTLTAYIVTSLLGYRKYQPNIDVQESIHFLESEFS
RGISDNYTLALITYALSSVGSPKAKEALNMLTWRAEQEGGMQFWVSSESKLSDSWQPRSL
DIEVAAYALLSHFLQFQTSEGIPIMRWLSRQRNSLGGFASTQDTTVALKALSEFAALMNT
ERTNIQVTVTGPSSPSPVKFLIDTHNRLLLQTAELAVVQPTAVNISANGFGFAICQLNVV
YNVKASGSSRRRRSIQNQEAFDLDVAVKENKDDLNHVDLNVCTSFSGPGRSGMALMEVNL
LSGFMVPSEAISLSETVKKVEYDHGKLNLYLDSVNETQFCVNIPAVRNFKVSNTQDASVS
IVDYYEPRRQAVRSYNSEVKLSSCDLCSDVQGCRPCEDGASGSHHHSSVIFIFCFKLLYF
MELWL
>Q3UJF0
MSQPERDNCSFDSVDKLTRTLQLAVHIPTFLLGLVLNLLAIRGFSAFLKKRKLDYIATSI
YMINLAVFDLLLVLSLPFKMVLPQVESPLPSFCTLVECLYFISMYGSVFTICFISLDRFL
AIQYPILASHLRSPRKTFGICCIIWMLVWIGSIPIYTFHREVERYKCFHNMSDVTWSASV
FFPLEIFGFLLPMGIMGFCSYRSIHILLRRPDSTEDWVQQRDTKGLGTKREPCIWTIATN
LVIFVVSFLPVHLGFFLQYLVRNRFILDCRMKQGISLFLQLSLCFSNINCCLDVFCYYFV
IKEFRMRIKAHRPSTIKLVNQDTMVSRG
>O95996
MASSVAPYEQLVRQVEALKAENSHLRQELRDNSSHLSKLETETSGMKEVLKHLQGKLEQE
ARVLVSSGQTEVLEQLKALQMDITSLYNLKFQPPTLGPEPAARTPEGSPVHGSGPSKDSF
GELSRATIRLLEELDRERCFLLNEIEKEEKEKLWYYSQLQGLSKRLDELPHVETQFSMQM
DLIRQQLEFEAQHIRSLMEERFGTSDEMVQRAQIRASRLEQIDKELLEAQDRVQQTEPQA
LLAVKSVPVDEDPETEVPTHPEDGTPQPGNSKVEVVFWLLSMLATRDQEDTARTLLAMSS
SPESCVAMRRSGCLPLLLQILHGTEAAAGGRAGAPGAPGAKDARMRANAALHNIVFSQPD
QGLARKEMRVLHVLEQIRAYCETCWDWLQARDGGPEGGGAGSAPIPIEPQICQATCAVMK
LSFDEEYRRAMNELGGLQAVAELLQVDYEMHKMTRDPLNLALRRYAGMTLTNLTFGDVAN
KATLCARRGCMEAIVAQLASDSEELHQVVSSILRNLSWRADINSKKVLREAGSVTALVQC
VLRATKESTLKSVLSALWNLSAHSTENKAAICQVDGALGFLVSTLTYKCQSNSLAIIESG
GGILRNVSSLVATREDYRQVLRDHNCLQTLLQHLTSHSLTIVSNACGTLWNLSARSARDQ
ELLWDLGAVGMLRNLVHSKHKMIAMGSAAALRNLLAHRPAKHQAAATAVSPGSCVPSLYV
RKQRALEAELDARHLAQALEHLEKQGPPAAEAATKKPLPPLRHLDGLAQDYASDSGCFDD
DDAPSSLAAAAATGEPASPAALSLFLGSPFLQGQALARTPPTRRGGKEAEKDTSGEAAVA
AKAKAKLALAVARIDQLVEDISALHTSSDDSFSLSSGDPGQEAPREGRAQSCSPCRGPEG
GRREAGSRAHPLLRLKAAHASLSNDSLNSGSASDGYCPREHMLPCPLAALASRREDPRCG
QPRPSRLDLDLPGCQAEPPAREATSADARVRTIKLSPTYQHVPLLEGASRAGAEPLAGPG
ISPGARKQAWLPADHLSKVPEKLAAAPLSVASKALQKLAAQEGPLSLSRCSSLSSLSSAG
RPGPSEGGDLDDSDSSLEGLEEAGPSEAELDSTWRAPGATSLPVAIPAPRRNRGRGLGVE
DATPSSSSENYVQETPLVLSRCSSVSSLGSFESPSIASSIPSEPCSGQGSGTISPSELPD
SPGQTMPPSRSKTPPLAPAPQGPPEATQFSLQWESYVKRFLDIADCRERCRLPSELDAGS
VRFTVEKPDENFSCASSLSALALHEHYVQQDVELRLLPSACPERGGGAGGAGLHFAGHRR

REEGPAPTGSRPRGAADQELELLRECLGAAVPARLRKVASALVPGRRALPVPVYMLVPAP
APAQEDDSCTDSAEGTPVNFSSAASLSDETLQGPPRDQPGGPAGRQRPTGRPTSARQAMG
HRHKAGGAGRSAEQSRGAGKNRAGLELPLGRPPSAPADKDGSKPGRTRGDGALQSLCLTT
PTEEAVYCFYGNDSDEEPPAAAPTPTHRRTSAIPRAFTRERPQGRKEAPAPSKAAPAAPP
PARTQPSLIADETPPCYSLSSSASSLSEPEPSEPPAVHPRGREPAVTKDPGPGGGRDSSP
SPRAAEELLQRCISSALPRRRPPVSGLRRRKPRATRLDERPAEGSRERGEEAAGSDRASD
LDSVEWRAIQEGANSIVTWLHQAAAATREASSESDSILSFVSGLSVGSTLQPPKHRKGRQ
AEGEMGSARRPEKRGAASVKTSGSPRSPAGPEKPRGTQKTTPGVPAVLRGRTVIYVPSPA
PRAQPKGTPGPRATPRKVAPPCLAQPAAPAKVPSPGQQRSRSLHRPAKTSELATLSQPPR
SATPPARLAKTPSSSSSQTSPASQPLPRKRPPVTQAAGALPGPGASPVPKTPARTLLAKQ
HKTQRSPVRIPFMQRPARRGPPPLARAVPEPGPRGRAGTEAGPGARGGRLGLVRVASALS
SGSESSDRSGFRRQLTFIKESPGLRRRRSELSSAESAASAPQGASPRRGRPALPAVFLCS
SRCEELRAAPRQGPAPARQRPPAARPSPGERPARRTTSESPSRLPVRAPAARPETVKRYA
SLPHISVARRPDGAVPAAPASADAARRSSDGEPRPLPRVAAPGTTWRRIRDEDVPHILRS
TLPATALPLRGSTPEDAPAGPPPRKTSDAVVQTEEVAAPKTNSSTSPSLETREPPGAPAG
GQLSLLGSDVDGPSLAKAPISAPFVHEGLGVAVGGFPASRHGSPSRSARVPPFNYVPSPM
VVAATTDSAAEKAPATASATLLE
>Q9H4E7
MALRKELLKSIWYAFTALDVEKSGKVSKSQLKVLSHNLYTVLHIPHDPVALEEHFRDDDD
GPVSSQGYMPYLNKYILDKVEEGAFVKEHFDELCWTLTAKKNYRADSNGNSMLSNQDAFR
LWCLFNFLSEDKYPLIMVPDEVEYLLKKVLSSMSLEVSLGELEELLAQEAQVAQTTGGLS
VWQFLELFNSGRCLRGVGRDTLSMAIHEVYQELIQDVLKQGYLWKRGHLRRNWAERWFQL
QPSCLCYFGSEECKEKRGIIPLDAHCCVEVLPDRDGKRCMFCVKTANRTYEMSASDTRQR
QEWTAAIQMAIRLQAEGKTSLHKDLKQKRREQREQRERRRAAKEEELLRLQQLQEEKERK
LQELELLQEAQRQAERLLQEEEERRRSQHRELQQALEGQLREAEQARASMQAEMELKEEE
AARQRQRIKELEEMQQRLQEALQLEVKARRDEESVRIAQTRLLEEEEEKLKQLMQLKEEQ
ERYIERAQQEKEELQQEMAQQSRSLQQAQQQLEEVRQNRQRADEDVEAAQRKLRQASTNV
KHWNVQMNRLMHPIEPGDKRPVTSSSFSGFQPPLLAHRDSSLKRLTRWGSQGNRTPSPNS
NEQQKSLNGGDEAPAPASTPQEDKLDPAPEN
>P21463
MRPGSLLQLTLLLALPRSLWGRGCTSPPCECHQEDDFRVTCKELHQIPSLPPSTQTLKLI
ETHLKTIPSLAFSSLPNISRIYLSIDATLQRLEPHSFYNLSKMTHIEIRNTRSLTYIDPD
ALTELPLLKFLGIFNTGLRIFPDLTKIYSTDVFFILEITDNPYMTSVPENAFQGLCNETL
TLKLYNNGFTSIQGHAFNGTKLDAVYLNKNKYLTAIDKDAFGGVYSGPTLLDVSSTSVTA
LPSKGLEHLKELIAKNTWTLKKLPLSLSFLHLTRADLSYPSHCCAFKNQKKIRGILESLM
CNESSIRNLRQRKSVNVMRGPVYQEYEEGLGDNHVGYKQNSKFQEGPSNSHYYVFFEEQE
DEIIGFGQELKNPQEETLQAFDSHYDYTVCGDNEDMVCTPKSDEFNPCEDIMGYKFLRIV
VWFVSPMALLGNVFVLFVLLTSHYKLTVPRFLMCNLAFADFCMGVYLLLIASVDLYTHTE
YYNHAIDWQTGPGCNTAGFFTVFASELSVYTLTVITLERWYAITFAMRLDRKIRLRHAYT
IMAGGWVSCFLLALLPMVGISSYAKVSICLPMDTDTPLALAYIALVLLLNVVAFVIVCSC
YVKIYITVRNPQYNPRDKDTKIAKRMAVLIFTDFMCMAPISFYALSALMNKPLITVTNSG
VLLVLFYPLNSCANPFLYAIFTKAFQRDVFILLSKFGLCKHQAQAYQAQRVCPNNNTGIQ
IQKIPQDTRQSLPNVQDTYEPLGSSHLTPKLQGRISEEYTQTAL
>P41930

MVANWVLALTRQFDPFMFMMVMGVGISSNILYSFPYPARWLRICSYIMFAIACLIFIAVQ
ALQILHLIVYIKEKSFREYFNDFFRNMKHNLFWGTYPMGLVTIINFLGALSKANTTKSPT
NARNLMIFVYVLWWYDLAVCLVIAWGISFLIWHDYYPLEGIGNYPSYNIKMASENMKSVL
LLDIIPLVVVASSCGTFTMSEIFFHAFNRNIQLITLVICALTWLHAIIFVFILIAIYFWS
LYINKIPPMTQVFTLFLLLGPMGQGSFGVLLLTDNIKKYAGKYYPTDNITREQEILTIAV
PWCFKILGMVSAMALLAMGYFFTVISVVSILSYYNKKEIENETGKVKRVYTFHKGFWGMT
FPMGTMSLGNEELYVQYNQYVPLYAFRVLGTIYGGVCVCWSILCLLCTLHEYSKKMLHAA
RKSSLFSESGTEKTTVSPYNSIESVEESNSALDFTRLA
>Q15109
MAAGTAVGAWVLVLSLWGAVVGAQNITARIGEPLVLKCKGAPKKPPQRLEWKLNTGRTEA
WKVLSPQGGGPWDSVARVLPNGSLFLPAVGIQDEGIFRCQAMNRNGKETKSNYRVRVYQI
PGKPEIVDSASELTAGVPNKVGTCVSEGSYPAGTLSWHLDGKPLVPNEKGVSVKEQTRRH
PETGLFTLQSELMVTPARGGDPRPTFSCSFSPGLPRHRALRTAPIQPRVWEPVPLEEVQL
VVEPEGGAVAPGGTVTLTCEVPAQPSPQIHWMKDGVPLPLPPSPVLILPEIGPQDQGTYS
CVATHSSHGPQESRAVSISIIEPGEEGPTAGSVGGSGLGTLALALGILGGLGTAALLIGV
ILWQRRQRRGEERKAPENQEEEEERAELNQSEEPEAGESSTGGP
>Q3E7D0
MSSSDPKPGPKPGPWPPTPESAAMPPSSWAKKTGFRPKFSGETTATDSSSGQLSLPVRAK
QQETQPDLEAGQTRLRPPPPVSAAVTNGETDKDKKEKPPPPPPGSVAVPVKDQPVKRRRD
SDGVVGRSNGPDGANGSGDPVRRPGRIEETVEVLPQSMDDDLVARNLHMKYGLRDTPGLV
PIGFYGLQHYLSMLGSLILVPLVIVPAMGGSHEEVANVVSTVLFVSGITTLLHTSFGSRL
PLIQGPSFVFLAPALAIINSPEFQGLNGNNNFKHIMRELQGAIIIGSAFQAVLGYSGLMS
LILRLVNPVVVAPTVAAVGLSFYSYGFPLVGKCLEIGVVQILLVIIFALYLRKISVLSHR
IFLIYAVPLSLAITWAAAFLLTETGAYTYKGCDPNVPVSNVVSTHCRKYMTRMKYCRVDT
SHALSSAPWFRFPYPLQWGVPLFNWKMAFVMCVVSVIASVDSVGSYHASSLLVASRPPTR
GVVSRAIGLEGFTSVLAGLWGTGTGSTTLTENVHTIAVTKMGSRRVVELGACVLVIFSLV
GKVGGFLASIPQVMVASLLCFMWAMFTALGLSNLRYSEAGSSRNIIIVGLSLFFSLSVPA
YFQQYGISPNSNLSVPSYYQPYIVSSHGPFKSQYKGMNYVMNTLLSMSMVIAFIMAVILD
NTVPGSKQERGVYVWSDSETATREPALAKDYELPFRVGRFFRWVKWVGI
>P63149
MSTPARRRLMRDFKRLQEDPPVGVSGAPSENNIMQWNAVIFGPEGTPFEDGTFKLVIEFS
EEYPNKPPTVRFLSKMFHPNVYADGSICLDILQNRWSPTYDVSSILTSIQSLLDEPNPNS
PANSQAAQLYQENKREYEKRVSAIVEQSWNDS
>Q8IWV1
MDGVTPTLSTIRGRTLESSTLHVTPRSLDRNKDQITNIFSGFAGLLAILLVVAVFCILWN
WNKRKKRQVPYLRVTVMPLLTLPQTRQRAKNIYDILPWRQEDLGRHESRSMRIFSTESLL
SRNSESPEHVPSQAGNAFQEHTAHIHATEYAVGIYDNAMVPQMCGNLTPSAHCINVRASR
DCASISSEDSHDYVNVPTAEEIAETLASTKSPSRNLFVLPSTQKLEFTEERDEGCGDAGD
CTSLYSPGAEDSDSLSNGEGSSQISNDYVNMTGLDLSAIQERQLWVAFQCCRDYENVPAA
DPSGSQQQAEKDVPSSNIGHVEDKTDDPGTHVQCVKRTFLASGDYADFQPFTQSEDSQMK
HREEMSNEDSSDYENVLTAKLGGRDSEQGPGTQLLPDE
>Q80WK2
MDHSAEKAAANAEVPQELLEEMLWYFRAEDAAPWNYSILVLAVLVVMTSMFLLRRSILAN
RNRKKQPQDKETPEDLHLDDSIMKENNSQVFLRETLISEKPDLAPGEPELKEKDSSLVFL

PDPQETES
>Q8TDS5
MLCHRGGQLIVPIIPLCPEHSCRGRRLQNLLSGPWPKQPMELHNLSSPSPSLSSSVLPPS
FSPSPSSAPSAFTTVGGSSGGPCHPTSSSLVSAFLAPILALEFVLGLVGNSLALFIFCIH
TRPWTSNTVFLVSLVAADFLLISNLPLRVDYYLLHETWRFGAAACKVNLFMLSTNRTASV
VFLTAIALNRYLKVVQPHHVLSRASVGAAARVAGGLWVGILLLNGHLLLSTFSGPSCLSY
RVGTKPSASLRWHQALYLLEFFLPLALILFAIVSIGLTIRNRGLGGQAGPQRAMRVLAMV
VAVYTICFLPSIIFGMASMVAFWLSACRSLDLCTQLFHGSLAFTYLNSVLDPVLYCFSSP
NFLHQSRALLGLTRGRQGPVSDESSYQPSRQWRYREASRKAEAIGKLKVQGEVSLEKEGS
SQG
>Q8IYR6
MGAAAAEAPLRLPAAPPLAFCCYTSVLLLFAFSLPGSRASNQPPGGGGGSGGDCPGGKGK
SINCSELNVRESDVRVCDESSCKYGGVCKEDGDGLKCACQFQCHTNYIPVCGSNGDTYQN
ECFLRRAACKHQKEITVIARGPCYSDNGSGSGEGEEEGSGAEVHRKHSKCGPCKYKAECD
EDAENVGCVCNIDCSGYSFNPVCASDGSSYNNPCFVREASCIKQEQIDIRHLGHCTDTDD
TSLLGKKDDGLQYRPDVKDASDQREDVYIGNHMPCPENLNGYCIHGKCEFIYSTQKASCR
CESGYTGQHCEKTDFSILYVVPSRQKLTHVLIAAIIGAVQIAIIVAIVMCITRKCPKNNR
GRRQKQNLGHFTSDTSSRMV
>Q8NHU3
MDIIETAKLEEHLENQPSDPTNTYARPAEPVEEENKNGNGKPKSLSSGLRKGTKKYPDYI
QIAMPTESRNKFPLEWWKTGIAFIYAVFNLVLTTVMITVVHERVPPKELSPPLPDKFFDY
IDRVKWAFSVSEINGIILVGLWITQWLFLRYKSIVGRRFCFIIGTLYLYRCITMYVTTLP
VPGMHFQCAPKLNGDSQAKVQRILRLISGGGLSITGSHILCGDFLFSGHTVTLTLTYLFI
KEYSPRHFWWYHLICWLLSAAGIICILVAHEHYTIDVIIAYYITTRLFWWYHSMANEKNL
KVSSQTNFLSRAWWFPIFYFFEKNVQGSIPCCFSWPLSWPPGCFKSSCKKYSRVQKIGED
NEKST
>Q9LDC0
MDESLEHQTQTHDQESEIVTEGSAVVHSEPSQEGNVPPKVDSEAEVLDEKVSKQIIKEGH
GSKPSKYSTCFLHYRAWTKNSQHKFEDTWHEQQPIELVLGKEKKELAGLAIGVASMKSGE
RALVHVGWELAYGKEGNFSFPNVPPMADLLYEVEVIGFDETKEGKARSDMTVEERIGAAD
RRKMDGNSLFKEEKLEEAMQQYEMAIAYMGDDFMFQLYGKYQDMALAVKNPCHLNIAACL
IKLKRYDEAIGHCNIVLTEEEKNPKALFRRGKAKAELGQMDSARDDFRKAQKYAPDDKAI
RRELRALAEQEKALYQKQKEMYKGIFKGKDEGGAKSKSLFWLIVLWQWFVSLFSRIFRRH
RVKAD
>Q9UU99
MKDSEHHHHGHNGFSKFLHKLGFGKSKGKSHKSNTSSIHERENTAAKGPASNVRPARPNV
STSSTSNEVRKSVPVGNPTVHTKTGSSSSPASKMRNTVNLQHIQAANQKTRNAEGERKVA
QRRVQSDKAEANDAAMSSSAPTVDVSEGNSAAEPKITPDDSDTPRLNVDMNDKINVDEAA
AKSDSKLNVDQINSTTESEKRVEKVNPNIANNPLKSPDAVAYSSETVSDEKQPTEHPVSA
NVPAKSEKAVCDENTKISLTNTEHYKFHSLRGDVEVVVGDLERDGRDTSLGDASVNEAAK
ETDVDSSRFISDEKAVTEDAMKTEHASNAPLTDERHFQFHSSEGDIEGIVGTMQRQRQSV
SSYDTIVPSQFYKQKVVQNSPPSLLSTDNKIPESGSDHPSSQDNSSKASLVENSQTQSST
PRKPLPTTTSPKVNPEPHSESISDTRPSTPRKVPPSTVPKMNPKLQGGNSVTAPSTPSKV
LPAMSPKVAPKFQGGRSSTAPSTPNKVLPAASAKAAPKLQEKAASFDIPNKSTIISTKST

VASPIKANENSPALKSKASFEFPKELDPDGTWNAPIPYPDANCPMAPTYRNLTSEQEEMY
EEVLKYCLELKEIPVASNSSKKTDLIELERLWLTRECILRYLRATKWHVSNAKKRIVDTL
VWRRHFGVNNMDPDEIQEENATGKQVLLGYDKDGRPCLYLYPARQNTKTSPLQIRHLVFS
LECAIDLMPPGVETLALLINFKSSSNRSNPSVGQGKEVLNILQTHYCERLGRALVINIPW
AVWGFFKLISPFIDPITREKLKFNEPLDRYVPKDQLDSNFGGSLHFEYHHEKYWPQLVEL
CKSRRLGILEKWRKMGSKIGTSEWDLKGGEEYVELMQQYVRPSLTNRSSSPTVTPTVNTD
RQPKTLTNSADELSPQKRRVENPKPVVKDEGPVSIVEDEESTPVVTKKEDSIPVAQSTKA
DAGLDAENDLLPKSGSGVSETPAFSHARDVSTASFSDAVSFITADSIE
>Q29106
MSSLPTSDGFNHQAHPSGQRPEIGSPPSLAHSVSASVCPFKPSDPDSIEPKAVKAVKALK
ASAEFQITFERKEQLPLQDPSDCASSADNAPANQTPAIPLQNSLKEAIVADNLEKSAEGS
TQGLKSHLHTRQEASLSVTTTRMQEPQRLIGEKGWHPEYQDPSQVNGLQQHEEPRNEQHE
VVQQNAPHDPEHLCNTGDLELLGERQQNQPKSVGLETAVRGDRPQQDVDLPGTEKNILPY
GCFGCSSSETFMEIDTVEQSLVAVLNSAGGQNTSVRNISASDLTVDNPLMEVETLKCNPS
SEFLSNPTSTQNLQLPESSVEMSGTNKEYGNHPSSLSLCGTCQPSVESAEESCSSITAAL
KELHELLVISSKPALENTSEEVTCRSEIVTEGQTDVKDLSERWTQSEHLTAAQNEQCSQV
SFYQATSVSVKTEELTDTSTDAGTEDVENITSSGPGDGLLVDKENVPRSRESVNESSLVT
LDSAKTSNQPHCTLGVEISPGLLAGEEGALNQTSEQTESLSSSFILVKDLGQGTQNPVTN
RPETRENVCPEAAGLRQEFEPPTSHPSSSPSFLAPLIFPAADIDRILRAGFTLQEALGAL
HRVGGNADLALLVLLAKNIVVPT
>A6BM72
MVLSLTGLIAFSFLQATLALNPEDPNVCSHWESYAVTVQESYAHPFDQIYYTRCTDILNW
FKCTRHRISYKTAYRRGLRTMYRRRSQCCPGYYENGDFCIPLCTEECVHGRCVSPDTCHC
EPGWGGPDCSSGCDSDHWGPHCSNRCQCQNGALCNPITGACVCAAGFRGWRCEELCAPGT
HGKGCQLPCQCRHGASCDPRAGECLCAPGYTGVYCEELCPPGSHGAHCELRCPCQNGGTC
HHITGECACPPGWTGAVCAQPCPPGTFGQNCSQDCPCHHGGQCDHVTGQCHCTAGYMGDR
CQEECPFGSFGFQCSQRCDCHNGGQCSPTTGACECEPGYKGPRCQERLCPEGLHGPGCTL
PCPCDADNTISCHPVTGACTCQPGWSGHHCNESCPVGYYGDGCQLPCTCQNGADCHSITG
GCTCAPGFMGEVCAVSCAAGTYGPNCSSICSCNNGGTCSPVDGSCTCKEGWQGLDCTLPC
PSGTWGLNCNESCTCANGAACSPIDGSCSCTPGWLGDTCELPCPDGTFGLNCSEHCDCSH
ADGCDPVTGHCCCLAGWTGIRCDSTCPPGRWGPNCSVSCSCENGGSCSPEDGSCECAPGF
RGPLCQRICPPGFYGHGCAQPCPLCVHSSRPCHHISGICECLPGFSGALCNQVCAGGYFG
QDCAQLCSCANNGTCSPIDGSCQCFPGWIGKDCSQACPPGFWGPACFHACSCHNGASCSA
EDGACHCTPGWTGLFCTQRCPAAFFGKDCGRVCQCQNGASCDHISGKCTCRTGFTGQHCE
QRCAPGTFGYGCQQLCECMNNSTCDHVTGTCYCSPGFKGIRCDQAALMMEELNPYTKISP
ALGAERHSVGAVTGIMLLLFLIVVLLGLFAWHRRRQKEKGRDLAPRVSYTPAMRMTSTDY
SLSGACGMDRRQNTYIMDKGFKDYMKESVCSSSTCSLNSSENPYATIKDPPILTCKLPES
SYVEMKSPVHMGSPYTDVPSLSTSNKNIYEVEPTVSVVQEGCGHNSSYIQNAYDLPRNSH
IPGHYDLLPVRQSPANGPSQDKQS
>O88407
MTQGKLSVANKAPGTEGQQQANGEKKDAPAVPSAPPSYEEATSGEGLKAGAFPQGPTAVP
LHPSWAYVDPSSSSGYEGGFPAGHHELFSTFSWDDQKVRQLFIRKVYTILLVQLLVTLAV
VALFTFCDVVKDYVQANPGWYWASYAVFFATYLTLACCSGPRRHFPWNLILLTIFTLSMA
YLTGMLSSYYNTTSVLLCLGITALVCLSVTIFSFQTKFDFTSCHGVLFVLLMTLFFSGLL

LAILLPFQYVPWLHAVYAVLGAGVFTLFLAFDTQLLMGNRRHSLSPEEYIFGALNIYLDI
IYIFTFFLQLFGTNRE
>Q9JKD3
MAEKVNNFPPLPKFIPLKPCFYQDFEADIPPQHLSLTKRLYYLWMLNSVTLAVNLVGCLA
WLIGGGGATNFGLAFLWLILFTPCSYVCWFRPIYKAFKTDSSFSFMAFFFTFMAQLVISI
IQAVGIPGWGVCGWIATISFFGTNIGSAVVMLIPTVMFTVVAVFSFIALSMVHKFYRGSG
GSFSKAQEEWTTGAWKNPHVQQAAQNAAMGAAQGAMNQPQTQYSATPNYTYSNEM
>P27105
MAEKRHTRDSEAQRLPDSFKDSPSKGLGPCGWILVAFSFLFTVITFPISIWMCIKIIKEY
ERAIIFRLGRILQGGAKGPGLFFILPCTDSFIKVDMRTISFDIPPQEILTKDSVTISVDG
VVYYRVQNATLAVANITNADSATRLLAQTTLRNVLGTKNLSQILSDREEIAHNMQSTLDD
ATDAWGIKVERVEIKDVKLPVQLQRAMAAEAEASREARAKVIAAEGEMNASRALKEASMV
ITESPAALQLRYLQTLTTIAAEKNSTIVFPLPIDMLQGIIGAKHSHLG
>P42702
MMDIYVCLKRPSWMVDNKRMRTASNFQWLLSTFILLYLMNQVNSQKKGAPHDLKCVTNNL
QVWNCSWKAPSGTGRGTDYEVCIENRSRSCYQLEKTSIKIPALSHGDYEITINSLHDFGS
STSKFTLNEQNVSLIPDTPEILNLSADFSTSTLYLKWNDRGSVFPHRSNVIWEIKVLRKE
SMELVKLVTHNTTLNGKDTLHHWSWASDMPLECAIHFVEIRCYIDNLHFSGLEEWSDWSP
VKNISWIPDSQTKVFPQDKVILVGSDITFCCVSQEKVLSALIGHTNCPLIHLDGENVAIK
IRNISVSASSGTNVVFTTEDNIFGTVIFAGYPPDTPQQLNCETHDLKEIICSWNPGRVTA
LVGPRATSYTLVESFSGKYVRLKRAEAPTNESYQLLFQMLPNQEIYNFTLNAHNPLGRSQ
STILVNITEKVYPHTPTSFKVKDINSTAVKLSWHLPGNFAKINFLCEIEIKKSNSVQEQR
NVTIKGVENSSYLVALDKLNPYTLYTFRIRCSTETFWKWSKWSNKKQHLTTEASPSKGPD
TWREWSSDGKNLIIYWKPLPINEANGKILSYNVSCSSDEETQSLSEIPDPQHKAEIRLDK
NDYIISVVAKNSVGSSPPSKIASMEIPNDDLKIEQVVGMGKGILLTWHYDPNMTCDYVIK
WCNSSRSEPCLMDWRKVPSNSTETVIESDEFRPGIRYNFFLYGCRNQGYQLLRSMIGYIE
ELAPIVAPNFTVEDTSADSILVKWEDIPVEELRGFLRGYLFYFGKGERDTSKMRVLESGR
SDIKVKNITDISQKTLRIADLQGKTSYHLVLRAYTDGGVGPEKSMYVVTKENSVGLIIAI
LIPVAVAVIVGVVTSILCYRKREWIKETFYPDIPNPENCKALQFQKSVCEGSSALKTLEM
NPCTPNNVEVLETRSAFPKIEDTEIISPVAERPEDRSDAEPENHVVVSYCPPIIEEEIPN
PAADEAGGTAQVIYIDVQSMYQPQAKPEEEQENDPVGGAGYKPQMHLPINSTVEDIAAEE
DLDKTAGYRPQANVNTWNLVSPDSPRSIDSNSEIVSFGSPCSINSRQFLIPPKDEDSPKS
NGGGWSFTNFFQNKPND
>Q05031
MIVNISAKMILSICFTFLSFFKATHAMDLDTTSKTSICDATALIQGGMLDYYEGTRYGGT
VGMFQSPYYWWHAGEAFGGMLENWFLCENDTYQELLYDALLAQTGSNYDYIPSNQTMVEG
NDDQGIWGITVMGAVERNFTDPGDGKPGWLAMVQAVFNTMYSRWDSEHCGGGLRWQIFTW
NSGYNYKNTVSNACLFQIAARLGRYTGNTTYLEVAEQVFDWLVDVGYVVLNDTANVFDGA
EIDTNCTDITKIEWTYNHGIVLGGLAYMYNATNGTGEWETSLTKILNGAKSYFFKDSIMY
ESACQDYGTCNTDQRTFKSIFSRMLGLTSVMAPFTRDTIDDLIKTSAEAAAKSCNGGTDG
HTCGLNWQKQTNDGYYGLGEQMSALEVIQNLLIHDRPAPYKEDNGGTSKGDANAGMNSST
TNVLQNNLNIKKGDRAGAAIITAVILSVLTGGAVWMLF
>O55207
MALSKGLRLLARLDPTGPSSVLLEARGRGDCLLFEAGAVATLAPEEKEVIKGLYGKPTDA

YGCLGELSLKSGGVPLSFLVLVTGCTSVGRIPDAEIYKITGTEFYPLQEEAKEEDRLPAL
KKILSSGVFYFAWPNDGACFDLTIRAQKQGDDCSEWGTSFFWNQLLHVPLRQHQVNCHDW
LLKVICGVVTIRTVYASHKQAKACLISRISCERAGARFLTRGVNDDGHVSNFVETEQAIY
MDDGVSSFVQIRGSVPLFWEQPGLQVGSHHLRLHRGLEANAPAFERHMVLLKEQYGQQVV
VNLLGSRGGEEVLNRAFKKLLWASCHAGDTPMINFDFHQFAKGRKLEKLENLLRPQLKLH
WDDFGVFAKGENVSPRFQKGTLRMNCLDCLDRTNTVQCFIALEVLHLQLESLGLNSKPIT
DRFVESFKAMWSLNGHGLSKVFTGSRALEGKAKVGKLKDGARSMSRTIQSNFFDGVKQEA
IKLLLVGDVYNEESTDKGRMLLDNTALLGLGSNKQNSLSGMLDGKATPRILKAMTERQSE
FTNFKRIQIAMGTWNVNGGKQFRSNLLGTTELTDWLLDAPQLSGAVDSQDDGGPADIFAV
GFEEMVELSAGNIVNASTTNRKMWGEQLQKAISRSHRYILLTSAQLVGVCLYIFVRPYHV
PFIRDVAIDTVKTGMGGKAGNKGAVGIRFQFHSTSFCFICSHLTAGQSQVKERNEDYREI
THKLSFPSGRNIFSHDYVFWCGDFNYRIDLTYEEVFYFVKRQDWKKLMEFDQLQLQKSSG
KIFKDFHEGTINFGPTYKYDVGSAAYDTSDKCRTPAWTDRVLWWRKKHPYDKTAGELNLL
DSDLDGDANIRHTWSPGTLKYYGRAELQASDHRPVLAIVEVEVQEVDVGARERVFQEVSS
VQGPLDATVIVNLQSPTLEERNEFPEDLRTELMQTLGNYGTIILVRINQGQMLVTFADSH
SALSVLDVDGMKVKGRAVKIRPKTKDWLEGLREELIRKRDSMAPVSPTANSCLLEENFDF
TSLDYESEGDVLEDDEDYLADEFGQPVVSDSELGGDDSSDTMSASTPASKSPALAKKKQH
PTYKDDADLMTLKLELEVAGNFRHRSPSRSLSVPNRPRPPHPPQRPPPPTGLMVKKSASD
ASISSGTHGQYSILQTAKLLPGAPQQPPKARTGISKPYNVKQIKTTNAQEAEAAIRCLLE
AGGGVPESAPGATPLRNQGSSKPEASLGPPVLPRRPVPRVPTMKKPTLRRTGKPMLPEEQ
CEQQPVHFTMASQEMNLETPPPITAPIPPVPKPRTFQPGRGVERRPSGGKPEPDDAPPVT
GAVELSSPEAPEAPSLAPKVPPRRKKSAPAAFHLQVLQSNSQLLQGLTCSSSSPSPPKPD
TPLLYPQMALGTSSAISPETDGPRVTEPEAASFHGDYPDPFWSLLHHPKLLNNNTWLSKS
SEPLDLGSRTPERTHTDSAQVNASVVERGLPPDHGGKDFSHWMAASNKDKRTTLGV
>Q4P9K9
MSALDEVAKLSQLTNITPDTIFSVLRDRFYAGLPYTALSDSILVSVNPYASSGNRNSDDT
LREYTSDYRQTNKQLRAATLPPHIFAHACNAYFYMRRTGQDQSLLMAGDTSSGKSEVRRL
ALRALIDLSVAPPGKKGSKLGVQIPSAEYILEALGNSRTLENSNASRFGKYTELQFSDSG
KLVGAKTLDYYLEKNRVVSAASSERNFHIFHYMVAGASDEEKQYLGIHDAASFRYLGQAS
RNMDTQDAAKFDRLKLAFKNVGFSKRNVASICQVLAAILHLGNIEFHYDRQRTQDSATIR
NPEVLDKVAEYLGISSKSLEEALTYKTKMIRNEVCTILLDADGASDHRDDLAKSLYSLLF
AWVNESLNEKLCRDDFDTFIGLLDLPGFQNLSKGNSLDQFCVNFACENLHRFMLRSVFEK
RRDEFADEGISHLSPEVPYFDNAETLRLMTNQPGGLIHIMDDQARRMPKKTDQTMIEAFG
KRWGNHPSFKVGPADRSGFSSFTISHYNSAVTYTSENLLEKNSEVVSTDFVSLLRGNPQE
SGKLRNDSSQSSGSTIPFIRGIFNTKVLKTQSHPKNDQTIVAAQQSVKPMRAPSTRRPNR
GNTIKRTNTIKKADDDDSDEDAADAADASTSKKNAVRCVAGDFRGALDLLLETLEDTKTW
FTLCLRPNDNQLPNQFEARVVKQQITTLGLSEMSRKLLNEYSVSMTYEEFCQRYADVPSL
QAVQMRDAVSGEAKQKFSAARQVMSWSDQEAVSGRVKVFLSHTAFRELEDELRAADAEEV
KNNEKRAQLDADAAARGESDPFSPVAVLADDYTRSRSNDFVGAYGDPFKERSSVALPLVG
RGAAGNEDDLEEVKSQYSGMSGTLARHSFVGGLSGAPSFVASEAYAPSRNMFADMGKNGL
NEKAGTAAFTEEPLGEVAEEVGVSSTRRKWVALTWAITFWIPSFILSRFRSLKRPDIRMA
WREKLAINLIIWFICACAVFVIVVLGNLICPKQHVYSPTEFASHKGDSSFTAIRGEVFDL
SNLVASHKTIVPVVPANSILAYAGEDATPIFPVQVNALCNGVDGNVSPWVQLSNENSTDK
HAQYHDFRSYHIDDARPDWYYESMWLLRSNYRVGFMGYTTDGIRDILSEGRAVAIYRGDI

YDVSDYIKQGNQGVLRAPDGFQAPANTNRKFMSDAIISLIAQNPGKDITKQLDNLPLDPV
VLDRQRVCLRNLYFIGKVDHRNSPQCRFAQYILLALSLFMVAILGFKFLAALQFGRARKP
EDHDKFVICQVPCYTEGEESMRKTINSLAALKYDDKRKLLFIICDGMIVGSGNDRPTPRI
VLDILGADPNLEPEALSFLSLGEGSKQHNMAKIYSGLYEHHGHVVPYIVVVKCGKPSERS
RPGNRGKRDSQLVLMRFLNKVHFGLPMNPMELEIYHQIKNVIGVNPSFYEYILQVDADTE
VEAMSLNRFISAFIRDKKVIGLCGETALSNAKASIITMLQVYEYYISHYLAKAFESLFGS
VTCLPGCFSMFRIRTPDTHRPLFIASQIVEDYAENRVDTLHTKNLLHLGEDRYLTTLVLK
HFGKYKTIFVRDCKAWTVAPDDWKVLLSQRRRWINSTVHNLVELIFTPGLCGFCLFSMRF
IVFIDLLSTIIAPVTVCYIVYLIVLVATANGTVPLTAIIMLAAIYGCQAVIFLLNRKFEM
IGWMIVYIIGIPIWSLFLPLYSFWHMDDFSWGNTRVVMGEKGQKVVLHEEGTFDPSEIPL
QTWTDYENELWERNSARSIGSIIEAARAENKSLGSRAGSQYAPSLYGQPMLPHNASFGHS
PSPSYGGTPSQFGAFAPGPGSQIGGSQIGAGAGYFPQDAARQSTYSIGGGYGGQAMSMYG
LPPSSSFGVPTGGSGFMPQPFNTTASMYGYPQQVAATQSIYGGSQLGFGGGFATAEQQQQ
QQQQQQAAGLSGSGGSKSPPREAVAGGLPSDSQIKLDIRSLIAESDLTTITKKQLRAKLE
QKYATSIESKKAFINSEIENVLSES
>Q7LHD1
MQFSTVASIAAIAAVASAASNITTATVTEESTTLVTITSCEDHVCSETVSPALVSTATVT
VNDVITQYTTWCPLPTTEAPKNTTSPAPTEKPTEKPTEKPTQQGSSTQTVTSYTGAAVKA
LPAAGALLAGAAALLL
>Q9P212
MTSEEMTASVLIPVTQRKVVSAQSAADESSEKVSDINISKAHTVRRSGETSHTISQLNKL
KEEPSGSNLPKILSIAREKIVSDENSNEKCWEKIMPDSAKNLNINCNNILRNHQHGLPQR
QFYEMYNSVAEEDLCLETGIPSPLERKVFPGIQLELDRPSMGISPLGNQSVIIETGRAHP
DSRRAVFHFHYEVDRRMSDTFCTLSENLILDDCGNCVPLPGGEEKQKKNYVAYTCKLMEL
AKNCDNKNEQLQCDHCDTLNDKYFCFEGSCEKVDMVYSGDSFCRKDFTDSQAAKTFLSHF
EDFPDNCDDVEEDAFKSKKERSTLLVRRFCKNDREVKKSVYTGTRAIVRTLPSGHIGLTA
WSYIDQKRNGPLLPCGRVMEPPSTVEIRQDGSQRLSEAQWYPIYNAVRREETENTVGSLL
HFLTKLPASETAHGRISVGPCLKQCVRDTVCEYRATLQRTSISQYITGSLLEATTSLGAR
SGLLSTFGGSTGRMMLKERQPGPSVANSNALPSSSAGISKELIDLQPLIQFPEEVASILM
EQEQTIYRRVLPVDYLCFLTRDLGTPECQSSLPCLKASISASILTTQNGEHNALEDLVMR
FNEVSSWVTWLILTAGSMEEKREVFSYLVHVAKCCWNMGNYNAVMEFLAGLRSRKVLKMW
QFMDQSDIETMRSLKDAMAQHESSCEYRKVVTRALHIPGCKVVPFCGVFLKELCEVLDGA
SGLMKLCPRYNSQEETLEFVADYSGQDNFLQRVGQNGLKNSEKESTVNSIFQVIRSCNRS
LETDEEDSPSEGNSSRKSSLKDKSRWQFIIGDLLDSDNDIFEQSKEYDSHGSEDSQKAFD
HGTELIPWYVLSIQADVHQFLLQGATVIHYDQDTHLSARCFLQLQPDNSTLTWVKPTTAS
PASSKAKLGVLNNTAEPGKFPLLGNAGLSSLTEGVLDLFAVKAVYMGHPGIDIHTVCVQN
KLGSMFLSETGVTLLYGLQTTDNRLLHFVAPKHTAKMLFSGLLELTRAVRKMRKFPDQRQ
QWLRKQYVSLYQEDGRYEGPTLAHAVELFGGRRWSARNPSPGTSAKNAEKPNMQRNNTLG
ISTTKKKKKILMRGESGEVTDDEMATRKAKMHKECRSRSGSDPQDINEQEESEVNAIANP
PNPLPSRRAHSLTTAGSPNLAAGTSSPIRPVSSPVLSSSNKSPSSAWSSSSWHGRIKGGM
KGFQSFMVSDSNMSFVEFVELFKSFSVRSRKDLKDLFDVYAVPCNRSGSESAPLYTNLTI
DENTSDLQPDLDLLTRNVSDLGLFIKSKQQLSDNQRQISDAIAAASIVTNGTGIESTSLG
IFGVGILQLNDFLVNCQGEHCTYDEILSIIQKFEPSISMCHQGLMSFEGFARFLMDKENF
ASKNDESQENIKELQLPLSYYYIESSHNTYLTGHQLKGESSVELYSQVLLQGCRSVELDC

WDGDDGMPIIYHGHTLTTKIPFKEVVEAIDRSAFINSDLPIIISIENHCSLPQQRKMAEI
FKTVFGEKLVTKFLFETDFSDDPMLPSPDQLRKKVLLKNKKLKAHQTPVDILKQKAHQLA
SMQVQAYNGGNANPRPANNEEEEDEEDEYDYDYESLSDDNILEDRPENKSCNDKLQFEYN
EEIPKRIKKADNSACNKGKVYDMELGEEFYLDQNKKESRQIAPELSDLVIYCQAVKFPGL
STLNASGSSRGKERKSRKSIFGNNPGRMSPGETASFNKTSGKSSCEGIRQTWEESSSPLN
PTTSLSAIIRTPKCYHISSLNENAAKRLCRRYSQKLTQHTACQLLRTYPAATRIDSSNPN
PLMFWLHGIQLVALNYQTDDLPLHLNAAMFEANGGCGYVLKPPVLWDKNCPMYQKFSPLE
RDLDSMDPAVYSLTIVSGQNVCPSNSMGSPCIEVDVLGMPLDSCHFRTKPIHRNTLNPMW
NEQFLFHVHFEDLVFLRFAVVENNSSAVTAQRIIPLKALKRGYRHLQLRNLHNEVLEISS
LFINSRRMEENSSGNTMSASSMFNTEERKCLQTHRVTVHGVPGPEPFTVFTINGGTKAKQ
LLQQILTNEQDIKPVTTDYFLMEEKYFISKEKNECRKQPFQRAIGPEEEIMQILSSWFPE
EGYMGRIVLKTQQENLEEKNIVQDDKEVILSSEEESFFVQVHDVSPEQPRTVIKAPRVST
AQDVIQQTLCKAKYSYSILSNPNPSDYVLLEEVVKDTTNKKTTTPKSSQRVLLDQECVFQ
AQSKWKGAGKFILKLKEQVQASREDKKKGISFASELKKLTKSTKQPRGLTSPSQLLTSES
IQTKEEKPVGGLSSSDTMDYRQ
>Q5VWK5
MNQVTIQWDAVIALYILFSWCHGGITNINCSGHIWVEPATIFKMGMNISIYCQAAIKNCQ
PRKLHFYKNGIKERFQITRINKTTARLWYKNFLEPHASMYCTAECPKHFQETLICGKDIS
SGYPPDIPDEVTCVIYEYSGNMTCTWNAGKLTYIDTKYVVHVKSLETEEEQQYLTSSYIN
ISTDSLQGGKKYLVWVQAANALGMEESKQLQIHLDDIVIPSAAVISRAETINATVPKTII
YWDSQTTIEKVSCEMRYKATTNQTWNVKEFDTNFTYVQQSEFYLEPNIKYVFQVRCQETG
KRYWQPWSSPFFHKTPETVPQVTSKAFQHDTWNSGLTVASISTGHLTSDNRGDIGLLLGM
IVFAVMLSILSLIGIFNRSFRTGIKRRILLLIPKWLYEDIPNMKNSNVVKMLQENSELMN
NNSSEQVLYVDPMITEIKEIFIPEHKPTDYKKENTGPLETRDYPQNSLFDNTTVVYIPDL
NTGYKPQISNFLPEGSHLSNNNEITSLTLKPPVDSLDSGNNPRLQKHPNFAFSVSSVNSL
SNTIFLGELSLILNQGECSSPDIQNSVEEETTMLLENDSPSETIPEQTLLPDEFVSCLGI
VNEELPSINTYFPQNILESHFNRISLLEK
>P51662
MAMVSEFLKQAWFIDNEEQDYINTVKTYKGGPGSAVSPYPAFNPSSDVAALHQAIMVKGV
DEATIIDILTKRNNAQRQQIKAAYLQEKGKPLDEVLKKALTGHLEEVVLALLKTPAQFDA
DELRAAMKGLGTDEDTLIEILASRNNKEIREINRVYREELKRDLAKDIASDTSGDFQKAL
LSLAKGDRSEDFGVNEDLADTDARALYEAGERRKGADVNVFTTILTTRSYLHLRRVFQKY
SKYSQHDMNKVLDLELKGDIEKCLTAIVQCATCKPAYFAEKLYQAMKGAGTRHKALIRIM
VSRSEVDMNDIKAFYQKKYGVSLCQAILDETKGDYEKILVALCGGN
>O08808
MEPSGGGLGPGRGTRDKKKGRSPDELPATGGDGGKHKKFLERFTSMRIKKEKEKPNSAHR
NSSASYGDDPTAQSLQDISDEQVLVLFEQMLVDMNLNEEKQQPLREKDIVIKREMVSQYL
HTSKAGMNQKESSRSAMMYIQELRSGLRDMHLLSCLESLRVSLNNNPVSWVQTFGAEGLA
SLLDILKRLHDEKEETSGNYDSRNQHEIIRCLKAFMNNKFGIKTMLETEEGILLLVRAMD
PAVPNMMIDAAKLLSALCILPQPEDMNERVLEAMTERAEMDEVERFQPLLDGLKSGTSIA
LKVGCLQLINALITPAEELDFRVHIRSELMRLGLHQVLQELREIENEDMKVQLCVFDEQG
DEDFFDLKGRLDDIRMEMDDFGEVFQIILNTVKDSKAEPHFLSILQHLLLVRNDYEARPQ
YYKLIEECVSQIVLHKNGTDPDFKCRHLQIDIERLVDQMIDKTKVEKSEAKATELEKKLD
SELTARHELQVEMKKMENDFEQKLQDLQGEKDALDSEKQQITAQKQDLEAEVSKLTGEVA

KLSKELEDAKNEMASLSAVVVAPSVSSSAAVPPAPPLPGDSGTVIPPPPPPPPLPGGVVP
PSPPLPPGTCIPPPPPLPGGACIPPPPQLPGSAAIPPPPPLPGVASIPPPPPLPGATAIP
PPPPLPGATAIPPPPPLPGGTGIPPPPPPLPGSVGVPPPPPLPGGPGLPPPPPPFPGAPG
IPPPPPGMGVPPPPPFGFGVPAAPVLPFGLTPKKVYKPEVQLRRPNWSKFVAEDLSQDCF
WTKVKEDRFENNELFAKLTLAFSAQTKTSKAKKDQEGGEEKKSVQKKKVKELKVLDSKTA
QNLSIFLGSFRMPYQEIKNVILEVNEAVLTESMIQNLIKQMPEPEQLKMLSELKEEYDDL
AESEQFGVVMGTVPRLRPRLNAILFKLQFSEQVENIKPEIVSVTAACEELRKSENFSSLL
ELTLLVGNYMNAGSRNAGAFGFNISFLCKLRDTKSADQKMTLLHFLAELCENDHPEVLKF
PDELAHVEKASRVSAENLQKSLDQMKKQIADVERDVQNFPAATDEKDKFVEKMTSFVKDA
QEQYNKLRMMHSNMETLYKELGDYFVFDPKKLSVEEFFMDLHNFRNMFLQAVKENQKRRE
TEEKMRRAKLAKEKAEKERLEKQQKREQLIDMNAEGDETGVMDSLLEALQSGAAFRRKRG
PRQVNRKAGCAVTSLLASELTKDDAMAPGPVKVPKKSEGVPTILEEAKELVGRAS
>P01832
MALFLLTCLLAVFSAATAQSSLLGPSSIFGPGEVNVLEGDSVSITCYYPTTSVTRHSRKF
WCREEESGRCVTLASTGYTSQEYSGRGKLTDFPDKGEFVVTVDQLTQNDSGSYKCGVGVN
GRGLDFGVNVLVSQKPEPDDVVYKQYESYTVTITCPFTYATRQLKKSFYKVEDGELVLII
DSSSKEAKDPRYKGRITLQIQSTTAKEFTVTIKHLQLNDAGQYVCQSGSDPTAEEQNVDL
RLLTPGLLYGNLGGSVTFECALDSEDANAVASLRQVRGGNVVIDSQGTIDPAFEGRILFT
KAENGHFSVVIAGLRKEDTGNYLCGVQSNGQSGDGPTQLRQLFVNEEIDVSRSPPVLKGF
PGGSVTIRCPYNPKRSDSHLQLYLWEGSQTRHLLVDSGEGLVQKDYTGRLALFEEPGNGT
FSVVLNQLTAEDEGFYWCVSDDDESLTTSVKLQIVDGEPSPTIDKFTAVQGEPVEITCHF
PCKYFSSEKYWCKWNDHGCEDLPTKLSSSGDLVKCNNNLVLTLTLDSVSEDDEGWYWCGA
KDGHEFEEVAAVRVELTEPAKVAVEPAKVPVDPAKAAPAPAEEKAKARCPVPRRRQWYPL
SRKLRTSCPEPRLLAEEVAVQSAEDPASGSRASVDASSASGQSGSAKVLISTLVPLGLVL
AAGAMAVAIARARHRRNVDRVSIGSYRTDISMSDLENSREFGAIDNPSACPDARETALGG
KDELATATESTVEIEEPKKAKRSSKEEADLAYSAFLLQSNTIAAEHQDGPKEA
>Q8TCU6
MEAPSGSEPGGDGAGDCAHPDPRAPGAAAPSSGPGPCAAARESERQLRLRLCVLNEILGT
ERDYVGTLRFLQSAFLHRIRQNVADSVEKGLTEENVKVLFSNIEDILEVHKDFLAALEYC
LHPEPQSQHELGNVFLKFKDKFCVYEEYCSNHEKALRLLVELNKIPTVRAFLLSCMLLGG
RKTTDIPLEGYLLSPIQRICKYPLLLKELAKRTPGKHPDHPAVQSALQAMKTVCSNINET
KRQMEKLEALEQLQSHIEGWEGSNLTDICTQLLLQGTLLKISAGNIQERAFFLFDNLLVY
CKRKSRVTGSKKSTKRTKSINGSLYIFRGRINTEVMEVENVEDGTADYHSNGYTVTNGWK
IHNTAKNKWFVCMAKTAEEKQKWLDAIIREREQRESLKLGMERDAYVMIAEKGEKLYHMM
MNKKVNLIKDRRRKLSTVPKCFLGNEFVAWLLEIGEISKTEEGVNLGQALLENGIIHHVS
DKHQFKNEQVMYRFRYDDGTYKARSELEDIMSKGVRLYCRLHSLYTPVIKDRDYHLKTYK
SVLPGSKLVDWLLAQGDCQTREEAVALGVGLCNNGFMHHVLEKSEFRDESQYFRFHADEE
MEGTSSKNKQLRNDFKLVENILAKRLLILPQEEDYGFDIEEKNKAVVVKSVQRGSLAEVA
GLQVGRKIYSINEDLVFLRPFSEVESILNQSFCSRRPLRLLVATKAKEIIKIPDQPDTLC
FQIRGAAPPYVYAVGRGSEAMAAGLCAGQCILKVNGSNVMNDGAPEVLEHFQAFRSRREE
ALGLYQWIYHTHEDAQEARASQEASTEDPSGEQAQEEDQADSAFPLLSLGPRLSLCEDSP
MVTLTVDNVHLEHGVVYEYVSTAGVRCHVLEKIVEPRGCFGLTAKILEAFAANDSVFVEN
CRRLMALSSAIVTMPHFEFRNICDTKLESIGQRIACYQEFAAQLKSRVSPPFKQAPLEPH
PLCGLDFCPTNCHINLMEVSYPKTTPSVGRSFSIRFGRKPSLIGLDPEQGHLNPMSYTQH

CITTMAAPSWKCLPAAEGDPQGQGLHDGSFGPASGTLGQEDRGLSFLLKQEDREIQDAYL
QLFTKLDVALKEMKQYVTQINRLLSTITEPTSGGSCDASLAEEASSLPLVSEESEMDRSD
HGGIKKVCFKVAEEDQEDSGHDTMSYRDSYSECNSNRDSVLSYTSVRSNSSYLGSDEMGS
GDELPCDMRIPSDKQDKLHGCLEHLFNQVDSINALLKGPVMSRAFEETKHFPMNHSLQEF
KQKEECTIRGRSLIQISIQEDPWNLPNSIKTLVDNIQRYVEDGKNQLLLALLKCTDTELQ
LRRDAIFCQALVAAVCTFSKQLLAALGYRYNNNGEYEESSRDASRKWLEQVAATGVLLHC
QSLLSPATVKEERTMLEDIWVTLSELDNVTFSFKQLDENYVANTNVFYHIEGSRQALKVI
FYLDSYHFSKLPSRLEGGASLRLHTALFTKVLENVEGLPSPGSQAAEDLQQDINAQSLEK
VQQYYRKLRAFYLERSNLPTDASTTAVKIDQLIRPINALDELCRLMKSFVHPKPGAAGSV
GAGLIPISSELCYRLGACQMVMCGTGMQRSTLSVSLEQAAILARSHGLLPKCIMQATDIM
RKQGPRVEILAKNLRVKDQMPQGAPRLYRLCQPPVDGDL
>P24394
MGWLCSGLLFPVSCLVLLQVASSGNMKVLQEPTCVSDYMSISTCEWKMNGPTNCSTELRL
LYQLVFLLSEAHTCIPENNGGAGCVCHLLMDDVVSADNYTLDLWAGQQLLWKGSFKPSEH
VKPRAPGNLTVHTNVSDTLLLTWSNPYPPDNYLYNHLTYAVNIWSENDPADFRIYNVTYL
EPSLRIAASTLKSGISYRARVRAWAQCYNTTWSEWSPSTKWHNSYREPFEQHLLLGVSVS
CIVILAVCLLCYVSITKIKKEWWDQIPNPARSRLVAIIIQDAQGSQWEKRSRGQEPAKCP
HWKNCLTKLLPCFLEHNMKRDEDPHKAAKEMPFQGSGKSAWCPVEISKTVLWPESISVVR
CVELFEAPVECEEEEEVEEEKGSFCASPESSRDDFQEGREGIVARLTESLFLDLLGEENG
GFCQQDMGESCLLPPSGSTSAHMPWDEFPSAGPKEAPPWGKEQPLHLEPSPPASPTQSPD
NLTCTETPLVIAGNPAYRSFSNSLSQSPCPRELGPDPLLARHLEEVEPEMPCVPQLSEPT
TVPQPEPETWEQILRRNVLQHGAAAAPVSAPTSGYQEFVHAVEQGGTQASAVVGLGPPGE
AGYKAFSSLLASSAVSPEKCGFGASSGEEGYKPFQDLIPGCPGDPAPVPVPLFTFGLDRE
PPRSPQSSHLPSSSPEHLGLEPGEKVEDMPKPPLPQEQATDPLVDSLGSGIVYSALTCHL
CGHLKQCHGQEDGGQTPVMASPCCGCCCGDRSSPPTTPLRAPDPSPGGVPLEASLCPASL
APSGISEKSKSSSSFHPAPGNAQSSSQTPKIVNFVSVGPTYMRVS
>P35001
MLCCMRRTKQVEKNEDGDQKIEQDGIKPEDKAHKAATKIQASFRGHITRKKLKGEKKADA
PASESEAADKKDEGPAGGRAENKESEASTATEASAADSAQLDEGSKDSSVPTEEKKGNGA
ADTGSEQPAPQAATPAASSEEKPAAAAETESATKASTDNSPSLKADEAKTKRSLNKPTCL
LLTPLATTTPAAEDATAKARLQPQMETVESSQTEEKTDAVEETKPTESAQQEEVKEEESK
ADQENA
>Q9W7F2
MPYELKKVFASLPQMERGVAKILAGDPKGNNFLYTNGKSVIIRNIDNPAIADIYTEHAHQ
AVVARYAPSGFYIASGDTSGKLRIWDTTQKEHLLKYEYQPFAGKIKDIAWTEDSKRIAVV
GEGREKFASVFLWDTGSSVGEITGNIKVINSVDIKQTRPYRLVTGSDDNCCAFLEGPPFK
FKFTMSDHSRFVNCVRFSPDGSKLASAGADGQIFLYDGKTGEKVCSLGGSKAHDGGIYAV
SWSPDGTQLLSASGDKTTKIWDVAANSAVTTFNLGSDVLDQQLGCLWQKDYLLSVSLSGY
INYLDKNNPARPLRVIKGHNKSIQCMTVNNSDGRSTIYTGSHDGHINYWDAETGENDTFT
GKGHTNQVSSMDLDGCNQLITCSMDDTLRYTSLISKDYSSSESVKMDVQPKCVAVGSGGY
VVTVCIGQIVLLKDKKKVFAIDSLDYEPEAVAIHKGSGTVAVGGADGKVHLYSIQGNSLK
DEGKTLPAKGAVTDLAYSHDGAFLAVTDANKVVTVFSVADGYSEKNSYYGHHAKALSVAW
SPDNEHFASSGMDMMVYVWTLSDPDTRIKMPDAHRLHHVSSLAWLDEHTLATVSHDACVK
QWTVTFK

>P26992
MAAPVPWACCAVLAAAAAVVYAQRHSPQEAPHVQYERLGSDVTLPCGTANWDAAVTWRVN
GTDLAPDLLNGSQLVLHGLELGHSGLYACFHRDSWHLRHQVLLHVGLPPREPVLSCRSNT
YPKGFYCSWHLPTPTYIPNTFNVTVLHGSKIMVCEKDPALKNRCHIRYMHLFSTIKYKVS
ISVSNALGHNATAITFDEFTIVKPDPPENVVARPVPSNPRRLEVTWQTPSTWPDPESFPL
KFFLRYRPLILDQWQHVELSDGTAHTITDAYAGKEYIIQVAAKDNEIGTWSDWSVAAHAT
PWTEEPRHLTTEAQAAETTTSTTSSLAPPPTTKICDPGELGSGGGPSAPFLVSVPITLAL
AAAAATASSLLI
>Q5H943
MNFYLLLASSILCALIVFWKYRRFQRNTGEMSSNSTALALVRPSSSGLINSNTDNNLAVY
DLSRDILNNFPHSIARQKRILVNLSMVENKLVELEHTLLSKGFRGASPHRKST
>Q5T1C6
MLRSCAARLRTLGALCLPPVGRRLPGSEPRPELRSFSSEEVILKDCSVPNPSWNKDLRLL
FDQFMKKCEDGSWKRLPSYKRTPTEWIQDFKTHFLDPKLMKEEQMSQAQLFTRSFDDGLG
FEYVMFYNDIEKRMVCLFQGGPYLEGPPGFIHGGAIATMIDATVGMCAMMAGGIVMTANL
NINYKRPIPLCSVVMINSQLDKVEGRKFFVSCNVQSVDEKTLYSEATSLFIKLNPAKSLT

>Q02596
MKFFTVLLFVSLAATSLALLPGSKDELQMKTQPTDAIPAAQSTPTSYTSEESTSSKDLSK
EPSIFREELISKDNVVIESTKPENQEAQDGLRSGSSQLEETTRPTTSAATTSEENLTKSS
QTVEEELGKIIEGFVTGAEDIISGASRITKS
>P48023
MQQPFNYPYPQIYWVDSSASSPWAPPGTVLPCPTSVPRRPGQRRPPPPPPPPPLPPPPPP
PPLPPLPLPPLKKRGNHSTGLCLLVMFFMVLVALVGLGLGMFQLFHLQKELAELRESTSQ
MHTASSLEKQIGHPSPPPEKKELRKVAHLTGKSNSRSMPLEWEDTYGIVLLSGVKYKKGG
LVINETGLYFVYSKVYFRGQSCNNLPLSHKVYMRNSKYPQDLVMMEGKMMSYCTTGQMWA
RSSYLGAVFNLTSADHLYVNVSELSLVNFEESQTFFGLYKL
>P50080
MKGEPKTYSMSDLSYYGEKAQQQNEKQQKQYVVRRNSTQSTSKQNVSVVLEDNASESNEL
PKGFILYASLIALALSLFLAALDIMIVSTIIEEVAKQFGSYSEIGWLFTGYSLPNALLAL
IWGRIATPIGFKETMLFAIVIFEIGSLISALANSMSMLIGGRVIAGVGGCGIQSLSFVIG
STLVEESQRGILIAVLSCSFAIASVVGPFLGGVFTSSVTWRWCFYVNLPIGGLAFFLFLF
FYNPGLSTFQETMDNIRKFPSQFIEIVRNVAYHLLKIKGFSKLNGWRKPFMELIFMYDII
EFVFCSAGFTCILLAFTFGGNRYAWNSASIIILFIIGIVLVVLAGIYDFLVFPKFNIVKA
TPHYQPLMSWTNIKKPGIFTVNIALFLTCAGYISQFTYIVQYFQLIYNDSAWRAAVHLVA
CIISTVVTAILCGAITDKTRQIKPIIVISSIFGVVGAGILTLLNNNANNSAHIGLLILPG
VAFGGLAQSSMLASQIQLDKKSPTFRSDFVSITTFNTFCKNLGQALGGVISNTVFSAAAI
KKLTKANIQLPDGTTVDNLVIYRQTNFDGSHSKLGNIISESLTDVFYMALGFYALSLIFA
VFASNKKVTASLR
>P98158
MERGAAAAAWMLLLAIAACLEPVSSQECGSGNFRCDNGYCIPASWRCDGTRDCLDDTDEI
GCPPRSCESGLFLCPAEGTCIPSSWVCDEDKDCSDGADEQQNCAGTTCSAQQMTCSNGQC
IPSEYRCDHVSDCPDGSDERNCHYPTCDQLTCANGACYNTSQRCDQKVDCRDSSDEANCT
TLCSQKEFECGSGECILRAYVCDHDNDCEDNSDERNCNYDTCGGHQFTCSNGQCINQNWV

CDGDDDCQDSGDEDGCESNQSHHRCYPREWACPGSGRCISIDKVCDGVPDCPEGDDENNV
TSGRTCGMGVCSVLNCEYQCHQTPFGGECFCPPGHIINSNDSRTCIDFDDCQIWGICDQK
CENRQGRHQCLCEEGYILERGQHCKSSDSFSAASVIFSNGRDLLVGDLHGRNFRILAESK
NRGMVMGVDFHYQKHRVFWTDPMQEKVFSTDINGLNTQEILNVSVDTPENLAVDWINNKL
YLVETKVNRIDVVNLEGNQRVTLITENLGHPRGIALDPTVGYLFFSDWGSLSGQPKVERA
FMDGSNRKDLVTTKVGWPAGITLDLVSKRVYWVDSRYDYIETVTYDGIQRKTVARGGSLV
PHPFGISLFEEHVFFTDWTKMAVMKASKFTETNPQVYHQSSLRPHGVTVYHALRQPNATN
PCGSNNGGCAQVCVLSHRTDNGGLGYRCKCEFGFELDDDEHRCVAVKNFLLFSSKTAVRG
IPFTLSTQEDVMVPVTGSPSFFVGIDFDAQHSTVFYSDLSKDIIYKQKIDGTGKEVITAN
RLESVECLTFDWISRNLYWTDGGLKSVTVLRLADKSRRQIISNLNNPRSIVVHPTAGYMF
LSDWFRPAKIMRAWSDGSHLMPIVNTSLGWPNGLAIDWSASRLYWVDAFFDKIEHSTLDG
LDRKRLGHVDQMTHPFGLTVFKDNVFITDWRLGAIIRVRKSDGGDMTVIRRGISSVMHVK
AYDADLQTGSNYCSQTTHANGDCSHFCFPVPNFQRVCGCPYGMKLQRDQMTCEGDPAREP
PTQQCGSLSFPCNNGKCVPSFFRCDGVDDCHDNSDEHQCGVFNNTCSPSAFACVRGGQCI
PGQWHCDRQNDCLDGSDEQNCPTHATSSTCPSTSFTCDNHVCIPKDWVCDTDNDCSDGSD
EKNCQASGTCQPTQFRCPDHRCISPLYVCDGDKDCADGSDEAGCVLNCTSAQFKCADGSS
CINSRYRCDGVYDCRDNSDEAGCPTRPPGMCHPDEFQCQGDGTCIPNTWECDGHPDCIHG
SDEHTGCVPKTCSPTHFLCDNGNCIYKAWICDGDNDCRDMSDEKDCPTQPFHCPSTQWQC
PGYSTCINLSALCDGVFDCPNGTDESPLCNQDSCSHFNGGCTHQCMQGPFGATCLCPLGY
QLANDTKTCEDINECDIPGFCSQHCVNMRGSFRCACDPEYTLESDGRTCKVTGSENPLLV
VASRDKIIVDNITAHTHNLYSLVQDVSFVVALDFDSVTGRVFWSDLLQGKTWSVFQNGTD
KRVVHDSGLSVTEMIAVDWIGRNLYWTDYALETIEVSKIDGSHRTVLISKNVTKPRGLAL
DPRMGDNVMFWSDWGHHPRIERASMDGTMRTVIVQEKIYWPCGLSIDYPNRLIYFMDAYL
DYIEFCDYDGHNRRQVIASDLVLHHPHALTLFEDFVYWTDRGTRQVMQANKWHGGNQSVV
MYSVHQPLGITAIHPSRQPPSRNPCASASCSHLCLLSAQAPRHYSCACPSGWNLSDDSVN
CVRGDQPFLMSVRDNIIFGISLDPEVKSNDAMVPISGIQHGYDVEFDDSEQFIYWVENPG
EIHRVKTDGSNRTVFAPLSLLGSSLGLALDWVSRNIYYTTPASRSIEVLTLKGDTRYGKT
LIANDGTPLGVGFPVGIAVDPARGKLYWSDHGTDSGVPAKIASANMDGTSLKILFTGNLQ
HLEVVTLDIQEQKLYWAVTSRGVIERGNVDGTERMILVHHLAHPWGLVVYGSFLYYSDEQ
YEVIERVDKSSGNNKVVLRDNVPYLRGLRVYHRRNAADSSNGCSNNPNACQQICLPVPGG
MFSCACASGFKLSPDGRSCSPYNSFMVVSMLPAVRGFSLELSDHSEAMVPVAGQGRNVLH
ADVDVANGFIYWCDFSSSVRSSNGIRRIKPDGSNFTNVVTYGIGANGIRGVALDWAAGNL
YFTNAFVYETLIEVLRINTTYRRVLLKVSVDMPRHIIVDPKHRYLFWADYGQKPKIERSF
LDCTNRTVLVSEGIVTPRGLAMDHDTGYIYWVDDSLDLIARIHLDGGESQVVRYGSRYPT
PYGITVFGESIIWVDRNLKKVFQASKQPGNTDPPVVIRDKINLLRDVTIFDEHAQPLSPA
ELNNNPCLQSNGGCSHFCFALPELPTPRCGCAFGTLGNDGKSCATSQEDFLIYSLNNSLR
SLHFDPRDHSLPFQVISVAGTAIALDYDRRNNRIFFTQKLNSLRGQISYVSLYSGSSSPT
VLLSNIGVTDGIAFDWINRRIYYSDFSNQTINSMAEDGSNRAVIARVSKPRAIVLDPCRG
YMYWTDWGTNAKIERATLGGNFRVPIVNTSLVWPNGLALDLETDLLYWADASLQKIERST
LTGTNREVVVSTAFHSFGLTVYGQYIYWTDLYTRKIYRANKYDGSDLVAMTTRLPTQPSG
ISTVVKTQRQQCSNPCDQFNGGCSHICAPGPNGAECQCPHEGNWYLANDNKYCVVDTGTR
CNQLQFTCLNGHCINQDWKCDNDNDCGDGSDELPTVCAFHTCRSTAFTCGNGRCVPYHYR
CDYYNDCGDNSDEAGCLFRNCNSTTEFTCSNGRCIPLSYVCNGINNCHDNDTSDEKNCPP
HTCPPDFTKCQTTNICVPRAFLCDGDNDCGDGSDENPIYCASHTCRSNEFQCLSPQRCIP

SYWFCDGEADCADGSDEPDTCGHSVNTCRASQFQCDNGRCISGNWVCDGDNDCGDMSDED
QRHHCELQNCSSTQFTCVNSRPPNRRCIPQYWVCDGDADCSDALDELQNCTMRTCSAGEF
SCANGRCVRQSFRCDRRNDCGDYSDERGCSYPPCHANQFTCQNGRCIPRFFVCDEDNDCG
DGSDEQEHLCHTPEPTCPLHQFRCDNGHCIEMGRVCNHVDDCSDNSDEKGCGINECLDSS
ISRCDHNCTDTITSFYCSCLPGYKLMSDKRSCVDIDECKESPQLCSQKCENVVGSYICKC
APGYIREPDGKSCRQNSNIEPYLIFSNRYYIRNLTTDGSSYSLILQGLGNVVALDFDRVE
KRLYWIDAEKQIIERMFLNKTNRETIINHRLRRAESLAVDWVSRKLYWLDAILDCLFVSD
LEGRHRKMIAQHCVDANNTFCFEHPRGIVLHPQRGHVYWADWGVHAYIGRIGMDGTNKSV
IISTKIEWPNAITIDYTNDLLYWADAHLGYIEFSDLEGHHRHTVYDGSLPHPFALTIFED
TVFWTDWNTRTVEKGNKYDGSGRVVLVNTTHKPFDIHVYHPYRQPIMSNPCGTNNGGCSH
LCLIKAGGRGFTCACPDDFQTVQLRDRTLCMPMCSSTQFLCGNNEKCIPIWWKCDGQKDC
SDGSDEPDLCPHRFCRLGQFQCRDGNCTSPQALCNARQDCADGSDEDRVLCEHHRCESNE
WQCANKRCIPQSWQCDSVNDCLDNSDEDTSHCASRTCRPGQFKCNNGRCIPQSWKCDVDN
DCGDYSDEPIDECTTAAYNCDNHTEFSCKTNYRCIPQWAVCNGFDDCRDNSDEQGCESVP
CHPSGDFRCANHHCIPLRWKCDGTDDCGDNSDEENCVPRECSESEFRCADQQCIPSRWVC
DQENDCGDNSDERDCEMKTCHPEHFQCTSGHCVPKALACDGRADCLDASDESACPTRFPN
GTYCPAAMFECKNHVCIQSFWICDGENDCVDGSDEEIHLCFNIPCESPQRFRCDNSRCVY
GHQLCNGVDDCGDGSDEKEEHCRKPTHKPCTDTEYKCSNGNCISQHYVCDNVNDCGDLSD
ETGCNLGDNRTCAENICEQNCTQLSSGGFICSCRPGFKPSTSDKNSCQDINECEEFGICP
QSCRNSKGSYECFCVDGFKSMSTHYGERCAADGSPPLLLLPENVRIRKYNTSSEKFSEYL
EEEEHIQTIDYDWDPEHIGLSVVYYTVLAQGSQFGAIKRAYIPNFESGSNNPIREVDLGL
KYLMQPDGLAVDWVGRHIYWSDAKSQRIEVATLDGRYRKWLITTQLDQPAAIAVNPKLGL
MFWTDQGKQPKIESAWMNGEHRSVLVSENLGWPNGLSIDYLNDDRVYWSDSKEDVIEAIK
YDGTDRRLIINEAMKPFSLDIFEDKLYWVAKEKGEVWRQNKFGKENKEKVLVVNPWLTQV
RIFHQLRYNQSVSNPCKQVCSHLCLLRPGGYSCACPQGSDFVTGSTVQCDAASELPVTMP
PPCRCMHGGNCYFDENELPKCKCSSGYSGEYCEVGLSRGIPPGTTMAVLLTFVIVIIVGA
LVLVGLFHYRKTGSLLPTLPKLPSLSSLAKPSENGNGVTFRSGADVNMDIGVSPFGPETI
IDRSMAMNEHFVMEVGKQPVIFENPMYAAKDNTSKVALAVQGPSTGAQVTVPENVENQNY
GRPIDPSEIVPEPKPASPGADEIQGKKWNIFKRKPKQTTNFENPIYAEMDSEVKDAVAVA
PPPSPSLPAKASKRNLTPGYTATEDTFKDTANLVKEDSDV
>Q09553
MRRVILPLVMTVTLCLAQEASEACTKALTDCENDLECQNRLAPLMAACSTNTCQPQCRSA
VLNVYQNKLGRILLRSDATCIPGRDELRTCNFLPAESTVHCSLGKLACEGDLQCNSKFGV
FMSECEADAARGACTDKCKTLLNQTIETSVGSVFSNCTCTARDDQLCTNLKDNLLGVCLK
NTPGVTLSPSDNSITDAPGGNDLADSSVGHGFNILSAISVYLLTVLVF
>P10675
MEGPRLTALMLLLLLTLAADRASAKGEKSLEYKIRDDPDLSQFYSWLEHNEVANSTLQLR
QVTVFAPTNLAFQNYKARDGDENIILYHMTNLAHSLDQLGHKVLSELDGNPPLWITRRRD
TIFVNNARVLTERSNYEAVNRHGKKQVLHVVDSVLEPVWSTSGGQLYNPDAFQFLNQSENL
DLGLHRVRSFRQRVFQNQKQNDFKLEGKHTFFIPVDEGFKPLPRPEKIDQKVIDGHIIPN
HVLFTSATPLDEEYETLAFTDMLRVVISFTMESDGKAHKPYVKSNTVIGDANHATGAVLA
EIVKANIPVKNGVVHLIQRPLMVVDNTVKQFLEGFEKEDGPLYKFYQVILDAGGDFINQI
TEMKDLTLFAPSNAAWSETTANNLLTDRKKFREILNLHIVAEKLSIESIVEQNVKQVPTM
ADRKNLYFNVVHGPAGNKTVTVEGGGVNATIVQPNIAATNGMVHIINKILGVPYTTVKEK

LRTDPMLNKTYHLGEMSDFNKMLDEKHTKFTYFVPRDLAWKKMEVRDPSAHRKLFMKEFT
YQVKQILERHLVISDRVYTMGALKKLASNTSSVLPTMRDHLRLRVRETEKSYYVEWQGEW
THIFRPDVECTNGIIHVMDYVFMKEGDIVVGGPDGAGQQVASFAVVASAHLVVLATVRWL
LH
>P30589
MAGYGHSTAGGFGSGSGSGPPGPQYMLPQYDEGDDPDADATPAGQGVRLLTNLDNSSYIS
VSEITSQSSHRDNIRPYVMPLLSQPFHTNASTQTHTRTQSPPFHSNTSPKTKQGHDEAVV
VNTPPAPFPSLSRSVPNKGPENQEHQGKKTDLETPPNSNQKEHAATTEEDDSDMGKAVTF
AGAPEILGSEDPQQQYAHLPHYPPPYSPFRPEEQDQFLMRQQQRQQQQQQQQQQQQQQQA
KAQPQQQGESQLREGGKLHKRKRLGQVKPLKLSAPPPTLALPTIPEGTSPRRASLREARK
KKQRSKQRLSNITEERDSTRSLTPPPSVHFGSPKRRNSEAARSHIIPNHSSRLRQAYEPS
IDARTYEPSLDTRTYEPSISDRRHMYEPSIDERSSYMDPPRIPPPDGGSYVSSYMGTESM
VSGHGRPWSPESATGYRVPPQGRYEPSEIDGHARPGTPGSSYGNARRPLPSAPAPLHYNS
PSRAASHYPRYHGGYADDVTVSMGPDDDRTDIFGPETDLSETRHLNDAYGFRSSQITLSE
DPHGTHARSRYDDEDDVSTTYSSNTGTSASGVDKFEHYGPIPEEGKHERRGVRPPQMSRK
EVQLINGELVLECKIPTILYSFLPRRDEVEFTHMRYTAVTCDPDDFVARGYKLRQNIGRT
ARETELFICVTMYNEDEFGFTRTMHAVMKNISHFCSRNKSRTWGADGWQKIVVCVVSDGR
EIIHPRTLDALAAMGVYQHGIAKNFVNQKAVQAHVYEYTTQVSLDSDLKFKGAEKGIVPC
QMIFCLKEKNQKKLNSHRWFFNAFGKALNPNVCILLDVGTRPGGTSLYHLWKAFDTDSNV
AGACGEIKAMKGRFGGNLLNPLVASQNFEYKMSNILDKPLESVFGYITVLPGALSAYRYH
ALQNDETGHGPLSQYFKGETLHGQHADVFTANMYLAEDRILCWELVAKRGERWVLKYVKG
CTGETDVPDTVPEFVSQRRRWLNGAFFAAVYSLVHFRQIWKTDHTFMRKALLHVEFLYHL
LQLLFTYFSLANFYLAFYFIAGGLADPHVDPFNSDGHVARIIFNILRYVCVLLICTQFIL
SLGNRPQGAKRMYLASMIIYAVIMVYTTFATIFIVVRQIQPSQKSDDKPDLELGNNVFTN
LIVSVASTLGLYFVMSFLYLDPWHMFTSAIQYFVLLPSYICTLQIYAFCNTHDVTWGTKG
DNVMRTDLGGAIGKGSTVELEMPSDQLDIDSGYDECLRNLRDRVMVPAVPVSEDQLQQDY
YKSVRTYMVVSWMVANATLAMAVSEAYGDSEIGDNFYLRFILWAVAALALFRALGSTTFA
AINLVSALVEGRVRLRLNMKGFRWIKEKWGDADVKGKFEGLGDRARGLARR
>Q15743
MGNITADNSSMSCTIDHTIHQTLAPVVYVTVLVVGFPANCLSLYFGYLQIKARNELGVYL
CNLTVADLFYICSLPFWLQYVLQHDNWSHGDLSCQVCGILLYENIYISVGFLCCISVDRY
LAVAHPFRFHQFRTLKAAVGVSVVIWAKELLTSIYFLMHEEVIEDENQHRVCFEHYPIQA
WQRAINYYRFLVGFLFPICLLLASYQGILRAVRRSHGTQKSRKDQIQRLVLSTVVIFLAC
FLPYHVLLLVRSVWEASCDFAKGVFNAYHFSLLLTSFNCVADPVLYCFVSETTHRDLARL
RGACLAFLTCSRTGRAREAYPLGAPEASGKSGAQGEEPELLTKLHPAFQTPNSPGSGGFP
TGRLA
>Q8GUQ5
MKAHKTVFNQHPLSLNKLFFVLLLIFFLPPASPAASVNGLYKDSQQLLSFKAALPPTPTL
LQNWLSSTGPCSFTGVSCKNSRVSSIDLSNTFLSVDFSLVTSYLLPLSNLESLVLKNANL
SGSLTSAAKSQCGVTLDSIDLAENTISGPISDISSFGVCSNLKSLNLSKNFLDPPGKEML
KAATFSLQVLDLSYNNISGFNLFPWVSSMGFVELEFFSLKGNKLAGSIPELDFKNLSYLD
LSANNFSTVFPSFKDCSNLQHLDLSSNKFYGDIGSSLSSCGKLSFLNLTNNQFVGLVPKL
PSESLQYLYLRGNDFQGVYPNQLADLCKTVVELDLSYNNFSGMVPESLGECSSLELVDIS
YNNFSGKLPVDTLSKLSNIKTMVLSFNKFVGGLPDSFSNLLKLETLDMSSNNLTGVIPSG

ICKDPMNNLKVLYLQNNLFKGPIPDSLSNCSQLVSLDLSFNYLTGSIPSSLGSLSKLKDL
ILWLNQLSGEIPQELMYLQALENLILDFNDLTGPIPASLSNCTKLNWISLSNNQLSGEIP
ASLGRLSNLAILKLGNNSISGNIPAELGNCQSLIWLDLNTNFLNGSIPPPLFKQSGNIAV
ALLTGKRYVYIKNDGSKECHGAGNLLEFGGIRQEQLDRISTRHPCNFTRVYRGITQPTFN
HNGSMIFLDLSYNKLEGSIPKELGAMYYLSILNLGHNDLSGMIPQQLGGLKNVAILDLSY
NRFNGTIPNSLTSLTLLGEIDLSNNNLSGMIPESAPFDTFPDYRFANNSLCGYPLPIPCS
SGPKSDANQHQKSHRRQASLAGSVAMGLLFSLFCIFGLIIVAIETKKRRRKKEAALEAYM
DGHSHSATANSAWKFTSAREALSINLAAFEKPLRKLTFADLLEATNGFHNDSLVGSGGFG
DVYKAQLKDGSVVAIKKLIHVSGQGDREFTAEMETIGKIKHRNLVPLLGYCKVGEERLLV
YEYMKYGSLEDVLHDRKKIGIKLNWPARRKIAIGAARGLAFLHHNCIPHIIHRDMKSSNV
LLDENLEARVSDFGMARLMSAMDTHLSVSTLAGTPGYVPPEYYQSFRCSTKGDVYSYGVV
LLELLTGKQPTDSADFGDNNLVGWVKLHAKGKITDVFDRELLKEDASIEIELLQHLKVAC
ACLDDRHWKRPTMIQVMAMFKEIQAGSGMDSTSTIGADDVNFSGVEGGIEMGINGSIKEG
NELSKHL
>Q8LCS8
MAEVKDQLEIKFRLNDGSDIGPKLFPDATTVATLKETVVAQWPRDKENGPKTVKDVKLIS
AGRILENNKTVGDCRSPVGNFSGAVTTMHVIIQHQVTEKEKKKKKPKGDLKQNKCVCLCF
GARC
>Q9NPG4
MMQLLQLLLGLLGPGGYLFLLGDCQEVTTLTVKYQVSEEVPSGTVIGKLSQELGREERRR
QAGAAFQVLQLPQALPIQVDSEEGLLSTGRRLDREQLCRQWDPCLVSFDVLATGDLALIH
VEIQVLDINDHQPRFPKGEQELEISESASLRTRIPLDRALDPDTGPNTLHTYTLSPSEHF
ALDVIVGPDETKHAELIVVKELDREIHSFFDLVLTAYDNGNPPKSGTSLVKVNVLDSNDN
SPAFAESSLALEIQEDAAPGTLLIKLTATDPDQGPNGEVEFFLSKHMPPEVLDTFSIDAK
TGQVILRRPLDYEKNPAYEVDVQARDLGPNPIPAHCKVLIKVLDVNDNIPSIHVTWASQP
SLVSEALPKDSFIALVMADDLDSGHNGLVHCWLSQELGHFRLKRTNGNTYMLLTNATLDR
EQWPKYTLTLLAQDQGLQPLSAKKQLSIQISDINDNAPVFEKSRYEVSTRENNLPSLHLI
TIKAHDADLGINGKVSYRIQDSPVAHLVAIDSNTGEVTAQRSLNYEEMAGFEFQVIAEDS
GQPMLASSVSVWVSLLDANDNAPEVVQPVLSDGKASLSVLVNASTGHLLVPIETPNGLGP
AGTDTPPLATHSSRPFLLTTIVARDADSGANGEPLYSIRSGNEAHLFILNPHTGQLFVNV
TNASSLIGSEWELEIVVEDQGSPPLQTRALLRVMFVTSVDHLRDSARKPGALSMSMLTVI
CLAVLLGIFGLILALFMSICRTEKKDNRAYNCREAESTYRQQPKRPQKHIQKADIHLVPV
LRGQAGEPCEVGQSHKDVDKEAMMEAGWDPCLQAPFHLTPTLYRTLRNQGNQGAPAESRE
VLQDTVNLLFNHPRQRNASRENLNLPEPQPATGQPRSRPLKVAGSPTGRLAGDQGSEEAP
QRPPASSATLRRQRHLNGKVSPEKESGPRQILRSLVRLSVAAFAERNPVEELTVDSPPVQ
QISQLLSLLHQGQFQPKPNHRGNKYLAKPGGSRSAIPDTDGPSARAGGQTDPEQEEGPLD
PEEDLSVKQLLEEELSSLLDPSTGLALDRLSAPDPAWMARLSLPLTTNYRDNVISPDAAA
TEEPRTFQTFGKAEAPELSPTGTRLASTFVSEMSSLLEMLLEQRSSMPVEAASEALRRLS
VCGRTLSLDLATSAASGMKVQGDPGGKTGTEGKSRGSSSSSRCL
>Q93XX5
MSACINGLCRAVTVSLLLLLLLSFSFSSACSNGNCQLLDSCSSATDCVSGLYCGDCPAVGR
SKPVCTRGQATSPTSIINGLPFNKYTWLMTHNAFSNANAPLLPGVERITFYNQEDTITNQ
LQNGVRGLMLDMYDFNNDIWLCHSLRGQCFNFTAFQPAINILREVEAFLSQNPTEIVTII
IEDYVHRPKGLSTLFANAGLDKYWFPVSKMPRKGEDWPTVTDMVQENHRLLVFTSVAAKE

DEEGVAYQWRYMVENESGDPGVKRGSCPNRKESQPLNSKSSSLFLMNYFPTYPVEKDACK
EHSAPLAEMVGTCLKSGGNRMPNFLAVNFYMRSDGGGVFEILDRMNGPVLCGCETLSACQ
PGAAYGSCKNVTVQTRTPSMDSTAGSNSGGSYSGSVQFSRSLASVAQLNNIVVFCFSLLP
LLIFLL
>O97148
MKTLLVLRISTVILVVLVIQKSYADILECDYFDTVDISAAQKLQNGSYLFEGLLVPAILT
GEYDFRILPDDSKQKVARHIRGCVCKLKPCVRFCCPHDHIMDNGVCYDNMSDEELAELDP
FLNVTLDDGSVSRRHFKNELIVQWDLPMPCDGMFYLDNREEQDKYTLFENGTFFRHFDRV
TLRKREYCLQHLTFADGNATSIRIAPHNCLIVPSITGQTVVMISSLICMVLTIAVYLFVK
KLQNLHGKCFICYMVCLFMGYLFLLLDLWQISISFCKPAGFLGYFFVMAAFFWLSVISLH
LWNTFRGSSHKANRFLFEHRFLAYNTYAWGMAVVLTGITVLADNIVENQDWNPRVGHEGH
CWIYTQAWSAMLYFYGPMVFLIAFNITMFILTAKRILGVKKDIQNFAHRQERKQKLNSDK
QTYTFFLRLFIIMGLSWSLEIGSYFSQSNQTWANVFLVADYLNWSQGIIIFILFVLKRST
WRLLQESIRGEGEEVNNSEEEISLENTTTRNVLL
>Q920E0
MCWPLLYPLMLGFSISPAECQTPSIYDDVESTREGQEASLRPTVELNESKSPDKPNPRGF
PGKPCANNSDTLELPASSEALLLGWVPTRLVPAIYGLVVVVGLPANGLALWVLATRVPRL
PSTILLMNLAVADLLLALVLPPRLVYHLRGQRWPFGEAACRVATAALYGHMYGSVLLLAA
VSLDRYLALVHSLRARALRGQRLTTILCLVAWLSAATLVLPLTFHRQTFLLAGSDRMLCH
DALPLAEQTSHWRPAFICLAVLGCFVPLLAMVLCYGATLRALAANGQRYSHAVRLTALVL
FSAVAAFTPSNVLLVLHYSNPSPEAWGNLYGAYVPSLALSTLNSCVDPFIYYYVSHEFRE
KVRAMLCRQLKASSSSQASREAGSRGTAICSSTLL
>Q4VC33
MAVQESAAQLSMTLKVQEYPTLKVPYETLNKRFRAAQKNIDRETSHVTMVVAELEKTLSS
CPAVDSVVSLLDGVVEKLSVLKRKAVESIQAEDESAKLCKRRIEHLKEHSSDQPAAASMW
KRKRMDRMMVEHLLRCGYYNTAVKLARQSGIEDLVNIEMFLTAKEVEESLERRETATCLA
WCHDNKSRLRKMKSCLEFSLRIQEFIELVRQNKRLDAVRHARKHFSQAEGSQLDEVRQVM
GMLAFPPDTHISPYKDLLDPARWRMLIQQFRYDNYRLHQLGNSSVFTLTLQAGLSAIKTP
QCYKEDGSSKSPDCPVCSRSLNKLAQPLPMAHCANSRLVCKISGDVMNENNPPMMLPNGY
VYGYNSLLSIRQDDKVVCPRTKEVFHFSQAEKVYIM
>Q62028
MVQWLAMLQLLWLQQLLLLGIHQGIAQDLTHIQEPSLEWRDKGIFIIQSESLKTCIQAGK
SVLTLENCKQPNEHMLWKWVSDDHLFNVGGSGCLGLNISALEQPLKLYECDSTLISLRWH
CDRKMIEGPLQYKVQVKSDNTVVARKQIHRWIAYTSSGGDICEHPSRDLYTLKGNAHGMP
CVFPFQFKGHWHHDCIREGQKEHLLWCATTSRYEEDEKWGFCPDPTSMKVFCDATWQRNG
SSRICYQFNLLSSLSWNQAHSSCLMQGGALLSIADEDEEDFIRKHLSKVVKEVWIGLNQL
DEKAGWQWSDGTPLSYLNWSQEITPGPFVEHHCGTLEVVSAAWRSRDCESTLPYICKRDL
NHTAQGILEKDSWKYHATHCDPDWTPFNRKCYKLKKDRKSWLGALHSCQSNDSVLMDVAS
LAEVEFLVSLLRDENASETWIGLSSNKIPVSFEWSSGSSVIFTNWYPLEPRILPNRRQLC
VSAEESDGRWKVKDCKERLFYICKKAGQVPADEQSGCPAGWERHGRFCYKIDTVLRSFEE
ASSGYYCSPALLTITSRFEQAFITSLISSVAEKDSYFWIALQDQNNTGEYTWKTVGQREP
VQYTYWNTRQPSNRGGCVVVRGGSSLGRWEVKDCSDFKAMSLCKTPVKIWEKTELEERWP
FHPCYMDWESATGLASCFKVFHSEKVLMKRSWREAEAFCEEFGAHLASFAHIEEENFVNE
LLHSKFNWTQERQFWIGFNRRNPLNAGSWAWSDGSPVVSSFLDNAYFEEDAKNCAVYKAN

KTLLPSNCASKHEWICRIPRDVRPKFPDWYQYDAPWLFYQNAEYLFHTHPAEWATFEFVC
GWLRSDFLTIYSAQEQEFIHSKIKGLTKYGVKWWIGLEEGGARDQIQWSNGSPVIFQNWD
KGREERVDSQRKRCVFISSITGLWGTENCSVPLPSICKRVKIWVIEKEKPPTQPGTCPKG
WLYFNYKCFLVTIPKDPRELKTWTGAQEFCVAKGGTLVSIKSELEQAFITMNLFGQTTNV
WIGLQSTNHEKWVNGKPLVYSNWSPSDIINIPSYNTTEFQKHIPLCALMSSNPNFHFTGK
WYFDDCGKEGYGFVCEKMQDTLEHHVNVSDTSAIPSTLEYGNRTYKIIRGNMTWYAAGKS
CRMHRAELASIPDAFHQAFLTVLLSRLGHTHWIGLSTTDNGQTFDWSDGTKSPFTYWKDE
ESAFLGDCAFADTNGRWHSTACESFLQGAICHVVTETKAFEHPGLCSETSVPWIKFKGNC
YSFSTVLDSRSFEDAHEFCKSEGSNLLAIRDAAENSFLLEELLAFGSSVQMVWLNAQFDN
NNKTLRWFDGTPTEQSNWGLRKPDMDHLKPHPCVVLRIPEGIWHFTPCEDKKGFICKMEA
GIPAVTAQPEKGLSHSIVPVTVTLTLIIALGIFMLCFWIYKQKSDIFQRLTGSRGSYYPT
LNFSTAHLEENILISDLEKNTNDEEVRDAPATESKRGHKGRPICISP

(3) 49 cell wall proteins ($\mathbb{S}_3$)
>P93349
MGVKGLLFSIVLINLSLLGLCGYPRKPVDVPFWKNYEPSWASHHIKYLSGGSTVDLVLDR
SSGAGFQSKKSYLFGHFSMKLKLVGGDSAGVVTAFYLSSNNAEHDEIDFEFLGNRTGQPY
ILQTNVFTGGKGDREQRIYLWFDPTKGYHSYSVLWNTFQIVIFVDDVPIRAFKNSKDLGV
KFPFNQPMKIYSSLWDADDWATRGGLEKTDWSNAPFTASYTSFHVDGCEAATPQEVQVCN
TKGMRWWDQKAFQDLDALQYRRLRWVRQKYTIYNYCTDRKRYPTLPPECTKDRDI
>P32478
MQYKKTLVASALAATTLAAYAPSEPWSTLTPTATYSGGVTDYASTFGIAVQPISTTSSAS
SAATTASSKAKRAASQIGDGQVQAATTTASVSTKSTAAAVSQIGDGQIQATTKTTAAAVS
QIGDGQIQATTKTTSAKTTAAAVSQISDGQIQATTTTLAPKSTAAAVSQIGDGQVQATTT
TLAPKSTAAAVSQIGDGQVQATTKTTAAAVSQIGDGQVQATTKTTAAAVSQIGDGQVQAT
TKTTAAAVSQIGDGQVQATTKTTAAAVSQITDGQVQATTKTTQAASQVSDGQVQATTATS
ASAAATSTDPVDAVSCKTSGTLEMNLKGGILTDGKGRIGSIVANRQFQFDGPPPQAGAIY
AAGWSITPDGNLAIGDNDVFYQCLSGTFYNLYDEHIGSQCTPVHLEAIDLIDC
>Q06100
MLYHLFVNNQIKLQDDFKAEAVATIRSSVFNSKGGTTVFNFLSAGENILLHISIRPGENA
IVFNSRTKGGAWGPEERVPYAGKFKGPNPSITVLDHGDRFQILFDNATAIYYTKRIKENA
AAIAYSAENSLFSSPVTVDIHGLLPPLPPA
>Q8GXV7
MKLTYQFFIFWFFLPFFAISGDDDYKNLIFKGCANQKSPDPTGVFSQNLKNLFTSLVSQS
SQSSFASVTSGTDNTTAVIGVFQCRGDLQNAQCYDCVSKIPKLVSKLCGGGRDDGNVVAA
RVHLAGCYIRYESSGFRQTSGTEMLFRVCGKKDSNDPGFVGKRETAFGMAENGVKTGSSG
GGGGGGGFYAGQYESVYVLGQCEGSLGNSDCGECVKDGFEKAKSECGESNSGQVYLQKCF
VSYSYYSHGVPNIEPLSGGEKRQHTERTIALAVGGVFVLGFVIVCLLVLRSAMKKKSNKY
DAY
>P80022
MASNSAFSLFLILLIITQCLSVLNAAKDFDFFYFVQQWPGSYCDTKQSCCYPTTGKPAAD
FGIHGLWPNNNDGTYPSNCDPNSPYDQSQISDLISSMQQNWPTLACPSGSGSTFWSHEWE
KHGTCAESVLTNQHAYFKKALDLKNQIDLLSILQGADIHPDGESYDLVNIRNAIKSAIGY
TPWIQCNVDQSGNSQLYQVYICVDGSGSSLIECPIFPGGKCGTSIEFPTF
>P23137

MGSKAFLFLGLCLAFFFLISSEVVAGELAETSNPMKLDGENGVDVDGRGGYNDVGGDGYY
GGGRGRGGGGYKRRGCRYGCCRKGYNGCKRCCSYAGEAMDKVTEAQPHN
>Q9S7Y7
MASSSSSLAFSLSLLLALILCFSPTQSYKTIGKGYRLVSIEESPDGGFIGYLQVKQKNKI
YGSDITTLRLFVKHETDSRLRVHITDAKQQRWEVPYNLLPREQPPQVGKVIGKSRKSPIT
VQEISGSELIFSYTTDPFTFAVKRRSNHETLFNTTSSLVFKDQYLEISTSLPKEASLYGL
GENSQANGIKLVPNEPYTLYTEDVSAINLNTDLYGSHPMYMDLRNVGGKAYAHAVLLLNS
NGMDVFYRGDSLTYKVIGGVFDFYFIAGPSPLNVVDQYTQLIGRPAPMPYWSLGFHQCRW
GYHNLSVVEDVVDNYKKAKIPLDVIWNDDDHMDGHKDFTLNPVAYPRAKLLAFLDKIHKI
GMKYIVINDPGIGVNASYGTFQRAMAADVFIKYEGKPFLAQVWPGPVYFPDFLNPKTVSW
WGDEIKRFHDLVPIDGLWIDMNEVSNFCSGLCTIPEGKQCPSGEGPGWVCCLDCKNITKT
RWDDPPYKINATGVVAPVGFKTIATSATHYNGVREYDAHSIYGFSETIATHKGLLNVQGK
RPFILSRSTFVGSGQYAAHWTGDNQGTWQSLQVSISTMLNFGIFGVPMVGSDICGFYPQP
TEELCNRWIEVGAFYPFSRDHANYYSPRQELYQWDTVADSARNALGMRYKILPFLYTLNY
EAHMTGAPIARPLFFSFPEYTECYGNSRQFLLGSSFMISPVLEQGKTEVEALFPPGSWYH
MFDMTQAVVSKNGKRVTLPAPLNFVNVHLYQNTILPTQQGGLISKDARTTPFSLVIAFPA
GASEGYATGKLYLDEDELPEMKLGNGQSTYVDFYASVGNGTMKMWSQVKEGKFALSKGWV
IEKVSVLGLRGAGQVSEIQINGSPMTKKIEVSSKEHTYVIGLEDEEENKSVMVEVRGLEM
LVGKDFNMSWKMGIN
>P15703
MRFSTTLATAATALFFTASQVSAIGELAFNLGVKNNDGTCKSTSDYETELQALKSYTSTV
KVYAASDCNTLQNLGPAAEAEGFTIFVGVWPTDDSHYAAEKAALQTYLPKIKESTVAGFL
VGSEALYRNDLTASQLSDKINDVRSVVADISDSDGKSYSGKQVGTVDSWNVLVAGYNSAV
IEASDFVMANAFSYWQGQTMQNASYSFFDDIMQALQVIQSTKGSTDITFWVGETGWPTDG
TNFESSYPSVDNAKQFWKEGICSMRAWGVNVIVFEAFDEDWKPNTSGTSDVEKHWGVFTS
SDNLKYSLDCDFS
>P53189
MISPISFLSSLLCLTYLTSALPILPKREVVTRVHTASTTNVVTDFYSTTTEVVIAPTVEF
LISDSVTFTTTLIPQGVNPTAEPTTTITKVLLKKAEMSTSSQPTSTLQPSTIPQSTSSFQ
AESTLQAVSTQQTAMSVSAGTSEDVQQLATTSTSISSSPSPTTTSTSTQNIVSGVGEANV
EGNTQSQHHSYQAAATSTLNQQTSTSIASQESTESTNTPTSSSTSSSTSSSTSSSTSSST
SSSTSSSTSSSTSSSTSSTQETAATTSEGSSSSSAAITSSPKAIAYSPYNDDGSCKSADA
VSSDLTLIKSKGISKIRVYGTDCNSFETVQPAAVKLGIKINQGLYITSSGVDSIDDSVTT
LIQYGQTNGWDVFDFITVGNEAINNGWCSVSDLISKISSVKSKLSEAGYSGQITTSEPPV
SFENNPDLCKKSDIDFVGINPHAYFDTSASAETAGTFVKGQVELIQGVCGTSNVFVTETG
YPSSGIQNGGNIPSTANQITAVQNILNEMDLDVTILSTYNDYWKAPGDYGIEQSFGVIEY
FP
>P53753
MQLYLTLLFLLSFVECSYISFISNNADEILETDLIETLSYATLTVGEPYVAQSVVVTRVS
AASHSPLSVSPKNRVSASPINSQDSDSNTRTAVQLSLSLSNYASQVSQKISAQTNNDPVT
VSNIYANDNSKSKSSVHNLSSVSGVASVMPSASTMRKVTTLLSQTASTSTSTLFSSSLSI
SGTQLNGTLLTSVSKGTIDPLVTQMPSYSSQETKIIPSSLTSNKTIYTISVRTNAATATG
EDSFIASTPASSTLFYPSNSTQDLVQTLASTTASPAYPSNRTQITLSPSVSLYSTTSPIY
PSNITENGSSPSPSLSSTVSPVYPSSSTGNILLSSLFSTVDSSSSPVSSTLDTIYVSSSM

QATISSSSSSRQTKTSSSSLSTSTSSTATTTENSSTTTIVNLFNAVSTDEPPTVFDRSPN
PMSLADGVSNDGPIQTNKFYTNLIVGSQESPAFVYPYSLWKYTSSSYGFAVQHTTVDQYS
YGGYDSSGNAEYLVNPLGIAHVVFSASNFDSSMTMQVDEMTLSSTRVVLSESNDSSNYLE
IPLVQGMGFATGIYHGSLNAKIGSSVGFNTIVSESSSNLAQGILKYRITLLNGVTWLCYV
IGPDDLTSTDFSLEVSSEYEIKASASVDGLIIQLAVAPSETDYEVFYDQAAGMYVTNFKL
QGVSDGSTATYEFSYTTQGESASGSTMIFALPHHESSFSDIMQDYYTGIQLASTTKGVMN
GYLTTSLQFSTSLNRQISWLPWSSQLGSNLLEYSKEQLQLLAEVANSELQVSISESISGL
NTYYLGKVIDKYSYILLTVSEIIQDEASTKSTLENIKSAFDILLQNEQTYPLIYDTKFNG
LVSSGDWGSTSTQYDFGNTYYNDHHFHYGYIIHAAAVIGYVDSKLNGTWAADNKDWVNSL
VRDVANPSEKDEYFAQSRMFDWFNGHSWAAGLYENGNGKNEESSSEDYNFAYAMKLWGAT
IGDQSMELRGDLMISIMKDAMNDYFYYQNDNTVEPEEIIGNKVSGILFDNIIDYTTYFGT
NTEYIHGIHMLPITPVSSNIRSETFVEEEWQTKIEPIIESIESGWTGILKLNQALFDPVD
SYAFFSDSTFDSSTYLDNGMSRTWALAFSGGLANSIA
>P26792
MGVTIRNRNYDHGSLPFLQSLLAILLVTTTTLHINGVEAFHEIHYNLQSVGAENVKQVHR
TGYHFQPKQNWINDPNGPMYYKGVYHLFYQYNPKGAVWGNIVWAHSVSTDLINWTPLEPA
IFPSKPFDKYGCRSGSATILPGNKPVILYTGIVEGPPKNVQVQNYAIPANLSDPYLRKWI
KPDNNPLVVANNGENATAFRDPTTAWLDKSGHWKMLVGSKRNRRGIAYLYRSKDFIKWTK
AKHPIHSQANTGMWECPDFFPVSLKGLNGLDTSVTGESVKHVLKVSLDLTRYEYYTVGTY
LTDKDRYIPDNTSVDGWAGLRYDYGNFYASKTFFDPSKNRRILWGWANESDSTAHDVAKG
WAGIQLIPRTLWLDPSGKQLMQWPIEELETLRGSKVKFSRKQDLSKGILVEVKGITAAQA
DVEVTFSFKSLAKREPFDPKWLEYDAEKICSLKGSTVQGGVGPFGLLTLASEKLEEYTPV
FFRVFKAQNTHKVLMCSDATRSSLKEGLYRPSFAGFVDVDLATDKKISLRSLIDNSVVES
FGAKGKTCISSRVYPTLAVYENAHLYVFNNGSETITVENLDAWSMKKPLRMN
>P11163
MRSLNTLLLSLFVAMSSGAPLLKIREEKNSSLPSIKIFGTGGTIASKGSTSATTAGYSVG
LTVNDLIEAVPSLAEKANLDYLQVSNVGSNSLNYTHLIPLYHGISEALASDDYAGAVVTH
GTDTMEETAFFLDLTINSEKPVCIAGAMRPATATSADGPMNLYQAVSIAASEKSLGRGTM
ITLNDRIASGFWTTKMNANSLDTFRADEQGYLGYFSNDDVEFYYPPVKPNGWQFFDISNL
TDPSEIPEVIILYSYQGLNPELIVKAVKDLGAKGIVLAGSGAGSWTATGSIVNEQLYEEY
GIPIVHSRRTADGTVPPDDAPEYAIGSGYLNPQKSRILLQLCLYSGYGMDQIRSVFSGVY
GG
>Q05538
MRLSEFTTLFLLFSVLLLSASAEQCGSQAGGALCASGLCCSKFGWCGNTNEYCGPGNCQS
QCPGGPGPSGDLGGVISNSMFDQMLNHRNDNACQGKNNFYSYNAFVTAAGSFPGFGTTGD
ITARKREIAAFLAQTSHETTGGWPTAPDGPYAWGYCFLREQGSPGDYCTPSSQWPCAPGR
KYFGRGPIQISHNYNYGPCGRAIGVDLLNNPDLVATDPVISFKSAIWFWMTPQSPKPSCH
DVITGRWQPSGADQAANRVPGFGVITNIINGGLECGHGSDSRVQDRIGFYRRYCGILGVS
PGENLDCGNQRSFGNGLLVDIM
>Q86L04
MQRTLSKVILNSGKNNLLKSSNLLNSNLLKATTTNIIGIKTINNNNNVNSIIGFKSLNKR
YFTSNTPKVEEEDDEIAPDEAIKAEEKIKETERVIGLSEKLSFQTETQKILHIVAESLYT
EKEVFIRELISNASDAIEKVRHTQLTNASMIEDASIPFEIKISTDEDNKTLIIQDSGIGM
TKDEMIKNLGKIGYSGSSDFIKKLGENPDKASIIGQFGVGFYSCFMVGHTIKIYTKSATP

GSKGYLWESDGTGSYSITEAEGVSRGTKIIIHLKPSSYEYSKKSIVENIIKKYSNFVGFP
IALNGTTVNTIKPLWTLNKNAISEEEHKEFYQFLSKSYDTPSYRVHFSTDTPLSIRSIFY
IPSQHMEKYGMGKMEPGVSLFSRKVLIQQKANGILPEWMRFVRGVVDSEDIPLNVSREHL
QDNGLIQRISSVLVKRILKHLNDEAKSDPEKFNVFMTEFGGFFKEGIITDFKWKDEISKL
LRFESSNGSTASKTDAVSLEQYVSRMKPEQKNIYFLSVPNRAVGLSSPYYEPFQLKDIEV
IFLYNAVDEFVLTNVGHFGDKKIVSVESKEAEEFLATNQDKKTETLSQDEIDKFLSWVST
VASDKVTQAKSTTRSISSPAIIIDHESANFRRMLKMVEPGKQHETPKQVVEFNMNHPIIL
KLVQQTESNPTIAKLVIDQVVDNAFVSAGLIEDNREMIPRINQLLDSLLTK
>P20075
MASRKDERAAKEERAQAAAELAAKELRDVNQDRERGIKVVEHKEEVSGGPGVIGSILKSV
QGTLGQAKEVVVGKAHDTAEVSRENTDYAYDKGREGGDVAAQKAEEAKEKAKMAKDTTMG
KAGEYKDYTAQKAEEAKEKAAQKAEETKEKAGEYKNYTAQKAGEAKDTTLGKAGEYKDYA
AQKAAEAKDTTAQKAAEAKEKTGEYKDYAAQKAAEAKVLAAQKAAEAKDTTGKDGEYKDY
AAQKAAEAKDATMQKTGEYKDYAAQKTAETKDATMEKAKEYKEYAAQKAAEAKDATMQKT
GEYKDYSAQKAAETKDATMEKTKEYKDYTAQKAAETKDATMEKAKEAKDTTVQKTGEYKD
YAAEKAKEGKDVTVEKAKEGKDTTVGKMTELKDSAADAARKAMDMFLGKKEEVKGKAGET
AEAAKEKYEDTEFAARKKMEELKLQEEGVKDEAKQRAEADRETAGDRGSAAKGTIFGAMG
SVKDAIVGKLTMPSDVVKDKQQQEAVIKVDETRPGAVAEALKAADQMHGQAFNDVGKMGD
EEVIVERKETRQGKM
>O13716
MKLVLFLVLLFSALINLTNADKMVVAHFIVGNTYPYTVSNWEEDIQDAIAVGIDGFALNM
GSDAWQVERIEDAYDAAASVSSDFKLFISFDMSIISADADFIEGVVRRFADKPNQLYYDG
KVFVSTFAGETDTFGYSDVSTGWDSAVKEPLASAGYPIYFVPSWTSLGQGALEESVADGF
LSWNAWPTTDADMNDNDDIGYQNLANSLGKLYVAPVSPWFYTHLSYKNWAYKSDWLIIDR
WNEMLSVQPDMIEVLTWNDYGESHYIGNIQGALPAGSEGYVDGFDHTAWRYLMSPYISAY
KLGLSEPYINFESLFYWYRPTPKSATATADSLSYPSGGDYMEDEIFVLVYLLQSAEVTVT
CGSTTQTFSGVPGVNQFTIPMETNASPSFTVARQGGTLASGTGPEIVDSLSIYNFNAYTG
VLYF
>Q9SYM4
MPGNKYNCSSSHIPLSRTERLLRDRELREKRKSNRARNPNDVAGSSENSENDLRLEGDSS
RQYVEQYLEGAAAAMAHDDACERQEVRPYNRQRLLVVANRLPVSAVRRGEDSWSLEISAG
GLVSALLGVKEFEARWIGWAGVNVPDEVGQKALSKALAEKRCIPVFLDEEIVHQYYNGYC
NNILWPLFHYLGLPQEDRLATTRSFQSQFAAYKKANQMFADVVNEHYEEGDVVWCHDYHL
MFLPKCLKEYNSKMKVGWFLHTPFPSSEIHRTLPSRSELLRSVLAADLVGFHTYDYARHF
VSACTRILGLEGTPEGVEDQGRLTRVAAFPIGIDSDRFIRALEVPEVIQHMKELKERFAG
RKVMLGVDRLDMIKGIPQKILAFEKFLEENANWRDKVVLLQIAVPTRTDVPEYQKLTSQV
HEIVGRINGRFGTLTAVPIHHLDRSLDFHALCALYAVTDVALVTSLRDGMNLVSYEFVAC
QEAKKGVLILSEFAGAAQSLGAGAILVNPWNITEVAASIGQALNMTAEEREKRHRHNFHH
VKTHTAQEWAETFVSELNDTVIEAQLRISKVPPELPQHDAIQRYSKSNNRLLILGFNATL
TEPVDNQGRRGDQIKEMDLNLHPELKGPLKALCSDPSTTIVVLSGSSRSVLDKNFGEYDM
WLAAENGMFLRLTNGEWMTTMPEHLNMEWVDSVKHVFKYFTERTPRSHFETRDTSLIWNY
KYADIEFGRLQARDLLQHLWTGPISNASVDVVQGSRSVEVRAVGVTKGAAIDRILGEIVH
SKSMTTPIDYVLCIGHFLGKDEDVYTFFEPELPSDMPAIARSRPSSDSGAKSSSGDRRPP
SKSTHNNNKSGSKSSSSSNSNNNNKSSQRSLQSERKSGSNHSLGNSRRPSPEKISWNVLD

LKGENYFSCAVGRTRTNARYLLGSPDDVVCFLEKLADTTSSP
>Q38913
MASFLVLAFSLAFVSQTTANYFYSSPPPPVKHYSPPPVYKSPPPPVKHYSPPPVYKSPPP
PVKHYSPPPVYKSPPPPVKYYSPPPVYKSPPPPVYKSPPPPVKHYSPPPVYKSPPPPVKH
YSPPPVYKSPPPPVKHYSPPPVYKSPPPPVKHYSPPPVYKSPPPPVKYYSPPPVYKSPPP
PVKHYSPPPVYKSPPPPVKYYSPPPVYKSPPPPVKHYSPPPVYKSPPPPVKYYSPPPVYK
SPPPPVHYSPPPVVYHSPPPPVHYSPPPVVYHSPPPPVHYSPPPVVYHSPPPPVHYSPPP
VVYHSPPPPVHYSPPPVVYHSPPPPKKHYEYKSPPPPVHYSPPTVYHSPPPPVHHYSPPH
QPYLYKSPPPPHY
>Q03464
MKMMVVVVVMMLSWLILKPPSTWAINTITFDVGNATINKYATFMKSIHNQAKDPTLKCYG
IPMLPNTNLTPKYLLVTLQDSSLKTITLMLKRNNLYVMGYADTYNGKCRYHIFKDISNTT
ERNDVMTTLCPNPSSRVGKNINYDSSYPALEKKVGRPRSQVQLGIQILNSGIGKIYGVDS
FTEKTEAEFLLVAIQMVSEAARFKYIENQVKTNFNRAFYPNAKVLNLEESWGKISTAIHN
AKNGALTSPLELKNANGSKWIVLRVDDIEPDVGLLKYVNGTCQATYQSAMFPHL
>P23776
MLSLKTLLCTLLTVSSVLATPVPARDPSSIQFVHEENKKRYYDYDHGSLGEPIRGVNIGG
WLLLEPYITPSLFEAFRTNDDNDEGIPVDEYHFCQYLGKDLAKSRLQSHWSTFYQEQDFA
NIASQGFNLVRIPIGYWAFQTLDDDPYVSGLQESYLDQAIGWARNNSLKVWVDLHGAAGS
QNGFDNSGLRDSYKFLEDSNLAVTTNVLNYILKKYSAEEYLDTVIGIELINEPLGPVLDM
DKMKNDYLAPAYEYLRNNIKSDQVIIIHDAFQPYNYWDDFMTENDGYWGVTIDHHHYQVF
ASDQLERSIDEHIKVACEWGTGVLNESHWTVCGEFAAALTDCTKWLNSVGFGARYDGSWV
NGDQTSSYIGSCANNDDIAYWSDERKENTRRYVEAQLDAFEMRGGWIIWCYKTESSLEWD
AQRLMFNGLFPQPLTDRKYPNQCGTISN
>P09351
MQVRNILVALVVVCFAVSEAATQAPTSPPDSSASAEQVAQLLNAPSELDRLKLLKDNQFV
FDFKNSKLGVTQSAGGKTVATSRTDFPAVIGHNIAMTVGFIEACGINLPHTHPRATEINF
IAKGRFQAGFFLENQATFIGHILEEGMATVFPQGAIHFEINLECEPAMFVAAFNNEDPGV
QTTASSFFGLPADVAAVSLNISSIQTVEDLAKYLPHNPAIAMKECMQRCGMTGSSESSES
SEDSSCSD
>O65351
MSSSFLSSTAFFLLLCLGFCHVSSSSSDQGTYIVHMAKSQMPSSFDLHSNWYDSSLRSIS
DSAELLYTYENAIHGFSTRLTQEEADSLMTQPGVISVLPEHRYELHTTRTPLFLGLDEHT
ADLFPEAGSYSDVVVGVLDTGVWPESKSYSDEGFGPIPSSWKGGCEAGTNFTASLCNRKL
IGARFFARGYESTMGPIDESKESRSPRDDDGHGTHTSSTAAGSVVEGASLLGYASGTARG
MAPRARVAVYKVCWLGGCFSSDILAAIDKAIADNVNVLSMSLGGGMSDYYRDGVAIGAFA
AMERGILVSCSAGNAGPSSSSLSNVAPWITTVGAGTLDRDFPALAILGNGKNFTGVSLFK
GEALPDKLLPFIYAGNASNATNGNLCMTGTLIPEKVKGKIVMCDRGINARVQKGDVVKAA
GGVGMILANTAANGEELVADAHLLPATTVGEKAGDIIRHYVTTDPNPTASISILGTVVGV
KPSPVVAAFSSRGPNSITPNILKPDLIAPGVNILAAWTGAAGPTGLASDSRRVEFNIISG
TSMSCPHVSGLAALLKSVHPEWSPAAIRSALMTTAYKTYKDGKPLLDIATGKPSTPFDHG
AGHVSPTTATNPGLIYDLTTEDYLGFLCALNYTSPQIRSVSRRNYTCDPSKSYSVADLNY
PSFAVNVDGVGAYKYTRTVTSVGGAGTYSVKVTSETTGVKISVEPAVLNFKEANEKKSYT
VTFTVDSSKPSGSNSFGSIEWSDGKHVVGSPVAISWT

>Q9C9H5
MTSLFFFVLLFSSLLISNGDANPNYKEALSKSLLFFQGQRSGPLPRGQQISWRASSGLSD
GSAAHVDLTGGYYDAGDNVKFNLPMAFTTTMLSWSALEYGKRMGPELENARVNIRWATDY
LLKCARATPGKLYVGVGDPNVDHKCWERPEDMDTPRTVYSVSASNPGSDVAAETAAALAA
ASMVFRKVDSKYSRLLLATAKDVMQFAIQYQGAYSDSLSSSVCPFYCSYSGYKDELMWGA
SWLLRATNNPYYANFIKSLGGGDQPDIFSWDNKYAGAYVLLSRRALLNKDSNFEQYKQAA
ENFICKILPDSPSSSTQYTQGGLMYKLPQSNLQYVTSITFLLTTYAKYMKATKHTFNCGS
SVIVPNALISLSKRQVDYILGDNPIKMSYMVGFSSNFPKRIHHRASSLPSHALRSQSLGC
NGGFQSFYTQNPNPNILTGAIVGGPNQNDGYPDQRDDYSHAEPATYINAAFVGPLAYFAA
GRST
>Q9M069
MALSISIYFLLIFLSHFPSSHAEPFIGVNYGQVADNLPPPSETVKLLQSTSIQKVRLYGA
DPAIIKALAGTGVGIVIGAANGDVPSLASDPNAATQWINSNVLPFYPASKIMLITVGNEI
LMSNDPNLVNQLLPAMQNVQKALEAVSLGGKIKVSTVNSMTVLGSSDPPSSGSFAAGYQT
GLKGILQFLSDTGSPFAINPYPFFAYQSDPRPETLAFCLFEPNAGRVDSKTGIKYTNMFD
AQVDAVHSALKSMGFEKVEIVVAETGWASRGDANEVGASVDNAKAYNGNLIAHLRSMVGT
PLMPGKPVDTYIFALYDENLKPGPSSERAFGLFKTDLSMVYDVGLAKSSSSSQVRY
>O04300
MASLPKPTPLLKDELDIVIPTIRNLDFLEMWRPFFEQYHLIIVQDGDPSKVIKVPEGFDY
ELYNRNDINRILGPKASCISFKDSACRCFGYMVSKKKYIYTIDDDCFVAKDPTGHEINAL
EQHIKNLLSPSTPFFFNTLYDPYREGTDFVRGYPFSLREGVPTAVSHGLWLNIPDYDAPT
QLVKPHERNTRFVDAVLTIPKGSLFPMCGMNLAFNRELIGPAMYFGLMGDGQPIGRYDDM
WAGWCIKVICDHLGYGVKTGLPYIWHSKASNPFVNLKKEYKGIFWQEEIIPFFQAATLSK
DCTSVQKCYIELSKQVKEKLGTIDPYFIKLADAMVTWVEAWDEINNNKSEETTSTKASEV
AATK
>P29060
MIKYSFLLTALVLFLRALKLEAGDIVIYWGQNGNEGSLADTCATNNYAIVNIAFLVVFGN
GQNPVLNLAGHCDPNAGACTGLSNDIRACQNQGIKVMLSLGGGAGSYFLSSADDARNVAN
YLWNNYLGGQSNTRPLGDAVLDGIDFDIEGGTTQHWDELAKTLSQFSQQRKVYLTAAPQC
PFPDTWLNGALSTGLFDYVWVQFYNNPPCQYSGGSADNLKNYWNQWNAIQAGKIFLGLPA
AQGAAGSGFIPSDVLVSQVLPLINGSPKYGGVMLWSKFYDNGYSSAIKANV
>Q01940
MRYSTVLAALALLGTSAVSVLAALPDQIDVKVKNLTPEDTIYDRTRQVFYQSNLYKGRIE
VYNPKTQSHFNVVIDGASSNGDGEQQMSGLSLLTHDNSKRLFAVMKNAKSFNFADQSSHG
ASSFHSFNLPLSENSKPVWSVNFEKVQDEFEKKAGKRPFGVVQSAQDRDGNSYVAFALGM
PAIARVSADGKTVSTFAWESGNGGQRPGYSGITFDPHSNKLIAFGGPRALTAFDVSKPYA
WPEPVKINGDFGTLSGTEKIVTVPVGNESVLVGARAPYAISFRSWDNWKSANIKKTKRSE
LQNSGFTAVADYYQGSEQGLYAVSAFFDNGAHGGRSDYPLYKLDNSILNF
>Q12355
MQLHSLIASTALLITSALAATSSSSSIPSSCTISSHATATAQSDLDKYSRCDTLVGNLTI
GGGLKTGALANVKEINGSLTIFNATNLTSFAADSLESITDSLNLQSLTILTSASFGSLQS
VDSIKLITLPAISSFTSNIKSANNIYISDTSLQSVDGFSALKKVNVFNVNNNKKLTSIKS
PVETVSDSLQFSFNGNQTKITFDDLVWANNISLTDVHSVSFANLQKINSSLGFINNSISS
LNFTKLNTIGQTFSIVSNDYLKNLSFSNLSTIGGALVVANNTGLQKIGGLDNLTTIGGTL

EVVGNFTSLNLDSLKSVKGGADVESKSSNFSCNALKALQKKGGIKGESFVCKNGASSTSV
KLSSTSKSQSSQTTAKVSKSSSKAEEKKFTSGDIKAAASASSVSSSGASSSSSKSSKGNA
AIMAPIGQTTPLVGLLTAIIMSIM
>Q9C168
MSNIISQAGQKAKEALTSAPSSKKVDDLKNEFKETDKSARLTTDYGVKQTTADDWLRIVS
DDKIGPSLLEDPFARERIMRFDHERIPERVVHARGSGAFGKFKVYESASDLTMAPVLTDT
SRETPVFVRFSTVLGSRGSADTVRDVRGFAVKFYTEEGNWDLVGNNIPVFFIQDAIKFPD
VIHAGKPEPHNEVPQAQSAHNNFWDFQFNHTEATHMFTWAMSDRAIPRSLRMMQGFGVNT
YTLINAQGKRHFVKFHWTPELGVHSLVWDEALKLAGQDPDFHRKDLWEAIENGAYPKWKF
GIQAIAEEDEHKFDFDILDATKIWPEDLVPVRYIGEMELNRNPDEFFPQTEQIAFCTSHV
VNGIGFSDDPLLQGRNFSYFDTQISRLGVNFQELPINRPVCPVMNFNRDGAMRHTISRGT
VNYYPNRFDACPPASLKEGGYLEYAQKVAGIKARARSAKFKEHFSQAQLFYNSMSPIEKQ
HMINAFGFELDHCEDPVVYGRMVQRLADIDLGLAQTIAEMVGGEAPTTTNHPNHGRKTIN
LSQTEFPPATPTIKSRRVAIIIADGYDNVAYDAAYAAISANQAIPLVIGPRRSKVTAANG
STVQPHHHLEGFRSTMVDAIFIPGGAKAAETLSKNGRALHWIREAFGHLKAIGATGEAVD
LVAKAIALPQVTVSSEAEVHESYGVVTLKKVKPESFTDAVKIAKGAAGFLGEFFYAIAQH
RNWDRELDGLHSMIAY
>Q9S8P4
MKLIAVLLLVVLATATTATAADADALQDLCVADYASVILVNGFACKPASNVTAEDFFSNL
LVKQGATNNTFGSLVTGANVQRIPGLNTLGVSMARIDYAPGGLNPPHTHPRATEMVFVLE
GQLDVGFITTTNQLIAKTIAKGETFVFPKGLVHFQKNNGWEPATVIAGFNSQLPGTVNIP
LTLFNATPPVPDNVLTKAFQIGTKEVQKIKSKFAPKK
>P53707
MSAGKYLLGTAALVGGVYYYDQYVQPILPRQQHQELAYQTQRVENKGSELNNKLTKKIEE
GKKFVNEKTESVTKQVKNSDVYQKLQLNTEDYKKHVEDAVDNDKNVFVVGIQKYIDFVNQ
LGEVKVQTGTTQYSTVSPNVEVKEKSIFGNWFDKSDNKVDQLKNDADKKINEAKDKAEST
KSDFFNWNSKKADELDKKANEAINWTNKQIDYASAEWHKHYEQAKGDWNKALDDLSKQWN
DSKKQLNGRFDTEKDRAIKGVEDAKSNFEKLSNDLANDASKNQKLKDAQDHFGKSLENLK
LFGDDVYNDFAKRFDDLFNRK
>P53616
MKLSATTLTAASLIGYSTIVSALPYAADIDTGCTTTAHGSHQHKRAVAVTYVYETVTVDK
NGQTVTPTSTEASSTVASTTTLISESSVTKSSSKVASSSESTEQIATTSSSAQTTLTSSE
TSTSESSVPISTSGSASTSSAASSATGSIYGDLADFSGPYEKFEDGTIPCGQFPSGQGVI
PISWLDEGGWSGVENTDTSTGGSCKEGSYCSYACQPGMSKTQWPSDQPSDGRSIGGLLCK
DGYLYRSNTDTDYLCEWGVDAAYVVSELSNDVAICRTDYPGTENMVIPTYVQAGDSLPLT
VVDQDTYYTWQGLKTSAQYYVNNAGISVEDACVWGSSSSGVGNWAPLNFGAGSSDGVAYL
SLIPNPNNGNALNFNVKIVAADDSSTVNGECIYENGSFSGGSDGCTVSVTAGKAKFVLYN

>Q42589
MAGVMKLACLLLACMIVAGPITSNAALSCGSVNSNLAACIGYVLQGGVIPPACCSGVKNL
NSIAKTTPDRQQACNCIQGAARALGSGLNAGRAAGIPKACGVNIPYKISTSTNCKTVR
>O59774
MRIWILIFFSFIKLVSSLQYTGNGVLALDFVAKTFPNQENQLEKRDYTYSPSGITSFPLD
LQSYTYYTTTLSIGRPSISYTVAIDLDMPYTWLTYYNVMAFNPAYLGIVNSGTQWSTDEL

RYFLCKKESDSCYFGNASSSFHFVTSPSTFFIRYDDNITVAGINVQDSLSYSHYQALPDF
QFGITLKEYVPSSMLPYKGVLGLAASTEINSIDYSDSISSFSPPTFLEQLVKEDILAYPA
FSMYLDNQGNGSLLLGAVDTSKYQGQFVALKQTKLTHYAVSIYSVQFLNSTFFSNYSIIT
DAYFQTRETYIYLPAELAYSVMDNAGAYLSEGYFALNCDEIDLEAALIFQFGCNSTIKVP
ISLLVIGQVSNICLLGIRPSTDSEIVLGLLFFRNAYTFYHQSQKMIAIGQAFYNATSNLS
AIVDQHIPGALTCSQYPTSVASTQLVQTSHFTSTSLSAVNISESVVYSYTSSSSMPSSAI
PSFNISLISQNAVANAGNSFSPLSAMVIMMMSAVFLGLGII
>P41797
MSKAVGIDLGTTYSCVAHFANDRVEIIANDQGNRTTPSFVAFTDTERLIGDAAKNQAAMN
PANTVFDAKRLIGRKFDDPEVINDAKHFPFKVIDKAGKPVIQVEYKGETKTFSPEEISSM
VLTKMKEIAEGYLGSTVKDAVVTVPAYFNDSQRQATKDAGTIAGLNVLRIINEPTAAAIA
YGLDKKGSRGEHNVLIFDLGGGTFDVSLLAIDEGIFEVKATAGDTHLGGEDFDNRLVNFF
IQEFKRKNKKDISTNQRALRRLRTACERAKRTLSSSAQTSIEIDSLYEGIDFYTSITRAR
FEELCADLFRSTLDPVGKVLADAKIDKSQVEEIVLVGGSTRIPKIQKLVSDFFNGKELNK
SINPDEAVAYGAAVQAAILTGDTSSKTQDILLLDVAPLSLGIETAGGIMTKLIPRNSTIP
TKKSETFSTYADNQPGVLIQVFEGERAKTKDNNLLGKFELSGIPPAPRGVPQIEVTFDID
ANGILNVSALEKGTGKTQKITITNDKGRLSKEEIDKMVSEAEKFKEEDEKEAARVQAKNQ
LESYAYSLKNTINDGEMKDKIGADDKEKLTKAIDETISWLDASQAASTEEYEDKRKELES
VANPIISGAYGAAGGAPGGAGGFPGAGGFPGGAPGAGGPGGATGGESSGPTVEEVD
>Q01682
MKLSGISLWLLAASIVHAGKSQFEAFENEFYFKDHLGTISVYHEPYFNGPTTSFPESCAI
KQVHLLQRHGSRNPTGDDTATDVSSAQYIDIFQNKLLNGSIPVNFSYPENPLYFVKHWTP
VIKAENADQLSSSGRIELFDLGRQVFERYYELFDTDVYDINTAAQERVVDSAEWFSYGMF
GDDMQNKTNFIVLPEDDSAGANSLAMYYSCPVYEDNNIDENTTEAAHTSWRNVFLKPIAN
RLNKYFDSGYNLTVSDVRSLYYICVYEIALRDNSDFCSLFTPSEFLNFEYDSDLDYAYWG
GPASEWASTLGGAYVNNLANNLRKGVNNASDRKVFLAFTHDSQIIPVEAALGFFPDITPE
HPLPTDKNIFTYSLKTSSFVPFAGNLITELFLCSDNKYYVRHLVNQQVYPLTDCGYGPSG
ASDGLCELSAYLNSSVRVNSTSNGIANFNSQCQAHSTNVTVYY
>Q40161
MHTKIHLPPCILLLLLFSLPSFNVVVGGDGESGNPFTPKGYLIRYWKKQISNDLPKPWFL
LNKASPLNAAQYATYTKLVADQNALTTQLHTFCSSANLMCAPDLSPSLEKHSGDIHFATY
SDKNFTNYGTNEPGIGVNTFKNYSEGENIPVNSFRRYGRGSPRDNKFDNYASDGNVIDQS
FNSYSTSTAGGSGKFTNYAANANDPNLHFTSYSDQGTGGVQKFTIYSQEANAGDQYFKSY
GKNGNGANGEFVSYGNDTNVIGSTFTNYGQTANGGDQKFTSYGFNGNVPENHFTNYGAGG
NGPSETFNSYRDQSNVGDDTFTTYVKDANGGEANFTNYGQSFNEGTDVFTTYGKGGNDPH
INFKTYGVNNTFKDYVKDTATFSNYHNKTSQVLASLMEVNGGKKVNNRWVEPGKFFREKM
LKSGTIMPMPDIKDKMPKRSFLPRVIASKLPFSTSKIAELKKIFHAGDESQVEKMIGDAL
SECERAPSAGETKRCVNSAEDMIDFATSVLGRNVVVRTTEDTKGSNGNIMIGSVKGINGG
KVTKSVSCHQTLYPYLLYYCHSVPKVRVYEADILDPNSKVKINHGVAICHVDTSSWGPSH
GAFVALGSGPGKIEVCHWIFENDMTWAIAD
>Q9P403
MQTSFVALLAVAASLASALPHGGNSYEASLPEPTNLPEPTKLPEPVEGPYKPKPPILPEP
IKDNYKPKTPILPEHVEGPYKPKLPEPTTGDPKNNTLPVPTCVDGKIKTHKVKSGESLTT
IAEKYDTGICNIAKLNNLADPNFVDLNQDLQIPTDACEKDNTSCIKPDGTATCVKDGKKD

GKDIYSVVSGDTLTSIAQALQITLQSLKDANPGVVPEHLNVGQKLNVPVC
>Q99109
MPSKIALSISVLTAALGMVSAAPHYEYKSYNAGHNVESVVENKLVDASDLTLGVDILKRT
DYSACKKYVSSYNAGYNVYSINENKLIDLSDATVKLSLLTNNGRPVKTWNKAPRYCLEYI
KSYNGGHNVIIDNENKLIDLSGLNLGIDILKRKAPEFSYYNPGHNVKSTTENKLVDASDL
LANVNILSKRGDKYSYYNGGYNVESDTENKLIDLSGLLANIDILKKRTGSQYSYYNGGHN
VESTNENKLVDASDLLANVDILKKRGDKYTYYNGGHNIESTNENKLIDLSDLTANVGVLD
DVHVNIGLLDKRTDKYSYYDGGHNIELTNENKLIDLSDLLANVDVLKKRTDKFSYYNGGH
NVESDTQNKLIDVSHLTAIVNALSKRTDKYSYYNGGHNVESTNENKLIDLSDLTALVNVL
SKRSLKGYGSKIHARDISEELYRRTNADEDCTCVKKSKPTPPKQTVTKPSADCDAPPAVT
PKPKPSTKPSPSPTTPPPSKDTSKPTTKPEPKPQPSDKPEPKPSDKPEPKPSDKPEPKPS
DKPEPTPSPKPTPPKVTCGTDEVYVDEGHNVVSKNSNKLLDLSNISINIGILDGLFGGKK
SKEVAADSTCTSKQGCCVSKSVYYYNGGNNVQSSNENSGIDLSGLNLGLDIL
>Q9M1G9
MGPSAHLISALGVIIMATMVAAYEPETYASPPPLYSSPLPEVEYKTPPLPYVDSSPPPTY
TPAPEVEYKSPPPPYVYSSPPPPTYSPSPKVDYKSPPPPYVYSSPPPPYYSPSPKVDYKS
PPPPYVYNSPPPPYYSPSPKVDYKSPPPPYVYSSPPPPYYSPSPKVEYKSPPPPYVYSSP
PPPYYSPSPKVDYKSPPPPYVYSSPPPPYYSPSPKVEYKSPPPPYVYSSPPPPYYSPSPK
VDYKSPPPPYVYSSPPPPYYSPSPKVDYKSPPPPYVYSSPPPPYYSPSPKVDYKSPPPPY
VYSSPPPPYYSPSPKVDYKSPPPPYVYSSPPPPYYSPSPKVDYKSPPPPYVYSSPPPPTY
SPSPKVDYKSPPPPYVYSSPPPPYYSPSPKVEYKSPPPPYVYSSPPPPTYSPSPKVYYKS
PPPPYVYSSPPPPYYSPSPKVYYKSPPPPYVYSSPPPPYYSPSPKVYYKSPPPPYVYSSP
PPPYYSPSPKVYYKSPPPPYVYSSPPPPYYSPSPKVYYKSPPPPYVYSSPPPPYYSPSPK
VHYKSPPPPYVYSSPPPPYYSPSPKVHYKSPPPPYVYNSPPPPYYSPSPKVYYKSPPPPY
VYSSPPPPYYSPSPKVYYKSPPPPYVYSSPPPPYYSPSPKVYYKSPPPPYYSPSPKVYYK
SPPHPHVCVCPPPPPCYSPSPKVVYKSPPPPYVYNSPPPPYYSPSPKVYYKSPPPPSYYS
PSPKVEYKSPPPPSYSPSPKTEY
>Q5MFV8
MIGKVVVSVASILLIVGVAIGVVAYINKNGDANLSPQMKAVRGICEATSDKASCVKTLEP
VKSDDPNKLIKAFMLATRDAITQSSNFTGKTEGNLGSGISPNNKAVLDYCKKVFMYALED
LSTIVEEMGEDLNQIGSKIDQLKQWLTGVYNYQTDCLDDIEEDDLRKTIGEGIASSKILT
SNAIDIFHTVVSAMAKLNLKVEDFKNMTGGIFAPSDKGAAPVNKGTPPVADDSPVADPDG
PARRLLEDIDETGIPTWVSGADRKLMTKAGRGSNDGGARIRATFVVAKDGSGQFKTVQQA
VNACPEKNPGRCIIHIKAGIYREQVIIPKKKNNIFMFGDGARKTVISYNRSVKLSPGTTT
SLSGTVQVESEGFMAKWIGFKNTAGPMGHQAVAIRVNGDRAVIFNCRFDGYQDTLYVNNG
RQFYRNIVVSGTVDFIFGKSATVIQNSLIVVRKGNKGQFNTVTADGNEKGLAMKIGIVLQ
NCRIVPDKKLAAERLIVESYLGRPWKKFSTTVIINSEIGDVIRPEGWKIWDGESFHKSCR
YVEYNNRGPGAITNRRVNWVKIARSAAEVNDFTVANWLGPINWIQEANVPVTLGL
>Q92211
MAIKIGINGFGRIGRLVLRVALGRKDIEVVAVNDPFIAPDYAAYMFKYDSTHGRYKGEVT
ASGDDLVIDGHKIKVFQERDPANIPWGKSGVDYVIESTGVFTKVEGAQKHIDAGAKKVII
TAPSADAPMFVVGVNEDKYTPDLKIISNASCTTNCLAPLAKVVNDTFGIEEGLMTTVHSI
TATQKTVDGPSHKDWRGGRTASGNIIPSSTGAAKAVGKVIPELNGKLTGMSLRLPTTDVS
VVDLTVRLKKAASYEEIAPAIKKASEGPLKGVLGYTEDAVVSTDFLGSSYSSIFDEKAGI

LLSPTFVKLISWYDNEYGYSTKVVDLLEHVA
>P05117
MVIQRNSILLLIIIFASSISTCRSNVIDDNLFKQVYDNILEQEFAHDFQAYLSYLSKNIE
SNNNIDKVDKNGIKVINVLSFGAKGDGKTYDNIAFEQAWNEACSSRTPVQFVVPKNKNYL
LKQITFSGPCRSSISVKIFGSLEASSKISDYKDRRLWIAFDSVQNLVVGGGGTINGNGQV
WWPSSCKINKSLPCRDAPTALTFWNCKNLKVNNLKSKNAQQIHIKFESCTNVVASNLMIN
ASAKSPNTDGVHVSNTQYIQISDTIIGTGDDCISIVSGSQNVQATNITCGPGHGISIGSL
GSGNSEAYVSNVTVNEAKIIGAENGVRIKTWQGGSGQASNIKFLNVEMQDVKYPIIIDQN
YCDRVEPCIQQFSAVQVKNVVYENIKGTSATKVAIKFDCSTNFPCEGIIMENINLVGESG
KPSEATCKNVHFNNAEHVTPHCTSLEISEDEALLYNY
>P93164
MPNDSVLSLFFFVTLFTCLLSATSHDDHIFLPSQLHDDDSVSCTATDPSLNYKPVIGILT
HPGDGASGRLSNATGVSYIAASYVKFVESGGARVIPLIYNESPENLNKKLDLVNGVLFTG
GWAVSGPYLDTLGNIFKKALERNDAGDHFPVIAFNLGGNLVIRIVSEQTDILEPFTASSL
PSSLVLWNEANAKGSLFQRFPSDLLTQLKTDCLVLHNHRYAISPRKLQYNTKLSDFFEIL
ATSGDRDGKTFVSTARGRKYPVTVNLWQPEKNAFEWATSLKAPHTEDAIRVTQSTANFFI
SEARKSTNTPDAQKVRDSLIYNYKPTFGGTAGKGYDQVYLFE
>Q9SU40
MDLFKILLLVFFVNISFCFAADPYSFYNFEVSYITASPLGVPQQVIAINGKFPGPTINVT
TNENLVVNVRNKLDEGLLLHWNGIQQRRVSWQDGVLGTNCPIPPKWNWTYEFQVKDQIGS
FFYFPSLHFQRASGGFGSFVVNPRAIIPVPFSTPDGDITVTIGDWYIRNHTALRKALDDG
KDLGMPDGVLINGKGPYRYNDTLVADGIDFETITVHPGKTYRLRVSNVGISTSLNFRIQG
HNLVLAESEGSYTVQQNYTSLDIHVGQSYSFLVTMDQNASSDYYIVASARVVNETIWRRV
TGVGILKYTNSKGKAKGQLPPGPQDEFDKTFSMNQARSIRWNVSASGARPNPQGSFKYGS
INVTDVYVLRNMPPVTISGKRRTTLNGISFKNPSTPIRLADKLKVKDVYKLDFPKRPLTG
PAKVATSIINGTYRGFMEVVLQNNDTKMQSYHMSGYAFFVVGMDYGEWTENSRGTYNKWD
GIARSTIQVYPGAWSAILISLDNPGAWNLRTENLDSWYLGQETYVRVVNPDENNKTEFGH
PDNVLYCGALSKLQKPQKVSSSASKSIGFTSLSMVVMALVMMMMLQH
>P52753
MQFSIIAISFLASLAMASPAKRGGGGGGSGSGSGSGSGSGSGGGSTTYTACSSTLYSEAQ
CCATDVLGVADLDCETVPETPTSASSFESICATSGRDAKCCTIPLLGQALLCQDPVGL
>P16933
MFARLPVVFLYAFVAFGALVAALPGGHPGTTTPPVTTTVTVTTPPSTTTIAAGGTCTTGS
LSCCNQVQSASSSPVTALLGLLGIVLSDLNVLVGISCSPLTVIGVGGSGCSAQTVCCENT
QFNGLINIGCTPINIL
>O13858
MSHASKNYNAQLTAAAASTALHGTKKIYNSNENDRAVNSFLGNRTSYGEAVNGSPQYDYM
RSRMSTLGSANQPMAPPSLRNRSSVYLPTPAGPPQIPVNTGKRYTLTSSSANYMTKSERQ
MRPPKSYDFPPSQQVRPSTSRSPSYASYNSEEVNFQSYQDPRLLPRTSQMYMPDNNYSPA
VKSPAAQRRSSSYMVPANSRGSPANYNTPYYPTAIPPPIEEYSPSVSLPTSPVAEESYNN
VQRSSTVRNNTTQKSVLKKPSRKMSPAYTSSYRQNSPSSQVPPVSKKHVIIYENKEGSSS
SESVYEDVFEDFDPSGSNQASLRSTSTIHYTPSSKRISVIPPNTSNIGSRVVSRSGQNNN
QPAQPGQYNQQSQPVQSYQSGQSTQHFQPVQPIQPVQSTQYYQPSSPVQPVQNGVPAPPM
QPVQSTQYYQPSSPVQPVQNVKPAQPAQPSLEDAAKRRVEEMLRQMDITPTASSTTANNA

YASAEPHPSAFPDDMNSVFSDSSFERERDSGRGRSTNLFSKFKSGRSRSKASGEAPYSYP
APPVPSVNNAGARLTLRDSGAAPEATYSLRQPSNHAYSEGRSYTFTGGQPPSVPTMPYGS
RFANDSDSMMGSTADFSSKKKGGKFKAFKKFFKMRF
>P46273
MSLSNKLSVKDLDVAGKRVFIRVDFNVPLDGKTITNNQRIVAALPTIKYVEEHKPKYIVL
ASHLGRPNGERNDKYSLAPVATELEKLLGQKVTFLNDCVGPEVTKAVENAKDGEIFLLEN
LRYHIEEEGSSKDKDGKKVKADPEAVKKFRQELTSLADVYINDAFGTAHRAHSSMVGLEV
PQRAAGFLMSKELEYFAKALENPERPFLAILGGAKVSDKIQLIDNLLDKVDMLIVGGGMA
FTFKKILNKMPIGDSLFDEAGAKNVEHLVEKAKKNNVELILPVDFVTADKFDKDAKTSSA
TDAEGIPDNWMGLDCGPKSVELFQQAVAKAKTIVWNGPPGVFEFEKFANGTKSLLDAAVK
SAENGNIVIIGGGDTATVAKKYGVVEKLSHVSTGGGASLELLEGKDLPGVVALSNKN

(4) 96 centrosome proteins ($\mathbb{S}_4$)

>Q96SN8
MMDLVLEEDVTVPGTLSGCSGLVPSVPDDLDGINPNAGLGNGVLPNVSEETVSPTRARNM
KDFENQITELKKENFNLKLRIYFLEERMQQEFHGPTEHIYKTNIELKVEVESLKRELQER
EQLLIKASKAVESLAEAGGSEIQRVKEDARKKVQQVEDLLTKRILLLEKDVTAAQAELEK
AFAGTETEKALRLRLESKLSEMKKMHEGDLAMALVLDEKDRLIEELKLSLKSKEALIQCL
KEEKSQMACPDENVSSGELRGLCAAPREEKERETEAAQMEHQKERNSFEERIQALEEDLR
EKEREIATEKKNSLKRDKAIQGLTMALKSKEKKVEELNSEIEKLSAAFAKAREALQKAQT
QEFQGSEDYETALSGKEALSAALRSQNLTKSTENHRLRRSIKKITQELSDLQQERERLEK
DLEEAHREKSKGDCTIRDLRNEVEKLRNEVNEREKAMENRYKSLLSESNKKLHNQEQVIK
HLTESTNQKDVLLQKFNEKDLEVIQQNCYLMAAEDLELRSEGLITEKCSSQQPPGSKTIF
SKEKKQSSDYEELIQVLKKEQDIYTHLVKSLQESDSINNLQAELNKIFALRKQLEQDVLS
YQNLRKTLEEQISEIRRREEESFSLYSDQTSYLSICLEENNRFQVEHFSQEELKKKVSDL
IQLVKELYTDNQHLKKTIFDLSCMGFQGNGFPDRLASTEQTELLASKEDEDTIKIGEDDE
INFLSDQHLQQSNEIMKDLSKGGCKNGYLRHTESKISDCDGAHAPGCLEEGAFINLLAPL
FNEKATLLLESRPDLLKVVRELLLGQLFLTEQEVSGEHLDGKTEKTPKQKGELVHFVQTN
SFSKPHDELKLSCEAQLVKAGEVPKVGLKDASVQTVATEGDLLRFKHEATREAWEEKPIN
TALSAEHRPENLHGVPGWQAALLSLPGITNREAKKSRLPILIKPSRSLGNMYRLPATQEV
VTQLQSQILELQGELKEFKTCNKQLHQKLILAEAVMEGRPTPDKTLLNAQPPVGAAYQDS
PGEQKGIKTTSSVWRDKEMDSDQQRSYEIDSEICPPDDLASLPSCKENPEDVLSPTSVAT
YLSSKSQPSAKVSVMGTDQSESINTSNETEYLKQKIHDLETELEGYQNFIFQLQKHSQCS
EAIITVLCGTEGAQDGLSKPKNGSDGEEMTFSSLHQVRYVKHVKILGPLAPEMIDSRVLE
NLKQQLEEQEYKLQKEQNLNMQLFSEIHNLQNKFRDLSPPRYDSLVQSQARELSLQRQQI
KDGHGICVISRQHMNTMIKAFEELLQASDVDYCVAEGFQEQLNQCAELLEKLEKLFLNGK
SVGVEMNTQNELMERIEEDNLTYQHLLPESPEPSASHALSDYETSEKSFFSRDQKQDNET
EKTSVMVNSFSQDLLMEHIQEIRTLRKRLEESIKTNEKLRKQLERQGSEFVQGSTSIFAS
GSELHSSLTSEIHFLRKQNQALNAMLIKGSRDKQKENDKLRESLSRKTVSLEHLQREYAS
VKEENERLQKEGSEKERHNQQLIQEVRCSGQELSRVQEEVKLRQQLLSQNDKLLQSLRVE
LKAYEKLDEEHRRLREASGEGWKGQDPFRDLHSLLMEIQALRLQLERSIETSSTLQSRLK
EQLARGAEKAQEGALTLAVQAVSIPEVPLQPDKHDGDKYPMESDNSFDLFDSSQAVTPKS
VSETPPLSGNDTDSLSCDSGSSATSTPCVSRLVTGHHLWASKNGRHVLGLIEDYEALLKQ
ISQGQRLLAEMDIQTQEAPSSTSQELGTKGPHPAPLSKFVSSVSTAKLTLEEAYRRLKLL
WRVSLPEDGQCPLHCEQIGEMKAEVTKLHKKLFEQEKKLQNTMKLLQLSKRQEKVIFDQL

VVTHKILRKARGNLELRPGGAHPGTCSPSRPGS
>Q96MT8
MEALLEGIQNRGHGGGFLTSCEAELQELMKQIDIMVAHKKSEWEGRTHALETCLKIREQE
LKSLRSQLDVTHKEVGMLHQQVEEHEKIKQEMTMEYKQELKKLHEELCILKRSYEKLQKK
QMREFRGNTKNHREDRSEIERLTAKIEEFRQKSLDWEKQRLIYQQQVSSLEAQRKALAEQ
SEIIQAQLVNRKQKLESVELSSQSEIQHLSSKLERANDTICANELEIERLTMRVNDLVGT
SMTVLQEQQQKEEKLRESEKLLEALQEEKRELKAALQSQENLIHEARIQKEKLQEKVKAT
NTQHAVEAIRPREESLAEKKYTSQGQGDLDSVLSQLNFTHTSEDLLQAEVTCLEGSLESV
SATCKQLSQELMEKYEELKRMEAHNNEYKAEIKKLKEQILQGEQSYSSALEGMKMEISHL
TQELHQRDITIASTKGSSSDMEKRLRAEMQKAEDKAVEHKEILDQLESLKLENRHLSEMV
MKLELGLHEAKEISLADLQENYIEALNKLVSENQQLQKDLMNTKSQLEISTQMCKKQNDR
IFKPTHSRTTEFKNTEFKPTHGQHRHDGIKTEHYKTDLHSPRGQASDSINPMSRVLSPLS
PQISPCSSTRSLTSYSLCKTHSLPSALDTNEANFSDTMSESMNDQEEFISSCSLPVSPLG
SIATRFLEEEELRSHHILERLDAHIEELKRESEKTVRQFTALK
>P91870
MEDDAPMNLCNEQFEEIEDSPIDDNDNESFYNADGDVELEEEEVHETPKNFKNGGRFKTN
MTNPKVNDLTTIEEKNEDLRSAASSRSASRPASVMSDKSFSSQFEFQSGGENAIEEYTNQ
VFADENKADLLFPETSKFMNGASPPKDKHHSWEPSIHHYDKQPPPDIQTNSPVFGNLNHR
KNKLIPQARAKPGANDNEIVERDNDENVPTTSDKSAFITSPMNSTNHDEKTSTPKRPTNR
KIGQYQGPNFDLSSIYVGSPQHQNTSISTGQQMPTSSYSHAHSETMMTNQTINESMVRRV
LNGNNKNQDLFAALEEARKRRAAQPSKPDFRINTTRTRVPIKPTSARHSGNVVSSTSNDN
TTAASSKDLTTSRKAMETFRQNASMADATNSNTASMTSILSTISTARTDISRSSRNHGGG
FSNTSVSTVIPANNGNVSLSHGRDGRDSVSSVRTMSRASSTSTVYAGSTFSGVSKPLRIH
AKRVAFGCVAVGETLRVEVEVENISDRQCLVRASTDSTTPVYQILDNKLTMVDPKKSIKF
QVSFSPSSVGRYQVIMSIEVPAQNFIHKIPMWGNGGIAKFVPTSPDLQQTINQSEYAMCT
SCAKRISFKISNSAGTRDGFAMIKVFDSAMRQLPDGCVAFFPAPGFIVKKKSDKRVDIRI
DSSYIDLHDENNFRTSSSLSTASTTSSFQRRILPGAKFFVHVVWGEETMRTRLRLLEVRT
GQHQLIDGHDFTSFQFSDEEVLRAVPVGFPAIKPEDRDLFAASYRSFFINFFTSTTEFRA
ATSRKKKEICSNDDSTLLETTAFRNQTFVNDVTIVPNTRFSNRK
>Q96CW5
MATPDQKSPNVLLQNLCCRILGRSEADVAQQFQYAVRVIGSNFAPTVERDEFLVAEKIKK
ELIRQRREADAALFSELHRKLHSQGVLKNKWSILYLLLSLSEDPRRQPSKVSSYATLFAQ
ALPRDAHSTPYYYARPQTLPLSYQDRSAQSAQSSGSVGSSGISSIGLCALSGPAPAPQSL
LPGQSNQAPGVGDCLRQQLGSRLAWTLTANQPSSQATTSKGVPSAVSRNMTRSRREGDTG
GTMEITEAALVRDILYVFQGIDGKNIKMNNTENCYKVEGKANLSRSLRDTAVRLSELGWL
HNKIRRYTDQRSLDRSFGLVGQSFCAALHQELREYYRLLSVLHSQLQLEDDQGVNLGLES
SLTLRRLLVWTYDPKIRLKTLAALVDHCQGRKGGELASAVHAYTKTGDPYMRSLVQHILS
LVSHPVLSFLYRWIYDGELEDTYHEFFVASDPTVKTDRLWHDKYTLRKSMIPSFMTMDQS
RKVLLIGKSINFLHQVCHDQTPTTKMIAVTKSAESPQDAADLFTDLENAFQGKIDAAYFE
TSKYLLDVLNKKYSLLDHMQAMRRYLLLGQGDFIRHLMDLLKPELVRPATTLYQHNLTGI
LETAVRATNAQFDSPEILRRLDVRLLEVSPGDTGWDVFSLDYHVDGPIATVFTRECMSHY
LRVFNFLWRAKRMEYILTDIRKGHMCNAKLLRNMPEFSGVLHQCHILASEMVHFIHQMQY
YITFEVLECSWDELWNKVQQAQDLDHIIAAHEVFLDTIISRCLLDSDSRALLNQLRAVFD
QIIELQNAQDAIYRAALEELQRRLQFEEKKKQREIEGQWGVTAAEEEEENKRIGEFKESI

PKMCSQLRILTHFYQGIVQQFLVLLTTSSDESLRFLSFRLDFNEHYKAREPRLRVSLGTR
GRRSSHT
>Q9Y3P9
MDDKASVGKISVSSDSVSTLNSEDFVLVSRQGDETPSTNNGSDDEKTGLKIVGNGSEQQL
QKELADVLMDPPMDDQPGEKELVKRSQLDGEGDGPLSNQLSASSTINPVPLVGLQKPEMS
LPVKPGQGDSEASSPFTPVADEDSVVFSKLTYLGCASVNAPRSEVEALRMMSILRSQCQI
SLDVTLSVPNVSEGIVRLLDPQTNTEIANYPIYKILFCVRGHDGTPESDCFAFTESHYNA
ELFRIHVFRCEIQEAVSRILYSFATAFRRSAKQTPLSATAAPQTPDSDIFTFSVSLEIKE
DDGKGYFSAVPKDKDRQCFKLRQGIDKKIVIYVQQTTNKELAIERCFGLLLSPGKDVRNS
DMHLLDLESMGKSSDGKSYVITGSWNPKSPHFQVVNEETPKDKVLFMTTAVDLVITEVQE
PVRFLLETKVRVCSPNERLFWPFSKRSTTENFFLKLKQIKQRERKNNTDTLYEVVCLESE
SERERRKTTASPSVRLPQSGSQSSVIPSPPEDDEEEDNDEPLLSGSGDVSKECAEKILET
WGELLSKWHLNLNVRPKQLSSLVRNGVPEALRGEVWQLLAGCHNNDHLVEKYRILITKES
PQDSAITRDINRTFPAHDYFKDTGGDGQDSLYKICKAYSVYDEEIGYCQGQSFLAAVLLL
HMPEEQAFSVLVKIMFDYGLRELFKQNFEDLHCKFYQLERLMQEYIPDLYNHFLDISLEA
HMYASQWFLTLFTAKFPLYMVFHIIDLLLCEGISVIFNVALGLLKTSKDDLLLTDFEGAL
KFFRVQLPKRYRSEENAKKLMELACNMKISQKKLKKYEKEYHTMREQQAQQEDPIERFER
ENRRLQEANMRLEQENDDLAHELVTSKIALRKDLDNAEEKADALNKELLMTKQKLIDAEE
EKRRLEEESAQLKEMCRRELDKAESEIKKNSSIIGDYKQICSQLSERLEKQQTANKVEIE
KIRQKVDDCERCREFFNKEGRVKGISSTKEVLDEDTDEEKETLKNQLREMELELAQTKLQ
LVEAECKIQDLEHHLGLALNEVQAAKKTWFNRTLSSIKTATGVQGKETC
>Q9ERR1
MDGEDIPDFSSLKEETAYWKELSLKYKQSFQEARDELVEFQEGSRELEAELEAQLVQAEQ
RNRDLQADNQRLKYEVEALKEKLEHQYAQSYKQVSVLEDDLSQTRAIKEQLHKYVRELEQ
ANDDLERAKRATIVSLEDFEQRLNQAIERNAFLESELDEKESLLVSVQRLKDEARDLRQE
LAVRERQQEVTRKSAPSSPTLDCEKMDSAVQASLSLPATPVGKGTENSFPSPKAIPNGFG
TSPLTPSARISALNIVGDLLRKVGALESKLAACRNFAKDQASRKSYVPGSVNCGVMNSNG
PECPRSGRATFFHKGAVNGFDPAPPPPGLGSSRPSSAPGMLPLSV
>Q23529
MLDLTNKESESSDNGNSKYEDSIDGREVGTSKPFKEERSLEDLQADLADMAVWMEGLDAT
KLPLNDPQLLCNGRAFSEVLHNVDKNFFTDGWLETMPENRTTNIMVFRSCTRKLWRKMFD
YVNHINRTVVSSRWTDIHERIDGIYESDLPAMVNLGMAVVTLAHIGKNAKRFVDYSKALT
STHKSMMSNVAKMVTTVIDEMPENPCFHEISELHGSQSELNSLSESSGKLNGNGSSERRS
NADQILVDAELEIERLRTETENQRKEIERLTKSFETAQHDMSSNSESGDISILEKQNEEL
RQKRRELEEKNLELDAAVDQFKGIVFELTNENDVLRRSDKERQRLQTVLDAAQSDLDEWK
TVANQYQKEAELSKQQDKEIKELLSQNKALKSRLDHHVKSATLEDANKNGIAQLRTQVGG
LTALNTELKASLDSKKRCVEQLEIQLIQHKEKVKELEDRKDELIEERNRLENQLIFKEAV
TPRSLHESMFEAGNLSFEPFSEKNTLPLEIENKRLTERIQELESLEPLKGELITLKSKNG
VLEEEKLFATKQIEELQQQIEDLQENLLKNQEHASGDVVGLKIQLEKAEVEAQQMREAKM
RAETNQAQVDEILKKRTAELEVNATALQKAKAVIDELEYNSRPVSEDSMTSVQAFKEMKE
ENEKLRQKVEKLEIELNTVTQGFEQENRLLTSASHQQVLNRSIDEVMSMRAHAGSEEPQT
LLDTQKMSGALPWRSLASETRRELPTAMASILVLGFLVFIAWMFININSALNAPPNA
>Q16584
MEPLKSLFLKSPLGSWNGSGSGGGGGGGGGRPEGSPKAAGYANPVWTALFDYEPSGQDEL

ALRKGDRVEVLSRDAAISGDEGWWAGQVGGQVGIFPSNYVSRGGGPPPCEVASFQELRLE
EVIGIGGFGKVYRGSWRGELVAVKAARQDPDEDISVTAESVRQEARLFAMLAHPNIIALK
AVCLEEPNLCLVMEYAAGGPLSRALAGRRVPPHVLVNWAVQIARGMHYLHCEALVPVIHR
DLKSNNILLLQPIESDDMEHKTLKITDFGLAREWHKTTQMSAAGTYAWMAPEVIKASTFS
KGSDVWSFGVLLWELLTGEVPYRGIDCLAVAYGVAVNKLTLPIPSTCPEPFAQLMADCWA
QDPHRRPDFASILQQLEALEAQVLREMPRDSFHSMQEGWKREIQGLFDELRAKEKELLSR
EEELTRAAREQRSQAEQLRRREHLLAQWELEVFERELTLLLQQVDRERPHVRRRRGTFKR
SKLRARDGGERISMPLDFKHRITVQASPGLDRRRNVFEVGPGDSPTFPRFRAIQLEPAEP
GQAWGRQSPRRLEDSSNGERRACWAWGPSSPKPGEAQNGRRRSRMDEATWYLDSDDSSPL
GSPSTPPALNGNPPRPSLEPEEPKRPVPAERGSSSGTPKLIQRALLRGTALLASLGLGRD
LQPPGGPGRERGESPTTPPTPTPAPCPTEPPPSPLICFSLKTPDSPPTPAPLLLDLGIPV
GQRSAKSPRREEEPRGGTVSPPPGTSRSAPGTPGTPRSPPLGLISRPRPSPLRSRIDPWS
FVSAGPRPSPLPSPQPAPRRAPWTLFPDSDPFWDSPPANPFQGGPQDCRAQTKDMGAQAP
WVPEAGP
>Q9H4X1
MKPPAAQGSPAAAAAAAPALDSAAAEDLSDALCEFDAVLADFASPFHERHFHYEEHLERM
KRRSSASVSDSSGFSDSESADSLYRNSFSFSDEKLNSPTDSTPALLSATVTPQKAKLGDT
KELEAFIADLDKTLASM
>Q8N4C6
MDEVEQDQHEARLKELFDSFDTTGTGSLGQEELTDLCHMLSLEEVAPVLQQTLLQDNLLG
RVHFDQFKEALILILSRTLSNEEHFQEPDCSLEAQPKYVRGGKRYGRRSLPEFQESVEEF
PEVTVIEPLDEEARPSHIPAGDCSEHWKTQRSEEYEAEGQLRFWNPDDLNASQSGSSPPQ
DWIEEKLQEVCEDLGITRDGHLNRKKLVSICEQYGLQNVDGEMLEEVFHNLDPDGTMSVE
DFFYGLFKNGKSLTPSASTPYRQLKRHLSMQSFDESGRRTTTSSAMTSTIGFRVFSCLDD
GMGHASVERILDTWQEEGIENSQEILKALDFSLDGNINLTELTLALENELLVTKNSIHQA
ALASFKAEIRHLLERVDQVVREKEKLRSDLDKAEKLKSLMASEVDDHHAAIERRNEYNLR
KLDEEYKERIAALKNELRKEREQILQQAGKQRLELEQEIEKAKTEENYIRDRLALSLKEN
SRLENELLENAEKLAEYENLTNKLQRNLENVLAEKFGDLDPSSAEFFLQEERLTQMRNEY
ERQCRVLQDQVDELQSELEEYRAQGRVLRLPLKNSPSEEVEANSGGIEPEHGLGSEECNP
LNMSIEAELVIEQMKEQHHRDICCLRLELEDKVRHYEKQLDETVVSCKKAQENMKQRHEN
ETHTLEKQISDLKNEIAELQGQAAVLKEAHHEATCRHEEEKKQLQVKLEEEKTHLQEKLR
LQHEMELKARLTQAQASFEREREGLQSSAWTEEKVRGLTQELEQFHQEQLTSLVEKHTLE
KEELRKELLEKHQRELQEGREKMETECNRRTSQIEAQFQSDCQKVTERCESALQSLEGRY
RQELKDLQEQQREEKSQWEFEKDELTQECAEAQELLKETLKREKTTSLVLTQEREMLEKT
YKEHLNSMVVERQQLLQDLEDLRNVSETQQSLLSDQILELKSSHKRELREREEVLCQAGA
SEQLASQRLERLEMEHDQERQEMMSKLLAMENIHKATCETADRERAEMSTEISRLQSKIK
EMQQATSPLSMLQSGCQVIGEEEVEGDGALSLLQQGEQLLEENGDVLLSLQRAHEQAVKE
NVKMATEISRLQQRLQKLEPGLVMSSCLDEPATEFFGNTAEQTEPFLQQNRTKQVEGVTR
RHVLSDLEDDEVRDLGSTGTSSVQRQEVKIEESEASVEGFSELENSEETRTESWELKNQI
SQLQEQLMMLCADCDRASEKKQDLLFDVSVLKKKLKMLERIPEASPKYKLLYEDVSREND
CLQEELRMMETRYDEALENNKELTAEVFRLQDELKKMEEVTETFLSLEKSYDEVKIENEE
LNVLVLRLQGKIEKLQESVVQRCDCCLWEASLENLEIEPDGNILQLNQTLEECVPRVRSV
HHVIEECKQENQYLEGNTQLLEKVKAHEIAWLHGTIQTHQERPRVQNQVILEENTTLLGF
QDKHFQHQATIAELELEKTKLQELTRKLKERVTILVKQKDVLSHGEKEEELKAMMHDLQI

TCSEMQQKVELLRYESEKLQQENSILRNEITTLNEEDSISNLKLGTLNGSQEEMWQKTET
VKQENAAVQKMVENLKKQISELKIKNQQLDLENTELSQKNSQNQEKLQELNQRLTEMLCQ
KEKEPGNSALEEREQEKFNLKEELERCKVQSSTLVSSLEAELSEVKIQTHIVQQENHLLK
DELEKMKQLHRCPDLSDFQQKISSVLSYNEKLLKEKEALSEELNSCVDKLAKSSLLEHRI
ATMKQEQKSWEHQSASLKSQLVASQEKVQNLEDTVQNVNLQMSRMKSDLRVTQQEKEALK
QEVMSLHKQLQNAGGKSWAPEIATHPSGLHNQQKRLSWDKLDHLMNEEQQLLWQENERLQ
TMVQNTKAELTHSREKVRQLESNLLPKHQKHLNPSGTMNPTEQEKLSLKRECDQFQKEQS
PANRKVSQMNSLEQELETIHLENEGLKKKQVKLDEQLMEMQHLRSTATPSPSPHAWDLQL
LQQQACPMVPREQFLQLQRQLLQAERINQHLQEELENRTSETNTPQGNQEQLVTVMEERM
IEVEQKLKLVKRLLQEKVNQLKEQVSLPGHLCSPTSHSSFNSSFTSLYCH
>Q91785
MPPGTKGDPSNVTQPLPCLPSAEEDAIKVFVRIRPPVEGTLTGVDGEQGLCLTALSSTTI
RLHSKPEPKMFTFDHVANVDTNQESVFSSVAKNIVESCMNGYNGTIFAYGQTGSGKTFTM
LGPSESDNFTHNLRGVIPRSFEYLFFLINREKEKAGEGKSFLCKCSFIEIYNEQIFDLLD
SASAGLFLREHIKKGVFVVGAVEQVVTSAAEAYQVLSMGWRNRRVASTSMNRESSRSHAV
FTVTIESMEKTNDLVNIRSSQLNLVDLAGSERQKDTQTEGVRLKEAGSINRSLSCLGQVI
TALVDVANGRQRHICYRDSKLTFLLRDSLGGNAKTFYIANVHPGSKCFGETLSTLQFAQR
AKLIKNKAVVNEDTQGNVSQLQAEVKKLKEQLSQLLSGQMPGDISVARVPSVGDNNMDYM
NNFIEAMMILEKSDREKKVLLQKVVQLEDLCNKKEKFIQSNKMIVKFREDHISRLEKAHK
EGRISLSNNEQDDFIAELKEEIRTLKEQVEHHPRVAKYALENHSLREENKRLHSLQSVKR
AQEVTAQMIAELEKAFLEVSVSEKDRQVAPMHSTPIQLDNNSLMSAARMRERMLQLESEL
ATSKQEYEEFKELTKKKQVEQESELQSLIKSNQHLENILEAIKANKRHEVSQLNRMHAAE
TIKNMTTPTKSYNLRSRLVPRLSPDAMPNGLMDTPKSGDVMDDIINEPIPPEMSEQAYEA
IAEELRIVQEQVTALQAKLDEEEGKNIRLQQQVNKLELCSTQIQELFNSERSNWNKEQQD
LIAQIKSLEKQKQENKSQEDVLKSEVHDLRVVLQSADRELGAVKGEYSLYREKQEKELSQ
LSARHMDVQLQLDNVRLEHETLLEEKRSLQDAFDNLEEVMKFEIDQLKQEISDSKHENET
LRAEFSNLLELLETEKERRQKLTSQLEEDKENKTKELLQVVDENMHLRKQCSELMTKCEQ
QVTELHGLEHSLTSSKEMIADLEKKNTADKEVVADLMNQIQVHRTTIIHKTESIDLLTRE
LEDIHSKYSIVLLAKEESKTVIEEQEKQIEELRECLERKQSADNIEKELLCDDLAHATEE
LEKLTEAFNKQEALLHTHEKELVEKEQQISELTNQVKLMTDLEISREQEKIRPASSNSSS
PVVLPETPRTPEGNPYDSEIANLQKRNTNLEILVSELNEERTSKNEEIIRLKMQLCETEN
MRLEIQNLQGMCKELKSQLENCNNVMKDSNDQKPSDMQDLKREIEKEVSERMEKGKATEH
ILKLQAELEETRNILCTKDHSLNELSKEIERTRSLEAKAFTEKEEIRSILEGKYEETEKL
SHELDMLRKQVLFLAEENGKILGHQNPNQKIQYLVKLKKENNKLLEEAEKLRIENLFLKE
SKKCEHCN
>Q9Y6D9
MEDLGENTMVLSTLRSLNNFISQRVEGGSGLDISTSAPGSLQMQYQQSMQLEERAEQIRS
KSHLIQVEREKMQMELSHKRARVELERAASTSARNYEREVDRNQELLTRIRQLQEREAGA
EEKMQEQLERNRQCQQNLDAASKRLREKEDSLAQAGETINALKGRISELQWSVMDQEMRV
KRLESEKQELQEQLDLQHKKCQEANQKIQELQASQEARADHEQQIKDLEQKLSLQEQDAA
IVKNMKSELVRLPRLERELKQLREESAHLREMRETNGLLQEELEGLQRKLGRQEKMQETL
VGLELENERLLAKLQSWERLDQTMGLSIRTPEDLSRFVVELQQRELALKDKNSAVTSSAR
GLEKARQQLQEELRQVSGQLLEERKKRETHEALARRLQKRVLLLTKERDGMRAILGSYDS
ELTPAEYSPQLTRRMREAEDMVQKVHSHSAEMEAQLSQALEELGGQKQRADMLEMELKML

KSQSSSAEQSFLFSREEADTLRLKVEELEGERSRLEEEKRMLEAQLERRALQGDYDQSRT
KVLHMSLNPTSVARQRLREDHSQLQAECERLRGLLRAMERGGTVPADLEAAAASLPSSKE
VAELKKQVESAELKNQRLKEVFQTKIQEFRKACYTLTGYQIDITTENQYRLTSLYAEHPG
DCLIFKATSPSGSKMQLLETEFSHTVGELIEVHLRRQDSIPAFLSSLTLELFSRQTVA
>Q68CZ1
MSGPTDETAGDLPVKDTGLNLFGMGGLQETSTTRTMKSRQAVSRVSREELEDRFLRLHDE
NILLKQHARKQEDKIKRMATKLIRLVNDKKRYERVGGGPKRLGRDVEMEEMIEQLQEKVH
ELEKQNETLKNRLISAKQQLQTQGYRQTPYNNVQSRINTGRRKANENAGLQECPRKGIKF
QDADVAETPHPMFTKYGNSLLEEARGEIRNLENVIQSQRGQIEELEHLAEILKTQLRRKE
NEIELSLLQLREQQATDQRSNIRDNVEMIKLHKQLVEKSNALSAMEGKFIQLQEKQRTLR
ISHDALMANGDELNMQLKEQRLKCCSLEKQLHSMKFSERRIEELQDRINDLEKERELLKE
NYDKLYDSAFSAAHEEQWKLKEQQLKVQIAQLETALKSDLTDKTEILDRLKTERDQNEKL
VQENRELQLQYLEQKQQLDELKKRIKLYNQENDINADELSEALLLIKAQKEQKNGDLSFL
VKVDSEINKDLERSMRELQATHAETVQELEKTRNMLIMQHKINKDYQMEVEAVTRKMENL
QQDYELKVEQYVHLLDIRAARIHKLEAQLKDIAYDTKQYKFKPEIMPDDSVDEFGETIHL
ERGENLFEIHINKVTFSSEVLQASGDKEPVTFCTYAFYDFELQTTPVVRGLHPEYNFTSQ
YLVHVNDLFLQYIQKNTITLEVHQAYSTEYETIAACQLKFHEILEKSGRIFCTASLIGTK
GDIPNFGTVEYWFRLRVPMDQAIRLYRERAKALGYITSNFKGPEHMQSLSQQAPKTAQLS
STDSTDGNLNELHITIRCCNHLQSRASHLQPHPYVVYKFFDFADHDTAIIPSSNDPQFDD
HMYFPVPMNMDLDRYLKSESLSFYVFDDSDTQENIYIGKVNVPLISLAHDRCISGIFELT
DHQKHPAGTIHVILKWKFAYLPPSGSITTEDLGNFIRSEEPEVVQRLPPASSVSTLVLAP
RPKPRQRLTPVDKKVSFVDIMPHQSDETSPPLEDRKEISPEVEHIPEIEINMLTVPHVPK
VSQEGSVDEVKENTEKMQQGKDDVSLLSEGQLAEQSLASSEDETEITEDLEPEVEEDMSA
SDSDDCIIPGPISKNIKQSLALSPGLGCSSAISAHCNFRLPGSSDFPASASQVDGITGAC
HHSQPSEKIRIEIIALSLNDSQVTMDDTIQRLFVECRFYSLPAEETPVSLPKPKSGQWVY
YNYSNVIYVDKENNKAKRDILKAILQKQEMPNRSLRFTVVSDPPEDEQDLECEDIGVAHV
DLADMFQEGRDLIEQNIDVFDARADGEGIGKLRVTVEALHALQSVYKQYRDDLEA
>Q9PVV4
MATGGGPPDEALSDQDLPNWSHESLDDRLNNMHWNGQKKGNRSAEKNKKKFVECDLRLTN
DISPESSPGVGRRRARTPHTFPHTRYVSQMSVPEQAELEKLKQKINFSDLDQRSIGSDSQ
GRATAANNKRQLADNRKPFNFLPSQLNTNKEKSKSPPKREASTRSLTKDFLASALNKDFL
SNSQAFLEEESKREPAIDSSQVVSRLVQIRDYITKASSLRDDLVQKKDLSVNVERLSNLI
EHLKFQEKSYLKFLQKMLASENEEEDVRTVDSAVGSGSVVESTLLTFDVPSEASDTTGVD
PRQEAKEELKNMKKQHALLTRMLQQQEQLRTLKGRQAALLALQHKAEQAIAKMDESVVTE
TTGSVSGLSLTSELNEELNDLIQRFHNQLHDSEDPPAPDNRRQAESLSLAREVYRSRNSS
TSDTPLEDKSPLFNNVGVLLEKKQKMDTLLGELHTLQDQQLNNTAFVASSVSPRRSTEQR
TLGSAVSSALTSDNRAARSPVTIGAYQTASVNESEDEENQNPAEKLKKLKEVRKRLNELR
ELVHYYEQTSDMMTDAMNENTKDEDETEDSEYDSEQEDAEPTTNIRNPQYRSSWAQMNIN
SNNQSGTNNRDERQLNTECEINNRSAVNLRSFNMPSALDCLYNIEHSDKEEDGNRELDDE
DAEDQGSRASLSSQNSVADDVQSVDFEQKFNRLVAAKQKLKQLQDLVAMYGDDSESEPVA
PERSFSGDQFPPEATTLKQQPNNTRPNVSKAQKDIALKEQAREKFYESKLQQQQRELSQL
QEERKKLIEIQEKIQTLRKACPDLQLSTSSAGTNPANRQNRQMTTTTSTPDVNTNGNIVV
PAMPDPEDSSSVDNEVWSEIRKHQILREDLRQRRKQLETLMAEHQRRQGNTETTSAASIR
SDDSDTQGLQQQTRTEKTMATWGGSTQCALEEEEEEEEVDDEECLSDVHQIDIEEDEQDN

TSCENNSYPQNSIRKTSFNGRSSKDGWKNQCPLSVEGNHRPSPKTRQQQNVSMRRQENYR
WMSELSHVEEKEHWQEQIDQIKKQLDYSTSICQTLMRDQQALSYLLQSMITTPYSVMPSN
VGASQVQLIMHQLNQCYTQLNWQQSNVLRLKQMLNDLLVQQPQHLQGESHQREDRGSSAP
PLTSPNIFPNFSFLPPTMNLLNMPAFGSIPNVVPGMNFNPVFPHGFENFTQNVASHTDTP
LQPHDQNTSGKTEYMAFPKPFESHRSNSTEKERNPPKKPDESEQGRRVWVENHQKSDQEK
KPACFGAGLSAGGASAKLAEESRKVKQFDEVSVESLSSMPDPVDPTTVTKMFKSRKASAQ
ASLASKDKTPKAKNKKRKVFHQKSKGIKSCGFQAASASSASEPNQTTCKHAQTEEVVVGN
VIKTSSAQRVEKESKATEMSSEAGSDVSMFETLRDTIYSEVATLISQNESRPHFLIELFH
ELQLLNTDYLRQKALFALQDIVTRHVSEGNAKKHETCTKALESTGWMASTSELTPSESLV
TTDDEMYAKNSDGPVCQEGEQNDGDNISSLSTSSNFEPFATDDLGNTVIHFDQALARMRE
YERMKSETENGLVADCCNNLNAAASSLEGTNDEARGRAQHSVDDASGIPCPYIDSKQLDR
QIKAIMKEVIPFLKEHMEEVCSPNLLTSIRRLVLTLTQQNDESKEFVKFFHKQLGSILQD
SLAKFSSKKLKDCGEDLLVEISEVLFNELAFFRLMQDLDNNSTAVQGTVSRKPEAVVVLE
SFTKEADKEEKTCPEENFSATRETDDEDKDKDETETAEENRNFDAEVLSGKSDISEEDDL
DESLPVSISFTKAETQALTNYGSGEDENEDEENYEFEARPVDVQTSLETSSEIADETEKE
EMEVRPEANIENEKALSLAVNVMGELSIHEDQAKSDCDVLAHPSLLLSNEKATDFSGTAL
VNNVKSPVDSPGTSGAGSSDTESPVLVNDFETGSGNLSQKSDEDDFVKVEDLPLKLSLPQ
EQIMKDIEEEENKNNLCDEILNINDEENGADQLAGDPLALKEPDSPAIHPA
>Q1MSJ5
MLFPLQVAAVTSSVRDDPLEHCVSPRTRARSPEICKMADNLDEFIEEQKARLAEDKAELE
SDPPYMEMKGKLSAKLSENSKILISMAKENIPPNSQQTRGSLGIDYGLSLPLGEDYERKK
HKLKEELRQDYRRYLTQERLKLERNKEYNQFLRGKEESSEKFRQVEKSTEPKSQRNKKPI
GQVKPDLTSQIQTSCENSEGPRKDVLTPSEAYEELLNQRRLEEDRYRQLDDEIELRNRRI
IKKANEEVGISNLKHQRFASKAGIPDRRFHRFNEDRVFDRRYHRPDQDPEVSEEMDERFR
YESDFDRRLSRVYTNDRMHRNKRGNMPPMEHDGDVIEQSNIRISSAENKSAPDNETSKSA
NQDTCSPFAGMLFGGEDRELIQRRKEKYRLELLEQMAEQQRNKRREKDLELRVAASGAQD
PEKSPDRLKQFSVAPRHFEEMIPPERPRIAFQTPLPPLSAPSVPPIPSVHPVPSQNEDLR
SGLSSALGEMVSPRIAPLPPPPLLPPLATNYRTPYDDAYYFYGSRNTFDPSLAYYGSGMM
GVQPAAYVSAPVTHQLAQPVVNTVGQNELKITSDQVINSGLIFEDKPKPSKQSLQSYQEA
LQQQIREREERRKKEREEKEEYEAKLEAEMRTYNPWGKGGGGAPLRDAKGNLITDLNRMH
RQNIDAYHNPDARTYEDKRAVVSLDPNLATSNAENLEDAANKSSGHMQTQSSPFARGNVF
GEPPTELQIKQQELYKNFLRFQIEEKKQREEAERERLRIAEEKEERRLAEQRARIQQEYE
EEQEKKREKEEEQRLKNEEHIRLAEERQKEAERKKKEEEGKYNLQLQHYCERDNLIGEET
KHMRQPSPIVPALQNKIASKLQRPPSVDSIIRSFIHESSMSRAQSPPVPARKNQLRAEEE
KKNVIMELSEMRKQLRSEERRLQERLLHMDSDDEIPIRKKERNPMDIFDMARHRLQAPVR
RQSPKGLDAATFQNVHDFNELKDRDSETRVDLKFMYLDPPRDHHTLEIQQQALLREQQKR
LNRIKMQEGAKVDLDAIPSAKVREQRMPRDDTSDFLKNSLLESDSAFIGAYGETYPAIED
DVLPPPSQLPSARERRRNKWKGLDIDSSRPNVAPDGLSLKSISSVNVDELRVRNEERMRR
LNEFHNKPINTDDESSLVDPDDIMKHIGDDGSNSVATEPWLRPGTSETLKRFMAEQLNQE
QQQIPGKPGTFTWQGLSTAHG
>Q9BV73
METRSPGLNNMKPQSLQLVLEEQVLALQQQMAENQAASWRKLKNSQEAQQRQATLVRKLQ
AKVLQYRSWCQELEKRLEATGGPIPQRWENVEEPNLDELLVRLEEEQQRCESLAEVNTQL
RLHMEKADVVNKALREDVEKLTVDWSRARDELMRKESQWQMEQEFFKGYLKGEHGRLLSL

WREVVTFRRHFLEMKSATDRDLMELKAEHVRLSGSLLTCCLRLTVGAQSREPNGSGRMDG
REPAQLLLLLAKTQELEKEAHERSQELIQLKSQGDLEKAELQDRVTELSALLTQSQKQNE
DYEKMIKALRETVEILETNHTELMEHEASLSRNAQEEKLSLQQVIKDITQVMVEEGDNIA
QGSGHENSLELDSSIFSQFDYQDADKALTLVRSVLTRRRQAVQDLRQQLAGCQEAVNLLQ
QQHDQWEEEGKALRQRLQKLTGERDTLAGQTVDLQGEVDSLSKERELLQKAREELRQQLE
VLEQEAWRLRRVNVELQLQGDSAQGQKEEQQEELHLAVRERERLQEMLMGLEAKQSESLS
ELITLREALESSHLEGELLRQEQTEVTAALARAEQSIAELSSSENTLKTEVADLRAAAVK
LSALNEALALDKVGLNQQLLQLEEENQSVCSRMEAAEQARNALQVDLAEAEKRREALWEK
NTHLEAQLQKAEEAGAELQADLRDIQEEKEEIQKKLSESRHQQEAATTQLEQLHQEAKRQ
EEVLARAVQEKEALVREKAALEVRLQAVERDRQDLAEQLQGLSSAKELLESSLFEAQQQN
SVIEVTKGQLEVQIQTVTQAKEVIQGEVRCLKLELDTERSQAEQERDAAARQLAQAEQEG
KTALEQQKAAHEKEVNQLREKWEKERSWHQQELAKALESLEREKMELEMRLKEQQTEMEA
IQAQREEERTQAESALCQMQLETEKERVSLLETLLQTQKELADASQQLERLRQDMKVQKL
KEQETTGILQTQLQEAQRELKEAARQHRDDLAALQEESSSLLQDKMDLQKQVEDLKSQLV
AQDDSQRLVEQEVQEKLRETQEYNRIQKELEREKASLTLSLMEKEQRLLVLQEADSIRQQ
ELSALRQDMQEAQGEQKELSAQMELLRQEVKEKEADFLAQEAQLLEELEASHITEQQLRA
SLWAQEAKAAQLQLRLRSTESQLEALAAEQQPGNQAQAQAQLASLYSALQQALGSVCESR
PELSGGGDSAPSVWGLEPDQNGARSLFKRGPLLTALSAEAVASALHKLHQDLWKTQQTRD
VLRDQVQKLEERLTDTEAEKSQVHTELQDLQRQLSQNQEEKSKWEGKQNSLESELMELHE
TMASLQSRLRRAELQRMEAQGERELLQAAKENLTAQVEHLQAAVVEARAQASAAGILEED
LRTARSALKLKNEEVESERERAQALQEQGELKVAQGKALQENLALLTQTLAEREEEVETL
RGQIQELEKQREMQKAALELLSLDLKKRNQEVDLQQEQIQELEKCRSVLEHLPMAVQERE
QKLTVQREQIRELEKDRETQRNVLEHQLLELEKKDQMIESQRGQVQDLKKQLVTLECLAL
ELEENHHKMECQQKLIKELEGQRETQRVALTHLTLDLEERSQELQAQSSQIHDLESHSTV
LARELQERDQEVKSQREQIEELQRQKEHLTQDLERRDQELMLQKERIQVLEDQRTRQTKI
LEEDLEQIKLSLRERGRELTTQRQLMQERAEEGKGPSKAQRGSLEHMKLILRDKEKEVEC
QQEHIHELQELKDQLEQQLQGLHRKVGETSLLLSQREQEIVVLQQQLQEAREQGELKEQS
LQSQLDEAQRALAQRDQELEALQQEQQQAQGQEERVKEKADALQGALEQAHMTLKERHGE
LQDHKEQARRLEEELAVEGRRVQALEEVLGDLRAESREQEKALLALQQQCAEQAQEHEVE
TRALQDSWLQAQAVLKERDQELEALRAESQSSRHQEEAARARAEALQEALGKAHAALQGK
EQHLLEQAELSRSLEASTATLQASLDACQAHSRQLEEALRIQEGEIQDQDLRYQEDVQQL
QQALAQRDEELRHQQEREQLLEKSLAQRVQENMIQEKQNLGQEREEEEIRGLHQSVRELQ
LTLAQKEQEILELRETQQRNNLEALPHSHKTSPMEEQSLKLDSLEPRLQRELERLQAALR
QTEAREIEWREKAQDLALSLAQTKASVSSLQEVAMFLQASVLERDSEQQRLQDELELTRR
ALEKERLHSPGATSTAELGSRGEQGVQLGEVSGVEAEPSPDGMEKQSWRQRLEHLQQAVA
RLEIDRSRLQRHNVQLRSTLEQVERERRKLKREAMRAAQAGSLEISKATASSPTQQDGRG
QKNSDAKCVAELQKEVVLLQAQLTLERKQKQDYITRSAQTSRELAGLHHSLSHSLLAVAQ
APEATVLEAETRRLDESLTQSLTSPGPVLLHPSPSTTQAASR
>Q9NS23
MSGEPELIELRELAPAGRAGKGRTRLERANALRIARGTACNPTRQLVPGRGHRFQPAGPA
THTWCDLCGDFIWGVVRKGLQCARLSADCKFTCHYRCRALVCLDCCGPRDLGWEPAVERD
TNVDEPVEWETPDLSQAEIEQKIKEYNAQINSNLFMSLNKDGSYTGFIKVQLKLVRPVSV
PSSKKPPSLQDARRGPGRGTSVRRRTSFYLPKDAVKHLHVLSRTRAREVIEALLRKFLVV
DDPRKFALFERAERHGQVYLRKLLDDEQPLRLRLLAGPSDKALSFVLKENDSGEVNWDAF

SMPELHNFLRILQREEEEHLRQILQKYSYCRQKIQEALHACPLG
>O95684
MAATAAAVVAEEDTELRDLLVQTLENSGVLNRIKAELRAAVFLALEEQEKVENKTPLVNE
SLKKFLNTKDGRLVASLVAEFLQFFNLDFTLAVFQPETSTLQGLEGRENLARDLGIIEAE
GTVGGPLLLEVIRRCQQKEKGPTTGEGALDLSDVHSPPKSPEGKTSAQTTPSKIPRYKGQ
GKKKTSGQKAGDKKANDEANQSDTSVSLSEPKSKSSLHLLSHETKIGSFLSNRTLDGKDK
AGLCPDEDDMEGDSFFDDPIPKPEKTYGLRKEPRKQAGSLASLSDAPPLKSGLSSLAGAP
SLKDSESKRGNTVLKDLKLISDKIGSLGLGTGEDDDYVDDFNSTSHRSEKSEISIGEEIE
EDLSVEIDDINTSDKLDDLTQDLTVSQLSDVADYLEDVA
>Q95ZG3
MKQNQPNLNVNLNNRLQQQQLNQVAQQQLQQQPLKNTKQNPINNQTNGILNNKPITTSSN
IMNSDNILNNNNNNNTNKPSSTFNFNITNTPPPPVNKQKPITSSSTSKPSNTTTTTTTTT
TTTKPPSNSLPSSSSSKVKVNSLTSQNNNNNNSNTNTNMTTQPPTFNNLIKPQSLPSTTG
MNEIKNTPTRTTTTTSTQHTKYIKEDIVVKENNNRISNMNNNNISGASGGTTFNSYNYLP
NNTLDNFINNSNTTTTTTNNNTNNNNNNNNNNNIINNIKQQHELELLQQKHIEEMKILQN
EQLNQLKKQQKKLNESDTDGSDDDDDDDDEYGENMNLSPRTLRELKNIQRLKNLEKFSPP
YLITKKTSPSIKTLPKFPEWTKERPFLNYGYLANISTNYTSLILPSKYEQTKIQLSSIPN
YFREQILIEDLLSTMIGIEGECIHIGVVQENEDDSLQSNLVSFVVEGEVDPSNNDLVQRI
LPLCSYYSFVNDFIQIRYAYEWGIINHSLCFSIDQLLKEYLVLVSQLEGQLKSERLSLQR
MWFYLQPTIKTFEILYRVTSEIMRCNSHGVQVINLLLKLQSKYSSDKKTDDLFHYLIKAT
TLPFLEMLDLWIHQGIIRDTYFEFMIEENTDLKRDNINRDFNDLYWEQRYQIRQDQIPKY
LQSHALKILTTGKYLNVVRECNQSISQQQQHNKSDNNNNNNNNVKLNSQQQLLLLKLNTT
QQQQQQQLSSTSASMIPTTIIEYSKNENVYIDKIEKAYDYASGILLNLLINERHLISRLK
AIKHYFLLCKGDFFSHFMDTTYDELKKPLDQINMVKMNSLLQLSLRTSSISEEDEFKDDL
ECEFIPYKLVDQLLNIININNFNNPTTTTTTNTSSNNITATTNTSSNNTTINTTVNTTLI
QSTSAHEVLKTNKLFGIESLAFNYNVGWPLSLIISRKSLIKYQIIFRHLFLCKHVEKVLV
DTWSQHQFRRSSYNKPGLSTLLSFTHLLRHRMIHFLQNLEYYMMLEVLEPNWNKMKNSIK
TSKTVDDVIRIHNDFLETCLTECMLTDTKLVNILMKFMSLCITFANFTNEMMQDEKNIDT
EQIKKTISSSENKFHAILKVLMDTLKSFSKTESNRHMIHLIHRLDYNGYYSHYFENNPNL
FKNKTSSSSNKSNNVSPPLEPRFSYQQQQQQQQQQQQQQQQQQQQQQQQQQQQQQQQQQQ
QQQQQQQQQQQQQQQQKQNQIQQQKQNQIQQQQQQSRGSTLPSNFSFQTALQQQINTTSA
TTTTSNQSPHTLIDDLKKRAYGILDSNNTKKTLERTSSSPALDNQSNSLPMTQSISQYAE
KLKNLRAVIKNDELK
>Q8TAM2
MSSEMEPLLLAWSYFRRRKFQLCADLCTQMLEKSPYDQAAWILKARALTEMVYIDEIDVD
QEGIAEMMLDENAIAQVPRPGTSLKLPGTNQTGGPSQAVRPITQAGRPITGFLRPSTQSG
RPGTMEQAIRTPRTAYTARPITSSSGRFVRLGTASMLTSPDGPFINLSRLNLTKYSQKPK
LAKALFEYIFHHENDVKTIHLEDVVLHLGIYPFLLRNKNHIEKNALDLAALSTEHSQYKD
WWWKVQIGKCYYRLGMYREAEKQFKSALKQQEMVDTFLYLAKVYVSLDQPVTALNLFKQG
LDKFPGEVTLLCGIARIYEEMNNMSSAAEYYKEVLKQDNTHVEAIACIGSNHFYSDQPEI
ALRFYRRLLQMGIYNGQLFNNLGLCCFYAQQYDMTLTSFERALSLAENEEEAADVWYNLG
HVAVGIGDTNLAHQCFRLALVNNNNHAEAYNNLAVLEMRKGHVEQARALLQTASSLAPHM
YEPHFNFATISDKIGDLQRSYVAAQKSEAAFPDHVDTQHLIKQLRQHFAML
>Q5JTW2

MIDSVKLRRDSAADFFSHYEYLCALQNSVPLPAVRACLREGVLDFNADRLRGVDWAPLLS
TLKINKDLPLVSIKSFFQPWLGDTGSDMNKFCRSRVPAIRYKDVTFQLCKALKGCLSISS
VLKNLELNGLILRERDLTILAKGLNKSASLVHLSLANCPIGDGGLEIICQGIKSSITLKT
VNFTGCNLTWQGADHMAKILKYQTMRRHEETWAESLRYRRPDLDCMAGLRRITLNCNTLI
GDLGACAFADSLSEDLWLRALDLQQCGLTNEGAKALLEALETNTTLVVLDIRKNPLIDHS
MMKAVIKKVLQNGRSAKSEYQWITSPSVKEPSKTAKQKRRTIILGSGHKGKATIRIGLAT
KKPVSSGRKHSLGKEYYAPAPLPPGVSGFLPWRTAERAKRHRGFPLIKTRDICNQLQQPG
FPVTVTVESPSSSEVEEVDDSSESVHEVPEKTSIEQEALQEKLEECLKQLKEERVIRLKV
DKRVSELEHENAQLRNINFSLSEALHAQSLTNMILDDEGVLGSIENSFQKFHAFLDLLKD
AGLGQLATMAGIDQSDFQLLGHPQMTSTVSNPPKEEKKALEDEKPEPKQNALGQMQNIQF
QKITGDARIPLPLDSFPVPVSTPEGLGTSSNNLGVPATEQRQESFEGFIARMCSPSPDAT
SGTGSQRKEEELSRNSRSSSEKKTKTESH
>Q9Y2Y0
MDALEGESFALSFSSASDAEFDAVVGYLEDIIMDDEFQLLQRNFMDKYYLEFEDTEENKL
IYTPIFNEYISLVEKYIEEQLLQRIPEFNMAAFTTTLQHHKDEVAGDIFDMLLTFTDFLA
FKEMFLDYRAEKEGRGLDLSSGLVVTSLCKSSSLPASQNNLRH
>Q9WU62
MGTTAPGPICLLDLCDQKLLDFVCNVDNKDFMWLKEIEEEAERMFIREFSNEPELMPKTP
SQKNRRKKRRVSNIQDENRDPVRKRLSRRKSRSSQVGTRHLRSKPVTIVEENGFPVLQRI
TRATAAAAAAAAAASVASASSSSTAGSPTVLTKKAVVEISTSERLSAELQLTKLKGSLPP
SPVSQGTLTSEEELTPKKSEAGKLDSVTVNSLKATPQSPKNRGVGEGRSVSKLKIARASW
GLQDSPGSTDSPWQERVLSPILLNNILPTTAKSPLGNIRSVRRSLISQDSQVPLASKYNL
VAKQENGSRRSSRRIAKKAGKEPEASARIICHSYLERLLNVEVPQNVGLEQEPVEVAEPE
EAEEEQEVSKNSGCPSKPRSATKIAISTPTSKPAAAGQTTTVEEQEAELDQTDGHREPPQ
SVRRKRSYKQAISEPDEEQLEDEELQPCQNKTPSPPCPANKVVRPLRTFLHTVQKNQMLM
TPTLASRSSVMKSFIKRNTPLRVDPKCSFVEKERQRLESLRRKEEAEQRRRQKVEEDKRR
RLEEVKLKREERLRKVLQARERVEQMKEEKKKQIEQKFAQIDEKTEKAKEERLAEKAKKK
ATAKKMEEVEARRKQEEEARRLRWLQQEEEERRHQEMLQRKKEEEQERRKAAEARRLAEQ
REQERRREQERREQERREQERREQERKEQERREQEQERLRAKREMQEREKALRLQKERLQ
KELEEKKRKEEQQRLAEQQLQEEQAKKAKEVAAARKVLNMTVDVQSPVCTSYQMTPQGPK
SIPKISVDDYGMDLNSDDSTDDESHPRKPIPSWAKGTQLSQAIVHQYYHPPNILELFGSI
LPLDLEDIFKKRKTRYHKRTSSAVWNSPPLKATMVPSSGD
>Q91572
MAFPLKDDLGRAKDCWGCPSDTPALSTCSNADIFRRINAMLDNSLDFTGVCTTPNTKGKC
EHLQDYQDTEGPAASRMLFSTSHEPLPRGLPDTNDLCLGLQSLSLTGWDRPWSTQDSEAG
GHSSTPTAAQSVFSMLNSPMGKPSPLGFLTFDPIGSDLMEKYPTPLLRSSRLDSRSILDS
RSSSPSDSDTSGFSSGSDHLSDLISSLRISPPLHFLPLGGGVSRDPLKLGIGSRLDQDHA
ALAAATVSPLGITKGWPSTSVWPSWDLLDSAEDPFSIEREARLHRQAAAVNEATCTWSGQ
LPPRNYKNPVYSCKVFLGGVPWDITETGLINTFRVFGALSVEWPGKDGKHPRCPPKGNMP
KGYVYLVFESEKSVRALLQACSQDLLSQDGLSEHYFKMSSRRMACKEVQVIPWVLADSNF
VRSPSQRLDPSKTVFVGALHGMLNAEALASIMNDLFGGVVYAGIDTDKHKYPIGSGRVTF
NNQRSYLKAVSAAFVEIKTAKFTKKVQIDPYLEDSVCQVCNAQPGPFFCRDQVCFKYFCR
SCWHWQHSMEILRHHRPLMRNQKSRDSS
>Q9UGJ1

MIHELLLALSGYPGSIFTWNKRSGLQVSQDFPFLHPSETSVLNRLCRLGTDYIRFTEFIE
QYTGHVQQQDHHPSQQGQGGLHGIYLRAFCTGLDSVLQPYRQALLDLEQEFLGDPHLSIS
HVNYFLDQFQLLFPSVMVVVEQIKSQKIHGCQILETVYKHSCGGLPPVRSALEKILAVCH
GVMYKQLSAWMLHGLLLDQHEEFFIKQGPSSGNVSAQPEEDEEDLGIGGLTGKQLRELQD
LRLIEEENMLAPSLKQFSLRVEILPSYIPVRVAEKILFVGESVQMFENQNVNLTRKGSIL
KNQEDTFAAELHRLKQQPLFSLVDFEQVVDRIRSTVAEHLWKLMVEESDLLGQLKIIKDF
YLLGRGELFQAFIDTAQHMLKTPPTAVTEHDVNVAFQQSAHKVLLDDDNLLPLLHLTIEY
HGKEHKADATQAREGPSRETSPREAPASGWAALGLSYKVQWPLHILFTPAVLEKYNVVFK
YLLSVRRVQAELQHCWALQMQRKHLKSNQTDAIKWRLRNHMAFLVDNLQYYLQVDVLESQ
FSQLLHQINSTRDFESIRLAHDHFLSNLLAQSFILLKPVFHCLNEILDLCHSFCSLVSQN
LGPLDERGAAQLSILVKGFSRQSSLLFKILSSVRNHQINSDLAQLLLRLDYNKYYTQAGG
TLGSFGM
>P23116
MPAYFQRPENALKRANEFLEVGKKQPALDVLYDVMKSKKHRTWQKIHEPIMLKYLELCVD
LRKSHLAKEGLYQYKNICQQVNIKSLEDVVRAYLKLAEEKTEAAKEESQQMVLDIEDLDN
IQTPESVLLSAVSGEDTQDRTDRLLLTPWVKFLWESYRQCLDLLRNNSRVERLYHDIAQQ
AFKFCLQYTRKAEFRKLCDNLRMHLSQIQRHHNQSTAINLNNPESQSMHLETRLVQLDSA
ISMELWQEAFKAVEDIHGLFSLSKKPPKPQLMANYYNKVSTVFWKSGNALFHASTLHRLY
HLSREMRKNLTQEEMQRMSTRVLLATLSIPITPERTDIARLLDMDGIIVEKQRRLATLLG
LQAPPTRIGLINDMVRFSVLQYVVPEVKDLYNWLEVEFNPLKLCERVTKVLNWVREQPEK
EPELQQYVPQLQNNTILRLLQQVAQIYQSIEFSRLTSLVPFVDAFQLERAIVDAARHCDL
QVRIDHTSRTLSFGSDLNYATREDAPVGPHLQSMPSEQIRNQLTAMSSVLAKAIEVIRPA
HILQEKEEQHQLAVNAYLKNSRKEHQRILARRQTIEERKERLESLNIQREKEELEQREAE
LQKVRKAEEERLRQEAKEREKERILQEHEQIKKKTVRERLEQIKKTELGAKAFKDIDIED
LEELDPDFIMAKQVEQLEKEKKELQERLKNQEKKIDYFERAKRLEEIPLIKSAYEEQRVK
DMDLWEQQEEERITTMQLEREKALEHKNRMSRMLEDRDLFVMRLKAARQSVYEEKLKQFE
ERLAEERHSRLEERKRQRKEERKITYYREKEEEEQRRAEEQMLKEREERERAERAKREEE
LREYQERVKKLEEVERKKRQRELEIEERERRREEERRLGDDPLSRKDSRWGDRDSEGTWR
KGPEADSEWRRGPPEKEWRRETRDDERPHRRDEDRLRRLGGDDEERESSLRPDDDRIPRR
GLDDDRGPRRGPDEDRFSRRGTDDDRPSWRNADDDRPPRRIGDDDRGSWRHTDDDRPPRR
GLDDERGSWRTADEDRGPRRGMDDDRGPRRGGADDERSSWRNADDDRGPRRGMDDDRGPR
RGLDDDRGPWRNAAEDRISRRGADDDRGPWRNMDDDRVPRRGDDARPGPWRPFVKPGGWR
EKEKAREESWGPPRESRPSEEREWDRDKEKDRDNQDREENDKDLERDRDRERDGDREDRF
RRPRDEGGWRRGPAEESSSWRDSSRRDDRDREDRRRDRDDRRDLRDLRDRRDLRDDRDRR
GPPLRSEREEASSWRRTDDRKDDRTEERDPPRRVPPPALSRDRERERERERGEKEKASWRA
EKDRESLRRTKNETDEDGWTTVRR
>Q66GS9
MTTAVERKYINIRKRLDQLGYRQTLTVECLPLVEKLFSDLVHTTESLRQSKLSAVKAEKE
SANFDFVLEPYKLENARLSRENNELYLELMKLREHSDQHVKELKTSLKKCARETADLKFL
NNQYAHKLKLLEKESKAKNERIQQLQEKNLHAVVQTPGGKKRSIAFRRQRMQIDEPVPPS
EVSSYPVPQPDDPYIADLLQVADNRIQELQQEVHQLQEKLAMMESGVRDYSKQIELRERE
IERLSVALDGGRSPDVLSLESRNKTNEKLIAHLNIQVDFLQQANKDLEKRIRELMETKET
VTSEVVNLSNKNEKLCQELTEIDQLAQQLERHKEELLETADKELGEAKKEIKRKLSEMQD
LEETMAKLQLELNLCQKEKERLSDELLVKSDLETVVHQLEQEKQRLSKKVESFAVTERQL

TLEVERMRLEHGIKRRDRSPSRLDTFLKGIEEERDYYKKELERLQHIIQRRSCSTSYSAR
EKSSIFRTPEKGDYNSEIHQITRERDELQRMLERFEKYMEDIQSNVKLLTAERDKLSVLY
NEAQEELSALRKESTQTTAPHNIVSLMEKEKELALSDLRRIMAEKEALREKLEHIEEVSL
FGKSELEKTIEHLTCVNHQLESEKYELKSKVLIMKETIESLENKLKVQAQKFSHVAGDSS
HQKTEVNSLRIVNEQLQRSVDDYQHRLSIKRGELESAQAQIKILEEKIDELNLKMTSQDE
EAHVMKKTIGVIDKEKDFLQETVDEKTEKIANLQENLANKEKAVAQMKIMISECESSVNQ
LKETLVNRDREINSLRRQLDAAHKELDEVGRSREIAFKENRRLQDDLATMARENQEISLE
LEAAVQEKEEMKSRVHKYITEVSRWESLMAAKEKENQDLLDRFQMLHNRAEDWEVKAHQA
EGESSSVRLELLSIDTERRHLRERVELLEKEIQEHINAHHAYESQISSMAKAMSRLEEEL
RHQEDEKATVLNDLSSLRELCIKLDSGKDIMTQQLNSKNLEFERVVVELENVKSESDLLK
KQLSNERHTVKNLESLLATNRDKEFHSHLTSHEKDTEIQLLKEKLTLSESKLTSQSRENT
MLRAKVAQLQTDYDALKRQISTERYERERAIQEMRRHGLATPPLSSTLRSPSHSPEHRNV

>O75161
MNDWHRIFTQNVLVPPHPQRARQPWKESTAFQCVLKWLDGPVIRQGVLEVLSEVECHLRV
SFFDVTYRHFFGRTWKTTVKPTKRPPSRIVFNEPLYFHTSLNHPHIVAVVEVVAEGKKRD
GSLQTLSCGFGILRIFSNQPDSPISASQDKRLRLYHGTPRALLHPLLQDPAEQNRHMTLI
ENCSLQYTLKPHPALEPAFHLLPENLLVSGLQQIPGLLPAHGESGDALRKPRLQKPITGH
LDDLFFTLYPSLEKFEEELLELHVQDHFQEGCGPLDGGALEILERRLRVGVHNGLGFVQR
PQVVVLVPEMDVALTRSASFSRKVVSSSKTSSGSQALVLRSRLRLPEMVGHPAFAVIFQL
EYVFSSPAGVDGNAASVTSLSNLACMHMVRWAVWNPLLEADSGRVTLPLQGGIQPNPSHC
LVYKVPSASMSSEEVKQVESGTLRFQFSLGSEEHLDAPTEPVSGPKVERRPSRKPPTSPS
SPPAPVPRVLAAPQNSPVGPGLSISQLAASPRSPTQHCLARPTSQLPHGSQASPAQAQEF
PLEAGISHLEADLSQTSLVLETSIAEQLQELPFTPLHAPIVVGTQTRSSAGQPSRASMVL
LQSSGFPEILDANKQPAEAVSATEPVTFNPQKEESDCLQSNEMVLQFLAFSRVAQDCRGT
SWPKTVYFTFQFYRFPPATTPRLQLVQLDEAGQPSSGALTHILVPVSRDGTFDAGSPGFQ
LRYMVGPGFLKPGERRCFARYLAVQTLQIDVWDGDSLLLIGSAAVQMKHLLRQGRPAVQA
SHELEVVATEYEQDNMVVSGDMLGFGRVKPIGVHSVVKGRLHLTLANVGHPCEQKVRGCS
TLPPSRSRVISNDGASRFSGGSLLTTGSSRRKHVVQAQKLADVDSELAAMLLTHARQGKG
PQDVSRESDATRRRKLERMRSVRLQEAGGDLGRRGTSVLAQQSVRTQHLRDLQVIAAYRE
RTKAESIASLLSLAITTEHTLHATLGVAEFFEFVLKNPHNTQHTVTVEIDNPELSVIVDS
QEWRDFKGAAGLHTPVEEDMFHLRGSLAPQLYLRPHETAHVPFKFQSFSAGQLAMVQASP
GLSNEKGMDAVSPWKSSAVPTKHAKVLFRASGGKPIAVLCLTVELQPHVVDQVFRFYHPE
LSFLKKAIRLPPWHTFPGAPVGMLGEDPPVHVRCSDPNVICETQNVGPGEPRDIFLKVAS
GPSPEIKDFFVIIYSDRWLATPTQTWQVYLHSLQRVDVSCVAGQLTRLSLVLRGTQTVRK
VRAFTSHPQELKTDPKGVFVLPPRGVQDLHVGVRPLRAGSRFVHLNLVDVDCHQLVASWL
VCLCCRQPLISKAFEIMLAAGEGKGVNKRITYTNPYPSRRTFHLHSDHPELLRFREDSFQ
VGGGETYTIGLQFAPSQRVGEEEILIYINDHEDKNEEAFCVKVIYQ
>P30305
MEVPQPEPAPGSALSPAGVCGGAQRPGHLPGLLLGSHGLLGSPVRAAASSPVTTLTQTMH
DLAGLGSETPKSQVGTLLFRSRSRLTHLSLSRRASESSLSSESSESSDAGLCMDSPSPMD
PHMAEQTFEQAIQAASRIIRNEQFAIRRFQSMPVRLLGHSPVLRNITNSQAPDGRRKSEA
GSGAASSSGEDKENDGFVFKMPWKPTHPSSTHALAEWASRREAFAQRPSSAPDLMCLSPD
RKMEVEELSPLALGRFSLTPAEGDTEEDDGFVDILESDLKDDDAVPPGMESLISAPLVKT

LEKEEEKDLVMYSKCQRLFRSPSMPCSVIRPILKRLERPQDRDTPVQNKRRRSVTPPEEQ
QEAEEPKARVLRSKSLCHDEIENLLDSDHRELIGDYSKAFLLQTVDGKHQDLKYISPETM
VALLTGKFSNIVDKFVIVDCRYPYEYEGGHIKTAVNLPLERDAESFLLKSPIAPCSLDKR
VILIFHCEFSSERGPRMCRFIRERDRAVNDYPSLYYPEMYILKGGYKEFFPQHPNFCEPQ
DYRPMNHEAFKDELKTFRLKTRSWAGERSRRELCSRLQDQ
>Q9UBK9
MATPPKRRAVEATGEKVLRYETFISDVLQRDLRKVLDHRDKVYEQLAKYLQLRNVIERLQ
EAKHSELYMQVDLGCNFFVDTVVPDTSRIYVALGYGFFLELTLAEALKFIDRKSSLLTEL
SNSLTKDSMNIKAHIHMLLEGLRELQGLQNFPEKPHH
>Q9UJW0
MASLLQSDRVLYLVQGEKKVRAPLSQLYFCRYCSELRSLECVSHEVDSHYCPSCLENMPS
AEAKLKKNRCANCFDCPGCMHTLSTRATSISTQLPDDPAKTTMKKAYYLACGFCRWTSRD
VGMADKSVASGGWQEPENPHTQRMNKLIEYYQQLAQKEKVERDRKKLARRRNYMPLAFSD
KYGLGTRLQRPRAGASISTLAGLSLKEGEDQKEIKIEPAQAVDEVEPLPEDYYTRPVNLT
EVTTLQQRLLQPDFQPVCASQLYPRHKHLLIKRSLRCRKCEHNLSKPEFNPTSIKFKIQL
VAVNYIPEVRIMSIPNLRYMKESQVLLTLTNPVENLTHVTLFECEEGDPDDINSTAKVVV
PPKELVLAGKDAAAEYDELAEPQDFQDDPDIIAFRKANKVGIFIKVTPQREEGEVTVCFK
MKHDFKNLAAPIRPIEESDQGTEVIWLTQHVELSLGPLLP
>Q80UF4
MAKSPGNSTLEDSLGQYQRSLRERANRSIHQLKCALREVDVTVEEDALDPSTSINVENED
TGVAWHELQHSHAVNQLKALLRQQTNKENETSPPRRRKLSPSRPSECDDGSMPTMHNLVP
IINDQSQYIHHLEAEVKFCKDELSGMKNRVQVVVLENERLQQELKSQRPEETLREQTFLD
ASGNMQNSWIMTREDSRVDEAAKRPFSHGDAETGKTASTGDANKWKLELERLKLTYEAKT
DLLESQLMLLRKDLAEYQKTCEDLKERLKHKESLLAASASSRVGGLCLKCAQHEAVLSQT
HSNVHIQTIERLTKERDDLMSVLVSVRSSLAEAQKRETSAYEQVKHAVQMTEEANFEKTK
ALIQCEQLKSELERQTERLEKELASQQEKRAVEKEMIKKEVAREREDAESKMLILSQNIA
KLEAQVEKVTREKTAAVSHLEEIQNHVASQEMDVTKVCGEMRFQLNKTKMEKDEVEKEHR
EYKAKSHKDLEMKVQEIEKLRLELSESEQHVEQEQQKAARARQECLRVTELLGEAERQLH
LTRLEKDSIQQSFSNEAKAQALQAQQREQELTQKIQQMETQHDKTESEQYLLLTSQNTFL
TKLKEECCLLAKKLEKVSLKSRSQIVRLSQEKRYLCDKLEKLQKRNDELEEQCIQHGRVH
ETMKERLRQLDKHGQATAQQLVQLLNKQNQLLLERQNLSEEVARLRAQLPSMPQSDC
>Q54I79
MSSVEFSNQPVVIDNGSGVIKAGFAGQDPPSHIFQSLVGNPKYKKVMGLNIENESTYFVG
DRINDWRGILKLKHPMDHGIVSNWSDMERVWTYTYDQLKIQPSEHPVLLTDVPNNPRLHR
ERAAQLFFETYNAPALYFSIPAVLSLYASGRTTGIVLDSGDGVTHVVPVFEGFALPHAIS
RIDIAGRDITEYLQHLLRRSGYNFKTSAEKEVVRIIKEKTCYVAHDPQKEEELLEPDSSS
SKPVQPQYTLPDGNVIELGAERFRAPEILFHPDIIGDESLGIHQCLDMSIRKSDLDLRKT
FYSNIILGGGSTLFQGFGDRLLNEVKKLAPKDIKIKITAPPERKYSAWMGGSILASLSTF
KDLWVTRQEYEEDGCSVIHRKIF
>Q9UNH5
MAAESGELIGACEFMKDRLYFATLRNRPKSTVNTHYFSIDEELVYENFYADFGPLNLAMV
YRYCCKLNKKLKSYSLSRKKIVHYTCFDQRKRANAAFLIGAYAVIYLKKTPEEAYRALLS
GSNPPYLPFRDASFGNCTYNLTILDCLQGIRKGLQHGFFDFETFDVDEYEHYERVENGDF
NWIVPGKFLAFSGPHPKSKIENGYPLHAPEAYFPYFKKHNVTAVVRLNKKIYEAKRFTDA

GFEHYDLFFIDGSTPSDNIVRRFLNICENTEGAIAVHCKAGLGRTGTLIACYVMKHYRFT
HAEIIAWIRICRPGSIIGPQQHFLEEKQASLWVQGDIFRSKLKNRPSSEGSINKILSGLD
DMSIGGNLSKTQNMERFGEDNLEDDDVEMKNGITQGDKLRALKSQRQPRTSPSCAFRSDD
TKGHPRAVSQPFRLSSSLQGSAVTLKTSKMALSPSATAKRINRTSLSSGATVRSFSINSR
LASSLGNLNAATDDPENKKTSSSSKAGFTASPFTNLLNGSSQPTTRNYPELNNNQYNRSS
NSNGGNLNSPPGPHSAKTEEHTTILRPSYTGLSSSSARFLSRSIPSLQSEYVHY
>Q61025
MAKDILGEAGLHFDELNKLRVLDPEVTQQTVELKEECKDFVDKIGQFQKIVGGLIELVDQ
LAKEAENEKMKAIGARNLLKSIAKQREAQQQQLQALIAEKKTQLERYRVEYEALCKVEAE
QNEFIDQFIFQK
>Q9Y6F1
MAPKPKPWVQTEGPEKKKGRQAGREEDPFRSTAEALKAIPAEKRIIRVDPTCPLSSNPGT
QVYEDYNCTLNQTNIENNNNKFYIIQLLQDSNRFFTCWNRWGRVGEVGQSKINHFTRLED
AKKDFEKKFREKTKNNWAERDHFVSHPGKYTLIEVQAEDEAQEAVVKVDRGPVRTVTKRV
QPCSLDPATQKLITNIFSKEMFKNTMALMDLDVKKMPLGKLSKQQIARGFEALEALEEAL
KGPTDGGQSLEELSSHFYTVIPHNFGHSQPPPINSPELLQAKKDMLLVLADIELAQALQA
VSEQEKTVEEVPHPLDRDYQLLKCQLQLLDSGAPEYKVIQTYLEQTGSNHRCPTLQHIWK
VNQEGEEDRFQAHSKLGNRKLLWHGTNMAVVAAILTSGLRIMPHSGGRVGKGIYFASENS
KSAGYVIGMKCGAHHVGYMFLGEVALGREHHINTDNPSLKSPPPGFDSVIARGHTEPDPT
QDTELELDGQQVVVPQGQPVPCPEFSSSTFSQSEYLIYQESQCRLRYLLEVHL
>Q8NEM0
MAAPILKDVVAYVEVWSSNGTENYSKTFTTQLVDMGAKVSKTFNKQVTHVIFKDGYQSTW
DKAQKRGVKLVSVLWVEKCRTAGAHIDESLFPAANMNEHLSSLIKKKRKCMQPKDFNFKT
PENDKRFQKKFEKMAKELQRQKTNLDDDVPILLFESNGSLIYTPTIEINSSHHSAMEKRL
QEMKEKRENLSPTSSQMIQQSHDNPSNSLCEAPLNISRDTLCSDEYFAGGLHSSFDDLCG
NSGCGNQERKLEGSINDIKSDVCISSLVLKANNIHSSPSFTHLDKSSPQKFLSNLSKEEI
NLQRNIAGKVVTPHQKQAAGMSQETFEEKYRLSPTLSSTKGHLLIHSRPRSSSVKRKRVS
HGSHSPPKEKCKRKRSTRRSIMPRLQLCRSEGRLQHVAGPALEALSCGESSYDDYFSPDN
LKERYSENLPPESQLPSSPAQLSCRSLSKKERTSIFEMSDFSCVGKKTRTVDITNFTAKT
ISSPRKTGNGEGRATSSCVTSAPEEALRCCRQAGKEDACPEGNGFSYTIEDPALPKGHDD
DLTPLEGSLEEMKEAVGLKSTQNKGTTSKISNSSEGEAQSEHEPCFIVDCNMETSTEEKE
NLPGGYSGSVKNRPTRHDVLDDSCDGFKDLIKPHEELKKSGRGKKPTRTLVMTSMPSEKQ
NVVIQVVDKLKGFSIAPDVCETTTHVLSGKPLRTLNVLLGIARGCWVLSYDWVLWSLELG
HWISEEPFELSHHFPAAPLCRSECHLSAGPYRGTLFADQPVMFVSPASSPPVAKLCELVH
LCGGRVSQVPRQASIVIGPYSGKKKATVKYLSEKWVLDSITQHKVCAPENYLLSQ
>Q7Z460
MEPRMESCLAQVLQKDVGKRLQVGQELIDYFSDKQKSADLEHDQTMLDKLVDGLATSWVN
SSNYKVVLLGMDILSALVTRLQDRFKAQIGTVLPSLIDRLGDAKDSVREQDQTLLLKIMD
QAANPQYVWDRMLGGFKHKNFRTREGICLCLIATLNASGAQTLTLSKIVPHICNLLGDPN
SQVRDAAINSLVEIYRHVGERVRADLSKKGLPQSRLNVIFTKFDEVQKSGNMIQSANDKN
FDDEDSVDGNRPSSASSTSSKAPPSSRRNVGMGTTRRLGSSTLGSKSSAAKEGAGAVDEE
DFIKAFDDVPVVQIYSSRDLEESINKIREILSDDKHDWEQRVNALKKIRSLLLAGAAEYD
NFFQHLRLLDGAFKLSAKDLRSQVVREACITLGHLSSVLGNKFDHGAEAIMPTIFNLIPN
SAKIMATSGVVAVRLIIRHTHIPRLIPVITSNCTSKSVAVRRRCFEFLDLLLQEWQTHSL

ERHISVLAETIKKGIHDADSEARIEARKCYWGFHSHFSREAEHLYHTLESSYQKALQSHL
KNSDSIVSLPQSDRSSSSSQESLNRPLSAKRSPTGSTTSRASTVSTKSVSTTGSLQRSRS
DIDVNAAASAKSKVSSSSGTTPFSSAAALPPGSYASLGRIRTRRQSSGSATNVASTPDNR
GRSRAKVVSQSQRSRSANPAGAGSRSSSPGKLLGSGYGGLTGGSSRGPPVTPSSEKRSKI
PRSQGCSRETSPNRIGLARSSRIPRPSMSQGCSRDTSRESSRDTSPARGFPPLDRFGLGQ
PGRIPGSVNAMRVLSTSTDLEAAVADALKKPVRRRYEPYGMYSDDDANSDASSVCSERSY
GSRNGGIPHYLRQTEDVAEVLNHCASSNWSERKEGLLGLQNLLKSQRTLSRVELKRLCEI
FTRMFADPHSKRVFSMFLETLVDFIIIHKDDLQDWLFVLLTQLLKKMGADLLGSVQAKVQ
KALDVTRDSFPFDQQFNILMRFIVDQTQTPNLKVKVAILKYIESLARQMDPTDFVNSSET
RLAVSRIITWTTEPKSSDVRKAAQIVLISLFELNTPEFTMLLGALPKTFQDGATKLLHNH
LKNSSNTSVGSPSNTIGRTPSRHTSSRTSPLTSPTNCSHGGLSPSRLWGWSADGLAKHPP
PFSQPNSIPTAPSHKALRRSYSPSMLDYDTENLNSEEIYSSLRGVTEAIEKFSFRSQEDL
NEPIKRDGKKECDIVSRDGGAASPATEGRGGSEVEGGRTALDNKTSLLNTQPPRAFPGPR
ARDYNPYPYSDAINTYDKTALKEAVFDDDMEQLRDVPIDHSDLVADLLKELSNHNERVEE
RKGALLELLKITREDSLGVWEEHFKTILLLLLETLGDKDHSIRALALRVLREILRNQPAR
FKNYAELTIMKTLEAHKDSHKEVVRAAEEAASTLASSIHPEQCIKVLCPIIQTADYPINL
AAIKMQTKVVERIAKESLLQLLVDIIPGLLQGYDNTESSVRKASVFCLVAIYSVIGEDLK
PHLAQLTGSKMKLLNLYIKRAQTTNSNSSSSSDVSTHS
>Q7TMK6
MSVDKAELCGSLLTWLQTFQVSPPCASPQDLSSGLAIAHVLNQIDPSWFNNEWLQGISED
SSPSWRLKVRKLEKILQSLVEYSKNVLGHPVSDQHLPDVSLIGEFSNPAELGKLLQLVLG
CAISCEKKQEYIQRIMTLEESVQHVVMEAIQELMTKDTPDSLSPENYGNFDTQSRRYYFL
SEEVEEGDHLQQHYLDLERQLLLLSEEKQNLAQENAALRERVGRSEVESAPGLTAKKLLL
LQSQLEQLQEENFRLESSREDDRLRCLELEREVAELQQRNQALTSLSQEAQALKDEMDEL
RQSSERARQLEATLNSCRRRLGELQELRRQVRQLEERNAGHAERTRQLEEELRRAGSLRA
QLEAQRRQVQELQGQWQEEAMKAEKWLFECRNLEEKCDLVTKEKERLLTERDSLREANEE
LRCAQLQPRGLAQADLSLDPTPSGLENLAAEILPAELRETLVRLQLENKRLCQQEAADRE
RQEELQRHLEEANRARHGLEAQQRLNQQQLSELRAQVEELQKALQEQGGKTEDPTLLKRK
LEDHLQKLHEADLELQRKREYIEELEPPTDSSTARRIEELQDSLQKKDADLRAMEERYRR
YVDKARTVIQTLEPKQRPPTVVSPEFHTLRSQLWERNLRIRQMEMDYEKSRRRQEQEEKL
LISAWYSMGMALEHRAGEEHAPAHAQSFLAQQRMATNARRGPLGRQALSLRPTDKH
>Q9NY27
MDVERLQEALKDFEKRGKKEVCPVLDQFLCHVAKTGETMIQWSQFKGYFIFKLEKVMDDF
RTSAPEPRGPPNPNVEYIPFDEMKERILKIVTGFNGIPFTIQRLCELLTDPRRNYTGTDK
FLRGVEKNVMVVSCVYPSSEKNNSNSLNRMNGVMFPGNSPSYTERSNINGPGTPRPLNRP
KVSLSAPMTTNGLPESTDSKEANLQQNEEKNHSDSSTSESEVSSVSPLKNKHPDEDAVEA
EGHEVKRLRFDKEGEVRETASQTTSSEISSVMVGETEASSSSQDKDKDSRCTRQHCTEED
EEEDEEEEEESFMTSREMIPERKNQEKESDDALTVNEETSEENNQMEESDVSQAEKDLLH
SEGSENEGPVSSSSSDCRETEELVGSNSSKTGEILSESSMENDDEATEVTDEPMEQD
>P34402
MASDENIGADGEQKPSRPFLRKGQGTARFRMPRNNKTSAGAPPTSELSSASSPSINVPRF
SLSNALPNSARTVDSGISNEDETRPPTTASLPMDQPSLSSSPENRLNPAPSVAEEHGHSG
QHAEEEEDNDTDEVSAMPSFVPDEPSTLVNSDHELSDDALKYKNAAAEFKAFERRMDSMR
SASTITTSLATPSSCAPSNSSEPPTRSTPIMNDLGVGPNNHNWPSSMQELSGISLETPQA

RPLGSNRINQLVRSEAQTGISLLQHHERPTVTAPLRRNDMMNSSRQNPQNGNVQDENRPE
HVYDQPIHVPGSSLDRQKLEIEIRRHRNLNIQLRDTIAHLDYAEESVHTTKRQLEEKISE
VNNFKKELIEEFKKCKKGVEEEFEKKFEKIKEDYDELYEKLKRDQRDLERDQKILKKGTG
ERNKEFTETIATLRDKLRASETKNAQYRQDIRVRDEKLKKKDEEIEKLQKDGNRLKSTLQ
TLEKRVKQLRTEKERDDKEKEMFAKVAMNRKTSNPVPPVLNQSVPISITSNGPSRHPSSS
SLTTFRKPSTSNRERGVSWADEPNEQSLEAVPQEFLMMPVKEMPGKFGKCTIYRDSLGET
SKVTDTIANGLLFEYSNGDLRWVNRQNAVNIYISAVDKTVRIDLPTYNISIIHTFQRQVE
VLRPGNNITLISIKRREVRTDLIYQNGMYKTEIFNRDGRYVTKDFSNQEVSRKYNPGTHT
YRDNQCRYVLVTDYNDFELVEPEFRLRWYQGDPTGLNNQYILKIIGRPECSEKTLRLEVN
LSTCEGTLETAEMIGDKRRKTTLFQWKK
>Q76N32
MALGEEKAEAEASEDTKAQSYGRGSCRERELDIPGPMSGEQPPRLEAEGGLISPVWGAEG
IPAPTCWIGTDPGGPSRAHQPQASDANREPVAERSEPALSGLPPATMGSGDLLLSGESQV
EKTKLSSSEEFPQTLSLPRTTTICSGHDADTEDDPSLADLPQALDLSQQPHSSGLSCLSQ
WKSVLSPGSAAQPSSCSISASSTGSSLQGHQERAEPRGGSLAKVSSSLEPVVPQEPSSVV
GLGPRPQWSPQPVFSGGDASGLGRRRLSFQAEYWACVLPDSLPPSPDRHSPLWNPNKEYE
DLLDYTYPLRPGPQLPKHLDSRVPADPVLQDSGVDLDSFSVSPASTLKSPTNVSPNCPPA
EATALPFSGPREPSLKQWPSRVPQKQGGMGLASWSQLASTPRAPGSRDARWERREPALRG
AKDRLTIGKHLDMGSPQLRTRDRGWPSPRPEREKRTSQSARRPTCTESRWKSEEEVESDD
EYLALPARLTQVSSLVSYLGSISTLVTLPTGDIKGQSPLEVSDSDGPASFPSSSSQSQLP
PGAALQGSGDPEGQNPCFLRSFVRAHDSAGEGSLGSSQALGVSSGLLKTRPSLPARLDRW
PFSDPDVEGQLPRKGGEQGKESLVQCVKTFCCQLEELICWLYNVADVTDHGTAARSNLTS
LKSSLQLYRQFKKDIDEHQSLTESVLQKGEILLQCLLENTPVLEDVLGRIAKQSGELESH
ADRLYDSILASLDMLAGCTLIPDKKPMAAMEHPCEGV
>Q99996
MEDEERQKKLEAGKAKIEELSLAFLVRQLAQFRQRKAQSDGQSPSKKQKKKRKTSSSKHD
VSAHHDLNIDQSQCNEMYINSSQRVESTVIPESTIMRTLHSGEITSHEQGFSVELESEIS
TTADDCSSEVNGCSFVMRTGKPTNLLREEEFGVDDSYSEQGAQDSPTHLEMMESELAGKQ
HEIEELNRELEEMRVTYGTEGLQQLQEFEAAIKQRDGIITQLTANLQQARREKDETMREF
LELTEQSQKLQIQFQQLQASETLRNSTHSSTAADLLQAKQQILTHQQQLEEQDHLLEDYQ
KKKEDFTMQISFLQEKIKVYEMEQDKKVENSNKEEIQEKETIIEELNTKIIEEEKKTLEL
KDKLTTADKLLGELQEQIVQKNQEIKNMKLELTNSKQKERQSSEEIKQLMGTVEELQKRN
HKDSQFETDIVQRMEQETQRKLEQLRAELDEMYGQQIVQMKQELIRQHMAQMEEMKTRHK
GEMENALRSYSNITVNEDQIKLMNVAINELNIKLQDTNSQKEKLKEELGLILEEKCALQR
QLEDLVEELSFSREQIQRARQTIAEQESKLNEAHKSLSTVEDLKAEIVSASESRKELELK
HEAEVTNYKIKLEMLEKEKNAVLDRMAESQEAELERLRTQLLFSHEEELSKLKEDLEIEH
RINIEKLKDNLGIHYKQQIDGLQNEMSQKIETMQFEKDNLITKQNQLILEISKLKDLQQS
LVNSKSEEMTLQINELQKEIEILRQEEKEKGTLEQEVQELQLKTELLEKQMKEKENDLQE
KFAQLEAENSILKDEKKTLEDMLKIHTPVSQEERLIFLDSIKSKSKDSVWEKEIEILIEE
NEDLKQQCIQLNEEIEKQRNTFSFAEKNFEVNYQELQEEYACLLKVKDDLEDSKNKQELE
YKSKLKALNEELHLQRINPTTVKMKSSVFDEDKTFVAETLEMGEVVEKDTTELMEKLEVT
KREKLELSQRLSDLSEQLKQKHGEISFLNEEVKSLKQEKEQVSLRCRELEIIINHNRAEN
VQSCDTQVSSLLDGVVTMTSRGAEGSVSKVNKSFGEESKIMVEDKVSFENMTVGEESKQE
QLILDHLPSVTKESSLRATQPSENDKLQKELNVLKSEQNDLRLQMEAQRICLSLVYSTHV

DQVREYMENEKDKALCSLKEELIFAQEEKIKELQKIHQLELQTMKTQETGDEGKPLHLLI
GKLQKAVSEECSYFLQTLCSVLGEYYTPALKCEVNAEDKENSGDYISENEDPELQDYRYE
VQDFQENMHTLLNKVTEEYNKLLVLQTRLSKIWGQQTDGMKLEFGEENLPKEETEFLSIH
SQMTNLEDIDVNHKSKLSSLQDLEKTKLEEQVQELESLISSLQQQLKETEQNYEAEIHCL
QKRLQAVSESTVPPSLPVDSVVITESDAQRTMYPGSCVKKNIDGTIEFSGEFGVKEETNI
VKLLEKQYQEQLEEEVAKVIVSMSIAFAQQTELSRISGGKENTASSKQAHAVCQQEQHYF
NEMKLSQDQIGFQTFETVDVKFKEEFKPLSKELGEHGKEILLSNSDPHDIPESKDCVLTI
SEEMFSKDKTFIVRQSIHDEISVSSMDASRQLMLNEEQLEDMRQELVRQYQEHQQATELL
RQAHMRQMERQREDQEQLQEEIKRLNRQLAQRSSIDNENLVSERERVLLEELEALKQLSL
AGREKLCCELRNSSTQTQNGNENQGEVEEQTFKEKELDRKPEDVPPEILSNERYALQKAN
NRLLKILLEVVKTTAAVEETIGRHVLGILDRSSKSQSSASLIWRSEAEASVKSCVHEEHT
RVTDESIPSYSGSDMPRNDINMWSKVTEEGTELSQRLVRSGFAGTEIDPENEELMLNISS
RLQAAVEKLLEAISETSSQLEHAKVTQTELMRESFRQKQEATESLKCQEELRERLHEESR
AREQLAVELSKAEGVIDGYADEKTLFERQIQEKTDIIDRLEQELLCASNRLQELEAEQQQ
IQEERELLSRQKEAMKAEAGPVEQQLLQETEKLMKEKLEVQCQAEKVRDDLQKQVKALEI
DVEEQVSRFIELEQEKNTELMDLRQQNQALEKQLEKMRKFLDEQAIDREHERDVFQQEIQ
KLEQQLKVVPRFQPISEHQTREVEQLANHLKEKTDKCSELLLSKEQLQRDIQERNEEIEK
LEFRVRELEQALLVSADTFQKVEDRKHFGAVEAKPELSLEVQLQAERDAIDRKEKEITNL
EEQLEQFREELENKNEEVQQLHMQLEIQKKESTTRLQELEQENKLFKDDMEKLGLAIKES
DAMSTQDQHVLFGKFAQIIQEKEVEIDQLNEQVTKLQQQLKITTDNKVIEEKNELIRDLE
TQIECLMSDQECVKRNREEEIEQLNEVIEKLQQELANIGQKTSMNAHSLSEEADSLKHQL
DVVIAEKLALEQQVETANEEMTFMKNVLKETNFKMNQLTQELFSLKRERESVEKIQSIPE
NSVNVAIDHLSKDKPELEVVLTEDALKSLENQTYFKSFEENGKGSIINLETRLLQLESTV
SAKDLELTQCYKQIKDMQEQGQFETEMLQKKIVNLQKIVEEKVAAALVSQIQLEAVQEYA
KFCQDNQTISSEPERTNIQNLNQLREDELGSDISALTLRISELESQVVEMHTSLILEKEQ
VEIAEKNVLEKEKKLLELQKLLEGNEKKQREKEKKRSPQDVEVLKTTTELFHSNEESGFF
NELEALRAESVATKAELASYKEKAEKLQEELLVKETNMTSLQKDLSQVRDHLAEAKEKLS
ILEKEDETEVQESKKACMFEPLPIKLSKSIASQTDGTLKISSSNQTPQILVKNAGIQINL
QSECSSEEVTEIISQFTEKIEKMQELHAAEILDMESRHISETETLKREHYVAVQLLKEEC
GTLKAVIQCLRSKEVFGFYNMCFSTLCDSGSDWGQGIYLTHSQGFDIASEGRGEESESAT
DSFPKKIKGLLRAVHNEGMQVLSLTESPYSDGEDHSIQQVSEPWLEERKAYINTISSLKD
LITKMQLQREAEVYDSSQSHESFSDWRGELLLALQQVFLEERSVLLAAFRTELTALGTTD
AVGLLNCLEQRIQEQGVEYQAAMECLQKADRRSLLSEIQALHAQMNGRKITLKREQESEK
PSQELLEYNIQQKQSQMLEMQVELSSMKDRATELQEQLSSEKMVVAELKSELAQTKLELE
TTLKAQHKHLKELEAFRLEVKDKTDEVHLLNDTLASEQKKSRELQWALEKEKAKLGRSEE
RDKEELEDLKFSLESQKQRNLQLNLLLEQQKQLLNESQQKIESQRMLYDAQLSEEQGRNL
ELQVLLESEKVRIREMSSTLDRERELHAQLQSSDGTGQSRPPLPSEDLLKELQKQLEEKH
SRIVELLNETEKYKLDSLQTRQQMEKDRQVHRKTLQTEQEANTEGQKKMHELQSKVEDLQ
RQLEEKRQQVYKLDLEGQRLQGIMQEFQKQELEREEKRESRRILYQNLNEPTTWSLTSDR
TRNWVLQQKIEGETKESNYAKLIEMNGGGTGCNHELEMIRQKLQCVASKLQVLPQKASER
LQFETADDEDFIWVQENIDEIILQLQKLTGQQGEEPSLVSPSTSCGSLTERLLRQNAELT
GHISQLTEEKNDLRNMVMKLEEQIRWYRQTGAGRDNSSRFSLNGGANIEAIIASEKEVWN
REKLTLQKSLKRAEAEVYKLKAELRNDSLLQTLSPDSEHVTLKRIYGKYLRAESFRKALI
YQKKYLLLLLGGFQECEDATLALLARMGGQPAFTDLEVITNRPKGFTRFRSAVRVSIAIS

RMKFLVRRWHRVTGSVSININRDGFGLNQGAEKTDSFYHSSGGLELYGEPRHTTYRSRSD
LDYIRSPLPFQNRYPGTPADFNPGSLACSQLQNYDPDRALTDYITRLEALQRRLGTIQSG
STTQFHAGMRR
>P11084
MAEISDLDRQIEQLLRCELIKESEVKALCAKAREILVEESNVQRVDSPVTVCGDIHGQFY
DLKELFRVGGDVPETNYLFMGDFVDRGFYSVETFLLLLALKVRYPDRITLIRGNHESRQI
TQVYGFYDECLRKYGSVTVWRYCTEIFDYLSLSAIIDGKIFCVHGGLSPSIQTLDQIRTI
DRKQEVPHDGPMCDLLWSDPEDTTGWGVSPRGAGYLFGSDVVAQFNAANDIDMICRAHQL
VMEGYKWHFNETVLTVWSAPNYCYRCGNVAAILELDEHLQKDFIIFEAAPQETRGIPSKK
PVADYFL
>Q7Z7A1
MKKGSQQKIFSKAKIPSSSHSPIPSSMSNMRSRSLSPLIGSETLPFHSGGQWCEQVEIAD
ENNMLLDYQDHKGADSHAGVRYITEALIKKLTKQDNLALIKSLNLSLSKDGGKKFKYIEN
LEKCVKLEVLNLSYNLIGKIEKLDKLLKLRELNLSYNKISKIEGIENMCNLQKLNLAGNE
IEHIPVWLGKKLKSLRVLNLKGNKISSLQDISKLKPLQDLISLILVENPVVTLPHYLQFT
IFHLRSLESLEGQPVTTQDRQEAFERFSLEEVERLERDLEKKMIETEELKSKQTRFLEEI
KNQDKLNKSLKEEAMLQKQSCEELKSDLNTKNELLKQKTIELTRACQKQYELEQELAFYK
IDAKFEPLNYYPSEYAEIDKAPDESPYIGKSRYKRNMFATESYIIDSAQAVQIKKMEPDE
QLRNDHMNLRGHTPLDTQLEDKEKKISAAQTRLSELHDEIEKAEQQILRATEEFKQLEEA
IQLKKISEAGKDLLYKQLSGRLQLVNKLRQEALDLELQMEKQKQEIAGKQKEIKDLQIAI
DSLDSKDPKHSHMKAQKSGKEQQLDIMNKQYQQLESRLDEILSRIAKETEEIKDLEEQLT
EGQIAANEALKKDLEGVISGLQEYLGTIKGQATQAQNECRKLRDEKETLLQRLTEVEQER
DQLEIVAMDAENMRKELAELESALQEQHEVNASLQQTQGDLSAYEAELEARLNLRDAEAN
QLKEELEKVTRLTQLEQSALQAELEKERQALKNALGKAQFSEEKEQENSELHAKLKHLQD
DNNLLKQQLKDFQNHLNHVVDGLVRPEEVAARVDELRRKLKLGTGEMNIHSPSDVLGKSL
ADLQKQFSEILARSKWERDEAQVRERKLQEEMALQQEKLATGQEEFRQACERALEARMNF
DKRQHEARIQQMENEIHYLQENLKSMEEIQGLTDLQLQEADEEKERILAQLRELEKKKKL
EDAKSQEQVFGLDKELKKLKKAVATSDKLATAELTIAKDQLKSLHGTVMKINQERAEELQ
EAERFSRKAAQAARDLTRAEAEIELLQNLLRQKGEQFRLEMEKTGVGTGANSQVLEIEKL
NETMERQRTEIARLQNVLDLTGSDNKGGFENVLEEIAELRREVSYQNDYISSMADPFKRR
GYWYFMPPPPSSKVSSHSSQATKDSGVGLKYSASTPVRKPRPGQQDGKEGSQPPPASGYW
VYSPIRSGLHKLFPSRDADSGGDSQEESELDDQEEPPFVPPPGYMMYTVLPDGSPVPQGM
ALYAPPPPLPNNSRPLTPGTVVYGPPPAGAPMVYGPPPPNFSIPFIPMGVLHCNVPEHHN
LENEVSRLEDIMQHLKSKKREERWMRASKRQSEKEMEELHHNIDDLLQEKKSLECEVEEL
HRTVQKRQQQKDFIDGNVESLMTELEIEKSLKHHEDIVDEIECIEKTLLKRRSELREADR
LLAEAESELSCTKEKTKNAVEKFTDAKRSLLQTESDAEELERRAQETAVNLVKADQQLRS
LQADAKDLEQHKIKQEEILKEINKIVAAKDSDFQCLSKKKEKLTEELQKLQKDIEMAERN
EDHHLQVLKESEVLLQAKRAELEKLKSQVTSQQQEMAVLDRQLGHKKEELHLLQGSMVQA
KADLQEALRLGETEVTEKCNHIREVKSLLEELSFQKGELNVQISERKTQLTLIKQEIEKE
EENLQVVLRQMSKHKTELKNILDMLQLENHELQGLKLQHDQRVSELEKTQVAVLEEKLEL
ENLQQISQQQKGEIEWQKQLLERDKREIERMTAESRALQSCVECLSKEKEDLQEKCDIWE
KKLAQTKRVLAAAEENSKMEQSNLEKLELNVRKLQQELDQLNRDKLSLHNDISAMQQQLQ
EKREAVNSLQEELANVQDHLNLAKQDLLHTTKHQDVLLSEQTRLQKDISEWANRFEDCQK
EEETKQQQLQVLQNEIEENKLKLVQQEMMFQRLQKERESEESKLETSKVTLKEQQHQLEK

ELTDQKSKLDQVLSKVLAAEERVRTLQEEERWCESLEKTLSQTKRQLSEREQQLVEKSGE
LLALQKEADSMRADFSLLRNQFLTERKKAEKQVASLKEALKIQRSQLEKNLLEQKQENSC
IQKEMATIELVAQDNHERARRLMKELNQMQYEYTELKKQMANQKDLERRQMEISDAMRTL
KSEVKDEIRTSLKNLNQFLPELPADLEAILERNENLEGELESLKENLPFTMNEGPFEEKL
NFSQVHIMDEHWRGEALREKLRHREDRLKAQLRHCMSKQAEVLIKGKRQTEGTLHSLRRQ
VDALGELVTSTSADSASSPSLSQLESSLTEDSQLGQNQEKNASAR
>Q96RK4
MAEERVATRTQFPVSTESQKPRQKKAPEFPILEKQNWLIHLHYIRKDYEACKAVIKEQLQ
ETQGLCEYAIYVQALIFRLEGNIQESLELFQTCAVLSPQSADNLKQVARSLFLLGKHKAA
IEVYNEAAKLNQKDWEISHNLGVCYIYLKQFNKAQDQLHNALNLNRHDLTYIMLGKIHLL
EGDLDKAIEVYKKAVEFSPENTELLTTLGLLYLQLGIYQKAFEHLGNALTYDPTNYKAIL
AAGSMMQTHGDFDVALTKYRVVACAVPESPPLWNNIGMCFFGKKKYVAAISCLKRANYLA
PFDWKILYNLGLVHLTMQQYASAFHFLSAAINFQPKMGELYMLLAVALTNLEDIENAKRA
YAEAVHLDKCNPLVNLNYAVLLYNQGEKKNALAQYQEMEKKVSLLKDNSSLEFDSEMVEM
AQKLGAALQVGEALVWTKPVKDPKSKHQTTSTSKPASFQQPLGSNQALGQAMSSAAAYRT
LPSGAGGTSQFTKPPSLPLEPEPAVESSPTETSEQIREK
>P34331
MNRLPNIAKPPQKSNQRKEKAPPEVPALIADKDRGTYYEKGRFLGKGGFAHCYELTNRAT
REVVAGKVVPKSMLVKQYQRDKVDNERILIHRELGHINIVKLFNFFEDNLNVYITLELCA
RRSLMELHKRRKAVTEPEARYFTHQIVDGVLYLHDLNIIHRDMKLGNLFLNDDLVVKIGD
FGLATTVNGDERKKTLCGTPNYIAPEVLNKAGHSFEVDIWAVGCILYILLFGQPPFESKS
LEETYSRIRHNNYTIPSIATQPAASLIRKMLDPEPTRRPTAKQVQRDGFFKSGFMPTRLP
VSCLTMVPKFGGHETSMMEENVAPRGVDARSQRPLNGRAGLSALPQHIVSNNADRERAQQ
QAAEATFREPEDAYLSQLFHQVAVLLEQRIPGLEEEEAALDGYQSPECLPVFWISKWVDY
SDKYGIGYQLCDNSVGVLFNDNSRIMLDQAGNELTYIEKSNKEHYFSMHSGEMPGLLNKK
VTLLKYFRSYMNDHLVKAGEGSEQRAGDDLARLPTLRVWFRTKSAIVLHLSNGTVQINFF
NDHVKMMMCPLMQAVTFIDQNKRMLTYKLNNLQRNGCPEKFLHRLKYAKTMIERLMSDAN
VVSQNPARQPDMPRSMAAARSASAGSRGPNQAASHLPQSASGSNIHPRR
>Q9UPV0
MAGRPLRIGDQLVLEEDYDETYIPSEQEILEFAREIGIDPIKEPELMWLAREGIVAPLPG
EWKPCQDITGDIYYFNFANGQSMWDHPCDEHYRSLVIQERAKLSTSGAIKKKKKKKEKKD
KKDRDPPKSSLALGSSLAPVHVPLGGLAPLRGLVDTPPSALRGSQSVSLGSSVESGRQLG
ELMLPSQGLKTSAYTKGLLGSIYEDKTALSLLGLGEETNEEDEEESDNQSVHSSSEPLRN
LHLDIGALGGDFEYEESLRTSQPEEKKDVSLDSDAAGPPTPCKPSSPGADSSLSSAVGKG
RQGSGARPGLPEKEENEKSEPKICRNLVTPKADPTGSEPAKASEKEAPEDTVDAGEEGSR
REEAAKEPKKKASALEEGSSDASQELEISEHMKEPQLSDSIASDPKSFHGLDFGFRSRIS
EHLLDVDVLSPVLGGACRQAQQPLGIEDKDDSQSSQDELQSKQSKGLEERLSPPLPHEER
AQSPPRSLATEEEPPQGPEGQPEWKEAEELGEDSAASLSLQLSLQREQAPSPPAACEKGK
EQHSQAEELGPGQEEAEDPEEKVAVSPTPPVSPEVRSTEPVAPPEQLSEAALKAMEEAVA
QVLEQDQRHLLESKQEKMQQLREKLCQEEEEEILRLHQQKEQSLSSLRERLQKAIEEEEA
RMREEESQRLSWLRAQVQSSTQADEDQIRAEQEASLQKLREELESQQKAERASLEQKNRQ
MLEQLKEEIEASEKSEQAALNAAKEKALQQLREQLEGERKEAVATLEKEHSAELERLCSS
LEAKHREVVSSLQKKIQEAQQKEEAQLQKCLGQVEHRVHQKSYHVAGYEHELSSLLREKR
QEVEGEHERRLDKMKEEHQQVMAKAREQYEAEERKQRAELLGHLTGELERLQRAHERELE

TVRQEQHKRLEDLRRRHREQERKLQDLELDLETRAKDVKARLALLEVQEETARREKQQLL
DVQRQVALKSEEATATHQQLEEAQKEHTHLLQSNQQLREILDELQARKLKLESQVDLLQA
QSQQLQKHFSSLEAEAQKKQHLLREVTVEENNASPHFEPDLHIEDLRKSLGTNQTKEVSS
SLSQSKEDLYLDSLSSHNVWHLLSAEGVALRSAKEFLVQQTRSMRRRQTALKAAQQHWRH
ELASAQEVAKDPPGIKALEDMRKNLEKETRHLDEMKSAMRKGHNLLKKKEEKLNQLESSL
WEEASDEGTLGGSPTKKAVTFDLSDMDSLSSESSESFSPPHREWWRQQRIDSTPSLTSRK
IHGLSHSLRQISSQLSSVLSILDSLNPQSPPPLLASMPAQLPPRDPKSTPTPTYYGSLAR
FSALSSATPTSTQWAWDSGQGPRLPSSVAQTVDDFLLEKWRKYFPSGIPLLSNSPTPLES
RLGYMSASEQLRLLQHSHSQVPEAGSTTFQGIIEANRRWLERVKNDPRLPLFSSTPKPKA
TLSLLQLGLDEHNRVKVYRF
>Q9GT24
MSGGKSGSRLSAYSHLKEIGKGGFGVVYAAQRENGEKVAIKRIDKKVPKNRVWTEIQTMK
ELKKSKYVVEFYEDFVEDGYTYIVMELCEGGSLQAYVREHGALDDATAVHVLRQLISAVS
FMHRVNVIHRDLSAGNVFIKDSKKKKMTVKLGDFGLATTLGRGETTCTIVGTPGFIAPQV
YDQEYTQSADVYSLGAVLYTMLTARNPPPKGLPPTCGMSPNAARLVEQMMDTDAKKRIPL
TQIVLSEFMYENTNENAVIFSREHSRDGRRQRSREPVRSSRDDRSRDGRALIRSSSQPAH
SGRAPLSNRPIHDRMPSTSSRGFDSERGRERDRDSGRGTVPPSREDRNRSQLWPIRMDRL
EGQRVCTAGGRYIVELDTRCRFEVAAQGNFVKRILIVEVDEMVQTVYVHRIPDRTVRGRN
GEEELITLTNNPFVYTSYSQMPKEVQNDYMRLQKMVAVTISGRVAKVTFRRPSQFPDAQA
QLMENGDLRIKLPRSVIVRKMDNGEIFNCIDGIATQKQAVSGITLTKVNEVYKYLIRFEQ
CLNGMDRGMVCFPIVFSAGTNMVGSSPSSLMPSGSSQTSRFPFSNLSNNQPSLVPHSAPF
LTKPTSSQRASSANVQRRVSTDENSSPSVAPSKYKIKIDPTTQKVRSIQATDGRVLRCST
SKADQFIFTDPAIRPDDQRFMRTDRVPDRASEMLHTLCERMRKLHQ
>Q9BTV5
MEEQREALRKIIKTLAVKNEEIQSFIYSLKQMLLNVEANSAKVQEDLEAEFQSLFSLLEE
LKEGMLMKIKQDRASRTYELQNQLAACTRALESSEELLETANQTLQAMDSEDFPQAAKQI
KDGVTMAPAFRLSLKAKVSDNMSHLMVDFAQERQMLQALKFLPVPSAPVIDLAESLVADN
CVTLVWRMPDEDSKIDHYVLEYRRTNFEGPPRLKEDQPWMVIEGIRQTEYTLTGLKFDMK
YMNFRVKACNKAVAGEFSEPVTLETPAFMFRLDASTSHQNLRVDDLSVEWDAMGGKVQDI
KAREKDGKGRTASPINSPARGTPSPKRMPSGRGGRDRFTAESYTVLGDTLIDGGEHYWEV
RYEPDSKAFGVGVAYRSLGRFEQLGKTAASWCLHVNNWLQVSFTAKHANKVKVLDAPVPD
CLGVHCDFHQGLLSFYNARTKQVLHTFKTRFTQPLLPAFTVWCGSFQVTTGLQVPSAVRC
LQKRGSATSSSNTSLT
>Q15691
MAVNVYSTSVTSDNLSRHDMLAWINESLQLNLTKIEQLCSGAAYCQFMDMLFPGSIALKK
VKFQAKLEHEYIQNFKILQAGFKRMGVDKIIPVDKLVKGKFQDNFEFVQWFKKFFDANYD
GKDYDPVAARQGQETAVAPSLVAPALNKPKKPLTSSSAAPQRPISTQRTAAAPKAGPGVV
RKNPGVGNGDDEAAELMQQVNVLKLTVEDLEKERDFYFGKLRNIELICQENEGENDPVLQ
RIVDILYATDEGFVIPDEGGPQEEQEEY
>Q5VT06
MRSSKSKEVPLPNPRNSQSKDTVQADITTSWDALSQTKAALRHIENKLEVAPTSTAVCDS
VMDTKKSSTSATRKISRKDGRYLDDSWVNAPISKSTKSRKEKSRSPLRATTLESNVKKNN
RVEFREPLVSYREIHGAPSNFSSSHLESKHVYCVDVNEEKTESGNWMIGSREERNIRSCD
FESSQSSVINDTVVRFLNDRPAIDALQNSECLIRMGASMRTEEEMPNRTKGSENNLKLSV

NNMAHDTDPKALRLTDSSPSSTSTSNSQRLDILKRRQHDVKLEKLKERIRKQWEHSEETN
GRGQKLGHIDHPVMVVNVDNSVTAKVRKVATAPPAPAYKGFNPSETKIRTPDGKVWQEAE
FQNMSRELYRDLALHFADDISIKEKPAEKSKEKKVVKPVRKVQKVAQLSSTECRTGSSHL
ISTSSWRDGQKLVKKILGPAPRMEPKEQRTASSDRGGRERTAKSGGHIGRAESDPRLDVL
HRHLQRNSERSRSKSRSENNIKKLASSLPDNKQEENTALNKDFLPIEIRGILDDLQLDST
AHTAKQDTVELQNQKSSAPVHAPRSHSPVKRKPDKITANEDPPVISKRRHYDTDEVRQYI
VRQQEERKRKQNEEKKAQKEATEQKNKRLQELYRKQKEAFTKVKNVPPSEPSATRRLQET
YSKLLLEKTLLEEPSHQHVTQETQAKPGYQPSGESDKENKVQERPPSASSSSDMSLSEPP
QPLARKDLMESTWMQPERLSPQVHHSQPQPFAGTAGSLLSHLLSLEHVGILHKDFESILP
TRKNHNMASRPLTFTPQPYVTSPAAYTDALLKPSASQYKSKLDRIEALKATAASLSSRIE
SEAKKLAGASINYGSAWNTEYDVQQAPQEDGPWTKAVTPPVKDDNEDVFSARIQKMLGSC
VSHATFDDDLPGVGNLSEFKKLPEMIRPQSAISSFRVRSPGPKPEGLLAQLCKRQTDSSS
SDMQACSQDKAKISLGSSIDSVSEGPLLSEGSLSEEEGDQDGQPLLKVAEILKEKEFCPG
ERNSYEPIKEFQKEAEKFLPLFGHIGGTQSKGPWEELAKGSPHSVINIFTKSYQLYGKGF
EDKLDRGTSTSRPLNATATPLSGVSYEDDFVSSPGTGTSTEKKSTLEPHSTLSPQEDHSN
RKSAYDPSSVDVTSQHSSGAQSAASSRSSTSSKGKKGKKEKTEWLDSFTGNVQNSLLDEE
KAERGSHQGKKSGTSSKLSVKDFEQTLDTDSTLEDLSGHSVSVSSDKGRSQKTPTSPLSP
SSQKSLQFDVAGTSSERSKSSVMPPTITGFKPNAPLTDLNPAASRTTTENMAPIPGSKRF
SPAGLHHRMAAELSYLNAIEESVRQLSDVERVRGISLAQQESVSLAQIIKAQQQRHERDL
ALLKLKAEQEALESQRQLEETRNKAAQVHAESLQQVVQSQREVTEVLQEATCKIAAQQSE
TARLTTDAARQICEMAELTRTHISDAVVASGAPLAILYDHQRQHLPDFVKQLRTRTETDR
KSPSVSLSQSKEGTLDSKHQKYSASYDSYSESSGYKNHDRRSSSGSSRQESPSVPSCKEN
EKKLNGEKIESSIDEQVQTAADDSLRSDSVPSLPDEKDSTSIATEYSLKFDESMTEDEIE
EQSFRSLLPSESHRRFNMEKRRGHHDDSDEEASPEKTTLSTAKELNMPFSGGQDSFSKFT
MEMVRQYMKEEEMRAAHQSSLLRLREKALKEKTKAELAWLEHQKKHLRDKGEDDKMPPLR
KKQRGLLLRLQQEKAEIKRLQEANKAARKERQLILKQQEEIEKIRQTTIKLQEKLKSAGE
SKLDSHSDDDTKDNKATSPGPTDLETRSPSPISISSSETSSIMQKLKKMRSRMDEKFLTK
REQKLMQRRQHAEELLEWKRRLDAEEAEIRQMEKQALAAWDKELIKPKTPKKELEDQRTE
QKEIASEEESPVPLYSHLNSESSIPEELGSPAVEYVPSESIGQEQPGSPDHSILTEEMIC
SQELESSTSPSKHSLPKSCTSVSKQESSKGSHRTGGQCHLPIKSHQHCYSWSDESLSMTQ
SETTSDQSDIEGRIRALKDELRKRKSVVNQLKKEQKKRQKERLKAQEASLIKQLESYDEF
IKKTEAELSQDLETSPTAKPQIKTLSSASEKPKIKPLTPLHRSETAKNWKSLTESERSRG
SLESIAEHVDASLSGSERSVSERSLSAYAKRVNEWDSRTEDFQTPSPVLRSSRKIREESG
DSLENVPALHLLKELNATSRILDMSDGKVGESSKKSEIKEIEYTKLKKSKIEDAFSKEGK
SDVLLKLVLEQGDSSEILSKKDLPLDSENVQKDLVGLAIENLHKSEEMLKERQSDQDMNH
SPNIQSGKDIHEQKNTKEKDLSWSEHLFAPKEIPYSEDFEVSSFKKEISAELYKDDFEVS
SLLSLRKDSQSCRDKPQPMRSSTSGATSFGSNEEISECLSEKSLSIHSNVHSDRLLELKS
PTELMKSKERSDVEHEQQVTESPSLASVPTADELFDFHIGDRVLIGNVQPGILRFKGETS
FAKGFWAGVELDKPEGNNNGTYDGIAYFECKEKHGIFAPPQKISHIPENFDDYVDINEDE
DCYSDERYQCYNQEQNDTEGPKDREKDVSEYFYEKSLPSVNDIEASVNRSRSLKIETDNV
QDISGVLEAHVHQQSSVDSQISSKENKDLISDATEKVSIAAEDDTLDNTFSEELEKQQQF
TEEEDNLYAEASEKLCTPLLDLLTREKNQLEAQLKSSLNEEKKSKQQLEKISLLTDSLLK
VFVKDTVNQLQQIKKTRDEKIQLSNQELLGDDQKKVTPQDLSQNVEEQSPSISGCFLSSE
LEDEKEEISSPDMCPRPESPVFGASGQEELAKRLAELELSREFLSALGDDQDWFDEDFGL

SSSHKIQKNKAEETIVPLMAEPKRVTQQPCETLLAVPHTAEEVEILVHNAAEELWKWKEL
GHDLHSISIPTKLLGCASKGLDIESTSKRVYKQAVFDLTKEIFEEIFAEDPNLNQPVWMK
PCRINSSYFRRVKNPNNLDEIKSFIASEVLKLFSLKKEPNHKTDWQKMMKFGRKKRDRVD
HILVQELHEEEAQWVNYDEDELCVKMQLADGIFETLIKDTIDVLNQISEKQGRMLLV
>A6H687
MGRFKGENRSQARWIMGGVSKGRGSGKSRKPRQAAFGQTGARVCPSSPQQDAVPRFRWPG
DAECASSTHTPTMSGCKLPMGLCPDMCPAAERARRERERRLHRLEVEPGGRGNAPRADPK
RTVKEYSRPAAGKPRPPPSLLRPPPVLLATVRYLAGEVAGRGDVSCAEVASFVADRLRAV
RLDLSLQGVDDADAATVLEAALATLLAVVARVRPEETRGAADPVLLQTQVQEGFGSLRRC
YARGKGPYPRQAAFQGLFLLYNLGSVEALQEVLQLPAALRACPPLQAALAVDAAFREDNH
ARLFRLLRTLPYLQSCAVQEHIGYARRKALARLSRALSTPKGQTLPLDFIEHFLALDGLQ
EARDLCQAHGLTLDKDRVVFLRGQYSEEGLPPPGAYHILVGNKLQGHTLEDVVMAEEGDI
HRPGSAA
>Q9D6T1
MTQSVVVQVGQCGNQIGCCFWDLALREHAAVNQKGIYDDAISSFFRNVDTRAVGDGGSIS
KGRISSLKARAVLIDMEEGVVNEILQGPLRDVFDSKQLITDISGSGNNWAVGHKVFGCLY
REQILEKLRKSAEQCDCLQCFFIIHSMGGGTGSGLGTFLLKVLEDEFPEVYRFVTAVYPS
SEDDVITSPYNSMLAMKELNEHADCVLPIDNQSLFDIISKIDLVVNSGKLGSAVKPKSLI
TSNMGAVKKHHKKPFDAMNNIVANLLLSLTSSARFEGSLNMDLNEISMNLVPFPKLHYLV
SSLTPLYTLADVNIPPRRLDQMFSDAFSKDHQLIQADPRHSLYLACALIVRGNVQISDLR
RNIERLKPALQFVSWNQEGWKTSLCSVPPVGHSHSLLALANNTCVKPTFMELRERFTRLY
KKKAHLHHYLQVDGMEESTFTEAVSSLSALIQEYSDLDATKSLPVPDVPRLSVAL
>Q9VC45
MSAFEITVTPSRLKQKKRAEGREPAVVVMAPFSAKAIVQFEDVPITKTARRQVRVLNPSD
DDIEVKVMKAIREEHNLSLEWMEHTVPARDEVSMELVWSPVLEVACKETLQLIDNRNFRK
EVMIILKSKSNQPVKNPRKFPTVGKTLQLKSPTGAGKTMKSVVSAAVQQKKRMSAAAAPP
SKQTWRVTAPSRPAAWAHPPPQAPLVEKNVYKTPQEEPVYISPQPRSLKENLSPMTPGNL
LDVIDNLRFTPLTETRGKGQATIFPDNLAAWPTPTLKGNVKSCANDMRPRRITPDDLEDQ
PATNKTFDVKHSETINISLDTLDCSRIDGQPHTPLNKTTTIVHATHTRALACIHEEEGPS
PPRTPTKSAIHDLKRDIKLVGSPLRKYSESMKDLSLLSPQTKYAIQGSMPNLNEMKIRSI
EQNRYYQEQQIQIKAKDLNSSSSSEASLAGQQEFLFNHSEILAQSSRFNLHEVGRKSVKG
SPVKNPHKRRSHELSFSDAPSNESLYRNETVAISPPKKQRVEDTTLPRSAAPANASARSS
SAHAWPHAQSKKFKLAQTMSLMKKPATPRKVRDTSIQPSVKLYDSELYMQTCINPDPFAA
TTTIDPFLASTMYLDEQAVDRHQADFKKWLNALVSIPADLDADLNNKIDVGKLFNEVRNK
ELVVAPTKEEQSMNYLTKYRLETLRKAAVELFFSEQMRLPCSKVAVYVNKQALRIRSDRN
LHLDVVMQRTILELLLCFNPLWLRLGLEVVFGEKIQMQSNRDIVGLSTFILNRLFRNKCE
EQRYSKAYTLTEEYAETIKKHSLQKILFLLLFLDQAKQKRIVKHNPCLFVKKSPHKETKD
ILLRFSSELLANIGDITRELRRLGYVLQHRQTFLDEFDYAFNNLAVDLRDGVRLTRVMEV
ILLRDDLTRQLRVPAISRLQRIFNVKLALGALGEANFQLGGDIAAQDIVDGHREKTLSLL
WQLIYKFRSPKFHAAATVLQKWWRRHWLHVVIQRRIRHKELMRRHRAATVIQAVFRGHQM
RKYVKLFKTERTQAAIILQKFTRRYLAQKQLYQSYHSIITIQRWWRAQQLGRQHRQRFVE
LREAAIFLQRIWRRRLFAKKLLAAAETARLQRSQKQQAAASYIQMQWRSYQLGRIQRQQF
LRQRDLIMFVQRRMRSKWSMLEQRKEFQQLKRAAINIQQRWRAKLSMRKCNADYLALRSS
VLKVQAYRKATIQMRIDRNHYYSLRKNVICLQQRLRAIMKMREQRENYLRLRNASILVQK

RYRMRQQMIQDRNAYLRTRKCIINVQRRWRATLQMRRERKNYLHLQTTTKRIQIKFRAKR
EMKKQRAEFLQLKKVTLVVQKRRRALLQMRKERQEYLHLREVTIKLQRRFHAQKSMRFMR
AKYRGTQAAVSCLQMHWRNHLLRKRERNSFLQLRQAAITLQRRYRARLNMIKQLKSYAQL
KQAAITIQTRYRAKKAMQKQVVLYQKQREAIIKVQRRYRGNLEMRKQIEVYQKQRQAVIR
LQKWWRSIRDMRLCKAGYRRIRLSSLSIQRKWRATVQARRQREIFLSTIRKVRLMQAFIR
ATLLMRQQRREFEMKRRAAVVIQRRFRARCAMLKARQDYQLIQSSVILVQRKFRANRSMK
QARQEFVQLRTIAVHLQQKFRGKRLMIEQRNCFQLLRCSMPGFQARARGFMARKRFQALM
TPEMMDLIRQKRAAKVIQRYWRGYLIRRRQKHQGLLDIRKRIAQLRQEAKAVNSVRCKVQ
EAVRFLRGRFIASDALAVLSRLDRLSRTVPHLLMWCSEFMSTFCYGIMAQAIRSEVDKQL
IERCSRIILNLARYNSTTVNTFQEGGLVTIAQMLLRWCDKDSEIFNTLCTLIWVFAHCPK
KRKIIHDYMTNPEAIYMVRETKKLVARKEKMKQNARKPPPMTSGRYKSQKINFTPCSLPS
LEPDFGIIRYSPYTFISSVYAFDTILCKLQIDMF
>O61585
MATKRAWKLQELVAHSSNVNCLALGPMSGRVMVTGGEDKKVNLWAVGKQNCIISLSGHTS
PVDSVKFNSSEELVVAGSQSGTMKIYDLEPAKIVRTLTGHRNSIRCMDFHPFGEFVASGS
TDTNVKLWDVRRKGCIYTYKGHSDQVNMIKFSPDGKWLVTASEDTTIKLWDLTMGKLFQE
FKNHTGGVTGIEFHPNEFLLASGSSDRTVQFWDLETFQLVSSTSPGASAVRSISFHPDGS
YLFCSSQDMLHAFGWEPIRCFDTFSVGWGKVADTVIASTQLIGASFNATNVSVYVADLSR
MSTTGIAQEPQSQPSKTPSGGAEEVPSKPLTASGRKNFVRERPHTTSSKQRQPDVKSEPE
RQSPTQDEGVKDDDATDIKDPDSYAKIFSPKTRVDHSPERNAQPFPAPLDVPGAQEPEPF
KHPPKPAAAAAVAPVSRAPAPSASDWQPAQANPAPNRVPAATKPVPAQEVAPSRKPDPIS
TIIPSDRNKPANLDMDAFLPPAHAQQAPRVNAPASRKQSDSERIEGLRKGHDSMCQVLSS
RHRNLDVVRAIWTAGDAKTSVESVVNMKDQAILVDILNIMLLKKSLWNLDMCVVVLPRLK
ELLSSKYENYVHTSCACLKLILKNFTSLFNQNIKCPPSGIDITREERYNKCSKCYSYLIA
TRGYVEEKQHVSGKLGSSFRELHLLLDQLE
>Q9UPN4
MKGTRAIGSVPERSPAGVDLSLTGLPPPVSRRPGSAATTKPIVRSVSVVTGSEQKRKVLE
ATGPGGSQAINNLRRSNSTTQVSQPRSGSPRPTEPTDFLMLFEGSPSGKKRPASLSTAPS
EKGATWNVLDDQPRGFTLPSNARSSSALDSPAGPRRKECTVALAPNFTANNRSNKGAVGN
CVTTMVHNRYTPSERAPPLKSSNQTAPSLNNIIKAATCEGSESSGFGKLPKNVSSATHSA
RNNTGGSTGLPRRKEVTEEEAERFIHQVNQAAVTIQRWYRHQVQRRGAGAARLEHLLQAK
REEQRQRSGEGTLLDLHQQKEAARRKAREEKARQARRAAIQELQQKRALRAQKASTAERG
PPENPRETRVPGMRQPAQELSPTPGGTAHQALKANNAGGGLPAAGPGDRCLPTSDSSPEP
QQPPEDRTQDVLAQDAAGDNLEMMAPSRGSAKSRGPLEELLHTLQLLEKEPDALPRPRTH
HRGRYAWASEVTTEDDASSLTADNLEKFGKLSAFPEPPEDGTLLSEAKLQSIMSFLDEME
KSGQDQLDSQQEGWVPEAGPGPLELGSEVSTSVMRLKLEVEEKKQAMLLLQRALAQQRDL
TARRVKETEKALSRQLQRQREHYEATIQRHLAFIDQLIEDKKVLSEKCEAVVAELKQEDQ
RCTERVAQAQAQHELEIKKLKELMSATEKARREKWISEKTKKIKEVTVRGLEPEIQKLIA
RHKQEVRRLKSLHEAELLQSDERASQRCLRQAEELREQLEREKEALGQQERERARQRFQQ
HLEQEQWALQQQRQRLYSEVAEERERLGQQAARQRAELEELRQQLEESSSALTRALRAEF
EKGREEQERRHQMELNTLKQQLELERQAWEAGRTRKEEAWLLNREQELREEIRKGRDKEI
ELVIHRLEADMALAKEESEKAAESRIKRLRDKYEAELSELEQSERKLQERCSELKGQLGE
AEGENLRLQGLVRQKERALEDAQAVNEQLSSERSNLAQVIRQEFEDRLAASEEETRQAKA
ELATLQARQQLELEEVHRRVKTALARKEEAVSSLRTQHEAAVKRADHLEELLEQHRRPTP

STK
>O76070
MDVFKKGFSIAKEGVVGAVEKTKQGVTEAAEKTKEGVMYVGAKTKENVVQSVTSVAEKTK
EQANAVSEAVVSSVNTVATKTVEEAENIAVTSGVVRKEDLRPSAPQQEGEASKEKEEVAE
EAQSGGD
>Q9BYV8
MSLRRHIGNPEYLMKRIPQNPRYQHIKSRLDTGNSMTKYTEKLEEIKKNYRYKKDELFKR
LKVTTFAQLIIQVASLSDQTLEVTAEEIQRLEDNDSAASDPDAETTARTNGKGNPGEQSP
SPEQFINNAGAGDSSRSTLQSVISGVGELDLDKGPVKKAEPHTKDKPYPDCPFLLLDVRD
RDSYQQCHIVGAYSYPIATLSRTMNPYSNDILEYKNAHGKIIILYDDDERLASQAATTMC
ERGFENLFMLSGGLKVLAQKFPEGLITGSLPASCQQALPPGSARKRSSPKGPPLPAENKW
RFTPEDLKKIEYYLEEEQGPADHPSRLNQANSSGRESKVPGARSAQNLPGGGPASHSNPR
SLSSGHLQGKPWK
>Q148B6
MSLLTSYEGLRHQIERLVRENEELKKLVRLIRENQELKSAIKTQAGGLCISGFTGGLGEA
AAGPPQHQGVFLPPASAAAKEPCSEDLGMVALAPLADMLNTPQLSPAAGSLVNPLAATLN
PLLSGQIPLLQNNQFANLVPCSMSNQLTNPTTVSPGVTLASSLGLPSTGPLNSQMTSPMT
VPPGTTLASSLGLTSTGSLTTSSRLVGPLAVSQSSPIMAPLAGTVAVSLSSPLLSSTATP
LGVAQNVVPNPINNIGQPETPRVRRAEPTRGNFSGTSAYAGPAPTSKVNDTRGSRVMEQS
RKNVVEMERKTPHRKSNKLPDNPRDTKQLVCERLVGEIAFQLDRRILSSIFPERVRLYGF
TVSNIPEKIIQASLNPSNHKLDEDLCQTLTQRYVSIMNKLQSLGYNGRVHPALTEQLVNE
YGILRERPELAASEGGCYTVDFLQRVLLETVHPSKLTDALLLLSCLHQLSHDDGKPMFIW

>Q6UVJ0
MSQVLFHQLVPLQVKCKDCEERRVSIRMSIELQSVSNPVHRKDLVIRLTDDTDPFFLYNL
VISEEDFQSLKFQQGLLVDFLAFPQKFIDLLQQCTQEHAKEIPRFLLQLVSPAAILDNSP
AFLNVVETNPFKHLTHLSLKLLPGNDVEIKKFLAGCLKCSKEEKLSLMQSLDDATKQLDF
TRKTLAEKKQELDKLRNEWASHTAALTNKHSQELTNEKEKALQAQVQYQQQHEQQKKDLE
ILHQQNIHQLQNRLSELEAANKDLTERKYKGDSTIRELKAKLSGVEEELQRTKQEVLSLR
RENSTLDVECHEKEKHVNQLQTKVAVLEQEIKDKDQLVLRTKEAFDTIQEQKVVLEENGE
KNQVQLGKLEATIKSLSAELLKANEIIKKLQGDLKTLMGKLKLKNTVTIQQEKLLAEKEE
KLQKEQKELQDVGQSLRIKEQEVCKLQEQLEATVKKLEESKQLLKNNEKLITWLNKELNE
NQLVRKQDVLGPSTTPPAHSSSNTIRSGISPNLNVVDGRLTYPTCGIGYPVSSAFAFQNT
FPHSISAKNTSHPGSGTKVQFNLQFTKPNASLGDVQSGATISMPCSTDKENGENVGLESK
YLKKREDSIPLRGLSQNLFSNSDHQRDGTLGALHTSSKPTALPSASSAYFPGQLPNS
>Q9VIF5
MEKLDKIRMSQKLSCWQHILTTLGTSSKTEQEWNTFFKGFLESWRKPYCIQTSCDPSIPL
RKELLVRPRKALQENPHGPGSTLPESPVFLEPINSTAPREHPSPSKSHSTGSTGCDPGNG
ERSPSVSNKNSKYKNPGNSKYAKIWKRSNNHTTSIFSKAQISKRRDKLSSTKKRPDTCAP
TDDSRKNREPRACAPNKNIFKTRETNAPNLTKNSCALPNVLILSPNSASKITQRGHSVGQ
TQDYKASPGKIIKRVPRYSLQCLKKKTEKVHNKIMDKPKNKQQPQTPPPFLLNNEYTESS
DDSDDQLPLSELSQKMKSNKLNTLFLSRNEDCSPAPEKVKLKGERPAQNKKEQLTWEPSI
LTNLTDLGKQVAEPLRKSVKKSAKQQKPRVRAPPQGKKTLPQLQTGLKTQDKQSTHEMIS
EQAKTISEASGQQTSQVQSSLSPNNIRNNSVKLISAKTLMPAQRSQDYSPNKMQVGQPLG

FAIELPSPVDGKEASVEIDTQLVHTSKFLENMTRPSTVEVHKFDLMDIFMGENEKLDYEY
DDDDVLSVAASWNGLDDENVPEDEPRKEAKTAEQLPKPEPSTETLKPIEKENAQKMQSIK
SFQIPKLNAKNLKTQPSVMRSIYENEELEKNKVLAKPAPPSLVHQPLAESNRNQRDEATA
ARRAKETFPVFAPLYRVAPESAATLVSANSQQVIPQVYFSTSNQDGVNWIKDVFGIRCMQ
SVDNKCISINCDHTMNSLGEVQKRLMRMDEDTLLSLYRQTIRSFFLFQTYYTSFVDIFKF
RNLWQYLLIMLVDCRLYKSISAPLLAHVYEALSKCGMQKEAVKRIMEHVWLPCKAHKYRD
LMLTTLNILSNANWEDYCDKLTQLDKDYNFEIPHKNLITILKSSVDCSDKFANALKLITL
HPNSIRTNETIMSILSNASKSYSYMHNESASASQGPPGAASFLAPPAAIQPPHTTVPNFG
YLPNPSFHYSNEYAININIHNFD
>O15078
MPPNINWKEIMKVDPDDLPRQEELADNLLISLSKVEVNELKSEKQENVIHLFRITQSLMK
MKAQEVELALEEVEKAGEEQAKFENQLKTKVMKLENELEMAQQSAGGRDTRFLRNEICQL
EKQLEQKDRELEDMEKELEKEKKVNEQLALRNEEAENENSKLRRENKRLKKKNEQLCQDI
IDYQKQIDSQKETLLSRRGEDSDYRSQLSKKNYELIQYLDEIQTLTEANEKIEVQNQEMR
KNLEESVQEMEKMTDEYNRMKAIVHQTDNVIDQLKKENDHYQLQVQELTDLLKSKNEEDD
PIMVAVNAKVEEWKLILSSKDDEIIEYQQMLHNLREKLKNAQLDADKSNVMALQQGIQER
DSQIKMLTEQVEQYTKEMEKNTCIIEDLKNELQRNKGASTLSQQTHMKIQSTLDILKEKT
KEAERTAELAEADAREKDKELVEALKRLKDYESGVYGLEDAVVEIKNCKNQIKIRDREIE
ILTKEINKLELKISDFLDENEALRERVGLEPKTMIDLTEFRNSKHLKQQQYRAENQILLK
EIESLEEERLDLKKKIRQMAQERGKRSATSGLTTEDLNLTENISQGDRISERKLDLLSLK
NMSEAQSKNEFLSRELIEKERDLERSRTVIAKFQNKLKELVEENKQLEEGMKEILQAIKE
MQKDPDVKGGETSLIIPSLERLVNAIESKNAEGIFDASLHLKAQVDQLTGRNEELRQELR
ESRKEAINYSQQLAKANLKIDHLEKETSLLRQSEGSNVVFKGIDLPDGIAPSSASIINSQ
NEYLIHLLQELENKEKKLKNLEDSLEDYNRKFAVIRHQQSLLYKEYLSEKETWKTESKTI
KEEKRKLEDQVQQDAIKVKEYNNLLNALQMDSDEMKKILAENSRKITVLQVNEKSLIRQY
TTLVELERQLRKENEKQKNELLSMEAEVCEKIGCLQRFKEMAIFKIAALQKVVDNSVSLS
ELELANKQYNELTAKYRDILQKDNMLVQRTSNLEHLECENISLKEQVESINKELEITKEK
LHTIEQAWEQETKLGNESSMDKAKKSITNSDIVSISKKITMLEMKELNERQRAEHCQKMY
EHLRTSLKQMEERNFELETKFAELTKINLDAQKVEQMLRDELADSVSKAVSDADRQRILE
LEKNEMELKVEVSKLREISDIARRQVEILNAQQQSRDKEVESLRMQLLDYQAQSDEKSLI
AKLHQHNVSLQLSEATALGKLESITSKLQKMEAYNLRLEQKLDEKEQALYYARLEGRNRA
KHLRQTIQSLRRQFSGALPLAQQEKFSKTMIQLQNDKLKIMQEMKNSQQEHRNMENKTLE
MELKLKGLEELISTLKDTKGAQKVINWHMKIEELRLQELKLNRELVKDKEEIKYLNNIIS
EYERTISSLEEEIVQQNKFHEERQMAWDQREVDLERQLDIFDRQQNEILNAAQKFEEATG
SIPDPSLPLPNQLEIALRKIKENIRIILETRATCKSLEEKLKEKESALRLAEQNILSRDK
VINELRLRLPATAEREKLIAELGRKEMEPKSHHTLKIAHQTIANMQARLNQKEEVLKKYQ
RLLEKAREEQREIVKKHEEDLHILHHRLELQADSSLNKFKQTAWDLMKQSPTPVPTNKHF
IRLAEMEQTVAEQDDSLSSLLVKLKKVSQDLERQREITELKVKEFENIKLQLQENHEDEV
KKVKAEVEDLKYLLDQSQKESQCLKSELQAQKEANSRAPTTTMRNLVERLKSQLALKEKQ
QKALSRALLELRAEMTAAAEERIISATSQKEAHLNVQQIVDRHTRELKTQVEDLNENLLK
LKEALKTSKNRENSLTDNLNDLNNELQKKQKAYNKILREKEEIDQENDELKRQIKRLTSG
LQGKPLTDNKQSLIEELQRKVKKLENQLEGKVEEVDLKPMKEKNAKEELIRWEEGKKWQA
KIEGIRNKLKEKEGEVFTLTKQLNTLKDLFAKADKEKLTLQRKLKTTGMTVDQVLGIRAL
ESEKELEELKKRNLDLENDILYMRAHQALPRDSVVEDLHLQNRYLQEKLHALEKQFSKDT

YSKPSISGIESDDHCQREQELQKENLKLSSENIELKFQLEQANKDLPRLKNQVRDLKEMC
EFLKKEKAEVQRKLGHVRGSGRSGKTIPELEKTIGLMKKVVEKVQRENEQLKKASGILTS
EKMANIEQENEKLKAELEKLKAHLGHQLSMHYESKTKGTEKIIAENERLRKELKKETDAA
EKLRIAKNNLEILNEKMTVQLEETGKRLQFAESRGPQLEGADSKSWKSIVVTRMYETKLK
ELETDIAKKNQSITDLKQLVKEATEREQKVNKYNEDLEQQIKILKHVPEGAETEQGLKRE
LQVLRLANHQLDKEKAELIHQIEANKDQSGAESTIPDADQLKEKIKDLETQLKMSDLEKQ
HLKEEIKKLKKELENFDPSFFEEIEDLKYNYKEEVKKNILLEEKVKKLSEQLGVELTSPV
AASEEFEDEEESPVNFPIY
>A3KGV1
MSASSSGGSPRFPSCGKNGVTSLTQKKVLRTPCGAPSVTVTKSHKRGMKGDTVNVRRSVR
VKTKVPWMPPGKSSARHVGCKWENPPHCLEITPPSSEKLVSVMRLSDLSTEDDDSGHCKM
NRYDKKIDSLMNAVGCLKSEVKMQKGERQMAKRFLEERKEELEEVAHELAETEHENTVLR
HNIERIKEEKDFTMLQKKHLQQEKECLMSKLVEAEMDGAAAAKQVMALKDTIGKLKTEKQ
MTCTDINTLTRQKELLLQKLSTFEETNRTLRDLLREQHCKEDSERLMEQQGTLLKRLAEA
DSEKARLLLLLQDKDKEVEELLQEIQCEKAQAKTASELSKSMESMRGHLQAQLRCKEAEN
SRLCMQIKNLERSGNQHKAEVEAIMEQLKELKQKGDRDKETLKKAIRAQKERAEKSEEYA
EQLHVQLADKDLYVAEALSTLESWRSRYNQVVKDKGDLELEIIVLNDRVTDLVNQQQSLE
EKMREDRDSLVERLHRQTAEYSAFKLENERLKASFAPMEDKLNQAHLEVQQLKASVKNYE
GMIDNYKSQVMKTRLEADEVAAQLERCDKENKMLKDEMNKEIEAARRQFQSQLADLQQLP
DILKITEAKLAECQDQLQGYERKNIDLTAIISDLRSRIEHQGDKLEMAREKHQASQKENK
QLSQKVDELERKLEATSAQNVEFLQVIAKREEAIHQAQLRLEEKTRECGSLARQLESAIE
DARRQVEQTKEQALSKERAAQSKILDLETQLSRTKTELGQLRRTRDDADRRYQSRLQDLK
DRLEQSESTNRSMQNYVQFLKASYANVFGDAPYTSSYLTSSPIRSRSPPA
>P45970
MSVSTSVVKSMLQLTNYFGSNFTPKTETISSLAELWERHGSMIASMMSCDTREFGTADKF
YEVIFRKIATTSLSFMTDSLEPKLAASGDSLEICKFYALFLEYMRKDHSGMLTRALEQAS
SENRVDTAVLLDMFLFFDNVYSESTADDWWACLRRDDEDQEASSGFRTPKRNNYSLTSLQ
TPTATARRLRTASSTARRSPIAEAVDSPTMKFMRSERELKQSKARIFGLEQMLGDLEDDK
TKLSAENRTLKLSNSDLKSDIAKLKGIAEEAQFRAEELSCDLEAKKDEVHNILQQLNESR
MTLRSEQRSLEEADIKKENLTAKLKTVTEDNGKLMKQARELRDLNDYEFARFRQQEQELT
ETLRATQDQMADLQEQLTGVEKIRASLKSENESLSASVEELSVASLRNKQDADNSKTMLS
EELARFEETVDKLRQEKLEALSMANSRIDALRLEHSEREKMMKSTNARLQADLDEERNEK
KRLMNLWNELNEKSLNVDKAVHKSNEEFQSMGSCLQNAKRQIEQLEVACKSKDDIIKFQE
EQNKRAANLIKNEKAIRDQASAQFAEKLAILKNSLAEKENETVTLKENFASVVMKHKAEL
EEKELFLQSRVDLIQRLEHEVADLREKESAEIKKSTWMGERIKIFQQAPMRGSRIRCGSP
DSLPDFLADNGPTITEEELRAQLATFPRKSIAPSVDDNEFDKGTPIGFKSNMYDGRESIC
SLDFFDRSSLQRSSMRSESIQLASPSSAGEFKQPFTPSGVTKERVGVLTARNEKVKPHLK
CSYASEVGSTNSPSADEENIKKSKKKNRRDSIFSAFSSKKQ
>O61308
MEGTCSEEENLAKRVTPTAVTSGTYGTHVVDTTDLALSDVVIGCGSTIYETSDSIGSDVM
LGLSSHGVSSVSGLDRDLNSFKKRIDANTEEQREHADMMVGLQRKIEEYRRRIVDAEKQV
SIQKANDDVSFSIKETADTWLPDMKSDAADYEWASRLDEERRRNDELHMQITQQQVDLQR
LQKYFEANMQEKEKIYQTREKNLAYYLNAEQRKMLDLWEELQRVRRQFADYKEQTERDLE
NQKNEVAKVTQSVGGMAGRLNTSSHGDSGLVQDVVLLEAMRRFRELQAVPVGASADDYNA

LMKKYEETVERVIELESHGDGSTAKMLSLEAELRRTKDKFIECSEFLRKLGDLAAGSYRG
DERTSKIVSLSPGGMSLPSEIYRSVRNILRNHDSEMQHIQRKLKNSDTQVCELTTRLEGT
EEARRRSDKQLVDAKREINIQQRAVDDANRELRRVEDRLHIMESEKIVAENARQQLEEEV
RRLTLQVDQSKADGERRVVEEGEIQKRIVEDEYRSMISELTRRMNAFQDENKRLKNDLGC
TKERLKNVEFEYNSTVRKLEDKDIALKHLEDTKLDLLKDLENQRTRYDAVTNELDTLQTT
FETSTKNIAQLEANIKEINLMRDEISKEKDSLAQKLADVTHKLEIETVRREDIQRSCVGH
SEDVEKQRLQIIEYEREVMALRRLNDELDTNVKTGQAKVTSLENSIISVQTEVTKLTTLN
DKLQKEKQSIMSSKQKADTDVDLLKEKLRKLEQECDKLKEENKALHEDEQIARQMCKEEA
SRIHLLERDLKDAMTEVEELKKQLQKMDEENSERLESVLRTKISSDTVDTSEIAEYTEVK
VKELREKYKADLERLQSNKDDLERRVQILEDELAERQRIVERQRTEMNDLKLEYQLESDR
LRAEMATVELKYQSEVEDERDQRSRDADSWKVTSEELRSKISFMEKMLEEAKHRETVLRE
EATEWEEKHDIISNESLKLRNEIERIRSDAEEDIQKWKKDVHMAQNELKNLERVCETLRS
QLTAANDRVASLNTTINEQTSKIRELNSHEHRLEEDLADSRATSSAIENDLGNATGRLRS
SEEHNAILQSENRKSKTEIEALKHQIDTIMNTKESCESEVERLKKKIVQTTTITKEQNEK
IEKLRIEHDHLERDYREKTKEVDRLKEVEKTFELKVNRARQELDEFSKKLIVTETERNAI
SGEAQKLDKEVQLVKEQLQYKSDEFHKALDELANAHRISEEGRVNAIHQLEARRFEIDDL
KSRLENSEQRLATLQQEYVNADKERGALNDAMRRFQATISRSVVAEEHVDVSTIETQMQK
LMSRIEAIERERNEYRDSLNRLKNRCSTSYSSVDRQETVYRTFEERVISAEDERRKVELK
LSSMKEMLKSQEEKLKQRDEERRNLKSNIVTFELEARAKDAQIRHLNDLLKRVQAELENS
QNDNRALRERQEQYETNRIHLEQRLPTDEGEPRVKALMAAFATERQSLSDSLKKLASQLQ
ISETKNADLRDDAERLKRDLLKAERVEEDLRRNLVEQTEIMRENQQLRSQLGVAQSDLAN
ASGRKQQLEGELAAVRAELRDHKQHLHDAISRIAELQRQLQDANAEKSRLTDRIIGLEKT
IGTLRNTETELRAQLSTAADERKALNSELEEMRRRIVQMESEKKDVDNQLEEVNKARIIM
TKKIEILETEKHSAELVISETASQREAIERSLNALERENKELYKNCAQLQQQIAQLEMDN
GERLIALTTKQKEDHEKFVAAVKAEKVQVERIVENRDRAQKSRIRQLENQLSMMREQLDN
ERARSHQMSERFIVNETNRRVSSSSFRLSGDAAGVAAATILHPQTDRLDYVFANRSALSS
YYTVPTEQHASRGKEAYRTSSTIKSSEGTTRESYTYQSRTVSSNIIEQANGMTSSASGEG
MSRAYPPTEVNQGDVTGRKSRPATRKQQMKSTFSE
>O94986
MSLDFGSVALPVQNEDEEYDEEDYEREKELQQLLTDLPHDMLDDDLSSPELQYSDCSEDG
TDGQPHHPEQLEMSWNEQMLPKSQSVNGPSCQGLEPYNKVTYKPYQSSAQNNGSPAQEIT
GSDTFEGLQQQFLGANENSAENMQIIQLQVLNKAKERQLENLIEKLNESERQIRYLNHQL
VIIKDEKDGLTLSLRESQKLFQNGKEREIQLEAQIKALETQIQALKVNEEQMIKKSRTTE
MALESLKQQLVDLHHSESLQRAREQHESIVMGLTKKYEEQVLSLQKNLDATVTALKEQED
ICSRLKDHVKQLERNQEAIKLEKTEIINKLTRSLEESQKQCAHLLQSGSVQEVAQLQFQL
QQAQKAHAMSANMNKALQEELTELKDEISLYESAAKLGIHPSDSEGELNIELTESYVDLG
IKKVNWKKSKVTSIVQEEDPNEELSKDEFILKLKAEVQRLLGSNSMKRHLVSQLQNDLKD
CHKKIEDLHQVKKDEKSIEVETKTDTSEKPKNQLWPESSTSDVVRDDILLLKNEIQVLQQ
QNQELKETEGKLRNTNQDLCNQMRQMVQDFDHDKQEAVDRCERTYQQHHEAMKTQIRESL
LAKHALEKQQLFEAYERTHLQLRSELDKLNKEVTAVQECYLEVCREKDNLELTLRKTTEK
EQQTQEKIKEKLIQQLEKEWQSKLDQTIKAMKKKTLDCGSQTDQVTTSDVISKKEMAIMI
EEQKCTIQQNLEQEKDIAIKGAMKKLEIELELKHCENITKQVEIAVQNAHQRWLGELPEL
AEYQALVKAEQKKWEEQHEVSVNKRISFAVSEAKEKWKSELENMRKNILPGKELEEKIHS
LQKELELKNEEVPVVIRAELAKARSEWNKEKQEEIHRIQEQNEQDYRQFLDDHRNKINEV

LAAAKEDFMKQKTELLLQKETELQTCLDQSRREWTMQEAKRIQLEIYQYEEDILTVLGVL
LSDTQKEHISDSEDKQLLEIMSTCSSKWMSVQYFEKLKGCIQKAFQDTLPLLVENADPEW
KKRNMAELSKDSASQGTGQGDPGPAAGHHAQPLALQATEAEADKKKVLEIKDLCCGHCFQ
ELEKAKQECQDLKGKLEKCCRHLQHLERKHKAVVEKIGEENNKVVEELIEENNDMKNKLE
ELQTLCKTPPRSLSAGAIENACLPCSGGALEELRGQYIKAVKKIKCDMLRYIQESKERAA
EMVKAEVLRERQETARKMRKYYLICLQQILQDDGKEGAEKKIMNAASKLATMAKLLETPI
SSKSQSKTTQSGMSK
>Q96RT8
MARHGPPWSRLDAQQERDVRELVRGVAGLQDEADPNFQLALNFAWSNFRFHRFLDVNSHK
IEKTIEGIYEKFVIHSDLSKAASWKRLTEEFLNAPLPSIKEIKTDAHYSILSLLLCLSDS
PSNSSYVETPRNKEVEKKDDFDWGKYLMEDEEMDIGPYMDTPNWSEESEEENDQQPLSRE
DSGIQVDRTPLEEQDQNRKLDPCISWKDEPDDRSWLEHHVVHQYWTARPSQFPHSLHLHS
NLAAVWDQHLYSSDPLYVPDDRVLVTETQVIRETLWLLSGVKKLFIFQLIDGKVTVRNNI
IVTHLTHSCLRSVLEQIAAYGQVVFRLQEFIDEVMGHSSESMLPGSGSVPKKSTEAPFRT
YQAFMWALYKYFISFKEELAEIEKCIINNDTTITLAIVVDKLAPRLSQLKVLHKVFSTGV
AEVPPDTRNVVRASHLLNTLYKAILEYDNVGEASEQTVSLLFSLWVETVRPYLQTVDEWI
VHGHLWDGAREFIIQRNKNVPVNHRDFWYATYTLYSVSEKTENEEKMSDNASASSGSDQG
PSSRQHTMVSFLKPVLKQIIMAGKSMQLLKNLQCAESTTCQAGARDAERKSLYTLFLESV
QSRLRHGEDSTPQVLTEQQATKENLMKMQSIAESHLELDDVHDPLLAINFARMYLEQSDF
HEKFAGGDVCVDRSSESVTCQTFELTLRSCLYPHIDKQYLDCCGNLMQTLKKDYRLVEYL
QAMRNFFLMEGGDTMYDFYTSIFDKIREKETWQNVSFLNVQLQEAVGQRYPEDSSRLSIS
FENVDTAKKKLPVHILDGLTLSYKVPWPVDIVISLECQKIYNQVFLLLLQIKWAKYSLDV
LLFGELVSTAEKPRLKEGLIHEQDTVAQFGPQKEPVRQQIHRMFLLRVKLMHFVNSLHNY
IMTRILHSTGLEFQHQVEEAKDLDQLIKIHYRYLSTIHDRCLLREKVSFVKEAIMKVLNL
ALMFADGWQAGLGTWRMESIEKMESDFKNCHMFLVTILNKAVCRGSFPHLESLALSLMAG
MEQS
>Q96IF1
MERLGEKASRLLEKFGRRKGESSRSGSDGTPGPGKGRLSGLGGPRKSGPRGATGGPGDEP
LEPAREQGSLDAERNQRGSFEAPRYEGSFPAGPPPTRALPLPQSLPPDFRLEPTAPALSP
RSSFASSSASDASKPSSPRGSLLLDGAGAGGAGGSRPCSNRTSGISMGYDQRHGSPLPAG
PCLFGPPLAGAPAGYSPGGVPSAYPELHAALDRLYAQRPAGFGCQESRHSYPPALGSPGA
LAGAGVGAAGPLERRGAQPGRHSVTGYGDCAVGARYQDELTALLRLTVGTGGREAGARGE
PSGIEPSGLEEPPGPFVPEAARARMREPEAREDYFGTCIKCNKGIYGQSNACQALDSLYH
TQCFVCCSCGRTLRCKAFYSVNGSVYCEEDYLFSGFQEAAEKCCVCGHLILEKILQAMGK
SYHPGCFRCIVCNKCLDGIPFTVDFSNQVYCVTDYHKNYAPKCAACGQPILPSEGCEDIV
RVISMDRDYHFECYHCEDCRMQLSDEEGCCCFPLDGHLLCHGCHMQRLNARQPPANYI
>Q6QNY1
MAAAAEGVLATRSDEPARDDAAVETAEEAKEPAEADITELCRDMFSKMATYLTGELTATS
EDYKLLENMNKLTSLKYLEMKDIAINISRNLKDLNQKYAGLQPYLDQINVIEEQVAALEQ
AAYKLDAYSKKLEAKYKKLEKR
>Q1ZXQ8
MGDEEAPSGSIEDRINHKNWKWRVSGLEELTTKFRNSIEGSGPLFNEWGPQFKKILADIN
PMSQERALEPLSAFIDRCDCVNKFAASYVGVLVEKLFASTRPRAKEKTIECLLLTIEADS
AEPVVEALLKGTSSTSPKILLASLAALTQALKTFGPKQIPVKLILKQFSPWFENRDKGIR

DQASELFIEIYRWIGKALIPLISEALTPIQLKALQDQFEKLPTDPAVPLKYTRSEAAKAL
ANASKGIQAKPEVVEEIDPYSLMTAVNILPKLTSEFYEGLQAKKWQERSEQMDKLVTILT
NTPKIETADFSELCKALKKILADVNVMIVQKAVVSIGLLADSLRGGFTSYVKPFITPILE
KFKEKKTSVLQSVHTTMDSLVGKSISLSDIIDELTATMQSKVPQIKQEVLVFICNSITNT
KKPADITKVTKQLTKIFMEALNDTDSNIRDNASKAFAALGGIIGERAMTPYLNQIDPIKA
KKIKDNMPAVATPVTITPQPLAPVDLKDIDLPVSSSNKKPAAATGNSKSSSTTTPTGRSS
NSSPLPPPPSSSDDIKNKLIGAGIVNNEIIEGLGKTQWKDRLQAVDDILENVKGLTADSI
NGMSESIIQLLCDKPSLKESNFQVLSSIFSIFIQCCKNDSNFTQQRCANSYLTTCIEKLT
DVKLKEISSELLFSTGESITPHAVFTSIYQFTSNHKNPKIIADSLVWIQQAIDEFGIGCC
SNGIQQLKPLLDYTKQCLESTNPDVKKSAIKLLCTIKINIGATLTDFLGDVKKPTMEVLD
REFQKIRDQKPPVPNRQWKGMPPPGSAPVQIEFPRVDISVKLTPAIITNLSDANWKTRSD
ALDEIERIIIDANRKIQPKLGGLIPALKNRLTDNNQKCTITTLNIIGMLSQAMGGQSFEK
HARLLIPGILLLLGDSKKPVRDAVISCMNVIVQSDLGFDVFIGSLAAPMIQESAFTRKES
LAWTIVNVTNMKAAPIPSEINTLAKGIISCLQDKSAEIRSLADNLLSILCTQIPLIEFKK
ELKHVKPASQPSIQTILDKYYQKTGQPIPPPSKTKQSTSSSSSSSSTTSQQSSTPSSPQP
IRQQQQQQQQQPTQPQQQQQQQQRRSILQSNNGASNCEFIIFDINGKMNRQKTNQIPSWH
FIEPTEEVVEILQDQVLQCFTEEFANLMFSSLPSNSQHMSDLMIGMIEQNPEAIISVLDI
LFRWITFKLFDTGLASQKRVLKILEILLNKLIDSEYSIGEYEASCLVPILLEKSGSATNE
QIKQIFKQSIQQLEELCLPNVLFRFAIEMVTSQNWRTRVEVLNVMASIIDKNGASVCGNL
KVVIPLITQNLNDSQSKQSSLLCLNKLYSHIKDECFKYSNISQQDKILIIGNNNNNNNNN
NNNNNNNNNNNVQQQQQQQQQQQQQQPRKSLSTDEMSTQLIGCLELLKNYSITKENIEHT
VEALKQFSGLMANGKLDDVFVNFAEEYFLVLTSILADTFPQVSKDATILRLCKYLIHTII
SILSNKVVAKQCNVRCLEIVLNETIKLYSLAESNSSKQGTESELSKAFNQILLRILQNCN
STILFSTLLQMMSRTDNDQSIQHPGKYNDLLLRCLLRATKSLTTPSILEELNVETVLSEI
NSFLKSNPSLDEITRKTTKTLTSELYQNRTNQVVKFTKDIISKGQQQKYQYLLNLLIELV
PKQFEDLINGNGNGNRISSGGSNNNNNNGGRVSSGGNNNNNSGRENINNININSSLDSPI
NDHHNSNNTTTTTTTRSRTSTGSSNSQQQQLPTTSANTTTTTTTTTTTGKTQSTLSTLKIN
KEPRDYSGKTDSQKKELLIEIFKKIGNKDLTLDGIHDLYFFIREYPDYDITPNLNSSSQQ
FQAYITRNLKKIKDSMDAPKDKDDEVVNYQERLKVIQNTFYNQHNQPSSQVVNNNNNNNN
NNNNNNNNNINNNNNNNNNNSGGNENIAPQLSNTASMASNTLQRLRTLNPDQNSGSNNNNS
HQNSPSTSSSNDLNSTVASLRQRLAQLTSKSNE
>P14635
MALRVTRNSKINAENKAKINMAGAKRVPTAPAATSKPGLRPRTALGDIGNKVSEQLQAKM
PMKKEAKPSATGKVIDKKLPKPLEKVPMLVPVPVSEPVPEPEPEPEPEPVKEEKLSPEPI
LVDTASPSPMETSGCAPAEEDLCQAFSDVILAVNDVDAEDGADPNLCSEYVKDIYAYLRQ
LEEEQAVRPKYLLGREVTGNMRAILIDWLVQVQMKFRLLQETMYMTVSIIDRFMQNNCVP
KKMLQLVGVTAMFIASKYEEMYPPEIGDFAFVTDNTYTKHQIRQMEMKILRALNFGLGRP
LPLHFLRRASKIGEVDVEQHTLAKYLMELTMLDYDMVHFPPSQIAAGAFCLALKILDNGE
WTPTLQHYLSYTEESLLPVMQHLAKNVVMVNQGLTKHMTVKNKYATSKHAKISTLPQLNS
ALVQDLAKAVAKV
>O61577
MSVDEICENTKMGREYALLGNYETSLVYYQGVLQQIQKLLTSVHEPQRKHQWQTIRQELS
QEYEHVKNITKTLNGFKSEPAAPEPAPNHRAAPFSHHQHAAKPAAAEPARDPDVWPPPTP
VDHRPSPPYQRAARKDPPRRSEPSKPANRAPGNDRGGRGPSDRRGDARSGGGGRGGARGS

DKDKNRGGKSDKDKKAPSGEEGDEKKFDPAGYDKDLVENLERDIVQRNPNVHWADIAGLT
EAKRLLEEAVVLPLWMPDYFKGIRRPWKGVLMVGPPGTGKTMLAKAVATECGTTFFNVSS
ASLTSKYHGESEKLVRLLFEMARFYAPSTIFIDEIDSICSKRGTGSEHEASRRVKSELLI
QMDGVSGPSAGEESSKMVMVLAATNFPWDIDEALRRRLEKRIYIPLPEIDGREQLLRINL
KEVPLADDIDLKSIAEKMDGYSGADITNVCRDASMMAMRRRIQGLRPEEIRHIPKEELNQ
PSTPADFLLALQKVSKSVGKEDLVKYMAWMEEFGSV
>Q5VYK3
MAAAAASASQDELNQLERVFLRLGHAETDEQLQNIISKFLPPVLLKLSSTQEGVRKKVME
LLVHLNKRIKSRPKIQLPVETLLVQYQDPAAVSFVTNFTIIYVKMGYPRLPVEKQCELAP
TLLTAMEGKPQPQQDSLMHLLIPTLFHMKYPVESSKSASPFNLAEKPKTVQLLLDFMLDV
LLMPYGYVLNESQSRQNSSSAQGSSSNSGGGSGIPQPPPGMSFYAAKRVIGDNPWTPEQL
EQCKLGIVKFIEAEQVPELEAVLHLVIASSDTRHSVATAADLELKSKQSLIDWNNPAIIN
KMYKVYLGDIPLKTKEGAVLKPELKRDPVSTRVKLKIVPHLLRSRQAAETFPANIQVVYD
GLFGTNTNSKLRTLSLQFVHHICITCPEIKIKPLGPMLLNGLTKLINEYKEDPKLLSMAY
SAVGKLSSRMPHLFTKDIALVQQLFEALCKEEPETRLAIQEALSMMVGAYSTLEGAQRTL
MEALVASYLIKPEVQVRQVAVKFASTVFPSDHIPSRYLLLLAAGDPREEVHGEAQRVLRC
LPGRNRKESTSEQMPSFPEMVYYIQEKASHRMKTPVKYMTGTTVLPFNPAAFGEIVLYLR
MCLAHSAGVVPTSQSLADMQDHAPAIGRYIRTLMSSGQMAPSSSNKSGETNPVQIYIGLL
QQLLAGVGGLPVMYCLLEAVSVYPEKLATKFVDKTEWIKSLMNNSKEEMRELAALFYSVV
VSTVSGNELKSMIEQLIKTTKDNHSPEIQHGSLLALGFTVGRYLAKKKMRMSEQQDLERN
ADTLPDQEELIQSATETIGSFLDSTSPLLAIAACTALGEIGRNGPLPIPSEGSGFTKLHL
VESLLSRIPSSKETNKMKERAIQTLGYFPVGDGDFPHQKLLLQGLMDSVEAKQIELQFTI
GEAITSAAIGTSSVAARDAWQMTEEEYTPPAGAKVNDVVPWVLDVILNKHIISPNPHVRQ
AACIWLLSLVRKLSTHKEVKSHLKEIQSAFVSVLSENDELSQDVASKGLGLVYELGNEQD
QQELVSTLVETLMTGKRVKHEVSGETVVFQGGALGKTPDGQGLSTYKELCSLASDLSQPD
LVYKFMNLANHHAMWNSRKGAAFGFNVIATRAGEQLAPFLPQLVPRLYRYQFDPNLGIRQ
AMTSIWNALVTDKSMVDKYLKEILQDLVKNLTSNMWRVRESSCLALNDLLRGRPLDDIID
KLPEIWETLFRVQDDIKESVRKAAELALKTLSKVCVKMCDPAKGAAGQRTIAALLPCLLD
KGMMSTVTEVRALSINTLVKISKSAGAMLKPHAPKLIPALLESLSVLEPQVLNYLSLRAT
EQEKAAMDSARLSAAKSSPMMETINMCLQYLDVSVLGELVPRLCELIRSGVGLGTKGGCA
SVIVSLTTQCPQDLTPYSGKLMSALLSGLTDRNSVIQKSCAFAMGHLVRTSRDSSTEKLL
QKLNGWYMEKEEPIYKTSCALTIHAIGRYSPDVLKNHAKEVLPLAFLGMHEIADEEKSEK
EECNLWTEVWQENVPGSFGGIRLYLQELITITQKALQSQSWKMKAQGAIAMASIAKQTSS
LVPPYLGMILTALLQGLAGRTWAGKEELLKAIACVVTACSAELEKSVPNQPSTNEILQAV
LKECSKENVKYKIVAISCAADILKATKEDRFQEFSNIVIPLIKKNSLESSGVRTTKNEEE
NEKEKELQLEYLLGAFESLGKAWPRNAETQRCYRQELCKLMCERLKLSTWKVQLGVLQSM
NAFFQGLMLLEEEHADPEALAEILLETCKSITYSLENKTYSSVRTEALSVIELLLKKLEE
SKQWECLTSECRVLLIESLATMEPDSRPELQEKAALLKKTLENLE
>Q8N137
MATSADSPSSPLGAEDLLSDSSEPPGLNQVSSEVTSQLYASLRLSRQAEATARAQLYLPS
TSPPHEGLDGFAQELSRSLSVGLEKNLKKKDGSKHIFEMESVRGQLQTMLQTSRDTAYRD
PLIPGAGSERREEDSFDSDSTATLLNTRPLQDLSPSSSAQALEELFPRYTSLRPGPPLNP
PDFQGLRDALDSEHTRRKHCERHIQSLQTRVLELQQQLAVAVAADRKKDTMIEQLDKTLA
RVVEGWNRHEAERTEVLRGLQEEHQAAELTRSKQQETVTRLEQSLSEAMEALNREQESAR

LQQRERETLEEERQALTLRLEAEQQRCCVLQEERDAARAGQLSEHRELETLRAALEEERQ
TWAQQEHQLKEHYQALQEESQAQLEREKEKSQREAQAAWETQHQLALVQSEVRRLEGELD
TARRERDALQLEMSLVQARYESQRIQLESELAVQLEQRVTERLAQAQESSLRQAASLREH
HRKQLQDLSGQHQQELASQLAQFKVEMAEREERQQQVAEDYELRLAREQARVCELQSGNQ
QLEEQRVELVERLQAMLQAHWDEANQLLSTTLPPPNPPAPPAGPSSPGPQEPEKEERRVW
TMPPMAVALKPVLQQSREARDELPGAPPVLCSSSSDLSLLLGPSFQSQHSFQPLEPKPDL
TSSTAGAFSALGAFHPDHRAERPFPEEDPGPDGEGLLKQGLPPAQLEGLKNFLHQLLETV
PQNNENPSVDLLPPKSGPLTVPSWEEAPQVPRIPPPVHKTKVPLAMASSLFRVPEPPSSH
SQGSGPSSGSPERGGDGLTFPRQLMEVSQLLRLYQARGWGALPAEDLLLYLKRLEHSGTD
GRGDNVPRRNTDSRLGEIPRKEIPSQAVPRRLATAPKTEKPPARKKSGHPAPSSMRSRGG
VWR
>Q96KG9
MWFFARDPVRDFPFELIPEPPEGGLPGPWALHRGRKKATGSPVSIFVYDVKPGAEEQTQV
AKAAFKRFKTLRHPNILAYIDGLETEKCLHVVTEAVTPLGIYLKARVEAGGLKELEISWG
LHQIVKALSFLVNDCSLIHNNVCMAAVFVDRAGEWKLGGLDYMYSAQGNGGGPPRKGIPE
LEQYDPPELADSSGRVVREKWSADMWRLGCLIWEVFNGPLPRAAALRNPGKIPKTLVPHY
CELVGANPKVRPNPARFLQNCRAPGGFMSNRFVETNLFLEEIQIKEPAEKQKFFQELSKS
LDAFPEDFCRHKVLPQLLTAFEFGNAGAVVLTPLFKVGKFLSAEEYQQKIIPVVVKMFSS
TDRAMRIRLLQQMEQFIQYLDEPTVNTQIFPHVVHGFLDTNPAIREQTVKSMLLLAPKLN
EANLNVELMKHFARLQAKDEQGPIRCNTTVCLGKIGSYLSASTRHRVLTSAFSRATRDPF
APSRVAGVLGFAATHNLYSMNDCAQKILPVLCGLTVDPEKSVRDQAFKAIRSFLSKLESV
SEDPTQLEEVEKDVHAASSPGMGGAAASWAGWAVTGVSSLTSKLIRSHPTTAPTETNIPQ
RPTPEGVPAPAPTPVPATPTTSGHWETQEEDKDTAEDSSTADRWDDEDWGSLEQEAESVL
AQQDDWSTGGQVSRASQVSNSDHKSSKSPESDWSSWEAEGSWEQGWQEPSSQEPPPDGTR
LASEYNWGGPESSDKGDPFATLSARPSTQPRPDSWGEDNWEGLETDSRQVKAELARKKRE
ERRREMEAKRAERKVAKGPMKLGARKLD
>P14270
MEAEGSSVPARAGSHEGSDSSGGAALKAPKHLWRHEQHHQYPLRQPQFRLLHPHHHLPPP
PPPSPQPQLQPPPPPPLPPPPPPPGATRGRYASSGASRVRHRGYSDTERYLYCRAMDRTS
YAVETGHRPGLKKSRMSWPSSFQGLRRFDVDNGTSAGRSPLDPMTSPGSGLILQANFVHS
QRRESFLYRSDSDYDLSPKSMSRNSSIASDIHGDDLIVTPFAQVLASLRTVRNNFAALTN
LQDRAPSKRSPMCNQPSINKATITEEAYQKLASETLEELDWCLDQLETLQTRHSVSEMAS
NKFKRMLNRELTHLSEMSRSGNQVSEYISNTFLDKQHEVEIPSPTQKEKEKKKRPMSQIS
GVKKLMHSSSLTNSCIPRFGVKTEQEDVLAKELEDVNKWGLHVFRIAELSGNRPLTVIMH
TIFQERDLLKTFKIPVDTLITYLMTLEDHYHADVAYHNNIHAADVVQSTHVLLSTPALEA
VFTDLEILAAIFASAIHDVDHPGVSNQFLINTNSELALMYNDSSVLENHHLAVGFKLLQE
ENCDIFQNLTKKQRQSLRKMAIDIVLATDMSKHMNLLADLKTMVETKKVTSSGVLLLDNY
SDRIQVLQNMVHCADLSNPTKPLQLYRQWTDRIMEEFFRQGDRERERGMEISPMCDKHNA
SVEKSQVGFIDYIVHPLWETWADLVHPDAQDILDTLEDNREWYQSTIPQSPSPAPDDQED
GRQGQTEKFQFELTLEEDGESDTEKDSGSQVEEDTSCSDSKTLCTQDSESTEIPLDEQVE
EEAVAEEESQPQTGVADDCCPDT
>Q24956
MNRAAIAAGKPSGSISTGKPRRKTRAEVSEEMKHEIREAFDLFDADRSGRIDFHELKVAM
RALGFDVKKEEIQRIMNEYDRDQLGEITFQDFEEVMIEKISNRDPTEEILKAFRLFDDDA

TGRISLKNLRRVAKELSENISDEELLAMIQEFDRDGDGEIDEEDFIAILRSTSAFS
>Q9NVX0
MAAANPWDPASAPNGAGLVLGHFIASGMVNQEMLNMSKKTVSCFVNFTRLQQITNIQAEI
YQKNLEIELLKLEKDTADVVHPFFLAQKCHTLQSMNNHLEAVLKEKRSLRQRLLKPMCQE
NLPIEAVYHRYMVHLLELAVTFIERLETHLETIRNIPHLAANLKKMNQALAKMDILVTET
EELAENILKWRKQQNEVSSCIPKILAEESYLYKHDIIMPPLPFTSKVHVQTINAK
>Q76I90
MEALTFPRYSPDDIVAYLRSHVLVGAEARNLTKADLFATLKPEVLHMIFIRILQKVYGIR
LEHFYMMPVNVDIVYPQIFEGFLPVCNLYIHMERFLPVCRVNDFQMSDVINPKAKRTARF
LSGILNFVHFRECRREAYLELQLNYKSAMEKHQQLETANQELEMKLEKLNTVPVEQQAEF
KQLSDDIQELEQLLSHDYRRKTAALQEVISQKKSDITERTRKLNELKVTMATLKEEQEQL
KSKIVESPEELKNYKELMKETVKKLKKSKQEVIEKYEGYRDLVEVLPSCQLEVQLYQKKM
ERQAANVERLASVLSEVRNLEDQLESAQIELKKGKTDEMSLKRLVTAKHERLSTAEIRIK
KKREDVEQYKHTVFEYFNRVQEKRGVVYDKVTAIQKEIQQMRFKIQQLNESAEEEEMKAK
EIYQNLKAGLEKRHDSLIKTAKNYAASREDKIAELQKGLLRVQSPGSSS
>Q94131
MAQTKPIAEQMAALNNSDDTSFAADRSNSLLNATCPARIQNSVDQRKINRSFNDSLSSGY
SGKWLRPKREALKITPLAQIDEAPATKRHSSAKDKHTEYKTRLCDAFRREGYCPYNDNCT
YAHGQDELRVPRRRQEYYSRDPPRERRDSRSRRDDVDTTINRSSSSASKHHDENRRPSNN
HGSSNRRQICHNFERGNCRYGPRCRFIHVEQMQHFNANATVYAPPSSDCPPPIAYYHHHP
QHQQQFLPFPMPYFLAPPPQAQQGAPFPVQYIPQQHDLMNSQPMYAPMAPTYYYQPINSN
GMPMMDVTIDPNATGGAFEVFPDGFFSQPPPTIIS
>Q8TAP6
MSLPPEKASELKQLIHQQLSKMDVHGRIREILAETIREELAPDQQHLSTEDLIKALRRRG
IIDDVMKELNFVTDSVEQELPSSPKQPICFDRQSTLKKTNIDPTRRYLYLQVLGGKAFLE
HLQEPEPLPGQVCSTFTLCLHYRNQRFRSKPVPCACEPDFHDGFLLEVHRESLGDGTRMA
DSTTMLSISDPIHMVLIKTDIFGETTLVASYFLEWRSVLGSENGVTSLTVELMGVGTESK
VSVGILNIKLEMYPPLNQTLSQEVVNTQLALERQKTAEKERLFLVYAKQWWREYLQIRPS
HNSRLVKIFAQDENGINRPVCSYVKPLRAGRLLDTPRQAARFVNVLGYERAPVIGGGGKQ
EQWCTLLAFLCRNKGDCEDHANLLCSLLLGYGLEAFVCVGTKAKGVPHAWVMTCGTDGAI
TFWESLTGHRYIHKPTNPDEPPVAEQPKPLYPYRTIGCVFNHQMFLGNCQPSDAVETCVF
DLNDESKWKPMSEEAIKSVCAPGATTSLPPFPPLCASTIDASVTSNEIEMQLRLLVSEHR
KDLGLTTVWEDQLSYLLSPALASYEFERTTSISAGNEEFQDAIRRAVPDGHTFKGFPIHF
VYRNARRAFATCLRSPFCEEIICCRGDQVRLAVRVRVFTYPESACAVWIMFACKYRSVL
>Q9UKA4
MATFRNNHMKTKASVRKSFSEDVFQSVKSLLQSQKELCSVTAEDCLQQDEHANLTEVTFL
GFNEETDAAHIQDLAAVSLELPDILNSLHFCSLNENEIICMKNINKPLDISSDPLNQSHP
SGMLCVMRVSPTSPRLRIDFIFSLLSKYATGIRYTLDTFLHQKHQLETTDEDDDDTNQSV
SSIEDDFVTAFEHLEEEETSKPYNDGMNITVLRSQCDAASQTVTGHHLETHDLKILISSG
QQKSLAKPSTSSVNVLGHKELPSVKTSVTTSISEPWTQRSFYRSSNASDKDSDLQKTFFS
SSPAYSSESECSSPSPVIFLDEEGYQKSLKAKLELPKIPVMKDDIEDSDSEVSEFFDSFD
QFDELEQTLETCLFNKDPVIGKSSQRKGHKHGKSCMNPQKFKFDRPALPANVRKPTPRKP
ESPYGNLCDAPDSPRPVKASREDSGLFSPIRSSAFSPLGGCTPAECFCQTDIGGDRIHEN
HDSVYYTYEDYAKSISCEVLGSVLRTHHTNTLSNINSIKHGENKTVTFKHGNLDQKNKSK

NKSLMIKDSIQKFAADLVEKSFGSAFKDLQKGVSSCTNALYHLAIKLTSSVLQMAFDELR
RQRAFSLKERAISGLANFLVSEALSNALKDLQYVKKQIFTNTVARFAADLAEELVFEGIM
EVCQFSYPQTPASPQCGSFDFEDKVVKLYAKDLSESVIQEAFIELSQVDVTFTTKAAVSV
STDNIKYVSAESVVPSTQAVTFSPSFHNQAIMVTKPVQEYKKEYTVQQALFCTSGIVTSI
PVPLAGSALLPYHISSTACQAKAHLSSDDSNSNGDSAQVHIATKNREEKAACLRNICLPS
EHNPGNQNDFKPTNDDIEMQSSSKLPNDPAIISNFSAAVVHTIVNETLESMTSLEVTKMV
DERTDYLTKSLKEKTPPFSHCDQAVLQCSEASSNKDMFADRLSKSIIKHSIDKSKSVIPN
IDKNAVYKESLPVSGEESQLTPEKSPKFPDSQNQLTHCSLSAAKDCVPECKVSMVHGSSL
ETLPSCPAVTGQKSDLKESAKDQPLKKHNLNSTSLEALSFGQENPFPHSHTFSSTALTCV
DGLHVEDKQKVRDRNVIPDTPPSTPLVPSRASSEWDIKKLTKKLKGELAKEFAPATPPST
PHNSSVGSLSENEQNTIEKEEFMLKLMRSLSEEVESSESGELPEVDVKSEHSGKKVQFAE
ALATHILSLATEMAASHLDNKIIQEPKVKNPCLNVQSQRSVSPTFLNPSDENLKTLCNFA
GDLAAEVITEAEKIAKVRNCMLFKQKKNSCYADGDEDYKVEEKLDIEAVVHPREVDPFIL
SLPPSSCMSGLMYKYPSCESVTDEYAGHLIQILKQEGGNSELIMDQYANRLAYRSVKSGL
QEAAKTTKVQCNSRMFPVPSSQVKTNKELLMFSNKEHHQEADKKRQSKRNEGYFCKNQTC
ERTLDPYRNEVSQLYSFSTSLVHSITKDAKEELTASLVGLPKSLTDSCLFEKSGYEEDNE
CHVTPELPKSLQPSSQNHRFYHSTGSLNGYGCGDNVVQAVEQYAKKVVDDTLELTLGSTV
FRVSETTKSADRVTYAEKLSPLTGQACRYCDLKELHNCTGNSSQHFFRQGSLASSKPASN
PKFSSRYQKSRIFHLSVPQIHVNLDKKAVLAEKIVAEAIEKAERELSSTSLAADSGIGQE
GASFAESLATETMTAAVTNVGHAVSSSKEIEDFQSTESVSSQQMNLSIGDDSTGSWSNLS
FEDEHQDESSSFHHLSESNGNSSSWSSLGLEGDLYEDNLSFPTSDSDGPDDKDEEHEDEV
EGLGQDGKTLLITNIDMEPCTVDPQLRIILQWLIASEAEVAELYFHDSANKEFMLLSKQL
QEKGWKVGDLLQAVLQYYEVMEKASSEERCKSLFDWLLENA
>O95835
MKRSEKPEGYRQMRPKTFPASNYTVSSRQMLQEIRESLRNLSKPSDAAKAEHNMSKMSTE
DPRQVRNPPKFGTHHKALQEIRNSLLPFANETNSSRSTSEVNPQMLQDLQAAGFDEDMVI
QALQKTNNRSIEAAIEFISKMSYQDPRREQMAAAAARPINASMKPGNVQQSVNRKQSWKG
SKESLVPQRHGPPLGESVAYHSESPNSQTDVGRPLSGSGISAFVQAHPSNGQRVNPPPPP
QVRSVTPPPPPRGQTPPPRGTTPPPPSWEPNSQTKRYSGNMEYVISRISPVPPGAWQEGY
PPPPLNTSPMNPPNQGQRGISSVPVGRQPIIMQSSSKFNFPSGRPGMQNGTGQTDFMIHQ
NVVPAGTVNRQPPPPYPLTAANGQSPSALQTGGSAAPSSYTNGSIPQSMMVPNRNSHNME
LYNISVPGLQTNWPQSSSAPAQSSPSSGHEIPTWQPNIPVRSNSFNNPLGNRASHSANSQ
PSATTVTAITPAPIQQPVKSMRVLKPELQTALAPTHPSWIPQPIQTVQPSPFPEGTASNV
TVMPPVAEAPNYQGPPPPYPKHLLHQNPSVPPYESISKPSKEDQPSLPKEDESEKSYENV
DSGDKEKKQITTSPITVRKNKKDEERRESRIQSYSPQAFKFFMEQHVENVLKSHQQRLHR
KKQLENEMMRVGLSQDAQDQMRKMLCQKESNYIRLKRAKMDKSMFVKIKTLGIGAFGEVC
LARKVDTKALYATKTLRKKDVLLRNQVAHVKAERDILAEADNEWVVRLYYSFQDKDNLYF
VMDYIPGGDMMSLLIRMGIFPESLARFYIAELTCAVESVHKMGFIHRDIKPDNILIDRDG
HIKLTDFGLCTGFRWTHDSKYYQSGDHPRQDSMDFSNEWGDPSSCRCGDRLKPLERRAAR
QHQRCLAHSLVGTPNYIAPEVLLRTGYTQLCDWWSVGVILFEMLVGQPPFLAQTPLETQM
KVINWQTSLHIPPQAKLSPEASDLIIKLCRGPEDRLGKNGADEIKAHPFFKTIDFSSDLR
QQSASYIPKITHPTDTSNFDPVDPDKLWSDDNEEENVNDTLNGWYKNGKHPEHAFYEFTF
RRFFDDNGYPYNYPKPIEYEYINSQGSEQQSDEDDQNTGSEIKNRDLVYV
>Q8TC76

MPTETLQTGSMVKPVSPAGTFTSAVPLRILNKGPDYFRRQAEPNPKRLSAVERLEADKAK
YVKSQEVINAKQEPVKPAVLAKPPVCPAAKRALGSPTLKVFGNHAKTESGVQRENLKLEI
LKNIINSSEGSSSGSGHKHSSRNWPPHRSEATDLHRHSFAESLKVYPTQGRRSPQEGGSH
VGRRLLEQSAESFLHVSHSSSDIRKVTSVKPLKAIPCSSSAPPLPPKPKIAAIASMKSPE
ADPVEPACGVSRRPSLQRSKSDLSDRYFRVDADVERFFNYCGLDPEELENLGMENFARAN
SDIISLNFRSASMISSDCEQSQDSNSDLRNDDSANDRVPYGISAIERNARIIKWLYSIKQ
ARESQKVSHV
>O95359
MGNENSTSDNQRTLSAQTPRSAQPPGNSQNIKRKQQDTPGSPDHRDASSIGSVGLGGFCT
ASESSASLDPCLVSPEVTEPRKDPQGARGPEGSLLPSPPPSQEREHPSSSMPFAECPPEG
CLASPAAAPEDGPQTQSPRREPAPNAPGDIAAAFPAERDSSTPYQEIAAVPSAGRERQPK
EEGQKSSFSFSSGIDQSPGMSPVPLREPMKAPLCGEGDQPGGFESQEKEAAGGFPPAESR
QGVASVQVTPEAPAAAQQGTESSAVLEKSPLKPMAPIPQDPAPRASDRERGQGEAPPQYL
TDDLEFLRACHLPRSNSGAAPEAEVNAASQESCQQPVGAYLPHAELPWGLPSPALVPEAG
GSGKEALDTIDVQGHPQTGMRGTKPNQVVCVAAGGQPEGGLPVSPEPSLLTPTEEAHPAS
SLASFPAAQIPIAVEEPGSSSRESVSKAGMPVSADAAKEVVDAGLVGLERQVSDLGSKGE
HPEGDPGEVPAPSPQERGEHLNTEQSHEVQPGVPPPPLPKEQSHEVQPGAPPPPLPKAPS
ESARGPPGPTDGAKVHEDSTSPAVAKEGSRSPGDSPGGKEEAPEPPDGGDPGNLQGEDSQ
AFSSKRDPEVGKDELSKPSSDAESRDHPSSHSAQPPRKGGAGHTDGPHSQTAEADASGLP
HKLGEEDPVLPPVPDGAGEPTVPEGAIWEGSGLQPKCPDTLQSREGLGRMESFLTLESEK
SDFPPTPVAEVAPKAQEGESTLEIRKMGSCDGEGLLTSPDQPRGPACDASRQEFHAGVPH
PPQGENLAADLGLTALILDQDQQGIPSCPGEGWIRGAASEWPLLSSEKHLQPSQAQPETS
IFDVLKEQAQPPENGKETSPSHPGFKDQGADSSQIHVPVEPQEDNNLPTHGGQEQALGSE
LQSQLPKGTLSDTPTSSPTDMVWESSLTEESELSAPTRQKLPALGEKRPEGACGDGQSSR
VSPPAADVLKDFSLAGNFSRKETCCTGQGPNKSQQALADALEEGSQHEEACQRHPGASEA
ADGCSPLWGLSKREMASGNTGEAPPCQPDSVALLDAVPCLPALAPASPGVTPTQDAPETE
ACDETQEGRQQPVPAPQQKMECWATSDAESPKLLASFPSAGEQGGEAGAAETGGSAGAGD
PGKQQAPEKPGEATLSCGLLQTEHCLTSGEEASTSALRESCQAEHPMASCQDALLPAREL
GGIPRSTMDFSTHQAVPDPKELLLSGPPEVAAPDTPYLHVDSAAQRGAEDSGVKAVSSAD
PRAPGESPCPVGEPPLALENAASLKLFAGSLAPLLQPGAAGGEIPAVQASSGSPKARTTE
GPVDSMPCLDRMPLLAKGKQATGEEKAATAPGAGAKASGEGMAGDAAGETEGSMERMGEP
SQDPKQGTSGGVDTSSEQIATLTGFPDFREHIAKIFEKPVLGALATPGEKAGAGRSAVGK
DLTRPLGPEKLLDGPPGVDVTLLPAPPARLQVEKKQQLAGEAEISHLALQDPASDKLLGP
AGLTWERNLPGAGVGKEMAGVPPTLREDERPEGPGAAWPGLEGQAYSQLERSRQELASGL
PSPAATQELPVERAAAFQVAPHSHGEEAVAQDRIPSGKQHQETSACDSPHGEDGPGDFAH
TGVPGHVPRSTCAPSPQREVLTVPEANSEPWTLDTLGGERRPGVTAGILEMRNALGNQST
PAPPTGEVADTPLEPGKVAGAAGEAEGDITLSTAETQACASGDLPEAGTTRTFSVVAGDL
VLPGSCQDPACSDKAPGMEGTAALHGDSPARPQQAKEQPGPERPIPAGDGKVCVSSPPEP
DETHDPKLQHLAPEELHTDRESPRPGPSMLPSVPKKDAPRVMDKVTSDETRGAEGTESSP
VADDIIQPAAPADLESPTLAASSYHGDVVGQVSTDLIAQSISPAAAHAGLPPSAAEHIVS
PSAPAGDRVEASTPSCPDPAKDLSRSSDSEEAFETPESTTPVKAPPAPPPPPPEVIPEPE
VSTQPPPEEPGCGSETVPVPDGPRSDSVEGSPFRPPSHSFSAVFDEDKPIASSGTYNLDF
DNIELVDTFQTLEPRASDAKNQEGKVNTRRKSTDSVPISKSTLSRSLSLQASDFDGASSS
GNPEAVALAPDAYSTGSSSASSTLKRTKKPRPPSLKKKQTTKKPTETPPVKETQQEPDEE

SLVPSGENLASETKTESAKTEGPSPALLEETPLEPAVGPKAACPLDSESAEGVVPPASGG
GRVQNSPPVGRKTLPLTTAPEAGEVTPSDSGGQEDSPAKGLSVRLEFDYSEDKSSWDNQQ
ENPPPTKKIGKKPVAKMPLRRPKMKKTPEKLDNTPASPPRSPAEPNDIPIAKGTYTFDID
KWDDPNFNPFSSTSKMQESPKLPQQSYNFDPDTCDESVDPFKTSSKTPSSPSKSPASFEI
PASAMEANGVDGDGLNKPAKKKKTPLKTDTFRVKKSPKRSPLSDPPSQDPTPAATPETPP
VISAVVHATDEEKLAVTNQKWTCMTVDLEADKQDYPQPSDLSTFVNETKFSSPTEELDYR
NSYEIEYMEKIGSSLPQDDDAPKKQALYLMFDTSQESPVKSSPVRMSESPTPCSGSSFEE
TEALVNTAAKNQHPVPRGLAPNQESHLQVPEKSSQKELEAMGLGTPSEAIEITAPEGSFA
SADALLSRLAHPVSLCGALDYLEPDLAEKNPPLFAQKLQEELEFAIMRIEALKLARQIAL
ASRSHQDAKREAAHPTDVSISKTALYSRIGTAEVEKPAGLLFQQPDLDSALQIARAEIIT
KEREVSEWKDKYEESRREVMEMRKIVAEYEKTIAQMIEDEQREKSVSHQTVQQLVLEKEQ
ALADLNSVEKSLADLFRRYEKMKEVLEGFRKNEEVLKRCAQEYLSRVKKEEQRYQALKVH
AEEKLDRANAEIAQVRGKAQQEQAAHQASLRKEQLRVDALERTLEQKNKEIEELTKICDE
LIAKMGKS
>P91349
MEDNSVLNEDSNLEHVEGQPRRSMSQPVLNVEGDKRTSSTSATQQQVLSGAFSSADVRSI
PIIQTWEENKALKTKITILRGELQMYQRRYSEAKEASQKRVKEVMDDYVDLKLGQENVQE
KMEQYKLMEEDLLAMQSRIETSEDNFARQMKEFEAQKHAMEERIKELELSATDANNTTVG
SFRGTLDDILKKNDPDFTLTSGYEERKINDLEAKLLSEIDKVAELEDHIQQLRQELDDQS
ARLADSENVRAQLEAATGQGILGAAGNAMVPNSTFMIGNGRESQTRDQLNYIDDLETKLA
DAKKENDKARQALVEYMNKCSKLEHEIRTMVKNSTFDSSSMLLGGQTSDELKIQIGKVNG
ELNVLRAENRELRIRCDQLTGGDGNLSISLGQSRLMAGIATNDVDSIGQGNETGGTSMRI
LPRESQLDDLEESKLPLMDTSSAVRNQQQFASMWEDFESVKDSLQNNHNDTLEGSFNSSM
PPPGRDATQSFLSQKSFKNSPIVMQKPKSLHLHLKSHQSEGAGEQIQNNSFSTKTASPHV
SQSHIPILHDMQQILDSSAMFLEGQHDVAVNVEQMQEKMSQIREALARLFERLKSSAALF
EEILERMGSSDPNADKIKKMKLAFETSINDKLNVSAILEAAEKDLHNMSLNFSILEKSIV
SQAAEASRRFTIAPDAEDVASSSLLNASYSPLFKFTSNSDIVEKLQNEVSELKNELEMAR
TRDMRSPLNGSSGRLSDVQINTNRMFEDLEVSEATLQKAKEENSTLKSQFAELEANLHQV
NSKLGEVRCELNEALARVDGEQETRVKAENALEEARQLISSLKHEENELKKTITDMGMRL
NEAKKSDEFLKSELSTALEEEKKSQNLADELSEELNGWRMRTKEAENKVEHASSEKSEML
ERIVHLETEMEKLSTSEIAADYCSTKMTERKKEIELAKYREDFENAAIVGLERISKEISE
LTKKTLKAKIIPSNISSIQLVCDELCRRLSREREQQHEYAKVMRDVNEKIEKLQLEKDAL
EHELKMMSSNNENVPPVGTSVSGMPTKTSNQKCAQPHYTSPTRQLLHESTMAVDAIVQKL
KKTHNMSGMGPELKETIGNVINESRVLRDFLHQKLILFKGIDMSNWKNETVDQLITDLGQ
LHQDNLMLEEQIKKYKKELKLTKSAIPTLGVEFQDRIKTEIGKIATDMGGAVKEIRKK
>O43303
MEEYEKFCEKSLARIQEASLSTESFLPAQSESISLIRFHGVAILSPLLNIEKRKEMQQEK
QKALDVEARKQVNRKKALLTRVQEILDNVQVRKAPNASDFDQWEMETVYSNSEVRNLNVP
ATFPNSFPSHTEHSTAAKLDKIAGILPLDNEDQCKTDGIDLARDSEGFNSPKQCDSSNIS
HVENEAFPKTSSATPQETLISDGPFSVNEQQDLPLLAEVIPDPYVMSLQNLMKKSKEYIE
REQSRRSLRGSINRIVNESHLDKEHDAVEVADCVKEKGQLTGKHCVSVIPDKPSLNKSNV
LLQGASTQASSMSMPVLASFSKVDIPIRTGHPTVLESNSDFKVIPTFVTENNVIKSLTGS
YAKLPSPEPSMSPKMHRRRSRTSSACHILINNPINACELSPKGKEQAMDLIIQDTDENTN
VPEIMPKLPTDLAGVCSSKVYVGKNTSEVKEDVVLGKSNQVCQSSGNHLENKVTHGLVTV

EGQLTSDERGAHIMNSTCAAMPKLHEPYASSQCIASPNFGTVSGLKPASMLEKNCSLQTE
LNKSYDVKNPSPLLMQNQNTRQQMDTPMVSCGNEQFLDNSFEKVKRRLDLDIDGLQKENC
PYVITSGITEQERQHLPEKRYPKGSGFVNKNKMLGTSSKESEELLKSKMLAFEEMRKRLE
EQHAQQLSLLIAEQEREQERLQKEIEEQEKMLKEKKAMTAEASELDINNAVELEWRKISD
SSLLETMLSQADSLHTSNSNSSGFTNSAMQYSFVSANEAPFYLWGSSTSGLTKLSVTRPF
GRAKTRWSQVFSLEIQAKFNKITAVAKGFLTRRLMQTDKLKQLRQTVKDTMEFIRSFQSE
APLKRGIVSAQDASLQERVLAQLRAALYGIHDIFFVMDAAERMSILHHDREVRKEKMLRQ
MDKMKSPRVALSAATQKSLDRKKYMKAAEMGMPNKKFLVKQNPSETRVLQPNQGQNAPVH
RLLSRQGTPKTSVKGVVQNRQKPSQSRVPNRVPVSGVYAGKIQRKRPNVATI
>P32744
MSRVISRSTPGGTCIVSKDDFLKSFEEVPKMEISSPSDFKEKLDQTIETLSKGQEDWNKR
MNKLKQIRSMVVHGEDVIGREQLLSQLVRLTDCLDLSVKDLRSQILREAAITCGFLFKRF
GTDVRQIAERCLPSAFAQVAVSTKVMATCGAVLTLFIVEFIQTKQIFTCIASYSTSKDKN
QRRQLCALLEIVLEHWNEKIKRTVLPQIGELIKAAICDADPETRVAGRKAFSKLDALHST
EADKLFASVDSSKQKMLRASDAASSSTSINSERGTAPFRSKLSAGSIGGIRNAPNISSKF
LAQRSASAIDTKQVTRMATSVSRTPNIRPMTTRTLSKIDTSPGGSKFARPTVGALGSRTS
SNLRARGSVPTSQPGSRNGSPPRRPSATEAFPAEMQRVKSNLGSNSFVSSLSAEEATKLQ
KAMNTAKESLRQPSRNDDDEFLLPKRPTPQKATPQKSALDTSRVEEVIRACSSTSANEKR
EGIKMLAGIVSEPNLSNAEIKSLGAVLNRLLGESTNQIVLESISSFVKTHHPRLSDWLKL
GLGKLFAKKGAEMTLNSKKQISTTISCILSSFDPTLQLKSTCELVCDPIHLMSPKSRVVL
LEYLNELLGKYMERGSSFNTKEMKATILKMFSWMADQRNEQLITPHGEKVLCSLFALNNA
DFSALFNDFNPDYRDWAYKVLQSHGHDQHVPQQDAVSEEACVRATISTTAAQIEDFVVSR
NLDMTPVKSPSTRAISSGFKRVDAEPLRPLSSEMNSQHRDEELSFNESFDRLKLNSTTHL
IDDTSEQSKYVASKLAQISGDMGAQQYEGLLSIQTMLCEGSFTLWEQNFAKLLIAVFDVL
SKSESDANKKVALRVLTKMCTSQASRLFDSTEMAICKVLDAAVNSQDGTMNVTADDCLKT
LATHLPLAKVVNISQLILNEEKAQEPKASLVLKMMTRLFEGLQADELSPVVDDLAPCVIK
SYDSPSSAVRKTAVYCLVAMVNKLGMKTMEPHLQNLSSGKLNLVQVYVNRAMSSSSHSHV

>Q20010
MNNYDDLPCSIYFKKPTVQEFVDQPRVFEDSEVPAFQEVLQLPQERTKPPVPSTQPIVAA
VEVAKKKSCLSAPKPRKEPPSHPALRQKTVAFGKTVNVSQTVEGTSRNSKKVLASTMSAQ
NTTTTEEQAAENWRDAMKTELQTIRTEIQEETARRQEELNAQNLVKMQELMSNFFQKITI
PKQQAIEPVEKDKENFHESPRQSRQQKPASKIASAREVIKRDGVIPPEALTIIEQRLRSD
PMFRQQIDNVLADAECDANRAAYSPPPPMSEVRYGSGVNPALMRETLTVERSIRYDNGLA
SIDSRQWTNERRDNRAPDSYRTYEPDQPCHSLYQKGQSISYYPSEAAGKTTARNNRTGYY
VEDSSDHEEDVVVNKRGQNYHEQAVPETPAERERRIREKYARRK
>Q8NHQ1
MFPVAPKPQDSSQPSDRLMTEKQQEEAEWESINVLLMMHGLKPLSLVKRTDLKDLIIFDK
QSSQRMRQNLKLLVEETSCQQNMIQELIETNQQLRNELQLEQSRAANQEQRANDLEQIME
SVKSKIGELEDESLSRACHQQNKIKDLQKEQKTLQVKCQHYKKKRTEQEETIASLQMEVC
RLKKEEEDRIVTQNRVFAYLCKRVPHTVLDRQLLCLIDYYESKIRKIHTQRQYKEDESQS
EEENDYRNLDASPTYKGLLMSLQNQLKESKSKIDALSSEKLNLQKDLETRPTQHELRLYK
QQVKKLEKALKKNVKLQELINHKKAEDTEKKDEPSKYNQQQALIDQRYFQVLCSINSIIH
NPRAPVIIYKQTKGGVQNFNKDLVQDCGFEHLVPVIEMWADQLTSLKDLYKSLKTLSAEL

VPWLNLKKQDENEGIKVEDLLFIVDTMLEEVENKEKDSNMPHFQTLQAIVSHFQKLFDVP
SLNGVYPRMNEVYTRLGEMNNAVRNLQELLELDSSSSLCVLVSTVGKLCRLINEDVNEQV
MQVLGPEDLQSIIYKLEEHEEFFPAFQAFTNDLLEILEIDDLDAIVPAVKKLKVLSY
>Q8TCU4
MEPEDLPWPGELEEEEEEEEEEEEEEEEEAAAAAAANVDDVVVVEEVEEEAGRELDSDSHY
GPQHLESIDDEEDEEAKAWLQAHPGRILPPLSPPQHRYSEGERTSLEKIVPLTCHVWQQI
VYQGNSRTQISDTNVVCLETTAQRGSGDDQKTESWHCLPQEMDSSQTLDTSQTRFNVRTE
DTEVTDFPSLEEGILTQSENQVKEPNRDLFCSPLLVIQDSFASPDLPLLTCLTQDQEFAP
DSLFHQSELSFAPLRGIPDKSEDTEWSSRPSEVSEALFQATAEVASDLASSRFSVSQHPL
IGSTAVGSQCPFLPSEQGNNEETISSVDELKIPKDCDRYDDLCSYMSWKTRKDTQWPENN
LADKDQVSVATSFDITDENIATKRSDHFDAARSYGQYWTQEDSSKQAETYLTKGLQGKVE
SDVITLDGLNENAVVCSERVAELQRKPTRESEYHSSDLRMLRMSPDTVPKAPKHLKAGDT
SKGGIAKVTQSNLKSGITTTPVDSDIGSHLSLSLEDLSQLAVSSLETTTGQHTDTLNQKT
LADTHLTEETLKVTAIPEPADQKTATPTVLSSSHSHRGKPSIFYQQGLPDSHLTEEALKV
SAAPGLADQTTGMSTLTSTSYSHREKPGTFYQQELPESNLTEEPLEVSAAPGPVEQKTGI
PTVSSTSHSHVEDLLFFYRQTLPDGHLTDQALKVSAVSGPADQKTGTATVLSTPHSHREK
PGIFYQQEFADSHQTEETLTKVSATPGPADQKTEIPAVQSSSYSQREKPSILYPQDLADS
HLPEEGLKVSAVAGPADQKTGLPTVPSSAYSHREKLLVFYQQALLDSHLPEEALKVSAVS
GPADGKTGTPAVTSTSSASSSLGEKPSAFYQQTLPNSHLTEEALKVSIVPGPGDQKTGIP
SAPSSFYSHREKPIIFSQQTLPDFLFPEEALKVSAVSVLAAQKTGTPTVSSNSHSHSEKS
SVFYQQELPDSDLPRESLKMSAIPGLTDQKTVPTPTVPSGSFSHREKPSIFYQQEWPDSY
ATEKALKVSTGPGPADQKTEIPAVQSSSYPQREKPSVLYPQVLSDSHLPEESLKVSAFPG
PADQMTDTPAVPSTFYSQREKPGIFYQQTLPESHLPKEALKISVAPGLADQKTGTPTVTS
TSYSQHREKPSIFHQQALPGTHIPEEAQKVSAVTGPGNQKTWIPRVLSTFYSQREKPGIF
YQQTLPGSHIPEEAQKVSPVLGPADQKTGTPTPTSASYSHTEKPGIFYQQVLPDNHPTEE
ALKISVASEPVDQTTGTPAVTSTSYSQYREKPSIFYQQSLPSSHLTEEAKNVSAVPGPAD
QKTVIPILPSTFYSHTEKPGVFYQQVLPHSHPTEEALKISVASEPVDQTTGTPTVTSTSY
SQHTEKPSIFYQQSLPGSHLTEEAKNVSAVPGPGDRKTGIPTLPSTFYSHTEKPGSFYQQ
VLPHSHLPEEALEVSVAPGPVDQTIGTPTVTSPSSSFGEKPIVIYKQAFPEGHLPEESLK
VSVAPGPVGQTTGAPTITSPSYSQHRAKSGSFYQLALLGSQIPEEALRVSSAPGPADQTT
GIPTITSTSYSFGEKPIVNYKQAFPDGHLPEEALKVSIVSGPTEKKTDIPAGPLGSSALG
EKPITFYRQALLDSPLNKEVVKVSAAPGPADQKTETLPVHSTSYSNRGKPVIFYQQTLSD
SHLPEEALKVPPVPGPDAQKTETPSVSSSLYSYREKPIVFYQQALPDSELTQEALKVSAV
PQPADQKTGLSTVTSSFYSHTEKPNISYQQELPDSHLTEEALKVSNVPGPADQKTGVSTV
TSTSYSHREKPIVSYQRELPHFTEAGLKILRVPGPADQKTGINILPSNSYPQREHSVISY
EQELPDLTEVTLKAIGVPGPADQKTGIQIASSSSYSNREKASIFHQQELPDVTEEALNVF
VVPGQGDRKTEIPTVPLSYYSRREKPSVISQQELPDSHLTEEALKVSPVSIPAEQKTGIP
IGLSSSYSHSHKEKLKISTVHIPDDQKTEFPAATLSSYSQIEKPKISTVIGPNDQKTPSQ
TAFHSSYSQTVKPNILFQQQLPDRDQSKGILKISAVPELTDVNTGKPVSLSSSYFHREKS
NIFSPQELPGSHVTEDVLKVSTIPGPAGQKTVLPTALPSSFSHREKPDIFYQKDLPDRHL
TEDALKISSALGQADQITGLQTVPSGTYSHGENHKLVSEHVQRLIDNLNSSDSSVSSNNV
LLNSQADDRVVINKPESAGFRDVGSEEIQDAENSAKTLKEIRTLLMEAENMALKRCNFPA
PLARFRDISDISFIQSKKVVCFKEPSSTGVSNGDLLHRQPFTEESPSSRCIQKDIGTQTN
LKCRRGIENWEFISSTTVRSPLQEAESKVSMALEETLRQYQAAKSVMRSEPEGCSGTIGN

KIIIPMMTVIKSDSSSDASDGNGSCSWDSNLPESLESVSDVLLNFFPYVSPKTSITDSRE
EEGVSESEDGGGSSVDSLAAHVKNLLQCESSLNHAKEILRNAEEEESRVRAHAWNMKFNL
AHDCGYSISELNEDDRRKVEEIKAELFGHGRTTDLSKGLQSPRGMGCKPEAVCSHIIIES
HEKGCFRTLTSEHPQLDRHPCAFRSAGPSEMTRGRQNPSSCRAKHVNLSASLDQNNSHFK
VWNSLQLKSHSPFQNFIPDEFKISKGLRMPFDEKMDPWLSELVEPAFVPPKEVDFHSSSQ
MPSPEPMKKFTTSITFSSHRHSKCISNSSVVKVGVTEGSQCTGASVGVFNSHFTEEQNPP
RDLKQKTSSPSSFKMHSNSQDKEVTILAEGRRQSQKLPVDFERSFQEEKPLERSDFTGSH
SEPSTRANCSNFKEIQISDNHTLISMGRPSSTLGVNRSSSRLGVKEKNVTITPDLPSCIF
LEQRELFEQSKAPRADDHVRKHHSPSPQHQDYVAPDLPSCIFLEQRELFEQCKAPYVDHQ
MRENHSPLPQGQDSIASDLPSPISLEQCQSKAPGVDDQMNKHHFPLPQGQDCVVEKNNQH
KPKSHISNINVEAKFNTVVSQSAPNHCTLAASASTPPSNRKALSCVHITLCPKTSSKLDS
GTLDERFHSLDAASKARMNSEFNFDLHTVSSRSLEPTSKLLTSKPVAQDQESLGFLGPKS
SLDFQVVQPSLPDSNTITQDLKTIPSQNSQIVTSRQIQVNISDFEGHSNPEGTPVFADRL
PEKMKTPLSAFSEKLSSDAVTQITTESPEKTLFSSEIFINAEDRGHEIIEPGNQKLRKAP
VKFASSSSVQQVTFSRGTDGQPLLLPYKPSGSTKMYYVPQLRQIPPSPDSKSDTTVESSH
SGSNDAIAPDFPAQVLGTRDDDLSATVNIKHKEGIYSKRVVTKASLPVGEKPLQNENADA
SVQVLITGDENLSDKKQQEIHSTRAVTEAAQAKEKESLQKDTADSSAAAAAEHSAQVGDP
EMKNLPDTKAITQKEEIHRKKTVPEEAWPNNKESLQINIEESECHSEFENTTRSVFRSAK
FYIHHPVHLPSDQDICHESLGKSVFMRHSWKDFFQHHPDKHREHMCLPLPYQNMDKTKTD
YTRIKSLSINVNLGNKEVMDTTKSQVRDYPKHNGQISDPQRDQKVTPEQTTQHTVSLNEL
WNKYRERQRQQRQPELGDRKELSLVDRLDRLAKILQNPITHSLQVSESTHDDSRGERSVK
EWSGRQQQRNKLQKKKRFKSLEKSHKNTGELKKSKVLSHHRAGRSNQIKIEQIKFDKYIL
SKQPGFNYISNTSSDCRPSEESELLTDTTTNILSGTTSTVESDILTQTDREVALHERSSS
VSTIDTARLIQAFGHERVCLSPRRIKLYSSITNQQRRYLEKRSKHSKKVLNTGHPLVTSE
HTRRRHIQVANHVISSDSISSSASSFLSSNSTFCNKQNVHMLNKGIQAGNLEIVNGAKKH
TRDVGITFPTPSSSEAKLEENSDVTSWSEEKREEKMLFTGYPEDRKLKKNKKNSHEGVSW
FVPVENVESRSKKENVPNTCGPGISWFEPITKTRPWREPLREQNCQGQHLDGRGYLAGPG
REAGRDLLKPFVRATLQESLQFHRPDFISRSGERIKRLKLIVQERKLQSMLQTERDALFN
IDRERQGHQNRMCPLPKRVFLAIQKNKPISKKEMIQRSKRIYEQLPEVQKKREEEKRKSE
YKSYRLRAQLYKKRVTNQLLGRKVPWD

>Q5SW79

MSLTSWFLVSSGGTRHRLPREMIFVGRDDCELMLQSRSVDKQHAVINYDASTDEHLVKDL
GSLNGTFVNDVRIPEQTYITLKLEDKLRFGYDTNLFTVVQGEMRVPEEALKHEKFTIQLQ
LSQKSSESELSKSASAKSIDSKVADAATEVQHKTTEALKSEEKAMDISAMPRGTPLYGQP
SWWGDDEVDEKRAFKTNGKPEEKNHEAGTSGCGIDAKQVEEQSAAANEEVLFPFCREPSY
FEIPTKEFQQPSQITESTIHEIPTKDTPSSHITGAGHASFTIEFDDSTPGKVTIRDHVTK
FTSDQRHKSKKSSPGTQDLLGIQTGMMAPENKVADWLAQNNPPQMLWERTEEDSKSIKSD
VPVYLKRLKGNKHDDGTQSDSENAGAHRRCSKRATLEEHLRRHHSEHKKLQKVQATEKHQ
DQAVTSSAHHRGGHGVPHGKLLKQKSEEPSVSIPFLQTALLRSSGSLGHRPSQEMDKMLK
NQATSATSEKDNDDDQSDKGTYTIELENPNSEEVEARKMIDKVFGVDDNQDYNRPVINEK
HKDLIKDWALSSAAAVMEERKPLTTSGFHHSEEGTSSSGSKRWVSQWASLAANHTRHDQE
ERIMEFSAPLPLENETEISESGMTVRSTGSATSLASQGERRRRTLPQLPNEEKSLESHRA
KVVTQRSEIGEKQDTELQEKETPTQVYQKDKQDADRPLSKMNRAVNGETLKTGGDNKTLL
HLGSSAPGKEKSETDKETSLVKQTLAKLQQQEQREEAQWTPTKLSSKNVSGQTDKCREET

FKQESQPPEKNSGHSTSKGDRVAQSESKRRKAEEILKSQTPKGGDKKESSKSLVRQGSFT
IEKPSPNIPIELIPHINKQTSSTPSSLALTSASRIRERSESLDPDSSMDTTLILKDTEAV
MAFLEAKLREDNKTDEGPDTPSYNRDNSISPESDVDTASTISLVTGETERKSTQKRKSFT
SLYKDRCSTGSPSKDVTKSSSSGAREKMEKKTKSRSTDVGSRADGRKFVQSSGRIRQPSV
DLTDDDQTSSVPHSAISDIMSSDQETYSCKPHGRTPLTSADEHVHSKLEGSKVTKSKTSP
VVSGSSSKSTTLPRPRPTRTSLLRRARLGEASDSELADADKASVASEVSTTSSTSKPPTG
RRNISRIDLLAQPRRTRLGSLSARSDSEATISRSSASSRTAEAIIRSGARLVPSDKFSPR
IRANSISRLSDSKVKSMTSAHGSASVNSRWRRFPTDYASTSEDEFGSNRNSPKHTRLRTS
PALKTTRLQSAGSAMPTSSSFKHRIKEQEDYIRDWTAHREEIARISQDLALIAREINDVA
GEIDSVTSSGTAPSTTVSTAATTPGSAIDTREELVDRVFDESLNFRKIPPLVHSKTPEGN
NGRSGDPRPQAAEPPDHLTITRRRTWSRDEVMGDNLLLSSVFQFSKKIRQSIDKTAGKIR
ILFKDKDRNWDDIESKLRAESEVPIVKTSSMEISSILQELKRVEKQLQAINAMIDPDGTL
EALNNMGFPSAMLPSPPKQKSSPVNNHHSPGQTPTLGQPEARALHPAAVSAAAEFENAES
EADFSIHFNRFNPDGEEEDVTVQE
>Q13042
MNLERLRKRVRQYLDQQQYQSALFWADKVASLSREEPQDIYWLAQCLYLTAQYHRAAHAL
RSRKLDKLYEACRYLAARCHYAAKEHQQALDVLDMEEPINKRLFEKYLKDESGFKDPSSD
WEMSQSSIKSSICLLRGKIYDALDNRTLATYSYKEALKLDVYCFEAFDLLTSHHMLTAQE
EKELLESLPLSKLCNEEQELLRFLFENKLKKYNKPSETVIPESVDGLQENLDVVVSLAER
HYYNCDFKMCYKLTSVVMEKDPFHASCLPVHIGTLVELNKANELFYLSHKLVDLYPSNPV
SWFAVGCYYLMVGHKNEHARRYLSKATTLEKTYGPAWIAYGHSFAVESEHDQAMAAYFTA
AQLMKGCHLPMLYIGLEYGLTNNSKLAERFFSQALSIAPEDPFVMHEVGVVAFQNGEWKT
AEKWFLDALEKIKAIGNEVTVDKWEPLLNNLGHVCRKLKKYAEALDYHRQALVLIPQNAS
TYSAIGYIHSLMGNFENAVDYFHTALGLRRDDTFSVTMLGHCIEMYIGDSEAYIGADIKD
KLKCYDFDVHTMKTLKNIISPPWDFREFEVEKQTAEETGLTPLETSRKTPDSRPSLEETF
EIEMNESDMMLETSMSDHST
>O75935
MAGLTDLQRLQARVEELERWVYGPGGARGSRKVADGLVKVQVALGNISSKRERVKILYKK
IEDLIKYLDPEYIDRIAIPDASKLQFILAEEQFILSQVALLEQVNALVPMLDSAHIKAVP
EHAARLQRLAQIHIQQQDQCVEITEESKALLEEYNKTTMLLSKQFVQWDELLCQLEAATQ
VKPAEE
>Q76I89
MRRSSTTSGSSGRQSTMVLRVQDSNKMGLQTPQTKDRGTFGKLSMSKTTSATSERKVSFF
GKRASGAGGSRNSQYGMFGTEKIKDPRPLHDKAFIQQCIKQLCEFLVENGYAHNVTIKSL
QSPSVKDFIKIFTFIYGFLCPSYELPDSKFEEEIPRVFKELGYPFPLSKSSMYTVGAPHT
WPQIVAALIWLTDCFKLYAAMRENPSSFDDGQNWGGETDDGIVHNKLFMDYVVKCYEHFM
KGGDTYEELDAEVQSKLKDLFNVDEFKIEGLAAENKRLHEEIARLEKERESEPDRLVSLR
KLRSSFQADVQKYQAYLANLESHTTILDQKMKSVNEEVETTEMEVEAMKQENARLQHIFD
NQKYSVADIERINHERNELQQTINKLTKELEAEEHQLWNEELKYARNKEAIETQLAEYHK
LARKLKLIPISAENSKGHDFEIHFNPEAGPNCLVKYRTQIKAPLMEIVNQTEEEIRKATQ
RKMSLEDTLEQVNAMVAEKKSSVKTLKEEAEKLDDLYHQKLKEAEEEEQKCANELELLEK
HKQLLESGVNEGLSEATNELHDIQRQYQIVMQTTTEESRKAGDNLNRLLEVITTHVVSIE
KYLDEQNSKIDRDYEEFMSEDLLSTLTGILDSYKKKAESI

(5) 385 chloroplast proteins ($\mathbb{S}_5$)

>Q9MTN3
MGSAKWDSRALSFYDTKKPGKPHTIKQHLHRRKGERGIRTLDNSLLRTMPVFKTGAINHS
AISPRDNFYYIFIPPNRTWPYQLIPSLSIDRIPTGYLSIVNLSRYTVQINA
>Q9TL05
MSGEVAQDQPPIRSPRSTRRSQEIVNSAITVQSSAKLDTDKSGAQADKVSSKKSRTTSPE
LLEDQVVSNESKTSKKKETKASQLFSKRKEIFSTRDLRFLKLRIASPTRIRSWGTHKLPN
GKQVGRITKAETINYRTYKPEMDGLFCERAFGPVKDWECHCGRTKGQERNKDGIPIPRVC
THCGVELRDSKIRRHRMGYIELVYPVVHIWYLKSIPSYLGVLLDKPRRELEAITYCTNYA
SSQDSMAFSASLLFPTTGSFRQSKDSMKWEYLNWFHIETYLGLKEATNSALVHYGKRIQD
SPIEVGLPLSEDPRQFSIGAQAIACQLRALNLRSVSRLLSRDLYIIDARETRFGALEEEE
MKRRSKLIRRLQLIHYFMQTKAQPEWMVIKALPVLPPDLRPIVQLEGGRFATTDLNDLYR
RVLNRNNRFMKLHKMVAPETLIRSEKRLLQEAVDGLFDNGKRGKPVLNSSNRPLKSLADA
LKGKQGRFRQNLLGKRVDYSGRSVIVVGPKLRLHQCGLPKEMALELFQPLVIRLLLKRKV
APNIRYAKKLMHHAVARGPENPTIIDAVVWDTLAAVVEGYPILLNRAPTLHRLGIQAFEP
VLINGRAIQLHPLVCTGFNADFDGDQMGVHIPLSAEARAEAKLLMLASHNLLSPATGQPI
VVPSQDMVLGWYYLTTENPWIESTEGLYFSGLSDVEHAYHQGQIHLHSIIWVRWGGESEG
SLGDEFEDNPLEIRIDANGHSWHIYSQYQLRYDDEGDLISQFIRTTTGRVVFNQLVHRHI
EWSLHDQVMEEIETEFPESVLPPDVMRCLVRLYSAHAIGERPAMLGLASPGTPSPGRACA
YPPRDDYADPNQIKEYLNRIGHW
>Q05728
MEARILQSSSSCYSSLYAVNRSRFSSVSSPKPFSVSFAQTTRTRTRVLSMSKKDGRTDKD
DDTDSLNYKDSGVDIDAGAELVKRIAKMAPGIGGFGGLFPLGDSYLVAGTDGVGTKLKLA
FETGIHDTIGIDLVAMSVNDIITSGAKPLFFLDYFATSRLDVDLAEKVIKGIVEGCRQSE
CALLGGETAEMPDFYAEGEYDLSGFAVGIVKKTSVINGKNIVAGDVLIGLPSSGVHSNGF
SLVRRVLARSNLSLKDALPGGSSTLGDALMAPTVIYVKQVLDMIEKGGVKGLAHITGGGF
TDNIPRVFPDGLGAVIHTDAWELPPLFKWIQQTGRIEDSEMRRTFNLGIGMVMVVSPEAA
SRILEEVKNGDYVAYRVGEVVNGEGVSYQ
>P51346
MNFLNKSLIRDSVVLQNRWKIIMKVSLNWLKELAKIEIIDAHSLANKLTQAGFEVEDVEM
VNIDGHKDYILDVTSTANRSDVLSMIGLSREVSALTQADIVKTADIPSAGLFDNISTIIS
DDSLLNCSYYVSAIMDNIVIQDSPKWLKNRLYSCGFISQNLIIDISNYIMLKWGQPINII
DLKKIADTNTESYIEITSNFCSSKQKSVKLDNKDIALSRDVLITQINNNITSIAGIGINQ
EFHVDQNTKLIFIEAAIFKEAVVRKSSRSIGIRTESSIRQERGLNIDNGRSAYRETLSLL
IELTHGKVKATFLKKETENSTLNIDISLKKIQDVLGPIKDNHTVRFLSFDEIENTLKSLQ
FKITKKDVKKFNVIIPSYRKYDVFREIDIVEEIARVYGYNQFQSKIPKIQFTKHPSSRRN
FIDQIRNILRNLGLTELVHYSLVKSKGEINLKNPLIKDYSTLRSSLLEGLINASAYNIKQ
SNQTVDGFEIGTVFNLKRNKIIETTKLAIILGGSLDIRSEWSEPAHSLNWYEAKGIIENF
FRKLNKSIQWVKRESSNDQINFIQNNKSATLTYNNDNIGLFGELNELTSSQFGFNTELFV
LEIDLDILQYSDPEINYLSYRIQPYSKYPCITRDLCIVIPKKMQINSLFQLLNQFNDNDL
ENMTLFDQYSNKLLGNGKKSIGLRFTYRSDHKTLTNLEIDNKQNELQKNIIKKLNLEIRK

>A1EA57
MAVPKKRTSMSKKRIRKNIWKKKTYFSIVHSYSLAKSRSFSSGNEHPKPKGFSGQQQQTN
K

>P05724
MQGLEYTLEPEQAKKCKTHQLVDLAKIDLNSWSQENVRPKTLLGRTSKKRLTSQFWYEFT
KKQKLKNALYYFVIYDITDNSILPVETLNQKNPLSTSFPFPFGRPLRGINFSGSCLHCLL
RVLK
>Q8LPT9
MSNSIGRNVLHQSLLCSTVFEHQSNRHSSGIPANSLFQAVSINQPAGASAARKSPLSTKF
YGTSLNARPKMAMGRHRPVLITPRAVLAVDSASELAGKFNLEGNVELQITVGAPTPGSLT
QVNIEISYSSNSLLLHWGAIRDKKEKWVLPSRPPDGTKILKNRALRTPFVSSGSKSLVKL
EIDDPAIEAVEFLILDEAQNKWFKNNGANFHVKLPSERSLIQNVSVPEDLVQTQAYLRWE
RKGKQIYTPEQEKEEYEAARTELLEEIVRGTSVEDLRAKLTNKNDRQEIKESSSHGTKNA
IPDDLVQIQSYIRWERAGKPNYSADQQLREFEEARKELQSELEKGISLDEIWKKITKGEI
QTKVSDQLKTKKYFRTERIQRKQRDFMQILNKHVAEPTEKKNISVEPKALTPVELFVGAT
EEQEGDSILNKKIYKLAGKELLVLVHKPGGKTKIHLATDGKEPLILHWALSKKAGEWLAP
PPSVLPAGSVLLSGSVETTFTTSSLADLPYQVQSIEIEIEEEGYVGMPSVLQSGGNWIKN
KGSDFYVDFSYESKQVQQDFGDGKGTAKALLEKIAGLEIEAQKSFMHRFNIAADLIQEAK
EAGELGFAGILVWMRFMATRQLIWNKNYNVKPREISKAQDRLTDLLQNVYISNPEYREIV
RMILSTVGRGGEGDVGQRIRDEILVIQRNNNCKGGMMEEWHQKLHNNTSPDDVIICQALI
DYIKSDFDISAYWKTLNDNGITKERLLSYDRAIHSEPNFRRDQKDGLLRDLGNYMRTLKA
VHSGADLESAITNCLGYRSEGQGFMVGVQINPIPNLPSGFPELLQFVSEHVEDRNVEALL
EGLLEARQEIRPLLCKHNDRLKDLLFLDIALESSVRTAIEKGYEELNEAGPEKIMYFVSL
ILENLALSLDDNEDLIYCLKGWSNALSMSKSKSDNWALFAKSVLDRTRLALAGKADWYQK
VLQPSAEYLGTLLSVDKWAVDIFTEEMIRAGSAAALSLLLNRLDPVLRKTASLGSWQVIS
PVEVFGYVAVVDELLAVQDKSYDQPTILLARRVKGEEEIPHGTVAVLTADMPDVLSHVSV
RARNCKVCFATCFDPNILADLQSNEGKMLHLKPTSADIAYSVVEGSELQDSSSANLKEED
GPSSSVALVKKQFAGRYAITSDEFTGELVGAKSRNIAYLKGKVPSWIGIPTSVALPFGVF
EKVLSDDINQAVAEKLQILKQKLGEEDHSALREIRETVLQMKAPNQLVQELKTEMKSSGM
PWPGDEGEQRWEQAWMAIKKVWASKWNERAFFSTRRVKLDHEYLCMAVLVQEIINADYAF
VIHTTNPSSGDSSEIYAEVVKGLGETLVGAYPGRALSFVCKKNDLKSPRVLGYPSKPIGL
FIRRSIIFRSDSNGEDLEGYAGAGLYDSVPMDEAEKVVLDYSSDHLITDGHFQQSILSSI
ARAGCEIEELFGSAQDIEGVVRDGKIYVVQTRPQM
>P0C306
MVYGYGKSNMPHPNRKRKGTDTQYDYWEELLVMVSGLYVLFCVFLVLFIFFDSFKQESNK
LELSGKEEKKKLNGENRLSRDIQNLLYIK
>Q3ZJ89
MTNKLDCLKLINFIEKSNLFTTNKIRDKKLLIAFSGGQDSSCLLTIFYILSKKWGFKLGA
VYCNHCWTDSPQSSLSAFDTLQMFDIPFYFVESPNSEPMKPEQKARDWRYSSFYTISKWE
DYDFVLTGHSLSDCVETVLFNLFRGSGLKGICSLKEDQKFSNLKTNDFFFQKTVCFLDPF
FYFSDKKGNPFFRKYRLFQSMSFLSIFPKFDFFAFGENCAIDKSNFPLKNKSKGKNLQFL
TSFDIKNWFGIKKICRFCFLDKIGQTGIKKKRGTNRVPILIFSRRVNKTTSPLALNFQTK
NKRVDRKSFLFRFSLAFALPLAFALRAKGEKVRKNALLNQIKPVSFFFKTKKDKTETNLF
HRYLQTIKKLQNRDKISSFITIFRPFILTLGNVKIKGKKIKNKNLPFQIKKLIRKKEKEK
GILWNSLSKLDNKDKLISNQIQKKGFKLIPKFILTKDKKSFSNFPPFIFDQLEKTFRKEK
VDTKPRNSSPQFEVLFNKLSQPSSDKKNVDFIVFRPLIKITRETLFLFSNNLKIPIYYDK
SNKDLNITRNYIRKVLVPLLKKINPRVEENIYKFSKIIEFYYQVFGDLKCPSDRFDIFNP

>P52410
MQALQSSSLRASPPNPLRLPSNRQSHQLITNARPLRRQQRSFISASASTVSAPKRETDPK
KRVVITGMGLVSVFGNDVDAYYEKLLSGESGISLIDRFDASKFPTRFGGQIRGFSSEGYI
DGKNERRLDDCLKYCIVAGKKALESANLGGDKLNTIDKRKAGVLVGTGMGGLTVFSEGVQ
NLIEKGHRRISPFFIPYAITNMGSALLAIDLGLMGPNYSISTACATSNYCFYAAANHIRR
GEADMMIAGGTEAAIIPIGLGGFVACRALSQRNDDPQTASRPWDKARDGFVMGEGAGVLV
MESLEHAMKRGAPIVAEYLGGAVNCDAHHMTDPRADGLGVSSCIERCLEDAGVSPEEVNY
INAHATSTLAGDLAEINAIKKVFKSTSGIKINATKSMIGHCLGAAGGLEAIATVKAINTG
WLHPSINQFNPEQAVDFDTVPNEKKQHEVDVAISNSFGFGGHNSVVAFSAFKP
>P31920
MVQKLRKKYKFKINTTSSRYYGSYNITRNWRILIRTNFQSLIEMDYSFIKKWHAIINDDK
ISFIMYTICKYVDFQKETDIFIKAYLELKKNCRPKYLEDLFSFDKNTIISIEKAKGDSPG
NVGGFIINCRDPEEELFWNRIPRYESKNNNCLSFKFITQYNLIEKARGNYFLPKIFLEVL
KKGPEEIDKEKRKTVLAKKIDNDEEIEKIEIKKEKQFEIKSFFEDSEFLAITEKYTKKCE
DLININNKGKKIRDLFCNYILELLILEINILNSQSKPKYSDVFICTYKKKRTIMK
>P51234
MLPNSILKKIYICNQKEKIISRSFLLKLFKSQIGYPKSLAQLNSALSSIMRWYSHKGYQW
ALIQMHYIEGSSSIIVNIDEGLISTIRTEYYTSSLERSSKSSYTNMIEKHLSIRIGHPIN
IIYLKRKINYLKKNKLVGNIIYSVERSRNNSSHLDLKFQIQELQDKELVLFGENLYKTSY
VIAFLSHLLQEPLYLANSWETYNIDQMSANRFKLHYYQPICNYQYINNKELADILIYLFS
KSTFQHIKNSRFNPLLNCDGETTSRCKLYLRNLSNASSYFTVSMHSLENTINLKLRYLNP
SLRISKKFTIQVVIQIIKKIASSCIFPSSIFLKNQKIFGQKFTNQYIFEGLMTYNITSCF
SMSEKIFLSRNVQTKYFVKNLQTVKFISTTKALIGSNDHWLKKQSKLLYRQFLVLWLKLY
YQNFDTLKWPTKGHLLEIESCYFTPFQESTFLNYHSQFHYNNLFFHKINIQHITHFSLPF
YFQSRINYILRNVLKVQSNLKMETLPILLCRAYFEDKVCKPFFNFSIRIRTEYQVPINNK
SRISFFCNYIRPFLKNSYQTCIIFQSSLINQKFISSLYQKLFYGLEIQLKLPINQIPPLS
IEYTINSGRKFCIYLYISHQQ
>Q9TLV9
MVRPLNDFHEAKVRIKLSSYSASLLNLSCNNVLQSIKESNTSVKGPVFLPTRRRVYCVLR
SPHVNKDSREHFEIRSHFRIIDISSCSSETIEMLMRLDLPTGVNIEIKT
>P32761
MSNFILKPGEKLPQDKLEELKKINDAVKKTKNFSKYLIDLRKLFQIDEVQVTSESKLFLA
GFLEGEASLNISTKKLATSKFGLVVDPEFNVTQHVNGVKVLYLALEVFKTGRIRHKSGSN
ATLVLTIDNRQSLEEKVIPFYEQYVVAFSSPEKVKRVANFKALLELFNNDAHQDLEQLVN
KILPIWDQMRKQQGQSNEGFPNLEAAQDFARNYKKGIK
>P05728
MIFYEIFQVSFYQNFLWEKSVRLLFKVQRRLFKVSYIHDKKNLYELQKLIFQSNCARLLA
IREVTQLSFNKKISGVDGKTTLNFLERFELNEYLRKNWNNWKPQSLRKRCVFDLNENLIS
DTISTISDRSWQVLIKFALEPVHEAFFHPFNFGFRYNVPIYKVQQAILLNLSNISFGSKK
RILKVELNCNFSIFNYDYLMKKLIAPRNIKLGIFRLLLELGFNLHFPENECQISTFSSLLL
NVMLNGVENVHNCVRYGYYMLFFLRPMDNEKILANQILSLLYTRGIKKNSSKFLLVSNTK
GFDFLGWHFKFSEKVKNGISAIPSLNNYQFFLNRVKRIVNNSNYGSVVKASKLYPVIKNW
REYHKYSDLRSLSYSLFFVKKHAFSAFNSESKQDFYSSKRLLFKSFLVSESFNTVSKKYN

FHILNCFSFGHLTFLSESINFFNKINLYFCVHCGMKCI
>Q9MUL5
MSNTRLTLFFTNIFQLLDSKIQNYSINFPIKILLLLLGFFIATVLATVFGQTGDWDVLVA
GILVAMIEILGNKMYSKKYISKKQVFDISFLSLIGINYIKIGLIFGLFVDAFKLGS
>O20118
MENATVPNDQNEDYIDLTKPMSKSYWKKMKVKGFYRWVLVRDFKKINDILNTNEIVPILV
SGAKTFTKSRFSAMRWSLNEFAFVDGFFFLLLFFFYKKKKRSKISNTSKRKKRTKKKRTR
NLGNLCFLRKNLASILFF
>P25851
MAATAATTTSSHLLLSSSRHVASSSQPSILSPRSLFSNNGKRAPTGVRNHQYASGVRCMA
VAADAAETKTAARKKSGYELQTLTGWLLRQEMKGEIDAELTIVMSSISLACKQIASLVQR
AGISNLTGVQGAVNIQGEDQKKLDVISNEVFSNCLRSSGRTGIIASEEEDVPVAVEESYS
GNYVVVFDPLDGSSNIDAAVSTGSIFGIYSPNDECIVDDSDDISALGSEEQRCIVNVCQP
GNNLLAAGYCMYSSSVIFVLTLGKGVFSFTLDPMYGEFVLTQENIEIPKAGRIYSFNEGN
YQMWDDKLKKYIDDLKDPGPTGKPYSARYIGSLVGDFHRTLLYGGIYGYPRDAKSKNGKL
RLLYECAPMSFIVEQAGGKGSDGHSRVLDIQPTEIHQRVPLYIGSTEEVEKLEKYLA
>Q9TLX3
MYNYTIYHPVKISGRGLYTGINSTITIYPELPHVGIYFLRTDLTDYPIVPIIPSTLLVLN
SKLSTVLGFHDDYSIMLIEHLMSAISLSQLTDLKIILEGPEIPILDGSSIIWLKLLQQAK
IYITSLSNCIIFKSSCTFFIQTKDTFIISFPYTSLAINTGIDFKNCLAIKSQWITILDLL
INRFELVGIASSRTFGDFNQINILINEGFFQGANLYNALAFQLGNWCNGPLRFICEPAKH
KVLDLIGDLRLLGVYNMFFLIGYKTNHFVNSQLVQFFKKLDK
>Q9MTP8
MDLLKKERKANEIKIMNYKESSGSKMNCKESSGSNSIDNMDGPVYNDRPMDGFFKLQIWI
HPLDILKMGWLNVKIHSLKFNK
>P34802
MASVTLGSWIVVHHHNHHHPSSILTKSRSRSCPITLTKPISFRSKRTVSSSSSIVSSSVV
TKEDNLRQSEPSSFDFMSYIITKAELVNKALDSAVPLREPLKIHEAMRYSLLAGGKRVRP
VLCIAACELVGGEESTAMPAACAVEMIHTMSLIHDDLPCMDNDDLRRGKPTNHKVFGEDV
AVLAGDALLSFAFEHLASATSSDVVSPVRVVRAVGELAKAIGTEGLVAGQVVDISSEGLD
LNDVGLEHLEFIHLHKTAALLEASAVLGAIVGGGSDDEIERLRKFARCIGLLFQVVDDIL
DVTKSSKELGKTAGKDLIADKLTYPKIMGLEKSREFAEKLNREARDQLLGFDSDKVAPLL
ALANYIAYRQN
>Q84KJ5
MAASSLSLSSPLSNPLRRFTLHHLHLSKKPLSSSSLFLCSAAKMNDHIHRVPALTEEEID
SVAIKTFERYALPSSSSVKRKGKGVTILWFRNDLRVLDNDALYKAWSSSDTILPVYCLDP
RLFHTTHFFNFPKTGALRGGFLMECLVDLRKNLMKRGLNLLIRSGKPEEILPSLAKDFGA
RTVFAHKETCSEEVDVERLVNQGLKRVGNSTKLELIWGSTMYHKDDLPFDVFDLPDVYTQ
FRKSVEAKCSIRSSTRIPLSLGPTPSVDDWGDVPTLEKLGVEPQEVTRGMRFVGGESAGV
GRVFEYFWKKDLLKVYKETRNGMLGPDYSTKFSPWLAFGCISPRFIYEEVQRYEKERVAN
NSTYWVLFELIWRDYFRFLSIKCGNSLFHLGGPRNVQGKWSQDQKLFESWRDAKTGYPLI
DANMKELSTTGFMSNRGRQIVCSFLVRDMGLDWRMGAEWFETCLLDYDPCSNYGNWTYGA
GVGNDPREDRYFSIPKQAQNYDPEGEYVAFWLQQLRRLPKEKRHWPGRLMYMDTVVPLKH
GNGPMAGGSKSGGGFRGSHSGRRSRHNGP

>Q6B8L8
MDNQNIKIYNHLKVHEIIYKTEELLNISDNRYKITIQIANRAKRKKYEDLDIIDDPTIKP
IIRSILEMVDEITQPEIISD
>Q9MUQ8
MINIEFGPSTILGIAVVCGGILLYITRTIKPEISRDHDIFFSSVALLIGGILIFQGWRLD
PILLFGQMLSTGTALCFIIESLKLRVPKENNKSLFSTQNKNNSNNRIIKFQPPSKYDINL
NQRGKKEKKNNNFQLKENSKYTKDSLVPSNWNDINISSIDYEKPIDYQKK
>P24846
MPYLQPPRPHPHPHPTSRLSRASPPSPFPFFPAGTSRSGRLQPVPVSGHSASRVSKGKFA
VAAVTLDDYLPMRSTEVKNRTSTDGIKSLRLITAVKTPYLPDGRFDLEAYDSLINTQING
GAEGVIVGGTTGEGHLMSWDEHIMLIGHTVNCFGANIKVIGNTGSNSTREAVHATEQGFA
VGMHAALHVNPYYGKTSTEGLISHFKEVLPMGPTIIYNVPSRTSQDIPPPVIEALSSYSN
MAGVKECVGHERVKCYTDKGISIWSGNDDECHDSRWKYGATGVISVASNLVPGLMHSLMF
EGENAALNEKLLPLMKWLFCEPNPIGLNTALAQLGVVRPVFRLPYTPLPLEKRVEFVRIV
EAIGRENFVGQKESRVLDDDDFVLISRY
>P05720
MALSNILLFKIEFYIFSSLLMIKKKLVFSILKLLQLTEKDSKILVNESLTNKNFSKLIEF
LDNYKVEKAKSITLQQLQSVLQNIKLNNSQKSEIIENIYSKLDAANHLIF
>P42738
MRSSCCSSAIGGFFDHRRELSTSTPISTLLPLPSTKSSFSVRCSLPQPSKPRSGTSSVHA
VMTLAGSLTGKKRVDESESLTLEGIRNSLIRQEDSIIFGLLERAKYCYNADTYDPTAFDM
DGFNGSLVEYMVKGTEKLHAKVGRFKSPDEHPFFPDDLPEPMLPPLQYPKVLHFAADSIN
INKKIWNMYFRDLVPRLVKKGDDGNYGSTAVCDAICLQCLSKRIHYGKFVAEAKFQASPE
AYESAIKAQDKDALMDMLTFPTVEDAIKKRVEMKTRTYGQEVKVGMEEKEEEEEEGNESH
VYKISPILVGDLYGDWIMPLTKEVQVEYLLRRLD
>Q06GS8
MVKLRLKRCGRKQRAVYRIVAIDVRSRREGRDLQKVGFYDPIKNQTYLNVPAVLYFLGKG
AQPTGTVHDISKKAEIFKELRVNQTIRVNQTKGGLHI
>Q9FWS4
MAVSLPNSFLQISPCVPSLQLRKPVMAAVKGGKQSVRRSSNTVVQITCRKKELHPEFHED
AKVYCNGELVMTTGGTKKEYVVDVWSGNHPFYLGNRSALMVDADQVEKFRKRFAGLSEIM
EIPVLKGEIIMPTKKSKGPKGKKK
>P29344
MASLAQQLAGGLRCPPLSNSNLSKPFSPKHTLKPRFSPIVSAVAVSNAQTRERQKLKQLF
EDAYERCRNAPMEGVSFTIDDFHTALDKYDFNSEMGSRVKGTVFCTDANGALVDITAKSS
AYLPLAEACIYRIKNVEEAGIIPGVREEFVIIGENEADDSLILSLRQIQYELAWERCRQL
QAEDVVVKGKIVGANKGGVVALVEGLRGFVPFSQISSKSSAEELLEKEIPLKFVEVDEEQ
SRLVMSNRKAMADSQAQLGIGSVVTGTVQSLKPYGAFIDIGGINGLLHVSQISHDRVSDI
ATVLQPGDTLKVMILSHDRERGRVSLSTKKLEPTPGDMIRNPKLVFEKAEEMAQTFRQRI
AQAEAMARADMLRFQPESGLTLSSDGILGPLTSDLPAEGLDLSVVPPAVES
>P49827
MGMSLQLLGINLRIKVILATSWLFENIAKVCGYPTKTLGMPIRTDVIPGYGLPIHQTRVP
PFANPTTFLEAVFGNIPQPSTIEKFYYESPQDGYYNFYIPHYRNVVFLPDWLSKWIQLHF
DLGIDTVGLETIRNTLFSMLVYFYFFAELRIMLSYFISINPYTRPWVYLISLTDWIYDIL

FHLGISKRVVLLGFPLLPILIHAALGNLIDSLNHLVFTMPFLPSEGEPGEFLIDGTARKV
LLFRYLPALWTSEPIPDRLREFWYTERPEIYQFMKKNYGHLDIDFLPDEILKQIYQFKHD
QIDPNSLTKNVEQFKVLGAQLISDSTIDFYQFSNCFVHKQEILFTFFSDYMDRLI
>Q9XIR4
MLLVSPACRGVYLQTIDPKPIDFSARASYALCFQIPTSIPKRECLMRLGTVFCFNQKHRE
QTSFKKRYVSTQNVDLPPILPKNKKKPYPIPFKQIQEEARKDKKLAQMGIEKQLDPPKNG
LLVPNLVPVADQVIDNWKLLIKGLAQLLHVVPVFACSECGAVHVANVGHNIRDCNGPTNS
QRRGSHSWVKGTINDVLIPVESYHMYDPFGRRIKHETRFEYERIPALVELCIQAGVEIPE
YPCRRRTQPIRMMGKRVIDRGGYHKEPEKPQTSSSLSSPLAELDTLGVFERYPPPTPEDI
PKIAQETMDAYEKVRLGVTKLMRKFTVKACGYCSEVHVGPWGHSVKLCGEFKHQWRDGKH
GWQDALVDEVFPPNYVWHVRDLKGNPLTGNLRRFYGKAPALVEICMHSGARVPQRYKAMM
RLDIIVPDSQEADMVA
>P51223
MQAKNNKPSDDFYDSADMQELAGETPIGWSATCLDQTICYYLDCDQEHFEDLDNSNHN
>P31921
MLIKVNFMEHYLLNKQFELASAIKSRNKILIHKLVSDVLVSYNSLCYAVYKTLKNSDDKS
SSNIQKKKKKKKNFQNLVDSLKCFVLNYDFYKIRCLNLYYLRNFIDCKDQFLHFHFLDIA
LQNLYSFIFFPFLESNLDKFTYGPRVFRSSIDAVKVLFLLGKQKKYSNYNKYLFCFAFNY
TIVKCFDAVFNSWVLSNVSFIDKSILSFWVKNGFDNFYSGDQFFFQKKNFEINRGTSLIF
LVIFNFVFMGMQFFLESSILSKFGFFSKFVLITDLNSVVILSSDLKTAKIVKSSLLIFFH
SRGICQNFRDNSIVDFFCKNCENKNFVFCGITFHYKLIHGEYKFVLSPILEKIKNVKKKV
LNLCKKFSKPLVLFDNIKPLMKSWFNSYKIIKNNYSFLKLSYWIVGKITKSIYLLYVNSN
FERGKFGIRKGRRSGRLYKNIAAQVVKRLYFLKDQQRFFINTIEGKIIFKGFSVGLSKKN
SIELKNFFSNSLKGKKFFATFGKNFF
>Q85FG9
MSIRLEDIDPVVVSMGPHHPSMHGVLRLVVALQGENVVDCELILGYLHRGMEKIAENRTT
SQYLPYVTRWDYLATMFTEAVTVNAPEKLANIQIPERASYIRVIMLELSRIASHLLWLGP
FLADIGAQTPFFYIFREREMIYDLFEAATGMRMMHNYFRIGGVAADLPYGWIDKCLDFCQ
YCLPKIYEYERLVTKNPIFLKRVQGVGFINRQEAINWGLSGPSLRASGVQWDLRKIDHYE
CYDKLDWQIQWQGEGDSLARYLVRIDEMKESINILRQALKLLPGGPYENLEGRRLVQKKD
KIDWNGFNYQFVGKKSSPTLKLPKQEHYVRVEAPKGELGIFLIGDDSVFPWRLKIRPPGF
INLQIVPQLIRGMKLADIVTILGSIDIIMGEVDR
>P05314
MASLPVNKIIPSSTTLLSSSNNNRRRNNSSIRCQKAVSPAAETAAVSPSVDAARLEPRVE
ERDGFWVLKEEFRSGINPAEKVKIEKDPMKLFIEDGISDLATLSMEEVDKSKHNKDDIDV
RLKWLGLFHRRKHHYGRFMMRLKLPNGVTTSEQTRYLASVIKKYGKDGCADVTTRQNWQI
RGVVLPDVPEIIKGLESVGLTSLQSGMDNVRNPVGNPLAGIDPHEIVDTRPFTNLISQFV
TANSRGNLSITNLPRKWNPCVIGSHDLYEHPHINDLAYMPATKNGKFGFNLLVGGFFSIK
RCEEAIPLDAWVSAEDVVPVCKAMLEAFRDLGFRGNRQKCRMMWLIDELGMEAFRGEVEK
RMPEQVLERASSEELVQKDWERREYLGVHPQKQQGLSFVGLHIPVGRLQADEMEELARIA
DVYGSGELRLTVEQNIIIPNVENSKIDSLLNEPLLKERYSPEPPILMKGLVACTGSQFCG
QAIIETKARALKVTEEVQRLVSVTRPVRMHWTGCPNSCGQVQVADIGFMGCMTRDENGKP
CEGADVFVGGRIGSDSHLGDIYKKAVPCKDLVPVVAEILINQFGAVPREREEAE
>Q9TL28

MPYIKHIETKRISARTYYGRFCVLPLPAGQGITLGNALRRILLGDLVGFAATSANLAGAS
HEFDTLPGIRESVLEILLNIKQLVFKQISSRPKDTNRFAMRASLNLTGPATVTAKDLVLP
SWMKVVDPSQYIATLASGASLEFEIQLSQGSGYRLRRTSLVPPGFTLPPPRDPLEPENDS
KSETKSKSKGKSKNTSTSDVQLADTDVNAQIIDTDSNSTETEKEAPHIPSMRDDHMTLHI
DAVFFPVTRVNYRVEEEVINGRRREELVIDIWTNGSLSPRKALDQAAVILIRMLASLQAP
PPLLIEPEKKPTTKTIAKEIALTPIESLDLSVRSFNCLKRANITNVGKLIAYTRQELLQL
KNFGTKSASEVVDVLNSRFKLALKGEEVTDQEQVVNQPSSQIATKKGARKKVNRASRPID
SKETRRSRNPVKSTASEVPEKMLRKSSKTKVKAPKSETLPKPSKSANLQQAEESLQVPKL
RRKSELSSSQNPEET
>P03938
MAYSSCLNRSLKPNKLLLRRIDGAIQVRSHVDRTFYSLVGSGRSGGGPPGLLSSRESIHP
LSVYGELSLEHRLRFVLNGKMEHLTTHLHRPRTTRSPLSFWGDGGIVPFEPFFHAFPGGL
EKAVINRTSLILPS
>P52334
MMRTVQLRTLRPCIRAQQQPVRAPTSVAAATATTPAPTKKCPFSLFAKLGGREAVEAAVD
KFYNKVVADPTVSVFFSKTDMKVQRSKQFAFLAYALGGAAEWKGKDMRTAHKDLVPHLTD
VHFQAVVRHLSDTLAELGVTPGDIADAMAVVASTKTEVLNMPRQQGAESNR
>Q5SD32
MTRVKRGSVARKRRKNILKLASGFQGAHSTIFRTGNQQIIKALASSYRDRGKRKRDFRRL
WITRINAAARNNGVSYTKFIRHLYKNQVSSNRKVLAQIAIFDTNSFSTILKKLSSGESIR
YRNFSPEFYSGEVRTP
>Q9ZST0
MAATAVTLPSSPAPFPVTTTASSSRNVRLLLRSPPPRRALRVAASAAADAPPKPAPPPTS
PSGIVLVDPTEAQKVHRLKAVYDQKVVPLITEEFGYTNVHQVPKVEKIVVNCGLGAEAGN
SKGLESAMKDLAMITGQWPVKTKAKKSVASFKIREGNTIGIAVTLRGRVMFNFLDRLINL
GLPRTMDFLGVNPNSFDGHGNFTIGLRDQGVFPEIPYEVGGKKNGMDVCIVTTAKTDNEA
LRLLTLLGMPFAEHIKSSVVIRKKRLKRHHFMSKGRGRR
>Q32S08
MNNYPWLTIITLFPISAGLLIPLIPNRGNNLIRWYALGICLIDFLLMTYVFGSQFDFYGQ
GIQLKEDISWINVIDFHWRVGVDGLSIALVLLTGFITTLATLAAWPVTRNPRLFYFLMLA
MYSGQLGLFLAQDLLLFFFMWELELIPVYLLLSMWGGRRRLYAATKFILYTAGGSIFLLA
AILTISLWGPNGPILDMETLSKQSYPLGLEILVYLGFLIAYAVKLPVFPFHTWLPDTHGE
AHYSTCMLLAGILLKMGGYGFIRINIEMLSDAHRIFAPWLVALGAGQIVYAALVSIAQKN
LKRRIAYSSVSHMGFVLIGAGSFSDLGLSGAILQMISHGLIGAGLFFLAGTTYDRSRTLI
LDDMGGWGSPLPKTFSTFTACAMASLALPGMSGFVAELMIFLGIVASSVYSPLFKAIITC
VEGIGIILTPIYLLSMVRKMFYGYNDISLKTLKDSLILDASPREVFIILSLLVPMLGIGF
YPDLTLQLWNQKAQEIVSLPSGNEKSLTLISYSYPVEKNFSLHH
>O82499
MLQLCSTFRPQLLLPCQFRFTNGVLIPQINYVASNSVVNIRPMIRCQRASGGRGGANRSK
PAKPQVKEGSNKTVIEGLVTESLPNGMFRVDLENGDNILGYICGKIRKNFIRILPGDKVK
VEMSVYDSTKGRIIFRMSSRD
>Q9SZ30
MEATAAPFSSIVSSRQNFSSSSSIRASSPASLFLSQKSIGNVNRKFKSPRSLSVRASSTS
DSVVTLLDYGAGNVRSIRNALRHLGFSIKDVQTPGDILNADRLIFPGVGAFAPAMDVLNR

TGMAEALCKYIENDRPFLGICLGLQLLFDSSEENGPVKGLGVIPGIVGRFDASAGIRVPH
IGWNALQVGKDSEILDDVGNRHVYFVHSYRAIPSDENKDWISSTCNYGESFISSIRRGNV
HAVQFHPEKSGEVGLSVLRRFLHPKLPATQKPMEGKASKLAKRVIACLDVRTNDKGDLVV
TKGDQYDVREQSNENEVRNLGKPVDLAGQYYKDGADEISFLNITGFRDFPLGDLPMIQVL
RQTSKNVFVPLTVGGGIRDFTDASGRYYSSLEVAAEYFRSGADKISIGSDAVSAAEEFIK
SGVKTGKSSLEQISRVYGNQAVVVSIDPRRVYVNHPDDVPYKVIRVTNPGPNGEEYAWYQ
CTVSGGREGRPIGAFELAKAVEELGAGEILLNCIDCDGQGKGFDIDLVKLISDSVGIPVI
ASSGAGTPDHFSEVFEKTNASAALAAGIFHRKEVPIQSVKEHLQEERIEVRI
>P31916
MYQSNLNLQKKVTSDLLYYSWLSLKCGWKYFFEIHNYCLFNSISRSWFKRTSSLIKKGFF
IYPTVPLKIKNFFLSCTKKNFNLLKFKIVENAFLIIIKNFFIYKIYVQSMNLIECIFNVT
SFSFMKPFFCKQCPNLLFSLTFFPFFKNDFLQKKKYFNSNKNFVKNFFSNEYFFSSSNSF
FSIKFWDSQIKNFMTLKIIKLFDYVHKVRLKNIFSKFTYDSFFFVEIDKMFNLNLVNISS
NLIYNSIENFGCSLLSSFFLNLYILEGDFFLDRFIFKICFKRNLFKTFFSFKKVSFFYQY
SLKNFIPLRLEKNFFVSSFLKEVNSGKYHNIDIFYLFNNKVFTVYEKNIYYVRYLNFLIF
GFLSSKNFIFFFKLKYLFFLRNKLYFNFREVQIFSSSNDKVIFLGVYIAYNKIYNFFEKL
RVNKKYFLNVFQKIITKHNTFLKALKNIFHYSRCFNFFKKNCYPNFYKKKNFSFFNFYTE
SFRILKFFDAFFINTHHFFLPVELITSTKLVNFQKYTMYSFDFYNQKLSILLKDILENFN
QFLSCSLVSIDLNLYNCLFEFKKHLVLLYNYYSPVYSFFSKRQRYKLNFNSFNYYSFSNF
SGQNRSFFSSKANQFRFFKFFVPFKVFLKKLRLLGFIHPFKFRPIGNVRLLLFEDKFILR
NFGFFVYSVLNWFSICENFSHLRFFVELIRESCFLTLCRKHNKMKLWSYSVYTFDLVFSK
SVYRTISFFPTRKFIFNLKRKSFLVDVRFNLDETIFLE
>Q9MUL6
MYFHILILNHTLMKKKSYSTYNLTIWQHIWTIILLFISISCIKQYNFSFQSFNRGEYSNF
TTFDSFNKKQVCNLISFDNALLKILLKNKICINTIEKQPIYKQIQICPDFCRLIFTSHSY
NPIGIINNVSDLSIIGFVDLYGNKWEEGSAFIAHKYFLENQKLPNIIGINNMNSYKLAQM
CFVLKNNKSTHSFVIDLRKEKNIILINNLGKVNIGNDFASLIKKLIFLKSFTSICKEGDD
ILSKIDINNLSRPIVQYSSYL
>Q8VY26
MASLITTKAMMSHHHVLSSTRITTLYSDNSIGDQQIKTKPQVPHRLFARRIFGVTRAVIN
SAAPSPLPEKEKVEGERRCHVAWTSVQQENWEGELTVQGKIPTWLNGTYLRNGPGLWNIG
DHDFRHLFDGYSTLVKLQFDGGRIFAAHRLLESDAYKAAKKHNRLCYREFSETPKSVIIN
KNPFSGIGEIVRLFSGESLTDNANTGVIKLGDGRVMCLTETQKGSILVDHETLETIGKFE
YDDVLSDHMIQSAHPIVTETEMWTLIPDLVKPGYRVVRMEAGSNKREVVGRVRCRSGSWG
PGWVHSFAVTENYVVIPEMPLRYSVKNLLRAEPTPLYKFEWCPQDGAFIHVMSKLTGEVV
ASVEVPAYVTFHFINAYEEDKNGDGKATVIIADCCEHNADTRILDMLRLDTLRSSHGHDV
LPDARIGRFRIPLDGSKYGKLETAVEAEKHGRAMDMCSINPLYLGQKYRYVYACGAQRPC
NFPNALSKVDIVEKKVVKNWHEHGMIPSEPFFVPRPGATHEDDGVVISIVSEENGGSFAIL
LDGSSFEEIARAKFPYGLPYGLHGCWIPKD
>O20142
MTHRFRNTTFMRYLSNSRFKKKLILRKIKVSGPFVIQRTVHPQSAHNPNENCGNFDQRKV
LGYFFKMFFYHNYNFVFTKLKLICLFLIISISSKKNFKLKVLILFFSIQKFTKSLDRNMS
LAFDCTHKRVT
>Q9FYA6

MERSAVASGFHRNYILCASRAATSTTRLHSLSSLRNFPSSSLRIRHCPSPISSNFIVSEV
SRNRRCDAVSSSTTDVTELAEIDWDKIDFGLKPTDYMYAMKCSRDGEFSQGQLQPFGNID
INPAAGVLNYGQGLFEGLKAYRKQDGNILLFRPEENAIRMRNGAERMCMPSPTVEQFVEA
VKTTVLANKRWIPPPGKGSLYIRPLLMGTGAVLGLAPAPEYTFLIFVSPVGNYFKEGVAP
INLIVETEFHRATPGGTGGVKTIGNYAAVLKAQSIAKAKGYSDVLYLDCLHKRYLEEVSS
CNIFIVKDNVISTPEIKGTILPGITRKSIIEVARSQGFKVEERNVTVDELVEADEVFCTG
TAVVLSPVGSITYKSQRFSYGEDGFGTVSKQLYTSLTSLQMGLSEDNMNWTVQLS
>Q9FN52
MASLLLTSSSMITTSCRSMVLRSGLPIGSSFPSLRLTRPYDKATLFVSCCSAESKKVATS
ATDLKPIMERRPEYIPNKLPHKNYVRVLDTTLRDGEQSPGAALTPPQKLEIARQLAKLRV
DIMEVGFPVSSEEEFEAIKTIAKTVGNEVDEETGYVPVICGIARCKKRDIEATWEALKYA
KRPRVMLFTSTSEIHMKYKLKKTKEEVIEMAVNSVKYAKSLGFKDIQFGCEDGGRTEKDF
ICKILGESIKAGATTVGFADTVGINMPQEFGELVAYVIENTPGADDIVFAIHCHNDLGVA
TANTISGICAGARQVEVTINGIGERSGNAPLEEVVMALKCRGESLMDGVYTKIDSRQIMA
TSKMVQEHTGMYVQPHKPIVGDNCFVHESGIHQDGILKNRSTYEILSPEDVGIVKSENSG
IVLGKLSGRHAVKDRLKELGYEISDEKFNDIFSRYRELTKDKKRITDADLKALVVNGAEI
SSEKLNSKGINDLMSSPQISAVV
>Q06FW2
MAKGKDNRGKVTLECTGCVQNRVNNKKKKKSAGIYRYLTQKNPKNTPSPLELRKFCPYCN
KHTIHGEIKE
>Q85FP0
MKVNYGSPAKSGPLHKNGEFYINKNCFLHPLLFLSEENFYLTDGKRRSHGADANLVFGAW
STVAVKRLIGSVRDPNLNFLKIYDSVSVRNLTDGLDVDLYLHPLLKMTYLILGIALFPKI
RAETSSKSKMLQSIHSMFLFLEDRFSKSNHILEADLPHNLHLETLIRLFRRQIKDVSFLH
LLRIVFRKRKIFCGKTFYSPGGGQDGSVDIPVRNFYIFEIDSLLLIPWKQVYKFRVNYLS
PIDSCNIIRKEIYASAYKFKWNKASIDYSFSRSLWIHYGRWRNKFLIASEGTHYFVKKML
YYLWILLKYHFHYRIKSNEPWIRKLLPTSCVSFLGYTLLAQLVSKNVRIETVTDLYISIL
GGKKFYPKIPNSIIITTLAKQRFCDFTGRPIGKSAWVTSTDDKIIDGYVQLWQVFSLYYG
ASMNQYRLRRLIFLLQMSCDSTLAGKHRSTIRLLRCKSNVEALNQILASRKFELSSSRRV
WRSSSIRSVLVQFTVLDIGL
>P05466
MAQVSRICNGVQNPSLISNLSKSSQRKSPLSVSLKTQQHPRAYPISSSWGLKKSGMTLIG
SELRPLKVMSSVSTAEKASEIVLQPIREISGLIKLPGSKSLSNRILLLAALSEGTTVVDN
LLNSDDINYMLDALKRLGLNVETDSENNRAVVEGCGGIFPASIDSKSDIELYLGNAGTAM
RPLTAAVTAAGGNASYVLDGVPRMRERPIGDLVVGLKQLGADVECTLGTNCPPVRVNANG
GLPGGKVKLSGSISSQYLTALLMSAPLALGDVEIEIVDKLISVPYVEMTLKLMERFGVSV
EHSDSWDRFFVKGGQKYKSPGNAYVEGDASSASYFLAGAAITGETVTVEGCGTTSLQGDV
KFAEVLEKMGCKVSWTENSVTVTGPPRDAFGMRHLRAIDVNMNKMPDVAMTLAVVALFAD
GPTTIRDVASWRVKETERMIAICTELRKLGATVEEGSDYCVITPPKKVKTAEIDTYDDHR
MAMAFSLAACADVPITINDPGCTRKTFPDYFQVLERITKH
>Q42533
MASSSFSVTSPAAAASVYAVTQTSSHFPIQNRSRRVSFRLSAKPKLRFLSKPSRSSYPVV
KAQSNKVSTGASSNAAKVDGPSSAEGKEKNSLKESSASSPELATEESISEFLTQVTTLVK
LVDSRDIVELQLKQLDCELVIRKKEALPQPQAPASYVMMQQPNQPSYAQQMAPPAAPAAA

APAPSTPASLPPPSPPTPAKSSLPTVKSPMAGTFYRSPAPGEPPFIKVGDKVQKGQVLCI
VEAMKLMNEIESDHTGTVVDIVAEDGKPVSLDTPLFVVQP
>Q39513
MVATAASSAFFPLPSADTSSRPGKLGNKPSSLSPLKPKSTPNGGLQVKANASAPPKINGS
PVGLKSGGLKTQEDAHSAPPPRTFINQLPDWSMLLAAITTVFLAAEKQWMMLDWKPKRPD
MLVDPFGLGSIVQDGLVFRQNFSIRSYEIGADRTASIETVMNHLQETALNHVKIAGLSND
GFGRTPEMYKRDLIWVVAKMQVMVNRYPTWGDTVEVNTWVAKSGKNGMRRDWLISDCNTG
EILTRASSVWVMMNQKTRRLSKIPDEVRNEIEPHFVDSPPVIEDDDRKLPKLDEKTADSI
RKGLTPRWNDLDVNQHVNNVKYIGWILESTPPEVLETQELCSLTLEYRRECGRESVLESL
TAMDPSGGGYGSQFQHLLRLEDGGEIVKGRTEWRPKNGVINGVVPTGESSPGDYS
>O80575
MKSLASPPCLRLIPTAHRQLNSRQSSSACYIHGGSSVNKSNNLSFSSSTSGFASPLAVEK
ELRSSFVQTAAVRHVTGSLIRGEGLRFAIVVARFNEVVTKLLLEGAIETFKKYSVREEDI
EVIWVPGSFEIGVVAQNLGKSGKFHAVLCIGAVIRGDTTHYDAVANSAASGVLSASINSG
VPCIFGVLTCEDMDQALNRSGGKAGNKGAETALTALEMASLFEHHLK
>Q9LS01
MASSSAAMSLESISMTTLNNLSRNHQSHRSSLLGFSRSFQNLGISSNGPDFSSRSRSTTS
KNLNVTRAFFWNWGKKTENSRPSKIQELNVYELNEGDRNSPAVLKLGKKPTELCLGDLVP
FTNKLYTGDLKKRVGITAGLCVLIQHVPEKSGDRFEASYSFYFGDYGHLSVQGQYLTYED
TFLAVTGGSGIFEGAYGQVKLRQLVYPTKLFYTFYLKGLANDLPLELTGTAVTPSKDVKP
APEAKAMEPSGVISNFTN
>Q42961
MASATASHTLCGIPATSSSTTNKAIAPSSARFLAKTPLRRLGFAGAAADSLFTNHVATKL
RSLKSSSKPIRGVASMAKKSVGDLTAAELKGKKVFVRADLNVPLDDNQNITDDTRIRAAV
PTIKHLMANGAKVILSSHLGRPKGVTPKYSLAPLVPRLSELLGIQVVKVEDCIGPEVEKL
VASLPEGGVLLLENVRFYKEEEKNEPEFAKKLASLADLYVNDAFGTAHRAHASTEGVTKF
LKPSVAGFLLQKELDYLVGAVSNPKRPFAAIVGGSKVSSKIGVIESLLEKCDILLLGGGM
IFTFYKAQGLSVGSSLVEEDKLELATSLLEKAKAKGVSLLLPSDVVIADKFAPDANSKIV
PASAIPDGWMGLDIGPDSVKTFNDALDTTKTVIWNGPMGVFEFDKFAVGTEAIAKKLADL
SGKGVTTIIGGGDSVAAVEKVGVASVMSHISTGGGASLELLEGKVLPGVIALDEADAPVA
V
>Q06801
MAIHTCFSLIPSSFSSPKLPYPKNTTFQSPIPKLSRPTFMFDRKGSFQNGTAAVPAVGED
FPIDYADWLPKRDPNDRRRAGILLHPTSFPGPYGIGDLGPQAFKFLDWLHLAGCSLWQVL
PLVPPGKRGNEDGSPYSGQDANCGNTLLISLEELVDDGLLKMEELPEPLPTDRVNYSTIS
EIKDPLITKAAKRLLSSEGELKDQLENFRRDPNISSWLEDAAYFAAIDNSVNTISWYDWP
EPLKNRHLAALEEVYQSEKDFIDIFIAQQFLFQRQWKKVRDYARSKGISIMGDMPIYVGY
HSADVWANKKQFLLNRKGFPLIVSGVPPDAFSETGQLWGSPLYDWKAMEKDGFSWWVRRI
QRATDLFDEFRIDHFRGFAGFWAVPSEEKIAILGRWKVGPGKPLFDAILQAVGKINIIAE
DLGVITEDVVQLRKSIEAPGMAVLQFAFGSDAENPHLPHNHEQNQVVYTGTHDNDTIRGW
WDTLPQEEKSNVLKYLSNIEEEEISRGLIEGAVSSVARIAIIPMQDVLGLGSDSRMNIPA
TQFGNWSWRIPSSTSFDNLDAEAKKLRDILATYGRL
>P46489
MAVVKLSPWANYSSSKSEIKSSSSSSSSKSSLSAYVINVSSSPRLSFYNPYPRRLHHQRL

SSPASIRCSVTSSDQIQAPLPAKQKPECFGVFCLTYDLKAEEETKSWKKIINVAVSGAAG
MISNHLLFKLASGEVFGPDQPISLKLLGSERSFAALEGVAMELEDSLYPLLRQVSIGIDP
YEIFQDAEWALLIGAKPRGPGMERADLLDINGQIFAEQGKALNAVASPNVKVMVVGNPCN
TNALICLKNAPNIPPKNFHALTRLDENRAKCQLALKAGVFYDKVSNVTIWGNHSTTQVPD
FLNAKIHGIPVTEVIRDRKWLEDEFTNMVQTRGGVLIKKWGRSSAASTAVSIVDAIRSLV
TPTPEGDWFSTGVYTNGNPYGIAEDIVFSMPCRSKGDGDYEFVKDVIFDDYLSKKIKKSE
DELLAEKKCVAHLTGEGIAVCDLPEDTMLPGEM
>Q1XDL5
MVNTLANELREGTTKSHSMAENVSFVKSFLGGVVDKKSYRKLVANLYFVYCAIEEELFSN
KNHPAIKPIYFTELNRKASLSEDLNYYYGSDWLDFIEPSPATKIYVDRIHTIGHKQPELL
VAHAYTRYLGDLSGGQILKKIARGAMNLSDSGGTKFYDFDQIKDDKLFKDQYRAALDMIP
LSDVQIQNIVSEANISFTLNMKMFEELNSSSLKIITMLIANTIQKFKAKYKSTLAMVD
>Q96255
MAATTNSFLVGSNNTQIPALKPKSSSQSFLHLSKPNTVNFVSKTKPVAVRCVASTTQVQD
GVRSGSVGSQERVFNFAAGPATLPENVLLKAQADLYNWRGSGMSVMEMSHRGKEFLSIIQ
KAESDLRQLLEIPQEYSVLFLQGGATTQFAALPLNLCKSDDTVDFVVTGSWGDKAVKEAK
KYCKTNVIWSGKSEKYTKVPSFEELEQTPDAKYLHICANETIHGVEFKDYPVPKNGFLVA
DMSSNFCSKPVDVSKFGVIYGGAQKNVGPSGVTIVIIRKDLIGNAQDITPVMLDYKIHDE
NSSLYNTPPCFGIYMCGLVFEDLLEQGGLKEVEKKNQRKADLLYNAIEESNGFFRCPVEK
SVRSLMNVPFTLEKSELEAEFIKEAAKEKMVQLKGHRSVGGMRASIYNAMPLAGVEKLVA
FMKDFQAKHA
>Q9TLX6
MYKRDTIAAIATCPNGGGVSILRLSGSKSIDVVKTVSLVSSNQCWHSHCILYGWIKDNED
QSFVDEVLILLMMAPRSYTAENVVEIHCHASIVLANEILRILVKQGVRLAKPGEFTMRAF
LNGRIGLSQVESVLKVIHSKTIASAKLAANTLRRGGSERIRRLKHTLSLLLADLEFHIDF
SDEFIDVDSIEDELRSTIQSSLLDIKDLISSYNKVSKLNEGTKVCIIGKPNVGKSSLLNA
IAKRECSIVTNFPGTTRDIVSFETMLGNTLVRLYDTAGIRQSVDEIEKIGISKTELFVDE
CQIVFFVLDAIQGLSSEDSVIFNKLNLMNKNFVILINKIDKKVQRKIDEIYETLKCSNRR
IIEVSAIKNIGLEKLNNCILDLSSKEDFDLPVHFSVNCKYLEILNNIYLILDELYTGSLN
KSVTSYDFIAVELRRVLQGLNQITGDEVVENNVLDAIFSKFCVGK
>Q9S7B5
MASSCLFNASVSSLNPKQDPIRRHRSTSLLRHRPVVISCTADGNNIKAPIETAVKPPHRT
EDNIRDEARRNRSNAVNPFSAKYVPFNAAPGSTESYSLDEIVYRSRSGGLLDVEHDMEAL
KRFDGAYWRDLFDSRVGKSTWPYGSGVWSKKEWVLPEIDDDDIVSAFEGNSNLFWAERFG
KQFLGMNDLWVKHCGISHTGSFKDLGMTVLVSQVNRLRKMKRPVVGVGCASTGDTSAALS
AYCASAGIPSIVFLPANKISMAQLVQPIANGAFVLSIDTDFDGCMKLIREITAELPIYLA
NSLNSLRLEGQKTAAIEILQQFDWQVPDWVIVPGGNLGNIYAFYKGFKMCQELGLVDRIP
RMVCAQAANANPLYLHYKSGWKDFKPMTASTTFASAIQIGDPVSIDRAVYALKKCNGIVE
EATEEELMDAMAQADSTGMFICPHTGVALTALFKLRNQGVIAPTDRTVVVSTAHGLKFTQ
SKIDYHSNAIPDMACRFSNPPVDVKADFGAVMDVLKSYLGSNTLTS
>A1E9W1
MIQPQTLLNVADNSGARKLMCIRVIGAAGNQRYARIGDVIIAVIKDAVPKMPLERSEVIR
AVIVRTRKEFKGDDGIIIRYDDNAAVIIDQKGNPKGTRVFGAVAEELRELNFTKIVSLAP
EVL

>Q06SG5
MARKGAPKKRLLLPDPIYNKVSVHMLVNRILKNGKKSIAYRIVYSVFRKISETMNQNPVE
VWEKALNNVKPRVEVKPRRRAGSIQQVPRVMPSAERARAIAIRWIVSACQKKAGKDMISK
LFSEISEASNKNGAAFRKKEELHKMALSNLMNSRRPDKIVKAITEQSEINDDVY
>P28255
MLIFILFVILKIILGVITEFCRIYLFALSIRVFLAWIVTINWYTQPYIVLKKLTDPYLNL
FRGTLPLILGMDFSSMLGFLFLECVIKLLESIYIEIIY
>Q85G60
MAVPKKRTPKSKTRSRKSQWMRKALKQLQKARTLAGRLAARQDQMQPTQMQPTQMQPN
>P42698
MLGGLYGDLPPPTDDEKPSGNSSSVWSSSTKMAPPTLRKPPAFAPPQTILRPLNKPKPIV
SAPYKPPPPSNSSQSVLIPANESAPSHQPALVGVTSSVIEEYDPARPNDYEEYKREKKRK
ATEAEMKREMDKRRQEDEERDKREREEREKERERDNSDPSRLNISGEEAWKRRAAMSGGG
SGGKGRSSSPPGNVDGFSIGKSETSGLGVGAGGQMTAAQRMMAKMGWKQGQGLGKSEQGI
TTPLMAKKTDRRAGVIVNASENKSSSAEKKVVKSVNINGEPTRVLLLRNMVGPGQVDDEL
EDEVGGECGKYGTVTRVLIFEITEPNFPVHEAVRIFVQFSRPEETTKALVDLDGRYFGGR
TVRATFYDEEKFSKNELAPVPGEIPGY
>P06258
MFLLYEYDFFWAFLIISILVPILAFLISGVLAPISKGPEKLSTYESGIEPMGDAWLQFRI
RYYMFALVFVVFDVETVFLYPWAMSFDVLGVSVFIEAFIFVLILIIGLVYAWRKGALEWS

>P23755
MSQSLEEKSVQERTRIKNSRYESGVIPYAKMGYWNPDYQVKDTDVLALFRVTPQPGVDPI
EAAAAVAGESSTATWTVVWTDLLTAADLYRAKAYKVDQVPNNPEQYFAYIAYELDLFEEG
SIANLTASIIGNVFGFKAVKALRLEDMRLPFAYIKTFQGPATGVILERERLDKFGRPLLG
CTTKPKLGLSGKNYGRVVYEALKGGLDFVKDDENINSQPFMRWRERYLFVMEAVNKAAAA
TGEVKGHYLNVTAATMEEMYARAQLAKELGSVIIMIDLVIGYTAIQTMAKWARDNDMILH
LHRAGNSTYSRQKNHGMNFRVICKWMRMAGVDHIHAGTVVGKLEGDPIITRGFYKTLLLP
KLERNLQEGLFFDMDWASLRKVMPVASGGIHAGQMHQLIHYLGEDVVLQFGGGTIGHPDG
IQSGATANRVALEAMILARNENRDFLTEGPEILREAAKNCGALRTALDLWKDITFNYTST
DTSDFVETPTANI
>P42804
MAVSSAFVGCPKLETLLNHHNLSPSSSSSSSVSQTPLGLNGVRVLPKNNRTRRGLIQKAR
CELSASSDSASNAASISALEQLKNSAADRYTKERSSIVVIGLSIHTAPVEMREKLAIPEA
EWPRAIAELCGLNHIEEAAVLSTCNRMEIYVLALSQHRGVKEVTEWMSKTSGIPVSEICQ
HRFLLYNKDATQHIFEVSAGLDSLVLGEGQILAQVKQVVKVGQGVNGFGRNISGLFKHAI
TVGKRVRTETNIASGAVSVSSAAVELALMKLPQSSNVSARMCVIGAGKMGKLVIKHLMAK
GCTKVVVVNRSEERVSAIREEMPGIEIIYRPLDEMLACASEADVVFTSTASETPLFLKEH
VENLPQASPEVGGLRHFVDISVPRNVGSCVGEVETARVYNVDDLKEVVAANKEDRMRKAM
EAQTIITEESTQFEAWRDSLETVPTIKKLRAYAERIRVAELEKCMSKMGDDINKKTTRAV
DDLSRGIVNRFLHGPMQHLRCDGSDSRTLSETLENMHALNRMYGLEKDILEEKLKAMAEQ
QQK
>O96805
MSHFTNLKTSFKNLFHLENALNKLEIPYKREKKAIDANNSKHHNVNLVIPQSNNYDITFN

WNGEEYELVLDASFWIQPYPVESFINKLSQHYANDVIITESQKIGFQPVKSKQHVDGSNT
ITLATLEFKK
>Q9M385
MAIPMSMAMATPTDSVSRVWSMSSLKSALPSTASLRLPSSSSRRPVTLRLPISSPSLPSF
SGLSPVNPLLSIGLPDWQSFENGFKIVDGGGRIYAMRHGRRVPKLNRPPDQRKALLRGLT
TQLLKHGRIKTTRARASAMRKFVDKMITLAKDGSLHKRRQALGYIYEKQIVHALFAEVPD
RYGERNGGYTRIIRTLPRRGDNAPMAYIELV
>Q9FV52
MASSVFLSSFSSSSSLQLCSSFHGEYLAPSRCFLGAPVTSSSLSLSGKKNSYSPRQFHVS
AKKVSGLEEAIRIRKMRELETKSKVRRNPPLRRGRVSPRLLVPDHIPRPPYVESGVLPDI
SSEFQIPGPEGIAKMRAACELAARVLNYAGTLVKPSVTTNEIDKAVHDMIIEAGAYPSPL
GYGGFPKSVCTSVNECMCHGIPDSRQLQSGDIINIDVTVYLDGYHGDTSRTFFCGEVDEG
FKRLVKVTEECLERGIAVCKDGASFKKIGKRISEHAEKFGYNVVERFVGHGVGPVFHSEP
LIYHYRNDEPGLMVEGQTFTIEPILTIGTTECVTWPDNWTTLTADGGVAAQFEHTILITR
TGSEILTKC
>P16881
MPFDRQPLLSGEKGMPATSLWLVGGAVIAAVCVIVNTSYNGTQLSVTARPIQAAVSQVSM
ARFAESGVSRGSGNRVSQAVPLMAASVGAESESRRWVASAILFPLSGLFAAVALKMAMMK
PKVAAVLPFTSEKDMKVWNPVNNKKFETFSYLPPLSDAQIAKQVDMIIAKGLSPCLEFAA
PENSFIANDNTVRFSGTAAGYYDNRYWTMWKLPMFGCTDASQVLREISECRRAYPQCYVR
LAAFDSVKQVQVISFVVQRPSGSSSSSWGMAAMTGEKDMKVWNPVNNKKFETFSYLPPLS
DAQIAKQVDMIIAKGLSPCLEFAAPENSFIANDNTVRFSGTAAGYYDNRYWTMWKLPMFG
CTDASQVLREISECRRAYPQCYVRLAAFDSVKQVQVISFVVQRPSGSSSSWGMAAMTGEK
DMKVWNPVNNKKFETFSYLPPLSDAQIAKQVDMIIAKGLSPCLEFAAPENSFIANDNTVR
FSGTAAGYYDNHYWTMWKLPMFGCTDASQVLREISECRRAYPQCYVRLAAFDSVKQVQVI
SFVVQRPSGSSSSSWGMAAMTGEKDMKVWNPVNNKKFETFSYLPPLSDAQIAKQVDMIIA
KGLSPCLEFAAPENSFIANDNTVRFSGTAAGYYDNRYWTMWKLPMFGCTDASQVLREISE
CRRAYPQCYVRLAAFDSVKQVQVISFVVQRPSGSSSSWGMAAMTGEKDMKVWNPVNNKKF
ETFSYLPPLSDAQIAKQVDMIIAKGLSPCLEFAAPENSFIANDNTVRFSGTAAGYYDNRY
WTMWKLPMFGCTDASQVLREISECRRAYPQCYVRLAAFDSVKQVQVISFVVQRPSGSSSS
SSWGMAAMTGEKEMKVWNPVNNKKFETFSYLPPLSDAQIAKQVDMIIAKGLSPCLEFAAP
ENSFIANDNTVRFSGTAAGYYDNRYWTMWKLPMFGCTDASQVLREISECRRAYPQCYVRL
AFDSVKQVQVISFVVQRPSGSSSSSWGMAAMTGEKDMKVWNPVNNKKFETFSYLPPLSDA
QIAKQVDMIIAKGLSPCLEFAAPENSFIANDNTVRFSGTAAGYYDNRYWTMWKLPMFGCT
DASQVLREISECRRAYPQCYVRLAAFDSVKQVQVISFVVQRPSGSSSSSWGMAAMTGEKE
MKVWNPVNNKKFETFSYLPPLSDAQIAKQVDMIIAKGLSPCLEFAAPENSFIANDNTVRF
SGTAAGYYDNRYWTMWKLPMFGCTDASQVLREISECRRAYPQCYVRLAAFDSVKQVQVIS
FVVQRPSSGGRSW
>Q32063
MTCTRTLLVLNRMLALKANFVLHSVKSYLNVSGVNKTLPAKGSAGVKNGFILSHLGCQ
>Q9ST69
MATTATTTPSATSLTTLHRRIPLFPTTTTLLSLSSSSKPLFLSLSSTRSFPTHLYCIKKD
DIDITFFEQDNPDEEITFDPPEKPEGYIPPRAVDEPPFESEEEIALAYEELYGAAYSGES
LLGNDVYAMDSKIKKATGFGSKSKKEKIRDGFEENVVQVRRVTKVVKGGKHMRFRAIVVV

GDKKGQVGVGVGKAKEVVSAVQKAAVDARRNIITVPMTKYLTFPHRNEADYGAARVMLRP
AAPGTGVIAGGAVRTVLEMAGVENALGKQLGSNNALNNARATIVAVQTMRQFSDVARDRG
IPMEELWK
>A2CI50
MAVRFLFEALQKAIDEEMEREKRVVLIGEDIGHYGGSYKVTQGLYGKYGKHRVIDTPIAE
YSFVGAAVGAAATGLIPVVEGMNMAFILLAYSQISNNMGMLCATSGGHFQVPMVLRGPGG
IGKQLGAEHSQRLESYFQSVPGLQIVTCSTPYNAKGLLKSAIRSKNPILFIEHVLLYNLK
GEVPDNDYLLPLEKAELVREGSDITVLTYSRQRYNVIQAVKVLVEEGYDPEVIDLISLKP
FDMETIGKSIQKTHKVLIVEECMMTGGISNVLQSLIIDNFFDALDAAPLILSSPNVPTPY
TGPLEEATVVQTIDIIESIEYGITGKPPKPRTAKK
>Q85CT7
MNIINLPELNYEVFFVFIELGLIFGSLGVVFLTNIVYSAFLLGLVFVCISFLYLLLDADF
VATAQILIYVGAVNILIVFAVMLINKPQSLQFLPSWTVGDTITLILCTSLFFLLISMILS
ISWSNIFSIAQSNKIGEQVLKSSVQGIGSSLLIDFLLPFELLSIVLLVALIGAITIARRE
KKVKLQKNRTLQVTKDSFIL
>P25306
MEFLCLAPTRSFSTNPKLTKSIPSDHTSTTSRIFTYQNMRGSTMRPLALPLKMSPIVSVP
DITAPVENVPAILPKVVPGELIVNKPTGGDSDELFQYLVDILASPVYDVAIESPLELAEK
LSDRLGVNFYIKREDKQRVFSFKLRGAYNMMSNLSREELDKGVITASAGNHAQGVALAGQ
RLNCVAKIVMPTTTPQIKIDAVRALGGDVVLYGKTFDEAQTHALELSEKDGLKYIPPFDD
PGVIKGQGTIGTEINRQLKDIHAVFIPVGGGGLIAGVATFFKQIAPNTKIIGVEPYGAAS
MTLSLHEGHRVKLSNVDTFADGVAVALVGEYTFAKCQELIDGMVLVANDGISAAIKDVYD
EGRNILETSGAVAIAGAAAYCEFYKIKNENIVAIASGANMDFSKLHKVTELAGLGSGKEA
LLATFMVEQQGSFKTFVGLVGSLNFTELTYRFTSERKNALILYRVNVDKESDLEKMIEDM
KSSNMTTLNLSHNELVVDHLKHLVGGSANISDEIFGEFIVPEKAETLKTFLDAFSPRWNI
TLCRYRNQGDINASLLMGFQVPQAEMDEFKNQADKLGYPYELDNYNEAFNLVVSE
>Q1XDD7
MNNKSLIFLAIKSLDIQNQISLTSNIFNLSFDIQLNQIIIDSQIKSDKDLKSILLKVLNS
IGNQSLDSNDLANNYKRRFRYYFSKMSFFRSLEKSIYENNILIDNLGITGLYLLYLIVEK
EDVFKLWLYYKNYTFDQLIRLIETKNNFYN
>Q96291
MASVASSTTLISSPSSRVFPAKSSLSSPSVSFLRTLSSPSASASLRSGFARRSSLSSTSR
RSFAVKAQADDLPLVGNKAPDFEAEAVFDQEFIKVKLSDYIGKKYVILFFYPLDFTFVCP
TEITAFSDRHSEFEKLNTEVLGVSVDSVFSHLAWVQTDRKSGGLGDLNYPLISDVTKSIS
KSFGVLIHDQGIALRGLFIIDKEGVIQHSTINNLGIGRSVDETMRTLQALQYIQENPDEV
CPAGWKPGEKSMKPDPKLSKEYFSAI
>P51311
MAIRLYRAYTPGTRNRTVSTFSEITTDKPEKSLINKHHFCKGRNNRGVITCRHKGGGHKQ
RYRLIDFKRNRHNIIAKVASIEYDPNRNARIALLHYLDGEKRYILHPRSLSVGAIVVSGP
MAPIEVGNALPLSTIPLGTAVHNIELRPYCGGQIVRSAGTYAQIVAKEGNFVTVKLPSSE
VRMIRKECYATIGQVGNIDASNITLGKAGRSRWLGKRPTVRGVVMNPVDHPHGGGGEGKS
PIGRSRPVTPWGKPALGVKTRNPNKYSNPYVLLVVNKVYLTYNLILKYNVEINT
>P12629
MATMACASSLTFPSAQTQKSFFGTNVKQTPVLSFPRPTVAAAVAVSARKSTSASTKCTEE

WRQLKEAVKKEFAIPHVPLDQRWMFTLEEATGPDIWNTTWYPKSADHVPTDKKWYVVDAT
DLILGRMASTIAIHIRGKNLASYTPSVDMGAFVIVVNADKVAVSGKKRTQKLYRRHSGRP
GGLKEETFDQLQKRIPERIIEHAVRGMLPKGRLGRYLFNHLKVYKGAEHPHQAQQPIDLP
LRDKRIRVEK
>P46416
MGSGCSSLSYSSSSTCNATVFSISSSPSSSSSLKLNPSSFLFQNPKTLRNQSPLRCGRSF
KMESQKPIFDLEKLDDEFVQKLVYDALVWSSLHGLVVGDKSYQKSGNVPGVGLMHAPIAL
LPTAFPEAYWKQACNVTPLFNELIDRVSLDGKFLQDSLSRTKKVDVFTSRLLDIHSKMLE
RNKKEDIRLGLHRFDYMLDEETNSLLQIEMNTISCSFPGLSRLVSQLHQSLLRSYGDQIG
IDSERVPINTSTIQFADALAKAWLEYSNPRAVVMVIVQPEERNMYDQHLLSSILREKHNI
VVIRKTLAEVEKEGSVQEDETLIVGGQAVAVVYFRSGYTPNDHPSESEWNARLLIEESSA
VKCPSIAYHLTGSKKIQQELAKPGVLERFLDNKEDIAKLRKCFAGLWSLDDSEIVKQAIE
KPGLFVMKPQREGGGNNIYGDDVRENLLRLQKEGEEGNAAYILMQRIFPKVSNMFLVREG
VYHKHQAISELGVYGAYLRSKDEVIVNEQSGYLMRTKIASSDEGGVAAGFGVLDSIYLI
>P51235
MEQLISFFDRSKGKWISQRTTYKLSNKQMNSLQSSIIIKPDQSSSNSQLIASLRWGEISR
QIIKNLNNQDFVNGNFKFHLRFTNQFNTKQVVTLCSITDKSLITFKTRYGSTTVDETYWF
ATDNLRLSTSIIKQCNFCVAVSFCSEIKI
>O19914
MILIIDNYDSFSYNLVQSVGEINSDLIVLRSDEIDVSKLQNLNIKHIIISPGPGHPDEYT
ICKQVVKFFAPYTPILGICLGHQIIATCYNASIKKSSPVFHGRASKIYCLKDKLFLGIHA
PFTAARYHSLVVDQDKRGLDLTTCLKTIAITREGVIMACKHRYYHFTYGIQFHPESMLTI
QGTKLLKNFLSLSYG
>P05729
MFLNGFLRRLTRKIKQHGYAFFVVSVDGIATGVKGSVKGSFSVASLDPMVMENIEKCAIL
HNEDPHIAYPHEQPFALPRKGKAKSVKGFAKSMKGKAKENSKPKDILFPTFLDYSSQTAY
IASILLNKYRIETFLLQNMAHLYRELIPCFDYDSWECVADALREKANEIFEANISRNLML
LILTGLYDNLVPHHQQEVFNKASNKFLKELEEKMDTSDPRVVIFLICLTMVLCAGGGFFS
CKRHSNKKRSKASYTPPVPDNYAIFNDLGKYKAKVPVFEWQNAHAHSLGIMPLPPNLVNE
MSSEEIKEAILEVPKEAAKGLVYGIPHFLIGSVVNSFTGVKYILMRVGTVVFFTIAGPFI
LLDYVKDRVREYYWFVTRNFSNFREFLFHAITSLLSLFPGGKMFQE
>O19888
MRNNIKSDIWSFHNKFRFDKKIILLLIRVSIKFNTKLVNYSRRPLFLDLLSVLTKKILFS
LAVKSFKDTFITLKIRNCSSLFLKHNSLLVFNSIILKLLKNFYDVLGFSSYEEKKFLILF
QNESLYKLCAFEFRDLTEIVFTFIIFGEIHDKDYSGKVCSNELNFRCLENFLIRVCDCVV
YFSIKYNYNSSILYALRGHSWIKSFKDKQRLFIYLLLSSYFHSIIDTTQKLNNSIFRGWI
VRGTYIDSISFEQFSLTSLKKLPKISLLVFLLINVIDTVIPQFNLFILFLRKAITFIFSD
LIIKTNLL
>Q9MTI5
MKRSLKNNPFVANPLLRKMEKLNRREDKILIRTWSRASTIILTMIGHTIAIHNGKEHLPI
YITDYMVGHKLGEFAPTINFHEHAKNDNKSRRSKMRIDY
>P80030
MAATAAASSLQMATTRPSISAASSKARTYVVGANPRNAYKIACTPHLSNLGCLRNDSALP
ASKKSFSFSTKAMSESSESKASSGLPIDLRGKRAFIAGIADDNGYGWAVAKSLAAAGAEI

LVGTWVPALNIFETSLRRGKFDQSRVLPDGSLMEIKKVYPLDAVFDNPEDVPEDVKANKR
YAGSSNWTVQEAAECVRQDFGSIDILVHSLANGPEVSKPLLETSRKGYLAAISASSYSFV
SLLSHFLPIMNPGGASISLTYIASERIIPGYGGGMSSAKAALESDTRVLAFEAGRKQNIR
VNTISAGPLGSRAAKAIGFIDTMIEYSYNNAPIQKTLTADEVGNAAAFLVSPLASAITGA
TIYVDNGLNSMGVALDSPVFKDLNK
>P22178
MISLNSSFLERSSVTGGSRTQSQSLRLSARRPVVTSMLNSNSLPERNVSVSVDSAVRDVN
APVAVEVDRSVGEKPFAAVGGGVEDMYGEDTATEDHYITPWSVSVASGYSLLRDPHHNKG
LAFTEKERDAHFLRGLLPPVVVNHDLQVKKMMHNIRQYQVPLQRYQAMMDLQQRNERLFY
KLLIENVEELLPIVYTPTVGEACQKYGSIFENSQGLFISLKDKGRILEILKNWPHKKIQV
IVVTDGERILGLGDLGCQGMGIPVGKLALYTALGGVRPSACLPITIDVGTNNEKLLNDDE
FYIGLKQKRAAGQEYAELMNEFMSAVKQNYGENLLIQFEDFANHNAFDLLEKYRTTHLVF
NDDIQGTASVVLGGLISALKLVGGSLADQKFLFLGAGEAGTGIAELIALEISKQTNIPLE
ESRKKVWLVDSKGLIVRSRLDSLQHFKKPWAHDHEPVNEFLDAIKTIRPTVLIGSSGTGQ
TFTKEVVETMSSLNEKPIILALSNPTSQSECTAEQAYTWSEGRAIFASGSPFKPVEYNGK
LYVSGQANNAYIFPGFGLGLIISGAIRVHDDMLLAASEAPAEQVTQEHFDKGLIFPPFTS
IRKISAHIAAKVAAKAYELGLASRLPQPENLVAYAESCMYSPKYRIYR
>Q9C5W3
MFLCFCPCHVPIMSRLSPATGISSRLRFSIGLSSDGRLIPFGFRFRRNDVPFKRRLRFVI
RAQLSEAFSPDLGLDSQAVKSRDTSNLPWIGPVPGDIAEVEAYCRIFRSAERLHGALMET
LCNPVTGECRVPYDFSPEEKPLLEDKIVSVLGCILSLLNKGRKEILSGRSSSMNSFNLDD
VGVAEESLPPLAVFRGEMKRCCESLHIALENYLTPDDERSGIVWRKLQKLKNVCYDAGFP
RSDNYPCQTLFANWDPIYSSNTKEDIDSYESEIAFWRGGQVTQEGLKWLIENGFKTIVDL
RAEIVKDTFYQTALDDAISLGKITVVQIPIDVRMAPKAEQVELFASIVSDSSKRPIYVHS
KEGVWRTSAMVSRWKQYMTRPITKEIPVSEESKRREVSETKLGSNAVVSGKGVPDEQTDK
VSEINEVDSRSASSQSKESGRFEGDTSASEFNMVSDPLKSQVPPGNIFSRKEMSKFLKSK
SIAPAGYLTNPSKILGTVPTPQFSYTGVTNGNQIVDKDSIRRLAETGNSNGTLLPTSSQS
LDFGNGKFSNGNVHASDNTNKSISDNRGNGFSAAPIAVPPSDNLSRAVGSHSVRESQTQR
NNSGSSSDSSDDEAGAIEGNMCASATGVVRVQSRKKAEMFLVRTDGVSCTREKVTESSLA
FTHPSTQQQMLLWKTTPKTVLLLKKLGQELMEEAKEAASFLYHQENMNVLVEPEVHDVFA
RIPGFGFVQTFYIQDTSDLHERVDFVACLGGDGVILHASNLFKGAVPPVVSFNLGSLGFL
TSHPFEDFRQDLKRVIHGNNTLDGVYITLRMRLRCEIYRKGKAMPGKVFDVLNEIVVDRG
SNPYLSKIECYEHDRLITKVQGDGVIVATPTGSTAYSTAAGGSMVHPNVPCMLFTPICPH
SLSFRPVILPDSAKLELKIPDDARSNAWVSFDGKRRQQLSRGDSVRIYMSQHPLPTVNKS
DQTGDWFRSLIRCLNWNERLDQKAL
>Q40545
MSQALNFFVSSSSRSPATFTISRPSVFPSTGSLRLLVKKSLRTLVVEASSAAASDLDEPQ
SSPVLVSENGSGGVLSSATQEYGRNAAPGTDSSSIEVDTVTEAELKENGFRSTRRTKLIC
TIGPATCGFEQLERLAEGGMNVARINMCHGTREWHRMVIERLRRLNEEKGFAVAIMMDTE
GSEIHMGDLGGASSAKAEDGEIWNFTVRSFDPPLPERTVTVNYDGFAEDVKVGDELLVDG
GMVRFEVIEKIGPDVKCLCTDPGLLLPRANLTFWRDGKLVRERNAMLPTISSKDWLDIDF
GIAEGVDFIAVSFVKSAEVIKHLKSYIQARARDSDISVIAKIESIDSLKNLEEIIQASDG
AMVARGDLGAQIPLEQVPSEQQKIVQICRQLNRPVIVASQLLESMIEYPIPTRAEVADVS
EAVRQRGDALMLSGESAMGQFPEKALTVLRSVSLRIERMWREQKRHEVIELPSIASSFSD

SISEEICNSAAKMANNLEVDALFVYTKNGHMASLLSRCRPDCPIFAFTTTTSVRRRLNLQ
WGLMPFRLSFSDDMESNLNKTFSLLKARGMIKSGDLIIAVSDMLQSIQVMNVP
>O82796
MEALTTSRVVPVQVPCRKLSSLFANFSCLELRRYPCRGLVSIMNHPKLLRPVTASVQPHE
LSTLGHEGNIVPSKEILDLWRSVEAVCFDVDSTVCVDEGIDELAEFCGAGKAVAEWTARA
MGGSVPFEEALAARLSLFKPSLSKVEEYLDKRPPRLSPGIEELVKKLRANNIDVYLISGG
FRQMINPVASILGIPRENIFANNLLFGNSGEFLGFDENEPTSRSGGKAKAVQQIRKGRLY
KTMAMIGDGATDLEARKPGGADLFICYAGVQLREAVAANADWLIFKFESLINSLD
>Q9MUM7
MILIKKFIVAIELFIFNRNNCRYILLCTDNKVKNIQTSLSICILCAISYALIKKIQQTFQ
LSSKFAASCS
>O48593
MAATFLPATSLRLTQNSTLRFLSFFTISNPSYSLFRPLRRRVLPPFDAFPANSRRRCFCT
AVSESLGSGDGNKVESYEKRFGSKVGEFRKKLRIAEVKGGADEGLSRVGQSLNIMGWVRT
LRSQSSVTFIEINDGSCLSNLQCVMTSDAEGYDQVESGSILTGASVSVQGTIVASQGTKQ
KVELKVEKIIVVGECDSSYPIQKKRVSREFLRTKAHLRPRTNTFGAVARVRNTLAYATHK
FFQESGFVWVASPIITASDCEGAGEQFCVTTLIPSSHENTDTSIDAIPKTKGGLIDWSQD
FFGKPAFLTVSGQLNGETYATALSDVYTFGPTFRAENSNTSRHLAEFWMIEPELAFADLD
DDMACATAYLQYVVKYVLDNCKEDMEFFDTWIEKGIIRRLSDVAEKEFLQLGYTDAIEIL
LKANKKFDFPVKWGLDLQSEHERYITEEAFGGRPVIIRDYPKEIKAFYMRENDDGKTVAA
MDMLVPRIGELIGGSQREERLEVLEARLDELKLNKESYWWYLDLRRYGSVPHAGFGLGFE
RLVQFVTGIDNIRDVIPFPRTPASAEF
>P05721
MFILGKSNYTNFIYGHLVIRHIKYKLFDDYKKLIKKLSSKLKTSNSTKANLGDNFSLTLP
TWPFFILSWFLHIRTLEFSSNGNKTANSISSVFFLFPKLLNVV
>P53536
MASMTMRFHPNSTAVTESVPRRGSVYGFIGYRSSSLFVRTNVIKYRSVKRNLEFRRRSAF
SVKCGSGNEAKQKVKDQEVQQEAKTSPSSFAPDTTSIVSSIKYHAEFTPLFSPEKFELPQ
AFIATAQSVRDALIINWNATYDYYEKLNVKQAYYLSMEFLQGRALLNAIGNLELTGPYAE
ALSQLSYKLEDVAHQEPDAALGNGGLGRLASCFLDSLATLNYPAWGYGLRYKYGLFKQRI
TKDGQEEVAEDWLEMGNPWEIVRNDVSYPVRFYGKVVSGSDGKKHWVGGEDIKAVAHDVP
IPGYKTRSTINLRLWSTKAASEEFDLNAFNSGRHTEASEALANAEKICYILYPGDESIEG
KTLRLKQQYTLCSASLQDIIARFERRSGASVNWEDFPEKVAVQMNDTHPTLCIPELMRIL
IDIKGLSWKDAWNITQRTVAYTNHTVLPEALEKWSMDLMEKLLPRHVEIIEMIDEELIRT
IIAEYGTADSDLLDKKLKEMRILENVELPAEFADILVKTKEATDISSEEVQISKEGGEEE
ETSKEGGEEEEEKEVGGGREEGDDGKEDEVEKAIAEKDGTVKSSIGDKKKKLPEPVPVPP
KLVRMANLCVVGGHAVNGVAEIHSEIVKDDVFNAFYKLWPEKFQNKTNGVTPRRWIRFCN
PDLSKIITQWIGTEDWILNTEKLAELRKFADNEDLQTQWREAKRNNKVKVAAFLRERTGY
SVSPDSMFDIQVKRIHEYKRQLLNIFGIVYRYKKMKEMNAAERKENFVPRVCIFGGKAFA
TYVQAKRIVKFITDVGATVNHDPEIGDLLKVIFVPDYNVSVAEMLIPASELSQHISTAGM
EASGTSNMKFAMNGCLQIGTLDGANVEIREEVGADNFFLFGAKAREIVGLRKERARGKFV
PDPRFEEVKKFVRSGVFGSYNYDELIGSLEGNEGFGRADYFLVGQDFPSYLECQEEVDKA
YRDQKKWTRMSILNTAGSSKFSSDRTIHEYAREIWNIEPVKLE
>Q9MTN4

MRELRIPTRKTNPIHNDVPENTTFLLLDQPSGEANTTGTLISKSDEVAINAKTANWKAIV
MLLILQATNKRIIPF
>P30222
MACKTLTCSASPLVSNGVVSATSRTNNKKTTTAPFSVCFPYSKCSVRKPASRLVAQATGD
NKDTSVDVHVSNNNQGGNNQGSAVERRPRRMALDVSPFGLLDPMSPMRTMRQMMDTMDRL
FEDTMTFPGSRNRGTGEIRAPWDIKDDENEIKMRFDMPGLSKEEVKVSVEDDVLVIKGEH
KKEESGKDDSWGRNYSSYDTRLSLPDNVDKDKVKAELKNGVLLISIPKTKVEKKVTDVEI
K
>O82782
MRTLSSQLYSNGGLTWFQKKNQSSLFIKHLRVSKPSRVQLISAVQFRPCIDIHKGKVKQI
VGSTLRDLKEDGSVLVTNFESDKSAEEYAKMYKEDGLTGGHVIMLGADPLSQAAAIGALH
AYPGGLQVGGGINSENCMSYIEEGASHVIVTSYVFNNGKIDLERLKDIVSIVGKQRLILD
LSCRKKDGRYAIVTDRWQKFSDVILDEKSLEFLGGFSDEFLVHGVDVEGKKLGIDEELVA
LLGNYSPIPVTYAGGVTVMDDVERIKDAGKGRVDVTVGSALDIFGGNLPYKDVVAWHHKQ
HSLH
>Q42588
MATCIDTCRTGNTQDDDSRFCCIKNFFRPGFSVNRKIHHTQIEDDDDVWIKMLEEAKSDV
KQEPILSNYYYASITSHRSLESALAHILSVKLSNLNLPSNTLFELFISVLEESPEIIEST
KQDLIAVKERDPACISYVHCFLGFKGFLACQAHRIAHTLWKQNRKIVALLIQNRVSESFA
VDIHPGAKIGKGILLDHATGVVIGETAVVGDNVSILHGVTLGGTGKQSGDRHPKIGDGVL
IGAGSCILGNITIGEGAKIGSGSVVVKDVPARTTAVGNPARLIGGKENPRKHDKIPCLTM
DQTSYLTEWSDYVI
>P37825
MYLGISGRSPYGSSVAVACQLPNINISGQIYLLLPPSEYINIGFNTRQWQYHCHWRPKFT
CPKGKEAVAVPLPLKFICPKGTSCQLPIFIYSEVYLYASEY
>P51359
MLQTKNSQEIKLNWYETVYLIKSDLNEDRTLCIINEYKSMLTNGGAKNIILQHRGRRHLS
YSIKDYRDGIYVQVNYEGNGKLVQSFEKSLRFDENIIRYLTNKQNKDNKIEG
>P49833
MDLLTKLLVFLIHIDQETILNFFGISDPNTIVINSDYQTIFENSELIPISTNVSLETLRI
IVQAIIDYSKTGLSLVDIENICLLITFIRFIILAVKYNIKTSFYICSISLFAAALWYFHA
KEVLLNWSGSIKGLGMKELSRARSSVIDDKNAYRLSYRSLIVTEFRKQLPGKFSFTDSEI
RSSNDFFSLKFALTKAAVRDEYRIDPISMVFSRLPKSLTPVTDKIYYTIFDNLLPRIVNQ
CIRTFKAMWPVTSWVYITRVWKQYCPYLIRWHWTFLQTYDWIEQYLFKLAFRIAIYAWSL
QREGIYDRPAVIAVGLRCMLYMEFLFVFYALIHALFSQYFYVPFLTENTELHVGPRLKIA
FIVVAIPIGKSIIHVGSLTD
>O78421
MKNDHYVFNFYFFRSKITSNHLIRLNTNYLKKTILFISSDIPETIYEKFYLQSFKLFIIY
YNINL
>P07505
MAAHTILASAPSHTTFSLISPFSSTPTNALSSSLQSSSFNGLSFKLSPTTQSLSLSTSAA
SKPLTIVAATKKAVAVLKGTSNVEGVVTLTQEDDGPTTVNVRISGLAPGKHGFHLHEFGD
TTNGCMSTGPHFNPDKKTHGAPEDEVRHAGDLGNIVANTDGVAEATIVDNQIPLTGPNSV
VGRALVVHELEDDLGKGGHELSPTTGNAGGRLACGVVGLTPV

>Q9TLX4
MTNIHSTAIVHPAASLGRNVVVGPYSIIGSDVSIGDYTRIGPHVVITGKTVIGCNNQILS
GCILGSVPQDLRYIDSELTGLYIGNNNLIRENVTVHRASGNGVTYIGNNNLIMVNCHVAH
DCQIRNNIIISNSVSLAGHVIIDSCVIIGGHAGLHQFVHVGALSMIAAMSKIEKNVLPFV
VVSGMPAITRTINLVGLKRYGISKTDINYIRLMLENLKVQPLAFDTYSIFKKFKSDNLLK
RNVAVQYFSDFLFNSFRARGFIPFKVPKK
>P51229
MCICINCNHIAECSTYHLIESRHKQVHINENPSFMPEFPVIHVNISSNSRINQIDWDLVE
CLSFVEKPNSWNLNNY
>P80484
MVRSGKKAVVLAAVAFCATSVVQKSHGFVPSPLRQRAAAAGAAAASAATMFAPAAFADEI
GDAAKKLGDASYAFAKEVDWNNGIFLQAPGKLQPLEALKAIDKMIVMGAAADPKLLKAAA
EAHHKAIGSVSGPNGVTSRADWDNVNAALGRVIASVPENMVMDVYDSVSKITDPKVPAYM
KSLVSGADAEKAYEGFLAFKDVVKKSQVTSAAGPATVPSGDKIGVAAQQLSEASYPFLKE
IDWLSDVYMKPLPGVSAQQSLKAIDKMIVMGAQADGNALKAAAEAHHKAIGSIDATGVTS
AADYAAVNAALGRVIASVPKSTVMDVYNAMAGATDTSIPLNMFSKVNPLDANAAAKAFYT
FKDVVQAAQR
>P31919
MASPSILKKYGKYFEYCPLEERMIELAKKGEIADAMLLFEKEKPSEFVYKGDAIEKRLRN
IYLSTRLGVKAKINFNDYVIPRDLRWMLDIYESYLNMGENKVFLILGGELRYLIDFFESY
LQFKGFYLLVVKEAKDLLRFRNTCHYDAIIFSDSSILEYQNVDELKNLFNSLETTLKVHN
RKNSVKVLLSPALPKAIMSSKPYKVLEQFFKEKGIEMEGILPYQLNADDKLLPPHFHNSE
MEKSKEYRELESKTKVYIQEFLKKANMNDENEGNDNQKNTN
>P36437
MKLAYWMYAGPAHIGVLRVSSSFKNVHAIMHAPLGDDYFNVMRSMLERERDFTPVTASIV
DRHVLARGSQEKVVENITRKNKEETPDLILLTPTCTSSILQEDLHNFVESALAKPVQIDE
HADHKVTQQSALSSVSPLLPLEENTLIVSELDKKLSPSSKLHINMPNICIPEGEGEGEQT
KNSIFVKSATLTNLSEEELLNQEHHTKTRNHSDVILADVNHYRVNELQAADRTLEQIVRY
YISQAQKQNCLNITKTAKPSVNIIGIFTLGFHNQHDCRELKRLFNDLGIQINEIIPEGGN
VHNLKKLPQAWFNFVPYREIGLMTAMYLKSEFNMPYVAITPMGLIDTAACIRSICKIITT
QLLNQTATVQEPSKFIYPKATSLEQTNILETSQKETILKDNPDSGNTLSTTVEEIETLFN
KYIDQQTRFVSQAAWFSRSIDCQNLTGKKAVVFGDATHSAAMTKLLAREMGIKVSCAGTY
CKHDADWFREQVSGFCDQVLITDDHTQVGDMIAQLEPAAIFGTQMERHVGKRLDIPCGVI
SAPVHIQNFPLGYRPFLGYEGTNQIADLVYNSFNLGMEDHLLQIFGGHDSENNSSIATHL
NTNNAINLAPGYLPEGEGSSRTSNVVSTISSEKKAIVWSPEGLAELNKVPGFVRGKVKRN
TEKYALQKNCSMITVEVMYAAKEALSA
>Q93ZN9
MSSTHQLVSSMISSSSSTFLAPSNFNLRTRNACLPMAKRVNTCKCVATPQEKIEYKTKVS
RNSNMSKLQAGYLFPEIARRRSAHLLKYPDAQVISLGIGDTTEPIPEVITSAMAKKAHEL
STIEGYSGYGAEQGAKPLRAAIAKTFYGGLGIGDDDVFVSDGAKCDISRLQVMFGSNVTI
AVQDPSYPAYVDSSVIMGQTGQFNTDVQKYGNIEYMRCTPENGFFPDLSTVGRTDIIFFC
SPNNPTGAAATREQLTQLVEFAKKNGSIIVYDSAYAMYMSDDNPRSIFEIPGAEEVAMET
ASFSKYAGFTGVRLGWTVIPKKLLYSDGFPVAKDFNRIICTCFNGASNISQAGALACLTP
EGLEAMHKVIGFYKENTNIIIDTFTSLGYDVYGGKNAPYVWVHFPNQSSWDVFAEILEKT

HVVTTPGSGFGPGGEGFVRVSAFGHRENILEACRRFKQLYK

>Q43349

MSASASSLSAFNPKSLPLCVSRPASVSVLPPSLSFKLHSDHLVSIFASSALKCSSPAEYP
SRFVRNVAVSSDFEVEEDDMFADGDDSAPVERNSFSPDLKLFVGNLSFNVDSAQLAQLFE
SAGNVEMVEVIYDKVTGRSRGFGFVTMSTAAEVEAAAQQFNGYEFEGRPLRVNAGPPPPK
REESFSRGPRSGGYGSERGGGYGSERGGGYGSERGGGYGSERGGGYGSQRSGGGYGGSQR
SSYGSGSGSGSGSGSGNRLYVGNLSWGVDDMALENLFNEQGKVVEARVIYDRDSGRSKGF
GFVTLSSSQEVQKAINSLNGADLDGRQIRVSEAEARPPRGQF

>P42757

MAFPIPARQLFIDGEWREPLLKNRIPIINPSTEEIIGDIPAATAEDVEVAVVAARKAFKR
NKGRDWAALWSHRAKYLRAIAAKITEKKDHFVKLETLDSGKPRDEAVLDIDDVATCFEYF
EYFAGQAEALDAKQKAPVTLPMERFKSHVLRQPIGVVGLISPWNYPLLMDTWKIAPALAA
GCTTVLKPSELASVTCLEFGEVCNEVGLPPGVLNILTGLGPDAGAPIVSHPDIDKVAFTG
SSATGSKIMASAAQLVKPVTLELGGKSPVIMFEDIDIETAVEWTLFGVFWTNGQICSATS
RLLVHESIAAEFVDRMVKWTKNIKISDPFEEGCRLGPVISKGQYDKIMKFISTAKSEGAT
ILCGGSRPEHLKKGYYIEPTIITDITTSMQIWKEEVFGPVICVKTFKTEDEAIELANDTE
YGLAGAVFSKDLERCERVTKALEVGAVWVNCSQPCFVHAPWGGVKRSGFGRELGEWGIEN
YLNIKQVTSDISDEPWGWYKSP

>P42732

MAQMVAMPVAHSLSLICNWAKSNPLSRNTLALPASNTPNKQSLSIRCARVGGVEIPANKR
IEYSLQYIHGIGRTRARQILVDLQMENKITKDMAEEELIILRDEVSKYMIEGDLRRFNAL
AIKRLKEIQCYRGVRHIQGLPCRGQRTKNNCRTLKGKKIAIAGKKKVSK

>Q06J20

MNFELKNFNTIRINKNQTILIKTTNLENSSKEDTYQFILKMLKAGVHFGHKIKKWNPKMS
PFIYQKLQGIHIIDIIQSYTYLKKACKLVYSKSLTDNYNTFLFVGTKKQSPIPNSIFYHA
SRCNSFYINNKWLSGMLTNWENTTRSINTLKYLKVYQKTNMFSKLSMKTHSIIDKKKNRL
EKYFGGIQNMTSLPSTVILVGQHNEITASKECRKLGINNITILDTDCNPEFVDLFIPAND
DSSSSLNLILGELQVAINIGRQKFCENLKTWKNSSSILKQI

>P31541

MMARALVQSTNILPSVAGERAGQFNGSRKDQRTVRMLCNVKCCSSRLNNFAGLRGCNALD
TLLVKSGETLHSKVAAATFVRRPRGCRFVPKAMFERFTEKAIKVIMLAQEEARRLGHNFV
GTEQILLGLIGEGTGIAAKVLKSMGINLKDARVEVEKIIGRGSGFIAVEIPFTPRAKRVL
ELSLEEARQLGHNYIGSEHLLLGLLREGEGVAARVLENLGADPTNIRTQVIRMVGESSEA
VGASVGGGTSGLKMPTLEEYGTNLTKLAEEGKLDPVVGRQAQIERVTQILGRRTKNNPCL
IGEPGVGKTAIAEGLAQRIANGDVPETIEGKKVITLDMGLLVAGTKYRGEFEERLKKLME
EIKQSDEIILFIDEVHTLIGAGAAEGAIDAANILKPALARGELQCIGATTLDEYRKHIEK
DPALERRFQPVKVPEPSVDETIQILKGLRERYEIHHKLHYTDEAIEAAAKLSHQYISDRF
LPDKAIDLIDEAGSRVRLRHAQLPEEARELEKELRQITKEKNEAVRGQDFEKAGELRDRE
MDLKAQISALIDKNKEKSKAESEAGDAAGPIVTEADIQHIVSSWTGIPVEKVSTDESDRL
LKMEETLHTRVIGQDEAVKAISRAIRRARVGLKNPNRPIASFIFSGPTGVGKSELAKSLA
TYYFGSEEAMIRLDMSEFMERHTVSKLIGSPPGYVGYTEGGQLTEAVRRRPYTVVLFDEI
EKAHPDVFNMMLQILEDGRLTDSKGRTVDFKNTLLIMTSNVGSSVIEKGGRRIGFDLDFD
EKDSSYNRIKSLVTEELKQYFRPEFLNRLSEMIVFRQLTKLEVKEIADIMLKEVFVRLKN
KEIELQVTERFRDRVVDEGYNPSYGARPLRRAIMRLLEDSMAEKMLAGEIKEGDSVIVDV

DSDGNVTVLNGTSGAPSDSAPEPILV

>Q93WX6

MEGVAMKLPSFPNAISIGHRSFSRVRCSSSLSVCSAAAASSATISTDSESVSLGHRVRKD
FRILHQEVNGSKLVYLDSAATSQKPAAVLDALQNYYEFYNSNVHRGIHYLSAKATDEFEL
ARKKVARFINASDSREIVFTRNATEAINLVAYSWGLSNLKPGDEVILTVAEHHSCIVPWQ
IVSQKTGAVLKFVTLNEDEVPDINKLRELISPKTKLVAVHHVSNVLASSLPIEEIVVWAH
DVGAKVLVDACQSVPHMVVDVQKLNADFLVASSHKMCGPTGIGFLYGKSDLLHSMPPFLG
GGEMISDVFLDHSTYAEPPSRFEAGTPAIGEAIALGAAVDYLSGIGMPKIHEYEVEIGKY
LYEKLSSLPDVRIYGPRPSESVHRGALCSFNVEGLHPTDLATFLDQQHGVAIRSGHHCAQ
PLHRYLGVNASARASLYFYNTKDDVDAFIVALADTVSFFNSFK

>Q9SMH4

MRTRAGAFFGKQRSTSPSGSSTSASRQWLRSSPGRTQRPAAHRVLAVCVRNPARPAADHP
LVSTTQAAAQRQARQWDSRSKPPQLQPQLRHTHVSRQAQRRQQQQVQQQAEAGALVVPTV
ITSFPEHLRPEVLANGVLLVDKPPHWEVPEVVAAVQRATGADKVASVAPLDARASGLMLL
CFGSATRLAPRVERAAKRYTGTLVLGGSSLSGDVRGGSFRAAQLPAEHLTDEDLREAAQG
LVTAAAGAPVHGAVATGHQGGGGLALRVLPRTWRLRQLPSSTEYYEERVEPSMRTLDMEL
LDFRVWRESALSHNDGAAGAEYDQHDQHKQELGRGLVWTRSPPHPRPVVLRFSALLVGRS
HVRSLIAMYGRRLRTAACLDDLRRTEIGSFNVEEAWPLEALVPVLQRHAH

>O78435

MITEFFERLFLSNKGSREDVKRRLKLVLAHDRSTLNASTLEKMREEILLVVSKYVELDTD
SLEFSIRTDSKMTALIANLPIRRILKDI

>O78459

MLKDIQIYELNERIKKKKLKTYNRNKLLKLLTYVERHKICIVLYFNLIKRQLIYIEFVFI
RN

>Q9SWI6

MATMSLAAASPLASIPRGIAAQAPCAAFLSIRLGGATATRFAGLAVASQPAERRAAAMVA
MAKREQELEEIRAMTTEQMEEEVVDLKGELFLLRLKRSARQEFKNSEFSRMRKRIARMLT
VKREREIEQGINKRLSRKLDRKWKQSIVVRPPPSLRGNKEE

>Q94IC4

MALSCSKVLSFYLSPVVGGGDVPKKLTFSSFLGLSKGVGGSRSSRVCAASNAPAPLAGVI
FEPFQELKKDYLAVPIAHNVXLARQNYADDSESAINEQINVEYNVSYVYHALFAYFDRDN
IALKGLAKFFKESSEEEREHAEQLIKYQNIRGGRVVLHPITSPPSEFEHSEKGDALYAME
LALSLEKLTNEKLLHVHSVAERNNDPQXADFIESEFLYEQVKSIKKIAEYVAQLRLVGKG
HGVWHFDQKLLHDEDHV

>Q9FY99

MAALSSSVTTRSYHSGYLASFSPVNGDRHRSLSFLSASPQGLNPLDLCVRFQRKSGRASV
FMQDGAIVTNSNSSESKTSLKGLKDEVLSALSQEAAKVGVESDGQSQSTVSITVVGASGD
LAKKKIFPALFALYYEGCLPEHFTIFGYSRSKMTDVELRNMVSKTLTCRIDKRANCGEKM
EEFLKRCFYHSGQYDSQEHFTELDKKLKEHEAGRISNRLFYLSIPPNIFVDAVKCASTSA
SSVNGWTRVIVEKPFGRDSETSAALTKSLKQYLEEDQIFRIDHYLGKELVENLSVLRFSN
LIFEPLWSRQYIRNVQFIFSEDFGTEGRGGYFDNYGIIRDIMQNHLLQILALFAMETPVS
LDAEDIRNEKVKVLRSMRPIRVEDVVIGQYKSHTKGGVTYPAYTDDKTVPKGSLTPTFAA
AALFIDNARWDGVPFLMKAGKALHTRSAEIRVQFRHVPGNLYNRNTGSDLDQATNELVIR
VQPDEAIYLKINNKVPGLGMRLDRSNLNLLYSARYSKEIPDAYERLLLDAIEGERRLFIR

SDELDAAWSLFTPLLKEIEEKKRIPEYYPYGSRGPVGAHYLAAKHKVQWGDVSIDQ
>Q2WGC1
MGGVENPVSTDKAIRLPERKQYSSNAEPNPSKTEVKRRIERFPRVRIVAINSHRLPAGAA
RGSAGSVTGRRVRRKRMITTLRLNHPIPLFPGKQNGFVS
>P94044
MSTATAPRLPAPRSGASYHYQTTAAPAANTLSFAGHARQAARASGPRLSSRFVASAAAVL
HKVKLVGPDGTEHEFEAPDDTYILEAAETAGVELPFSCRAGSCSTCAGRMSAGEVDQSEG
SFLDDGQMAEGYLLTCISYPKADCVIHTHKEEDLY
>O78431
MKFNNLKISLELLFLYAQIKVKLTHLKHYSTRCSTLSLITVFPLDLLNYLSLIIIDVYYE
ICINYPNNFWLDSTSFDSRFLFILQNIEKKILRHNNYELLTKSINFKNYNLFLSSANLIT
HEDEKLFIWFFHILSEILIHKHKSSTISVKLLSIVGVHLIVKTLLIFLLLINNQSVVNSI
FLSDSKIYHPQFINFNFWWQNLNQLVLRRIYSLYPIYVIKNNVLTTNYVFLPYFNSYLLQ
SNLTTWFSKLLK
>P93184
MLTATKPLVGGACAAPSSSARRRTFVVPEARRKPGNGRRTSVSKVGSTSTSTTTTTTTTL
SADSNGAAVGTVTRPDVHVQDRTHATEMKATVTVHMSKAAGVRDFLYDLILKTWLHVDLV
SSELDPQTGQEREPISGAVKHSGRVDDEWDMYEATFKVPASFGPIGAVQVTNYHHSEMLL
GDIEVFPTGQEESAVTFHCKSWIDPSHCTPDKRVFFPAHSYLPSQTPKGVEGLRKRELEI
LRGTGCGERKEHDRIYDYDVYNDLGNPDDDNNPTTRPVLGGKEHPYPRRCRTGRPRSKKD
PFSEERSHKEHIYVPRDEAFTERKMGAFDTKKFMSQLHALTTGLKTAKHKSQSFPSLSAI
DQLYDDNFRNQPVQPEGGKLRFVIDLLETELLHLFKLEGAAFLEGIRRVFKFETPEIHDR
DKFAWFRDEEFARQTIAGMNPMSIQLVTEFPIKSNLDEATYGPADSLITKEVVEEQIRRV
MTADEAVQNKKLFMLDYHDLLLPYVHKVRKLDGTTLYGSRALFFLTADGTLRPIAIELTR
PKSKKKPQWRQVFTPGCDGSVTGSWLWQLAKAHILAHDAGVHQLVSHWLRTHACTEPYII
AANRQLSQMHPVYRLLHPHFRFTMEINAQARAMLINAGGIIEGSFVPGEYSLELSSVAYD
QQWRFDMEALPEDLIRRGMAVRNPNGELELAIEDYPYANDGLLVWDAIKQWALTYVQHYY
PCAADIVDDEELQAWWTEVRTKGHADKQDEPWWPELDSHENLAQTLATIMWVTSGHHAAV
NFGQYPMAGYIPNRPTMARRNMPTEIGGDDMRDFVEAPEKVLLDTFPSQYQSAIVLAILD
LLSTHSSDEEYMGTHEEPAWTKDGVINQAFEEFKESTRKIVEQVDEWNNDPDRKNRHGAG
MVPYVLLRPSDGDPTDGDPTDEKMVMEMGIPNSISI
>P30397
MFSFFYRFNWNFIKWNLVDNTVSLTQHKIYLASKKSDIFLVKNKQRRFFKSFYAHIFSVR
NCFEKFPSLQSYFYSSKDKFFLVSLLSSKRNLFLHGFFYNIKKAFLFYSYDFFLLYKLSF
SLLLTSTLLPYINDINPCFIKDYYFFVEGNFFDFEQILIDVFKYSYYFKVENFKFLYFAR
FSWIYKNFPLDVNFLKNFLDRKNLVFFKSLFLIIFNFIFNGLNFFFQENFFISYKVYYLF
YIDKFYFFFKFPYDFFICKKIIISFFSLRGIILNININSFYRFETFVFNFFIFDFLRFGS
NILLHINKKHIQFYKLSLKMIVKMFLKKSVFFLVNLLNKKILDCLNRGFFCFNNNFLFLE
LDLYLYRLLWRYIKKLHSRKTRTWIYSKYWKFFSGIWRFFITDIKTGNFLFLKSHLYSSK
YFYSYRNMKFKISNTLNIFNLYNKGKLERMIFEKFKYKFSPNFIVLYNNQRGLCFFCKKS
IYSNRFVILNIKKWNFSFFENLILIHFYCNNFNQLQ
>Q3ZJ93
MQTTQNRLKTKRYASFFLNPKLNRPIQKKGWIKKADFNQLTDSLCKQSLKAYNEKLYSPL
SQFKIPDFLNIQRSSFRQFLKSGLITEFKNCKSITNSNQTFEVFFYAEHYKLNRPKWTPK

QAILKKKSYSSQLYLPIQLSNYQTEEVHLQWVLLANLPLMTKNGHFILNGCPRVLMNQIV
RSPGVYFRKVVKNQKTVYSADFIAQRGSWLRLEVDTKKGDIWAKLKKTPKIPIFTFLRAL
GLTLPILNNSIDFAKLRYLLQFKEESKRSKKIKDFLGPGINNSLDIVEFRSLLKSLKKYM
KPKPAKKSSKKQKTKKIKKWLTPFVCTTYFQSLLELCQKLYPQKKFLELDPKLAQKFLFR
KFQNPRTYDLSILGREKINKKLGISIPLEKTILTSQDVLFACLFLMDLLQGLVISDDIDD
LKNRKIKPSGELIQTQLSTGLIRLEKIIREKLKQPKQDSDSFSNLFSVKPLNQAFRDFFG
TNPLSQLMDQTNALSEITHKRRLSSLGPGGINRETAGMAIRGIHPTHYGRICPIETPEGQ
NAGLVNSFTIFSHLNTKSFIETPFYKMYKGFILKNKNPFLFSSDQERNLVLAPGDIQTSL
FHFLPPGVPIPSRQFKEFKRVSRNEINFIAVSPIQMISIATSLIPFLEHNDGNRALMGSN
MQRQAVPTVRPSKPIVGTGLESRVISDVGHGLQVQKSGFVSYVDGNKIIIYSKKRIDQPF
FFDTKKLHFPKKKVPFLNLDLQQQNTCRSSFKKKLVKSANIFTFKEKQLYQLSKIKNKKQ
RVCFEKSLKSQDFSSLLNFLTLFNIKTKVQAEKKASVQDASFILVPKKNLDLEVNKRIFL
PVFLKKRNEYNFLNYYKLTERTLYSGFSILPFKLTNKGNYFNNKQISCLSNSLFLNDFLG
QFHLLKFFSTKIFYSKLSRNFKPFISKRSLVLINKFSHQKLLFSYCKIILIHFYTKFNKK
TPKNFLLNTKLSSKLMKRKDFSHFFRKDEKTMPIQNLTNFKSAHLHSQTAVKGVCQQIRS
ARKTQIANPFWYNSFPLFLIKSSPLTPLLFRRSFVPKVFNPLGLHFAYQQSKGPKHVSRT
FKTLDRNEQKFSYNQKFQKLNFIFVKPYKFKRKNSPFAPFARSSKARGSAKAIFSQAQRL
WGEKVFLVQNKENLPQTLGSCKIMTNQFYLKFSLLLKQQKKNFSNKKTGMENQNCQNSLT
SQKNGFQCLKKMEPLHFPTKKSSISTLSFVKKILPYFQHPLLNLTTQNKIKNKLMIFKKP
FLFWSACFADKQSLRSILFSLKKSQKVTTLKKTKVKKKFEIVYNENKKKYFQKINKNFFL
PLLCRTRNLNFASNSLLFQISYPVAVNQSNLIQRNLISQELKHNSKRFIKTLLKIKPFVG
KFVFPFCPICLKRKASIETQSFVKKSDKNFQFCFCFSRKKQKTLKPLLRIKSSDKMNTLK
PLQKFSLLENFKVPPQSSLTARDKILRSTIGTKGLNDSLSMKSTDGEYAINDKKRRVEII
NSSSLFYNNLIPQNYILNNYHRSNQDTYMIHRPLVQEGQWVEKGDILADSSVSVQGELSI
GQNILVGYIPWEGYNFEDAVLISERLIFDDLYTSLHIERYEVEIRDTQFGLEQITNQIPN
EKGEYDYIDSNGIAKIGTWVKEGDILVGKIAPIGQKKLTAYENLLYDILNKEIPKTRDTS
LRVPRGVTGRVIHVEIVETTKKKGNDSSIFAKSLKFPKKQKLKRVTSPEYWYPVNTKKDQ
KVQSFSFKNKSEKLFLTPVQSYFNKEAKTKNPKLNSPFFYLNKRNPDFNLKRNKRIAKEM
YFFNKNEKIKLSTLQIKIKGLEKKEFSFPKILKARFSLLQKAQTANLKIEKFLDDKFWNN
VNYSNKNFSRNGSQKENSNADGLQFYNPLKKTKTKVLQPMEKTSFSGPQKVHIYLAEKRK
LQIGDKIAGRHGNKGIISNILPRQDMPYLSDGTPLDIVLNPLGVPSRMNVGQILECLLGM
AGYYLKQNFKIQPFDEIYGCEASRSLVYSKLYEARIKTGQDWLFNANHPGKTRLFDGRTG
DCFKQPVTVGVAYILKLIHLVDDKIHARSTGPYSLVTQQPLRGRSKQGGQRVGEMEVWAL
EGFGAAYILQEILTIKSDDLKGRNQVMNSILNNKPVNFGLPESFKVLVRELQSLCLDIAI
YRYRENGTPTKININNFW
>Q9TM37
MFSKLEAIIRPFKLEEVKVRLMTFGVPGITVTNVMGCGKQIGGIERSKGVEYDSELIEKV
KIEIVVMNEHIDELIEIIINAVWTGEIGDGKIFVSPVSSVIRIRTQDKDLDAI
>A0T0C2
MAKKITALIKLALPASKATPAPPVGPALGQHGVNIAAFCKEYNARTNDKAGLIIPVEISV
YEDRSYTFVLKTPPASVLLANAAKIKKGSSNPNTVSVGSITKTQLEEIANIKLPDLNTTK
ISSAMKIVEGTARNMGISILD
>Q9TLR8
MVLNANHLQTKKTIKETLLAPRFYTTDFDEISRYDISSNFEEIEAIVNEFRSDYNKKHFI

RDEEFDRHWSQIDAQTKQMFVEFLERSCTAEFSGFLLYKELARKLKEKMPVLAEGFLLMS
RDEARHAGFLNKAMADFNLTLDLGFMTKNRRYTFFKPKFIFYATYLSEKIGYWRYITIYR
HLEKHPEHRIYPIFKFFESWCQDENRHGDFFAAIVKSQPYLLEGFISKLWCRFFLLAVFA
TMYLNDCQRGEFYKSIGLDPRQFDIYVIQKTNESAAKLFPTVLDLDNPRFFECLDKCACY
NKLLLEIDDREQPFFNQVLNVFYKFYLYFLLVLNFIMLYFLRTVNCNSFTRMVK
>Q9S7W5
MNIVSWKDANDEVAGGATTREREVKEDQEETEVRATSGKTVIKKQPTSISSSSSSWMKS
KDPRIVRVSRAFGGKDRHSKVCTLRGLRDRRVRLSVPTAIQLYDLQERLGVDQPSKAVDW
LLDAAKEEIDELPPLPISPENFSIFNHHQSFLNLGQRPGQDPTQLGFKINGCVQKSTTTS
REENDREKGENDVVYTNNHHVGSYGTYHNLEHHHHHHQHLSLQADYHSHQLHSLVPFPSQ
ILVCPMTTSPTTTTIQSLFPSSSSAGSGTMETLDPRQMVSHFQMPLMGNSSSSSSQNIST
LYSLLHGSSSNNGGRDIDNRMSSVQFNRTNSTTTANMSRHLGSERCTSRGSDHHM
>P0C310
MTKDETLLVFTLVVSSVSVFLFGILLFMVLISATRDFRERTKSKLVKIMIWAGIVVITFA
IAVRIYPIFIFLLKERIKPLVEALYDKLPWIWEVSLSRYWDRLIDFLDRYLWACAQRIQT
GIRKQKGEFVVTFSCRVKKRLYARAIEVGIHLSLLSNLFWILKTTLAVGYRLLWVLYYII
SFEGFLGSFRLYLVYFGFYCLLFSGKWLRTSEDRGERQAQISGILLRGMLIECAFSVLCL
EEDSNLHAL
>P05725
MNTKYNKEFLLYLAGFVDGDGSIIAQIKPNQSYKFKHQLSLAFQVTQKTQRRWFLDKLVD
EIGVGYVRDRGSVSDYILSEIKPLHNFLTQLQPFLKLKQKQANLVLKIIWRLPSAKESPD
KFLEVCTWVDQIAALNDSKTRKTTSETVRAVLDSLSEKKKSSP
>Q1XDB5
MRTRYKNTYKEFLGACFLGSVTLLSISLWNIINQAGKNHSYKAFIEFDSAYGIQEGTAVR
LRGLPVGKVVGISQSSNSILTSIEIKSSSTIIPKTSLIETNQTGLLNDTVIDIIPLSKLS
IDYSSIKAGPLSGACDNSQIICNLNYLKGERGLNYDDLIRATTRISQRFDDPKLFYGLYY
LIGNMIKLSNNFVDLTEQIAYMSLFIRLQLENLKEQ
>P51342
MNNHYLNQGCSFEKVSKNILKKNSHCNPWLSFFNQQDSEHQIFSLKQNDIIIFDVNSKLY
IILAGCLIVKKVFRNRKKIILNVLTSEDSFGHTQFASNRFYYEVEALDVAQVLSIEYSII
MNICQNYPNFSVFLVNHLLLCSIKANHFMEIISHKSITNRLISLLLLLSEHNGISQNNGI
LIDLTITHKVLAQIIGSNRVSITRIISKLIHTKFISMQKKKVIIHDPILLSQRFSNR
>P51237
MNKCNLLVQILQCKSVKTTSNENQIIKLKARFKKRNKVVAIDLLIWNRQSLEIFKLLKKF
DYIIIEGKLHRSETTDIKLMLNQQKDLVFSTSRIFKYKSLLKNKDIDLFIK
>P51208
MEIILFDNKINSRFNKVTSTNYAAKLDQTSEIWLFNCIENTQHIFLKSQLKLSQITKIFI
SGTSFKYAAGLPGLLSSLTLSGKINTVSIYGPNSLKAYLQACTKYSQTNFSFSVNFHAVS
YGKLVSGQSYTVICLPLSSNKLLYGFTILKKQQQGVFNLKKAITLNISQGPIYGELKGKH
NFLSPDGYYLHGEDFSSSTTLGNKISIPVVLKYSRIISEMHWLSSYTVKSNTYPHQQATK
YLLSNIETNVMNYQVSQDNNLIE
>Q42463
MASICTSNFHFLCRKNNSSPISHHLLLSPSSLSFSRCGGLRLCRCAAVKTGSEGGGIRSD
NAELLRKPVISTELETTSESEELVKEESDDEVGKKSGDGEGWVDWEDQILEDTVPLVGFV

RMILHSGKYAIGDRLSPDHQRTILQRLLPYHPECDKKIGPGVDYITVGYHPDFENSRCLF
IVRKDGETVDFSYWKCIKGLIRKNYPLYADSFILRHFRKRRRND
>Q9ZS97
MASLLANGISSFSPQPTSDSSKSPKGFHPKPESLKFPSPKSLNPTRPIFKLRADVGIDSR
PIGASESSSSGTSTVSSTDKLQQYFQNLDYDKKYGFVEDIDSFTIPKGLSEETIRLISKL
KEEPDWMLEFRFKAYAKFLKLEEPKWSDNRYPSINFQDMCYYSAPKKKPTLNSLDEVDPQ
LLEYFDKLGVPLTEQKRLANVAVDAVIDSVSIATTHRKTLEKSGVIFCSISEAIREYPDL
IKKYLGRVVPSDDNYYAALNSAVFSDGSFCYIPKNTRCPMPISTYFRINAMETGQFERTL
IVAEEGSFVEYLEGCTAPSYDTNQLHAAVVELYCGKGAEIKYSTVQNWYAGDEQGKGGIY
NFVTKRGLCAGDRSKISWTQVETGSAITWKYPSVVLEGDDSVGEFYSVALTNNYQQADTG
TKMIHKGKNTKSRIISKGISAGHSRNCYRGLVQVQSKAEGAKNTSTCDSMLIGDKAAANT
YPYIQVKNPSAKVEHEASTSKIGEDQLFYFQQRGIDHERALAAMISGFCRDVFNKLPDEF
GAEVNQLMSIKLEGSVG
>P13446
MYCGRYETIGETRGNSLNVFIGAAAGFVAAVALINSGLATSFYSTPVRAVPQVIVPSSLA
ASSQLPVVPKETNIQVNSAQILYPDSTVKGQERTITILGVCSFLSASLFYIWKQFGMKAR
TTKPADLQEVSGGRIWSLASTTGSNIGAGKTVRVATRKSPLAMWQAEFIQSELERLWPGI
TVELQPMSTRGDKILDSPLAKVGGKGLFVKELETALLENRSDIAVHSTKDVPMELPEGLV
LGVICKRHDPCDAIVFPKGSNLKSLEDLPHGARVGTSSLRRQCQLLLKRPDLKFLELRGN
VNTRLAKLDSGDYDAIILAAAGLKRLGFSDRVLPGETNIIDPNVMCPAAGQGALSIELRT
NDPEIAALLEPLHHIPDAVTVACERAMNRRLNGGCQVPISGFAQLKDGQLRMEARVGSVT
GKGPLIIQSKTFRLPWSGRTWPQLQKESEALGVEVADMLLADGAQAYLDEAYASRTLGWA

>P49535
MTKISTAKTFFINGQEYYTTNTINLHDLLNYFDFNSSLLVLEYNNFICNKKNWEKIMISN
NDKIEIVTIVGGG
>A0T0Y7
MSRIGKLPIKLPTTVEISHQSDATVQVINVKGKFGSLQRTLPEELKIEQIHNDNGSSLIV
SFENQTRTNKSLQGLYRTLINNMVIGVTEQFVIILTLQGVGYRASVQGKSLVLNLGYSHP
VEIDIPEGITVEVTQNTTLNIKACDKEQLGLFAAKVRAWRPPEPYKGKGILYKNEQILRK
AGKSGKK
>P0C471
MKKILFSMFYSILVGEEPDSVFLKKEGKQNQVKMIWVAPSSCAKDLTISEGTGATFLFNF
HSRVSYLFPRPFLRPRNMKWTNSFS
>P51354
MQLQINVASHPLIQHWSGILENNNNPGTILRTACSELGKWITYEIMREWLVTETVSIKDN
TTISLISNHYKYIIVIIMPYGFILAEGARALLPTANIALVSYNNSIDNISDKLDSFTKIL
VLDLFLDEIIMTSVLEELIKKGAILNNIKIACLECGSSQLNKLGQKWSTLEIYTTKVNSI
TDNSEATKEQVLKDKFFA
>Q84Y95
MALRSVKTPTLITSVAVVSSSVTNKPHSIRFSLKPTSALVVHNHQLSFYGSNLKLKPTKF
RCSASALTPQLKDTLEKLVNSEKVVLFMKGTRDFPMCGFSNTVVQILKNLNVPFEDVNIL
ENEMLRQGLKEYSNWPTFPQLYIGGEFFGGCDITLEAFKTGELQEEVEKAMCS
>Q9TL10

MLVVGNRIVDVSRHTSIGTIVIATVDKLAPGLGIAFVSWTHGQGKGGLPSTKKHYGILPL
RSWRGRGPLDFATTHELTGENLILQHGDFVLVQIVQDGNHAKVHLVSGHIALTTSRLVVW
PGLSSKDWIFSHQIGQKINNYLHVRKLVSYMRRDQILCTGPQRWMKEQYALEHQWENFVL
EFIEQPTGISYLTSMTEKFVSPVCAEWFQHPLSVWIIGCNLQIRESMTKWMITHVPHKSR
HIEITTLDAWKNWYNLHRAAIVQPQIPLRSGGTMIIEFTEIGWSFDINSGIGLEIGSKTC
ANEEAIYAIAQQILLRSMHGFILIDFIGDIDLEKLRVNLIQFTSLLEQDSYHIRIISISA
DGLVCVIRHRRSKLI
>Q10712
MATLRVSSLFASSSSSLHSNPSVFTKYQSSPKWAFSFPVTPLCSKRSKRIVHCIAGDTLG
LTRPNESDAPKISIGAKDTAVVQWQGDLLAIGATENDMARDENSKFKNPLLQQLDSELNG
LLSAASSEEDFSGKSGQSVNLRFPGGRITLVGLGSSASSPTSYHSLGQAAAAAAKSSQAR
NIAVALASTDGLSAESKINSASAIATGVVLGSFEDNRFRSESKKSTLESLDILGLGTGPE
IERKIKYAEHVCAGVILGRELVNAPANIVTPAVLAEEAKKIASTYSDVISVNILDAEQCK
ELKMGAYLAVAAAATENPPYFIHLCFKTPTKERKTKLALVGKGLTFDSGGYNLKVGARSR
IELMKNDMGGAAAVLGAAKALGEIRPSRVEVHFIVAACENMISAEGMRPGDIVTASNGKT
IEVNNTDAEGRLTLADALIYACNQGVEKIIDLATLTGAIMVALGPSVAGAFTPNDDLARE
VVEAAEASGEKLWRMPMEESYWESMKSGVADMINTGPGNGGAITGALFLKQFVDEKVQWL
HLDVAGPVWSDEKKNATGYGVSTLVEWVLRN
>Q06J39
MENKLFSCIESRLRGKRNYYSRFYIGPFLKNQAFLYSNVLRRVLLSDSSNIVITAVNIVG
AKHEYSLLPGVRESSLDLLLNLKELVFVKDIYTKFNKSYFAYLKSNGPKIIRCSDIILPN
TIYAVDSTQYIATISNNKSLILKMKLTANLLNNFYSNLNYELNSLLSYYTNDILEKTNFS
LIIDPNFSCVNKVNSSLISLDNLENYNDSISLEVWTNSSYSPRIIIQNSIKSMVNLFLSI
YDTSFIDIKLENSDNFLENHLVSLSNRATFFKKSFFNICTEFNTVKYKTSFDKNLTLISI
DELDLSIYSKFLLKRHNILTLYDLFKIDKRVLERFYNISYKTLQSIERKMVKYGTVNK
>Q3ZJ90
MFIKKKKKVSSFEFSSLRSTFSFVHWAKLKHQTSSIIENHSDQISPIQIDFYNRPFEKG
RLKALVSWSISYFGEKKTVDLVENLKSIGYAYATKAGISLGIDDLKIPPSKKDYITKAEQ
ILEFTNQDVKKGYLTSIEYFSKVIETWNKTSESLKEEVISNFKKTDELNPVFIMAFSGAR
GNISQVRQLTSMRGLMSDPQGRIINFPIQSNFREGLTLTEYLISCYGARKGVVDTALRTA
TSGYLTRRLVDVAHHVIVRGFNCGTKKGLYLSDLKKGDKILLSLKNRIIGRRLAEDIYNS
KKQLIATKNQEISSNLSSLITKNKTSIFVRSPLTCQDKNYVCQLCYGWSLANHRLVPSGE
AIGIIAAQSIGEPGTQLTMRTFHTGGVFSGEVTDEIKAPFRGIVFFNTSIPGKLIRTTYG
QIAFLTKQESFLTLIPENPKQQWLDSSKKLGFSKQKEPDFQKDSLFLQKKVEFKIPAYTL
LFVKNNQLVEKNQVLGEASTFLTRQNQSIESYQTIYSEFSGEVKFQHSKGVQVLKKELDF
KIDDQELSSVLKNKLRKLKSLFNPSANSSTGEFWILSAQKQTISKPVNLLVKPGDFLHQK
ALLYLAKKASYSNYFETIALDPSHNLPTLPLPILFYDFGVNSLAKNLNKNAFFNLALLNE
QLYLRKGNLFLMQDRSTQKKANRSTIKILPSLQKQTLQNFKKVCSDLPFVSRSLFPISES
SRDKLNSIQNGVKLQSTIVGKHRFLSLNIIFTQIAKKKFFKMNLNSEVKIKSLKQFDLNH
ESNEMKQIGVFNKDKTPTGGFGIQEIFYTSFESKLKTSPLFLLAQFSSDKTLIDLTKQNV
QDFKFKNLNYQYFHPFLVLASDPPFCVSEKTLHFMQSCGQSLNEIDPNPKIETFFSKSAP
SFLDKNRRNKRFKSLKKTMEKNHLLSLYYFLTQPLSDDNDDTIMNSFNRKSKLNKFSTKR
LNKKQIVFGKLNHHFFADKGGDDFHFKNFIHGGLSFKEVCEVDLYLKERVEVFNWKIATL
GTPVSDIITKAEKFVLFHSPTNACGSFILEQPLKNPCFYAFGKIGFLRGKTDYGQPNKIF

SSNLFNLKKGWKTKTEGRFTNRPNKQKFKSFINSVEKSGPLNKKIFQNEGPFALNDFTKI
QPIVGNQLNENFFTNAAELLKKRPGAAKQEFQLTSFLEKFINRHNRLFWFPKDQNILNFA
DQQSKGPTNLLFQRKFLKDLTLFSDGDPSFSCRSFVFQTKFYLSKKLFENLPRHNFENYD
IISGPINFQEFEILQKIPFSFYPLSSRSTQKFGPFFILPREVTKKQSFQIFKYPLLFELK
TYSPTFFSTVFFFIRNFGPKFHKKRERKLIKAIFRPLPRLIGFSTLKSPLDSGQTTTEKA
GLQKPVYRFIKKSELFFSIFDYQILKNYRRLASNLILKQNGLNLLNYSPILNSLLKSALL
FNIASAKKNSVFEKLGLKISAAYSESKTFGIATEKNSLNSNSVVLFQKKQSGFLIRELNP
FHNTLGSPFFDRSKKVDNPQSSLTFDRPQSANERKQILKKARQKLRLFPLNLNEKKNRFS
SVTLDLLRDQTTLHKMQSCGEAESGNLKTKETLFKKVKRENKKITEIFTFCPFCPQLKSK
GKRKSKGDQLFQEPCNLNLGENFLSCLPFGFEYPVKARRRLVKEQRLPFSLSLILPDKLN
YYQSLTSFPFSKQMGNRLLKNLTQLNFLNVGCIRLSQLDSKILRSDSFNPQRKELKSKKF
NNFSINELSDSFMNLGWTGFSQKNLILKYLDTDQIFKKGGPLKSNLQDSQLVECFFKTKK
VHFLFISKLLKESSLNSFFYNFYYSGSSFNSFADIQKFENKASFFQTKKKLYNFEKKDQW
QKKMSFLFFIQNKKTHLFLPNSKGFFLKKKKDISCERFSTNVILSKSQSKNETKELKKAS
LANQNLKKHSTISTENALKNKMNQSFSFSHFNKEKKPLSDCRAQTKGAIQKFSTKLPPTQ
TGWIFAILNPKIYLNKHNCVEFAGNSNLSDISFDNYVTLTCFLTIRFVNTFFELHANVDF
WLKTLNTFHLGLLKQSSFDFKASKIKLQKTPFLFPSFPPNYIELVFVKKAILRRVAKKDS
TFCNLYSFENFLLDKRFQKFKTHFNLQSHLKLAPSFCVDLQGSKGLENFAWTGKLRSQSQ
ASWILETNLFSPCFADKQSKSPLLEFENKKCPAFAKGKLQKSKIEKFEFTYNDIKNKLVY
FNKSFFLTSDQRPVFQRSFPKKIHRKTLFITHPSIIWKSRFESSLKSLTFFKMKAKQNGT
GSLSFFEKKSLETSLNKTSVLFKKVLAIKTFLLFSFSSLNLVSGKLNFLSSVDQNKKSNL
KIQSIFENFAYQQSKGSNEINFSRSAALHSMQSFVFSEKLRKTLSLRISNFHKIQKYANQ
NLESGIGFFSFFQKSLSIVFFDSAYHFSPQNTSTKDSFEIKKENKTVINNYCRFSNFKLL
KLPVFKNRFLNKYSLFKSFLNYLSYSFDVKTSKQILVRLPLLKETCFHFNKNSRFKPKLL
ILNQANSQQLLATCFVQPYSEFSNSSFVFNSKSAHLHSQTAVKHRQNFEKKSKIIFDERK
TFSFISSSTQVLFVSKKATHYLNENFRSQNYKKKTYDFIDNANVLKNRFFERLSPVEFHR
KREGFLSKDQKQMTFKYQNMQGGLIPALDSTSTFAPFARSSKARGSAKAIFSQAQRLWGE
ESFINDKQKSIRNQIIFAKNSRFKNLLILNNKNENEKLFYLNLKKKVSEQSTMNFFVPAL
YKKLFYTKQSISKFLEVKIQPNLQIQWTFFNSNISKHEKQQKFLLPLFDETFNIQGSNLK
NGLNFGMLSLSYSTLDPFFECLKKRVNSSWFFNGKQTFKKKKKIAKEGAFFNHSFFLDAK
KSKQLNKKIQKKFTKRLQTLNFSEIKKGFFISEKFKTRLSCLIKKPFLISTFFLSYRLKK
PKLALNFNYQSLGNNSKKFSLIRLNSIDFNLSKSQRGWFHNQNVSKQFRFFKHNRSVNLF
QIHFDFENSCDPCFAMQKQTTSSKPVLFLYNLKPLKTDFFQKGFQTTSQLLFKHINHSIV
PLKDDANHLSSFLNQANFRGAFEPKAKTIADKLISQNVAITKPNLPKSNFSSLKGEVFFV
TNSRQFKLVDLSVFKKSSREIQLLTNLDLITFRIKNRNFPSKHIEEQKPNLIEKQLAQSK
NKLQIYIGQLLRYGKEISPGIGLNQSGQILILQSNKLVLRYAKPFLLASGGICDLVQGDF
VKNQSPLLNLKYKSLKTEDIVQGIPKIEQLFEARENFQDELGINNLLKNKFLVYKTLYHP
KEAVRKSFEFIQHYIIDGIQYVYQSQGVNISDKHIEIIVKQMTSKVRILEPRNSGLLRGD
VVDLDWIERINLDILTGKKAQYEPIVLGITKASLDRRGFISAASFQETIKVLTKATILQR
RDYLRGLKENVILGHLINSGTGSTLYSILKEKKSNFLNRFLQ
>Q8VYN6
MDALSQAISSGISVPYKNNSSSLVPSHGLTSLILRKSRSPVNPSSRSRVSVRASEIQHSK
TSASSIDLSDPDWKLKYEKDFEQRFSIPHITDVLPDAEAIRSTFCLKMRSPTEDFVGGYP
SDEEWHGYINNNDRVLLKVISYSSPTSAGAECLDHDCSWVEQWIHRAGPREKIYFRPEEV

KAAIITCGGLCPGLNDVIRHIVITLEIYGVKNIVGIPFGYRGFSDKDLTEMPLSRKVVQN
IHLSGGSLLGVSRGGPSVSEIVDSMEERGINMLFVLGGNGTHAGANAIHNECRKRKIKVA
VVGVPKTIDNDILHMDKTFGFDTAVEEAQRAINSAYIEAHSAYHGIGVVKLMGRNSGFIA
MQASLASGQVDICLIPEVPFNLHGPNGVLKHLKYLIETKGSAVICVAEGAGQNFLEKTNA
KDASGNAVLGDFGVYIQQETKKYFKEISTPIDVKYIDPTYMIRAVRANASDGILCTVLGQ
NAVHGAFAGYSGITVGIINTHYAYLPITEVIAYPKSVDPNSRMWHRCLTSTGQPDFI
>P49235
MAPLLAAAMNHAAAHPGLRSHLVGPNNESFSRHHLPSSSPQSSKRRCNLSFTTRSARVGS
QNGVQMLSPSEIPQRDWFPSDFTFGAATSAYQIEGAWNEDGKGESNWDHFCHNHPERILD
GSNSDIGANSYHMYKTDVRLLKEMGMDAYRFSISWPRILPKGTKEGGINPDGIKYYRNLI
NLLLENGIEPYVTIFHWDVPQALEEKYGGFLDKSHKSIVEDYTYFAKVCFDNFGDKVKNW
LTFNEPQTFTSFSYGTGVFAPGRCSPGLDCAYPTGNSLVEPYTAGHNILLAHAEAVDLYN
KHYKRDDTRIGLAFDVMGRVPYGTSFLDKQAEERSWDINLGWFLEPVVRGDYPFSMRSLA
RERLPFFKDEQKEKLAGSYNMLGLNYYTSRFSKNIDISPNYSPVLNTDDAYASQEVNGPD
GKPIGPPMGNPWIYMYPEGLKDLLMIMKNKYGNPPIYITENGIGDVDTKETPLPMEAALN
DYKRLDYIQRHIATLKESIDLGSNVQGYFAWSLLDNFEWFAGFTERYGIVYVDRNNNCTR
YMKESAKWLKEFNTAKKPSKKILTPA
>P41347
MTSTVTTTVGCGGLPVRPLSTATRGRPRRCAVRAQAAGADASNDKSVEVMRKFSEQYARR
SNTFFCADKTVTAVVIKGLADHRDTLGAPLCPCRHYDDKAAEVAQGFWNCPCVPMRERKE
CHCMLFLTPDNDFAGKDQVISFEEIKEATSKF
>Q9MTN0
MEPEISTDSCRVKKNMKSKKPAVPISSLSNLNIRIVDIVMILWARSTYFLFFFLLISHF
>P31561
MIFTMYFFRNFVFLHSFMNYIIKVKSILINRYSLATCNAILLYFFQTNYYKIYLLVNTSL
SFYNLIFFLRNFLRIVKKKNDPKVFLLALLIISKNYIQYALYCNFLFQVGNRTLKVIKSV
EISKNEILKNDPSLKALEFWKKIKIFYSKNFISKIAVIMVISFSGYCAFNFAFKFFGVLK
ESSKILASFFSFNHDQKNDNSEDNRSEDDLKSSQDPVNIPSIEQSFKVEDFSIFNPDFWS
TDFDKILLEEGYDLEGSLSTLWLTPRTFIVMKRIVQTGFYLSMCVANAYAIAETVKRGQP
VSNDMLQFNPTLQTGFVYPYGQSMSAPGQIPGGIWAERLFLKKKCLLHKI
>P48337
MKYLKIYVLRSRLLKDGNLAFFKVKNLQFFDKNFFVTEIRRYLLLDLESWNITNVMFFVT
NNLKNTSTFHVVHNFLGLEELKQSLLEITKKFKLLRFKLCSKSYQGNKFFAVLNAFEVKD
FLSGDIIFPPHLKLLNSRELLFTKISFNITLKILLKIEAKKASFQKPIKRLNLIIEKDEF
FDNYMIFDLTTDGSVTPFNAFIKALQSSNLVTLTNA
>P29463
MESRVLTGGATAIRGGLPLLRKPAAVMKFTTAAHAISRDFPAGAVTAKPVGPLIAGPNLI
WGRQLRPAILLETSPKRESIKPCSAAASSSAGSSDSSGDAKVGFFNKATLTTGFFFFMWY
FLNVIFNILNKKIYNYFPYPYFVSVIHLAVGVVYCLVSWGVGLPKRAPIDSTQLKLLTPV
AFCHALGHVTSNVSFAAVRVSFTHTVKALEPFFNAAASQFILGQQIPLALWLSLAPVVLG
VSMASLTELSFNWLGFTSAMISNISFTYRSIYSKKAMTDMDSTNVYAYISIIALIFCLPP
AIFIEGPQLLQHGFNDAIAKVGLTKFVTDLFWVGMFYHLYNQVATNTLERVAPLTHAVGN
VLKRVFVIGFSIVIFGNKISTQTGIGTCIAIAGVAIYSFIKAKMEEEKRQKKAA
>Q00865

MGFVDMDYDIISYSDQMPLAQHYSYFIIYSPSKSRFHVIPYKDDEYLNRFYWLQISLGFS
IDASNISSQYSANPTINLSLILD
>Q00497
MEARVSQSLQLSSWINSDKVVRKPSGLLRFSEKWNEKPRHRVVVSCHLQPRKAAHSDRRV
QLKVSCSPQNVQASVLESGCFSASIDEIETLKNKAEEVEEYLDGRCVYLVGMMGCGKTTV
GRILAETLGYSFFDCDRLIEQAVGGITVAEIFELRGESFFRDNETEVLHKLSLMHRLVVS
TGGGAVVRPINWRHMHKGISVWLDVPLEALAKRITTEGTKSRPLLHEESGDVYDTTLKRL
TTLMETRGENYANASARVSLENIALKREKDVCHITPAEITLEVLIQIENFLKTQKSVVVL

>P48496
MAVVSTSLASQITNPNSAVSTQFSGLRRSFLKLENSVSTQSSFFQNVDSHLRLSSSSRRC
PRGVVAMAGSGKFFVGGNWKCNGTKESITKLVSDLNSATLEADVDVVVAPPFVYIDQVKS
SLTGRVEISAQNCWIGKGGAFTGEISVEQLKDLGCQWVILGHSERRHVIGEQNEFIGKKA
AYALNQGVGVIACIGELLEEREAGKTFDVCYQQLKAFADALPSWDNVVVAYEPVWAIGTG
KVASPDQAQEVHVAVRDWLKKNVSEEVASKTRIIYGGSVNGGNCAELAKQEDIDGFLVGG
ASLKGPEFATIVNSVTAKKVAA
>Q07510
MATSYGFFSPSVPSSLNNKISPSLGINGSGFCSHLGISKRVFCSSIEASEKHAAAGVSSS
ESRVSRLVNRGCKLVGCGSAVPKLQISNDDLSKFVETSDEWIATRTGIRQRHVLSGKDSL
VDLAAEAARNALQMANVNPDDIDLILMCTSTPEDLFGSAPQVQRALGCSRTPLSYDITAA
CSGFMLGLVSAACHVRGGGFKNVLVIGADALSRFVDWTDRGTCILFGDAAGAVVVQACDS
EEDGMFAFDLHSDGGGGRHLNASLLNDETDAAIGNNGAVTGFPPKRPSYSCINMNGKEVF
RFAVRCVPQSIEAALQKAGLTSSNIDWLLLHQANQRIIDAVATRLEVPSERVLSNLANYG
NTSAASIPLALDEAVRSGKVKPGNIIATSGFGAGLTWGSSIIRWG
>P36443
MTLALMQSMGKLKFLHCLLPLPFRDVPFPLRASKLRDFNAINKFVPLRDVSGSCEVLILY
INIECLHTPKEDVSGSGTATAILILRRSSGGPTATKIYLPEDVLPTAEANEF
>P30956
MATMVAGISLRGPVMSSHRTFSVTKRASLPQSKLSSELSFVTSQLSGLKISSTHFISSSA
PLSVPFKPSLQPVARRICPFTGKKSNRANKVSHSNHKTKKLQFVNLQYKRIWWEAGKRYV
KLRLSTKAIKTIEKNGLDAVAKKAGIDLSKK
>Q6B8Q1
MTYKNILDKLVGKWIHQRTSYFIHNQQIDYHQEEIKLKQIDNIYISTKDNNSLYQYELSN
KINNQKIYYIFSKQEESEFGKLHKITNDEIKYYRFTIHTHNCIKIESVRENISYNEYIYL
INNRFKITISILKRGQKYLATSFISEIKIFN
>Q85FL6
MVLTSDHLDKKNIRKIEYKGEDSFSNSMSKLPLQGGMSDSIFMASISDFSNWSRLSSLWP
LLYGTSCCFIEFASLIGSRFDFDRYGLVPRSSPRQADLIVTAGTITMKMAPSLIRLYEQM
PEPKYVIAMGACTITGGMFSTDSYSTVRGVDKLIPVDIYLPGCPPKPEAIMDAVTKLRKK
IARNRFVNRASRPIRTKYFSISHQLNLVPGTCAGKYNWDRENCGTKLAPANFSENCQKFA
NEHDELKSAI
>Q20EV4
MAYSVSRLVCSDGSIFSSNTNFETPFQPRVFHPSTSSEINERPDLNLLEGTPRTIVVTSG
KGGVGKTTATANLGMSIARLGYRVVLVDADIGLRNLDLLLGLENRVLYTAMDILDGQCRL

DQALIRDKRWKNLSLLAISKNRQRYNVTRKRMNMLIESLQKQGYDYILIDCPAGIDVGFI
NAVSPAKEAIIVTTPEITSIRDADRVAGLLESNGIYNVKLLVNRVRSEMIQQNDMMSVRD
VQEMLGIPLLGAIPEDNHVIISTNRGEPLVLKKKLTLSGIAFENAARRLIGKQDYFIDLQ
TPYRNVFQRFQQFLGF
>A4GYN8
MPTIKQLIRNTRQPIRNVTKSPALGGCPQRRGTCTRVYSGSKGDLSVNFSTITPKKPNSA
LRKVARVRLTSGFEITAYIPGIGHNSQEHSVVLVRGGRVKDLPGVRYHIVRGTLDAVGVK
DRQQGRSQYGVKKPK
>Q1XDM0
MSYPKKVQLPLTNYNSTGLNLNKSNLLVLYEDMLLGRNFEDMCAQMYYKGKMFGFVHLYN
GQEAVSTGVIKLLNPTDYVCSTYRDHVHALSKGVPSKNVMAELFGKETGCSKGRGGSMHI
FSAPHNFLGGFAFIAEGIPVATGAAFQSIYRQQVLKETEDLRVTACFFGDGTTNNGQFFE
CLNMAVLWKLPIIFVVENNQWAIGMAHHRSSSIPEIHKKAEAFGLPGIEVDGMDVLAVRQ
AAKQAVQRARQGDGPTLIEALTYRFRGHSLADPDELRSRQEKEAWVARDPIKKLKKYILD
NEIANIGELNEIQNAVKTELEQAVKFAISSPEPNMSELKRYLFADN
>O19889
MTHNKTIKKVYIVGAGPGDPELLTLKAQRVLREADVVIYDALVNPEILQHCKPNAEIIKV
GKRRNNHSFSQLQIASLLIDRAKKGERVIRLKGGDPLIFGRLGEEILELFSKNIEIEIIP
GITSAFAVAADMKFPLTHRQLGSSITFLTGEEFYEKTTNKLKWERIIWGADTIIVYMILY
NLPKIIKKFLSVGYSAEKPIVLVQWASLKKKNFLIGTIGTIIHQVIESKFGPPSLAIIGE
VIEISSIIQKL
>Q9FLN4
MAMATSMSLNLIGAFKGLSLSSTSSFLRGDLSFSPKTSFTVTLPLENLQAPIPLTIESAH
KKGAGSTKNGRDSPGQRLGVKIYGDQVAKPGAIIVRQRGTKFHAGKNVGIGKDHTIFSLI
DGLVKFEKFGPDRKKISVYPREIVPENPNSYRARKRENFRLQREKKKARRENYSYTLPTP
ELVLASASVDDAEANPEC
>P0C308
MGAFPSPPPWGWSTGFITTPLTTGRLPSQHLDPALPKLFWFTPTLPTCPTVAKQFWDTKR
TSPDGNLKVADLPSFAISFATAPAALANCPPLPRVISMLCMAVPKGISVEVDSSFLSKNP
FPNCTSFFQSIRLSRCI
>O04130
MAFSSSCSSVKAVNSRWTSPSPSPSSRFAVLPAFLHRRYATSVKLTAISAALKTVEQTTL
TEDNRFSTVGSDSDEYNPTLPKPRILVTEKLGEAGVNLLREFGDVDCSYDLSPEDLKKKV
AESDALIVRSGTKVTREVFEAAKGRLKVVGRAGVGIDNVDLQAATEHGCLVVNAPTANTV
AAAEHGIALLASMARNVAQADASIKAGKWERSKYVGVSLVGKTLAVMGFGKVGTEVARRA
KGLGMTVISHDPYAPADRARALGVDLVSFDQAISTADFVSLHMPLTPATKKVFNDETFSK
MKKGVRLINVARGGVIDEDALVRALDAGIVAQAALDVFCEEPPSKDSRLIQHENVTVTPH
LGASTKEAQEGVAIEIAEAVAGALKGELSATAVNAPMVAPEVLSELTPYIVLAEKLGRLA
VQLASGGKGVQSIRVVYRSARDRDDLDTRLLRAMITKGIIEPISDSYVNLVNADFIAKQK
GLRISEERMVVDSSPEYPVDSIQVQILNVESNFAGAVSDAGDISIEGKVKYGVPHLTCVG
SFGVDVSLEGNLILCRQVDQPGMIGQVGNILGEQNVNVNFMSVGRTVLRKQAIMAIGVDE
EPDNKTLERIGGVSAIEEFVFLKL
>P94026
MASVFSVHPLPSSSFLCPLKTTKSRTKHHQTFYTYQKTILINSLQLTELDPKIPQPVQTF

WQWLCKEGVVTTKTPVKPGIVPEGLGLVAKRDIAKGETVLQVPKRFWINPDAVAESEIGN
VCSGLKPWISVALFLLREKWRDDSKWKYYMDVLPKSTDSTIYWSEEELSEIQGTQLLSTT
MSVKDYVQNEFQKVEEEVILRNKQLFPFPITLDDFFWAFGILRSRAFSRLRNQNLILVPF
ADLTNHNARVTTEDHAHEVRGPAGLFSWDLLFSLRSPLKLKAGDQLFIQYDLNKSNADMA
LDYGFIEPSSARDAFTLTLEISESDEFYGDKLDIAETNGIGETAYFDIKIGQSLPPTMIP
YLRLVALGGTDAFLLESIFRNSVWGHLGLPVSRANEELICKVVRDACKSALSGYHTTIEE
DEKLMEEGNLSTRLQIAVGIRLGEKRVLKQIDDIFRERELELDELEYYGERRLKDLGLVG
EQGDIIFWEPK
>Q32065
MTFLNHYTYLFSIPEKQADKVSGILRLAQARPIETLQNERINKQLNAFLKTYKFEKLITN
YKKMQSFIPNNSLNGNKTNSSTNKLYATSLNVFPENPPLMVRKAVSDEADKFSKFTYSKV
QVVTNNLNNGMNSKEFIKANNLKPSLRAAESLVLNHLTYNKFKENLYFKTNNIQPTKSKS
TSLFFLNILSNSKPRTCSDFLSSPKIRKTWFRNTAWSLQTQQHRSSNGINLSLQLPYALG
PSVPAGASGQNMYELPVAQSSSRFGTYYFLQKLLSKYLDVWNASADNGSVLSNSENIKLN
FSMVSLLDSKMAIQTPNSLYFVFTQLNQKTFLSYWLLPVAGLALLTPTLLTLTGQSVSVQ
KFNSFINKKTDMMVLSNTEMPSKSFGTPTLFGTSVEIYLPNSYMPKGEGESGINRVNSSI
NAVKKNTVTANLVLDSESQEVATSFQNDLISIKYCFNNLYNYISNKTALSTKNLFLFSAI
KSNATKHKRTQSFFSVENTTTLGNNSNFVKGHFKSSINAFSSYLPSTNVHSMIPLTSLPY
LKAISPLYSKFMIDHSLKFITPKTTLKLLQHKLNKSPKQMYTKTQNFTGLRDLRALNSFS
FGQVNFRTNHFLHSNSRPLNHYNQALKLINGYEQYKNNLQINCNKTLDLNTKNKLVYQVH
KSHLFNQKCSQIVYKQSLYNRDLCTIRGTGTKVVDYFSHGDKLSNKNGIVLDYFVYSNLL
FDNKTNTIINKDGKQNITKLKLNLTKTTVPFKTLIKKYTSINSLVANEQTRNNLNLGLIH
FNGHLSVVSNANLLTGRPVKFIYYKFDKRLNSYLIYVNQNLKKFIQLNNNFLKPKPLSHQ
KNKPVEDFNQYATNNSSPPKTNVFEKSFVEDSSLRKPLTSLRGSKQFLNSLTILFKHQKM
FKKKTLKAHKWHSDTQGIFRKHTNSSFGSANFSNGPEESSLSTRLHIQKKRKAKKQRLET
RRQKKRTRFFPRPVWLRSRMFLNFLTERNKYYLNSTITKQGFSLPSKDVVTTKLDWLKED
MRPSSLGAYQYKSLLTQKAGNKFQRQSFTEVVSTMEYINGIHKALNNSIFNKIVRKSLLS
SSQNPLKLRLVANYSKMQFMHRVKLPFYRTLKHSEGTKNLANKKQNLRDIKIKANYNNFK
SQKANNQPQQNDKDKDKDTMFRDFWVWSYNNTQTNAFNQNLWWLLPNLTTKQSNLEFLTS
TYPTAKETQRAKEEIHGNSIPTASKNQIALIRLNWALNKTNINTFTDYSKRNNLWTTQKL
RNQSKNNKTKSLEKQFITNWEKFFLNKNLNIFSKKIISKVKQKKQKLNYMTSYLNVQSEH
NVKIFHNSWWTHLNIKNLVNNQDMVIPVREGYFSVGNFNSEFINSAIIKSINNKTLVENY
VYSPSSEKETMQLLLMSSSILLHLCAIISLVSISQVRCFVKFHLILLYKLSNVYNAILNQ
LSNKLQKNLPIYNNINKLNSRYFYMNHQKSQIKQRKKLLTYFSLTLLKKQFVTVKPLQIR
NFASIKNQSSNNSNLTYTDMLPLSLRANKFRGSKYDISIREEEGQSAHIKPSKSMYAKLN
ILSLKTIFLKQLLMNKKPSALPSNVGLKSNRETQKSQLIQRIKTKELQISLKKNIIGFSK
VTKNHILKILFNVIEVFQTAVRNISSFFEKPAEFTTTWIAYGFLVEWSSDFITIIPENVD
IYIWNVFSKIYRTIPLSFISTTLGPASTVFDPVTNSTIPIQMGNFNYQKMVAFPILLSLS
HLLHRRILYLFDTLFSTITQPDTDLIARQEKGTLFWDIWADFLVTAADYYNVNVAALSTI
KAEQNSLIENISNDFDNLTMSSKKPFFMPNKGVSNIKNIFWIKKLKEPQLPESIVQNREV
FVRERKRTLKGLFNIYAPQEETLWNNPTSPKNLSDEKISFKLFNQLNLQLFAEKNKIKPY
FEAYFSTTQQKTNIMQSAFPEANLNRWSVNQFITYQSWHSHNGSNNSNGDLFIDYHPPKT
FSHIPALKYNSILQQPIGSLVCQIYSGLFNKQISKNILLVNPKTTSNNLVDYNVLLIQAL
AGETEMKIITDNAQRYALVNRGFAIGIKLLREVFDAIALNTPCIFLLEDIHAIGERRPML

ISDFGGGMSDDNGSFKEDFFGSQRDEVHEKNQVVYQLTRHAITHYKKPFKGDYSLAIPTN
LYVTDLFLKLPTQSISNLTNVENHNLSIKNKIQHNGTQSLTETKRNLGGDINKNSYLQLT
QFTKTLAPPSTSPFSVLLLKEEKRLKPNKIVEELPWTSLPGEQLATKPRTSYSVRAKVAM
LAELSLSNLSAKLDMITDLLVIIDSVRSNKGFVVFATTDIPHVLDPALRRPGRLDETICL
PNIHTSNILNFTKNYEIFKSAKDTSNFGKKIILNEMQNLTTTSTQRDMYLSCLPTNNQTH
KTKREGVLTMNLKDYNILLNQVYFAEGTGGILNSQMHKDSLQKSLNFALISHSKKLKELN
VSKLIGSNGTVSQGNVDQLGVFAGQIVNKQKKSLQQHLPNSKKSFKKKYKDKAIIYYEVG
KFVLNYFLNNQLTQSSIIDKPVSVTNKQTNDITIFGNDFLNLKTINYLSLYNSKNKILLQ
LMLIFGGKISQLLSSKNLVKSLKQASINSYMVEEESGSISSAGMPLGQTHLLPKALSVLA
KPMIFSDGYNNQNLKTATTLLLSFIHKRYLYRKNLIVPKLLSFADGNILDEPPSPPFSSL
LIPAKRFENYKRFFRDTLTGDKMGQRKSQITLLEKLQYHMQLRSIKQLNATFSSQENLDF
QSNAALTSQKLDTLMSLSTNNLLQNPTNINWYYQNRILKRHGQYLTNQWWNGQLSEHNAE
TVFLSDIDWRSSFIKNKNINITKSKNLYRLTQQKNNTDGLDVLLDFPDTDQYYNPKRRRW
LLNNGSWNFWFNFDKLYSEEIVTTWILESLIQTYKYLHKNTELLDFVTNKFITLGYIAPE
NANLQNISGFPSQSELLSTKEIILTNSFKRF
>O20163
MKKDSTIISTCNEFSLSSVKTKRVYKNNHLLCSVSGGQDSIVSFFFLVNFLYLSKNFKLI
KSKLSSFSVASKSAEKKRCFDCFFSLKKNKKINALINKEKKLSRLDLVFCQHFWQTQNFF
CVEFLFQLTFFFDIPYTFVLSKNILVSENRARGWRKKTFSRLLKIQKLSTIVTGQTKTDT
AEKSITNLLRGTSPKSFSLITKANSKTTSSFFCFRLFRPKTFYLTLTGNKRERKVNRRIS
FSFYRTQKKDSKKLRETRAPFFIEMASFSAFFGNTFQKKKSSNSFLLYKTRLSFSFCFSG
ETREHSINSLKPLQNRNRYHISKLVKFYKFPLTIDATNFSLNFSRNKIRHLFIPFIRFLF
KLKFETLLLNFLYLVNLEHEQIENENLELKRVLNFLRINYLKKNFFSSNRRSFFVIQTGH
KNNNVFPTKKKEKRWFYCNEIGISKIRKQCKIHEDPILETSLVIYFTSVLTTSRKYSLLQ
NLLYNYREIEINFRQISKLENSFW
>P49532
MNDFWTNVFRYPRFFISSLIGLLLVILSPFKNLFKIAKLRIFVILFILLFFLGIYFVLVN
MTGL
>Q1ACG0
MLSPRKTKFRKHHRGRMKGISTRGNSIVFGKFALQALEPSWITSRQIEAGRRSMTRYARR
GGKIWIRIFPDKPVTMRPAETRMGSGKGSPEYWVAVVKPGRILYEMDGVPENIARAAMKI
AAYKMPNKTQFLIRDNKDLIDSKI
>Q9SE20
MATSSAYLSCPATSATGKKHVFPNGSPGFLVFGGTRLSNRLVTRKSVIRADLDSMVSDMS
TNAPKGLFPPEPEHYRGPKLKVAIIGAGLAGMSTAVELLDQGHEVDIYESRTFIGGKVGS
FVDRRGNHIEMGLHVFFGCYNNLFRLLKKVGAEKNLLVKEHTHTFVNKGGEIGELDFRFP
VGAPLHGINAFLSTNQLKIYDKARNAVALALSPVVRALVDPDGALQQIRDLDNVSFSEWF
LSKGGTRASIQRMWDPVAYALGFIDCDNMSARCMLTIFALFATKTEASLLRMLKGSPDVY
LSGPIKKYIMDKGGRFHLRWGCREVLYETSSDGSMYVSGLAMSKATQKKIVKADAYVAAC
DVPGIKRLVPQKWRELEFFDNIYKLVGVPVVTVQLRYNGWVTELQDLERSRQLKRAAGLD
NLLYTPDADFSCFADLALASPDDYYIEGQGSLLQCVLTPGDPYMPLSNDEIIKRVTKQVL
ALFPSSQGLEVTWSSVLKIGQSLYREGPGKDPFRPDQKTPVENFFLAGSYTKQDYIDSME
GATLSGRQASAYICNVGEQLMALRKKITAAELNDISKGVSLSDELSLV
>Q6B950

MFELYLIIITCLLLLVCYLITNSLVSIYTQIRVINNIEPIKDKISLGNDYILYMTRLYMK
RKKWLYCITMLEFYINQIKIHETEITGEYYNCIGSCYQAIKMYKIAKKYYLKAYKRTPSR
QHILKNLANIYNTVGDIKNAKKIYQRLT
>O20120
MVTLSDLESNASSSVTKNEVNTSSLVNGNGVLIITENVNKEHSVENQLASSKTEEQTLKI
SKKSNLNPAQKSSTFGLENTSPVIRPTSVFTKSTVLSTENNLVSFEVDFMNFFSALNFFL
ESLVYFVVNDFSS
>Q8L785
MAILHFSLPLIVSFLRPHASPRFFLLPRSLSQSPFLSRRRFHRTSAVSSAAVHHQSYRNP
DDDVTRAVSVPTFQQAIQRLQEYWASVGCAVMQPSNTEVGAGTMNPCTFLRVLGPEPWNV
AYVEPSIRPDDSRYGENPNRLQRHTQFQVILKPDPGNSQQLFINSLSALGIDVTAHDIRF
VEDNWESPVLGAWGLGWEIWMDGMEITQFTYFQQAGSLPLSPVSVEITYGLERIIMLLQE
VDHFKKILYADGITYGELFLENEKEMSSYYLEHASVDRLQKHFDYFDEEARSLLALGLPI
PAYDQLLKTSHAFNILDARGFIGVTERARYFGRMRSLARQCAQLWLATRESLGHPLGVAS
EPVPPVCHRAALEKVAEKVSEDPRSFIIEIGTEEMPPQDVINASEQLRVLVLELLENQRL
RHGAVKAFGTPRRLVVLVDAMSSKQLEEEVEVRGPPASKAFDDEGNPTKAAEGFSRRYGV
PLEKLYRKVSGKTEYVHARVTEPARLALEVLSEDLPGILAKISFPKSMRWNSSVMFSRPI
RWVMALHGDLVVPFSFAGISSGNVSCGLRNTASASLLVQNAESYEDTMRNSGINIEIEER
KKIILEKSNALAKSVSGRLVVPQNLLNEVANLVEAPVPLIGKFKESFLELPEELLTIVMQ
KHQKYFSIIDESGQLLPYFIAVANGAINEDVVKKGNEAVLRARYEDAKFFYEVDTRKRFS
EFRDQLQGILFHEKLGTMLDKMNRLKKMVSKLCLALKIDEDLLPVVEDAASLAMSDLATA
VVTEFTALSGIMARHYALRDGYSEQIAEALLEITLPRFSGDVIPKTDAGMVLAIGDRLDS
LVGLFAAGCQPSSTNDPFGLRRISYGLVQILVEKDKNVNFKRVLELAASVQPTKVEANTV
EDVYQFVTRRLEQLLVDNGVSPEVVRSVLAERGNNPCLAARTAYKTEKLSKGEMFPKIVE
AYSRPTRIVRGKDVGVGVEVDENAFETPQERTLWSTYTSIKDRIHTGIEIEDFTEISMQL
VEPLEDFFNNVFVMVEEERVRKNRLALLNNIANLPKGVIDLSFLPGF
>P05722
MYWSLTACSAVLFCLRDSLIFLADDVNPERGNEGVAVGTGGIPGRGIPPLITANGIGLYT
FTSIGLLVPFTLGLKTQNQQIKIVVKSKISETNKLVIFCCLKHIISRKARSWLNDSR
>P34811
MAAESSLRVATPTLCNLNGSQRRPTTTTLSPLRFMGFRPRPSSHSLTSSSLSHFFGSTRI
HSNSSSSYSSISRQHAPRRNFSVFAMSADGDAKRSVPLKDYRNIGIMAHIDAGKTTTTER
ILYYTGRNYKIGEVHEGTATMDWMEQEQERGITITSAATTTFWNKHRINIIDTPGHVDFT
LEVERALRVLDGAICLFDSVAGVEPQSETVWRQADKYGVPRICFVNKMDRLGANFYRTRD
MIVTNLGAKPLVIQLPIGSEDNFKGVIDLVRNKAIVWSGEELGAKFDIVDIPEDLQEQAQ
DYRAQMIENIVEFDDQAMENYLEGIEPDEETIKKLIRKGTISASFVPVMCGSAFKNKGVQ
PLLDAVVDYLPSPLDLPAMKGSDPENPEATIERLASDDEPFAGLAFKIMSDPFVGSLTFV
RVYAGKLGAGSYVLNANKGKKERIGRLLEMHANSRDDVKVALAGDIIALAGLKDTITGET
LCDPDNPIVLERMDFPDPVIKVAIEPKTKADVDKMATGLIKLAQEDPSFHFSRDEEINQT
VIEGMGELHLEIIVDRLKREFKVEANVGAPQVNYRESISKISEVKYVHKKQSGGQGQFAD
ITVRFEPMDPGSGYEFKSEIKGGAVPREYIPGVMKGLEECMSNGVLAGFPVVDVRAVLTD
GSYHDVDSSVLAFQLAARGAFREGIRKAGPRMLEPIMKVEVVTPEEHLGDVIGDLNSRRG
QINSFGDKPGGLKVVDSLVPLAEMFQYVSTLRGMTKGRASYTMQLAMFDVVPQHIQNQLA
TKEQEVAA

>P26302
MAFCSPHTTTSLRSPCTTIPNSGFRQNQVIFFTTRSSRRSNTRHGARTFQVSCAVEQPIV
IGLAADSGCGKSTFMRRLTSVFGGAAEPPKGGNPDSNTLISDTTTVICLDDYHSLDRTGR
KEKGVTALDPKANDFDLMYEQVKAIKEGKAIEKPIYNHVTGLLDPAELIQPPKIFVIEGL
HPMYDERVRELLDFSIYLDISNEVKFAWKIQRDMAERGHSLESIKASIEARKPDFDAFID
PQKQYADAVIEVLPTQLIPDDNEGKVLRVKLIMKEGIKFFNPVYLFDEGSTINWIPCGRK
LTCSYPGIKFSYGPDTYFGQEVSVLEMDGQFDRLDELIYVESHLSNLSTKFYGEVTQQML
KHADFPGSNNGTGLFQTIVGLKIRDLYEQIIAERAGVPAEAAKV
>P52423
MEAQQIISRFCPKSSLAPSIPMVKQPFSLNFPPLHSLSSYPFLQSQNLGFPTGALHAISF
VHKEVCSSSWRIWCSKSSSSTAEPEEDHEVRAQVTVRRKKLAVFVSGGGSNFRSIHEASK
KGSLHGDVTVLVTNKSECGGAQYARNNGIPVILFPKAKDEPKGLSPCDLVDTLRKFEVDF
VLLAGYLKLIPVELIRAFERSIFNIHPSLLPAFGGKGYYGMKVHKAVIASGARFSGPTIH
FVDEHYDTGRILAQRVVPVLANDTAEELAARVLNEEHQLYVEVVEALCEERIVWRKDGVP
LIQSRENPNEFL
>Q9MUN5
MICKNSIVTSSSKLFYKMFFIAISIEILEYNYIYPLCIEIAQHSGNIKIALCLKEILQKK
KYNLILLKIASIVISNFLSTNTYSFIYTPYDVEILFSNLLLEKYEKDKEMSYMIDVFTKL
SHLKQNIIYPYLFNQLKHDCNKKHIFLLLKKIDYTDISTFTHSIFIK
>P51335
MLNFRAKCTTYSLEEYTRYSKHLILPQIKLEGQERLKQSSILCVGAGGLGSPALIYLAAS
GIGKIGIVDNDIIDISNLQRQILYTVNDIGLSKAYIAKKKILEINPTCNVQIFNTRLQSI
NAIEIIRQYDIIIDGTDNFGSRYIISDSCLELNKIHIYGAIFQFEGQVSTFNYQGGPKYR
DFHNNIETENNPEDTCSNAGVLGLLPGIIGTLQATEAIKIILGYKSVLSGIILKYNAMTI
SFEKFKIIHTQFILSQPKKKIKSLLVGNSSYPVQEIDVIELQNELYRNSFKYIILDVRSK
EEYEESHLDKAVNLPIKDMKKRYYSDLNLQDKISFIYCSVDSRSIFAYNFLRKQEFKVIR
VKGGLSSWTNIIGNEKLYVKSC
>Q32S00
MKLHKKKLNITMTKQFIQKFPSNTVGSGGKTLLSKFQENSGLTETQIYILTQRVARLSSH
LKNHNKDYSSQRGLRKLLGKRKRLLAYLSNEDVERYENLLIQLGIRGLKKI
>P36177
MVRSSCLQCDQPSQGSNSTFGCGGPLAAVCATGLLVLVLYSPSSQTANWSAQGISTKALY
PAVPVPSTLLPGSAPAKHQLHVWRAHAMSEATTNNSFKQSLFGYNAISSIWLQLAGVAAT
FFAFGALMAAVTQRKEIAVFSASGQAAEPEGAEPLKRPFFPSPAAKPKPLFSTPANSFSNI
FQAPPSLRTDSTYGRGPRSTSFTDISNWPSNNALRNPQSVIDIGGGVDFLGDRSPGNPFT
RLRGSPSSTLSNLGMGLGLGLGKGKGFGKGFGKGRGFPVEEEVEEEQEVLSWADRRRALA
DPDAPPMNEDIKYPQLRLVRAVPGGRDEKLGVMSRQEALELAEAEDIDLVLVSIDTDPPV
AKLVNYSKLKYESEKKKKDSHKKGKVKEVKELKVSHKIGQHDYDVRVKQARKFLEGGHRI
KVSMEFKGRENQFVEIGRAVMKRFQNDLADMGKADAVPKKLGTRLILNLAPAGEALKVIA
ERRAERDRKAAAEEEGEGDDLDFVDENEDEDVEGEGEEEEAEELEEETAEGTEVPTRS
>Q9LER7
MALLCFNSLPSLSSLSSSSSSRLLQSPSFASPVLSLKPNAVESKNRVSLSAYSLNSSHGR
IVVKAAASGVDGAEPESKEEPKTVVAAVPVDKLPLESKEAKEKLLLELRLKMKLAKKIRL
RRKRLVRKRKMRKKGRWPPSKMKKNKNV

>P19593
MKRIKLKYRKLKRQQFIKVEKFNIGIDMVECHRWLENKQESIACAKREGNIVLVEKLAQE
IVNSSFGRAVAVQTVASSKGSRSPGLSRESFKTNKNYVAMMATLEQITSNPHKYKATPLS
RIYIPKRDGSARPLSIPSYTDRCLQALYKLAIEPMAEEVADLSSYGFRPMRNVSWAVGRV
LNGLNNPLANYQYVVEIDIKGCVDNINHQFISQVTPFIPKKILWAWLKCGYIERNSNTLQ
PTTTGVPQGGIISPLIMNLTLDGLEFHIYKKIQKSSSQSKGNTYCRYADDMVILTTTEET
ALIALPAVKEFLAVRGLEVKLAKTTIKNIINDRNGFEFLSFRFRKVYRRNRKRLTSQVGI
PISAIKNFRKNIKAISKTRKSLDTINDEINAVFRDCGYYYRFAHTSGYVFSSLGYWLWKQ
FYKHCYKRTKDKFDKANHTKINEIVLSSYFKPIGSGLSTKPFVIDKKGKPHSLFSIRSIE
YCPPTYTDSARNAFIFEDSVTLTKVNMRSKSSWKRVILEKWGPCCGLCRKNLEINSIPYE
LHHILPKRFGGKDTPNNMVLLCKSPCHQLVSSSIQKADVSEIQNYISLGILEIPMDYLEN
LKTSQSSY
>Q96529
MSLSSLTLDSNPRFAVGGPYHRRYPPLHHPRSFVSCSAKRPAVSASLSVAADSAATESLG
RIGSLSQVSGVLGCQWGDEGKGKLVDILAQHFDIVARCQGGANAGHTIYNSEGKKFALHL
VPSGILNEDTTCVIGNGVVVHLPGLFKEIDGLESNGVSCKGRILVSDRAHLLFDFHQEVD
GLRESELAKSFIGTTKRGIGPAYSSKVIRNGIRVGDLRHMDTLPQKLDLLLSDAAARFQG
FKYTPEMLREEVEAYKRYADRLEPYITDTVHFINDSISQKKKVLVEGGQATMLDIDFGTY
PFVTSSSPSAGGICTGLGIAPSVVGDLIGVVKAYTTRVGSGPFPTENLGTGGDLLRLAGQ
EFGTTTGRPRRCGWLDIVALKFSCQINGFASLNLTKLDVLSDLNEIQLGVAYKRSDGTPV
KSFPGDLRLLEELHVEYEVLPGWKSDISSVRNYSDLPKAAQQYVERIEELVGVPIHYIGI
GPGRDALIYK
>Q9CAF5
MAGVNLQLRHAYSIAQFVPTVSSPPPLPTQRVRLGTSPSRVLLCNLRANSAAAPILRTTR
RSVIVSASSVSSAVDSDSLVEDRDDVGRIPLLEVRDLRAVIAESRQEILKGVNLVVYEGE
VHAVMGKNGSGKSTFSKVLVGHPDYEVTGGSIVFKGQNLLDMEPEDRSLAGLFMSFQSPV
EIPGVSNMDFLNMAFNARKRKLGQPELDPIQFYSHLVSKLEVVNMKTDFLNRNVNEGFSG
GERKRNEILQLAVLGAELAILDEIDSGLDVDALQDVAKAVNGLLTPKNSVLMITHYQRLL
DYIKPTLIHIMENGRIIKTGDNSLAKLLEKEGYKAISG
>Q1XDQ4
MKNKIQLKINYALVLSAATAVYGYKSYQPTINTLLIKDEENWPFFSKRQTAFIPIIVLKK
NHQNP
>O24163
MTTTPIANHPNIFTHQSSSSPLAFLNRTSFIPFSSISKRNSVNCNGWRTRCSVAKDYTVP
SSAVDGGPAAELDCVIVGAGISGLCIAQVMSANYPNLMVTEARDRAGGNITTVERDGYLW
EEGPNSFQPSDPMLTMAVDCGLKDDLVLGDPNAPRFVLWKGKLRPVPSKLTDLAFFDLMS
IPGKLRAGFGAIGLRPSPPGHEESVEQFVRRNLGGEVFERLIEPFCSGVYAGDPSKLSMK
AAFGKVWKLEETGGSIIGGTFKAIKERSSTPKAPRDPRLPKPKGQTVGSFRKGLRMLPDA
ISARLGSKLKLSWKLSSITKSEKGGYHLTYETPEGVVSLQSRSIVMTVPSYVASNILRPL
SVAAADALSNFYYPPVGAVTITYPQEAIRDERLVDGELKGFGQLHPRTQGVETLGTIYSS
SLFPNRAPKGRVLLLNYIGGAKNPEILSKTESQLVEVVDRDLRKMLIKPKAQDPLVVGVR
VWPQAIPQFLVGHLDTLSTAKAAMNDNGLEGLFLGGNYVSGVALGRCVEGAYEVASEVTG
FLSRYAYK
>P51211

MTNTIVISCLHNIAAILYCGQIQKLVVANAHYQVSDIYLGTVDKIFSGINAAFVDLGKNE
YSGFIHISDTGPLKKKYYINNITNILTIRQKILVQIIKEPTLNKGPRLTANITLSGRYIV
LMPFSQAICISRKIYDEDERHYLKALAILIKPPTMGLLFRPSAIGIDEEIILSELTNLKE
QWYFIQKSAINNCAPVLLYKDEDIVKKVIRDFYDNNTKNIVIDSNLGLKQLNYYINTWQC
NFSSTIPSLQLYSSNKCILDTFRINQAISRALIPKVDLILGGYMFIETLEAFTIIDVNSG
SFNNSTSARETVLKTNCSAATEIAYQLKIRNIAGVIIIDFIDMESQRDQLQLLEHFDKEL
SLDDAKPQIVQLSELGLVELTRRRKGKSLYELVSNDSNYFHFFIQLEQLDSIKPSSYKSK
RLSSSVKSWLFSEIDMINRVFFKKSNLYRLSNFYYARNLYIIYSDFTVNKNIQLAARYKL
MYSLQNIQILPSTWYFNFLDIHTNNITNYLS
>P11673
MCLLYTHFYHLILNTRRVDSSFFVFSFHLYFLNDNKGMSPLCLSLVGSGLHLSVMHLLFH
VFLFLFDSFLRP
>P46666
MLIVLFRSKDIRGGRFVRPILIFRTKRSWILFRIGPERRREAEMPTDLCLFSNSPDPIVP
VFGTSSAKVTEWVSHQSNPFDKSGVILDIIFYIYRNIIE
>P59287
MALPSAAMQSNPEKLNLFHRLSSLPTTSLEYGNNRFPFFSSSAKSHFKKPTQACLSSTTH
QEVRPLAYFPPTVWGNRFASLTFNPSEFESYDERVIVLKKKVKDILISSTSDSVETVILI
DLLCRLGVSYHFENDIEELLSKIFNSQPDLVDEKECDLYTAAIVFRVFRQHGFKMSSDVF
SKFKDSDGKFKESLRGDAKGMLSLFEASHLSVHGEDILEEAFAFTKDYLQSSAVELFPNL
KRHITNALEQPFHSGVPRLEARKFIDLYEADIECRNETLLEFAKLDYNRVQLLHQQELCQ
FSKWWKDLNLASDIPYARDRMAEIFFWAVAMYFEPDYAHTRMIIAKVVLLISLIDDTIDA
YATMEETHILAEAVARWDMSCLEKLPDYMKVIYKLLLNTFSEFEKELTAEGKSYSVKYGR
EAFQELVRGYYLEAVWRDEGKIPSFDDYLYNGSMTTGLPLVSTASFMGVQEITGLNEFQW
LETNPKLSYASGAFIRLVNDLTSHVTEQQRGHVASCIDCYMNQHGVSKDEAVKILQKMAT
DCWKEINEECMRQSQVSVGHLMRIVNLARLTDVSYKYGDGYTDSQQLKQFVKGLFVDPIS
I
>Q3ZJ77
MIKIIIKETFSFKSVLMHFLKEKVSNNKISKKNKIMPSYTLLSCIDSRVENLTKFYGRFE
LGPFAPGQALTVANALRRSLLSQLPGTSITLVEVRGASNEYEIITGVRESILDILLNLKQ
IVLTSDFEIFSPQIGFLSVEGPGVIRANDLKLPSFIYAVDPNQYIATLSNSGRLNMKFLI
CCGKNYITYNPNDSQYFEWLSLLKKSKPLIKSNSPKLNKGNINSEIDSDISIDKKNDEVI
FFQNANTRVVDSQTALKKGLVLKNTLRINKSSFLINSNYQSFFPKTQQKAFSIFRKNSKT
FLSTMGFYKEWKKEREFLKKDLYKNQEDFNKSYDFQETKKKLQTKLIKNFETPKKKLFKV
QNSANPFNKAFIPLTEEKVDYDSDFNLDHKSTKIGYFPIDAIFMPINRVNYLIESTEDIK
LKIKDRVILEVWTNGSIHPRHAIHKAAKALIQLFLPLQQIRTNLFLISDNSHFEEGQDKI
KKRIDNFKKEALLKANPQIQANPQLKKRNFDHRLLELDIANLELTARPYSCLKLANINTI
EDLISYSQEDLLSIKNFGRRSLIEVQKALQLMKLTLK
>O19886
MEKFKLLKLGKPATLLLEDGTVFRGKSFSNPQTVTGEIVFNTGMTGYQEIFTDPSYNEQL
VVLTYPEIGNTGTNLDDYESLKIQAKGIILKNLSNAYSNFRAMRSLLSFLKRHKVVEIHD
IDTRALVLHIRNNKTMLAVISNETYNLTFKEAKATIKYGKHTLNSNLASRVSTSLDYYQW
MQPLNRKLKFGYSIIRSFNSAINKKLHTSKKLRIIVLDLGVKFNILRYLKSFECEVIIVN
YKSTYQQVMKFHPDGIVISNGPGDPAKINRVVVRVNKFINKKIPILGICLGHQILARCFE

AKTFKLKFGHRGLNHPVGINKRMEITSQNHGFAVNFEFLKSNKFYANHLNLNDGTLAGIS
SQNFPLISVQYHPEGSPGPHDSNYLFKYWVYIIYKSKSS
>O49937
MATSTSSSLSLSFFSSSLFSSKSRNFSSKPILKLPSSSHSQTSLSLSIKSELIPLPILNF
SGEKVGETFLNLKTAPPEKARAVVHRGLITHLQNKRRGTASTLTRAEVRGGGRKPYPQKK
TGRARRGSQGSPLRPGGGVIFGPKPRDWTIKMNKKERRLALSTAIASAVGNSFVVEEFAE
NFEKPKTKDFIAAMQRWGLDPAEKSLFFLMDLVENVEKSGRNIRTLKLLTPRSLNLFDVL
NAEKLVFTEGTIQYLNQRYGVDTLEDEDEEEEEEEEGEEVDDGVEDGTPEPAE
>P51248
MTLQISAQNVKALRDKTGAGMMDCKKALEASNGNEEKALDSLRQKGLASANKKSGRTAIE
GLLESYIHTGGRIGVLVEVNCETDFVARRPEFQKLAKDIAMQIAACPNVEYVSMMHISDE
TISLEKRIEAGRDDIKDKPAEMIEKIVEGRIKKRLKELSLLDQMFIRNQDITIEDLINQN
IALLGENIKIRRFVRFILGGGEENTKANFADEVADILNKK
>Q6JJ61
MAAYSAAALSWSSSSLLGNYKLSKNAPLGTAKLVERTSNFTIVCQKKTKKIRKIILKEDV
QELGKKGQLMNVRAGYYRNYLLPMGMAQIVTPQLLKEMKIEEERIEAEKKRVKEEAQQLA
LIFETVGGFKVKRKGGKGKQIFGSVTAQDLVDIIKAQLQREVDKRIVSLPEIRETGAYTA
ELKLHPEVTARVQVIVSAN
>O78425
MEQKNKKNTIPWIWGFNVYAENWNGRLAMISFLLIICVEFITNKNVLDLVKLN
>P49829
MNISSLIEDGVYPFEKDKVKQNYQKGNFEPIFQTFNVKYDKTLSARTKLILKTWKGKAFY
YVNEDILYLLNIDQVEQDFLLKILHSMGIFLQNATSASFFDIYIYDINITEVPYYKSQKL
ENGTLLDSLTFTLKCYVTPPPEKPEIPW
>P29102
MAAALQTNIRPVKFPATLRALTKQSSPAPFRVRCAAASPGKKRYNITLLPGDGIGPEVIS
IAKNVLQQAGSLEGLEFSFQEMPVGGAALDLVGVPLPEETVSAAKESDAVLLGAIGGYKW
DKNEKHLKPETGLLQLRAGLKVFANLRPATVLPQLVDASTLKREVAEGVDLMVVRELTGG
IYFGVPRGIKTNENGEEVGYNTEVYAAHEIDRIARVAFETARKRRGKLCSVDKANVLDAS
ILWRRRVTALAAEYPDVELSHMYVDNAAMQLVRDPKQFDTIVTNNIFGDILSDEASMITG
SIGMLPSASLSDSGPGLFEPIHGSAPDIAGQDKANPLATILSAAMLLKYGLGEEKAAKRI
EDAVLGALNKGFRTGDIYSAGTKLVGCKEMGEEVLKSVDSHVQASV
>Q9MUR1
MAQLENNSTLLVRENELKGLVSNWLIEMKLMHRPLGFDYQGVETLEVKAQNLTSVAIALY
AYGFNYLRSQCAYDVSPGGDLASVYHLTKVDDNADQPQEVCIKVFVPRTKPIIPSVFWIW
KTADFQERESYDMFGIYYEGHPHLKRILMPEHWIGWPLRKDYITPDFYELQDAY
>Q9SCX9
MRFRSFFFSSSIFSLSHSRSPSLSSSRFSSLSAAMSPALEKSRQGNGGCNDDSKSKVTVV
GSGNWGSVAAKLIASNALKLPSFHDEVRMWVFEEVLPNGEKLNDVINKTNENVKYLPGIK
LGRNVVADPDLENAVKDANMLVFVTPHQFMDGICKKLDGKITGDVEAISLVKGMEVKKEG
PCMISSLISKQLGINCCVLMGANIANEIAVEKFSEATVGYRGSREIADTWVQLFSTPYFM
VTPVHDVEGVELCGTLKNVVAIAAGFVDGLEMGNNTKAAIMRIGLREMKALSKLLFPSVK
DSTFFESCGVADVITTCLGGRNRRVAEAFAKSRGKRSFDELEAEMLQGQKLQGVSTAREV
YEVLKHCGWLEMFPLFSTVHQICTGRLQPEAIVQYRENKL

>P51218
MYCSQAITQTSRLPVSNNFYCKKTFFSSVNSNYQNLSQMQSWSSLKVLAFGLPDLHSIPV
NLNNSFQEAIVISTINDNNIFADVTNSWIKMHKDKNHYSLFYKEIFKALLTKQIHVIDSP
IGSKDIDNKIILIQQYIWKKGLKSPQFNSIARLNKNVDISNISTKFIMEQLSTSPVFVVK
NGLNEIILGHPLSRIRRTAFNQIASSLSNLFHQPIATSPISNGLFFFHPDDAIEFKNFVQ
SKAPEACKDMDIKIQPVGLHVAYKMNRNFSSDIQFRFVPDFKEVGDLIFRHQQAKNLHFH
ENQYYGKNFFQGQPIYMIQPIIIKHRNGRISIIKFTGANDDREVVFTNLEAANKSWANFI
KRTPYLKRLKKPNLLVYNLESFLKDKEVLHKADLTKFIVVTNKSAYITTKESVTLPYHNG
LSKHLELNVKPKLFFVKLWIRRLLFTLTYE
>P30722
MAKVVGIDLGTTNSVVAVMEGGKPTVITNSEGGTTTPSVVAYAKNGDLLVGQIAKRQAVI
NSENTFYSVKRFIGRPSKEVSDELRQTPYKIEDSEGKIRLKCPNLNKNFAAEEISAQVLR
KLVNDANKYLGEKVEKAVITVPAYFNDSQRQATKDAGKIAGLEVLRIINEPTAASLAYGL
DKKDNETILVFDLGGGTFDVSILEVGDGVFEVLSTSGDTRLGGDDFDEKIVKWLLNEFEK
EEKFSLKGDSQALQRLTEAAEKAKIELSSLSQTEINLPFITANENGAKHIEKTLTGEKFE
SLCSDLFDRCRIPVENALKDAKLKPNQIDEVVLVGGSTRIPAVKKLVKDILGKEPNETVN
PDEVVAIGAAIQAGVLSGEVKDILLLDVTPLSLGVETLGGVTTKIIPRNTTVPTKKSEIF
STAVDNQPNVEIHVLQGEREFARDNKSLGTFRLDGILPAPRGIPQIEVTFDIDANGILSV
TAQDKGTSKQQSITISGASTLPKEEVEKMVKEAEQNAAADKEKGENIRVKNEADLYCYQA
EKQISELPEALVNENQSLIKESKETVEMLKENIKKEDYDKIKENLKKLQEKLMEIGQKAY
AKKEPLKDEDSNKAGSQDDFIDADFTESK
>P53797
MVVAILRVVSAIEIPIRLGFSEANWRFSSPKYDNLGRKKSRLSVYSLYTTSKYACVGFEA
ENNGKFLIRSSLVANPAGEATISSEQKVYDVVLKQAALVKDQTKSSRKSTDVKPDIVLPG
TVYLLKDAYDRCGEVCAEYAKTFYLGTLLMTPERRRAIWAIYVWCRRTDELVDGHNASHI
TPSALDRWEARLEDLFAGRPYDMFDAALSDTVSRFPVDIQPFMDMVEGMRMDLKKSRYKN
FDELYLYCYYVAGTVGLMSVPVMGIAPESLAEAESVYNAALALGIANQLTNILRDVGEDA
RRGRIYLPQDELAEAGLSDEDVFTGKVTDKWRSFMKRQIKRARTFFEQAEKGVTELSQAS
RWPVWASLLLYRQILDEIEANDYNNFTKRAYVSKVKRLAALPLAYGKSLLIPLSLRPPSL
SKA
>O78458
MSFLLPFLYVSGLSLILLFILVFIISQIWSRRTADLRLVSIQEKIRNAKADGKDFYELGV
IFLSKKLYDQAIINFRYALNLWELDDKTGLANLYNTIGFTYTQIAQYDLALFYYEKALLH
EPNYLVTLKNIGFIYEKTNNLVKAKDIYLQILKYDVDNKFATDKLNLLTGRLIRDDRI
>O19892
MTDLPFTLDQLRILRAILIQGSFKKAATSLYISQPAVSSHVHNIEKQLNIQLFDRSHRNA
QLTEAGQLLLKYGGRILALCEETCRALEDLQNLQCGNLIIGASQTTGTYLMPKLIGLFRQ
KYPQISVQLQVHSTRRISWSVANGQLDLAIIGGEVPKELTEILEVRNFVEDELTLILPPS
HPFSKFSCIQKEDLYRLRFIALDKNSTIRKVIDKILNRNGIDSSRLKTEMELSSIEAIKN
AVQSGLGASFVSISAIDKELKLQLLNRVNIDKIKIKRMLSIITNYSRYRSKASETFCHEI
LGLLVNLPFKNAPSTEK
>P08889
MPPCSSKSILSTKSSMFILVSFALLRFIFYFVEFYRLVIGMNNLEIKRMNQKIVRNSERW
EDPFDRIISFHYIDNFKKLFIL

>Q8S9K3
MNNSTRLISLFSPHPPPLFLLRGLYISRIANLRRFHRRAFPPSSVASTNLCSFRPLVSLP
PLIPTFPIGRFYNHQVRVSAADFVPSYHNQQLPEWTELLQSLSKAGYFSDSGSISGLESE
FFPGFPDELLRPALACLALARERPELLEMLSRRDVEVLVENGKPFLFKTGPDSLKRMSLY
LRSGLQGIGKLMDMEKASTVDLMRLILSYVVDVASSEESKQHNKEIMESSVRSLLSQIAK
MSLRPPESNVHDTMQNQYSDRDGQGVRSFQNNVEMKRGDWICSRCSGMNFARNVKCFQCD
EARPKRQLTGSEWECPQCDFYNYGRNVACLRCDCKRPRDSSLNSANSDYSSDPELERRLV
ENEKKAQRWLSKVAQGGSDANSVDTDEDFPEIMPLRKGVNRYVVSTRKPPLERRLANTEN
RVATDGNSKRSDDNALGSKTTRSLNEILGSSSSLTSRSDDKNVSSRRFESSQGINTDFVP
FVPLPSDMFAKKPKEETQIGLIDNIQVDGFSGGNQNVYQEDKSDANHSGKETDRLEKEDH
KSEEPARWFKRVTELHNVSDLESAIPQEISPEKMPMRKGENRFVVSRKKDRSLTSPAYKR
PEDSDFVPFVPFPPDYFAKEKQPKESIDTLPAPATENVSQVVQQEPREPSINKSDTVAVK
IRNGKSLEGSLVKESDLLDMSEEAKAERWFKRVAEIKNISELSEIPDEDFPSIMPMRKGV
NRFVVSKRKTPLERRLTSQRHQRNPHITNSDPTGKGDK
>P51379
MMKKKVHPIVLAILDGWGHSNSHQGNAIKIAKTPTIDSLLETYPNTLLVASGEEVGLPKG
QMGNSEVGHTTIGGGRVVEQELVKIGNSIENKSFFNNVQLNCACDHAISTQTSLHLIGLC
SNGGVHSHLDHLLALIHVAESKKVPNLYIHLITDGRDTNSNSAKSFIKLIADHIEKKSFV
IISTISGRYYAMDRDFRWSRTKAAYTVLTSDNSDITNQPLNYNDLIDHYYNKGISDEFIP
PSRINTGSIKDGDAIIFFNFRPDRMRQLVQALVQKPFNCFLTKSLSHLHVLTFTNYDSSL
NTALAFPPNTLNNFLGETISKYGLKQFRVAETEKYAHVTYFFNGGAEEPFSGEDRELVSS
PSVTTYDLSPDMSAELVTQKSISAINKGIYSCIVINYANADMLGHTGKLKETIRSIETVD
NCIALLFDAVSQSNGTLIITADHGNAECMLTNEGTSCTSHTTNLVPFILIEGEQATISGH
GGQVEFRKNGSLADVAPTILDILNLKKPPEMTGKSLIINSKYETRNLDKTFIEL
>P82411
MSVSAIFGARVVTIPSVLRTSSVDGRTVKLQPSTGGSCGGGVITIECSSRPQKKGTAHHM
KTRPKKTARWDIKRGPAVYPPLPPLPAEWTIVSSAVDEADSSSSTTSSSAEIAQSA
>Q06R99
MAKGTRFINFRRYGRFGSRRYRRFGSHKSVRKIPRGVIHIQTSFHNTIVTVTDGRGQVVS
WSSAGTCRFKGPRKRTPFAAQIVAADAIGPVVDQGLQRAKIKLKGTGRGRDAALRTISKS
GIGFTSVQDVTPVPHNGCRPPNKKRQ
>Q9MUQ5
MNKFFSINIILWALIGLNFHILSYSLKISISNFPWQWPQEGLETYMIHTDYYLGSILFIS
FLGGRFASLISQLAYQIIQINIVQLIIFFSKNLHISFLLPNILNQLINLDTNRGTITEYT
CIQDHIINNGWLSYFVVNLISYDLFENTLNQYNTQAIDSFFNKSVIFLIRLYIMNILFSL
LTSNSLNEWIMYSIKDNFLLLPNQLIIICSVVLLVFLLQFCVY
>P19167
MFYFISRKFYDQFKYGILTDKTNKLLKNNVYTFDVDIQMSKRQFKDLIETAFSVKITSVN
SYVKSSKYYRSNNFEGMKKYYKRMFIKLNDLETIPFFSCL
>P30524
MSSMQFSSVLPLEGKACVSPVRREGSACERLKIGDSSSIRHERASRRMCNGGARGPAATG
AQCVLTSDASPADTLVLRTSFRRNYADPNEVAAVILGGGTGTQLFPLTSTRATPAVPIGG
CYRLIDIPMSNCFNSGINKIFVMTQFNSASLNRHIHRTYLGGGINFTDGSVEVLAATQMP
GEAAGWFRGTADAVRKFIWVLEDYYKHKSIEHILILSGDQLYRMDYMELVQKHVDDNADI

TLSCAPVGESRASEYGLVKFDSSGRVIQFSEKPKGDDLEAMKVDTSFLNFAIDDPAKYPY
IASMGVYVFKRDVLLNLLKSRYAELHDFGSEILPRALHDHNVQAYVFTDYWEDIGTIRSF
FDANMALCEQPPKFEFYDPKTPFFTSPRYLPPTKSDKCRIKEAIISHGCFLRECKIEHSI
IGVRSRLNSGSELKNAMMMGADSYETEDEISRLMSEGKVPIGVGENTKISNCIIDMNARI
GRDVVISNKEGVQEADRPEEGYYIRSGIVVIQKNATIKDGTVV
>P13564
MQVRRDDDGAGGCAGDAVPGGGEGQDGVPARQPAGRVWGVSRAARATSGFKVLALGPETT
GVIQRMQQLLDMDTTPFTDKIIAEYIWVGGSGIDLRSKSRTISKPVEDPSELPKWNYDGS
STGQAPGEDSEVILYPQAIFKDPFRGGNNILVICDTYTPQGEPIPTNKRHMAAQIFSDPK
VTSQVPWFGIEQEYTLMQRDVNWPLGWPVGGYPGPQGPYYCAVGSDKSFGRDISDAHYKA
CLYAGIEISGTNGEVMPGQWEYQVGPSVGIDAGDHIWASRYILERITEQAGVVLTLDPKP
IQGDWNGAGCHTNYSTLSMREDGGFDVIKKAILNLSLRHDLHIAAYGEGNERRLTGLHET
ASISDFSWGVANRGCSIRVGRDTEAKGKGYLEDRRPASNMDPYTVTALLAETTILWEPTL
EAEALAAKKLALKV
>P24226
MSFDLSRLSLTSSPRLSFLTRTATKKGFVRCSMKSYRLSELSFSQVENLKARPRIDFSSI
FTTVNPIIDAVRSKGDTAVKEYTERFDKVQLNKVVEDVSELDIPELDSAVKEAFDVAYDN
IYAFHFAQMSTEKSVENMKGVRCKRVSRSIGSVGLYVPGGTAVLPSTALMLAIPAQIAGC
KTVVLATPPTKEGSICKEVLYCAKRAGVTHILKAGGAQAIAAMAWGTDSCPKVEKIFGPG
NQYVTAAKMILQNSEAMVSIDMPAGPSEVLVIADEHASPVYIAADLLSQAEHGPDSQVVL
VVVGDGVNLKAIEEEIAKQCKSLPRGEFASKALSHSFTVFARDMIEAITFSNLYAPEHLI
INVKDAEKWEGLIENAGSVFIGPWTPESVGDYASGTNHVLPTYGYARMYSGVSLDSFLKF
MTVQSLTEEGLRNLGPYVATMAEIEGLDAHKRAVTLRLKDIEAKQTQTK
>Q9MUP3
MYFQNIDALIFSKLLDTLLNIFFLLFFLVLGAEFTLYIINVYFPNLLLYGNALGIKFVLS
ILGSLIAFPIGLVFQNNYKNFLNYINNFTVDIFFTSLLSIIGALFIANLYFSFLDISSIL
KEFLFIKYLLRLLIQTILCYSGFILVTFHSDYLLNILVPEMAEAIFSYNKIVRPSITRVL
HTSYIINHNIIAIFQSQFVEGLVLIPKSTIQELQILNNSKNFKDKYLGRLGFSRLNELKY
NYFDQIILESSLTNCHENVLFKYNKTYNNEQQNIVLMSSCSNNIKESNISQLLISNLFPL
IININKLIELLKLPYLSGESFYLQIVRKGKRLGQGIGYLEDGTMVIVNAGSKYIGQKIKV
IVKKVWQKSTGKILFTQPIKNS
>P23408
MALSLAINVPPVRDNLLRPQPFQSQFRPNLLKFPIPSSARIRCSSSSSFNDISLKTVTPD
NKNSFVCRFNATQLEVQETNQPYAETYAVGRHIRMSADKARRVVDQIRGRPYEASLMVLE
LMPYRACEAIIKIVFSAGANASHNLGLSKSSLFISKAEVNEGKTLKRVRARAQGRANQIL
KRTCHITITVRGLPDESDKEENSS
>Q06SJ3
MSRYLGPRLRITRRLGHLSGLTRKKPAFKPLNPVNPFGPRKIIPPGEHGRNKSFKKKPYE
SCEYDYLIRLKLKQRLRFHYGLTERQLVRYVQQAKKIKGSTGRVLLRLLEMRLDNIVFRL
HMAPTIKAARQLISHGHILINKKKVTIPSYQCEPKDIITVAPKIISMELVSRFLSEFDRE
KSRYDRILQILEFGRKGVTMPTKNLKTSSKTLSKNSQSRKYVKNEKRAKIQSLKIGAVLN
VTINHRGRKEADAISYGFGKMIVIHPFFVGKQSINKNVRVIIYKKSKNNKILYTYPANPF
YLHLENLRKLNSLDVAKLFSHSNNQISSKFNKKPLKKSITRKSKRSISALILMNGAKMLP
KTNLERRKRVNKDSATNLTENKQIKKEFSPSVFVRIVAAKCLNSKSKQKLLNVSSFQSSL

KKFNPSFSKDPVAYREKYVYTKTSRALRLFGTRFYATILKNTRDVKNSSFIERKKFDSRI
IGKTPKVNLTKTTRKAEAKAPDFYSSIFKKSSDNQSQNFLNAELLKRESQNSSFSSTFNN
SNKVEFSNGASLPVTFKSGKVNNTLIPKLNSNKLADQSVNAQLMNVPALFKQYKNIFSKL
SSLKNQKNFKKPKYTNLRSKILTNFLFESKNLSLNLNHFHNFIFVVFSKFCKVLVSKTNL
NFKIDNILSISQNFKEFFKHRNFEAKSIDSKKFSMDILLFSLKLKHFTKLNNKNVGTEIG
KTREFELSSSILDKKIQNDISLLLLLDKMKTHLQTSLNFVNQFFSSEILMNGIRPYFSSI
FSFVLKSEELVKTILTNVINSIKTVLTCSKLTTLKISQQSFLGSAASEKLEKTESFFLEN
NLIEKLVNLNSNQSNTLYKKIINFAMVSIPKAVFLVDYSENQISLLNKYSLYSAKKQKIQ
MVKTLNFLKRYNLINYSNFENFKQMIQLNIKNHQTILVHFKDSILNKSKLNLRRNKLSPD
SELVLNLEQKLLINQTKCKISLLESLKSNSKLSLFSQFNQNCNQILCRTKIFLNILNKKN
SLFQKLNLLKDFHLIKGKDYESLKNLLNNQFQLLQKLKSLVSIVDFPQSVSQSSLLNRVL
NKNLEKITETFGSISTLWKVLILQKQNKLNVTESVKQNILQNISVAKAEFTQTVLITILN
KTDLSAFIKHYQSSKINNFTFISKLHKINLLDKLNKLNLLDDTVFVQMFSDIREKLAVKK
LKQTNVVLTKFLEFGTMSKLSQTELLSLDSFIQTAFLNSNSLTNNIQHLGHKERDNFVRS
SSFWKKFSFLLTPSLIKKGLENLKTLNSISSSQYSYLILKLQNLYDQNKTLNLGLNKQYV
HEKLRIIKTLLVLIAKNVNITTLTSLNLFKNTTWGSKTLLNLGLNRQKELKLMSGLLISQ
KIKYSSSFQKWEKDEFSFLMTNYIENQYNYLISKILMRGILTKEEAYFYSRENCQDYLTE
KLSFLHSETKQILTSIKLYLLRYQFMVQNNKNSSFSPWNLRFQEENFSLKSSISERTLNS
SRIFSLKKQKSVLLDKTMYQKLKNFLQKEILSKNMKSLLENKHFILAYLNKELKAKLFYK
VSRLQRKNTLLTETEIYEFSNTFKKLTASNLSVKLATNLSTLIAPTNLSTNFTKKIFKIY
KSTNIPVYYNLLSNDTTFLDNKIDNVYLSKVLNKLKLQLNNNKLDLLMVNYTNDKLLRLR
AKSSMYQTFAKLAFIENGMILNKHLFPVRSAKSVYLKQFNKNLKFVIYLYNLNMLRSRDI
LSVQQYEQFKDNYENIFKLIKRKTFVISILNKRKKWKFINYGTYQTLFKNISKNLTTKIL
SLFQQSTEKNFFQKEKVNKQVQYSLEVEALVQQTLEQLVNSSESPNHSLTSLIYRFIEKS
IVFTHPSSNSLKQLTIENKTVIKPVIKKALQRLISLQKKLKSLGPWSSKEKIMLQNKQKT
LILQSLVSYLQKQETPFNSNFKTVSNKFKQISMFQTLSFAQRKTVLNKLCLNTYFTPNLQ
TISNAKDLSNEKKSRALALSGQLTNLQFKLQKEYLTKLNVSLLKAEKNESDLISFNESIL
LMLQKTTGVDLTNYKQFSRIVTNNSNIDKGSLAKEIYTTKLLFEFIRQNLMDNYRKDKFN
FELKQVSKFQKSQFLTNILFKGGSFSSRLSLKKCQNNLIKLKISKLLNHIFSVMPFVNNQ
TTTTGFISYDHIDTLVSFGIISSTMANVLNKKINVQIQKQKLRKTLLSLQNLQKMTFTSS
NKFNRLIYSSILMDVFSRLYELKKAKTITERKYVLIKQKLKIFSLFSALNYKLLDLKEKG
SISSSKALELKNQIIQKISQKMKKVKTFAKFKQSLKALKGENFSSRLSYLPKDLDSQKTL
EKAKAFEFNPSLLKILKSRGRWARVTVKQLVKQKLLTTKQQEKLQTIVDKQNLMKMKKLR
RLVSVFVYCRQIVETQRNTVANNSNYEPLMQNVISSVLKSFNGPWKRVLLNLLYKQNFIS
ENLFLSYLTNNTQKTKSSKTKVISLNNNVDTTYTKTKLKRLLTMYYKQICKLRQFQTNRE
ILKGEFEQKLSAILSSVILVLEKGGLDAFKVIYNTKWVNELCQSNLKINNQKNKNSQISS
AIREFVSKYNFLKDQYLNTYKKLQLNDKMKLFKMYKTNLLQELVNLEKTIKSPNAQSFEK
SEILSISRLEQLKMQGLISTKICNKLTVMLNNSLQRLIKLDRLFALQNLYLVTSEAKAIS
ENASYMNTGAVLEANSSKVQIQTESNIIQNNEQYIKLKQKIFQSYFRYEYKHIEKSVIKQ
KLLLQRLDSYNLKQKTTKQYKTNLRRKKSKLLSSEQFNSFFQQLLNFLDSRYKSAGRNRR
NPRINSIIRRLNQKLSFDKTLTKKFGDHLQTFIDKRFGPALPIPPHLELKRWKIKTSKLQ
SKQKLNLKYFILPVGIVRDLAPRRSVGLPILERLIVEYYSRN
>Q9M4G5
MAMESALTSTRVSIPSLCSGISSSHHHHRSLSFLNFPKLSSFKYSFRTISPVPFVVSASS

VSPSSPSTSVAQSQDLKIKSVPTKPIEGQKTGTSGLRKKVKVFMQDNYLANWIQALFNSL
PLEDYKNGLLVLGGDGRYFNREAAQIIIKIAAGNGVGKILVGKDGILSTQAVSAVIRKRE
ANGGFIMSASHNPGGPEYDWGIKFNYSSGQPAPESITDKIYGNTLSISEIKIADIPDVDL
SQLGVTKYGNFSVEVVDPVADYLELMENVFDFSLIRSLVSRPDFRFVFDAMHAVTGAYAK
PIFVDKLGASLESIANGVPLEDFGHGHPDPNLTYAEDLVNILYGENGPDFGAASDGDGDR
NMILGRSFFVTPSDSVAIIAAQCQYAIHYFQSGPKGLARSMPTSGSLDRVAQKLNLPFFE
VPTGWKFFGNLMDAGKLSICGEESFGTGSDHIREKDGIWAVLAWLSILAYRNKDKKSGEK
LVSVADVVKDHWATYGRNFFSRYDYEECESEGANNMIEYLRDLISKSKAGDKYGSYSLDF
ADDFAYTDPVDGSVASKQGVRFVFSDGSRIIFRLSGTGSAGATVRIYIEQFEPDVSKHDM
DAQIALKPLIDLALSVSKLKDFTGREKPTVIT
>P12859
MATHAALASTRIPTNTRFPSKTSHSFPSQCASKRLEVGEFSGLKSTSCISYVHSARDSSF
YDVVAAQLTSKANGSTAVKGVTVAKLKVAINGFGRIGRNFLRCWHGRKDSPLEVIVVNDS
GGVKNASHLLKYDSMLGTFKAEVKILNNETITVDGKPIKVVSSRDPLKLPWAELGIDIVI
EGTGVFVDGPGAGKHIQAGAKKVIITAPAKGADIPTYVIGVNEQDYGHEVADIISNASCT
TNCLAPFAKVLDEEFGIVKGTMTTTHSYTGDQRLLDASHRDLRRARAAALNIVPTSTGAA
KAVSLVLPQLKGKLNGIALRVPTPNVSVVDLVVNVAKKGISAEDVNAAFRKAAEGPLKGI
LDVCDVPLVSVDFRCSDVSTTIDSSLTMVMGDDMVKVVAWYDNEWGYSQRVVDLAHLVAN
KWPGTPKVGSGDPLEDFCETNPADEECKVYE
>Q9TLT2
MKNVDFCTFGVKLGMTQILDNFGNLVPVTIVSQPKPCVITRVTTSLNSQQKLIIQIGCYS
DFRANKPTIGYFKKQSVPNLRYLHEFQTYKNNEYQVGKSLDLKELDQQLVQVSAYTIGKG
FAGYQKKHKFSRGAMSHGSKSHRRPGSIGAGTTPGRVFPGVKMAGRLGFSRRSIKNLLVL
KVDLTNKLFLLKGSVPGKYGNLLLVSLKKTNYFLD
>Q6B8R9
MSKNVSAIKKNQVSLRNKRKNKSYKSAIKTLIKKYINSLDELMDLNNTYVCTLKLSAVYK
KIDQAVRKGVLHKNQGSRKKSMLAKAMKISISRIKTI
>Q9MTM7
MLWEIRYTLRLFMERLRIIRSYLGQATPCPLTSLLYRFFLRFCFEKCYLCFIHSFHIMDH
EFKMVGLTLPFQNLKQFSENLSAPLLTLQSIPFSEYSKKIQQFSFIRTYFLIDLILSMNA
GIPIQ
>Q5YLB4
MALLKPFPLQHSLRCMASRFLLHSHYHTHTLSFSSISLSRPSIVLNPRVINKLRRLSDAI
LIRNMVSLRAFMSSSTTTEAFQENTKSKGYGSEQIQVLEGLDPVRKRPGMYIGSTGPRGL
HHLVYEILDNAVDEAQAGFATKIDVVLHADNSVSIADNGRGIPTELHPVTKKSSLETVLT
VLHAGGKFGGSSSGYNVSGGLHGVGLSVVNALSQALEVTIWRDGKEYQQKYSRGKPITTL
ICHDLPVEMRDRQGTAIRFWPDKEVFTTEMQFDYNTIAGRIRELAFLNPELTIALKKEDI
DPEKIQCNEYFYAGGLVEYVKWLNADKKPLHDVLGFRKEADGITIDMALQWCSDAYSDTM
LGYANSIRTIDGGTHIDGVKAALTRILNNLGKKSKTIKEKDISLSGEHVREGLTCVISVK
VPNPEFEGQTKTRLGNPEVRKVVDQSVQEYLTEYLELHPDVLDSILSKSLNALKAALAAK
RARELVRQKSVLKSSSLPGKLADCSATNPEEAEIFIVEGDSAGGSAKQGRDRRFQAILPL
RGKILNIERKDEAAMYKNEEIQNLILGLGLGVKGEDFKKEALRYHKIIILTDADVDGAHI
RTLLLTFFFRYQRALFEEGCIYVGVPPLYKVERGKQVYYCYDDAELKKVQRSFPSNASYN
IQRFKGLGEMMPAQLWETTMNPETRLLKQLVVEDAAEANVVFSSLMGSRVDIRKQLIQNS

ASMMNLEQLDI
>Q93YW0
MPSLSTPPSQNLAFSPAASATSSRLTPSSKRSFYPHRLPDPTALCRCSSSSGSNSSSSSS
SDDNPRWDSAIQDVLKSAIKRFDSVLSWYATLDNDDGEQGSENVEKIDDDWDWDRWKKHF
DQVDDQDRLLSVLKSQLNRAIKREDYEDAARLKVAIAATATNDAVGKVMSTFYRALLEER
YKDAVYLRDKAGAGLVGWWSGISEDVKDPFGLIVQITAEHGRYVARSYNPRQLSTSAAGA
PLFEIFLTLDGKGNYKKQAVYLKWKEIFPDVPTMPSRTLTPGRFLTSPGRKEDTGNLAVE
SSEDEESDNSDDDSDLLEESSGFQSFLRDMIPGVKVKVMKVTAPGRVDKDFISKVIEQIA
DEEDEENDLDIEDIDVEDDTKAEIDEKNADIELESVTDEIIDNNGGREIAVKFVIGDIVD
RLSGNQPLKESLRSPANLESVENSSFYLRLEKDLNVKESKGVEGTTLVDGKGSRQSRRRI
ENIMGDLAKSIEKEKKISVKMLKDVGELLSLTLSQAQNRQQLSGLTKFRRIDVTPSLDPL
DGLYIGAHGLYTSEVIHLKRKFGQWKGGKESKKPTDIEFYEYVEAVKLTGDPYVPAGKVA
FRAKIGRRYELPHKGLIPEEFGVIARYKGQGRLADPGFRNPRWVDGELVILDGKYVKGGP
VVGFVYWAPEYHFVMFFNRLRLQA
>Q8L6J3
MSSTKTPISLTIKLNQFTDKPTGLDINRYHNSPIMWRNIIKQLSSRTPQKLLFSSKNRTY
SFLGFGQDSVFKDNTKFRSLIPISCSNIVMGFQNLGEYLPGDEFLSRPLLKNQVNSNDFC
CRKSYASVAEAVAVSSTDAEEDVSVVDEVQELLTELKKEEKKQFAFRRRKQRMLTSGMGH
RKYQTLKRRQVKVETEAWEQAAKEYKELLFDMCEQKLAPNLPYVKSLFLGWFEPLRDKIA
EEQELCSQGKSKAAYAKYLYQLPADMMAVITMHKLMGLLMTGGDHGTARVVQAALVIGDA
IEQEVRIHNFLEKTKKQKAEKDKQKEDGEHVTQEQEKLRKKVTNLMKKQKLRAVGQIVRR
QDDSKPWGQDAKAKVGSRLIELLLQTAYIQPPANQLAVDPPDIRPAFLHSVRTVAKETKS
ASRRYGIIQCDELVFKGLERTARHMVIPYMPMLVPPVKWTGYDKGGHLYLPSYVMRTHGA
RQQREAVKRASRNQLQPVFEALDTLGSTKWRINKRVLSVIDRIWAGGGRLADLVDRDDAP
LPEEPDTEDEALRTKWRWKVKSVKKENRERHSQRCDIELKLAVARKMKDEEGFFYPHNVD
FRGRAYPMHPHLNHLGSDICRGVLVFAEGRPLGESGLRWLKIHLANLFAGGVEKLSLEGR
IAFTENHMDDIFDSADKPLEGRRWWLNAEDPFQCLAVCINLSEAVRSSSPETSISHIPVH
QDGSCNGLQHYAALGRDELGAAAVNLVAGEKPADVYSGIAARVLDIMKRDAQRDPAEFPD
AVRARALVNQVDRKLVKQTVMTSVYGVTYIGARDQIKRRLKERGAIADDSELFGAACYAA
KVTLTALGEMFEAARSIMTWLGECAKIIASENEPVRWTTPLGLPVVQPYRKIGRHLIKTS
LQILTLQQETEKVMVKRQRTAFPPNFIHSLDGSHMMMTAVACRRAGLNFAGVHDSYWTHA
CDVDKLNRILREKFVELYETPILEKLLESFQTSYPTLLFPPLPERGDFDLRDVLESPYFF
N
>P24493
MASTFNIPCNAGTIKNFNNSQRNLGFSSNLGINFAKTRFSNCGDSGRIPSQLVVRASERR
DNLTQQKTGLSIEECEAAVVAGNAPSAPPVPPTPKAPSGTPSVSPLSLGRRPRRNRTSPV
FRAAFQETTLSPANVVYPLFIHEGEEDTPIGAMPGCYRLGWRHGLVEEVAKARDVVVNSI
VVFPKPDALKSPTGDEAYNENGLVPRTIRMLKDKFPDLIIYTDVALDPYYYDGHDGIVTQ
HGVIMNDETVHQLCKQAVAQARAGADVVSPSDMMDGRVGAIRAALDAEGYSNVSIMSYTA
KYASSFYGPFREALDSNPRFGDKKTYQMNPANYREALIETQEDESEGADILLVKPGLPYL
DIIRLLRDNSDLPIAAYQVSGEYSMIKAGGVLKMIDEEKVMLESLLCLRRAGADIILTYF
ALQAARCLCGEKR
>Q94KU5
MATLFTVTTTSRPFPANPSKTFSPSISLKPNALSFSLTHHRPPRPLRFSKIRSSLPSESD

SEPEGGYSITDEWGEQPAEPESPPDNAPSAVSDEWGEKSESVPEESVTRFAESDPPTNED
EWEEREADDGVDKTWELKRCLADTVYGTELGFRAGSEVRAEVLEIVNQLEALNPTQAPVE
NPELLDGNWVLLYTAFSELLPLLAAGSTPLLKVKSISQSIDTKSLSIDNSTTLSSPFADF
SFSATASFEVRTPSRIEVSFKEGTLKPPEIKSSVDLPESVGVFGQEINLSFLKQSLNPLQ
DVAANISRAISGQPPLKLPFPGNRGSSWLLTTYLDKDLRISRGDGGLFVLAREGSSLLEL

>Q6B8M3
MTIRKALSLDFLKPILELEYQIQQLSKISTYQQVVLDQELVSFKEQLSILKYEVFQSLTP
LQRLHLVRQADRPTTLDYIPYIMSEWLELHGDRGGKDDPALVGGIGKLNNYTVIFVGHQR
GKDTKDNVLRNFGMASPGGYRKALRLMQHANQFNFPILTFIDTPGAWAGIDAETLGQGEA
IAVNLREMFSFDVPIICTILGEGGSGGALGIGIGDKILMLEYAVYTVATPEACAAILWKD
SKRSLDAAEALKITSADLKVLGVIDKVVKEPIGGSQSNPSQAAYILKESLISELHFLTQL
LPSKRKNLRYSKFRKIGTFYEGSEDYFG
>P28759
MASLGGLQNVSGINFLIKEGPKVNAKFELKPPPYPLNGLEPVMSQQTLEFHWGKHHKTYV
ENLKKQVVGTELDGKSLEEIIVTSYNKGDILPAFNNAAQVWNHDFFWECMKPGGGGKPSG
ELLELIERDFGSFVKFLDEFKAAAATQFGSGWAWLAYRARKFDGENVANPPSPDEDNKLV
VLKSPNAVNPLVWGGYYPLLTIDVWEHAYYLDFQNRRPDYISVFMDKLVSWDAVSSRLEQ
AKALITSA
>P51203
MNNYWPNSQGPILNKEVAELLVRTSVKINKKLTNCSNEVLILDVFRTEVKRNLLKIILAE
LEKILLEIYTSNLDLSDVTNLTESILIDLFHRSIKRFCRLYELDYNSICQNVHDINYLMY
DYKMLLQILIIQLIFGSSSKVKKTFNLFAENIPIKQVEIFLENYLIQLSNIIAHILIQNF
ETVQETNASYLCNVKFLSDRKLEKLKNNLVWNTLIQNYIERPRAIYESRYKVWVFYQDGL
NCQYVYACRSTELYTLSSAQVLVTFLLEIQDFFIPKIKSTVFLIGQFIIYIGQNIFNQIM
RTFLEIIRRSSNFKKQSSSL
>Q9SUI6
MASFATIAAVQPSAAVKGLGGSSLAGAKLFIKPSRQSFKTKSTRAGAVVAKYGDKSVYFD
LEDLGNTTGQWDVYGSDAPSPYNPLQSKFFETFAAPFTKRGLLLKFLILGGGSLLTYVSA
NSTGDVLPIKRGPQEPPKLGPRGKL
>P35006
MGRGLSTKKFYFKIGVQLKSNWNPLIDKLISILKLNSFYSSFVLRLIKLCLTKYLLLYAQ
QH
>P30924
MEINFKVLSKPIRGSFPSFSPKVSSGASRNKICFPSQHSTGLKFGSQERSWDISSTPKSR
VRKDERMKHSSAISAVLTDDNSTMAPLEEDVKTENIGLLNLDPTLEPYLDHFRHRMKRYV
DQKMLIEKYEGPLEEFAQGYLKFGFNREDGCIVYREWAPAAQEDEVIGDFNGWNGSNHMM
EKDQFGVWSIRIPDVDSKPVIPHNSRVKFRFKHGNGVWVDRIPAWIKYATADATKFAAPY
DGVYWDPPPSERYHFKYPRPPKPRAPRIYEAHVGMSSSEPRVNSYREFADDVLPRIKANN
YNTVQLMAIMEHSYYGSFGYHVTNFFAVSSRYGNPEDLKYLIDKAHSLGLQVLVDVVHSH
ASNNVTDGLNGFDIGQGSQESYFHAGERGYHKLWDSRLFNYANWEVLRFLLSNLRWWLEE
YNFDGFRFDGITSMLYVHHGINMGFTGNYNEYFSEATDVDAVVYLMLANNLIHKIFPDAT
VIAEDVSGMPGLGRPVSEGGIGFDYRLAMAIPDKWIDYLKNKNDEDWSMKEVTSSLTNRR
YTEKCIAYAESHDQSIVGDKTIAFLLMDKEMYSGMSCLTDASPVVDRGIALHKMIHFFTM

ALGGEGYLNFMGNEFGHPEWIDFPREGNNWSYDKCRRQWNLADSEHLRYKFMNAFDRAMN
SLDEKFSFLASGKQIVSSMDDDNKVVVFERGDLVFVFNFHPKNTYEGYKVGCDLPGKYRV
ALDSDAWEFGGHGRTGHDVDHFTSPEGIPGVPETNFNGRQIPSKCCLLREHVWLITELMN
ACQKLKITRQTFVVSYYQQPISRRVTRNLKIRYLQISVTLTNACQKLKFTRQTFLVSYYQ
QPILRRVTRKLKDSLSTNIST
>Q9SR43
MALSMEFGFSIGSCFKAPNPPVLISASPNKINFTLRRRKKRFLLRVSAVSYKEFAESALE
ETRKRIVLEPSHLQEKYSSMTGLDGKTELQMLAFKSSKIRLLRSMAIENETMQVFDFAGF
MEPEYDTPIFCANFFTSTNVNIVVLDLNPLHQLTDQTDYQDKYYNKIMSIYHKYAETFPW
GGKLTGESIKFFSPLVMWTRFSSSKEKHKALFSAFLEYYQAWLEMTIQVREEMEPSHVRA
NCEAQHKYLTWRAQKDPGHGLLKRLVGEAKAKELLRDFLFNGVDELGTKTFIDYFPEYQT
EDGTVSDKRSIIGKSYETRPWDLTGQFIG
>O78502
MSETLNLQIPSPTFEGSTGGWLRAAETEEKYAITWTSPKEQVFEMPTGGAAIMRKGENLL
YLARKEQCLALGTQVKTSFKITDYKIYRIFPSGEVQYLHPKDGVFPEKVNPGRIGVGNVS
HSIGKNLNPAQIKFTNKSFCD
>P08927
MASTFSATTSSCNLSSSAAISSFPLAAGKRNANKVVLPRKNRNVKVSAMAKELHFNKDGS
AIKKLQNGVNKLADLVGVTLGPKGRNVVLESKYGSPKIVNDGVTVAKEVELEDPVENIGA
KLVRQAAAKTNDLAGDGTTTSVVLAQGLIAEGVKVVAAGANPVLITRGIEKTSKALVAEL
KKMSKEVEDSELADVAAVSAGNNHEVGNMIAEALSKVGRKGVVTLEEGKSAENSLYVVEG
MQFDRGYISPYFVTDSEKMTVEFENCKLLLVDKKITNARDLINILEDAIRSGFPIVIIAE
DIEQEALATLVVNKLRGSLKIAALKAPGFGERKSQYLDDIAILTGGTVIREEVGLTLDKA
DKEVLGNAAKVVLTKDTTTIVGDGSTQEAVNKRVSQIKNQIEAAEQEYEKEKLSERIAKL
SGGVAVIQVGAQTETELKEKKLRVEDALNATKAAVEEGIVVGGGCTLLRLASKVDAIKDT
LANDEEKVGADIVKRALSYPLKLIAKNAGVNGSVVSEKVLSSDNPKYGYNAATGKYEDLM
AAGIIDPTKVVRCCLEHASSVAKTFLMSDCVVVEIKEPESAPVGNPMDNSGYGNI
>P24613
MASATLAFSCSSLCATLKLPQNLNPLLLNVPPLSKPFSGVVSPPSLSRLSLLPVAAKRRR
FQEIPEELKAEFEEFQRPPNQKPQLSDVLPDDFQAPEPGTPEYNDIINQFLPKKGPPPPR
EEIFAVVVIGSRQYIVIPGRWIYTQRLKGATVNDKIVLNKVLLVGTKASTYIGTPIVTNA
AVHAVVEEQLLDDKVIVFKYKKKKNYRRNIGHRQPITRIKITGITGYEDYPASTLEAEVE
AKEEAEAEAEAEAVPV
>O78439
MNNFELILLYYGKKIFMSFVKDFKLLLKARYPIIYINTYEEERVEYVIKSAIQSFTNRAV
YSWDFIEGYCNNPNDNGYAIRNPLQALEFIDKFTLETPLLVLLKDFHLFLNDISVSRKLR
NLAKSLRNQSKTIVIIASDLTIPLNLSDSITILHFPLPKNSEIKKELLRIQESLGYSLPE
HSLDNLVKASQGLSLEKIRRVLAKIIATYKEINVESLDLVFKEKQQLISQTQILEFYNPV
TKISDIGGLNELKSWLKFRAASFSKRAEEYGLPIPKGLLLVGIQGTGKSLTAKSIANEWN
LPLLKLDVGRLFGGIIGESEARVRQMIQFAETISPCILWIDEIDKAFTGSNFNNDSGTTK
RVFGTFITWLSEKKSPVFVVATANNIDSLPPELLRKGRFDEVFFIGLPDLKERECIFKVH
LKKIRPKSWSTYDTEILSAQSNRFSGAEIEESIYEAMHIAFNENREFTTSDILNSLKRVV
PLAYTSQKNIEELEDWASAGKIRLAS
>P51282

MLIGQKVRIKYSKSKIANDIAVRVGDIGVIRGIKFINNQCVTIIVEFENHTRLWMFREEL
IYLNK
>P51217
MEENMNFITLTEPAARQIKILKSNFDNEVCLRIGVKQGGCSGMSYSMNFESVSSLRETDE
VLMLDSFLVACDPKSLLYLYGLSLDYSSELIGGGFQFSNPNASQTCGCGKSFSG
>P18823
MINEDPSSLTDMDNNIDSWKNNSENSSYSHADSLADVSNIDNLLSDKIFSIRDSNSNIYD
IYYAYDTNDTNITKYKWTNNINRCIESYLRSQICEDIDFNSDICDKVQRTIIILIRSTND
TNDISDTNDISDTNDTNDTNAIYDPFDISDTNDTNEIYDPFFILDINDTNDTNDIYGIYD
PDDIYETNIKDICERYSEIYPRNREKSTFVPIDYSDPNCMEKLARLWVQCETCYGLNFKQ
FFRPKMNICEHCGEHLKMSSSDRIDLSIDRDTWNPMDEDMVSVDPIKFDSIKELGSEEES
SKDRLDEDMLSPDPIELDSEEESSKDRVDSEEEKDQSYIDRLDSYQEKTGLPETVQTGTD
QREEIHPLFEDIMNQLDLYLQTAKNRVDSEEEKDQSYIDRLDSYQEKTGLPEAVQTGTGQ
LNGIPLALAVMDSEFIAGSMGCVVGEKITRLIEYATNLLLPLIIVCASGGARMQEGSLSL
MQMAKISSALYNYQINQKLFYVAILTSPTTGGVTASFGMLGDIIIAEPNATIAFAGKRVI
EQLLNKEVPEGSQSADLLFDRGLLDAVVPRHLLKEFLTELFQFHGFVPLT
>P29214
MNKSQLISKIAYYTKYSKTDIEKIITSMLEIIVDTVATGEKVTLVGFGSFEARERKAREG
RNPRTGEKLFLPASRIPTFSVGNFFRNKVNKTF
>Q01292
MAATAATTFSLSSSSSTSAAASKALKQSPKPSALNLGFLGSSSTIKACRSLKAARVLPSG
ANGGGSALSAQMVSAPSINTPSATTFDFDSSVFKKEKVTLSGHDEYIVRGGRNLFPLLPD
AFKGIKQIGVIGWGSQAPAQAQNLKDSLTEAKSDVVVKIGLRKGSNSFAEARAAGFSEEN
GTLGDMWETISGSDLVLLLISDSAQADNYEKVFSHMKPNSILGLSHGFLLGHLQSLGQDF
PKNISVIAVCPKGMGPSVRRLYVQGKEVNGAGINSSFAVHQDVDGRATDVALGWSIALGS
PFTFATTLEQEYKSDIFGERGILLGAVHGIVECLFRRYTESGMSEDLAYKNTVECITGVI
SKTISTKGMLALYNSLSEEGKKDFQAAYSASYYPSMDILYECYEDVASGSEIRSVVLAGR
RFYEKEGLPAFPMGKIDQTRMWKVGEKVRSVRPAGDLGPLYPFTAGVYVALMMAQIEILR
KKGHSYSEIINESVIEAVDSLNPFMHARGVSFMVDNCSTTARLGSRKWAPRFDYILSQQA
LVAVDNGAPINQDLISNFLSDPVHEAIGVCAQLRPSVDISVTADADFVRPELRQA
>Q944I4
MAVAISGSSLISSTLQHYNNRIYIRDYPIPCHSSNPICNSFNFKRRSFSPSSPKFNDHVV
NPSSSYLSSKLSPIRTHSSFAACGCSWIQDNSMVHDYATTTNGTSKRCSALPTTNTVDVS
SVSDLFEFICSGPLVNKIGITPQRVGQSIDKWLLYGSQLCRLFQLNELKLTIPQKARLYH
YYIPVFIWCEDQIALHNSKFKDGDDVPPLVIGFSAPQGCGKTTLVFALDYLFKTTKKKSA
TISVDDFYLTAEGQAELRKKNPGNALLEYRGNAGSHDLKLSVETLEALSKLTKEGLKMKV
PRYNKSAYSGRGDRADSSTWPEVEGPLSVILFEGWMLGFKPLPADVVKAVDPQLEVVNKN
LEAYYDAWDKYIDAWVVIKIQDPSYVYRWRLQAEIAMRQDGQAGMSDEEVNDFVSRYLPA
YKAYLPTLYAEGPSGSDPDRVLAIDIDEERNPILAN
>O78411
MKVTKFYNLKAEKHLIIQILVEKDFFLQIYTKIDTQVFYFQSHQILYRSINILHTKKVLI
NLPNLLSLLHSELVSETVINYLITLFNDRDNFRILNINDSLKVLLDNFIRRELQNSCAKI
VSLTFNFNLSVETLLQKSNLLISNINTYNKKVSLKSISQLLLETILEIDKKTNRSTHVLT
GFFDLDHILVGLQKSDLIIIAGRPSMGKTAFMLSLVRNVADIQSFPIVIFSLEMSSKQLI

YRLISNETNIATSRLREGNISISEWEILNRAMTILSNLNIYLDDENNLDVLDIQSKLASL
QQIYGDIGLIVIDYLQLLQYKEKSSQRHIELSYITRYLKLIAKDFNLPLVVLSQLSRNVE
FRLNKRPILSDLKESGCLSYATNQPYFLKSDNVNFSKLTSLKVSNHYILSATLELLIPFQ
YNRIYPIVSLIKRELQTGYKVVYELDFYISVIVSTVEHYVLTLNGWKRILELTVDDLVAT
LDIQYLIYNNTEVDLFSSNVIFSSVINLICMNRINVYDFWIPKTNNFFVNALLVHNSIEQ
DADVVLFLYRDSYYQHNLKQSNIDMCEVIVAKHRHGTIGMVNLIFNPNTVSFMNLIKES
>P31918
MFLKDIHQFFLGFVITNTIIVIIGQINLQFKNSYNIVSGLKVQQLDSIKYYCKVLTHDSS
CLFFRNCLAVIDSIIVNIDEYLSLSPFKRREKFLQDVESLNNPSVPARLFINPKKDFILI
HRKGVSLKIRSTFQMYSIYKFNVSVSKNAAIFNTLRPSDFISFLEYYSDMNINEILINVL
NNYGLNDISLFLVKKIFYLFDSDKIFFNEFIGVDSVPLEYFLLFLSKKYIIRARKESPLK
IYTDFEDFYIHGSFKNILKMQQQKIKQNLGFKTKSNFFTGI
>O65837
MECVGVQNVGAMAVLTRPRLNRWSGGELCQEKSIFLAYEQYESKCNSSSGSDSCVVDKED
FADEEDYIKAGGSQLVFVQMQQKKDMDQQSKLSDELRQISAGQTVLDLVVIGCGPAGLAL
AAESAKLGLNVGLVGPDLPFTNNYGVWEDEFKDLGLQACIEHVWRDTIVYLDDDEPILIG
RAYGRVSRHFLHEELLKRCVEAGVLYLNSKVDRIVEATNGQSLVECEGDVVIPCRFVTVA
SGAASGKFLQYELGSPRVSVQTAYGVEVEVDNNPFDPSLMVFMDYRDYLRHDAQSLEAKY
PTFLYAMPMSPTRVFFEETCLASKDAMPFDLLKKKLMLRLNTLGVRIKEIYEEEWSYIPV
GGSLPNTEQKTLAFGAAASMVHPATGYSVVRSLSEAPKCASVLANILRQHYSKNMLTSSS
IPSISTQAWNTLWPQERKRQRSFFLFGLALILQLDIEGIRSFFRAFFRVPKWMWQGFLGS
SLSSADLMLFAFYMFIIAPNDMRKGLIRHLLSDPTGATLIRTYLTF
>Q8WHX7
MKFANIFALEHLLTLGAYLFCIGFYGLITSQNMIKALMCLELVLNAVNINFVTFSNYFDT
QHIKGEIFVLFIMAIAAAEAAIGLAIVLTIYRDRKSIRIDQFNLLK
>P48447
MNKNYNSYLGLSLEQEFKLKLYSQIVETLNEKQKXRLLLEILKYMLLNDNILKYLIKYTN
LK
>Q06463
MNIQLIKNFNPILKINTQSSNIKLNKIIPNEWLFILMNTGSFTQNLNSLLISDININISQ
KYNYNQPQQFHNTRTVWLANNNNQRFAFAQSRWIIKNNYTNYLNLLNNKPIGKSFIINET
DLHKKIEEIYCGHSYILEKNFQSQQLIWGRKYTLLYNKKSFIVIQEYFSPNLTKFLKFH
>Q42545
MAIIPLAQLNELTISSSSSSFLTKSISSHSLHSSCICASSRISQFRGGFSKRRSDSTRSK
SMRLRCSFSPMESARIKVIGVGGGGNNAVNRMISSGLQSVDFYAINTDSQALLQSSAENP
LQIGELLTRGLGTGGNPLLGEQAAEESKDAIANALKGSDLVFITAGMGGGTGSGAAPVVA
QISKDAGYLTVGVVTYPFSFEGRKRSLQALEAIEKLQKNVDTLIVIPNDRLLDIADEQTP
LQDAFLLADDVLRQGVQGISDIITIPGLVNVDFADVKAVMKDSGTAMLGVGVSSSKNRAE
EAAEQATLAPLIGSSIQSATGVVYNITGGKDITLQEVNRVSQVVTSLADPSANIIFGAVV
DDRYTGEIHVTIIATGFSQSFQKTLLTDPRAAKLLDKMGSSGQQENKGMSLPHQKQSPST
ISTKSSSPRRLFF
>Q40147
MAAVNGVGLSWPSKLTKNQTPKWGFSPSHRRCNPSSSSSATIRMTASVDEKKKTFTLEKS
EEAFSKAKELMPGGVNSPVRAFKSVGGQPIIIDSVKGSRMRDIDGNEYIDYVGSWGPAII

GHADDEVLAALAETMKKGTSFGAPCLLENTLAEMVISAVPSIEMVRFVNSGTEACMGVLR
LARAFTCRPKIIKFEGCYHGHADPFLVKAGSGVATLGLPDSPGVPKAATIDTLTAPYNDI
SAVESLFEEHKGEIAAVILEPVVGNAGFIPPKLEFLAAIRKITKENDALLIFDEVMTGFR
LAYGGAQEYFGITPDLTTLGKIIGGGLPVGAYGGRRDIMEMVAPAGPMYQAGTLSGNPLA
MTAGIHTLKRLQGQGTYEHLDKITAELTQGILDAGKKTGHAMCGGSIRGMFGFFFADGPI
YNFSDAKKSDTEKFGRFYRGMLEEGVYFAPSQFEAGFTSLAHTPEDIQRTVAAAEKVLKQ
I
>Q9M4W3
MAMATSFYCSTAIPSKKTNQNRENFLCSPVGGSKTTPSYIRLSTRQSRTLSLVVSAAASG
AAVEAEPKFAAVTPSKILSFRVGHGFDLHRLEPGYPLIIGGINIPHDRGCEAHSDGDVLL
HCVVDAILGALGLPDIGQIFPDTDPKWKGAPSSVFIKEAVRLMDEAGYELGNLDATLILQ
RPKVSPHKEAIRQNLCQLLGADPCVVNLKAKTHEKVDSLGENRSIAAHTVVLLMRK
>Q8L770
MASCLQASMNSLLPRSSSFSPHPPLSSNSSGRRNLKTFRYAFRAKASAKIPMPPINPKDP
FLSTLASIAANSPEKLLNRPVNADVPPYLDIFDSPQLMSSPAQVERSVAYNEHRPRTPPP
DLPSMLLDGRIVYIGMPLVPAVTELVVAELMYLQWLDPKEPIYIYINSTGTTRDDGETVG
MESEGFAIYDSLMQLKNEVHTVCVGAAIGQACLLLSAGTKGKRFMMPHAKAMIQQPRVPS
SGLMPASDVLIRAKEVITNRDILVELLSKHTGNSVETVANVMRRPYYMDAPKAKEFGVID
RILWRGQEKIIADVVPSEEFDKNAGIKSVV
>P48338
MSIDRILKQFLYKSIKPWQNKNIDDINQGEIYNIITNKSLTDNTINSVKRSFQNRLNENV
NLTNVRKSYKKIKFVSFSLTKFYSNENNVFFVKLKIEIFVCDSFNTSLIRDFLNLITSED
ILWIMYSVEPVILIHTDSKDKILQETYLKLECKAVKSQLYLNIYVIFDEKRVWTKNLVES
FFSTIKNGKILNISNVPKLGGLCFCYVLNNLLLKANKKNQLAEIKIYQHTKYEIPSKYKI
KFINESPLFLREYTGSLNFLYILLSMLLDNITNK
>Q7XJM2
MSLPIPPKFLPPLKSPPIHHHQTPPPLAPPRAAISISIPDTGLGRTGTILDESTSSAFRD
YQSLFVSQRSETIEPVVIKPIEGSIPVNFPSGTYYLAGPGLFTDDHGSTVHPLDGHGYLR
AFHIDGNKRKATFTAKYVKTEAKKEEHDPVTDTWRFTHRGPFSVLKGGKRFGNTKVMKNV
ANTSVLKWAGRLLCLWEGGEPYEIESGSLDTVGRFNVENNGCESCDDDDSSDRDLSGHDI
WDTAADLLKPILQGVFKMPPKRFLSHYKVDGRRKRLLTVTCNAEDMLLPRSNFTFCEYDS
EFKLIQTKEFKIDDHMMIHDWAFTDTHYILFANRVKLNPIGSIAAMCGMSPMVSALSLNP
SNESSPIYILPRFSDKYSRGGRDWRVPVEVSSQLWLIHSGNAYETREDNGDLKIQIQASA
CSYRWFDFQKMFGYDWQSNKLDPSVMNLNRGDDKLLPHLVKVSMTLDSTGNCNSCDVEPL
NGWNKPSDFPVINSSWSGKKNKYMYSAASSGTRSELPHFPFDMVVKFDLDSNLVRTWSTG
ARRFVGEPMFVPKNSVEEGEEEDDGYIVVVEYAVSVERCYLVILDAKKIGESDAVVSRLE
VPRNLTFPMGFHGLWASD
>Q06SI4
MGQKIHPYGYRVGITQPHSARWFANTYQYPQYVFEDFLLRQSLFKNLPVENLPRKRTEDK
SETKLVQIKIERLIRNTIKIKLYVTSPENASELFEHEALTKSPKKDFGTTNSNKRNLQTN
LESKKDRNTLRLNIQKRLLNKKLLQLQILKLQNMQSKLVTMKNLLDGSQNTNKKVFSSSV
FVQKNETETIVNNYPFYLNNIAINYSIGKTLIEGIKDCLNMNLDGKKKVKYLFYFLQLKK
QLSKTLLYQLDVYQKVEKQKLVNFTGLNKSEEQISAFQNTINLRLFKLKALSLKWTNHSR
HLTNVLVSIQSKLTLELETLVSKLDQNKTNLNQKCATLINILKFKYMLRKNNFNKLELFV

LNQKIEEIKLKWFQSLTIAGTVQAETDSQSDFYSSKKILLELNMISCEKQKMLTLLTKFK
FSLNFYNTHLEKTKRIILAGLFLLSKFGWKKEIFNGLNTFLSQSVLIIKKLETHLGKNYK
ILQERIIVKINHMTILESTIKQNLNLLQILKIAQDHNTNRDKIKVSQLYKNLFKVFYLTS
LSSTPAFHETLTNKTFLYDLVSESLDKYALLISQKVINLNSSLICYSEEKTTQIPVYNDK
FLNILTREYLSNLIRLFKNDSINYYKSKALYQFSLALLNQRYKLNKIIEMKIKNLLLQIK
PEEKTKNTFLVSIRDLNISLKSNTLFIKQLETWLTLLKKKLTENTATNRYLLQARIHKIN
CYLKKIIPVKITENNLVSTIEKNFIFVNYNILKQKLLAELLQMQQTIILAKKEEWEKEES
LSFPPSTSFFTKTNISIGNLNQTLQILQNRILDLQGRFNQENQQKTAGKTVNTDLSPILQ
VENKTNVSKTLQLKTTLQQLLKTNYENSFIVLQTRRQVYNSIINRLGGHRKFGKSRAGEK
ENIWKNPYETAEYLDFKLKKVNNSTDTNSLTTLLETIKQKIYERKPIKLMYTRKQKFLAK
KTIKKKLKHFLVLMALKNNYTNLSSLKNIYDVKTFAKLCENLTNLQTMPKVSVIELVKVH
QPKQYAVCLANFVVENLEKRFSFRSTMKRASEQAMSTANVKGIKIQISGRLNGAEIARTE
WLRDGVVPLQTLRANIDYSYKTAKTIYGILGVKVWLFKDTPANMNS
>Q9MUM6
MSLQVPVAKEDNRESNSHLATLSGNLYTRMNLPHEPRARAGIVFFMEKQSNLALHSEFLL
HVIIN
>O20159
MATQPSENTPKVITEKKIFLPTEKKINLFNIIDRFNNHLKKLKEKLQEAHFQKQANKLTS
KEQELKDNDDGKSKSKNENDSTSPEKKDVLTGTGVALPGLIQLAGGFTVSITGNAGTAQL
GGRSSRNGNQRQSISRRIIIRSENQRTQNLTESVESVHTSQKKNEVRQALMISAIKKATH
GTGVNQKKPEEAVRINAEASRKIKPEVNYNLKIFLAEKTLTSKEKKEAAKLIKKINHEIS
EWLTKFFTTRTGRQRELQKNLIWSLIKKFLPKVIGLERSGNHVHAYFMLSKLYGLLRMAC
LKVISQKKTILSRRNKMPKERIRVKDEIIRLEKEIKHLTNPAEIGLKEKQLLGQKEELDS
LNAEDVLFQGFEIKLTPTESQFWDEACLTEKQEISRQLHYDNWEDYKKNLTETKTQKRIY
HNILSKLITLGTKKENKNLLEEHFVFFVTKGNFYIRAFLEIQFAHLFGAARRGENEPEDS
SVQGTNSHLNLVYLETFSHSISCLYEMGGYIANVELTTGAILKLLSQSLDSLLLSSSNSS
VDERNSYVKTKASLQSLKQLLLEYCNFHNDPESFDVNTTQELKQRLKDLSSVSLSKKKEL
ELYKELLKAFKLCFQDIMNALSEVLKKLEKRIPEKTFTELKELFLLHQSHLIKLKQVCGA
LSSLFGFFLIELKSLWQLPLDEYIGVFIGSEPRKK
>P27456
MNQAMATPLSLSCCSPTLTRSTLFFTKTFPFSRSFSTPLPLSTKTLISLSPPHRTFAVRA
ESQNGADPARQYDFDLFTIGAGSGGVRASRFASNFGASSAVCELPFSTISSDTTGGVGGT
CVIRGCVPKKLLVYASKFSHEFEESNGFGWRYDSEPKHDWSSLIANKNAELQRLTGIYKN
TLKNAGVKLIEGRGKIVDAHTVDVDGKLYSAKHILVSVGGRPFIPDIPGKEYAIDSDAAL
DLPSKPQKIAIVGGGYIALEFAGIFNGLKSEVHVFIRQKKVLRGFDEEIRDFVAENMALR
GIEFHTEESPVAITKAADGSLSLKTNKGTEEGFSHIMFATGRSPNTKDLGLESVGVKVAK
DGSIEVDEYSQTSVPSIWAIGDATNRVNLTPVALMEGVALAKTLFQNEPTKPDYRAIPSA
VFSQPPIGGVGLTEEQAAEQYGDIDVFTANFRPMKATLSGLPDRVFMKLIVSAETNVVLG
LHMCGEDAAEIAQGFAVGIKAGLTKADFDATVGIHPTAAEEFVTMRTPTRKVRKNQASQG
KSDSKAKAVAGS
>P46314
MNLRNNKLHSSFIQRKAIKVISGINNFNVGQIFKIIHACEISKATYVDVARNPKIVSFIK
SISSIPVCVSSIDPRALYESVLAGADLVEIGNFDCFYTNGVYLSNDQIIAIVKQVKYLLP
YTDICVTIPHILKLHEQIYLAQKLESLGINLLQTEGSITNFSNEKLLEAKKINDNILYST

SMACCALSSVYSISKVVSLPVIASSGINGISASVALSYGASGIGIRSSVSKLGNIIDMSN
YIDEMIFSTSKKLYDIDQNNLLYMATSNLSLNKCSII
>P51336
MSHTIVTEKCIGVAECVNACPVSCIHKGEGKNTINKDWYWIDFAACIDCSICIQVCPTKG
AILDKEEPSLQRKLR
>O47027
MLNKKPPYLILSLIMIKTQKSKQTAVRKLIPGNVISLKITAMGANNVGINEFTFGIPVLV
PNAKLGETVQAKVLKILASKKIAIAKLIKVVTKTNATETNNLAALTPGATLDVTITKLGP
NSTGIADLGNNYNNVNKLIVKKSAITIGEKVTVLVTRVKNGYAFGVATVSTQRLNQVSTS
LTQNSFKGTKFTVVLPKNAKRYLKHLVFKVTTATAQNLSVNGFEQTLFKKGAVGLDTRIN
NQNGSAGNLTTVPTNTILFVKPNLGAKLGDKVQIQIIKFASIENGTLTYNVAIAKIIKLN
PLSTPQKKAFVRSSLRQMLKSGMHYGEKAIKCNARMKNYVWTRKKGTDTKVEARPLIKKG
RNLINLLKTRRCLTKALAQLTKYAAKGKTFLFVGTKKAASGLVARAALFSKKAFFVNTRW
LGGMLTNWKTILKSISKIRPILKEKQMIIKDILEKRQTIKARLIQKALLLRKKSKLMLKK
GRLLIQMLKQNNSNSTSGKQAVRFLFTEKTNLLNTKRKEFVSKGILLLEKRQQLVVKRQE
LITQSQTLKSKAIQLTNTISQFIKQFNLFT
>P51348
MAKIQAIRGTKDILPDEQLYWQFIHEKVASLLKYANYKEIKTPIFENSDLYDRGIGENTD
IVNKEMYRFNDRSNRDITLRPEGTAGVVRAFIENRMDVQNRLQKLWYSGPMFRYERPQSG
RQRQFHQLGIEFIGSSDARADAEIIHLAMNIFNDLSVNDLQLDINSIGKAEDRNHYQAKL
QEYLEQYYDDLDTDSQNRLSSNPIRILDTKNKYTQTILNDSPRISDFLSLESEKHFDDVC
DYLTLLNVPYKINTQLVRGLDYYNDTAFEIKILKSQGQDTLCGGGRYDSLIHQLGGNKTP
AVGCAIGLERLLLVAKDNILLPHMSIDCYIVSKGIKARKIGIIITQFLRNQLVKTELDIS
SSNFGKQLKQAHKKRAIACLILGDNEIQQETITIKWMYTQEQENMSIIEFKNKISYLKKK
IAFNKKFNSY
>O20130
MKKEFLHFLAFLITFVFCVKTQQVVKILRSRTMIDFGLEFQKAQIEMHNNPEDRVRFVDA
VNLCLPASDTFFQNPDRENKFSENYSIKLIDTTPHVSEIVLQTFVKEILSLSNINTEDTL
GKETLGKETYRLSNLLKKSKFGMESHHRIQNNSEGPNTFENGLGISTPKHGIAHFADAIQ
RFLDHKGNSSSNTRAAGARVEAIREALNDRDAINKSEEARKAREEVFIPSEPSKPSIASK
RSSASKSTKS
>O22886
MSILQVSTSSLSSSTLLSISPRKSLSSTKSCRIVRCSVEGTTVTERKVSATSEPLLLRAV
KGEVVDRPPVWLMRQAGRYMKSYQTLCEKYPSFRDRSENADLVVEISLQPWKVFKPDGVI
LFSDILTPLSGMNIPFDIVKGKGPIIFNPPQSAADVAQVREFVPEESVPYVGEALRRLRN
EVNNEAAVLGFVGAPFTLSSYVIEGGSSKNFTQIKRLAFSQPKVLHALLQKFTTSMITYI
RYQADSGAQAVQIFDSWATELSPVDFEEFSLPYLKQIVEAVKQTHPNLPLILYASGSGGL
LERLARTGVDVVSLDWTVDMAEGRDRLGRDIAVQGNVDPGVLFGSKEFITSRIHDTVKKA
GRDKHILNLGHGIKVGTPEENVAHFFEVAQEIRY
>P51202
MKNQIQVQLSKLKETSNSENAIKKQLEIIETIEYNDYLQLKKLAEIFHYRITKPEYISNC
VDGLIYEKLLNSQNSEIIKFTSKLCPKGIVPLKSNKQMDYQDLQILLVQNNLIAADKLTQ
QKLIKLAGVDEKSRNWLYFTDIKKIPVEDLQTIDQLWNAHSKGKFGFFVQRQIWLALEKN
WEQFWYKIGWEINRVPCRYPEEFHWNSTGPKGHLPLCNQLRGVQVLSALFSHKAWDNY

>P49536
MNYASFIIKIIKKPKQSFFEKEISLTEISGKLYQIRNKKKIEIPVSLSLWGNLAYDTMQY
YHINDYVIVEGYVSLRDKISSGYQIPVDKHIEVSGFKIYPFLLDSIQLTKMDK
>Q85AT7
MNTYAIIDTGGEQLRVEPGRFYDMRHFTLLNPSILDSNTKVLIYRVLMIHHESNIALGDS
WLEDATIKGRVLHSHFKDKITIYKMRSKKKMRRKLGYRLNLARFVVDSICFDGKEFYK
>O78452
MLYYVGADLDSTYKFACFIMKQVKFNDLVKNIKVQINASNIVSFSSKRLVSFA
>Q8MA04
MERNKNYYINVFHKQAENDLNFNKFYFYFFQETFYCLAYKQVSYKFLQNNVFIKKKKKFS
FFNLKRTIRSMRNQNYKESFFFIEQKKLSKKLLFSKLFYELLQEILKCILEISFKIKKNP
KISSYSTMSSIHGPFISFEENLIYFPVAIQSYLPNRVHPELIVRILRSYNLDVNLFHFLR
NIVHNGLYILSPPFLLNFGFRSLSTIFFNIYAYEIDLGFLSFLKLQKDLPQKYQTELDIF
SSSRKITFYFKNYSDFNNLKKIDQIERKYNNIIDYGPIYYLRYSNILLISLNLIKKNANF
FQLIYIRFFHLRFHFLFAKNLVKKISFNQDQIFFLGFLVFFFYTKIQIRIKLKKCLVNFI
KLEKKICINVPIKLLIIFLSKNGFCDISGNSKSKLSWSVLQDIEIIEKFRRLWLTISGYY
SGSSNKYCLKIVLYILRYSCAKTLACKHKMSLKKIWKKYTLNLSVTLKFQTGKKKFLSFS
HFKDFHKDEKSWQLNLKETNSIVYTFWN
>Q32001
MSLTQQQKDLIFGSLLGDGNLQTGSVGRTWRYRALHKSEHQTYLFHKYEILKPLCGENTL
PTESIVFDERTNKEVKRWFFNTLTNPSLKFFADMFYTYDQNTQKWVKDVPVKVQTFLTPQ
ALAYFYIDDGALKWLNKSNAMQICTESFSQGGTIRIQKALKTLYNIDTTLTKKTLQDGRI
GYRIAIPEASSGAFREVIKPFLVDCMRYKVSDGNKGHL
>Q8SL92
MKLVHILFVKKKSNLNSFTTVFEISLNTKYKNLFLLGNIFRQFLLGSFEGLVINEKRFYV
SHTNSLYKDFYLVNEFSLLEEVLEPFYDVWTVFDNLRFKEKFSLSKRYYARLLTYGSGEI
NSKDIIVPSNLSLLENKSLFTLITDSLCIDVILQILSKNGSPFNGVERVNYILENSNSGN
INYNKLYLDISTRYFLSPMEALFECFRKTNLALSKM
>Q9TLR9
MNIRKNSKSVSKRFKVTSNKLILYKPASKSHLQEKKTVSRKKRLCRVKRIKVVDSKSIKL
RYMF
>Q9MS98
MKHTLSVLVEDESGVLTRISGLFARRGFNIDSLAVGPAEKAGISRITMVIRGDNRIIEQI
TKHLYKLVNVLKIQDITNIPCVERELMLIKVKTGENKRNEILEIANIFRARVVDLSESFI
ILEITGDPGKIAAIEKLLEKYGICEIARTGKIALTRESKINTEILRLNTMINNDKIRGGG
T
>Q7YKY4
MNLISKKNELKQGFYPLFFLEEFYIRVLIHMNHNHILKIGNIKQSKLIHVNRICHYLLIK
RLIRQIRKQSHKYNGISEFPNYETEFYFQYKNRFYNLMIENVFLLILQMIWQHQKTRKND
PCILIHRSIQSTFPFLENKIIHCIWIIHGNIQLFHTIQQLNFLFLLLYERIRDKSFLHLL
KNIFNLKKELLIEAFYCDKFHLIELSMFFRNLYINEFDSFIVYHIVKTWKLAYLLNPSQA
IDDSSFIQKNHILLNIKRKQKSLPLVSWLANRSFYSLYGNIHYVRRDLSFLMAIQAGKHI
SRFWKYNSINFLQLKLGFPCSLDVLYLKSLFNQDFLFLGYRIVNKLWKKNFKIRAVSWYS
PILFFFKGRRISTKMPVLNLIHRLSVMHLCNLEGYPIHKAAWSVFNDKQIMNIFSNLLRN

IILYYSGCSNRSDLGKIQYILEFSCMKTLAFKHKSSIRSTWTQYKKHVSLLSLVKNRHKN
GKTSVDLYFLFQKTNKLWLLDLSKIQDSLACFIFID
>A1E9Z1
MPNILSLTCICFNSVLYPTTSFFFAKLPEAYAIFNPIVDVMPVIPLFFFLLAFVWQAAVS
FR
>P56350
MVELNFTQKQKQFLKDRKSLKSLVNQHLASDLGETSLSQYLDEFQILYNRFFCFYFFSYG
ENVFCSEEIFSSQLKNGFFPTKTKDEILSSPALNVIFSEKDFLLLKNIRIFLGMDSNNFF
LTEELFN
>Q38802
MSLQYHVLNSIPSTTFLSSTKTTISSSFLTISGSPLNVARDKSRSGSIHCSKLRTQEYIN
SQEVQHDLPLIHEWQQLQGEDAPQISVGSNSNAFKEAVKSVKTILRNLTDGEITISAYDT
AWVALIDAGDKTPAFPSAVKWIAENQLSDGSWGDAYLFSYHDRLINTLACVVALRSWNLF
PHQCNKGITFFRENIGKLEDENDEHMPIGFEVAFPSLLEIARGINIDVPYDSPVLKDIYA
KKELKLTRIPKEIMHKIPTTLLHSLEGMRDLDWEKLLKLQSQDGSFLFSPSSTAFAFMQT
RDSNCLEYLRNAVKRFNGGVPNVFPVDLFEHIWIVDRLQRLGISRYFEEEIKECLDYVHR
YWTDNGICWARCSHVQDIDDTAMAFRLLRQHGYQVSADVFKNFEKEGEFFCFVGQSNQAV
TGMFNLYRASQLAFPREEILKNAKEFSYNYLLEKREREELIDKWIIMKDLPGEIGFALEI
PWYASLPRVETRFYIDQYGGENDVWIGKTLYRMPYVNNNGYLELAKQDYNNCQAQHQLEW
DIFQKWYEENRLSEWGVRRSELLECYYLAAATIFESERSHERMVWAKSSVLVKAISSSFG
ESSDSRRSFSDQFHEYIANARRSDHHFNDRNMRLDRPGSVQASRLAGVLIGTLNQMSFDL
FMSHGRDVNNLLYLSWGDWMEKWKLYGDEGEGELMVKMIILMKNNDLTNFFTHTHFVRLA
EIINRICLPRQYLKARRNDEKEKTIKSMEKEMGKMVELALSESDTFRDVSITFLDVAKAF
YYFALCGDHLQTHISKVLFQKV
>Q85A69
MSILVYEVSKSLGNLKVLDRVSLYVRKVSLVALLGPSGSGKSSLLRIIAGLDSPDYGSVW
LHGTDMTNTSTQYRHMAFVFQHYALFKNMTVYENISFGLRLRGFSYQKIRNKVNDLLDCL
RISDIVSEYPGKLSGGQKQRVALARSLAIKSDFLLLDEPFGALDGELRRHLSKWLKRYLK
DNGITTIMVTHDQKEAISMADEIVVLKQGRFLQQGRSKNLYDEPIDYFVGIFSGSFIEFP
QLEESLDAPLGSSSSSSSSTKKSMEKDFTPFIPDLIWSQIFTNQSIHHYHFFLRPHELYL
ESQIDLKAIPVQIKKIIYKRTFVQLDLSITPSSWNITIPIGYQAFRKLNIQSFVQKLYIK
PRNQVYLRAYPKKKNIISKQI
>A1E9S2
MAKKSLIQREKKRHKLEQKYHLIRRSSKKKIRSKVSPLSLSEKTKMQEKLQSLPRNSAPT
RLHRRCFLTGRPRANYRDFGLSGHILREMVYACLLPGATRSSW
>P92959
MATMSALQSSFTSLSLSPSSSFLGQRLISPISLSVTSPVKPAENPCLVLAKLKRWERKEC
KPNSLPILHKMHVKFGDTVKVISGRDKGKIGEVTKIFTHNSTIVIKDVNLKTKHMKSREE
GEPGQIVKIEAPIHSSNVMLYSKEKDVVSRVGHKVLEDGQKVRYLIKTGELIDTIEKWKL
LKEAKDKETTQVAVTSAS
>P51216
MYRNMLLYPSPRLNNKQKQYLKDISILNQISYKLSLDSKSYFVITINILDQVINVEQFCF
NSKGQLYSIFFKGKTAKLLCHTILNSNHINILLSNQHVAYLARELVKSELGILFNQQYIQ
D

>Q9SHI1
MPSLASLVSLGGACASVSGTSSSDASYALVKRVSLSRRSVKGTKKWLCRYSVSSSTTTTT
ADFIADQNNNSVSIDSNSFRGSKDGDDSEVVLKQTPKPVLKPPVARVERGLGVNTAPWSK
DLSNGGKFDGEEERNKVIESLGEVLDKAEKLEIPKPGNKEGGEAVKPSQPSANSSNSRNG
SYANASDGGTRKTKTMKSVWRKGDAVAAVQKVVKESPKIFNRGVQTEPRTREEGEVNAKA
GTPLAPPQPPFRPQPPVRPQPMLQGKPMVAPPVKKSPILKDLGMAAKPLVSEEVDSSVKS
KERKPILVDKFASKKKGVDPAASQAVLAPTKPGKGPPSNKFRVEHRNKKNASASPRRRIV
AEDDGDDDASISRSGRKGRKWSKASRKAVRLQAAKDAAPVKAEILEVEEEGMSIEDLAYN
LAIGEGDILGYLYSKGIRPDGVHTLDREMVKMICRDYDVEVLDADSVKVEEMAKKRQTFD
EEDLDKLEDRPPVITIMGHVDHGKTTLLDYIRKSKVAASEAGGITQGIGAYKVSVPVDGK
LQSCVFLDTPGHEAFGAMRARGARVTDIAIIVVAADDGIRPQTNEAIAHAKAAAVPIVIA
INKIDKEGASPDRVMQELSSIGLMPEDWGGDVPMVQISALKGENVDDLLETVMLVAELQE
LKANPHRNAKGIVIEAGLDKAKGPFATFIVQKGTLKRGDVVVCGEAFGKVRALFDHSGER
VDEAGPSIPVQVIGLNNVPIAGDEFEIVSSLDVAREMAEARAVSLRDERISAKAGDGKVT
LSSLASAVSAKKMSGLDLHQLNIILKVDVQGSIEAVRQALQVLPQENVTLKFLLQATGDV
SNSDVDLASASEAIVFGFNVKASGSVKKAAENKGVEIRLYRVIYELIDDVRNAMEGLLES
VEEQIPIGSAEVRATFSSGSGRVAGCMVNEGKFVKDCGIRVVRKGKTVHVGVLDSLKRVK
ENVKEVSAGLECGIGMDDYDDWIEGDIIEAFNAVQKRRTLEEASASMSAAIEEAGV
>Q06RB0
MEKSERPLIKKKRPFRKKKRSFRKRRSPIESGHQIDFTNTSLLNQFISQQGKILPRKVTR
LTLKQQRFMTSAIKQARILSLLPFVFDDKKVKKKQKEEFQKKQKEEFQKKQKEEFQKKQK
EEFQKKQKEEFQKKQKEEFQKKQFQKKEFQRTKSTARTTNEKQTNEKQTKSTARTTNEKQ
TKSTARTTNEKQTKSTARTTNEKQTKSTARTTNEKQTKSNDRTTDLRTRKK
>Q85WU2
MIRVLGLLWDPLSSWVEVSGPIILFGLYYGFIATLPFGPSKIYSMRSFFLGETLYGIIAI
SGSITGQLIVFLSMYYSPIYAALWKPHAITLLVIPYTFCRVFRSLEKPSSPESTHPMNSI
KNPKILSLFMGGLILQLLNPILLANPVLTRLVNLFLFRYSDNISFMISSFCGWLGGHILF
INLTKLVSLRLVSFRIERNSPIDHTSLRRYIHQTFSVLLISYFSFYLGRSPLIFHRKKKD
NKKKDRSAAMAKKDRSVAMAKKNCSVAMVKKDRSVAEDEDRSVAMAKKGRSVAEDEDRSE
PLAMVMVQEARSVSFIAKKARSVAEDKDPEDEHRSVAMAKKARSVAEDKDPEDEHRSVAM
AKKDRSVAEDEDRSVAMAKKDRSVAEDEDRSVAEDEDRSVAEDEDRSVAEDEDRSVAEDE
DRSVAEDEDRFVAEDEDRSVAEDEDRSVAEDEDRSVAEDEDPEDEDRSVPREQKKLKGLP
ISWFKQPCPIKFFDPDRIYQPIRYIGNSPFHCLKPVMRTEVSQYFFGAYSSDGKKRISFT
LLPSVLALGEKLGKYRDLLDTSCLSEDPYHRWNHTMKRRRDSLENEFSDRVKALSHGSPA
ENVIERRVKFSNSQGDSFTEMYDPLLNGALRGTIDQFESPKMLNDLIISIISNLSDFIEI
PIKDCKEGFPNDQFGYGYHISEHWQELEHKSFRLPWEPLPTDTFRSLVPSTKSSRRGKVE
PISKRLYPLAERIIPNKAKKIFEEYLSNIDSSFGKLIYPKDLLEMQIQEIYTKDDDSLIH
FLWLEIAFRVAYMDLAPEIASCNERIYTNSLVNIYNRIDGRNVAPTGTVKWELILSLFTT
KQIFLFESLAQHEWTILRNCRGNVSTDDSTQTKDFIDLYGKILLNEPLQFREIKKHLPRW
PSDLMRAERDGDGDDRGPIQISTSRIRTRKVKSKLTLDFGKERIVLKRYHAESDYRRSLI
RGSMRAKRRKIMIWKRVQPNVHSLFFLRRMEIPTYPKDYYDTFDSGRVNQEQIQKEVREK
IHRGKYLDPVLHNTTLAEQVCLAWPEVHYFRGLILVAQSNIRKNIILPSLIIAKNIGRIL
LFKAPEFAQDWEEMKEELHIKCGSDGTEFSKKGFPDKWYKYGMQIKLIFPFRLKPWHSQS
KKRLRLRWSYLTTLGFETDIPFGDPKPKLGIFSEFFQPIFKKVKKGLKKGLKKGLKKGLK

RELILFKKVKRRLMGSTGIRRVSRVRYIDKSELNDRIQNKLLSETETTPMGSANDSSEVN
DQFGYETQTINVKDPDDWTTTMKERIESIAIINSSPITGMSLMDSEIHTGSKGSFNILGS
TLKKRLVQIRRIPGRFRNKSVQLIRKRFYSMKLMKLFLKRMDRYLLLSVIHFIGSNIKFW
IRSAWIRSTGNIATIYGRIFIINNDISKINRGKITNYYSINEKRKDFEIRPDRNMLSMSQ
AYVFHKIWQIGAIDRSYSKYFLKYRAQTSYPLIKKKIKELLDIQRILDHEKPQDLKENDW
KQWLRCFDRYKLSPQIWSRINPQKWRNRVNKQCTCYEERFIPYEKKKDYIFATVIEPSLG
LLRKMNKRYRYDLLSYSYLNSTKELDILNLTKDSDILNLTKDSDILNLTKDSDILNLTKD
SDILNLTKDSDTLKIEKRRSGLDLKFWLFPELSGKENIYDNSKFIPGYSILSEAQERKKI
EEKEREERIKVIEERIGIIRSDVQNKKVEEKQTGTGEVEEKQTGKVKQKQFGLKVEDQKT
DGKKKTNQVLVQHKILKDIGEEDISDLAGMCRILIEIEDPTKFMQIYEENINLNLMFLII
YEDLKGYEKYINARDSNANDINANDINANDINAKDSNANDINAKDSNANDINANDSNAKD
SNANDINAKDINAKDSNANDINAKDSNADVPKKKEDEIPKTVVSSEPYRLSSIVNDNLLI
YKIVSMWLKSEKIKRAGLGDDKNILSSFNLEDILLPKRRREFRILNRFDLENDHVGFFNG
KSIQNDEELMGRDQHLSVDTTQRIKRFLWPSYRLEDLLCMNRYWFNTNDGSRSAMLRIRM
YPLNVN
>Q32RJ9
MLTNPIFYLTNSNTSKASTHSNTNSKSLFNTKREDLQILYKLNQLLIEKSILHPYQRILI
AVSGGQDSICLLNILNKLRSTWHWKLGIVHCDHKWYLDSALQAIHVSRLAANMQIDLYQA
LTTQSVNNEQLARNWRYELIRHVAICHNFSVIVTGHTASDRVETLIYNLIRGSGISGLQS
ISWKRKLNSMLTIRMRSVEEQIILYSKHVKCYQNRENLLEDKKNVDIYLIRPLLNVSRME
LKHLVQNWKLSIWSDCSNQNISICRNRIRHQLLPYLRQYFHPKIDYVLNSCTEVMYTETL
YLDSIARYVLSKALSFISMPLNDQTCVKLDFELLRAVPLAIQRRILKYFLDVITHTSVSF
KHVEQVRIACLITTNSSNQFAKDSMNFTDQLLINRSIYLPGKKILQVINARFLIICNNIT

>Q9SCC7
MIAFISNYITFKTNRTYKNNICQLHCQSLNDNDIEARKIKEEAERRKQQAERNRMQKQMK
IDRLNAIPEDAEAGTVEEFMYKDGVKEILEKLDNDLVGLVPVKSRVREIAALLVVDKLRR
NLGLDTSVPSLHMCFTGAPGTGKTTVAMRMGQILQRMGYCRSGHLVVATRDDLVGQYVGH
TAPKTKEVIKKAMGGVLLIDEAYYLYNASNDRDYGQESIEILLNVMEENREDLVVVLAGY
KDRMDKFFSFIPGMSSRVGNHIEFPNYEAEELLSIAKVMCRDLEYEMSKDAEPIFFEYIK
KRMTMPYFSNARTVRNAVDRARMRAAIRLFNQATSGNSNGLVSKKQLMTLEKEDFVSVEE
LIARGDNAIVE
>P09754
MDFYNICCKYLKEIELTREDYSADRNSPQYNLFCSHHINPKFNKGGDEPENLVLLHYFEH
AYVHLFRWLITDNPRDLGGFTSAMNAKERRELQIEKRRENPPKLTLPPLPPPAEERKKPA
KTAKAIRAGKAVGSKYQLQSLKRANPFTMFMANLVLKFINNNGIEVIHKPSDNIKDVSVN
SASAIGRALNNVYPTETLTNSPDGISKLLKGTNKIVQNWRIDSLFIDDFEYEITQEVLLK
YQDLFETLTTYFSENQELSILELTKSMADIRNSNPKEIALIQKMFTFIKKWSFLIKSLSE
GKADDEIDLPTD
>Q8HSW1
MAAMIRLFRSSSSSSSNSISLISRSLSTAAASETVKSQSYPHNPHSTSVDPKAKTVQWVF
LGCPGVGKGTYASRLSTLLGVPHIATGDLVRDELKSSGPLSKQLAEIVNQGKLVSDEIIL
NLLSKRLESGEAKGEAGFILDGFPRTVRQAEILTEVTDIDLVVNLKLPERVLVEKCLGRR
ICSECGKNFNVASIDVAGENGAPRISMARLNPPFTVCFKLITRADDTEAIVKERLSIYWD

KSQPVEDFYRSQGKLLEFDLPGGIPESWPKLLEVLNLDEQEYKLSPAA
>O20173
MNKKESILREFALLCYFFIVYLLLPKNAKHPLADKSFLDPHPVARVSPFGSRPPFLLMVP
STSVPNASLESGQSSTISPRRQKESSIPVLQASGSVSANKTFQSTESSALHQPKSSSSER
TGKIITRQRPHSGPTSPRVTPGELLPTLEALKLQEALDFAQLKLKTRQVRYTINLRSQKL
LDTICELNDEKRAEVEEQAAYIFTLQKELVETGYEKVSSYNFTYSRLKQKAEELKYASKK
EVAQLVNSL
>P82412
MLSMAVQPNINAIAKPSIYQSPKLSLKPFKTPAFANPKPFFSSPSFSQLKKKNNWSLFVA
PETISDVAIMGNEVDIDDDLLVNKEKLKVLVKPMDKPRLVLKFIWMEKNIGLALDQTIPG
HGTVPLSPYYFWPRKDAWEELKVLLENKPWISQKQMIILLNQATDIINLWQQSGGNLAS
>P38365
MTTGVAVMSSATAASTATATAAATARIPLFLSRNNSSATVCSTLRCRTITRTRTRARLAI
CCEVALKSDSSTGFDSSSSSPPEEDEELKKNLEKVGCKVKVKSPLKVYHVPKLPEVELTP
DMVGVIKQYVGFWKGKYISPNYPFKVEYRIDVPDRGSVKLVVHLKEEEFEIIAE
>P0C586
MSSAVVASSTTFLVALASSASRGGPRRGRVVGVAAPPALLYDGRAGRLALRAPPPPRPRP
RRRDAGVVRRADDGENEAAVERAGEDDEEEEEFSSGAWQPPRSRRGGVGKVLKRRGTVPP
VGRYGSGGDAARVRGAAAPAPAPTQDAASSKNGALLSGRDDDTPASRNGSVVTGADKPAA
ATPPVTITKLPAPDSPVILPSVDKPQPEFVIPDATAPAPPPPGSNPRSSAPLPKPDNSEF
AEDKSAKVVESAPKPKATRSSPIPAVEEETWDFKKYFDLNEPDAAEDGDDDDDWADSDAS
DSEIDQDDDSGPLAGENVMNVIVVAAECSPWCKTGGLGDVAGALPKALARRGHRVMVVVP
RYGDYAEAQDVGIRKYYKAAGQDLEVKYFHAFIDGVDFVFIDAPLFRHRQDDIYGGNRQE
IMKRMILFCKAAVEVPWHVPCGGVPYGDGNLVFLANDWHTALLPVYLKAYYRDNGMMQYT
RSVLVIHNIAYQGRGPVDEFPYMELPEHYLDHFKLYDPVGGEHANIFGAGLKMADRVVTV
SPGYLWELKTTEGGWGLHDIIRENDWKMNGIVNGIDYREWNPEVDVHLQSDGYANYTVAS
LDSGKPRCKAALQRELGLEVRDDVPLIGFIGRLDGQKGVDIIGDAMPWIAGQDVQLVLLG
SGRRDLEVMLQRFEAQHNSKVRGWVGFSVKMAHRITAGADVLVMPSRFEPCGLNQLYAMA
YGTVPVVHAVGGLRDTVSAFDPFEDTGLGWTFDRAEPHKLIEALGHCLETYRKYKESWRG
LQVRGMSQDLSWDHAAELYEEVLVKAKYQW
>P25873
MATPLSISSNPLTSRHCYRLHLSSTSFKGNVSVLGANPSQILSLKLNQTLKTRNQQQFAR
PLVVVSQTAATSSAVVAPERFRLDNLGPQPGSRKKQKRKGRGISAGQGASCGFGMRGQKS
RSGPGIMRGFEGGQTALYRRLPKLRGIAGGMRSGLPKYLPVNIKDIETAGFQEGDEVSLE
TLKQKGLINPSGRERKLPLKILGTGELSMKLTFKARAFSTQAKEKLEASGCTLTVLPGRK
KWVKPSVAKNQARADEYFAKKRAAAAEAATSEPAASA
>P36211
MAATTLSIATTIRSSSFSSGLASAHHFPSRPLSIEFPFSFGVSSSSTLSHRAIYLHPISA
VKTPKKIKKIGSEISSLTLEESRILVDYVQDKFGVSILFSAPAAAALPPPLDNGGATASV
ERQTTFDVVINDVPRGNRIAVITAIRAMTSLSLSESKELIEGFPKKFKEGVTKDEAEEDK
TQLEEAGAKVSIV
>Q9MUR2
MSPIIINSVRLISSLLTIVFIMLPKVDTFSKIMKNKKILLNWQENISTLSIIKWISIILF
LSSHCALFITL

>P49079
MRSLTVASRHPGAAFSTRRRPLLHPAAAGRDSTFQRCWRWEKTQDSSFGSSLRTSRLPRT
VHGDILKNLLAPTAGAVSVEQAEAIADLPKGDMWSVHKFGGTCMGTSERIHNVADIVLRD
PSERKLVVVSAMSKVTDMMYNLVNKAQSRDDSYIAVLDEVFDKHMTTAKDLLAGEDLARF
LSQLHADISNLKAMLRAIYIAGHATESFSDFVVGHGELWSAQMLSYAIQKSGTPCSWMDT
REVLVVNPSGANQVDPDYLESEKRLEKWFSRCPAETIIATGFIASTPENIPTTLKRDGSD
FSAAIIGSLVKARQVTIWTDVDGVFSADPRKVSEAVILSTLSYQEAWEMSYFGANVLHPR
TIIPVMKYNIPIVIRNIFNTSAPGTMICQQPANENGDLEACVKAFATIDKLALVNVEGTG
MAGVPGTANAIFGAVKDVGANVIMISQASSEHSVCFAVPEKEVALVSAALHARFREALAA
GRLSKVEVIHNCSILATVGLRMASTPGVSATLFDALAKANINVRAIAQGCSEYNITIVLK
QEDCVRALRAAHSRFFLSKTTLAVGIIGPGLIGRTLLNQLKDQAAVLKENMNIDLRVMGI
AGSRTMLLSDIGVDLTQWKEKLQTEAEPANLDKFVHHLSENHFFPNRVLVDCTADTSVAS
HYYDWLKKGIHVITPNKKANSGPLDRYLKLRTLQRASYTHYFYEATVGAGLPIISTLRGL
LETGDKILRIEGIFSGTLSYIFNNFEGARTFSDVVAEAKKAGYTEPDPRDDLSGTDVARK
VIILARESGLGLELSDIPVRSLVPEALKSCTSADEYMQKLPSFDEDWARERKNAEAAGEV
LRYVGVVDVVSKKGQVELRAYKRDHPFAQLSGSDNIIAFTTSRYKDQPLIVRGPGAGAEV
TAGGVFCDILRLSSYLGAPS
>Q20EX8
MRNTVLNTLNAFPTQLGSNKSTLSSKIFHSYLGVLRPPTPLNTSMEVLGEGAHQNREAKR
SSLRFEFCSALKECQKGKVPFGSSLRLEAAENLTFECETGNYHTFCPISCVRWLYQQIAD
SFFLVIGTKTCGYFLQNAMGVMIFAEPRYAMAELEEGDIAAQLNDYKELKRLCLQIKHDR
NPSVIVWIGTCTTEIIKMDLENLAKLIEAELKVPIVVARANGLDYAFTQGEDTVLASLVN
RCPSSHESSLDSMKLPSGGREKQINDVNTSKPEGYLSEVISLTSNGDDINKKSCTKPVPK
KSLVLFGSVPNSVQTQLTLELAKQGINVDGWLPSRYSELPVLNKDVYVCGINPFLSRTAT
SLMRRRKCHLISAPFPIGPDGTRRWIEKICTVLNTDKSSTSLEEVQKNLQQREEKVWKSL
QSYLDLVKKKSVFFMGDNLLEISLARFFIRCGMIVYEIGIPYMDRRYQAAELALLEQTCL
EMNVPLPRIVEKPDNYNQIQRIRELQPDIVVTGLAHSNPLEARGVTTKWSTEFTFAQIHG
FANSRDVLELITRPVRRNQNLDALGFTSLVKN
>Q9XJ27
MASITNLASSLSSLSFSSQVSQRPNTISFPRANSVFALPAKSARRASLSITATVSAPPEE
EEIVELKKYVKSRLPGGFAAQKIIGTGRRKCAIARVVLQEGTGKVIINYRDAKEYLQGNP
LWLQYVKVPLVTLGYENSYDIFVKAHGGGLSGQAQAITLGVARALLKVSADHRSPLKKEG
LLTRDARVVERKKAGLKKARKAPQFSKR
>P51329
MSRFWNNVVKFPRFLVSVILGLILTIISPFFVLFRKPSTSFFFIISFTGLLVVLTAIIKK
MLNIECC
>Q3BAI2
MQVVRIFTDMSISPSLSPRQCPDRYAFRAGRNLPDKEFRYLRTVLVTAAVHRGFGRRLPC
HQVTNFLDLPALGRRQPPYMVLRLCGDLCFW
>Q05753
MQSLSTPHTISLLLPRTSPSRLSPSLHSLAFPTRLRSLSYSSQTSILPDAGDDFIVGDCL
VYEDGVFEDPYLDKEVTQVAKQERKKNRRGGAKRLDESEIEPENLVPEEWRDIQAEVNLT
KKDKRKIAQEMEFGVRVEKKRQGLIPLRKVDLNDFLTYKEAKLAQLRPVILDKPGNFSDD
SGASSDGETAVSSPSERVAPKNPRWAVYGKGFDHVAKFFNSDKYDPSDKKSDGPRKLLSK

EEKFMLNSRNPDLAVATSKKWLPLHTLAACGEFYLVDSLLKHNLDINATDVGGLTVLHRA
IIGKKQAITNYLLRESANPFVLDDEGATLMHYAVQTASAPTIKLLLLYNADINAQDRDGW
TPLHVAVQARRSDIVKLLLIKGADIEVKNKDGLTPLGLCLYLGREIRTYEVMKLLKEFPL
SRHKKRLVTTDEDIE
>P02902
MAHCLAAVSSFSPSAVRRRLSSQVANVVSSRSSVSFHSRQMSFVSISSRPSSLRFKICCA
AMGEAQAKKETVDKVCMIVKKQLAVPDGTPVTAESKFSELGADSLDTVEIVMGLEEEFNI
TVDETSAQDIATVQDAANLIEKLVTEKTA
>O46903
MSIKERLGLVISDKMDKTVVVSIANRVTHKRYGKIVTKTKKYKVHDPNNNCQVGDLILIN
ETRPLSKTKRWMFKEIKQKSLKLDKDTIGE
>P12347
MCLPFFTSINSSGLFKAVSWRIIESFLLELFFIFYSSFSWSSSLSSSEGTGAGTGTGTGT
GTGTGTGTGTGTGTGTGTGTGTGTGTGTGTGTGTGTGTGTGTGTGTGTGTGTGTGTGTGT
GTGTGTGTGTGTGTGTGTIWEGELVFFYAECKGYPKIKGVFWVVSFFYDTAKNV
>P09856
MALHLSLSHQSWTSPAHPITSSDPTRSSVPGTGLSRRVDFLGSCKINGVFVVKRKDRRRM
RGGEVRASMEQALGTQEMEAIVGKVTEVNKDTFWPIVKAAGDKPVVLDMFTQWCGPCKAM
APKYEKLAEEYLDVIFLKLDCNQENKTLAKELGIRVVPTFKILKENSVVGEVTGAKYDKL
LEAIQAARSS
>P23326
MAMASATATLSFKTPSLSLSPPSTRCSAAQGISLTHFNKQLKSTLNLSSSSSISSSKVQP
IVLKNKRISTVDSSVSTSSPSFTVFAAKGYKMKTHKASAKRFRVTGKGKIVRRRAGKQHL
LAKKNTKRKNRLSKLIQVDRSDYDNVIGALPYLKVNRKV
>Q9TLT0
MNNKSIRTQILIEALPYIQQFRGAIFVIKYGGAAMKDLISKERLIADIVFLSCIGLKLVC
VHGGGPEINFWLNKMNVESKFHDGIRITDELTMQVVEMVLAGKINKELVSLLNNKGVKGV
GLCGKDGTILTARKAQCGSIDMGLVGEIKSVDPYLILTLLKENYIPVIASVGADETGKTY
NINADFVAGEIAASLGAEKLILVTNTSGILADVSQPESLIRDTNIMQLRQLLSRGIISKG
MIPKVNCSIRSLAQGVRAVHIIDGTKPHSLLLEVLTNNGIGTRFLYKQSILLQLNCFSFS
NVSI
>Q52TG1
MEEFKKYFERDSYWQQHFLYPLLFQEYIYALAHIHNHGLNGSIFYESGEVLGYDNKSSLI
LVKRLILRMYQQNFLIHSSNESHQNGFVGHKNPFFSQMISGGFAVIVEIPFSLPSLIEKK
KKEITKSQNLRSIHSIFPFLVDQFSHLNYVSDILIPYPIHLEILIQIIQSWIQDVPSLHL
LRFFLYEYHNWNSFITRKKSLSTFGKENPRFFWFLYNSYVSEYESVFCFLRKHSSYLLST
SYRNLIERTHFYGKIEHLPVVCSNDFHKNLQLFKDPCMHYVRYQGKAILASKGTSLLMKK
WKWYLVNFWECNFSFWFQPYRIHINQLSNSSFLFWGYFSIVLINPLPVRSQMLGYSCLID
NTLTKKLETLVPIIPLIGSLSKAKFCNVSGHPASKPIWTDLSDSDIIDRFVRICRTLSHY
HSGSSKKQSLYRMKYILRLSCARTLARKHKTTVRAFFQRLGSGFLEEFFTEEQKFLSLVL
PRTSLASGSDRVYKERIWYLDIIRINDLVNYSRLAMELCTGNITKRIEKRSKIAFLFSTD
MLKCSCSGS
>P56809
MLLLKHGRIEILDQNTMYGWYELPKQEFLNSEQPELLLTTSKKFPLIKDGNPLENQKYAC

RIKLLLLSVPITNQLNN
>P49534
MSLLIIGGTGTLGRQVVLQALTKGYQVRCLVRNFRKANFLKEWGAELIYGDLSRPETIPP
CLQGITAVIDTSTSRPSDLDTLKQVDWDGKCALIEAAQAANVKHFVFCSSQNVEQFLNIP
LMEMKFGIETKLQQSNIPYTVFRLAGFYQGLIEQYAIPVLENLPILVTNENTCVSYMDTQ
DIAKFCLRSLQLPETKNRTFVLGGQKGWVSSEIINLCEQLAGQSAKVNKIPLFLLKLVSQ
IFGFFEWGQNISDRLAFAEILNVENDFSKSTFDLYKTFKVDDSEITQLDDYFLEYFIRLL
KRLRDINFEDIQKQKNLII
>Q948J9
MELLNGVETLVSGIHHHHRTNAKRNRLVRSVKILNSGNHEIPRKCLCFDLYDKLVPYKKA
WSWQKSIVEEKKTLIDRNQDCADTVILLQHSPVYTMGTASTEDYLNFDIKDAPFNVYRTE
RGGEVTYHGPGQLVMYPIINLRNHEMDLHWYLRMLEEIVIRVLSSTFSIKASRLDGLTGV
WVGNQKVAAIGIRVSKWITYHGLALNVTTDLTPFNWIVPCGIRDRKVGNIKGLLEDGEHG
MVDDLRLIDIVHESLLKEFSEAFQLQIEKQTVSDPNIL
>O48917
MAHLLSASCPSVISLSSSSSKNSVKPFVSGQTFFNAQLLSRSSLKGLLFQEKKPRKSCVF
RATAVPITQQAPPETSTNNSSSKPKRVMVIGGDGYCGWATALHLSKKNYEVCIVDNLVRR
LFDHQLGLESLTPIASIHDRISRWKALTGKSIELYVGDICDFEFLAESFKSFEPDSVVHF
GEQRSAPYSMIDRSRAVYTQHNNVIGTLNVLFAIKEFGEECHLVKLGTMGEYGTPNIDIE
EGYITITHNGRTDTLPYPKQASSFYHLSKVHDSHNIAFTCKAWGIRATDLNQGVVYGVKT
DETEMHEELRNRLDYDAVFGTALNRFCVQAAVGHPLTVYGKGGQTRGYLDIRDTVQCVEI
AIANPAKAGEFRVFNQFTEQFSVNELASLVTKAGSKLGLDVKKMTVPNPRVEAEEHYYNA
KHTKLMELGLEPHYLSDSLLDSLLNFAVQFKDRVDTKQIMPSVSWKKIGVKTKSMTT
>P28257
MDNLRRKEKILIADDESSIRRILETRLSIIGYEVLTAPDGRSALFLFHKEHPNLVVLDVM
MPKIDGYGVCQEIRKESDIPIIMLTALGDVTDRITGLELGADDYVVKPFSPKELEARIRC
VLRRVDKFYFSNTFTNSGIINIGFLKIDINRKQVYKNEERIRLTGMEFNLLELLISNSGE
PLSRTTILEEVWGYTPERHLDTRVVDVHISRLRAKLEDDPSNPELILTSRGTGYLFQRIM
EINKNYNPIIQIQKI
>Q9LE95
MATAAPPSLSLCYASSSFQYQQDPSFQTHFKPLLLNSSLCRLTLNQRERSCLKWVDFTSQ
KQSPKSVSFRVLAAVAAEAEVADMEEEEGESGGVATLPSPTKPKKGKAALPLKSDRTRSK
RFLEIQKLREIKQEYDLKTALSLMKQMSSTKFVETAEAHFRLNIDPKYNDQQLRATVSLP
KGTGKTVKIAVLAQGDKIDEAKAAGADIVGGEELIEQIKGGFMDFDKLIATSDMMAKVAS
LGRILGPRGLMPTPKAGTVTPNVAQAVEEFKKGKVEFRVDKTGIVHIPFGKLNFEEEDLL
INLFATIKSVETNKPTGAKGVYWKSAHISSSMGPSIRLNIREMLDYKPPSNA
>Q43295
MIAAGAKSLLGLSMASPKGIFDSNSMSNSRSVVVVRACVSMDGSQTLSHNKNGSIPEVKS
INGHTGQKQGPLSTVGNSTNIKWHECSVEKVDRQRLLDQKGCVIWVTGLSGSGKSTLACA
LNQMLYQKGKLCYILDGDNVRHGLNRDLSFKAEDRAENIRRVGEVAKLFADAGIICIASL
ISPYRTDRDACRSLLPEGDFVEVFMDVPLSVCEARDPKGLYKLARAGKIKGFTGIDDPYE
PPLNCEISLGREGGTSPIEMAEKVVGYLDNKGYLQA
>P27793
MASSLSTKPFLSGSRRRSTTDGSGWSYFQTSDLRQLSNQSVQISVRRQTAPLKLVVQASG

SSFGKVFQVTTYGESHGGGVGCVIDGCPPRFPISEADIQSDLDRRRPGQSRITTPRKETD
TCKIYSGVADGFTTGSPIHISVPNTDQRGNDYSEMAKAYRPSHADATYDFKYGVRSVQGG
GRSSARETIGRVAAGALAKKILKAYAGTEVLAYVSQAHKVVLPEGLVDHETLSLEQIESN
IVRCPDSEYAEKMIAAIDAVRVKGDSVGGVVTCIMRNVPRGLGSPVFDKLEAELAKACMS
LPATKGFEFGSGFSGTFLTGSEHNDEFYTDENGRIRTRTNRSGGIQGGISNGEIINMRIA
FKPTSTIGKKQNTVTREREEIELIARGRHDPCVVPRAVPMVEAMVALVLLDQLMLQHAQG
NLFSINPALQEPLSETVSSAAASLQGV
>A2YMU2
MPPLHAVSPAAAAAPPRALSSAARVPQRPGCVPERPNILSSSTNFMSLRAGPMRFYSRPL
ILQNSDKRAVLRHATIEEIEAEKSVIEDQARERMEKAIETVQNNFNTVRTGRANPAMLDR
IEVEYYGTPVNLKSIAQINTPDATSLLIQPYDKSSLKLIEKTIVAANLGVTPSNDGEVIR
VTVPPLTSDRRKELAKTVAKLAEEGKVAIRNIRRDAIKAYDKLEKEKKLSEDNVKDLSAD
LQKVTDEYMKKIEAIQKQKEQELMKI
>P55217
MAVSSFQCPTIFSSSSISGFQCRSDPDLVGSPVGGSSRRRVHASAGISSSFTGDAGLSSR
ILRFPPNFVRQLSIKARRNCSNIGVAQIVAAKWSNNPSSALPSAAAAAATSSASAVSSAA
SAAAASSAAAAPVAAAPPVVLKSVDEEVVVAEEGIREKIGSVQLTDSKHSFLSSDGSLTV
HAGERLGRGIVTDAITTPVVNTSAYFFKKTAELIDFKEKRSVSFEYGRYGNPTTVVLEDK
ISALEGAESTLVMASGMCASTVMLLALVPAGGHIVTTTDCYRKTRIFMENFLPKLGITVT
VIDPADIAGLEAAVNEFKVSLFFTESPTNPFLRCVDIELVSKICHKRGTLVCIDGTFATP
LNQKALALGADLVVHSATKYIGGHNDVLAGCICGSLKLVSEIRNLHHVLGGTLNPNAAYL
IIRGMKTLHLRVQQQNSTAFRMAEILEAHPKVSHVYYPGLPSHPEHELAKRQMTGFGGVV
SFEIDGDIETTIKFVDSLKIPYIAPSFGGCESIVDQPAIMSYWDLPQEERLKYGIKDNLV
RFSFGVEDFEDVKADILQALEAI
>O78417
MDKELKKEKIKLILIEPEEHLGYLLSEFFYQQNFVTYISESFIHLTDIINLHNPDIIIID
DSQNLETYKLSNIYLPIILLTTRGLKNDRMKIHKLGFDAYMLKPFDPDELIAIISNLIYK
KRNIKELQFIKHRISNLNLKLKYRETSFSYINLTVKEKEVFKFIQDGLTNKQISNVMGIT
QRSVEKYVTKIFEKLKIQNRIQILSYFN
>Q9MV14
MAAMVDAKPAASVQGTPLLATATLPVFTRGIYSTKRITLETSSPSSPPPPKPLIIVTPAG
KGTFNVILFLHGTSLSNKSYSKIFDHIASHGFIVVAPQLYTSIPPPSATNELNSAAEVAE
WLPQGLQQNLPENTEANVSLVAVMGHSRGGQTAFALSLRYGFGAVIGLDPVAGTSKTTGL
DPSILSFDSFDFSIPVTVIGTGLGGVARCITACAPEGANHEEFFNRCKNSSRAHFVATDY
GHMDILDDNPSDVKSWALSKYFCKNGNESRDPMRRCVSGIVVAFLKDFFYGDAEDFRQIL
KDPSFAPIKLDSVEYIDASSMLTTTHVKV
>O80439
MASLILGAPPRVTVALPSSRLSSSHSETAGVSLSCFTHQFSLSTSSSSSIPLVYCGRGDR
KTAKGKRFNHSFGNARPRNKSKGRGPERVPVPPAPPRKDKFENDEKIKIDIDESLFSN
>O19908
MCGIIGYVGEGSCRDVLINGLDKLSYRGYDSAGIAFIKNSKINVVRSKGRIEKLKEKIND
NFQKFEIGNIGIGHTRWATHGEPTEINAHPHLDAEGQFAVVQNGVIENYVQLKNYLTVNG
TYFLSDTDAEVIPHLIAYKQKHLKLQIVEAILCALSELKGNFSTVIIARDMPDSIFVYQN
KTALTLGKGSNFYSVSSDPIALIPYTKNFIQLHDRELGIISISQLAIYNKGKFTYPSRRF

KANLNDLITNKASFDSYTLKEIHDQKKVLRNLIISTLQSEKSIDESGQLHLEYKKIKNFQ
IIACGSSFNAALVGKVILEKLIRIPVHVYYGSEFKTNLPPLLPCTLTIAVSQSGETGDML
SAIEIEKSRRKFQNTVYKPYLLSITNKNYSSITKKTAQSIDLKAGIEIGVAATKTFTAQT
LSFYLLALKLAEHKFTLRKKEINKHLDEIRNLPKAIAHLLIKDESSIKWLSKQLKEISKC
FYIGKGLNLGSALEGALKLKEISYIHCDGYAAGEIKHGPIALVENNTLIITITDPEQSQE
STFASSQEAKARGAVLLAITHIEDSSIYQTFDFIIKIPKISQICASITSSVSLQLFAYYM
AYYKGNDIDKPRNLAKSVTVE
>Q6B909
MKNKITVILKKNLANLGSIGTVVKVSSGYAFNYLIPRDFAQLATQGSLKHHKMFLDMKQQ
KLDEIQDKLQASAQKFNKISKISVKKKVGNNNQIFGSVNDKEIISKLFSITGEKLEKKNI
YLPNIKALGIYNLNIIFTSDIQVSMKLQILPFFA
>P42379
MPIGVPRIIYCWGEELPAQWTDIYNFIFRRRMVFLMQYLDDELCNQICGLLINIHMEDRS
KELEKKEIERSGLFKGGPKTQKGGTGAGETGASSIQNKKSNSSSFEDLLAADEDLGIDEN
NTLEQYTLQKITMEWLNWNAQFFDYSDEPYLFYLAEMLSKDFNKGDARMLFSNNNKFSMP
FSQMLNTGSMSDPRRPQSTNGANWNSSEQNNSLDIYSPFRMLANFEAQDYDFKQINPSLA
SKEEVFKLFNNTILKNGGQRNNNMSKLLTELAQRNWENKTNSQENLYKSTEKALSQRNLR
KEYIKDRTLNNYSSDPFNTKGYVNAQGASTGPSPRTRGMHADGSLNYLDFYSYNDSYNDF
KTAPRGKQAERAFQEEESKKVFVIINSFGGSVGNGITVHDALQFIKAGSLTLALGVAASA
ASLALAGGTIGERYVTEGCHVMIHQPECLTSDHTVLTTRGWIPIADVTLDDKVAVLDNNT
GEMSYQNPQKVHKYDYEGPMYEVKTAGVDLFVTPNHRMYVNTTNNTTNQNYNLVEASSIF
GKKVRYKNDAIWNKTDYQFILPETATLTGHTNKISSTPAIQPEMNAWLTFFGLWIANGHT
TKIAEKTAENNQQKQRYKVILTQVKEDVCDIIEQTLNKLGFNFIRSGKDYTIENKQLWSY
LNPFDNGALNKYLPDWVWELSSQQCKILLNSLCLGNCLFTKNDDTLHYFSTSERFANDVS
RLALHAGTTSTIQLEAAPSNLYDTIIGLPVEVNTTLWRVIINQSSFYSYSTDKSSALNLS
NNVACYVNAQSALTLEQNSQKINKNTLVLTKNNVKSQTMHSQRAERVDTALLTQKELDNS
LNHEILINKNPGTSQLECVVNPEVNNTSTNDRFVYYKGPVYCLTGPNNVFYVQRNGKAVW
TGNSSIQGQASDIWIDSQEIMKIRLDVAEIYSLATYRPRHKILRDLDRDFYLTATETIHY
GLADEIASNEVMQEIIEMTSKVWDYHDTKQQRLLESRDSTTSGADTQSQN
>Q9LWB5
MATTAPNLHSLSSSFAFSNPSSNVSATSFTFQIPNKKAQISCISSKKLHTQKSFNFHDAV
TPMNKPSFGRDLMVAQATEAVAPTTEEAATSQPKTSKKAKKLKYPRRILDVYQILQSPII
TEAAIKNIADENSLLFTVDVRADKKMIREAISNFFGVKVRKVNTLIRPDGTKKAYIMLNK
EYNASELAKKIGIFPGGN
>Q9T052
MAYHPVYNETMSMGGGSSNEFGQWLDKQLVPFDTSSGSLRVELLHGNLDIWVKEAKHLPN
MDGFHNTLVGGMFFGLGRRNHKVDGENSSKITSDPYVTVSISGAVIGRTFVISNSENPVW
MQHFDVPVAHSAAKVHFVVKDSDIIGSQIIGAVEIPTEQLCSGNRIEGLFPILNSRGKPC
KQGAVLSLSIQYIPMERMRLYQKGVGFGVECVGVPGTYFPLRKGGRVTLYQDAHVDDGTL
PSVHLDGGIQYRHGKCWEDMADAIRRARRLIYITGWSVFHPVRLVRRNNDPTQGTLGELL
KVKSQEGVRVLVLVWDDPTSRSLLGFSTKGLMNTSDEETRRFFKHSSVQVLLCPRYGGKG
HSFIKKSEVETIYTHHQKTMIVDAEAAQNRRKIVAFVGGLDLCNGRFDTPKHPLFRTLKT
IHKDDFHNPNFVTTADDGPREPWHDLHSKIDGPAAYDVLANFEERWMKASKPRGIGRLRT
SSDDSLLRLDRIPDIMGLSEASSANDNDPESWHVQVFRSIDSSSVKGFPKDPKEATGRNL

LCGKNILIDMSIHAAYVKAIRSAQHFIYIENQYFLGSSFNWDSNKNLGANNLIPMEIALK
IANKIRAREKFAAYIVIPMWPEGAPTSNPIQRILYWQHKTMQMMYQTIYKALVEVGLDGQ
LEPQDFLNFFCLGTREVGTREVPDGTVSVYNSPRKPPQLNAAQVQALKSRRFMIYVHSKG
MVVDDEFVLIGSANINQRSLEGTRDTEIAMGGYQPHHSWAKKGSRPRGQIFGYRMSLWAE
HLGFLEQEFEEPENMECVRRVRQLSELNWRQYAAEEVTEMPGHLLKYPVQVDRTGKVSSL
PGYETFPDLGGKIIGSFLVVEENLTI
>P51273
MNLYNNQCPVPLEQQPVNEYNSLKNSWFFCWPTLSSHSYNKKITITLIATCFLVSPVLLS
IFPIAKLPLKFFFSEFIISSLITCFILIRLYLGWSYVVKRLMSATVFYEESGWYDGQIWV
KPSEILVKDRFIGLYEVFPLLNKIKNTLSCLSLMTTAPAILFFYF
>Q42736
MMSSLFVEGMLLKSADESCLPAKGKQRRTGDLRRLNHHRQPAFVRWICRRKLSGVSRIEF
HSGGLTPPRAVLNPVSPPVTTTKKRVFTFGKGRSEGNKDMKSLLGGKGANLAEMASIGLS
VPPGLTISTEACEEYQQNGKKLPPGLWDEILEGLRYVQKEMSASLGDPSKPLLLSVRSGA
AISMPGMMDTVLNLGLNDEVVAGLAGKSGARFAYDSYRRFLDMFGNVVMGIPHSLFDEKL
EEMKAEKGVHLDTDLTAADLKDLVEQYKNVYVEAKGEKFPTDPKKQLELAVNAVFDSWDS
PRANKYRSINQITGLKGTAVNIQCMVFGNMGNTSGTGVLFTRNPSTGEKKLYGEFLVNAQ
GEDVVAGIRTPEDLATMETCMPEAYRELVENCKILERHYKDMMDIEFTVQENRLWMLQCR
TGKRTGKGAVRIAVDMVNEGLIDTRTAIKRVETQHLDQLLHPQFENPSAYKSHVVATGLP
ASPGAAVGQVVFSAEDAETWHAQGKSAILVRTETSPEDVGGMHAAAGILTARGGMTSHAA
VVARGWGKCCVSGCADIRVNDDMKVLTIGDRVIKEGDWLSLNGSTGEVILGKQLLAPPAM
SNDLETFMSWADQVRRLKVMANADTPNDALTARNNGAQGIGLCRTEHMFFASDERIKAVR
KMIMAVTPEQRKAALDLLLPYQRSDFEGIFRAMDGLPVTIRLLDPPLHEFLPEGDLEHIV
NELAVDTGMSEDEIYSKIEKLSEVNPMLGFRGCRLGISYPELTEMQVRAIFQAAVSMNNQ
GVTVIPEIMVPLVGTPQELRHQIGVIRGVAANVFAEMGLTMDYKVGTMIEIPRAALIAEE
IAKEAEFFSFGTNDLTQMTFGYSRDDVGKFLQIYLSQGILQHDPFEVLDQKGVGQLIKMA
TEKGRAANPNLKVGICGEHGGEPSSVAFFDGVGLDYVSCSPFRVPIARLAAAQVVV
>Q06FN2
MTNNKNFADWDSLECKDLHNDLLYGRFALSPLTAKESRLLKKGLREALLTGILCLRFTHA
KIQNACKNLNLMNIVGIQESLDEILKNFGKIILTGKLEEFVGKGPFVAILDVRGPLNAMA
VDIELPPGIKVEIETQHIATITEPIPFVVELRIELVSSTSKGETGITDEEGFSIDPNPPI
QKVNSSIQGYEYGGQTFQTLFIEILSTSPTVPNKALLLVSMKIMNLFQIVLQAKYLDYKE
LEKGIHVGVFCVSALRAEQSKWIKTILEDALYMVGGRKHQGPLTDEEDDSIDSNFTPVQN
LDCRIESYEEEGQTFQRLFLEIWTKSPTEPQEALWEASAKILELFSLFLQTSKENEKDLK
QIIKDWTENKRKHQEVLRLLDSEESGSIGWITKMKLAYMHMTLLSMNAMYILLVHRLKPD
YDRYNSITDQIVQELRASLNKLREIQKGEYSEQRILVHSIAQEIEAALQKYETTYKLDDF
VIKAKKDMITMIWDKERADLESSLIRWESDEDYLNLKKMNPVEMDRSFAELQYQETLRKE
QDEQSSQQQKDQMEKRRWERQNREREREKRGNREF
>Q1XDT3
MTTYYFALASQNFLLVQEPLEEVFRERVNYYQSNNKAIDFWLIPNPSFLEKPEMISFKNL
VPKDAVAIISTNPIFINWLKLRIGYICIGQFEDNLQLSEESLNITVLTDKI
>P09753
MSRKKTIKDYENLAATRNHEVISVSNKETPSQGDITLLCKTCNKEFTTTTISYQNARKTG
CPHCKATSASLYWTGRARTKTPEQAKKNAEIKEHINKTRKEKGKAFANIKNKEDLKEKLT

NDLYLPNGEKNAYNDFILKRLNDPVTGKMMEKHHIIPLHAGGPDEKWNLISLTPEDHIEA
HNLRYLVYNETGDKNTIKFRNKTPNVTDQISKAKALGNETRRAQGTGIYEPGMSSKAGKI
GGSVKSVEKDLKQSTKMTSGVYDALYNGSRWKHTKTNTEIVIPPNTIVKMPQLVEKLIEA
LPPCEEKTRLAGAKLTTATSALARVIKGKNEGGRSSYFGWSICKE
>Q93Y52
MALAIRSSLRAAAMGRKAFRQAVPVRVAPAQRVRSVTTASAEITAYSVEEKGPKDSLEYR
MFFKQGAKEVSCWHEIPLYAGDGHLHYICEIPKETSAKMEVATDEPRTPIKQDVKKGKLR
FYPYNINWNYGMLPQTWEDPGHTDATLGAAGDNDPVDVVEIGAAAAKRGGVYKVKPVGVL
AMIDDGELDWKVIAISADDPKAALCNDVEDVEKHFPGEIQKVLEWFRDYKIPDGKPANKF
GYDNKCMNKEFTLNVIKETHEAYVKLKSGARANSEELSLI
>O20133
MRAKSIAYVWSTATQSLTPEQCEAMIKLREPLNVVFDQGMRNVQLILKKQDHFDELMTTY
ETCQTAEKNFHETTFLPAKANLDTFVGSQKARKEAANHFKKIKSQSIEYTKEANQAFTAA
MKYDQNHIVPAKNALQKIETCIINEVEDKLEEILNPSSITEKTLKGKTQRQKTNAALINR
IKRRNTAFNNHDNNIINSFHGPSSFEQISIYYNLPIDRSFQKMVQKQLEKIKVLPN
>Q06SG0
MINDTISDMLTRIRNASIVRKKSVLIPYTKMNLKIAEILEKEGYILSFEINTNKAISITK
KTSETNLRMLKIYLKYYKKYKTNFYKLNVIKNKKALSDFKNLIKLLKGKQGNKKTPCITN
LKRISKPGLRIYVNHKELPRVLAGTGIYILSTSKGILTDREARFRGIGGEVLCSVW
>Q9XFS9
MMTLNSLSPAESKAISFLDTSRFNPIPKLSGGFSLRRRNQGRGFGKGVKCSVKVQQQQQP
PPAWPGRAVPEAPRQSWDGPKPISIVGSTGSIGTQTLDIVAENPDKFRVVALAAGSNVTL
LADQVRRFKPALVAVRNESLINELKEALADLDYKLEIIPGEQGVIEVARHPEAVTVVTGI
VGCAGLKPTVAAIEAGKDIALANKETLIAGGPFVLPLANKHNVKILPADSEHSAIFQCIQ
GLPEGALRKIILTASGGAFRDWPVEKLKEVKVADALKHPNWNMGKKITVDSATLFNKGLE
VIEAHYLFGAEYDDIEIVIHPQSIIHSMIETQDSSVLAQLGWPDMRLPILYTMSWPDRVP
CSEVTWPRLDLCKLGSLTFKKPDNVKYPSMDLAYAAGRAGGTMTGVLSAANEKAVEMFID
EKISYLDIFKVVELTCDKHRNELVTSPSLEEIVHYDLWAREYAANVQLSSGARPVHA
>P52420
MSSLCASNCYPSSSSINLFSNNNNPTKPFLLSLRFASSNSLPFVAPLKFSTTNHVLSNSR
FSSNRIQRRLFLLRCVSEESQPSLSIGNGGSEERVNVLVIGGGGREHALCHALKRSPSCD
SVLCAPGNAGISSSGDATCVPDLDISDSLAVISFCQKWNVGLVVVGPEVPLVAGLANDLV
KAGILTFGPSSQAAALEGSKNFMKNLCHKYNIPTAKYKTFSDASAAKEYIQEQGAPIVIK
ADGLAAGKGVTVAMELEEAFEAVDSMLVKGVFGSAGCQVVVEEFLEGEEASFFALVDGEN
AIPLESAQDHKRVGDGDTGPNTGGMGAYSPAPVLTKELQDFVMESIIHPTVKGMAEEGCK
FVGVLFAGLMIEKKSGLPKLIEFNVRFGDPECQVLMMRLESDLAKVLLAACKGELSGVSL
DWSKDSAMVVVMASNGYPGSYEKGSIIKNLEEAERVAPGVKVFHAGTGLDSEGNVVATGG
RVLGVTAKGKDLEEARERAYSAVQQINWPGGFFRHDIGWRALRQKQVATKEE
>P51281
MLIADDLEKLLEILPHFVREPLKQHSNRKNLIEVVMDLGRRPEARFPGNPEYLSQRSISW
QDLDYCVKKVGNFSGDNRAGIEKTLHRISSMRNREGSIIGLTCRVGRAVFGTISIIRDLL
EQGDSILLLGKPGVGKTTAVREIARVLSDEMEKRVVIIDTSNEIAGDGDIPHPAIGRARR
MQVAQPDLQHQVMIEAVENHMPEVIIIDEIGTELEALAARTIAERGVQLVGTAHGNYLES
LIKNPTLADLIGGIQYVTLGDDEAKRRGTQKSILERKAAPAFQIAIEIHDRKAWIVHEKV

EETIDQILQGHQPFVQKRQIQDNGRILIKCYPSQSTEVLSTNSSSLQKMSSLKQKTHFLQ
QREVKNKTFDLNKLENRDTSLLSTTINTPVNINNHSFQVEASIQYLYAYSLSWQHITSVI
SALDLPIILTKEIEKSDAILALRSQVKQNTKLRQIAKSRQIIIYTIQNSTVPQITRALRK
ILNINTSSDLNWVKLCKSKKFYEIQALQEAKLAIEIIILNENSIVQLTPRSAYIRKMQHN
LIDNYQLRARSFGEEPYRKLRIYPE
>Q94FY8
MNLAVAAALPSVTPRTGVVLPRSSRRHCPRGVVPRAASSSVSSFTSPSAAAAPIYTPTPQ
DRSLRTPHSGYHFDGTARPFFEGWYFKVSIPECRQSFCFMYSVENPLFRDGMSDLDKLLY
RPRFTGVGAQILGADDKYICQFSEKSNNFWGSRHELMLGNTFISNKESTPPQGEVPPQDF
SRRVLEGLQVTPIWHQGFIRDDGRSNYVPNVQTARWEYSTRPVYGWGDVKSKQLSTAGWL
AAFPFFEPHWQICMARGLSTGWIEWDGERFEFENAPSYSEKNWGGGFPRKWYWIQCNVFP
GASGEVSLTAAGGLRKIGLGDTYESPSLIGIHYEGQFFEFVPWTGTVSWDIGLWGLWKMS
GENKTHLVEIEATTAESGTALRAPTIEAGLVPACKDTCYGDLRLQLWEKKYDGSKGEMIL
DATSNMAALEVGGGPWFNGWKGTTVVNEVVNNIVGTPVDVESLLPIPFLKPPGL
>Q38732
MATINLSLLPKTLTPNSKTLAPLLSILSTSSLSFLPCTRPHPIKSRSAAYPTVRALTDGE
YSSRRNNNNNNSGEERETIMLPGCDYNHWLIVMEFPKDPAPTREQMIDTYLNTLATVLGS
MEEAKKNMYAFSTTTYTGFQCTVTEETSEKFKGLPGVLWVLPDSYIDVKNKDYGGDKYVN
GEIIPCQYPTYQPKQSRSSKYKSKAYVRQRDGPPAEQRRPKQEATPESST
>Q9TKU9
MAVPKKRKSKSRANSQNHVWKREIVKQARRAVSLAKALLGGNTNFLLVSPGPTTPIKPNP
KKQTGRRPRSQRRRT
>O78420
MRHLFKLLFLYMQLNLLFYIPNKKSFLQLGEEKVYLTFQNSNTNLNIPYDSLLHKLNSDK
NVFSKESYDNWSSFRQLIIDKYGRIQGILTESSKNSNLKNSIQLNNYLLQKTIYYEFLCI
EFQLY
>Q9MTP2
MKFRVKGKSVDFKNREGSSPSIPKKRSFDSLPINPYFVLFVLLSVQNSLSFFYSFYSFPK
GCKRLFFSINPRLVLNMLHVQKNFVLARDQHLNDSQSIISILGLPLKKSFFFFFIFLSKK
ALGLGKTFHTFFVFLLIDIERSHLVKCQGSG
>P05723
MICFKQQKITSLFVSLILLLTTILICWFCVLSPKVNGTNSPIEVNVYNPIPLAVMRGGIP
LPGMPPVPTATPSLPRSGFTSSAKKIKESRKQKSTALQAVKDQYILRVARL
>P28645
MALNLNPVSTPFQCRRLPSFSPRQTPSRRSPKFFMASTLSSSSPKEAESLKKPFSPPREV
HVQVTHSMPQEKIEIFKSLEGWAEENLLVHLKPVEKCWQPQDYLPDPASEDFRDQVKEIQ
ERAKEIPDDLYVVLVGDMITEEALPTYQTMLNTLDGAKDETGASPTSWAVWTRAWTAEEN
RHGDLLNKYLYLSGRVDMRSIEKTIQYLIGSGMDPRTENNPYLGFVYTSFQERATFVSHG
NSARLAKEHGDLKMAQICGIIASDEKRHETAYTKIVEKLFEIDPDATVLAFADMMKKKIS
MPAHLMYDGRDDNLFDHFSAVAQRLGVYTAKDYADILEFLVGRWEVEKLTGLSSEGQKAQ
DYVCSLPPRIRRLEERARERAKQAPSMPFSWIFDRQVKL
>P51382
MIINIHINFFMTTISSVFKKLDKQCALIPFITAGDPDLVSTSKALKILDQHGADIIELGL
PYSDPLADGPIIQAASSRALKQSINLNNILDMVNITSSNIVAPIVLFTYYNPVLNLGINN

FISAISRAGIKGLLIPDLPIEESDYIISVCKLFNIELIFLLSPTSSIERINKIVEQAPGC
IYLVSTTGVTGQKPELTGKLKRLTETIKKMTQKPIILGFGISTAEQIKEIKGWNINGIVI
GSAFVKRLSSDFPEEGLRKIKDFCTTAKSIIIS
>O20143
MIKKHFKKITEHFSLVKISAILVGVVCGLRVYCSLNNKGAANLNFSQDEFFFKPTVAQIA
HESRVSKPMSHFFDEANTGNSNEERKVMFLSPINPATSSASPKKFKWVNTISIGPSLSLN
QNSVSSSVNTSPAREKISEIREKVKEFGEELASNDLVPTQEDVRGWEDLGNRYLEYLELK
LKPQLEQHNLAGFQFVNYGNLAERAKDMLANNTLCGLKVVKTNGESKLISISQFNKIFTI
LGENQLELEHHTISTLQDVRLMAQGVQQKQAVAMRLEPTLQNLLTTLRNQISNFEKTENW
TIEPSFKNLMVYTAPSPFRLQGSMCCHLGEKSLSLGVGETTFTQFSESNPHFGLITGWNF
GPKFSFGQETGLVSFSGYGIHAFCGLGFRI
>Q6B949
MMAFWKIFFKHHNIFSSNLNNQLVNQDAADKILLVKYLEGRGTIVNVYLSFNSYVNLHDL
EKLCDSVGWVRRPLKKVKIAIDNSFVTASLFYEQNKKKFLIGFARATSDTSFNATIWDVV
IHPDFQGQGLGKMLMAQIIKQLRYEDINTITLFADPQVVNFYKHLGFITDPDGVKGMFWY
PL
>Q9MTM9
MGFDSVASGLTGLEPAASALTGRCSDQLNYNPREIKKYTYSYNCILTISI
>P48269
MNFVNLEQIENSLRNATFCMLFLTTFLYWFYTAFYSTNPQQIINPLSLTNIKTNFTYPDG
FQANNINVSTTYLPINPVLNTEREEQPEANGTNGLLGVSSLVVNLKSIAIPRIMMGVSNL
LLVLLLLVRWEKSGHFPLSNLYESLMFLAWCCTFLYLLYCTSFTLLVEKMLGSLIAPCSL
LMNAFATFSLPKEMQQASPLVPALQSNWLMMHVTVMIISYATLIIGSLLSILFLILFKHK
KGTPKKYDNFINNLDALSYRIIGLGFPFLTIGILSGAVWANEAWGSYWSWDPKETWALLT
WLVFAIYLHTRLTKGWEGEKPAIIAAVGFLVVWFCYLGVNLIGEGLHSYGFFN
>Q9CAF6
MTPVLCHSTASIPNPNSLMSLSSTLRLSSSLLRRSFFRFPLTDPLCRLRRTEPSATRFFS
SRTPRSGKFVVGAGKRGDEQVKEESGANNGGLVVSGDESRIVPFELHKEATESYMSYALS
VLLGRALPDVRDGLKPVHRRILFAMHELGMSSKKPYKKCARVVGEVLGKFHPHGDTAVYD
SLVRMAQSFSLRCPLIQGHGNFGSIDADPPAAMRYTECRLDPLAEAVLLSDLDQDTVDFV
ANFDNSQKEPAVLPARLPALLLNGASGIAVGMATNIPPHNLGELVDVLCALIHNPEATLQ
ELLEYMPAPDFPTGGIIMGNLGVLDAYRTGRGRVVVRGKAEVELLDPKTKRNAVIITEIP
YQTNKATLVQKIAELVENKTLEGISDIRDESDRNGMRVVIELKRGGDPALVLNNLYRHTA
LQSSFSCNMVGICDGEPKLMGLKELLQAFIDFRCSVVERRARFKLSHAQQRKHIIEGIVV
GLDNVDEVIELITKASSHSSATAALQSEYGLSEKQAEAILEITLRRLTALERKKFTDESS
SLTEQITKLEQLLSTRTNILKLIEQEAIELKDRFSSPRRSMLEDSDSGDLEDIDVIPNEE
MLMAVSEKGYVKRMKADTFNLQHRGTIGKSVGKLRVDDAMSDFLVCHAHDHVLFFSDRGI
VYSTRAYKIPECSRNAAGTPLVQILSMSEGERVTSIVPVSEFAEDRYLLMLTVNGCIKKV
SLKLFSGIRSTGIIAIQLNSGDELKWVRCCSSDDLVAMASQNGMVALSTCDGVRTLSRNT
KGVTAMRLKNEDKIASMDIIPASLRKDMEEKSEDASLVKQSTGPWLLFVCENGYGKRVPL
SSFRRSRLNRVGLSGYKFAEDDRLAAVFVVGYSLAEDGESDEQVVLVSQSGTVNRIKVRD
ISIQSRRARGVILMRLDHAGKIQSASLISAADEEETEGTLSNEAVEAVSL
>Q02073
MAATHLTSTSSLTINTLPSFEGLRSASGISKINVSVAYPSFTSRSFRGLVVRAASITTSK

YTSVKPLGDRVLIKTKIVEEKTTSGIFLPTAAQKKPQSGEVVAIGSGKKVGDKKLPVAVK
TGAEVVYSKYTGTEIEVDGSSHLIVKEDDIIGILETDDVKDLKPLNDRLLIKVAEVENKT
SGGLLLAESSKEKPSFGTVVATGPGVLDEEGNRIPLPVCSGNTVLYSKYAGNDFKGVDGS
DYMVLRVSDVMAVLS
>P17597
MAAATTTTTTSSSISFSTKPSPSSSKSPLPISRFSLPFSLNPNKSSSSSRRRGIKSSSPS
SISAVLNTTTNVTTTPSPTKPTKPETFISRFAPDQPRKGADILVEALERQGVETVFAYPG
GASMEIHQALTRSSSIRNVLPRHEQGGVFAAEGYARSSGKPGICIATSGPGATNLVSGLA
DALLDSVPLVAITGQVPRRMIGTDAFQETPIVEVTRSITKHNYLVMDVEDIPRIIEEAFF
LATSGRPGPVLVDVPKDIQQQLAIPNWEQAMRLPGYMSRMPKPPEDSHLEQIVRLISESK
KPVLYVGGGCLNSSDELGRFVELTGIPVASTLMGLGSYPCDDELSLHMLGMHGTVYANYA
VEHSDLLLAFGVRFDDRVTGKLEAFASRAKIVHIDIDSAEIGKNKTPHVSVCGDVKLALQ
GMNKVLENRAEELKLDFGVWRNELNVQKQKFPLSFKTFGEAIPPQYAIKVLDELTDGKAI
ISTGVGQHQMWAAQFYNYKKPRQWLSSGGLGAMGFGLPAAIGASVANPDAIVVDIDGDGS
FIMNVQELATIRVENLPVKVLLLNNQHLGMVMQWEDRFYKANRAHTFLGDPAQEDEIFPN
MLLFAAACGIPAARVTKKADLREAIQTMLDTPGPYLLDVICPHQEHVLPMIPSGGTFNDV
ITEGDGRIKY
>P82244
MATLSLLSTGVGAAITNRTPSASLTFITGSRTTNKRVSFNGGSARSGSLHCSFLAPSSSL
SSNFSGLSLGLDLTSNTGVSTDRCRRFVVRAGKAALCLTKRSRSRKSLARTHGFRLRMST
TSGRALLKRRRAKGRKILCTKTNPSSGKRASP
>Q1XDC4
MKRQLNLRFNRKDIRWYLLRLFSNLQFSIILLLLIAIFSTIGTVIEQNKESSFYQTQYTL
SNEYYNILNWKNIELFGFNHVYTTWWFLSLLFIFSLSLFTCSISRQIPSLQNARRWHFYK
NPNQFKKFTGSQEIKTTQLNLLASCLQNYNYHIFQQGKSIYGYKGLLGRLAPIFVHGSII
LLLTGSVLGLVSGFSAQEMVPSGELFRLQNIISSGKFSYIPQEFSARVNDFNIEYNPNKS
ISQFFSDISILNSEGKELKRSTIYVNKPLEFHGLTIYQTDWDIIAIRVRINNGNILQIPL
KSVLLPNNNKIWIGVLFQEKESQLSVVLSDLQGQATIYNKNGKNILSINIGEKYIINNST
ITFLNTIASTGLQIKNDPGIPIVYASFFFLITSISVSYISYSQIWIVEKNRHFYIGGVTN
RAQLMFEEELLKISKMSSSI
>Q01572
MRPIHICLNVRFLFFRPIYACNIDFITLAFAVRKSLFDDPLNPLAIFKTLFLSYSSYTTS
FNSHESPCIKSFTNSTLKINIYKILVNLPYLQK
>O20162
MVTEFRDAWEARYGLVPGACCLKNSVTFSSLESGQGLERNPNQEALADRKNKKCQFKNEY
VACHATVFPHFVNPENPKAVEFLKNHPLNIPVNMDMTFGPLKDTPAFCYGGHKLGLCNSL
YDSIIYDDTYQGKGEHNLKKFFSEAIATCFLYQNAIKPINYKPKNMKHSVIGVSLYY
>A2Y3Q5
MWSSSQASTRGVIEVGRVEAGPSHFPKRPAPRNSSRVNLSRTYAIKSCSVSSRTGLCLGQ
CYHKKSSACKCKLGWSSQPLSSLRHHLRVHSSASEAVLTSQSDFTKLLVGNEKIGVLLLN
LGGPETLDDVQPFLFNLFADPDIIRLPRLFRFLQKPLAQFISVVRAPKSKEGYASIGGGS
PLRQITDAQAEALRKALCDKDIPAKVYVGMRYWHPFTEEAIEQIKRDGITKLVVLPLYPQ
FSISTSGSSLRLLEGIFREDEYLVNMQHTVIPSWYQREGYIKAMATLIEKELRTFSEPQK
VMIFFSAHGVPLAYVEEAGDPYKAEMEECVDLIMEELEKRGITNSCTLAYQSRVGPVEWL

RPYTDETIIELGQKGVKSLLAVPISFVSEHIETLEEIDVEYKELALESGIKHWGRVPALG
CEPTFITDLADAVIESLPYVGAMAVSNLEARQPLVPLGSVEELLAAYDSKRDELPPPVTV
WEWGWTKSAETWNGRAAMLAVLALLVLEVTTGEGFLHQWGILPLFH
>Q8SAY0
MLASPALAGARAFAATVSGSLGIPIPAISAPSPSQARRRASLVVVAKVKVSTPQADRIAR
HVRLRKKVSGTTERPRLSVFRSNKHLYAQVIDDTKSCTLVSASTMHKSLSKDLEYSAGPT
VEVAQKIGEVIAKSCLEKGITKVVFDRGGFLYHGRIKALADAARENGLDF
>P16096
MASASLLKTSPVLDNPEFLKGQTLRIPSVAGVRFTPSGSSSLTVRASSYADELVKTAKTV
ASPGRGILAMDESNATCGKRLASIGLENTEANRQAYRTLLISAPGLGQYVSGAILFEETL
YQSTTDGKKMVDVLIEQGIVPGIKVDKGWLPLPGSNDESWCQGLDGLACRSAAYYQQGAR
FAKWRTVVSIPNGPSALAVKEAAWGLARYAAITQDNGLDPILEPEIMLDGEHGIDRTFRV
AQQVWAEVFFNLAENNVLLEGSSLKPSMVGPGALSARKGPPEQVADYPLKLLHRRRGPVV
PGIMVLSGGQSEVEATLNLNAMNQSPNPWHVSFSYARALQNTCLKTWVEGQENVKAQDFA
CAKSNSLAQLGKYTGEGESEERKKDMFVKATLTY
>Q9MTN9
MFVGPTILLSSDLDKQGYQISNKVRLLIPRRSASSSMPPIGTHGAGLTCNT
>O80333
MPKRSNGSLVVRCAVSVVRFSKENVSCDLASENFTFSRDSFPVVSTVLRVSEAVYRMAAV
ASLGSALSVSSAALSQNVSVSNNATKESAFLGLRMGEVAKFGGALLSVSTVAANLKSKPG
VLSVNAVTAPAETMNKPSSKKTATKSTCIITGASSGLGLATAKALADTGEWHVIMACRDF
LKAERAARSVGIPKDSYTVIHCDLASFDSVRAFVDNFRRTERQLDVLVCNAAVYFPTDKE
PKFSAEGFELSVGTNHMGHFLLARLLMEDLQKAKDSLKRMIIVGSITGNSNTVAGNVPPK
ANLGHLRGLAGGLNGVNSSSMIDGGEFDGAKAYKDSKVCNMFTMQEFHRRYHAETGITFS
SLYPGCIAETGLFRNHVTLFRTLFPPFQKYITKGYVSEEEAGKRMAQVVSDPKLSKSGVY
WSWNKDSGSFENELSEEASNPEKAKRLWELSERLSGLV
>Q9MTM6
MNLQAHLIVNGSNLSGLLAIGTEKPHFVFSIYRENRKESNKREGKFFYISVLRYYTYNAR
KGWGQCKKRRKSFGPGMKSNGIQG
>P46248
MASLMLSLGSTSLLPREINKDKLKLGTSASNPFLKAKSFSRVTMTVAVKPSRFEGITMAP
PDPILGVSEAFKADTNGMKLNLGVGAYRTEELQPYVLNVVKKAENLMLERGDNKEYLPIE
GLAAFNKATAELLFGAGHPVIKEQRVATIQGLSGTGSLRLAAALIERYFPGAKVVISSPT
WGNHKNIFNDAKVPWSEYRYYDPKTIGLDFEGMIADIKEAPEGSFILLHGCAHNPTGIDP
TPEQWVKIADVIQEKNHIPFFDVAYQGFASGSLDEDAASVRLFAERGMEFFVAQSYSKNL
GLYAERIGAINVVCSSADAATRVKSQLKRIARPMYSNPPVHGARIVANVVGDVTMFSEWK
AEMEMMAGRIKTVRQELYDSLVSKDKSGKDWSFILKQIGMFSFTGLNKAQSDNMTDKWHV
YMTKDGRISLAGLSLAKCEYLADAIIDSYHNVS
>P27608
MALSSSSTTNSLLPNKSQLVQNQSLLPSPLKNVSFTTNSTKPVRFVQPISAIHSSDSSKN
PIVSDKPSSKPSPPAATVTAAATTVTKTEWTVESWKSKKALQLPEYPNQEELQSVLKTIE
EFPPIVFAGEARSLEERLGEAAMGRAFLLQGGDCAESFKEFNANNIRDTFRILLQMGAVL
MFGGQMPVIKVGRMAGQFAKPRSDNFEEKNGVKLPSYRGDNVNGDAFDAKSRTPDPQRLI
RAYCQSAATLNLLRAFATGGYAAMQRINQWNLDFTEHSEQGDRYRELANRVDEALGFMAA

AGLTVDHPIMKTTEFWTSHECLLLPYEQSLTRLDSTSGLYYDCSAHFIWVGERTRQLDGA
HVEFLRGVANPLGIKVSDKMDPSALVKLIEILNPDNKAGRITIITRMGAENMRVKLPHLI
RAVRRAGQIVTWVSDPMHGNTIKAPCGLKTRPFDSIRAEVRAFFDVHEQEGSHPGGVHLE
MTGQNVTECIGGSRTVTFDDLSSRYHTHCDPRLNASQSLELAFIIAERLRKRRLGSQNVL
GQ
>Q9TM10
MQTIAIKDFIDIKYIKQDQISEIIISRPQVLNAFRPRTINEIIAAFYDSREDSSIGVVIL
SGHGSRAFCVGGDQKIRSKTGYIDEKGRSSLNVLELQRIIRTFPKPVIAKVSGYAVGGGQ
ILNMMCDLTIASENAVLGQSGPKVGSFDAGYGSAYMARIIGQKKARELWFTCYQYDAFEA
YKMGLVNWVVKIEDLDSYAIKLATDILNKSPLAIRFLKSSLNADCDGQSGLQELAGYSTM
LFYMSAEGQEGHRAFLENREPDFSKFNS
>Q43064
MTASSSLFSCSMHMEVLTPKISKWPKDFVSCHSKISYVETNYLKSTCYPISRFLCINNLS
KCDKMIKTRQRDGIHCFSEGQKFQLDDVIEAQQFDRDILNAIFEIARDMENIERNSPESQ
ILKGYLMATLFYEPSTRTRLSFESAMRRLGGEVLTTENAREFSSAAKGETLEDTIRTVEG
YSDLIVLRHFESGAARRAAAIAGIPIVNAGDGPGQHPSQALLDVYTIEREIGKLDGIKVG
LVGDLANGRTVRSLAYLLAKYKDVKIYFVSPEVVKMKDDIKDYLTSKGVDWEESSDLVEV
ASECDVVYQTRIQKERFGERLDLYEKARGKFIVNQNILNAMQRHAVIMHPLPRLDEITVD
VDADPRAAYFRQAKYGLYIRMALLKLLLVGW
>Q06J65
MKKYNKSFLIDVNCETLKVRQAFPFTALIGQTNMKLALILNVIDPKIGGVMSMGDRGTGK
STIVRSLVDLLPNIQVVSEDPFNSDPIDYDLMSQEVKEAKKSGEKIYTTSIKTPMVDLPL
GATEDRVCGTIDIEKALIDGTKAFEPGLLAKANRGILYVDEVNLLDDHLVDILLDSAAGG
WNTVEREGISIVHPARFILIGSGNPEEGELRPQLLDRFGMHVRIKTIKDPLSRVKIVERR
SNFDKSAESFLKNYEYLQDFLKNRIVNAQGSLKDIKIDYQYKVNIAELCSNLNIDGLRGD
IVTNRAVKAFVALNSRKTVLDKDVFTIMSLCLTHRLKKNPLESIDSSQNIVTIFKDIFGY
SDLTA
>P82413
MASKVLPQALLVIPSNHSLQCPPLKKQLGFPIDSNRRFSLSSNCRSNLMVSRASSNLFSS
NFSSIFSFPARNSFVVRSEAEDSSDAPAESVAVVAEEELPVESEAEAEERPPRQQRVKLG
DIMGILNKKAVHAAEELRPVPGIRTGDIVQIRLEVPENKRRLSVYKGIVISRQNAGIHTT
IRIRRIIAGVGVEIVFPLYSPNIKEIKVVSHRKVRKARLYYLRDKLPRLSTFK

(6) 79 cyanelle proteins ($\mathbb{S}_6$)
>P48331
MKTTKSFIIIWTFIGFLLNLLALFTPFELPLFDIADKTNTTYLRMGISFQVASNLFISCV
SGATPAKYAQILYLIIGIFGEPLYMTGNSYDMTQDPSWGYVIGSFGASERAGEKAFEKKL
SLLNILISSYSGLFVIHAYGAIGLLLHSKSWEHWKSYLIMYSLIPLPSQLVMIFLTSILA
FLFRKILD
>P48326
MTFDYTNSFYNYDQYNNKKFDIVVLKPLKEEEIEYYLMQSLDALRINKAVVFNFEHIDYP
LAQRILDSLAGATYALGGDKTCIREKLYFFVPKTVKLQAPNNETPSFF
>P48135
MGQKEQTLAYGTGRRKTSVAKVRLIPGKGEILINSKPANLYLQYNLSYLAAVKSPLESLG
LANEYNVIVNTQGGGLTGQSEAIRLGLARALCQISLTHRPLLKSDHHLTRDARAKERKKY

GLHKARKAPQYSKR
>P48119
MTQLTDTSLIPFDLLEIQKASFKWFFEEGLIEEIENFSPVIDLRGNFEVHFYLKNYELEA
PTFTLEEAKQRDLTYAAQLYIPVELRDLETGIVKQDRIFFGEIPLMTDRCTFLINGVERV
IINQIIRSPGIYYSVNTDEQGTRTYDVTIISNRGAWLKFEVDKDDLIWMRIDKTHRLPCH
IFLKALNLSESEILSALTHPEFLQKTIYEHGDCTEEEALIEFHRQLRPGEPPNSIQGRYL
LYSRFFDPKRYDLGFVGRYKLNKKLNLNVSPNIRILTTQDILEILNYLINLQFGMGQIDD
IDHLGNRRVKAVGELLQSQLRIGLNRLERLIHDRLSMLTTKSFKKRKKLTTLINAKPINE
CLKEFFGSSQLSQFMDQTNPLAELIHKRRLTILGPGGLSRDRAGCAVRDIHPSHYGRICP
IETPEGQNAGLVGSLTTHAHLNQYGFIETPFYKVEKGYIQKELGIIYLTADEEDQYRIAA
ADVLINETNKIIGEDISVRYRQEFITTKIDQIDFIAISPLQTFSVSTSLIPFLEHDDANR
ALMGSNMQRQAVPLLHAEKPLVGTGLEFQVAKDSRRVLINKSEGLVKRVTGDHICIQTDT
GKEINYTLIKYQRSNQDTCITQRPIVFEGERVKKGQILADDTATDKGELALGQNLLVAYM
PWEGYNYEDAILINERLVYEDVYTSIHIEKYETETRQTKLGIEEITREIPNVNEYSLRNL
DEKGIVSVGSWIKGGDILVGKVAPKAESDQPPEGKLLKAIFGEKNRDVRDTSLRMPSGEK
GRIVDVRIFIREFGEMLYPGANTANTIVRIYIAQKRKIQVGDKMAGRHGNKGIISKILPR
QDMPYLPDGTPIDIILNPLGVPSRMNVGQVYECLLGWAAEHLGVRFKLIPFDERFGKQAS
RTFIHEKLKEAKELTNKNWLFNSNHPGKIQLFDGRTGESFDNPIMVGKAYMLKLVHLVDD
KIHARSTGPYSLITQQPLGGKAQQGGQRFGEMEVWALEAFGAAYTLQEILTIKSDDMLGR
NEALKAIVKGKAIPRPGIPESFKVLMRELQALCLDVVAYKIQNGDQDECEALAIDLSGSL
ATPIDNIIKEVIQQEELGEELTS
>P48134
MQVITLQKPSKKITYRTTYILKPSLTEEEITKEIEDYQKLLIDNGAEDIIIENTGKCRLA
YLINKQNDGIYFQITYRGANTLVNILQRRMRLSINTLRYQTFKV
>P17009
MASNQQLKIRIQLRSYDSSLLENSCEQIIEAAKRTDATAVGPIPLPTKKKIYCVLRSPHV
DKDSREHFEIRVHRRIIDLYLPSSKTIDTLTRLDLPAGVDVEVKL
>P23405
MIQPQSYLTAADNSGARKLMCIRVLGGGNRRYARIGDVIVAVVKDGIPNIPIKKSDTVKA
VIVRTRKELKRDNGMNICFDDNAAVIINADGNPRGTRVFGPVARELRDKNFTKIISLAPE
VL
>P48100
MNQINNSTLTFQCETGNYHTFCPISCVAWLYQKIEDSFFLVIGTKTCGYFLQNSMGVMIF
AEPRYAMAELEEGDISAQINDYEELKRLCTQIKNDRNPSVIVFIGTCTTEIIKMDLEGIA
PKLEAEIGIPIVVARANGLDYTFTQGEDTVLASLIQRCPSKDRETEKINKNNSSLFPSLS
IFSKNKNESNLSQDKPNLPLVLFGSLPNSLTNQLEHELEKQNIKISGWLPTKNYKELPVI
HEGDYVCGVNPYLARTATNLIRRRKCKLISAPFPIGPDGTRAWIEKICSIFNIEPTGLDE
REKAVWDSLENYLPLVKGKSVFFMGDNLLELSIARFLIRCGMIVPEIGIPYLHKRYQEAE
IKLLEDTCRKMQVPTPLIIEKPDNYEELKRIEQYRPDLVITGMANANPLEARGINTKWSV
EFTFAQIHGFSNARDILELVTRSLRRKNYIQQLGWKELVE
>P48125
MKKRSRRMQELVGKVEEQLYEPLEAIQLLKQISTAKFIETVEVHCRLGIDPKYNDQQLRA
TVTLPNGTGQTLRVAVITRGEKINEASTAGADLVGAEELIDEIQNGRLDFDCLIATPDMM
PQVAKLGRVLGPRGLMPSPKGGTVTFDLAQAINDFKAGKLEFRNDRTGIVHVLFGKANFS

EEALLGNLKAVQEAVDRNRPSGVKGKYWKSIYITSTMSPSIEVDYNLLRDLKLTENT
>P48254
MPIGYPLVKAMDKDRFISYFLINNALLNERVIFLCNYEDATDESIYIGMLLYLESENSQK
PVSFYINSSITFPNLCFGLYDTILQIKADIVTICLGLAGGMSSLILAAGTKGQRFALPNS
RIMMQEPLIDGGVNGQATDLAIEAKELMDTKEILINLYHERTGQPKPVIEKDLQRPRYFS
AQAAKEYGFIDSLLMASNG
>P48275
MSHLTQVQTNIVNEEALKKALNALNIKWQDGPQNIKNLYGQEQTVDILIKQKNNFDIGFV
KEELEYKLIADLQYWEQPYTVETFLQQLKQQYAYQIILDETINKGFEKLEEEYDKDGSIR
LVVQRWRY
>P48124
MSSKTDEILEQLKGLTLIEAADLVKAIEEAFGVDASAPVGGGMVMMAPAAGGGADAAEEK
SAFDLVLEEVPADKKIAVLKVVRSLTNLGLKEAKDLVEATPKVVKEGASKDELKQLKKNL
KQLEQK
>P24312
MSSQARTQTRAGFKAGVKDYRLTYYTPEYTPKETDILAAFRMTPQPGVPPEECAAAVAAE
SSTGTWTTVWTDGLTSLDRYKGRSYGFEPVPGEENQYICYVAYPLDLFEEGSVTNMLTSI
VGNVFGFKALRALRLEDLRIPVGYSKTFQGPPHGITVERDKLNKYGRALLGCTIKPKLGL
SAKNYGRAVYECLRGGLDFTKDDENVNSQPFMRWRDRFLYVMDAIKKSQAETGEIKGHYL
NATAPTTEEMIKRAEFAAELDAPIIMHDYITAGFTSNTTLARWCRDNGPLLHIHRAMHAV
IDRQKNHGIHFRVLAKTLRMSGGDHLHSGTVVGKLEGDRAGTLGFVDLMRDDHIEQDRSR
GIFFTQDWASMPGVMPVASGGIHIWHMPALVDIFGDDSCLQFGGGTLGHPWGNAPGAVAN
RVALEACVQARNEGRNLAREGNEIIREAARFSPELAAACEVWKEIKFEFETIDTI
>P48078
MSLSEQAILEQDKNIVSNIVQDILCEQISIEKTELNLDLYIYEDLKIDSLDLVEIIKQIE
EKFDIKIDDSKILYMNTLEEFIDFTLQTIYMKHGFEYLQNKQF
>P23406
MLSPRRTKFRKQQRGRMKGISTRGNNLVFGDFGLQALEPAWITSRQIEASRRAINRYVRR
GGKIWIRIFPDKPVTMRPAETRMGSGKGAPEYWVAIVKPGRVIFEINGVSQEMAKAAFRI
ATFKLPIKTKFISSRV
>P48255
MSTEKTKILEVKNLKAQVDGTEILKGVNLTINSGEIHAIMGPNGSGKSTFSKILAGHPAY
QVTGGEILFKNKNLLELEPEERARAGVFLAFQYPIEIAGVSNIDFLRLAYNNRRKEEGLT
ELDPLTFYSIVKEKLNVVKMDPHFLNRNVNEGFSGGEKKRNEILQMALLNPSLAILDETD
SGLDIDALRIVAEGVNQLSNKENSIILITHYQRLLDYIVPDYIHVMQNGRILKTGGAELA
KELEIKGYDWLNELEMVKK
>P15760
MSVYRRRLSPLKPNQVIDYQDVELLRTFITDQGKILPRRVTGLTAKQQRAVTKAIKQARV
LALLPFVNRES
>P23404
MASKERVGVVIRNPQEKTVIVAVNNRVRHNKYSKIIIRTKKYQVHDHSHICKLGDEVKIS
EVKPISKTKRWIISEVLSSTVNPEKFGD
>P48323
MLFASNAIILKIKQRNSNSYIYTLYTYEEGLKEVIIFTNQKILCSNLKLYRIYFVLFKKG

APWDQIQRLYPLFFYPKIEQNPLKMIVLEYLSELIIYQLYSPENCTFIYFIFHYTLFQLN
ICSNNMILPIFISSLYSLLKFLGWEPELSNCVYTGESLNSENSNLTDSKIGFSASYGGIV
KLNVLPKQEYIGFFNKDELFIFTNDN
>P18062
MQLRVERKFETFSYLPPLNDQQIARQLQYALSNGYSPAIEFSFTGKAEDLVWTLWKLPLF
GAQSPEEVLSEIQACKQQFPNAYIRVVAFDSIRQVQTLMFLVYKPL
>P48132
MAIVTLDEMLEAGVHFGHQSRRWNPKMSQYIFTERNGIHIIDLVQTAQLLAEAYDFLRIS
AEEGKKVLFIGTKRQAANIIAQEATRCGAFYINQRWLGGTLTNWTTIQSRVEYLKELEMR
EESGALDLLPKKEAAILRRQLEKLRKSLGGIQTMRHIPDIVVIVDQKRENNAVQECKKLN
IPIIALLDTNSDPDVVDIPIPGNDDAIRSIKLIIGKLADAILEGSHTKTSSAE
>P48128
MTTYAIIDIAGKQLWVEPKRYYSVNKINVEIGKKIALQRILLLSKIGEKIKIGQPFLNGI
SINAIILRHFLAPKVIVYKMQPKKKTRRKRGHRQHLTSFLINDF
>P48118
MTQFQIECVESKVESSCNQYGRFIVEPLEPGQGITIGNSLRRVLLSDIEGSAITAVRIAG
VNHEFCSIPGVKEDVLELLLNLKNIVLKSYTNEPQIGRLKIQGACKVTTANFELPSEVEI
IHGTQYVATVAENAILEMEFKIEKGRGYKIIDKKQTENSIDFLPIDAIFMPVRKVNYMVE
EIFLRNSEIKERLILEIWRNGSVTPQDSISQAAEVLLNLFNPFKKINFENTDDSTEQTED
KISQIPIEELQLSVRAYNCLKRAQIHSIGDLLNVSQEELLEIRNFGQKSAKEVIDALQKR
LGISLPKRKTNKKEN
>P17245
MARQKFDGNKPHVNIGTIGHVDHGKTTLTAAITTALASQGKGKARKYDEIDAAPEEKARG
ITINTAHVEYETEKRHYAHVDCPGHADYVKNMITGAAQMDGAILVVSAADGPMPQTREHI
LLAKQVGVPNMVVFLNKEDQIDDADLLELVELEVRELLSKYDFPGDQIPFVSGSALLALE
SLSSNPKLMRGEDKWVDKILALMDAVDEYIPTPERPIDKSFLMAIEDVFSITGRGTVATG
RIERGAIKVGETVELVGLKDTKSTTVTGLEMFQKTLEEGMAGDNIGILLRGVQKTDIERG
MVLAKPGSITPHTQFESEVYVLTKDEGGRHTPFFSGYRPQFYVRTTDVTGSIDAFTADDG
SNAEMVMPGDRIKMTVSLVHPIAIEQGMRFAIREGGRTIGAGVVSKILK
>P14810
MYKLKTRKAAAKRYKAVGNKKISRRKAFRSHLLQKKSTNRKRQLSQVVIASPGDTKKIYL
MLPYL
>P17292
MSRRSTAKKRLILPDPIYNSRLVTLLINHMLKDGKKSIARSFIYEALKIIEEKKGSDPLE
VLEQAVRNSTPLIEVKARRIGGSTYQVPMEVRVDRGITLALRVVNSFSLQRLGKTIAVKL
ANELIDAANETGNTIKKREEMHRMAEANKAFVHYRY
>P48332
MEFENQKFLVILITPMVLVLALDDFYVEEEFVINDKKLLRLVIEHIKEKGQIQKRKTQMP
LIIHNTRYVLRHIKKYT
>P48101
MKNNNRPIFPFTAIVGQEEMKLALLLNIVDPKIGGVMIMGDRGTGKSTTIRALADLLPEI
DIVANDPFNSHPTDIELMSDNVRQLKENGEEISLIQKKVPMIDLPLGATEDRVCGTIDIE
KALTEGVKAFEPGLLAQANRGILYVDEVNLLDDHLVDILLDSAASGWNTVEREGISIRHP
ARFVLVGSGNPEEGELRPQLLDRFGMHAEIRTVKDPTLRVQIVEERSEFDRSPEDFLQEY

KLQQEVLRQRIINAQQQLNNVQLNYEIKVKISQVCSELDVDGLRGDIVTNRAAKALAAFE
GRDEVTVDDVLRIITLCLRHRLRKDPLEEIDSGQKVEKVFQRIFSNL
>P48140
MANTKSAIKRIKTIERNRIRNCAYKSVVKTFIKKYLKVLSDYTNAPNSNGVENIQTTLGI
VYTKIDKAVKRGVYHSNKAARMKSKLALKYNVIKK
>P48271
MKVEELPFSLEQLRILKAIATEGSFKKAAESLYMTQPAISLQIQTLEKKLNIALFDRSGR
RALMTEAGHLLLRYGDCILALCEETCHALEDLRNLQGGTLVIGASQTTGTYLMPRLIGLF
RQRYPQVTVKLHVHSTRRISWNIANGQVDLAVIGGEVPHELQESLTITPYAEDELVLILP
KSHPFAKLESIQKEDLYRLKFIALDAQSAIRKVIDKVLAQNGIDSNLFRVEMELNSIESI
KNAVQSGLGAAFVSVSAIAKELELGILHWCRIENITIKRMLSIITNPNRYHSQAARIFSE
EILTMFSLQSPEVKKRALSIIQNT
>P31605
MNNKIDNFLKQKKLIKVISGLNNFNTTHVIKIAKAASKTNASFIDIAAAPKLVEKVKKEV
PNLPICVSAIKPELFVPCVKAGAELIEIGNFDSLYNQGYKINFSDVLSLVKQTRSLLPDT
PLSVTIPYLLPLNLQLELAYRLEDLNVDLIQTEGKINKITSLLDNRNIETILPTLASTYL
IANNVTIPVICASGLTISTIELPFKLNASGIGIGNAVSKLNSTEEMINLLNEISTRINYS
TVL
>P15766
MSIGILGTKLGMTQIFDEAGNAIPVTIIQAGPCPITQIKTTATDGYNAIQVGYRETKEKN
LTKAQLGHLQKTNNSALRVLQEFSIESSDSIEVEKPITVELFNDNDIVNIQGYSIGRGFS
GYQKRHNFARGPMSHGSKNHRLPGSIGAGSTPGRVYPGTRMAGRKGDSKITIRGLKIVKV
DSERSLLIVKGSVPGKPGGLLTITQVKKV
>P48329
MGLNYNQEDFMGLDRFFQDAVSHNNTDANAASSIEVEMYECDCMYPTFAEIARRSGQPEI
GAMFDAIAKEEGMHAQLLTKLYSELEVKDSAETLEAKRLVSTIESQIDAVASDSRGLRRA
LETALEVETIESQKTYPAFAKLAAEQGNMEVATAFEAIVKSETKHANWVKRALENLLEVA

>P48279
MSILVIGATGTLGRQIVRSALDEGYQVRCLVRNLRKAAFLKEWGAKLIWGDLSQPESLLP
ALTGIRVIIDTSTSRPTDPAGVYQVDLKGKKALIDAAKAMKIEKFIFFSILNSEKYSQVP
LMRIKTVTEELLKESGLNYTIFKLCGFFQGLIGQYAVPILDQQTVWITTESTSIAYMDTI
DIARFTLRSLVLKETNNRVFPLVGTRSWNSADIIQLCERLSGQNAKVTRVPIAFLELARN
TSCFFEWGWNIADRLAFTEVLSKSQFFNSSMDEVYKIFKIESTSTTILESYLQEYFSRIL
KRLKEINSQQSQKKKTSDLFV
>P48126
MAKKVIAIIKIVLPAGKATPAPPVGPALGPYGVSSVNFCKEYNAKTADKGGLVIPAEITV
YDDRSYSFVLKTPPASVLIAKAASVEKGSGEPNRKKVGSITKAQLEQIAKVKFPDLNTQN
LEAASRIVEGTARNMGITITD
>P15761
MARSLKKGPFIAHHLLKKVELLNTSGKTEVIKTWSRASTILPMMVGHTIAVHNGRQHLPV
FITDQMVGHKLGEFAPTRTFKGHTKSDKKARR
>P48120
MSENLFSKKKIFFNQTIGKKEVKNVIAWAFTSYGAARTAYLVEQLKDLGFHYATKAGISL

SVEDLLIPPLKDSLLQTAENEIKATLNRYLLGEITEVERFQKVIDIWHRTSETLKDEVVD
YFKSTDPLNSLYMMSFSGARGNLSQVHQLVGMRGLMADPQGEIIDFPIRSNFKEGLTTTE
YLISSYGARKGVVDTALRTADSGYLTRRLVDIAQEVIIREIDCETPRGVILTALKENGKI
LISLKDRLVGRVLLHNLYHPKTHSLIAQKNESISVLLADDIIKAGLKEVWIRSPLTCKAT
RSVCQYCYGWNLAHGRLVELGEAVGIIAAQSIGEPGTQLTMRTFHTGGVFTAEVAKQITA
PFPGRIYYPLNTTFREIRTRYGDNAWWVENNAKLRLEGENGKRVSFNLTQGSIIRIKDQS
WVETNDLLAEIASTAPNTRRAKEKTTRELRTDIAGEVYFQNLEIDEAGGQVDTAGGSIWI
LGGNVYNLSSNFDVILKNGDFILNGSILARTQFISQYGGYVRLTDDNLTVEVIPASLKIQ
NAKIFIDETDQPCLHFKNNETFELKIQNETQLKHGQVVAERYLTTEMGGIIRYADLDAKT
TNKNGYEITKSGSLLWIPEETHEVNTEAKNVLVKNGQYIPSGTQLIKNKKIRNKHSGVVE
LVHKKNFVIEIIIKPGIVLKIKNNVSFSKKAKRFIQPGEYLFSKFVVEDLRYLDYIITST
GIILLLRPVVEYKVESPKTIIRDDKNHLKISSIQSILYKDGERIKSVQGIELFKIQLKLQ
VRKGLKHLPIKTNFIPSSQNSFELEFLIIESILPKNDKYFDSTVLESLAKAQTKILVKNN
QLVKKGELIAQIEIISINQGEIRWIGDNTAKQIRRLLLITEKQIVTIPIQNLTKLKNKNL
NLGNFIRAGEEIEETIKIPNSGQIIEITSTYIRLRISRPYLISARAIIRVLTGDLVQSGE
SLALLVFERAKTGDIIQGLPRIEELLEARKPKDNCVLSTHPGFTQLYYSETIELKIKSLD
KINTLLELQPGVFPTVTNNQFIEAGAPLSEGDISVHEILEIFFNLYFKNRVNCLSLADAI
HLSLQKIQQFLVSEVQSVYQSQGIDISDKHIEIIIKQMTNKVKIEEGGDTTLLPNELIEF
QQIEKMNEKFSTSNGQLASYTPILLGITKSSLNTQSFISAASFQETTRVLAKAAVEGKID
QLRGLKENVIIGNLIPAGTGFSAYNDNAVFQNEDIESIEVKTSVLNSPAESDTNSDLDDI
ILNKD
>P48324
MGKPNLKDQIKQLYLVLLEEIKIFILTIKLVISKKNVWIISYYHFLKKVVVSKKQILNLS
RLLLRFTLLSIFILFVLDLISKYITAPILTGIFNFIFKILISLVNLLYKAIYWIYIIFAI
FLHFMNYLLVLLELTLQPIFEWIEIFFFNSLIYFLKLIFLLYSNFEKGILIFLMWLKSRI
KK
>P14809
MTRVKRGNVARKRRKKILKLASGFRGAHSRLFRVANQQVMKALRYAYNDRNKRKRDFRAL
WIARINASARLEGMTYSKLMGSLKKLNIILNRKSLSQLAIYDKDAFMEILKTIP
>P48129
MARQCELTGKKANNGYTVSHSHRRTKCLQKANLQTKRIWSPTLKRWLKLQVSTKVIKDLK
RKSIDALLKI
>P48261
MILLIDNYDSFTYNLAQYLSELNIKVLVKRNDKITLDEIKNLNIQGIIISPCPGGPEDSG
ISQGIIKYLGNQIPILGVCLGHQTIGHVFGGKIIKAPKLIHGKPSIIFHDGKGVFQNLKN
PITATRYHSLIIEKESCPDELEITAWTEDGLIMGIQHKKYKQLQGIQFHPESILTESGKQ
ILQNFINCLN
>P14808
MSRIGKRLINIPSQVTVSIKDQVFSVKGPKGELSKQIPYGIQVVQQNDHLVVERVAESLL
ARKLHGLCRTLVSNLVQGVFQGFERRLEIQGVGYRAQMDGKKLVLNIGFSHPVVIEPPTE
IQLQVENNTNIIIKGIDKELVGKLAAEIRAVRPPEPYKGKGIRYLGENVKRKVGKAGKK
>P15768
MATEVKAIAKYVRTSPQKVRRILDQIRGKSYKEAVMLLSVMPYKACSIILKIVDSAAANA
QVTKGFNKNKLIISKTFVDKGPTLKRFRPRAQGRGYKILKPTCHITVQVQDQSL

>P14807
MVQRLKTVYEQEVIKQLMTRFQYKNIHEVPKLKKITVNRGLGEAAQNAKILEASVKEITE
ITGQKAIVTRAKKAIAGFKLRQDMPIGVMVTLRGDYMYAFLDRLINLSLPRIRDFRGITA
KSFDGRGNYNLGLKEQLIFPEVDYDGIEQIRGMDISIVTTAKTDQEGLALLKSLGMPFAE
S
>P48136
MARQIKRSGTTKQKKNIPVGVAHIQSTFNNTIISITSPTGEVIAWASAGSSGFKGARKGT
PFAAQTAAENSARQAMEQGMRQIEVIISGPGSGREMAIKALQATGLEISLIRDITPVPHN
GCRPPKRRRV
>P48133
MSRYKGPSLRIIRRLGELPGLTRKVVKKRKYPPGQHGQKSRKRSEYAIRLEEKQKLRYNY
GLTERQLVQCVRRAKQMKGSTGQILLQLLEMRLDNIVFRLGMAPTIPASRQLVNHGHICV
NNKVVSIPSYQCKPGDIITVKERNASKKIVETNLLFPGLANLPSHLEFDKSKLQGKVNDI
IQREWVALEVNELLIVEFYSR
>P48335
MLYLRDHQPVAVLFDDNFEKIQGVVDRNGYVFKNVTPQQITTIEFLLNKKLLKVRGFKNI
IIRWAALYLLICDSQTKIYEVILEKGHDITFGTEAGYVFFGEYINMDQLRKRFFYMDHLH
NLWREGNLFSEVHVNLTKSAHCYIDLTENKTPILSMLDDVSSFRIKDDNYRWPSQLKDE
>P48274
MCICVNCDLLVFCSLYQTIEILHKESLSFELDQIVEFEPRKSNIIVNINSSENSIKHEWD
VISCESFVLNLSKYNVS
>P15769
MARAKGVRILVTLECTECRSNGERLGGGVSRYATKKNRRNTPNRLELNKFCPYCKKHVLH
REIK
>P48138
MAKKSMIERDKKRENLMNKYLVKRQQLKTRLNETDSVEEKLLLNQELQKLPRNSAPSRVR
IRCWLTGRPRGNYRDFGVSRHVLREMAHQGLLPGVTKSSW
>P14826
MVNDTIADMLTGIRNANLAKHKVARVKATKITRCLANVLKEEGLIQNFEEIENNLQNELL
ISLKYKGKKRQPIITALKRISKPGLRGYANHKELPRVLGGLGIAILSTSSGIMTDQTARH
KGCGGEVLCYIW
>P23407
MKLTRKQATQRRHRRVRRKVFGTSERPRLAVFRSHQHIYAQIIDDTQHRTLAASSTLEPS
VKNSELSSTSTCAASAIVGQLIAKKALEKGITQVVFDRGGKIYHGRVRTLAEAAREAGLQ
F
>P31179
MSIFLKNKQFENITSQEQTKNNYKQLINDIQTLKKDLNAIILAHYYQEPDIQDVADFLGD
SLGLAREAAKTNADIIVFAGVHFMAETAKILNPEKMVLLPDLNAGCSLADSCPPEIFSEF
KKAHSDHLVISYINCSASIKAMSDIICTSANAVDIVNKIPLTQPILFAPDQNLGRYVISK
TGRDLLLWPGSCIVHETFSEKKIFEFQSLYPTAEVIAHPECEPTILKHANYIGSTTSLLQ
YVKNSKKTTFIVITEPGIIHQMKKSCPEKQFLALPTVSGCACNECPHMRLNTLEKLYLAM
KTRSPQIEIPESILLNAKKPIERMLEMSN
>P48336
MQNSNWYKFFKNIKPEIISFSFLVSLLWFIRSPLKNTTELLEFEVEPKTIPFNQEFLNQV

YEKAKHKYIWNYSPTQIIHKLEKLINTRIRI
>P48259
MEINKKKILVVDDEISIRRILETRLSMIGYEVVTAADGEEALTIFQLEHPNLVVLDVMMP
KLDGYGVCQELRKESDIPIIMLTALGDVADRITGLELGADDYVVKPFSPKELEARIRSLL
RRTENPTLSTTSTNGENLQIGFLKIDINKRQVFKNGERIRLTGMEFSLLELLISKMGEPF
SRAQILQEVWGYTPERHIDTRVVDVHISRLRSKLEENPSNPDLILTARGIGYLFQNTSN
>P48127
MHTQQLISQIESQYLKDNLPEICVGDTVKVGVLIQEEDNKNSGEKERIQFYEGVVISLSK
LKNINGTIRVRRILQGIGIERTFLVHSPLIKSINIIRRSKVRRAKLYYLRTLTGKATRLK
QRLIKHYRLIY
>P48137
MARIAGVDLPRDKRVEIGLTYIYGIGLTRSKEILSKIELDPDTRVRDLPDNAIAQLREII
ETDYQVEGDLRRFESMNIKRLADIGCYRGRRHRLNLPVRGQRTRTNARTRRGARQTVAGK
KKAPSK
>P48139
MVKLRLKRYGRKGQVTYRIVAMNNLSRRDGKAIEELGFYNPRTNESSLNIANIKRRIEQG
AQPTNTVRYILAKANIL
>P48099
MKLAYWMYTGPAHIGTLRIASSFKNVHAIMHAPLGDDYFNVMRSMLERERNYTPVTTSVV
DRNVLARGSQEKVIDNILRKDKEEQPDLIVLTPTCTSSILQEDLQNFVEQASLNSMADVL
LADVNHYRINELQACDKTLEQIVRFYIEKNRASLTLEKVKTLLPSVNLIGISSLGFHNNH
DTRELKKLFELYEIILNCSLPQGTTVKTLINLPKAWLNILPYRELGLLTSKYLNKEFGLL
SLVILPMGNINLNRFLKKLLFSLKLENNTITKVINIKLLKLLNLNWIKKEAIKSKLKRKK
AIIFGSSNHVATLTKLLSKEIGLEIILCGTYCKSESKWFSEQVQNYCNKILITEDHTMIS
NEISKLKPDVIFGTQMERHIGKRLGIPCGVISSPIHIQNFPLSYKPMVGYEGVKTIMDLL
FNSLNLKDRKNSFTLFNEII
>P48276
MCTFCPIPTEQQPLNEYQILNNSVLFNWPSQKLKIYFFYLFTIYSIGFLLTFLITFYNDL
FIVHPVNIFVHGIIGGNFVLILDLLRLYLGWSYICQRLLSATVSYEESGWYDGQIWVKSS
EVLIQDRLIGIYQVRPVLNRLKQTLGVVILILGFTLLIQ
>Q37106
MGKIVGIDLGTTNSVVAVMEGGKPTVITNAEGFRTTPSVVAYTKTGDRLVGQIAKRQAVL
NPGNTFYSVKRFIGRKFDEVTEEAKQVPYKVTGTQNVRIECPALQTSFTPEEISAQVLRK
LAEDASKYIGEPVTQAVITVPAYFNDSQRQATKDAGKIAGLEVLRIINEPTAASLAYGLD
KKKNETILVFDLGGGTFDVSILEVGDGVFEVLSTSGDTHLGGDDFDKKLVDFFIEEFQKL
ENINLREDQQALQRLTEAAEKAKIELSSVTQTEITLPFLSLTPSGPKHFDTTLNRAKFEE
LCSTLIDRCRIPVETALKDAKLTKEDIDEVVLVGGSTRIPAVRELVQKVLDKVPKQSVNP
DEVVAVGAAVQAGVLAGEVKDIVLLDVTPLSLGVETLGGVMSKIIVRNTTIPTQKSELFS
TATDGQTNVEIHVLQGERGFAKDNKSLGTFRLDGIAPAPRGVPQIEVTFDIDANGILSVT
AKDKGTGKEKSITITGASTLSQDEVERMVQEAEKNAAQDKENRQKIELRNQAESLINQAE
RQLKEFKLEPNVQAPIEQTIQQLQTALKEDNLEQVQSLCQELQNSLLEIGKQVYSKTETT
TPNKNEEDVIDASFSEEK
>P48273
MSNFFTNVLRYPLFLISFSLGIFLTFFRWIEPITKKPLNLIVLIISFGTVLMFLNLTFKA

MLDLN
>P48077
MNIIVVGLSHKTAPVDFREKLSIPKVRIGEAIRELCNYPHIEEVAILSTCNRLEIYLLTS
DTYQGIREATQFLADSSDLSLPELRQHLFILLHQDAVMHLMRVTAGLDSLIIGEGQILSQ
VKQCYQLGQQYQGIGPVLNNIFKQAISAGKRVRTETQISTGAVSISSAAVELAQIKKQDL
RTANITILGAGKMSRLLVQHLLSKRVKDINIVNRSVERAKLLVDQFKEANINIYNLSELK
TILQNSDIVFTGTSSQEPIITPELINDCDNLPSELMLFDIAVPRNVDPNVSQFDNIKVFN
VDDLKVVVSQNQQTRRKMAKAAEILLEEELSAFNIWWGSLEAIPTINKLREKAEIIRVKE
LEKAISRLGNEFVSDHQEIVESLTRGIVNKILHDPMVQLRAQQDIEIRGRALKILQTLFN
LDTIKNGMSPTL
>P48358
MTNFQLISDNLKEEKIEFEILWIGQSELFYKGIESTLIDKYWQFLLLNDGSLTKQLQLLK
NKKIKRKLLEESPINFTTSFISYLTQPIKIPIIQRNIFLYSNEVEPLIYATSWWSENTIN
SFFLDKEQPIWSNLAQLKIEFYRDLRKILLINSKSLEKQFNKKGPFWSRYYLIWYNNFPI
TIIFEIFSPIIKV
>Q37757
MAKQILYHESARRALEKGMDILAEAVAVTLGPKGRNVVLEKKFGAPQIVNDGVTIAKEIE
LEDHIENTGVALIRQAASKTNDVAGDGTTTATVLAYAMVKEGMRNVAAGANPMAIKRGID
RATQKVVETIAAHAKPVEDSRAITQVASISAGNDDEVGKMIADAIEKVGKEGVISLEEGK
STTTELEITEGMRFDKGYISPYFVTDPERMEVIQKDAMFLITDKKITLVQDLVPILEQVA
RARKPLFIIAEDIEKEALATLVVNKLRGNLNVSAVKAPGFGDRRKAMMQDIAVLTNTQVI
SEQTGLRLDSIKLDVLGKARQVNITKDYTTVIAEGNEEGVSARCEQIRRQIEETDSAYEK
EKLQERLAKLAGGVAVIKVGAATETEMKDKKLRLEDAINATKAAVEEGIVPGGGTTLAHL
AIELENWVQSNLEHEELIGGMIVARSLTAPLKRIVENAGQNGAVIAEQVKDKPFNIGYNA
ANAQFVDMFEAGIVDPAKVTRSGLQNAASIAGMVLTTECIVVDKAESQQKAASVNRNSFD
Y
>P17294
MPTIQQLIRSKRTKIEKKTKSPALKACPQRRGVCTRVYTTTPKKPNSALRKVARVRLTSG
FEVTAYIPGIGHNLQEHSVVLVRGGRVKDLPGVRYHIVRGALDAAGVKDRRQSRSKYGAK
RPKA
>P48257
MNISTLQLILETFSFFIYFILTFLFFAQAIFKNHWLKTFNFSGMLIANVSIFLLLITRWI
TAGYFPISNLYESLFFLVWGLNTIWLFLYKNNMRIQLLDNSVSLLLTLLVGFLHFILPQE
MREISPLVPALKSNWLMMHVSVMMISYATLMIGSLLSIIYLYFLYRVDKLLNKINAVNLM
TSNQNSNSLIYNLSFNTLLNSSKSMEYQILLKDLSLIKLSKRLDNLSYRLITFGFPLLTI
GIIAGAVWANEAWGSYWSWDPKETWALITWLIFAIYLHTRIIKGWQGKKAAMVASLGFFI
IWICYLGVNLLGKGIHSYGWFF
>P31171
MASITNILAPVENELDLLTKNLKKLVGSGHPILSAASEHLFSASGKRPRPAIVLLISKAT
MENEIITSRHRRLAEITEIIHTASLVHDDILDESDVRRGIPTVHSDFGTKIAILAGDFLF
AQSSWYLANLESLEVVKLISKVITDFAEGEIRRGLNQFKVDLTLEEYLEKSFYKTASLLA
ASSKAAALLSHVDLTVANDLYNYGRHLGLAFQIVDDILDFTSSTEELGKPSCSDLKKGNL
TAPVLFALEQNSELIPLIQRQFSEPKDFEYTLQIVEETKAIEKTRELAMEHAQVAIQCLE
NLPPSSSKEALKLITKYVLERLY

>P15764
MAIRSYKAYTPGTRNRTISEFSEITKSEPEKSLTFLKHRKKGRNNRGIITTAHKGGGSKR
LYRIIDFKRDLKLVPAKVAAIEYDPNRNARIALLHYQNGEKGYILHARGLAVGNMVYSGP
NAPIEVGNSLPLSEIPLATEIHNIELTPGKGGQLVRSAGSSAQLLAKEGNYVTLRLPSGE
MRFVRKECYATIGQIGNAEISNISIGKAGRNRWLGIRPTVRGVVKNPVDHPHGGGEGRAP
IGRSTPVTPWGKPALGRRTRRTKKYSDNLIIRRRK
>P48262
MINSKNNSPIEISIIDYQMGNLYSVCKGIERSGGIPKIINSAEEIKKATALILPGVGSFD
PAMKQLKKQDLINPIKDAISEKKPFLGICLGLHLLFEESEEGIESGLGILSGSVKRIKNE
PEITIPHMGWNQLQLTNPKCYLWDGLTKYPWVYFVHSYFAQPKNPNVIAAAVTHGTQQVT
AAIQYENISAVQFHPEKSASIGLHMLNNFVQQSCN
>P42080
MPKHEQYFDYVKINLASPERIRQWGERILKNGEIVGKITKPETINYRTLKPEMDGLFCEK
IFGPVKDWECHCGKYKSIFYRGVICERCGVEITESQVRRHRMGYIELAAPVTHIWYLKGI
PSYISILLNKKVKEIEQVVYFNAYVVLNPGKSDLLNYGVILTEDDEKWPYIEEKLYTKEL
MDVELGIGAEAIQRLLSDLDLQAEAKTIREILISNSDKKKNTQKRAKLIKKLRIINNFIA
TKAKPEWMILSLIPVIPPDLRPMVQLDGGRFATSDLNDLYRRVINRNNRLLRLQEVFAPE
IVVRNEKRILQEAVDALIDNGRRGRIVVGAKNRPLKSLSDIIEGKQGRFRQNLLGKRVDY
SGRSVIVVGLQLKLYQCGLPWEMAIELFQPFVIHRLIHQGLVNNIKAAKKLIQSNDPIIR
DILEEVIQNHPVLLNRAPTLHRLSIQAFEPILVEGRAIQLHPLVCPAFNADFDGDQMAVH
VPLSLEAQTEARLLMLASNNLLSPATGQPIVTPSQDMVLGCYYLTVDNLKNQKGKGSYFI
NSEDVLIAYEQQRIDLHSYIWVRFDTFSFDDILEEKNIQQVLYKREIDEFGTVIKVYNTR
RIREDKDGFCLTQYIITTAGRVLFNKVVQKALIMEI
>P48260
MVNTQSPKNSGLENLVNQPYKYGLPLIFEIETISKGLTEETIRLISEKKNEPQFMLEFRL
QAYRKWLEMSNEPEWAHLNYPKINYQDMVYYSAPKQKKKLQSLDEVDPTLLETFEKLGIP
LTEQKRLANVAVDAIFDSVSVATTFKEELAKEGVIFCPISEAVQKYPDLIKKYLGSVVST
SDNYFSCLNAAVFSDGSFCYIPKNVRCPLELSTYFRINNGESGQFERTLIVADEGSYVSY
LEGCTAPQFDTNQLHAAVVELVALDNAEIKYSTVQNWYAGDENGKGGIYNFVTKRGLCAG
KNSKISWTQVETGSAITWKYPSCVLLGDNSIGEFYSVALTNRYQQADTGTKMIHIGKNTR
SRIISKGISAGHSQNSYRGLVKIGPKAVGARNYSQCDSLLIGDNSQANTFPHLQIKNPTA
KVEHEASTSKIGEEQIFYFLQRGINAEEAISLIISGFCREVFNNLPMEFALEADKLLGLK
LEGSVG
>P23402
MANRQKMSKTRDKKPDWQERVVQIRRVSKVVKGGKKLSFRAIVVIGNERGQVGVGIGKAS
DVINAVKKAAADGKKHVVEVPLTRSNSIPHPIDGIGGAARVIMRPSAEGTGVIAGGAVRT
VLELAGVRNILAKQLGSNNPLNNARAAMNALSRLKTFSQFAKDRGVIAE
>P23401
MGQKIHPIGFRLGITQKHRSCWFANPKQYPTLLQEDHLIRQYIEKNLSNAGIAQIYIQRK
ADRIELELRTARPGVVVGRGGRGIEVLRKGLKDLLGEQKQIRINVIEVKQIDAEAALIGE
FITQQLERRVAFRRIVRKAITRAQRRGIEGIKIQISGRLNGAEIARSEWSREGRVPLQTL
RAEIDYSYKRAQTIYGVLGVKVWIFKGEVIPGNPTEISE
>Q37761
MATVTLNVKTVRPLGERVLVKVSQSEEKTAGGILLPDTVKEKPQIGEIIAEGPGRRNDDG

SFQPLEVTVNSKVLYSKYAGTDIKLENEEYVLLSEKDILAIIA
>P48110
MKLAVYGKGGIGKSTTSCNISVALAKRGKKVLQIGCDPKHDSTFTLTGHLIPTIIDTLQE
KDFHYEDIWPEDVIYKGYAGVDCVEAGGPPAGAGCGGYVVGETVKLLKELNAFDEYDIIL
FDVLGDVVCGGFASPLNYADYCLIVTDNGFDALFAANRIAASVREKARTHQLRLAGLIGN
RTTKSDLIEKYTENVPIPILQLLPLIEDIRISRVKGKTLFEMSESNPELSPICDYYLNIA
DQILAKPEGIIPNEVLDRDLFTLLSDFYLNMDSSESSNLTLV
>P17007
MAVYKVRLICEEQGLDTTIECPDDEYILDAAEEQGIDLPYSCRAGACSTCAGKVVEGTVD
QSDQSFLDDAQLAAGYVLTCVAYPSSDCTVKTHQEESLY
>P48277
MDNFLFSFYIIFFSLIVLALISFLIQEIQKSRFLDLEITRLRKKIYQEKATEKDYNNLAS
IYIKKKLYSQAQKEFLKILENKNLDKSQLALIYNSLGYICSAQEQYELAIEYYKKALFYI
PDFILARINLARIFELKNLKTEAINMYQEVLIFDPKNQLAKRKLSIIENLIL
>P48327
MIEFSLLTKITLSFTILFLILSIISIIKGWKNRFQFIGVTSFSALLTISFFVFTFVPFTH
KLIPGAEKYKVVFDDGANQIVIAVSSKLNQDQLVATLEQAASDLFSPGRLSRNNNSFIIK
ARALSDSIGTTNNSLYLGELDKILDSNTNDFETKIKIFNDSTL

(7) 2186 cytoplasm proteins ($\mathbb{S}_7$)
>O04016
MAAAAFVPIPTDTYKLGFIGAGKMAESIARGVVKSGVLPASRIRTAHLGSARREAFESFG
VKVLDRNDQVVEDSDVIIFSVKPQIVKEVVLQLRPLLSEKQLLVSIVAGVKLKELEDWAG
HSRFIRVMPNTPAAVGEAASVMSLGATATGEDGELITKLFGAIGKIWKADEKLFDAVTGL
SGSGPAYIFLAIEALADGGVAAGLPRELALGLASQTVLGAASMVARGGKHPGQLKDDVAS
AGGTTIAGIHELEKGGFRGTLMNAVVSATKRSQEIFKR
>Q91VB4
MVRLYNLHPFGSQQVVPCQWEPEQVCCGGSDALFVAAGCKVEAFAVQGEELCRQRCAFST
LGRVLRMAYSEAGDYLVAIEEKNKTIFLRAYVNWRSKRSDNSRVCIRMVGHNVEASFCES
FRDQMSIIEMPMSEAPLCFSCCPVKGDLLVGCTNKLVLFTLKYDIINEEFSILNFERSLI
IHIDNITPVEISFCVGYVAVMSDLEVLLLKLESDPIHGESVDHHPQETSNPLKEAEGVSN
ETSQLESEDFVICLKPMELIGEKCEQSGISVKLESTGLEDEKVKYLRVRHLLYRRFAPDI
SSYVLSDNIKLHSLQLLPIHQSGFHPDENDLSPKKEMPNLFCFFSLPHVGYLYMVVKSVE
LMSVYWYPEKSQQAVLTPQFLHVITSQSLQCFTVRCSAAVAHEEDLYMDTTLKACPPVSM
DVCALRIQLFIGLKAICHFKNHIILLTKAEPEAIPERRESPKKLISRKDASVRSGTPHVA
EAAWNLYLVNTTAPVQLYKEMVDYSNSYKTVKTESCLHLLSEAHLLVRAALMDGSQLEPA
EKAELLEAFKESCGHLGDCYSRLTTEQSHLALPYYKMSGLSLAEVLARVDWTEESESQKY
ERGLVFYINHSLYENLDEELSKELAAKVAQIFHMAEPKQLPHVLSSPSMKNIDPLTALHY
LRKLDSCGVSPVLVTLTKAAVALKMGDLDMYRNEMKSHSEMKLVYGFILEPRLLIQQWKG
QIVPTELAIDLKETQPGLLVASVLGLQKNDKIGIVETDSFFKVLCGKDEDAVPQLLIDFW
EAQLVACLPNVVLEELFFKLISQYVWRLSERRCPDTVPLRTAEDLINACSHYGLVNPWVH
VLTTSDSLADKNYTDDLLKLQSLICSPSLDVASIIPFLEPLSEDTVAGLSAHALCHTRLQ
EYEQCIDTLLERCPEAVIAYANQELKEDHWILWWKKLLPELCQRVKSGGERSHLHLSLLK
ETLSVIAVGLDLRDFLNVLPEDGAAAFFLPYLLFCSRKKSLT
>P50091

MPSVCHTSPIEKIIQQGHRIQNDSLIPSKRTKLAHTELTAHYATEDSHVEKHFLHNGSNF
DGIDNVRYQNQPSPLTFITPNNTVDSSDWVPQFSSMKIDDSLEFSSEYKRLYSNYESQQR
LNSSRQHLPFKNCMIRKTSCTYPPQKTLRQQRQGNRDNPTDAFQFDAEFQVLEREIQKER
YEPITRRDEKWFDQDQSELQRIATDIVKCCTPPPSSASSSTLSSSVESKLSESKFIQLM
RNISSGDVTLKKNADGNSASELFSSNNGELVGNRHIFVKDEIHKDILD
>P0C0X0
MDSKTPVTLAKVIKVLGRTGSRGGVTQVRVEFLEDTSRTIVRNVKGPVRENDILVLMESE
REARRLR
>Q9UT48
MSDTTSLNVPELESPPIERVELESLLVMNIIKHCRDSFPNMGTIGQLVGIDIDGVLQVSS
SFESPSVLENEESAVNKSVSGKARQAHTEAMLNRLQYIGAVTGHVGWYLGAYVSSFLSSP
FFVETQYAYQKANPNSIAFLYDLSQSSNGTLYMRAYQLTPEFMAAHEEKTWTASSLNSHN
LTPSNVIRELPIVIHNSHLATCLLHSLSEPPTPASTLTAEAALEDCESNLPLTETFSNFE
VSLGTRYRKNIELLLESTDEFHYEQGNLGFHQRQLAREQAKIQQWIAKRKAENANRAAEN
LQPLPLDDWKRIFKLPAEPRLLDSLLISSQIMKSTQIDEQSSAFLSKLAGVRNAYAS
>P38913
MQLSKAAEMCYEITNSYLHIDQKSQIIASTQEAIRLTRKYLLSEIFVRWSPLNGEISFSY
NGGKDCQVLLLLYLSCLWEYFFIKAQNSQFDFEFQSFPMQRLPTVFIDQEETFPTLENFV
LETSERYCLSLYESQRQSGASVNMADAFRDFIKIYPETEAIVIGIRHTDPFGEALKPIQR
TDSNWPDFMRLQPLLHWDLTNIWSFLLYSNEPICGLYGKGFTSIGGINNSLPNPHLRKDS
NNPALHFEWEIIHAFGKDAEGERSSAINTSPISVVDKERFSKYHDNYYPGWYLVDDTLER
AGRIKN
>Q8WZK3
MSTLSYVSPDALKGWLMESPNEISIIDVRDYDYEGERIPGSVRIPSDTFLASVDQHVDDL
MKKRSLIVHCTYSQVRGPKAARVLSEILRNRITESKEKLSLSQKEKLFQNLPTVYILHGG
FSAWKRRYGGQQGLIEYD
>Q91785
MPPGTKGDPSNVTQPLPCLPSAEEDAIKVFVRIRPPVEGTLTGVDGEQGLCLTALSSTTI
RLHSKPEPKMFTFDHVANVDTNQESVFSSVAKNIVESCMNGYNGTIFAYGQTGSGKTFTM
LGPSESDNFTHNLRGVIPRSFEYLFFLINREKEKAGEGKSFLCKCSFIEIYNEQIFDLLD
SASAGLFLREHIKKGVFVVGAVEQVVTSAAEAYQVLSMGWRNRRVASTSMNRESSRSHAV
FTVTIESMEKTNDLVNIRSSQLNLVDLAGSERQKDTQTEGVRLKEAGSINRSLSCLGQVI
TALVDVANGRQRHICYRDSKLTFLLRDSLGGNAKTFYIANVHPGSKCFGETLSTLQFAQR
AKLIKNKAVVNEDTQGNVSQLQAEVKKLKEQLSQLLSGQMPGDISVARVPSVGDNNMDYM
NNFIEAMMILEKSDREKKVLLQKVVQLEDLCNKKEKFIQSNKMIVKFREDHISRLEKAHK
EGRISLSNNEQDDFIAELKEEIRTLKEQVEHHPRVAKYALENHSLREENKRLHSLQSVKR
AQEVTAQMIAELEKAFLEVSVSEKDRQVAPMHSTPIQLDNNSLMSAARMRERMLQLESEL
ATSKQEYEEFKELTKKKQVEQESELQSLIKSNQHLENILEAIKANKRHEVSQLNRMHAAE
TIKNMTTPTKSYNLRSRLVPRLSPDAMPNGLMDTPKSGDVMDDIINEPIPPEMSEQAYEA
IAEELRIVQEQVTALQAKLDEEEGKNIRLQQQVNKLELCSTQIQELFNSERSNWNKEQQD
LIAQIKSLEKQKQENKSQEDVLKSEVHDLRVVLQSADRELGAVKGEYSLYREKQEKELSQ
LSARHMDVQLQLDNVRLEHETLLEEKRSLQDAFDNLEEVMKFEIDQLKQEISDSKHENET
LRAEFSNLLELLETEKERRQKLTSQLEEDKENKTKELLQVVDENMHLRKQCSELMTKCEQ
QVTELHGLEHSLTSSKEMIADLEKKNTADKEVVADLMNQIQVHRTTIIHKTESIDLLTRE

LEDIHSKYSIVLLAKEESKTVIEEQEKQIEELRECLERKQSADNIEKELLCDDLAHATEE
LEKLTEAFNKQEALLHTHEKELVEKEQQISELTNQVKLMTDLEISREQEKIRPASSNSSS
PVVLPETPRTPEGNPYDSEIANLQKRNTNLEILVSELNEERTSKNEEIIRLKMQLCETEN
MRLEIQNLQGMCKELKSQLENCNNVMKDSNDQKPSDMQDLKREIEKEVSERMEKGKATEH
ILKLQAELEETRNILCTKDHSLNELSKEIERTRSLEAKAFTEKEEIRSILEGKYEETEKL
SHELDMLRKQVLFLAEENGKILGHQNPNQKIQYLVKLKKENNKLLEEAEKLRIENLFLKE
SKKCEHCN
>P63158
MGKGDPKKPRGKMSSYAFFVQTCREEHKKKHPDASVNFSEFSKKCSERWKTMSAKEKGKF
EDMAKADKARYEREMKTYIPPKGETKKKFKDPNAPKRPPSAFFLFCSEYRPKIKGEHPGL
SIGDVAKKLGEMWNNTAADDKQPYEKKAAKLKEKYEKDIAAYRAKGKPDAAKKGVVKAEK
SKKKKEEEDDEEDEEDEEEEEEEEEDEDEEEDDDDE
>P07666
MEQLLAQLHTCSELIAEGYSSTGNIGWLNEFCATFLDFASDLKARLPEVAPSGANLDVET
IFLCLTQVVTCITHLERTISMEAPHMTRQHFLDRLDWCLRRLLVSLTQLEGNVTPVKNLE
DHSFVELMDLALDHLDDYMEKLAQQRNNSLHILEESFTEDTYQLASIVNHIVRHALAFAN
VAIHSDKKALTALCETLLAECATFHEEAGEPNSGHRKLEALSLERALYALESFLNEALLH
LLFVSLIDLENASVEKLKDALQRDPAGAQELISAFDTNMDRIQQIGVLAIAFSQDIKTKT
IVRSCLASLESLDACIVPALQLPESTSSAHHAEVLQEHFNQELLIFRNVIHEIIDSCSLI
NNYLDMLGERIHVQDKSHLKLIVQRGGVVVDHFRLPVNYSGLSEDGKRVHKDLILILREC
QAVVNLDVPVDPKRIVKRLKILYSVLAKLRDLICRDNLEPDSSVASEAQVPSSATRTFVR
SSRSFGKRHRSFVKQTGNCSVFGPQDSLAESGHSESDLISFQITEILRLD
>Q08119
MTEIIDLDLVDDFIKKPMVKQQKNQSSKPRVKRRGQLTFDDFRNIKIVEEPVVLSHNSSI
DESLDAATQNTKKREKYEGTCDEEMKTKEMEANMASKYSNIKTHSNDTNKVESISEHTTS
NNRPLNTLNWSPNIPLRYSDFAKFMSDETVTESNWAPPLCTPLPYAGDVMKILSFIVKFK
WVFSDDLLNLSFQDVEIGLELKVAGHSAKNIRICQDKMNLLFCSLLRLLFCSEKRADNQT
HRNFTLKRFLSLKNPYGKLVGKLRSLIQEWGLPKEWRGNSDILSTLNFNGGGLLTMEPLD
RIILLRCMIDWNCSYSALFHNEIQRLTHLKGDTGFNHQTFHASRFAMCGANNILDSCEVL
CSLMSQKLENRKKRKPSDKGKLSKINSQMKFLKGVRKSLSEKVTTDRLRAAVKINEEWGE
YFANEFTHTPIDDPTVDEIYKLRTSEFMIARIPRVGDFYLPPFWIGNECSSVNTSYSFND
MSTYLNYFVKFKEEGTKILPAKTAQNENKCQLKLIYRNTPACIRNLQFNDVHFAEVPHWF
EVAGDSNSLSNFIEYLESLSSLTENDTDDTKKGIDNLIEFLKIFSIFINETIQRITAAPT
GSTEGRHLRTSSQRRTTVHYSSDVNGDVSEESENEVDIDVSDDYDSEYLSEENTLTRKGE
DRTDKSFGKRELHNGAKDCDRNCDDIEIFSEPVRQLQDNSREKRSLRRNARKGL
>P53082
MTGERIEKVKINDEFAKSHFLTTQWRETKRQRHYKMPVTEQGLRERIESAIPQVYHIIVT
DLSYGCGQSFDIVVVSDFFQGKSKLMRSRAVNKAVKEELQEIHAFSCKCYTEEEWSKIVV

>Q92837
MPCRREEEEEAGEEAEGEEEEEDSFLLLQQSVALGSSGEVDRLVAQIGETLQLDAAQHSP
ASPCGPPGAPLRAPGPLAAAVPADKARSPAVPLLLPPALAETVGPAPPGVLRCALGDRGR
VRGRAAPYCVAELATGPSALSPLPPQADLDGPPGAGKQGIPQPLSGPCRRGWLRGAAASR
RLQQRRGSQPETRTGDDDPHRLLQQLVLSGNLIKEAVRRLHSRRLQLRAKLPQRPLLGPL

SAPVHEPPSPRSPRAACSDPGASGRAQLRTGDGVLVPGS
>O18388
MTSDIAMQLIAILEKTVSPDKNELLSAKNFLEQAAASNLPEFLKALSEILVNTANSAVAR
MAAGLQLKNHLTSKDEKVSQQYQDRWHQFPSEIRELIKNNILAALGTENTRPSCAAQCVA
YVAVIELPINRWPMLIQTLVNKVVSEGSSEMHRESALEAIGYICQDIRFGVMENQSNDVL
TAIIHGMRKVEPSNHVRLAATTALHNSLEFTKSNFEKDMERNFIMEVVCEATQCQDSQIC
VAALQCLVKIMTLYYQYMEPYMAQALFPITLAAMKSDNDAVALQGIEFWSNVCDEEIDLA
IESQEATDQGRAPQRVSKHYARGALQFLTPVLVEKLTKQDECDDEDTWSPAKAASVCLMV
LATCCEDEIVPHVLPFIKENIESPNWRFRDAAVMTFGSVLNGLETNTLKPLVEQAMPTLI
RLMYDSSVIVRDTIAWTFGRICDIIPEAAINETYLQTLLECFVKSLKSEPRVAANVCWAF
IGLSDAAWEAAVTNDGETPETYALSPYFEYIITQLLETTDRSDGAQANLRCAAYQALMDM
IKNSPLDCYLVVQRTTLVILERLNQVMQMETQINNHSDRHQFNDLQSLLCATLQSVLRKV
HEQDAPQISDAIMTALLTMFNSSAGKSGVVQEEAFLAVSTLVELLGAQFAKYMPAFKDFL
VMGLKNFQEYQVCCAAVGLTGDIFRALKDLMVPYSNEIMTVLINNLTEPTIHRTVKPQVL
SAFGDIALSIGNHFLPYLSMVLDMLRVASNLQTDANNFDMNEYINELRESILEAYTGIIQ
GLKGVDQTAHTDVMHMEPHLMHIISFIKRIAQEGDVSDSMLASAAGFIGDLCTSFGPRLY
PLLDDAIITQFLAEGKRSKAQRTKMLCTWAVKEIKKINTQVITQ
>Q12090
MGSLLRPVDLVNQPLGFQERYKILQKLFKQLQKAYAHTKGTNIDLERLATRLEVHVAKNS
LSGQSYKFNMSILLRDVLKYKGDLSKIKINGRPLKGAKPHLSSIGNANSITTKSKAMEAL
KALILDSKVLEKNGYIVKEMQNKTNDDNSTQLYAPCLRCSSNFKKTDIMEKTLCRYHPLK
RIYNRDTKNHQYPCCGETTDSVSFLRLGCKTFFHHVFRGESYDELCKISKFSSTDDIDGV
ENVLSLDCEMAFTSLGYEMIRLTIVDFFTGKTLFDHVIQPIGDIVDLNSDFSGVHEIDRT
NCPTYKEALDVFLSENLINKNSILIGHGLENDLNVMRLFHNKVIDTAILYSRTKFKVSLK
NLAFEVLSRKIQNGEHDSSQDAIATMDVVKVKIGISPSQNKWEK
>P41252
MSNKMLQQVPENINFPAEEEKILEFWTEFNCFQECLKQSKHKPKFTFYDGPPFATGLPHY
GHILAGTIKDIVTRYAHQSGFHVDRRFGWDCHGLPVEYEIDKTLGIRGPEDVAKMGITEY
NNQCRAIVMRYSAEWKSTVSRLGRWIDFDNDYKTLYPQFMESVWWVFKQLYDKGLVYRGV
KVMPFSTACNTPLSNFESHQNYKDVQDPSVFVTFPLEEDETVSLVAWTTTPWTLPSNLAV
CVNPEMQYVKIKDVARGRLLILMEARLSALYKLESDYEILERFPGAYLKGKKYRPLFDYF
LKCKENGAFTVLVDNYVKEEEGTGVVHQAPYFGAEDYRVCMDFNIIRKDSLPVCPVDASG
CFTTEVTDFAGQYVKDADKSIIRTLKEQGRLLVATTFTHSYPFCWRSDTPLIYKAVPSWF
VRVENMVDQLLRNNDLCYWVPELVREKRFGNWLKDARDWTISRNRYWGTPIPLWVSDDFE
EVVCIGSVAELEELSGAKISDLHRESVDHLTIPSRCGKGSLHRISEVFDCWFESGSMPYA
QVHYPFENKREFEDAFPADFIAEGIDQTRGWFYTLLVLATALFGQPPFKNVIVNGLVLAS
DGQKMSKRKKNYPDPVSIIQKYGADALRLYLINSPVVRAENLRFKEEGVRDVLKDVLLPW
YNAYRFLIQNVLRLQKEEEIEFLYNENTVRESPNITDRWILSFMQSLIGFFETEMAAYRL
YTVVPRLVKFVDILTNWYVRMNRRRLKGENGMEDCVMALETLFSVLLSLCRLMAPYTPFL
TELMYQNLKVLIDPVSVQDKDTLSIHYLMLPRVREELIDKKTESAVSQMQSVIELGRVIR
DRKTIPIKYPLKEIVVIHQDPEALKDIKSLEKYIIEELNVRKVTLSTDKNKYGIRLRAEP
DHMVLGKRLKGAFKAVMTSIKQLSSEELEQFQKTGTIVVEGHELHDEDIRLMYTFDQATG
GTAQFEAHSDAQALVLLDVTPDQSMVDEGMAREVINRIQKLRKKCNLVPTDEITVYYKAK
SEGTYLNSVIESHTEFIFTTIKAPLKPYPVSPSDKVLIQEKTQLKGSELEITLTRGSSLP

GPACAYVNLNICANGSEQGGVLLLENPKGDNRLDLLKLKSVVTSIFGVKNTELAVFHDET
EIQNQTDLLSLSGKTLCVTAGSAPSLINSSSTLLCQYINLQLLNAKPQECLMGTVGTLLL
ENPLGQNGLTHQGLLYEAAKVFGLRSRKLKLFLNETQTQEITEDIPVKTLNMKTVYVSVL
PTTADF
>O14319
MASEQSNPRLPRRPPYMAEKARATLKEAFPNTDDAIIRAVLAASGYKLEPAFNALLGLSD
PQVAEEMEQAETSYAYDTAAHDDPVQRQLEEDERCARELANRYNSHRPERRRKTNNDRRN
YPPQNRTAKPNDNDGDDYSFFEDDLPVIKDTFMRGFQSFKQRSMEWVENIASKFDGEEED
DDDEKYSAPSKIYPSPRRSTAATLESAYEERPPSLPRRKPSRPGTAITLPPYESDPHMLN
EKDFERLRLESSSSPMMRRSSLNSNRRSVESSSSAAFVEGQSFILDSNGAIEVANSAFAL
DDSDLESAYNEELEMKKDTSKPTASTKEVVVEKKPDESRKQAARTLETVSEEQMGSSNAK
SKVLTSEPKDSTSVEAEKTETDEPAVGKGASDVSDTAEISEKTEAKNADSEANLEEKSDV
GEEKESKDENNKASLHKDVEEKDTKITNEDTGKTETDVKAKETDSIEANDKDEKTDSKET
EDKVEETESKEADVKAKETDSIEVDDKEEKTDSKETADKVEQTDSKDTNEKPAKDDNKEA
NEKAEKVDSKDVKEKIEEAADLQNSGKET
>P16181
MAEQTEKAFLKQPKVFLSSKKSGKGKRPGKGGNRFWKNIGLGFKTPREAIDGAYVDKKCP
FTGTVSIRGRILAGTCHSAKMQRTIIVRRDYLHFVKKYQRYEKRHSNIPAHVSPCFRVKE
GDHIIIGQCRPLSKTVRFNVLKVIPAGSSSSFGKKAFTGM
>O94682
MLEQSESHAFINNAPKEDRIQVKFEQLFESLPLPLRAEEALSKLRHDSARLMILKTSDPT
LNMSTYSIEDSPMGFECLKYNLSDNNKLLSQNNYRLPDYLEEDEIVSYTFSKTGPTTSKN
KHPSHSNTIRSPPYKVKKESCHTEINNVSNVSTESINVIDASRGYSPYTSVDSLSVSKNR
SFISLEESASNQYDAAEAFYFNADSSSPLRKLSPIELPVTPIRRKTPTINPNSELKRLQT
FGKLILHKGSRRR
>P79065
MFPLGSVVEVITGERGFVRYAGEVENRKGVYVGLELLPEFAEFGKNRGVVDGREYFKTKN
NEKTGIFVPFDKCKLASSISSSPSPKIDGTAASIGMGFPPMSPNLQSSIPRLTNVSSSSN
LSMNTISSTALTPTEKILQKRIEDLLYERQNHQQQLEEVLATVDQLQSLVTNFNDQQDEV
DELRERITLKEERIQQMRNEASQRRFEFKTTIECLEESSNRAIETYENRIAELEAQLEMY
MSGKSEDDLLFSLQQERDYALNQVEILQERVDTLMKQKANSSTANEKLSHMESSSPTLTN
ASFESPKRGKGSNDLPENHPQRRQTLEFYEIEIEVLREKVEKLQALSDEKDFYISKLEKS
LDRNDTTPVPSDEKLSNYAAEKENLVSRISELEHTIEQLTINNERDNERMSPAEFELETT
QEVEENDSDSHDDEETWCEVCETNNHSLQECPTVFGSTDEA
>P36159
MFTFIPITHPTSDTKHPLLLVQSAHGEKYFFGKIGEGSQRSLTENKIRISKLKDIFLTGE
LNWSDIGGLPGMILTIADQGKSNLVLHYGNDILNYIVSTWRYFVFRFGIDLNDHIMKDKE
VYKDKIIAVKSFNVLKNGGEDRLGVFDSFQKGVLRSIVAKMFPKHAPTDRYDPSSDPHLN
VELPDLDAKVEVSTNYEISFSPVRGKFKVEEAIKLGVPKGPLFAKLTKGQTITLDNGIVV
TPEQVLENERHFAKVLILDIPDDLYLNAFVEKFKDYDCAELGMVYYFLGDEVTINDNLFA
FIDIFEKNNYGKVNHMISHNKISPNTISFFGSALTTLKLKALQVNNYNLPKTDRVFSKDF
YDRFDTPLSRGTSMCKSQEEPLNTIIEKDNIHIFSQNKTVTFEPFRMNEEPMKCNINGEV
ADFSWQEIFEEHVKPLEFPLADVDTVINNQLHVDNFNNSAEKKKHVEIITLGTGSALPSK
YRNVVSTLVKVPFTDADGNTINRNIMLDAGENTLGTIHRMFSQLAVKSIFQDLKMIYLSH

LHADHHLGIISVLNEWYKYNKDDETSYIYVVTPWQYHKFVNEWLVLENKEILKRIKYISC
EHFINDSFVRMQTQSVPLAEFNEILKENSNQESNRKLELDRDSSYRDVDLIRQMYEDLSI
EYFQTCRAIHCDWAYSNSITFRMDENNEHNTFKVSYSGDTRPNIEKFSLEIGYNSDLLIH
EATLENQLLEDAVKKKHCTINEAIGVSNKMNARKLILTHFSQRYPKLPQLDNNIDVMARE
FCFAFDSMIVDYEKIGEQQRIFPLLNKAFVEEKEEEEDVDDVESVQDLEVKLKKHKKN
>Q9P7X6
MENGLGNRSNKLIRDSKRTQYLDYLPPYQADTVNDVVNEIRAADRESLGILQNVTHEASQ
PFQPQDHPSEAAALLMFHSSSIYNKRCLMAYHNLRLQRLRQYCWSGGKRMESCLDTSLST
YERDYLTRYSELLAAYKGAWSELDLTGSLVPPKNLFIDVRVLKDVGDIETEYGTINLTKN
SQLHVRATDVERLIAQGFLAKL
>P48407
MSVGMGIDLEAFRKSQRADGFASILAIGTANPPNVVDQSTYPDYYFRVTNNEDNTDLKDK
FKRICERSAIKKRHMYLTEEILKKNPELCAFLEVPSLDTRQAMLAAEVPRLGKEAAEKAI
EEWGQPKSRITHLIFCTTTTPDLPGADFEVAKLLGLHPSVKRVGVFQHGCFAGGTVLRLA
KDLAENNRGARVLVVCSENTAVTFRGPSETHLDGLVGLALFGDGASALIVGADPIPQVEK
PCFEIVWTAQTVVPNSDGAISGKLREVGLTFQLKGAVPDLISTNIEKCLVEAFSQFNISD
WNQLFWIAHPGGHAILDQVEASLNLDPTKLRATRHVMSEYGNMSSACVHFILDETRKASR
QNGCSTSGGGFQMGVLFGFGPGLTVETVVLKSIPFP
>Q06685
MSGIKKEPIESDEVPQQETKNNLPSAPSEMSPLFLNKNTQKAMQSIAPILEGFSPKTSAS
ENMSLKLPPPGIQDDHSEENLTVHDTLQRTISTALGNGNNTNTVTTSGLKKADSESKSEA
DPEGLSNSNIVNDADNINSISKTGSPHLPQGTMDAEQTNMGTNSVPTSSASSRKSSTSHP
KPRLPKVGKIGVCAMDAKVLSKPMRHILNRLIEHGEFETVIFGDKVILDERIENWPTCDF
LISFFSSGFPLDKAIKYVKLRKPFIINDLIMQKILWDRRLCLQVLEAYNVPTPPRLEISR
DGGPRANEELRAKLREHGVEVKPVEEPEWKMVDDDTLEVDGKTMTKPFVEKPVDGEDHNI
YIYYHSKNGGGGRRLFRKVGNKSSEFDPTLVHPRTEGSYIYEQFMDTDNFEDVKAYTIGE
NFCHAETRKSPVVDGIVRRNTHGKEVRYITELSDEEKTIAGKVSKAFSQMICGFDLLRVS
GKSYVIDVNGFSFVKDNKAYYDSCANILRSTFIEAKKKMDMEKKNLPIIREEKEQKWVFK
GLAIIIRHADRTPKQKFKHSFTSPIFISLLKGHKEEVVIRNVNDLKIVLQALRIALDEKA
GNPAKIKVLANALEKKLNFPGTKIQLKPVLNKENEVEKVQFILKWGGEPTHSAKYQATEL
GEQMRQDFDLLNKSILQNIKIFSSSERRVLHTAQYWTRALFGADELGSDEISIRKDLLDD
SNAAKDLMDKVKKKLKPLLREGKEAPPQFAWPSKMPEPYLVIKRVVELMNYHKKIMDNNF
AKKDVNSMQTRWCTSEDPSLFKERWDKLFKEFNNAEKVDPSKISELYDTMKYDALHNRQF
LENIFDPGLPNEAIADELGSHSLVDRYPINVLAKNNFKIIDSHSMNNSGKNSSNSVGSLG
WVLESGKTSTARNPKSSSQFDEPRFMQLRELYKLAKVLFDFICPKEYGISDAEKLDIGLL
TSLPLAKQILNDIGDMKNRETPACVAYFTKESHIYTLLNIIYESGIPMRIARNALPELDY
LSQITFELYESTDASGQKSHSIRLKMSPGCHTQDPLDVQLDDRHYISCIPKISLTKHLDM
DYVQQKLRNKFTRVIMPPKFTPVNITSPNLSFQKRKTRRKSVSVEKLKRPASSGSSSSTS
VNKTLD
>Q99933
MAQRGGARRPRGDRERLGSRLRALRPGREPRQSEPPAQRGPPPSRRPPARSTASGHDRPT
RGAAAGARRPRMKKKTRRRSTRSEELTRSEELTLSEEATWSEEATQSEEATQGEEMNRSQ
EVTRDEESTRSEEVTREEMAAAGLTVTVTHSNEKHDLHVTSQQGSSEPVVQDLAQVVEEV
IGVPQSFQKLIFKGKSLKEMETPLSALGIQDGCRVMLIGKKNSPQEEVELKKLKHLEKSV

EKIADQLEELNKELTGIQQGFLPKDLQAEALCKLDRRVKATIEQFMKILEEIDTLILPEN
FKDSRLKRKGLVKKVQAFLAECDTVEQNICQETERLQSTNFALAE
>O15150
MTSQITSNSPVKWKRRTRHDTCGKITLFALQKTMYFDNLSTKKHNLDNLELERSCFEISE
TMDSSLSIITYSMSVHSLQKNGEPMKQEKQQFIKTDWTIDMINRRQFEKYAIDILTATKD
IHRGGWVERIDNKAWQYVRVYENGDIEVLITLQIIRFHKYFDTPVWLQFNPNHLLPADYV
QLDHVMKYVDHSHLTRADLANDIYNINLQRYDFGLFGVTRDIYRSLSGDLETRYWGRRKS
ERQIRLYDKMREMKKHGKADDIPDGITDWWRLEFQFRGGKVESWQEEVMDKMQSFHVLAV
DDNDDLSEIDKAILARVNADKFDFKRVGKRYAAKIRKMVRENVGFDTTVAELSLKTFNEQ
KDELQRQLDSMLAKYNIGAQTEEMTAYFEEELKQTGNLDFSVVESESALERNVIRNIAKT
WREENSLTHRTNTNS
>O44406
MSADEPSPEDEKYLESLRDLLKISQEFDASNAKQNDEPEKTAVEVESAETRTDESEKSID
IPREQQLLPSERVEPLKSMVEPEYVKKVIRQMDTMTAEQLKQALMKIKVSTGGNKKTLRK
RVAQYYRKENALLNRKMEPNADKTARFFDYLIAIDFECTCVEIIYDYPHEIIELPAVLID
VREMKIISEFRTYVRPVRNPKLSEFCMQFTKIAQETVDAAPYFREALQRLYTWMRKFNLG
QKNSRFAFVTDGPHDMWKFMQFQCLLSNIRMPHMFRSFINIKKTFKEKFNGLIKGNGKSG
IENMLERLDLSFVGNKHSGLDDATNIAAIAIQMMKLKIELRINQKCSYKENQRSAARKDE
ERELEDAANVDLTSVDISRRDFQLWMRRLPLKLSSVTRREFINEEYLDCDSCDDLTDDKN
DEAAFQEKMAIREYLENKQTEDFAKIAAERGIFKIGEIKSYQTARPIIEDDDVDVESEEE
DYGTEFEMLEVVERMPPVSSTLHTEVDLDAVWERDGGSDSERENLSNAPSLHEFPSSSTS
SPHATSEHVTSSSPLHIDDDVDRVLNAPPKNSLASSSNRSSF
>Q14315
MMNNSGYSDAGLGLGDETDEMPSTEKDLAEDAPWKKIQQNTFTRWCNEHLKCVGKRLTDL
QRDLSDGLRLIALLEVLSQKRMYRKFHPRPNFRQMKLENVSVALEFLEREHIKLVSIDSK
AIVDGNLKLILGLIWTLILHYSISMPMWEDEDDEDARKQTPKQRLLGWIQNKVPQLPITN
FNRDWQDGKALGALVDNCAPGLCPDWEAWDPNQPVENSREAMQQADDWLGVPQVIAPEEI
VDPNVDEHSVMTYLSQFPKAKLKPGAPVRSKQLNPKKAIAYGPGIEPQGNTVLQPAHFTV
QTVDAGVGEVLVYIEDPEGHTEEAKVVPNNDKDRTYAVSYVPKVAGLHKVTVLFAGQNIE
RSPFEVNVGMALGDANKVSARGPGLEPVGNVANKPTYFDIYTAGAGTGDVAVVIVDPQGR
RDTVEVALEDKGDSTFRCTYRPAMEGPHTVHVAFAGAPITRSPFPVHVSEACNPNACRAS
GRGLQPKGVRVKEVADFKVFTKGAGSGELKVTVKGPKGTEEPVKVREAGDGVFECEYYPV
VPGKYVVTITWGGYAIPRSPFEVQVSPEAGVQKVRAWGPGLETGQVGKSADFVVEAIGTE
VGTLGFSIEGPSQAKIECDDKGDGSCDVRYWPTEPGEYAVHVICDDEDIRDSPFIAHILP
APPDCFPDKVKAFGPGLEPTGCIVDKPAEFTIDARAAGKGDLKLYAQDADGCPIDIKVIP
NGNGTFRCSYVPTKPIKHTIIISWGGVNVPKSPFRVNVGEGSHPERVKVYGPGVEKTGLK
ANEPTYFTVDCSEAGQGDVSIGIKCAPGVVGPAEADIDFDIIKNDNDTFTVKYTPPGAGR
YTIMVLFANQEIPASPFHIKVDPSHDASKVKAEGPGLNRTGVEVGKPTHFTVLTKGAGKA
KLDVQFAGTAKGEVVRDFEIIDNHDYSYTVKYTAVQQGNMAVTVTYGGDPVPKSPFVVNV
APPLDLSKIKVQGLNSKVAVGQEQAFSVNTRGAGGQGQLDVRMTSPSRRPIPCKLEPGGG
AEAQAVRYMPPEEGPYKVDITYDGHPVPGSPFAVEGVLPPDPSKVCAYGPGLKGGLVGTP
APFSIDTKGAGTGGLGLTVEGPCEAKIECQDNGDGSCAVSYLPTEPGEYTINILFAEAHI
PGSPFKATIRPVFDPSKVRASGPGLERGKVGEAATFTVDCSEAGEAELTIEILSDAGVKA
EVLIHNNADGTYHITYSPAFPGTYTITIKYGGHPVPKFPTRVHVQPAVDTSGVKVSGPGV

EPHGVLREVTTEFTVDARSLTATGGNHVTARVLNPSGAKTDTYVTDNGDGTYRVQYTAYE
EGVHLVEVLYDEVAVPKSPFRVGVTEGCDPTRVRAFGPGLEGGLVNKANRFTVETRGAGT
GGLGLAIEGPSEAKMSCKDNKDGSCTVEYIPFTPGDYDVNITFGGRPIPGSPFRVPVKDV
VDPGKVKCSGPGLGAGVRARVPQTFTVDCSQAGRAPLQVAVLGPTGVAEPVEVRDNGDGT
HTVHYTPATDGPYTVAVKYADQEVPRSPFKIKVLPAHDASKVRASGPGLNASGIPASLPV
EFTIDARDAGEGLLTVQILGPEGKPKKANIRDNGDGTYAVSYLPDMSGRYTITIKYGGDE
IPYSPFRIHALPTGDASKCLVTVSIGGHGLGACLGPRIQIGQETVITVDAKAAGEGKVTC
TVSTPDGAELDVDVVENHDGTFDIYYTAPEPGKYVITIRFGGEHIPNSPFHVLACDPLPH
EEEPSEVPQLRQPYAPPRPGARPTHWATEEPVVPVEPMESMLRPFNLVIPFAVQKGELTG
EVRMPSGKTARPNITDNKDGTITVRYAPTEKGLHQMGIKYDGNHIPGSPLQFYVDAINSR
HVSAYGPGLSHGMVNKPATFTIVTKDAGEGGLSLAVEGPSKAEITCKDNKDGTCTVSYLP
TAPGDYSIIVRFDDKHIPGSPFTAKITGDDSMRTSQLNVGTSTDVSLKITESDLSQLTAS
IRAPSGNEEPCLLKRLPNRHIGISFTPKEVGEHVVSVRKSGKHVTNSPFKILVGPSEIGD
ASKVRVWGKGLSEGHTFQVAEFIVDTRNAGYGGLGLSIEGPSKVDINCEDMEDGTCKVTY
CPTEPGTYIINIKFADKHVPGSPFTVKVTGEGRMKESITRRRQAPSIATIGSTCDLNLKI
PGNWFQMVSAQERLTRTFTRSSHTYTRTERTEISKTRGGETKPEVRVEESTQVGGDPFPA
VFGDFLGRERLGSFGSITRQQEGEASSQDMTAQVTSPSGKVEAAEIVEGEDSAYSVRFVP
QEMGPHTVAVKYRGQHVPGSPFQFTVGPLGEGGAHKVRAGRAGLERGVAGVPAEFSIWTR
EAGAGGLSIAVEGPSKAEIAFEDRKDGSCGVSYVVQEPGDYEVSIKFNDEHIPDSPFVVP
VASLSDDARRLTVTSLQETGLKVNQPASFAVQLNGARGVIDARVHTPSGAVEECYVSELD
SDKHTIRFIPHENGVHSIDVKFNGAHIPGSPFKIRVGEQSQAGDPGLVSAYGPGLEGGTT
GVSSEFIVNTLNAGSGALSVTIDGPSKVQLDCRECPEGHVVTYTPMAPGNYLIAIKYGGP
QHIVGSPFKAKVTGPRLSGGHSLHETSTVLVETVTKSSSSRGSSYSSIPKFSSDASKVVT
RGPGLSQAFVGQKNSFTVDCSKAGTNMMMVGVHGPKTPCEEVYVKHMGNRVYNVTYTVKE
KGDYILIVKWGDESVPGSPFKVKVP
>O43008
MLTSKHEFSLFLNTPAINSKSEKEVISEGPLTTSNEIDVTFSESFSSHLINLRRVPSVNS
IIEQEKKGLTKISPSILNQNIAYKEKRQQLFSVLYEETVGYVSMDTLCIAISLLDRCFTV
KPTIPTTSFKIYAIGCLFIAFKLTSDYSVAKKSFCENLAPSLSTKNLEKYEKIVLALLNF
DIYVISLPSVESFLTPLIFQHVFFKSLPSESCDQMMVEWQYLLVEVMKG
>Q9UBN7
MTSTGQDSTTTRQRRSRQNPQSPPQDSSVTSKRNIKKGAVPRSIPNLAEVKKKGKMKKLG
QAMEEDLIVGLQGMDLNLEAEALAGTGLVLDEQLNEFHCLWDDSFPEGPERLHAIKEQLI
QEGLLDRCVSFQARFAEKEELMLVHSLEYIDLMETTQYMNEGELRVLADTYDSVYLHPNS
YSCACLASGSVLRLVDAVLGAEIRNGMAIIRPPGHHAQHSLMDGYCMFNHVAVAARYAQQ
KHRIRRVLIVDWDVHHGQGTQFTFDQDPSVLYFSIHRYEQGRFWPHLKASNWSTTGFGQG
QGYTINVPWNQVGMRDADYIAAFLHVLLPVALEFQPQLVLVAAGFDALQGDPKGEMAATP
AGFAQLTHLLMGLAGGKLILSLEGGYNLRALAEGVSASLHTLLGDPCPMLESPGAPCRSA
QASVSCALEALEPFWEVLVRSTETVERDNMEEDNVEESEEEGPWEPPVLPILTWPVLQSR
TGLVYDQNMMNHCNLWDSHHPEVPQRILRIMCRLEELGLAGRCLTLTPRPATEAELLTCH
SAEYVGHLRATEKMKTRELHRESSNFDSIYICPSTFACAQLATGAACRLVEAVLSGEVLN
GAAVVRPPGHHAEQDAACGFCFFNSVAVAARHAQTISGHALRILIVDWDVHHGNGTQHMF
EDDPSVLYVSLHRYDHGTFFPMGDEGASSQIGRAAGTGFTVNVAWNGPRMGDADYLAAWH
RLVLPIAYEFNPELVLVSAGFDAARGDPLGGCQVSPEGYAHLTHLLMGLASGRIILILEG

GYNLTSISESMAACTRSLLGDPPPLLTLPRPPLSGALASITETIQVHRRYWRSLRVMKVE
DREGPSSSKLVTKKAPQPAKPRLAERMTTREKKVLEAGMGKVTSASFGEESTPGQTNSET
AVVALTQDQPSEAATGGATLAQTISEAAIGGAMLGQTTSEEAVGGATPDQTTSEETVGGA
ILDQTTSEDAVGGATLGQTTSEEAVGGATLAQTISEAAMEGATLDQTTSEEAPGGTELIQ
TPLASSTDHQTPPTSPVQGTTPQISPSTLIGSLRTLELGSESQGASESQAPGEENLLGEA
AGGQDMADSMLMQGSRGLTDQAIFYAVTPLPWCPHLVAVCPIPAAGLDVTQPCGDCGTIQ
ENWVCLSCYQVYCGRYINGHMLQHHGNSGHPLVLSYIDLSAWCYYCQAYVHHQALLDVKN
IAHQNKFGEDMPHPH
>Q08378
MDGASAEQDGLQEDRSHSGPSSLPEAPLKPPGPLVPPDQQDKVQCAEVNRASTEGESPDG
PGQGGLCQNGPTPPFPDPPSSLDPTTSPVGPDASPGVAGFHDNLRKSQGTSAEGSVRKEA
LQSLRLSLPMQETQLCSTDSPLPLEKEEQVRLQARKWLEEQLKQYRVKRQQERSSQPATK
TRLFSTLDPELMLNPENLPRASTLAMTKEYSFLRTSVPRGPKVGSLGLPAHPREKKTSKS
SKIRSLADYRTEDSNAGNSGGNVPAPDSTKGSLKQNRSSAASVVSEISLSPDTDDRLENT
SLAGDSVSEVDGNDSDSSSYSSASTRGTYGILSKTVGTQDTPYMVNGQEIPADTLGQFPS
IKDVLQAAAAEHQDQGQEVNGEVRSRRDSICSSVSLESSAAETQEEMLQVLKEKMRLEGQ
LEALSLEASQALKEKAELQAQLAALSTKLQAQVECSHSSQQRQDSLSSEVDTLKQSCWDL
ERAMTDLQNMLEAKNASLASSNNDLQVAEEQYQRLMAKVEDMQRSMLSKDNTVHDLRQQM
TALQSQLQQVQLERTTLTSKLKASQAEISSLQSVRQWYQQQLALAQEARVRLQGEMAHIQ
VGQMTQAGLLEHLKLENVSLSQQLTETQHRSMKEKGRIAAQLQGIEADMLDQEAAFMQIQ
EAKTMVEEDLQRRLEEFEGERERLQRMADSAASLEQQLEQVKLTLLQRDQQLEALQQEHL
DLMKQLTLTQEALQSREQSLDALQTHYDELQARLGELQGEAASREDTICLLQNEKIILEA
ALQAAKSGKEELDRGARRLEEGTEETSETLEKLREELAIKSGQVEHLQQETAALKKQMQK
IKEQFLQQKVMVEAYRRDATSKDQLISELKATRKRLDSELKELRQELMQVHGEKRTAEAE
LSRLHREVAQVRQHMADLEGHLQSAQKERDEMETHLQSLQFDKEQMVAVTEANEALKKQI
EELQQEARKAITEQKQKMRRLGSDLTSAQKEMKTKHKAYENAVGILSRRLQEALAAKEAA
DAELGQLRAQGGSSDSSLALHERIQALEAELQAVSHSKTLLEKELQEVIALTSQELEESR
EKVLELEDELQESRGFRKKIKRLEESNKKLALELEHEKGKLTGLGQSNAALREHNSILET
ALAKREADLVQLNLQVQAVLQRKEEEDRQMKHLVQALQASLEKEKEKVNSLKEQVAAAKV
EAGHNRRHFKAASLELSEVKKELQAKEHLVQKLQAEADDLQIREGKHSQEIAQFQAELAE
ARAQLQLLQKQLDEQLSKQPVGNQEMENLKWEVDQKEREIQSLKQQLDLTEQQGRKELEG
LQQLLQNVKSELEMAQEDLSMTQKDKFMLQAKVSELKNNMKTLLQQNQQLKLDLRRGAAK
TRKEPKGEASSSNPATPIKIPDCPVPASLLEELLRPPPAVSKEPLKNLNSCLQQLKQEMD
SLQRQMEEHALTVHESLSSWTPLEPATASPVPPGGHAGPRGDPQRHSQSRASKEGPGE
>Q06625
MNRSLLLRLSDTGEPITSCSYGKGVLTLPPIPLPKDAPKDQPLYTVKLLVSAGSPVARDG
LVWTNCPPDHNTPFKRDKFYKKIIHSSFHEDDCIDLNVYAPGSYCFYLSFRNDNEKLETT
RKYYFVALPMLYINDQFLPLNSIALQSVVSKWLGSDWEPILSKIAAKNYNMVHFTPLQER
GESNSPYSIYDQLQFDQEHFKSPEDVKNLVEHIHRDLNMLSLTDIVFNHTANNSPWLVEH
PEAGYNHITAPHLISAIELDQELLNFSRNLKSWGYPTELKNIEDLFKIMDGIKVHVLGSL
KLWEYYAVNVQTALRDIKAHWNDESNESYSFPENIKDISSDFVKLASFVKDNVTEPNFGT
LGERNSNRINVPKFIQLLKLINDGGSDDSESSLATAQNILNEVNLPLYREYDDDVSEILE
QLFNRIKYLRLDDGGPKQGPVTVDVPLTEPYFTRFKGKDGTDYALANNGWIWNGNPLVDF
ASQNSRAYLRREVIVWGDCVKLRYGKSPEDSPYLWERMSKYIEMNAKIFDGFRIDNCHST

PIHVGEYFLDLARKYNPNLYVVAELFSGSETLDCLFVERLGISSLIREAMQAWSEEELSR
LVHKHGGRPIGSYKFVPMDDFSYPADINLNEEHCFNDSNDNSIRCVSEIMIPKILTATPP
HALFMDCTHDNETPFEKRTVEDTLPNAALVALCSSAIGSVYGYDEIFPHLLNLVTEKRHY
DISTPTGSPSIGITKVKATLNSIRTSIGEKAYDIEDSEMHVHHQGQYITFHRMDVKSGKG
WYLIARMKFSDNDDPNETLPPVVLNQSTCSLRFSYALERVGDEIPNDDKFIKGIPTKLKE
LEGFDISYDDSKKISTIKLPNEFPQGSIAIFETQQNGVDESLDHFIRSGALKATSSLTLE
SINSVLYRSEPEEYDVSAGEGGAYIIPNFGKPVYCGLQGWVSVLRKIVFYNDLAHPLSAN
LRNGHWALDYTISRLNYYSDEAGINEVQNWLRSRFDRVKKLPSYLVPSYFALIIGILYGC
CRLKAIQLMSRNIGKSTLFVQSLSMTSIQMVSRMKSTSILPGENVPSMAAGLPHFSVNYM
RCWGRDVFISLRGMLLTTGRFDEAKAHILAFAKTLKHGLIPNLLDAGRNPRYNARDAAWF
FLQAVQDYVYIVPDGEKILQEQVTRRFPLDDTYIPVDDPRAFSYSSTLEEIIYEILSRHA
KGIKFREANAGPNLDRVMTDKGFNVEIHVDWSTGLIHGGSQYNCGTWMDKMGESEKAGSV
GIPGTPRDGAAIEINGLLKSALRFVIELKNKGLFKFSDVETQDGGRIDFTEWNQLLQDNF
EKRYYVPEDPSQDADYDVSAKLGVNRRGIYRDLYKSGKPYEDYQLRPNFAIAMTVAPELF
VPEHAIKAITIADEVLRGPVGMRTLDPSDYNYRPYYNNGEDSDDFATSKGRNYHQGPEWV
WLYGYFLRAFHHFHFKTSPRCQNAAKEKPSSYLYQQLYYRLKGHRKWIFESVWAGLTELT
NKDGEVCNDSSPTQAWSSACLLDLFYDLWDAYEDDS

>Q8W2B8

MACINGENRDFSSSSSLSSLPMIVSRNFSARDDGETGDEFPFERIFPVYARGTLNPVADP
VLLDFTNSSYDPIWDSIREEAKLEAEEEPVLSSFLYASILSHDCLEQALSFVLANRLQNP
TLLATQLMDIFCNVMVHDRGIQSSIRLDVQAFKDRDPACLSYSSAILHLKGYLALQAYRV
AHKLWKQGRKLLALALQSRVSEVFGIDIHPAARIGKGILLDHGTGVVIGETAVIGDRVSI
LHGVTLGGTGKETGDRHPNIGDGALLGACVTILGNIKIGAGAMVAAGSLVLKDVPSHSMV
AGNPAKLIGFVDEQDPSMTMEHDATREFFQNVAVAYRETIPNGSSVSGSCRERRH

>Q9NZN9

MDAALLLNVEGVKKTILHGGTGELPNFITGSRVIFHFRTMKCDEERTVIDDSRQVGQPMH
IIIGNMFKLEVWEILLTSMRVHEVAEFWCDTIHTGVYPILSRSLRQMAQGKDPTEWHVHT
CGLANMFAYHTLGYEDLDELQKEPQPLVFVIELLQVDAPSDYQRETWNLSNHEKMKAVPV
LHGEGNRLFKLGRYEEASSKYQEAIICLRNLQTKEKPWEVQWLKLEKMINTLILNYCQCL
LKKEEYYEVLEHTSDILRHHPGIVKAYYVRARAHAEVWNEAEAKADLQKVLELEPSMQKA
VRRELRLLENRMAEKQEEERLRCRNMLSQGATQPPAEPPTEPPAQSSTEPPAEPPTAPSA
ELSAGPPAEPATEPPPSPGHSLQH

>Q9URV1

MGLGFSSKKQLPAYCGPLPVGSLVLELSVPEEFRCEYKTIEHKLRTVKVRIFYPLDPTKD
VEPRTDELWLPFHEGIPEVAKGFRWWLLRAFASGLTNLALPVYKGELFHPPNNGKLPVFI
FSHGLVGSRNVYSSLCGTIASYGIVVLAMEHRDNSAIISTVRDPLHPEEPPYVVQYREIS
DFYADATVVLQNERLLFRQQEIQIALQMIRNINDLGTPDENLPFLCSVDSSFYNSVFQSM
KGNLNTAQGELIVAGHSFGAATCAFISGSSTKSLYNDYMFHTEFKCSILYDIWMLPVRQL
HLSTMRYPTLMIISYEFRRFVDNFQALESWLVNKDSENQNAGESADEKMSVVPLKKYSHV
FVYDGTVHANQSDLPILLPRMVLRVLKGKFEADPYEALRINTRSSVQFLRENHVENVQGD
NDPSSLQTNIIPGWERIM

>P49327

MEEVVIAGMSGKLPESENLQEFWDNLIGGVDMVTDDDRRWKAGLYGLPRRSGKLKDLSRF
DASFFGVHPKQAHTMDPQLRLLLEVTYEAIVDGGINPDSLRGTHTGVWVGVSGSETSEAL

SRDPETLVGYSMVGCQRAMMANRLSFFFDFRGPSIALDTACSSSLMALQNAYQAIHSGQC
PAAIVGGINVLLKPNTSVQFLRLGMLSPEGTCKAFDTAGNGYCRSEGVVAVLLTKKSLAR
RVYATILNAGTNTDGFKEQGVTFPSGDIQEQLIRSLYQSAGVAPESFEYIEAHGTGTKVG
DPQELNGITRALCATRQEPLLIGSTKSNMGHPEPASGLAALAKVLLSLEHGLWAPNLHFH
SPNPEIPALLDGRLQVVDQPLPVRGGNVGINSFGFGGSNVHIILRPNTQPPPAPAPHATL
PRLLRASGRTPEAVQKLLEQGLRHSQDLAFLSMLNDIAAVPATAMPFRGYAVLGGERGGP
EVQQVPAGERPLWFICSGMGTQWRGMGLSLMRLDRFRDSILRSDEAVKPFGLKVSQLLLS
TDESTFDDIVHSFVSLTAIQIGLIDLLSCMGLRPDGIVGHSLGEVACGYADGCLSQEEAV
LAAYWRGQCIKEAHLPPGAMAAVGLSWEECKQRCPPGVVPACHNSKDTVTISGPQAPVFE
FVEQLRKEGVFAKEVRTGGMAFHSYFMEAIAPPLLQELKKVIREPKPRSARWLSTSIPEA
QWHSSLARTSSAEYNVNNLVSPVLFQEALWHVPEHAVVLEIAPHALLQAVLKRGLKPSCT
IIPLMKKDHRDNLEFFLAGIGRLHLSGIDANPNALFPPVEFPAPRGTPLISPLIKWDHSL
AWDVPAAEDFPNGSGSPSAAIYNIDTSSESPDHYLVDHTLDGRVLFPATGYLSIVWKTLA
RALGLGVEQLPVVFEDVVLHQATILPKTGTVSLEVRLLEASRAFEVSENGNLVVSGKVYQ
WDDPDPRLFDHPESPTPNPTEPLFLAQAEVYKELRLRGYDYGPHFQGILEASLEGDSGRL
LWKDNWVSFMDTMLQMSILGSAKHGLYLPTRVTAIHIDPATHRQKLYTLQDKAQVADVVV
SRWLRVTVAGGVHISGLHTESAPRRQQEQQVPILEKFCFTPHTEEGCLSERAALQEELQL
CKGLVQALQTTVTQQGLKMVVPGLDGAQIPRDPSQQELPRLLSAACRLQLNGNLQLELAQ
VLAQERPKLPEDPLLSGLLDSPALKACLDTAVENMPSLKMKVVEVLAGHGHLYSRIPGLL
SPHPLLQLSYTATDRHPQALEAAQAELQQHDVAQGQWDPADPAPSALGSADLLVCNCAVA
ALGDPASALSNMVAALREGGFLLLHTLLRGHPLGDIVAFLTSTEPQYGQGILSQDAWESL
FSRVSLRLVGLKKSFYGSTLFLCRRPTPQDSPIFLPVDDTSFRWVESLKGILADEDSSRP
VWLKAINCATSGVVGLVNCLRREPGGNRLRCVLLSNLSSTSHVPEVDPGSAELQKVLQGD
LVMNVYRDGAWGAFRHFLLEEDKPEEPTAHAFVSTLTRGDLSSIRWVCSSLRHAQPTCPG
AQLCTVYYASLNFRDIMLATGKLSPDAIPGKWTSQDSLLGMEFSGRDASGKRVMGLVPAK
GLATSVLLSPDFLWDVPSNWTLEEAASVPVVYSTAYYALVVRGRVRPGETLLIHSGSGGV
GQAAIAIALSLGCRVFTTVGSAEKRAYLQARFPQLDSTSFANSRDTSFEQHVLWHTGGKG
VDLVLNSLAEEKLQASVRCLATHGRFLEIGKFDLSQNHPLGMAIFLKNVTFHGVLLDAFF
NESSADWREVWALVQAGIRDGVVRPLKCTVFHGAQVEDAFRYMAQGKHIGKVVVQVLAEE
PEAVLKGAKPKLMSAISKTFCPAHKSYIIAGGLGGFGLELAQWLIQRGVQKLVLTSRSGI
RTGYQAKQVRRWRRQGVQVQVSTSNISSLEGARGLIAEAAQLGPVGGVFNLAVVLRDGLL
ENQTPEFFQDVCKPKYSGTLNLDRVTREACPELDYFVVFSSVSCGRGNAGQSNYGFANSA
MERICEKRRHEGLPGLAVQWGAIGDVGILVETMSTNDTIVSGTLPQRMASCLEVLDLFLN
QPHMVLSSFVLAEKAAAYRDRDSQRDLVEAVAHILGIRDLAAVNLDSSLADLGLDSLMSV
EVRQTLERELNLVLSVREVRQLTLRKLQELSSKADEASELACPTPKEDGLAQQQTQLNLR
SLLVNPEGPTLMRLNSVQSSERPLFLVHPIEGSTTVFHSLASRLSIPTYGLQCTRAAPLD
SIHSLAAYYIDCIRQVQPEGPYRVAGYSYGACVAFEMCSQLQAQQSPAPTHNSLFLFDGS
PTYVLAYTQSYRAKLTPGCEAEAETEAICFFVQQFTDMEHNRVLEALLPLKGLEERVAAA
VDLIIKSHQGLDRQELSFAARSFYYKLRAAEQYTPKAKYHGNVMLLRAKTGGAYGEDLGA
DYNLSQVCDGKVSVHVIEGDHRTLLEGSGLESIISIIHSSLAEPRVSVREG
>Q9R257
MLGMIRNSLFGSVETWPWQVLSTGGKEDVSYEERACEGGKFATVEVTDKPVDEALREAMP
KIMKYVGGTNDKGVGMGMTVPVSFAVFPNEDGSLQKKLKVWFRIPNQFQGSPPAPSDESV
KIEEREGITVYSTQFGGYAKEADYVAHATQLRTTLEGTPATYQGDVYYCAGYDPPMKPYG

RRNEVWLVKA
>Q32NN2
MVGEMETKEKPKPTPDYLMQLMNDKKLMSSLPNFCGIFTHLERLLDEEISRVRKDMYNDT
MNSSSNEKRTSELPDGIGPIVQLQEKLYVPVKEYPDFNFVGRILGPRGLTAKQLEAETGC
KIMVRGKGSMRDKKKEEQNRGKPNWEHLNEDLHVLITVEDAQNRAELKLKRAVEEVKKLL
VPAAEGEDSLKKMQLMELAILNGTYRDANLKSPALAFSLAATGQAPRIITGPAPVLSPAA
LRTPTPAGHTLMPLIRQIQTAVMPNGTPHPTATLMQQAPEGGLIYTPYEYPYTLAPATSI
LEYPIEASGVLGAVATKVRRHDMRVHPYQRIVTADRAATGN
>Q9H2G9
MTTKNLETKVTVTSSPIRGAGDGMETEEPPKSVEVTSGVQSRKHHSLQSPWKKAVPSESP
GVLQLGKMLTEKAMEVKAVRILVPKAAITHDIPNKNTKVKSLGHHKGEFLGQSEGVIEPN
KELSEVKNVLEKLKNSERRLLQDKEGLSNQLRVQTEVNRELKKLLVASVGDDLQYHFERL
AREKNQLILENEALGRNTAQLSEQLERMSIQCDVWRSKFLASRVMADELTNSRAALQRQN
RDAHGAIQDLLSEREQFRQEMIATQKLLEELLVSLQWGREQTYSPSVQPHSTAELALTNH
KLAKAVNSHLLGNVGINNQKKIPSTVEFCSTPAEKMAETVLRILDPVTCKESSPDNPFFE
SSPTTLLATKKNIGRFHPYTRYENITFNCCNHCRGELIAL
>P81900
MSSDSSRRIQVPEELKEVLLQFSISFLVEQPPDVIDYAVEYFTKLQSERPSVSHTDQSTD
DQLSVNSQDADAEPPVMASSRRKSVFAEAYDPEADDDDDGATAVFPKTDEQRARLVESVK
NVLLFRSLEKEQMNQVLDAMFERKVQPGDFIIRQGDDGDNFYVIESGVYKVYINDKHINT
YNHTGLFGELALLYNMPRAATVQAETSGLLWAMDRQTFRRILLKSAFRKRKMYEELLNSV
PMLKALQNYERMNLADALVSKSYDNGERIIKQGDAADGMYFIEEGTVSVRMDQDDAEVEI
SQLGKGQYFGELALVTHRPRAASVYATGGVVKLAFLDVKAFERLLGPCMDIMKRNIDDYE
SQLVKIFGSKNNITDTR
>A2A884
MDPDQSIKGTKKADGSPRKRLTKGEAIQTSVSSSAPYPGSGTTAPSESATQELLATQPFS
GPSQEKTGQQQKPARRPSIEASVHISQLPQHPLTPAFMSPGKPEHLLEGSTWQLVDPMRP
GPSGSFVAPGLHPQSQLLPSHASILPPEELPGIPKVFVPRPSQVSLKPAEEAHKKERKPQ
KPGKYICQYCSRPCAKPSVLQKHIRSHTGERPYPCGPCGFSFKTKSNLYKHRKSHAHRIK
AGLASGSSSEMYPPGLEMERIPGEEFEEPTEGESTDSEEETGAASGPSTDVLPKPKHPLL
SSSLYSSGSHGSSQERCSLSQSSTGPSLEDPAPFAEASSEHPLSHKPEDTHTIKQKLALR
LSERKKLIEEQTFLSPGSKGSTESGYFSRSESAEQQVSPPNTNAKSYAEIIFGKCGRIGQ
RTSMLASTSTQPLLPLSSEDKPSLVPLSVPRTQVIEHITKLITINEAVVDTSEIDSVKPR
RSSLTRRSSVESPKSSLYRDSLSSHGEKTKQEQSLLSLQHPPSSTHPVPLLRSHSMPSAA
CTISTHHHTFRGSYSFDDHVADPEVPSRNTPVFTSHPRMLKRQPAIELPLGGEYSSEEPG
PSSKDPTSKPSDEPEPKESDLTKKTKKGFKTKGANYECTICGARYKKRDNYEAHKKYYCS
ELQITKAHSVGAHEVEKTQAEPEPWSQMMHYKLGATLELTPLRKRRKEKSLGDEEEPPAF
GSPGPSETAHNRPLGSTKSPAEASKSAPSLEGPTSFQPRTPKPGAGSEPGKERRTMSKEI
SVIQHTSSFEKSDPPEQPSGLEEDKPPAQFSSPPPAPHGRSAHSLQPRLVRQPNIQVPEI
LVTEEPDRPDTEPEPPPKEPEKTEEFQWPQRSQTLAQLPAEKLPPKKKRLRLAEMAQSSG
ESSFESSVPLSRSPSQESSISLSGSSRSASFDREDHGKAEAPGPFSDTRSKTLGSHMLTV
PSHHPHAREMRRSASEQSPNVPHSSHMTETRSKSFDYGSLSPTGPSLAVPAAPPPPAAPP
ERRKCFLVRQASLNRPPEAELEAVPKGKQESSEEPAASKPSTKSSVPQISVGTTQGGPSG
GKSQMQDRPPLGSSPPYTEALQVFQPLGTQLPPPASLFSLQQLLPQEQEQSSEFFPTQAM

AGLLSSPYSMPPLPPSLFQAPPLPLQPTVLHPSQLHLPQLLPHAADIPFQQPPSFLPMPC
PAPSTLSGYFLPLQSQFALQLPGEIESHLPPVKTSLPPLATGPPGPSSSTEYSSDIQLPP
VTPQATSPAPTSAPPLALPACPDAMVSLVVPVRIQTHMPSYGSAMYTTLSQILVTQSPGS
PASTALTKYEEPSSKSMTVCEADVYEAEPGPSSISKEQNRGYQTPYLRVPERKGTSLSSE
GILSLEGCSSTASGSKRVLSPAGSLELTMETQQQKRVKEEEASKADEKLELVSTCSVVLT
STEDRKKTEKPHVGGQGRSRREAETLSSLSSDVSDPKELSPLSHSTLSHGTAPGSEALKE
YAQPSSKAHRRGLPPMSVKKEDPKEQTDLPPLAPPSSLPLSDTSPKPAKLQEGTDSKKVL
QFPSLHTTTNVSWCYLNYIKPNHIQHADRRSSVYAGWCISLYNPNLPGVSTKAALSLLRS
KQKVSKETYTMATAPHPEAGRLVPSNSRKPRMTEVHLPSLVSPESQKDPARVEKEEKQGK
AEEGTPTSKRGEPARVKIFEGGYKSNEEYIYVRGRGRGRYVCEECGIRCKKPSMLKKHIR
THTDVRPYVCKHCHFAFKTKGNLTKHMKSKAHSKKCQETGVLEELEAEEGTSDDLHQDSE
GQEGAEAVEEHQFSDLEDSDSDSDLDEDEEEEEEEEESQDELSGPCSEAAPPCLPPTLQE
NSSPVEGPQAPDSTSDEVPQGSSISEATHLTASSCSTPSRGTQGLPRLGLAPLEKDMSSA
PSPKATSPRRPWSPSKEAGSRPSLTRKHSLTKNDSSPQQCSPAREAQASVTSTPGPQMGP
GRDLGPHLCGSPRLELSCLTPYPIGREAPAGLERATDTGTPRYSPTRRWSLGQAESPPQT
VLPGKWALAGPCSPSADKSGLGLGPVPRALLQPVPLPHTLLSRSPETCTSAWRKTESRSP
SAGPAPLFPRPFSAPHDFHGHLPSRSEENLFSHLPLHSQLLSRAPCPLIPIGGIQMVQAR
PGAQPTVLPGPCAAWVSGFSGGGSDLTGAREAQERSRWSPTESPSASVSPVAKVSKFTLS
SELEEERTGRGPGRPPDWEPHRAEAPPGPMGTHSPCSPQLPQGHQVAPSWRGLLGSPHTL
ANLKASSFPPLDRSSSMDCLAETSTYSPPRSRNLSGEPRTRQGSPELLGRGELRTPLFLP
KGSGPPSI
>Q9UTQ7
MPSFRDDHIIVIQTGSLYHRATFGLAESLEPPTIRVRTRVGKNDNGEYVFTNKEDINMED
PNVKTDETKVETSESTSEQPSNSNVTEEKNMGSSFHSECKVDDFTPLPNEIQPIQRGRVV
DWEALKAFWKHLYSLLLKDPNDTTFRYPVCLVIPTYWSLYDRELATQFFFEECQVPGFTI
AYEPLMGLYAIGILHGLVIDIGYEKTDITPILDGQIIFTATQQLPLGGRYMTQHLQNLLR
ESLPTLKSSGQYVSKEDITELFAEQVKCSEIAQVVRDEQDTAKDPVKALTSTNNVEEEDP
AEDIGKIIASGQTRAYLAQKEREKNGESEKDEKPDNTDVEFQTIAIEPLGDCIVGRERFK
ICEPIFHRPSNETVSLPEAIYITIKNYAANYKRRNDLWENIIVLGCGSRIRGFRECLIQQ
LQFKYQLSGSLEPYYAAQGTDSILGMTTMPPTPYPALINPCKILPDYFPSWKPDAGTNAM
FEELAFLGGSIVAKTSFNESVSSHYVTLEEYAQHGPTAIHTKQ
>Q14134
MEAADASRSNGSSPEARDARSPSGPSGSLENGTKADGKDAKTTNGHGGEAAEGKSLGSAL
KPGEGRSALFAGNEWRRPIIQFVESGDDKNSNYFSMDSMEGKRSPYAGLQLGAAKKPPVT
FAEKGELRKSIFSESRKPTVSIMEPGETRRNSYPRADTGLFSRSKSGSEEVLCDSCIGNK
QKAVKSCLVCQASFCELHLKPHLEGAAFRDHQLLEPIRDFEARKCPVHGKTMELFCQTDQ
TCICYLCMFQEHKNHSTVTVEEAKAEKETELSLQKEQLQLKIIEIEDEAEKWQKEKDRIK
SFTTNEKAILEQNFRDLVRDLEKQKEEVRAALEQREQDAVDQVKVIMDALDERAKVLHED
KQTREQLHSISDSVLFLQEFGALMSNYSLPPPLPTYHVLLEGEGLGQSLGNFKDDLLNVC
MRHVEKMCKADLSRNFIERNHMENGGDHRYVNNYTNSFGGEWSAPDTMKRYSMYLTPKGG
VRTSYQPSSPGRFTKETTQKNFNNLYGTKGNYTSRVWEYSSSIQNSDNDLPVVQGSSSFS
LKGYPSLMRSQSPKAQPQTWKSGKQTMLSHYRPFYVNKGNGIGSNEAP
>P41127
MKHNNVIPNGHFKKHWQNYVKTWFNQPARKTRRRIARQKKAVKIFPRPTSGPLRPVVHGQ

TLKYNMKVRTGKGFTLEELKAAGIPKKLAPTIGIAVDHRRKNRSLEGLQTNVQRLKTYKT
KLVIFPRRARKVKAGDSTPEELANATQVQGDYLPIVREKPTMELVKLTSEMKSFKAFDKI
RLERTNKRHAGARAKRAAEAEKEEKK
>P00336
MATLKEKLIAPVAEEETTIPNNKITVVGVGQVGMACAISILGKSLTDELALVDVLEDKLK
GEMMDLQHGSLFLQTPKIVADKDYSVTANSKIVVVTAGVRQQEGESRLNLVQRNVNVFKF
IIPQIVKYSPDCIIIVVSNPVDILTYVTWKLSGLPKHRVIGSGCNLDSARFRYLMAEKLG
VHPSSCHGWILGEHGDSSVAVWSGVNVAGVSLQELNPEMGTDNDSENWKEVHKMVVESAY
EVIKLKGYTNWAIGLSVADLIESMLKNLSRIHPVSTMVQGMYGIENEVFLSLPCVLNARG
LTSVINQKLKDDEVAQLKNSADTLWGIQKDLKDL
>Q07812
MDGSGEQPRGGGPTSSEQIMKTGALLLQGFIQDRAGRMGGEAPELALDPVPQDASTKKLS
ECLKRIGDELDSNMELQRMIAAVDTDSPREVFFRVAADMFSDGNFNWGRVVALFYFASKL
VLKALCTKVPELIRTIMGWTLDFLRERLLGWIQDQGGWDGLLSYFGTPTWQTVTIFVAGV
LTASLTIWKKMG
>Q9NRM7
MRPKTFPATTYSGNSRQRLQEIREGLKQPSKSSVQGLPAGPNSDTSLDAKVLGSKDATRQ
QQQMRATPKFGPYQKALREIRYSLLPFANESGTSAAAEVNRQMLQELVNAGCDQEMAGRA
LKQTGSRSIEAALEYISKMGYLDPRNEQIVRVIKQTSPGKGLMPTPVTRRPSFEGTGDSF
ASYHQLSGTPYEGPSFGADGPTALEEMPRPYVDYLFPGVGPHGPGHQHQHPPKGYGASVE
AAGAHFPLQGAHYGRPHLLVPGEPLGYGVQRSPSFQSKTPPETGGYASLPTKGQGGPPGA
GLAFPPPAAGLYVPHPHHKQAGPAAHQLHVLGSRSQVFASDSPPQSLLTPSRNSLNVDLY
ELSSTSVQQWPAATLARRDSLQKPGLEAPPRAHVAFRPDCPVPSRTNSFNSHQPRPGPPG
KAEPSLPAPNTVTAVTAAHILHPVKSVRVLRPEPQTAVGPSHPAWVPAPAPAPAPAPAPA
AEGLDAKEEHALALGGAGAFPLDVEYGGPDRRCPPPPYPKHLLLRSKSEQYDLDSLCAGM
EQSLRAGPNEPEGGDKSRKSAKGDKGGKDKKQIQTSPVPVRKNSRDEEKRESRIKSYSPY
AFKFFMEQHVENVIKTYQQKVNRRLQLEQEMAKAGLCEAEQEQMRKILYQKESNYNRLKR
AKMDKSMFVKIKTLGIGAFGEVCLACKVDTHALYAMKTLRKKDVLNRNQVVHVKAERDIL
AEADNEWVVKLYYSFQDKDSLYFVMDYIPGGDMMSLLIRMEVFPEHLARFYIAELTLAIE
SVHKMGFIHRDIKPDNILIDLDGHIKLTDFGLCTGFRWTHNSKYYQKGSHVRQDSMEPSD
LWDDVSNCRCGDRLKTLEQRARKQHQRCLAHSLVGTPNYIAPEVLLRKGYTQLCDWWSVG
VILFEMLVGQPPFLAPTPTETQLKVINWENTLHIPAQVKLSPEARDLITKLCCSADHRLG
RNGADDLKAHPFFSAIDFSSDIRKQPAPYVPTISHPMDTSNFDPVDEESPWNDASEGSTK
AWDTLTSPNNKHPEHAFYEFTFRRFFDDNGYPFRCPKPSGAEASQAESSDLESSDLVDQT
EGCQPVYV
>Q56A24
MVLILGRRLNREDLGVRDSPATKRKVFEMDPKSLTGHEYFDFSSGSSHAENILQIFNEFR
DSRLFTDVIICVEGKEFPCHRAVLSACSSYFRAMFCNDHRESREMLVEINGILAEAMECF
LQYVYTGKVKITTENVQYLFETSSLFQISVLRDACAKFLEEQLDPCNCLGIQRFADTHSL
KTLFTKCKTFALQTFEDVSQHEEFLELDKDELIDYICSDELVIGKEEMVFEAVMRWVYRA
VDLRRPLLHELLTHVRLPLLHPNYFVQTVEVDQLIQNSPECYQLLHEARRYHILGNEMMS
PRTRPRRSTGYSEVIVVVGGCERVGGFNLPYTECYDPVTGEWKSLAKLPEFTKSEYAVCA
LRNDILVSGGRINSRDVWIYNSQLNIWIRVASLNKGRWRHKMAVLLGKVYVVGGYDGQNR
LSSVECYDSFSNRWTEVAPLKEAVSSPAVTSCIGKLFVIGGGPDDNTCSDKVQSYDPETN

SWLLRAAIPIAKRCITAVSLNNLIYVAGGLTKAVYCYDPVEDYWMHVQNTFSRQENCGMS
VCNGKIYILGGRRENGEATDTILCYDPATSIITGVAAMPRPVSYHGCVTIHRYNEKCFKL

>P47147
MEYIKIAKVSNVVLHRRGTATQGTLHLTTHHLIFESPQLSTEFWFPYPLIYGVHKNPGST
LLSKLTSTNQIQLEGTDSQNYKLYQGKDLWSFVNIKVIGKDYAVFSLDFGGDLHLQARKV
YDSILNLTVLSNITQLYAFIYISNNLERKLPSPDSWDIYDPIKEFRRQGLDSKDETCPWR
LSTVNEHYEFCPTYPSKLFVPRSTSDILLKHASKFRSQKRIPVLTYHHKATDCNILRSSQ
PLPGLINQRSIQDEKLVWESFNSFCNKDIRRTKHVIVDARPRTNALAQMALGGGTENMDN
YNFFLADNNMGVDKSLKLPTVTRLFLGIDNIHIVSNTAAYMTEVICQGGDLNLPLEQNLI
RSQKFSNWLKLNTLILKSVDMLLKSIIFNHSNVLVHCSDGWDRTSQVVSLLEICLDPFYR
TFEGFMILVEKDWCSFGHRFLERSGHLNSDIRFHDNTMHSNFNDVDTNGDDLDIGVNTQD
DYAEDDEGGEDETNLINLSRISKKFNENFKLNKKSLKFVSPVFQQFLDCVYQLLTQNPDL
FEFNERFLRRLVYHLYSCQYGTFLSNSEKEKFQQNLPNKTKSVWDYFRSRRKQFINPNFI
QRKRSGMNEHDQNLEEEEKVEWISPDLKKVQWWWQLYGRKDSEMNDELRHKRDSVPISVD
KKSKEHSNSDGGKGLNLSIFGFDMFNRK
>Q9H869
MEEEASRSAAATNPGSRLTRWPPPDKREGSAVDPGKRRSLAATPSSSLPCTLIALGLRHE
KEANELMEDLFETFQDEMGFSNMEDDGPEEEERVAEPQANFNTPQALRFEELLANLLNEQ
HQIAKELFEQLKMKKPSAKQQKEVEKVKPQCKEVHQTLILDPAQRKRLQQQMQQHVQLLT
QIHLLATCNPNLNPEASSTRICLKELGTFAQSSIALHHQYNPKFQTLFQPCNLMGAMQLI
EDFSTHVSIDCSPHKTVKKTANEFPCLPKQVAWILATSKVFMYPELLPVCSLKAKNPQDK
ILFTKAEDNKYLLTCKTARQLTVRIKNLNMNRAPDNIIKFYKKTKQLPVLGKCCEEIQPH
QWKPPIEREEHRLPFWLKASLPSIQEELRHMADGAREVGNMTGTTEINSDQGLEKDNSEL
GSETRYPLLLPKGVVLKLKPVADRFPKKAWRQKRSSVLKPLLIQPSPSLQPSFNPGKTPA
QSTHSEAPPSKMVLRIPHPIQPATVLQTVPGVPPLGVSGGESFESPAALPAMPPEARTSF
PLSESQTLLSSAPVPKVMMPSPASSMFRKPYVRRRPSKRRGARAFRCIKPAPVIHPASVI
FTVPATTVKIVSLGGGCNMIQPVNAAVAQSPQTIPIATLLVNPTSFPCPLNQPLVASSVS
PLIVSGNSVNLPIPSTPEDKAHMNVDIACAVADGENAFQGLEPKLEPQELSPLSATVFPK
VEHSPGPPPVDKQCQEGLSENSAYRWTVVKTEEGRQALEPLPQGIQESLNNSSPGDLEEV
VKMEPEDATEEISGFL
>Q8T626
MTAELKASDTEMTKPEERHLLQLLAQLEKVPHHQLEQKFVAYFKKVGKGDNQSNCTLEDF
ATYFLGALRQSTKRYYYSQNSETPVFPLTPIHQLKPPVADVVKEEEQQQLNESSILSRSS
CSMSSTSNTTPAPQQPQGRRSTPGSGAQFCSTPNRSGGGGAGGGHSICLGNFLVNTPIQS
HQRSKKKITPQKQQQNQHASLGSGTGAGATPQSKPRRRVLPMTISKNVSASSSFGDTSSF
SNENNFWRISQSSEIFDRSQEAALEMEARKFLLLKKQEIKSEAPVNVSQQERTPEDEVFP
EEAISLEGVANANHLQLLSTIYSLLMDLNLVPNVLRELSFVVHLLNVRDFGQSPVKSYVP
SALEGLTQHKSCVYFAAKLLENQKKLLLQLDKRTLSVLLQNERLSLLPQGVVQELETHCQ
RRQDSTTPFAMDTSSSSQQNVYYHVENDSRDNFPSQNEFGAFKSQRDLFYKALKRWEVSH
LNRGFNFPSELGPRIMDIYKKSEHPVNMTHLAKLFVNQLLISATETTESPEELGLKLDPL
RHNKLAQRLVTSSSSVEGQFPRSQAFFRDFIAVCSSVAFLVHLKLELFVQLMRNNDSTFD
LLQLTDDVAVEEQSAQQGPYIVRVQTMANMLILAKFLGYVTVMPFSGTTQHGNPTPPYLC
PQQLQLRNHFRPDFNLREILERSMRQGKLLITLPWLVQYLAMLDLVSLHLPDAVATLELL

YGLYADIRMAKLQPAAVFIARSCIGWLLDAQPQLVNGYYNYRAQEAGVGSISAIVDICLK
DLSCHDKSHAPLLDELLPVACPFLQEFRVSITPSRQAKSGRFRYITTRLEQLQQNSSSIS
KDAIVASELSPAEQQQRKLVDAYLQSQNASTRRLIEFVTERTFKCVVKDAQQEILLPSKA
SADAKVNEIKSTMLEEVFQELHEIFQDARENACQRWKEHVHQMLDRRIEHSLDGLLPSST
NAVLRSTYAHLIRVQAQTQLQQWLQSSVLQSTFYHGDLQELATKVCNCNRNKADAAAAGG
GGSSELQLSPDIGFSLSEFLYQLQQWLHCLSLRPEYVGSQEDLAELLRKAQKAVLLPKMP
TVFYHLIGSGLVHLLQLLITRKPNFLDKDIISASCSVWRSQQLMDSKASPGIFDGLISIS
FVQEMANSADSFRMLETILRSMLQSGAIRADHLNELFMPLFAENWSPKVWCMLSELLQQL
SLSGKDSEAHYASGDSPEDEAKSHLFMEMLADLSRDLDNF
>P39008
MQSMNVQPRVLAVGGEQFFSQRQASEQHQQQNMGPQVYSPKVNRARMFPQGMPVNTINGS
VNQEMNNAYLLKQKNEPLLTQQQQQQQQQQQPFNIGTPVSVASLPPGLNVLQQQQQQQQQ
QQQQQQGVGLNRPLASQLPKHLTNQSMPPIFLPPPNYLFVRDVWKSNLYSEFAVIRQLVS
QYNHVSISTEFVGTLARPIGTFRSKVDYHYQTMRANVDFLNPIQLGLSLSDANGNKPDNG
PSTWQFNFEFDPKKEIMSTESLELLRKSGINFEKHENLGIDVFEFSQLLMDSGLMMDDSV
TWITYHAAYDLGFLINILMNDSMPNNKEDFEWWVHQYMPNFYDLNLVYKIIQEFKNPQLQ
QSSQQQQQQYSLTTLADELGLPRFSIFTTTGGQSLLMLLSFCQLSKLSMHKFPNGTDFA
KYQGVIYGIDGDQ
>Q96PM9
MQPPLDLKQILPFPLEPAPTLGLFSNYSTMDPVQKAVLSHTFGGPLLKTKRPVISCNICQ
IRFNSQSQAEAHYKGNRHARRVKGIEAAKTRGREPGVREPGDPAPPGSTPTNGDGVAPRP
VSMENGLGPAPGSPEKQPGSPSPPSIPETGQGVTKGEGGTPAPASLPGGSKEEEEKAKRL
LYCALCKVAVNSLSQLEAHNKGTKHKTILEARSGLGPIKAYPRLGPPTPGEPEAPAQDRT
FHCEICNVKVNSEVQLKQHISSRRHRDGVAGKPNPLLSRHKKSRGAGELAGTLTFSKELP
KSLAGGLLPSPLAVAAVMAAAAGSPLSLRPAPAAPLLQGPPITHPLLHPAPGPIRTAHGP
ILFSPY
>O60200
MGNIMSASFAPECTDLKTKYDSCFNEWYSEKFLKGKSVENECSKQWYAYTTCVNAALVKQ
GIKPALDEAREEAPFENGGKLKEVDK
>Q07930
MFHRRKRPYNTRNYGHDDKKFKSQYIDIMPDFSPSGLLELESNNKEGIALKHVEPQDAIS
PDNYMDMLGLEARDRTMYELVIYRKNDKDKGPWKRYDLNGRSCYLVGRELGHSLDTDLDD
RTEIVVADIGIPEETSSKQHCVIQFRNVRGILKCYVMDLDSSNGTCLNNVVIPGARYIEL
RSGDVLTLSEFEEDNDYELIFMNV
>P70062
MPQLNSGGGDELGANDELIRFKDEGEQEEKSPGEGSAEGDLADVKSSLVNESENHSSDSD
SEVERRPPPREAFEKHRDYLTEALRRQQDAAFFKGPPYAGYPFLMIPDLGGHYLPNGALS
PSARTYLQMKWPLLDSPSTAGLKDARSPSPAHLSNKVPVVQHPHHMHPLTPLITYSNEHF
SPGTPPGHLSPEIDPKTGIPRPPHPSELSPYYPLSPGAVGQIPHPLGWLVPPQGQPMYSI
PPGGFRHPYPALAMNASMSSLVSSRFSPHMVPPPHHSLHTSGIPHPAIVSPIVKQEPSSG
NISPNLHTKSNMIVKKEEEKKPHIKKPLNAFMLYMKEMRAKVVAECTLKESAAINQILGR
RWHSLSREEQAKYYELARKERQLHSQLYPSWSARDNYGKRKKRKREKQSPEMETHTKTKK
MCVQHLPADKSCDSPASSHGSMLDSPATPSAALASPAAPAATHSEQAQPLSLTTKPEARA
QLSLSHSAAFLASKSPSSSSFSGHLSSPVGSPLLSRPIPLTSSILSPSGVFPSALQALPL

LQAQPLSLVTKSSD
>O16844
MEIPIQVAVRIFPHRELKDLLRSFGPTEPKKDAQAVDEGADSKDSEAQVPAAEKDNPSIS
ETDPNGNAEQDSAADSKTIPDANGNDSGQKDYPDSAYCVQAIPISASALGLPSALPGGDP
MDSIAAGLIQVGPHTVPVTHALPSSSSQEQVYHQTVFPLITLFLEGFDASVVTYGQRGQG
KSYTLYGNVQDPTLTDSTEGVVQLCVRDIFSHISLHPERTYAINVGFVEICGGDVCDLLG
MGNIHCTNVDAVFHWLQVGLSARQSLPAHTLFTLTLEQQWVSKEGLLQHRLSTASFSDLC
GTERCGDQPPGRPLDAGLCMLEQVISTLTDPGLMYGVNGNIPYGQTTLTTLLKDSFGGRA
QTLVILCVSPLEEHLPETLGNLQFAFKVQCVRNFVIMNTYSDDNTMIVQPAEPVPESNSS
AGPLSQAGPGDNFGLQFAASQWSKLVTNAEGLFSKLIDSKLITEVEKEQIEEWLFLKQEC
EECLSSTEAMRQQKQLVPILEAEEPEDVNSEAANSESPNSDNENDTDNESHRPDLDDKIE
SLMEEFRDKTDALILEKHAEYLSKHPKAVMQSQDREIEAQPPEENGDDRKVSIGSRRRSV
QPGASLSTAELAMLNRVASQQPPPPIDPESVVDPLESSSGEGIRQAALAAAAATAPIEQL
QKKLRKLVAEIEGKQRQLREIEETIQVKQNIIAELVKNSDTRSHAKQRFHKKRAKLEAEC
DKAKKQLGKALVQGRDQSEIERWTTIIGHLERRLEDLSSMKHIAGESGQKVKKLQQSVGE
SRKQADDLQKKLRKECKLRCQMEAELAKLRESRETGKELVKAQGSPEQQGRQLKAVQARI
THLNHILREKSDNLEEQPGPEQQETLRHEIRNLRGTRDLLLEERCHLDRKLKRDKVLTQK
EERKLLECDEAIEAIDAAIEFKNEMITGHRSIDTSDRIQREKGEQMLMARLNRLSTEEMR
TLLYKYFTKVIDLRDSSRKLELQLVQLERERDAWEWKERVLSNAVRQARLEGERNAVLLQ
RQHEMKLTLMLRHMAEETSASSASYGERALAPACVAPPVQASSDFDYDHFYKGGGNPSKA
LIKAPKPMPTGSALDKYKDKEQRSGRNIFAKFHVLTRYASAAAAGSSGSTAEESTALIES
TTTATATTTSTTTTGAVGKVKDKALVSFRPEQLKRLMPAPTATKVTRQKNKIIIQDASRR
N
>O75533
MAKIAKTHEDIEAQIREIQGKKAALDEAQGVGLDSTGYYDQEIYGGSDSRFAGYVTSIAA
TELEDDDDDYSSSTSLLGQKKPGYHAPVALLNDIPQSTEQYDPFAEHRPPKIADREDEYK
KHRRTMIISPERLDPFADGGKTPDPKMNVRTYMDVMREQHLTKEEREIRQQLAEKAKAGE
LKVVNGAAASQPPSKRKRRWDQTADQTPGATPKKLSSWDQAETPGHTPSLRWDETPGRAK
GSETPGATPGSKIWDPTPSHTPAGAATPGRGDTPGHATPGHGGATSSARKNRWDETPKTE
RDTPGHGSGWAETPRTDRGGDSIGETPTPGASKRKSRWDETPASQMGGSTPVLTPGKTPI
GTPAMNMATPTPGHIMSMTPEQLQAWRWEREIDERNRPLSDEELDAMFPEGYKVLPPPAG
YVPIRTPARKLTATPTPLGGMTGFHMQTEDRTMKSVNDQPSGNLPFLKPDDIQYFDKLLV
DVDESTLSPEEQKERKIMKLLLKIKNGTPPMRKAALRQITDKAREFGAGPLFNQILPLLM
SPTLEDQERHLLVKVIDRILYKLDDLVRPYVHKILVVIEPLLIDEDYYARVEGLEIISNL
AKAAGLATMISTMRPDIDNMDEYVRNTTARAFAVVASALGIPSLLPFLKAVCKSKKSWQA
RHTGIKIVQQIAILMGCAILPHLRSLVEIIEHGLVDEQQKVRTISALAIAALAEAATPYG
IESFDSVLKPLWKGIRQHRGKGLAAFLKAIGYLIPLMDAEYANYYTREVMLILIREFQSP
DEEMKKIVLKVVKQCCGTDGVEANYIKTEILPPFFKHFWQHRMALDRRNYRQLVDTTVEL
ANKVGAAEIISRIVDDLKDEAEQYRKMVMETIEKIMGNLGAADIDHKLEEQLIDGILYAF
QEQTTEDSVMLNGFGTVVNALGKRVKPYLPQICGTVLWRLNNKSAKVRQQAADLISRTAV
VMKTCQEEKLMGHLGVVLYEYLGEEYPEVLGSILGALKAIVNVIGMHKMTPPIKDLLPRL
TPILKNRHEKVQENCIDLVGRIADRGAEYVSAREWMRICFELLELLKAHKKAIRRATVNT
FGYIAKAIGPHDVLATLLNNLKVQERQNRVCTTVAIAIVAETCSPFTVLPALMNEYRVPE
LNVQNGVLKSLSFLFEYIGEMGKDYIYAVTPLLEDALMDRDLVHRQTASAVVQHMSLGVY

GFGCEDSLNHLLNYVWPNVFETSPHVIQAVMGALEGLRVAIGPCRMLQYCLQGLFHPARK
VRDVYWKIYNSIYIGSQDALIAHYPRIYNDDKNTYIRYELDYIL
>O94111
MASEKKDKLEPQIKSVDMSEDMQQEAVEVAIEAMEKYHIEKDIAQYIKREFDSRKGATWH
CVVGRNFGSFVTHETKHFIYFYLGHCAILLFKTQ
>Q64096
MSDCWCFIFCKEHVRSNPLSPQHDGASREEADHQVDVSDGIRLVPDKAEATAATASDEIM
HQDIVPLCAADIQEQLKKRFAYLSGGRGQDGSPVITFPDYPAFSEIPDKEFQNVMTYLTS
IPSLQDAGIGFILVIDRRQDKWTSVKASVLRIAASFPANLQLVLVLRPTGFFQRTLSDIA
FKFNRDEFKMKVPVMMLSSVPELHGYIDKSQLTEDLGGTLDYCHSRWLCHRTAIESFALM
VKQTAQMLQAFGTELAETELPNDVQSTSLVLSAHTEKKAKVKEDLQLALKEGNSILESLR
EPLAESAAHSVNQDQLDNQATVQRLLAQLNETEAAFDEFWAKHQQKLEQCLQLRHFEQGF
REVKTTLDSMSQKIAAFTDVGNSLAHVQHLLKDLTAFEEKSSVAVDKARALSLEGQQLIE
NRHYAVDSIHPKCEELQHLCDHFASEVTRRRGLLSKSLELHSLLETSMKWSDEGIFLLAS
QPVDKCQSQDGAEASFQEIEKFLETGAENKIQELNEIYKEYECILNQDLLEHVQKVFQKQ
ESTEEMFHRRQASLKKLAAKQTRPVQPVAPRPEALTKSPSPSPGSWRSSENSSSEGNALR
RGPYRRAKSEMSEPRQGRTSSTGEEEESLAILRRHVMNELLDTERAYVEELLCVLEGYAA
EMDNPLMAHLISTGLQNKKNILFGNMEEIYHFHNRNIPAGVESCIDCPELVGRCFLERME
EFQIYEKYCQNKPRSESLWRQCSDCPFFQECQKKLDHKLSLDSYLLKPVQRITKYQLLLK
EMLKYSKHCEGAEDLQEALSSILGILKAVNDSMHLIAITGYDGNLGDLGKLLMQGSFSVW
TDHKKGHTKVKELARFKPMQRHLFLHEKAVLFCKKREENGEGYEKAPSYSYKQSLNMTAV
GITENVKGDTKKFEIWYNAREEVYIIQAPTPEIKAAWVNEIRKVLTSQLQACREASQHRA
LEQSHSLPLPTPSSTSPTKGNTRNVKKLEDRKTDPLSLEGYVSSSLPKPPEKGKGWSKTS
HSLEAPEEDGGWSSAEELINSSDAEEDGGVGPKKLVPGKYTVVMDDEKGGPDTLAMRSGD
MVEVVEEGAEGLWYVRDLTSSKEGWVPASSLSTLLGKSSSAQCLSSSGKIHCARQLCPEP
AEILSPEPV
>P34730
MSQTREDSVYLAKLAEQAERYEEMVENMKAVASSGQELSVEERNLLSVAYKNVIGARRAS
WRIVSSIEQKEESKEKSEHQVELIRSYRSKIETELTKISDDILSVLDSHLIPSATTGESK
VFYYKMKGDYHRYLAEFSSGDAREKATNSSLEAYKTASEIATTELPPTHPIRLGLALNFS
VFYYEIQNSPDKACHLAKQAFDDAIAELDTLSEESYKDSTLIMQLLRDNLTLWTSDISES
GQEDQQQQQQQQQQQQQQQQQAPAEQTQGEPTK
>Q9Y238
METRSSKTRRSLASRTNECQGTMWAPTSPPAGSSSPSQPTWKSSLYSSLAYSEAFHYSFA
ARPRRLTQLALAQRPEPQLLRLRPSSLRTQDISHLLTGVFRNLYSAEVIGDEVSASLIKA
RGSENERHEEFVDQLQQIRELYKQRLDEFEMLERHITQAQARAIAENERVMSQAGVQDLE
SLVRLPPVKSVSRWCIDSELLRKHHLISPEDYYTDTVPFHSAPKGISLPGCSKLTFSCEK
RSVQKKELNKKLEDSCRKKLAEFEDELDHTVDSLTWNLTPKAKERTREPLKKASQPRNKN
WMNHLRVPQRELDRLLLARMESRNHFLKNPRFFPPNTRYGGKSLVFPPKKPAPIGEFQST
EPEQSCADTPVFLAKPPIGFFTDYEIGPVYEMVIALQNTTTTSRYLRVLPPSTPYFALGL
GMFPGKGGMVAPGMTCQYIVQFFPDCLGDFDDFILVETQSAHTLLIPLQARRPPPVLTLS
PVLDCGYCLIGGVKMTRFICKNVGFSVGRFCIMPKTSWPPLSFKAIATVGFVEQPPFGIL
PSVFELAPGHAILVEVLFSPKSLGKAEQTFIIMCDNCQIKELVTIGIGQLIALDLIYISG
EKSQPDPGELTDLTAQHFIRFEPENLRSTARKQLIIRNATHVELAFYWQIMKPNLQPLMP

GETFSMDSIKCYPDKETAFSIMPRKGVLSPHTDHEFILSFSPHELRDFHSVLQMVLEEVP
EPVSSEAESLGHSSYSVDDVIVLEIEVKGSVEPFQVLLEPYALIIPGENYIGINVKKAFK
MWNNSKSPIRYLWGKISDCHIIEVEPGTGVIEPSEVGDFELNFTGGVPGPTSQDLLCEIE
DSPSPVVLHIEAVFKGPALIINVSALQFGLLRLGQKATNSIQIRNVSQLPATWRMKESPV
SLQERPEDVSPFDIEPSSGQLHSLGECRVDITLEALHCQHLETVLELEVENGAWSYLPVY
AEVQKPHVYLQSSQVEVRNLYLGVPTKTTITLINGTLLPTQFHWGKLLGHQAEFCMVTVS
PKHGLLGPSEECQLKLELTAHTQEELTHLALPCHVSGMKKPLVLGISGKPQGLQVAITIS
KESSDCSTEQWPGHPKELRLDFGSAVPLRTRVTRQLILTNRSPIRTRFSLKFEYFGSPQN
SLSKKTSLPNMPPALLKTVRMQEHLAKREQLDFMESMLSHGKGAAFFPHFSQGMLGPYQQ
LCIDITGCANMWGEYWDNLICTVGDLLPEVIPVHMAAVGCPISSLRTTSYTIDQAQKEPA
MRFGTQVSGGDTVTRTLRLNNSSPCDIRLDWETYVPEDKEDRLVELLVFYGPPFPLRDQA
GNELVCPDTPEGGCLLWSPGPSSSSEFSHETDSSVEGSSSASNRVAQKLISVILQAHEGV
PSGHLYCISPKQVVVPAGGSSTIYISFTPMVLSPEILHKVECTGYALGFMSLDSKVEREI
PRKRHRLQDFAVGPLKLDLHSYVRPAQLSVELDYGGSMEFQCQASDLIPEQPCSGVLSEL
VTTHHLKLTNTTEIPHYFRLMVSRPFSVSQDGASQDHRAPGPGQKQECEEETASADKQLV
LQAQENMLVNVSFSLSLELLSYQKLPADQTLPGVDIQQSASGEREMVFTQNLLLEYTNQT
TQVVPLRAVVAVPELQLSTSWVDFGTCFVSQQRVREVYLMNLSGCRSYWTMLMGQQEPAK
AAVAFRVSPNSGLLEARSANAPPTSIALQVFFTARSSELYESTMVVEGVLGEKSCTLRLR
GQGSYDERYMLPHQP
>P40554
MVNVKVEFLGGLDAIFGKQRVHKIKMDKEDPVTVGDLIDHIVSTMINNPNDVSIFIEDDS
IRPGIITLINDTDWELEGEKDYILEDGDIISFTSTLHGG
>Q9UBW5
MAEGKAGGAAGLFAKQVQKKFSRAQEKVLQKLGKAVETKDERFEQSASNFYQQQAEGHKL
YKDLKNFLSAVKVMHESSKRVSETLQEIYSSEWDGHEELKAIVWNNDLLWEDYEEKLADQ
AVRTMEIYVAQFSEIKERIAKRGRKLVDYDSARHHLEAVQNAKKKDEAKTAKAEEEFNKA
QTVFEDLNQELLEELPILYNSRIGCYVTIFQNISNLRDVFYREMSKLNHNLYEVMSKLEK
QHSNKVFVVKGLSSSSRRSLVISPPVRTATVSSPLTSPTSPSTLSLKSESESVSATEDLA
PDAAQGEDNSEIKELLEEEEIEKEGSEASSSEEDEPLPACNGPAQAQPSPTTERAKSQEE
VLPSSTTPSPGGALSPSGQPSSSATEVVLRTRTASEGSEQPKKRASIQRTSAPPSRPPPP
RATASPRPSSGNIPSSPTASGGGSPTSPRASLGTGTASPRTSLEVSPNPEPPEKPVRTPE
AKENENIHNQNPEELCTSPTLMTSQVASEPGEAKKMEDKEKDNKLISANSSEGQDQLQVS
MVPENNNLTAPEPQEEVSTSENPQL
>P49963
MATAVPMKKNWSPSMKHNDMERWICIYPAYINRKKTRQEGRRLPKENCVDNPSYIEIRDV
LSVSNLQFLMENKKYCRENSSEMEFRGRVRVQLRNVDGTLYNNDFPTRESIMLHIASKIP
QLKTRQNKSGDSYHQQSQPQSNASGSGGGGGGKKGKGKRR
>P53738
MKFLTTNFLKCSVKACDTSNDNFPLQYDGSKCQLVQDESIEFNPEFLLNIVDRVDWPAVL
TVAAELGNNALPPTKPSFPSSIQELTDDDMAILNDLHTLLLQTSIAEGEMKCRNCGHIYY
IKNGIPNLLLPPHLV
>O16785
MSTLGRSKTPSRDEPKKPGVFEKLSGTLSRKKKAPEDEHGNQGGAHHATDEDEVLELELE
GREALDQSLVPVLARNIWLEEGEIRRYLTKETARDQKLAQVVDLLIYWLNEELADQRIVV

RHLQEDLFDGQIIQKLLEKLEQIRIEVPEVSQSEEGQRQKLQIVVQTANRILGQPREQEK
WSADLIHQKDFTAIIQLLVLLALHYRAPVRFPDNVVANVVVAQKEHGQVKTHRITEQITT
VQTELAPKGTRDAFDTLFDYGPDKLAHVKTSLLAFCNKHLNKINLEVSDLDNQFQDGVFL
VLLVGLLEGYFVPLYHFNLQVQSHEEKVKNVQFAFKLMEDTGLEKPRSRVQDIANGDVKS
TLRLLHLLFTKYKHI
>P23228
MPGSLPVNTESCWPKDVGIVALEIYFPSQYVDQTELEKYDGVDAGKYTIGLGQSKMGFCS
DREDINSLCLTVVQKLMERNSLSYDCIGRLEVGTETIIDKSKSVKTVLMQLFEESGNTDV
EGIDTTNACYGGTAALFNAINWIESSSWDGRYALVVAGDIAVYATGNARPTGGAGAVAML
VGSNAPLIFERGLRGTHMQHAYDFYKPDMVSEYPVVDGKLSIQCYLSALDRCYSVYRNKI
HAQWQKEGTDRGFTLNDFGFMIFHSPYCKLVQKSVARLLLNDFLSDQNAETANGVFSGLE
AFRDVKLEDTYFDRDVEKAFMKASAELFNQKTKASLLVSNQNGNMYTPSVYGCLASLLAQ
YSPEHLAGQRISEFSYGSGFAATLYSIRVTQDATPGSALDKITASLSDLKARLDSRKCIA
PDVFAENMKIRQETHHLANYIPQCSVEDLFEGTWYLVRVDEKHRRTYARRPVMGDGPLEA
GVEVVHPGIVHEHIPSPAKKVPRIPATTESEGVTVAISNGVH
>Q99257
MSGFHNVGNINMMAQQQMQQNRIKISVRNWQNATMNDLINFISRNARVAVYDAHVEGPLV
IGYVNSKAEAESLMKWNGVRFAGSNLKFELLDNNGASAGTSDTISFLRGVLLKRYDPQTK
LLNLGALHSDPELIQKGVFSSISTQSKMFPAMMKLASTEKSLIVESVNLADNQLKDISAI
STLAQTFPNLKNLCLANNQIFRFRSLEVWKNKFKDLRELLMTNNPITTDKLYRTEMLRLF
PKLVVLDNVIVRDEQKLQTVYSLPMKIQQFFFENDALGQSSTDFATNFLNLWDNNREQLL
NLYSPQSQFSVSVDSTIPPSTVTDSDQTPAFGYYMSSSRNISKVSSEKSIQQRLSIGQES
INSIFKTLPKTKHHLQEQPNEYSMETISYPQINGFVITLHGFFEETGKPELESNKKTGKN
NYQKNRRYNHGYNSTSNNKLSKKSFDRTWVIVPMNNSVIIASDLLTVRAYSTGAWKTASI
AIAQPPQQQASVLPQVASMNPNITTPPQPQPSVVPGGMSIPGAPQGAMVMAPTLQLPPDV
QSRLNPVQLELLNKLHLETKLNAEYTFMLAEQSNWNYEVAIKGFQSSMNGIPREAFVQF
>Q06411
MEDSDSNTDKKFFFKKRRIDSYNYSDEEDNNSSMNSDMTYTNDALKTSSGNAPTISKLTK
TYGIGAKLLSSMGYVAGKGLGKDGSGITTPIETQSRPMHNAGLGMFSNTNSSNYHSENED
YLSSEDEVVEGIEQVKFNKTSTEVLGEALLNDSGDMTIVRTLRELRLAGVQLPESILKEL
DPLNAVPKPKKDVVVEILQELLGIEKSLEAIRQRTSPLEVQVKEYYGQERLLSELEVTLR
DESKHVSLYDKIGAILKLSDDELIDRLTSCLLRKELLIEFDLDHLEKPNDILDELTQIIE
LLAYRMDTTSKFLNRTQTTIFKVIYPKLKKFWEGFDMTKSKIDSAITLLLDFQQVLSFIG
CKEHIMEEFVYPKLLQELDNWELHDEVDHVSPRIWVLDFMVLIDDKIKDTIVDKIEAKFF
AYCKNWYHRESFCITNSDIIFIKELICERRYYKILCKEFLPKFLDELWERHNDPIYELED
WKEKQEWKEKDSGFFYFMKKLRSYTHYFHPKQYELMMRGTFNNINKILYQWHLYSTVEDL
HKSKWWLNWLMNTVFEHSLPTEIELSEIRKSYNIFAMSHRYHLDKSTLDEDFDLRQGLRN
LMETQVIDDISQSEQEPTYTVQNIPLGKVSSSFKDVVEDYCLEKGYLISKIPNRYTQLPY
GRDQDCIVPLFEIRNGKKKMEVALKHDILWVEDSSGTFKPIYLWALDL
>P47844
MPSDRRVALVTGANKGVGFAITRALCRLFSGDVLLTAQDEAQGQAAVQQLQAEGLSPRFH
QLDITDLQSIRALRDFLRRAYGGLNVLVNNAVIAFKMEDTTPFHIQAEVTMKTNFDGTRD
VCTELLPLMRPGGRVVNVSSMTCLRALKSCSPELQQKFRSETITEEELVGLMKKFVEDTK
KGVHQTEGWPDTAYGVTKMGVTVLSRIQARHLSEHRGGDKILVNACCPGWVRTDMGGPNA

TKSPEEGAETPVYLALLPPDAEGPHGQFVMDKKVEQW

>Q64559

MKLLARTLYLWEVGRQVASWSLTSGQECLVLRETWWASMRAVRTRAVHHKPGHCIAMGRI
MRPDDANVAGNVHGGTILKMIEEAGVIISTRHCNSQNGERCVAALARVERTDFLSPMCIG
EVAHVSAEITYTSKHSVEVQVHVLSENILTGTKKLTNKATLWYVPLSLKNVDKVLEVPPI
VYLRQEQEEEGRKRYEAQKLERMETKWRNGDIVQPILNPEPNTVSYSQSSLIHLVGPSDC
TLHGFVHGGVTMKLMDEVAGIVAARHCKTNIVTASVDAINFHDKIRKGCVITISGRMTFT
SNKSMEIEVLVDADPVVDNSQKRYRAASAFFTYVSLNQEGKPLPVPQLVPETEDEKKRFE
EGKGRYLQMKAKRQGHTEPQP

>O60155

MSVDSLVSCLEQLITDDLKLVEHQEVSNGATWASALQSTKDVPSHALTKTIVLKPKTAKS
QTVVPIILAALETTSTPSGIAAKAVGSKEARMAAADLVEEVFGIPPTDVGIFSVNKENAS
KVHVVLDAALIQHNGLLAFHPSSSAKTVFVSPAAVQTYLKSVGVNPIIVDFSAPGSATAP
SKPAAQKKKAEPSKNDAAIENAALIGITVRKDADFPNWYQQVLTKSDMIEYYDISGCYIL
KPWSYSIWEAIQGWFDKEIKKLGVRNGYFPLFVSSKVLEKEKDHVEGFAPEVAWVTRAGT
SELDEPIAIRPTSETVMYPYYAKWIRSHRDLPLKLNQWNSVVRWEFKNPQPFLRTREFLW
QEGHTAHMTLEGATEEVHQILDLYARIYTDLLAVPVIKGVKSENEKFAGGMFTTTVEGYI
PTTGRGIQGATSHCLGQNFSKMFNIVVEDPNAEIGPTGERPKLFVWQNSWGLSTRTIGVA
VMVHGDDKGLKLPPAIALVQSVVVPCGITNKTTDQERNEIEGFCSKLADRLNAADIRTEA
DLRAYTPGYKFSHWEMKGVPLRLEYGPNDAKKNQVTAVRRDTFEKIPVPLNNLEKGVSDL
LAKIQTNMYETAKAERDAHVVKVKEWADFVPALNKKNIVMIPWCNTTECEKEIKKNSARQ
VNGDEPEDEKAPSMGAKSLCIPLEQPSGEDAIIEGTTKCAGCGNLAKVWGLFGRSY

>Q76I89

MRRSSTTSGSSGRQSTMVLRVQDSNKMGLQTPQTKDRGTFGKLSMSKTTSATSERKVSFF
GKRASGAGGSRNSQYGMFGTEKIKDPRPLHDKAFIQQCIKQLCEFLVENGYAHNVTIKSL
QSPSVKDFIKIFTFIYGFLCPSYELPDSKFEEEIPRVFKELGYPFPLSKSSMYTVGAPHT
WPQIVAALIWLTDCFKLYAAMRENPSSFDDGQNWGGETDDGIVHNKLFMDYVVKCYEHFM
KGGDTYEELDAEVQSKLKDLFNVDEFKIEGLAAENKRLHEEIARLEKERESEPDRLVSLR
KLRSSFQADVQKYQAYLANLESHTTILDQKMKSVNEEVETTEMEVEAMKQENARLQHIFD
NQKYSVADIERINHERNELQQTINKLTKELEAEEHQLWNEELKYARNKEAIETQLAEYHK
LARKLKLIPISAENSKGHDFEIHFNPEAGPNCLVKYRTQIKAPLMEIVNQTEEEIRKATQ
RKMSLEDTLEQVNAMVAEKKSSVKTLKEEAEKLDDLYHQKLKEAEEEEQKCANELELLEK
HKQLLESGVNEGLSEATNELHDIQRQYQIVMQTTTEESRKAGDNLNRLLEVITTHVVSIE
KYLDEQNSKIDRDYEEFMSEDLLSTLTGILDSYKKKAESI

>Q9Y5Z9

MAASQVLGEKINILSGETVKAGDRDPLGNDCPEQDRLPQRSWRQKCASYVLALRPWSFSA
SLTPVALGSALAYRSHGVLDPRLLVGCAVAVLAVHGAGNLVNTYYDFSKGIDHKKSDDRT
LVDRILEPQDVVRFGVFLYTLGCVCAACLYYLSPLKLEHLALIYFGGLSGSFLYTGGIGF
KYVALGDLIILITFGPLAVMFAYAIQVGSLAIFPLVYAIPLALSTEAILHSNNTRDMESD
REAGIVTLAILIGPTFSYILYNTLLFLPYLVFSILATHCTISLALPLLTIPMAFSLERQF
RSQAFNKLPQRTAKLNLLLGLFYVFGIILAPAGSLPKI

>Q8K0T2

MPSETLWEIAKAEVEKRRSHGSEGDGAEIGEKSVFFIGSKNGGKTTIILRCLDRDESAKP
TLALEYTYGRKTKGHNTPKDIAHFWELGGGTSLLDLISIPITVDTLRTFSIVLVLDLSKP

NDLWSTMENLLQATKSHVDKVIMKLGKTSSKASAEMRQRMWSVVQKDHPDRELIDPFPIP
LVIIGSKYDIFQDFDPEKRKVICKTLRFVAHYYGASLMFTSKSEALLLKIRGVINQLAFG
IDKSKSICVDQNKPLFITAGLDSLCQIGSPPVPDSDIGKLQAHSPMELWKKVYDKLFPPK
STGTLKAVQDPARDPQYAESEVDEMRVQKDQELEHYKRSSSKTWKQIELDS
>P10826
MTTSGHACPVPAVNGHMTHYPATPYPLLFPPVIGGLSLPPLHGLHGHPPPSGCSTPSPAT
IETQSTSSEELVPSPPSPLPPPRVYKPCFVCQDKSSGYHYGVSACEGCKGFFRRSIQKNM
IYTCHRDKNCVINKVTRNRCQYCRLQKCFEVGMSKESVRNDRNKKKKETSKQECTESYEM
TAELDDLTEKIRKAHQETFPSLCQLGKYTTNSSADHRVRLDLGLWDKFSELATKCIIKIV
EFAKRLPGFTGLTIADQITLLKAACLDILILRICTRYTPEQDTMTFSDGLTLNRTQMHNA
GFGPLTDLVFTFANQLLPLEMDDTETGLLSAICLICGDRQDLEEPTKVDKLQEPLLEALK
IYIRKRRPSKPHMFPKILMKITDLRSISAKGAERVITLKMEIPGSMPPLIQEMLENSEGH
EPLTPSSSGNTAEHSPSISPSSVENSGVSQSPLVQ
>Q9H4X1
MKPPAAQGSPAAAAAAAPALDSAAAEDLSDALCEFDAVLADFASPFHERHFHYEEHLERM
KRRSSASVSDSSGFSDSESADSLYRNSFSFSDEKLNSPTDSTPALLSATVTPQKAKLGDT
KELEAFIADLDKTLASM
>Q9UTE1
MSNTGSARTEEFTKGTGYEATKPNNDTDDDAKQNYPSKEPHMKLGDYTEETSFVDDSIFK
REELTPDRNSPANDVEKMEVPKPTPQWMKLKDEGKLGKHRKNRLRRPGRFPVRQESTIDI
PGLTVSKRTENDSNNASYGIREKLIIILVGIPATGKSYIGSKLSRYYNWLKYNCRFFSVG
DKRREEGASTYSMSADFFDIKNEETFKFRENVALETLEDLLHWMIHENGVIGILDATNST
HERRKHLYDRISKEADIGIMFLESLCTDDILFEENIKLKIKGPDYEGYDTESALKDLRER
VDLYKKYYEPLDERDEQLPFLQYVKVINVGIKVVTHNIEGFLAGQAVYFMLNLNIQKRQI
WLTRPGESLDTVAGRIGGDASLTPIGKQYAQDLANFMDRQRVLWQLRYTNDLASTNKRFS
LSEASSFNVWSSVRKRAIETIEFFNPDSYNVKKIRLLNDLNLGSREGLTLREFSEKYPDE
FDVIKRKDYAYRFSGQGGESYLDVIHRLQPLIVEIERSSGNILVVSHRIVSNILMTYFLN
YHPEDIIDVGLPLHTLFCIESDRYGTTCMAYRYDAANRQFIKDPMFDLRKRT
>Q9Z2K5
MNTTPGTVGSDPVILATAGYDHTVRFWQAHSGICTRTVQHQDSQVNALEITPDRSMIAAT
GYQHIRMYDLNSNNPNPIISYDGVSKNIASVGFHEDGRWMYTGGEDCTARIWDLRSRNLQ
CQRIFQVNAPINCVCLHPNQAELIVGDQSGTSHIWDLKTDHNEQLIPEPEFSITSAHIDP
DASYMAAVNSAGNCFVWNLTGGIGDEVTQLIPKTKIPAHTRYALQCRFSPDSTLLATCSA
DQTCKIWRTSNFSLMTELSIKSSNPGESSRGWMWGCAFSGDSQYIVTASSDNLARLWCVE
TGEIKREYGGHQKAVVCLAFNDSVLG
>P35790
MKTKFCTGGEAEPSPLGLLLSCGSGSAAPAPGVGQQRDAASDLESKQLGGQQPPLALPPP
PPLPLPLPLPQPPPPQPPADEQPEPRTRRRAYLWCKEFLPGAWRGLREDEFHISVIRGGL
SNMLFQCSLPDTTATLGDEPRKVLLRLYGAILQVRSCNKEGSEQAQKENEFQGAEAMVLE
SVMFAILAERSLGPKLYGIFPQGRLEQFIPSRRLDTEELGLPDISAEIAEKMATFHGMKM
PFNKEPKWLFGTMEKYLKEVLRIKFTEESRIKKLHKLLSYNLPLELENLRSLLESTPSPV
VFCHNDCQEGNILLLEGRENSEKQKLMLIDFEYSSYNYRGFDIGNHFCEWMYDYSYEKYP
FFRANIRKYPTKKQQLHFISSYLPAFQNDFENLSTEEKSIIKEEMLLEVNRFALASHFLW
GQWSIVQAKISSIEFGYMDYAQARFDAYFHQKRKLGV

>Q62523
MAAPRPPPAISVSVSAPAFYAPQKKFAPVVAPKPKVNPFRPGDSEPPVAAGAQRAQMGRV
GEIPPPPPEDFPLPPPPLIGEGDDSEGALGGAFPPPPPPMIEEPFPPAPLEEDIFPSPPP
PLEEEGGPEAPTQLPPQPREKVCSIDLEIDSLSSLLDDMTKNDPFKARVSSGYVPPPVAT
PFVPKPSTKPAPGGTAPLPPWKTPSSSQPPPQPQRKPQVQLHVQPQAKPHVQPQPVSSAN
TQPRGPLSQAPTPAPKFAPVAPKFTPVVSKFSPGAPSGPGPQPIKKWCLRMPPSSVSTGS
PQPPSFTYAQQKEKPLVQEKQHPQPPPAQNQNQVRSPGGPGPLTLKEVEELEQLTQQLMQ
DMEHPQRQSVAVNESCGKCNQPLARAQPAVRALGQLFHITCFTCHQCQQQLQGQQFYSLE
GAPYCEGCYTDTLEKCNTCGQPITDRMLRATGKAYHPQCFTCVVCACPLEGTSFIVDQAN
QPHSVPDYHKQYAPRCSVCSEPIMPEPGRDETVRVVALDKNFHMKCYKCEDCGKPLSIEA
DDNGCFPLDGHVLCRKCHSARAQT
>Q9P3A9
MASFGELSFLSEISNIHKSNKKHEIQSAGTVALDNGVESPYYICVQVENQGIEVYHMKDE
RLFASCPLPEKTRFSCSPIYIKEGNWHYIWTCTSLKSNGEWKILLWKFNDLEEESEVVYR
DISNQQIFALHFISSTGQLVIVFRNGKIAFLDPEDDKVHMSASVNESATLLQSMYVPSQA
NPIDAVRLASNEASGTNNNPKEIEMNSDTTSSVPKSGSTSFSANVTSSTSMVYLLYSVHV
DKIIQYYVDSFSVSERRLVNTRAVVLKEVQAPSHILMSKDSTSIYTISLEGLSIYSLHDE
SKSYMLIKILHFQSISKIEHIELISDNFLLIQHDSQLSLWDITFGTIQDVYDLKQKPTIL
TFTCYKSSLKKMNQNSQLTGYIAVLLKKGLAIVPYTLPIKMLLADAVGKRTSKIGKLRGT
NELIGEGVLTKSKNGPSMRDQLLKNIQLQDHSLRDELISLRSLAEQKNTIEFDAKFLGVV
ERYQNQYANNCKILKTSSVLPIPFAHAIESLLFSLDEENELQVSCAASGTLNYLIRHRLF
SYSTLVQKGFSGSVFDCLYKFQKDIAFNFLERTSDISAYEIACAIKTAINSSKVKLLRSA
LARLSLFDSTTSREALLLAFVPEDFDSLFKTLGNLVVDSNLASVKFNLETYIYCLSVVLD
AMGVGGVASSSENLEAARILYNDLQEKLTNLTAMSLVLPAISEIVKRKKDVHAERVQFYA
NPQPKAIVDDMGDLATLLKKDFLSEKRKGKSQRARGKEIDMAIGKYTVERLEI
>Q05541
MSSIDNDSDVDLTEDLAVAKIVKENPVARKMVRYILSRGESQNSIITRNKLQSVIHEAAR
EENIAKPSFSKMFMDINAILYNVYGFELQGLPSKNNMNAGGNGSNSNTNKSMPEPLGHRA
QKFILLNNVPHSKNFDDFKILQSAHTYEELIVTGEYIGDDIASGTSNTLESKLSTDRDLV
YKGVLSVILCIVFFSKNNILHQELIKFLETFGIPSDGSKIAILNITIEDLIKSLEKREYI
VRLEEKSDTDGEVISYRIGRRTQAELGLESLEKLVQEIMGLEKEQTKSLHDDIIKSIGDS
YSI
>Q02770
MSSNIEPQTTAKCILYTTKGNIAIELWAKECPETCKRFLSMLSDGTFTNGEFKELKPTQW
LMFNANSTGEYRTVAEEKNPRIRFNRDGLLGWDRRRNTWFITVLADSKHVLNDCNVFGKI
VGKSIYIFREILGGEIEASSRDNDVKRFMYPAVLKDVEITIPFFEDIFGSKRRLEDNEKK
EQEPAKKLVKSAKVKMVYEDEQEDDDGDVQKLKPRKRMILPAWIKDDSRSEGIKLDASLD
QPQEALIREKTELHDNVDEATTKETESQENIKEEPMDKRERETLAMLSKFQERIKNKNIL
K
>O14134
MTSSVLIQGYEEDDVLKLLQELLDAETSQSCADVGKKIAQLFSNDNPLVTLKTTGFLDGL
ERAARNKKSGFHREAAMIGFATVIKNLGTPSEVVFLPYLPTILDSFSDRGEVVRQAAKMA
AQALLDCLPAGAVETRLIPSLISYLDDSSIKWPSKVAALQLLGSLASSSPKAVADYMAAL
IPCIKERMHDTKPEISRAAITCMLNLCSVVENNDIIPHIPKLVDCMAHPETLEACIKDLS

ATTFVATVESVALAVLVPILKRALAQRSQSMLRLTVIITDNLCKLVPDPAEASDFLPELI
PDVERIAQTAAMPEVRALASHALTTLNKAAAAQAAKAANNSEKQALDSACKELREAVLKN
TSVPHELANSIIDYVCDALAALYKSNNFDKDKWTSQLGVLYLSPLVGEELASQISSKIYD
DLHAFYKSLNSVDGISNLTIEEEELVNTDFSLAYGGRLLLSHTNLHLYRGHRYGVVGHNG
CGKSTLLRAIGDYKVENFPSPDEVKTCFVAHSLQGEDTSMAILDFVAQDKALLTMNVTRQ
EAADALHSVGFTAEMQENPVASLSGGWKMKLELARAMLQKADILLLDEPTNHLDVANIAW
LEAYLTSQKNITCLIVSHDSSFLDHVCTDIIHYEGVKNQAKKLGYYQGNLSAFVKVKPEA
KSYYTLTATNEKFVFPPPGILTGVRSNTRLILKMTNASYTYPNAKKKSLDNVTVGLSLSS
RVAILGPNGAGKSTLIKVLIGEVIPQEGKVFKHPNLRVGYVAQHAFHHLDQHLEKTPSQY
IQWRYAGGQDREVSEKESRKLTEEDRAQLQRDITVNGERRRVEALIGRQKLKKSFQYEIK
WFGKPHKYNTWVSREILLENGFQKFVQAFDDMESSREGLGFRELIPEDIRAHFEDVGLPG
DIADYSPISSLSGGQKVKVVIAACLWNNPQLLVLDEPTNFLDRDALGGLAVAIRDWEGGV
VMISHNEEFVSALCPEHWHVEAGKVTGKGKTAVDDGKFEDLSEKDLKKIEAKATKKKKLT
RNEIKAKERRARERELAWLQSPKGTEKPKSFFSDDEE
>Q12483
MKQFGLAAFDELKDGKYNDVNKTILEKQSVELRDQLMVFQERLVEFAKKHNSELQASPEF
RSKFMHMCSSIGIDPLSLFDRDKHLFTVNDFYYEVCLKVIEICRQTKDMNGGVISFQELE
KVHFRKLNVGLDDLEKSIDMLKSLECFEIFQIRGKKFLRSVPNELTSDQTKILEICSILG
YSSISLLKANLGWEAVRSKSALDEMVANGLLWIDYQGGAEALYWDPSWITRQL
>P49591
MVLDLDLFRVDKGGDPALIRETQEKRFKDPGLVDQLVKADSEWRRCRFRADNLNKLKNLC
SKTIGEKMKKKEPVGDDESVPENVLSFDDLTADALANLKVSQIKKVRLLIDEAILKCDAE
RIKLEAERFENLREIGNLLHPSVPISNDEDVDNKVERIWGDCTVRKKYSHVDLVVMVDGF
EGEKGAVVAGSRGYFLKGVLVFLEQALIQYALRTLGSRGYIPIYTPFFMRKEVMQEVAQL
SQFDEELYKVIGKGSEKSDDNSYDEKYLIATSEQPIAALHRDEWLRPEDLPIKYAGLSTC
FRQEVGSHGRDTRGIFRVHQFEKIEQFVYSSPHDNKSWEMFEEMITTAEEFYQSLGIPYH
IVNIVSGSLNHAASKKLDLEAWFPGSGAFRELVSCSNCTDYQARRLRIRYGQTKKMMDKV
EFVHMLNATMCATTRTICAILENYQTEKGITVPEKLKEFMPPGLQELIPFVKPAPIEQEP
SKKQKKQHEGSKKKAAARDVTLENRLQNMEVTDA
>O35973
MSGPLEGADGGGDPRPGEPFCPGGVPSPGAPQHRPCPGPSLADDTDANSNGSSGNESNGP
ESRGASQRSSHSSSSGNGKDSALLETTESSKSTNSQSPSPPSSSIAYSLLSASSEQDNPS
TSGCSSEQSARARTQKELMTALRELKLRLPPERRGKGRSGTLATLQYALACVKQVQANQE
YYQQWSLEEGEPCAMDMSTYTLEELEHITSEYTLRNQDTFSVAVSFLTGRIVYISEQAGV
LLRCKRDVFRGARFSELLAPQDVGVFYGSTTPSRLPTWGTGTSAGSGLKDFTQEKSVFCR
IRGGPDRDPGPRYQPFRLTPYVTKIRVSDGAPAQPCCLLIAERIHSGYEAPRIPPDKRIF
TTRHTPSCLFQDVDERAAPLLGYLPQDLLGAPVLLFLHPEDRPLMLAIHKKILQLAGQPF
DHSPIRFCARNGEYVTMDTSWAGFVHPWSRKVAFVLGRHKVRTAPLNEDVFTPPAPSPAP
SLDSDIQELSEQIHRLLLQPVHSSSPTGLCGVGPLMSPGPLHSPGSSSDSNGGDAEGPGP
PAPVTFQQICKDVHLVKHQGQQLFIESRAKPPPRPRLLATGTFKAKVLPCQSPNPELEVA
PVPDQASLALAPEEPERKETSGCSYQQINCLDSILRYLESCNIPSTTKRKCASSSSYTAS
SASDDDKQRAGPVPVGAKKDPSSAMLSGEGATPRKEPVVGGTLSPLALANKAESVVSVTS
QCSFSSTIVHVGDKKPPESDIIMMEDLPGLAPGPAPSPAPSPTVAPDPTPDAYRPVGLTK
AVLSLHTQKEEQAFLNRFRDLGRLRGLDTSSVAPSAPGCHHGPIPPGRRHHCRSKAKRSR

HHHHQTPRPETPCYVSHPSPVPSSGPWPPPPATTPFPAMVQPYPLPVFSPRGGPQPLPPA
PTSVSPATFPSPLVTPMVALVLPNYLFPTPPSYPYGVSQAPVEGPPTPASHSPSPSLPPP
PLSPPHRPDSPLFNSRCSSPLQLNLLQLEESPRTEGGAAAGGPGSSAGPLPPSEETAEPE
ARLVEVTESSNQDALSGSSDLLELLLQEDSRSGTGSAASGSLGSGLGSGSGSGSHEGGST
SASITRSSQSSHTSKYFGSIDSSEAEAGAARARTEPGDQVIKCVLQDPIWLLMANADQRV
MMTYQVPSRDAASVLKQDRERLRAMQKQQPRFSEDQRRELGAVHSWVRKGQLPRALDVMA
CVDCGSSVQDPGHSDDPLFSELDGLGLEPMEEGGGEGGGCGVGGGGGDGGEEAQTQIGAK
GSSSQDSAMEEEEQGGGSSSPALPAEENSTS
>P36118
MAIKKRNKIRLPSGSPEEVGIDGSAHKPMQQIKPLVSNDSEDDDNDICVLQPIKFKKVPK
RDITFDGEQAIKEDNSHYEDLYHSKKNTNASTRNKDDLLILNMEDLMEGNHHLLSDSSEA
GSSSEGEHISSIPTRGEIAKLKAQKSLSRRKISESDVTTERDYVKLLDSEDKREIMETIR
LNGGLKRNNEKEITNFSDDEMQGFQDEMLALTDNQIAIQKDSKRKIIEKAINEVPYRTNE
EWETQLLSKGNINKSNEKIITPLPVLFPDDDESGNSIERINEMVSKICLQRKKVEMRLQA
LEKTKIDLEKSKASLINKLIGN
>Q6ULP2
MEPDIIRMYSSSPPPLDNGAEDDDDDEFGEFGGFSEVSPSGVGFVDFDTPDYTRPKEEFV
PSNHFMPIHEFSENVDSLTSFKSIKNGNDKDITAELSAPVKGQSDVLLSTTSKEIISSEM
LATSIDGMERPGNLNKVVEQRQNVGTLESFSPGDFRTNMNVVHQNKQLESCNGEKPPCLE
ILTNGFAVLETVNPQGTDDLDNVADSKGRKPLSTHSTEYNLDSVPSPAEEFADFATFSKK
ERIQLEEIECAVLNDREALTIRENNKINRVNELNSVKEVALGRSLDNKGDTDGEDQVCVS
EISIVTNRGFSVEKQGLPTLQQDEFLQSGVQSKAWSLVDSADNSEAIRREQCKTEEKLDL
LTSKCAHLCMDSVKTSDDEVGSPKEESRKFTNFQSPNIDPTEENDLDDSLSVKNGDSSND
FVTCNDINEDDFGDFGDFGSASGSTPPFVTGTQDSMSDATFEESSEHFPHFSEPGDDFGE
FGDINAVSCQEETILTKSDLKQTSDNLSEECQLARKSSGTGTEPVAKLKNGQEGEIGHFD
SVPNIQDDCNGFQDSDDFADFSSAGPSQVVDWNAFEDEQKDSCSWAAFGDQQATESHHRK
EAWQSHRTDENIDTPGTPKTHSVPSATSKGAVASGHLQESATSVQTALLNRLERIFEACF
PSILVPDAEEEVTSLKHLLETSTLPIKTREALPESGELLDVWTELQDIHDAHGLRYQWGG
SHSNKKLLSSLGIDTRNILFTGNKKQPVIVPMYAAGLGMLEPTKEPLKPLSAAEKIASIG
QTATMSPDMNTCTSDQFQESLPPVQFDWSSSGLTNPLDASGGSTLLNLDFFGPVDDSSSS
SSTTIPGVDPELYELTTSKLEISTSSLKVTDAFARLMSTVEKTSTSTSRKPKREEHLSEE
AIKVIAGLPDLTFMHAKVLMFPATLTPSTSSQEKADG
>P53150
MSQLTEFISCIPVVNEEQNEEDERGLCKIQIEDGAMLETLDENSLSGLRIEKMLVSEGTG
IFSKSSFGINDLRIFTGENIDEESKKYVWYELLKMLTGHKVYIASLDEKVVFTKWTCRMQ
DDEVWKVVMELESSAIIRKIAELTLHPVKKGEIDLFEMADKLYKDICCVNDSYRNIKESD
SSNRNRVEQLARERELLDKLLETRDERTRAMMVTLLNEKKKKIRELHEILRQNNIKLSDD
DVLDSALINTEVQKPISELNSPGKRMKRRKTVVEPQNLQKKLKDTSRRRANRKISNQSVI
KMEDDDFDDFQFFGLSKRPIITAKDKLSEKYDDITSFGDDTQSISFESDSSSDVQKHLVS
LEDNGIQISAGRSDEDYGDISGSESETDASAGEKKSSNHSEQSGNDREPCLQTESETDIE
T
>P47118
MSNTWDDVWASDSDVETERSPDLVKLRENHSKRGYLDGIVSSKEEKLQEGFNDGFPTGAK
LGKQVGIIMGILLGLRTRFGDEDEDLSKAYIDAQKELRINKVLSKSIFDPNFDLQEKHPL

ITKWTDIANTYCEKYHVPSIQ
>Q9HC16
MKPHFRNTVERMYRDTFSYNFYNRPILSRRNTVWLCYEVKTKGPSRPPLDAKIFRGQVYS
ELKYHPEMRFFHWFSKWRKLHRDQEYEVTWYISWSPCTKCTRDMATFLAEDPKVTLTIFV
ARLYYFWDPDYQEALRSLCQKRDGPRATMKIMNYDEFQHCWSKFVYSQRELFEPWNNLPK
YYILLHIMLGEILRHSMDPPTFTFNFNNEPWVRGRHETYLCYEVERMHNDTWVLLNQRRG
FLCNQAPHKHGFLEGRHAELCFLDVIPFWKLDLDQDYRVTCFTSWSPCFSCAQEMAKFIS
KNKHVSLCIFTARIYDDQGRCQEGLRTLAEAGAKISIMTYSEFKHCWDTFVDHQGCPFQP
WDGLDEHSQDLSGRLRAILQNQEN
>Q9P791
MSQDQKPKVLEVSNLENQDAKWTSLKKIRWQDEKGKIRFWEMAERTTRSEANVDAVAILA
IVPIDGSPHVLCQKQFRPPIGKFCIEIPAGLVDSKESCEDAAIRELREETGYVGTVMDST
TVMYNDPGLTNANLKIILADIDMSKPENQNPQQQLDDGEYIENFPIKLSSLQEELFSLEK
KGFAIDVRLSTFALGLHAGLKYLSS
>Q9Y6R0
MSRSAAASGGPRRPERHLPPAPCGAPGPPETCRTEPDGAGTMNKLRQSLRRRKPAYVPEA
SRPHQWQADEDAVRKGTCSFPVRYLGHVEVEESRGMHVCEDAVKKLKAMGRKSVKSVLWV
SADGLRVVDDKTKDLLVDQTIEKVSFCAPDRNLDKAFSYICRDGTTRRWICHCFLALKDS
GERLSHAVGCAFAACLERKQRREKECGVTAAFDASRTSFAREGSFRLSGGGRPAEREAPD
KKKAEAAAAPTVAPGPAQPGHVSPTPATTSPGEKGEAGTPVAAGTTAAAIPRRHAPLEQL
VRQGSFRGFPALSQKNSPFKRQLSLRLNELPSTLQRRTDFQVKGTVPEMEPPGAGDSDSI
NALCTQISSSFASAGAPAPGPPPATTGTSAWGEPSVPPAAAFQPGHKRTPSEAERWLEEV
SQVAKAQQQQQQQQQQQQQQQQQQQQQAASVAPVPTMPPALQPFPAPVGPFDAAPAQVAVF
LPPPHMQPPFVPAYPGLGYPPMPRVPVVGITPSQMVANAFCSAAQLQPQPATLLGKAGAF
PPPAIPSAPGSQARPRPNGAPWPPEPAPAPAPELDPFEAQWAALEGKATVEKPSNPFSGD
LQKTFEIEL
>O35156
MSRFVQDLSKAMSQDGASQFQEVILQELELSVKKELEKILTTATSHEYEHTKKDLDGFRK
LYHRFLQEKGPSVDWGKIQRPPEDSIQPYEKIKARGLPDNISSVLNKLVVVKLNGGLGTS
MGCKGPKSLIGVRNENTFLDLTVQQIEHLNKSYNTDVPLVLMNSFNTDEDTKKILQKYNH
CRVKIYTFNQSRYPRINKESLLPVAKDVSSSGESTEAWYPPGHGDIYASFYNSGLLDTFL
EEGKEYIFVSNIDNLGATVDLYILNHLMNPPNGKRCEFVMEVTNKTRADVKGGTLTQYEG
KLRLVEIAQVPKAHVDEFKSVSKFKIFNTNNLWISLAAVKRLQEQNAIDMEIIVNPKTLD
GGLNVIQLETAVGAAIKSFENSLGINVPRSRFLPVKTTSDLLLVMSNLYSLNAGSLTMSE
KREFPTVPLVKLGSSFTKVQDYLRRFESIPDMLELDHLTVSGDVTFGKNVSLKGTVIIIA
NHGDRIDIPPGAVLENKIVSGNLRILDH
>O74348
MSNVEQPFMQGIDDGWNLKNYLNIQSTSLCLDKLCNIYTQGQDENEDAKELQAIKIKIEW
CKDTSVLLSSICVMLLDSIHDFLVQQGKITKESISKRDYSHEITIFNFKYAQQEMQNEKL
YHFAKLLEPALESLKVTNNHITHATIVGQVSGSEHNLSTAIEQVDVIVNYFYDSSEKFLE
LGNKVQTLGKAKNKKHWLGVYQSFGKASVDQIQKQLECYNVRMMELNKTDENNESAICES
QASSKEDERSDKTTSSSKKKSFTKLISNSRLNLWNKQ
>O08918
MKDLGAKHLAGGEGVQLFGLLNFYLEQEQRYQPREKGLILMEATPENDNTLCSRLRNAKV

EDLRSLTNFFGSGTETFVLAVNILDRFLALMKVKPKHLSCIGVCCFLLAARLAEEEGDVP
PTHDVIRISQCKCTASDIKRMEKIISEKLHYELEATTALNFLHLYHAIVFCHTSERKEIL
SLDKLEAQLKACNCRVVFSKARPSVLALCLLNLEIETIKSVELLEILLLVKKHLKLSDTE
FFYWRELVSKCLAEYSSPRCCKPDLKKLVWIVSRRTAQNLHSSYYSVPELPTIPEGGCFD
GSESEDSGEDMSCGEESLSSSPPSDQECTFFFDFQVAQTLCFPP
>P07263
MLSRSLNKVVTSIKSSSIIRMSSATAAATSAPTANAANALKASKAPKKGKLQVSLKTPKG
TKDWADSDMVIREAIFSTLSGLFKKHGGVTIDTPVFELREILAGKYGEDSKLIYNLEDQG
GELCSLRYDLTVPFARYVAMNNIQSIKRYHIAKVYRRDQPAMTKGRMREFYQCDFDVAGT
FESMVPDSECLSILVEGLTSLGIKDFKIKLNHRKILDGIFQIAGVKDEDVRKISSAVDKL
DKSPWEAVKKEMTEEKGQSEETADKIGEYVKLNGSLKEIHAVLSADANITSNEKAKQGLD
DIATLMKYTEAFDIDSFISFDLSLARGLDYYTGLIYEVVTSASAPPENASELKKKAKSAE
DASEFVGVGSIAAGGRYDNLVNMFSEASGKKSTQIPCVGISFGVERIFSLIKQRINSSTT
IKPTATQVFVMAFGGGKDWTGYLPERMKVTKQLWDAGIEAEYVYKAKANPRKQFDAAEKA
GCHIAVILGKEEYLEGKLRVKRLGQEFADDDGELVSAADIVPIVQEKLSQIHEDGLNEVT
RLIKGL
>Q08971
MSTFNAETADNLEDIEKQFAVVAVEQAETYWKLLTSVPGSKLRLTKFDDEIYENFMERFP
EYKDVERVKKFTEEELKTKEAKERWRKFFTIFEKKIEDYNFGTLLRTDASAEYGQFTTCF
VVRLQFYAFEIARNKHGLNDWIVGQK
>Q9P7D5
MFSGKVRAFIDEELFHSNRNNSSDGLSLDTPLAIHTPAKGFDADLSPQSLYDLHTVTTPV
TPLAPDEWDFSLDQSSGVIPSPSSFLSDHNNNNLFSDDTISRQYSNTDDINPSDFGGQCA
ILDSQNFTLSNASTKSKWSFTKHGSNTPSDSSSPLCNSSKRVVGMLRRFLPSSRMVRLSK
AHQPLRIPTTGVSLDSADLTPLSVSTSHLNHPSTSNSPDPLYSASQPPSIKTDASPVDIK
NMDAAEKLKKIDLLLEEILQLDSAYDAAERRMIESGWSSVDEIRDVHNKRLDAWSEWKQK
LLPLKKCC
>O74526
MPSDKPVFDIGSQAPERSDSESPSSRSIGSGTPAPVRKGLSKFKNSFLSRKNSSQIKSPS
DYKSSAHEQRVNHTTDSMAHVPGNNSPLQTPQKSPPRQKHTAPATPIPVSASRHHKPHHS
GLKNLLEKAMHPGHKSNANSPTSESPSKGFGSFINNHILHKNTSSHPSSPVNGKSSDIHK
SQSYQHLKNSPPNSRTARKPVPRRANSASHNLGSTKSPNGNAKESLSRSAELPSKAKPME
INNGYRKKPSPLSPNSSIRNREGGNGSYFDGPLTASPTPSSPTGTPNSMSKSPSLSSLAS
TGASYRPGPSKPLVSRVRDNYANTSYESWPHSTEFDMFTYAVSGSLKLTPQGTGFDCINP
ANPFSPGYSGKSSMKSDDNVGSSANTAPNSPTSANSSEGNQGNGPTTYPIKPPTNISEIP
RKLKSGFIPPYAKRVVPRLSAKYKLVDETKDMGSGATAVIRIVTLKNPKENEKNLRFAVK
AYRRKADDETDGQYIAKLASEWLVQCRMEHPNVVKSYDLCIDSHIFPLYSDTWCAVMDFC
PRGDLLSLIEDRHDRLGKKDFECMIKQILRGLNYIHSQGIAHRDIKPENILISEYGVLRI
TDFGACDVLCNPGDDITAVESKSMGIFGSDPYMAPEILTPGSYNAFFADMWSTAIVLHCL
YFRTYPFRKASQNDQLYAKYCKAWREYNLICDVQNIRISKTLPYFKPVNDLPMHMQRLFF
CLANPTAEQRITAQEALNLPFVQEIECCSVDDCTCTHDAPEECLEWANPPVQKLSTPHNH
L
>Q9UT39
MDIGILSSLKPAQSFRDNSLGSFINSIDYSDSGEYVATTCSADDTVQIYDALDPKQVHTI

TCFETGIEVARFTHHDHNLLLSTTKGNKDIQYVSIYDNKRISYFSGHTDIVSSIEVSPIE
DQFVSTANDKTLKLWKMNQSSRCLGNLDLPSLGIPAYDPTGLVFAVACHSLSRIFLYDVR
NYGSDPFSTFTIDDSRYLSRFSFPPMMPEWKHMEFSNDGKCILLSTRANVHYILDAFSGD
VLSRLEDFQELPFSNNFHGGSTTFVPQGNFVIGSADDRTLNVWNLRHTFHHKGKTRPPEH
RIVSQSIINPGLVKYNPRYDQLLTAGSQLVFWLPEKYALTS
>P38861
MEFTPIDPHQHQNAATLLCCNCGTPIDGSTGLVMCYDCIKLTVDITQGIPREANISFCRN
CERFLQPPGQWIRAELESRELLAICLRRLKGLTFVRLVDASFIWTEPHSRRIRIKLTVQG
EAMTNTIIQQTFEVEYIVIAMQCPDCARSYTTNTWRATVQIRQKVPHKRTFLFLEQLILK
HNAHVDTISISEAKDGLDFFYAQKNHAVKMIDFLNAVVPIKHKKSEELISQDTHTGASTY
KFSYSVEIVPICKDDLVVLPKKLAKSMGNISQFVLCSKISNTVQFMDPTTLQTADLSPSV
YWRAPFNALADVTQLVEFIVLDVDSTGISRGNRVLADITVARTSDLGVNDQVYYVRSHLG
GICHAGDSVMGYFIANSNYNSDLFDGLNIDYVPDVVLVKKLYQRKSKKSRHWKLKRMAKE
HKDIDASLDYNSRAQKQEMERAEKDYELFLQELEEDAELRQSVNLYKNREANVPPEEHEM
DEDEDEDAPQINIDELLDELDEMTLEDGVENTPVESQQ
>Q06559
MNANLPISKKRKFVSDGIFKAELNEFLTRELAEDGYSGVEVRVTPSRTEIIIMATKTQQV
LGEKGRRIRELTAMVQKRFNFETGRIELYAEKVAARGLCAIAQAESLRYKLTGGLAVRRA
CYGVLRYIMESGAKGCEVVVSGKLRGQRAKSMKFVDGLMIHSGDPCNDYVETATRHVLLR
QGVLGIKVKVMLPYDPKNKIGPKKPLPDNVSVVEPKEEKIYETPETEYKIPPPSKPLDDL
SEAKVL
>Q06705
MSFDRQLTEDQEVVLKQIWTHLFHLWQVPVDGTHIFPNNSLHSSSTPAKKKKSSWFSKLQ
SSDHTQDSSEAAEAAHLYEKGKIHKALANLDPQTTKKQFWHDIKNETPDATILKFIRARK
WNADKTIAMLGHDLYWRKDTINKIINGGERAVYENNETGVIKNLELQKATIQGYDNDMRP
VILVRPRLHHSSDQTEQELEKFSLLVIEQSKLFFKENYPASTTILFDLNGFSMSNMDYAP
VKFLITCFEAHYPESLGHLLIHKAPWIFNPIWNIIKNWLDPVVASKIVFTKNIDELHKFI
QPQYIPRYLGGENDNDLDHYTPPDGSLDVHLKDTETRAMIEKEREELVEQFLTVTAQWIE
HQPLNDPAYIQLQEKRVQLSTALCENYSKLDPYIRSRSVYDYNGSLKV
>O94668
MNSALKAIIELCIEEDLLPKSLDVLEYLLSTTSIPIPACYIRMCITLLVHPEYPTSSNIQ
ESCNWILQQVVENRSKINFDAWSFENDLSDNDMPKENYLKIFSNQCLFTQDVDYWDLIAY
MSSRPPDLERWMSLMDIWLRILEIDAEENGSALMKKYMGEDLCELQDAIRICFSCFTLPQ
QTMESPFATSMSKAPAKTESRGSNSFEGTSRLANLGAKLLCLIWAMLPKGRFFVVHLTDA
FHFLSKLDERFSKLNAKQQDLFFGNLGASNLRYCLSQYILMKGIGIGINIRHTRCEFTKQ
LTQLLPTSLPKNKIRQKLHLRAWVSFLGSALRESDRNFDKELFYTTLKQLYTMYQQTELV
GIDRPMAILKDMLVLLDISKKLSLDKIV
>P36152
MSQYKTGLLLIHPAVTTTPELVENTKAQAASKKVKFVDQFLINKLNDGSITLENAKYETV
HYLTPEAQTDIKFPKKLISVLADSLKPNGSLIGLSDIYKVDALINGFEIINEPDYCWIKM
DSSKLNQTVSIPLKKKKTNNTKLQSGSKLPTFKKASSSTSNLPSFKKADHSRQPIVKETD
SFKPPSFKMTTEPKVYRVVDDLIEDSDDDDFSSDSSKAQYFDQVDTSDDSIEEEELIDED
GSGKSMITMITCGKSKTKKKKACKDCTCGMKEQEENEINDIRSQQDKVVKFTEDELTEID
FTIDGKKVGGCGSCSLGDAFRCSGCPYLGLPAFKPGQPINLDSISDDL

>O74192
MSSKHWDYKKDLVLSHLAGLCQSNPHVRLIESERVVISAENQEDKITLISGGGSGHEPLH
AGFVTKDGLLDAAVAGFIFASPSTKQIFSAIKAKPSKKGTLIIVKNYTGDILHFGLAAEK
AKAEGLNAELLIVQDDVSVGKAKNGLVGRRGLAGTSLVHKILGAKAYLQKDNLELHQLVT
FGEKVVANLVTIGASLDHVTIPARANKQEEDDSDDEHGYEVLKHDEFEIGMGIHNEPGIK
KSSPIPTVDELVAELLEYLLSTTDKDRNYVQFDKNDEVVLLINNLGGTSVLELYAIQNIV
VDQLASKYSIKPVRIFTGTFTTSLDGPGFSITLLNATKTGDKDILKFLDHKTSAPGWNSN
ISDWSGRVDNFIVAAPEIDEGDSSSKVSVDAKLYADLLESGVKKVISKEPKITLYDTVAG
DGDCGETLANGSNAILKALAEGKLDLKDGVKSLVQITDIVETAMGGTSGGLYSIFISALA
KSLKEKELSEGAYTLTLETISGSLQAALQSLFKYTRARTGDRTLIDALEPFVKEFAKSKD
LKLANKAAHDGAEATRKLEAKFGRASYVAEEEFKQFESEGGLPDPGAIGLAALISGITDA
YFKSETKL
>Q01766
MHFKDLGLHDYTLKNLMYENNCCKFYDAVDENNISYVLKFVPSDVTSEGDTFPFVDRFQV
KEGVFLVYSSNDFGKEGTDYFTYTGSGGNEVHISGTSSEAGIKPQFIETCHPKHLKRGTK
EQEDINSSTSKKSAVINNFSGEKTPNPRPQSSNISERETYVGILNVKCKNKNSSKIRSEK
LVSSVIETKHTPGLASILSKEGTTYPNNADGKHISIVNPSSKIYHSSHKQIVKTPIPKSG
LSPIERCPFNGQNIKCYSPRPLDHESPQRDFNNNFQLRILKSSVLQRRQSTQNS
>P20435
MSDYEEAFNDGNENFEDFDVEHFSDEETYEEKPQFKDGETTDANGKTIVTGGNGPEDFQQ
HEQIRRKTLKEKAIPKDQRATTPYMTKYERARILGTRALQISMNAPVFVDLEGETDPLRI
AMKELAEKKIPLVIRRYLPDGSFEDWSVEELIVDL
>P28290
MDRPLSSSAEAEEELEWQVASRRRKAWAKCRSSWQASETEDLSTEATTQDEEEDEEEDLP
GAQLPAAGGRGNVPNEKIAIWLKDCRTPLGASLDEQSSSTLKGVLVRNGGSFEDDLSLGA
EANHLHESDAQIENCNNILAKERRLQFHQKGRSMNSTGSGKSSGTVSSVSELLELYEEDP
EEILYNLGFGRDEPDIASKIPSRFFNSSSFAKGIDIKVFLSAQMQRMEVENPNYALTSRF
RQIEVLTTVANAFSSLYSQVSGTPLQRIGSMSSVTSNKETDPPPPLTRSNTANRLMKTLS
KLNLCVDKTEKGESSSPSPSAEKGKILNVSVIEESGNKNDQKSQKIMKKKESSSMLATVK
EEVSGSSAAVTENADSDRISDEANSNFNQGTENEQSKETQSHESKLGEESGIVESKLDSD
FNISSHSELENSSELKSVHISTPEKEPCAPLTIPSIRNIMTQQKDSFEMEEVQSTEGEAP
HVPATYQLGLTKSKRDHLLRTASQHSDSSGFAEDSTDCLSLNHLQVQESLQAMGSSADSC
DSETTVTSLGEDLATPTAQDQPYFNESEEESLVPLQKGLEKAAAVADKRKSGSQDFPQCN
TIENTGTKQSTCSPGDHIIEITEVEEDLFPAETVELLREASAESDVGKSSSESEFTQYTTH
HILKSLASIEAKCSDMSSENTTGPPSSMDRVNTALQRAQMKVCSLSNQRMGRSLLKSKDL
LKQRYLFAKAGYPLRRSQSLPTTLLSPVRVVSSVNVRLSPGKETRCSPPSFTYKYTPEEE
QELEKRVMEHDGQSLVKSTIFISPSSVKKEEAPQSEAPRVEECHHGRTPTCSRLAPPPMS
QSTCSLHSIHSEWQERPLCEHTRTLSTHSVPNISGATCSAFASPFGCPYSHRHATYPYRV
CSVNPPSAIEMQLRRVLHDIRNSLQNLSQYPMMRGPDPAAAPYSTQKSSVLPLYENTFQE
LQVMRRSLNLFRTQMMDLELAMLRQQTMVYHHMTEEERFEVDQLQGLRNSVRMELQDLEL
QLEERLLGLEEQLRAVRMPSPFRSSALMGMCGSRSADNLSCPSPLNVMEPVTELMQEQSY
LKSELGLGLGEMGFEIPPGESSESVFSQATSESSSVCSGPSHANRRTGVPSTASVGKSKT
PLVARKKVFRASVALTPTAPSRTGSVQTPPDLESSEEVDAAEGAPEVVGPKSEVEEGHGK
LPSMPAAEEMHKNVEQDELQQVIREIKESIVGEIRREIVSGLLAAVSSSKASNSKQDYH

>O35244
MPGGLLLGDEAPNFEANTTIGHIRFHDFLGDSWGILFSHPRDFTPVCTTELGRAAKLAPE
FAKRNVKLIALSIDSVEDHFAWSKDINAYNGAAPTEKLPFPIIDDKDRDLAILLGMLDPA
EKDEKGMPVTARVVFIFGPDKKLKLSILYPATTGRNFDEILRVVDSLQLTASNPVATPVD
WKKGESVMVLPTLPEEEAKQLFPKGVFTKELPSGKKYLRYTPQP
>Q9EPK5
MNPSSVPHPLPPPGQQVIHVTQDLDTDLEALFNSVMNPKPSSWRKKILPESFFKEPDSGS
HSRQSSTDSSGGHPGPRLAGGAQHVRSHSSPASLQLGTGAGAAGGPAQQHAHLRQQSYDV
TDELPLPPGWEMTFTATGQRYFLNHIEKITTWQDPRKVMNQPLNHVNLHPSITSTSVPQR
SMAVSQPNLAMNHQHQQVVATSLSPQNHPTQNQPTGLMSVPNALTTQQQQQQKLRLQRIQ
MERERIRMRQEELMRQEAALCRQLPMETETMAPVNTPAMSTDMRSVTNSSSDPFLNGGPY
HSREQSTDSGLGLGCYSVPTTPEDFLSNMDEMDTGENSGQTPMTVNPQQTRFPDFLDCLP
GTNVDLGTLESEDLIPLFNDVESALNKSEPFLTWL
>O94510
MASLTTALPNKAVVAHFMMGLTYNYAQSDFQNDIQNAISLGLDGFVLNFGNDSWMMSKLT
LMYNAADALNLQFLLYLNLDMSEMSTVPASTLVTYVQTFANRGHQARINNNVVVGTFLGQ
DINFGQSSVNQGWQVAFKNALASAGINIFFMPTWPLDASTIYQTYPVADGFCKWNCWPYY
TSSPTSDAEDLVYIQNSKATNKKYMATVSPIFYTHFTSKNYSFFSEGLWFTRWMQLIKDQ
PNYVQVLTWNDYGESTYIGPTNYAADFPVIGSNSHEWVDSFTHAPLSYSLPLFIQMYKQN
TTGLPSNFSGISQLYVTYRVHSKNATASSDSIPRPDNYQNSSDVISVISFAKSSYTLRVS
VNGTVLGTTNVNAGVQSANVSFIVNNTAAAGLPLFQILNGTTVIAQGYGPLNILGNNSVV
LYNFNFCTTRISW
>O42650
MVEELKNDAQTNEEESEYSDFSDGSDNDYFREDDIDWNQASSNYSARQENFGNNSSKINS
VNDHVSTLSRYVNNIKLNDRFEAEGFRTRGLLILHTFTFSCLIVLTNKDKSSIKDKSDRA
TSEQVLDPRTRMILLKLINNGTISEINGCISTGKEANVYHATNEDGKHFAIKIYKTSILV
FKDRDRYVSGEFRFRHGYNKRNPRKMVRLWAEKEIRNLKRVAAAGIPCPEPILLKQHVLL
MSFLGDKKGWAYPKLKDIDMTPGEATKLYQLVARNMRILFHVCHLVHADLSEYNLLYHKG
KVYFIDVSQSVEHDHPQSIDFLRMDILNISTFFRRLNAGCLSLPQLFKFITEEGSCEKEA
MKTRLNAIYEEEPTTEEYEEEFLKTYVPRTLDEVYDIDRDTEIVNAGGVNSLVYKHLLNT
DFQKLDLNDTTKNQNDQILPNETSESDDDANSISSMENEEERTSDSKSSAKQGKGNGRAK
ETPEEKRARKKKTKEDKAEKRKSKIPKYEKKRKLKQSGRKK
>Q9NS73
MAAATELNRPSSGDRNLERRCRPNLSREVLYEIFRSLHTLVGQLDLRDDVVKITIDWNKL
QSLSAFQPALLFSALEQHILYLQPFLAKLQSPIKEENTTAVEEIGRTEMGNKNEVNDKFS
IGDLQEEEKHKESDLRDVKKTQIHFDPEVVQIKAGKAEIDRRISAFIERKQAEINENNVR
EFCNVIDCNQENSCARTDAIFTPYPGFKSHVKVSRVVNTYGPQTRPEGIPGSGHKPNSML
RDCGNQAVEERLQNIEAHLRLQTGGPVPRDIYQRIKKLEDKILELEGISPEYFQSVSFSG
KRRKVQPPQQNYSLAELDEKISALKQALLRKSREAESMATHHLP
>Q7KNF2
MADEDITLNEDQLLESLEETNGEQETEIATEVEEEGSMQIDPELEAIKARVKEMEEEAEK
IKQMQSEVDKQMAGGSTTGLATVPLSLEEKQEIDTRSVYVGNVDYGASAEELEAHFHGCG
TINRVTILCNKADGHPKGFAYIEFGSKEFVETALAMNETLFRGRQIKVMSKRTNRPGLST
TNRFARGSFRGRGARVSRACCHSTFRGARRAMGYRGRANYYAPY

>O94244
MSEEVVQDAPLENNELNAEIDLQKTIQEEYKLWKQNVPFLYDLVITHALEWPSLTIQWLP
DKKTIPGTDYSIQRLILGTHTSGNDQNYLQIASVQLPNFDEDTTEFTPSTIRRAQATGSY
TIEISQKIPHDGDVNRARYMPQKPEIIATMGEGGNAYIFDTTCHDALTTGEALPQAVLKG
HTAEGFGLCWNPNLPGNLATGAEDQVICLWDVQTQSFTSSETKVISPIAKYHRHTDIVND
VQFHPQHEALLASVSDDCTLQIHDTRLNPEEEAPKVIQAHSKAINAVAINPFNDYLLATA
SADKTVALWDLRNPYQRLHTLEGHEDEVYGLEWSPHDEPILASSSTDRRVCIWDLEKIGE
EQTPEDAEDGSPELLFMHGGHTNRISEFSWCPNERWVVGSLADDNILQIWSPSRVIWGRD
HVQVSPRDLE
>Q00684
MRRSVYLDNTIEFLRGRVYLGAYDYTPEDTDELVFFTVEDAIFYNSFHLDFGPMNIGHLY
RFAVIFHEILNDPENANKAVVFYSSASTRQRANAACMLCCYMILVQAWTPHQVLQPLAQV
DPPFMPFRDAGYSNADFEITIQDVVYGVWRAKEKGLIDLHSFNLESYEKYEHVEFGDFNV
LTPDFIAFASPQEDHPKGYLATKSSHLNQPFKSVLNFFANNNVQLVVRLNSHLYNKKHFE
DIGIQHLDLIFEDGTCPDLSIVKNFVGAAETIIKRGGKIAVHCKAGLGRTGCLIGAHLIY
TYGFTANECIGFLRFIRPGMVVGPQQHWLYLHQNDFREWKYTTRISLKPSEAIGGLYPLI
SLEEYRLQKKKLKDDKRVAQNNIEGELRDLTMTPPSNGHGALSARNSSQPSTANNGSNSF
KSSAVPQTSPGQPRKGQNGSNTIEDINNNRNPTSHANRKVVIESNNSDDESMQDTNGTSN
HYPKVSRKKNDISSASSSRMEDNEPSATNINNAADDTILRQLLPKNRRVTSGRRTTSAAG
GIRKISGSIKK
>Q8BHK3
MSVTKSARSPQVATPLNLDLPESAKKLQSQDPSPANGSSSESSKKTKGITGFQTLVHLVK
GNMGTGILGLPLAVKNAGILMGPLSLLVMGLIACHCMHILVRCAQRFCHRLNKPFMDYGD
TVMHGLAFSPNAWLQNHAHWGRRVVSFFLIVTQLGFCCVYIVFLADNLKQVVEAVNSTTI
SCHKNETVVLTPTMDSRLYMLSFLPVLGLLVFVRNLRVLTIFSLLANISMLVSLVIIAQY
IIQEIPDASQLPLVASWKTYPLFFGTAIFSFESIGVVLPLENKMKDARGFPTILSLGMSI
ITTLYIAIGALGYLRFGDDIKASITLNLPNCWLYQSVKLLYVVGILCTYALQFYVPAEII
IPLAVSQVSKRWALPVDLSIRLALVCLTCMLAILIPRLDLVLSLVGSVSSSALALIIPPL
LEVVTYYGEGISPLTVTKDALISILGFMGFVVGTYQALDELIKSGNSPALSNSTMFIQ
>Q9P4X2
MYFLILFSSNAELGEVLRTMRSVEDRFNKRFHYPWTFYGMDEFTEYFMSTTSTIASGHCE
YGTKGITYTNHLQFILTISYLVHWDLSQVKPLVENNRLYQQRADALAQENLSYVYSPLFH
SFQDWVVRSLLYHPQLEYDYVWRIEPGLKLVCEEKKDIFSTFKDSDIVFTNHVCERKHSG
IYAMEEAIEEYKILNTQGDFSNIWVYSNNYTYCKYWPFNEILSLKQIRHNQTYTNLINYL
LGSGGTYYHRWTESDILSAAFGVLRARANHMESVGFFLNDDVHYCPETIPYTGRCACL
>P43565
MFNRSNTAGGSQAMKEGLGINKLSPISSNSNPSSLTSSNYEKYLQLATEKNPCMILELEL
DGKVRYGSPQWNTITGVADDSGSSPTYIADLILGSDQDKGVFQKATDMLLMNDDTSCTIT
FKIKAADYEGSAGCDDESTITTLEARGILIRDGHTQLPSHTMWIVKPRTNDWSDFYANED
AQDDMVIQLSDNCDDIDIQLPEEFAKTLGFGAKIFVQYLKRIRLEMIIDEFNLPLPKMEL
CRVCENFVPVWWLETHSQSCVCEHRTESLIQLLHDNLLEQQAILANFTKDSEYKGSQIQV
RSNNFLNQVLDSLRELCQDAIDINPSEMVPDLYHSLSTFPQDNGNNNNNNNNNNNNNNAL
LDQFPIQKDTVSLNSYFQFSPRTNHNIQNVTSWQSRFFLNDDQDPGLALLIHDTLDLARK
KVDAVLRLDNAMTYSLKIKNEVNNYVVQLIREQIEINKHAILTHPMNLRSSSIFHSPLPQ

IHSQQPEAENLIYSSSTPLQVQHDQCASFEAPSKSHLEPIPFPVSSIEETPTANDIRHPS
PLPRSCSNTVMKLPTPRRKLDSNGLFSDAYLNADIIPNPSIESTISIDRDNNTNSRGSSM
KQYGIGEATDSRTSNSERPSSSSSRLGIRSRSITPRQKIEYSHVDNDDRTNEMLSRDKDS
LQPQPSVDTTITSSTQATTTGTKTNSNNSTNSVLPKLMTSISLTPRRGSPSFGNLASHSM
QQTNSFKLIHDKSPISSPFTFSKDFLTPEQHPSNIARTDSINNAMLTSPNMPLSPLLLAT
NQTVKSPTPSIKDYDILKPISKGAYGSVYLARKKLTGDYFAIKVLRKSDMIAKNQVTNVK
SERAIMMVQSDKPYVARLFASFQNKDNLFLVMEYLPGGDLATLIKMMGYLPDQWAKQYLT
EIVVGVNDMHQNGIIHHDLKPENLLIDNAGHVKLTDFGLSRAGLIRRHKFVPHKSSLSIS
STLPIDNPANNFTMNNNNSNHSQLSTPDSFTSDHKQYNRSKKSSLGQQYEHSEYSSTSNS
HSMTPTPSTNTVVYPSYYRGKDRSHGSSNIDLPASLRRSESQLSFSLLDISRSSTPPLAN
PTNSNANNIMRRKSLTENKSFSNDLLSSDAIAATNTNINSNNNISLSPAPSDLALFYPDD
SKQNKKFFGTPDYLAPETIEGKGEDNKQCDWWSVGCIFFELLLGYPPFHAETPDAVFKKI
LSGVIQWPEFKNEEEEREFLTPEAKDLIEKLLVVDPAKRLGAKGIQEIKDHPYFKNVDWD
HVYDEEASFVPTIDNPEDTDYFDLRGAELQDFGDDIENDNANILFGKHGINTDVSELSAA
NLSPPLNHKNILSRKLSMSNTTNRSSNNSNSSVHDFGAHTPVNKLSIASVLESVPQETGY
ITPNGTGTTTTSAKNSPNLKNLSLAIPPHMRDRRSSKLNDSQTEFGSFNFRNLSALDKAN
KDAINRLKSEHFSEQPGVHRRTSSASLMGSSSDGSVSTPGSNASNTTSGGKLKIHKPTIS
GSPSTFGTFPKTFLRSDSFSTRSYSPERSISIDSSTLSRKGSIIGDNQQTTANSSDSPTM
TKFKSPLSPANTTTVSSYFSRQRVLSKSFSQRTNSSDLSAEESDRLQAISRVNSLRNRRR
SGRKSSSTSEIGYHMDVLVCEPIPIHRYRVTKDLENLGCTVVSVGAGDELVSRATSGVSF
DLIMTALKLPKLGAIDIVQLLKQTNGANSTTPIVAITNYFQEAATSRVFDDVLEKPVKLD
ELKKLVAKYALKKSQEDEEHTILSDSDETH
>Q12338
MTKDAVNLDAYTVSFMPFYTEYQGPTEEFKDYKFEDTIYFRGKELKREKSATPSSSDNTT
SNTFSNGAILSGNTITGKIVSVNNYEREGTDRNELARLQELISLIDVINQ
>Q9UKT7
MKRGGRDSDRNSSEEGTAEKSKKLRTTNEHSQTCDWGNLLQDIILQVFKYLPLLDRAHAS
QVCRNWNQVFHMPDLWRCFEFELNQPATSYLKATHPELIKQIIKRHSNHLQYVSFKVDSS
KESAEAACDILSQLVNCSLKTLGLISTARPSFMDLPKSHFISALTVVFVNSKSLSSLKID
DTPVDDPSLKVLVANNSDTLKLLKMSSCPHVSPAGILCVADQCHGLRELALNYHLLSDEL
LLALSSEKHVRLEHLRIDVVSENPGQTHFHTIQKSSWDAFIRHSPKVNLVMYFFLYEEEF
DPFFRYEIPATHLYFGRSVSKDVLGRVGMTCPRLVELVVCANGLRPLDEELIRIAERCKN
LSAIGLGECEVSCSAFVEFVKMCGGRLSQLSIMEEVLIPDQKYSLEQIHWEVSKHLGRVW
FPDMMPTW
>Q9VVR2
MYNDEVRTPQALKNRYITNVNGLKCRARRNSHSNSSNSSAASATPTNGGKENGKYSPQMS
GNVCTPPPKRLHKVRNPFEGAMADRLHLPLIASPSLFRSRTPQLSSTQFEWNIDEVSQLK
PADVEPHETQFHDSPDPEQESKAQLAISAFFKESLIVPSPVDCPLRKQRIILNCSEDNTP
ISNKSRRMRDCEVQTELTLPPILPKALEDALRPYFQPHLAGRLSGRSKSSGGPDIFNSSM
RRKLFDLHNVIVLGEQDTAEPSRSMVGSSPQGKQTMFAGRLSDSASGESSFGCLSPIRNL
CGLPPGTPDNGTCSGKRKLLMHELELPSPIAPSEHLSRRLVHSKVEISVTEQHDTLSERT
ALKFTPDRSSSPMGGGLEHSDCSINQRVRRLRVNSTRQVVIETGDQPLFEETEGEEEEAE
SEDDEEADAMQLSTVSFNCSSSNSDTPRGHKRHRSAQRKNLSQSFSANLEEEADQTQGGA
GSIQPAEPPSVAMPQQGARIPLYRADSGFNETSSTTFAFSQDLPLDVSMACCSTPSTRS

>Q07440
MAESELMHIHSLAEHYLQYVLQVPAFESAPSQACRVLQRVAFSVQKEVEKNLKSYLDDFH
VESIDTARIIFNQVMEKEFEDGIINWGRIVTIFAFGGVLLKKLPQEQIALDVCAYKQVSS
FVAEFIMNNTGEWIRQNGGWEDGFIKKFEPKSGWLTFLQMTGQIWEMLFLLK
>Q96301
MVGLEDDTERERSPVVENGFSNGSRSSSSSAGVLSPSRKVTQGNDTLSYANILRARNKFA
DALALYEAMLEKDSKNVEAHIGKGICLQTQNKGNLAFDCFSEAIRLDPHNACALTHCGIL
HKEEGRLVEAAESYQKALMADASYKPAAECLAIVLTDLGTSLKLAGNTQEGIQKYYEALK
IDPHYAPAYYNLGVVYSEMMQYDNALSCYEKAALERPMYAEAYCNMGVIYKNRGDLEMAI
TCYERCLAVSPNFEIAKNNMAIALTDLGTKVKLEGDVTQGVAYYKKALYYNWHYADAMYN
LGVAYGEMLKFDMAIVFYELAFHFNPHCAEACNNLGVLYKDRDNLDKAVECYQMALSIKP
NFAQSLNNLGVVYTVQGKMDAAASMIEKAILANPTYAEAFNNLGVLYRDAGNITMAIDAY
EECLKIDPDSRNAGQNRLLAMNYINEGLDDKLFEAHRDWGWRFTRLHPQYTSWDNLKDPE
RPITIGYISPDFFTHSVSYFIEAPLTHHDYTKYKVVVYSAVVKADAKTYRFRDKVLKKGG
VWKDIYGIDEKKIASMVREDKIDILVELTGHTANNKLGTMACRPAPVQVTWIGYPNTTGL
PTVDYRITDSLADPPDTKQKQVEELVRLPDCFLCYTPSPEAGPVCPTPALSNGFVTFGSF
NNLAKITPKVLQVWARILCAVPNSRLVVKCKPFCCDSIRQRFLTTLEQLGLESKRVDLLP
LILFNHDHMQAYSLMDISLDTFPYAGTTTTCESLYMGVPCVTMAGSVHAHNVGVSLLTKV
GLGHLVAKNEDEYVQLSVDLASDVTALSKLRMSLRDLMAGSPVCNGPSFAVGLESAYRNM
WKKYCKGEVPSLRRMEMLQKEVHDDPLISKDLGPSRVSVTGEATPSLKANGSAPVPSSLP
TQSPQLSKRMDSTS
>Q9HBG6
MRAVLTWRDKAEHCINDIAFKPDGTQLILAAGSRLLVYDTSDGTLLQPLKGHKDTVYCVA
YAKDGKRFASGSADKSVIIWTSKLEGILKYTHNDAIQCVSYNPITHQLASCSSSDFGLWS
PEQKSVSKHKSSSKIICCSWTNDGQYLALGMFNGIISIRNKNGEEKVKIERPGGSLSPIW
SICWNPSSRWESFWMNRENEDAEDVIVNRYIQEIPSTLKSAVYSSQGSEAEEEEPEEEDD
SPRDDNLEERNDILAVADWGQKVSFYQLSGKQTGKDRALNFDPCCISYFTKGEYILLGGS
DKQVSLFTKDGVRLGTVGEQNSWVWTCQAKPDSNYVVVGCQDGTISFYQLIFSTVHGLYK
DRYAYRDSMTDVIVQHLITEQKVRIKCKELVKKIAIYRNRLAIQLPEKILIYELYSEDLS
DMHYRVKEKIIKKFECNLLVVCANHIILCQEKRLQCLSFSGVKEREWQMESLIRYIKVIG
GPPGREGLLVGLKNGQILKIFVDNLFAIVLLKQATAVRCLDMSASRKKLAVVDENDTCLV
YDIDTKELLFQEPNANSVAWNTQCEDMLCFSGGGYLNIKASTFPVHRQKLQGFVVGYNGS
KIFCLHVFSISAVEVPQSAPMYQYLDRKLFKEAYQIACLGVTDTDWRELAMEALEGLDFE
TAKKAFIRVQDLRYLELISSIEERKKRGETNNDLFLADVFSYQGKFHEAAKLYKRSGHEN
LALEMYTDLCMFEYAKDFLGSGDPKETKMLITKQADWARNIKEPKAAVEMYISAGEHVKA
IEICGDHGWVDMLIDIARKLDKAEREPLLLCATYLKKLDSPGYAAETYLKMGDLKSLVQL
HVETQRWDEAFALGEKHPEFKDDIYMPYAQWLAENDRFEEAQKAFHKAGRQREAVQVLEQ
LTNNAVAESRFNDAAYYYWMLSMQCLDIAQDPAQKDTMLGKFYHFQRLAELYHGYHAIHR
HTEDPFSVHRPETLFNISRFLLHSLPKDTPSGISKVRKILFTLAKQSKALGAYRLARHAY
DKLRGLYIPARFQKSIELGTLTIRAKPFHDSEELVPLCYRCSTNNPLLNNLGNVCINCRQ
PFIFSASSYDVLHLVEFYLEEGITDEEAISLIDLEVLRPKRDDRQLEIANNSSQILRLVE
TKDSIGDEDPFTAKLSFEQGGSEFVPVVVSRLVLRSMSRRDVLIKRWPPPLRWQYFRSLL
PDASITMCPSCFQMFHSEDYELLVLQHGCCPYCRRCKDDPGP
>P39428

MASSSAPDENEFQFGCPPAPCQDPSEPRVLCCTACLSENLRDDEDRICPKCRADNLHPVS
PGSPLTQEKVHSDVAEAEIMCPFAGVGCSFKGSPQSMQEHEATSQSSHLYLLLAVLKEWK
SSPGSNLGSAPMALERNLSELQLQAAVEATGDLEVDCYRAPCCESQEELALQHLVKEKLL
AQLEEKLRVFANIVAVLNKEVEASHLALAASIHQSQLDREHLLSLEQRVVELQQTLAQKD
QVLGKLEHSLRLMEEASFDGTFLWKITNVTKRCHESVCGRTVSLFSPAFYTAKYGYKLCL
RLYLNGDGSGKKTHLSLFIVIMRGEYDALLPWPFRNKVTFMLLDQNNREHAIDAFRPDLS
SASFQRPQSETNVASGCPLFFPLSKLQSPKHAYVKDDTMFLKCIVDTSA
>P19414
MLSARSAIKRPIVRGLATVSNLTRDSKVNQNLLEDHSFINYKQNVETLDIVRKRLNRPFT
YAEKILYGHLDDPHGQDIQRGVSYLKLRPDRVACQDATAQMAILQFMSAGLPQVAKPVTV
HCDHLIQAQVGGEKDLKRAIDLNKEVYDFLASATAKYNMGFWKPGSGIIHQIVLENYAFP
GALIIGTDSHTPNAGGLGQLAIGVGGADAVDVMAGRPWELKAPKILGVKLTGKMNGWTSP
KDIILKLAGITTVKGGTGKIVEYFGDGVDTFSATGMGTICNMGAEIGATTSVFPFNKSMI
EYLEATGRGKIADFAKLYHKDLLSADKDAEYDEVVEIDLNTLEPYINGPFTPDLATPVSK
MKEVAVANNWPLDVRVGLIGSCTNSSYEDMSRSASIVKDAAAHGLKSKTIFTVTPGSEQI
RATIERDGQLETFKEFGGIVLANACGPCIGQWDRRDIKKGDKNTIVSSYNRNFTSRNDGN
PQTHAFVASPELVTAFAIAGDLRFNPLTDKLKDKDGNEFMLKPPHGDGLPQRGYDAGENT
YQAPPADRSTVEVKVSPTSDRLQLLKPFKPWDGKDAKDMPILIKAVGKTTTDHISMAGPW
LKYRGHLENISNNYMIGAINAENKKANCVKNVYTGEYKGVPDTARDYRDQGIKWVVIGDE
NFGEGSSREHAALEPRFLGGFAIITKSFARIHETNLKKQGLLPLNFKNPADYDKINPDDR
IDILGLAELAPGKPVTMRVHPKNGKPWDAVLTHTFNDEQIEWFKYGSALNKIKADEKK
>Q9BWU0
MLAPLRNAPGREGATSPSPPTDATGSLGEWDVDRNVKTEGWVSKERISKLHRLRMADILS
QSETLASQDLSGDFKKPALPVSPAARSKAPASSSSNPEEVQKEGPTALQDSNSGEPDIPP
PQPDCGDFRSLQEEQSRPPTAVSSPGGPARAPPYQEPPWGGPATAPYSLETLKGGTILGT
RSLKGTSYCLFGRLSGCDVCLEHPSVSRYHAVLQHRASGPDGECDSNGPGFYLYDLGSTH
GTFLNKTRIPPRTYCRVHVGHVVRFGGSTRLFILQGPEEDREAESELTVTQLKELRKQQQ
ILLEKKMLGEDSDEEEEMDTSERKINAGSQDDEMGCTWGMGEDAVEDDAEENPIVLEFQQ
EREAFYIKDPKKALQGFFDREGEELEYEFDEQGHSTWLCRVRLPVDDSTGKQLVAEAIHS
GKKKEAMIQCSLEACRILDTLGLLRQEAVSRKRKAKNWEDEDFYDSDDDTFLDRTGLIEK
KRLNRMKKAGKIDEKPETFESLVAKLNDAERELSEISERLKASSQVLSESPSQDSLDAFM
SEMKSGSTLDGVSRKKLHLRTFELRKEQQRLKGLIKIVKPAEIPELKKTETQTTGAENKA
KKLTLPLFGAMKGGSKFKLKTGTVGKLPPKRPELPPTLMRMKDEPEVEEEEEEEEEEEKE
KEEHEKKKLEDGSLSRPQPEIEPEAAVQEMRPPTDLTHFKETQTHENMSQLSEEEQNKDY
QDCSKTTSLCAGPSASKNEYEKSRGELKKKKTPGPGKLPPTLSSKYPEDDPDYCVWVPPE
GQSGDGRTHLNDKYGY
>Q9NQT5
MAEPASVAAESLAGSRARAARTVLGQVVLPGEELLLPEQEDAEGPGGAVERPLSLNARAC
SRVRVVCGPGLRRCGDRLLVTKCGRLRHKEPGSGSGGGVYWVDSQQKRYVPVKGDHVIGI
VTAKSGDIFKVDVGGSEPASLSYLSFEGATKRNRPNVQVGDLIYGQFVVANKDMEPEMVC
IDSCGRANGMGVIGQDGLLFKVTLGLIRKLLAPDCEIIQEVGKLYPLEIVFGMNGRIWVK
AKTIQQTLILANILEACEHMTSDQRKQIFSRLAES
>Q12418
MLLDVNTNHTLMHDAHVHEHCLIKSIRDDGALHSWSDSSKVFYPKSFYATATNKKNNKLA

SASMNKTATSNRTVSDEIYFHSTKPQFDGQGSAERTRTLTKRNSFKRTRILKARDDSELL
NENRSSLMTPSLSSVMSQVRKTNSAKTLSGECPIHEGHLTQSIKRKFSEEAQSDCSSLSS
SKLHPLTDDIADAVDLQTPAIGDEVLAEPVVPKMKIININDLDLFDDWEVKDLVDIFPPV
YERRPRSSSALSLVSASSDAKLRPTSVDFQIIDKKGGKTSRRKSRSKSTTENMIYENDLV
ELEQWPSASPSPETDGSIASSELLPNKRIRQKSLNTNFLKLYSIETSCKRKSILPEVEVD
DHLLKQLTYSEIRSLEIKKEPNVSTNDIKLALITRKKLWSDMVHETRNDLFGDSTPWNLH
FVATTSNTEPSQGRESASEHATADLKSSLVRVHSDVKPWFNNGGTMLKPCGKLNLGKVTN
KTSAPTREIQYVVKGWCDSRFL
>Q04418
MDLLGDIVEKDTSDSVESNDNGTLSTNNCGTGFPELYKPKKISSWKERLREKRAQKKKTS
GKDAEKQQTSTDAPLSEAKSIHNENIKVLQGMSDEQIVQEREDLYNSLDPKLIAKLLKNI
NKRAKDENNTPLFAEIEGASGTWVGGNKQGIYDLPPLDDEDVDVALEIRPMLGKDAKHVQ
FEEAGKEKDVEGEGKTNDDVDDIAPLDFQMAQCIDHMKNEELFKDVHFIKEESQNEINLE
KLDINDPNFNDKLHEKYFPDLPKEVDKLKWMQPVQQKTDKNYIIEDVSECRFDFNGDLVP
PTRQIDSTIHSGLHHHSDSPELAGYTIVELEHLARSTFPSQRCIAIQTLGRILYKLGQKS
YYQLVPEIDADTYKEDGSISNVMDKIYSMFWDLIKDGKVIESLEISSDEKFTRNLSVRNY
AIDALWLWKQGGGDFRTKK
>Q9P7V3
MNKSIFIQLQDQIDKEHSIREKLTAEVDLLDEKLRVLQLLLANCEQSRNENLQEKEHGLT
LEDLENQEEILEALEIIKSKTRGLAELASNFPYYKYNGVWDRSIQKVVYLYLLASWTGRL
DKSLRPTYSLLSLSEVGQILQVPVFPEESTFHLSIEQYLHAVLSLCSELARQSVNSVISG
NYHIPFEALNTIQKVHSSFQVLSLKNDSLRRHFDGLKYDLKRSEDVVYDLRIHKLV
>Q9JMH3
MATCPVLQKETLFRTGVHAYRIPALLYLKKQKTLLAFAEKRASKTDEHAELIVLRRGSYN
EATNRVKWQPEEVVTQAQLEGHRSMNPCPLYDKQTKTLFLFFIAVPGRVSEHHQLHTKVN
VTRLCCVSSTDHGRTWSPIQDLTETTIGSTHQEWATFAVGPGHCLQLRNPAGSLLVPAYA
YRKLHPAQKPTPFAFCFISLDHGHTWKLGNFVAENSLECQVAEVGTGAQRMVYLNARSFL
GARVQAQSPNDGLDFQDNRVVSKLVEPPHGCHGSVVAFHNPISKPHALDTWLLYTHPTDS
RNRTNLGVYLNQMPLDPTAWSEPTLLAMGICAYSDLQNMGQGPDGSPQFGCLYESGNYEE
IIFLIFTLKQAFPTVFDAQ
>Q03088
MSSSSLRVLAIGNNPNILFYTSRFQLAKNIDLYHVNDSKSCQFEIETEYYGKDRFELENH
FTSIEHLTEALSSKSSEAVFDIIIMSAPSLQELSSLASKLTSIIDSNTKIFLESSGFIQL
EPFVKLSMESPHVNVFSILTDLDIRQIGPNHFKHFPSTAKENTIYLGESKSSTEKYSSGV
ITLLTTFEKLFAKLFSNIKINLCNFSSIEFLSQQWKLAISRICFDPLLIMFEQENPSDLD
QQIIAKPLISGLVTEIITVAKTMGARLNSSHDNENSLLSLWKNSYHSTNKPPALVYHFIH
QTTPLNIDILLLQTILLADDFGIKTPYLEFLYSVLSQFERLNSGKSKWFIRSDEKTQILQ
SLQKSQKNESALQTQITSLQGQISKLRQELLMQAKQHEMETNELKEKHQVALKAQAQAQA
QAQSQAQTSIEALTPTEATNQSDTNEYKATGTPNLRDIEDMALYSVNYGDSPVRSPPPVV
SSQPQMNSPLSSHSQTFGENNGTNDKLLQERELQLRKKELELQERELEFQKRALQQQRFN
NSNNSIPRKPSFPQLQQSANVRSNSRGMHGTNGAMSQPASAGNFVDPISSSIAAYDPQQP
PSLPLQQPQQSVQVQPFHSHSIKPTSRKNRNSNMPNIGNPSSINMSDFGRPPNNSSQTRL
NSMPTHSIVNQNRLRSQQSKNKLNMPHATNPNNTFNQVPAPSLNNHVPTQRQFSSSTMIE
VTNNNNKVNNSSSNPDISTNSVVHNAMQFTNTNNNTSSTVDINDPKNIAPPPTTSVSAPS

TPTLSSSSQMANMASPSTDNGDNEEKNGGKKKRFGLFKKKNKSKK
>Q9VVV9
MAGNIVKWWKHKILGGYKQFSVQECTTDSEELMYHQVRASSSCSAPPDLLLVSERDNNIQ
LRSPVVNIITTPPGNASGAGSKQQSHHQTNHHSSGRSHPGHTAHPQDVSSGGSHSKHLRI
SSTSNGKHGKYSNMQQQLPQDEDVVDAAATMQQQQHTGHAHSRHLHHHKEERIRLEEFTC
DVSVEGGKSSQPLQFSFTFYDLDGHHGKITKDDIVGIVYTIYESIGKSVVVPHCGSKTIN
VRLTVSPEGKSKSQPVVPVPVAAGFSSSHASKLKKLPTGLAAMSKPLAGGGVGSGGASAL
TTSAGNRRQHRYRPRKLIKSDDEDDDSNSEKEKDAAHAPAADQPSGSGTKATGKSHHHQS
QSARYHQKNNSRAEQCCTEQNTPDNGHNTYENMLNLKCCKPEVDQVDCPSHRQHHQSHPN
HQMRQQDIYMKQATQRVKMLRRARKQKYQDHCLETRQRSLSVGNDSACPNRHLQLQQPPV
GHPQPQSLNHKSASGSPPLGVGGGGDMMLDGVQLRQPRPHSLTPHQHQQQNQQQQQQQRK
SAECWKSALNRNDLISIIRESMEKNRLCFQLNGKPQANVSPIRQPAAQQQPQQQQRQRCN
TGSKIPTLITNHSPVAQQSPLSCSPPTAEPTTPSIPAAPPAIEVNGQQHHPTHPTHPSHH
NHHEHPQPHIPIYHQQLAINPAVLAAQQTHNTAHNKLNLCGYDSFLHATICGGGAAAHSP
PATPSNVATVQPIPKKSQKNLLQGYQRLEQSQQQQQQQRSSKDYKNYGNLIYAKLSEQLQ
QKDREQRRQRHKQQQHQMLQDQPKDASRSEQRPPTSNSSSAGSKIYGDAVECAHLLASEE
EDLPPSPQLTSTPSKVVSTDTLIDLNDDVGEAVAEAVTEGGKQSLEAEESGQQVEVELDT
SASSSMIHRYVHEHIHHHYHHFKEQQDV
>O95379
MHSEAEESKEVATDVFNSKNLAVQAQKKILGKMVSKSIATTLIDDTSSEVLDELYRVTRE
YTQNKKEAEKIIKNLIKTVIKLAILYRNNQFNQDELALMEKFKKKVHQLAMTVVSFHQVD
YTFDRNVLSRLLNECREMLHQIIQRHLTAKSHGRVNNVFDHFSDCEFLAALYNPFGNFKP
HLQKLCDGINKMLDEENI
>Q9P7K5
MGFTSVEPPDFFDWTARAIFLLSTLRSRRNAKQIERENALLDQIKELLSIAIIQCHERNI
PSIDLPPSFFNYVCEQLPRHPFAKIASSYIDTHTLFFQISISSFLCRFSNCSEDEIRLFK
QHQSLDSFLLSLFDLLCSHHFSVEFTSISLIIKSSQWLFYTLSDYAHESLTGVEADITTK
FLSTLADTWPEFEEQCTLSKHLFLLGDFSLISYVKVLCGVFNIPNYNAFILSVEDLLPEN
ISFAAVADFQLSKYRAINDNSVLRSDDFSSKAVFDKYVERECYYSENAISKIFQNYPDLA
VLFEELQYAEGNNDLLSAILNTNKYTLRPDDCAPYFYRLMDLCLRYCYSQSNEIGTNVLW
SMTTSFINIWRIHPGTCCCVVLSIYWPYLEDKLLKFSDLGDIFEYFFSLIHDCDYAIWSV
HDEHQFSELLAFYHRRALNDLVEELGSCPRSSDSCRAILDILNNYIFSCPLFLVTPEELE
EQYTDFFKKLKEKYGEFFQNLLKKELTGKPESDLEGLRLVGVQLQATYENLKSNFSARIF
NQLSVHSFFAEATCHLFLEKLPTITKTYVYSNCKEIPDHILEMINALYQQTRALVACTGY
LSSDYPKIYEAFSGTFVYRNLSLIIDKGLANVLQTVLRESFIPKRSRFYSDSTKMILAPL
QDCYDFLQKIDWPSSKDANRFYLHLGKLSENYIHYYVDSMYALFIYSTDIPLKAEPLVPD
LPVKQLKEKASHFKRHSGDPNFLKLYRESFTLINTVSLVSGWLKMLHQRIDYKILTEKVM
HFKDMEVPSATVIISIKMAHLLVTPKAPYSFSVLLQDPESEFCVHTRDVYDELVPNWKET
YQMEITNVKRFKLIIYQRNIHTGLETVYGHATLEVDPDKLKQGHGKDVWLDITNKGQILI
GLRVLLKRETPNYYCFAQMEYLTVTEVKMLNVLMISLSKFIRNSLRNSGLMDFVRYLDGD
ENDNAEAETKEKMDELQARCEKEQQSLLAELDDFFYWIYVNLLPKPLFYLLSRLWFGLLE
TFRDILVPPVTDSTLRRIPLNKRELKMVYSWLQLFYDFFRNFQDIAPFDFLNTDEYKSVM
AIKDYYFVKSKDVKRVSS
>P31166

MQTIIISPLVSHRLCLARAVPCNRLLNNHHRAPPSIRLSNHRSTTSLRLFSSAAASRDSE
MATEDVQDPRIAKIASSIRVIPDFPKPGIMFQDITTLLLDTEAFKDTIALFVDRYKDKGI
SVVAGVEARGFIFGPPIALAIGAKFVPMRKPKKLPGKVISEEYSLEYGTDTIEMHVGAVE
PGERAIIIDDLIATGGTLAAAIRLLERVGVKIVECACVIELPELKGKEKLGETSLFVLVK
SAA
>P25332
MGITVIGSLNYDLDTFTDRLPNAGETFRANHFETHAGGKGLNQAAAIGKLKNPSSRYSVR
MIGNVGNDTFGKQLKDTLSDCGVDITHVGTYEGINTGTATILIEEKAGGQNRILIVEGAN
SKTIYDPKQLCEIFPEGKEEEEYVVFQHEIPDPLSIIKWIHANRPNFQIVYNPSPFKAMP
KKDWELVDLLVVNEIEGLQIVESVFDNELVEEIREKIKDDFLGEYRKICELLYEKLMNRK
KRGIVVMTLGSRGVLFCSHESPEVQFLPAIQNVSVVDTTGAGDTFLGGLVTQLYQGETLS
TAIKFSTLASSLTIQRKGAAESMPLYKDVQKDA
>Q9H2X6
MAPVYEGMASHVQVFSPHTLQSSAFCSVKKLKIEPSSNWDMTGYGSHSKVYSQSKNIPLS
QPATTTVSTSLPVPNPSLPYEQTIVFPGSTGHIVVTSASSTSVTGQVLGGPHNLMRRSTV
SLLDTYQKCGLKRKSEEIENTSSVQIIEEHPPMIQNNASGATVATATTSTATSKNSGSNS
EGDYQLVQHEVLCSMTNTYEVLEFLGRGTFGQVVKCWKRGTNEIVAIKILKNHPSYARQG
QIEVSILARLSTESADDYNFVRAYECFQHKNHTCLVFEMLEQNLYDFLKQNKFSPLPLKY
IRPVLQQVATALMKLKSLGLIHADLKPENIMLVDPSRQPYRVKVIDFGSASHVSKAVCST
YLQSRYYRAPEIILGLPFCEAIDMWSLGCVIAELFLGWPLYPGASEYDQIRYISQTQGLP
AEYLLSAGTKTTRFFNRDTDSPYPLWRLKTPDDHEAETGIKSKEARKYIFNCLDDMAQVN
MTTDLEGSDMLVEKADRREFIDLLKKMLTIDADKRITPIETLNHPFVTMTHLLDFPHSTH
VKSCFQNMEICKRRVNMYDTVNQSKTPFITHVAPSTSTNLTMTFNNQLTTVHNQAPSSTS
ATISLANPEVSILNYPSTLYQPSAASMAAVAQRSMPLQTGTAQICARPDPFQQALIVCPP
GFQGLQASPSKHAGYSVRMENAVPIVTQAPGAQPLQIQPGLLAQQAWPSGTQQILLPPAW
QQLTGVATHTSVQHATVIPETMAGTQQLADWRNTHAHGSHYNPIMQQPALLTGHVTLPAA
QPLNVGVAHVMRQQPTSTTSSRKSKQHQSSVRNVSTCEVSSSQAISSPQRSKRVKENTPP
RCAMVHSSPACSTSVTCGWGDVASSTTRERQRQTIVIPDTPSPTVSVITISSDTDEEEEQ
KHAPTSTVSKQRKNVISCVTVHDSPYSDSSSNTSPYSVQQRAGHNNANAFDTKGSLENHC
TGNPRTIIVPPLKTQASEVLVECDSLVPVNTSHHSSSYKSKSSSNVTSTSGHSSGSSSGA
ITYRQQRPGPHFQQQQPLNLSQAQQHITTDRTGSHRRQQAYITPTMAQAPYSFPHNSPSH
GTVHPHLAAAAAAAHLPTQPHLYTYTAPAALGSTGTVAHLVASQGSARHTVQHTAYPASI
VHQVPVSMGPRVLPSPTIHPSQYPAQFAHQTYISASPASTVYTGYPLSPAKVNQYPYI
>Q9NAN2
MSYNGSYHQNHHSTLQVKSKFDSEWRRFSIPMHSASGVSYDGFRSLVEKLHHLESVQFTL
CYNSTGGDLLPITNDDNLRKSFESARPLLRLLIQRRGESWEEKYGYGTDSDKRWKGISSL
MAQKPPKRSYSISNPEDFRQVSAIIDVDIVPEAHRRVRLCKHGQERPLGFYIRDGTSVRV
TERGVVKVSGIFISRLVDGGLAESTGLLGVNDEVLEVNGIEVLGKTLDQVTDMMVANAHN
LIITVKPANQRNTLSRGPSQQGTPNASEMSAATAAATGGIQRPMKMNGSSDGSYHPKQHD
ANDSDSGED
>P53867
MQRWLQLWKMDLVQKVSHGVFEGSSEEPAALMNHDYIVLGEVYPERDEESGAEQCEQDCR
YRGEAVSDGFLSSLFGREISSYTKEFLLDVQSRVNFTYRTRFVPIARAPDGPSPLSLNLL
VRTNPISTIEDYIANPDCFNTDIGWGCMIRTGQSLLGNALQILHLGRDFRVNGNESLERE

SKFVNWFNDTPEAPFSLHNFVSAGTELSDKRPGEWFGPAATARSIQSLIYGFPECGIDDC
IVSVSSGDIYENEVEKVFAENPNSRILFLLGVKLGINAVNESYRESICGILSSTQSVGIA
GGRPSSSLYFFGYQGNEFLHFDPHIPQPAVEDSFVESCHTSKFGKLQLSEMDPSMLIGIL
IKGEKDWQQWKLEVAESAIINVLAKRMDDFDVSCSMDDVESVSSNSMKKDASNNENLGVL
EGDYVDIGAIFPHTTNTEDVDEYDCFQDIHCKKQKIVVMGNTHTVNANLTDYEVEGVLVE
KETVGIHSPIDEKC
>P25367
MDTDKLISEAESHFSQGNHAEAVAKLTSAAQSNPNDEQMSTIESLIQKIAGYVMDNRSGG
SDASQDRAAGGGSSFMNTLMADSKGSSQTQLGKLALLATVMTHSSNKGSSNRGFDVGTVM
SMLSGSGGGSQSMGASGLAALASQFFKSGNNSQGQGQGQGQGQGQGQGQGQGSFTALASL
ASSFMNSNNNNQQGQNQSSGGSSFGALASMASSFMHSNNNQNSNNSQQGYNQSYQNGNQN
SQGYNNQQYQGGNGGYQQQQGQSGGAFSSLASMAQSYLGGGQTQSNQQQYNQQGQNNQQQ
YQQQGQNYQHQQQGQQQQQGHSSSFSALASMASSYLGNNSNSNSSYGGQQQANEYGRPQQ
NGQQQSNEYGRPQYGGNQNSNGQHESFNFSGNFSQQNNNGNQNRY
>Q9NWZ8
MAAVKASTSKATRPWYSHPVYARYWQHYHQAMAWMQSHHNAYRKAVESCFNLPWYLPSAL
LPQSSYDNEAAYPQSFYDHHVAWQDYPCSSSHFRRSGQHPRYSSRIQASTKEDQALSKEE
EMETESDAEVECDLSNMEITEELRQYFAETERHREERRRQQQLDAERLDSYVNADHDLYC
NTRRSVEAPTERPGERRQAEMKRLYGDSAAKIQAMEAAVQLSFDKHCDRKQPKYWPVIPL
KF
>Q9P7K9
MSSTLTCYCPGDGSLLGEVKLFNKSDIDQSIILAEEAQKEWKSTSFAERRNFLKALKENI
IRNQDKYAEIACKDTGKTLVDAAFGEILVTLEKINWTLANGEQSLRPTKRPNSLLTSYKG
GYVKYEPLGVIAALVSWNYPLHNALGPIISALFAGNAIVVKGSELTAWSTHQYCEMVRSL
LQSMGHSPELVQCITCLPDVADHLTSHSGIKHITFIGSQPIAKLVAASAAKQLTPLCLEL
GGKDPCILTDDHRLEEILSIVMRGVFQSAGQNCIGIERIIALDGVYDTIITKLYNRISTM
RLGMYTQNDVDMGAMVSNNRFDHLESLIQDAVSKGARLVYGGHRFQHPKYPKGNYFLPTL
LVDATNEMKIAQEECFAPIALVFRAKSPEHALEIANGTEFGLGASVFGRDKQLCQYFTDN
LETGMVAVNDFGAFYLLQMPFGGCKKSGYGRFAGYEGLRGICNSKAIAYDRFSAIHTGIP
PAVDYPIPDSQKAWQFVRGLMGTVYGAWISLVPNVYQLWRNS
>Q9JLJ3
MSTGTFVVSQPLNYRGGARVEPVDASGTEKAFEPATGREIATFKCSGEKEVNLAVENAKA
AFKIWSKKSGLERCQVLLEAARIIKERRDEIAIMETINNGKSIFEARLDVDTSWQCLEYY
AGLAASMAGEHIQLPGGSFGYTRREPLGVCLGIGAWNYPFQIACWKSAPALACGNAMIFK
PSPFTPVSALLLAEIYTKAGAPNGLFNVVQGGAATGQFLCQHRDVAKVSFTGSVPTGMKI
MEMAAKGIKPITLELGGKSPLIIFSDCNMKNAVKGALLANFLTQGQVCCNGTRVFVQKEI
ADAFTKEVVRQTQRIKIGDPLLEDTRMGPLINAPHLERVLGFVRSAKEQGATVLCGGEPY
APEDPKLKHGYYMTPCILTNCTDDMTCVKEEIFGPVMSILTFETEAEVLERANDTTFGLA
AGVFTRDIQRAHRVAAELQAGTCYINNYNVSPVELPFGGYKKSGFGRENGRVTIEYYSQL
KTVCVEMGDVESAF
>O94305
MADLYTKDWTDVKDKPVARVVFIHGFGEHVNAYPEFFEALNERNIEVYTFDQRGFGHSRK
GGPKKQGCTGGWSLVFPDLDYQILRASDTELPLFLWGHSMGGGLALRYGISGTHRHKLAG
VIAQAPMLRCHPDTEPNFLLRKALTLVSKVHPNFLFDSDVQSQHITRDEAVNQRLQDDPL

VSSVGSLQVFSDMLNRGTKTIELAPQFFLPLLITHGTDDNVTCSDSSKEFYENAGTKDKT
YQSYPGFYHSLHIEKKPEVYEYLDKVAAWIYEHSKPSETVKSEQETAVEHPKPTATTSAP
SASPTGVPVEEESHKATSDAVPPAEAKPEPVPASAAERAPTSESTTVPETIVASTTKVIS
EPAPRVTTAATADIVTNK
>Q8CIQ7
MWTPTEEEKYGVVICSFRGSVPQGLVLEIGETVQILEKCEGWYRGVSTKKPNVKGLFPAN
YIHLKKAIVSNRGQYETVVPLEDSIVTEVTTTLQEWASLWKQLYVKHKVDLFYKLRHVMN
ELIDLRRQLLSGHLTQDQVREVKRHITVRLDWGNEHLGLDLVPRKDFEVVDSDQISVSDL
YKMHLSSRQSVQQSTSQVDTMRPRHGETCRMPVPYHFFFSLKSFTYNTIGEDSDVFFSLY
DMREGKQISERFLVRLNKNGGPRNPEKIERMCALFTDLSSKDMKRDLYIVAHVIRIGRML
LNDSKKGPAHLHYRRPYGCAVLSILDVLQSLTELKEEKDFVLKVYTCNNESEWTQIHENI
IRKSSTKYSAPSASHGLIISLQLFRGDMEQIRRENPMIFNRGLAITRKLGFPDVIMPGDI
RNDLYLTLEKGDFERGGKSVQKNIEVTMYVLYADGEILKDCISLGSGEPNRSSYHSFVLY
HSNSPRWGEIIKLPIPIDRFRGSHLRFEFRHCSTKDKGEKKLFGFAFSPLMRDDGTTLSD
DIHELYVYKCDENSTFNNHALYLGLPCCKEDYNGCPNIPSSLIFQRSKESFFISTQLSST
KLTQNVDLLALLKWKAFPDRIMDILGRLRHVSGEEIVKFLQDILDTLFVILDDNTEKYGL
LVFQSLVFIINLLRDIKYFHFRPVMDTYIQKHFAGALAYKELIRCLKWYMDCSAELIRQD
HIQEAMRALEYLFKFIVQSRILYSRATCGMEEEQFRSSIQELFQSIRFVLSLDSRNSETL
LFTQAALLNSFPTIFDELLQMFTVQEVAEFVRGTLGSMPSTVHIGQSMDVVKLQSIARTV
DSRLFSFSESRRILLPVVLHHIHLHLRQQKELLICSGILGSIFSIVKTSSLEADVMEEVE
MMVESLLDVLLQTLLTIMSKSHAQEAVRGHCPVTAEITGEYVSCLLSLLRQMCDTHFQHL
LDNFQSKDELKEFLLKIFCVFRNLMKMSVFPRDWMVMRLLTSNIIVTTVQYLSSALHKNF
TETDFDFKVWNSYFSLAVLFINQPSLQLEIITSAKRKKILDKYGDMRVMMAYELFSMWQN
LGEHKIHFIPGMIGPFLGVTLVPQPEVRNIMIPIFHDMMDWEQRKNGNFKQVEAELIDKL
DSMVSEGKGDESYRELFGLLTQLFGPYPSLLEKVEQETWRETGISFVTSVTRLMERLLDY
RDCMKGEETENKKVGCTVNLMNFYKSEINKEEMYIRYIHKLCDMHLQAENYTEAAFTLLL
YCELLQWEDRPLREFLHYPSQTEWQRKEGLCRKIIHYFNKGKSWEFGIPLCRELACQYES
LYDYQSLSWIRKMEASYYDNIIEQQRLEPEFFRVGFYGRKFPFFLRNKEYVCRGHDYERL
EAFQQRMLSEFPQAVAMQHPNHPDDAILQCDAQYLQIYAVTPIPDYVDVLQMDRVPDRVK
SFYRVNNVRKFRYDRPFHKGPKDKDNEFKSLWIERTTLTLTHSLPGISRWFEVERRELVE
VSPLENAIQVVENKNQELRALISQYQHKQVHGNINLLSMCLNGVIDAAVNGGIARYQEAF
FDKDYITKHPGDAEKISQLKELMQEQVHVLGVGLAVHEKFVHPEMRPLHKKLIDQFQMMR
ASLYHEFPGLDKLSPACSGTSTPRGNVLASHSPMSPENIKMTHRHSPMNLMGTGRHSSSS
LSSHASSEAGNMMMMGDNSMGEAPEDLYHHMQLAYHNPRYQGSVTNVSVLSSSQASPSSS
SLSSTHSAPSQMITSAPSSTRGSPSLPDKYRHAREMMLLLPTHRDRPSSAMYPAAILENG
QPPNFQRALFQQVVGACKPCSDPNLSMAEKGHYSLHFDAFHHPLGDTPPALPARTLRKSP
LHPIPASPTSPQSGLDGSNSTLSGSASSGVSSLSESNFGHSSEAPPRTDTMDSMPSQAWN
GDEGLEPPYLPVHYSLSESAVLDAIKSQPCRSHSAPGCVLPQDPMDPPALPPKPYHPRLP
ALEHDEGMLLREEAERPRGLHRKASLPPGSVKEEQARLAWEHGRGEQ
>O42657
MLGRLKDQLNMTLAQGQEEAKNRRRQFQEEDQLRRNNKSSNKLSQNEEDAKNMDSVVQKL
NELQNNVVAFQKLLQEKTPLSSIQDLEGFREFMENLEHRYEMTVSEVRRLSHEVNDLQTD
RENLKHQFEDQIEKLNSEISNQNSLILQKKDELEKSIQRCSELEEKINSLESAQSIEQEV
ISSLKDDKTVETKNDVPEVSRPSTDTIGVSSALSKKKKKRNRKNQKKKSTKQNIEATTEN

DALSESISTPDIQKVAQSEDVDAEQDTVADSKEEERRDIANDDLIVNANAKEPMHHFSFT
DLDTLLNWPKYVFHHHKIHLADTIPLVFLDLKPKEYTVDLETPKLVEELTKQLHVAESTL
KENSEKFKQNSESLKSRVDNLNDYITKLQNEIDECRRNLLWAESSCETIREENQKNIKKL
NDAESLKSRLLQSRTQMQTELDSYITSNSQLKDEITSLKQTVSESEAERKRLFSSAQEKQ
LQMKETVNKLTSLQEQNNEFDRQLKEQEEDLQNKEEELTELRKLLREQTQDSQKLRLLVE
QLELERQDLKQAGENHYSNLSSDYETQIKSLESSLTNSQAECVSFQEKINELNSQIDELK
LKLNEANKKYQELAISFENSNVKTQSVEPDNGLSLEALKNENQTLLKNLEDSTARYEHLQ
KSFKNVFNQLRKQQPSNHGRNSSVSRSSSSVEVNSKHPGSDDMLIDKEYTRNILFQFLEQ
RDRRPEIVNLLSILLDLSEEQKQKLLSVKY
>Q9Y817
MPLAFTNPGTPLKRTAAYDNVQWNQNRILTFGTRYTVLIIVNNYESYQNIQFGESIGAVA
LDQRTGFLAIAHGQVLDIYKPADSLKWTHHYNYITQLSEDITQLSWGYNFDLLLCASSYT
KLINFSEDKPRLVWSQNRISDSFLSSTISSDACFIASIETKNPLAKVWQRTSPQGTVSSC
DDFSYLVHPCRVRFSQWSKHLHPDASGSTPFLSTVGYDNVLRIWQVGSRFGSQLMHLSCF
LNLRKYYKSDQPPYCCFIDKDDFTIALAHAISRHSGQVHKDRILNRLLALASGQPHIFLL
FTFECLYIFSLVIDQETLSSFELINQVPTNFPKGALQPDGFIPIYRTSKHEFYDYNIILF
FQNSAREYMFSLTDFFYSDFELRVSYDFYGHDYPIKSLTRTSNGHGIVSRDVNRICMFQS
YISQDGKNQLICRFRLLLGESDFILPLYAGEYAAVLNLNSLKLWHCNEAIPPTLLCTCRQ
VPTSPIITFFILPVQNNDNSALCALTEGGHAWFWSVSNEAHDGSAVLRFIDRVNFTKNLN
IASAVDVMGWSSTLNLSSLSSFDQEVFQSISKDGLLQTWMARVLGDDKAEISEISKVQTS
IKSATMIKGSTSKKVAVVSENNRRLSIFDTRSSEFSEKEEFSSVFDDYGPINDLDWTSTP
NSHSILAVGFLHDVILLCQNRRSYMNDIPCWSRIRRYNLLEYTNSLISDSSWLDNGTLVT
AIGNGLFYFDNSLPEDQSLLFPASLKRSAKNIFDLAYQLNGILPVFHPQLLQQLLLRNQP
FLFTNILLRFYLCLKDDVDMHFLLNMDCSEFYCCDEEISKDLLIKSLSQNSAEILNPAEL
DFEETSVSKHLGLYIDEMIKKLTALLKVKKVKTLTRSSQFLLLNLIEAFNKARALRDSLD
LNGKRYCIMLNQYVLSKHQRQLTSLPTREMAWAFHSSNKAVLLDHTKKLHGKPLLWNAVE
EYAIPFWLNEQLLKDVFLELSRNHYAEIDDRNPENVSLYHIALHKINVFRDLWRLASWHK
ESARTVKLLSNDFSKKKWKVVASKNAFALLSKHRYFYASAFFLLADSCYDAAKVCIRNLK
SISLAIAMTRVYEGDDGPTLKRLINEYVLPLAVSQNDRWLTNWSFTILKQEKKALQSLVS
PIRSLVSDNDFIQLYTSDKNDQEIHENKDSQRNSTNEPVRNKFPSDDPAFILLYECLRSS
NVQIDESLEYRFVLHNANVYCQLGCDLIALGLLRNWKFQKNPATAHLETEDYMVTDKQSA
VEKTHDSPKLGKTLMGQDIRPTPDDVPEFNESAFSFE
>Q9HDV1
MISLSLLYRILTLPIILVGTTILYFTIGTNFPHDELRHNLLSTLFCSSMLHLSKGLTVKD
VRIFFHDSIGSTLLKNRKKLNSENELPNYGEKFTHKYDNQDMPDSVWLAKVNGMTKSDPI
ILHLHGGMMALPYDKVILVGLSNLYKLFSTTMNRPPSILLVDYSLVSQGYTYPKQVRECL
NVYQVLISKGFRNITVLGESAGGTLILSFLYQISELSKLNKVVWPKGVALISPWLDLTNA
KKIGSYRANDGLDVICYETLNRFGKAYVNNEESLFTSSVVNINMNCDISIWSKIPPIQDG
KVLVLFGENEVFRDEILSWTSKIGLLKAYPNRVLMDKQGIHIGLFLEESPSIGPGMTNLD
IWKKKFSVNSLYTFLRETFEE
>Q5PQN5
MSTSLNYKSFSKEQQTMDNLEKQLICPICLEMFTKPVVILPCQHNLCRKCASDIFQASNP
YLPTRGGTTVASGGRFRCPSCRHEVVLDRHGVYGLQRNLLVENIIDIYKQESTRPEKKLD
QPMCEEHEEERINIYCLNCEVPTCSLCKVFGAHKDCQVAPLTHVFQRQKSELSDGIAVLV

GSNDRVQGVISQLEDTCKTIEECCRKQKQDLCEKFDHLYSILEERKTEMTQAITRTQEEK
LEHVRTLIRKYSDHLENVSKLVESGIQFMDEPEMAVFLQNAKTLLQKITEASKAFQMEKI
EQGYEIMNNFTVNLNREEKIIREIDFSREEEDEDDEGEVDEEGEGEDAVEVEEAENVQVA
SSGEEETLEKAAEPSELAAEIQAASGPLLASSPDSVSSLPPAADVLVTQGEVVPTDSQQT
TQSETSGPSAAETADPLFYPSWYKGQSRKMISNPPHTPGGEGLSQIGPSAAEDSSVQSAE
VAEAAANEQAAVSGKESSSTAATSQIGFEASSPQGQAAALGSGGGADSEPARHVFSFSWL
NSLNE
>Q9UJW0
MASLLQSDRVLYLVQGEKKVRAPLSQLYFCRYCSELRSLECVSHEVDSHYCPSCLENMPS
AEAKLKKNRCANCFDCPGCMHTLSTRATSISTQLPDDPAKTTMKKAYYLACGFCRWTSRD
VGMADKSVASGGWQEPENPHTQRMNKLIEYYQQLAQKEKVERDRKKLARRRNYMPLAFSD
KYGLGTRLQRPRAGASISTLAGLSLKEGEDQKEIKIEPAQAVDEVEPLPEDYYTRPVNLT
EVTTLQQRLLQPDFQPVCASQLYPRHKHLLIKRSLRCRKCEHNLSKPEFNPTSIKFKIQL
VAVNYIPEVRIMSIPNLRYMKESQVLLTLTNPVENLTHVTLFECEEGDPDDINSTAKVVV
PPKELVLAGKDAAAEYDELAEPQDFQDDPDIIAFRKANKVGIFIKVTPQREEGEVTVCFK
MKHDFKNLAAPIRPIEESDQGTEVIWLTQHVELSLGPLLP
>P81274
MREDHSFHVRYRMEASCLELALEGERLCKSGDCRAGVSFFEAAVQVGTEDLKTLSAIYSQ
LGNAYFYLHDYAKALEYHHHDLTLARTIGDQLGEAKASGNLGNTLKVLGNFDEAIVCCQR
HLDISRELNDKVGEARALYNLGNVYHAKGKSFGCPGPQDVGEFPEEVRDALQAAVDFYEE
NLSLVTALGDRAAQGRAFGNLGNTHYLLGNFRDAVIAHEQRLLIAKEFGDKAAERRAYSN
LGNAYIFLGEFETASEYYKKTLLLARQLKDRAVEAQSCYSLGNTYTLLQDYEKAIDYHLK
HLAIAQELNDRIGEGRACWSLGNAYTALGNHDQAMHFAEKHLEISREVGDKSGELTARLN
LSDLQMVLGLSYSTNNSIMSENTEIDSSLNGVRPKLGRRHSMENMELMKLTPEKVQNWNS
EILAKQKPLIAKPSAKLLFVNRLKGKKYKTNSSTKVLQDASNSIDHRIPNSQRKISADTI
GDEGFFDLLSRFQSNRMDDQRCCLQEKNCHTASTTTSSTPPKMMLKTSSVPVVSPNTDEF
LDLLASSQSRRLDDQRASFSNLPGLRLTQNSQSVLSHLMTNDNKEADEDFFDILVKCQGS
RLDDQRCAPPPATTKGPTVPDEDFFSLILRSQGKRMDEQRVLLQRDQNRDTDFGLKDFLQ
NNALLEFKNSGKKSADH
>P28159
MKSYSQFNLNAAAPPAIAYETTVVNPNGSPLDPHQQQQQQSQDMPHFGLPGPQPPSSQQQ
QQQLQVHHQQQQQQQQQQQQQQHQQQMQMSLLPGPYRPHIEEKKLTRDAMEKYMRERNDM
VIVILHAKVAQKSYGNEKRFFCPPPCIYLFGSGWRRRYEEMLQQGEGEQGAQLCAFIGIG
SSDQDMQQLDLNGKQYCAAKTLFISDSDKRKHFMLSVKMFYGNGHDIGVFNSKRIKVISK
PSKKKQSLKNADLCIASGTNVALFNRLRSQTVSTRYLHVENGHFHASSTQWGAFTIHLLD
DNESESEEFQVRDGYIHYGATVKLVCSVTGMALPRLIIRKVDKQMALLEADDPVSQLHKC
AFYMKDTDRMYLCLSQEKIIQFQATPCPKEPNKEMINDGACWTIISTDKAEYQFYEGMGP
VASPVTPVPIVNSLNLNGGGDVAMLELSGDNFTPHLQVWFGDVEAETMYRCTETLLCVVP
EISQFRGEWLWVRQPTQVPISLVRNDGIIYATGLTFTYTPEPGPRPHCNTQAEDVMRARQ
NNNNNNITSISNNNNSNNAGSPAAGGGLQQQQQQHQALPSISEVQWNSHGSGLS
>Q9WV48
MTHSPATSEDEERHSASECPEGGSESDSSPDGPGRGPQGTRGRGSGAPGNLASTRGLQGR
SMSVPDDAHFSMMVFRIGIPDLHQTKCLRFNPDATIWTAKQQVLCALSESLQDVLNYGLF
QPATSGRDANFLEEERLLREYPQSFEKGVPYLEFRYKTRVYKQTNLDEKQLAKLHTKTGL

KKFLEYVQLGTSDKVARLLDKGLDPNYHDSDSGETPLTLAAQTEGSVEVIRTLCLGGAHI
DFRARDGMTALHKAACARHCLALTALLDLGGSPNYKDRRGLTPLFHTAMVGGDPRCCELL
LYNRAQLGIADENGWQEIHQACQRGHSQHLEHLLFYGAEPGAQNASGNTALHICALYNKE
TCARILLYRGANKDVKNNNGQTPFQVAVIAGNFELGELIRNHREQDVVPFQESPKYAARR
RGPPGAGLTVPPALLRANSDTSMALPDWMVFSAPGASSSGTPGPTSGPQGQSQPSAPSTK
LSSGTLRSASSPRGARARSPSRGRHPEDAKRQPRGRPSSSGTPRDGPAGGTGGSGGPGGS
LGSRGRRRKLYSAVPGRSFMAVKSYQAQGEGEISLSKGEKIKVLSIGEGGFWEGQVKGRV
GWFPSDCLEEVANRSQEGRQESRSDKAKRLFRHYTVGSYDSFDAPSLIDGIDSGSDYIIK
EKTVLLQKKDSEGFGFVLRGAKAQTPIEEFTPTPAFPALQYLESVDEGGVAWRAGLRMGD
FLIEVNGQNVVKVGHRQVVNMIRQGGNTLMVKVVMVTRHPDMDEAVHKKASQQAKRLPPP
AISLRSKSMTSELEEMVSPWKKKIEYEQQPAAVPSMEKKRTVYQMALNKLDEILAAAQQT
ISASESPGPGGLASLGKHRPKGFFATESSFDPHHRSQPSYDRPSFLPPGPGLMLRQKSIG
AAEDDRPYLAPPAMKFSRSLSVPGSEDIPPPPTTSPPEPPYSTPPAPSSSGRLTPSPRGG
PFNPSSGGPLPASSPSSFDGPSPPDTRGGGREKSLYHSAALPPAHHHPPHHHHHHAPPPQ
PHHHHAHPPHPPEMETGGSPDDPPPRLALGPQPSLRGWRGGGPSPTSGAPSPSHHSSSGG
SSGPTQAPALRYFQLPPRAASAAMYVPARSGRGRKGPLVKQTKVEGEPQKGSIPSASSPT
SPALPRSEPPPAGPSEKNSIPIPTIIIKAPSTSSSGRSSQGSSTEAEPPTQPDGAGGGGS
SPSPAPATSPVPPSPSPVPTPASPSGPATLDFTSQFGAALVGAARREGGWQNEARRRSTL
FLSTDAGDEDGGDSGLGPGGPPGPRLRHSKSIDEGMFSAEPYLRLESGGSSGGYGAYAAG
SRAYGGSGSSSAFTSFLPPRPLVHPLTGKALDPASPLGLALAARERALKESSEGGGTPQP
PPRPPSPRYDAPPPTLHHHSPHSPHSPHARHEPVLRLWGDPARRELGYRAGLGSQEKALT
ASPPAARRSLLHRLPPTAPGVGPLLLQLGPEPPTPHPGVSKAWRTAAPEEPERLPLHVRF
LENCQARPPPAGTRGSSTEDGPGVPPPSPRRVLPTSPTSPRGNEENGLPLLVLPPPAPSV
DVDDGEFLFAEPLPPPLEFSNSFEKPESPLTPGPPHPLPDPPSPATPLPAAPPPAVAAAP
PTLDSTASSLTSYDSEVATLTQGAPAAPGDPPAPGPPAPAAPAPPAPQPGPDPPPGTDSG
IEEVDSRSSSDHPLETISSASTLSSLSAEGGGNTGGVAGGGAGVASGTELLDTYVAYLDG
QAFGGSGTPGPPYPPQLMTPSKLRGRALGTSGNLRPGPSGGLRDPVTPTSPTVSVTGAGT
DGLLALSACPGPSTAGVAGGPVAVEPEVPPVPLPAASSLPRKLLPWEEGPGPPPPPLPGP
LSQPQASALATVKASIISELSSKLQQFGGSSTAGGALPWARGGSGGSTDSHHGGASYIPE
RTSSLQRQRLSEDSQTSLLSKPSSSIFQNWPKPPLPPLPTGSGVSSSTAAPGATSPSAS
SASASTRHLQGVEFEMRPPLLRRAPSPSLLPASDHKVSPAPRPSSLPILPSGPIYPGLFD
IRSSPTGGAGGSTDPFAPVFVPPHPGISGGLGGALSGASRSLSPTRLLSLPPDKPFGAKP
LGFWTKFDVADWLEWLGLSEHRAQFLDHEIDGSHLPALTKEDYVDLGVTRVGHRMNIDRA
LKFFLER
>P48200
MDAPKAGYAFEYLIETLNDSSHKKFFDVSKLGTKYDVLPYSIRVLLEAAVRNCDGFLMKK
EDVMNILDWKTKQSNVEVPFFPARVLLQDFTGIPAMVDFAAMREAVKTLGGDPEKVHPAC
PTDLTVDHSLQIDFSKCAIQNAPNPGGGDLQKAGKLSPLKVQPKKLPCRGQTTCRGSCDS
GELGRNSGTFSSQIENTPILCPFHLQPVPEPETVLKNQEVEFGRNRERLQFFKWSSRVLK
NVAVIPPGTGMAHQINLEYLSRVVFEEKDLLFPDSVVGTDSHITMVNGLGILGWGVGGIE
TEAVMLGLPVSLTLPEVVGCELTGSSNPFVTSIDVVLGITKHLRQVGVAGKFVEFFGSGV
SQLSIVDRTTIANMCPEYGAILSFFPVDNVTLKHLEHTGFSKAKLESMETYLKAVKLFRN
DQNSSGEPEYSQVIQINLNSIVPSVSGPKRPQDRVAVTDMKSDFQACLNEKVGFKGFQIA
AEKQKDIVSIHYEGSEYKLSHGSVVIAAVISCTNNCNPSVMLAAGLLAKKAVEAGLRVKP

YIRTSLSPGSGMVTHYLSSSGVLPYLSKLGFEIVGYGCSTCVGNTAPLSDAVLNAVKQGD
LVTCGILSGNKNFEGRLCDCVRANYLASPPLVVAYAIAGTVNIDFQTEPLGTDPTGKNIY
LHDIWPSREEVHRVEEEHVILSMFKALKDKIEMGNKRWNSLEAPDSVLFPWDLKSTYIRC
PSFFDKLTKEPIALQAIENAHVLLYLGDSVTTDHISPAGSIARNSAAAKYLTNRGLTPRE
FNSYGARRGNDAVMTRGTFANIKLFNKFIGKPAPKTIHFPSGQTLDVFEAAELYQKEGIP
LIILAGKKYGSGNSRDWAAKGPYLLGVKAVLAESYEKIHKDHLIGIGIAPLQFLPGENAD
SLGLSGRETFSLTFPEELSPGITLNIQTSTGKVFSVIASFEDDVEITLYKHGGLLNFVAR
KFS
>Q9UN86
MVMEKPSPLLVGREFVRQYYTLLNKAPEYLHRFYGRNSSYVHGGVDASGKPQEAVYGQND
IHHKVLSLNFSECHTKIRHVDAHATLSDGVVVQVMGLLSNSGQPERKFMQTFVLAPEGSV
PNKFYVHNDMFRYEDEVFGDSEPELDEESEDEVEEEQEERQPSPEPVQENANSGYYEAHP
VTNGIEEPLEESSHEPEPEPESETKTEELKPQVEEKNLEELEEKSTTPPPAEPVSLPQEP
PKAFSWASVTSKNLPPSGTVSSSGIPPHVKAPVSQPRVEAKPEVQSQPPRVREQRPRERP
GFPPRGPRPGRGDMEQNDSDNRRIIRYPDSHQLFVGNLPHDIDENELKEFFMSFGNVVEL
RINTKGVGGKLPNFGFVVFDDSEPVQRILIAKPIMFRGEVRLNVEEKKTRAARERETRGG
GDDRRDIRRNDRGPGGPRGIVGGGMMRDRDGRGPPPRGGMAQKLGSGRGTGQMEGRFTGQ
RR
>O94475
MVVQDKWKRKATLAYKKKHDIHDAPVKPRKKNLGNNEWRFKNAGEYFDEDNNEEEYPSLE
SLKTVGNGDTIQDEEEDFEYSKLQARPLTGLGISNQKPKSKVRTIQREEVADMLESVEHE
KSIQEFRKRYNVQKPKVNISHAEAEEDIDSFLESMDQSAPPSITEDKGENYISSNHSSMH
ISRNDSRNYMSEKPNKDQSWLDELLR
>O13706
MLKNIKNRLGEDDIVNSLDGITALSQEWIDKVVDAINEGHVSESDERESRKLGEKMNVNS
QKLKTSSPPKVVRKNKRHHTTVKSVLSDSDLDAEFTDGSEAYEDD
>O13046
MPAIKKNMRYGHPEGHTDVCFDDSGNFLVTCGSDGDIRIWESLDDDDPKSISIGEKAYSF
ALKNGKVVTAASNNAIQLHTFPDGEPDGILTRFTTNANHVVFNTDGTRIAAGSGDFLVKV
LQVEDSTQQKTLRGHSAPVLSVSFDPKDIYLASASCDGSVRIWKISDQTCEAVLPLLEKC
NDVFNAKSICRLAWQPKSGKFVAIPVGKAVHLYDRDSLKNICTLSDDFITQPVNIVAWSP
CGQYLVAGSVDGCIVAWNIATKACLERIKHEKGYTICALAWHPHLPQIAYTDNEGNLGLL
EDVCQGDVKQPSAKVSSAETKDYDELFDGDDDEDFLNGDMIGHEGAVNDEDDDDNFTALT
GRPRNRGAIFDDDISSDVPSLKLVGNENPVVEDDQASSVQNFTSVASVKLSYNGPMPTPQ
QKPFQSGSTPVHLMHRFMVWNSVGVIRCYNDEQDNAIDVEFHDTSIHHAIHLTNSLNHTL
ADVSQEAVLLACETTEELASKLQCLHFSSWDTSKEWMVDMPKGENIQAICLGQGWVACAT
SALLIRIFSVGGVQKELISLFGPVVCMASHGEQLIVVYHRGMGFDGDQCLGVQLLELGKK
KKQVLHGDPLPLSRKSYLSWLGFTAEGSPCYVDSEGIVRLLNRSLGDTWVPICNTREHCK
GKSDHYWVVGIHENPQQVRCIPCKGSRFPPTLPRPAVAVLPFNLPYCQITTEKGQMEEQY
WRSQIFSNHSDYLSKHGYECDENFKAEAQKMQQELLMKMFALSCKLEREFRCMELAEFMT
QNVMNLAIKYASRSKRLILAQRLSEMALEKAAEQASAEQNEEEDEEEEDFRSRLTAGYSR
TATEWGDSRAKPVKQDQYEENNEEEMEEEEKEQEEALESNTPTANPFNKSVKTPDVSESK
LGAILSSNQGRVNPFKVSASQKSPAFASNSSRSTSILDNMGKFPRKPSASGSPSTSKSDS
VIIKPLAPKAKSKQGQATLFHSVQAKPIAKKTTEEKKAVPTPPKAAADIAENKKPKTGFQ

LWLDENRPSILSENAGLDESEIIKEGMSRFRMLTSEERMLWTEKAKGDYPGEDGADAKKR
KRPEQENMASNGCPQENTDSGIAAAKKHKPLGQSANNKLSAFAFKKE
>Q08491
MSLLEQLARKRIEKSKGLLSADQSHSTSKSASLLERLHKNRETKDNNAETKRKDLKTLLA
KDKVKRSDFTPNQHSVSLSLKLSALKKSNSDLEKQGKSVTLDSKENELPTKRKSPDDKLN
LEESWKAIKEMNHYCFLKNDPCINQTDDFAFTNFIIKDKKNSLSTSIPLSSQNSSFLSLK
KHNNELLGIFVPCNLPKTTRKVAIENFNRPSPDDIIQSAQLNAFNEKLENLNIKSVPKAE
KKEPINLQTPPTESIDIHSFIATHPLNLTCLFLGDTNAGKSTLLGHLLYDLNEISMSSMR
ELQKKSSNLDPSSSNSFKVILDNTKTERENGFSMFKKVIQVENDLLPPSSTLTLIDTPGS
IKYFNKETLNSILTFDPEVYVLVIDCNYDSWEKSLDGPNNQIYEILKVISYLNKNSACKK
HLIILLNKADLISWDKHRLEMIQSELNYVLKENFQWTDAEFQFIPCSGLLGSNLNKTENI
TKSKYKSEFDSINYVPEWYEGPTFFSQLYLLVEHNMNKIETTLEEPFVGTILQSSVLQPI
AEINYVSLKVLINSGYIQSGQTIEIHTQYEDFHYYGIVSRMKNSKQILETNTKNNISVGL
NPDILEVLVKIHNTEDFTKKQFHIRKGDIIIHSRKTNTLSPNLPNTLKLLALRLIKLSIQ
THALSDPVDLGSELLLYHNLTHNAVKLVKILGTNDISINPNQSLIVEVEIIEPDFALNVI
DSKYITNNIVLTSIDHKVIAVGRIACQ
>Q07617
MTTKDYPSLWGFGTTKTFKIPIEHLDFKYIEKCSDVKHLEKILCVLRSGEEGYYPELTEF
CEKHLQALAPESRALRKDKPAATAASFTAEEWEKIDGDIKSWVSEIKKEEDKMHFHETET
FPAMKDNLPPVRGSNSCLHVGKEKYSKRPTKKKTPRDYAEWDKFDVEKECLKIDEDYKEK
TVIDKSRLSKIETRIDTAGLTEKEKDFLATREKEKGNEAFNSGDYEEAVMYYTRSISALP
TVVAYNNRAQAEIKLQNWNSAFQDCEKVLELEPGNVKALLRRATTYKHQNKLREATEDLS
KVLDVEPDNDLAKKTLSEVERDLKNSEAASETQTKGKRMVIQEIENSEDEEGKSGRKHED
GGGDKKPAEPAGAARAAQPCVMGNIQKKLTGKAEGGKRPARGAPQRGQTPEAGADKRSPR
RASAAAAAGGGATGHPGGGQGAENPAGLKSQGNELFRSGQFAEAAGKYSAAIALLEPAGS
EIADDLSILYSNRAACYLKEGNCSGCIQDCNRALELHPFSMKPLLRRAMAYETLEQYGKA
YVDYKTVLQIDCGLQLANDSVNRLSRILMELDGPNWREKLSPIPAVPASVPLQAWHPAKE
MISKQAGDSSSHRQQGITDEKTFKALKEEGNQCVNDKNYKDALSKYSECLKINNTECAIY
TNRALCYLKLCQFEEAKQDCDQALQLADGNVKAFYRRALAHKGLKNYQKSLIDLNKVILL
DPSIIEAKMELEEVTRLLNLKDKTAPFNKEKERRKIEIQEVNEGKEEPGRPAGEVSTGCL
ASEKGGKSSRSPEDPEKLPIAKPNNAYEFGQIINALSTRKDKEACAHLLAITAPKDLPMF
LSNKLEGDTFLLLIQSLKNNLIEKDPSLVYQHLLYLSKAERFKMMLTLISKGQKELIEQL
FEDLSDTPNNHFTLEDIQALKRQYEL
>O14030
MANSCLLSADNSSSSTTSNPNVAPPISTLPDPVATISSSSSSHLDMGAIHPPHHSSLPPH
MGVDPSTMADAHNAHSSLTPPQSGYSSMPSLPYLQQPFQIPSQRFSRQQQSHPFPAAQHA
VNGQPQALYPFIYQSRNVPMGSTMFASSNQSAAHPDGNNALPMDNTHANISYMQSSQSMP
VNSYSYDRYTPNQPSYLESKPGNHQPSYTSEQPMYSTASVPQQISNGPTAVNGLPMNSYT
PHSNHLHSPSPNSNSGPTDSLSAPNSTSSPSMAHANGASFASQYPSLNKSIFPASYSSSA
EDGQNMQAPAHAYMQSSIYGVNQEQKSEYPSNLSMQSSMSIKDPSQLQRIHLYPQHSQYD
PNGMTMRDHYSERIEPEAKPSDETLTVRSSRDLSVHNVGTLPVLSAAAATQAAMPHTMGP
SAHDSASAPSPHMQSQQALPYQYYNPLPAMADPAQNVPQQLPPPIHSHLSDDQHIQYSYP
NTFVNRFPQNIHHPSANLLDASAALNPVQNPLLMPQQNHEHSPLVRSDAALHDHGPLLPV
YPDVDSRFV

>P63300
MALAVRVVYCGAUGYKPKYLQLKEKLEHEFPGCLDICGEGTPQVTGFFEVTVAGKLVHSK
KRGDGYVDTESKFRKLVTAIKAALAQCQ
>Q8RY95
MDEVGAQVAAPMFIHQSLGRKRDLYYPMSNRLVQSQPQRRDEWNSKMWDWDSRRFEAKPV
DVEVQEFDLTLRNRSGEERGLDLNLGSGLTAVEETTTTTQNVRPNKKVRSGSPGGNYPMC
QVDNCTEDLSHAKDYHRRHKVCEVHSKATKALVGKQMQRFCQQCSRFHLLSEFDEGKRSC
RRRLAGHNRRRRKTTQPEEVASGVVVPGNHDTTNNTANANMDLMALLTALACAQGKNAVK
PPVGSPAVPDREQLLQILNKINALPLPMDLVSKLNNIGSLARKNMDHPTVNPQNDMNGAS
PSTMDLLAVLSTTLGSSSPDALAILSQGGFGNKDSEKTKLSSYENGVTTNLEKRTFGFSS
VGGERSSSSNQSPSQDSDSRGQDTRSSLSLQLFTSSPEDESRPTVASSRKYYSSASSNPV
EDRSPSSSPVMQELFPLQASPETMRSKNHKNSSPRTGCLPLELFGASNRGAADPNFKGFG
QQSGYASSGSDYSPPSLNSDAQDRTGKIVFKLLDKDPSQLPGTLRSEIYNWLSNIPSEME
SYIRPGCVVLSVYVAMSPAAWEQLEQKLLQRLGVLLQNSPSDFWRNARFIVNTGRQLASH
KNGKVRCSKSWRTWNSPELISVSPVAVVAGEETSLVVRGRSLTNDGISIRCTHMGSYMAM
EVTRAVCRQTIFDELNVNSFKVQNVHPGFLGRCFIEVENGFRGDSFPLIIANASICKELN
RLGEEFHPKSQDMTEEQAQSSNRGPTSREEVLCFLNELGWLFQKNQTSELREQSDFSLAR
FKFLLVCSVERDYCALIRTLLDMLVERNLVNDELNREALDMLAEIQLLNRAVKRKSTKMV
ELLIHYLVNPLTLSSSRKFVFLPNITGPGGITPLHLAACTSGSDDMIDLLTNDPQEIGLS
SWNTLRDATGQTPYSYAAIRNNHNYNSLVARKLADKRNKQVSLNIEHEVVDQTGLSKRLS
LEMNKSSSSCASCATVALKYQRRVSGSQRLFPTPIIHSMLAVATVCVCVCVFMHAFPIVR
QGSHFSWGGLDYGSI
>Q14449
MTTSLQDGQSAASRAAARDSPLAAQVCGAAQGRGDAHDLAPAPWLHARALLPLPDGTRGC
AADRRKKKDLDVPEMPSIPNPFPELCCSPFTSVLSADLFPKANSRKKQVIKVYSEDETSR
ALDVPSDITARDVCQLLILKNHYIDDHSWTLFEHLPHIGVERTIEDHELVIEVLSNWGIE
EENKLYFRKNYAKYEFFKNPMYFFPEHMVSFATETNGEISPTQILQMFLSSSTYPEIHGF
LHAKEQGKKSWKKIYFFLRRSGLYFSTKGTSKEPRHLQFFSEFGNSDIYVSLAGKKKHGA
PTNYGFCFKPNKAGGPRDLKMLCAEEEQSRTCWVTAIRLLKYGMQLYQNYMHPYQGRSGC
SSQSISPMRSISENSLVAMDFSGQKSRVIENPTEALSVAVEEGLAWRKKGCLRLGTHGSP
TASSQSSATNMAIHRSQPWFHHKISRDEAQRLIIQQGLVDGVFLVRDSQSNPKTFVLSMS
HGQKIKHFQIIPVEDDGEMFHTLDDGHTRFTDLIQLVEFYQLNKGVLPCKLKHYCARIAL

>Q05905
MPALLKRLLFQVGPHPNERTFTLSSVSTDGHYISLRPFVKPSGDELSFPFEWAFAGTNET
VKANDQGNGVVTQDFNFWLDTNVYLNVPNTHRGEVNTTWKNWDSGCVEETGAVYPFGADK
ESVSFRELWQPVDPSREDLVIVSPNNEKFSSNARSIVLKVTDEAYDGLVIVIGRWIQGFL
SQKNNNTIEGLNFIRLLEKDSGKSEFLLSYGKEVNKIPQSYENLKKGSTVTSNGLNWEVI
EYHA
>Q56P03
MNRLPDDYDPYAVEEPSDEEPALSSSEDEVDVLLHGTPDQKRKLIRECLTGESESSSEDE
FEKEMEAELNSTMKTMEDKLSSLGTGSSSGNGKVATAPTRYYDDIYFDSDSEDEDRAVQV
TKKKKKKQHKIPTNDELLYDPEKDNRDQAWVDAQRRGYHGLGPQRSRQQQPVPNSDAVLN
CPACMTTLCLDCQRHESYKTQYRAMFVMNCSINKEEVLRYKASENRKKRRVHKKMRSNQE

DAAEKAETDVEEIYHPVMCTECSTEVAVYDKDEVFHFFNVLASHS
>Q02819
MPTSVPRGAPFLLLPPLLMLSAVLAVPVDRAAPPQEDSQATETPDTGLYYHRYLQEVINV
LETDGHFREKLQAANAEDIKSGKLSQELDFVSHNVRTKLDELKRQEVSRLRMLLKAKMDA
KQEPNLQVDHMNLLKQFEHLDPQNQHTFEARDLELLIQTATRDLAQYDAAHHEEFKRYEM
LKEHERRRYLESLGEEQRKEAERKLQEQQRRHREHPKVNVPGSQAQLKEVWEELDGLDPN
RFNPKTFFILHDINSDGVLDEQELEALFTKELEKVYDPKNEEDDMREMEEERLRMREHVM
KNVDTNQDRLVTLEEFLASTQRKEFGDTGEGWKTVEMSPAYTEEELKRFEEELAAREAEL
NARAQRLSQETEALGRSQDRLEAQKRELQQAVLQMEQRKQQLQEQSAPPSKPDGQLQFRA
DTDDAPVPAPAGDQKDVPASEKKVPEQPPELPQLDSQHL
>Q9UKT4
MSRRPCSCALRPPRCSCSASPSAVTAAGRPRPSDSCKEESSTLSVKMKCDFNCNHVHSGL
KLVKPDDIGRLVSYTPAYLEGSCKDCIKDYERLSCIGSPIVSPRIVQLETESKRLHNKEN
QHVQQTLNSTNEIEALETSRLYEDSGYSSFSLQSGLSEHEEGSLLEENFGDSLQSCLLQI
QSPDQYPNKNLLPVLHFEKVVCSTLKKNAKRNPKVDREMLKEIIARGNFRLQNIIGRKMG
LECVDILSELFRRGLRHVLATILAQLSDMDLINVSKVSTTWKKILEDDKGAFQLYSKAIQ
RVTENNNKFSPHASTREYVMFRTPLASVQKSAAQTSLKKDAQTKLSNQGDQKGSTYSRHN
EFSEVAKTLKKNESLKACIRCNSPAKYDCYLQRATCKREGCGFDYCTKCLCNYHTTKDCS
DGKLLKASCKIGPLPGTKKSKKNLRRL
>O75874
MSKKISGGSVVEMQGDEMTRIIWELIKEKLIFPYVELDLHSYDLGIENRDATNDQVTKDA
AEAIKKHNVGVKCATITPDEKRVEEFKLKQMWKSPNGTIRNILGGTVFREAIICKNIPRL
VSGWVKPIIIGRHAYGDQYRATDFVVPGPGKVEITYTPSDGTQKVTYLVHNFEEGGGVAM
GMYNQDKSIEDFAHSSFQMALSKGWPLYLSTKNTILKKYDGRFKDIFQEIYDKQYKSQFE
AQKIWYEHRLIDDMVAQAMKSEGGFIWACKNYDGDVQSDSVAQGYGSLGMMTSVLVCPDG
KTVEAEAAHGTVTRHYRMYQKGQETSTNPIASIFAWTRGLAHRAKLDNNKELAFFANALE
EVSIETIEAGFMTKDLAACIKGLPNVQRSDYLNTFEFMDKLGENLKIKLAQAKL
>P48240
MNVQDRRRLLGPAAAKPMAFSNTTTHVPEKKSTDLTPKGNESEQELSLHTGFIENCNGSA
LVEARSLGHQTSLITAVYGPRSIRGSFTSQGTISIQLKNGLLEKYNTNELKEVSSFLMGI
FNSVVNLSRYPKSGIDIFVYLTYDKDLTNNPQDDDSQSKMMSSQISSLIPHCITSITLAL
ADAGIELVDMAGAGEANGTVVSFIKNGEEIVGFWKDDGDDEDLLECLDRCKEQYNRYRDL
MISCLMNQET
>O13800
MNQVTQFSGNSANLMLYWILKSYYSRFDRIMWIDTLGTFNPAALPLPCLEKTMLVRSFDA
QGLKDAVDELESNLKSSEDQAYALCIDSFSNPIGLLMAQGNISFAHAFMMTLGRKCRILT
RKFRLAVYLSTSLVYIKQLHLSKPALGNSWPFCLDHSYILEDQQHNKCLVHCNQSRKDLL
GCSQLFLLLKGSFTHEYLTIDFYNGTPVSVSSKLHVSPSLYHSSTLNSPS
>O74405
MSNKQNIESEVSWVKSKGAFVHPSLEFSVIPDAGSCVLANNDINENTVLLKLPPNILINK
RTCSRYSFRDKLTSFQFLSWLISEDVHSNLEISPYYTKALPQGFSFHPVTLTSDHPLWSI
LPDEVRNSLLERKNVMAFDYEQVKKFVSVDQPTFQWGWLCVNTRCLYYDTGSKNTEDHLT
LAPIFEYFNHSPEAQTALINTRGTITIKSTRRIDKGEQIFLCYGPHGNDKLFTEYGFCLS
NNPNISIQLDRFIEFDKWQQSFLQDHGYWNDYTCSLHGASFRTLVGVRTLLVSPSEKLND

ASYDQTRRVLQYINGFSDGSRDRQDVEDYLKKVLQELLCEAEECKEKVKGISDGSYVFIC
AEQLWKDRIMCCQYLMEHSFE
>Q16531
MSYNYVVTAQKPTAVNGCVTGHFTSAEDLNLLIAKNTRLEIYVVTAEGLRPVKEVGMYGK
IAVMELFRPKGESKDLLFILTAKYNACILEYKQSGESIDIITRAHGNVQDRIGRPSETGI
IGIIDPECRMIGLRLYDGLFKVIPLDRDNKELKAFNIRLEELHVIDVKFLYGCQAPTICF
VYQDPQGRHVKTYEVSLREKEFNKGPWKQENVEAEASMVIAVPEPFGGAIIIGQESITYH
NGDKYLAIAPPIIKQSTIVCHNRVDPNGSRYLLGDMEGRLFMLLLEKEEQMDGTVTLKDL
RVELLGETSIAECLTYLDNGVVFVGSRLGDSQLVKLNVDSNEQGSYVVAMETFTNLGPIV
DMCVVDLERQGQGQLVTCSGAFKEGSLRIIRNGIGIHEHASIDLPGIKGLWPLRSDPNRE
TDDTLVLSFVGQTRVLMLNGEEVEETELMGFVDDQQTFFCGNVAHQQLIQITSASVRLVS
QEPKALVSEWKEPQAKNISVASCNSSQVVVAVGRALYYLQIHPQELRQISHTEMEHEVAC
LDITPLGDSNGLSPLCAIGLWTDISARILKLPSFELLHKEMLGGEIIPRSILMTTFESSH
YLLCALGDGALFYFGLNIETGLLSDRKKVTLGTQPTVLRTFRSLSTTNVFACSDRPTVIY
SSNHKLVFSNVNLKEVNYMCPLNSDGYPDSLALANNSTLTIGTIDEIQKLHIRTVPLYES
PRKICYQEVSQCFGVLSSRIEVQDTSGGTTALRPSASTQALSSSVSSSKLFSSSTAPHET
SFGEEVEVHNLLIIDQHTFEVLHAHQFLQNEYALSLVSCKLGKDPNTYFIVGTAMVYPEE
AEPKQGRIVVFQYSDGKLQTVAEKEVKGAVYSMVEFNGKLLASINSTVRLYEWTTEKELR
TECNHYNNIMALYLKTKGDFILVGDLMRSVLLLAYKPMEGNFEEIARDFNPNWMSAVEIL
DDDNFLGAENAFNLFVCQKDSAATTDEERQHLQEVGLFHLGEFVNVFCHGSLVMQNLGET
STPTQGSVLFGTVNGMIGLVTSLSESWYNLLLDMQNRLNKVIKSVGKIEHSFWRSFHTER
KTEPATGFIDGDLIESFLDISRPKMQEVVANLQYDDGSGMKREATADDLIKVVEELTRIH

>P78759
MKGLRSKFVKALSLKDEQGSHKNGHSKSHYISKNGSYVETDDVKHTDTHHSSKHELKKLK
SHFLKDTLKHKRNHHANSNNEKHENSDKKIHTTVLASGHEDSDYSTFLPVIETSKVKDAN
HFPPNYPEPKNDSVSSNIDEFPNDSISSASFLSVPQSTPPYLVSQPTPLNKFLAGAENVD
IPHSLRPVPRREHSSQFQVSEKRTLVRLPSFDDVHTSEREELFIKKLEQCNIIFDFNDPS
SDLASKEIKREALLQMIDYVSENRGISSASLFPYVVNTFSLNVFRPISPALNDYSSDMFA
LDDEPFLEPAWPHLEEVYLLFIKFLESPDFRASKAKSLVDRRFFNRLLALFDTEDPRERE
LLKTTLHRIYGKFLNLRSYIRKSMNNVFLQFIYEREKFHGIAELLEILGSIINGFAVPLK
EEHKIFLSKVLIPLHQTKSVFLYHPQLTYCIVQFIDKDPSLTKAVLTGILKYWPRINSFK
ELLFLNEIEDIFEVLEPSEFVNIMSPLFQQLARSISSMHFQVAERALCLWSNEYFTSLVS
QNVVTLLPIIYPSLYKTANEHWNSTIQAIACNVLQIFVDMDADFFNGLVEDYKQAIIKQE
EVMIIRKQQWCQIEALAAENKPTDYLR
>Q3HNM7
MRRPPGDGDSTGEGPGNWGLWGAQESRRLCCVGPDRCGQALLQIGINMMALPGGRHLDSV
PLQEQRLHFMQVDSVQRWMEDLKLMTECECMCVLQAKPISLEEDTQGDLILAGGPGPGDP
LQLLLKRGWVISTELRRIGQKLAQDRWARVHSMSVRLTCHARSMVSEYSTISRTASQEMG
QAEKLLMEKCSELSAVTERCLQVENEHVLKSMKACVSETLSLLGEHFGQLLELALTREVQ
ALVRKIDTSDNIYITESTTGNLFGLTQEGAPLCRIIAKEGGVVALFKVCRQDSFRCLYPQ
ALRTLASICCVEEGVHQLEKVDGILCLADILTDESHSEATRAEAAAVVAQVTSPHLSFTQ
HLTSFLENMEEIVTALIKLCQEASSGEVFLLASAALANITFFDKMACEMLLQLNAIRVLL
EACGDKQRVDTPYTRDQIVTILANMSVLEQCGSDIIQENGVQLIMGMLSEKPRSGTPAEV

AACERVQQKAAVTLARLCRDPDVAQEAVRLSCMSRLIELCRSPSERNSSDAVLVACLAAL
RRLAGVCPEGLQDSDFQQLVQPRLVDSFLLCSNMEESFV
>Q08696
MSLCRCSSICLRRPILRSFNFLNKYIDQSQHSGGLSSFQRLSFSNTGKNESTDDSNQIRC
DADKTAADKKKQKQKEEEQAKIREYKRECLKVQKRVLAEEIRCSGEKDRILIQGMMKCLT
DGMKKACTKIAKAKLIADKELAVQCAALSKKDKVKALLKKCEREKSKEKECNQNSPAEGD
KDRTKKGKTKGKSGGGNKKRSTKENRAKKGKKLVKNRFTQKLEHCIKSEWADVCECRQNF
TEDERKRLAASYKCMGTKIKSICRKRVIAEMCEAAGYVKSSEPKKKGKKKKNDEKKEKEL
EREILKEQAEEEAKIRGVVKEVKKKCKEKALKKKCKDLGRKMKEEAEKKKCAALAKKQKE
EDEKKACKELAKKKKEADEKKKCEEAANKEKKAAEKKKCEKAAKERKEAAEKKKCEEAAK
KEKEAAERKKCEELAKNIKKAAEKKKCKEAAKKEKEAAERKKCEELAKKIKKAAEKKKCE
ETAKKGKEVAERKKCEELAKKIKKAEIKKKCKKLAKKEKETAEKKKCEKAAKKRKEAAEK
KKCEKAAKKRKEAAEKKKCEKSAKKRKEAAEKKKCEKAAKERKEAAEKKKCEEAAKKEKE
VAERKKCEELAKKIKKAAEKKKCKEAAKKEKEAAEREKCGELAKKIKKAAEKKKCKKLAK
KEKETAEKKKCEKAAKKRKEAAEKKKCAEAAKKEKEAAEKKKCEEAAKKEKEAAERKKCE
ELAKKIKKAAEKKKCKKLAKKKKAGEKNKLKKGNKKGKKALKEKKKCRELAKKKAAEKKK
CKEAAKKEKEAAEKKKCEKTAKKRKEEAEKKKCEKTAKKRKEAAEKKKCEKAAKKRKEEA
EKKKCEKTAKKRKETAEKKKCEKAAKKRKQAAEKKKCEKAAKKRKEAAEKKKCAEAAKKE
KELAEKKKCEEAAKKEKEVAERKKCEELAKKIKKAAEKKKCKKLAKKEKKAGEKNKLKKK
AGKGKKKCKKLGKKSKRAAEKKKCAEAAKKEKEAATKKKCEERAKKQKEAAEKKQCEERA
KKLKEAAEQKQCEERAKKLKEAAEKKQCEERAKKLKEAAEQKQCEERAKKLKEAAEKKQC
EERAKKEKEAAEKKQCEERAKKLKEAAEKKQCEERAKKEKEAAEKKRCEEAAKREKEAAE
KKKCAEAAKKEKEATEKQKCAEAAKKEKEAAEKKKCAEAAKREKEAAQKKKCADLAKKEQ
EPAEMKKCEEAAKKEKEAAEKQKCAKAAKKEKEAAEKKKCAEAAKKEQEAAEKKKCAEAA
KKEKEAEKKRKCEKAEKAAALKRQCAKLVIRAKEAALRKKCAIIAKKAKMAAEKKECEKL
AKKAKEAIEWKKCAKLAKKKREAEKKKCAKLAKKEKEAAEKKKRCKDLAKNKKKGHKKKG
RNENRKKRTDC
>Q9H4D5
MSLPSGHTTGHTDQVVQRRARCWDIYQRRFSSRSEPVNPGMHSSSHQQQDGDAAMHGAHM
DSPVRYTPYTISPYNRKGSFRKQDQTHVNMEREQKPPERRMEGNMPDGTLGSWFKITVPF
GIKYNEKWLLNLIQNECSVPFVPVEFHYENMHASFFVENASIAYALKNVSGKIWDEDNEK
ISIFVNPAGIPHFVHRELKSEKVEQIKLAMNQQCDVSQEALDIQRLPFYPDMVNRDTKMA
SNPRKCMAASLDVHEENIPTVMSAGEMDKWKGIEPGEKCADRSPVCTTFSDTSSNINSIL
ELFPKLLCLDGQQSPRATLCGTEAHKRLPTCKGSFFGSEMLKNLVLQFLQQYYLIYDSGD
RQGLLSAYHDEACFSLSIPFNPEDSAPSSFCKFFKDSRNIKILKDPYLRGELLKHTKLDI
VDSLSALPKTQHDLSSFLVDMWYQTEWMLCFSVNGVFKEVEGQSQGSVLAFTRTFIATPG
SSSSLCIVNDKLFVRDTSHQGTQSALFTLVPTAFSSSVPAFSQEQQKMLPS
>Q9I7U4
MQRQNPNPYQQQNQQHQQVQQFSSQEYSHSSQEQHQEQRISRTEQHVQRSQVTTQRQVQQ
HHGGSIGGAYVPPSLTHVYAQGDISPPVFEQIFKNARFAQGGNALFEGRLRGNPKPFVTW
TRKGAPLLESQKFRMSYNEATGDVSLLINQIGPGDEGEYTCTARNQYGEAICSVYIQPEG
APMPALQPIQNLEKNIYSNGYSYTSIEEEFRVDTFEYRLLREVSFREAITRRSGYEQDSQ
LSQELDRNQGPAQAPQISQKPRSSKLIEGSDAVFTARVGSNPKPRLTWFHNGQRLVASQK
YEISYSSGVATLRVKNATARDGGHYTLLAENLQGCVVSSAVLAVEPAAETAYEPKPVDVM

AEQLEAGKALPPAFVKAFGDREITEGRMTRFDCRVTGNPYPEVFWLINGRQVRDDASHKI
LVNESGSHSLMITNVTRLDAGAVQCLARNKAGEVAIEAQLNVLEKEQVVAPQFVQRFSTM
TVREGEPITMSANAIGTPQPRITWQKDGVQISSTAERFVGIDGGATCLEIPRVTANDAGW
YQCTAQNIAGSTANRARLYVEVPREQPNYEQRRLNLPRPTKVIEPEPIPGPEIIYLRHVE
RAKPHLRPGEEDRVYPPPQFIIPLQNVQQTEGGRVHMEARIEPVGDPTMVVEWYLNGRPL
AASARATSVFKFGFIALDLLSIMGHDSGEYMCRVTNASGVAESRAILSVVQRPSIEQSSQ
NPNSLQYINQLEDYSRYQRTESIDEQLNQAPQFIRPLRDLGEFEEGKNVHFEAQVTPVND
PSMRVEWYKDGLPITASSRITAIFNFGYVSLNILHLRAEDAGTYTVRAVNRIGEAISQSS
IRVHSRSQVTADLGIPEQQRYIEKVEELEDYRKSQQRRHVQEAAEAIAPPQFKTPIQNQL
DLREHAHAHFEARLEPVGDSTMRVEWLKDGQPLEASSRITTYHNFGYVALTIKQLTIYDA
GTYTCRAYNAMGQDTTVAQLTVISKNEIVSESQHPGGLQKIQHLEDSSRYGRREEEETYI
TQAPRFLGPLKGTTKILEGQRAHFEARVEPQSDLGLVIEWYHNGRSITAANRIQTYYDFG
YVALDISQVRAEDAGVYLVVARNKLGEAQQQATMIVETRSSIDTSSMHRGLYEKTQNLEN
KPFVEPQYDIEEISKSKPVFVTPLSDPKPIHDGKNIHLECRLEPMGDPTMRVEWFHNGRP
VTVGSRFRTYYDFGFVALDIIKATAADSGEYTVRATNHLGTAHTSACVRVIDHTDVVTET
QNEQSLEQIQLLEDSRRRHHQEEDITIMQAPQFTRGLHNIETIEGTNVHLECRLQPVGDP
SMRIEWFVNGKPVKTGHRFRPAYEFDYVALDLLGCYAIDSGVYTCQARNQLGEAVTSCSV
RIIAKNDLILETQNESGLQKIQYLEDSTRHRRSEFVDEVVNIRPRFLTHPKSLTNTREGG
HAHFECKIEPVTDPNLKVEWFKNGRPITVGHRFRPIHDFGYVALDIVHLIAEDSGVYTCR
AVNLIGSDETQVELQCRSGEQIVTVTQNEAGLEQIHYLEDRSRYTRREEIDESTKQAPVF
TTSLKNVEIKENQRAHFECRLIPVSDPSMRVEWYHNNLPLKSGSRFTETNNFGFVALDIM
STLPEDAGTYTCRAYNAVGEAITSAVAVVHTKKSIYLESQHETALPRLQHLEDGSKRQRI
SVQDEFVSQAPVFTMPVRDVRVAENQAVHFEARLIPVGDPKLTVEWLRNGQPIEASNRTT
TMHDFGYVALNMKYVNPEDSGTYTCRAVNELGQAVTSASLIVQSKTSIQLETQHEAAMHK
IHQLEDHSRYQRREEEEYTVTTAPVFVTKLIGPSNLVEGQSAHYECRIEPYPDPNLKVEW
FHNGKPLSTGHRFRTTYDFGFAALDILTVYAEDSGEYTCRVTNNLGEAINSIVLNVTSRS
SIIHETQHEEALTKIQHLEDTSRFQRKTDEEQFHAERPQFGRPLRNAKVNEGAPVHLEAT
LIPVNDPTMKVEWYCNGRPIQTGHRFKTTYDFGFVALDILYAHAEDTGTYMCKAKNAIGE
AVTTCAVNVTANKTLDLDTLDAQRLEKIRQLETYAPPPKPVVEEKGQKPIFLTPLSNLEH
LKEGEHAHLECRVEPINDPNLKIEWFCNGKQLPTGHRYRTTHDFGYVALDILYVYGEDTG
TYICKATNQLGEAVNTCNVRVLNRRSMILDTQHPDALEKIQKLESKVPNARTEVGDAPIS
PPHFTAELRGSTEIYEGQTAHFEAQVAPVHDPNLRIEFYHNGKPLPSASRFHITFDFGYV
SLDITHAVAEDAGEYSVRAVNALGQAVSSTNLRVIPRGTIISDTQHPEGLEKIRKLESTA
PHQRQEPETPGTRQRPVFTQPLQNIDRINEHQTAHFEARLIPVGDPNLKVEWYRNEKIIE
DSSRITKQHDFGFVSLDISHIRKEDEGVYMCRAVNPLGEAVTTASMRVVSEASIQMDTQH
PDSISRIHQLEKPLAPRPTEPERLFEKPIFTQLLTGPSELWEGTHAHFEARVVPVGDPSL
KFEWFINGVELQMGSRLRTTHDFGFVTLDITAVVPEDAGVYMCRAYNAAGEAVSSTAMKV
KTKSNIDGQPLIPESWEAIRLKEAAMNRVPEMFVDSTPQQAPVFTTHLQSYDKLHEGQHV
LLEAQVEPRADPNLRIEWFKNGISLTTGSRIRSTFDFGLVTLSINGLRADDSAIYTCKAT
NQVGEAVSTSSLKIEDRHWLQAESLHPDSLPRIGELEAPKEGRPEAPEPTYETPVFITHL
NNIECKESDNVRFECNVEPARDPTMSIEWFYNGQPLQAAAKFKSIYDFGYCALDLTNSYA
ENSGVYTCKATNSKGSATTSGTLKCTGGKTMFLDTQHPQGEAGLEAVQETEEELANRYTS
KTTKPETQYPPPVWTKPLQAEFHLSEAQPIHLEANVEPKEDPNLFIEWYFNGKMLNHGSR
FKMTSEFGFVTMDMIEVYARDQGIYTCKAYNKAGEAFTSTTIFCSSKENIIESTQHPKGA

EGLEQIQDLEDSLRKDGSKPEQPDLGIPPRFTTEFVNIADIGEGELAHFEANLIPVGDQS
MVIEWFYNGKVLEASHRVRTIYAFGTVALEVLGTKIEDTGTYTCRATNKHGTAEISCNLE
CVDKPRGQKPRFTSHIQPLEGLKDGQSAHFECTLIPVNDPDLKVEWYHNGKLMRHSNRIK
TVSDFGYVVLDISYLQDHDSGEYVCRAWNKYGEDFTRTTLNCGGRGGVFYDSLQPDSLQR
IRELECPQGQQADTSAPLVAEPPKFITQIVDVTKLVEGQSAHFEARLTPITDPDLVVEWY
FNGKKLPHGHRFRTFHDFGIVILDILYCYEENSGVYEARARNKYGEDVTRASLKCASKSS
LILDSQLPRGMEGGLEKIANLEYSMVRTREETTEETKGKAPVFTVPLENIENLREGENAH
FEARITPADDPKLKVEWYWNGRPLKAGSRFRTFCDFGFVILEISPVYPEDSGEYSCRAIN
EYGEAVTTATMKIQGKRSIIMESQLPKGMEGTIDRIAELEGLGSRSTEFVPDDDTGKPPE
FITSPFDMVIGENALAHFECRLQPINDPSMRVDWFHNGKALWAGSRIKTINDFGFVILEI
AGCYQRDSGLYTCKATNKHGEATVSCKLQVKGRQGIVMEPQLPSNFRTGTESLQKLEETM
HKREELVTEDEQPNPPKFTEEIKDNLDVPEGGPIHFDCRVEPVGDPTMRIEWFYNGHVMA
TGSRVHQLNDFGFIALDVDYIYARDSGEYTCRATNKWGTATTSAKVTCKGKHNIVYESQL
PEGMTSEKLKELERGRIPEAPKVVEEVFGPPKFTTQITSVTVDEAEAVRFECQVEPKTDP
SLRVEWYRNGKPLPSGHRYRNIFDMGFVSLDILYVYGEDSGEYVCRAINNYGEDRTRATV
SCKKLPTILLQNQVPRGMKRSDALTQMEATIKKYTSEVHLTEDDLFDPDRKQPPRFVTQI
KEQLTLTEMAVTKFECQLAPVGDPNMKVEWFFNGKPLLHKNRFQPIYDFGYVAMNFGWVY
PEDSGEYVCRATNLYGKDETRAIIKVSGKPGIVYDSQLPAHMQSIDRIREMEASWQVVPD
EVDPDAKPRTKPVFVSKLEPQTVEEGDPARFCVRVTGHPRPRVMWLINGHTVVHGSRYKL
TNDGMFHLDVPKTRQYDTGKVEVIARNSVGESIATTELKVVARSDDYRNVLKNSPRPWYD
YELAAYQKERQENELEKVFDERKQVLSEQSSHTLKGVEHLKPKQYKPPTPDWQQNVKAKK
SEDYYNKLQTLETEQLLKETNLRRDTHQYAIPGEKVVSSSQAKGMAQSYEENLQEKTSTT
EVQAAPPKGIAQPSESSVHGREVHMNKQQQVQKEIQGDLEITRKITATETTEVEHKGTIQ
ERVVQGPVKPAKAPVFTKKIQPCRVFENEQAKFEVEFEGEPNPTVKWYRESFPIQNSPDL
QIHTFSGKSILIIRQVFVEDSAVFSCVAENRGGTAKCSANLVVEERRRAGKGGIQPPSFV
TTIQSTTVATGQLARFDAKVTGTRPLDVYWLKNGMKIQPSIKFKMLEEDSVHTLLIIEPF
AEDSGRYECVAVNAAGEARCDGDCIVQSPSKPEKPTTPGSEKAPHIVEQLKSQTVEEGSK
VIFRCRVDGKPTPTARWMRGENFVKPSRYFQMSRQGEYYQLVISEAFPEDEGTYKCVAEN
KLGSIQTSAQLKVRPIENLDAPPTITALKDVSVTEGMPAQFKTTVTGKVKATSVQWFREG
QLIPETPDFQMIFDGNSAVLLIGTTYEEDSGIFTVRVTSSTGQVESSAKLTVKKRRISAF
QLRTIDSAEDESSSSGREDSAPESPHAFQPGQQPGQQFGQFLGVNGQGQHQGRSRQKKPK
VRSKSLQPATKVIPWRKSSRPTRGRSLDKGVFLPGFKPEPVKSWTEETINLKATPIEKKK
PAPKLEAAKVVLKSIKTERDQGIMSLGATLEQIIAGKTEKEAIPWITMREKLKAVESVQQ
QLNKFDLDEVYLQPLEGQIE
>Q9HDU4
MTGDYKEYKGYALITGGAGFIGSNFLDYAVDKYPDFHFTCIDKLSYVSNYTTVFLSKVLN
QPNFRFLEMDLATNYKFLYQFMVEDSEINKITHIINFAAESSVDRSFIDPLYFTKNNILS
TQNLLECVRILLGKKEELRNRLNFVHVSTDEVYGEQDENASVDEKSKLNPTSPYAASKAA
VDLIIQSYRYSYKISVTVIRANNVYGPRQYEEKLIPMTLGKLKKFINQKSQKIMQDKITL
HGDGLHKRKYLHIYDFINAIDLVWMKQGSEVYHSTLESKMSGQIFNIGSDDEIDNLSLVK
FICDYFLYRKLSLKNLDYSKYITFVQDRNYNDSRYSLNYEKIKSLGWRPQIPLETGLRKL
IDEYY
>Q9HDW0
MQLQSPFGDGISCECLNIRVLGIPNETKHQWIFVPRDLIKIKIYSLLQICKAENCSAVAC

RGCNLCILAVQGNIEISEEPQKLFQEENVKVYIYDSAISLSSVRASFPISELGIRMDIIK
ARAEPREDEIRASFSSLVNKEIKRTVEELLLSKRSTNRLSLLAFMRNQQLNYEAYETKLL
EDASTIEKSLEKEVKTIYDSNIASPKESSDAIDADHAMIDESRSTQRRKSKPKKHVAFTD
EYQVAFSDKPGKYFNQPMQYSKQFKLDNPDYEASSDSLNDIENLSTLTFRSDEELEFDFD
TNNINNNKSGDSLEMSTTIPSDEENEDFTSKVDAMEIHSGSLPLNIDSSPIFTNHSPSSS
LSSESSFEASPSSFVDRKNRWIQMLKKADEHSRSIQARSMGYVLSDDLDNSKAFRPYKQS
FLAQGWKSLN
>Q68SB1
MANPKEKTPMCLVNELARFHSIQPQYKLLNESGPAHSKMFSVQLSLGEQTWESEGSSIKK
AQQAVANKALTESTLPKPVQKPPKSNVNNNPGSITPTVELNGLAMKRGEPAIYRPLDPKP
FPNYRANYNFRGMYNQRYHCPMPKIFYVQLTVGNNEFFGEGKTRQAARHNAAMKALQALQ
NEPIPEKSPQNGESGKEMDDDKDANKSEISLVFEIALKRNMPVSFEVIKESGPPHMKSFV
TRVSVGEFSAEGEGNSKKLSKKRAATTVLQELKKLPPLPVIEKPKLFFKKRPKTIIKAGP
EYGQGMNPISRLAQIQQARKEKEPDYVLLSERGMPRRREFVMQVKVGNEVATGTGPNKKI
AKKNAAEAMLLQLGYKASTSLQDQLDKTGENKGWSGPKPGFPEPANNTPKGILHLSPDVY
QEMEASRHRVTSGTTLGYLSPKDMNQPSSSFFSVESPSPTSSAPAARELLMNGTSPAAEA
IGLKGSSPTPPCSSVQPSKQLEYLARIQGFQAALSALKQFSEQGLESIDGVVNVENGSLE
KQAKHLREKADNNQANPGSITQDCKKSKSVI
>P05740
MARYGATSTNPAKSASARGSYLRVSFKNTRETAQAINGWELTKAQKYLEQVLDHQRAIPF
RRFNSSIGRTAQGKEFGVTKARWPAKSVKFVQGLLQNAAANAEAKGLDATKLYVSHIQVN
QAPKQRRRTYRAHGRINKYESSPSHIELVVTEKEEAVAKAAEKKVVRLTSRQRGRIAAQK
RIAA
>P02886
MSTQGLVQLLANAQCHLRTSTNYNGVHTQFNSALNYKNNGTNTIDGSEAWCSSIVDTNQY
IVAGCEVPRTFMCVALQGRGDADQWVTSYKIRYSLDNVSWFEYRNGAAVTGVTDRNTVVN
HFFDTPIRARSIAIHPLTWNGHISLRCEFYTQPVQSSVTQVGADIYTGDNCALNTGSGKR
EVVVPVKFQFEFATLPKVALNFDQIDCTDATNQTRIGVQPRNITTKGFDCVFYTWNENKV
YSLRADYIATALE
>Q96IV0
MAAAALGSSSGSASPAVAELCQNTPETFLEASKLLLTYADNILRNPNDEKYRSIRIGNTA
FSTRLLPVRGAVECLFEMGFEEGETHLIFPKKASVEQLQKIRDLIAIERSSRLDGSNKSH
KVKSSQQPAASTQLPTTPSSNPSGLNQHTRNRQGQSSDPPSASTVAADSAILEVLQSNIQ
HVLVYENPALQEKALACIPVQELKRKSQEKLSRARKLDKGINISDEDFLLLELLHWFKEE
FFHWVNNVLCSKCGGQTRSRDRSLLPSDDELKWGAKEVEDHYCDACQFSNRFPRYNNPEK
LLETRCGRCGEWANCFTLCCRAVGFEARYVWDYTDHVWTEVYSPSQQRWLHCDACEDVCD
KPLLYEIGWGKKLSYVIAFSKDEVVDVTWRYSCKHEEVIARRTKVKEALLRDTINGLNKQ
RQLFLSENRRKELLQRIIVELVEFISPKTPKPGELGGRISGSVAWRVARGEMGLQRKETL
FIPCENEKISKQLHLCYNIVKDRYVRVSNNNQTISGWENGVWKMESIFRKVETDWHMVYL
ARKEGSSFAYISWKFECGSVGLKVDSISIRTSSQTFQTGTVEWKLRSDTAQVELTGDNSL
HSYADFSGATEVILEAELSRGDGDVAWQHTQLFRQSLNDHEENCLEIIIKFSDL
>O14727
MDAKARNCLLQHREALEKDIKTSYIMDHMISDGFLTISEEEKVRNEPTQQQRAAMLIKMI
LKKDNDSYVSFYNALLHEGYKDLAALLHDGIPVVSSSSGKDSVSGITSYVRTVLCEGGVP

QRPVVFVTRKKLVNAIQQKLSKLKGEPGWVTIHGMAGCGKSVLAAEAVRDHSLLEGCFPG
GVHWVSVGKQDKSGLLMKLQNLCTRLDQDESFSQRLPLNIEEAKDRLRILMLRKHPRSLL
ILDDVWDSWVLKAFDSQCQILLTTRDKSVTDSVMGPKYVVPVESSLGKEKGLEILSLFVN
MKKADLPEQAHSIIKECKGSPLVVSLIGALLRDFPNRWEYYLKQLQNKQFKRIRKSSSYD
YEALDEAMSISVEMLREDIKDYYTDLSILQKDVKVPTKVLCILWDMETEEVEDILQEFVN
KSLLFCDRNGKSFRYYLHDLQVDFLTEKNCSQLQDLHKKIITQFQRYHQPHTLSPDQEDC
MYWYNFLAYHMASAKMHKELCALMFSLDWIKAKTELVGPAHLIHEFVEYRHILDEKDCAV
SENFQEFLSLNGHLLGRQPFPNIVQLGLCEPETSEVYQQAKLQAKQEVDNGMLYLEWINK
KNITNLSRLVVRPHTDAVYHACFSEDGQRIASCGADKTLQVFKAETGEKLLEIKAHEDEV
LCCAFSTDDRFIATCSVDKKVKIWNSMTGELVHTYDEHSEQVNCCHFTNSSHHLLLATGS
SDCFLKLWDLNQKECRNTMFGHTNSVNHCRFSPDDKLLASCSADGTLKLWDATSANERKS
INVKQFFLNLEDPQEDMEVIVKCCSWSADGARIMVAAKNKIFLFDIHTSGLLGEIHTGHH
STIQYCDFSPQNHLAVVALSQYCVELWNTDSRSKVADCRGHLSWVHGVMFSPDGSSFLTS
SDDQTIRLWETKKVCKNSAVMLKQEVDVVFQENEVMVLAVDHIRRLQLINGRTGQIDYLT
EAQVSCCCLSPHLQYIAFGDENGAIEILELVNNRIFQSRFQHKKTVWHIQFTADEKTLIS
SSDDAEIQVWNWQLDKCIFLRGHQETVKDFRLLKNSRLLSWSFDGTVKVWNIITGNKEKD
FVCHQGTVLSCDISHDATKFSSTSADKTAKIWSFDLLLPLHELRGHNGCVRCSAFSVDST
LLATGDDNGEIRIWNVSNGELLHLCAPLSEEGAATHGGWVTDLCFSPDGKMLISAGGYIK
WWNVVTGESSQTFYTNGTNLKKIHVSPDFKTYVTVDNLGILYILQTLE
>Q8TCD5
MARSVRVLVDMDGVLADFEAGLLRGFRRRFPEEPHVPLEQRRGFLAREQYRALRPDLADK
VASVYEAPGFFLDLEPIPGALDAVREMNDLPDTQVFICTSPLLKYHHCVGEKYRWVEQHL
GPQFVERIILTRDKTVVLGDLLIDDKDTVRGQEETPSWEHILFTCCHNRHLVLPPTRRRL
LSWSDNWREILDSKRGAAQRE
>P40547
MTVAYSLENLKKISNSLVGDQLAKVDYFLAPKCQIFQCLLSIEQSDGVELKNAKLDLLYT
LLHLEPQQRDIVGTYYFDIVSAIYKSMSLASSFTKNNSSTNYKYIKLLNLCAGVYPNCGF
PDLQYLQNGFIQLVNHKFLRSKCKIDEVVTIIELLKLFLLVDEKNCSDFNKSKFMEEERE
VTETSHYQDFKMAESLEHIIVKISSKYLDQISLKYIVRLKVSRPASPSSVKNDPFDNKGV
DCTRAIPKKINISNMYDSSLLSLALLLYLRYHYMIPGDRKLRNDATFKMFVLGLLKSNDV
NIRCVALKFLLQPYFTEDKKWEDTRTLEKILPYLVKSFNYDPLPWWFDPFDMLDSLIVLY
NEITPMNNPVLTTLAHTNVIFCILSRFAQCLSLPQHNEATLKTTTKFIKICASFAASDEK
YRLLLLNDTLLLNHLEYGLESHITLIQDFISLKDEIKETTTESHSMCLPPIYDHDFVAAW
LLLLKSFSRSVSALRTTLKRNKIAQLLLQILSKTYTLTKECYFAGQDFMKPEIMIMGITL
GSICNFVVEFSNLQSFMLRNGIIDIIEKMLTDPLFNSKKAWDDNEDERRIALQGIPVHEV
KANSLWVLRHLMYNCQNEEKFQLLAKIPMNLILDFINDPCWAVQAQCFQLLRNLTCNSRK
IVNILLEKFKDVEYKIDPQTGNKISIGSTYLFEFLAKKMRLLNPLDTQQKKAMEGILYII
VNLAAVNENKKQLVIEQDEILNIMSEILVETTTDSSSNGNDSNLKLACLWVLNNLLWNSS
VSHYTQYAIENGLEPGHSPSDSENPQSTVTIGYNESVAGGYSRGKYYDEPDGDDSSSNAN
DDEDDDNDEGDDEGDEFVRTPAAKGSTSNVQVTRATVERCRKLVEVGLYDLVRKNITDES
LSVREKARTLLYHMDLLLKVK
>Q99JE6
MAGARPGVHALQLEPPTVVETLRRGSKFIKWDEEASSRNLVTLRLDPNGFFLYWTGPNME
VDTLDISSIRDTRTGRYARLPKDPKIREVLGFGGPDTRLEEKLMTVVAGPDPVNTTFLNF

MAVQDDTVKVWSEELFKLAMNILAQNAPEHVLRKAYTKLKLQVNQDGRIPVKNILKMFSA
DKKRVETALICGLNFNRSESIRPDEFSLEIFERFLNKLLLRPDIDKILLEIGAKGKPYLT
LEQLMDFINQKQRDPRLNEVLYPPLRSSQARLLIEKYEPNKQFLERDQMSMEGFSRYLGG
EENGILPLEALDLSMDMTQPLSAYFINSSHNTYLTAGQLAGTSSVEMYRQALLWGCRCVE
LDVWKGRPPEEEPFITHGFTMTTEVPLRDVLEAIAETAFKTSPYPVILSFENHVDSAKQQ
AKMAEYCRSIFGEALLIDPLDKYPLSAGTPLPSPQDLMGRILVKNKKRHRPSTGVPDSSV
AKRPLEQSNSALSESSAATEPSSPQLGSPSSDSCPGLSNGEEVGLEKTSLEPQKSLGEEG
LNRGPNVLMPDRDREDEEEDEEEEETTDPKKPTTDEGTASSEVNATEEMSTLVNYVEPVK
FKSFEASRKRNKCFEMSSFVETKAMEQLTKSPMEFVEYNKQQLSRIYPKGTRVDSSNYMP
QLFWNVGCQLVALNFQTLDLPMQLNAGVFEYNGRSGYLLKPEFMRRPDKSFDPFTEVIVD
GIVANALRVKVISGQFLSDRKVGIYVEVDMFGLPVDTRRKYRTRTSQGNSFNPVWDEEPF
DFPKVVLPTLASLRIAAFEEGGRFVGHRILPVSAIRSGYHYVCLRNEANQPLCLPALLIY
TEASDYIPDDHQDYAEALINPIKHVSLMDQRAKQLAALIGESEAQASTEMCQETPSQQQG
SQLSSNPVPNPLDDSPRWPPGPTTSPTSTSLSSPGQRDDLIASILSEVTPTPLEELRSHK
AMVKLRSRQDRDLRELHKKHQRKAVALTRRLLDGLAQARAEGKCRPSSSALSRATNVEDV
KEEEKEAARQYREFQNRQVQSLLELREAQADAETERRLEHLKQAQQRLREVVLDAHTTQF
KRLKELNEREKKELQKILDRKRNNSISEAKTREKHKKEVELTEINRRHITESVNSIRRLE
EAQKQRHERLLAGQQQVLQQLVEEEPKLVAQLTQECQEQRERLPQEIRRCLLGETSEGLG
DGPLVACASNGHAAGSGGHQSGADSESQEENTQL
>O36019
MPRLNTQLPRMYSAPPGHSKAVSTELNKDLSSVGGRSAKLGQVIHHCFYKTGLIILESRL
NVFGTSRPRESSKNNKWFNLEIVETELYAEQFKIWKNIELSPSRKIPPMVLHTYLDISDL
SKNQTLSVSDGTHSHAINFNNMSTMKIVLERWIVNLDGEALSTPLELAVLYKKLVVLFRS
LYTYTHLMPLWKLKSKIHKLRAHGTSLKVGCALSTDDVLSNDFLPISAPISSSLGSSIAT
FSFSPVGTPAGDFRISVQYRKNCHFEVHDSDALLSNQLLSADKHQLAASNNSQDFEDGKQ
YDQPPPSFATRLAKQSDPNSLLQSEIQHLASIESITAQAAPLVTIHPFKSPSLSASPGSN
FDNMSISPKVAVNRYIHRGPSATSLNKFSMISDAASKSRAKLPPLTSGSLKLNTLDISNT
PNLRRFSSSFGPRERKESFSSRNRLPLVNHPIRSIFKHNVSENPITDHSEHAVYDSEFAS
KDDLSGFIQLLDSHAHHLNASEGSKSSGSFPGKVQTLTSGISPVAHPHNSLGSSNEIFDI
DTYNHSIDNSGSRFTEAVKHNLGNSSHSIMRHHTLGTLRSRPSFSEKSTFPAPLTSISQA
STFQGDNRSPSTVIPHTQTEVPSANDTSKQLASLHDMRKSQSPICARSATSAGLPRFEYH
TSLSKSLEHSSTPASLQATKTPSPSFVLEPGIPQEYKKHFDNLSEERRQCLTPSTPTYEY
YNEHNPNYDDDLLFTMTDMTLEPHDVSAIRLGSPKSDD
>P29452
MADKILRAKRKQFINSVSIGTINGLLDELLEKRVLNQEEMDKIKLANITAMDKARDLCDH
VSKKGPQASQIFITYICNEDCYLAGILELQSAPSAETFVATEDSKGGHPSSSETKEEQNK
EDGTFPGLTGTLKFCPLEKAQKLWKENPSEIYPIMNTTTRTRLALIICNTEFQHLSPRVG
AQVDLREMKLLLEDLGYTVKVKENLTALEMVKEVKEFAACPEHKTSDSTFLVFMSHGIQE
GICGTTYSNEVSDILKVDTIFQMMNTLKCPSLKDKPKVIIIQACRGEKQGVVLLKDSVRD
SEEDFLTDAIFEDDGIKKAHIEKDFIAFCSSTPDNVSWRHPVRGSLFIESLIKHMKEYAW
SCDLEDIFRKVRFSFEQPEFRLQMPTADRVTLTKRFYLFPGH
>Q13485
MDNMSITNTPTSNDACLSIVHSLMCHRQGGESETFAKRAIESLVKKLKEKKDELDSLITA
ITTNGAHPSKCVTIQRTLDGRLQVAGRKGFPHVIYARLWRWPDLHKNELKHVKYCQYAFD

LKCDSVCVNPYHYERVVSPGIDLSGLTLQSNAPSSMMVKDEYVHDFEGQPSLSTEGHSIQ
TIQHPPSNRASTETYSTPALLAPSESNATSTANFPNIPVASTSQPASILGGSHSEGLLQI
ASGPQPGQQQNGFTGQPATYHHNSTTTWTGSRTAPYTPNLPHHQNGHLQHHPPMPPHPGH
YWPVHNELAFQPPISNHPAPEYWCSIAYFEMDVQVGETFKVPSSCPIVTVDGYVDPSGGD
RFCLGQLSNVHRTEAIERARLHIGKGVQLECKGEGDVWVRCLSDHAVFVQSYYLDREAGR
APGDAVHKIYPSAYIKVFDLRQCHRQMQQQAATAQAAAAAQAAAVAGNIPGPGSVGGIAP
AISLSAAAGIGVDDLRRLCILRMSFVKGWGPDYPRQSIKETPCWIEIHLHRALQLLDEVL
HTMPIADPQPLD
>Q9BUP3
MAETEALSKLREDFRMQNKSVFILGASGETGRVLLKEILEQGLFSKVTLIGRRKLTFDEE
AYKNVNQEVVDFEKLDDYASAFQGHDVGFCCLGTTRGKAGAEGFVRVDRDYVLKSAELAK
AGGCKHFNLLSSKGADKSSNFLYLQVKGEVEAKVEELKFDRYSVFRPGVLLCDRQESRPG
EWLVRKFFGSLPDSWARGHSVPVVTVVRAMLNNVVRPRDKQMELLENKAIHDLGKAHGSL
KP
>P40005
MEQRNNVFQAKYNEYKQILEELQTKIIELGHDKDEHTIVIKTLKDAEPTRKCYRMIGGAL
VESDVQTSLPILETKKENIEGTISKMKETLIQTAKEFEKWKKDNKIQVVKN
>Q9BRV8
MSCTIEKILTDAKTLLERLREHDAAAESLVDQSAALHRRVAAMREAGTALPDQYQEDASD
MKDMSKYKPHILLSQENTQIRDLQQENRELWISLEEHQDALELIMSKYRKQMLQLMVAKK
AVDAEPVLKAHQSHSAEIESQIDRICEMGEVMRKAVQVDDDQFCKIQEKLAQLELENKEL
RELLSISSESLQARKENSMDTASQAIK
>Q76N89
MLLHLCSVKNLYQNRFLGLAAMASPSRNSQSRRRCKEPLRYSYNPDQFHNMDLRGGPHDG
VTIPRSTSDTDLVTSDSRSTLMVSSSYYSIGHSQDLVIHWDIKEEVDAGDWIGMYLIDEV
LSENFLDYKNRGVNGSHRGQIIWKIDASSYFVEPETKICFKYYHGVSGALRATTPSVTVK
NSAAPIFKSIGADETVQGQGSRRLISFSLSDFQAMGLKKGMFFNPDPYLKISIQPGKHSI
FPALPHHGQERRSKIIGNTVNPIWQAEQFSFVSLPTDVLEIEVKDKFAKSRPIIKRFLGK
LSMPVQRLLERHAIGDRVVSYTLGRRLPTDHVSGQLQFRFEITSSIHPDDEEISLSTEPE
SAQIQDSPMNNLMESGSGEPRSEAPESSESWKPEQLGEGSVPDRPGNQSIELSRPAEEAA
VITEAGDQGMVSVGPEGAGELLAQVQKDIQPAPSAEELAEQLDLGEEASALLLEDGEAPA
STKEEPLEEEATTQSRAGREEEEKEQEEEGDVSTLEQGEGRLQLRASVKRKSRPCSLPVS
ELETVIASACGDPETPRTHYIRIHTLLHSMPSAQGGSAAEEEDGAEEESTLKDSSEKDGL
SEVDTVAADPSALEEDREEPEGATPGTAHPGHSGGHFPSLANGAAQDGDTHPSTGSESDS
SPRQGGDHSCEGCDASCCSPSCYSSSCYSTSCYSSSCYSASCYSPSCYNGNRFASHTRFS
SVDSAKISESTVFSSQDDEEEENSAFESVPDSMQSPELDPESTNGAGPWQDELAAPSGHV
ERSPEGLESPVAGPSNRREGECPILHNSQPVSQLPSLRPEHHHYPTIDEPLPPNWEARID
SHGRVFYVDHVNRTTTWQRPTAAATPDGMRRSGSIQQMEQLNRRYQNIQRTIATERSEED
SGSQSCEQAPAGGGGGGGSDSEAESSQSSLDLRREGSLSPVNSQKITLLLQSPAVKFITN
PEFFTVLHANYSAYRVFTSSTCLKHMILKVRRDARNFERYQHNRDLVNFINMFADTRLEL
PRGWEIKTDQQGKSFFVDHNSRATTFIDPRIPLQNGRLPNHLTHRQHLQRLRSYSAGEAS
EVSRNRGASLLARPGHSLVAAIRSQHQHESLPLAYNDKIVAFLRQPNIFEMLQERQPSLA
RNHTLREKIHYIRTEGNHGLEKLSCDADLVILLSLFEEEIMSYVPLQAAFHPGYSFSPRC
SPCSSPQNSPGLQRASARAPSPYRRDFEAKLRNFYRKLEAKGFGQGPGKIKLIIRRDHLL

EGTFNQVMAYSRKELQRNKLYVTFVGEEGLDYSGPSREFFFLLSQELFNPYYGLFEYSAN
DTYTVQISPMSAFVENHLEWFRFSGRILGLALIHQYLLDAFFTRPFYKALLRLPCDLSDL
EYLDEEFHQSLQWMKDNNITDILDLTFTVNEEVFGQVTERELKSGGANTQVTEKNKKEYI
ERMVKWRVERGVVQQTEALVRGFYEVVDSRLVSVFDARELELVIAGTAEIDLNDWRNNTE
YRGGYHDGHLVIRWFWAAVERFNNEQRLRLLQFVTGTSSVPYEGFAALRGSNGLRRFCIE
KWGKITSLPRAHTCFNRLDLPPYPSYSMLYEKLLTAVEETSTFGLE
>Q07527
MVGGALICKYLPREEQLKLISDLIQNDSLEEVLELIETSPLDITTDSNIETPIFEKITEQ
VIAYASIDGEAREMFRSSRAEMNKALRTSAQLLCCLPSVWHKFQVWMSYRLNDIISENYK
HLFNDNFGKKIVQPFFDSFAEEQNANIKHENLHLDILSLLHYLEVVYLFDECKNGISSKC
LDFIIVPLLGCNSEEIADSCSKLMRWHIKYLSKCCNTDSNFDKLIWTFIKQLYAEGSQQA
WKQKNSLSFLLRFLLAAELSPELITYIKTDAYWRHIQTELDNDVHEHRKLALSILKLTIQ
KLSSHGITLQTTFYKCNDLANIEMLGSWKKFTTLYEMIALDTSLNQIQAAKQDIIKIFDN
EHLHHSWGLILLSTGLKSSMESVRKYMMTLMFSITNMSAFSSNLPLLTKTLLPAAMSAHY
FDVKGVSCPHGEKLSLFVNNLLSQTTEGISDILFEILKLLVEKGTSFDPSRIYLSYGILV
FFQNNKQKTINSDHLSLIRKLYDFAAEEEVLETTIQTIYLKFLLYIDPSVSASELLFTLV
SHIKLKGGTYKYVEPLFEDYRDLAVSHFDDLQAKENLTTNIGKDTIFDLLASIIFDFKDI
DITPDFLIEVAKSKQDIPVYTSKAVTFLTQLLSGEPSNGYTYENATALLSYPNFTISTWK
SINVNNLFKSVMEKFSLDKFKFFAEIYQKTYECRFDTIELNFNDLLSLYEMVKKSANQCS
RESFKVKDSAYSSYFELLNTFLKTYALNRDSSEGNDDELHILLRLVDENINKDNGNYLGN
LAVCKLLYFIIDSYIHCSTSVSDDDIFIVKFIFEKFSFIWECINSERLVLKERDLHLMLI
KGLFHPVILYFGSNQYIDTLTSKLEEHAQTIISLSYSRRSLLPLLGSQLRVFMKFYGKLL
REDVNYWWLINIIVGVFKQPQMDVNLYKLKPVISSLFDHKLNNYYIKGDELYEKVYGPDE
ILARVSIIDSILYANDQLKIRLIEKVTEKTNALYAIKRTDGAEALQRLLQWQLLLLSLLT
TNEKKLSETSMIRILKSIEDESSPLVRVYKEWFISSKVVDYYKTGNPKFAEDYLFSLLED
HSKPVFVVSAEKICFMVLKDLRNDEKKYGFTQLLDRFICTLVPNAASNKPLVRHFSNSLI
ISLWPTFKAYLSDHTLRNIIENLYSNAKKTQIFGQYRAGDANIWDLKGDRKLTNMFGGVL
KKVTDHDCPYISESVFEKYLQEKDIVPIGTDERSLWLDKRDTNTESVNNANISCDTSPLQ
TKSGAWETVLDLDNKKSNDVVTRSELIVVSSLVDKPPNLGGICRLCDVLGVGLLTVQDIK
VKNHPQFKNVAVTADRWMPMEEVALDEIASFMKEKKKEGYTLIGLEQTDKSVKLDNNFQF
PKKSLILLGTEAFGIPGTLLSELDLCLEIQQFGVIRSMNIQTATAVIVHSYTVQHM
>Q9BXF6
MALVRGAEPAAGPSRWLPTHVQVTVLRARGLRGKSSGAGSTSDAYTVIQVGREKYSTSVV
EKTHGCPEWREECSFELPPGALDGLLRAQEADAGPAPWAASSAAACELVLTTMHRSLIGV
DKFLGQATVALDEVFGAGRAQHTQWYKLHSKPGKKEKERGEIEVTIQFTRNNLSASMFDL
SMKDKPRSPFSKIRDKMKGKKKYDLESASAILPSSAIEDPDLGSLGKMGKAKGFFLRNKL
RKSSLTQSNTSLGSDSTLSSASGSLAYQGPGAELLTRSPSRSSWLSTEGGRDSAQSPKLF
THKRTYSDEANQMRVAPPRALLDLQGHLDAASRSSLCVNGSHIYNEEPQGPVRHRSSISG
SLPSSGSLQAVSSRFSEEGPRSTDDTWPRGSRSNSSSEAVLGQEELSAQAKVLAPGASHP
GEEEGARLPEGKPVQVATPIVASSEAVAEKEGARKEERKPRMGLFHHHHQGLSRSELGRR
SSLGEKGGPILGASPHHSSSGEEKAKSSWFGLREAKDPTQKPSPHPVKPLSAAPVEGSPD
RKQSRSSLSIALSSGLEKLKTVTSGSIQPVTQAPQAGQMVDTKRLKDSAVLDQSAKYYHL
THDELISLLLQRERELSQRDEHVQELESYIDRLLVRIMETSPTLLQIPPGPPK
>Q9Y5L0

MEGAKPTLQLVYQAVQALYHDPDPSGKERASFWLGELQRSVHAWEISDQLLQIRQDVESC
YFAAQTMKMKIQTSFYELPTDSHASLRDSLLTHIQNLKDLSPVIVTQLALAIADLALQMP
SWKGCVQTLVEKYSNDVTSLPFLLEILTVLPEEVHSRSLRIGANRRTEIIEDLAFYSSTV
VSLLMTCVEKAGTDEKMLMKVFRCLGSWFNLGVLDSNFMANNKLLALLFEVLQQDKTSSN
LHEAASDCVCSALYAIENVETNLPLAMQLFQGVLTLETAYHMAVAREDLDKVLNYCRIFT
ELCETFLEKIVCTPGQGLGDLRTLELLLICAGHPQYEVVEISFNFWYRLGEHLYKTNDEV
IHGIFKAYIQRLLHALARHCQLEPDHEGVPEETDDFGEFRMRVSDLVKDLIFLIGSMECF
AQLYSTLKEGNPPWEVTEAVLFIMAAIAKSVDPENNPTLVEVLEGVVRLPETVHTAVRYT
SIELVGEMSEVVDRNPQFLDPVLGYLMKGLCEKPLASAAAKAIHNICSVCRDHMAQHFNG
LLEIARSLDSFLLSPEAAVGLLKGTALVLARLPLDKITECLSELCSVQVMALKKLLSQEP
SNGISSDPTVFLDRLAVIFRHTNPIVENGQTHPCQKVIQEIWPVLSETLNKHRADNRIVE
RCCRCLRFAVRCVGKGSAALLQPLVTQMVNVYHVHQHSCFLYLGSILVDEYGMEEGCRQG
LLDMLQALCIPTFQLLEQQNGLQNHPDTVDDLFRLATRFIQRSPVTLLRSQVVIPILQWA
IASTTLDHRDANCSVMRFLRDLIHTGVANDHEEDFELRKELIGQVMNQLGQQLVSQLLHT
CCFCLPPYTLPDVAEVLWEIMQVDRPTFCRWLENSLKGLPKETTVGAVTVTHKQLTDFHK
QVTSAEECKQVCWALRDFTRLFR
>O43504
MEATLEQHLEDTMKNPSIVGVLCTDSQGLNLGCRGTLSDEHAGVISVLAQQAAKLTSDPT
DIPVVCLESDNGNIMIQKHDGITVAVHKMAS
>P11353
MPAPQDPRNLPIRQQMEALIRRKQAEITQGLESIDTVKFHADTWTRGNDGGGGTSMVIQD
GTTFEKGGVNVSVVYGQLSPAAVSAMKADHKNLRLPEDPKTGLPVTDGVKFFACGLSMVI
HPVNPHAPTTHLNYRYFETWNQDGTPQTWWFGGGADLTPSYLYEEDGQLFHQLHKDALDK
HDTALYPRFKKWCDEYFYITHRKETRGIGGIFFDDYDERDPQEILKMVEDCFDAFLPSYL
TIVKRRKDMPYTKEEQQWQAIRRGRYVEFNLIYDRGTQFGLRTPGSRVESILMSLPEHAS
WLYNHHPAPGSREAKLLEVTTKPREWVK
>Q99M63
MSIEIESSDVIRLIMQYLKENSLHRALATFTEETTVSLNTVDSIESFVADINSGHWDTVL
QAIQSLKLPDKTLIDLYEQVVLELIELRELGAARSLLRQTDPMIMLKQTQPERYIHLENL
LARSYFDPREAYPDGSSKEKRRAAIAQALAGEVSVVPPSRLMALLGQALKWQQHQGLLPP
GMTIDLFRGKAAVKDVEEEKFPTQLSRHIKFGQKSHVECARFSPDGQYLVTGSVDGFIEV
WNFTTGKIRKDLKYQAQDNFMMMDDAVLCMCFSRDTEMLATGAQDGKIKVWKIQSGQCLR
RFERAHSKGVTCLSFSKDSSQILSASFDQTIRIHGLKSGKTLKEFRGHSSFVNEATFTQD
GHYIISASSDGTVKIWNMKTTECSNTFKSLGSTAGTDITVNSVILLPKNPEHFVVCNRSN
TVVIMNMQGQIVRSFSSGKREGGDFVCCALSPRGEWIYCVGEDFVLYCFSTVTGKLERTL
TVHEKDVIGIAHHPHQNLIATYSEDGLLKLWKP
>O60089
MVWRTIAGSCSLSAYHHTAKQSIDKYKSVLEEERKKQLLRTETGLLRQSSSRSSSLLDQR
IRQITAKDKTVNLLRKTLNRPLLHEVDYHGKSLEILDMLLQSRNSMISSLRSELQEKNQK
DKKKEVNKLEEKMTNAKEPNVNVPSMKASSAISVETPETIESEASVDHKEASKIIKDVEK
EE
>P38835
MALPIEGKLSMANNRIERLKSPSSSSTCSMDEVLITSSNNSSSICLETMRQLPREGVSGQ
INIIKETAASSSSHAALFIKQDLYEHIDPLPAYPPSYDLVNPNKEVRFPIFGDTAPCPKS

SLPPLYAPAVYELTLISLKLERLSPYEISSNRSWRNFIIEINSTQLNFYHIDESLTKHIR
NYSSGETKSEKEDRIHSDLVHRSDQSQHLHHRLFTLPTRSASEFKKADQERISYRVKRDR
SRYLTDEALYKSFTLQNARFGIPTDYTKKSFVLRMSCESEQFLLRFSHIDDMIDWSMYLS
IGISVSLDLEVREYPDYRIVPRRRRRRRRRRRRRRHTHRSESSMGSFSQRFIRSNSRPDL
IQRYSTGSSTNNNTTIRERSNTFTAGLLDHYCTGLSKTPTEALISSAASGESSDNSTLGS
TRSLSGCSASRSIASRSLKFKIKNFFRPKNSSRTEKLHRLRSNSSNLNSVIETEEDDEHH
ESSGGDHPEPGVPVNTTIKVERPMHRNRAISMPQRQSLRRAISEEVVPIKFPNSTVGESV
HSPSPIEHLSVDGCEIMLQSQNAVMKEELRSVASNLVANERDEASIRPKPQSSSIYLSGL
APNGESATDLSQSSRSLCLTNRDAEINDDESATETDEDENDGETDEYAGDDTNDDTDDSN
IGYAYGSESDYSCLIEERIRNRRRASSTLSCFSNIPYGTDDIKWKPAIKEISRRRYLRDS
LKCIKPFLDSNDCLGKVIYIPVSGPTFETSNKIHFSNRQSLQKQKNHFLKGFIVGPTALI
ELNCKNKNAIVGTTKDAEDHGEDDGDGDDGEDDDDDDDDDDDDDDDDEDDDDDDDDDDDD
DDDDDGQITA
>P13231
MGNRAFKSHHGHFLSAEGEAVKTHHGHHDHHTHFHVENHGGKVALKTHCGKYLSIGDHKQ
VYLSHHLHGDHSLFHLEHHGGKVSIKGHHHHYISADHHGHVSTKEHHDHDTTFEEIII
>Q9ESJ7
MSLQPAPASRVFMELVPWADRGRENHPISAAEAQPIGRRPHVAEAHPGAREVHVSGAAEV
SASPDRALVTVRVSSTKEVSAEAKKSVCRRLDYITQSLQQQGFQAENVTVTKNIRRVENA
YHMEAEVCITFTEFGKMQNICNFLVEKLDSSVVISPPEFYHTPGSVENLRRQACLVAVEN
AWRKAQEVCDLVGQTLGKPLLIKEEETKDWEGQTDDHQLSRLPGTLTVQQKIKSATIHAA
SKVFITFEVKGKEKKKKHL
>Q9N425
MTTFFNKLFTGASSSSSAAASSGMDLGSMESLCAAINELCSEKHVSENVGELAAGIVRKN
KELFEKKSNDVEAFLLHCSPNVGSAAMVAAIKGMFDTSAAKNNETGTDRAVELLNHYVDE
NNFVGGHLKLVPEIIFPLLRDVGLYCLEKKNKPEIGQRIIMKALGSMFPRNGSNAPNVLT
SAHGVLFACALETKDYASVEPFIDLHVDEIANENCIQDQEKSDNRERDFGDVFLGRMKKG
AAFGSQPVAHNPHLHPKYVLDYLYNGACILIELKRFEDALFLLEICVGMPAFSVQDQHLD
SFKKYVLISLILKGKVDVTENGDKSAIRHFKTKSPEYKQFSEIRFSRSSNTHSAVESLVK
SAKDRLRKDGNTEIAKYLVVEMKKKTIMSLTRMFTSIRISEIEELAFLKSRDQVTELIGQ
LVEEQRITVRIDGDMVFWTELSPVPSKNDVENKIRIVDHLNTLLHEKNTTMKAGSGRMRP
SVMYNEDEGLSMPPTESKFFS
>O75334
MMCEVMPTINEDTPMSQRGSQSSGSDSDSHFEQLMVNMLDERDRLLDTLRETQESLSLAQ
QRLQDVIYDRDSLQRQLNSALPQDIESLTGGLAGSKGADPPEFAALTKELNACREQLLEK
EEEISELKAERNNTRLLLEHLECLVSRHERSLRMTVVKRQAQSPSGVSSEVEVLKALKSL
FEHHKALDEKVRERLRVSLERVSALEEELAAANQEIVALREQNVHIQRKMASSEGSTESE
HLEGMEPGQKVHEKRLSNGSIDSTDETSQIVELQELLEKQNYEMAQMKERLAALSSRVGE
VEQEAETARKDLIKTEEMNTKYQRDIREAMAQKEDMEERITTLEKRYLSAQRESTSIHDM
NDKLENELANKEAILRQMEEKNRQLQERLELAEEKLQQTMRKAETLPEVEAELAQRIAAL
TKAEETHGNIEERMRHLEGQLEEKNQELQRARQREKMNEEHNKRLSDTVDRLLTESNERL
QLHLKERMAALEEKNVLIQESETFRKNLEESLHDKESLAEEIEKLRSELDQLKMRTGSLI
EPTIPRTHLDTSAELRYSVGSLVDSQSDYRTTKVIRRPRRGRMGVRRDEPKVKSLGDHEW
NRTQQIGVLSSHPFESDTEMSDIDDDDRETIFSSMDLLSPSGHSDAQTLAMMLQEQLDAI

NKEIRLIQEEKESTELRAEEIENRVASVSLEGLNLAMVHPGTSITASVTASSLASSSPPS
GHSTPKLTPRSPAREMDRMGVMTLPSDLRKHRRKIAVVEEDGREDKATIKCETSPPPTPR
ALRMTHTLPSSYHNDARSSLSVSLEPESLGLGSANSSQDSLHKAPKKKGIKSSIGRLFGK
KEKARLGQLRGFMETEAAAQESLGLGKLGTQAEKDRRLKKKHELLEEARRKGLPFAQWDG
PTVVAWLELWLGMPAWYVAACRANVKSGAIMSALSDTEIQREIGISNPLHRLKLRLAIQE
MVSLTSPSAPPTSRTPSGNVWVTHEEMENLAAPAKTKESEEGSWAQCPVFLQTLAYGDMN
HEWIGNEWLPSLGLPQYRSYFMECLVDARMLDHLTKKDLRVHLKMVDSFHRTSLQYGIMC
LKRLNYDRKELERRREASQHEIKDVLVWSNDRVIRWIQAIGLREYANNILESGVHGSLIA
LDENFDYSSLALLLQIPTQNTQARQILEREYNNLLALGTERRLDESDDKNFRRGSTWRRQ
FPPREVHGISMMPGSSETLPAGFRLTTTSGQSRKMTTDVASSRLQRLDNSTVRTYSC
>Q7M6U3
MSRGAPFPVPCPVLLGTFTDDSLEAQLHEYAKQGNCVKLKKILKKGVCVDAVNTQGQSAL
FVAALLGHVKLVDVLVDYGSDPNHRCFDGSTPVHAAAFSGNQWILSKLLTAGGDLRLHDE
KGRNPQAWALTAGKDRSTQMVEFMQRCTSHMKAIIQGFSYDLLKKIDSPQRLIGSPPWFG
SLIQGSPNSSPNRQLKPGIISAQNIYSFGFGKFYLTSGMQLTYPGSLPVIGEKEVVQADD
EPTFSFFSGPYMVMTNLVWNRSRVTVKELNLPTRPHCSRLRLADLLIAEQEHSSNLRHPN
LLQLMAVCLSRDLEKIRLVYERITVGTLFSVLHERRSQFPVLHMEVIVHLLLQVADALIY
LHSRGFIHRSLSSYAVHIVSAGEARLTNLEYLTESQDSGAHRNVTRMPLPTQLYNWAAPE
VVLQKAATVKSDIYSFSVIIQEILTDSIPWNGLDGSLVKETIALGNYLEADVRLPEPYYD
IVKSGIHAKQKNRTMNLQDIRYILKNDLKEFIGAQKTQPTESPRGQSYEPHPDVNICLGL
TSEYQKDPPDLDIKELKEMGSQPHSPTDHSFLTVKPTLAPQTLDSSLSAQKPDNANVPSP
PAACLAEEVRSPTASQDSLCSFEINEIYSGCLTLGTDKEEECLGTAASPEGDRPNQGDEL
PSLEEELDKMERELHCFCEEDKSISEVDTDLLFEDDDWQSDSLGSLNLPEPTREAKGKTS
SWSKTDEYVSKCVLNLKISQVMMQQSAEWLRKLEQEVEELEWAQKELDSQCSSLRDASLK
FANAKFQPAVGPPSLAYLPPVMQLPGLKQPENGGTWLTLARSPGNEREFQEGHFSKKPEK
LSACGWKPFTQVSEESRGDCSELNNQLPTLRGPGKQSTGEQLPSTQEARESLEKNTNQNS
RSMASVSSEIYATKSRNNEDNGEAHLKWRLAVKEMAEKAVSGQLLLPPWNPQSSAPFESK
VENESTPLPRPPIRGPESTEWQHILEYQRENDEPKGNTKFGKMDNSDCDKNKHSRWTGLQ
RFTGIRYPFFRNHEQPEQNEASQASCDTSVGTEKFYSTSSPIGDDFERFQDSFAQRQGYV
EENFQIREIFEKNAEILTKPQFQAIQCAEDKQDETLGETPKELKEKNTSLTDIQDLSSIT
YDQDGYFKETSYKTPKLKHAPTSASTPLSPESISSAASHYEDCLENTTFHVKRGSTFCWN
GQEAMRTLSAKFTTVRERAKSLESLLASSKSLPAKLTDSKRLCMLSETGSSNVSAAFVTS
THATKRKSLPRELAEATSQQHLDELPPPAQELLDEIEQLKQQQVSSLASHENTARDLSVT
NKDKKHLEEQETNSSKDSSFLSSREIQDLEDTERAHSSLDEDLERFLQSPEENTALLDPT
KGSTREKKNKDQDVVEQKRKKKESIKPERRESDSSLGTLEEDELKPCFWKRLGWSEPSRI
IVLDQSDLSD
>Q02936
MRHIAHTQRCLSRLTSLVALLLIVLPMVFSPAHSCGPGRGLGRHRARNLYPLVLKQTIPN
LSEYTNSASGPLEGVIRRDSPKFKDLVPNYNRDILFRDEEGTGADRLMSKRCKEKLNVLA
YSVMNEWPGIRLLVTESWDEDYHHGQESLHYEGRAVTIATSDRDQSKYGMLARLAVEAGF
DWVSYVSRRHIYCSVKSDSSISSHVHGCFTPESTALLESGVRKPLGELSIGDRVLSMTAN
GQAVYSEVILFMDRNLEQMQNFVQLHTDGGAVLTVTPAHLVSVWQPESQKLTFVFADRIE
EKNQVLVRDVETGELRPQRVVKVGSVRSKGVVAPLTREGTIVVNSVAASCYAVINSQSLA
HWGLAPMRLLSTLEAWLPAKEQLHSSPKVVSSAQQQNGIHWYANALYKVKDYVLPQSWRH

D
>Q920K5
MGCGGSRADAIEPRYYESWTRETESTWLTYTDSDALPSAAATDSGPEAGGLHAGVLEDGP
SSNGVLRPAAPGGIANPEKKMNCGTQCPNSQSLSSGPLTQKQNGLWTTEAKRDAKRMSAR
EVAISVTENIRQMDRSKRVTKNCIN
>O59679
MSNPRMFQSRGKRVVSDSLPIANSTHTLEDTLKLRPDWTIALSQHETETRSTRTSCSTTT
ESTIAMRSKQKAIISVLEEFRDSEAAYVHDLHVAQRYYADRLSDRVKKSEWKDVFEIFLV
LCKQASLFEIEMHKSLNDEINYILDDQDACLKPKPSVAQLFLSWLPKLSAVYGRYCVIQE
NIGKKVEKWMKNSSISEYLQECDSMAKIESNSWNLDSFLVKPVQRFLKYPLLLNQLYRSA
SLGIISDYVLLGEACHKSEIASQRMNELKRRRDIIITALDSVSNSQEVLLLSTDSIDKKI
AKLQNSTNIFYVPEHEPILAFVHQLSSSYTNLLNLRSAICDWLKFSRYHYLKFFTFVEAY
SVFCKDTKSADKWALISVALDNIAKGAVLRLTEQCQTSVLRPISNGILFFRNPLCVTDVW
IKKATAFSKRRQSQVFEEDLESFPLLSNCLLEELPLFLEMARNVTDECILAFAQIQATFY
DTIQKVLEPVVAKFNLTDHQDIPSIESIMDFTSLRSSMESSPKSK
>Q9US06
MQENRRQDYILRVRYHNPLPPPPFPPKLINIPNPVKQYALPNFVSTLVQEKKIPIENDIE
LGMPLDLAGITGFFEGDTSWMHSDLSSVNLDPIDRSLLKVAGGSGSTHLEVPFLRRTEYI
SSEVARAASNRGNLRLTASTSKALAEQRGRSLREVPKQLEAINESFDVVQQPLEQLKHPT
KPDLKPVSAWNLLPNTSMAGIQHLMLRVADDLSERSHSYSSLVNLQEGHNLTKRHEVALF
MPSSAEGEEFLSYYLPSEETAEEIQAKVNDASADVHEPFVYNHFRNFDASMHVNSTGLED
LCLTFHTDKDHPEANQVLYTPIYARSTLKRRHVRAPVSLDAVDGIELSLRDLNDEESLQL
KRARYDTFGLGNIKDLEEEEEKLRSVEGSLNEELSEEEKPAESREQLESAEQTNGVKPET
QAQNMSASESQANSPAPPVEEGNTQPSPVEQLQNEED
>O74733
MSTIFQDLVDSTVIKNAVQGQFTSLVSNNLVVSKVNSLHLFEIEKIQKDESSFPLDDSLQ
NEFSTSIIDESQAFMETNMHLIRTNEQTTYVLRLVSQVKVFGTITEISALKGKGSNGCDL
LIMLTDYAKVSTLEWDMQSQSFVTNSLHYYEDVKSSNICSSHTPTQLLVDPDSDCCLLRF
LTDMMAIIPYPANEDLDMEEAAIENSKISSSYAYKPSFVLASSQLDASISRILDVKFLYG
YREPTLAILYSPEQTSTVTLPLRKDTVLFSLVTLDLEQRASAVITTIQSLPYDIYASVSI
PTPLGGSLLLGGNELIYVDSAGRTVGIGVNSYYSKCTDFPLQDQSDFNLELEGTIAIPLT
SSKTETPFVVLVHTSGQFFYLDFLLDGKSVKGLSLQALDLEINDDFLKSGITCAVPAGEN
LVFLGSQTTDSYLLRWSRRTTNEEVRLDEGDDTLYGTNDAEMDDMLDIYETDESVGSKRK
IAYENGPLRLEICDVLTNIGPITDFAVGKAGSYSYFPQDNHGPLELVGTAGADGAGGLVV
FRRNIFPLIAGEFQFDGCEALWTVSISGKLRNMKSRIQAQYSNPELETYLVLSKEKESFI
FLAGETFDEVQHSDFSKDSKTLNVGSLLSGMRMVQICPTSLRVYDSNLRLTQLFNFSKKQ
IVVSTSICDPCIIVVFLGGGIALYKMDLKSQRLIKTDLQNRLSDVKTASLVSPDSSALFA
KLFTYNETLNAKGQIANGMNDSASETDLDIQPNHKTSNNDQMGYDQSVSADDVPEVDNTI
VTEKNVSNLDQESLEKHPILFALTDEGKLKVYNLADFSLLMECDVFDLPPTLFNGMESER
TYFNKESSQELVELLVADLGDDFKEPHLFLRSRLNEITVYKAFLYSNTDKHKNLLAFAKV
PQETMTREFQANVGTPRDAESTMEKKASSSVDHLKMTALEVVGNHSAVFVTGRKPFLILS
TLHSNAKFFPISSNIPILSVAPFHAHHAPQGYIYVDENSFIRICKFQEDFEYDNKWPYKK
VSLGKQINGIAYHPTKMVYAVGSAVPIEFKVTDEDGNEPYAITDDNDYLPMANTGSLDLV
SPLTWTVIDSYEFQQFEIPLSVALVNLEVSETTKLRKPYIAVGTSITKGEDIAVRGSTYL

FEIIDVVPQPGRPETRHKLKLVTREEIKGTVAVVCEVDGYLLSGQGQKVIVRALEDEDHL
VGVSFIDLGSYTLSAKCLRNLLLFGDVRQNVTFVGFAEEPYRMTLFSKGQEALNVSAADF
LVQGENLYFVVADTSGNLRLLAYDPENPESHSGERLVTRGDFHIGNVITAMTILPKEKKH
QNAEYGYDTGDDFSCVMVNSDGGLQMLVPISDRVYRRLNIIQNYLANRVNTIGGLNPKSY
RLITSPSNLTNPTRRILDGMLIDYFTYMSVAHRHEMAHKCGVPVSTIMNDLVELDEALSY
M
>Q12139
MHGKELAGRLRKRENDNDLSPNSSSSPAERFRCPHPECNKTFSRQEHLSRHKLNHWPKEI
YVCSYVLPTTNAPCNKTFVRKDLLIRHEKRHSKVKNRLSRPSKDQISSSNKDFSKNAPYN
PSEVPLSTQSGTSTINLIKNSVNPPPSITQESKFRPFLQQAQQPQQVQQSQQPQQIQQLQ
QLQFPQQLRAPLQQPMLQQQMHPQQASPTFPSYDPRIRNNGQNGNQFFNLIFDNRTGVNG
FEVDAANNNGNGNDQNMNINPAVQQQRYQDRNFASSSYQQPLQPLTQDQQQEQYFQQQKL
AQQQQQQQQQQQQQQQLPPQNPFGDPLTSSSSGANLSVMQDLFSTNFLNSDPLQSFMQEL
SEAPQVSIEDTFSDKNTIPPNEKPVQQDEGFQNPPVMFELPQDNIKIPKAQPKFNDNPST
SVKDNLSSQKLNINELKRRSSKDSGVGNNSSLNYKEQLRHSMKSVPSFFHPDPLTKYKIS
KEKCQEMFSFVPELRYVSIESIHKSLKSFWLNFHPQYGLLHKPSFHVDKQPAILNLALIM
TGASFLGSEYREQISDPICGPLRWIIFSHADFQPPSKTYIIQSLLLVEGYEKTSTNRYLH
ERSFLHHGTTIQLLRRTPSLGGHPLMVKTGKTSGENSIQDPQEVYKRWIDFEMLKRIAFY
AFYMDTTHAVVFGYWNLFINSNQIQLTLPCPDQVWESYDLSYETLMEHGYGSTKRDENNT
FLSALMQLMKNVIQILRNNNIRRNKVNNGGIESTPTDLESTTDWNIQSLFGKKILLAGII
SILFQCQEEVNGDYFITNFRGGITDHLGLSWKDILSFAMNYWLHEVQKSCTDPKACRIST
PSEETLTNRKIDEDNGDGLCDDDLDLLSSDNPSNCKIPVIHISQIVLRILHHDYYIYAGA
PWRMNVPIGRDEYDMISRRILQFAKDPYNGGVAVVYAFQFLFEMFIIKENNVPTVVKSYN
INSDPVITRPYAIALTSLLIWSCNFALHGCEVSIWDNTEASKDECFQPDDSNGGNILGNT
DNNGSTIANNNLKEKNNYIPIESFEVYLLRMYRNLYVDSSLDVVSFQNDVWAKASLLQHI
SNTHFLCGMMQFMRDIFNKSYWDLGREFGKLFDNCLERSLGKTSPTCHNMFDV
>Q12000
MPPKKGKQAQAAGKKKDNVDKTFGMKNKNRSTKVQKYIKQVQSQSDPKKEEMRLKKLEEK
KRREAEEAERRALFNPVADQRVRAGVDPKSMVCALFKLGNCNKGAKCKFSHDLNVGRRME
KKDLYQDTRSEKENDTMDNWDEEKLRKVILSKHGNPKTTTDKVCKYFIEAVENGKYGWFW
ICPNGGDKCMYRHSLPEGFVLKTNEQKRLERESLEKQPKITLEEFIETERGKLDKSKLTP
ITIANFAQWKKDHVIAKINAEKKLSSKRKPTGREIILKMSAENKSFETDNADMPDDVTQG
SAWDLTEFTDALKKADHQDDGGIKDYGDGSNPTFDIKKANSATLA
>P34078
MSKKFSSKNSQRYVVVHRPHDDPSFYDTDASAHVLVPVSNPNKTSPEADLRKKDVSSTKP
KGRRAHVGEAALYGINFDDSEYDYTQHLKPIGLDPENSIFIASKGNEQKVEKKNIEDLFI
EPKYRRDEIEKDDALPVFQRGMAKPEYLLHQQDTTDEIRGFKPDMNPALREVLEALEDEA
YVVNDDVVVEDISKKTQLQGDNYGEEEKEDDIFAQLLGSGEAKDEDEFEDEFDEWDIDNV
ENFEDENYVKEMAQFDNIENLEDLENIDYQADVRRFQKDNSILEKHNSDDEFSNAGLDSV
NPSEEEDVLGELPSIQDKSKTGKKKRKSRQKKGAMSDVSGFSMSSSAIARTETMTVLDDQ
YDQIINGYENYEEELEEDEEQNYQPFDMSAERSDFESMLDDFLDNYELESGGRKLAKKDK
EIERLKEAADEVSKGKLSQRRNRERQEKKKLEKVTNTLSSLKF
>P21818
MAKTAMAYKEKMKELSMLSLICSCFYPEPRNINIYTYDDMEVKQINKRASGQAFELILKP

PSPISEAPRTLASPKKKDLSLEEIQKKLEAAEGRRKSQEAQVLKQLAEKREHEREVLQKA
LEENNNFSKMAEEKLILKMEQIKENREANLAAIIERLQEKERHAAEVRRNKELQVELSG
>P93836
MGHQNAAVSENQNHDDGAASSPGFKLVGFSKFVRKNPKSDKFKVKRFHHIEFWCGDATNV
ARRFSWGLGMRFSAKSDLSTGNMVHASYLLTSGDLRFLFTAPYSPSLSAGEIKPTTTASI
PSFDHGSCRSFFSSHGLGVRAVAIEVEDAESAFSISVANGAIPSSPPIVLNEAVTIAEVK
LYGDVVLRYVSYKAEDTEKSEFLPGFERVEDASSFPLDYGIRRLDHAVGNVPELGPALTY
VAGFTGFHQFAEFTADDVGTAESGLNSAVLASNDEMVLLPINEPVHGTKRKSQIQTYLEH
NEGAGLQHLALMSEDIFRTLREMRKRSSIGGFDFMPSPPPTYYQNLKKRVGDVLSDDQIK
ECEELGILVDRDDQGTLLQIFTKPLGDRPTIFIEIIQRVGCMMKDEEGKAYQSGGCGGFG
KGNFSELFKSIEEYEKTLEAKQLVG
>O13601
MARTAFHDEFVDYYTILGAESTSSYVEIRQQYLKLVLRYHPDRNPGREAEVLPQFQLIQK
AHEVLKDPKLRELFDQRRLLEAGRPDGVLRFRPKKSGPKNDISTKVASKVSVTMATKFAE
KKKKQDRENVDSKDNNITNFSLHRSFSASGKMEKNNSFKEVSTSKSYISSGYLHPKTSPI
FKKNGYATENVVDPISSSPRFKGPNYNKFNAKLYLESLREKRRTYTPLSEISNGLNSNGV
ENSSITKSSPRSSSSSNNERFKDTSEESIIFTSPNTPEHPSVYQTDITPEIKLEHSDNNS
PSKPEIPFRHPTSKPLPPKPLSRSKSSSLSRNQTRSQLNDLSAENDSTSNSTEYDDQLQS
ILRSLAIEGDDDEVAKVLPKPPSVPTIQAPIPPEAPRNLTNASVDSYLNSFEMYQRRWSS
YSIIYTQYAFQWQIFKNKCFQLDLMNTPGQSRLIDNWKEGSQAIQLFYAYEQMHLRALEE
LQSLKESLFASFGI
>P49028
MSTEDFYLRYYVGHKGKFGHEFLEFEFRPDGKLRYANNSNYKNDTMIRKEAFVHQSVMEE
LKRIIIDSEIMQEDDLPWPPPDRVGRQELEIVIGDEHISFTTSKTGSLVDVNRSKDPEGL
RCFYYLVQDLKCLVFSLIGLHFKIKPI
>Q5JVS0
MKGALGSPVAAAGAAMQESFGCVVANRFHQLLDDESDPFDILREAERRRQQQLQRKRRDE
AAAAAGAGPRGGRSPAGASGHRAGAGGRRESQKERKSLPAPVAQRPDSPGGGLQAPGQKR
TPRRGEQQGWNDSRGPEGMLERAERRSYREYRPYETERQADFTAEKFPDEKPGDRFDRDR
PLRGRGGPRGGMRGRGRGGPGNRVFDAFDQRGKREFERYGGNDKIAVRTEDNMGGCGVRT
WGSGKDTSDVEPTAPMEEPTVVEESQGTPEEESPAKVPELEVEEETQVQEMTLDEWKNLQ
EQTRPKPEFNIRKPESTVPSKAVVIHKSKYRDDMVKDDYEDDSHVFRKPANDITSQLEIN
FGNLPRPGRGARGGTRGGRGRIRRAENYGPRAEVVMQDVAPNPDDPEDFPALS
>O14185
MTSQLEKEAREWIEETLHTKLNAQLDLLDQLQSGVILCRICKEALGANIRYKESNMPFVQ
MENISAFINYAQQVVHVPSQDMFQTSDLFERRNDEQVLRSIHSFSRYAAKMFPGKVRGLG
PKLAEKKPRVFSAQQQREFREGVNSLQYGSFDMPTQGTEKIAFSRRRDPTGNMY
>P49965
MANEKAFNLQSAYSDLNKACSAQAYDKIINISGKILTKFPGETKAFQCKVVALIRAEKYE
DCLSFLKKNPTLSSHVIFEKAYVEYRLNRLTEAAKTLESEEASDSKVQELKAQVLYRKGD
FAGAYAYLRTVIRNSQDDYSEERLANLTAVAAAESCFNNANLDLDVNPQMYEGKFNLACY
HLGRGDCYLASRSLDDAENTCNLCLSEDPELTEEERNEELAPIRVQRAYILQLNKEEEEA
NQVYQSVIRQRASDPALLAVAANNIVCINQDQNIFDSRKRIKMASTDGLQFKLFSRQRTD
MLINQALFYWYTNQMEACTAKLRTVSQEELSPRALLLSATQLIKEKNVDKAVLLLQSYLS

NFAGSQIDAEIPLALAQLNLRRTSANNLGTGSPQPKNALNVAQMLENILPQHLIHSPGVL
STRIALYLLASSGENAVQTRPDSMKQIVNCIESTLHYYEELGEQNEIYSHLLDNCAAFLL
QQGEAKLAAELLEKQLARLESNICQEKQNSLIKQVLVARLVRAYAQFDRPKAEQTCKSLQ
AKESLSEADVDTLETTFLYGAKSLKRLGKPSEPTDTSDKGKSKRSQIKKSISDIPSSEDP
GAQPVISRPKRKKRKVRLPKNYQPGVMPDPNRWLPRRERTHYRGKRRDKRFAPTRGPQGQ
ITGESEWDAAIRSPKVKIPEDGSAGSTPKQMSNTAKQQQKKGRKKGR
>Q9P783
MSKRAAEETVEFNSKNGPGQRGTVADNVDTEMGEFEDAYEDEIESEEEYIEADGEKDNGM
DEEEQNDAQPSKIPWLPGGKINADEKLVADPSVYEMLHNIQVKWPFLSFDILQDSLGEER
RAWPHQMYLVGGSQALDSNDNELTVMKLSQLYKTQHDENDDASDNSDVEEDPILEHKSIS
TKGACNRVRSARRPANSSKESLLASFHETGKVHIWDIAPHLRSLDSPGVMVSRKENSPLY
TVNRHKTEGYALDWSPFEYSLLSGDNANEIFLTKYSNGGWQTDSSPFLSHTAAVEDLQWS
PSEKNVFSSCSCDGTFRIWDVRNKQKTSALTVNAHPGVDVNVLSWNTRVPNLLATGADNG
VWSVWDLRSLKSSSSVATPVASFKWHRAPIYSIEWHPNEDSVIGVVGADNQISLWDLSVE
LDEEEQDSRAAEGLQDVPPQLMFIHMGQQEIKEMHWHRQIPGTIVSTAMTGINVFKTITF

>Q06673
MGNTDSKSSSILLNHCIALVRPEDADASSPSRTSSPSPSLSVDADPLSLNLSIFKLDSGP
DVEALFSDKPNVPLDTVFNDFYLDFISVDVQDFSINSSFKKILHIISSLNPPNFNNLIVF
LSLYIILSANSLPASRTGLHSSRLINAIKTLSILIPIYFDRVKSSTQDHYDVFWATQHEI
EGLPLQNIPLGERLLLAILKLAFQDNFTTAVTAHPSELWEIGILTNSNKYRSLLNMHHQW
HLFANRLLLLRLLAALFSSDLYTSGGKQDINMFLVYWCTQMPKDKSIQFTSSLLNCTMRF
ILNNNKDFQSLKANFFSSDATASNWQTLYFQFVQSCLHVLNLSMSYKAQDNVITIFLTQL
QREYDLKLILSSFIKIFKYPIDLAIEQESNIFNFTNNKHIDASRRRAVSTSSHDNSSSSH
ASLPSSSSAAYHTKPQTKPQLPEIHPLLIPMTILMTNLIDCNKCFQNYFADKFASRFIIF
SIYYLKYYDYSSLSSSSSTTRSNSSTTSNGTSNDTSNERSIVELNENSVSQILLPLLNHL
LLILTSKKLVLFKMLQTFNLNYYTNNLPNFYKLSNINGDINNLTFRDFTVIQLSNLILDN
IKFNLQPNPIFYELIYNLLPINDEILTSSHKNDDSHDDLILLSAKKKSASPSAATSSHTS
SSKLSYNAAMSLLYVLSKSSNKVYLTTYATPVFKTKDIPYMISPGFKMDLLALLLRSITI
FFTLYFDDAENLLFAMVRHQSITHQINDSINSISKALDMNPNLNSHIMTLKQMGFNRKVQ
WKDFYQFEEITDLPQVNLYSSANQQHQNQQQGQNDNRGQNQNEDPGQENESPTPYLLFNP
ASLENETPGTVKHFSSTNHDKNYQVIAFIDFKSDSNLNLQHQLEYWPHRPQWPTPLTFTH
KCKNPKYENFNEVWSGTVYLQILLRVIKQILSKVPEIPRIKSVQYFETLSKLSALRSDIL
TTIHPRLPLDVRRLTTFQPLSMHTNDKLLMWFHIATWANIFTQTSFKYEETFSHELRQFE
SLLDISIDECEGNTISKPTTDRLGYIRRSRGQSSVSLERTISAGSGVSTPTMALNRTKSN
GSGNLMNYFFQNTAQNHFQHLRSSSSSSSITLEKTTSNSSSIRTRPNSHHVAPETNNNNS
TNGNSNNSSNGGFSFFKWKWGGNNSNGGSDDTKASQRDPNVSTSIITDNLNSYMFEEEIS
PGVVNNIIENNIWVGTDIRLFKIANFRKESFSFLEMTSSFFKKFKFINSDNDNYNNNEFD
DNTQLRYTSRGLYR
>Q8QZY3
MEEPSEKVDPMKDPETPQKKDEEDALDDTDVLQPETLVKVMKKLTLNPGVKRSARRRSLR
NRIAAVPVENKSEKIRREVQSAFPKRRVRTLLSVLKDPIAKMRRLVRIEQRQKRLEGNEF
ERDSEPFRCLCTFCHYQRWDPSENAKIGKN
>P53060

MSEEEAHKTVEVDDVGVQLDEGDEEDLLEYDDELVEEQPSDARIRNVAETLMKSELPKVT
VEYKDTTFLLFTSDDKNESNNPIICENAALYQRPMGEFMESIRKFMGNRFGRLAFATKEL
VLQLKSLDLTLFEDNVYNNHISFSDVYTIFKILKERSESNFETDIPTHLAIELSTRPRFV
SRYNALVELTESSATLKNIKPFSNDETHPLIVDDNDQYTHQNTSEVIVMDIDDDVGEDSE
D
>Q96GX9
MSGCDAREGDCCSRRCGAQDKEHPRYLIPELCKQFYHLGWVTGTGGGISLKHGDEIYIAP
SGVQKERIQPEDMFVCDINEKDISGPSPSKKLKKSQCTPLFMNAYTMRGAGAVIHTHSKA
AVMATLLFPGREFKITHQEMIKGIKKCTSGGYYRYDDMLVVPIIENTPEEKDLKDRMAHA
MNEYPDSCAVLVRRHGVYVWGETWEKAKTMCECYDYLFDIAVSMKKVGLDPSQLPVGENG
IV
>O13688
MNASNNISKFPDLDNSSKLIDHILDSDDSEELDELPDISSLVPSARAQSRKQYLKNDSSN
SSTYRWNIDLLSSTATIDDSVAKRRKLAVQNLLQYDSTQTFQTGDEIDELIGKSVGSNVL
NVLRSNPIYDDDLRYEYCSNSKARVPDWNTLKAECLKDNDLEFNEGIIPTTFGDLLSAKL
VPLDIALSICSLQFFRSLGDTTCSEWIANLEKIFYSYKSSSNNLNQIVRFIFETTADMIG
IDLAKRQVPIQLERTSASENLKSNLKIKVINFLKCCGTLYRFSDDTVRFEMIQDACRILI
DNQVGSFCKWQFSQFMELPISLNPDFLISNIHKVSESPRVWVTILSSLSRSCQKFRKKIA
FTLFVGKQSKNDDSDFSSLCQRLDEISASCNNDYTTLLYQIRTFGYAVDEKHFKTNERLE
CLLEKLRKIDLTISGSTDHLLLSRCEVKDCIHRLFMVLYYLNTNSAPELERIIESDLPNN
NKQKDRYFKDKTSNLSMKENKSFSAKKVKKGKKKNKRQAYKR
>Q09884
MDISSFLLPQLLRKYQQDVYNIASKQNTLLVMRTGAGKTLLAVKLIKQKLEEQILIQESN
LEHKKISVFLVNKVPLVFQQAEYIRSQLPAKVGMFYGELSIEMSEQLLTNIILKYNVIVI
TADLFYLFLARGFLSINDLNLIIFDECHHAIGNDAYARIMNDFYHRAKAVLSKKHFTLPR
IFGMTASPFTGKKGNLYHRLYQWEQLFDSKAHVVSENELADYFCLPEESYVMYSNKLVVP
PSDSIIKKCEETLQGCKLISRAVKTALAETIDMGLWFGEQVWLYLVDFVETKRLKKKALG
KQLSDDEELAIDRLKIFVEDWKNNKYSDNGPRIPVFDSTDVTDKVFKLLELLKATYRKSD
SVRTVIFVERKATAFTLSLFMKTLNLPNIRAHSFIGHGPSDQGEFSMTFRRQKDTLHKFK
TGKYNVLIATAVAEEGIDVPSCNLVIRFNICRTVTQYVQSRGRARAMASKFLIFLNTEEL
LIHERILHEEKNLKFALSELSNSNIFDSLVCEERERVTDDIVYEVGETGALLTGLYAVSL
LYNFCNTLSRDVYTRYYPTFTAQPCLSGWYCFEVELPKACKVPAAQGSPAKSIRKAKQNA
AFIMCLDLIRMGLIDKHLKPLDFRRKIADLETLEEDELKDEGYIETYERYVPKSWMKVPE
DITRCFVSLLYTDANEGDNHIFHPLVFVQAHSFPKIDSFILNSTVGPRVKIVLETIEDSF
KIDSHLLELLKKSTRYLLQFGLSTSLEQQIPTPYWLAPLNLSCTDYRFLENLIDVDTIQN
FFKLPEPVQNVTDLQSDTVLLVNPQSIYEQYAFEGFVNSEFMIPAKKKDKAPSALCKKLP
LRLNYSLWGNRAKSIPKSQQVRSFYINDLYILPVSRHLKNSALLIPSILYHIENLLVASS
FIEHFRLDCKIDTACQALTSAESQLNFDYDRLEFYGDCFLKLGASITVFLKFPDTQEYQL
HFNRKKIISNCNLYKVAIDCELPKYALSTPLEIRHWCPYGFQKSTSDKCRYAVLQKLSVK
RIADMVEASIGACLLDSGLDSALKICKSLSVGLLDISNWDEWNNYFDLNTYADSLRNVQF
PYSSYIEETIGYSFKNKKLLHLAFIHPSMMSQQGIYENYQQLEFLGDAVLDYIIVQYLYK
KYPNATSGELTDYKSFYVCNKSLSYIGFVLNLHKYIQHESAAMCDAIFEYQELIEAFRET
ASENPWFWFEIDSPKFISDTLEAMICAIFLDSGFSLQSLQFVLPLFLNSLGDATHTKAKG
DIEHKVYQLLKDQGCEDFGTKCVIEEVKSSHKTLLNTELHLTKYYGFSFFRHGNIVAYGK

SRKVANAKYIMKQRLLKLLEDKSNLLLYSCNCKFSKKKPSDEQIKGDGKVKSLT
>P20065
MLLPATMSDKPDMAEIEKFDKSKLKKTETQEKNPLPSKETIEQEKQAGES
>Q70J99
MATLLSHPQQRPPFLRQAIKIRRRRVRDLQDPPPQMAPEIQPPSHHFSPEQRALLYEDAL
YTVLHRLGHPEPNHVTEASELLRYLQEAFHVEPEEHQQTLQRVRELEKPIFCLKATVKQA
KGILGKDVSGFSDPYCLLGIEQGVGVPGGSPGSRHRQKAVVRHTIPEEETHRTQVITQTL
NPVWDETFILEFEDITNASFHLDMWDLDTVESVRQKLGELTDLHGLRRIFKEARKDKGQD
DFLGNVVLRLQDLRCREDQWYPLEPRTETYPDRGQCHLQFQLIHKRRATSASRSQPSYTV
HLHLLQQLVSHEVTQHEAGSTSWDGSLSPQAATVLFLHATQKDLSDFHQSMAQWLAYSRL
YQSLEFPSSCLLHPITSIEYQWIQGRLKAEQQEELAASFSSLLTYGLSLIRRFRSVFPLS
VSDSPARLQSLLRVLVQMCKMKAFGELCPNTAPLPQLVTEALQTGTTEWFHLKQQHHQPM
VQGIPEAGKALLGLVQDVIGDLHQCQRTWDKIFHNTLKIHLFSMAFRELQWLVAKRVQDH
TTVVGDVVSPEMGESLFQLYISLKELCQLRMSSSERDGVLALDNFHRWFQPAIPSWLQKT
YNEALARVQRAVQMDELVPLGELTKHSTSAVDLSTCFAQISHTARQLDWPDPEEAFMITV
KFVEDTCRLALVYCSLIKARARELSSGQKDQGQAANMLCVVVNDMEQLRLVIGKLPAQLA
WEALEQRVGAVLEQGQLQNTLHAQLQSALAGLGHEIRTGVRTLAEQLEVGIAKHIQKLVG
VRESVLPEDAILPLMKFLEVELCYMNTNLVQENFSSLLTLLWTHTLTVLVEAAASQRSSS
LASNRLKIALQNLEICFHAEGCGLPPKALHTATFQALQRDLELQAASSRELIRKYFCSRI
QQQAETTSEELGAVTVKASYRASEQKLRVELLSASSLLPLDSNGSSDPFVQLTLEPRHEF
PELAARETQKHKKDLHPLFDETFEFLVPAEPCRKAGACLLLTVLDYDTLGADDLEGEAFL
PLREVPGLSGSEEPGEVPQTRLPLTYPAPNGDPILQLLEGRKGDREAQVFVRLRRHRAKQ
ASQHALRPAP
>O94561
MDAPRRSSRLAAKIANVLDSKGTIIPEAAPVMLKKPAKDESVDSTIQVGDVIPDITLPDE
DGTSIRLRDITANKGLVIFAYPKASTPGCTKQGCGFRDNYPKIQASDYEVLGLSFDTSKA
QKAFKDKQNFPYHLLSDPKGELIKKLGAEKPGGGKLFRSHWIFEKGTGKCIVKEIDISPL
VSVDKAFAVITDSEP
>Q03933
MKQSSNVPAFLSKLWTLVEETHTNEFITWSQNGQSFLVLDEQRFAKEILPKYFKHNNMAS
FVRQLNMYGFRKVVHIDSGIVKQERDGPVEFQHPYFKQGQDDLLENIKRKVSSSKPEENK
IRQEDLTKIISSAQKVQIKQETIESRLSELKSENESLWKEVSELRAKHAQQQQVIRKIVQ
FIVTLVQNNQLVSLKRKRPLLLNTNGAQKKNLFQHIVKEPTDNHHHKVPHSRTEGLKPRE
RISDDIIIYDVTDDNADEENIPVIPETNEDVISDPSNCSQYPDIVIVEDDNEDEYAPVIQ
SGEQNEPARESLSSGSDGSSPLMSSAVQLNGSSSLTSEDPVTMMDSILNDNINLLGKVEL
LDYLDSIDCSLEDFQAMLSGRQFSIDPDLLVDLFTSSVQMNPTDYINNTKSENKGLETTK
NNVVQPVSEEGRKSKSKPDKQLIQYTAFPLLAFLDGNPASSVEQASTTASSEVLSSVDKP
IEVDELLDSSLDPEPTQSKLVRLEPLTEAEASEATLFYLCELAPAPLDSDMPLLDS
>P39729
MSTTVEKIKAIEDEMARTQKNKATSFHLGQLKAKLAKLRRELLTSASSGSGGGAGIGFDV
ARTGVASVGFVGFPSVGKSTLLSKLTGTESEAAEYEFTTLVTVPGVIRYKGAKIQMLDLP
GIIDGAKDGRGRGKQVIAVARTCNLLFIILDVNKPLHHKQIIEKELEGVGIRLNKTPPDI
LIKKKEKGGISITNTVPLTHLGNDEIRAVMSEYRINSAEIAFRCDATVDDLIDVLEASSR
RYMPAIYVLNKIDSLSIEELELLYRIPNAVPISSGQDWNLDELLQVMWDRLNLVRIYTKP

KGQIPDFTDPVVLRSDRCSVKDFCNQIHKSLVDDFRNALVYGSSVKHQPQYVGLSHILED
EDVVTILKK
>Q9UBT7
MAASPGPAGVGGAGAVYGSGSSGFALDSGLEIKTRSVEQTLLPLVSQITTLINHKDNTKK
SDKTLQAIQRVGQAVNLAVGRFVKVGEAIANENWDLKEEINIACIEAKQAGETIAALTDI
TNLNHLESDGQITIFTDKTGVIKAARLLLSSVTKVLLLADRVVIKQIITSRNKVLATMER
LEKVNSFQEFVQIFSQFGNEMVEFAHLSGDRQNDLKDEKKKAKMAAARAVLEKCTMMLLT
ASKTCLRHPNCESAHKNKEGVFDRMKVALDKVIEIVTDCKPNGETDISSISIFTGIKEFK
MNIEALRENLYFQSKENLSVTLEVILERMEDFTDSAYTSHEHRERILELSTQARMELQQL
ISVWIQAQSKKTKSIAEELELSILKISHSLNELKKELHSTATQLAADLLKYHADHVVLKA
LKLTGVEGNLEALAEYACKLSEQKEQLVETCRLLRHISGTEPLEITCIHAEETFQVTGQQ
IISAAETLTLHPSSKIAKENLDVFCEAWESQISDMSTLLREINDVFEGRRGEKYGYLSLP
KPMKNNANLKSLKPDKPDSEEQAKIAKLGLKLGLLTSDADCEIEKWEDQENEIVQYGRNM
SSMAYSLYLFTRGEGPLKTSQDLIHQLEVFAAEGLKLTSSVQAFSKQLKDDDKLMLLLEI
NKLIPLCHQLQTVTKTSLQNKVFLKVDKCITKTRSMMALLVQLLSLCYKLLKKLQMENNG
WVSVTNKDTMDSKT
>Q9UQ80
MSGEDEQQEQTIAEDLVVTKYKMGGDIANRVLRSLVEASSSGVSVLSLCEKGDAMIMEET
GKIFKKEKEMKKGIAFPTSISVNNCVCHFSPLKSDQDYILKEGDLVKIDLGVHVDGFIAN
VAHTFVVDVAQGTQVTGRKADVIKAAHLCAEAALRLVKPGNQNTQVTEAWNKVAHSFNCT
PIEGMLSHQLKQHVIDGEKTIIQNPTDQQKKDHEKAEFEVHEVYAVDVLVSSGEGKAKDA
GQRTTIYKRDPSKQYGLKMKTSRAFFSEVERRFDAMPFTLRAFEDEKKARMGVVECAKHE
LLQPFNVLYEKEGEFVAQFKFTVLLMPNGPMRITSGPFEPDLYKSEMEVQDAELKALLQS
SASRKTQKKKKKKASKTAENATSGETLEENEAGD
>Q9Z2A5
MASWSAPSPSLVEYFEGQTSFQCGYCKNKLGSRSYGMWAHSMTVQDYQDLIDRGWRRSGK
YVYKPVMDQTCCPQYTIRCHPLQFQPSKSHKKVLKKMLKFLAKGEISKGNCEDEPMDSTV
EDAVDGDFALINKLDIKCDLKTLSDLKGSIESEEKEKEKSIKKEGSKEFIHPQSIEEKLG
SGEPSHPIKVHIGPKPGKGADLSKPPCRKAREMRKERQRLKRMQQASAAASEAQGQPVCL
LPKAKSNQPKSLEDLIFQSLPENASHKLEVRVVRSSPPSPQFRATFQESYQVYKRYQMVV
HKDPPDKPTVSQFTRFLCSSPLEAEHPADGPECGYGSFHQQYWLDGKIIAVGVLDILPYC
VFSVYLYYDPDYSFLSLGVYSALREIAFTRQLHEKTSQLSYYYMGFYIHSCPKMRYKGQY
RPSDLLCPETYVWVPIEQCLPSLDNSKYCRFNQDPEAEDEGRSKELDRLRVFHRRSAMPY
GVYKNHQEDPSEEAGVLEYANLVGQKCSERMLLFRH
>O14146
MSSKRRQALRDFYNLPGSNSSSSTVSTTRKPSSSNTVSPVEETALDKVDPEFSDPNFDSN
AYVSKFMEKATPAELLQRDKSLVASIQGLSVDHRSLVYNHYKHLLEASDAINSFCKNLNT
LLPALNDAYQACPKPTRKNGNNDTYSAPQSFNGNEFSAP
>Q14914
MVRTKTWTLKKHFVGYPTNSDFELKTAELPPLKNGEVLLEALFLTVDPYMRVAAKRLKEG
DTMMGQQVAKVVESKNVALPKGTIVLASPGWTTHSISDGKDLEKLLTEWPDTIPLSLALG
TVGMPGLTAYFGLLEICGVKGGETVMVNAAAGAVGSVVGQIAKLKGCKVVGAVGSDEKVA
YLQKLGFDVVFNYKTVESLEETLKKASPDGYDCYFDNVGGEFSNTVIGQMKKFGRIAICG
AISTYNRTGPLPPGPPPEIVIYQELRMEAFVVYRWQGDARQKALKDLLKWVLEGKIQYKE

YIIEGFENMPAAFMGMLKGDNLGKTIVKA
>Q80WG7
MSESEEISEFGYIMELLAKGKVTIKNIEKELICPACKELFTHPLILPCQHSVCHKCVKEL
LLSLDDSFNDVASDSSNQSSPRLRLTSPSMDKIDKINRPGWKRNSLTPRPTTFPCPGCEH
DVDLGERGVSGLFRNFTLETIVERYRQAARAATAIMCDLCKPPPQESTKSCMDCSASYCN
ECFKIYHPWGTVKAQHEYVGPTTNFRPKVLMCPEHETERINMYCELCRRPVCHLCKLGGN
HSNHRVTTMSSAYKTLKEKLSKDIDFLIGKESQVKSQISELNLLMKETECNGERAKEEAL
AHFEKLFEILEDRKSSVLKAIDASKKLRLDKFHTQMEEYQGLLENNGLVGYAQEVLKETD
QSCFVQTAKQLHLRIQKATESLKSFRPAAQASFEDYVVNISKQTEVLGELSFFSSGIDIP
EINEEQSKVYNNALIDWHHPEKDKADSYVLEYRKINRDEEMISWNEIEVHGTSKVVSNLE
SNSPYAFRVRAYRGSICSPCSRELILHTPPAPVFSFLFDEKCGYNTEHLLLNLKRDRVES
RAGFNVLLAAERIQVGHYTSLDYIIGDVGVTKGKHFWACRVEPYSYLVKVGVASSDKLQE
WLRSPRDAASPRYEQDSGHDSGSEDACFDSSQPFTLVTIGMKKFFIPKSPTSSNEPENRV
LPMPTSIGIFLDCDKGKVSFYDMDHMKCLYERQVDCSHTMYPAFALMGSGGIQLEEAITA
KYLEYEEDV
>O59719
MSTWPIDAISKKPSVSLDYGIIGACQLLNPSGYHRAEKVENNEWTFARYDSYTGVELVDL
DKRWDILSLNPNYKLPKLTELPAEATNSTRKATSSSFGLSVCRYVPRNLFSTLAKESQLA
SSAESIYNPHILNTFCLGKTAPSDKNRYTTTECLACSKISNETAENLLFLCLSSPWIFRA
SPTLQNTATFEEFPFYLNVSAFEFAEDPIWKFSQPVRQLVFSNGEPSLLFALSSCELVIF
QVTYDLMFLDEPNTPGCLSAIPLRFLYADDVYNSDSFANVSVHPTDNSFFLTTSSSGRWT
IWQFLDDGYRECFGSSFKESLQQALCSAPTSSIAYHEEEINPDNTIYRAKWEEYFGGVIL
HNAFFILHVSLGEVPDFHLLFHCSSDVRILQVVNESMPIKSEFFILTTESVLWMDIQHPQ
KPLLEWKHNRKLDPTLKITVTATFSQNIYVSVYSQMNGVVQQIHFSKDRALPVSGSHPFL
LLNEVQVPIRSLIIQPCYFFESSEFDRQGPFDSPFWSAIIDKADGSLSLHILCEKSSLKI
YNLEELTGSSQVAMKFKTITPSVNTSEDDSANDQEIISTPNSDFQQLSLSRLYHVLCSNR
NKKNTITLDEFASKVPDFVEAFDSINHDVIITLSELYDGFPLKGTFTNVAEIISHLEGTE
LSFYFPYMFGINYNNASFFYTSVDSIASLIHRRWESNTDKSVEIPCFQSSQRRSIKNVIH
YLFLSSIGVSREKIFDPSLSRKEIDSTDLLTFIKPFYDTEGLELNEDVRNILENWEIGKI
SSTYVIGDSAVETEDPSSSQFSDFYISQQQSSVLPSSQIATVFSQEDVPNFSSLYSESSS
QTIPIMSQVVSGKYGSRPSKKKKKRSGF
>Q03154
MTSKGPEEEHPSVTLFRQYLRIRTVQPKPDYGAAVAFFEETARQLGLGCQKVEVAPGYVV
TVLTWPGTNPTLSSILLNSHTDVVPVFKEHWSHDPFEAFKDSEGYIYARGAQDMKCVSIQ
YLEAVRRLKVEGHRFPRTIHMTFVPDEEVGGHQGMELFVQRPEFHALRAGFALDEGIANP
TDAFTVFYSERSPWWVRVTSTGRPGHASRFMEDTAAEKLHKVVNSILAFREKEWQRLQSN
PHLKEGSVTSVNLTKLEGGVAYNVIPATMSASFDFRVAPDVDFKAFEEQLQSWCQAAGEG
VTLEFAQKWMHPQVTPTDDSNPWWAAFSRVCKDMNLTLEPEIMPAATDNRYIRAVGVPAL
GFSPMNRTPVLLHDHDERLHEAVFLRGVDIYTRLLPALASVPALPSDS
>Q9QYB1
MALSMPLNGLKEEDKEPLIELFVKAGSDGESIGNCPFSQRLFMILWLKGVVFSVTTVDLK
RKPADLQNLAPGTHPPFITFNSEVKTDVNKIEEFLEEVLCPPKYLKLSPKHPESNTAGMD
IFAKFSAYIKNSRPEANEALERGLLKTLQKLDEYLNSPLPDEIDENSMEDIKFSTRRFLD
GDEMTLADCNLLPKLHIVKVVAKKYRNFDIPKGMTGIWRYLTNAYSRDEFTNTCPSDKEV

EIAYSDVAKRLTK
>Q96A58
MAKSAEVKLAIFGRAGVGKSALVVRFLTKRFIWEYDPTLESTYRHQATIDDEVVSMEILD
TAGQEDTIQREGHMRWGEGFVLVYDITDRGSFEEVLPLKNILDEIKKPKNVTLILVGNKA
DLDHSRQVSTEEGEKLATELACAFYECSACTGEGNITEIFYELCREVRRRRMVQGKTRRR
SSTTHVKQAINKMLTKISS
>P04792
MTERRVPFSLLRGPSWDPFRDWYPHSRLFDQAFGLPRLPEEWSQWLGGSSWPGYVRPLPP
AAIESPAVAAPAYSRALSRQLSSGVSEIRHTADRWRVSLDVNHFAPDELTVKTKDGVVEI
TGKHEERQDEHGYISRCFTRKYTLPPGVDPTQVSSSLSPEGTLTVEAPMPKLATQSNEIT
IPVTFESRAQLGGPEAAKSDETAAK
>Q9UTB3
MPASKYAGWSRDQLISRILELDGKLQKNTNSLPNKEIEGKNEVIKNKNKLDNGNEIEPQH
LSQELVLQHALQDASSLPKLKPVRPFQLEKTVFVAFKFAYCGWNYNGLAYQLEPTPLPTV
EGKVFEALLRAHLITDPSSCSFSRCGRTDKGVSAMGQVISLNVRHSERRPIIYCDVLNRL
LPADIRITGYAFPPEGFDARFSCKQRHYKYLFTKDYPGGSLDIDRMNEAAALYLGEHDFR
NFCKIDASKQITNYHRRILSSKVICIDPSTGLYAFDLQGTAFLWHQVRCMMAILFLIGQK
LEPASLINDLLDIKKVPTKPIYDMASEYPLILYDCDFDNIEWTLPTDSPTTAPRIAKHTY
ETIYHQWHSLRIREQIASFMLDIADHNVKRYGKSEESSSTMNVGEGLLKRTKKYIPILSR
QRQESVEVVNERYKRKKGLKDSDVKSENIK
>Q10223
MLQFSFQNCKTHTCYMNTSVNEHLDQDESFLRILIDTRKKIRSSYFKPQTFDEFVHCLAR
VRAMKRLVSICSNFDEEDNWNGVWNTLVVDGDSHASAMIDYEGLLDKCSLSRNLLELIND
YAEYTTQVLPFRKIPSSDNLDSSLNLTVASPKSNLYYRYSSESPKYAKILDCPDEILQLI
FSYCYDASYIEKLPFAFSYRKQRHTLIHDLPNTCLRFKKILSPRNVSFWKRLLKVHKKNP
NSAVTCSESINTSAVAKFTDIPTIIPIFLDPSYQSYAAKTIHSDTSSLASSIIQTGDGTQ
SCDESTYIELACNLVGRCVLCNRIPKLTKFKDCITSFYRPSVATNICDQCLEAIIDFYEP
VSRYKFDVMKDRLGVGYISRPGQKFPSWLSEHVQLARDEEKAFKSIYHSQGEFARSLFRL
FRAQLAELCEQ
>Q12008
MTASTPSNVMTLFLLRHGQSELNHENIFCGWIDAKLTEKGKEQARHSAELIEQYCKANNL
RLPQIGYTSRLIRTQQTIETMCEEFKLKPQLQVVYDFNKIKLGDEFGSDDKDNMKIPILQ
TWRLNERHYGSWQGQRKPNVLKEYGKDKYMFIRRDYEGKPPPVDLDREMIQQENEKGSST
GYEFKEPNRQIKYELECSNHDIVLPDSESLREVVYRLNPFLQNVILKLANQYDESSCLIV
GHGSSVRSLLKILEGISDDDIKNVDIPNGIPLVVELDKNNGLKFIRKFYLDPESAKINAE
KVRNEGFIKNP
>Q8TC76
MPTETLQTGSMVKPVSPAGTFTSAVPLRILNKGPDYFRRQAEPNPKRLSAVERLEADKAK
YVKSQEVINAKQEPVKPAVLAKPPVCPAAKRALGSPTLKVFGNHAKTESGVQRENLKLEI
LKNIINSSEGSSSGSGHKHSSRNWPPHRSEATDLHRHSFAESLKVYPTQGRRSPQEGGSH
VGRRLLEQSAESFLHVSHSSSDIRKVTSVKPLKAIPCSSSAPPLPPKPKIAAIASMKSPE
ADPVEPACGVSRRPSLQRSKSDLSDRYFRVDADVERFFNYCGLDPEELENLGMENFARAN
SDIISLNFRSASMISSDCEQSQDSNSDLRNDDSANDRVPYGISAIERNARIIKWLYSIKQ
ARESQKVSHV

>P53859
MACNFQFPEIAYPGKLICPQYGTENKDGEDIIFNYVPGPGTKLIQYEHNGRTLEAITATL
VGTVRCEEEKKTDQEEEREGTDQSTEEEKSVDASPNDVTRRTVKNILVSVLPGTEKGRKT
NKYANNDFANNLPKEGDIVLTRVTRLSLQRANVEILAVEDKPSPIDSGIGSNGSGIVAAG
GGSGAATFSVSQASSDLGETFRGIIRSQDVRSTDRDRVKVIECFKPGDIVRAQVLSLGDG
TNYYLTTARNDLGVVFARAANGAGGLMYATDWQMMTSPVTGATEKRKCAKPF
>O08573
MALFSAQSPYINPIIPFTGPIQGGLQEGLQVTLQGTTKSFAQRFVVNFQNSFNGNDIAFH
FNPRFEEGGYVVCNTKQNGQWGPEERKMQMPFQKGMPFELCFLVQRSEFKVMVNKKFFVQ
YQHRVPYHLVDTIAVSGCLKLSFITFQNSAAPVQHVFSTLQFSQPVQFPRTPKGRKQKTQ
NFRPAHQAPMAQTTIHMVHSTPGQMFSTPGIPPVVYPTPAYTIPFYTPIPNGLYPSKSIM
ISGNVLPDATRFHINLRCGGDIAFHLNPRFNENAVVRNTQINNSWGQEERSLLGRMPFSR
GQSFSVWIICEGHCFKVAVNGQHMCEYYHRLKNLQDINTLEVAGDIQLTHVQT
>Q9UT23
MSDDSFSSDEDWDELDTQVVDKIENEYHNNTIGLNGYSVDEYFDANDSNRYRLQHELDES
AAQQWVYPINVSFRDYQFNIVQKALFENVLVALPTGLGKTFIAAVVMMNYLRWFPKSYIV
FMAPTKPLVTQQMEACYKITGIPKSQTAELSGHVPVTTRNQYYQSRNVFFVTPQTILNDI
KHGICDRTRISCLVIDEAHRSTGNYAYVEVVHLLSLSNKNFRILALSATPGNKLEAIQNV
IDSLHISRIEIRTENSIDISQYVQKKEVDFFPVDLSAEITDIRDRFSSILEPMLQKLNKG
NYYRIQNAKDITSFTVVQAKQAFLAMSGQNFPANQKWDILNTFDALATFAYPLNLLLNHG
IRPFYQKLREVEEECFVGRSGYKKRIINHENYRPLMDDIEILLRDQSFVGHPKLEHLERI
VTEYFEKEQTKDTRIMIFVEIRSSAEEILRFLGKFYPNVRPAIFIGQSAVRKAAGMSQKL
QNETVKQFQKGEVNTLIATSIGEEGLDIGEVDMIICYDASASPIRMLQRMGRTGRKRKGY
IYMLLTRGKEEAKWERAKDAYRTLQDNIVSGRGLSLSEKSYRILPEKFRPVCDKRVIEIP
KENEEVVVAPKKVQLRTKIKKKFFMPENALNGFITASALGKPKRALAKSEESPFEICPVT
YSIEQEKKLEKYKRVCLRGLDIHRNRRLSQLSVTGRIPHSLATKSIHSFLKHLNTIDSQK
AQEWRREINNQFQVSNINSTDRDTKQPKMHDFRQPLHPNPMTTLKRKGQHNSFSYDKSTL
FYDNNNNLEEDLPDVNISISSRNEEASKTKPFDDRQQRLQQLVEKRKRMKGMLI
>O74331
MNAAEWVAELKRKSQIESFKEQKGLVYDDHIEKVLYSGSVNGTLLVIEGNSCSGKTELLY
HLASNVLLRSSQELVLIVSSEWDWSIKRLTFILHERLISSRGVSQTCKCNCAVKLENEST
VHLQNAAREDGTDEDINSNSVNDVSLSSGETMLQFPEHEEQHECNREMEQLYESASSTVY
PCSFWEDAERKIDAQCAILWPMEFSGVVESIPQSTKDLLRIWKEAKIQMHEDFRCNKTEF
NEACFDASSSRLGCILMDGLSTFYWQLRLERGYTQVYADLQNRLCTSSKLFGCPVVCTNW
LLNNKQHLPIQCKRFRSERRANARFYLENCVSKLGETFYLSVKGVQTKTEMASPYSQQEM
EKV
>O13760
MTCVNVCFFLFPPCHRNKITEADKSLVDLLIPSLCCSLAVFPSIPLINTHSNLCLFSNFS
HSCFLFCTHPDTLPTSLSINPKKLSLSFSFPLSQKRPFPNFLHPFTGSELSLFRCLLLFF
FFLLFFLSFSFSFSFLFFLSQIFIVYFSSFPILHFLFFFFLCVCVFLSFLFSLSHLLSLA
ILFLPLLLRVFSTLSRLPRLFCLCLQKKRRVLIPFAFTSFRKIASLPCVC
>Q10360
MSNPPTRPTLYNLVNADGELSYRARDLVQDFSVPIPHELPDPDLVHSIQDFATEYMLATG
KKSFECLDESALLGLGYLVTEWMDAMVTDCLEEFEER

>O94660
MTLAAVAQLNSSGSILKNLAICKELISQAAAKGAKCIFFPEASDFIAHNSDEAIELTNHP
DCSKFIRDVRESATKHSIFVNICVHEPSKVKNKLLNSSLFIEPLHGEIISRYSKAHLFDV
EIKNGPTLKESNTTLRGEAILPPCKTPLGKVGSAICFDIRFPEQAIKLRNMGAHIITYPS
AFTEKTGAAHWEVLLRARALDSQCYVIAPAQGGKHNEKRASYGHSMIVDPWGTVIAQYSD
ISSPNGLIFADLDLNLVDHVRTYIPLLRRNDLYPTI
>Q9C0X0
MLRYNFRYLHNVLSHTVVFCFTEVSLHIVKQLGVESYACTVHASRFQKSYSASIDYCQRF
LFSSNLKHNYVNLNIIFTSCIFRSLREFLRFYIAAIMCFL
>P02400
MKYLAAYLLLVQGGNAAPSAADIKAVVESVGAEVDEARINELLSSLEGKGSLEEIIAEGQ
KKFATVPTGGASSAAAGAAGAAAGGDAAEEEKEEEAKEESDDDMGFGLFD
>O82803
MAEEVEEERLKYLDFVRAAGVYAVDSFSTLYLYAKDISGPLKPGVDTIENVVKTVVTPVY
YIPLEAVKFVDKTVDVSVTSLDGVVPPVIKQVSAQTYSVAQDAPRIVLDVASSVFNTGVQ
EGAKALYANLEPKAEQYAVITWRALNKLPLVPQVANVVVPTAVYFSEKYNDVVRGTTEQG
YRVSSYLPLLPTEKITKVFGDEAS
>Q9Z327
MLGTHFPPPPLGPSEGRAAPCTFQIPDGSYRCLALEAEESSSEDGLQGEVRLVDLEEEGT
SKSRVNHGTPPLSRAPAIIQPSSCCREARGGFQHSDKPSREWDVVQARKVMTASVSSSPV
PRVAQKPALGRSTSFTEKDLKEAKERSQRIAAQLTTPPSSNSRGVQLFNRRRQRVNEFTL
ESRGQRSPKLSQEALQTGHPSSPIGHAPGLSVNPTSPSKPGSPKHSSSQSPSRGVPGHIM
EGYSEEASLLRHLEKVASEEEEVPLVVYLKENAALLTANGLHLSQNRETQQSSPNPPETE
EVPSPAADINQNPSSPNATLTTAASNSHHNQPTADVNQNPPATITPVQQNSSETQCSPNG
TLDSKPNTPSADDGQRPVPAEEVRSSILLIDKVSAPPSAASPFSREATPLSSSGPPAADL
MSSSLLIGMQPSTLVASAEQEVSGHAAVTTPTKVYSEVHLTLAKPASVVNRTARPFGMQS
PGTTSQIEQSPMMGRRHFGEKAWAPPASSMADRSPQPQRHIMARSPMVERRLVGQRSPVV
ERRPLGNFTPPPTYAETLSTAPVASQVRSPPSYSTLYPSSDPKPPHLKGQVVPANKTGIL
EESMARRGSRKSMFTFVEKPKVTPNPDLLDLVQTADEKRRQRDQGEVGMEDEPFALGAEA
SNFQQEPIARDRSGPAAAEETVPEWASCLKSPRIQAKPKPKPNQNLSEASGKGAELYARR
QSRMEKYVIESSGHAELARCPSPTMSLPSSWKYTTNAPGGFRVASLSPARTPPASLYHGY
LPENGVLRPEPTKQQPHQMRPSLYALSPVKEPAKISSRATSSRASSRTVSPRAASPAKPS
SLDLVPNLPRAGLPPSPALPRPSRSSPGLYNAPVQDSLQPTAVSPTYSSDISPVSPSRAW
SPRAKQAPRPSFSTRNAGIEAQVWKPSFCFK
>Q9U5W9
MASDGEDISVTPAESVTSATDTEEEDIDSPLMQSELHSDEEQPDVEEVPLTTEESEMDEL
IKQLEDYSPTIPDALTMHILKTAGFCTVDPKIVRLVSVSAQKFISDIANDALQHCKTRTT
NIQHSSGHSSSKDKKNPKDRKYTLAMEDLVPALADHGITMRKPQYFV
>Q06677
MSDPFAHLLTSLKNKDSASASKETTPQSSNSPSITGSAVADVARTDKSPNDSLHSISAPP
LIPSPKVDFSAPPLVPTNSTTKSNTANNTPPSALANTDDDFNQLFGMGTVTTTDTIQKPD
EDYYGSKEDHLYNGDDALVDEVKDMEIARLMSLGLSIEEATEFYENDVTYERYLEILKSK
QKERNDLAIRKKESGIKMEKSGLSNIVGTDSNNLFSMATDFFNKGKKLVDQWTSFPPEAN
DRLNNYSKTHDKVEDYDLPQVNDSPNRILFEDNEVVENLPPADNPDQDLLTDFETKIDIT

KRTAPDVSHSSSPTSGILIEENSRRNEPLIEDSLLDFSEGNLTNSKSNEDSTLFNENSNT
DSTIPISDIELSGYNEFKAKGTSLFKNGDYINSLQEYEKSLNTLPLNHPLRIIALSNIIA
SQLKIGEYSKSIENSSMALELFPSSKAKWKNKISNSDPERSFNDIWPKIMIRRAESFEHL
ESFKKALETYQELIKKNFFDDKIMQGKRRCQDFINPPPVKKSMPVKKKTTTTSPATKKQN
LTASSSNSPISVDSTSEIKKRELENAKLALYDKVFEKISSWKDGKDDDIRHLLANLSSLL
TWCNWKDVSMQDLVMPKRVKITYMKAVAKTHPDKIPESLSLENKMIAENIFSTLSIAWDK
FKLQNDIN
>P42861
MSDYFSKLKEHVVESTEINGCTPSIATATFNAPYEVARKTKMLGVTDSSLLNLPAWKRLQ
SLYEKYGNDSILSHFEKDHQRFQRYSIEIDLHSDDNFLFLDYSKSHINDEIKDALVALAE
ERGVRAFAKAMFDGQRVNSTENRAVLHVALRNRSNRPIIVDGKDVMSDVNNVLAQMKDFT
ERVRSGEWKGQTGKSIYNIVNIGIGGSDLGPVMVTEALKPFSKRDLHCFFVSNVDGTHMA
EVLKQVNLEETIFIIASKTFTTQETLTNAMSARNALMSYLKENGISTDGAVAKHFVALST
NTEKVREFGIDTVNMFAFWDWVGGRYSVWSAIGLSVMLSIGYDNFVEFLTGAHVMDNHFA
STPTEQNLPMMLALVGIWYNNFFGSETQAVLPYDQYLWRLPAYLQQLDMESNGKGVTKKS
GAVAVQTGPIVFGEAGTNGQHAFYQLIHQGTKIIPCDFIGCVQTQNRVGDHHRTLMSNFF
AQTEALMVGKNAEEVRQELVKSGMSGDAIENMIPHKTFTGSRPSNSILVNALTPRALGAI
IAMYEHKVLVQGAIWGINSYDQWGVELGKVLAKSILPQLKSGNIVSDHDGSTNGLINMFN
TRAHL
>P19037
MSLIPSIFGGRRSNVFDPFSQDLWDPFEGFFTPSSALANASTARDVAAFTNARVDWKETP
EAHVFKADLPGLKKEEVKVEVEDKNVLQISGERSKENEEKNDKWHRVERASGKFMRRFRL
PENAKMEEVKATMENGVLTVVVPKAPEKKPQVKSIDISGAN
>Q02932
MASSLNELNLVQVLEQASNPQHIRSDVQKLAEQQLRQWETQAGFHYLLQSIYLNLSNSLQ
IRWLAVIQFKNGVDKYWRSTRINAIPKDEKASIRGRLFEMIDEQNNQLCIQNAQASARIA
RLDFPVEWPTLFEDLENLLNDEIIRKDSVKIYNILMHINQIVKVLGTARIGRCRPAMQSK
VPLILPLIVRIYLQSFEEWTTSSNLNYEDLSSLQVSYLALKVLRRIICEGYDRPQTDQSV
CDFIKLSVSHFEMLISNHENFKKFDIYEKFIKCLGKLYFNLVTGSPANFILLPCSTQILI
TYTRLIFDKAPKVYRENSDVTGDFWEQTAIRGLLILKRVINFIHKKGAITLKARSDKLTI
DASINKINTEFLNENLITRLVDTLMEWYLRLRPTELENWFMDPEEWINEQMATSYEYQIR
PCAENVFQDLMNTFSELLVPYLLKKIENDASKLSNSLDDFLRKDAIYASFQLSASAVSEM
VDFDRLLIQVFLPEATNTNISGDELRIIRRRVALIINEWSTVKCSEESKSLCYKLFTNFL
TDEDDKVVLLTTVQTVRTMVDDWNFNKDTFQPFLTENVHLLLRKILPSVSLTETRLYVLN
TLSDIIIQTKPLISRDLLVEILQIIPNLWEIATNNASEAILANALLRLLRNLVSSLGSQS
HLTWDIAIPVVALACDPSSMQYQLLSEDGYELWGMLLQNFSSHDQEFDDKFVELVPFLKY
GIETHTEILPTLLEIIKSYALILNPVDFFSNNTFQDIFKQMSKYLLKLREDSFQLVLEIW
EILILSNESDYENLLLQKFYETGVLSALFDAIFLEEAPSSYLCSQIIQIIARISYVNPDA
LMTFLATYHDNLPTSNENARMPESIRKIVSKDQTYDSVVNKLLTGWIVCFRDIFDPKFKK
VHILGISSLLRTGLVPILTEFSSIASLWIEMLEEINETNRGDCEKYHLNDIVTEQSIAFH
PLTAEQLRYHQLCKNNDPVHNISLKDFISQSMEYLESHLGVERYQEFLKTINPSLLENLQ
MFLSIQPQEARP
>Q02872
MKEASDREEAPKMVEKNYSTGFRKAHGEKDQSVTKPISLDGRTGEVIVRKSTGKTKIRKG

QTEEEYTQQLQHYFEVEQGPVRTKVGWMDEVDPLVEIREGKYDISNKHQRQVLSGFCHRL
FYQCKYKECLDLSTYFLGLFEPFNVKNKMKRELEELEYMIERCRGHVL
>P30656
MQAIADSFSVPNRLVKELQYDNEQNLESDFVTGASQFQRLAPSLTVPPIASPQQFLRAHT
DDSRNPDCKIKIAHGTTTLAFRFQGGIIVAVDSRATAGNWVASQTVKKVIEINPFLLGTM
AGGAADCQFWETWLGSQCRLHELREKERISVAAASKILSNLVYQYKGAGLSMGTMICGYT
RKEGPTIYYVDSDGTRLKGDIFCVGSGQTFAYGVLDSNYKWDLSVEDALYLGKRSILAAA
HRDAYSGGSVNLYHVTEDGWIYHGNHDVGELFWKVKEEEGSFNNVIG
>Q08748
MNRTVSTLSSTVSDVSVEIPSICNVINTELPTSDVYLYTLKLILLDYINEPRFKEAALLS
NRTGTSRVLSDKTNHQQTQHGKKLVVDKQDDMSERDIVQATLRILKGKLAQISGNKNLAP
NEMHWKSIVKMYYSMLDSSSADTFSKMGQMEEVVGYFTNIASNELKKMTIKNSRDELFSE
VAYFIDLVIDVLPDSCANIIKRLLDYKINLKKGETTVKKKRAASPATVPQYRSISGSTIS
NKQPSFKVQDISHMKYFMQLFETDETKLHQDVMAVKDDCTNPIFCGELRYLRKKIKKDNG
TLTASDFSSDREYNLWKNYELLEIANLMDRFEIGEKVTSHGNRLIPKDAKSVFVRLIGLV
LKKECSNAVNAINLSQEALFFFHKSARYWRIEYPSTISSLVYSAANLSVLGDEELNIPIT
ENLFSVIRNKYLCSEDNLDPSAWNAQDRYLWAANLFHTTDQSMRTINNLLTAIFSGTKPK
FSPVLSFYYSNIVGDPVMEFYETQSVAVKKYWIKLFKKTLFKASEDYFVSLLQDMLKANA
IEIQNVQNLVETIIEAIKAIQKRYNKPLLDEISLPRQCAVFLCEVYGSDSLNLIKTAEKS
TMKMTGQKLGPIDALDMYDVLKELRQIYLQVKPKGKFFFNLENYFIKYLTRLCDDVSRNV
QKVIKSSLESENWQPVNDQDHFSRSVLDIFKMINESTSMLEKFGWQNEFQLAQMITVILK
AFSDGMLSYSAQLMELIQRDLQEGDEPSYSLESSDTRSSLSLNNANVNHEKSRSSRLFED
LKNVVKSTPKMVAPAPYQFKKRTCVLLNDLDKTLFLLESFEEKADPSKISSVIAQYHSSH
NLEDNGKSFDDQNMKQVYTLRIIGAENIKGFSKTGLSNTYVSMRNITLQREIGTTKIVAR
SITPKWDEEFVFESPFGKSNDIMFTIWHHPHSRLKNLAEDDLCGKANMKFTPRKLKDDGF
PIDFSLTLNPQGTLYCQISLESEKIDAVSSMGRIYRSFSRSRDRAINLIVNKFSDFIAFA
FSRTTLKTVCGHHGSALASDEAVYDAILPLFDYLNANLNILASELSQRLLFMVMLRAWNL
VLENADLLLLPALNSAKVNILRSAKKSLWENTLSTTKTVSGYGRPLTQAEIEAVFKWLDA
LCVDFFHNKGEGPPLAELKNEYYQNILLIPAFYDKSVSELKDEVQRLIPLYEEYLRWFYL
KKTPITFTNKSAGTISRKKSLVANIVKEPKEQLERDAEVMNIILRILIAKGQHDYVHRIL
HDRKELVNTMKNRRAVSRAVTPTGKKGRN
>Q9NX74
MILNSLSLCYHNKLILAPMVRVGTLPMRLLALDYGADIVYCEELIDLKMIQCKRVVNEVL
STVDFVAPDDRVVFRTCEREQNRVVFQMGTSDAERALAVARLVENDVAGIDVNMGCPKQY
STKGGMGAALLSDPDKIEKILSTLVKGTRRPVTCKIRILPSLEDTLSLVKRIERTGIAAI
AVHGRKREERPQHPVSCEVIKAIADTLSIPVIANGGSHDHIQQYSDIEDFRQATAASSVM
VARAAMWNPSIFLKEGLRPLEEVMQKYIRYAVQYDNHYTNTKYCLCQMLREQLESPQGRL
LHAAQSSREICEAFGLGAFYEETTQELDAQQARLSAKTSEQTGEPAEDTSGVIKMAVKFD
RRAYPAQITPKMCLLEWCRREKLAQPVYETVQRPLDRLFSSIVTVAEQKYQSTLWDKSKK
LAEQAAAIVCLRSQGLPEGRLGEESPSLHKRKREAPDQDPGGPRAQELAQPGDLCKKPFV
ALGSGEESPLEGW
>Q00978
MASGRARCTRKLRNWVVEQVESGQFPGVCWDDTAKTMFRIPWKHAGKQDFREDQDAAFFK
AWAIFKGKYKEGDTGGPAVWKTRLRCALNKSSEFKEVPERGRMDVAEPYKVYQLLPPGIV

SGQPGTQKVPSKRQHSSVSSERKEEEDAMQNCTLSPSVLQDSLNNEEEGASGGAVHSDIG
SSSSSSSPEPQEVTDTTEAPFQGDQRSLEFLLPPEPDYSLLLTFIYNGRVVGEAQVQSLD
CRLVAEPSGSESSMEQVLFPKPGPLEPTQRLLSQLERGILVASNPRGLFVQRLCPIPISW
NAPQAPPGPGPHLLPSNECVELFRTAYFCRDLVRYFQGLGPPPKFQVTLNFWEESHGSSH
TPQNLITVKMEQAFARYLLEQTPEQQAAILSLV
>Q8K450
MAAPSGVPPLRVLEELGIGLSPTGEVTEAVTSEGAYYLEQVTITETSEDECEYEEIPDDN
FSIPEGEEDLEKAIHIIGEQARDIHILEQQTILPARNVMQEAIEDFLCNFLIKMGMTRTL
DCFQAEWYELIQKKGSDFKGLGNVPDVYSQVMLLETENKNLKKELKHFKQAAEKAKEDLL
RTQKERDFHRMHHKRIVQEKNKLIADLKGLKLHYASYEPTIRVLHEKHHALLKEKMLTSL
ERDRAVGKISGLQATLKNIDMGHIQVPVIKGSYESASITRESGDRAGHSCEKENSSEGPT
QKSLREAREEVGYKSKLKNEKKDSEFPVDMQPDPNVTSCTENVSAAKFDYKLNNIFRLHE
LPVSCIVMHPCRDYLISCSEDRLWKMVGLPQGNVLLTGSGHTDWLSGCCFHPSGSKLATS
SGDSTIKLWDLNKGECTLTLEGHNHAVWSCTWHSCGDFVASASLDMTSKIWDVNSERCRY
TLYGHTDSVNSIEFFPFSNILLTASADKTLSVWDARTGKCEQSLYGHMHSVNDATFTPRG
HIIASCDARGVTKLWDFRKLIPIVSIDVGPSSGNEVNFDQSGRVLAQASANGIIHLLDLK
SGQIHKLVGHESEVHSVVFSHLGENLYSGGSDGTIRLWI
>Q9P2M1
MKLTSEKLPKNPFYASVSQYAAKNQKFFQWKKEKTDYTHANLVDKALQLLKERILKGDTL
AYFLRGQLYFEEGWYEEALEQFEEIKEKDHQATYQLGVMYYDGLGTTLDAEKGVDYMKKI
LDSPCPKARHLKFAAAYNLGRAYYEGKGVKRSNEEAERLWLIAADNGNPKASVKAQSMLG
LYYSTKEPKELEKAFYWHSEACGNGNLESQGALGLMYLYGQGIRQDTEAALQCLREAAER
GNVYAQGNLVEYYYKMKFFTKCVAFSKRIADYDEVHDIPMIAQVTDCLPEFIGRGMAMAS
FYHARCLQLGLGITRDETTAKHYYSKACRLNPALADELHSLLIRQRI
>Q8WNR4
MARNVTKRNKRCRGHQKAIYKKKSHSSSESGLRNYSLYINRVLKEVVPQKGISSRTIDII
NTMINDMFERISTEACNLMYYRKRCTLTPEDIEKAVYLLLPEKLAKYAVAFGKEAVQRYV
RS
>Q03920
MLPTPYVKCDYDKVYEPAEDSFLILDCLEKEHDFLKQKFGNRLAIVCEIGSGSGIVTTFL
MQNKIIPQENSIHLAVDINPWALEATLDTAKLNSCKSSFLEVIQADLNSSIRNNQVDVLI
FNPPYVPAECVPDVPGSREEADQWLDLALLGGKDGMAITDKLLRQLEQILSPDGVAYILF
CARNKPKEVIKRFVDTYKWNVKLIETRKAGWEVLSVYSFTR
>Q07655
MSLVHNQTNLNESKFLIERAFSSSSETVPLSKEATYPMPTAYSFSAVRSNSETNIKRENP
QGFAKEPIMTSMLHNLTMSTGKGNGNDVNSLAPHDVDVGPYCLLLRNLPKDITLRECYCI
FSLATGVSSIELKRDDREPFNDNEKVVVVKFGSLSLVTHYANILNSKSEIFGPSFPFRSH
IDVVNEQTQLPVSFQEHVSSGTTNSSPKNYQLSSSAQNEIQNQSFNTISYGKTSSSPLGP
SAAKPRPSLLSERSLRFSFNDPFGLETISQRKESVPFLRNSISQHDLSNVTTTPVPAGMP
PQKDAGKSLLLLEKDEINESIWNGDELVNDVGNSSFGASLQEPPMSSTPVMEWNASSTAN
IPLFQLSSQENHQSNLLPPSHHSISQDVPHIQSQPNLNNSGVIHSATSLPHYHLLNQINA
STKTQSIQQSVSNVPSNLDLNLQTENGHPQSSAPNGSSIFNNQKVNQGFLVSEQDTSTIS
RQKECSSTASASAFSKNNETNVAGSTTISQADLSLLAKVPPPANPADQNPPCNTLYVGNL
PPDATEQELRQLFSNQQGFRRLSFRNKMNSHGHGNGHGHGPICFVEFEDVSFATRALAEL

YGSQLPHPRPSLNNKGGIRLSFSKNPLGVRGSNSRSKSGYSFNGSYGKS
>O43592
MDEQALLGLNPNADSDFRQRALAYFEQLKISPDAWQVCAEALAQRTYSDDHVKFFCFQVL
EHQVKYKYSELTTVQQQLIRETLISWLQAQMLNPQPEKTFIRNKAAQVFALLFVTEYLTK
WPKFFFDILSVVDLNPRGVDLYLRILMAIDSELVDRDVVHTSEEARRNTLIKDTMREQCI
PNLVESWYQILQNYQFTNSEVTCQCLEVVGAYVSWIDLSLIANDRFINMLLGHMSIEVLR
EEACDCLFEVVNKGMDPVDKMKLVESLCQVLQSAGFFSIDQEEDVDFLARFSKLVNGMGQ
SLIVSWSKLIKNGDIKNAQEALQAIETKVALMLQLLIHEDDDISSNIIGFCYDYLHILKQ
LTVLSDQQKANVEAIMLAVMKKLTYDEEYNFENEGEDEAMFVEYRKQLKLLLDRLAQVSP
ELLLASVRRVFSSTLQNWQTTRFMEVEVAIRLLYMLAEALPVSHGAHFSGDVSKASALQD
MMRTLVTSGVSSYQHTSVTLEFFETVVRYEKFFTVEPQHIPCVLMAFLDHRGLRHSSAKV
RSRTAYLFSRFVKSLNKQMNPFIEDILNRIQDLLELSPPENGHQSLLSSDDQLFIYETAG
VLIVNSEYPAERKQALMRNLLTPLMEKFKILLEKLMLAQDEERQASLADCLNHAVGFASR
TSKAFSNKQTVKQCGCSEVYLDCLQTFLPALSCPLQKDILRSGVRTFLHRMIICLEEEVL
PFIPSASEHMLKDCEAKDLQEFIPLINQITAKFKIQVSPFLQQMFMPLLHAIFEVLLRPA
EENDQSAALEKQMLRRSYFAFLQTVTGSGMSEVIANQGAENVERVLVTVIQGAVEYPDPI
AQKTCFIILSKLVELWGGKDGPVGFADFVYKHIVPACFLAPLKQTFDLADAQTVLALSEC
AVTLKTIHLKRGPECVQYLQQEYLPSLQVAPEIIQEFCQALQQPDAKVFKNYLKVFFQRA
KP
>Q9BW27
MEELDGEPTVTLIPGVNSKKNQMYFDWGPGEMLVCETSFNKKEKSEMVPSCPFIYIIRKD
VDVYSQILRKLFNESHGIFLGLQRIDEELTGKSRKSQLVRVSKNYRSVIRACMEEMHQVA
IAAKDPANGRQFSSQVSILSAMELIWNLCEILFIEVAPAGPLLLHLLDWVRLHVCEVDSL
SADVLGSENPSKHDSFWNLVTILVLQGRLDEARQMLSKEADASPASAGICRIMGDLMRTM
PILSPGNTQTLTELELKWQHWHEECERYLQDSTFATSPHLESLLKIMLGDEAALLEQKEL
LSNWYHFLVTRLLYSNPTVKPIDLHYYAQSSLDLFLGGESSPEPLDNILLAAFEFDIHQV
IKECSIALSNWWFVAHLTDLLDHCKLLQSHNLYFGSNMREFLLLEYASGLFAHPSLWQLG
VDYFDYCPELGRVSLELHIERIPLNTEQKALKVLRICEQRQMTEQVRSICKILAMKAVRN
NRLGSALSWSIRAKDAAFATLVSDRFLRDYCERGCFSDLDLIDNLGPAMMLSDRLTFLGK
YREFHRMYGEKRFADAASLLLSLMTSRIAPRSFWMTLLTDALPLLEQKQVIFSAEQTYEL
MRCLEDLTSRRPVHGESDTEQLQDDDIETTKVEMLRLSLARNLARAIIREGSLEGS
>Q9BPX3
MGAERRLLSIKEAFRLAQQPHQNQAKLVVALSRTYRTMDDKTVFHEEFIHYLKYVMVVYK
REPAVERVIEFAAKFVTSFHQSDMEDDEEEEDGGLLNYLFTFLLKSHEANSNAVRFRVCL
LINKLLGSMPENAQIDDDVFDKINKAMLIRLKDKIPNVRIQAVLALSRLQDPKDDECPVV
NAYATLIENDSNPEVRRAVLSCIAPSAKTLPKIVGRTKDVKEAVRKLAYQVLAEKVHMRA
MSIAQRVMLLQQGLNDRSDAVKQAMQKHLLQGWLRFSEGNILELLHRLDVENSSEVAVSV
LNALFSITPLSELVGLCKNNDGRKLIPVETLTPEIALYWCALCEYLKSKGDEGEEFLEQI
LPEPVVYADYLLSYIQSIPVVNEEHRGDFSYIGNLMTKEFIGQQLILIIKSLDTSEEGGR
KKLLAVLQEILILPTIPISLVSFLVERLLHIIIDDNKRTQIVTEIISEIRAPIVTVGVNN
DPADVRKKELKMAEIKVKLIEAKEALENCITLQDFNRASELKEEIKALEDARINLLKETE
QLEIKEVHIEKNDAETLQKCLILCYELLKQMSISTGLSATMNGIIESLILPGIISIHPVV
RNLAVLCLGCCGLQNQDFARKHFVLLLQVLQIDDVTIKISALKAIFDQLMTFGIEPFKTK
KIKTLHCEGTEINSDDEQESKEVEETATAKNVLKLLSDFLDSEVSELRTGAAEGLAKLMF

SGLLVSSRILSRLILLWYNPVTEEDVQLRHCLGVFFPVFAYASRTNQECFEEAFLPTLQT
LANAPASSPLAEIDITNVAELLVDLTRPSGLNPQAKTSQDYQALTVHDNLAMKICNEILT
SPCSPEIRVYTKALSSLELSSHLAKDLLVLLNEILEQVKDRTCLRALEKIKIQLEKGNKE
FGDQAEAAQDATLTTTTFQNEDEKNKEVYMTPLRGVKATQASKSTQLKTNRGQRKVTVSA
RTNRRCQTAEADSESDHEVPEPESEMKMRLPRRAKTAALEKSKLNLAQFLNEDLS
>P89886
MFKKFTREDVHSRSKVKSSIQRTLKAKLVKQYPKIEDVIDELIPKKSQIELIKCEDKIQL
YSVDGEVLFFQKFDELIPSLKLVHKFPEAYPTVQVDRGAIKFVLSGANIMCPGLTSAGAD
LPPAPGYEKGTIVVINAENKENALAIGELMMGTEEIKSVNKGHSIELIHHLGDPLWNFSV
E
>P23356
MQLKPMEINPEMLNKVLTRLGVAGQWRFEDVLGLEEESLGSVPAPACALLLLFPLTAQRC
FKLGREAASRFHHPDYPGRLFILLVSQHENFRKKQIEELKGQEVSPKVYFMKQTIGNSCG
TIGLIHAVANNQDKLEFEDGSVLKQFLSETEKLSPEDRAKCFEKNEAIQAAHDAVAQEGQ
CRVDDKVNFHFILFNNVDGHLYELDGRMPFPVNHGTSSEDSLLQDAAKVCREFTEREQGE
VRFSAVALCKAA
>Q8TAX9
MFSVFEEITRIVVKEMDAGGDMIAVRSLVDADRFRCFHLVGEKRTFFGCRHYTTGLTLMD
ILDTDGDKWLDELDSGLQGQKAEFQILDNVDSTGELIVRLPKEITISGSFQGFHHQKIKI
SENRISQQYLATLENRKLKRELPFSFRSINTRENLYLVTETLETVKEETLKSDRQYKFWS
QISQGHLSYKHKGQREVTIPPNRVLSYRVKQLVFPNKETMSAGLDIHFRGKTKSFPEGKS
LGSEDSRNMKEKLEDMESVLKDLTEEKRKDVLNSLAKCLGKEDIRQDLEQRVSEVLISGE
LHMEDPDKPLLSSLFNAAGVLVEARAKAILDFLDALLELSEEQQFVAEALEKGTLPLLKD
QVKSVMEQNWDELASSPPDMDYDPEARILCALYVVVSILLELAEGPTSVSS
>Q7Z417
MEEKPGQPQPQHHHSHHHPHHHPQQQQQQPHHHHHYYFYNHSHNHHHHHHHQQPHQYLQH
GAEGSPKAQPKPLKHEQKHTLQQHQETPKKKTGYGELNGNAGEREISLKNLSSDEATNPI
SRVLNGNQQVVDTSLKQTVKANTFGKAGIKTKNFIQKNSMDKKNGKSYENKSGENQSVDK
SDTIPIPNGVVTNNSGYITNGYMGKGADNDGSGSESGYTTPKKRKARRNSAKGCENLNIV
QDKIMQQETSVPTLKQGLETFKPDYSEQKGNRVDGSKPIWKYETGPGGTSRGKPAVGDML
RKSSDSKPGVSSKKFDDRPKGKHASAVASKEDSWTLFKPPPVFPVDNSSAKIVPKISYAS
KVKENLNKTIQNSSVSPTSSSSSSSSTGETQTQSSSRLSQVPMSALKSVTSANFSNGPVL
AGTDGNVYPPGGQPLLTTAANTLTPISSGTDSVLQDMSLTSAAVEQIKTSLFIYPSNMQT
MLLSTAQVDLPSQTDQQNLGDIFQNQWGLSFINEPSAGPETVTGKSSEHKVMEVTFQGEY
PATLVSQGAEIIPSGTEHPVFPKAYELEKRTSPQVLGSILKSGTTSESGALSLEPSHIGD
LQKADTSSQGALVFLSKDYEIESQNPLASPTNTLLGSAKEQRYQRGLERNDSWGSFDLRA
AIVYHTKEMESIWNLQKQDPKRIITYNEAMDSPDQ
>Q9UBB6
MSCCDLAAAGQLGKASIMASDCEPALNQAEGRNPTLERYLGALREAKNDSEQFAALLLVT
KAVKAGDIDAKTRRRIFDAVGFTFPNRLLTTKEAPDGCPDHVLRALGVALLACFCSDPEL
AAHPQVLNKIPILSTFLTARGDPDDAARRSMIDDTYQCLTAVAGTPRGPRHLIAGGTVSA
LCQAYLGHGYGFDQALALLVGLLAAAETQCWKEAEPDLLAVLRGLSEDFQKAEDASKFEL
CQLLPLFLPPTTVPPECYRDLQAGLARILGSKLSSWQRNPALKLAARLAHACGSDWIPAG
SSGSKFLALLVNLACVEVRLALEETGTEVKEDVVTACYALMELGIQECTRCEQSLLKEPQ

KVQLVSVMKEAIGAVIHYLLQVGSEKQKEPFVFASVRILGAWLAEETSSLRKEVCQLLPF
LVRYAKTLYEEAEEANDLSQQVANLAISPTTPGPTWPGDALRLLLPGWCHLTVEDGPREI
LIKEGAPSLLCKYFLQQWELTSPGHDTSVLPDSVEIGLQTCCHIFLNLVVTAPGLIKRDA
CFTSLMNTLMTSLPALVQQQGRLLLAANVATLGLLMARLLSTSPALQGTPASRGFFAAAI
LFLSQSHVARATPGSDQAVLALSPEYEGIWADLQELWFLGMQAFTGCVPLLPWLAPAALR
SRWPQELLQLLGSVSPNSVKPEMVAAYQGVLVELARANRLCREAMRLQAGEETASHYRMA
ALEQCLSEP
>O94565
MGYKQLIYKLNHPPLRRIDERNDPLFSNGCKNVHLEAQLHPRMNATFVMLARNSDLPGVV
SSINSLEKHFNRHFNYPYTFLNDEPFDEKFKETILKLTSANVEFGTLEKDTFGFPGNVDV
DAAREAIASQGDRGIMYGDTESYHHMCRFFSGFFYKHPLLLKYQWYWRVEPDVAFTCDIS
YDPFYYMEENGKIYGYVVALKELEDTVPNLFRYTSAYRRNNNLTSNMWKFFLDAPKKENY
DISRKDPTVGLSFSSHLNAMIDSSYSAETSSMEGESYNMCHFWSNFEIANFKFFRNEQYE
NFFRTMDATGGFWTERWGDAPFHSLAAGLFLSKEQVHYFRDLGYRHSDIHHCGQSLGCNC
ECIPELSEIESTSGGCVTQWVNLIGDGFLDE
>O60178
MKFGCVQFFPKLGKVNENIVHLRQLLDQHSEALQSVKLLVFPEMCLTGYNFKNSESIQPF
LENVTSNHCPSIQFAQEVSEQYRCYTIIGFPEFQNSNGISTLYNSTALISPKKELLNVYH
KHFLFETDKSWATEGKGFSFEPCIPELGPISMAICMDINPYDFKAPFEKFEYANFILREL
EHQQMVSSNVSRPIICLSMAWLVSDDKVIDASLPDIKNLHYWTTRLSPLINSNTDAIVLV
ANRWGKENDLNFSGTSCIMELSQGRAILHGVLKAAENGIVVGELEK
>Q86SQ0
MEEHSYIQKELDLQNGSLEEDSVVHSVENDSQNMMESLSPKKYSSSLRFKANGDYSGSYL
TLSQPVPAKRSPSPLGTSVRSSPSLAKIQGSKQFSYDGTDKNIPMKPPTPLLNTTSSLSG
YPLGRADFDHYTGRDSERALRLSEKPPYSKYSSRHKSHDNVYSLGGLEGRKASGSLLAMW
NGSSLSDAGPPPISRSGAASMPSSPKQARKMSIQDSLALQPKLTRHKELASENINLRTRK
YSSSSLSHMGAYSRSLPRLYRATENQLTPLSLPPRNSLGNSKRTKLGEKDLPHSVIDNDN
YLNFSSLSSGALPYKTSASEGNPYVSSTLSVPASPRVARKMLLASTSSCASDDFDQASYV
GTNPSHSLLAGESDRVFATRRNFSCGSVEFDEADLESLRQASGTPQPALRERKSSISSIS
GRDDLMDYHRRQREERLREQEMERLERQRLETILSLCAEYTKPDSRLSTGTTVEDVQKIN
KELEKLQLSDEESVFEEALMSPDTRYRCHRKDSLPDADLASCGSLSQSSASFFTPRSTRN
DELLSDLTRTPPPPSSTFPKASSESSYLSILPKTPEGISEEQRSQELAAMEETRIVILNN
LEELKQKIKDINDQMDESFRELDMECALLDGEQKSETTELMKEKEILDHLNRKIAELEKN
IVGEKTKEKVKLDAEREKLERLQELYSEQKTQLDNCPESMREQLQQQLKRDADLLDVESK
HFEDLEFQQLEHESRLDEEKENLTQQLLREVAEYQRNIVSRKEKISALKKQANHIVQQAQ
REQDHFVKEKNNLIMMLQREKENLCNLEKKYSSLSGGKGFPVNPNTLKEGYISVNEINEP
CGNSTNLSPSTQFPADADAVATEPATAVLASQPQSKEHFRSLEERKKQHKEGLYLSDTLP
RKKTTSSISPHFSSATMGRSITPKAHLPLGQSNSCGSVLPPSLAAMAKDSESRRMLRGYN
HQQMSEGHRQKSEFYNRTASESNVYLNSFHYPDHSYKDQAFDTLSLDSSDSMETSISACS
PDNISSASTSNIARIEEMERLLKQAHAEKTRLLESREREMEAKKRALEEEKRRREILEKR
LQEETSQRQKLIEKEVKIRERQRAQARPLTRYLPVRKEDFDLRSHVETAGHNIDTCYHVS
ITEKTCRGFLIKMGGKIKTWKKRWFVFDRNKRTFSYYADKHETKLKGVIYFQAIEEVYYD
HLKNANKSPNPLLTFSVKTHDRIYYMVAPSPEAMRIWMDVIVTGAEGYTHFLL
>Q40577

MASAAVANYEEEIVRPVADFSPSLWGDQFLSFSIKNQVAEKYAKEIEALKEQTRNMLLAT
GMKLADTLNLIDTIERLGISYHFEKEIDDILDQIYNQNSNCNDLCTSALQFRLLRQHGFN
ISPEIFSKFQDENGKFKESLASDVLGLLNLYEASHVRTHADDILEDALAFSTIHLESAAP
HLKSPLREQVTHALEQCLHKGVPRVETRFFISSIYDKEQSKNNVLLRFAKLDFNLLQMLH
KQELAQVSRWWKDLDFVTTLPYARDRVVECYFWALGVYFEPQYSQARVMLVKTISMISIV
DDTFDAYGTVKELEAYTDAIQRWDINEIDRLPDYMKISYKAILDLYKDYEKELSSAGRSH
IVCHAIERMKEVVRNYNVESTWFIEGYTPPVSEYLSNALATTTYYYLATTSYLGMKSATE
QDFEWLSKNPKILEASVIICRVIDDTATYEVEKSRGQIATGIECCMRDYGISTKEAMAKF
QNMAETAWKDINEGLLRPTPVSTEFLTPILNLARIVEVTYIHNLDGYTHPEKVLKPHIIN
LLVDSIKI
>Q9BQ95
MSWVQATLLARGLCRAWGGTCGAALTGTSISQVPRRLPRGLHCSAAAHSSEQSLVPSPPE
PRQRPTKALVPFEDLFGQAPGGERDKASFLQTVQKFAEHSVRKRGHIDFIYLALRKMREY
GVERDLAVYNQLLNIFPKEVFRPRNIIQRIFVHYPRQQECGIAVLEQMENHGVMPNKETE
FLLIQIFGRKSYPMLKLVRLKLWFPRFMNVNPFPVPRDLPQDPVELAMFGLRHMEPDLSA
RVTIYQVPLPKDSTGAADPPQPHIVGIQSPDQQAALARHNPARPVFVEGPFSLWLRNKCV
YYHILRADLLPPEEREVEETPEEWNLYYPMQLDLEYVRSGWDNYEFDINEVEEGPVFAMC
MAGAHDQATMAKWIQGLQETNPTLAQIPVVFRLAGSTRELQTSSAGLEEPPLPEDHQEED
DNLQRQQQGQS
>O74560
MPPVRAEKKRKTDLIEQVCVTNKAGELVDLEDVLEYGPYSLTGILSSEKDEQEPLFDESI
VLGYSIKISPVWTYNLKDVPEKETMSIWIVTPQRRYGILSPSSEYKAIYEQISEKNRLFY
LIKTKFKDDMISGTLEDYDNYIEVLKEKLELPSCFQAILLVQKHIRFLLTQMVATSSLHV
WSESPFFIRIRSSYEHLILQINKNIYNARQERKKSKLSSNNPSDNNTTMKSSLNQALTLI
NLPEQPFSISSPTATPQLGVVKRTSPLRFPLNDIWLSGLRIVDPNIESISLWKRIQVSTS
PKHQRYISLQEVCSVIAQQLQITNLEALNKLSSHGETLLQIMHTAFTWRGTKLFNDIKHA
IGFRSSVQQARSQFRGYCYDYLFMHCNNGEKTSLHLLRTLICMKLDFSNAQLAAKILFHF
LLFDIGSGLSGSDYTYEQYINHSAVAFSFTEEIFEKNFVTVLPDFVKLFSISFGYWPAFS
FYDELLKLLRNKYPKVYSSTPNLCDQVWLDRTNLFPCNRSTRSTLPYRPTKLLDLASASS
CLSKKETDFKQDTGLYSYNLEKVEALKVSPDLQTGIWSCPVQNCLYFAVCDNPYKPSQVI
YDHLLGHVDSKFIFKTPSNSVRSFTNKLEHIMYNIN
>P05085
MGISSKNGPKKMGRAKTFTGCWTCRGRKVKCDLRHPHCQRCEKSNLPCGGYDIKLRWSKP
MQFDPYGVPIPQNSPATTTNLSGSVDEPQYQRRNIDFVRYDEEYVYHEDMDDELTMLHTP
PIEKISDNKTWIIKKFGVFKGTDKIDKQYAPRKKRNRKRVAKSLESSASISLSSLPSSST
ISFPIRHIEDKLRNKGHVKTGILSANDGVPPTPNLLDYDWNNLNITGYEWISSELRDDAL
LSAVTLQGHHLGHTQPQEISLEENSNVVSGEEHVNAKEHGCAFEADNQGSSTLPNKAASA
NDKLYQQNLKLLFQKNSSNSEEPDPQALIDDVFVNIEPRSLPASDLNKITLAPPNEESRM
PKSMLELTSYSSDLPPELVDIIPKTDLTVHGLARFLLNHYFNNVADKMTVVVLEKNPWKT
LYFPRALMALGDLAGLGQSSNSRNALLNALLAVSCFHLQSKYPRNYKLQKYFLGLGIELR
NQASNFLRLCLNTKSSIPEKYKDVLTAILSMNSIDVVWGTMADCQDHLALCEDFVESRMK
LRPNISEKTKTLHRIFSFLKLIQDSTALDKVRAKEIVILPSEEDDNYKPLDTSNATTSSS
EPRVDVVQEGLFREALNENDGKIHIEFVKEPITNVSADSTPSSTTPPIFTNIATESYYNK
SDISKLVSKTDENIIGTDSLYGLPNSLILLFSDCVRIVRHNEYYNLTYLPVPRKFNELSL

NFEKRLLKWKSEWNFHQENSEGKSFINSTAEALYHHTMSFYFSLIIYYFTMARSLNCQFL
QNYVAKVLDHLNAMEELVDQKKVKIVPLIWQGFMAGCACTDENRQQEFRRWAAKLAESGV
GSYWGARQVMLEVWRRRKEDEPGDNWYSVYKDWEMNLMLS
>P55830
MAVGKNKGLSKGGKKGGKKKVVDPFSRKDWYDVKAPNMFQTRQIGKTLVNRTQGQRIASD
YLKGRVFEVSLADLQKDIDPERSFRKFRLIAEDVQDRNVLCNFHGMDLTTDKYRSMVKKW
QTLIEAIVEAKTVDGYLLRVFCIGFTAKDQQSQRKTCYAQQSQVRKIRARMTDIITNEVS
GADLKQLVNKLALDSIAKDIEKSCQRIYPLHDVYIRKVKVLKKPRFDVSKLLELHGDGGG
KSVEAVVSSEGAVIDRPEGYEPPVQESV
>P39741
MAGVKAYELRTKSKEQLASQLVDLKKELAELKVQKLSRPSLPKIKTVRKSIACVLTVINE
QQREAVRQLYKGKKYQPKDLRAKKTRALRRALTKFEASQVTEKQRKKQIAFPQRKYAIKA

>P36889
MADYKVKDISLAEWGRKAIELAENEMPGLMELRREYGPSQPLKGAKIAGCLHMTVQTAVL
IETLKALGADVRWSSCNIFSTQDNAAAAIAKAGVPVFAWKGETDEEYEWCIAQTVKGFSG
DGLPNMILDDGGDLTNLVIDHHPELVPKIFGISEETTTGVKNLYKRLSKGNLPMCAINVN
DSVTKSKFDNLYGCRESLVDGMKRATDVMIAGKTCCVCGYGDVGKGCAAALRAFGARVVV
TEVDPINALQASMEGYQVALVEDVMADAHIFVTTTGNDDIITSEHFPHMRDDAIVCNIGH
FDTEIQVGWLEANAKEHVEIKPQVDRYTMENGRHIILLAKGRLVNLGCASGHPSFVMSNS
FTNQVLAQIELWSNRDNGKYPRGDKAGVFFLPKALDEKVAALHLAHVGAKLTKLTPKQAE
YINCPVNGPFKPDHYRY
>P56628
MAPNTSRKQKVIKTLTVDVSSPTENGVFDPASYSKYLIDHIKVDGAVGNLGNAIEVTEDG
SIVTVVSSAKFSGKYLKYLTKKYLKKNQLRDWIRFVSIRQNQYKLVFYQVTPEDADEEED
DE
>Q9H3U1
MTVSGPGTPEPRPATPGASSVEQLRKEGNELFKCGDYGGALAAYTQALGLDATPQDQAVL
HRNRAACHLKLEDYDKAETEASKAIEKDGGDVKALYRRSQALEKLGRLDQAVLDLQRCVS
LEPKNKVFQEALRNIGGQIQEKVRYMSSTDAKVEQMFQILLDPEEKGTEKKQKASQNLVV
LAREDAGAEKIFRSNGVQLLQRLLDMGETDLMLAALRTLVGICSEHQSRTVATLSILGTR
RVVSILGVESQAVSLAACHLLQVMFDALKEGVKKGFRGKEGAIIVDPARELKVLISNLLD
LLTEVGVSGQGRDNALTLLIKAVPRKSLKDPNNSLTLWVIDQGLKKILEVGGSLQDPPGE
LAVTANSRMSASILLSKLFDDLKCDAERENFHRLCENYIKSWFEGQGLAGKLRAIQTVSC
LLQGPCDAGNRALELSGVMESVIALCASEQEEEQLVAVEALIHAAGKAKRASFITANGVS
LLKDLYKCSEKDSIRIRALVGLCKLGSAGGTDFSMKQFAEGSTLKLAKQCRKWLCNDQID
AGTRRWAVEGLAYLTFDADVKEEFVEDAAALKALFQLSRLEERSVLFAVASALVNCTNSY
DYEEPDPKMVELAKYAKQHVPEQHPKDKPSFVRARVKKLLAAGVVSAMVCMVKTESPVLT
SSCRELLSRVFLALVEEVEDRGTVVAQGGGRALIPLALEGTDVGQTKAAQALAKLTITSN
PEMTFPGERIYEVVRPLVSLLHLNCSGLQNFEALMALTNLAGISERLRQKILKEKAVPMI
EGYMFEEHEMIRRAATECMCNLAMSKEVQDLFEAQGNDRLKLLVLYSGEDDELLQRAAAG
GLAMLTSMRPTLCSRIPQVTTHWLEILQALLLSSNQELQHRGAVVVLNMVEASREIASTL
MESEMMEILSVLAKGDHSPVTRAAAACLDKAVEYGLIQPNQDGE
>O09130

MAEPLRGRGPRSRGGRGARRARGARGRCPRARQSPARLIPDTVLVDLVSDSDEEVLEVAD
PVEVPVARLPAPAKPEQDSDSDSEGAAEGPAGAPRTLVRRRRRRLLDPGEAPVVPVYSGK
VQSSLNLIPDNSSLLKLCPSEPEDEADLTNSGSSPSEDDALPSGSPWRKKLRKKCEKEEK
KMEEFPDQDISPLPQPSSRNKSRKHTEALQKLREVNKRLQDLRSCLSPKQHQSPALQSTD
DEVVLVEGPVLPQSSRLFTLKIRCRADLVRLPVRMSEPLQNVVDHMANHLGVSPNRILLL
FGESELSPTATPSTLKLGVADIIDCVVLASSSEATETSQELRLRVQGKEKHQMLEISLSP
DSPLKVLMSHYEEAMGLSGHKLSFFFDGTKLSGKELPADLGLESGDLIEVWG
>Q00202
MLGAMAEEAVAPVAVPTTQEQPTSQPAAAQVTTVTSPSVTATAAAATAAVASPQANGNAA
SPVAPASSTSRPAEELTCMWQGCSEKLPTPESLYEHVCERHVGRKSTNNLNLTCQWGSCR
TTTVKRDHITSHIRVHVPLKPHKCDFCGKAFKRPQDLKKHVKTHADDSVLVRSPEPGSRN
PDMMFGGNGKGYAAAHYFEPALNPVPSQGYAHGPPQYYQAHHAPQPSNPSYGNVYYALNT
GPEPHQASYESKKRGYDALNEFFGDLKRRQFDPNSYAAVGQRLLSLQNLSLPVLTAAPLP
EYQAMPAPVAVASGPYGGGPHPAPAYHLPPMSNVRTKNDLINIDQFLQQMQDTIYENDDN
VAAAGVAQPGAHYIHNGISYRTTHSPPTQLPSAHATTQTTAGPIISNTSAHSPSSSTPAL
TPPSSAQSYTSGRSPISLPSAHRVSPPHESGSSMYPRLPSATDGMTSGYTAASSAAPPST
LGGIFDNDERRRYTGGTLQRARPASRAASESMDLSSDDKESGERTPKQISASLIDPALHS
GSPGEDDVTRTAKAATEVAERSDVQSEWVEKVRLIEYLRNYIANRLERGEFSDDSEQEQD
QEQEQDQEQEQDQEQGQDRVSRSPVSKADVDMEGVERDSLPRSPRTVPIKTDGESAEDSV
MYPTLRGLDEDGDSKMPS
>Q12134
MQQTKFGKMYLDHDSVVEYSEDEIVEADRITLGYKKRLSMIENQMRHLLEDFSLDVQQIE
PILADLQKYYDAFLQLLQKRNKSLQCKRSTHQPVPSPMNSQTSTNAKVNLSGKLMKFQLN
SVQKFDEENILRILQNKIEFEHYFQIDKGKKQKVLLLAVYQCLNGPTRLHKVLNIEGIIH
NNSIRTILGKQVSSSKWTVFLYDVKLVLLAHRQDVPNLETSKMIVRYGDLFPCALYFKDH
TAY
>P38840
MTAGSAPPVDYTSLKKNFQPFLSRRVENRSLKSFWDASDISDDVIELAGGMPNERFFPIE
SMDLKISKVPFNDNPKWHNSFTTAHLDLGSPSELPIARSFQYAETKGLPPLLHFVKDFVS
RINRPAFSDETESNWDVILSGGSNDSMFKVFETICDESTTVMIEEFTFTPAMSNVEATGA
KVIPIKMNLTFDRESQGIDVEYLTQLLDNWSTGPYKDLNKPRVLYTIATGQNPTGMSVPQ
WKREKIYQLAQRHDFLIVEDDPYGYLYFPSYNPQEPLENPYHSSDLTTERYLNDFLMKSF
LTLDTDARVIRLETFSKIFAPGLRLSFIVANKFLLQKILDLADITTRAPSGTSQAIVYST
IKAMAESNLSSSLSMKEAMFEGWIRWIMQIASKYNHRKNLTLKALYETESYQAGQFTVME
PSAGMFIIIKINWGNFDRPDDLPQQMDILDKFLLKNGVKLVLGYKMAVCPNYSKQNSDFL
RLTIAYARDDDQLIEASKRIGSGIKEFFDNYKS
>P82891
MGKRSQKSSVLMVCVGNLCRSPIAEAVMRDLVARAGLQGEWHVESAGIEDWHSGHQPDER
ALNVLARHNIEYNGKARVLAPEDFLEFDYIFAMDLSNLAALRRMAPKGTTAKLLILGNFG
LKPDERIIEDPYYDIGEASFEEIYRQCSIACRNFLKQARLKQIM
>Q9Y7J5
MENPSPTRTTNLQRIRSLYRLQEDKNNLNIPEPINGKVQEIDTKESLENVDRYGNYRSDF
NVNNLGFYKVSKDPTLDKPNPAKRLGNKLIGHDDMLKYVDESIFMVVPGESHDRNEISAK
ELSRIQKWKSMCSIHLVSGNQLHSFRKTRKLILRTFKGIPDCWRSIAWWSFLVDSLPDKK

LIDKYYQLNEQICDYDVQIDLDVPRTAATHFLFRKRYIGGQRLLFRVLHAVALYIPRVGY
VQGMASIAATLLIYYPEEQAFIMMVNLLENRGMGDLFSSGFDTLLKAFDMLKHELSFTQS
GRHLAEIGAEPSAFATRWYLTVFHQCVPFHTQLRIWDLLFLLGGSKGQTVRLLQATSLAV
IQGMWDTLIDADFEVVMQALSGVIPIQNDNALLARIQLFWEKMPSENSSKSKRN
>Q12502
MAFSRLTSTHQSNHNGYSNSNKKGQSLPLTLSIDVESPPCVLYGSAMESSGAVLSGLFTV
TVVDPYSSAEDKSLKNTESNVSTTSKSLKRKSTFGSALSSRLSSLSASTSNISPSTSSTS
ISHSPTPANLRIMAGYTKITITSVTLSLVQKIHFHKPFVPNISSMQTCMNCKTKITNMKS
WEIQSNTQDLSVGSHSYPFSYLIPGSVPCSSSLGATAETQVKYELIAVVTYIDPHRNSFS
SGHSTPRKEGSSSKKRLLQLAMPIAVTRSIPRGPDKNSLRVFPPTELTAAAVLPNVVYPK
STFPLEMKLDGVSSGDRRWRMRKLSWRIEETTRVKAHACPVHKHELRQLEEQVKIKESEK
SKKPRSHIKRYGELGPQIRVAVNSLENMPSQRLPGEPGREQAPNSSGPASTGNVGLDDEN
PVNEDEEDQPGSEFIHPSDDALRQELLMQQQRARQQQLQQELKNNSSLFTEEVRIISKGE
MKSGWKTDFDNNGKIELVTEIDCMGLNSGVSNPVMHASTLQTPSTGNKKPSINVACDIQD
PNLGLYVSHILAVEIVVAEETLQYANGQPIRKPNSKNKKETNNNTMNVHNPDQRLAELSP
IFANRNTPKVRRMGPEDITPVNSNKSNHSTNKEKASNGASNSNIVSVPTGAARVLRMQFR
LTVTERSGLGISWDEEVPPIYQDVELLSPPCYELSINNGIKNKLYSTMSTPVRSEDDFVG
GSDEDIGNYESQGLEPGPNVQEVTITQNKLTIPPTAHHYQPASSSQRSLTTVQSPPLESV
VSVQGSVPFRGHVLTPHSTRDIRIQNFSDFLDSNRITQ
>P53537
MGFKVETNGGDGSLVSAKVPPLANPLAEKPDEIASNISYHAQYTPHFSPFKFQLQQAYYA
TAESVRDRLIQQWNETYLHFHKVDPKQTYYLSMEFLQGRALTNAIGNLNIQDAYADALRK
FGLELEEITEQEKDAALGNGGLGRLASCFLDSMATLNLPAWGYGLRYRYGLFKQIITKEG
QEEVAEDWLEKFSPWEIVRHDVLYPIRFFGQVEVNPDGSRQWIGGEVIQALAYDVPIPGY
QTKNTISLRLWEAKACADDFDLFLFNDGQLESASVLHSRAQQICSVLYPGDATEGGKLLR
LKQQYFLCSASLQDIISRFKERRQGPWNWSEFPTKVAVQLNDTHPTLSIPELMRLLMDDE
GLGWDEAWAVTSKTVAYTNHTVLPEALEKWSQPVMWKLLPRHMEIIEEIDRRFVALISKT
RLDLEDEVSNMRILDNNLQKPVVRMANLCVVSSHTVNGVAQLHSDILKSELFASYVSIWP
TKFQNKTNGITPRRWINFCSPELSRIITKWLKTDKWVTNLDLLTGLREFADNEDLQAEWL
SAKRANKQRLAQYVLQVTGENIDPDSLFDIQVKRIHEYKRQLLNILGVIYRYKKLKEMSP
EERKSTTARTVMIGGKAFATYTNAKRIVKLVDDVGSVVNSDPEVNSYLKVVFVPNYNVSV
AEVLIPGSELSQHISTAGMEASGTSNMKFALNRVLIIGTLDGANVEIREEIGEENFFLFG
ATADEVPRLRKERENGLFKPDPRFEEAKKFIRSGVFGSYDYNPLLDSLEGNSGYGRGDYF
LVGYDFPSYMDAQEKVDEAYRDKKRWLKMSILSTAGSGKFSSDRTIAQYAKEIWNIEECR
VP
>Q9C0E2
MMAAALGPPEVIAQLENAAKVLMAPPSMVNNEQRQHAEHIFLSFRKSKSPFAVCKHILET
SKVDYVLFQAATAIMEAVVREWILLEKGSIESLRTFLLTYVLQRPNLQKYVREQILLAVA
VIVKRGSLDKSIDCKSIFHEVSQLISSGNPTVQTLACSILTALLSEFSSSSKTSNIGLSM
EFHGNCKRVFQEEDLRQIFMLTVEVLQEFSRRENLNAQMSSVFQRYLALANQVLSWNFLP
PNLGRHYIAMFESSQNVLLKPTESWRETLLDSRVMELFFTVHRKIREDSDMAQDSLQCLA
QLASLHGPIFPDEGSQVDYLAHFIEGLLNTINGIEIEDSEAVGISSIISNLITVFPRNVL
TAIPSELFSSFVNCLTHLTCSFGRSAALEEVLDKDDMVYMEAYDKLLESWLTLVQDDKHF
HKGFFTQHAVQVFNSYIQCHLAAPDGTRNLTANGVASREEEEISELQEDDRDQFSDQLAS

VGMLGRIAAEHCIPLLTSLLEERVTRLHGQLQRHQQQLLASPGSSTVDNKMLDDLYEDIH
WLILVTGYLLADDTQGETPLIPPEIMEYSIKHSSEVDINTTLQILGSPGEKASSIPGYNR
TDSVIRLLSAILRVSEVESRAIRADLTHLLSPQMGKDIVWFLKRWAKTYLLVDEKLYDQI
SLPFSTAFGADTEGSQWIIGYLLQKVISNLSVWSSEQDLANDTVQLLVTLVERRERANLV
IQCENWWNLAKQFASRSPPLNFLSSPVQRTLMKALVLGGFAHMDTETKQQYWTEVLQPLQ
QRFLRVINQENFQQMCQQEEVKQEITATLEALCGIAEATQIDNVAILFNFLMDFLTNCIG
LMEVYKNTPETVNLIIEVFVEVAHKQICYLGESKAMNLYEACLTLLQVYSKNNLGRQRID
VTAEEEQYQDLLLIMELLTNLLSKEFIDFSDTDEVFRGHEPGQAANRSVSAADVVLYGVN
LILPLMSQDLLKFPTLCNQYYKLITFICEIFPEKIPQLPEDLFKSLMYSLELGMTSMSSE
VCQLCLEALTPLAEQCAKAQETDSPLFLATRHFLKLVFDMLVLQKHNTEMTTAAGEAFYT
LVCLHQAEYSELVETLLSSQQDPVIYQRLADAFNKLTASSTPPTLDRKQKMAFLKSLEEF
MANVGGLLCVK
>Q12429
MKFIALISGGKDSFYNIFHCLKNNHELIALGNIYPKESEEQELDSFMFQTVGHDLIDYYS
KCIGVPLFRRSILRNTSNNVELNYTATQDDEIEELFELLRTVKDKIPDLEAVSVGAILSS
YQRTRVENVCSRLGLVVLSYLWQRDQAELMGEMCLMSKDVNNVENDTNSGNKFDARIIKV
AAIGLNEKHLGMSLPMMQPVLQKLNQLYQVHICGEGGEFETMVLDAPFFQHGYLELIDIV
KCSDGEVHNARLKVKFQPRNLSKSFLLNQLDQLPVPSIFGNNWQDLTQNLPKQQAKTGEQ
RFENHMSNALPQTTINKTNDKLYISNLQSRKSETVEKQSEDIFTELADILHSNQIPRNHI
LSASLLIRDMSNFGKINKIYNEFLDLSKYGPLPPSRACVGSKCLPEDCHVQLSVVVDVKN
TGKEKINKNKGGLHVQGRSYWAPCNIGPYSQSTWLNDDANQVSFISGQIGLVPQSMEILG
TPLTDQIVLALQHFDTLCETIGAQEKLLMTCYISDESVLDSVIKTWAFYCSNMNHRSDLW
MDKSDDVEKCLVLVKISELPRGAVAEFGGVTCKRLIVDDNDSDKKEREENDDVSTVFQKL
NLNIEGFHNTTVSAFGYNRNFITGFVDSREELELILEKTPKSAQITLYYNPKEIITFHHH
IGYYPVEKLFDYRGKEHRFGLHIRS
>Q14152
MPAYFQRPENALKRANEFLEVGKKQPALDVLYDVMKSKKHRTWQKIHEPIMLKYLELCVD
LRKSHLAKEGLYQYKNICQQVNIKSLEDVVRAYLKMAEEKTEAAKEESQQMVLDIEDLDN
IQTPESVLLSAVSGEDTQDRTDRLLLTPWVKFLWESYRQCLDLLRNNSRVERLYHDIAQQ
AFKFCLQYTRKAEFRKLCDNLRMHLSQIQRHHNQSTAINLNNPESQSMHLETRLVQLDSA
ISMELWQEAFKAVEDIHGLFSLSKKPPKPQLMANYYNKVSTVFWKSGNALFHASTLHRLY
HLSREMRKNLTQDEMQRMSTRVLLATLSIPITPERTDIARLLDMDGIIVEKQRRLATLLG
LQAPPTRIGLINDMVRFNVLQYVVPEVKDLYNWLEVEFNPLKLCERVTKVLNWVREQPEK
EPELQQYVPQLQNNTILRLLQQVSQIYQSIEFSRLTSLVPFVDAFQLERAIVDAARHCDL
QVRIDHTSRTLSFGSDLNYATREDAPIGPHLQSMPSEQIRNQLTAMSSVLAKALEVIKPA
HILQEKEEQHQLAVTAYLKNSRKEHQRILARRQTIEERKERLESLNIQREKEELEQREAE
LQKVRKAEEERLRQEAKEREKERILQEHEQIKKKTVRERLEQIKKTELGAKAFKDIDIED
LEELDPDFIMAKQVEQLEKEKKELQERLKNQEKKIDYFERAKRLEEIPLIKSAYEEQRIK
DMDLWEQQEEERITTMQLEREKALEHKNRMSRMLEDRDLFVMRLKAARQSVYEEKLKQFE
ERLAEERHNRLEERKRQRKEERRITYYREKEEEEQRRAEEQMLKEREERERAERAKREEE
LREYQERVKKLEEVERKKRQRELEIEERERRREEERRLGDSSLSRKDSRWGDRDSEGTWR
KGPEADSEWRRGPPEKEWRRGEGRDEDRSHRRDEERPRRLGDDEDREPSLRPDDDRVPRR
GMDDDRGPRRGPEEDRFSRRGADDDRPSWRNTDDDRPPRRIADEDRGNWRHADDDRPPRR
GLDEDRGSWRTADEDRGPRRGMDDDRGPRRGGADDERSSWRNADDDRGPRRGLDDDRGPR

RGMDDDRGPRRGMDDDRGPRRGMDDDRGPRRGLDDDRGPWRNADDDRIPRRGAEDDRGPW
RNMDDDRLSRRADDDRFPRRGDDSRPGPWRPLVKPGGWREKEKAREESWGPPRESRPSEE
REWDREKERDRDNQDREENDKDPERERDRERDVDREDRFRRPRDEGGWRRGPAEESSSWR
DSSRRDDRDRDDRRRERDDRRDLRERRDLRDDRDRRGPPLRSEREEVSSWRRADDRKDDR
VEERDPPRRVPPPALSRDRERDRDREREGEKEKASWRAEKDRESLRRTKNETDEDGWTTV
RR
>Q960N3
MFVEMSVCDSPNKNSKLDKKSLTPFKKVRRKNWKQEAAYKSDTSKGQEVSYVGERFIPNR
FERENIEFNLKYIGKRKERDILETGVTLTASYWRQSGFISNINRTFGIGERRLFQFSSQQ
GTRSRVVDNDSADSDWPCNPRARPYAIQNATHEMPGICSPVDYNMMDWSSGGMVAMSSGQ
DVMLWRNLDESTMVFSVESPTSLKYSPDGKHLAIGCMDRNYPVLDLWEVRSPTEFLVSYR
KLFFKSMGYISCIEWSHDGKEVICGTQCGVIIVLAMPTLNTLMQLREHRHTVKKMKFAPT
HKYFASSDTDGKIFIFDAVLKVRLLKLDGRSIVFDWHPWTGEDLAVAERSPASIFIFNIP
RRQFVASYRRRDDRIVIKTLTYSKITGELLVNVIRRDDADLAVCEILVLASLNRVVDLMS
HQDRGTLFLMWNPDGTKIATGGLDDTFSLWNFFPTYKREAILRKQEQKAKDKCSSLSLYK
GIR
>O14262
MIPSKNIQKIKLVTFDAFGTILHLSKPVPIVYSEVAQKYGVHATIDEIEHNTFKDFSEKH
KNHGKKSGLNPHDWWIKVIEHSFPTPVPAEMAEELWSYFSKKTGYTIHPLLIDFLKRNKE
ERKYIIGIISNTDERIRTVLEDYGIDHLIDIYAFSYDVGFEKPSREIFDYAMEKAVKLLG
QEIQPEECMHLGDDLIKDVSAARNIQWNAEYCDIKTNFLKYFEQK
>P15494
MGVFNYETETTSVIPAARLFKAFILDGDNLFPKVAPQAISSVENIEGNGGPGTIKKISFP
EGFPFKYVKDRVDEVDHTNFKYNYSVIEGGPIGDTLEKISNEIKIVATPDGGSILKISNK
YHTKGDHEVKAEQVKASKEMGETLLRAVESYLLAHSDAYN
>Q9NHX0
MAEEIEVEETEDFHDMDFNDEEPSCSGGAVQARTERFVVKKWVAHAMWGWDVAVDNCAIC
RNHIMNLCIECQADPNANQDECTVAWGECNHAFHYHCIARWLKTRLVCPLDNKEWVYQKY
GR
>Q99MD9
MATESTAAAAIAAELVSADKIEDAPAPSTSADKMESLDVDSEAKKLLGLGQKHLVMGDIP
AAVNAFQEAASLLGKKYGETANECGEAFFFYGKSLLELARMENGVLGNALEGVHVEEEEG
EKTEDESLVENNDNVDEEAREELREQVYDAMGEKEAKKAEGKSLTKPETDKEQESEVEKG
GREDMDISEPEEKLQETVEPTSKQLTESSEEAKEAAIPGLNEDEVASGKTEQESLCTEKG
KSISGAYVQNKEFRETVEEGEEIISLEKKPKETSEDQPIRAAEKQGTLMKVVEIEAEIDP
QVKSADVGGEEPKDQVATSESELGKAVLMELSGQDVEASPVVAAEAGAEVSEKPGQEITV
IPNNGPVVGQSTVGDQTPSEPQTSAERLTETKDGSSVEEVKAELVPEQEEAMLPVEESEA
AGDGVETKVAQKATEKAPEDKFKIAANEETPERDEQMKEGEETEGSEEEDRENDKAEETP
NESVLEKKSLQENEEEEIGNLELAWDMLDLAKIIFKRQETKEAQLYAAQAHLKLGEVSVE
SENYIQAVEEFQACLSLQEQYLEAHDRLLAETHYQLGLAYGYNSQYDEAVAQFGKSIDVI
EKRMAVLHEQMKEAEGSFTEYEKEIEELKELLPEIREKIEDAKESQRSGNVAELALKATL
VESSTSGFTPSGAGASVSMIASRKPTDGASSSNCVTDISHLVRKKRKPEEESPRKDDAKK
AKQEPEVNGGSGDAVSSGKEVSENMEAEAENQAESQTAEGTVESAATIKSTAC
>Q9UTC3

MDYYDIDSILSENQKVPCTSTVSIPGLGHEGRMVPTGSKVELPFWLAEVLAINSFVSIHM
PAPFSSVVRNALKANPNSVSIRDITTHYYHFAEKMLHLISDDSLVQISLNTLRSRAMLIA
DASLNPQGALQQNSQFIEGLDDFEKHILRVSHNAHRSLINWQNSTS
>P38732
MITQTELDNCLQWAQNNGAFIDPKISFRITEDAGVSAFVNEKFSPKPDQALIRVPETLLI
TSQQALSEFSQAANERSLLNSVTQLYLSKLKFGTDAVHLKSFYKPYLDVLPLHLPQPYFW
STDEVMNLHGTDVYLTMRDTLNKLVKEWRMLFQALSIEHSSQDKQFLSLFQENKDSAVVP
LEQFCAHINGCKLEDSEWNSFVAYLWSYCIFNSRAFPRVILGRAGTDRTNLNEGFLYPIV
DLLNHKNDVPVRWEMNEQNELCFMSQTTTFSAQDELFNNYGNISNEKCLLNYGFWDSSNK
FDFSRLTLKLPSTLVSGLPVDFNKSGNFVTDDGETTILQFSLKISEPLPPVLLALFAYLS
KLKSEETPTVRSVLEGIDQLTSVVSQRLLFYKNFKIKTSSTQKLRPHVIKLIKLYYQDNK
KILNATTEKLSVLQKKIYSNNKEFSLSFKTIFKNDKIFANSLLLVFGAINYEDLITKDCL
NDALLLWIVKLINDKSNNQGGFIKQTFKEVSDSIVIEKEDVMEFLPFYKKYFPNLSERIP
EIYSVGDWGIRQFIVADTAIDRLVWIRKSNKEPIFLMKKAYDLQI
>P51843
MAGENHQWQGSILYNMLMSAKQTRAAPEAPETRLVDQCWGCSCGDEPGVGREGLLGGRNV
ALLYRCCFCGKDHPRQGSILYSMLTSAKQTYAAPKAPEATLGPCWGCSCGSDPGVGRAGL
PGGRPVALLYRCCFCGEDHPRQGSILYSLLTSSKQTHVAPAAPEARPGGAWWDRSYFAQR
PGGKEALPGGRATALLYRCCFCGEDHPQQGSTLYCVPTSTNQAQAAPEERPRAPWWDTSS
GALRPVALKSPQVVCEAASAGLLKTLRFVKYLPCFQVLPLDQQLVLVRNCWASLLMLELA
QDRLQFETVEVSEPSMLQKILTTRRRETGGNEPLPVPTLQHHLAPPAEARKVPSASQVQA
IKCFLSKCWSLNISTKEYAYLKGTVLFNPDVPGLQCVKYIQGLQWGTQQILSEHTRMTHQ
GPHDRFIELNSTLFLLRFINANVIAELFFRPIIGTVSMDDMMLEMLCTKI
>P28650
MSGTRASNDRPPGTGGVKRGRLQQEAAATGSRVTVVLGAQWGDEGKGKVVDLLATDADIV
SRCQGGNNAGHTVVVDGKEYDFHLLPSGIINTKAVSFIGNGVVIHLPGLFEEAEKNEKKG
LKDWEKRLIISDRAHLVFDFHQAVDGLQEVQRQAQEGKNIGTTKKGIGPTYSSKAARTGL
RICDLLSDFDEFSARFKNLAHQHQSMFPTLEIDVEGQLKRLKGFAERIRPMVRDGVYFMY
EALHGPPKKVLVEGANAALLDIDFGTYPFVTSSNCTVGGVCTGLGIPPQNIGDVYGVVKA
YTTRVGIGAFPTEQINEIGDLLQNRGHEWGVTTGRKRRCGWLDLMILRYAHMVNGFTALA
LTKLDILDVLSEIKVGISYKLNGKRIPYFPANQEILQKVEVEYETLPGWKADTTGARKWE
DLPPQAQSYVRFVENHMGVAVKWVGVGKSRESMIQLF
>Q9VHR8
MFWLRGLQRSRQQLNRLCHFRSRLNYGTSPSIRSVQPSISRSLGFCSSLVRYSCSTNLPQ
PAEMASKTAHFVLPNTQPIADLDCKSAFENLTEKEKLYAHHFSQASWDGGLIALIQSSPE
APLIFSLLHRIFLAEKIPVLRAKALEAGVSADDFTAFLVYACGVFANAGNYKGMGDSKIV
PNLSEKQFETIVKASAAYSDEPRVGKIFEKVKNLIYALESRNEILGFAPDGITTYWSDNC
TKEDSEIVNAWLTSKRIEPYMCRTFKIVENGQTVYDVKLGSVAESTQDGITLPLEEYNGN
KFRVTRGDYQKLLQRVNQHLLQAQKYAANENESKMIEHYVRSFEQGSLDEHKNGSRWWIK
DKGPVIETYIGFIETYRDPAGGRAEFEGFVAMVNKESSAKFSELVNRAEKLIEYLPWTEP
YEKDSYLKPDFTSLDVLTFAGSGVPAGINIPNYDEIRQDEGFKNVSLGNVLANINRKDPI
PFLTEEDQTLMKEYKVKAFEVQVGLHELLGHGSGKLFRIDENGVYNFDKENTKNLVTGEP
ITKWYLPGETYDTKFGAIGSSYEECRAEAVGLYLSLQRDILEIFGFKDKAEQDNIIYVNW
LSLIWNGMGVALEMFNPKSKLWLQAHSRARFVIMKVLLEAGEGLVKVEETEKGKNLLLTV

DRSKIDTVGRKALGDFLTKLQVYKSTADIEAASKMYEHYSKVDESGSHPWAKWRDICLAH
KKPRMILVQANTAIGQDQKVQLKTYEPTHEGYIQSWVERYPNTDIDDLLESIVEKDKKYF
PTAFNN
>Q8IWE5
MEPGEVKDRILENISLSVKKLQSYFAACEDEIPAIRNHDKVLQRLCEHLDHALLYGLQDL
SSGYWVLVVHFTRREAIKQIEVLQHVATNLGRSRAWLYLALNENSLESYLRLFQENLGLL
HKYYVKNALVCSHDHLTLFLTLVSGLEFIRFELDLDAPYLDLAPYMPDYYKPQYLLDFED
RLPSSVHGSDSLSLNSFNSVTSTNLEWDDSAIAPSSEDYDFGDVFPAVPSVPSTDWEDGD
LTDTVSGPRSTASDLTSSKASTRSPTQRQNPFNEEPAETVSSSDTTPVHTTSQEKEEAQA
LDPPDACTELEVIRVTKKKKIGKKKKSRSDEEASPLHPACSQKKCAKQGDGDSRNGSPSL
GRDSPDTMLASPQEEGEGPSSTTESSERSEPGLLIPEMKDTSMERLGQPLSKVIDQLNGQ
LDPSTWCSRAEPPDQSFRTGSPGDAPERPPLCDFSEGLSAPMDFYRFTVESPSTVTSGGG
HHDPAGLGQPLHVPSSPEAAGQEEEGGGGEGQTPRPLEDTTREAQELEAQLSLVREGPVS
EPEPGTQEVLCQLKRDQPSPCLSSAEDSGVDEGQGSPSEMVHSSEFRVDNNHLLLLMIHV
FRENEEQLFKMIRMSTGHMEGNLQLLYVLLTDCYVYLLRKGATEKPYLVEEAVSYNELDY
VSVGLDQQTVKLVCTNRRKQFLLDTADVALAEFFLASLKSAMIKGCREPPYPSILTDATM
EKLALAKFVAQESKCEASAVTVRFYGLVHWEDPTDESLGPTPCHCSPPEGTITKEGMLHY
KAGTSYLGKEHWKTCFVVLSNGILYQYPDRTDVIPLLSVNMGGEQCGGCRRANTTDRPHA
FQVILSDRPCLELSAESEAEMAEWMQHLCQAVSKGVIPQGVAPSPCIPCCLVLTDDRLFT
CHEDCQTSFFRSLGTAKLGDISAVSTEPGKEYCVLEFSQDSQQLLPPWVIYLSCTSELDR
LLSALNSGWKTIYQVDLPHTAIQEASNKKKFEDALSLIHSAWQRSDSLCRGRASRDPWC
>Q95PZ0
MALNAPSGSCSSKVLLHPLVIMQMSEHYSRTKVQQGPTVKKVFGAILGRQNGRQVEAINS
FVLKMETEEMAEPVTFSTEHLLQRADQYLEVFPELQVIGLYCAGEDDNLTPEEKPLLSKL
TNAVRNSEKAGQIDATLFLKLNSITAGTTRKLPLFAFEADVTDQEKHKPIEWILVSEESE
RVGVNHIAKLSTKHGKDGKSVGKKHAEAQDAAMSMLQNRVDLIVAYLEKVQDGTLQPNFE
ILKEANLLAQKLKTIDRYAAEFTDSFEKEEKTMTVFSLMPRLTTLLGNMQNVWNKLSAQR
ADLLADDGFHGKSTSRWAHPVRFKSQHLGRPQQADDDDYFDDEDLENDMSGPRRKIHAAD
SPAGSRRRRVPPRAMNFLGRNSGMQAATDEMELSGQEENMGSNYIPDVPRPSATAHNESD
ESSQAS
>P38414
MASLLFGRGQKLKGTVILMQKNVLDINALTAAQSPSGIIGGAFGVVGSIAGSIIDTATAF
LGRSVRLRLISATVADASGKGKVSKEAFLEGLLTSIPTLGDKQSAFSVHFEWDSNMGTPG
AFYIENFMQGGEFFLVSLTLDDVPNVGSIKFACNSWIYNDKKYQSDRIFFANKTYLPSAT
PAPLVSYRQEELKTLRGDGTGERQEWDRIYDYDVYNDLGAPDQKATLGRPVLGGSSTLPY
PRRGRTGRKKTVKEPQSESRSDTVYLPRDEAFGHVKSSDFLVYILKSASQNIVPQLRSVV
TLQLNNPEFNTFEDVRSLYDGGIKLPTDVLSKISPIPLFSELFRSDGEAALKFPPPKVIQ
VDHSAWMTDEEFAREMIAGVNPHIIKEVLSFPIKSKLDSQLYGDNTSKITKEHLEPNLGG
VTVEGAIQTNRLFTPDHHDALFPYLRKINATATKAYATRTVLFLQDNGTLKPLAIELSTP
HPDGDSFGPVSKVYLPASEGVEASIWLLAKAFVVVNDSCYHQLVSHWLNTHAVVEPFIIA
TNRHLSVVHPIHKLLLPHYRDTMNINALARNVLVNAEGIIESTFLWGNYAMEMSAVVYKD
WVFPDQGLPNDLIKRGVAVKDPSSPHGVRLLIEDYPYASDGLEIWAAIKSWVEEYVNFYY
KSDAAIAQDAELQAFWKELVEVGHGDLKSATWWFKMQNRKELIEACSILIWIASALHAAV
NFGQYPYGGYILNRPTKSRKFMPEKGTPEYDDLAKNYEKAYLRTITPKNDTLTDLTIIEV

LSRHASDEQYLGERIEGDDWTTDSVPKEAFKRFGKKLAEIEEKLTQRNNDESLRNRYGPV
KMPYTLLYPSSEEGLTCRGIPNSISI
>Q12098
MELQRAQRNLKFLQNEDYVNVTDQTNLNGESQNAYSLGMETQVPEMQFSLSSDDDSIGTQ
VKSVTAQKSPMTQETTKNDTERNKDVDKSCNPVSTSHPDLGGSNIEENIFINTQIQSRLD
DAEEETNLKLKLEKFKYSFKSSNADDTHSNANVTAKRRPAIRKANSKLKTKPKTKRDPKI
IKNITDFNINNYERSRTASLLKQLSGKHKKVLDIIKTQNEGNSDKPPRARNNKGEKATFD
TYSEQEWKDIMKLLLQKFPQSEETDLNEVQKFLYGSEKSSNSLDNQESSQQRLWTASQLP
PELPDEAIQPEQEERIRDTQSAVNFLSLSQVMDDKSEIMKDEESIIISRGDSTSSQEYGN
GLEPQQPVGNVVGEDIELAVGTRINAFSLTDYKACKPMSVEVSRRCENSTDNDYDNISIV
SDTTDETSTLFPLDQYRYVFIENDERPPLATDTIGSTQFFTPNTSPLDGIIDLTQESFKA
VRSLISPLKVENNKTGVTSQASNQVQVPATRTPTIIPQKNLTTTLKTEEEKNNIGSSIRV
KLLQESVVKLNPKLVKHNFYRVEANDSEEEETEFDDQFCIADIQLVDSSKISTKDSTQNP
TTSNDIIDTSAASSIASPEKFCEIMMSQSMKELRQSLKTVGLKPMRTKVEIIQSLQTASQ
ILSTANPDNKGEHGGVANFSKIEIFDHLTELIEAFPDFLERIYTFEPIPLNELIEKLFSA
EPFVSQIDEMTIREWADVQGICLRNDKK
>P48760
MSWARSRLCSTLSLAAVSARGATTEGPARRGMSAGPAPQEPGMEYQDAVRTLNTLQTNAS
YLEQVKRQRSDPQAQLEAMEMYLARSGLQVEDLNRLNIIHVTGTKGKGSTCAFTERILRN
YGLKTGFFRSPHMVQVRDRIRINGKPISPELFTKHFWCLYNQLEEFKDDSHVSMPSYFRF
LTLMAFHVFLQEKVDLAVVEVGIGGAFDCTNIIRKPVVCGVSSLGIDHTSLLGDTVEKIA
WQKGGIFKPGVPAFTVVQPEGPLAVLRDRAQQIGCPLYLCPPLEALEEVGLPLSLGLEGA
HQRSNAALALQLAHCWLERQDHQDIQELKVSRPSIRWQLPLAPVFRPTPHMRRGLRDTVW
PGRTQILQRGPLTWYLDGAHTTSSVQACVHWYRQSLERSKRTDGGSEVHILLFNSTGDRD
SAALLKLLQPCQFDYAVFCPNVTEVSSIGNADQQNFTVTLDQVLLRCLQHQQHWNGLAEK
QASSNLWSSCGPDPAGPGSLLLAPHPPQPTRTSSLVFSCISHALLWISQGRDPIFQPQSL
PRNLLNHPTANSGASILREAAAIHVLVTGSLHLVGGVLKLLDPSMSQ
>P05787
MSIRVTQKSYKVSTSGPRAFSSRSYTSGPGSRISSSSFSRVGSSNFRGGLGGGYGGASGM
GGITAVTVNQSLLSPLVLEVDPNIQAVRTQEKEQIKTLNNKFASFIDKVRFLEQQNKMLE
TKWSLLQQQKTARSNMDNMFESYINNLRRQLETLGQEKLKLEAELGNMQGLVEDFKNKYE
DEINKRTEMENEFVLIKKDVDEAYMNKVELESRLEGLTDEINFLRQLYEEEIRELQSQIS
DTSVVLSMDNSRSLDMDSIIAEVKAQYEDIANRSRAEAESMYQIKYEELQSLAGKHGDDL
RRTKTEISEMNRNISRLQAEIEGLKGQRASLEAAIADAEQRGELAIKDANAKLSELEAAL
QRAKQDMARQLREYQELMNVKLALDIEIATYRKLLEGEESRLESGMQNMSIHTKTTSGYA
GGLSSAYGGLTSPGLSYSLGSSFGSGAGSSSFSRTSSSRAVVVKKIETRDGKLVSESSDV
LPK
>Q9GS00
MQNELLDDPMDTGAPAAEAAAADEPLPPAHGIDREVTPKPAGNPDLMQQSLDNPAIPMHL
IEKRDDRRESCDDGYSLTVNESAIDIESLACSYDSNAFFLRARFIARHCPILRADAYISL
INYLKEHTTDITHYVAFFNELESELARKEFKNRQLNFQIPLRDQKWIEENGATWQSTTDQ
LQAEYKRHKDEGVKESTRRAMEDLFQHYMMAGKIDEAIRLYSRGIRDYCTQLKHSINMWI
NWMEVAICANDWGKLDTVTNTAYRSLKDADDAEKNSQQSQQAPPQRGENAPYMVERDPNA
PIQTLTNRQLIETALAKCLAAQVLLKLRNKRYSQVVETILQIKTECLQSKWFVTSSDLGI

YGMLSAMATMSRADLKLQVSGNGTFRKLLESEPQLIELLGSYTSSRFGRCFEIMRSVKPR
LLLDPFISRNVDELFEKIRQKCVLQYLQPYSTIKMATMAEAVGMSSAELQLSLLELIEQK
HVSLKIDQNEGIVRILDERDENAILKRVNVTCDRATQKAKSLLWKTTLAGANIHSISDKE
TRPKRKNQKESAKFDRNFGGIDVDEDPRGIAGPSGLSDDFNIAYDQQPQQQVQYLEDLGD
I
>Q23985
MASSAGSAASGSVVPGGGGSAASSCATMALSTAGSGGPPVNHAHAVCVWEFESRGKWLPY
SPAVSQHLERAHAKKLTRVMLSDADPSLEQYYVNVRTMTQESEAETAGSGLLTIGVRRMF
YAPSSPAGKGTKWEWSGGSADSNNDWRPYNMHVQCIIEDAWARGEQTLDLCNTHIGLPYT
INFCNLTQLRQPSGPMRSIRRTQQAPYPLVKLTPQQANQLKSNSASVSSQYNTLPKLGDT
KSLHRVPMTRQQHPLPTSHQVQQQQHQHQHQQQQQQQHHHQHQQQQHQQQQQHQMQHHQI
HHQTAPRKPPKKHSEISTTNLRQILNNLNIFSSSTKHQSNMSTAASASSSSSSASLHHAN
HLSHAHFSHAKNMLTASMNSHHSRCSEGSLQSQRSSRMGSHRSRSRTRTSDTDTNSVKSH
RRRPSVDTVSTYLSHESKESLRSRNFAISVNDLLDCSLGSDEVFVPSLPPSSLGERAPVP
PPLPLHPRQQQQQQQQQQQLQMQQQQQAQQQQQQSIAGSIVGVDPASDMISRFVKVVEPP
LWPNAQPCPMCMEELVHSAQNPAISLSRCQHLMHLQCLNGMIIAQQNEMNKNLFIECPVC
GIVYGEKVGNQPIGSMSWSIISKNLPGHEGQNTIQIVYDIASGLQTEEHPHPGRAFFAVG
FPRICYLPDCPLGRKVLRFLKIAFDRRLLFSIGRSVTTGREDVVIWNSVDHKTQFNMFPD
PTYLQRTMQQLVHLGVTD
>Q13085
MDEPSPLAQPLELNQHSRFIIGSVSEDNSEDEISNLVKLDLLEEKEGSLSPASVGSDTLS
DLGISSLQDGLALHIRSSMSGLHLVKQGRDRKKIDSQRDFTVASPAEFVTRFGGNKVIEK
VLIANNGIAAVKCMRSIRRWSYEMFRNERAIRFVVMVTPEDLKANAEYIKMADHYVPVPG
GPNNNNYANVELILDIAKRIPVQAVWAGWGHASENPKLPELLLKNGIAFMGPPSQAMWAL
GDKIASSIVAQTAGIPTLPWSGSGLRVDWQENDFSKRILNVPQELYEKGYVKDVDDGLQA
AEEVGYPVMIKASEGGGGKGIRKVNNADDFPNLFRQVQAEVPGSPIFVMRLAKQSRHLEV
QILADQYGNAISLFGRDCSVQRRHQKIIEEAPATIATPAVFEHMEQCAVKLAKMVGYVSA
GTVEYLYSQDGSFYFLELNPRLQVEHPCTEMVADVNLPAAQLQIAMGIPLYRIKDIRMMY
GVSPWGDSPIDFEDSAHVPCPRGHVIAARITSENPDEGFKPSSGTVQELNFRSNKNVWGY
FSVAAAGGLHEFADSQFGHCFSWGENREEAISNMVVALKELSIRGDFRTTVEYLIKLLET
ESFQMNRIDTGWLDRLIAEKVQAERPDTMLGVVCGALHVADVSLRNSVSNFLHSLERGQV
LPAHTLLNTVDVELIYEGVKYVLKVTRQSPNSYVVIMNGSCVEVDVHRLSDGGLLLSYDG
SSYTTYMKEEVDRYRITIGNKTCVFEKENDPSVMRSPSAGKLIQYIVEDGGHVFAGQCYA
EIEVMKMVMTLTAVESGCIHYVKRPGAALDPGCVLAKMQLDNPSKVQQAELHTGSLPRIQ
STALRGEKLHRVFHYVLDNLVNVMNGYCLPDPFFSSKVKDWVERLMKTLRDPSLPLLELQ
DIMTSVSGRIPPNVEKSIKKEMAQYASNITSVLCQFPSQQIANILDSHAATLNRKSEREV
FFMNTQSIVQLVQRYRSGIRGHMKAVVMDLLRQYLRVETQFQNGHYDKCVFALREENKSD
MNTVLNYIFSHAQVTKKNLLVTMLIDQLCGRDPTLTDELLNILTELTQLSKTTNAKVALR
ARQVLIASHLPSYELRHNQVESIFLSAIDMYGHQFCIENLQKLILSETSIFDVLPNFFYH
SNQVVRMAALEVYVRRAYIAYELNSVQHRQLKDNTCVVEFQFMLPTSHPNRGNIPTLNRM
SFSSNLNHYGMTHVASVSDVLLDNSFTPPCQRMGGMVSFRTFEDFVRIFDEVMGCFSDSP
PQSPTFPEAGHTSLYDEDKVPRDEPIHILNVAIKTDCDIEDDRLAAMFREFTQQNKATLV
DHGIRRLTFLVAQKDFRKQVNYEVDRRFHREFPKFFTFRARDKFEEDRIYRHLEPALAFQ
LELNRMRNFDLTAIPCANHKMHLYLGAAKVEVGTEVTDYRFFVRAIIRHSDLVTKEASFE

YLQNEGERLLLEAMDELEVAFNNTNVRTDCNHIFLNFVPTVIMDPSKIEESVRSMVMRYG
SRLWKLRVLQAELKINIRLTPTGKAIPIRLFLTNESGYYLDISLYKEVTDSRTAQIMFQA
YGDKQGPLHGMLINTPYVTKDLLQSKRFQAQSLGTTYIYDIPEMFRQSLIKLWESMSTQA
FLPSPPLPSDMLTYTELVLDDQGQLVHMNRLPGGNEIGMVAWKMTFKSPEYPEGRDIIVI
GNDITYRIGSFGPQEDLLFLRASELARAEGIPRIYVSANSGARIGLAEEIRHMFHVAWVD
PEDPYKGYRYLYLTPQDYKRVSALNSVHCEHVEDEGESRYKITDIIGKEEGIGPENLRGS
GMIAGESSLAYNEIITISLVTCRAIGIGAYLVRLGQRTIQVENSHLILTGAGALNKVLGR
EVYTSNNQLGGIQIMHNNGVTHCTVCDDFEGVFTVLHWLSYMPKSVHSSVPLLNSKDPID
RIIEFVPTKTPYDPRWMLAGRPHPTQKGQWLSGFFDYGSFSEIMQPWAQTVVVGRARLGG
IPVGVVAVETRTVELSIPADPANLDSEAKIIQQAGQVWFPDSAFKTYQAIKDFNREGLPL
MVFANWRGFSGGMKDMYDQVLKFGAYIVDGLRECCQPVLVYIPPQAELRGGSWVVIDSSI
NPRHMEMYADRESRGSVLEPEGTVEIKFRRKDLVKTMRRVDPVYIHLAERLGTPELSTAE
RKELENKLKEREEFLIPIYHQVAVQFADLHDTPGRMQEKGVISDILDWKTSRTFFYWRLR
RLLLEDLVKKKIHNANPELTDGQIQAMLRRWFVEVEGTVKAYVWDNNKDLAEWLEKQLTE
EDGVHSVIEENIKCISRDYVLKQIRSLVQANPEVAMDSIIHMTQHISPTQRAEVIRILST
MDSPST
>Q3TZW7
MMSKVKQVIQDLTVEKDKGGEASRHSEEESHLLEEVETKNPGGYVKRKVRRLVCNFRWPT
PNRHVDHSEGREDAGRFVGQETEAKRKSQEQWMRFSKWFLTPESDSHYNLGLIP
>P49166
MGKGTPSFGKRHNKSHTLCNRCGRRSFHVQKKTCSSCGYPAAKTRSYNWGAKAKRRHTTG
TGRMRYLKHVSRRFKNGFQTGSASKASA
>Q43139
MDHAADAHRTDLMTITRHVLNEQSRNPESRGDFTILLSHIVLGCKFVASAVNKAGLAQLI
GLAGETNVQGEEQKKLDVLSNEVFVKALVSSGRTCVLVSEEDEETTFVDPKLRGKYCVCF
DPLDGSSNIDCGVSIGTIFGIYMIKDKDNVTLSDVLQPGKDMLAAGYCMYGSSCTLVLST
GTGVNGFTLDPSLGEFILTHPDIKIPKKGKIYSVNEGNAKNWDVPVAKFVEKCKYPKDGS
PPKSLRYIGSMVADVHRTLLYGGVFLYPADQKSPNGKLRVLYEVFPMSFLMEQAGGQSFT
GKERALDLVPTKIHERSPIFLGSYDDVEEIKALYAEQAKSSSA
>P12962
MIKYTIDELFQLKPSLTLEVNFDAVEFRAIIEKVKQLQHLKEEEFNSHHVGHFGRRRSSH
HHGRPKIKHNKPKVTTDSDGWCTFEAKKKGSGEDDEEETETTPTSTVPVATIAQETLKVK
PNNKNISSNRPADTRDIVADKPILGFNAFAALESEDEDDEA
>O94315
MSKTAASSAVDASQAGTVKVGDMVVNRMGFGAMRVTGDGIWDEPKDKEACIATLKRLPEL
NINFIDTADSYGPEVSENLLREALYPYKGLIIATKGGLVRTGPNEWHPCGAPKFLRQEVL
MSMRRLGVKQIDLWQLHRIDPKVPRKDQFSEIAAMKKEGLIRHVGLSEVTVDDIKEAEQY
FPVVSVQNLFNLVNRKNEKVLEYCEQKGIAFIPWYPLASGALAKPGTILDAVSKDLDRST
SQIALSWVLQRSPVMLPIPGTSKVDHLEENVKAAGIQLSSEVFAKLDEEGKSEDAKRQEE
EKKKSS
>P28870
MSEELPQIEIVQEGDNTTFAKPGDTVTIHYDGKLTNGKEFDSSRKRGKPFTCTVGVGQVI
KGWDISLTNNYGKGGANLPKISKGTKAILTIPPNLAYGPRGIPPIIGPNETLVFEVELLG
VNGQ

>Q53ET0
MATSGANGPGSATASASNPRKFSEKIALQKQRQAEETAAFEEVMMDIGSTRLQAQKLRLA
YTRSSHYGGSLPNVNQIGSGLAEFQSPLHSPLDSSRSTRHHGLVERVQRDPRRMVSPLRR
YTRHIDSSPYSPAYLSPPPESSWRRTMAWGNFPAEKGQLFRLPSALNRTSSDSALHTSVM
NPSPQDTYPGPTPPSILPSRRGGILDGEMDPKVPAIEENLLDDKHLLKPWDAKKLSSSSS
RPRSCEVPGINIFPSPDQPANVPVLPPAMNTGGSLPDLTNLHFPPPLPTPLDPEETAYPS
LSGGNSTSNLTHTMTHLGISRGMGLPGPGYDAPGLHSPLSHPSLQSSLSNPNLQASLSSPQ
PQLQGSHSHPSLPASSLARHVLPTTSLGHPSLSAPALSSSSSSSSTSSPVLGAPSYPAST
PGASPHHRRVPLSPLSLLAGPADARRSQQQLPKQFSPTMSPTLSSITQGVPLDTSKLSTD
QRLPPYPYSSPSLVLPTQPHTPKSLQQPGLPSQSCSVQSSGGQPPGRQSHYGTPYPPGPS
GHGQQSYHRPMSDFNLGNLEQFSMESPSASLVLDPPGFSEGPGFLGGEGPMGGPQDPHTF
NHQNLTHCSRHGSGPNIILTGDSSPGFSKEIAAALAGVPGFEVSAAGLELGLGLEDELRM
EPLGLEGLNMLSDPCALLPDPAVEESFRSDRLQ
>Q8WVN8
MSVSGLKAELKFLASIFDKNHERFRIVSWKLDELHCQFLVPQQGSPHSLPPPLTLHCNIT
ESYPSSSPIWFVDSEDPNLTSVLERLEDTKNNNLLRQQLKWLICELCSLYNLPKHLDVEM
LDQPLPTGQNGTTEEVTSEEEEEEEEMAEDIEDLDHYEMKEEEPISGKKSEDEGIEKENL
AILEKIRKTQRQDHLNGAVSGSVQASDRLMKELRDIYRSQSYKTGIYSVELINDSLYDWH
VKLQKVDPDSPLHSDLQILKEKEGIEYILLNFSFKDNFPFDPPFVRVVLPVLSGGYVLGG
GALCMELLTKQGWSSAYSIESVIMQINATLVKGKARVQFGANKNQYNLARAQQSYNSIVQ
IHEKNGWYTPPKEDG
>Q8IUG5
MAISSRLALWEQKIREEDKSPPPSSPPPLFSVIPGGFIKQLVRGTEKEAKEARQRKQLAV
ASPEREIPEISISQPNSKSSSGTRSGSQQISQDDQSSSPGSSDILGKESEGSRSPDPEQM
TSINGEKAQELGSSATPTKKTVPFKRGVRRGDVLLMVAKLDPDSAKPEKTHPHDAPPCKT
SPPATDTGKEKKGETSRTPCGSQASTEILAPKAEKTRTGGLGDPGQGTVALKKGEEGQSI
VGKGLGTPKTTELKEAEPQGKDRQGTRPQAQGPGEGVRPGKAEKEGAEPTNTVEKGNVSK
DVGSEGKHVRPQIPGRKWGGFLGRRSKWDGPQNKKDKEGVLLSKAEKTGEPQTQMEKTSQ
VQGELGDDLRMGEKAGELRSTTGKAGESWDKKEKMGQPQGKSGNAGEARSQTEKGCEAPK
EVSTMVESPAAPGKGGWPGSRGQEAEEPCSRAGDGAGALETELEGPSQPALEKDAERPRI
RKENQDGPAPQEEGKGGQSRDSDQAPEDRWYEAEKVWLAQKDGFTLATVLKPDEGTADLP
AGRVRLWIDADKTITEVDEEHVHRANPPELDQVEDLASLISVNESSVLNTLLQRYKAQLL
HTCTGPDLIVLQPRGPSVPSAGKVPKGRRDGLPAHIGSMAQRAYWALLNQRRDQSIVALG
RSGAGKTTCCEQVLEHLVGMAGSVDGRVSVEKIRATFTVLRAFGSVSMAHSRSATRFSMV
MSLDFNATGRITAAQLQTMLLEKSRVARQPEGESNFLVFSQMLAGLDLDLRTELNLHQMA
DSSSFGMGVWSKPEDKQKAAAAFAQLQGAMEMLGISESEQRAVWRVLAAIYHLGAAGACK
VGRKQFMRFEWANYAAEALGCEYEELNTATFKHHLRQIIQQMTFGPSRWGLEDEETSSGL
KMTGVDCVEGMASGLYQELFAAVVSLINRSFSSHHLSMASIMVVDSPGFQNPRHQGKDRA
ATFEELCHNYAHERLQLLFYQRTFVSTLQRYQEEGVPVQFDLPDPSPGTTVAVVDQNPSQ
VRLPAGGGAQDARGLFWVLDEEVHVEGSSDSVVLERLCAAFEKKGAGTEGSSALRTCEQP
LQCEIFHQLGWDPVRYDLTGWLHRAKPNLSALDAPQVLHQSKREELRSLFQARAKLPPVC
RAVAGLEGTSQQALQRSRMVRRTFASSLAAVRRKAPCSQIKLQMDALTSMIKRSRLHFIH
CLVPNPVVESRSGQESPPPPQPGRDKPGAGGPLALDIPALRVQLAGFHILEALRLHRTGY
ADHMGLTRFRRQFQVLDAPLLKKLMSTSEGIDERKAVEELLETLDLEKKAVAVGHSQVFL

KAGVISRLEKQREKLVSQSIVLFQAACKGFLSRQEFKKLKIRRLAAQCIQKNVAVFLAVK
DWPWWQLLGSLQPLLSATIGTEQLRAKEEELTTLRRKLEKSEKLRNELRQNTDLLESKIA
DLTSDLADERFKGDVACQVLESERAERLQAFREVQELKSKHEQVQKKLGDVNKQLEEAQQ
KIQLNDLERNPTGGADEWQMRFDCAQMENEFLRKRLQQCEERLDSELTARKELEQKLGEL
QSAYDGAKKMAHQLKRKCHHLTCDLEDTCVLLENQQSRNHELEKKQKKFDLQLAQALGES
VFEKGLREKVTQENTSVRWELGQLQQQLKQKEQEASQLKQQVEMLQDHKRELLGSPSLGE
NCVAGLKERLWKLESSALEQQKIQSQQENTIKQLEQLRQRFELEIERMKQMHQKDREDQE
EELEDVRQSCQKRLHQLEMQLEQEYEEKQMVLHEKQDLEGLIGTLCDQIGHRDFDVEKRL
RRDLRRTHALLSDVQLLLGTMEDGKTSVSKEELEKVHSQLEQSEAKCEEALKTQKVLTAD
LESMHSELENMTRNKSLVDEQLYRLQFEKADLLKRIDEDQDDLNELMQKHKDLIAQSAAD
IGQIQELQLQLEEAKKEKHKLQEQLQVAQMRIEYLEQSTVDRAIVSRQEAVICDLENKTE
FQKVQIKRFEVLVIRLRDSLIKMGEELSQAATSESQQRESSQYYQRRLEELKADMEELVQ
REAEASRRCMELEKYVEELAAVRQTLQTDLETSIRRIADLQAALEEVASSDSDTESVQTA
VDCGSSGRKEMDNVSILSSQPEGSLQSWLSCTLSLATDTMRTPSRQSATSSRILSPRINE
EAGDTERTQSALALSRARSTNVHSKTSGDKPVSPHFVRRQKYCHFGDGEVLAVQRKSTER
LEPASSPLASRSTNTSPLSREKLPSPSAALSEFVEGLRRKRAQRGQGSTLGLEDWPTLPI
YQTTGASTLRRGRAGSDEGNLSLRVGAKSPLEIEGAAGGLLRSTSLKCISSDGVGGTTLL
PEKSKTQFSSCESLLESRPSMGRKLSSPTTPRDMLLSPTLRPRRRCLESSVDDAGCPDLG
KEPLVFQNRQFAHLMEEPLGSDPFSWKLPSLDYERKTKVDFDDFLPAIRKPQTPTSLAGS
AKGGQDGSQRSSIHFETEEANRSFLSGIKTILKKSPEPKEDPAHLSDSSSSSGSIVSFKS
ADSIKSRPGIPRLAGDGGERTSPERREPGTGRKDDDVASIMKKYLQK
>O09035
MSQVEFEMACASLKQLKGPVSDQEKLLVYSFYKQATQGDCNIPVPPATDVRAKAKYEAWM
VNKGMSKMDAMRIYIAKVEELKKKEPC
>Q09902
MGKLVKHCHTSLHSELVILFYAKNRFCISIDHLRPLKSHRTHGHYCLLNFSLRENKNYLI
IVYLPIEGFSANHMCISHGTSFNIK
>Q86W56
MNAGPGCEPCTKRPRWGAATTSPAASDARSFPSRQRRVLDPKDAHVQFRVPPSSPACVPG
RAGQHRGSATSLVFKQKTITSWMDTKGIKTAESESLDSKENNNTRIESMMSSVQKDNFYQ
HNVEKLENVSQLSLDKSPTEKSTQYLNQHQTAAMCKWQNEGKHTEQLLESEPQTVTLVPE
QFSNANIDRSPQNDDHSDTDSEENRDNQQFLTTVKLANAKQTTEDEQAREAKSHQKCSKS
CDPGEDCASCQQDEIDVVPESPLSDVGSEDVGTGPKNDNKLTRQESCLGNSPPFEKESEP
ESPMDVDNSKNSCQDSEADEETSPGFDEQEDGSSSQTANKPSRFQARDADIEFRKRYSTK
GGEVRLHFQFEGGESRTGMNDLNAKLPGNISSLNVECRNSKQHGKKDSKITDHFMRLPKA
EDRRKEQWETKHQRTERKIPKYVPPHLSPDKKWLGTPIEEMRRMPRCGIRLPLLRPSANH
TVTIRVDLLRAGEVPKPFPTHYKDLWDNKHVKMPCSEQNLYPVEDENGERTAGSRWELIQ
TALLNKFTRPQNLKDAILKYNVAYSKKWDFTALIDFWDKVLEEAEAQHLYQSILPDMVKI
ALCLPNICTQPIPLLKQKMNHSITMSQEQIASLLANAFFCTFPRRNAKMKSEYSSYPDIN
FNRLFEGRSSRKPEKLKTLFCYFRRVTEKKPTGLVTFTRQSLEDFPEWERCEKPLTRLHV
TYEGTIEENGQGMLQVDFANRFVGGGVTSAGLVQEEIRFLINPELIISRLFTEVLDHNEC
LIITGTEQYSEYTGYAETYRWSRSHEDGSERDDWQRRCTEIVAIDALHFRRYLDQFVPEK
MRRELNKAYCGFLRPGVSSENLSAVATGNWGCGAFGGDARLKALIQILAAAAAERDVVYF
TFGDSELMRDIYSMHIFLTERKLTVGDVYKLLLRYYNEECRNCSTPGPDIKLYPFIYHAV

ESCAETADHSGQRTGT
>Q11120
MGDTQQPTVLDRFAPHALDTSEAERLSREYHKNGPYNHVVIRPLINDTLLRNVRKELMEN
IHFTEKVTDIYRVWQTGDLANLDGLDKKEQNMLKYLRQVRDALYSEEFRSHVQKITGCGP
LSASKKDLSVNVYSKGCHLMNHDDVIGTRCISYILYLVEPDEGWKPEYGGALRLFPTLQP
SFPAADFCHSIPPQWNQLSFFRVKPGHSFHDVEEVYVDKPRMAISGWFHYPQPGEPGYDA
NYCDNTVSTLQSLTMKQMDLELPRFSYYPVVKPEPLSKQDTDILSNYINPLYLTPDGIEK
LSKRFFQDSVIVLVEFLNQEFANTLLKRIIDAERQPTPMHSSEVSFPWKTAIPPHKHRYL
YLDHEEFGPDIILPMDLQRLPAFQRWIQLVSGLPLRSFHQVGRRFRPGSDFTLATTNDTA
LLEATLCLSPGTGIANTDNGAYDIYMIGDEDPDVDAAVYRGDQQDDSILLSLPAAWNVFS
LVYRDEGVLQFVKYVSRQAESSRWDIYSQWNPVAE
>P38874
MASSSHNPVILLKRILSLTESSPFILCLDSIAQTSYKLIQEFVHQSKSKGNEYPIVYISF
ETVNKPSYCTQFIDATQMDFVHLVKQIISYLPAATATQAKKHMVIIDSLNYISTEYITRF
LSEIASPHCTMVATYHKDIKDENRTVIPDWNNNYPDKLTLLQFMATTIVDIDVVLTGTLD
TEEVSELLNEFRIPRGLNNDIFQLRLVNKRKSGRSLEYDFIVNSNTHEYELLSTTKQEEE
SSSNGLETPEMLQGLTTFNLGTSNKQKLAKDQVALPFLEAQSFGQGGAIVYEYEKDDDYD
EEDPYEDPF
>O48626
MPEIDALFESINVRDLLAGHDLNDPTTPLSAPDLRLLINRLESHSLRIKSKVQSYLVAHH
SDFSELFSLCQDTVSRTRLISDDVSDVLQLVSDRPIDVEIRSVVDEITEKTKEVKLKRES
LDLVNAIVGICEALQETKEALKNGRFRFAAERIRELKVVLRIGEEEDGEPVAYALLRKEW
SNCFDEIQEVLAKFMENAVRFELDSSRIRIKYQLSVGETAGIALSTVLEAMEVIGILDYG
LAKAADSIFKHVITPAVTHASTFAAVEDLCKSAGEVTEATLRLEQSSDHKFEDVDGDAMY
SGILKVVKFICSSLCFGNVTWIHSFGRLTWPRISELIISKFLSKVVPEDASKLADFQKII
ERTSQFEAALKELNFVSSSDAESRLSKYAEDVEVHFASRKKIEILAKARNLLLQCNFTIP
QDIAMRNAKHIVCLLFSSERCVVSEAASQLMNLVHKTLEDVCVSSARVASEFYNAARDSI
LLYEAVVPVKLEKQLDGLNEAAVLLHNDCLYLFEEILGLAFEYRASFPSSIKEYAVFADI
APRFKLMAEEVLQKQVHLVISSLREAIDSADGFQNTHQIKQFKSAEFSIDQVVFSLKNVH
MIWEPVLRPKTYKQSMCAVLESVFRRIARDILLLDDMAADETFELQKLIYLMLKNLSSVL
DSVRSADETSRPLDDIIPSLRKTRKLAELLDMPLMSITSAWESGELFRCNFTRTEVQDFI
KAIFTDSPLRKECLWRIDEVNQ
>Q24211
MLKLPKGLKKKKKKSKKDQELFTEEELEQYKRDLKAKQEAAATKSDAGESDGASSDVEAH
HEPVAFNSGLGSGSSSSILNAQQQLSDQNQGAAGGDEEWAKFKALTSGVDSILHKTQDEL
DRIKKESFYQRLPSAAEKKKQKEEEAARLEAEQQEREKQRLGQIEAQRDKLAEAVVQLSE
SEEEAGDYEADDIFATDYIEAITSGELQLAVVPDSPVLAEDGPDPFDTAYAEKVIVGADR
AKGNKKLVSLGAAVEVLSGRVDREHAVALANPKRKLRKGIQNLLLSESIELADSEAELLA
ATSNAEPQHNLLDDLDEELSESSVPIDLSVSLHLHLIKHKQPVEEEEELEQKGRENQLLN
PDLSEFDSLKDEEDDEFAELAAESLTKKEEVTVVSQVVLPVAQLPTEAFEAGSWAEFEEQ
SGQEPGKPKRPPPPVRPPTGPHIVPGAIYVSEDEEENPEDDPFNTNYAEQVIKKTTVLEE
DDDFDPRAEEHATEPPFLAAPQRDLLAGSATDLSQVVPAPLAPTLSVDQEAEDFDPFDTS
AVSALVQPKSTELRFLERELLNYSGLDGVTLKHSLSDQDFDPRADQKEPAAPQVKLEQKE
TDFDTAQRKSSLSLNIQAKSVGFLVPASDLLGAGNELGASKKPLTPYYAPSDNRLQERER

EAEDVDPFDTSHVPEAKLSDIELKHIEKDLISVPANLRHSLSDPDFDPRAPPTPVPAEVL
LAVEENINIKVLTPAQDRKKLTNSGGSGKSEEDIDPFDTSIAANLQPGQTELKLLENELL
PETKTLVTDVLDVQSDAQELGLGDKVLTPSTHSRPSLPAQDIDPFDTSIAENLAPGEAEI
KLLESELIER
>P24322
MEHVTMAVTSSSPGPAPLSLLSNNDDFIAPFNINTKFPSAIVPPRTSSNQPISVAIPSNR
ISSAGLAATQQAQTRKRKASVAQISLPSMLPTSFSPYTMAPQPPQPPPNPDRFATEDFFS
PSRRTWSEEKEKVLTGPYDYLNGHPGKDIRSQMVKAFDAWLDVPSESLEVITKVISMLHT
ASLLVDDVEDNSVLRRGFPVAHSIFGIPQTINTSNYVYFYALQELQKLKNPKAVSIFSEE
LLNLHRGQGMDLFWRDTLTCPTEDDYLEMVSNKTGGLFRLGIKLMQAESRSPVDCVPLVN
IIGLIFQIADDYHNLWNREYTANKGMCEDLTEGKFSFPVIHSIRSNPSNMQLLNILKQKT
GDEEVKRYAVAYMESTGSFEYTRKVIKVLVDRARQMTEDIDDGRGKSGGIHKILDRIMLH
QEENVAQKNGKKE
>Q9USY3
MSVKESLSLLNSMQGNVKIGNVEPAKGNEGYVDNAGYVDCTKSYFEATKSLKEEQLVCDP
KFTLLDSISAFEIMEPKMDSGIDYQPLRVDFSRDLSYLEILALMDLIVSAEKEWHYGSPL
SESLLCSAHVFSICKSPISQVGDSGFSSGSGRNTTDIVLFPFVLAVIKCCDIVHREFLMG
NLYDEEDISSFSYHMSFLQNYPIEKLNYLLQSSIEYLASEVIKFSAELRQIIEGILNRIQ
LRIGILRVYERSDIKTTIDALHLIKNLVPEIQNTVSVVDSSIKESILKQYWDFRVQAQLV
ATAPVRNIPPTGIEHSYQRILYFADDMLLILNSHTLASSLAVYQFCLDFTRLNRTPEPYV
RSSLQALITANNAVNLRDQPTSYMLECIREFSGLPSNFYNPNTRTVIEKNSISSAYGPLV
ESLIAHSTNIMVDLVRICSHNPCRFRRNLINLLPEITVAHFEAEALDLKFVAKSLPSNGP
FSSFIYHVKLNAIEHILLSSFEQKLHQPYQWPHFFAVLDHVFSIHQTHLELHGKDRNTPP
MAKTFVTYLHRILNAIKETYSGYLLLTVLCMRLNIIKTPSFTLDEKIQESYYMAHYRPLI
NLRQPKPLLRSEADCIIKNLQNFSTDDLIIKSNEKFTAAKNSLINVIKSGFEQNEFINPY
FLQTNYLKNLLCCCITNLVSLAILSKDHSANLKIVEIPGNPLPSLSRT
>Q9Y314
MTRHGKNCTAGAVYTYHEKKKDTAASGYGTQNIRLSRDAVKDFDCCCLSLQPCHDPVVTP
DGYLYEREAILEYILHQKKEIARQMKAYEKQRGTRREEQKELQRAASQDHVRGFLEKESA
IVSRPLNPFTAKALSGTSPDDVQPGPSVGPPSKDKDKVLPSFWIPSLTPEAKATKLEKPS
RTVTCPMSGKPLRMSDLTPVHFTPLDSSVDRVGLITRSERYVCAVTRDSLSNATPCAVLR
PSGAVVTLECVEKLIRKDMVDPVTGDKLTDRDIIVLQRGGTGFAGSGVKLQAEKSRPVMQ
A
>O94665
MIVIEPTILYEAAALYTRYTANGGDSNDVVALLVGPIVQTLSIPSSTSSAPLVTSVREIL
ELACNDAVLDKSFLNERLEHCKLQDFPLVGCLVLGNRVDHRVRSIAHQLQEATAFPYLML
FPSNFDLTQLKLFRLPDFNNKDQFWKEEWSTEQYEIAENPSGYASALHTLTQLDDGSETN
AINVSTQKFAIQKLQSCAMRVREQLEYNPTTFASRDLSSISYICRLLDTMDLPSDTFSHQ
KSQASFLCALQQILRCTELIEKVYSNGS
>O74758
MTHNTMLQRFNGLLEPTHLYIVSVVAYSACNRPDKISGIAKEAMEHVGPSIYPKLREALV
KSSPLVGFPRTINSLREMTTNIPPPFPDEFARAADADIDTGKRGKIYFEKTYGKVTQRVL
RSMQSSSLDLANIALDYAYGKVLSFNEVVSPLETSLMIIAALVPQDVNPQLRGHLKGALN
HGATKEQVMSARNIALEISKECGIQFKGDIETL

>Q06682
MSEGKVDEFMAITGADDAAIATQFIEMADGNLNTAISLFFENGGAALLSSNNTPTPSNST
PMAPTSVDSDADAQLAERLQREAYQQQQPDQDYVRPPDEARHEVLTETSGFPISYGGIGG
RFEPLHRVNDMFDEGRPESIFNQRLDDTNTNTYINDNSSDSLDSEEENDDDEYEYVEEPV
IELDEDGNIKEYTKLVRKPKTISKEQKLALLFRPPFSIMSKLDLDAAKQKARAKQKWIMI
NIQDSGIFQCQALNRDLWSSRPVKTIIKENFVFLQYQYESRNAQPYLQFYHLNNKDDLPH
IAILDPITGERVKQWNRVVPIPEQFISEINEFLASFSLDPKVPNPTVNEPLPKVDPTTLT
EEQQMELAIKESLNNNSSKSNQEEVPSTGEEQKRVQEPDPFSTIEARVHPEPPNKPGITT
RIQIRTGDGSRLVRRFNALEDTVRTIYEVIKTEMDGFADSRFTLNDHQREDLIDKLNMTI
ADAGLKNSSLLLEKLDPEIE
>Q09773
MKPSENEKIHPVRHSKYEDKSKLPFETIPDPVLKYVKRARDAELQPQLAKVSQKLFADIS
TATAAEKLQFTVPRRARFEINDPRNPTFSMMALQDIISVLEYRRNKNPTSTAHICIDKKG
KEAVSYSWDKILNRAKQFASVAQNHVGLKPGTRVILYYRKSEASEYIISIFGCLLLGIVV
IPLSPSSSSESLKLVVNEEKVRVAFTTEATYRIFIKDTEVNNAKSLAWWKSNDFTNYKFE
QIKQYRMRANDTVLIDYTFSSSTYNDIKAVYTTKTFLSQMRNLTSSMITNPARQAGWKLH
NFEDSKDVLITNFHPSMPLGLIMGVFNSVFAGYCTIFCDEEVLKTPGLLAYLITKYRCTY
SLFDYAGLKQTVYNYQEDPKSTLSFKKNYTPNLSSLKLCMVECEVVDPEFNIIVSDRWLQ
PLGTNNSKEVITPILCLRKFGGIPISFKDWMISYTNRQKHERQMADTCYQEILVLKSSIE
KEKVEIVPFLDIRKYSPNEVLCMSPFWYPIPEASVAVVNEDSKNICKVGEVGEIWVYSDC
LPKLKAASVINNPQGEQLTDYENNQENKFEKNNSFMDSGLKGFLHNEKIFVLGNKDEQIR
QYRKTMVADKLMEEKYVHYSHYLIKKIMKYVPEIFDGVAFEIEIDKAVYSVLVLESPIIK
RSLIRNNRLKGRDLFSELVKITESSFQVTQDIFKLDVLSILIAPYKSLPRSRYMGIQAIN
TNKCKLAFLSGNLPVSYVRFFMDSALPKSKINLNNSKGIWSKDASKHHREIISLNVKQKM
DPYKHQNKVSKARENLLNHFTILDFLKTKSAKTPTSPAILTFNAIEKTKVELTWAHFELK
VASHVEYMQLSVKAKARSHILLLYYDPLEFLISIHSCFHLGVIPIPFQIREISQLIGEIE
EFTKIAKAFNVEAILVDHKVLISLKSRDISNHFQQTCIDLNMKAPKIFETTGIPISKRAK
KALNLITPGELNNKGKVALISINKLEDGSIIPTQFSHSSLLAFCHEQKEFVIGNEEKPII
GGIEFSSGMGLLHTALLGVYAGVPTLLIKQENLCNNGSLLFEAIEQNSLSKVMIPLNICQ
KSFSTAQGCNSIVINSTLSSIIVPCYDRPISSRVNSIIEDIARIGLAPNKVKLAYSHPIN
PFVCWNADSSMEKMKDYFDANQLRSGLVNVREDVLRNRQPLLYGSGTSTLYNEICIVHPE
EKYICQEGEIGEIWINGKHGSYCENNELNSGCELLVQETLDKKSYSRTGQLGFIHHLKKK
DQNMEKVPVLYTLDFIWNTLELNGLNHSVKDIEETIELVHPNICTDGCILFQASGSVVIL
LEVHSQQKFASLIPIIVATALAAHEIILDCVAFVPKGTFLRRPTGEKRRADILKQWTGGD
LKHMTSYLIRQDFLLNEDFVGTELIGTTDSYDYSDENLIINSSQLNLL
>Q4KWH8
MADLEVYKNLSPEKVERCMSVMQSGTQMIKLKRGTKGLVRLFYLDEHRTRLRWRPSRKSE
KAKILIDSIYKVTEGRQSEIFHRQAEGNFDPSCCFTIYHGNHMESLDLITSNPEEARTWI
TGLKYLMAGISDEDSLAKRQRTHDQWVKQTFEEADKNGDGLLNIEEIHQLMHKLNVNLPR
RKVRQMFQEADTDENQGTLTFEEFCVFYKMMSLRRDLYLLLLSYSDKKDHLTVEELAQFL
KVEQKMNNVTTDYCLDIIKKFEVSEENKVKNVLGIEGFTNFMRSPACDIFNPLHHEVYQD
MDQPLCNYYIASSHNTYLTGDQLLSQSKVDMYARVLQEGCRCVEVDCWDGPDGEPVVHHG
YTLTSKILFRDVVETINKHAFVKNEFPVILSIENHCSIQQQRKIAQYLKGIFGDKLDLSS
VDTGECKQLPSPQSLKGKILVKGKKLPYHLGDDAEEGEVSDEDSADEIEDECKFKLHYSN

GTTEHQVESFIRKKLESLLKESQIRDKEDPDSFTVRALLKATHEGLNAHLKQSPDVKESG
KKSHGRSLMTNFGKHKKTTKSRSKSYSTDDEEDTQQSTGKEGGQLYRLGRRRKTMKLCRE
LSDLVVYTNSVAAQDIVDDGTTGNVLSFSETRAHQVVQQKSEQFMIYNQKQLTRIYPSAY
RIDSSNFNPLPYWNAGCQLVALNYQSEGRMMQLNRAKFKANGNCGYVLKPQQMCKGTFNP
FSGDPLPANPKKQLILKVISGQQLPKPPDSMFGDRGEIIDPFVEVEIIGLPVDCCKDQTR
VVDDNGFNPVWEETLTFTVHMPEIALVRFLVWDHDPIGRDFVGQRTVTFSSLVPGYRHVY
LEGLTEASIFVHITINEIYGKWSPLILNPSYTILHFLGATKNRQLQGLKGLFNKNPRHSS
SENNSHYVRKRSIGDRILRRTASAPAKGRKKSKMGFQEMVEIKDSVSEATRDQDGVLRRT
TRSLQARPVSMPVDRNLLGALSLPVSETAKDIEGKENSLAEDKDGRRKGKASIKDPHFLN
FNKKLSSSSSALLHKDTSQGDTIVSTAHMSVTGEQLGMSSPRGGRTTSNATSNCQENPCP
SKSLSPKQHLAPDPVVNPTQDLHGVKIKEKGNPEDFVEGKSILSGSVLSHSNLEIKNLEG
NRGKGRAATSFSLSDVSMLCSDIPDLHSTAILQESVISHLIDNVTLTNENEPGSSISALI
GQFDETNNQALTVVSHLHNTSVMSGHCPLPSLGLKMPIKHGFCKGKSKSSFLCSSPELIA
LSSSETTKHATNTVYETTCTPISKTKPDDDLSSKAKTAALESNLPGSPNTSRGWLPKSPT
KGEDWETLKSCSPASSPDLTLEDVIADPTLCFNSGESSLVEIDGESENLSLTTCEYRREG
TSQLASPLKLKYNQGVVEHFQRGLRNGYCKETLRPSVPEIFNNIQDVKTQSISYLAYQGA
GFVHNHFSDSDAKMFQTCVPQQSSAQDMHVPVPKQLAHLPLPALKLPSPCKSKSLGDLTS
EDIACNFESKYQCISKSFVTTGIRDKKGVTVKTKSLEPIDALTEQLRKLVSFDQEDNCQV
LYSKQDANQLPRALVRKLSSRSQSRVRNIASRAKEKQEANKQKVPNPSNGAGVVLRNKPS
APTPAVNRHSTGSYIAGYLKNTKGGGLEGRGIPEGACTALHYGHVDQFCSDNSVLQTEPS
SDDKPEIYFLLRL
>Q9Z2Y3
MGEQPIFSTRAHVFQIDPNTKKNWVPTSKHAVTVSYFYDSTRNVYRIISLDGSKAIINST
ITPNMTFTKTSQKFGQWADSRANTVYGLGFSSEHHLSKFAEKFQEFKEAARLAKEKSQEK
MELTSTPSQESAGGDLQSPLTPESINGTDDERTPDVTQNSEPRAEPTQNALPFPHSAGDR
TQALSHASSAISKHWEAELATLKGNNAKLTAALLESTANVKQWKQQLAAYQEEAERLHKR
VTELECVSSQANAVHSHKTELNQTVQELEETLKVKEEEIERLKQEIDNARELQEQRDSLT
QKLQEVEIRNKDLEGQLSDLEQRLEKSQNEQEAFRSNLKTLLEILDGKIFELTELRDNLA
KLLECS
>Q9H7D7
MQANGAGGGGGGGGGGGGGGGGGGQGQTPELACLSAQNGESSPSSSSSAGDLAHANGLL
PSAPSAASNNSNSLNVNNGVPGGAAAASSATVAAASATTAASSSLATPELGSSLKKKKRL
SQSDEDVIRLIGQHLNGLGLNQTVDLLMQESGCRLEHPSATKFRNHVMEGDWDKAENDLN
ELKPLVHSPHAIVVRGALEISQTLLGIIVRMKFLLLQQKYLEYLEDGKVLEALQVLRCEL
TPLKYNTERIHVLSGYLMCSHAEDLRAKAEWEGKGTASRSKLLDKLQTYLPPSVMLPPRR
LQTLLRQAVELQRDRCLYHNTKLDNNLDSVSLLIDHVCSRRQFPCYTQQILTEHCNEVWF
CKFSNDGTKLATGSKDTTVIIWQVDPDTHLLKLLKTLEGHAYGVSYIAWSPDDNYLVACG
PDDCSELWLWNVQTGELRTKMSQSHEDSLTSVAWNPDGKRFVTGGQRGQFYQCDLDGNLL
DSWEGVRVQCLWCLSDGKTVLASDTHQRIRGYNFEDLTDRNIVQEDHPIMSFTISKNGRL
ALLNVATQGVHLWDLQDRVLVRKYQGVTQGFYTIHSCFGGHNEDFIASGSEDHKVYIWHK
RSELPIAELTGHTRTVNCVSWNPQIPSMMASASDDGTVRIWGPAPFIDHQNIEEECSSMD
S
>O94392
MTQKNTGPRRRTAVACDRCRRRKIRCTGSDIPGQPCLACQKAHADCHFSTTSWRACRKKS

APLHCPSTNGKTNVLPSYASTPSLSPMTSSHFPYASDGTLISATNHCDDCSYNPHYLPVN
SNGSSPSHTSSLDSIPGYASGSGPFKTPYRPVAVSESSSRSSFSMGSSEFASSTWSQSPM
QSSLYVSPSSQPLDRFSPASFPSKETLTSSSLSSSVPRSVSLSNFADSNTSNYPESSLLP
AAKVGNNGFVGSNVLGSMSYSNVEDSVRFQTDSVSQMSSKSLEHLPMLSEMTLSSSASFG
ASVSPKSTPGSNSTGAAVDTNSVHSNGGSDLDYSSYLTDSSAVDSPSLDADEVLRSFNLA
NDPIGAVDPLTLEIDQGSGSQLLGQQSFANDSLNAKQSSGKLGPLPPQSFNSSDYISFDE
PSRYLSDDASLWPDQYVDKVQPLATMKTCNPAVFSSNTSLDDMFFFIRDFDEDHPIQTAY

>Q96FF9
MSGRRTRSGGAAQRSGPRAPSPTKPLRRSQRKSGSELPSILPEIWPKTPSAAAVRKPIVL
KRIVAHAVEVPAVQSPRRSPRISFFLEKENEPPGRELTKEDLFKTHSVPATPTSTPVPNP
EAESSSKEGELDARDLEMSKKVRRSYSRLETLGSASTSTPGRRSCFGFEGLLGAEDLSGV
SPVVCSKLTEVPRVCAKPWAPDMTLPGISPPPEKQKRKKKMPEILKTELDEWAAAMNAE
FEAAEQFDLLVE
>P26784
MSVEPVVVIDGKGHLVGRLASVVAKQLLNGQKIVVVRAEELNISGEFFRNKLKYHDFLRK
ATAFNKTRGPFHFRAPSRIFYKALRGMVSHKTARGKAALERLKVFEGIPPPYDKKKRVVV
PQALRVLRLKPGRKYTTLGKLSTSVGWKYEDVVAKLEAKRKVSSAEYYAKKRAFTKKVAS
ANATAAESDVAKQLAALGY
>Q673G8
MLGRKIPGSGVLGAGAGMDRGRTGERLHAALAGLQELHFLRDKQSAMVHWALTLNRDQPD
PSKQENVSKEELRLEATLSLLKQQLTRLRRQDVGLKTHLQQLDQQITELKLDVCKASTEH
LESDSRPSSGFYELSDGGSGSLSNSCTSVYSESLSSSSQTSLLPLLSTSYASHGRSSCGQ
TGVSRRCSADESTAQSDAPRSGVKLGSSLIRTATARADRARQRPVSTGDLDRMIGPGFGA
FKSTDVKSSTPCSSPQNPSVDPKYQSNLVSSNGTEVYRYPSPLHAVALQSPIFSCTSDQG
SSVALDEMPEEETQNLNEESTISSSVGYINKLLQRSSSRVNLLSSIKRIETVSGTHEQIP
RSQEMIYGSLNGPQLLGSLQQLSMPLENALETGKTSALNNNQKQMDPPCLGSNFKEVEVQ
MLHHGKHPESSLDLQKNNFPINNTAGKVGAESDNGASEKRSGHFPKDLSVVQKPVERKSS
FTSGREGSRASCHDKSSIPQSEFVHAQFVPAGSQRVKVRQADKKTKSVKLRKKSSEKPSA
KKQHQKPLSREFCTKNRTDLKQSGSCRGKVTYLEESQAQSCSDCSCNGLINSHCIQNNHQ
QIPSSKSTKSRKAPEPVYHPLDHAKKKPSSRKWPSSSEIPLPPTLHTQRSKEMLNSRKVA
MVRSVSARPRSGHWGCPPPRALPHSLSTSSYFSYLESRYPAAPVSSRHPPRCESEFSEYS
AECASLFHSTIAASSDGEMSDYTTNRFGDSESSQGSQTASESDSSLSLDEEDLLEEEEED
EGGLVWAQAAMGTTAAGFSLQQHHRSESAACRIKASRALKKKIRRFQPASLKVMTLV
>Q12168
MCYSRQAIPPPVPNRPGGTTNRGPPPLPPRANVQPPVCSSENSSKPRENRVAGESLRTPS
SSNPLADSQVNSDNIFQSPVLSNLKAPPSVFNKVQHPVPKPNIDDQSVDPLETNKFYTNM
LLDDNTQPIWTHPYSIWFSRDPELFGLAANHTLASQRVFDTTTNPPRFYFNPTNIKSFVF
KAREFVSSNDIKLEFRDMKHMSMCLLMSLSSSQFIEFPLVQGMGFVTAIYHDLGFELRSA
VGFRSLERISVNERYGKYNIQLENNRNWILYLTSPDYSFPQDFQISLLDSNTIISSHKIN
GLICQLSADSVPSIDMAAGCYPVYCDLSGQTVDEHFTNYRFNYTVAGYSQSGTTLMYALP
HHKAAFTPEMQEREIASSLDSTVKGLMTGYLTNSFDMQVQVPQELGFEPVALSLNKKADY
SQEKLSKIREAAVQEVQLSDPQQESNIDSMYFSGKILAKYAWILYVTHYILHDENLTKEL
LSKLTIAMERFISNQQVLPLNYDVSWKGIISSGSSSQDFGNSYYNDHHFHYSYHVITAAI

ISLVDSDLSGVTNNSWLENNRDWVECLIRDYSGVDNDDPYFPQFRSFDWFNGHSWAKGLF
PSGDGKDEESTSEDVNSCYAIKLWGLVTGNSKLTDIANLQLGIMRNVFQSYFLYESNNTV
QPKEFIGNKVSGILFENKIDHATYFGMEPQYIHMIHAIPITSASSWVRTPNFVKEEWEEK
MQPIIDQVNDGWKGIIMLNMALLDPKFSYDFFSQPDFNRNFLDNGQSLTWSLAYSGAFS
>P46783
MLMPKKNRIAIYELLFKEGVMVAKKDVHMPKHPELADKNVPNLHVMKAMQSLKSRGYVKE
QFAWRHFYWYLTNEGIQYLRDYLHLPPEIVPATLRRSRPETGRPRPKGLEGERPARLTRG
EADRDTYRRSAVPPGADKKAEAGAGSATEFQFRGGFGRGRGQPPQ
>Q920N1
MAEEVWMGTWRPHRPRGPIMALYSSPGPKYLIPPTTGFVKHTPTKLRAPAYSFRGAPMLL
AENCSPGPRYSVNPKILKTGKDLGPAYSILGRYHTKTLLTPGPGDYFPEKSTKYVFDSAP
SHSISARTKTFRVDSTPGPAAYMLPVVMGPHTVGKVSQPSFSIKGRSKLGSFSDDLHKTP
GPAAYRQTEVQVTKFKAPQYTMAARVEPPGDKTLKPGPGAHSPEKVTLNKPCAPTVTFGI
KHSDYMTPLVVDVE
>P37261
MSPTGNYLNAITNRRTIYNLKPELPQGVGLDDVKRTVHVILKNTPTAFNSQVNRAVIIVG
DTHKRIWDAVASAMPTAEAKKRPESCRDEAYGSVIFFTDEGPTEKLQRDFPALAAAFPTC
AAHTTGAVQIQSWTALELLGLGANLQHYNDYVKSALPQDVPIAWTVQSQLVFGVPTALPE
EKTFINNVINVYH
>P21964
MPEAPPLLLAAVLLGLVLLVVLLLLLRHWGWGLCLIGWNEFILQPIHNLLMGDTKEQRIL
NHVLQHAEPGNAQSVLEAIDTYCEQKEWAMNVGDKKGKIVDAVIQEHQPSVLLELGAYCG
YSAVRMARLLSPGARLITIEINPDCAAITQRMVDFAGVKDKVTLVVGASQDIIPQLKKKY
DVDTLDMVFLDHWKDRYLPDTLLLEECGLLRKGTVLLADNVICPGAPDFLAHVRGSSCFE
CTHYQSFLEYREVVDGLEKAIYKGPGSEAGP
>Q12453
MNFSSIFKSISNFQFPYTIEETAITETALWQCFDGTRKADSLPVTVFKAKRSPENESLIL
NAVHKSKILKIPGLCTVLETFDSDPQSTFIVTERVVPFPWDNLGSLSQNKFGVELGISQL
LATLGFLKNFVLGTLSKDSVFINIKGEWVLFGLELCSSKEGLSAFEFASRARSYYNIIGS
QLPCEDPNTIDSMGLGLLIKSLMAPSCLPKDWIVNVNMISDGKITIENFRKRLENTETWR
SNPLINFYQELRELHIKDPQGKLVVMSNLENLYLESREIFRNLTPGMIENFIIPELCEII
KLLMTQSISSAASPIGMNFNASHKLVPFLAIVLDLTSETNTFPVGFNDLITQSFKLPDRQ
VRFLLLIYLPKLIGPLSKSEISSRIYPHFIQGLTDSDATLRLQTLKTIPCIVSCLTERQL
NNELLRFLAKTQVDSDVEIRTWTVIIISKISTILSTSVGNRSNILATAFTKSLKDPQVKP
RLAALYGLEKSIELFDVNTIANKILTVIAPGLLDKSPIVRGRAKILFEEYLEKLEKEAQL
IQTNDSTADSEDVKDIDFENYGCDEEDMNKEDNLLAAQFLNNLRLNSPSATTPSNITESE
IDSAQDGSGWDDLSDTDGFITNGTTESFDETTNPVTTASTPKLFGKPIKINKSWNDELND
DGWIQDESGPSKVPQKHTRPQNSTLAKSIAPSSRLSIKKKKTTILAPRNIASNSTVTTKS
SLSNKTARSKPISSIRGSVTKKGNVDGWDDDGDSDSWDTNW
>P38115
MSSSVASTENIVENMLHPKTTEIYFSLNNGVRIPALGLGTANPHEKLAETKQAVKAAIKA
GYRHIDTAWAYETEPFVGEAIKELLEDGSIKREDLFITTKVWPVLWDEVDRSLNESLKAL
GLEYVDLLLQHWPLCFEKIKDPKGISGLVKTPVDDSGKTMYAADGDYLETYKQLEKIYLD
PNDHRVRAIGVSNFSIEYLERLIKECRVKPTVNQVETHPHLPQMELRKFCFMHDILLTAY

SPLGSHGAPNLKIPLVKKLAEKYNVTGNDLLISYHIRQGTIVIPRSLNPVRISSSIEFAS
LTKDELQELNDFGEKYPVRFIDEPFAAILPEFTGNGPNLDNLKY
>P25604
MSANGKISVPEAVVNWLFKVIQPIYNDGRTTFHDSLALLDNFHSLRPRTRVFTHSDGTPQ
LLLSIYGTISTGEDGSSPHSIPVIMWVPSMYPVKPPFISINLENFDMNTISSSLPIQEYI
DSNGWIALPILHCWDPAAMNLIMVVQELMSLLHEPPQDQAPSLPPKPNTQLQQEQNTPPL
PPKPKSPHLKPPLPPPPPPQPASNALDLMDMDNTDISPTNHHEMLQNLQTVVNELYREDV
DYVADKILTRQTVMQESIARFHEIIAIDKNHLRAVEQAIEQTMHSLNAQIDVLTANRAKV
QQFSSTSHVDDEDVNSIAVAKTDGLNQLYNLVAQDYALTDTIECLSRMLHRGTIPLDTFV
KQGRELARQQFLVRWHIQRITSPLS
>Q641K1
MSKLKVVGEKSLTNSSRVVGLLAQLEKINTDSTESDTARYVTSKILHLAQSQEKTRREMT
TKGSTGMEVLLSTLENTKDLQTVLNILSILIELVSSGGGRRASFLVAKGGSQILLQLLMN
ASKDSPPHEEVMVQTHSILAKIGPKDKKFGVKARVNGALTVTLNLVKQHFQNYRLVLPCL
QLLRVYSTNSVNSVSLGKNGVVELMFKIIGPFSKKNSGLMKVALDTLAALLKSKTNARRA
VDRGYVQVLLTIYVDWHRHDNRHRNMLIRKGILQSLKSVTNIKLGRKAFIDANGMKILYN
TSQECLAVRTLDPLVNTSSLIMRKCFPKNRLPLPTIKSSFHFQLPIIPVTGPVAQLYSLP
PEVDDVVDESDDNDDIDLEVENELENEDDLDQSFKNDDIETDINKLRPQQVPGRTIEELK
MYEHLFPELVDDFQDYELISKEPKPFVFEGKARGPIVVPTAGEEVPGNSGSVKKGVVMKE
RASPKGEEAKEDPKGHDRTLPQQLGGQSRVAPSAHSFNNDLVKALDRITLQNVPSQVASG
LNAGMRKDFGLPLTVLSCTKACPHVAKCGSTLFEGRTVHLGKLCCTGVETEDDEDTESHS
STEQAPSVEASDGPTLHDPDLYIEIVKNTKSVPEYSEVAYPDYFGHIPPPFKEPILERPY
GVQRTKIAQDIERLIHQNDIIDRVVYDLDNPTYTTPEEGDTLKFNSKFESGNLRKVIQIR
KSEYDLILNSDINSNHYHQWFYFEVSGMRPGVAYRFNIINCEKSNSQFNYGMQPLMYSVQ
EALNARPWWIRMGTDICYYKNHFSRSSVAAGGQKGKSYYTITFTVNFPHKDDVCYFAYHY
PYTYSTLQMHLQKLESAHNPQQIYFRKDVLCETLSGNICPLVTITAMPESNYYEHICQFR
TRPYIFLSARVHPGETNASWVMKGTLEYLMSNSPTAQSLRESYIFKIVPMLNPDGVINGN
HRCSLSGEDLNRQWQSPNPELHPTIYHAKGLLQYLAAVKRLPLVYCDYHGHSRKKNVFMY
GCSIKETVWHTHDNSASCDIVEDMGYRTLPKILSHIAPAFCMSSCSFVVEKSKESTARVV
VWREIGVQRSYTMESTLCGCDQGRYKGLQIGTRELEEMGAKFCVGLLRLKRLTSSLEYNL
PSNLLDFENDLIESSCKVTSPTTYVLDEDEPRFLEEVDYSAESNDELDVELAENTGDYEP
SAQEEALSDSEVSRTHLI
>Q86XR7
MGIGKSKINSCPLSLSWGKRHSVDTSPGYHESDSKKSEDLSLCNVAEHSNTTEGPTGKQE
GAQSVEEMFEEEAEEEVFLKFVILHAEDDTDEALRVQNLLQDDFGIKPGIIFAEMPCGRQ
HLQNLDDAVNGSAWTILLLTENFLRDTWCNFQFYTSLMNSVNRQHKYNSVIPMRPLNNPL
PRERTPFALQTINALEEESRGFPTQVERIFQESVYKTQQTIWKETRNMVQRQFIA
>Q9CQ20
MMQICDTYNQKHSLFNAMNRFIGAVNNMDQTVMVPSLLRDVPLSEPEIDEVSVEVGGSGG
CLEERTTPAPSPGSANESFFAPSRDMYSHYVLLKSIRNDIEWGVLHQPSSPPAGSEESTW
KPKDILVGLSHLESADAGEEDLEQQFHYHLRGLHTVLSKLTRKANILTNRYKQEIGFSNW
GH
>O74854
MKPSEAREHAIRKIKEYLERRHKQQFKALFGCLTINIYLKIFTLISTPDLCNCRLVCRKF

QQLCDYNSIYVKKLLLMNAWDVTLSRKLYYESQDGMSQANMSSNTSKGFHLQSSDKKYAD
SDRPKLSSSSLLSLPDQKIPFPVDYSTVQGKQVVLTVLDCVSQRVEFARLDYIKVWRALA
PIYRNLAYASNTIDPIAFSAFRTPEEQSKVLKYLIRFGNSYPYGTYRQAMQNAILDMASL
FEQACLDEFELGLHSRNLDLLRKFSHVLHDFSGPNAYVSMYLAKQTDFVRSFFHFDPYSL
FISNNLEEIHINWNILESVVNDTIKLLESESKFSEATLPEPELVQVPYAKDILGNSLKDY
VISICEHIGEEETELFLVFISGFYGLCKKFFSIPNGPALVDTIFQPQIDIFISQELHYFK
TVGWSLVDQWDQKLEEKEDATECFFYKNVSQNTAKNNFLETFKNVMLLPVSLFTIPSENN
SASNLAEKAIEQKEEEDPELSKLDAARFVPANIYVSKDRLKHLPTTELAAQAAVLDSKLE
GISTMFSLELALKIVHLCKVSLARAKVFMGTSVPQDDDIKGLSKDLFVQLLRELGQGHLK
HGFDRAIEHLSSFDPRRDFSSNTVEPVVKFLELINVGDMIQQMMDSFFNEEMSPICVKDD
MFDPAIAEKKKFEQLLDERAAFGLHKGINVLIEHADFLLETKTPMNLFSDQTIGSITNTI
EPTAAAKNVVQFLGFHMRILVGRADHEILDVFYKEVGMRLFDSLTRYIKSHKFSVDGGLK
LLSDCNLYYEFIHSLHQSSLLPYFKTLKEIAHLFIIDGKNAEEIGKLATDTSRFSSAFHT
EEVYEILHSRIDWLNIKYEVDKVIHGLACCIM
>O14329
MTQNHNIPTAIQIQNPINNNVSVTISDQLPKPSANNPNLLSVDTRPTHRKGHHHKHSLSH
QYFLPPKNRQPLEIPASYPIPTFKETFAILTFPQKLKLTSSILFFLVAVGVLLSGDATIL
LTLSCSLIVEGVLIIINVWRETLDSFLVWRHTCLRYPFGMQQMELLVDFSFSILLIFLGM
NLLKEPAEHAIEDWGNLHHAGDHEEETVHIHLTISLFASAIISGFALLLDHPSAHIRELN
SRFFHGLTLVPSLILVLLLSLGYQVGSFLSHLLSLTIAVTALVNGFSIAKSLALMLLLTY
SNKEKVFECVSLIKEDTRIDQLNYAAIWQPHYNTCIANIGLTVSGGEREQAAVREDIIRI
IQKTVGSIFGAGVQPKWEISVDIQRA
>Q8VIJ5
MVQKESQAALEERESERNANPASVSGASLEPSAAPAPGEDNPSGAGAAAGTGAAGGARRF
LCGVVEGFYGRPWVMEQRKELFRRLQKWELNTYLYAPKDDYKHRMFWREMYSVEEAEQLM
TLISAAREYEIEFIYAISPGLDITFSNPKEVSTLKRKLDQVSQFGCRSFALLFDDIDHNM
CAADKEVFSSFAHAQVSITNEIYQYLGEPETFLFCPTEYCGTFCYPSVSQSPYLRTVGEK
LLPGIEVLWTGPKVVSKEIPVESIEEVSKIIKRAPVIWDNIHANDYDQKRLFLGPYKGRS
TELIPRLKGVLTNPNCEFEANYVAIHTLATWYKSNMNGVRKDVVMTDSEDSTVSIQIKLE
NEGSDEDIETDVLYSPQMALKLALTEWLQEFGVPHQYSSRQVAHSGAKTSVVDGTPLVAA
PSLNATTVVTTVYQEPIMSQGAALSGEPSALTKEEEKKQPDEEPMDMVVEKQEESEHKSD
NQILTEIVEAKMAEELKPMDTDKESIAESKSPEMSMQEDCINDIAPMQTDEQANKEQFVP
GPNEKPLYAAEPVTLEDLQLLADLFYLPYEHGPKGAQMLREFQWLRANSSVVSVNCKGKD
SEKIEEWRSRAAKFEEMCALVMGMFTRLSNCANRTILYDMYSYVWDIKSIMSMVKSFVQW
LGCRSHSSAQFLIGDQEPWAFRGGLAGEFQRLLPIDGANDLFFQPPPLTPTSKVYTIRPY
FPKDEASVYKICREMYDDGVGLPFQSQPDLIGDKLVGGLLSLSLDYCFVLEDEDGICGYA
LGTVDVTPFIKKCKISWIPFMQEKYTKPNGDKELSEAEKIMLSFHEEQEVLPETFLANFP
SLIKMDIHKKVTDPSVAKSMMACLLSSLKANGSRGAFCEVRPDDKRILEFYSKLGCFEIA
KMEGFPKDVVILGRSL
>O43602
MDSFTTVSHVGMCEETDASFNVFSPKFQFDRSHCQSLRFHQNMELDFGHFDERDKTSRNM
RGSRMNGLPSPTHSAHCSFYRTRTLQALSNEKKAKKVRFYRNGDRYFKGIVYAVSSDRFR
SFDALLADLTRSLSDNINLPQGVRYIYTIDGSRKIGSMDELEEGESYVCSSDNFFKKVEY
TKNVNPNWSVNVKTSANMKAPQSLASSNSAQARENKDFVRPKLVTIIRSGVKPRKAVRVL

LNKKTAHSFEQVLTDITEAIKLETGVVKKLYTLDGKQVTCLHDFFGDDDVFIACGPEKFR
YAQDDFSLDENECRVMKGNPSATAGPKASPTPQKTSAKSPGPMRRSKSPADSANGTSSSQ
LSTPKSKQSPISTPTSPGSLRKHKDLYLPLSLDDSDSLGDSM
>Q9UUI2
MSSRRNDYHYDGNDHQYRSPLKSNVDQNSFYESSYRSRQSYHQRTKTPRSSYDSPSSSTN
SKEHNSPYHYRVPSNNSTRASFGAASTDTNVELPKINLPDSSLSSKLQSCKSACENSSQS
LLNVEQQYAQQVHFWEKIRTDIYREGLRSDAAVKSLNDFVNNVSF
>P40316
MMPANEDKENNIVYTGNESSGINFPQTPAHLLKRSHSNILKPPVRLDQLKRDANSNNGNT
LKYIQGGKEVSPTKRLHTHAQQQGRLPLAAKDNNRSKSFIFPETSNQSKDADLPQLQNTL
SIRKNDQLRKLSQISRSRSRANHNDLLSNSRKLQKYGSVLGYNALPKMKSLVLKDLADSG
KNEESSDDDEGNEDSESKLGKKLQSALLKQDSSDGENELNGGLGLFNEQGGLQQLIKNST
KNEQKTKNDKSDKTDDYDIEIAPQRQEPLPYVPEGYSPFQQDDIEKLKTFNSPYKLDLED
EDDTPDKVDLLPLEQIDEEGEKDETECITRNQEEGAALPLLSKNFKEVAAVPTMELVYSE
EGLDPEELEDLVT
>Q9HD40
MNRESFAAGERLVSPAYVRQGCEARRSHEHLIRLLLEKGKCPENGWDESTLELFLHELAI
MDSNNFLGNCGVGEREGRVASALVARRHYRFIHGIGRSGDISAVQPKAAGSSLLNKITNS
LVLDIIKLAGVHTVANCFVVPMATGMSLTLCFLTLRHKRPKAKYIIWPRIDQKSCFKSMI
TAGFEPVVIENVLEGDELRTDLKAVEAKVQELGPDCILCIHSTTSCFAPRVPDRLEELAV
ICANYDIPHIVNNAYGVQSSKCMHLIQQGARVGRIDAFVQSLDKNFMVPVGGAIIAGFND
SFIQEISKMYPGRASASPSLDVLITLLSLGSNGYKKLLKERKEMFSYLSNQIKKLSEAYN
ERLLHTPHNPISLAMTLKTLDEHRDKAVTQLGSMLFTRQVSGARVVPLGSMQTVSGYTFR
GFMSHTNNYPCAYLNAASAIGMKMQDVDLFIKRLDRCLKAVRKERSKESDDNYDKTEDVD
IEEMALKLDNVLLDTYQDASS
>P40763
MAQWNQLQQLDTRYLEQLHQLYSDSFPMELRQFLAPWIESQDWAYAASKESHATLVFHNL
LGEIDQQYSRFLQESNVLYQHNLRRIKQFLQSRYLEKPMEIARIVARCLWEESRLLQTAA
TAAQQGGQANHPTAAVVTEKQQMLEQHLQDVRKRVQDLEQKMKVVENLQDDFDFNYKTLK
SQGDMQDLNGNNQSVTRQKMQQLEQMLTALDQMRRSIVSELAGLLSAMEYVQKTLTDEEL
ADWKRRQQIACIGGPPNICLDRLENWITSLAESQLQTRQQIKKLEELQQKVSYKGDPIVQ
HRPMLEERIVELFRNLMKSAFVVERQPCMPMHPDRPLVIKTGVQFTTKVRLLVKFPELNY
QLKIKVCIDKDSGDVAALRGSRKFNILGTNTKVMNMEESNNGSLSAEFKHLTLREQRCGN
GGRANCDASLIVTEELHLITFETEVYHQGLKIDLETHSLPVVVISNICQMPNAWASILWY
NMLTNNPKNVNFFTKPPIGTWDQVAEVLSWQFSSTTKRGLSIEQLTTLAEKLLGPGVNYS
GCQITWAKFCKENMAGKGFSFWVWLDNIIDLVKKYILALWNEGYIMGFISKERERAILST
KPPGTFLLRFSESSKEGGVTFTWVEKDISGKTQIQSVEPYTKQQLNNMSFAEIIMGYKIM
DATNILVSPLVYLYPDIPKEEAFGKYCRPESQEHPEADPGSAAPYLKTKFICVTPTTCSN
TIDLPMSPRTLDSLMQFGNNGEGAEPSAGGQFESLTFDMELTSECATSPM
>P05735
MANLRTQKRLAASVVGVGKRKVWLDPNETSEIAQANSRNAIRKLVKNGTIVKKAVTVHSK
SRTRAHAQSKREGRHSGYGKRKGTREARLPSQVVWIRRLRVLRRLLAKYRDAGKIDKHLY
HVLYKESKGNAFKHKRALVEHIIQAKADAQREKALNEEAEARRLKNRAARDRRAQRVAEK
RDALLKEDA

>Q920Q8
MIPNGYLMFEDENFIESSVAKLNALRKSGQFCDVRLQVCGHEMLAHRAVLACCSPYLFEI
FNSDSDPHGVSHVKLDDLNPEAVEVLLNYAYTAQLKADKELVKDVYSAAKKLKMDRVKQV
CGDYLLSRMDVTSCISYRNFASCMGDSRLLNKVDAYIQEHLLQISEEEEFLKLPRLKLEV
MLEDNVCLPSNGKLYTKVINWVQRSIWENGDSLEELMEEVQTLYYSADHKLLDGNPLDGQ
AEVFGSDDDHIQFVQKKPPRENGHKQISGSSTGCLSSPNASMQSPKHEWKIVASEKTSNN
TYLCLAVLDSTFCVIFLHGRNSPQSSPTSTPKLSKSLSFEMQPDELLEKPMSPMQYARSG
LGTAEMNGKLIAAGGYNREECLRTVECYDPHTDHWSFLAPMRTPRARFQMAVLMGQLYVV
GGSNGHSDDLSCGEMYDPNIDDWTPVPELRTNRCNAGVCALNGKLYIVGGSDPYGQKGLK
NCDVFDPVTKSWTSCAPLNIRRHQSAVCELGGYLYIIGGAESWNCLNTVERYNPENNTWT
LIAPMNVARRGAGVAVLDGKLFVGGGFDGSHAISCVEMYDPTRNEWKMMGNMTSPRSNAG
ITTVGNTIYAVGGFDGNEFLNTVEVYNPQSNEWSPYTKIFQF
>O73622
MALKAEDSFDIYSQLIQSFCRYAENTTVSESQSPAFNAQKEFQTCQDHACCTHSEAHTHI
LMQQWQLLEEQWEYIDHLKTDVAALKQLLHGFMNSLSGTDSGMEGTNHFLPPHQNPTLLK
DEEIVASALNRPSVDINGFEENITTGAQIHAAFTKSPKKMPATSTPRKSEVLWSCSPTLA
TDGYFMLPDIILNPLDGKKLVSMLRSSNYEPHRFAELLFQHHVPHSLFQLWANKVNFDGS
RGKLGLPRNLMIDILHQTSKRFVLGPKEKRKIKTRLNLLLRTRQDRAWWDVGL
>Q8NFZ5
MSRDPGSGGWEEAPRAAAALCTLYHEAGQRLRRLQDQLAARDALIARLRARLAALEGDAA
PSLVDALLEQVARFREQLRRQEGGAAEAQMRQEIERLTERLEEKEREMQQLLSQPQHERE
KEVVLLRRSMAEGERARAASDVLCRSLANETHQLRRTLTATAHMCQHLAKCLDERQHAQR
NVGERSPDQSEHTDGHTSVQSVIEKLQEENRLLKQKVTHVEDLNAKWQRYNASRDEYVRG
LHAQLRGLQIPHEPELMRKEISRLNRQLEEKINDCAEVKQELAASRTARDAALERVQMLE
QQILAYKDDFMSERADRERAQSRIQELEEKVASLLHQVSWRQDSREPDAGRIHAGSKTAK
YLAADALELMVPGGWRPGTGSQQPEPPAEGGHPGAAQRGQGDLQCPHCLQCFSDEQGEEL
LRHVAECCQ
>Q8IUH3
MDEAGSSASGGGFRPGVDSLDEPPNSRIFLVISKYTPESVLRERFSPFGDIQDIWVVRDK
HTKESKGIAFVKFARSSQACRAMEEMHGQCLGPNDTKPIKVFIAQSRSSGSHRDVEDEEL
TRIFVMIPKSYTEEDLREKFKVYGDIEYCSIIKNKVTGESKGLGYVRYLKPSQAAQAIEN
CDRSFRAILAEPKNKASESSEQDYYSNMRQEALGHEPRVNMFPFVGEQQSEFSSFDKNDS
RGQEAISKRLSVVSRVPFTEEQLFSIFDIVPGLEYCEVQRDPYSNYGHGVVQYFNVASAI
YAKYKLHGFQYPPGNRIGVSFIDDGSNATDLLRKMATQMVAAQLASMVWNNPSQQQFMQF
GGSSGSQLPQIQTDVVLPSCKKKAPAETPVKERLFIVFNPHPLPLDVLEDIFCRFGNLIE
VYLVSGKNVGYAKYADRISANDAIATLHGKILNGVRLKVMLADSPREESNKRQRTY
>Q09722
MEGSDFILVVDSSVEEQVEELAMYLDNLEANTDKNVLALCREYLASENVKEVLNLFLTRL
PLLAQAPEKELEPILAVFINLIQESAAFEDHVSKFCQALEQIADQNNNLTPAILSVLSIL
FNTAVKERQHARLSILTSVVTLTTRYSLFSTLAPNLKYFPDWLKEAGVSVSDHRAFNIFV
SKAIQSYDDEQSFAFLLEAVKMDNSTADEAVRELVQRAVNSPKYFFFDDIVTLPPVQQLE
QSTLQLLGILSGGMTDDYVSWVAENHAHCQHQKFDEDAIARKMKLLTIASLATQAPNNTL
SYGDVAKSLKIDENEVELWIIDVIRAGLVEGRMSQLTKTLSIHRSSYRVFGKHEWVALHE
KLAKWGSSLRYMLQVMEQPLSSFTIASSKKGNRDGSAVTASE

>Q12018
MSETLPRSDDLEATWNFIEPGINQILGNEKNQASTSKRVYKILSPTMYMEVYTAIYNYCV
NKSRSSGHFSTDSRTGQSTILVGSEIYEKLKNYLKNYILNFKQSNSETFLQFYVKRWKRF
TIGAIFLNHAFDYMNRYWVQKERSDGKRHIFDVNTLCLMTWKEVMFDPSKDVLINELLDQ
VTLGREGQIIQRSNISTAIKSLVALGIDPQDLKKLNLNVYIQVFEKPFLKKTQEYYTQYT
NDYLEKHSVTEYIFEAHEIIKREEKAMTIYWDDHTKKPLSMALNKVLITDHIEKLENEFV
VLLDARDIEKITSLYALIRRDFTLIPRMASVFENYVKKTGENEISSLLAMHKHNIMKNEN
ANPKKLALMTAHSLSPKDYIKKLLEVHDIFSKIFNESFPDDIPLAKALDNACGAFININE
FALPAGSPKSATSKTSEMLAKYSDILLKKATKPEVASDMSDEDIITIFKYLTDKDAFETH
YRRLFAKRLIHGTSTSAEDEENIIQRLQAANSMEYTGKITKMFQDIRLSKILEDDFAVAL
KNEPDYSKAKYPDLQPFVLAENMWPFSYQEVEFKLPKELVPSHEKLKESYSQKHNGRILK
WLWPLCRGELKADIGKPGRMPFNFTVTLFQMAILLLYNDADVLTLENIQEGTSLTIQHIA
AAMVPFIKFKLIQQVPPGLDALVKPETQFKLSRPYKALKTNINFASGVKNDILQSLSGGG
HDNHGNKLGNKRLTEDERIEKELNTERQIFLEACIVRIMKAKRNLPHTTLVNECIAQSHQ
RFNAKVSMVKRAIDSLIQKGYLQRGDDGESYAYLA
>P22531
MSYQQQQCKQPCQPPPVCPTPKCPEPCPPPKCPEPCPPPKCPQPCPPQQCQQKCPPVTPS
PPCQPKCPPKSK
>P08536
MPAPHGGILQDLIARDALKKNELLSEAQSSDILVWNLTPRQLCDIELILNGGFSPLTGFL
NENDYSSVVTDSRLADGTLWTIPITLDVDEAFANQIKPDTRIALFQDDEIPIAILTVQDV
YKPNKTIEAEKVFRGDPEHPAISYLFNVAGDYYVGGSLEAIQLPQHYDYPGLRKTPAQLR
LEFQSRQWDRVVAFQTRNPMHRAHRELTVRAAREANAKVLIHPVVGLTKPGDIDHHTRVR
VYQEIIKRYPNGIAFLSLLPLAMRMSGDREAVWHAIIRKNYGASHFIVGRDHAGPGKNSK
GVDFYGPYDAQELVESYKHELDIEVVPFRMVTYLPDEDRYAPIDQIDTTKTRTLNISGTE
LRRRLRVGGEIPEWFSYPEVVKILRESNPPRPKQGFSIVLGNSLTVSREQLSIALLSTFL
QFGGGRYYKIFEHNNKTELLSLIQDFIGSGSGLIIPNQWEDDKDSVVGKQNVYLLDTSSS
ADIQLESADEPISHIVQKVVLFLEDNGFFVF
>Q60I27
MCNPEEAALLRLEEVFSATLAHVNSLVLQPLLPAAPDPSDPWGRECLRLLQQLHKSSQQL
WEVTEESLHSLQERLRYPDSTGLESLLLLRGADRVLQAHIEYIESYTSCMVVQAFQKAAK
RRSEYWRGQRKALRQLLSGVSSEGSVGASLGQALHQPLAHHVQQYVLLLLSLGDTIGEHH
PTRELVVNAVTLFGNLQSFMKQELDQAVATQALWHTLRGRLRDVLCTPAHRLLQDSQDVP
VTVAPLRAERVLLFDDALVLLQGHNVHTFDLKLVWVDPGQDGCTFHLLTPEEEFSFCAKD
SQGQAVWQWKVTWAVHQALHGKKDFPVLGAGLEPSQPPDCRCAEYTFQAEGRLCQATYEG
EWCRGRPHGKGTLKWPDGRNHVGNFCQGLEHGFGIRLLPQASEDKFDCYKCHWREGSMCG
YGICEYSTDEVYKGYFQEGLRHGFGVLESGPQAPQPFRYTGHWERGQRSGYGIEEDGDRG
ERYIGMWQAGQRHGPGVMVTQAGVCYQGTFQADKTVGPGILLSEDDSLYEGTFTRDLTLM
GKGKVTFPNGFTLEGSFGSGAGRGLHTQGVLDTAALPPDPSSTCKRQLGVGAFPVESRWQ
GVYSPFRDFVCAGCPRDLQEALLGFDVQSSRELRRSQDYLSCERTHPEDSVGSMEDILEE
LLQHREPKALQLYLRKALSNSLHPLGKLLRTLMLTFQATYAGVGANKHLQELAQEEVKQH
AQELWAAYRGLLRVALERKGQALEEDEDTETRDLQVHGLVLPLMLPSFYSELFTLYLLLH
EREDSFYSQGIANLSLFPDTQLLEFLDVQKHLWPLKDLTLTSNQRYSLVRDKCFLSATEC
LQKIMTTVDPREKLEVLERTYGEIEGTVSRVLGREYKLPMDDLLPLLIYVVSRARIQHLG

AEIHLIRDMMDPNHTGGLYDFLLTALESCYEHIQKEDMRLHRLPGHWHSRELW

>P54661

MEEIKETVEKKNKEKKYLNDYKIISEGNNIKVLQKKDFKPKFSLVSYKNYEQNFDFENAK
IVSTNFAIDRHIKMKNPPDPFVFIKMPSSKLVQSQLKLYYENEGYKDIPVWITPCSAFMR
YIKSYPIVGTVTETMEKKKGFTNRFLSTSETKASVSVGFFGCESSMEVTSGYEYEDTVTS
EETRSWSQTLNEGSYIVYQNVLVYAYIIYGGSYKPYIDQMNKFNPGLNIKFFREDKCIFF
VPINRDDAFTLRYQDNTWDPVEYDVLIDYLGQNQDKWFSGGLP

>Q01630

MFPFLAPIRSANHVEQQGTKYYVEITFLADSPYSRAPSSTGTEKSVIPTLRNLDDLETVS
NKSRSSEGVNKLGSRPNSRSSAIVKKTSVSSLPTSARSEGKSSPIPVKDAIPAPARHKNK
GLEMSSAMMELFGGGGSTSPAPSKRENPADAPSDRVVSNAKLSQPGPEWFEGFEQMDMTD
IELPPDPNCPAEKVLRGEKSTPDFDSDWQEAKEDVLDPQSYPKSFNPAESLPGPDIGAGV
DDEEEPEAEAQEMEEPQYESKVDEKDDDDNGSSSSKKGHPSEDGDSTRNGETPTDRRNSG
AIEETADGESSAQSAAEKKNSGDDGNGGGGEMSILLVKNAQIVNDDAIFVADILIEDGII
QNVAPNLEAPEGAEVLDAAGKLALPAGIDVYTQVTDSSVDDLSTGCKSAIAGGTGTIVEV
VRPRGAESVVSAVKRVKNQLEKSGISCHVALSVAITDFCEQEMSELVKNEGINSFVLDGV
SLTDDKLLELFEHVKRLGALIRVVPENKSIVAMLEKKMLKLGVTGPEGFPQSRPESLEAD
RVSGVCVLGNLASCPISIVQVSSADSLAAIEKARASGALAHAEIASAAVTADGSALFSQD
LRFASAHLTDVPLRRGAPDRMIGALSTQPLVVCTSGHRPVNSATRVAAKDFAIAQKGSTG
AEERMAVVWERAVRSGRIDAMRFVAVTSTNAAKMFNMYPKKGRIAVGADADLVIWDASGK
RVLESSRAQSSQENSMYDGLTVHSVVTATIVGGKIAYQNGEVREAPVAGGFLRLSPNSPY
LFSMVGQRDKFANVERVEREASSQQQKPQQNGHHKNSGDFDRNRTKVMESSIDFGGSAAN
RPRNPPGGRTTGFW

>Q12179

MHGPTSKAISRNVRSVKRPRRAPRPVVSTQAMNKLSNVTLSAEQEKLRERVLSFMRSNLS
QYKSDWKHPAMFVIQGDAGTGKSVILNSLFNEIQKLSQFSPSSEDILHGTHNYLVVNHPE
MLKLYIRISDSFKYISKSSLERPTSLINNLQKRKVMADVVIVDEAHLLATSKDAFKRFYG
ENHLKDLMSLCKVLVLVYDDKQALRMGSYWDEGSNNGATLKDFYNEIPPKSRDWYTLKQQ
FRVAAPQNVLNWIDQISVAGKIPPIESVLSKGNADCADDKIKNFDFKIWDDCGAMYEAIK
EKDRQYGQCRMLSTYDFPYRLDGKDYYVECGDNFKVRWDRYTPREVTPWSERCDTIDEVG
SVYTIQGFDLNYAGVILGRSIGYDAANDCIKLRPELYDDRAGFTKKKNIHNAEDVKQKII
MNSINVLLTRGVRGLYVYAYDPELRERLLRPSKK

>Q02642

MPIDQEKLAKLQKLSANNKVGGTRRKLNKKAGSSAGANKDDTKLQSQLAKLHAVTIDNVA
EANFFKDDGKVMHFNKVGVQVAAQHNTSVFYGLPQEKNLQDLFPGIISQLGPEAIQALSQ
LAAQMEKHEAKAPADAEKKDEAIPELVEGQTFDADVE

>Q7TPV4

MAEMKSPTKAEPATPAEAAQSDRHSLLEHSREFLDFFWDIAKPDQETRLRATEKLLEYLR
TRPNDSEMKYALKRLITGLGVGREAARPCYSLALAQLLQSFEDIPLCDILDQIQEKYSLQ
AMNKAMMRPSLFANLFGVLALFQSGRLVKDKEALMKSVQLLKILSQHPNHLQGQPIKALV
DILSEVPESMFQEILPKVLKGNMKVILRSPKYLELFLLAKQRVPTKLESLMGSVDLFSED
NIPSLVNILKVAANSVKKEHKLPNVALDLLRLALKESRFELFWKKVLEEGLLKNPSWTSS
YMCFRLLGASLPLLSEEQLQLVMRGDLIRHFGENMVISKPQNLFKIIPEISTYVGTFLEG
CQDDPKRQLTMMVAFTTITNQGLPVMPTFWRVTRFLNAEALQSYVAWLRDMFLQPDLNSL

VDFSTANQKRAQDASLNVPERAVFRLRKWIIHRLVSLVDHLHLEKDEAVVEQIARFCLFH
AFFKTKKATPQIPETKQHFSFPLDDRNRGVFVSAFFSLLQTLSVKFRQTPDLAENGKPWT
YRLVQLADMLLNHNRNVTSVTSLTTQQRQAWDQMMSTLKELEARSSETRAIAFQHLLLLV
GLHIFKSPAESCDVLGDIQTCIKKSMEQNPRRSRSRAKASQEPVWVEVMVEILLSLLAQP
SNLMRQVVRSVFGHICPHLTPRCLQLILAVLSPVTNEDEDDNVVVTDDADEKQLQHGEDE
DSDNEDNKNSESDMDSEDGEESEEEDRDKDVDPGFRQQLMEVLKAGNALGGVDNEEEEEL
GDEAMMALDQNLASLFKEQKMRIQARNEEKNKLQKEKKLRRDFQIRALDLIEVLVTKQPE
HPLILELLEPLLNVIQHSMRSKGSTKQEQDLLHKTARIFMHHLCRARRYCHEVGPCAEAL
HAQVERLVQQAGSQADASVALYYFNASLYLLRVLKGNTNKRHQDGHKLHGADTEDSEDQA
ANCLDLDFVTRVYSASLESLLTKRNSSLTVPMFLSLFSRYPVICKNLLPVLAQHVAGPSR
PRHQAQACLMLQKTLSARELRVCFEDPEWEQLITQLLGKATQTLQTLGEAQSKGEHQKEL
SILELLNTLLRTVNHEKLSVDLTAPLGVLQSKQQKLQQSLQQGNHSSGSNRLYDLYWQAM
RMLGVQRPKSEKKNAKDIPSDTQSPVSTKRKKKGFLPETKKRKKLKSEGTTPEKNAASQQ
DAVTEGAMPAATGKDQPPSTGKKKRKRVKASTPSQVNGITGAKSPAPSNPTLSPSTPAKT
PKLQKKKEKLSQVNGATPVSPIEPESKKHHQEALSTKEVIRKSPHPQSALPKKRARLSLV
SRSPSLLQSGVKKRRVASRRVQTP
>Q6Q6R3
MSWTCPRCQQPVYFAEKVSSLGKNWHRFCLKCERCHSILSPGGHAEHNGRPYCHKPCYGA
LFGPRGVNIGGVGCYLYNLPTPPPASRISLSPSNFSPPRPRTGLSRAKKRPPYLKTFTGE
TSLCPGCGDPVFFAEKVMSLGKIGTTLPEVPTCRKTLTAGSHAEHDGMPYCHIPCYGYLF
GPKGGQSDPRHWTYENVWTTSLVVRHLDPSPIPSDLHPLANKALGDRALSFRCEHWRCGL
LHL
>P38720
MSADFGLIGLAVMGQNLILNAADHGFTVCAYNRTQSKVDHFLANEAKGKSIIGATSIEDF
ISKLKRPRKVMLLVKAGAPVDALINQIVPLLEKGDIIIDGGNSHFPDSNRRYEELKKKGI
LFVGSGVSGGEEGARYGPSLMPGGSEEAWPHIKNIFQSISAKSDGEPCCEWVGPAGAGHY
VKMVHNGIEYGDMQLICEAYDIMKRLGGFTDKEISDVFAKWNNGVLDSFLVEITRDILKF
DDVDGKPLVEKIMDTAGQKGTGKWTAINALDLGMPVTLIGEAVFARCLSALKNERIRASK
VLPGPEVPKDAVKDREQFVDDLEQALYASKIISYAQGFMLIREAAATYGWKLNNPAIALM
WRGGCIIRSVFLGQITKAYREEPDLENLLFNKFFADAVTKAQSGWRKSIALATTYGIPTP
AFSTALSFYDGYRSERLPANLLQAQRDYFGAHTFRVLPECASDNLPVDKDIHINWTGHGG
NVSSSTYQA
>O13290
MDKNDNSQCQLSTVSDEILIFLKKLLSLSVSDNLDDICETIALQSTFVDTFRSFINDEYY
TVIYLYGSPCLTSSPTSPDSCTFGNMTFQWTSLLQDVFSGTSAFAIFKRFPLTDTPLTLQ
NMLYINQLPILKGGHKSNEIPERPINAIFLYTKYVMSCYFTAYLAMESVDDRSTIDLNSP
SKGKELELTCQKFADFERSFTFFCREYQNSDTILQHHPLILSTIKHAEENNLDLSVRLLP
SKVLSDSEFYKSLSNLVNVWLKTTRSLIKLFHDQISKTALEEFNFWQFYYRSLSRLNDQL
HSRPVLFVLDILAFGKRFHTIASFNSETNIQCFVDKVGKIDALFKEISLDIFLSSSTLES
LQLSAALLYSTFSKKWRNTGYPETRVLDFINFITEDLLKLISRLLPALGALSLSNVDFSH
RTAVSSDILSLCYIRLKDFLRISGSLKEEQSYYGLKNSIKQIKAFENKLKYIQSFHEKHQ
QLIGALSEVYGLTHLTELEILEHLNKKEHVFNILTVFKDLQSLNVLDISLKGVNAWNSLE
TSYYNCMTVLEDEVIAQLKSLLQYSKTSSQMFTTLMRFQPLFFRTRVRTSISDCLHLLVN
RIKQELDLLKTRFTEDVSDTELIAMNELRNLPMASSAIIWATQLKNRLHEYTKNINIIFG

EDWNNFPDGFELKVECITLQKRLDTNLIFTNWINDVSSRNLNFDFDSKIFYLTQSESAES
PLRLSVSIDFDPCSFCKEIRTLAHLGYNIPSQLMELASCLQRIQLIAMCLIDSVQSFNDV
SFEISKTEEERFLLQEYELAVRQHIVTGLFISWNDFIVGNLSTPPKCAIGKRNFLKNIHP
NVENYAYQFSSLTSLLMNKRNAISHTYMQIQEQLFQLDICEYSGDIFLTIQRKLQDLIDL
LYVNGYSNLPPFVRALNLRFQDLLISRCRKFLSFFKTTILTSGNENNDLKSKFSSDMYEK
LRGFLKPTNLTIQRNIIEFDPPVYRKKEDSIYLLDMCLQSVVNIPLLSIKTTAQRNCTLI
GFFPVINRLESEILGIFESLLFHFDSILGYQNYWKKVEPFLNLNSLNLLLKSELFQLDQC
YSLSLYLIHLKSEVDEIGKVTDFKIFSVNNTEFKSQVYLYLREWINALFDRFTFLLGKES
EHLLNELDDTHSSLSTVDFTVNNTESLINSLKIFKKAGCYKLNVEHKIITYQNYEMTFND
CDAFSEFNFSLLKDITSKWKDLLESFECRRLKLENNKDEILRNFSEFAKRVNTETLSLIS
EWCASSLSLIKANYDEFSSTVDDFLFRFSKATEQCLMVKYIKKDLEIEIEESCDFSIQTE
EIHLYKKFKDVISSNLEFIVEIKNTRWKLFDTATLSVQTTHQINALESVHTSFQHFKLFT
NTKQSLNQLKDCTLLLQKLKSCPLKPVHWISLFEITKSTEQLDFEKLLVSDILGIDLQAH
ESFITTLLNSAVVEANLENQFNEVHSFWKNSYFSFKSFKGRNYIVVGCQELIDAVEKNMD
SLNLIKTSRHFKDGDMNITDLQSKMKIIVKFLNIWKEIQQIWTHLSAIFYESTYIQQLLP
ELAASFFNSSKTYMHLVTLLKERSYLYKVSNIPSLLESAAKLSTTLEDSKKSLLKYFELQ
RHKISRLYFLGDDDLMELISNPCDPFVINKQIIKLYPGIRSLIVDTENTNINGCTTNEGN
ELLFDNPICLLDNTQPLHWISSLEPFLKATLFQLFSTSFQQIRDFYYNKSRNVFCKEWFL
RYPSQITLLSLRCTLCHEIETGIADCCLDAVFNFINDGISSLVLLADENELSIKKKVTLM
FNELLHFKETVGLLCKNSFNNYFWSREVKAFYREDHDDEAVVIKMFSLEFIYAFEYSELD
DPIVYTDLTRNCFSVLLHSIASNLGGSPIGPAGTGKTETVKAVSAYLGKNVFVFNCDNAF
NYKTIQRILSGLAQIGTYICFDEFNRLDSGTLSAISYDIQRIQSLVSHSDGLCQSPILLD
APTIFVTMNPGYLGRFKLPSNLKKLFRPIWMGSPDNKKICEILFLSFGFKESSLLSQVLD
SFFLCCSGSLSNCLHYDFGLRAMKVVIKAAKRIKGFLKKKNTICQELEILWYAIREVLYP
SLIYQDIPLFFKAEESYFNFPAVKANAFIDPDNFEVNIEQTLSKNFFGNNQYLKLKIMQL
YQMSEAYNGIILLGKTGSGKSQIFRILQSALLNIGIDCIVYVISPKALTKESLFGSMNMD
TREWTDGVFTKLLRKTRDSCYYKRYMFVFDDELSPEWVEAMNSLLDDNKTLTLSNGERIA
LQPYVKIFFEADSVASLTRATISRCGLICISNIDDNILSSTDKMLSFTSGATNYPLGSSN
DEFSTVFSKVLTDEVMMNLISSCYKFSVDLQHIMNFTKQRFFTTFYSLLDQTKLFTRSSN
ITESLSFKELCNYLKKKICYILAWCCTGDTDAKSRERFTHWLMQNASVDLPEIKDFEHVS
ILDFDVSLETQSWYPIAGKTLKSSALKYAGNTVIPTLDTVRYAEFLNFSLTKNRCVIFCG
PPGSGKSMLMLGTLRSRQDVEVIALNFSISTSSKSVVSFLEQSTVYYRSTGMTIMCPKNH
EKVLVLFCDEINLPRSRNCLAEDVICFLRHMLEHQGFWHPLHKEWVTIKNIFVCGACNPS
TDIGRNDFPERFLRRTVLIFVDYPESYSLVTIYNALLEKSALINQYKTIILNIVKASVKF
YQVLRENFKSSTQGYVYTPRDLTRWLISFKNYAESYAETNNLSLIKVWYHEACRVLLDRL
VSQKECSWGMTELQKVIVTDFGEFEVSVIFEKQIIFTDILKNGLEFLDFASLRPKLESLY
KKFYSSHPNNTLVFVDETITHILRFHRILNNSGMHALLQGSVGLGQKAVVEFVCWLNSFS
LFELQKNQTYSIEDFEDNLKSILILAGTTNCKACLAINESIAGVPGFLDLLNNLLTNSEV
SNFFDQNDWAEIKKNLNKLNEFQPLKFDSEESVTEIFMNNVFQNLCVVFYVYTSADVDFQ
TNSLSPALLNRCTIDYYHSWDYHSMLQIANEVLQETISLNALDHDNPNLKNIKGSSIYDA
VAQAVVNTHTSIVWEFKHLGKTSYFSCLHFIRFLNTFCLIFGRDANKLSKEKSRIENGFK
KIKETSQGIDKFKEALSDQQNVLFSKTKTANDRLQCIIQTKQAVEAKKVYSLQAEASLQK
KSFLLNEKKNSVMKEVSYAKPAVIEARKSVSDIKKAHLIELRSLSRPPMAIRITMEVVCK
LLGFSATDWKNVQQLLKRDDFIPKILNYNLEKELSINLRRKIEQDYFSNPIFTFDSVNRA

SKACGPLLLWIKSICNYSKVLEKLEPLNSEVDRLKLEQKNAEECIQETIAACKDLDEKLL
QLQEEYASMISEIHSMELQMDEVKCKMQRSIEVITDLSIERNEWSGFLNLYPKRMWNLVG
ESLMEASFVVYAGNLDPSMRIFLRNKCEPIISSFGFPISKSAVRTNIERCVQTSIESKYY
KNLTDYSLENIYIIQENKSPLLIIDPSSQILDILPSLYKGKASDLISFSNKSFQNQIKLA
LLSGSAIIIKDAELWDVSIEPLLKPEFFTGSGEVQTTFAKDTITITLPLNIIFFSEVQSN
ELENKASKFMNVVNFTLSISLLETQMLKSVISVQEPGVFKQKDNCFTLKLSIERQIRSLQ
EQLLKTLCSSNENIVGTDEIVVLLKNLKEKHETIRLAYSESQSINRKVDELIRRYKLSIK
SFLSVVVVFQHFISLKKSYSFSFNFIWSTFHQMLNVVLENRNQDFKSLIMDALRDLIRRC
FLYIFPEDRVLFLFLLMFFFFPKETESLRKLLIVNGKTLELEQSYLNFFETCSDSNERGG
LESLFFKTHASNIQNFCTEVLANTHCEEDCLKLLYDLWSSAFKVEFSNIKYDFLKIINDE
SESRMPTIVYLMENCEIDSLLQNAKIPQNIKKLTVSLGSAENESLADSYLKLASTEPLWL
FINNIHLSTPWAEKLPSKMSNHLHKNSRIVCLSEIHNQLPHQLLCISRSIVFNKQTSFKN
NLLNLLELLPTMTHTLPHNRFRLFFFLSWLHATLAEIYCFTCSSWKEPCYFDDSDFYFGT
KILCNILYRNVHLEEFSWGTFKDLLLNVVYGPKVSASSDFIALDKILKRLIAQFKTQISS
NILLTDNFKFILPYEITFSSAKEVIGQLPDEIPPGWLDIPENSKRKRTDIYFSMCI
>Q71A39
MEQNILNSILDKFGDGDQERSDIRHNDSGDENDNHSDHEGNHGNNECCEGNEDGDKEYEV
DVEDMTDEQYAQFIQEQQEPKIKSGGNTGVKGVLSDYAEHREKQKQKYLQKKYETQKMLE
KMCFTTRDQPPPTEEENQLDSDDDDLERIRKARMEQWKSKQQITSDVKKPEKKVFGYFKQ
IDSSQYIHEIDNEPPNVFVIIHLFQNYIPECVLLNQQLGQLAVKYRYIKFLKILSKEAKE
NYHDEALPSLLVYIGGKLLVSFVPLTEELGRNFDQEDLELLLSSYDIIPNPMKAKNSNWE
TSLSRKRPESDDDNDD
>Q20010
MNNYDDLPCSIYFKKPTVQEFVDQPRVFEDSEVPAFQEVLQLPQERTKPPVPSTQPIVAA
VEVAKKKSCLSAPKPRKEPPSHPALRQKTVAFGKTVNVSQTVEGTSRNSKKVLASTMSAQ
NTTTTEEQAAENWRDAMKTELQTIRTEIQEETARRQEELNAQNLVKMQELMSNFFQKITI
PKQQAIEPVEKDKENFHESPRQSRQQKPASKIASAREVIKRDGVIPPEALTIIEQRLRSD
PMFRQQIDNVLADAECDANRAAYSPPPPMSEVRYGSGVNPALMRETLTVERSIRYDNGLA
SIDSRQWTNERRDNRAPDSYRTYEPDQPCHSLYQKGQSISYYPSEAAGKTTARNNRTGYY
VEDSSDHEEDVVVNKRGQNYHEQAVPETPAERERRIREKYARRK
>Q63042
MRTQQKRDIKFREDCPQDREELGRNTWAFLHTLAAYYPDMPTPEQQQDMAQFIHIFSKFY
PCEECAEDIRKRIDRSQPDTSTRVSFSQWLCRLHNEVNRKLGKPDFDCSRVDERWRDGWK
DGSCD
>O74417
MSQVIQYSEADLLYLSKSPLIKKPENLPEWILPEAMKADRERHIRQEKEMKRHDDDGRQY
QSDRKFAKSKHDDIILGPPKLSFASSNENGRSVGRHDDLLSLGNVYNGVASLRYRNGASV
SRSSSIGHSGSTAPWSSVGRHNRKKDNEHRDEMEGLEKVMKQRAGNTGPLVANSTEEFEA
WKRMMKASSGEAGAGNVITPGVTSTTGAPSGKASLSRAASNSSTSARAGISVDSLFGKRS
AAQAMATVVPSVSTPGGTPPRFASPALTVESMVHAAPSTTTSSRFSKFFNGLAGPETSKT
STPNMSNNPSPRVANTVVSPAPIPTIGSATIDKDAEGFQRVLAMLGRQASSTTGSPKVAL
SQMPQVNVNPSNQDLASVKQPSGFSPPSAVAPPPGLGNHNFSNDDSSFFRSLMTSDTRSV
PPPPGFSTNAPKAVKSPEISAPPGLYRGLSSGASIPSAPPGFGYQQPSFPYSPGFPPSAY
NQNRTGYGFPDTSRPPH

>P25386

MDIIQGLIQQPKIQSVDETIPTLCDRVENSTLISDRRSAVLGLKAFSRQYRESVIASGLK
PLLNTLKRDYMDEDSVKAILETILILFIRGDGHDDLTRGWISQQSRLQNGKYPSPLVMKQ
EKEQVDQFSLWIADALTQSEDLIHLLVEFWEIDNFHIRLYTIQLLEAVMATRPLKARSAL
ISLPTSISTMVSLLDDMHEPIRDEAILLLMAVVNDSPHVQKLVAFENIFERLFSIIEEEG
GLRGSLVVNDCLSLINNILKYNTSNQTLFLETGNLPKLAHLLSEPISQDEVFFWNDQRIV
NINTALDIVSLTVEPGNTVTTKHQNALLDSSVLMVVLRLAFFHNIPKKVRPVALLTAANM
VRSNEHAQLEFSKIDVPYFDPSLPVNSTANGGPIKLIPVVSILINWMLYANSVHTFDTRV
ACSRLLKAYFMDNFDLQRDFLLKQVQLCNNSTNNVGDNAKENGGSNKSDKESDSDKDTDG
KDGTEYEGSFKANLFEVLLNYDAELNLNPFKLFFTTDIFMFFFQQDHKYSEELREITRNV
TTGNDLEDEEPLKAIQTISELLTTSLTAADIRIPISYLTFLIYWLFGDFKATNDFLSDKS
VIKSLLSFSYQIQDEDVTIKCLVTMLLGVAYEFSSKESPFPRKEYFEFITKTLGKDNYAS
RIKQFKKDSYFSKVDMNEDSILTPELDETGLPKVYFSTYFIQLFNENIYRIRTALSHDPD
EEPINKISFEEVEKLQRQCTKLKGEITSLQTETESTHENLTEKLIALTNEHKELDEKYQI
LNSSHSSLKENFSILETELKNVRDSLDEMTQLRDVLETKDKENQTALLEYKSTIHKQEDS
IKTLEKGLETILSQKKKAEDGINKMGKDLFALSREMQAVEENCKNLQKEKDKSNVNHQKE
TKSLKEDIAAKITEIKAINENLEEMKIQCNNLSKEKEHISKELVEYKSRFQSHDNLVAKL
TEKLKSLANNYKDMQAENESLIKAVEESKNESSIQLSNLQNKIDSMSQEKENFQIERGSI
EKNIEQLKKTISDLEQTKEEIISKSDSSKDEYESQISLLKEKLETATTANDENVNKISEL
TKTREELEAELAAYKNLKNELETKLETSEKALKEVKENEEHLKEEKIQLEKEATETKQQL
NSLRANLESLEKEHEDLAAQLKKYEEQIANKERQYNEEISQLNDEITSTQQENESIKKKN
DELEGEVKAMKSTSEEQSNLKKSEIDALNLQIKELKKKNETNEASLLESIKSVESETVKI
KELQDECNFKEKEVSELEDKLKASEDKNSKYLELQKESEKIKEELDAKTTELKIQLEKIT
NLSKAKEKSESELSRLKKTSSEERKNAEEQLEKLKNEIQIKNQAFEKERKLLNEGSSTIT
QEYSEKINTLEDELIRLQNENELKAKEIDNTRSELEKVSLSNDELLEEKQNTIKSLQDEI
LSYKDKITRNDEKLLSIERDNKRDLESLKEQLRAAQESKAKVEEGLKKLEEESSKEKAEL
EKSKEMMKKLESTIESNETELKSSMETIRKSDEKLEQSKKSAEEDIKNLQHEKSDLISRI
NESEKDIEELKSKLRIEAKSGSELETVKQELNNAQEKIRINAEENTVLKSKLEDIERELK
DKQAEIKSNQEEKELLTSRLKELEQELDSTQQKAQKSEEERRAEVRKFQVEKSQLDEKAM
LLETKYNDLVNKEQAWKRDEDTVKKTTDSQRQEIEKLAKELDNLKAENSKLKEANEDRSE
IDDLMLLVTDLDEKNAKYRSKLKDLGVEISSDEEDDEEDDEEDEEEGQVA

>Q3E7B7

MARGNQRDLARQKNLKKQKDMAKNQKKSGDPKKRMESDAEILRQKQAAADARREAEKLEK
LKAEKTRR

>Q6K1E7

MAAPARTPRSRILGCSSMLRFLRNLVGSKGGSKSTNKPLTRSQPSSSWEQDVVSPMMGHQ
GGRGRKEPRAKVHSAASSNGKREPPPRVLSAAPSNPRHDAFELGTGDSGSQTLTSKDVPK
LRAQGVEVTSVPLRGTWEVLEQLPEKKGEEEEPVGEVSGASDREHFGQALETEQGCLQWV
PGPLALTPGAFIKEEEDEHCPIEFGDLKPSSCKVGSTPWNYLLGLYKQLQKSAMAKAQRP
AAPQLALKDGLPHEEKGEREEAVDESCPKWCAPRASSDESCPKWCAPRASTYQSPLQKKF
RSTDTVGFVESELKKILSVQREARLWKVGNPEGRELLTQPDITLEEAGMVDGQHLLLEEM
DEMGNWPPPD

>Q9NPJ8

MATSLDFKTYVDQACRAAEEFVNIYYETMDKRRRALTRLYLDKATLIWNGNAVSGLDALN

NFFDTLPSSEFQVNMLDCQPVHEQATQSQTTVLVVTSGTVKFDGNKQHFFNQNFLLTAQS
TPNNTVWKIASDCFRFQDWSSS
>Q9HGN4
MSEIITSLQTELSNRHANMVSEAVLAFSEHHDSALHSYFLNARVKDVILESLRTTEEKLR
ALDKLQLDASKNLSNLLVEYNSINAELESVNSKISSISSQEYALEEFQALSQNFEKNLVQ
HRRNSQNKLKCAMEVNIKTNYIVILLTSINLKKLKMIETSTHKAILDNF
>Q01532
MLPTSVSRSLYLKTFRSHLLRAPQIVLKRMSSSIDISKINSWNKEFQSDLTHQLATTVLK
NYNADDALLNKTRLQKQDNRVFNTVVSTDSTPVTNQKSSGRCWLFAATNQLRLNVLSELN
LKEFELSQAYLFFYDKLEKANYFLDQIVSSADQDIDSRLVQYLLAAPTEDGGQYSMFLNL
VKKYGLIPKDLYGDLPYSTTASRKWNSLLTTKLREFAETLRTALKERSADDSIIVTLREQ
MQREIFRLMSLFMDIPPVQPNEQFTWEYVDKDKKIHTIKSTPLEFASKYAKLDPSTPVSL
INDPRHPYGKLIKIDRLGNVLGGDAVIYLNVDNETLSKLVVKRLQNNKAVFFGSHTPKFM
DKKTGVMDIELWNYPAIGYNLPQQKASRIRYHESLMTHAMLITGCHVDETSKLPLRYRVE
NSWGKDSGKDGLYVMTQKYFEEYCFQIVVDINELPKELASKFTSGKEEPIVLPIWDPMGA
LAK
>Q9U1K1
MTEHQAEEQADTPPTKVKATPTPTPSGKFKDAKEDAFLSTSPDSANGDAQHKLPADQLAM
SSSAHPQQAGQARPLILQAFHRCSSPEQCVTLHDILDSFKAPLSEDQAWALIHQFAGLYH
QVAVQAHTCAADYEAALPTGFELHFHRDGSVHFSGPDQLTPKEQLQQEQIPLPPQHDVIV
DQPDHSASSSGDSSVINRAFDNSNHHHHHQHHHPPLVVSHRKIISELAEIVYTALDYNLP
EDEECQVSQELENLFNFMTADETDDDCIDEGIDEGDKRWDDESEEERNDTKELEHIIETC
RNHIKTTLPENHYRAVCRALVTETIELRVFLQQVLNNEAGAEKLIKASESSATTQQELAK
LGFNDWARFWVQVIDELRRGVRLKKSNHERTPIEYELTPYEILMGDIRAKKYQLRKVMVN
GDIPPRVKKDAHAMILEFIRSRPPLKKASDRQLGPPRMCEPSPREQLMESIRKGKELKQI
TPPEAPTLRERVLPSANSTLSRSRQRLIKVDFSKFQDDDLFYDENSISSSHSTAATHQHH
PHFAEMHRCSQPKMPPYPFGGYMVPSQARQDCRETASLMRPRRTMEPAKQVPPPEEPSFT
KDEYHKFYDTALESYDLATQCESRRASLRRHTIVGCQSNLDETHSMPPTRPESRQSDDVS
KETPKRSPAEQTHPSDEGSSTSSLGPWNKSFMDKQTWMERGDDRLSVTLAEIVHIRSVMT
KAELEGLPMDVRVKEDVEKRRVCFLCLRTRFSFFGPWGIQCKLCQRTVCAKCYTKMRIPS
EHFRNVPLVLISPSLLSSPASSSTPSPSHHAQQAHSSSTGNIMDDQFPKSLIERLLRSES
DRKTRSTVGSAPSSPKHQRSNMSTPGISVGPGASSSSAAATGQAVEALHDQATMSSSYSA
AMRPSGVHQQQKQHYNNAMSRSMEGPRSLPVHSPAYRPLSNNSTLERKSRFSRGFNLFSS
GSHLAQTQEQKENLRGEQVTVCNDCQGLVNEITSSVKQKRSSARNRTIQNLTLDLTPVWK

>P35401
MGKTERKKELLELFEYEKIKGDVSYSSIMKKAGGNGKGFLDRYFALHRNYILYYKLGKSS
LKPDDKQEPQGYINLMDCNPDDTKEIAPLMFQISHKHRTYIVKAKDESSMKQFLTLLIAR
IRSLEKIDIDKLGCTVVVLTKVKKFREVLTNPLILPDRVSPEMAEEWVKQMKNYNASFNL
ADPFIKQVEQISEFCRGEVKEYIDWFGGPEGPRLAMIRCEETVLSNWVEYINKTSSEITT
YQDNRFFREDFKDIAVHLKNMTTFIDCYNDYMIHCRKYNNNKPNTKFLEEKQTFKEYIEK
FIPKVASCNDVSLNQFYDRSLIQSSDGIVTINTSGIKKTLINQSNIISITSTTTTTTTTT
TTTCSMPNMSNLIHSLDHTNLNIIDLNHSKSQQQLHPPPSPHHQHLHHQIVSNSKDFNIS
VSSNNFNDGNSEFPNLDINCDFDLTSASNLSSPILSSEVPSNVVDPIGSGQGGGGSGGGG

VTAVTEEAINEKWHFDCNTSMIFKPPSEDGRNEGSNMSTSSITSKMSLSLNGGFDMKWVY
QCGYFKSKNMGSISWNGKHWCWSHPRTSYKIKYIWDPTKQSFLNIPFKSRVGATGGGSVP
SSQSTNNLQSSTSSMSSLSSSSTSTTKRSHPTTLYPDYQFKDNLLTPIIIEGRHQPSLTL
IDSPLTIPNACLLTIAMTQYIQDALIHLSLGPKVLSSK
>Q06628
MVAEEDIEKQVLQLIDSFFLKTTLLICSTESSRYQSSTENIFLFDDTWFEDHSELVSELP
EIISKWSHYDGRKELPPLVVETYLDLRQLNSSHLVRLKDHEGHLWNVCKGTKKQEIVMER
WLIELDNSSPTFKSYSEDETDVNELSKQLVLLFRYLLTLIQLLPTTELYQLLIKSYNGPQ
NEGSSNPITSTGPLVSIRTCVLDGSKPILSKGRIGLSKPIINTYSNALNESNLPAHLDQK
KITPVWTKFGLLRVSVSYRRDWKFEINNTNDELFSARHASVSHNSQGPQNQPEQEGQSDQ
DIGKRQPQFQQQQQPQQQQQQQQQQQRQHQVQTQQQRQIPDRRSLSLSPCTRANSFEPQS
WQKKVYPISRPVQPFKVGSIGSQSASRNPSNSSFFNQPPVHRPSMSSNYGPQMNIEGTSV
GSTSKYSSSFGNIRRHSSVKTTENAEKVSKAVKSPLQPQESQEDLMDFVKLLEEKPDLTI
KKTSGNNPPNINISDSLIRYQNLKPSNDLLSEDLSVSLSMDPNHTYHRGRSDSHSPLPSI
SPSMHYGSLNSRMSQGANASHLIARGGGNSSTSALNSRRNSLDKSSNKQGMSGLPPIFGG
ESTSYHHDNKIQKYNQLGVEEDDDDENDRLLNQMGNSATKFKSSISPRSIDSISSSFIKS
RIPIRQPYHYSQPTTAPFQAQAKFHKPANKLIDNGNRSNSNNNNHNGNDAVGVMHNDEDD
QDDDLVFFMSDMNLSKEG
>P78358
MQAEGRGTGGSTGDADGPGGPGIPDGPGGNAGGPGEAGATGGRGPRGAGAARASGPGGGA
PRGPHGGAASGLNGCCRCGARGPESRLLEFYLAMPFATPMEAELARRSLAQDAPPLPVPG
VLLKEFTVSGNILTIRLTAADHRQLQLSISSCLQQLSLLMWITQCFLPVFLAQPPSGQRR

>Q5T7V8
MSWAAVLAVAAARFGHFWGCRWPGPMAQGWAGFSEEELRRLKQTKDPFEPQRRLPAKKSR
QQLQREKALVEQSQKLGLQDGSTSLLPEQLLSAPKQRVNVQKPPFSSPTLPSHFTLTSPV
GDGQPQGIESQPKELGLENSHDGHNNVEILPPKPDCKLEKKKVELQEKSRWEVLQQEQRL
MEEKNKRKKALLAKAIAERSKRTQAETMKLKRIQKELQALDDMVSADIGILRNRIDQASL
DYSYARKRFDRAEAEYIAAKLDIQRKTEIKEQLTEHLCTIIQQNELRKAKKLEELMQQLD
VEADEETLELEVEVERLLHEQEVESRRPVVRLERPFQPAEESVTLEFAKENRKCQEQAVS
PKVDDQCGNSSSIPFLSPNCPNQEGNDISAALAT
>Q99188
MASVPSLCDILIPLEKNSRSDGDAESSNTVLIQLRKGHHERMRSPYTIQKFYKFLKRAHC
EENLEFFEKAHQFLQLKQNRSISEEKLLEVWNKSLYIKYIAVDSPKECNFSQDTREIFEK
CFANNEVPADVDVLCAISHVMGLLMDGYHRFVSSVNEKKYSATYAHNDSATEQDLKNEST
TSFSSLGVEDISEDRNSYLKKPDINGLSTIIQETSANTTNESQCSDRTSRPSESSSSLNT
TSSTYKNTSTRNLQKPQNTGILNSGKGLLQKLNFVKKRKSFKQPSGVICSHYNSNVQNRL
KGQNSHQQR
>Q63285
MAGSGCAWGAEPPRFLEAFGRLWQVQSRLGSGSSASVYRVRCCGTPGSPPGALKQFLPPG
TTGAAASAAEYGFRKERAALEQLQGHRNIVTLYGVFTIHFSPNVPSRCLLLELLDVSVSE
LLVYSSHQGCSMWMIQHCARDVLEALAFLHHEGYVHADLKPRNILWSAENECFKLIDFGL
SFKEGNQDVKYIQTDGYRAPEAELQNCLAQAGLQSDTECTSAVDLWSLGIILLEMFSGMK
LKHTVRSQEWKANSSAIIDHIFASKAVVNAAIPAYHLRDLIKSMLHDDPSRRIPAEMALC

SPFFSIPFAPHIEDLVMLPTPVLRLLNVLDDDYLENEDEYEDVVEDVKEECQKYGPVVSL
LVPKENPGRGQVFVEYANAGDSKAAQKLLTGRMFDGKFVVATFYPLSAYKRGYLYQTLL
>Q90Z16
MSSKPQIRPAENGEHCRSKMENGMDSMAPPTLSTYTPEEMVQQMKELITENNELKEAMKL
HNQAMKDRYEELSIWREKQKEEREFYETKFKEAKQCLLAKCVENEQLQQQLQSLKEREEG
AEMEGCATPEKEARQLKSKVQRLQAEKADLLAIISELQVKLNIASAEDSFVEIGMNEEVN
RTARENQDNSSEMASNIAVYIRSKSADESKNLESEELTVSQLLCCLRNETQRREKLEKEL
QDHKERLSKMENETSNCLESGTQTNQEEESSEAIGSEVESLKKQICALFKELQEAHEKLK
EAELIQKKLQEKCQTLEKVNSAAATELEEKQQLIYTIKKLELQVESVQAEVKLEQAKTQD
EKTRYSSLQDAYNKLLAELTEAMKTISEMKVKEHDRVDKVVVEELNAKVLLAEQALAAKQ
LQMDEMKQLIAKQEEDLETMAVLRAQMEVYCSDFHAERAAREKIHEEKEQLAVQLAYLLK
EQQNLEDLGRSSLAEMQNRHGARAPDREHSPRLVQRGTGSQEWPEQRNISIYSCPKCEEI
LPDLDTLQIHVMDCIN
>P48524
MAKDLNDSGFPPKRKPLLRPQRSDFTANSSTTMNVNANTRGRGRQKQEGGKGSSRSPSLH
SPKSWIRSASATGILGLRRPELAHSHSHAPSTGTPAGGNRSPLRRSTANATPVETGRSLT
DGDINNVVDVLPSFEMYNTLHRHIPQGNVDPDRHDFPPSYQEANNSTATGAAGSSADLSH
QSLSTDALGATRSSSTSNLENLIPLRTEHHSIAAHQSTAVDEDSLDIPPILDDLNDTDNI
FIDKLYTLPKMSTPIEITIKTTKHAPIPHVKPEEESILKEYTSGDLIHGFITIENKSQAN
LKFEMFYVTLESYISIIDKVKSKRTIKRFLRMVDLSASWSYSKIALGSGVDFIPADVDYD
GSVFGLNNSRVLEPGVKYKKFFIFKLPLQLLDVTCKQEHFSHCLLPPSFGIDKYRNNCKY
SGIKVNRVLGCGHLGTKGSPILTNDMSDDNLSINYTIDARIVGKDQKASKLYIMKEREYN
LRVIPFGFDANVVGERTTMSQLNDITKLVQERLDALRKIFQRLEKKEPITNRDIHGADLS
GTIDDSIESDSQEILQRKLDQLHIKNRNNYLVNYNDLKLGHDLDNGRSGNSGHNTDTSRA
WGPFVESELKYKLKNKSNSSSFLNFSHFLNSSSSSMSSSSNAGKNNHDLTGNKERTGLIL
VKAKIPKQGLPYWAPSLLRKTNVFESKSKHDQENWVRLSELIPEDVKKPLEKLDLQLTCI
ESDNSLPHDPPEIQSITTELICITAKSDNSIPIKLNSELLMNKEKLTSIKALYDDFHSKI
CEYETKFNKNFLELNELYNMNRGDRRPKELKFTDFITSQLFNDIESICNLKVSVHNLSNI
FKKQVSTLKQHSKHALSEDSISHTGNGSSSSPSSASLTPVTSSSKSSLFLPSGSSSTSLK
FTDQIVHKWVRIAPLQYKRDINVNLEFNKDIKETLIPSFESCLCCRFYCVRVMIKFENHL
GVAKIDIPISVRQVTK
>Q05874
MSDIESLGEAAGLFEEPEDFLPPPPKPHFAEYQRSHITKESKSDVKDIKLRLVGTSPLWG
HLLWNAGIYTANHLDSHPELIKGKTVLELGAAAALPSVICALNGAQMVVSTDYPDPDLMQ
NIDYNIKSNVPEDFNNVSTEGYIWGNDYSPLLAHIEKIGNNNGKFDLIILSDLVFNHTEH
HKLLQTTKDLLAEKGQALVVFSPHRPKLLEKDLEFFELAKNEFHLVPQLIEMVNWKPMFD
EDEETIEVRSRVYAYYLTHEK
>P00958
MSFLISFDKSKKHPAHLQLANNLKIALALEYASKNLKPEVDNDNAAMELRNTKEPFLLFD
ANAILRYVMDDFEGQTSDKYQFALASLQNLLYHKELPQQHVEVLTNKAIENYLVELKEPL
TTTDLILFANVYALNSSLVHSKFPELPSKVHNAVALAKKHVPRDSSSFKNIGAVKIQADL
TVKPKDSEILPKPNERNILITSALPYVNNVPHLGNIIGSVLSADIFARYCKGRNYNALFI
CGTDEYGTATETKALEEGVTPRQLCDKYHKIHSDVYKWFQIGFDYFGRTTTDKQTEIAQH
IFTKLNSNGYLEEQSMKQLYCPVHNSYLADRYVEGECPKCHYDDARGDQCDKCGALLDPF

ELINPRCKLDDASPEPKYSDHIFLSLDKLESQISEWVEKASEEGNWSKNSKTITQSWLKD
GLKPRCITRDLVWGTPVPLEKYKDKVLYVWFDATIGYVSITSNYTKEWKQWWNNPEHVSL
YQFMGKDNVPFHTVVFPGSQLGTEENWTMLHHLNTTEYLQYENGKFSKSRGVGVFGNNAQ
DSGISPSVWRYYLASVRPESSDSHFSWDDFVARNNSELLANLGNFVNRLIKFVNAKYNGV
VPKFDPKKVSNYDGLVKDINEILSNYVKEMELGHERRGLEIAMSLSARGNQFLQENKLDN
TLFSQSPEKSDAVVAVGLNIIYAVSSIITPYMPEIGEKINKMLNAPALKIDDRFHLAILE
GHNINKAEYLFQRIDEKKIDEWRAKYGGQQV
>P38768
MADFLLRPIKQRHRNEDKYVSVDAADGSVSKIEPIADFVIKTKLLSANGPEKLQDGRKVF
INVCHSPLVPKPEVDFNARIVFPLIIQNEWEIPIITSCYRMDHDKKGQECYVWDCCINSD
CSRWICDDIQLREILVEWCLESCEIRDSVVLCRDRIAFPKMKKKGAELPALEVLNDELHQ
DYKAKMHKIIEEEAGDPMSILRGRNDDGDDNNDPDDGTLPPLFPIENKISGAKIEEIDKN
EIAHRNLKQAPAPAPAPHEQQEDVPEYEVKMKRFKGAAYKLRILIENKAPNSKPDRFSPS
YNFAENILYINGKLSIPLPRDIVVNAADIKIFHIRKERTLYIYI
>Q10357
MFEVEYLIKDCDDVNKNTLEQEFQDLNIEDWKWDAATGQLIVKGSVSPSKVLRRLENATS
KPILIRGASNKESGVSILYEANEDITQIPKVYGLCRFIPTEEKIFLDLIATQLLPNREYT
GLVTISGDISRGLKSAGDSLVTLFNANSNEQGKIVLDKEVSGSLPNWIGHCFVLKCVDDS
DSATMGIISRSAGLGQNTKQICACTGKSLWTEHAELKSVNEGSSCCSKKDSSPSEKPSCC
SQEKKSCCSSKKPSCCSQEKKGCCSTEKTSCCSQEKKSCCTSEKPSCCSNGKSTVCA
>Q9ULJ6
MNSMDRHIQQTNDRLQCIKQHLQNPANFHNAATELLDWCGDPRAFQRPFEQSLMGCLTVV
SRVAAQQGFDLDLGYRLLAVCAANRDKFTPKSAALLSSWCEELGRLLLLRHQKSRQSDPP
GKLPMQPPLSSMSSMKPTLSHSDGSFPYDSVPWQQNTNQPPGSLSVVTTVWGVTNTSQSQ
VLGNPMANANNPMNPGGNPMASGMTTSNPGLNSPQFAGQQQQFSAKAGPAQPYIQQSMYG
RPNYPGSGGFGASYPGGPNAPAGMGIPPHTRPPADFTQPAAAAAAAAVAAAAATATATAT
ATVAALQETQNKDINQYGPMGPTQAYNSQFMNQPGPRGPASMGGSMNPASMAAGMTPSGM
SGPPMGMNQPRPPGISPFGTHGQRMPQQTYPGPRPQSLPIQNIKRPYPGEPNYGNQQYGP
NSQFPTQPGQYPAPNPPRPLTSPNYPGQRMPSQPSSGQYPPPTVNMGQYYKPEQFNGQNN
TFSGSSYSNYSQGNVNRPPRPVPVANYPHSPVPGNPTPPMTPGSSIPPYLSPSQDVKPPF
PPDIKPNMSALPPPPANHNDELRLTFPVRDGVVLEPFRLEHNLAVSNHVFHLRPTVHQTL
MWRSDLELQFKCYHHEDRQMNTNWPASVQVSVNATPLTIERGDNKTSHKPLHLKHVCQPG
RNTIQITVTACCCSHLFVLQLVHRPSVRSVLQGLLKKRLLPAEHCITKIKRNFSSVAASS
GNTTLNGEDGVEQTAIKVSLKCPITFRRIQLPARGHDCKHVQCFDLESYLQLNCERGTWR
CPVCNKTALLEGLEVDQYMWGILNAIQHSEFEEVTIDPTCSWRPVPIKSDLHIKDDPDGI
PSKRFKTMSPSQMIMPNVMEMIAALGPGPSPYPLPPPPGGTNSNDYSSQGNNYQGHGNFD
FPHGNPGGTSMNDFMHGPPQLSHPPDMPNNMAALEKPLSHPMQETMPHAGSSDQPHPSIQ
QGLHVPHPSSQSGPPLHHSGAPPPPPSQPPRQPPQAAPSSHPHSDLTFNPSSALEGQAGA
QGASDMPEPSLDLLPELTNPDELLSYLDPPDLPSNSNDDLLSLFENN
>Q9UUA8
MCSVEKIVRLRHNLLCHVRIHFKTKEDLDIRAFLFSLQRELHAVAFLNQGSIRTLYEKRV
VDITSESVQSHAIIETARNFALPNTAEEEENMDRKPELQLSFKGSMTSKIIHIYVQPFDS

>Q99395

MMPYNTPPNIQEPMNFASSNPFGIIPDALSFQNFKYDRLQQQQQQQQQQQQNRTASSLQQ
PQQQQPISPPLFLVGAGTSENSNLNKNANTSTIPPLLFSRSSQHYVVPDIDHSSIIYKNN
ICKSFKDDLFFCPRSLLSLEEQQACEKMDRLTAEQMSLYHQNTQSSSNPGSMSSSPPNSA
SSIFNSRPKFNPYTSQSFNPLESVQE
>P13696
MPVDLSKWSGPLSLQEVDERPQHPLQVKYGGAEVDELGKVLTPTQVKNRPTSITWDGLDP
GKLYTLVLTDPDAPSRKDPKYREWHHFLVVNMKGNNISSGTVLSDYVGSGPPKGTGLHRY
VWLVYEQEGPLKCDEPILSNRSGDHRGKFKVASFRKKYELGAPVAGTCYQAEWDDYVPKL
YEQLSGK
>O14122
MPPEAKRIVVKGFDPRKSRQRQETYYVTMIDRLNMALQVVMAGLGLKTGYQELYSGVENL
TRADQASRCFNILQHHMSSGIQLLKDSAESFIQLEGTETDTNACTVVVGCWNKWLERVEI
VQNIFYYMDKTFLSHHPDYPTIEELSLSLFREKLMAVKNIQIPFLNSLLQSFENLHSSKS
TDHAYLQDAMLMLHRTEMYSSVFVPMYLVMLSRFYDTESSQKIQELPLEEYLEYAMSSLE
REDAYVEKFDIVRDKKSIRETVQRCLITSHLDTLTKGISQFIEKRDAHSCKLLYALLQFN
HETEYLIQPWSDCLVDVGFKLVNDESKDDTLVQELLSFHKFLQVVVDESFLHDETLSYAM
RKAFETFINGAKGSQREAPARLIAKYIDYLLRVGEQASGGKPLKEVFSEILDLFRYIASK
DIFEAYYKLDIAKRLLLNKSASAQNELMLLDMLKKTCGSQFTHSLEGMFRDVNISKEFTS
SFRHSKAAHNLHRDLYVNVLSQAYWPSYPESHIRLPDDMQQDLDCFEKFYLSKQVGKKIS
WYASLGHCIVKARFPLGNKELSISLFQACVLLQFNNCLGGEGISYQDLKKSTELSDIDLT
RTLQSLSCARIRPLVMVPKSKKPSPDTMFYVNEKFTDKLYRVKINQIYLKEERQENSDVQ
EQVVRDRQFELQASIVRVMKQKEKMKHDDLVQYVINNVKDRGIPLVSDVKTAIEKLLEKE
YLEREDNDIYTYVT
>Q9BYN0
MGLRAGGTLGRAGAGRGAPEGPGPSGGAQGGSIHSGRIAAVHNVPLSVLIRPLPSVLDPA
KVQSLVDTIREDPDSVPPIDVLWIKGAQGGDYFYSFGGCHRYAAYQQLQRETIPAKLVQS
TLSDLRVYLGASTPDLQ
>Q03262
MLQGILETVPSDLKDPISLWFKQDRNPKTIEEVTALCKKSDWNELHKRFDSRIQFGTAGL
RSQMQAGFSRMNTLVVIQASQGLATYVRQQFPDNLVAVVGHDHRFHSKEFARATAAAFLL
KGFKVHYLNPDHEFVHTPLVPFAVDKLKASVGVMITASHNPKMDNGYKVYYSNGCQIIPP
HDHAISDSIDANLEPWANVWDFDDVLNKALKQGKLMYSREEMLKLYLEEVSKNLVEINPL
KLEVKAKPWFVYTPMHGVGFDIFSTIVKKTLCLVEGKDYLCVPEQQNPDPSFPTVGFPNP
EEKGALDIGINLAEKHDIDLLVANDPDADRFSVAVKDMQSGEWRQLTGNEIGFLFAFYEY
QKYKSMDKEFQHVHPLAMLNSTVSSQMIKKMAEIEGFHYEDTLTGFKWIGNRAILLEKKG
YYVPFGFEEAIGYMFPAMEHDKDGISASIVFLQAYCKWKIDHNLDPLNVLENGFKKYGVF
KEYNGYYVVPNPTVTKDIFDYIRNVYTPEGASYPSSIGEEIEVLYYRDLTTGYQSDTINH
KPTLPVDPTSQMITVSARPSNGSENEHIRFTIRGSGTEPKLKVYIEACANEEQRASFLAK
LTWNVLRREWFRPDEMNIVTKF
>P54577
MGDAPSPEEKLHLITRNLQEVLGEEKLKEILKERELKIYWGTATTGKPHVAYFVPMSKIA
DFLKAGCEVTILFADLHAYLDNMKAPWELLELRVSYYENVIKAMLESIGVPLEKLKFIKG
TDYQLSKEYTLDVYRLSSVVTQHDSKKAGAEVVKQVEHPLLSGLLYPGLQALDEEYLKVD
AQFGGIDQRKIFTFAEKYLPALGYSKRVHLMNPMVPGLTGSKMSSSEEESKIDLLDRKED

VKKKLKKAFCEPGNVENNGVLSFIKHVLFPLKSEFVILRDEKWGGNKTYTAYVDLEKDFA
AEVVHPGDLKNSVEVALNKLLDPIREKFNTPALKKLASAAYPDPSKQKPMAKGPAKNSEP
EEVIPSRLDIRVGKIITVEKHPDADSLYVEKIDVGEAEPRTVVSGLVQFVPKEELQDRLV
VVLCNLKPQKMRGVESQGMLLCASIEGINRQVEPLDPPAGSAPGEHVFVKGYEKGQPDEE
LKPKKKVFEKLQADFKISEECIAQWKQTNFMTKLGSISCKSLKGGNIS
>Q7KRY6
MPRVAKPKAAAPAKKVVSAKKAKSKLYKMPEKVKEGTVFTDLAKGQWRIGPSIGVGGFGE
IYAACKVGEKNYDAVVKCEPHGNGPLFVEMHFYLRNAKLEDIKQFMQKHGLKSLGMPYIL
ANGSVEVNGEKHRFIVMPRYGSDLTKFLEQNGKRLPEGTVYRLAIQMLDVYQYMHSNGYV
HADLKAANILLGLEKGGAAQAYLVDFGLASHFVTGDFKPDPKKMHNGTIEYTSRDAHLGV
PTRRADLEILGYNLIEWLGAELPWVTQKLLAVPPKVQKAKEAFMDNIGESLKTLFPKGVP
PPIGDFMKYVSKLTHNQEPDYDKCRSWFSSALKQLKIPNNGDLDFKMKPQTSSNNNLSPP
GTSKAATARKAKKIDSPVLNSSLDEKISASEDDEEEEEKSHRKKTAKKVTPSARNAKVSP
LKRVADSSPPSQKRVKTEPKSTPRERATPKASPKPRSTPKASPKPQTPTAARLRTPNAKI
NFSPSISLRGRPGGKTVINDDLTPQPRSKKTYEFNFELDVSMDANVIVNVKRKKKADQDK
ATAVDSRTPSSRSALASSSKEEASPVTRVNLRKVNGHGDSSTPGRSPRTPAVTVRKYQG
>O89050
MAAGGAVAVAPECRLLPYALHKWSSFSSTYLPENILVDKPNDQSSRWSSESNYPPQYLIL
KLERPAIVQNITFGKYEKTHVCNLKKFKVFGGMNEENMTELLSSGLKNDYNKETFTLKHK
IDEQMFPCRFIKIVPLLSWGPSFNFSIWYVELSGIDDPDIVQPCLNWYSKYREQEAIRLC
LKHFRQHNYTEAFESLQKKTKIALEHPMLTDMHDKLVLKGDFDACEELIEKAVNDGLFNQ
YISQQEYKPRWSQIIPKSTKGDGEDNRPGMRGGHQMVIDVQTETVYLFGGWDGTQDLADF
WAYSVKENQWTCISRDTEKENGPSARSCHKMCIDIQRRQIYTLGRYLDSSVRNSKSLKSD
FYRYDIDTNTWMLLSEDTAADGGPKLVFDHQMCMDSEKHMIYTFGGRILTCNGSVDDSRA
SEPQFSGLFAFNCQCQTWKLLREDSCNAGPEDIQSRIGHCMLFHSKNRCLYVFGGQRSKT
YLNDFFSYDVDSDHVDIISDGTKKDSGMVPMTGFTQRATIDPELNEIHVLSGLSKDKEKR
EENVRNSFWIYDIVRNSWSCVYKNDQATKDNLSKSLQEEEPCPRFAHQLVYDELHKVHYL
FGGNPGKSCSPKMRLDDFWSLKLCRPSKDYLLRHCKYLIRKHRFEEKAQMDPLSALKYLQ
NDLYITVDHSDPEETKEFQLLASALFKSGSDFTALGFSDVDHTYAQRTQLFDTLVNFFPD
SMTPPKGNLVDLITL
>P15624
MPTVSVNKQQLFDLLGKNYTSQEFDELCFEFGMEMDEDTTEEALKTGEEPELKLDISANR
YDLLCIEGISQSLNEYLERKERPDYKLSKPTTKLIIDKSTEQIRPFATAAVLRNIKLNEK
SYASFIALQDKLHANLCRNRSLVAMGTHDLDSIEGPFHYRALPPKDIKFVPLNQTQEFTG
DKLIEFYKSPEQKNNIGRYVHIIEDSPVFPVIMDSKDRVCSLPPLINSEHSKISVNTRNI
LIDITATDKTKAEIVLNILTTMFSRYCDEPFTVEPVEIVSEHNGQSRLAPNFNDRIMDVS
IKYINSCLGLDQSADEIAHCLKKMSLHAVQSKEDKDILHVDIPVTRPDILHACDIMEDAA
VGYGFNNLPKGEKLSNANFIAKPLPINKVSDIFRVASSQATWVEVLPLTLCSHDENFKFL
RQSDNGDLAVKLANPKTLEYQVVRTTLLPGILKTVKENRKHSLPIKVFETGDVVFKDDKL
ERKAYNERHWAAIYVGKNSGFEIIQGLLGKIMQTFRTEWIADYGAAASGRGYWIEEDDSV
KTYFPGRGAKVMFRSKEGAEPKQIGHLGVLHPEVMMNFDVPFAASFVEVNAEVFL
>P21266
MSCESSMVLGYWDIRGLAHAIRLLLEFTDTSYEEKRYTCGEAPDYDRSQWLDVKFKLDLD
FPNLPYLLDGKNKITQSNAILRYIARKHNMCGETEEEKIRVDIIENQVMDFRTQLIRLCY

SSDHEKLKPQYLEELPGQLKQFSMFLGKFSWFAGEKLTFVDFLTYDILDQNRIFDPKCLD
EFPNLKAFMCRFEALEKIAAYLQSDQFCKMPINNKMAQWGNKPVC
>P40063
MYSNHNLNSDDCCFDWNEEKAAELQRTGVSFDRSLTPQSLRTSTRRLSEENKQQSGTMHI
DTSPSVVSDIISSRRDRSQDFFGPHSSSPIAPSERQRADQRSRLESMRLTRRRDKMTKVR
GGLEKMEEMIMQGEHLREMQRLKQEAQKNALPSDMAEYMEWQNNEDLEDDELLAFIEKQE
TYKNELEHFLNNANKNVYENNSYPNSHT
>P21524
MYVYKRDGRKEPVQFDKITARISRLCYGLDPKHIDAVKVTQRIISGVYEGVTTIELDNLA
AETCAYMTTVHPDYATLAARIAISNLHKQTTKQFSKVVEDLYRYVNAATGKPAPMISDDV
YNIVMENKDKLNSAIVYDRDFQYSYFGFKTLERSYLLRINGQVAERPQHLIMRVALGIHG
RDIEAALETYNLMSLKYFTHASPTLFNAGTPKPQMSSCFLVAMKEDSIEGIYDTLKECAL
ISKTAGGIGLHIHNIRSTGSYIAGTNGTSNGLIPMIRVFNNTARYVDQGGNKRPGAFALY
LEPWHADIFDFIDIRKNHGKEEIRARDLFPALWIPDLFMKRVEENGTWTLFSPTSAPGLS
DCYGDEFEALYTRYEKEGRGKTIKAQKLWYSILEAQTETGTPFVVYKDACNRKSNQKNLG
VIKSSNLCCEIVEYSAPDETAVCNLASVALPAFIETSEDGKTSTYNFKKLHEIAKVVTRN
LNRVIDRNYYPVEEARKSNMRHRPIALGVQGLADTFMLLRLPFDSEEARLLNIQIFETIY
HASMEASCELAQKDGPYETFQGSPASQGILQFDMWDQKPYGMWDWDTLRKDIMKHGVRNS
LTMAPMPTASTSQILGYNECFEPVTSNMYSRRVLSGEFQVVNPYLLRDLVDLGIWDEGMK
QYLITQNGSIQGLPNVPQELKDLYKTVWEISQKTIINMAADRSVYIDQSHSLNLFLRAPT
MGKLTSMHFYGWKKGLKTGMYYLRTQAASAAIQFTIDQKIADQATENVADISNLKRPSYM
PSSASYAASDFVPAAVTANATIPSLDSSSEASREASPAPTGSHSLTKGMAELNVQESKVE
VPEVPAPTKNEEKAAPIVDDEETEFDIYNSKVIACAIDNPEACEMCSG
>P0C2H9
MAGLKDVVTREYTINLHKRLHGVSFKKRAPRAVKEIKKFAKLHMGTEDVRLAPELNQAIW
KRGVKGVEYRLRLRISRKRNEEEDAKNPLFSYVEPVLVASAKGLQTVVVEEDA
>P19889
MVRENKAAWKAQYFIKVVELFDEFPKCFIVGADNVGSKQMQNIRTSLRGLAVVLMGKNTM
MRKAIRGHLENNPQLEKLLPHIKGNVGFVFTKGDLAEVRDKLLESKVRAPARPGAIAPLH
VIIPAQNTGLGPEKTSFFQALSIPTKISKGTIEIINDVPILKPGDKVGASEATLLNMLNI
SPFSYGLIVNQVYDSGSIFSPEILDIKPEDLRAKFQQGVANLAAVCLSVGYPTIASAPHS
IANGFKNLLAIAATTEVEFKEATTIKEYIKDPSKFAAAASASAAPAAGGATEKKEEAKKP
ESESEEEDDDMGFGLFD
>O74522
MADFMDIEPSSHSAKASQYESSAPASSSLGNSHPNESLDYYIYDYFVKHNFEEAAQAFLR
ESKIQIPKSSSSTAFSPSNNNAPSPFPPKNSSLASPSKISESISGDRLYNHMSSAPSPNK
KEETNVVHANEDISLDKRQSFGSSSLPPSEVSINVPEGFLVEWFNIFWDVFSARVSRVNS
TPIQLYDPSTQRQMARPMSNLQASQPVPSSTFSRSAVVPNPSLPLNPSVLQGQVMNNPTI
PKGTPSTSIEGAKTSIPPSHAMQNPHNSFPASADRLQKNHPVQSSNFNPYTPAPSITVPP
NYIPNTAMMGPSYSSFGDTDPRTYPAGMGPNPTAARNGFYPPTPAQIHQLKAQQQHLQRQ
SKQMSEPAPINMKSNKDQQLQYVDFRGVGSGADLQKQQWNKSTSAEGLQPNGLVMRNFGD
VRHQKLPTSSPPSQHPPVGQIPSQYLPYQAGLKVPGNTPIPVKQVGGMPLQSPLPVSMKP
SADDHSRATPTRSVEAPTLPSYAPRHPTQANGSRYMNPSTSRMTPQSPYMQNYYRPHAQM
QDQNNMMSYMLSQQKAMEIAKSREMAIQRNTQTLSSGNQPPQQSGPNPNEFSMSMDPANM

QQGNHALSDYHMQLMLLEEQNKKRLMMARQEQGTGSLSPQSYMNSRYSVDVGKEHMSMIP
NQTAPMIQPNVPVSANSPAQANTPAADSTKSGTIQPTNRNGEGLSYSPHQQFSPSAPQAE
KLSRSMSPFVSQQQQLNQPVSNKPDGLTQNKEVTGMPLNKEELTNPAFPQSRTSWMMPQS
FDTSSLNAPGAKDSSSFSSLHAQPGKSGIATMGVADNTIRTTERSTFSEIMKDSPSAHAS
PGAKTSPNASRAPEPTGGTNSISQDTTQSLQMQSNSVNSSSMVDASKSKEKSGGDTSALD
SNAKNEPTAAKPISKLEDDALFNDSAGNAFGFSSKTDSNVEMLNDFDFESFLNDAGADSA
SVYY
>O94664
MPRRAVLFRGNSFISFSSISSGNESSAHGDASLGQADKPLTVDVLDAWTTQFTKKQTRVF
HEAYVSAYIDLQQKYARLERLQEQILNLERSLLNYKSLKSNSTTTVATVPSEKRYFFRRK
PKTSSTTNSLSSSSSSVSHMNQDANPNSTVLTFTAVPYDSCLPAPDRHTPSSASSRASET
GTTSPQSMRNQISLLYSKVDQVKTEIASAQAVMHDLLRTYPGSESLQQSTHTQF
>Q9P6I7
MSNLPIYNFIRKLPKCEHHVHLEGCLSPDLVFRLAKKNGITLPSDDAAYTTPSTLLASYE
HFGCLDDFLRYYYIAVSVLIEASDFEALAYEYFSIAHSQGVHHAEVFFDPQTHTSRGISY
DVVVSGFSAACERANRDFGMSTNLIMCFLRHLPSEAAHETFAEALKRNDFENGIVAGVGL
DSSEVDFPPELFQEVYKLAAEKGIRRTGHAGEEGDPSYIRSGLDNLSLQRIDHGIRLVED
KELMKRVAEENIMLTMCPLSNLKLRCVNSIAELPVREFLEAGVPFSINCDDPAYFGGYTL
ENYFAIQKHFNLTVKEWVFIANAAINGSWISGKRKEELLSSVQKCVKEYTAEIQQPKTLE
TAVEVQA
>Q05584
MQVKSIKMRWESGGVNYCYLLSDSKNKKSWLIDPAEPPEVLPELTEDEKISVEAIVNTHH
HYDHADGNADILKYLKEKNPTSKVEVIGGSKDCPKVTIIPENLKKLHLGDLEITCIRTPC
HTRDSICYYVKDPTTDERCIFTGDTLFTAGCGRFFEGTGEEMDIALNNSILETVGRQNWS
KTRVYPGHEYTSDNVKFVRKIYPQVGENKALDELEQFCSKHEVTAGRFTLKDEVEFNPFM
RLEDPKVQKAAGDTNNSWDRAQIMDKLRAMKNRM
>Q9H257
MSDYENDDECWSVLEGFRVTLTSVIDPSRITPYLRQCKVLNPDDEEQVLSDPNLVIRKRK
VGVLLDILQRTGHKGYVAFLESLELYYPQLYKKVTGKEPARVFSMIIDASGESGLTQLLM
TEVMKLQKKVQDLTALLSSKDDFIKELRVKDSLLRKHQERVQRLKEECEAGSRELKRCKE
ENYDLAMRLAHQSEEKGAALMRNRDLQLEIDQLKHSLMKAEDDCKVERKHTLKLRHAMEQ
RPSQELLWELQQEKALLQARVQELEASVQEGKLDRSSPYIQVLEEDWRQALRDHQEQANT
IFSLRKDLRQGEARRLRCMEEKEMFELQCLALRKDSKMYKDRIEAILLQMEEVAIERDQA
IATREELHAQHARGLQEKDALRKQVRELGEKADELQLQVFQCEAQLLAVEGRLRRQQLET
LVLSSDLEDGSPRRSQELSLPQDLEDTQLSDKGCLAGGGSPKQPFAALHQEQVLRNPHDA
GLSSGEPPEKERRRLKESFENYRRKRALRKMQKGWRQGEEDRENTTGSDNTDTEGS
>P02412
MGIDIRHNKDRKVRRKEPKSQDIYLRLLVKLYRFLARRTNSSFNRVVLKRLFMSRTNRPP
LSMSRLIRKMKLQGRENKTAVVVGYITDDVRIHDIPKLKVCALKITSGDRSRILKSGGQI
MTFDQLALAAPKGQNTVLLSGPRKAREVYRHFGKAPGTPHSRTKPYVLSKGRKFERARGR
RASRGYKN
>Q8CJ27
MATMQAASCPEERGRRARPDPEAGDPSPPVLLLSHFCGVPFLCFGDVRVGTSRTRSLVLH
NPHEEPLQVELSLLRAAGQGFSVAPNRCELKPKEKLTISVTWTPLREGGVREIVTFLVND

FLKHQAILLGNAEEPKKKKRSLWNTSKKIPASSKHTKRTSKNQHFNESFTISQKDRIRSP
LQPCENLAMSECSSPTENKVPTPSISPIRECQSETCLPLFLRESTAYSSLHESENTQNLK
VQDASISQTFDFNEEVANETFINPISVCHQSEGDRKLTLAPNCSSPLNSTQTQIHFLSPD
SFVNNRYTSDNDLKSMKNVLSDTFRKDPAESVCLESQTVHEVCQTILSPDSFLNDNYGLK
KGLNFKSVNPVLSPTQFVKDSMGHVGQQTGKSNEASQDWRINEGLAYTPECQHAQTPSSR
SEKQNPVEVKPHKYDFTKQKPKTCEFQDAFCHQSKQPHKRRPILSATVTKRKPTNAREKL
PEINKPDAKRCLEGLVGQRGKEVGSLREKGFHPSLPVVEPGVSKALSYRDEVTPATVVVA
RKRKSHGTVGDANGKVAAEEWMDMCEVKRIHFSPLESTPSTVARTTKKEGLTSKRISSLE
RSGLKKKMDSSILKTPLSKTKKKRRSIVAVAQSHLTFIKPLKAAIPRHPMPFAAKNMFYD
ERWKEKQEQGFTWWLNYILTPDDFTVKTNVSKVNAASLVLGAESQHKISVPKAPTKEEVS
LRAYTASCRLNRLRRTACSLFTSEKMVKAIKKVEIEIEVGRLLVRKDRHLWKDIGQRQKV
LNWLLSYNPLWLRIGLETVFGELIPLADNSDVTGLAMFILNRLLWNPDIAAEYRHPTVPL
LFRDGHEAALSKFTLKKLLLLICFLDHAKISRLIDHDPCLFCKDAEFKASKELLLAFSRD
FLSGEGDLSRHLSFLGLPVSHVQTPLDEFDFAVTNLAVDLQCGVRLVRTVELLTQNWNLS
DKLRIPAISRVQKMHNVDLVLQVLKSRGVPLTDEHGSAISSKDVVDRHREKTLGLLWKIA
LAFQVDISLNLDQLKEEIDFLKHTHSIKRAMSALTCPSQAITNKQRDKRISGNFERYGDS
VQLLMDWVNAVCAFYNKKVENFTVSFSDGRVLCYLIHHYHPCYVPFDAICQRTSQSVACA
QTGSVVLNSSSESEGGCLDLSLEALDHESTPEMYKELLENEKKNFHLVRSAARDLGGIPA
MIHHSDMSNTIPDEKVVITYLSFLCARLLDLRKEIRAARLIQTTWRKYKLKRDLKHHQER
DKAARVIQSVVLNFLSRRRLQKNVSAALVIQKCWRRVSAQRKLRMLKNEKLAKLQNKSAV
LIQAYWRRYSTRKRFLRLKHYSVILQSRIRMKIALTSYKRYLWATVTIQRHWRAYLSGKR
DQQIFRKLKSSSLVIQFMFRRWKRRKLQLQTKAAVTLQRAFREWHLRKQIRERSAVVIQS
WYRMHRELQKYIYIRSCVIVIQRRVRCFQAQKLYKRRKDAILTLQKHYRARQKGKLAHAD
YLQKRAATIRLQAAFRGMKARHSYRLQIGAACVLQSYWRMRQERVRFLNLKKMVIKLQAH
IRKYQQLQKYKKIKKAAITIQTHFRASISARRVLASYQKTRSSVIVLQSACRGMQARKAF
RHALASVIKIQSYYRAYICRKTFQNFKNATIKLQSIVKMKQSRKQYLQIRAAALFIQRWY
RSQKLASQKRKEYIQVRESCIKLQSHFRGCLVRKQLRLQCKAAISLQSYFRMRTARQRYL
KMCKAALVIQSFYCAYRAQISQRKNFLQVKRAAICLQAAYRGCKVRRQIKQQSTAAVTIQ
RVFRGHSQRMKYQTMLQSAVKIQRWYRAQKVAYDMRIQFLKTREAVVCLQSAYRGWQVRQ
QLRRQHEAAVKIQSTFRMAVAQQQYKLLRAAAAVIQQHVRARAAGKRQHLAYIQLRHAAL
VFQAAWKGKMLRRQIARQHQCAALIQSYYRMHIQRRKWSIMKTAALQIQLCYRAYKVGKE
QRHLYLKTKAAVVTLQSAYRGMKVRKRVAECHKAAVTIQSKFRAYRTQKKYTTYRTSAIV
IQRWYRNIKITTQQHQEYLNLRRAAVQVQAAYRGIRVRRRIQHMHMAATLIEAMFKMRQS
RVRYLKMRTAALIIQVRYRAYYLGKIQHEKYLRTLKAIKTLQAGVRGARVRRTVRKMHFA
ATLIQSHFRGHRQQTYFHRLRKAATMVQQRYRAVKEGSAEFQRYSRLRRSVLLIQAAFRG
LRTRRHLKAMHLAATLIQRRFRTFAMRRKFLSLRKTAIWIQRQYRARLYAKYSRQQLLLE
KAVIKIQSSYRGWVVRKRVQKMHRAATVIQATFRMHGAYMRYQHLKRASVVIQVHTAAEL
QRQKHAAVILQAAVRGMKTRSHLKTMHSSATLIQSQFRAFIVRRRFIALRKAAIFVQRKF
RATLYAKHKLHQFLQLRKAAITIQSSYRRLMVQKKLQEMHRAAALIQATFRMHRTYVAFH
IWKCASIRIQQCYRTYRTIKLQKEKLIREEQHSAAVLIQSTYRMYRQRCFYQQRRWAAKV
IQKTYRANKRRQDLLYVCKEETPLLQMHFQGLNTAKQGRQQHGAAMITQKHFRAFKARRL
MEAERGFQAGCRKYKAKKYLSKVEAACRIQAWYRRWRAHKKYLTLLKAVNIIEGYLSAQL
ARRRFLKMRAAAIIIQRKWRATLSVRGARENLKRHRAACVIQAHFRGYQARQSFLQQRSA
VLIIQRHVRAMVAAKQERIKYIKLKKSTVVVQALVRGWLVRKRVSEQKAKTRLFHFTAAA

YCHMCALKIQRAYRLHVTLRNAKKHMDSVIFIQRWFRKRLQRKRFIEQYHKILSTRREAH
ACWLQQDRAASVIQKAVRRFLLCRRQEKITSCATRIQALWRGYSWRKKNDHTEIKAIRRS
LRAVSTTVEEENKLYRRTERALHHLLTYKHLSAILDALKHLEVVTRLSPLCCENMAESGA
VSTIFVVIRSCNRSVPCMEVVGYAVQVLLNVAKYDKTIAAVYEAENCVDTLLELLQVYRE
KPGDRVAEKSASIFTRTCCLLAVLLKTEQCAFDAQSRSKVTDRIYRLYKFTVPKHKVNTQ
GLFDKQKQNSCVGFPCIPERTMKTRLVSRLKPQWVLRRDNVEEITNSLQAIQLVMDTLGI
SY
>P15700
MTAATTSQPAFSPDQVSVIFVLGGPGAGKGTQCEKLVKDYSFVHLSAGDLLRAEQGRAGS
QYGELIKNCIKEGQIVPQEITLALLRNAISDNVKANKHKFLIDGFPRKMDQAISFERDIV
ESKFILFFDCPEDIMLERLLERGKTSGRSDDNIESIKKRFNTFKETSMPVIEYFETKSKV
VRVRCDRSVEDVYKDVQDAIRDSL
>P11245
MDIEAYFERIGYKNSRNKLDLETLTDILEHQIRAVPFENLNMHCGQAMELGLEAIFDHIV
RRNRGGWCLQVNQLLYWALTTIGFQTTMLGGYFYIPPVNKYSTGMVHLLLQVTIDGRNYI
VDAGSGSSSQMWQPLELISGKDQPQVPCIFCLTEERGIWYLDQIRREQYITNKEFLNSHL
LPKKKHQKIYLFTLEPRTIEDFESMNTYLQTSPTSSFITTSFCSLQTPEGVYCLVGFILT
YRKFNYKDNTDLVEFKTLTEEEVEEVLKNIFKISLGRNLVPKPGDGSLTI
>Q9UTH3
MSQDVFKHLTSKQVQRTRARSFGASFERPLASFLPKKENKNLLSNAARVKLLTFSSSSPY
SFSYSPVLSRDANDGYIPLDTSSRANLWESVTDYDLQHANEPLVGNYYISTEALGDGLNR
SPFTINPEKRRSLSDISYVPIPHKEHSNWPVHCSSQAIVTTSNTTFSIWSANDYFCLLFG
YAGSQLNRHSVFDIFPKSFSSHLSNLIVSFPIDNEHERILFCGDVFPVITVDGVRLMDFW
VKEKQGKLIWILEFVEESYIDIKLQDGVAVDERTEQPLTSDLLPSRLPKTWDQRLYLTTK
TTEGYYCPSMIYPLSKSSFQYIIFHYAAGLLFINSDFKIISLNEALFESMLGYSDLLTKD
ISLIFPDFKLVLQQLADSHALDPGRVVSEIHVRHAYRTCTADKMKKADYPYLIHSDGNII
QIDCQIISVSPHSVKSNEPAFGVWLIFDSVDNRASDFVRSMRSSVILEEVVISDESEEEE
DLSADEDYVDSEWEVVPHNIASYTTIKELGIGAYGQVKLATYKSNKVHEVILKSISKSRI
LLDSWMRDKDLGTVPMEISILHFLKAHSHPNIVKMITFFEDNENYYLLTEPQKPGIDLFD
YIELKPSISEKESKAIFFQIALAVAHLHSFDIIHRDIKDENVILEGNGCARLIDFGSSSL
TKNGPFDTFRGTVGFAAPELLRGEKYLGKEQDIWALGILLYTIVYRENPYYNIEEILDAK
LRIPFELSKDNVDLICRMLDRNVHDRITIEETLQHHWFDDIRYLDTSHIRIPLSS
>Q2QSB9
MPNVVDPPQISFAAKDMDLTEWEGDILAVLVTETDVSKATSSSSRFTNAAAALAKLDGEL
GGLLSEASAEEEFAGRAGQSVALRLPTAPGLHGFKRVCLVGVGNNMPSSAAACRSTGETI
AAVAKSAQARSAAVALASPPPGWVQGEDLRLNAAAAVASGVVLGLHEDRRYKSDSKKVHL
KQVDLIGFGSGQEMGRKLQYANHVSSAVIFAKELVNSPANVLTPAVLAEEASNIASSYSD
VLTATILDEEKCRELKMGSYLAVAAASANPPHFIHLCYKPPGGNVKRKLAIVGKGLTFDR
FYLSLDNLLIVTKFVCIGGYNIKIGAVTTIELMKKDMGGSAALFGAAKALGQIKPPGVEV
HFISAACENMISGTGMRPGDIVTASNGKTIEVDNTDAEGRLTLADALVYACKLGVDKIID
LATLTGYCRIALGPSIAGILTPSDELDKEVAAAYEASGEKFWRLPLEESYWEQMKSSVAD
MLNTGSPLGGAITAGLFLKQFVDEKVKWMHVDMAGPVWNYKKQEATGFGVSTLVEWVLIN
SS
>P51116

MGGLASGGDVEPGLPVEVRGSNGAFYKGFVKDVHEDSVTIFFENNWQSERQIPFGDVRLP
PPADYNKEITEGDEVEVYSRANEQEPCGWWLARVRMMKGDFYVIEYAACDATYNEIVTLE
RLRPVNPNPLATKGSFFKVTMAVPEDLREACSNENVHKEFKKALGANCIFLNITNSELFI
LSTTEAPVKRASLLGDMHFRSLRTKLLLMSRNEEATKHLETSKQLAAAFQEEFTVREDLM
GLAIGTHGANIQQARKVPGVTAIELGEETCTFRIYGETPEACRQARSYLEFSEDSVQVPR
NLVGKVIGKNGKVIQEIVDKSGVVRVRVEGDNDKKNPREEGMVPFIFVGTRENISNAQAL
LEYHLSYLQEVEQLRLERLQIDEQLRQIGLGFRPPGSGRGSGGSDKAGYSTDESSSSSLH
ATRTYGGSYGGRGRGRRTGGPAYGPSSDVSTASETESEKREEPNRAGPGDRDPPTRGEES
RRRPTGGRGRGPPPAPRPTSRYNSSSISSVLKDPDSNPYSLLDTSEPEPPVDSEPGEPPP
ASARRRRSRRRRTDEDRTVMDGGLESDGPNMTENGLEDESRPQRRNRSRRRRNRGNRTDG
SISGDRQPVTVADYISRAESQSRQRPPLERTKPSEDSLSGQKGDSVSKLPKGPSENGELS
APLELGSMVNGVS
>P41227
MNIRNARPEDLMNMQHCNLLCLPENYQMKYYFYHGLSWPQLSYIAEDENGKIVGYVLAKM
EEDPDDVPHGHITSLAVKRSHRRLGLAQKLMDQASRAMIENFNAKYVSLHVRKSNRAALH
LYSNTLNFQISEVEPKYYADGEDAYAMKRDLTQMADELRRHLELKEKGRHVVLGAIENKV
ESKGNSPPSSGEACREEKGLAAEDSGGDSKDLSEVSETTESTDVKDSSEASDSAS
>P57678
MDLGPLNICEEMTILHGGFLLAEQLFHPKALAELTKSDWERVGRPIVEALREISSAAAHS
QPFAWKKKALIIIWAKVLQPHPVTPSDTETRWQEDLFFSVGNMIPTINHTILFELLKSLE
ASGLFIQLLMALPTTICHAELERFLEHVTVDTSAEDVAFFLDIWWEVMKHKGHPQDPLLS
QFSAMAHKYLPALDEFPHPPKRLRSDPDACPTMPLLAMLLRGLTQIQSRILGPGRKCCAL
ANLADMLTVFALTEDDPQEVSATVYLDKLATVISVWNSDTQNPYHQQALAEKVKEAERDV
SLTSLAKLPSETIFVGCEFLHHLLREWGEELQAVLRSSQGTSYDSYRLCDSLTSFSQNAT
LYLNRTSLSKEDRQVVSELAECVRDFLRKTSTVLKNRALEDITASIAMAVIQQKMDRHME
VCYIFASEKKWAFSDEWVACLGSNRALFREPDLVLRLLETVIDVSTADRAIPESQIRQVI
HLILECYADLSLPGKNKVLAGILRSWGRKGLSEKLLAYVEGFQEDLNTTFNQLTQSASEQ
GLAKAVASVARLVIVHPEVTVKKMCSLAVVNLGTHKFLAQILTAFPALRFVEVQGPNSSA
TFMVSCLKETVWMKFSTPKEEKQFLELLNCLMSPVKPQGIPVAALLEPDEVLKEFVLPFL
RLDVEEVDLSLRIFIQTLEANACREEYWLQTCSPFPLLFSLCQLLDRFSKYWPLPKEKRC
LSLDRKDLAIHILELLCEIVSANAETFSPDVWIKSLSWLHRKLEQLDWTVGLRLKSFFEG
HFKCEVPATLFEICKLSEDEWTSQAHPGYGAGTGLLAWMECCCVSSGISERMLSLLVVDV
GNPEEVRLFSKGFLVALVQVMPWCSPQEWQRLHQLTRRLLEKQLLHVPYSLEYIQFVPLL
NLKPFAQELQLSVLFLRTFQFLCSHSCRNWLPLEGWNHVVKLLCGSLTRLLDSVRAIQAA
GPWVQGPEQDLTQEALFVYTQVFCHALHIMAMLHPEVCEPLYVLALETLTCYETLSKTNP
SVSSLLQRAHEQRFLKSIAEGIGPEERRQTLLQKMSSF
>Q9USP4
MKEMINLDSSSEPSIVTKQFSVEECLSKLKEQHCYVPMRTIGRLRLRKSSDQTEKKCWKE
KLMKIRSELEELWEQSMMYEEQKELTQLGEMLDRLWDKHINSEGSKSETISDTAISGNDD
TMEKRLEQFSDDTLQDTLETEKNLNSKTSESLKSPTLSYPFDLDSLDKRIFKLESKIGYA
DEPLSELLNKCMEKLEIVEQDPQFWQSRIESWKQLLAKDFLKHHERNLCSIEKQTTLKNS
SLKELCTEEDIVIMLEICSSQLPFVEQYMPILPLLLERLKSLQNMHTDAAEAISSWQGSK
DVMMTMQSELNEWKNTVERLDHSKFYTQSVEEMRRLSDTVTQLEKRVLKLQ
>P30533

MAPRRVRSFLRGLPALLLLLLFLGPWPAASHGGKYSREKNQPKPSPKRESGEEFRMEKLN
QLWEKAQRLHLPPVRLAELHADLKIQERDELAWKKLKLDGLDEDGEKEARLIRNLNVILA
KYGLDGKKDARQVTSNSLSGTQEDGLDDPRLEKLWHKAKTSGKFSGEELDKLWREFLHHK
EKVHEYNVLLETLSRTEEIHENVISPSDLSDIKGSVLHSRHTELKEKLRSINQGLDRLRR
VSHQGYSTEAEFEEPRVIDLWDLAQSANLTDKELEAFREELKHFEAKIEKHNHYQKQLEI
AHEKLRHAESVGDGERVSRSREKHALLEGRTKELGYTVKKHLQDLSGRISRARHNEL
>Q03016
MITNTEFDVPVDWLYKGKSRRKTNTKPSRPSTSPASSSSTSSSKNGDNSTSGNRSSNDKP
RARSSSVSNAALCNTEKPDLKRNDGNTSASDTDNIPLLTPINSGNRSDSADIDNPATVDA
IDLIDNDDNGSSTQFVRKKRSTSISNAVVSSKPRLASSAINATASSSVGKGKHPPISSPS
NATLKRSNSTSGEKTKRSIFGSLFSKRSTSSSASTAKKPLPVVNTSTTENESGGIKAVAT
PDPRVKEISSPMRGVAPTASKPQTPILPSPALAVKDLSTVSLKRVSFAVDKFESDPPQQL
PSRTPKKGNILIPDDMISEVPSISVGISSSNQSAKSTNSNIKGPLYTKKSKEYILALENQ
KLALREAAKHQQEAHFAANRIAFEVANFKTASDAGGKLTEKSSEGTITKQREEVSPPNVE
ADRELENNKLAENLSKAGIDKPIHMHEHYFKEPDQDKYQDGHSIENNEVTLDVIYTRCCH
LREILPIPSTLRQVKDKTAPLQILKFLNPKPTLIDILSFCDFITIAPIHTIVFDNVALNQ
DMFRIIISALVNSTVLDKLSLRNVRIDQDGWKLLCKFLLLNKSLNKLDISQTKIKSDLAE
SLYRHNMDWNLFTDVLSQRSHKPIEELLFNGIQFSKIPYSCFARLLTSFATQKNFPESGI
RLGLAGATTSNISQDCLKFIFNWMSQYNVQGVDLAFNDLSTMIKPMVGKLSALSYDNLRY
FILNSTNISTSYDLALLLKYLSKLPNLIFLDLSNLSQCFPDILPYMYKYLPRFPNLKRIH
LDSNNLTLKELAVVCNILIKCKSLSHVSMTNQNVENFYLMNGTDSPVQQTNTDGDLDSSS
TLDVKGQFAKNSFSSTLYAFARDSPNLIGLDFDYDLISEEIQSRIALCLMRNMKRTMDST
FQLDELDSQDDLLFDGSLVTMTAESVLEKLNLLSDKSTKVKKDTTKRYLLKKYIEKFHIL
HHNVQHTIDTMFEKRKSGELPLQEKENLVRLLLLEQNLCNILELFSHNPNLNDVLGSSRD
DSKESVDSSEDSKLPALKHVESGYHVPEEKIQPENDVITARPHLMATDSGKTIDVFTGKP
LVFKHTSSSTSVGCKKQEEEEGELHKWGFFVQQQRSLYPENESTRQTPFASGDTPINTET
AGKSTSSPSVSTSNNETATTSLFSPANPKILPKIPSGAVLRSAIMKAKGIDSIDDLIQNV
NSNNIELENIYGESIQNSASTFTPGVDSDVSAPNTDKGSVETLPAVSTDDPNCEVKVTAT
YDKLLNNLSMERSIRL
>O18601
MTIKNNNNNNTLKHLQAFPPLKNDNLLRAARGEVVDRVPVWVMRQAGRYLPEFQELRKQH
DFFTVCRTPELACEVTMQPLRRFDLDASIIFSDILVIPQALGLTVEMHAGVGPVLPQPIC
TPEDLKRLTPDGALSRLTYVGDAITMMRHKLDGRVPLIGFTGAPWTLMGYMIEGGGSKTM
SKAKAWLTNYPEDTKLFLILLTDVIVDYLEMQVIAGAQMLQVFESSAEHLSKEEFLLGSE
PYLRRIRDDLVDRLTKKVIPAVPLVSSYPNNIFTLLTYLISLLIIFAKGAGHSLKEQSEL
GYDVIGLDWTVDPVEARAVVGPNITLQGNLDPQSMYCEPNELRSLATEMVHKCGKSRYIA
NVGHGITPQTPITSMEVLVEAAHNAL
>P20135
MGLELYLDLLSQPCRSIYIFARTNNIPFEFKHVELFKDSVLGKKPAAASGAERPRTGPSN
SEGDGKISLLKKVPVLKDGDFTLAECTAILLYLSRKYNTPDHWYPSDIKKRAQVDEYLSW
HHANIRANAPKTMWIKVLIPLFTGQPQPSEKLQEVMEGLSTSLKQFEERFLQDKAFIIGS
EISLADLVAIVELMQPVGVGCDIFEDRPRLMEWRRRVEEAVGKELFFQAHEMILNIKELS
NIQIDPQLKEHLAPVLMKMLK
>O13905

MDLYDYMAPTTTTEEDDEEGYEKLKEEMGSALNNLTNGKFGLFWNSMKEKSENFLDDTKG
KASSGMQQLKSQLEENIPVNSAMENLRKVEETAGSFWSGFGSTVNGFLDQALQISPKEDD
VSTPTHEASSSVFLNRHERQLLELVQNENTFTQIISEPSHGITFESWEKEISIDGKTEEI
SLLLEEYPDLRKQMESLVPSEVSYDDFWKRFFWHKEVVQPIKAIQSGNDEEEIFSWGDER
SDEEESDNEQVNDEKKQSSEEDTTENNSAAEVIDETVNDLESAVSKGLQIETQPASHDGE
VDGEVKEEEENKVSSSSNIEASQSSLEVKDEANRKVDDDDEDDDDWE
>Q9UUK0
MASKVDSSHEFPSQIKRCLSSSKYIQLATCFHDQPHSSLMTFTYLPAGSAAPYEVEDCII
LSTGENSKKYFNISSNPRVSLLVHDWTTNRQETDPDASSLYTLLYKMNQAQFSNTSVTLN
GLATVLPKNSKEEEFFREKHLNTNDKGNTKQYVEGEDMRIIKIKLESARICDQRLNNVQK
WNARGDETPF
>P53197
MSTNLNPFMNNTPSSSPLKGSESKRVSKRPISSSSSASLLSSPSRRSRPSTVYGDRYIPS
RTDIDFNSIVSISSMASVPALNPSSTEDQVEYQKERQAHETYNTLLKNELFGEMLSKDTV
GSESSIDRIKNTRPSTRGNVHAENTTRHGYELERVSTPPPEAAGLEEFSPHSTPVTPRRL
FTSQQDEITRPSSNSVRGASLLTYQQRKGRRLSAASLLQSQFFDSMSPVRPDSKQLLLSP
GKQFRQIAKVPYRVLDAPSLADDFYYSLIDWSSTDVLAVALGKSIFLTDNNTGDVVHLCD
TENEYTSLSWIGAGSHLAVGQANGLVEIYDVMKRKCIRTLSGHIDRVACLSWNNNHVLTSG
SRDHRILHRDVRMPDPFFETIESHTQEVCGLKWNVADNKLASGGNDNVVHVYEGTSKSPI
LTFDEHKAAVKAMAWSPHKRGVLATGGGTADRRLKIWNVNTSIKMSDIDSGSQICNMVWS
KNTNELVTSHGYSKYNLTLWDCNSMDPIAILKGHSFRVLHLTLSNDGTTVVSGAGDETLR
YWKLFDKPKAKVQPNSLIFDAFNQIR
>Q9Y6Z7
MNGFASLLRRNQFILLVLFLLQIQSLGLDIDSRPTAEVCATHTISPGPKGDDGEKGDPGE
EGKHGKVGRMGPKGIKGELGDMGDQGNIGKTGPIGKKGDKGEKGLLGIPGEKGKAGTVCD
CGRYRKFVGQLDISIARLKTSMKFVKNVIAGIRETEEKFYYIVQEEKNYRESLTHCRIRG
GMLAMPKDEAANTLIADYVAKSGFFRVFIGVNDLEREGQYMFTDNTPLQNYSNWNEGEPS
DPYGHEDCVEMLSSGRWNDTECHLTMYFVCEFIKKKK
>O60112
MDDPNSYDVIIVGTNLRNSILSAALSWANQRVLHIDENSFYGEIDGSLTLRDLEQINEKI
KKVDSSQILNDNGSHKSPLKRFEVQFLNKDLIPKNKGSVIQFHPQEIFASSELVKLLSET
KIYKYLLLKPARSFRLLTSNEEWIKVPESRADIFNNKNLSLASKRIVMRFMKFVSNIADE
QNQNLVKEWESKPFYKFLEEVFQLSGAIEESIIYGLCQSLSKDIPTKDALDTVLKYFHSF
GMYGDYSYLLAMYGTGSELCQGFCRSSAVMGGTFMLGQAIDKIDESKIVLKDGSTLSAKK
IVSSVDEGKLPHQQIQQRYLLVKGDCQQLFQEDGFFAALDASLIHFSPFSISENFGNSVQ
AKLYGSGSGDCPEGYCIWYLKTLNDSPCNEVFNLATKKLCSFSGCDDLEIIVQVDETLNQ
LRHIDYDDTLHSAKSLFYEILGQNNTFLQREGLFDEDDE
>O74957
MSYKPSSEIALRPGYGGLGKQITLKANFFQIISLPNETINQYHVIVGDGSRVPRKQSQLI
WNSKEVKQYFGSSWMNSVYDGRSMCWSKGDIADGTIKVNIGSESHPREIEFSIQKSSKIN
LHTLSQFVNSKYSSDPQVLSSIMFLDLLLKKKPSETLFGFMHSFFTGENGVSLGGGVEAW
KGFYQSIRPNQGFMSVNVDISSSAFWRNDSLLQILMEYTDCSNVRDLTRFDLKRLSRKFR
FLKVTCQHRNNVGTDLANRVYSIEGFSSKSASDSFFVRRLNGEEQKISVAEYFLENHNVR
LQYPNLPCILVKNGAMLPIEFCFVVKGQRYTAKLNSDQTANMIRFAVQRPFERVQQIDDF

VHQMDWDTDPYLTQYGMKIQKKMLEVPARVLETPSIRYGGDCIERPVSGRWNLRGKRFLD
PPRAPIRSWAVMCFTSTRRLPMRGIENFLQTYVQTLTSLGINFVMKKPPVLYADIRGSVE
ELCITLYKKAEQVGNAPPDYLFFILDKNSPEPYGSIKRVCNTMLGVPSQCAISKHILQSK
PQYCANLGMKINVKVGGINCSLIPKSNPLGNVPTLILGGDVYHPGVGATGVSIASIVASV
DLNGCKYTAVSRSQPRHQEVIEGMKDIVVYLLQGFRAMTKQQPQRIIYFRDGTSEGQFLS
VINDELSQIKEACHSLSPKYNPKILVCTTQKRHHARFFIKNKSDGDRNGNPLPGTIIEKH
VTHPYQYDFYLISHPSLQGVSVPVHYTVLHDEIQMPPDQFQTLCYNLCYVYARATSAVSL
VPPVYYAHLVSNLARYQDVTADDTFVETSEASMDQEVKPLLALSSKLKTKMWYM
>Q04233
MQKSVRVGDYFDNDDNGLWSWYLTNLRLGDFEELIGNQLKYTLLKRFLNSHFYGDNNISA
RPNKKILLVSIPENVHEDISILEIFLKDYFHLEKLEHIQISKLTHSHCYNHENHYLLTDN
LNNFQDPTFLEFASTSWQVQKNSKALNNNNRNSIPPPTISSSKASNGKLESNVSDDQWSN
INTQTSTATRTNTNTRTLTSPDTVDINVTSVNSQSNNNDTPQDNENEVDEEDATSSIVLN
FSHSRTVDSKPNRLPKIFPSYTNEDYTPSHSEIMSIDSFAGEDVSSTYPGQDLSLTTARR
EDESGQDEVEDHYSRVSHDLGDESIDQASYSMESSVSYTSYSSSSNSSSAHYSLSSSSRG
NPKRENIDHTNATYVSELSSITSSIDNLTTSTTPEEEDNLIHHNYDAQGYGSGEDDGEEV
YDDEDLSSSDYSVLSILPSISICDSLGYFRLVLQSILIQDPDTKEIFTAIRQSNNKPTMA
SVTDDWLLYDSNFSMNNLQILTLQDLLDIKRSFPKILFYTMVIVTNSGKQVEEEFKNPNY
DNREGISKEQPLDSELSLTNDPQQYFPTAYNNGYNDYIDDEDDEDDGDDASLSEQSGPQM
YIPTRMESNVTTAHRSIRTVNSIGEWAFNRHNSVTKIDKSNSNELDNSKTGESTVLSSEP
HPMTQLSNSNTTSSNFSHSLKTKNSHKPNSKGNNESNSKNELKKIKSSINAMSAVERSKS
LPLPTLLKSLSGIDNPTHATNKDRKRWKFQMNRFKNHKNSGSAGTDKSQRCAIM
>Q21067
MSTYAAYTTSTYQGRGLASGTYASGFGQLVSGMSSAGAICTTQIRDAREREKREIGLLND
RLADYIEKVRFLEAQNRCLSHDIDILRNGFSGGGHVSGLFDAEINQAKHILEQTTAHRST
FERDITGLSAEIEVFRKKWLDAVNAVKAHREDHDVDLDRLAKIEAEISLFKRKIRIVEED
VIRIRRENDGIYNEIARIKQLTHNEIALKNERSLNVSDLLQRINLLQTENNVRIEQELVF
IRRDTTADNRDYFRHELQAAIRDIRADYEAISIRNRQDIEVWYREQIRKIQDESVRVNPD
LYKEELISIRTTVTNVRSRLAEVEGRNFFLERLIDDLKNNEEAKFFEISLAERDAQIATL
RDQCTELSIQMEKLCDNEISLRAEIERYRILLNGANVTTYTSNTHGSGSGIAVGGVVGGS
TRVISQTTRTHSSSNTSYSNVPASRGGYSISGNVGGISVGGTIGSHGASAHATGGIIGSG
VQAHRGSVSSLITDKPRDRVHDEKGVDQSGRHFHSWYLGTISINQVTPSYIELKNICKIR
RVDVGGFRIEQSVNGQVLGSAQINVPLILDPQEVVRFNHRHGKYLGQFFMDVDAFDNSTV
ARTSMYNYTEPHEERAWFVYLD
>Q8N2I9
MKRRASDRGAGETSARAKALGSGISGNNAKRAGPFILGPRLGNSPVPSIVQCLARKDGTD
DFYQLKILTLEERGDQGIESQEERQGKMLLHTEYSLLSLLHTQDGVVHHHGLFQDRTCEI
VEDTESSRMVKKMKKRICLVLDCLCAHDFSDKTADLINLQHYVIKEKRLSERETVVIFYD
VVRVVEALHQKNIVHRDLKLGNMVLNKRTHRITITNFCLGKHLVSEGDLLKDQRGSPAYI
SPDVLSGRPYRGKPSDMWALGVVLFTMLYGQFPFYDSIPQELFRKIKAAEYTIPEDGRVS
ENTVCLIRKLLVLDPQQRLAAADVLEALSAIIASWQSLSSLSGPLQVVPDIDDQMSNADS
SQEAKVTEECSQYEFENYMRQQLLLAEEKSSIHDARSWVPKRQFGSAPPVRRLGHDAQPM
TSLDTAILAQRYLRK
>O73817

MSIMSYNGGAVMAMKGKQCVAIAADRRFGVQAQMVTTDFQKIFPMGDRLYIGLAGLATDV
QTVSQRLKFRLNLYELKEGRQIKPKTFMSMVSNLLYERRFGPYYIEPVIAGLDPKTFEPF
ICSLDLIGCPMVTEDFVVSGTCSEQMYGMCESLWEPDMEPEDLFETISQAMLNAVDRDAV
SGMGVVVQVIEKDKITTRTLKARMD
>O60136
MRVLVAETGREDNVSVHSREVSVNGSDSGTGNDAYKLETDDEHPPATPVRGLSTRSQKRP
FRPLTIQQDEDVENDTGMNTEYNDDNSSLVNTPRDSTTYAETNSPNTEKLAGRDDDRSLL
NLIDRGEIDSKRQRHYVPVHCKRMPKKEITLNQKLYVSQVIGPADAPKSKTIFGKRIMQW
EPSQFKESVWASEISKSGKYLATAGKDAIIRVWKVIETPERRETLLKEGPQSCGRFFTPS
SIFEPEPVLECVGHNAEVLSISWSKNDFLLTSSADRTVRLWHPKSTKSLAVFRHNEIVTC
VAFHPIDDRYFVSGSLDCKIQLWSILRHKILHWTELEYVVSTICFYPDGESIVVGMFYGL
CAIYETKNLQYVSSWLIHHSPSRNKKCRVTGLQCASIIPNDSKSDNVVLVSTNDNAIRLY
NTVTHALVLKLSDAHRVNGRVLSRLSEDNSYIISGSDTNEVVLWHIPNKVLINASRKRSV
ILHLPTETFKVCSERLTSTIIAPARTAVEAKESEHDRSLIAEGYNLVKPHSHRSYGTPPV
HPIDIIIATNMAGMTIVLCVDYDLGNSHHGFRNSKFGHFVKRLLRT
>Q84KG5
MGEVAKEEVEERRSIVAVNPQPSKGLVSSAVDLIEKAVVYLFHDKSKPCHYLSGNFAPVV
DETPPCPDLPVRGHLPECLNGEFVRVGPNPKFMPVAGYHWFDGDGMIHGMRIKDGKATYA
SRYVKTSRLKQEEYFEGPKFMKIGDLKGFFGLFMVQMQLLRAKLKVIDVSYGVGTGNTAL
IYHHGKLLALSEADKPYVVKVLEDGDLQTLGLLDYDKRLSHSFTAHPKVDPFTDEMFTFG
YAHTPPYVTYRVISKDGVMRDPVPITIPASVMMHDFAITENYSIFMDLPLYFQPKEMVKG
GKLIFSFDATKKARFGVLPRYAKDDSLIRWFELPNCFIFHNANAWEEGDEVVLITCRLEN
PDLDMVNGAVKEKLENFKNELYEMRFNMKTGAASQKQLSVSAVDFPRINESYTTRKQRYV
YGTILDNITKVKGIIKFDLHAEPEAGKKKLEVGGNVQGIFDLGPGRYGSEAVFVPRERGI
KSEEDDGYLIFFVHDENTGKSEVNVIDAKTMSAEPVAVVELPNRVPYGFHAFFVNEEQLQ
WQQTDV
>Q03441
MVDSNDDQPERHALLHNDIQTSNNSRPSLVQKKYLPTPQDTRKSRASYTGSAMINPTSKQ
SRTGSGAQRTSRTAQKLKLLPEEPFQRDSERLTDLKNQEVYSQVNRIKDKPARRDAEKLG
KAHRHLLPRSTAYCTASSYNMKELVRWLKDCRKLHHTHPKLFDECLYTPFIYNDWRGDKR
FEDEDVIRLDDEGGEIIVSDKHPDLFIFEYGVVVMWGFTEREEKAFLNDIEKFEKEKLAE
EDIQVEEFNYYVTKSYQPRIYNDFITLRDGSNYMVKLSISHAIAQSVKISLFEELVDNTI
EDTQDIPQEIAYSGKVSMSKEDIMKSIGELFILRININLHGSVLDSPEIMWSEPQLEPIY
QATRGYLEINQRVSLLNQRLEVISDLLQMLKEQLGHSHEEYLEFIVILLVGVEVLISVIN
IVVDMLASQH
>Q9Y7N7
MAKLSVNDLNDFLSPGAVCIKPAQVKKQESKNDIRIDGDAYYEVTKDTGETSELGIASIS
LNDCLACSGCITSAETVLVNLQSYQEVLKHLESRKSQEILYVSLSPQVRANLAAYYGLSL
QEIQAVLEMVFIGKLGFHAILDTNASREIVLQQCAQEFCNSWLQSRAHKNQNQVTNSVVN
EHPLIPHSTSQISGVHSNTSSNSGINENAVLPILSSSCPGWICYVEKTHSNLIPNLSRVR
SPQQACGRILKDWAVQQFSMQRNDVWHLSLMPCFDKKLEASRDEFSENGVRDVDSVLTPK
ELVEMFKFLRIDPIELTKNPIPFQQSTDAIPFWYPRITYEEQIGSSSGGYMGYVLSYAAK
MLFGIDDVGPYVSMNNKNGDLTEYTLRHPETNEQLISMATCYGFRNIQNLVRRVHGNSSV
RKGRVLLKKRVRSNAQNPTEEPSRYDYVEVMACPGGCINGGGQLPFPSVERIVSARDWMQ

QVEKLYYEPGTRSVDQSAVSYMLEQWVKDPTLTPKFLHTSYRAVQTDNDNPLLLANKW
>Q86UV5
MAPRLQLEKAAWRWAETVRPEEVSQEHIETAYRIWLEPCIRGVCRRNCKGNPNCLVGIGE
HIWLGEIDENSFHNIDDPNCERRKKNSFVGLTNLGATCYVNTFLQVWFLNLELRQALYLC
PSTCSDYMLGDGIQEEKDYEPQTICEHLQYLFALLQNSNRRYIDPSGFVKALGLDTGQQQ
DAQEFSKLFMSLLEDTLSKQKNPDVRNIVQQQFCGEYAYVTVCNQCGRESKLLSKFYELE
LNIQGHKQLTDCISEFLKEEKLEGDNRYFCENCQSKQNATRKIRLLSLPCTLNLQLMRFV
FDRQTGHKKKLNTYIGFSEILDMEPYVEHKGGSYVYELSAVLIHRGVSAYSGHYIAHVKD
PQSGEWYKFNDEDIEKMEGKKLQLGIEEDLAEPSKSQTRKPKCGKGTHCSRNAYMLVYRL
QTQEKPNTTVQVPAFLQELVDRDNSKFEEWCIEMAEMRKQSVDKGKAKHEEVKELYQRLP
AGAEPYEFVSLEWLQKWLDESTPTKPIDNHACLCSHDKLHPDKISIMKRISEYAADIFYS
RYGGGPRLTVKALCKECVVERCRILRLKNQLNEDYKTVNNLLKAAVKGSDGFWVGKSSLR
SWRQLALEQLDEQDGDAEQSNGKMNGSTLNKDESKEERKEEEELNFNEDILCPHGELCIS
ENERRLVSKEAWSKLQQYFPKAPEFPSYKECCSQCKILEREGEENEALHKMIANEQKTSL
PNLFQDKNRPCLSNWPEDTDVLYIVSQFFVEEWRKFVRKPTRCSPVSSVGNSALLCPHGG
LMFTFASMTKEDSKLIALIWPSEWQMIQKLFVVDHVIKITRIEVGDVNPSETQYISEPKL
CPECREGLLCQQQRDLREYTQATIYVHKVVDNKKVMKDSAPELNVSSSETEEDKEEAKPD
GEKDPDFNQSNGGTKRQKISHQNYIAYQKQVIRRSMRHRKVRGEKALLVSANQTLKELKI
QIMHAFSVAPFDQNLSIDGKILSDDCATLGTLGVIPESVILLKADEPIADYAAMDDVMQV
CMPEEGFKGTGLLGH
>O94431
MAENNVYGHEMKKHFMLDPDYVNVNNGSCGTESLAVYNKHVQLLKEAQSKPDFMCNAYMP
MYMEATRNEVAKLIGADSSNIVFCNSATDGISTVLLTFPWEQNDEILMLNVAYPTCTYAA
DFAKNQHNLRLDVIDVGVEIDEDLFLKEVEQRFLQSKPRAFICDILSSMPVILFPWEKVV
KLCKKYNIVSIIDGAHAIGHIPMNLANVDPDFLFTNAHKWLNSPAACTVLYVSAKNHNLI
EALPLSYGYGLREKESIAVDTLTNRFVNSFKQDLPKFIAVGEAIKFRKSIGGEEKIQQYC
HEIALKGAEIISKELGTSFIKPPYPVAMVNVEVPLRNIPSIETQKVFWPKYNTFLRFMEF
KGKFYTRLSGAVYLEESDFYYIAKVIKDFCSL
>Q8IVS8
MAAALQVLPRLARAPLHPLLWRGSVARLASSMALAEQARQLFESAVGAVLPGPMLHRALS
LDPGGRQLKVRDRNFQLRQNLYLVGFGKAVLGMAAAAEELLGQHLVQGVISVPKGIRAAM
ERAGKQEMLLKPHSRVQVFEGAEDNLPDRDALRAALAIQQLAEGLTADDLLLVLISGGGS
ALLPAPIPPVTLEEKQTLTRLLAARGATIQELNTIRKALSQLKGGGLAQAAYPAQVVSLI
LSDVVGDPVEVIASGPTVASSHNVQDCLHILNRYGLRAALPRSVKTVLSRADSDPHGPHT
CGHVLNVIIGSNVLALAEAQRQAEALGYQAVVLSAAMQGDVKSMAQFYGLLAHVARTRLT
PSMAGASVEEDAQLHELAAELQIPDLQLEEALETMAWGRGPVCLLAGGEPTVQLQGSGRG
GRNQELALRVGAELRRWPLGPIDVLFLSGGTDGQDGPTEAAGAWVTPELASQAAAEGLDI
ATFLAHNDSHTFFCCLQGGAHLLHTGMTGTNVMDTHLLFLRPR
>Q9P305
MSEDLSPTSSRVDLSNPHGFTKEGVDLSKLSPQELKLYKMYGKLPSKKDLLRHKMQDRQY
FDSGDYALKKAGVIKSDDVIVNNSSNNLPVTNPSGLRESIIRRRMSSSSGGDSISRQGSI
SSGPPPRSPNK
>Q9VF87
MTEKITLADALSNVEVLDELSLPDEQPCIEAQPCSIIYKANFDTNFEDRNGFVTGIAKYI

EEATTHANLNVLLDEGQKHAVMLYTWRCCSRAIPQPKSNEQPNRVEIYEKTVEVLAPEVN
KLLNFMYFQRKAIEAFSGEVKRLCHAEKRKDFVSEAYLLTLGKFINMFAVLDELKNMKSS
VKNDYSTYRRAAQFLKVMSDSHTLQESQNLSMFLATQNKIRDTVKDTLEKIVGYEDLLSD
VVNICVHMFETKMYLTPEEKHMLVKVMGFGLFLMDSDACNINKLDQKKKIRLDRIDRIFK
NLEVVPLFGDMQIAPFNYIKRSKHFDSSKWPLSSSNAISPQADLMVHLPQIREDHVKYIS
ELARYTNEVTTTVKENPSDAENRITADLALRGLQLLSEWTSVVTELYSWKLLHPTDHHQN
KECPVEAEEYERATRYNYTSEEKFALIEVIAMIKGLQVLMARIETVLCEAIRRNIYSELQ
DFVQLSLREPLRKAVKNKKDLIRSIIMSVRETSADWQKGYEPTDDPVAKGKKDPDGGFRI
QVPRLNVGPSSTQLYMVRTMLESLIADKSGGKRTLRKDIDGNCLLQIDTFHKTSFYWSYL
LNFSDTLQKCCDLSQLWYREFYLEMTMGRKVNKCLVRHQHNEECKDLITMEKRIQFPIEM
SMPWILTDHILQTKEPSMMEFVLYPLDLYNDSAYYALTVFRKQFLYDEVEAEVNLCFDQF
VYKLSEQIFAHYKQLAGSIFLDKRFRLECEVLGFNFQSYPRNNRYETLLKQRHVQLLGRS
IDLNKLITQRINANMHKSIELAISRFEGNDITGIVELEGLLEANRICHKLLSKYLALDNF
DGMVKEANHNVLAPYGRITLHVFVELNYDFLVNYCYNAATNRFIRTKVNLSSSQAIQREK
PPQMSHYYLWGSKQLNAAYSTQYGQYTGFVGSPHFHAMCRLLGYQGIAVVMDIILKDIVK
PLIQGSLLQFTKTLMIAMPKSCKLPRCEYGSPGVLSYYQAHLTDIVQYPDAKTELFQSFR
EFGNSIIFCLLIEQALSQEEVCDLLHAALFQNIFPRPFCKENEKPEAKQKRLEAQFANLQ
IVSNVEKIGTAKQAMIAREGDLLTRERLCCGLSIFEVILNRVKSYLDDPVWCGPPPANGI
IHVDECSEFHRLWSALQFVYCIPVRGTEYTIEELFGEGLNWAGCVMIVLLGQQRRFEALD
FCYHILRVQRVDGKDEDVKGIQLKRMVDRIRRFQVLNSQIFSILNKYLKGGDGEGSNVEH
VRCFPPPQHPSVISSSSHYQDPQKLRQSINN
>Q12291
MLLMRRFAFLTSSVYFKYIPIYSQYHYSSQFPINMNPKKVAQLPVHNKSTLPPQEIIDLF
KITFLEELYPKDQDNEKSPLTEQIQAVKSDLYNRDYNAAFNNDSKRIAYCCRWSPSRATS
YASVFAHFPELLKIIRCEIDDKDSNVLCIGGGAGGELVALASIFTLSRDFSSKFASALKI
DNEVNKKPRNLNIQLVDIADWSTVVEKLTATIKSKWLYGDSEAESFNVNFTHKDCLQMTE
PQDIKIYQGLDLITLLFTTNELFTQKKVESIKFLQRLNENCAPGCHLLILESAGSYSHIT
INNKKFPIQFLIDTILVGNRKDKGTTGPWSLVSENDSIWYRMDPKLDYSIPLENMRFFYR
LYVKN
>P45432
MERDEEASGPMMEMCTNGGEETSNRRPIISGEPLDIEAYAALYKGRTKIMRLLFIANHCG
GNHALQFDALRMAYDEIKKGENTQLFREVVNKIGNRLGEKYGMDLAWCEAVDRRAEQKKV
KLENELSSYRTNLIKESIRMGYNDFGDFYYACGMLGDAFKNYIRTRDYCTTTKHIIHMCM
NAILVSIEMGQFTHVTSYVNKAEQNPETLEPMVNAKLRCASGLAHLELKKYKLAARKFLD
VNPELGNSYNEVIAPQDIATYGGLCALASFDRSELKQKVIDNINFRNFLELVPDVRELIN
DFYSSRYASCLEYLASLKSNLLLDIHLHDHVDTLYDQIRKKALIQYTLPFVSVDLSRMAD
AFKTSVSGLEKELEALITDNQIQARIDSHNKILYARHADQRNATFQKVLQMGNEFDRDVR
AMLLRANLLKHEYHARSARKL
>P50111
MSNRDNESMLRTTSSDKAIASQRDKRKSEVLIAAQSLDNEIRSVKNLKRLSIGSMDLLID
PELDIKFGGESSGRRSWSGTTSSSASMPSDTTTVNNTRYSDPTPLENLHGRGNSGIESSN
KTKQGNYLGIKKGVHSPSRKLNANVLKKNLLWVPANQHPNVKPDNFLELVQDTLQNIQLS
DNGEDNDGNSNENNDIEDNGEDKESQSYENKENNTINLNRGLSRHGNASLIRRPSTLRRS
YTEFDDNEDDDNKGDSASETVNKVEERISKIKERPVSLRDITEELTKISNSAGLTDNDAI

TLARTLSMAGSYSDKKDQPQPEGHYDEGDIGFSTSQANTLDDGEFASNMPINNTMTWPER
SSLRRSRFNTYRIRSQEQEKEVEQSVDEMKNDDEERLKLTKNTIKVEIDPHKSPFRQQDE
DSENMSSPGSIGDFQDIYNHYRQSSGEWEQEMGIEKEAEEVPVKVRNDTVEQDLELREGT
TDMVKPSATDDNKETKRHRRRNGWTWLNNKMSREDDNEENQGDDENEENVDSQRMELDNS
KKHYISLFNGGEKTEVSNKEEMNNSSTSTATSQTRQKIEKTFANLFRRKPHHKHDASSSP
SSSPSSSPSIPNNDAVHVRVRKSKKLGNKSGREPVEPIVLRNRPRPHRHHHSRHGSQKIS
VKTLKDSQPQQQIPLQPQLEGAIEIEKKEESDSESLPQLQPAVSVSSTKSNSRDREEEEA
KKKNKKRSNTTEISNQQHSKHVQKENTDEQKAQLQAPAQEQVQTSVPVQASAPVQNSAPV
QTSAPVEASAQTQAPAAPPLKHTSILPPRKLTFADVKKPDKPNSPVQFTDSAFGFPLPLL
TVSTVIMFDHRLPINVERAIYRLSHLKLSNSKRGLREQVLLSNFMYAYLNLVNHTLYMEQ
VAHDKEQQQQQQQQP
>Q9USU3
MSAQHLHSCKFYRLPLEIIPLICRFLSVQDIQSFIRVFPSFQTILDSSNDLFWKKKNYEL
RIRRNRLLRGSYAAGVSSSAMNGFVNGTQISSTPAEREYYSKSDEIKNICGLPPGPMKVE
QIGHAIDHLVSETHTVDHLGSSIKASFFHIDDVPLEWISSICMQVQSFFSPAAREAISSL
KSRTTTSNLLLSLFVGILFEDDLWYFFNTLYMLSSTSAIEFAYFLDSIFTVVRTDYEHYR
DPLSQTLITSCTRIHNIVAVIESPYDEPNTKGLTSEQKMIVECQLNPGEVLKVKAFAGTG
KTKALLEFAKSRPKDKILYVAFNKAAKEDAELRFPFNVKCSTMHGLAYGAILAQADLPQA
KLERQLSNSTIASLLSLQVAFPKANRKNNPGTPSASLVASHIMFTLNRFMHSTDWQLGFR
HISKRSLEVTKLSKEKLLAYTKKLWSLIVNFEYTHAPLIPDAYMKLLHLYEFPNIFSKYD
YILFDEAQDFTPCMVDLIYRQKHARIVIVGDAHQCIYGFRGANACAFNENLYPSTKQLCL
TKSFRFGNSVAKYANFLLSLKGENVKLKGVQNDHAYWSSASNPNNVSGAFRFFPHTIIFR
TNKELILQSIRLSVSLPKEIPIAILGSMRKKAFQLLRSGSELAHGQRPSHPKLKDFSSWG
EFEVHVKNSAEEDAELALVYDMADELFSESFLSRLDNCEKRLMDSKDDGDNGIILATAHQ
SKGLEWDNVQLGNDFRPKFDSVSFSRIGSSRYLQEEINILYVALTRAKKRLILNDTITKL
YALECGLVRFAGGILTEDQLQPGKVALFVDWQIDKFSFFYETPAEGYNLLVEANEKSVWD
IFFGVLSGAWQNYIANTSERLKRSMLFIENQLFAVHDQ
>O94730
MGKKEKVNALEFSVNKGIFTKTRLKSLYSDFTSLFIKNPEGFLANVNTWREAIETSAWSG
KLNSRIILVFDETFESAFSSPALGRPLSLGAVAAYWIQQGVWLRKQDFLNDCKKGNLVRE
DGFSIFSILRWSFQKLGFQNQASSILSNRSPSGQYVIRKNIEKLACLVHNEAMRRCSSYT
SAIYTWDFFQDTFGSLYWEEGKLNKDEMECLLNWMCYQKHVLIFDSKIIKFLPNSQIDAQ
LASIDKSIDGSVADLIQARASIAQRSEFLNEELEQLSQVLNQAVKKGEKTIAITYLRRKK
ILSKDLERKVSSRLQLDTIISNIDNAVDNKILLIAMSSGSEALDAILAQMGGTEKVEDVL
ENVNDTLARSEEIDATIQTYNPQNIDLEDEAVEKEWQDLVAEEQKVEDIVSTLGNVSLKT
PSDTFTLTNTDSDKKTSKPEKIQAELVEQ
>P12617
MRGLRRGLSRLGPRLGPWAVPRSLRRVLRAAGPWRGQSSAGSVSERGGASMEEVLSRSVP
LLPPYETKEKAPPPAERRSAEFVRYYRGLEAGSRRAELLGCLARDFGADHGRVAEFSAKV
LQAREQEREQGALLQAEDRVRYYLTPRYRALFQHLGRLEGGLRFLVELRGDLVEGLAAKA
VDGPHVKEMSGVLKNMLSEWFSTGFLNLERVTWQSPCEVLQKISDSEAVHPVRNWVDLKR
RVGPYRRCYFFSHCAIPGEPLIILHVALTSDISSSIQSIVKDVESLETEDAEKITTAIFY
SISLAQQGLQGVELGNHLIKRVVKELQKDLPQIEAFSSLSPIPGFTKWLVGLLSSQTKEL
GRNELFTESERQEISEITEDSTTETLKKLLTNSEWVKSEKLVKALHSPLMRLCAWYLYGE

KHRGYALNPVANFHLQNGAELWRINWMGDTSPRGIAASCGMMVNYRYFLEDTASNSAAYL
GTKHIKASEQVLSFVSQFQQNSKL
>Q00341
MSSVAVLTQESFAEHRSGLVPQQIKVATLNSEEESDPPTYKDAFPPLPEKAACLESAQEP
AGAWGNKIRPIKASVITQVFHVPLEERKYKDMNQFGEGEQAKICLEIMQRTGAHLELSLA
KDQGLSIMVSGKLDAVMKARKDIVARLQTQASATVAIPKEHHRFVIGKNGEKLQDLELKT
ATKIQIPRPDDPSNQIKITGTKEGIEKARHEVLLISAEQDKRAVERLEVEKAFHPFIAGP
YNRLVGEIMQETGTRINIPPPSVNRTEIVFTGEKEQLAQAVARIKKIYEEKKKKTTTIAV
EVKKSQHKYVIGPKGNSLQEILERTGVSVEIPPSDSISETVILRGEPEKLGQALTEVYAK
ANSFTVSSVAAPSWLHRFIIGKKGQNLAKITQQMPKVHIEFTEGEDKITLEGPTEDVNVA
QEQIEGMVKDLINRMDYVEINIDHKFHRHLIGKSGANINRIKDQYKVSVRIPPDSEKSNL
IRIEGDPQGVQQAKRELLELASRMENERTKDLIIEQRFHRTIIGQKGERIREIRDKFPEV
IINFPDPAQKSDIVQLRGPKNEVEKCTKYMQKMVADLVENSYSISVPIFKQFHKNIIGKG
GANIKKIREESNTKIDLPAENSNSETIIITGKRANCEAARSRILSIQKDLANIAEVEVSI
PAKLHNSLIGTKGRLIRSIMEECGGVHIHFPVEGSGSDTVVIRGPSSDVEKAKKQLLHLA
EEKQTKSFTVDIRAKPEYHKFLIGKGGGKIRKVRDSTGARVIFPAAEDKDQDLITIIGKE
DAVREAQKELEALIQNLDNVVEDSMLVDPKHHRHFVIRRGQVLREIAEEYGGVMVSFPRS
GTQSDKVTLKGAKDCVEAAKKRIQEIIEDLEAQVTLECAIPQKFHRSVMGPKGSRIQQIT
RDFSVQIKFPDREENAVHSTEPVVQENGDEAGEGREAKDCDPGSPRRCDIIIISGRKEKC
EAAKEALEALVPVTIEVEVPFDLHRYVIGQKGSGIRKMMDEFEVNIHVPAPELQSDIIAI
TGLAANLDRAKAGLLERVKELQAEQEDRALRSFKLSVTVDPKYHPKIIGRKGAVITQIRL
EHDVNIQFPDKDDGNQPQDQITITGYEKNTEAARDAILRIVGELEQMVSEDVPLDHRVHA
RIIGARGKAIRKIMDEFKVDIRFPQSGAPDPNCVTVTGLPENVEEAIDHILNLEEEYLAD
VVDSEALQVYMKPPAHEEAKAPSRGFVVRDAPWTASSSEKAPDMSSSEEFPSFGAQVAPK
TLPWGPKR
>P15265
MSDPSKTNQCPPPCCPPKPCCPPKPCCPQKPPCCPKSPCCPPKSPCCPPKPCPCPPPCPC
PCPATCPCPLKPPCCPQKCSCCPKKCTCCPQPPPCCAQPTCCSSENKTESDSDTSGQTLE
KGSQSPQSPPGAQGNWNQKKSNK
>Q07688
MSTIPSEIINWTILNEIISMDDDDSDFSKGLIIQFIDQAQTTFAQMQRQLDGEKNLTELD
NLGHFLKGSSAALGLQRIAWVCERIQNLGRKMEHFFPNKTELVNTLSDKSIINGINIDED
DEEIKIQVDDKDENSIYLILIAKALNQSRLEFKLARIELSKYYNTNL
>O55137
MEATLNLEPSGRSCWDEPLSIAVRGLAPEQPVTLRSVLRDEKGALFRAHARYRADSHGEL
DLARTPALGGSFSGLEPMGLLWAMEPDRPFWRLVKRDVQTPFVVELEVLDGHEPDGGQRL
AHAVHERHFLAPGVRRVPVREGRVRATLFLPPEPGPFPGIIDLFGVGGGLLEYRASLLAG
KGFAVMALAYYNYDDLPKNMETMHMEYFEEAVNYLRSHPEVKGPGIGLLGISKGGELGLA
MASFLKGITAAVVINGSVAAVGNTISYKDETIPPVTILRNQVKMTKDGLKDVVDALQSPL
VDKKSFIPVERSDTTFLFLVGQDDHNWKSEFYADEISKRLQAHGKEKPQIICYPAAGHYI
EPPYFPLCSAGMHLLVGANITFGGEPKPHAMAQLDAWQQLQTFFHKQLGSECLHVSPKI
>Q99222
MLRRELNNSISNRSIENESFPFERPNVSYIISAEFDNKLGPILKHQYPKDIPGFNQFSHE
QRNGNTSVSMNLASLMIPSSIERNPGKQDITVFTLYYNKFTQNYQLFPVPKDPRFSFNLH

HREQSDGSVTNSIYYDAENHQDAKNNRYTIVLEDDELECQEVQNNQKAIDNEPLFFINVA
NTVLDTTNDRGAVIKSIAIGTPLKTFFAFKNIIVLVLDLYMKAPTQAAATDILLDCFNML
NSIDLTLINDIHSKSSIQEVLHSIHDESIITKVFLDPDSTLKKLFCINGFDTKDKYGNIV
TFHDQLIQYHFTRFQPKTLPPFLLKIPLQFNMIRREPIYIENDYNELVLKFLDKFVPYLL
KAGQKVNAWKLVINSTKLSKEDLCAFILSLANITATYASDPQSYFKGNAALIFPYMDISL
VDGLRAYVASNSDFVGCFAIIGTANPIFRYQLDIWDYYYDVDEGVFYENNSPEKEKPDTV
AEVKIGPNPLRKIFNRPHFSTNAVNESQVNLGQKLFSLLIDEYHDSDTIMSVLRRLNVLQ
LENLLDALKRREIPPNIALKDEYIMFYKDFFIFPEFFDYFTLHSIELLSNLDNCLFSLGN
TCQLFSTEQIYSQLSQILDIVKELFRMVSVSRTNIEKFLNACLNYSPFKILPTAQLHGDN
ISRWSFESEVRQGFDNFNSYMGIEKDPHGVIVSAIDLFTQIYSFDILAFFLTFITKESGQ
DLPFTKSLSRRRTYLTRIAQSSSLRQFLQLSTRPNIRILGGNGQGTGNSNYPEFTNASSV
ISPKLRASPLLERRASKICYAITKLLYRLECHPIGMALLKKYLHNQLREAYLESKRHFIS
KKGDSTNTSSTIASSSFAGASVPLSSNESGMLNGLKQINEQQESTLETTQKED
>Q9JJN6
MNREGAPGKSPEEMYIQQKVRVLLMLRKMGSNLTASEEEFLRTYAGVVSSQLSQLPQHSI
DQGAEDVVMAFSRSETEDRRQ
>Q9UT17
MDSANKNTKIFAEQLGNDFTISVDDEDMEMEYPLRSSTPTKVIDDQREILLQKASRISKL
DEALCELNNESNGSIDLSIDSFIPSYDEFLHKKLLNEDTGHESKVSDQVKQVISTPMSTC
FEQWFEGEEQSSTGNNQDISYSSVNSLSPKRRGISLMSIDANKLSPKKTSPNSGYITSPL
RHPILLSETEPGTPTKNDSPAYIGLPSQPNSITTLNNTNQKFQVPDNDKPPMSSLNELSA
SYKPIVFDSSQHKTQGTDNNDSFYHDDTNCIHLSRLEKIRELLTFVQLELKYYVEPLKKE
LQETKTLLAQKEEEISNLKDKLQNAGLS
>P53238
MCAKKLKYAAGDDFVRYATPKEAMEETRREFEKEKQRQQQIKVTQAQTPNTRVHSAPIPL
QTQYNKNRAENGHHSYGSPQSYSPRHTKTPVDPRYNVIAQKPAGRPIPPAPTHYNNLNTS
AQRIASSPPPLIHNQAVPAQLLKKVAPASFDSREDVRDMQVATQLFHNHDVKGKNRLTAE
ELQNLLQNDDNSHFCISSVDALINLFGASRFGTVNQAEFIALYKRVKSWRKVYVDNDING
SLTISVSEFHNSLQELGYLIPFEVSEKTFDQYAEFINRNGTGKELKFDKFVEALVWLMRL
TKLFRKFDTNQEGIATIQYKDFIYATLYLGRFLPH
>Q02754
MQQDIVNDHQEEAQGWKWEQIKEIIESGELARLKRSRQMTDKYHEHKKRTAGLDMNQYVL
QKLGWSLDEPQLENAAAKAFSSSTLYAVRANDFPYNFEPGVVHLVLWSKVALPVHSPDKA
VREAARARMNAFLQAQPLLRPLLSSGHVAWFVNYPELQSVARIFHAHVLLFFPRERYSAE
QVKTTVDDILSHGFEPLA
>P46952
MERRLGVRAWVKENRGSFQPPVCNKLMHQEQLKVMFVGGPNTRKDYHIEEGEEVFYQLEG
DMVLRVLEQGKHRDVVIRQGEIFLLPARVPHSPQRFANTVGLVVERRRLETELDGLRYYV
GDTMDVLFEKWFYCKDLGTQLAPIIQEFFSSEQYRTGKPIPDQLLKEPPFPLSTRSIMEP
MSLDAWLDSHHRELQAGTPLSLFGDTYETQVIAYGQGSSEGLRQNVDVWLWQLEGSSVVT
MGGRRLSLAPDDSLLVLAGTSYAWERTQGSVALSVTQDPACKKPLG
>Q00196
MVHSSMGAPEIRMSKPLEAEKQSLDSPSEHTDTERNGPDINHQNPQNKASPFSVSPTGPS
TKIKAEDPSGDSAPAAPPPPQPAQPHLPQAQLMLTGSQLAGDIQQLLQLQQLVLVPGHHL

QPPAQFLLPQAQQSQPGLLPTPNLFQLPQQTQGALLTSQPRAGLPTQPPKCLEPPSHPEE
PSDLEELEQFARTFKQRRIKLGFTQGDVGLAMGKLYGNDFSQTTISRFEALNLSFKNMCK
LKPLLEKWLNDAETMSVDSSLPSPNQLSSPSLGFDGLPGRRRKKRTSIETNVRFALEKSF
LANQKPTSEEILLIAEQLHMEKEVIRVWFCNRRQKEKRINPCSAAPMLPSPGKPTSYSPH
LVTPQGGAGTLPLSQASSSLSTTVTTLSSAVGTLHPSRTAGGGGGGGGAAPPLNSIPSVT
PPPPATTNSTNPSPQGSHSAIGLSGLNPSAGPGLWWNPAPYQP
>Q02767
MQKHNIKLNQNQDISQLFHDEVPLFDNSITSKDKEVIETLSEIYSIVITLDHVEKAYLKD
SIDDTQYTNTVDKLLKQFKVYLNSQNKEEINKHFQSIEAFCDTYNITASNAITRLERGIP
ITAEHAISTTTSAPSGDNKQSSSSDKKFNAKYVAEATGNFITVMDALKLNYNAKDQLHPL
LAELLISINRVTRDDFENRSKLIDWIVRINKLSIGDTLTETQIRELLFDLELAYKSFYAL
LD
>Q42539
MKQFWSPSSINKNKAMVENLQNHGIVTSDEVAKAMEAVDRGVFVTDRSSAYVDSPMSIGY
NVTISAPHMHAMCLQLLEKHLKPGMRVLDVGSGTGYLTACFAVMVGTEGRAIGVEHIPEL
VASSVKNIEASAASPFLKEGSLAVHVGDGRQGWAEFAPYDAIHVGAAAPEIPEALIDQLK
PGGRLVIPVGNIFQDLQVVDKNSDGSVSIKDETSVRYVPLTSREAQLRGD
>P39108
MLRYHMQGFSGYGVQYSPFFDNRLAVAAGSNFGLVGNGKLFILEIDRSGRIVEVNSFLTQ
DCLFDLAWNESHENQVLVAQGDGTLRLFDTTFKEFPIAIFKEHEREVFSCNWNLVNRQNF
LSSSWDGSIKIWSPLRKQSLMTLTPRPLEITKMVDPLNAIILKKKSFTGISKNRNCVYQA
QFSPHDQNLVLSCSGNSYASLFDIRLPSGKNQNNFLVHSGLEALTCDFNKYRPYVVATGG
VDNAIRIWDIRMLNKNESATIKRTVPGQLHNSSCINEIPNAHGLAIRKVTWSPHHSNILM
SASYDMTCRIWRDLSNDGAKETYKTNSTDATKGSIFNFTQHSEFVFGADWSLWGKPGYVA
STAWDGNLFVWNGLG
>Q9US01
MDLEELQKIIQEEQIRCEREIAEAAEARNSSNSLIVVDEYSKESEDVLENGLEHVQSNEF
TESEDYKSIERSLAVTEAVMARFEKEMGSLRDQMNFFHQILDNDNLDGFLTGSSKPC
>O15392
MGAPTLPPAWQPFLKDHRISTFKNWPFLEGCACTPERMAEAGFIHCPTENEPDLAQCFFC
FKELEGWEPDDDPIEEHKKHSSGCAFLSVKKQFEELTLGEFLKLDRERAKNKIAKETNNK
KKEFEETAEKVRRAIEQLAAMD
>Q08954
MKGTGGVVVGTQNPVRDYNHSTDEEYQRLRRLADEAYKKRDQLSHESQTAYQQGDKKLAH
ELSEKSKAQLKTAEDFNMQAAEYVFVENNADSSSNEIDLHGLYVKEALFILQKRIKFAID
HNEPQLNVIVGKGLHSQNGIAKLKPSIEEFCAKHGIRNHLEKGNSGVLVLELQGVQMQMD
GPAVNAPTNQYNAQPHPQYNNNGGQPQGQAQNYNNSGNDNKDSTLTSIFKIFCNCIQSLA

>O65735
MSNFKSKYHDELIANAAYIGTPGKGILAADESTGTIGKRLASINVENVETNRRALRELLF
TAPNVLQYLSGVILFEETLYQSTAAGKPFVDVLNEAGVLPGIKVDKGTVELAGTDGETTT
QGLDGLGARCAKYYEAGARFAKWRAVLKIGPNEPSLSILSIENAYGLARYAVICQENGLV
PIVELEILVDGSHDIHKCAAITERVLAATYKALSDHHVLLEGTLLKPNMVTPGSDSPKVA
PEVVAEHTVRALQRTVPAAVPAVVFLSGGQSEEEATVNLNAINQVKGKKPWTLSFSFGRA

LQQSTLKAWSGKEENVKNAQDALLTRAKANSEATLGTYKGNSQLGEGASESLHVKDYKY
>Q86WG5
MARLADYFIVVGYDHEKPGSGEGLGKIIQRFPQKDWDDTPFPQGIELFCQPGGWQLSRER
KQPTFFVVVLTDIDSDRHYCSCLTFYEAEINLQGTKKEEIEGEAKVSGLIQPAEVFAPKS
LVLVSRLYYPEIFRACLGLIYTVYVDSLNVSLESLIANLCACLVPAAGGSQKLFSLGAGD
RQLIQTPLHDSLPITGTSVALLFQQLGIQNVLSLFCAVLTENKVLFHSASFQRLSDACRA
LESLMFPLKYSYPYIPILPAQLLEVLSSPTPFIIGVHSVFKTDVHELLDVIIADLDGGTI
KIPECIHLSSLPEPLLHQTQSALSLILHPDLEVADHAFPPPRTALSHSKMLDKEVRAVFL
RLFAQLFQGYRSCLQLIRIHAEPVIHFHKTAFLGQRGLVENDFLTKVLSGMAFAGFVSER
GPPYRSCDLFDELVAFEVERIKVEENNPVKMIKHVRELAEQLFKNENPNPHMAFQKVPRP
TEGSHLRVHILPFPEINEARVQELIQENVAKNQNAPPATRIEKKCVVPAGPPVVSIMDKV
TTVFNSAQRLEVVRNCISFIFENKILETEKTLPAALRALKGKAARQCLTDELGLHVQQNR
AILDHQQFDYIIRMMNCTLQDCSSLEEYNIAAALLPLTSAFYRKLAPGVSQFAYTCVQDH
PIWTNQQFWETTFYNAVQEQVRSLYLSAKEDNHAPHLKQKDKLPDDHYQEKTAMDLAAEQ
LRLWPTLSKSTQQELVQHEESTVFSQAIHFANLMVNLLVPLDTSKNKLLRTSAPGDWESG
SNSIVTNSIAGSVAESYDTESGFEDSENTDIANSVVRFITRFIDKVCTESGVTQDHIKSL
HCMIPGIVAMHIETLEAVHRESRRLPPIQKPKILRPALLPGEEIVCEGLRVLLDPDGREE
ATGGLLGGPQLLPAEGALFLTTYRILFRGTPHDQLVGEQTVVRSFPIASITKEKKITMQN
QLQQNMQEGLQITSASFQLIKVAFDEEVSPEVVEIFKKQLMKFRYPQSIFSTFAFAAGQT
TPQIILPKQKEKNTSFRTFSKTIVKGAKRAGKMTIGRQYLLKKKTGTIVEERVNRPGWNE
DDDVSVSDESELPTSTTLKASEKSTMEQLVEKACFRDYQRLGLGTISGSSSRSRPEYFRI
TASNRMYSLCRSYPGLLVVPQAVQDSSLPRVARCYRHNRLPVVCWKNSRSGTLLLRSGGF
HGKGVVGLFKSQNSPQAAPTSSLESSSSIEQEKYLQALLNAVSVHQKLRGNSTLTVRPAF
ALSPGVWASLRSSTRLISSPTSFIDVGARLAGKDHSASFSNSSYLQNQLLKRQAALYIFG
EKSQLRNFKVEFALNCEFVPVEFHEIRQVKASFKKLMRACIPSTIPTDSEVTFLKALGDS
EWFPQLHRIMQLAVVVSEVLENGSSVLVCLEEGWDITAQVTSLVQLLSDPFYRTLEGFQM
LVEKEWLSFGHKFSQRSSLTLNCQGSGFAPVFLQFLDCVHQVHNQYPTEFEFNLYYLKFL
AFHYVSNRFKTFLLDSDYERLEHGTLFDDKGEKHAKKGVCIWECIDRMHKRSPIFFNYLY
SPLEIEALKPNVNVSSLKKWDYYIEETLSTGPSYDWMMLTPKHFPSEDSDLAGEAGPRSQ
RRTVWPCYDDVSCTQPDALTSLFSEIEKLEHKLNQAPEKWQQLWERVTVDLKEEPRTDRS
QRHLSRSPGIVSTNLPSYQKRSLLHLPDSSMGEEQNSSISPSNGVERRAATLYSQYTSKN
DENRSFEGTLYKRGALLKGWKPRWFVLDVTKHQLRYYDSGEDTSCKGHIDLAEVEMVIPA
GPSMGAPKHTSDKAFFDLKTSKRVYNFCAQDGQSAQQWMDKIQSCISDA
>Q9P6L9
MEENTAQKQSNATGLQRHVSGRWISNVPSYEGNEWSSEESESEDKESNLTSSENIGKNPE
RELNSDGTDRIIEEEEEEDDIENEASSFSEQSENENYDFSYPGTFVYRKAASSSHETLAT
KVVSADESARLWAEKRRFLLQKLGKDPNSKPIDYKSSMASSSTRSSQSSQVSAIQPQSQD
DNRVSDIRQMENRRELNVKRRSVSSPAEDLAIRPLSFSRYKKAEEIPESLPPIKGRDSFS
SVRELNESNVFARAPNKNRFSFASVAETGSGSNFSRATSSRSSKTRSVVYEPNHRHTSFF
GDTLSNLSSSLNDKKSPNDDHAVLDSELMEQTLPDSAEAVEIANNVKEDIVSTANDDANE
LDNTSEKATLETSSKPVTEQAIEQSEVVQEVEEGDTNMTTNTILKPTDEIINKGYAYSIT
SSHSNHSNEKFEQIPSPDEKLSRTDIDTHVKQLQTTKSTDSLSIPFVESPQQNPEEELSD
SLSNDFGIDAEKEKDENLSKPEHHPSITSVNSPFLYSPSKQPSGELDEENLKLRLQDSDV
DDEVATIRSLKQNNATNSEVETVTNDPSFSQQPGTHSRILRNFDAISSIDSIPDSFSDSA

VDLPVDSSKYTLVGKTQSNSNLLQDNAEKHISKSNLEKVDVKGDTSPNSVEQGIEPQAAS
VVGSPRSSLVSNNSLEEGALLKNSESLESIYGYLSEEPDPALTKLNTEDPFWTKGSSDDE
HTIASVEQDVYPYKTSLPTIVEATTSHTESSNSKAPAIEETTTTKVEVVAAHDAVSISSN
SSTHKSHSEPLNRDELEVAALSRGSLPEPPKPNKQIVVQEEDQRDTLSLKTSTTGLSSHS
KSAENNSTQQSTTSPSINSGASADAVSSGISKKADNSETNLNYFATLDAFVKSPLMSFDG
LGELGSTDQRYNFFNQKIQEYSAYDSGLDNWIQFCLEKDGEAPKEAPPPPRSEPVSTTTK
LAKRITQPSLSPARSTVTITHNIATEAKKKSKSAANDILHLFKKKAKSEQSNAKSSSSSI
KESKKMKTLFGKKLGFKS
>O13781
MKPAHTFSLIFSLLFVINPCYSATLPLAIRRELTWGQVNILHTTDIHGWLAGHARDKRYS
GDLGDFASFVQHMKHLAKQKDVDLLLVDTGDLHDGTGLSDLTDPPGSWTDSLLANMDYDI
LVAGNHELYLSTVANDTYHNFIPLWEGRYLAANIDIYDEHVSSFVPFGDRYAIFSTPHNV
KILAFGFVNSFSGATDTVRVLPVSQFSLQPWWNDMVSNKDIDLILIPAHVPVHNASELDI
VLSELRIHFPTTPIQVLGGHSHIRDYAIYDEKAVGIESGRYCETVGWVSINGIPSFKSLV
KLSGISSLMGVESYLAERLRFIGESIRRSSQQIFSSLQSAKSASVSFSRSYIDWNPEGFM
FHSKTKKSSFNTSLGEFISNGIYEARKALGLLTPIGCSPKKFAFSEVPFNDSNSIYHLFQ
SELFPKIVVNESRHHIPHYIIVNSGGIRGGLNSGAFGLDEVFQVCPFKSNIFYVLKDVPW
SITKYLPQALQHSGYLIAEDTLQINTPQVSDAKFEDFYEHSIRKTYGYTTHDDLGDDGDD
TAHLTTPHYEPLRFICSQVGFDDSFSGDDKQVVDVVAPSFVIPRLDDILNKIADKPLYSP
DDWELYFTRPDGKHSMTDLLPLYADLYWDHDCLYK
>P38094
MATLSSLRHLIQTHPLIDNHAHNLLSQSAACKYAKYPFEQIISEAQGVALANAPSTLSFH
RAASQLATLYQSSSSDWDSVRAARDQSVQRDYEGLIRKCLEGTQVLLLDDLLTENDVELF
DWHDRFTASATKRIVRIEALAASVLSQIVHGGPVPQDSSDLSAFQTLWESFSRNFSALVS
DAIADPAVVGFKSVICYRTGLDVQPTDDRDTERLIRSFARTISQAAVSTPRVEDKPLNDW
LVRQTLNLLKAAKVTQPNKPLQLHTGLGDNDINLLKSNPAHLQSLIAQYPEVDFVLLHSS
YPYTREAGYLACVYPNVYLDLGEVFPMVSRDAQESILRESLEIVPSTRLLWSTDGHFFPE
TFWLANRQFRDALEKVFVDYVQNGDYTIEQAMQAAADILFHNSNRLYELNEQPPSAALSS
GHQTVSRISSTDLLEKFIRSNPGVKYVWTQFIDYTATVRVRMFPVMEFAKIVRKQRRLGI
SMATFWMLQDDEVVGGSTTGQFYLIPDLSTLSPNVGIDSKSATVMTWWKSEQGESLEECP
RTNLLNINNKLKDEFGIQATCGFEIEVVFLKPTTDPSTGEEDWAPSVTNHSWSQMTRETR
RMLPLLEEIAETLASIGIHLQQFHAESAPGQFEFILPPDNPVAAVDTLIKSRQVIANIVE
KHGLRATLYPRPYPSAAGTASHAHVSISPSTKEESFLAGVLQHYPAVLAFTLSGDASYDR
VKSGIWAGSEWVTWGTQNREAPIRKISPGHWEIKSLDGLANMYLAMAAFLAAGYTGVKEN
LPLTIKDCPYDAASLPESERAALGITTKLPNTLAKSLAALESDEILRSLLGENLVEDYII
VKRAESKKLSAMDEKARRKWLVERY
>Q06410
MNEADVTKFVNNARKTLTDAQLLCSSANLRIVDIKKKLSSWQLSISKLNFLIVGLRQQGK
FLYTILKEGIGTKLIQKQWNQAVLVVLVDEMKYWQYEITSKVQRLDGIVNELSISEKDDT
DPSKLGDYISRDNVNLLNDKLKEVPVIERQIENIKLQYENMVRKVNKELIDTKLTDVTQK
FQSKFGIDNLMETNVAEQFSRELTDLEKDLAEIMNSLTQHFDKTLLLQDKKIDNDEREEL
FKVVQGDDKELYNIFKTLHEVIDDVDKTILNLGQFLQAKIKEKTELHSEVSEIINDFNRN
LEYLLIFKDISNLIDSFKNSCTQDIQTTKELCEFYDNFEESYGNLVLEAKRRKDVANRMK
TILKDCEKQLQNLDAQDQEERQNFIAENGTYLPETIWPGKIDDFSSLYTLNYNVKNP

>Q13330
MAANMYRVGDYVYFENSSSNPYLIRRIEELNKTANGNVEAKVVCFYRRRDISSTLIALAD
KHATLSVCYKAGPGADNGEEGEIEEEMENPEMVDLPEKLKHQLRHRELFLSRQLESLPAT
HIRGKCSVTLLNETESLKSYLEREDFFFYSLVYDPQQKTLLADKGEIRVGNRYQADITDL
LKEGEEDGRDQSRLETQVWEAHNPLTDKQIDQFLVVARSVGTFARALDCSSSVRQPSLHM
SAAAASRDITLFHAMDTLHKNIYDISKAISALVPQGGPVLCRDEMEEWSASEANLFEEAL
EKYGKDFTDIQQDFLPWKSLTSIIEYYYMWKTTDRYVQQKRLKAAEAESKLKQVYIPNYN
KPNPNQISVNNVKAGVVNGTGAPGQSPGAGRACESCYTTQSYQWYSWGPPNMQCRLCASC
WTYWKKYGGLKMPTRLDGERPGPNRSNMSPHGLPARSSGSPKFAMKTRQAFYLHTTKLTR
IARRLCREILRPWHAARNPYLPINSAAIKAECTARLPEASQSPLVLKQAVRKPLEAVLRY
LETHPRPPKPDPVKSVSSVLSSLTPAKVAPVINNGSPTILGKRSYEQHNGVDGNMKKRLL
MPSRGLANHGQTRHMGPSRNLLLNGKSYPTKVRLIRGGSLPPVKRRRMNWIDAPGDVFYM
PKEETRKIRKLLSSSETKRAARRPYKPIALRQSQALPPRPPPPAPVNDEPIVIED
>Q04429
MENLCPPPPSQMKDFSTPPRNRHRHKRSFAISGDFEFLKQPASAPVLPSAYDSPTFENTP
RRVSGMSVTMPDESQNESALLNSPSPRFFISEASTYSSPIKGVPDAIINLDDVLINKPKM
CRSHRKTKSVPVKLDEFYSSHKCSSVPELTINEEIDEDDTNPQLLEPVKPLSSTSLSTDM
NEDKKMTLKNARSHNSLKIQAQKQRYYNSARYLPLNSEDRATDPQILTKQSSVTSLFSSR
SITPVSCNINNAGRINAISGNYLDDVLYDLDTPATTLIQDIDNLQTSINERVRLSPQSSS
IKKYFSKDGKSVSSFNFQSQECDMVSFTEDFAHVTSLSSSILDSEKQTDDEEEESIPEEI
LRGEPLHVYNETSGSDKSVILPTKQKSAPINKDSKHSSTQYEEKSFKKNRKFKIFAKLFC
TRK
>O55239
MESGFTSKDTYLSHFNPRDYLEKYYSFGSRHCAENEILRHLLKNLFKIFCLGAVKGELLI
DIGSGPTIYQLLSACESFTEIIVSDYTDQNLWELQKWLKKEPGAFDWSPVVTYVCDLEGN
RMKGPEKEEKLRRAIKQVLKCDVTQSQPLGGVSLPPADCLLSTLCLDAACPDLPAYRTAL
RNLGSLLKPGGFLVMVDALKSSYYMIGEQKFSSLPLGWETVRDAVEEAGYTIEQFEVISQ
NYSSTTSNNEGLFSLVGRKPGRSE
>P38238
MGKSSKDKRDLYYRKAKEQGYRARSAFKLLQLNDQFHFLDDPNLKRVVDLCAAPGSWSQV
LSRKLFDESPSSDKEDRKIVSVDLQPMSPIPHVTTLQADITHPKTLARILKLFGNEKADF
VCSDGAPDVTGLHDLDEYVQQQLIMSALQLTACILKKGGTFVAKIFRGRDIDMLYSQLGY
LFDKIVCAKPRSSRGTSLEAFIVCLGYNPPSNWTPKLDVNTSVDEFFQGCFLNKLCISDK
LSHWNEEERNIAEFMACGSLQSFDSDATYHDLPSSVAGTSSSLDPVQSPTNPPYKKALEL
KRSGKLTRSV
>Q61818
MQSFRERCGFHGKQQNYPQTSQETSRLENYRQPGQAGLSCDRQRLLAKDYYSPQPYTGYE
GGTGTPSGTVATAAADKYHRGSKSLQGRPAFPSYVQDSSPYPGRYSGEEGLQTWGGPQPP
PPQPQPLPGAVSKYEENLMKKTVVPPPNRQYPEQGPQLPFRTHSLHVPPPQPQQPLAYPK
LQRQKPQNDLASPLPFPQGSHFPQHSQSFPTSSTYAPTVQGGGQGAHSYKSCTAPSAQPH
DRPMSANANLAPGQRVQNLHAYQPGRLGYEQQQQALQGRHHTQETLHYQNLAKYQHYGQQ
GQGYCPPDTAVRTPEQYYQTFSPSSSHSPARSVGRSPSYSSTPSPLMPNLENFPYSQQPL
STGAFPTGITDHSHFMPLLNPSPTDAASSVDPQAGNCKPLQKEKLPDNLLSDLSLQSLTA
LTSQVENISNTVQQLLLSKATMPQKKGVKNLVSRTPEQHKSQHCSPEGSGYSAEPAGTPL

SEPPSSTPQSTHAEPQDTDYLSGSEDPLERSFLYCSQARGSPARVNSNSKAKPESVSTCS
VTSPDDMSTKSDDSFQSLHSTLPLDSFSKFVAGERDCPRLLLSALAQEDLASEILGLQEA
IVEKADKAWAEASSLPKDNGKPPFSLENHGACLDTVAKTSWSQPGEPETLPEPLQLDKGG
STKDFSPGLFEDPSVAFATTDPKKTSSPLSFGTKPLLGTATPDPTTAAFDCFPDTPTASS
VDGANPFAWPEENLGDACPRWGLHPGELTKGLEQGAKASDGVGKADAHEASACMGFQEDH
AIGKPAAALSGDFKQQEAEGVKEEVGGLLQCPEVAKADQWLEESRHCCSSTDFGDLPLLP
PPGRKEDLEAEEEYSSLCELLGSPEQRPSLQDPLSPKAPLMCTKEEAEEALDTKAGWVSP
CHLSGEPAVLLGPSVGAQSKVQSWFESSLSHMKPGEEGPEMERAPGSSGTSQGSLAPKPN
KPAVPEGPIAKKEPVPRGKSLRSRRVHRGLPEAEDSPCRVPALPKDLLLPESCTGPPQGQ
AEGAGAPGRGLSEGLPRMCTRSLTALSEPQTPGPPGLTTTPTPPDKLGGKQRAAFKSGKR
VGKPSPKAASSPSNPAALPVASDSSPMGSKTKEPDSPSMPGKDQRSMVLRSRTKPQQVFH
AKRRRPSESRIPDCRATKKLPANNHLPTAFKVSSGPQKEGRMSQRVKVPKPGTGNKLSDR
PLHTLKRKSAFMAPVPAKKRSLILRSNNGSGGDGREERAESSPGLLRRMASPQRARPRGS
GEPPPPPPLEPPAACMGLSTQSSLPSAVRTKVLPPRKGRGLKLEAIVQKITSPGLKKLAC
RVAGAPPGTPRSPALPERRPGGSPAGAEEGLGGMGQMLPAASGADPLCRNPASRSLKGKL
LNSKKLSSAADCPKAEAFMSPETLPSLGTARAPKKRSRKGRTGTLGPSKGPLEKRPCPGQ
PLLLAPHDRASSTQGGGEDNSSGGGKKPKTEELGPASQPPEGRPCQPQTRAQKQPGQASY
SSYSKRKRLSRGRGKTAHASPCKGRATRRRQQQVLPLDPAEPEIRLKYISSCKRLRADSR
TPAFSPFVRVEKRDAYTTICTVVNSPGDEPKPHWKPSSSAASSSTSSSSLEPAGASLTTF
PGGSVLQQRPSLPLSSTMHLGPVVSKALSTSCLVCCLCQNPANFKDLGDLCGPYYPEHCL
PKKKPKLKEKARLEGTLEEASLPLERTLKGLECSASTTAAAPTTATITTPTALGRLSRPD
GPADPAKQGPLRTSARGLSRRLQSCYCCDGQGDGGEEVAQADKSRKHECSKEAPTEPGGD
TQEHWVHEACAVWTSGVYLVAGKLFGLQEAMKVAVDMPCTSCHEPGATISCSYKGCIHTY
HYPCANDTGCTFIEENFTLKCPKHKRLPL
>Q10325
MPATILKSTASSKTLLIIKFDTNSSRYPFYKKHLLELNSESTFLGLLTKGQADTSITRLN
QDDVRLFEKAKTVADRTKDVAYSFSDPILSTQLRTPPPQPTSIRYLYFGTYRIKPWYTSP
YPEEYSCAKNLYICESCLKYMNSDHVLQRHKMKCSWSYPPGDEIYRDKNISIFEVDGQRQ
PIYCQNLCLLAKMFLHSKMLYYDVEPFLFYVLTEFDGQECKVIGYFSKEKRSASDYNVSC
ILTLPIYQRRGYGVFLIDFSYLLTQVEGKLGSPEKPLSDLGLVTYRSYWKMRVAKALLEI
TTPISINAIAKSTSMVCDDVISTLESLSVFKYDPLKKKYVLQLKRDELENVYKAWNIKHP
QRVNPKLLRWTPYLGEEQISNLLLKENILIPLPQKRLLDNSHHLDSV
>Q06494
MSVADLKNNIHKLDTGYGLMSLTWRAEPIPQSQAFEAMHRVVELSRERGHKAFFNVGEFY
GPDFINLSYVHDFFAKYPDLRKDVVISCKGGADNATLTPRGSHDDVVQSVKNSVSAIGGY
IDIFEVARIDTSLCTKGEVYPYESFEALAEMISEGVIGGISLSEVNEEQIRAIHKDWGKF
LTCVEVELSLFSNDILHNGIAKTCAELGLSIICYSPLGRGLLTGQLKSNADIPEGDFRKS
LKRFSDESLKKNLTLVRFLQEEIVDKRPQNNSITLAQLALGWVKHWNKVPEYSGAKFIPI
PSGSSISKVNENFDEQKTKLTDQEFNAINKYLTTFHTVGDRYEMA
>Q96KQ4
MMPMILTVFLSNNEQILTEVPITPETTCRDVVEFCKEPGEGSCHLAEVWRGNERPIPFDH
MMYEHLQIWGPRREEVKFFLRHEDSPTENSEQGGRQTQEQRTQRNVINVPGDKRTEYGVG
NPRVELTLSELQDMAARQQQQIENQQQMLVAKEQRLHFLKQQERRQQSISENEKLQKLK
ERVEAQENKLKKIRAMRGQVDYSKIMNGNLSAEIERFSAMFQEKKQEVQTAILRVDQLSQ

QLEDLKKGKLNGFQSYNGKLTGPAAVELKRLYQELQIRNQLNQEQNSKLQQQKELLNKRN
MEVAMMDKRISELRERLYGKKIQLNRVNGTSSPQSPLSTSGRVAAVGPYIQVPSAGSFPV
LGDPIKPQSLSIASNAAHGRSKSANDGNWPTLKQNSSSSVKPVQVAGADWKDPSVEGSVK
QGTVSSQPVPFSALGPTEKPGIEIGKVPPPIPGVGKQLPPSYGTYPSPTPLGPGSTSSLE
RRKEGSLPRPSAGLPSRQRPTLLPATGSTPQPGSSQQIQQRISVPPSPTYPPAGPPAFPA
GDSKPELPLTVAIRPFLADKGSRPQSPRKGPQTVNSSSIYSMYLQQATPPKNYQPAAHSA
LNKSVKAVYGKPVLPSGSTSPSPLPFLHGSLSTGTPQPQPPSESTEKEPEQDGPAAPADG
STVESLPRPLSPTKLTPIVHSPLRYQSDADLEALRRKLANAPRPLKKRSSITEPEGPGGP
NIQKLLYQRFNTLAGGMEGTPFYQPSPSQDFMGTLADVDNGNTNANGNLEELPPAQPTAP
LPAEPAPSSDANDNELPSPEPEELICPQTTHQTAEPAEDNNNNVATVPTTEQIPSPVAEA
PSPGEEQVPPAPLPPASHPPATSTNKRTNLKKPNSERTGHGLRVRFNPLALLLDASLEGE
FDLVQRIIYEVEDPSKPNDEGITPLHNAVCAGHHHIVKFLLDFGVNVNAADSDGWTPLHC
AASCNSVHLCKQLVESGAAIFASTISDIETAADKCEEMEEGYIQCSQFLYGVQEKLGVMN
KGVAYALWDYEAQNSDELSFHEGDALTILRRKDESETEWWWARLGDREGYVPKNLLGLYP
RIKPRQRTLA
>Q9P7L4
MLSRAGKVVCIGRNYAAHIRELNNPFPTKPFFFLKPTSAIVEPGHGNLIIPPDVSAHYEV
ELGLIMKDRLPARRPVSSNSWLDSIGAYFVGIDMTARNIQNEAKKKGLPWSFAKGYDTFL
PVGPIIPKHLIPDPHNVILELSLNGKVVQKDSTSLMLNKIPKIFSSITEAMSLNPGDLVL
TGTPKGVGPVVPGDILSARLLTAQEQEIIPSKFEIKAEKCD
>Q99707
MSPALQDLSQPEGLKKTLRDEINAILQKRIMVLDGGMGTMIQREKLNEEHFRGQEFKDHA
RPLKGNNDILSITQPDVIYQIHKEYLLAGADIIETNTFSSTSIAQADYGLEHLAYRMNMC
SAGVARKAAEEVTLQTGIKRFVAGALGPTNKTLSVSPSVERPDYRNITFDELVEAYQEQA
KGLLDGGVDILLIETIFDTANAKAALFALQNLFEEKYAPRPIFISGTIVDKSGRTLSGQT
GEGFVISVSHGEPLCIGLNCALGAAEMRPFIEIIGKCTTAYVLCYPNAGLPNTFGDYDET
PSMMAKHLKDFAMDGLVNIVGGCCGSTPDHIREIAEAVKNCKPRVPPATAFEGHMLLSGL
EPFRIGPYTNFVNIGERCNVAGSRKFAKLIMAGNYEEALCVAKVQVEMGAQVLDVNMDDG
MLDGPSAMTRFCNLIASEPDIAKVPLCIDSSNFAVIEAGLKCCQGKCIVNSISLKEGEDD
FLEKARKIKKYGAAMVVMAFDEEGQATETDTKIRVCTRAYHLLVKKLGFNPNDIIFDPNI
LTIGTGMEEHNLYAINFIHATKVIKETLPGARISGGLSNLSFSFRGMEAIREAMHGVFLY
HAIKSGMDMGIVNAGNLPVYDDIHKELLQLCEDLIWNKDPEATEKLLRYAQTQGTGGKKV
IQTDEWRNGPVEERLEYALVKGIEKHIIEDTEEARLNQKKYPRPLNIIEGPLMNGMKIVG
DLFGAGKMFLPQVIKSARVMKKAVGHLIPFMEKEREETRVLNGTVEEEDPYQGTIVLATV
KGDVHDIGKNIVGVVLGCNNFRVIDLGVMTPCDKILKAALDHKADIIGLSGLITPSLDEM
IFVAKEMERLAIRIPLLIGGATTSKTHTAVKIAPRYSAPVIHVLDASKSVVVCSQLLDEN
LKDEYFEEIMEEYEDIRQDHYESLKERRYLPLSQARKSGFQMDWLSEPHPVKPTFIGTQV
FEDYDLQKLVDYIDWKPFFDVWQLRGKYPNRGFPKIFNDKTVGGEARKVYDDAHNMLNTL
ISQKKLRARGVVGFWPAQSIQDDIHLYAEAAVPQAAEPIATFYGLRQQAEKDSASTEPYY
CLSDFIAPLHSGIRDYLGLFAVACFGVEELSKAYEDDGDDYSSIMVKALGDRLAEAFAEE
LHERVRRELWAYCGSEQLDVADLRRLRYKGIRPAPGYPSQPDHTEKLTMWRLADIEQSTG
IRLTESLAMAPASAVSGLYFSNLKSKYFAVGKISKDQVEDYALRKNISVAEVEKWLGPIL
GYDTD
>Q9P0U3

MDDIADRMRMDAGEVTLVNHNSVFKTHLLPQTGFPEDQLSLSDQQILSSRQGHLDRSFTC
STRSAAYNPSYYSDNPSSDSFLGSGDLRTFGQSANGQWRNSTPSSSSSLQKSRNSRSLYL
ETRKTSSGLSNSFAGKSNHHCHVSAYEKSFPIKPVPSPSWSGSCRRSLLSPKKTQRRHVS
TAEETVQEEEREIYRQLLQMVTGKQFTIAKPTTHFPLHLSRCLSSSKNTLKDSLFKNGNS
CASQIIGSDTSSSGSASILTNQEQLSHSVYSLSSYTPDVAFGSKDSGTLHHPHHHHSVPH
QPDNLAASNTQSEGSDSVILLKVKDSQTPTPSSTFFQAELWIKELTSVYDSRARERLRQI
EEQKALALQLQNQRLQEREHSVHDSVELHLRVPLEKEIPVTVVQETQKKGHKLTDSEDEF
PEITEEMEKEIKNVFRNGNQDEVLSEAFRLTITRKDIQTLNHLNWLNDEIINFYMNMLME
RSKEKGLPSVHAFNTFFFTKLKTAGYQAVKRWTKKVDVFSVDILLVPIHLGVHWCLAVVD
FRKKNITYYDSMGGINNEACRILLQYLKQESIDKKRKEFDTNGWQLFSKKSQIPQQMNGS
DCGMFACKYADCITKDRPINFTQQHMPYFRKRMVWEILHRKLL
>P29295
MDLRVGRKFRIGRKIGSGSFGDIYHGTNLISGEEVAIKLESIRSRHPQLDYESRVYRYLS
GGVGIPFIRWFGREGEYNAMVIDLLGPSLEDLFNYCHRRFSFKTVIMLALQMFCRIQYIH
GRSFIHRDIKPDNFLMGVGRRGSTVHVIDFGLSKKYRDFNTHRHIPYRENKSLTGTARYA
SVNTHLGIEQSRRDDLESLGYVLIYFCKGSLPWQGLKATTKKQKYDRIMEKKLNVSVETL
CSGLPLEFQEYMAYCKNLKFDEKPDYLFLARLFKDLSIKLEYHNDHLFDWTMLRYTKAMV
EKQRDLLIEKGDLNANSNAASASNSTDNKSETFNKIKLLAMKKFPTHFHYYKNEDKHNPS
PEEIKQQTILNNNAASSLPEELLNALDKGMENLRQQQPQQQVQSSQPQPQPQQLQQQPNG
QRPNYYPEPLLQQQQRDSQEQQQQVPMATTRATQYPPQINSNNFNTNQASVPPQMRSNPQ
QPPQDKPAGQSIWL
>Q9ERD6
MDLMNGQASSVTIAATVSEKSSSSESLSEKGSELKKSFDAVVFDVLKVTPEEYAGQITLM
DVPVFKAIQPDELSSCGWNKKEKYSSAPNAVAFTRRFNHVSFWVVREILHAQTLKIRAEV
LSHYIKTAKKLYELNNLHALMAVVSGLQSAPIFRLTKTWALLSRKDKTTFEKLEYVMSKE
DNYKRLRDYISSLKMTPCIPYLGIYLSDLTYIDSAYPSTGSILENEQRSNLMNNILRIIS
DLQQSCEYDIPILPHVQKYLNSVQYIEELQKFVEDDNYKLSLKIEPGASTPRSAASREDL
AGPDIGASPQGGRKSSAAAAAAAAAEGALLPQTPPSPRNLIPHGHRKCHSLGYNFIHKMN
TAEFKSATFPNAGPRHLLDDSVMEPHAPSRGQAESSTLSSGISIGSSDGSELSEETSWPA
FERNRLYHSLGPVTRVPRNGYRSHTKASSSAESEDLAVHLYPGAVTIQGVLRRKTLLKEG
KKPTVASWTKYWAALCGTQLFYYAAKSLKATERKHFKSTSNKNVSVVGWMVMMADDPEHP
DLFLLTDSEKGNSYKFQAGSRMNAMLWFKHLSAACQSNKQQVPTNLMTFE
>Q53I77
MASEEASLRALESLMSEFFHNGTSNERKREIESLLNNFAQQLGAWRFCFYFLSQSQNDYV
LMYSLSVFENMINKMWLGVPSQEKMEIRNSLPKLLLSQHKSLPSFICNKLCKVIVDMGRQ
DWPMFYHDFFTNILQLIQTPSTTPLGLIMLKTASEELACPREDLIVARKEELRKLLLEQV
PTVLDLLTGVLESIWDKHSITAATPPPSPTASDTDDLLSNLIHTPNLTKQLSQPPPSLEA
ESERVCALALECLSHLFSWIPLSASITPSLLTTIFQFARLGCDARSRQTNSVTTNTTASV
VNGRSSSPPTAPARDLARLGVLAMSCINELMCKNCVPLEFQEYLLRVCQQTFYLLQRITR
ETNAHSVRSRFEELDESYVEKFTDFLRLFVSVHLRRIESNAQFPLLEFLTLLFKYTFHQP
TREGYLSCLDIWAQFLDYLTNKIRNRLEDRDAIIGRYEDALVLLLNEVLNRIQFRYNQTQ
LEELDDETLDDDQQTEWQRYLRHSLEVVAKIMDLLPTHAFSKLFAALQENLNVYLGLQRC
LVTNGNDQRLNVTAENDCRRLHCSLRDLSSLLQAVGRLAEYFIGDMFGARFNDALTVVER
LVEVTLYGSRIKLYNMETAVPSVLKPDLIDVHAQSLAALQAYSHWLARYYSEVQRQNPEQ

FISIISTAMEALPPLISTKVQEKLLLSACHLLVSIATTVRPMFLLNIPSVQKVFSRVTDS
SAQRLPEEAQVLLCRALSNVLLLPWPNVPEGEQQWAERSSHHSNLLNALTRDYRLLKGSS
LPQRKGQLEATKRVICQTLGVLRDIVENISGEGTKSRQICYQSLQESAQLSLTLFPAYIH
QSDVTEEMLSFFLALFQGLRVQMGAPFTEQIIQTFLNMFTREQLAESILQEGSAGCHVVE
KFLKILQVVVQEPGQSFKPFLPSILSLCMEQLYPIIAERPSPDVKAELFELLFQLLHHNW
RYFYRSSVLASVHRDGSDEPMENQAQFIVVMQAFGQSFLQPDIHIFRQNLSYLETLNSKH
KLYHKKLFQTGMLPQFVSVLLQVLIHKSHDLLQEEIGIAVYNMASVDFSTFFSTFLPEFL
TGCQGLDTSQKSVLARNFKMERDLPSFTQSVHRLVNDLRYYRLCNDSLPPGTVKL
>Q03785
MMMFHNCRINNYLITSQIGEGAYGLVYRALDIRTDRQYAIKAVVQSYGVSKEADMGNDKI
HKNSVKLQKKLAKLFKESKNVVRVPSIDLESIENMSEEDFKKLPHYKEISLHLRVHHHKN
IVTIHEVLQSAVCTFIVMDYYPTDLFTSIVDNRHFVTNGLLVKKVFLQICSALNYCHEHG
IYHCDIKPENLLLDTEDNVFLCDFGLSTTSTYIKPNVCIGSSYYMPPERISFDGRVSSSK
SGGHKLGKVCPSCNGDLWSLGIILINLTCIRNPWLKADKTEDNTYYYFTKDPNILKQILP
LSDDFYSLLSKILQVNPKNRMSLQELMKEVSSITSFTNEGPLSKVPPLSKSVYEKFVSPV
DNTNENLSPKSYVYMHDSKAAKNLSYTSSSEEEDGIKEGIDDDNGSRSGSFGTLDTDTGL
HSSFTSTSCESDNECSKISNKFSLFEKKFNELRMSSSSLTN
>P32849
MSHIEQEERKRFFNDDLDTSETSLNFKSENKESFLFANSHNDDDDDVVVSVSDTTEGEGD
RSIVPVRREIEEEGQNQFITELLRIIPEMPKDLVMELNEKFGSQEEGLSLALSHYFDHNS
GTSISKIPSSPNQLNTLSDTSNSTLSPSSFHPKRRRIYGFRNQTRLEDKVTWKRFIGALQ
VTGMATRPTVRPLKYGSQMKLKRSSEEISATKVYDSRGRKKASMASLVRIFDIQYDREIG
RVSEDIAQILYPLLSSHEISFEVTLIFCDNKRLSIGDSFILQLDCFLTSLIFEERNDGES
LMKRRRTEGGNKREKDNGNFGRTLTETDEELESRSKRLALLKLFDKLRLKPILDEQKALE
KHKIELNSDPEIIDLDNDEICSNQVTEVHNNLRDTQHEEETMNLNQLKTFYKAAQSSESL
KSLPETEPSRDVFKLELRNYQKQGLTWMLRREQEFAKAASDGEASETGANMINPLWKQFK
WPNDMSWAAQNLQQDHVNVEDGIFFYANLHSGEFSLAKPILKTMIKGGILSDEMGLGKTV
AAYSLVLSCPHDSDVVDKKLFDIENTAVSDNLPSTWQDNKKPYASKTTLIVVPMSLLTQW
SNEFTKANNSPDMYHEVYYGGNVSSLKTLLTKTKTPPTVVLTTYGIVQNEWTKHSKGRMT
DEDVNISSGLFSVNFYRIIIDEGHNIRNRTTVTSKAVMALQGKCKWVLTGTPIINRLDDL
YSLVKFLELDPWRQINYWKTFVSTPFESKNYKQAFDVVNAILEPVLLRRTKQMKDKDGKP
LVELPPKEVVIKRLPFSKSQDLLYKFLLDKAEVSVKSGIARGDLLKKYSTILVHILRLRQ
VCCHPGLIGSQDENDEDLSKNNKLVTEQTVELDSLMRVVSERFDNSFSKEELDAMIQRLK
VKYPDNKSFQSLECSICTTEPMDLDKALFTECGHSFCEKCLFEYIEFQNSKNLGLKCPNC
RNQIDACRLLALVQTNSNSKNLEFKPYSPASKSSKITALLKELQLLQDSSAGEQVVIFSQ
FSTYLDILEKELTHTFSKDVAKIYKFDGRLSLKERTSVLADFAVKDYSRQKILLLSLKAG
GVGLNLTCASHAYMMDPWWSPSMEDQAIDRLHRIGQTNSVKVMRFIIQDSIEEKMLRIQE
KKRTIGEAMDTDEDERRKRRIEEIQMLFE
>Q07963
MEDSDLSITNIRDFLTELPKLAKCEYSETTSYLLWKTLNLRLKHSDNDINWRSLVSILNS
EAWENEKYRDILNGRKWRTLEFENDHHSVGNMHIGTACTRLCFPSETIYYCFTCSTNPLY
EICELCFDKEKHVNHSYVAKVVMRPEGRICHCGDPFAFNDPSDAFKCKNELNNIPISNDN
SNVTDDENVISLLNYVLDFLIDVTVSYKEEAEAHSSERKASSLMHPNQNSITDDIMEKHE
CEPLVNDENFVFFDNNWSNTRKEAHMEWAIQIEEEECNVHYMDLASTITRILNTPVEYAI

SITKALEDSHDVVTVLQSENFFEIDQIAKEFQKENIVVHVRKADDIFKRKLTDDLTDWLY
SLCFKAATSLQNKYALRISMLDVWYSHFSKMRVSPTNTNPDFSKINLLGGFLISNEDSDE
SWFKPWSLENIEDERISKILTNYNERLIRAHSPNTVSHFYNFYGSRFQYIIINSINILSK
KSKFKMLKIMASLFSLRDESRKFLAAQYIDVYLSVLYDAVASDAKECQVTLMSILGQYTF
QDPSIANMTISSGFIERTIRFAFTLMAFNPEDLMSYLPISLYNGFKLPTETIRNRRTIIC
FKDLCTIMSANTVPEELLSNEAIFNAIIESFSEFSNVLPLKRETKEHVEVENFDFSAFYF
FFSSILIMTDGYTRSISLVKDAAFRKQIVLKLLDVAQTREFESLTNSRKAISPDNASTNE
NDSNKATLSTVRETICNYVAETINFQVGVNTQYFFNPMSYLFKFVIQWSQCGRYEPIPAS
LTNYINLFEVFQDKQKALYISESALSTLVLIGQINVGFWVRNGTPITHQARMYTKYSMRE
FTYISDIFNVQFSMAMCNPDELMVTYLSRWGLKHWANGVPMYDYPDTETTVAVVNECILL
LIQLLTEVRSLVMKSSKEGFERTFKSEIIHALCFDTCSYAQIVNCIPEHITKHPSFDIYL
EKYANYTSPVSLTDNGIFVLKEKYKDEIDPYYIGLSSSRRYDVEKNIRLNMANLKKMKYE
DTFVPAKKVKDLLKNTLFSGLYSISSVNTFGLFLKNTLDHIIKYDYDNLLPRVVHLIHLC
VVNNLNEFMGILWHEYAIVDTEFCHYHSIGSILYYCLLKDNFSESHGKIREIFRYLMETA
PHVNVNSYLREQTTSYTPGILWPTKEDKSHKDKEFERKKHLARLRKKKLMKKLAQQQMKF
MENNSVDTSDISTPRTTSPSLSPTRINAENSSNTINSCCDDDCVFCKMPKDDDVFVYFSY
QERNICDHGIDFTNPTDVNRINSLFSGKQTKDSAIQENPQDDDGTRLKFTSCEPVLRACG
HGSHTKCLSGHMKSIRGIQNQTTKNIPLSYGSGLIYCPVCNSLSNSFLPKTNDIDKRTSS
QFFMCIEKRSEAEENLDPMSSICIKAAMILGDLQGKKVTTIEDAYKVVNSVFINTISNTE
LRLRSHKKEGKIVNMERISSQCILTLHLVCELKSFIYKKFVNSKTFSSEISRKIWNWNEF
LIKGNNVNLLLYMSQNFDNIDGGKTPQPPNLCIYEMFKRRFHQLLLLLARDMMRVNFYKD
CRNKIKISSNGSEEPSTSFSYLFNTFKKYVDLFKPDDVRFDFTSLEKIKDFICSLLLESL
SIFCRRTFLLFNIQYDDDGDGDNNNNRSNNFMDVKQREIELIFRYFKLPNLTHFLKDFFY
NELTQNIERYNDGNDNLRIQQVIYDMVQNINTRAYPSPEHIQLIELPLNLSKFSLDNDEI
SNKCDKYEIAVCLLCGQKCHIQKSIALQGYLQGECTDHMRNGCEITSAYGVFLMTGTNAI
YLSYGKRGTFYAAPYLSKYGETNEDYKFGTPVYLNRARYANLANEIVFGNMIPHIVFRLT
DGSADLGGWETM
>Q7RTR2
MRKQEVRTGREAGQGHGTGSPAEQVKALMDLLAGKGSQGSQAPQALDRTPDAPLGPCSND
SRIQRHRKALLSKVGGGPELGGPWHRLASLLLVEGLTDLQLREHDFTQVEATRGGGHPAR
TVALDRLFLPLSRVSVPPRVSITIGVAGMGKTTLVRHFVRLWAHGQVGKDFSLVLPLTFR
DLNTHEKLCADRLICSVFPHVGEPSLAVAVPARALLILDGLDECRTPLDFSNTVACTDPK
KEIPVDHLITNIIRGNLFPEVSIWITSRPSASGQIPGGLVDRMTEIRGFNEEEIKVCLEQ
MFPEDQALLGWMLSQVQADRALYLMCTVPAFCRLTGMALGHLWRSRTGPQDAELWPPRTL
CELYSWYFRMALSGEGQEKGKASPRIEQVAHGGRKMVGTLGRLAFHGLLKKKYVFYEQDM
KAFGVDLALLQGAPCSCFLQREETLASSVAYCFTHLSLQEFVAAAYYYGASRRAIFDLFT
ESGVSWPRLGFLTHFRSAAQRAMQAEDGRLDVFLRFLSGLLSPRVNALLAGSLLAQGEHQ
AYRTQVAELLQGCLRPDAAVCARAINVLHCLHELQHTELARSVEEAMESGALARLTGPAH
RAALAYLLQVSDACAQEANLSLSLSQGVLQSLLPQLLYCRKLRLDTNQFQDPVMELLGSV
LSGKDCRIQKISLAENQISNKGAKALARSLLVNRSLTSLDLRGNSIGPQGAKALADALKI
NRTLTSLSLQGNTVRDDGARSMAEALASNRTLSMLHLQKNSIGPMGAQRMADALKQNRSL
KELMFSSNSIGDGGAKALAEALKVNQGLESLDLQSNSISDAGVAALMGALCTNQTLLSLS
LRENSISPEGAQAIAHALCANSTLKNLDLTANLLHDQGARAIAVAVRENRTLTSLHLQWN
FIQAGAAQALGQALQLNRSLTSLDLQENAIGDDGACAVARALKVNTALTALYLQVASIGA

SGAQVLGEALAVNRTLEILDLRGNAIGVAGAKALANALKVNSSLRRLNLQENSLGMDGAI
CIATALSGNHRLQHINLQGNHIGDSGARMISEAIKTNAPTCTVEM
>Q02880
MAKSGGCGAGAGVGGGNGALTWVTLFDQNNAAKKEESETANKNDSSKKLSVERVYQKKTQ
LEHILLRPDTYIGSVEPLTQFMWVYDEDVGMNCREVTFVPGLYKIFDEILVNAADNKQRD
KNMTCIKVSIDPESNIISIWNNGKGIPVVEHKVEKVYVPALIFGQLLTSSNYDDDEKKVT
GGRNGYGAKLCNIFSTKFTVETACKEYKHSFKQTWMNNMMKTSEAKIKHFDGEDYTCITF
QPDLSKFKMEKLDKDIVALMTRRAYDLAGSCRGVKVMFNGKKLPVNGFRSYVDLYVKDKL
DETGVALKVIHELANERWDVCLTLSEKGFQQISFVNSIATTKGGRHVDYVVDQVVGKLIE
VVKKKNKAGVSVKPFQVKNHIWVFINCLIENPTFDSQTKENMTLQPKSFGSKCQLSEKFF
KAASNCGIVESILNWVKFKAQTQLNKKCSSVKYSKIKGIPKLDDANDAGGKHSLECTLIL
TEGDSAKSLAVSGLGVIGRDRYGVFPLRGKILNVREASHKQIMENAEINNIIKIVGLQYK
KSYDDAESLKTLRYGKIMIMTDQDQDGSHIKGLLINFIHHNWPSLLKHGFLEEFITPIVK
ASKNKQELSFYSIPEFDEWKKHIENQKAWKIKYYKGLGTSTAKEAKEYFADMERHRILFR
YAGPEDDAAITLAFSKKKIDDRKEWLTNFMEDRRQRRLHGLPEQFLYGTATKHLTYNDFI
NKELILFSNSDNERSIPSLVDGFKPGQRKVLFTCFKRNDKREVKVAQLAGSVAEMSAYHH
GEQALMMTIVNLAQNFVGSNNINLLQPIGQFGTRLHGGKDAASPRYIFTMLSTLARLLFP
AVDDNLLKFLYDDNQRVEPEWYIPIIPMVLINGAEGIGTGWACKLPNYDAREIVNNVRRM
LDGLDPHPMLPNYKNFKGTIQELGQNQYAVSGEIFVVDRNTVEITELPVRTWTQVYKEQV
LEPMLNGTDKTPALISDYKEYHTDTTVKFVVKMTEEKLAQAEAAGLHKVFKLQTTLTCNS
MVLFDHMGCLKKYETVQDILKEFFDLRLSYYGLRKEWLVGMLGAESTKLNNQARFILEKI
QGKITIENRSKKDLIQMLVQRGYESDPVKAWKEAQEKAAEEDETQNQHDDSSSDSGTPSG
PDFNYILNMSLWSLTKEKVEELIKQRDAKGREVNDLKRKSPSDLWKEDLAAFVEELDKVE
SQEREDVLAGMSGKAIKGKVGKPKVKKLQLEETMPSPYGRRIIPEITAMKADASKKLLKK
KKGDLDTAAVKVEFDEEFSGAPVEGAGEEALTPSVPINKGPKPKREKKEPGTRVRKTPTS
SGKPSAKKVKKRNPWSDDESKSESDLEETEPVVIPRDSLLRRAAAERPKYTFDFSEEEDD
DADDDDDDNNDLEELKVKASPITNDGEDEFVPSDGLDKDEYTFSPGKSKATPEKSLHDKK
SQDFGNLFSFPSYSQKSEDDSAKFDSNEEDSASVFSPSFGLKQTDKVPSKTVAAKKGKPS
SDTVPKPKRAPKQKKVVEAVNSDSDSEFGIPKKTTTPKGKGRGAKKRKASGSENEGDYNP
GRKTSKTTSKKPKKTSFDQDSDVDIFPSDFPTEPPSLPRTGRARKEVKYFAESDEEEDDV
DFAMFN
>Q03471
MATSTETISSLAQPFVHLENPINSPLVKETIRPRNDTTITPPPTQWSYLCHPRVKEVQDE
VDGYFLENWKFPSFKAVRTFLDAKFSEVTCLYFPLALDDRIHFACRLLTVLFLIDDVLEH
MSFADGEAYNNRLIPISRGDVLPDRTKPEEFILYDLWESMRAHDAELANEVLEPTFVFMR
AQTDRARLSIHELGHYLEYREKDVGKALLSALMRFSMGLRLSADELQDMKALEANCAKQL
SVVNDIYSYDKEEEASRTGHKEGAFLCSAVKVLAEESKLGIPATKRVLWSMTREWETVHD
EIVAEKIASPDGCSEAAKAYMKGLEYQMSGNEQWSKTTRRYN
>Q9VAW5
MSAKQSGSPKAGTSPLDKRVLQPKAQQQQQQTKQTASQNNNAQNTASNKKTQQQQQPQQG
ATTTTQTTTAAAAAATAAAAATAATTSAAAATSSAAAVPAATTETATTTTVDASQADALA
KVVVKDKKKVNQKASDFSNVGDWPTLIGGTSGSGKATSNEPKRNPTKKQQSAKTAAAVAA
SSNTTSSEVATESNVAGTTSSSNSNPSSSTTTTNTTTNSQATTAPVASTSHDAKAQRDAE
PAANSAAGTTGTAAGPALTKKIPKHKWRPLPIDLAKSSRPKPIGGRPNRRFSDDIADQRR

PPRVYHDRQPGAGGAGVESRHPHAGRHPYGSRPATATSERVDSWRSSSSTTTAAFDEQRS
GAAGGAGAAGTGVGSATRGPRRYRTPYRGGRQGRGGFSRQGPGRPTYRIPRHLLASGEYA
NYLPADAAGADSQSSYVLMGTHYFGNVPAAYIELDANSIKEAIKKQVEYYFSVDNLTGDF
FLRRKMDPEGYIPVTLIASFHRVLALTTDVAVIVNAIKESDKLELFEGYKVRTKTTPTTW
PITEVPEVNEGEPKAIGTLEQEQLEQNDGQEKLEEQTEADSPPPILTSAMATKPLNSIPP
PPMPRNPQNLVPKMLQDKQQSRSSTIAALNSVNAISALTQQVEGGAAELAGHLSGLAESV
KPKSTSTPDKRNAASAGNGAGSAAALVAEPEGIWKEVKRRSKTNAIKENATTPPQQQQPP
LSQTLNNNNDNVKTNNTSSKSKSSSNNAPSNASSSATVCVTTNNASSATKATTKTTTTST
ATTTTNNNIKSGNAAYSKTHSKSSSKTAAPPSAQCHAEKEELDFQFDEELMDPLPPGTGR
INNFTENFSDDDESDYEFADRDINKLLIVAQVGRAPKHEGYDRTADFTSRTKITQDLENI
INDGLVNYEEDLWTTTNVVADYKTVNVISQADFEKLAGGRNKSVLPPQVVPPPPPFEEDL
DETLVGDTTLNSTLNNTLKSRRARFYAAPNSHSIDPRTPRKRKLRHTANPPVEAHVGWLL
DTVEHRPRTTSMGSSAGTSPTASSYGSFGSSVPQSLPVFQHPSHALLKENNFTQQAYHKY
HSRCLKERRRLGYGQSQEMNTLYRFWSFFLRENFNKSMYNEFRSLALEDAGNGFRYGLEC
LFRFFSYGLEKKFRPNIYQDFQDETIADYETGQLYGLEKFWAFLKYYKNGEKLEVQPKLA
EYLKSFKNIEDFRVVEPEINEMLQGVGSLNPGRQLNRHRSVSESDGTAVIAAGGRRLNTT
ITNRSDYVGRLLQQQHQQQQQQQHHQYQQGYGGYNQQQNRRRTGSFGSTTVRIRSGSLGN
KPQVANRNQGSQHELRRGGSNSGLAPHKRQQQQKPKPGAGSQTGSTRATTSAAATATTAA
SAATSTAATPAVTVSSGSSSSKK
>P24309
MTKLRIAVQGCCHGQLNQIYKEVSRIHAKTPIDLLIILGDFQSIRDGQDFKSIAIPPKYQ
RLGDFISYYNNEIEAPVPTIFIGGNHESMRHLMLLPHGGYVAKNIFYMGYSNVIWFKGIR
IGSLSGIWKEWDFNKQRPDWNDLENNNWKANIRNLYHVRISDIAPLFMIKHRIDIMLSHD
WPNGVVYHGDTKHLLKLKPFFEQDIKEGKLGSPVTWQLLRDLRPQWWLSAHLHVRFMASI
KHNKRSHEPPNKSTSKTKKNNNEIDLDLSSDEDERSGIMNCQEENEYDSKYGETRFLALD
KCLPRRRWLEILEIEPDTSHASWKDENHRMFWDPEFINNLVICQKNKNLLSNKPFNSVNW
IELSQSNREEGRDIDWENYAIPAYTLDIQKDEVRQTKAFISKFMT
>Q86DA5
MLPNRKPPRPSPSFQSLNNNNQRFPLRRSLKIAQAHSLPTVPSEDAKDDDLITPELSTDF
DFREIQHRYLMALQNEQDGETTSTDDAFFELDDDDDLSSPSVPGSPVDPPSISVPLPPKS
APPCPTQPAPLTNGDLYPTILSNGTPIPNGRITPALSTVSVSLIHEARLQQSLSTPCNGS
EEEMHNGQVRKESEYRRFKSEGSTAGASLPAAEKTHMDELSPVDQRSTSGTARFLIQQDS
VVNPSTKMSNTEQVAMMHTLKTKLSKYQAMMDKAFEEIAKVEDANIIEGCTIVRKLMRKV
WNTPKVSADLANALCDYLRDRDYFDKLIKMFISPNTAACDQVRMECGKVLEECTSSANLE
YIVNKSYTKKIMIVAMKLNKTPDQQRLSLSLIGNLFKHSNAVSLSLIETDVIDHIILTFK
RAPECPDILRHAALALANILLYTCFEGKKKIIQKKIPEWLFFLASQADDVTRYYACIAVC
TIVSVKEFEPLVRKSDTMKLVEPFLQVHDPATFARDYHKYAQGNTKEWLERLLPMLQPSR
RREARSVAAFHFTLEATIKKEQNKLDVFQEIGAIQALKEVASSPDEVAAKFASEALTVIG
EEVPYKLAQQVPGWTCADVQYWVKKIGFEEYVEKFAKQMVDGDLLLQLTENDLKHDVGMI
SGLHRKRFLRELQTLKVAADYSSVDESNLDNFLMGLSPELSVYTYQMLTNGVNRSLLSSL
TDEMMQNACGITNPIHRLKLTQAFETAKHPDDVEVAMLSKQIDVFISYRRSTGNQLASLI
KVLLQLRGYRVFIDVDKLYAGKFDSSLLKNIQAAKHFILVLTPNSLDRLLNDDNCEDWVH
KELKCAFEHQKNIIPIFDTAFEFPTKEDQIPNDIRMITKYNGVKWVHDYQDACMAKVVRF
ITGELNRTTPTTKEMPSISRKTTQQRWQTTNTVSRTGPSRSIGGPRMEPPTPTCKLLKHR

HLVYYLIFLVFSVTPTGSQERATSTRRKIQPSASTTSDRN
>P47979
MVYSPMSRPQVPLAFRQWPPYNYKSDPLVNSKLSQSTTSVNAGPSLISPSFLDSYANSSS
LLHKPSTNLGSLKTSSLLASDEVFPSSVMPQLRPLDSSLSVSPEMDGWPWHHNSSSNPQG
YAWTPSLLSSNATSYLHSGSSPHGNTSNHPSPISSLESLPSRSSTGSGSLDFSGLANLHD
DSKSLAMSLNMAGVPVSVDENSQTSFPFVHGQPESTMSRKPKLCVQSKSMTNIRNSVAKP
SLVRQSHSAGVIPIKPTASNASIRNAPSNLSKQFSPSGNSPLTEASKPFVPQPSAAGDFR
QAKGSASHPHGSGSSNGVAPNGKRALYKTEPCKNWQISGTCRYGSKCQFAHGNQELKEPP
RHPKYKSERCRSFMMYGYCPYGLRCCFLHDESNAQKSATIKQSP
>Q05515
MLKWIKGGISAVTGMAEPEYGKDYIHSVADRVKNKQPYRETSREDFFWQAPDHTNVESVI
FYFSDLKTGIFGFAQVIHSNIIGLHTASQFTFRIFDSKNPEDLNIWTSTKLENFYIEGPN
FYADNLSVELSEDGESYHIQSSVCDLSVVDLHIRRLTPGAKIGDDPATYYGNNINEPWGS
MRHVFWPRNACHGTIKVKKEVIPESDEEESSADEDDNEDEDEESGDSEEESGSEEESDSE
EVEITYEDRTITFKEEDPAISTFIMAFQGMKPHHAAKAWNFMFFHSEKYSAVLMEFTTPK
SYANTKISAGIITDDKEVLAMTTNNLVEHLNSEIDSVGWKVPKDIKITFKGINTKVKDEQ
LESENGTEQALQGEDEKEDEKEDEEEEEYKNVAEENKICAVVEGPLNNLVERIDVMGEIP
SFVKNIVSGVAGTKPFIYQYADPKSSTLQINGGEKIHGVAWTEVTFISESDVISEESYNE
A
>P70670
MPGEATETVPATEQELPQPQAETAVLPMSSALKVAAVGQPGPTPPSSLGPQQSPIVTAHQ
PSPLPSSVSSTPFEVPFAQPITAETALPSGTAPPTPTFLPHLIGPPISPAALALASPMIG
LAQKGARSSSAPLSLVALAPHSVQKSSVCPPHPLTSPPSAAGAELGALTASIPPLEPKTS
TSQVPSQGTLNLKGTAPCPPDVVRAFPSHLENPLASVQPGLMSCPQTLSNTSPVKGVPIS
SALTQSRLSLNLKGPVSPPARNTAAPSIPLAPSTSLGCHLPLLHHSSVDSPIQPPGQSGL
AVSNPTSVGHSGIAASCPPERCVVPALPSRLLAVDSGAAPSDDKGSSAVTNELCSPPGSS
NVAGTSLSPKASLVPKGSNVALQPLVTQVPASQKTGLKEIPVSCIGATHHALDNPSAISV
APATHVPPPTSSGLVSSKDPASPVTSLVVPAAHKQFPAPPASATLGVPVSPLPATEGLKN
LPISALVNVGAPVSPAQAGLPTRKDTTLQPLAPIALKESPSSQSASSLEVLSEDTVTKKT
TGGPAPVVRPAIAGVATTTSLRADSPPAVIRADSCVSPNTVSQPLKRSVTDPAMAPRTAK
NTAPSTTSPLVPLASEGCPVASSMALSPQNASVSETALALSPEIPKSVPFPDPPLAEISF
SNARKVDAVSHMESSGSSRQGHPDASVTAKGTVVCLADSSLDTSVSASKGSALSGASSPL
YPLEVSFLPEAGLAVQGPKGSLNKLSPTPPSSKGAPVPSTGAPPSPKGAPIVPTESSISS
KQVPAEILPSPQKTPEVTASRLISAVQSPKVDPIMSDVTPTSPKKTSATAVPKDTSATLS
LKSVPAVTSLSPPKAPVAPSNEATIVPTEIPTSLKNALAAATPKETLATSIPKVTSPSPQ
KTPKSVSLKGAPAMTSKKATEIAASKDVSPSQFPKEVPLLQHVPPTSPPKSPVSDTLSGA
LTSPPPKGPPATLAETPTYPKKSPKPAASKKTPATPSPEGVTAVPLEIPPCSKKAPKTAA
PKESSATSSSKRAPKTAVSKEIPSKGVTAVPLEISLPLKETSKSATPGEKSASSPKRSPK
TAGPKETPPGGVTAVPPEISLPPKETPQNATPNESLAASSQKRSPKTSVPKETPPGGVTA
MPLEIPSAPQKAPKTAVPKQIPTPEDAVTILAGSPLSPKKASKTAAPKEAPATPSVGVIA
VSGEISPSPKKTSKTAAPKENSATLPPKRSPKTAAPKETPATSSEGVTAVPSEISPSPPT
PASKGVPVTLTPKGAPNALAESPASPKKVPKTAAPEETSTTPSPQKIPKVAGPKEASATP
PSKKTPKTAVPKETSAPSEGVTAVPLEIPPSPRKAPKTAAPKETPAPSPEGATTAPVQIP
PSPRKGSKKAGSKETPTTPSPEGVTAAPLEIPISSKKTSKMASPKETLVTPSSKKLSQTV

GPKETSLEGATAVPLEIPPSHKKAPKTVDPKQVPLTPSPKDAPTTLAESPSSPKKAPKTA
APPSERVTTVPPEKPATPQKASGTTASKVPVPAETQEVAVSSRETPVTPAVPPVKNPSSH
KKTSKTIELKEAPATLPPSPTKSPKIPSSKKAPRTSAPKEFPASPSIKPVTTSLAQTAPP
SLQKAPSTTIPKENLAAPAVLPVSSKSPAAPARASASLSPATAAPQTAPKEATTIPSCKK
AAATETPIETSTAPSLEGAPKETSETSVSKVLMSSPPKKASSSKRASTLPATTLPSLKEA
SVLSPTATSSGKDSHISPVSDACSTGTTTPQASEKLPSKKGPTAFTEMLAAPAPESALAI
TAPIQKSPGANSNSASSPKCPDPSSKKDTKGLPSAVALAPQTVPVEKDTSKAIETLLVSP
AKGSDCLHSPKGPVGSQVATPLAAFTSDKVPPEAVSASVAPKPAPAASLTLAPSPVAPLP
PKQPLLESAPGSVLESPSKLPVPAEEDELPPLIPPEAVSGGEPFQPILVNMPAPKPAGTP
APAPSAKQPVLKNNKGSGTESDSDESVPELEEQDSTQTATQQAQLAAAAEIDEEPVSKAK
QSRSEKKARKAMSKLGLRQVTGVTRVTIRKSKNILFVITKPDVYKSPASDTYIVFGEAKI
EDLSQQAQLAAAEKFKVQGEAVSNIQENTQTPTVQEESEEEEVDETGVEVKDIELVMSQA
NVSRAKAVRALKNNSNDIVNAIMELTM
>P40057
MEELICTYPYHSNLFMFLFLFFCPSKRARRGHPKFLFTLCYKSNHLIPKLLPPSLFTKRV
MLNPSSHPPSPDFPTGSSASPRVKLRPSTLWAPPLTVSSDFAASSSSTAPVTVTDKPVTP
AVSKRYQP
>P25359
MAESTTLETIEIHPITFPPEVLARISPELSLQRHLSLGIRPCLRKYEEFRDVAIENNTLS
RYADAGNIDTKNNILGSNVLKSGKTIVITSITGGIIEETSAAIKDLDDFGEEELFEVTKE
EDIIANYASVYPVVEVERGRVGACTDEEMTISQKLHDSILHSRILPKKALKVKAGVRSAN
EDGTFSVLYPDELEDDTLNETNLKMKRKWSYVLYAKIVVLSRTGPVFDLCWNSLMYALQS
VKLPRAFIDERASDLRMTIRTRGRSATIRETYEIICDQTKSVPLMINAKNIAFASNYGIV
ELDPECQLQNSDNSEEEEVDIDMDKLNTVLIADLDTEAEETSIHSTISILAAPSGNYKQL
TLVGGGAKITPEMIKRSLLLSRVRADDLSTRFNI
>Q12830
MVSEEEEEEDGDAEETQDSEDDEEDEMEEDDDDSDYPEEMEDDDDDASYCTESSFRSHST
YSSTPGRRKPRVHRPRSPILEEKDIPPLEFPKSSEDLMVPNEHIMNVIAIYEVLRNFGTV
LRLSPFRFEDFCAALVSQEQCTLMAEMHVVLLKAVLREEDTSNTTFGPADLKDSVNSTLY
FIDGMTWPEVLRVYCESDKEYHHVLPYQEAEDYPYGPVENKIKVLQFLVDQFLTTNIARE
ELMSEGVIQYDDHCRVCHKLGDLLCCETCSAVYHLECVKPPLEEVPEDEWQCEVCVAHKV
PGVTDCVAEIQKNKPYIRHEPIGYDRSRRKYWFLNRRLIIEEDTENENEKKIWYYSTKVQ
LAELIDCLDKDYWEAELCKILEEMREEIHRHMDITEDLTNKARGSNKSFLAAANEEILES
IRAKKGDIDNVKSPEETEKDKNETENDSKDAEKNREEFEDQSLEKDSDDKTPDDDPEQGK
SEEPTEVGDKGNSVSANLGDNTTNATSEETSPSEGRSPVGCLSETPDSSNMAEKKVASEL
PQDVPEEPNKTCESSNTSATTTSIQPNLENSNSSSELNSSQSESAKAADDPENGERESHT
PVSIQEEIVGDFKSEKSNGELSESPGAGKGASGSTRIITRLRNPDSKLSQLKSQQVAAAA
HEANKLFKEGKEVLVVNSQGEISRLSTKKEVIMKGNINNYFKLGQEGKYRVYHNQYSTNS
FALNKHQHREDHDKRRHLAHKFCLTPAGEFKWNGSVHGSKVLTISTLRLTITQLENNIPS
SFLHPNWASHRANWIKAVQMCSKPREFALALAILECAVKPVVMLPIWRESLGHTRLHRMT
SIEREEKEKVKKKEKKQEEEETMQQATWVKYTFPVKHQVWKQKGEEYRVTGYGGWSWISK
THVYRFVPKLPGNTNVNYRKSLEGTKNNMDENMDESDKRKCSRSPKKIKIEPDSEKDEVK
GSDAAKGADQNEMDISKITEKKDQDVKELLDSDSDKPCKEEPMEVDDDMKTESHVNCQES
SQVDVVNVSEGFHLRTSYKKKTKSSKLDGLLERRIKQFTLEEKQRLEKIKLEGGIKGIGK

TSTNSSKNLSESPVITKAKEGCQSDSMRQEQSPNANNDQPEDLIQGCSESDSSVLRMSDP
SHTTNKLYPKDRVLDDVSIRSPETKCPKQNSIENDIEEKVSDLASRGQEPSKSKTKGNDF
FIDDSKLASADDIGTLICKNKKPLIQEESDTIVSSSKSALHSSVPKSTNDRDATPLSRAM
DFEGKLGCDSESNSTLENSSDTVSIQDSSEEDMIVQNSNESISEQFRTREQDVEVLEPLK
CELVSGESTGNCEDRLPVKGTEANGKKPSQQKKLEERPVNKCSDQIKLKNTTDKKNNENR
ESEKKGQRTSTFQINGKDNKPKIYLKGECLKEISESRVVSGNVEPKVNNINKIIPENDIK
SLTVKESAIRPFINGDVIMEDFNERNSSETKSHLLSSSDAEGNYRDSLETLPSTKESDST
QTTTPSASCPESNSVNQVEDMEIETSEVKKVTSSPITSEEESNLSNDFIDENGLPINKNE
NVNGESKRKTVITEVTTMTSTVATESKTVIKVEKGDKQTVVSSTENCAKSTVTTTTTTVT
KLSTPSTGGSVDIISVKEQSKTVVTTTVTDSLTTTGGTLVTSMTVSKEYSTRDKVKLMKF
SRPKKTRSGTALPSYRKFVTKSTKKSIFVLPNDDLKKLARKGGIREVPYFNYNAKPALDI
WPYPSPRPTFGITWRYRLQTVKSLAGVSLMLRLLWASLRWDDMAAKVPPGGGSTRTETSE
TEITTTEIIKRRDVGPYGIRFEYCIRKIICPIGVPETPKETPTPQRKGLRSSALRPKRPE
TPKQTGPVIIETWVAEEELELWEIRAFAERVEKEKAQAVEQQAKKRLEQQKPTVIATSTT
SPTSSTTSTISPAQKVMVAPISGSVTTGTKMVLTTKVGSPATVTFQQNKNFHQTFATWVK
QGQSNSGVVQVQQKVLGIIPSSTGTSQQTFTSFQPRTATVTIRPNTSGSGGTTSNSQVIT
GPQIRPGMTVIRTPLQQSTLGKAIIRTPVMVQPGAPQQVMTQIIRGQPVSTAVSAPNTVS
STPGQKSLTSATSTSNIQSSASQPPRPQQGQVKLTMAQLTQLTQGHGGNQGLTVVIQGQG
QTTGQLQLIPQGVTVLPGPGQQLMQAAMPNGTVQRFLFTPLATTATTASTTTTTVSTTAA
GTGEQRQSKLSPQMQVHQDKTLPPAQSSSVGPAKAQPQTAQPSARPQPQTQPQSPAQPEV
QTQPEVQTQTTVSSHVPSEAQPTHAQSSKPQVAAQSQPQSNVQGQSPVRVQSPSQTRIRP
STPSQLSPGQQSQVQTTTSQPIPIQPHTSLQIPSQGQPQSQPQVQSSTQTLSSGQTLNQV
SVSSPSRPQLQIQQPQPQVIAVPQLQQQVQVLSQIQSQVVAQIQAQQSGVPQQIKLQLPI
QIQQSSAVQTHQIQNVVTVQAASVQEQLQRVQQLRDQQQKKKQQQIEIKREHTLQASNQS
EIIQKQVVMKHNAVIEHLKQKKSMTPAEREENQRMIVCNQVMKYILDKIDKEEKQAAKKR
KREESVEQKRSKQNATKLSALLFKHKEQLRAEILKKRALLDKDLQIEVQEELKRDLKIKK
EKDLMQLAQATAVAAPCPPVTPAPPAPPAPPPSPPPPPAVQHTGLLSTPTLPAASQKRKR
EEEKDSSSKSKKKKMISTTSKETKKDTKLYCICKTPYDESKFYIGCDRCQNWYHGRCVGI
LQSEAELIDEYVCPQCQSTEDAMTVLTPLTEKDYEGLKRVLRSLQAHKMAWPFLEPVDPN
DAPDYYGVIKEPMDLATMEERVQRRYYEKLTEFVADMTKIFDNCRYYNPSDSPFYQCAEV
LESFFVQKLKGFKASRSHNNKLQSTAS
>Q9XG54
MENKVVEEKQVDKIPLMSPCKMGKFELCHRVVLAPLTRQRSYGYIPQPHAILHYSQRSTN
GGLLIGEATVISETGIGYKDVPGIWTKEQVEAWKPIVDAVHAKGGIFFCQIWHVGRVSNK
DFQPNGEDPISCTDRGLTPQIRSNGIDIAHFTRPRRLTTDEIPQIVNEFRVAARNAIEAG
FDGVEIHGAHGYLIDQFMKDQVNDRSDKYGGSLENRCRFALEIVEAVANEIGSDRVGIRI
SPFAHYNEAGDTNPTALGLYMVESLNKYDLAYCHVVEPRMKTAWEKIECTESLVPMRKAY
KGTFIVAGGYDREDGNRALIEDRADLVAYGRLFISNPDLPKRFELNAPLNKYNRDTFYTS
DPIVGYTDYPFLETMT
>Q10271
MFRKKKSRVLQRDVNFGNCLEDMNLQITNDFYLRKLNAPNELYEFQVTKDKEYNEKFYFA
VLDHVYSWTLQNSLLTRISIPLESSPSEPHSWIYATRNWQTNENGIVIIIQSMSHPLIWS
NRNLREGDVRRATVLDVIAKCTEKKLSVVIFSPSALLWDQSINFPRVCANLFNNVYQNLD
NPLFHLSGTSNH

>O60477
MNWRFVELLYFLFIWGRISVQPSHQEPAGTDQHVSKEFDWLISDRGPFHHSRSYLSFVER
HRQGFTTRYKIYREFARWKVRNTAIERRDLVRHPVPLMPEFQRSIRLLGRRPTTQQFIDT
IIKKYGTHLLISATLGGEEALTMYMDKSRLDRKSGNATQSVEALHQLASSYFVDRDGTMR
RLHEIQISTGAIKVTETRTGPLGCNSYDNLDSVSSVLLQSTESKLHLQGLQIIFPQYLQE
KFVQSALSYIMCNGEGEYLCQNSQCRCQCAEEFPQCNCPITDIQIMEYTLANMAKSWAEA
YKDLENSDEFKSFMKRLPSNHFLTIGSIHQHWGNDWDLQNRYKLLQSATEAQRQKIQRTA
RKLFGLSVRCRHNPNHQLPRERTIQQWLARVQSLLYCNENGFWGTFLESQRSCVCHGSTT
LCQRPIPCVIGGNNSCAMCSLANISLCGSCNKGYKLYRGRCEPQNVDSERSEQFISFETD
LDFQDLELKYLLQKMDSRLYVHTTFISNEIRLDTFFDPRWRKRMSLTLKSNKNRMDFIHM
VIGMSMRICQMRNSSLDPMFFVYVNPFSGSHSEGWNMPFGEFGYPRWEKIRLQNSQCYNW
TLLLGNRWKTFFETVHIYLRSRTRLPTLLRNETGQGPVDLSDPSKRQFYIKISDVQVFGY
SLRFNADLLRSAVQQVNQSYTQGGQFYSSSSVMLLLLDIRDRINRLAPPVAPGKPQLDLF
SCMLKHRLKLTNSEIIRVNHALDLYNTEILKQSDQMTAKLC
>Q9ZNR6
MADQAMTDQDQGAVTLYSGTAITDAKKNHPFSVKVGLAQVLRGGAIVEVSSVNQAKLAES
AGACSVIVSDPVRSRGGVRRMPDPVLIKEVKRAVSVPVMARARVGHFVEAQILESLAVDY
IDESEIISVADDDHFINKHNFRSPFICGCRDTGEALRRIREGAAMIRIQGDLTATGNIAE
TVKNVRSLMGEVRVLNNMDDDEVFTFAKKISAPYDLVAQTKQMGRVPVVQFASGGITTPA
DAALMMQLGCDGVFVGSEVFDGPDPFKKLRSIVQAVQHYNDPHVLAEMSSGLENAMESLN
VRGDRIQDFGQGSV
>Q49MG5
MSDEVFSTTLAYTKSPKVTKRTTFQDELIRAITARSARQRSSEYSDDFDSDEIVSLGDFS
DTSADENSVNKKMNDFHISDDEEKNPSKLLFLKTNKSNGNITKDEPVCAIKNEEEMAPDG
CEDIVVKSFSESQNKDEEFEKDKIKMKPKPRILSIKSTSSAENNSLDTDDHFKPSPRPRS
MLKKKSHMEEKDGLEDKETALSEELELHSAPSSLPTPNGIQLEAEKKAFSENLDPEDSCL
TSLASSSLKQILGDSFSPGSEGNASGKDPNEEITENHNSLKSDENKENSFSADHVTTAVE
KSKESQVTADDLEEEKAKAELIMDDDRTVDPLLSKSQSILISTSATASSKKTIEDRNIKN
KKSTNNRASSASARLMTSEFLKKSSSKRRTPSTTTSSHYLGTLKVLDQKPSQKQSIEPDR
ADNIRAAVYQEWLEKKNVYLHEMHRIKRIESENLRIQNEQKKAAKREEALASFEAWKAMK
EKEAKKIAAKKRLEEKNKKKTEEENAARKGEALQAFEKWKEKKMEYLKEKNRKEREYERA
KKQKEEETVAEKKKDNLTAVEKWNEKKEAFFKQKEKEKINEKRKEELKRAEKKDKDKQAI
NEYEKWLENKEKQERIERKQKKRHSFLESEALPPWSPPSRTVFAKVF
>Q9USV1
MSQTINSELNENATSQCKEDLKVSLSESDLRDSQGQLGIENPPKCNNSGNHSDNLGFIEQ
SETVHPENEKALTPDLRDTKIHTSLPITTPFSKKRAREAKNILLKPFKSPLRQTASPQVA
DTNLKPSLAVTNLNSDETNTSSEPVTSPLRTTPNSIKRQKRLFKSPISNCLNPKSDPEIT
QLLSRRLKLEKEVRNLQEQLITAETARKVEAKNEDKDLQTLIQKWKNAAQQAAEVLFKPM
AERIRLAGGVTQSFRIEEGENKGQIQEVRTEFTMSMFLNQFGVPVHLMSFDEENGDWKS
>Q9Y7R1
MSAAWRKKITNAHRNECLQVYRSLLRSCDEIQNRPLGEKLSKIVKARTRHYQNVGNAYKA
EALLKDAKKQCETLKSALNGKLPEVNIESHMLSKYRKYLNEGGKQGDVGITKPHTKTKKL
KSNSIFNYVRNGEVLYHLVSTTGGDTFLRPKFWPQSQRISGMLKKRITMKENKHRLIQSM
SCMLRHAKLEDEFMKAYISEEDVGYADEIHKCIQMVLSDLDSIYKRESKNWHIVSQFYNM

LELEAARQKLENRYLNFWKVSRRSSP
>Q12125
MVPAESNAVQAKLAKTLQRFENKIKAGDYYEAHQTLRTIANRYVRSKSYEHAIELISQGA
LSFLKAKQGGSGTDLIFYLLEVYDLAEVKVDDISVARLVRLIAELDPSEPNLKDVITGMN
NWSIKFSEYKFGDPYLHNTIGSKLLEGDFVYEAERYFMLGTHDSMIKYVDLLWDWLCQVD
DIEDSTVAEFFSRLVFNYLFISNISFAHESKDIFLERFIEKFHPKYEKIDKNGYEIVFFE
DYSDLNFLQLLLITCQTKDKSYFLNLKNHYLDFSQAYKSELEFLGQEYFNIVAPKQTNFL
QDMMSGFLGGSK
>Q12494
MDTSHEIHDKIPDTLREQQQHLRQKESEGCITTLKDLNVPETKKLSSVLHGRKASTYLRI
FRDDECLADNNNGVDSNNGGSVTCADKITRSEATPKSVPEGLQVSEKKNNPDTLSSSLSS
FILSNHEEPAIKPNKHVAHRNNITETGQGSGEDIAKQQSHQPQVLHHQTSLKPIQNVDEG
CISPKSTYQESLHGISEDLTLKPVSSATYYPHKSKADSGYEEKDKMENDIDTIQPATINC
ASGIATLPSSYNRHTFKVKTYSTLSQSLRQENVNNRSNEKKPQQFVPHSESIKEKPNTFE
QDKEGEQADEEEDEGDNEHREYPLAVELKPFTNRVGGHTAIFRFSKRAVCKALVNRENRW
YENIELCHKELLQFMPRYIGVLNVRQHFQSKDDFLSDLDQENNGKNDTSNENKDIEVNHN
NNDDIALNTEPTGTPLTHIHSFPLEHSSRQVLEKEHPEIESVHPHVKRSLSSSNQPSLLP
EVVLNDNRHIIPESLWYKYSDSPNSAPNDSYFSSSSSHNSCSFGERGNTNKLKRRDSGST
MINTELKNLVIREVFAPKCFRRKRNSNTTTMGNHNARLGSSPSFLTQKSRASSHDASNTS
MKTLGDSSSQASLQMDDSKVNPNLQDPFLKKSLHEKISNALDGSHSVMDLKQFHKNEQIK
HKNSFCNSLSPILTATNSRDDGEFATSPNYISNAQDGVFDMDEDTGNETINMDNHGCHLD
SGKNMIIKSLAYNVSNDYSHHDIESITFEETSHTIVSKFILLEDLTRNMNKPCALDLKMG
TRQYGVDAKRAKQLSQRAKCLKTTSRRLGVRICGLKVWNKDYYITRDKYFGRRVKVGWQF
ARVLARFLYDGKTIESLIRQIPRLIKQLDTLYSEIFNLKGYRLYGASLLLMYDGDANKSN
SKRKKAANVKVNLIDFARCVTKEDAMECMDKFRIPPKSPNIEDKGFLRGVKSLRFYLLLI
WNYLTSDMPLIFDEVEMNDMISEEADSNSFTSATGSKINFNSKWDWLDEFDKEDEEMYND
PNSKLRQKWRKYELIFDAEPRYNDDAQVSD
>Q9Y7P6
MSEEDTSKLRILSVGSNAISAFISWRLSESKACHTTLIWRNRCESVLSEGIRIRSSVFGS
TKWKPDVVAPTVEQLAMNSEPFDYIFVCLKILPSVYNLDTAIKEVVTPGHTCIVLNTTGI
VGAEKELQHAFPNNPVLSFVLPDQFAQRGPLQFEHTTFAADSAKSVIYVGLTEEEDDVPD
SVQDAMIETLTLTLEAGGVSCDFLSKIQKKQWETGVGHMCFYPLSIINDEPNLALMYRLK
SFAKVIDGLMDEAFSIAQAQGCEFEPEKLDVLKRHIVNRMLATPRPSYPYQDYIAHRPLE
VAVLLGYPVEIAKELGVSVPRMETLLALFDAKNKRNLTVRAGTPQSSPNFNPAMRRSPVG
AASRSPSRSTIGISNRIGSVDDLLNTRQFTSSPIGGSMPKGPNSIYKIPSASMVNLSSPL
VTSPSGLNPTGRPSRFGGRVRGNPLTMNKAGSVSDLLSTSTNMASSDALETASMIGVPSA
MPPNSFDMLTLTQRRNRRNNQSSSPPAPSDRRYTMGARRPVQTRGMTDSVIPILEDPMST
LYDTSRYPTRNSKPATPKASRPPSIASTVHRRMD
>Q9R109
MLPCCYKSITYKEQEDLTLRPHCCLPCSCLPCSCLQCSESLGGLQVGRSTAQEKDHSQLK
ELYSAGNLTVLSTDPLLHQDPVQLDFHFRLTPHSSAHWHGLLCDHRLFLDIPYQALDQGN
RESLTATLEYVEEKTNVDSVFVNFQIDRKDRGALLRAFSYMGFEVVRPDHPALPPWDNVI
FMVYPLERDLGHPGQ
>P24521

MSELRAFSAPGKALLAGGYLVLDTKYEAFVVGLSARMHAVAHPYGSLQGSDKFEVRVKSK
QFKDGEWLYHISPKSGFIPVSIGGSKNPFIEKVIANVFSYFKPNMDDYCNRNLFVIDIFS
DDAYHSQEDSVTEHRGNRRLSFHSHRIEEVPKTGLGSSAGLVTVLTTALASFFVSDLENN
VDKYREVIHNLAQVAHCQAQGKIGSGFDVAAAAYGSIRYRRFPPALISNLPDIGSATYGS
KLAHLVDEEDWNITIKSNHLPSGLTLWMGDIKNGSETVKLVQKVKNWYDSHMPESLKIYT
ELDHANSRFMDGLSKLDRLHETHDDYSDQIFESLERNDCTCQKYPEITEVRDAVATIRRS
FRKITKESGADIEPPVQTSLLDDCQTLKGVLTCLIPGAGGYDAIAVITKQDVDLRAQTAN
DKRFSKVQWLDVTQADWGVRKEKDPETYLDK
>Q9H497
MLRGPWRQLWLFFLLLLPGAPEPRGASRPWEGTDEPGSAWAWPGFQRLQEQLRAAGALSK
RYWTLFSCQVWPDDCDEDEEAATGPLGWRLPLLGQRYLDLLTTWYCSFKDCCPRGDCRIS
NNFTGLEWDLNVRLHGQHLVQQLVLRTVRGYLETPQPEKALALSFHGWSGTGKNFVARML
VENLYRDGLMSDCVRMFIATFHFPHPKYVDLYKEQLMSQIRETQQLCHQTLFIFDEAEKL
HPGLLEVLGPHLERRAPEGHRAESPWTIFLFLSNLRGDIINEVVLKLLKAGWSREEITME
HLEPHLQAEIVETIDNGFGHSRLVKENLIDYFIPFLPLEYRHVRLCARDAFLSQELLYKE
ETLDEIAQMMVYVPKEEQLFSSQGCKSISQRINYFLS
>O74867
MDSIELIPGLPGPGRRLLQLLKDLPSNPSVIVREIPDAIDAFRESITPATRPSCLGLLDT
ILSCFELPIPTEPIMKALGKLLEPMSWADLKKFGIESYLVTGLEHPSTDVEIFCLHLIRR
ANWEKKEIGEPLFEAIMACINSRSIAVSEEATVLLFDMADFPYYFNLIIKRFTFVDYEIM
NSTLRVRWLTLFAKLSTKSPEYLEELIKDDKLVINGDNKDILLQINFVDVLGIMLEAPFT
YEYISSEKTKYLDTIAQDYKGSADSYTDHIGLKFLPRLCELHPDAINSLDEKHQLFDAVR
ERMKTNDATSILLYGVFLSNTVVTQALIKKYGIEDEANRYVPRWLTRRFLMDEPGMSSFA
HALLQPDHNLWMQLWRILPPNTLSTLVNTASSPIPRSRQLAYQCLLHIAQNSPIQIASEG
FAIRHLLEAEGDHETCLLRFQVLKTMMESPHGGPKLPFGRYREDILKRLQQGPIVSGAST
LKVAAETA
>O18734
MAAQRLGKRVLSKLQSPSRARGPGGSPGGLQKRHARVTVKYDRRELQRRLDVEKWIDGRL
EELYRGREADMPDEVNIDELLELESEEERSRKIQGLLKSCTNPTENFVQELLVKLRGLHK
QPGLRQPSPSGDGSLSPRQDRARTAPP
>Q3E841
MKSSIPITEVLPRAVGSLTFDENYNLLDTSGVAKVIEKSPIAEIIRKSNAELGRLGYSVY
EDAQYIGHAFKKAGHFIVYFTPKNKNREGVVPPVGITN
>Q08887
MDRDISYQQNYTSTGATATSSRQPSTDNNADTNFLKVMSEFKYNFNSPLPTTTQFPTPYS
SNQYQQTQDHFANTDAHNSSSNESSLVENSILPHHQQIQQQQQQQQQQQQQQALGSLVP
PAVTRTDTSETLDDINVQPSSVLQFGNSLPSEFLVASPEQFKEFLLDSPSTNFNFFHKTP
AKTPLRFVTDSNGAQQSTTENPGQQQNVFSNVDLNNLLKSNGKTPSSSCTGAFSRTPLSK
IDMNLMFNQPLPTSPSKRFSSLSLTPYGRKILNDVGTPYAKALISSNSALVDFQKARKDI
TTNATSIGLENANNILQRTPLRSNNKKLFIKTPQDTINSTSTLTKDNENKQDIYGSSPTT
IQLNSSITKSISKLDNSRIPLLASRSDNILDSNVDDQLFDLGLTRLPLSPTPNCNSLHST
TTGTSALQIPELPKMGSFRSDTGINPISSSNTVSFKSKSGNNNSKGRIKKNGKKPSKFQI
IVANIDQFNQDTSSSSLSSSLNASSSAGNSNSNVTKKRASKLKRSQSLLSDSGSKSQARK
SCNSKSNGNLFNSQ

>O04147
MEEVKKDVYSVWALPDEESEPRFKKLMEALRSEFTGPRFVPHVTVAVSAYLTADEAKKMF
ESACDGLKAYTATVDRVSTGTFFFQCVFLLLQTTPEVMEAGEHCKNHFNCSTTTPYMPHL
SLLYAELTEEEKKNAQEKAYTLDSSLDGLSFRLNRLALCKTDTEDKTLETWETVAVCNLN
P
>O43012
MPTVGLTGPLCSGKDAVVEYLETKHGFNAIFRLPQLNEDGEYIYRTGDLVLGSVDDLISY
LTPRWRERFVINGIHSPRLLSALLKRPFFLLVYIDAPIMLRFNRYKTYVILQCQHVVANF
KFIVIILHAMCSVYSSLANTTLEEFCSIQDAAAFQSDNAGTRHRALANLLINNDSNIKLH
LWEKLQKADLLNPNRFRPSWDSYFMEMASLAAKRSNCMKRRVGCVLVRGNRVIATGYNGT
PRGATNCNEGGCPRCNSASSCGKELDTCLCLHAEENALLEAGRERVGNNAILYCDTCPCL
TCSVKITQLGIKEVVYHTSYNMDSHTASLLQAAGVQLRQYIPPENSIF
>Q9WTW1
MLAVSLKWRLGVVRRRPKDDGPYSKGGKDTAGTDGALVCRRQSIPEEFRGITMVELIKRE
GSTLGLTISGGTDKDGKPRVSNLRPGGLAARSDLLNVGDYIRSVNGIRLTRLRHDEIITL
LKNVGERVVLEVEYELPPPAPENNPRIISKTVDVSLYKEGNSFGFVLRGGAHEDLHKSRP
LVLTYVRPGGPADREGSLKVGDRLLSIDGIPLHGASHATAIATLQQCSHEALFQVEYDVA
TPDTVANASGPLVVEIAKTPGSALGISLTTGSHRNKPAITIDRIKPASVVDRSGALHAGD
HILAIDGTSTEHCSLVEATKLLASVTEKVRLEILPAPQSRRPLKPPEAVRIQRSEQLHHW
DPCVPSCHSPRPSHCRAPTWAPGGQDQSRSVSSTPFSSPTMNPAFPCANASTLPRGPMSP
RTTAGRRRQRRKEHRSSLSLASSTVGPGGQIVHTETTEVVLCGDPLSGFGLQLQGGIFAT
ETLSSPPLVRFIEPDSPAERCGLLQVGDRVLAINGIATEDGTMEEANQLLRDAALARKVV
LEIEFDVAESVIPSSGTFHVKLPKRRGVELGITISSASRKRGEPLIISDIKKGSVAHRTG
TLEPGDKLLAIDNIRLDHCPMEYAVQILRQCEDLVKLKIRKDEDNSDEQESSGAVSYTVE
LKRYGGPLGITISGTEEPFDPIIISGLTKRGLAERTGAIHVGDRILAINSVSLKGRPLSE
AIHLLQVAGETVTLKIKKQLDRPLLPRQSGSLSEASDVDEDPPEALKGGLLTTHFSPAVP
SVDSAVESWGSSATEGGFGGSGSYTPQVAVRSVTPQEWRSSRLKSSPPPLEPRRTSYTPG
PTDESFPEEEEGDWEPPMSPAPGPAREEGFWRVLGEALEDLESCGQSELLRELEASIMTG
TVQSVAVDGRPGSRPWRRSREVGTSPEDLQELLLPTPLEMHRVTLHKDPVRNDFGFSVSD
GLLEKGVYVHTVRIDGPAQHGGLQPFDRLLQVNHVRTRDFDCCLAVPLLAEAGDILELVV
SRNPLAQSRRTPGAPGPSSPQMI
>Q9QYW3
MVMAEGTAVLRRNRPGTKAQDFYNWPDESFDEMDSTLAVQQYIQQNIRADCSNIDKILEP
PEGQDEGVWKYEHLRQFCLELNGLAVKLQSECHPDTCTQMTATEQWIFLCAAHKTPKECP
AIDYTRHTLDGAACLLNSNKYFPSRVSIKESSVAKLGSVCRRIYRIFSHAYFHHRQIFDE
YENETFLCHRFTKFVMKYNLMSKDNLIVPILEEEVQNSVSGESEA
>O13758
MDALSHESGYSNAYVDELKRKSRQTPSEFTKKEEDSSTKESELQTRSSPPLPVNTSLEWN
MLNQGIPEEAMIKELKDREGRKRNIAMMTDNYISLETGDQLMLAQNHKEEKLLQTEDEIQ
DEGYSGFENYVEESEKLQEIYHHSSESLRTRSIQMAVDQKNMEMELDDEDIAEPIQSWEH
TQIKKGAFGESPAFTNGLSVKLPNILTMDEQIQRLKEAIASEKLQQEERSQIIKSLMEEE
LEINEQEEKIKHSFIDLDKTLLNNLTKSKS
>P51662
MAMVSEFLKQAWFIDNEEQDYINTVKTYKGGPGSAVSPYPAFNPSSDVAALHQAIMVKGV

DEATIIDILTKRNNAQRQQIKAAYLQEKGKPLDEVLKKALTGHLEEVVLALLKTPAQFDA
DELRAAMKGLGTDEDTLIEILASRNNKEIREINRVYREELKRDLAKDIASDTSGDFQKAL
LSLAKGDRSEDFGVNEDLADTDARALYEAGERRKGADVNVFTTILTTRSYLHLRRVFQKY
SKYSQHDMNKVLDLELKGDIEKCLTAIVQCATCKPAYFAEKLYQAMKGAGTRHKALIRIM
VSRSEVDMNDIKAFYQKKYGVSLCQAILDETKGDYEKILVALCGGN
>P00564
MPFGNTHNKFKLNYKPQEEYPDLSKHNNHMAKVLTPDLYNKLRDKETPSGFTLDDVIQTG
VDNPGHPFIMTVGCVAGDEESYTVFKDLFDPIIQDRHGGYKPTDKHKTDLNHENLKGGDD
LDPNYVLSSRVRTGRSIKGYTLPPHCSRGERRAVEKLSVEALNSLTGEFKGKYYPLKSMT
EQEQQQLIDDHFLFDKPVSPLLLASGMARDWPDARGIWHNDNKSFLVWVNEEDHLRVISM
EKGGNMKEVFRRFCVGLQKIEEIFKKAGHPFMWNEHLGYVLTCPSNLGTGLRGGVHVKLA
NLSKHPKFEEILTRLRLQKRGTGGVDTAAVGAVFDISNADRLGSSEVEQVQLVVDGVKLM
VEMEKKLEKGQSIDDMIPAQK
>O08785
MVFTVSCSKMSSIVDRDDSSIFDGLVEEDDKDKAKRVSRNKSEKKRRDQFNVLIKELGSM
LPGNARKMDKSTVLQKSIDFLRKHKETTAQSDASEIRQDWKPTFLSNEEFTQLMLEALDG
FFLAIMTDGSIIYVSESVTSLLEHLPSDLVDQSIFNFIPEGEHSEVYKILSTHLLESDSL
TPEYLKSKNQLEFCCHMLRGTIDPKEPSTYEYVRFIGNFKSLTSVSTSTHNGFEGTIQRT
HRPSYEDRVCFVATVRLATPQFIKEMCTVEEPNEEFTSRHSLEWKFLFLDHRAPPIIGYL
PFEVLGTSGYDYYHVDDLENLAKCHEHLMQYGKGKSCYYRFLTKGQQWIWLQTHYYITYH
QWNSRPEFIVCTHTVVSYAEVRAERRRELGIEESLPETAADKSQDSGSDNRINTVSLKEA
LERFDHSPTPSASSRSSRKSSHTAVSDPSSTPTKIPTDTSTPPRQHLPAHEKMTQRRSSF
SSQSINSQSVGPSLTQPAMSQAANLPIPQGMSQFQFSAQLGAMQHLKDQLEQRTRMIEAN
IHRQQEELRKIQEQLQMVHGQGLQMFLQQSNPGLNFGSVQLSSGNSNIQQLTPVNMQGQV
VPANQVQSGHISTGQHMIQQQTLQSTSTQQSQQSVMSGHSQQTSLPSQTPSTLTAPLYNT
MVISQPAAGSMVQIPSSMPQNSTQSATVTTFTQDRQIRFSQGQQLVTKLVTAPVACGAVM
VPSTMLMGQVVTAYPTFATQQQQAQTLSVTQQQQQQQQPPQQQQQQQQSSQEQQLPSVQ
QPAQAQLGQPPQQFLQTSRLLHGNPSTQLILSAAFPLQQSTFPPSHHQQHQPQQQQQLPR
HRTDSLTDPSKVQPQ
>O14227
MAVSLNDFISVKLKRYSRESPWERLRVPYRNKKQKKWASVHNNEAQLHSANKRNDNCLIQ
RSSTWRLGDMITLVIKDIPVTWLSNEGGKLYNLWEPLHDYGTIEFMKINEPLNGQTSTTA
IVQFAPPPKVPFWEPNGKINVKGVDLAVQIDITAHRSHISRQVFSKNSFRSDQLVKIPLS
SFKLGQVYDERIVPLFGVDCGITVTESNLLVYFNFKKLCVLFDASFDKQIETFRLDFDFH
SIIGDVGTDYYDDHISLVFRFRFSPLIFRKSKNATESRVQTFWTASHLWRRHYDILPFNV
SPTTASPIELLNCHNAPIGRCNVLVLSFSIRDESDKDDIAFLLHNLEKFNLKSQLDKVVF
HLVPDYKHRCSLINDKEIEEEIAYLLQACLSKNLLSEIDLPIILANLKKLSKERAKKFLR
LILTSKTALINPSELDFTKSFVFYDLSSASSIHIKKLYVTPTTLRIVEDSLEAGNRVIRN
FKDFANRFMRVQITDEYYKQKIRGGSDGFRNEKLYSRIQQLLTYGIKVGNQIYEFLAFGN
SQLREHGAYFFASGSDLNAKQIREWMGDFSEINSVSKYAARMGQCFSTTKEINRFCVDIS
LQDDIVRNNHCFTDGVGMASLSVIRRLSLEVKNHDMFPSAFQFRMGGYKGVLSLAPPTKL
EYHQGNLVFPRRSQDKFKSFHSTLEVIKISRFSNAHLNMQLITLLEGLGVEKTVFLELTR
SQLSKMNESINSKQKSILMLRDNVDEYHSTLIIADFIQAGFLERDDAFTENLLNLYYEWV
LRLIKEKQKVSVPKGAYLLGVADETGTLKGHYDDAVLSVPEIFIQITDTSTSFGSYSTGK

LKTRVIVGLCIVARNPSLHPGDVRVCKAVRCDELMHLKNVIVFPTTGDRSIPAMCSGGDL
DGDEYTVIWDQRLLPKIVNYPPLLESSPKKSIDFLEGKPLIDSVKEFFVNYIKYDSLGLI
SNAWKAWAHDHDNNPEGIFGNVCLELAEMHSKAVDFAKSGVACKMQAKYHPKRYPDFMQK
TKTRSFRSETAVGKIFRYAARFQRESGRPATYNPIMNTVYDPCMKLPRFKTEYLNVAEEV
KKHYDNDLRSIMARFDISTEYEVYTAFILFKDDLAKTVNEYGLREEVSFQFDLLKKKYTQ
EYLEKCALSNQSAFDSSEYEERINSAVAATYDVTYDQRVKSVGNGTTEVLISFPYLFSSR
LCQLSRKAMLTANNF
>P49902
MSTSWSDRLQNAADMPANMDKHALKKYRREAYHRVFVNRSLAMEKIKCFGFDMDYTLAVY
KSPEYESLGFELTVERLVSIGYPQELLSFAYDSTFPTRGLVFDTLYGNLLKVDAYGNLLV
CAHGFNFIRGPETREQYPNKFIQRDDTERFYILNTLFNLPETYLLACLVDFFTNCPRYTS
CETGFKDGDLFMSYRSMFQDVRDAVDWVHYKGSLKEKTVENLEKYVVKDGKLPLLLSRMK
EVGKVFLATNSDYKYTDKIMTYLFDFPHGPKPGSSHRPWQSYFDLILVDARKPLFFGEGT
VLRQVDTKTGKLKIGTYTGPLQHGIVYSGGSSDTICDLLGAKGKDILYIGDHIFGDILKS
KKRQGWRTFLVIPELAQELHVWTDKSSLFEELQSLDIFLAELYKHLDSSSNERPDISSIQ
RRIKKVTHDMDMCYGMMGSLFRSGSRQTLFASQVMRYADLYAASFINLLYYPFSYLFRAA
HVLMPHESTVEHTHVDINEMESPLATRNRTSVDFKDTDYKRHQLTRSISEIKPPNLFPLA
PQEITHCHDEDDDEEEEEEEE
>Q8CG72
MAVAAAAAATAMSAAGGGGASAARSISRFRGCLAGALLGDCVGAVYEAHDTVSLASVLSH
VESLEPDPGTPGSARTETLYYTDDTAMTRALVQSLLAKEAFDEVDMAHRFAQEYKKDPDR
GYGAGVITVFKKLLNPKCRDVYEPARAQFNGKGSYGNGGAMRVAGISLAYSSVQDVQKFA
RLSAQLTHASSLGYNGAILQALAVHLALQGVSSSEHFLEQLLGHMEELEGDAQSVLDAKE
LGMEERPYSSRLKKVGELLDQDVVSREEVVSELGNGIAAFESVPTAIYCFLRCMEPHPEI
PSTFNSLQRTLIYSISLGGDTDTIATMAGAIAGAYYGMEQVPESWQQSCEGFEETDVLAQ
SLHRVFQESS
>Q05948
MAVAIKKEKTKFAPVKEVLSEKDHANYTKFQDTSKLEWFCRTSNHKKFKSHSLLKAVRNP
TETRIETQTLYFTDLTNGKCGLIQLLYSSVMGGIYKGFQLNFKIFKASSEENSEEDIDIW
ESFKIDNIKDFDTLKVESDNVTFHFVPLENSSSSGFAQLLIKIDIPKGSTSCLLKDLKVD
ITVNLQEGFIINPDGSNYYLDKSISLEELAKRDSSSTSRKMIRHVFVPRGFCNGTISYKK
NDKPVKLDLKDTPMLYLDAVQGLIPNKAASKWNFLCFNGEKRSMMCIEFTTTKEYGSTTV
TIWAVSDKDKILEVGSSVNDHAVKFPSTKEDKQNGWKYPTSISFPRGFEESNLRLVNRYD
IMSELPAFIRSIAENLANMKPFIYQFCQKSKFDDDEGVSIIESTFIN
>P53032
MSTSITVRNRDRSLPPLLLPNVSLLEKDIRRKGTQNVGITDPELLSTTWTRKRAFPTDEL
LGGYKRLKPAAADSNECAIGIATVTPPPTLPVSAIVPPPQNYTPPLFEYHPHALASMVNE
DANASCTQMSIISRSTSNSTTSSATSTSSISKRQRSGPSCDKCRLKKIKCNAKIEILLQD
DTIMPMISNKLRYVLTPDDIRLYRGTLLRNIAIPDDVIEGTGSRKLIKHIDKLVLLTPCL
PCIKKKHSSSSTNFPKNDKCTFSKGFTRADINISSKISLKFKDKTIYDITYDDYKSIDF
>P11075
MSEQNSVVNAEKGDGEISSNVETASSVNPSVKPQNAIKEEAKETNGEDQKCKGPENAGST
AETKETSNDATNGMKTPEETEDTNDKRHDDEGEDGDEDEDEDEDEDEDNGDEDDEDVDSS
SSETSSEDGEDSESVSGESTESSSGEDEESDESDGNTSNSSSGDESGSEEEEEEEEEEEE

EENAGEPAIAHQDSVPTNDSTAPRSTHTRNISLSSNGSNTNSTIILVKTTLETILNDKDI
KKNSNAQKAIERTLQKFKEFDPQTTNNPHYVDSILVFEALRASCRTKSSKVQSLALDCLS
KLFSFRSLDETLLVNPPDSLASNDQRQDAADGITPPPKQKIIDAAIDTISDCFQGEGTDD
RVELQIVRALSSCILEEDSSSLCHGASLLKAIRTIYNVFVFSLNPSNQGIAQATLTQIIS
SVYDKIDLKQSTSSAVSLSTKNHQQQSAIELSEASENAETPAPLTLENMDKLNDDEERLM
DAQQPDSIAITNQDLAVKDAFLVFRVMAKICAKPLETELDMRSHAVRSKLLSLHIIYSII
KDHIDVFLSHNIFLPGKERVCFIDSIRQYLRLVLSRNAASPLAPVFEVTLEIMWLLIANL
RADFVKEIPVFLTEIYFPISELTTSTSQQKRYFLSVIQRICNDPRTLVEFYLNYDCNPGM
PNVMEITVDYLTRLALTRVEITQTQRSYYDEQISKSLSTYNFSQLPLLTSSNLSSSPDVG
QVNLLFPLDFALKMVSLNCIVSVLRSLSSWAHKALNPNTHTANKVLLNTTSSARQESRSS
LSNDVRSSIMTSNDDFKPTYEDEESRSLSSQNIDADDPTQFENLKLRKTALSECIAIFNN
KPKKAIPVLIKKGFLKDDSPISIAKWLLETEGLDMAAVGDYLGEGDDKNIAIMHAFVDEF
DFTGMSIVDALRSFLQSFRLPGEGQKIDRFMLKFAERFVDQNPGVFSKADTAYVLSYSLI
MLNTDLHSSQIKNKMSLQEFLENNEGIDNGRDLPRDFLEGLFNEIANNEIKLISEQHQAM
LSGDTNLVQQQQSAFNFFNSRDLTREAYNQVSKEISSKTELVFKNLNKNKGGPDVYYAAS
HVEHVKSIFETLWMSFLAALTPPFKDYDDIDTTNKCLEGLKISIKIASTFRINDARTSFV
GALVQFCNLQNLEEIKVKNVNAMVILLEVALSEGNYLEGSWKDILLVVSQMERLQLISKG
IDRDTVPDVAQARVANPRVSYESSRSNNTSFFDVWGKKATPTELAQEKHHNQTLSPEISK
FISSSELVVLMDNIFTKSSELSGNAIVDFIKALTAVSLEEIESSENASTPRMFSLQKMVD
VCYYNMDRIKLEWTPLWAVMGKAFNKIATNSNLAVVFFAIDSLRQLSMRFLDIEELSGFE
FQHDFLKPFEYTVQNSGNTEVQEMIIECFRNFILTKSESIKSGWKPILESLQYTARSSTE
SIVLKTQLLVSNDIVTNHFENVFSQEDAFSELVGVFREITKNKRFQKLSLHALESLRKMT
QNVADICFYNENKTEEERKHNDALLRGKDIFQDVWFPMLFCFNDTIMTAEDLEVRSRALN
YMFDALVAYGGKFNDDFWEKICKKLLFPIFGVLSKHWEVNQFNSHDDLSVWLSTTLIQAL
RNLIALFTHYFESLNRMLDGFLGLLVSCICQENDTIARIGRSCLQQLILQNVSKFNEYHW
NQIGDVFDKLFDLTTANELFDYDPLQQGRKSSVSHHQTTNDTSQHSDDDSNDRRENDSNI
SETVERAHQEESSEDVGGDMVETLNGQTKLNNGNSVPTVKDELNPKPASLSIPKKTKHMK
RNESNEDIRRRINIKNSIVVKCVLQLLMIELLNELFENEDFAHCIPYKEAIRITRLLEKS
YEFSRDFNEDYGLRTRLVEARVVDKIPNLLKQETSAAAVLLDIMFQLYLNDDEKKADLIT
RLITICIQVVEGYVSLDDRTMERSINAWRSVIVEILQGYYEFDDEDFRLYCPAMYALVIQ
ILDKSVPTELRHAIKQFLSRVGELYLSTD
>Q868H8
MRFKKSLTCIDMHTEGEAARIVTSGLPHIPGSNMAEKKAYLQENMDYLRRGIMLEPRGHD
DMFGAFLFDPIEEGADLGIVFMDTGGYLNMCGHNSIAAVTAAVETGILSVPAKATNVPVV
LDTPAGLVRGTAHLQSGTESEVSNASIINVPSFLYQQDVVIVLPKPYGEVRVDIAFGGNF
FAIVPAEHLGIDISVQNLSRLQEAGELLRTEINRSVKVQHPQLPHINTVDCVEIYGNATN
PEAKYKNVVIFGNRQADRSPCGTGTSAKMATLYAKGQLRIGETFVYESILGSLFQGRVLG
EERIPGVKVPVTKDAEEGMLVVTTEITGKAFIMGFNTMLFDPTDPFLNGFTLKR
>Q3MKQ1
MESKVEQGVKNLNMENDHQEKEEKEEKPQDANKREPVVALPFEAGEYYVPRGSRRRFRVR
QPIAHYRWDLMHRVGEPQGRMREENVQRFGEDMRQLMEKLRERQLSHSLRAVSTDPPHHD
HHDEFCLMP
>Q4U925
MDQMEVPDNDKNRKYPTKDLKIILPKTYADVNSKKGPEYWDYENITLKWNVPDSYEIVRK

IGRGKFSEVFEGLNTVTKDKCVIKILKPVKKKKIKREIKILQNLRGGPNIIKLLDIVKDP
QSRTPSLIFEHVNNTDFKTLYPTLTIQDIKYYIYQLLKAMNYCHSQGIMHRDIKPHNVMI
DHEKKILRLIDWGLAEFYHPEQEYSVRVATRYYKGPELLVDMRYYDYSLDIWSIGCMLAG
IIFKKEPFFYGHDNYDQLVKIAKVLGTEDLHRYFEKYGLKFAPAYQEILGNHSKKPWTKF
VHHENQHLVSPEVMDLLDRMLVYDHTKRITPLEAMEHPFFNEIKNNSV
>Q42484
MDFISSLIVGCAQVLCESMNMAERRGHKTDLRQAITDLETAIGDLKAIRDDLTLRIQQDG
LEGRSCSNRAREWLSAVQVTETKTALLLVRFRRREQRTRMRRRYLSCFGCADYKLCKKVS
AILKSIGELRERSEAIKTDGGSIQVTCREIPIKSVVGNTTMMEQVLEFLSEEEERGIIGV
YGPGGVGKTTLMQSINNELITKGHQYDVLIWVQMSREFGECTIQQAVGARLGLSWDEKET
GENRALKIYRALRQKRFLLLLDDVWEEIDLEKTGVPRPDRENKCKVMFTTRSIALCNNMG
AEYKLRVEFLEKKHAWELFCSKVWRKDLLESSSIRRLAEIIVSKCGGLPLALITLGGAMA
HRETEEEWIHASEVLTRFPAEMKGMNYVFALLKFSYDNLESDLLRSCFLYCALFPEEHSI
EIEQLVEYWVGEGFLTSSHGVNTIYKGYFLIGDLKAACLLETGDEKTQVKMHNVVRSFAL
WMASEQGTYKELILVEPSMGHTEAPKAENWRQALVISLLDNRIQTLPEKLICPKLTTLML
QQNSSLKKIPTGFFMHMPVLRVLDLSFTSITEIPLSIKYLVELYHLSMSGTKISVLPQEL
GNLRKLKHLDLQRTQFLQTIPRDAICWLSKLEVLNLYYSYAGWELQSFGEDEAEELGFAD
LEYLENLTTLGITVLSLETLKTLFEFGALHKHIQHLHVEECNELLYFNLPSLTNHGRNLR
RLSIKSCHDLEYLVTPADFENDWLPSLEVLTLHSLHNLTRVWGNSVSQDCLRNIRCINIS
HCNKLKNVSWVQKLPKLEVIELFDCREIEELISEHESPSVEDPTLFPSLKTLRTRDLPEL
NSILPSRFSFQKVETLVITNCPRVKKLPFQERRTQMNLPTVYCEEKWWKALEKDQPNEEL
CYLPRFVPN
>P98158
MERGAAAAAWMLLLAIAACLEPVSSQECGSGNFRCDNGYCIPASWRCDGTRDCLDDTDEI
GCPPRSCESGLFLCPAEGTCIPSSWVCDEDKDCSDGADEQQNCAGTTCSAQQMTCSNGQC
IPSEYRCDHVSDCPDGSDERNCHYPTCDQLTCANGACYNTSQRCDQKVDCRDSSDEANCT
TLCSQKEFECGSGECILRAYVCDHDNDCEDNSDERNCNYDTCGGHQFTCSNGQCINQNWV
CDGDDDCQDSGDEDGCESNQSHHRCYPREWACPGSGRCISIDKVCDGVPDCPEGDDENNV
TSGRTCGMGVCSVLNCEYQCHQTPFGGECFCPPGHIINSNDSRTCIDFDDCQIWGICDQK
CENRQGRHQCLCEEGYILERGQHCKSSDSFSAASVIFSNGRDLLVGDLHGRNFRILAESK
NRGMVMGVDFHYQKHRVFWTDPMQEKVFSTDINGLNTQEILNVSVDTPENLAVDWINNKL
YLVETKVNRIDVVNLEGNQRVTLITENLGHPRGIALDPTVGYLFFSDWGSLSGQPKVERA
FMDGSNRKDLVTTKVGWPAGITLDLVSKRVYWVDSRYDYIETVTYDGIQRKTVARGGSLV
PHPFGISLFEEHVFFTDWTKMAVMKASKFTETNPQVYHQSSLRPHGVTVYHALRQPNATN
PCGSNNGGCAQVCVLSHRTDNGGLGYRCKCEFGFELDDDEHRCVAVKNFLLFSSKTAVRG
IPFTLSTQEDVMVPVTGSPSFFVGIDFDAQHSTVFYSDLSKDIIYKQKIDGTGKEVITAN
RLESVECLTFDWISRNLYWTDGGLKSVTVLRLADKSRRQIISNLNNPRSIVVHPTAGYMF
LSDWFRPAKIMRAWSDGSHLMPIVNTSLGWPNGLAIDWSASRLYWVDAFFDKIEHSTLDG
LDRKRLGHVDQMTHPFGLTVFKDNVFITDWRLGAIIRVRKSDGGDMTVIRRGISSVMHVK
AYDADLQTGSNYCSQTTHANGDCSHFCFPVPNFQRVCGCPYGMKLQRDQMTCEGDPAREP
PTQQCGSLSFPCNNGKCVPSFFRCDGVDDCHDNSDEHQCGVFNNTCSPSAFACVRGGQCI
PGQWHCDRQNDCLDGSDEQNCPTHATSSTCPSTSFTCDNHVCIPKDWVCDTDNDCSDGSD
EKNCQASGTCQPTQFRCPDHRCISPLYVCDGDKDCADGSDEAGCVLNCTSAQFKCADGSS
CINSRYRCDGVYDCRDNSDEAGCPTRPPGMCHPDEFQCQGDGTCIPNTWECDGHPDCIHG

SDEHTGCVPKTCSPTHFLCDNGNCIYKAWICDGDNDCRDMSDEKDCPTQPFHCPSTQWQC
PGYSTCINLSALCDGVFDCPNGTDESPLCNQDSCSHFNGGCTHQCMQGPFGATCLCPLGY
QLANDTKTCEDINECDIPGFCSQHCVNMRGSFRCACDPEYTLESDGRTCKVTGSENPLLV
VASRDKIIVDNITAHTHNLYSLVQDVSFVVALDFDSVTGRVFWSDLLQGKTWSVFQNGTD
KRVVHDSGLSVTEMIAVDWIGRNLYWTDYALETIEVSKIDGSHRTVLISKNVTKPRGLAL
DPRMGDNVMFWSDWGHHPRIERASMDGTMRTVIVQEKIYWPCGLSIDYPNRLIYFMDAYL
DYIEFCDYDGHNRRQVIASDLVLHHPHALTLFEDFVYWTDRGTRQVMQANKWHGGNQSVV
MYSVHQPLGITAIHPSRQPPSRNPCASASCSHLCLLSAQAPRHYSCACPSGWNLSDDSVN
CVRGDQPFLMSVRDNIIFGISLDPEVKSNDAMVPISGIQHGYDVEFDDSEQFIYWVENPG
EIHRVKTDGSNRTVFAPLSLLGSSLGLALDWVSRNIYYTTPASRSIEVLTLKGDTRYGKT
LIANDGTPLGVGFPVGIAVDPARGKLYWSDHGTDSGVPAKIASANMDGTSLKILFTGNLQ
HLEVVTLDIQEQKLYWAVTSRGVIERGNVDGTERMILVHHLAHPWGLVVYGSFLYYSDEQ
YEVIERVDKSSGNNKVVLRDNVPYLRGLRVYHRRNAADSSNGCSNNPNACQQICLPVPGG
MFSCACASGFKLSPDGRSCSPYNSFMVVSMLPAVRGFSLELSDHSEAMVPVAGQGRNVLH
ADVDVANGFIYWCDFSSSVRSSNGIRRIKPDGSNFTNVVTYGIGANGIRGVALDWAAGNL
YFTNAFVYETLIEVLRINTTYRRVLLKVSVDMPRHIIVDPKHRYLFWADYGQKPKIERSF
LDCTNRTVLVSEGIVTPRGLAMDHDTGYIYWVDDSLDLIARIHLDGGESQVVRYGSRYPT
PYGITVFGESIIWVDRNLKKVFQASKQPGNTDPPVVIRDKINLLRDVTIFDEHAQPLSPA
ELNNNPCLQSNGGCSHFCFALPELPTPRCGCAFGTLGNDGKSCATSQEDFLIYSLNNSLR
SLHFDPRDHSLPFQVISVAGTAIALDYDRRNNRIFFTQKLNSLRGQISYVSLYSGSSSPT
VLLSNIGVTDGIAFDWINRRIYYSDFSNQTINSMAEDGSNRAVIARVSKPRAIVLDPCRG
YMYWTDWGTNAKIERATLGGNFRVPIVNTSLVWPNGLALDLETDLLYWADASLQKIERST
LTGTNREVVVSTAFHSFGLTVYGQYIYWTDLYTRKIYRANKYDGSDLVAMTTRLPTQPSG
ISTVVKTQRQQCSNPCDQFNGGCSHICAPGPNGAECQCPHEGNWYLANDNKYCVVDTGTR
CNQLQFTCLNGHCINQDWKCDNDNDCGDGSDELPTVCAFHTCRSTAFTCGNGRCVPYHYR
CDYYNDCGDNSDEAGCLFRNCNSTTEFTCSNGRCIPLSYVCNGINNCHDNDTSDEKNCPP
HTCPPDFTKCQTTNICVPRAFLCDGDNDCGDGSDENPIYCASHTCRSNEFQCLSPQRCIP
SYWFCDGEADCADGSDEPDTCGHSVNTCRASQFQCDNGRCISGNWVCDGDNDCGDMSDED
QRHHCELQNCSSTQFTCVNSRPPNRRCIPQYWVCDGDADCSDALDELQNCTMRTCSAGEF
SCANGRCVRQSFRCDRRNDCGDYSDERGCSYPPCHANQFTCQNGRCIPRFFVCDEDNDCG
DGSDEQEHLCHTPEPTCPLHQFRCDNGHCIEMGRVCNHVDDCSDNSDEKGCGINECLDSS
ISRCDHNCTDTITSFYCSCLPGYKLMSDKRSCVDIDECKESPQLCSQKCENVVGSYICKC
APGYIREPDGKSCRQNSNIEPYLIFSNRYYIRNLTTDGSSYSLILQGLGNVVALDFDRVE
KRLYWIDAEKQIIERMFLNKTNRETIINHRLRRAESLAVDWVSRKLYWLDAILDCLFVSD
LEGRHRKMIAQHCVDANNTFCFEHPRGIVLHPQRGHVYWADWGVHAYIGRIGMDGTNKSV
IISTKIEWPNAITIDYTNDLLYWADAHLGYIEFSDLEGHHRHTVYDGSLPHPFALTIFED
TVFWTDWNTRTVEKGNKYDGSGRVVLVNTTHKPFDIHVYHPYRQPIMSNPCGTNNGGCSH
LCLIKAGGRGFTCACPDDFQTVQLRDRTLCMPMCSSTQFLCGNNEKCIPIWWKCDGQKDC
SDGSDEPDLCPHRFCRLGQFQCRDGNCTSPQALCNARQDCADGSDEDRVLCEHHRCESNE
WQCANKRCIPQSWQCDSVNDCLDNSDEDTSHCASRTCRPGQFKCNNGRCIPQSWKCDVDN
DCGDYSDEPIDECTTAAYNCDNHTEFSCKTNYRCIPQWAVCNGFDDCRDNSDEQGCESVP
CHPSGDFRCANHHCIPLRWKCDGTDDCGDNSDEENCVPRECSESEFRCADQQCIPSRWVC
DQENDCGDNSDERDCEMKTCHPEHFQCTSGHCVPKALACDGRADCLDASDESACPTRFPN
GTYCPAAMFECKNHVCIQSFWICDGENDCVDGSDEEIHLCFNIPCESPQRFRCDNSRCVY

GHQLCNGVDDCGDGSDEKEEHCRKPTHKPCTDTEYKCSNGNCISQHYVCDNVNDCGDLSD
ETGCNLGDNRTCAENICEQNCTQLSSGGFICSCRPGFKPSTSDKNSCQDINECEEFGICP
QSCRNSKGSYECFCVDGFKSMSTHYGERCAADGSPPLLLLPENVRIRKYNTSSEKFSEYL
EEEEHIQTIDYDWDPEHIGLSVVYYTVLAQGSQFGAIKRAYIPNFESGSNNPIREVDLGL
KYLMQPDGLAVDWVGRHIYWSDAKSQRIEVATLDGRYRKWLITTQLDQPAAIAVNPKLGL
MFWTDQGKQPKIESAWMNGEHRSVLVSENLGWPNGLSIDYLNDDRVYWSDSKEDVIEAIK
YDGTDRRLIINEAMKPFSLDIFEDKLYWVAKEKGEVWRQNKFGKENKEKVLVVNPWLTQV
RIFHQLRYNQSVSNPCKQVCSHLCLLRPGGYSCACPQGSDFVTGSTVQCDAASELPVTMP
PPCRCMHGGNCYFDENELPKCKCSSGYSGEYCEVGLSRGIPPGTTMAVLLTFVIVIIVGA
LVLVGLFHYRKTGSLLPTLPKLPSLSSLAKPSENGNGVTFRSGADVNMDIGVSPFGPETI
IDRSMAMNEHFVMEVGKQPVIFENPMYAAKDNTSKVALAVQGPSTGAQVTVPENVENQNY
GRPIDPSEIVPEPKPASPGADEIQGKKWNIFKRKPKQTTNFENPIYAEMDSEVKDAVAVA
PPPSPSLPAKASKRNLTPGYTATEDTFKDTANLVKEDSDV
>Q12380
MNDIKQLLWNGELNVLVSIDPSFLMKGSPREIAVLRIRVPRETYLVNYMPLIWNKIKSFL
SFDPLTDSEKYFWFEHNKTPIPWNYPVGVLFDCLAGKSATFTTSFENQVKDVLTFLRIHL
VMGDSLPPTIIPIASSKTQAEKFWFHQWKQVCFILNGSSKAIMSLSVNEARKFWGSVITR
NFQDFIEISNKISSSRPRHIPLIIQTSRTSGTFRISQPTISMTGVNPTLKDIEGDILDVK
EGINGNDVMVICQGIEIPWHMLLYDLYSKLRSFDGFLYITLVPIKGGDKASSEL
>Q9C1W6
MSDREEKLAAAKQRLLEVKKKKRQAKSKNSVAEDNVDRESNDGNNQVNEPTGNDNTQVVE
NTEDISASNVVSAEGAEASTGDASTQSPETSENVVKNSVDESVAEKPEKEDLAVIESEDK
AAKPDGEIKKNVETEVTSRSTSSQEKDELEKQVKTLHDLNEQKDKKIKELKERINDLTYD
YETLKANADDSEGKQTLVSKREAALEEFQSKLLIRENEINKRELKMNGKEDDLKKREKDL
ENRLLKVEEHEKSLNERATKLSEANENFNNRFKEFEEREKSAIKQNKEQSSEGSKTANQT
HEQKELINSLEKKVSDIALEKQLLEEAVERYKLAMVEFAECQRRLKELEAMHFEQPGSVT
EKLFPDPTNFEVIDNVSIDLTLTGFPGAHGIPFSV
>O74354
MRTTLRLHIIKDGEQDNQFMILFDPSSSISLLKEKVQETYKSLYPFESNINIRNIKNEES
YDIPNEYLVGEIFPTNSKVIVESFSSPLKKLDGTMINFKEKNIQHDLDGVENDFATVQSA
SNGVHAINGKRTHPDESENPRKLPKKNFVEAIDANSPGFVYRPTSIRDRAYSISSNHDNE
STLTEGIALKEIESPDKDRKADGIVNLSVTQEEDDNHQSFNSSLTPSQPTTYNRANFFSI
NDASSDSSSDAPLRTLSSPSRLRMKDNDRKYLVEHSPAALIKESETIDGIDDKSLRSSTR
EVSVESPNEDSVNDDSSSDVSDEKETEAKHEIRAPAIIVRETSSHPSTAVPSENDTTESE
NDTLSESSTTSISSSPSENSDTSDDLTKVDSPNKSLVNDNVSAKHDKESENGKSKFPPPS
QTLVTTSTISAAGNEPSDEIGSENDSDSDSDSDSSVPLSQLQKKSQQRNSVSHEIQNRGT
KGSPKEPKAKPSTERPETHRTLSYSRLSELSKTFSPEIREPSLTKKTAVSMQESKEEGRS
DESSESEESGSSSDESDNSEKEDRSNPIPVEKRASTVLNTKKKRKAKRNSALAGLAALV
>P10857
MADTAAVAKGVENLSVSASKTKELKGTEKRDTLIEIEKRYQQKWEQEGVFEVDAPSTAEF
PLDAITPDELRQKHPKFFGTIAYPYMNGRLHAGHAFSFSKIEYHTGFARMQGKRALFPQG
YHCTGLPIKASADKLVKEIEMFGQEFERYKEDEVVEGAAPAAAAAPKTKEDLTKFNAKKG
KTVAKTGGAKYQFQILKSLGIPVSEIHKFADPQYWLHYFPPECKKDLTNFGARIDWRRQF
VTTDANPYYDAFVRWQMNRLLELNKIKFGKRYTIYSIKDGQPCMDHDRSEGEGVLPQEYT

ALKLKVTEWAPKAAEALKGKLPEGANVYLCPATLRPETMYGQVCCFVGPALKYGVFKAAE
NEYFVITERAAKNMAYQGIFEKEGVIEKAADIVGSDLIGTLVNAPLSVHKEVYVLPMDTV
LATKGTGVVTSVPSDSPDDCAMMTELAKKPEFYGIQKEWAEKEIVSVIKTPTSDLLAPYL
VKKLKINSPKDAKQLLEAKELAYKEGFYQGIMNYGDFKGEKVETAKPKVRQQLIDAGDAF
AYSEPENKVVSRSGDECSVALMDQWYIDYGEDSWRTVLYDYVENKDGKGINTYYADTQHA
FKGVIGWLKQWACARTYGLGSKLPWDPNFLVESLSDSTVYMAYYTVAHWLHRDLFGREKG
KGNIGADQMIDEVWDYIFCRTELSDHLVTKSGIPKETLDSMRREFQYFYPLDIRVSGKDL
IPNHLTFWLYNHIALFPREYWPKSVRANGHLQLNGEKMSKSTGNFMTLDDVVKKYGADAA
RVALADAGDGISDSNFVEDVADNTILRFYTNKEWIEETLKDESLRTGELNSFQDALFDNE
MNALVNEARKHYEETSYKLALKAAHYDFLNARDMYREACAAAGIPLHKDLVTKYIRLQAL
VITPIAPHWADYVWQECLGEPKSIQFARWPEVPAANPALTAARDYVRTTSSAINSAEAAQ
LKKMAKGRQSDFDPKKPKKLTIFATENFPTWQAKYIDLLSEVWDAATGTQKIDDKELNGR
IAKMGEMKKAMPFVQALKKRLKDGEPAEQILSRKLSFDEKATLLAMIPGLKRTAGLESVQ
VVLVEEGSKTGKDLTNGGAEIEVTAPMAEAALPGQPSFFFTNV
>Q9GRF8
MPSFADLTTENGLVELNKFVSDKTYIVGFVPSSADVQAFNLVKTAPCATKYPHAARWFNT
IASYSAAEQGQFEKVTETVTIAAPAAPKADDDVDLFGSDDEDDEEYERQLEERRKKAMEH
KKPKETVIAKSSILLDVKPWDDETDMVELEKCVRSIEMDGLVWGASKLVAVGYGIKKLVI
NLVVEDLKVSTDELEEKIKDFEDYVQSVDVAAFNKI
>P62024
MDYPKMDYFLDVESAHRLLDVESAQRFFYSQGAQARRATLLLPPTLMAASSEDDIDRRPI
RRVRSKSDTPYLAEARISFNLGAAEEVERLAAMRSDSLVPGTHTPPIRRRSKFANLGRIF
KPWKWRKKKSEKFKHTSAALERKISMRQSREELIKRGVLKEIYDKDGELSISNEDDSLTN
GQSLSSSQLSLPALSEMEPVPMPRDPCSYEVLQASDIMDGPDPGAPVKLPCLPVKLSPPL
PPKKVLICMPVGGPELSLASYAAQKSSQQAVAQHHHTVLPSQMQHQLQYGSHGQHLPSST
GTLPMHPSGCRMIDELNKTLAMTMQRLESSEQRVPCSTSYHSSGLHSSDGVTKAGPLGLP
EIRQVPTVVIECDDNKENVPHEPDYEDSSCLYAREEEEEEEDEDDDASLYTSSLAMKVCR
KDSLAIKLSNRPSKRELEEKNILPRQTDEERLELRQQIGTKLTRRLSQRPTAEELEQRNI
LKPRNEQEEQEEKREIKRRLTRKLSQRPTVEELRERKILIRFSDYVEVADAQDYDRRADK
PWTRLTAADKAAIRKELNEFKSTEMEVHELSRHLTRFHRP
>Q8BKT7
MSSESSKKRKPKVIRSDGTPTEGKRNRSDTEQEGKYYSEEAEVDLRDPGRDYELYKYTCQ
ELQRLMAEIQDLKSKGSKDVAIEIEERRIQSCVHFMTLKKLNRLAHIRLKKGRDQTHEAK
QKVDAYHLQLQNLLYEVMHLQKEITKCLEFKSKHEEIDLVSLEEFYSEAPPSISKAEITM
GDPHQQTLARLDWELEQRKRLAEKYRECLSNKEKILKEIEVKRDYLSSLQPRLNSIMQAS
LPVQEYLFMPFDQAHKQYETARHLPPPLYVLFVQATAYGQACDKTLSVAIEGSVDEAKAL
FKPPEDSQDDESDSDAEEEQTTKRRRPTLGVQLDDKRKEMLKRHPLSVLLDLKCKDNSVL
HLTFYYLMNLNIMTVKAKVTTAVELITPISAGDLLSPDSVLSCLYPGDHGKKTPNPANQY
QFDKVGILTLRDYVLELGHPYLWVQKLGGLHFPKEQPQQTVMPDHSQSASHMETTMKLLK
TRVQSRLALHKQFASLEHGIVPVTSDCQDLFPAKVVSRLVKWVIITHEDYMELHFTKDIV
EAGLAGDTNLYYLALIERGTAKLQAAVVLNPGYSSIPPVFRLCLNWKGEKTNSNDDNIRA
MESEVNVCYKELCGPRPSHQLLTNQLQRLCVLLDVYLETESHDDSFEGPKEFPQEKMCLR
LFRGPSRMKPFKYNHPQGFFSHR
>Q06149

MDGSHFPMKSTTGEPVSSGKKGKRRKVIKSCAFCRKRKLKCSQARPMCQQCVIRKLPQCV
YTEEFNYPLSNTELFEQVPNVALVQKIENLQTLLKENDNNNAKPVYCRSSENPLRSLRTS
VLGDNGSRYVFGPTSWKTLSLFEQNKFQTEFQNLWKVLKPLPECTKSQLNENDVVADLPS
FPQMESCIKSFFAGPLFGILHIFNQDDILSLLDRLFIRDTTDKNLVILLDLQGNAKDKYN
LGIVLQILCLGYYNQDIPSSVSHFLHTLSAASLSSSSSNFVEKLQFFLLSYISVMINCTD
GVWDATQGVDLINELCQGCISLGLNDIDKWYLNESEETKQNLRCIWFWALFLDVSTSYDI
GNPPSISDDLLDLSIFTAQNFQSPSIDFRRVKLMHDFLDVSRFTTREIHKREMNEKLTTF
SLRLIEFIQSNFSPIEHYTNSVYYSDIDPFDILILSRSLSIVASIYNIEMIIAQQSRIID
KNRMVQFLLISISVCVNTMVFHFKEPINDQENVLTEGLKLSIILINPLLIRIVSQVYSLA
FNRLIFREKAFLFLIDLDTGKKIQFIKYEEENFDELLTGFDVRTDKFLSFSGTIIRFYEI
IDSIFAVNERNKRLLKAVSNFYQLTSTLAFERVSRVLFDKASQARIETEKIWLKKGINME
HFSDLMIEDFINDVWKTFKEISKDLWSIDKKKFYKQYHFDL
>P53890
MGLDQDKIKKRLSQIEIDINQMNQMIDENLQLVEPAEDEAVEDNVKDTGVVDAVKVAETA
LFSGNDGADSNPGDSAQVEEHKTAQVHIPTENEANKSTDDPSQLSVTQPFIAKEQITHTA
IAIGDSYNSFVANSAGNEKAKDSCTENKEDGTVNIDQNRGEADVEIIENNDDEWEDEKSD
VEEGRVDKGTEENSEIESFKSPMPQNNTLGGENKLDAELVLDKFSSANKDLDIQPQTIVV
GGDNEYNHESSRLADQTPHDDNSENCPNRSGGSTPLDSQTKIFIPKKNSKEDGTNINHFN
SDGDGQKKMANFETRRPTNPFRVISVSSNSNSRNGSRKSSLNKYDSPVSSPITSASELGS
IAKLEKRHDYLSMKCIKLQKEIDYLNKMNAQGSLSMEDGKRLHRAVVKLQEYLDKKTKEK
YEVGVLLSRHLRKQIDRGENGQFWIGTK
>Q96QK1
MPTTQQSPQDEQEKLLDEAIQAVKVQSFQMKRCLDKNKLMDALKHASNMLGELRTSMLSP
KSYYELYMAISDELHYLEVYLTDEFAKGRKVADLYELVQYAGNIIPRLYLLITVGVVYVK
SFPQSRKDILKDLVEMCRGVQHPLRGLFLRNYLLQCTRNILPDEGEPTDEETTGDISDSM
DFVLLNFAEMNKLWVRMQHQGHSRDREKRERERQELRILVGTNLVRLSQLEGVNVERYKQ
IVLTGILEQVVNCRDALAQEYLMECIIQVFPDEFHLQTLNPFLRACAELHQNVNVKNIII
ALIDRLALFAHREDGPGIPADIKLFDIFSQQVATVIQSRQDMPSEDVVSLQVSLINLAMK
CYPDRVDYVDKVLETTVEIFNKLNLEHIATSSAVSKELTRLLKIPVDTYNNILTVLKLKH
FHPLFEYFDYESRKSMSCYVLSNVLDYNTEIVSQDQVDSIMNLVSTLIQDQPDQPVEDPD
PEDFADEQSLVGRFIHLLRSEDPDQQYLILNTARKHFGAGGNQRIRFTLPPLVFAAYQLA
FRYKENSKVDDKWEKKCQKIFSFAHQTISALIKAELAELPLRLFLQGALAAGEIGFENHE
TVAYEFMSQAFSLYEDEISDSKAQLAAITLIIGTFERMKCFSEENHEPLRTQCALAASKL
LKKPDQGRAVSTCAHLFWSGRNTDKNGEELHGGKRVMECLKKALKIANQCMDPSLQVQLF
IEILNRYIYFYEKENDAVTIQVLNQLIQKIREDLPNLESSEETEQINKHFHNTLEHLRLR
RESPESEGPIYEGLIL
>Q8LPU4
MALKQKDTDAAATATGTKKRRRVFFSDTDAGVEANECMKVFLVWNPGEVSSVDCTAIQPF
DLNHFFGEDGKIYGYKNLKINVWISAKSFHGYADVSFDETSDGGKGITDLKPVLQNIFGE
NLVEKEEFLHTFSKECEYIRTAVTNGSAIKHDGSYESDPAVEIVRVELQGAAAFLYSRLV
PLVLLLVEGSTPIDIGEHGWEMLLVVKKATQEAGSKFELLGFAAVHNFYHYPESIRLRIS
QILVLPPYQGEGHGLGLLEAINYIAQSENIYDVTIESPSDYLQYVRSSIDCLRLLMFDPI
KPALGAIVLSLKETNLSKRAQSLRMVPPADLMETVRQKLKINKKQFLRCWEILVFLSLDS
QDHKSMDNFRACIYDRMKGEILGSASGTNRKRLLQMPTSFNKEASFAVYWTQEIEDEDEQ

TVEQQPEDLKTQEQQLNELVDIQIEEIAGVAKNVTSRCKDKMTELVVQ
>Q8GWK1
MEDDMWCVSSSGSSRSYRSETAAKYQSGPYQDLEEFEEVDDDIAVEYPCPFCASDYDLVE
LCHHIDEEHRHEANNGICPVCSKRVKMHMVDHITSHHRDVLKSEQKEMSYREDPYLSDKY
LQPHLDELPPSMNHHQHPSKHVSDQFLSFINNSALPNQTKLVLPDSSVEDKNPIKDSSAA
KEGTSSCPLSDSDKLEKAKKCEFVQGLLSSAMFDDECDSSE
>Q9U1M8
MEDDDTLNGEYFQPVEDMITLPILTEESLLLNLKMRYKKKEIYTYTGSILVAVNPYEILP
IYTADIVKSYFAKSRNLMLPHIFAVSDAAFTNMIEEGKNQSIIISGESGAGKTESTKLII
QYLAARTNRHSQVEQMIVESSPILEAFGNAKTIRNNNSSRFGKFIEIQFNREGHISGARI
INYLLEKSRISHQASSERNYHIFYQLLAGASDELKEKLKLGEPEDYHYLSQSGCIRIENI
NDVEDFEHVKYAMNVLGLPEDKQFTIFSIVSAVLHIGNLKFEKSEKTQGAEGSEVSNKDT
LKIIAQLLSVDPVKLETCLTIRHVLIRGQNFVIPLKVNEAEDTRDSLAKALYGNVFNWLV
VFINSKIHKPQKNSTFIGVLDIFGFENFKKNSFEQFCINFANEKLQQHFNQHIFKLEQEE
YEKEKINWSKIVYNDNQECLDLIEKRPLGILSLLDEESRFPQATDLTYLDKLHTNHEKHP
YYEKPRRSKNTFVVKHYAGEVHYDTQGFLDKNKDTVSDDLSSLLQGSKSKFIIELFTPPR
EEGDDSDKGREKKKTTAGQTFKTQLQSLINILSSTQPHYVRCIKPNTTKEPAVYDRELIQ
AQLRYAGMMETIRIRKLGYPIRHTHKEFRDRYLILDYRARSTDHKQTCAGLINLLSGTGG
LERDEWQLGNTKVFIRDHQYLKLEELRKLKLLKKVTLIQSVWRMYRCKKRYQQIRASAKI
LGAAMLSHSSRRDFQEQRQAVQRIKGFFKMLTYQKQFKIIQINLRIVQNNIRSFIARRHS
RNAVLLKRDRNARMLEIQREKDEEERNRQEKEERDRQEKEDKEKETADRRQLQEEQKRRE
EELRAKREEEELKKLEEKKSQLKELNQIDELSSLERMLKEQQDKNINELDDFVNSLEAFS
FEGGVDDSQPYSFNHKMYEMSPEALDKISITDLLQGLKQTVRSVTKFEVDESKFELPPGI
ENVLKRAPGIKRQASSFLPGQPIPDVYSSPQYPVDEADDDDSNNNYINSNNGDLPLPTSQ
SSDFSLPPPPSSSSMDFGLPPPPPSSSSGGTYSLPPMPVFDFGMIDPILGAPPPPPSTSD
STSPSATATGNNTPNSSSASASQSTNQVNPQPTVSVVELPQILNDEEISLYSFYDYANKN
FNIEKLKQKDDIFSYQKSHIKSSLLVHSDAEQTKVAVEIFSKVLHYMNSNPLVSKKDPAD
FYSPVKFILTKGLAIESLRDEIYCQLIKQSTSNPIQDLNIRVWELIHFTCSTFPPTRKLI
KYFAAYLKTTIQQSDVSKSVKDSAQASYFILQRFTLNGARKQVPSVTELESIKENRPIFV
RITATDGSLKGLHIDSATTCQESSNDLSQRSRMRVNSKENGFTIIESFNGIERDIAPTDK
LCDVLSKVENLQATLSSKIQVNFKFVFKKKLFFDNITNNVPTTSINVENEFYYHQLFNDL
FNSNYCKDQDYQISIGSLKLQFESSDYTDEIRAWLPGNGRGKYFTTDIEKNRFDDFINKY
KSHKGLSPEDAKKQMVQLLEKHPLANCSLVVCEHQSESLPYPKNFVLALNVNGINIYDPA
TSKMLESVKYSNQSQQNLKSDDKSVSIILENKSTLQAFTGDVQKLVSLIKEYSLYLRNNA
KYARALKDYNVSDTSLLPFKRNDIITITFKDQENKWFMGQLNGKEGSFPVDHVEILLSDV
PPPQPVHPVATLSPPMSPTIPNITNTPPPPPSISDSMSPPPQVGMLPPPPPPSVMGSTKP
IEIPSLGIPPPPPSSSNSSVPNSPIGSPMMGIPPPPPTISVHSLSNSGNSTPPPPLPSLS
TPPTLSTPPPISSPPNFRSSLRVSMLNTSNDGGDNSSDDPSKRLTVSPAIGTDSQLAQWA
STRFRSFKRASTLNQQQATLKRKAPVDPNTAFYFNKDPIKESLIEMEAKLSKKAIKNFSE
IMMWMGDYPIPKGQTASLVIQSIISRGIENHELRDEIYCQAYRQTNKNPKVESAKKGFEL
IYFLSITFSPSDSLLQPFMEQLMSRNIAIQSSSPQLASLIAVCIEKLESHPIPSYQQRKM
GPSATEIQSFRSNLENGDISTCKIRFIDQSTKLAKINTYTTIREITDTVCRQYGISQQSI
KMFGISAVNETAGISKVVSETDMIYDVLARWEQSEEKGEFYFQVRRRFFLDDVNKILDQE
HLWTDDDICFELTYCQIRDEWMKGLYTNVNEKDSSIIAAILIQLLYPNQSKLVLTKEVVR

QVLPDQILNSQNIKVWISMIESQIFELVSQTPEYLKLMFINLIGSKSPLFGCTLFNIQQK
ENPPKAWLAINKKGVSIFDPHTKESKNFWTFQSISNVAFTDDTFCIMTGNLMKPIKQTFT
TDEHSSIASVYQFYSSQ
>P36408
MSSDISEKIQQARRDAESMKEQIRANRDVMNDTTLKTFTRDLPGLPKMEGKIKVRRNLKG
HLAKIYAMHWAEDNVHLVSASQDGKLLVWDGLTTNKVHAIPLRSSWVMTCAYSPTANFVA
CGGLDNICSIYNLRSREQPIRVCRELNSHTGYLSCCRFLNDRQIVTSSGDMTCILWDVEN
GTKITEFSDHNGDVMSVSVSPDKNYFISGACDATAKLWDLRSGKCVQTFTGHEADINAVQ
YFPNGLSFGTGSDDASCRLFDIRADRELMQYTHDNILCGITSVGFSFSGRFLFAGYDDFT
CNVWDTLKGERVLSLTGHGNRVSCLGVPTDGMALCTGSWDSLLKIWA
>O08837
MPRIMIKGGVWRNTEDEILKAAVMKYGKNQWSRIASLLHRKSAKQCKARWYEWLDPSIKK
TEWSREEEEKLLHLAKLMPTQWRTIAPIIGRTAAQCLEHYEFLLDKTAQRDNEEETTDDP
RKLKPGEIDPNPETKPARPDPIDMDEDELEMLSEARARLANTQGKKAKRKAREKQLEEAR
RLAALQKRRELRAAGIEIQKKRKKKRGVDYNAEIPFEKKPALGFYDTSEENYQALDADFR
KLRQQDLDGELRSEKEGRDRKKDKQHLKRKKESDLPSAILQTSGVSEFTKKRSKLVLPAP
QISDAELQEVVKVGQASEVARQTAEESGITNSASSTLLSEYNVTNNSIALRTPRTPASQD
RILQEAQNLMALTNVDTPLKGGLNTPLHESDFSGVTPQRQVVQTPNTVLSTPFRTPSNGA
EGLTPRSGTTPKPVTNATPGRTPLRDKLNINPEDGMADYSDPSYVKQMERESREHLRLGL
LGLPAPKNDFEIVLPENAEKELEEREMDDTYIEDAADVDARKQAIRDAERVKEMKRMHKA
VQKDLPRPSEVNETILRPLNVEPPLTDLQKSEELIKKEMITMLHYDLLHHPYEPSGNKKG
KNVGFATNNSEHITYLEHSPYEKFSKEDLKKAQDVLVQEMEVVKQGMSHGELSSEAYNQV
WEECYSQVLYLPAQSRYTRANLASKKDRIESLEKRLEINRGHMTTEAKRAAKMEKKMKIL
LGGYQSRAMGLLKQLNDLWDQIEQAHLELRTFEELKKHEDSAIPRRLECLKEDVQRQQER
EKELQQRYADLLMEKETLQAKF
>Q15287
MDLSGVKKKSLLGVKENNKKSSTRAPSPTKRKDRSDEKSKDRSKDKGATKESSEKDRGRD
KTRKRRSASSGSSSTRSRSSSTSSSGSSTSTGSSSGSSSSSASSRSGSSSTSRSSSSSSS
SGSPSPSRRRHDNRRRSRSKSKPPKRDEKERKRRSPSPKPTKVHIGRLTRNVTKDHIMEI
FSTYGKIKMIDMPVERMHPHLSKGYAYVEFENPDEAEKALKHMDGGQIDGQEITATAVLA
PWPRPPPRRFSPPRRMLPPPPMWRRSPPRMRRRSRSPRRRSPVRRRSRSPGRRRHRSRSS
SNSSR
>Q9PVV4
MATGGGPPDEALSDQDLPNWSHESLDDRLNNMHWNGQKKGNRSAEKNKKKFVECDLRLTN
DISPESSPGVGRRRARTPHTFPHTRYVSQMSVPEQAELEKLKQKINFSDLDQRSIGSDSQ
GRATAANNKRQLADNRKPFNFLPSQLNTNKEKSKSPPKREASTRSLTKDFLASALNKDFL
SNSQAFLEEESKREPAIDSSQVVSRLVQIRDYITKASSLRDDLVQKKDLSVNVERLSNLI
EHLKFQEKSYLKFLQKMLASENEEEDVRTVDSAVGSGSVVESTLLTFDVPSEASDTTGVD
PRQEAKEELKNMKKQHALLTRMLQQQEQLRTLKGRQAALLALQHKAEQAIAKMDESVVTE
TTGSVSGLSLTSELNEELNDLIQRFHNQLHDSEDPPAPDNRRQAESLSLAREVYRSRNSS
TSDTPLEDKSPLFNNVGVLLEKKQKMDTLLGELHTLQDQQLNNTAFVASSVSPRRSTEQR
TLGSAVSSALTSDNRAARSPVTIGAYQTASVNESEDEENQNPAEKLKKLKEVRKRLNELR
ELVHYYEQTSDMMTDAMNENTKDEDETEDSEYDSEQEDAEPTTNIRNPQYRSSWAQMNIN
SNNQSGTNNRDERQLNTECEINNRSAVNLRSFNMPSALDCLYNIEHSDKEEDGNRELDDE

DAEDQGSRASLSSQNSVADDVQSVDFEQKFNRLVAAKQKLKQLQDLVAMYGDDSESEPVA
PERSFSGDQFPPEATTLKQQPNNTRPNVSKAQKDIALKEQAREKFYESKLQQQQRELSQL
QEERKKLIEIQEKIQTLRKACPDLQLSTSSAGTNPANRQNRQMTTTTSTPDVNTNGNIVV
PAMPDPEDSSSVDNEVWSEIRKHQILREDLRQRRKQLETLMAEHQRRQGNTETTSAASIR
SDDSDTQGLQQQTRTEKTMATWGGSTQCALEEEEEEEEVDDEECLSDVHQIDIEEDEQDN
TSCENNSYPQNSIRKTSFNGRSSKDGWKNQCPLSVEGNHRPSPKTRQQQNVSMRRQENYR
WMSELSHVEEKEHWQEQIDQIKKQLDYSTSICQTLMRDQQALSYLLQSMITTPYSVMPSN
VGASQVQLIMHQLNQCYTQLNWQQSNVLRLKQMLNDLLVQQPQHLQGESHQREDRGSSAP
PLTSPNIFPNFSFLPPTMNLLNMPAFGSIPNVVPGMNFNPVFPHGFENFTQNVASHTDTP
LQPHDQNTSGKTEYMAFPKPFESHRSNSTEKERNPPKKPDESEQGRRVWVENHQKSDQEK
KPACFGAGLSAGGASAKLAEESRKVKQFDEVSVESLSSMPDPVDPTTVTKMFKSRKASAQ
ASLASKDKTPKAKNKKRKVFHQKSKGIKSCGFQAASASSASEPNQTTCKHAQTEEVVVGN
VIKTSSAQRVEKESKATEMSSEAGSDVSMFETLRDTIYSEVATLISQNESRPHFLIELFH
ELQLLNTDYLRQKALFALQDIVTRHVSEGNAKKHETCTKALESTGWMASTSELTPSESLV
TTDDEMYAKNSDGPVCQEGEQNDGDNISSLSTSSNFEPFATDDLGNTVIHFDQALARMRE
YERMKSETENGLVADCCNNLNAAASSLEGTNDEARGRAQHSVDDASGIPCPYIDSKQLDR
QIKAIMKEVIPFLKEHMEEVCSPNLLTSIRRLVLTLTQQNDESKEFVKFFHKQLGSILQD
SLAKFSSKKLKDCGEDLLVEISEVLFNELAFFRLMQDLDNNSTAVQGTVSRKPEAVVVLE
SFTKEADKEEKTCPEENFSATRETDDEDKDKDETETAEENRNFDAEVLSGKSDISEEDDL
DESLPVSISFTKAETQALTNYGSGEDENEDEENYEFEARPVDVQTSLETSSEIADETEKE
EMEVRPEANIENEKALSLAVNVMGELSIHEDQAKSDCDVLAHPSLLLSNEKATDFSGTAL
VNNVKSPVDSPGTSGAGSSDTESPVLVNDFETGSGNLSQKSDEDDFVKVEDLPLKLSLPQ
EQIMKDIEEEENKNNLCDEILNINDEENGADQLAGDPLALKEPDSPAIHPA
>Q15042
MAADSEPESEVFEITDFTTASEWERFISKVEEVLNDWKLIGNSLGKPLEKGIFTSGTWEE
KSDEISFADFKFSVTHHYLVQESTDKEGKDELLEDVVPQSMQDLLGMNNDFPPRAHCLVR
WYGLREFVVIAPAAHSDAVLSESKCNLLLSSVSIALGNTGCQVPLFVQIHHKWRRMYVGE
CQGPGVRTDFEMVHLRKVPNQYTHLSGLLDIFKSKIGCPLTPLPPVSIAIRFTYVLQDWQ
QYFWPQQPPDIDALVGGEVGGLEFGKLPFGACEDPISELHLATTWPHLTEGIIVDNDVYS
DLDPIQAPHWSVRVRKAENPQCLLGDFVTEFFKICRRKESTDEILGRSAFEEEGKETADI
THALSKLTEPASVPIHKLSVSNMVHTAKKKIRKHRGVEESPLNNDVLNTILLFLFPDAVS
EKPLDGTTSTDNNNPPSESEDYNLYNQFKSAPSDSLTYKLALCLCMINFYHGGLKGVAHL
WQEFVLEMRFRWENNFLIPGLASGPPDLRCCLLHQKLQMLNCCIERKKARDEGKKTSASD
VTNIYPGDAGKAGDQLVPDNLKETDKEKGEVGKSWDSWSDSEEEFFECLSDTEELKGNGQ
ESGKKGGPKEMANLRPEGRLYQHGKLTLLHNGEPLYIPVTQEPAPMTEDLLEEQSEVLAK
LGTSAEGAHLRARMQSACLLSDMESFKAANPGCSLEDFVRWYSPRDYIEEEVIDEKGNVV
LKGELSARMKIPSNMWVEAWETAKPIPARRQRRLFDDTREAEKVLHYLAIQKPADLARHL
LPCVIHAAVLKVKEEESLENISSVKKIIKQIISHSSKVLHFPNPEDKKLEEIIHQITNVE
ALIARARSLKAKFGTEKCEQEEEKEDLERFVSCLLEQPEVLVTGAGRGHAGRIIHKLFVN
AQRAAAMTPPEEELKRMGSPEERRQNSVSDFPPPAGREFILRTTVPRPAPYSKALPQRMY
SVLTKEDFRLAGAFSSDTSFF
>Q21341
MSADYSSEKFKATHLFDEILWHFRSNLSLKTNRRGLATAENSFSGKEAVDFLMIEMPRII
PNNVPERDKMQKFLEFMMDMNVISEAFPKKVKQRRPFSESRIYLFMKTLDELKHPKPRSR

RSASFSGARKSAKVAQPASPAATMHRPPKARLPRRLSRSNGNIDKAGIDNSSGGVENHGF
DDHKDDEIPKKQRTPKILNRSLESICTEEYTEKREVSEMKEKVYDWLPFFKSRRNHTKVN
QPTRRSASLDRNHCVLEQEKAEAALRSQKVTTPPIREAPKDVVFMHPQGPLPAVPSRYQN
HRTSIAGSNPALLSRGRMYESIMRRSSVVPVADSVQANNECSFWKTELLGRLEQIYDRTL
PCEWASKVDGYDIQWNMIEIDHADGIVKSRCQGLQPDYPQTVIQFMDYLVRYPFVTHKKM
DTGLEYNVNRIFITLVNRLEDLNAPLQFDECSLIVNLLCKIDSFAAMLDNGPARRWSKVM
ISSSASSIEEAGLMVDGFSRDLPACGIRASKYRRRALSPFDNRVNLEIQDEKSYEIREQW
LIEAIQLVLLSLPTSRRRKLHKFVTFIQSIETNAVFDLADPSNGSSNNREAAIIGLWTGV
CSKCRKQQGMLITAVLLANFQSLFAVPVEFIEQVKRLECEEKDRYSGPRYAKITRSRNDW
QPPVPDKPQASPAVFKKPLREVACEKTKKGLFTRLLRK
>Q9DCR4
MAEMAELCELYEESNELQMDVLPGEGYMEVGRGARGPAPEEGPMEEEAGPAAARAQRGLF
PEAGADLEGDEFDDWEDDYEFPEEERWSGAMHRVSAALEEANKVFLRTARAGDALDGGFQ
ARCEKSPFDQLAFIEELFSLMVVNRLTEELGCDEIIDRELMLTREEETT
>P32469
MLYLIGLGLSYKSDITVRGLEAIKKCSRVYLEHYTSILMAASQEELESYYGKEIILADRE
LVETGSKQILNNADKEDVAFLVVGDPFGATTHTDLVLRAKREAIPVEIIHNASVMNAVGA
CGLQLYNFGQTVSMVFFTDNWRPDSWYDKIWENRKIGLHTLVLLDIKVKEQSIENMARGR
LIYEPPRYMSIAQCCEQLLEIEEKRGTKAYTPDTPAVAISRLGSSSQSFKSGTISELANY
DSGEPLHSLVILGRQCHELELEYLLEFADDKEKFGKDVANDQEYFKPAAWVPPTEDDSDE

>O14135
MDIKYNELVNQFAPGAKQITIKKIRKKGNGIFSLNRYTSGTVLLEVPLENIICRKTVEQF
RNSCDKFASIATLEEWNDMSFRTQAMLFLCYLWLGIQPRTNKWDKFLTVLPLSINTPAQW
PEKEVYSLQGTSIFNPVCVKRKILQQEWLSLNQRYSDSWPSKITLPKWVHADALFHSRCL
ESPFKDPVLAPVIDLCNHSSKSNAKWSFSEDAMQLYLDKDIDENEEVTINYGSEKGSAEF
LFSYGFLPEPEGDRITNVMKLLIPEDSNDSLDLAKRRSCKTPPMIEFVSDSSGELWWHAP
FLFFSVLNVEDFTNFKMVCDESKAQTVDWEFEGQKCSVEDLPKLVQLSPKRDLYILRVFC
LAEQLADAALNTNIENMYNPTERRSESVELLKRESFLLKKVLLYLRDVISKLLKSKVVVE
FIHSQTIES
>Q8TCG2
MEDPSEPDRLASADGGSPEEEEDGEREPLLPRIAWAHPRRGAPGSAVRLLDAAGEEGEAG
DEELPLPPGDVGVSRSSSAELDRSRPAVSVTIGTSEMNAFLDDPEFADIMLRAEQAIEVG
IFPERISQGSSGSYFVKDPKRKIIGVFKPKSEEPYGQLNPKWTKYVHKVCCPCCFGRGCL
IPNQGYLSEAGAYLVDNKLHLSIVPKTKVVWLVSETFNYNAIDRAKSRGKKYALEKVPKV
GRKFHRIGLPPKIGSFQLFVEGYKEAEYWLRKFEADPLPENIRKQFQSQFERLVILDYII
RNTDRGNDNWLVRYEKQKCEKEIDHKESKWIDDEEFLIKIAAIDNGLAFPFKHPDEWRAY
PFHWAWLPQAKVPFSEEIRNLILPYISDMNFVQDLCEDLYELFKTDKGFDKATFESQMSV
MRGQILNLTQALRDGKSPFQLVQIPCVIVERSQGGSQGRIVHLSNSFTQTVNCRKPFFSS
W
>O13629
MLEAVTNPKSSLATFSKQLNGLLQAKVVVCKSVNPYFNLALENYLYENSTAKHCLLLYTN
SPSVIIGRNQNPWVEANVKLCRDNFVNIIRRKSGGGTVFHDFGNLNYSVLMNREEFSHTE
NASIMIQALRNLGVHARLNQRHDIVLAQSQRKISGSAYKISRNRCYHHGTMLLNSDLEGV

REYLRSPSTGILSKGVSSTRSPVSNTKLLKAEFIKQVISCFLLHKSHSTTTKPLSKPRAS
SKRLYDIEPKSVITLEQNDLLGVPSILKAVNELQSWEWTFGQTPSFKQHLESTELSVSMD
ISVVHGRLEKVIFSTPNATLEHELSSIPWTGLCYESGFANTFLISGIHSKEAISILKWIS
DSI
>O13992
MNEEDTDFAWLHNNSAEHLRFLSHRIFIGPIPSNFIHSTSGFFNKRFQNYTKRQICYNVA
SPNEPPIDFSFLMHKSTDENPDTPSNLDSPSTQNVGSTNNTRASQSLLRRSSSFFRRRHR
KNGTHASTDNNPFSESSTLQPQTAERTSQQAVRSAITETTNPSVSVQNSNSTSTSSAAMI
IPHRDSQNSLEIAPLISPESQLSSLHPSSSRRHLISTPHVNRGTQFKRSSSCRNSRQPLL
SGVDKHLTSNFTDANLIIKQSVVLARIESTFTVLPSDYNDSAAQRVPRKTISPWSQCLLV
ARQTDVENSIRLDFISKKLRKRLNGDVVHNLDVPHDKSYKSNYLFSVVLSPHQASWNIYN
SFDNSMVLWCPYGKNKTLICLLNFQSSLLSFEWISIISRALLFSPRPSLLISVPAFHIHL
RLNFPCFKDTTRPHTNETFVTTDDITQLSRTSTLSLSTASPRLVHDLVMKSWDISEDQFV
ESCLGVLEVNPEWSGIVKTWSKSHTLGLCWRMYDRLEWINSFSSLKYVGLLAAKDLYQLE
LRPKLHYPNHVTFRDGSKMDEPTPVEGYLIRLTSSTGRKTRYGRMFHKELYFAIFNNFLF
AIQPDSVLPLSMLSKSLNLDDKLPFLSNNENDKYVYEFDPFKIANCRASGLNETIDSSVR
ESFLLLLQAERKRELDMLTIADSFLDLSRVESVCPVEDVEERNIFEITMTNGMKLVFQSY
CERTRNLWINKITEVASYWKQRLFLDLQEYHDVRETNINILHIEQSIEPDVACYLNHWEV
AGCVASTLIYHYCSMLGCRVIRMQGTLFKKKDALFEKCFAILIPGQLVFFQDATRTKFGK
LCTKTHYRKRYSISLKNAFIYTGLSTMDEFARGPNDPQPHISRLPRCYEDGWQSFDRDDM
LSFVIWSSGGVDYDLRPHHSVPDTAYGMAKDKLSKPKRFMFLARTRQERDVWAKRISKEI
NRGHS
>Q9HEQ9
MSAEETVTQNAPNTVQDQVEASVDAAVHASAEQSNTPAQQADDFRVFVGRLSTSTKKSEI
RSLFETVGTVRKVTIPFRRVRRGTRLVPSGIAFVTFNNQEDVDKAIETLNGKTLDDREIV
VQKARPVQEQPIKDRKKSKNKNGEEPETSTSVENAESAKGSSDENEANTATAPSSNEANG
VDKKQNEIKGKGGSGKNKAKPLPPNSIYVSGLSVTLTNEGLKEMFDAYNPTRARIAVRSL
PPYIIRRIKLRGEQRRGRGFGFVSFANAEDQSRAIEEMNGKQVGDLTLVVKSAVFREDKQ
NDENEKNENEPIEASESAPTNVSDSTEPKASEVDSEEKSGVTASAITA
>P10383
MDMQVQRPITSGSRQAPDPYDQYLESRGLYRKHTARDASSLFRVIAEQMYDTQMLHYEIR
LECVRFMTLKRRIFEKEIPGDFDSYMQDMSKPKTYGTMTELRAMSCLYRRNVILYEPYNM
GTSVVFNRRYAENFRVFFNNENHFDSVYDVEYIERAAICQSIAFKLLYQKLFKLPDVSFA
VEIMLHPHTFNWDRFNVEFDDKGYMVRIHCTDGRVFKLDLPGDTNCILENYKLCNFHSTN
GNQSINARKGGRLEIKNQEERKASGSSGHEPNDLLPMCPNRLESCVRQLLDDGISPFPYK
VAKSMDPYMYRNIEFDCWNDMRKEAKLYNVYINDYNFKVGAKCKVELPNETEMYTCHVQN
ISKDKNYCHVFVERIGKEIVVPYESLHPLPPDEYRPWSLPFRYHRQMPRLPLPKYAGKAN
KSSKWKKNKLFEMDQYFEHSKCDLMPYMPVDNCYQGVHIQDDEQRDHNDPEQNDQNPTTE
QRDREEPQAQKQHQRTKASRVQPQNSSSSQNQEVSGSAAPPPTQYMNYVPMIPSRPGHLP
PPWPASPMAIAEEFPFPISGTPHPPPTEGCVYMPFGGYGPPPPGAVALSGPHPFMPLPSP
PLNVTGIGEPRRSLHPNGEDLPVDMVTLRYFYNMGVDLHWRMSHHTPPDELGMFGYHQQN
NTDQQAGRTVVIGATEDNLTAVESTPPPSPEVANATEQSPLEKSAYAKRNLNSVKVRGKR
PEQLQDIKDSLGPAAFLPTPTPSPSSNGSQFSFYTTPSPHHHLITPPRLLQPPPPPPIFY
HKAGPPQLGGAAQGQTPYAWGMPAPVVSPYEVINNYNMDPSAQPQQQQPATLQPAPLSVQ

SQPAAVYAATRHH

>P41057

MAHENVWFSHPRRYGKGSRQCRVCSSHTGLIRKYGLNICRQCFREKANDIGFNKFR

>Q02753

MGKSHGYRSRTRYMFQRDFRKHGAVHLSTYLKVYKVGDIVDIKANGSIQKGMPHKFYQGK
TGVVYNVTKSSVGVIINKMVGNRYLEKRLNLRVEHIKHSKCRQEFLERVKANAAKRAEAK
AQGVAVQLKRQPAQPRESRIVSTEGNVPQTLAPVPYETFI

>Q9BZR8

MCSTSGCDLEEIPLDDDDLNTIEFKILAYYTRHHVFKSTPALFSPKLLRTRSLSQRGLGN
CSANESWTEVSWPCRNSQSSEKAINLGKKKSSWKAFFGVVEKEDSQSTPAKVSAQGQRTL
EYQDSHSQQWSRCLSNVEQCLEHEAVDPKVISIANRVAEIVYSWPPPQATQAGGFKSKEI
FVTEGLSFQLQGHVPVASSSKKDEEEQILAKIVELLKYSGDQLERKLKKDKALMGHFQDG
LSYSVFKTITDQVLMGVDPRGESEVKAQGFKAALVIDVTAKLTAIDNHPMNRVLGFGTKY
LKENFSPWIQQHGGWEKILGISHEEVD

>Q14749

MVDSVYRTRSLGVAAEGLPDQYADGEAARVWQLYIGDTRSRTAEYKAWLLGLLRQHGCQR
VLDVACGTGVDSIMLVEEGFSVTSVDASDKMLKYALKERWNRRHEPAFDKWVIEEANWMT
LDKDVPQSAEGGFDAVICLGNSFAHLPDCKGDQSEHRLALKNIASMVRAGGLLVIDHRNY
DHILSTGCAPPGKNIYYKSDLTKDVTTSVLIVNNKAHMVTLDYTVQVPGAGQDGSPGLSK
FRLSYYPHCLASFTELLQAAFGGKCQHSVLGDFKPYKPGQTYIPCYFIHVLKRTD

>Q9Y751

MSQLRPRDSSAGKSSSPRRTDRNPESIDVNSKGGSEGMTSGLVPGTAPHKEDETEAEDDI
DCGRLITSKSLATMLTTASMYAGVSQLDDDAIESSALDEPIPYVPYEEDAESVMSESTVG
VDTVRDTDSSEQINPEIDGLVALRKKQQHTLSKFELSVVRKRCSQLSLAGSSSGSTRRNS
FTLDNNETTRAVKLCEKLKTTFDLSDDDEFVNDYPCWLLHEVFLQGHIYITSRYLLYFAF
LPKRDSTVTMSGALSIRSSTTMRFSVRRWAVLKGNYFRLYANSTERYFPSLNIDLRFLLK
VELSNPNLEENKPTVFKLITEARTHYFQADSLDNARSWVTDLRKHIFTAKNSGGHVTIKV
PLENILDLSIETLFEASQTLKLKVLESEESYAIDDYYFMFFNNGDQVLDSIRQSMKALGI
ELTDSDSSESDSDVSGSETNGRSTHRKSKLSRSLSVLTPIPRGIGSIYKPIKSSASGIIG
AIKKPQKSATRPFSSVVETVVPNDNDSELKQDHAGDAPKDSEEPSTKPSNWSAKSLVQGF
LSTTSSISQSMLFASPMHYNNQLFIERGEEDPYFVTNKEEREVAQSRFRKHFSLPDSEEL
LASYFCHFQKNIPVYGKVYLGTTCICYRSLFPGTNTTMILPYSDIENVYNLKGFRFGYSG
LVIVIRAHEELFFEFGSNESRDDCDLFLLKQLDFTKSHKNAHSEQKRKRNDSIKLAESVQ
LADARLRYFETRIESDIGREVPIILEENQYSTSEIRSKRRYKFVLLTIGSRGDVQPYISL
AKGLLAENHKVKIVTHEEFKPWVESYGIEFATIAGNPAELMSLMVTHKSLSVGFLKEAKE
KFTGWIGELLQSSWDACQDADVLIESPSAMAGIHIAEKLQIPYFRAFTMPWTRTRAYPHA
FVVPEQKRGGSYNYLTHIIFENVFWKGISGEVNKWREQVLMLPKTNLERLEQNKVPFLYN
VSPTVFPPSMDFPHWVKVVGYWFLDEGEADSYDPPKPLLEFMEKAKTDGKKLVYIGFGSI
VVSDPKQLTEAVIDAVLSADVRCILNKGWSDRLGKQTGVEVELPEEIYNSGNVPHDWLFG
KIDASVHHGGSGTTGATLRAGIPTIIKPFFGDQFFYANRVEDIGVGIGLRKLNSKSLSKA
IKEVTTNTRIIEKAKEIGKQIQSENGVSAAIRCLYQEMEYAKKLSKSKQKYWDNQSEDIS
DDSVSGSWFEV

>Q8NG50

MAELVPFAVPIESDKTLLVWELSSGPTAEALHHSLFTAFSQFGLLYSVRVFPNAAVAHPG

FYAVIKFYSARAAHRAQKACDRKQLFQKSPVKVRLGTRHKAVQHQALALNSSKCQELANY
YFGFNGCSKRIIKLQELSDLEERENEDSMVPLPKQSLKFFCALEVVLPSCDCRSPGIGLV
EEPMDKVEEGPLSFLMKRKTAQKLAIQKALSDAFQKLLIVVLESGKIAVEYRPSEDIVGV
RCEEELHGLIQVPCSPWKQYGQEEEGYLSDFSLEEEEFRLPELD
>Q9NXR7
MSPEVALNRISPMLSPFISSVVRNGKVGLDATNCLRITDLKSGCTSLTPGPNCDRFKLHI
PYAGETLKWDIIFNAQYPELPPDFIFGEDAEFLPDPSALQNLASWNPSNPECLLLVVKEL
VQQYHQFQCSRLRESSRLMFEYQTLLEEPQYGENMEIYAGKKNNWTGEFSARFLLKLPVD
FSNIPTYLLKDVNEDPGEDVALLSVSFEDTEATQVYPKLYLSPRIEHALGGSSALHIPAF
PGGGCLIDYVPQVCHLLTNKVQYVIQGYHKRREYIAAFLSHFGTGVVEYDAEGFTKLTLL
LMWKDFCFLVHIDLPLFFPRDQPTLTFQSVYHFTNSGQLYSQAQKNYPYSPRWDGNEMAK
RAKGCQGSRDACSPWEQVLAFAVAKTGCKLLQPQRNWPSSRGPPWRASEGERTAQ
>Q9H2D6
MEEVPGDALCEHFEANILTQNRCQNCFHPEEAHGARYQELRSPSGAEVPYCDLPRCPPAP
EDPLSASTSGCQSVVDPGLRPGPKRGPSPSAGLPEEGPTAAPRSRSRELEAVPYLEGLTT
SLCGSCNEDPGSDPTSSPDSATPDDTSNSSSVDWDTVERQEEEAPSWDELAVMIPRRPRE
GPRADSSQRAPSLLTRSPVGGDAAGQKKEDTGGGGRSAGQHWARLRGESGLSLERHRSTL
TQASSMTPHSGPRSTTSQASPAQRDTAQAASTREIPRASSPHRITQRDTSRASSTQQEIS
RASSTQQETSRASSTQEDTPRASSTQEDTPRASSTQWNTPRASSPSRSTQLDNPRTSSTQ
QDNPQTSFPTCTPQRENPRTPCVQQDDPRASSPNRTTQRENSRTSCAQRDNPKASRTSSP
NRATRDNPRTSCAQRDNPRASSPSRATRDNPTTSCAQRDNPRASRTSSPNRATRDNPRTS
CAQRDNPRASSPSRATRDNPTTSCAQRDNPRASRTSSPNRATRDNPRTSCAQRDNPRASS
PNRAARDNPTTSCAQRDNPRASRTSSPNRATRDNPRTSCAQRDNPRASSPNRATRDNPTT
SCAQRDNPRASRTSSPNRATRDNPRTSCAQRDNPRASSPNRTTQQDSPRTSCARRDDPRA
SSPNRTIQQENPRTSCALRDNPRASSPSRTIQQENPRTSCAQRDDPRASSPNRTTQQENP
RTSCARRDNPRASSRNRTIQRDNPRTSCAQRDNPRASSPNRTIQQENLRTSCTRQDNPRT
SSPNRATRDNPRTSCAQRDNLRASSPIRATQQDNPRTCIQQNIPRSSSTQQDNPKTSCTK
RDNLRPTCTQRDRTQSFSFQRDNPGTSSSQCCTQKENLRPSSPHRSTQWNNPRNSSPHRT
NKDIPWASFPLRPTQSDGPRTSSPSRSKQSEVPWASIALRPTQGDRPQTSSPSRPAQHDP
PQSSFGPTQYNLPSRATSSSHNPGHQSTSRTSSPVYPAAYGAPLTSPEPSQPPCAVCIGH
RDAPRASSPPRYLQHDPFPFFPEPRAPESEPPHHEPPYIPPAVCIGHRDAPRASSPPRHT
QFDPFPFLPDTSDAEHQCQSPQHEPLQLPAPVCIGYRDAPRASSPPRQAPEPSLLFQDLP
RASTESLVPSMDSLHECPHIPTPVCIGHRDAPSFSSPPRQAPEPSLFFQDPPGTSMESLA
PSTDSLHGSPVLIPQVCIGHRDAPRASSPPRHPPSDLAFLAPSPSPGSSGGSRGSAPPGE
TRHNLEREEYTVLADLPPPRRLAQRQPGPQAQCSSGGRTHSPGRAEVERLFGQERRKSEA
AGAFQAQDEGRSQQPSQGQSQLLRRQSSPAPSRQVTMLPAKQAELTRRSQAEPPHPWSPE
KRPEGDRQLQGSPLPPRTSARTPERELRTQRPLESGQAGPRQPLGVWQSQEEPPGSQGPH
RHLERSWSSQEGGLGPGGWWGCGEPSLGAAKAPEGAWGGTSREYKESWGQPEAWEEKPTH
ELPRELGKRSPLTSPPENWGGPAESSQSWHSGTPTAVGWGAEGACPYPRGSERRPELDWR
DLLGLLRAPGEGVWARVPSLDWEGLLELLQARLPRKDPAGHRDDLARALGPELGPPGTND
VPEQESHSQPEGWAEATPVNGHSPALQSQSPVQLPSPACTSTQWPKIKVTRGPATATLAG
LEQTGPLGSRSTAKGPSLPELQFQPEEPEESEPSRGQDPLTDQKQADSADKRPAEGKAGS
PLKGRLVTSWRMPGDRPTLFNPFLLSLGVLRWRRPDLLNFKKGWMSILDEPGEPPSPSLT
TTSTSQWKKHWFVLTDSSLKYYRDSTAEEADELDGEIDLRSCTDVTEYAVQRNYGFQIHT

KDAVYTLSAMTSGIRRNWIEALRKTVRPTSAPDVTKLSDSNKENALHSYSTQKGPLKAGE
QRAGSEVISRGGPRKADGQRQALDYVELSPLTQASPQRARTPARTPDRLAKQEELERDLA
QRSEERRKWFEATDSRTPEVPAGEGPRRGLGAPLTEDQQNRLSEEIEKKWQELEKLPLRE
NKRVPLTALLNQSRGERRGPPSDGHEALEKEVQALRAQLEAWRLQGEAPQSALRSQEDGH
IPPGYISQEACERSLAEMESSHQQVMEELQRHHERELQRLQQEKEWLLAEETAATASAIE
AMKKAYQEELSRELSKTRSLQQGPDGLRKQHQSDVEALKRELQVLSEQYSQKCLEIGALM
RQAEEREHTLRRCQQEGQELLRHNQELHGRLSEEIDQLRGFIASQGMGNGCGRSNERSSC
ELEVLLRVKENELQYLKKEVQCLRDELQMMQKDKRFTSGKYQDVYVELSHIKTRSEREIE
QLKEHLRLAMAALQEKESMRNSLAE
>Q99996
MEDEERQKKLEAGKAKIEELSLAFLVRQLAQFRQRKAQSDGQSPSKKQKKKRKTSSSKHD
VSAHHDLNIDQSQCNEMYINSSQRVESTVIPESTIMRTLHSGEITSHEQGFSVELESEIS
TTADDCSSEVNGCSFVMRTGKPTNLLREEEFGVDDSYSEQGAQDSPTHLEMMESELAGKQ
HEIEELNRELEEMRVTYGTEGLQQLQEFEAAIKQRDGIITQLTANLQQARREKDETMREF
LELTEQSQKLQIQFQQLQASETLRNSTHSSTAADLLQAKQQILTHQQQLEEQDHLLEDYQ
KKKEDFTMQISFLQEKIKVYEMEQDKKVENSNKEEIQEKETIIEELNTKIIEEEKKTLEL
KDKLTTADKLLGELQEQIVQKNQEIKNMKLELTNSKQKERQSSEEIKQLMGTVEELQKRN
HKDSQFETDIVQRMEQETQRKLEQLRAELDEMYGQQIVQMKQELIRQHMAQMEEMKTRHK
GEMENALRSYSNITVNEDQIKLMNVAINELNIKLQDTNSQKEKLKEELGLILEEKCALQR
QLEDLVEELSFSREQIQRARQTIAEQESKLNEAHKSLSTVEDLKAEIVSASESRKELELK
HEAEVTNYKIKLEMLEKEKNAVLDRMAESQEAELERLRTQLLFSHEEELSKLKEDLEIEH
RINIEKLKDNLGIHYKQQIDGLQNEMSQKIETMQFEKDNLITKQNQLILEISKLKDLQQS
LVNSKSEEMTLQINELQKEIEILRQEEKEKGTLEQEVQELQLKTELLEKQMKEKENDLQE
KFAQLEAENSILKDEKKTLEDMLKIHTPVSQEERLIFLDSIKSKSKDSVWEKEIEILIEE
NEDLKQQCIQLNEEIEKQRNTFSFAEKNFEVNYQELQEEYACLLKVKDDLEDSKNKQELE
YKSKLKALNEELHLQRINPTTVKMKSSVFDEDKTFVAETLEMGEVVEKDTTELMEKLEVT
KREKLELSQRLSDLSEQLKQKHGEISFLNEEVKSLKQEKEQVSLRCRELEIIINHNRAEN
VQSCDTQVSSLLDGVVTMTSRGAEGSVSKVNKSFGEESKIMVEDKVSFENMTVGEESKQE
QLILDHLPSVTKESSLRATQPSENDKLQKELNVLKSEQNDLRLQMEAQRICLSLVYSTHV
DQVREYMENEKDKALCSLKEELIFAQEEKIKELQKIHQLELQTMKTQETGDEGKPLHLLI
GKLQKAVSEECSYFLQTLCSVLGEYYTPALKCEVNAEDKENSGDYISENEDPELQDYRYE
VQDFQENMHTLLNKVTEEYNKLLVLQTRLSKIWGQQTDGMKLEFGEENLPKEETEFLSIH
SQMTNLEDIDVNHKSKLSSLQDLEKTKLEEQVQELESLISSLQQQLKETEQNYEAEIHCL
QKRLQAVSESTVPPSLPVDSVVITESDAQRTMYPGSCVKKNIDGTIEFSGEFGVKEETNI
VKLLEKQYQEQLEEEVAKVIVSMSIAFAQQTELSRISGGKENTASSKQAHAVCQQEQHYF
NEMKLSQDQIGFQTFETVDVKFKEEFKPLSKELGEHGKEILLSNSDPHDIPESKDCVLTI
SEEMFSKDKTFIVRQSIHDEISVSSMDASRQLMLNEEQLEDMRQELVRQYQEHQQATELL
RQAHMRQMERQREDQEQLQEEIKRLNRQLAQRSSIDNENLVSERERVLLEELEALKQLSL
AGREKLCCELRNSSTQTQNGNENQGEVEEQTFKEKELDRKPEDVPPEILSNERYALQKAN
NRLLKILLEVVKTTAAVEETIGRHVLGILDRSSKSQSSASLIWRSEAEASVKSCVHEEHT
RVTDESIPSYSGSDMPRNDINMWSKVTEEGTELSQRLVRSGFAGTEIDPENEELMLNISS
RLQAAVEKLLEAISETSSQLEHAKVTQTELMRESFRQKQEATESLKCQEELRERLHEESR
AREQLAVELSKAEGVIDGYADEKTLFERQIQEKTDIIDRLEQELLCASNRLQELEAEQQQ
IQEERELLSRQKEAMKAEAGPVEQQLLQETEKLMKEKLEVQCQAEKVRDDLQKQVKALEI

DVEEQVSRFIELEQEKNTELMDLRQQNQALEKQLEKMRKFLDEQAIDREHERDVFQQEIQ
KLEQQLKVVPRFQPISEHQTREVEQLANHLKEKTDKCSELLLSKEQLQRDIQERNEEIEK
LEFRVRELEQALLVSADTFQKVEDRKHFGAVEAKPELSLEVQLQAERDAIDRKEKEITNL
EEQLEQFREELENKNEEVQQLHMQLEIQKKESTTRLQELEQENKLFKDDMEKLGLAIKES
DAMSTQDQHVLFGKFAQIIQEKEVEIDQLNEQVTKLQQQLKITTDNKVIEEKNELIRDLE
TQIECLMSDQECVKRNREEEIEQLNEVIEKLQQELANIGQKTSMNAHSLSEEADSLKHQL
DVVIAEKLALEQQVETANEEMTFMKNVLKETNFKMNQLTQELFSLKRERESVEKIQSIPE
NSVNVAIDHLSKDKPELEVVLTEDALKSLENQTYFKSFEENGKGSIINLETRLLQLESTV
SAKDLELTQCYKQIKDMQEQGQFETEMLQKKIVNLQKIVEEKVAAALVSQIQLEAVQEYA
KFCQDNQTISSEPERTNIQNLNQLREDELGSDISALTLRISELESQVVEMHTSLILEKEQ
VEIAEKNVLEKEKKLLELQKLLEGNEKKQREKEKKRSPQDVEVLKTTTELFHSNEESGFF
NELEALRAESVATKAELASYKEKAEKLQEELLVKETNMTSLQKDLSQVRDHLAEAKEKLS
ILEKEDETEVQESKKACMFEPLPIKLSKSIASQTDGTLKISSSNQTPQILVKNAGIQINL
QSECSSEEVTEIISQFTEKIEKMQELHAAEILDMESRHISETETLKREHYVAVQLLKEEC
GTLKAVIQCLRSKEVFGFYNMCFSTLCDSGSDWGQGIYLTHSQGFDIASEGRGEESESAT
DSFPKKIKGLLRAVHNEGMQVLSLTESPYSDGEDHSIQQVSEPWLEERKAYINTISSLKD
LITKMQLQREAEVYDSSQSHESFSDWRGELLLALQQVFLEERSVLLAAFRTELTALGTTD
AVGLLNCLEQRIQEQGVEYQAAMECLQKADRRSLLSEIQALHAQMNGRKITLKREQESEK
PSQELLEYNIQQKQSQMLEMQVELSSMKDRATELQEQLSSEKMVVAELKSELAQTKLELE
TTLKAQHKHLKELEAFRLEVKDKTDEVHLLNDTLASEQKKSRELQWALEKEKAKLGRSEE
RDKEELEDLKFSLESQKQRNLQLNLLLEQQKQLLNESQQKIESQRMLYDAQLSEEQGRNL
ELQVLLESEKVRIREMSSTLDRERELHAQLQSSDGTGQSRPPLPSEDLLKELQKQLEEKH
SRIVELLNETEKYKLDSLQTRQQMEKDRQVHRKTLQTEQEANTEGQKKMHELQSKVEDLQ
RQLEEKRQQVYKLDLEGQRLQGIMQEFQKQELEREEKRESRRILYQNLNEPTTWSLTSDR
TRNWVLQQKIEGETKESNYAKLIEMNGGGTGCNHELEMIRQKLQCVASKLQVLPQKASER
LQFETADDEDFIWVQENIDEIILQLQKLTGQQGEEPSLVSPSTSCGSLTERLLRQNAELT
GHISQLTEEKNDLRNMVMKLEEQIRWYRQTGAGRDNSSRFSLNGGANIEAIIASEKEVWN
REKLTLQKSLKRAEAEVYKLKAELRNDSLLQTLSPDSEHVTLKRIYGKYLRAESFRKALI
YQKKYLLLLLGGFQECEDATLALLARMGGQPAFTDLEVITNRPKGFTRFRSAVRVSIAIS
RMKFLVRRWHRVTGSVSININRDGFGLNQGAEKTDSFYHSSGGLELYGEPRHTTYRSRSD
LDYIRSPLPFQNRYPGTPADFNPGSLACSQLQNYDPDRALTDYITRLEALQRRLGTIQSG
STTQFHAGMRR
>Q08686
MPLFDLISPKAFVKLVASEKVHRIVPVDATWYLPSWKLDNKVDFLTKPRIPNSIFFDIDA
ISDKKSPYPHMFPTKKVFDDAMSNLGVQKDDILVVYDRVGNFSSPRCAWTLGVMGHPKVY
LLNNFNQYREFKYPLDSSKVAAFSPYPKSHYESSESFQDKEIVDYEEMFQLVKSGELAKK
FNAFDARSLGRFEGTEPEPRSDIPSGHIPGTQPLPYGSLLDPETKTYPEAGEAIHATLEK
ALKDFHCTLDPSKPTICSCGTGVSGVIIKTALELAGVPNVRLYDGSWTEWVLKSGPEWIA
ENRD
>P43612
MSFWPFGQNLNHSNINKILDEYFHVLHELERINPSVGKAIPAIFNNVQERGTSDSLDSIP
EEYSHGDEVKTARGDQKSRFEKDDQQERYEKEEEERSMNSSESSTTSFSSGSTSKTDLDE
EDISNATAPMMVTTKNLDNSFIERMLVETELLNELSRQNKTLLDFICFGFFFDKKTNKKV
NNMEYLVDQLMECISKIKTATTVDLNNLIDYQEQQQLDDSSQEDVYVESDTEQEEEKEDD

NNSNNKKRRKRGSSSFGNDDINNNDDDDDANEDDESAYLTKATIISEIFSLDIWLISESL
VKNQSYLNKIWSIINQPNFNSENSPLVPIFLKINQNLLLTRQDQYLNFIRTERSFVDDML
KHVDISLLMDFFLKIISTDKIESPTGIIELVYDQNLISKCLSFLNNKESPADIQACVGDF
LKALIAISANAPLDDISIGPNSLTRQLASPESIAKLVDIMINQRGAALNTTVSIVIELIR
KNNSDYDQVNLLTTTIKTHPPSNRDPIYLGYLLRKFSNHLSDFFQIILDIENDANIPLHE
NQLHEKFKPLGFERFKVVELIAELLHCSNMGLMNSKRAERIARRRDKVRSQLSHHLQDAL
NDLSIEEKEQLKTKHSPTRDTDHDLKNNNGKIDNDNNDNDDESDYGDEIDESFEIPYINM
KQTIKLRTDPTVGDLFKIKLYDTRIVSKIMELFLTHPWNNFWHNVIFDIIQQIFNGRMDF
SYNSFLVLSLFNLKSSYQFMTDIVISDEKGTDVSRFSPVIRDPNFDFKITTDFILRGYQD
SYKFYELRKMNLGYMGHIVLIAEEVVKFSKLYKVELISPDIQVILQTEEWQYYSEEVLNE
TRMMYSKILGGGSYIDDGNGNIIPQLPDNTTVLTPNGDASNNNEILDSDTGSSNGTSGGG
QLINVESLEEQLSLSTESDLHNKLREMLINRAQEDVDNKNTENGVFILGPPEDKNSNSNI
NNTNHNSNNSNNNDNNDNNDNDNDNTRNYNEDADNDNDYDHE
>Q9ULA0
MQVAMNGKARKEAVQTAAKELLKFVNRSPSPFHAVAECRNRLLQAGFSELKETEKWNIKP
ESKYFMTRNSSTIIAFAVGGQYVPGNGFSLIGAHTDSPCLRVKRRSRRSQVGFQQVGVET
YGGGIWSTWFDRDLTLAGRVIVKCPTSGRLEQQLVHVERPILRIPHLAIHLQRNINENFG
PNTEMHLVPILATAIQEELEKGTPEPGPLNAVDERHHSVLMSLLCAHLGLSPKDIVEMEL
CLADTQPAVLGGAYDEFIFAPRLDNLHSCFCALQALIDSCAGPGSLATEPHVRMVTLYDN
EEVGSESAQGAQSLLTELVLRRISASCQHPTAFEEAIPKSFMISADMAHAVHPNYLDKHE
ENHRPLFHKGPVIKVNSKQRYASNAVSEALIREVANKVKVPLQDLMVRNDTPCGTTIGPI
LASRLGLRVLDLGSPQLAMHSIREMACTTGVLQTLTLFKGFFELFPSLSHNLLVD
>P31384
MNDPSLLGYPNVGPQQQQQQQQQQHAGLLGKGTPNALQQQLHMNQLTGIPPPGLMNNSDV
HTSSNNNSRQLLDQLANGNANMLNMNMDNNNNNNNNNNNNNNNGGGSGVMMNASTAAVNS
IGMVPTVGTPVNINVNASNPLLHPHLDDPSLLNNPIWKLQLHLAAVSAQSLGQPNIYARQ
NAMKKYLATQQAQQAQQQAQQQAQQQVPGPFGPGPQAAPPALQPTDFQQSHIAEASKSLV
DCTKQALMEMADTLTDSKTAKKQQPTGDSTPSGTATNSAVSTPLTPKIELFANGKDEANQ
ALLQHKKLSQYSIDEDDDIENRMVMPKDSKYDDQLWHALDLSNLQIFNISANIFKYDFLT
RLYLNGNSLTELPAEIKNLSNLRVLDLSHNRLTSLPAELGSCFQLKYFYFFDNMVTTLPW
EFGNLCNLQFLGVEGNPLEKQFLKILTEKSVTGLIFYLRDNRPEIPLPHERRFIEINTDG
EPQREYDSLQQSTEHLATDLAKRTFTVLSYNTLCQHYATPKMYRYTPSWALSWDYRRNKL
KEQLLSYDSDLLCLQEVESKTFEEYWVPLLDKHGYTGIFHAKARAKTMHSKDSKKVDGCC
IFFKRDQFKLITKDAMDFSGAWMKHKKFQRTEDYLNRAMNKDNVALFLKLQHIPSGDTIW
AVTTHLHWDPKFNDVKTFQVGVLLDHLETLLKEETSHNFRQDIKKFPVLICGDFNSYINS
AVYELINTGRVQIHQEGNGRDFGYMSEKNFSHNLALKSSYNCIGELPFTNFTPSFTDVID
YIWFSTHALRVRGLLGEVDPEYESKFIGFPNDKFPSDHIPLLARFEFMKTNTGSKKV
>P38862
MSSERVLSYAPAFKSFLDTSFFQELSRLKLDVLKLDSTCQPLTVNLDLHNIPKSADQVPL
FLTNRSFEKHNNKRTNEVPLQGSIFNFNVLDEFKNLDKQLFLHQRALECWEDGIKDINKC
VSFVIISFADLKKYRFYYWLGVPCFQRPSSTVLHVRPEPSLKGLFSKCQKWFDVNYSKWV
CILDADDEIVNYDKCIIRKTKVLAIRDTSTMENVPSALTKNFLSVLQYDVPDLIDFKLLI
IRQNEGSFALNATFASIDPQSSSSNPDMKVSGWERNVQGKLAPRVVDLSSLLDPLKIADQ
SVDLNLKLMKWRILPDLNLDIIKNTKVLLLGAGTLGCYVSRALIAWGVRKITFVDNGTVS

YSNPVRQALYNFEDCGKPKAELAAASLKRIFPLMDATGVKLSIPMIGHKLVNEEAQHKDF
DRLRALIKEHDIIFLLVDSRESRWLPSLLSNIENKTVINAALGFDSYLVMRHGNRDEQSS
KQLGCYFCHDVVAPTDSLTDRTLDQMCTVTRPGVAMMASSLAVELMTSLLQTKYSGSETT
VLGDIPHQIRGFLHNFSILKLETPAYEHCPACSPKVIEAFTDLGWEFVKKALEHPLYLEE
ISGLSVIKQEVERLGNDVFEWEDDESDEIA
>Q8WXE9
MTTLDHVIATHQSEWVSFNEEPPFPAHSQGGTEEHLPGLSSSPDQSESSSGENHVVDGGS
QDHSHSEQDDSSEKMGLISEAASPPGSPEQPPPDLASAISNWVQFEDDTPWASTSPPHQE
TAETALPLTMPCWTCPSFDSLGRCPLTSESSWTTHSEDTSSPSFGCSYTDLQLINAEEQT
SGQASGADSTDNSSSLQEDEEVEMEAISWQASSPAMNGHPAPPVTSARFPSWVTFDDNEV
SCPLPPVTSPLKPNTPPSASVIPDVPYNSMGSFKKRDRPKSTLMNFSKVQKLDISSLNRT
PSVTEASPWRATNPFLNETLQDVQPSPINPFSAFFEEQERRSQNSSISSTTGKSQRDSLI
VIYQDAISFDDSSKTQSHSDAVEKLKQLQIDDPDHFGSATLPDDDPVAWIELDAHPPGSA
RSQPRDGWPMMLRIPEKKNIMSSRHWGPIFVKLTDTGYLQLYYEQGLEKPFREFKLEICH
EISEPRLQNYDENGRIHSLRIDRVTYKEKKKYQPKPAVAHTAEREQVIKLGTTNYDDFLS
FIHAVQDRLMDLPVLSMDLSTVGLNYLEEEITVDVRDEFSGIVSKGDNQILQHHVLTRIH
ILSFLSGLAECRLGLNDILVKGNEIVLRQDIMPTTTTKWIKLHECRFHGCVDEDVFHNSR
VILFNPLDACRFELMRFRTVFAEKTLPFTLRTATSVNGAEVEVQSWLRMSTGFSANRDPL
TQVPCENVMIRYPVPSEWVKNFRRESVLGEKSLKAKVNRGASFGSTSVSGSEPVMRVTLG
TAKYEHAFNSIVWRINRLPDKNSASGHPHCFFCHLELGSDREVPSRFANHVNVEFSMPTT
SASKASVRSISVEDKTDVRKWVNYSAHYSYQVALGSIWLMLPTPFVHPTTLPLLFLLAML
TMFAW
>P38260
MEKLLQWSIANSQGDKEAMARAGQPDPKLLQQLFGGGGPDDPTLMKESMAVIMNPEVDLE
TKLVAFDNFEMLIENLDNANNIENLKLWEPLLDVLVQTKDEELRAAALSIIGTAVQNNLD
SQNNFMKYDNGLRSLIEIASDKTKPLDVRTKAFYALSNLIRNHKDISEKFFKLNGLDCIA
PVLSDNTAKPKLKMRAIALLTAYLSSVKIDENIISVLRKDGVIESTIECLSDESNLNIID
RVLSFLSHLISSGIKFNEQELHKLNEGYKHIEPLKDRLNEDDYLAVKYVL
>Q9Y266
MGGEQEEERFDGMLLAMAQQHEGGVQELVNTFFSFLRRKTDFFIGGEEGMAEKLITQTFS
HHNQLAQKTRREKRARQEAERREKAERAARLAKEAKSETSGPQIKELTDEEAERLQLEID
QKKDAENHEAQLKNGSLDSPGKQDTEEDEEEDEKDKGKLKPNLGNGADLPNYRWTQTLSE
LDLAVPFCVNFRLKGKDMVVDIQRRHLRVGLKGQPAIIDGELYNEVKVEESSWLIEDGKV
VTVHLEKINKMEWWSRLVSSDPEINTKKINPENSKLSDLDSETRSMVEKMMYDQRQKSMG
LPTSDEQKKQEILKKFMDQHPEMDFSKAKFN
>P40121
MYTAIPQSGSPFPGSVQDPGLHVWRVEKLKPVPVAQENQGVFFSGDSYLVLHNGPEEVSH
LHLWIGQQSSRDEQGACAVLAVHLNTLLGERPVQHREVQGNESDLFMSYFPRGLKYQEGG
VESAFHKTSTGAPAAIKKLYQVKGKKNIRATERALNWDSFNTGDCFILDLGQNIFAWCGG
KSNILERNKARDLALAIRDSERQGKAQVEIVTDGEEPAEMIQVLGPKPALKEGNPEEDLT
ADKANAQAAALYKVSDATGQMNLTKVADSSPFALELLISDDCFVLDNGLCGKIYIWKGRK
ANEKERQAALQVAEGFISRMQYAPNTQVEILPQGRESPIFKQFFKDWK
>O13999
MRPTSGCSKDDTIQKQNRRHNTVDNKQEKLPLSIEIFLNKQINKISFDTIRSKQNCRLKE

IYCRLKIRCRLKKKFIKSLSKKIISYHFISFHTIVVLLLLPPFSHLLVLVYPSVFTTAFY
HQKWALRLNPCLPTYFFHRQRQCVTLLIRNANENMRARRVNSVMLTKPKQFLFLLEFITL
FIFTYCL
>P60484
MTAIIKEIVSRNKRRYQEDGFDLDLTYIYPNIIAMGFPAERLEGVYRNNIDDVVRFLDSK
HKNHYKIYNLCAERHYDTAKFNCRVAQYPFEDHNPPQLELIKPFCEDLDQWLSEDDNHVA
AIHCKAGKGRTGVMICAYLLHRGKFLKAQEALDFYGEVRTRDKKGVTIPSQRRYVYYYSY
LLKNHLDYRPVALLFHKMMFETIPMFSGGTCNPQFVVCQLKVKIYSSNSGPTRREDKFMY
FEFPQPLPVCGDIKVEFFHKQNKMLKKDKMFHFWVNTFFIPGPEETSEKVENGSLCDQEI
DSICSIERADNDKEYLVLTLTKNDLDKANKDKANRYFSPNFKVKLYFTKTVEEPSNPEAS
SSTSVTPDVSDNEPDHYRYSDTTDSDPENEPFDEDQHTQITKV
>O94513
MGSELKSTSPLAVKYDLSQKIMQHLDRHLIFPLLEFLSLRQTHDPKELLQAKYDLLKDTN
MTDYVANLWTNLHGGHTDEDMANAFAEKRRSVLQELSELEEEVQGILGVLENPDLIAALR
QDKGQNLQHLQEHYNITPERIAVLYKFAQFQYNCGNYGGASDLLYHFRAFSKDPELNASA
TWGKFASEILTVDWDGAMEELGKLREMVDSKSFKDSAVQLRNRTWLLHWSLFPLFNHANG
CDTLCDLFFYTPYLNTIQTSCPWLLRYLTVAVVTNQNNANQKPRNPRQSYQRRMRDLVRI
ISQENYEYSDPVTSFISALYTEVDFEKAQHCLRECEEVLKTDFFLVSLCDHFLEGARKLL
AEAYCRIHSVISVDVLANKLEMDSAQLIQLVENRNNPSVAAASNVAADQSTEDESIESTS
TNVVADDLITEAETATEAEEPEPEVQFGFKAKLDGESIIIEHPTYSAFQQIIDRTKSLSF
ESQNLEQSLAKSISELKHATV
>Q9P7F2
MAYYPHLPYHHHHHTSVPGERLSDPLDPLSADELKLAVEIIRHEYPSKHFAFNVVTLEEP
PKAKYLHWKYSKEDAHKPERIALAVLLEKGVPGILEARVNLTKAEVIQIEHITGVCPILT
ADMLVNTEQIVRKDPAVIEQCILSGVPPDQMDHVYCDPWTIGYDERYGNTRRMQQAMMYY
RSNEDDSQYSHPLDFCPIIDTEDQKVVAIDIPPVRRPLSKHKHSNFNKKDIEAELGKMRE
VKPISVTQPEGVNFRMKGRYIEWQNFRMHIGFNYREGIVLSDISYNDNGHIRPLFYRMSI
AEMVVPYGNPEHPHQRKHAFDLGEYGAGYLTNPLALGCDCKGVIHYLDAHFVNNTGEVET
VKNAICIHEEDDGVLFKHSDFRDKFRTTISARGIRLVISQIFTAANYEYMVYWIFHMDGV
IECELKLTGILNTYAMNEGEDLKGWGTQVYPQVNAHNHQHLFCLRLNPMLDGYSNSVAVV
DGVSGPGEPGSKENYYGNAFTTERTVPKTVKEAICDYNSDTSRTWDICNPNKLHPYSGKP
VSYKLVSRETPRLMARPGSLVSNRAGFARHHIHVTPYKDGQIYPAGDYVPQTSGEPTKGL
PEWIAEEPDASVDNTDIVVWHTFGITHFPAPEDFPLMPAEPIRLLLRPRNFFLRNPALDV
PPSKNVTTSEVKQAHHHGNLHMMDMMKSLTDATSEFAFGEKFCEKHKGDHFF
>P32643
MSTFSASDFNSERYSSSRPSYPSDFYKMIDEYHDGERKLLVDVGCGPGTATLQMAQELKP
FEQIIGSDLSATMIKTAEVIKEGSPDTYKNVSFKISSSDDFKFLGADSVDKQKIDMITAV
ECAHWFDFEKFQRSAYANLRKDGTIAIWGYADPIFPDYPEFDDLMIEVPYGKQGLGPYWE
QPGRSRLRNMLKDSHLDPELFHDIQVSYFCAEDVRDKVKLHQHTKKPLLIRKQVTLVEFA
DYVRTWSAYHQWKQDPKNKDKEDVADWFIKESLRRRPELSTNTKIEVVWNTFYKLGKRV
>Q9HDX2
MFRNYNWFDAKEPISYESEIYAKGLKFQRPQITVDGRHWEQLAVERMTKDAAGYVYGCAG
KRETYDKNMESFKKWSIIPNRLIKSGFPDLSTTVFGQKYPFPIALAPVGVQKIFNPEGES
GSCAAATREHIPYIISTASATSFEDIEKASGPGERWYQLYWPSNDHQDITISLLNRAKKT

GCRVLIVTLDTFILGWRPSDMDNGYDPFLNPDSIGVEHGFSDPVFRKQFKEKHGVEVEEN
MLEAAKEFAGIVFPGISHDWEDLKFLRKHWDGPIVLKGIMNVPDAKKAVEYGMQGIVVSN
HGGRQQDGGVASLTMLPKIVDAVGDKLDVLFDSGVRSGADIAKALALGAKMVLIGRPYVY
GLALEGSSGVSHVIRCLLGDLELTLHLSGIVSVKPKDLNRDVLYKEE
>Q9P799
MISNSSIEDRINKNEAYIAKFFEIFSKNDKIRFHGVSLDRKALDSFMVRNNFKKRFVSGI
NLPILIRLHFYKKLFTILWLTVRTVALLSHARNIQCLIQESDGDAKDVARSIYITTAEFR
NYLLTEKGFYVTTRSISHFRAILDSIRYPFGKDHASKNMELLPITYDLSFCTVFHGYLTS
ILEAYEFFRGVFKKYKAPLSSNFIAIGKTTDESNFSQLHHYFQKFDERIRRILHLISQEL
DGYCRIAKQ
>Q9P7S3
MSNNSRLPENGVQSGNGEDAELKKSMRIIEREIPDSLLEKIPEAEDYIALQDLERKLDSL
IVRKRFDLQDSLSRNSHKTRILRMYIHSTVANQSWQQKGENQENNSGDINSLPIPEWTLH
IEGRLLVNPDDEDDKAFELAPFTNFFRKIAIQILRSDDLYPSGNYVEWNKLPDNSNTSNG
ITVTRKGDQSVDVKIMLYPEEHPERYKLSKAFANILGIREGTRPDIVSYLWQYIKFHRLQ
DMEEKRLINCDKALRDLFEADRLYFPRIPELMNRFLEPIDPIVIPYTINVSEHTVEKVTI
FDIRINTEDPRHSQIRSFLATMMSQDKIRSIDDKLTELIQAITYSQSKYDFMKKFSESPI
EFINEWIESQSRDLEIVLDGTNMNYAEKRSADYYQQPWVHESAFHYLNLLNSKKQQSVLN
ASAKK
>P38042
MAVNPELAPFTLSRGIPSFDDQALSTIIQLQDCIQQAIQQLNYSTAEFLAELLYAECSIL
DKSSVVYWSDAVYLYALSLFLNKSYHTAFQISKEFKEYHLGIAYIFGRCALQLSQGVNEAI
LTLLSIINVFSSNSSNTRINMVLNSNLVHIPDLATLNCLLGNLYMKLDHSKEGAFYHSEA
LAINPYLWESYEAICKMRATVDLKRVFFDIAGKKSNSHNNNAASSFPSTSLSHFEPRSQP
SLYSKTNKNGNNNINNNVNTLFQSSNSPPSTSASSFSSIQHFSRSQQQQANTSIRTCQNK
NTQTPKNPAINSKTSSALPNNISMNLVSPSSKQPTISSLAKVYNRNKLLTTPPSKLLNND
RNHQNNNNNNNNNNNNNNNNNNNNNNNNIINKTTFKTPRNLYSSTGRLTTSKKNPRSLII
SNSILTSDYQITLPEIMYNFALILRSSSQYNSFKAIRLFESQIPSHIKDTMPWCLVQLGK
LHFEIINYDMSLKYFNRLKDLQPARVKDMEIFSTLLWHLHDKVKSSNLANGLMDTMPNKP
ETWCCIGNLLSLQKDHDAAIKAFEKATQLDPNFAYAYTLQGHEHSSNDSSDSAKTCYRKA
LACDPQHYNAYYGLGTSAMKLGQYEEALLYFEKARSINPVNVVLICCCGGSLEKLGYKEK
ALQYYELACHLQPTSSLSKYKMGQLLYSMTRYNVALQTFEELVKLVPDDATAHYLLGQTY
RIVGRKKDAIKELTVAMNLDPKGNQVIIDELQKCHMQE
>Q86TG7
MTERRRDELSEEINNLREKVMKQSEENNNLQSQVQKLTEENTTLREQVEPTPEDEDDDIE
LRGAAAAAAPPPPIEEECPEDLPEKFDGNPDMLAPFMAQCQIFMEKSTRDFSVDRVRVCF
VTSMMTGRAARWASAKLERSHYLMHNYPAFMMEMKHVFEDPQRREVAKRKIRRLRQGMGS
VIDYSNAFQMIAQDLDWNEPALIDQYHEGLSDHIQEELSHLEVAKSLSALIGQCIHIERR
LARAAAARKPRSPPRALVLPHIASHHQVDPTEPVGGARMRLTQEEKERRRKLNLCLYCGT
GGHYADNCPAKASKSSPAGKLPGPAVEGPSATGPEIIRSPQDDASSPHLQVMLQIHLPGR
HTLFVRAMIDSGASGNFIDHEYVAQNGIPLRIKDWPILVEAIDGRPIASGPVVHETHDLI
VDLGDHREVLSFDVTQSPFFPVVLGVRWLSTHDPNITWSTRSIVFDSEYCRYHCRMYSPI
PPSLPPPAPQPPLYYPVDGYRVYQPVRYYYVQNVYTPVDEHVYPDHRLVDPHIEMIPGAH
SIPSGHVYSLSEPEMAALRDFVARNVKDGLITPTIAPNGAQVLQVKRGWKLQVSYDCRAP

NNFTIQNQYPRLSIPNLEDQAHLATYTEFVPQIPGYQTYPTYAAYPTYPVGFAWYPVGRD
GQGRSLYVPVMITWNPHWYRQPPVPQYPPPQPPPPPPPPPPPPSYSTL
>Q28175
MSSQVEHPAGGYKKLFETVEELSSPLTAHVTGRIPLWLTGSLLRCGPGLFEVGSEPFYHL
FDGQALLHKFDFKEGHVTYHRRFIRTDAYVRAMTEKRIVITEFGTCAFPDPCKNIFSRFF
SYFRGVEVTDNALVNIYPVGEDYYACTETNFITKVNPETLETIKQVDLCNYVSVNGATAH
PHIENDGTVYNIGNCFGKNFSIAYNIVKIPPLQADKEDPISKSEIVVQFPCSDRFKPSYV
HSFGLTPNYIVFVETPVKINLFKFLSSWSLWGANYMDCFESNETMGVWLHIADKKRKKYI
NNKYRTSPFNLFHHINTYEDHEFLIVDLCCWKGFEFVYNYSYLANLRENWEEVKKNARKA
PQPEVRRYVLPLNIDKADTGKNLVTLPNTTATAILCSDETIWLEPEVLFSGPRQAFEFPQ
INYQKYGGKPYTYAYGLGLNHFVPDRLCKLNVKTKETWVWQEPDSYPSEPIFVSHPDALE
EDDGVVLSVVVSPGAGQKPAYLLILNAKDLSEVARAEVEINIPVTFHGLFKKS
>P40384
MNSNDKKKVVRSWIEQFVHDFVEQLSKPTKDSVNVALKRRKHNSWNGSLDSKANERQKVK
VFSFPRNETTIAQLFRVLDCVHEAVISDTVITKRDIYYRDVDLFKRQTVVDELLGDISNT
IGCSRSDLNVEASAKGLVFGSIHIALENGTVITATKPLLISHHRISSITSTAKWVLVIEK
EAVFQTLTEEALADTIIVTAKGFPDLMTRKFLVKLAKALPDAKFFGIFDWDPHGLCIYSC
FKYGSNAYSHEPHSQLRNLQLLGPLYEDIFNKNQEFSLKLNKRDIKMITTLLQFEGFQKE
PVVREQLQRMLFIQKKAEIQAILEFPSWIKGKLADADKSGKHSVR
>Q3BBV0
MVVSAGPWSSEKAETNILEINEKLRPQLAENKQQFRNLKEKCFVTQLAGFLANRQKKYKY
EECKDLIKFMLRNERQFKEEKLAEQLKQAEELRQYKVLVHSQERELTQLREKLREGRDAS
RSLNQHLQALLTPDEPDKSQGQDLQEQLAEGCRLAQHLVQKLSPENDEDEDEDVQVEEAE
KVLESSAPREVQKAEESKVPEDSLEECAITCSNSHGPCDSNQPHKNINITFEEDKVNSTL
VVDRESSHDECQDAVNILPVPGPTSSATNVSMVVSAGPLSSEKAEMNILEMNEKLRPQLA
EKKQQFRNLKEKCFVTQLAGFLANQQNKYKYEECEDLIKSMLRNERQFKEEKLAEQLKQA
EELRQYKVLVHSQERELTQLREKLREGRDASRSLNQHLQALLTPDEPDKSQGQDLQEQLA
EGCRLAQHLVQKLSPENDEDEDEDVQVEEAEKVLESSAPREVQKAEESKVPEDSLEECAI
TCSNSHGPCDSNQPHKNINITFEEDKVNSALVVDRESSHDECQDAVNILPVPGPTSSATN
VSMVVSAGPLSSEKAEMNILEMNEKLRPQLAEKKQQFRNLKEKCFVTQLACFLANQQNKY
KYEECKDLIKSVLRNERQFKEEKLAEQLKQAEELRQYKVLVHSQERELTQLREKLREGRD
ASRSLNQHLQALLTPDEPDKSQGQDLQEQLAEGCRLAQQLFQKLSPENDNDHDEDVQVEV
AEKVQKSSAPREMQKAEEKEVPEDSLEECAITCSNSHGPYDSNQPHRKTKITFEEDKVDS
TLIGSSSHVEWEDAVHIIPENESDDEEEEEKGPVSPRNLQESEEEEVPQESWDEGYSTLS
IPPEMLASYQSYSGTFHSLEEQQVCMAVDIGGHRWDQVKKEDQEATGPRLSRELLDEKGP
EVLQDSLDRCYSTPSGYLELTDSCQPYRSAFYILEQQRVGWALDMDEIEKYQEVEEDQDP
SCPRLSRELLDEKEPEVLQDSLDRCYSTPSGYLELPDLGQPYRSAVYSLEEQYLGLALDV
DRIKKDQEEEEDQGPPCPRLSRELLEAVEPEVLQDSLDRCYSTPSSCLEQPDSCLPYGSS
FYALEEKHVGFSLDVGEIEKKGKGKKRRGRRSTKKRRRRGRKEGEEDQNPPCPRLSGMLM
EVEEPEVLQDSLDRCYSTPSMYFELPDSFQHYRSVFYSFEEQHISFALDVDNRFLTLMGT
SLHLVFQMGVIFPQ
>Q99MI1
MYGSARSVGKVEPSSQSPGRSPRLPRSPRLGHRRTNSTGGSSGNSVGGGSGKTLSMENIQ
SLNAAYATSGPMYLSDHENVGAETPKSTMTLGRSGGRLPYGVRMTAMGSSPNIASSGVAS

DTIAFGEHHLPPVSMASTVPHSLRQARDNTIMDLQTQLKEVLRENDLLRKDVEVKESKLS
SSMNSIKTFWSPELKKERALRKDEASKITIWKEQYRVVQEENQHMQMTIQALQDELRIQR
DLNQLFQQDSSSRTGEPCVAELTEENFQRLHAEHERQAKELFLLRKTLEEMELRIETQKQ
TLNARDESIKKLLEMLQSKGLSAKATEEDHERTRRLAEAEMHVHHLESLLEQKEKENNML
REEMHRRFENAPDSAKTKALQTVIEMKDSKISSMERGLRDLEEEIQMLKSNGALSSEERE
EEMKQMEVYRSHSKFMKNKVEQLKEELSSKDAQGEELKKRAAGLQSEIGQVKQELSRKDT
ELLALQTKLETLTNQFSDSKQHIEVLKESLTAKEQRAAILQTEVDALRLRLEEKETMLNK
KTKQIQDMAEEKGTQAGEIHDLKDMLDVKERKVNVLQKKIENLQEQLRDKEKQMSSLKER
VKSLQADTTNTDTALTTLEEALADKERTIERLKEQRDRDEREKQEEIDTYKKDLKDLREK
VSLLQGDLSEKEASLLDIKEHASSLASSGLKKDSRLKTLEIALEQKKEECLKMESQLKKA
HEATLEARASPEMSDRIQQLEREISRYKDESSKAQTEVDRLLEILKEVENEKNDKDKKIA
ELESLTSRQVKDQNKKVANLKHKEQVEKKKSAQMLEEARRREDSLSDSSQQLQDSLRKKD
DRIEELEEALRESVQITAEREMVLAQEESARTNAEKQVEELLMAMEKVKQELESMKAKLS
STQQSLAEKETHLTNLRAERRKHLEEVLEMKQEALLAAISEKDANIALLELSSSKKKTQE
EVAALKREKDRLVQQLKQQTQNRMKLMADNYEDDHFRSSRSNQTNHKPSPDQIIQPLLEL
DQNRSKLKLYIGHLTALCHDRDPLILRGLTPPASYNADGEQAAWENELQQMTQEQLQNEL
EKVEGDNAELQEFANTILQQIADHCPDILEQVVNALEESS
>Q12156
MSNLYKIGTETRNKIKKFRTSTARTDSIKALSIKIEPKPSYEIIVDEDEQEELDEIEDLS
ELAEILPDNSPRFVLTAYPTTTKDGFKQTPLVLVYWKPMTVVSQEWKMLYAGALEMIREE
CGTFKLIEVSSGLEDDSDVEELREQLENC
>Q10197
MEEEESLEGIISIDESLITSRLSQILDDVLDDRSSSHSVEKLKDVAVKYLQFCQFQPTLL
DKLLSKYVPNLASYLLKVKNIGKCNSITVILYQFCKIRGYKAVRVLFPVGVQYIKELYTL
LNESSNNTWHFHYIVLLWLSQALNTPFPLNSLDDSLDVKKTIYTIAIKYLENSGIDKEAS
CLVLSRLFSRDDGLDLLLGFLHHCESSWFKRSIFYKIGCLFSLSSFLKICPRNDCLQTVD
VAFQFLNVAREDLVGQENSALRKLLCKCYTRLGIVLLPVNSSPNWKYSISNPDSFFQLPD
DSNEEVHIYLEVIVDFLLSSVSDIDSFVRWSAAKGLAKIISRLPWNLAEQVIDAIIELMT
ENMFLNPIENTVNISITSPLVWHGAILFFAKLAGAGLIKYSKCLHILPLIEVGLSYEVRY
GTRVTGQSIRDASCYFVWSFYHCYSKSAIEGLQTNLILCLLQTVLFDNEINVRRAATAAL
FEVIGRHASIPDGLSLISHINYVSVTDISNCYGDLCMKVAHFPQFRSCVFQRLFTNLQHW
DVKVQQLSAFSLRQLSIKYPKELSIYLPPILDYLSVGNADFIFGYTIGLASIIGGFLSIS
FPFDINRIHDLLSHKNLLSLKKFSRQQQTKIILGILKGIQQIFANDIRVDRAFFSEAFSV
IIAAIDLQEETIIKDISDAYSVLVKFDDMEETLEVLLDYIRKCSTSKEARIVYIILQNLP
NISFRYQKKICKLLLDIYPQLHSIDYQAPVANALQNIIPFTYEKTESIEEFVKELLQVCS
NYLTDTRGDVGSWIRKPAMKAISSLLVKDSSGKKLSEDIVWCCISYIIRQTFDKIDSLRG
LAYQALEQIRVHYLIRRCEALTNIINRISFRNNPNMDGEVLNELNISLLEIPNLRLQAFY
GITVFTADGFGSDLAVKCFEFYLSYVYQLEDSFKKSNSRYGKRDLLQLYIDILSSEDEIA
RFYFPIMKSFTSLLAYGCFTDFQNVKGYVFLHFVQNTNDISMSKAIFIVQRRALTCKSPG
GLSAILELYRTLFLSKNELLRHHALKYTANLLLNPIEKVRYQAADTLLYAKSIGLLTFLP
NELNQKLLTLDWFVPVSQNATFVKQLRNIIQKQIDKLIADR
>P87054
MNVGDLKSANDLYESVRSIEGVTGVHLFRIRDAGILKSTMIPELDALTTISLDELNKSPT
VFTKNLLQGCGGTDLDVPAELKRLVIFVCNILESCNEFTDLGSARLLRLTFDSCQLVIMY

DSLFVVAITKDET
>Q12245
MLVKTISRTIESESGFLQPTLDVIATLPADDRSKKIPISLVVGFKQEASLNSSSSLSCYY
YAIPLMRDRHINLKSGGSNVVGIPLLDTKDDRIRDMARHMATIISERFNRPCYVTWSSLP
SEDPSMLVANHLYILKKCLDLLKTELGE
>Q9P7M9
MDKKEGDQVHHDKTLLQDLSLPENVASEKRLPIDDVQCVNSPMLSPALDIEPKPNVVGTA
ERLPKVRCVVRARIPTVKGTEIFLHLYKNDFDNKEHLAIVFGQQIRSDSLDAVQEGETEM
DRMVRGAYVGTLYPGRKSSYIDVNSSKTTEEQKEEARKTNFVPLVRIHSECYTGETAWSA
RCDCGEQLDEAARLISEEGNGVIIYLRQEGRGIGLSEKLKAYNLQDLGNDTVQANLLLQH
PADGRSYAVATAMLMDLGQRDIRLLTNNPEKVNSIEGPNKEVRVVERISMIPKSWRGEKG
IESAEVVLYLKTKIEKMGHLLDLPDS
>Q12400
MSNDEINQNEEKVKRTPPLPPVPEGMSKKQWKKMCKRQRWEENKAKYNAERRVKKKRLRH
ERSAKIQEYIDRGEEVPQELIREPRINVNQTDSGIEIILDCSFDELMNDKEIVSLSNQVT
RAYSANRRANHFAEIKVAPFDKRLKQRFETTLKNTNYENWNHFKFLPDDKIMFGDEHISK
DKIVYLTADTEEKLEKLEPGMRYIVGGIVDKNRYKELCLKKAQKMGIPTRRLPIDEYINL
EGRRVLTTTHVVQLMLKYFDDHNWKNAFESVLPPRKLDAEAKSASSSPAPKDT
>P47072
MGILDSLKKPVNIKSSLPKFSRSATSINLSSHPVSSRSFLELPPELFSITKPIFKLLQAH
ANKIYFQSSDAEAVWNVKDSSGHVFEAESISLLGSHIIITNSSVQVADVAIIDSPSNINQ
CEISSVGEFLQFNNGQLSITCNDFGLLEKFKRLCMISIFEFISIYKALTGTVISSYGLRM
SDMHIILNSPFNFKDWCEVYLDGQGWVKVWCHIDKVSKTNNSKSSSDNDAKGKYQIRFYR
DDKSTSSKNCVFFIPDNEYVQDIFFYNINAAEPSKNMNDFFQGLQMIKLVGNVRFCSDTD
FNDVVDSGSIYSSANNGSGDSSSTALNNESPNTTPKSRTFFSPKGHRRNSSHVSSLTSRS
TKKPITNFTTRTNGLLIRPLPHHGVHHLEAMIRFIIPLMDCARLYGRPVQFKTERTDINS
LMFGLPKLPSVDYFAEEEIAHLMTQEFNPLKEKDTDDTMALTMSRFSSYLQERMTKVSKR
NTELNFRTFSDVMGMYNTTRDHSKLNCMSDKDNSVKEFSLSDKSNVSSETTNMMNQLQVN
AHEYKSSMCERPIVASTSPIA
>Q9LN63
MTSDGATSTSAAAAAAAMATRRKPSWRERENNRRRERRRRAVAAKIYTGLRAQGNYNLPK
HCDNNEVLKALCSEAGWVVEEDGTTYRKGHKPLPGDMAGSSSRATPYSSHNQSPLSSTFD
SPILSYQVSPSSSSFPSPSRVGDPHNISTIFPFLRNGGIPSSLPPLRISNSAPVTPPVSS
PTSRNPKPLPTWESFTKQSMSMAAKQSMTSLNYPFYAVSAPASPTHHRQFHAPATIPECD
ESDSSTVDSGHWISFQKFAQQQPFSASMVPTSPTFNLVKPAPQQLSPNTAAIQEIGQSSE
FKFENSQVKPWEGERIHDVAMEDLELTLGNGKAHS
>P07806
MNKWLNTLSKTFTFRLLNCHYRRSLPLCQNFSLKKSLTHNQVRFFKMSDLDNLPPVDPKT
GEVIINPLKEDGSPKTPKEIEKEKKKAEKLLKFAAKQAKKNAAATTGASQKKPKKKKEVE
PIPEFIDKTVPGEKKILVSLDDPALKAYNPANVESSWYDWWIKTGVFEPEFTADGKVKPE
GVFCIPAPPPNVTGALHIGHALTIAIQDSLIRYNRMKGKTVLFLPGFDHAGIATQSVVEK
QIWAKDRKTRHDYGREAFVGKVWEWKEEYHSRIKNQIQKLGASYDWSREAFTLSPELTKS
VEEAFVRLHDEGVIYRASRLVNWSVKLNTAISNLEVENKDVKSRTLLSVPGYDEKVEFGV
LTSFAYPVIGSDEKLIIATTRPETIFGDTAVAVHPDDDRYKHLHGKFIQHPFLPRKIPII

TDKEAVDMEFGTGAVKITPAHDQNDYNTGKRHNLEFINILTDDGLLNEECGPEWQGMKRF
DARKKVIEQLKEKNLYVGQEDNEMTIPTCSRSGDIIEPLLKPQWWVSQSEMAKDAIKVVR
DGQITITPKSSEAEYFHWLGNIQDWCISRQLWWGHRCPVYFINIEGEEHDRIDGDYWVAG
RSMEEAEKKAAAKYPNSKFTLEQDEDVLDTWFSSGLWPFSTLGWPEKTKDMETFYPFSML
ETGWDILFFWVTRMILLGLKLTGSVPFKEVFCHSLVRDAQGRKMSKSLGNVIDPLDVITG
IKLDDLHAKLLQGNLDPREVEKAKIGQKESYPNGIPQCGTDAMRFALCAYTTGGRDINLD
ILRVEGYRKFCNKIYQATKFALMRLGDDYQPPATEGLSGNESLVEKWILHKLTETSKIVN
EALDKRDFLTSTSSIYEFWYLICDVYIENSKYLIQEGSAIEKKSAKDTLYILLDNALKLI
HPFMPFISEEMWQRLPKRSTEKAASIVKASYPVYVSEYDDVKSANAYDLVLNITKEARSL
LSEYNILKNGKVFVESNHEEYFKTAEDQKDSIVSLIKAIDEVTVVRDASEIPEGCVLQSV
NPEVNVHLLVKGHVDIDAEIAKVQKKLEKAKKSKNGIEQTINSKDYETKANTQAKEANKS
KLDNTVAEIEGLEATIENLKRLKL
>Q2M3C7
MDGNSLLSVPSNLESSRMYDVLEPQQGRGCGSSGSGPGNSITACKKVLRSNSLLESTDYW
LQNQRMPCQIGFVEDKSENCASVCFVNLDVNKDECSTEHLQQKLVNVSPDLPKLISSMNV
QQPKENEIVVLSGLASGNLQADFEVSQCPWLPDICLVQCARGNRPNSTNCIIFEINKFLI
GLELVQERQLHLETNILKLEDDTNCSLSSIEEDFLTASEHLEEESEVDESRNDYENINVS
ANVLESKQLKGATQVEWNCNKEKWLYALEDKYINKYPTPLIKTERSPENLTKNTALQSLD
PSAKPSQWKREAVGNGRQATHYYHSEAFKGQMEKSQALYIPKDAYFSMMDKDVPSACAVA
EQRSNLNPGDHEDTRNALPPRQDGEVTTGKYATNLAESVLQDAFIRLSQSQSTLPQESAV
SVSVGSSLLPSCYSTKDTVVSRSWNELPKIVVVQSPDGSDAAPQPGISSWPEMEVSVETS
SILSGENSSRQPQSALEVALACAATVIGTISSPQATERLKMEQVVSNFPPGSSGALQTQA
PQGLKEPSINEYSFPSALCGMTQVASAVAVCGLGEREEVTCSVAPSGSLPPAAEASEAMP
PLCGLASMELGKEAIAKGLLKEAALVLTRPNTYSSIGDFLDSMNRRIMETASKSQTLCSE
NVVRNELAHTLSNVILRHSIDEVHHKNMIIDPNDNRHSSEILDTLMESTNQLLLDVICFT
FKKMSHIVRLGECPAVLSKETIRRRETEPSCQPSDPGASQAWTKATESSSSSPLSNSHNT
SLVINNLVDGMYSKQDKGGVRPGLFKNPTLQSQLSRSHRVPDSSTATTSSKEIYLKGIAG
EDTKSPHHSENECRASSEGQRSPTVSQSRSGSQEAEESIHPNTQEKYNCATSRINEVQVN
LSLLGDDLLLPAQSTLQTKHPDIYCITDFAEELADTVVSMATEIAAICLDNSSGKQPWFC
AWKRGSEFLMTPNVPCRSLKRKKESQGSGTAVRKHKPPRLSEIKRKTDEHPELKEKLMNR
VVDESMNLEDVPDSVNLFANEVAAKIMNLTEFSMVDGMWQAQGYPRNRLLSGDRWSRLKA
SSCESIPEEDSEARAYVNSLGLMSTLSQPVSRASSVSKQSSCESITDEFSRFMVNQMENE
GRGFELLLDYYAGKNASSILNSAMQQACRKSDHLSVRPSCPSKQSSTESITEEFYRYMLR
DIERDSRESASSRRSSQDWTAGLLSPSLRSPVCHRQSSMPDSRSPCSRLTVNVPIKANSL
DGFAQNCPQDFLSVQPVSSASSSGLCKSDSCLYRRGGTDHITNMLIHETWASSIEALMRK
NKIIVDDAEEADTEPVSGGSPSQAEKCANRLAASRMCSGPTLLVQESLDCPRKDSVTECK
QPPVSSLSKTASLTNHSPLDSKKETSSCQDPVPINHKRRSLCSREVPLIQIETDQREACA
GEPEPFLSKSSLLEEAEGHSNDKNIPDVVRGGDTAVSACQIHSDSLDTRDVPEAEASTEA
RAPDEAPNPPSSSEESTGSWTQLANEEDNPDDTSSFLQLSERSMSNGNSSATSSLGIMDL
DIYQESMPSSPMINELVEEKKILKGQSESTEAPASGPPTGTASPQRSLLVINFDLEPECP
DAELRATLQWIAASELGIPTIYFKKSQENRIEKFLDVVQLVHRKSWKVGDIFHAVVQYCK
MHEEQKDGRLSLFDWLLELG
>O94518
MDKNIKREIEIKRAKLLLLKNKENCTDAVSSSNKEGPKQISEDQLSNFLCKILKPTNLTP

QTYSLESKSSNLVSDCELKISSVYQSYSSTFNVSNHVPSISKTVNIRETSKHLKHSTKAP
KCSLPTSALEIDHLNNFLHSSAKILDRALCDQSNQLFTDYTVKKKSKKNKSQLEENGLNH
LFTFQDEKITLNSVVNSISYSSFFEELLITSYAKPKEALRTRGLAIVWNQRWKNSPESVL
KARSEITVCKPSPFHPQLIAGGAYNGQVFLWDLRQGQYPVSFTTIISGGHLEPVTDITYI
NNPPSNNIVTCSTDGLVHIWEPDMFSRPSETICLSSQVDSSSQCIPATCLSFIPENNMEF
LVGAEDGKLQRGYRSDYSETKAVQPSNVSYEGHNVFISGIDVMTSNSQNVFLEKNKDFAL
TSSFDWTVRLWQCSPSRNQHELVPSNDLDEQVIINSCKTFTHKAMVFDVKWCVSEPCCFA
SVDALGNLNLWDLQKDVEAPVTSDIPDGKPLNKIAWQPEKRNLACGGLNGNVHIYKHLSP
NLAN
>Q9UMX1
MAELRPSGAPGPTAPPAPGPTAPPAFASLFPPGLHAIYGECRRLYPDQPNPLQVTAIVKY
WLGGPDPLDYVSMYRNVGSPSANIPEHWHYISFGLSDLYGDNRVHEFTGTDGPSGFGFEL
TFRLKRETGESAPPTWPAELMQGLARYVFQSENTFCSGDHVSWHSPLDNSESRIQHMLLT
EDPQMQPVQTPFGVVTFLQIVGVCTEELHSAQQWNGQGILELLRTVPIAGGPWLITDMRR
GETIFEIDPHLQERVDKGIETDGSNLSGVSAKCAWDDLSRPPEDDEDSRSICIGTQPRRL
SGKDTEQIRETLRRGLEINSKPVLPPINPQRQNGLAHDRAPSRKDSLESDSSTAIIPHEL
IRTRQLESVHLKFNQESGALIPLCLRGRLLHGRHFTYKSITGDMAITFVSTGVEGAFATE
EHPYAAHGPWLQILLTEEFVEKMLEDLEDLTSPEEFKLPKEYSWPEKKLKVSILPDVVFD
SPLH
>O59760
MIPEACLFDMDGLLVDTESIYTKSTNIILKRYNKGPFSMEVKAKMMGRTSKEASRIFLDW
SGIDLTCEEYIALQRETQAELWRHTKPLPGVMNLLSKLKSLNIPIALATSSDTHNFEKKS
AHLSHLFDHFDGNIITGDDPRLPVGRGKPHPDIWFIALKMINDKRKAQGQAEILPENCLV
FEDSITGVQSGRAAGMKVVWVPDVNILPFFSLSPEQAADKHITKVLSLENFDVTKV
>Q12288
MTVKKIAILYTDEDNEWSKPWGNFVDMAIKLLEQTRKLECIAEDVEYEVFHVQKNVFPQL
SDLQKDEYLGIYITGSKYDSFDNEIEWIMKLRSFLNEMLTSKTEYPPVAGICFGHQVIAA
ALGSSVGRNPKGFEGGVVSLKLNSVGQKLFGAQELNLSEVHSDCVFDVPEGYQNWASSEK
CQNQGFYRQNRVLTFQGHPEFNSDVAQKGLLKSQDKLTLEEFNRYERQCQELDNNGIQAA
RNIWRLFLQKI
>Q9HGL9
MAGGHKKNEATRQTDEVIQQKYDESLSKHKNRCELSLTFEYKLSNKLRARLKAAFFKVDH
GWEDQTLDQVETLVRKENHVHRRSRSINDLDSSKLRRPHHPNSIMGSNTGKLRASSFTRL
VSPREGFAQLPYKKPVSPYSSSSAVSSTSSSFDRTSPPWLNYVEPHTELPRLPFELSHAS
TASAKSPLFGMNYHISRPEETDESLRLGTANLSRIRKSTSLPFPTAYPLSADDCKAAEVM
LTLVNSMPSKKP
>O62479
MTSKIALFDQTLIASLLQPLSLNQPDFKAYKTKVKLKISEQRNETSGEKELKFEISRSDD
FEFLFSETLNNEKYQILARDHDLTVDFDAFPKVIIQHLLCKNIVKNLEEDGEVDARKKAG
YHSIADPGKPTEINIILDAEKNFCSFELFSKTPISKGKIFSIKLHAVRGDHLISHLLKIC
SSQAVKLSTFYKSADELASLRQKCGDLEKQVEKLSGVKEEFEEMSEKFKELEDEVELVKE
ERENIRLLVEDKEDEVADLKQDTESLQKQLEENQEELEIVGNMLREEQGKVDQLQKRNVA
HQKEIGKLRAELGTAQRNLEKADQLLKRNSQQQNQQSLDMRKLGELEADLKEKDSMVESL
TETIGILRKELENEKLKAAENMDSFEKLSMENENLKEKIAHYRAQRFSPAPSGLPGLQTG

LTNRLTPSFKPVLGPHTPYGANLNSRTPFRDNTTLNFQNSTIATPHAFRFNSQLIADETT
GSSVTNTPPAQR
>P53200
MSDSDSDSDYELTLSANALAALEEFKREEQQHQEAFQKLYDETDEDFQKKKKEEGMKLFK
EDWQLSQFWYSDDTAAILADAILEGADENTVIAIVSAPSVYAAIQKKPTNEIPTEHIYLF
EFDKRFELLAGRDHFFFYDYNKPLDFSDEIKGKVDRLLIDPPFLNEDCQTKSSITAKCLL
APNDNSKTKKGVFKHRLISCTGERMSEVISKVYSDTRITTFLPEHSNGLSNEFRCYANFE
CSSWKFAS
>P87052
MPIIRSLTRGKSIRLLQKQSTKRTNPCVYLEAKAVEQTPATEFNSILQEIISTEYNYSVD
LKKTLNEIIMPIEGQSMEREAIGICTCFKTLNQLHTKLYQQMISSGSPVYSLSQSLPAFR
DVYHMYTINLSGISAIEKIISDPSSCNSQLLIPLQHLLRYPIHLARVCKMLRKYPFLNGD
FRMAVKTLDDFRELCSYIDQEKAREESYQVLSALCGNQGVAVDIPRHIKFKGSVIPATSF
IYDFNACVFCDDGLVVWTRYVKKIEMKAVSIEQCLRVDELSNSILVCTLNMKGKLLNRHL
APQTMAASVFLKWYHERAGISNGKENKLNRLKVVNAKT
>P01039
MDPGTTGIVGGVSEAKPATPEIQEVADKVKRQLEEKTNEKYEKFKVVEYKSQVVAGQILF
MKVDVGNGRFLHMKVLRGLSGDDDLKLLDYQTNKTKNDELTDF
>Q5JY77
MTGAEIESGAQVKPEKKPGEEVVGGAEIENDVPLVVRPKVRTQAQIMPGARPKNKSKVMP
GASTKVETSAVGGARPKSKAKAIPVSRFKEEAQMWAQPRFGAERLSKTERNSQTNIIASP
LVSTDSVLVAKTKYLSEDRELVNTDTESFPRRKAHYQAGFQPSFRSKEETNMGSWCCPRP
TSKQEASPNSDFKWVDKSVSSLFWSGDEVTAKFHPGNRVKDSNRSMHMANQEANTMSRSQ
TNQELYIASSSGSEDESVKTPWFWARDKTNTWSGPREDPNSRSRFRSKKEVYVESSSGSE
HEDHLESWFGAGKEAKFRSKMRAGKEANNRARHRAKREACIDFMPGSIDVIKKESCFWPE
ENANTFSRPMIKKEARARAMTKEEAKTKARARAKQEARSEEEALIGTWFWATDESSMADE
ASIESSLQVEDESIIGSWFWTEEEASMGTGASSKSRPRTDGERIGDSLFGAREKTSMKTG
AEATSESILAADDEQVIIGSWFWAGEEVNQEAEEETIFGSWFWVIDAASVESGVGVSCES
RTRSEEEEVIGPWFWSGEQVDIEAGIGEEARPGAEEETIFGSWFWAENQTYMDCRAETSC
DTMQGAEEEEPIIGSWFWTRVEACVEGDVNSKSSLEDKEEAMIPCFGAKEEVSMKHGTGV
RCRFMAGAEETNNKSCFWAEKEPCMYPAGGGSWKSRPEEEEDIVNSWFWSRKYTKPEAII
GSWLWATEESNIDGTGEKAKLLTEEETIINSWFWKEDEAISEATDREESRPEAEEGDIIG
SWFWAGEEDRLEPAAETREEDRLAAEKEGIVGSWFGAREETIRREAGSCSKSSPKAEEEE
VIIGSWFWEEEASPEAVAGVGFESKPGTEEEEITVGSWFWPEEEASIQAGSQAVEEMESE
TEEETIFGSWFWDGKEVSEEAGPCCVSKPEDDEEMIVESWFWSRDKAIKETGTVATCESK
PENEEGAIVGSWFEAEDEVDNRTDNGSNCGSRTLADEDEAIVGSWFWAGDEAHFESNPSP
VFRAICRSTCSVEQEPDPSRRPQSWEEVTVQFKPGPWGRVGFPSISPFRFPKEAASLFCE
MFGGKPRNMVLSPEGEDQESLLQPDQPSPEFPFQYDPSYRSVQEIREHLRAKESTEPESS
SCNCIQCELKIGSEEFEELLLLMEKIRDPFIHEISKIAMGMRSASQFTRDFIRDSGVVSL
IETLLNYPSSRVRTSFLENMIRMAPPYPNLNIIQTYICKVCEETLAYSVDSPEQLSGIRM
IRHLTTTTDYHTLVANYMSGFLSLLATGNAKTRFHVLKMLLNLSENLFMTKELLSAEAVS
EFIGLFNREETNDNIQIVLAIFENIGNNIKKETVFSDDDFNIEPLISAFHKVEKFAKELQ
GKTDNQNDPEGDQEN
>O14512

MVFRNVGRPPEEEDVEAAPEPGPSELLCPRHRCALDPKALPPGLALERTWGPAAGLEAQL
AALGLGQPAGPGVKTVGGGCCPCPCPPQPPPPQPQPPAAAPQAGEDPTETSDALLVLEGL
ESEAESLETNSCSEEELSSPGRGGGGGGRLLLQPPGPELPPVPFPLQDLVPLGRLSRGEQ
QQQQQQQPPPPPPPPGPLRPLAGPSRKGSFKIRLSRLFRTKSCNGGSGGGDGTGKRPSGE
LAASAASLTDMGGSAGRELDAGRKPKLTRTQSAFSPVSFSPLFTGETVSLVDVDISQRGL
TSPHPPTPPPPPRRSLSLLDDISGTLPTSVLVAPMGSSLQSFPLPPPPPPHAPDAFPRIA
PIRAAESLHSQPPQHLQCPLYRPDSSSFAASLRELEKCGWYWGPMNWEDAEMKLKGKPDG
SFLVRDSSDPRYILSLSFRSQGITHHTRMEHYRGTFSLWCHPKFEDRCQSVVEFIKRAIM
HSKNGKFLYFLRSRVPGLPPTPVQLLYPVSRFSNVKSLQHLCRFRIRQLVRIDHIPDLPL
PKPLISYIRKFYYYDPQEEVYLSLKEAQLISKQKQEVEPST
>Q9UBW8
MSAEVKVTGQNQEQFLLLAKSAKGAALATLIHQVLEAPGVYYVFGELLDMPNVRELAESDF
ASTFRLLTVFAYGTYADYLAEARNLPPLTEAQKNKLRHLSVVTLAAKVKCIPYAVLLEAL
ALRNVRQLEDLVIEAVYADVLRGSLDQRNQRLEVDYSIGRDIQRQDLSAIARTLQEWCVG
CEVVLSGIEEQVSRANQHKEQQLGLKQQIESEVANLKKTIKVTTAAAAAATSQDPEQHLT
ELREPAPGTNQRQPSKKASKGKGLRGSAKIWSKSN
>Q12180
MENQGGDYSPNGFSNSASNMNAVFNNEITGRSDISNVNHQTGTPRLVPETQIWSMPVPDQ
LMTMPNRENTLMTGSTIGPNIPMNVAYPNTIYSPTEHQSQFQTQQNRDISTMMEHTNSND
MSGSGKNLKKRVSKACDHCRKRKIRCDEVDQQTKKCSNCIKFQLPCTFKHRDEILKKKRK
LEIKHHATPGESLQTSNSISNPVASSSVPNSGRFELLNGNSPLESNIIDKVSNIQNNLNK
KMNSKIEKLDRKMSYIIDSVARLEWLLDKAVKKQEGKYKEKNNLPKPARKIYSTALLTAQ
KLYWFKQSLGVKASNEEFLSPISEILSISLKWYATQMKKFMDLSSPAFFSSEIILYSLPP
KKQAKRLLENFHATLLSSVTGIISLKECLDLAEKYYSESGEKLTYPEHLLLNVCLCSGAS
ATQSIIRGDSKFLRKDRYDPTSQELKKIENVALLNAMYYYHKLSTICSGTRTLQALLLLN
RYFQLTYDTELANCILGTAIRLAVDMELNRKSSYKSLDFEEAIRRRRMWWHCFCTDKLYS
LMLSRPPIVGERDMDMLTDQNYYEVIKTNILPDLIDKKEDLDKITDVNSALNVVVNFCQH
ISLFISYYVSKLVSIESKIYSTCFAVRSTLDLSFDAMLDKIKDLNDSLNNWRDNLHVSMK
LKSYKQYLSVLYAQKSQENPALSFEIACSRVLNCHFRALYSKVILSMMTTSLLIDNERLY
KGSRHDIPQLFILFSSQYLNASKEMLQLFQGINYQAHMYNEVMYQFSTAMFVLFFYVVDN
MNDLKKKGEVKEIIDILKKSYDRLVGENDEQLLFDNVKWNTLIVFYSHFLKYVLQRYHAL
NDSTSIFDSKPYDETITKVIMHSRKIKDETVDQLIMSLKSYGSLHSLQKGNEADLADDGL
NTNDISSEDFAEEAPINLFGELSVEILKLLKSHSPISNFGDLSPSSNRKGISDDSSLYPI
RSDLTSLVYPIHSSDTGDTLSSGLETPENSNFNSDSGIKEDFEAFRALLPLGKLIYDRDY
SFVNTFRDYE
>A6BLY7
MSLRFSGGSRHVGIQSGSLRPPSGGAGFAGSSVAGGSVAGSGFSWALGGTLGSAPGGSHA
TGALGNVSGVCFIGSEGGLLSGNEKVTMQNLNNRLASYLDNVKALEEANSELERKIKTWH
EKYGPGSCRGLDRDYSKYHLTIEDLKSKIISSTAANANIILQIDNARLAADDFRLKYENE
LTLHQNVEADINGLRRVLDELTLCRTDQELQYESLSEEMTYLKKNHEEEMKVLQCAAGGN
VNVEMNAAPGVDLTVLLNNMRAEYEALAEQNRRDAEAWFQEKSATLQQQISNDLGAATSA
RTELTELKRSLQTLEIELQSLSATKHSLECSLAETEGNYCSQLAQIQAQISALEEQLHQV
RTETEGQKLEHEQLLDIKAHLEKEIETYCRLIDGDENSCSVSKGFESGTSGNSPKDVSKT
TLVKTVVEEIDQRGKVLSSRIHSIEEKMSKMSNGKAEQRVPF

>Q61081
MVDYSVWDHIEVSDDEDETHPNIDTASLFRWRHQARVERMEQFQKEKEELDRGCRECKRK
VAECQRKLKELEVAESDGQVELERLRAEAQQLRKEERSWEQKLEDMRKKEKNMPWNVDTL
SKDGFSKSMVNTKPEKAEEDSEEAREQKHKTFVEKYEKQIKHFGMLHRWDDSQKYLSDNV
HLVCEETANYLVIWCIDLEVEEKCALMEQVAHQTMVMQFILELAKSLKVDPRACFRQFFT
KIKTADHQYMEGFKYELEAFKERVRGRAKLRIEKAMKEYEEEERKKRLGPGGLDPVEVYE
SLPEELQKCFDVKDVQMLQDAISKMDPTDAKYHMQRCIDSGLWVPNSKSGEAKEGEEAGP
GDPLLEAVPKAGNEKDVSA
>P25693
MSNYEALLKFNRKAVSKEMVQYLASTTASIIKIKKTNSMIDIALPAPPLTKFINRLIKHS
NVQTPTLMATSVYLAKLRSIIPSNVYGIETTRHRIFLGCLILAAKTLNDSSPLNKHWAEY
TDGLLILREVNTIERELLEYFDWDVTISTDDLITCLSPFLKPIKEEQLYKSQRDCRTLKN
FSAQEKDIVNKTSISHSRSSSNMSIPSLASTSTLSTLESRRSNLSNYSNRIRTLPELHES
NNISDKFSPRTYNIDSKHDNKENRPIPTIKPFNFSKARPVILKTGLNKQIIKEDTKVKKS
NWSNYFKS
>P40337
MPRRAENWDEAEVGAEEAGVEEYGPEEDGGEESGAEESGPEESGPEELGAEEEMEAGRPR
PVLRSVNSREPSQVIFCNRSPRVVLPVWLNFDGEPQPYPTLPPGTGRRIHSYRGHLWLFR
DAGTHDGLLVNQTELFVPSLNVDGQPIFANITLPVYTLKERCLQVVRSLVKPENYRRLDI
VRSLYEDLEDHPNVQKDLERLTQERIAHQRMGD
>P32827
MGKGKPRGLNSARKLRVHRRNNRWAENNYKKRLLGTAFKSSPFGGSSHAKGIVLEKLGIE
SKQPNSAIRKCVRVQLIKNGKKVTAFVPNDGCLNFVDENDEVLLAGFGRKGKAKGDIPGV
RFKVVKVSGVSLLALWKEKKEKPRS
>P22935
MPNFSGNWKIIRSENFEEMLKALGVNMMMRKIAVAAASKPAVEIKQENDTFYIKTSTTVR
TTEINFKIGEEFEEQTVDGRPCKSLVKWESGNKMVCEQRLLKGEGPKTSWSRELTNDGEL
ILTMTADDVVCTRVYVRE
>Q06263
MASNAARVVATAKDFDKVGLGIIGYYLQLYAVELILSEEDRSQEMTALATELLDTIEAFK
KEIGGESEAEDSDKSLHVMNTLIHDQEKAKIYMLNFTMSLYNEKLKQLKDGPWDVMLKRS
LWCCIDLFSCILHLWKENISETSTNSLQKRIKYCKIYLSKLAKGEIGSSDEKTLDYADFA
DDSEEIKDEDVDHQTSDLENNNNDKVEGLAPKDQTTSYEPVDEVPEFIDDADSVNEEEQT
VDKNEDAITKDEQQVVKKEVDLTRPSAPSEPAAAEHKSYTKDELTKIMDRASKIEQIQKL
AKYAISALNYEDLPTAKDELTKALDLLNSI
>P20449
MSDTKRDPADLLASLKIDNEKEDTSEVSTKETVKSQPEKTADSIKPAEKLVPKVEEKKTK
QEDSNLISSEYEVKVKLADIQADPNSPLYSAKSFDELGLAPELLKGIYAMKFQKPSKIQE
RALPLLLHNPPRNMIAQSQSGTGKTAAFSLTMLTRVNPEDASPQAICLAPSRELARQTLE
VVQEMGKFTKITSQLIVPDSFEKNKQINAQVIVGTPGTVLDLMRRKLMQLQKIKIFVLDE
ADNMLDQQGLGDQCIRVKRFLPKDTQLVLFSATFADAVRQYAKKIVPNANTLELQTNEVN
VDAIKQLYMDCKNEADKFDVLTELYGLMTIGSSIIFVATKKTANVLYGKLKSEGHEVSIL
HGDLQTQERDRLIDDFREGRSKVLITTNVLARGIDIPTVSMVVNYDLPTLANGQADPATY
IHRIGRTGRFGRKGVAISFVHDKNSFNILSAIQKYFGDIEMTRVPTDDWDEVEKIVKKVL

KD
>Q9URY5
MSSVEVNRENADVANTNRQANLAEGYEIKELNESQKQYIRSSIPILESSGVNLTKAFYQK
MLGNYPEVLPYFNKAHQISLSQPRILAFALLNYAKNIDDLTSLSAFMDQIVVKHVGLQIK
AEHYPIVGHCLLSTMQELLPSDVATPAFLEAWTTAYGNLAKILIDSEKKVYQSQPWNGFV
EFKVTELINESSDVKSVYLGPKDPAFRISHAHPGQYVSVLWEIPGLSHKTLREYSLSNRV
DTCRNQFRISVRRVAGGVVSNFVHDNLKVGDIVGVSPPAGNFVYKRSEENVNRPLLCFAG
GIGITPLIPIIETALLDGRKVNFCYSSRNYVSRPFKQWLEQLKLKYKENLKLKEFFSEES
SVTKEQIVDEVMTRIINEEDLEKLDLSECDIYMLGPNNYMRFVKQELVKLGVEPNKVQSE
FFGPYIP
>P25028
MSFSNVLIMRQPDEGKCHICKRVFCCGKCRQKHQFKAHAIAVREPLGLRSAGGGIIEHRH
QMESGATTIYVFCPICERRPLLLREEMHGELLAHIETCHLPLRCRKCQRNYTRVDDLREF
SKCVDQQQSCTDVTGATETSKATLKKAANSTAISTQTSPSVTPISLINMRWKAKSRVTHE
EFISDSVSSIRNLSSFSNSSIRRSIGQLGVNPSETMEKGKVIRSTSTPLHVESVFAKPKE
PITFNASTGGHVSSIYHEEPSPTPESNPVQQQQQQQQPLQQRAWKMGARNKMSAATPLRQ
VMSKSIQKAFVEHGGMMVHQPPSAVVQRRVRLDLSEHSSHEAAGSSALDLRLSPAMRRTQ
SESSASEVNSGSSSSYSTSRNADLCKRQFLLSAQKLTTESIIITRTNSSSQKTSSTVYNS
CESVEIIRSTSESAEVCHVPAITPIRVTGAGINKKQIKFETPPKSSQQMRSNGEGDETKD
QFFTPEPGTPEIPERRHRQAIVPRQLSGEFSPKKDKPKEKGLAVMALISPPLQQPRVRPP
LRECRQQRVYSGVQDVGEPEVVDAEEEDEVFRPTNASTCNDKKLEAPNSGRLWSLMSSMM
RLPASLRGEREKDRDRDRDSDKENAGSGSLIRRCASIAGSLVRPSARDSSMEDQQCLKRK
RTQTLDSQYCSPLSPSSSSKRYRIRPREPIERMRRQ
>Q9UTR7
MTKETHENHLRTHNGIFPVSLLSTQARQLLNFKLKSPSYYLLNSFKLLVKSEGNNQSTTA
DVTLTKSPNAYHASSAREEKDLQVSRRDTCFYCSRKIIKCICNEEHVGIESVQLKLILLL
CQNLPNVTTNAKKYFSSLADGHNFTLTLYKFSLDNQTFSQLLSRFKSFATLTELLQVHNV
MLQVNFSFQARQLNTEIQLRRCHSLEKTWKFLFGDYELPDVLKDMESSNSDWSDTSLKRI
AYLCSLVEELKLHSSIMNDKYVCLVSKHNNALEDKFNSEAARQLLQKSLSIVASNLKQAE
NKTISYEEKLSIAQNSINEIQTQNRDLKLETEKLQDQIKALLERNQSLQEALETVKNDEK
NLREMNANYETEMKEARQKLNNKEALISHYDDDFRAKELKISRLSESLREKAGLLEFQSS
VSEQRDLLYQEQIQSSIKDMENVFRKNEYLMEELNELKNNLEVESSKVLRLDEEMKCLKD
EQLSQFDTVFSLTDERDGLQKDLKNTKGNLDDEIGRSAFLKSQIRDQELTIEKLHDSLET
LSQTNNSLQCEISEKNAELNSVNSKLSEGRAHLETANKENEILKQQLELSESKLASLLNS
YQSFINKKEHLYSFLQLVEPSFAKSDSSNATESQISESVRKGISIFNLLFIVYKNVCSQA
GINPSTKLEDLDEHTLSDELTYITKKFVQKDQEYQTKEIELRNYKITLQSLLEDKLIGVN
TDCRSPSCSDFEQLGQESENNTSISGRVSKLVKSFNDSSSISNNTKISITKSPSGEKVSV
FKEMSDIALRDMDKNRKLLGENVDVRNIVVQKDESLNIDLQNNAVVPELHFKEGMVYDSL
ENAYTYLAESKRMLANELQMKQEDLEKVILELEAYKEIFLEEKQIPCEEFMPGKNAKSEK
SLRSVFQEQLMRETKRVRKLEKVNSELKLHCFELSERLREREHTLQQTFGDK
>P34108
MREIVHIQAGQCGNQIGAKFWEVISDEHGVDPTGAYHGDSDLQLERINVYYNEATGGRYV
PRAILMDLEPGTMDSVRAGPYGQIFRPDNFVFGQTGAGNNWAKGHYTEGAELIDSVLDVV
RKEAESCDCLQGFQIAHSLGGGTGSGMGTLLISKIREEYPDRMMMTFSVFPSPKVSDTVV

EPYNATLSVHQLVENADEVMCIDNEALYDICFRTLKLTTPTFGDLNHLVSIVMSGVTCCL
RFPGQLNSDLRKLAVNLIPFPRLHFFLIGFAPLTSRGSQQYRALTVPELTQQMFDAKNMM
AASDPRHGRYLTASAMFRGRMSTKEVDEQMLNVQNKNSSYFVEWIPNNIKSSVCDIPPRG
LKMAATFIGNSTAIQEMFKRVSEQFTAMFRRKAFLHWYTGEGMDEMEFTEAESNMNDLVS
EYQQYQDATAEEEGEFDENEGAEGEEQPADY
>Q12230
MHRTYSLRNQRAPTAAELQAPPPPPSSTKSKFFGKASIASSFRKNAAGNFGPELARKLSQ
LVKTEKGVLRAMEVVASERREAAKQLSLWGADNDDDVSDVTDKLGVLIYELGELQDQFID
KYDQYRVTLKSIRNIEASVQPSRDRKEKITDEIAHLKYKDPQSTKIPVLEQELVRAEAES
LVAEAQLSNITREKLKAAYSYMFDSLRELSEKFALIAGYGKALLELLDDSPVTPGEARPA
YDGYEASRQIIMDAESALESWTLDMAAVKPTLSFHQTVDDVYEDEDGEEEEEPEIQNGDI
PGQVVEEEEVEWTTEVPVDDEAHEADHHVSQNGHTSGSENI
>Q6SJ96
MASAPWPERVPRLLAPRLPSYPPPPPTVGLRSMEQEETYLELYLDQCAAQDGLAPPRSPL
FSPVVPYDMYILNASNPDTAFNSNPEVKETSGDFSSVDLSFLPDEVTQENKDQPVISKHE
TEENSESQSPQSRLPSPSEQDVGLGLNSSSLSNSHSQLHPGDTDSVQPSPEKPNSDSLSL
ASITPMTPMTPISECCGIVPQLQNIVSTVNLACKLDLKKIALHAKNAEYNPKRFAAVIMR
IREPRTTALIFSSGKMVCTGAKSEEQSRLAARKYARVVQKLGFPARFLDFKIQNMVGSCD
VRFPIRLEGLVLTHQQFSSYEPELFPGLIYRMVKPRIVLLIFVSGKVVLTGAKERSEIYE
AFENIYPILKGFKKA
>P51858
MSRSNRQKEYKCGDLVFAKMKGYPHWPARIDEMPEAAVKSTANKYQVFFFGTHETAFLGP
KDLFPYEESKEKFGKPNKRKGFSEGLWEIENNPTVKASGYQSSQKKSCVEEPEPEPEAAE
GDGDKKGNAEGSSDEEGKLVIDEPAKEKNEKGALKRRAGDLLEDSPKRPKEAENPEGEEK
EAATLEVERPLPMEVEKNSTPSEPGSGRGPPQEEEEEEDEEEEATKEDAEAPGIRDHESL

>Q9UU84
MTEELFSNSETCSLSDPLTSKTLSSIENPFDDPYILSEESEILTIKTDVLRPNILNRKSS
FYPEADAPVRKSNLQVKKIFENQVLNCDGIKVAITIPDTSLIAGSLLHGNIWLSYEGAKN
VETGVWIKEMFLDFYGILNFKGHSEPFYSISEKYSFCNLKTSALILSKAASASIGNKGLF
LKCSCKVGFPFSFIVPLDIGPGTYHSSKLELLYYISSTLTLTSLDQNIRCTRCTIPKRVI
TSMHNNIAELYNPISCIKSLPYLSLEEAKTTLEVHTDRSLFFSGQIIDFTICYTHNHHRR
IHNIKARLLETHIFHPNTQNHYGGYCMNQAEETFASCGYLKRYQKTKTKTRAKSTWKIAN
KSVYSNLAPTLKNTIHAQIKIPEFCRSVNLKDQLKIDYTLEVCFSAFFKPRILSIQIPIT
ILHSWN
>Q18066
MASLAPFFKISDSILADESFDDLEINNHFKCTSKDLNEYPRTSTPLPGEITSIYSLPRSY
NNQNLCTRSKPTTFISQNQQKGMTEKPKHSEYFLRLMKTRQEMQKKLTAKNGSNKSVQQY
LRKLPPSTSKEVTIIPVKMKPDDNSVYISKELSDRTDTKLPKVSTKEVEKFGEQSDDEER
HERSHIASDFNQKTPQSTHATSNHRKRIFIGRTGAKQQHVSKKATESELPYNIDVSTSNT
TNATEALKLLKRPLLLPSKDPRSSKMPRLDLNQHQFRCKQYIVHSQFTCTQIEKTFKCRV
GTSTESEDLDDYYFDAMPDGKAPHFPQQPVARQNDDGSLELECFVDANPTPQVKWYYDNK
EVENSGRFSANLANKGSDSYSAILTIKELADADAGAYRCAIVNPHGKGNANFNLKLTGFS
SPTFVEKPQISSRDDGQVMVMEFRAKSILEPTFVWQKLVGGGAEEIIANSDRIKAVKKLE

AGNVYYSALEIKEPTKDKDAGQFICTVKNESGKLTATFTVKFEVPEGAPSFTRKPQILQQ
TSAGGEPAICFDIGYSARMNPQVTWISPKSKKMKESSRIKFKTNDEGNGNFTAQLELTNY
KAKDSGTYTCNIKNDAGEANVELTLNIEGPLDDYADDSEN
>Q05778
MLFKQWNDLPEPKHLLDLPEISKNLQSLEVCPVPKVEFPQDLDVPQYSTAVITTKIMNPL
FPKNLLQLTSIGEIKTTLTVKSPSLPQSSGKHSWNYDENFPNEVDPDQKNDTADETVYGF
SFPIYSFGKTLLFSMEENFISISPIFGNMISRSIISQLAQFSPDIIVIGTSDKIASMKVM
TENECTLQPPEFITGFIGSVLTQLIVGPSKGLKFKCLVAPSEGPNGFEKLSLSDMGSLVD
LCGQWLGFEPSRYSEECYRLWRCDSAAIGAQSGLYI
>Q6PEM8
MEGRVSSVGSPHSFLNAPVLFRGPVEPLVFLANFALVLQGPLTTQYLWHRFSTELGYNGT
RHRENCGNQSADPLMKEVETLTSHWTLYMNVGGFLVGLFWSTLLGAWSDRVGRRPLLVLA
SLGLLLQAVVSIFVVQLELHVGFFVLGRALCALLGDFNGLLAASFASVADVSSNHSRTFR
MALLEACIGVAGTLASLLGGHWLRAQGYANPFWLALALLIVMALYAAFCFGETVKEPKST
RLFTLRHHRSIARLYVVPAPEKSRMHLALYSLAIFVVVTVHFGAQDILTLYELSAPLCWD
SKLIGYGSAAQHLPYLTSLLGLRLLQFCLADTWVAEIGLAFNILGMVVFAFATITPLMFT
GYGLLFLSLVTTPVIRAKLSKLVSESEQGALFSAVACVNSLAMLMASGIFNSIYPATLNF
MKGFPFLLGAGLLFIPAILIGVLEKVNPHPEFQQFPQSP
>P53866
MAKRHSHYQGSRRRHARGSNSKKAGRGNAKGIQGRKIKKKPTPTNSWHNSSIPLGEGDLD
DVGADFNPGRAFISPKTIEDYYFGRDAKSRSMKMGGLRPGNRYDSSTDLQAGRAAFRKRP
MQFVKAKEVYDPSHNMIQKLRAKNETKNSEEIVEREADVFEEPGKMTSDVEYINNEDSEN
EDDDSQNSPSTDHSLSSNESKVEDGDLFFVDEEAQQSPDLTKIKRVCIEEIARPREVAIE
FDPILTIGKVELSVSEGNESKEISVDVPNKGNKTYHPFAGYISNVLHGMHTSDSDNDELD
YEIETENNSEPLYESSASSEVDQGFNYVGQRHNSRADNNLLPSPSPQLTEDIKCLSINGT
KTFEGNNDNLPSPASEELEFGFKEEDFVINTNDIVVSNIRMGGVDNSYYLRCYRLLGDYD
FHWIDQDLLTDFVVDELGLPEDRLPAYLNFIKNSLIPKIEPAEPTYSDIPISDSSDEGDS
YEGDSYEDDEDMASSVVHSDIEEGLDDLIAYTLKHDTERFKTFETKSLETKGKGKKKKLL
IDDALALDTETLETLQSKFSKRIETKAKKRKAKEDFIDQENRNSNDMLKKYPYGLHIQNI
KDEFESFLSRNNDRLTFPPLDPHGNKTVMKIAKHYNMKSSKIGKANHTSVVVEKIKKTKW
SSPNYSLIDQLMRQRPVFMRIDIRRPREEQAAFERTKTIRGKFHVKEGEIVGQNAPEIGN
ENIGRRMLEKLGWKSGEGLGIQGNKGISEPIFAKIKKNRSGLRHSES
>P28005
MVRLKSRYILFEIIFPPTDTNVEESVSKADILLSHHRASPADVSIKSILQEIRRSLSLNL
GDYGSAKCNSLLQLKYFSNKTSTGIIRCHREDCDLVIMALMLMSKIGDVDGLIVNPVKVS
GTIKKIEQFAMRRNSKILNIIKCSQSSHLSDNDFIINDFKKIGRENENENEDD
>Q08965
MEQSNKQHRKAKEKNTAKKKLHTQGHNAKAFAVAAPGKMARTMQRSSDVNERKLHVPMVD
RTPEDDPPPFIVAVVGPPGTGKTTLIRSLVRRMTKSTLNDIQGPITVVSGKHRRLTFLEC
PADDLNAMIDIAKIADLVLLLIDGNFGFEMETMEFLNIAQHHGMPRVLGVATHLDLFKSQ
STLRASKKRLKHRFWTEVYQGAKLFYLSGVINGRYPDREILNLSRFISVMKFRPLKWRNE
HPYMLADRFTDLTHPELIETQGLQIDRKVAIYGYLHGTPLPSAPGTRVHIAGVGDFSVAQ
IEKLPDPCPTPFYQQKLDDFEREKMKEEAKANGEITTASTTRRRKRLDDKDKLIYAPMSD
VGGVLMDKDAVYIDIGKKNEEPSFVPGQERGEGEKLMTGLQSVEQSIAEKFDGVGLQLFS

NGTELHEVADHEGMDVESGEESIEDDEGKSKGRTSLRKPRIYGKPVQEEDADIDNLPSDE
EPYTNDDDVQDSEPRMVEIDFNNTGEQGAEKLALETDSEFEESEDEFSWERTAANKLKKT
ESKKRTWNIGKLIYMDNISPEECIRRWRGEDDDSKDESDIEEDVDDDFFRKKDGTVTKEG
NKDHAVDLEKFVPYFDTFEKLAKKWKSVDAIKERFLGAGILGNDNKTKSDSNEGGEELYG
DFEDLEDGNPSEQAEDNSDKESEDEDENEDTNGDDDNSFTNFDAEEKKDLTMEQEREMNA
AKKEKLRAQFEIEEGENFKEDDENNEYDTWYELQKAKISKQLEINNIEYQEMTPEQRQRI
EGFKAGSYVRIVFEKVPMEFVKNFNPKFPIVMGGLLPTEIKFGIVKARLRRHRWHKKILK
TNDPLVLSLGWRRFQTLPIYTTTDSRTRTRMLKYTPEHTYCNAAFYGPLCSPNTPFCGVQ
IVANSDTGNGFRIAATGIVEEIDVNIEIVKKLKLVGFPYKIFKNTAFIKDMFSSAMEVAR
FEGAQIKTVSGIRGEIKRALSKPEGHYRAAFEDKILMSDIVILRSWYPVRVKKFYNPVTS
LLLKEKTEWKGLRLTGQIRAAMNLETPSNPDSAYHKIERVERHFNGLKVPKAVQKELPFK
SQIHQMKPQKKKTYMAKRAVVLGGDEKKARSFIQKVLTISKAKDSKRKEQKASQRKERLK
KLAKMEEEKSQRDKEKKKEYFAQNGKRTTMGGDDESRPRKMRR
>Q3E846
MGLFSFDGGKKESQPPNTRSQRKLCWESRDAFFQCLDKADILDAMDPKNSKSIKSHCKVE
NEKFEENCAHSWIKYFKEKRVIDFKREQTIKRIEQEAKQRERNQ
>Q03305
MSELHALLTFPERPISQSYYVPKLQHFLKSGIPATYTLEQVAAFEHEEKNRNGDKEFRES
TDDNKTSNTTPLHVLARSLPLDIKDEELQVVMDMMNILFEYGAGWNFIDYEDKTVGDLFL
ERNQSRESPLYRRLVEAGVSAELLLRKLNGGDVEFLDTDELIGIEPEESVQTAVDGQKEE
SVGSDDDATAANQQVYLKTELEYKDDALITKENKDGVMMDWETKIMELASETLFPDPEAT
NSATILNIGFGMGIIDTFIQARKPYRHYICEAHPDVLAKMKMDGWYEKDNVVILEGRWQD
TLNNLLDKGEVFFDGIYYDTFSEHYQDILDLYDVVVGLIKPEGVFSFFNGLGADRSLCYD
VYKEIVEIDVATYGMKCDYTRYSLDEQLPDWNDVKRSYFNCNYYYHPRITFA
>P17695
METNFSFDSNLIVIIIITLFATRIIAKRFLSTPKMVSQETVAHVKDLIGQKEVFVAAKTY
CPYCKATLSTLFQELNVPKSKALVLELDEMSNGSEIQDALEEISGQKTVPNVYINGKHIG
GNSDLETLKKNGKLAEILKPVFQ
>P97440
MACRPRSPPGYGSRRDGGASPRSPARWSLGRKRRADGRDRKPEDSEEGELQTADHRPESF
TTPEGHKPRSRCSDWASAVEEDEMRTRVNKEIARYKRKLLINDFGRERKSSSGSSDSKES
MSSVPADVETDESVLMRRQKQINYGKNTIAYDRYIKEVPRHLRQPGIHPRTPNKFKKYSR
RSWDQQIKLWKVALHFWDPPAEEGCDLQEIQPVDLGEMETEFTESSSESQTSSQDNFDVY
AGTPTKVRHVDCQVEDEFDLEACLTEPLKDFSAMS
>Q07555
MELRSRRSAEAYLVTPEEPAKNKSERSIESNERVGTREAKSENTSVFSPAYSDTATTDSS
KKVDDNEYYNFTSHFMPSLKNTRELENTILNLIQRIKEGDDETLVSEKDLILSVLNRSLA
STSHWKLQAQLSELRATSEGRYAVETNLLKKEVEFLKNKTPKTNESASSAELRPLLERPL
KRKLSLPGLAQRPLSTGARLEGGYGGVSPNSWKTKVPKLPLPASRPSLNLSPQKVPTGTD
KVEEDTKIDTLELVENNKPHPRMRRRSDNPATNEYVRVFHLEKKEPKSRKK
>O95208
MTTSSIRRQMKNIVNNYSEAEIKVREATSNDPWGPSSSLMTEIADLTYNVVAFSEIMSMV
WKRLNDHGKNWRHVYKALTLLDYLIKTGSERVAQQCRENIFAIQTLKDFQYIDRDGKDQG
INVREKSKQLVALLKDEERLKAERAQALKTKERMAQVATGMGSNQITFGRGSSQPNLSTS

HSEQEYGKAGGSPASYHGSPEASLCPQHRTGAPLGQSEELQPLSQRHPFLPHLGLASRPN
GDWSQPCLTCDRAARASTSPRVSSELEQARPQTSGEEELQLQLALAMSREVAEQEERLRR
GDDLRLQMALEESRRDTVKIPKKKEHGSLPQQTTLLDLMDALPSSGPAAQKAEPWGPSAS
TNQTNPWGGPAAPASTSDPWPSFGTKPAASIDPWGVPTGATAQSVPKNSDPWAASQQPAS
SAGKRASDAWGAVSTTKPVSVSGSFELFSNLNGTIKDDFSEFDNLRTSKKTAESVTSLPS
QNNGTTSPDPFESQPLTVASSKPSSARKTPESFLGPNAALVNLDSLVTRPAPPAQSLNPF
LAPGAPANSAPVNPFQVNQPQPLTLNQLRGSPVLGTSTSFGPGPGVESMAVASMTSAAPQ
PALGATGSSLTPLGPAMMNMVGSVGIPPSAAQATGTTNPFLL
>Q9P6M8
MNNSKLSVPGQYEDESSTIYVGNIDSRLSDEEIIKHFSKYGQVESIFRRLLDSFYPKEKA
KPFKPPKNGVQYGFVKFVNAESIEEVLKDAKGMTLGQRKLTIKARVVNPAKLTDKKFKPN
DTSITANVFNPSIQNNTDEENVKPGLKQSQIKEFIPNVEERNWETVRGINSRNPKPETTL
TIPSLGLEDISMQVVPNAFASTLPLSILNLPAEVTPIDLYNHFKQAGVVKGTAVSQFLDQ
RGFRYGEVIMDSVESCQNAIEKLNNVPYKGSILEVSIKNKASSSVKSIPTTPTGESLWPF
PSENANKTQINIENATCSKMTWIMGSPTKEKSQWGSVSTTGVSNQQNHPAAWNPDNKPQS
IVHWDSLRESSPSIPNSPIDPSNLYVKNLDDTVITCKSQLEDLFSPFGSILSSMLACYPN
SGISKGYGFVAFRQIEAAVRAKDTLNGMMVGKKRIFVCFAERKSDRIRRLQAFFANKPTS
EQTAQQDNKALFVKPERSSTVTIRKPIESSTNKISENPTTLSSKVENKNEPKTGENKEPS
QTNEYVNCKQENKELSGQLSGNLDIKKEAGKLSHDGEQGNLLKPLVFHANTKLNNRGSAK
MGSTATNLKKIQDMLHNKKLSNAYFVPRARATTCTTLTYVTVEPSHFQDEDCNESTNMLS
LVGYMDSYPEATSNIQKMNSYHIKDSNKENFLSTTKVNNNSPTTIQLTQI
>Q92331
MDYEDNLEAPVWDELNHEGDKTQSLVSNSIESIGQISTNEERKDNELLETTASFADKIDL
DSAPEWKDPGLSVAGNPQLEEHDNSKADDLINSLAPEQDPIADLKNSTTQFIATRESGGA
LFTGNANSPLVFDDTIYDANTSPNTSKSISGRRSGKPRILFDSARAQRNSKRNHSLKAKR
TTASDDTIKTPFTDPLKKAEKENEFVEEPLDDRNERRENNEGKFTASVEKNILEQVDRPL
YNLPQTGANISSPAEVEENSEKFGKTKIGSKVPPTEKAVAFKVEVKDPVKVGELTSIHVE
YTVISESSLLELKYAQVSRRYRDFRWLYRQLQNNHWGKVIPPPPEKQSVGSFKENFIENR
RFQMESMLKKICQDPVLQKDKDFLLFLTSDDFSSESKKRAFLTGSGAINDSNDLSEVRIS
EIQLLGAEDAAEVLKNGGIDAESHKGFMSISFSSLPKYNEADEFFIEKKQKIDELEDNLK
KLSKSLEMVDTSRNTLAASTEEFSSMVETLASLNVSEPNSELLNNFADVHKSIKSSLERS
SLQETLTMGVMLDDYIRSLASVKAIFNQRSKLGYFLVVIENDMNKKHSQLGKLGQNIHSE
KFREMRKEFQTLERRYNLTKKQWQAVGDKIKDEFQGFSTDKIREFRNGMEISLEAAIESQ
KECIELWETFYQTNL
>Q4AE70
MAAAAATAVGPGAGSAGVAGPGGAGPCATVSVFPGARLLTIGDANGEIQRHAEQQALRLE
VRAGPDAAGIALYSHEDVCVFKCSVSRETECSRVGRQSFIITLGCNSVLIQFATPHDFCS
FYNILKTCRGHTLERSVFSERTEESSAVQYFQFYGYLSQQQNMMQDYVRTGTYQRAILQN
HTDFKDKIVLDVGCGSGILSFFAAQAGARKIYAVEASTMAQHAEVLVKSNNLTDRIVVIP
GKVEEVSLPEQVDIIISEPMGYMLFNERMLESYLHAKKYLKPSGNMFPTIGDVHLAPFTD
EQLYMEQFTKANFWYQPSFHGVDLSALRGAAVDEYFRQPVVDTFDIRILMAKSVKYTVNF
LEAKEGDLHRIEIPFKFHMLHSGLVHGLAFWFDVAFIGSIMTVWLSTAPTEPLTHWYQVR
CLFQSPLFAKAGDTLSGTCLLIANKRQSYDISIVAQVDQTGSKSSNLLDLKNPFFRYTGT
TPSPPPGSHYTSPSENMWNTGSTYNLSSGVAVAGMPTAYDLSSVIAGGSSVGHNNLIPLA

NTGIVNHTHSRMGSIMSTGIVQGNRVAGPWAGDLPPGLRTRSSYQWGPGRLRGHAGSSVP
MTCPTGSSGAQGGGGSSSAHYAVNNQFTMGGPAISMASPMSIPTNTMHYGS
>Q07953
MPINQPSGQIKLTNVSLVRLKKARKRFEVACYQNKVQDYRKGIEKDLDEVLQIHQVFMNV
SKGLVANKEDLQKCFGTTNVDDVIEEIMHKGEIQLSEKERQLMLNKVNNEMLTIVSAKCI
NPVSKKRYPPTMIHKALQELKFSPVINKPAKLQALEAIKLLVSKQIIPIVRAKMKVKVAI
SEPSRQPELIEKISKLIASSPGESTKPELDPWTCTGLIDPVNYRDLMTLCDKKGTVQVLD
MAVIDNTTHN
>Q9USK7
MPEMDKSNDFFEENPCPNSLGDVSSLIEDFINLQSNSQNIAFVTSGGTLVPLEQNTVRFI
DNFSAGNRGAASAEYFCKSGYSVIFLYRNYSLMPFTRRYEGPWTNLFERSNGSQEKPWIV
KQEFQSTVFNALNELDTYTKHHRLIMIPYTTLTEYLWYLKAIAERLQIIASRALFYLAAA
VSDFHIPPKELSEHKIQSGSGSNKLEVKMHPVPKFLRFLVDTWAPEASIISFKLETDPSI
LIDKARVALQRYNHQLVIANLLSTRKRSVAFVTSKTVEWLHLSDDDIVKKVEIEQYIVSR
AISLHKERIQEFAKAP
>O94829
MERREEQPGAAGAGAAPALDFTVENVEKALHQLYYDPNIENKNLAQKWLMQAQVSPQAWH
FSWQLLQPDKVPEIQYFGASALHIKISRYWSDIPTDQYESLKAQLFTQITRFASGSKIVL
TRLCVALASLALSMMPDAWPCAVADMVRLFQAEDSPVDGQGRCLALLELLTVLPEEFQTS
RLPQYRKGLVRTSLAVECGAVFPLLEQLLQQPSSPSCVRQKVLKCFSSWVQLEVPLQDCE
ALIQAAFAALQDSELFDSSVEAIVNAISQPDAQRYVNTLLKLIPLVLGLQEQLRQAVQNG
DMETSHGICRIAVALGENHSRALLDQVEHWQSFLALVNMIMFCTGIPGHYPVNETTSSLT
LTFWYTLQDDILSFEAEKQAVYQQVYRPVYFQLVDVLLHKAQFPSDEEYGFWSSDEKEQF
RIYRVDISDTLMYVYEMLGAELLSNLYDKLGRLLTSSEEPYSWQHTEALLYGFQSIAETI
DVNYSDVVPGLIGLIPRISISNVQLADTVMFTIGALSEWLADHPVMINSVLPLVLHALGN
PELSVSSVSTLKKICRECKYDLPPYAANIVAVSQDVLMKQIHKTSQCMWLMQALGFLLSA
LQVEEILKNLHSLISPYIQQLEKLAEEIPNPSNKLAIVHILGLLSNLFTTLDISHHEDDH
EGPELRKLPVPQGPNPVVVVLQQVFQLIQKVLSKWLNDAQVVEAVCAIFEKSVKTLLDDF
APMVPQLCEMLGRMYSTIPQASALDLTRQLVHIFAHEPAHFPPIEALFLLVTSVTLTLFQ
QGPRDHPDIVDSFMQLLAQALKRKPDLFLCERLDVKAVFQCAVLALKFPEAPTVKASCGF
FTELLPRCGEVESVGKVVQEDGRMLLIAVLEAIGGQASRSLMDCFADILFALNKHCFSLL
SMWIKEALQPPGFPSARLSPEQKDTFSQQILRERVNKRRVKEMVKEFTLLCRGLHGTDYT
ADY
>Q9P7N2
MSIKFFLLVSRQGKVRLAKWFNTLSIKERAKIIRDVSSLVITRKPKMCNFVEYKGEKIVY
RRYASLFFVCGIEQDDNELIILEVIHKFVECLDKYFGNVCELDLIFNFEKAYYVMEELLL
AGELQESSKTNVLSAVLAGDAESEADAQQDSLQKLVGSVKKR
>Q02864
MSSRFARSNGNPNHIRKRNHSPDPIGIDNYKRKRLIIDLENLSLNDKGPKNGHADDNNLI
HNNIVFTDAIDDKVLKEIIKCSTSKRGDNDLFYDKIWERLREKRLQIIKWVDYKEIAYLS
WWKWFHNQMTSKYTYDGEADTDVEMMAVDTDVDMDA
>O15169
MNIQEQGFPLDLGASFTEDAPRPPVPGEEGELVSTDPRPASYSFCSGKGVGIKGETSTAT
PRRSDLDLGYEPEGSASPTPPYLKWAESLHSLLDDQDGISLFRTFLKQEGCADLLDFWFA

CTGFRKLEPCDSNEEKRLKLARAIYRKYILDNNGIVSRQTKPATKSFIKGCIMKQLIDPA
MFDQAQTEIQATMEENTYPSFLKSDIYLEYTRTGSESPKVCSDQSSGSGTGKGISGYLPT
LNEDEEWKCDQDMDEDDGRDAAPPGRLPQKLLLETAAPRVSSSRRYSEGREFRYGSWREP
VNPYYVNAGYALAPATSANDSEQQSLSSDADTLSLTDSSVDGIPPYRIRKQHRREMQESV
QVNGRVPLPHIPRTYRVPKEVRVEPQKFAEELIHRLEAVQRTREAEEKLEERLKRVRMEE
EGEDGDPSSGPPGPCHKLPPAPAWHHFPPRCVDMGCAGLRDAHEENPESILDEHVQRVLR
TPGRQSPGPGHRSPDSGHVAKMPVALGGAASGHGKHVPKSGAKLDAAGLHHHRHVHHHVH
HSTARPKEQVEAEATRRAQSSFAWGLEPHSHGARSRGYSESVGAAPNASDGLAHSGKVGV
ACKRNAKKAESGKSASTEVPGASEDAEKNQKIMQWIIEGEKEISRHRRTGHGSSGTRKPQ
PHENSRPLSLEHPWAGPQLRTSVQPSHLFIQDPTMPPHPAPNPLTQLEEARRRLEEEEKR
ASRAPSKQRYVQEVMRRGRACVRPACAPVLHVVPAVSDMELSETETRSQRKVGGGSAQPC
DSIVVAYYFCGEPIPYRTLVRGRAVTLGQFKELLTKKGSYRYYFKKVSDEFDCGVVFEEV
REDEAVLPVFEEKIIGKVEKVD
>P38737
MSISSDEAKEKQLVEKAELRLAIADSPQKFETNLQTFLPPLLLKLASPHASVRTAVFSAL
KNLISRINTLPQVQLPVRALIVQAKEPNLAAQQDSTNVRLYSLLLASKGIDRLSLQDRQQ
LLPLVVSNISCLTGTVAARMFHILLKLILEWVAPQESSHEQEEFVQFLQLDNDGFSFLMR
QFTRFFLLVPSKQVQVSQQPLSRGYTCPGLSLTDVAFFTYDAGVTFNKEQLNKFKKAIFQ
FVCRGMAATQTIEQSPRMIELMEFLCVVSTDSTNLSDDAAQFMKRFPMPYENEEFITFLQ
TLYIGNTANGRPPVKAILQEKILSILNRSHFATTKAECISLICSIGLHSSEYKLRSLTLS
FIRHVAKLNYKNLNPASSSPSSTDFSTCIVSLIRNNLHAEGWPKLQLGPQTPAFNTAILQ
RQLQYETLGDILKRDFELVSDLSYIEFLFESLKNDLPQFRSSIQESLLSLVGHLSILPQQ
SKLKLKNLLRKNLSIDEQQREDNNDAVNSIMALKFVSIKFTNAAFPFHDPEARLFNIWGT
VRTNRFDIIEESFKGLQPFWFRVNNASINTSATVKTSDLLGSHLSETEFPPFREFLQVLI
DQLDSEAASITRKSLNNAVRFSKQCLISNAIYGKKTMVIQDEDWSVRIDKALELDDTVVS
RVNEMVQGMNDDIFIRYLTLLSNEFTATNSKGEQIAIFPYQDPIFGSVLLTLLNFVSNNV
LRRLEILVPDLYHLVIMKFQSLSDNDLAVCATIIGIISTAIADSTHVKRITKIAQSQTMA
ETYVASYVVPRLYLKDQTNHIESDSILNLLNILTTHLSHPGTNKDMILKLVCQVTKFGLL
LQVSAQERKDFLKKVMDTIQDKLINDVTAIQTWSYLSLYSTDLENSSLFQEKLLETNVSK
QNDFLFSVGESLSVVAGKWSSKYLIKQIDIPNFNVEIMQQKFPATNVTTILDEIFSGCDS
TKPSLRKASCIWLLSYIQYLGHLPEVSSKCNDIHLRFMRFLADRDEFIQDSAARGLSLVY
EIGGSDLKESMVKGLLKSFTESTAGSASTSATGISGSVSEETELFEPGVLNTGDGSISTY
KDILNLASEVGDPALVYKFMSLAKSSALWSSRKGIAFGLGAIMSKSSLEELLLKDQQTAK
KLIPKLYRYRFDPFQAVSRSMTDIWNTLIPESSLTISLYFNDILDELLCGMANKEWRVRE
ASTSALLQLIQSQPQEKFSEKMLKIWTMAFRTMDDIKDSVREVGTKFTTVLAKILARSID
VEKGVNPTKSKEILDNILPFLWGPHGLNSDAEEVRNFALTTLIDLVKHSPGAIKPFTPKL
IYDFITLFSSIEPQVINYLALNAANYNIDANVIDTQRKNGVTNSPLFQTIEKLINNSDDC
MMEEIINVVIKASRKSVGLPSKVASSLVIIILVKRYSIEMKPYSGKLLKVCLTMFEDRNE
SVNIAFAISMGYLFKVSALDKCIKYSEKLITKYFEPTSTENNKKVVGTAIDSILNYAKSE
FDNVASVFMPLIFIACNDEDKDLETLYNKIWTEASSSGAGTVKLYLPEILNVLCVNIKSN
DFSIRKTCAKSVIQLCGGINDSIPYPQIVKLFDISREALSGRSWDGKEHIVAALVSLTEK
FSQTVADNNDLQESINHVMYTEVSRKSMKYVKKILPLYARYINVNPQEETITFLIEKAKE
MIRLLGSESDDSEGSIKQTSDESTIKRIKPNTEITQKSSKENIENEEYVINLLKVSVDIC
NNSKSRYPMNLLEFIIDEIAYLFHNDRIIHTWRTQLAASEIGISIVGRFSTISSADFIQN

VGRLWDQTFPINCNKETIENVKLQMIKFGGLIIQKIPSLQNNIEENLRLLNSIDSTSRIE
LELKNIGL
>P25443
MSAPEAQQQKRGGFGGRNRGRPNRRGPRNTEEKGWVPVTKLGRLVKAGKITTIEEIFLHS
LPVKEFQIIDTLLPGLQDEVMNIKPVQKQTRAGQRTRFKAVVVVGDSNGHVGLGIKTAKE
VAGAIRAGIIIAKLSVIPIRRGYWGTNLGQPHSLATKTTGKCGSVTVRLIPAPRGSGIVA
SPAVKKLLQLAGVEDVYTQSNGKTRTLENTLKAAFVAIGNTYGFLTPNLWAEQPLPVSPL
DIYSDEASAQKKRF
>O75170
MFWKFDLNTTSHVDKLLDKEHVTLQELMDEDDILQECKAQNQKLLDFLCRQQCMEELVSL
ITQDPPLDMEEKVRFKYPNTACELLTCDVPQISDRLGGDESLLSLLYDFLDHEPPLNPLL
ASFFSKTIGNLIARKTEQVITFLKKKDKFISLVLKHIGTSALMDLLLRLVSCVEPAGLRQ
DVLHWLNEEKVIQRLVELIHPSQDEDRQSNASQTLCDIVRLGRDQGSQLQEALEPDPLLT
ALESQDCVEQLLKNMFDGDRTESCLVSGTQVLLTLLETRRVGTEGLVDSFSQGLERSYAV
SSSVLHGIEPRLKDFHQLLLNPPKKKAILTTIGVLEEPLGNARLHGARLMAALLHTNTPS
INQELCRLNTMDLLLDLFFKYTWNNFLHFQVELCIAAILSHAAREERTEASGSESRVEPP
HENGNRSLETPQPAASLPDNTMVTHLFQKCCLVQRILEAWEANDHTQAAGGMRRGNMGHL
TRIANAVVQNLERGPVQTHISEVIRGLPADCRGRWESFVEETLTETNRRNTVDLVSTHHL
HSSSEDEDIEGAFPNELSLQQAFSDYQIQQMTANFVDQFGFNDEEFADQDDNINAPFDRI
AEINFNIDADEDSPSAALFEACCSDRIQPFDDDEDEDIWEDSDTRCAARVMARPRFGAPH
ASESCSKNGPERGGQDGKASLEAHRDAPGAGAPPAPGKKEAPPVEGDSEGAMWTAVFDEP
ANSTPTAPGVVRDVGSSVWAAGTSAPEEKGWAKFTDFQPFCCSESGPRCSSPVDTECSHA
EGSRSQGPEKASQASYFAVSPASPCAWNVCVTRKAPLLASDSSSSGGSHSEDGDQKAASA
MDAVSRGPGREAPPLPTVARTEEAVGRVGCADSRLLSPACPAPKEVTAAPAVAVPPEATV
AITTALSKAGPAIPTPAVSSALAVAVPLGPIMAVTAAPAMVATLGTVTKDGKTDAPPEGA
ALNGPV
>Q93015
MELILSTSPAELTLDPACQPKLPLDSTCQPEMTFNPGPTELTLDPEHQPEETPAPSLAEL
TLEPVHRRPELLDACADLINDQWPRSRTSRLHSLGQSSDAFPLCLMLLSPHPTLEAAPVV
VGHARLSRVLNQPQSLLVETVVVARALRGRGFGRRLMEGLEVFARARGFRKLHLTTHDQV
HFYTHLGYQLGEPVQGLVFTSRRLPATLLNAFPTAPSPRPPRKAPNLTAQAAPRGPKGPP
LPPPPPLPECLTISPPVPSGPPSKSLLETQYQNVRGRPIFWMEKDI
>O14140
MSRAALPSLENLEDDDEFEDFATENWPMKDTELDTGDDTLWENNWDDEDIGDDDFSVQLQ
AELKKKGVAAN
>O59834
MSSTELSEKDLAYLREAIKVSQQARDEGQHPFGCIIVDENDNVIMSAGNRVPDGDVTQHA
ETRAVGLITKTRRDLEKCTLYTSTEPCAMCSGAIFWSGIRRMIFGLSNENLIKLTQKSGE
CPPLYINSRDILGAASHPIEVVGPYIEDEAIIPHKGFWDGGR
>P12272
MQRRLVQQWSVAVFLLSYAVPSCGRSVEGLSRRLKRAVSEHQLLHDKGKSIQDLRRRFFL
HHLIAEIHTAEIRATSEVSPNSKPSPNTKNHPVRFGSDDEGRYLTQETNKVETYKEQPLK
TPGKKKKGKPGKRKEQEKKKRRTRSAWLDSGVTGSGLEGDHLSDTSTTSLELDSRRH
>O00410

MAAAAAEQQQFYLLLGNLLSPDNVVRKQAEETYENIPGQSKITFLLQAIRNTTAAEEARQ
MAAVLLRRLLSSAFDEVYPALPSDVQTAIKSELLMIIQMETQSSMRKKVCDIAAELARNL
IDEDGNNQWPEGLKFLFDSVSSQNVGLREAALHIFWNFPGIFGNQQQHYLDVIKRMLVQC
MQDQEHPSIRTLSARATAAFILANEHNVALFKHFADLLPGFLQAVNDSCYQNDDSVLKSL
VEIADTVPKYLRPHLEATLQLSLKLCGDTSLNNMQRQLALEVIVTLSETAAAMLRKHTNI
VAQTIPQMLAMMVDLEEDEDWANADELEDDDFDSNAVAGESALDRMACGLGGKLVLPMIK
EHIMQMLQNPDWKYRHAGLMALSAIGEGCHQQMEGILNEIVNFVLLFLQDPHPRVRYAAC
NAVGQMATDFAPGFQKKFHEKVIAALLQTMEDQGNQRVQAHAAAALINFTEDCPKSLLIP
YLDNLVKHLHSIMVLKLQELIQKGTKLVLEQVVTSIASVADTAEEKFVPYYDLFMPSLKH
IVENAVQKELRLLRGKTIECISLIGLAVGKEKFMQDASDVMQLLLKTQTDFNDMEDDDPQ
ISYMISAWARMCKILGKEFQQYLPVVMGPLMKTASIKPEVALLDTQDMENMSDDDGWEFV
NLGDQQSFGIKTAGLEEKSTACQMLVCYAKELKEGFVEYTEQVVKLMVPLLKFYFHDGVR
VAAAESMPLLLECARVRGPEYLTQMWHFMCDALIKAIGTEPDSDVLSEIMHSFAKCIEVM
GDGCLNNEHFEELGGILKAKLEEHFKNQELRQVKRQDEDYDEQVEESLQDEDDNDVYILT
KVSDILHSIFSSYKEKVLPWFEQLLPLIVNLICPHRPWPDRQWGLCIFDDVIEHCSPASF
KYAEYFLRPMLQYVCDNSPEVRQAAAYGLGVMAQYGGDNYRPFCTEALPLLVRVIQSADS
KTKENVNATENCISAVGKIMKFKPDCVNVEEVLPHWLSWLPLHEDKEEAVQTFNYLCDLI
ESNHPIVLGPNNTNLPKIFSIIAEGEMHEAIKHEDPCAKRLANVVRQVQTSGGLWTECIA
QLSPEQQAAIQELLNSA
>P32461
MEVAPALSTTQSDVAFQKVETHEIDRSSYLGPCYNSDELMQLISAYYNVEPLVGYLEQHP
EYQNVTLQFPDDLIKDSSLIVRLLQSKFPHGKIKFWVLADTAYSACCVDEVAAEHVHAEV
VVHFGDACLNAIQNLPVVYSFGTPFLDLALVVENFQRAFPDLSSKICLMANAPFSKHLSQ
LYNILKGDLHYTNIIYSQVNTSAVEEKFVTILDTFHVPEDVDQVGVFEKNSVLFGQHDKA
DNISPEDYHLFHLTTPQDPRLLYLSTVFQSVHIFDPALPGMVTGPFPSLMRRYKYMHVAR
TAGCIGILVNTLSLRNTRETINELVKLIKTREKKHYLFVVGKPNVAKLANFEDIDIWCIL
GCSQSGIIVDQFNEFYKPIITPYELNLALSEEVTWTGKWVVDFRDAIDEIEQNLGGQDTI
SASTTSDEPEFDVVRGRYTSTSRPLRALTHLELEAADDDDSKQLTTRHTASGAVIKGTVS
TSASALQNRSWKGLGSDFDSTEVDNTGADIEEGISGVARGYGFDREDAMKKENK
>O14744
MAAMAVGGAGGSRVSSGRDLNCVPEIADTLGAVAKQGFDFLCMPVFHPRFKREFIQEPAK
NRPGPQTRSDLLLSGRDWNTLIVGKLSPWIRPDSKVEKIRRNSEAAMLQELNFGAYLGLP
AFLLPLNQEDNTNLARVLTNHIHTGHHSSMFWMRVPLVAPEDLRDDIIENAPTTHTEEYS
GEEKTWMWWHNFRTLCDYSKRIAVALEIGADLPSNHVIDRWLGEPIKAAILPTSIFLTNK
KGFPVLSKMHQRLIFRLLKLEVQFIITGTNHHSEKEFCSYLQYLEYLSQNRPPPNAYELF
AKGYEDYLQSPLQPLMDNLESQTYEVFEKDPIKYSQYQQAIYKCLLDRVPEEEKDTNVQV
LMVLGAGRGPLVNASLRAAKQADRRIKLYAVEKNPNAVVTLENWQFEEWGSQVTVVSSDM
REWVAPEKADIIVSELLGSFADNELSPECLDGAQHFLKDDGVSIPGEYTSFLAPISSSKL
YNEVRACREKDRDPEAQFEMPYVVRLHNFHQLSAPQPCFTFSHPNRDPMIDNNRYCTLEF
PVEVNTVLHGFAGYFETVLYQDITLSIRPETHSPGMFSWFPILFPIKQPITVREGQTICV
RFWRCSNSKKVWYEWAVTAPVCSAIHNPTGRSYTIGL
>Q6ZVD7
MARPVQLAPGSLALVLCRLEAQKAAGAAEEPGGRAVFRAFRRANARCFWNARLARAASRL
AFQGWLRRGVLLVRAPPACLQVLRDAWRRRALRPPRGFRIRAVGDVFPVQMNPITQSQFV

PLGEVLCCAISDMNTAQIVVTQESLLERLMKHYPGIAIPSEDILYTTLGTLIKERKIYHT
GEGYFIVTPQTYFITNTTTQENKRMLPSDESRLMPASMTYLVSMESCAESAQENAAPISH
CQSCQCFRDMHTQDVQEAPVAAEVTRKSHRGLGESVSWVQNGAVSVSAEHHICESTKPLP
YTRDKEKGKKFGFSLLWRSLSRKEKPKTEHSSFSAQFPPEEWPVRDEDDLDNIPRDVEHE
IIKRINPILTVDNLIKHTVLMQKYEEQKKYNSQGTSTDMLTIGHKYPSKEGVKKRQGLSA
KPQGQGHSRRDRHKARNQGSEFQPGSIRLEKHPKLPATQPIPRIKSPNEMVGQKPLGEIT
TVLGSHLIYKKRISNPFQGLSHRGSTISKGHKIQKTSDLKPSQTGPKEKPFQKPRSLDSS
RIFDGKAKEPYAEQPNDKMEAESIYINDPTVKPINDDFRGHLFSHPQQSMLQNDGKCCPF
MESMLRYEVYGGENEVIPEVLRKSHSHFDKLGETKQTPHSLPSRGASFSDRTPSACRLVD
NTIHQFQNLGLLDYPVGVNPLRQAARQDKDSEELLRKGFVQDAETTSLENEQLSNDDQAL
YQNEVEDDDGACSSLYLEEDDISENDDLRQMLPGHSQYSFTGGSQGNHLGKQKVIERSLT
EYNSTMERVESQVLKRNECYKPTGLHATPGESQEPNLSAESCGLNSGAQFGFNYEEEPSV
AKCVQASAPADERIFDYYSARKASFEAEVIQDTIGDTGKKPASWSQSPQNQEMRKHFPQK
FQLFNTSHMPVLAQDVQYEHSHLEGTENHSMAGDSGIDSPRTQSLGSNNSVILDGLKRRQ
NFLQNVEGTKSSQPLTSNSLLPLTPVINV
>P32189
MAASKKAVLGPLVGAVDQGTSSTRFLVFNSKTAELLSHHQVEIKQEFPREGWVEQDPKEI
LHSVYECIEKTCEKLGQLNIDISNIKAIGVSNQRETTVVWDKITGEPLYNAVVWLDLRTQ
STVESLSKRIPGNNNFVKSKTGLPLSTYFSAVKLRWLLDNVRKVQKAVEEKRALFGTIDS
WLIWSLTGGVNGGVHCTDVTNASRTMLFNIHSLEWDKQLCEFFGIPMEILPNVRSSSEIY
GLMKAGALEGVPISGCLGDQSAALVGQMCFQIGQAKNTYGTGCFLLCNTGHKCVFSDHGL
LTTVAYKLGRDKPVYYALEGSVAIAGAVIRWLRDNLGIIKTSEEIEKLAKEVGTSYGCYF
VPAFSGLYAPYWEPSARGIICGLTQFTNKCHIAFAALEAVCFQTREILDAMNRDCGIPLS
HLQVDGGMTSNKILMQLQADILYIPVVKPSMPETTALGAAMAAGAAEGVGVWSLEPEDLS
AVTMERFEPQINAEESEIRYSTWKKAVMKSMGWVTTQSPESGIP
>P36156
MSKQWASGTNGAFKRQVSSFRETISKQHPIYKPAKGRYWLYVSLACPWAHRTLITRALKG
LTSVIGCSVVHWHLDEKGWRFLDMEKQLEDSEDFLEHWHDVAGGIRTAKEDSSKSFAEIK
NDSQRFMVDATNEPHYGYKRISDLYYKSDPQYSARFTVPVLWDLETQTIVNNESSEIIRI
LNSSAFDEFVDDDHKKTDLVPAQLKTQIDDFNSWVYDSINNGVYKTGFAEKAEVYESEVN
NVFEHLDKVEKILSDKYSKLKAKYGEEDRQKILGEFFTVGDQLTEADIRLYTTVIRFDPV
YVQHFKCNFTSIRAGYPFIHLWVRNLYWNYDAFRYTTDFDHIKLHYTRSHTRINPLGITP
LGPKPDIRPL
>Q6PRD7
MGTSSTDSQQAGHRRCSTSNTSAENLTCLSLPGSPGKTAPLPGPAQAGAGQPLPKGCAAV
KAEVGIPAPHTSQEVRIHIRRLLSWAAPGACGLRSTPCALPQALPQARPCPGRWFFPGCS
LPTGGAQTILSLWTWRHFLNWALQQREENSGRARRVPPVPRTAPVSKGEGSHPPQNSNGE
KVKTITPDVGLHQSLTSDPTVAVLRAKRAPEAHPPRSCSGSLTARVCHMGVCQGQGDTED
GRMTLMG
>Q06349
MSTEQDAVLGLAKDLEGINLLTVPNLERGHQSKLCKEKTTSDSSSSRKPSQQRDNYRKRR
PKLICIPYTSFLHTGMHNFLTKPPRDIFHESKEVALFTNGRAYTILRKDLIPNLKESIAE
LYESSLLEAKKRKVPYLGHDLFANIDEFVPMTISELDSVSPCFSYIENWILDNPGKDFKI
GKKFTVVTTRHHIVDLTMHLFNRRNRQTSLIVTYMGAGLLSFCRNVKKDSQMSKEGIYSN

DPNMKKICYSGFEFENWVTENSKVADLTGSKCPIFSLVESKLSEEIGLLIRCEMDAFNPV
SETNTELKCFAPLSMHNSNHRRKLLKTWVQTGLLPNSDIMIGLRDSHSGQLLDIQWYSRD
LLCKKFNHPGLPTNKKELNYNAQIAVEWCHYCIEAICKLVEANISDYSSTKPESFEIGID
TNNAIVITKLKTTPRNVELFGM
>O15287
MSRQTTSVGSSCLDLWREKNDRLVRQAKVAQNSGLTLRRQQLAQDALEGLRGLLHSLQGL
PAAVPVLPLELTVTCNFIILRASLAQGFTEDQAQDIQRSLERVLETQEQQGPRLEQGLRE
LWDSVLRASCLLPELLSALHRLVGLQAALWLSADRLGDLALLLETLNGSQSGASKDLLLL
LKTWSPPAEELDAPLTLQDAQGLKDVLLTAFAYRQGLQELITGNPDKALSSLHEAASGLC
PRPVLVQVYTALGSCHRKMGNPQRALLYLVAALKEGSAWGPPLLEASRLYQQLGDTTAEL
ESLELLVEALNVPCSSKAPQFLIEVELLLPPPDLASPLHCGTQSQTKHILASRCLQTGRA
GDAAEHYLDLLALLLDSSEPRFSPPPSPPGPCMPEVFLEAAVALIQAGRAQDALTLCEEL
LSRTSSLLPKMSRLWEDARKGTKELPYCPLWVSATHLLQGQAWVQLGAQKVAISEFSRCL
ELLFRATPEEKEQGAAFNCEQGCKSDAALQQLRAAALISRGLEWVASGQDTKALQDFLLS
VQMCPGNRDTYFHLLQTLKRLDRRDEATALWWRLEAQTKGSHEDALWSLPLYLESYLSWI
RPSDRDAFLEEFRTSLPKSCDL
>P09353
MSVCKGVSGNPAKGEVFLYKHVNFQGDSWKVTGNVYDFRSVSGLNDVVSSVKVGPNTKAF
IFKDDRFNGNFIRLEESSQVTDLTTRNLNDAISSIIVATFESA
>P62627
MAEVEETLKRLQSQKGVQGIIVVNTEGIPIKSTMDNPTTTQYANLMHNFILKARSTVREI
DPQNDLTFLRIRSKKNEIMVAPDKDYFLIVIQNPTE
>Q9UKA4
MATFRNNHMKTKASVRKSFSEDVFQSVKSLLQSQKELCSVTAEDCLQQDEHANLTEVTFL
GFNEETDAAHIQDLAAVSLELPDILNSLHFCSLNENEIICMKNINKPLDISSDPLNQSHP
SGMLCVMRVSPTSPRLRIDFIFSLLSKYATGIRYTLDTFLHQKHQLETTDEDDDDTNQSV
SSIEDDFVTAFEHLEEEETSKPYNDGMNITVLRSQCDAASQTVTGHHLETHDLKILISSG
QQKSLAKPSTSSVNVLGHKELPSVKTSVTTSISEPWTQRSFYRSSNASDKDSDLQKTFFS
SSPAYSSESECSSPSPVIFLDEEGYQKSLKAKLELPKIPVMKDDIEDSDSEVSEFFDSFD
QFDELEQTLETCLFNKDPVIGKSSQRKGHKHGKSCMNPQKFKFDRPALPANVRKPTPRKP
ESPYGNLCDAPDSPRPVKASREDSGLFSPIRSSAFSPLGGCTPAECFCQTDIGGDRIHEN
HDSVYYTYEDYAKSISCEVLGSVLRTHHTNTLSNINSIKHGENKTVTFKHGNLDQKNKSK
NKSLMIKDSIQKFAADLVEKSFGSAFKDLQKGVSSCTNALYHLAIKLTSSVLQMAFDELR
RQRAFSLKERAISGLANFLVSEALSNALKDLQYVKKQIFTNTVARFAADLAEELVFEGIM
EVCQFSYPQTPASPQCGSFDFEDKVVKLYAKDLSESVIQEAFIELSQVDVTFTTKAAVSV
STDNIKYVSAESVVPSTQAVTFSPSFHNQAIMVTKPVQEYKKEYTVQQALFCTSGIVTSI
PVPLAGSALLPYHISSTACQAKAHLSSDDSNSNGDSAQVHIATKNREEKAACLRNICLPS
EHNPGNQNDFKPTNDDIEMQSSSKLPNDPAIISNFSAAVVHTIVNETLESMTSLEVTKMV
DERTDYLTKSLKEKTPPFSHCDQAVLQCSEASSNKDMFADRLSKSIIKHSIDKSKSVIPN
IDKNAVYKESLPVSGEESQLTPEKSPKFPDSQNQLTHCSLSAAKDCVPECKVSMVHGSSL
ETLPSCPAVTGQKSDLKESAKDQPLKKHNLNSTSLEALSFGQENPFPHSHTFSSTALTCV
DGLHVEDKQKVRDRNVIPDTPPSTPLVPSRASSEWDIKKLTKKLKGELAKEFAPATPPST
PHNSSVGSLSENEQNTIEKEEFMLKLMRSLSEEVESSESGELPEVDVKSEHSGKKVQFAE
ALATHILSLATEMAASHLDNKIIQEPKVKNPCLNVQSQRSVSPTFLNPSDENLKTLCNFA

GDLAAEVITEAEKIAKVRNCMLFKQKKNSCYADGDEDYKVEEKLDIEAVVHPREVDPFIL
SLPPSSCMSGLMYKYPSCESVTDEYAGHLIQILKQEGGNSELIMDQYANRLAYRSVKSGL
QEAAKTTKVQCNSRMFPVPSSQVKTNKELLMFSNKEHHQEADKKRQSKRNEGYFCKNQTC
ERTLDPYRNEVSQLYSFSTSLVHSITKDAKEELTASLVGLPKSLTDSCLFEKSGYEEDNE
CHVTPELPKSLQPSSQNHRFYHSTGSLNGYGCGDNVVQAVEQYAKKVVDDTLELTLGSTV
FRVSETTKSADRVTYAEKLSPLTGQACRYCDLKELHNCTGNSSQHFFRQGSLASSKPASN
PKFSSRYQKSRIFHLSVPQIHVNLDKKAVLAEKIVAEAIEKAERELSSTSLAADSGIGQE
GASFAESLATETMTAAVTNVGHAVSSSKEIEDFQSTESVSSQQMNLSIGDDSTGSWSNLS
FEDEHQDESSSFHHLSESNGNSSSWSSLGLEGDLYEDNLSFPTSDSDGPDDKDEEHEDEV
EGLGQDGKTLLITNIDMEPCTVDPQLRIILQWLIASEAEVAELYFHDSANKEFMLLSKQL
QEKGWKVGDLLQAVLQYYEVMEKASSEERCKSLFDWLLENA
>Q8C0E2
MSFFGFGQSVEVEILLNDAESRKRAEHKTEDGKKEKYFLFYDGETVSGKVSLSLKNPNKR
LEHQGIKIEFIGQIELYYDRGNHHEFVSLVKDLARPGEITQSQAFDFEFTHVEKPYESYT
GQNVKLRYFLRATISRRLNDVVKEMDIVVHTLSTYPELNSSIKMEVGIEDCLHIEFEYNK
SKYHLKDVIVGKIYFLLVRIKIKHMEIDIIKRETTGTGPNVYHENDTIAKYEIMDGAPVR
GESIPIRLFLAGYELTPTMRDINKKFSVRYYLNLVLIDEEERRYFKQQEVVLWRKGDIVR
KSMSHQAAIASQRFEGTTSLGEVRTPGQLSDNNSRQ
>Q8IQG1
MNWAISNLTGGNRVSRAILVRYSSVPLADATNDGSSTAPQTPTASTPRPSSSHSSLSSTF
AATFLHASSRHIPGRAVPRQNANGQNGGKGNASGAGGGAGGGGAGGASGGTGGTGAQAAG
QLSLPLDVEETVTCFCRKARRKERDGDQNSTDTKLYLEESVLERKLPEADLKALVDLPAG
LDYNEWLASHTLALFEHVNLVYGTISEFCTQSGCADMTGPGNRTYLWFDEKGKKTRVAAP
QYIDYVMTFTQKTVSDESIFPTKYANEFPGSFESIARKILRLQFHVIAHLYAAHFREIAL
LGLHTHLNLTFAHLTALHRRFNLIDEKETDVLRDLEVALRLTDDTGGCQDATSTTSSVHD
HSHSGDLQHQSLQQQQHHNSSSNSTSSAEAFHVNSQSNNGSTSASASVSLIDGDAVAPP
ICTQPEAGAGCKPAGSSGLLGGILGDLTSGEFGDTTRYCTSAVPQAAAAAGAGVGGTAIG
ATDAAALNNGAGALHLNFSNNNNNNHNLNHHHHHHHHHGHHGHHHAAQQQQQHSGLIQCN
AAGGGGNATGVATGGATAAASSTTTA
>Q9UBG3
MPQLLQNINGIIEAFRRYARTEGNCTALTRGELKRLLEQEFADVIVKPHDPATVDEVLRL
LDEDHTGTVEFKEFLVLVFKVAQACFKTLSESAEGACGSQESGSLHSGASQELGEGQRSG
TEVGRAGKGQHYEGSSHRQSQQGSRGQNRPGVQTQGQATGSAWVSSYDRQAESQSQERIS
PQIQLSGQTEQTQKAGEGKRNQTTEMRPERQPQTREQDRAHQTGETVTGSGTQTQAGATQ
TVEQDSSHQTGRTSKQTQEATNDQNRGTETHGQGRSQTSQAVTGGHAQIQAGTHTQTPTQ
TVEQDSSHQTGSTSTQTQESTNGQNRGTEIHGQGRSQTSQAVTGGHTQIQAGSHTETVEQ
DRSQTVSHGGAREQGQTQTQPGSGQRWMQVSNPEAGETVPGGQAQTGASTESGRQEWSST
HPRRCVTEGQGDRQPTVVGEEWVDDHSRETVILRLDQGNLHTSVSSAQGQDAAQSEEKRG
ITARELYSYLRSTKP
>Q792Q4
MVCEKCEKKLGRVITPDTWKDGARNTTESGGRKLNENKALTSKKARFDPYGKNKFSTCRI
CKSSVHQPGSHYCQGCAYKKGICAMCGKKVLDTKNYKQTSV
>P80196
MMKSQKKLLIKIIVVQCLLVLCVTSQDFDFFYFVQQWPASYCDTRRSCCYPTTGKPDEDF

SIHGLWPNYKDGKWPQNCDRESSLDESEFSDLISTMEKNWPSLACPSSDGLKFWSHEWLK
HGTCSALNQHAYFQTALDFKTKSNLLQNLNNAGIKPRNGDYYGVESIKKAIEKGVGHTPF
IECNVDSQGNHQLYQVYLCVDSSASKFIDCPIFPHGGKCGSKIEFPSFSTNDDHDEF
>P53030
MSKITSSQVREHVKELLKYSNETKKRNFLETVELQVGLKNYDPQRDKRFSGSLKLPNCPR
PNMSICIFGDAFDVDRAKSCGVDAMSVDDLKKLNKNKKLIKKLSKKYNAFIASEVLIKQV
PRLLGPQLSKAGKFPTPVSHNDDLYGKVTDVRSTIKFQLKKVLCLAVAVGNVEMEEDVLV
NQILMSVNFFVSLLKKNWQNVGSLVVKSSMGPAFRLY
>Q9VXY7
MASAEQKHATGNGTTGNGTAMNDVDQPKDAKDLLPHLESLERIIKLPVVNAAWDKSQDVY
GKVKGKNRVFEWALTAAEDCVTRAVTTAAPFVTKLDRPIAYVDQTLVKGIDKLEVKAPII
KDTPQEIYNQAKSKVIDVVQPHLERVVKFKAAGQQKAASLKDLAWQKANEVLATQYGSLA
VNGVDTTTALAERLLEYYFPKCESDVEEDNDDKQNAVVQNGKSSENDMPVPASEDPVLHT
VQTVGRLSNKISRRVYRNVSRQIKQVQKGNINDYLSSLIAALKLHQYINFINSSMGTNVE
QSGGSSSDACSPFGTTTSTTTTTTTSSTSNNKPVVALPHVAKSKRAPAVSSQ
>P53307
MYMIVVKYLYALCSSFFDCILNFNETVFGPSHRAFNPNNIIFIVDFQNFNILDSNMLVSH
MTRHLSSWQYPTWVLVLTIRTTVSMHNRCTMRCSQTLESIPFHHTSKTFAFAD
>Q9P3U1
MGMGSKLDVSSSLNSSSSNMDRDRSESPKRHVNGSNSLKRGLHFDGEHTERDPHLGNGQK
YTQQERRVYVGNLSYQVRWFELKEFMGQVGNVLNCEILNLPNGLSKGCAIIEYSTAEEAR
TAIKTLSNQKFMGRLVYIREDREQNARFGSSSVSPSASSNGKDSEPDRQLFVGNVSSISN
PLYSFPRTLLPYNVRWQDLKDLFRQAGSVIRADIQMNQEGRSRGIGIVVMSSMKEAMHAI
QMLHNTDFMGRTLEVRLDRFAHHKIEDLVYHAYPHGPCSDCIYVGNLPWATSDRNLLDLF
TDIGSVIRARIAYEPTGRSKGFGVVQFENENDAASSIEKLNGYRYGGRPLQLSYAHYATP
LPAVPTVLSGLPSVAYTPPVMHIPTTNSPLTPQSFTNSFLGYLPISMPVANMPAIAPISV
ALPENNIEHSLEPS
>Q52EB3
MADRSARRARPGDDSVGQFVIGAEIGKGSFAQVYMGKHKVSGAAVAIKSVELARLNKKLK
ENLYGEINILKTLRHPHIVALHDCVESATHINLMMEYCELGDLSLFIKKREKLSTNPATH
DMARKYPNVPNSGLNEVVIRHFLKQLSSALKFLRESNLVHRDVKPQNLLLLPSPEFREVN
KLARPILTASQDSLVPVAGLASLPMLKLADFGFARVLPSTSLAETLCGSPLYMAPEILRY
ERYDAKADLWSVGTVLFEMIVGRPPFRASNHVELLRKIEAAEDVIKFPRETTISSEMKGL
TRALLKRNPVERISFENFFAHPVIISSIPGLVEDDIPKPEASEQRSSSKDTRAASKSDDP
IASPRKYSFRRHPTDNDQIRDQFRRVEPPSSAAESAPSRQTSAFSGIAAEARKQAAAEAS
ARTGQSSRNEPGDNLVPRRPQAQPSTSAPSKPGLYEERRRGISNASLNRSNRESSSPTSA
ALANDSARAPPQQTSRRVQAEEREKAAQDVAFERDYVVVEKKHVEVNAFADEMAANPRLG
GQGTPLSPKSGQIVRRATQQGNPTSTTGAIPAAPSRTMQIAQGTQRHYHERGTSLSASPG
STASFITKAIQDASLRLLGIKYPAGLTKGASPPELYNPYPAYPTPSPPVGLISSGKQSTP
VDEDARVAQLIEEHATRSDVVYGFAEVKYKQLVPLAPSAEHGLGGTTLEDMPTGEEDVLT
VEAIVSLSEEALVLYVKALTLLAKSMDIASLWWARKNRGDQANNALSSTRDSVNTQTLSL
RINSAVQWVRSRFNEVLEKAEVVRLRLMDAQKRLPDDHPSHPSNHPQGSESVNGASAEGV
FLTVGVTAEKLMYDRALEMSRTAAINEITNEDLAGCEISYVTAIRMLEAVLDNDEDAPKR
RLSANMDDSGGDGEDGHAEINADDQQAVQKMIHMIRSRLTSVRSKVRMISNASKAQQQSQ

PQSLIRRRSGDVTPRSVPSYSS
>P41262
MSSGLTGPQKAALKSSWSRFMDNAVTNGTNFYMDLFKAYPDTLTPFKSLFEDVSFNQMTD
HPTMKAQALVFCDGMSSFVDNLDDHEVLVVLLQKMAKLHFNRGIRIKELRDGYGVLLRYL
EDHCHVEGSTKNAWEDFIAYICRVQGDFMKERL
>Q06704
MFKQLSQIGKNLTDELAKGLADDMSPTPSEQQIEDDKSGLPKEIQAKLRKFEKYEQKYPL
LLSAYKNEKLKSEKLEAVEKILAENTPISNIDDAVDTLPAFFQDLNNKNNLLNDEIKRLT
KQNSEIPESASSETLKDKEEEFLKKEQNYKNDIDDLKKKMEALNIELDTVQKEKNDTVSG
LREKIVALENILKEEREAKKQKEEVSISELKEELAIKNHSLEDSRMKITELEQNLSSKST
IMEEKSSELAELNITLKEKERKLSELEKKMKELPKAISHQNVGNNNRRKKNRNKGKKNKG
GITTGDISEEETVDNSINTEEYDKLKENLQELQEKYKDCEDWKQKYEDIEAELKDAKELE
NSQLEKSAKELETLNTELIDTKKSLKEKNSELEEVRDMLRTVGNELVDAKDEIKESSSKQ
NEEVKTVKLELDDLRHKNATMIEAYEAKNTELRSKIELLSKKVEHLKNLCTEKEKEQTTS
QNKVAKLNEEISQLTYEKSNITKELTSLRTSYKQKEKTVSYLEEQVKQFSEQKDVAEKST
EQLRKDHAKISNRLDLLKKENETLHNDIAKNSNSYEEYLKENGKLSERLNILQEKYNTLQ
NVKSNSNEHIDSIKRQCEELNVKLKESTKKILSLEDELNEYANIVQDKTREANTLRRLVS
DSQTDDSSKQKELENKLAYLTDEKNKLEAELDLQTSRKATELQEWKHTVTELKSEIHALK
LREEGLKSEVDALKHVNNDIKRKTQATSDDSDQLEQITSNLKLSLSKADEKNFELQSANE
KLLNLNNELNKKFDRLLKNYRSLSSQLNALKERQYSDKSGRVSRSGSIGTLANANIDSSP
ANNSNPTKLEKIRSSSSLELDSEKNEKIAYIKNVLLGFLEHKEQRNQLLPVISMLLQLDS
TDEKRLVMSLK
>O42845
MIESELATSRWSLMLEADIATQTTRSLTNELSFIVAGLRPSTKESVLHFLELIFINLKYQ
SKKWLYSLVICKSLIALLGRKRLSANVRKIVRFLNVIICVIGLWKGLSAMSGKNTFINGL
QSYLISETALPELGSFQELSTSSLGSFRMFQQIAVGFVDALFCTRIPASLWIKYKEYTTS
AETTVPQECGLCMMCVQRGDERVAITTPYTTDCGHTYCYACIMSRLKLVNNVSCPICKHR
IRFALPDQTMG
>P36132
MVNLNTIPPKNGRDYYIALGLEGSANKLGVGIVKHPLLPKHANSDLSYDCEAEMLSNIRD
TYVTPPGEGFLPRDTARHHRNWCIRLIKQALAEADIKSPTLDIDVICFTKGPGMGAPLHS
VVIAARTCSLLWDVPLVGVNHCIGHIEMGREITKAQNPVVLYVSGGNTQVIAYSEKRYRI
FGETLDIAIGNCLDRFARTLKIPNEPSPGYNIEQLAKKAPHKENLVELPYTVKGMDLSMS
GILASIDLLAKDLFKGNKKNKILFDKTTGEQKVTVEDLCYSLQENLFAMLVEITERAMAH
VNSNQVLIVGGVGCNVRLQEMMAQMCKDRANGQVHATDNRFCIDNGVMIAQAGLLEYRMG
GIVKDFSETVVTQKFRTDEVYAAWRD
>P70195
MAAVSVFQPPVGGFSFDNCRRNAVLEADFAKKGFKLPKARKTGTTIAGVVYKDGIVLGAD
TRATEGMVVADKNCSKIHFISPNIYCCGAGTAADTDMTTQLISSNLELHSLTTGRLPRVV
TANRMLKQMLFRYQGYIGAALVLGGVDVTGPHLYSIYPHGSTDKLPYVTMGSGSLAAMAV
FEDKFRPDMEEEEAKKLVSEAIAAGIFNDLGSGSNIDLCVISKSKLDFLRPFSVPNKKGT
RLGRYRCEKGTTAVLTEKVTPLEIEVLEETVQTMDTS
>Q9UJ41
MVVVTGREPDSRRQDGAMSSSDAEDDFLEPATPTATQAGHALPLLPQERCAEFPALRGPP

TQGACSSCVQRGPVLCHRAPPGAAGEHAATEGREGAPSVSGTHALLQRPLGADCGDRPAA
CGPAEGPLCQAQVVSRKKMSLKSERRGIHVDQSDLLCKKGCGYYGNPAWQGFCSKCWREE
YHKARQKQIQEDWELAERVLLCCPGWSAMVQFQLTATSASWAQVILLLQPPKWLGLQKLQ
REEEEAFASSQSSQGAQSLTFSKFEEKKTNEKTRKVTTVKKFFSASSRVGSKKEIQEAKA
PSPSINRQTSIETDRVSKEFIEFLKTFHKTGQEIYKQTKLFLEGMHYKRDLSIEEQSECA
QDFYHNVAERMQTRGKERRFHHVGQAGLELLTSGDPPASASQSAGNTGVEPPHPAVPPER
VEKIMDQIEKYIMTRLYKYVFCPETTDDEKKDLAIQKRIRALRWVTPQMLCVPVNEDIPE
VSDMVVKAITDIIEMDSKRVPRDKLACITKCSKHIFNAIKITKNEPASADDFLPTLIYIV
LKGNPPRLQSNIQYITRFCNPSRLMTGEDGYYFTNLCCAVAFIEKLDAQSLNLSQEDFDR
YMSGQTSPRKQEAESWSPDACLGVKQMYKNLDLLSQLNERQERIMNEAKKLEKDLIDWTD
GIAREVQDIVEKYPLEIKPPNQPLAAIDSENVENDKLPPPLQPQVYAG
>Q01846
MPKFPQFRLILVLFYLISMIQWSVITFSLGFFLNVCIFAYFVFFKSLPDLPKPQPRFVDI
VPESSNTVDVDKELKSVEGLIQDGNAQIGKELESIVNLIIKDFVQPWFTKIDKNSDAEFL
KVIKWRLLQTLLVVKDKLMKNDSASLIVLKLLPIFNKHFSTFCDAREAVLSDLTLERHKS
ANIDLQIAVEFNKNYKIHKSLSLKPNALQKEIEKSIRKTVIGLLPHLFDNDELDSLLVFT
LMTEVLTTCIISPLIFKFTDPDSWNLRIVSLSQNYFEEKHKVHKIRRMLSKELQDHRKVM
NDVANKDVGEPSSEKLELNAEYTGKQFEHYLNQLDSLLDLSDIKYVAYSLALKIYQLKEN
EHLTKENLKYKKRLLLSLNLIESKLSFPGSEIDTASKKLAREANYPDLNMDNGIVLKEMA
SFLTSITLKDIVDDSEFLPFFESFLGSVPETQGSTFLEYSQTIESFKNPLEDATSEDIIS
GYSGISTMQLQEISSKFFHNNNLQNMKLLDEGLVKNIILFRNSFQINNDEDTFILARKSV
LLLQTEAIKYLDDRFLPLFKKTPSFLKMLSTSHIISTDIYAHFLSRIGGVNNPEQNKIIK
DNVKTDFMNPVRIFANPGITDALDNIVNGSGSKPHKSRISSNPRYSQLFGSENDNIFKDK
LFDDENDNTSEISVVEDQLDHPRNMEKVSVSSGNSGLNPSQFYGSNNFRDNIASLTISID
QIEKELELLRHLILKADLTNNQMQLKILKKSQRTLLKELEMKELLKQQYMVQENGNSLFR
KTKIYIRSYFSENSSNGLKEITYYIINIHHFNNGQVSSWDMARRYNEFFELNTYLKKNFR
DLMRQLQDLFPSKVKMSLKYHVTKTLLYEERKQKLEKYLRELLSISEICEDNIFRRFLTD
PTPFKLNKEYMHDDILEEPLHEPIGSSNSTSNSSSVVDLQSSEDGGELNFYEDERHFFTD
SGYPFYSQNKSFVKQICDLFISLFALNKANAGWLRGRAIITVLQQLLGSTIEKYIKVSIQ
KLRSEDQVFEAIVTFKNMLWGDNGLFERKRNETAEATRSEGERLRTEQLALTSLQRLFAD
TCGRVVGLRDSHEAAGRVHAMLQNPYLNASLLLEALDAILLDIICND
>O74389
MPSLNQEETPKKTKNPYRKGHSRRQSRHSIPSDELNSNSSKSLNNDRISRTIENSLQKET
NSDNKIKKLSKTKSNPTNPKSEEDPSQICQSFDESNIITPSKYLFPLSKDYSPNEGLFQT
DMNEKNPHDLWNPTVPMAYLSRSLQGLTISKINETEQKFPLSSVHAYVEIGEGMFSMEIV
TEQLRLLGATVHASLQMTEENPVSHAVLYDCSQETLKKISSAQVPIVCVSPKWVLACYSS
KYLVDEEMYLVDPQDILQTKCLQTDDSELLNPLDPFTDTQMIQDPSDAFFFDTQAENLEP
FVNGSPDSHCFNADSFLDQTLSDDYEFGLITCSDSNESSMSNDAQRENNPETISQISLSP
TADLFSSFKITTDLEMTHLESAKKKYLAYKPLIGSPLKKKVSLIDF
>Q99J83
MTDDKDVLRDVWFGRIPTCFTLYQDEITEREAEPYYLLLPRVSYLTLVTDKVKKHFQKVM
RQEDVSEIWFEYEGTPLKWHYPIGLLFDLLASSSALPWNITVHFKSFPEKDLLHCPSKDA
VEAHFMSCMKEADALKHKSQVINEMQKKDHKQLWMGLQNDRFDQFWAINRKLMEYPPEEN
GFRYIPFRIYQTTTERPFIQKLFRPVAADGQLHTLGDLLREVCPSAVAPEDGEKRSQVMI

HGIEPMLETPLQWLSEHLSYPDNFLHISIVPQPTD
>Q9Y239
MEEQGHSEMEIIPSESHPHIQLLKSNRELLVTHIRNTQCLVDNLLKNDYFSAEDAEIVCA
CPTQPDKVRKILDLVQSKGEEVSEFFLYLLQQLADAYVDLRPWLLEIGFSPSLLTQSKVV
VNTDPVSRYTQQLRHHLGRDSKFVLCYAQKEELLLEEIYMDTIMELVGFSNESLGSLNSL
ACLLDHTTGILNEQGETIFILGDAGVGKSMLLQRLQSLWATGRLDAGVKFFFHFRCRMFS
CFKESDRLCLQDLLFKHYCYPERDPEEVFAFLLRFPHVALFTFDGLDELHSDLDLSRVPD
SSCPWEPAHPLVLLANLLSGKLLKGASKLLTARTGIEVPRQFLRKKVLLRGFSPSHLRAY
ARRMFPERALQDRLLSQLEANPNLCSLCSVPLFCWIIFRCFQHFRAAFEGSPQLPDCTMT
LTDVFLLVTEVHLNRMQPSSLVQRNTRSPVETLHAGRDTLCSLGQVAHRGMEKSLFVFTQ
EEVQASGLQERDMQLGFLRALPELGPGGDQQSYEFFHLTLQAFFTAFFLVLDDRVGTQEL
LRFFQEWMPPAGAATTSCYPPFLPFQCLQGSGPAREDLFKNKDHFQFTNLFLCGLLSKAK
QKLLRHLVPAAALRRKRKALWAHLFSSLRGYLKSLPRVQESFNQVQAMPTFIWMLRCIY
ETQSQKVGQLAARGICANYLKLTYCNACSADCSALSFVLHHFPKRLALDLDNNNLNDYGV
RELQPCFSRLTVLRLSVNQITDGGVKVLSEELTKYKIVTYLGLYNNQITDVGARYVTKIL
DECKGLTHLKLGKNKITSEGGKYLALAVKNSKSISEVGMWGNQVGDEGAKAFAEALRNHP
SLTTLSLASNGISTEGGKSLARALQQNTSLEILWLTQNELNDEVAESLAEMLKVNQTLKH
LWLIQNQITAKGTAQLADALQSNTGITEICLNGNLIKPEEAKVYEDEKRIICF
>Q9Y4X5
MDSDEGYNYEFDEDEECSEEDSGAEEEEDEDDDEPDDDTLDLGEVELVEPGLGVGGERDG
LLCGETGGGGGSALGPGGGGGGGGGGGGGGPGHEQEEDYRYEVLTAEQILQHMVECIREV
NEVIQNPATITRILLSHFNWDKEKLMERYFDGNLEKLFAECHVINPSKKSRTRQMNTRSS
AQDMPCQICYLNYPNSYFTGLECGHKFCMQCWSEYLTTKIMEEGMGQTISCPAHGCDILV
DDNTVMRLITDSKVKLKYQHLITNSFVECNRLLKWCPAPDCHHVVKVQYPDAKPVRCKCG
RQFCFNCGENWHDPVKCKWLKKWIKKCDDDSETSNWIAANTKECPKCHVTIEKDGGCNHM
VCRNQNCKAEFCWVCLGPWEPHGSAWYNCNRYNEDDAKAARDAQERSRAALQRYLFYCNR
YMNHMQSLRFEHKLYAQVKQKMEEMQQHNMSWIEVQFLKKAVDVLCQCRATLMYTYVFAF
YLKKNNQSIIFENNQADLENATEVLSGYLERDISQDSLQDIKQKVQDKYRYCESRRRVLL
QHVHEGYEKDLWEYIED
>P25963
MFQAAERPQEWAMEGPRDGLKKERLLDDRHDSGLDSMKDEEYEQMVKELQEIRLEPQEVP
RGSEPWKQQLTEDGDSFLHLAIIHEEKALTMEVIRQVKGDLAFLNFQNNLQQTPLHLAVI
TNQPEIAEALLGAGCDPELRDFRGNTPLHLACEQGCLASVGVLTQSCTTPHLHSILKATN
YNGHTCLHLASIHGYLGIVELLVSLGADVNAQEPCNGRTALHLAVDLQNPDLVSLLLKCG
ADVNRVTYQGYSPYQLTWGRPSTRIQQQLGQLTLENLQMLPESEDEESYDTESEFTEFTE
DELPYDDCVFGGQRLTL
>Q12454
MTLVTPLQFSTVQPNLYRGSYPREINLPFLRTLRLKYILSLTPEPLSTDPLMVKFCEENN
IKTIHIKCQSERKADKTKPKIKRKKKTVPIEYDVVVRCVKFLIDKGHYPCYMHCTNGELI
ISLVVACMRKFSYWSTVSILNEFLVYNSSINIHERNFIENFNSEIEVDDLDIKDKVPWIT
VRYIARTATESKDELRVDDANASEKVARVSSVSNSLPKLKFHSM
>Q9HDV6
MSLNEIAHDQPVENGPLYSEKQDHTAVDYALHILFTQFVRLSEQKISFLSRYHANEGKNA
EPVRFNVGEQEAILLLKKGEDNEFDRCIQALVALASSKPVAVIESLLCWRKVRVDITSSS

GTPRVVNERRSSISIYILCRVLTEIAETIPSNALEESTVSCLLECVFHQLLSAKNLPVSS
SYFSLANWESFAFLVGSMSRFNFVMVSDRFIEEIEHLEKSGCDSRQKETVLVHLLRAMRY
LRLQLYPTTLLEESIAFLQSLTSFFMKANTALKIEYAYLMEQLLRPLISRATFEVNIPAW
SRTIETIYPVVLKMCTKTKYWNVFFPFCCTLLCLSPKQFFLKHWISSLDAAFFRVKDRRL
RNTGLPHLSRVVWTYSNQYKEEPSIMNSNISNILKSGFSIGKKFSVVPFATLEELDGYAQ
IVRVVGAHFPELVIKEILTPLSTDAFTENIGPEKLMIVVRSVYYILHDMKYKKSDSTIFE
YIEFEFVDLHEVAVGTPLQQFVHNLSQKLLFLVFQLSTNTNCYLDSKNATSLLALYINAL
KVFPCLMGKLEQRVIDAYVKCLNCSNKIIHTVCHNSLIYFSSGLKMSKSVISCLSRKLVK
GSEYLLRTYHEVIRIWISQQEAVIEKRNSVLSNKSCASSESNTPASVKQDYDDIQSWTII
EEIQSLGVLHLSSPSVGIRKFAVALLNDVKQLNTEYLMLSSDKVEVGDIYSEPTIVDVLK
DCDSSILSVESHLPTAAERSRLRKFINDGTKDMLLKLATSNSGVDISIWYNVFPRFIKVC
FERFPTTMALLRNTVCEKLPSITMQLLSRIESSELNFNLKSAVKNDNFPEFLLVQWKLYL
IVACCTITYTSNVDYSHTDLRRTLTAVQSGNHLRGLQETIKITSAESLFSMVLPLIFTEF
SPIREAVVFAIGCINVNAFPQLVRSLKPYISVLKQDHQEFIFAGLNFPSVKRRNKPDLLL
RSEIAHIFAMTSHLLLHELLNEDREALSVISEFLKDLKGFLSTPNVQADEKYLKLRCYFS
QLLEKVLLVQNLHPSSEVLPFSGRASCWKLLDEWTGFGPARAITKNREELMRAHIRGNNK
DIRERDKLLASFEAGKQNLEYLAIKAMIALCTAKLQQELTEGVFLFDIEILLNWFTAVFG
SPSKAIVGLARKGLTALLLENSSNEVLLQKVINRCFSKEISQTISNYYFLSLSEMLIQIN
PNNLSKPKLLPLCLVNLSTNNLSVRLKAFELLGNFHLNNFTMTALMEFKTFLESSNPALY
LKPQYLFSVQLASDFTEDSFTLTSECLRYFNYGHQHRRGLVTVLLPWLQNLELKMDVENK
TFDSFTAVILIDLIEVTAKFTNDLPNEIEALWTSLALSRHKSNWTVILFFLMQQCYQRKT
FSFVDCTRQITIYLAKTELLSDLYSTLLSFVCPANVSNENQEVYKFSLEDLSSTYVANLE
DLFPSEKNHISYSPCQLSLLLLMDILPNPSIITVTDDVATILHAAFIQFDHYSRIVQEQC
QQIFQYVVRKVLAMEGRLDYDDAYFDFNVESSLITGLRGKTKKEDDLVKYHNMILKTIEL
LSSSYPELKQIWGEVALSWATTCPSRRLACNSFQLFRSLLPDFDARMLLEIISRLVGTIS
DETSYLRDYSVEILRTFNSYVLVMNSDDLLSYSQILWTAIACLTTIHEDEFIESVKIAYA
YFLRVEETDSATQQIMETFPQNWVGDYQGLQILILKGFRSTNSFEITMNFFLLLMDFKDN
DLVGVGYLRILSCLLVSLPAMVYTYEHSDPITFDLSVFCHKLATLASQFNDDALIELLDT
YLKRKFRSIKDFLKHTVSYLYSYYFEDYELEIVSTLTMFLSNNLTWFRKSTLDVLKELFP
LIDFQKPIYSEHGLGIVSPLLRLLPTGYAMEALSLLTDSVLHVSAPTDMQTLKLLMVDPK
LKNSDDRLAGFFEIPDEDGWYEPNSEYAAAITKSNVHAVFYSCSTTEASVSTPEVRFHAD
EVSNYPRHVPTDSHGSLDENSLGELVTTLHSLDVFFAEDRDEELIQPDVAVDPKLDITSE
DYDDRIATILSGSLRRQKQGLMAYETESFRDYSPNEEIDASAKLPMDMPPVRVRKSSFIS
KLKPHYFMDAESYYPSLKALGNSAEHRLSLDEIRGAAELKQNKNWNSMLDQSVSMINEDS
VEDHETHENYYHLRTMFQGSESNESFTDTTRSRGWH
>O42868
MAPKTNLQISVICASNQNRSMEAHNVLKNAGYQVDSFGTGSAVRLPGPSIDKPNIYQFGY
PYDEIYKELEAQDSRLYTANGLLKMLDRNRRIKRAPCRWQDQDSIYNIVITCEERCYDAI
CEDLYRRGETLNRPVYLINVDIKDNHEEASVGGKAILDLVNKLTEAQDKLEELFPSIMAD
FQSNHPKLPVLYTIHFF
>P38427
MALIVASLFLPYQPQFELDTSLPENSQVDSSLVNIQAMANDQQQQRALSNNISQESLVAP
APEQGVPPAISRSATRSPSAFNRASSTTNTATLDDLVSSDIFMENLTANATTSHTPTSKT
MLKPRKNGSVERFFSPSSNIPTDRIASPIQHEHDSGSRIASPIQQQQQDPTTNLLKNVNK

SLLVHSLLNNTSQTSLEGPNNHIVTPKSRAGNRPTSAATSLVNRTKQGSASSGSSGSSAP
PSIKRITPHLTASAAKQRPLLAKQPSNLKYSELADISSSETSSQHNESDPDDLTTAPDEE
YVSDLEMDDAKQDYKVPKFGGYSNKSKLKKYALLRSSQELFSRLPWSIVPSIKGNGAMKN
AINTAVLENIIPHRHVKWVGTVGIPTDEIPENILANISDSLKDKYDSYPVLTDDDTFKAA
YKNYCKQILWPTLHYQIPDNPNSKAFEDHSWKFYRNLNQRFADAIVKIYKKGDTIWIHDY
HLMLVPQMVRDVLPFAKIGFTLHVSFPSSEVFRCLAQREKILEGLTGADFVGFQTREYAR
HFLQTSNRLLMADVVHDEELKYNGRVVSVRFTPVGIDAFDLQSQLKDGSVMQWRQLIRER
WQGKKLIVCRDQFDRIRGIHKKLLAYEKFLVENPEYVEKSTLIQICIGSSKDVELERQIM
IVVDRINSLSTNISISQPVVFLHQDLDFSQYLALSSEADLFVVSSLREGMNLTCHEFIVC
SEDKNAPLLLSEFTGSASLLNDGAIIINPWDTKNFSQAILKGLEMPFDKRRPQWKKLMKD
IINNDSTNWIKTSLQDIHISWQFNQEGSKIFKLNTKTLMEDYQSSKKRMFVFNIAEPPSS
RMISILNDMTSKGNIVYIMNSFPKPILENLYSRVQNIGLIAENGAYVSLNGVWYNIVDQV
DWRNDVAKILEDKVERLPGSYYKINESMIKFHTENAEDQDRVASVIGDAITHINTVFDHR
GIHAYVYKNVVSVQQVGLSLSAAQFLFRFYNSASDPLDTSSGQITNIQTPSQQNPSDQEQ
QPPASPTVSMNHIDFACVSGSSSPVLEPLFKLVNDEASEGQVKAGHAIVYGDATSTYAKE
HVNGLNELFTIISRIIED
>P32873
MTDNLTTTHGGSTTLELLAQYNDHRSKKDKSIEHIEKGTCSGKERNPSYDEIFTENIKLK
LQVQEYETEIESLEKVIDMLQKNREASLEVVLEQVQNDSRDSYVNDQSFVLPPRSAERKA
HIKSLNLPIPTLSPPLQQGSDVALETSVTPTVPQIGVTSNTSISRKHLQNMILNDEIEAN
SSFSSPKIINRSVSSPTKIHSEQLASPAASVTYTTSRITIKSPNKGSKSPLQERLRSPQN
PNRMTAVINNHLHSPLKASTSNNLDELTESKSQQLTNDAIQKNDRVYSSITSSAYTTGTP
TSAAKSPSSLLEVKEGENKALGFSPASKEKLDDFTQLLDSSFGEEDLVNTDSKDPLSIKS
TINESLPPPPAPPTFFSPTSSGNIKNSTPLSSHLASPVILNKKDDNFGAQSAKNLKKPVL
TSSLPNLSTKLSTTSQNASLPPNPPVESSSKQKQLGETASIHSTNTLNTFSSTPQGSLKT
LRRPHASSVSTVKSVAQSLKSDIPLFVQPEDFGTIQIEVLSTLYRDNEDDLSILIAIIDR
KSGKEMFKFSKSIHKVRELDVYMKSHVPDLPLPTLPDRQLFQTLSPTKVDTRKNILNQYY
TSIFSVPEFPKNVGLKIAQFISTDTVMTPPMMDDNVKDGSLLLRRPKTLTGNSTWRVRYG
ILRDDVLQLFDKNQLTETIKLRQSSIELIPNLPEDRFGTRNGFLITEHKKSGLSTSTKYY
ICTETSKERELWLSAFSDYIDPSQSLSLSSSRNANDTDSASHLSAGTHHSKFGNATISAT
DTPSYVTDLTQEYNNNNNISNSSNNIANSDGIDSNPSSHSNFLASSSGNAEEEKDSRRAK
MRSLFPFKKLTGPASAMNHIGITISNDSDSPTSPDSIIKSPSKKLMEVSSSSNSSTGPHV
STAIFGSSLETCLRLSSHKYQNVYDLPSVVYRCLEYLYKNRGIQEEGIFRLSGSSTVIKT
LQERFDKEYDVDLCRYNESIEAKDDEASPSLYIGVNTVSGLLKLYLRKLPHLLFGDEQFL
SFKRVVDENHNNPVQISLGFKELIESGLVPHANLSLMYALFELLVRINENSKFNKMNLRN
LCIVFSPTLNIPISMLQPFITDFACIFQGGEPVKEEEREKVDIHIPQV
>Q9H299
MSGLRVYSTSVTGSREIKSQQSEVTRILDGKRIQYQLVDISQDNALRDEMRALAGNPKAT
PPQIVNGDQYCGDYELFVEAVEQNTLQEFLKLA
>P35195
MPKIFCLADVCMVPIGTDSASISDFVALIEKKIRESPLKSTLHSAGTTIEGPWDDVMGLI
GEIHEYGHEKGYVRVHTDIRVGTRTDKHQTAQDKIDVVLKKISQ
>P46970
MDITELLQCFACTLDHNAAVRTNAETHLKNASKVPGFLGACLDIIAADEVPENIKLSASL

YFKNKITYGWSAGARQGSNELLDSHVDPDEKPVVKDMLIKTMVSVSKTSPRCIRVLKSAL
TVIISEDYPSKKWGNLLPNSLELLANEDITVTYVGLLCLAEIFRTYRWKNNDERQDLEEL
ILNYFPALLNYGANVLFQDGKYMNNEQIGELVKLIIKIYKFVSYHDLPFTLQRSESFTPW
ACFFVSIIQQPLPQEVLSISDIEVRSKNPWVKCKKWALANLYRLFQRYASTSLTRKFQYD
EFKQMYCEEFLTQFLQVVFDQIEKWGTGQLWLSDECLYYILNFVEQCVVQKTTWKLVGPH
YNVILQHVIFPLLKPTAETLEAFDNDPQEYINRNMDFWDVGYSPDLAALALLTTCVTKRG
KTTLQPTLEFMVSTLQSAVGDYNNIMLDNALQIESCLRIFSSIIDRLITKDSPFASEMEK
FILTYVLPFFKSQYGFLQSRVCDICSKLGSMDFKDPVITSTIYEGVMNCLNNSSNSLPVE
LTAALALQTFISDDQFNMKLSEHVVPTMQKLLSLSNDFESDVISGVMQDFVEQFAEQLQP
FGVELMNTLVQQFLKLAIDLHETSNLDPDSFTNVDSIPDESDKQMAALGILSTTISILLS
FENSPEILKNLEQSFYPAAEFILKNDIEDFYRECCEFVENSTFLLRDITPISWKILELIG
ECNRKPDSMVSYYLSDFMLALNNILIYGRNELKKNEFYTKIIFEIYQKAVTAEDNSLDDL
RVVFDLSQELVLALDDSLPQQYRERLLADVVGSILTQKNELKTNVVFSVTAFNVVISNMI
TEPLITLQYLKQQGCLEIFFQTWITDYIPNYKRCYDIKLSVLALLKIILKLESNDYSVLN
LENLVPQLGSIVTQLASRLPTALRQLANQRKEFSSSGFEEDTKWDENFLDVGDDDENDDE
GDLTEKYLELIKNRADSLDFVDGYDAKETFDDLEEDPLTGSILDTVDVYKVFKESIANLQ
HVDSNRYQGILRHLTPADQELFMGIMNA
>Q5U5M8
MESSQGRRRRPGTVVPGEAAETDSELSASSSEEELYLGPSGPTRGRPTGLRVAGEAAETD
SEPEPEPTVVPVDLPPLVVQRDPAETWGTEETPAMAPARSLLQLRLAESQTRLDHDVAAA
VSGVYRRAGRDVAALAGRLAAAQATGLAAAHSVRLARGDLCALAERLDIVAGCRLLPDIR
GVPGMEPEQDPGPRA
>O42914
MSVASKNLFDLLGEETPAATTTEKKTAASRDKKRSDSPPVPRELVAQSTTSRKRDPNQPT
PRERTVNKKADQPRRRRQAPQGNEAFAREGKEARANNAAHPVDATGAPSNRRNARARRGR
EFDRHSQTGRVDTKKATERGWGDLVNSAANPDVAENEGNTPSGAQTPAAEEENVKTLDEY
LSERKSAAKPVGRTVEKLENATKVEKSAPEELFASLKKSASQKKSAAKESKPKKVLLDIE
QTFTARPARGGRPNRAPRRGPSETASKTQQAPPTLSETDFPALA
>Q9SMZ4
MNSNGHEEEKKLGNGVVGILAETVNKWERRTPLTPSHCARLLHGGKDRTGISRIVVQPSA
KRIHHDALYEDVGCEISDDLSDCGLILGIKQPELEMILPERAYAFFSHTHKAQKENMPLL
DKILSERVTLCDYELIVGDHGKRLLAFGKYAGRAGLVDFLHGLGQRKLILGYSTPFLSLG
ASYMYSSLAAAKAAVISVGEEIASQGLPLGICPLVFVFTGTGNVSLGAQEIFKLLPHTFV
EPSKLPELFVKDKGISQNGISTKRVYQVYGCIITSQDMVEHKDPSKSFDKADYYAHPEHY
NPVFHEKISPYTSVLVNCMYWEKRFPCLLSTKQLQDLTKKGLPLVGICDITCDIGGSIEF
VNRATLIDSPFFRFNPSNNSYYDDMDGDGVLCMAVDILPTEFAKEASQHFGDILSGFVGS
LASMTEISDLPAHLKRACISYRGELTSLYEYIPRMRKSNPEEAQDNIIANGVSSQRTFNI
LVSLSGHLFDKFLINEALDMIEAAGGSFHLAKCELGQSADAESYSELEVGADDKRVLDQI
IDSLTRLANPNEDYISPHREANKISLKIGKVQQENEIKEKPEMTKKSGVLILGAGRVCRP
AADFLASVRTISSQQWYKTYFGADSEEKTDVHVIVASLYLKDAKETVEGISDVEAVRLDV
SDSESLLKYVSQVDVVLSLLPASCHAVVAKTCIELKKHLVTASYVDDETSMLHEKAKSAG
ITILGEMGLDPGIDHMMAMKMINDAHIKKGKVKSFTSYCGGLPSPAAANNPLAYKFSWNP
AGAIRAGQNPAKYKSNGDIIHVDGKNLYDSAARFRVPNLPAFALECFPNRDSLVYGEHYG
IESEATTIFRGTLRYEGFSMIMATLSKLGFFDSEANQVLSTGKRITFGALLSNILNKDAD

NESEPLAGEEEISKRIIKLGHSKETAAKAAKTIVFLGFNEEREVPSLCKSVFDATCYLME
EKLAYSGNEQDMVLLHHEVEVEFLESKRIEKHTATLLEFGDIKNGQTTTAMAKTVGIPAA
IGALLLIEDKIKTRGVLRPLEAEVYLPALDILQAYGIKLMEKAE
>P30044
MGLAGVCALRRSAGYILVGGAGGQSAAAAARRCSEGEWASGGVRSFSRAAAAMAPIKVGD
AIPAVEVFEGEPGNKVNLAELFKGKKGVLFGVPGAFTPGCSKTHLPGFVEQAEALKAKGV
QVVACLSVNDAFVTGEWGRAHKAEGKVRLLADPTGAFGKETDLLLDDSLVSIFGNRRLKR
FSMVVQDGIVKALNVEPDGTGLTCSLAPNIISQL
>P97305
MTTANCGAHDELDFKLVFGEDGAPAPPPPGSRPADLEPDDCASIYIFNVDPPPSTLTTPL
CLPHHGLPSHSSVLSPSFQLQSHKNYEGTCEIPESKYSPLGGPKPFECPSIQFTSISPNC
QQELDAHEDDLQINDPEREFLERPSRDHLYLPLEPSYRESSLSPSPASSISSRSWFSDAS
SCESLSHIYDDVDSELNEAAARFTLGSPLTSPGGSPGGCPGEESWHQQYGSGHSLSPRQS
PCHSPRSSITDENWLSPRPASGPSSRPTSPCGKRRHSSAEVCYAGSLSPHHSPVPSPGHS
PRGSVTEDTWLTAPVHTGSGLSPAPFPFQYCVETDIPLKTRKTSEDQAAILPGKLEICSD
DQGNLSPSRETSVDDGLGSQYPLKKDSSGDQFLSVPSPFTWSKPKPGHTPIFRTSSLPPL
DWPLPTHFGQCELKIEVQPKTHHRAHYETEGSRGAVKASTGGHPVVKLLGYSEKPINLQM
FIGTADDRYLRPHAFYQVHRITGKTVATASQEIIIASTKVLEIPLLPENNMSASIDCAGI
LKLRNSDIELRKGETDIGRKNTRVRLVFRVHIPQPSGKVLSLQIASIPVECSQRSAQELP
HIEKYSINSCSVNGGHEMIVTGSNFLPESKIIFLEKGQDGGPHWEVEGKIIREKCQGAHI
VLEVPPYHNPAVTSAVQVHFYLCNGKRKKSQSQRFTYTPVLMKQEQREDTDLPSVPSLPV
PHSAQAQRPSSETGHPHDRAMSAPGGLLCQVQPAYTSMVASTHLPQLQCRDEGAGKEQHI
ATSSVMHQPFQVTPTSPIGSSYQSIQTSMYNGPTCLPVNPASSQEFDPVLFQQDAALSSL
VNLGCQPLSPIPFHSSNSDATGHLLAHSPHSVQTPPHLQSMGYHCSNAGQTALSSPVADQ
ITGQPSSHLQPITYCPSHPGSATAASPAASHPLASSPISGPSSPQLQPMPYQSPSSGTAS
SPSPTTRMHSGQHSTQAQSTGQGGLSVPSSLVCHSLCDPASFPPGGATVSIKPEPEDQEP
NFATIGLQDITLDDVNEIIGRDMSQISVSQATEVMRDTPLPGPASPDLMTSHSAH
>Q9UUE5
MESSDEVDLPVYHLQNAPIENGKLNDWRSKGRTVAFKLHPFLRKGLAYINNKEFDENTLK
SDKFEEVESLVYEFSTPSTLIEPNYLLTAWHELWEELQDTYMTPILDSEANLLLLTQFYG
KISSLFRSKIIQVLEELQSRINEGEKDCQPVLDSLWEVESAWRCAEAIYFPPSSPYTLST
GILDWVNAYDPQPIADDGLEIMAYRIPYQHPEFWPYVNKTAIRGLFEQTISCLEMSGLTK
EWPVLKETVDELIDILRYSPCTHQKRIRSVSDFERRWKLWRSRLANLRHVVKKHRDIDSE
VLDDFVVLLDILNGNKEVIMLSCAHWQEYFSALAFLYGPLDCKNPEDISLLYQLATGEDS
KFYVNGTIEYEQICVNLCSNEPLNAIKHAYLLDLGLAVHLADLLSKSGHLRDYITEEYPI
TLREHLILEYGQCVLESRNLWQTSFAYWKCVADSGYQRIKACIPYVPLSDVDAKETALQL
CKQLKLRDEAQLVLTHWADELIARNHYGEALIALDNAANYSALNRVTWELFDICIAEKKS
FSPDKDELLYELFSSPKACTPTLASIISPAATIHQYFFYLQHKKELNASELLVGLLTMVD
FPSSRFPKLLELLHEFLNNPLQSNSTDFKLSLVNVYDCIAVLQDQQSTVKDQQLLLSIHE
RLSSAISWYFLHLKK
>O94614
MDSASSMRNRSPLGVPEFSYSSQSVNNIRGIKSRNRSSIGHSITDPQVASRINAFYLSNV
QSSIPFETDAIGPAYGIGDMIAPEMNDTQSLSASVTNMKKENFYSRPNVSSSSILLTIKR
ATSSQETKRDRPLPNIRNSAPSATRSHSTPCVAPGYLRTSNEAADVVFPHEEAHFSNHNP

KPNNGSPLQKQVVADLPFPLPVSDEEQLDWIRANEDLVHSQDIDEALEWAEYVLRFTQSH
LPYLQTYESENLHEINYLESMCENALYKIREFSELENAKAMYFDAYVYETGAFDVESDIQ
RAWDLYSSSANLGYTRSLYRLGVLLEDQGNLEEAVEYFEKGVSENDSACCWRLSLLILEG
MLDGVGEYAHRHASGLELLERSADTADADVPSGLYSHALVNLHEHPGLVDLGSENIRVPI
DEATALKSFAKAAFLGHSSAQLRMGAVYEFGKYGCPVVPRYSLFYYSAAAKRGETEADLA
VAKWYLNGSDGIPVDEDLAFMHAERASMAGNANAQFLMGYLFDTRGNTEQATYWYNEAAK
AGHSEAIERLALLENQIQEPEPENITSSQYPNQDVIKEIPVTASETSPPHAPAVSSTPVT
SAPPVSQTKVTKVSVPKKTSKKFLIKHNKCIIS
>P27058
MGTPSYDIKNKGDDMQEEPKVKLHHEKGGDEKEKIIEKETPSQDINNKDTISSYVLRDDT
QEIPKMEHEEGGYVKEKIVEKETISQYIIKIEGDDDAQEKLKVEYEEEEYEKEKIVEKET
PSQDINNKGDDAQEKPKVEHEEGDDKETPSQDIIKMEGEGALEITKVVCEKIIVREDLAV
QSKPPSKRDPPKMQTDNNKL
>Q9VMA3
MDPMKEEAIELNGHAMQMAEAEQENGAGALKIATNAGATDRPAHQQLPLPVEDQQDEVLT
PAEKGKFEYPPPPPPPPTPVQAPLATKATALNASQEHDDDEANSEKWEDPCAPPPPPPLPT
SAFLATGLGYLKLPAFKLKDALEKAITKLEANKRTLKASPESSRSIKNKNVVALEMLPRR
SNPETIGDGSMLASTSTAVMLQTKKPAVIVEMERRCKIINLLAKQNQILESISGEAIPMH
GPSKHLHEDEGLTLQVLSARASTPYTQPSSMLSCTAVSCDLEHDSPRKQVASKEAVPEQQ
SSQVQQKRPPSTGIHKPGSLRAPKAVRPTTAPVVSSKPVKSYTRSRLMDIRNGMFNALMH
RSKESFVMPRIATCDDIELEGRLRRMNIWRTSDGTRFRTRSTTANLNMNNNNNNECMPAF
FKNKNKPNLISDESIIQSQPPQPQTEFQDPAIVNQRRIGSGRLNHSKWGYNDEDYHSYHN
GKSQHMEEVNSKNSKNMTVLQFFDNGEISSQPQRRPNTPVMGMSINRSENDTLHSNESSE
DLSRANENYVKRVMSGFLVVSKPKSRDVEDRHHRRYRNQNEEPEWFSCGPTSRLDTIELC
GFDEDEEKMLKEGNKNHGLGETERETSKQKMDHKYKWTHAEPMGRSKYMPKHDTNNNHNV
ENMNNVMATEHQQQKEEKRPGSGRSFQFDKFNQSQQNYESSSYVNHQQPPQTQPQQMQQQ
SNTNTNNSKFMSFFANEGNSSSSSLNEFFKQAINQGHGNNPEQPKSLGHIGQMPSVDQLE
AKWRRNSLNNVGETANKQTDNFQKLIGSLSSAKPQSQAVGYDAISNFIMQQQQYQQQQQK
QHLIIQQQQQHTAFLASLQLKAILGRADTQLLLLRLTKGEISKHGLLVQLANPRLTDMDR
EAITAVLQFTNTQQQQQQHKQQLDMLSSTVIASQLQNLHNLAIVQQTLAARQQPQHNPQT
QAPHQLSQEDLQAHANVIMRNAVMKRKIEEQTSKLINGGAKHQAQQQYLNRGQQRQARPD
ANSNALLHALISGGGNNHASGYPMNGQPQKHHSNLRFGDNQNFQSFESNQPHFATQYKQQ
YQQSQQQHPHQQPQQLNSLHQNNAGAVNSFNKAQMQAQSAISMLPNSGDEFH
>Q9Y7M8
MYTCPKTCTLTLKFFIDRFHTDYRLSKLAISFFDQKKNKMADSIPVKKKGSAKGPPSFVT
PEYIEKQRQKKLEKMAKKAARKPIAPPSNAPPEFNTDFIKREMLSIPNYAPFETEKSALD
ITIMTYNVLAQTNIRRSMFPHSGEALKWKNRSRMLANELTYYSPTLGCMQEVDAEFVPNF
YKKLLGGLGYELHFIKGEGKTHGIMIFWKSSLFKKVQDLTIYYDDHDELPGRMNTKNIGC
CVRLERVDDPSRGLFLATTHLFWHPYGSYERLRQGAILVKEVNKMAQSHPSWPVFIAGDF
NTEPFDTNFPALTTRPLSICQRATDIIERSMNYVFGESELEEKNASTKTENDSNEDDKEE
CQSSSTSSVPESTASTPKKRILHVQNDYVPHYRSFYQQHEQNPVLFSLYSVGYKLVHPEN
AKNTFDHPAFTNWAHAYQGHLDYIFVMNRDTSLQTPENQVVEGIKLKALLRVPLPSEMKE
AEPLEGRYPSDHVALMANVQIV
>P29547

MSQGTLYANFRIRTWVPRGLVKALKLDVKVVTPDAAAEQFARDFPLKKVPAFVGPKGYKL
TEAMAINYYLVKLSQDDKMKTQLLGADDDLNAQAQIIRWQSLANSDLCIQIANTIVPLKG
GAPYNKKSVDSAMDAVDKIVDIFENRLKNYTYLATENISLADLVAASIFTRYFESLFGTE
WRAQHPAIVRWFNTVRASPFLKDEYKDFKFADKPLSPPQKKKEKKAPAAAPAASKKKEEA
KPAATETETSSKKPKHPLELLGKSTFVLDDWKRKYSNEDTRPVALPWFWEHYNPEEYSLW
KVTYKYNDELTLTFMSNNLVGGFFNRLSASTKYMFGCLVVYGENNNNGIVGAVMVRGQDY
VPAFDVAPDWESYDYAKLDPTNDDDKEFINNMWAWDKPVSVNGEPKEIVDGKVLK
>P40069
MDQQFLSQLEQTLHAITSGVGLKEATKTLQTQFYTQPTTLPALIHILQNGSDDSLKQLAG
VEARKLVSKHWNAIDESTRASIKTSLLQTAFSEPKENVRHSNARVIASIGTEELDGNKWP
DLVPNLIQTASGEDVQTRQTAIFILFSLLEDFTSSLSGHIDDFLALFSQTINDPSSLEIR
SLSAQALNHVSALIEEQETINPVQAQKFAASIPSVVNVLDAVIKADDTMNAKLIFNCLND
FLLLDSQLTGNFIVDLIKLSLQIAVNSEIDEDVRVFALQFIISSLSYRKSKVSQSKLGPE
ITVAALKVACEEIDVDDELNNEDETGENEENTPSSSAIRLLAFASSELPPSQVASVIVEH
IPAMLQSANVFERRAILLAISVAVTGSPDYILSQFDKIIPATINGLKDTEPIVKLAALKC
IHQLTTDLQDEVAKFHEEYLPLIIDIIDSAKNIVIYNYATVALDGLLEFIAYDAIAKYLD
PLMNKLFYMLESNESSKLRCAVVSAIGSAAFAAGSAFIPYFKTSVHYLEKFIQNCSQIEG
MSEDDIELRANTFENISTMARAVRSDAFAEFAEPLVNSAYEAIKTDSARLRESGYAFIAN
LAKVYGENFAPFLKTILPEIFKTLELDEYQFNFDGDAEDLAAFADSANEEELQNKFTVNT
GISYEKEVASAALSELALGTKEHFLPYVEQSLKVLNEQVDESYGLRETALNTIWNVVKSV
LLASKVEPESYPKGIPASSYVNADVLAVIQAARETSMGNLSDEFETSMVITVMEDFANMI
KQFGAIIIMDNGDSSMLEALCMQVLSVLKGTHTCQTIDIEEDVPRDEELDASETEATLQD
VALEVLVSLSQALAGDFAKVFDNFRPVVFGLFQSKSKNKRSSAVGAASELALGMKEQNPF
VHEMLEALVIRLTSDKSLEVRGNAAYGVGLLCEYASMDISAVYEPVLKALYELLSAADQK
ALAAEDDEATREIIDRAYANASGCVARMALKNSALVPLEQTVPALLAHLPLNTGFEEYNP
IFELIMKLYQENSPVITNETPRIIEIFSAVFTKENDRIKLEKESTLGREENMERLKQFQT
EEMKHKVIELLKYLNTTYNGIVAQNPVLAAVIA
>P22265
MLHEALMLEIYRQALNAGALPTARPRSTESANSSERCPSHDSNSSEHGGGAGSGGVGHRL
DAAALSTGVMPGEGPTTLHSSFPAVPQSLPSQPPSMEAYLHMVAAAAQQYGFPLAAAAAA
GAGPRLPLPLANEAAAPFKLPPQASPTASSNNSEALDFRTNLYGRAESAEPPASEGEEEE
FDDGANNPLDLSVGTRKRGHESEPQLGHIQVKKMFKSDSPPANSVASPSASQLLPGVNPY
LAAVAAANIFRAGQFPDWNSKNDLVVDPLEKMSDIVKGGASGMGTKEKMHSSKATTPQAA
SQPPKSPVQPTPNQNSESGGGSGGGAAGSGAVTKARHNIWQSHWQNKGVASSVFRCVWCK
QSFPTLEALTTHMKDSKHCGVNVPPFGNLPSNNPQPQHHHPTPPPPPQNHNLRKHSSGSA
SNHSPSANVKNAFQYRGDPPTPLPRKLVRGQNVWLGKGVEQAMQILKCMRCGESFRSLGE
MTKHMQETQHYTNILSQEQSISIKSGNANANSDAKESHNSLSSEESRTLSAVLTCKVCDK
AFNSLGDLSNHMAKNNHYAEPLLQSAGARKRPAPKKREKSLPVRKLLEMKGGSGTTQEDH
SNEKTSVQGKPGLGPGGGDKNDAALFAERMRQYITGVKAPEEIAKVAAAQLLAKNKSPEL
VEQKNGGSAKAAGASSVLSAIEQMFTTSFDTPPRHASLPASSPSNSSTKNTSPVASSILK
RLGIDETVDYNKPLIDTNDPYYQHYRYTSSERSGSECSAEARPRLDAPTPEKQQQGGGHD
EESSKPAIKQEREAESKPVKMEIKSEFVDEPNEAEETSKMEAAVVNGSATNNNNNIVERS
SPKTPSSAASPQTRLLPPRSPAESQRSVTPKSPASSHKSYDGSSEGTKKFPSDSLNALSS
MFDSLGSSGAGANSRAKLAAAAAAGGSESPENLTAGGNSLAALRQFCVKKEKTA

>Q8NFG4
MNAIVALCHFCELHGPRTLFCTEVLHAPLPQGDGNEDSPGQGEQAEEEEGGIQMNSRMRA
HSPAEGASVESSSPGPKKSDMCEGCRSLAAGHPGYISHDKETSIKYVSHQHPSHPQLFSI
VRQACVRSLSCEVCPGREGPIFFGDEQHGFVFSHTFFIKDSLARGFQRWYSIITIMMDRI
YLINSWPFLLGKVRGIIDELQGKALKVFEAEQFGCPQRAQRMNTAFTPFLHQRNGNAARS
LTSLTSDDNLWACLHTSFAWLLKACGSRLTEKLLEGAPTEDTLVQMEKLADLEEESESWD
NSEAEEEEKAPVLPESTEGRELTQGPAESSSLSGCGSWQPRKLPVFKSLRHMRQVLGAPS
FRMLAWHVLMGNQVIWKSRDVDLVQSAFEVLRTMLPVGCVRIIPYSSQYEEAYRCNFLGL
SPHVQIPPHVLSSEFAVIVEVHAAARSTLHPVGCEDDQSLSKYEFVVTSGSPVAADRVGP
TILNKIEAALTNQNLSVDVVDQCLVCLKEEWMNKVKVLFKFTKVDSRPKEDTQKLLSILG
ASEEDNVKLLKFWMTGLSKTYKSHLMSTVRSPTASESRN
>O13755
MRCLTIYTWTFRRLPFIPSTNSASFFSTLRFNMSTANNTQAIVIGGGLAGLSATNTILDL
GGNVLLLDKNTAFGGNSVKAASGINAAPTQLQFDQHVSDSVNTFYNDSILSAKSKAKPEL
LRTLTSKSSSAVDWLSERFGLQMDQLSRLAGHSEPRTHRGTHPDYPFKPLAFVLVDQTEK
FAASHPDRLQIKKNARVTRLLTNPNHDKVFGVEYMDLSDKSNHTVYGPVVLATGGYAADY
SDDSLLKLYHPEALSLSTTNGPYCTGDGHKMVMSIGGSTVDLDLVQIHPTGFVDPKDPTA
LTKFLAAEALRGSGAVLLTSQGRRFCDELGYRDYVTGEMMKLKSPVYLVLNSAAAEEVAN
FIKFYSFKGLMKKMKAEELCSTLNCTKDELASTFSEYNRAAKGEIPDEFGRKYFGKTPLE
LTDTFTVGEVVPVLHYTMGGVQVDTQSRVLSTNGNVIDGLFAAGEIVGGIHGENRLGGSS
LLACVVFGRLAGQGASSTMLRRFIASSTSTASS
>O43609
MDPQNQHGSGSSLVVIQQPSLDSRQRLDYEREIQPTAILSLDQIKAIRGSNEYTEGPSVV
KRPAPRTAPRQEKHERTHEIIPINVNNNYEHRHTSHLGHAVLPSNARGPILSRSTSTGSA
ASSGSNSSASSEQGLLGRSPPTRPVPGHRSERAIRTQPKQLIVDDLKGSLKEDLTQHKFI
CEQCGKCKCGECTAPRTLPSCLACNRQCLCSAESMVEYGTCMCLVKGIFYHCSNDDEGDS
YSDNPCSCSQSHCCSRYLCMGAMSLFLPCLLCYPPAKGCLKLCRRCYDWIHRPGCRCKNS
NTVYCKLESCPSRGQGKPS
>O46640
MDNSRKYLLNFKGCNFERTLVDMKILEKLDDFEIRDDDVFVITYPKSGTVWTQQILSLIY
FEGHRNRTEKWDTLDRVPFLEYNIRKVDIENRPSPRLFASHLPYYLAPKSLKNNKAKIIY
VYRNPKDVLISFFHFSNMVVKLEASNTLENFMEKFLDGKVVGSIWFDHIRGWYEHKNDFN
ILFMMYEDMKKDLRSSILKISSFLEKDLSEEEVDAIVRQATFENMKFIPQANYNNILSNE
IGRRHNEGAFLRKGAVGDWKHHMTVEQSERFDRIFQEEMKDFPLKFIWDLNDEANSNHSA
K
>O13895
MEEVVHYFLEGNMPVAQFREALALHYTNEKELFEQAKRCLNICCGSNNFAKRNDVLFSLL
DVAVSISSLELRKELISELYVPVQSLEEAPSEYLVSCCLQLATIYEAEQNFELLCSSLEA
VEKHGHFENDLEQLLLLRIRLGDAYLKLGKAEKAILTVRTSIPLAFKVSNDQLLMELQLC
NARALDETGQFLEAAKCYYRVLQYKVPGNELIYRENLCSVAQCLLLAIPSPIVLQFLQEI
SLQPSVREIPFYSLVEKYLKRKFIGKEDGAFLLPFLLPHQVIHMNRLIEDGRHFLETNIL
FLSEFFEVSSTSILAKHFKLSEEQVDTVVADMVIQERLNASIDQCEGYITFLPEYGKANN
LPNYVNKIATVLQHYGS
>Q96WW2

MEEKENVNLVEKSNYVALENTREIDVFDEFLNAIGNENTITPVYADSSLTHLRKKSYTKV
VHDCTYALVINPYDKKVIWRRGLAYLRLGHPHLANRDWEHSLELDPNNTYIQKSLHRLKE
VYYIYRECAETWQLRHLRVASSQQLPVGLRKQYPNIIRGKIWWKKVHDNCQLCGQQCELK
KENLSAMRSMLYMANTYAKDDTENHSPSAQIGIESSEDELENKITKGEHSLLVPEELYRS
NYPCPQNIDQFLYMIKVLSAPCLYIETFSFPISTINQLFKAHGMSVEQLNLFLKSIHYIG
LCSRFCKQWSDKARSLMQALSGLPWFSFVVQHCLHITAAQILLHIPDIQEEEFRNWHVSK
KPINNTDLSSEFEIAEIPINCYT
>P05754
MGISRDSRHKRSATGAKRAQFRKKRKFELGRQPANTKIGAKRIHSVRTRGGNKKYRALRI
ETGNFSWASEGISKKTRIAGVVYHPSNNELVRTNTLTKAAIVQIDATPFRQWFEAHYGQT
LGKKKNVKEEETVAKSKNAERKWAARAASAKIESSVESQFSAGRLYACISSRPGQSGRCD
GYILEGEELAFYLRRLTAKK
>Q12849
MAGTRWVLGALLRGCGCNCSSCRRTGAACLPFYSAAGSIPSGVSGRRRLLLLLGAAAAAS
YPALRASLLPQSLAAAAAVPTRSYSQESKTTYLEDLPPPPEYELAPSKLEEEVDDVFLIR
AQGLPWSCTMEDVLNFFSDCRIRNGENGIHFLLNRDGKRRGDALIEMESEQDVQKALEKH
RMYMGQRYVEVYEINNEDVDALMKSLQVKSSPVVNDGVVRLRGLPYSCNEKDIVDFFAGL
NIVDITFVMDYRGRRKTGEAYVQFEEPEMANQALLKHREEIGNRYIEIFPSRRNEVRTHV
GSYKGKKIASFPTAKYITEPEMVFEEHEVNEDIQPMTAFESEKEIELPKEVPEKLPEAAD
FGTTSSLHFVHMRGLPFQANAQDIINFFAPLKPVRITMEYSSSGKATGEADVHFETHEDA
VAAMLKDRSHVHHRYIELFLNSCPKGK
>O95429
MSALRRSGYGPSDGPSYGRYYGPGGGDVPVHPPPPLYPLRPEPPQPPISWRVRGGGPAET
TWLGEGGGGDGYYPSGGAWPEPGRAGGSHQEQPPYPSYNSNYWNSTARSRAPYPSTYPVR
PELQGQSLNSYTNGAYGPTYPPGPGANTASYSGAYYAPGYTQTSYSTEVPSTYRSSGNSP
TPVSRWIYPQQDCQTEAPPLRGQVPGYPPSQNPGMTLPHYPYGDGNRSVPQSGPTVRPQE
DAWASPGAYGMGGRYPWPSSAPSAPPGNLYMTESTSPWPSSGSPQSPPSPPVQQPKDSSY
PYSQSDQSMNRHNFPCSVHQYESSGTVNNDDSDLLDSQVQYSAEPQLYGNATSDHPNNQD
QSSSLPEECVPSDESTPPSIKKIIHVLEKVQYLEQEVEEFVGKKTDKAYWLLEEMLTKEL
LELDSVETGGQDSVRQARKEAVCKIQAILEKLEKKGL
>Q07084
MLNSALLWKVWLRIDNSTDEVNQPIAVQFDEIDTVDDLKSRFFQKLSSTRWREINDNASI
AIGLYAPKFDNQADNTSSNNTNDNSCRSKSNGAGSGANLSVNSNTKSSVSPTAGSFGLSK
DLAKDRNVLQHPKPTQKRGALYDAFAAVPTVAATTNVDFPPNEAPMLSPQRPYSTSPKQF
PATTKSPLLRFASVSPYPKFHSDNQIMASAGLTYVSPHNKNKYTRPLIRKGLNFTTESVN
DCTYKIIFEPDELAINIYKELFGTMGSQPASQPLLIFSNVNLRQDVPPLDILNVVDYVPT
NEEISQQKTQPTDHGAVGVFHLDDHISPGEQGLKQTIGDKADLKGKDGNSSPQEFKLITD
EEQLRRASQELKDEEKDAESPWQAILLLPKGYKGGVDFRNKPVAHTDSSFNNEDTITHSE
LEVNTGSPSQESGSLNEAGIGITQPMSEVQRRKEDVTPASPILTSSQTPHYSNSLYNAPF
AVSSPPDPLPNLFTTTSEKVFPKINVLIVEDNVINQAILGSFLRKHKISYKLAKNGQEAV
NIWKEGGLHLIFMDLQLPVLSGIEAAKQIRDFEKQNGIGIQKSLNNSHSNLEKGTSKRFS
QAPVIIVALTASNSQMDKRKALLSGCNDYLTKPVNLHWLSKKITEWGCMQALIDFDSWKQ
GESRMTDSVLVKSPQKPIAPSNPHSFKQATSMTPTHSPVRKNSNLSPTQIEL
>P43638

MDSKDTQKLLKEHRIPCIDVGWLVRPSASTSKSSRPGKSESKANSVAPDIQMDTARPPVF
ETSVDSSSSILSSNDKGRRHSVAASLLMDNQRANAGSTSVPTNIPPPRGRSKSVVETNLS
NVEADSGHHHHHRHHHHTEDAPAPKKVGFFKSLFGHRKKDQEQQEKERERKERSPSPTHV
DRGAAIRRERTATISAESPPPLQYNAPPSYNDTVVPLTRSKTESEVYYENHPQSYYHGRM
RTYHSPEEGKVDGTSPADDHNYGGSRPDPRLMDFLRYYKSKDYKLAAFKEGNFIKSSASP
TTKKNRRASFSLHNDKPQPAKSLAHQKFDAKGRPIPPHPDAPKLPSAFRKKHPSNASIVD
TVDSNSDVSSSAQNNNQTPSSHKFGAFLRKVTSYGNNNNNSTNASSLSANVNNPDTSSTS
LWSSSSMEFDPSKITTVPGLENIRPLKHVSFATNTYFNDPPQQICSKNPRKGEVEVKPNG
SVVIHRLTPQERKKIMESTSLGVVVGGTGQLKLLNPEEDDANAKSKEEMAPQKQNEVEAH
DEEDNNSQRRNIVMAAAEAAAEARAKEAPNELKRIVTNNEEEVTVSKTASHLTIDKPMIS
RRGASTSSLASMVSSDTNGTNADDEGEILPPPSLKIPHDIVYTRCCHLREILPIPATLKQ
LKKGSTDPIPILQLRNPRPSMVEIWSFSDFLSIAPVLCLSLDGVQLTVQMLRIILSSLVY
KQHFQKLSLRNTPLDEEGWKVLCYFVSKAKSLHSIDLTMVPSIKTNVQKPSKSSLKSKIL
RMQCNLENRSDMNWDLLTASIALMGGLEEIVISGAKMNSAQFKNFILVACIATERLGLAY
NGLSKSQCDDLAKWMVQSKVTGLDVGFNDLNGKLSSFTDAVLGKIQKANEKNVFKFLSLN
GTNLRVNEHDTFENNEVLKLISVLCYLENLKFLDISNNPAIFPHCVPTLIDFLPVFVNLV
RLHIDYNNLSSTSVVMLAEILPMCSRLNYFSMLGTELDLASSKALAEAVRKSSSLMTLDV
DYVYMPENIKEKISLYALRNIQGELKRVNSDDKDIKDSQFSSLQDQLSLLLTEKADNSEH
YNKMVENFMAKIALARIKISKVVHDLFDLKLNGQLNLEGKEALIRLCFIEASLERGCDLL
KQRHNNTLKSPEAVSKSRKGGNQAQPNSESCQRMLLSSSILQNSDHIALMPFGSAIVEKS
SPDAEDAVEFREGDDSNVNHEDVPANDQQFRDEVDIKNKYSIIKRELEHEKLVGGGDLPV
DKEILNRAAQSLDSDQIKEFLLKNDVSTILGVIDELHSQGYHLHHIFKKQGNQEETAFRT
KDEQQSSQSNDSSANASPTTDPISTGSNTSRTNDNAHIPPTDAPGFDKFMNNAEENAIDA
AYDDVLDKIQDARNSSTK
>Q2XU92
MTQEKKAEDLERGTDATSAVPRLWSTHCDGEVLLRLSKHGPGHETPMTIPELFQESAERF
SAYPALASKNGKKWDTLTFSQYYEMCRKAAKSLIKLGLQRFQCVGILGFNSVEWVVTALG
TILAGGLCVGIYATNSAEACQYVIQQANVSILIVENDQQLQKILLIPPDKMETVKAIVQY
KLPLMESMANLYSWNDFMELGNDIPNIQLDRVILSQKANQCAVILYTSGTTGTPKGVLLS
HDNITWTAGAMSQEMEINRVSGKQNTIVSYLPLSHIAAQLTDIWIPIKIGALTFFAQPDA
LRGTLVYTLQEVKPTLFMGVPRIWEKMQDTIKENVARSSRLRKKAFAWAKMLGLKVNTKR
MLGKRDIPMNYRMAKALVFAKVRTSLGLDNCHAFFSSASPLSQDVSEFFLSLDIPIGEIY
GMSECSGPHTVSNKSVYRVLSCGKVLSGCKNMLYNQNKEGVGEVCMWGRHVFMGYLNKEE
ATLEALDENGWLHSGDIGRLDSHDFLYITGRIKEILITAGGENVSPIPIETLVKEKIPII
SHAMLVGDKAKFLCMLLTLKCETDRKSGEPLNKLSVEAKSFCQMLGSQATTVSDILKSRD
QVVYTAIQYGIDIVNQQAMSDSHRIRKWIILEKDFSIQGGELGPTSKLKRSVITQKYKAQ
IDSMYLS
>P14000
MKSAPFLFLLGLLGLVTAQTQDPALLDLLRENPDLLSLLLQSNEHRAPLVKPNVVLLVAD
HMGSGDLTSYGHPTQEAGFIDKMAAEGLRFTNGYVGDAVCTPSRSAIMTGRLPVRIGTFG
ETRVFLPWTKTGLPKSELTIAEAMKEAGYATGMVGKWHLGINENSSTDGAHLPFNHGFDF
VGHNLPFTNSWSCDDTGLHKDFPDSQRCYLYVNATLVSQPYQHKGLTQLFTDDALGFIED
NHADPFFLYVAFAHMHTSLFSSDDFSCTSRRGRYGDNLLEMHDAVQKIVDKLEENNISEN
TIIFFISDHGPHREYCEEGGDASIFRGGKSHSWEGGHRIPYIVYWPGTISPGISNEIVTS

MDIIATAADLGGTTLPTDRIYDGKSIKDVLLEGSASPHSSFFYYCKDNLMAVRVGKYKAH
FRTQRVRSQDEYGLECAGGFPLEDYFDCNDCEGDCVTEHDPPLLFDLHRDPGEAYPLEAC
GHEDVFLTVKSTVEEHKAALVKGTPLLDSFDHSIVPCCNPANGCICNYVHEPGMPECYQD
QVATAARHYRP
>P07377
MSLKERKSINECDLKGKKVLIRVDFNVPLDDGNITNDYRIRSALPAVQKVLTEGGSCVLM
SHLGRPKGVSMAEGKELGSAGGIPGFEQKATLKPVAKALSELLSRPVTFAPDCLNAADVV
SKMSPGDVVLLENVRFYKEEGSKKSTEEREAMAKILSSYGDVYISDAFGTAHRDSATMTG
IPKILGNGAAGYLMEKEISYFAKVLGNPPRPLVAIVGGAKVSDKIQLLDNMLQRIDYLLI
GGAMAYTFLKAQGYSIGISMCEESKLEFARSLLKKAEDRKVQIILPIDHVCHTEFKAVDS
PLITEDQNIPEGHMALDIGPKTIEKYVQTIGKCKSAIWNGPMGVFEMVPYSKGTFAIAKA
MGRGTHEHGLMSIIGGGDSASAAELSGEAKRMSHVSTGGGASLELLEGKTLPGVTVLDDK
E
>Q10303
MHISTGMVRASISNTLVTLTIEGNNDRYQVILDKNVNAKQFVKTCWRNKKPLIVKEGKIF
VDKGFPFDPCRTFLEALYEKYSYSSPLPSSVEFKSGKQVEFCGFEKIQSKQRDLKSLRVI
ILDNYRIEDIEIEYEYSKILPEVIDLDLSRNLFHEFFPILKLCSQLPSLRNLTLDSNLFS
NFISSNTVLLIPHLTQLSVNGCGLNSKDVQWITETFPSLEVLYLEANEIILSKATSFKNL
QFLQTLSLANNLNLYSADGYAVDVFQGINNLNLSSTSLADVAELPVHTLHKLTFLDISEN
NIRDIRSLDHLRTLENLKHLRITLSYFNKPTDIAKLLVIARIPSLVKLNDVNISPNERLD
AELYYTSCIRKLVIDNEIKDVEELTKIEPFWEEIWKSHELPSIQFHLEASINDWTSGILK
NRITKGIKISSINSGATMLLKLHYNWKLLDIKALISYHFAIPVHTSTFVFASSERDVSFS
PKTVRDDQKRLFELPFTCTFIDVYAKESGNV
>Q9P7R3
MSDRKRVYGPSVSVPPYFEEPEQPVFTRTRDVDRCRKIYLKLGWATKAVGSSYFESEKIK
IACTVSGPRPSKTFAFRSSAKLNCEFRLSPFSTSVRQGHVQTVEEKSYSQMIEAAISPSI
LLHLYPKSSIDVYIQVIESDGALATLAAAISCASSAIADANIDCIDLVTGSSVLFNPNTD
EYWIDPDYVDERARAAKGSVVMGYMASLGHVTQVWERGTCSPSRLSFLTEKCIKNAKDTR
LVINHALLLEKSKSEDP
>Q8BQP9
MSSAPNGRKKRPSRSTRSSIFQISKPPLQSGDWERRGSGSESAHKTQRALDDCKMLVQEF
NTQVALYRELVISIGDVSVSCPSLRAEMHKTRTKGCEMARQAHQKLAAISGPEDGEIHPE
ICRLYIQLQCCLEMYTTEMLKSICLLGSLQFHRKGKEASGGAKNLDSKIEENAETPALED
SLSSPLESQQQCWQVATDIENTERDMREMKNLLSKLRETMPLPLKNQDDSSLLNLTPYPM
VRRRKRRFFGLCCLVSS
>O42889
MPVSVSDSFVFRNIFGDAEIRKIWSDENRTQEYLNWEAALARAEASLGIIPKYAGEEIQR
VCKVENIDFSKLEEETINIGYPVLGVVHQLANLCSGDSGKYCHWGATTQDVTDSATVRQM
IDSFKIIKGYLEKAIELATVLVIKHRETTMAGRSNLQQAVPITFGFKMARFVATLRRHHQ
RLCELLPRVSVLEFGGACGTLASLENKGLMVQQKLAEELGLLQPEIAWHTERDRIAEAGC
FLGMLTGTLAKFATDIKLLMQTEVAEVFEPFKANRGSSSTMPQKRNPISCVYITASTSFV
RQGVAALLDAMVEDHERATGAWEIEWIVLPDVFVHTVGTLKQTVFLLEGLEVHPSRMNEN
LSITNGLIVSEAVMMALAPKLGRDEAHDLVYRLCHLSIQENKPLVELLLAERQVTNYLSK
EEVTSLLNPENYLGSTFEMIERALQIP

>Q86UA6
MAESLRSPRRSLYKLVGSPPWKEAFRQRCLERMRNSRDRLLNRYRQAGSSGPGNSQNSFL
VQEVMEEEWNALQSVENCPEDLAQLEELIDMAVLEEIQQELIKQEQSIISEYEKSLQFDE
KCLSIMLAEWEANPLICPVCTKYNLRITSGVVVCQCGLSIPSHSSELTEQKLRACLEGSI
NEHSAHCPHTPEFSVTGGTEEKSSLLMSCLACDTWAVIL
>Q17390
MYSLKPKVVEFDKVWVQLRPSIIDIINLRPITNVQWHHKFSDVYDICVSIPTPLSERLYN
EVKACIQEHVRQKRQDIVDVDPDLLLQEYHKMWRVFHEGAIFIHRLFGYLNKQFVKQKRC
TDLDNFAQYAAFLQIPDVKEIGCLALEIWKEDLVKTILPQLVKLLLIAIDNDRKGNFPHI
ANEVSGVINSFVKMEETDFDVVPAEGARYKARESTTAFYQESFEKPLLTDTEQYYSALAQ
KMLTDLSCSEYMEQVIVLLEQEEMRAKKYLHESSVEKVITLCQKVMIKAHKDKLHAVCHD
LITNEENKDLRNMYRLLKPIQAGLSVMVKEFEEYVKKKGLEAVSRLTGENVPQQFVENVL
RVYNKFNDMKTAVFMDDGEFSSGLDKALQGVVNSKEPGQSVPKASERLARYTDGLLKKST
KGLSETDLEAKLDSAIVIFRYIEDKDIFQKFYSKMLANRLIASTSISMDAEELMINKLKQ
ACGYEFTSKLSRMFTDIGLSQELSNNFDKHIADIKTVQPDVKFVPTQTMILQAGSWPLNA
PQLSTNSNNQTAQDVANFHLPRILQPVIQEFEKFYTGKHNGRKLTWLFNMSQGDVRLTYL
DKQYVAQMYVYQMAALLCFERRDAILVKDIGEEIGVSGDYLLKTIRTILDVTLLTCDDQN
LTADSLVRLNMSMTSKRMKFRLQAPQVNKAVEKEQEAVANTFQVSQDRKYYMECAIVRIM
KTRKVLKHNALVTEIMDQTKGRFSPDVPFIKKSIEDLIEKMYIQRTDQNDEYQYLA
>Q04522
MTIKVHGNPRSTATQRVLVALYEKHLEFEFVPIDMGAGGHKQPSYLALNPFGQVPALEDG
EIKLFESRAITKYLAYTHDHQNEGTSLIHKEKHEMAAQLVWEEVEAHQFDPVASKLAWEL
VFKGIFGMQTDTTVVEENEAKLAKVLDVYEARLTESEYLGANDSFTLVDLHHLPLLGYLM
GTQVKKLFEERAHVSAWCKKILARPSWEKTLALQKQA
>O14929
MAGFGAMEKFLVEYKSAVEKKLAEYKCNTNTAIELKLVRFPEDLENDIRTFFPEYTHQLF
GDDETAFGYKGLKILLYYIAGSLSTMFRVEYASKVDENFDCVEADDVEGKIRQIIPPGFC
TNTNDFLSLLEKEVDFKPFGTLLHTYSVLSPTGGENFTFQIYKADMTCRGFREYHERLQT
FLMWFIETASFIDVDDERWHYFLVFEKYNKDGATLFATVGYMTVYNYYVYPDKTRPRVSQ
MLILTPFQGQGHGAQLLETVHRYYTEFPTVLDITAEDPSKSYVKLRDFVLVKLCQDLPCF
SREKLMQGFNEDMAIEAQQKFKINKQHARRVYEILRLLVTDMSDAEQYRSYRLDIKRRLI
SPYKKKQRDLAKMRKCLRPEELTNQMNQIEISMQHEQLEESFQELVEDYRRVIERLAQE
>P52286
MVTSNVVLVSGEGERFTVDKKIAERSLLLKNYLNDMHDSNLQNNSDSESDSDSETNHKSK
DNNNGDDDDEDDDEIVMPVPNVRSSVLQKVIEWAEHHRDSNFPDEDDDDSRKSAPVDSWD
REFLKVDQEMLYEIILAANYLNIKPLLDAGCKVVAEMIRGRSPEEIRRTFNIVNDFTPEE
EAAIRRENEWAEDR
>Q710D7
MISSKPRLVVPYGLKTLLEGVSRAILKINPPNITQFAAVYFKELIVFREGNTSLDIKDLV
KQFHQIKVEKWSEGTTQEKEPECMEEQVETSVVSQEPTRMEKSTDTEEDNIAGPLFMNKT
TQFPSVHAEVLLEPEETPEAACGGSPKPSTPKAVTPPSSPSPAAVSQEFAYVPADPAQFA
AQMLGNVSSIHSDQSDVLMVDVATSMPVFSEEVLSSEAAEDARVAIPSVYSAEVVALQVL
SQTSVHVDLGPKPKDDEAEPTTASSFPLQDEQDPPAYDQAPEVPLQADIEVTSFVHVSSI
YNNEPVIEGVTYVEQIPEHIVIPFTDHVASLKDNEPPDSPIPVACDTGMSEKTVGSVSLA

QLEVESHYSSVHMEAEASVLFSDTSLKGQPAQFPDAGGSTKAVGSEKPLHLEVEFTALVP
GNSGQEESQGSSAAQEMEVKLVLSGEAATAVLSAASVRAAGGSPTPVPEGLTEPELEPEL
EAALEQGLMKPDAETTTV
>P40208
MTISTLSNETTKSGSCSGQGKNGGKDFTYGKKCFTKEEWKEQVAKYSAMGELYANKTIHY
PLKIQPNSSGGSQDEGFATIQTTPIEPTLPRLLLNYFVSMAYEDSSIRMAKELGFIRNNK
DIAVFNDLYKIKERFHIKHLIKLGRINEAMEEINSIFGLEVLEETFNATGSYTGRTDRQQ
QQQQQQFDIDGDLHFKLLLLNLIEMIRSHHQENITKDSNDFILNLIQYSQNKLAIKASS
SVKKMQELELAMTLLLFPLSDSADSGSIKLPKSLQNLYSISLRSKIADLVNEKLLKFIHP
RIQFEISNNNSKFPDLLNSDKKIITQNFTVYNNNLVNGSNGTKITHISSDQPINEKMSSN
EVTAAANSVWLNQRDGNVGTGSAATTFHNLENKNYWNQTSELLSSSNGKEKGLEFNNYYS
SEFPYEPRLTQIMKLWCWCENQLHHNQIGVPRVEN
>Q8N668
MAAGELEGGKPLSGLLNALAQDTFHGYPGITEELLRSQLYPEVPPEEFRPFLAKMRGILK
SIASADMDFNQLEAFLTAQTKKQGGITSDQAAVISKFWKSHKTKIRESLMNQSRWNSGLR
GLSWRVDGKSQSRHSAQIHTPVAIIELELGKYGQESEFLCLEFDEVKVNQILKTLSEVEE
SISTLISQPN
>P34087
MFFIKDLSLNITLHPSFFGPRMKQYLKTKLLEEVEGSCTGKFGYILCVLDYDNIDIQRGR
ILPTDGSAEFNVKYRAVVFKPFKGEVVDGTVVSCSQHGFEVQVGPMKVFVTKHLMPQDLT
FNAGSNPPSYQSSEDVITIKSRIRVKIEGCISQVSSIHAIGSIKEDYLGAI
>Q9UHB6
MESSPFNRRQWTSLSLRVTAKELSLVNKNKSSAIVEIFSKYQKAAEETNMEKKRSNTENL
SQHFRKGTLTVLKKKWENPGLGAESHTDSLRNSSTEIRHRADHPPAEVTSHAASGAKADQ
EEQIHPRSRLRSPPEALVQGRYPHIKDGEDLKDHSTESKKMENCLGESRHEVEKSEISEN
TDASGKIEKYNVPLNRLKMMFEKGEPTQTKILRAQSRSASGRKISENSYSLDDLEIGPGQ
LSSSTFDSEKNESRRNLELPRLSETSIKDRMAKYQAAVSKQSSSTNYTNELKASGGEIKI
HKMEQKENVPPGPEVCITHQEGEKISANENSLAVRSTPAEDDSRDSQVKSEVQQPVHPKP
LSPDSRASSLSESSPPKAMKKFQAPARETCVECQKTVYPMERLLANQQVFHISCFRCSYC
NNKLSLGTYASLHGRIYCKPHFNQLFKSKGNYDEGFGHRPHKDLWASKNENEEILERPAQ
LANARETPHSPGVEDAPIAKVGVLAASMEAKASSQQEKEDKPAETKKLRIAWPPPTELGS
SGSALEEGIKMSKPKWPPEDEISKPEVPEDVDLDLKKLRRSSSLKERSRPFTVAASFQST
SVKSPKTVSPPIRKGWSMSEQSEESVGGRVAERKQVENAKASKKNGNVGKTTWQNKESKG
ETGKRSKEGHSLEMENENLVENGADSDEDDNSFLKQQSPQEPKSLNWSSFVDNTFAEEFT
TQNQKSQDVELWEGEVVKELSVEEQIKRNRYYDEDEDEE
>P40165
MSTLKVVSSKLAAEIDKELMGPQIGFTLQQLMELAGFSVAQAVCRQFPLRGKTETEKGKH
VFVIAGPGNNGGDGLVCARHLKLFGYNPVVFYPKRSERTEFYKQLVHQLNFFKVPVLSQD
EGNWLEYLKPEKTLCIVDAIFGFSFKPPMREPFKGIVEELCKVQNIIPIVSVDVPTGWDV
DKGPISQPSINPAVLVSLTVPKPCSSHIRENQTTHYVGGRFIPRDFANKFGFEPFGYEST
DQILKL
>Q9FML6
MAINEPEFATAVAVATKKRTFKKFSFRGFNVDALLKMSNVDLAKLFNARVRRRFYRGLKK
QPLILIKKLRRAKKEASDENKMKPEVVKTHLRNMIIVPEMIGSVVGVHNGKKFNEIVIKP

EMIGHYLAEFSMTCKKVNHHRPRICGCCCFRRSTRFIPLR
>P53930
MAPTISKRIKTLSVSRPIIYGNTAKKMGSVKPPNAPAEHTHLWTIFVRGPQNEDISYFIK
KVVFKLHDTYPNPVRSIEAPPFELTETGWGEFDINIKVYFVEEANEKVLNFYHRLRLHPY
ANPVPNSDNGNEQNTTDHNSKDAEVSSVYFDEIVFNEPNEEFFKILMSRPGNLLPSNKTD
DCVYSKQLEQEEIDRIEIGIEKVDKEIDELKQKLENLVKQEAINGS
>P53107
MDEILAKAGSQAVTFAIKSGISIASTYALKTITNFVVQIPKDDARRIDQLKFKLESRMAI
VSSAIDLIKLVAARGNTNLQITLRLTKDLKEEIDRFDEKINEMTQKVEGSRSAKTQNEAI
KAVENYIKDLLLRIEEITPFINLSLTTSGANLNSALPYQLSPGLLLKASDFVSENNRKYE
KAMKSNEKGTGDKEILKVQVGPTFEVTLFSIFYNLTSENNGQSGIVWKEDMKRAKARIYR
LNSTGRKYDYFMKIEQDFNDGRYHEDDDKEDTPQELAIDLNHIKKLFFSVSGKLLRLEEQ
DSPVLVLKIDRSDDKENESSEGDKGLLDDITWYAVSGYEAIEEDEEEDEEEDEEEGKDGE
ERKEEEEEENKLEDKDSSITLLEYIIRLTSLQSNDQKSILEVSDERLSIYLNDENTNSRK
DRISNSTIEETEKKLKNLKL
>Q06698
MAKKTKNNSKSSTPVNDVPTTAGKKKAKGKKGQEPEPEDDKRAKQQSNRAKVTSTASWTG
KLPHTILHETCQKRKWNKVEYDMKKIGDKGFIAIAVLSFTDPKTKETLTARMNDPTYDKA
SGKGLVIPQETPIEARHMASTIALYRIAYNTNLHMMLPPNHRKTWYALDDFRKDNLKTDE
KRINKLFDLDPFKTMVEDRKLKAQREKEQVAQNNQAQKEQVARTILSSHGGISSSGKDRQ
ERKVASHKNSHNPSLVRFPKKVWENSIFVDLDESSRQLIETSLKEKIDWQAKKISHKNET
IAENREDLKAKLLTLQFRPKHVEEAMLYKDPLSFLLFNLPEDDLPPFFHKKKGDTKNKVE
ITNLPLSTRMIVERLTEIGVSSDEALLALQQNDMNENEAAGFLTREILPTLNSNTNEPVS
ETESIECWNQELESLESIYEGCVMDAKEDSHYTLNLIEKLKIKLKVYRTKNYPASLPGIV
VSTFDKNYKLPDYIKKQILTRLLHYLQEGNLIGDMLVYHIYEWLKENISKIIDNPGPLIP
DSDSKGAINKRNISNGKRSINNSSSRKFTKTTISEDTLSVLREEYTKRIKSSEYKSMQLV
REQLPAWKKQKVIIDIINKNEVVLITGETGSGKSTQVVQFILDFLQKEKGDFGKTKIVCT
QPRRISAIGLAERVSDERCVTCGEEVGYVIRGVNKTKASTRIKFMTTGVLVRLLQNARTM
LENTIVVIDEVHERSIDTDLIVTLMKNLLHRVRGMKIVLMSATVNVDLFKKFFPGLATCH
IEGRTFPITDYFLEDILSDLDFKIKREKALSYDDDSVDERNNDDQYLKPRADSKFFTSGQ
INYDLLCQVVEYVHKRLKAANDNGSIIVFLPGVGEINKCCNLLANKSNEADFMVLPLHSA
LTPEDQKRVFKKYHGKRKVVVSTNIAETSITIDDCVATIDTGRAKSMFYNPKDNTTKLIE
SFISKAEVKQRRGRAGRVREGLSYKLFSKNLYENDMISMPIPEIKRIPLESLYLSVKAMG
IKDVKAFLSTALDAPPLPALQKAERILTTIGLVDESDKSLTQLGQFISLMPVMDSKHGKL
LIYGILFGCTDISVLLVSILGIGVLPFIGGFENREKIKKLLCKYESRGDLFAVLEIVRDY
FKIKDSSIKRKYLRDNLLSYNKINEIKSSTAQYYSILKDVGFLPMDYKVGSISDLNRNER
NFDILRAILTGAFYPHIARVQLPDVKYLSTSSGAVEKDPEAKMIKYWIRSEEYQDKLEEY
KTKISQETQKVDLEDLPLPATRAFIHPSSVLFSTNSVNLEDAKLLSEVDGPISRQSKIPT
VVKYPFVLFTTSQVTNKLYLRDLTPTTTLSLLLFGGAISYDIGGTIHSPGIVVDNWLPIR
TWCKNGVLIKELRTQLDEAIRKKLESPDYAKKSQIDNSGADKTLKIVEKIIASEQ
>P23643
MVKKKTNNDKGKEVKENEGKLDIDSESSPHERENDKKKTEDDSLRATESEETNTHNANPN
ETVRADKFSQEESRPIEDSPHTDKNTAQESCQPSSAEDNVINTDITSLNEKTSTNDEQEK
GLPLKISEGPFTISTLLDNVPSDLIYTCCEAYENHIFLGTTTGDLLHYFELERGNYMLVS

QTKFDAESNSKIDKILLLPKVEGALILCDNELVLFILPEFAPRPNTTRLKGISDVVICNF
SRSSKAYRIYAFHAEGVRLLKISADSLVLTKAFNFKLIDKACAHEETLMVSKLNSYELIN
LKSSQVIPLFRISETDEDLEPIITSFNEQSEFLVCSGGGSYDSGAMALVVNHHGDIIKGT
IVLKNYPRNVIVEFPYIIAESAFQSVDIYSALPSEKSQLLQSITTSGSDLKISKSDNVFT
NTNNSEEFKEKIFNKLRLEPLTHSDNKFRIERERAFVEESYEEKTSLIVYNNLGIHLLVP
TPMVLRFTSCEESEIDNIEDQLKKLAKKDLTKFEHIEAKYLMSLLLFLMTLHYDHIEDEV
MKKWCDFSDKVDIRILFYMFGWKVYSEIWCFHGLINIVERLKSLKLTNKCENILKMLLMM
KNELKKKNKTGLLTNDFDDIMKTIDITLFKLRLEKKETITVDMFERESYDEIIREINLHD
DKLPRIELLIEIYKEKGEYLKALNLLREAGDYISLVSFIEENLKKLPEDYIKERIADDLL
LTLKQGDENTEECAIKKVLKILDMACINKNDFLNKIPAEETSLKVSFIEQLGVQNSNDSK
FLFNYYLAKLREIINQSNIWSILGDFIKEYKDDFAYDKTDITNFIHIKLKHSLQCENFSK
YYEKCENLKSENEKDDEFINFTFDEISKIDKEHILTLLFFPNELTNWVSSEELLKIYLSF
NDFRSVEKYIGKQNLVAVMKQYLDISSLNYSVELVTNLLQRNFELLDDTDIQLKILETIP
SVFPVQTISELLLKVLIKYQEKKEESNLRKCLLKNQISISDELSRNFDSQG
>Q61337
MGTPKQPSLAPAHALGLRKSDPGIRSLGSDAGGRRWRPAAQSMFQIPEFEPSEQEDASAT
DRGLGPSLTEDQPGPYLAPGLLGSNIHQQGRAATNSHHGGAGAMETRSRHSSYPAGTEED
EGMEEELSPFRGRSRSAPPNLWAAQRYGRELRRMSDEFEGSFKGLPRPKSAGTATQMRQS
AGWTRIIQSWWDRNLGKGGSTPSQ
>O88593
MLFACALLALLGLATSCSFIVPRSEWRALPSECSSRLGHPVRYVVISHTAGSFCNSPDSC
EQQARNVQHYHKNELGWCDVAYNFLIGEDGHVYEGRGWNIKGDHTGPIWNPMSIGITFMG
NFMDRVPAKRALRAALNLLECGVSRGFLRSNYEVKGHRDVQSTLSPGDQLYQVIQSWEHY
RE
>P06208
MVKESIIALAEHAASRASRVIPPVKLAYKNMLKDPSSKYKPFNAPKLSNRKWPDNRITRA
PRWLSTDLRDGNQSLPDPMSVEQKKEYFHKLVNIGFKEIEVSFPSASQTDFDFTRYAVEN
APDDVSIQCLVQSREHLIKRTVEALTGAKKATIHTYLATSDMFREIVFNMSREEAISKAV
EATKLVRKLTKDDPSQQATRWSYEFSPECFSDTPGEFAVEICEAVKKAWEPTEENPIIFN
LPATVEVASPNVYADQIEYFATHITEREKVCISTHCHNDRGCGVAATELGMLAGADRVEG
CLFGNGERTGNVDLVTVAMNMYTQGVSPNLDFSDLTSVLDVVERCNKIPVSQRAPYGGDL
VVCAFSGSHQDAIKKGFNLQNKKRAQGETQWRIPYLPLDPKDIGRDYEAVIRVNSQSGKG
GAAWVILRSLGLDLPRNMQIEFSSAVQDHADSLGRELKSDEISKLFKEAYNYNDEQYQAI
SLVNYNVEKFGTERRVFTGQVKVGDQIVDIEGTGNGPISSLVDALSNLLNVRFAVANYTE
HSLGSGSSTQAASYIHLSYRRNADNEKAYKWGVGVSEDVGDSSVRAIFATINNIIHSGDV
SIPSLAEVEGKNAAASGSA
>Q09760
MQRSAKLKIRAPRKFSANQVDFATHWEVLQRAIGDIFQKSTSQLSFEELYRNAYILVLHK
YGEKLYNHVQDVIRSRLKEETVPAIYKNYDASLLGNALLDIRKNDSYSTSWSRSLEAAHR
FLSSLVNSWKDHIVSMQMISSVLKYLDKVYSKSADKVPVNENGIYIFREVVLLNSFEIGE
KCVETILILVYLERKGNTINRPLINDCLDMLNSLPSENKKETLYDVLFAPKFLSYTRNFY
EIESSTVIGVFGVVEYLKKAEKRFEEEKERSKNYLFTKIASPLLSVVEDELLSKHLDDLL
ENQSTGFFSMIDSSNFEGLQLVYESFSRVELGVKSLKKYLAKYVAHHGKLINETTSQALE
GKMAVGRLSSNATMATLWVQKVLALWDRLNTIISTTMDADRSILNSLSDAFVTFVDGYTR

APEYISLFIDDNLKKDARKAIEGSIEATLQNSVTLFRFISEKDVFEKYYKTHLAKRLLNN
RSISSDAELGMISRLKQEAGNVFTQKLEGMFNDMNLSQELLQEYKHNSALQSAKPALDLN
VSILASTFWPIDLSPHKIKCNFPKVLLAQIDQFTDFYLSKHTGRKLLWYPSMGSADVRVN
FKDRKYDLNVSTIASVILLLFQDLKENQCLIFEEILEKTNIEVGDLKRNLQSLACAKYKI
LLKDPKGREVNAGDKFYFNENFVSNLARIKISTVAQTRVEDDSERKRTLEKVDESRKHQA
DACIVRVMKDRKVCEHNQLMAEVTRQLNPRFHPSPMMIKRRIEALIEREYLQRQADNGRI
YEYLA
>Q8N7A1
MADSQLFCVAEERSGHCAVVDGNFLYVWGGYVSIEDNEVYLPNDEIWTYDIDSGLWRMHL
MEGELPASMSGSCGACINGKLYIFGGYDDKGYSNRLYFVNLRTRDETYIWEKITDFEGQP
PTPRDKLSCWVYKDRLIYFGGYGCRRHSELQDCFDVHDASWEEQIFWGWHNDVHIFDTKT
QTWFQPEIKGGVPPQPRAAHTCAVLGNKGYIFGGRVLQTRMNDLHYLNLDTWTWSGRITI
NGESPKHRSWHTLTPIADDKLFLCGGLSADNIPLSDGWIHNVTTNCWKQLTHLPKTRPRL
WHTACLGKENEIMVFGGSKDDLLALDTGHCNDLLIFQTQPYSLLRSCLDCIGKNSIMLES
QISLLPPKLLQQVLKKITFWAAANHREEQRVQKEETENKYQWISSN
>P47897
MAALDSLSLFTSLGLSEQKARETLKNSALSAQLREAATQAQQTLGSTIDKATGILLYGLA
SRLRDTRRLSFLVSYIASKKIHTEPQLSAALEYVRSHPLDPIDTVDFERECGVGVIVTPE
QIEEAVEAAINRHRPQLLVERYHFNMGLLMGEARAVLKWADGKMIKNEVDMQVLHLLGPK
LEADLEKKFKVAKARLEETDRRTAKDVVENGETADQTLSLMEQLRGEALKFHKPGENYKT
PGYVVTPHTMNLLKQHLEITGGQVRTRFPPEPNGILHIGHAKAINFNFGYAKANNGICFL
RFDDTNPEKEEAKFFTAICDMVAWLGYTPYKVTYASDYFDQLYAWAVELIRRGLAYVCHQ
RGEELKGHNTLPSPWRDRPMEESLLLFEAMRKGKFSEGEATLRMKLVMEDGKMDPVAYRV
KYTPHHRTGDKWCIYPTYDYTHCLCDSIEHITHSLCTKEFQARRSSYFWLCNALDVYCPV
QWEYGRLNLHYAVVSKRKILQLVATGAVRDWDDPRLFTLTALRRRGFPPEAINNFCARVG
VTVAQTTMEPHLLEACVRDVLNDTAPRAMAVLESLRVIITNFPAAKSLDIQVPNFPADET
KGFHQVPFAPIVFIERTDFKEEPEPGFKRLAWGQPVGLRHTGYVIELQHVVKGPSGCVES
LEVTCRRADAGEKPKAFIHWVSQPLMCEVRLYERLFQHKNPEDPTEVPGGFLSDLNLASL
HVVDAALVDCSVALAKPFDKFQFERLGYFSVDPDSHQGKLVFNRTVTLKEDPGKV
>P40465
MEYEDLELITIWPSPTKNKLCQFIKQNLSKEHVVTQLFFIDATSSFPLSQFQKLVPPTLP
ENVRIYENIRINTCLDLEELSAITVKLLQILSMNKINAQRGTEDAVTEPLKIILYINGLE
VMFRNSQFKSSPQRSHELLRDTLLKLRVMGNDENENASIRTLLEFPKEQLLDYYLKKNNN
TRTSSVRSKRRRIKNGDSLAEYIWKYYADSLFE
>Q02979
MKFGKTFANHRIPEWSSQYVGYKSLKKMIKEITRLQEDIYRAHNKNSYDEGRPPTKMRDS
SNSAQNYLDSPKIQKLLASFFFAVDRDIEKVDTFYNSQYAEYKKRFERLLSSNQFNEIKS
TLVVDANKEDAVAQTLLTKDTREMNMLLKGTSQASRLSYHKDDLIEIQSILAELRKQFRN
LKWYAELNKRAFGKILKKLDKKVGTNQQMSTMKTRILPLQFANDSLITKDLSLLKTIWEQ
VTFRINSYERVMRSTSPNANANDNTEFFKIICVFIEEDDSKGLIRELTNLYSELSLIPTR
IMISVLNKAALSKSLACIDAILKVIPSLNDSEDINRRNFFHHHIIAIGKLIRKQEILSRK
KKSQPSKYTNSEGEIVTDLRTLHTTLSAPAESDSITEEEKSSACTLSYILEELPIHLRPC
LFQHDNYKRTPLHYSCQYGLSEVTKLIIKLMKEWNIWNEIPIDDVSAFGDAESLTPLHLC
VLGAHPKTTEVLLQSLDPNVKLKSSSLLHLATEWNNYPLLHVLLSSKRFDINYQDNELHE

TPLYLACRLNFFEAAVCLLYNGADLEIREKLFGWTAIFVAAAEGFTDIVKLLIANNANFD
IEDEGGWTPMEHAVLRGHLHIADMVQIRDELVTHPHSQLNSGSEEKEPLNEISAGELNER
NENGNGGNKGSLGKLAGPIKSYGHRFLDNNESLILITLGSNDTRNKSPSISLSSEALAKV
IGLETDCALSLVISCNDSIDKSSVILDLPLDDNVDAVDFKVPFKVDYSHTLYFDIVPTYG
TRSLETHNRIDCQKNNNNYVMARGVSMLNKSYSSVGVNRSILNGSVTVPIIANHTLEILG
TLKFEYIIITPFEHPQLPLERTETYWKSLVSTRVIGHRGLGKNNPNKSLQIGENTVESFI
MAASLGASYVEFDVQLTKDNVPVVYHDFLVAETGVDIPMHELTLEQFLDLNNADKEHIQR
GAGHSPHHVNGADTALQKYRGRSVDDSDVSTLRRAWDLHDNDPNGKSNNAHWSDNRMRLT
KTFKKNNFKGNARGHSIASSFVTLKELFKKIPANVGFNIECKFPMLDEAEEEELGQIMME
MNHWVDTVLKVVFDNANGRDIIFSSFHPDICIMLSLKQPVIPILFLTEGGSEQMADLRAS
SLQNGIRFAKKWNLLGIVSAAAPILKAPRLVQVVKSNGLVCVTYGVDNNDPENASIQIEA
GVDAVIVDSVLAIRRGLTKKNEK
>Q9USR3
MDCIDKSIRRESLRKLGCLPDHGWNLFVQSSNRYSKLLEADSKLFKAQRIEDTVLSICEV
TLENKASENALTLFQQLRFYLLNYLFLEDTSEYKSVLCSLDESVYPNLLPLTANICPCIL
KALLALVRQHRELSSDIVKFLNSIFLALTRILNTPHSSNNNPTHTKTTIFEKVFYASHLG
FFFEEIADACISALPQDACLWAMEVCNTILVSDLPCKLIDLHNDSFLHHSNYYAGFGLSY
YLYLSSMKAYINSSGCLWYEDATSFWDIVKHRPSSPEEKPFLTQFQSILNLSDKFLSKTG
TLRLQEKLGTSCNLFFVLVLKLALLSSSYIGFVPYTYSDWINVLLGSISEDPELLPHLIE
LMASLACVFPTHFQFAMKRLRLIAIYAPRFDDGNVTYAQLASRKLAQLFNYSSRDTAITS
IYQFANLLSPADTPDSYLAAPKERLSISDNMSSSSSQTATVNTISNYLNVIDSVREIALT
VNDEKIYGLAISLLIQKFSRKFDSRVSTELVRTLADISTKANDRDFSILVQFYSIQNKML
VKNGDEALTIAICDSRCLIARGTESNSFKRSDLLKSLLEEIIHIGIVRDTPAPELKGYYF
GMSKAVQALVECVAGTDWSSLPKEEMYPALFRDMWFTLVINRFRYSVDLGETLEVDLENI
AKNSPLLVFEDFGSNFESNLELNTVLRTHIEHSVISQIKSELLVRVPNIDLKPLSTSEIC
FLSAVLLLESLRCKSNKLSALVEYLLDPSLRDSQLPQSIRAIALYNLTSFVESLSKERRV
ASSTIDEFQKLLCLCCNRVDCVRQLALECMNYIMETLPHLLGIKEILFSVLELSSLLWKA
RTEECTDQYVPQLLYKSNKLNLSVILSDIYSCREEVLFQFNRHARSWIQNSSKAIPYQVK
NLLESYLADFVDFDDLEVVELGRSLAVELGTRIVSSDRDNFVLPAFGNWTPDTSSEFMAE
YTIRQRYSHVDNSILNIEGDMSTDRIDLILNEKSKLFETHLAALKSLEDDILQGNTVSPQ
RLRNEVRKAAAHAVQEPIFQFSVLARKIVRIPFLDFSPSSFKLGITLWNWMMNQVPSFSS
FLISNIIRNWKNEIVTEKRGYFSTAKSKSPLALPMTYSPTERASFLSYKNKVMSQMIPHL
LLLQLIAGNFEGFWYGDRQTAKLIVHFMKFVLKKITSMEVNLNVLTRELHFKFVSFGLRI
AENLLNSPLGSRFYNLCVDAGLCWFSGMPNWTYGADKLHVAAEISVMRSLRDKLDSFLLR
YPLKVSTTLKQKLLIILLNNEMYMLYTWLTPVLHGRNVRMVEPIPDSDAASSPITLEMLQ
VAWNVSPNIVLYAPKRFQNAPLKQMALNFVIANPFTSVKHEVALEYLFEHYPSGEFPIDR
KFILYWEPMYPVSGPVMFMPNVKWNPQLLQYTMRSMESYPVSVTFFYVPQIVQSLRYDSM
GYAESFILETSGTSQLFAHQILWNMKANLYKDEAATVPDSIKPILDRVMDKMINSLSGED
KQFYEREFTFFNEVTSISGKLKPFIRKSKPEKKAKIDEEMKKIKLDVGVYLPSNPDGVIV
GIDRKSGKPLQSHAKAPFMATFKIRKEKLVDADPEELAVNGTEEEAGDSAKKQTYEVWQS
AIFKVGDDCRQDVLTLQLIAMFKNIFNSVGLDVYLFPYRVTATNPGCGVIDVLPNCISRD
MLGREAVNGLYDYFRTKFGDEDSIAFQKARSNFVQSMAAYSVITYLLQFKDRHNGNIMID
DQGHILHIDFGFIFDIAPGGITFESAPFKLTTEMIAVMGGSNKSQPFQWFQELCVKAFLA
CRPYAHYICQAVEVMLDSGLPCFKGQLTITHCLERFALNLNERQASTFMLHLIEQSYANK

RTLMYDQFQKATNGIPY
>O13641
MEKESEIQSLPPTRRYTISLALPISSLNVAYNLQLKTSFVWKISRIVSLYGIDEIILLED
PEYVQNTQVHTLSSDAYLKDPTKFLTDLLCYFETPFFMRKELFPLNPHLKYTSCFPLLPL
RNDKASTVNIEFPYREGIVTHPSPQAKNKYIINAGLSHNVIVSSPSVLAPRTRVTVRLKA
QSPNEEGQLQGDIVSFSAPREKGGHYWGYKVRSCLSSQLCKSSPYKGGYDFVVQINSQTS
AITSKELEASLPSSFRHAVLVLQDNVVQKELVDSTAKSISFNPFPVSFLNDPIPEEILLA
AMTRLSDMLLMHGRR
>Q8WUX9
MWSPEREAEAPAGGDPAGLLPPEWEEDEERMSFLFSAFKRSREVNSTDWDSKMGFWAPLV
LSHSRRQGVVRLRLRDLQEAFQRKGSVPLGLATVLQDLLRRGELQRESDFMASVDSSWIS
WGVGVFLLKPLKWTLSNMLGDNKVPAEEVLVAVELLKEKAEEVYRLYQNSPLSSHPVVAL
SELSTLCANSCPDERTFYLVLLQLQKEKRVTVLEQNGEKIVKFARGPRAKVSPVNDVDVG
VYQLMQSEQLLSRKVESLSQEAERCKEEARRACRAGKKQLALRSLKAKQRTEKRIEALHA
KLDTVQGILDRIYASQTDQMVFNAYQAGVGALKLSMKDVTVEKAESLVDQIQELCDTQDE
VSQTLAGGVTNGLDFDSEELEKELDILLQDTTKEPLDLPDNPRNRHFTNSVPNPRISDAE
LEAELEKLSLSEGGLVPSSKSPKRQLEPTLKPL
>Q9FV49
MAIGNPEVATMGKENTEAESSNGNESQLSSDLTKSLDLAEVKEDEKDNNQEEEDGLKAEA
STKKKKKKSKSKKKKSSLQQTDPPSIPVLELFPSGDFPQGEIQQYNDDNLWRTTSEEKRE
MERLQKPIYNSLRQAAEVHRQVRKYMRSILKPGMLMIDLCETLENTVRKLISENGLQAGI
AFPTGCSLNNVAAHWTPNSGDKTVLQYDDVMKLDFGTHIDGHIVDSAFTVAFNPMFDPLL
AASRDATYTGIKEAGVDVRLCDVGAAVQEVMESYEVEINGKVYQVKSIRNLNGHSIGRYQ
IHAEKSVPNVRGGEQTKMEEGELYAIETFGSTGKGYVREDLECSHYMKNYDVGHVPLRLP
RAKQLLATINKNFSTLAFCRRYLDRLGETKYLMALKNLCDSGIIEPCPPVCDVKGSYISQ
FEHTILLRPTCKEIISKGDDY
>Q6P0E1
MYNMMETELKPPAPQPNTGGTGNTNSSGNNQKNSPDRIKRPMNAFMVWSRGQRRKMAQEN
PKMHNSEISKRLGAEWKLLSESEKRPFIDEAKRLRALHMKEHPDYKYRPRRKTKTLMKKD
KYTLPGGLLAPGGNGMGAGVGVGAGLGAGVNQRMDSYAHMNGWTNGGYGMMQEQLGYPQH
PSLNAHNTAQMQPMHRYDMSALQYNSMTNSQTYMNGSPTYSMSYSQQSTPGMTLGSMGSV
VKSESSSSPPVVTSSSHSRAGQCQTGDLRDMISMYLPGAEVQDQSAQSRLHMSQHYQSAP
VPGTTINGTIPLSHM
>O88958
MKLIILEHYSQASEWAAKYIRNRIIQFNPGPDKYFTLGLPTGSTPLGCYQKLIEYYKNGD
LSFQYVKTFNMDEYVGLPRDHPESYHSFMWDNFFKHIDIHPENTHILDGNAADLQAECDA
FEEKIQAAGGIELFVGGIGPDGHIAFNEPGSSLVSRTRVKTLAMDTILANARFFDGDLAK
VPTMALTVGVGTVMDAKEVMILITGAHKAFALYKAIEEGVNHMWTVSAFQQHPRTVFVCD
EDATLELKVKTVKYFKGLMLVHNKLVDPLYSIKEKEIQKSQSAKKPYSD
>O43137
MSSALYKQSTNFTHSTGSFLQSAPVELTTVSGYQEFLKKQEKKNYEIQTVLSEDKSHGYV
LKDGEVIANIIGEAKDYLLDLAGQA
>Q06251
MELPSINSTTSISDNQELRNYYDKLLFKNNSGKSLADLPGKMADFNDNSAAAHPRSRVDF

INGYIGFREDKQSLLGQKNTKRASFSAFADEGRKQSEMSINGKSPNLSLFSFEFNGTPTQ
DRKPYKQDYLNVMNTSPNNILSPLNNSSQKYYPQKQQQQQQQQQQQQQQSIFDPGRRSSY
ISDALIHGNAATQQPQYSQPVYINNNPSLQVPYTAPSEYTQQQQYSSPFNARRNTQPVLN
LHPAAAPTNDAGLAVVDGKNLTSSKELHDLYLDCGSNYFASDKVYKFIDSIKGTLRGDNV
SASSSRIIEFLDFLKNCNLNYNPQSDAFISTAVSNASSTGAAKSKNSTSMHLHYKPLVLV
SLKNGKLELLSKPQTATLILKRGDLVIIDGDRGKDLVLVVEPVVDINLALFINFLKKKIH
FDSLITNSQQHFPNDQFIKTLVDTTNGKPVAHELNPKLYDIIELTQLIIPSKQVLRFATP
WESSTNLHNKFQDELKALHIAQLKLRSLNNNNSGGGLNIKILNAEFQFDRKKLTFYYICQ
ERNDFRDLIKELFKFYKTRIWLCAIPNNLSIDSKFYDSNKFEWEMYQDMMSHYSMDNTGI
VVAPELNRLKLDDFQIGVYMELVKVLFG
>O94636
MSVYLLHLASTHAHFHLPELETLAKIENVEFWLIPKSEYLNVAFKSDKSRNQALFSHAIK
EFQDSISPNDATTQNPFMLAVVNSNEDARRWIRRSIFCKGIYEIYCIGDSFTRLHEKMKE
LNPAPWDSFKHNSSYKFTFETFGTRRTMKEQLSIISDFEYMQLQGPVSMHNPQHVFTVLE
NRRNNVEGPKVYFGHWCGSGSRDAIDTFDLKQRSYIGITSFDAELSLVTAQMAMAAPGKL
IYDPFVGTGSFLYTCSFFGAHTLGSDIDGRQMRGKNGRSIKSNFRQYKLSPFFLDTFTGD
VTNCPLRKNLLLDAIVCDPPYGVRAGAKKIAKCSQRPPKESSSTGNHYPKLEQYQISDMV
YDIICFASDRLVDGGRLVLWLPTITEEYGIDDIPSHPYLSLIYNSIQPFTHWSRRLLTFQ
RLPRAHDSKSLNLLPKINNKTPSHHNFREKVS
>Q8TE02
MTPSEGARAGTGRELEMLDSLLALGGLVLLRDSVEWEGRSLLKALVKKSALCGEQVHILG
CEVSEEEFREGFDSDINNRLVYHDFFRDPLNWSKTEEAFPGGPLGALRAMCKRTDPVPVT
IALDSLSWLLLRLPCTTLCQVLHAVSHQDSCPGDSSSVGKVSVLGLLHEELHGPGPVGAL
SSLAQTEVTLGGTMGQASAHILCRRPRQRPTDQTQWFSILPDFSLDLQEGPSVESQPYSD
PHIPPVDPTTHLTFNLHLSKKEREARDSLILPFQFSSEKQQALLRPRPGQATSHIFYEPD
AYDDLDQEDPDDDLDI
>Q54XQ2
MKTSKDSSNSNSNNNNNNNNNNNNNNNNNNNGLNGTYSSKSLSPPTSPKQMSGNSIISNST
GNLSSGSGSGSSSGGNKKFINRSFNTFIDFIKKPARRNSKAHNTPPPYPEVDTGFGYFLE
LDSKPPKPFDEKDDPIHNSTGSTDSWEGDLNSSGGGGKQPQQTQQQSSGENLNNSSDRNN
NSNENNQIVDDNSNVFNKGVVVQTVPMTEIGLFPVSLMMAQQRAEHPINVEDIESDVQTC
SIPNSKPLYSSLGCSTNSSNSGSSISAPNITFQSFGISPVNNNNNNNNNSNNNSNSNSNS
NSNSNNNNNNNNNNNNNNNNNNNNNNNNNNNSTVNSNNSSLNNSPRYLNSSSSPRSMQHLSS
KITTTTTTTTTTTTTTSDDNNGNTNNNISNNNNIINNSNNNSNSNNNNNNNINNTNHKFK
TMEKYFSSKENFPIPFPKLKDELGVIIENSSNKGYVGGNKGSEDRRKKIEQKKKQVPAPE
MKATKEKFIETITDPTTLNSFRSFMENTQSNENLEFFLEVKRFNTIQDQVLLKHTCDDIW
RRFFDDLAVTQLCVESSLKKLINNRRENPTHSMFNEVLDLLLDDIVCDAFRNYISSPFNP
EWKSEFKKKFTNNTYSTTTQPINNFNNTNNNNNNNCSTPPNNYSSSPIKQSNINNNNNNA
SSSNIASSSNVNNNNNNSNGSNTSSSHHRERLDNIKGNRERVDSNGKERSIDNKDILSLL
ESNLSNHSNSSSNSNGKDKDKDKDKNENTTDNSNNNNNSNNNLNNLIIKNNFNNILQSPQ
IVSKINEIFKDSNNSQLSISITLDNDEDPTIFHLDESDMESLIEEVVKDNISVHTEISYS
DVSIHKWIASGSSGRVYNGQYKGKDVAIKVLGPEVCVHFDLNEFKREVALMSIFKHDNLA
RCLGAGQYDDKYFHLTEYCHNGSLFSYLRDQRNNISFGQRLHFALGIARGMRYLHSMSII
HRDLKSMNILLTKRLKIKIVDFGTSRVANKYNMTTHVGTQAWMAPEIFTSRTYTNKVDVY

SYAIILFEIFTRKSAYDENANINIPNMVMKGERPELPKDMQTSISNIIKKCWQQKPSNRP
SFIKIVAYLESIIYPSVSNSLGLVASTSFSSSALWSGQILAQPKN
>Q8WXD5
MSEWMKKGPLEWQDYIYKEVRVTASEKNEYKGWVLTTDPVSANIVLVNFLEDGSMSVTGI
MGHAVQTVETMNEGDHRVREKLMHLFTSGDCKAYSPEDLEERKNSLKKWLEKNHIPITEQ
GDAPRTLCVAGVLTIDPPYGPENCSSSNEIILSRVQDLIEGHLTASQ
>Q9FVL0
MGTLDTKGFTEEQEALVVKSWNAMKKNSAELGLKLFLKIFEIAPSAQKLFSFLKDSKVPL
EQNTKLKPHAMSVFLMTCESAVQLRKSGKVTVRESSLKKLGANHFKYGVVDEHFEVTKFA
LLETIKEAVPEMWSPAMKNAWGEAYDQLVNAIKSEMKPSS
>Q91437
MATLFLDDGSSFKGRLFGASSTVSGEVVFQTGMVGYPEALTDPSYLSQILVLTYPLIGNY
GIPKDEEDEHGLSKWFESAKIHAAALVIGENSQNPSHWSSVRSLDQRLKEHGIPALEGID
TRSLTKKIREKGTLLGKLVIDGTDENSLPYDDPNKRHLVKEVSIKEPKVYHPSGNVKIMA
VDCGMKYNQIRSLCKRGAAVTVVPWDYLFDSNEFDGLFISNGPGDPEYCQQTINNVKKAI
SEEKPKPLFGICLGHQILSLAIGAKTYKMKYGNRGHNQPCIHEGTQRCFYTSQNHGFAVE
PCSLPRDWSVLFTNANDQSNEGIIHNSKPLFSVQFHPEHKAGPTDLVDLFDIFLECARDV
KLGVNLDKTVKGRVISHYSFKNGTENSKTPPGRIQPHKVLILGSGGLSIGQAGEFDYSGS
QAIKALKEENVQSVLINPNIATVQTSKGLADKVYFLPITPEYVTQVIMNERPDGILLTFG
GQTALNCGVELQKRGVLEKYHVRVLGTPVSSIEMTEDRKIFVEKMAEINEYVVPSEAAFT
LEQAQGAAERLGYPVLVRAAFALGGLGSGFAQNKEELVTLVTQAFAHTSQILVDKSLKGW
KEIEYEVVRDAYDNCITVCNMENVDPLGIHTGESIVVAPSQTLNDKEYNLLRTTAIKVIR
HLGVVGECNIQYALSPESEQYFIIEVNARLSRSSALASKATGYPLAYVAAKLALGIPLPV
LRNSVTNSTTANYEPSLDYCVVKVPRWDLSKFLRLSTKIGSSMKSVGEVMAIGRNFEEAF
QKALRMVDENCVGFDHTLKPASDEELETPTDKRIFVLAAALRAGYEIDRLYELTKIDKWF
LHKMKNIVEYSLKLSELYMKDEVPRHDLLKVKRLGFSDKQIAMAIQSTELAVRRLRQEWK
ILPVVKQIDTVAAEWPAQTNYLYLTYNGEGHDLDFTKPHVMVIGSGVYRIGSSVEFDWCA
VRCIQQLRKMGYKTRMVNYNPETVSTDYDMCDRLYFDEISFEVVMDIYELENPEGIILSM
GGQLPNNIAMDLHRQQCRILGTSPESIDTAENRFKFSRMLDTIGISQPRWKELSDTESSK
QFCTKVGYPCLIRPSYVLSGVAMNVAYSDNDLEKFLSSAVAVSKEHPVVISKFIQEAKEI
DVDAVACDGVVIAVAISEHVENAGVHSGDATLVTPPQDLNQKTTERIKAIVHAIGQELQA
TGPFNLQLIAKDDQLKVIECNVRVSRSFPFVSKTLGVDMIALATKVIMGEEVEPVGLMTG
TGVVGVKVPQFSFSRLAGADVVLGVEMTSTGEVACFGENRYEAYLKAMLSTGFKIPKKNI
LLSIGSYKNKSELLSTVQSLEQLGYNLYASLGTADFYTEHGVKIKAVDWPFEDTDNGCPL
KERHRNIMDYLEENHFDLVINLSMRNSGGRRLSSFVTKGYRTRRLAVDYSVPLIIDIKCT
KLFVEALRLVGDTPPVKTHIDSMSSHKLIRLPGLIDVHVHLREPGGTHKEDFASGTAAAL
AGGVTMVCAMPNTNPAITDQTSFALVQKLATAGARCDFALFLGASSDNADVLPLISNSAA
GLKMYLNDTFSTLKMDNVSLWMEHFEKWPKHLPIVVHAERQTVAAILMVAQLYQRPVHIC
HVARKEEIQIIRAAKQKGVQVTCEVAPHHLFLNEEDLESIGHGKGQVRPMLSTKEDVNAL
WENLDVIDCFATDHAPHSVEEKNSDSPPPGYPGLETMLPLLLTAVSEGRLTIDDLVKRLY
ENPRKIFSLPVQENTYVEVDLEQEWIIPSYMQFTKSKWTPFEGKKVKGRVRRVVLRGEVA
YIDGQVLVPPGYGQDVRAWPLGVPLPPPPTTVKTPEHSKPTQTETVRTRTASPRRLASSG
PAVDARFHLPPRIHRCSDPGLPNAEGEYKEKPVKKFIEQDTVSQDGYIYPPPVSRLLSPQ
NLAAQAVPHPYSLLLHPFVGQHILSVKRFTKDQLSHLFNVAHNLRLTVQKDRSLDILKGK

VMASMFYEVSTRTSSSFRAAMHRLGGSVIHFSEATSSVQKGESLLDSVQTMSCYVDVVVL
RHPEPGAVELAAKHSRKPIINAGDGVGEHPTQALLDIFTIREELGTVNGMTITMVGDLKH
GRTVHSLAYLLTLYRVNLRYVTPRNLRMPPNIIRFLASRGIKQEEFDSLEEALPDTDVLY
MTRIQKERFASEEEYEACFGQFILTPHIMTKGKKKMVVMHPLPRVNEVSVEVDSDPRAAY
FRQAENGMYVRMALLATVLGKF
>P20433
MNVSTSTFQTRRRRLKKVEEEENAATLQLGQEFQLKQINHQGEEEELIALNLSEARLVIK
EALVERRRAFKRSQKKHKKKHLKHENANDETTAVEDEDDDLDEDDVNADDDDFMHSETRE
KELESIDVLLEQTTGGNNKDLKNTMQYLTNFSRFRDQETVGAVIQLLKSTGLHPFEVAQL
GSLACDTADEAKTLIPSLNNKISDDELERILKELSNLETLY
>P12830
MGPWSRSLSALLLLLQVSSWLCQEPEPCHPGFDAESYTFTVPRRHLERGRVLGRVNFEDC
TGRQRTAYFSLDTRFKVGTDGVITVKRPLRFHNPQIHFLVYAWDSTYRKFSTKVTLNTVG
HHHRPPPHQASVSGIQAELLTFPNSSPGLRRQKRDWVIPPISCPENEKGPFPKNLVQIKS
NKDKEGKVFYSITGQGADTPPVGVFIIERETGWLKVTEPLDRERIATYTLFSHAVSSNGN
AVEDPMEILITVTDQNDNKPEFTQEVFKGSVMEGALPGTSVMEVTATDADDDVNTYNAAI
AYTILSQDPELPDKNMFTINRNTGVISVVTTGLDRESFPTYTLVVQAADLQGEGLSTTAT
AVITVTDTNDNPPIFNPTTYKGQVPENEANVVITTLKVTDADAPNTPAWEAVYTILNDDG
GQFVVTTNPVNNDGILKTAKGLDFEAKQQYILHVAVTNVVPFEVSLTTSTATVTVDVLDV
NEAPIFVPPEKRVEVSEDFGVGQEITSYTAQEPDTFMEQKITYRIWRDTANWLEINPDTG
AISTRAELDREDFEHVKNSTYTALIIATDNGSPVATGTGTLLLILSDVNDNAPIPEPRTI
FFCERNPKPQVINIIDADLPPNTSPFTAELTHGASANWTIQYNDPTQESIILKPKMALEV
GDYKINLKLMDNQNKDQVTTLEVSVCDCEGAAGVCRKAQPVEAGLQIPAILGILGGILAL
LILILLLLLFLRRRAVVKEPLLPPEDDTRDNVYYYDEEGGGEEDQDFDLSQLHRGLDARP
EVTRNDVAPTLMSVPRYLPRPANPDEIGNFIDENLKAADTDPTAPPYDSLLVFDYEGSGS
EAASLSSLNSSESDKDQDYDYLNEWGNRFKKLADMYGGGEDD
>P02405
MVNVPKTRKTYCKGKTCRKHTQHKVTQYKAGKASLFAQGKRRYDRKQSGFGGQTKPVFHK
KAKTTKKVVLRLECVKCKTRAQLTLKRCKHFELGGEKKQKGQALQF
>Q04949
MDNCNAWDKLLSQNESTINSTETATITAIIYSPSSKTLHQFINICFPEGSNSILDTTLIN
YATIGWTNDLKENYSVDVYTLIRNTDDALDLLKPFLQEHSSKVRWLILLDWTLNDQKLWL
NELSYAFNKIKQLNDDNEFSVWCLNSGEILNLQRHTTVWQSVHIDFILQTLRSFCYFNDS
SLFYICEDHTEEKREEAQRLKYQELLKHFCEDRDMKDHIEMVKRSEILIPKGCDSIGLIK
TVDERFEPTEVKEQHFLARYMDFIPTIDKIREDRKTTSGIDLDKLYPLEVFKVNIQEELG
KMFAKYRENSRI
>P42858
MATLEKLMKAFESLKSFQQQQQQQQQQQQQQQQQQQQQQQPPPPPPPPPPPQLPQPPPQA
QPLLPQPQPPPPPPPPPPGPAVAEEPLHRPKKELSATKKDRVNHCLTICENIVAQSVRNS
PEFQKLLGIAMELFLLCSDDAESDVRMVADECLNKVIKALMDSNLPRLQLELYKEIKKNG
APRSLRAALWRFAELAHLVRPQKCRPYLVNLLPCLTRTSKRPEESVQETLAAAVPKIMAS
FGNFANDNEIKVLLKAFIANLKSSSPTIRRTAAGSAVSICQHSRRTQYFYSWLLNVLLGL
LVPVEDEHSTLLILGVLLTLRYLVPLLQQQVKDTSLKGSFGVTRKEMEVSPSAEQLVQVY
ELTLHHTQHQDHNVVTGALELLQQLFRTPPPELLQTLTAVGGIGQLTAAKEESGGRSRSG

SIVELIAGGGSSCSPVLSRKQKGKVLLGEEEALEDDSESRSDVSSSALTASVKDEISGEL
AASSGVSTPGSAGHDIITEQPRSQHTLQADSVDLASCDLTSSATDGDEEDILSHSSSQVS
AVPSDPAMDLNDGTQASSPISDSSQTTTEGPDSAVTPSDSSEIVLDGTDNQYLGLQIGQP
QDEDEEATGILPDEASEAFRNSSMALQQAHLLKNMSHCRQPSDSSVDKFVLRDEATEPGD
QENKPCRIKGDIGQSTDDDSAPLVHCVRLLSASFLLTGGKNVLVPDRDVRVSVKALALSC
VGAAVALHPESFFSKLYKVPLDTTEYPEEQYVSDILNYIDHGDPQVRGATAILCGTLICS
ILSRSRFHVGDWMGTIRTLTGNTFSLADCIPLLRKTLKDESSVTCKLACTAVRNCVMSLC
SSSYSELGLQLIIDVLTLRNSSYWLVRTELLETLAEIDFRLVSFLEAKAENLHRGAHHYT
GLLKLQERVLNNVVIHLLGDEDPRVRHVAAASLIRLVPKLFYKCDQGQADPVVAVARDQS
SVYLKLLMHETQPPSHFSVSTITRIYRGYNLLPSITDVTMENNLSRVIAAVSHELITSTT
RALTFGCCEALCLLSTAFPVCIWSLGWHCGVPPLSASDESRKSCTVGMATMILTLLSSAW
FPLDLSAHQDALILAGNLLAASAPKSLRSSWASEEEANPAATKQEEVWPALGDRALVPMV
EQLFSHLLKVINICAHVLDDVAPGPAIKAALPSLTNPPSLSPIRRKGKEKEPGEQASVPL
SPKKGSEASAASRQSDTSGPVTTSKSSSLGSFYHLPSYLKLHDVLKATHANYKVTLDLQN
STEKFGGFLRSALDVLSQILELATLQDIGKCVEEILGYLKSCFSREPMMATVCVQQLLKT
LFGTNLASQFDGLSSNPSKSQGRAQRLGSSSVRPGLYHYCFMAPYTHFTQALADASLRNM
VQAEQENDTSGWFDVLQKVSTQLKTNLTSVTKNRADKNAIHNHIRLFEPLVIKALKQYTT
TTCVQLQKQVLDLLAQLVQLRVNYCLLDSDQVFIGFVLKQFEYIEVGQFRESEAIIPNIF
FFLVLLSYERYHSKQIIGIPKIIQLCDGIMASGRKAVTHAIPALQPIVHDLFVLRGTNKA
DAGKELETQKEVVVSMLLRLIQYHQVLEMFILVLQQCHKENEDKWKRLSRQIADIILPML
AKQQMHIDSHEALGVLNTLFEILAPSSLRPVDMLLRSMFVTPNTMASVSTVQLWISGILA
ILRVLISQSTEDIVLSRIQELSFSPYLISCTVINRLRDGDSTSTLEEHSEGKQIKNLPEE
TFSRFLLQLVGILLEDIVTKQLKVEMSEQQHTFYCQELGTLLMCLIHIFKSGMFRRITAA
ATRLFRSDGCGGSFYTLDSLNLRARSMITTHPALVLLWCQILLLVNHTDYRWWAEVQQTP
KRHSLSSTKLLSPQMSGEEEDSDLAAKLGMCNREIVRRGALILFCDYVCQNLHDSEHLTW
LIVNHIQDLISLSHEPPVQDFISAVHRNSAASGLFIQAIQSRCENLSTPTMLKKTLQCLE
GIHLSQSGAVLTLYVDRLLCTPFRVLARMVDILACRRVEMLLAANLQSSMAQLPMEELNR
IQEYLQSSGLAQRHQRLYSLLDRFRLSTMQDSLSPSPPVSSHPLDGDGHVSLETVSPDKD
WYVHLVKSQCWTRSDSALLEGAELVNRIPAEDMNAFMMNSEFNLSLLAPCLSLGMSEISG
GQKSALFEAAREVTLARVSGTVQQLPAVHHVFQPELPAEPAAYWSKLNDLFGDAALYQSL
PTLARALAQYLVVVSKLPSHLHLPPEKEKDIVKFVVATLEALSWHLIHEQIPLSLDLQAG
LDCCCLALQLPGLWSVVSSTEFVTHACSLIYCVHFILEAVAVQPGEQLLSPERRTNTPKA
ISEEEEEVDPNTQNPKYITAACEMVAEMVESLQSVLALGHKRNSGVPAFLTPLLRNIIIS
LARLPLVNSYTRVPPLVWKLGWSPKPGGDFGTAFPEIPVEFLQEKEVFKEFIYRINTLGW
TSRTQFEETWATLLGVLVTQPLVMEQEESPPEEDTERTQINVLAVQAITSLVLSAMTVPV
AGNPAVSCLEQQPRNKPLKALDTRFGRKLSIIRGIVEQEIQAMVSKRENIATHHLYQAWD
PVPSLSPATTGALISHEKLLLQINPERELGSMSYKLGQVSIHSVWLGNSITPLREEEWDE
EEEEEADAPAPSSPPTSPVNSRKHRAGVDIHSCSQFLLELYSRWILPSSSARRTPAILIS
EVVRSLLVVSDLFTERNQFELMYVTLTELRRVHPSEDEILAQYLVPATCKAAAVLGMDKA
VAEPVSRLLESTLRSSHLPSRVGALHGVLYVLECDLLDDTAKQLIPVISDYLLSNLKGIA
HCVNIHSQQHVLVMCATAFYLIENYPLDVGPEFSASIIQMCGVMLSGSEESTPSIIYHCA
LRGLERLLLSEQLSRLDAESLVKLSVDRVNVHSPHRAMAALGLMLTCMYTGKEKVSPGRT
SDPNPAAPDSESVIVAMERVSVLFDRIRKGFPCEARVVARILPQFLDDFFPPQDIMNKVI
GEFLSNQQPYPQFMATVVYKVFQTLHSTGQSSMVRDWVMLSLSNFTQRAPVAMATWSLSC

FFVSASTSPWVAAILPHVISRMGKLEQVDVNLFCLVATDFYRHQIEEELDRRAFQSVLEV
VAAPGSPYHRLLTCLRNVHKVTTC
>P20424
MVLADLGKRINSAVNNAISNTQDDFTTSVDVMLKGIVTALLESDVNIALVSKLRNNIRSQ
LLSENRSEKSTTNAQTKKLIQKTVFDELCKLVTCEGSEEKAFVPKKRKTNIIMFVGLQGS
GKTTSCTKLAVYYSKRGFKVGLVCADTFRAGAFDQLKQNAIRARIPFYGSYTETDPAKVA
EEGINKFKKEKFDIIIVDTSGRHHQEEELFQEMIEISNVIKPNQTIMVLDASIGQAAEQQ
SKAFKESSDFGAIILTKMDGHARGGGAISAVAATNTPIIFIGTGEHIHDLEKFSPKSFIS
KLLGIGDIESLFEQLQTVSNKEDAKATMENIQKGKFTLLDFKKQMQTIMKMGPLSNIAQM
IPGMSNMMNQVGEEETSQKMKKMVYVLDSMTKEELESDGRMFIEEPTRMVRVAKGSGTSV
FEVEMILMQQQMMARMAQTATQQQPGAPGANARMPGMPNMPGMPNMPGMPNMPGMPKVTP
QMMQQAQQKLKQNPGLMQNMMNMFGGGMGGGMGGGMPDMNEMMKMMQDPQMQQMAKQFGM
G
>O14043
MTELHPDEQHVFLQCLNKMQKELDLSFERYERLFKTTQELQEKVKFSSSRFYNKDSTYSK
DQGLAKESAALELQKLKQENVLLRKDLNNLEQISDCYAKGMDDIMKHVATYTATMNERMN
ACHKEYIKMYEALASERNSLYKINEQNVQSLNKVKEWLLKSLEEEP
>Q96EY5
MDPVPGTDSAPLAGLAWSSASAPPPRGFSAISCTVEGAPASFGKSFAQKSGYFLCLSSLG
SLENPQENVVADIQIVVDKSPLPLGFSPVCDPMDSKASVSKKKRMCVKLLPLGATDTAVF
DVRLSGKTKTVPGYLRIGDMGGFAIWCKKAKAPRPVPKPRGLSRDMQGLSLDAASQPSKG
GLLERTASRLGSRASTLRRNDSIYEASSLYGISAMDGVPFTLHPRFEGKSCSPLAFSAFG
DLTIKSLADIEEEYNYGFVVEKTAAARLPPSVS
>Q920B0
MTEGRHCQVHLLDDRRLELLVQPKLLSRELLDLVASHFNLKEKEYFGITFIDDTGQENWL
QLDHRVLEHDLPKKPGPTLLHFAVRFYIESISFLKDKNTVELFFLNAKACVHKGQIEVDS
ETIFKLAALVLQESKGDYTSDENARKDLKTLPVFPTKTLQEHPSLAYCEDRVIEHYLKIK
GLTRGQAVVQYMKIVEALPTYGVHYYAVKDKQGLPWWLGISYKGIGQYDLQDKVKPRKLF
QWKQLENLYFREKKFAVEVHDPRRISVSRRTFGQSGLFVQTWYANSSLIKSIWVMAISQH
QFYLDRKQSKAKIPSARSLDDIAMDLTETGTQRGSKLVTLEAKSQFIMASNGSLISSGSQ
DSEGMEEQKREKILELKKKEKLLQEKLLQKVEELKKICLREAELTGRMPKEYPLNIGEKP
PQVRRRVGTTFKLDDNLLPTEEDPALQELESNFLIQQKLVEAAKKLASEPDLCKTVKKKR
KQDYTDAVKRLQEIENSINEYRIRCGKKPSQKAAVVPPEDIIPSESSSLSDTTTYDDPND
SFTLAGQRPSSVPHSPRILPPKSLGIERIHFRKSSINEQFMDTRHSREMLSTHSSPYKTL
ERRPQGGRSMPTTPVLTRNAYSSSHLEPDSSSQHCRQRSGSLESQSHLLSEMDSDKPFFT
LSKSQRSSSTEILDDGSSYTSQSSSEYYCVTPAASPYYTTQTLDTRARGRRRSKKHSVST
SNSGSMPNLAQKDPLRNGVYSKGQDPPPSGYYIAGYPPYAECDLYYSGGYVYENDTEGQY
SVNPSYRSSAHYGYDRQRDYSRSFHEDEVDRVPHNPYATLRLPRKAAVKSEHITKNIHKA
LVAEHLRGWYQRASGQKDQGHSPQTSFDSDRGSQRCLGFAGLQVPCSPSSRASSYSSVSS
TNASGNWRTQLTIGLSEYENPVHSPYTSYYGSIYNPLSSPSRQYAETTPLDGTDGSQLED
NLEGSEQRLFWHEDSKPGTLV
>P32844
MSSDPLQQVCDLIKGDLSLERVRDIKEQLLKEKSVVEYQLNKESDKYYGEVEESLKLLNL
SKNSVTSIKQQINEVNKLGNDNRFAINRYDILFRATKLYETVNTTSSIYDRIYNFVALME

HIERLLVAELAEDALETGCPHLLEIHFLLTSARDFQEQVVVMAKEATEDAQRTVMKLFSR
LSGIISKFDKLLDGLTYDIVEMARAEQISLAIRLFKIYDLEEREDLRIEAIRNIIKKKEI
EIEKSSIKKLPNSKNTARLQDETPKVIEYPTNKGLYQEIMSGTISTRTAPRGYKHFLING
INNSISEMFGEMREKYVGDQKFDVLDNMDWIFNELIIVKEHIANCCPPHWNIFEVYFDQY
YKELHSLITDLVESEPETIIILDILAFDKTFQDTLKQDFGFTKSEVKSVIGDKEKETLFK
DYLNLIVVKMTEWIGNLEKAEFDVFLERSTPPHSDSDGLLFLDGTKTCFQMFTQQVEVAA
GTNQAKILVGVVERFSDLLTKRQKNWISKISEEIKKQINYNHKYDIDPESITPEDECPGG
LVEYLIAVSNDQMKAADYAVAISSKYGKLVSKVYEKQITNHLEGTLDGFAEVAQCSSLGL
ITLMFDDLRKPYQEIFSKTWYMGSQAQQIADTLDEYLLDIKPQMNSVLFVNFIDNVIGET
IIKFLTALSFEHSFKNKNNKFLEAMKRDFEIFYQLFVKVLDGNESKDTLITQNFTVMEFF
MDLSCEPIDSILDIWQKYLEVYWDSRIDLLVGILKCRKDVSSSERKKIVQQATEMLHEYR
RNMEANGVDREPTLMRRFVLEFEKQ
>Q08949
MSFKATITESGKQNIWFRAIYVLSTIQDDIKITVTTNELIAWSMNETDTTLCQVRFQKSF
FEEYEFKPHEIVFGENGVQVIEDTYGNSHKLYSFRVNGRHLTTISRKPDGDGIKSFTIAV
NNTSTCPESLANRLIVVIEMDSLIVKEYCPQFQPIKYDPIIINLKYKRRFLDVFGTAASD
RNPQEPLDPKLLDVFTNTERELTSALFNEEVESDIRKRNQLTAADEINYICCNSTLLKNF
LDNCNVNVTDEVKLEINVHRLSITAFTKAVYGKNNDLLRNALSMSNTISTLDLEHYCLFT
TIEDEKQDKRSHSKRREHMKSIIFKLKDFKNFITIGPSWKTTQDGNDNISLWFCHPGDPI
LMQMQKPGVKLELVEVTDSNINDDILEGKFIKTAISGSKEEAGLKDNKESCESPLKSKTA
LKRENLPHSVAGTRNSPLKVSYLTPDNGSTVAKTYRNNTARKLFVEEQSQSTNYEQDKRF
RQASSVHMNMNREQSFDIGTTHEVACPRNESNSLKRSIADICNETEDPTQQSTFAKRADT
TVTWGKALPAADDEVSCSNIDRKGMLKKEKLKHMQGLLNSQNDTSNHKKQDNKEMEDGLG
LTQVEKPRGIFD
>Q9CQ84
MFLTVKLLLGRRCSLKVSGKESVATLKKLVSQHLQVPEEQQHLLFRGQLLADDKYLSDYS
IGPNASINVIMRPPEDAALDKTHQTQPLWLQLGQVLDKHFGAKDAKTVLGFLRQEHEERL
QRLSLEALEQLVGQLLAQQQLDELAEEKEAPAVASELEQNNGGGGGGGGTGGEGGGKKEE
EEGEEADQ
>O35235
MRRASRDYGKYLRSSEEMGSGPGVPHEGPLHPAPSAPAPAPPPAASRSMFLALLGLGLGQ
VVCSIALFLYFRAQMDPNRISEDSTHCFYRILRLHENAGLQDSTLESEDTLPDSCRRMKQ
AFQGAVQKELQHIVGPQRFSGAPAMMEGSWLDVAQRGKPEAQPFAHLTINAASIPSGSHK
VTLSSWYHDRGWAKISNMTLSNGKLRVNQDGFYYLYANICFRHHETSGSVPTDYLQLMVY
VVKTSIKIPSSHNLMKGGSTKNWSGNSEFHFYSINVGGFFKLRAGEEISIQVSNPSLLDP
DQDATYFGAFKVQDID
>O74424
MHDSSWTEADILGVCSFLDIPKTKIDPLLQVDAFTSILIPLISKSKDYESIKNDRIVTEV
NYEQQLRNSEKKLLQSNERYDLLEDERKLLENELSQIKEYLREKSSSYDTVLHDCSSLKS
VNEALKQAQDQNLKQTAQLQNLLSDKEKEVEKKITIIKDLKDALASSTHQVLELQHTQQE
KASLQTNYEFELQKLTQKNSILENNNTWLSRELQGVNDKLLSLHQEASLEKSQLSSQLSD
AVLEKDALQRKVSSLSQQFTESNLRYQNIVAELSEMRKQYEFSQVSFEKEISSQKQISEL
WMEKCEDCSLRLKELQNSNGELEKLLEAAQSSFEEQLESHKEAEASLKSQINFLEKEVSS
LESQLKLANERLRHYDEIEISDMSELKYSNLLNNSMKGFKGQSSVSDLYSERLYYKQKYE

QTCQEVERLQRSYNHVMEEANLQHPLVKEQFKRFAHMQREIVAMSEQYQKSLEDCQKAKS
RYEQLETLFKDKCTENKHYEQETKDLARQVQVLLHELDLCENGIVLGVDSRKKINSYVEK
SLTEDETDTDQIISSRLVVFRNIRELQQQNQNLLSAVHELADRMEKDEKPDLDGAEIQEE
TLIKANETIDQLTKMLEEVSDQLRYSLKERDFFRSLVQENEKLLDMAPATPNSKLNTNLI
EQTSYQRSLIRLEQLTNELESLKSISRNKEKKFEEAISSLQLEKSNIQLQLTSLTSERSL
ALEKLNDLEKSLVLSERSKDELDESYKSLQEQLASKKIEVQNVSSQLSICNSQLEQSNHI
VDNLKSENLLLTSVKDKLKADLSNLESKLSSLQQDNFHMKAQIESSNQEYTATVDSMNSR
ILELSNDLRVANSKLSECSDDVRRLTLQNSFDLREHQTLVLQLQSNITELKQDITLQRTV
RNQLEIQTTELKERLKFMEERQENLQSKLIAANKDTTQNPDNVEVEAISIELERTKEKLR
MAELEKSNIQQKYLASEKTLEMMNETHEQFKHLVESEISTREEKITSLRSELLDLNKRVE
VLKEEKESSSKELAKQLEDAVREKDSALSFKKDYEKIRSDADRVITSLKEDIEKERSLMK
ECHSNYESEIVSHGRTTQKLRDLRTEFDEVNTKYLKLKANFEQQHSGLSGAEKDWNIQRK
AMEDEISSLKDYILGLENQNKLLHSQFDSLSQQITVLQQNSSENLNISANLEAVQDNDLR
ELVSYLRHEKEIMDNKYELTILDNRGLNQQVKSLQSTVDSLQLELNRLQSLPVSNDQTDT
PIISGSQEVQLLYESNSVLRKDNDAKLGKIQELEKEVEKLNASLNPLQTEINELKAEIGA
KTASLNLMKEYNSRWKLRFQSVLNKYERVDPTQLEELKKNCEALEKEKQELETKLQETAK
ETDTFKQQVNSLNEEVENLKKEVEQANTKNTRLAAAWNEKCENLKKSSLTRFAHLKQELT
NKNKELTSKNAENEAMQKEIESLKDSNHQLQESASSDAEQITKEQFEQLKSEKERTEKEL
ADSKNELEHLQSEAVDADGKTEISNLEKEIHELRSDKEGLVQQVQNLSAELAALREHSPT
QGSLENADEIARLRSQLESTKQYYEKEKETEILAARSELVAEKEKTKEELENQLNEKSQR
IKELEEQAQKNSSENTHDNIDDMIKQQVEEKLKENSANFDVKLKKVVAETEFRSKAKISV
YEKKTRDLQNKITQLEETIENLNKQLSNPEKTDESTSSVTETKPVTSKPTASKADVGQNA
TEASSAKREPSGKSLSARLQGTGKQKGVQRPAVSRPVPMKPDSGKLSITGASKRIATSKN
AAQNAKELSSTAKSGSLKRQRDDANKGGSSSNQKKAK
>P33751
MLLFPRRTNIAFFKTTGIFANFPLLGRTITTSPSFLTHKLSKEVTRASTSPPRPKRIVVA
ITGATGVALGIRLLQVLKELSVETHLVISKWGAATMKYETDWEPHDVAALATKTYSVRDV
SACISSGSFQHDGMIVVPCSMKSLAAIRIGFTEDLITRAADVSIKENRKLLLVTRETPLS
SIHLENMLSLCRAGVIIFPPVPAFYTRPKSLHDLLEQSVGRILDCFGIHADTFPRWEGIK
SK
>Q04004
MEDKLDRYYTNVLSNAEKDKHTTVDSDDKSSGEENLDELLNELDRELDEDHEFLSAYRSE
RLQQISDHLKQVKKNVEDDGYGRLQCIDNEADAIQICTKTTMVVIHFELETFGKCQYMNE
KLENLAKRYLTTRFIKVNVQTCPFLVNKLNIKVLPFVVGYKNGLEKVRYVGFSKLGNDPN
GFDIRRLEQSLAHSGVIEDTFEIRKHSSVNTERFASTNHDRSESDSDLDI
>Q08229
MSEEREENGISRATLNTQRLSAMIDSLNNEKDDRLFPSPTTTRTMITEEKADQSDVFKPP
SRLLRSPAGDVSLPPGDNRSSMISNYSGIIQEGVEVSYVVKNRQQTQERRTSKDSNSLYS
LKEPVSKNELPSLPMLPSEATLTKHLSDNQSTKSNTNADEIVIKPVTNAKPVGRFNSNTS
KKVEGRGSLKLLSSPLRQEKVMRSSIGSGNLASESGSSTYNTKFHQSIQEQLEEEEEGNV
SDKLSIVSSVIPELYTTTNEAPKAINPIRSETNDYNPTIPPRSKDRPRSRLFIEEGDGEG
DLLTEEILPTPVQPGGHYKNSSQISTVSEQKSESYYSAATSMPPEEETYLTRPLPSTPNE
DSRVTSNLKRDDTLKAIHDRANHTSTSTNKQDDDMYEDIIEETPKKTKLKKDTKKKLNKK
KSVKELRSFDIDTLNQLLSVTKGTLIGSEFAQLGMKIEEKRALERLVDSLSRLTADMVLD

PDRYEEGLKRLDKATKALEGF
>P70033
MDTWEDDRVDQRACLICGDRATGLHYGIISCEGCKGFFKRSICNKRVYRCSRDKNCVMSR
KQRNRCQYCRLLKCLQMGMNRKAIREDGMPGGRNKSIGPVQISDEEVERIMSGQEFEEEA
NTSWSNNGDSDHSSPGNAVSESNQPSPVSTPSSSRSMELNGFSSLRDQYLGTPGPTHYQY
LPHLFSFSAHPTIIPAQSRSLDPQSHTLINQLLTAEDIEPLNTPMLIEDGYKVTQSELFA
LLCRLADELLFRQITWVKKLPFFCDLSIKDYTCLLSTTWQELILLSSITTYSKQIFGDLT
DVTSKYSPSEDELHRFSEDGMEVMERLIYLFRKFSQLKVSNEEYVCMKAINFLNQDIQGI
SSASQVEQLNKRYWYVCQDFTEYRYPHQPNRFPDLMMCLPEVRYIAGKLVNVPLEQLPLL
FKAFLHSCKTIVTKE
>Q06340
MTGDSRSISEPSINLDPDNTSFSDENSDDFFMDNSYDIDEIDHSDESNRQSVIVDSKVTV
PPSKHSTLTLSDSEDSDAKEQHQSLSRSSSKNVNIEDITEPKPDKPSGRTRGRSVMKESV
VEINSSESDLDEDKNFPRSRSRSRSSIRSISPAGKYKRQKSSLLYTYDENDDFFKELAKE
AKKSTTISKESTPDQRKRVYNIKFLSKLEGTINKAVQVKVLGKYEFSKILPAALDGLMKS
YKIPKVMKDIYKVENVTLYWNNAKLLTFMTCNSLHIPQDFENEVSDIDVTIVSKEYEKNF
EATLESKLKEEEAALLIKERQEMERKLEKKRNEQEESEYREFESELKNVEETQEIKENDT
VMNTKLLQEGGSLSGNSSSMEEVMRIALMGQDNKKIYVHVRRSTPFSKIAEYYRIQKQLP
QKTRVKLLFDHDELDMNECIADQDMEDEDMVDVIID
>P19880
MSVSTAKRSLDVVSPGSLAEFEGSKSRHDEIENEHRRTGTRDGEDSEQPKKKGSKTSKKQ
DLDPETKQKRTAQNRAAQRAFRERKERKMKELEKKVQSLESIQQQNEVEATFLRDQLITL
VNELKKYRPETRNDSKVLEYLARRDPNLHFSKNNVNHSNSEPIDTPNDDIQENVKQKMNF
TFQYPLDNDNDNDNSKNVGKQLPSPNDPSHSAPMPINQTQKKLSDATDSSSATLDSLSNS
NDVLNNTPNSSTSMDWLDNVIYTNRFVSGDDGSNSKTKNLDSNMFSNDFNFENQFDEQVS
EFCSKMNQVCGTRQCPIPKKPISALDKEVFASSSILSSNSPALTNTWESHSNITDNTPAN
VIATDATKYENSFSGFGRLGFDMSANHYVVNDNSTGSTDSTGSTGNKNKKNNNNSDDVLP
FISESPFDMNQVTNFFSPGSTGIGNNAASNTNPSLLQSSKEDIPFINANLAFPDDNSTNI
QLQPFSESQSQNKFDYDMFFRDSSKEGNNLFGEFLEDDDDDKKAANMSDDESSLIKNQLI
NEEPELPKQYLQSVPGNESEISQKNGSSLQNADKINNGNDNDNDNDVVPSKEGSLLRCSE
IWDRITTHPKYSDIDVDGLCSELMAKAKCSERGVVINAEDVQLALNKHMN
>O02751
MEEFDSKDISTSKDEDCVPLGGECHEDDINELVKEDEVDGEEETQKTKGTKRKAESILAR
KRKQGRLSLDQEEEEDASRESGGRIIEKEDAAAEQEKGAESEDARQEEADVLASSVSDAE
PKSELPPSTQTKTGEETEETSSSNLVKVEELEKPKKAEEVKLTKSPLAGEEVRFLTQQGR
LSGRTSEDEPRRSEGVQHATGEERRADTNTSSKNEAAGQKWKGQSAVDVSGDESKLRCCK
EEYCIGTWNVRSMNPGKLDVVKQEMERINIDILGISELKWTGMGELNSDDHYIYYCGQQS
LRRNGVALIVNKRVRNAIIGCNLKNDRMISVRFQGKPFNLTVIQVYAPTPYAEEGEVYRF
YEDLQHLLEITPKIDVLFIIGDWNAKVGSQEIPGITGRFGLGMQNEAGRRLIEFCHHNRL
VITNTLFQQPSRRLYTWTSPDGRYRDQIDYIICRQRWRSSVQSAKTRPGADCGSDHKLLI
AKFRLKLKIIPKTTRPFRVTNEEDATNEEAKSVLKQNEKEKPEANVPSTVSSVPGGSGMT
KEVGETSQEAKSVFKQDEKDKPQANVPSSVPSLPAGSGPEKCDLEKKKDCNN
>Q12330
MSNKVKTKAMVPPINCIFNFLQQQTPVTIWLFEQIGIRIKGKIVGFDEFMNVVIDEAVEI

PVNSADGKEDVEKGTPLGKILLKGDNITLITSAD
>Q06608
MAWTSTLPAHLLNLIKNSKYVHVATCSKDCIPSVALMNYIYVPGEKLFGQTDNKNDYIIF
VSPQDTQKFYNIKENPKVALLFHDWIIANNLSVGKESISGTPTPTSIPHEEQRQSELLNL
LQELNQAELNQMSASIGGETEIVNPESEESKYYKDLILKANPDAKAFIFEKNTAVVKVRI
DNARVSNNENRTMFLSKGKS
>O09486
MQQNTRYLFASEAVSRGHPDKACDQVSDRVLDYCLQAEKKCKKASRVALETTIKGNVVGL
FGEVTCQKTFPTISWFRELVTEIGYSREDLDLDPTTCSVHINVRGQEAEIRGVVHNQERA
AKETLGAGDQGLMFGYATDETPERCPCLWFLLRRFKLALRSKFEEARKKKIELAVAKGIK
VSQVDATPELRDTIDFWWLHTDAKSQVIIEYEDDSGALKPITARVAVLSVQHSKFVTHDQ
YEDRLPQLVKGVLDEYGMHSEATEYLINIKQKTSGYGWTVGGPNADAGTTGRKIIVDTYG
GHGAHGGGAFSGKDPSKVDRSAAYYARYVAQNLVRAKLCRRVLVQVSYVIGKPEPLNIYV
NTYGTGTHSDSELLEIINKNFDFRPGFIIEELDLLNPDRIKYVETAYHGHFGRPEFPWEQ
EKTLTL
>Q9VCI3
MATATSGSGLHLEAIDRIGSIPLVESSVKRVETIYDKVKNNNRLFSWYFETAEATISAAY
ETIQPAVKLFEPSIQRLDNVMCKSLDILEQRIPLVYLPPEMMYWNTKEYMSDHLVRPVLK
RADSVKQIGNAVLESPLTTYAAERIDGAFTVGDKFVDKYLVPIQTDQDQTDVKKLVSALN
DNFSPIRQQLNRMFGHKSPQEDDNEAVPDERGAIKAIHHGQRFSRKLKRRLTQRTIAEAR
ALKKQSKEAIHVLFYAAELIATDPKQAVQKAKELWVYLSADEPENQARPATLEQLIVLLT
RESARRVVHLVNFSAHVAANIPRNLAHTTTEVAHHIIYINHRIITISRLDKVKTISKEEA
ESLFKRMLAFYGSLQGLTNAYLERVASFLSGRMEAEKVTGSDGGNSNHRSSRRRQDPNHY
SATHNNINGVY
>P38281
MAFLNIFKQKRGDEASQLSAKGREEISQSIKICKSDDAANEHSCSGDCKTEIEEGEQAFA
KLKIEHETPLLNSSKTPKIHFVVPTSQIDWQHDACLEDPKSVQYKISQWCDKNSAKFSNV
GTGKTLNCAVSSLPKDIMDIDVMRGTKNNVLILPYFIWLNDLRSDDVEATLDGLVPDLLD
ENISREKLLETRPNVAVARERAFVFICSHTTRDKRCGITAPYLKKVFDSKLQEHGLYRDN
SDYRAEGVKIAFVNHVGGHKFAANVQIYLRNPNTLIWLGRVTPTIVPSIVEHLIVPEEPT
LPFPEKVRCIKKYQSW
>Q13112
MKVITCEIAWHNKEPVYSLDFQHGTAGRIHRLASAGVDTNVRIWKVEKGPDGKAIVEFLS
NLARHTKAVNVVRFSPTGEILASGGDDAVILLWKVNDNKEPEQIAFQDEDEAQLNKENWT
VVKTLRGHLEDVYDICWATDGNLMASASVDNTAIIWDVSKGQKISIFNEHKSYVQGVTWD
PLGQYVATLSCDRVLRVYSIQKKRVAFNVSKMLSGIGAEGEARSYRMFHDDSMKSFFRRL
SFTPDGSLLLTPAGCVESGENVMNTTYVFSRKNLKRPIAHLPCPGKATLAVRCCPVYFEL
RPVVETGVELMSLPYRLVFAVASEDSVLLYDTQQSFPFGYVSNIHYHTLSDISWSSDGAF
LAISSTDGYCSFVTFEKDELGIPLKEKPVLNMRTPDTAKKTKSQTHRGSSPGPRPVEGTP
ASRTQDPSSPGTTPPQARQAPAPTVIRDPPSITPAVKSPLPGPSEEKTLQPSSQNTKAHP
SRRVTLNTLQAWSKTTPRRINLTPLKTDTPPSSVPTSVISTPSTEEIQSETPGDAQGSPP
ELKRPRLDENKGGTESLDP
>Q96PM5
MAATAREDGASGQERGQRGCEHYDRGCLLKAPCCDKLYTCRLCHDNNEDHQLDRFKVKEV

QCINCEKIQHAQQTCEECSTLFGEYYCDICHLFDKDKKQYHCENCGICRIGPKEDFFHCL
KCNLCLAMNLQGRHKCIENVSRQNCPICLEDIHTSRVVAHVLPCGHLLHRTCYEEMLKEG
YRCPLCMHSALDMTRYWRQLDDEVAQTPMPSEYQNMTVDILCNDCNGRSTVQFHILGMKC
KICESYNTAQAGGRRISLDQQ
>Q9H2M9
MACSIVQFCYFQDLQAARDFLFPHLREEILSGALRRDPSKSTDWEDDGWGAWEENEPQEP
EEEGNTCKTQKTSWLQDCVLSLSPTNDLMVIAREQKAVFLVPKWKYSDKGKEEMQFAVGW
SGSLNVEEGECVTSALCIPLASQKRSSTGRPDWTCIVVGFTSGYVRFYTENGVLLLAQLL
NEDPVLQLKCRTYEIPRHPGVTEQNEELSILYPAAIVTIDGFSLFQSLRACRNQVAKAAA
SGNENIQPPPLAYKKWGLQDIDTIIDHASVGIMTLSPFDQMKTASNIGGFNAAIKNSPPA
MSQYITVGSNPFTGFFYALEGSTQPLLSHVALAVASKLTSALFNAASGWLGWKSKHEEEA
VQKQKPKVEPATPLAVRFGLPDSRRHGESICLSPCNTLAAVTDDFGRVILLDVARGIAIR
MWKGYRDAQIGWIQTVEDLHERVPEKADFSPFGNSQGPSRVAQFLVIYAPRRGILEVWST
QQGPRVGAFNVGKHCRLLYPGYKIMGLNNVTSQSWQPQTYQICLVDPVSGSVKTVNVPFH
LALSDKKSERAKDMHLVKKLAALLKTKSPNLDLVETEIKELILDIKYPATKKQALESILA
SERLPFSCLRNITQTLMDTLKSQELESVDEGLLQFCANKLKLLQLYESVSQLNSLDFHLD
TPFSDNDLALLLRLDEKELLKLQALLEKYKQENTRTNVRFSDDKDGVLPVKTFLEYLEYE
KDVLNIKKISEEEYVALGSFFFWKCLHGESSTEDMCHTLESAGLSPQLLLSLLLSVWLSK
EKDILDKPQSICCLHTMLSLLSKMKVAIDETWDSQSVSPWWQQMRTACIQSENNGAALLS
AHVGHSVAAQISNNMTEKKFSQTVLGADSEALTDSWEALSLDTEYWKLLLKQLEDCLILQ
TLLHSKGNTQTSKVSSLQAEPLPRLSVKKLLEGGKGGIADSVAKWIFKQDFSPEVLKLAN
EERDAENPDEPKEGVNRSFLEVSEMEMDLGAIPDLLHLAYEQFPCSLELDVLHAHCCWEY
VVQWNKDPEEARFFVRSIEHLKQIFNAHVQNGIALMMWNTFLVKRFSAATYLMDKVGKSP
KDRLCRRDVGMSDTAMTSFLGSCLDLLQILMEADVSRDEIQVPVLDTEDAWLSVEGPISI
VELALEQKHIHYPLVEHHSILCSILYAVMRFSLKTVKPLSLFDSKGKNAFFKDLTSIQLL
PSGEMDPNFISVRQQFLLKVVSAAVQAQHSATKVKDPTEEATPTPFGKDQDWPALAVDLA
HHLQVSEDVVRRHYVGELYNYGVDHLGEEAILQVHDKEVLASQLLVLTGQRLAHALLHTQ
TKEGMELLARLPPTLCTWLKAMDPQDLQNTEVPIATTAKLVNKVIELLPEKHGQYGLALH
LIEAVEAISLPSL
>Q96RS0
MCCEKWSRVAEMFLFTEEREDCKILCLCSRAFVEDRKLYNLGLKGYYIRDSGNNSGDQAT
EEEEGGYSCGTAESHDSKGIGLDESELDSEAELMRSMGLPLQFGRITAHKDFEVSMNTRN
KVKIKKKKHQKKYLDEIVQESWRKEYEEDDILASDDPSSIEQYENTRTYELQSKKDTETE
NPPVENTLSPKLEITEKWEKYWNEYGGGLLWQSWQEKHPGQALSSEPWNFPDTKEEWEQH
YSQLYWYYLEQFQYWEAQGWTFDASQSCDTDTYTSKTEADDKNDEKCMKVDLVSFPSSPI
MVDNDSSGTSDKDHSEILDGISNIKLNSEEVTQSQLDSCTSHDGHQQLSEVSSKRECPAS
GQSEPRNGGTNEESNSSGNTNTDPPAEDSQKSSGANTSKDRPHASGTDGDESEEDPPEHK
PSKLKRSHELDIDENPASDFDDSGSLLGFKYGSGQKYGGIPNFSHRQVRYLEKNVKLKSK
YLDMRRQIKMKNKHIFFTKESEKPFFKKSKILSKVEKFLTWVNKPMDEEASQESSSHDNV
HDASTSSDSEEQDMSVKKGDDLLETNNPEPEKCQSVSSAGELETENYERDSLLATVPDEQ
DCVTQEVPDSRQAETEAEVKKKKNKKKNKKVNGLPPEIAAVPELAKYWAQRYRLFSRFDD
GIKLDREGWFSVTPEKIAEHIAGRVSQSFKCDVVVDAFCGVGGNTIQFALTGMRVIAIDI
DPVKIALARNNAEVYGIADKIEFICGDFLLLASFLKADVVFLSPPWGGPDYATAETFDIR
TMMSPDGFEIFRLSKKITNNIVYFLPRNADIDQVASLAGPGGQVEIEQNFLNNKLKTITA

YFGDLIRRPASET
>P25637
MDDDHEQLVEELEAVEAIYPDLLSKKQEDGSIIVVKVPQHEYMTLQISFPTHYPSEEAPN
VIEVGVCTSLAKRDLYDTKYLQHLFQEVMDSVFHRGSVCLFDFLTELDGVLYVEPEEETE
PVQQSDIPTDPFEGWTASDPITDRGSTFMAFAAHVTSEEQAFAMLDLLKTDSKMRKANHV
MSAWRIKQDGSAATYQDSDDDGETAAGSRMLHLITIMDVWNVIVVVARWFGGAHIGPDRF
KHINSTAREAVVRAGFDS
>Q9UNE2
MADTIFGSGNDQWVCPNDRQLALRAKLQTGWSVHTYQTEKQRRKQHLSPAEVEAILQVIQ
RAERLDVLEQQRIGRLVERLETMRRNVMGNGLSQCLLCGEVLGFLGSSSVFCKDCRKKVC
TKCGIEASPGQKRPLWLCKICSEQREVWKRSGAWFYKGLPKYILPLKTPGRADDPHFRPL
PTEPAEREPRSSETSRIYTWARGRVVSSDSDSDSDLSSSSLEDRLPSTGVRDRKGDKPWK
ESGGSVEAPRMGFTHPPGHLSGCQSSLASGETGTGSADPPGGPRPGLTRRAPVKDTPGRA
PAADAAPAGPSSCLG
>P40976
MSQTAPSDTEYNQRLERWSERLKSQTISHLPTDYSRPVPSRLVEAVFERTLPEDAKTALI
KVYVAAQAKGILVTPFNILLTIFIILVSRMTGDEDISIGTSSENAIPFVLRTFIQPSDSF
LDLLAKVCDLEKEGSSDAVDFSDLINFLNAKLSKKDDPRKTLVHLRFYNAPDAPSENFLS
TTGLDVDLTVLVSVKKPSDQLTSLRSQFTFPDLQLKLIYNQLLFSESRVNIVADQLLKLV
VSASKDVTGPIGALDLMTPTQMNVLPDPTVDLDWSGYRGAIQDIFASNAAKFPDRECIVV
TPSVTIDAPVTSYTYRQIDESSNILAHHLVKNGIERGDVVMVYAYRGVDLVVAVMGVLKA
GATFSVIDPAYPPARQIIYLSVAKPRALVVLEDAGVLSPTVVEYVEKSLELKTYVPALKL
AKDGSLTGGSVSKGADDILQHVLHLKSEQTGVVVGPDSTPTLSFTSGSEGIPKGVKGRHF
SLAYYFDWMAQEFNLSESDRFTMLSGIAHDPIQRDIFTPLFLGASLIVPTAEDIGTPGQL
AQWANKYKVTVTHLTPAMGQLLAAQADEPIPSLHHAFFVGDILTKRDCLRLQVLANNVNV
VNMYGTTETQRSVSYFVVPARSQDQTFLESQKDVIPAGRGMKNVQLLVINRFDTNKICGI
GEVGEIYLRAGGLAEGYLGNDELTSKKFLKSWFADPSKFVDRTPENAPWKPYWFGIRDRM
YRSGDLGRYLPTGNVECSGRADDQIKIRGFRIELGEINTHLSRHPNVRENITLVRRDKDE
EPTLVAYIVPQGLNKDDFDSATESEDIVVNGLKKYRKLIHDIREYLKTKLPSYAIPSVIV
PLHKMPLNPNGKIDKPALPFPDTSQLAAASRSHSKHGVDETLTATERDIRDIWLRIIPHA
TDVNKKASFFDIGGHSILATRLIFELRKKFAVNVPLGLVFSEPTIEGLAKEIERMKSGEM
ISVMDIGKEETREPEIEYGKDALDLVDLIPKEFPTSKDLGIDEPKTVFLTGANGYLGVFI
LRDLMTRSSNLKVIALVRASSEEHGLKRLKDSCTAYGVWDESWAQKISVVNGDLALENWG
IEERKWNKLTEVVDYVIHNGALVHWVYPYSKLRGPNVMGTITALKLCSLGKGKSLSFVSS
TSTVDTEYYVNLSNEITSKGGNGIPESDPLQGSSKDLHTGYGQSKWVSEYLVRQAGLRGL
RGVVVRPGYILGDSKSGAINTDDFLVRMVKGCIELGLYPNINNTVNMVPADHVARVVTAS
AFHPEQGVIVAHVTSHPRLRFNQFLGTLSTFGFNTKLSEYVNWRIALERFVINESHDSAL
YPLLHFVLDNLPANTKAPELDDTNTREILKRDASWTNVDVSNGAAILEHEMGLYLSYLVA
IGFLPKPTLEGKKLPEVKINEATLEKLASAGGRGGAPTH
>P21242
MTSIGTGYDLSNSVFSPDGRNFQVEYAVKAVENGTTSIGIKCNDGVVFAVEKLITSKLLV
PQKNVKIQVVDRHIGCVYSGLIPDGRHLVNRGREEAASFKKLYKTPIPIPAFADRLGQYV
QAHTLYNSVRPFGVSTIFGGVDKNGAHLYMLEPSGSYWGYKGAATGKGRQSAKAELEKLV
DHHPEGLSAREAVKQAAKIIYLAHEDNKEKDFELEISWCSLSETNGLHKFVKGDLLQEAI

DFAQKEINGDDDEDEDDSDNVMSSDDENAPVATNANATTDQEGDIHLE
>P47130
MDVDIGCYFEEKRYDDKLLDFIRYDVKTPKKTKYILQRPTATDEESVRLQRFYQLGVDLK
LKYSKRRSLKKQGRIKNATEELLRLANEQLKLFNRIVERETNWIIYPLWVMAKQLIRLAN
ESSELNKDSIEECGRTIHRSFTICLNDRNPRLNENKKIGCYMFANLEFSIYHRLSNKDMI
KNLVKVLESRVNARDIPPLNKSLAMEHKSQVVLYNYYLGQYYGCLENDHERGFFHLNEAL
LQCPMLYVESTGKFVLQGQMEKIMILLVPLALLTKRLYPHWDHPVIAGVITRSKRLSQVY
PTLVRSVISGNLSLYEATAASHERFFLSQGLHVVITLLREVVFTRLVQRCWQWGNDRKSI
MPLKILLATKQHDSSANEDEEEQLDALECRLASAIASGLLRAYLSHSNRCIVFSKKEPFP
HSK
>Q13895
MPKFKAARGVGGQEKHAPLADQILAGNAVRAGVREKRRGRGTGEAEEEYVGPRLSRRILQ
QARQQQEELEAEHGTGDKPAAPRERTTRLGPRMPQDGSDDEDEEWPTLEKAATMTAAGHH
AEVVVDPEDERAIEMFMNKNPPARRTLADIIMEKLTEKQTEVETVMSEVSGFPMPQLDPR
VLEVYRGVREVLSKYRSGKLPKAFKIIPALSNWEQILYVTEPEAWTAAAMYQATRIFASN
LKERMAQRFYNLVLLPRVRDDVAEYKRLNFHLYMALKKALFKPGAWFKGILIPLCESGTC
TLREAIIVGSIITKCSIPVLHSSAAMLKIAEMEYSGANSIFLRLLLDKKYALPYRVLDAL
VFHFLGFRTEKRELPVLWHQCLLTLVQRYKADLATDQKEALLELLRLQPHPQLSPEIRRE
LQSAVPRDVEDVPITVE
>Q16665
MEGAGGANDKKKISSERRKEKSRDAARSRRSKESEVFYELAHQLPLPHNVSSHLDKASVM
RLTISYLRVRKLLDAGDLDIEDDMKAQMNCFYLKALDGFVMVLTDDGDMIYISDNVNKYM
GLTQFELTGHSVFDFTHPCDHEEMREMLTHRNGLVKKGKEQNTQRSFFLRMKCTLTSRGR
TMNIKSATWKVLHCTGHIHVYDTNSNQPQCGYKKPPMTCLVLICEPIPHPSNIEIPLDSK
TFLSRHSLDMKFSYCDERITELMGYEPEELLGRSIYEYYHALDSDHLTKTHHDMFTKGQV
TTGQYRMLAKRGGYVWVETQATVIYNTKNSQPQCIVCVNYVVSGIIQHDLIFSLQQTECV
LKPVESSDMKMTQLFTKVESEDTSSLFDKLKKEPDALTLLAPAAGDTIISLDFGSNDTET
DDQQLEEVPLYNDVMLPSPNEKLQNINLAMSPLPTAETPKPLRSSADPALNQEVALKLEP
NPESLELSFTMPQIQDQTPSPSDGSTRQSSPEPNSPSEYCFYVDSDMVNEFKLELVEKLF
AEDTEAKNPFSTQDTDLDLEMLAPYIPMDDDFQLRSFDQLSPLESSSASPESASPQSTVT
VFQQTQIQEPTANATTTTATTDELKTVTKDRMEDIKILIASPSPTHIHKETTSATSSPYR
DTQSRTASPNRAGKGVIEQTEKSHPRSPNVLSVALSQRTTVPEEELNPKILALQNAQRKR
KMEHDGSLFQAVGIGTLLQQPDDHAATTSLSWKRVKGCKSSEQNGMEQKTIILIPSDLAC
RLLGQSMDESGLPQLTSYDCEVNAPIQGSRNLLQGEELLRALDQVN
>O00453
MLSRNDDICIYGGLGLGGLLLLAVVLLSACLCWLHRRVKRLERSWTQGSSEQELHYASLQ
RLPVPSSEGPDLRGRDKRGTKEDPRADYACIAENKPT
>Q9WUL6
MAVMEVACPGTPGSAVGQQKELAKAKEKTQSLGKKQSCIFKLEAVEKSPVFCGKWEILND
VITKGTAKDGSEGGPPAISIIAQAECENSQEFSPTFSERIFIAGSQQYSQSESLDQIPNN
VAHATEGKMARVCRRGKRHGKARKKRRKKRSKSLAQAGVALAKPLPRTPEQESCTIPVQE
DESPLGNLYARNVSQFTKPLGGPGLGHLCFKKQDEGLRPVLPRPELHKLISPLQCLNHVW
KLHHPQATGPRPHPTHPFPYSGMPHPFPFYPLEPWKPYMLDSAVLDKLAGVSGQRPLPGP
PHLSQLAHGDSQKPLPGPHLESSCPSRGALEKVPVEEYLVHALQGSVSSGQAHSLASLAK

TWSSGSAKLQRLGPETEDNEGVLLTEKLKPVDYEYREEVHWMTHQPRVGRGSFGEVHRMK
DKQTGFQCAVKKVRLEVFRVEELVACAGLSSPRIVPLYGAVREGPWVNIFMELLEGGSLG
QLIKQMGCLPEDRALYYLGQALEGLEYLHTRRILHGDVKADNVLLSSDGSRAALCDFGHA
LCLQPDGLGKSLLTGDYIPGTETHMAPEVVMGKPCDAKVDIWSSCCMMLHMLNGCHPWTQ
YFRGPLCLKIASEPPPIREIPPSCAPLTAQAIQEGLRKEPVHRASAMELRRKVGKALQEV
GGLKSPWKGEYKEPRPPPQDQATCHQTLPTPPRENPPAKANTDGAPEPQPPLPPEPPEPS
KAPALNLSKEESGTWEPLPLSSLDPATAKGPSFPDRRATLPELELQQLEIELFLNSLSQP
FSLEEQEQILSCLSIDSLSLSDDSEKNPSKASQSSRDTLSSGVHSWNSQAEARTCSCSTA
LARGRPTDIPSYFNGVKVQIQSLNGEHLHIREFHRVKVGDIATGISSQIPATAFSLVTKD
GQPVCYDMEVPDSGIDLQCTLAPDGSFAWTWRVKHGQLENRP
>O77460
MLAKITRSSFYASRAVGRLSGSIPTSPAALASNCRYIQIERKRTKSHEMALYETVEKGAK
NSPSYSLYFKNKCGNVISPMHDIPLYANEEKTIYNMVVEVPRWTNAKMEISLKTPMNPIK
QDIKKGKLRFVANCFPHKGYIWNYGALPQTWENPDHIEPSTGCKGDNDPIDVIEIGYRVA
KRGDVLKVKVLGTIALIDEGETDWKIIAIDVNDPLASKVNDIADVDQYFPGLLRATVEWF
KIYKIPDGKPENQFAFNGDAKNADFANTIIAETHKFWQNLVHQSPASGSISTTNITNRNS
EHVIPKEEAEKILAEAPDGGQVEEVSDTVDTWHFIHLK
>P25355
MPMNNFLDEFNLFDSIITMMKNDPCCVEDYEPIVENLNRIFQRTFNDEEHRKSMANSQLF
WERLRDTLEAMLLPASLNENSSIPYTRTVRGLILMMRNLAAENQEIPQKLLLQNLVIRGF
LHATSEYVVDTPLIKHLYIACLTCLFNIQQNYSTVDMTTFPALLQFLQYPYGIKLEDGEE
EEHFWLPYLFLFKTYLNNDEFSNEFFRDNDTPQKDYYCVRDRIFFDIVTAKFIQDQENSF
LIEKGRNYLDDSKLEITSIDLSVLECISKSLTTASFGKYLNGLEERQPGKFTTLLQILQL
VVTSKEDWNTYELTAIMSWCYPILQRLACKDIPAFFNKSCNDYAPSVAIQLHSTLLSCLD
IISDLCKFNHVRKFLISYDSVKILVSLLDTFQKNLLRINFLKGNGDTVNEIKITDHEGNK
IEDRLLIFNRVNTNESFIRADNFPHCKLVIIEILASLVYAHPEIQDQIRELGGLALILSN
CVIDDNDPFIKERSIVCLKFLLKNNAKNQEYVKKMEAQDVVQDDALSKAGFEISVEKGGK
VRLVSKEEDPGNENSEIISIDED
>Q9NQL2
MSQVLGKPQPQDEDDAEEEEEEDELVGLADYGDGPDSSDADPDSGTEEGVLDFSDPFSTE
VKPRILLMGLRRSGKSSIQKVVFHKMSPNETLFLESTNKICREDVSNSSFVNFQIWDFPG
QIDFFDPTFDYEMIFRGTGALIFVIDSQDDYMEALARLHLTVTRAYKVNTDINFEVFIHK
VDGLSDDHKIETQRDIHQRANDDLADAGLEKIHLSFYLTSIYDHSIFEAFSKVVQKLIPQ
LPTLENLLNIFISNSGIEKAFLFDVVSKIYIATDSTPVDMQTYELCCDMIDVVIDISCIY
GLKEDGAGTPYDKESTAIIKLNNTTVLYLKEVTKFLALVCFVREESFERKGLIDYNFHCF
RKAIHEVFEVRMKVVKSRKVQNRLQKKKRATPNGTPRVLL
>O75935
MAGLTDLQRLQARVEELERWVYGPGGARGSRKVADGLVKVQVALGNISSKRERVKILYKK
IEDLIKYLDPEYIDRIAIPDASKLQFILAEEQFILSQVALLEQVNALVPMLDSAHIKAVP
EHAARLQRLAQIHIQQQDQCVEITEESKALLEEYNKTTMLLSKQFVQWDELLCQLEAATQ
VKPAEE
>P35630
MKGLAMLGIGRIGWIEKKIPECGPLDALVRPLALAPCTSDTHTVWAGAIGDRHDMILGHE
AVGQIVKVGSLVKRLKVGDKVIVPAITPDWGEEESQRGYPMHSGGMLGGWKFSNFKDGVF

SEVFHVNEADANLALLPRDIKPEDAVMLSDMVTTGFHGAELANIKLGDTVCVIGIGPVGL
MSVAGANHLGAGRIFAVGSRKHCCDIALEYGATDIINYKNGDIVEQILKATDGKGVDKVV
IAGGDVHTFAQAVKMIKPGSDIGNVNYLGEGDNIDIPRSEWGVGMGHKHIHGGLTPGGRV
RMEKLASLISTGKLDTSKLITHRFEGLEKVEDALMLMKNKPADLIKPVVRIHYDDEDTLH

>Q12447
MASSSSTLPLHMYIRPLIIEDLKQILNLESQGFPPNERASEEIISFRLINCPELCSGLFI
REIEGKEVKKETLIGHIMGTKIPHEYITIESMGKLQVESSNHIGIHSVVIKPEYQKKNLA
TLLLTDYIQKLSNQEIGNKIVLIAHEPLIPFYERVGFKIIAENTNVAKDKNFAEQKWIDM
ERELIKEEYDN
>Q7Z203
MINIEDISKSSNQSEEKQLKSTSSKPKYSFAAKSLFKGSNNITPYYLSTSNTFQCVASES
IQTWLLSDDGHIFTSSGNFVLDVSSGGYFVELVQLNSNSKTQIWTIDTTNNKIQNQGNGK
YLDIDNLNICVAPLNGNATQQWTTFRRAPIPTGNWGYFQSKQMDSNNNYWGLSVLNNSKS
YNTSVVMNKVQAKSKGQIWQMTNDGHILSRLDGSLVLDIGPSINGSTTNYYLDTNVYKAN
DLKQQWGINENNQIFNQYYPNLCIGFVGELGVDSTVNCVLAQPSSASDINFQWITNPTYS
LNEIVSEVPEPFPAYTSGDLLASYQYLSNIATSNFTDDIRSLYTGINVSLQSFLSIVTNA
TCPSSIHSTEDFSNVQNQIKTELIYAIDVRLVFENYSGFYSKLFSQGSSNLTNLANLINV
DMSSNQMVNANYTDAITSVFYSLISEIPVGGSIIANIGQSAVEWGELISQSNYQGASTYQ
VELSQLYSHLNTNYENEMANAQSMKDTILQDWGMMSKTYALCFLPTNDPSSLNINGLNFE
KISDVASVAYEIAMIQMLLPTTYQIYFTPAGYWVPYSDGDFAYSDNSGTYIMATIEYSNS
YPPKELTDKLWNNGVSKQEFFWSAYGWNLATSLTYYNMANKFGNIYKLAFPTIKNFTGVP
MQFVMTNDGDNLCNFPVKTHFAKFFSIYYSCGDLGHHYFDIAVNDINDNKVANFTVDINL
EAIEGSYVSIKTGSLVVQPGYAVGNPICNQGSYSLMFSASILIPIYKSE
>Q9H5N1
MAAAAPVAADDDERRRRPGAALEDSRSQEGANGEAESGELSRLRAELAGALAEMETMKAV
AEVSESTKAEAVAAVQRQCQEEVASLQAILKDSISSYEAQITALKQERQQQQQDCEEKER
ELGRLKQLLSRAYPLDSLEKQMEKAHEDSEKLREIVLPMEKEIEELKAKLLRAEELIQEI
QRRPRHAPSLHGSTELLPLSRDPSPPLEPLEELSGDGGPAAEAFAHNCDDSASISSFSLG
GGVGSSSSLPQSRQGLSPEQEETASLVSTGTLVPEGIYLPPPGYQLVPDTQWEQLQTEGR
QLQKDLESVSRERDELQEGLRRSNEDCAKQMQVLLAQVQNSEQLLRTLQGTVSQAQERVQ
LQMAELVTTHKCLHHEVKRLNEENQGLRAEQLPSSAPQGSQQEQGEEESLPSSVPELQQL
LCCTRQEARARLQAQEHGAERLRIEIVTLREALEEETVARASLEGQLRVQREETEVLEAS
LCSLRTEMERVQQEQSKAQLPDLLSEQRAKVLRLQAELETSEQVQRDFVRLSQALQVRLE
RIRQAETLEQVRSIMDEAPLTDVRDIKDT
>P97864
MTDDQDCAAELEKVDSSSEDGVDAKPDRSSIISSILLKKKRNASAGPVRTGRDRVPTYLY
RMDFQKMGKCIIINNKNFDKATGMDVRNGTDKDAGALFKCFQNLGFEVTVHNDCSCAKMQ
DLLRKASEEDHSNSACFACVLLSHGEEDLIYGKDGVTPIKDLTAHFRGDRCKTLLEKPKL
FFIQACRGTELDDGIQADSGPINDIDANPRNKIPVEADFLFAYSTVPGYYSWRNPGKGSW
FVQALCSILNEHGKDLEIMQILTRVNDRVARHFESQSDDPRFNEKKQIPCMVSMLTKELY
FSR
>P50109
MGTNNTSNNNGTTKKMSLEEFLGNDTLGESVWDEEDINLDAISNTTNIDILKQTKAGEHQ

RDGHQQHPHGGHGPMNRSRFSNAGPFGGGSMGDFANHHHPLQHQQGPPYIVKFSDLPPRF
SNFDIEDLFQAKFTKFIKFKLFWEINKNPSISTLKSGSIFDQNFKRDSKVAFVELYTSRD
MDKILNYWTTPLKEIYHITTAPAEFEDFKDYSTKVKLLTDPKDDAGKPFITKTQRSKSNP
FGSAKPVDTQSKILDIEEKMENLHVEDTTTLRASLIPSSDSMATTATGSKITILKKQTPT
EEESHSATPTPKPLSYSEVVERSVVNETSKKGTPLSKLDSPALELQSKPDKSDEFKGGDE
QGFEKGGDDKAQLDVSNDKDKGSETDVDKQFTFKNVEREHSMSRTKYNGNHNNNNGNFRG
SNRYRGGPNGSSYKGGHNNRGNRGGYRGGSSYNNNNNNTNDNNNNNNNSSSNNNNGSRYH
DRQNNEEGLTSDSSLDASGNKKNDFTNSTSNTQQYSIFKPASGFLGQGNNDSIRNNGRGN
YNSSGMNGGSRGRGFGRGRGFGRGAYNNRGSRGGRGSSGNYSNYNNRTTDMPL
>Q03308
MKNPSFDWERLKDVFYRSRAIGELKWPTQYEEFKCALSLTVIAVEIQDFIQVYNYFGQLL
GKINLQRIHEDIIKFEFDKDEKLILVTKSSIKIVKGWSPLTIESVPLQDPTIDTIWDYHN
GIMLLAKSRDIYKLNGNEWELLYENKDKKYNLLTKNHWSCNDDSIILLDVDHVYQVSTSN
GALLKLITDSSWHKVTISSRGFICLYNMKDNKLQIFRDPARILMEHNLDSTPDDICWCGN
DTVACSFEDEIKLYGPDGLYVTFWYPFTVTNLRAEVDGLKVITTEKIYFLSRVQPQTSNI
FRIGSTEPGAMLVDSFSLLEDHAPKAIEILKNFVLEKGVLDCIAAAIDEFEPKLQKMLLN
AASYGKASLQYKSFDASIFVNACNTIKLLNCFRSFGIFLTVEEYRCISLKGVIDRLLKYH
RYYECIQICKLANERFLLGYVFTEWAKDKIKGSPDMEDDELLDKIKSRLSVIDMTDTLQM
VAVAKVAYLEGRFQLSRNLALLEKNEEARIEQLYNLDDDSIALKECIKVQNYSLTISLLI
ALSKKLTNSQLTKLLIIDMFNNPLYLYYMRMDKAYLYDFYRQTDRFIDLAHVLLQQGKEQ
QSLHSFLPQIKDLYSQVQNSEVVNNTIEQLQRQEKLWIYQESLGKRFAISFTNMTLDQTL
SKLIETGQDKQVKEIVKKFKISEKKLYHLKCKTLVEAKKFDELLQFAQSRKSPIGYMPFY
TYLKSRGHMDKASPYVNMIPGLSYQEKKKLYVECRGFRDAIQLAGKEKDIPGLKEIYNII
PPNEPELKALANETMSRI
>Q23972
MKYATGTDNAMTSGISGQTNNSNSASNEMQPTTSTPTAAHKEATSTATTTATYANGNPNS
NANPSQSQPSNALFCEQVTTVTNLFEKWNDCERTVVMYALLKRLRYPSLKFLQYSIDSNL
TQNLGTSQTNLSSVVIDINANNPVYLQNLLNAYKTFQPCDLLDAMSSSSSDKDSMPCYGS
DFQITTSAQCDERKLYARKEDILHEVLNMLPLLKPGNEEAKLIYLTLIPVAVKDTMQQIV
PTELVQQIFSYLLIHPAITSEDRRSLNIWLRHLEDHIQAAAAGLTNRSYFLQPSPQLVAG
GSSTGSGSCSSSATSSSTASCSSVASSSLCPASGSRSSRTNDWQTIAPPSKQLQNKLAGD
WRGNGGGSSSGSINPLCDNLNGITLNELASSQNSLGLSLEGSSSLVNGVVAGAGSMLGIA
GGDDHDTSFSKNGTEILDFDPVTADMGEACSLASSSLCGRNGGNPVEDRSQPPPNLQQQL
LQPPPYASILMGNVGDQFGEINRWSLDSKIAALKTRRSNSLTTQTISSCSSSSNSSVITV
NDNCSNSTENLAQFANKPRSFSLSIEHQRGALMNSGSDTRLDEFKPNYIKFHTRNVGMSG
IGLWLKSLRLHKYIELFKNMTYEEMLLITEDFLQSVGVTKGASHKLALCIDKLKERANIL
NRVEQELLSGQMELSTAVEELTNIVLTPMKPLESPGPPEENIGLRFLKVIDIVTNTLQQD
PYAVQDDETLGVLMWILDRSIHNEAFMNHASQLKDLKFKLSKMKISMVPKMHHVKPAGVG
PNNGNINKPRWNGKTRKCDTKNGSNDRINNRKNSNDMLNFSLNCLPHPLPHHSQQAPPPL
PQFDYNGYGGGPSHQPQYKSSSYPSFMGNPQQQPPPPPSSKSHHHPQQMQQMLQQHNHFP
ALPQQTPPQSHRRSLNNLILVAGGPQQPQQLIFKPGQGVLTNNGSNDNLVLERNQQSQQQ
QQQRKLSGGVSSAEQQPKKTMAAVVMENLAKFDQHFTLF
>P54623
MNSNRTSSSHNSRNLKILNASFDVPRPPGGGNSPLPSQGRSVRELEEQMSALRKENFNLK

LRIYFLEEGQPGARADSSTESLSKQLIDAKIEIATLRKTVDVKMELLKDAARAISHHEEL
QRKADIDSQAIIDELQEQIHAYQMAESGGQPVENIAKTRKMLRLESEVQRLEEELVNIEA
RNVAARNELEFMLAERLESLTACEGKIQELAIKNSELVERLEKETASAESSNEAIDSLKV
ELEACRKENQDLVTSIRTLKHDMKRQVRSMKEAANTMDVQRQSILLLEATIKRKEKSCGS
MQKNVLNYEALIAKLNAELETMRQQNVYFRELSENLQQKEVRQLDRGVAIVQPMRMTADA
GRFVWQSGTIVAQEPLPLERQSAAATSNVSVSAVRLSSGPPPDQTYPVNGHDRAKYGLLQ
LFAAKLDWISAVPLALGHLALKIILLAMRLHSCLQNVHIPLRANRDLGAQLADKICELQE
AQEKLKERERIHEQACRTIQKLMQKLSSQEKEIKKLNQENEQSANKENDCAKTVISPSSS
GRSMSDNEASSQEMSTNLRVRYELKINEQEEKIKQLQTEVKKKTANLQNLVNKELWEKNR
EVERLTKLLANQQKTLPQISEESAGEADLQQSFTEAEYMRALERNKLLQRKVDVLFQRLA
DDQQNSAVIGQLRLELQQARTEVETADKWRLECVDVCSVLTNRLEELAGFLNSLLKHKDV
LGVLAADRRNAMRKAVDRSLDLSKSLNMTLNITATSLADQSLAQLCNLSEILYTEGDASH
KTFNSHEELHAATSMAPTVENLKAENKALKKELEKRRSSEGQRKERRSLPLPSQQFDNQS
ESEAWSEPDRKVSLARIGLDETSNSLAAPEQAISESESEGRTCATRQDRNRNSERIAQLE
EQIAQKDERMLNVQCQMVELDNRYKQEQLRCLDITQQLEQLRAINEALTADLHAIGSHEE
ERMVELQRQLELKNQQIDQLKLAHSTLTADSQITEMELQALQQQMQEIEQLHADSVETLQ
SQLQKLKLDAVQQLEEHERLHREALERDWVALTTYQEQAQQLLELQRSLDYHQENEKELK
QTLVENELATRALKKQLDESTLQASKAVMERTKAYNDKLQLEKRSEELRLQLEALKEEHQ
KLLQKRSNSSDVSQSGYTSEEVAVPMGPPSGQATTCKQAAAAVLGQRVNTSSPDLGIESD
AGRISSVEVSNAQRAMLKTVEMKTEGSASPKAKSEESTSPDSKSNVATGAATVHDCAKVD
LENAELRRKLIRTKRAFEDTYEKLRMANKAKAQVEKDIKNQILKTHNVLRNVRSNMENEL

>P53942
MVPPTVEASLESPYTKSYFSPVPSALLEQNDSPIIMGIDEAGRGPVLGPMVYAVAYSTQK
YQDETIIPNYEFDDSKKLTDPIRRMLFSKIYQDNEELTQIGYATTCITPLDISRGMSKFP
PTRNYNLNEQAHDVTMALIDGVIKQNVKLSHVYVDTVGPPASYQKKLEQRFPGVKFTVAK
KADSLYCMVSVASVVAKVTRDILVESLKRDPDEILGSGYPSDPKTVAWLKRNQTSLMGWP
ANMVRFSWQTCQTLLDDASKNSIPIKWEEQYMDSRKNAAQKTKQLQLQMVAKPVRRKRLR
TLDNWYR
>Q12449
MVVNNPNNWHWVDKNCIGWAKEYFKQKLVGVEAGSVKDKKYAKIKSVSSIEGDCEVNQRK
GKVISLFDLKITVLIEGHVDSKDGSALPFEGSINVPEVAFDSEASSYQFDISIFKETSEL
SEAKPLIRSELLPKLRQIFQQFGKDLLATHGNDIQVPESQVKSNYTRGNQKSSFTEIKDS
ASKPKKNALPSSTSTSAPVSSTNKVPQNGSGNSTSIYLEPTFNVPSSELYETFLDKQRIL
AWTRSAQFFNSGPKLETKEKFELFGGNVISELVSCEKDKKLVFHWKLKDWSAPFNSTIEM
TFHESQEFHETKLQVKWTGIPVGEEDRVRANFEEYYVRSIKLTFGFGAVL
>P53958
MSTFNSYSQPKESNDNSHNNVNKSKSLLDIIFGTNVSEWAFSENALMKAMDLKIEQEKTK
QQYYKLENLNRSIELFKLASSSGLPINQIHKLFNTDHGVPASSPMKAGGNQPHNNTEGTQ
SSENLPRLNGSMKSLKPLNMNTVSPTPMSRQPSPYKFPASSSTGGISHSTVTNVQRRANS
PARIGASAVAALNDNISIKEEDVARRIPSGTKSQESPLNKKPTSLHSRNLSLPIGKFTNP
NIPSTMTSILSFNRDQQQPLSQPLPPPPQQQQDLHTHNLHTIPRKPGMVQKKHRRARSTS
SFGVIDLSIIDEAKEKQVQRSPSPIHSNVSVALTSHDKPIESNMKEQPNMLQSVREGRQV
HDDLDDRTCSESSSRNESPVRTITKDNSVGKILNST

>Q96HS1
MAFRQALQLAACGLAGGSAAVLFSAVAVGKPRAGGDAEPRPAEPPAWAGGARPGPGVWDP
NWDRREPLSLINVRKRNVESGEEELASKLDHYKAKATRHIFLIRHSQYHVDGSLEKDRTL
TPLGREQAELTGLRLASLGLKFNKIVHSSMTRAIETTDIISRHLPGVCKVSTDLLREGAP
IEPDPPVSHWKPEAVQYYEDGARIEAAFRNYIHRADARQEEDSYEIFICHANVIRYIVCR
ALQFPPEGWLRLSLNNGSITHLVIRPNGRVALRTLGDTGFMPPDKITRS
>Q08444
MTENQTAHVRALILDATPLITQSYTHYQNYAQSFYTTPTVFQEIKDAQARKNLEIWQSLG
TLKLVHPSENSIAKVSTFAKLTGDYSVLSANDLHILALTYELEIKLNNGDWRLRKKPGDA
LDASKADVGTDGKQKLTEDNKKEEDSESVPKKKNKRRGGKKQKAKREAREAREAENANLE
LESKAEEHVEEAGSKEQICNDENIKESSDLNEVFEDADDDGDWITPENLTEAIIKDSGED
TTGSLGVEASEEDRHVALNRPENQVALATGDFAVQNVALQMNLNLMNFMSGLKIKRIRNY
MLRCHACFKIFPLPKDGKPKHFCASCGGQGTLLRCAVSVDSRTGNVTPHLKSNFQWNNRG
NRYSVASPLSKNSQKRYGKKGHVHSKPQENVILREDQKEYEKVIKQEEWTRRHNEKILNN
WIGGGSADNYISPFAITGLKQHNVRIGKGRYVNSSKRRS
>Q9NHD5
MTRSSIELGDVTPHNIKQLKKLNTVVFPVSYNDKFYVDVLEAGELAKLAYYNDIVVGAVC
CRIDNTENQRRLYIMTLGCLSPYRRLGIGTVMFEHIMNFAEKDGNFDSIFLHVQINNNGA
IEFYKKFGFEIVDTKEQYYKRIEPADAHVLQKTLRRTAPNSNSTATSTTANSNSRSKARQ
FTFV
>P79106
MGVNQSVSFPPVTGPHLVGCGDVMEGQSLQGSFFRLFYPCQEAEETSEQPLWIPRYEYCA
GLAEYLKFNKRWGGLLFNLGVGSCRLPVSWNGPFKTKDSGYPLIIFSHGMGAFRTVYSAF
CMELASRGFVVAVPEHRDGSAAATCFCKQTPEENQPDNEALKEEWIPHRQIEEGEKEFYV
RNYQVHQRVSECVRVLKILQEVTAGQAVLNILPGGLDLMTLKGGIDVSRVAVMGHSFGGA
TAILALAKEMQFRCAVALDAWMFPLEHDFYPTARGPIFFINAEKFQTVETVNLMKKICDQ
HHQSRIITVLGSVHRSLTDFVFVAGNWISKFFSSHTRGSLDPYEGQETVVRAMLAFLQKH
LDLKEDYDQWNNFIEGIGPSLTPGAPHHLSSL
>Q9BRA2
MARYEEVSVSGFEEFHRAVEQHNGKTIFAYFTGSKDAGGKSWCPDCVQAEPVVREGLKHI
SEGCVFIYCQVGEKPYWKDPNNDFRKNLKVTAVPTLLKYGTPQKLVESECLQANLVEMLF
SED
>Q16635
MPLHVKWPFPAVPPLTWTLASSVVMGLVGTYSCFWTKYMNHLTVHNREVLYELIEKRGPA
TPLITVSNHQSCMDDPHLWGILKLRHIWNLKLMRWTPAAADICFTKELHSHFFSLGKCVP
VCRGAEFFQAENEGKGVLDTGRHMPGAGKRREKGDGVYQKGMDFILEKLNHGDWVHIFPE
GKVNMSSEFLRFKWGIGRLIAECHLNPIILPLWHVGMNDVLPNSPPYFPRFGQKITVLIG
KPFSALPVLERLRAENKSAVEMRKALTDFIQEEFQHLKTQAEQLHNHLQPGR
>Q8LBI1
MVFVKSTKSNAYFKRYQVKFRRRRDGKTDYRARIRLINQDKNKYNTPKYRFVVRFTNKDI
VAQIVSASIAGDIVKASAYAHELPQYGLTVGLTNYAAAYCTGLLLARRVLKMLEMDDEYE
GNVEATGEDFSVEPTDSRRPFRALLDVGLIRTTTGNRVFGALKGALDGGLDIPHSDKRFA
GFHKENKQLDAEIHRNYIYGGHVSNYMKLLGEDEPEKLQTHFSAYIKKGVEAESIEELYK
KVHAAIRADPNPKKTVKPAPKQHKRYNLKKLTYEERKNKLIERVKALNGAGGDDDDEDDE

E
>Q12982
MEGVELKEEWQDEDFPIPLPEDDSIEADILAITGPEDQPGSLEVNGNKVRKKLMAPDISL
TLDPSDGSVLSDDLDESGEIDLDGLDTPSENSNEFEWEDDLPKPKTTEVIRKGSITEYTA
AEEKEDGRRWRMFRIGEQDHRVDMKAIEPYKKVISHGGYYGDGLNAIVVFAVCFMPESSQ
PNYRYLMDNLFKYVIGTLELLVAENYMIVYLNGATTRRKMPSLGWLRKCYQQIDRRLRKN
LKSLIIVHPSWFIRTLLAVTRPFISSKFSQKIRYVFNLAELAELVPMEYVGIPECIKQVD
QELNGKQDEPKNEQ
>Q9VCA8
MNNDAKNHESDDLNVRSTAYFNQQTTTNQPKAPATSKNNTGSGSGSNNNNNNTNQNPNRQ
LNHNLPRIAAARQSIAAALLKNSGRKILTAKNEPLTTTESSGVLTNTPLPSNSRLKVNNN
NNTNNTAKMSGTSSSQSSATPTPPTASSSTTTTTTTNISTGGGGSGSSGGGGGSTTVIAN
PASVTNTGAGSAAKFRAAVASAPSPALPATNAPANATAAAAIAAIATAPAPSSSSSSSSS
SKKTRAAVAALKRQVALQQQQPVTGNAPNMTSKDSAHLKFATTTLLMGAAAAAADSNAGA
ALGGSGAGGSGSSSSVGAVGGARMALNPAVDMANAAVLLKQKLKDAAAAASASASNRSAT
SSMSSTASSLSSSAGIVNAISSALQNIITPDTDTDTEFYPQPVTTDLSESEEESVSEDDI
PESDPDSCPHEGEVREDEDETEEESEDSDESEGEEEEEDEEEIDVLQDNDADDEEIDDED
EEEDAPEVSSFLLDANNKRSSNISALLEAAANEKAPVLRHATHAIDETKQALTKMRCASS
PRDKNSGFSRSLVAACTDNDVNTVKRLLCKGNVNLNDAAASTDDGESLLSMACSAGYYEL
AQVLLAMSAAQVEDKGQKDSTPLMEAASAGHLDIVKLLLNHNADVNAHCATGNTPLMFAC
AGGQVDVVKVLLKHGANVEEQNENGHTPLMEAASAGHVEVAKVLLEHGAGINTHSNEFKE
SALTLACYKGHLDMVRFLLQAGADQEHKTDEMHTALMEASMDGHVEVARLLLDSGAQVNM
PTDSFESPLTLAACGGHVELATLLIERGANIEEVNDEGYTPLMEAAREGHEEMVALLLSK
GANINATTEETQETALTLACCGGFMEVAAFLIKEGANLELGASTPLMEASQEGHTDLVSF
LLKKKANVHAETQTGDTALTHACENGHTDAAGVLLSYGAELEHESEGGRTPLMKACRAGH
LCTVKFLIQKGANVNKQTTSNDHTALSLACAGGHQSVVELLLKNNADPFHKLKDNSTMLI
EASKGGHTRVVELLFRYPNISPTENAASANVTQAAPTSNQPGPNQMRQKIMKQQLQHQLQ
QLNAPPGLHELSEAARASNQQHFHQQQFSSAGNGSSNIVAMGTGDFLDAGELQLTATAGM
SAGAGTSTTGSETGMEEYGEVGGIDLTTLGAQQQEGLIAKSRLFHLQQQQQQQQQQQQQQ
QQQQQQQQQQQQQQQQPPAAGQHQLVPCKHFDLDMEHINSLQPPQKAPPAPPVLFHTVCQ
QPVMQQQQQQLQPGQLKLKAMLPNRNRALKTAEVVEFIDCPVDQQQPGEQVRTQPLGEDG
KTPQFACAGEDPRLQRRRGFMPELKKGELPPESSSSDPNELALKGADNNQPVPTALDNSA
CAQIPARNSGGAITHSSEVLQSTAISDRPKVKATNKNNRKQAAAAAAAAAAAAAAAAAAA
QHAQQVLPNPMVSIYNNLHLQHLQHPHLQFQQQLQLHHQRVAGLDNAAAAAAAAASSANM
AYSISPASPLPSPTGSGNYVDQQLQQQSMDVALQRKTAMDDFRGMLETAVNGPRGRKDLA
LNTPQLNFFKDGWHMVGVHNFFGDQPKSPTETPPEMEETTMSSPTEADRLGSEPRAEMKN
LATLCSAAAAAAAVAAVNKDQVEISSDLESECEDDAEGGAGADCEENTLPPEPIELAAAL
REDGIIVEEEEDDEEEDDDDEEQDTNSGEVDKLNYDDEDAEVDNDGEVDYIDEDEGGGEG
EEEEDDADDDEFFLDEPDSDQGTGNNNNNSKSGASSLPLKQRKMATRLENLILNSQTVCD
FPPELSNSELVHVLPQISNLKAAANSNAALNSVLQQQLAAASAAAAHAKASVVHQKQQHG
EGDQQCEDDGSASASELYSGLEHFANDGEMEDIFQELASSLNYPELAEFSLNQMCKGRFA
GNWAQSSGKWTGQEQLVGVVRSPGLINPGDVPQDAQRQANLVLLDYPMQQNIQLEQRLLD
AEEMHLQQHQQTPLSLLPFTDEQQQQLHHQALSNASDFQQHQQLALENDPELKQQLQQNS
NARIIKAVAAQHQQQPPTNFVYNVESGDKNAPPVQLLFQLPPHMAQHQAQQQQGVGEPLT

EQQQQQLHAEQAHLFQHRTGGQRPPTQSELEQVAQELLLQRSGQVPAGAPVVGVQAIPLK
QKHFNLHPPPCPPTCVQHQVATQTHPASVVVPQPAVGYTQFALQASQQQQMQQNELSIWP
MATPTPAPSSGVSSTKSMPGGIAKKAIDKQSRKERRCVVRQTPAGIQENTKLHLQPQVAT
AQQQFLVQNQLAVATTVSLDKTIEIDSETESNHDTALTLACAGGHEELVELLINRGANIE
HRDKKGFTPLILAATAGHDKVVDILLKHSAELEAQSERTKDTPLSLACSGGRYEVVELLL
SVGANKEHRNVSDYTPLSLAASGGYVNIIKLLLSHGAEINSRTGSKLGISPLMLAAMNGH
TPAVKLLLDQGSDINAQIETNRNTALTLACFQGRHEVVSLLLDRRANVEHRAKTGLTPLM
EAASGGYIEVGRVLLDKGADVNAAPVPTSRDTALTIAADKGHQKFVELLLSRNASVEVKN
KKGNSPLWLAAHGGHLSVVELLYDHNADIDSQDNRRVSCLMAAFRKGHTKIVKWMVQYVS
QFPSDQEMIRFIGTISDKELIDKCFDCMKILRSAKEAQAVKANKNASILLEELDLERTRE
ESRKAAAARRRERKKKKMEKKEEKRRQQQGNGPGGDDMQGDDDDASDKDDDSDKDDEDE
EAAPAAAREEGDSGIDQGSCSSGDTKGARFGGSQSAQAAEAAANSVSTNSQGKKNKKQAK
NKVLISVEPTQPVITSNSVLKGVCAKKHPAVEVVKQPPATQQAAPLKRQLDVKKEEPALK
KKEEKNSSSSSSSKREKENLAPKEVALPAKQQPSSSSKLQSSESASNINSSTATNTSSAN
TTRKEVAKPASQTASATTLNPAKRTEVDGWKEVVRKSSAQQTTAVGASGAPLPVTATSSA
TSVQHHPHHHLANSSSNSSSSLTTSTTTAASSVPEMTCKKVQVPVNAISRVIGRGGSNIN
AIRATTGAHIEVEKQGKNQSERCITIKGLTDATKQAHMLILALIKDPDVDILQMLPRINS
SIKQASSGGASTPMSVGTWDNRTAAGVNAYTFSSAASTTSTSSSSSASSTTPAGASYSNA
HKQHQQQPQSVKGPSGRSSTSVKSNGSSTKVSASSGSGSRSGRAGSSYLAQQQPGRSSGG
GSSNGVIKSKSESSSKSLPAAQKSSTTLGKSSTVSPGAQNFAKAAAIGQSSPKKAEGGAT
SAVVTSAGGRSSGVVAPFGRGKPVAGQGGPAATAASNVAQLGSVSGNSNILAGPIGTFNV
ADVAAVNAAAAAGAAAATNSNVKPIAPIAPPSKRVGSPTQVQQQHQTQQQQQQQLPQPAP
VPGPQPQQQPLQQQQQQQAPQQQPQQPNQQQQPQTSQQNLVINTNLLNDLMAASAANTTS
DSFSAQLAAKLSSAYSLFSDYQQSQWGKLGDPGIGGGAGAVGDGLPQADASKAPGYNRNI
LSSPVGSSKASSNHSTSPPVGNVIQQQQQQQPQSSQQALNIITSGPGGPATAPARSPMVS
ANEGNPAVGQPSMNGTQGLGETAPAHSPGVIKPPTATVPIQRHVPMPISAPEAGAPPTFG
AIGSNPASGNNSAAAQAAAAAAASAMIDRQQQNLQNLQTLQNLQRMVGASQQQQPQQQLN
YPMDPTSSFIVDANNVLRLNPRVIFPQGNTKPPQPPPQGGTQSNVFGGNPGRQPPGTGAR
QPGGAAAQRWYGGTLEYPSYTGRDMLHLENGAGGMAGMGSPSAMSPNHDDIRKMPRPIGT
ERAASWKYNNFNVGGPSLNMEDALASVLPPWAHELKAQPPGLQQPPPPPQSQQQQQQPLN
WLKQQPQQQQYRAYNNGPYPQQQQQHEPMNMPMDYHNMQAPPNMSQQQQQHVNLMPSYGY
QHFVGAPGAVDISAHMPDKMEVWDHHDKHMPWTNYTTNWSN
>P05738
MKYIQTEQQIEVPEGVTVSIKSRIVKVVGPRGTLTKNLKHIDVTFTKVNNQLIKVAVHNG
GRKHVAALRTVKSLVDNMITGVTKGYKYKMRYVYAHFPINVNIVEKDGAKFIEVRNFLGD
KKIRNVPVRDGVTIEFSTNVKDEIVLSGNSVEDVSQNAADLQQICRVRNKDIRKFLDGIY
VSHKGFITEDL
>Q6V1X1
MWKRSEQMKIKSGKCNMAAAMETEQLGVEIFETADCEENIESQDRPKLEPFYVERYSWSQ
LKKLLADTRKYHGYMMAKAPHDFMFVKRNDPDGPHSDRIYYLAMSGENRENTLFYSEIPK
TINRAAVLMLSWKPLLDLFQATLDYGMYSREEELLRERKRIGTVGIASYDYHQGSGTFLF
QAGSGIYHVKDGGPQGFTQQPLRPNLVETSCPNIRMDPKLCPADPDWIAFIHSNDIWISN
IVTREERRLTYVHNELANMEEDARSAGVATFVLQEEFDRYSGYWWCPKAETTPSGGKILR
ILYEENDESEVEIIHVTSPMLETRRADSFRYPKTGTANPKVTFKMSEIMIDAEGRIIDVI

DKELIQPFEILFEGVEYIARAGWTPEGKYAWSILLDRSQTRLQIVLISPELFIPVEDDVM
ERQRLIESVPDSVTPLIIYEETTDIWINIHDIFHVFPQSHEEEIEFIFASECKTGFRHLY
KITSILKESKYKRSSGGLPAPSDFKCPIKEEIAITSGEWEVLGRHGSNIQVDEVRRLVYF
EGTKDSPLEHHLYVVSYVNPGEVTRLTDRGYSHSCCISQHCDFFISKYSNQKNPHCVSLY
KLSSPEDDPTCKTKEFWATILDSAGPLPDYTPPEIFSFESTTGFTLYGMLYKPHDLQPGK
KYPTVLFIYGGPQVQLVNNRFKGVKYFRLNTLASLGYVVVVIDNRGSCHRGLKFEGAFKY
KMGQIEIDDQVEGLQYLASRYDFIDLDRVGIHGWSYGGYLSLMALMQRSDIFRVAIAGAP
VTLWIFYDTGYTERYMGHPDQNEQGYYLGSVAMQAEKFPSEPNRLLLLHGFLDENVHFAH
TSILLSFLVRAGKPYDLQIYPQERHSIRVPESGEHYELHLLHYLQENLGSRIAALKVI
>Q9LG26
MRRHKRWPLRSLVCSFSSSAAETVTTSTAASATAAFPLKHVTRSNFETTLNDLRSLVKAA
DFVAIDLEMTGVTSAPWRDSLEFDRYDVRYLKVKDSAEKFAVVQFGVCPFRWDSRTQSFV
SYPHNFFVFPRQELTFDPPAHEFLCQTTSMDFLAKYQFDFNTCIHEGISYLSRREEEEAS
KRLKMLHGEDGIDSSGETEELKLVRLADVLFAARMEKLLNEWRSGLLHGGNASSEFPRIS
NGSNQSMETVFHHMRPALSLKGFTSHQLRVLNSVLRKHFGDLVYIHSNDKSSSSRDIVVY
TDSDSDKENLMKEAKDERKRLAERKIQSAIGFRQVIDLLASEKKLIVGHNCFLDIAHVYS
KFVGPLPSTAEKFVASINSHFPYIVDTKILLNVNPMLHQRMKKSSTSLSSAFSSLCPQIE
FSSRSSDSFLQQRVNIDVEIDNVRCSNWNAGGKHEAGYDAFMTGCIFAQACNHLGFDFKQ
HSQLDDFAQNEKLEKYINRLYLSWTRGDIIDLRTGHSNADNWRVSKFKYENIVLIWNFPR
KLKARGIKECICKAFGSASVTSVYHVDDSAVFVLFKNSELVWDFLALKRQLESSDGPVSV
LHPLSKILEGGNTGAADYEAYKEICSSHVSEVMFSDQAETVGVKSRTRPNAQCETETREE
NTVTVTHKASDLIDAFLANRVEVETATSN
>Q12068
MSVFVSGANGFIAQHIVDLLLKEDYKVIGSARSQEKAENLTEAFGNNPKFSMEVVPDISK
LDAFDHVFQKHGKDIKIVLHTASPFCFDITDSERDLLIPAVNGVKGILHSIKKYAADSVE
RVVLTSSYAAVFDMAKENDKSLTFNEESWNPATWESCQSDPVNAYCGSKKFAEKAAWEFL
EENRDSVKFELTAVNPVYVFGPQMFDKDVKKHLNTSCELVNSLMHLSPEDKIPELFGGYI
DVRDVAKAHLVAFQKRETIGQRLIVSEARFTMQDVLDILNEDFPVLKGNIPVGKPGSGAT
HNTLGATLDNKKSKKLLGFKFRNLKETIDDTASQILKFEGRI
>Q99383
MSSDEEDFNDIYGDDKPTTTEEVKKEEEQNKAGSGTSQLDQLAALQALSSSLNKLNNPNS
NNSSSNNSNQDTSSSKQDGTANDKEGSNEDTKNEKKQESATSANANANASSAGPSGLPWE
QLQQTMSQFQQPSSQSPPQQQVTQTKEERSKADLSKESCKMFIGGLNWDTTEDNLREYFG
KYGTVTDLKIMKDPATGRSRGFGFLSFEKPSSVDEVVKTQHILDGKVIDPKRAIPRDEQD
KTGKIFVGGIGPDVRPKEFEEFFSQWGTIIDAQLMLDKDTGQSRGFGFVTYDSADAVDRV
CQNKFIDFKDRKIEIKRAEPRHMQQKSSNNGGNNGGNNMNRRGGNFGNQGDFNQMYQNPM
MGGYNPMMNPQAMTDYYQKMQEYYQQMQKQTGMDYTQMYQQQMQQMAMMMPGFAMPPNAM
TLNQPQQDSNATQGSPAPSDSDNNKSNDVQTIGNTSNTDSGSPPLNLPNGPKGPSQYNDD
HNSGYGYNRDRGDRDRNDRDRDYNHRSGGNHRRNGRGGRGGYNRRNNGYHPYNR
>Q9URU9
MNFRPEQQYILEKPGILLSFEQLRINFKHILRHLEHESHVINSTLTTLISQENASMDEKI
EKIDSLLSRVSTVKKKMKHLHDCEALFIKQTKSRLLFMNRLQGIRDMESADFLDWSRVRL
NRLVADYMMANGYHGAAALLCKDSQLENLVDLGIYKRYQLIHDSILQQELKEVLSWCSEH
RAILKKNNSTLELEVRLQRFIELIKSKKLCQAIAFAKAHFGTWANEHPARLQLAAALLAF

PEFTNGSPYSLLLSDDRWEYLASLFTSNFTAVHNIPSVPLLHALLAAGLSSLKTPLCYLD
ANDDNPLALQSQTVKQCPVCTPCLNDLGKALPYAHITQSAIVDSLTGEGLDSDNCPVAFP
NGRVYGIQSLISWNEANGTREGFLRDPYSGKEFPFQLLRKVYVV
>P32644
MDFQCRTCSQAYDAEQMMKHLSSTRHKTVFDTSNDEDICCEECQDKNIHQLQIIRFGGED
MVLLCNSCFRKEYSETERPSTSYSLQNGSILKFWEKYVKVRECCCDECGEESNLNANRNG
EVLCDKCLPKSNRAKDFVSEKSGRFLYIYLGLNETQNSTRKPRKKGGRRVGRGKKGRKGA
KIKKEKKETFEAKISRIAYEVKKENSTIQSSSSSNLRNFKGFKAVESDPVVAAKVSKSET
SRSNPGPSNRNKGKGNKANHKKNSGNGIGKEKERKTNIRNNVRNSQPIPEDRKNTNSHVT
TNSGGKGKNESVDKHQLPQPKALNGNGSGSTNTTGLKKGKKDHAGQKTKGNDKTGNKNPR
EAKLNSAGRKNALGKKSNNQPNKGTSRWTIGSDTESSREPSISPNENTTSITKSRNRNKK
ASKPTLNEKSKTTTMPKKLETKNQEKNNGKTKDGKLIYEEGEPLTRYNTFKSTLSYPDLN
TYLNDYSFALFLEQKLENEFVQNFNILWPRNEKDTAFIINVEKNNNSELEKLLPANLLAL
GRPAFNERQPFFFCTQDEQKVWYIFIKELSIQRGKYVLLVELFSWNNLSLPTKNGSSQFK
LLPTSAQTSRILFAMTRITNPKFIDLLLGQKPIKEIYFDNRLKFSSDKLNRSQKTAVEHV
LNNSITILQGPPGTGKTSTIEEIIIQVIERFHAFPILCVAASNIAIDNIAEKIMENRPQI
KILRILSKKKEQQYSDDHPLGEICLHNIVYKNLSPDMQVVANKTRRGEMISKSEDTKFYK
EKNRVTNKVVSQSQIIFTTNIAAGGRELKVIKECPVVIMDEATQSSEASTLVPLSLPGIR
NFVFVGDEKQLSSFSNIPQLETSLFERVLSNGTYKNPLMLDTQYRMHPKISEFPIKKIYN
GELKDGVTDEQKAWPGVQHPLFFYQCDLGPESRVRSTQRDIVGFTYENKHECVEIVKIIQ
ILMLDKKVPLEEIGVITPYSAQRDLLSDILTKNVVINPKQISMQQEYDEIELFNAAGSQG
TAGSLQNNVINIINGLHVATVDSFQGHEKSFIIFSCVRNNTENKIGFLRDKRRLNVALTR
AKHGLIVVGNKNVLRKGDPLWKDYITYLEEQEVIFTDLTAY
>Q9NYS0
MGKGCKVVVCGLLSVGKTAILEQLLYGNHTIGMEDCETMEDVYMASVETDRGVKEQLHLY
DTRGLQEGVELPKHYFSFADGFVLVYSVNNLESFQRVELLKKEIDKFKDKKEVAIVVLGN
KIDLSEQRQVDAEVAQQWAKSEKVRLWEVTVTDRKTLIEPFTLLASKLSQPQSKSSFPLP
GRKNKGNSNSEN
>A2ZDX9
MEHQGQHGHVTSRVDEYGNPVGTGAGHGQMGTAGMGTHGTTGGMGTHGTTGGMGTHGTTG
TGGGQFQPMREEHKTGGVLQRSGSSSSSSSEDDGMGGRRKKGIKEKIKEKLPGGNKGEQQ
HAMGGTGGAYGQQGHGTGMTTGTTGAHGTTTTDTGEKKGIMDKIKEKLPGQH
>A2WWV5
MAGSGVLEVHLVDAKGLTGNDFLGEIGKIDPYVVVQYRSQERKSSVARDQGKNPSWNEVF
KFQINSTAATGQHKLFLRLMDHDTFSRDDFLGEATINVTDLISLGMEHGTWEMSESKHRV
VLADKTYHGEIRVSLTFTASAKAQDHAEQVGGWAHSFRQ
>Q9R1K7
MSIVTVQLGQCGNQIGFEVFDALFRDSHCSQGLCSKRDNEAYQASCRERFFREEENGVPV
ARAVLVDMEPKVINQTLSKAAQSGRWNYGQHTSFCQKQGSGNNWAYGYSVHGPKHEESIM
NLIQTEVEKCDSLSGFFIIMSMAGGTGSGLGAFVTQKLQDQYSSSLKMNQIIWPYGTGEV
IVQNYNSILTLSHLYRSSDALLIHENDAVHKICAKRMNIKQISFRDLNQVLAHQLGSVFQ
PTYSEDSSFHYRRNPLGDLMEHLVPHPEFKMLGVRNIPQMSAASLAYSAFTWAGLLKHLR
QMLISSAKMEEGINWQVRPPLTGLPPIGKASAHKEHHFNTSLANLVVLRGREVHSADVEG
FKDPALYTSWLEPVDAFSVWKTQRAFDKYEKSAALVSNSQLLVKPLDMIVGKAWNMFSSK

AFIHQYTKFGMEEEDFLDSFALLEQVVASYGSLGP
>P48151
MATVTSESEFQHMLRVCNTNLDGRRKVPYALTGIKGCGRRYAYLVCKRAGIDVNKRAGLM
TPAEIEKIVDILTTQLNYKIPVWFLNRQKDNKDGKDSQLIANAVETRLREDIEALKKMRA
HRGLRHYWGLRVRGQNTKTTGRRGRTVGVSRTKRA
>P78847
MDVWESQHFSALLYLLQCIESNPSNPNVSSRLLIQCLESYSKDFLKFLALDPANANSRKK
LESGEVELGGVINKVNEQFIQLSLTLSTQLNLDEIQCASLLQRGIEASQNLDRTPVQAAL
YFFFLAREQLLECLESLTRVVGLKDLESDISTALKSYLQSLCENGNNLVKTCIDTIPILD
NKVSEILKSEAGGQILGVSEVVDFQEFIRLSHEAHVAELETISVILYQLAKVDLFQNSHF
ESLLVMLRKYDSPNKNAVLILPTLYAFIDKVLEVEYLPDQKVQLRSNSVEILQKIHQAII
QSPSQDWRSSQFKNILGIWWVTRLNATCKQIEKVPSFIDYETTIKNAANEIIQNGVFSDM
ITLLVYPFRQSETEGMEWAFAFKSRSRITVNWSLIRPFIASIIFSELRSFAQAFVSYMPD
ILKTLRLLEEDRYLTNTTYPTSIPGEQIEEYFPFEEFYYLLSSIYTYNVSWISDFWDDIE
SDMYGFLTWSMGSQIPGIITAFTLLLASLCKNTTSASKIYELFSEPIPEVGHLESLMITS
PSWSYIFNVFRYYISHLKPVQTVVTSSGLARVHTDPSEIDTDSALILQAYILLFSSVVRQ
DAQIASTFCENQDLNPIATLFELLECRLPDSVRICIVRALESLAHLSTGSFNNALWTALD
NWFVSSVLFDVDGGLAPMSIPAISKRSLTKPVTSCGPLLNNIRRLTVNLEMKISFVNLLT
SLTRNKSELNVNLTFPENLGASYRTPGVQPYVDYVVETFVASSTQWRLMRDVGSIRLQYA
CLQYMLAVLDGLNIDLLLYSRILSSKVRDNLQNNNLHVYLTRHPAISLLEALYTESVYSG
LFDLVEYGFDQLEDVSVPKTIVITVSASLCILRNVLSLQRVLFKNVVPYIAELGISKYIL
DLTISRRAYKEVFMTRISSIVHLALLVGSRHKCFLRSAIEILSYLVDAEGFMNKRNPDEN
KLLCSIIRTANESKRIIFGFIRTFESQFLTLLSTNDESSLILKLLLNNLKQSGGVYSLAL
LILGFDISSTNVITLRDQPGYVGSRVSLMNSLLDFIEGRTIVNGIWETPVIMVQALEIVA
FLCSCPLTSEVTLSVIRARPGLLVKMVDGEPILTQQVIQNLGGFSSEEVSMVSRCIRSRT
QIMNMLATEIHYAASVGQNKYLNEYVASLIRTNEKTHSTELSQKESGFKILEFMDILRID
PQSITFELPNIPGFNLNMFITRFDRGHSDFQFDTERVLKIYRLFFEAEMTSLGGSAEEKY
SWLEQQNSKLKELAQRLNTFNAHVVLLQDIHGCLTAWARLTGLLVDDCNEVISDVHFDDF
IWEVLRLVLPGVTVHNLGTQGTVSVTSSVIETILPHALRRIGALKPEELGKSTYFMEGIH
DVIVGLLKGIQCQQSDESIRENLYGSLLSILTVFQKHTSANGGDKVDPVFKDAFQKLITP
NLLTSQFFDVLTKDALYTNGSCWELSVIILNFLHHVSPDISTHLYKYYLRRNFVSSFIDA
FSRAFSELLSSNKDVLEVLSGLEAGQCLLITFAQNKLTVHSVLTFDYIKLMVQMLLQLCK
QGGIQYLKPPVQRLMIQLLQIFILVLMRVTLKEISNKDKLLLQSIFLLSRKLQDSVQTGA
DQALSPEMNTVKKYVETISRLLDLYRD
>P35997
MVLVQDLLHPTAASEARKHKLKTLVQGPRSYFLDVKCPGCLNITTVFSHAQTAVTCESCS
TILCTPTGGKAKLSEGTSFRRK
>Q06108
MSDYFTFPKQENGGISKQPATPGSTRSSSRNLELPKNYRSFGGSSDELASMYSADSQYLM
DMIPDSLTLKNEPASGNTQMNGPDGKENKDIKLDEYILPKTDPRSPYYINMPIPKKLPKS
EGKARAKQKVNRADPSDLDVENIYETSGEFVREYPTDILIDRFHKWKKILKSLIAYFREA
AYSQEQIARINYQMKNAVKFAFLTDLEDETNKLVDPSISKLPTKKPQPVPLAAQKLDSKY
DTDVEQPQSIQSVPSEEVASASSGFMKFGSGSIQDIQVILKKYHLSLGSQQYKISKEILA
YIIPKLTDLRKDLTTKMKEIKELNGDFKTNIGEHIKITSRLLNKYIASVKLLDEASTSGD

KQGEKLKPKHDPYLLKLQLDLQLKRQLLEENYLREAFLNLQSAALQLEKIVYSKIQSALQ
RYSALIDSEARLMIKNLCHELQQGILSRPPAVEWDNFVSHHPTCLMNLKSTDPPPQPRRL
SDIVYPNMKSPLAKCIRVGYLLKKTESSKSFTKGYFVLTTNYLHEFKSSDFFLDSKSPRS
KNKPVVEQSDISRVNKDGTNAGSHPSSKGTQDPKLTKRRKGLSSSNLYPISSLSLNDCSL
KDSTDSTFVLQGYASYHSPEDTCTKESSTTSDLACPTKTLASNKGKHQRTPSALSMVSVP
KFLKSSSVPKEQKKAKEEANINKKSICEKRVEWTFKIFSASLEPTPEESKNFKKWVQDIK
ALTSFNSTQERSNFIEEKILKSRNHNNGKSSQRSKNSTYITPVDSFVNLSEKVTPSSSVT
TLNTRKRANRPRYIDIPKSANMNAGAMNSVYRSKVNTPAIDENGNLAIVGETKNSAPQNG
MSYTIRTPCKSPYSPYTGEGMLYNRSADNLMASSSRKASAPGEVPQIAVSNHGDEAIIPA
SAYSDSSHKSSRASSVASIHNQRVDFYPSPLMNLPGVSPSCLALDGNANGYFGIPLNCNS
EARRGSDLSPFEMESPLFEENRTQNCSGSRKSSACHIPHQCGPRKEGNDSRLIYGNEKGA
SQSRLTLKEPLTSKGVEAPYSSLKKTYSAENVPLTSTVSNDKSLHSRKEGSTNTVPATSA
SSK
>O14323
MEPCEDKCKRGLSLNFFEEDGPSLRLNSDSLDFLARDFKVEGMSQDNFDQKTKLYITEES
LQKEVNIFLTKIYIRESEREPPQSHNSVFQLLSKIRNSVPNVSAFRNNLSILSKELSFFS
FARHIHNRRLCWAEFIYCIRRGIKAIFKTTVQFLPTRLAKIFEKKASEVLKDNLLQTCNS
KREGEVCDVKEPAVASSESSDCFNDMQELNNIVDLRDYSNSRFQQNRLLDRNLKGWIQGE
SEKALKGRRTTKRNDKENYNYPDFSNDNELLFSLATLIVENNPKKENIIPKYYLRYLQRL
SRTEINKEIIEIEKLELEVVQFQMSIANLINTQVEVTNTIEELGLRCRPLPNENE
>P39999
MTALFCLELRTNIFLIMNDCIIINYWKGFIFSFHSYFFPFRFESSLRAHYPGKRNYSDFS
VIPLPYYIDVRSFHICESQHIIALPLQIPLPYRMLIRMYPV
>Q9ERG2
MDELAGGGGGGQGMAAPPRPQQGPGGNLSLPPGANGAPGGGGPPAAEAAGPPAGPELSRP
QQYTIPGILHYIQHEWARFEMERAHWEVERAELQARIAFLQGERKGQENLKKDLVRRIKM
LEYALKQERAKYHKLKYGTELNQGDLKMPTFESEETKDVEAPPAPQNSQLTWKQGRQLLR
QYLQEVGYTDTILDVRSQRVRSLLGLSNSEPNGSVEAKNLEQILNGGESPKQKGQEIKRP
PGDVLETFNFLENADDSDEEENDMIEGIPEGKDKLRIHKHKIGNEGLAADLTDDPDTEEA
LKEFDFLVTAEDGEGAGEARSSGDGTEWDKDDLSPTAEVWDVDQGLISKLKEQYKKERKG
KKGVKRVNRTNLCDMITDLGDDELPHIPSGIINQSRSASTRMADHEGARAEEAEPITFPS
GGGKSFIMGSDDVLLSVLGLGDLADLTVTNDADYSYDLPANKDAFRKTWNPKYTLRSHFD
GVRALAFHPVEPVLVTASEDHTLKLWNLQKTVPAKKSASLDVEPIYTFRAHIGPVLSLAI
SSNGEQCFSGGIDATIQWWNMPSPNVDPYDTYESNVLAGTLVAHTDAVWGLAYSGIKNQL
LSCSADGTIRLWNPQEKLPCVCTYNGDKEHGIPTSVDFIGCDPAHMVTSFNTGSAVIYDL
ETSQSLVMLSSQVDSGLQSSNHINRVVSHPTLPVTITAHEDRHIKFFDNKTGKMIHSMVA
HLDAVTSLAVDPNGIYLMSGSHDCSIRLWNLDSKTCVQEITAHRKKLDESIYDVAFHPSK
AYIASAGADALAKVFV
>Q96KS0
MDSPCQPQPLSQALPQLPGSSSEPLEPEPGRARMGVESYLPCPLLPSYHCPGVPSEASAG
SGTPRATATSTTASPLRDGFGGQDGGELRPLQSEGAAALVTKGCQRLAAQGARPEAPKRK
WAEDGGDAPSPSKRPWARQENQEAEREGGMSCSCSSGSGEASAGLMEEALPSAPERLALD
YIVPCMRYYGICVKDSFLGAALGGRVLAEVEALKRGGRLRDGQLVSQRAIPPRSIRGDQI
AWVEGHEPGCRSIGALMAHVDAVIRHCAGRLGSYVINGRTKAMVACYPGNGLGYVRHVDN

PHGDGRCITCIYYLNQNWDVKVHGGLLQIFPEGRPVVANIEPLFDRLLIFWSDRRNPHEV
KPAYATRYAITVWYFDAKERAAAKDKYQLASGQKGVQVPVSQPPTPT
>P97767
MEMQDLTSPHSRLSGSSESPSGPKLDSSHINSTSMTPNGTEVKTEPMSSSEIASTAADGS
LDSFSGSALGSSSFSPRPAHPFSPPQIYPSKSYPHILPTPSSQTMAAYGQTQFTTGMQQA
TAYATYPQPGQPYGISSYGALWAGIKTESGLSQSQSPGQTGFLSYGTSFGTPQPGQAPYS
YQMQGSSFTTSSGLYSGNNSLTNSSGFNSSQQDYPSYPGFGQGQYAQYYNSSPYPAHYMT
SSNTSPTTPSTNATYQLQEPPSGVTSQAVTDPTAEYSTIHSPSTPIKETDSERLRRGSDG
KSRGRGRRNNNPSPPPDSDLERVLLWDLDETIIVFHSLLTGSYANRYGRDPPTSVSLGLR
MEEMIFNLADTHLFFNDLEECDQVHIDDVSSDDNGQDLSTYNFGRDGFPAAATSANLCLA
TGVRGGVDWMRKLAFRYRRVKEIYNTYKNKVGGLLGPAKREAWLQLRAEIEALTDSWLTL
ALKALSLIHSRTNCVNILVTTTQLSPALAKVLLYGLGIVFPIENIYSATKIGKESCFERI
IQRFGRKVVYLLIGDGVEEEQGAKKHAMPFWRVSSHSDLMALHHALELEYL
>P50748
MWNDIELLTNDDTGSGYLSVGSRKEHGTALYQVDLLVKISSEKASLNPKIQACSLSDGFI
IVADQSVILLDSICRSLQLHLVFDTEVDVVGLCQEGKFLLVGERSGNLHLIHVTSKQTLL
TNAFVQKANDENRRTYQNLVIEKDGSNEGTYYMLLLTYSGFFCITNLQLLKIQQAIENVD
FSTAKKLQGQIKSSFISTENYHTLGCLSLVAGDLASEVPVIIGGTGNCAFSKWEPDSSKK
GMTVKNLIDAEIIKGAKKFQLIDNLLFVLDTDNVLSLWDIYTLTPVWNWPSLHVEEFLLT
TEADSPSSVTWQGITNLKLIALTASANKKMKNLMVYSLPTMEILYSLEVSSVSSLVQTGI
STDTIYLLEGVCKNDPKLSEDSVSVLVLRCLTEALPENRLSRLLHKHRFAEAESFAIQFG
LDVELVYKVKSNHILEKLALSSVDASEQTEWQQLVDDAKENLHKIQDDEFVVNYCLKAQW
ITYETTQEMLNYAKTRLLKKEDKTALIYSDGLKEVLRAHAKLTTFYGAFGPEKFSGSSWI
EFLNNEDDLKDIFLQLKEGNLVCAQYLWLRHRANFESRFDVKMLESLLNSMSASVSLQKL
CPWFKNDVIPFVRRTVPEGQIILAKWLEQAARNLELTDKANWPENGLQLAEIFFTAEKTD
ELGLASSWHWISLKDYQNTEEVCQLRTLVNNLRELITLHRKYNCKLALSDFEKENTTTIV
FRMFDKVLAPELIPSILEKFIRVYMREHDLQEEELLLLYIEDLLNRCSSKSTSLFETAWE
AKAMAVIACLSDTDLIFDAVLKIMYAAVVPWSAAVEQLVKQHLEMDHPKVKLLQESYKLM
EMKKLLRGYGIREVNLLNKEIMRVVRYILKQDVPSSLEDALKVAQAFMLSDDEIYSLRII
DLIDREQGEDCLLLLKSLPPAEAEKTAERVIIWARLALQEEPDHSKEGKAWRMSVAKTSV
DILKILCDIQKDNLQKKDECEEMLKLFKEVASLQENFEVFLSFEDYSNSSLVADLREQHI
KAHEVAQAKHKPGSTPEPIAAEVRSPSMESKLHRQALALQMSKQELEAELTLRALKDGNI
KTALKKCSDLFKYHCNADTGKLLFLTCQKLCQMLADNVPVTVPVGLNLPSMIHDLASQAA
TICSPDFLLDALELCKHTLMAVELSRQCQMDDCGILMKASFGTHKDPYEEWSYSDFFSED
GIVLESQMVLPVIYELISSLVPLAESKRYPLESTSLPYCSLNEGDGLVLPVINSISALLQ
NLQESSQWELALRFVVGSFGTCLQHSVSNFMNATLSEKLFGETTLVKSRHVVMELKEKAV
IFIRENATTLLHKVFNCRLVDLDLALGYCTLLPQKDVFENLWKLIDKAWQNYDKILAISL
VGSELASLYQEIEMGLKFRELSTDAQWGIRLGKLGISFQPVFRQHFLTKKDLIKALVENI
DMDTSLILEYCSTFQLDCDAVLQLFIETLLHNTNAGQGQGDASMDSAKRRHPKLLAKALE
MVPLLTSTKDLVISLSGILHKLDPYDYEMIEVVLKVIERADEKITNININQALSILKHLK
SYRRISPPVDLEYQYMLEHVITLPSAAQTRLPFHLIFFGTAQNFWKILSTELSEESFPTL
LLISKLMKFSLDTLYVSTAKHVFEKKLKPKLLKLTQAKSSTLINKEITKITQTIESCLLS
IVNPEWAVAIAISLAQDIPEGSFKISALKFCLYLAERWLQNIPSQDEKREKAEALLKKLH
IQYRRSGTEAVLIAHKLNTEEYLRVIGKPAHLIVSLYEHPSINQRIQNSSGTDYPDIHAA

AKEIAEVNEINLEKVWDMLLEKWLCPSTKPGEKPSELFELQEDEALRRVQYLLLSRPIDY
SSRMLFVFATSTTTTLGMHQLTFAHRTRALQCLFYLADKETIESLFKKPIEEVKSYLRCI
TFLASFETLNIPITYELFCSSPKEGMIKGLWKNHSHESMAVRLVTELCLEYKIYDLQLWN
GLLQKLLGFNMIPYLRKVLKAISSIHSLWQVPYFSKAWQRVIQIPLLSASCPLSPDQLSD
CSESLIAVLECPVSGDLDLIGVARQYIQLELPAFALACLMLMPHSEKRHQQIKNFLGSCD
PQVILKQLEEHMNTGQLAGFSHQIRSLILNNIINKKEFGILAKTKYFQMLKMHAMNTNNI
TELVNYLANDLSLDEASVLITEYSKHCGKPVPPDTAPCEILKMFLSGLS
>Q9USJ6
MTLPEKLLKITPKILVIMGSVRSKRLCPTIATWVGEMGKRETNFDYEKVDLTDWPLSMSD
EPGLPIMGIDVYTQEHTKAWGSKIAGADGFVFVTPQYNGGYPAILKNALDHLYHEWNGKP
LLIVSYGGHGGGDCASQLKHVAGFLKMRVAPTMPALTLPRDKIVQGVVDPAVEFTKHLGE
LKKAFGEFSQLFESNPERKP
>P78406
MSLFGTTSGFGTSGTSMFGSATTDNHNPMKDIEVTSSPDDSIGCLSFSPPTLPGNFLIAG
SWANDVRCWEVQDSGQTIPKAQQMHTGPVLDVCWSDDGSKVFTASCDKTAKMWDLSSNQA
IQIAQHDAPVKTIHWIKAPNYSCVMTGSWDKTLKFWDTRSSNPMMVLQLPERCYCADVIY
PMAVVATAERGLIVYQLENQPSEFRRIESPLKHQHRCVAIFKDKQNKPTGFALGSIEGRV
AIHYINPPNPAKDNFTFKCHRSNGTNTSAPQDIYAVNGIAFHPVHGTLATVGSDGRFSFW
DKDARTKLKTSEQLDQPISACCFNHNGNIFAYASSYDWSKGHEFYNPQKKNYIFLRNAAE
ELKPRNKK
>P08153
MDTSNSWFDASKVQSLNFDLQTNSYYSNARGSDPSSYAIEGEYKTLATDDLGNILNLNYG
ETNEVIMNEINDLNLPLGPLSDEKSVKVSTFSELIGNDWQSMNFDLENNSREVTLNATSL
LNENRLNQDSGMTVYQKTMSDKPHDEKKISMADNLLSTINKSEINKGFDRNLGELLLQQQ
QELREQLRAQQEANKKLELELKQTQYKQQQLQATLENSDGPQFLSPKRKISPASENVEDV
YANSLSPMISPPMSNTSFTGSPSRRNNRQKYCLQRKNSSGTVGPLCFQELNEGFNDSLIS
PKKIRSNPNENLSSKTKFITPFTPKSRVSSATSNSANITPNNLRLDFKINVEDQESEYSE
KPLGLGIELLGKPGPSPTKSVSLKSASVDIMPTIPGSVNNTPSVNKVSLSSSYIDQYTPR
GKQLHFSSISENALGINAATPHLKPPSQQARHREGVFNDLDPNVLTKNTDNEGDDNEENE
PESRFVISETPSPVLKSQSKYEGRSPQFGTHIKEINTYTTNSPSKITRKLTTLPRGSIDK
YVKEMPDKTFECLFPGCTKTFKRRYNIRSHIQTHLEDRPYSCDHPGCDKAFVRNHDLIRH
KKSHQEKAYACPCGKKFNREDALVVHRSRMICSGGKKYENVVIKRSPRKRGRPRKDGTSS
VSSSPIKENINKDHNGQLMFKLEDQLRRERSYDGNGTGIMVSPMKTNQR
>Q8IX03
MPRPELPLPEGWEEARDFDGKVYYIDHTNRTTSWIDPRDRYTKPLTFADCISDELPLGWE
EAYDPQVGDYFIDHNTKTTQIEDPRVQWRREQEHMLKDYLVVAQEALSAQKEIYQVKQQR
LELAQQEYQQLHAVWEHKLGSQVSLVSGSSSSSKYDPEILKAEIATAKSRVNKLKREMVH
LQHELQFKERGFQTLKKIDKKMSDAQGSYKLDEAQAVLRETKAIKKAITCGEKEKQDLIK
SLAMLKDGFRTDRGSHSDLWSSSSSLESSSFPLPKQYLDVSSQTDISGSFGINSNNQLAE
KVRLRLRYEEAKRRIANLKIQLAKLDSEAWPGVLDSERDRLILINEKEELLKEMRFISPR
KWTQGEVEQLEMARKRLEKDLQAARDTQSKALTERLKLNSKRNQLVRELEEATRQVATLH
SQLKSLSSSMQSLSSGSSPGSLTSSRGSLVASSLDSSTSASFTDLYYDPFEQLDSELQSK
VEFLLLEGATGFRPSGCITTIHEDEVAKTQKAEGGGRLQALRSLSGTPKSMTSLSPRSSL
SSPSPPCSPLMADPLLAGDAFLNSLEFEDPELSATLCELSLGNSAQERYRLEEPGTEGKQ

LGQAVNTAQGCGLKVACVSAAVSDESVAGDSGVYEASVQRLGASEAAAFDSDESEAVGAT
RIQIALKYDEKNKQFAILIIQLSNLSALLQQQDQKVNIRVAVLPCSESTTCLFRTRPLDA
SDTLVFNEVFWVSMSYPALHQKTLRVDVCTTDRSHLEECLGGAQISLAEVCRSGERSTRW
YNLLSYKYLKKQSRELKPVGVMAPASGPASTDAVSALLEQTAVELEKRQEGRSSTQTLED
SWRYEETSENEAVAEEEEEEVEEEEGEEDVFTEKASPDMDGYPALKVDKETNTETPAPSP
TVVRPKDRRVGTPSQGPFLRGSTIIRSKTFSPGPQSQYVCRLNRSDSDSSTLSKKPPFVR
NSLERRSVRMKRPSSVKSLRSERLIRTSLDLELDLQATRTWHSQLTQEISVLKELKEQLE
QAKSHGEKELPQWLREDERFRLLLRMLEKRQMDRAEHKGELQTDKMMRAAAKDVHRLRGQ
SCKEPPEVQSFREKMAFFTRPRMNIPALSADDV
>Q12087
MAKVHGSLARAGKVKSQTPKVEKTEKPKKPKGRAYKRLLYTRRFVNVTLVNGKRRMNPGP
SVQ
>P35194
MAKQRQTTKSSKRYRYSSFKARIDDLKIEPARNLEKRVHDYVESSHFLASFDQWKEINLS
AKFTEFAAEIEHDVQTLPQILYHDKKIFNSLVSFINFHDEFSLQPLLDLLAQFCHDLGPD
FLKFYEEAIKTLINLLDAAIEFESSNVFEWGFNCLAYIFKYLSKFLVKKLVLTCDLLIPL
LSHSKEYLSRFSAEALSFLVRKCPVSNLREFVRSVFEKLEGDDEQTNLYEGLLILFTESM
TSTQETLHSKAKAIMSVLLHEALTKSSPERSVSLLSDIWMNISKYASIESLLPVYEVMYQ
DFNDSLDATNIDRILKVLTTIVFSESGRKIPDWNKITILIERIMSQSENCASLSQDKVAF
LFALFIRNSDVKTLTLFHQKLFNYALTNISDCFLEFFQFALRLSYERVFSFNGLKFLQLF
LKKNWQSQGKKIALFFLEVDDKPELQKVREVNFPEEFILSIRDFFVTAEINDSNDLFEIY
WRAIIFKYSKLQNTEIIIPLLERIFSTFASPDNFTKDMVGTLLKIYRKEDDASGNNLLKT
ILDNYENYKESLNFLRGWNKLVSNLHPSESLKGLMSHYPSLLLSLTDNFMLPDGKIRYET
LELMKTLMILQGMQVPDLLSSCMVIEEIPLTLQNARDLTIRIKNVGAEFGKTKTDKLVSS
FFLKYLFGLLTVRFSPVWTGVFDTLPNVYTKDEALVWKLVLSFIKLPDENQNLDYYQPLL
EDGANKVLWDSSVVRLRDTIDTFSHIWSKYSTQNTSIISTTIERRGNTTYPILIRNQALK
VMLSIPQVAENHFVDIAPFVYNDFKTYKDEEDMENERVITGSWTEVDRNVFLKTLSKFKN
IKNVYSATELHDHLMVLLGSRNTDVQKLALDALLAYKNPTLNKYRDNLKNLLDDTLFKDE
ITTFLTENGSQSIKAEDEKVVMPYVLRIFFGRAQVPPTSGQKRSRKIAVISVLPNFKKPY
INDFLSLASERLDYNYFFGNSHQINSSKATLKTIRRMTGFVNIVNSTLSVLRTNFPLHTN
SVLQPLIYSIAMAYYVLDTESTEEVHLRKMASNLRQQGLKCLSSVFEFVGNTFDWSTSME
DIYAVVVKPRISHFSDENLQQPSSLLRLFLYWAHNPSLYQFLYYDEFATATALMDTISNQ
HVKEAVIGPIIEAADSIIRNPVNDDHYVDLVTLICTSCLKILPSLYVKLSDSNSISTFLN
LLVSITEMGFIQDDHVRSRLISSLISILKGKLKKLQENDTQKILKILKLIVFNYNCSWSD
IEELYTTISSLFKTFDERNLRVSLTELFIELGRKVPELESISKLVADLNSYSSSRMHEYD
FPRILSTFKGLIEDGYKSYSELEWLPLLFTFLHFINNKEELALRTNASHAIMKFIDFINE
KPNLNEASKSISMLKDILLPNIRIGLRDSLEEVQSEYVSVLSYMVKNTKYFTDFEDMAIL
LYNGDEEADFFTNVNHIQLHRRQRAIKRLGEHAHQLKDNSISHYLIPMIEHYVFSDDERY
RNIGNETQIAIGGLAQHMSWNQYKALLRRYISMLKTKPNQMKQAVQLIVQLSVPLRETLR
IVRDGAESKLTLSKFPSNLDEPSNFIKQELYPTLSKILGTRDDETIIERMPIAEALVNIV
LGLTNDDITNFLPSILTNICQVLRSKSEELRDAVRVTLGKISIILGAEYLVFVIKELMAT
LKRGSQIHVLSYTVHYILKSMHGVLKHSDLDTSSSMIVKIIMENIFGFAGEEKDSENYHT
KVKEIKSNKSYDAGEILASNISLTEFGTLLSPVKALLMVRINLRNQNKLSELLRRYLLGL
NHNSDSESESILKFCHQLFQESEMSNSPQIPKKKVKDQVDEKEDFFLVNLESKSYTINSN

SLLLNSTLQKFALDLLRNVITRHRSFLTVSHLEGFIPFLRDSLLSENEGVVISTLRILIT
LIRLDFSDESSEIFKNCARKVLNIIKVSPSTSSELCQMGLKFLSAFIRHTDSTLKDTALS
YVLGRVLPDLNEPSRQGLAFNFLKALVSKHIMLPELYDIADTTREIMVTNHSKEIRDVSR
SVYYQFLMEYDQSKGRLEKQFKFMVDNLQYPTESGRQSVMELINLIITKANPALLSKLSS
SFFLALVNVSFNDDAPRCREMASVLISTMLPKLENKDLEIVEKYIAAWLKQVDNASFLNL
GLRTYKVYLKSIGFEHTIELDELAIKRIRYILSDTSVGSEHQWDLVYSALNTFSSYMEAT
ESVYKHGFKDIWDGIITCLLYPHSWVRQSAANLVHQLIANKDKLEISLTNLEIQTIATRI
LHQLGAPSIPENLANVSIKTLVNISILWKEQRTPFIMDVSKQTGEDLKYTTAIDYMVTRI
GGIIRSDEHRMDSFMSKKACIQLLALLVQVLDEDEVIAEGEKILLPLYGYLETYYSRAVD
EEQEELRTLSNECLKILEDKLQVSDFTKIYTAVKQTVLERRKERRSKRAILAVNAPQISA
DKKLRKHARSREKRKHEKDENGYYQRRNKRKRA
>Q9FK12
MDEYIDFRPLKYTEHKTSMTKYTKKSSEKLSGGKSLKKVSICYTDPDATDSSSDEDEEDF
LFPRRRVKRFVNEITVEPSCNNVVTGVSMKDRKRLSSSSDETQSPASSRQRPNNKVSVSG
QIKKFRGVRQRPWGKWAAEIRDPEQRRRIWLGTFETAEEAAVVYDNAAIRLRGPDALTNF
SIPPQEEEEEEEPEPVIEEKPVIMTTPTPTTSSSESTEEDLQHLSSPTSVLNHRSEEIQQ
VQQPFKSAKPEPGVSNAPWWHTGFNTGLGESDDSFPLDTPFLDNYFNESPPEMSIFDQPM
DQIFCENDDIFNDMLFLGGETMNIEDELTSSSIKDMGSTFSDFDDSLISDLLVA
>Q96GN5
MELATRYQIPKEVADIFNAPSDDEEFVGFRDDVPMETLSSEESCDSFDSLESGKQQDVRF
HSKYFTEELRRIFIEDTDSETEDFAGFTQSDLNGKTNPEVMVVESDLSDDGKASLVSEEE
EDEEEDKATPRRSRSRRSSIGLRVAFQFPTKKLANKPDKNSSSEQLFSSARLQNEKKTIL
ERKKDCRQVIQREDSTSESEDDSRDESQESSDALLKRTMNIKENKAMLAQLLAELNSMPD
FFPVRTPTSASRKKTVRRAFSEGQITRRMNPTRSARPPEKFALENFTVSAAKFAEEFYSF
RRRKTIGGKCREYRRRHRISSFRPVEDITEEDLENVAITVRDKIYDKVLGNTCHQCRQKT
IDTKTVCRNQGCCGVRGQFCGPCLRNRYGEDVRSALLDPDWVCPPCRGICNCSYCRKRDG
RCATGILIHLAKFYGYDNVKEYLESLQKELVEDN
>O74759
MQTFRLTSTGRNILRPDELAFQPREEIPYKSFHPDLQIDEPLEILEGDHTQYAGLRDSLV
TYKSENSYVLKALLNAKIENVKPVGVQTENINPQEKKFGYKTAKQLDWSPDEYFKFVAIH
PYSKTSFPVSYDLDELDTMWLTYYNEFQLSSNSEWENVSKEFLEIVLTIIEREWLYLEAW
MPKIEPVRVEDELDGRCVICNEAECENSNAIVFCDNCNTSVHQNCYGIPFVPEGQWFCKK
CLLAPHEVICCAFCPDRDGAFCTTLDGRWCHTICAIAIPEISFHDTSRLDLVRNIASIPK
SRWKLVCCICKLRWGTCVQCSDKNCYAAYHITCARRAGFFYKIYSHSASYDSVDMETYCD
KHTPPDYLNGLMKRLFPLAELYYKRMATDVPLNFQATKAPDFVPEGPWKSHPLPAFIVDK
VTKVLLSYNVKRQDLPSIVTDICKFYHMKRRSRKDAPLLKSQLLMDSLENLPVRASKDRV
RSLEVAKALQDQYQSLLTLVESTAKRQLLKCQLSNLRKKFLNLNYFPAQRLLQDTLVKII
DLDVDGLFNMPLDNGWIGWVELKRQVFSYQIGSISSLEKKLEPIWDVDGVIQCIDDMEQL
TAMVQFAQKTEGEVKKLFIKAKIYFESLSLDERGNLKVPSLGINGLEYDNWPGLNELEMS
QLDIPSQGNLKSLHDFIEGLDLNEKIGKFPISMFQNQVAQFSTIEIPKMSGRANGMHNFH
SEDVTGQSNHALPNSVTKKNGTKQPYTKNSLPFNERITRSKAKKNYS
>P48592
MASKENIADNMEKFSLKSPSKKILTDSTNNVRKMSIGHEANGQLAKESSTVNGIGKSANS
LMEKSVTPFDPSLEPLLRENPRRFVIFPIQYHDIWQMYKKAEASFWTVEEVDLSKDLTDW

HRLKDDERHFISHVLAFFAASDGIVNENLVERFSQEVQITEARCFYGFQIAMENVHSEMY
SVLIDTYIRDPHQREYLFNAIETMPAVKRKADWALSWISSKSANFGERIIAFAAVEGIFF
SGSFASIFWLKKRGLMPGLTFSNELISRDEGLHCDFAVLMFQHLVQRPKRERIIEIIRDA
VAIEQEFLTDALPVNLIGMNCDLMSQYIEFVADRLLVELGVGKIYNTKNPFNFMEMISLD
GKTNFFEKKVGEYQRMGVVSNPLDNVFTLDADF
>P18297
MEGGRLGCAVCVLTGASRGFGRALAPQLAGLLSPGSVLLLSARSDSMLRQLKEELCTQQP
GLQVVLAAADLGTESGVQQLLSAVRELPRPERLQRLLLINNAGTLGDVSKGFLNINDLAE
VNNYWALNLTSMLCLTTGTLNAFSNSPGLSKTVVNISSLCALQPFKGWGLYCAGKAARDM
LYQVLAVEEPSVRVLSYAPGPLDTNMQQLARETSMDPELRSRLQKLNSEGELVDCGTSAQ
KLLSLLQRDTFQSGAHVDFYDI
>P20618
MLSSTAMYSAPGRDLGMEPHRAAGPLQLRFSPYVFNGGTILAIAGEDFAIVASDTRLSEG
FSIHTRDSPKCYKLTDKTVIGCSGFHGDCLTLTKIIEARLKMYKHSNNKAMTTGAIAAML
STILYSRRFFPYYVYNIIGGLDEEGKGAVYSFDPVGSYQRDSFKAGGSASAMLQPLLDNQ
VGFKNMQNVEHVPLSLDRAMRLVKDVFISAAERDVYTGDALRICIVTKEGIREETVSLRK
D
>P25294
MVKETKLYDLLGVSPSANEQELKKGYRKAALKYHPDKPTGDTEKFKEISEAFEILNDPQK
REIYDQYGLEAARSGGPSFGPGGPGGAGGAGGFPGGAGGFSGGHAFSNEDAFNIFSQFFG
GSSPFGGADDSGFSFSSYPSGGGAGMGGMPGGMGGMHGGMGGMPGGFRSASSSPTYPEEE
TVQVNLPVSLEDLFVGKKKSFKIGRKGPHGASEKTQIDIQLKPGWKAGTKITYKNQGDYN
PQTGRRKTLQFVIQEKSHPNFKRDGDDLIYTLPLSFKESLLGFSKTIQTIDGRTLPLSRV
QPVQPSQTSTYPGQGMPTPKNPSQRGNLIVKYKVDYPISLNDAQKRAIDENF
>P04573
MAAPKARALGPEEKDECMKIFDIFDRNAENIAPVSDTMDMLTKLGQTYTKRETEAIMKEA
RGPKGDKKNIGPEEWLTLCSKWVRQDDEEEILRAFKVFDANGDGVIDFDEFKFIMQKVGE
EPLTDAEVEEAMKEADEDGNGVIDIPEFMDLIKKSKNALKES
>P54005
MGSISRYLLKKAADGLKDEQRLKIEMSDSKSVPECFHFNRERRMPIAEINGEDGFFMFPS
QQSLENFENTKKYSNELSPDAIGIPLFQIINCTLPFGKRGHSNTVVGNVPYYKIFKFILR
TADEPPPYTVAKIVCSNNGLILYKVPLYDIYKNVSQANVTYSFVGTTSTEPNLLAMAHRE
GHRDLDTKVNNLNLRWHVTYSPVVTNDHYKLILLADYEVNRLDEDVIRAAKNKMSIDQKD
QKVQRFVAAHYTREFETSLFRWVAQEGHLILGEYSTDQGSFGLNNIPPLTEELGCQSLLI
HYIEYMKRQRKKIAKEARRQNKRNVANTTNMNMNLM
>Q45VK7
MAGSLGDVRKLFLFTTTQNYFGLRPELWDQPPLSNCPEVNNFLDDGNQMLLRVQRSEAGL
AFSNTIDFDDAKDKVLVFFKLRPEVITDGNLHNNILVSSMLESPINSLYQAVRQVFAPML
LKDQEWSRNFDPKLQNLLSELEAGLGIVLRKSDTNLPKLKLKEDDTRGILTPSDEFQFWI
EQAHRGSKQISKERASYFKELFETISREFYNLDSLSLLEVVDLVETTRDVVDDVWRQTEH
DHYPESRMLHLLDVIGGSFGRFVQKKLGSLKLWEDPYYLVKENLKAGISICEQWVIVCSH
LTGQVWQRYVPHPWKSGKYFPETLDKLGKRLEEVLAIRTIHEKLLYFLPASEERIVCLSR
VFEPFTGVNPVQYNPYTEPLWKAAVSQYEKIIAPAEQKIAGKLKNYISEIQDSPQQLLQA
FLKYKELVKRPTISKELMLERETLLARLGDSAKDFRLDFENRCRGIPGDPSGPLSGKNLS

EVVNNIVWVRQLELKVDDTIKIAEALLSDLSGFRSFHRSAEDLLDQFKLYEQEQFDDWSR
EVQSGLSDSRSGLCIEANSRIMELDPNDGALKVHYSDRLVILLREVRQLSALGFVIPAKI
QQVANVAQKFCKQAIILKQVAHFYNSIDQQMIQSQRPMMLQSALAFEQIIKNSKAGSGGK
SQITWDNPKELEGYIQKLQNAAERLATENRRLRKWHTTFCEKVVILMNIDLLRQQQRWKD
GLQELRTGLATVAAQGFQASDMRAWRQHWNHQLYKALEHQYQVGLEALNENLPEINVDLT
YKQGRLQFRPPFEEIRAKYYREMKRFIGIPNQFKGVGEAGDESIFSVMIDRNASGFLTIY
SKAEDLFRRLSAVLHQHKEWVVIGQVDMEALVEKNLSTVHDWEKNFKALKIKGKEVERLP
SAVKVDCLNINCSPVKTVIDDLIQKLFDLLVLSLKKSIQTHIHEIDTFVTEAMKVLTVIP
QSVEEIGDTNVQYSNLQDRRPEILPLFQEAEDKNRLLRTVAGGGVETVSNLRAKWDKFEL
MMESHQLMIKDQIEVMKGNVKSRLQIYYQELDKFKARWDQLKPGDDIIETGQQNTMDQSA
KSIKEKKIEFDDLEVIRKKLVDDCHHFGLEEPNFSLAYSISKDIESCAQIWALYEEFQQG
LQDMAKEDWITYRAKIYLFEEFLINWHERLRKIEEHSVMTVKLQSEVDRYKMIIPILKYV
RGEHLSPDHWLDLFRLLGLPRGTSLEKLLFGDLLRVADTIVEKASELKDLNSRAQGEVTI
REALRELDLWGVGAVFSLIDYEDSQNHTIKLIKDWKDIVNQVGDNRCLLQSLKDSPYYKG
FEDKVSIWERKLAQLDEYLQNLNHIQRKWVYLEPIFGRGALPKEQTRFNKVDEDFRSIMM
DIRKDSRVTTLTTHAGIRNTLLTILDQLQRCQKSLNEFLEEKRSAFPRFYFIGDDDLLEI
LGQSTNPSVIQSHLKKLFAGINSVCFDEESKHITAMKSLEGEVVPFKSKVLLSNNVEAWL
NDLALEMKQTLKQLLKECVTAGRSSQGAIDPSLFPSQILCLAEQIKFTEDVENAIKDHSL
HQIEAQLVAKLERYTSVDTSSEDPGNSESGILELKLKALILDIIHNIDIVKQLNQVQVHT
TDDWAWKKQVRFYMKSDHTCYVQMVDSELQYTYEYQGNAPKLVYTPLTDKCYLTLTQAMK
MGLGGNPYGPAGTGKTESVKALGGLLGRQVLVFNCDEGIDVKSMGRIFVGLVKCGAWGCF
DEFNRLEEAVLSAVSMQIQTIQDALKNHRTVCELLGKEVEINANSGIFITMNPAGKGYGG
RQKLPDNLKQLFRPVAMSRPDNDLIAEVILYSEGFKDAKELGRKLVAIFNLSRELLTPQQ
HYDWGLRALKTVLRGSGNLLRQLKKNSTKQDVNENHIVVQALRLNTMSKFTFADCTRFDA
LIKDVFPGIDFKEVEYDELSSALKQVFEEANYEVIPNQMKKALELYEQLRQRTGVVIVGP
SGAGKSTLWRMLRAALCKIGKVVKQYTMNPKAMPRHQLLGHIDMDTREWSDGVLTNSARQ
VVREPQDVSSWIICDGDIDPEWIESLNSVLDDNRLLTMPSGERIQFGPNVNFVFETHDLS
CASPATISRMGMIFLSDEETDLNSLIKSWLRNQPLEYRSNLENWIGDYFSKALQWVLKQN
DYVVETSLVGTVMNGLSHLHGCKYHDQFIINLIRGLGGNLNMKSRLEFTKEVFNWARETP
PDSHRPMDTYYDCDRGQLASYMLKKPESLTADDFSNGHILPVIQTPDMQRGLDYFKPWLS
SDTKQPFILVGPEGCGKGMLLRYAFSQLRSTEIATIHCSAQTTSRHLLQKLSQTCMVIST
NTGRVYRPKDCERLVLYLKDINLPKLDKWGTSTLVAFLQQVLTYQGFYDENLEWVGLENI
QIVASMSAGGRLGRHKLTTRFTSIVRLCAIDYPEREQLQTIYGAYLEAVLHKNLKNHSIW
GSSSKIYLLAGSMVQVYEQVRAKFTVDEYSHYFFTPCILTQWVLGLFRYDLEGGSSNHPL
DYVLEIVAYEARRLFRDKIVGVKELHLFDNILTSVLQGDWGSDILDNMADSFYVTWGAHH
VSGGKTAPGQPLPPHGKPLGKLTSADLKDVIKKGLIHYGRDNQNLDILLFQEVLEYMSRI
DRVLSFPGGSLLLAGRSGVGRRTVTSLVSHMHGAVLFSPKISRGYEPKQFRNDLKHVLQL
AGIEAQQVVLLLEDYQFVHPTFLEMINSLLASGEVPGLYTLEELEPLLLPLKDQASQDGF
FGPVFNYFTYRIQQNLHIVLIMDSANLNFIVNCESNPALHKKCQVLWMEGWSDSSMKKIP
EMLFSETDGEEKYEKKRKDEKKRNSVDPDFIKSFLLIHESCKAYGATPSRYMTFLHVYSA
ISSSKKKELLKRQSHLQAGVSKLNEAKALVDELNRKAGEQSILLRIKQDEADSALQEITV
SMQDASEQKTELERLKQRIAEEVVKIEERKSKIDDELKEVQPLVNEAKLAVGNIRPESLS
EIRSLRMPPDVIRDILEGVLRLMGIFDTSWVSMKSFLAKRGVREDIATFDARNIPKEIRE
SVEELLFKNKASFDPKNAKRASTAAAPLAAWVKANVQYSHVLERIQPLETEQSGLELNLK

KTEDRKRKLEDLLNSVGQKVSELKEKFQSRTSEAAKLEAEVSKAQETIKAAEVLISQLDR
EHRRWNAQVAEIAEELATLPKRAQLAAAFITYLSAAPEGLRKNCLEEWTKAAGLEKFDLR
RFLCTESEQLIWKSEGLPSDDLSIENALVILQSRVCPFLIDPSSQATEWLKTHLKDSHLE
VINQQDSNFITALELAVRFGKTLIIQEMDGVEPVLYPLLRRDLVAQGPRYVVQIGDKIID
YNEDFRLFLSTRNPNPFIPPDAASIVTEVNFTTTRSGLRGQLLALTIQHEKPDLEEQKTK
LLQQEEDKKIQLARLEESLLETLATSQGNILENKDLIESLNQTKASSALIQDSLKESYKL
QISLDQERDAYLPLAESASKMYFIISDLSKINNMYRFSLASFLRLFQRALQNKQDSENTE
ERIQCLVNSLKHMVYEYICRCLFKADQLMFALHFVRGMHPELFQENEWDTFTGVVVGDML
RKADSQQRIRDQLPSWIDQERSWAVATLKISLPSLYQTLCLEDGAFWRTYYHHSMCEQEF
PSILAKKVSLFQQVLVVQALRPDRLQSAMALFACKALGLKELSPLPLNLKRLYKETLEIE
PILIIISPGADPSQELQELASAERSSECYHQVAMGQGQADLAIQMLKECARNGDWLCLKN
LHLVVSWLPVLEKELNTLQPKDSFRLWLTAEVHPNFTPILLQSSLKITYESPPGLKKNLM
RTYESWTPEQISKRDNIHRAHALFSLAWFHAACQERRNYIPQGWTKFYEFSLSDLRAGYH
VIDRLFDGTKDVQWEFVHGLLENSIYGGRVDNYFDLRVLQSYLKQFFNSSIIDVLNQRNK
KSIFPYSISLPNSCSILDYRAVIEKLPEDDKPSFFGLPANIARSSQRMISSQVISQLRIL
GRSVTAGCKFDREIWSNELSPVLNLWKKLNQNSNLIHQKVSPPNDRQGSPILSFIILEQF
NAIRLVQSVHQSLAALSKVIRGTTLLSSEVQKLASALLNQKCPLTWQSRWEGPEDPLQYL
RGLVARTLAIQNWVEKAEKQVLLADTLDLSELFHPDTFLNALRQETARATGCSVDSLKFV
ASWKGRLQEAKLQIKISGLLLEGCSFDGNRLSENQHDSPSVSSVLPCYMGWTPQGSYGPY
SPDECISLPVYTSAERERVVTNIDVPCGGNQDQWIQCGAALFLKNQ

>O13795

MFVPRQLNVRKIKAFTGKENNSIADGNNNKLKDEHYKHNEASKEPSHSISGGLMLNQQDR
QLIEPLNPDFLSAVDSILEIYFHRERQKEKVHLAFLIQQDDFWKGIRPNPTQNNLKYALS
YVTNALFHFDNSSHMVIRNENIVLPLDIPLYDRIIYVEPVPATLSNKSLLLAGKLRKYLK
EFLPYVDAIGTPEGYAFVILYKKVDQSALSKLPVPPGWVLLTRKEWTNREEKYFENQLHL
VKASSSDVSNSSNSFPENRYPKLTKVEKQMTKSVSKTSQTDKDEDNLDFTKNLLTRIKNL
HPLTNKSTIHSLLSYVFSRQTQNIACEPMYIDYRKDETEAIIRWKTPLHAETCINAFRTQ
ERKQNSHDDIRAHRKKGSSRPFLIAELITGEEEKNYWRMLKK

>Q9ES00

MEELSADEIRRRRLARLAGGQTSQPTTPLTSPQRENPPGPPIAASAPGPSQSLGLNVHNM
TPATSPIGAAGVAHRSQSSEGVSSLSSSPSNSLETQSQSLSRSQSMDIDGVSCEKSMSQV
DVDSGIENMEVDENDRREKRSLSDKEPSSGPEVSEEQALQLVCKIFRVSWKDRDRDVIFL
SSLSAQFKQNPKEVFSDFKDLIGQILMEVLMMSTQTRDENPFASLTATSQPIATAARSPD
RNLMLNTGSSSGTSPMFCNMGSFSTSSLSSLGASGGASNWDSYSDHFTIETCKETDMLNY
LIECFDRVGIEEKKAPKMCSQPAVSQLLSNIRSQCISHTALVLQGSLTQPRSLQQPSFLV
PYMLCRNLPYGFIQELVRTTHQDEEVFKQIFIPILQGLALAAKECSLDSDYFKYPLMALG
ELCETKFGKTHPMCNLVASLPLWLPKSLSPGSGRELQRLSYLGAFFSFSVFAEDDAKVVE
KYFSGPAITLENTRVVSQSLQHYLELGRQELFKILHSILLNGETREAALSYMAALVNANM
KKAQMQADDRLVSTDGFMLNLLWVLQQLSTKIKLETVDPTYIFHPRCRITLPNDETRINA
TMEDVNERLTELYGDQPPFSEPKFPTECFFLTLHAHHLSILPSCRRYIRRLRAIRELNRT
VEDLKNNESQWKDSPLATRHREMLKRCKTQLKKLVRCKACADAGLLDESFLRRCLNFYGL
LIQLMLRILDPAYPDVTLPLNSEVPKVFAALPEFYVEDVAEFLFFIVQYSPQVLYEPCTQ
DIVMFLVVMLCNQNYIRNPYLVAKLVEVMFMTNPSVQPRTQKFFEMIENHPLSTKLLVPS
LMKFYTDVEHTGATSEFYDKFTIRYHISTIFKSLWQNIAHHGTFMEEFNSGKQFVRYINM

LINDTTFLLDESLESLKRIHEVQEEMKNKEQWDQLPRDQQQARQSQLAQDERVSRSYLAL
ATETVDMFHLLTKQVQKPFLRPELGPRLAAMLNFNLQQLCGPKCRDLKVENPEKYGFEPK
KLLDQLTDIYLQLDCARFAKAIADDQRSYSKELFEEVISKMRKAGIKSTIAIEKFKLLAE
KVEEIVAKNARAEIDYSDAPDEFRDPLMDTLMTDPVRLPSGTVMDRSIILRHLLNSPTDP
FNRQMLTESMLEPVPELKEQIQAWMREKQSSDH
>Q9USS4
MDKKIADLLQELQSSSNVTSNQLENVDKSSIPLSKARKPSDFELNNSIEDAEKIKTYPAA
LRYIFALNSRHPEKLQKLRKMKAMQERQERNWSLERQRLVQHFESKTKLQEILKPLSSDP
KLKQPSNCLNDQTNNDSAQTLDDNRALQEFDEQVVQLTNRMYKEQLAILADLKIPLFLSV
NNDEELSEDQKRLLQLLQDLLGA
>Q08969
MSNLLNKFADKLHGNDHDERYEDDNDDQTRQQRHEKHQQREFRNQGSKADPYGEENQGNF
PQRQQPQSNLGGNTQFGGNDFQQQTTDYTAGTGGGTYTQTYRETNTQGQLDDDEDDDFLT
SGQQQKQGRTRGAQSNRYQSSNIGSGRRDLSGSGNDEYDDDSGNQGVW
>P43619
MPVYEHLLPVNGAWRQDVTNWLSEDVPSFDFGGYVVGSDLKEANLYCKQDGMLCGVPFAQ
EVFNQCELQVEWLFKEGSFLEPSKNDSGKIVVAKITGPAKNILLAERTALNILSRSSGIA
TASHKIISLARSTGYKGTIAGTRKTTPGLRRLEKYSMLVGGCDTHRYDLSSMVMLKDNHI
WATGSITNAVKNARAVCGFAVKIEVECLSEDEATEAIEAGADVIMLDNFKGDGLKMCAQS
LKNKWNGKKHFLLECSGGLNLDNLEEYLCDDIDIYSTSSIHQGTPVIDFSLKLAH
>Q02875
MNPINSLAFDLHSVKLADANSDTAALSNSNTPTMNNAALLQRPSSIMDSIGVQRVPSPFV
PGSNAISGASTVPFNAYDAEITGSPLQISANQENNSAFSAASSNLHMNASSPSVLNKPSS
TFPNVAPYLYNATGPAPNVGNQPPPPGIESQWKYIDSNGNIQGPFGTNNMSQWYQGGYFT
PTLQICRLATSPEPFGVNDRFIRLGELTTLVNNYQDPFVAFDFIVIRALNAVPLVAPTSS
EKQKVESRDLIPVADVHSDDFTYEEILGLKFEDGSYYHETQVWVPVDGRHITKVDRIPKI
SAYTAPLSTTSSRSNKTTSSHEEKVPSHEEASPEEQEVFSEEGRTVSNITNEEESIVKNP
TKQEEESRGSEKEQNILDQVQPEIEEVDRKDVISTADEPKSKDTPQMTSEEQKRFAKAEL
MAQKLLEEQQRQEEEKKRREEQRKLKKEKKLKQKQKKEEEKLKKKKKEEGKLEKEKQKEL
LNNILTGDTETPSSENTATSITTNLAPWANKKPEGAVYNQISSALEDLKKENSSKKEKKP
NRTQLDREQALKLQKEILSSAQIPKTQTGSAWGIKPQQPIKVDIKGELMKDSTKINSQSK
INKANNGDIKPDSTFIEEQKKLWEQVQKKTKKFNRASSLDDFISRTPSPSSSALNSSNTS
NAWTTVSSKSTTHIASTMPVAGNQSKSYISLDTLRSSGGLSTATKTKMSDKSKQIGSSTS
IPTLKARQVKPSRIPAYPGNASVSKRQEFLRWCRSQLKLNTGVQPDNVLEMLLSLPPGSE
SKEIIADTIYSYSSTMDGRRFATDFIKKRLECEEEINDPLSWSEVLAMPEGSSEDWEFQV
VGKKKGKRF
>P08405
MESQQLSQHSPNSHGSACASVTSKEVHTNQDPLDVSASKTEECEKASTKANSQQTTTPAS
SAVPENPHHASPQPASVPPPQNGPYPQQCMMTQNQANPSGWSFYGHPSMIPYTPYQMSPM
YFPPGPQSQFPQYPSSVGTPLSTPSPESGNTFTDSSSADSDMTSTKKYVRPPPMLTSPND
FPNWVKTYIKFLQNSNLGGIIPTVNGKPVRQITDDELTFLYNTFQIFAPSQFLPTWVKDI
LSVDYTDIMKILSKSIEKMQSDTQEANDIVTLANLQYNGSTPADAFETKVTNIIDRLNNN
GIHINNKVACQLIMRGLSGEYKFLRYTRHRHLNMTVAELFLDIHAIYEEQQGSRNSKPNY
RRNPSDEKNDSRSYTNTTKPKVIARNPQKTNNSKSKTARAHNVSTSNNSPSTDNDSISKS

TTEPIQLNNKHDLHLRPETY
>Q9NQZ3
MSAANPETPNSTISREASTQSSSAAASQGWVLPEGKIVPNTVFVGGIDARMDETEIGSCF
GRYGSVKEVKIITNRTGVSKGYGFVSFVNDVDVQKIVGSQIHFHGKKLKLGPAIRKQKLC
ARHVQPRPLVVNPPPPPQFQNVWRNPNTETYLQPQITPNPVTQHVQSAANPETPNSTISR
EASTQSSSAAASQGWVLPEGKIVPNTVFVGGIDARMDETEIGSCFGRYGSVKEVKIITNR
TGVSKGYGFVSFVNDVDVQKIVGSQIHFHGKKLKLGPAIRKQKLCARHVQPRPLVVNPPP
PPQFQNVWRNPNTETYLQPQITPNPVTQHVQSAANPETPNSTISREASTQSSSAAASQGW
VLPEGKIVPNTVFVGGIDARMDETEIGSCFGRYGSVKEVKIITNRTGVSKGYGFVSFVND
VDVQKIVGSQIHFHGKKLKLGPAIRKQKLCARHVQPRPLVVNPPPPPQFQNVWRNPNTET
YLQPQITPNPVTQHVQAYSAYPHSPGQVITGCQLLVYNYQEYPTYPDSAFQVTTGYQLPV
YNYQPFPAYPRSPFQVTAGYQLPVYNYQAFPAYPNSPFQVATGYQFPVYNYQPFPAYPSS
PFQVTAGYQLPVYNYQAFPAYPNSPFQVATGYQFPVYNYQAFPAYPNSPVQVTTGYQLPV
YNYQAFPAYPSSPFQVTTGYQLPVYNYQAFPAYPNSAVQVTTGYQFHVYNYQMPPQCPVG
EQRRNLWTEAYKWWYLVCLIQRRD
>Q2L4W6
MNGIITPQKQKQLMSSPSRDPLSTTELSTPTSQTTVDVNDTKKSEGLDSTIILLTPGTSP
NATPGSSELGLSKKPNSIKNNTYSTAAQAAYLNRRAADQSTVMTTDPISYANNNINNGTL
PQQNAYYANSYYPSYYAQSQAISNNRPGVSGFRPAFNSVAPCYGSQWQTHQSVHPYHVNN
THQYLKPYVQNVYPQMPSLNQPGLHIVNQPTYLAPVPSATVPTNSVSLSMPSFSQGQKNI
PAAINQEMSVGTTKENTNYLSQLVGLHPAIPPAIPSMFPMSHDNKKSNMESTSRTRNVYI
RGLPPNTSDENLLLYTNRFGKVSSSKAIIDMETNLCKGYGFACFEEEKSALICISAMTLC
GYQCSFAKESFSARLQSLQDTESTNLYISNLPLHWNESDISTLFKPSKIISNRVLRDSKE
QSRGVGFARMQDRKTAEDIINKFNNFVLDPALPPLQIRFADSTDQKKFKGQTQKRRLWRA
REYSVLTKGMTANNAFSKVEEFANNSMPSKVGMYVPLESNTVYQHSPTYDTYGWQSMYPS
YSVYPSNYENSRSTTPYHAHPATSAAN
>Q9NWX6
MWGACKVKVHDSLATISITLRRYLRLGATMAKSKFEYVRDFEADDTCLAHCWVVVRLDGR
NFHRFAEKHNFAKPNDSRALQLMTKCAQTVMEELEDIVIAYGQSDEYSFVFKRKTNWFKR
RASKFMTHVASQFASSYVFYWRDYFEDQPLLYPPGFDGRVVVYPSNQTLKDYLSWRQADC
HINNLYNTVFWALIQQSGLTPVQAQGRLQGTLAADKNEILFSEFNINYNNELPMYRKGTV
LIWQKVDEVMTKEIKLPTEMEGKKMAVTRTRTKPVPLHCDIIGDAFWKEHPEILDEDS
>P80534
MVKVGVNGFGRIGRLVTRAAFNSGKVDIVAINDPFIDLNYMVYMFKYDSTHGKFKVDIVA
INDPFIDLNYMVYMFKYDSTHGKFKGTVKAENGKLVINGHAITIFQERDPSKIKWGDAGA
EYVVESTGVFTTMEKAGAHLKGGAKRVIISAPSRDAPMFVMGVNHEKYDKSLKIVSNASC
TTNCLAPLAKVIHDNFGIVEGLMTTVHAITATQKTVDGPSAKLWRDGAGAAQNIIPASTG
AAKAVGKVIPELNGKLTGMAFRVPTANVSVVDLTCRLEKPAKYDDIKRVVKQACDGPLKG
MLGYTEHQVVSSDFNGDSHSSTFDAGAGIALNDHFVKLVSWYDNEFGYSNRVVDLMVHMA
SKE
>Q61191
MASAVSPANLPAVLLQPRWKRVVGWSGPVPRPRHGHRAVAIKELIVVFGGGNEGIVDELH
VYNTATNQWFIPAVRGDIPPGCAAYGFVCDGTRLLVFGGMVEYGKYSNDLYELQASRWEW
KRLKAKTPKNGPPPCPRLGHSFSLVGNKCYLFGGLANDSEDPKNNIPRYLNDLYILELRP

GSGVVAWDIPITYGVLPPPRESHTAVVYTEKDNKKSKLVIYGGMSGCRLGDLWTLDIETL
TWNKPSLSGVAPLPRSLHSATTIGNKMYVFGGWVPLVMDDVKVATHEKEWKCTNTLACLN
LDTMAWETILMDTLEDNIPRARAGHCAVAINTRLYIWSGRDGYRKAWNNQVCCKDLWYLE
TEKPPPPARVQLVRANTNSLEVSWGAVATADSYLLQLQKYDIPATAATATSPTPNPVPSV
PANPPKSPAPAAAAPAVQPLTQVGITLVPQAATAPPSTTTIQVLPTVPGSSISVPTAART
QGRPAVLKVTGPQATTGTPLVTMRPASQAGKAPVTVTSLPASVRMVVPTQSAQGTVIGSN
PQMSGMAALAAAAAATQKIPPSSAPTAWSVPAGTTIVKTVAVTPGTTTLPATVKVASSPV
MVSNPATRMLKTAAAQVGTSVSSAANTSTRPIITVHKSGTVTVAQQAQVVTTVVGGVTKT
ITLVKSPISVPGGSALISNLGKVMSVVQTKPVQTSAVTGQASTGPVTQIIQTKGPLPAGT
ILKLVTSADGKPTTIITTTQASGAGTKPTILGISSVSPSTTKPGTTTIIKTIPMSAIITQ
AGATGVTSSPGIKSPITIITTKVMTSGTGAPAKIITAVPKIATGHGQQGVTQVVLKGAPG
QPGTILRTVPMGGVRLVTPVTVSAVKPAVTTLVVKGTTGVTTLGTVTGTVSTSLAGAGAH
STSASLATPITTLGTIATLSSQVINPTAITVSAAQTTLTAAGGLTTPTITMQPVSQPTQV
TLITAPSGVEAQPVHDLPVSILASPTTEQPTATVTIADSGQGDVQPGTVTLVCSNPPCET
HETGTTNTATTTVVANLGGHPQPTQVQFVCDRQETAASLVTSAVGQQNGNVVRVCSNPPC
ETHETGTTNTATTATSNMAGQHGSSNPPCETHETGTTSTATTAMSSMGTGQQRDTRRTTN
TPTVVRITVAPGALERVQGTVKPQCQTQQTNMTTTTMTVQATGAPCSAGPLLRPSVALES
GSHSPAFVQLALPSVRVGLSGPSSKDMPTARQPETYHTYTTNTPTTTRSIMVAGELGAAR
VVPTSTYESLQASSPSSTMTMTALEALLCPSATVTQVCSNPPCETHETGTTNTATTSNAG
SAQRVCSNPPCETHETGTTHTATTATSNGGAGQPEGGQQPASGHPCETHQTTSTGTTMSV
SVGTLIPDATSSHGTLESGLEVVAVPTVTSQAGSTLLASFPTQRVCSNPPCETHETGTTH
TATTVTSNMSSNQDPPPAASDQGEVASTQGDSTNITSASAITTSVSSTLPRAVTTVTQST
PVPGPSVPPPEELQVSPGPRQQLPPRQLLQSASTPLMGESTEVLSASQTPELQAAVDLSS
TGDPSSGQEPTTSAVVATVVVQPPPPTQSEVDQLSLPQELMAEAQAGTTTLMVTGLTPEE
LAVTAAAEAAAQAAATEEAQALAIQAVLQAAQQAVMGTGEPMDTSEAAAAVTQAELGHLS
AEGQEGQATTIPIVLTQQELAALVQQQQQLQEAQAQAQQQHHLPTEALAPADSLNDPSIE
SNCLNELASAVPSTVALLPSTATESLAPSNTFVAPQPVVASPAKMQAAATLTEVANGIES
LGVKPDLPPPPSKAPVKKENQWFDVGVIKGTSVMVTHYFLPPDDAVQSDDDSGTVPDYNQ
LKKQELQPGTAYKFRVAGINACGRGPFSEISAFKTCLPGFPGAPCAIKISKSPDGAHLTW
EPPSVTSGKIIEYSVYLAIQSSQASGEPKSSTPAQLAFMRVYCGPSPSCLVQSSSLSNAH
IDYTTKPAIIFRIAARNEKGYGPATQVRWLQETSKDSSGTKPASKRPMSSPEMKSAPKKS
KADGQ
>Q9ERE4
MTSLTQRSSGLVQRRTEASRNAADKERAAGGGGGNGEDEAQSRRDEQDDDDKGDSKETRL
TLMEEVLLLGLKDREGYTSFWNDCISSGLRGCMLIELALRGRLQLEACGMRRKSLLTRKV
ICKSDAPTGDVLLDEALKHVKETQPPETVQNWIELLSGETWNPLKLHYQLRNVRERLAKN
LVEKGVLTTEKQNFLLFDMTTHPLTNNNIKQRLIKKVQEAVLDKWVNDPHRMDKRLLALI
YLAHASDVLENAFAPLLDEQYDLATKRVRQLLDLDPEVECLKANTNEVLWAVVAAFTK
>Q12882
MAPVLSKDSADIESILALNPRTQTHATLCSTSAKKLDKKHWKRNPDKNCFNCEKLENNFD
DIKHTTLGERGALREAMRCLKCADAPCQKSCPTNLDIKSFITSIANKNYYGAAKMIFSDN
PLGLTCGMVCPTSDLCVGGCNLYATEEGPINIGGLQQFATEVFKAMSIPQIRNPSLPPPE
KMSEAYSAKIALFGAGPASISCASFLARLGYSDITIFEKQEYVGGLSTSEIPQFRLPYDV
VNFEIELMKDLGVKIICGKSLSVNEMTLSTLKEKGYKAAFIGIGLPEPNKDAIFQGLTQD

QGFYTSKDFLPLVAKGSKAGMCACHSPLPSIRGVVIVLGAGDTAFDCATSALRCGARRVF
IVFRKGFVNIRAVPEEMELAKEEKCEFLPFLSPRKVIVKGGRIVAMQFVRTEQDETGKWN
EDEDQMVHLKADVVISAFGSVLSDPKVKEALSPIKFNRWGLPEVDPETMQTSEAWVFAGG
DVVGLANTTVESVNDGKQASWYIHKYVQSQYGASVSAKPELPLFYTPIDLVDISVEMAGL
KFINPFGLASATPATSTSMIRRAFEAGWGFALTKTFSLDKDIVTNVSPRIIRGTTSGPMY
GPGQSSFLNIELISEKTAAYWCQSVTELKADFPDNIVIASIMCSYNKNDWTELAKKSEDS
GADALELNLSCPHGMGERGMGLACGQDPELVRNICRWVRQAVQIPFFAKLTPNVTDIVSI
ARAAKEGGANGVTATNTVSGLMGLKSDGTPWPAVGIAKRTTYGGVSGTAIRPIALRAVTS
IARALPGFPILATGGIDSAESGLQFLHSGASVLQVCSAIQNQDFTVIEDYCTGLKALLYL
KSIEELQDWDGQSPATVSHQKGKPVPRIAELMDKKLPSFGPYLEQRKKIIAENKIRLKEQ
NVAFSPLKRNCFIPKRPIPTIKDVIGKALQYLGTFGELSNVEQVVAMIDEEMCINCGKCY
MTCNDSGYQAIQFDPETHLPTITDTCTGCTLCLSVCPIVDCIKMVSRTTPYEPKRGVPLS
VNPVC
>P38932
MNLFDVADFYINKIVTSQSKLSVANVNEHQRIKVLLLDKNTTPTISLCATQSELLKHEIY
LVERIENEQREVSRHLRCLVYVKPTEETLQHLLRELRNPRYGEYQIFFSNIVSKSQLERL
AESDDLEAVTKVEEIFQDFFILNQDLFSFDLQPREFLSNKLVWSEGGLTKCTNSLVSVLL
SLKIKPDIRYEGASKICERLAKEVSYEIGKNERTFFDFPVMDSTPVLLILDRNTDPITPL
LQPWTYQSMINEYIGIKRNIVDLSKVPRIDKDLEKVTLSSKQDAFFRDTMYLNFGELGDK
VKQYVTTYKDKTQTNSQINSIEDIKNFIEKYPEFRKLSGNVAKHMAIVGELDRQLKIKNI
WEISEIEQNLSAHDANEEDFSDLIKLLQNEAVDKYYKLKLACIYSLNNQTSSDKIRQLVE
ILSQQLPPEDVNFFHKFKSLFSRQDKMTQSNHDKDDILTELARRFNSRMNSKSNTAENVY
MQHIPEISSLLTDLSKNALFRDRFKEIDTQGHRVIGNQQSKDIPQDVILFVIGGVTYEEA
RLVHDFNGTMNNRMRVVLGGTSILSTKEYMDSIRSAK
>P39732
MVDVQKRKKLLAKAAASASIPAIKGSVPLDSYDIKIIQYKNALYKLNELNRLLNVLVPHL
KKKRDNDESYKIIPLVNFILSLCEGPIFNVSPVLAKRYHLLCRFQLIKLSEVQQRLSTNF
IDVEGWMFPEEVPLDHYKSCIYNNSLQWKILNSLSCIAQNAIKIYNAKLRQILLERDAYK
ARSLPFDTSIIEDLLNPVEMTLILDLAVLINDPVRDKSTHSFYKLQWQVMEKLNSCVHSK
IFPILRTYYNQLQKFSETRPTSLSNLQKDLPHWEWTLHRIYTFHLRVFSVLCVIISFSRQ
IFLPNKQHFLDIKTRLSSENVYHYDLIICELMALLSPECDDVTALFELQENLKFWTQTAR
TDNNSSRTPIFHLQPGLVVELFNNHICKIIPKLRSIMGLLSNWMDCWKYIEKNYKTFDET
NDLRENLKEKLERDKALYLEVKNAKSKLKKKPSITKLPASSSPSPSPTSSASPSRQASLE
SIRTRARAHLASNSSRSPSVSPVRTTFNNKNAETKKSVVSPEKRKLINGRRPRSSSLQSY
TNKQQTSYLNSTRHPSIAPPSKLNNQRSNSLQSSTMTLNQKIVQDTVRHLMNKSASTPNP
SASSSLAPSPKVSSINNTSSGKSSSTLIANSSDTLAIETLTLDPESNSSELSIKRVRFAG
VPPMTEAENPKPTKVGWYKKPAVLHYPPIPASAMIKPLQHKSKYNTLRQEEGFTFRKSLR
DGLEWENGESGSETTMMPFGIEIKESTGHRIASKIRSKLR
>Q06002
MYRRSYVFQTRKEQYERAEEGRAAEPDRLAEARAAAPNLAALQGLGERVAAHVQRARALE
QRHAVLRRQLDAFQRLDELAGPEDALARHVEGNRQRARDLAAERTRLERQGAEAQRALDE
FRSKYENECECQLLLKEMLERLNKEADEALLRNLRLQIEAQFLQGDISAAKDRYKKNLLE
IQTYVTILQQIIQTTPQAAAITSGMRREKLLTEREAAALQCQLEDGREMICLLQAQRTEL
QAQTAALEQAIRDAHECYDDEIQLYNEQIDTLRKEIEEAERSLERSSYDCRQLVVVQQTL

RNELDRYHRIIENEGNRLSSAFIETPITLYTASHGASLSPRHGGKDLTRAVQDITAAKPR
LKGLPKNLPRKKEMVAKDRADEILEETLLRGPEDMKPGRVVIKEEGESKLEPGDEEASPP
TQEGAPEDVPDGGKISKAFEKLGKMIKEKVKGPKEPEPPADLYTKGRYVMVSGDGSFVDP
GFCVFSVPAKGGVVVSKGDDSVPPDSGVEPSPQQPEPPLEEGQGPPQEKEDGLKEEGGPP
EGKGEPPEGKGDSVKEEGGPPEGKGDGVKEEGGPPEGKGDGVKEEGGPPEGKGDGVKKEG
EPPEGKGEGLKEEEGPLQEKEDGQSPTPHPADKGDEKNAKELKGLQGKQDDQKEEGARGP
CPMVAPGPEGPSTPRSQGPQVILGGSEGHGARSGSRLARSPPRKLAYEKVEVMESIEKFS
TESIQTYEETAVIVETMIEKTKANKKKLGEKGSSSA
>Q70KF4
MESGDSGLAAQGFLGWGADEEVAQELETEEESEGEGEETAAESEEEPDARLSDEDEEGKT
KQECIVSDPSFSMVAVQREDSGITWETNSSRSSTPWASGESQTSGICSLEGSALTSPPGS
VSFIMDEVKRTRKRTQKSKRGSPSLRRKGSKKRNSLESQDVLTNQEDGPSISESPVLNIE
NEKSSIGTYDKTRRKKTASNTPPITGAIYKEHKPLVLKPVYIGTVQYKIKMFNSVKEELI
PLQFYGTLPKGYVIKEIHYRRGKDSSISLEPDLSNGGSNIVPQRKLAQSPEEDKVRELAP
PWRGALSKGSRTSLFSHEEQKKTYADSNLNVPSSTEHAFPSSARNDTADQEENLSLPQMM
PQQPADESKTHRMEPPSIPATMVLERAKEELEQNAQGKESSEDDASVLTGSADDVQQEGL
VSVNHSMPWEAEKESLETGPPRPAPAIQEKFEPDMEGLEPISTEKTEQASEYVTSSEPIV
HREEEHAPEPIVHREEEHAPEPIVHREEEHAPEPESIVHREEEHAPESIVHREEEHAPEP
VPIVHREEEHAPEPESIVHREEEHAPEPIVHRDKGHALEPIVHREEEHAPEPIVHRDEGH
APEPIVHREEEHVPEPESIVRKGEEHAPEPIVHREEEQVPEPESIVHREEEHAPEPIVHR
EEEQVPEPESIVHREEEHAPEPMVLREEHAPEPIVRREEEHAPEPIVHREEEHAPEPMVH
RKAQQLERGVETSTPITDITEPEDSSLEEEIIELDYPESPLASKETSPSPLSPEVEHRKE
PILPTQMTFTPERITLSEEEREENESVSTDSAFVSEYSVLQDLNHTPEKLEVEAVSVSDV
KSSNEPAVFSEDDEERESYSPAMTSVSEQSLSPSTTEKTSAIQSPLFSTVSPVLSGDEAS
ENVCHSPESESAAEYSVPAHAQELLLKTGDHKLPLKSQRVSEPIIQAEDEKEDIGLLPPA
ALSQAVLSEDESLGSGSFASDSKLPFKPSVSQNATRESPQKTIDDMPQFKPRGLSDPATL
LEEEKEAIGVGLSSSNEVSAVECALPPQTTELLSESHAPPPWAISSEQVVQSEEGSRDQQ
RGSFSSTPELGHTSLLLKGASSPTGLSEQGQEEDNIGPLSPDSAFASEFSFSPYPTQELE
KRELGRDSPLCLTSPSEQTVLSEEDTEEADLFSPDSASQVSIPPYRIAETEQNKVEPDEL
LPTRSAPDYPYFSEADEEEAGSSVVTLVPEHSEPSQEREESSPCRPVFEDLSLPPSADKT
GQAETMSDVPTISTSVSEYLILARQAKTQASLEPEAEDLVPPPTSGWEKRDAKSSLPAVT
IAASSSALSSVVKEETTSVLPTSQPSVSPESTCVLKPEQEPTAPLTLTSADEQMALPRVG
REKAVLDSQEATAHKSQDQTPEPRLPNVPGSGMKYSVLSDLGDEPKADVKLNLAPTVTSE
LEQRMLSKNEPEVAKPHSPPEETSISGPKVLSAVKTEVKQESKITRELPAASSGRERGAE
HSPPVPPALPALTEETGKDTEASSSATTVPVTKLDSNSTKLGRDEVLTDPSLASPVEHPG
LKGIGKSELGSGLPLPSMSASEVLRPEPKLPVNSGVEVEREDNEPPPLQVSPTSKPTVPN
DKHEEITRSPDSENLVSDDLAPTLLAFRHEMNRQAEETSSPVPGSFLSGEQELIKLPPEP
EKHKQLSEVPTAGSELIDSRDRDRSLGIEPVKPIGTEPGPSILEKGPAELQRRGKEQEEN
RKLPVPASAPLETASFDLPIEQKEPKRTLHEGQAVEVPDESSSSADKPELGVKQLAEKKE
NLEQPKPFVTTERASVTGSKVKESLISPKDNIWMLEKPDGLVNQHEDRKPGTGQLESSES
TDLMSEKLGAASLDTDHTSETRNQETSKAPVSGEKLSQEPRRVQSKAVDDSEEGRKLASG
NVEVLTQSKSVPAVKAKATPQPPETPEVTQKPSEKSLVTEQGLPAEKGKKGISSFKSWMS
SLLFGSSIPDSKVSDNEDLETRPGPSVEKAVPAIEPKGTVPAEVNIAEKPAVHSLPEVTV
KLAEEPKGVSVKSSISQDLKEKLTFLSNEDVLKQPKSNSENYGQKELPGFSEGMGESLAT

SVGDKHPGIHPCSPMGEKVGMEEAQNMAPLHITESQRRQKPEVSPPSMWNISARKEEPSS
DHKETWLSSSDVVDRMPQKPKSAQSAFTRMNSEEPASMILPVESKGSLSDLGEDRLRQEM
PKPTSLEHCEEEVERPTEEKDGWETRSFSLAGKRGLAEKQEIMAPLELRENEAVGELQRM
PESRPFKLEESKAAERLEQRISPTEKLMEKPSKTLALDRREKEVQEWVFSEGEKQEYPPA
AMPVPGASAVSLDKAQPHLLAKPTPVVEKPEHIVTEVYPEIRERKAAETQPHPQEEGKTL
VEKTKVSRVESPHGEETDGHSLTQEGNLELEKSGESRVDLKEERRRFVMPELPLGASVAA
EDGSVQPRPLSKDAARASDMTDETKHLGTPPTQPSAVEPQTLVLGTSVEHAVKKQETWSD
RPTVHTFQTSKDDTEEMLKQSVLISKHHLEAVEDVHRNEPPSSAASNYAQFMLSASEISA
DGVPPMGGTAQEPEGTSVKDEEFSVTSKPAGLSEDQKSAFSIISEGCEILNIHAPAFIPS
VDQEESEQMQDKLQYLEEKASFKSISVHDEKKAAASHKTQKSKLEVPDRKITSLKENKTK
ETHKTKEEIATDSGMGDFTPIQPTVSGEEDYFEKYTLIDYNLSPGSGKQKSTVEESSEEA
TKTLTSFPESSAEQALDHEYNLVKLDESFYGPEKDDSKLSHAEMQKSLAIQKPDDRNAPK
GISRDVDSRSPGMPLFDVEEGVLSKRQIFPTTPKAVNPELLEEPPALSFFYKDLYEGACG
EKNEGETASEGDSVDSETSFPRRHSDTDDGPGMYFEKYILKDDILHDESVTQEDQGQGLE
EKPVGEEDSQQLRVAEREIRRKPETSFWEKNLEEQHKVVGREGEPTGHMETLDEAAMQQK
APITEQVRAVTQKMSYAVPFQDTRCVLESEPSSQGNEAGNASPDVNLNVPVQVSFPEEES
AAGATYAPEVLQERLVPSVSREERLHNTPVQDEYDFVGSLNQEAASQAILPEEPGSESSP
KEVLSQGSESFEHIREQELTSEGEPRMSASQEVWDRTEDQSARESVTAKTQKEPKKTQAE
SYCYTCKSLVSEMDKALDIHKDHEVSALDTAISAVKVQLGEFLENLQEKSLRIEAFVSEI
ESFFNTIEEKCSKNEKRLEMQNEEMMKRVLAQYDEKAQSFEEVKKKKMEFLHDQMVHFLQ
SMDTAKDTLETIVREAEELDETVFLASFEEINERLLSAMESTASLENMPAAFSLFEHYDD
SSARSDQMLKQVAVPQPPRLEPQEPSSATSTTIAVYWSVNKEDVVDSFQVYCVEEPQDDQ
EINELVEEYRLTVKESCCIFEDLEPDRCYQVWVMAVNFTGCSLPSERAIFRTAPSTPVIH
VEDCTVCWNTATVRWRPANPEATETYTLEYCRQHSPEGEGLRSFSGIKGHQLKVNLPPND
NYFFYVRATNASGTSEQSEAALISTRGTRFLLLRETAHPALQISANGTVISFSERRRLTE
IPSVLGEELPACGQHYWETTVADSPAYRLGICTSSAVRAGALGQGETSWYMHCSEPQRYT
FFYSGIVSEVHATERPARVGILLDYTNQRLLFINAESGQLLFIVRHRFNEGVHPAFALEK
PGRCTLHLGLEPPDSVRHK
>Q9USR0
MNSFLLAREWGQEPNSSFRRQLLQHRLQRLALNPDVKIKRRHGAGILGSVNALSIDNTIH
QLMVSGGANSSINVWDLQNIDQKEDEDLILDTLNAVPARTSHKFGITDLHWFPFDNGIFT
SSSFDHTLKVWDVSTLQEAYTFTMEDMIYSHAWSPIASHCLIATAYRSSSIRLCDMQSGS
YTHSLSGHTGNVLAVDWCPKNEFVLASGSADGTCRLWDIRKVSSSFACMDLHNKYLPSNQ
TNISHYGTVNGLAWTSDARYLASCGTDDRIRVWNMESGRNTLREFGPIIHNQTTSFAVHP
CMIQPSMDSDVFVLFPNDDGSLALLNLLEGSFVRRLSTHSLKRINCAAYRPDFEQCFTGD
MNGNIYMWSPKALKSPTKISDLETRRNIIQEVYDSLINIPVTYQ
>P51854
MADAEARAEFPEEARPDRGTLQVLQDMASRLRIHSIRATCSTSSGHPTSCSSSSEIMSVL
FFYIMRYKQSDPENPDNDRFVLAKRLSFVDVATGWLGQGLGVACGMAYTGKYFDRASYRV
FCLMSDGESSEGSVWEAMAFASYYSLDNLVAIFDVNRLGHSGALPAEHCINIYQRRCEAF
GWNTYVVDGRDVEALCQVFWQASQVKHKPTAVVAKTFKGRGTPSIEDAESWHAKPMPRER
ADAIIKLIESQIQTSRNLDPQPPIEDSPEVNITDVRMTSPPDYRVGDKIATRKACGLALA
KLGYANNRVVVLDGDTRYSTFSEIFNKEYPERFIECFMAEQNMVSVALGCASRGRTIAFA
STFAAFLTRAFDHIRIGGLAESNINIIGSHCGVSVGDDGASQMALEDIAMFRTIPKCTIF

YPTDAVSTEHAVALAANAKGMCFIRTTRPETMVIYTPQERFEIGQAKVLRHCVSDKVTVI
GAGITVYEALAAADELSKQDIFIRVIDLFTIKPLDVATIVSSAKATEGRIITVEDHYPQG
GIGEAVCAAVSMDPDIQVHSLAVSGVPQSGKSEELLDMYGISARHIIVAVKCMLLN
>O95154
MSRQLSRARPATVLGAMEMGRRMDAPTSAAVTRAFLERGHTEIDTAFVYSEGQSETILGG
LGLRLGGSDCRVKIDTKAIPLFGNSLKPDSLRFQLETSLKRLQCPRVDLFYLHMPDHSTP
VEETLRACHQLHQEGKFVELGLSNYAAWEVAEICTLCKSNGWILPTVYQGMYNAITRQVE
TELFPCLRHFGLRFYAFNPLAGGLLTGKYKYEDKNGKQPVGRFFGNTWAEMYRNRYWKEH
HFEGIALVEKALQAAYGASAPSMTSATLRWMYHHSQLQGAHGDAVILGMSSLEQLEQNLA
AAEEGPLEPAVVDAFNQAWHLVTHECPNYFR
>O60064
MISSAVILVGGPSRGTRFRPLSFDVPKPLFKIGGREMIYHHLAALSKIESVKDVFLVGFY
DESVFKDFINEVASHFPSFNRIKYLREYNCLGTGGGLYHFRDQILKGHTSNVFVMHADVC
CSFPLQELLNVHHEKKALVTLMATKVSKEDASNFGCLVEEPSTGRVLHYVDKPSSYLSNI
ISCGIYIFDASIFDEIKKAYERRLEEVEKQLRSLDEGMEDYLSLETDVLAPLCSDSSKAI
YAYNTPEFWRQIKTAGSAVPANSLYLQKAYHDGTLPKPDTEAEIIQPVFIHPNAIVSKGA
KIGPNVSIGARVRIEDGARIRNSIIQEDCEISANAVVLHSILSRHCKIGKWSRVEGSPTL
PSQHSTTIMRNSVKVQAITVMGADCIVHDEVRVQNCLVLPHKEIKVGLVGEIVM
>Q3E7A7
MLGMIRWVVEGTLVAMLLSAIRRETGMIFFYNQYQLGGWIHRYLSWGEMCYTRTLKMVKR
SKFFRKQLNEDGFGRINDSGPKRRGRDQSQYSSRFVELD
>Q12020
MSNFKNFTLNSFEDYYGKPSETPKMEEEKLEVTNVNASSSKKVHKSKKSTSKYDQKNVFR
NSMTGIAQILPTKPVKIIEQNIDFANPKSFDLLQSTHTICFNKRINTTNTKLNVETHTSS
DIDNDILHVGAPTDLGGNSNDEAETRQLRKFRWSNNKEKSLCEKLTVIYWALLLHTTKRA
SKRRPILCHQMIAEFFNRVYKEKSRVPITSRYIRDNLVAWVTQGKELHEKGWVGDAKTGD
LQEQFNIATVKLYESAEDGRLSIGKDKPFREENTGSDSLVRAEEDSTAVTNENGHISSEK
NLKKDRRESIRNQILTLDLNDEDFFQNVMKVLSAIDEPELRQYVIVISELVSMEMDDGKT
VREKLRDVELNINRLQVDIKEIKEMLVTLINK
>O74653
MASQRFVIALHSFPGKSSDELPLVEGRKYLLIKMDEEFGDGWWEGEDEQGNRGIFPASHV
ELISDERSDSSDSRRGKEDFSISTAEVTRSSLSSSRSTSSRSDKDSEKLYSNNSLSSSHS
SILNGPLDSLSKPSVPSNFNSMFPSSKQEGPSPLLDNQPSSDLSNFNTIDADYNNASAST
SAPATSASLKKVLSAEDSVRETITDIETALQNMSTSASRTPNDSSPLPYIENRPASSLAV
SEKIQNVPNWSTEEVVEWLMNAGLGSVAPNFAENEITGEILLGLDSNVLKELNITSFGKR
FEVLRKIQQLKDSYEQSLYEEYPQFAEPISVSQSSDSSSSIPKKSNDEAGGSPSKSSPTR
PGFNDYVNRPTSVMPSLSNMIVSPDLDSSPSTDWNQYVIPPLATPSSRNSKSTQSAVPEN
VSRFDSNEPSATSPILKRSSPTDSISQNSGLPSRLTEPISSPSTSSIDVDKEGTSFPGLP
YHSSKGNLYAPQPSSNVPTKFTGGASESSSVPPRPIPSAMKGKAPASAISIEALEELDPP
KITTIDGESPSSISSRLPSSNLEQGSSSSVTKSPESMPDPSAKASSPVTSKGVSINEKSA
VNNYATPLSKPQPKDTKGSKLGNTFVAPSPAASLPASPPVGTELKTRPTLRSVASSPLNK
EPIGKRKSKRDIFGRQKVLPTGISEGLSNIPAKEAIKTADCHGWMRKRSDRYGVWKSRYF
VLKGTRLSYYHSLNDASEKGLIDMTSHRVTKTDDIVLSGGKTAIKLIPPAPGAAKAAVMF
TPPKVHYFTCENNEELHRWSSAFLKATVERDMSVPVLTTSRMPTISLSKAKELRTRPPSL

LIDDENEANLTSSIGLKKNAKQKNKKSSKQK
>Q9H8T0
MNPFWSMSTSSVRKRSEGEEKTLTGDVKTSPPRTAPKKQLPSIPKNALPITKPTSPAPAA
QSTNGTHASYGPFYLEYSLLAEFTLVVKQKLPGVYVQPSYRSALMWFGVIFIRHGLYQDG
VFKFTVYIPDNYPDGDCPRLVFDIPVFHPLVDPTSGELDVKRAFAKWRRNHNHIWQVLMY
ARRVFYKIDTASPLNPEAAVLYEKDIQLFKSKVVDSVKVCTARLFDQPKIEDPYAISFSP
WNPSVHDEAREKMLTQKKPEEQHNKSVHVAGLSWVKPGSVQPFSKEEKTVAT
>P79005
MISVQSTETIQKVLENEGDQQFDKEVVQNSDSNIETGQISDSLTKAVEERAETESSSNLS
NFTTSESESSKPAYCFNSHSQNMAEGSISIPVISHSMNVENEVSTAEGQDSRTGESENDQ
NAMIVRSIWDIEKASLLVNDCQNIANMAEQKVMMVSAIFSESSKDIVNPESFSERLGKET
VKDLYEFNEQLTTKYGLEFRTIFFSYIRKYDAYWCLFEDLEKPLKSIQFFTGLIDLLDNT
NKHLTLRSIVLDALLSADEEDSFYGDALVLFEELVIRYFGTDSNPSIDASEFILSCLPYT
SLDALNVVCGQVWKSQKICDFLKSTIGNTSNSPLQLRASFPAFVNAVIHFLLEFKNVRRL
ERKDLSVKGMLYDSDSQQILNRLRERVSGSTAQSADEASGHESDASEDTFSERTLGLNSI
DNTEISEVVSLGLVSSALDKITGLLSADNLSETVSQARDFSHTLSKSLKSRAKSLSQKEA
ANRSKLIAKRGDNLRREASLSSEQDDLSEDFPPVRESDEQESRSGGRSSAMRVSIERSAA
RSGTRRSQGNPYEGYRTRRKWTDEEENELYEMISQHGCCWSKIIHIQKLENGPLKTFGPT
QIKDKARLIKARFMKQNRLQELYSKSLNWKNVTVGQAYCELHKIPYIEATPPLLREELVN
YQS
>P05318
MSTESALSYAALILADSEIEISSEKLLTLTNAANVPDENIWADIFAKALDGQNLKDLLVN
FSAGAAAPAGVAGGVAGGEAGEAEAEKEEEEAKEESDDDMGFGLFD
>Q29AK2
MGLPPLQTQPGTFLVALGLCCWLLATANAHNCQHQHPKAHEVVHGVRIQLADSDDDDAAG
SSDAAVHSVRRRSVTAEQPLRILLVYDDSVYRLEEEKFNLINDTVLPEAVQFWEQALMVR
ETKGIIRLNRKCDSTQVYVKNGHTHCIDQCKATTMCGEVQVPNEHLDVCRVCNATGQNCR
IDSNTQPGDGIQNADFVFYVSARQTQRCFKGLTVAYAAHCQQEAALDRPIAGHANLCPES
ISTKPQELQTLISTVKHEILHALGFSVSLYAFFRDDEGRPRTPRKSETGKPFLNEKLQIH
QWSNETIRKVVRENWSVRGGHVNKEVDMMVTPRVVAEARAHFDCDKLEGAELEDQGGEGT
ALTHWEKRILENEAMTGTHTQSPVFSRITLALMEDSGWYRANYSMATPLSWGKGLGCSFA
MRSCKDWIQMNHARGRSIHPFCSKVKQDPLQTECTDDRNSVALCNLIRHDYELPKSYQNF
DSLQNVKSGEEGFYGGSVSLADHCPYIQEFTWRSKNIIVRGSHCRFVENNPKPEKNFALE
SYGHGSKCFDHSEAMWEERSCHQTREWQHWGSGCYKYTCFDGRLHILVGNYTYKCSFPGQ
KLSIRIAANGWLHKGAIMCPPCHELCGSHFAAQGKQCRPGEEADPLNKYPRDNLACGAES
RQQRSVAIISAALLLAAGLRLGHS
>Q9P7P9
MSIKLGYLFKLPSYKTSSISPEVIVDLEKFLNLCEGSCSQCNTPFCVDVGPRNAIIQLPN
GADVLNLNPNFQAIFECCSKNSLTGVQIFGMYNDGTYELRSFCPVHGVNEDPANGSGAGS
VGVFFALNNPSIISSDFAHLLFNQGKILGRNALIRVAIKLSANGLYDIHVGGGSKICISG
TAEI
>O88466
MVRERKCILCHIVYGSKKEMDEHMRSMLHHRELENLKGRDISHECRVCRVTEVGLSAYAK
HISGQLHKDNVDAQEREDDGKEEEEEEYFDKELVQLIQERKEQSRQDEPPSNSQEVNSDD

RQPQWRREDRIPYQDRESYSQPPRHHRGPPQRDWKWEKDGFNSTRKNSFPHSLRNSGGPR
GSSVWHKGATRGSSTWFLNHSNSGGGWHSNNGMVDWNYNGTGRNSSWHSEGTGGFPSWHM
NNSNGNWKSSVRGTNSWNYNGLGDKFQQGRNRNPNYQMEDMTKMWNKKSNKPSKYSQERC
KWQRQDRDKAAKYRSPPEGYASDTFPSEGLLEFNFEQRESQTTKQTDTAASKINGKNGTK
ARDKFRRWTPYPSQKTLDLQSALKEVIGSKSDTLEKPLFNFSLITAGLRKPVDKTSNPPV
IKTQKAGPPGSPSHKAISDGTAFCEVARACSITEQSEPHQKSNKIPLLKSPLLPLPTPKS
GPHKQNLKNRSKNKETKSFPSGDHSHLLNTSTLEGSHGSSYTSKSLGLCPRVLKENKTVS
GTQKEPDEKLNNTSQKAQDTVLQCPKTLQNPLPTTPKRMENDAKESSVEESAKDSLSIES
QPHSAGNSAMTSDAENHGIKSEGVASLTTEVVSCSTHTVDKEQGSQIPGTPENLSTSPRN
STVLQKEAEVQVSAATSPHSGLLLDLKTSLEDAQDNNLVKSDGPFETESFEDTSLDTELQ
KPDLNNQPPGTLLPELSKLGFPASLQRDLSRHISLKSKTGTHLPEPNLNSARRIRNVSGH
RKNETEKESGLKPTLRQILNASRRNVNWEQVIQQVTKKKQELGKGLPRFGIEMVPLVQNE
QEVLDLDEEPDLSSLEGFQWEGVSIPSSSGLARKRSLSESSVVMDRAPVYSFFTGEGTGK
ENEAQQSPSPNTALSAAQSQKTAMYLEQEVAPLTPSVGTGERVGNIPTQRRHSAQLPSGH
IMPVMHSARDLHSQERSTPLSERHAQESTGEGNSLSSNASSGHAVSSLADAATDSSCTSG
AEQTDGHSIRKKRRATGDGSSPELPSLERKNKRRKIKGKKERSQVDQLLTISLREEELSK
SLQCMDNKLLQARAALQTAYVEVQRLLVLKQQITVEMSALRTHRIQILQGLQETYEPPEH
PDQAPCSLISREQRNSRSQTSFETALLPAPFFPGFLDPPPSHASLPSPGNPLQITMSTFQ
AHGTAPDSSVQIKQEPMSPEQEGNMNALPQGCASNVSKELLQTNRVVDDGSSVYPAIPAV
IAPESTENCQEVSKDLNFSVEQGNSRSKGNSPSCQSPDLPGINRGEETAKGSSGSEACSS
SFLRLSFTPETPAEKETQSPADQPEQQAESTLASAETRGSKKKKKLRKKKTLRATHVPEN
SDTEQDVFTAKPARKVKTAKAAKGAKVTTSQTGQEQGTARDEPDSDSSLEVLEVTNPQLE
VVAIDTSESGDEKPDSPSKKDAWIAAEQNPIETSRSGCDEVSSTSELGTRYKDGVPVSVA
ETQTVISIKASKHSSEISSEPGDDEEPTEGSFEGHQAAVNAIQIFGNFLYTCSADTTVRV
YNLVSRKCVGVFEGHTSKVNCLLVTHTSGKSSVLYTGSSDHTIRCYNIKTRECMEQLQLE
DRVLCLHNRWRTLYAGLANGTVVTFDIKNNKRQEIFECHGPRAVSCLATAQEGARKLLVV
GSYDCTISVRDARNGLLLRTLEGHSKTVLCMKVVNDLVFSGSSDQSVHAHNIHTGELVRI
YKGHNHAVTVVNILGKVMVTACLDKFVRVYELQSHDRLQVYGGHKDMIMCMTIHKSVIYT
GCYDGSIQAVRLNLMQNYRCWWYGCTLIFGVVDHLKQHLLTDHTNPNFQTLKCRWRNCDA
FFTARKGSKQDVAGHIERHAEDDSKIDS
>Q09772
MKRRATFQCPLIESTIKHQSTNYTKTETATTSHEVSENANEHKGKSNIDINKIAYYNVVW
RKITMKKHKTWEGDGFLVREDSNLTLYNSDFTRLGSCHSRVPQIKEGELLRISGKEISIE
KEISAESYEAGTHIDDSLRDVHMPEMKRKFKPPLRPNTMYQTISALPKPRHDPTVLGALV
MSRPKTWDPRTHVDVVIDPFLSKHLYSHQREGVSFLYDCLLGMEGKCGYSAILADEMGLG
KTLQTITVVWTLLKQSYYANRSSTINNAMVVAPVTLLKNWENEFYNWLGHERIHVYIARC
AEDFQEFTSNKTYSIIITGYETVCTYLRNYGCGIDIDLLICDEAHRLKSMSSQTWITLNK
LKTRKRLLLTGTPLQNDLSEYFSMVNFIIPGSLGTPNSFKAQYERPILRSRSMNASSRDI
SLGAARLQRLFEFTSNFTLRRKANILAKHLPPRTDIVLFIKPTHQQENVYGHVLDGFKSS
VDQKGYYLKILTRLSKICNSTILLRNEKENFLSTELQDKHVFEQENMLLSSSKLQILAAL
LKSFQRGCQKAVIVSQYKETLELIELFLSILHVRFCKLLGSTPFSERDLIVHNFNTSSFK
EFSVLLLSSKAGGCGLNLTGSTRLIIYEPSWNPAQDLQALSRIYRSGQKRPVCIYTFLSS
GMLDERIFIRQNTKQGLSSSFIDSDASQKKNFFTGEDIKTLFSYSKTETCLTYELAFDSD
EIDSLTKKKDPLTKIDHTIDSPNTKEKDKEISSWKKASEYMDTNLGKKCPENAFQGWTWQ

FPNDLSIMNGVEDYIPLEPLPINCLMHKKFE
>P24482
MFGSGNVLPVKIQPPLLRPLAYRVLSRKYGLSIKSDGLSALAEFVGTNIGANWRQGPATI
KFLEQFAAVWKQQERGLFIDQSGVKEVIQEMKEREKVEWSHEHPIQHEENILGRTDDDEN
NSDDEMPIAADSSLQNVSLSSPMRQPTERDEYKQPFKPESSKALDWRDYFKVINASQQQR
FSYNPHKMQFIFVPNKKQNGLGGIAGFLPDIEDKVQMFLTRYYLTNDRVMRNENFQNSDM
FNPLSSMVSLQNELSNTNRQQQSSSMSITPIKNLLGRDAQNFLLLGLLNKNFKGNWSLED
PSGSVEIDISQTIPTQGHYYVPGCMVLVEGIYYSVGNKFHVTSMTLPPGERREITLETIG
NLDLLGIHGISNNNFIARLDKDLKIRLHLLEKELTDHKFVILGANLFLDDLKIMTALSKI
LQKLNDDPPTLLIWQGSFTSVPVFASMSSRNISSSTQFKNNFDALATLLSRFDNLTENTT
MIFIPGPNDLWGSMVSLGASGTLPQDPIPSAFTKKINKVCKNVVWSSNPTRIAYLSQEIV
IFRDDLSGRFKRHRLEFPFNESEDVYTENDNMMSKDTDIVPIDELVKEPDQLPQKVQETR
KLVKTILDQGHLSPFLDSLRPISWDLDHTLTLCPIPSTMVLCDTTSAQFDLTYNGCKVIN
PGSFIHNRRARYMEYVPSSKKTIQEEIYI
>P36105
MSTDSIVKASNWRLVEVGRVVLIKKGQSAGKLAAIVEIIDQKKVLIDGPKAGVPRQAINL
GQVVLTPLTFALPRGARTATVSKKWAAAAVCEKWAASSWAKKIAQRERRAALTDFERFQV
MVLRKQKRYTVKKALAKA
>Q04751
MRSSVYSENTYNCIRTSKEHLTERRRVAMAPMFQHFLNLCVEKFPESIEHKDTDGNGNFT
TAILEREIIYIPEDDTDSIDSVDSLKCINYKLHKSRGDQVLDACVQLIDKHLGAKYRRAS
RIMYGNRKPWKANKLAEMKSAGLVYVCYWDNGVLGAFTSFMLTEETGLVEGDALHEVSVP
VIYLYEVHVASAHRGHGIGRRLLEHALCDGVARHTRRMCDNFFGVALTVFSDNTRARRLY
EALGFYRAPGSPAPASPTIRHTRHGGGRVVVPCDPLYYVYCLHMP
>Q15025
MEGRGPYRIYDPGGSVPSGEASAAFERLVKENSRLKEKMQGIKMLGELLEESQMEATRLR
QKAEELVKDNELLPPPSPSLGSFDPLAELTGKDSNVTASPTAPACPSDKPAPVQKPPSSG
TSSEFEVVTPEEQNSPESSSHANAMALGPLPREDGNLMLHLQRLETTLSVCAEEPDHGQL
FTHLGRMALEFNRLASKVHKNEQRTSILQTLCEQLRKENEALKAKLDKGLEQRDQAAERL
REENLELKKLLMSNGNKEGASGRPGSPKMEGTGKKAVAGQQQASVTAGKVPEVVALGAAE
KKVKMLEQQRSELLEVNKQWDQHFRSMKQQYEQKITELRQKLADLQKQVTDLEAEREQKQ
RDFDRKLLLAKSKIEMEETDKEQLTAEAKELRQKVKYLQDQLSPLTRQREYQEKEIQRLN
KALEEALSIQTPPSSPPTAFGSPEGAGALLRKQELVTQNELLKQQVKIFEEDFQRERSDR
ERMNEEKEELKKQVEKLQAQVTLSNAQLKAFKDEEKAREALRQQKRKAKASGERYHVEPH
PEHLCGAYPYAYPPMPAMVPHHGFEDWSQIRYPPPPMAMEHPPPLPNSRLFHLPEYTWRL
PCGGVRNPNQSSQVMDPPTARPTEPESPKNDREGPQ
>Q9NR19
MGLPEERVRSGSGSRGQEEAGAGGRARSWSPPPEVSRSAHVPSLQRYRELHRRSVEEPRE
FWGDIAKEFYWKTPCPGPFLRYNFDVTKGKIFIEWMKGATTNICYNVLDRNVHEKKLGDK
VAFYWEGNEPGETTQITYHQLLVQVCQFSNVLRKQGIQKGDRVAIYMPMIPELVVAMLAC
ARIGALHSIVFAGFSSESLCERILDSSCSLLITTDAFYRGEKLVNLKELADEALQKCQEK
GFPVRCCIVVKHLGRAELGMGDSTSQSPPIKRSCPDVQISWNQGIDLWWHELMQEAGDEC
EPEWCDAEDPLFILYTSGSTGKPKGVVHTVGGYMLYVATTFKYVFDFHAEDVFWCTADIG
WITGHSYVTYGPLANGATSVLFEGIPTYPDVNRLWSIVDKYKVTKFYTAPTAIRLLMKFG

DEPVTKHSRASLQVLGTVGEPINPEAWLWYHRVVGAQRCPIVDTFWQTETGGHMLTPLPG
ATPMKPGSATFPFFGVAPAILNESGEELEGEAEGYLVFKQPWPGIMRTVYGNHERFETTY
FKKFPGYYVTGDGCQRDQDGYYWITGRIDDMLNVSGHLLSTAEVESALVEHEAVAEAAVV
GHPHPVKGECLYCFVTLCDGHTFSPKLTEELKKQIREKIGPIATPDYIQNAPGLPKTRSG
KIMRRVLRKIAQNDHDLGDMSTVADPSVISHLFSHRCLTIQ
>Q09196
MSTDDSPYKQAFSLFDRHGTGRIPKTSIGDLLRACGQNPTLAEITEIESTLPAEVDMEQF
LQVLNRPNGFDMPGDPEEFVKGFQVFDKDATGMIGVGELRYVLTSLGEKLSNEEMDELLK
GVPVKDGMVNYHDFVQMILAN
>P52734
MHGHRVPGGPGPSDPERSAANTPGAAPLACADSDPGALEPGLPVSRGSGTALGGPLDPQF
VGPSDASLGAPPSSRVLPCGPSPQHHRALRFSYHLEGSQPRPGLHQGNRILVKSLSLDPG
QSLEPHPEGPQRLRSDPGPPTEIPGPRPSPLKRAPGPKPQVPPKPSYLQMPRVLPPPEPI
PPPPSRPLPADPRVAKGLVPRAEASTSSAAVSSLIEKFEREPVIVASDRPAPGPCPVPPE
PAMLPQPPPQPTGSQLPEGEASRCLFLLAPGPRDGEKVPNRDSGIDSISSPSNSEETCFV
SDDGPPIHSLCPGPPALASMPVALADPHRPGSQEVDSDLEEEEEEEEEEKEREIPVPPME
RQESVELTVQQKVFHIANELLQTEKAYVSRLHLLDQVFCARLLEEARNRSSFPADVVHGI
FSNICSIYCFHQQFLLPELEKRMEEWDRYPRIGDILQKLAPFLKMYGEYVKNFDRAVELV
NTWTERSTQFKVIIHEVQKEEACRNLTLQHHMLEPVQRIPRYELLLKDYLLKLPHGSPDS
KDAQKSLELIATAAEHSNAAIRKMERMHKLLKVYELLGGEEDIVSPTKELIKEGHILKLS
AKNGTTQDRYLILFNDRLLYCVPRLRLLGQKFTVRARIDVDGMELKESSNLNMPRTFLVS
GKQRSLELQARTEEEKKDWVQAINSTLLKHEQTLETFKLLNSTNRDDEDTPPNSPNVDLG
KRAPTPIREKEVTMCMRCQEPFNSITKRRHHCKACGHVVCGKCSEFRARLIYDNNRSNRV
CTDCYVALHGAPGSSPACSQHTPQRRRSILEKQASVAAENSVICSFLHYMEKGGKGWHKA
WFVVPENEPLVLYIYGAPQDVKAQRSLPLIGFEVGPPEAGERPDRRHVFKITQSHLSWYF
SPETEELQRRWMAVLGRAGRGDTFCPGPTLSEDKEMEETPVAASGATAEPPEASQTRDKT

>Q04377
MRRETVGEFSSDDDDDILLELGTRPPRFTQIPPSSAALQTQIPTTLEVTTTTLNNKQSKN
DNQLVNQLNKAQGEASMLRDKINFLNIEREKEKNIQAVKVNELQVKHLQELAKLKQELQK
LEDEKKFLQMEARGKSKREVITNVKPPSTTLSTNTNTITPDSSSVAIEAKPQSPQSKKRK
ISDNLLKKNMVPLNPNRIIPDETSLFLESILLHQIIGADLSTIEILNRLKLDYITEFKFK
NFVIAKGAPIGKSIVSLLLRCKKTLTLDRFIDTLLEDIAVLIKEISVHPNESKLAVPFLV
ALMYQIVQFRPSATHNLALKDCFLFICDLIRIYHHVLKVPIHESNMNLHVEPQIFQYELI
DYLIISYSFDLLEGILRVLQSHPKQTYMEFFDENILKSFEFVYKLALTISYKPMVNVIFS
AVEVVNIITSIILNMDNSSDLKSLISGSWWRDCITRLYALLEKEIKSGDVYNENVDTTTL
HMSKYHDFFGLIRNIGDNELGGLISKLIYTDRLQSVPRVISKEDIGMDSDKFTAPIIGYK
MEKWLLKLKDEVLNIFENLLMIYGDDATIVNGEMLIHSSKFLSREQALMIERYVGQDSPN
LDLRCHLIEHTLTIIYRLWKDHFKQLREEQIKQVESQLIMSLWRFLVCQTETVTANEREM
RDHRHLVDSLHDLTIKDQASYYEDAFEDLPEYIEEELKMQLNKRTGRIMQVKYDEKFQEM
ARTILESKSFDLTTLEEADSLYISMGL
>Q9Y7Q6
MRIPVIRSPLEIRDTERKGRGVFALEPIPAQTCIEISPVLMFSKEEYEQHGQYTVLNEYT
YVWSEGKQGLALGLGSMFNHDRHPNVYWKKDNRNNYISYYTLREIKTNEELCISYGDHLW

FEDEASSASRISPNEENEDFPLQNISL
>O23627
MRIFSTFVFHRRQQIFNLRQFQTTTILRNPISIAPIQIPMDATEQSLRQSLSEKSSSVEA
QGNAVRALKASRAAKPEIDAAIEQLNKLKLEKSTVEKELQSIISSSGNGSLNREAFRKAV
VNTLERRLFYIPSFKIYSGVAGLFDYGPPGCAIKSNVLSFWRQHFILEENMLEVDCPCVT
PEVVLKASGHVDKFTDLMVKDEKTGTCYRADHLLKDYCTEKLEKDLTISAEKAAELKDVL
AVMEDFSPEQLGAKIREYGITAPDTKNPLSDPYPFNLMFQTSIGPSGLIPGYMRPETAQG
IFVNFKDLYYYNGKKLPFAAAQIGQAFRNEISPRQGLLRVREFTLAEIEHFVDPENKSHP
KFSDVAKLEFLMFPREEQMSGQSAKKLCLGEAVAKGTVNNETLGYFIGRVYLFLTRLGID
KERLRFRQHLANEMAHYAADCWDAEIESSYGWIECVGIADRSAYDLRAHSDKSGTPLVAE
EKFAEPKEVEKLVITPVKKELGLAFKGNQKNVVESLEAMNEEEAMEMKATLESKGEVEFY
VCTLKKSVNIKKNMVSISKEKKKEHQRVFTPSVIEPSFGIGRIIYCLYEHCFSTRPSKAG
DEQLNLFRFPPLVAPIKCTVFPLVQNQQFEEVAKVISKELASVGISHKIDITGTSIGKRY
ARTDELGVPFAITVDSDTSVTIRERDSKDQVRVTLKEAASVVSSVSEGKMTWQDVWATFP
HHSSAAADE
>O74910
MTNSSPRVKRRTDTQYLHSVSSKLPKVDFDTNNEEFFEEEFEIYDPFYRAELPCPKPSLS
ISKHSIAKVPSNVNKRLELQLLLTSGTFLPNSRPYLSERVRKHTHLLSNSITGDDKPSLI
HVDFTPEECFILQEAKLKFGPVNSVQFNDAYSTHISPKLPGRAYEDCQKFEIDNPSLSPV
DKHGAIILRTYKKNKKLLPDYLKSFYNAGSSYFQREQVHQLMDGESVFFLKPWKHFNETS
GDTVCVAYNPLCEKFALGSTAQDGAYNRLGNLWIGDFHSETIQSLESHYKLNQVGEKEYS
TISDLCFSKGNLFLYTGAFDNAVKVWDMEGNLCGIFNAPTDYIHKLALSDDDLLAVACKN
GYGYLLSTDNSTGEILTSANLIYPEALEKGYSASLIEFSNFLGRSSDKVIIGYDSFHTSN
NRGCLALFDASTASFVQKFNTADEAFTSLYMHPSQVGFVASSNTLSNGRVYYLDTRMYKV
CLNFTTTQKDINHATISNSGILVTSSGTDNQTFVWDSRKPDKPLSLLKHGKTKMIHFDGA
NEEEVDAGINMAQWQPKGNLFVTGGSDGIVKVWDLRLNNPFIQNFTEMNSAITYGGFSED
ASKLTVCCVGGDVNMYSLGNDNGNKFGEFRIIENRLLT
>P32769
MAYSDYSDGADDMPDFHDEGEFDDYLNDDEYDLMNEVFPTLKAQLQDYQGWDNLSLKLAL
FDNNFDLESTLAELKKTLKKKKTPKKPIAAANGSANVTQKLANISISQQRPNDRLPDWLD
EEESEGERNGEEANDEKTVQRYYKTTVPTKPKKPHDISAFVKSALPHLSFVVLGHVDAGK
STLMGRLLYDLNIVNQSQLRKLQRESETMGKSSFKFAWIMDQTNEERERGVTVSICTSHF
STHRANFTIVDAPGHRDFVPNAIMGISQADMAILCVDCSTNAFESGFDLDGQTKEHMLLA
SSLGIHNLIIAMNKMDNVDWSQQRFEEIKSKLLPYLVDIGFFEDNINWVPISGFSGEGVY
KIEYTDEVRQWYNGPNLMSTLENAAFKISKENEGINKDDPFLFSVLEIIPSKKTSNDLAL
VSGKLESGSIQPGESLTIYPSEQSCIVDKIQVGSQQGQSTNHEETDVAIKGDFVTLKLRK
AYPEDIQNGDLAASVDYSSIHSAQCFVLELTTFDMNRPLLPGTPFILFIGVKEQPARIKR
LISFIDKGNTASKKKIRHLGSKQRAFVEIELIEVKRWIPLLTAHENDRLGRVVLRKDGRT
IAAGKISEITQ
>P49915
MALCNGDSKLENAGGDLKDGHHHYEGAVVILDAGAQYGKVIDRRVRELFVQSEIFPLETP
AFAIKEQGFRAIIISGGPNSVYAEDAPWFDPAIFTIGKPVLGICYGMQMMNKVFGGTVHK
KSVREDGVFNISVDNTCSLFRGLQKEEVVLLTHGDSVDKVADGFKVVARSGNIVAGIANE
SKKLYGAQFHPEVGLTENGKVILKNFLYDIAGCSGTFTVQNRELECIREIKERVGTSKVL

VLLSGGVDSTVCTALLNRALNQEQVIAVHIDNGFMRKRESQSVEEALKKLGIQVKVINAA
HSFYNGTTTLPISDEDRTPRKRISKTLNMTTSPEEKRKIIGDTFVKIANEVIGEMNLKPE
EVFLAQGTLRPDLIESASLVASGKAELIKTHHNDTELIRKLREEGKVIEPLKDFHKDEVR
ILGRELGLPEELVSRHPFPGPGLAIRVICAEEPYICKDFPETNNILKIVADFSASVKKPH
TLLQRVKACTTEEDQEKLMQITSLHSLNAFLLPIKTVGVQGDCRSYSYVCGISSKDEPDW
ESLIFLARLIPRMCHNVNRVVYIFGPPVKEPPTDVTPTFLTTGVLSTLRQADFEAHNILR
ESGYAGKISQMPVILTPLHFDRDPLQKQPSCQRSVVIRTFITSDFMTGIPATPGNEIPVE
VVLKMVTEIKKIPGISRIMYDLTSKPPGTTEWE
>P30657
MNHDPFSWGRPADSTYGAYNTQIANAGASPMVNTQQPIVTGTSVISMKYDNGVIIAADNL
GSYGSLLRFNGVERLIPVGDNTVVGISGDISDMQHIERLLKDLVTENAYDNPLADAEEAL
EPSYIFEYLATVMYQRRSKMNPLWNAIIVAGVQSNGDQFLRYVNLLGVTYSSPTLATGFG
AHMANPLLRKVVDRESDIPKTTVQVAEEAIVNAMRVLYYRDARSSRNFSLAIIDKNTGLT
FKKNLQVENMKWDFAKDIKGYGTQKI
>O60870
MGKSDFLTPKAIANRIKSKGLQKLRWYCQMCQKQCRDENGFKCHCMSESHQRQLLLASEN
PQQFMDYFSEEFRNDFLELLRRRFGTKRVHNNIVYNEYISHREHIHMNATQWETLTDFTK
WLGREGLCKVDETPKGWYIQYIDRDPETIRRQLELEKKKKQDLDDEEKTAKFIEEQVRRG
LEGKEQEVPTFTELSRENDEEKVTFNLSKGACSSSGATSSKSSTLGPSALKTIGSSASVK
RKESSQSSTQSKEKKKKKSALDEIMEIEEEKKRTARTDYWLQPEIIVKIITKKLGEKYHK
KKAIVKEVIDKYTAVVKMIDSGDKLKLDQTHLETVIPAPGKRILVLNGGYRGNEGTLESI
NEKTFSATIVIETGPLKGRRVEGIQYEDISKLA
>Q9UHB9
MAAEKQVPGGGGGGGSGGGGGSGGGGSGGGRGAGGEENKENERPSAGSKANKEFGDSLSL
EILQIIKESQQQHGLRHGDFQRYRGYCSRRQRRLRKTLNFKMGNRHKFTGKKVTEELLTD
NRYLLLVLMDAERAWSYAMQLKQEANTEPRKRFHLLSRLRKAVKHAEELERLCESNRVDA
KTKLEAQAYTAYLSGMLRFEHQEWKAAIEAFNKCKTIYEKLASAFTEEQAVLYNQRVEEI
SPNIRYCAYNIGDQSAINELMQMRLRSGGTEGLLAEKLEALITQTRAKQAATMSEVEWRG
RTVPVKIDKVRIFLLGLADNEAAIVQAESEETKERLFESMLSECRDAIQVVREELKPDQK
QRDYILEGEPGKVSNLQYLHSYLTYIKLSTAIKRNENMAKGLQRALLQQQPEDDSKRSPR
PQDLIRLYDIILQNLVELLQLPGLEEDKAFQKEIGLKTLVFKAYRCFFIAQSYVLVKKWS
EALVLYDRVLKYANEVNSDAGAFKNSLKDLPDVQELITQVRSEKCSLQAAAILDANDAHQ
TETSSSQVKDNKPLVERFETFCLDPSLVTKQANLVHFPPGFQPIPCKPLFFDLALNHVAF
PPLEDKLEQKTKSGLTGYIKGIFGFRS
>O00562
MLIKEYHILLPMSLDEYQVAQLYMIQKKSREESSGEGSGVEILANRPYTDGPGGSGQYTH
KVYHVGSHIPGWFRALLPKAALQVEEESWNAYPYTRTRYTCPFVEKFSIEIETYYLPDGG
QQPNVFNLSGAERRQRILDTIDIVRDAVAPGEYKAEEDPRLYHSVKTGRGPLSDDWARTA
AQTGPLMCAYKLCKVEFRYWGMQAKIEQFIHDVGLRRVMLRAHRQAWCWQDEWTELSMAD
IRALEEETARMLAQRMAKCNTGSEGSEAQPPGKPSTEARSAASNTGTPDGPEAPPGPDAS
PDASFGKQWSSSSRSSYSSQHGGAVSPQSLSEWRMQNIARDSENSSEEEFFDAHEGFSDS
EEVFPKEMTKWNSNDFIDAFASPVEAEGTPEPGAEAAKGIEDGAQAPRDSEGLDGAGELG
AEACAVHALFLILHSGNILDSGPGDANSKQADVQTLSSAFEAVTRIHFPEALGHVALRLV
PCPPICAAAYALVSNLSPYSHDGDSLSRSQDHIPLAALPLLATSSSRYQGAVATVIARTN

QAYSAFLRSPEGAGFCGQVALIGDGVGGILGFDALCHSANAGTGSRGSSRRGSMNNELLS
PEFGPVRDPLADGVEGLGRGSPEPSALPPQRIPSDMASPEPEGSQNSLQAAPATTSSWEP
RRASTAFCPPAASSEAPDGPSSTARLDFKVSGFFLFGSPLGLVLALRKTVMPALEAAQMR
PACEQIYNLFHAADPCASRLEPLLAPKFQAIAPLTVPRYQKFPLGDGSSLLLADTLQTHS
SLFLEELEMLVPSTPTSTSGAFWKGSELATDPPAQPAAPSTTSEVVKILERWWGTKRIDY
SLYCPEALTAFPTVTLPHLFHASYWESADVVAFILRQVIEKERPQLAECEEPSIYSPAFP
REKWQRKRTQVKIRNVTSNHRASDTVVCEGRPQVLSGRFMYGPLDVVTLTGEKVDVYIMT
QPLSGKWIHFGTEVTNSSGRLTFPVPPERALGIGVYPVRMVVRGDHTYAECCLTVVARGT
EAVVFSIDGSFTASVSIMGSDPKVRAGAVDVVRHWQDSGYLIVYVTGRPDMQKHRVVAWL
SQHNFPHGVVSFCDGLTHDPLRQKAMFLQSLVQEVELNIVAGYGSPKDVAVYAALGLSPS
QTYIVGRAVRKLQAQCQFLSDGYVAHLGQLEAGSHSHASSGPPRAALGKSSYGVAAPVDF
LRKQSQLLRSRGPSQAEREGPGTPPTTLARGKARSISLKLDSEE
>Q96AQ7
MEYAMKSLSLLYPKSLSRHVSVRTSVVTQQLLSEPSPKAPRARPCRVSTADRSVRKGIMA
YSLEDLLLKVRDTLMLADKPFFLVLEEDGTTVETEEYFQALAGDTVFMVLQKGQKWQPPS
EQGTRHPLSLSHKPAKKIDVARVTFDLYKLNPQDFIGCLNVKATFYDTYSLSYDLHCCGA
KRIMKEAFRWALFSMQATGHVLLGTSCYLQQLLDATEEGQPPKGKASSLIPTCLKILQ
>O94525
MDPHWKRHDSSNIPTQPSPSASPKSNKPSSAQRFGNLPWVAPDVIQSQIEYLQALQMQQS
LSESENYLQPNFFPFQSGPFSKSRRENTLLPRLNPLAPIGARQPNPSIPQQFSKPINESG
TGTMGPAVGELTSPVMKNRAESIFSPVTESFEAFTQGMQTTPQRAGAGVSTATSHTRRRS
SAGTDPFSPVSPSNPNFLTPLKPIDGNQEWQQSPLESPLSMHSLQESLHSVEPESPIFQQ
APQIPLSNASQQSYINHSSDGASLPQSSFLNFNSSGKRVSVSAAAQQHKNLFLPYLPQAS
LPKFIGTGKLLVGTLHINRKNRSDAYVITDVLDEPIFICGSKDRNRTLEGDLVAVELLDV
NEIMQTKREKEEKKIRRNLSLSGSSKYSVNSKAKMMSISTPMALGMNRGVLSFERSIEKR
KNDYEVTGQSLSFVDDVSLTPDSAPKYAGHVVAVLNRPSGETCSGTLALYRPNSLALKNQ
SSHRRNSSTSSGETGKGPKIVWFKPSDKRIPLIAISSDQVPPTFFTNNDDFKDKVFLAGI
KRWPTTSLHPFGTLYESIGTIGDPKVEYKAILHDFSCHTYDFPESLSHCVKRLPTPIPAE
ELQKRYNLRDKLTFMIGTRDYALHIDTGSIDGVITLGIHVADVAYYVKQDMPLDDEAANR
VSEVQLLQGSVPFLPKKVSEEISLIEGHDCLTISIVVQLDLKSGAVLQCSLGPSVIHPSS
FISLESAQANLQTNEALQLVDASAHVLTKLRLKTDKKLDLQSLYCFEFCDGQIPNIQNIN
MSIFEAFPIACSLQQIEHWVNEKVASHLMSVFPSKVILRKNQRPSDFASLVAVSELTGVN
LSASSTPSEIMTEIATTKDKRTKMLLQLTLRRMCNESEYTIGTSNAKDVSHFVFSCPYYT
HFCHPTRRYIDICVQRQLREAFDGRPDFSKDYRSLLSITQSCNTLSNFYRNAQEKSLHAY
LCQLLHSINLRSGLVKHKAFVLAVDQEYIDIVIYEFGLERRISLDLLPLSNCDFNEQKHE
LYLSWRTNASFFYASSSLEIDTPCFNDFKKKFLDSNGMCKQICDMMDAQVRDLANAAESQ
ESFYSKARGNDSTSKTAKSSSGNQDISGDGKLHSLRMKEPEVVQTIRPGQEVSVLIFADT
SATYSLSLITLQSPLSS
>Q14674
MRSFKRVNFGTLLSSQKEAEELLPDLKEFLSNPPAGFPSSRSDAERRQACDAILRACNQQ
LTAKLACPRHLGSLLELAELACDGYLVSTPQRPPLYLERILFVLLRNAAAQGSPEVTLRL
AQPLHACLVQCSREAAPQDYEAVARGSFSLLWKGAEALLERRAAFAARLKALSFLVLLED
ESTPCEVPHFASPTACRAVAAHQLFDASGHGLNEADADFLDDLLSRHVIRALVGERGSSS
GLLSPQRALCLLELTLEHCRRFCWSRHHDKAISAVEKAHSYLRNTNLAPSLQLCQLGVKL

LQVGEEGPQAVAKLLIKASAVLSKSMEAPSPPLRALYESCQFFLSGLERGTKRRYRLDAI
LSLFAFLGGYCSLLQQLRDDGVYGGSSKQQQSFLQMYFQGLHLYTVVVYDFAQGCQIVDL
ADLTQLVDSCKSTVVWMLEALEGLSGQELTDHMGMTASYTSNLAYSFYSHKLYAEACAIS
EPLCQHLGLVKPGTYPEVPPEKLHRCFRLQVESLKKLGKQAQGCKMVILWLAALQPCSPE
HMAEPVTFWVRVKMDAARAGDKELQLKTLRDSLSGWDPETLALLLREELQAYKAVRADTG
QERFNIICDLLELSPEETPAGAWARATHLVELAQVLCYHDFTQQTNCSALDAIREALQLL
DSVRPEAQARDQLLDDKAQALLWLYICTLEAKIQEGIERDRRAQAPGNLEEFEVNDLNYE
DKLQEDRFLYSNIAFNLAADAAQSKCLDQALALWKELLTKGQAPAVRCLQQTAASLQILA
ALYQLVAKPMQALEVLLLLRIVSERLKDHSKAAGSSCHITQLLLTLGCPSYAQLHLEEAA
SSLKHLDQTTDTYLLLSLTCDLLRSQLYWTHQKVTKGVSLLLSVLRDPALQKSSKAWYLL
RVQVLQLVAAYLSLPSNNLSHSLWEQLCAQGWQTPEIALIDSHKLLRSIILLLMGSDILS
TQKAAVETSFLDYGENLVQKWQVLSEVLSCSEKLVCHLGRLGSVSEAKAFCLEALKLTTK
LQIPRQCALFLVLKGELELARNDIDLCQSDLQQVLFLLESCTEFGGVTQHLDSVKKVHLQ
KGKQQAQVPCPPQLPEEELFLRGPALELVATVAKEPGPIAPSTNSSPVLKTKPQPIPNFL
SHSPTCDCSLCASPVLTAVCLRWVLVTAGVRLAMGHQAQGLDLLQVVLKGCPEAAERLTQ
ALQASLNHKTPPSLVPSLLDEILAQAYTLLALEGLNQPSNESLQKVLQSGLKFVAARIPH
LEPWRASLLLIWALTKLGGLSCCTTQLFASSWGWQPPLIKSVPGSEPSKTQGQKRSGRGR
QKLASAPLSLNNTSQKGLEGRGLPCTPKPPDRIRQAGPHVPFTVFEEVCPTESKPEVPQA
PRVQQRVQTRLKVNFSDDSDLEDPVSAEAWLAEEPKRRGTASRGRGRARKGLSLKTDAVV
APGSAPGNPGLNGRSRRAKKVASRHCEERRPQRASDQARPGPEIMRTIPEEELTDNWRKM
SFEILRGSDGEDSASGGKTPAPGPEAASGEWELLRLDSSKKKLPSPCPDKESDKDLGPRL
QLPSAPVATGLSTLDSICDSLSVAFRGISHCPPSGLYAHLCRFLALCLGHRDPYATAFLV
TESVSITCRHQLLTHLHRQLSKAQKHRGSLEIADQLQGLSLQEMPGDVPLARIQRLFSFR
ALESGHFPQPEKESFQERLALIPSGVTVCVLALATLQPGTVGNTLLLTRLEKDSPPVSVQ
IPTGQNKLHLRSVLNEFDAIQKAQKENSSCTDKREWWTGRLALDHRMEVLIASLEKSVLG
CWKGLLLPSSEEPGPAQEASRLQELLQDCGWKYPDRTLLKIMLSGAGALTPQDIQALAYG
LCPTQPERAQELLNEAVGRLQGLTVPSNSHLVLVLDKDLQKLPWESMPSLQALPVTRLPS
FRFLLSYSIIKEYGASPVLSQGVDPRSTFYVLNPHNNLSSTEEQFRANFSSEAGWRGVVG
EVPRPEQVQEALTKHDLYIYAGHGAGARFLDGQAVLRLSCRAVALLFGCSSAALAVHGNL
EGAGIVLKYIMAGCPLFLGNLWDVTDRDIDRYTEALLQGWLGAGPGAPLLYYVNQARQAP
RLKYLIGAAPIAYGLPVSLR
>P32917
MMETPTDNIVSPFHNFGSSTQYSGTLSRTPNQIIELEKPSTLSPLSRGKKWTEKLARFQR
SSAKKKRFSPSPISSSTFSFSPKSRVTSSNSSGNEDGNLMNTPSTVSTDYLPQHPHRTSS
LPRPNSNLFHASNSNLSRANEPPRAENLSDNIPPKVAPFGYPIQRTSIKKSFLNASCTLC
DEPISNRRKGEKIIELACGHLSHQECLIISFGTTSKADVRALFPFCTKCKKDTNKAVQCI
PENDELKDILISDFLIHKIPDSELSITPQSRFPPYSPLLPPFGLSYTPVERQTIYSQAPS
LNPNLILAAPPKERNQIPQKKSNYTFLHSPLGHRRIPSGANSILADTSVALSANDSISAV
SNSVRAKDDETKTTLPLLRSYFIQILLNNFQEELQDWRIDGDYGLLRLVDKLMISKDGQR
YIQCWCFLFEDAFVIAEVDNDVDVLEIRLKNLEVFTPIANLRMTTLEASVLKCTLNKQHC
ADLSDLYIVQNINSDESTTVQKWISGILNQDFVFNEDNITSTLPILPIIKNFSKDVGNGR
HETSTFLGLINPNKVVEVGNVHDNDTVIIRRGFTLNSGECSRQSTVDSIQSVLTTISSIL
SLKREKPDNLAIILQIDFTKLKEEDSLIVVYNSLKALTIKFARLQFCFVDRNNYVLDYGS
VLHKIDSLDSISNLKSKSSSTQFSPIWLKNTLYPENIHEHLGIVAVSNSNMEAKKSILFQ

DYRCFTSFGRRRPNELKIKVGYLNVDYSDKIDELVEASSWTFVLETLCYSFGLSFDEHDD
DDEEDNDDSTDNELDNSSGSLSDAESTTTIHIDSPFDNENATANMVNDRNLLTEGEHSNI
ENLETVASSVQPALIPNIRFSLHSEEEGTNENENENDMPVLLLSDMDKGIDGITRRSSFS
SLIESGNNNCPLHMDYI
>O74859
MSVHKTNDAFKVLMNSAKEPIVEDIPKKYRKQSFRDNLKVYIESPESYKNVIYYDDDVVL
VRDMFPKSKMHLLLMTRDPHLTHVHPLEIMMKHRSLVEKLVSYVQGDLSGLIFDEARNCL
SQQLTNEALCNYIKVGFHAGPSMNNLHLHIMTLDHVSPSLKNSAHYISFTSPFFVKIDTP
TSNLPTRGTLTSLFQEDLKCWRCGETFGRHFTKLKAHLQEEYDDWLDKSVSM
>Q92985
MALAPERAAPRVLFGEWLLGEISSGCYEGLQWLDEARTCFRVPWKHFARKDLSEADARIF
KAWAVARGRWPPSSRGGGPPPEAETAERAGWKTNFRCALRSTRRFVMLRDNSGDPADPHK
VYALSRELCWREGPGTDQTEAEAPAAVPPPQGGPPGPFLAHTHAGLQAPGPLPAPAGDKG
DLLLQAVQQSCLADHLLTASWGADPVPTKAPGEGQEGLPLTGACAGGPGLPAGELYGWAV
ETTPSPGPQPAALTTGEAAAPESPHQAEPYLSPSPSACTAVQEPSPGALDVTIMYKGRTV
LQKVVGHPSCTFLYGPPDPAVRATDPQQVAFPSPAELPDQKQLRYTEELLRHVAPGLHLE
LRGPQLWARRMGKCKVYWEVGGPPGSASPSTPACLLPRNCDTPIFDFRVFFQELVEFRAR
QRRGSPRYTIYLGFGQDLSAGRPKEKSLVLVKLEPWLCRVHLEGTQREGVSSLDSSSLSL
CLSSANSLYDDIECFLMELEQPA
>P87145
MDNLLVRGLTHKLYDKRKATAYELERVVKGYLENDETEKIRAVISQLANDFVYSPARGPN
ATFGGLIGLAAVAIALGPKIDSYMESILLPVLYCFNDSDSKIRYYACESMYNIGKVAKGE
VFRYFNLIFDVLCKLFADTEITVKNGAELLDRLIKDIVMQQAATYMSSAEDIKNFKEGPV
SSSIQDVPVMSTEQPRMHTFSLSELVPLLSERLYVINPNTRMFLVSWIRLLDSIPDLEFI
SYLPFLLDGLMNYLSDPNESIRIVTSNCLYDFLREIQKIAKVKYHILQRDEESEPDFFDS
MVRRNMSDAELKEISDYVESSLRDGSFILEAHIQIDYKRILEIIIDHLGSSVPLIQEKAL
KWLFEFIYIAPKDVLLQIPKVLENLLPLMSNDENMRQSAKDLSQNLVILVSKIMDIEFSG
SETNNKDNSLSVDFRSLIEVLQKLLSNDNEETRLCALEWVLLLQRRTGGKLINMHDPIFQ
TLLLQLSDPSDLVVSRTLELLAHIAISHKSVNLVPFLKSLLQMFAEDRKFLNSRGNLIIR
QLCNYIEGERVYTSFAGILETEENLELASIMVEVLNNNLFTAPELYDLRKKLKQSAPKLQ
NIFTTLYTAWCHNSIAVFSLCLLSQNYEHAANLLSVFAEIEFNIDMLIQLDKLVQLIESP
VFTYMRLQLLEPEKYPYLHKALYGILMLLPQSSAFRTLRDRLQCSSTPRTNTILANERLP
RSRRDDPYWTDLLERLKAVQLSHQNNYREPIRATRLALAGALPSTPTATTISTTTSASGI
TTTASNSRDSFITRLPPTAALSTGARKKPKQ
>Q8IZH2
MGVPKFYRWISERYPCLSEVVKEHQIPEFDNLYLDMNGIIHQCSHPNDDDVHFRISDDKI
FTDIFHYLEVLFRIIKPRKVFFMAVDGVAPRAKMNQQRGRRFRSAKEAEDKIKKAIEKGE
TLPTEARFDSNCITPGTEFMARLHEHLKYFVNMKISTDKSWQGVTIYFSGHETPGEGEHK
IMEFIRSEKAKPDHDPNTRHCLYGLDADLIMLGLTSHEAHFSLLREEVRFGGKKTQRVCA
PEETTFHLLHLSLMREYIDYEFSVLKEKITFKYDIERIIDDWILMGFLVGNDFIPHLPHL
HINHDALPLLYGTYVTILPELGGYINESGHLNLPRFEKYLVKLSDFDREHFSEVFVDLKW
FESKVGNKYLNEAAGVAAEEARNYKEKKKLKGQENSLCWTALDKNEGEMITSKDNLEDET
EDDDLFETEFRQYKRTYYMTKMGVDVVSDDFLADQAACYVQAIQWILHYYYHGVQSWSWY
YPYHYAPFLSDIHNISTLKIHFELGKPFKPFEQLLAVLPAASKNLLPACYQHLMTNEDSP

IIEYYPPDFKTDLNGKQQEWEAVVLIPFIDEKRLLEAMETCNHSLKKEERKRNQHSECLM
CWYDRDTEFIYPSPWPEKFPAIERCCTRYKIISLDAWRVDINKNKITRIDQKALYFCGFP
TLKHIRHKFFLKKSGVQVFQQSSRGENMMLEILVDAESDELTVENVASSVLGKSVFVNWP
HLEEARVVAVSDGETKFYLEEPPGTQKLYSGRTAPPSKVVHLGDKEQSNWAKEVQGISEH
YLRRKGIIINETSAVVYAQLLTGRKYQINQNGEVRLEKQWSKQVVPFVYQTIVKDIRAFD
SRFSNIKTLDDLFPLRSMVFMLGTPYYGCTGEVQDSGDVITEGRIRVIFSIPCEPNLDAL
IQNQHKYSIKYNPGYVLASRLGVSGYLVSRFTGSIFIGRGSRRNPHGDHKANVGLNLKFN
KKNEEVPGYTKKVGSEWMYSSAAEQLLAEYLERAPELFSYIAKNSQEDVFYEDDIWPGEN
ENGAEKVQEIITWLKGHPVSTLSRSSCDLQILDAAIVEKIEEEVEKCKQRKNNKKVRVTV
KPHLLYRPLEQQHGVIPDRDAEFCLFDRVVNVRENFSVPVGLRGTIIGIKGANREADVLF
EVLFDEEFPGGLTIRCSPGRGYRLPTSALVNLSHGSRSETGNQKLTAIVKPQPAVHQHSS
SSSVSSGHLGALNHSPQSLFVPTQVPTKDDDEFCNIWQSLQGSGKMQYFQPTIQEKGAVL
PQEISQVNQHHKSGFNDNSVKYQQRKHDPHRKFKEECKSPKAECWSQKMSNKQPNSGIEN
FLASLNISKENEVQSSHHGEPPSEEHLSPQSFAMGTRMLKEILKIDGSNTVDHKNEIKQI
ANEIPVSSNRRDEYGLPSQPKQNKKLASYMNKPHSANEYHNVQSMDNMCWPAPSQIPPVS
TPVTELSRICSLVGMPQPDFSFLRMPQTMTVCQVKLSNGLLVHGPQCHSENEAKEKAALF
ALQQLGSLGMNFPLPSQVFANYPSAVPPGTIPPAFPPPTGWDHYGSNYALGAANIMPSSS
HLFGSMPWGPSVPVPGKPFHHTLYSGTMPMAGGIPGGVHNQFIPLQVTKKRVANKKNFEN
KEAQSSQATPVQTSQPDSSNIVKVSPRESSSASLKSSPIAQPASSFQVETASQGHSISHH
KSTPISSSRRKSRKLAVNFGVSKPSE
>Q04178
MSANDKQYISYNNVHQLCQVSAERIKNFKPDLIIAIGGGGFIPARILRTFLKEPGVPTIR
IFAIILSLYEDLNSVGSEVEEVGVKVSRTQWIDYEQCKLDLVGKNVLIVDEVDDTRTTLH
YALSELEKDAAEQAKAKGIDTEKSPEMKTNFGIFVLHDKQKPKKADLPAEMLNDKNRYFA
AKTVPDKWYAYPWESTDIVFHTRMAIEQGNDIFIPEQEHKQ
>Q9D0S4
MADPSEHVGLGGPRSPARPEPPPTRFHQVHGANIRMDPSGTRATRVESFAHGVCFSREPL
APGQVFLVEIEEKELGWCGHLRLGLTALDPASLAAVPEFSLPDLVSLGHSWVFAITRHHN
RVPREGQPEAEAAVPSGPQALLVEPYLRIEQFRIPRDRLVGRSRPGLYSHLLDQLYEQNV
LPPTARRSRLGVLFCPREDGTADMHIIINGEDMGPSARGLPAAQPLYAVVDVFASTKSVR
LVQLEYGLPSLQTLCRLVIHKRVVHRLAIDVLHLPKGLKDFCKYE
>P32626
MVIGQKVLLARIPKMGDNYSTYLLDIEGTVCPISFVKETLFPYFTNKVPQLVQQDTRDSP
VSNILSQFHIDNKEQLQAHILELVAKDVKDPILKQLQGYVWAHGYESGQIKAPVYADAID
FIKRKKRVFIYSSGSVKAQKLLFGYVQDPNAPAHDSLDLNSYIDGYFDINTSGKKTETQS
YANILRDIGAKASEVLFLSDNPLELDAAAGVGIATGLASRPGNAPVPDGQKYQVYKNFET
L
>P42845
MSQPQMSPEKEQELASKILHRAELAQMTRQLKLGLSNVPSTKRKQDSTTKKRSGEDAEDV
DEDHKTLLEAISPAKKPLHDDTNKMTVISPVKFVEKPNTPPSSRQRKAEDRSQQIKPRKE
DTPSTPRASATPIILPHASSHYQRPHDKNFMTPKRNNNNSSNHSNNNNNIKKKAAGSKDA
PQDSDNTAGADLLMYLATSPYNKSSHHGTPMAVRMPTTPRSYHYASQLSLNGNTASTSND
AVRFSHIKPSASSPQSTFKSNLLPNFPDESLMDSPSLYLSNNNGSVQATLSPQQRRKPTT
NTLHPPSNVPTTPSRELNGTNFNLLRTPNFNMGDYLHNLFSPSPRVPAQQGASNTSASIP

SVPAMVPGSSSNTSAIATAAISSHTTNNFLDMNANGIPLIVGPGTDRIGEGESIDDKLTD

>P87314
MKIKKIPWLGHFDDRGHRLSIFSIHIHPDGSRIATGGLDGTIRIWSTEAINRENENENEN
EDLPKQLCCMSTHTGTVTSVRFSPNGQYLASGSDDRVVIIWHKEEAIPGLGSTFGSGEKH
TENWRSYRRLLGHDNDIQDLCWSYDSQLVVSVGLDSSIIVWNGTTFERLKRIEAHQSHVK
GITFDPAGKYFATESDDRTIKVWRVSDFSIEKTITGPFNNSPLSTYFRRPSWSPDGKHIA
APNAMNGPVSCVSIIERGTWTSEINLIGHEGPVEVTAFNPKLFRDKNDKLVCILACGGQD
RSLSIWSSALPRPLLSCQNVFQKSIGDVCWSPDGLSLFLCSYDGNVLVCTFEKEEFGDMV
SDEEISKALAKYGHGRHGIVLPESAKQLELEETAYAILKKPSSLSTTDPTLVPQSSSTPK
SAQKTPQKLPAFLPNRLTAETVDTNKLTASKEQIASPKRPGPSDNGNEIPTKFVQKVTIT
KEGKKRVAPQLLTTLSATPSTSRLASTQLQHTGSSQLPPQQFSQPINSLPKGGVPILIVG
NKTKVNHENDESDQALQEEKIEEGLLKNYYSSLIDSSTSISNINFEAPRYKTNIVHSLNN
EQKYVLEVKNGTSEKNPTRIVALENGNTKWMDYLPRPVILVTGSIHFWSIACDDGSLHLY
SLTGSRLLPPIMIESKASFLHCNNAYLLCISSSGMVYAWNVVNKTALFTANSLAPILSRV
SNNVTIENNSTDIPHVVIASISKEGVPSVTLSTGETYVYSSTMLCWQRITEPWWAIGSRE
WDSSGLLQSNTQTESQPLKIYEHRTNNVLMDSGRGKLLQKMVADAITEEGYDDFETIVTI
NHLENKIASARLLKLDDEFLVTSEVYVRLLMHHGLWQKLEEFLGELRTQTKCSIKLSGRE
VVAKMLVVLRQAVQTDNEFDRANKLIEKYAST
>O14455
MAVKTGIAIGLNKGKKVTQMTPAPKISYKKGAASNRTKFVRSLVREIAGLSPYERRLIDL
IRNSGEKRARKVAKKRLGSFTRAKAKVEEMNNIIAASRRH
>Q9NRJ1
MGECPHLVDVRLGHRSLATGPEQSDICHTGSEARWTTTWYGSLSFSRHKYKMLADLTPGV
EMSCRHWARWLTPVIPALWKAEAGGLPELRSSRPAWTTW
>Q9BZI7
MKEEKEHRPKEKRVTLLTPAGATGSGGGTSGDSSKGEDKQDRNKEKKEALSKVVIRRLPP
TLTKEQLQEHLQPMPEHDYFEFFSNDTSLYPHMYARAYINFKNQEDIILFRDRFDGYVFL
DNKGQEYPAIVEFAPFQKAAKKKTKKRDTKVGTIDDDPEYRKFLESYATDNEKMTSTPET
LLEEIEAKNRELIAKKTTPLLSFLKNKQRMREEKREERRRREIERKRQREEERRKWKEEE
KRKRKDIEKLKKIDRIPERDKLKDEPKIKVHRFLLQAVNQKNLLKKPEKGDEKELDKREK
AKKLDKENLSDERASGQSCTLPKRSDSELKDEKPKRPEDESGRDYREREREYERDQERIL
RERERLKRQEEERRRQKERYEKEKTFKRKEEEMKKEKDTLRDKGKKAESTESIGSSEKTE
KKEEVVKRDRIRNKDRPAMQLYQPGARSRNRLCPPDDSTKSGDSAAERKQESGISHRKEG
GEE
>P36138
MSNSHHTSQGRRNKLSVWVKKIINTTTTTNASVSSSKPRRGTRAGPTRVKRAELDPDGTT
ISSSLRPLVDRNSLHSSESDDEGDRRVAWDEPPTGKVRQQQQQQQQQNDNASVIPLVSF
CSSSVKSSTFSDIHSIQSTRPTIFSNRTFETNSSVLAIPPQSILDRSRTLPPSNASNTTT
RRP
>Q12802
MKLNPQQAPLYGDCVVTVLLAEEDKAEDDVVFYLVFLGSTLRHCTSTRKVSSDTLETIAP
GHDCCETVKVQLCASKEGLPVFVVAEEDFHFVQDEAYDAAQFLATSAGNQQALNFTRFLD
QSGPPSGDVNSLDKKLVLAFRHLKLPTEWNVLGTDQSLHDAGPRETLMHFAVRLGLLRLT

WFLLQKPGGRGALSIHNQEGATPVSLALERGYHKLHQLLTEENAGEPDSWSSLSYEIPYG
DCSVRHHRELDIYTLTSESDSHHEHPFPGDGCTGPIFKLMNIQQQLMKTNLKQMDSLMPL
MMTAQDPSSAPETDGQFLPCAPEPTDPQRLSSSEETESTQCCPGSPVAQTESPCDLSSIV
EEENTDRSCRKKNKGVERKGEEVEPAPIVDSGTVSDQDSCLQSLPDCGVKGTEGLSSCGN
RNEETGTKSSGMPTDQESLSSGDAVLQRDLVMEPGTAQYSSGGELGGISTTNVSTPDTAG
EMEHGLMNPDATVWKNVLQGGESTKERFENSNIGTAGASDVHVTSKPVDKISVPNCAPAA
SSLDGNKPAESSLAFSNEETSTEKTAETETSRSREESADAPVDQNSVVIPAAAKDKISDG
LEPYTLLAAGIGEAMSPSDLALLGLEEDVMPHQNSETNSSHAQSQKGKSSPICSTTGDDK
LCADSACQQNTVTSSGDLVAKLCDNIVSESESTTARQPSSQDPPDASHCEDPQAHTVTSD
PVRDTQERADFCPFKVVDNKGQRKDVKLDKPLTNMLEVVSHPHPVVPKMEKELVPDQAVI
SDSTFSLANSPGSESVTKDDALSFVPSQKEKGTATPELHTATDYRDGPDGNSNEPDTRPL
EDRAVGLSTSSTAAELQHGMGNTSLTGLGGEHEGPAPPAIPEALNIKGNTDSSLQSVGKA
TLALDSVLTEEGKLLVVSESSAAQEQDKDKAVTCSSIKENALSSGTLQEEQRTPPPGQDT
QQFHEKSISADCAKDKALQLSNSPGASSAFLKAETEHNKEVAPQVSLLTQGGAAQSLVPP
GASLATESRQEALGAEHNSSALLPCLLPDGSDGSDALNCSQPSPLDVGVKNTQSQGKTSA
CEVSGDVTVDVTGVNALQGMAEPRRENISHNTQDILIPNVLLSQEKNAVLGLPVALQDKA
VTDPQGVGTPEMIPLDWEKGKLEGADHSCTMGDAEEAQIDDEAHPVLLQPVAKELPTDME
LSAHDDGAPAGVREVMRAPPSGRERSTPSLPCMVSAQDAPLPKGADLIEEAASRIVDAVI
EQVKAAGALLTEGEACHMSLSSPELGPLTKGLESAFTEKVSTFPPGESLPMGSTPEEATG
SLAGCFAGREEPEKIILPVQGPEPAAEMPDVKAEDEVDFRASSISEEVAVGSIAATLKMK
QGPMTQAINRENWCTIEPCPDAASLLASKQSPECENFLDVGLGRECTSKQGVLKRESGSD
SDLFHSPSDDMDSIIFPKPEEEHLACDITGSSSSTDDTASLDRHSSHGSDVSLSQILKPN
RSRDRQSLDGFYSHGMGAEGRESESEPADPGDVEEEEMDSITEVPANCSVLRSSMRSLSP
FRRHSWGPGKNAASDAEMNHRSSMRVLGDVVRRPPIHRRSFSLEGLTGGAGVGNKPSSSL
EVSSANAEELRHPFSGEERVDSLVSLSEEDLESDQREHRMFDQQICHRSKQQGFNYCTSA
ISSPLTKSISLMTISHPGLDNSRPFHSTFHNTSANLTESITEENYNFLPHSPSKKDSEWK
SGTKVSRTFSYIKNKMSSSKKSKEKEKEKDKIKEKEKDSKDKEKDKKTVNGHTFSSIPVV
GPISCSQCMKPFTNKDAYTCANCSAFVHKGCRESLASCAKVKMKQPKGSLQAHDTSSLPT
VIMRNKPSQPKERPRSAVLLVDETATTPIFANRRSQQSVSLSKSVSIQNITGVGNDENMS
NTWKFLSHSTDSLNKISKVNESTESLTDEGVGTDMNEGQLLGDFEIESKQLEAESWSRII
DSKFLKQQKKDVVKRQEVIYELMQTEFHHVRTLKIMSGVYSQGMMADLLFEQQMVEKLFP
CLDELISIHSQFFQRILERKKESLVDKSEKNFLIKRIGDVLVNQFSGENAERLKKTYGKF
CGQHNQSVNYFKDLYAKDKRFQAFVKKKMSSSVVRRLGIPECILLVTQRITKYPVLFQRI
LQCTKDNEVEQEDLAQSLSLVKDVIGAVDSKVASYEKKVRLNEIYTKTDSKSIMRMKSGQ
MFAKEDLKRKKLVRDGSVFLKNAAGRLKEVQAVLLTDILVFLQEKDQKYIFASLDQKSTV
ISLKKLIVREVAHEEKGLFLISMGMTDPEMVEVHASSKEERNSWIQIIQDTINTLNRDED
EGIPSENEEEKKMLDTRARELKEQLHQKDQKILLLLEEKEMIFRDMAECSTPLPEDCSPT
HSPRVLFRSNTEEALKGGPLMKSAINEVEILQGLVSGNLGGTLGPTVSSPIEQDVVGPVS
LPRRAETFGGFDSHQMNASKGGEKEEGDDGQDLRRTESDSGLKKGGNANLVFMLKRNSEQ
VVQSVVHLYELLSALQGVVLQQDSYIEDQKLVLSERALTRSLSRPSSLIEQEKQRSLEKQ
RQDLANLQKQQAQYLEEKRRREREWEARERELREREALLAQREEEVQQGQQDLEKEREEL
QQKKGTYQYDLERLRAAQKQLEREQEQLRREAERLSQRQTERDLCQVSHPHTKLMRIPSF
FPSPEEPPSPSAPSIAKSGSLDSELSVSPKRNSISRTHKDKGPFHILSSTSQTNKGPEGQ
SQAPASTSASTRLFGLTKPKEKKEKKKKNKTSRSQPGDGPASEVSAEGEEIFC

>O74562
MDTRGYSETKKGRYPVGKKSLESPNGYSNYGTSMDNELSSEYASRGMHIGDLENLVNEIE
DEWKDLGREDYQPISTALELLDDSSFGRDYKSFLNVYDRISAALQTIAHTHKDDFTRGIS
AYGEIMEGIQKCNSRIIALKQSLEASQECIGNTNSKELQQTLARSSQYKKVISVLKELNE
ANQLFDNFHTLVDSKQYYHASDLIRRVWDELSRSDFDGILVVEQFKSRMTGLLSHLEDIL
SEELVSITFLKDAVAYPIVSYCSPNPLRETSNPYFLRDFLKNNANTSTLGQSEQLRYLEE
ALSLKLSDCLKMDYGRDSLRDIRIVLESLNLLGKLPNAISSLKSRTSAEMFTTVDSTSRA
IVNKYSLGNNVSTVNPFSKSLYDIGLHAETDREHTMISEFLTNLFTKLRCVLMHYRGISE
FMTKLETKTPKHASSSHKSSIMSVNSDPTSPKVSKFDTSDSTFPFDTLLQAFESEIRLML
KDYLISKEEYIENSGNFVVGTEMSIYNLPGENEEDKLFDVTNEIAVENKSNAFYARINEL
VNEKAPELILNKSNASVSTIELFSGSSKEIVRLAGHVVFVGPSVFHASSVLPQTVFFLED
SVSILKNPNIPPQFAVNFMKEFLRGSYIPQLYKFMSSHFDTIMKDVGAFQLHRDWKIYSK
IPIFKCHVAIVQYFHDLQDYLPIVALNLVEFYELLHTLLVRFRNHCSDYLSDLCRTAVLK
EYKHVNEDTEDVDDTVRVKLLHDDVTYPQFIKFLKQKNPSLEGLNELCRMENKRLLQYED
RAITSEVKLPVSVLSKDSDLVNSVSYLHNSMEWFLQRCFSRFMNGSRRMNVLQQNQANFG
GDFLPIDNLLGNNSDLMKGYVTVCIELYTDFYKSAYKEVFDSLQRLQFDALLLIRMEVRL
QYIHSINQSVNLPDYVVEYRGRPDASIMALNSTIVTTNLKLETCLNEWERRFVFQGLSEL
VDSSLYSIFYKIESMNRGSCLQMLKNMSAMIQILKTVKEIHGDVEFPKSSRVFGIYQNGA
KKIIEHFIAAPKKELLPDVKQMVRIYYQRLMKDAKRNGRDDLYRQYQKKIGSVLTQFDNT
VGGARKNP
>Q9UUF3
MPIYNILRKVIHLAKFAKSKLISITSDRDQISMSIPEVSSHDSCLNDCHSVNSEEQAKPV
CCLELNAHKDGIKVVDTSNDASTFSNSPLVPDNFGVVYPGIIYRSACPRASNFNFLESLH
IRTIISLRQEEYSEEDLHYFTKHHINYYHIAMPGSKHRKNDCISSSSNPDISDVDDLVRK
TLQLLLNKENWPVLLHCSRGKHRTGIVIGCLRALMNWPVGNRLQEYISFSHPKEREVDEE
YIQNFSSDPSLKSSLNDLKRYISDSSSELADVVLSSESPTVQAATVNETCRSPGS
>P39979
MKKTPDPSPPFASTKNVGMSNEEPEKMVNDRIVVKAIEPKDEEAWNKLWKEYQGFQKTVM
PPEVATTTFARFIDPTVKLWGALAFDTETGDAIGFAHYLNHLTSWHVEEVVYMNDLYVTE
RARVKGVGRKLIEFVYSRADELGTPAVYWVTDHYNHRAQLLYTKVAYKTDKVLYKRNGY
>Q96AX9
MGWKPSEARGQSQSFQASGLQPRSLKAARRATGRPDRSRAARPTTDPSAHRSRAAPPNMD
PDPQAGVQVGMRVVRGVDWKWGQQDGGEGGVGTVVELGRHGSPSTPDRTVVVQWDQGTRT
NYRAGYQGAHDLLLYDNAQIGVRHPNIICDCCKKHGLRGMRWKCRVCLDYDLCTQCYMHN
KHELAHAFDRYETAHSRPVTLSPRQGLPRIPLRGIFQGAKVVRGPDWEWGSQDGGEGKPG
RVVDIRGWDVETGRSVASVTWADGTTNVYRVGHKGKVDLKCVGEAAGGFYYKDHLPRLGK
PAELQRRVSADSQPFQHGDKVKCLLDTDVLREMQEGHGGWNPRMAEFIGQTGTVHRITDR
GDVRVQFNHETRWTFHPGALTKHHSFWVGDVVRVIGDLDTVKRLQAGHGEWTDDMAPALG
RVGKVVKVFGDGNLRVAVAGQRWTFSPSCLVAYRPEEDANLDVAERARENKSSLSVALDK
LRAQKSDPEHPGRLVVEVALGNAARALDLLRRRPEQVDTKNQGRTALQVAAYLGQVELIR
LLLQARAGVDLPDDEGNTALHYAALGNQPEATRVLLSAGCRADAINSTQSTALHVAVQRG
FLEVVRALCERGCDVNLPDAHSDTPLHSAISAGTGASGIVEVLTEVPNIDVTATNSQGFT
LLHHASLKGHALAVRKILARARQLVDAKKEDGFTALHLAALNNHREVAQILIREGRCDVN
VRNRKLQSPLHLAVQQAHVGLVPLLVDAGCSVNAEDEEGDTALHVALQRHQLLPLVADGA

GGDPGPLQLLSRLQASGLPGSAELTVGAAVACFLALEGADVSYTNHRGRSPLDLAAEGRV
LKALQGCAQRFRERQAGGGAAPGPRQTLGTPNTVTNLHVGAAPGPEAAECLVCSELALLV
LFSPCQHRTVCEECARRMKKCIRCQVVVSKKLRPDGSEVASAAPAPGPPRQLVEELQSRY
RQMEERITCPICIDSHIRLVFQCGHGACAPCGSALSACPICRQPIRDRIQIFV
>Q9P5M9
MAKVTGLPEFEDLHTKRKWMLNHMAAAFRMFGRNGYNEGTAGHVTVRDPIDENTFWINPL
EVPFSLMKPSDLVHINSDGEIIGGSKMKYNTSGFAIHYEMHKVRPEVICVCHVHSIYGKA
FSALGKPLDMLNTDCCVFYNNHGIYFDMDDVISMPEEGRRTAKGLADYKAVLVQNHGIMT
VGTTVDEAAYLLSLMERSCQIQLLIDSATKVGERKHVHPTRAKAIRENADNPVGLYTAQQ
PNFLYEVACSNGELEII
>Q7Z3T8
MDSYFKAAVSDLDKLLDDFEQNPDEQDYLQDVQNAYDSNHCSVSSELASSQRTSLLPKDQ
ECVNSCASSETSYGTNESSLNEKTLKGLTSIQNEKNVTGLDLLSSVDGGTSDEIQPLYMG
RCSKPICDLISDMGNLVHATNSEEDIKKLLPDDFKSNADSLIGLDLSSVSDTPCVSSTDH
DSDTVREQQNDTSSELQNREIGGIKELGIKVDTTLSDSYNYSGTENLKDKKIFNQLESIV
DFNMSSALTRQSSKMFHAKDKLQHKSQPCGLLKDVGLVKEEVDVAVITAAECLKEEGKTS
ALTCSLPKNEDLCLNDSNSRDENFKLPDFSFQEDKTVIKQSAQEDSKSLDLKDNDVIQDS
SSALHVSSKDVPSSLSCLPASGSMCGSLIESKARGDFLPQHEHKDNIQDAVTIHEEIQNS
VVLGGEPFKENDLLKQEKCKSILLQSLIEGMEDRKIDPDQTVIRAESLDGGDTSSTVVES
QEGLSGTHVPESSDCCEGFINTFSSNDMDGQDLDYFNIDEGAKSGPLISDAELDAFLTEQ
YLQTTNIKSFEENVNDSKSQMNQIDMKGLDDGNINNIYFNAEAGAIGESHGINIICETVD
KQNTIENGLSLGEKSTIPVQQGLPTSKSEITNQLSVSDINSQSVGGARPKQLFSLPSRTR
SSKDLNKPDVPDTIESEPSTADTVVPITCAIDSTADPQVSFNSNYIDIESNSEGGSSFVT
ANEDSVPENTCKEGLVLGQKQPTWVPDSEAPNCMNCQVKFTFTKRRHHCRACGKVFCGVC
CNRKCKLQYLEKEARVCVVCYETISKAQAFERMMSPTGSNLKSNHSDECTTVQPPQENQT
SSIPSPATLPVSALKQPGVEGLCSKEQKRVWFADGILPNGEVADTTKLSSGSKRCSEDFS
PLSPDVPMTVNTVDHSHSTTVEKPNNETGDITRNEIIQSPISQVPSVEKLSMNTGNEGLP
TSGSFTLDDDVFAETEEPSSPTGVLVNSNLPIASISDYRLLCDINKYVCNKISLLPNDED
SLPPLLVASGEKGSVPVVEEHPSHEQIILLLEGEGFHPVTFVLNANLLVNVKFIFYSSDK
YWYFSTNGLHGLGQAEIIILLLCLPNEDTIPKDIFRLFITIYKDALKGKYIENLDNITFT
ESFLSSKDHGGFLFITPTFQKLDDLSLPSNPFLCGILIQKLEIPWAKVFPMRLMLRLGAE
YKAYPAPLTSIRGRKPLFGEIGHTIMNLLVDLRNYQYTLHNIDQLLIHMEMGKSCIKIPR
KKYSDVMKVLNSSNEHVISIGASFSTEADSHLVCIQNDGIYETQANSATGHPRKVTGASF
VVFNGALKTSSGFLAKSSIVEDGLMVQITPETMNGLRLALREQKDFKITCGKVDAVDLRE
YVDICWVDAEEKGNKGVISSVDGISLQGFPSEKIKLEADFETDEKIVKCTEVFYFLKDQD
LSILSTSYQFAKEIAMACSAALCPHLKTLKSNGMNKIGLRVSIDTDMVEFQAGSEGQLLP
QHYLNDLDSALIPVIHGGTSNSSLPLEIELVFFIIEHLF
>P0C0T4
MPPKQQLSKAAKAAAALAGGKKSKKKWSKKSMKDRAQHAVILDQEKYDRILKEVPTYRYV
SVSVLVDRLKIGGSLARIALRHLEKEGIIKPISKHSKQAIYTRAAASE
>P70583
MPLLCALLRSRLQPSLLRSLTLTSAQSLSCGGSRGVRTWSARTGPGACADPAVSVSKRAR
AEDDASLRFVRLSEHATAPTRGSARAAGYDLYSAYDYTIPSMEKALVKTDIQIAVPSGCY
GRVAPRSGLAVKHFIDVGAGVIDEDYRGNVGVVLFNFGKEKFEVKKGDRIAQLICERILY

PDLEEVQTLDNTERGSGGFGSTGKN
>P25007
MVSFCATLIRQFRHRSAAAFQIAESAILANKSITLASSACSVNRGQLQFGIQIVREYSKA
SKMSTLPRVFFDMTADNEPLGRIVMELRSDVVPKTAENFRALCTGEKGFGYKGSIFHRVI
PNFMCQGGDFTNHNGTGGKSIYGNKFPDENFELKHTGSGILSMANAGANTNGSQFFICTV
KTAWLDNKHVVFGEVVEGLDVVKKIESYGSQSGKTSKKIIVANSGSL
>P91124
MAAQFYERNTSGMNADRFMTRLTDESTVNTMQRHYWTARQFIRTKLGKKEDEHLEASDNE
LDTCLNLYRSVHGTSFQLLNNVDNYANFLLDETLVQNVLGKYLKEKGKIDKTEAVGRILI
AVGRSLLFSSHRLNAARIGVSTFYNKLSVFVERAIGDCSQTIEAVQMCRTEYRGSLLWMK
KTSEELDPEVDGSMEKFREAQTTVKSNKERLDRLKTDTLQKVDLLSASRSNLLSYVLTHY
QNELYEYYSKTSRAFETLAENINCYNNYDFEILSHLATGTKPERERKSEKEESAKTSQPR
GNEEELKNLLFGRESPQFGEEEVQDESRSQCDSPLIEDVDDERRKTGDLLDLESAASIAF
PIGPLATLFDTSSFVPPILPPPKPNAVSDDILSLFDGNKANSSGKEASATTMDWQSLIDG
FDRENEDNLL
>P70444
MDSEVSNGSGLGAEHITDLLVFGFLQSSGCTRQELEVLGRELPVQAYWEADLEDELQTDG
SQASRSFNQGRIEPDSESQEEIIHNIARHLAQIGDEMDHNIQPTLVRQLAAQFMNGSLSE
EDKRNCLAKALDEVKTAFPRDMENDKAMLIMTMLLAKKVASHAPSLLRDVFHTTVNFINQ
NLFSYVRNLVRNEMD
>P34611
METQLSVQLGLDSLLTDFGLESVMNKQQQLFANMGLSDIGAPTPSTAIPVPNAHLHPSMV
AGSDPSNPVVGFGFGSPSSTTSSSPPLSNSPTIEQQQHAQLTAMMQGIMSNNNVAVSNGS
GVQVASVPAVHCSGCKSNETATSFCQDCNANLCDNCTMAHKFMHCFADHRVVSLTTPGTG
SSSSSTSSSSSASSTSSHQVPSLGGKQSPDSMMLGSGKRSVLCLQHRASELVFFCVSCNL
AICRDCTVSDHPSGTHQYELIADVADKQMLKMEQLIADARSKHADMLDMFKQVDNKQQVL
TASLHNAHAQLEETVSNLINVIQDQKKTLAKDIDNAFAAKQIQLTMVDKRIQSMADKLSQ
TIEFSRRLMSFASPAEVMVFKQLLDTRLQLFLGFNPDTSGVLMTPCEIEYLGAAGLFNNS
ASTVSQLLGSVHGGSPINNAPAANDFLMPQAGLAPIGRAQSRIIPIEQNQLARSPPHHIA
GSLPMNAYSDSNLLRPNKDFGGSSQSLGPFNPLGASVPGAAADPFSSQYDKWSLGVEPSV
GGLLEGGNVDEEKFQTLFPPSRSQIKRQKMIYHCKFGEFGVMEGQFTEPSGVAVNGQGDI
VVADTNNHRIQVFDKEGRFKFQFGECGKRDGQLLYPNRVAVNRTTGDFVVTERSPTHQIQ
VYNQYGQFLRKFGANILQHPRGVCVDSKGRIIVVECKVMRVIIFDMFGNILQKFSCSRYL
EFPNGVCTNDKNEILISDNRAHCIKVFSYEGQYLRQIGGEGVTNYPIGVGINSLGEVVVA
DNHNNFNLTVFSQDGTMIGALESRVKHAQCFDVALVDDGSVVLASKDYRLYLYRFLPATS
GQSTSSASSQI
>P83326
MAFSYCSSSLFVSLLLVILFISPLSQRPSVKAENHLISEICPKTRNPSLCLQALESDPRS
ASKDLKGLGQFSIDIAQASAKQTSKIIASLTNQATDPKLKGRYETCSENYADAIDSLGQA
KQFLTSGDYNSLNIYASAAFDGAGTCEDSFEGPPNIPTQLHQADLKLEDLCDIVLVISNL
LPGSK
>P09367
MAAQESLHVKTPLRDSMALSKVAGTSVFLKMDSSQPSGSFKIRGIGHLCKMKAKQGCKHF
VCSSVVQIWGSRMRGRSHSGDEQPHVRSQALLPDTPSPLTAGNAGMATAYAARRLGLPAT

IVVPSTTPALTIERLKNEGATVEVVGEMLDEAIQLAKALEKNNPGWVYISPFDDPLIWEG
HTSLVKELKETLSAKPGAIVLSVGGGGLLCGVVQGLREVGWEDVPIIAMETFGAHSFHAA
VKEGKLVTLPKITSVAKALGVNTVGAQTLKLFYEHPIFSEVISDQEAVTAIEKFVDDEKI
LVEPACGAALAAVYSGVVCRLQAEGRLQTPLASLVVIVCGGSNISLAQLQALKAQLGLNE
LLK
>Q09874
MDNVQEHDPDTQEHNNETQNHKQEDHSNSYQTRTIPFIEPLSREYQKRIILCQTVNGPCP
IIALSNALILKSNVDRPFELPKKRYITPDELTEYLVEFAKAYGLCKNQQSLQDKLTSMHF
GQQLNPCLYDIEKFEYGHEIFCTFGVRLVHGWILSDDMGLSDEDLSYLRKLEYYEKVADT
FAERRSLLEMQEPLTEQQQDFLNNSTCVDKVMENRYTMQFLTNAGLKKILELVGPGEIVV
VFRSSHFSTMYSNPDSFAQFTLVTDSGYARTGEDVVWETFDSQTVETGNGELCAANFIPA
VYVLNQRKEEKKKRAKDDEQYAKRLAKEEEERGKKETPKKASNTPRRNKSNTQKSRKQSE
NCLIS
>Q19203
MDDDIFKEARKERDDFEELMNACDLAKMSVKNNEMVHGLETFGINGESSENGNKEKPKEI
MKVVAPTVEAYVGSSSAQTPTKSSGGALDGSDQQEVRQDGTSVQKDDNGFVFYKCRFCGL
TFNFMNTLRAHERIHDVSQPYVCGKCGDSFEFACQLEYHAAQHSEIDGYKCECGRTFFSY
TEMLYHKHTDDPLELIGAPETTTIKVSKKRVLPVSEQDLPQPAFVTEGYEPKHPLRVYND
VRSKPYICEYCSKSYSDSRGLAYHMYSHRGEKYFNPRASRYMMGREGVGYTDSRSYYLFP
RTSGYVTPRF
>P17079
MPPKFDPNEVKYLYLRAVGGEVGASAALAPKIGPLGLSPKKVGEDIAKATKEFKGIKVTV
QLKIQNRQAAASVVPSASSLVITALKEPPRDRKKDKNVKHSGNIQLDEIIEIARQMRDKS
FGRTLASVTKEILGTAQSVGCRVDFKNPHDIIEGINAGEIEIPEN
>Q8IUQ0
MGPVSLLPKYQKLNTWNGDLAKMTHLQAGLSPETIEKARLELNENPDVLHQDIQQVRDMI
ITRPDIGFLRTDDAFILRFLRARKFHQADAFRLLAQYFQYRQLNLDMFKNFKADDPGIKR
ALIDGFPGVLENRDHYGRKILLLFAANWDQSRNSFTDILRAILLSLEVLIEDPELQINGF
ILIIDWSNFSFKQASKLTPSILKLAIEGLQDSFPARFGGVHFVNQPWYIHALYTLIKPFL
KDKTRKRIFLHGNNLNSLHQLIHPEFLPSEFGGTLPPYDMGTWARTLLGPDYSDENDYTH
TSYNAMHVKHTSSNLERECSPKLMKRSQSVVEAGTLKHEEKGENENTQPLLALD
>P46948
MSRLEIYSPEGLRLDGRRWNELRRFESSINTHPHAADGSSYMEQGNNKIITLVKGPKEPR
LKSQMDTSKALLNVSVNITKFSKFERSKSSHKNERRVLEIQTSLVRMFEKNVMLNIYPRT
VIDIEIHVLEQDGGIMGSLINGITLALIDAGISMFDYISGISVGLYDTTPLLDTNSLEEN
AMSTVTLGVVGKSEKLSLLLVEDKIPLDRLENVLAIGIAGAHRVRDLMDEELRKHAQKRV
SNASAR
>P40106
MGLTTKPLSLKVNAALFDVDGTIIISQPAIAAFWRDFGKDKPYFDAEHVIQVSHGWRTFD
AIAKFAPDFANEEYVNKLEAEIPVKYGEKSIEVPGAVKLCNALNALPKEKWAVATSGTRD
MAQKWFEHLGIRRPKYFITANDVKQGKPHPEPYLKGRNGLGYPINEQDPSKSKVVVFEDA
PAGIAAGKAAGCKIIGIATTFDLDFLKEKGCDIIVKNHESIRVGGYNAETDEVEFIFDDY
LYAKDDLLKW
>Q04410

MFRIAKNLVRTFEQSVQDTLALSQDSSNLDAFFQSIPPNLLSAQLESPVDAVSEGVKHTN
VNETLSGLRIVWVDEMQFQLQSFFDYIVGFNDDPVPVVSNQHGFSYPDYRRITSIFNEHC
GRTLKVNIWSAKGGTFRDEYISIISKESDDLDDVSLNHDERRPSSGEAHQFQALGFKVQW
TPLIASTFTYHILNVNIPDGPAQSAGLIPDEDYIIGCQDGLLATGGETLLQDIVRSRANY
DLVLYVYNKVSDCVRPITVHIGPDGRLGCNVGYGFLHRIPTVKHCPQQAQQQGQDDNPVP
VPVPVESETAFVPSAFTAPPVPTKKKSKNKKGTQPLAMDDYFNEGRDKSSTAAKSAESDI
LAPPPQKQSSSD
>Q03771
MSELEATIRQAKEALAENNAKKALKILKPFKSSLKKENANNVILNEVFADAYLDNGQVEK
AYPILARACELDPEGQVGGPDKFFTMGQIMGGQDGVSIITRGIMNISNTGGEMLTNVQVE
KIVGGLLSVIEIWMTDLCMEPNAEEQCEELIQKAMELTEGKSPETWSTLGSIKISQQKFG
EAYEAFSQAWNFFELKKQEIGSGINENGDTTQKAGLQSEYVDLLQPLLSLTKMCLEVGAY
EVALKVIAAVRDIDEDNIEGYYLEGFTYYLMSKLEIFKLNNPEVSLRPENIYEFNQLIQE
VPLDLSHEPISQLIYDSRLALSFALQAGVNADSKDEIVQELLGGANALLQEIGGPLDPSE
LTQIKKGDLVNENEDLEELDIEEEYSD
>Q9P0M2
MSEEFEANTMDSLVDMPFATVDIQDDCGITDEPQINLKRSQENEWVKSDQVKKRKKKRKD
YQPNYFLSIPITNKEIIKGIKILQNAIIQQDERLAKAMVSDGSFHITLLVMQLLNEDEVN
IGIDALLELKPFIEELLQGKHLTLPFQGIGTFGNQVGFVKLAEGDHVNSLLEIAETANRT
FQEKGILVGESRSFKPHLTFMKLSKSPWLRKNGVKKIDPDLYEKFISHRFGEEILYRIDL
CSMLKKKQSNGYYHCESSIVIGEKNGGEPDDAELVRLSKRLVENAVLKAVQQYLEETQNK
NKPGEGSSVKTEAADQNGNDNENNRK
>O95997
MATLIYVDKENGEPGTRVVAKDGLKLGSGPSIKALDGRSQVSTPRFGKTFDAPPALPKAT
RKALGTVNRATEKSVKTKGPLKQKQPSFSAKKMTEKTVKAKSSVPASDDAYPEIEKFFPF
NPLDFESFDLPEEHQIAHLPLSGVPLMILDEERELEKLFQLGPPSPVKMPSPPWESNLLQ
SPSSILSTLDVELPPVCCDIDI
>P32357
MNTVPFTSAPIEVTIGIDQYSFNVKENQPFHGIKDIPIGHVHVIHFQHADNSSMRYGYWF
DCRMGNFYIQYDPKDGLYKMMEERDGAKFENIVHNFKERQMMVSYPKIDEDDTWYNLTEF
VQMDKIRKIVRKDENQFSYVDSSMTTVQENELLKSSLQKAGSKMEAKNEDDPAHSLNYTV
INFKSREAIRPGHEMEDFLDKSYYLNTVMLQGIFKNSSNYFGELQFAFLNAMFFGNYGSS
LQWHAMIELICSSATVPKHMLDKLDEILYYQIKTLPEQYSDILLNERVWNICLYSSFQKN
SLHNTEKIMENKYPELLGKDNEDDALIYGISDEERDDEDDEHNPTIVGGLYYQRP
>Q96PN6
MNTPKEEFQDWPIVRIAAHLPDLIVYGHFSPERPFMDYFDGVLMFVDISGFTAMTEKFSS
AMYMDRGAEQLVEILNYHISAIVEKVLIFGGDILKFAGDALLALWRVERKQLKNIITVVI
KCSLEIHGLFETQEWEEGLDIRVKIGLAAGHISMLVFGDETHSHFLVIGQAVDDVRLAQN
MAQMNDVILSPNCWQLCDRSMIEIESVPDQRAVKVNFLKPPPNFNFDEFFTKCTTFMHYY
PSGEHKNLLRLACTLKPDPELEMSLQKYVMESILKQIDNKQLQGYLSELRPVTIVFVNLM
FEDQDKAEEIGPAIQDAYMHITSVLKIFQGQINKVFMFDKGCSFLCVFGFPGEKVPDELT
HALECAMDIFDFCSQVHKIQTVSIGVASGIVFCGIVGHTVRHEYTVIGQKVNLAARMMMY
YPGIVTCDSVTYNGSNLPAYFFKELPKKVMKGVADSGPLYQYWGRTEKVMFGMACLICNR
KEDYPLLGRNKEINYFMYTMKKFLISNSSQVLMYEGLPGYGKSQILMKIEYLAQGKNHRI

IAISLNKISFHQTFYTIQMFMANVLGLDTCKHYKERQTNLRNKVMTLLDEKFYCLLNDIF
HVQFPISREISRMSTLKKQKQLEILFMKILKLIVKEERIIFIIDEAQFVDSTSWRFMEKL
IRTLPIFIIMSLCPFVNIPCAAARAVIKNRNTTYIVVGAVQPNDISNKICLDLNVSCISK
ELDSYLGEGSCGIPFYCEELLKNLEHHEVLVFQQTESEEKTNRTWNNLFKYSIKLTEKLN
MVTLHSDKESEEVCHLTSGVRLKNLSPPTSLKEISLIQLDSMRLSHQMLVRCAAIIGLTF
TTELLFEILPCWNMKMMIKTLATLVESNIFYCFRNGKELQKALKQNDPSFEVHYRSLSLK
PSEGMDHGEEEQLRELENEVIECHRIRFCNPMMQKTAYELWLKDQRKAMHLKCARFLEED
AHRCDHCRGRDFIPYHHFTVNIRLNALDMDAIKKMAMSHGFKTEEKLILSNSEIPETSAF
FPENRSPEEIREKILNFFDHVLTKMKTSDEDIIPLESCQCEEILEIVILPLAHHFLALGE
NDKALYYFLEIASAYLIFCDNYMAYMYLNEGQKLLKTLKKDKSWSQTFESATFYSLKGEV
CFNMGQIVLAKKMLRKALKLLNRIFPYNLISLFLHIHVEKNRHFHYVNRQAQESPPPGKK
RLAQLYRQTVCLSLLWRIYSYSYLFHCKYYAHLAVMMQMNTALETQNCFQIIKAYLDYSL
YHHLAGYKGVWFKYEVMAMEHIFNLPLKGEGIEIVAYVAETLVFNKLIMGHLDLAIELGS
RALQMWALLQNPNRHYQSLCRLSRCLLLNSRYPQLIQVLGRLWELSVTQEHIFSKAFFYF
VCLDILLYSGFVYRTFEECLEFIHQYENNRILKFHSGLLLGLYSSVAIWYARLQEWDNFY
KFSNRAKNLLPRRTMTLTYYDGISRYMEGQVLHLQKQIKEQSENAQASGEELLKNLENLV
AQNTTGPVFCPRLYHLMAYVCILMGDGQKCGLFLNTALRLSETQGNILEKCWLNMNKESW
YSTSELKEDQWLQTILSLPSWEKIVAGRVNIQDLQKNKFLMRANTVDNHF
>Q9H814
MALEVGDMEDGQLSDSDSDMTVAPSDRPLQLPKVLGGDSAMRAFQNTATACAPVSHYRAV
ESVDSSEESFSDSDDDSCLWKRKRQKCFNPPPKPEPFQFGQSSQKPPVAGGKKINNIWGA
VLQEQNQDAVATELGILGMEGTIDRSRQSETYNYLLAKKLRKESQEHTKDLDKELDEYMH
GGKKMGSKEEENGQGHLKRKRPVKDRLGNRPEMNYKGRYEITAEDSQEKVADEISFRLQE
PKKDLIARVVRIIGNKKAIELLMETAEVEQNGGLFIMNGSRRRTPGGVFLNLLKNTPSIS
EEQIKDIFYIENQKEYENKKAARKRRTQVLGKKMKQAIKSLNFQEDDDTSRETFASDTNE
ALASLDESQEGHAEAKLEAEEAIEVDHSHDLDIF
>P53396
MSAKAISEQTGKELLYKFICTTSAIQNRFKYARVTPDTDWARLLQDHPWLLSQNLVVKPD
QLIKRRGKLGLVGVNLTLDGVKSWLKPRLGQEATVGKATGFLKNFLIEPFVPHSQAEEFY
VCIYATREGDYVLFHHEGGVDVGDVDAKAQKLLVGVDEKLNPEDIKKHLLVHAPEDKKEI
LASFISGLFNFYEDLYFTYLEINPLVVTKDGVYVLDLAAKVDATADYICKVKWGDIEFPP
PFGREAYPEEAYIADLDAKSGASLKLTLLNPKGRIWTMVAGGGASVVYSDTICDLGGVNE
LANYGEYSGAPSEQQTYDYAKTILSLMTREKHPDGKILIIGGSIANFTNVAATFKGIVRA
IRDYQGPLKEHEVTIFVRRGGPNYQEGLRVMGEVGKTTGIPIHVFGTETHMTAIVGMALG
HRPIPNQPPTAAHTANFLLNASGSTSTPAPSRTASFSESRADEVAPAKKAKPAMPQDSVP
SPRSLQGKSTTLFSRHTKAIVWGMQTRAVQGMLDFDYVCSRDEPSVAAMVYPFTGDHKQK
FYWGHKEILIPVFKNMADAMRKHPEVDVLINFASLRSAYDSTMETMNYAQIRTIAIIAEG
IPEALTRKLIKKADQKGVTIIGPATVGGIKPGCFKIGNTGGMLDNILASKLYRPGSVAYV
SRSGGMSNELNNIISRTTDGVYEGVAIGGDRYPGSTFMDHVLRYQDTPGVKMIVVLGEIG
GTEEYKICRGIKEGRLTKPIVCWCIGTCATMFSSEVQFGHAGACANQASETAVAKNQALK
EAGVFVPRSFDELGEIIQSVYEDLVANGVIVPAQEVPPPTVPMDYSWARELGLIRKPASF
MTSICDERGQELIYAGMPITEVFKEEMGIGGVLGLLWFQKRLPKYSCQFIEMCLMVTADH
GPAVSGAHNTIICARAGKDLVSSLTSGLLTIGDRFGGALDAAAKMFSKAFDSGIIPMEFV
NKMKKEGKLIMGIGHRVKSINNPDMRVQILKDYVRQHFPATPLLDYALEVEKITTSKKPN

LILNVDGLIGVAFVDMLRNCGSFTREEADEYIDIGALNGIFVLGRSMGFIGHYLDQKRLK
QGLYRHPWDDISYVLPEHMSM
>O23979
MSQEDSTSAAAAQQPTSRPAPKLNERILSSLSRRGGGAHPWHDLEIGPGAPAVFNVVVEI
TKGSKVKYELDKKTGLIKVDRVLYSSVVYPHNYGFIPRTLCEDNDPMDVLVLMQEPVIPG
SFLRARAIGLMPMIDQGEKDDKIIAVCADDPEYRHYSTSVSLLPRLQEIKRLEDYKKNEN
KEVAVDAFLPATTAREAIQYSMDLYAQYILQSLRQ
>Q9UT60
MTSMSGASPVIHWGFLGAGSIAAVFAKDLVGVPERHKVQHEIVAVATRDSEHRASSFAKN
HCAPCKPKAYGSYEELVKDDKVDIVYISSTHPQHYEVVKLALLNDKAVLCEKPLTINYPE
ALELVELARARNLFFAEGFWIRFYPIVKAAKTLLHEDRVCGDHFRLFVDFSQDFRFRELP
SESRLRTVSLGAGVLLDMGVYPLTWSRLLLYDDPKNEKQEPTVSSNALTFEDHNGDIGDY
TTAVTLVFPKTESIAMLCTSMDRGKMSDDFLKLDGENGNQLFISGDCYRPQSIKLIRASG
ETEVFDFSFDDATGFFYEQDAVAECLLKNMKEAPEIPHEETLKMMQLTDQIRRQINVTYP
ADLRYTTA
>Q06338
MVQAIKLNDLKNRKRKNVEEENGSDESEIDISSTDSENEEEQNGEEEIVNIDFDFFGGNP
EVDFHALKNLLRQLFGPQESTRIQLSSLADLILGSPTTTIKTDGKESDPYCFLSFVDFKA
NHLSDYVKYLQKVDMRLSTFFKTMIDSGNKNCALVLSERLINMPPEVVPPLYKITLEDVA
TALGDDKHYDFYIIVTRKYEVNFDTDDDTDSGKRNKNKDERSKKRVKADEVDYFHEEDRF
FEKYAKIHFESEAKKGVISSYMILDHEGLVKSIDELETEISTW
>Q8K4G5
MPSLLGLKCLGKLCSSEIGKVPSPERASLRNSHRRLLIEDLSVPETPDPAHRRRGTVIHL
VYLYSAGCGPPELRFSSYDPSVAHPQDPHHSSEKPVIHCHKCGEPCKGEVLRVQTKHFHI
KCFTCKVCGCDLAQGGFFIKNGDYLCTLDYQRMYGTRCHGCGEFVEGEVVTALGKTYHPN
CFACTICKRPFPPGDRVTFNGRDCLCQLCAQPMSSSPKEASCSSNCAGCGRDIKNGQALL
ALDKQWHLGCFKCKSCGKVLTGEYISKDGSPYCEKDYQGLFGVKCEACHQFITGKVLEAG
DKHYHPSCARCSRCNQMFTEGEEMYLQGSTVWHPDCKQSTKTEEKLRPPNIPRSSSDFFY
PKSLIRRTGRSPALQLLSPPCLTNSNKNPRQPTRTSSESIYSRPGSSIPGSPGHTIYAKV
DNEILDYKDLAAIPKVKAIYDIERPDLITYEPFYTSGYEDKQERQSLGESPRTLSPTPSA
EGYQDVRDRMIHRSTSQGSINSPVYSRHSYTPTTSRSPQHFHRPELLSPGVHRWSPLRTS
SFSSTHSDSRPNPPFRHHFLPHVKGNEPSSGRNSPLPYRPDSRPLTPTYAQAPKHFHVPD
QGINIYRKPPIYKQHAALAAQSKASEDIIKFSKFPAAQAPDPNEIPKIETDHWPGPPSLA
AVGTDPRRRSSGREEDEEELLRRRQLQEEQLMKLNSGLGQLILKEEMEKESRERASLASR
YDSPLHSASHAPSSKTSSLPGYGKNGLHRPVSTDFAQYNSYGDISGGVRDYQTLPDGHMP
AVRMDRGVSMPNMLEPKIFPYEMLMVTNRGRNKILRDVDRTRLERHLAPEVFWEIFGMSI
QEFDKLPLWRRNDMKKKAKLF
>P25298
MSSSTTPDLLYPSADKVAEPSDNIHGDELRLRERIKDNPTNILSYFQLIQYLETQESYAK
VREVYEQFHNTFPFYSPAWTLQLKGELARDEFETVEKILAQCLSGKLENNDLSLWSTYLD
YIRRKNNLITGGQEARAVIVKAFQLVMQKCAIFEPKSSSFWNEYLNFLEQWKPFNKWEEQ
QRIDMLREFYKKMLCVPFDNLEKMWNRYTQWEQEINSLTARKFIGELSAEYMKARSLYQE
WLNVTNGLKRASPINLRTANKKNIPQPGTSDSNIQQLQIWLNWIKWERENKLMLSEDMLS
QRISYVYKQGIQYMIFSAEMWYDYSMYISENSDRQNILYTALLANPDSPSLTFKLSECYE

LDNDSESVSNCFDKCTQTLLSQYKKIASDVNSGEDNNTEYEQELLYKQREKLTFVFCVYM
NTMKRISGLSAARTVFGKCRKLKRILTHDVYVENAYLEFQNQNDYKTAFKVLELGLKYFQ
NDGVYINKYLDFLIFLNKDSQIKTLFETSVEKVQDLTQLKEIYKKMISYESKFGNLNNVY
SLEKRFFERFPQENLIEVFTSRYQIQNSNLIKKLELTYMYNEEEDSYFSSGNGDGHHGSY
NMSSSDRKRLMEETGNNGNFSNKKFKRDSELPTEVLDLLSVIPKRQYFNTNLLDAQKLVN
FLNDQVEIPTVESTKSG
>Q9P6M1
MIVLHHLKNSRSTRIVWMLEELKVPYEIKVYDRVDGRAPPAYTKLSPLGKSPIVVDDGVT
YIESAAILEHLVRKYGPSFKPSEEDVAELEKYELWMHFSEASLMPFIWASHVLDLSVNMT
PIFFRYIVRQFVNGIKSKYLSKETFLNLDYIDNHLASNEYFAGEQFTAADPQMCFPIFAA
QRDYLSQKPYKNIKRWMRVVSDRPACRIAAEKVEDNTLTLFSDVERYSHPPTPPPEQVRS
DE
>Q91572
MAFPLKDDLGRAKDCWGCPSDTPALSTCSNADIFRRINAMLDNSLDFTGVCTTPNTKGKC
EHLQDYQDTEGPAASRMLFSTSHEPLPRGLPDTNDLCLGLQSLSLTGWDRPWSTQDSEAG
GHSSTPTAAQSVFSMLNSPMGKPSPLGFLTFDPIGSDLMEKYPTPLLRSSRLDSRSILDS
RSSSPSDSDTSGFSSGSDHLSDLISSLRISPPLHFLPLGGGVSRDPLKLGIGSRLDQDHA
ALAAATVSPLGITKGWPSTSVWPSWDLLDSAEDPFSIEREARLHRQAAAVNEATCTWSGQ
LPPRNYKNPVYSCKVFLGGVPWDITETGLINTFRVFGALSVEWPGKDGKHPRCPPKGNMP
KGYVYLVFESEKSVRALLQACSQDLLSQDGLSEHYFKMSSRRMACKEVQVIPWVLADSNF
VRSPSQRLDPSKTVFVGALHGMLNAEALASIMNDLFGGVVYAGIDTDKHKYPIGSGRVTF
NNQRSYLKAVSAAFVEIKTAKFTKKVQIDPYLEDSVCQVCNAQPGPFFCRDQVCFKYFCR
SCWHWQHSMEILRHHRPLMRNQKSRDSS
>P00332
MTIPDKQLAAVFHTHGGPENVKFEEVPVAEPGQDEVLVNIKYTGVCHTDLHALQGDWPLP
AKMPLIGGHEGAGVVVKVGAGVTRLKIGDRVGVKWMNSSCGNCEYCMKAEETICPHIQLS
GYTVDGTFQHYCIANATHATIIPESVPLEVAAPIMCAGITCYRALKESKVGPGEWICIPG
AGGGLGHLAVQYAKAMAMRVVAIDTGDDKAELVKSFGAEVFLDFKKEADMIEAVKAATNG
GAHGTLVLSTSPKSYEQAAGFARPGSTMVTVSMPAGAKLGADIFWLTVKMLKICGSHVGN
RIDSIEALEYVSRGLVKPYYKVQPFSTLPDVYRLMHENKIAGRIVLDLSK
>P14735
MRYRLAWLLHPALPSTFRSVLGARLPPPERLCGFQKKTYSKMNNPAIKRIGNHITKSPED
KREYRGLELANGIKVLLMSDPTTDKSSAALDVHIGSLSDPPNIAGLSHFCEHMLFLGTKK
YPKENEYSQFLSEHAGSSNAFTSGEHTNYYFDVSHEHLEGALDRFAQFFLCPLFDESCKD
REVNAVDSEHEKNVMNDAWRLFQLEKATGNPKHPFSKFGTGNKYTLETRPNQEGIDVRQE
LLKFHSAYYSSNLMAVCVLGRESLDDLTNLVVKLFSEVENKNVPLPEFPEHPFQEEHLKQ
LYKIVPIKDIRNLYVTFPIPDLQKYYKSNPGHYLGHLIGHEGPGSLLSELKSKGWVNTLV
GGQKEGARGFMFFIINVDLTEEGLLHVEDIILHMFQYIQKLRAEGPQEWVFQECKDLNAV
AFRFKDKERPRGYTSKIAGILHYYPLEEVLTAEYLLEEFRPDLIEMVLDKLRPENVRVAI
VSKSFEGKTDRTEEWYGTQYKQEAIPDEVIKKWQNADLNGKFKLPTKNEFIPTNFEILPL
EKEATPYPALIKDTVMSKLWFKQDDKKKKPKACLNFEFFSPFAYVDPLHCNMAYLYLELL
KDSLNEYAYAAELAGLSYDLQNTIYGMYLSVKGYNDKQPILLKKIIEKMATFEIDEKRFE
IIKEAYMRSLNNFRAEQPHQHAMYYLRLLMTEVAWTKDELKEALDDVTLPRLKAFIPQLL
SRLHIEALLHGNITKQAALGIMQMVEDTLIEHAHTKPLLPSQLVRYREVQLPDRGWFVYQ

QRNEVHNNCGIEIYYQTDMQSTSENMFLELFCQIISEPCFNTLRTKEQLGYIVFSGPRRA
NGIQSLRFIIQSEKPPHYLESRVEAFLITMEKSIEDMTEEAFQKHIQALAIRRLDKPKKL
SAECAKYWGEIISQQYNFDRDNTEVAYLKTLTKEDIIKFYKEMLAVDAPRRHKVSVHVLA
REMDSCPVVGEFPCQNDINLSQAPALPQPEVIQNMTEFKRGLPLFPLVKPHINFMAAKL
>P70470
MCGNNMSAPMPAVVPAARKATAAVIFLHGLGDTGHGWAEAFAGIKSSHIKYICPHAPVMP
VTLNMSMMMPSWFDIIGLSPDSQEDESGIKQAAETVKALIDQEVKNGIPSNRIILGGFSQ
GGALSLYTALTTQQKLAGVTALSCWLPLRASFSQGPINSANRDISVLQCHGDCDPLVPLM
FGSLTVERLKGLVNPANVTFKVYEGMMHSSCQQEMMDVKYFIDKLLPPID
>P37254
MLSDTIDTKQQQQQLHVLFIDSYDSFTYNVVRLIEQQTDISPGVNAVHVTTVHSDTFQSM
DQLLPLLPLFDAIVVGPGPGNPNNGAQDMGIISELFENANGKLDEVPILGICLGFQAMCL
AQGADVSELNTIKHGQVYEMHLNDAARACGLFSGYPDTFKSTRYHSLHVNAEGIDTLLPL
CTTEDENGILLMSAQTKNKPWFGVQYHPESCCSELGGLLVSNFLKLSFINNVKTGRWEKK
KLNGEFSDILSRLDRTIDRDPIYKVKEKYPKGEDTTYVKQFEVSEDPKLTFEICNIIREE
KFVMSSSVISENTGEWSIIALPNSASQVFTHYGAMKKTTVHYWQDSEISYTLLKKCLDGQ
DSDLPGSLEVIHEDKSQFWITLGKFMENKIIDNHREIPFIGGLVGILGYEIGQYIACGRC
NDDENSLVPDAKLVFINNSIVINHKQGKLYCISLDNTFPVALEQSLRDSFVRKKNIKQSL
SWPKYLPEEIDFIITMPDKLDYAKAFKKCQDYMHKGDSYEMCLTTQTKVVPSAVIEPWRI
FQTLVQRNPAPFSSFFEFKDIIPRQDETPPVLCFLSTSPERFLKWDADTCELRPIKGTVK
KGPQMNLAKATRILKTPKEFGENLMILDLIRNDLYELVPDVRVEEFMSVQEYATVYQLVS
VVKAHGLTSASKKTRYSGIDVLKHSLPPGSMTGAPKKITVQLLQDKIESKLNKHVNGGAR
GVYSGVTGYWSVNSNGDWSVNIRCMYSYNGGTSWQLGAGGAITVLSTLDGELEEMYNKLE
SNLQIFM
>P33287
MVAQQQGKFTVGDYLAERLAQVGVRHHFVVPGDYNLILLDKLQAHPDLKEVGCANELNCS
LAAEGYARANGISACVVTYSVGALSAFNGTGSAYAENLPLVLISGSPNTNDPSQYHILHH
TLGHPDYTYQYEMAKKITCCAVAIPRAIDAPRLIDRALRAAILARKPCYIEIPTNLAGAT
CVRPGPISAITDPITSDKSALEAAAKCAAEYLDGKLKPVILVGPKAGRAGSEKELIEFAE
AMGCAVALQPAAKGMFPEDHKQFVGIFWGQVSSDAADAMVHWADAMICVGAVFNDYSTVG
WTAVPNIPLMTVDMDHVTFPGAHFSRVRMCEFLSHLATQVTFNDSTMIEYKRLKPDPPHV
HTAEREEPLSRKEISRQVQEMLTDKTSLFVDTGDSWFNGIQLKLPPGAKFEIEMQWGHIG
WSIPAAFGYALRHPDRHTIVLVGDGSFQVTAQEVSQMVRFKVPITIMLINNRGYTIEVEI
HDGSYNKIKNWDYAMLVEAFNSTDGHAKGLLANTAGELADAIKVAESHKEGPTLIECTID
QDDCSKELITWGHYVAAANARPPRNMSVQE
>Q9D552
METNSNNFGELQELKDMATLAKLLARAPFLESQYYFRNRAVDSFRKFENDAAVMIQSWFR
GCQVRAYMRHLNRVVTIIQKWWRSYLGRKFYQLVVEAAYYTMKMNLYNEMAVRIQRRWRG
FRIRKYCFNYYYLKEYLRAVSETNDAIREALEEFAEMKEREERKVLLEREEKQKDYQARK
MHYLLSTKQISGIYNSPFREHPDPWELRLQKAKPLGHQKYTAEKGKTSQSPSNWLACTSV
HSFPQSESLPPISRKRCQGPFRDINEVLEQRYKPLEPTLRVAEPINHLRLAREAFKQEER
MRNVQDKMFLPFSSYHKKEKYIPMIHSSSAYNSDSYGQKHFRSQDSKKWISDKDFQTVLP
SFQLFSKYGKLYSKAGEIV
>Q60575

MSGASVKVAVRVRPFNSRETSKESKCIIQMQGNSTSIINPKNPKEAPKSFSFDYSYWSHT
SPEDPCFASQNRVYNDIGKEMLLHAFEGYNVCIFAYGQTGAGKSYTMMGKQEESQAGIIP
QLCEELFEKINDNCNEEMSYSVEVSYMEIYCERVRDLLNPKNKGNLRVREHPLLGPYVED
LSKLAVTSYTDIADLMDAGNKARTVAATNMNETSSRSHAVFTIVFTQKKQDPETNLSTEK
VSKISLVDLAGSERADSTGAKGTRLKEGANINKSLTTLGKVISALAEVDNCTSKSKKKKK
TDFIPYRDSVLTWLLRENLGGNSRTAMVAALSPADINYDETLSTLRYADRAKQIKCNAVI
NEDPNAKLVRELKEEVTRLKDLLRAQGLGDIIDIDPLIDDYSGSGGKYLKDFQNNKHRYL
LASENQRPGNFSTASMGSLTSSPSSCSLNSQVGLTSVTSIQERIMSTPGGEEAIERLKES
EKIIAELNETWEEKLRKTEAIRMEREALLAEMGVAIREDGGTLGVFSPKKTPHLVNLNED
PLMSECLLYYIKDGITRVGQADAERRQDIVLSGAHIKEEHCLFRSERSNTGEVIVTLEPC
ERSETYVNGKRVAHPVQLRSGNRIIMGKNHVFRFNHPEQARAEREKTPSAETPSEPVDWT
FAQRELLEKQGIDMKQEMEKRLQEMEILYKKEKEEADLLLEQQRLDYESKLQALQRQVET
RSLAAETTEEEEEEEEVPWTQHEFELAQWAFRKWKSHQFTSLRDLLWGNAVYLKEANAIS
VELKKKVQFQFVLLTDTLYSPVPPELLPSEMEKTHEDRPFPRTVVAVEVQDLKNGATHYW
SLDKLKQRLDLMREMYDRAGEVASSAQDDSETTMTGSDPFYDRFHWFKLVGSSPIFHGCV
NERLADRTPSPTFSTADSDITELADEQQDAMEDFDDEAFVDDTGSDAGTEEGSELFSDGH
DPFYDRSPWFILVGRAFVYLSNLLYPVPLIHRVAIVSEKGEVRGFLRVAVQAIAADEEAP
DYGSGIRQSGTAKISFDNEYFNQSDFSSAAMTRSGLSLEELRIVEGQGQSSEVISPPEEV
NRMNDLDLKSGTLLDGKMVMEGFSEEIGNHLKLGSAFTFRVTVLQASGILPEYADIFCQF
NFLHRHDEAFSTEPLKNNGRGSPLGFYHVQNIAVEVTESFVDYIKTKPIVFEVFGHYQQH
PLHLQGQDLNSPPQPSRRFFPPPMPLSKPVPATKLNTMNKTTLGQSMSKYDLLVWFEISE
LEPTGEYIPAVVDHTAGLPCQGTFLLHQGIQRRITVTIIHEKGSELHWKDVRELVVGRIR
NKPEVDEAAVDAVLSLNIISAKSLKAAHSSSRTFYRFEAVWDSSLHNSLLLNRVTPYGEK
IYMTLSAYLELDHCIQPAVITKDVCMVFYSRDAKISPPRSLRNLFGSGYSKSPDSNRVTG
IYELSLCKMADTGSPGMQRRRRKVLDTSVAYVRGEENLAGWRPRGDSLILEHQWELEKLE
LLHEVEKTRHFLLLRERLGDSVPKSLSDSLSPSLSSGTLSTSTSISSQISTTTFESAITP
SESSGYDSADVESLVDREKELATKCLQLLTHTFNREFSQVHGSISDCKLSDISPIGRDPS
VSSFSSSTLTPSSTCPSLVDSRSSSMDQKTPEANSRASSPCQEFEQFQIVPTVETPYLAR
AGKNEFLNLVPDIEEVRAGSVVSKKGYLHFKEPLSSNWAKHFVVVRRPYVFIYNSDKDPV
ERGIINLSTAQVEYSEDQQAMVKTPNTFAVCTKHRGVLLQALNDKDMNDWLYAFNPLLAG
TIRSKLSRRCPSQPKY

>P52302

MSLKLMAFVCALLLLCTLTHVLSAATTVRPYKFGFTIDEQQHRAEKRDERGIIMGEFGFI
TADGIYHVTVYATDEEGKFRIISMKSYPYAGPVGSKSVPVTTTPKVLLPAAPVALPKYNF
NSEACSGCFLKKSPPKTEIRTLSQPLAPVQPGKPDGSSDIGLNVQLPFRESIAQTVASRL
GLLQDTLQTSNTNTNAPNTKTIELGLNIAMSTYYTTKNAVTGHVSTQTSNSQTPSANTKI
DYNVGVVEKASPPVYRPLNIRLNEDVMRQAITYGNTIPGHVPLPNQPLVETSLLPASQVK
IFALDGNAKVPLASNIQSVAQHPNAGLNAKVPLASNIQSVAQHPNAGLNAKVPLASNIQS
VAQHPNAGLKNINRSGVSSAKTLANTKTRPPHTFNPHQTPLLSSATAPGISGVTANTPTG
NVPSNGGGIAAGKAPGNPQAGGSGGIIGAGAPGGRKVSAGGIGSGSAIGGVSGGSKASGN
GGAIGSGSAIGGGATGSKASGFGFGSNIGGGVSGSKPSGFGSESKIGGPDSGSKALGFGS
GSKIGGGITGTKASGFGGEIGSGRGSASSATGDLYKFKYILDYNGHEETGGRNGDKQGSY
FAIGEDAVQRTIEYIANEFGFQPHVSWRKLDAKEALPEENSLKHYEFKWFNQE

>Q99741

MPQTRSQAQATISFPKRKLSRALNKAKNSSDAKLEPTNVQTVTCSPRVKALPLSPRKRLG
DDNLCNTPHLPPCSPPKQGKKENGPPHSHTLKGRRLVFDNQLTIKSPSKRELAKVHQNKI
LSSVRKSQEITTNSEQRCPLKKESACVRLFKQEGTCYQQAKLVLNTAVPDRLPAREREMD
VIRNFLREHICGKKAGSLYLSGAPGTGKTACLSRILQDLKKELKGFKTIMLNCMSLRTAQ
AVFPAIAQEICQEEVSRPAGKDMMRKLEKHMTAEKGPMIVLVLDEMDQLDSKGQDVLYTL
FEWPWLSNSHLVLIGIANTLDLTDRILPRLQAREKCKPQLLNFPPYTRNQIVTILQDRLN
QVSRDQVLDNAAVQFCARKVSAVSGDVRKALDVCRRAIEIVESDVKSQTILKPLSECKSP
SEPLIPKRVGLIHISQVISEVDGNRMTLSQEGAQDSFPLQQKILVCSLMLLIRQLKIKEV
TLGKLYEAYSKVCRKQQVAAVDQSECLSLSGLLEARGILGLKRNKETRLTKVFFKIEEKE
IEHALKDKALIGNILATGLP
>Q9USR6
MKINQNTTVDCGNLILVPYQKCHVLKYHNWMKNEELQELTCSEPLTLDEEYQMQASWSTD
EDKLTFIVLLNENDEAKKPSILDHVKAHSVESMIGDVNMFLTEEYADGIEEFDDSPSDAN
ATNATKESEVHIVGELELMIAEPQNRRKGYGTKIVDAFLHYVESSGIAKNKQILKYRVKV
GSQNKPSIRLFKKLGFSQVKYNAYFDHVELELMRTS
>Q09777
MPALHNPSSPPPSYEAVTSYRNGNSIDSGDKRQQCSRLMKITSNGRPYSKDFLELFSTMV
ISTNFSRNRYRFSYVENSCTLLQLLSTLENLQLSQVNRIKSRCGSKVLKSTTKFTIPKTA
AKCLCNTFLNARLLQIVNNPSARKFSNEKCLLQLTRKGYSVVSEFLQHNGHNSQAELYAS
KWNHSAPVIVSISRFSSTDQILKDSSFCEMLLVRMLGSVHEIGKSKNPLLVPVYSVSSPS
PKDSLSKVTKYQMFGIDIAEWLMCNTMLLDWSEMETVASDLLIHSYIAYENNSETPLKFS
YAKGVSYFLTGKGIATLGWTKNVSNNKLINKINEVEKGSTNKEILETILRKPNLQTYFFE
FLKKNFCDENQRFYSEVCEFNDYFSHANETNDHEAIRESFAHACGIYNCFLSSNAPNAVN
LPSDLYEKITNHMALAMEVEPLNEWLQLIHILLLEAQTAVLDLMAGDSLLKFLELNGGLG
I
>Q9BZM1
MALLSRPALTLLLLLMAAVVRCQEQAQTTDWRATLKTIRNGVHKIDTYLNAALDLLGGED
GLCQYKCSDGSKPFPRYGYKPSPPNGCGSPLFGVHLNIGIPSLTKCCNQHDRCYETCGKS
KNDCDEEFQYCLSKICRDVQKTLGLTQHVQACETTVELLFDSVIHLGCKPYLDSQRAACR
CHYEEKTDL
>Q05791
MSSQEYTTFIDIPVTRAQVEHCSYSFWSSLYPKYVPKSIVLKSLPKKFIQYLEQDGIKLP
QEENSRSVYTEEIIRNEDNDYSDWEDDEDTATEFVQEVEPLIDFPELHQKLKDALNELGA
VAPKLNWSAPRDATWILPNNTMKCNEVNELYLLLNASNYIMHDLQRAFKGCVDGDDIKGL
KFDLVLRQWCDMNPALEFRVFVKNAHIVGATQRDLNYYDYLDELSDTFKDLIDEIVHDVV
LPKFPDKSFVLDVYIPRPFNKIFIVDINPFARKTDSLLFSWNEIAAIAPPKNDVEDYELR
LVTRHNTGRFASKEHSENHVPQDLVEASLNPEAIRELTQKWKELLSQQAKEESSDSENET

>P15330
MFPNQNNGAAPGQGPAVDGQQSLNYNGLPAQQQQQLAQSTKNVRKKPYVKITEQPAGKAL
RFRYECEGRSAGSIPGVNSTPENKTYPTIEIVGYKGRAVVVVSCVTKDTPYRPHPHNLVG
KEGCKKGVCTLEINSETMRAVFSNLGIQCVKKKDIEAALKAREEIRVDPFKTGFSHRFQP
SSIDLNSVRLCFQVFMESEQKGRFTSPLPPVVSEPIFDKKAMSDLVICRLCSCSATVFGN
TQIILLCEKVAKEDISVRFFEEKNGQSVWEAFGDFQHTDVHKQTAITFKTPRYHTLDITE

PAKVFIQLRRPSDGVTSEALPFEYVPMDSGKHTFWNLHRHLKRKPDEDLFQQILRLDAKR
EVQPPTIEVIDLDTPKIDVQREIPSEMEFNHEESQQSEPALEQEQSVQQEQYTQEQSLQQ
EQYTQEQSLQQEQYLQQLEQQQSFQLEEPMQQDQELPAQQSFDQAIDHLPDHTSDHIPED
MEAADAHAEAEAHRLRSEQEKEIDTIIDEKVRELEQLDLGQQLEPRPLTANDKITEWMKS
SEIEQQVHEPSPTAEADVLDSALEISKADKTLDELLETVAELDEIYTDFKVQRDTYKNTI
QNELAGLQGRAPLQVEDSFDDAATYTSLQIAFKNPVLIPMDDIMPPTPPMSQCAPEDAHQ
HYDPVEVNSQARKPETPMRPVPPVPPAILTIQYPPEEDKLPPLPPKRIRKQDSNAENRSI
EANTVQTKPSTGESPLNKRLPPAPKNPNFNTLPRQKKPGFFSKLFSRRKSKPDLAQGQEN
SSILDSKANSREPSIGHFNMQDPMRASLRSSKSAAPFISNPAPAKSSPVKAKKPGSKLTK
PVGRSVSSVSGKRPAYLNADVVHIPLKGDSVNSLPQQQRTEGYSQSSTISVGAGLDRRTA
SALQLADIPISEGGMELVAIADRQSLHNLVSSIEGHFNVQLDPNLDLTEAEHFALYTSIP
PLAAASEFDETSAYYAPVDAGEILTPDEVAKRLAAANGI
>O59672
MEQVIQNNCSEVDPHVLSYCTGYANDAIKDVDEASDHTISLIRNLLLDAGAREEAVQLIQ
NELEKQFKEREEHLDTLQTNGLVRLKETIRMDSQSEISSTMGLVGEKMELDAVKGRKVES
RVDRRKLEKAERKIRAKWEKRTIKAEYESSKLVQPEQKSYEEFYMAVNPLDLSGSNQGKS
KDIKIDGIDLAFAGHRILTGASLTLAQGRRYGLTGRNGIGKSTLLRALSRREIAIPTHIT
ILHVEQEMTGDDTPALQSVLDADVWRKYLIQDQEKITNRLSTIEKELEELSKDQTADQAI
SRRLERERDELDLRLLDIQNKLSEMDSDRAESRAATILAGLGFTQEMQSHATKTFSGGWR
MRLSLARALFCQPDLLLLDEPSNMLDVPSIAFLSEYLQTYKNIVLVVSHDRSFLNEVATD
IIHQHSERLDYYKGNFSQFYATREERCKNQLREYEKQMEYRKHLQSFIDKFRYNAAKSSE
AQSRIKKLEKLPILEKPQTEEEVEFEFPPVEKISPPILQMSDVNFEYVPGHPILKHVDID
VQMDSRIGVVGPNGAGKSTMLKLLIEQLHPTSGIVSRHPRLRIAYFAQHHVDTLDLNLNA
LSFLAKTFPGKGEEEYRRHLGAFGVSGPLALQKMITLSGGQKSRVAFACLGLQNPHILIL
DEPTNHLDMESMDALTRAVKRFQGGVILVSHDVDFLDKTCTSIWQCDHNVVSKFDGTISQ
YKKFCLSQQPTMTIKT
>O42647
MDFNKIGDENVVFQIFKLEMLIMSEFPSFIEEKDKLLCLNVLANLLGGIFENEDKIIPNY
KEAVIAALFERLLLSSNQKSTSKSNQMIKLLNNSLAHNNGLPSQRCNHDSKLVYMSLVQN
NGLPGQRSNYVCCPLCMEQHCIELLTYQLFAKWRNFPRIDFSPVLQGNRGPRFFYTCFLC
NSTFLASA
>Q10331
MHQNALNNATLNVLGHKKNGSSSIQELIEMDEEEAEMNQNVVCIGPNETAVSVELGDEYE
KFNIITSLKKDNLFSKDGLLYAYYELCQEKFEKCLKEDDEEWIELWDLESRTWDLIQRLY
SFRLSEQQGHIQSHAFSSRAVLEEEYYSQNPEAFENNIVFNWARDNSSDPPSIEIRGNRW
FYTREDIKMKNRGGSRFSSNISGTIVSNLDPDADIRDDKRLDERDDNFERQFFHTAFCLF
RSGSFEELLELCRRTGNHWRSASLQGILEYRDNLIDDVLQSETSGNKRKELLRRSCLALT
KNKRIDSYERALYGALCGDLNSVLDVCTTWEDAMWAYYNSMTQYNLDVYLSSKAPQTETQ
LPPVDSGLGLTPELIFNSLSNSSIASIQEEASHPLIKLQTHIICNKISEILSSAHIQLEA
IRTGNAPESGDLVTPPLLRILTHIILFLKISGLAVDEYTSDSIIQAYIELLASAKKVNLV
PLYIQYLSNQVQYEAYSRFLILVDEESARSEQLQLAKKYSLDINHAALLAVEYVYDEVVS
VSPEEVHTVYLKSIEEPVEPSYKKLICTLEWLLITSQTDELLRFANLVYRFFLSIGELNS
AYDLYTHIPSDALNTLSSSDGEPENDSKFRDAYELMNYRALCRALKFYQEWEELSKQEVF
EDSAVTKASTSYKVWKKKLNFASRKCVKSFTDLLQANWLHPSTLELKPDDDPLLYKTLMT

IRNLYVPELILGLHNVYFSLEDYDSCFALANEVASEDLKLYHCLIKSGRLVEYVSYLGKA
GECSLSTPNGLFSL
>P53808
MAGAACCFSDEQFREACAELQKPALTGADWQLLVEASGITIYRLLDQPSGLYEYKVFGVL
EGCSPALLTDVYMDLDYRKQWDQYVKELYEKESDEQMVAYWEVKYPFPLSNRDYVYTRQR
RDLDVDRRKIYVVLAQSISAPQFPEKSGVIRVKQYKQSLAIESDGKKGSRVFMYYFDNPG
GQIPSWLINWAAKNGVPNFLKDMVKACQNYHKKT
>Q5XPI4
MASKGAGMSFSRKSYRLTSDAEKSRVTGIVQEKLLNDYLNRIFSSSEHAPPAATSRKPLN
FQNLPEHLDQLLQVDNEEEESQGQVEGRLGPSTVVLDHTGGFEGLLLVDDDLLGVIGHSN
FGTIRSTTCVYKGKWLYEVLISSQGLMQIGWCTISCRFNQEEGVGDTHNSYAYDGNRVRK
WNVTTTNYGKAWAAGDIVSCLIDLDDGTLSFCLNGVSLGTAFENLSRGLGMAYFPAISLS
FKESVAFNFGSRPLRYPVAGYRPLQDPPSADLVRAQRLLGCFRAVLSVELDPVEGRLLDK
ESSKWRLRGQPTVLLTLAHIFHHFAPLLRKVYLVEAVLMSFLLGIVEKGTPTQAQSVVHQ
VLDLLWLFMEDYEVQDCLKQLMMSLLRLYRFSPIVPDLGLQIHYLRLTIAILRHEKSRKF
LLSNVLFDVLRSVVFFYIKSPLRVEEAGLQELIPTTWWPHCSSREGKESTEMKEETAEER
LRRRAYERGCQRLRKRIEVVEELQVQILKLLLDNKDDNGGEASRYIFLTKFRKFLQENAS
GRGNMPMLCPPEYMVCFLHRLISALRYYWDEYKASNPHASFSEEAYIPPQVFYNGKVDYF
DLQRLGGLLSHLRKTLKDDLASKANIVIDPLELQSTAMDDLDEDEEPAPAMAQRPMQALA
VGGPLPLPRPGWLSSPTLGRANRFLSTAAVSLMTPRRPLSTSEKVKVRTLSVEQRTREDI
EGSHWNEGLLLGRPPEEPEQPLTENSLLEVLDGAVMMYNLSVHQQLGKMVGVSDDVNEYA
MALRDTEDKLRRCPKRRKDILAELTKSQKVFSEKLDHLSRRLAWVHATVYSQEKMLDIYW
LLRVCLRTIEHGDRTGSLFAFMPEFYLSVAINSYSALKNYFGPVHSMEELPGYEETLTRL
AAILAKHFADARIVGTDIRDSLMQALASYVCYPHSLRAVERIPEEQRIAMVRNLLAPYEQ
RPWAQTNWILVRLWRGCGFGYRYTRLPHLLKTKLEDANLPSLQKPCPSTLLQQHMADLLQ
QGPDVAPSFLNSVLNQLNWAFSEFIGMIQEIQQAAERLERNFVDSRQLKVCATCFDLSVS
LLRVLEMTITLVPEIFLDWTRPTSEMLLRRLAQLLNQVLNRVTAERNLFDRVVTLRLPGL
ESVDHYPILVAVTGILVQLLVRGPASEREQATSVLLADPCFQLRSICYLLGQPEPPAPGT
ALPAPDRKRFSLQSYADYISADELAQVEQMLAHLTSASAQAAAASLPTSEEDLCPICYAH
PISAVFQPCGHKSCKACINQHLMNNKDCFFCKTTIVSVEDWEKGANTSTTSSAA
>O74461
MERLATRSSHDDPYSRSSLPTSNAINSNHESNGSTFSYVQSLRRAKATVWSDIGRVAPLH
SSPSIKSSSQNGKSSSKGLGGMRSRVFSSQHHGVYHTRPASLHSRTMAPQHTILTPRLSA
TEGKDDDEDELVISTSNTAPTYISMIESSRASSTHSGTAPSIMGMSIHSRADSRAETTQS
DGFESRSGSPTHDIQSYLVNRRSSSSESSDEDSAEEGMKRLVITNMGDNDEFDSD
>P38011
MASNEVLVLRGTLEGHNGWVTSLATSAGQPNLLLSASRDKTLISWKLTGDDQKFGVPVRS
FKGHSHIVQDCTLTADGAYALSASWDKTLRLWDVATGETYQRFVGHKSDVMSVDIDKKAS
MIISGSRDKTIKVWTIKGQCLATLLGHNDWVSQVRVVPNEKADDDSVTIISAGNDKMVKA
WNLNQFQIEADFIGHNSNINTLTASPDGTLIASAGKDGEIMLWNLAAKKAMYTLSAQDEV
FSLAFSPNRYWLAAATATGIKVFSLDPQYLVDDLRPEFAGYSKAAEPHAVSLAWSADGQT
LFAGYTDNVIRVWQVMTAN
>Q80VL0
MSRRRISCKDLGHADCQGWLYKKKEKGTFLSNKWKKFWVVLKGSSLYWYGNQLAEKADGF

VNLPDFTVERASECKKKNAFKINHPQIKTFYFAAENLQEMNMWLNKLGFAVTHKESTTKD
EECYSESEQEDPETAVEAPPPPSASATSSPVAARRASSSSPKRRETSCSFSSLENTVKAP
SRFSSSGSKERQSWLDIVNSSPATEDVGHPLSFAVQVHTLASSEASSCRVSENSSTTPES
GCLNSLSSDDTSSLNNSQDHLTVPDRASGSRMTDRDEIKSSEDDEMEKLYKSLEQASLSP
LGDRRPSTKKELRKSFVKRCKNPSINEKLHKIRTLNSTLKCKEHDLAMINQLLDDPKLTA
RKYREWKVMNTLLIQDIYQQQVPQDPEVTPQEIMNPTSSDCVENSL
>P54105
MSFLKSFPPPGPAEGLLRQQPDTEAVLNGKGLGTGTLYIAESRLSWLDGSGLGFSLEYPT
ISLHALSRDRSDCLGEHLYVMVNAKFEEESKEPVADEEEEDSDDDVEPITEFRFVPSDKS
ALEAMFTAMCECQALHPDPEDEDSDDYDGEEYDVEAHEQGQGDIPTFYTYEEGLSHLTAE
GQATLERLEGMLSQSVSSQYNMAGVRTEDSIRDYEDGMEVDTTPTVAGQFEDADVDH
>Q9H467
MELERIVSAALLAFVQTHLPEADLSGLDEVIFSYVLGVLEDLGPSGPSEENFDMEAFTEM
MEAYVPGFAHIPRGTIGDMMQKLSGQLSDARNKENLQPQSSGVQGQVPISPEPLQRPEML
KEETRSSAAAAADTQDEATGAEEELLPGVDVLLEVFPTCSVEQAQWVLAKARGDLEEAVQ
MLVEGKEEGPAAWEGPNQDLPRRLRGPQKDELKSFILQKYMMVDSAEDQKIHRPMAPKEA
PKKLIRYIDNQVVSTKGERFKDVRNPEAEEMKATYINLKPARKYRFH
>P23226
MEHHEDNAQLDNYLQNRLAESLQICGGAGEHNPHLADATGGNGCAPGIAPSKSDEVDGEE
DEEWKYIHEVRQSEKLQQEKLPLTKETGNGFGPGRDSDNQVHGNGAAAVFNLYEEDVEVI
KNDGDFSTNSNTTTSTDEVVARQAQEPNQLPEQLQQQQQIESQGVHEDPRQEDEDEHSSV
ATTYGTSSLSENNSSPLDQEEVVMVAQTVGQEQLVDFDNKENSYFVKNLEENHSQLNPNA
VAFVPGVGSQSSSPLPAAEDPLPGVQPRPFLPGGTLDDLVAESPRKEFARINMDGIAVPD
EREFDIEADMRPHELEQESDTFGAGHLEMQLLNGIGTADQAALRDVLDHGPETSVDMELP
LDQVPNDADIMKQSIYAEHNSSIEDILNSVQPLPIQTCDDKELIHVEEKEHVSKSPSTEE
LQFQSDFPNNQESHTLFNNTEQDPMQASFYLEHTSQKAQEGCQEQMQLPAECSDIFADQS
LLLDTSAPQLSSEADSPVAKLELESQQAGIVDITPSPLSSTAEKHLVEDTKELVEEYTLD
PESHFFGVVSSQAPLQLFGKHTLPSIIHSCKHRVASEQNDEENAVFESVSGYETQNFDEI
SSPPEGINPFAQPFTPAHLVIEQANTMMEDVGGMPIPASEDFAICDKVASKSSNEVEDHR
SEQQAFVKEELLHPVGDVVAQVENLGTEKNFVVEEERLPISVSDEIPLSSASKEKLLPDT
TDEQLLTSALEEKLRSVAPEESVSTAADGQSISQFDEYVIASNKPLEDILEPEKDVEVAK
SLSEKTSVATVAGGAVVGATKTHSATKAGSTAASAKSKTETLVMKKTTASSTSVYGANKS
AAPRPSTARLGIKSTSIATKTSTTSSLTGNPRKSLSSNVGSTVKPPTKLSGTRPATAPVS
KVTLGAKTITNKPTASGTASDNVTRTTLRPLVSTNARRPATSGTGSVASSTARRPVTNAK
GSAPGSAASTKVRPAATMTAPVKPKVLSPRSTISSTTTVRKVPSTSTPSFSTRSPNKQQS
NGLGKNTSSTTTSTATATITKSFTARSAPKFTHSASLTYNNGSTSRRLLVPGSSSTTTTS
SLRKSSPLKAAPGKAASKPLTPQSKDGTAKSSPAVLKARNSTLMGGEGVAPSNECVPTPN
GQINAEEVVKLKGKGLAEEVKIIEEQKLHEQDVPAHNAEVPLLDF
>Q10326
MSRSICTLTCILPTFSMDYWNQECVELSQVENLFTELASRLELLEMNSKNVLYRLTIGTN
PDRNWEAIAFIREFFDSAKHGYVIPKHEIRNRIRLLSEASLISCLRWLLHRIPGGVITWS
TYKLFDEAETRANYPVRGFDIFMKHATKHSCHFNILKCFLKLLMSLSAKLAVSSNSSTVS
SLELVSQIASIWAFDWPMMNLQETFIYWDRCTNACLRLLLCYIRYTNKSSETGFSCLPSA
LQSQLQSFNYPPSLKKLNDAKAHVFTFSMNYYMTRDPLEMIQIVTKMNIPDHLTATLPRS

SDDIQNDCLFALRQVSKRSSYHSVFSAQESAWTDFIKNGFDHPILPTCTQETVYSLLTGN
DTACVYPFPNKPYRLPDVDFEIFSHCSFESLTTVTTNTNIWWVWAESRCTEIPESKRTVF
PNCTMLIDKTGRLIILQQTVPQKPVALTASSNKRKNRIFGKIRRSFKRILKPRKINKTVK
IMSNSQRRSCQSVLSEGNRTILLHLADQMNACSLQTKSRESIKTLKLIEEKDEYWEPETA
GYFNTIVGWADQRKSLYDEAVIKINHSNMLNTLPPTSQGATSTTVSSASSNFLSSSCTPI
DDTNSVTGSTLSCSFDEMKLSDKIDDANSLKDDDFIQGSKKDFFEMNLNHSSYQNKDELK
PFQLLVKHAFKPPSYRLIRPPLRDWQSSDTLSSEMSKSISSSRSSPFSLEQTISKIQNKL

>P20398
MSLQQLKRKSLRDGWLMDGVVPSPGAEIDSPLFQTESKIQQLEKELESLQMQQLRLENPA
AVQPEAKAIQTPFLNGEKIQQGGGQAGDAKEVTAGQANNTHGIIHEEQPTKEDQDMGTVL
PIPAPRGKTVPKEDENQANPELKVDMEHQKVELVDLIQECPVENQTVEHVEKNKPHLDQE
HTEKNQDGQHGILEFLTQDQQLGNPNLQHLDRYLITEVTVKHFEKNQAHPGQEKNQDEQH
GILKYLTQDQQNENPNLGHLDQYLITEITLQSNPLENISVHDQTQSTSDQNMETKLPTDI
PQQKESQSEGKIQTKDQGPEFELLYKDHSHEKGTTDQTQHQELLPSSIEPKEENPKAQDE
KLDHHNESVSTVHEQKEVHDMDPRQLSTHQKSLSISEDQNQGSVSLSDPQNQDQSLALPE
KGLEETPQLNLSHEVQCEEPSLMEQISISLLQSMEQNQEGADQTPKSVALEELSELLSSD
MIKQSVLLSKNLEESPSTASTEETQETMCQAVIIPDLKKENSDPEVVQESSSHEPMSSTI
AQSSSAEGNSSPESRPLLQKSQGTDSQQGGNTATQQEERRKKKTCQCCVVM
>O74428
MERPSLSRRTSSSTVSTDGEGVYSRSTKERKRNFIVNSRLKRGGGHVRVNRSGRLGSVTM
RPSALTRAHSQNPNSSLVNNSSAVSHLTKQRSLNDLHELNGPKHVAKNGLIPLTQRKPNC
VWDDAPVDNDSTAGNLDSDSALPTPSVTTNEAADSSRASSPVTRVVAVHDNKKKIINSNI
SNAPPFNNTDVQASARPPAAGQDDSAADASTTKSSPVHNEVMAEPLPHSNNREVTQATNQ
PKWQIHSGSDIASEPPTLSRGNSLSLLANRKVPSTNVKKSQAELYDLGSSTSRTQQKLLI
QRASSKFDIVEDDMDTNPSKRFSNPHTKHIMDLVRTQYRNVLRTRELIPEFLEKIRSSYS
NNQNFDSQNAFNTSAAGTAGTREETISNGQNGIVASAETSKKDDGVQSASLNASMSARSH
ARQRSIHVPKTRKDTDYESIHQKLLQLWSQG
>Q9UGK8
MEREPSASEAAPAAAALFAWGANSYGQLGLGHKEDVLLPQQLNDFCKPRSVRRITGGGGH
SAVVTDGGDLFVCGLNKDGQLGLGHTEDIPYFTPCKSLFGCPIQQVACGWDFTIMLTENG
QVLSCGSNSFGQLGVPHGPRRCVVPQAIELHKEKVVCIAAGLRHAVAATASGIVFQWGTG
LASCGRRLCPGQTLPLFFTAKEPSRVTGLENSKAMCVLAGSDHSASLTDAGEVYVWGSNK
HGQLANEAAFLPVPQKIEAHCFQNEKVTAIWSGWTHLVAQTETGKMFTWGRADYGQLGRK
LETYEGWKLEKQDSFLPCSRPPNSMPSSPHCLTGATEVSCGSEHNLAIIGGVCYSWGWNE
HGMCGDGTEANVWAPKPVQALLSSSGLLVGCGAGHSLALCQLPAHPALVQDPKVTYLSPD
AIEDTESQKAMDKERNWKERQSETSTQSQSDWSRNGGL
>O55103
MEARSRSAEELRRAELVEIIVETEAQTGVSGFNVAGGGKEGIFVRELREDSPAAKSLSLQ
EGDQLLSARVFFENFKYEDALRLLQCAEPYKVSFCLKRTVPTGDLALRPGTVSGYEMKGP
RAKVAKLNIQSLAPVKKKKMVTGALGTPADLAPVDVEFSFPKFSRLRRGLKAEAVKGPVP
AAPARRRLQLPRLRVREVAEEAQVARMAAAAPPPRKAKAEAEAATGAGFTAPQIELVGPR
LPSAEVGVPQVSVPKGTPSTEAASGFALHLPTLGLGAPAAPAVEPPATGIQVPQVELPTL
PSLPTLPTLPCLDTQEGAAVVKVPTLDVAAPSMGVDLALPGAEVEAQGEVPEVALKMPRL

SFPRFGIRGKEATEAKVVKGSPEAKAKGPRLRMPTFGLSLLEPRPSGPEAVAESKLKLPT
LKMPSFGIGVAGPEVKAPTGPEVKLPKVPEVKLPKVPEAAIPDVQLPEVQLPKMSDMKLP
KIPEMVVPDVRLPEVQLPKVPEMKVPEMKLPKWPEMAVPDVHLPDVQLPKVPEMKLPKVP
EMAVPDVHLPDVQLPKVPEMKLPEMKLPKVPEMAVPDVRLPEVQLPKVSEVKLPKMPEMA
VPDVHLPELQLPKMSEVKLPKMPEMAVPDVRLPEVQLPKVSEMKLPKMPEMTMPDIRLPE
VQLPKVPDIKLPEMKLPEIKLPKVPDMAVPDVPLPELQLPKVSDIRLPEMQVSQVPEVQL
PKMPEMKLSKVPEVQRKSAGAEQAKGTEFSFKLPKMTMPKLGKVGKPGEASIEVPDKLMT
LPCLQPEVGTEASHVGVPSLSLPSVELDLPGALGLEGQVQEAVPGKVEKPEGPRVAVGVG
EVGFRVPSVEIVTPQLPTVEVEKEQLEMVEMKVKPSSKFSLPKFGLSGPKAVKGEVEGPG
RATKLKVSKFTISLPKARAGTEAEAKGAGEAGLLPALDLSIPQLSLDAQLPSGKVEVADS
KPKSSRFALPKFGVKGRDSEADVLVAGEAELEGKGWGWDGKVKMPKLKMPSFGLSRGKEA
ETQDGRVSPGEKLEAIAGQLKIPAVELVTPGAQETEKVTSGVKPSGLQVSTTGQVVAEGQ
ESVQRVSTLGISLPQVELASFGEAGPEIVAPSAEGTAGSRVQVPQVMLELPGTQVAGGDL
LVGEGIFKMPTVTVPQLELDVGLGHEAQAGEAAKSEGGIKLKLPTLGTGSRGEGVEPQGP
EAQRTFHLSLPDVELTSPVSSHAEYQVVEGDGDGGHKLKVRLPLFGLAKAKEGIEVGEKV
KSPKLRLPRVGFSQSESVSGEGSPSPEEEEEGSGEGASSRRGRVRVRLPRVGLASPSKVS
KGQEGDATSKSPVGEKSPKFRFPRVSLSPKARSGSRDREEGGFRVRLPSVGFSETAVPGS
TRIEGTQAAAI

>Q80SU3

MFPASTFHPCPHPYPQATKAGDGWRFGARGCRPAPPSFLPGYRQLMAAEYVDSHQRAQLM
ALLSRMGPRSVSSRDAAVQVNPRRDASVQCSLGRRTLQPAGCRASPDARSGSCQPRGHAG
AGRSPRSWQTVAPFSSVTFCGLSSSLEVAGGRQTPTKGEGSPASSGTREPEPREVAARKA
VPQPRSEEGDVQAAGQAGWEQQPPPEDRNSVAAMQSEPGSEEPCPAAEMAQDPGDSDAPR
DQASPQSTEQDKERLRFQFLEQKYGYYHCKDCKIRWESAYVWCVQGTSKVYFKQFCRVCE
KSYNPYRVEDITCQSCKRTRCACPVRLRHVDPKRPHRQDLCGRCKDKRLSCDSTFSFKYI
I

>O75363

MGNQMSVPQRVEDQENEPEAETYQDNASALNGVPVVVSTHTVQHLEEVDLGISVKTDNVA
TSSPETTEISAVADANGKNLGKEAKPEAPAAKSRFFLMLSRPVPGRTGDQAADSSLGSVK
LDVSSNKAPANKDPSESWTLPVAAGPGQDTDKTPGHAPAQDKVLSAARDPTLLPPETGGA
GGEAPSKPKDSSFFDKFFKLDKGQEKVPGDSQQEAKRAEHQDKVDEVPGLSGQSDDVPAG
KDIVDGKEKEGQELGTADCSVPGDPEGLETAKDDSQAAAIAENNNSIMSFFKTLVSPNKA
ETKKDPEDTGAEKSPTTSADLKSDKANFTSQETQGAGKNSKGCNPSGHTQSVTTPEPAKE
GTKEKSGPTSLPLGKLFWKKSVKEDSVPTGAEENVVCESPVEIIKSKEVESALQTVDLNE
GDAAPEPTEAKLKREESKPRTSLMAFLRQMSVKGDGGITHSEEINGKDSSCQTSDSTEKT
ITPPEPEPTGAPQKGKEGSSKDKKSAAEMNKQKSNKQEAKEPAQCTEQATVDTNSLQNGD
KLQKRPEKRQQSLGGFFKGLGPKRMLDAQVQTDPVSIGPVGKSK

>Q9NRN7

MVFPAKRFCLVPSMEGVRWAFSCGTWLPSRAEWLLAVRSIQPEEKERIGQFVFARDAKAA
MAGRLMIRKLVAEKLNIPWNHIRLQRTAKGKPVLAKDSSNPYPNFNFNISHQGDYAVLAA
EPELQVGIDIMKTSFPGRGSIPEFFHIMKRKFTNKEWETIRSFKDEWTQLDMFYRNWALK
ESFIKAIGVGLGFELQRLEFDLSPLNLDIGQVYKETRLFLDGEEEKEWAFEESKIDEHHF
VAVALRKPDGSRHQDVPSQDDSKPTQRQFTILNFNDLMSSAVPMTPEDPSFWDCFCFTEE
IPIRNGTKS

>O94183
MKFLSTAAALLVCLAPVSTTARSLDFFKSSQSPIQAQAKSVPGNNPLEYCNDPSGDILDI
KQVDLSPNPPLPGKTLAITASGTLREKIEDGAYVLLEVKYGLITLVRQTADLCEQLVNVE
LKCPLGPGDMTLTKQVDLPKQIPPGKYTVQADVFNSDGEHITCLKALNIEFKGPF
>P53323
MTQEFIDKVSSYLTPDVDIAPISQGAEAIVFTTTTHPYLPRAKDSHQKYIIKYRPPKRYR
HPQIDQALTKHRTLNESRLLAKLYLIPGLCVPQLIACDPYNGFIWLEFLGEDLPGGHGFS
NLKNFLWMHDQDPYSDLVATTLRKVGRQIGLLHWNDYCHGDLTSSNIVLVRDGARWTPHL
IDFGLGSVSNLVEDKGVDLYVLERAILSTHSKHAEKYNAWIMEGFEEVYREQGAKGAKKL
KEVTKRFEEVRLRGRKRSMLG
>Q8NEL9
MNYPGRGSPRSPEHNGRGGGGGAWELGSDARPAFGGGVCCFEHLPGGDPDDGDVPLALLR
GEPGLHLAPGTDDHNHHLALDPCLSDENYDFSSAESGSSLRYYSEGESGGGGSSLSLHPP
QQPPLVPTNSGGGGATGGSPGERKRTRLGGPAARHRYEVVTELGPEEVRWFYKEDKKTWK
PFIGYDSLRIELAFRTLLQTTGARPQGGDRDGDHVCSPTGPASSSGEDDDEDRACGFCQS
TTGHEPEMVELVNIEPVCVRGGLYEVDVTQGECYPVYWNQADKIPVMRGQWFIDGTWQPL
EEEESNLIEQEHLNCFRGQQMQENFDIEVSKSIDGKDAVHSFKLSRNHVDWHSVDEVYLY
SDATTSKIARTVTQKLGFSKASSSGTRLHRGYVEEATLEDKPSQTTHIVFVVHGIGQKMD
QGRIIKNTAMMREAARKIEERHFSNHATHVEFLPVEWRSKLTLDGDTVDSITPDKVRGLR
DMLNSSAMDIMYYTSPLYRDELVKGLQQELNRLYSLFCSRNPDFEEKGGKVSIVSHSLGC
VITYDIMTGWNPVRLYEQLLQKEEELPDERWMSYEERHLLDELYITKRRLKEIEERLHGL
KASSMTQTPALKFKVENFFCMGSPLAVFLALRGIRPGNTGSQDHILPREICNRLLNIFHP
TDPVAYRLEPLILKHYSNISPVQIHWYNTSNPLPYEHMKPSFLNPAKEPTSVSENEGIST
IPSPVTSPVLSRRHYGESITNIGKASILGAASIGKGLGGMLFSRFGRSSTTQSSETSKDS
MEDEKKPVASPSATTVGTQTLPHSSSGFLDSAYFRLQESFFNLPQLLFPENVMQNKDNAL
VELDHRIDFELREGLVESRYWSAVTSHTAYWSSLDVALFLLTFMYKHEHDDDAKPNLDPI

>P50119
MAFTGKFEMESEKNYDEFMKLLGLPSDVVEKSRNIKIVTEIKQDGQDFTWSHHYSGGQIM
TNKFTIGKESEIQTFGGKKFKAVVNMEGGKVVANFPNYQHTSEIKGDKLVEVSSIGGVTY
ERVSKRLA
>Q8TFH5
MQDRFLKENTEIINLSSSIHPNRDSYLDSQSDPLNQNLYNIETENVKDLNIEDVDYYEKL
QNFKIVDENIDPGLRTYSKRSVGVNNTFQNPCNRKIEGYIKEIERLSNSNKNLQAAVLQM
AVSDTDDPRLKEEYKQTEKELLREISGNHKSKILKLEEELNDLKHSMKEMQLYMTKIIDK
AMNNASLENLFSLDENNTKIHDK
>O60099
MRNLNTELDFFVSFSALKEENISQLKVYWAQSNLVELYYGESFYINIVCSRPIVDENVTT
WPENEGFRIEGTLLESLVESVTSASNYPKVNAFSLMCSGIPSFIWVEDDKQYALWACLAQ
LDSYSYPNHKLEIHVYKHILPDLPQSSEVDRNSETEGTREENSNTSDWDEQNEYVSEVEG
DYNLLGSLSNDIQFRSNPPVISGAYVFGESDVANELKDPSRKTKTRKKVFECSVPVVLKL
RSYALNESNQYITSIVSPDSSCENIRILGFKAETNAATISLFAPKTPEKLQITLSSSDVF
TIIHLLQIPDNIKHIELQCFCKVIVAQKLSETLVESMPFTYKHPPVLITRRTPTKTHTKQ
LSSSGNVSAAQSISSLNLLEISASSPSYVPSGSSFSVRANLYNPLDFSIDLLIRIPLYCY

DDCLTNKEHSTSKSEEKSSELLKYPNGHTISPGIIALSTKLYTGSIFPKCEKDFDLHFLA
YKPGSYDLSSISIEDVNHVVHKPRKLSKELQIIVT
>P24298
MASSTGDRSQAVRHGLRAKVLTLDGMNPRVRRVEYAVRGPIVQRALELEQELRQGVKKPF
TEVIRANIGDAQAMGQRPITFLRQVLALCVNPDLLSSPNFPDDAKKRAERILQACGGHSL
GAYSVSSGIQLIREDVARYIERRDGGIPADPNNVFLSTGASDAIVTVLKLLVAGEGHTRT
GVLIPIPQYPLYSATLAELGAVQVDYYLDEERAWALDVAELHRALGQARDHCRPRALCVI
NPGNPTGQVQTRECIEAVIRFAFEERLFLLADEVYQDNVYAAGSQFHSFKKVLMEMGPPY
AGQQELASFHSTSKGYMGECGFRGGYVEVVNMDAAVQQQMLKLMSVRLCPPVPGQALLDL
VVSPPAPTDPSFAQFQAEKQAVLAELAAKAKLTEQVFNEAPGISCNPVQGAMYSFPRVQL
PPRAVERAQELGLAPDMFFCLRLLEETGICVVPGSGFGQREGTYHFRMTILPPLEKLRLL
LEKLSRFHAKFTLEYS
>Q08886
MPQASTFGSHSLEAHPLHIQPAVHIKLSKEERSHYREQYDSLKYISNYVSVFDQALSDNI
DSRIRKENEALLKKYYESRKPFTFTSFRQGSVISSSDSSTGFTERTKTYCFLNDFVSNCV
NEVDPYTLKMTVRNRNTALNMENLDDERKSKDDIYDFEDNTDDECNAKCHGAFHYSSERL
EILRSRSTISYFKYYKKLLTVDLRDSDVLKRHNLWMPMITRRFRFLLVSSSKPEDVRLTT
PIPTFSESDLDIFKNKTCPLFINGTDCVPRSYDTFSGSSVIASIFSEYKLPSLSYHCSVE
LNDQLFIVGGLMACHRYDEEAPDLKDFYVDGIKNLPPPLIPELINNPSMIPNPHLYCFSL
TSSRLTRPDISGYIPPPLVCTQGCKLTERHIFLYGGFEIKSETQVDDKGRYFIRKRAFLN
NTGYILDTVTFNFSKIELVAPPYQFAIYNNFSPRFGHMQASISNSNNNVSNENTTTSAKG
RRSISPYRQGNGDHKIDDLVGSPGSTDYLEDDAIPPVTNPRSTDSLSSKHCSTATHICSS
VNTILIFGGYSQTGDDKYEAMNDMWKINIPVVSRGKRNYYKFADTVTATKIPIIDDPELW
PSRRAFSACCVPDYFTKDVEPIETRLLRNLKNDFSIDLEIRPGNKPSQPLFPNIPHSRKE
KKSGRDSMHISNSNNSTSEDTSSKSTRNTTSSPPTSPKHTPPLNPSKKCASIGRTIAFHG
GSDGYDVCSDMWWFDFDSETWTKIDLYAKTQEESDGLVPINLCMVGHSMTTVGHKVVLIG
GLRQGDVDRIYRDETLPEEVISGVPLGSGVINVVDLNTQCLQGCKLIRNDGDTKESVIMD
PHVGTPHQVLAVAGTIELVKGTMTLIGGVVAGREDISSLYLRGAVLQFILPSMNLAN
>Q9SWI1
MVTLTPSSASTPKTSFDFMKNNNSHSSLYVSSSSYLSSKEDALVTTKKLMEPSKTLNMSI
NPKQEEFGDEKKMVKKAPEDPEIGVFGAEKYFNGDMDSDQGSSVLSLTNPEVERTVVDSK
QSAKKSTGTPSVRSESSWNSQSVLLQNKLVNSCNSSFKEKKNSNGQIQKVTNNKKSFLAN
LGCKCACSDGDSVDVEEKTSVKRSADPNISVITMRSSADMNTELIKIQKQEELSQRKSLE
VFGSPVAIEKKSSVVQKKLPLPPWKSRTEEDDTKSEGSDSSSDLFEIEGLTGNPKPFLTR
QGSDPASPTCYAPSEVSVEWSIVTASAADFSVMSECATSPVRRNRPTQIPRIPITAKSAP
QRRKSSSSSGGNGFLMSCKSHKSVMVSGDLDRRSSMNKTQPSYVPRFPMETTKPKSFETR
RRISNSSISHTQSSLLYSQ
>Q8TFG6
MVMQERNSNEDNKVNHGIDNIYVLELDLDGSVLFASTNFSQITGSLELTGKPAALLSSDQ
EIFNAAIEQLLDDDSHSVKIHTVISKLPSLEENVFVQDEEMELQSKSVELDCVGVLIRDP
LTSRPSHTLWVLQPLKPTRITRQEIGQQLTETLGFGAQLLAQHLEKLQTVPSTDSLPPFE
TVLCRVCDHEIQNWYFEHHTELCLLIHHAEARAQEANDLLNEQRNALQSLLDSLDDQIEP
DVSYLGVPLAAVLPSSITSSAKSNSRSSLSQKIRNYISNMLFDAISYTDSCLAIHLPFIP
ESTTREDNQPFSEIRLLSPASEVFNAKTLSWCLPHIDDPGLQLMFENTDALVKKKLDAIN

RLSNIIYYSERVRCEIEDQVQTIIEQSIQVDGYDEPLSTTTPTLIEPIQETLMTQSPIIE
CEPFNTVKPSVSPEEVHDISQFNHRNDPPITAASVDSSNSFSVHRSSTNHSSTNSGSPNL
SRRNNLAIPIASRRKSVSAVNTLYGVGSSYTSESFPFSKLTVPVERNSFRETESPKPFLS
RQIGISTLSSNISSGKGTPSIQDYEIIKPISKGTFGTVYLSRKNTTGEIYAIKVLRKVDM
ISKNQVANVKAERAVLMAQEESAFVAKLYYAFQSRDYLYLVMEFMNGGDCASLLKSLYTI
PESWAKIYIAEVALGLEHLHRLGIIHRDIKPDNILMSITGHLKLADFGLSQLGLTTRQLR
LQKGKNNILSPPSFQSPTALGDPGDNIASSPLILPTSVSAFSYDEKSQKQKTELATFTTY
KEDDTTTTTRTSIDSISSKYLESPVDSQKVNRTPNLQSVPFFRQPDAPKRFVGTPDYLAP
ETLRGSTQDDMVDWWALGCVLFEFLFGYPPFHAETPEKVFENILANNIAWPDLEMYPCSE
EALDLINGFLQPNPERRLGFSDINEIKEHPFFNGINWDDIFSHEAPFIPAPETPLDTAYF
DSRGAGAAESNMSSSVNSGEEVSKDNNVSQERGSQFLRSSHGRSRERSTSARRSRRFSEA
NSEFDEFGPFSYKNLSVLERANRNAIEKIRSEIAGKLHISPPDPHIGYTPGSDMPSAKLY
DQQLTLSPSLMTNQGSNFSSTDSTPRKSINSSDVESRSKTDGPKSMHDLIKQLHMRKHSS
HTNQSTGSSESDDLFNLDLPISNLETSYPFKIEEGQASPLSSPLSKTPPFFSSSVPLKAL
ICVSKLNLFSELIKLLKSYKFQVSIVTDEDKMLRTLMADEKFSIIFLQLDLTRVSGVSIL
KIVRSSNCANRNTPAIALTPTRIDINAAIPRMFDGRLYLPINAFLLRGYIARLCNK
>O75419
MFVSDFRKEFYEVVQSQRVLLFVASDVDALCACKILQALFQCDHVQYTLVPVSGWQELET
AFLEHKEQFHYFILINCGANVDLLDILQPDEDTIFFVCDTHRPVNVVNVYNDTQIKLLIK
QDDDLEVPAYEDIFRDEEEDEEHSGNDSDGSEPSEKRTRLEEEIVEQTMRRRQRREWEAR
RRDILFDYEQYEYHGTSSAMVMFELAWMLSKDLNDMLWWAIVGLTDQWVQDKITQMKYVT
DVGVLQRHVSRHNHRNEDEENTLSVDCTRISFEYDLRLVLYQHWSLHDSLCNTSYTAARF
KLWSVHGQKRLQEFLADMGLPLKQVKQKFQAMDISLKENLREMIEESANKFGMKDMRVQT
FSIHFGFKHKFLASDVVFATMSLMESPEKDGSGTDHFIQALDSLSRSNLDKLYHGLELAK
KQLRATQQTIASCLCTNLVISQGPFLYCSLMEGTPDVMLFSRPASLSLLSKHLLKSFVCS
TKNRRCKLLPLVMAAPLSMEHGTVTVVGIPPETDSSDRKNFFGRAFEKAAESTSSRMLHN
HFDLSVIELKAEDRSKFLDALISLLS
>O02696
MQPGATTCTEDRIQHALERCLHGLSLSRRSTSWSAGLCLNCWSLQELVSRDPGHFLILLE
QILQKTREVQEKGTYDLLAPLALLFYSTVLCTPHFPPDSDLLLKAARTYHRFLTWPVPYC
SICQELLTFIDAELKAPGISYQRLVRAEQGLSTRSHRSSTVTVLLLNPVEVQAEFLDVAD
KLSTPGPSPHSAYITLLLHAFQATFGAHCDLSGLHRRLQSKTLAELEAIFTETAEAQELA
SGIGDAAEARQWLRTKLQAVGEKAGFPGVLDTAKPGKLRTIPIPVARCYTYSWNQDSFDI
LQEILLKEQELLQPEILDDEEDEDEEDEEEDLDADGHCAERDSVLSTGSAASHASTLSLA
SSQASGPTLSRQLLTSFVSGLSDGVDSGYMEDIEESAYERPRRPGGHERRGHRRPGQKFN
RIYKLFKSTSQMVLRRDSRSLEGSPDSGPPLRRAGSLCSPLDSPTLPPSRAQGSRSLPQP
KLSPQLPGWLLAPASRHQRRRPFLSGDEDPKASTLRVVVFGSDRISGKVVRAYSNLRRLE
NNRPLLTRFFKLQFFYVPVKRSRGTGTPTSPAPRSQTPPLPTDAPRHPGPAELGAAPWEE
STNDISHYLGMLDPWYERNVLGLMHLPPEVLCQSLKAEPRPLEGSPAQLPILADMLLYYC
RFAARPVLLQVYQTELTFITGEKTTEIFIHSLELGHSAATRAIKASGPGSKRLGIDGDRE
AVPLTLQIIYSKGAISGRSRWSNMEKLCTSVNLSKACRQQEELDSSTEALTLNLTEVVKR
QTPKSKKGFNQISTSQIKVDKVQIIGSNSCPFAVCLDQDERKILQSVIRCEVSPCYKPEK
SSLCPPPQRPSYPPAPATPDLCSLLCLPIMTFSGALP
>P70331

MSQVSGQCPSRCDAPHGVPSAALDPAQTMSLLPGLEVARSTHPVEASSEEGFPEEAAPSM
PHDSGLRAQQALNSIDLDVPTEAVTCQPQGNPQGCTPLLPNGSSHDHLSEPGSAGHAGNG
ALGGSKAHRKLQTHPSLGSQAGRKSRGSARSASQVPLQAQEGKAPAVRIHRQTASPTCCL
RNAQLSGTALRSLRLESQGHRELNNKTLSQSNNKKPGVAAHAAIIPALTRPKQNCHDPSL
LPGTHGVGKEF
>O88712
MGSSHLLNKGLPLGVRPPIMNGPMHPRPLVALLDGRDCTVEMPILKDVATVAFCDAQSTQ
EIHEKVLNEAVGALMYHTITLTREDLEKFKALRIIVRIGSGFDNIDIKSAGDLGIAVCNV
PAASVEETADSTLCHILNLYRRTTWLHQALREGTRVQSVEQIREVASGAARIRGETLGII
GLGRVGQAVALRAKAFGFNVLFYDPYLSDGIERALGLQRVSTLQDLLFHSDCVTLHCGLN
EHNHHLINDFTVKQMRQGAFLVNTARGGLVDEKALAQALKEGRIRGAALDVHESEPFSFS
QGPLKDAPNLICTPHAAWYSEQASIEMREEAAREIRRAITGRIPDSLKNCVNKDHLTAAT
HWASMDPAVVHPELNGAAYSRYPPGVVSVAPTGIPAAVEGIVPSAMSLSHGLPPVAHPPH
APSPGQTVKPEADRDHTSDQL
>P08523
MAEDGPQKQQLDMPLVLDQDLTKQMRLRVESLKQRGEKKQDGEKLLRPAESVYRLDFIQQ
QKLQFDHWNVVLDKPGKVTITGTSQNWTPDLTNLMTRQLLDPAAIFWRKEDSDAMDWNEA
DALEFGERLSDLAKIRKVMYFLITFGEGVEPANLKASVVFNQL
>Q96Q83
MEEKRRRARVQGAWAAPVKSQAIAQPATTAKSHLHQKPGQTWKNKEHHLSDREFVFKEPQ
QVVRRAPEPRVIDREGVYEISLSPTGVSRVCLYPGFVDVKEADWILEQLCQDVPWKQRTG
IREDITYQQPRLTAWYGELPYTYSRITMEPNPHWHPVLRTLKNRIEENTGHTFNSLLCNL
YRNEKDSVDWHSDDEPSLGRCPIIASLSFGATRTFEMRKKPPPEENGDYTYVERVKIPLD
HGTLLIMEGATQADWQHRVPKEYHSREPRVNLTFRTVYPDPRGAPW
>P40157
MNILKKFMESGNKPELITIPSGQFNLLRSKNSPKAALECIYNNATLSVRKIGKFDYELAV
YRVEDDSEGGTGDEAENFEDDTISVLSTQSKKKEEEWSVEISDKIMFHKTWDKQGNVALV
WENLRGDEQDEKVQFVVAADVSFSDVEQFIQTVYRCQFEVRNKKSSLTASADDLKEIEHR
STRLFVQDDDDELDSSSDDFQDAKDTSFEHEKESEILERTPSPLKKVPEGEYCCLVMSSL
YMYDPIQEKFILQEPVVKVAIIDTGKYEFWLAIEGKDNRLGTQVAPNINPTFELATDAFL
FNYTLQNITLSYMLKFKDLDKCIQFRFAWVKCLWMTLNKETWTDVPEKEKDYILDSSSVP
LEKQFDDILHIDDRSNEERDKESSESENDSEDEDDENDHSKRIISSEAFEEPRRATSKGN
SSLTVAFRNNRSYVTRDNRIGVFKTDDEDDSLEFVAAIKNISNLGGKSIDPHKPMLYMED
RNLILTDGENENKLYKMDIERGKVIEEWSTGDKNVVQYGPTKKFDQMTPEQTIVGVSQKG
VFKIDPRINGKNKIAVDESKDYVGKYNFSSIGTTESGYIAIGSEKGDIKLYDRLGIRAKT
AIPSLGQAIKFITTSADGKWLLATCESTLLLMDLKIKDGKNAGNIGFLKSFPASENVKTY
VLKIRPEHSASILTYTKKPIRFTKAYFNTGIGQQEQTIVTSTGPYAISWSLKGILNQDGS
NNYPYRIRRYNADVVADNFEFGSDKKVIVALKDDVSLSKVKSFKQPSKGVLMPSASLQDF
YG
>Q8IZY5
MVTLLPIEGQEIHFFEILESECVLYTGWIERASGSSIYPEAKARLPLEALLGSNKEPMLP
KETVLSLKRYNLGSSAMKRNVPGHVLQRPSYLTRIQVTLLCNSSAEAL
>Q9BRQ8
MGSQVSVESGALHVVIVGGGFGGIAAASQLQALNVPFMLVDMKDSFHHNVAALRASVETG

FAKKTFISYSVTFKDNFRQGLVVGIDLKNQMVLLQGGEALPFSHLILATGSTGPFPGKFN
EVSSQQAAIQAYEDMVRQVQRSRFIVVVGGGSAGVEMAAEIKTEYPEKEVTLIHSQVALA
DKELLPSVRQEVKEILLRKGVQLLLSERVSNLEELPLNEYREYIKVQTDKGTEVATNLVI
LCTGIKINSSAYRKAFESRLASSGALRVNEHLQVEGHSNVYAIGDCADVRTPKMAYLAGL
HANIAVANIVNSVKQRPLQAYKPGALTFLLSMGRNDGVGQISGFYVGRLMVRLTKSRDLF
VSTSWKTMRQSPP
>O74472
MRIRSATPSLILLVIAIVFFVLAICTPPLANNLTLGKYGDVRFGVFGYCLNSNCSKPLVG
YNSDYLDEHAKDGFRTSVIVRQRASYGLVIVPVSACICLISTIMTIFAHIGAIARSPGFF
NVIGTITFFNIFITAIAFVICVITFVPHIQWPSWLVLANVGIQLIVLLLLLVARRQATRL
QAKHLRRATSGSLGYNPYSLQNSSNIFSTSSRKGDLPKFSDYSAEKPMYDTISEDDGLKR
GGSVSKLKPTFSNDSRSLSSYAPTVREPVPVPKSNSGFRFPFMRNKPAEQAPENPFRDPE
NPFKDPASAPAPNPWSINDVQANNDKKPSRFSWGRS
>Q15003
MGPPGPALPATMNNSSSETRGHPHSASSPSERVFPMPLPRKAPLNIPGTPVLEDFPQNDD
EKERLQRRRSRVFDLQFSTDSPRLLASPSSRSIDISATIPKFTNTQITEHYSTCIKLSTE
NKITTKNAFGLHLIDFMSEILKQKDTEPTNFKVAAGTLDASTKIYAVRVDAVHADVYRVL
GGLGKDAPSLEEVEGHVADGSATEMGTTKKAVKPKKKHLHRTIEQNINNLNVSEADRKCE
IDPMFQKTAASFDECSTAGVFLSTLHCQDYRSELLFPSDVQTLSTGEPLELPELGCVEMT
DLKAPLQQCAEDRQICPSLAGFQFTQWDSETHNESVSALVDKFKKNDQVFDINAEVDESD
CGDFPDGSLGDDFDANDEPDHTAVGDHEEFRSWKEPCQVQSCQEEMISLGDGDIRTMCPL
LSMKPGEYSYFSPRTMSMWAGPDHWRFRPRRKQDAPSQSENKKKSTKKDFEIDFEDDIDF
DVYFRKTKAATILTKSTLENQNWRATTLPTDFNYNVDTLVQLHLKPGTRLLKMAQGHRVE
TEHYEEIEDYDYNNPNDTSNFCPGLQAADSDDEDLDDLFVGPVGNSDLSPYPCHPPKTAQ
QNGDTPEAQGLDITTYGESNLVAEPQKVNKIEIHYAKTAKKMDMKKLKQSMWSLLTALSG
KEADAEANHREAGKEAALAEVADEKMLSGLTKDLQRSLPPVMAQNLSIPLAFACLLHLAN
EKNLKLEGTEDLSDVLVRQGD
>P40048
MKDSVDCPSILPTDRTSVLSETSTLVGSSSHVYSRHAPMNSYHNSMNSNIYHSPKASSPL
VSYKTSSPVLLKRATAPVLPSFKPKEQRYNKPQGCSLITAVELGKIIETLPDEKVLLLDV
RPFTEHAKSIITNSIHVCLPSTLLRRKNFTFSKLLDNLTPSEQSVLKSKLAIDNLRIIIY
DSTANQTESSVSLPCYGIASKLIEFDTNVKKTVSILMCGFPQFKILFPDHINTNTFNSDC
ISSAEPKSPKTNLMNSLHNTAPHMTATTPLSSPQMNLKLKVPDDSRSDHSNFSSSPSPRN
VLSDSPMSSSSPISALFKFQLPAPQTNINQMFKFSQNEEIMGLETYLSAVNIKEEHERWY
NNDSAKKSLQNFQFPKNQNSLEKDTNKDKLGFQIRYENLSKNYEKEVIDSVIPEWFQHLM
SIPKIELVSQFQKLDFLEKRRLNHSVSFRKKENSFILEKPSSYPEQLTSTSSSTIMPPKF
PDVNKVQKRSHSQPIFTQYSKYKSMLSLESDSDSESDDVIISSGVELGAKNRYKDIFPYE
HSRVILKKGLQSSKGIKHSHSTSDGGILDNYINANYLSLPRFSVEQNSSFQTTTTTTRRV
RYIATQAPMPSTVHDFYTCILNNGVPLVLSLTNDFENGIEKCYRYWQEGNYNGIHVKLLE
KKILKMPSTTSMRKNTMGTQNSSLYSAGVQGNSSNYSTDNDNDNDNNNNNNNNSNIPVTA
AACDDDDDDDDDAILIRQILLTYHDQEKPYELLQIQVKNWPDLGTLLNPISILQAINVKN
HIIDTLFARNYYQNDQLPTILVHCSAGCGRTGTLCTIDSILSNFEMFEMLQKEFVKLKYP
AKLFDPISWTINIFRKERISMVQNINQFIFIYDCLLFYFRLRLDDITERTDGDGSNKDNI
SLSALIEQIEKLEILQTFVDDKLKELPQ

>Q9QY78

MSWSPSLPTQTCGAWEMKERLGTGGFGNVIRWHNQVTGEQIAIKQCRQELSPKNRDRWCL
EIQIMRRLNHPNVVAARDVPEGMQNLAPNDLPLLAMEYCQGGDLRRYLNQFENCCGLREG
AILTLLSDIASALRYLHENRIIHRDLKPENIVLQQGEKRLIHKIIDLGYAKELDQGSLCT
SFVGTLQYLAPELLEQQKYTVTVDYWSFGTLAFECITGFRPFLPNWQPVQWHSKVRQKSE
VDIVVSEDLNGTVKFSSSSPFPNNLNSVLAERLEKWLQLMLTWQPRQRGVDPQYGPNGCF
RALDDILNLKLVHILNMVTGTIHTYPVMEDESLQSLKTRIREDTGILETDQELLQEAGLV
LLPDKPATQCISDSKTNEGLTLDMDLVFLFDNSKMSYETQITPRPQPESVSCVLQEPKRN
LSFFQMRKVWGQVWHSIQTLKEDCNRLQQGQRAAMMNLLRNNSCLSKMKNAMASTAQQLK
AKLDFFKTSIQIDLEKYREQTEFGITSDKLLLAWREMEQAVEQCGRENDVKVLVERMMAL
QTDIVDLQRSPMGRKQGGTLDDLEEQARELYRRLREKPRDQRTEGDSQDMVRLLLQAIQS
FEKKVRVIYSQLSKTVVCKQKALELLPKVEEVVRLMNEDEKTVVRLQEKRQKELWNLLKI
ACSKVRGPVSGSPDSMNVSRLSHPGHLMSQPSSACDSLPDSDKKSEELVAEAHALCSRLE
SALQDTVKQQDRSFTTLDWSWLQMEDEERCGLEQACD

>O13759

MSIDCLYRRSSLFDTSFVPLHSSIPATSKMSASNSDVNAPISPVVDEGKSELVSPTLERL
VAPFNCSPSSTPLQDVAGVGSKMSDTLWMGDLEPWMDATFIQQLWASLNEPVNVKVMRSK
ASSSETLISYCFVQFSSSAAAERALMKYNNTMIPGAHCTFKLNWATGGGIQHNNFVSRDP
EFSIFVGDLLPTTEDSDLFMTFRSIYPSCTSAKIIVDPVTGLSRKYGFVRFSSEKEQQHA
LMHMQGYLCQGRPLRISVASPKSRASIAADSALGIVPTSTSNRQPNQDLCSMDPLNTTVF
VGGLASNLSEKDLQVCFQPFGRILNIKIPFGKGCGFVQYSEKSAAEKAINTMQGALVGTS
HIRLAWGHNTLPVSALSQSQSQVSDEGFDRTLSANQIFGMNQSVIGANSGSSNSSGSSLK
SAPVSPRTAAAQSLLPNSVVSSINGMNSVNFSTISPPPLSRSASISPTLSGSGSGLTPLS
SHFPSAATGLVGGQVYPQSSVLQSSKINGSAKVQPSVKLPEWLQPFSGNNHNSFATQDLL
TRVSSLKLVDDEQPASLNGSAFQARASRPWNLGRERQSSLIDLRHELEQNENGLEKSGFG
LNLRGRLPPRSYSTFNCTGQYLQPSLRLSRDS

>O74915

MSHMDSERLLFKRYLQRLFITRILTLIRLDLSLFRRYNTNTIVGSVAYETLVDTVCDVSS
NSTFVVKCNRQYPCTRCLKYGEACTYDERDNRKKRHSLSYLHSLESQLARLESFIMTLKN
SDPEGRDEMLKSVVFSDHLTEPNDQEVKSEGPIEYPVFLNVQDSKTVSFYGPTSAYDLSL
PDITKDNKTTWNFQASYSPMVSECLKLFFRYQYSQFLFVYRESFLSDYYYNFHDGFYCTE
HLIYAICAIGASMSDDENISRHSKSYYDASWQKLLEFGLDRSHLTSVQCLLCLGYYDIGM
GNTSLGWLLSGLAFRMGQDLGFQLNPENWYIDNSPAISSADSDIRRRVFWGSYVADKFIG
FIMGRPTMLKRSDASIPGSNQLPEFAGLEEFKLNVTDYMSLTDFSVCDAVALFVDLSDIA
DSILLNMFSPTSKNRATNINCVLSNLGKYNLELMNWHYKLPDTISWRTIDLKKDRIPNLC
AVSLYYHLIRICLNRPFLSRKEVTANDLTPKTICTDSINEIVTVIRAHRTANGLRYSTLY
IVYAAIVSCSVILLLRDMCTDSELLTLNNDMMFFIEVLKECSQTWKLAQRSIVLIENTLK
GKTNSQSTSEFVSPISDTENGSSSQQVSEAKDIVEPSDVLDELQKFDLLPENDDNFQTFQ
AFYGGPPIILSPNLYEKKLNEKTL

>P38623

MLKIKALFSKKKPDQADLSQESKKPFKGKTRSSGTNNKDVSQITSSPKKSFQDKNIVQYP
SVVADDHHMKSLTDELVTTIDSDSSPSDNITTENVETVTSVPAIDVHENSEGQLSSDPLI
SDESLSEQSEIISDIQDDSTDDDNMEDEIPEKSFLEQKELIGYKLINKIGEGAFSKVFRA
IPAKNSSHEFLTKNYKAVAIKVIKKADLSSINGDHRKKDKGKDSTKTSSRDQALKEVALH

KTVSAGCSQIVAFIDFQETDSYYYIIQELLTGGEIFGEIVRLTYFSEDLSRHVIKQLALA
VKHMHSLGVVHRDIKPENLLFEPIEFTPSIKPKLRKSDDPQTKADEGIFTPGVGGGGIGI
VKLADFGLSKQIFSKNTKTPCGTVGYTAPEVVKDEHYSMKVDMWGIGCVLYTMLCGFPPF
YDEKIDTLTEKISRGEYTFLKPWWDEISAGAKNAVSKLLELEPSKRYDIDQFLDDPWLNT
FDCLPKEGESSQKKAGTSERRHPHKKQFQLFQRDSSLLFSPAAVAMRDAFDIGNAVKRTE
EDRMGTRGGLGSLAEDEELEDSYSGAQGDEQLEQNMFQLTLDTSTILQRRKKVQENDVGP
TIPISATIRE
>Q8X1T0
MKVYINVYDLMPDSPVNKLAWTLGLGIYHTGLVLEGKEYAFGAHEIPGSTGVFATMPRPP
LEGCRWRCSIALPNCTLPKPDVDRILIRLSQEFTGLSYSLLERNCNHFTNAAAIELTGSP
IPSFLNRISRIGLAFPTITNALLQHGQKNTSDVDDSSDSSSDVDEETLIVSKSKKAHKDI
PKFSAPPPSADLNNLITDSLP
>Q8N140
MKMDVSVRAAGCSDDLSSGEADVDPKLLELTADEEKCRSIRRQYRQLMYCVRQNREDIVS
SANNSLTEALEEANVLFDGVSRTREAALDARFLVMASDLGKEKAKQLNSDMNFFNQLAFC
DFLFLFVGLNWMEGDPDKLSDCDDSIALSFWKAIEKEATSWMVKAETFHFVFGSFKLERS
APKPRLEHQKKVRKMEENGNMPTKLQKLDLSSYPEATEKNVERILGLLQTYFRKYPDTPV
SYFEFVIDPNSFSRTVENIFYVSFIVRDGFARIRLDEDRLPILEPMNVNQMGEGNDSSCH
GRKQGVISLTLQEWKNIVAAFEISEAMITYSSY
>Q7Z2X4
MFSLPLSLPLCEDTAFLPSKCCSSHKTIKQARTLIMIFLASGTHFQTMLKSKLNVLTLKK
EPLPAVIFHEPEAIELCTTTPLMKTRTHSGCKVTYLGKVSTTGMQFLSGCTEKPVIELWK
KHTLAREDVFPANALLEIRPFQVWLHHLDHKGEATVHMDTFQVARIAYCTADHNVSPNIF
AWVYREINDDLSYQMDCHAVECESKLEAKKLAHAMMEAFRKTFHSMKSDGRIHSNSSSEE
VSQELESDDG
>O94632
MTEIENIGALEVLFSPESIEQSLKRCQLPSTLLYDEKGLRLFDEITNLKEYYLYESELDI
LKKFSDSIANQLLSPDLPNTVIELGCGNMRKTKLLLDAFEKKGCDVHFYALDLNEAELQK
GLQELRQTTNYQHVKVSGICGCFERLLQCLDRFRSEPNSRISMLYLGASIGNFDRKSAAS
FLRSFASRLNIHDNLLISFDHRNKAELVQLAYDDPYRITEKFEKNILASVNAVFGENLFD
ENDWEYKSVYDEDLGVHRAYLQAKNEVTVIKGPMFFQFKPSHLILIEESWKNSDQECRQI
IEKGDFKLVSKYESTIADYSTYVITKQFPAMLQLPLQPCPSLAEWDALRKVWLFITNKLL
NKDNMYTAWIPLRHPPIFYIGHVPVFNDIYLTKIVKNKATANKKHFWEWFQRGIDPDIED
PSKCHWHSEVPESWPSPDQLREYEKESWEYHIVKLCKAMDELSTSEKRILWLCYEHVAMH
VETTLYIYVQSFQNANQTVSICGSLPEPAEKLTKAPLWVNVPETEIAVGMPLTTQYTSVG
SNLQSSDLSAHENTDELFYFAWDNEKPMRKKLVSSFSIANRPISNGEYLDFINKKSKTER
VYPKQWAEIDGTLYIRTMYGLLPLDDYLGWPVMTSYDDLNNYASSQGCRLPTEDELNCFY
DRVLERTDEPYVSTEGKATGFQQLHPLALSDNSSNQIFTGAWEWTSTVLEKHEDFEPEEL
YPDYTRDFFDGKHNVVLGGSFATATRISNRRSFRNFYQAGYKYAWIGARLVKN
>P01095
MTKNFIVTLKKNTPDVEAKKFLDSVHHAGGSIVHEFDIIKGYTIKVPDVLHLNKLKEKHN
DVIENVEEDKEVHTN
>Q9DD02
MFGCLVAGRLVQTAAQQVAEDKFVFDLPDYENINHVVVFMLGTIPFPEGMGGSVYFSYPD

SNGVPVWQLLGFVTNGKPSAIFKISGLKSGEGSQHPFGAMNIVRTPSVAQIGISVELLDS
LAQQTPVGSAAVSSVDSFTQFTQKMLDNFYNFASSFALSQAQMTPNPSEMFIPANVVLKW
YENFQRRLAQNPLFWKT
>Q9V3B4
MTAGRHWSYDSLKQIAIDGCTENVEQLKLIFVEEIGSRRRSDCIRTIEDLIDCLERADEL
SEYNVEPLRRISGNMPQLIEALSAYTKPENILGHPVNLYQELRLAEELRQQLRIAPASQN
AQPSVSELAAAVPPTAIQNYATPAAFTDHKRTMVFKKISEELGRYWRRLGRSAGIGEGQM
DTIEERYPHDLKSQILRLLQLIEEDDCHDPKHFLLRLCRALGDCGRNDLRKRVEQIMSH
>O59787
MSNHEEEIEELAQRIGKIEVGDTKSDIYQKVLNLIDIERYEHALKIIEKYLGETDAVYER
AYCAFQLGKEDFSLEDKQFLQHLQAQKAYRLSDFSKALKIYEHLENDLPEQRADIRVNML
AAASQLPGLQLNNAVSLDDQDSVFNLATRYLTIGDWNQAIELLSSSLEKLENSDSNSEDH
KSQINLCRLQLFFAYLQAGDNEKASKESLKISKDCLDETSQAIFVNNLISMSIDNPYISF
RDLHGTNLEKALSSLLASQKKQFIRNLALLDMAVGKQRSVRKEKKRNPEESIYFSTILLR
EETKSLISPKKLPGYLENLFKSDSDNIVVALLLMQHKISNGNFRGALSIYQKLRTSLEAS
QSLSVLYSPGLVGLGDALHYKIQSTGFKSQLLHEAANYWRKQQSCEAKLLLCTRSLLAHL
DERAPVSTIQDDMSVIDDLLQWKGPISELVSCKVAALCYLDKEIESGMDKYLVPTKDLIT
GIDVDDIEIRGVPVSAAIGPIKRSVEANSSNSSKKTRKRRKPTPKSFNPKATPDPQRWIP
KRDRTNVKIKSKGKSMQGGVA
>P47142
MSALPPVYSFPPLYTRQPNSLTRRQQISTWIDIISQYCKTKKIWYMSVDGTVINDNELDS
GSTDNDDSKKISKNLFNNEDIQRSVSQVFIDEIWSQMTKEGKCLPIDQSGRRSSNTTTTR
YFILWKSLDSWASLILQWFEDSGKLNQVITLYELSEGDETVNWEFHRMPESLLYYCLKPL
CDRNRATMLKDENDKVIAIKVV
>P97784
MGVNAVHWFRKGLRLHDNPALKECIQGADTIRCVYILDPWFAGSSNVGINRWRFLLQCLE
DLDANLRKLNSRLFVIRGQPADVFPRLFKEWNITKLSIEYDSEPFGKERDAAIKKLATEA
GVEVIVRISHTLYDLDKIIELNGGQPPLTYKRFQTLVSKMEPLEMPADTITSDVIGKCMT
PLSDDHDEKYGVPSLEELGFDTDGLSSAVWPGGETEALTRLERHLERKAWVANFERPRMN
ANSLLASPTGLSPYLRFGCLSCRLFYFKLTDLYKKVKKNSSPPLSLYGQLLWREFFYTAA
TNNPRFDKMEGNPICVQIPWDKNPEALAKWAEGRTGFPWIDAIMTQLRQEGWIHHLARHA
VACFLTRGDLWISWEEGMKVFEELLLDADWSINAGSWMWLSCSSFFQQFFHCYCPVGFGR
RTDPNGDYIRRYLPVLRGFPAKYIYDPWNAPEGIQKVAKCLIGVNYPKPMVNHAEASRLN
IERMKQIYQQLSRYRGLGLLASVPSNSNGNGGLMGYAPGENVPSCSSSGNGGLMGYAPGE
NVPSCSGGNCSQGSGILHYAHGDSQQTHSLKQGRSSAGTGLSSGKRPSQEEDAQSVGPKV
QRQSSN
>Q60446
MSVVGLDVGSQSCYIAVARAGGIETIANEFSDRCTPSVISFGPKNRTIGVAAKNQQITHA
NNTVSSFKRFHGRAFSDPFIQKEKESLSYDLVPMKNGGVGIKVMYMDEEHLFSVEQITAM
LLTKLKETAENNLKKPVTDCVISVPSFFTDAERRSVLDAAQIVGLNCLRLMNDMTAVALN
YGIYKQDLPNADEKPQGSGVCGHGPSSFQVSACAFNKGKLKVLGTAFDPFLGGKNFDEKL
VEHFCAEFKTKYKLDAKSKIRALLRLHQECEKLKKLMSSNSTDLPLNIECFMNDKDVSAK
MNRSQFEELCAELLQKIEVPLHSLMEQTHLKTEDVSAIEIVGGATRIPAVKERIAKFFGK
DVSTTLNADEAVARGCALQCAILSPAFKVREFSVTDAVPFPISLVWNHDSEETEGVHEVF

SRNHAAPFSKVLTFLRRGPFELEAFYSDPQGVPYPEAKIGRFVVQNVSAQKDGEKSKVKV
KVRVNTHGIFTISTASMVEKVPTEEDDGSSVEADMECPNQKPAESSDVDKNSQQDNSEAG
TQPQVQTDGQQTSQSPPSPELPSEENKIPDADKANEKKVDQPPEAKKPKIKVVNVELPVE
ANLVWQLGRDLLNMYIETEGKMIMQDKLEKERNDAKNAVEECVYEFRDKLCGPYEKFICQ
QEHEKFLRLLTETEDWLYEEGEDQAKQAYIDKLEELMKMGNPVKVRFQEAEERPKVLEEL
GQRLQHYAKIAADFRSKDEKYNHIDESEMKKVEKSVNEVMEWMNNVMNAQAKRSLDQDPV
VRTHEIRAKVKELNNVCEPVVNQPKPKIESPKLERTPNGPNLDKKEDLEGKDNFGAEAPH
QNGECHPNEKGSVNMDLD
>Q8BP71
MAEGGQAQQQPPQLGPGAAARGMKRESEVELPVPGAGADGPEPGLSKRPRTEEAADGGMQ
NEPLTPGYHGFPARDGQGNQEPTTTPDAMVQPFTTIPFPPPPQNGIPTEYGVPHTQDYAG
QTSEHNLTLYGSTQPHGEQSSNSPSNQNGSLTQTEGGAQTDGQQSQTQSSENSESKSTPK
RLHVSNIPFRFRDPDLRQMFGQFGKILDVEIIFNERGSKGFGFVTFENSADADRAREKLH
GTVVEGRKIEVNNATARVMTNKKMVTPYANGWKLSPVVGAVYGPELYAASSFQADVSLGN
EAAVPLSGRGGINTYIPLISLPLVPGFPYPTAATTAAAFRGAHLRGRGRTVYGAVRAVPP
TAIPAYPGVVYQDGFYGADLYGGYAAYRYAQPATATAATAAAAAAAAYSDGYGRVYTADP
YHALAPAASYGVGAVASLYRGGYSRFAPY
>P22031
ELVSEFCLKKERVCEDSSLTISCPEGEGIVIYDAIYGRKRGEVCPGLFGAFTKNRKCRSS
NSQQVVENSCEGKSSCTVLASNSVFGDPCPGTAKYLAVTYICSFL
>Q09152
MSVSCCCRNLGKTIKKAIPSHHLHLRSLGGSLYRRRIQSSSMETDLKSTFLNVYSVLKSD
LLHDPSFEFTNESRLWVDRMLDYNVRGGKLNRGLSVVDSFKLLKQGNDLTEQEVFLSCAL
GWCIEWLQAYFLVLDDIMDNSVTRRGQPCWFRVPQVGMVAINDGILLRNHIHRILKKHFR
DKPYYVDLVDLFNEVELQTACGQMIDLITTFEGEKDLAKYSLSIHRRIVQYKTAYYSFYL
PVACALLMAGENLENHIDVKNVLVDMGIYFQVQDDYLDCFADPETLGKIGTDIEDFKCSW
LVVKALERCSEEQTKILYENYGKPDPSNVAKVKDLYKELDLEGVFMEYESKSYEKLTGAI
EGHQSKAIQAVLKSFLAKIYKRQK
>O13965
MTVGTLSVVSSTASDTASHVSDTRKRQYQRDEALRKKIISELGKKSGNFESPVRKIRRNG
EPGTVDSAALDPALTVHMQSLVTETAQLMAAKRQNCVLVVDDDEQLAGIVTATDIATRCV
GAGLNARQTLIADIMSTSPLCITSDTRFDDALLLMIEHKFRHLPVVSDGGPDGSAGDEGD
VIGIINMRACLREPLNRIARQQEAAQKLVEALEGAQEEIENKSVSGNTNSSSVSGNHAAE
FLEYVESLKKKASGLEIMSLIDSSEEPFLVGTRTTVAEATESMARSGVSAVLVMDNGAVS
GVFTAHDVVLRVLAAGLDPYRSSVIRVMTPHPDCALASLRVSTALERMIEGKFSNLPVVD
ESDAIIGMLSLFHLATAIEQTPEEEEEVFDQAENDAGIEPSNGFEDQQQQLLGNSNEVVE
NYDVNPPLPLNPLPSNTQQSESTYEYSARQLPKPPVQAWQNENLSSNNKPQEYVGVENDY
NFSNNPPTAMSEQSFHPSVSQKPMDTPENGSNSFAASPYLQPYNSASQLAPSYVGSLPQY
HGNPSFVEQALQDLVQPTDSASQIFPLNPQSPSQFTIKYRSIAGRVHRLRLDGINSVSDL
RTAVEEREKEQLVTLTYIDDEGDVVELVSDSDLREAILLARRRGLPRLEVRGVAAFTNHL
ESSHPPISTVDSSIGSASVVEKGVANSIVDIHQPTAKADKGNSKKPIYIGIVSSSIVILA
VSMWYLRRKR
>P15245
MTKYSESYCDVLIVGAGPAGLMAARVLSEYVRQKPDLKVRIIDKRSTKVYNGQADGLQCR

TLESLKNLGLADKILSEANDMSTIALYNPDENGHIRRTDRIPDTLPGISRYHQVVLHQGR
IERHILDSIAEISDTRIKVERPLIPEKMEIDSSKAEDPEAYPVTMTLRYMSDHESTPLQF
GHKTENSLFHSNLQTQEEEDANYRLPEGKEAGEIETVHCKYVIGCDGGHSWVRRTLGFEM
IGEQTDYIWGVLDAVPASNFPDIRSPCAIHSAESGSIMIIPRENNLVRFYVQLQARAEKG
GRVDRTKFTPEVVIANAKKIFHPYTFDVQQLDWFTAYHIGQRVTEKFSKDERVFIAGDAC
HTHSPKAGQGMNTSMMDTYNLGWKLGLVLTGRAKRDILKTYEEERHAFAQALIDFDHQFS
RLFSGRPAKDVADEMGVSMDVFKEAFVKGNEFASGTAINYDENLVTDKKSSKQELAKNCV
VGTRFKSQPVVRHSEGLWMHFGDRLVTDGRFRIIVFAGKATDATQMSRIKKFSAYLDSEN
SVISLYTPKVSDRNSRIDVITIHSCHRDDIEMHDFPAPALHPKWQYDFIYADCDSWHHPH
PKSYQAWGVDETKGAVVVVRPDGYTSLVTDLEGTAEIDRYFSGILVEPKEKSGAQTEADW
TKSTA
>P17076
MAPGKKVAPAPFGAKSTKSNKTRNPLTHSTPKNFGIGQAVQPKRNLSRYVKWPEYVRVQR
QKKILSIRLKVPPTIAQFQYTLDRNTAAETFKLFNKYRPETAAEKKERLTKEAAAVAEGK
SKQDASPKPYAVKYGLNHVVALIENKKAKLVLIANDVDPIELVVFLPALCKKMGVPYAIV
KGKARLGTLVNQKTSAVAALTEVRAEDEAALAKLVSTIDANFADKYDEVKKHWGGGILGN
KAQAKMDKRAKNSDSA
>O09171
MAPIAGKKAKRGILERLNAGEVVIGDGGFVFALEKRGYVKAGPWTPEAAVEHPEAVRQLH
REFLRAGSNVMQTFTFYASEDKLENRGNYVAEKISGQKVNEAACDIARQVADEGDALVAG
GVSQTPSYLSCKSETEVKKIFHQQLEVFMKKNVDFLIAEYFEHVEEAVWAVEALKTSGKP
IAATMCIGPEGDLHGVSPGECAVRLVKAGAAIVGVNCHFDPSTSLQTIKLMKEGLEAARL
KAYLMSHALAYHTPDCGKQGFIDLPEFPFGLEPRVATRWDIQKYAREAYNLGVRYIGGCC
GFEPYHIRAIAEELAPERGFLPPASEKHGSWGSGLDMHTKPWIRARARKEYWQNLRIASG
RPYNPSMSKPDAWGVTKGAAELMQQKEATTEQQLRALFEKQKFKSAQ
>Q53HL2
MAPRKGSSRVAKTNSLRRRKLASFLKDFDREVEIRIKQIESDRQNLLKEVDNLYNIEILR
LPKALREMNWLDYFALGGNKQALEEAATADLDITEINKLTAEAIQTPLKSAKTRKVIQVD
EMIVEEEEEEENERKNLQTARVKRCPPSKKRTQSIQGKGKGKRSSRANTVTPAVGRLEVS
MVKPTPGLTPRFDSRVFKTPGLRTPAAGERIYNISGNGSPLADSKEIFLTVPVGGGESLR
LLASDLQRHSIAQLDPEALGNIKKLSNRLAQICSSIRTHK
>Q61097
MDRAALRAAAMGEKKEGGGGGAAADGGAGAAVSRALQQCGQLQKLIDISIGSLRGLRTKC
SVSNDLTQQEIRTLEAKLVKYICKQQQSKLSVTPSDRTAELNSYPRFSDWLYIFNVRPEV
VQEIPQELTLDALLEMDEAKAKEMLRRWGASTEECSRLQQALTCLRKVTGLGGEHKMDSG
WSSTDARDSSLGPPMDMLSSLGRAGASTQGPRSISVSALPASDSPVPGLSEGLSDSCIPL
HTSGRLTPRALHSFITPPTTPQLRRHAKLKPPRTPPPPSRKVFQLLPSFPTLTRSKSHES
QLGNRIDDVTPMKFELPHGSPQLVRRDIGLSVTHRFSTKSWLSQVCNVCQKSMIFGVKCK
HCRLKCHNKCTKEAPACRITFLPLARLRRTESVPSDINNPVDRAAEPHFGTLPKALTKKE
HPPAMNLDSSSNPSSTTSSTPSSPAPFLTSSNPSSATTPPNPSPGQRDSRFSFPDISACS
QAAPLSSTADSTRLDDQPKTDVLGVHEAEAEEPEAGKSEAEDDEEDEVDDLPSSRRPWRG
PISRKASQTSVYLQEWDIPFEQVELGEPIGQGRWGRVHRGRWHGEVAIRLLEMDGHNQDH
LKLFKKEVMNYRQTRHENVVLFMGACMNPPHLAIITSFCKGRTLHSFVRDPKTSLDINKT
RQIAQEIIKGMGYLHAKGIVHKDLKSKNVFYDNGKVVITDFGLFGISGVVREERRENQLK

LSHDWLCYLAPEIVREMIPGRDEDQLPFSKAADVYAFGTVWYELQARDWPFKHQPAEALI
WQIGSGEGVRRVLASVSLGKEVGEILSACWAFDLQERPSFSLLMDMLERLPKLNRRLSHP
GHFWKSADINSSKVMPRFERFGLGTLESGNPKM
>P00390
MALLPRALSAGAGPSWRRAARAFRGFLLLLPEPAALTRALSRAMACRQEPQPQGPPPAAG
AVASYDYLVIGGGSGGLASARRAAELGARAAVVESHKLGGTCVNVGCVPKKVMWNTAVHS
EFMHDHADYGFPSCEGKFNWRVIKEKRDAYVSRLNAIYQNNLTKSHIEIIRGHAAFTSDP
KPTIEVSGKKYTAPHILIATGGMPSTPHESQIPGASLGITSDGFFQLEELPGRSVIVGAG
YIAVEMAGILSALGSKTSLMIRHDKVLRSFDSMISTNCTEELENAGVEVLKFSQVKEVKK
TLSGLEVSMVTAVPGRLPVMTMIPDVDCLLWAIGRVPNTKDLSLNKLGIQTDDKGHIIVD
EFQNTNVKGIYAVGDVCGKALLTPVAIAAGRKLAHRLFEYKEDSKLDYNNIPTVVFSHPP
IGTVGLTEDEAIHKYGIENVKTYSTSFTPMYHAVTKRKTKCVMKMVCANKEEKVVGIHMQ
GLGCDEMLQGFAVAVKMGATKADFDNTVAIHPTSSEELVTLR
>Q05583
MASINLIKSLKLYKEKIWSFDFSQGILATGSTDRKIKLVSVKYDDFTLIDVLDETAHKKA
IRSVAWRPHTSLLAAGSFDSTVSIWAKEESADRTFEMDLLAIIEGHENEVKGVAWSNDGY
YLATCSRDKSVWIWETDESGEEYECISVLQEHSQDVKHVIWHPSEALLASSSYDDTVRIW
KDYDDDWECVAVLNGHEGTVWSSDFDKTEGVFRLCSGSDDSTVRVWKYMGDDEDDQQEWV
CEAILPDVHKRQVYNVAWGFNGLIASVGADGVLAVYEEVDGEWKVFAKRALCHGVYEINV
VKWLELNGKTILATGGDDGIVNFWSLEKAA
>Q9UUH5
MDQYRDLPKEDPDTAIEYESGVDEESSLKIEKFRVLIFGKDNSQDDLVKSVFNLSKLSEK
EKMPFGIKCESEENPNFESYHSKVGKKSCKRFMRWLKANAFAEERDIDCIWYQLNLLDIS
DEDILCLQYLQGCLIPIVSIIYDFNEFLSPFTKSFLSKIGESNTITVKGMPLADVLVFRK
KVLKLVSQLSFMTDRKELVYTTCLALEDDDAYTNFVSAQQEDSSCKFYGGILIGVSHCKY
AAAGASYPLPIPASGLLAQKLAIGLLCKDIIKLWNLCPESSILTKEILLELPKASDVAKS
IGKMILTSMFVPLWLLKGIWEAPATAKIIIGSIADVTLPVQRMYYFIKAGGKLDVPTIHA
FYQHYKEFVRPKCLEHIAEITTFNVVTAFSSEAVYQEAIRLLDRFRYVSKSSEEDLIVPS
FVVNDLITYD
>P51521
MPKIFLIKNRLHQQQQRLLESQNLLQHKNQDDERLVPPLSPSGSGSGPSPTPTSQPPPEP
QGQGQQVLGQVPDSDQQPLSLTRKRFHHRRHYFGQSRHSLDHLNQNQSPNPNANANPNQI
QNPAELEVECATGQVQNENFAAELLQRLTPNTATTAQNNIVNNLVNNSRAATSVLATKDC
TIENSPISIPKNQRAEDEEEQEDQEKEKPAEREREKSDERTEQVEKEERVEREEEEDDEV
DVGVEAPRPRFYNTGVVLTQAQRKEYPQEPKDLSLTIAKSSPASPHIHSDSESDSDSDGG
CKLIVDEKPPLPVIKPLSLRLRSTPPPADQRPSPPPPRDPAPAVRCSVIQRAPQSQLPTS
RAGFLLPPLDQLGPEQQEPIDYHVPKRRSPSYDSDEELNARRLERARQVREARRRSTILA
ARVLLAQSQRLNPRLVRSLPGILAAAAGHGRNSSSSSGAAGQGFQSSGFGSQNSGSGSSS
GNQNAGSGAGSPGSGAGGGGGMGGGRDGRGNYGPNSPPTGALPPFYESLKSGQQSTASNN
TGQSPGANHSHFNANPANFLQNAAAAAYIMSAGSGGGGCTGNGGGGASGPGGGPSANSGG
GGGGGGGNGYINCGGVGGPNNSLDGNNLLNFASVSNYNESNSKFHNHHHHHQHNNNNNNN
GGQTSMMGHPFYGGNPSAYGIILKDEPDIEYDEAKIDIGTFAQNIIQATMGSSGQFNASA
YEDAIMSDLASSGQCPNGAVDPLQFTATLMLSSQTDHLLEQLSDAVDLSSFLQRSCVDDE
ESTSPRQDFELVSTPSLTPDSVTPVEQHNTNTTQLDVLHENLLTQLTHNIVRGGSNQQQQ

HHQQHGVQQQQQQQHSVQQQQQHNVQQQHGVQQQHVQQQPPPSYQHATRGLMMQQQPQHG
GYQQQAAIMSQQQQQLLSQQQQQSHHQQQQQQQHAAAYQQHNIYAQQQQQQQQQHHQQQQ
QQQHHHFHHQQQQQPQPQSHHSHHHGHGHDNSNMSLPSPTAAAAAAAAAAAAAAAAAAHL
QRPMSSSSSSGGTNSSNSSGGSSNSPLLDANAAAAAAAALLDTKPLIQSLGLPPDLQLEF
VNGGHGIKNPLAVENAHGGHHRIRNIDCIDDLSKHGHHSQHQQQQGSPQQQNMQQSVQQQ
SVQQQQSLQQQQQQQHHQHHSNSSASSNASSHGSAEALCMGSSGGANEDSSSGNNKFVCR
VCMKTFSLQRLLNRHMKCHSDIKRYLCTFCGKGFNDTFDLKRHTRTHTGVRPYKCNLCEK
SFTQRCSLESHCQKVHSVQHQYAYKERRAKMYVCEECGHTTCEPEVHYLHLKNNHPFSPA
LLKFYDKRHFKFTNSQFANNLLGQLPMPVHN
>Q08689
MGRDICTLDNVYANNLGMLTKLAHVTVPNLYQDAFFSALFAEDSLVAKNKKPSSKKDVHF
TQMAYYSEIPVGGLVAKLVPKKQNELSLKGIQIEFLGVLPNYRHKSIGSKLLKFAEDKCS
ECHQHNVFVYLPAVDDLTKQWFIAHGFEQVGETVNNFIKGVNGDEQDAILLKKHIS
>P21580
MAEQVLPQALYLSNMRKAVKIRERTPEDIFKPTNGIIHHFKTMHRYTLEMFRTCQFCPQF
REIIHKALIDRNIQATLESQKKLNWCREVRKLVALKTNGDGNCLMHATSQYMWGVQDTDL
VLRKALFSTLKETDTRNFKFRWQLESLKSQEFVETGLCYDTRNWNDEWDNLIKMASTDTP
MARSGLQYNSLEEIHIFVLCNILRRPIIVISDKMLRSLESGSNFAPLKVGGIYLPLHWPA
QECYRYPIVLGYDSHHFVPLVTLKDSGPEIRAVPLVNRDRGRFEDLKVHFLTDPENEMKE
KLLKEYLMVIEIPVQGWDHGTTHLINAAKLDEANLPKEINLVDDYFELVQHEYKKWQENS
EQGRREGHAQNPMEPSVPQLSLMDVKCETPNCPFFMSVNTQPLCHECSERRQKNQNKLPK
LNSKPGPEGLPGMALGASRGEAYEPLAWNPEESTGGPHSAPPTAPSPFLFSETTAMKCRS
PGCPFTLNVQHNGFCERCHNARQLHASHAPDHTRHLDPGKCQACLQDVTRTFNGICSTCF
KRTTAEASSSLSTSLPPSCHQRSKSDPSRLVRSPSPHSCHRAGNDAPAGCLSQAARTPGD
RTGTSKCRKAGCVYFGTPENKGFCTLCFIEYRENKHFAAASGKVSPTASRFQNTIPCLGR
ECGTLGSTMFEGYCQKCFIEAQNQRFHEAKRTEEQLRSSQRRDVPRTTQSTSRPKCARAS
CKNILACRSEELCMECQHPNQRMGPGAHRGEPAPEDPPKQRCRAPACDHFGNAKCNGYCN
ECFQFKQMYG
>Q9P7W7
MGRANGKRRNHRKNRMNPIQKKGFLEQEIPLNNSEAALPLLSRLDNPEIKERSWACSAIS
NIIASCTEGRLYLLKNGLVSKLIDRISDDSVEVVVEATGALRNLAIEEGYSICMDMYRKN
VLAPLQIWQTKIVQTLDEATEGKNVLETYEDRSTFSCLCAIAENISSLLVNLGETTSQVI
KVLNQKNTLVFLSRLLIPEAKIPQSVQEMALQAFFTLTDDNDDGIITWIQNSSDFALKTI
NQIYLYFSYPSCLVRVYSIGIIYNIYQSGFVNKKMESVVKGISLFDSFIPEALPILSDLL
PSEENYRNLVRQVYDKDTYFKTKKDDLTLSSELLVIPATLELISSMTSLLQSLADGTDEL
EEQEDSLMEDEDLSYMDDMSNVVNEDENLIIDEIPSNTPQKGNFKLVEYMLDHVLPKVIT
YCVVAFEFSSEEIPSNLSNYFQEVGDRTIECLNNISWSCNSVFVESSEAFTRWKLSAGKI
LQWIFQTIFLRLGLGVWPFSSEGFTTACSLLWSVSKPFPAEIQVLSVDDISTLILFSSTH
GSLEAQSRLLGAFGSLGRCGNIQINQLLGQTLMSCVIASDPNPLLAVEALNAIFDVYGDK
SYPYDAPVFKGSGYLSQLSEALPRLKNMVKKIDRRREKHLRFRAEEALENLESFIDYKHA
EYAS
>O13822
MSVVCSCLLPTPSTDFVNQRNMPIPIIAHIAQFKYEHLITHWAQYTKAAIAVSTIAAFKF
WTSGRTTTWERKMNGMVVMVTGGSSGIGQVVVEKLASLGAQVVILLRTEPDQFTVDYIMD

LRKRTKNQLIYTEVCDLSSMLSVRKFATKWIDCTPIRRLDMIVLCSGVLLPPFMDRQTTE
EGVELQWATNFLGPYQLLRILRPVIYGQPGHREVRIVAATCSSYILGNIDFNDLDLSNHP
YPRKSPWKVVGNAKLALMTYLYDFQKKAEAHERPDKMPCNLHTIMANPGVVRTPGFRRVV
SFGKVWGLFLYLLLWPFWWLLLKGTIHGAQSFFHAICSPEFASITQPVLVNECSIVEYSR
KEITDPEFAEKLIKAADAQIDEVEKQYKKKKIKKSKK
>P41056
MAESHRLYVKGKHLSYQRSKRVNNPNVSLIKIEGVATPQEAQFYLGKRIAYVYRASKEVR
GSKIRVMWGKVTRTHGNSGVVRATFRNNLPAKTFGASVRIFLYPSNI
>P41911
MLAVRRLTRYTFLKRTHPVLYTRRAYKILPSRSTFLRRSLLQTQLHSKMTAHTNIKQHKH
CHEDHPIRRSDSAVSIVHLKRAPFKVTVIGSGNWGTTIAKVIAENTELHSHIFEPEVRMW
VFDEKIGDENLTDIINTRHQNVKYLPNIDLPHNLVADPDLLHSIKGADILVFNIPHQFLP
NIVKQLQGHVAPHVRAISCLKGFELGSKGVQLLSSYVTDELGIQCGALSGANLAPEVAKE
HWSETTVAYQLPKDYQGDGKDVDHKILKLLFHRPYFHVNVIDDVAGISIAGALKNVVALA
CGFVEGMGWGNNASAAIQRLGLGEIIKFGRMFFPESKVETYYQESAGVADLITTCSGGRN
VKVATYMAKTGKSALEAEKELLNGQSAQGIITCREVHEWLQTCELTQEFPLFEAVYQIVY
NNVRMEDLPEMIEELDIDDE
>O95271
MAASRRSQHHHHHHQQQLQPAPGASAPPPPPPPPLSPGLAPGTTPASPTASGLAPFASPR
HGLALPEGDGSRDPPDRPRSPDPVDGTSCCSTTSTICTVAAAPVVPAVSTSSAAGVAPNP
AGSGSNNSPSSSSSPTSSSSSSPSSPGSSLAESPEAAGVSSTAPLGPGAAGPGTGVPAVS
GALRELLEACRNGDVSRVKRLVDAANVNAKDMAGRKSSPLHFAAGFGRKDVVEHLLQMGA
NVHARDDGGLIPLHNACSFGHAEVVSLLLCQGADPNARDNWNYTPLHEAAIKGKIDVCIV
LLQHGADPNIRNTDGKSALDLADPSAKAVLTGEYKKDELLEAARSGNEEKLMALLTPLNV
NCHASDGRKSTPLHLAAGYNRVRIVQLLLQHGADVHAKDKGGLVPLHNACSYGHYEVTEL
LLKHGACVNAMDLWQFTPLHEAASKNRVEVCSLLLSHGADPTLVNCHGKSAVDMAPTPEL
RERLTYEFKGHSLLQAAREADLAKVKKTLALEIINFKQPQSHETALHCAVASLHPKRKQV
TELLLRKGANVNEKNKDFMTPLHVAAERAHNDVMEVLHKHGAKMNALDTLGQTALHRAAL
AGHLQTCRLLLSYGSDPSIISLQGFTAAQMGNEAVQQILSESTPIRTSDVDYRLLEASKA
GDLETVKQLCSSQNVNCRDLEGRHSTPLHFAAGYNRVSVVEYLLHHGADVHAKDKGGLVP
LHNACSYGHYEVAELLVRHGASVNVADLWKFTPLHEAAAKGKYEICKLLLKHGADPTKKN
RDGNTPLDLVKEGDTDIQDLLKGDAALLDAAKKGCLARVQKLCTPENINCRDTQGRNSTP
LHLAAGYNNLEVAEYLLEHGADVNAQDKGGLIPLHNAASYGHVDIAALLIKYNTCVNATD
KWAFTPLHEAAQKGRTQLCALLLAHGADPTMKNQEGQTPLDLATADDIRALLIDAMPPEA
LPTCFKPQATVVSASLISPASTPSCLSAASSIDNLTGPLAELAVGGASNAGDGAAGTERK
EGEVAGLDMNISQFLKSLGLEHLRDIFETEQITLDVLADMGHEELKEIGINAYGHRHKLI
KGVERLLGGQQGTNPYLTFHCVNQGTILLDLAPEDKEYQSVEEEMQSTIREHRDGGNAGG
IFNRYNVIRIQKVVNKKLRERFCHRQKEVSEENHNHHNERMLFHGSPFINAIIHKGFDER
HAYIGGMFGAGIYFAENSSKSNQYVYGIGGGTGCPTHKDRSCYICHRQMLFCRVTLGKSF
LQFSTMKMAHAPPGHHSVIGRPSVNGLAYAEYVIYRGEQAYPEYLITYQIMKPEAPSQTA
TAAEQKT
>Q12734
MQSTVPIAIASNGNKRDVVQNVSAGDEGDILQRLARNREMISTSLSPQKSSGFSGRRRSS
SVRDALSSFFGTGNSPTSSMDDYSNLMNRNYSTASTAMCRGNSFPSDVGTKAYNITGSYQ

PDRHRNSVPYTTIDQLHTRQDTGLRRESDPVAAKQISSNNDIVRSFITHHASNSTMFINR
VLSDYLADRGFIKQTPLYNKKSVLEISIATSAESVFLPTTKSDETEYLSLIHGSLNQART
QPVGSTNTAESDFLPSCPTMDTLNENNDLSLFPLHTQRTSPSNTARTGNAMDTSNSDRAS
PASNNNTTDADSFVASGNNNPMNNNNSPARNRHPNSHSRSLPNAWNSQMPSFSFALIFSL
NKSTTLSDIKVELTSNVRVVWFNGLPPTKNVNEECYNIGSLDWTLNADNFNLFIPQGAKS
PLDIVENHSNNRKLKVLQKLSMRKRRSFSNKAVLRENILNNLNASNSTNKLNAGVYVFTI
PIVLASRIPESLYYPSARVSYSLRLATKLKDEHTQLVASRPRSSSISSPQKLRSYSCSDS
YEYSQIDDTIEGETYNNDKNSTGKIAFPSSWLKSAKGRLKRNNSNGRSDNNGASSSGLAM
QHDSEDTINLQYPLNLVRTPPEISVTTANKPLYINKVWENCLSYEISFAQKYVPLNGEIP
ITIKVAPLVKSLSVKRIRVSCREKISYRSKDYQYDFDQLDPLASDPCNPYHMRYLVRKKK
DRSLPLFEVASKCTSGPSIREEVVTNTVDDNLLAYTSSKENNKDIPFSESFTVKTKLKFP
KYCEVDATKAASLPPYGIDLFDPIKDPTQSENTSNNGNVLGFLVGRPNRASKTVHKIPQD
KNHNEVNDTNGNSNTSLQTSSNVPIQHYTRLNKPRRGLYLDSMHFKNIQCSHKLEIVLRV
SKTDSGSSKIIRHYEVIVDTPIYLISDLCNTSNIDLPTYDMATTESSKVLPPTFEEATSV
SASPRSSVSYYPDDISMQQLNLSRSTSLANGYLSTLHPKTTAVSDSSNGAPIRDQQEQQA
RPLRTEDYALQMGNENNAYSNMDGLLSQDIFEQETAATLFKRDIVTMNFNNNIFTPRYSP
RTFTNTDYNYNDNDNNDNDTEGPGPIIHPGPEPPRYDEISS
>Q03266
MSLMDNWKTDMESYDEGGLVANPNFEVLATFRYDPGFARQSASKKEIFETPDPRLGLRDE
DIRQQIINEDYSSYLRVREVNSGGDLLENIQHPDAWKHDCKTIVCQRVEDMLQVIYERFF
LLDEQYQRIRIALSYFKIDFSTSLNDLLKLLVENLINCKEGNSEYHEKIQKMINERQCYK
MRVLVSKTGDIRIEAIPMPMEPILKLTTDYDSVSTYFIKTMLNGFLIDSTINWDVVVSSE
PLNASAFTSFKTTSRDHYARARVRMQTAINNLRGSEPTSSVSQCEILFSNKSGLLMEGSI
TNVAVIQKDPNGSKKYVTPRLATGCLCGTMRHYLLRLGLIEEGDIDIGSLTVGNEVLLFN
GVMGCIKGTVKTKY
>Q8WXC3
MGTKREAILKVLENLTPEELKKFKMKLGTVPLREGFERIPRGALGQLDIVDLTDKLVASY
YEDYAAELVVAVLRDMRMLEEAARLQRAA
>Q09697
MISSFSNGFWSKDYATGVKKLFDCLDNGVEENEQVKNLLKLYKEANEEFGEKLQEITKEC
LKGKKPENTEDGATSNKAFEGLRSEIANQGKQHIRIAKDLETLIIAPFSKMSIDHSQKLQ
TSQQVLTNQIKSYEKKYYTLKKTKSAYYNKCRNLEDYEEESKESNETTSEAITDLTTVSS
PQQQSLLENDDDLIQLGFMEFRPEELKEVLAQVLQEIPLQDYRVPILGTYPNTCSGNIIV
SWLQENLPVPTLVAAEAFGQDLIAQGFLRHMGVGGSFVNSTNFHYQWKDKAFQFAGLNSV
DSLVENAKALPLVGEYLSDYISHRKLYSSETQSQRLKREVLDANKIYSESVVDLDKCRTL
VEETIADHLQFLQKCETDRVLYYKDFFMDLSTIISNFLPSMKLLADQIVVYQEIIQPESD
IRYILESAATGPFLPRVEIYEDYYNDIKDQIFGVDVEFLSHRDKKRVPIIVSTILSYLDL
LYPTLASDEVRQNIWLVNSPLSSVHQLREALNHSSSVTKEVLSQYTPSVVIGVLKLYFLE
LPDSIVPSSAFELIRSIYMNHSNDTPYRLRLLQNLLSQLRRVNLATLSAIITHLNRLITL
TPNKETFTINLANSLSLCISRPATWNLGIQHDKHPTKFMEDLLTYGPSIFEELRKLNSSK
RVSDRVLYQSSATPRSTDVSPTRPDSISSVRSHTAVESPRSSFEELQPSEIPAESEFTLE
NVPTSLIRSSYALNTRKTRRNFSHSSASNESAAIFIDQDAKIVNEAVASRDSSLSGS
>O94506
MSFPTDMETNEILDQLDKIDERNEDILLKSLKASKCTYKPWSREEFLRRLLTYRSRWAYV

NDPQIGEINCCLNGWLCESNNILVCDVCRNKINLTALQQVDAENDSLNELPEKTKERLEV
SLKEEHQDNCLWRLHKFPPDIYHLSVSAELVQVGRRFNSLSTRLVSTHLPEEMTLKRLEK
VANKIRVDWEKEDAAVLLGIALTGWSEQVPGRLYVCNYCHRRLGVWNLQSEGQDFDVLEE
HKKSCPWVIPQPFTDLLGWQQIFELLCKESIFQSTTKTMDVSQYTDYTFSLLQGLR
>Q56B11
MAAAVLSGPTTGSPAGAPGGPGGLSAAGSGPRLRLLLLESVSGLLQPRTGSHVAPVHPPI
QWAPYLPGLMCLLRLHGTAGGAQNLSALGALVNLSNAHLSSIKTRFEGLCLLSLLVGESP
TELFQQHCVSWLRSIQQVLQSQDSPPTMELAVAILRDLLRYASQLPTLFRDISTNHLPGL
LTSLLGLRPQCEQSALEGMKACVTYFPRACGFLKGKLASFFLSRLDSLNPQLQQLACECY
SRLPSLGAGFSQGLKHTENWEQELHSLLTSLHSLLGSLFEETETAPVQSEGPGVEMLLSP
SEDDNTHVLLQLWQRFSGLARCLGLMLSSEFGAPVSVPVQEILDLICRILGISSKNINLL
GDGPLRLLLLPSLHLEALDLLSALILACGGRLLRFGALISRLLPQVLNTWSTGRDALAPG
QERPYSTIRTKVYAILELWVQVCGASAGMLQGGASGEALLTHLLSDISPPADALKLCSTR
GSSDGGLQSGKPSAPKKLKLDMGEALAPPSQRKGDRNADSDVCAAALRGLSRTILMCGPL
VKEETHRRLHDLVLPLVMSVQQGEVLGSSPYNSSCCRLELYRLLLALLLAPSPRCPPPLS
CALKAFSLGQWEDSLEVSSFCSEALVTCSALTHPRVPPLQSSGPACPTPAPVPPPEAPSS
FRAPAFHTPGPMPSIGALPSPGPVPSAGPIPTVGSMSSAGSVPSTGPVPSRPGPPATANH
LGLAVPGLVSVPPRLLPGSENHRAGSGEDPVLAPSGTPPPSIPPDETFGGRVPRPAFVHY
DKEEASDVEISLESDSDDSVVIVPEGLPSLPPPPSGTPPPVAPIGPPTASPPVPAKEDSE
ELPATPGPLPPPPPPPPPVSGPVTLPPPQLVPEGTPGGGGPTAMEEDLTVININSSDEEE
EEEEEEEEEDEDVEEEDFEEEEEDEEEYFEEEEEEEEFEEEFEEEEGELEEEEEEEEEEL
EEVEDVEFGSAGEVEEGGPPPPTLPPALPPTDSPKVQPEAEPEPGLLLEVEEPGAEDGPG
PEIAPTLAPEVLPSQEEVEREGESPTAGPPQELVEEESSAPPTLLEEGTEGGGDKVPPPP
ETPAQEEMETETEASAPQGKEQDDTAAMLADFIDCPPDDEKPPPATEPDS
>Q9US35
MASNVDKFSSRFYLNVNKEELKKEYVGKTIQQVPTPGFVIDEAIFEKNCNRMLDRASDIG
VTFRAHVKTHKTIEGTLLQLGDGRTKAVVVSTLMEGFSLIPLILEGKIDDLLYGLPVAKS
RLPELYELSKIVPHLRLMIDNPKQLDILREFTSTLPDDAKPWSIFVKIDMGTHRAGVTND
SQVVKDLISTILSDKSLFDLFGFYCHAGHSYASRSIDAASEFLCAEIDAANTAAKFATSI
DPSLKLTLSVGATPTAHSVSPKVKELLPTLSGKLEVHAGNYPMNDVQQMITKCISQADVA
DYVFAEVISNYPGRNGEPGEVLVNAGVIAMSRETSPEGDFGIVITPGFESFYVDRLSQEH
GILKSKDPKATLPDASQVLCIIPNHSCITAAAFPWYYITKGSDVITDIWVPWKGW
>Q03010
MSTLDIAEDNKIKNEEFKIWKKSIPSLYQHISSLKPIFGSGVDESPSTLRSIVFTNDSSC
NKSKGVLSVPLLYSQGSEIFEVDCIVPLGLHYKKPESISEPLVQPDYTMESQKVEQTVLI
PKWEFKGETIAKMIYVDNSEINVKVIALSTNGSLAWFREGVKSPVYTMMEPSTSLSSASS
GNQNKPCVDFAISNDSKTLTVTKEKHLDNENATIKLIDNSGKIGEVLRTIPVPGIKNIQE
IKFLNNQIFATCSDDGIIRFWGNEIGKKPLWILNDSLDGKTTCFAASPFVDTLFMTGTSG
GALKVWDIRAVIALGDADAELNINQGHNKVNELFKVHHFYSEQVSKIEFSSISPMEVVTI
GGLGNVYHWNFEPVFAIYNEIHEDFQGIISDELEAESMAFYHTEGCRREIGENNKVNTVA
YHKYIEDLVATVDSDGLLTVYKPFTGKVLDGSREVGAAKS
>Q54BD4
MSNNNPKKRPLDAISNTFEVKQEEPEFSSDGFNTTNDDLMSLMTFLDNGTGQQNQQNQQN
QQPQPQPQPQPQLPQPQSQQPIYNSNTTTVTVKTEGIATSPLSNASSPISTNNNINNNTN

NNNNNNNNNNNNNNNNNNNNNNINTATPPAIGVQQNSNIPYSYPIYTDVTGQQTQHQQNI
GQNSVNIDPYFQTIDGAQIQQQQLLQQQLQPIQQVNNPQIDQAQIQQQQAQQIQQQQAQI
QAQQAQIQQQQLEQQHLQQQQFQFQQQQQQQQQQQQQQQQQQQQQQQQQQQQQQQQHHQQ
QQQQQQHQQQQQHQQQQQHQQHQQQQHQQHQQIHQNHQNQLNHQNQLNNQNQLNNQNQLN
NQNQLNHQNQLNQPTQPQMQQIELPILHQLPVEPQHLHPIPTHINNNGNNNNNNNNNGSN
SSNSSNGIGSPDDIIEPNILSSIQHCNNNNLPLPDHLLINTPYGNVLQPHQQIINECLKL
HLAQKEQLDKMKIVQKQVLAHPQKETFQMLDNEQNTLKKQIDAEITSLQQIDQTFVLSPP
EIRNVIFLLHELTIQSIQLELYHEELQLLVRPQNPPPTIAALVVIEQPFPMVITKCKPLE
DDPVVVQLLCGTRTELQMIGKVRATMIVENQQGSKTSSSPKTIETEVVSMDETNRLAKYH
LKFLNGTRKNPVTLKFGMQVQVVGGTAVNIESPPTSPFIVITNECQYEESDGTLLKKDSF
GNNNEIPWASYANKLQRHFLRATRQDFMKPTRYLSRHELMYIHHQFFGSKPMIPQSSFDS
FWIWFGKGLQKLRYQRHVCSMWQSGLIYGFISRQSVEEALRNEEQGTFLIRFSERHAGHF
AVGYKVDDPDPEKRIRHYLVKADDTAGAKKTLPDFLSECPQFTKILQLTIDVSTGEPRLR
NFPKDVVLEPYYSKRETLPATNGYDSLPTIL
>Q86VQ3
MDVDKELGMESVKAGASGKPEMRLGTQEETSEGDANESSLLVLSSNVPLLALEFLEIAQA
KEKAFLPMVSHTFHMRTEESDASQEGDDLPKSSANTSHPKQDDSPKSSEETIQPKEGDIP
KAPEETIQSKKEDLPKSSEKAIQPKESNIPKSSAKPIQPKLGNIPKASVKPSQPKEGDIP
KAPEETIQSKKEDLPKSSEEAIQPKEGDIPKSSAKPIQPKLGNIAKTSVKPSQPKESDIP
KSPEETIQPKEGDIPKSSAKPIQPKLGNIPKASVKPSQPKEGDISKSPEEAIQPKEGDLP
KSLEEAIQPKEGDIPKSPEEAIQPKEGDIPKSLEEAIQPKKGDIPKSPEETIQPKKDDIP
KSPEEAIQPKEGDIPKSPKQAIQPKEGDIPKSLEEAIPPKEIDIPKSPEETIQPKEDDSP
KSLEEATPSKEGDILKPEEETMEFPEGDKVKVILSKEDFEASLKEAGERLVAVDFSATWC
GPCRTIRPFFHALSVKHEDVVFLEVDADNCEEVVRECAIMCVPTFQFYKKEEKVDELCGA
LKEKLEAVIAELK
>P56932
MAGAGGGGCPTGGNDFQWCFSQVKGAVDEDVAEADIISTVEFNYSGDLLATGDKGGRVVI
FQREQENKGRAHSRGEYNVYSTFQSHEPEFDYLKSLEIEEKINKIRWLPQQNAAHFLLST
NDKTIKLWKISERDKRAEGYNLKDEDGRLRDPFRITALRVPILKPMDLMVEASPRRIFAN
AHTYHINSISVNSDHETYLSADDLRINLWHLEITDRSFNIVDIKPANMEELTEVITAAEF
HPHQCNVFVYSSSKGTIRLCDMRSSALCDRHAKFFEEPEDPSSRSFFSEIISSISDVKFS
HSGRYMMTRDYLSVKVWDLNMEGRPVETHHVHEYLRSKLCSLYENDCIFDKFECCWNGSD
SAIMTGSYNNFFRMFDRNTRRDVTLEASRENSKPRASLKPRKVCSGGKRKKDEISVDSLD
FNKKILHTAWHPMESIIAVAATNNLYIFQDKIN
>O55207
MALSKGLRLLARLDPTGPSSVLLEARGRGDCLLFEAGAVATLAPEEKEVIKGLYGKPTDA
YGCLGELSLKSGGVPLSFLVLVTGCTSVGRIPDAEIYKITGTEFYPLQEEAKEEDRLPAL
KKILSSGVFYFAWPNDGACFDLTIRAQKQGDDCSEWGTSFFWNQLLHVPLRQHQVNCHDW
LLKVICGVVTIRTVYASHKQAKACLISRISCERAGARFLTRGVNDDGHVSNFVETEQAIY
MDDGVSSFVQIRGSVPLFWEQPGLQVGSHHLRLHRGLEANAPAFERHMVLLKEQYGQQVV
VNLLGSRGGEEVLNRAFKKLLWASCHAGDTPMINFDFHQFAKGRKLEKLENLLRPQLKLH
WDDFGVFAKGENVSPRFQKGTLRMNCLDCLDRTNTVQCFIALEVLHLQLESLGLNSKPIT
DRFVESFKAMWSLNGHGLSKVFTGSRALEGKAKVGKLKDGARSMSRTIQSNFFDGVKQEA
IKLLLVGDVYNEESTDKGRMLLDNTALLGLGSNKQNSLSGMLDGKATPRILKAMTERQSE

FTNFKRIQIAMGTWNVNGGKQFRSNLLGTTELTDWLLDAPQLSGAVDSQDDGGPADIFAV
GFEEMVELSAGNIVNASTTNRKMWGEQLQKAISRSHRYILLTSAQLVGVCLYIFVRPYHV
PFIRDVAIDTVKTGMGGKAGNKGAVGIRFQFHSTSFCFICSHLTAGQSQVKERNEDYREI
THKLSFPSGRNIFSHDYVFWCGDFNYRIDLTYEEVFYFVKRQDWKKLMEFDQLQLQKSSG
KIFKDFHEGTINFGPTYKYDVGSAAYDTSDKCRTPAWTDRVLWWRKKHPYDKTAGELNLL
DSDLDGDANIRHTWSPGTLKYYGRAELQASDHRPVLAIVEVEVQEVDVGARERVFQEVSS
VQGPLDATVIVNLQSPTLEERNEFPEDLRTELMQTLGNYGTIILVRINQGQMLVTFADSH
SALSVLDVDGMKVKGRAVKIRPKTKDWLEGLREELIRKRDSMAPVSPTANSCLLEENFDF
TSLDYESEGDVLEDDEDYLADEFGQPVVSDSELGGDDSSDTMSASTPASKSPALAKKKQH
PTYKDDADLMTLKLELEVAGNFRHRSPSRSLSVPNRPRPPHPPQRPPPPTGLMVKKSASD
ASISSGTHGQYSILQTAKLLPGAPQQPPKARTGISKPYNVKQIKTTNAQEAEAAIRCLLE
AGGGVPESAPGATPLRNQGSSKPEASLGPPVLPRRPVPRVPTMKKPTLRRTGKPMLPEEQ
CEQQPVHFTMASQEMNLETPPPITAPIPPVPKPRTFQPGRGVERRPSGGKPEPDDAPPVT
GAVELSSPEAPEAPSLAPKVPPRRKKSAPAAFHLQVLQSNSQLLQGLTCSSSSPSPPKPD
TPLLYPQMALGTSSAISPETDGPRVTEPEAASFHGDYPDPFWSLLHHPKLLNNNTWLSKS
SEPLDLGSRTPERTHTDSAQVNASVVERGLPPDHGGKDFSHWMAASNKDKRTTLGV
>P06100
MEKFGLKALVPLLKLEDKELSSTYDHSMTLGADLSSMLYSLGIPRDSQDHRVLDTFQSPW
AETSRSEVEPRFFTPESFTNIPGVLQSTVTPPCFNSIQNDQQRVALFQDETLFFLFYKHP
GTVIQELTYLELRKRNWRYHKTLKAWLTKDPMMEPIVSADGLSERGSYVFFDPQRWEKCQ
RDFLLFYNAIM
>O95347
MHIKSIILEGFKSYAQRTEVNGFDPLFNAITGLNGSGKSNILDSICFLLGISNLSQVRAS
NLQDLVYKNGQAGITKASVSITFDNSDKKQSPLGFEVHDEITVTRQVVIGGRNKYLINGV
NANNTRVQDLFCSVGLNVNNPHFLIMQGRITKVLNMKPPEILSMIEEAAGTRMYEYKKIA
AQKTIEKKEAKLKEIKTILEEEITPTIQKLKEERSSYLEYQKVMREIEHLSRLYIAYQFL
LAEDTKVRSAEELKEMQDKVIKLQEELSENDKKIKALNHEIEELEKRKDKETGVILRSLE
DALAEAQRVNTKSQSAFDLKKKNLACEESKRKELEKNMVEDSKTLAAKEKEVKKITDGLH
ALQEASNKDAEALAAAQQHFNAVSAGLSSNEDGAEATLAGQMMACKNDISKAQTEAKQAQ
MKLKHAQQELKNKQAEVKKMDSGYRKDQEALEAVKRLKEKLEAEMKKLNYEENKEESLLE
KRRQLSRDIGRLKETYEALLARFPNLRFAYKDPEKNWNRNCVKGLVASLISVKDTSATTA
LELVAGERLYNVVVDTEVTGKKLLERGELKRRYTIIPLNKISARCIAPETLRVAQNLVGP
DNVHVALSLVEYKPELQKAMEFVFGTTFVCDNMDNAKKVAFDKRIMTRTVTLGGDVFDPH
GTLSGGARSQAASILTKFQELKDVQDELRIKENELRALEEELAGLKNTAEKYRQLKQQWE
MKTEEADLLQTKLQQSSYHKQQEELDALKKTIEESEETLKNTKEIQRKAEEKYEVLENKM
KNAEAERERELKDAQKKLDCAKTKADASSKKMKEKQQEVEAITLELEELKREHTSYKQQL
EAVNEAIKSYESQIEVMAAEVAKNKESVNKAQEEVTKQKEVITAQDTVIKLNMQKWQNTR
SKTMILSLKIKELDHHISKHKREAEDGAAKVSKMLKDYDWINAERHLFGQPNSAYDFKTN
NPKEAGQRLQKLQEMKEKLGRNVNMRAMNVLTEAEERCNDLMKKKRIVENDKSKILTTIE
DLDQKKNQALNIAWQKVNKDFGSIFSTLLPGANAMLAPPEGQTVLDGLEFKVALGNTWKE
NLTELSGGQRSLVALSLILSMLLFKPAPIYILDEVDAALDLSHTQNIGQMLRTHFTHSQF
IVVSLKEGMFNNANVLFKTKFVDGVSTVARFTQCQNGKISKEAKSKAKPPKGAHVEV
>Q8IWT3
MVGERHAGDLMVPLGPRLQAYPEELIRQRPGHDGHPEYLIRWSVLKCGEVGKVGVEEGKA

EHILMWLSAPEVYANCPGLLGERALSKGLQHEPAGVSGSFPRDPGGLDEVAMGEMEADVQ
ALVRRAARQLAESGTPSLTAAVLHTIHVLSAYASIGPLTGVFRETGALDLLMHMLCNPEP
QIRRSAGKMLQALAAHDAGSRAHVLLSLSQQDGIEQHMDFDSRYTLLELFAETTSSEEHC
MAFEGIHLPQIPGKLLFSLVKRYLCVTSLLDQLNSSPELGAGDQSSPCATREKSRGQREL
EFSMAVGNLISELVRSMGWARNLSEQGMSPPRPTRSIFQPYISGPSLLLPTIVTTPRRQG
WVFRQRSEFSSRSGYGEYVQQTLQPGMRVRMLDDYEEISAGDEGEFRQSNNGIPPVQVFW
QSTGRTYWVHWHMLEILGPEEATEDKASAAVEKGAGATVLGTAFPSWDWNPMDGLYPLPY
LQPEPQKNERVGYLTQAEWWELLFFIKKLDLCEQQPIFQNLWKNLDETLGEKALGEISVS
VEMAESLLQVLSSRFEGSTLNDLLNSQIYTKYGLLSNEPSSSSTSRNHSCTPDPEEESKS
EASFSEEETESLKAKAEAPKTEAEPTKTRTETPMAQSDSQLFNQLLVTEGMTLPTEMKEA
ASEMARALRGPGPRSSLDQHVAAVVATVQISSLDTNLQLSGLSALSQAVEEVTERDHPLV
RPDRSLREKLVKMLVELLTNQVGEKMVVVQALRLLYLLMTKHEWRPLFAREGGIYAVLVC
MQEYKTSVLVQQAGLAALKMLAVASSSEIPTFVTGRDSIHSLFDAQMTREIFASIDSATR
PGSESLLLTVPAAVILMLNTEGCSSAARNGLLLLNLLLCNHHTLGDQIITQELRDTLFRH
SGIAPRTEPMPTTRTILMMLLNRYSEPPGSPERAALETPIIQGQDGSPELLIRSLVGGPS
AELLLDLERVLCREGSPGGAVRPLLKRLQQETQPFLLLLRTLDAPGPNKTLLLSVLRVIT
RLLDFPEAMVLPWHEVLEPCLNCLSGPSSDSEIVQELTCFLHRLASMHKDYAVVLCCLGA
KEILSKVLDKHSAQLLLGCELRDLVTECEKYAQLYSNLTSSILAGCIQMVLGQIEDHRRT
HQPINIPFFDVFLRHLCQGSSVEVKEDKCWEKVEVSSNPHRASKLTDHNPKTYWESNGST
GSHYITLHMHRGVLVRQLTLLVASEDSSYMPARVVVFGGDSTSCIGTELNTVNVMPSASR
VILLENLNRFWPIIQIRIKRCQQGGIDTRVRGVEVLGPKPTFWPLFREQLCRRTCLFYTI
RAQAWSRDIAEDHRRLLQLCPRLNRVLRHEQNFADRFLPDDEAAQALGKTCWEALVSPLV
QNITSPDAEGVSALGWLLDQYLEQRETSRNPLSRAASFASRVRRLCHLLVHVEPPPGPSP
EPSTRPFSKNSKGRDRSPAPSPVLPSSSLRNITQCWLSVVQEQVSRFLAAAWRAPDFVPR
YCKLYEHLQRAGSELFGPRAAFMLALRSGFSGALLQQSFLTAAHMSEQFARYIDQQIQGG
LIGGAPGVEMLGQLQRHLEPIMVLSGLELATTFEHFYQHYMADRLLSFGSSWLEGAVLEQ
IGLCFPNRLPQLMLQSLSTSEELQRQFHLFQLQRLDKLFLEQEDEEEKRLEEEEEEEEEE
EAEKELFIEDPSPAISILVLSPRCWPVSPLCYLYHPRKCLPTEFCDALDRFSSFYSQSQN
HPVLDMGPHRRLQWTWLGRAELQFGKQILHVSTVQMWLLLKFNQTEEVSVETLLKDSDLS
PELLLQALVPLTSGNGPLTLHEGQDFPHGGVLRLHEPGPQRSGEALWLIPPQAYLNVEKD
EGRTLEQKRNLLSCLLVRILKAHGEKGLHIDQLVCLVLEAWQKGPNPPGTLGHTVAGGVA
CTSTDVLSCILHLLGQGYVKRRDDRPQILMYAAPEPMGPCRGQADVPFCGSQSETSKPSP
EAVATLASLQLPAGRTMSPQEVEGLMKQTVRQVQETLNLEPDVAQHLLAHSHWGAEQLLQ
SYSEDPEPLLLAAGLCVHQAQAVPVRPDHCPVCVSPLGCDDDLPSLCCMHYCCKSCWNEY
LTTRIEQNLVLNCTCPIADCPAQPTGAFIRAIVSSPEVISKYEKALLRGYVESCSNLTWC
TNPQGCDRILCRQGLGCGTTCSKCGWASCFNCSFPEAHYPASCGHMSQWVDDGGYYDGMS
VEAQSKHLAKLISKRCPSCQAPIEKNEGCLHMTCAKCNHGFCWRCLKSWKPNHKDYYNCS
AMVSKAARQEKRFQDYNERCTFHHQAREFAVNLRNRVSAIHEVPPPRSFTFLNDACQGLE
QARKVLAYACVYSFYSQDAEYMDVVEQQTENLELHTNALQILLEETLLRCRDLASSLRLL
RADCLSTGMELLRRIQERLLAILQHSAQDFRVGLQSPSVEAWEAKGPNMPGSQPQASSGP
EAEEEEEDDEDDVPEWQQDEFDEELDNDSFSYDESENLDQETFFFGDEEEDEDEAYD
>Q8R5J9
MDVNLAPLRAWDDFFPGSDRFARPDFRDISKWNNRVVSNLLYYQTNYLVVAAMMISVVGF
LSPFNMILGGVIVVLVFMGFVWAAHNKDILRRMKKQYPTAFVMVVMLASYFLISMFGGVM

VFVFGITLPLLLMFIHASLRLRNLKNKLENKMEGIGLKKTPMGIILDALEQQEDNINKFA
DYISKARE
>Q6WQI6
MGNWGLGIAPWVDGESELEFRRLGMQGPLEALRRREWNTQRASFSFSFLIALSPHTVDYC
HSYELFNRRWHGHVLATQRPSLFILMLV
>Q04341
MSDILDEIVIEDVVANCPQEFLQYHKCIRDNEENPGKCKDGRMILSTCIREKVPSVKSIM
SECSEPMKKYDQCIRDNMGTRTINENCLGFLQDLRKCAELQVKNKNIKPSINGVNLELIK
D
>O94308
MSLNLLVNQEELKRYLDEYGVWSKIFRALFVARNSKPLRSFCVHYAIKELKEKTYNLELY
QSLFEEFQDCFENEQLDVEWVESVTFHRKQNLEQLRRELKAYKNNLIRESIRAAQLDLAS
FFADVGQFDSALRSYAKVREYCTNAGQIAHLSLELMRISIWIGNYSHVLAFGSRAKSTVS
AAMELTSPIYAYCGLANFCLGDYEEALAHFLKVETDTSDGIITKTDISVYISLCALACWD
HKRLIQELERNEEFNALSDLDPSLRRCLQAKCNRKYSLLLDTLQQNAQDYSLDMYLAPQL
TNLFSLIRERSLLDYLIPYSALPFSKIAVDFHIDENFIEKNLLEIIEAKKLNGKVDSLQK
RVYIEPSSEPENFEDIKNIAQTSLLYSKALYLQTLAAMNTENTDDEAPVIAQTNITAEDS
QN
>O95391
MSATVVDAVNAAPLSGSKEMSLEEPKKMTREDWRKKKELEEQRKLGNAPAEVDEEGKDIN
PHIPQYISSVPWYIDPSKRPTLKHQRPQPEKQKQFSSSGEWYKRGVKENSIITKYRKGAC
ENCGAMTHKKKDCFERPRRVGAKFTGTNIAPDEHVQPQLMFDYDGKRDRWNGYNPEEHMK
IVEEYAKVDLAKRTLKAQKLQEELASGKLVEQANSPKHQWGEEEPNSQTEKDHNSEDEDE
DKYADDIDMPGQNFDSKRRITVRNLRIREDIAKYLRNLDPNSAYYDPKTRAMRENPYANA
GKNPDEVSYAGDNFVRYTGDTISMAQTQLFAWEAYDKGSEVHLQADPTKLELLYKSFKVK
KEDFKEQQKESILEKYGGQEHLDAPPAELLLAQTEDYVEYSRHGTVIKGQERAVACSKYE
EDVKIHNHTHIWGSYWKEGRWGYKCCHSFFKYSYCTGEAGKEIVNSEECIINEITGEESV
KKPQTLMELHQEKLKEEKKKKKKKKKKHRKSSSDSDDEEKKHEKLKKALNAEEARLLHVK
ETMQIDERKRPYNSMYETREPTEEEMEAYRMKRQRPDDPMASFLGQ
>P32454
MPIVRWLLLKSAVRGSSLIGKAHPCLRSIAAHPRYLSNVYSPPAGVSRSLRINVMWKQSK
LTPPRFVKIMNRRPLFTETSHACAKCQKTSQLLNKTPNREILPDNVVPLHYDLTVEPDFK
TFKFEGSVKIELKINNPAIDTVTLNTVDTDIHSAKIGDVTSSEIISEEEQQVTTFAFPKG
TMSSFKGNAFLDIKFTGILNDNMAGFYRAKYEDKLTGETKYMATTQMEPTDARRAFPCFD
EPNLKASFAITLVSDPSLTHLSNMDVKNEYVKDGKKVTLFNTTPKMSTYLVAFIVAELKY
VESKNFRIPVRVYATPGNEKHGQFAADLTAKTLAFFEKTFGIQYPLPKMDNVAVHEFSAG
AMENWGLVTYRVVDLLLDKDNSTLDRIQRVAEVVQHELAHQWFGNLVTMDWWEGLWLNEG
FATWMSWYSCNEFQPEWKVWEQYVTDTLQHALSLDSLRSSHPIEVPVKKADEINQIFDAI
SYSKGASLLRMISKWLGEETFIKGVSQYLNKFKYGNAKTEDLWDALADASGKDVRSVMNI
WTKKVGFPVISVSEDGNGKITFRQNRYLSTADVKPDEDKTIYPVFLALKTKNGVDSSVVL
SERSKTIELEDPTFFKVNSEQSGIYITSYTDERWAKLGQQADLLSVEDRVGLVADVKTLS
ASGYTSTTNFLNLVSKWNNEKSFVVWDQIINSISSMKSTWLFEPKETQDALDNFTKQLIS
GMTHHLGWEFKSSDSFSTQRLKVTMFGAACAARDADVEKAALKMFTDYCSGNKEAIPALI
KPIVFNTVARVGGAENYEKVYKIYLDPISNDEKLAALRSLGRFKEPKLLERTLGYLFDGT

VLNQDIYIPMQGMRAHQEGVEALWNWVKKNWDELVKRLPPGLSMLGSVVTLGTSGFTSMQ
KIDEIKKFFATKSTKGFDQSLAQSLDTITSKAQWG
>O43061
MRLYFSAFRHPKRLGLCLLITLSIILLGWQCVLLYNHSPEIQRSVYTLTGLAPSSKMAFV
TMLTVRAANGENEVENTQQDWYYNSTRLLVHRLVKFKPTKSKYPVVVLAMKGIDQWKLDQ
LQEDGAIVKVVDPLYAHEVVDDVNDIALLDSRWSMMFTKLRVFEMYEYDRICFLDSDILP
IKKMDKVFDVHQLSYSKDSVLFPPTLFYKPRRSIFWRRFTEEFAAYGLTRDDLYPYVFAA
VSDPGMWHETPPPFKDYFNAGLFVFKPLKAHYKRLMALARFPKLYDNANMMEQSLLNFAY
NSAGAFPWESLDWTFNGLWARKNDLPYLKAVHGKHWQPEGSLGYDEDTSKLWWDAFQEMQ
TYHDQQRNADLY
>Q6NZG4
MNKRRKFLFASVLAVQNSNFIYPSCQRCFSKIILESKRFTCPKCGSSGDTGSTNYRYKLS
LKVAESSKLFFITVFGSCLDTFFGLTATGLHRYLGDSIKNLETLDSGRTQSLLTTAVEKC
FVGQSFVFGVTNFGDVCGHDSDSSNFQQPCCKHRGEVRTLVASQIILPSPHVKGFTVIAY
LHPLLHKKPHCGSQEHSSQSLTSDDSDSDLNNIQGSGNTSWFSESSVGEDFFRYWEPSLE
LTSSDSQVTSSDDFPASKQIMASGTPNQNRHCISVSEVTSSKNCHDSLQSLWSLHSCMDK
NNTTGKLGEELGLPSPVCNSCHESRLSDSHFFPSQMQEPFEEHNLECYSKAEKNDYSQYD
IACYQHHEVNTTPILQQRSSAFSLSSLKPEETANPSQNSDSLIWDDLPLSESLNKFLAVV
ESEVAVTEIDAKNRKQGMDKSIDKCHKNYSRLSLTPWRNTRALNTSCFSLRSSQAMMKDS
GKEALFSNCKSNSSPHIQKESKADKTEAAVSIAGSRSDISEDFLPDTCSSALFTSSKDRE
AFITQKRTSGVLQQRNEISCRPSTSISDCSDLRSRYFTGCGQKLHSGTKGKLAIQNCSKK
YSDASDLHKLENKHRWPKKEDDNFTICRKLTYPLETLCSSPESINTSKEMPYRPSNNNLT
PSSSGDHEGSYNASADLFDYIAKDKDIGTEITKIPQDNLLPLEVPCTENYPINENRGQPS
QKPSLQSISPSRYSRPRSQSDSECDFEESQDFVPCSQSTPVAGFHQRIHGLNGASKILPS
IYSNPNANYKNRKNSPLTGQYQATSACSKNVKAFRQKPESPFVSSLTQANVFNHCPAAES
LGNDVDEWVPPTTKKVFISDILGFRVMGLRKCLDLHYSPDPKELPRKKLIKVTHKTNI
>Q15021
MAPQMYEFHLPLSPEELLKSGGVNQYVVQEVLSIKHLPPQLRAFQAAFRAQGPLAMLQHF
DTIYSILHHFRSIDPGLKEDTLEFLIKVVSRHSQELPAILDDTTLSGSDRNAHLNALKMN
CYALIRLLESFETMASQTNLVDLDLGGKGKKARTKAAHGFDWEEERQPILQLLTQLLQLD
IRHLWNHSIIEEEFVSLVTGCCYRLLENPTINHQKNRPTREAITHLLGVALTRYNHMLSA
TVKIIQMLQHFEHLAPVLVAAVSLWATDYGMKSIVGEIVREIGQKCPQELSRDPSGTKGF
AAFLTELAERVPAILMSSMCILLDHLDGENYMMRNAVLAAMAEMVLQVLSGDQLEAAARD
TRDQFLDTLQAHGHDVNSFVRSRVLQLFTRIVQQKALPLTRFQAVVALAVGRLADKSVLV
CKNAIQLLASFLANNPFSCKLSDADLAGPLQKETQKLQEMRAQRRTAAASAVLDPEEEWE
AMLPELKSTLQQLLQLPQGEEEIPEQIANTETTEDVKGRIYQLLAKASYKKAIILTREAT
GHFQESEPFSHIDPEESEETRLLNILGLIFKGPAASTQEKNPRESTGNMVTGQTVCKNKP
NMSDPEESRGNDELVKQEMLVQYLQDAYSFSRKITEAIGIISKMMYENTTTVVQEVIEFF
VMVFQFGVPQALFGVRRMLPLIWSKEPGVREAVLNAYRQLYLNPKGDSARAKAQALIQNL
SLLLVDASVGTIQCLEEILCEFVQKDELKPAVTQLLWERATEKVACCPLERCSSVMLLGM
MARGKPEIVGSNLDTLVSIGLDEKFPQDYRLAQQVCHAIANISDRRKPSLGKRHPPFRLP
QEHRLFERLRETVTKGFVHPDPLWIPFKEVAVTLIYQLAEGPEVICAQILQGCAKQALEK
LEEKRTSQEDPKESPAMLPTFLLMNLLSLAGDVALQQLVHLEQAVSGELCRRRVLREEQE
HKTKDPKEKNTSSETTMEEELGLVGATADDTEAELIRGICEMELLDGKQTLAAFVPLLLK

VCNNPGLYSNPDLSAAASLALGKFCMISATFCDSQLRLLFTMLEKSPLPIVRSNLMVATG
DLAIRFPNLVDPWTPHLYARLRDPAQQVRKTAGLVMTHLILKDMVKVKGQVSEMAVLLID
PEPQIAALAKNFFNELSHKGNAIYNLLPDIISRLSDPELGVEEEPFHTIMKQLLSYITKD
KQTESLVEKLCQRFRTSRTERQQRDLAYCVSQLPLTERGLRKMLDNFDCFGDKLSDESIF
SAFLSVVGKLRRGAKPEGKAIIDEFEQKLRACHTRGLDGIKELEIGQAGSQRAPSAKKPS
SGSRYQPLASTASDNDFVTPEPRRTTRRHPNTQQRASKKKPKVVFSSDESSEEDLSAEMT
EDETPKKTTPILRASARRHRS
>O74342
MPEKDWIGEFGPSKFKSPIDKLNQLLPESNDPSILYETSQHLLSEFDKTLNDSLRVLNQQ
INQVSEVLPRLPTMISALDRESKRLFESCEKMDPEPSALMPLQELEKIRSNIQLTISQID
NLTP
>P07900
MPEETQTQDQPMEEEEVETFAFQAEIAQLMSLIINTFYSNKEIFLRELISNSSDALDKIR
YESLTDPSKLDSGKELHINLIPNKQDRTLTIVDTGIGMTKADLINNLGTIAKSGTKAFME
ALQAGADISMIGQFGVGFYSAYLVAEKVTVITKHNDDEQYAWESSAGGSFTVRTDTGEPM
GRGTKVILHLKEDQTEYLEERRIKEIVKKHSQFIGYPITLFVEKERDKEVSDDEAEEKED
KEEEKEKEEKESEDKPEIEDVGSDEEEEKKDGDKKKKKKIKEKYIDQEELNKTKPIWTRN
PDDITNEEYGEFYKSLTNDWEDHLAVKHFSVEGQLEFRALLFVPRRAPFDLFENRKKKNN
IKLYVRRVFIMDNCEELIPEYLNFIRGVVDSEDLPLNISREMLQQSKILKVIRKNLVKKC
LELFTELAEDKENYKKFYEQFSKNIKLGIHEDSQNRKKLSELLRYYTSASGDEMVSLKDY
CTRMKENQKHIYYITGETKDQVANSAFVERLRKHGLEVIYMIEPIDEYCVQQLKEFEGKT
LVSVTKEGLELPEDEEEKKKQEEKKTKFENLCKIMKDILEKKVEKVVVSNRLVTSPCCIV
TSTYGWTANMERIMKAQALRDNSTMGYMAAKKHLEINPDHSIIETLRQKAEADKNDKSVK
DLVILLYETALLSSGFSLEDPQTHANRIYRMIKLGLGIDEDDPTADDTSAAVTEEMPPLE
GDDDTSRMEEVD
>Q99247
MNQLTVSYGLISPDYCTSQDAHILPITKILYPDIPGKNYFLTSGRDGSIILHKNTQLSNE
PETAATTIKNDAIRMQVHSDWASDLIHVNMKNSDPSAGDTFISVSHDFSIVLISVNAQLT
TWDKKIIGDHDDYIKCIVPIHYEMSNDYELEEQEGGPDNVHDGINNGIVVDEQNNFLFVT
GGLDRKIKLWCLSSGPEKMATLLHTFDNAQSNDTGSIYSMSPIIPKYSFDDNQTSRPFDF
VAGDCNGDLIFYSCKYRKEVIRIQNAHRTNIKVVRTLDDSTRLISTSSDGVINVWDLNCR
HDQTTGALQLPKKIGSWSWDSSIWCVQGTSLDKLYFGDSQGNVMRANLSSYEDAKLTRIF
KPDHHHHHHHHHEHEEQNISTTDAKVKKYGGILDIALLPNEKLLFSFCTDSNLNVLDLTN
NHFSVNEGGFALTRSSLLTNRRHVITENTKGQMQRWDIVSCELLNTFDSSEGSFDDIVMK
YTSKEILSHWCTVSVKVGMLFVKINPKFLKTEVYGSALKDYQVVNNIEINSDERYNLGKI
VINSLFNEFISYEVQKDKLLRKKIFSLKKKDLTNSLTLDTGYNSESKKNNKDKKRKSTFK
ISSTLSIGNTNSSGTPPNSAPATPVMAETIVLEEQPLLQSASDKAIDDSLELVQPLPASK
KPYFRTQSSGSLLSRKFKSFRSTSGRATTGLNTPEEPKGILPDTPHVINDDSAFPQAINT
TQQSKDATPESMLWNHPFKLEQKLSAISSQDLPSNNTHNKLRSSENSRANSTSTLEGNEK
KKPEFMPDLLEQIQESYKQQYMNTSSLKYLTKRLPVTKIIKASSCPIIRVKSATLVLVHL
WKEGSCGGRVLFSTLLPPSHVDNETVSGGKENSKPPDDEEVDLQAVDDDKLGKYDLIDGE
LGSRLNRRQIFEQLEENLPYWFAKALFRDIKTVEEQPKLNFLIMPWSSVGGSEAAGNENK
KKFISASDTTESSGNDSSDSSLGNGNEAVSPSTQQQFHNMLKFGRPKTSEQELNPTDLPR
ISEANVKLVAPGMIRVKKIKLYVADRFETKTPEMKAKMEPSLWLDLLCRGQVLDNDMTLN

TVRTLYWKSQGDIVLEYRRKVHNSPLVHEVNGNEGK
>P49631
MAKRTKKVGITGKYGVRYGSSLRRQVKKLEIQQHARYDCSFCGKKTVKRGAAGIWTCSCC
KKTVAGGAYTVSTAAAATVRSTIRRLREMVEA
>O13600
MQQGHFTSNSYHSKTLNSSSLPVSSKFSHTNDPDVEGVDNDSFSKALCNKDLDPSSTIRS
VISSLLDSLQDKALQQSPNTSLSKLLLEDQDFGSLWLSLSFIFCNYWQPAMLILDSKSIH
PLPLTDGEVLHRWECEVDSNNKTTIDLKYWYCSCSQFSYNAFNSSRSFDEPMPKNEQETW
GGRCLSHHPTICSHILAASILRACHNLVI
>P78955
MRFKTRISNLYTLTRLVQALDKIGRFCWLRLMPETVNFVIVPDFRMTQVWSVLEVETIFE
DYVVQSNADNVINLEVPIDNFYKALRSAANASDSTVRLSKKNNQPLLSLSTTWSGRAFGS
NIVTHNIPVRVLSQSYVSVIKEPTAPEPDCHIFLPQLNFLRHVVDKYKSLSDRIIMSANM
SGELQLSVNIPSARVSTKWKGLENPELDPSQVEDISRHPSQTRAPEEFVHMRLDSKDLVN
MLKISSVAKRVIACFCEGHALVLYVYITDPEDEHTAVLTYYISTYVD
>O60664
MSADGAEADGSTQVTVEEPVQQPSVVDRVASMPLISSTCDMVSAAYASTKESYPHIKTVC
DAAEKGVRTLTAAAVSGAQPILSKLEPQIASASEYAHRGLDKLEENLPILQQPTEKVLAD
TKELVSSKVSGAQEMVSSAKDTVATQLSEAVDATRGAVQSGVDKTKSVVTGGVQSVMGSR
LGQMVLSGVDTVLGKSEEWADNHLPLTDAELARIATSLDGFDVASVQQQRQEQSYFVRLG
SLSERLRQHAYEHSLGKLRATKQRAQEALLQLSQALSLMETVKQGVDQKLVEGQEKLHQM
WLSWNQKQLQGPEKEPPKPEQVESRALTMFRDIAQQLQATCTSLGSSIQGLPTNVKDQVQ
QARRQVEDLQATFSSIHSFQDLSSSILAQSRERVASAREALDHMVEYVAQNTPVTWLVGP
FAPGITEKAPEEKK
>Q09HN1
METLKSENKKRVLPSWMTAPVDEKRELSVKTPKRKKIAAGQVGLATRAPVMKTVYCMNEA
EMVDVALGILIEGRKQEEPTLVAPDKPQPSPPYSASPHTSSPGSSSEKEDSGNRWPALGL
SPSHGPEAADSPCSRSPEEEEEDALKYVREIFFS
>Q8WUN7
MGGCVGAQHDSSGSLNENSEGTGVALGRNQPLKKEKPKWKSDYPMTDGQLRSKRDEFWDT
APAFEGRKEIWDALKAAAHAFESNDHELAQAIIDGANITLPHGALTECYDELGNRYQLPV
YCLAPPINMIEEKSDIETLDIPEPPPNSGYECQLRLRLSTGKDLKLVVRSTDTVFHMKRR
LHAAEGVEPGSQRWFFSGRPLTDKMKFEELKIPKDYVVQVIVSQPVQNPTPVEN
>Q8K2Y9
MEEEGKKGKKPGIVSPFKRVFLKGEKSRDKKAHEKVTERRPLHTVVLALPERVEPDRLLS
DYIEKEVKYLGQLTSIPGYLNPSSRTEILHFIDKAKRSHQLPGHLTQEHDAVLSLSAYNV
KLAWRDGEDIILRVPIHDIAAVSYVRDDAAHLVVLKTAQDPGISPSQSLCAESSRGLSAG
SLSESAVGPVEACCLVIMATESKVAAEELCSLLSQVFQIVYTESTIDFLDRAIFDGASTP
THHLSLHSDDSSTKVDMKDSYDADASTFCFPDSGDVGGLPPLPFCMQTSPHSKTVSESEL
STSATELLQDYMLTLRTKLSSQEIQQFAALLHEYRNGASIHEFCISLRQLYGDSRKFLLL
GLRPFIPEKDSQHFENFLETIGVKDGRGIITDSFGRHRRALSTTSTSTINGNRTTGSPDD
RSAPSEGDEWDRMISDISSDIEALGCSMDQDSA
>P33280
MSPSLVDNHAAAYIAAPSSAKAPMIQKPGNTFGMSSPIESKFLSQPRDLGIVAVGFSGGQ

CKPGVDAAPSALIESGLLTQLREELGYRLHGDDEVHLYTDLVPKEDPPHRNMKNPRAVSN
VTKRIAEQVHSHAKEGRLVLTLGGDHSIAIGTIAGSAKAIKERLGREIAVIWVDAHADIN
TPETSGSGNIHGMPVSFLTGLASEDKEEFFGWLKPDHLLSVKKLVYIGLRDVDPGEKRIL
RENGIKAFSMHDIDKHGIGRVMEMALGHIGNDTPIHLSFDVDALDPMWAPSTGTPVRGGL
TLREGDFICECVHETGSLVAVDLVEVNPTLAAPNDVGAHETVRAGCSLVRCALGESLL
>P53933
MNSQGYDESSSSTAATSGPTSGDPRMGKKQRFMNLIRTTKDVYIPNLTSSISQKTMDGIR
STTNSFEGYNDLPMELPHNTTITYFPTYTTTNLVDPDGLSAPRKDFETTVRCAVSYPGNP
TSRRNRWLLSLCKQYLRTGTAEADVAPVVPPHLEEDSGDLNDSQSSIESSLSSKSENRYS
HMGIQEEDVLNERIQGFLSKKVPNTPVVVDLLPKDKLRGDTASFFGTTDSYGNLLIKAET
DFLPSKINITLDTPIEGHADPISETFPANYVSPYGIGLISDIDDTIKHTGVTGDRRSMFR
NVFIHDVQSWVIDGVPLWYKTLHDVADVDFFYVSNSPIQTFTLLKQYICANFPPGPIFLK
QYSGNFFSTIMTSSANRKIQPIANILKDFPKKKFILVGDSGEHDLEAYTTTALQFPNQIL
AIYIRCCSNSMSDVPSHDEEVMNEVNNIIELQQRPMQMTKSTVRTRRRPPPPPIPSTQKP
SLTEEQTESIRMSRRNKDENNAKRVAPPPLPNRQLPNLDANTYYVPSSQNDYGMYGAFMD
KKADEWKRRVMDSIQKLSNQDTTLMFFSDPALSLEDSIRRIREKYSN
>P31007
MPVKKQEAHRALELLEDYHARLSEPQDRALRIAIERVIRIFKSRLFQALLDIQEFYELTL
LDDSKSIQQKTAETLQIATKWEKDGQAVKIADFIKSSNLNRNCAYEFNNDASSNQTNQSA
LNQNPIANNVSAQAQAEALSRTFKSELEEILNQRMRIESDTENAKEPTVEQQQKQQQAQQ
RSSRSPQQQNPQQQQGSKSRSGSQTVNGDDSWLYEDIQLERGNSGLGFSIAGGTDNPHIG
TDTSIYITKLISGGAAAADGRLSINDIIVSVNDVSVVDVPHASAVDALKKAGNVVKLHVK
RKRGTATTPAAGSAAGDARDSAASGPKVIEIDLVKGGKGLGFSIAGGIGNQHIPGDNGIY
VTKLMDGGAAQVDGRLSIGDKLIAVRTNGSEKNLENVTHELAVATLKSITDKVTLIIGKT
QHLTTSASGGGGGGLSSGQQLSQSQSQLATSQSQSQVHQQQHATPMVNSQSTEPGSRYAS
TNVLAAVPPGTPRAVSTEDITREPRTITIQKGPQGLGFNIVGGEDGQGIYVSFILAGGPA
DLGSELKRGDQLLSVNNVNLTHATHEEAAQALKTSGGVVTLLAQYRPEEYNRFEARIQEL
KQQAALGAGGSGTLLRTTQKRSLYVRALFDYDPNRDDGLPSRGLPFKHGDILHVTNASDD
EWWQARRVLGDNEDEQIGIVPSKRRWERKMRARDRSVKFQGHAAANNNLDKQSTLDRKKK
NFTFSRKFPFMKSRDEKNEDGSDQEPFMLCYTQDDANAEGASEENVLSYEAVQRLSINYT
RPVIILGPLKDRINDDLISEYPDKFGSCVPHTTRPKREYEVDGRDYHFVSSREQMERDIQ
NHLFIEAGQYNDNLYGTSVASVREVAEKGKHCILDVSGNAIKRLQVAQLYPVAVFIKPKS
VDSVMEMNRRMTEEQAKKTYERAIKMEQEFGEYFTGVVQGDTIEEIYSKVKSMIWSQSGP
TIWVPSKESL
>Q9EQ10
MAVLNQMSVLDMIKAFRRNWRAHCNSERTTLCGPDSMLLALQLSMAENNKQHHGEFTVCL
SDVLLTWKYFLHEKLNLPIENMKVVDHYEDIRKTYDDFLKNSNTLDLIDVYKKCSIMTSN
CEKHMISSIQLRDFLSGTECAVNDENNPHSPASPVKCSQNDKQVRLLAKKIFLSYLSLLV
NSKNDLALAQILNTPDRGLGREAFTNLKHAAREKQLSMFLMATSFIRTLELGGKGYAPPP
SDPLWAHVKGLSAFVNFIDKLNEILGEVPNPSLAGGRILSAIRTQLIKGHSSGEPFYKAV
EEVVQDLNLRIKNIINSQEGVTVSATNISPVPPKSFAINHDTAYCGRDAVKILLVLLDED
AASAPTRNKAELLYNDQSTAPHGGASVLTLFRSPTQVKSTPVKPLRERIHTSLQAEKNKQ
MRQTLIRSQFACTYKDDCIMSKSKWNVNSSSKPLSALRLENDVSEGAQPSVGKARLETSS
ENVHVDRSKDDKGPRKSTKRLAKSKQPGVREDIHCDQGDELYQPKNVKILKVPSDSHRK

AGGKLAPGARGNRCTTKDKLIPGQTKLTRFFML
>P14796
IIEPSLKALASKYNCDKSVCRKCYARLPPRATNCRKRKCGHTNQLRPKKKLK
>Q9HDX7
MSSSKIKELREGLMSSSKWSMAPGMMMQQNQPRPATTTPPISRGNEDADEGLNTARSFSS
FQDLKGMSGDAKEQESATLVHLTKGRAHPRSRRPPRQISIDSAKKEETKNTGSTKAADTK
SSVEATISPLKDTKSPSNASVFPIKPTESKTSTTKDSETAKEEDDASSSTTKAVEATTSK
ASSAHTDTLATSASNSDRGASTPEMVVKAEKREGSTSPIPYSSLSIAERIKQAQNTPFLE
SKVLPQNNETSDEENVDVKPAAGTVKNVMQAFLQPATPAKDTASKEPSKSSQQPVRTKPR
IAQSPFLAQDAKENGGNVEVSSPLSFSASKSPAAVDSSTKTPTEQVNVVSKQAPTTSSTS
VISPDPLQTAAPSANVNEVIASLESKVVTRRTGSGNNYRVGLRNVSGSERTKSLSKESPV
EPEKPALPDATSSSTPTTENKESWTNQGIKSSQQRSANASPATSPSNQASIHASFTKESS
THSSPSFTLESLFSGAPRVVELTRFSQPSPACSRALLARWKEEYRTSIH
>Q4V7Y7
MAAPSPTKTTWKLQEIVAHGCSVSSVVLGRSSGRLVATGGDDCRVHLWSVNKPNCIMSLT
GHTTPVESVRFNNSEELIVAGSQSGSLRIWDLEAAKILRTLMGHKANVSSLDFHPYGEFV
ASGSLDTNIKLWDVRRKGCVFRYKGHTQAVRCLRFSPDGKWLASASDDHSVKLWDLTAGK
MMAELSEHKGPVNIIEFHPNEYLLASGSADRTVRFWDLEKFQLVGCTEGETIPVRAILFS
NDGGCIFCGGKDSLRVYGWEPDQCFDTVPVGWGKVSDLAICNNQLIGVSSAQSNISSFVV
DLTRVKMTGCAPQGPVPAEIPISQPAPTGTSLRRIYERPSTTCSKPNRVSPTSDDEEKES
RAEIQNPEDYKEIFQPKNAISRTPPRNSEPFPAPPEDDISIVKEAVAPTPDVVTPATSNK
KNTEQLQRPPVAASTPIVCQEPSPVPAPQSKPPVISAARNEPIGLKAADFLPAVKSSSPT
EVVDDEAVSQIRKGHDTMCMVLTSRMRNLDTVRAVWSSGDIKTSIDSAVAINDLSVVVDL
LNIINQKASLWKLDLCMTVLPQIEKMLQSKYESYVQTGCISLKLILQRFLPLITDILAAP
PSVGVDISREERLSKCKLCYKQLRILSPLVKSKASQSGRYGSAFRELHLLMSGLE
>P98171
MAAHGKLRRERGLQAEYETQVKEMRWQLSEQLRCLELQGELRRELLQELAEFMRRRAEVE
LEYSRGLEKLAERFSSRGGRLGSSREHQSFRKEPSLLSPLHCWAVLLQHTRQQSRESAAL
SEVLAGPLAQRLSHIAEDVGRLVKKSRDLEQQLQDELLEVVSELQTAKKTYQAYHMESVN
AEAKLREAERQEEKRAGRSVPTTTAGATEAGPLRKSSLKKGGRLVEKRQAKFMEHKLKCT
KARNEYLLSLASVNAAVSNYYLHDVLDLMDCCDTGFHLALGQVLRSYTAAESRTQASQVQ
GLGSLEEAVEALDPPGDKAKVLEVHATVFCPPLRFDYHPHDGDEVAEICVEMELRDEILP
RAQNIQSRLDRQTIETEEVNKTLKATLQALLEVVASDDGDVLDSFQTSPSTESLKSTSSD
PGSRQAGRRRGQQQETETFYLTKLQEYLSGRSILAKLQAKHEKLQEALQRGDKEEQEVSW
TQYTQRKFQKSRQPRPSSQYNQRLFGGDMEKFIQSSGQPVPLVVESCIRFINLNGLQHEG
IFRVSGAQLRVSEIRDAFERGEDPLVEGCTAHDLDSVAGVLKLYFRSLEPPLFPPDLFGE
LLASSELEATAERVEHVSRLLWRLPAPVLVVLRYLFTFLNHLAQYSDENMMDPYNLAVCF
GPTLLPVPAGQDPVALQGRVNQLVQTLIVQPDRVFPPLTSLPGPVYEKCMAPPSASCLGD
AQLESLGADNEPELEAEMPAQEDDLEGVVEAVACFAYTGRTAQELSFRRGDVLRLHERAS
SDWWRGEHNGMRGLIPHKYITLPAGTEKQVVGAGLQTAGESGSSPEGLLASELVHRPEPC
TSPEAMGPSGHRRRCLVPASPEQHVEVDKAVAQNMDSVFKELLGKTSVRQGLGPASTTSP
SPGPRSPKAPPSSRLGRNKGFSRGPGAPASPSASHPQGLDTTPKPH
>P38285
MKLERVSSNGSFKRGRDIQSLESPCTRPLKKMSPSPSFTSLKMEKPFKDIVRKYGGHLHQ

SSYNPGSSKVELVRPDLSLKTDQSFLQSSVQTTPNKKSCNEYLSTPEATPLKNTATENAW
ATSRVVSASSLSIVTPTEIKNILVDEFSELKLGQPLTAQHQRSHAVFEIPEIVENIIKMI
VSLESANIPKERPCLRRNPQSYEHSLLMYKDEERAKKAWSAAQQLRDPPLVGHKEKKQGA
LFSCMMVNRLWLNVTRPFLFKSLHFKSVHNFKEFLRTSQETTQVMRPSHFILHKLHQVTQ
PDIERLSRMECQNLKWLEFYVCPRITPPLSWFDNLHKLEKLIIPGNKNIDDNFLLRLSQS
IPNLKHLVLRACDNVSDSGVVCIALNCPKLKTFNIGRHRRGNLITSVSLVALGKYTQVET
VGFAGCDVDDAGIWEFARLNGKNVERLSLNSCRLLTDYSLPILFALNSFPNLAVLEIRNL
DKITDVRHFVKYNLWKKSLDAPILIEACERITKLIDQEENRVKRINSLVALKDMTAWVNA
DDEIENNVD

>P25822

MKFLGGNDDRNGRGGVGVGTDAIVGSRGGVSQDAADAAGAAAAAAVGYVFQQRPSPGGVG
VGVGGVGGGVPGVGAVGSTLHEAAAAEYAAHFAQKQQQTRWACGDDGHGIDNPDKWKYNP
PMNPANAAPGGPPGNGSNGGPGAIGTIGMGSGLGGGGGGGAGGGNNGGSGTNGGLHHQSM
AAAAANMAAMQQAAALAKHNHMISQAAAAVAAQQQHQHPHQQHPQQQQQQQQAQNQGHPH
HLMGGGNGLGNGNGLGIQHPGQQQQQQQQQQQQQHPGQYNANLLNHAAALGHMSSYAQSG
GSMYDHHGGAMHPGMNGGMPKQQPLGPPGAGGPQDYVYMGGQTTVPMGAAMMPPQNQYMN
SSAVAAANRNAAITTSTAKKLWEKSDGKGVSSSTPGGPLHPLQIPGIGDPSSVWKDHTWS
TQGENILVPPPSRAYAHGGASDTSNSGNAGILSPRDSTCAKVVEYVFSGSPTNKDSSLSG
LEPHLRNLKFDDNDKSRDDKEKANSPFDTNGLKKDDQVTNSNGVVNGIDDDKGFNRTPGS
RQPSPAEESQPRPPNLLFPPLPFNHMLMDHGQGMGGGLGGVVGSGNGVGGGSGGGGAGGA
YAAHQQMAAQMSQLQPPMMNGVGGGMPMAAQSPMLNHQAAGPNHMESPGNLLQQQNFDVQ
QLFRSQNPGLAAVATNAAAAAAAAAAAATSAASAAAAVGAPPVPNGSLQQSQQQQQQQQQ
QQQQQMHMAAASQQFLAAQQQAQNAAYAAQQATSYVINPGQEAAPYMGMIAAAQMPYYGV
APWGMYPGNLIPQQGTQPRRPLTPSQQGAENQPYQVIPAFLDHTGSLLMGGPRTGTPMRL
VSPAPVLVPPGATRAGPPPPQGPQLYQPQPQTAQQNLYSQQNGSSVGGLALNTSSLTGRR
DSFDRSTSAFSPSTMDYTSSGVAAAANAVNSTVAQAAAAAAAAAAARGKWPGAMSGAASG
AYGALGAGNASASPLGAPITPPPSAQSCLLGSRAPGAESRQRQQQQQQLAAVGLPATAAA
AQAAVAAAANNMFGSNSSIFSNPLAIPGTAAVAAAAAAAAAANSRQVAATAAAAAAVAAA
AGGVGGAPQPGRSRLLEDFRNQRYPNLQLRDLANHIVEFSQDQHGSRFIQQKLERATAAE
KQMVFSEILAAAYSLMTDVFGNYVIQKFFEFGTPEQKNTLGMQVKGHVLQLALQMYGCRV
IQKALESISPEQQQEIVHELDGHVLKCVKDQNGNHVVQKCIECVDPVALQFIINAFKGQV
YSLSTHPYGCRVIQRILEHCTAEQTTPILDELHEHTEQLIQDQYGNYVIQHVLEHGKQED
KSILINSVRGKVLVLSQHKFASNVVEKCVTHATRGERTGLIDEVCTFNDNALHVMMKDQY
ANYVVQKMIDVSEPTQLKKLMTKIRPHMAALRKYTYGKHINAKLEKYYMKITNPITVGTG
AGGVPAASSAAAVSSGATSASVTACTSGSSTTTTSTTNSLASPTICSVQENGSAMVVEPS
SPDASESSSSVVSGAVNSSLGPIGPPTNGNVVL

>O43264

MASFVTEVLAHSGRLEKEDLGTRISRLTRRVEEIKGEVCNMISKKYSEFLPSMQSAQGLI
TQVDKLSEDIDLLKSRIESEVRRDLHVSTGEFTDLKQQLERDSVVLSLLKQLQEFSTAIE
EYNCALTEKKYVTGAQRLEEAQKCLKLLKSRKCFDLKILKSLSMELTIQKQNILYHLGEE
WQKLIVWKFPPSKDTSSLESYLQTELHLYTEQSHKEEKTPMPPISSVLLAFSVLGELHSK
LKSFGQMLLKYILRPLASCPSLHAVIESQPNIVIIRFESIMTNLEYPSPSEVFTKIRLVL
EVLQKQLLDLPLDTDLENEKTSTVPLAEMLGDMIWEDLSECLIKNCLVYSIPTNSSKLQQ
YEEIIQSTEEFENALKEMRFLKGDTTDLLKYARNINSHFANKKCQDVIVAARNLMTSEIH

NTVKIIPDSKINVPELPTPDEDNKLEVQKVSNTQYHEVMNLEPENTLDQHSFSLPTCRIS
ESVKKLMELAYQTLLEATTSSDQCAVQLFYSVRNIFHLFHDVVPTYHKENLQKLPQLAAI
HHNNCMYIAHHLLTLGHQFRLRLAPILCDGTATFVDLVPGFRRLGTECFLAQMRAQKGEL
LERLSSARNFSNMDDEENYSAASKAVRQVLHQLKRLGIVWQDVLPVNIYCKAMGTLLNTA
ISEVIGKITALEDISTEDGDRLYSLCKTVMDEGPQVFAPLSEESKNKKYQEEVPVYVPKW
MPFKELMMMLQASLQEIGDRWADGKGPLAAAFSSSEVKALIRALFQNTERRAAALAKIK
>Q12277
MSLSVAEKSYLYDSLASTPSIRPDGRLPHQFRPIEIFTDFLPSSNGSSRIIASDGSECIV
SIKSKVVDHHVENELLQVDVDIAGQRDDALVVETITSLLNKVLKSGSGVDSSKLQLTKKY
SFKIFVDVLVISSHSHPVSLISFAIYSALNSTYLPKLISAFDDLEVEELPTFHDYDMVKL
DINPPLVFILAVVGNNMLLDPAANESEVANNGLIISWSNGKITSPIRSVALNDSNVKSFK
PHLLKQGLAMVEKYAPDVVRSLENL
>O00443
MAQISSNSGFKECPSSHPEPTRAKDVDKEEALQMEAEALAKLQKDRQVTDNQRGFELSSS
TRKKAQVYNKQDYDLMVFPESDSQKRALDIDVEKLTQAELEKLLLDDSFETKKTPVLPVT
PILSPSFSAQLYFRPTIQRGQWPPGLPGPSTYALPSIYPSTYSKQAAFQNGFNPRMPTFP
STEPIYLSLPGQSPYFSYPLTPATPFHPQGSLPIYRPVVSTDMAKLFDKIASTSEFLKNG
KARTDLEITDSKVSNLQVSPKSEDISKFDWLDLDPLSKPKVDNVEVLDHEEEKNVSSLLA
KDPWDAVLLEERSTANCHLERKVNGKSLSVATVTRSQSLNIRTTQLAKAQGHISQKDPNG
TSSLPTGSSLLQEVEVQNEEMAAFCRSITKLKTKFPYTNHRTNPGYLLSPVTAQRNICGE
NASVKVSIDIEGFQLPVTFTCDVSSTVEIIIMQALCWVHDDLNQVDVGSYVLKVCGQEEV
LQNNHCLGSHEHIQNCRKWDTEIRLQLLTFSAMCQNLARTAEDDETPVDLNKHLYQIEKP
CKEAMTRHPVEELLDSYHNQVELALQIENQHRAVDQVIKAVRKICSALDGVETLAITESV
KKLKRAVNLPRSKTADVTSLFGGEDTSRSSTRGSLNPENPVQVSINQLTAAIYDLLRLHA
NSGRSPTDCAQSSKSVKEAWTTTEQLQFTIFAAHGISSNWVSNYEKYYLICSLSHNGKDL
FKPIQSKKVGTYKNFFYLIKWDELIIFPIQISQLPLESVLHLTLFGILNQSSGSSPDSNK
QRKGPEALGKVSLPLFDFKRFLTCGTKLLYLWTSSHTNSVPGTVTKKGYVMERIVLQVDF
PSPAFDIIYTTPQVDRSIIQQHNLETLENDIKGKLLDILHKDSSLGLSKEDKAFLWEKRY
YCFKHPNCLPKILASAPNWKWVNLAKTYSLLHQWPALYPLIALELLDSKFADQEVRSLAV
TWIEAISDDELTDLLPQFVQALKYEIYLNSSLVQFLLSRALGNIQIAHNLYWLLKDALHD
VQFSTRYEHVLGALLSVGGKRLREELLKQTKLVQLLGGVAEKVRQASGSARQVVLQRSME
RVQSFFQKNKCRLPLKPSLVAKELNIKSCSFFSSNAVPLKVTMVNADPMGEEINVMFKVG
EDLRQDMLALQMIKIMDKIWLKEGLDLRMVIFKCLSTGRDRGMVELVPASDTLRKIQVEY
GVTGSFKDKPLAEWLRKYNPSEEEYEKASENFIYSCAGCCVATYVLGICDRHNDNIMLRS
TGHMFHIDFGKFLGHAQMFGSFKRDRAPFVLTSDMAYVINGGEKPTIRFQLFVDLCCQAY
NLIRKQTNLFLNLLSLMIPSGLPELTSIQDLKYVRDALQPQTTDAEATIFFTRLIESSLG
SIATKFNFFIHNLAQLRFSGLPSNDEPILSFSPKTYSFRQDGRIKEVSVFTYHKKYNPDK
HYIYVVRILREGQIEPSFVFRTFDEFQELHNKLSIIFPLWKLPGFPNRMVLGRTHIKDVA
AKRKIELNSYLQSLMNASTDVAECDLVCTFFHPLLRDEKAEGIARSADAGSFSPTPGQIG
GAVKLSISYRNGTLFIMVMHIKDLVTEDGADPNPYVKTYLLPDNHKTSKRKTKISRKTRN
PTFNEMLVYSGYSKETLRQRELQLSVLSAESLRENFFLGGVTLPLKDFNLSKETVKWYQL
TAATYL
>Q6IA86
MVAPVLETSHVFCCPNRVRGVLNWSSGPRGLLAFGTSCSVVLYDPLKRVVVTNLNGHTAR

VNCIQWICKQDGSPSTELVSGGSDNQVIHWEIEDNQLLKAVHLQGHEGPVYAVHAVYQRR
TSDPALCTLIVSAAADSAVRLWSKKGPEVMCLQTLNFGNGFALALCLSFLPNTDVPILAC
GNDDCRIHIFAQQNDQFQKVLSLCGHEDWIRGVEWAAFGRDLFLASCSQDCLIRIWKLYI
KSTSLETQDDDNIRLKENTFTIENESVKIAFAVTLETVLAGHENWVNAVHWQPVFYKDGV
LQQPVRLLSASMDKTMILWAPDEESGVWLEQVRVGEVGGNTLGFYDCQFNEDGSMIIAHA
FHGALHLWKQNTVNPREWTPEIVISGHFDGVQDLVWDPEGEFIITVGTDQTTRLFAPWKR
KDQSQVTWHEIARPQIHGYDLKCLAMINRFQFVSGADEKVLRVFSAPRNFVENFCAITGQ
SLNHVLCNQDSDLPEGATVPALGLSNKAVFQGDIASQPSDEEELLTSTGFEYQQVAFQPS
ILTEPPTEDHLLQNTLWPEVQKLYGHGYEIFCVTCNSSKTLLASACKAAKKEHAAIILWN
TTSWKQVQNLVFHSLTVTQMAFSPNEKFLLAVSRDRTWSLWKKQDTISPEFEPVFSLFAF
TNKITSVHSRIIWSCDWSPDSKYFFTGSRDKKVVVWGECDSTDDCIEHNIGPCSSVLDVG
GAVTAVSVCPVLHPSQRYVVAVGLECGKICLYTWKKTDQVPEINDWTHCVETSQSQSHTL
AIRKLCWKNCSGKTEQKEAEGAEWLHFASCGEDHTVKIHRVNKCAL
>Q9UQB8
MSLSRSEEMHRLTENVYKTIMEQFNPSLRNFIAMGKNYEKALAGVTYAAKGYFDALVKMG
ELASESQGSKELGDVLFQMAEVHRQIQNQLEEMLKSFHNELLTQLEQKVELDSRYLSAAL
KKYQTEQRSKGDALDKCQAELKKLRKKSQGSKNPQKYSDKELQYIDAISNKQGELENYVS
DGYKTALTEERRRFCFLVEKQCAVAKNSAAYHSKGKELLAQKLPLWQQACADPSKIPERA
VQLMQQVASNGATLPSALSASKSNLVISDPIPGAKPLPVPPELAPFVGRMSAQESTPIMN
GVTGPDGEDYSPWADRKAAQPKSLSPPQSQSKLSDSYSNTLPVRKSVTPKNSYATTENKT
LPRSSSMAAGLERNGRMRVKAIFSHAAGDNSTLLSFKEGDLITLLVPEARDGWHYGESEK
TKMRGWFPFSYTRVLDSDGSDRLHMSLQQGKSSSTGNLLDKDDLAIPPPDYGAASRAFPA
QTASGFKQRPYSVAVPAFSQGLDDYGARSMSRNPFAHVQLKPTVTNDRCDLSAQGPEGRE
HGDGSARTLAGR
>Q22560
MGDEFRPSHEERSKMYKSNVRDQNEMRRKRREDEVQIRKNRRDEKFERNRQITVQRSLSH
EETSELLKSVADGLQSMQETTIHEALTVLHENLNNTVWTIHVLVKVQILHKLSDVYCNRV
ISQTTRLLISRTLLKISGIDEVKYERYSSDDRCIQSLVFNISTYGSSEDILCDTFQSIAC
FIIRSITYRNLALDCAIVSELIDASTINMSIILHRSLMWLVFLFCEKLDRCSPHVDEIAP
LLEIISNGIQSTDAMVQTDAASSCASLAEWPPIYHYMSDLKLCSKLVANLRNDKGNARPK
VKAGINSIIQATGYFTEEMIDAGLLEVLKGFVNVSYMSQEVCFIISNICVEGEQTIDKLI
SSGVLREVARVMEASEYRSRREAAFVICHCCASANQKHLEYVVELGMLSAFTDLLTCMDV
SLVSYILDAIYLLLQFGEMRLLPDNSNPVAIKLEEIGCREKLEFLCESQSVDIHARAYTI
IDRFYVDDDAPLNDDPFAGYQRNNIDDTIEKMIREPIMDQPFSF
>Q64289
MTKSYSESGLMGEPQPQGPPSWTDECLSSQDEEHEADKKEDELEAMNAEEDSLRNGGEEE
DEDEDLEEEEEEEEEEDDQKPKRRGPKKKKMTKARLERFKLRRMKANARERNRMHGLNAA
LDNLRKVVPCYSKTQKLSKIETLRLAKNYIWALSEILRSGKSPDLVSFVQTLCKGLSQPT
TNLVAGCLQLNPRTFLPEQNPDMPPHLPTASASFPVHPYSYQSPGLPSPPYGTMDSSHVF
HVKPPPHAYSAALEPFFESPLTDCTSPSFDGPLSPPLSINGNFSFKHEPSTEFEKNYAFT
MHYPAATLAGPQSHGSIFSSGAAAPRCEIPIDNIMSFDSHSHHERVMSAQLNAIFHD
>Q95V11
MEQEREIVFSLSPEETSELKNGVNLISRSEKEKFVIYKDPTAENTVEEPATSHMYKACLN
KCKHQGGTFIKDIEDGDNCILRCTKHGWKLDAKTMRYVNPPDSFSQQQLVPEYNEDGSLD

IVELKPPQPWETDKRDPMPLEVGEVQITYFTHACIEIKLGDLIMFTDPWLIGPAFARGWW
LMHEPPADWLDRLAKADLIYISHLHSDHLNYPTLELLSQRNPDIPIYVGDTSMPVFVRLE
QSGVKLNNIHIKKFGKWIEINKDTRFMIMMDGVHPDMDTCALIDYKGHLILDTVDCTNPN
GGRLPIGVDMMLSDFAGGASGFPMTFSGGKYTEEWKAEFVKRERRKLLYYKMQQVRDVAP
TVYCPFAGYFVEAHPSDHYIRSTNTKNDPDALNALINKYSPNIKTWSPSPGAVLDLKKAI
QGDRDFITDPPRGTQKFKDSWDFEKYVNAINKNIEEEIFSYPEWIQFYYKWTGFKNYNLV
IRMVERDDDFCPVVGGYDFMVDFVGEEPTFPTERPAREHSYLEMENRIGVHRETVRQGLF
WDDLYIGFNNRISREPDTFHYLFWNHMQILLPRTDPDWEGFLRDMKTEGAPQKAIWNPSQ
ATPAVEAKDPSSDSKDSATKPGTHWNYERLLRPLGIVVALVGVGVAIWKSESK
>O94590
MTLLELQKELFLSKINSVECATKWKVLIVDTKTADIINHFITIHSLLEEKIAAVEILENP
RTPNSSFEALYILHSEEKLVDCILKDEEYDKRYPGIHIVFLDMVKEPLINKLRTSRIASK
IRTVQVAYLDFTSLESRYFQVHDSFSGLRLYHPSNAAIIRQELSKVAHGIFSVCVSLGIS
PNIRCYYPKNAPHASKTMSFILANQLSEIVEEYCSKHPGYHEAASKSTCLIVDRSLDTAA
PFLHEFTYQAMIHDLLPIKNEQYPYEILGPQGTEKRTGKLDDDDLVYTTIRHMHMRDAIE
KLMKDFNQFCIDNTLFLDKERATSLNDMRSMLAGLSDFQELRDQYSLHLTMAQECMNIFE
KQQLNLIGAIEQDLSTGSNVEGKVPRSVLSELLPLLDEGNAEESTKIRLLLLYIIYRDGI
ILQDLFRLFRHSNLSTSREQIFQNLEQLGTRVIKNLTDQSSKRKEVANSLPAGEDVYELS
RYVPTLKVVLENLIQDKLDPELFPYVRNTTPQTEVSMEQTSLRSSRPSWTRSRSMASKLP
REKMLVFVAGGTTFSELRTCYELSDKYNKDIYIGSTVCYSPNEWLDFFSKFQQPREALKF
PEDKPRHIPCLEARPSPNRIQEQPNIDVPSTRYENTSSSDAVSIHKDEKKSKGKSHKLGK
LMMSSKKDKDKSKEKVPTYGTSDKKKKKRFGVF
>Q12509
METPPIVIDNGSYEIKFGPSTNKKPFRALNALAKDKFGTSYLSNHIKNIKDISSITFRRP
HELGQLTLWELESCIWDYCLFNPSEFDGFDLKEGKGHHLVASESCMTLPELSKHADQVIF
EEYEFDSLFKSPVAVFVPFTKSYKGEMRTISGKDEDIDIVRGNSDSTNSTSSESKNAQDS
GSDYHDFQLVIDSGFNCTWIIPVLKGIPYYKAVKKLDIGGRFLTGLLKETLSFRHYNMMD
ETILVNNIKEQCLFVSPVSYFDSFKTKDKHALEYVLPDFQTSFLGYVRNPRKENVPLPED
AQIITLTDELFTIPETFFHPEISQITKPGIVEAILESLSMLPEIVRPLMVGNIVCTGGNF
NLPNFAQRLAAELQRQLPTDWTCHVSVPEGDCALFGWEVMSQFAKTDSYRKARVTREEYY
EHGPDWCTKHRFGYQNWI
>O94701
MSKNPLGTLVVRIWKAKNLPNKALVGKQSPYCVCRVGEVVKRTQTDKRSGQEPSWNAVLE
FNIPSESYHIMKITVFHEGFRKHPHLIGDTVLSFEKAMKEELQSEWYELKNEFQFAGELS
VQFKFIPTDPLYFDRASSKPVLQFPYSSVAALTPVPKKPSKPSKPRKKVPVSHPLPPTPP
SREEHVSVPRESSLFTYEDDPLPSFPSPYMVDDYYTQDVFVSDNVNDYSYGVQNPTNPRL
SVEDYDANHSSLPPVPPPHLILPTASSSQIFH
>Q9P7K3
MATKVEEINKLKKKNLHFRQCSADSLSNSSMQFDLSGLNTPVGELATSNLPSPAHPPFGE
LHQESRTSNSSAMHIENVVASRLMYNEVANGSFALEPKNILVVTKPRKHSLVYKTAEITK
YILTIGTPETKVYVDMRLARSKRFSAHNIAKEANTDIDRIKYWNPYICLIKPSIFDLAIT
IGDNSTLLYTSWLFQKIGPPVLSFSDDDVPGFLTHFSLSNYQQHLYQVLTQNVSLRFCSR
LQCSFHKYDEKTKQYSLASTTYSLDEILISRGEHPFISNLNVYNNSELMTVVQADGLVVA
TPTGSTNISANAGGSLVHPALNAILVTPVCPHTLSFRPIILPDYNVLNVEIPLDSRSSAF

FSVDRHESVEMHRGDYLSIVTSHYPFTTIQNPGYQWTKVLEDKFNWNVRERQKPFSRKPS
LSDVKDTSDDKFDITDNSYCREFSADIDG
>O94684
MDDEGARKVDEKCPLCQADRYLNPNMKLLINPECYHKMCESCVDRIFTTGPAQCPTPGCN
KILRKAKFREQTFEDAQIEREVDVRKRISRIFNKGQQEFDSLQAYNDYLEEVEILTFNLI
YKIDVEETEEKVKQYEKQNRDSIAANSARAAAEARILAQNEILLKRQKQEAREAAIREHQ
KEKERREQVEQQIIFDLATSGKDPNKIIQLSDSLKKQQENIASSVSNISRSSSILLSDVQ
QVAEDTTPFSPLAGEKDGSKYFSYSKNTYQDLYLEKVSHEPGRKCGFRIEDCHLRCLYEA
FSGIDYDLESLKKLEVAS
>P11986
MTEDNIAPITSVKVVTDKCTYKDNELLTKYSYENAVVTKTASGRFDVTPTVQDYVFKLDL
KKPEKLGIMLIGLGGNNGSTLVASVLANKHNVEFQTKEGVKQPNYFGSMTQCSTLKLGID
AEGNDVYAPFNSLLPMVSPNDFVVSGWDINNADLYEAMQRSQVLEYDLQQRLKAKMSLVK
PLPSIYYPDFIAANQDERANNCINLDEKGNVTTRGKWTHLQRIRRDIQNFKEENALDKVI
VLWTANTERYVEVSPGVNDTMENLLQSIKNDHEEIAPSTIFAAASILEGVPYINGSPQNT
FVPGLVQLAEHEGTFIAGDDLKSGQTKLKSVLAQFLVDAGIKPVSIASYNHLGNNDGYNL
SAPKQFRSKEISKSSVIDDIIASNDILYNDKLGKKVDHCIVIKYMKPVGDSKVAMDEYYS
ELMLGGHNRISIHNVCEDSLLATPLIIDLLVMTEFCTRVSYKKVDPVKEDAGKFENFYPV
LTFLSYWLKAPLTRPGFHPVNGLNKQRTALENFLRLLIGLPSQNELRFEERLL
>Q9NS86
MGETMSKRLKLHLGGEAEMEERAFVNPFPDYEAAAGALLASGAAEETGCVRPPATTDEPG
LPFHQDGKIIHNFIRRIQTKIKDLLQQMEEGLKTADPHDCSAYTGWTGIALLYLQLYRVT
CDQTYLLRSLDYVKRTLRNLNGRRVTFLCGDAGPLAVGAVIYHKLRSDCESQECVTKLLQ
LQRSVVCQESDLPDELLYGRAGYLYALLYLNTEIGPGTVCESAIKEVVNAIIESGKTLSR
EERKTERCPLLYQWHRKQYVGAAHGMAGIYYMLMQPAAKVDQETLTEMVKPSIDYVRHKK
FRSGNYPSSLSNETDRLVHWCHGAPGVIHMLMQAYKVFKEEKYLKEAMECSDVIWQRGLL
RKGYGICHGTAGNGYSFLSLYRLTQDKKYLYRACKFAEWCLDYGAHGCRIPDRPYSLFEG
MAGAIHFLSDVLGPETSRFPAFELDSSKRD
>Q04005
MNTNQSNPNTDLTDDANIEHTLHRLLTQANNHFDDTVKIDGQSLDLGKDLEQVMMDNLDC
TDIFDSDIASQKHLTLESLFNDEHNTDSSTLLEMQRSANDSLVGIDLDRHKKGYTGKASL
DKSTNQNNVHKPDKEQKNYKIDKPTIKKKKSLLKTTNEPMLSPASLSPSSSLASSDANES
HLKIESMITDITSKIDSARQDIVSATKPAKFTNEFTISQISEMKARIINTHKLLLNFNFI
KEGYARSCIQLKKSMDSLKDSEIHRAHLLVENDDLKQQILELTQKLNEKSSKES
>Q96CS2
MEPQEERETQVAAWLKKIFGDHPIPQYEVNPRTTEILHHLSERNRVRDRDVYLVIEDLKQ
KASEYESEAKYLQDLLMESVNFSPANLSSTGSRYLNALVDSAVALETKDTSLASFIPAVN
DLTSDLFRTKSKSEEIKIELEKLEKNLTATLVLEKCLQEDVKKAELHLSTERAKVDNRRQ
NMDFLKAKSEEFRFGIKAAEEQLSARGMDASLSHQSLVALSEKLARLKQQTIPLKKKLES
YLDLMPNPSLAQVKIEEAKRELDSIEAELTRRVDMMEL
>P61457
MAGKAHRLSAEERDQLLPNLRAVGWNELEGRDAIFKQFHFKDFNRAFGFMTRVALQAEKL
DHHPEWFNVYNKVHITLSTHECAGLSERDINLASFIEQVAVSMT
>Q86UT5

MVTPSPPGNHSLSLEAPRLHTASDLLGNHSLGLPLITALVGSRDRRGRVFSPVPVPLPTN
PTTQHPTRQKLPSTLSGHRVCQAHGEPVLGLCPLLPLFCCPPHPPDPWSLERPRFCLLSK
EEGKSFGFHLQQELGRAGHVVCRVDPGTSAQRQGLQEGDRILAVNNDVVEHEDYAVVVRR
IRASSPRVLLTVLARHAHDVARAQLGEDAHLCPTLGPGVRPRLCHIVKDEGGFGFSVTHG
NQGPFWLVLSTGGAAERAGVPPGARLLEVNGVSVEKFTHNQLTRKLWQSGQQVTLLVAGP
EVEEQCRQLGLPLAAPLAEGWALPTKPRCLHLEKGPQGFGFLLREEKGLDGRPGQFLWEV
DPGLPAKKAGMQAGDRLVAVAGESVEGLGHEETVSRIQGQGSCVSLTVVDPEADRFFSMV
RLSPLLFLENTEAPASPQGSSSASLVETEDPSLEDTSVPSVPLGSRQCFLYPGPGGSYGF
RLSCVASGPRLFISQVTPGGSAARAGLQVGDVILEVNGYPVGGQNDLERLQQLPEAEPPL
CLKLAARSLRGLEAWIPPGAAEDWALASDLL
>Q9U1H9
MPKRGGGSSQRYNNNVGNGGGRYNAPEDFDDFDVEDRQRRKDRNKRRVSFKPSQCLHNKK
DIKLRPEDLRRWDEDDDMSDMTTAVKDRPTSRRRGSPIPRGKFGKLMPNSFGWYQVTLQN
AQIYEKETLLSALLAAMSPHVFIPQYWRVERNCVIFFTDDYEAAERIQHLGKNGHLPDGY
RLMPRVRSGIPLVAIDDAFKEKMKVTMAKRYNIQTKALDLSRFHADPDLKQVFCPLFRQN
VMGAAIDIMCDNIPDLEALNLNDNSISSMEAFKGVEKRLPNLKILYLGDNKIPSLAHLVV
LRNLSILELVLKNNPCRSRYKDSQQFISEVRRKFPKLVKLDGETLEPQITFDLSEQGRLL
ETKASYLCDVAGAEVVRQFLDQYFRIFDSGNRQALLDAYHEKAMLSISMPSASQAGRLNS
FWKFNRNLRRLLNGEENRTRNLKYGRLACVSTLDEWPKTQHDRRTFTVDLTIYNTSMMVF
TVTGLFKELNDETNNPASMELYDVRHFARTYVVVPQNNGFCIRNETIFITNATHEQVREF
KRSQHQPAPGAMPSTSSAVTSPQAGAAAGLQGRLNALGVATGPVAILSGDPLAATAPVNS
GSAAISTTAVAPGAQDESTKMQMIEAMSAQSQMNVIWSRKCLEETNWDFNHAAFVFEKLF
KENKIPPEAFMK
>Q9UUJ4
MNGTNTEDNSKQILIQTMYTYDSSGETLKIAIAWKIILKKPKGKNIKDYIEALRKGIEDQ
EHCEKYASTLLEPRPKTKKDVVLKNSNVTECVALKAKPFSKKIEDMDIFLLTNVHENLQE
KRQTSGSLAHLDIEYTFNGLFRFLKCTADIKLKQTKVYEGADFLRIKTLFEEIFMFLKRD
CKSPLVLTRLVELGDYVLDLIIITQSIMQNNANNGTGVISRAKFLEFYVFLEQLIFNKLS
FASVEQLEKLLDQIVKRMKICFTYCKNDNPSIRLLYSECFFSYAEIYFPCLHSFDAQLSS
AASKCVQILRDIITNEELQTDKQELSKSAYSAPSILLIGLKDMLFPEDIS
>Q95YL4
MYGYGYTPAVVAPTVMSFSFVPPQAFQQCWFYSLYTQIQQAQLYEMQSWFMRVDANRSGT
ISSGELQYLNIGGTPLGIETATKLIKVFDHNKNGQIDFYEYAALHQFINNLYRCFVANDR
NFSGTIDANEIYNALITSGFQLPFPTVNYLFLKLSPSGYGLLFTQFLNLCATVALTRSLF
EWNDPMRTGVVHLNLAQLYDIIALV
>Q9NQE9
MAEEQVNRSAGLAPDCEASATAETTVSSVGTCEAAGKSPEPKDYDSTCVFCRIAGRQDPG
TELLHCENEDLICFKDIKPAATHHYLVVPKKHIGNCRTLRKDQVELVENMVTVGKTILER
NNFTDFTNVRMGFHMPPFCSISHLHLHVLAPVDQLGFLSKLVYRVNSYWFITADHLIEKL
RT
>Q8TDR2
METGKDGARRGTQSPERKRRSPVPRAPSTKLRPAAAARAMDPVAAEAPGEAFLARRRPEG
GGGSARPRYSLLAEIGRGSYGVVYEAVAGRSGARVAVKKIRCDAPENVELALAEFWALTS
LKRRHQNVVQFEECVLQRNGLAQRMSHGNKSSQLYLRLVETSLKGERILGYAEEPCYLWF

VMEFCEGGDLNQYVLSRRPDPATNKSFMLQLTSAIAFLHKNHIVHRDLKPDNILITERSG
TPILKVADFGLSKVCAGLAPRGKEGNQDNKNVNVNKYWLSSACGSDFYMAPEVWEGHYTA
KADIFALGIIIWAMIERITFIDSETKKELLGTYIKQGTEIVPVGEALLENPKMELHIPQK
RRTSMSEGIKQLLKDMLAANPQDRPDAFELETRMDQVTCAA
>Q99321
MGKTADNHGPVRSETAREGRENQVYSPVTGARLVAGCICLTPDKKQVLMITSSAHKKRWI
VPKGGVEKDEPNYETTAQRETWEEAGCIGKIVANLGTVEDMRPPKDWNKDIKQFENSRKD
SEVAKHPPRTEFHFYELEIENLLDKFPECHKRHRKLYSYTEAKQNLIDAKRPELLEALNR
SAIIKDDK
>P23542
MSATLFNNIELLPPDALFGIKQRYGQDQRATKVDLGIGAYRDDNGKPWVLPSVKAAEKLI
HNDSSYNHEYLGITGLPSLTSNAAKIIFGTQSDAFQEDRVISVQSLSGTGALHISAKFFS
KFFPDKLVYLSKPTWANHMAIFENQGLKTATYPYWANETKSLDLNGFLNAIQKAPEGSIF
VLHSCAHNPTGLDPTSEQWVQIVDAIASKNHIALFDTAYQGFATGDLDKDAYAVRLGVEK
LSTVSPVFVCQSFAKNAGMYGERVGCFHLALTKQAQNKTIKPAVTSQLAKIIRSEVSNPP
AYGAKIVAKLLETPELTEQWHKDMVTMSSRITKMRHALRDHLVKLGTPGNWDHIVNQCGM
FSFTGLTPQMVKRLEETHAVYLVASGRASIAGLNQGNVEYVAKAIDEVVRFYTIEAKL
>O74496
MVVLAVSIVNRGGKAIISRQFREMSRVRVESLLSSFPALVSEKSQNTTVESDNVRFVYQP
LDELYIVLITNLQSNILQDIDTLHLLSQVVTSICSSLEEREILEYAFEIFTAFDEATSLG
YRDNVSLTQIKTYLEMESHEEKIQEIVSRNKEIEATEERKRRIKQLELQKKEAARRAAQN
LPSADAYESIGYQTVNTTFATSNVEDESAMESYHAAAKASSAPKAKGMQLGKKKNTSLLY

>Q96JC1
MHDAFEPVPILEKLPLQIDCLAAWEEWLLVGTKQGHLLLYRIRKDVVPADVASPESGSCN
RFEVTLEKSNKNFSKKIQQIHVVSQFKILVSLLENNIYVHDLLTFQQITTVSKAKGASLF
TCDLQHTETGEEVLRMCVAVKKKLQLYFWKDREFHELQGDFSVPDVPKSMAWCENSICVG
FKRDYYLIRVDGKGSIKELFPTGKQLEPLVAPLADGKVAVGQDDLTVVLNEEGICTQKCA
LNWTDIPVAMEHQPPYIIAVLPRYVEIRTFEPRLLVQSIELQRPRFITSGGSNIIYVASN
HFVWRLIPVPMATQIQQLLQDKQFELALQLAEMKDDSDSEKQQQIHHIKNLYAFNLFCQK
RFDESMQVFAKLGTDPTHVMGLYPDLLPTDYRKQLQYPNPLPVLSGAELEKAHLALIDYL
TQKRSQLVKKLNDSDHQSSTSPLMEGTPTIKSKKKLLQIIDTTLLKCYLHTNVALVAPLL
RLENNHCHIEESEHVLKKAHKYSELIILYEKKGLHEKALQVLVDQSKKANSPLKGHERTV
QYLQHLGTENLHLIFSYSVWVLRDFPEDGLKIFTEDLPEVESLPRDRVLGFLIENFKGLA
IPYLEHIIHVWEETGSRFHNCLIQLYCEKVQGLMKEYLLSFPAGKTPVPAGEEEGELGEY
RQKLLMFLEISSYYDPGRLICDFPFDGLLEERALLLGRMGKHEQALFIYVHILKDTRMAE
EYCHKHYDRNKDGNKDVYLSLLRMYLSPPSIHCLGPIKLELLEPKANLQAALQVLELHHS
KLDTTKALNLLPANTQINDIRIFLEKVLEENAQKKRFNQVLKNLLHAEFLRVQEERILHQ
QVKCIITEEKVCMVCKKKIGNSAFARYPNGVVVHYFCSKEVNPADT
>Q05930
MTKRRNLFMVGSSFTIDHLPPEIWLCISKLVGTSDLHNLCLINRRLYLTITSDEIWKRRC
YDRWINRESLDILTGNDYDSIPVSQWYSYYLRRAKWENKIFCLLWGLTEETNPQHFREKY
LHILQFRHYKLATFLHRIIKQGYIPDKRPLDLITYANYLLKNVRHKYVFPLFYPTNAAEL
KNLNNMASRDAEMIYLRLSAIDTSFDDLLDAREFILNGICSDLLQKYKKIEEFLKLRPVT

RVSKLISISTDYLDCFTQPHDSVGQTNDRATGRELHREDFMLLRVYSREGRGYKTIILAI
IQAITKRYNVDSYLARDHLVVSEPDFPDGRAFVTVNEDFQPYIFDKEDLLSVWSNNFHNA
ENFESTVLPALLEPISIQHLLTEFFRELLRCKPRPFEGYPNRAHGLRDMFPYGKVEVPRD
VTMYFAFIYDLFDGMFESGMTSLRGQMLRDLLNYVNANNFGDLNIIIGQNALKEPNDCWS
NKRDYVLLDDNNKIGYFYTDIETEDTLCALNQYEVDGKVFITTIDILGDIRVRLAEGLTP
FQGDNDKLWESFSSVVPRTDWGLFFKGYDKERRRMQLNPYIEEKLSNLANDEQPLHNL
>P05747
MAKSKNHTAHNQTRKAHRNGIKKPKTYKYPSLKGVDPKFRRNHKHALHGTAKALAAAKK
>Q9SLN5
MASESDASSIATLSCARCEKPAHLQCPKCIDLKLPREQASFCTQECFKAAWSSHKSVHVK
AQLSSIGDQNSDLISQGWLYCVKKGQARTPKLPHFDWTGPLKQYPISTKRVVPAEIEKPD
WAIDGTPKVEPNSDLQHVVEIKTPEQIQRMRETCKIAREVLDAAARVIHPGVTTDEIDRV
VHEATIAAGGYPSPLNYYFFPKSCCTSVNEVICHGIPDARKLEDGDIVNVDVTVCYKGCH
GDLNETYFVGNVDEASRQLVKCTYECLEKAIAIVKPGVRFREIGEIVNRHATMSGLSVVR
SYCGHGIGDLFHCAPNIPHYARNKAVGVMKAGQTFTIEPMINAGGWRDRTWPDGWTAVTA
DGKRSAQFEHTLLVTETGVEVLTARLPSSPDVYPWLTK
>Q13316
MKISILLMFLWGLSCALPVTRYQNNESEDSEEWKGHLAQAPTPPLESSESSEGSKVSSEE
QANEDPSDSTQSEEGLGSDDHQYIYRLAGGFSRSTGKGGDDKDDDEDDSGDDTFGDDDSG
PGPKDRQEGGNSRLGSDEDSDDTIQASEESAPQGQDSAQDTTSESRELDNEDRVDSKPEG
GDSTQESESEEHWVGGGSDGESSHGDGSELDDEGMQSDDPESIRSERGNSRMNSAGMKSK
ESGENSEQANTQDSGGSQLLEHPSRKIFRKSRISEEDDRSELDDNNTMEEVKSDSTENSN
SRDTGLSQPRRDSKGDSQEDSKENLSQEESQNVDGPSSESSQEANLSSQENSSESQEEVV
SESRGDNPDPTTSYVEDQEDSDSSEEDSSHTLSHSKSESREEQADSESSESLNFSEESPE
SPEDENSSSQEGLQSHSSSAESQSEESHSEEDDSDSQDSSRSKEDSNSTESKSSSEEDGQ
LKNIEIESRKLTVDAYHNKPIGDQDDNDCQDGY
>Q92413
MTSNGTNGSATAYHASSTQEAIQAENDFAAHNYHPLPVVFARAQGTSVWDPEGRHYLDFL
SAYSAVNQGHCHPKLVAALVDQASRLTLSSRAFYNDVFPKFAEMVTKYFGFDMVLPMNTG
AEAVETGIKIARKWGYKVKGIPENEAIILSAENNFHGRTMAAISLSSDPESRENYGPYVP
NIGCTIPGTEKPITYNDKAALREAFEKAGSNLAAFLVEPIQGEAGIIVPDDDYLQLARSL
CDQHNVLLICDEIQTGIARTGKLLCHEWSGIKPDMVLLGKAISGGMYPVSCVLGRKDVML
TVEPGTHGSTYGGNPLACAVAIRALEVVQEENMVERAEKLGQAFRSGLEAIQNPIIQTVR
GKGLLNAIVIDESKTNGHTAWDLCMLMKEKGLLAKPTHQNIIRLAPPLVITEEEIAKALE
IIKAAVAELPNLKGAAEDKVVPPPEKKVKITLEN
>Q12416
MSLRTPKRSRTSDEQEQEQEQEQVQNPDTHVNNEHQQRPGPTTLLSTPVRLKNGFGTPSP
PSPPGITKSITKSRRRPSTTSLQGIFMSPVNKRRVGITAHGRVYDHNDDGHESESEDDEN
EEENENQKKYDGHVSMPLLPPTTPKSRRSEVFLSPSPRLRSPPTAARRSTGERPIREISH
TLRTRLNYALVKLQNGWTDKTLPELETELAPAVQTPPRRYHNRFPDSADAGTSAHTAFLQ
ALGGHPPREEATAVETLMLLSSPTKKQQHRPVPATSAGEPTDETEPESDTEVETS
>Q17353
MSASSTSSSSTSCPEGGEPSGSCKSSDEGESTLKKRMQQYGIASGYANSSISTLDRSQYQ
SLPLNGTRRVTVQFGRMKIVVPWKESDQTVGQLADAALLRYKKARGMANEDRIHVHRLEC

ASDGGILDMDDVLEEVFDLNYDQILAITDEANGGSTTPTYSQIQKQQHHYAQPLPYARKF
DGGPSTPIASAFGSVTVNHQAHRAASPYNVGFARSNSRDFAPQPTHSKERRDSVVEVSSF
DQIPQSGLRVSTPKPSRQSEDVIDGKPMNQPILRSSLRTEASGSRTEEATPVKQSRVTLS
PEVEKKLAEQDERKSERRKHYDKNPGRFARGSDRKSRITDALLDARDRIADQLESQNPAE
ETKSQMIRVKIDQGPMPGTSLVTFPPIPEKSENEKQLGIEVNAVFDESSELPGTSEPTKL
SSVQIMKIEDGGRIAKDGRIRVGDCIVAIDGKPVDQMSIIRVRASISDLAAVTSRPVTLI
INRSLESFLEQESSAKPIQSALQQANTQYIGHTTVVELIKSSNGFGFTVTGRETAKGERL
FYIGTVKPYGVALGHLKSGDRLLEINGTPTGQWTQSEIVEKLKETMVGEKIKFLVSRVSQ
SAIMSTSASSENKENEETLKVVEEEKIPQKLPLPALMTPPVPKDTPALSPSGASRFEIVI
PFINGSSSAGLGVSLKARVSKKSNGSKVDCGIFIKNVMHGGAAFKEGGLRVDDRIVGVED
IDLEPLDNREAQAALAKKLKEVGMISSNVRLTISRYNECNPGQISRDLSRITVDASSPSP
SSRMSSHTAPDSLLPSPATRGTSSSGADSSHSRQSSASSAVPAVPARLTERDSIVSDGTS
RNDESELPDSADPFNREGLGRKSLSEKRGMGAAADPQHIKLFQDIKHQRQNSAPTSSTQK
RSKSQPRSSSQRNYRSPMKLVDLPTTAAASASTNSQNLDDSDMLNRRSQSMESINRPVES
ILRGTGQIPTGSSSKVQFMQAASPDQHPFPPGAALLRLKNEESRSRDKSRRKSMGNPFSA
MRNFFGFGSKSRDASPEKTPTESVQLRSVERPKSIIDERNNGSSERAPPPLPPHQSQRRG
SGGNVFVDYGEPYGLIPQYPHNTTSGYESYADSELYDRYAAHRYHPRGGPIIDEDEYIYR
QQSTSGNSPINTSSYVNYGLPASNAYHVGSRIPPQTSSGSISKTSGAMRRVYPAEYDEDV
AYHQQIPQQSTRYQQGSGSGRGNADYHHMFNSWFAYTGGGAVGAAPVIKSSYGSSPVRIA
AASAIERGESFVVEPVSGSSASATDRRGRSTSSGAVASGSSSTGFQYAAKEKYADARSGK
FNGGSTRLFIPRHGGGLSAAAFATNFGGEAYETRGGGAGGSPSQYRRRDQGPPHRFPQY
>O13824
MSREEQIKKLNQYLDNRELAFRAKDGDKNIFHTESQLDSSLKKNTAFMKRCKSSLTSENY
DSFIKEIKTLSLKKFIPEITAAIVEGMMKCKATKDILSSVKIVWALNLRFSTAFTGPMLA
NLYCALYPNPGYSLCHESYFELKQNENEVSEKDRSSHLLKVRPLLRFLIEFWLNGVVGTP
EDFVSYLPSTDSNDKKFRKPWFEEQNLKKPLVVLLFNDLMDTRFGFLLLPVLTSLVRTFS
CELFTTEDFEDKETLELVNRLNPVVWRTYLRKSLNSYVDKLEVYCQKRKSLFEELNKQYQ
EQSIIRADPNNEKFQRLANFSKSIESEFSSYASLSEVLNRKASEDLLELNFMEKASSGTN
SVFNASGERSESANVETAQVWDDREQYFFYEVFPNFNEGSIAEMKSSIYESSQEGIRSSS
ENNKKEDDLKDSTGDLNTTQVSSRVDNFLLKLPSMVSLELTNEMALEFYDLNTKASRNRL
IKALCTIPRTSSFLVPYYVRLARILSQLSSEFSTSLVDHARHSFKRMIHRKAKHEYDTRL
LIVRYISELTKFQLMPFHMVFECYKLCINEFTPFDLEVLALLLESCGRFLLRYPETKLQM
QSFLEAIQKKKLASALASQDQLVLENALHFVNPPKRGIIVSKKKSLKEEFLYDLIQIRLK
DDNVFPTLLLLRKFDWKDDYQILYNTIMEVWNIKYNSLNALARLLSALYKFHPEFCIHVI
DDTLESLFSAVNNSDHVEKQKRLAQARFISELCVIHMLDVRAITNFLFHLLPLEKFESFL
TMKASTLTNINNDMFRLRLIVVVLQTCGPSIIRSKTKKTMLTYLLAYQCYFLIQPEMPLD
MLYEFEDVIGYVRPSMKVYMHYEEARNALTERLQAISDDWEEDDTRPVFQGANDGDISSN
EESVYLPEDISDESETDEESSGLEESDLLDSEDEDIDNEMQLSRELDEEFERLTNESLLT
RMHEKNPGFDVPLPLRASSLGSPYVTRNEESASESSHVMFTLLTKRGNKQRSQYLEIPSH
SSLVRSTKNQQTEEIMERKRVKEMVLNFE
>O95721
MSAYPKSYNPFDDDGEDEGARPAPWRDARDLPDGPDAPADRQQYLRQEVLRRAEATAAST
SRSLALMYESEKVGVASSEELARQRGVLERTEKMVDKMDQDLKISQKHINSIKSVFGGLV
NYFKSKPVETPPEQNGTLTSQPNNRLKEAISTSKEQEAKYQASHPNLRKLDDTDPVPRGA

GSAMSTDAYPKNPHLRAYHQKIDSNLDELSMGLGRLKDIALGMQTEIEEQDDILDRLTTK
VDKLDVNIKSTERKVRQL
>O14893
MRRAELAGLKTMAWVPAESAVEELMPRLLPVEPCDLTEGFDPSVPPRTPQEYLRRVQIEA
AQCPDVVVAQIDPKKLKRKQSVNISLSGCQPAPEGYSPTLQWQQQQVAQFSTVRQNVNKH
RSHWKSQQLDSNVTMPKSEDEEGWKKFCLGEKLCADGAVGPATNESPGIDYVQIGFPPLL
SIVSRMNQATVTSVLEYLSNWFGERDFTPELGRWLYALLACLEKPLLPEAHSLIRQLARR
CSEVRLLVDSKDDERVPALNLLICLVSRYFDQRDLADEPS
>Q9ES73
MAQKPDGGAGLRGFQAEASVEDSALLVQTLMEAIQISEAPPTSQATAAASGPNASPQSSQ
PPTANEKADTEVSAAAARPKTGFKAQNTTTKGPNDYSQARNAKEMPKNQPKVAFKSQNAT
SKGPHAASDFSHAASTGKSAAKKSEMAFKGQNTTTKAGPSATYNFTQSPSANEMTNNQPK
TAKAWNDTTKIPGADAQTQNVNQAKMADVGTSAGISETDGAAAQTSADGSQAQNVESRTI
IRGKRTRKINNLNVEENSNGDQRRASLASGNWRSAPVPVTTQNPPGAPPNVLWQTPLAWQ
NPSGWQNQTARQTPPARQSPPARQTPSAWQNPVAWQNPVIWPNPVIWQNPVIWPNPIVWP
GPIVWPNPMAWQSTPGWQSPPSWQAPPSWQSPQDWQGPPDWQLPPDWSMPPDWSFPSDWP
FPPDWIPADWPIPPDWQNLRPSPNLRSSPNSRASQNQGPPQPRDVALLQERANKLVKYLM
LKDYTKVPIKRSEMLRDIIREYTDVYPEIIERACFVLEKKFGIQLKEIDKEEHLYILIST
PESLAGILGTTKDTPKLGLLLVILGIIFMNGNRATEAVLWEALRKMGLRPGVRHPLLGDL
RKLLTYEFVKQKYLDYRRVPNSNPPEYEFLWGLRSYHETSKMKVLRFIAEVQKRDPRDWT
AQFMEAADEALDALDAAAAEAEARAEARNRMGIGDEAVSGPWSWDDIEFELLTWDEEGDF
GDPWSRIPFTFWARYHQNARSRFPQAFTGPIIGPSGTATANFAANFGAIGFFWVE
>Q06265
MKETPLSNCERRFLLRAIEEKKRLDGRQTYDYRNIRISFGTDYGCCIVELGKTRVLGQVS
CELVSPKLNRATEGILFFNLELSQMAAPAFEPGRQSDLLVKLNRLMERCLRNSKCIDTES
LCVVAGEKVWQIRVDLHLLNHDGNIIDAASIAAIVALCHFRRPDVSVQGDEVTLYTPEER
DPVPLSIHHMPICVSFAFFQQGTYLLVDPNEREERVMDGLLVIAMNKHREICTIQSSGGI
MLLKDQVLRCSKIAGVKVAEITELILKALENDQKVRKEGGKFGFAESIANQRITAFKMEK
APIDTSDVEEKAEEIIAEAEPPSEVVSTPVLWTPGTAQIGEGVENSWGDLEDSEKEDDEG
GGDQAIILDGIKMDTGVEVSDIGSQDAPIILSDSEEEEMIILEPDKNPKKIRTQTTSAKQ
EKAPSKKPVKRRKKKRAAN
>Q9JIL5
MYAAVEHGPVLCSDSNILCLSWKGRVPKSEKEKPVCRRRYYEEGWLATGNGRGVVGVTFT
SSHCRRDRSTPQRINFNLRGHNSEVVLVRWNEPYQKLATCDADGGIFVWIQYEGRWSVEL
VNDRGAQVSDFTWSHDGTQALISYRDGFVLVGSVSGQRHWSSEINLESQITCGIWTPDDQ
QVLFGTADGQVIVMDCHGRMLAHVLLHESDGILSMSWNYPIFLVEDSSESDTDSDDYSPP
QDGPAAYPIPVQNTKPLLTVSFTSGDISLMNNYDDLSPTVIRSGLKEVVAQWCTQGDLLA
VAGMEQQAQLSELPNGPLLKSAMVKFYNVRGEHIFTLDTLVQRPIISICWGHRDSRLLMA
SGPALYVVRVEHRVSSLQLLCQQAIASTLREDKDVNKLTLPPRLCSYLSTAFIPTIKPPI
PDPNNMRDFVSYPSAGNERLHCTMKRTEDDPEVGGPCYTLYLEYLGGLVPILKGRRISKL
RPEFVIMDPRTDSKSDEIYGNSLISTVIDSCNCSDSSDIELSDDWAAKKSPKISRSSKSP
KLPRISIEARKSPKLPRAAQEISRSPRLPMRKPSMGSPSLTRREFPFEDITQHNYLAQVT
SNIWGTKFKIVGLAAFLPTNLGAVIYKTSLLHLQPRQMTIYLPEVRKISMDYINLPVFNP
NVFSEDEDDLPVTGASGVPENNPPCTVNIPIAPIHSSAQALSPTQSIGLVQSLLANQNVQ

LDVLTNQTTAVGSAEHAGDAATQYPVSSRYSNPGQVIFGGVEMGRIIQNPPQLPLPPPPP
PPPQAPMQLSAVDHGDRDHEHLQKSAKALRPVPQLAAEGDAVVFSAPQEVQVAKMNPPPP
YPGTIPAAPTTAAPPPPLPPPQPPVDVCLKKGDFSLYPTAAHYQPPLGYERITTFDSSGN
VEEVCRPRTRMLCSQNTYTLPGPGSSATLRLTATEKKVPQPCTSATLNRLTVPRYSIPTG
DPPPYPEIASQLAQGRSAAQRLDNSLIHATLRRNNREVALKMAQLADSSRAPLQPLAKPK
GGAAGAVAQLPARPPPALYTCSQCSGAGPSSQSGAALAHAISTSPLASQSSYNLLSPPDT
SRDRTDYVNSAFTEDEALSQHCQLEKPLRHPPLPEAAVTMKRPPPYQWDPMLGEDVWVPQ
ERTAQPTVPNPLKLSPLMLGQGQHLDVARVPFVPPKSPSSPTATFPTGYGMGMPYPGSYN
NPSLPGVQAPCSPKDALSQAQFAQQESAVVLQPAYPPSLSYCTLPPTYPGSSTCSSVQLP
PIALHPWNSYSTCPPMQNTQGTLPPKPHLVVEKPLVSPPPAELQSHMGTEVMVETADNFQ
EVLSLTESPVPQRTEKFGKKNRKRLDSRAEEGSVQAITEGKVKKDARTLSDFNSLISSPR
LGREKKKVKSQKDQLKSKKLNKTNEFQDSSESEPELFISGDELMNQSQGSKKGWKSKRSL
RTASELEEFKCRKASEKEDGRLGSQGFVYVMANKQPLWNEATQVYQLDFGGRVTQESAKN
FQIELEGRQVMQFGRIDGNAYILDFQYPFSAVQAFAVALANVTQRLK
>Q9UT53
MNGDNFANSFPKNPLLSRNSSSSNVIQYSDEFSPDEDWLNEHAAKEHEERIRRPSVNRAW
QKNSTSGGPSVSLEKREADVASLGEVMDLEEVPRGITRQARQLNEYIFPKHRFRNHLVDE
GKIPLVLVACGSFSPITYLHLRMFEMATDTIQEQTNMELVAGYFSPVNDHYKKEGLAPAY
HRVRMCELACERTSSWLMVDAWESLQPSYTCTARVLDHFDEEINQKRGGITLSDGTKRPC
KIMLLAGGDLIASMGEPGVWSDKDLHHILGKFGCCIVERTGSDVWAFLLAHDIMFAYRGN
ILVIKQLIYNDISSTKVRLFIRRGMSIRYLLPNSVIQYIERYALYRDAEPVKTIFYQSPF
VRMEP
>O14015
MANRFTSSDQTQETHGHHVDKHSFSGRQRHPSMGVHSFNEYMNEYPEAGPLVQEEEDDME
SSSYHATQEDIGDDDSRSESTRNRSSQHTGRVNFSSLGGLGSIFGGKKSRSKSANGTQKK
NANNNEEQLDEEEQNDPYLDRDISLLGSTI
>P10551
MSQNVYIVSTARTPIGSFQGSLSSKTAVELGAAALKGALAKVPELDASKDFDEIIFGNVL
SANLGQAPARQVALTAGLGNHIVATTVNKVCASAMKAIILGAQSIKCGNADVVVAGGCES
MTNAPYYMPAARGGAKFGQTVLIDGVERDGLNDAYDGLAMGVHAEKCARDWDITRDQQDS
FAIESYQKSQQSQKEGKFDNEIVPVTIKGFRGKPDTQVTNDEEPARLHVEKLKSARTVFQ
RENGTVTAANASPINDGAAAIILVSERVLKEKNLKPLAIVKGWGEAAHLPADFTWAPSLA
VPKALKHAGIEDINSVDYFEFNEAFSVVGLVNTKILKLDPSKVNVYGGAVALGHPLGCSG
ARVVVTLLSILQQEGGKIGVAAICNGGGGASSVVIEKL
>O74914
MASEGKVLLVASSYYGPFYPDGMNTGVHFAELLIPYQVFREAGYEVQLTSETGKCKFDDH
SIKKSALGEVERDAFDNKDNEFWYALKDIKPADKINYKEFCIMFIAGGHAAMFDLPHATN
LQTLAQQIYASNGVLAAVCHGPVMLPFVDDTKSPEGRSVVYGKKVTAFNSTGELVMGVSS
ALRERNMQDLNSLFREAGAEFVDPPTPMSDFTQVDGRIVTGVNPMSAKSTAEAAIKVSQS
LRKT
>Q9VXH6
MASKYERILSDCRSKNVLWEDPDFPAVQSSVFYYQTPPFTFQWKRIMDLADSGSGAVAAN
SSAAPVFLNETAEFDVVPGKMGDRWLVSCLGLLSSLRNLFYRVVPADQTLASAHGVFRFR
LWWCGEWVEVLVDDRLPTINGRLAFMQPQASNCFWAALLEKAIAKLHGSYEALKYGTRSD

GLTDLLGGVVRQMPILADNIRPQTLKELLTTTCIVTCLADKSATVAKKNLAERMPNGILV
NVNYRLSSLDKVKTLMGDSVQLVCLKDTFSSKPFGEKTHFLGDWSPMSKTWERVSQVERA
RLIRQLGPGEFWLSFCDFVEIFSTMEVVYLDTETSNDEEMLKSRPLHWKMKMHQGQWKRG
VTAGGCRNHESFHINPQLLISVQDEQDLVIALNQHTAVEPKVIGFTMYTWDGEYMLSECL
QKDFFKNHVSYLNSDYGNTRHVSYHTHLEAGHYVLIPTTYEPAEEAHFTVRILGTGSFRL
SCLETQTMILLDPFPALKSTDAERCGGPKVKSVCQYEPVYMQLADENKTINCFELHELLE
ACLPNDYIKGCANIDICRQVIALQDRSGSGRITFQQFKTFMVNLKSWQGVFKMYTKEKAG
ILRAERLRDALCDIGFQLSTDIMNCLIQRYIRKDGTLRLSDFVSAVIHLTTAFNQFHLKN
YGQVNVIEVHLHDWIKSILSC
>P51580
MDGTRTSLDIEEYSDTEVQKNQVLTLEEWQDKWVNGKTAFHQEQGHQLLKKHLDTFLKGK
SGLRVFFPLCGKAVEMKWFADRGHSVVGVEISELGIQEFFTEQNLSYSEEPITEIPGTKV
FKSSSGNISLYCCSIFDLPRTNIGKFDMIWDRGALVAINPGDRKCYADTMFSLLGKKFQY
LLCVLSYDPTKHPGPPFYVPHAEIERLFGKICNIRCLEKVDAFEERHKSWGIDCLFEKLY
LLTEK
>Q8R080
MDAGSKKEDFLLLEDEKFDFDLSLSSSSTNEDDEVFFGPVGHKERCIAASLDLNRRVPGQ
PLAPGSGSPCTLSPLTGEKFVEVYKEAHLLALQIESHSRREVAQAATPQNPVNQGKETFV
QDSQLKVSLFEKEQKRDRSPMSLKRETFCLPSSRVQPPMGEPQLLASPGLLSSPVSAGPA
QTQSNQGLPCSSQPLPRESSTSQPPSQAGPQKRITSKLQPPRALPVRGRNLHLATEKLKK
EVPASIQRTKLVNEKGSQSDVLQDKPSTAPDAASREGHPGKRSLPIPGKLGLKKTLLKPP
GYTGNLTRKSSTSGSASSLESGVYRSSVAGKAKSSEQRSSIPASGSQRRTSTSKSGRIGP
AASRQALPAAPARVFGRQANKADAAQTVAEQPKVPTLSPLTQQPQTPEQRGPRLDPDTET
PQLNKTVSIKRRDSYLSCKTEAVSTTTNPFKVPQFSVGESPGGVTPKFSRTHRLQSWTPA
SRVVSSTPVRRSSGTTPQGLPGSMRTPLSTRRMSVLPTPASRRLSSLPLMAPQSMPRALV
SPLCVPARRLSSEPRRRSTVRAELTQESSGSGSGGQAQGLSSDESSSPPSSVPQALNFSP
EKSASPPPQGSSTGAAQGEAEPPEDTLPSEVHGGGCSHTPSEGLLLDLKLDQLTITPEAG
GRDLADCPLIDFSNTPESNTALGPSSWPLIDLIMNTPDMGRNDVGKPAKAELGQLIDLGS
PLIQLSPEADKENVDSPLLKF
>O60185
MSLQAIIYNEDKLLVLDQLLLPYERKYISVSNVEDAFAVIKKMQVRGAPAIAIVAALSVA
VGLKHLSEDADAKEYVINSFEHLKQSRPTAVNLFETAKFMQGIALQEGKNCRNKIIQEAE
RMLVKDLEDNHNIGTAGRKFLLQHYKDKDKLTVLTHCNTGSLATSGYGTALGIIRSLHES
GNLEHAYCTETRPYNQGSRLTAFELVHDKIPATLVTDSTVASIMHKIDAIVVGADRVTRN
GDTANKIGTYNLAILAKHFGKPFIVAAPFSSIDTSLASGSQITIEQRPPIEMTTITGPII
TDSNSRNLEDPKRVRISIAAPGIDVYSPAFDVTPANLITAIATEKGFYTQQTNNKYDFS
>P48566
MQNDQQRFSLQNRTVLAHPYKRLGGAFTVKSPSVPNFHDKMHSDHSSSDSALVNGSFRAN
DHRSVEPSCLGQASPSEHDGNLSVIDLYGDEVESQRAEGEDDDDNNGDNGNEDLEEVHSD
DLDLVPDDDNRQRVELEGAASATSANSNGINNTHFDRYGFKKQNNYISEAEYDKWWVEYS
QYCVRRKHKWQLLLEKSGLPVTDDSPSRFPSKSERLKRYVRKGIPAEWRGNAWWHFARGQ
EKLNKNKGVYSQLLRKMKQIKKQNPNEKQVQDLDIIERDLNRTFPDNIHFQSSLHNKEGP
PIIKSLRRVLVAFSLYNPKIGYCQSMNFLAGLLLLFLDEERAFWMLVIITSRYLPGVHNI
NLEGVNIDQGVLMLCVKEYIPEVWSYIKPSIDHHQKNNKTFSPSNKKVLFNMQKNEFLYR

LPPITLCTASWFMSCFVGVVPIETTLRIWDCLFYEESHFLFKVSLAVLKLSEHDLSKIKP
RNNSLNYSWGSNLNQRGGSMGQEDSDMEIFQVIQTFPKTLLNPNEIFEKIIFKRRFNLNR
LDQDEIDRCRKFVAAQRLKFKTYGELLGNSTSEADLPINDNTDNKGIHITSDAVNEALSS
EVYGFKKSLAGVHWNNSIKEKVKQMRKKKDKGD
>P52580
MASEKSKILVVGGTGYLGRHVVAASARLGHPTSALVRDTAPSDPAKAALLKSFQDAGVTL
LKGDLYDQASLVSAVKGADVVISVLGSMQIADQSRLVDAIKEAGNVKRFFPSEFGLDVDR
TGIVEPAKSILGAKVGIRRATEAAGIPYTYAVAGFFAGFGLPKVGQVLAPGPPADKAVVL
GDGDTKAVFVEEGDIATYTVLAADDPRAENKVLYIKPPANTLSHNELLSLWEKKTGKTFR
REYVPEEAVLKQIQESPIPLNIILAIGHAAFVRGEQTGFEIDPAKGVDASELYPDVKYTT
VDEYLNRFL
>P11915
MPSVALNSPRLPRVFVVGVGMTKFMKPGGENSRDYPDLAKEAGQKALADRQIPYSAVEQA
CVGYVYGESTCGQRAIYHSLGLTGIPIINVNNNCSTGSTALFMAQQLVQGGLANCVLALG
FEKMEKGSLGTKYSDRSNPLEKHIDVLINKYGMSACPFAPQLFGSAGKEHMETYGTKVEH
FAKIGWKNHKHSVNNPYSQFQDEYSLDEIMKSRPVFDFLTVLQCCPTSDGAAAAIVSSEE
FVQKHGLQSKAVEIVAQEMVTDMPSTFEEKSVIKMVGYDMSKEAARKCYEKSGLGPSDVD
VIELHDCFSTNELLTYEALGLCPEGQGGALVDRGDNTYGGKWVINPSGGLISKGHPLGAT
GLAQCAELCWQLRGEAGKRQVPGAKVALQHNLGLGGAAVVTLYRMGFPEAASSFRTHQIS
AAPTSSAGDGFKANLIFKEIEKKLEEEGEEFVKKIGGIFAFKVKDGPGGKEATWVVDVKN
GKGSVLPDSDKKADCTITMADSDLLALMTGKMNPQSAFFQGKLKIAGNMGLAMKLQSLQL
QPDKAKL
>P40070
MLPLYLLTNAKGQQMQIELKNGEIIQGILTNVDNWMNLTLSNVTEYSEESAINSEDNAES
SKAVKLNEIYIRGTFIKFIKLQDNIIDKVKQQINSNNNSNSNGPGHKRYYNNRDSNNNRG
NYNRRNNNNGNSNRRPYSQNRQYNNSNSSNINNSINSINSNNQNMNNGLGGSVQHHFNSS
SPQKVEF
>O94560
MKSNLSLKRQDEKRADKEPNLYTLQGVIQYLQYEAFKNERDHNLWEIERAELKIRVAQLE
RERAKLEQSLSFQQRRAEMLEKSLRELRKDKNISVKDLDEFHLLDKPAANNTKADAEACL
LKSKNYIKKSLQEIVYLTNMQPNVSWNVLQEPTRGIKVPKESNNTQQNNQFVMEPSQNKG
NVNDANFFEVEYARNENMNKLSSSELISDDLLEDEIMKPLSSGESLPKKEEEVTKSPSFT
LDDSVASNEQTLAQLNIESPVDQKKSKAKKKKEQKWTLKFESDKGTSTQCITHQPLPGST
PSFASGTENGVINVWRLDEDSNDNSMGIIKPHLTFYGHEGPVLCVCVPKATHHIFSGGHD
GTIRCWSLPANQTSDSISKILTGSTIFQGHEDCVWELFCHEVKDNNPILLSLSSDGTVRG
WKYTGEQLFKIRCDSKQPLSMSVTDSRIAIAYNDGNVRFYDLDTQILVSQMRIAGNSAIG
NPAVKDRINKIVWKNGNPDRLYSLHENGMVRVFDVKSEELLAEKSISKVSLTGIAFAVNR
PEFAISASDGRVFFLRQDDKLSTLESLPSREAQEEITDLSDILWINSPVDKLEHLIVGCK
ERISVYDRKYLP
>O74875
MLSIFKNLLGTSEEDGTTQEANSKDTKGLKEERKRKKRKNKYKIPPGHTQQDWDALVASG
KNLSGVESPISVTAEELAKHCSPDDCWMAIRGKVYNVTAYLPYHPVGPKKILKHSGVDAT
KPYLKHHDWVNEEELLKTSFVGYLV
>P07280

MPGVSVRDVAAQDFINAYASFLQRQGKLEVPGYVDIVKTSSGNEMPPQDAEGWFYKRAAS
VARHIYMRKQVGVGKLNKLYGGAKSRGVRPYKHIDASGSINRKVLQALEKIGIVEISPKG
GRRISENGQRDLDRIAAQTLEEDE
>Q6PHQ8
MVMKATVDDDASGWELGVPEKMEKSSTSWVDITQDFEDACRELKLGELLHDKLFGLFEAM
SAIEMMDPKMDAGMIGNQVNRKVLNFEQAIKDGTIKIKDLSLPELIGIMDTCFCCLITWL
EGHSLAQTVFTCLYIHNPDFIEDPAMKAFALGILKICDIAREKVNKAAVFEEEDFQSMTY
GFKMANSVTDLRVTGMLKDVEDDLQRRVKSTRSRQGEERDPEVELEHQQCLAAFSRVKFT
RVLLTVLIAFTKKETSAVAEAQKLMVQAADLLSAIHTSLHHGIQAQNGTTKGDHPIMMGF
EPLVNQRLLPPTFPRYAKIIKREEMVNYFSRLIDRIKTVCEVVNLPNLHCILDFFCEFSE
QSPCVLSRSLLQTTFLVDNKKVFGTHLMQDMVKDALRSFVSPPVLSPKCCLYNNHQAKDC
IDSFVTHCVRPFCSLVQIHGHNRARQRDKLGHILEEFATLQDEAEKVDAALHTMLLKQEP
QRQHLACLGTWVLYHNLRIMIQYLLSGFELELYSMHEYYYIYWYLSEFLYAWLMSTLSRA
DGSQMAEERIMEEQQKGRSSKKTKKKKKVRPLSREITMSQAYQNMCAGMFKTMVAFDMDG
KVRKPKFELDSEQVRYEHRFAPFNSVMTPPPVHYLQFKEMSDLNKYSPPPQSPELYVAAS
KHFQQAKMILENIPNPDREVSRILKVAKPNFVVMKLLAGGHKKESKVPPEFDFSVHKYFP
VVKLV
>O13978
MTNTVTIELKIGYKYAAEVVKAVLGVILFHRQFSTVPARTIDVLDITVPTLVGAELNEQL
ATKAAEFIDTIRNEAGANGQMILLLYERSPKKSWFGKGNTIPWEQWILHTTILEEGDSYQ
ESSLSLEAAVEQIVQAVNLRSLSYLPPVAMDSGNYPYEIVTPTSTEGWGSLLKRMIIENV
SGGD
>P42226
MSLWGLVSKMPPEKVQRLYVDFPQHLRHLLGDWLESQPWEFLVGSDAFCCNLASALLSDT
VQHLQASVGEQGEGSTILQHISTLESIYQRDPLKLVATFRQILQGEKKAVMEQFRHLPMP
FHWKQEELKFKTGLRRLQHRVGEIHLLREALQKGAEAGQVSLHSLIETPANGTGPSEALA
MLLQETTGELEAAKALVLKRIQIWKRQQQLAGNGAPFEESLAPLQERCESLVDIYSQLQQ
EVGAAGGELEPKTRASLTGRLDEVLRTLVTSCFLVEKQPPQVLKTQTKFQAGVRFLLGLR
FLGAPAKPPLVRADMVTEKQARELSVPQGPGAGAESTGEIINNTVPLENSIPGNCCSALF
KNLLLKKIKRCERKGTESVTEEKCAVLFSASFTLGPGKLPIQLQALSLPLVVIVHGNQDN
NAKATILWDNAFSEMDRVPFVVAERVPWEKMCETLNLKFMAEVGTNRGLLPEHFLFLAQK
IFNDNSLSMEAFQHRSVSWSQFNKEILLGRGFTFWQWFDGVLDLTKRCLRSYWSDRLIIG
FISKQYVTSLLLNEPDGTFLLRFSDSEIGGITIAHVIRGQDGSPQIENIQPFSAKDLSIR
SLGDRIRDLAQLKNLYPKKPKDEAFRSHYKPEQMGKDGRGYVPATIKMTVERDQPLPTPE
LQMPTMVPSYDLGMAPDSSMSMQLGPDMVPQVYPPHSHSIPPYQGLSPEESVNVLSAFQE
PHLQMPPSLGQMSLPFDQPHPQGLLPCQPQEHAVSSPDPLLCSDVTMVEDSCLSQPVTAF
PQGTWIGEDIFPPLLPPTEQDLTKLLLEGQGESGGGSLGAQPLLQPSHYGQSGISMSHMD
LRANPSW
>Q1W1G1
MEAPTLSELEGLRYSELQKLAKTAGLKANLKADKLLKALKVHFYPESKDESPDYDGGSSL
TDTDELNSSQEKDEPVSVSFVTHRRGRGRKPLKNYDTPKDEFLTVSVGTGTESLASETDN
TQDQNCLESKKKKVSPPTIDNKHRKRSRSEDTSKQNNSETTEKRQKKASDITSVPSAGKI
PRYAGRLSKPESKPSTPNFKKLHEAHFKKMESIDKYMERKQKRLDTVSSSIQEMKMLTKK
SNLLKLVEKTPVSDIKKPVKSRLSLLSSLPPTTGASPSRTPTNQRRSGRFSAANKSILFD

RSGFKPSVLSSSKMNVRFSEATKDNEHKRSLIKTPARKSSSFLAITPESEPRQMLPNVKK
TPARKSLSVLAVTPESEPKQMLPSVKKNEPMATPEKAKKTDLNTTIQPSTVILESTCPQN
KEIAITPFKFTAQTTETPNTNKKGRFDLQASLSRPLGYQPHKGKLKPWGGSEENKCGSNN
NVSVLKNNFKQPHLQTREDRRKQHEQDRKGKRDQTLGTRRGVPVQ
>A2A870
MTGQCCEELQRAPKPRMALRTKKGLKGSIEDVLGDLLGDDTTPPEKPAEPASHAKDTASS
PQWQASKAKFLPKDSVEGLAGADAEASSVSDADPQVFLQNMKDLDSMDDDLFGRMKSHQP
SGKGAAKGPGKEGPSNHKPAGTLTANEKGYTMPTKKPPPSSSKTGLQYKKFSFEDFEDPL
AGLLSDEEEETATKLPAVERKPAPKSPGAAAGQGPSVPLTPGDTPIRKKELLFDEGDDIM
TTLGFEDSPKAERKKTGDQEGPLPARSKLDELLGRGTAAKLLTRPGTGERREFQLDKKYQ
KMGGEESVPARDKEDSWDDETLTFGAYKPTVASSEGRQSRRQSVSRFLGEGGPDPKGESL
GFKQSSPPASSPIHPRKGGADWLGLKDNDLDLLSPSPVQKAQQEDSPMTPSLLPPTNQPS
APEPQSAPTGLPSAAKPPAKGARPSLKASQASSPKASEEKEDDWLSHVISQKKSQNLARE
ERAGPPKDLASLGSLGQTPSGSLPVAQVLEQAPAGEASKPTTQGMAAVRPGVTGSSMSWS
QATTVLPVDDPKKGAASASGDFSSREPAVYIPHSQEPTGLSVPIQTLLPESMMQSLLPGS
GYQKQLLAAQGQLQSSTAQLQVELLQSQTKLSELEAQVRKLELERAQHRMLLESLQQRHQ
ADLELIEDAHRSRIKVLETSYQQREEQLRREKEVLSAQHASYCREAEQARAELVAQHQRQ
MAMAEQERDQEVARLRELQQASILEMRKDHEHQLQRLKMLKDQEIDAVTSATSHTRSLNG
IIEQMEKFSSSLNTLSSRVEASHLTTSQQRELGIRQQDEQLRALQERLGRQQRDMEEERN
RLQEVIGKMEVRLSEQSRLLEQERWRVAAEKTKAESAQRTLEEQRKIMVQQIAMEREELE
RAKSALLEEQKSVMNKCGEERRRLAAEWAEYFTQQKLSKERAEREAERAMHADSQREGTI
ISLTKEQAELTVRACELRAKEEKLLAEREALERERQELRLEKDRLHKASLRLQARAQEVE
HMSKVASKKYEEGEQALQEAQQMQNEQQGRLQVVQRQQEWLRQQEQRVHQEHLSLAQQRL
QLDRVRQEVPASLPGLPPRVQGPAASSRDAVQAPASSSPQCSQPAAAQVPTHLLAKLLLL
KHTAEEDHDFLENEQFFLETLKKAPYNMAYHSA
>P38710
MTIDLASIEKFLCELATEKVGPIIKSKSGTQKDYDLKTGSRSVDIVTAIDKQVEKLIWES
VKTQYPTFKFIGEESYVKGETVITDDPTFIIDPIDGTTNFVHDFPFSCTSLGLTVNKEPV
VGVIYNPHINLLVSASKGNGMRVNNKDYDYKSKLESMGSLILNKSVVALQPGSAREGKNF
QTKMATYEKLLSCDYGFVHGFRNLGSSAMTMAYIAMGYLDSYWDGGCYSWDVCAGWCILK
EVGGRVVGANPGEWSIDVDNRTYLAVRGTINNESDEQTKYITDFWNCVDGHLKYD
>Q05789
MSTLAEVYTIIEDAEQECRKGDFTNAKAKYQEAIEVLGPQNENLSQNKLSSDVTQAIDLL
KQDITAKIQELELLIEKQSSEENNIGMVNNNMLIGSVILNNKSPINGISNARNWDNPAYQ
DTLSPINDPLLMSILNRLQFNLNNDIQLKTEGGKNSKNSEMKINLRLEQFKKELVLYEQK
KFKEYGMKIDEITKENKKLANEIGRLRERWDSLVESAKQRRDKQKN
>P11708
MSEPIRVLVTGAAGQIAYSLLYSIGNGSVFGKDQPIILVLLDITPMMGVLDGVLMELQDC
ALPLLKDVIATDKEEIAFKDLDVAILVGSMPRRDGMERKDLLKANVKIFKCQGAALDKYA
KKSVKVIVVGNPANTNCLTASKSAPSIPKENFSCLTRLDHNRAKAQIALKLGVTSDDVKN
VIIWGNHSSTQYPDVNHAKVKLQAKEVGVYEAVKDDSWLKGEFITTVQQRGAAVIKARKL
SSAMSAAKAICDHVRDIWFGTPEGEFVSMGIISDGNSYGVPDDLLYSFPVTIKDKTWKIV
EGLPINDFSREKMDLTAKELAEEKETAFEFLSSA
>Q9JKC9

MALRPGAGASGAAGAGTGPGGAGSFMFPVAGGMRPPQGLIPMQQQGFPMVSVMQPNMQGM
MGMNYSSQMSQGPIAMQAGIPMGPMPAAGVPFLGQPPFLGMRPAAPQYTPDMQKQFAEEQ
QKRFEQQQKLLEEERKRRQFEEQKQKLRLLSSVKPKTGEKNRDDALEAIKGNLDGFSRDA
KMHPTPASHPKKPDCPTSSHSTKTVSPSSAFLGEDEFSGFMQGPVELPTCGPSSTAQPFQ
SFLPSTPLGQLHTQKAGAQPLPPGQAPVSFAVHGVHGQIPCLSAASASHSMQKAGPSLEE
KLLVSCDISASGQEHIKLSSPEAGHRAVVPGSSKNSPGLMAHNGGAVDGCVSGPTTAVAE
KTSDQNLSKEESGVGVFPSQDPVQPRMPPWIYNESLVPDAYKKILETTMTPTGIDTAKLY
PILMSSGLPRETLGQIWALANRTTPGKLTKEELYTVLAMVAVTQRGVPAMSPDTLNQFPA
APIPTLSGFPMTLPTPVSQPTAMTSGPAGSIPLSLGQPIMGINLVGPVGGAAAPTSSGFM
PAYPSNQVGKTEEDDFQDFQDASKSGSIDDSFTDFQEVPASSKTSNSQHGNSAPSLLIPL
PGTKASTDKYAVFKGISAEKPSENPASFGESGDKYSAFRELEPTADSKPLGESFAEFRST
GTDDGFTDFKTADSVSPLEPPTKDSFPSAFASGAAQQTQTQVKTPLNLADLDMFSSVDCS
GEKPVPFSAAFSTSKSVSSRPQPAGSAAAPASLASTKASSLADDFGEFNLFGEYSNPASV
GEQDDFADFMAFGNSSIPSEPKADDKYEALREEGSPGALSTSTVEGAHNPPVSSSKYDVF
KQLSLEGAGLAIEEFKENTPSTKSDGDFADFHSSKFSSTSSDKSLGEKAVAFRHAKEDSA
SVKSLDLPSIGGSSVGKEDSEDALSVQFDMKLADVGGDLKHVMSDSSLDLPTVSGQHPPA
AGSALASEDALPETPFPAFASFKDMMPQTTEQKEYESGDFQDFTRQDMPMVDRSQENTCP
SPASSVASHETPKEGADDFGEFQSEKPKISKFDFLVANSQSKMKSSEEMIKSELATFDLS
VQGSHKRSLSLGDKEISRSSPSPALEQPFRDRSNTLSERAALPVIRDKYKDLTGEVEENE
RYAYEWQRCLGSALDVIKKANDTLNGISSSAVCTEVIQSAQGMEYLLGVVEVYRVTKRVE
LGIKATAVCSEKLQQLLKDIDKVWNNLIGFMSLTTLTPDENSLDFSSCMLRPGIKNAQEL
ACGVCLLNVDSRSRKEETPAEEQPKKAFNSETDSFKLAYGGHQYHASCANFWINCVEPKP
PGLLLPDLL
>P04637
MEEPQSDPSVEPPLSQETFSDLWKLLPENNVLSPLPSQAMDDLMLSPDDIEQWFTEDPGP
DEAPRMPEAAPPVAPAPAAPTPAAPAPAPSWPLSSSVPSQKTYQGSYGFRLGFLHSGTAK
SVTCTYSPALNKMFCQLAKTCPVQLWVDSTPPPGTRVRAMAIYKQSQHMTEVVRRCPHHE
RCSDSDGLAPPQHLIRVEGNLRVEYLDDRNTFRHSVVVPYEPPEVGSDCTTIHYNYMCNS
SCMGGMNRRPILTIITLEDSSGNLLGRNSFEVRVCACPGRDRRTEEENLRKKGEPHHELP
PGSTKRALPNNTSSSPQPKKKPLDGEYFTLQIRGRERFEMFRELNEALELKDAQAGKEPG
GSRAHSSHLKSKKGQSTSRHKKLMFKTEGPDSD
>Q9NT62
MQNVINTVKGKALEVAEYLTPVLKESKFKETGVITPEEFVAAGDHLVHHCPTWQWATGEE
LKVKAYLPTGKQFLVTKNVPCYKRCKQMEYSDELEAIIEEDDGDGGWVDTYHNTGITGIT
EAVKEITLENKDNIRLQDCSALCEEEEDEDEGEAADMEEYEESGLLETDEATLDTRKIVE
ACKAKTDAGGEDAILQTRTYDLYITYDKYYQTPRLWLFGYDEQRQPLTVEHMYEDISQDH
VKKTVTIENHPHLPPPPMCSVHPCRHAEVMKKIIETVAEGGGELGVHMYLLIFLKFVQAV
IPTIEYDYTRHFTM
>Q06148
MDSELKGQQLSDAEWCVKKINGEGNCLLLFLPMSSPTTIVMIVLVSLERLVPYVFKLSQT
QLSQQCQSQGFTDSISLNLIKLKLMDILQAPQEINQIGLVDSNLVFSFDVSADITVSINS
VPSHVTKDMFYMILQSLCMLLLKLVNLSTQYHYVQRDILNEKQKCLDFLLISLRDLDGGS
KVISQWAPENSKNYESLQQCTDDDIIKKLLHKGKFQHQEFLADSLKTLLSLRNKFQDVSR
FEESGELNKKERVRFPAVNHFYNDDFELQADPTNEARPNSRGKIKPKTDFKPKSRESSTS

SQLRLENFSESEATPEKTKSSSSLVEEYPQKKRKFGKVRIKN
>P08566
MVQLAKVPILGNDIIHVGYNIHDHLVETIIKHCPSSTYVICNDTNLSKVPYYQQLVLEFK
ASLPEGSRLLTYVVKPGETSKSRETKAQLEDYLLVEGCTRDTVMVAIGGGVIGDMIGFVA
STFMRGVRVVQVPTSLLAMVDSSIGGKTAIDTPLGKNFIGAFWQPKFVLVDIKWLETLAK
REFINGMAEVIKTACIWNADEFTRLESNASLFLNVVNGAKNVKVTNQLTNEIDEISNTDI
EAMLDHTYKLVLESIKVKAEVVSSDERESSLRNLLNFGHSIGHAYEAILTPQALHGECVS
IGMVKEAELSRYFGILSPTQVARLSKILVAYGLPVSPDEKWFKELTLHKKTPLDILLKKM
SIDKKNEGSKKKVVILESIGKCYGDSAQFVSDEDLRFILTDETLVYPFKDIPADQQKVVI
PPGSKSISNRALILAALGEGQCKIKNLLHSDDTKHMLTAVHELKGATISWEDNGETVVVE
GHGGSTLSACADPLYLGNAGTASRFLTSLAALVNSTSSQKYIVLTGNARMQQRPIAPLVD
SLRANGTKIEYLNNEGSLPIKVYTDSVFKGGRIELAATVSSQYVSSILMCAPYAEEPVTL
ALVGGKPISKLYVDMTIKMMEKFGINVETSTTEPYTYYIPKGHYINPSEYVIESDASSAT
YPLAFAAMTGTTVTVPNIGFESLQGDARFARDVLKPMGCKITQTATSTTVSGPPVGTLKP
LKHVDMEPMTDAFLTACVVAAISHDSDPNSANTTTIEGIANQRVKECNRILAMATELAKF
GVKTTELPDGIQVHGLNSIKDLKVPSDSSGPVGVCTYDDHRVAMSFSLLAGMVNSQNERD
EVANPVRILERHCTGKTWPGWWDVLHSELGAKLDGAEPLECTSKKNSKKSVVIIGMRAAG
KTTISKWCASALGYKLVDLDELFEQQHNNQSVKQFVVENGWEKFREEETRIFKEVIQNYG
DDGYVFSTGGGIVESAESRKALKDFASSGGYVLHLHRDIEETIVFLQSDPSRPAYVEEIR
EVWNRREGWYKECSNFSFFAPHCSAEAEFQALRRSFSKYIATITGVREIEIPSGRSAFVC
LTFDDLTEQTENLTPICYGCEAVEVRVDHLANYSADFVSKQLSILRKATDSIPIIFTVRT
MKQGGNFPDEEFKTLRELYDIALKNGVEFLDLELTLPTDIQYEVINKRGNTKIIGSHHDF
QGLYSWDDAEWENRFNQALTLDVDVVKFVGTAVNFEDNLRLEHFRDTHKNKPLIAVNMTS
KGSISRVLNNVLTPVTSDLLPNSAAPGQLTVAQINKMYTSMGGIEPKELFVVGKPIGHSR
SPILHNTGYEILGLPHKFDKFETESAQLVKEKLLDGNKNFGGAAVTIPLKLDIMQYMDEL
TDAAKVIGAVNTVIPLGNKKFKGDNTDWLGIRNALINNGVPEYVGHTAGLVIGAGGTSRA
ALYALHSLGCKKIFIINRTTSKLKPLIESLPSEFNIIGIESTKSIEEIKEHVGVAVSCVP
ADKPLDDELLSKLERFLVKGAHAAFVPTLLEAAYKPSVTPVMTISQDKYQWHVVPGSQML
VHQGVAQFEKWTGFKGPFKAIFDAVTKE
>O57406
MNGTMDHPDHPDPDSIKMFVGQVPRSWSEKELRELFEQYGAVYEINVLRDRSQNPPQSKG
CCFITFYTRKAALEAQNALHNMKVLPGMHHPIQMKPADSEKNNAVEDRKLFIGMVSKNCN
ENDIRAMFSPFGQIEECRILRGPDGMSRGCAFVTFTTRSMAQMAIKSMHQAQTMEGCSSP
IVVKFADTQKDKEQKRMTQQLQQQMQQLNAASMWGNLTGLNSLAPQYLALLQQTASSGNL
NSLSGLHPMGAEYGTGMTSGLNAIQLQNLAALAAAASAAQNTPSAGAALTSSSSPLSILT
SSGSSPSSNNSSINTMASLGALQTLAGATAGLNVNSLAGMAAFNGGLGSSLSNGTGSTME
ALSQAYSGIQQYAAAALPSLYNQSLLSQQGLGAAGSQKEGPEGANLFIYHLPQEFGDQDL
LQMFMPFGNVVSSKVFIDKQTNLSKCFGFVSYDNPVSAQAAIQSMNGFQIGMKRLKVQLK
RSKNDSKPY
>P30568
MAKDMTLLWGSGSPPCWRVMIVLEEKNLQAYNSKLLSFEKGEHKSAEVMSMNPRGQLPSF
KHGSKVLNESYAACMYLESQFKSQGNKLIPDCPAEQAMMYQRMFEGLTLAQKMADVIYYS
WKVPEAERHDSAVKRNKENLSTELKLWEEYLQKTSGSFVAGKSFSLADVSVFPGVAYLFR
FGLTEERYPQLTAYYNSLKERPSIKASWPPTWLESPQGQDMLKDV

>Q26648
MLRNSVITPTPPAKNLASFVTKPTTDIMGYHDNTTRLSNRIDDIETWRETLEKTLADVDE
EIRKLEEDKDLAERALEAKALPLDVASECKTLRDGRRDNDVVDDLANSEVGKEIDVIEGI
KDALQAKVSSAFEQLCLLQEARQQLHADLRDKTEAKKIDTYCHDLTISSPDICYQPNSTR
TPKGSTTPQTWEDFSRYNKDRADAEMRSSQRLREAIHSTVAQTDNDLEAQRQATEFALRK
RIHEMKRAKDEDEWQKKNTEEEIAKQERNIRELEQAIKDKENPLKLAMTRLENRTYRPNV
ELCRDNAQYGLVNEVHEIQDSIKALEKKLQDAHNARDACEKQLYRINKDLELKNNSLDLD
NKCMQVREKLTTGPVTQTMNNLSTFKTDRELYTAQRSLLA
>Q8N427
MASKKREVQLQTVINNQSLWDEMLQNKGLTVIDVYQAWCGPCRAMQPLFRKLKNELNEDE
ILHFAVAEADNIVTLQPFRDKCEPVFLFSVNGKIIEKIQGANAPLVNKKVINLIDEERKI
AAGEMARPQYPEIPLVDSDSEVSEESPCESVQELYSIAIIKPDAVISKKVLEIKRKITKA
GFIIEAEHKTVLTEEQVVNFYSRIADQCDFEEFVSFMTSGLSYILVVSQGSKHNPPSEET
EPQTDTEPNERSEDQPEVEAQVTPGMMKNKQDSLQEYLERQHLAQLCDIEEDAANVAKFM
DAFFPDFKKMKSMKLEKTLALLRPNLFHERKDDVLRIIKDEDFKILEQRQVVLSEKEAQA
LCKEYENEDYFNKLIENMTSGPSLALVLLRDNGLQYWKQLLGPRTVEEAIEYFPESLCAQ
FAMDSLPVNQLYGSDSLETAEREIQHFFPLQSTLGLIKPHATSEQREQILKIVKEAGFDL
TQVKKMFLTPEQIEKIYPKVTGKDFYKDLLEMLSVGPSMVMILTKWNAVAEWRRLMGPTD
PEEAKLLSPDSIRAQFGISKLKNIVHGASNAYEAKEVVNRLFEDPEEN
>P47181
MNTDSHNLSEPYNIGGQKYINMKKKEDLGVCQPGLTQKAFTVEDKFDYKAIIEKMEVYGL
CVVKNFIETSRCDEILKEIEPHFYRYESWQGSPFPKETTVATRSVLHSSTVLKDVVCDRM
FCDISKHFLNEENYFAAGKVINKCTSDIQLNSGIVYKVGAGASDQGYHREDIVHHTTHQA
CERFQYGTETMVGLGVAFTDMNKENGSTRMIVGSHLWGPHDSCGNFDKRMEFHVNVAKGD
AVLFLGSLYHAASANRTSQDRVAGYFFMTKSYLKPEENLHLGTDLRVFKGLPLEALQLLG
LGISEPFCGHIDYKSPGHLISSSLFENDIEKGYYGETIRVNYGSTQ
>Q8LAD0
MTVGVLALQGSFNEHIAALRRLGVQGVEIRKADQLLTVSSLIIPGGESTTMAKLAEYHNL
FPALREFVKMGKPVWGTCAGLIFLADRAVGQKEGGQELVGGLDCTVHRNFFGSQIQSFEA
DILVPQLTSQEGGPETYRGVFIRAPAVLDVGPDVEVLADYPVPSNKVLYSSSTVQIQEED
ALPETKVIVAVKQGNLLATAFHPELTADTRWHSYFIKMTKEIEQGASSSSSKTIVSVGET
SAGPEPAKPDLPIFQ
>Q5XXA6
MRVNEKYSTLPAEDRSVHIINICAIEDIGYLPSEGTLLNSLSVDPDAECKYGLYFRDGRR
KVDYILVYHHKRPSGNRTLVRRVQHSDTPSGARSVKQDHPLPGKGASLDAGSGEPPMDYH
EDDKRFRREEYEGNLLEAGLELERDEDTKIHGVGFVKIHAPWNVLCREAEFLKLKMPTKK
MYHINETRGLLKKINSVLQKITDPIQPKVAEHRPQTMKRLSYPFSREKQHLFDLSDKDSF
FDSKTRSTIVYEILKRTTCTKAKYSMGITSLLANGVYAAAYPLHDGDYNGENVEFNDRKL
LYEEWARYGVFYKYQPIDLVRKYFGEKIGLYFAWLGVYTQMLIPASIVGIIVFLYGCATM
DENIPSMEMCDQRHNITMCPLCDKTCSYWKMSSACATARASHLFDNPATVFFSVFMALWA
ATFMEHWKRKQMRLNYRWDLTGFEEEEEAVKDHPRAEYEARVLEKSLKKESRNKEKRRHI
PEESTNKWKQRVKTAMAGVKLTDKVKLTWRDRFPAYLTNLVSIIFMIAVTFAIVLGVIIY
RISMAAALAMNSSPSVRSNIRVTVTATAVIINLVVIILLDEVYGCIARWLTKIEVPKTEK
SFEERLIFKAFLLKFVNSYTPIFYVAFFKGRFVGRPGDYVYIFRSFRMEECAPGGCLMEL

CIQLSIIMLGKQLIQNNLFEIGIPKMKKLIRYLKLKQQSPPDHEECVKRKQRYEVDYNLE
PFAGLTPEYMEMIIQFGFVTLFVASFPLAPLFALLNNIIEIRLDAKKFVTELRRPVAVRA
KDIGIWYNILRGIGKLAVIINAFVISFTSDFIPRLVYLYMYSKNGTMHGFVNHTLSSFNV
SDFQNGTAPNDPLDLGYEVQICRYKDYREPPWSENKYDISKDFWAVLAARLAFVIVFQNL
VMFMSDFVDWVIPDIPKDISQQIHKEKVLMVELFMREEQDKQQLLETWMEKERQKDEPPC
NHHNTKACPDSLGSPAPSHAYHGGVL
>Q9P779
MLKCTIKNEQIETLRSGDTFVSYEIETESDLPVFEDKKFSVRRRYKDFEMLHNILSHDYN
GYAIPPLPRKYTVSSFSGGSLSPIFIARRMQSLQTFLDRCSTHPVISNSMHMYQFLENNS
WKSYYHNAWMQSENTKSKGNNVSGGIESSIQNLDPYAQSLYETAKQLLQNADTDLSKLEK
TCVQYMNSVQNFPTDIPVPSNLSISNLDVVSVEFKRLKRNSIFLINSFHSKVITSIQDLE
DYMVVFKSLIKSREQKVKQFEHFQQIVQSNSNNPDQSSRSDPNFVEATPVVQQTPELKPS
PNTTIRTSSLFSIPKFFKKKRYSLGQDDANPMELLQLSFQELCIFNEKLEQELNFLRERI
DVEMRKTLQMVCDCHVEYFSGILEQHAVKE
>P36102
MDKINPDWAKDIPCRNITIYGYCKKEKEGCPFKHSDNTTATTINDVPPPIDVGEATTPTM
TSVPKFNAKVSASFTPMTVGSDSLTTVTNTTSAATNATGNIAMAATSATASTVNPMINPI
VNSSLVNNNNNNSNISISIPTTASSSNYDPFNAPIFTPSSTSSIHTNANAHSFPFPSIAN
SGGININATDDNSNNMSMANNVPPPMQPPPIESSNLKYPRIYPPPHSLLQYHLYAPEQPS
SLKSLLKPNERSADQLFIPNNIREDLTKKNLSILQVFPSSGKVIPSIVQDYFNLVPLNFN
NNDFLNKTTLFKVFSNYDGKAYVLKRLPNIDKSMNPNKISKIYQIWSKINCTNLIKFRDI
FQTTKFGDLSICLVFDYYPNSLSLYDYHFVNFPKFPITNNYLWIYLVQLTNVINSIHSQN
LSIGNTLNWRKVLITGDPGRIKLSHCNFMDLLFNDDTDTVVSSGGSTIEGQQQLDYKYLG
ELLFNLSINIENSNNNTAPKEYRLEEITPQSIDDMRQIDDKFKDVLKYLISDNGDSKKSI
HDLTSHFYDKMFMVLESSQTYTEYMESVLSRELENGRLFRLVNKLNCIFGRIESRIDINW
SESGTKFPIILFYDYVFHQVDSNGKPIMDLTHVLRCLNKLDAGIQEKLMLVTPDELNCII
ISYKELKDLIESTFRSITQ
>Q9P6K4
MLHFLFHSGSSSNRNSSPKESYELLHGLDKQYQSTKDVTFRLVLVQDIGDRKKTVLFDSN
HVDGQKGDSVLRDSANAPLTDLMFGAIPISHKGTTTKLHILHPPNPATRSYMLTQLFQIN
THGTVVNSSHETIASATSLFENSSSNFSEDPNKPNSSDAFESNKEDSPLLKSFNDSAIPE
NAANLSSSSKNMKDSTLSSQKARSNTSSSFLTPLHEQLESRCALHTAAKDPFRSKNSLRC
NRGHSPLSSQQILPAISNNTSEKPDSNNCGFLLPSNSTSIKDLKNVKKGNRLNSPPFITI
PQSIKNTNSNFLLSSPSLFSDTRTRPASYALALIITVPYEYDEIVHPVSTYYTMLSNFTL
SLQKEIDERIRNLLFVSLSSGGDNKNDTGIPLIQSSSSKVGFGPYALSKDLITAKSFHKC
ILLLKTGFSAPLIKPSVFSGSKWVENMRLLTDLCKSPAQKCLFSNLLTATRKFCLERQKD
DVTFKVLLQSSKAPIARRFLYLLAPLMRPSIAQCSDTLLNPIQLYPNSGILSSSSLSTSF
GCPSVSGSLRVPSYDMKINDSCKAIDIHSEKPSFADSPRKTSLRNYLSSSWRLKFMRSSY
QNNETDPLNPTSGSFLRQPMQYSSPSGVSESAASSFLDIENIDEYLESAENMKYLPRSTV
GPGGMLHVDLLETNAKQESEATTSTVPPSPSQVGFLKALHPSFDLQAAPPNSYVSFSDDD
FISATLLYMLEDVSRNKSQLLAEKKHLKSQLMVANLDTYSLDCYEIHEFPSEWENDYAPF
LLKEHHKVIGETYVSSFDIQQGCFNVIKRRLSSYKWDKSDDSFVSEVLKGDLKEVLRVCS
HC
>Q5I2A0

MAGVSPAVFGCPDVTLGRNTAVREVQENVTSVDSLTLVSSNTDFAFSLYRKLVLKNPDEN
VVFSPFSICTALALLSLGAKSNTLKEILEGLKFNLTETPEPDIHQGFRYLLDLLSQPGNQ
VQISTGSALFIEKHLQILAEFKEKARALYQAEAFTADFQQPLKATKLINDYVSNHTQGKI
KELISGLKESTLMVLVNYIYFKGKWKNPFDPNDTFKSEFYLDEKRSVIVSMMKTGYLTTP
YFRDEELSCTVVELKYTGNASAMFILPDQGRMQQVEASLQPETLRKWKNSLKPRMIHELR
LPKFSISTDYSLEHILPELGIREVFSTQADLSAITGTKDLRVSQVVHKAVLDVAETGTEA
AAATGMAGVGCCAVFDFLEIFFNRPFLMIISDTKAHIALFMAKVTNPERSMNFPNGEGAS
SQRLESKRLCFGDPLCLIGQ
>Q07831
MKAIVFVLIFAVAFAVTLRQGPIVCNLCTGLINTLENLLTTKGADKVKDYIDSLCNKASG
FIATLCTKVLDFGVDKLIQLIEDKVDANAICAKIHAC
>P38219
MPPKKQVEEKKVLLGRPGNNLKAGIVGLANVGKSTFFQAITRCPLGNPANYPFATIDPEE
ARVIVPSPRFDKLCEIYKKTASEVPAHLTVYDIAGLTKGASAGEGLGNAFLSHIRSVDSI
YQVVRCFDDAEIIHVEGDVDPVRDLEIINQELRLKDIEFAQKALEGAEKIAKRGGQSLEV
KQKKEEMDLITKIIKLLESGQRVANHSWTSKEVEIINSMFLLTAKPCIYLINLSERDYIR
KKNKHLLRIKEWVDKYSPGDLIIPFSVSLEERLSHMSPEDAEEELKKLQTISALPKIITT
MRQKLDLISFFTCGPDEVREWTIRRGTKAPQAAGVIHNDLMNTFILAQVMKCEDVFEYKD
DSAIKAAGKLMQKGKDYVVEDGDIIYFRAGAGKN
>Q9P6Q0
MTNSDDDLFSEKSTSSDTQQVQNILELEAKIPDILSSAGKCIEAIQLNNSLEDFRKYSKE
FLETVEFISTGLRRQALELEKAEVPVVSLQPKKRYASTPLSNLIFDQSSKLIPKYC
>Q9Y4B6
MTTVVVHVDSKAELTTLLEQWEKEHGSGQDMVPILTRMSQLIEKETEEYRKGDPDPFDDR
HPGRADPECMLGHLLRILFKNDDFMNALVNAYVMTSREPPLNTAACRLLLDIMPGLETAV
VFQEKEGIVENLFKWAREADQPLRTYSTGLLGGAMENQDIAANYRDENSQLVAIVLRRLR
ELQLQEVALRQENKRPSPRKLSSEPLLPLDEEAVDMDYGDMAVDVVDGDQEEASGDMEIS
FHLDSGHKTSSRVNSTTKPEDGGLKKNKSAKQGDRENFRKAKQKLGFSSSDPDRMFVELS
NSSWSEMSPWVIGTNYTLYPMTPAIEQRLILQYLTPLGEYQELLPIFMQLGSRELMMFYI
DLKQTNDVLLTFEALKHLASLLLHNKFATEFVAHGGVQKLLEIPRPSMAATGVSMCLYYL
SYNQDAMERVCMHPHNVLSDVVNYTLWLMECSHASGCCHATMFFSICFSFRAVLELFDRY
DGLRRLVNLISTLEILNLEDQGALLSDDEIFASRQTGKHTCMALRKYFEAHLAIKLEQVK
QSLQRTEGGILVHPQPPYKACSYTHEQIVEMMEFLIEYGPAQLYWEPAEVFLKLSCVQLL
LQLISIACNWKTYYARNDTVRFALDVLAILTVVPKIQLQLAESVDVLDEAGSTVSTVGIS
IILGVAEGEFFIHDAEIQKSALQIIINCVCGPDNRISSIGKFISGTPRRKLPQNPKSSEH
TLAKMWNVVQSNNGIKVLLSLLSIKMPITDADQIRALACKALVGLSRSSTVRQIISKLPL
FSSCQIQQLMKEPVLQDKRSDHVKFCKYAAELIERVSGKPLLIGTDVSLARLQKADVVAQ
SRISFPEKELLLLIRNHLISKGLGETATVLTKEADLPMTAASHSSAFTPVTAAASPVSLP
RTPRIANGIATRLGSHAAVGASAPSAPTAHPQPRPPQGPLALPGPSYAGNSPLIGRISFI
RERPSPCNGRKIRVLRQKSDHGAYSQSPAIKKQLDRHLPSPPTLDSIITEYLREQHARCK
NPVATCPPFSLFTPHQCPEPKQRRQAPINFTSRLNRRASFPKYGGVDGGCFDRHLIFSRF
RPISVFREANEDESGFTCCAFSARERFLMLGTCTGQLKLYNVFSGQEEASYNCHNSAITH
LEPSRDGSLLLTSATWSQPLSALWGMKSVFDMKHSFTEDHYVEFSKHSQDRVIGTKGDIA
HIYDIQTGNKLLTLFNPDLANNYKRNCATFNPTDDLVLNDGVLWDVRSAQAIHKFDKFNM

NISGVFHPNGLEVIINTEIWDLRTFHLLHTVPALDQCRVVFNHTGTVMYGAMLQADDEDD
LMEERMKSPFGSSFRTFNATDYKPIATIDVKRNIFDLCTDTKDCYLAVIENQGSMDALNM
DTVCRLYEVGRQRLAEDEDEEEDQEEEEQEEEDDDEDDDDTDDLDELDTDQLLEAELEED
DNNENAGEDGDNDFSPSDEELANLLEEGEDGEDEDSDADEEVELILGDTDSSDNSDLEDD
IILSLNE
>P40454
MSLDRVDWPHATFSTPVKRIFDTQTTLDFQSSLAIHRIKYHLHKYTTLISHCSDPDPHAT
ASSIAMVNGLMGVLDKLAHLIDETPPLPGPRRYGNLACREWHHKLDERLPQWLQEMLPSE
YHEVVPELQYYLGNSFGSSTRLDYGTGHELSFMATVAALDMLGMFPHMRGADVFLLFNKY
YTIMRRLILTYTLEPAGSHGVWGLDDHFHLVYILGSSQWQLLDAQAPLQPREILDKSLVR
EYKDTNFYCQGINFINEVKMGPFEEHSPILYDIAVTVPRWSKVCKGLLKMYSVEVLKKFP
VVQHFWFGTGFFPWVNIQNGTDLPVFEEKEEESIEQANAGSPGREQTSTRFPTSTSMPPP
GVPPSGNNINYLLSHQNQSHRNQTSFSRDRLRR
>O94266
MPYNPLYESLSNQFDPSRIETVLDPMGYTIKRRALPVSLTIAGSDCSGGAGIQADLKTMT
SLGVYGMSAITCLVAENAGGVDSVEEMSPAFVESQIDCCIRDIPCHVVKTGMLGSPEIVK
AVARSAKKFNFSKLVVDPVMVATSGDSLVTKDIVSVLNEELLPLTYLVTPNIPEAIVLAK
NQGLDISNINSVSDMERCAAVIHKLGPKHVLLKGGHMPVNNLGLKSSDDEDLRVVDILYD
GNRFYHFSSSYLKKGEVHGTGCTLSSAIASFLAWEHSLTEAVQFGIDYVHGAITHSPPIN
NCSTNILNHMTRLRIVPFAPGHFIEYILSHPQVVPAWKEYINHKFTNMLAKGTLPLPAFQ
DYLKQDYLYLVNFARAYSLKGYKENTFPNILEAAQSVIHVIEEKELHVSMCSSYGVSLQD
LKSCEESPACTAYSRYILDTGAAQDVAALDFVQAPCLIGYYVIAARLMKEPFRNPQGPYQ
KWVDNYFCEDYLSAVRRGCRQIEEIVLKLSPERIQELIEIFIRATKFETLFWETPYYEYV
TKQNLEDKEFS
>Q8NFH8
MEAAAAAAAAAAAAAAAAGGGCGSGPPPLLLSEGEQQCYSELFARCAGAAGGGPGSGPPEA
ARVAPGTATAAAGPVADLFRASQLPAETLHQITELCGAKRVGYFGPTQFYIALKLIAAAQ
SGLPVRIESIKCELPLPRFMMSKNDGEIRFGNPAELHGTKVQIPYLTTEKNSFKRMDDED
KQQETQSPTMSPLASPPSSPPHYQRVPLSHGYSKLRSSAEQMHPAPYEARQPLVQPEGSS
SGGPGTKPLRHQASLIRSFSVERELQDNSSYPDEPWRITEEQREYYVNQFRSLQPDPSSF
ISGSVAKNFFTKSKLSIPELSYIWELSDADCDGALTLPEFCAAFHLIVARKNGYPLPEGL
PPTLQPEYLQAAFPKPKWDCQLFDSYSESLPANQQPRDLNRMEKTSVKDMADLPVPNQDV
TSDDKQALKSTINEALPKDVSEDPATPKDSNSLKARPRSRSYSSTSIEEAMKRGEDPPTP
PPRPQKTHSRASSLDLNKVFQPSVPATKSGLLPPPPALPPRPCPSQSEQVSEAELLPQLS
RAPSQAAESSPAKKDVLYSQPPSKPIRRKFRPENQATENQEPSTAASGPASAATMKPHPT
VQKQSSKQKKAIQTAIRKNKEANAVLARLNSELQQQLKEVHQERIALENQLEQLRPVTVL

>Q9P2D0
MSSPMPDCTSKCRSLKHALDVLSVVTKGSENQIKAFLSSHCYNAATIKDVFGRNALHLVS
SCGKKGVLDWLIQKGVDLLVKDKESGWTALHRSIFYGHIDCVWSLLKHGVSLYIQDKEGL
SALDLVMKDRPTHVVFKNTDPTDVYTWGDNTNFTLGHGSQNSKHHPELVDLFSRSGIYIK
QVVLCKFHSVFLSQKGQVYTCGHGPGGRLGHGDEQTCLVPRLVEGLNGHNCSQVAAAKDH
TVVLTEDGCVYTFGLNIFHQLGIIPPPSSCNVPRQIQAKYLKGRTIIGVAAGRFHTVLWT
REAVYTMGLNGGQLGCLLDPNGEKCVTAPRQVSALHHKDIALSLVAASDGATVCVTTRGD

IYLLADYQCKKMASKQLNLKKVLVSGGHMEYKVDPEHLKENGGQKICILAMDGAGRVFCW
RSVNSSLKQCRWAYPRQVFISDIALNRNEILFVTQDGEGFRGRWFEEKRKSSEKKEILSN
LHNSSSDVSYVSDINSVYERIRLEKLTFAHRAVSVSTDPSGCNFAILQSDPKTSLYEIPA
VSSSSFFEEFGKLLREADEMDSIHDVTFQVGNRLFPAHKYILAVHSDFFQKLFLSDGNTS
EFTDIYQKDEDSAGCHLFVVEKVHPDMFEYLLQFIYTDTCDFLTHGFKPRIHLNKNPEEY
QGTLNSHLNKVNFHEDDNQKSAFEVYKSNQAQTVSERQKSKPKSCKKGKNIREDDPVRML
QTVAKKFDFSNLSSRLDGVRFENEKINVIAKNTGNKLKLSQKKCSFLCDVTMKSVDGKEF
PCHKCVLCARLEYFHSMLSSSWIEASSCAALEMPIHSDILKVILDYLYTDEAVVIKESQN
VDFICSVLVVADQLLITRLKEICEVALTEKLTLKNAAMLLEFAAMYSAKQLKLSCLQFIG
LNMAALLEARSLDVLSDGVLKDLSEFYRKMIPAMDRRVITPYQDGPDISYLEVEDGDIFL
KEEINMEQNHSETMFKKAKTKAKKKPRKRSDSSGGYNLSDIIQSPSSTGLLKSGKTNSVE
SLPELLTSDSEGSYAGVGSPRDLQSPDFTTGFHSDKIEAKVKPYVNGTSPVYSREDLKPW
EKSPILKISAPQPIPSNRIDTTSSASWVAGSFSPVSPPVVDLRTIMEIEESRQKCGATPK
SHLGKTVSHGVKLSQKQRKMIALTTKENNSGMNSMETVLFTPSKAPKPVNAWASSLHSVS
SKSFRDFLLEEKKSVTSHSSGDHVKKVSFKGIENSQAPKIVRCSTHGTPGPEGNHISDLP
LLDSPNPWLSSSVTAPSMVAPVTFASIVEEELQQEAALIRSREKPLALIQIEEHAIQDLL
VFYEAFGNPEEFVIVERTPQGPLAVPMWNKHGC
>Q53QV2
MSIYFPIHCPDYLRSAKMTEVMMNTQPMEEIGLSPRKDGLSYQIFPDPSDFDRCCKLKDR
LPSIVVEPTEGEVESGELRWPPEEFLVQEDEQDNCEETAKENKEQ
>P48998
MSHQHTVPVTVPAVVQESLKTVCSPAQTQQEQTKQPTPYPAQCQVFTDTQEKGFPKHEEK
EANPVKDLPEQESEHHQQPGPQKQQLQVKKPEQELQEQELHSEKQPQEPQGLLCLGQQQQ
REPQEQEQHLRQHQQPQQESQGQGLCLGQQQDVLAPQELHMGQHQKEKLQEPELPLGQQQ
KTPEEQELILGEKQQKLHLVERHQEPQEQELHHGQKQKQQQPQEQELQLVQHQKQKQHEP
ELCLRKQQQQESHERELHLGKQQQQESHEPELHLGKQQHQESHEPELHLGKQQHQESCEP
ELHLGEQQHQEQQQHQESCEPELHLGKQQHQETQESELQLGKQQKPHEPDMVLDPKEKQK
LHDPELHLGKQQHQESQESELQVEKKQHEKSPEPELHLGKQQELHEPDMTEDQKEKQSLH
EPELHLGKQQESHEPDMTEDQKEKQSLYEPELHLGKQQEQQIEYEGYQRSKSLNQLLKQE
KASRGQELDDSHLEQEKELLDQRLDQELVNKDEQLERKKHKLENLTQKEKQIKQLVPSTD
RVQETQPIQPVKEDSLTTKKQQHSHEVQ
>Q14764
MATEEFIIRIPPYHYIHVLDQNSNVSRVEVGPKTYIRQDNERVLFAPMRMVTVPPRHYCT
VANPVSRDAQGLVLFDVTGQVRLRHADLEIRLAQDPFPLYPGEVLEKDITPLQVVLPNTA
LHLKALLDFEDKDGDKVVAGDEWLFEGPGTYIPRKEVEVVEIIQATIIRQNQALRLRARK
ECWDRDGKERVTGEEWLVTTVGAYLPAVFEEVLDLVDAVILTEKTALHLRARRNFRDFRG
VSRRTGEEWLVTVQDTEAHVPDVHEEVLGVVPITTLGPHNYCVILDPVGPDGKNQLGQKR
VVKGEKSFFLQPGEQLEQGIQDVYVLSEQQGLLLRALQPLEEGEDEEKVSHQAGDHWLIR
GPLEYVPSAKVEVVEERQAIPLDENEGIYVQDVKTGKVRAVIGSTYMLTQDEVLWEKELP
PGVEELLNKGQDPLADRGEKDTAKSLQPLAPRNKTRVVSYRVPHNAAVQVYDYREKRARV
VFGPELVSLGPEEQFTVLSLSAGRPKRPHARRALCLLLGPDFFTDVITIETADHARLQLQ
LAYNWHFEVNDRKDPQETAKLFSVPDFVGDACKAIASRVRGAVASVTFDDFHKNSARIIR
TAVFGFETSEAKGPDGMALPRPRDQAVFPQNGLVVSSVDVQSVEPVDQRTRDALQRSVQL
AIEITTNSQEAAAKHEAQRLEQEARGRLERQKILDQSEAEKARKELLELEALSMAVESTG

TAKAEAESRAEAARIEGEGSVLQAKLKAQALAIETEAELQRVQKVRELELVYARAQLELE
VSKAQQLAEVEVKKFKQMTEAIGPSTIRDLAVAGPEMQVKLLQSLGLKSTLITDGSTPIN
LFNTAFGLLGMGPEGQPLGRRVASGPSPGEGISPQSAQAPQAPGDNHVVPVLR
>Q9USL5
MKLIQKNIEKNGSGWITMCPEEPEDMWHLYNILQVGDQLKASTVRRVVKVGATGSTSGSR
VVMKLRILVENMDFDTKAAQLHIKGRTTEYHPEVKMGSYHTLDLELHRNFTLYKNEWDAF
ALDRVDAACNPSRNAEIGAVVLDEGLANICLITDYMTILRQRIDQVIPRKRRGDSSAYQK
GLDKFYDSVFQSINSEFDFDKLKVVILASPGFVARGLYDYIFSMAVKLDLKQIVKSKNKF
VILHSSTGHIHSLNEILKDPAVESKLADTKYVQEIRVLNKFYDVMNEDDRKAWYGPNHVL
KAFELGAIGELLISDSLFRSSDIATRKKWVSLVEGVKEINCPVYIFSSLHESGKQLDLLS
GIAAILTYPVDEEDISEDEEDEESQNFEHS
>Q02606
MLLERLHKRLHAGSSRRSQENKDKNCKPEDALPIQPEAQHQTQDPQPLLNCDYDDMIAFD
RNLSTPVFTPVMTPINNSSSNQAKSSDASYFPPYLNANRTRQNSASSLASSVSDFAQNFK
QHTFYNNNAQFTSFTPQFVGLLLEVYQNTCSDPTITPFDTTNPPSGILNRVAKAAIQQSE
LQQLDIGCDRNSWLLTLVRQRLLQEVRKDGYLSRNTSLTSLPPPPPPQFSEMLRVPSPFV
NADITDPIPLSNTNSNPNVSSTTSLTNTLNWYSLQRSNVSMKNRNGSSQYISELQPQPIL
ARTNSNNSVSNSNAFSLLTPTPTTDSAFNFNIALLSRQRSNIISSPLASTRLPTANVSTE
ESSILPNESLKLKRDLLRLKR
>Q12515
MATFNPQNEMENQARVQEYKVSTGRGGAGNIHKSMSKPSPVLLPLKSNSKTVANNNNNGS
NQEKVPRFAIGRGGAGNIFHDPHLTRSAQQLDSNDNINYNDVINDIDDYISPITSDMVDE
GGSNPVTNTRSRISATRSHQSLHATTSSPNNNAPIVVGRGGAGNIFFNKKKVASNGGNEE
DEIRGGNIEDEDTINANEDNLFVVTSNGNALAAIKSTSKKPKNKLKGKSAPEKFAIGRGG
AGNIISPKSSRNTINHNLNDDDEDKFNLKDDNGKEKKKKKKKKSGFFSSLKTMFN
>Q9P7U2
MSSLSPELYGCLGNSGLKVSKLILGCMSYGKKEYWEDWVLEDEEEVFKIMKAAYDAGIRT
FDTANCYSAGVSEELVGKFIRKYEIPRSSIVILSKCFFPVRKDLIKIFGDLSSRGVHFLD
SPELANQCGLSRKHIFDAVEDSVKRLGTYIDVLQIHRYDPHVSAEEVMRALNDVVESGKV
RYIGASTMRCYQFIELQNTAEKHGWHKFISMQNYHNLLYREEEREMIPYCQKTGVGLIPW
SPLARGLLTRSIDANEETIRSKTDLYTRALEFGAGYKAILSRVEELAKKYNVSMATLATA
WSLHKGDYPIVGISKVERLKDALAAVELKLSEEDIKYLEEPYCPVPIQGEI
>P21127
MGDEKDSWKVKTLDEILQEKKRRKEQEEKAEIKRLKNSDDRDSKRDSLEEGELRDHRMEI
TIRNSPYRREDSMEDRGEEDDSLAIKPPQQMSRKEKVHHRKDEKRKEKRRHRSHSAEGGK
HARVKEKEREHERRKRHREEQDKARREWERQKRREMAREHSRRERDRLEQLERKRERERK
MREQQKEQREQKERERRAEERRKEREARREVSAHHRTMREDYSDKVKASHWSRSPPRPPR
ERFELGDGRKPGEARPARAQKPAQLKEEKMEERDLLSDLQDISDSERKTSSAESSSAESG
SGSEEEEEEEEEEEEEGSTSEESEEEEEEEEEEEEETGSNSEEASEQSAEEVSEEEMSED
EERENENHLLVVPESRFDRDSGESEEAEEEVGEGTPQSSALTEGDYVPDSPALSPIELKQ
ELPKYLPALQGCRSVEEFQCLNRIEEGTYGVVYRAKDKKTDEIVALKRLKMEKEKEGFPI
TSLREINTILKAQHPNIVTVREIVVGSNMDKIYIVMNYVEHDLKSLMETMKQPFLPGEVK
TLMIQLLRGVKHLHDNWILHRDLKTSNLLLSHAGILKVGDFGLAREYGSPLKAYTPVVVT
LWYRAPELLLGAKEYSTAVDMWSVGCIFGELLTQKPLFPGKSEIDQINKVFKDLGTPSEK

IWPGYSELPAVKKMTFSEHPYNNLRKRFGALLSDQGFDLMNKFLTYFPGRRISAEDGLKH
EYFRETPLPIDPSMFPTWPAKSEQQRVKRGTSPRPPEGGLGYSQLGDDDLKETGFHLTTT
NQGASAAGPGFSLKF
>Q91WZ8
MLETLRERLLSVQQDFTSGLKTLSDKSREAKVKGKPRTAPRLPKYSAGLELLSRYEDAWA
ALHRRAKECADAGELVDSEVVMLSAHWEKKRTSLNELQGQLQQLPALLQDLESLMASLAH
LETSFEEVENHLLHLEDLCGQCELERHKQAQAQHLESYKKSKRKELEAFKAELDTEHTQK
ALEMEHTQQLKLKERQKFFEEAFQQDMEQYLSTGYLQIAERREPMGSMSSMEVNVDVLEQ
MDLMDISDQEALDVFLNSGGEDNIVMSPGVEMESNPNQNEMSLQIPSPSESASQPPASPS
ACTDLDTADAPLIQSDEEEVQVDTALVTLHTDRKSTPGVSDDSDQCDSTQDI
>Q05924
MIRLPRLYQRYLLYLVVFVVIALFYFLQAPRVEEHIGFDLALPISHVDNLWFQNKGLEGF
SNDDKLVVNIGYDECFHIGRFYEGCFNRHELKSTLTDGHQYLQRKRIHKDLRGSFGRRWF
GKSEYLYYDVLYPALVDYFGSNLEKLNVEAVTGISKYPKDKSLPFMDVSITFEPISIELL
QKRSYISDINILFGVDCIQPIANWTLQKEFPLVKYRYSEPAYLTYKFVGTRPVDTGAQRL
QETDEGKFKIVQLADLHLGVGESECIDEYPKHEACKADPKTETFVQQVLDIEKPQLVVFT
GDQIMGDRSIQDSETVLLKAVAPVIARKIPWAMVWGNHDDEGSLTRWQLSEIASVLPYSL
FKFSPHDTHDNTFGVGNYIYQIFSNNDTEVPVGTLYFLDSHKYSTVGKIYPGYDWIKESQ
WKYIEDYHDVNLKFKTGLSMAFFHIPLPEYLNIESKTHPGEKNPLIGMYKEGVTAPKYNS
EGITTLDRLSVDVVSCGHDHCNDYCLRDDSTPNKIWLCYGGGGGEGGYAGYGGTERRIRI
YEINVNENNIHTWKRLNGSPKEIFDFQSMLDGNSPESV
>Q12108
MGQSSSISSSNEEGSSHSKKFTNSKDILAYFNNKAQQQVTIPELVSFKGNLQIEDLNTPI
SHKALCNSLYFPQNHAMIVGIVTNMLRVLSNFPLMKSSYEPITGYGLLKCILLLNRARCA
KFLKTKSYDQLKLLFISLSLQKTDKEELSEESENDGNKELTIKQIITGFDDVDTEMLCIP
ADFMLQFLTWLLILTVDCPTTNSKLDNTETHDQWGNFKVSALNLLRTMNPDVVGDIESHS
ITFQQFSTAIRTVMPNLLKPLENLMEHFFYLQHDLVDHDTNLSSIQDSKVMTPALLAQLS
TGLPKELFIHKLQSLYIGRKSGFSMRSLQAKVFKWMAPSILVVSGMRITNSEEYAAEKNP
RYRHFLEEFPKLKESDQMMDASHLNKRKTTFAVYIDDPWKVTNKDYFGDLNTRIIEISPR
QDIYKVNQKGTIYFNTIGGGIGIGDKQPLIKPASKRYIPGNVSLTFDSTLEFAVFRNTGY
GGSLDPGLLSMERKEENSPYELHFLIQDVEVWGCGGEKELEEQIKQLEWEEAESKRRQQI
NLRSLGEDRALLEMAGLVGQHQGGGSM
>Q03002
MSYTNKRHTYYGGFTNDLSDTFQYPQRTDEQRRKHVTFGPYILGSTLGEGEFGKVKLGWP
KNFSNSSNSTFDFPKQVAIKLIKRDSISNDYRKEVKIYREINALKHLSHPNIVKLEEVLQ
NSRYIGIVLEYACGGEFYKYIQKKRRLKEMNACRLFSQLISGVHYIHSKGLVHRDLKLEN
LLLDKNENLVITDFGFVNEFCSRNELMKTSCGSPCYAAPELVISAEPYEARKADIWSCGV
ILYAILAGYLPWDDDPNNPEGSDIGRLYNYINSTPLKFPDYILPIPRDLLRRMLVSDPKK
RINLKQIKKHEWLKPHSSFLSITPDEWDKLNNTQSVFRLAKPRRRYGSRPQSSCSTSSLG
SRSDKRDSLVIDSTLITFPAPPQESQNHIITRPASIASDQRLSPIRRSNRHNRSNSAASV
ALQAVVNADREYVLSHEQSLSPVQNIRQTTGNMTASLSPPPAISPGDIIIETTPIKRNTI
SGSSIVPSLEEESSTTMQTSKIQPNNMASSQNHQYNKNKTQNSLQSAKNFYRTSSSSHTK
PRPTSYHPGSYTTPPYNSNTLSIYEINEKAKSSASSQTLNQRDTSPFDSTPYLALDTCIT
SSSSIESSPKLITHGQFSVAKPSVDLQSVSGDLIKYKRDADVVTRIYDEKYKQKRKSLRY

SGIFSDISCDTVTEESDELRPPESPLQQHEGQESIDKAKTEDTSEKGSKSSNIAKATAQK
HVNNHLERSLNEAESTKKRFSFLSLYSYDTSKSSLYSSMDSKRKPSPPSQRRPKKDDSYQ
TNSKNHYITASNMQTSHQVSKDLPAPTMVQNKCTLETKKAVRSNRSSIMVSEVNKASVDN
KAAQSPEHSTAKRVLGFFKRRSMKI
>Q9XFA2
MASPNGGVTTYDYHDSDSAAPVNAQTIEELHSLQRKAATTTKDGASPLQSISASLASLAR
EYGPNLVKGDPEATKGAPPVPIKHQQPSAAAATIAASDSSLKFTHVLYNLSPAELYEQAF
GQKKSSFITSTGALATLSGAKTGRSPRDKRVVKDETTSQELWWGKGSPNIEMDERQFVIN
RERALDYLNSLDKVYVNDQFLNWDSENRIKVRIITSRAYHALFMHNMCIRPTEEELESFG
TPDFTIYNAGEFPANRYANYMTSSTSINISLARREMVILGTQYAGEMKKGLFGVMHYLMP
KRGILSLHSGCNMGKEGDVALFFGLSGTGKTTLSTDHNRLLIGDDEHCWSDNGVSNIEGG
CYAKCIDLSQEKEPDIWNAIKFGTVLENVVFNERTREVDYADKSITENTRAAYPIEFIPN
AKIPCVGPHPKNVILLACDAYGVLPPVSKLNLAQTMYHFISGYTAIVAGTEDGVKEPTAT
FSACFGAAFIMYHPTKYAAMLAEKMQKYGATGWLVNTGWSGGRYGVGNRIKLPYTRKIID
AIHSGELLNASYKKTEVFGLEIPTAINGVPSEILGPVNTWTDKAAYKETLLKLAGLFKNN
FEVFASYKIGNNNSLTEQILAAAPNF
>Q07266
MAGVSFSGHRLELLAAYEEVIREESAADWALYTYEDGSDDLKLAASGEGGLQELSGHFEN
QKVMYGFCSVKDSQAALPKYVLINWVGEDVPDARKCACASHVAKVAEFFQGVDVIVNASS
VEDIDAGAIGQRLSNGLARLSSPVLHRLRLREDENAEPVGTTYQKTDAAVEMKRINREQF
WEQAKKEEELRKEEERKKALDARLRFEQERMEQERQEQEERERRYREREQQIEEHRRKQQ
SLEAEEAKRRLKDQSIFGDQRDEEEESQMKKSESEVEEAAAIIAQRPDNPREFFRQQERV
ASASGGSCDAPSPFNHRPGRPYCPFIKASDSGPSSSSSSSSSPPRTPFPYITCHRTPNLS
SSLPCSHLDSHRRMAPTPIPTRSPSDSSTASTPITEQIERALDEVTSSQPPPPPPPPPPA
QEAQESAPRLDGEEVCKEAKVAAAPQVWAGCAEEPPRAQEPPLLQSSPTEDLMCTESPEQ
AVLAASPEPDASVTSVADAHAADTIETTTATTATTIADNVTPAAASLIDLWPGNGEEAST
PQAEPRVPTPPSGAEASLAEVPLLNEAAQEPLPPVGEGCANLLNFDELPEPPATFCDPEE
EAEGEPLAASQVLTMPSALEEVDQVLEQELEPEPHLLTNGETTQKEGTQQASEGYFSQSQ
EEEFAQSEEPCAKAPPPVFYNKPPEIDITCWDADPVPEEEEGFEGGD
>O43933
MWGSDRLAGAGGGGAAVTVAFTNARDCFLHLPRRLVAQLHLLQNQAIEVVWSHQPAFLSW
VEGRHFSDQGENVAEINRQVGQKLGLSNGGQVFLKPCSHVVSCQQVEVEPLSADDWEILE
LHAVSLEQHLLDQIRIVFPKAIFPVWVDQQTYIFIQIVALIPAASYGRLETDTKLLIQPK
TRRAKENTFSKADAEYKKLHSYGRDQKGMMKELQTKQLQSNTVGITESNENESEIPVDSS
SVASLWTMIGSIFSFQSEKKQETSWGLTEINAFKNMQSKVVPLDNIFRVCKSQPPSIYNA
SATSVFHKHCAIHVFPWDQEYFDVEPSFTVTYGKLVKLLSPKQQQSKTKQNVLSPEKEKQ
MSEPLDQKKIRSDHNEEDEKACVLQVVWNGLEELNNAIKYTKNVEVLHLGKVWIPDDLRK
RLNIEMHAVVRITPVEVTPKIPRSLKLQPRENLPKDISEEDIKTVFYSWLQQSTTTMLPL
VISEEEFIKLETKDGLKEFSLSIVHSWEKEKDKNIFLLSPNLLQKTTIQVLLDPMVKEEN
SEEIDFILPFLKLSSLGGVNSLGVSSLEHITHSLLGRPLSRQLMSLVAGLRNGALLLTGG
KGSGKSTLAKAICKEAFDKLDAHVERVDCKALRGKRLENIQKTLEVAFSEAVWMQPSVVL
LDDLDLIAGLPAVPEHEHSPDAVQSQRLAHALNDMIKEFISMGSLVALIATSQSQQSLHP
LLVSAQGVHIFQCVQHIQPPNQEQRCEILCNVIKNKLDCDINKFTDLDLQHVAKETGGFV
ARDFTVLVDRAIHSRLSRQSISTREKLVLTTLDFQKALRGFLPASLRSVNLHKPRDLGWD

KIGGLHEVRQILMDTIQLPAKYPELFANLPIRQRTGILLYGPPGTGKTLLAGVIARESRM
NFISVKGPELLSKYIGASEQAVRDIFIRAQAAKPCILFFDEFESIAPRRGHDNTGVTDRV
VNQLLTQLDGVEGLQGVYVLAATSRPDLIDPALLRPGRLDKCVYCPPPDQVSRLEILNVL
SDSLPLADDVDLQHVASVTDSFTGADLKALLYNAQLEALHGMLLSSGLQDGSSSSDSDLS
LSSMVFLNHSSGSDDSAGDGECGLDQSLVSLEMSEILPDESKFNMYRLYFGSSYESELGN
GTSSDLSSQCLSAPSSMTQDLPGVPGKDQLFSQPPVLRTASQEGCQELTQEQRDQLRADI
SIIKGRYRSQSGEDESMNQPGPIKTRLAISQSHLMTALGHTRPSISEDDWKNFAELYESF
QNPKRRKNQSGTMFRPGQKVTLA
>Q9Y7K1
MGFPLEKSEDEWKKELGPEKYRIMRQKGTEHPGAGRFTHQFPKQGVFVCAACKELLYKAS
TKFESHCGWPAFFDNLPGKVKRIEDNSYGMHRVEAVCANCGGHLGHIFKGEGYSNPTDER
HCINSASLEFHNEATNDN
>Q99547
MAAERKTKLSKNLLRMKFMQRGLDSETKKQLEEEEKKIISEEHWYLDLPELKEKESFIIE
EQSFLLCEDLLYGRMSFRGFNPEVEKLMLQMNAKHKAEEVEDETVELDVSDEEMARRYET
LVGTIGKKFARKRDHANYEEDENGDITPIKAKKMFLKPQD
>O35309
MDADKDNIKQACDERSAEMDDMRGEQSMGLVHEIMSENKELDEEIKKLEAELQSDAREFQ
IKENVPEKKLKLTSVESPKDGCHFSNSSCSFQVSSQILYELQEGQALITFEKEEVAQNVI
SMGNHVVQMEGTPVKVSAHPVPLNTGVRFQVHVDISKMKINVTGIPDELSEEQTRDKLEL
SFCKSRNGGGEVESVDYDRKSRSAVITFVETGVVDKILKKKTYPLYMNQKCHSVAVSPCI
ERCLEKYQVFSAVSKKTVLLTGLEGIPVDEETGEDLLNIHFQRKNNGGGEVEVVKCSLDQ
SFAAYFKEEARETI
>O95453
MEIIRSNFKSNLHKVYQAIEEADFFAIDGEFSGISDGPSVSALTNGFDTPEERYQKLKKH
SMDFLLFQFGLCTFKYDYTDSKYITKSFNFYVFPKPFNRSSPDVKFVCQSSSIDFLASQG
FDFNKVFRNGIPYLNQEEERQLREQYDEKRSQANGAGALSYVSPNTSKCPVTIPEDQKKF
IDQVVEKIEDLLQSEENKNLDLEPCTGFQRKLIYQTLSWKYPKGIHVETLETEKKERYIV
ISKVDEEERKRREQQKHAKEQEELNDAVGFSRVIHAIANSGKLVIGHNMLLDVMHTVHQF
YCPLPADLSEFKEMTTCVFPRLLDTKLMASTQPFKDIINNTSLAELEKRLKETPFNPPKV
ESAEGFPSYDTASEQLHEAGYDAYITGLCFISMANYLGSFLSPPKIHVSARSKLIEPFFN
KLFLMRVMDIPYLNLEGPDLQPKRDHVLHVTFPKEWKTSDLYQLFSAFGNIQISWIDDTS
AFVSLSQPEQVKIAVNTSKYAESYRIQTYAEYMGRKQEEKQIKRKWTEDSWKEADSKRLN
PQCIPYTLQNHYYRNNSFTAPSTVGKRNLSPSQEEAGLEDGVSGEISDTELEQTDSCAEP
LSEGRKKAKKLKRMKKELSPAGSISKNSPATLFEVPDTW
>Q08956
MGIPMQIYQDGKGVQFYHTRYQNVFDERASKYGNYTVNNDYPQLPDTIKEHIDQLTFSNV
GEDGGDVGNYSEEDDDGDEEKELEDVFRSNRGLEFVRINNYFTTHDLQSFKSFRNFNSKY
WIFYSNQAEDKKLLLYDFNGQHLIFIKQQFYGQLNLLLSDAIICMDCNFGYNSNTIQILV
GFQNGKLLKLNCDLNGNVNNHLLLKDPSTSSHQSHLSILNVWAGLLPHFVVSFSLKDGLL
ITSLDHQQSNGSFQSFHTNIDLPVDLRTTTNVKSVLNFPQFTLYKGNDMIFHCKNLLGSD
ASTLNKEINFMLKIDEDVQKIDYLLKTNHILLETNMRYLSIPTRDPIENSNSSPPVSDSE
VYPIFYKTQELHVHASGTGRQIANNGKYIFITEQHLYGTALSVYKYSISFKRWLFVGYSD
IRAKYGIRSVKDLFVGNCPSVNSPVLTILTDDNNIQTILLK

>P07954
MYRALRLLARSRPLVRAPAAALASAPGLGGAAVPSFWPPNAARMASQNSFRIEYDTFGEL
KVPNDKYYGAQTVRSTMNFKIGGVTERMPTPVIKAFGILKRAAAEVNQDYGLDPKIANAI
MKAADEVAEGKLNDHFPLVVWQTGSGTQTNMNVNEVISNRAIEMLGGELGSKIPVHPNDH
VNKSQSSNDTFPTAMHIAAAIEVHEVLLPGLQKLHDALDAKSKEFAQIIKIGRTHTQDAV
PLTLGQEFSGYVQQVKYAMTRIKAAMPRIYELAAGGTAVGTGLNTRIGFAEKVAAKVAAL
TGLPFVTAPNKFEALAAHDALVELSGAMNTTACSLMKIANDIRFLGSGPRSGLGELILPE
NEPGSSIMPGKVNPTQCEAMTMVAAQVMGNHVAVTVGGSNGHFELNVFKPMMIKNVLHSA
RLLGDASVSFTENCVVGIQANTERINKLMNESLMLVTALNPHIGYDKAAKIAKTAHKNGS
TLKETAIELGYLTAEQFDEWVKPKDMLGPK
>P46947
MALHQYLSETYGPTKPKNKTKKKKKESKSDANSDKTSLIVKERLSTLQQEQEKSGVASFS
KFDKQKSKNIWKNLETNELSHAITHPSASSITGNESKNDLKEIRAQEPLVTVADKSKTRK
TIYRDAQGHKIQEDSKIDDSSFSRSKYEDEKAAEREQYLKNLNMGDVQKLGINVDAHDKK
KNQTASSLTIEDPAITFTHDKERTVKTSLLGRKLYDKPAPENRFAIMPGSRWDGVHRSNG
FEEKWFAKQNEINEKKVQSYTLQEDY
>O70311
MAEDSESAASQQSLELDDQDTCGIDGDNEEETEHAKGSPGGDLGAKKKKKKQKRKKEKPN
SGGTKSDSASDSQEIKIQQSSKHNAIWQQISAGAAMGGDTMEGEWIDLRMYHKNPTIPIQ
KLQDIQRAMELLSACQGPARNIDEATKRRYQFWDTQPVPKLNEVITSHGAIEPDKDNIRQ
EPYSLPQGFMWDTLDLSNAEVLKELYTLLNENYVEDDDNMFRFDYSPEFLLWALRPPGWL
LQWHCGVRVSSNKKLVGFISAIPANIRIYDSVKRMVEINFLCVHKKLRSKRVAPVLIREI
TRRVNLEGIFQAVYTAGVVLPKPVATCRYWHRSLNPRKLVEVKFSHLSRNMTLQRTMKLY
RLPDVTKTSGLRPMEPKDIKAVRELINIYLKQFHLAPVMDDAEVAHWFLPREHIIDTFVV
ENPSGKLTDFLSFYTLPSTVMHHPAHKSLKAAYSFYNIHTETPLLDLMNDALIIAKLKGF
DVFNALDLMENKTFLEKLKFGIGDGNLQYYLYNWRCPGTDSEKVGLVLQ
>Q13868
MAMEMRLPVARKPLSERLGRDTKKHLVVPGDTITTDTGFMRGHGTYMGEEKLIASVAGSV
ERVNKLICVKALKTRYIGEVGDIVVGRITEVQQKRWKVETNSRLDSVLLLSSMNLPGGEL
RRRSAEDELAMRGFLQEGDLISAEVQAVFSDGAVSLHTRSLKYGKLGQGVLVQVSPSLVK
RQKTHFHDLPCGASVILGNNGFIWIYPTPEHKEEEAGGFIANLEPVSLADREVISRLRNC
IISLVTQRMMLYDTSILYCYEASLPHQIKDILKPEIMEEIVMETRQRLLEQEG
>O46560
MQAGSWVVGGGDSDSRVLSIQSHVVRGYVGNRAATFPLQVLGFEVDAVNSVQFSNHTGYA
HWKGQVLNSDELHALYEGLKLNNVNQYDYVLTGYTRDKSFLAMVVDIVRELKQQNPRLVY
VCDPVMGDKWDGEGSMYVPEDLLPVYREKVVPVADIITPNQFEAELLTGRRIHSEEEALA
VMDMLHAMGPDTVVITSSDLPSPRGKDYLIALGSQRTRSPDGSVATQRIRMEICKVDAVF
VGTGDLFAAMLLAWTHKHPNNLKVACEKTVSAMHHVLRRTIQCAKAKAGEGLKPSPAQLE
LRMVQSKRDIEDPEVVVQATVL
>Q13387
MADRAEMFSLSTFHSLSPPGCRPPQDISLEEFDDEDLSEITDDCGLGLSYDSDHCEKDSL
SLGRSEQPHPICSFQDDFQEFEMIDDNEEEDDEDEEEEEEEEEGDGEGQEGGDPGSEAPA
PGPLIPSPSVEEPHKHRPTTLRLTTLGAQDSLNNNGGFDLVRPASWQETALCSPAPEALR
ELPGPLPATDTGPGGAQSPVRPGCDCEGNRPAEPPAPGGTSPSSDPGIEADLRSRSSGGR

GGRRSSQELSSPGSDSEDAGGARLGRMISSISETELELSSDGGSSSSGRSSHLTNSIEEA
SSPASEPEPPREPPRRPAFLPVGPDDTNSEYESGSESEPDLSEDADSPWLLSNLVSRMIS
EGSSPIRCPGQCLSPAPRPPGEPVSPAGGAAQDSQDPEAAAGPGGVELVDMETLCAPPPP
APAAPRPGPAQPGPCLFLSNPTRDTITPLWAAPGRAARPGRACSAACSEEEDEEDDEEEE
DAEDSAGSPGGRGTGPSAPRDASLVYDAVKYTLVVDEHTQLELVSLRRCAGLGHDSEEDS
GGEASEEEAGAALLGGGQVSGDTSPDSPDLTFSKKFLNVFVNSTSRSSSTESFGLFSCLV
NGEEREQTHRAVFRFIPRHPDELELDVDDPVLVEAEEDDFWFRGFNMRTGERGVFPAFYA
HAVPGPAKDLLGSKRSPCWVERFDVQFLGSVEVPCHQGNGILCAAMQKIATARKLTVHLR
PPASCDLEISLRGVKLSLSGGGPEFQRCSHFFQMKNISFCGCHPRNSCYFGFITKHPLLS
RFACHVFVSQESMRPVAQSVGRAFLEYYQEHLAYACPTEDIYLE
>O14713
MFRKGKKRHSSSSSQSSEISTKSKSVDSSLGGLSRSSTVASLDTDSTKSSGQSNNNSDTC
AEFRIKYVGAIEKLKLSEGKGLEGPLDLINYIDVAQQDGKLPFVPPEEEFIMGVSKYGIK
VSTSDQYDVLHRHALYLIIRMVCYDDGLGAGKSLLALKTTDASNEEYSLWVYQCNSLEQA
QAICKVLSTAFDSVLTSEKP
>P26307
MEASSGSSPPHSQENPPEHGGDMGGAPAEEIGGEAADDFMFAEDTFPSLPDFPCLSSPSS
STFSSNSSSNSSSAYTNTAGRAGGEPSEPASAGEGFDALDDIDQLLDFASLSMPWDSEPF
PGVSMMLENAMSAPPQPVGDGMSEEKAVPEGTTGGEEACMDASEGEELPRFFMEWLTSNR
ENISAEDLRGIRLRRSTIEAAAARLGGGRQGTMQLLKLILTWVQNHHLQRKRPRDVMEEE
AGLHVQLPSPVANPPGYEFPAGGQDMAAGGGTSWMPHQQAFTPPAAYGGDAVYPSAAGQQ
YSFHQGPSTSSVVVNSQPFSPPPVGDMHGANMAWPQQYVPFPPPGASTGSYPMPQPFSPG
FGGQYAGAGAGHLSVAPQRMAGVEASATKEARKKRMARQRRLSCLQQQRSQQLSLGQIQT
SVHLQEPSPRSTHSGPVTPSAGGWGFWSPSSQQQVQNPLSKSNSSRAPPSSLEAAAAAPQ
TKPAPAGARQDDIHHRLAAASDKRQGAKADKNLRFLLQKVLKQSDVGSLGRIVLPKKEAE
VHLPELKTRDGISIPMEDIGTSRVWNMRYRFWPNNKSRMYLLENTGEFVRSNELQEGDFI
VIYSDVKSGKYLIRGVKVRPPPAQEQGSGSSGGGKHRPLCPAGPERAAAAGAPEDAVVDG
VSGACKGRSPEGVRRVRQQGAGAMSQMAVSI
>Q7Z4G1
MEASSEPPLDAKSDVTNQLVDFQWKLGMAVSSDTCRSLKYPYVAVMLKVADHSGQVKTKC
FEMTIPQFQNFYRQFKEIAAVIETV
>Q9UTG3
MFRRNTLTPGKGRNSLDMFSVNDFMTWIDGMKKTSDENGCSPFTLKPIKSTSDRKRRRHS
RMSIVNVWNQSPTSYRDQLLHFNHESDVSIDDTSEVAGEETFGQIERFEYLLSSDGDGDG
DVEIENPEVDDYQVSEHDFGLEDDFNGDGCQQMIEIESDEAISEEEEDLSNENKSESQLG
ESFSFNQDNTFVNYASPASITEEFVEPKDSQFNHDVNLMQGSAPGYSTVEPEDNFASEIQ
TNAPEVHLNYENSDYTEDHIDLLDHHFCDLSEISKFNHQHSGKPDHPSLVSNASLAPFIV
EGNGIKNGLLHYNMETAETDESYTDLDDTFARLKNLSQRHTNHSTDHHDDTVDSHLLHSF
VSAENANEPTNDVDDNSLQEQVADASQFVSFLETTKTVATSNLRSTKRKLSEILENGQSG
DCSLTDTNVDFDISEKQASGNSRSMIPLRKKR
>P54688
MPAILSRVAPRTFNLVGSRLMASAARLETVPREEIHKEYDRKKTFYHRDLEIQLAGPTQL
KTKPLDPTKLKFGHTYADYMMTCDWDAERGWHHPKIEPIGELKIHPGAKVLHYASELFEG
MKAYRGIDNKIRMFRPEMNMARMKRTALRAALPDFDSEEMINVLTELLRLDQEWVPNSDV

CSLYLRPTLIGTDPTLGVGCATEAKMFVITGPVGAYYSTGFQPVSLLADSRFIRAFPGGV
GAYKMGCNYAPTIWVGKEAASKNCQQVLWLYGENEDLTEVGTMNIFLFWKNEEGDMELIT
PPLHRGLILPGVTRDSLLELGREWGEYKVTERTLNMEEVKKALSEKRLYEMFGSGTACVV
SPVGKILYHNKVTDEYEELHIPTMSSKFGVMQKFYNTINDIQYGRIIKDGWMRDI
>Q9QZU4
MAVNDCFSLTYPHNPHPGDLIEVFRPCYQHWALYLGDGYVINIAPIDGIRSSFTSAKSVF
STKALVKMQLLKDVVGNDTYRINNKYDTTYPPLPVEEVIQRSEFPIGQEVAYDLLVNNCE
HFVTLLRYGEGVSEQANRAIGTIGLVAAGIDIFTFLGLFPKRQRTKY
>P41569
MGRVIRAQRKGEGSVFTAHTKKRKGEPKLRALDYAERHGYLKGTVKQIIHDPGRGAPLAV
VHFRDPYKFNVRKQLFIAAEGMYTGQFVYCGKRAQLQIGNVLPIGLMPEGTIVCNLEEKT
GDRGKLARTSGNYASVIAHNPDTKRTRVKLPSGAKKVLPSANRAMVGIVAGGGRIDKPIL
KAGRAYRKYKVKRNCWPKVRGVAMNPVEHPHGGGNHQHIGKASTVKRGTPPGRKVGLIAA
RRTGRIRGGKGDEKFKEKEKK
>Q9P2W6
MGRTWCGMWRRRRPGRRSAVPRWPHLSSQSGVEPPDRWTGTPGWPSRDQEAPGSMMPPAA
AQPSAHGALVPPATAHEPVDHPALHWLACCCCLSLPGQLPLAIRLGWDLDLEAGPSSGKL
CPRARRWQPLPS
>P87241
MQIPEKYLSSVDNVEEETQILFALSKQKDADLGMLDSKQEQIALTIGNKPIKVKQSLQSL
HQSRGSTGSVLWKTSVKVVPWLLQQSWFMNSLTPKTSILELGSGISGLAGILLSPFVGNY
VASDKQLYLKKIRENLDQNNASDVEVHELDWKSTPYPKDWTFDFLDYVLFFDCIYNPHLN
AHLVSCLASLAERYPGMQCLFAQELRDQETLVDFLERVRPYFEVDLIKMEEINKTSVASS
TNLPPANMSLFIMKPYNHEEYMLKALNEAKKCEPTDSAFCVGAVIVQNGKIVSTGYSRER
PGNTHAEECAIEKFMLKNPTDSLEGAIMYSTMEPCSKRLSKKVSCTDLIVKQKFSTVVLG
SLEPDIFVKCEGVDLLKKAGIVVIEKLTFQDDCLREAVRGHPPKH
>Q9Y697
MLLRAAWRRAAVAVTAAPGPKPAAPTRGLRLRVGDRAPQSAVPADTAAAPEVGPVLRPLY
MDVQATTPLDPRVLDAMLPYLINYYGNPHSRTHAYGWESEAAMERARQQVASLIGADPRE
IIFTSGATESNNIAIKGVARFYRSRKKHLITTQTEHKCVLDSCRSLEAEGFQVTYLPVQK
SGIIDLKELEAAIQPDTSLVSVMTVNNEIGVKQPIAEIGRICSSRKVYFHTDAAQAVGKI
PLDVNDMKIDLMSISGHKIYGPKGVGAIYIRRRPRVRVEALQSGGGQERGMRSGTVPTPL
VVGLGAACEVAQQEMEYDHKRISKLSERLIQNIMKSLPDVVMNGDPKHHYPGCINLSFAY
VEGESLLMALKDVALSSGSACTSASLEPSYVLRAIGTDEDLAHSSIRFGIGRFTTEEEVD
YTVEKCIQHVKRLREMSPLWEMVQDGIDLKSIKWTQH
>Q9H089
MGRRRAPAGGSLGRALMRHQTQRSRSHRHTDSWLHTSELNDGYDWGRLNLQSVTEQSSLD
DFLATAELAGTEFVAEKLNIKFVPAEARTGLLSFEESQRIKKLHEENKQFLCIPRRPNWN
QNTTPEELKQAEKDNFLEWRRQLVRLEEEQKLILTPFERNLDFWRQLWRVIERSDIVVQI
VDARNPLLFRCEDLECYVKEMDANKENVILINKADLLTAEQRSAWAMYFEKEDVKVIFWS
ALAGAIPLNGDSEEEANRDDRQSNTTKFGHSSFDQAEISHSESEHLPARDSPSLSENPTT
DEDDSEYEDCPEEEEDDWQTCSEEDGPKEEDCSQDWKESSTADSEARSRKTPQKRQIHNF
SHLVSKQELLELFKELHTGRKVKDGQLTVGLVGYPNVGKSSTINTIMGNKKVSVSATPGH
TKHFQTLYVEPGLCLCDCPGLVMPSFVSTKAEMTCSGILPIDQMRDHVPPVSLVCQNIPR

HVLEATYGINIITPREDEDPHRPPTSEELLTAYGYMRGFMTAHGQPDQPRSARYILKDYV
SGKLLYCHPPPGRDPVTFQHQHQRLLENKMNSDEIKMQLGRNKKAKQIENIVDKTFFHQE
NVRALTKGVQAVMGYKPGSGVVTASTASSENGAGKPWKKHGNRNKKEKSRRLYKHLDM
>Q7RTN6
MSFLVSKPERIRRWVSEKFIVEGLRDLELFGEQPPGDTRRKTNDASSESIASFSKQEVMS
SFLPEGGCYELLTVIGKGFEDLMTVNLARYKPTGEYVTVRRINLEACSNEMVTFLQGELH
VSKLFNHPNIVPYRATFIADNELWVVTSFMAYGSAKDLICTHFMDGMNELAIAYILQGVL
KALDYIHHMGYVHRSVKASHILISVDGKVYLSGLRSNLSMISHGQRQRVVHDFPKYSVKV
LPWLSPEVLQQNLQGYDAKSDIYSVGITACELANGHVPFKDMPATQMLLEKLNGTVPCLL
DTSTIPAEELTMSPSRSVANSGLSDSLTTSTPRPSNGDSPSHPYHRTFSPHFHHFVEQCL
QRNPDARPSASTLLNHSFFKQIKRRASEALPELLRPVTPITNFEGSQSQDHSGIFGLVTN
LEELEVDDWEF
>O43324
MAAAAELSLLEKSLGLSKGNKYSAQGERQIPVLQTNNGPSLTGLTTIAAHLVKQANKEYL
LGSTAEEKAIVQQWLEYRVTQVDGHSSKNDIHTLLKDLNSYLEDKVYLTGYNFTLADILL
YYGLHRFIVDLTVQEKEKYLNVSRWFCHIQHYPGIRQHLSSVVFIKNRLYTNSH
>Q12044
MANQKQMRTQILITDIPSGKFTSKWPTQLEKTLFKEQFPNLQSHLQYYTPLPFLNRIIII
FDNEDDTLQVFKFLQELLAKENSGPMKLFVTESLLNNQHPRSRSTDDAVSLQDNNLALLE
DHRNKPLLSINTDPGVTGVDSSSLNKGGSSLSPDKSSLESPTMLKLSTDSKPFSYQEPLP
KLSRSSSSTSNLSLNRSSQTSLPSQLENKDKSASGTKCLFASKPLGLTIDTSTRSNAASC
TENDVNATASNPPKSPSITVNEFFH
>Q9U489
MATIVPCSLEKEEGAPSGPRRLQTEIDVDANDSGNELSMGGSSSEGDSMSHHRGEHSPNH
HHQDNHLGSGPPPPQFTGSLFDTPPSMIQSPQQQPQFQFNTGFGLGLPQDSFRCSVCSKS
STIGVLPFVCAHKTCQSCYQMTPSSYDRRACKLCGAVSTATANFTSQMYLSPTLPSPPRG
ALMSDCSTPTMNNHINSSTPLHQPRAFSFSLSGMPGSPSPVMGARMPSSAGGLMMRPIGF
PDSDSSLTSWSPLQQPSQLSINNLSSIGGHQQQSPMLMQNVFDSLAVNDDTPVFSPLSPT
NTSMHMPPSLMASPDVPKHSATIAPPRNSMCSTPRLQLATPMSSQSQQTFPIPSPLGSQP
QQQQPMGPIQCQGCESKISFAYCMQCQEALCIHCVQAHQRVRATKQHAFVELQQLMATLM
SRAVQPQQAQQYTQNVGGSVRQALGSVGSGDVFFSGHVSGVENDSIGSGESSPRSSSVCG
THDSVIIGICENCPHSVLLCAICVAQHPGKHRVQPLGDIRVAVGEVVNESQLLQWQCEKT
GDTIKQIIDGIVTNATTAENEIRAAFDTHVNALEERRKELLKRVETVKNLKLSVLISQAE
SLQSKQIDLQQAIQTATKLMDSSDCDEMVLRQVFEKLASCQMGNEGTEPNNNILNVLMLA
CQVNEDDRLKFTAPQDGILLNKARQFGNIESGPCAKNSSIVGDSFKKAIRERQTVIYVQL
RDACGDLLSSSIAATQPTSQALLPHQEPHSHLEQAMPTSDVQAFVISPDGSTVEVTMTPR
ENGIVALSYYPSIEGSYTLNILVKGTPISGCPTTMDIRRGRNYDEIAAKGPILTFGKEGS
GDGELCRPWGICVDQRGRVIVADRSNNRVQIFDKDGNFISKFGTSGNRPGQFDRPAGITT
NSLNNIVVADKDNHRVQVFDENGMFLLKFGDRGRAVGYFNYPWGVATNSHNAIAVSDTRN
HRVQIFTPQGQFVRKCGFDSAYFFKNLDSPRGLCYLPDGQLLITDFNNHRLAVLSPRNMS
EMKVYGSEGDGDGMFVRPQGVVIDPEGHILVCDSRNNRVQVFASDDMRFIGSFGLGPVPN
SGFQMPQELPAPYSSLGGPFGAPAFSSAPTPLTPSPRQLLDRPTDLAVGPDGRIYVVDFG
NNCIRVF
>O94374

MESRYLSDRRSRFKSKGVFKADELRRQREEQQIEIRKQKREESLNKRRNLNAVLQNDIDV
EEEADQSQVQMEQQMKDEFPKLTADVMSDDIELQLGAVTKFRKYLSKETHPPIDQVIACG
VVDRFVQFLESEHHLLQFEAAWALTNIASGTTDQTRIVVDSGAVPRFIQLLSSPEKDVRE
QVVWALGNIAGDSSACRDYVLGNGVLQPLLNILQSSASDVSMLRNATWTLSNLCRGKNPP
PNWSTISVAVPILAKLLYSEDVEIIVDACWAISYLSDGPNEKIGAILDVGCAPRLVELLS
SPSVNIQTPALRSVGNIVTGTDAQTQIIIDCGALNAFPSLLSHQKENIRKEACWTISNIT
AGNTQQIQAIIESNLIPPLVHLLSYADYKTKKEACWAISNATSGGLGQPDQIRYLVSQGV
IKPLCDMLNGSDNKIIQVALDAIENILKVGEMDRTMDLENINQYAVYVEEAGGMDMIHDL
QSSGNNDIYLKAYSIIEKYFSDEDAVEDLAPETENGAFTFGNGPQAQGEFKFDSQDMAM
>P18963
MNQSDPQDKKNFPMEYSLTKHLFFDRLLLVLPIESNLKTYADVEADSVFNSCRSIILNIA
ITKDLNPIIENTLGLIDLIVQDEEITSDNITDDIAHSILVLLRLLSDVFEYYWDQNNDFK
KIRNDNYKPGFSSHRPNFHTSRPKHTRINPALATMLLCKISKLKFNTRTLKVLQNMSHHL
SGSATISKSSILPDSQEFLQKRNYPAYTEKIDLTIDYIQRFISASNHVEFTKCVKTKVVA
PLLISHTSTELGVVNHLDLFGCEYLTDKNLLAYLDILQHLSSYMKRTIFHSLLLYYASKA
FLFWIMARPKEYVKIYNNLISSDYNSPSSSSDNGGSNNSDKTSISQLVSLLFDDVYSTFS
VSSLLTNVNNDHHYHLHHSSSSSKTTNTNSPNSISKTSIKQSSVNASGNVSPSQFSTGND
ASPTSPMASLSSPLNTNILGYPLSPITSTLGQANTSTSTTAATTKTDADTPSTMNTNNNN
NNNNSANLNNIPQRIFSLDDISSFNSSRKSLNLDDSNSLFLWDTSQHSNASMTNTNMHAG
VNNSQSQNDQSSLNYMENIMELYSNYTGSELSSHTAILRFLVVLTLLDSEVYDEMNSNSY
RKISEPIMNINPKDSNTSSWGSASKNPSIRHLTHGLKKLTLQQGRKRNVKFLTYLIRNLN
GGQFVSDVSLIDSIRSILFLMTMTSSISQIDSNIASVIFSKRFYNLLGQNLEVGTNWNSA
TANTFISHCVERNPLTHRRLQLEFFASGLQLDSDLFLRHLQLEKELNHIDLPKISLYTEG
FRVFFHLVSTKKLHEDIAEKTSSVLKRLFCIIADILLKATPYFDDNVTKIIASILDGHIL
DQFDAARTLSNDDHVSFDAATSVYTEPTEIIHNSSDASLVSSLSQSPLSINSGSNITNTR
TWDIQSILPTLSNRSSASDLSLSNILTNPLEAQQNNNANLLAHRLSGVPTTKRYASPNDS
ERSRQSPYSSPPQLQQSDLPSPLSVLSSSAGFSSNHSITATPTILKNIKSPKPNKTKKIA
DDKQLKQPSYSRVILSDNDEARKIMMNIFSIFKRMTNWFIRPDANTEFPKTFTDIIKPLF
VSILDSNQRLQVTARAFIEIPLSYIATFEDIDNDLDPRVLNDHYLLCTYAVTLFASSLFD
LKLENAKREMLLDIIVKFQRVRSYLSNLAEKHNLVQAIITTERLTLPLLVGAVGSGIFIS
LYCSRGNTPRLIKISCCEFLRSLRFYQKYVGALDQYSIYNIDFIDAMAQDNFTASGSVAL
QRRLRNNILTYIKGSDSILLDSMDVIYKKWFYFSCSKSVTQEELVDFRSLAGILASMSGI
LSDMQELEKSKSAPDNEGDSLSFESRNPAYEVHKSLKLELTKKMNFFISKQCQWLNNPNL
LTRENSRDILSIELHPLSFNLLFNNLGLKIDELMSIDLSKSHEDSSFVLLEQIIIIIRTI
LKRDDDEKIMLLFSTDLLDAVDKLIEIVEKISIKSSKYYKGIIQMSKMFRAFEHSEKNLG
ISNHFHLKNKWLKLVIGWFKLSINKDYDFENLSRPLREMDLQKRDEDFLYIDTSIESAKA
LAYLTHNVPLEIPPSSSKEDWNRSSTVSFGNHFTILLKGLEKSADLNQFPVSLRHKISIL
NENVIIALTNLSNANVNVSLKFTLPMGYSPNKDIRIAFLRVFIDIVTNYPVNPEKHEMDK
MLAIDDFLKYIIKNPILAFFGSLACSPADVDLYAGGFLNAFDTRNASHILVTELLKQEIK
RAARSDDILRRNSCATRALSLYTRSRGNKYLIKTLRPVLQGIVDNKESFEIDKMKPGSEN
SEKMLDLFEKYMTRLIDAITSSIDDFPIELVDICKTIYNAASVNFPEYAYIAVGSFVFLR
FIGPALVSPDSENIIIVTHAHDRKPFITLAKVIQSLANGRENIFKKDILVSKEEFLKTCS
DKIFNFLSELCKIPTNNFTVNVREDPTPISFDYSFLHKFFYLNEFTIRKEIINESKLPGE
FSFLKNTVMLNDKILGVLGQPSMEIKNEIPPFVVENREKYPSLYEFMSRYAFKKVDMKEE

EEDNAPFVHEAMTLDGIQIIVVTFTNCEYNNFVMDSLVYKVLQIYARMWCSKHYVVIDCT
TFYGGKANFQKLTTLFFSLIPEQASSNCMGCYYFNVNKSFMDQWASSYTVENPYLVTTIP
RCFINSNTDQSLIKSLGLSGRSLEVLKDVRVTLHDITLYDKEKKKFCPVSLKIGNKYFQV
LHEIPQLYKVTVSNRTFSIKFNNVYKISNLISVDVSNTTGVSSEFTLSLDNEEKLVFCSP
KYLEIVKMFYYAQLKMEEDFGTDFSNDISFSTSSSAVNASYCNVKEVGEIISHLSLVILV
GLFNEDDLVKNISYNLLVATQEAFNLDFGTRLHKSPETYVPDDTTTFLALIFKAFSESST
ELTPYIWKYMLDGLENDVIPQEHIPTVVCSLSYWVPNLYEHVYLANDEEGPEAISRIIYS
LIRLTVKEPNFTTAYLQQIWFLLALDGRLTNVIVEEIVSHALDRDSENRDWMKAVSILTS
FPTTEIACQVIEKLINMIKSFLPSLAVEASAHSWSELTILSKISVSIFFESPLLSQMYLP
EILFAVSLLIDVGPSEIRVSLYELLMNVCHSLTNNESLPERNRKNLDIVCATFARQKLNF
ISGFSQEKGRVLPNFAASSFSSKFGTLDLFTKNIMLLMEYGSISEGAQWEAKYKKYLMDA
IFGHRSFFSARAMMILGIMSKSHTSLFLCKELLVETMKVFAEPVVDDEQMFIIIAHVFTY
SKIVEGLDPSSELMKELFWLATICVESPHPLLFEGGLLFMVNCLKRLYTVHLQLGFDGKS
LAKKLMESRNFAATLLAKLESYNGCIWNEDNFPHIILGFIANGLSIPVVKGAALDCLQAL
FKNTYYERKSNPKSSDYLCYLFLLHLVLSPEQLSTLLLEVGFEDELVPLNNTLKVPLTLI
NWLSSDSDKSNIVLYQGALLFSCVMSDEPCKFRFALLMRYLLKVNPICVFRFYTLTRKEF
RRLSTLEQSSEAVAVSFELIGMLVTHSEFNYLEEFNDEMVELLKKRGLSVVKPLDIFDQE
HIEKLKGEGEHQVAIYERKRLATMILARMSCS
>P97849
MRTPGAGTASVASLGLLWLLGLPWTWSAAAAFGVYVGSGGWRFLRIVCKTARRDLFGLSV
LIRVRLELRRHRRAGDTIPRIFQAVAQRQPERLALVDASSGICWTFAQLDTYSNAVANLF
LQLGFAPGDVVAVFLEGRPEFVGLWLGLAKAGVVAALLNVNLRREPLAFCLGTSAAKALI
YGGEMAAAVAEVSEQLGKSLLKFCSGDLGPESVLPDTQLLDPMLAEAPTTPLAQAPGKGM
DDRLFYIYTSGTTGLPKAAIVVHSRYYRIAAFGHHSYSMRANDVLYDCLPLYHSAGNIMG
VGQCIIYGLTVVLRKKFSASRFWDDCVKYNCTVVQYIGEICRYLLRQPVRDVERRHRVRL
AVGNGLRPAIWEEFTQGFGVRQIGEFYGATECNCSIANMDGKVGSCGFNSRILTHVYPIR
LVKVNEDTMEPLRDSQGLCIPCQPGEPGLLVGQINQQDPLRRFDGYVSDSATNKKIAHSV
FRKGDSAYLSGDVLVMDELGYMYFRDRSGDTFRWRGENVSTTEVEAVLSRLLGQTDVAVY
GVAVPGVEGKSGMAAIADPHNQLDPNSMYQELQKVLASYAQPIFLRLLPQVDTTGTFKIQ
KTRLQREGFDPRQTSDRLFFLDLKQGRYLPLDERVHARICAGDFSL
>O96935
MKLTKGCAYKYIIFTVLILANILYDNKKRCMIKKNLRISSCGIISRLLKSNSNYNSFNKN
YNFTSAISELQFSNFWNLDILQKDIFSNIHNNKNKPQSYIIHKRLMSEKGDNNNNNHQNN
NGNDNKKRLGSVVNNEENTCSDKRMKPFEEGHGITQVDKMNNNSDHLQQNGVMNLNSNNV
ENNNNNNSVVVKKNEPKIHYRKDYKPSGFIINNVTLNINIHDNETIVRSVLDMDISKHNV
GEDLVFDGVGLKINEISINNKKLVEGEEYTYDNEFLTIFSKFVPKSKFAFSSEVIIHPET
NYALTGLYKSKNIIVSQCEATGFRRITFFIDRPDMMAKYDVTVTADKEKYPVLLSNGDKV
NEFEIPGGRHGARFNDPHLKPCYLFAVVAGDLKHLSATYITKYTKKKVELYVFSEEKYVS
KLQWALECLKKSMAFDEDYFGLEYDLSRLNLVAVSDFNVGAMENKGLNIFNANSLLASKK
NSIDFSYARILTVVGHEYFHNYTGNRVTLRDWFQLTLKEGLTVHRENLFSEEMTKTVTTR
LSHVDLLRSVQFLEDSSPLSHPIRPESYVSMENFYTTTVYDKGSEVMRMYLTILGEEYYK
KGFDIYIKKNDGNTATCEDFNYAMEQAYKMKKADNSANLNQYLLWFSQSGTPHVSFKYNY
DAEKKQYSIHVNQYTKPDENQKEKKPLFIPISVGLINPENGKEMISQTTLELTKESDTFV
FNNIAVKPIPSLFRGFSAPVYIEDNLTDEERILLLKYDSDAFVRYNSCTNIYMKQILMNY

NEFLKAKNEKLESFNLTPVNAQFIDAIKYLLEDPHADAGFKSYIVSLPQDRYIINFVSNL
DTDVLADTKEYIYKQIGDKLNDVYYKMFKSLEAKADDLTYFNDESHVDFDQMNMRTLRNT
LLSLLSKAQYPNILNEIIEHSKSPYPSNWLTSLSVSAYFDKYFELYDKTYKLSKDDELLL
QEWLKTVSRSDRKDIYEILKKLENEVLKDSKNPNDIRAVYLPFTNNLRRFHDISGKGYKL
IAEVITKTDKFNPMVATQLCEPFKLWNKLDTKRQELMLNEMNTMLQEPNISNNLKEYLLR
LTNKL
>Q99315
MSFMDQIPGGGNYPKLPVECLPNFPIQPSLTFRGRNDSHKLKNFISEIMLNMSMISWPND
ASRIVYCRRHLLNPAAQWANDFVQEQGILEITFDTFIQGLYQHFYKPPDINKIFNAITQL
SEAKLGIERLNQRFRKIWDRMPPDFMTEKAAIMTYTRLLTKETYNIVRMHKPETLKDAME
EAYQTTALTERFFPGFELDADGDTIIGATTHLQEEYDSDYDSEDNLTQNGYVHTVRTRRS
YNKPMSNHRNRRNNNPSREECIKNRLCFYCKKEGHRLNECRARKAVLTDLELESKDQQTP
FIKTLPIVHYIAIPEMDNTAEKTIKIQNTKVKTLFDSGSPTSFIRRDIVELLKYEIYETP
PLRFRGFVATKSAVTSEAVTIDLKINDLHITLAAYILDNMDYQLLIGNPILRRYPKILHT
VLNTRESPDSLKPKTYRSETVNNVRTYSAGNRGNPRNIKLSFAPTILEATDPKSAGNRGD
SRTKTLSLATTTPAAIDPLTTLDNPGSTQSTFAQFPIPEEASILEEDGKYSNVVSTIQSV
EPNATDHSNKDTFCTLPVWLQQKYREIIRNDLPPRPADINNIPVKHDIEIKPGARLPRLQ
PYHVTEKNEQEINKIVQKLLDNKFIVPSKSPCSSPVVLVPKKDGTFRLCVDYRTLNKATI
SDPFPLPRIDNLLSRIGNAQIFTTLDLHSGYHQIPMEPKDRYKTAFVTPSGKYEYTVMPF
GLVNAPSTFARYMADTFRDLRFVNVYLDDILIFSESPEEHWKHLDTVLERLKNENLIVKK
KKCKFASEETEFLGYSIGIQKIAPLQHKCAAIRDFPTPKTVKQAQRFLGMINYYRRFIPN
CSKIAQPIQLFICDKSQWTEKQDKAIDKLKDALCNSPVLVPFNNKANYRLTTDASKDGIG
AVLEEVDNKNKLVGVVGYFSKSLESAQKNYPAGELELLGIIKALHHFRYMLHGKHFTLRT
DHISLLSLQNKNEPARRVQRWLDDLATYDFTLEYLAGPKNVVADAISRAVYTITPETSRP
IDTESWKSYYKSDPLCSAVLIHMKELTQHNVTPEDMSAFRSYQKKLELSETFRKNYSLED
EMIYYQDRLVVPIKQQNAVMRLYHDHTLFGGHFGVTVTLAKISPIYYWPKLQHSIIQYIR
TCVQCQLIKSHRPRLHGLLQPLPIAEGRWLDISMDFVTGLPPTSNNLNMILVVVDRFSKR
AHFIATRKTLDATQLIDLLFRYIFSYHGFPRTITSDRDVRMTADKYQELTKRLGIKSTMS
SANHPQTDGQSERTIQTLNRLLRAYASTNIQNWHVYLPQIEFVYNSTPTRTLGKSPFEID
LGYLPNTPAIKSDDEVNARSFTAVELAKHLKALTIQTKEQLEHAQIEMETNNNQRRKPLL
LNIGDHVLVHRDAYFKKGAYMKVQQIYVGPFRVVKKINDNAYELDLNSHKKKHRVINVQF
LKKFVYRPDAYPKNKPISSTERIKRAHEVTALIGIDTTHKTYLCHMQDVDPTLSVEYSEA
EFCQIPERTRRSILANFRQLYETQDNPEREEDVVSQNEICQYDNTSP
>Q16719
MEPSSLELPADTVQRIAAELKCHPTDERVALHLDEEDKLRHFRECFYIPKIQDLPPVDLS
LVNKDENAIYFLGNSLGLQPKMVKTYLEEELDKWAKIAAYGHEVGKRPWITGDESIVGLM
KDIVGANEKEIALMNALTVNLHLLMLSFFKPTPKRYKILLEAKAFPSDHYAIESQLQLHG
LNIEESMRMIKPREGEETLRIEDILEVIEKEGDSIAVILFSGVHFYTGQHFNIPAITKAG
QAKGCYVGFDLAHAVGNVELYLHDWGVDFACWCSYKYLNAGAGGIAGAFIHEKHAHTIKP
ALVGWFGHELSTRFKMDNKLQLIPGVCGFRISNPPILLVCSLHASLEIFKQATMKALRKK
SVLLTGYLEYLIKHNYGKDKAATKKPVVNIITPSHVEERGCQLTITFSVPNKDVFQELEK
RGVVCDKRNPNGIRVAPVPLYNSFHDVYKFTNLLTSILDSAETKN
>P52920
MTEILPHVNDEVLPAEYELNQPEPEHCPGPESDMAGKSDACGGCANKEICESLPKGPDPD

IPLITDNLSGIEHKILVLSGKGGVGKSTFAAMLSWALSADEDLQVGAMDLDICGPSLPHM
LGCIKETVHESNSGWTPVYVTDNLATMSIQYMLPEDDSAIIWRGSKKNLLIKKFLKDVDW
DKLDYLVIDTPPGTSDEHISINKYMRESGIDGALVVTTPQEVALLDVRKEIDFCKKAGIN
ILGLVENMSGFVCPNCKGESQIFKATTGGGEALCKELGIKFLGSVPLDPRIGKSCDMGES
FLDNYPDSPASSAVLNVVEALRDAVGDV
>Q7Z995
MDFFWERMLNDPQNHFSDDQKEFLNHAKYRVKRNTIFGMLVGFSLPLYLARNKKVTPIRL
YATSIIGGLAGNGVAQITTLAYNLSAIRQRDDGFEILRKIQDSLIRQRTTMRQGRFPSSS
SEFPPKNSKYQLPGSMPNTGASSSQDPFTNSQSTEKEDAMYSKDNGFEDRSKPASAWEAI
RNRNRNTGNNSFPFYEEDSSVKSTDSAFSGQENSEAFPSRTSNLGSEQLDEPISHEQEAF
DQLIWNDSSSSK
>Q08202
MFAAIASGNPLQLSVEVPNSNGLQHTIVLSRTKPKLYSHITLFILPNVTFPQDYIATVYF
KLSPQEEFKLFGYLSSEKPSAIFKVQIPSSKKDAGDTSDGLGEIDMDVDDGSGAADPFTD
TNGSSSNNISELIIGISIEPREQGMMKLEEWKASMNAEAQKNNSLILSRPNLGIIRNITT
AGQLAQVYPSLTQELAAKIVQHAYNYLSGFLDAQGNVPIKRFDTWWDKFRNRLANDGTFL
DEVTKN
>Q8IWJ2
MKQEVEDSVTKMGDAHKELEQSHINYVKEIENLKNELMAVRSKYSEDKANLQKQLEEAMN
TQLELSEQLKFQNNSEDNVKKLQEEIEKIRPGFEEQILYLQKQLDATTDEKKETVTQLQN
IIEANSQHYQKNINSLQEELLQLKAIHQEEVKELMCQIEASAKEHEAEINKLNELKENLV
KQCEASEKNIQKKYECELENLRKATSNANQDNQICSILLQENTFVEQVVNEKVKHLEDTL
KELESQHSILKDEVTYMNNLKLKLEMDAQHIKDEFFHEREDLEFKINELLLAKEEQGCVI
EKLKSELAGLNKQFCYTVEQHNREVQSLKEQHQKEISELNETFLSDSEKEKLTLMFEIQG
LKEQCENLQQEKQEAILNYESLREIMEILQTELGESAGKISQEFESMKQQQASDVHELQQ
KLRTAFTEKDALLETVNRLQGENEKLLSQQELVPELENTIKNLQEKNGVYLLSLSQRDTM
LKELEGKINSLTEEKDDFINKLKNSHEEMDNFHKKCEREERLILELGKKVEQTIQYNSEL
EQKVNELTGGLEETLKEKDQNDQKLEKLMVQMKVLSEDKEVLSAEVKSLYEENNKLSSEK
KQLSRDLEVFLSQKEDVILKEHITQLEKKLQLMVEEQDNLNKLLENEQVQKLFVKTQLYG
FLKEMGSEVSEDSEEKDVVNVLQAVGESLAKINEEKCNLAFQRDEKVLELEKEIKCLQEE
SVVQCEELKSLLRDYEQEKVLLRKELEEIQSEKEALQSDLLEMKNANEKTRLENQNLLIQ
VEEVSQTCSKSEIHNEKEKCFIKEHENLKPLLEQKELRDRRAELILLKDSLAKSPSVKND
PLSSVKELEEKIENLEKECKEKEEKINKIKLVAVKAKKELDSSRKETQTVKEELESLRSE
KDQLSASMRDLIQGAESYKNLLLEYEKQSEQLDVEKERANNFEHRIEDLTRQLRNSTLQC
ETINSDNEDLLARIETLQSNAKLLEVQILEVQRAKAMVDKELEAEKLQKEQKIKEHATTV
NELEELQVQLQKQKKQLQKTMQELELVKKDAQQTTLMNMEIADYERLMKELNQKLTNKNN
KIEDLEQEIKIQKQKQETLQEEITSLQSSVQQYEEKNTKIKQLLVKTKKELADSKQAETD
HLILQASLKGELEASQQQVEVYKIQLAEITSEKHKIHEHLKTSAEQHQRTLSAYQQRVTA
LQEECRAAKAEQATVTSEFESYKVRVHNVLKQQKNKSMSQAETEGAKQEREHLEMLIDQL
KIKLQDSQNNLQINVSELQTLQSEHDTLLERHNKMLQETVSKEAELREKLCSIQSENMMM
KSEHTQTVSQLTSQNEVLRNSFRDQVRHLQEEHRKTVETLQQQLSKMEAQLFQLKNEPTT
RSPVSSQQSLKNLRERRNTDLPLLDMHTVTREEGEGMETTDTESVSSASTYTQSLEQLLN
SPETKLEPPLWHAEFTKEELVQKLSSTTKSADHLNGLLRETEATNAILMEQIKLLKSEIR
RLERNQEREKSAANLEYLKNVLLQFIFLKPGSERERLLPVINTMLQLSPEEKGKLAAVAQ

GEEENASRSSGWASYLHSWSGLR
>Q9BTV5
MEEQREALRKIIKTLAVKNEEIQSFIYSLKQMLLNVEANSAKVQEDLEAEFQSLFSLLEE
LKEGMLMKIKQDRASRTYELQNQLAACTRALESSEELLETANQTLQAMDSEDFPQAAKQI
KDGVTMAPAFRLSLKAKVSDNMSHLMVDFAQERQMLQALKFLPVPSAPVIDLAESLVADN
CVTLVWRMPDEDSKIDHYVLEYRRTNFEGPPRLKEDQPWMVIEGIRQTEYTLTGLKFDMK
YMNFRVKACNKAVAGEFSEPVTLETPAFMFRLDASTSHQNLRVDDLSVEWDAMGGKVQDI
KAREKDGKGRTASPINSPARGTPSPKRMPSGRGGRDRFTAESYTVLGDTLIDGGEHYWEV
RYEPDSKAFGVGVAYRSLGRFEQLGKTAASWCLHVNNWLQVSFTAKHANKVKVLDAPVPD
CLGVHCDFHQGLLSFYNARTKQVLHTFKTRFTQPLLPAFTVWCGSFQVTTGLQVPSAVRC
LQKRGSATSSSNTSLT
>O16797
MSHRKFSAPRHGSMAFYPKKRSARHRGKVKAFPKDDASKPVHLTCFIGYKAGMTHIVREA
DRPGSKINKKEVVEAVTVLETPPMIVVGAVGYIETPFGLRALVNVWAQHLSEECRRRFYK
NWYKSKKKAFTKASKKWTDDLGKKSIENDFRKMLRYCKVIRVIAHSQIRLIKQRQKKAHV
MEIQLNGGSIEDKVKWAREHLEKPIQVSNVFGQDEMIDCVGVTKGKGFKGVTSRWHTKKL
PRKTHKGLRKVACIGAWHPSRVSTTVARAGQKGYHHRTEINKKIYRIGAGIHTKDGKVIK
NNASTEYDLTDKSITPMGGFPHYGEVNNDFVMIKGCCIGSKKRIITLRKSLLKHTKRSAL
EQIKLKFIDTSSKMGHGRFQTPADKLAFMGPLKKDRLKEEAAATTAAAAAATTTSA
>Q9PTG8
MSLQIINDENAGNDITAEKFDLLLNPQTTGRPSILRPSQKDNLPPKPALKSTKVTFQTPM
RDPQTLRIMTPSVANKPENVFLLEDCTQALEQLHLSLPSSCGPNPVEINVSVSNNQPDSE
ELPVRTTGAYSIDFDNFDDINPFKSKTQMLNSPIKADLPSLTENIETTTPVVPADEASKQ
MVSLNLSAANLDSPVTVQFSSESGYIGVSEKTALDDTLPLSESGIKDLQGLNASSNNIEE
PVPLDKDICSNNEKDDATMADSTCEGTSDAHTSSNNIEEPVTLDKDICSSNEKDNAAVAY
STCEGTSDAQSPLPIPKSSYSFDPDQFDMMNPFKTGGSKLQNSPAGKKQTPPSADLNTAK
TEPVKLEFNFGDGDVSERKPPPKKLGKRPLLKTAAKKPSPKPEIASEKQEQQTAKPSEDE
AIVPKASYKFDWEKFDDPNFNPFGCGGSKISSSPKGQKIANEQPSACTQGSKPEAECTAS
DMAPAENADEKDHGEIEPSQDSGAAEDRSQAEDQSVALSKVEVPHEQTTDCSPVENETQP
EVSLINEEPSQKEVEHTSSDMTPPEINGTDSEFKLATEADFLLAADMDFKPASEIFSEGF
RQPVEIDYLENFGTNSFKESVLRKQSLYLKFDPLLRESPKKSAAGINLLPSVPLKCSSDL
FGAIPEANFPLIPSIENEEKPKGLDLLGTFTVADTALLIVDAPSSVAVPNPFLSTSDAIV
EMLKYSQKDMDAAIETVRLEVQEKDLEVLEWKTKHEKLYLEYVEMGKIIAEFEGTITQIL
EDSQRQKETAKLELNKVLQEKQQVQVDLNSMETSFSELFKRLEKQKEALEGYRKNEEALK
KCVEDYLVRIKKEEQRYQALKAHAEEKLNRANEEIAHVRSKAKSEATRLQATLRKEQMKI
QSLERSLEQKSKENDELTKICDDLILKMEKI
>Q12199
MSKRNTPPLRSSGINTIQINAAREMHAQTVRARRMPMPTSGITTPSVQPTAAPATPPRHI
CNNPNNPQCLHCGSVIIPSPRATLPLEDNPSISINDWTISSRKKPILNSQELDIWENEKL
KGLTLPEMIFGNNYIRIENSKQHWSIEFNALDALKEVQLQDSGIRVAYSNDWINSKKRQN
STNGAQRFTNDVNDDSLNIIHKYDWTYTTRYKGTESSPESKFRLDNDQKLPLDKLAVHDK
ILFYDDMILFEDELADNGISILNVKIRVMNERLLLLSRFFLRVDDVLVRVYDTRIYVEFD
ENVVIRESKEFEGKYQDVLAKHRLSQSHDPKAALRDSNWVAQNTPMIKRQCEIIQF
>P28272

MTASLTTKFLNNTYENPFMNASGVHCMTTQELDELANSKAGAFITKSATTLEREGNPEPR
YISVPLGSINSMGLPNEGIDYYLSYVLNRQKNYPDAPAIFFSVAGMSIDENLNLLRKIQD
SEFNGITELNLSCPNVPGKPQVAYDFDLTKETLEKVFAFFKKPLGVKLPPYFDFAHFDIM
AKILNEFPLAYVNSINSIGNGLFIDVEKESVVVKPKNGFGGIGGEYVKPTALANVRAFYT
RLRPEIKVIGTGGIKSGKDAFEHLLCGASMLQIGTELQKEGVKIFERIEKELKDIMEAKG
YTSIDQFRGKLNSI
>Q13191
MANSMNGRNPGGRGGNPRKGRILGIIDAIQDAVGPPKQAAADRRTVEKTWKLMDKVVRLC
QNPKLQLKNSPPYILDILPDTYQHLRLILSKYDDNQKLAQLSENEYFKIYIDSLMKKSKR
AIRLFKEGKERMYEEQSQDRRNLTKLSLIFSHMLAEIKAIFPNGQFQGDNFRITKADAAE
FWRKFFGDKTIVPWKVFRQCLHEVHQISSGLEAMALKSTIDLTCNDYISVFEFDIFTRLF
QPWGSILRNWNFLAVTHPGYMAFLTYDEVKARLQKYSTKPGSYIFRLSCTRLGQWAIGYV
TGDGNILQTIPHNKPLFQALIDGSREGFYLYPDGRSYNPDLTGLCEPTPHDHIKVTQEQY
ELYCEMGSTFQLCKICAENDKDVKIEPCGHLMCTSCLTAWQESDGQGCPFCRCEIKGTEP
IIVDPFDPRDEGSRCCSIIDPFGMPMLDLDDDDDREESLMMNRLANVRKCTDRQNSPVTS
PGSSPLAQRRKPQPDPLQIPHLSLPPVPPRLDLIQKGIVRSPCGSPTGSPKSSPCMVRKQ
DKPLPAPPPPLRDPPPPPPERPPPIPPDNRLSRHIHHVESVPSRDPPMPLEAWCPRDVFG
TNQLVGCRLLGEGSPKPGITASSNVNGRHSRVGSDPVLMRKHRRHDLPLEGAKVFSNGHL
GSEEYDVPPRLSPPPPVTTLLPSIKCTGPLANSLSEKTRDPVEEDDDEYKIPSSHPVSLN
SQPSHCHNVKPPVRSCDNGHCMLNGTHGPSSEKKSNIPDLSIYLKGDVFDSASDPVPLPP
ARPPTRDNPKHGSSLNRTPSDYDLLIPPLGEDAFDALPPSLPPPPPPARHSLIEHSKPPG
SSSRPSSGQDLFLLPSDPFVDLASGQVPLPPARRLPGENVKTNRTSQDYDQLPSCSDGSQ
APARPPKPRPRRTAPEIHHRKPHGPEAALENVDAKIAKLMGEGYAFEEVKRALEIAQNNV
EVARSILREFAFPPPVSPRLNL
>Q9BEC7
MASPKKKLQGLVAATITPMTEHGEINFSVIGQYVDYLVEVQGVKNIFVNGTTGEGLSLSI
SERCQVAEEWVTKGRNKLDQIVIHVGALSLKESQELAQHAAKIGADGIAVIAPFFLKPWN
KDNLINFLKEVAAAAPALPFYYYHIPALTGVKIRAEELLDGIQDKIPTFQGLKFSDTDLL
DFGQCVDQNHQRQFAFLFGVDEQLLSALVMGATGAVGSTYNYLGRKTNQMLEAFERKDFS
SALNHQFCIQRFINFVVKLGFGVSQTKAIMTLVSGIPMGPPRLPLQKASREFTDNAKAKL
KSLDVLSFTDLKDGNLEAC
>P22133
MPHSVTPSIEQDSLKIAILGAAGGIGQSLSLLLKAQLQYQLKESNRSVTHIHLALYDVNQ
EAINGVTADLSHIDTPISVSSHSPAGGIENCLHNASIVVIPAGVPRKPGMTRDDLFNVNA
GIISQLGDSIAECCDLSKVFVLVISNPVNSLVPVMVSNILKNHPQSRNSGIERRIMGVTK
LDIVRASTFLREINIESGLTPRVNSMPDVPVIGGHSGETIIPLFSQSNFLSRLNEDQLKY
LIHRVQYGGDEVVKAKNGKGSATLSMAHAGYKCVVQFVSLLLGNIEQIHGTYYVPLKDAN
NFPIAPGADQLLPLVDGADYFAIPLTITTKGVSYVDYDIVNRMNDMERNQMLPICVSQLK
KNIDKGLEFVASRSASS
>Q03048
MSRSGVAVADESLTAFNDLKLGKKYKFILFGLNDAKTEIVVKETSTDPSYDAFLEKLPEN
DCLYAIYDFEYEINGNEGKRSKIVFFTWSPDTAPVRSKMVYASSKDALRRALNGVSTDVQ
GTDFSEVSYDSVLERVSRGAGSH
>Q0KL02

MKAMDVLPILKEKVAYLSGGRDKRGGPILTFPARSNHDRIRQEDLRRLISYLACIPSEEV
CKRGFTVIVDMRGSKWDSIKPLLKILQESFPCCIHIALIIKPDNFWQKQRTNFGSSKFEF
ETNMVSLEGLTKVVDPSQLTPEFDGCLEYNHEEWIEIRVAFEEYISNAAHMLSRLEELQD
VLAKKELPQDLEGARNMIDEHSQLKKKVIKAPIEDLDLEGQKLLQRIQSSDSFPKKNSGS
GNADLQNLLPKVSTMLDRLHSTRQHLHQMWHVRKLKLDQCFQLRLFEQDAEKMFDWITHN
KGLFLNSYTEIGTSHPHAMELQTQHNHFAMNCMNVYVNINRIMSVANRLVESGHYASQQI
KQIANQLEQEWKAFAAALDERSTLLDMSSIFHQKAEKYMSNVDSWCKACGEVDLPSELQD
LEDAIHHHQGIYEHITLAYSEVSQDGKSLLDKLQRPLTPGSSDSLTASANYSKAVHHVLD
VIHEVLHHQRQLENIWQHRKVRLHQRLQLCVFQQDVQQVLDWIENHGEAFLSKHTGVGKS
LHRARALQKRHEDFEEVAQNTYTNADKLLEAAEQLAQTGECDPEEIYQAAHQLEDRIQDF
VRRVEQRKILLDMSVSFHTHVKELWTWLEELQKELLDDVYAESVEAVQDLIKRFGQQQQT
TLQVTVNVIKEGEDLIQQLRDSAISSNKTPHNSSINHIETVLQQLDEAQSQMEELFQERK
IKLELFLQLRIFERDAIDIISDLESWNDELSQQMNDFDTEDLTIAEQRLQHHADKALTMN
NLTFDVIHQGQDLLQYVNEVQASGVELLCDRDVDMATRVQDLLEFLHEKQQELDLAAEQH
RKHLEQCVQLRHLQAEVKQVLGWIRNGESMLNAGLITASSLQEAEQLQREHEQFQHAIEK
THQSALQVQQKAEAMLQANHYDMDMIRDCAEKVASHWQQLMLKMEDRLKLVNASVAFYKT
SEQVCSVLESLEQEYKREEDWCGGADKLGPNSETDHVTPMISKHLEQKEAFLKACTLARR
NADVFLKYLHRNSVSMPGMVTHIKAPEQQVKNILNELFQRENRVLHYWTMRKRRLDQYQQ
YVVFERSAKQALEWIHDNGEFYLSTHTSTGSSIQHTQELLKEHEEFQITAKQTKERVKLL
IQLADGFCEKGHAHAAEIKKCVTAVDKRYRDFSLRMEKYRTSLEKALGISSDSNKSSKSL
QLDIIPASIPGSEVKLRDAAHELNEEKRKSARRKEFIMAELIQTEKAYVRDLRECMDTYL
WEMTSGVEEIPPGIVNKELIIFGNMQEIYEFHNNIFLKELEKYEQLPEDVGHCFVTWADK
FQMYVTYCKNKPDSTQLILEHAGSYFDEIQQRHGLANSISSYLIKPVQRITKYQLLLKEL
LTCCEEGKGEIKDGLEVMLSVPKRANDAMHLSMLEGFDENIESQGELILQESFQVWDPKT
LIRKGRERHLFLFEMSLVFSKEVKDSSGRSKYLYKSKLFTSELGVTEHVEGDPCKFALWV
GRTPTSDNKIVLKASSIENKQDWIKHIREVIQERTVHLRGALKEPIHIPKTAPAARQKGR
RDGEDLDSQGDGSSQPDTISIASRTSQNTLDSDKLSGGCELTVVIHDFTACNSNELTIRR
GQTVEVLERPHDKPDWCLVRTTDRSPAAEGLVPCGSLCIAHSRSSMEMEGIFNHKDSLSV
SSNDASPPASVASLQPHMIGAQSSPGPKRPGNTLRKWLTSPVRRLSSGKADGHAKKLAHK
HKKSREVRKSADAGSQKDSDDSAATPQDETIEERGRNEGLSSGTLSKSSSSGMQSCGEEE
GEEGADAVPLPPPMAIQQHSLLQPDSQDDKASSRLLVRPTSSETPSAAELVSAIEELVKS
KMALEDRPSSLLVDQGDSSSPSFNPSDNSLLSSSSPIDEMEERKCSSLKRRHYVLQELVE
TERDYVRDLGCVVEGYMALMKEDGVPDDMKGKDKIVFGNIHQIYDWHRDFFLGELEKCLE
DPEKLGSLFVKHERRLHMYIVYCQNKPKSEHIVSEYIDTFFEDLKQRLGHRLQLTDLLIK
PVQRIMKYQLLLKDFLKYSKKASLDTSELEKAVEVMCIVPKRCNDMMNVGRLQGFDGKIV
AQGKLLLQDTFLVTDQDAGLLPRCKERRVFLFEQIVIFSEPLDKKKGFSMPGFLFKNSIK
VSCLCLEENVESDPCKFALTSRTGDAVETFVLHSSSPSVRQTWIHEINQILENQRNFLNA
LTSPIEYQRNHSGGGGSGSGGSSGGGGGSGGSGASSGGSSSHGSGPSSCSSGPSSSRSRP
SRIPQPVRHHPPMLVSSAASSQAEADKMSGMSAPSPSLPTPSSSLALEASLGQPSRLPLS
GDSEGHERETEPIPKMKVMESPRKAPGSTSGTSQDGNTKDARGNLGSLPLGKTRPGAVSP
LNSPLSTTFPSPFGKEAFPPSSPLQKGGSFWSSIPASPASRPSSFTFPGDSDSLQRQTHR
HAAPSKDTDRMSTCSSASEQSVQSTQSNGEGSSSSNISTMLVTHEYTAVKEDEINVYQGE
VVQILASNQQNMFLVFRAATDQCPAAEGWIPGFVLGHTSAVIMENPDGTLKKSTSWHTAL
RLRKKSEKKDKDGKRDGKLENGYRKPREGLSNKVSVKLLNPNYIYDVPPEFVIPLSEVTC

ETGETVVFRCRVCGRPKASITWKGPEHNTLNNDDHYSISYSDIGEATLKIIGVSTEDDGI
YTCIAVNDMGSASSSASLRVLGPGSDGIVVTWKDNFDAFYSEVAELGRGRFAVVKKCDQK
GTKRAVATKFVNKKLMKRDQVTHELGILQNLQHPLLVSLLDTFETPTSYVLVLEMADQGR
LLDCVVRWGSLTEGKVRAHLGEVLEAVRYLHNCRIAHLDLKPENILVDQSLAKPTIKLAD
FGDAVQLNTTYYIHQLLGNPEFAAPEIILGNPVSLTADTWSVGVLTYVLLSGVSPFLDDS
VEETCLNICRLDFSFPEDYFQGVSQKAKEFVCFLLQEDPAKRPSAALALQEQWLQAGNGS
GKGTGVLDTSRLTSFIERRKHQNDVRPIRSIKNFLQSRLLPRV
>Q02733
MGGEDEILSTMEDFAAVYDVLVIGCGPGGFTAAMQASQAGLLTACVDQRASLGGAYLVDG
AVPSKTLLYESYLYRLLQQQELIEQRGTRLFPAKFDMQAAQSALKHNIEELGNVYKRELS
KNNVTVYKGTAAFKDPHHVEIAQRGMKPFIVEAKYIVVATGSAVIQCPGVAIDNDKIISS
DKALSLDYIPSRFTIMGGGTIGLEIACIFNNLGSRVTIVESQSEICQNMDNELASATKTL
LQCQGIAFLLDTRVQLAEADAAGQLNITLLNKVSKKTYVHHCDVLMVSIGRRPLLKGLDI
SSIGLDERDFVENVDVQTQSLLKYPHIKPIGDVTLGPMLALKAEEQAIRAIQSIGCTGSD
GTSNCGFPPNVLYCQPQIGWVGYTEEGLAKARIPYQKGRVLFSQNVRYNTLLPREENTTV
SPFIKVLIDSRDMKILGVHMINDDANELLSQASMAVSLGLTAHDVCKVPFPHPSLSESFK
QAVQLAMANGTSPGVHVRE
>P50542
MAMRELVEAECGGANPLMKLAGHFTQDKALRQEGLRPGPWPPGAPASEAASKPLGVASED
ELVAEFLQDQNAPLVSRAPQTFKMDDLLAEMQQIEQSNFRQAPQRAPGVADLALSENWAQ
EFLAAGDAVDVTQDYNETDWSQEFISEVTDPLSVSPARWAEEYLEQSEEKLWLGEPEGTA
TDRWYDEYHPEEDLQHTASDFVAKVDDPKLANSEFLKFVRQIGEGQVSLESGAGSGRAQA
EQWAAEFIQQQGTSDAWVDQFTRPVNTSALDMEFERAKSAIESDVDFWDKLQAELEEMAK
RDAEAHPWLSDYDDLTSATYDKGYQFEEENPLRDHPQPFEEGLRRLQEGDLPNAVLLFEA
AVQQDPKHMEAWQYLGTTQAENEQELLAISALRRCLELKPDNQTALMALAVSFTNESLQR
QACETLRDWLRYTPAYAHLVTPAEEGAGGAGLGPSKRILGSLLSDSLFLEVKELFLAAVR
LDPTSIDPDVQCGLGVLFNLSGEYDKAVDCFTAALSVRPNDYLLWNKLGATLANGNQSEE
AVAAYRRALELQPGYIRSRYNLGISCINLGAHREAVEHFLEALNMQRKSRGPRGEGGAMS
ENIWSTLRLALSMLGQSDAYGAADARDLSTLLTMFGLPQ
>P32588
MSENNEEQHQQQQQQPVAVETPSAVEAPASADPSSEQSVAVEGNSEQAEDNQGENDPSV
VPANAITGGRETSDRVLYVGNLDKAITEDILKQYFQVGGPIANIKIMIDKNNKNVNYAFV
EYHQSHDANIALQTLNGKQIENNIVKINWAFQSQQSSSDDTFNLFVGDLNVNVDDETLRN
AFKDFPSYLSGHVMWDMQTGSSRGYGFVSFTSQDDAQNAMDSMQGQDLNGRPLRINWAAK
RDNNNNNNYQQRRNYGNNNRGGFRQYNNNNNNNMNMGMNMNMNMNMNNSRGMPPSSMGMP
IGAMPLPSQGQPQQSQTIGLPPQVNPQAVDHIIRSAPPRVTTAYIGNIPHFATEADLIPL
FQNFGFILDFKHYPEKGCCFIKYDTHEQAAVCIVALANFPFQGRNLRTGWGKERSNFMPQ
QQQQGGQPLIMNDQQQPVMSEQQQQQQQQQQQ
>O74542
MTVSEGIARIQAFRKQVPLVEKNENITFFNLSFQPPMNSIVANTINEYVDWGLYNPNPKP
LWQQKAEETRELVAKYLNASSSDSIAFTRDTTEGLNLFQRSMHFQPGDNVVLLDGEHPNH
GFGWISLQEAGLEVRLIPSKDIYYADASTFEPYVDEKTKAIGISSVMFHSGQLNNVKDIC
NKFRPENIHVLVDMTQQVGLSSIDVQELNVSACAFACHKGLSCPTGLGVLYVAPDVLPQL
KNVPPIVGAGAIENLDANLLVNLNPIYFSTARRFEHLNKSLITTLCLNNYLSFLMDIGFE

NIEQYLRELGKSLVDELSKLGVTIIGSKDPKKRSTHSYVAKILNPEWDAFLKNEGVYASQ
YRDGIRLSLGLYNNAADISRLANTIKKGVLNKIPFN
>O94530
METKIQTVDTRLISFEKPSNDSEHPFEHTRVSIPPSETRSALENAANILRNTDYPVAFPT
ETVYGLGADARRTEAVLSIYKAKNRPADNPLIVHVASLDQLRRLLLSAYPKAKSEVKNQA
HDSEEIIPKVYLPLIKKFWPGPLSILLPVVDEANPPVSPIVTAGQKTFAVRMPQHPVALA
LISISDSPLAAPSANASTRPSPTLAKHVYNDLQGKIPLILDGGACGVGVESTVVNGLCDP
PVILRPGGISLEEIQSSGGAWERTKVFVAKKSDMETDFIPQTPGMKYRHYSPTAKVLLFV
NYTESDAYGVFEKYLSEQGITKEKQKIGVLCSKRWNEESFPSHCPFVFLHMGRDGHEITK
NLFAQLRDLDLQGVDFVLVEGVSEENEGLAIMNRLGKAASVVFEGPSH
>Q6ZVD8
MKRNGSRNCLNRRSRFGSRERDWLREDVKRGCVYLYGADTTTATTTTTTSSSSSSSSSSS
DLHLVLCTVETPASEICAGEGRESLYLQLHGDLVRRLEPTERPLQIVYDYLSRLGFDDPV
RIQEEATNPDLGCMIRFYGEKPCHMDRLDRILLSGIYNVRKGKTQLHKWAERLVVLCGTC
LIVSSVKDCQTGKMHILPLVGGKIEEVKRRQYSLAFSSAGAQAQTYHVSFETLAEYQRWQ
RQASKVVSQRISTVDLSCYSLEEVPEHLFYSQDITYLNLRHNFMQLERPGGLDTLYKFSQ
LKGLNLSHNKLGLFPILLCEISTLTELNLSCNGFHDLPSQIGNLLNLQTLCLDGNFLTTL
PEELGNLQQLSSLGISFNNFSQIPEVYEKLTMLDRVVMAGNCLEVLNLGVLNRMNHIKHV
DLRMNHLKTMVIENLEGNKHITHVDLRDNRLTDLDLSSLCSLEQLHCGRNQLRELTLSGF
SLRTLYASSNRLTAVNVYPVPSLLTFLDLSRNLLECVPDWACEAKKIEVLDVSYNLLTEV
PVRILSSLSLRKLMLGHNHVQNLPTLVEHIPLEVLDLQHNALTRLPDTLFSKALNLRYLN
ASANSLESLPSACTGEESLSMLQLLYLTNNLLTDQCIPVLVGHLHLRILHLANNQLQTFP
ASKLNKLEQLEELNLSGNKLKTIPTTIANCKRLHTLVAHSNNISIFPEILQLPQIQFVDL
SCNDLTEILIPEALPATLQDLDLTGNTNLVLEHKTLDIFSHITTLKIDQKPLPTTDSTVT
STFWSHGLAEMAGQRNKLCVSALAMDSFAEGVGAVYGMFDGDRNEELPRLLQCTMADVLL
EEVQQSTNDTVFMANTFLVSHRKLGMAGQKLGSSALLCYIRPDTADPASSFSLTVANVGT
CQAVLCRGGKPVPLSKVFSLEQDPEEAQRVKDQKAIITEDNKVNGVTCCTRMLGCTYLYP
WILPKPHISSTPLTIQDELLILGNKALWEHLSYTEAVNAVRHVQDPLAAAKKLCTLAQSY
GCQDNVGAMVVYLNIGEEGCTCEMNGLTLPGPVGFASTTTIKDAPKPATPSSSSGIASEF
SSEMSTSEVSSEVGSTASDEHNAGGLDTALLPRPERRCSLHPTPTSGLFQRQPSSATFSS
NQSDNGLDSDDDQPVEGVITNGSKVEVEVDIHCCRGRDLENSPPLIESSPTLCSEEHARG
SCFGIRRQNSVNSGMLLPMSKDRMELQKSPSTSCLYGKKLSNGSIVPLEDSLNLIEVATE
VPKRKTGYFAAPTQMEPEDQFVVPHDLEEEVKEQMKQHQDSRLEPEPHEEDRTEPPEEFD
TAL
>Q99LZ3
MTEVLDLHGQDSDGGSEEMVLTPAELIEKLEQAWMNEKFAPELLESKAEIVECVMEQLEH
MEENLRRAKKGDLKVSIHRMEMERIRYVLSSYLRCRLMKIEKFFPHILEKEKVRSEGEPS
SLSPEEFVFAKEYMDHTETHFKNVALKHMPPNLQKVDLLRAVPKPDLDSYVFLRVKERQE
NILVEPEADEQRDYVIDLEVGSQHLIRYKTIAPLVASGAVQLI
>P55265
MNPRQGYSLSGYYTHPFQGYEHRQLRYQQPGPGSSPSSFLLKQIEFLKGQLPEAPVIGKQ
TPSLPPSLPGLRPRFPVLLASSTRGRQVDIRGVPRGVHLGSQGLQRGFQHPSPRGRSLPQ
RGVDCLSSHFQELSIYQDQEQRILKFLEELGEGKATTAHDLSGKLGTPKKEINRVLYSLA
KKGKLQKEAGTPPLWKIAVSTQAWNQHSGVVRPDGHSQGAPNSDPSLEPEDRNSTSVSED

LLEPFIAVSAQAWNQHSGVVRPDSHSQGSPNSDPGLEPEDSNSTSALEDPLEFLDMAEIK
EKICDYLFNVSDSSALNLAKNIGLTKARDINAVLIDMERQGDVYRQGTTPPIWHLTDKKR
ERMQIKRNTNSVPETAPAAIPETRRNAEFLTCNIPTSNASNNMVTTEKVENGQEPVIKLE
NRQEARPEPARLKPPVHYNGPSKAGYVDFENGQWATDDIPDDLNSIRAAPGEFRAIMEMP
SFYSHGLPRCSPYKKLTECQLKNPISGLLEYAQFASQTCEFNMIEQSGPPHEPRFKFQVV
INGREFPPAEAGSKKVAKQDAAMKAMTILLEEAKAKDSGKSEESSHYSTEKESEKTAESQ
TPTPSATSFFSGKSPVTTLLECMHKLGNSCEFRLLSKEGPAHEPKFQYCVAVGAQTFPSV
SAPSKKVAKQMAAEEAMKALHGEATNSMASDNQPEGMISESLDNLESMMPNKVRKIGELV
RYLNTNPVGGLLEYARSHGFAAEFKLVDQSGPPHEPKFVYQAKVGGRWFPAVCAHSKKQG
KQEAADAALRVLIGENEKAERMGFTEVTPVTGASLRRTMLLLSRSPEAQPKTLPLTGSTF
HDQIAMLSHRCFNTLTNSFQPSLLGRKILAAIIMKKDSEDMGVVVSLGTGNRCVKGDSLS
LKGETVNDCHAEIISRRGFIRFLYSELMKYNSQTAKDSIFEPAKGGEKLQIKKTVSFHLY
ISTAPCGDGALFDKSCSDRAMESTESRHYPVFENPKQGKLRTKVENGEGTIPVESSDIVP
TWDGIRLGERLRTMSCSDKILRWNVLGLQGALLTHFLQPIYLKSVTLGYLFSQGHLTRAI
CCRVTRDGSAFEDGLRHPFIVNHPKVGRVSIYDSKRQSGKTKETSVNWCLADGYDLEILD
GTRGTVDGPRNELSRVSKKNIFLLFKKLCSFRYRRDLLRLSYGEAKKAARDYETAKNYFK
KGLKDMGYGNWISKPQEEKNFYLCPV
>O59722
MSEVETPTAASPTFPVETSHRLDTLHTSSTEQIIKDSENVVHTTLKLPTSTLSREFWMKD
ERTNNCSLCETEFTLFRRKHHCRICGKIICKYCLKEAPGFIFRLQGSIKVCRPCASILVN
NYSRSQLFNHSLNESKNRDLTEQHPFVTLDELNSNDQVLSSFGDLSSTFEMPNNIHPPEV
APMIAIPSSRSNYDSPGWAHHSIFLDWSKRNLDSNVINVEDSESGKYNALTITNSYDAGP
SSVSTDYRPVNFGKVPSYSKLRKNKAFSSAKVSDMYLSADERNRLEDFSKGDRGLSFVNL
SPNIKATSYDRLSTVINEPFISRSSSLTDERGLADSGNSYHHFSDSDDESLFNDGLGLSF
HANSAIIKQRQQNVASIQRYGNESYLSNFLKAFLPKTVCDYLFPSSTIPDGLPALIENFN
ARVDKVNHPGGTEEPLPYQGKSRASSVVTSSKSTCILPPWILFSDSFNQLVCTFLGKLLF
QMLNDEGVDSPMQWVLCLPKILLKMALDLGPDIRSGDDIDVRSYVKIKKIPGGSIQDCFL
VNGVLFSKKASSKSMDRSLRRPRIALLTFSLDYACDEQRILSLDLIISQQEEYIINLVNR
ICMLKPNLVFAQGQIPSIALKYFEEHGVIAFHGLKESVLYDIARCCRADIISSIDKLSLC
PRLGTCGRFQLRTYVVDENKGLRKTFAILDRCSERLGCTIVLRGADYNQLSKVKKIVELV
VLIAYHIKLECALLRDKFVNMPELFETTYQSLSRKSLPSFASTAADKEKSQNHEKKSLNS
DNQSLRPLENENQSVSSTQGSNSPLELINNLPASDDYSSITKALKTRFLTFSPFLSKPLP
RLLNQVNYYQFIRNKLLKDVKLHPYSPTGSFVMKQSENDNVEESYEESYKFFCIDERYHF
LEKQWTLYYSHSKLMFSPFSSQRIILLYSIINKETSVPCIGPERCLLEFYRETDCTLGQY
IEDSCLNTNVSCGGEYCKTNDMLWHYRSYVHGNSRISVFLESFSCPVPGLEEKIIMWSYC
KFCKKNTHITVMSEETWKYSFGKYLEFMFYNSQIRDRFEFCDHSVMAQHVHYFGYCNMAL
RFQRDLIEIFELFVPSVTLRNNPSYIKELKEKEYKRLKGVIEKCLSSVASRINQIKCDWV
TDPEKFESCTSEISKFRTLLSSDYTELYSEFDSIYLNSSTSDYLSLNSILRVLQGKMVKW
EQRFLDYQRLYLPSYKELSKIAAAQIKKVFLERPLSQTPLDLPETLENTQIDIYPSFKTE
STDDQLEKVTQTNVASNKRVAPYADSMANVGSPESDCFSVATSSDIPKANIDFTNDISTQ
NTFPASPVSNSGFSRQTYPNISQRQGVNMLSHKRKSASTSDRRFVNASSTSGMNMPISSS
ISAKISSIQNSTKYSPRKPIPAKDVRVSSLVRRFEELSLQLQEKQKRDEELIKARRKRAL
PVVPSKPVVEVFNDLNEAFDDENSEDENGINDTKENRATESNFSGVDSMSKERENVSSNE
DNSPEAFEDIFGILFKNESGLEEQQNLEPSSQMDKEGSKLPTSGPLADKTSVYRILSAFW

NEWNSLNPPPFEFPLQPTEHMFSDSNVIIREDEPSSLISFTLSSPDYLSKMVEIEDSMDE
ALTNQGLQGSTQFKIENLMLKPTGTHLKYQFEEGSARLSCKVFFAEQFSALRRACGCEET
FVTSLARCSLWESSGGKSGSAFLKTFDKKYILKVLSRLESDSLLNFAPAYFDYISKVFFH
ELPTALTKIFGFYRVDIRNPTTGTICKTDIMIMENVFYDECPSRIFDLKGSMRNRHVEST
GKVDEVLLDENLVELIYESPIFVSEQLKSLLHSCLWNDTLFLSKLNIMDYSLIVGIDYTK
KELYVGIIDFIRTYTWDKKLESWVKEKGLVGRGPEPTIVTPKQYKNRFRKAMDCYILASQ
DFETGEGFKFCE
>Q9Y3M8
MFSQVPRTPASGCYYLNSMTPEGQEMYLRFDQTTRRSPYRMSRILARHQLVTKIQQEIEA
KEACDWLRAAGFPQYAQLYEDSQFPINIVAVKNDHDFLEKDLVEPLCRRLNTLNKCASMK
LDVNFQRKKGDDSDEEDLCISNKWTFQRTSRRWSRVDDLYTLLPRGDRNGSPGGTGMRNT
TSSESVLTDLSEPEVCSIHSESSGGSDSRSQPGQCCTDNPVMLDAPLVSSSLPQPPRDVL
NHPFHPKNEKPTRARAKSFLKRMETLRGKGAHGRHKGSGRTGGLVISGPMLQQEPESFKA
MQCIQIPNGDLQNSPPPACRKGLPCSGKSSGESSPSEHSSSGVSTPCLKERKCHEANKRG
GMYLEDLDVLAGTALPDAGDQSRMHEFHSQENLVVHIPKDHKPGTFPKALSIESLSPTDS
SNGVNWRTGSISLGREQVPGAREPRLMASCHRASRVSIYDNVPGSHLYASTGDLLDLEKD
DLFPHLDDILQHVNGLQEVVDDWSKDVLPELQTHDTLVGEPGLSTFPSPNQITLDFEGNS
VSEGRTTPSDVERDVTSLNESEPPGVRDRRDSGVGASLTRPNRRLRWNSFQLSHQPRPAP
ASPHISSQTASQLSLLQRFSLLRLTAIMEKHSMSNKHGWTWSVPKFMKRMKVPDYKDKAV
FGVPLIVHVQRTGQPLPQSIQQALRYLRSNCLDQVGLFRKSGVKSRIHALRQMNENFPEN
VNYEDQSAYDVADMVKQFFRDLPEPLFTNKLSETFLHIYQYVSKEQRLQAVQAAILLLAD
ENREVLQTLLCFLNDVVNLVEENQMTPMNLAVCLAPSLFHLNLLKKESSPRVIQKKYATG
KPDQKDLNENLAAAQGLAHMIMECDRLFEVPHELVAQSRNSYVEAEIHVPTLEELGTQLE
ESGATFHTYLNHLIQGLQKEAKEKFKGWVTCSSTDNTDLAFKKVGDGNPLKLWKASVEVE
APPSVVLNRVLRERHLWDEDFVQWKVVETLDRQTEIYQYVLNSMAPHPSRDFVVLRTWKT
DLPKGMCTLVSLSVEHEEAQLLGGVRAVVMDSQYLIEPCGSGKSRLTHICRIDLKGHSPE
WYSKGFGHLCAAEVARIRNSFQPLIAEGPETKI
>Q8VHT6
MAAPRDAEIHKDVQNYYGNVLKTSADLQTNACVTPAKGVPEYIRKSLQNVHEEVISRYYG
CGLVVPEHLENCRILDLGSGSGRDCYVLSQLVGQKGHITGIDMTKVQVEVAKAYLEYHTE
KFGFQTPNVTFLHGQIEMLAEAGIQKESYDIVISNCVINLVPDKQKVLREVYQVLKYGGE
LYFSDVYASLEVSEDIKSHKVLWGECLGGALYWKDLAVIAKKIGFCPPRLVTANIITVGN
KELERVLGDCRFVSATFRLFKLPKTEPAGRCQVVYNGGIMGHEKELIFDANFTFKEGEAV
EVDEETAAILRNSRFAHDFLFTPVEASLLAPQTKVIIRDPFKLAEESDKMKPRCAPEGTG
GCCGKRKSC
>P00435
MCAAQRSAAALAAAAPRTVYAFSARPLAGGEPFNLSSLRGKVLLIENVASLUGTTVRDYT
QMNDLQRRLGPRGLVVLGFPCNQFGHQENAKNEEILNCLKYVRPGGGFEPNFMLFEKCEV
NGEKAHPLFAFLREVLPTPSDDATALMTDPKFITWSPVCRNDVSWNFEKFLVGPDGVPVR
RYSRRFLTIDIEPDIETLLSQGASA
>P0C0V8
MENDKGQLVELYVPRKCSATNRIIKADDHASVQINVAKVDEEGRAIPGEYVTYALSGYVR
SRGESDDSLNRLAQNDGLLKNVWSYSR
>P37880

MDGLVAQCSARLLQQEKEIKSLTAEIDRLKNCGHLEDSPSLDQLREENLKLKYRLNILQR
SLQAEKRRRPTKNMININSRLQDVFGCAIKAAYPDLDNPPLVVTPSQQPKFGDYQCNSAM
GISQMLKAKEQKVNPRGIAENITKHLPNNEYIDRVEIAGPGFINVHLRKDFVSEQLTNLL
VNGIQLPALGENKKVIVDFSSPNIAKEMHVGHLRSTIIGESMSRLFEFAGYDVLRLNHVG
DWGTQFGMLIAHLQDQFPDYLTVSPPIGDLQAFYKESKKRFDTEEEFKKRAYQCVVSLQS
KDPDFIKAWNLICDVSRAEFNKIYDALDITLIERGESFYQDRMKDIVKEFEDKGYVQVDD
GRKIVFVPGCSIPLTIVKSDGGFTYDTSDLAAIKQRLFEEKANKIIYVVDNGQAVHFQTI
FAAAQMIGWYDPKVTQVTHVGFGVVLGEDKKKFKTRSGETVRLVDLLGEGLKRSMDKLKE
KERDKVLTEEELTAAQTSIAYGCIKYADLSHNRLNDYIFSFDKMLDDRGNTAAYLLYAFT
RIRSIARLANVDEEMLQKAARETKIVLDHEKEWKLGRCILRFPEILQKMLDDLFLHTLCD
YIYELATTFTEFYDSCYCVEKDRQTGKVLKVNMWRMLLCEAVAAVMAKGFDILGIKPVQR
M
>O74411
MRLSSRFYLFTSFTAWSVVIAGATPAVCGAPEYIIETQNDLDVLSNCQVVNGSIIMNTSS
ATSLNFNRIETITGDLIIRNNNYLASVALTSISSVGGELRFEKLTRLASVYAPQLANVGQ
LTMRILPNLQGIRFDKGIKKAHKLTIEDTQLSTLAGISLNTTDTMVIVNNNYLREVDMPY
LESVESKFYVSYNAREISVTLPRLKTVGDMTIQRVAHLQLSNLEEVKGFLGFLNSTLQDI
SCPNITRIGQSLFFIGNQELTSLNFKQLESIGGTFMIANSAALAQIKGYDKLKTVAGSID
FTGNFSSVELPKLRDVKGGLNIQTTSSDFTCPFRQSDGIIKGKSFVCRGSVDNPRQSKES
SFDDDDFDELLGNGTIKGSKNSTSPVKQSGAAKVDSRPFRLVTFFLVLVSGFAHLL
>P48034
MEGGSELLFYVNGRKVTEKNVDPETMLLPYLRKKLRLTGTKYGCGGGGCGACTVMISRYN
PITKKIRHYPANACLTPICSLYGAAVTTVEGIGSTKTRIHPVQERIAKCHGTQCGFCTPG
MVMSLYTLLRNHPEPTLTQLNDALGGNLCRCTGYRPIINACKTFCKTSGCCQSKENGVCC
LDQGMNGLPEFEEGNETSLKLFSEEEFLPLDPTQELIFPPELMTMAEKKTQKTRIFGSDR
MTWISPVTLKELLEAKVKYPQAPVVMGNTSVGPDMKFKGIFHPVIISPDRIEELSVVNYT
DNGLTLGAAVSLAEVKDILANVTRKLPEEKTQMYHALLKHLETLAGPQIRNMASLGGHIV
SRHPDSDLNPLLAVGNCTLNLLSKEGRRQIPLNEQFLRKCPSADLKPEEILISVNIPYSR
KWEFVSAFRQAQRQQNALAIVNSGMRVCFGKGDGIIRELSIAYGGVGPTTILANNSCQKL
IGRPWNEEMLDAACRLILDEVSLPGSAPGGRVEFKRTLIVSFLFKFYLEVSQILKGMDLV
HYPSLASKYESALEDLHSRHYWSTLKYQNADLKQLSQDPIGHPIMHLSGIKHATGEAIYC
DDMPVVDRELFLTFVTSSRAHAKIVSIDVSAALSLPGVVDILTGEHLPGINTTFGFLTDA
DQLLSTDEVSCVGQLVCAVIADSEVQARRAAQQVKIVYQDLEPVILTIEEAIQNKSFFEP
ERKLEYGNVDEAFKMVDQILEGEIHMGGQEHFYMETQSMLVVPKGEDREIDVYVSAQFPK
YIQDITASVLKVSANKVMCHVKRVGGAFGGKVTKTGVLAAITAFAANKHGRPVRCILERG
EDILITGGRHPYLGKYKAGFMNDGRILALDMEHYNNAGAFLDESLFVIEMGLLKLENAYK
FPNLRCRGWACRTNLPSNTALRGFGFPQAGLITEACITEVAAKCGLPPEKVRMINMYKEI
DQTPYKQEINTKNLTQCWKECMATSSYTLRKAAVEKFNSENYWKKKGLAMVPLKYPIGLG
SVAAGQAAALVHIYLDGSVLVTHGGIEMGQGVHTKMIQVVSRELRMPLSSIHLRGTSTET
IPNTNPSGGSVVADLNGLAVKDACQTLLKRLKPIISKNPKGTWKDWAQAAFNESISLSAT
GYFRGYESNINWETGEGHPFEYFVYGAACSEVEIDCLTGAHKNIRTDIVMDVGYSINPAL
DVGQIEGAFIQGMGLYTIEELNYSPQGVLYTRGPNQYKIPAICDIPMELHISFLPPSENS
NTLYSSKGLGESGIFLGCSVFFAIHDAIRAARQERGLPGPLRLNSPLTPEKIRMACEDKF
TKMIPRDEPGSYVPWSVPI

>P41005

MVNTDNKENEPPNMEKAHMDSSNALYRVQRPLQRRPLQELSIELVKPSQTITVKKSKKST
NSSSYFAQLHAASGQNPPPSVHSSHKQPSKARSPNPLLSMR

>Q06152

MEESKTKRKEDRIDLKNTPPQKKSKRDSTNDETARTSLRSIMPRGYKMMENMGYKEGETL
GSNESALKEPIKVEINTKRRGIRAEKPDPSTMNDMQMSEQRFIKRESEMKNNKRLKKIWY
RIQKVAFEMMGDSDLYNPGEDPRDFNVLWRSYVMQLNEEVAKNDLNNHSNNDNEDKNNMI
PMVNESLEASPAIKGEKFGRPSTLIDCDTSIIGSRITKDTELAELEELSIEKRITKLNIF
LRSEKYYCFFCGIKYKDEGDLYEHCPGVNEDDHK

>Q8VED2

MEEGPAVGTLSREVSTEEAEPLGAAWSGDSGHVSQSHSSASGPWDDDGPEDAPGRDLPLL
RRAASGYASSLLPSAGPRPEVEALDASLEELLAKVDEFVGMLDMIRGDSSHVVGEGVPRI
HAKAAEMRRIYGRIDKLEAFVRMIGSSVARMEEQVAKAEAELGTFPRAFRRLLHTISVPA
LFRSAPSGPQRAAYEPPVLFRTEDHFPGCGDRPQL

>P53080

MSTDTMYFNSSRLLPSAGRNKTNNLIKQKTRNNRARGNAAKNANNNNYITDIPPPQTLPN
GQKPNFGHSSNKKPSFNQKKHSPPSSPSSTTTLGKKNRQNNKETPRQNNKDDTRLLSQNL
KNLLLNQKQSPHGSQGIIPMGCNGSAKKLSHSYAGSTFATNGPREAKNLPKPSFL

>O70305

MRSSTAAVQRPAAGDPEPRRPAGWAARRSLPRTARRGGRGGAVAYPSAGPPPRGPGAPPR
GPRSPPCASDCFGSNGHGASRPGSRRLLGVCGPPRPFVVVLLALAPAATPARACPPGVRA
SPPRSGVSSSARPAPGCPRPACEPVYGPLTMSLKPQPQPPAPATGRKPGGGLLSSPGAAP
ASAAVTSASVVPAPAAPVASSSAAAGGGRPGLGRGRNSSKGLPQPTISFDGIYANVRMVH
ILTSVVGSKCEVQVKNGGIYEGVFKTYSPKCDLVLDAAHEKSTESSSGPKREEIMESVLF
KCSDFVVVQFKDTDSSYARRDAFTDSALSAKVNGEHKEKDLEPWDAGELTASEELELEND
VSNGWDPNDMFRYNEENYGVVSTYDSSLSSYTVPLERDNSEEFLKREARANQLAEEIESS
AQYKARVALENDDRSEEEKYTAVQRNCSDREGHGPNTRDNKYIPPGQRNREVLSWGSGRQ
SSPRMGQPGPGSMPSRAASHTSDFNPNAGSDQRVVNGGVPWPSPCPSHSSRPPSRYQSGP
NSLPPRAATHTRPPSRPPSRPSRPPSHPSAHGSPAPVSTMPKRMSSEGPPRMSPKAQRHP
RNHRVSAGRGSMSSGLEFVSHNPPSEAAAPPVARTSPAGGTWSSVVSGVPRLSPKTHRPR
SPRQSSIGNSPSGPVLASPQAGIIPAEAVSMPVPAASPTPASPASNRALTPSIEAKDSRL
QDQRQNSPAGSKENVKASETSPSFSKADNKGMSPVVSEHRKQIDDLKKFKNDFRLQPSST
SESMDQLLSKNREGEKSRDLIKDKTEASAKDSFIDSSSSSSNCTSGSSKTNSPSISPSML
SNAEHKRGPEVTSQGVQTSSPACKQEKDDREEKKDTTEQVRKSTLNPNAKEFNPRSFSQP
KPSTTPTSPRPQAQPSPSMVGHQQPAPVYTQPVCFAPNMMYPVPVSPGVQPLYPIPMTPM
PVNQAKTYRAGKVPNMPQQRQDQHHQSTMMHPASAAGPPIVATPPAYSTQYVAYSPQQFP
NQPLVQHVPHYQSQHPHVYSPVIQGNARMMAPPAHAQPGLVSSSAAQFGAHEQTHAMYAC
PKLPYNKETSPSFYFAISTGSLAQQYAHPNAALHPHTPHPQPSATPTGQQQSQHGGSHPA
PSPVQHHQHQAAQALHLASPQQQSAIYHAGLAPTPPSMTPASNTQSPQSSFPAAQQTVFT
IHPSHVQPAYTTPPHMAHVPQAHVQSGMVPSHPTAHAPMMLMTTQPPGPKAALAQSALQP
IPVSTTAHFPYMTHPSVQAHHQQQL

>Q9Y6Q9

MSGLGENLDPLASDSRKRKLPCDTPGQGLTCSGEKRRREQESKYIEELAELISANLSDID
NFNVKPDKCAILKETVRQIRQIKEQGKTISNDDDVQKADVSSTGQGVIDKDSLGPLLLQA

LDGFLFVVNRDGNIVFVSENVTQYLQYKQEDLVNTSVYNILHEEDRKDFLKNLPKSTVNG
VSWTNETQRQKSHTFNCRMLMKTPHDILEDINASPEMRQRYETMQCFALSQPRAMMEEGE
DLQSCMICVARRITTGERTFPSNPESFITRHDLSGKVVNIDTNSLRSSMRPGFEDIIRRC
IQRFFSLNDGQSWSQKRHYQEAYLNGHAETPVYRFSLADGTIVTAQTKSKLFRNPVTNDR
HGFVSTHFLQREQNGYRPNPNPVGQGIRPPMAGCNSSVGGMSMSPNQGLQMPSSRAYGLA
DPSTTGQMSGARYGGSSNIASLTPGPGMQSPSSYQNNNYGLNMSSPPHGSPGLAPNQQNI
MISPRNRGSPKIASHQFSPVAGVHSPMASSGNTGNHSFSSSSLSALQAISEGVGTSLLST
LSSPGPKLDNSPNMNITQPSKVSNQDSKSPLGFYCDQNPVESSMCQSNSRDHLSDKESKE
SSVEGAENQRGPLESKGHKKLLQLLTCSSDDRGHSSLTNSPLDSSCKESSVSVTSPSGVS
SSTSGGVSSTSNMHGSLLQEKHRILHKLLQNGNSPAEVAKITAEATGKDTSSITSCGDGN
VVKQEQLSPKKKENNALLRYLLDRDDPSDALSKELQPQVEGVDNKMSQCTSSTIPSSSQE
KDPKIKTETSEEGSGDLDNLDAILGDLTSSDFYNNSISSNGSHLGTKQQVFQGTNSLGLK
SSQSVQSIRPPYNRAVSLDSPVSVGSSPPVKNISAFPMLPKQPMLGGNPRMMDSQENYGS
SMGGPNRNVTVTQTPSSGDWGLPNSKAGRMEPMNSNSMGRPGGDYNTSLPRPALGGSIPT
LPLRSNSIPGARPVLQQQQQMLQMRPGEIPMGMGANPYGQAAASNQLGSWPDGMLSMEQV
SHGTQNRPLLRNSLDDLVGPPSNLEGQSDERALLDQLHTLLSNTDATGLEEIDRALGIPE
LVNQGQALEPKQDAFQGQEAAVMMDQKAGLYGQTYPAQGPPMQGGFHLQGQSPSFNSMMN
QMNQQGNFPLQGMHPRANIMRPRTNTPKQLRMQLQQRLQGQQFLNQSRQALELKMENPTA
GGAAVMRPMMQPQVSSQQGFLNAQMVAQRSRELLSHHFRQQRVAMMMQQQQQQQQQQQQQ
QQQQQQQQQQQQQQQQQTQAFSPPPNVTASPSMDGLLAGPTMPQAPPQQFPYQPNYGMGQQ
PDPAFGRVSSPPNAMMSSRMGPSQNPMMQHPQAASIYQSSEMKGWPSGNLARNSSFSQQQ
FAHQGNPAVYSMVHMNGSSGHMGQMNMNPMPMSGMPMGPDQKYC
>A2AQ25
MEESEGQKCEPNLPPSGDSRQMPQQGRSNLHVTSQEDAACRRPRERLSNGNARAQVSKPA
RNIPRRHTLGGPRSSKEILGMQPSEMDRKREAFLEHLKQKYPHHATAIMGHQERLRDQTK
SPKLSHSPQPPNLGDPVEHLSETSGDSLEAMSEGEVPSPFARGSRTRASLPVVRSANQTK
ERSLGVLYLQYGDETKQLRMPNEVTSTDTIRALFVSAFPQQLTMKMLESPSVAIYIKDDS
RNVYYELNDVRNIQDRSLLKVYNKDPSHAFNHMTKAVNGDMRMQREIVYARGDGLVAPRP
GSVAHPPHVIPNSPPSTPVPHSLPPSPSRIPYGGSRPMAIPGNATIPRDRLSSLPVSRSI
SPSPSAILERRDVKPDEDMSSKNLVMFRNEGFYADPYLYHEGRMSIASSHGGHPLDVPDH
VIAYHRTAIRSASAYCSPSLQAEMHMEQSLYRQKSRKYPDSHLPTLGSKTPPASPHRVGD
LRMIDLHPHLNTHGPPHTLQPDRASPSRQSFKKEPGTLVYIEKPRNTSGLSSLVDLGPPL
VEKQGFAYSTTTIPKDRETRERMQAMEKQIASLTGLVQSALFKGPITSSSKEASSEKMVK
ATANRNQADGAGTAHVSAGKVLGSVEFSLPPSQPLPAGTSPIHTSLLDMRRNVAELRLQL
QQMRQLQLQNQEILRAMMKKAELEISNKVKETMKRLEDPVQRQRTLVEQERQKYLHEEER
IVKKLCELEDFVEDLKKDSSSTGRVVTLKDVEDGAFLLRQVGEAVATLKGEFPTLQNKMR
AVLRIEVEAVRFLKEEPHKLDSLLKRVRSMTDVLTMLRRHVTDGLLKGTDASQAAQYVAM
EKATAAEVLKHQEETAHAPGQPLHCSTGSPGDVKSEVVPLSTMTVHHVQSSPVVMQPSQH
SSALMNPAQNLPGGTRPHTASPPAITQEVTSAQSAPGPQSPQTPVNGSSMQSLFIEEIHS
VSAKNRAVSIEKAEKKWEEKRQNLEHYNGKEFEKLLEEAQANIMKSIPNLEMPPASSPVS
KGDAAGDKLELSEDSPNSEQELDKIGGKSPPPPPPPPRRSYLPGSGLTTTRSGDVVYTGR
SMSKVSSEDPGPTPQTRATKCPPEEPASAWAPSPPPVPAPSSKEEEEEEEEGDKIMAELQ
AFQKCSFMDVNPNSHAEQSRANSHLKDTRAGATAPPKEKKNLEFYHEDVRKSDVECENGP
QVESQKVTAGALRPSGPPKWERVMVDSISDTSRTSECRADTFTEENATPNKSLFRDSRNY

SQKNVPKVSFSSSGLNSLEGEINKGPNVSGLQCAIPDLENQKLNFGKTKEIGQQGQENAD
KSHIPLPTRSAEFSIHDVKTQDQDVPVTGYGQVVLRSKVGRHANMNMNEDGESTPSSPSE
EHTATDNIAFMITKTAVQVLSSGEVHDIVSQKGQDVQTVNIDGRKETASQHEGTEGEEPV
VCLDKKPVIIIFDEPMDIRSAYKRLSTIFEECDEELERMLTEEKIEEEEEDENEDSGVRT
SSQMSCEQVDSRSDRMGQKAETQSQPHVLSAELLTPGVQGVRKAEQRKLSSADSPDSGNK
CGMVDDQFESPKKKFKFKFPKKQLAALTQAIRTGTKTGKKTLQVVVYEEEEEDGTLKQHK
EAKRFEITRSQPEDALKTMARRQEQLSPEGTLPASRTDEIRKSTYRTLDSLEQTIKQLEN
TISEMSPRALVDTSCSSNRDCGASLPHMAQEVSPRSLLVLDEVPPAPEPPTSISPASRKG
SSTTPQTSRMPVPMTSKNRPGSLDKASKQSKLQDPRQYRQANGSAKKAGGDCKPTSPSLP
ASKIPALSPSSGKSSSLPSASGDSSNLPNAPATKPSIASTPLSPQAGRSAHSASLIPSVS
NGSLKFQSPPHAGKGHHHLSFALQTQNGRAAPTTSSSSSPPSPASPTSLNQGARGIRTIH
TPSLASYKAQNGSSSKATPSTAKETS
>P38707
MSSLYIKEATGVDELTTAGSQDHPFKTPAYALFASQQKSDATEPKLFVFKTEDNEYQEIS
ASALKKARKGCDGLKKKAVKQKEQELKKQQKEAENAAKQLSALNITIKEDESLPAAIKTR
IYDSYSKVGQRVKVSGWIHRLRSNKKVIFVVLRDGSGFIQCVLSGDLALAQQTLDLTLES
TVTLYGTIVKLPEGKTAPGGVELNVDYYEVVGLAPGGEDSFTNKIAEGSDPSLLLDQRHL
ALRGDALSAVMKVRAALLKSVRRVYDEEHLTEVTPPCMVQTQVEGGSTLFKMNYYGEEAY
LTQSSQLYLETCLASLGDVYTIQESFRAEKSHTRRHLSEYTHIEAELAFLTFDDLLQHIE
TLIVKSVQYVLEDPIAGPLVKQLNPNFKAPKAPFMRLQYKDAITWLNEHDIKNEEGEDFK
FGDDIAEAAERKMTDTIGVPIFLTRFPVEIKSFYMKRCSDDPRVTESVDVLMPNVGEITG
GSMRIDDMDELMAGFKREGIDTDAYYWFIDQRKYGTCPHGGYGIGTERILAWLCDRFTVR
DCSLYPRFSGRCKP
>P70460
MSETVICSSRATVMLYDDSNKRWLPAGTGPQAFSRVQIYHNPTANSFRVVGRKMQPDQQV
VINCAIIRGVKYNQATPIFHQWRDARQVWGLNFGSKEDAIQFATGMANALEALEGGGPPP
APAPPAWSAQNGPSPEELEQQKRQPEHMERRVSNAGGPPAPPAGGPPPPPGPPPPPGPPP
PPGLPSSGVSGAGHGAGAAPPPAPPLPTAQGPNSGGSGAPGLAAAIAGAKLRKVSKQEEA
SGGPLAPKAENSRSTGGGLMEEMNAMLARRRKATQVGEKPPKDESASEESEARLPAQSEP
VRRPWEKNSTTLPRMKSSSSVTTSEAHPSTPCSSDDSDLERVKQELLEEVRKELQKMKEE
IIEVFVQELRKRGSP
>Q9URV3
MKKENWQKIENVYLLGKKIDKAKRMANKGESNTNMLSTEENIVKAKAVSRKKPVDVSYAG
LARHESHVDTDYHGQALNTDNQDPKNYQARSFVKENELYESSQDCGHSRLAQILSEENQI
KRLPQSDNTTTERIIENNPNYSNKPSKLKRALSGENSVFSIHQRNTLSPSSNLPRIPSDL
YDIKQFLENLDARSFKSDMDLKRSQNMRRRATGYPAIVVPFLNGSLPQADLPPLRTIEDI
DNLTREQCLTFIQGYGIPIDSSDTVYLKEKLRDAIGMRAFTDMSFEMNSFHLESPR
>P40413
MAARPQQPPMEMPDLSNAIVAQDEMGRPFIIVKDQGNKKRQHGLEAKKSHILAARSVASI
IKTSLGPRGLDKILISPDGEITITNDGATILSQMELDNEIAKLLVQLSKSQDDEIGDGTT
GVVVLASALLDQALELIQKGIHPIKIANGFDEAAKLAISKLEETCDDISASNDELFRDFL
LRAAKTSLGSKIVSKDHDRFAEMAVEAVINVMDKDRKDVDFDLIKMQGRVGGSISDSKLI
NGVILDKDFSHPQMPKCVLPKEGSDGVKLAILTCPFEPPKPKTKHKLDISSVEEYQKLQT
YEQDKFKEMIDDVKKAGADVVICQWGFDDEANHLLLQNDLPAVRWVGGQELEHIAISTNG

RIVPRFQDLSKDKLGTCSRIYEQEFGTTKDRMLIIEQSKETKTVTCFVRGSNKMIVDEAE
RALHDSLCVVRNLVKDSRVVYGGGAAEVTMSLAVSEEADKQRGIDQYAFRGFAQALDTIP
MTLAENSGLDPIGTLSTLKSKQLKEKISNIGVDCLGYGSNDMKELFVVDPFIGKKQQILL
ATQLCRMILKIDNVIISGKDEY
>P48570
MTAAKPNPYAAKPGDYLSNVNNFQLIDSTLREGEQFANAFFDTEKKIEIARALDDFGVDY
IELTSPVASEQSRKDCEAICKLGLKAKILTHIRCHMDDAKVAVETGVDGVDVVIGTSKFL
RQYSHGKDMNYIAKSAVEVIEFVKSKGIEIRFSSEDSFRSDLVDLLNIYKTVDKIGVNRV
GIADTVGCANPRQVYELIRTLKSVVSCDIECHFHNDTGCAIANAYTALEGGARLIDVSVL
GIGERNGITPLGGLMARMIVAAPDYVKSKYKLHKIRDIENLVADAVEVNIPFNNPITGFC
AFTHKAGIHAKAILANPSTYEILDPHDFGMKRYIHFANRLTGWNAIKARVDQLNLNLTDD
QIKEVTAKIKKLGDVRSLNIDDVDSIIKNFHAEVSTPQVLSAKKNKKNDSDVPELATIPA
AKRTKPSA
>Q64008
MNILAPVRRDRVLAELPQCLKKEAALHVRKDFHPRVTCACQEHRTGTVGFKISKVIVVGD
LSVGKTCLINRFCKDTFDKNYKATIGVDFEMERFEVLGVPFSLQLWDTAGQERFKCIAST
YYRGAQAIIIVFNLNDVASLEHTKQWLTDALKENDPSNVLLFLVGSKKDLSTPAQYSLME
KDALKVAQEIKAEYWAVSSLTGENVREFFFRVAALTFEANVLADVEKSGARHIADVVRIN
SDDKNLYLTASKKKATCCP
>Q96DU7
MRRCPCRGSLNEAEAGALPAAARMGLEAPRGGRRRQPGQQRPGPGAGAPAGRPEGGGPWA
RTEGSSLHSEPERAGLGPAPGTESPQAEFWTDGQTEPAAAGLGVETERPKQKTEPDRSSL
RTHLEWSWSELETTCLWTETGTDGLWTDPHRSDLQFQPEEASPWTQPGVHGPWTELETHG
SQTQPERVKSWADNLWTHQNSSSLQTHPEGACPSKEPSADGSWKELYTDGSRTQQDIEGP
WTEPYTDGSQKKQDTEAARKQPGTGGFQIQQDTDGSWTQPSTDGSQTAPGTDCLLGEPED
GPLEEPEPGELLTHLYSHLKCSPLCPVPRLIITPETPEPEAQPVGPPSRVEGGSGGFSSA
SSFDESEDDVVAGGGGASDPEDRSGSKPWKKLKTVLKYSPFVVSFRKHYPWVQLSGHAGN
FQAGEDGRILKRFCQCEQRSLEQLMKDPLRPFVPAYYGMVLQDGQTFNQMEDLLADFEGP
SIMDCKMGSRTYLEEELVKARERPRPRKDMYEKMVAVDPGAPTPEEHAQGAVTKPRYMQW
RETMSSTSTLGFRIEGIKKADGTCNTNFKKTQALEQVTKVLEDFVDGDHVILQKYVACLE
ELREALEISPFFKTHEVVGSSLLFVHDHTGLAKVWMIDFGKTVALPDHQTLSHRLPWAEG
NREDGYLWGLDNMICLLQGLAQS
>Q8TD19
MSVLGEYERHCDSINSDFGSESGGCGDSSPGPSASQGPRAGGGAAEQEELHYIPIRVLGR
GAFGEATLYRRTEDDSLVVWKEVDLTRLSEKERRDALNEIVILALLQHDNIIAYYNHFMD
NTTLLIELEYCNGGNLYDKILRQKDKLFEEEMVVWYLFQIVSAVSCIHKAGILHRDIKTL
NIFLTKANLIKLGDYGLAKKLNSEYSMAETLVGTPYYMSPELCQGVKYNFKSDIWAVGCV
IFELLTLKRTFDATNPLNLCVKIVQGIRAMEVDSSQYSLELIQMVHSCLDQDPEQRPTAD
ELLDRPLLRKRRREMEEKVTLLNAPTKRPRSSTVTEAPIAVVTSRTSEVYVWGGGKSTPQ
KLDVIKSGCSARQVCAGNTHFAVVTVEKELYTWVNMQGGTKLHGQLGHGDKASYRQPKHV
EKLQGKAIRQVSCGDDFTVCVTDEGQLYAFGSDYYGCMGVDKVAGPEVLEPMQLNFFLSN
PVEQVSCGDNHVVVLTRNKEVYSWGCGEYGRLGLDSEEDYYTPQKVDVPKALIIVAVQCG
CDGTFLLTQSGKVLACGLNEFNKLGLNQCMSGIINHEAYHEVPYTTSFTLAKQLSFYKIR
TIAPGKTHTAAIDERGRLLTFGCNKCGQLGVGNYKKRLGINLLGGPLGGKQVIRVSCGDE

FTIAATDDNHIFAWGNGGNGRLAMTPTERPHGSDICTSWPRPIFGSLHHVPDLSCRGWHT
ILIVEKVLNSKTIRSNSSGLSIGTVFQSSSPGGGGGGGGGEEEDSQQESETPDPSGGFRG
TMEADRGMEGLISPTEAMGNSNGASSSCPGWLRKELENAEFIPMPDSPSPLSAAFSESEK
DTLPYEELQGLKVASEAPLEHKPQVEASSPRLNPAVTCAGKGTPLTPPACACSSLQVEVE
RLQGLVLKCLAEQQKLQQENLQIFTQLQKLNKKLEGGQQVGMHSKGTQTAKEEMEMDPKP
DLDSDSWCLLGTDSCRPSL
>Q9NQI0
MGDEDWEAEINPHMSSYVPIFEKDRYSGENGDNFNRTPASSSEMDDGPSRRDHFMKSGFA
SGRNFGNRDAGECNKRDNTSTMGGFGVGKSFGNRGFSNSRFEDGDSSGFWRESSNDCEDN
PTRNRGFSKRGGYRDGNNSEASGPYRRGGRGSFRGCRGGFGLGSPNNDLDPDECMQRTGG
LFGSRRPVLSGTGNGDTSQSRSGSGSERGGYKGLNEEVITGSGKNSWKSEAEGGESSDTQ
GPKVTYIPPPPPEDEDSIFAHYQTGINFDKYDTILVEVSGHDAPPAILTFEEANLCQTLN
NNIAKAGYTKLTPVQKYSIPIILAGRDLMACAQTGSGKTAAFLLPILAHMMHDGITASRF
KELQEPECIIVAPTRELVNQIYLEARKFSFGTCVRAVVIYGGTQLGHSIRQIVQGCNILC
ATPGRLMDIIGKEKIGLKQIKYLVLDEADRMLDMGFGPEMKKLISCPGMPSKEQRQTLMF
SATFPEEIQRLAAEFLKSNYLFVAVGQVGGACRDVQQTVLQVGQFSKREKLVEILRNIGD
ERTMVFVETKKKADFIATFLCQEKISTTSIHGDREQREREQALGDFRFGKCPVLVATSVA
ARGLDIENVQHVINFDLPSTIDEYVHRIGRTGRCGNTGRAISFFDLESDNHLAQPLVKVL
TDAQQDVPAWLEEIAFSTYIPGFSGSTRGNVFASVDTRKGKSTLNTAGFSSSQAPNPVDD
ESWD
>Q12342
MILDPLSPNIENHTQDEIIEFWEKTESIANIPKENLDESHVNSSLVAYLKFATDSYKVFI
NTDRDLYRMSLILLESSLFEFKKEFCLSKLQSLLNIDLLEMNMKFIIVYILLCEAKKNVY
SLEIMLKFQGFTVFYNTLYTQFAYLSKYGKERTVASKHQYNSNNSSTGTSLDSLDRSLTD
IDLGIIDEMKQISTVLMDLLFQIMKYCKCVIANLQIVDDFFVYYMMESMRSDTMDDMFNN
AEFKLLLALNEQYMMFAKEYDIENKVYKYLINGSVSRCFTELLLLKFNRASDPPLQIMMC
KIIYLILTPRGDYSPMNFFYTNDLRVLIDVLIRELQNISEDEEVLRNTLLRVLIPLLKNT
QLSKTHYRKDDLNKLLNYLSTLDNICVDSPALHEHQVTVALSRKCLQQIPWLETPSTPSD
GGSSVSSNNTSRNSSIVALGTPDNQNILARKGHLYSNRELDVSAESLTKRKAKAPPPPPP
PPPSRKCGTPK
>O35129
MAQNLKDLAGRLPAGPRGMGTALKLLLGAGAVAYGVRESVFTVEGGHRAIFFNRIGGVQQ
DTILAEGLHFRIPWFQYPIIYDIRARPRKISSPTGSKDLQMVNISLRVLSRPNAQELPSM
YQRLGLDYEERVLPSIVNEVLKSVVAKFNASQLITQRAQVSLLIRRELTERAKDFSLILD
DVAITELSFSREYTAAVEAKQVAQQEAQRAQFLVEKAKQEQRQKIVQAEGEAEAAKMLGE
ALSKNPGYIKLRKIRAAQNISKTIATSQNRIYLTADNLVLNLQDESFTRGSDSLIKGKK
>P13693
MIIYRDLISHDEMFSDIYKIREIADGLCLEVEGKMVSRTEGNIDDSLIGGNASAEGPEGE
GTESTVITGVDIVMNHHLQETSFTKEAYKKYIKDYMKSIKGKLEEQRPERVKPFMTGAAE
QIKHILANFKNYQFFIGENMNPDGMVALLDYREDGVTPYMIFFKDGLEMEKC
>Q64514
MATAATEEPFPFHGLLPKKETGASSFLCRYPEYDGRGVLIAVLDTGVDPGAPGMQVTTDG
KPKIIDIIDTTGSGDVNTATEVEPKDGEIIGLSGRVLKIPANWTNPLGKYHIGIKNGYDF
YPKALKERIQKERKEKIWDPIHRVALAEACRKQEEFDIANNGSSQANKLIKEELQSQVEL

LNSFEKKYSDPGPVYDCLVWHDGETWRACVDSNENGDLSKCAVLRNYKEAQEYSSFGTAE
MLNYSVNIYDDGNLLSIVTSGGAHGTHVASIAAGHFPEEPERNGVAPGAQILSIKIGDTR
LSTMETGTGLIRAMIEVINHKCDLVNYSYGEATHWPNSGRICEVINEAVWKHNTIYVSSA
GNNGPCLSTVGCPGGTTSSVIGVGAYVSPDMMVAEYSLREKLPANQYTWSSRGPSADGAL
GVSISAPGGAIASVPNWTLRGTQLMNGTSMSSPNACGGIALVLSGLKANNVDYTVHSVRR
ALENTAIKADNIEVFAQGHGIIQVDKAYDYLIQNTSFANRLGFTVTVGNNRGIYLRDPVQ
VAAPSDHGVGIEPVFPENTENSEKISFQLHLALTSNSSWVQCPSHLELMNQCRHINIRVD
PRGLREGLHYTEVCGYDIASPNAGPLFRVPITAVIAAKVNESSHYDLAFTDVHFKPGQIR
RHFVEVPEGATWAEVTVCSCSSEVSAKFVLHAVQLVKQRAYRSHEFYKFCSLPEKGTLIE
AFPVLGGKAIEFCIARWWASLSDVNIDYTISFHGIVCTAPQLNIHASEGINRFDVQSSLK
YEDLAPCITLKSWVQTLRPVNAKTRPLGSRDVLPNNRQLYEMVLTYSFHQPKSGEVTPSC
PLLCELLYESEFDSQLWIIFDQNKRQMGSGDAYPHQYSLKLEKGDYTIRLQIRHEQISDL
DRLKDLPFIVSHRLSNTLSLDIHENHSLALLGKKKSSSLTLPPKYNQPFFVTSLPDDKIP
KGAGPGCYLAGSLTLSKTELGKKAGQSAAKRQGKFKKDVIPVHYYLIPPPTKIKNGSKDK
EKDSEKEKDLKEEFTEALRDLKIQWMTKLDSTDIYNELKETYPAYLPLYVARLHQLDAEK
ERMKRLNEIVDAANAVISHIDQTALAVYIAMKTDPRPDAATIKNDMDKQKSTLIDALCRK
GCALADHLLHTQPHDGAAAGDAEAKEEEGESTMESLSETYWETTKWTDLFDTKVLIFAYK
HALVNKMYGRGLKFATKLVEEKPTKENWKNCIQLMKLLGWTHCASFTENWLPIMYPPDYC
VF
>Q7LKZ6
MQESSQEEPVHYVQWFYLSNYDLYDETSSMELTGAQSWRRFLNYDNNALEAKYNEIITEE
VSQEPNQKNVIVGISGLHVVNLPTLTLKPLYWPSASKNDTLRVVRGLWMYEDTGLPVINE
LSDMLEEGYQEVKPYAVNWDNVLKEEEEGKDSQDDPSVLSHKRNKSSKELIDAMQWPLKP
TSSGKYVFYEDGYRALICQGGLLSYFSKGTQSRRTSIKGMPVVRDFPYFEDDGFNGPKQV
TDLFLVVHGIGQKRSETEERFLFTKTCNVFRSLIQIQKNIMKEDPLIRNDYEPQLLPICW
RNKLNFNSYIKPVAGDEGRVEDEEFEENRFSIEDIEIDSIPAVRRLLGDVMSDIPYYMSH
HKESIIKSVIREANRVYHLWKDCNPYFLENKGRIFIIGHSLGSTVVFDILSLQPTFVKEI
TIDDDESCFIFDTNGFFCFGGPVGFFLHLNQQSIIPRRGRGSSRYEINKNFINSDVPKSY
TYDGYDRYGCLAVDSFYNIYNHLDPVAMRLNPTVDLSFSKGIHPTRILFTRKSSSILRMI
SHSNTDPVELRSYNQSLQQNKNNEPTAVVPLEPEQTVELETRNFRREERAKWRMQELNEN
SQLDYVFTSAHGVISNKYLSMLSAHSSYWSSEDLACFLVVETGRNFGIANSIEQFRGKHM
PRIFKGDSS
>Q91WM1
MRSIRSFANDDRHVMVKHSTIYPSPEELEAVQNMVSTVECALKHVSDWLDETNKGTKPEG
ETEVKKDEAVENYSKDQGGRTLCGVMRIGLVAKGLLIKDDMDLELVLMCKDKPTETLLNT
VKDNLPIQIQKLTEEKYQVEQCINEASIIIRNTKEPTLTLKVILTSPLIRDELEKKDGEN
VMMKDPPDLLDRQKCLNALASLRHAKWFQARANGLKSCVIVLRILRDLCNRVPTWAPLKG
WPLELICEKSIGTCNRPLGAGEALRRVMECLASGILLPGGPGLHDPCERDPTDALSYMTT
QQKEDITHSAQHALRLSAFGQIYKVLEMDPLPSSKPFQKYSWSVTDKEGAGSSALKRPFE
DGLGDDKDPNKKMKRNLRKILDSKAIDLMNALMRLNQIRPGLQYKLLSQSGPVHAPVFTM
SVDVDGTTYEASGPSKKTAKLHVAVKVLQAMGYPTGFDADIECISSDEKSDNESKNDTVS
SNSSNNTGNSTTETSSTLEVRTQGPILTASGKNPVMELNEKRRGLKYELISETGGSHDKR
FVMEVEVDGQKFRGAGPNKKVAKASAALAALEKLFSGPNAANNKKKKIIPQAKGVVNTAV
SAAVQAVRGRGRGTLTRGAFVGATAAPGYIAPGYGTPYGYSTAAPAYGLPKRMVLLPVMK

FPTYPVPHYSFF
>Q8WXI4
MIQNVGNHLRRGLASVFSNRTSRKSALRAGNDSAMADGEGYRNPTEVQMSQLVLPCHTNQ
RGELSVGQLLKWIDTTACLSAERHAGCPCVTASMDDIYFEHTISVGQVVNIKAKVNRAFN
SSMEVGIQVASEDLCSEKQWNVCKALATFVARREITKVKLKQITPRTEEEKMEHSVAAER
RRMRLVYADTIKDLLANCAIQGDLESRDCSRMVPAEKTRVESVELVLPPHANHQGNTFGG
QIMAWMENVATIAASRLCRAHPTLKAIEMFHFRGPSQVGDRLVLKAIVNNAFKHSMEVGV
CVEAYRQEAETHRRHINSAFMTFVVLDADDQPQLLPWIRPQPGDGERRYREASARKKIRL
DRKYIVSCKQTEVPLSVPWDPSNQVYLSYNNVSSLKMLVAKDNWVLSSEISQVRLYTLED
DKFLSFHMEMVVHVDAAQAFLLLSDLRQRPEWDKHYRSVELVQQVDEDDAIYHVTSPALG
GHTKPQDFVILASRRKPCDNGDPYVIALRSVTLPTHRETPEYRRGETLCSGFCLWREGDQ
LTKCCWVRVSLTELVSASGFYSWGLESRSKGRRSDGWNGKLAGGHLSTLKAIPVAKINSR
FGYLQDT
>Q15121
MAEYGTLLQDLTNNITLEDLEQLKSACKEDIPSEKSEEITTGSAWFSFLESHNKLDKDNL
SYIEHIFEISRRPDLLTMVVDYRTRVLKISEEDELDTKLTRIPSAKKYKDIIRQPSEEEI
IKLAPPPKKA
>Q01984
MASFMRSLFSDHSRYVESFRRFLNNSTEHQCMQEFMDKKLPGIIARIGETKAEIKILSIG
GGAGEIDLQILSKVQAQYPGICINNEVVEPNAEQIVKYKELVAKTSNMENIKFAWHKETS
SEYQKRVVEEDEEPPKWDFIHMIQMLYYVKDIPATLKFFHGLLAANAKILIIILVSGTSGW
EKLWKKYGFRLPRDDLCQYVTSSDLAQILDDLGIKYECYDLLSTMDITDCFIDGNENGDL
LWDFLTETCNFIKTAPLDLKEEIMKDLQEPEFSVKKEGKVLFNNNLSFIVVEANV
>Q9BYI3
MFTSEKGVVEEWLSEFKTLPETSLPNYATNLKDKSSLVSSLYKVIQEPQSELLEPVCHQL
FEFYRSGEEQLLQFTLQFLPELIWCYLAVSASRNVHSSGCIEALLLGVYNLEIVDKQGHT
KVLSFTIPSLSKPSVYHEPSSIGSMALTESALSQHGLSKVVYSGPHPQREMLTAQNRFEV
LTFLLLCYNAALTYMPSVSLQSLCQICSRICVCGYPRQHVRKYKGISSRIPVSSGFMVQM
LTGIYFAFYNGEWDLAQKALDDIIYRAQLELYPEPLLVANAIKASLPHGPMKSNKEGTRC
IQVEITPTSSRISRNAVTSMSIRGHRWKRHGNTELTGQEELMEISEVDEGFYSRAASSTS
QSGLSNSSHNCSNKPSIGKNHRRSGGSKTGGKEKETTGESCKDHFARKQTQRAQSENLEL
LSLKRLTLTTSQSLPKPSSHGLAKTAATVFSKSFEQVSGVTVPHNPSSAVGCGAGTDANR
FSACSLQEEKLIYVSERTELPMKHQSGQQRPPSISITLSTD
>O14170
MSLSRCPTDNSSSRINSSVPLINSSSPATPPESFDPQVFPSSLIHGDNLLPQDDQIASDP
RSESNSCNGNTSSSLPCTDSYQYPLKHSCTPSFLRKFNESIENVSYKCLDHSPPDSVPGD
FSISLVPQRNFLYSHSSLPPKIISIDRNNRIKLDNSISSNSDNFPPSPKVDTSNTVSPGS
KPISEDLEDLNLQSIVQTFEDLPEGIQSYAFFQLLRSCNRQSMRLLLNECEPLLKKDILS
NLPFSIVQSILLNLDIHSFLSCRLVSPTWNRILDVHTSYWKHMFSLFGFQINENDWKYAN
PNLNRPPFLHNDQISDDYFPEIFKRHFLNRKRWLFPSIPPSHLSFPIHVPNFMITSLLLH
KDRIITTSGSGTIQIHNAITGVLEARLEGHKEGVWAVKIHENTLVSGSIDKTVRVWNIEK
AKCTHIFRGHISIIRCLEILVPSRLIRHGVEIVEPDQPYIVSGSRDHTLRVWKLPKNTDP
PYLPDNTNSIDRWEKNPYFVHTLIGHTDSVRTISGYGDILVSGSYDSSIRIWRVSTGECL
YHLRGHSLRIYSVLYEPERNICISGSMDKSIRVWDLSTGTCKYVLEGHDAFVTLLNVFQN

RLISGSADSTIRIWDLNTGKPLMVLPSNSGYISSFVSDEHKIISGNDGSVKLWDVRTGKL
LRFLLTDLTKIWHVDFDAMRCVAAVQRDDQAYLEVINFSGSRP
>Q10447
MLSNSTFHEHHAKSHFHNNACQSNASSSACRASEDHLVSSFPNDSIIDLQPSRPAPEPPK
KKFGYYARRLSGHFLSLIHGSGNSTRSPPFHLQNQKSNGQSEVWHSSDDSGSPKRLNRSR
SSKEDMYRRRSLHGLPSLSRRNSKKSSTLSRSISLHLRSESAPISLPIHLYKSYSYNHSP
SSLPTVLNSQALSSPPVPTTPDEVSTNRLSSSTSSMNCRNLVPDNFNISIRPNTTNYRSS
IQENSNGNRDSISPSAYDAPLLHNVDTQSIDGFVSVASHFSSASTAESLDDGHSATTIQQ
GDVSSYPLSRSVSTPVPMSPISISPAKPSPQSPKLSQSAVGHPSSSIPAAAMHKVSYSDD
LMRFVAREKYYLQIVDCLCTQKDPLFFYTDFTKICQQDTVGTYVARQTLDKEVVVIKRFD
ISAVTHRRLLLEELQRLSGLSHKNLIRYNESFWYLNNIWSVFEYKDPSTKLSALIPKYFF
SELNIASICYEISSGLAFLHNSGIAHHNLTTECIYLTKSSCLKIGNYAFSSPYIERQTNR
GAVSHVPDWLIEKNYKEGFMKDVKSLGLVALEIFQGQPNFFRKSIQSIQLTPNANVLVNR
VRGLISQEFKEFLLQTLQAETLQGPNINMLLETSSFLEKRQTLNFEICLNNLNLRERKAS
RYSYL
>P40326
MRVFRHNSMVYTRFMGIFWDCLVLDLKPLYSLDGSVTIKDGGGGYTVENDMFVCSALFPL
LKGGKRKINGKKRLSHRENTLVVAKHQIGRQEEMAVKCQTKHTFTGLI
>P26782
MSDAVTIRTRKVISNPLLARKQFVVDVLHPNRANVSKDELREKLAEVYKAEKDAVSVFGF
RTQFGGGKSVGFGLVYNSVAEAKKFEPTYRLVRYGLAEKVEKASRQQRKQKKNRDKKIFG
TGKRLAKKVARRNAD
>Q6J5K9
MSSEASGSITIKEEASTSGTATNIPVESSETEAKKPEIGADKAESQSETTEGGKAAETTE
NHLKRLQNLRKELSYLSETDWMYESLDKKAAHKFFTNPDRNREDILESLDRENSFLDQKL
MEEKMDQQLKANPNEINGVLSNKIAELTHGLSEMDVSKESSCTARKGVITSLDGDRGVID
KDVLFETKVAEDIILDLHVGCVVEYLTFTTGEAMRVVKVKSILEHSWEDTSQKEIEKAVD
NLKNEKPTFFNTETRSVLGLISQRLASSIDVETEYGQLTVELDNIEMNFIPTNGDRVRLE
CNIQLDDGFVDKQGEILEVTKLFPTRIQEGEKCIVERVYVHMVVLGPETYILKTDLPTGT
DLHLGDIVLADLIECQYSKFTRRAIKITPLEKNFGATKLTQQSSMGSSGSGKAVTVTGVN
RFITAELWQKESVSLKLTNNLNRTLRLESITVCNDSESQLSVVSPLESKEISSGSEITVT
FEIHTQFLGEAIEKYVLNFDLLKVRRVFTVIVCKTKEEVAEAEKRMIAAEALMAPGRNSQ
ERSRFYANQVWCNKVDVIPGQQIVTKRRFVALRLGCFEVPKELRQICLTSERRQEMIKAI
EQHYSFLTEPLSIKTYMHRFRLLLHLEEIECFVNFRNYDRDRAHFLRDGEFLTLQIENLA
ERRPSLVIGDTLRVINPWSDPDSQTTKSYEGIIHKVLFDRILLKFHSSFQEKYNGEDYRL
EFYFSRYSFRKQHHAISKIVGVMGEDFLFPSKVTKRENPQLDVYMKDDDMYLYDSKLEWY
NQSLNSIQKRAVFNILRGEAENIPYVLFGPPGSGKTMTLIETLLQLVRNLPGARILVGTP
SNSSADLVTKRLIDSKALLQGDFIRLVSYNQVEKDLIPPEIMSYCATSDVGAVGSCEDKM
MVTESGLKLRCQAKFIGTHRITISTCTTLGNFLQLGFPAGHFTHVLFDEAGQCTEPETMV
PIVMLTKKRSQVVLSGDPRQLQSIVTSRIASKMGFSISFLERLLERSPYRKDLQRFPESS
GYNPLVLTKLLYNYRALPSIMSIYSRLFYDDELIPVLSEKDSRESRLLSKLRCVFESEKD
IPQAHGTFFYGIIGENRQNNDSPSWFNPQEVREVFLMTIALYRANVTADQIGIITPYQKQ
VKMLRSMFIGTDVVMPKIGSVEEFQGQERDIILISTVRSSEEILRMDARFSLGFVRCSKR
LNVAVSRARAMMIIFGNPHLLAVDECWRQLILFCVKNNAYFGCDLPQMVINQKDEVPVCL

ETFVPSLNTTDDLN
>Q9D2N4
MIEDSGKRGNTMAERRQLFAEMRAQDLDRIRLSTYRTACKLRFVQKKCNLHLVDIWNVIE
ALRENALNNLDPNIELNVARLEAVLSTIFYQLNKRMPTTHQIHVEQSISLLLNFLLAAFD
PEGHGKISVFAVKMALATLCGGKIMDKLRYIFSMISDSSGVMVYGRYDQFLREVLKLPTA
VFEGPSFGYTEQSARSCFSQQKKVTLNGFLDTLMSDPPPQCLVWLPLLHRLANVENVFHP
VECSYCHSESIMGFRYRCQQCHNYQLCQDCFWRGHAGGSHSNQHQMKEYTSWKSPAKKLT
NALSKSLSCASSREPLHPMFPDQPEKPLNLAHIVPPRPVTSMNDTLFSHSVPSSGSPFIT
RSSPPKDSEVEQNKMLARAAPAFLKGRGIQYSLNVADRLADEHVLIGLYVNMLRNDPPCM
LESSNRLDEEHRLIARYAARLAAESSSSQPTQQRSAPDISFTIDANKQQRQLIAELENKN
REILQEIQRLRVEHEQASQPTPEKAQQNPTLLAELRLLRQRKDELEQRMSALQESRRELM
VQLEGLMKLLKEEELKQGTQGASSPRSSPSHTISRPIPMPIRSASACPTPTHTPQDSLTG
VGGDVQEAFAQSSRRNLRSDLLVAADSITNTMSSLVKELNSEVASETESTVDSEFSRPQF
EDLAPSPTSEKAFLAQIHSRKPGYIHGGAASTTHGDMVPEDGDPYTQPEDGNYENESVRQ
LENELQLEEYLKQKLQDEAYQVSLQG
>Q95ZG3
MKQNQPNLNVNLNNRLQQQQLNQVAQQQLQQQPLKNTKQNPINNQTNGILNNKPITTSSN
IMNSDNILNNNNNNNTNKPSSTFNFNITNTPPPPVNKQKPITSSSTSKPSNTTTTTTTTT
TTTKPPSNSLPSSSSSKVKVNSLTSQNNNNNNSNTNTNMTTQPPTFNNLIKPQSLPSTTG
MNEIKNTPTRTTTTTSTQHTKYIKEDIVVKENNNRISNMNNNNISGASGGTTFNSYNYLP
NNTLDNFINNSNTTTTTTNNNTNNNNNNNNNNNIINNIKQQHELELLQQKHIEEMKILQN
EQLNQLKKQQKKLNESDTDGSDDDDDDDDEYGENMNLSPRTLRELKNIQRLKNLEKFSPP
YLITKKTSPSIKTLPKFPEWTKERPFLNYGYLANISTNYTSLILPSKYEQTKIQLSSIPN
YFREQILIEDLLSTMIGIEGECIHIGVVQENEDDSLQSNLVSFVVEGEVDPSNNDLVQRI
LPLCSYYSFVNDFIQIRYAYEWGIINHSLCFSIDQLLKEYLVLVSQLEGQLKSERLSLQR
MWFYLQPTIKTFEILYRVTSEIMRCNSHGVQVINLLLKLQSKYSSDKKTDDLFHYLIKAT
TLPFLEMLDLWIHQGIIRDTYFEFMIEENTDLKRDNINRDFNDLYWEQRYQIRQDQIPKY
LQSHALKILTTGKYLNVVRECNQSISQQQQHNKSDNNNNNNNNVKLNSQQQLLLLKLNTT
QQQQQQQLSSTSASMIPTTIIEYSKNENVYIDKIEKAYDYASGILLNLLINERHLISRLK
AIKHYFLLCKGDFFSHFMDTTYDELKKPLDQINMVKMNSLLQLSLRTSSISEEDEFKDDL
ECEFIPYKLVDQLLNIININNFNNPTTTTTTNTSSNNITATTNTSSNNTTINTTVNTTLI
QSTSAHEVLKTNKLFGIESLAFNYNVGWPLSLIISRKSLIKYQIIFRHLFLCKHVEKVLV
DTWSQHQFRRSSYNKPGLSTLLSFTHLLRHRMIHFLQNLEYYMMLEVLEPNWNKMKNSIK
TSKTVDDVIRIHNDFLETCLTECMLTDTKLVNILMKFMSLCITFANFTNEMMQDEKNIDT
EQIKKTISSSENKFHAILKVLMDTLKSFSKTESNRHMIHLIHRLDYNGYYSHYFENNPNL
FKNKTSSSSNKSNNVSPPLEPRFSYQQQQQQQQQQQQQQQQQQQQQQQQQQQQQQQQQQQ
QQQQQQQQQQQQQQQQKQNQIQQQKQNQIQQQQQQSRGSTLPSNFSFQTALQQQINTTSA
TTTTSNQSPHTLIDDLKKRAYGILDSNNTKKTLERTSSSPALDNQSNSLPMTQSISQYAE
KLKNLRAVIKNDELK
>Q06523
MSGYFSGFSLNKITDSIATAAHKTQDTLNNALANANVNLNDPQTRLSIKSRTRFVQESLG
TVSDISKLPPQYQFLEKKSDSLEKVCKRILLVSKTFEVEGYDYPPNLTESISDWWSLNKD
GWFGSKKSESSTKKKGSNHDDAFLPRSFAQAISKAAVDCECEFQNLEHNEKAELKKKKES
IKTAQTTEAQGADHNEEDEEDEEDEEDDEDLSNLIKVFDSWSTCYKNIDEGKAEMDSMMV

KEFNKKLETLINQDFKKVHDLRKKVEESRLKFDTMRYEVKAKEAELEAKKAEATGEAHSK
DVSAKDISANTTTSFDETPSTEDEKPKSEGAEEESKKEANEPTVDDVADRKEDLKSNKVN
DEPPIEESEDNKLLEKLEDEFVSNTTAAVETMEEITDSSEILGLIKLFQNFQLVYFRQCV
QEVEANLKVLNGLEI
>Q8CDG1
MDPVRPLFRGPTPVHPSQCVRMPGCWPQAPRPLEPAWGRAGPAGRGLVFRKPEDSSPPLQ
PVQKDSVGLVSMFRGMGLDTAFRPPSKREVPPLGRGVLGRGLSANMVRKDREEPRSSLPD
PSVLAAGDSKLAEASVGWSRMLGRGSSEVSLLPLGRAASSIGRGMDKPPSAFGLTARDPP
RLPQPPALSPTSLHSADPPPVLTMERKEKELLVKQGSKGTPQSLGLNLIKIQCHNEAVYQ
YHVTFSPSVECKSMRFGMLKDHQSVTGNVTAFDGSILYLPVKLQQVVELKSQRKTDDAEI
SIKIQLTKILEPCSDLCIPFYNVVFRRVMKLLDMKLVGRNFYDPTSAMVLQQHRLQIWPG
YAASIRRTDGGLFLLADVSHKVIRNDSVLDVMHAIYQQNKEHFQDECSKLLVGSIVITRY
NNRTYRIDDVDWNKTPKDSFVMSDGKEITFLEYYSKNYGITVKEDDQPLLIHRPSERQNN
HGMLLKGEILLLPELSFMTGIPEKMKKDFRAMKDLTQQINLSPKQHHGALECLLQRISQN
ETASNELTRWGLSLHKDVHKIEGRLLPMERINLRNTSFVTSEDLNWVKEVTRDASILTIP
MHFWALFYPKRAMDQARELVNMLEKIAGPIGMRISPPAWVELKDDRIETYIRTIQSLLGV
EGKIQMVVCIIMGTRDDLYGAIKKLCCVQSPVPSQVINVRTIGQPTRLRSVAQKILLQMN
CKLGGELWGVDIPLKQLMVIGMDVYHDPSRGMRSVVGFVASINLTLTKWYSRVVFQMPHQ
EIVDSLKLCLVGSLKKYYEVNHCLPEKIVVYRDGVSDGQLKTVANYEIPQLQKCFEAFDN
YHPKMVVFVVQKKISTNLYLAAPDHFVTPSPGTVVDHTITSCEWVDFYLLAHHVRQGCGI
PTHYICVLNTANLSPDHMQRLTFKLCHMYWNWPGTIRVPAPCKYAHKLAFLSGQILHHEP
AIQLCGNLFFL
>O75884
MASPSKAVIVPGNGGGDVTTHGWYGWVKKELEKIPGFQCLAKNMPDPITARESIWLPFME
TELHCDEKTIIIGHSSGAIAAMRYAETHRVYAIVLVSAYTSDLGDENERASGYFTRPWQW
EKIKANCPYIVQFGSTDDPFLPWKEQQEVADRLETKLHKFTDCGHFQNTEFHELITVVKS
LLKVPA
>Q08732
MPNLLSRNPFHGHHNDHHHDRENSSNNPPQLIRSSKSFLNFIGRKQSNDSLRSEKSTDSM
KSTTTTTNYTTTNLNNNTHSHSNATSISTNNYNNNYETNHHHNISHGLHDYTSPASPKQT
HSMAELKRFFRPSVNKKLSMSQLRSKKHSTHSPPPSKSTSTVNLNNHYRAQHPHGFTDHY
AHTQSAIPPSTDSILSLSNNINIYHDDCILAQKYGKLGKLLGSGAGGSVKVLVRPTDGAT
FAVKEFRPRKPNESVKEYAKKCTAEFCIGSTLHHPNVIETVDVFSDSKQNKYYEVMEYCP
IDFFAVVMTGKMSRGEINCCLKQLTEGVKYLHSMGLAHRDLKLDNCVMTSQGILKLIDFG
SAVVFRYPFEDGVTMAHGIVGSDPYLAPEVITSTKSYDPQCVDIWSIGIIYCCMVLKRFP
WKAPRDSDDNFRLYCMPDDIEHDYVESARHHEELLKERKEKRQRFLNHSDCSAINQQQPA
HESNLKTVQNQVPNTPASIQGKSDNKPDIVEEETEENKEDDSNNDKESTPDNDKESTIDI
KISKNENKSTVVSANPKKVDADADADCDANGDSNGRVDCKANSDCNDKTDCNANNDCSNE
SDCNAKVDTNVNTAANANPDMVPQNNPQQQQQQQQQQQQQQQQQQQQHHHHQHQNQDKAH
SIASDNKSSQQHRGPHHKKIIHGPYRLLRLLPHASRPIMSRILQVDPKKRATLDDIFNDE
WFAAIAACTMDSKNKVIRAPGHHHTLVREENAHLETYKV
>Q03769
MDSLSKKIQNLGIHDIRNAARFAQNVIVQYEPYQIDIRRATNTDAWGPTPKHLAKVLRNR
YQVPLYLMTEYTLKRLVDHIATRPKNLYEKARKDYVNYGSEWRVVLKCLVVIEFLLLNVD

TGDELNQIRSCLLTHKHILTREIAQFKVKFSNDGKMEIHERGIRKKGELILQYLEDSQFL
KKERAKNKKNALKIRQQGESSIYNANQISTSASYDNIDDDEFDADADGFDSEMDANNVTN
FNVPVETEANSNTRRRSHMEEQRRQRREILREQIKNKEQQRKRKQQQDSIPDLIDLDDST
STTNNITIDNGNNDNKNNNINSNSDDDDDEFGDFQSETSPDTTAPKTSNSKIDDLLDWDG
PKSDTDTTAAAQTSLPFAEKKQQKARPQATKDKSKGNDAFSDLFSYSKSLV
>Q9GZT8
MLSSCVRPVPTTVRFVDSLICNSSRSFMDLKALLSSLNDFASLSFAESWDNVGLLVEPSP
PHTVNTLFLTNDLTEEVMEEVLQKKADLILSYHPPIFRPMKRITWNTWKERLVIRALENR
VGIYSPHTAYDAAPQGVNNWLAKGLGACTSRPIHPSKAPNYPTEGNHRVEFNVNYTQDLD
KVMSAVKGIDGVSVTSFSARTGNEEQTRINLNCTQKALMQVVDFLSRNKQLYQKTEILSL
EKPLLLHTGMGRLCTLDESVSLATMIDRIKRHLKLSHIRLALGVGRTLESQVKVVALCAG
SGSSVLQGVEADLYLTGEMSHHDTLDAASQGINVILCEHSNTERGFLSDLRDMLDSHLEN
KINIILSETDRDPLQVV
>Q10277
MVVSSPSVSLLHSPVEKLSAQLEKTTLLQDIPPGSLSENDNSTTFIKPPLETASSSTPIP
SSSSSGVLNPSSVRGKPVACLFVASLNSSRSEEELTATVKDYFQQWGPLLHVKVLKDWLQ
RPYSFVQFQNTDDASKALSEAQNTILDGRHIRIERAKVNRTIRISSAPHQPYITKKDIDN
LLEPYGEVEDVTEIPDQSAFLVRFVYRDEAIAAYTALKHSAWPVLWAENVTYQNGHYKKK
GSSPFSPPNAHSRRRKSQGKDQSNTPVIKAPAPIPFSVSSDPPSTMGRSNSAVQSPSYFA
HSLVNSTEFSTPNESLSSLPSILPSIPSLESGKAELPTDGSFEQPGYPMNPSMMFAAMPP
PIDPYSIFVGQLDPVNCTHYLLVDLFSKYGKVIDCKIIHQSKKPAFAFLRFDSQQAAYAA
VCGKTRSPHQKKPLRVEFRQLRPMQQFSPQYQYPSYPYPMFPAPFSPPRNAMMPIPAPMD
QFSTFHQSMATLPPGAVPTSIPQSYYPIYSPEMAMPQSYSPMYYTHNPPMDGN
>Q12383
MLQDNNGPAVKRAKPSERLQCEYFMEKKKRRCGMTRSSQNLYCSEHLNLMKKAANSQVHN
KNGSEAEKERERVPCPLDPNHTVWADQLKKHLKKCNKTKLSHLNDDKPYYEPGYNGENGL
LSSSVKIDITAEHLVQSIELLYKVFEGESMDELPLRQLNNKLMSLKRFPQLPSNTKHAVQ
QSSLIENLVDAGAFERPESLNFIEFGCGRAEFSRYVSLYLLTQLTSLPAEHSGSNSNEFV
LIDRATNRMKFDKKIKDDFSEIKSNSPSKPISCPSIKRIKIDIRDLKMDPILKSTPGDDI
QYVCISKHLCGVATDLTLRCIGNSSILHGDDNNGCNPKLKAICIAMCCRHVCDYGDYVNR
SYVTSLVEKYRAHGSILTYETFFRVLTKLCSWGTCGRKPGTAITDIVNVVESFEGAEPYT
ITIKERENIGLMARRVIDEGRLVYVKEKFTEFNAELIRYVESDVSLENVAMLVYKK
>O14618
MASDSGNQGTLCTLEFAVQMTCQSCVDAVRKSLQGVAGVQDVEVHLEDQMVLVHTTLPSQ
EVQALLEGTGRQAVLKGMGSGQLQNLGAAVAILGGPGTVQGVVRFLQLTPERCLIEGTID
GLEPGLHGLHVHQYGDLTNNCNSCGNHFNPDGASHGGPQDSDRHRGDLGNVRADADGRAI
FRMEDEQLKVWDVIGRSLIIDEGEDDLGRGGHPLSKITGNSGERLACGIIARSAGLFQNP
KQICSCDGLTIWEERGRPIAGKGRKESAQPPAHL
>P20695
MPKNKKRNAPHRGGGGGGGSGAATSAATTGGPHRTVQPFSDEDASIETMSHCSGYSDPSS
FAEDGPEVLDEEGTQEDLEYKLKGLIDLTLDKSAKTRQAALEGVKNALSSKVLYEFVLER
RMTLTDSIERCLKKGKSDGQRAAAALASVLCIQLGPGLESEEILKTLGPILKKIICDGTA
SIQARQTCATCFGVCCFIATDDITELYSTLECLEGIFTKSYLKEKDTNVPCSTPNTVLHI
SSLLAWTLLLTICPISEVKKKLELHFHKLPSLLSCDDVNMRIAAGESLALLFELARGMES

DFFYEDMDSLTQMLRALATDGNKHRAKVDKRKQRSVFRDVLRAVEERDFPTETVKFGPER
MYIDSWVKKHTYDTFKEALGSGMQYHLQTNEFLRNVFELGPPVMLDAATLKTMKIPRFER
HLYNSAAFKARTKARSKCRDKRADVGEFF
>Q10719
MMTASFKGIRISKPIVKEISSFGSLTNIDLHTDPYEKVEAKALSRSRLKNQSVKKTDLRI
TNDYSSLFVSIENKKNTIPDIHCNNLSKRPVGFTDSYVPGEALLCPDDPNLVNELFDDVL
KRLREEKSDSGEQFHWDANVTLSQENKWSFITGYLSRNDKFLKISSNDSELNVKASVISY
IEKLSCTPLKLKYVESLAVALRTESVTWVRYFLLMGGHIVLAKILQAIHEKKHLKSPDIT
LEIAILKSMRCIIGQKIGTDFYLSNKYPIDSVVHCLLSTKLLVRKLAAELLTFLCYSEKP
NGINAIMKCMKNFANLKVESMNVFDLWLSQWKRTLLEKVVTEEKKVLEEAALHDKITIEY
IISQMLLINAFLENIPSKTALLQFKESLRIGNFKSILLILKKINDEGVLKQLEKCVKLVS
LDTANEKHFLKHTPNSAAHQSLLNTNMFNDANFEFMVKEHIKNFLKLLKEHNNPVRIIKL
LDCLVLTLQADKGNGNSNNDIDCFYKELKIGNEQGNNAPGYSSVTSGYGTTNSKKVVASY
DNTDDNEYSVSKSELYATGDTNNTTNQGYENSERKYVESSKYETDLKNIFDCLCLLFPSE
FDPQSSFSAEKIFSKLKHLLTRTRDSYISEDTKILRLLNRSSSKLQDRDQEYLISKNNVN
GNGNRDWVQPVNTTSSVSAFASPRIKPKLLFQPNQDEKLKLCKLDLAKANSITLKSEPSS
AVSTHSFEVHLSMPGTQIKSANLAEKMETSKHKVFNPKRIDVVSDLPLDYRKSYYGRFSI
TDTKRFSKIENMRIKEVIDGNPFKAPPPAPLPPPAPPLPTAMSSLQKFEKNDSQIFRKTI
IIPENISIDDIFKFCSGSESEVYASKIPGELCNPSKRLKQLHWKRLEVPFEKTLWNIVVA
DPYLLTLKLTAEGIFKQLEDCYPLREVTVSNKKVKEYTGFMPVDLQQMVSIRLHRFNSLT
PIEIAKKFFHCDHDIMELVDFFNDRKFFRQEGLKKQLKPYMSSRNEEVMEVSEKLLELSR
FDQIYTLIVVDIDTYYEKRMAALKIKSFLANNFRDFRRQIRKLHCASLELKSSLHFKYFL
NLVLHIGNFMNDAPRRAKGYRLESLLRASMIKNDKTGLTLLHTIEKIVRTHFPQLEAFLV
DLKAIPEISRFNLEQLEQDCNDICERMKNVEKDFSNEGIFSNHKALHPDDHICEVMVPWI
PSGKSMVDELNSDITELKTTLKTTLLMYGENPDEPTSSARFFNNLNEIILEYKKASTVNQ
KMEKEEELAFLRLQALKASVKSNNAENSGSSKNEEVSATMENLISQLQKGLCDDSLSNKT
DCTESSKQTIETLMNYENDKQTLEGSNKKRQTVVLKAECMLKQLENNNELKR
>P32628
MVSLTFKNFKKEKVPLDLEPSNTILETKTKLAQSISCEESQIKLIYSGKVLQDSKTVSEC
GLKDGDQVVFMVSQKKSTKTKVTEPPIAPESATTPGRENSTEASPSTDASAAPAATAPEG
SQPQEEQTATTERTESASTPGFVVGTERNETIERIMEMGYQREEVERALRAAFNNPDRAV
EYLLMGIPENLRQPEPQQQTAAAAEQPSTAATTAEQPAEDDLFAQAAQGGNASSGALGTT
GGATDAAQGGPPGSIGLTVEDLLSLRQVVSGNPEALAPLLENISARYPQLREHIMANPEV
FVSMLLEAVGDNMQDVMEGADDMVEGEDIEVTGEAAAAGLGQGEGEGSFQVDYTPEDDQA
ISRLCELGFERDLVIQVYFACDKNEEAAANILFSDHAD
>P50747
MEDRLHMDNGLVPQKIVSVHLQDSTLKEVKDQVSNKQAQILEPKPEPSLEIKPEQDGMEH
VGRDDPKALGEEPKQRRGSASGSEPAGDSDRGGGPVEHYHLHLSSCHECLELENSTIESV
KFASAENIPDLPYDYSSSLESVADETSPEREGRRVNLTGKAPNILLYVGSDSQEALGRFH
EVRSVLADCVDIDSYILYHLLEDSALRDPWTDNCLLLVIATRESIPEDLYQKFMAYLSQG
GKVLGLSSSFTFGGFQVTSKGALHKTVQNLVFSKADQSEVKLSVLSSGCRYQEGPVRLSP
GRLQGHLENEDKDRMIVHVPFGTRGGEAVLCQVHLELPPSSNIVQTPEDFNLLKSSNFRR
YEVLREILTTLGLSCDMKQVPALTPLYLLSAAEEIRDPLMQWLGKHVDSEGEIKSGQLSL
RFVSSYVSEVEITPSCIPVVTNMEAFSSEHFNLEIYRQNLQTKQLGKVILFAEVTPTTMR

LLDGLMFQTPQEMGLIVIAARQTEGKGRGGNVWLSPVGCALSTLLISIPLRSQLGQRIPF
VQHLMSVAVVEAVRSIPEYQDINLRVKWPNDIYYSDLMKIGGVLVNSTLMGETFYILIGC
GFNVTNSNPTICINDLITEYNKQHKAELKPLRADYLIARVVTVLEKLIKEFQDKGPNSVL
PLYYRYWVHSGQQVHLGSAEGPKVSIVGLDDSGFLQVHQEGGEVVTVHPDGNSFDMLRNL
ILPKRR
>Q9JLN5
MERPSPCGSWLVGCLFTIAVFQPPVQVLGDAGKVYIAPLRDTANLPCPLFLWPNMVLSEM
RWYRPGHLPRTQAVHVFRDGQDRDEDLMPEYKGRTALVRDAHKESYILQISNVRLEDRGL
YQCQVWVGNSSREDNVTLQVAVLGSDPYIHVKGYDAGWIELLCQSVGWFPKPWTEWRDTT
GRALLSLSEVHSLDENGLFRTAVSSRIRDNALGNVSCTIHNEALGQEKTTAMIIGAPERG
SLSSPAVALSVVLPVLGLLILLGIWLICKQKKSKEKLLYEQAMEVENLLEDHAKEKGRLH
KALKKLRSELKLKRAAANAGWRRARLHFVAVTLDPDTAHPKLILSEDRRCVRLGDRKRPV
PDNPERFDFVVSVLGSEYFTTGCHYWEVYVGEKTKWILGVCSESVSRKGKVTASPANGHW
LVRQSRGNEYEALTSPQTSFRLKESPKCVGIFLDYEAGIISFYNVTDKSHIFTFTHSFSS
PLRPFFEPCLHDEGKNTAPLIICTELQKSEESIVPKQEGKDRANGDVSLKMNPSLLSPQG
SELFLLNDTWPSNLGPALKGLKVPSL
>Q8CJF7
MQDLTAQVTSDLLHFPEVTIEALGEDEITLESVLRGKFAAGKNGLACLACGPQLEVVNSL
TGERLSAYRFSGVNEQPPVVLAVKEFSWHKRTGLLIGLEEADGSVLCLYDLGISRVVKAV
VLPGRVTAIEPIINHGGASASTQHLHPSLRWLFGVAAVVTDVGQILLIDLCLDDLSCSQN
EVEASDLEVITGIPAEVPHIRERVMREGRHLCFQLVSPLGVAISTLSYINRTNQLAVGFS
DGYLALWNMKSMKREYYTQLEGGRVPVHAVAFQEPENDPRNCCYLWAVQSTQDSEGDVLS
LHLLQLAFGDRKCLASGQILYEGLEYCEERYTLDLAGGTFPLRGQTSNTKLLGCQSIERF
PSHGDREESMREALSPDTSVSVFTWQVNIYGQGKPSVYLGLFDINRWYHAQMPDSLRSGE
SLHNCSYFALWSLDSVVSRTSPHHILDILVHERSLNRGVPPSYPPPEQFFNPSTFNFDAT
CLLDSGVIHVTCAGFQKETLTFLKKSGPTLNEVIPDSYNRCLVAGLLSPRLIDIQPSSLS
QEEQLEAILSAAIQTSSLGLLTGYIRTWIIEEQPNSAANLRFVLEWTWNKVVLTKEEFDR
LCVPLFDGSCRFIDPQTIQSIQQCHLLLSNLSTVLSCFAMEAQGITERGLVDLSNKHMVT
QLLCQYAHMVLWFCHSGLLPEGLDDALQLSRLRYNYPVIQNYYTSRRQKSERSPRGKWNH
DCLMIDGLVSQLGDEVEKLWKRDEGGTGRYPPASIHALLDIYLLDNITEASKHAITIYLL
LDIMYSFPNKTDTPIESFPTAFAISWGQVKLVQGFWLLDHNDYENGLDLLFHPVTAKPAS
WQHSKIIEAFMSQGEHKQALRYLQTMKPTVSSSNEVILHLTVLLFNRCMVEAWNLLRQNS
NRVNIEELLKHAYEVCQEMGLMEDLLKLPFTNTEQECLVKFLQSSTSVENHEFLLVHHLQ
RANYISALKLNQILKNNLMSDRDPRLRERSVTRNSILDQYGKILPRVQRKLAVERAKPYH
LSTSSVFHEVSRPKPLSAFPKKAITGTVLTRSTFISNVLSKIGEVWASHEPRNGVSLFNS
PKTEQPSPVVHSFPHPELPEAFVGTPISNTSQRISRLLDLVVHPVPQPSQCLEFIQQSPT
RSPLCLLSSSLPLSSQFKRPHQNTSRPSELLLLETPLIVKKAKSLALSATSSGFAEFTPP
SILRSGFRTTPLASPSLSPGRSLTPPFRVKETRISFMEEGMNTHWTDRATDDRNTKAFVS
TSFHKCGLPAETEWMKTSDKNTYFPLDVPAKGPQKVVAESLATHSGRLEKLDVSKEDSTA
STRSDQTSLEYHDAPSPEDLEGAVFVSPKPASSSTELTTNSTLQTERDNDKDAFKSEGAP
SPVKKQIGTGDAAVEAFSELSRLDPVERAEASFAVSSVCEGETSTSNSKTSVLDGIVPIE
SRTSILTADHKESVANTVADVESSGSTSSKCPVTSERSLGQKLTLNLKEDEIEAHVPKEN
VGLPEESPRISAAPSDTHEIHLIGCENLEVQNSEEEAKNLSFDELYPLGAEKLEYNLSTI
EQQFCDLPDDKDSAECDAAEVDGELFVAQSNFTLILEGEEGEAEASDSAAPNMLPKATKE

KPVCHREPHNQERVTDLPSAVTADQESHKVETLPYVPEPVKVAIAENLLDVIKDTRSKEA
TPVAAGEAGDEDGAVIVSKAAHSSRLTNSTPKTVKEPHAETVNTSQNDDMVSSRTLTRRQ
HALSLNVTSEQEPSAVATPKKRTRKIKETPESSERTCSDLKVAPENQLTAQSPPAPRRGK
KKDVSQGTLPSSGAVEPEPEPQGTPGRLRLRTQPPEPAAEETPSRTKVRLSSVRKGTPRR
LKKSVENGQSTEILDDLKGSEAASHDGTVTELRNANLEDTQNMEYKQDEHSDQQLPLKRK
RVREREVSVSSVTEEPKLDSSQLPLQTGLDVPATPRKRGRPRKVVPLEADGGTTGKEQTS
PQKKDVPVVRRSTRNTPARNVSTLEKSVLVPNKEAALVVTSKRRPTKKSAEESSKDPSAA
VSDLAGGAAHTESADRRDGLLAAAALTPSAQGTRTRSRRTMLLTDISEPKTEPLFPPPSV
KVPKKKSKAENMEAAAQLKELVSDLSSQFVVSPPALRTRQKSISNTSKLLGELESDPKPL
EIIEQKPKRSRTVKTRASRNTGKGSSWSPPPVEIKLVSPLASPVDEIKTGKPRKTAEIAG
KTLGRGRKKPSSFPKQILRRKML
>P93804
MGLFTVTKKATTPFDGQKPGTSGLRKKVTVFQQPHYLQNFVQSTFNALPVDQVRGATIVV
SGDGRYFSKDAVQIITKMAAANGVRRVWVGQNSLMSTPAVSAVIRERVGADGSKATGAFI
LTASHNPGGPKEDFGIKYNMGNGGPAPESVTDKIFSNTTTISEYLISEDLPDVDISVVGV
TSFSGPEGPFDVDVFDSSVDYIKLMKTIFDFEAIKKLLTSPKFTFCYDALHGVAGAYAKH
IFVEELGADESSLLNCVPKEDFGGGHPDPNLTYAKELVERMGLGKSSSNVEPPEFGAAAD
GDADRNMILGKRFFVTPSDSVAIIAANAVQSIPYFASGLKGVARSMPTSAALDVVAKNLN
LKFFEVPTGWKFFGNLMDAGMCSICGEESFGTGSDHIREKDGIWAVLAWLSIIAFKNKDN
LGGDKLVTVEDIVRQHWATYGRHYYTRYDYENVDAGAAKELMANLVSMQSSLSDVNKLVK
EIRSDVSEVVAADEFEYKDPVDGSVSKHQGIRYLFGDGSRLVFRLSGTGSVGATIRVYIE
QYERDSSKTGRDSQDALAPLVDVALKLSKMQEYTGRSAPTVIT
>P47122
MSLSTIICIGMAGSGKTTFMQRLNSHLRAEKTPPYVINLDPAVLRVPYGANIDIRDSIKY
KKVMENYQLGPNGAIVTSLNLFSTKIDQVIRLVEQKKDKFQNCIIDTPGQIECFVWSASG
AIITESFASSFPTVIAYIVDTPRNSSPTTFMSNMLYACSILYKTKLPMIVVFNKTDVCKA
DFAKEWMTDFESFQAAIKEDQDLNGDNGLGSGYMSSLVNSMSLMLEEFYSQLDVVGVSSF
TGDGFDEFMQCVDKKVDEYDQYYKQEREKALNLKKKKEEMRKQKSLNGLMKDLGLNEKSS
AAASDNDSIDAISDLEEDANDGLVDRDEDEGVEREYTFPGEERTKGEVNENSAPDLQRRY
QEAMQQVGKTASSETAENIAKYIRN
>P42577
MSVSQARQNYHAESEAGINRQINMELYASYSYQSMAYYFDRDDVALPGFHKFFKHQSEEE
REHAEKLMKYQNKRGGRIVLQDIKKPDRDEWGTGLEAMQVALQLEKSVNQSLLDLHKLCT
SHDDAQMADFLESEFLEEQVKSIKELSDYITNLKRVGPGLGEYIFDKETLSSSS
>P07270
MGRTTSEGIHGFVDDLEPKSSILDKVGDFITVNTKRHDGREDFNEQNDELNSQENHNSSE
NGNENENEQDSLALDDLDRAFELVEGMDMDWMMPSHAHHSPATTATIKPRLLYSPLIHTQ
SAVPVTISPNLVATATSTTSANKVTKNKSNSSPYLNKRRGKPGPDSATSLFELPDSVIPT
PKPKPKPKQYPKVILPSNSTRRVSPVTAKTSSSAEGVVVASESPVIAPHGSSHSRSLSKR
RSSGALVDDDKRESHKHAEQARRNRLAVALHELASLIPAEWKQQNVSAAPSKATTVEAAC
RYIRHLQQNVST
>P14605
MDQSKRLLKSAVPNPPEHFKTGISWLDDLDEKDDDSATSVNYDIPEITEANLCNDSHEAL
SPCTQPVGNSGRPVEAFKTYPSTPAVPSKSVLFHFYEPDENFSLSDTGRTKSDTALAARE

SSEKSEVPRDTRSAGIKPYKENNSSNCAISKEAGLRRLIDKDRESFDKNLNQSFTNLTFP
EPISDDSDSVEFQRDSLNNNWPASLEGSIHELPRNSDDDGIPASAAHILDLDYHRDSYDS
PWKKFLPYPSILSDDSWKAPESWGTSLPTEAIPKQVFTTRFFARPSLGNRKKEFFLRVYR
DDRTSVSFICPIGIQTHEVIKLLARLFFLPSSANFYLLLIQFNTERILLPHEQPCIIFER
LLSLFGCKVTSDEEINEEDNYSVARLVFTTMDIGADVLRKFSEKKITANLDISRSNLEVI
PVKIYPYAHELISLNVSHNLSLDLPLDFMERCVKLKRLDISNNLRSPRGKPITALRQLEV
LNMSRNDIYELDPLIFSGLSRNSLKELNIANNKLFFLPHSTRYLVNLTYLDLSYNNFVTF
PLIITELSQLETLNFSHNLLSQISSKIGSLVKLKHLYLQFNDLSNRLPQEIGLLKNLETI
DLSYNAITNIASLSECPKLNSINVACNLLSFYEYSNPSATFIDFSFCPLTTIDPAFSYSN
LVYFDISHAKLIGLKDSVIETLVNVETVKVNYNHFTSISDAISAMQNLKYLSCTNCEMSY
VSPNLGKLKHLVHLDLHANNIKIFPEEVWQVSSLKVVNLSSNILEKIKLPVATSKKLTRT
ISQLKIMRTLSGNPVSSLSSQEFVMPTVEELYLVDNRLGNDCFTALEYFKCLKVLNLSYN
YLTEIPSKFFQNFSDLKHLFVSGNELANLSISSTAQVLLETLYANGNRLSSFPKNEALSK
SLRFLDISTNNLQNLAVEKAEKKSLTKLPQLEYLNLSGNTWFRFSEHEDTNFTKSYLKNL
KFLSIMDLNTKFSNAPSDVLNHFIQRNSPQPNILRYGVCGYLSRSIPVISACELVVNNFL
HPQSSLYCVLDSDISAGKNNRVLKFVYDNLASCLAHEINAADSSSEQICNALRRGFLRLN
KKLGNVIHYDLRKSSEGDVDSNYVTTMNISEKGYSMDSSCLDIGVSIILVYVRDTRAFVA
NVGTSMAIMSTRNDSEPTTLSVMHDVYNRDEIRRIVDSCGFISGEIKSTTTRAIGRLSQF
PGVQAVPYVNVQYLSELNEFIILANQEFWSVLSKRTVIDVVRANRHSPLLASTKLRDYAI
AYGAEKNVLVVIVELNGLFEENSLNFNQLRGDEKTLAISEKNDNMSFVQDLPDDSSLARM
NREVSPPKGCIAMVFTDIKNSTLLWERHPIAMRSAIKTHNTIMRRQLRATGGYEVKTEGD
AFMVCFQTVPAALLWCFSVQLQLLSADWPNEIVESVQGRLVLGSKNEVLYRGLSVRIGVN
YGVTVSELDPITRRMDYYGPVVNRTSRVVSVADGGQIAVSAEVVSVLNQLDSETMSSEKT
NVNEMEVRALKQIGYIIHNLGEFKLKGLDTTEMISLVYPVQLQGRLERLIKSRSLGTPTA
LPETQTYTPVRSRSNSLRPMLARLSDSKSVHGEEGGSGKRSVSSLRNVSPSESTGGYEGC
IFDDQQYQLLYELCERLEDHAAILHGFPEPPPCDTGLAAPVNQAEEYSLFYRLTLRIENT
IYCVSQMLGHTG
>O42854
MSSFPTRVVALYPYRSSFSDDLEFDPGQVIDVVSNLDGDWYTGTYVDSDGNRKIGSFPKD
FTEPAEDAVFVERASEMALHQPTPTSAVHSRNSSLGYAPSITRSIKSISNNTEHLGADTE
SYLSANDFIDSTSEALTKIVDVDTLSAPFGNDSNSRPHSLKNVEKLHTFSAPYTISEETP
SCSTENDSLPLTATHTITGGEDAATGAAVTNTTTTHITTSTNTSTVIPSNPNSVFLVDCT
HSQCPTDLPNIATTQHSLRYLDNASASAITVLERTHPAASSTMATESSHQSPSADSQAEE
LSKSQRVAKDDDPFVVSNTANSDEPASSSKPAKPLTDLNRAFSQRLNLDPQKPGKSQGEI
SEQEEDEYDDAESDEMHSPYSTHEPESEPEDQDEPSEKDDENKDVEEEQEQEQEEEQIDP
EEAKRIALRERMAKMSGGIGMHVFGLPGLAAPIGRKNTLRRTPAKSSEEAKSTTNDSSPP
KDSSSTSTQPTEQSNAQQAPSPKEEERPLPSEPSQNQPAEYRDTPDTPRNIMPLPGLMSA
DQPIKVTEPSNDADKAIVAEGPNNEEETKGPVIPETQETSEQQVHKTPSPEKQKVLSPPP
IITNFDKETLASNEAHEAVPQKPSAPQVTRLMAPQDSSSVVTPSPTSLLDPARAVRKVID
GIDPPKEAGAGATADVESAANSPITPPRTWHSPDFTSKSFEPIERKLPSRISEVTEDSID
EDKQNEVDPSTSARALPPPGLRFGKVDTLASLAHDDLDDLPAVPRIFSPPPLPKTPSGEF
GDNEFMFPKKSNRVRGHQSRPSTGSQLRNVVPVSIVTSGGRPALPDEMASPSSSIGHPLP
SPPPADFNSLNVDFYEPHSYLESPAPEPQPSYEEESFNATVIHAPTPSTATFQGHPTISN
VATPPLKQDVTESKASPVADASATHQSSTGLTQEITQLGSNMRLPTKLTRPSNDGRKASG

PRPAAPPSIPPPLPVSNILSSPTSEPPKDHPPSAPLSKPVSTSPAAPLARVPPVPKLSSK
APPVPLPSADAPPIPVPSTAPPVPIPTSTPPVPKSSSGAPSAPPPVPAPSSEIPSIPAPS
GAPPVPAPSGIPPVPKPSVAAPPVPKPSVAVPPVPAPSGAPPVPKPSVAAPPVPVPSGAP
PVPKPSVAAPPVPAPSGAPPVPKPSVAAPPVPAPSSGIPPVPKPAAGVPPVPPPSEAPPV
PKPSVGVPPVPPPSTAPPVPTPSAGLPPVPVPTAKAPPVPAPSSEAPSVSTPRSSVPSPH
SNASPSPTSSSMASAAPARTSVSRSKSKAERHETSTSSRKSSKSGEHHHHHNEGHADSSS
TRTSLAHQDSRKSLHRHLSRSSSRASKKPSIVSTTGPFNESFSAKPVEPCASEKWWLNST
AVPKSVVQMNDSVLYMIKEGITGQDKKYKSVHILFPDYSQTVLTATFNPHNQNITQLSQL
QLAPPAQPSKARLDEEYACYGSTILKKARAYQGSMVGDGSAFTFVNSVMSILAHNLEPIN
KQTFGGVIYKNVGNVTVQQIGEIRPGDIVTFDKAKFSGQKGTLRSKYSLEVGKPMHYGII
SEWDVSKLKIRVLEQGRESKKVSVASYKFGDLKSGEVTVWRVMRRSWLGWN
>Q8R5G7
MAAPQDLDIAVWLALVHLEQYADTFRRHGLATAGAAQHLGHEELRHLGISATGHRKRILR
LLRAGSAEGFLDSHLDNTMEPTPSPAPDAQPPKPVPKPRTVFGLSNPATAQRPGLSPIFW
DPEVSRNSECTQRSSPLLPSSSEHPSVPNTMEMMPNAIYFGLDLRGRAQAAQDVTPDSSQ
ATVPTPAFRPTTGTVHIMDPGCLYYGVQPVGIPGASDRRDGRGVCQERAEHRQDLETRED
AGYASLELPGDSILSLPTQDAETSDDLISPYASFSSTADRPVPLLSGWLDKLSPQGNYVF
QRRFVQFNGRSLMYFGSDKDPFPKGVIPLTAIEMTRSSKDNKFQVITGQRVFVFRTESEA
QRDLWCSTLQSCLKEQRLLGHPRPPHPPRPLRTGTLELRGHKAKVFAALIPGELALYKSE
QAFSLGIGICFIELQGCSVRETKSRSFDLLTPHRCFSFTAESGGARQSWAAALQEAVTET
LSDYEVAEKVWSNPANRHCADCRASRPDWAAVNLGVVICKQCAGQHRALGSGISKVQSLK
LDTSVWSNEIVQLFIVLGNDRANCFWAGALPPGEGLHPDSAPGPRGEFISRKYKLGLFRK
PHPRHPDHSQLLQALCAAMAGPNLLKNMAQLLCVETSEGEEPLSPSALNGSLLSLLPSDS
PGVYNEVVVPATYRGFLYCGSISNKAGAPPLRRGRDAPPRLWCVLGAALEMFASESSPEP
LSLLQPQDIVCLGVSPPPADPGDLDRFPFSFELILTGGRIQHFATDGADSLEAWISAVGK
WFSPLSCHQLLGPGLLRMGRLWLRSPSHAGLAPGLWLSGFGLLRGDHLFLCPAPGPGPPA
PEDMVHLRRLQEISVVSAADTPDKKEHLVLVETGRTLYLQGEGRLDFAAWNTAIGGAAGG
GGTGLQEQQMSRGDIPIIVDACISFVTQHGLRLEGVYRKGGARARSLRLLAEFRRDARSV
KLRPREHFVEDVTDTLKRFFRELDDPVTSARLLPRWREAAELSQKNQRLEKYKEVISCLP
RVNRRTLATLIGHLYRVQKCASLNQMCTRNLALLFAPSVFQTDGRGEHEVRVLQELIDGY
ISVFDIDSDQAAQIDLEVSLITTWKDVQLSQAGDLIMEVYIEQQLPDNCVTLKVSPTLTA
EELTNQVLEMRGAASGTDLWVTFEILEHGELERPLHPKEKVLEQALQWCQLPEPCSASLL
LRKVSMAHAGCLFTGVRRESPRVGLLRCREEPPRLLGNRFQERFFLVRGRCLLLLKEKKS
SKPEREWSLEGAKVYLGIRKKLKPPTLWGFTLILEKMHLCLSCMDEEEMWDWTTSILKAQ
HDDQQSVVLRRRSSSDLARQKFGTMPLLPIRGDDSGATLLSANQTLRRLHNRRTLSMFFP
MKSPQGSVEEQDELEEPVYEEPVYEEVGAFPELTKDTTFSSTWEWSAKSDPSLTSQRSFD
QPPLSKASMLGHEERIPDPPPGPPSKSSSQARGSLEEQLLQELNNLILRKGEPASCPESS
SQPTSPQAPSPTSLPTPTPSLPTQPPCTSNPPSSQPLT
>P07277
MSLPFLTSAPGKVIIFGEHSAVYNKPAVAASVSALRTYLLISESSAPDTIELDFPDISFN
HKWSINDFNAITEDQVNSQKLAKAQQATDGLSQELVSLLDPLLAQLSESFHYHAAFCFLY
MFVCLCPHAKNIKFSLKSTLPIGAGLGSSASISVSLALAMAYLGGLIGSNDLEKLSENDK
HIVNQWAFIGEKCIHGTPSGIDNAVATYGNALLFEKDSHNGTINTNNFKFLDDFPAIPMI
LTYTRIPRSTKDLVARVRVLVTEKFPEVMKPILDAMGECALQGLEIMTKLSKCKGTDDEA

VETNNELYEQLLELIRINHGLLVSIGVSHPGLELIKNLSDDLRIGSTKLTGAGGGGCSLT
LLRRDITQEQIDSFKKKLQDDFSYETFETDLGGTGCCLLSAKNLNKDLKIKSLVFQLFEN
KTTTKQQIDDLLLPGNTNLPWTS
>Q99497
MASKRALVILAKGAEEMETVIPVDVMRRAGIKVTVAGLAGKDPVQCSRDVVICPDASLED
AKKEGPYDVVVLPGGNLGAQNLSESAAVKEILKEQENRKGLIAAICAGPTALLAHEIGFG
SKVTTHPLAKDKMMNGGHYTYSENRVEKDGLILTSRGPGTSFEFALAIVEALNGKEVAAQ
VKAPLVLKD
>Q05926
MSAFVTKAEEMIKSHPYFQLSASWCPDCVYANSIWNKLNVQDKVFVFDIGSLPRNEQEKW
RIAFQKVVGSRNLPTIVVNGKFWGTESQLHRFEAKGTLEEELTKIGLLP
>P40463
MDTSNHNQDHDSHVAAQRENDNNYMPPSPSMSESSMIFERNVEDPSYLYKTVSNNAANSL
SRQSSRTSLFNHNNSSNRNFHNLSQRSSAVNLHLQPSRTNESIASYQTYNPDFVVQTPLD
HRRTLENFVPPALDAGCSIVTDDTTGLDDVDMVYSRRPSTIGLDRALGRTRSLSSQSFDN
ETSPAHPRSPNDHGSRLLRFYSYADMLSDDNNNNVSNATSTSSTANPLRRPPMQGHYSFS
SSLLNSPSHLPSPPSASASPPQHMNFTNPFIISRRYSNTTINNANGGTSAGSTTGAALSR
SPSNQQYLLKQQRSPSGSARSRRNSNRPGSAANIMIGKPKSKFHMESSGSEGFSSEEEDN
TMIERDKLNLKQKLQSQLAQPPSIANMVNDNHNNTNKHKNTINNNIKNSPAFTNSNPSSK
SNSNSTITSMNPDTTK
>Q9QZM0
MAENGESSGPPRPSRGPAAAPGAASPPAEPKIIKVTVKTPKEKEEFAVPENSTVQQFKEA
ISKRFKSQTDQLVLIFAGKILKDQDTLMQHGIHDGLTVHLVIKSQNRPQGQATTQPSTTA
GTSTTTTTTTTAAAPAATTSSAPRSSSTPTTTNSSSFGLGSLSSLSNLGLNSPNFTELRN
QMQQQLLASPEMMIQIMENPFVQSMLSNPDLMRQLIMANPQMQQLIQRNPEISHLLNNPD
IMRQTLEIARNPAMMQEMMRNQDLALSNLESIPGGYNALRRMYTDIQEPMLNAAQEQFGG
NPFATVGSSSTSGEGTQPSRTENRDPLPNPWAPPPTTQTAATTTTTTTTSSGSGSGSSSS
STTAGNTMAAANYVASIFSTPGMQSLLQQITENPQLIQNMLSAPYMRSMMQSLSQNPDMA
AQMMLSSPLFTSNPQLQEQMRPQLPNFLQQMQNPETIAAMSNPRAMQALMQIQQGLQTLA
TEAPGLIPSFAPGVGMGVLGTAITPVGPVTPIGPIGPIVPFTPIGPIGPIGPTGPASSPG
STGTGIPPATTVSSSAPTETISPTSESGPNQQFIQQMVQALTGGSPPQPPNPEVRFQQQL
EQLNAMGFLNREANLQALIATGGDINAAIERLLGSQPS
>O59748
MAENENHVLSIRMSHPYYSEKEISRILSTRDPKENNLRMQAFAWISTLSKTLKFPVRTSG
LAMLLYSRFQLFFPVNEIPLLECATACLVVASKIEDTAKKFRDILLAHYLQKHPGSEVDA
HSQKRILGLERMTLELICFDFRVRHPHNYMVKFAKSLKFSSSTASIAWNVCTDAYKTYTM
LKYPAHIVAVASISIACKLQQLPQPIIPRSFFAPPALTEAVIADILDLYMHYQPHTCIGN
MYTTEKLLGLCVDFQRAQKNSGRPQKPPQIDPHSSSLADEYRESNKRLQESKESCARFIL
DCDRKYFNTEFEKRMLEERRNKGTV
>Q10434
MEKEAIYKLIRESPPGEVNQVVHDIRDIGLSDEEAIHEQLKLYHEDYNSSVSISDDEKVI
ISADNRLEGNRYYDQVLQKSFTINYETMEAENVEDYTEAIKIPDEIVKQIKKVASDHYLS
DVTFGIIKKSDEVESFTIVLVSSKYNPKNYWNGSWRCICNYNVSEKKLEGRSHIRVHYYE
DGNVWLDASRPISATVEETSKLYEVLAQVENGIQQSFNVELSSLNDKKFKELRRQLPVTR

QKINWENVSGIRMRNT
>Q15650
MAVAGAVSGEPLVHWCTQQLRKTFGLDVSEEIIQYVLSIESAEEIREYVTDLLQGNEGKK
GQFIEELITKWQKNDQELISDPLQQCFKKDEILDGQKSGDHLKRGRKKGRNRQEVPAFTE
PDTTAEVKTPFDLAKAQENSNSVKKKTKFVNLYTREGQDRLAVLLPGRHPCDCLGQKHKL
INNCLICGRIVCEQEGSGPCLFCGTLVCTHEEQDILQRDSNKSQKLLKKLMSGVENSGKV
DISTKDLLPHQELRIKSGLEKAIKHKDKLLEFDRTSIRRTQVIDDESDYFASDSNQWLSK
LERETLQKREEELRELRHASRLSKKVTIDFAGRKILEEENSLAEYHSRLDETIQAIANGT
LNQPLTKLDRSSEEPLGVLVNPNMYQSPPQWVDHTGAASQKKAFRSSGFGLEFNSFQHQL
RIQDQEFQEGFDGGWCLSVHQPWASLLVRGIKRVEGRSWYTPHRGRLWIAATAKKPSPQE
VSELQATYRLLRGKDVEFPNDYPSGCLLGCVDLIDCLSQKQFKEQFPDISQESDSPFVFI
CKNPQEMVVKFPIKGNPKIWKLDSKIHQGAKKGLMKQNKAV
>Q04235
MSDETMPVEFLVSDKRLLKTIKVKLETNGLFVTPIYSDNDNKVIKSSIEDLNHPLAVEIN
NIAGVKARFHESGNLERSEGHLKHQSNSITEFTKSFLKDHGLANDKIFLSHLLDHLPLKY
TIYPPVVLFNNSTVRSFNHPIWQKAFQLKLFDPNEYYRELLCFLSPGKPSKGTSLHPNNR
LLTHLAINNPITEADVLRRPFNIQPLYGKLIDDSILDDNDNTLWENPSQEQLNSSIWCKV
IQNGVTQIWSPVFTMFSRGNIKEKKRVLTTFPDICNNDVVDLYAGIGYFTFSYLTKGART
LFAFELNPWSVEGLKRGLKANGFNKSGNCHVFQESNEMCVQRLTEFLSQNPGFRLRIRHI
NLGLLPSSKQGWPLAIKLIYLQGASLEKVTMHIHENVHIDAIEDGSFEKNVIVELDAINE
SIALIRNRGIKLQFVRSKLERIKTFAPDIWHVCVDVDVIVST
>Q9Y2T7
MSEVEAAAGATAVPAATVPATAAGVVAVVVPVPAGEPQKGGGAGGGGGAASGPAAGTPSA
PGSRTPGNPATAVSGTPAPPARSQADKPVLAIQVLGTVKWFNVRNGYGFINRNDTKEDVF
VHQTAIKRNNPRKFLRSVGDGETVEFDVVEGEKGAEATNVTGPGGVPVKGSRYAPNRRKS
RRFIPRPPSVAPPPMVAEIPSAGTGPGSKGERAEDSGQRPRRWCPPPFFYRRRFVRGPRP
PNQQQPIEGTDRVEPKETAPLEGHQQQGDERVPPPRFRPRYRRPFRPRPRQQPTTEGGDG
ETKPSQGPADGSRPEPQRPRNRPYFQRRRQQAPGPQQAPGPRQPAAPETSAPVNSGDPTT
TILE
>Q90Z72
MQLRKMQTLKKEHGSVDTSSNVDKIMVLKSTLAEVSEELSTNEDILLTEASSGKSKSSAC
RRKREFIPDEKKDAMYWEKRRKNNEAAKRSREKRRLNDLVLENKLIALGEENATLKAELL
SLKLKFGLISSASYAQEIQKLSSSTTVYFQDYQSSKPNINSFVDEHEPSVVGSSCISVIK
HSPQSSMSDMSEMPSVEHTQGSRIQSNCRSPENKFQIIKQEPIELEREPRDDRGSYKASI
YPNYMGTTFNMYSHSPPLLQVNRSSSNSPRTSETDDGVVGKSSDGEDEQQVPKGPIHSPV
EHKNVHATVKVPEVNSSALPHKLRIKAKAMQVKVEAMDNDYDATQKLSSPIDMSSKRHFE
LEKHGAQNLVHSSHTPFSVQVTNIQDWSLKPELWHQKELNVKIQNGCKTGVVEIKDNVYN
VSESENLYLKQGIANLSAEVASLKRLITTQQISASDSG
>Q9QXL2
MLGAADESSVRVAVRIRPQLAKEKIEGCHICTSVTPGEPQVFLGKDKAFTFDYVFDIDSQ
QEQIYTQCIEKLIEGCFEGYNATVFAYGQTGAGKTYTMGTGFDVNIMEEEQGIISRAVRH
LFKSIDEKKTSAIKNGLPPPEFKVNAQFLELYNEEVLDLFDTTRDIDAKNKKSNIRIHED
STGGIYTVGVTTRTVNTEPEMMQCLKLGALSRTTASTQMNVQSSRSHAIFTIHVCQTRVC
PQTDAENATDNKLISESSPMNEFETLTAKFHFVDLAGSERLKRTGATGERAKEGISINCG

LLALGNVISALGDKSKRATHVPYRDSKLTRLLQDSLGGNSQTIMIACVSPSDRDFMETLN
TLKYANRARNIKNKVMVNQDRASQQINALRSEITRLQMELMEYKTGKRIIDEEGVESIND
MFHENAMLQTENNNLRVRIKAMQETVDALRARITQLVSEQANQVLARAGEGNEEISNMIH
SYIKEIEDLRAKLLESEAVNENLRKNLTRATARSPYFSASSAFSPTILSSDKETIEIIDL
AKKDLEKLKRKEKKKKKRLQKLEESGREERSVAGKDDNADTDQEKKEEKGVSEKENNELD
VEENQEVSDHEDEEEEEEDEEEEDDIEGEESSDESDSESDEKANYQADLANITCEIAIKQ
KLIDELENSQKRLQTLKKQYEEKLMMLQHKIRDTQLERDQVLQNLGSVESYSEEKAKKVK
CEYEKKLHAMNKELQRLQTAQKEHARLLKNQSQYEKQLKKLQQDVMEMKKTKVRLMKQMK
EEQEKARLTESRRNREIAQLKKDQRKRDHQLRLLEAQKRNQEVVLRRKTEEVTALRRQVR
PMSDKVAGKVTRKLSSSESPAPDTGSSAASGEADTSRPGTQQKMRIPVARVQALPTPTTN
GTRKKYQRKGFTGRVFTSKTARMKWQLLERRVTDIIMQKMTISNMEADMNRLLRQREELT
KRREKLSKRREKIVKESGEGDKSVANIIEEMESLTANIDYINDSIADCQANIMQMEEAKE
EGETLDVTAVINACTLTEARYLLDHFLSMGINKGLQAAQKEAQIKVLEGRLKQTEITSAT
QNQLLFHMLKEKAELNPELDALLGHALQDLDGAPPENEEDSSEEDGPLHSPGSEGSTLSS
DLMKLCGEVKPKNKARRRTTTQMELLYADSSEVASDTSAGDASLSGPLAPVAEGQEIGMN
TETSGTSARDKELLAPSGLPSKIGSISRQSSLSEKKVPEPSPVTRRKAYEKADKPKAKEH
KHSDSGASETSLSPPSSPPSRPRNELNVFNRLTVPQGTPSVQQDKSDESDSSLSEVHRGI
INPFPACKGVRASPLQCVHIAEGHTKAVLCVDSTDDLLFTGSKDRTCKVWNLVTGQEIMS
LGVHPNNVVSVKYCNYTSLVFTVSTSYIKVWDIRESAKCIRTLTSSGQVTLGEACSASTS
RTVAIPSGESQINQIALNPTGTFLYAASGNAVRMWDLKRFQSTGKLTGHLGPVMCLTVDQ
ISNGQDLIITGSKDHYIKMFDVTEGALGTVSPTHNFEPPHYDGIEALAIQGDNLFSGSRD
NGIKKWDLAQKGLLQQVPNAHKDWVCALGLVPGHPVLLSGCRGGILKLWNVDTFVPVGEM
RGHDSPINAICVNSTHVFTAADDRTVRIWKAHNLQDGQLSDTGDLGEDIASN
>Q9P6N1
MPSIILYCSWNQDRGFLSIGSENGYQVYRSNPFTLCFSKKANGASICEMLYESSLLAFVN
ISPESTRLLKLVDIKRDIVLCRIFYPSPVLSVRFTWNRLVVLIKGSIYVYNLKNMELINT
LNTSKGNVIAFAVHENYVAYNSPTNPGDIYLASLDTAIPVTLIHCHSSAVQVVDFHPRGH
LIATASAKGTVIRVITTSDGELVTELRRGYIPASIVSISFHPVEPFLACASENGTIHVFK
ISKQPSDPNSSPTSSVTVSSSWSKYLTSNVAKVWDTRKEFATAKIPEASFYGKIIFSSSG
PHIQVASYSGHYYRFAVNLKNGGNCALLERYIFDD
>Q12242
MMDNQAVKSLLEMGIPHEVAVDALQRTGGNLEAAVNFIFSNELPEQAEMGEENDGSQPRI
SENKIVAGTKPCDVPNNGDQDIDMPDVSGVDVDYDDDEDITDERSGSNSTSGCRVTAQNY
DRYSISETSIPPPSYSIVQHNEFKSNVGDPTVVLPLPLNSLIESYFGLFALLTAVYFPHV
FLKPDFKDLNYRADWFKGSSFTEPKYRLAYCEAEDGSTTSEIVLASGPNEGLQPHLLWQL
QKLISVVNTRKCERAFVSAKVFTSSLEPQLRSKLADSEHLYEVLPAFIKSLAVDLEMCPG
IRDRETRSLFISSALHTPNKNEPPMETFLSLFHFLPEEYDSNLYKMFNVLLYPEEEEEEE
DVIRGGEQEEARYVEPENTLKEVAPVLTILFNELETNTESVSLPNGVDIPLEFYPQLYTK
QCKDQLIRHIISKRKQARTRSRCLLQEINELKSYQGKNISTILESTLAYLQTIPDDANNE
AAKQIASLKDTLNSARAAKMEEYKDLASKLHGEWNLSHPETHIINTAKQLGLIENPYILT
MAALSPYSYFIRSRNGAWSWIQSNTLGTEFKVKKCSSPSVVQEAIKHGTKYASETPLMFI
YCEEGKIPTEEVVAEALKSNSGCLKFAEDDQNSLKTLRSQFFDGMGDPEQATNNINNGND
NDNDDDIDSDN
>Q9JIM4

MLEVQSSNHGCERQAPTTSPASSAGHAVEVRPGLYLGGAAAVAGPDYLREAGITAVLTVD
SEPAFPAGAGFEGLQSLFVPALDKPETDLLSHLDRCVAFIGQARSEGRAVLVHCHAGVSR
SVAVVTAFIMKTEQLTFEKAYENLQTIKPEAKMNEGFEWQLKLYEAMGHEVHTSSAVYKQ
YRLQKVTEKYPELRNLPRELFAVDPTTVSQGLKDDILYKCRKCRRSLFRRSSILDHSEGS
GPVAFAHKRTGLSSVLTTGNQAQCTSYFIEPVQWMESALLGVMDGQLLCPKCSAKLGSFN
WYGEQCSCGRWITPAFQIHKNRVDEVKTLPALGSQTKKP
>Q12229
MDIFRHTFGNNDDSFIRIPGAFREEPPADLNGRTEDQNSNTNEPTQSRDGRLKSILHFLF
QAPLIVLYYLLNFIVRSSRLLKPLLRLHGFYQRKHNRLLDHSSQLHRLLENLENEAQAVT
CSEGNGNNDDGSNTDSTSNNESSGVQFSFGSLYNPENGTFSKSIMQNSYTELLDACSEQV
KFGVIYLHDPLLDNHMDYVNKILCSEAFVNMIRKYQVLLWYGDVTTSEGLQVSNALKIRQ
YPLLGIISLKAEKKIELIARVEGSISNYKAQDLEAIFSKNYSRLIQLRQQRQNIEMQRLI
RQQQDSRYQDSLRRDQQRESERLEQTQREQMEREHQRIENQWLLWRKSQLKPEPSSDKDA
SKVAIRLENGQRLVRKFDASLPTEEIYAFVELQLHDMLNSENDTLPVYQPANYQHQYSFK
LITPVPRRELDLSTKISDVSGIYPSGNIVMERLDE
>Q06409
MSQQDSQRWLPTDRLIYGVLVKSFLPLQRYPELVYENSNYANVYVGAEVYVFEESVDKKW
CRAYQCLRPFPEEFISNMNSANDVLPDVKPKVVIFPRKYVHFEAEKAVSTMPFFKAPSAE
DFKPLISKECESRSFCDSLYVSSTDDISTGKPRKTPRPPFPFFRYQKRSFKDEMGPILSL
ISSHVYSMYSIGEFSIYRKMIKLYYDLDTIRFRLSMNLTTEAEKINLIRAATSLRTKIAK
FLSSTYRKNKLIANSTPRNPDPYGFEGIFARDIDTGELLSYEIDKLRTLVSSSMLCGLTN
NFPTVPVVESDDESSSNGLFGTVRSSILVNLKDLAWDPSISDPKYQDLSICVYLRTKDEV
LTESFTMTKSSNMESALDEIPAMLFKNILETIVHKNKVYLVVVLKETIAITTETAPEISS
YNISTEESSSHSPFSPFNSSTENKIDHVKKGLAAGVINISPVFKFYNGLSVANKAQRFNL
YLYSSDSSDSQNFNSSKDADLGWGGLINKIIKDSSEGVSVNPRAVSLSVTVKEIIGKQEA
EKVLSTSLVPIRSIPTYFYDTMFSQAERIYLNLGRVSLYGLPAADTNIENVTVQISCRNK
AVKFCKNKLEERSGDWKFVSVRPNESIGESIRIEGVENMNEDETLRVLVYLNGFLMAKSN
IHIKKKNEIIEYRKGTVFQIMSSKSVPLIHLELEASYFGRRYNINPAITNFLVLQTKNVE
FDQQLKEHYSVTLKQLNNVSFKDLLKHFDTILAHYLLLLESVNEATDKKGPSSSLPNIVF
SEFVKFLNLMLTHQENSRYWFNRLYKKVMSKELECPNVAPILIKHMTTIFDRSHSSWTRT
GTAICRTILYIIVLAIGSSHSDEMPNFSHFFRSLHKFLMLADEPIMADQILLIESIPSML
ETMTNHCKVEDLVRFAIGLFECCQEKEMNQKMYSRPLSVREEEYLNTKFNCLLKLINKKV
LQNYLTNTESVDKLRLQFLSKTLEWLLTPYTPGDDKCFHVESLRLVNSVFITIIEDYKFD
MLQRNLIRLLPYLCKSFVHLRRYCKKARLMRPRRVFTMLFPREIPCNYIPVDSIVNDEVV
VEVLLELAIIICEITKIASSRFPSYQSFSEIINLCDKDTLFQSNFYSRQITNENVYTITK
TVFLFFKQDWFPGMKWLGVSALLGRSSLILLSLCKDYIIENNSPSPSKESEKRVDMRLWA
EYVKVILLVSNHKSASLTKLAITPRKAVYLISGDLKKISAYILNECWDALATGHYNITYA
KKYGLGALSDCQFELFVHNQFLIREIFIFAFHRHIDATRICCKILWGLGLNFWRIFGSLQ
PAVNACIPELFSAYQIGKLRLNDYELERFVSCLFFMMHVPDSDTFFPACMDFLRDLLGFL
HIVNEIYKIPNQEEFDDDRTARHIEMFEYLLEANRPELFHKMIYDLFIHFIQKKDFVQAA
LSLELLAGTYAWDSNDTLEAISFPPLPEQSSFERKEYLLKESARNFSRGQKPEKALAVYK
DLIKAYDEINYDLNGLAFVHDQIAGIYTRLQSIDRLVPTYFKVSFMGFGFPKSLRNKSFV
FEGLPFEHITSMHDRLLRSYHGSNIVHSQEEVDMLLMNPPMGKYIHVASVEPCLSISDNY
NSSDKKSSINNKVRMYIENRDLRTFSNSRRLPGAKGVTDLWVEEYTYHTMNTFPTLMNRS

EIVKVTKSKLSPLENAIRSLQVKIQELYGLENMCNKTLKDHGDVNDLFTELSTNITGTIS
APVNGGISQYKAFLEPSTSKQFSTDDLGRLTLAFDELVAVLGRCLTLHAELLPSKDLKPS
HDLLVRLFEENFAEEIERYSRTLSEANRSRNNMITARIISHKNPNKKASFSGRDHHTSGS
NHSQFVLEHSDSFGPNSLLFGKYLTRTLSHSSTTSSLDKSGIVSGTSSTFLAGSQPNTNT
DSQHKHDYSHSG
>Q6Q0C0
MSSGKSARYNRFSGGPSNLPTPDVTTGTRMETTFGPAFSAVTTITKADGTSTYKQHCRTP
SSSSTLAYSPRDEEDSMPPISTPRRSDSAISVRSLHSESSMSLRSTFSLPEEEEEPEPLV
FAEQPSVKLCCQLCCSVFKDPVITTCGHTFCRRCALKSEKCPVDNVKLTVVVNNIAVAEQ
IGELFIHCRHGCRVAGSGKPPIFEVDPRGCPFTIKLSARKDHEGSCDYRPVRCPNNPSCP
PLLRMNLEAHLKECEHIKCPHSKYGCTFIGNQDTYETHLETCRFEGLKEFLQQTDDRFHE
MHVALAQKDQEIAFLRSMLGKLSEKIDQLEKSLELKFDVLDENQSKLSEDLMEFRRDASM
LNDELSHINARLNMGILGSYDPQQIFKCKGTFVGHQGPVWCLCVYSMGDLLFSGSSDKTI
KVWDTCTTYKCQKTLEGHDGIVLALCIQGCKLYSGSADCTIIVWDIQNLQKVNTIRAHDN
PVCTLVSSHNVLFSGSLKAIKVWDIVGTELKLKKELTGLNHWVRALVAAQSYLYSGSYQT
IKIWDIRTLDCIHVLQTSGGSVYSIAVTNHHIVCGTYENLIHVWDIESKEQVRTLTGHVG
TVYALAVISTPDQTKVFSASYDRSLRVWSMDNMICTQTLLRHQGSVTALAVSRGRLFSGA
VDSTVKVWTC
>Q9EQH4
MADTAAGPGGSGTRPGSKQSTNPADNYHLARRRTLQVVVSSLLTEAGFESAEKASVETLT
EMLQSYISEIGRSAKSYCEHTARTQPTLSDIVVTLVEMGFNVDTLPAYAKRSQRMVITAP
PVTNQPVTPKALTAGQNRPHPPHIPSHFPEFPDPHTYIKTPTYREPVSDYQILREKAASQ
RRDVERALTRFMAKTGETQSLFKDDVSTFPLIAARPFTIPYLTALLPSELEIQQMEETDS
SEQEEQTDTENNALHISTDDSGAEKESASVLQQSSSLSGSRNGEESVIDNPYLRPVKKPK
IRRKKSLS
>P55060
MELSDANLQTLTEYLKKTLDPDPAIRRPAEKFLESVEGNQNYPLLLLTLLEKSQDNVIKV
CASVTFKNYIKRNWRIVEDEPNKICEADRVAIKANIVHLMLSSPEQIQKQLSDAISIIGR
EDFPQKWPDLLTEMVNRFQSGDFHVINGVLRTAHSLFKRYRHEFKSNELWTEIKLVLDAF
ALPLTNLFKATIELCSTHANDASALRILFSSLILISKLFYSLNFQDLPEFFEDNMETWMN
NFHTLLTLDNKLLQTDDEEEAGLLELLKSQICDNAALYAQKYDEEFQRYLPRFVTAIWNL
LVTTGQEVKYDLLVSNAIQFLASVCERPHYKNLFEDQNTLTSICEKVIVPNMEFRAADEE
AFEDNSEEYIRRDLEGSDIDTRRRAACDLVRGLCKFFEGPVTGIFSGYVNSMLQEYAKNP
SVNWKHKDAAIYLVTSLASKAQTQKHGITQANELVNLTEFFVNHILPDLKSANVNEFPVL
KADGIKYIMIFRNQVPKEHLLVSIPLLINHLQAESIVVHTYAAHALERLFTMRGPNNATL
FTAAEIAPFVEILLTNLFKALTLPGSSENEYIMKAIMRSFSLLQEAIIPYIPTLITQLTQ
KLLAVSKNPSKPHFNHYMFEAICLSIRITCKANPAAVVNFEEALFLVFTEILQNDVQEFI
PYVFQVMSLLLETHKNDIPSSYMALFPHLLQPVLWERTGNIPALVRLLQAFLERGSNTIA
SAAADKIPGLLGVFQKLIASKANDHQGFYLLNSIIEHMPPESVDQYRKQIFILLFQRLQN
SKTTKFIKSFLVFINLYCIKYGALALQEIFDGIQPKMFGMVLEKIIIPEIQKVSGNVEKK
ICAVGITKLLTECPPMMDTEYTKLWTPLLQSLIGLFELPEDDTIPDEEHFIDIEDTPGYQ
TAFSQLAFAGKKEHDPVGQMVNNPKIHLAQSLHKLSTACPGRVPSMVSTSLNAEALQYLQ
GYLQAASVTLL
>O14131

MSENVYLNEALNVVDQCFKTFFDKYTDRMGSAFACSGTIDKDHIFLVWSVAVYAEAMADS
LRYTSKFERKFEHVFQALKKYWSPVFNACCAFHYFEGNDDVYYDDNAQVAIGLATAGYYT
TNSSRRDHYVSRAESIISLIINKGWNQQRGGIAWHTRTTGPPWTNLNACSTSMSAVAALR
LALALDDPNKKQHLVSFAWNCVRWIQENLLDENHIVCDGLSLKDGKWVLDKARFTYNTGT
TMTAMSLLMGLGEFTKGLQDIEKLPTYLEDMARGALDTNGPLYDQSCNGSFKVWSDNTFF
AQHLSEGLMTFSYAMPSSALAKPAQKMVLDQADFLMKYLRIPKEGLYYRNFGLYKLSPEL
TASFNKFFNANKQFQPDKDERIQQEGPVEQRPLCPTLIGSAGAARMLFSAAEIVNRNNPS
SGESTTLPQPSHGKKDKDCVIS
>Q09728
MVVINNVKMACMKCIRGHRSSTCKHNDRELFPIRPKGRPISQCEKCRIARITRHLHVKCT
CNSRKKGSKCSTSSTTDLDSSSASNSSCSIPSSISEKLLPRDNVKTHCPKRSASCCGKKP
DVMPLKINLESQTDFMGMPLQSQRPHSESYRMLPEPEKFKSEYGYPSQFLPIEKLTSNVA
YPPNYNNYLKSPYQQPTNFPPEIQYNYSHSPQHSIQEAEEAAVYGPPVYRSGYQILYNNN
TDSIAAAAATHDLYPQPDVPLTFAMLADGNYVPLPSSTNTYGPSNSYGYEININESTNHV
DSSYLPHPIQLSNYFTLPSSCAQADAACQCGDNCECLGCLTHPNNATTLAALNHISALEK
ETISHTDLHHTFKHEVNSSNNYELTNDELAASSPLYTSSSVPPSHITTGST
>P14270
MEAEGSSVPARAGSHEGSDSSGGAALKAPKHLWRHEQHHQYPLRQPQFRLLHPHHHLPPP
PPPSPQPQLQPPPPPPLPPPPPPPGATRGRYASSGASRVRHRGYSDTERYLYCRAMDRTS
YAVETGHRPGLKKSRMSWPSSFQGLRRFDVDNGTSAGRSPLDPMTSPGSGLILQANFVHS
QRRESFLYRSDSDYDLSPKSMSRNSSIASDIHGDDLIVTPFAQVLASLRTVRNNFAALTN
LQDRAPSKRSPMCNQPSINKATITEEAYQKLASETLEELDWCLDQLETLQTRHSVSEMAS
NKFKRMLNRELTHLSEMSRSGNQVSEYISNTFLDKQHEVEIPSPTQKEKEKKKRPMSQIS
GVKKLMHSSSLTNSCIPRFGVKTEQEDVLAKELEDVNKWGLHVFRIAELSGNRPLTVIMH
TIFQERDLLKTFKIPVDTLITYLMTLEDHYHADVAYHNNIHAADVVQSTHVLLSTPALEA
VFTDLEILAAIFASAIHDVDHPGVSNQFLINTNSELALMYNDSSVLENHHLAVGFKLLQE
ENCDIFQNLTKKQRQSLRKMAIDIVLATDMSKHMNLLADLKTMVETKKVTSSGVLLLDNY
SDRIQVLQNMVHCADLSNPTKPLQLYRQWTDRIMEEFFRQGDRERERGMEISPMCDKHNA
SVEKSQVGFIDYIVHPLWETWADLVHPDAQDILDTLEDNREWYQSTIPQSPSPAPDDQED
GRQGQTEKFQFELTLEEDGESDTEKDSGSQVEEDTSCSDSKTLCTQDSESTEIPLDEQVE
EEAVAEEESQPQTGVADDCCPDT
>Q13574
METFFRRHFRGKVPGPGEGQRRPSSVGLPTGKARRRSPAGQASSSLAQRRRSSAQLQGCL
LSCGVRAQGSSRRRSSTVPPSCNPRFIVDKVLTPQPTTVGAQLLGAPLLLTGLVGMNEEE
GVQEDVVAEASSAIQPGTKTPGPPPPRGAQPLLPLPRYVRRASSHCCPADAVYDHALWGL
HGYYRRLSQRRPSGQHPGPGGRRASGTTAGTMLPTRVRPLSRRRQVALRRKAAGPQAWSA
LLAKAITKSGLQHLAPPPPTPGAPCSESERQIRSTVDWSESATYGEHIWFETNVSGDFCY
VGEQYCVARMLKSVSRRKCAACKIVVHTPCIEQLEKINFRCKPSFRESGSRNVREPTFVR
HHWVHRRRQDGKCRHCGKGFQQKFTFHSKEIVAISCSWCKQAYHSKVSCFMLQQIEEPCS
LGVHAAVVIPPTWILRARRPQNTLKASKKKKRASFKRKSSKKGPEEGRWRPFIIRPTPSP
LMKPLLVFVNPKSGGNQGAKIIQSFLWYLNPRQVFDLSQGGPKEALEMYRKVHNLRILAC
GGDGTVGWILSTLDQLRLKPPPPVAILPLGTGNDLARTLNWGGGYTDEPVSKILSHVEEG
NVVQLDRWDLHAEPNPEAGPEDRDEGATDRLPLDVFNNYFSLGFDAHVTLEFHESREANP
EKFNSRFRNKMFYAGTAFSDFLMGSSKDLAKHIRVVCDGMDLTPKIQDLKPQCVVFLNIP

RYCAGTMPWGHPGEHHDFEPQRHDDGYLEVIGFTMTSLAALQVGGHGERLTQCREVVLTT
SKAIPVQVDGEPCKLAASRIRIALRNQATMVQKAKRRSAAPLHSDQQPVPEQLRIQVSRV
SMHDYEALHYDKEQLKEASVPLGTVVVPGDSDLELCRAHIERLQQEPDGAGAKSPTCQKL
SPKWCFLDATTASRFYRIDRAQEHLNYVTEIAQDEIYILDPELLGASARPDLPTPTSPLP
TSPCSPTPRSLQGDAAPPQGEELIEAAKRNDFCKLQELHRAGGDLMHRDEQSRTLLHHAV
STGSKDVVRYLLDHAPPEILDAVEENGETCLHQAAALGQRTICHYIVEAGASLMKTDQQG
DTPRQRAEKAQDTELAAYLENRQHYQMIQREDQETAV
>Q9H8E8
MDSSIHLSSLISRHDDEATRTSTSEGLEEGEVEGETLLIVESEDQASVDLSHDQSGDSLN
SDEGDVSWMEEQLSYFCDKCQKWIPASQLREQLSYLKGDNFFRFTCSDCSADGKEQYERL
KLTWQQVVMLAMYNLSLEGSGRQGYFRWKEDICAFIEKHWTFLLGNRKKTSTWWSTVAGC
LSVGSPMYFRSGAQEFGEPGWWKLVHNKPPTMKPEGEKLSASTLKIKAASKPTLDPIITV
EGLRKRASRNPVESAMELKEKRSRTQEAKDIRRAQKEAAGFLDRSTSSTPVKFISRGRRP
DVILEKGEVIDFSSLSSSDRTPLTSPSPSPSLDFSAPGTPASHSATPSLLSEADLIPDVM
PPQALFHDDDEMEGDGVIDPGMEYVPPPAGSVASGPVVGGRKKVRGPEQIKQEVESEEEK
PDRMDIDSEDTDSNTSLQTRAREKRKPQLEKDTKPKEPRYTPVSIYEEKLLLKRLEACPG
AVAMTPEARRLKRKLIVRQAKRDRGLPLFDLDQVVNAALLLVDGIYGAKEGGISRLPAGQ
ATYRTTCQDFRILDRYQTSLPSRKGFRHQTTKFLYRLVGSEDMAVDQSIVSPYTSRILKP
YIRRDYETKPPKLQLLSQIRSHLHRSDPHWTPEPDAPLDYCYVRPNHIPTINSMCQEFFW
PGIDLSECLQYPDFSVVVLYKKVIIAFGFMVPDVKYNEAYISFLFVHPEWRRAGIATFMI
YHLIQTCMGKDVTLHVSASNPAMLLYQKFGFKTEEYVLDFYDKYYPLESTECKHAFFLRL
RR
>Q53EL6
MDVENEQILNVNPADPDNLSDSLFSGDEENAGTEEVKNEINGNWISASSINEARINAKAK
RRLRKNSSRDSGRGDSVSDSGSDALRSGLTVPTSPKGRLLDRRSRSGKGRGLPKKGGAGG
KGVWGTPGQVYDVEEVDVKDPNYDDDQENCVYETVVLPLDERAFEKTLTPIIQEYFEHGD
TNEVAEMLRDLNLGEMKSGVPVLAVSLALEGKASHREMTSKLLSDLCGTVMSTTDVEKSF
DKLLKDLPELALDTPRAPQLVGQFIARAVGDGILCNTYIDSYKGTVDCVQARAALDKATV
LLSMSKGGKRKDSVWGSGGGQQSVNHLVKEIDMLLKEYLLSGDISEAEHCLKELEVPHFH
HELVYEAIIMVLESTGESTFKMILDLLKSLWKSSTITVDQMKRGYERIYNEIPDINLDVP
HSYSVLERFVEECFQAGIISKQLRDLCPSRGRKRFVSEGDGGRLKPESY
>Q03898
MSNKSNRRSLRDIGNTIGRNNIPSDKDNVFVRLSMSPLRTTSQKEFLKPPMRISPNKTDG
MKHSIQVTPRRIMSPECLKGYVSKETQSLDRPQFKNSNKNVKIQNSDHITNIIFPTSPTK
LTFSNENKIGGDGSLTRIRARFKNGLMSPERIQQQQQQHILPSDAKSNTDLCSNTELKDA
PFENDLPRAKLKGKNLLVELKKEEEDVGNGIESLTKSNTKLNSMLANEGKIHKASFQKSV
KFKLPDNIVTEETVELKEIKDLLLQMLRRQREIESRLSNIELQLTEIPKHK
>Q94464
MDQLIPVINKLQDVFNTLGSDPLDLPQIVVVGSQSSGKSSVLENIVGRDFLPRGSGIVTR
RPLILQLTHLPIADDGSQTQEWGEFLHKPNDMFYDFSEIREEIIRDTDRMTGKNKGISAQ
PINLKIYSPHVVNLTLVDLPGITKVPVGDQPTDIEQQIRRMVMAYIKKQNAIIVAVTPAN
TDLANSDALQLAKEVDPEGKRTIGVITKLDLMDKGTDAMEVLTGRVIPLTLGFIGVINRS
QEDIIAKKSIRESLKSEILYFKNHPIYKSIANRSGTAYLSKTLNKLLMFHIRDTLPDLKV
KVSKMLSDVQGELSTYGDPLYDTKNSQGALLLQIITIFSSNFKDAIDGKLTDLSNNELYG

GARISYIFNEIYSHCVNNIDPLEGISLNDIRTTMRNATGPRAALFIPEISFELLVKKQVV
RLEEPSAQCVEYVYDELQRIVSQLEAKELSRFINLKARVIEVVNNLLQKHKVPTKTMIEH
LIKIETAFINTSHPDFVGGEGIFESLYKKQQLQQQNHLQQLQDQYQQQQQQQQQQQQQNG
INNNQKGDNGNMNVNQQNMNQQNMNQQNQSTNPFLQQQQQGQNKYPGGPPAQQQPNQQPN
QLNKGPQNMPPNQSKPSSIPQNGPNNNNNNNNNNNRQDHQQGSFFSSFFRASPDPSLGQY
GGANNSNNSNNPTSPINSSSNSGNNYNTFGGQQSSSSSSQQLQQSSQSQYKTSYNNNNNS
SSNNSSYNRYQDDFYGRGDKLNQVPSIIKAPDDLTSKEKFETELIRELLISYFNIVKKNV
KDSVPKSIMHFLVNQSKEHIQNELVAALYKEELFDELLEESPQISSKRKSCKAMIEILRK
ANEIINEIRDFRN
>Q9C5X3
MSYSDSDSSSHGGEYKNFRQITRERLLYEMLRSAKTGSSKSTWKVLIMDKLTVKIMSYAC
KMADITQEGVSLVEDIFRRRQPLPSMDAIYFIQPTKENVIMFLSDMSGKSPLYKKAFVFF
SSPVSKELVGHIKKDSSVLPRIGALREMNLEFFAIDSQGFITDHERALEDLFGDEETSRK
GDACLNVMASRIATVFASLREFPAVRYRAAKSLDASTMTTLRDLIPTKLAAGIWNCLAKH
KQSIENFPQTETCELLILDRSIDQIAPVIHEWTYDAMCHDLLNMEGNKYVHVIPSKSGGQ
PEKKDVLLEEHDPIWLELRHAHIADASERLHDKMTNFLSKNKAAQLQGKRDGAELSTRDL
QKMVQALPQYSEQIDKLSLHVEIARKLNDLIREQGLRELGQLEQDLVFGDAGMKDVIKYL
STQEEASREGKLRLLMILATIYPEKFEGEKGQNLMKLAKLSSDDMTAVNNMSLLGSAVDA
KKNTPGGFTLKFDLHKKKRAVRKERQEEAAWQLSRFYPMIEELIEKLSKGELPKEDFPCM
NDPSPSFHGSTSLSSAASSSQGQAAQSMRSRRTPTWAKPRGSDDGYSSDSVLRHASSDFR
KMGQRIFVFIVGGATRSELKVCHKLSTKLKREVILGSTSLDDPPQFITKLKLLTANDDLS
LDDLQI
>P38348
MSEKETNYVENLLTQLENELNEDNLPEDINTLLRKCSLNLVTVVSLPDMDVKPLLATIKR
FLTSNVSYDSLNYDYLLDVVDKLVPMADFDDVLEVYSAEDLVKALRSEIDPLKVAACRVI
ENSQPKGLFATSNIIDILLDILFDEKVENDKLITAIEKALERLSTDELIRRRLFDNNLPY
LVSVKGRMETVSFVRLIDFLTIEFQFISGPEFKDIIFCFTKEEILKSVEDILVFIELVNY
YTKFLLEIRNQDKYWALRHVKKILPVFAQLFEDTENYPDVRAFSTNCLLQLFAEVSRIEE
DEYSLFKTMDKDSLKIGSEAKLITEWLELINPQYLVKYHKDVVENYFHVSGYSIGMLRNL
SADEECFNAIRNKFSAEIVLRLPYLEQMQVVETLTRYEYTSKFLLNEMPKVMGSLIGDGS
AGAIIDLETVHYRNSALRNLLDKGEEKLSVWYEPLLREYSKAVNGKNYSTGSETKIADCR

>Q9V3D5
MLDRCEMPIQLDNEKLRRDVRLSGSRLDLPQLCNGSRRLDGHNNHVAANENTVTTTSLNG
NGNGNGNSNSNNNNNIGSPVSSSTTNSSNGGNERGSSTKSNSSSGSGSSGNSASSTGSGE
LKCNTPMTPSELVKKFRNYLTDLEFEELKVYKEVWYFGQHASKNYNKPAPTANTTNLGYD
DDNGNYKIIEHDHIAFRYEILEVIGKGSFGQVIRALDHKTNTHVAIKIIRNKKRFLNQAV
VELNILDELREKDADGSHNVIHMLDYTYFRKHLCITFELMSLNLYELIKKNNYNGFSMSL
IRRFCNSIVKCLRLLYKENIIHCDLKPENILLKQRGSSSIKVIDFGSSCYVDRKIYTYIQ
SRFYRSPEVILGLQYGTAIDMWSLGCILAELYTGFPLFPGENEVEQLACIMEVLGLPPKV
LISVARRRRLFFDSRDAPRCITNTKGRKRSPGSKSLAHILHCQDRYFIDFLQRCLEWDPA
ERMTPDEAAHHEFLQPSASSRHRSCRMSSSSSSSGLNSVSQKSSCYSFSEISPGTNGPVV
ASITSTTAVHNAAIATTTKSRQQPPSQSHGHAQSNGHLPDIKLSASDKYNSMQKVAVRSK
ITSSVSDLESVQQYSLHRIYGGVGSGSTHHVSSAATRKHLPGTGSGIVGAMSSKYGSSTL

AHNHHNVTHHNASTATIATTTHHHHHHGGQQQQQSSSGASTMAMSHSQSTGDVSDRAIFG
RA
>Q9W4X9
MEEEAPRFNVLEEAFNGNGNGCANVEATQSAILKVLTRVNRFQMRVRKHIEDNYTEFLPN
NTSPDIFLEESGSLNREIHDMLENLGSEGLDALDEANVKMAGNGRQLREILLGLGVSEHV
LRIDELFQCVEEAKATKDYLVLLDLVGRLRAFIYGDDSVDGDAQVATPEVRRIFKALECY
ETIKVKYHVQAYMLQQSLQERFDRLVQLQCKSFPTSRCVTLQVSRDQTQLQDIVQALFQE
PYNPARLCEFLLDNCIEPVIMRPVMADYSEEADGGTYVRLSLSYATKEPSSAHVRPNYKQ
VLENLRLLLHTLAGINCSVSRDQHVFGIIGDHVKDKMLKLLVDECLIPAVPESTEEYQTS
TLCEDVAQLEQLLVDSFIINPEQDRALGQFVEKYETYYRNRMYRRVLETAREIIQRDLQD
MVLVAPNNHSAEVANDPFLFPRCMISKSAQDFVKLMDRILRQPTDKLGDQEADPIAGVIS
IMLHTYINEVPKVHRKLLESIPQQAVLFHNNCMFFTHWVAQHANKGIESLAALAKTLQAT
GQQHFRVQVDYQSSILMGIMQEFEFESTHTLGSGPLKLVRQCLRQLELLKNVWANVLPET
VYNATFCELINTFVAELIRRVFTLRDISAQMACELSDLIDVVLQRAPTLFREPNEVVQVL
SWLKLQQLKAMLNASLMEITELWGDGVGPLTASYKSDEIKHLIRALFQDTDWRAKAITQI
V
>Q12433
MMSPAQDKLQHQHHNPNSSSSSSSKMTNVYQVTTPKSPQDLENNMDEPFKMDTATSNPDK
DSENTQRLKYECAKGEIQNVLNLHIMLNHKHVRHLRRNVQKVNAKLALLETLHKDTGLLN
KIERTYQLKIKQHQQHSVLGGHFHDSTATENTNASNYNLSYPVLSDYNINCQPLSSSSNR
NLSTTRIPHHHYHTRSKSNGLLLEPSALRPANSNIIDYRLTGSKSLSEAITKPTPVSLPH
SNSDGISSPRSSSISPLDEQPGFQILPFKPSQMHLNHRRNYSSTCLTSNSGIIGKTENNE
PIFRRYDGILVIITCSKCDRSGFTSAQGIVNHTRLKHSKLYSSQPLAVLNNQKLLPNDKQ
DPEILSKFKKLNLDPNKDYLPSDIAIPKPQSPINHSENHTRAPKTVKNTPHLEKLYQNKE
DFKKLIDMVNETPDDLNEYLKQREIQLRYQKEQEEESSKSDDEASYVPSPSLSATATTTT
TTDPPSPPVLSSSLQRKLLRKRKLSLNSSTPMEDLPLRERLRANPTDKKPRKAALLTNEL
EGPDPAAKSSSYYNLRSKSRLRGSHT
>Q9BXW4
MPPPQKIPSVRPFKQRKSLAIRQEEVAGIRAKFPNKIPVVVERYPRETFLPPLDKTKFLV
PQELTMTQFLSIIRSRMVLRATEAFYLLVNNKSLVSMSATMAEIYRDYKDEDGFVYMTYA
SQETFGCLESAAPRDGSSLEDRPCNPL
>Q08415
MFRSAAALSVHLMWPLWGRKAGASLTRCLHQSLTMTKRLQARRLDGIDQNLWVEFGKLTK
EYDVVNLGQGFPDFSPPDFATQAFQQATSGNFMLNQYTRAFGYPPLTNVLASFFGKLLGQ
EMDPLTNVLVTVGAYGALFTRFQALVDEGDEVIIMEPAFDCYEPMTMMAGGCPVFVTLKP
SPAPKGKLGASNDWQLDPAELASKFTPRTKILVLNTPNNPLGKVFSRMELELVANLCQQH
DVVCISDEVYQWLVYDGHQHVSIASLPGMWDRTLTIGSAGKSFSATGWKVGWVMGPDNIM
KHLRTVHQNSIFHCPTQAQAAVAQCFEREQQHFGQPSSYFLQLPQAMELNRDHMIRSLQS
VGLKLWISQGSYFLIADISDFKSKMPDLPGAEDEPYDRRFAKWMIKNMGLVGIPVSTFFS
RPHQKDFDHYIRFCFVKDKATLQAMDERLRKWKELQP
>P19236
MLWLLVLTAPWLGGSVPISPDPGLRHEQVGIVGGCKVPARRYPWQVSLRFHGMGSGQWQH
ICGGSLIHPQWVLTAAHCVELEGLEAATLRVQVGQLRLYDHDQLCNVTEIIRHPNFNMSW
YGWDSADIALLKLEAPLTLSEDVNLVSLPSPSLIVPPGMLCWVTGWGDIADHTPLPPPYH

LQEVEVPIVGNRECNCHYQTILEQDDEVIKQDMLCAGSEGHDSCQMDSGGPLVCRWKCTW
IQVGVVSWGYGCGYNLPGVYARVTSYVSWIHQHIPLSPGP
>Q02521
MSKFSLKLGSKTLKKNISKKTKKKNSLQKANLFDWDDAETASLSHKPQSKIKIQSIDKFD
LDEESSSKKKLVIKLSENADTKKNDAPLVEYVTEKEYNEVPVEEFGDALLRGMGWESDSE
QDSKGDKTQSRNKDVSNVSQIHPDGLGIGAKLNKAINVEEASFMPVVKIDKITGTKVDDD
KKNKS
>O94246
MIQNEKKHLILYTGDVTKNLKSGIWNASRIKSNLELVKALENVKLTKFTGDGDENLKKNI
KVLVPVNEKPQKLDGKQEEYEIIVKLFFLDGENIDIKKREETLSQVFYNLHMLFGIDFVS
TLVVSFPHITFLKESGNSSSNEIYDSIDEIPPQEIQSWVDTWKLLEEKVGEGKIGTLGVS
EFGVNELQRLISSVNVVPESTQINIGQNCKLPNDLLNFADRHHLKLFFHSDPSALLSESE
ITSVIHKACPEIPNPARVDWVIRYTILTRHTAVIHQKGYIVQSTYTE
>O13897
MKSATKSKILCIHGYAESGELFSVKLRALRERMADSVDFYFPTGPIELDKAKDELNGSGF
DALSTVFSSSPASHRRGWWRINEYADTKQLEPTKAFEYLASYIKEHGPFDGILGFSQGTN
LAANLAALVTIPKYQEYFSQPPFRFALFFSGYFRPLLMDGAVHATKLDLPTLHLLGKYDT
VLSTETSTTLVRACKDAQVLFHPAAHQIPAPHAYVEPAADFIDFFSREDWPIISKHISLI
VPTKKVNTTSAQTSLNNVEHDLISKIMSRSFKGGINVVVSDLQMFNEYKRIFGPKVLSLV
VTDGQEPDTYSENVHYTPNFKGALAYLEAYQNSIGRIYVIGDKKLLTLGMLCRCTKRIIA
ITDAEDKFISQSSAQQVSGSLPFLEHSKEWLKAKSSQIRQWTGQSRLKHMSQDNSGEPVT
MKLQMWERL
>P53072
MGEKRNRNGKDANSQNRKKFKVSSGFLDPGTSGIYATCSRRHERQAAQELQLLFEEKFQE
LYGDIKEGEDESENDEKKDLSIEDQIKKELQELKGEETGKDLSSGETKKKDPLAFIDLNC
ECVTFCKTRKPIVPEEFVLSIMKDLADPKNMVKRTRYVQKLTPITYSCNAKMEQLIKLAN
LVIGPHFHDPSNVKKNYKFAVEVTRRNFNTIERMDIINQVVKLVNKEGSEFNHTVDLKNY
DKLILVECFKSNIGMCVVDGDYKTKYRKYNVQQLYESKFRKDEDKSVKQ
>P25375
MRLLLCKNWFASPVISPLLYTRSLYSMANTTSFPIAPQAPPNWSFTPSDISGKTNEIINN
SNNFYDSMSKVESPSVSNFVEPFMKFENELGPIINQLTFLQHVSSDKEIRDASVNSSMKL
DELNIDLSLRHDIFLQFARVWQDVQSKADSVERETFKYVEKSYKDYIHSGLELDEGNRLK
IKEIKKKISVNSINFSKNLGEQKEYITFTKEQLEGVPDSILTQFETIKSDKDSNETLYKV
TFKYPDIFPVMKLASSAQTRKQAFLADQNKVPENEAILLDTLKLRDELASLLGYDTYANY
NLYDKMAEDSTTVMNFLNDLKDKLIPLGRKELQVLQDMKAEDVKKLNQGADPNYYIWDHR
YYDNKYLLENFNVDLEKISEYFPLEATITGMLEIYETLFNLKFIETKDSQNKSVWHDDVK
QIAVWNMDDPKSPNFVGWIYFDLHPRDGKYGHAANFGLSSSFMIDDTTRSYPVTALVCNF
SKSTKDKPSLLKHNEIVTFFHELGHGIHDLVGQNKESRFNGPGSVPWDFVEAPSQMLEFW
TWNKNELINLSSHYKTGEKIPESLINSLIKTKHVNGALFTLRQLHFGLFDMKVHTCKDLQ
NLSICDTWNQLRQDISLISNGGTLSKGYDSFGHIMSDSYSAGYYGYLWAEVFATDMYHTK
FAKDPLNAKNGIQYRDIVLARGGLYDINDNLKEFLGREPSKDAFLKELGLQN
>O13974
MSSLRLRLKYENQSAVETVEANATVGSFLDLVAAKFSLPRNSIALKFGFPPQDIPLVNSD
VPLSTLVSSGQQILVLKNAATSFSTNEPAKPPIPNAATKPTFPPQTEISNPPAVSHQSKN

TSQDPPYVSTPIGDIALRVMPDDNSCLFRALSKPLGFSPYELREIVANQVLSNPDIYSTA
ILGKPSIEYASWIRKETSWGGYIELSILSSHFGVEICSVDVKTGRVDSYNPQPATGQRTY
IVYSGIHYDLAALAAVLWDTDVDVVLFDASDVTITPYVQQLASLLKNMHYYTDTASFSIR
CTICGTGLVGEKDATAHALATGHTQFGEY
>P49914
MAAAAVSSAKRSLRGELKQRLRAMSAEERLRQSRVLSQKVIAHSEYQKSKRISIFLSMQD
EIETEEIIKDIFQRGKICFIPRYRFQSNHMDMVRIESPEEISLLPKTSWNIPQPGEGDVR
EEALSTGGLDLIFMPGLGFDKHGNRLGRGKGYYDAYLKRCLQHQEVKPYTLALAFKEQIC
LQVPVNENDMKVDEVLYEDSSTA
>Q8TEQ6
MGQEPRTLPPSPNWYCARCSDAVPGGLFGFAARTSVFLVRVGPGAGESPGTPPFRVIGEL
VGHTERVSGFTFSHHPGQYNLCATSSDDGTVKIWDVETKTVVTEHALHQHTISTLHWSPR
VKDLIVSGDEKGVVFCYWFNRNDSQHLFIEPRTIFCLTCSPHHEDLVAIGYKDGIVVIID
ISKKGEVIHRLRGHDDEIHSIAWCPLPGEDCLSINQEETSEEAEITNGNAVAQAPVTKGC
YLATGSKDQTIRIWSCSRGRGVMILKLPFLKRRGGGIDPTVKERLWLTLHWPSNQPTQLV
SSCFGGELLQWDLTQSWRRKYTLFSASSEGQNHSRIVFNLCPLQTEDDKQLLLSTSMDRD
VKCWDIATLECSWTLPSLGGFAYSLAFSSVDIGSLAIGVGDGMIRVWNTLSIKNNYDVKN
FWQGVKSKVTALCWHPTKEGCLAFGTDDGKVGLYDTYSNKPPQISSTYHKKTVYTLAWGP
PVPPMSLGGEGDRPSLALYSCGGEGIVLQHNPWKLSGEAFDINKLIRDTNSIKYKLPVHT
EISWKADGKIMALGNEDGSIEIFQIPNLKLICTIQQHHKLVNTISWHHEHGSQPELSYLM
ASGSNNAVIYVHNLKTVIESSPESPVTITEPYRTLSGHTAKITSVAWSPHHDGRLVSASY
DGTAQVWDALREEPLCNFRGHQGRLLCVAWSPLDPDCIYSGADDFCVHKWLTSMQDHSRP
PQGKKSIELEKKRLSQPKAKPKKKKKPTLRTPVKLESIDGNEEESMKENSGPVENGVSDQ
EGEEQAREPELPCGLAPAVSREPVICTPVSSGFEKSKVTINNKVILLKKEPPKEKPETLI
KKRKARSLLPLSTSLDHRSKEELHQDCLVLATAKHSRELNEDVSADVEERFHLGLFTDRA
TLYRMIDIEGKGHLENGHPELFHQLMLWKGDLKGVLQTAAERGELTDNLVAMAPAAGYHV
WLWAVEAFAKQLCFQDQYVKAASHLLSIHKVYEAVELLKSNHFYREAIAIAKARLRPEDP
VLKDLYLSWGTVLERDGHYAVAAKCYLGATCAYDAAKVLAKKGDAASLRTAAELAAIVGE
DELSASLALRCAQELLLANNWVGAQEALQLHESLQGQRLVFCLLELLSRHLEEKQLSEGK
SSSSYHTWNTGTEGPFVERVTAVWKSIFSLDTPEQYQEAFQKLQNIKYPSATNNTPAKQL
LLHICHDLTLAVLSQQMASWDEAVQALLRAVVRSYDSGSFTIMQEVYSAFLPDGCDHLRD
KLGDHQSPATPAFKSLEAFFLYGRLYEFWWSLSRPCPNSSVWVRAGHRTLSVEPSQQLDT
ASTEETDPETSQPEPNRPSELDLRLTEEGERMLSTFKELFSEKHASLQNSQRTVAEVQET
LAEMIRQHQKSQLCKSTANGPDKNEPEVEAEQPLCSSQSQCKEEKNEPLSLPELTKRLTE
ANQRMAKFPESIKAWPFPDVLECCLVLLLIRSHFPGCLAQEMQQQAQELLQKYGNTKTYR
RHCQTFCM
>P06754
MDAIKKKMQAMKVDKDGALERALVCEQEARDANTRAEKAEEEARQLQKKIQTVENELDQT
QEALTLVTGKLEEKNKALQNKKKTTKMTTSIPQGTLLDVLKKKMRQTKEEMEKYKDECEE
FHKRLQLEVVRREEAESEVAALNRRIQLLEEDLERSEERLGSATAKLSEASQAADESERA
RKILENRALADEERMDALENQLKEARFLAEEADKKYDEVARKLAMVEADLERAEERAEQG
ENKIVELEEELRVVGNNLKSLEVSEEKANQREEEYKNQIKTLNTRLKEAEARAEFAERSV
QKLQKEVDRLEDDLVLEKERYKDIGDDLDTAFVELILKE
>P29461

MDRIAQQYRNGKRDNNGNRMASSAISEKGHIQVNQTRTPGQMPVYRGETINLSNLPQNQI
KPCKDLDDVNIRRNNSNRHSKILLLDLCAGPNTNSFLGNTNAKDITVLSLPLPSTLVKRS
NYPFENLLKNYLGSDEKYIEFTKIIKDYDIFIFSDSFSRISSCLKTTFCLIEKFKKFICH
FFPSPYLKFFLLEGSLNDSKAPSLGKNKKNCILPKLDLNLNVNLTSRSTLNLRINIPPPN
DSNKIFLQSLKKDLIHYSPNSLQKFFQFNMPADLAPNDTILPNWLKFCSVKENEKVILKK
LFNNFETLENFEMQRLEKCLKFKKKPLHQKQLSQKQRGPQSTDDSKLYSLTSLQRQYKSS
LKSNIQKNQKLKLIIPKNNTSSSPSPLSSDDTIMSPINDYELTEGIQSFTKNRYSNILPY
EHSRVKLPHSPKPPAVSEASTTETKTDKSYPMCPVDAKNHSCKPNDYINANYLKLTQINP
DFKYIATQAPLPSTMDDFWKVITLNKVKVIISLNSDDELNLRKWDIYWNNLSYSNHTIKL
QNTWENICNINGCVLRVFQVKKTAPQNDNISQDCDLPHNGDLTSITMAVSEPFIVYQLQY
KNWLDSCGVDMNDIIKLHKVKNSLLFNPQSFITSLEKDVCKPDLIDDNNSELHLDTANSS
PLLVHCSAGCGRTGVFVTLDFLLSILSPTTNHSNKIDVWNMTQDLIFIIVNELRKQRISM
VQNLTQYIACYEALLNYFALQKQIKNALPC
>Q92624
MAAVELEWIPETLYNTAISAVVDNYIRSRRDIRSLPENIQFDVYYKLYQQGRLCQLGSEF
CELEVFAKVLRALDKRHLLHHCFQALMDHGVKVASVLAYSFSRRCSYIAESDAAVKEKAI
QVGFVLGGFLSDAGWYSDAEKVFLSCLQLCTLHDEMLHWFRAVECCVRLLHVRNGNCKYH
LGEETFKLAQTYMDKLSKHGQQANKAALYGELCALLFAKSHYDEAYKWCIEAMKEITAGL
PVKVVVDVLRQASKACVVKREFKKAEQLIKHAVYLARDHFGSKHPKYSDTLLDYGFYLLN
VDNICQSVAIYQAALDIRQSVFGGKNIHVATAHEDLAYSSYVHQYSSGKFDNALFHAERA
IGIITHILPEDHLLLASSKRVKALILEEIAIDCHNKETEQRLLQEAHDLHLSSLQLAKKA
FGEFNVQTAKHYGNLGRLYQSMRKFKEAEEMHIKAIQIKEQLLGQEDYEVALSVGHLASL
YNYDMNQYENAEKLYLRSIAIGKKLFGEGYSGLEYDYRGLIKLYNSIGNYEKVFEYHNVL
SNWNRLRDRQYSVTDALEDVSTSPQSTEEVVQSFLISQNVEGPSC
>P38632
MALNDNPIPKSVPLHPKSGKYFHNLHARDLSNIYQQCYKQIDETINQLVDSTSPSTIGIE
EQVADITSTYKLLSTYESESNSFDEHIKDLKKNFKQSSDACPQIDLSTWDKYRTGELTAP
KLSELYLNMPTPEPATMVNNTDTLKILKVLPYIWNDPTCVIPDLQNPADEDDLQIEGGKI
ELTCPITCKPYEAPLISRKCNHVFDRDGIQNYLQGYTTRDCPQAACSQVVSMRDFVRDPI
MELRCKIAKMKESQEQDKRSSQAIDVL
>P41910
MKFIDELDIERVNQTLNFETNDCKIVGSCDIFTTKAVASDRKLYKTIDQHLDTILQENEN
YNATLQQQLAAPETNQSPCSSPFYSNRRDSNSFWEQKRRISFSEYNSNNNTNNSNGNSSN
NNNYSGPNGSSPATFPKSAKLNDQNLKELVSNYDSGSMSSSSLDSSSKNDERIRRRSSSS
ISSFKSGKSSNNNYSSGTATNNVNKRRKSSINERPSNLSLGPFGPINEPSSRKIFAYLIA
ILNASYPDHDFSSVEPTDFVKTSLKTFISKFENTLYSLGRQPEEWVWEVINSHMTLSDCV
LFQYSPSNSFLEDEPGYLWNLIGFLYNRKRKRVAYLYLICSRLNSSTGEVEDALAKKPQG
KLIIDDGSNEYEGEYDFTYDENVIDDKSDQEESLQ
>Q02866
MARQLFTPPITNPRFDPNQSIRESYKNTTGGMQFQQNLHEDQNDNERSSCDGDENSTTGE
RLENNKSPILTKQEIDEALNTVTNLPPELSKLIDIFIDDLKQPKYVRPLSVLQLSSLFQS
FYIKFDKASFQHVSSANNNGYYFSGGGSSSFLAAKETLSSGLSGIFGRSRSSSGNSLMRP
RRSSSLFSNESISNSTNATQMLSPEEIKKQLKINELNNMKIEKYMELCERDVFKKILIVG
TSVSSPNKMKTFKPHQLQTFKVGNLFRNSVEFTEYNKLLNEKILCLSKLSTMNKINLIKF

LSLNNGIDPEPKFEEIKDILYEFTYHSISPCEKIKALLKLHEIMTYSQEMSNDDYLSLLI
YYIITIVPRDIFLNAEFIRLFRYKKKLVETESFALTNLEAALVFVEGLTKNDFSNELQDK
LTVNESKILENSISSRVSLPSKTAIMHKNNGNNGSNLGDIVTPTIQRPDVTRSNSYDGFR
TVFDSSLKNIIGKIRSYTPPHPNNTSNNNLHSSNNLNIPRSSSQLSMELSNRDTTEMSRD
GSRSTSSSSRSSASLEHGNREFTGDLTVTASINGADKKEFQKSWKKYKGYKFEDLTICEL
RDLFEIYQKMMQ
>Q04409
MSFENLHKVNAEALEDAVVEICSSLQVDAAKLDELTAYFIECMEKGLNNTSVGEEKTVDK
GLPMIPTYVTSLPNGTERGVLLAADLGGTHFRVCSVTLNGDGTFDMQQLKSKIPEEYLND
KDVTSEELFSYLGRRTRAFVRKHHPELLKSTGENIKPLKMGFTFSYPVDQTSLSSGTLIR
WTKSFKIEDTVGKDVVRLYQEQLDIQGLSMINVVALTNDTVGTFLSHCYTSGSRPSSAGE
ISEPVIGCIFGTGTNGCYMEDIENIKKLPDELRTRLLHEGKTQMCINIEWGSFDNELKHL
SATKYDIDIDQKFSPNPGYHLFEKRISGMYLGELLRNILVDLHARGLILGQYRNYDQLPH
RLKTPFQLCSEVLSRIEIDDSTNLRETELSFLQSLRLPTTFEERKAIQNLVRSITRRSAY
LAAVPIAAILIKTNALNKRYHGEVEIGFDGYVIEYYPGFRSMLRHALALSPIGTEGERKI
HLRLAKDGSGVGAALCALVA
>P52758
MSSLIRRVISTAKAPGAIGPYSQAVLVDRTIYISGQIGMDPSSGQLVSGGVAEEAKQALK
NMGEILKAAGCDFTNVVKTTVLLADINDFNTVNEIYKQYFKSNFPARAAYQVAALPKGSR
IEIEAVAIQGPLTTASL
>Q12314
MKYIIEHMEEGFSEWVILEYSQILREVGAENLILSSLPESTTEKDIPQRLLKLGLRWTTK
DLKGINEDFKDLELLKDGRVCLLDPRATIDLQPEDATKFDYFVFGGILGDHPPRDRTKEL
KTAYPNLLISRRLGDKQMTTDTAIRTTQLIIKDRIAFEDIKFIDYPEFRFNKNEATEMPF
RYVLDKEGKPILPEGMLDLIKKDSAQSLDDLLM
>O94378
MERIQENDPIIKSADESKESPAETLSSIFKRPKIKSSSLNKAYLGKAGSVNGASNTSTNQ
ISSLKVDVSSPPSTAPGSAGSTPKTTPVSLNAGVPPSNTGANITTNSNNNAQTISSSSQS
VGHIDNPSGLGTHGKVRLSIKLSTGGSGGSSVTGKRTAPGNPWAIRSAERLASNPTSIGT
SSPESIDNNSNNKKSAPSLYAAAAASAARHSMSAAYASFTGTVYDSNGNVIKGLDTSSSL
SSSSHPSHEALSTIDTKNLQALAASAAPSSCRQQPVDEERQDRVSKISKHFQDSGTPVSE
EVSPTRLPSPTSARRASHLSEGKWDEIDDEDDDDWGSTIEFEDGTTVVIDAKTRRYEPVK
RQDTLASNHVPSAPSNESVSSAAPSKIPVPEEASINEHSNITDEAVSLPEPVYPNAPTSA
VSVHNPTISDEAEPTERAFLKKEKPQISKEKKNPNVTHEEFVEKSTALPRHINKVEIVSD
SSESFAHKNNEKRFDKEASGRINDLRAEQQQIMLEARQKAIERRRNEEEELARSRERARK
KAEEIKISSSKMNSSPTITNERSSNVESSKKVEAIYTKEPQSITNSPEGLHSNQETTEID
PSSPRDDSTPLSWRSHSHPIPPVQVSPLFTSDKNDPLQLNPQSDDSTSSPKHGKSRSIDE
VILSIKDVIFDNNLYKQRSYQSGVAANAHLPHRVPNEHGGVSRIGNSNALGQPVSSSSSY
SHSKGRDTRSSSYVVSSETSSLKSSRLILSHANSVRIQRSKGDVILNLHLPKVNPERFLP
TKSLKLEEMKPLEEKVPIYRSSSKQPLRIHPTHPLRVTVCLPGSKTIQVARRSFRNSMNA
NRYDPQRRERIDSGLWRAKN
>P04370
MGNHSGKRELSAEKASKDGEIHRGEAGKKRSVGKLSQTASEDSDVFGEADAIQNNGTSAE
DTAVTDSKHTADPKNNWQGAHPADPGNRPHLIRLFSRDAPGREDNTFKDRPSESDELQTI

QEDPTAASGGLDVMASQKRPSQRSKYLATASTMDHARHGFLPRHRDTGILDSIGRFFSGD
RGAPKRGSGKDSHTRTTHYGSLPQKSQHGRTQDENPVVHFFKNIVTPRTPPPSQGKGGRD
SRSGSPMARR
>P41812
MSGSLSRGNGGKKVLNKNQLLKRNRIRNARSIRAEAVAASSTKTGTPSDLSESGSKLNVD
QFISSRQFEVKQLQLAMHNSKAASSTRIFQALPRKLRRRTASHNVRRIPKRMRNRALREM
RKSDQQDVLKGSSASSRKAHGLNAKQLYKARMSIKLLRLASKSTSMKLSMPPEVTSSNCH
VRQKIKTLKRMIKESSTANPNIKLLNNRMGSYDCTGVNELAPIPKGRVKYTKRQKHFAWL
PTHIWNAKRSHMMKRWGYQMVWAPTQKCFKLTHRLGGDTCSSDGALCMDSSYIGTIIVKD
KSNDSEGDFLKSIIGKLTAERANLRKYREGQVLFQGLIYSFNEENGEDSTKPLGPCDVFW
VQKDTAIIRLHPSIYTQVFNILLQHKEKLTVQDCRYSLASVTLKGAKALESLASCLRSTE
YSKSFEQFKMVSMITDHNALPQRCTFAFEAIDPRHLAAPKKLNDSQRKTVNSDDILSLHE
NYPQDEINAVFNELCDPESRTQSYNNQNTLKEISARRYKLLTATPNSINKTTVPFKESDD
PSIPLVIIRRLKTRDWIVVLPWFWLLPLWHLLNRIPRMYHIGLRQFQQIQYENKQLYFPD
DYPFTQLGYIENSFYKKEASKTKWDRKPMGKRINFEKIKDIHNTKLPAYSGEIGDFFSSD
WRFLQILRNGIDYLQRNDKTLELMDSKKTGQFNAQGVRDINCVNDVLEFCKDYEAKTKAM
SLSIEENIPVALCKNRKCQFRTPDSISVNSSSFSLTFFPRCIIAVSCTLLERGHPKDNAR
IYQVPEKDLEHWLQLAKGVYRPNGRKDHDLKIPLPEVHDLIGFITSGTYHLNCGNGMGIG
FIDHHAAIRQPTRYVLIRNVGTNTYRLGEWSKISV
>Q9NKV0
MSLFGALMGDFDDDLGLMNNHMNHTMNAMNMQMRSMNRLMNSFMPDPFMQVSPFDQGFQQ
NALMERPQMPAMPAMGLFGMPMMPNFNRLLNADIGGNSGASFCQSTVMTMSSGPDGRPQI
YQASTSTKTGPGGVRETRRTVQDSRTGVKKMAIGHHIGERAHIIEKEQDMRSGQLEERQE
FINLEEGEAEQFDREFTSRASRGAVQSRHHAGGMQAIMPARPAAHTSTLTIEPVEDDDDD
DDDCVIQEQQPVRSSAGRHYSSAPTAPQNSGNIATAAAPTPTSSPTTYDTSNGNNNNYVS
SRRSYLRNGHGHSLATPRRPLRTPPSSPLATVSTSPSIHPHPYAANPRRQQRAVKHFHTE
DEAASSYKAVKRGKKK
>Q9H4A3
MSGGAAEKQSSTPGSLFLSPPAPAPKNGSSSDSSVGEKLGAAAADAVTGRTEEYRRRRHT
MDKDSRGAAATTTTTEHRFFRRSVICDSNATALELPGLPLSLPQPSIPAAVPQSAPPEPH
REETVTATATSQVAQQPPAAAAPGEQAVAGPAPSTVPSSTSKDRPVSQPSLVGSKEEPPP
ARSGSGGGSAKEPQEERSQQQDDIEELETKAVGMSNDGRFLKFDIEIGRGSFKTVYKGLD
TETTVEVAWCELQDRKLTKSERQRFKEEAEMLKGLQHPNIVRFYDSWESTVKGKKCIVLV
TELMTSGTLKTYLKRFKVMKIKVLRSWCRQILKGLQFLHTRTPPIIHRDLKCDNIFITGP
TGSVKIGDLGLATLKRASFAKSVIGTPEFMAPEMYEEKYDESVDVYAFGMCMLEMATSEY
PYSECQNAAQIYRRVTSGVKPASFDKVAIPEVKEIIEGCIRQNKDERYSIKDLLNHAFFQ
EETGVRVELAEEDDGEKIAIKLWLRIEDIKKLKGKYKDNEAIEFSFDLERDVPEDVAQEM
VESGYVCEGDHKTMAKAIKDRVSLIKRKREQRQLVREEQEKKKQEESSLKQQVEQSSASQ
TGIKQLPSASTGIPTASTTSASVSTQVEPEEPEADQHQQLQYQQPSISVLSDGTVDSGQG
SSVFTESRVSSQQTVSYGSQHEQAHSTGTVPGHIPSTVQAQSQPHGVYPPSSVAQGQSQG
QPSSSSLTGVSSSQPIQHPQQQQGIQQTAPPQQTVQYSLSQTSTSSEATTAQPVSQPQAP
QVLPQVSAGKQLPVSQPVPTIQGEPQIPVATQPSVVPVHSGAHFLPVGQPLPTPLLPQYP
VSQIPISTPHVSTAQTGFSSLPITMAAGITQPLLTLASSATTAAIPGVSTVVPSQLPTLL
QPVTQLPSQVHPQLLQPAVQSMGIPANLGQAAEVPLSSGDVLYQGFPPRLPPQYPGDSNI

APSSNVASVCIHSTVLSPPMPTEVLATPGYFPTVVQPYVESNLLVPMGGVGGQVQVSQPG
GSLAQAPTTSSQQAVLESTQGVSQVAPAEPVAVAQPQATQPTTLASSVDSAHSDVASGMS
DGNENVPSSSGRHEGRTTKRHYRKSVRSRSRHEKTSRPKLRILNVSNKGDRVVECQLETH
NRKMVTFKFDLDGDNPEEIATIMVNNDFILAIERESFVDQVREIIEKADEMLSEDVSVEP
EGDQGLESLQGKDDYGFSGSQKLEGEFKQPIPASSMPQQIGIPTSSLTQVVHSAGRRFIV
SPVPESRLRESKVFPSEITDTVAASTAQSPGMNLSHSASSLSLQQAFSELRRAQMTEGPN
TAPPNFSHTGPTFPVVPPFLSSIAGVPTTAAATAPVPATSSPPNDISTSVIQSEVTVPTE
EGIAGVATSTGVVTSGGLPIPPVSESPVLSSVVSSITIPAVVSISTTSPSLQVPTSTSEI
VVSSTALYPSVTVSATSASAGGSTATPGPKPPAVVSQQAAGSTTVGATLTSVSTTTSFPS
TASQLSIQLSSSTSTPTLAETVVVSAHSLDKTSHSSTTGLAFSLSAPSSSSSPGAGVSSY
ISQPGGLHPLVIPSVIASTPILPQAAGPTSTPLLPQVPSIPPLVQPVANVPAVQQTLIHS
QPQPALLPNQPHTHCPEVDSDTQPKAPGIDDIKTLEEKLRSLFSEHSSSGAQHASVSLET
SLVIESTVTPGIPTTAVAPSKLLTSTTSTCLPPTNLPLGTVALPVTPVVTPGQVSTPVST
TTSGVKPGTAPSKPPLTKAPVLPVGTELPAGTLPSEQLPPFPGPSLTQSQQPLEDLDAQL
RRTLSPEIITVTSAVGPVSMAAPTAITEAGTQPQKGVSQVKEGPVLATSSGAGVFKMGRF
QVSVAADGAQKEGKNKSEDAKSVHFESSTSESSVLSSSSPESTLVKPEPNGITIPGISSD
VPESAHKTTASEAKSDTGQPTKVGRFQVTTTANKVGRFSVSKTEDKITDTKKEGPVASPP
FMDLEQAVLPAVIPKKEKPELSEPSHLNGPSSDPEAAFLSRDVDDGSGSPHSPHQLSSKS
LPSQNLSQSLSNSFNSSYMSSDNESDIEDEDLKLELRRLRDKHLKEIQDLQSRQKHEIES
LYTKLGKVPPAVIIPPAAPLSGRRRRPTKSKGSKSSRSSSLGNKSPQLSGNLSGQSAASV
LHPQQTLHPPGNIPESGQNQLLQPLKPSPSSDNLYSAFTSDGAISVPSLSAPGQGTSSTN
TVGATVNSQAAQAQPPAMTSSRKGTFTDDLHKLVDNWARDAMNLSGRRGSKGHMNYEGPG
MARKFSAPGQLCISMTSNLGGSAPISAASATSLGHFTKSMCPPQQYGFPATPFGAQWSGT
GGPAPQPLGQFQPVGTASLQNFNISNLQKSISNPPGSNLRTT
>Q9BMM0
MVESIAAGQVGDNKHIEMWKIKRLINKLENCKGNGTSMVSLIIPPKEDINKSGKLLVGEL
SAAQNIKSRITRQSVITAITSTKEKLKLYRQTPTNGLCIYCGVILMEDGKTEKKINFDFE
PFRPINQFMYFCGGKFQTEPLTTLLADDDKFGFIIVDGNGALYATLQGNSREILQKITVE
LPKKHRKGGQSSVRFARLREEKRHNYLRKVAELAGSNFITNDKPNVTGLVLAGNAGFKNE
LSETDMLDKRLLPIIVSIVDVSYGGENGLNEAITLSADALTNVKFVAEKKLVSTFFEQIS
LDTGMIVFGVQDTMKALELGAVETILLFEELEITRYVIKNPVKGDTRTLFLNPTQQKDSK
YFKDQASGLDMDVIAEDQLAEWLCHNYQNYGAQVEFITDKSQEGYQFVKGFGGIGGFLRY
KVDMEDALGDVGDGGDDFDPDTDFI
>Q02884
MSFRKRGEILNDRGSGLRGPLLRGPPRTSSTPLRTGNRRAPGNVPLSDTTARLKKLNIAD
ESKTKMGLDSSHVGVRPSPATSQPTTSTGSADLDSILGHMGLPLGNSVLVEEQSTTEFHS
ILGKLFAAQGIVHNRISDSSADKTRNGDTHVIVLSLNQMFAKELPGIYKGSRKQMKKNLI
SEEESKVTVQNLNETQRSTPSRYKDLKIAWKYKLADEKRLGSPDRDDIQQNSEYKDYNHQ
FDITTRLMPAPIASELTFIAPTQPVSTILSQIEQTIKRNDKKLIRIVIPSLLHPAMYPPK
MFESSEIIGLMHGVRSLVKKYYERVVLFASISIDIITPPLLVLLRNMFDSVINLEPFNQE
MTEFLERVYKSQPGKIQHGLVHILKLPVFTDRGEMRVLKSEWAFKNGRKKFEIEQWGIPV
DDAEGSAASEQSHSHSHSDEISHNIPAKKTKISLDY
>P53256
MSVQAEIGILDHVDGSSEFVSQDTKVICSVTGPIEPKARQELPTQLALEIIVRPAKGVAT

TREKVLEDKLRAVLTPLITRHCYPRQLCQITCQILESGEDEAEFSLRELSCCINAAFLAL
VDAGIALNSMCASIPIAIIKDTSDIIVDPTAEQLKISLSVHTLALEFVNGGKVVKNVLLL
DSNGDFNEDQLFSLLELGEQKCQELVTNIRRIIQDNISPRLVV
>Q8GWZ6
METTVKNSSSDGEWKVVLPSKGRQGRRRKPKPKGQAEEEEQPWKSDDLEIDPQRQARLKQ
KMEISLKKIESSSFYTAFLEQLKSPEVSNQIRLVLGSETQLQMVMYGIGSIESYESPRFQ
LSIAILMKREFDWVGDNIEVFDPVLSATESSYLESLGCSVLSVNEQARREALKPTLFFMP
HCEANLYSNLLQANWRMDRLSKIALFGNSFQMYEEQVSFDAEVICATKRIIAAQRVTSEF
AIETESDDYFAAFHDSSWHFFSSGIDSELPLFVSD
>Q8CJ99
MATEIDLLRDQNVKLNDILRQHQIEHIFRDPAMQNSMSKGGRGDTLTNSVNDQSALPPLI
AEYEKHLEELNRQLTYYQKHMGEMKLQLETVITENERLHSKLKDAVEKQLEALPFGTGIG
NDICADDETVRNLQEQLQIANQEKNWAVQLWQTASQELESVQKLYQEHMTEAQIHVFENR
KQKDQLNNFQQLTKKLHVANENIEMTNHHFLKTVTEQNMEIEKLRKQLRQAKLDLRVAVT
KVEELTKVTEGLQEQMLKKEEDIMSAQGKEEASDRRVQQLQSSIKQLESRLCIAIQEANV
LKTGKTQLEKQIKELQAKCSESENEKYEAISRARDSMQLLEEANIKQNQILLEEKQKEVE
REKMKKTISHLIQDAAIKARKEVESTKKQYEVLILQLKEELSALQMDCDEKQGQIDRAIR
GKRAVEEELEKIYREGKQDEGDYRKLEEMHQRCLAAERSKDDLQLRLKTAENRIKQLEIN
SSEEISRSHEMIQKLQTVLESERENCGFVSEQRLKLQQENEQLQKETEDLRKVALEAQKK
AKLKVSTMEHQFSIKEHGFEVQLREMEDSNRNSIVELRHLLAAQQKTANRWKEETKKLTE
SAEMRISSLKSELSRQKLHTQELLSQLEMANEKVAENEKLILEHQEKANRLQRRLSQAEE
RAASASQQLSVITVQRRKAASMMNLENI
>P39079
MSLQLLNPKAESLRRDAALKVNVTSAEGLQSVLETNLGPKGTLKMLVDGAGNIKLTKDGK
VLLTEMQIQSPTAVLIARAAAAQDEITGDGTTTVVCLVGELLRQAHRFIQEGVHPRIITD
GFEIARKESMKFLDEFKISKTNLSNDREFLLQVARSSLLTKVDADLTEVLTPIVTDAVLS
VYDAQADNLDLHMVEIMQMQHLSPKDTTFIKGLVLDHGGRHPDMPTRVKNAYVLILNVSL
EYEKTEVNSGFFYSSADQRDKLAASERKFVDAKLKKIIDLKNEVCGMDPDKGFVIINQKG
IDPMSLDVFAKHNILALRRAKRRNMERLQLVTGGEAQNSVEDLSPQILGFSGLVYQETIG
EEKFTYVTENTDPKSCTILIKGSTHYALAQTKDAVRDGLRAVANVLKDKNIIPGAGAFYI
ALSRYLRSANMNKLGAKGKTKTGIEAFAEALLVIPKTLVKNSGFDPLDVLAMVEDELDDA
QDSDETRYVGVDLNIGDSCDPTIEGIWDSYRVLRNAITGATGIASNLLLCDELLRAGRST
LKETPQ
>Q9Y7P8
MHSVCSIFLSCSHRVIQAKHPPFPLFHSYFHIPDFLSFVFPFVASPPLAFARRKLDHVPK
KFARSIGPFLLIVFLFFNLFPTFFFLPFFPDTTKRPNLAD
>Q8CGU1
MEESSLSRAPSRGGVNFLNVARTYIPNTKVECHYTLPPGTMPSASDWIGIFKVEAACVRD
YHTFVWSSVPESTTDGSPTHASVQFQASYLPKPGAQLYQFRYVNRQGRVCGQSPPFQFRE
PRPMDELVTLEEADGGSDILLVVPKATVLQNQLDESQQERNDLMQLKLQLEDQVTELRSR
VQELEAALATARQEHSELTEQYKGLSRSHGELSEERDILSQQQGDHVARILELEDDIQTM
SDKVLMKEVELDRVRDTVKALTREQEKLLRQLKEFQADKEQSEAELQTVREENCCLNTEL
EEAKSRQEEQGAQVQRLKDKLAHMKDTLGQAQQKVAELEPLKEQLRGVQELAASSQQKAA
LLGEELASAAGARDRTIAELHRSRLEVAEVNGRLAELSLHMKEEKCQWSKERTGLLQSME

AEKDKILKLSAEILRLEKTVQEERTQSHVFKTELAREKDSSLVQLSESKRELTELRSALR
VLQKEKEQLQTEKQELLEYMRKLEARLEKVADEKWTEDAATEDEEATAGLSCPASLTDSE
DESPEDMRLPSYGLCESGNTSSSPPGPREPSSLVVINQPAPIAPQFSGPGEASSSDSEAE
DEKSVLMAAVQSGGEEASLLLPELGSAFYDVASAFTVSSLSEASPGVPANPPWKECPICK
ERFPAESDKDALEGHMDGHFFFSTQDPFTFE
>O14111
MPFTKSCRSANTLRKGIKNGKLILKIIVNDVCCWVLIKVTNKQIDNVESCLSGDESNDSK
KSSASFYMKLKYGSYRALADNILSTDENRKEDIAVFDVPLPNGLQIDTFTLCLYRKSKWK
KQIVGEAYIGIQALLLSTNPDETCKYPVISPPSGRNKENQSPSHQICNLSLKWIIYDPED
ADADSKTLAKAWLQQIKMNQTSIDPMSNISKSLKELEVDNVESDLEDSSFIAEPDSSIPP
SESSVSISTDTGKETPPSKSKKSSNQPYVSIGEGNSDLLGFVFLEIISVSNLPPLKNVFR
TGFDMDPFVITAFSKNIFRTKWLRHNLNPVYNEKFLFEVGAFESNYDLVFKVVDHDKMSL
NDSIAVGSFNVQSIINSSAQVDPETGLYSFNIETSSPSQDTSSKAEDSPTVQKIADDFSS
AVGKDLRTDIIEQIIPLTLCCKHDFSTPRDVKLSFKAMFFPIAALRQKFWRVMLAQYGDI
EDGHIGKLGMYAVLDTLGSNIPNSMVDDIYTELSSKNHDDTSDSITVDEAVICLERLVDL
VCHQDQQATQTPQSPSSNEESGPGTPTQTSDQYEDSEDSRNFPSKLYLVYLSNCPLCLKF
KLSKVNQQKATVHLATCASHDWKRVDRLMMTSYVSLNQAQRRWFSKAFAKVVYGSSKVGS
TSATTLVQNRQTGQIQEEKMNAYVRIGIRLLYRGIRNRRIEGSKVKKILRSLTLKQGMKY
DSPISVKEIKPFIRFFDLNMNEVDMPVGGFKTFNEFFYRKLKPGSRPCAFPDNPDILVSP
ADSRIVAYECIEKATTYWIKGTEFTVERLLGYSNEAQRFVGGSICISRLAPQDYHRFHSP
VNGCIGPITKIEGQYYTVNPMAIRSYLDVFGENVRVLIPIDSNEFGKVMLVAVGAMMVGS
TVLTVDEGKIVQRSDELGYFKFGGSTVITLFEPNVTSFDEDLLRNSKTKIETLVKMGERI
GQKIDPNKPTDAEDHSKSDS
>Q9USN5
MYLTKQIPHYSRIHTTQLLTVVNHRTIPFKIRGSFSNCRRYSNFATVPLLERPFKNKSKH
NTARASRPFSTQKMLLTKSHPPNMYKDSVFFGYRPIVFSGKQDQKSKVIQCLRTERKTIP
DDLVEDEVDRFYNKLGLDDYYFQMEPVSIIADHIEIIYAAKIAAHASHAKEELNIHVKNE
NEDLAIYLDSSPVTQPELDQSSAVEESISTRYLDPFKLTDPTAYRVESFTSVTNIDRTST
ENSNIYTYFVTKCDFVDNPKKDASPDVPTDIASVSDKTFLEKASDNTIEMYQDVMNSVLT
RFGPVVRLFDYQGRSEIRLVVGYRRGSIFQYFPSLSKLFRYYGLHSTRTYVEQFSNGVTI
ISYNFKPELFKNAAVTSINELFSQITREASLLYCLPSTDFQPLFVSEKLSIQEVTYAHCV
RIFCEHVMNKLGPEYSSLSAILDHSNNIHAEILETIKRRLSTLAFTRTKIHDTIMQYPGL
VHTLFEQFYLEHAINHNSTPHLHRAKSATSLADEASTYSITPMSATALMDLIQKTCTNEE
DVSVMEMFVKFNTHLLKTNFFQTTKVALSFRFDPSFLDSTQYKDPLYAMIMSIGNEFRGF
HLRFRDVARGGIRLIKSANPEAFGLNARGLFDENYNLAKTQMLKNKDIPEGGAKGVILLG
KDCQDKPELAFMKYIDSIIDLLIVNKSQPLVDKLGKPEILFMGPDENTADLVNWATIHAH
RRNAPWWKSFFTGKKPTMGGIPHDKYGMTSLSVRCYVEGIYKKLNITDPSKLTKVQTGGP
DGDLGSNEIKLSNEKYIAVIDGSGVLYDPAGLDRTELLRLADERKTIDHFDAGKLSPEGY
RVLVKDTNLKLPNGEIVRNGTIFRNTAHLRYKADTFVPCGGRPNAININNVEQLIDDHGR
PAFKYLVEGANLFITQDAKSVLEKAGVIVIRDASANKGGVTSSSLEVLASLSFDDASFKE
NMCVHDGKVPTFYADYVNEVKRIIQRNANLEFEAIWKGHSENKIPYTSLSNHLSTEIVKL
DHDIYNYEKLWADVGFRNAVLRASIPKTLQAKIGLEKMLERIPESYLRAIFSTYLASRFV
YQHVVSSDPFAFFDYISTEMKMLKDA
>Q9ERS5

MPYVDRQNRICGFLDIEDNENSGKFLRRYFILDTQANCLLWYMDNPQNLAVGAGAVGSLQ
LTYISKVSIATPKQKPKTPFCFVINALSQRYFLQANDQKDLKDWVEALNQASKITVPKAG
TVPLATEVLKNLTAPPTLEKKPQVAYKTEIIGGVVVQTPISQNGGDGQEGCEPGTHAFLR
RSQSYIPTSGCRPSTGPPLIKSGYCVKQGNVRKSWKRRFFALDDFTICYFKCEQDREPLR
TIPLKDVLKTHECLVKSGDLLMRDNLFEIITTSRTFYVQADSPEDMHSWIEGIGAAVQAL
KCHPREPSFSRSISLTRPGSSTLTSAPNSILSRRRPPAEEKRGLCKAPSVASSWQPWTPV
PQAEEKPLSVEHAPEDSLFMPNPGESTATGVLASSRVRHRSEPQHPKEKPFVFNLDDENI
RTSDV
>O95487
MSAPAGSSHPAASARIPPKFGGSAVSGAAAPAGPGAGPAPHQQNGPAQNQMQVPSGYGLH
HQNYIAPSGHYSQGPGKMTSLPLDTQCGDYYSALYTVPTQNVTPNTVNQQPGAQQLYSRG
PPAPHIVGSTLGSFQGAASSASHLHTSASQPYSSFVNHYNSPAMYSASSSVASQGFPSTC
GHYAMSTVSNAAYPSVSYPSLPAGDTYGQMFTSQNAPTVRPVKDNSFSGQNTAISHPSPL
PPLPSQQHHQQQSLSGYSTLTWSSPGLPSTQDNLIRNHTGSLAVANNNPTITVADSLSCP
VMQNVQPPKSSPVVSTVLSGSSGSSSTRTPPTANHPVEPVTSVTQPSELLQQKGVQYGEY
VNNQASSAPTPLSSTSDDEEEEEEDEEAGVDSSSTTSSASPMPNSYDALEGGSYPDMLSS
SASSPAPDPAPEPDPASAPAPASAPAPVVPQPSKMAKPLAMAIQHFSLVIRMLQHHLFLE
YSPSNPVYSGFQQYPQQYPGVNQLSSSIGGLSLQSSPQPESLRPVNLTQERNILPMTPVW
APVPNLNADLKKLNCSPDSFRCTLTNIPQTQALLNKAKLPLGLLLHPFRDLTQLPVITSN
TIVRCRSCRTYINPFVSFIDQRRWKCNLCYRVNDVPEEFMYNPLTRSYGEPHKRPEVQNS
TVEFIASSDYMLRPPQPAVYLFVLDVSHNAVEAGYLTILCQSLLENLDKLPGDSRTRIGF
MTFDSTIHFYNLQEGLSQPQMLIVSDIDDVFLPTPDSLLVNLYESKELIKDLLNALPNMF
TNTRETHSALGPALQAAFKLMSPTGGRVSVFQTQLPSLGAGLLQSREDPNQRSSTKVVQH
LGPATDFYKKLALDCSGQQTAVDLFLLSSQYSDLASLACMSKYSAGCIYYYPSFHYTHNP
SQAEKLQKDLKRYLTRKIGFEAVMRIRCTKGLSMHTFHGNFFVRSTDLLSLANINPDAGF
AVQLSIEESLTDTSLVCFQTALLYTSSKGERRIRVHTLCLPVVSSLADVYAGVDVQAAIC
LLANMAVDRSVSSSLSDARDALVNAVVDSLSAYGSTVSNLQHSALMAPSSLKLFPLYVLA
LLKQKAFRTGTSTRLDDRVYAMCQIKSQPLVHLMKMIHPNLYRIDRLTDEGAVHVNDRIV
PQPPLQKLSAEKLTREGAFLMDCGSVFYIWVGKGCDNNFIEDVLGYTNFASIPQKMTHLP
ELDTLSSERARSFITWLRDSRPLSPILHIVKDESPAKAEFFQHLIEDRTEAAFSYYEFLL
HVQQQICK
>Q96JB8
MIQSDKGADPPDKKDMKLSTATNPQNGLSQILRLVLQELSLFYSRDVNGVCLLYDLLHSP
WLQALLKIYDCLQEFKEKKLVPATPHAQVLSYEVVELLRETPTSPEIQELRQMLQAPHFK
ALLSAHDTIAQKDFEPLLPPLPDNIPESEEAMRIVCLVKNQQPLGATIKRHEMTGDILVA
RIIHGGLAERSGLLYAGDKLVEVNGVSVEGLDPEQVIHILAMSRGTIMFKVVPVSDPPVN
SQQMVYYVRAMTEYWPQEDPDIPCMDAGLPFQKGDILQIVDQNDALWWQARKISDPATCAG
LVPSNHLLKRKQREFWWSQPYQPHTCLKSTLSISMEEEDDMKIDEKCVEADEETFESEEL
SEDKEEFVGYGQKFFIAGFRRSMRLCRRKSHLSPLHASVCCTGSCYSAVGAPYEEVVRYQ
RRPSDKYRLIVLMGPSGVGVNELRRQLIEFNPSHFQSAVPHTTRTKKSYEMNGREYHYVS
KETFENLIYSHRMLEYGEYKGHLYGTSVDAVQTVLVEGKICVMDLEPQDIQGVRTHELKP
YVIFIKPSNMRCMKQSRKNAKVITDYYVDMKFKDEDLQEMENLAQRMETQFGQFFDHVIV
NDSLHDACAQLLSAIQKAQEEPQWVPATWISSDTESQ
>P15303

MDFETNEDINGVRFTWNVFPSTRSDANSNVVPVGCLYTPLKEYDELNVAPYNPVVCSGPH
CKSILNPYCVIDPRNSSWSCPICNSRNHLPPQYTNLSQENMPLELQSTTIEYITNKPVTV
PPIFFFVVDLTSETENLDSLKESIITSLSLLPPNALIGLITYGNVVQLHDLSSETIDRCN
VFRGDREYQLEALTEMLTGQKPTGPGGAASHLPNAMNKVTPFSLNRFFLPLEQVEFKLNQ
LLENLSPDQWSVPAGHRPLRATGSALNIASLLLQGCYKNIPARIILFASGPGTVAPGLIV
NSELKDPLRSHHDIDSDHAQHYKKACKFYNQIAQRVAANGHTVDIFAGCYDQIGMSEMKQ
LTDSTGGVLLLTDAFSTAIFKQSYLRLFAKDEEGYLKMAFNGNMAVKTSKDLKVQGLIGH
ASAVKKTDANNISESEIGIGATSTWKMASLSPYHSYAIFFEIANTAANSNPMMSAPGSAD
RPHLAYTQFITTYQHSSGTNRIRVTTVANQLLPFGTPAIAASFDQEAAAVLMARIAVHKA
ETDDGADVIRWLDRTLIKLCQKYADYNKDDPQSFRLAPNFSLYPQFTYYLRRSQFLSVFN
NSPDETAFYRHIFTREDTTNSLIMIQPTLTSFSMEDDPQPVLLDSISVKPNTILLLDTFF
FILIYHGEQIAQWRKAGYQDDPQYADFKALLEEPKLEAAELLVDRFPLPRFIDTEAGGSQ
ARFLLSKLNPSDNYQDMARGGSTIVLTDDVSLQNFMTHLQQVAVSGQA
>P31209
MPSTDLKKQADAAVESDVNTNNEAVESSTKEESSNTPSTETQPEKKAEEPEAAAEPSEST
STPTNASSVATPSGTAPTSASLYVGELDPSVTEAMLFELFNSIGPVASIRVCRDAVTRRS
LGYAYVNFHNMEDGEKALDELNYTLIKGRPCRIMWSQRDPSLRKMGTGNVFIKNLDPAID
NKALHDTFSAFGKILSCKVAVDELGNAKGYGFVHFDSVESANAAIEHVNGMLLNDKKVYV
GHHVSRRERQSKVEALKANFTNVYIKNLDTEITEQEFSDLFGQFGEITSLSLVKDQNDKP
RGFGFVNYANHECAQKAVDELNDKEYKGKKLYVGRAQKKHEREEELRKRYEQMKLEKMNK
YQGVNLFIKNLQDEVDDERLKAEFSAFGTITSAKIMTDEQGKSKGFGFVCYTTPEEANKA
VTEMNQRMLAGKPLYVALAQRKEVRRSQLEAQIQARNQFRLQQQVAAAAGIPAVQYGATG
PLIYGPGGYPIPAAVNGRGMPMVPGHNGPMPMYPGMPTQFPAGGPAPGYPGMNARGPVPA
QGRPMMMPGSVPSAGPAEAEAVPAVPGMPERFTAADLAAVPEESRKQVLGELLYPKVFVR
EEKLSGKITGMLLEMPNSELLELLEDDSALNERVNEAIGVLQEFVDQEPGFTE
>O13563
MSMSSTVIKFRAGVCEYNEDSRLCTPIPVQGEIEIKPNEEEELGFWDFEWRPTEKPVGRE
LDPISLILIPGETMWVPIKSSKSGRIFALVFSSNERYFFWLQEKNSGNLPLNELSAKDKE
IYNKMIGVLNNSSESDEEESNDEKQKAQDVDVSMQD
>P35520
MPSETPQAEVGPTGCPHRSGPHSAKGSLEKGSPEDKEAKEPLWIRPDAPSRCTWQLGRPA
SESPHHHTAPAKSPKILPDILKKIGDTPMVRINKIGKKFGLKCELLAKCEFFNAGGSVKD
RISLRMIEDAERDGTLKPGDTIIEPTSGNTGIGLALAAAVRGYRCIIVMPEKMSSEKVDV
LRALGAEIVRTPTNARFDSPESHVGVAWRLKNEIPNSHILDQYRNASNPLAHYDTTADEI
LQQCDGKLDMLVASVGTGGTITGIARKLKEKCPGCRIIGVDPEGSILAEPEELNQTEQTT
YEVEGIGYDFIPTVLDRTVVDKWFKSNDEEAFTFARMLIAQEGLLCGGSAGSTVAVAVKA
AQELQEGQRCVVILPDSVRNYMTKFLSDRWMLQKGFLKEEDLTEKKPWWWHLRVQELGLS
APLTVLPTITCGHTIEILREKGFDQAPVVDEAGVILGMVTLGNMLSSLLAGKVQPSDQVG
KVIYKQFKQIRLTDTLGRLSHILEMDHFALVVHEQIQYHSTGKSSQRQMVFGVVTAIDLL
NFVAAQERDQK
>Q99PP9
MAELDLIAPGPLTGVTAHPLAPLGPDPVSAIPVEKEDADPLSKSGEETQEQGHDPAELGA
PGEEDQILCDFCLGASRVRAVKSCLTCMVNYCEEHLRPHQENSKLHSHQLTEPAKDQDLR
TCPAHHSPLVSFCHTHQQCICQECGEGEHRGDSTVSLDAARRNKEVDLRCMQLDLEQKLK

LNENAIARLQANHKSVLVSVSEVKVVAEEKFGELLAAVRKAQADVMVFLEEKEQAALNQV
NSIKTHLEHRSLEMEKSKQELERLAAISNTVLFLEEYCKLKKTEDTASPSIYIGLKDKLS
GIRKVITDSTLNLIQLLESYKEKLQEFSREEEYDIRTQVSAIVQRKYRTSKPEPRTRDEF
LQYACDITFDPDTAHRYLRLQEDNRKVTNTTPWEHPYPDLPSRFLHWRQVLSQQSLYLHR
YYFEVELSGGGTYVGLTCKGIDRKGEERNSCISGNSFSWSIHWNGKEFTAWHSDTETPLK
VSPFRRLGIYVNFPGGILSFYGVEYDAMTLIHKFDCKFSEPVYAAFWLSKKENAIRIVDL
GEEPEKPAGSSVEAAP
>P47089
MLREVIYCGICSYPPEYCEFSGKLKRCKVWLSENHADLYAKLYGTDDNTQEVEAVTNKLA
ESSIGEAREEKLEKDLLKIQKKQENREQRELAKKLSSKVIIKREARTKRKFIVAISGLEV
FDIDMKKLAKTFASRFATGCSVSKNAEKKEEVVIQGDVMDEVETYIHSLLEEKGLKDVKV
ETIDAKKKKKPAAEGAAK
>P49758
MAQGSGDQRAVGVADPEESSPNMIVYCKIEDIITKMQDDKTGGVPIRTVKSFLSKIPSVV
TGTDIVQWLMKNLSIEDPVEAIHLGSLIAAQGYIFPISDHVLTMKDDGTFYRFQAPYFWP
SNCWEPENTDYAIYLCKRTMQNKARLELADYEAENLARLQRAFAKKWEFIFMQAEAQVKI
DRKKDKTERKILDSQERAFWDVHRPVPGCLNTTEMDIRKCRRLKNPQKVKKSVYGVTEES
QAQSPVHVLSQPIRKTTKEDIRKQITFLNAQIDRHCLKMSKVAESLIAYTEQYVEYDPLI
TPAEPSNPWISEEVALWDIEMSKDPSQQRVKRWGFSFDEILKDQVGRDQFLRFLESEFSS
ENLRFWLAVQDLKKQPLQDVAKRVEEIWQEFLAPGAPSAINLDSHSYEITSQNVKDGGRY
TFEDAQEHIYKLMKSDSYARFPPVKCLPGFAAGQEEGKVAGGQAPHGPDAVPPEPSSPQS
RAWARGPHRQAAALHICGQSFLTRRLGHCEGERVRAMKGDGGETRWVNGQTRKKESTEPG
LGPVEAPVYSPLFFVQLYSNTPLAKRP
>Q5VZK9
MTEESSDVPRELIESIKDVIGRKIKISVKKKVKLEVKGDKVENKVLVLTSCRAFLVTARI
PTKLELTFSYLEIHGVVCSKSAQMIVETEKCSISMKMASPEDVSEVLAHIGTCLRKIFPG
LSPVRIMKKVSMEPSERLASLQALWDSQTVAEQGPCGGFSQMYACVCDWLGFSYREEVQW
DVDTIYLTQDTRELNLQDFSHLDHRDLIPIIAALEYNQWFTKLSSKDLKLSTDVCEQILR
VVSRSNRLEELVLENAGLRTDFAQKLASALAHNPNSGLHTINLAGNPLEDRGVSSLSIQF
AKLPKGLKHLNLSKTSLSPKGVNSLSQSLSANPLTASTLVHLDLSGNVLRGDDLSHMYNF
LAQPNAIVHLDLSNTECSLDMVCGALLRGCLQYLAVLNLSRTVFSHRKGKEVPPSFKQFF
SSSLALMHINLSGTKLSPEPLKALLLGLACNHNLKGVSLDLSNCELRSGGAQVLEGCIAE
IHNITSLDISDNGLESDLSTLIVWLSKNRSIQHLALGKNFNNMKSKNLTPVLDNLVQMIQ
DEESPLQSLSLADSKLKTEVTIIINALGSNTSLTKVDISGNGMGDMGAKMLAKALQINTK
LRTVIWDKNNITAQGFQDIAVAMEKNYTLRFMPIPMYDASQALKTNPEKTEDALQKIENY
LLRNHETRKYLQEQAYRLQQGIVTSTTQQMIDRICVKVQDHLNSLRNCGGDAIQEDLKSA
ERLMRDAKNSKTLLPNLYHVGGASWAGASGLLSSPIQETLESMAGEVTRVVDEQLKALLE
SMVDAAENLCPNVMKKAHIRQDLIHASTEKISIPRTFVKNVLLEQSGIDILNKISEVKLT
VASFLSDRIVDEILDALSHCHHKLADHFSRRGKTLPQQESLEIELAEEKPVKRSIITVEE
LTEIERLEDLDTCMMTPKSKRKSIHSRMLRPVSRAFEMEFDLDKALEEVPIHIEDPPFPS
LRQEKRSSGFISELPSEEGKKLEHFTKLRPKRNKKQQPTQAAVCAANIVSQDGEQNGLMG
RVDEGVDEFFTKKVTKMDSKKWSTRGSESHELNEGGDEKKKRDSRKSSGFLNLIKSRSKS
ERPPTILMTEEPSSPKGAVRSPPVDCPRKDTKAAEHNGNSERIEEIKTPDSFEESQGEEI
GKVERSDSKSSPQAGRRYGVQVMGSGLLAEMKAKQEKRAACAQKKLGNDAVSQDSSSPAL

SGVERSDGGGAVPKLHPGLPENRFGLGTPEKNTKAEPKAEAGSRSRSSSSTPTSPKPLLQ
SPKPSLAARPVIPQKPRTASRPDDIPDSPSSPKVALLPPVLKKVPSDKERDGQSSPQPSP
RTFSQEVSRRSWGQQAQEYQEQKQRSSSKDGHQGSKSNDSGEEAEKEFIFV
>Q96TC7
MSRLGALGGARAGLGLLLGTAAGLGFLCLLYSQRWKRTQRHGRSQSLPNSLDYTQTSDPG
RHVMLLRAVPGGAGDASVLPSLPREGQEKVLDRLDFVLTSLVALRREVEELRSSLRGLAG
EIVGEVRCHMEENQRVARRRRFPFVRERSDSTGSSSVYFTASSGATFTDAESEGGYTTAN
AESDNERDSDKESEDGEDEVSCETVKMGRKDSLDLEEEAASGASSALEAGGSSGLEDVLP
LLQQADELHRGDEQGKREGFQLLLNNKLVYGSRQDFLWRLARAYSDMCELTEEVSEKKSY
ALDGKEEAEAALEKGDESADCHLWYAVLCGQLAEHESIQRRIQSGFSFKEHVDKAIALQP
ENPMAHFLLGRWCYQVSHLSWLEKKTATALLESPLSATVEDALQSFLKAEELQPGFSKAG
RVYISKCYRELGKNSEARWWMKLALELPDVTKEDLAIQKDLEELEVILRD
>P87242
MNAAKSLSIVPFLDCLSRLKTTPRTGWLYHGIEKPESIADHMYRMGILTMLCNDPSINKE
RCLKIAVVHDMAESIVGDITPHENVSKEEKHRMESEAMVSITQQLIPLNLSLQAEEIKEL
FLEYESASTPEAKFVKDIDKFEMIAQMFEYERKFNGEKDLSQFTWAGKLIQHPLVKGWLN
DVLQEREQFWASVRQKKL
>Q04930
MLLSAPVNSTVRRKPHSPNKKKPKETGTAASFSSSSSTVVLSSNNDGSFDALWDPSISKA
SDFESSYISAKRLKPESSNRQKKKNSYKYSREENTNEVEEKTSLGSSSKTEADNIFNDQL
TSAGNTTYVSNKRDVNFGANSAVVLLGLPTSKSESHRQYHSPSASTTNEDEEDIGVDILV
DNHIDSCETVSINNNRGITHQYPETESDVDFDEAVILTPMDGTDKGVKNPRPLEKKYSSS
CFEDRTPLNLDDGHFSECNHFSTLDVSSFFHLNEHVHKIDEVELDGPDRTFSLDNVAINT
RKKDIDCLYNSSREDLSNLTCSSEGPRNDSYDSDYNIDEVTYRDDESTDEDESLPTPDRK
RKKIGHKACEILDSKRIGIKVPKLYVWSLSDKPFSVIDGLCTKSLYPLSDDINTPESLSS
CSSSVSSRENQKGDATFDNDAMIADLLNIGGLEVEKASNGHIELIGE
>Q9ES54
MAESIIIRVQSPDGVKRITATKRETAATFLKKVAKEFGFQNNGFSVYINRNKTGEITASS
SKSLHLLKIKHGDLLFLFPSSLAGPSSEMETSTSVGLKAFGAPHVVEDEIDQYLSKQDGK
IYRSRDPQLCRHGPLGKCVHCVPLEPFDEDYLNHLEPPVKHMSFHAYIRKLTGGADKGKF
VALENISCKIKSGCEGHLPWPNGICTKCQPSAITLNRQKYRHVDNIMFENHTVADRFLDF
WRKTGNQHFGYLYGRYTEHKDIPLGIRAEVAAIYEPPQIGTQNSLELLEDPKAEVVDEIA
SKLGLRKVGWIFTDLVSEDTRKGTVRYSRNKDTYFLSSEECITAGDFQNKHPNICRLSPD
GHFGSKFVTAVATGGPDNQVHFEGYQVSNQCMALVRDECLLPCKDAPELGYAKESSSEQY
VPDVFYKDIDKFGNEITQLARPLPVEYLIIDITTTFPKDPVYTFSISQNPFPIENRDVLG
ETQDFHSLATYLSQNTSSVFLDTISDFHLLLFLVTNEVMPLQDSISLLLEAVRTRNEELA
QTWKKSEQWATIEQLCSTVGVQLPGLHEFGAVGGSARAATSAMWACQHCTFMNQPGTGHC
EMCSLPRT
>Q04179
MTVSKIPIWLDCDPGHDDAIAILLGCFHPAFNLLGISTCFGNAPPENTDYNARSLLTAMG
KAQAIPVYKGAQRPWKREPHYAPDIHGISGLDGTSLLPKPTFEARTDKTYIEAIEEAILA
NNGEISFVSTGALTTLATVFRCKPYLKKSVKYISIMGGGLHGLGNCNPNLSAEFNVWIDP
DAANYIFRDPDVKDKCIVVPLNLTHKAIATYKVNEMIYNEKNNSKLRELFLELFQFFAHT
YKDMQGFESGPPIHDPVALMPLLEFYGWDPSSAVGFRYKRMDISCIDDVFNENSGKIIIE

KEYPNDSDVGTIIGLDLNIQYFWDQIFEALNRADKMSTIG
>O95456
MAATFFGEVVKAPCRAGTEDEEEEEEGRRETPEDREVRLQLARKREVRLLRRQTKTSLEV
SLLEKYPCSKFIIAIGNNAVAFLSSFVMNSGVWEEVGCAKLWNEWCRTTDTTHLSSTEAF
CVFYHLKSNPSVFLCQCSCYVAEDQQYQWLEKVFGSCPRKNMQITILTCRHVTDYKTSES
TGSLPSPFLRALKTQNFKDSACCPLLEQPNIVHDLPAAVLSYCQVWKIPAILYLCYTDVM
KLDLITVEAFKPILSTRSLKGLVKNIPQSTEILKKLMTTNEIQSNIYT
>O14960
MFSTKALLLAGLISTALAGPWANICAGKSSNEIRTCDRHGCGQYSAQRSQRPHQGVDVLC
SAGSTVYAPFTGMIVGQEKPYQNKNAINNGVRISGRGFCVKMFYIKPIKYKGPIKKGEKL
GTLLPLQKVYPGIQSHVHIENCDSSDPTAYL
>P56216
MSKTQEFRPLTLPPKLSLSDFNEFIQDIIRIVGSENVEVISSKDQIVDGSYMKPTHTHDP
HHVMDQDYFLASAIVAPRNVADVQSIVGLANKFSFPLWPISIGRNSGYGGAAPRVSGSVV
LDMGKNMNRVLEVNVEGAYCVVEPGVTYHDLHNYLEANNLRDKLWLDVPDLGGGSVLGNA
VERGVGYTPYGDHWMMHSGMEVVLANGELLRTGMGALPDPKRPETMGLKPEDQPWSKIAH
LFPYGFGPYIDGLFSQSNMGIVTKIGIWLMPNPRGYQSYLITLPKDGDLKQAVDIIRPLR
LGMALQNVPTIRHILLDAAVLGDKRSYSSRTEPLSDEELDKIAKQLNLGRWNFYGALYGP
EPIRRVLWETIKDAFSAIPGVKFYFPEDTPENSVLRVRDKTMQGIPTYDELKWIDWLPNG
AHLFFSPIAKVSGEDAMMQYAVTKKRCQEAGLDFIGTFTVGMREMHHIVCIVFNKKDLIQ
KRKVQWLMRTLIDDCAANGWGEYRTHLAFMDQIMETYNWNNSSFLRFNEVLKNAVDPNGI
IAPGKSGVWPSQYSHVTWKL
>Q15020
MATAAETSASEPEAESKAGPKADGEEDEVKAARTRRKVLSRAVAAATYKTMGPAWDQQEE
GVSESDGDEYAMASSAESSPGEYEWEYDEEEEKNQLEIERLEEQLSINVYDYNCHVDLIR
LLRLEGELTKVRMARQKMSEIFPLTEELWLEWLHDEISMAQDGLDREHVYDLFEKAVKDY
ICPNIWLEYGQYSVGGIGQKGGLEKVRSVFERALSSVGLHMTKGLALWEAYREFESAIVE
AARLEKVHSLFRRQLAIPLYDMEATFAEYEEWSEDPIPESVIQNYNKALQQLEKYKPYEE
ALLQAEAPRLAEYQAYIDFEMKIGDPARIQLIFERALVENCLVPDLWIRYSQYLDRQLKV
KDLVLSVHNRAIRNCPWTVALWSRYLLAMERHGVDHQVISVTFEKALNAGFIQATDYVEI
WQAYLDYLRRRVDFKQDSSKELEELRAAFTRALEYLKQEVEERFNESGDPSCVIMQNWAR
IEARLCNNMQKARELWDSIMTRGNAKYANMWLEYYNLERAHGDTQHCRKALHRAVQCTSD
YPEHVCEVLLTMERTEGSLEDWDIAVQKTETRLARVNEQRMKAAEKEAALVQQEEEKAEQ
RKRARAEKKALKKKKKIRGPEKRGADEDDEKEWGDDEEEQPSKRRRVENSIPAAGETQNV
EVAAGPAGKCAAVDVEPPSKQKEKAASLKRDMPKVLHDSSKDSITVFVSNLPYSMQEPDT
KLRPLFEACGEVVQIRPIFSNRGDFRGYCYVEFKEEKSALQALEMDRKSVEGRPMFVSPC
VDKSKNPDFKVFRYSTSLEKHKLFISGLPFSCTKEELEEICKAHGTVKDLRLVTNRAGKP
KGLAYVEYENESQASQAVMKMDGMTIKENIIKVAISNPPQRKVPEKPETRKAPGGPMLLP
QTYGARGKGRTQLSLLPRALQRPSAAAPQAENGPAAAPAVAAPAATEAPKMSNADFAKLF
LRK
>Q03862
MALAISHEDTQILLKDKNILQESVLNKYRTAGQIAQTALKYVTSLINDSYHSKTTQRQLT
VPELCLLTDSFILTRLEQYYKNKVNERGIAIPTTIDIDQISGGWCPEIDDTQNLLNWNKG
KDSTFASSVTGTLRPGDLVKITLGVHIDGYTSEVSHTMVIYPVDETKPILQPTGPLLGGK

ADAVAAAHIAMETVVALLACALTPEKLPASLGGTSSGITGQLIRTIVDTIARSYNCGVVP
GSRVRRIRRFLAGQNEGIVAEREYKGVVWTESHQEADLLSNTDAKDLTVVDRGQSTPFTN
VSAIPSDDFVVQSGEVYLIDLKMASLEHCTKKGLVTLETVDSYTGKSHKAGELIARPGAY
VRDFAQTHILKLKTSRQLLTKIDKQGVYPFKLSHLSSNFPFVHENEEELQSLKKDLKSFR
LGMSEISNNYLCVESPIQIARWVPWDHILKATNPNGNLSYDATSTLTLPGHELPLPKLGV
SAIKLKSLMNSTKESISLPVARECNTIVLCDSSVSTTDRPELLRLTGGSKTCQPSWIHSQ
HELNPQDSIVQGIFQLATLAKDKRFGLLLKETQPMKQKSVETSNGGVEETMKM
>Q07915
MRIYQCHFCSSPCYPGHGIMFVRNDAKEFRFCRSKCHKAFKQRRNPRKLKWTKAFRKAAG
KELAVDSTLTFAQRRNVPVRYNRELVATTLKAMARIEEIRQKRERAFYKNRMRGNKEKDF
LRDKKLVESNPELLRIREVEIARKLAKEQERAESVSEQEESEEEEEDMEIDSDEEEEEQL
EKQKILLKNRRRNTKKIAF
>P42036
MSKRKTKEPKVENVTLGPAVREGEQVFGVVHVFASFNDTFIHVTDLSGRETLVRITGGMK
VKADRDESSPYAAMLAAQDVAQRCKELGITAIHVKLRATGGNKTKTPGPGAQSALRALAR
SGMKIGRIEDVTPIPTDSTRRKGGRRGRRL
>Q9N428
MFSPDTNSEFYDDFYTKYNTNMAPTTSTFNAQPRLSLDSGLGLTNDSFYSTDLNETHLMD
ISAGNASTSFQSQPPPQLESSPPTKWARIRTPPNRKKKISHEGPNQEALPPGFNLFSPPS
RKKIKTAFSSPPPKSMKTPDSLRKSIRISSPSPFKVTFSKTPLKLSNNENVTGIHIGRSG
TYYNKQVTGSASKRCLLPSKPDGFTFLGSPGNSDVLDFPLQTTFEGFGDLDTPAKINAAL
EVNNDDSMDYYQYAGMIETSSSLATYTNSTGPLVENHARNLKSVPISRTASRNLMKISRK
SVEEPVPEPQAVHVEPVIEKPTKISAKKEKEQKKSAAKEAALKEAKEKEMRIWKLAPFET
PKKEVPLYSGRWLVISTGRTLAQQELFSDAKSFFKANPPPAPRAPQAPELASGPRRIPTI
QRVTLFKHRYRSPRD
>Q9ESN9
MMEIQMDEGGGVVVYQDDYCSGSVMSERVSGLAGSIYREFERLIHCYDEEVVKELMPLVV
NVLENLDSVLSENQEHEVELELLREDNEQLLTQYEREKALRKQAEEKFIEFEDALEQEKK
ELQIQVEHYEFQTRQLELKAKNYADQISRLEERESEMKKEYNALHQRHTEMIQTYVEHIE
RSKMQQVGGSGQTESSLPGRSRKERPTSLNVFPLADGMVRAQMGGKLVPAGDHWHLSDLG
QLQSSSSYQCPNDEMSESGQSSAAATPSTTGTKSNTPTSSVPSAAVTPLNESLQPLGDYV
SVTKNNKQAREKRNSRNMEVQVTQEMRNVSIGMGSSDEWSDVQDIIDSTPELDVCPETRL
ERTGSSPTQGIVNKAFGINTDSLYHELSTAGSEVIGDVDEGADLLGEFSVRDDFFGMGKE
VGNLLLENSQLLETKNALNVVKNDLIAKVDQLSGEQEVLKGELEAAKQAKVKLENRIKEL
EEELKRVKSEAVTARREPREEVEDVSSYLCTELDKIPMAQRRRFTRVEMARVLMERNQYK
ERLMELQEAVRWTEMIRASREHPSVQEKKKSTIWQFFSRLFSSSSSPPPAKRSYPSVNIH
YKSPTAAGFSQRRSHALCQISAGSRPLEFFPDDDCTSSARREQKREQYRQVREHVRNDDG
RLQACGWSLPAKYKQLSPNGGQEDTRMKNVPVPVYCRPLVEKDPSTKLWCAAGVNLSGWK
PHEEDSSNGPKPVPGRDPLTCDREGEGEPKSTHPSPEKKKAKETPEADATSSRVWILTST
LTTSKVVIIDANQPGTIVDQFTVCNAHVLCISSIPAASDSDYPPGEMFLDSDVNPEDSGA
DGVLAGITLVGCATRCNVPRSNCSSRGDTPVLDKGQGDVATTANGKVNPSQSTEEATEAT
EVPDPGPSESEATTVRPGPLTEHVFTDPAPTPSSSTQPASENGSESNGTIVQPQVEPSGE
LSTTTSSAAPTMWLGAQNGWLYVHSAVANWKKCLHSIKLKDSVLSLVHVKGRVLVALADG
TLAIFHRGEDGQWDLSNYHLMDLGHPHHSIRCMAVVNDRVWCGYKNKVHVIQPKTMQIEK

SFDAHPRRESQVRQLAWIGDGVWVSIRLDSTLRLYHAHTHQHLQDVDIEPYVSKMLGTGK
LGFSFVRITALLIAGNRLWVGTGNGVVISIPLTETVVLHRGQLLGLRANKTSPTSGEGTR
PGGIIHVYGDDSSDKAASSFIPYCSMAQAQLCFHGHRDAVKFFVSVPGNVLATLNGSVLD
SPSEGPGPAAPAADAEGQKLKNALVLSGGEGYIDFRIGDGEDDETEECAGDVNQTKPSLS
KAERSHIIVWQVSYTPE
>P47164
MISRTIGESIPPNTKHAVSVCLPTWEATVGYEEGESSIINSLTTGYPRFFIHKSIKKLCE
ILSAKYSMEDEACLCFPSYKVANRCREFIKVKTGLSTKVRILQLCTPKPMNQEEKLWRRE
CKITVVFVDQEIFPVMKQYWQHSGEIVSSRMAEYILHELQVKDNLKKMETVDNGKKFMTE
DENRVNEEYIETRFGRNLNFLAADKAKYLIRKRIATKVVEKIDSEGLSDLFSFEHYNESN
GPFNVGSGEALDDDQLNSDIPAETITSMGESGSNSTFENTATDDLKFHVNPNTDVYLFPS
GMASIFTAHRLLLNFDAKRLSRSSSRQDKLIGYGPPFKKTVMFGFPYTDTLSILRKFNHT
HFLGQGDSTSMNALKNILHSGEQILAVFIEAPSNPLLKMGDLQELKRLSDLYSFYIVVDE
TVGGFVNIDVLPYADIVCSSLTKIFSGDSNVIAGSLVLNPRGKIYEFARKFMKTEDGYED
CLWCEDALCLERNSRDFVERTIKVNTNTDILLKRVLLPQVGKLFKKIYYPSLTSEDTKRN
YDSVMSTKDGGYGGLFSLTFFNIEEAKKFFNNLELCKGPSLGTNFTLACPYAIIAHYQEL
DEVAQYGVETNLVRVSVGLENSDVLCNVFQRAIEKALGE
>O00471
MATTAELFEEPFVADEYIERLVWRTPGGGSRGGPEAFDPKRLLEEFVNHIQELQIMDERI
QRKVEKLEQQCQKEAKEFAKKVQELQKSNQVAFQHFQELDEHISYVATKVCHLGDQLEGV
NTPRQRAVEAQKLMKYFNEFLDGELKSDVFTNSEKIKEAADIIQKLHLIAQELPFDRFSE
VKSKIASKYHDLECQLIQEFTSAQRRGEISRMREVAAVLLHFKGYSHCVDVYIKQCQEGA
YLRNDIFEDAGILCQRVNKQVGDIFSNPETVLAKLIQNVFEIKLQSFVKEQLEECRKSDA
EQYLKNLYDLYTRTTNLSSKLMEFNLGTDKQTFLSKLIKSIFISYLENYIEVETGYLKSR
SAMILQRYYDSKNHQKRSIGTGGIQDLKERIRQRTNLPLGPSIDTHGETFLSQEVVVNLL
QETKQAFERCHRLSDPSDLPRNAFRIFTILVEFLCIEHIDYALETGLAGIPSSDSRNANL
YFLDVVQQANTIFHLFDKQFNDHLMPLISSSPKLSECLQKKKEIIEQMEMKLDTGIDRTL
NCMIGQMKHILAAEQKKTDFKPEDENNVLIQYTNACVKVCAYVRKQVEKIKNSMDGKNVD
TVLMELGVRFHRLIYEHLQQYSYSCMGGMLAICDVAEYRKCAKDFKIPMVLHLFDTLHAL
CNLLVVAPDNLKQVCSGEQLANLDKNILHSFVQLRADYRSARLARHFS
>Q8BG60
MVMFKKIKSFEVVFNDPEKVYGSGEKVAGRVIVEVCEVTRVKAVRILACGVAKVLWMQGS
QQCKQTLDYLRYEDTLLLEEQPTAGENEMVIMRPGNKYEYKFGFELPQGPLGTSFKGKYG
CVDYWVKAFLDRPSQPTQEAKKNFEVMDLVDVNTPDLMAPVSAKKEKKVSCMFIPDGRVS
VSARIDRKGFCEGDDISIHADFENTCSRIVVPKAAIVARHTYLANGQTKVFTQKLSSVRG
NHIISGTCASWRGKSLRVQKIRPSILGCNILKVEYSLLIYVSVPGSKKVILDLPLVIGSR
SGLSSRTSSMASRTSSEMSWIDLNIPDTPEAPPCYMDIIPEDHRLESPTTPLLDDVDDSQ
DSPIFMYAPEFQFMPPPTYTEVDPCVLNNNNNNNNVQ
>Q92540
MSLQSAQYLRQAEVLKADMTDSKLGPAEVWTSRQALQDLYQKMLVTDLEYALDKKVEQDL
WNHAFKNQITTLQGQAKNRANPNRSEVQANLSLFLEAASGFYTQLLQELCTVFNVDLPCR
VKSSQLGIISNKQTHTSAIVKPQSSSCSYICQHCLVHLGDIARYRNQTSQAESYYRHAAQ
LVPSNGQPYNQLAILASSKGDHLTTIFYYCRSIAVKFPFPAASTNLQKALSKALESRDEV
KTKWGVSDFIKAFIKFHGHVYLSKSLEKLSPLREKLEEQFKRLLFQKAFNSQQLVHVTVI

NLFQLHHLRDFSNETEQHTYSQDEQLCWTQLLALFMSFLGILCKCPLQNESQEESYNAYP
LPAVKVSMDWLRLRPRVFQEAVVDERQYIWPWLISLLNSFHPHEEDLSSISATPLPEEFE
LQGFLALRPSFRNLDFSKGHQGITGDKEGQQRRIRQQRLISIGKWIADNQPRLIQCENEV
GKLLFITEIPELILEDPSEAKENLILQETSVIESLAADGSPGLKSVLSTSRNLSNNCDTG
EKPVVTFKENIKTREVNRDQGRSFPPKEVRRDYSKGITVTKNDGKKDNNKRKTETKKCTL
EKLQETGKQNVAVQVKSQTELRKTPVSEARKTPVTQTPTQASNSQFIPIHHPGAFPPLPS
RPGFPPPTYVIPPPVAFSMGSGYTFPAGVSVPGTFLQPTAHSPAGNQVQAGKQSHIPYSQ
QRPSGPGPMNQGPQQSQPPSQQPLTSLPAQPTAQSTSQLQVQALTQQQQSPTKAVPALGK
SPPHHSGFQQYQQADASKQLWNPPQVQGPLGKIMPVKQPYYLQTQDPIKLFEPSLQPPVM
QQQPLEKKMKPFPMEPYNHNPSEVKVPEFYWDSSYSMADNRSVMAQQANIDRRGKRSPGV
FRPEQDPVPRMPFEKSLLEKPSELMSHSSSFLSLTGFSLNQERYPNNSMFNEVYGKNLTS
SSKAELSPSMAPQETSLYSLFEGTPWSPSLPASSDHSTPASQSPHSSNPSSLPSSPPTHN
HNSVPFSNFGPIGTPDNRDRRTADRWKTDKPAMGGFGIDYLSATSSSESSWHQASTPSGT
WTGHGPSMEDSSAVLMESLKSIWSSSMMHPGPSALEQLLMQQKQKQQRGQGTMNPPH
>Q04170
MSFENKLPTPLENNDAKGHMVCTLNKTTDARRAAETLSIAFSNSPAFHFICKKILNIPLA
EKVPTRTITTDIISPFLDSPYGEISEVNTFDAVAVWSLPPHVPKARSNDAKFNKDFIDDL
NARVKQVIPNGINYYYLFCIGKNLNEKGIRGSVRTIFEEYKRRADEENCAIVLEAIAEHA
KSVYEYFGFRNYMTFKYGECEVDSNGNCDPNGEGFTAYLMIYHKDGNKVLKE
>Q8NEZ2
MSWLFPLTKSASSSAAGSPGGLTSLQQQKQRLIESLRNSHSSIAEIQKDVEYRLPFTINN
LTININILLPPQFPQEKPVISVYPPIRHHLMDKQGVYVTSPLVNNFTMHSDLGKIIQSLL
DEFWKNPPVLAPTSTAFPYLYSNPSGMSPYASQGFPFLPPYPPQEANRSITSLSVADTVS
SSTTSHTTAKPAAPSFGVLSNLPLPIPTVDASIPTSQNGFGYKMPDVPDAFPELSELSVS
QLTDMNEQEEVLLEQFLTLPQLKQIITDKDDLVKSIEELARKNLLLEPSLEAKRQTVLDK
YELLTQMKSTFEKKMQRQHELSESCSASALQARLKVAAHEAEEESDNIAEDFLEGKMEID
DFLSSFMEKRTICHCRRAKEEKLQQAIAMHSQFHAPL
>Q24523
MAENQSAASKDSGHQQQHQQQQQQHQQHQQPLATTSVTAASTTSVLANQSPTNSQASSP
ENSQEALPLLRRQQSAAAATVAAAAATVAATTSGTSQQQQQQHRNSISNMFDRTVNAKFK
PASSNAGPGNNPVRRNSMLTPSRGVTIGGTGGNIRKLTKVSSLTSNHHFAVCYPPSNIYQ
NSNNAGSNSALQRTTSESLRLNMMSRVAAGATPTTVSRASSNSSLATSTSTSLAPKSSSS
SGGSNSTPQQQQQQLVSSNNSSSSSNNSFTKASSPNNNGARSVGGAATSAATGTTAAAGS
HHHQPHHHHHHHHHHHQHHNHQQQQQQQTSLSQGHASLTVAGGSASAGGGGGGGSGSSSG
TAAGGTNRKPKTTSSFEITSVTVGHPKLNAAGDTGDESADDLDESHTDDNSRITDLENET
PSMSEDTFSKEEVYYANNALSTNAPVIPTSSQYGLVVVDPIAPSLGQTIQNVQVNVSDNI
INVVSGAVTPGGTKKKDDIKETQHRSERFKVVKIESTEPFKRGRWMCMDYLDHSSVGNGG
NNNEKTGSSTSEAHAATTDGGAAGVGAGSEAPAHKTTQSMILPPTQKLNENHLEANSTDA
NWNYAEQQQQQQQQQQQQQTIVGNALTKTLPVALRNVSRSSSVTRSPNATVEFLSPNLLA
QQQQQQQQLFDSVNANAASSPNPAGDPNNMDYARTAAMQLHQTLQQLKQREDAMDVPPGA
GGYANYQNGGDSAVGAASNNNSAAAATGESQLSTSYVEQQQQQQQPLSPAPLTPQAAPTF
AAVAAGQSPNFQLEQQQQQQQATSQIDGIVPQPFNPQQQQQQTPQQSTAQQAAAAANATS
AVTAPPPQQTSNTSNAAVTTGQGQTMPLLSHMTSYEQQQPNLGAAAAAAAAGGTAATSVA
APQAIPTLQLQSAPSTIADPQQLMVPQQQQQQQHQEEQQQQPQQQQQPLPPANIASASAN

NSNLNLTNTNVVATGEATTNALTLTDEQATAALAAAFATGAAAAATGATSAAAATQQQIQ
QLQQQPNAESETESASGTSAVAIDNKIEQAMDLVKSHLMIAVREEVEVLKERISELMDKI
NKLELENSILKSNIPQETLQQLQLQLQLAAPPATPAIQAAPAVQSVVAPAAAGQAVQQQA
AGAVAVTGVATSPASAVVPTSIPNGSAENGSSAVESAAVSVEQQVQQVTSAAAAAASVVT
ANGPMS
>Q500Y9
MAIRKLLLLLKPIDPYPFLQTEGASLIKNPQVLQYLESRCKVHKNAIKFCQEILSKKPVE
WKPISRNDLSHPIRDVDMVITVGGDGTLLHASHFIDDSVPVLGVNSDPTQAHEVEELSDQ
FDASRSTGHLCAATVENFEQVLDDILFGRVVPAKVSRISLKLNSETLLSHALNDILIAQP
CPAAVSRFSFKIKNKDGASSPKTVNCRSSGLRICTAAGSTAAMQSAGGFVMPMLSRDLQF
MVREPISPGSTASLMHSTFKPDQFMDVNWYSDHGTIYIDGCQVQHSVQLGDTIEISSDAP
VLNVFLSHGISQIRSRY
>P29952
MSNKLFRLDAGYQQYDWGKIGSSSAVAQFAAHSDPSVQIEQDKPYAELWMGTHSKMPSYN
HESKESLRDIISKNPSAMLGKDIIDKFHATNELPFLFKVLSIEKVLSIQAHPDKALGKIL
HAQDPKNYPDDNHKPEMAIAVTDFEGFCGFKPLQEIADELKRIPELRNIVGEETSRNFIE
NIQPSAQKGSPEDEQNKKLLQAVFSRVMNASDDKIKIQARSLVERSKNSPSDFNKPDLPE
LIQRLNKQFPDDVGLFCGCLLLNHCRLNAGEAIFLRAKDPHAYISGDIMECMAASDNVVR
AGFTPKFKDVKNLVSMLTYTYDPVEKQKMQPLKFDRSSGNGKSVLYNPPIEEFAVLETTF
DEKLGQRHFEGVDGPSILITTKGNGYIKADGQKLKAEPGFVFFIAPHLPVDLEAEDEAFT
TYRAFVEPN
>P41738
MSSGANITYASRKRRKPVQKTVKPVPAEGIKSNPSKRHRDRLNTELDRLASLLPFPQDVI
NKLDKLSVLRLSVSYLRAKSFFDVALKSTPADRSRGQDQCRAQVRDWQDLQEGEFLLQAL
NGFVLVVTADALVFYASSTIQDYLGFQQSDVIHQSVYELIHTEDRAEFQRQLHWALNPSQ
CTDSAQGVDETHGLPQPAVYYTPDQLPPENTAFMERCFRCRLRCLLDNSSGFLAMNFQGR
LKYLHGQNKKGKDGALLPPQLALFAIATPLQPPSILEIRTKNFIFRTKHKLDFTPIGCDA
KGQLILGYTEVELCNKGSGYQFIHAADMLHCAESHIRMIKTGESGMTVFRLLAKHSRWRW
VQSNARLIYRNGRPDYIIATQRPLTDEEGREHLQKRSMTLPFMFATGEAVLYEISSPFSP
IMDPLPIRTKSNTSRKDWAPQSTPSKDSFHPNSLMSALIQQDESIYLCPPSSPAPLDSHF
LMDSMSECGSWQGSFAVASNEALLKHEEIRHTQDVNLTLSGGPSELFPDNKNNDLYSIMR
NLGIDFEDIRSMQNEEFFRTDSSGEVDFKDIDITDEILTYVQDSLNNSTLLNSACQQQPV
SQHLSCMLQERLQLEQQQQLQQQHPTQTLEPQRQLCQVEVPQHELGQKTKHMQVNGMFAS
WNPAPPVSFSCPQQERKHYSLFSGLQGTAQEFPYKSEVDSMPYTQNFAPCNQSLLPEHSK
GTQLDFPGRDFERSLHPNASNLEDFVSCLQVPENQRHGINSQSAMVSPQAYYAGAMSMYQ
CQAGPQHTPVDQMQYSPEIPGSQAFLSKFQSPSILNEAYSADLSSIGHLQTAAHLPRLAE
AQPLPDITPSGFL
>Q08162
MSVPAIAPRRKRLADGLSVTQKVFVRSRNGGATKIVREHYLRSDIPCLSRSCTKCPQIVV
PDAQNELPKFILSDSPLELSAPIGKHYVVLDTNVVLQAIDLLENPNCFFDVIVPQIVLDE
VRNKSYPVYTRLRTLCRDSDDHKRFIVFHNEFSEHTFVERLPNETINDRNDRAIRKTCQW
YSEHLKPYDINVVLVTNDRLNREAATKEVESNIITKSLVQYIELLPNADDIRDSIPQMDS
FDKDLERDTFSDFTFPEYYSTARVMGGLKNGVLYQGNIQISEYNFLEGSVSLPRFSKPVL
IVGQKNLNRAFNGDQVIVELLPQSEWKAPSSIVLDSEHFDVNDNPDIEAGDDDDNNESSS

NTTVISDKQRRLLAKDAMIAQRSKKIQPTAKVVYIQRRSWRQYVGQLAPSSVDPQSSSTQ
NVFVILMDKCLPKVRIRTRRAAELLDKRIVISIDSWPTTHKYPLGHFVRDLGTIESAQAE
TEALLLEHDVEYRPFSKKVLECLPAEGHDWKAPTKLDDPEAVSKDPLLTKRKDLRDKLIC
SIDPPGCVDIDDALHAKKLPNGNWEVGVHIADVTHFVKPGTALDAEGAARGTSVYLVDKR
IDMLPMLLGTDLCSLKPYVDRFAFSVIWELDDSANIVNVNFMKSVIRSREAFSYEQAQLR
IDDKTQNDELTMGMRALLKLSVKLKQKRLEAGALNLASPEVKVHMDSETSDPNEVEIKKL
LATNSLVEEFMLLANISVARKIYDAFPQTAMLRRHAAPPSTNFEILNEMLNTRKNMSISL
ESSKALADSLDRCVDPEDPYFNTLVRIMSTRCMMAAQYFYSGAYSYPDFRHYGLAVDIYT
HFTSPIRRYCDVVAHRQLAGAIGYEPLSLTHRDKNKMDMICRNINRKHRNAQFAGRASIE
YYVGQVMRNNESTETGYVIKVFNNGIVVLVPKFGVEGLIRLDNLTEDPNSAAFDEVEYKL
TFVPTNSDKPRDVYVFDKVEVQVRSVMDPITSKRKAELLLK
>P05737
MAAEKILTPESQLKKSKAQQKTAEQVAAERAARKAANKEKRAIILERNAAYQKEYETAER
NIIQAKRDAKAAGSYYVEAQHKLVFVVRIKGINKIPPKPRKVLQLLRLTRINSGTFVKVT
KATLELLKLIEPYVAYGYPSYSTIRQLVYKRGFGKINKQRVPLSDNAIIEANLGKYGILS
IDDLIHEIITVGPHFKQANNFLWPFKLSNPSGGWGVPRKFKHFIQGGSFGNREEFINKLV
KSMN
>O60086
MFARRCGRLANRFVRLKSTSATSPITYKALHANSPLPRCRIIEPPRATVPEAVSNIIMST
PFNRVQRPKRHVFNCLVQNEPGVLSRLSGILAARGFNIDSLVVCATEVENLSRMTIVLRG
ADEVVEQAKRQIEDIVSVWAVLDYTGTSMVERELLLAKVSLLGPDHFQEHFERSEKVAES
TNAKAKSDGEGVMNANAALQLRASQLAAINQLTTLFHGRVADISTETIILELTATPDRVD
NFLSLLRPYGVLEACRTGTSAMTRAPHSNEVTEEAEDDVEVEEVFLPPG
>P08879
MAANKERTFIMVKPDGVQRGLVGKIIERFEQKGFKLVALKFTWASKELLEKHYADLSARP
FFPGLVNYMNSGPVVPMVWEGLNVVKTGRQMLGATNPADSLPGTIRGDFCIQVGRNIIHG
SDAVESAEKEIALWFNEKELVTWTPAAKDWIYE
>P38315
MEPIDDILFEVTDAFKTQKEDLLELVTLIDIYGEQVNQEGSYEEKTRFIETLNTLLEDNP
STTGEIGWDLPKGLLKFLSKDNVDVNGRLGTNMIVQGVMKCFYAISIQGEPKKCLITGLE
LLSSLCSKDFSKSDQQNKEDFVDKKANTLPPEGVIENSSNRKDFPSYGESKSSNEFFLKL
KSYILFEFIGASLKRISTLFPSKYLGAAVSTIEKFVYSHADTFEDALFLLRRVYTFCRNY
IPPDPPKDIQLNEDFTREMFDKVVEEESELQVRLLRRLCTFGISTPIKTVTTNADVKYYC
ALNQQKFELSAYYTEYLELFCRYYQMAFSLDVDIEGEFQNVIKECRIIYKSVPQEISAVN
DEAKLVLERMVYKLAYTFEVQKAAKEKNVGLDYNGVILFSGIHYLETNQHLVKEMNITDA
IYLYLRFTTPSLYSKVYYNVAVESVSRYWLWYAITTEPLEDVKKELKNLSVFVTKTLLHV
LLQKNCIQVNQQLRMITFTLLTRLLCLIPEKVAFEFILDVLKTSPLPLAKTSVLCVFKDL
SRRRISTKDNDSETDLIVEKLSKLKVNDSNKAQQSNIRHYIQLDSSKMKAVHDCCLQTIQ
DSFTADAKKSDILLLLTYLNIFIVLKKTWDEDLLKIVCSKIDSNLKSVEPDKLPKYKEIV
DKNESLNDYFTGIK
>Q12123
MGSQDLASLIGRFKYVRVLDSNPHTKVISLLGSIDGKDAVLTAEKTHFIFDETVRRPSQS
GRSTPIFFHREIDEYSFLNGITDLKELTSNDIYYWGLSVLKQHILHNPTAKVNLIWPASQ
FHIKGYDQQDLHVVRETPDMYRNIVVPFIQEMCTSERMKWVNNILYEGAEDDRVVYKEYS

SRNKEDGFVILPDMKWDGINIDSLYLVAIVYRDDIKSLRDLNPNHRDWLIRLNKKIKTII
PQHYDYNVNPDELRVFIHYQPSYYHFHVHIVNIRHPGVGEERGSGMTILLEDVIEALGFL
GPEGYMKKTLTYVIGENHDLWKKGFKEEVEKQLKHDGIATSPEKGSGFNTNLG
>Q56WK6
MAQEEVQKSADVAAAPVVKEKPITDKEVTIPTPVAEKEEVAAPVSDEKAVPEKEVTPEKE
APAAEAEKSVSVKEEETVVVAEKVVVLTAEEVQKKALEEFKELVREALNKREFTAPVTPV
KEEKTEEKKTEEETKEEEKTEEKKEETTTEVKVEEEKPAVPAAEEEKSSEAAPVETKSEE
KPEEKAEVTTEKASSAEEDGTKTVEAIEESIVSVSPPESAVAPVVVETVAVAEAEPVEPE
EVSIWGVPLLQDERSDVILTKFLRARDFKVKEALTMLKNTVQWRKENKIDELVESGEEVS
EFEKMVFAHGVDKEGHVVIYSSYGEFQNKELFSDKEKLNKFLSWRIQLQEKCVRAIDFSN
PEAKSSFVFVSDFRNAPGLGKRALWQFIRRAVKQFEDNYPEFAAKELFINVPWWYIPYYK
TFGSIITSPRTRSKMVLAGPSKSADTIFKYIAPEQVPVKYGGLSKDTPLTEETITEAIVK
PAANYTIELPASEACTLSWELRVLGADVSYGAQFEPTTEGSYAVIVSKTRKIGSTDEPVI
TDSFKVGEPGKIVITIDNQTSKKKKVLYRFKTQ
>Q9Y2Y0
MDALEGESFALSFSSASDAEFDAVVGYLEDIIMDDEFQLLQRNFMDKYYLEFEDTEENKL
IYTPIFNEYISLVEKYIEEQLLQRIPEFNMAAFTTTLQHHKDEVAGDIFDMLLTFTDFLA
FKEMFLDYRAEKEGRGLDLSSGLVVTSLCKSSSLPASQNNLRH
>Q9HGQ0
MTALMNHIYIDNPLISNSTNNVTHELLIDLHELYNDGEISRIVLLRTLVTQSADDATWII
NLTDDVLNGLPLLKKRDRYTTQCHSTNMASTYDCDTGANAVGARGGATLAADYRGDWGGG
VMLYKPLVVKACLTEI
>Q9WU49
MSSEPPPPPQPPTHQTSIGLLDTPRARDRSPSPLRGNVVPSPLPTRRTRTFSATVRASQG
PVYKGVCKCFCRSKGHGFITPADGGPDIFLHISDVEGEYVPVEGDEVTYKMCSIPPKNEK
LQAVEVVITHLAPGTKHETWSGHVISS
>Q9UKD1
MATPDVSVHMEEVVVVTTPDTAVDGSGVEGVKTVLVTTNLAPHGGDLTEDNMETENAAAA
AAAAFTASSQLKEAVLVKMAEEGENLEAEIVYPITCGDSRANLIWRKFVCPGINVKCVQY
DEHVISPKEFVHLAGKSTLKDWKRAIRMNGIMLRKIMDSGELDFYQHDKVCSNTCRSTKI
DLSGARVSLSSPTSAEYIPLTPAAADVNGSPATITIETCEDPGDWTAAIGDDTFTFWRGL
KDAGLLDEVIQEFHQELVETMRGLQQRVQDPPLQLRDAVLLNNIVQNFGMLDLVKKVLAS
HKCQMDRSREQYARDLAALEQQCDEHRRRAKELKHKSQHLSNVLMTLTPVSLPPPVKRPR
LARATSGPAAMASQVLTQSAQLALGPGVPVPQLTSVPLGKVVSTLPSTVLGKGSLQAPPA
SSPASPLLGGYTVLASSGSTYPSTVEIHPDASSLTVLSTAAVQDGSTVFKVVSPLQLLTL
PGLGPTLQNVAQASPGSSTIVTVPAGAAPGPEEHTATIEVAAMAEDHERK
>P13007
MEFDELMQCPYDKNHMIRPSRFPYHLVKCRENNRAAAKILATCPYNARHRVPKQELDLHM
ASCEYRVTMEPISAAFSHQKVETSTWQSPPCEEVWETDEDPVSRPKPFILNDFTPSQPFN
MSEGDGNMPYTGISSNYRPEVQPMNSVMQVKQNQPEPEPFTSSERNYDPRSKEPPNPKQP
AVNGYKPATTNTNPWCRQTGGSRGAAPPKLGAKSSDEGPRNKEFPTPKANLMNEYVPVAA
NANPWCRQPGGSSAASEPLGVDSFDEWPCLGRQPWVRK
>Q06053
MEQNAEKRSIVGDDNSTVKRQDTSPSKGIAHIKPEYIVPLKQNENQKVAIYDEEMSSDRM

TNEFAGGTNKKNKNGRGKKRGQNKNRDNRQVKEQNVLCPRLIHGDISKCSFGDNCRFVHD
INLYLSTKKPEVESNIFPSCPVFNSLGFCPMGFKCRFLSSHLNKEDNILISKKEIDPDAQ
TIWSVKGEVNHISPERKLDLIKRRFPFTKSNEILEIIDSFQQECRDSMKPEEEVESTPQL
KKQDPDVEQPVAPQVEQRNKELSEHRMKQREVYLKYKDTRYFAQEKKPLDLYHKKIVSPL
TTVGNLPYRRLMRKLGADVTYSEMALAVPLIQGTNSEWALPKAHTSEFPGFGVQVACSKA
WQAAKAAEALANSVSEISEINLNSGCPIDLLYRQGSGSALLDNPARMIRCLNAMNYVSKD
IPITVKIRTGTKEGHPIAEGLVKRLVNETDVAAITLHGRSRQQRYTKSADWDYVSQVADT
LRSAEADFIETEQGKEGRDSKNRIQFVGNGDVNNFEDWYRYLNGNENIDSVMVARGALIK
PWIFEEVESQQYLDKTSTERLDILRDYAQFSMEHWGTDEYGISQCRRFFCEFMSFFHRYV
PMGICERYPVKLNERPPNWCGRDELETLMGSTDVNDWIKLSDLFFGKTDENFVFVPKHKS
SSYANRDS
>O14183
MDNKKGGLFKIAKKLRNGTKVWARAGYFKCKLLKITTTGAPCLFAGRLKRRYDATFQSMV
EIFRQEIANDVTCGRVETALLYFVNKFFRSVGCFGKGKEREVKTDRQRDTGSGEQRIRLE
RDTETLYQSQLRINQVNGWMSGWMQISLILQ
>Q8R2H7
MSLSQNAIFKSQTGEENLMSSNHRDSESITDVCSNEDLPEVELVNLLEEQLPQYKLRVDS
LFLYENQDWSQSSHQQQDASETLSPVLAEETFRYMILGTDRVEQMTKTYNDIDMVTHLLA
ERDRDLELAARIGQALLKRNHVLSEQNESLEEQLGQAFDQVNQLQHELSKKEELLRIVSI
ASEESETDSSCSTPLRFNESFSLSQGLLQLDMMHEKLKELEEENMALRSKACHIKTETFT
YEEKEQKLINDCVNELRETNAQMSRMTEELSGKSDELLRYQEEISSLLSQIVDLQHKLKE
HVIEKEELRLHLQASKDAQRQLTMELHELQDRNMECLGMLHESQEEIKELRNKAGPSAHL
CFSQAYGVFAGESLAAEIEGTMRKKLSLDEESVFKQKAQQKRVFDTVKVANDTRGRSVTF
PVLLPIPGSNRSSVIMTAKPFESGVQQTEDKTLPNQGSSTEVPGNSHPRDPPGLPEDSDL
ATALHRLSLRRQNYLSEKQFFAEEWERKLQILAEQEEEVSSCEALTENLASFCTDQSETT
ELGSAGCLRGFMPEKLQIVKPLEGSQTLHHWQQLAQPNLGTILDPRPGVITKGFTQMPKD
AVYHISDLEEDEEVGITFQVQQPLQLEQKPAPPPPVTGIFLPPMTSAGGPVSVATSNPGK
CLSFTNSTFTFTTCRILHPSDITQVTPSSGFPSLSCGSSAGSASNTAVNSPAASYRLSIG
ESITNRRDSTITFSSTRSLAKLLQERGISAKVYHSPASENPLLQLRPKALATPSTPPNSP
SQSPCSSPVPFEPRVHVSENFLASRPAETFLQEMYGLRPSRAPPDVGQLKMNLVDRLKRL
GIARVVKTPVPRENGKSREAEMGLQKPDSAVYLNSGGSLLGGLRRNQSLPVMMGSFGAPV
CTTSPKMGILKED
>Q60664
MLCVKGPPEQEPEDGALDVTRGCQCPLPTEDSILGQELLDCTRMNEDQSTDENGAGHFYS
ESPSQLREYLTQPSSEQTSSSESTVTSSESGSDILHMASGDLDCKPLCEKEEEARAASAM
QGTSLAPAAYGDYTSVGVAKAASQLEAGEELRTTENGGKGSAPGETEISMPPKASVKLVN
FQQSENTSANEKEVEAEFLRLSLGLKCDWFTLEKRVKLEERSRDLAEENLKKEITNCLKL
LESLTPLCEEDNQAQEIVKKLEKSIVLLSQCTARVASRAEMLGAINQESRVSRAVEVMIQ
HVENLKRMYAKEHAELEDLKQALLQNDRSFNSLPDEDDCQIKKRSSSLNSKPSSLRRVTI
ASLPRNLGNVGLVSGMENNDRFSRRSSSWRILGTKQGEHRPSLHRFISTYSWADAEDERS
DVKARDAPEPQGEEAVERTRKPSLSERRSSTLAWDRGTICSSVASWVTHLQASFRRANRA
LWLTGLIIILIAALMSFLTGQLFQTAVEAAPTQEGDSWLSLEHILWPFTRLGHDGPPPV
>Q9URY7
MAPRTIVKVDLNKPAWEQPNLHNRWHPDIPFAESIDEGETVKIECLDWTGGQIKNDDSAE

DIKNVDLTRIHYLSGPFEIKGAEPGDVLVVEIQDVQPLENQPWGYSGIFAKENGGGFLDE
HYPKAAKAVFDFEGIFCSSRHIPGVRFPGLIHPGIIGTAPSKEILAEWNRREGALVAENP
HSTHVMAQLPNASYAFAGILADEKLSATVASEGARTIPGRPENGGNCDIKNLSRGSKVFL
PVHVPGAKLSIGDLHFSQGDGEISFCGAIEMAGSITIKCKILKNGISDLAMKSPMYLPGP
VEPHFSPSRYLTFEGFSVDESGKQHYLCTTTAYRQTCLRVIEYFRRFGYNDYQLYLLLSC
APIQGHVAGIVDIPNSCTTIGVPMDIFEFDVSPNGKAKIIDLGSCAFANS
>P11171
MTTEKSLVTEAENSQHQQKEEGEEAINSGQQEPQQEESCQTAAEGDNWCEQKLKASNGDT
PTHEDLTKNKERTSESRGLSRLFSSFLKRPKSQVSEEEGKEVESDKEKGEGGQKEIEFGT
SLDEEIILKAPIAAPEPELKTDPSLDLHSLSSAETQPAQEELREDPDFEIKEGEGLEECS
KIEVKEESPQSKAETELKASQKPIRKHRNMHCKVSLLDDTVYECVVEKHAKGQDLLKRVC
EHLNLLEEDYFGLAIWDNATSKTWLDSAKEIKKQVRGVPWNFTFNVKFYPPDPAQLTEDI
TRYYLCLQLRQDIVAGRLPCSFATLALLGSYTIQSELGDYDPELHGVDYVSDFKLAPNQT
KELEEKVMELHKSYRSMTPAQADLEFLENAKKLSMYGVDLHKAKDLEGVDIILGVCSSGL
LVYKDKLRINRFPWPKVLKISYKRSSFFIKIRPGEQEQYESTIGFKLPSYRAAKKLWKVC
VEHHTFFRLTSTDTIPKSKFLALGSKFRYSGRTQAQTRQASALIDRPAPHFERTASKRAS
RSLDGAAAVDSADRSPRPTSAPAITQGQVAEGGVLDASAKKTVVPKAQKETVKAEVKKED
EPPEQAEPEPTEAWKVEKTHIEVTVPTSNGDQTQKLAEKTEDLIRMRKKKRERLDGENIY
IRHSNLMLEDLDKSQEEIKKHHASISELKKNFMESVPEPRPSEWDKRLSTHSPFRTLNIN
GQIPTGEGPPLVKTQTVTISDNANAVKSEIPTKDVPIVHTETKTITYEAAQTDDNSGDLD
PGVLLTAQTITSETPSSTTTTQITKTVKGGISETRIEKRIVITGDADIDHDQVLVQAIKE
AKEQHPDMSVTKVVVHQETEIADE
>Q7YXU4
MNLKPPTSKVELRIKCHKILDKDTLSKSDPRASVYEKDRAGQFRLIGKTETIQNQLNPEF
KTPIVIDYRFEEIQVLKFEIHDVDKNDEDFIGDASCTLTSILSKPGQTVCLQLLTKSGKH
AGSMTVIAEEIKNTLQTIKFNLIGKKFDKKDLFGAGCDPYLIISRKVPSTNTFVKIYESQ
VQKGTLNPVFSGIEMKLEELCGGDMQREIKFEFYDWDRIGKHDYIGEFHTNAQELLQPNQ
AFNVINSHKQEKKSGYKNSGTVSVSDAVIEREYNFLEYIMGGCQMNLIVGIDCTASNGDS
NDPNSLHYKNAQGLNQYANAICSVGNVIVPYTTTPLIPVYGFGGIMPGQSEVSHCFPMTL
NASNTLCCGVNGVLDCYYDNISKIQLHGPTYFAPLINMAARYASQGQSQSNQKYTILMII
TDGEILDADNTIDAIVKSSGLPLSIIIVGVGNANFTNMNILDGDDAALQSGGVRAERDIV
QFVAMRDYLNRPNELAAEVLREIPTQFLSFMKKYKFRPNPPPPPPPVIIGAPPQYDNPTT
TTTATSPSTGIDLNKGSNVGLNLTKTESSPSPSGGAGIDLNKGSVVDITKGVSNVSLEKN

>P25171
MPRRKALTNNNNAGEAEQQPPKAKRARIAFHLELPKRRTVLGNVLVCGNGDVGQLGLGED
ILERKRLSPVAGIPDAVDISAGGMHNLVLTKSGDIYSFGCNDEGALGRDTSEDGSESKPD
LIDLPGKALCISAGDSHSACLLEDGRVFAWGSFRDSHGNMGLTIDGNKRTPIDLMEGTVC
CSIASGADHLVILTTAGKVFTVGCAEQGQLGRLSERSISGEGRRGKRDLLRPTQLIITRA
KPFEAIWATNYCTFMRESQTQVIWATGLNNFKQLAHETKGKEFALTPIKTELKDIRHIAG
GQHHTVILTTDLKCSVVGRPEYGRLGLGDVKDVVEKPTIVKKLTEKIVSVGCGEVCSYAV
TIDGKLYSWGSGVNNQLGVGDGDDELEPIVVVSKNTQGKHMLLASGGGQHAIFLVKADKQ
DQKENVPVKVSGSSSISKKDKTPPQDNVDKEAENVDKQEQKENLPAKASTSSKKNKTPPQ
DNADKEAENVDKQEQKENLPAKASTSSKKIKTPPQDDAAEEVEEESAQEPTPKKAKKPAA

KRGGKKT
>Q8BMI0
MGPRKKSAKVCVMDSEVAEEMTADEEKDYMNQLSHEVLCHIFRYLPLQDIMCMECLSRKL
KEAVTLYLRVVRVVDLCAGRWWEYMPSGFTDSSFLTLLKKMPDVEQLYGLHPRYLERRRV
RGQEAFSIPGVLEALQACPNLVGVETSHLELVESIWTYMPHVHILGKFRNRNGAFPIPPE
NKLKIPIGAKIQTLHLVGVNVPEIPCIPMLRHLYMKWVRLTKPQPFKDFLCISLRTFVMR
NCAGPTNSLKYVPLVTGLASARNLEHLEMVRVPFLGGLIQHVVEDSWRSGGFRNLHTIVL
GACKNALEVDLGYLIITAARRLHEVRIQPSLTKDGVFSALKMAELEFPQFETLHLGYVDE
FLLQSRMANADLVKYGLADVVENPGIITDIGMKAVNEVFSCIKYLAIYNCPHLHNPYNWI
SDHSRWMRLVDINLVRCHALKLDSFGQFVELLPSLEFISLDQMFREPPKGCARVGLSAGT
GIGVSSALVSNQNSNNDNDNNAPNNNANLHDNNHHHPDDSDDDNDFRPDLQAGEAQFAAD
ALNEMEDMVQEDGELVAESGNGMPAHNREVLPVDADEEQAGPSGLQRVVKPTPIADHDSE
SDDEEDSLELQEVWAPKNGTRRYSEREEKTGDSGQSRETAVSGKGKTPLRKRCNNSHQTG
QAKPFPLEESSCEKGCQVTSEQIKADMKAARDVSEKKKSKDVYPSCSSSSSSTAASTAGN
ASSPSTASQSPDFARTVTSSGSSEPSPPEVDVSRQCVCSPGGSEDSEAMEEGDAESSVCP
RCCCLRPQESQRRTGRCSDEERPSTSRACVVNGADGTRSAFSFRTLPQGGSSGPAHDERT
NGSGCGATGEDRRGSSQPESCDVQSNEDYPRRPLTRARSRLSHVPLISESEVAKTKPCHA
MKRKRTADKSTSTSDPVIEDDHVQVLVLKSKNLVGVTMTNCGITDLVLKDCPKMMFIHAT
RCRVLKHLKVENAPIVNRFDYAQCKKLNMDQVLDQILRMPPERNRIIYLRPMQQVDTLTL
EQKLFSGPYPYHICIIHEFSNPPNVRNKVRIRNWMDTIANINQELIKYEFFLEATRTEED
LKKYPKYPWGREIYTLEGVVDGAPYSMISDFPWLRSLRTAEPNSFARYDFEDDEESTIYA
PRRKGQLSADICMETIGEEISEMRQMKRGIFQRVVAIFIHYCDVNGEPVEDDYI
>P35207
MSEGFSSSSIQELYQSLKEITNNADVELFEDRITKLDFESTDEPKHANDIIKDRFLRPSN
ALPWSLLDMVQDVPHTSSPEDCSGKLDYKELLKVPDPINRTSYQFKRTGLEGKISGYKEE
VDLKEVANANASNSLSITRSINHNQNSVRGSTAQLPFTPGGIPMKSVKTDSEQNGSSTMA
NATKLLHKDGQGLFDIPEGMNRGIKPMDSPAENEDQNGQFKELKQLNEIDNELDIRIEAN
EAKLKEEEKSAKSISEEIMEEATEETTADNADDAEIDELLPIGIDFGRTKPVSKSVPVKK
EWAHVVDLNHKIENFDELIPNPARSWPFELDTFQKEAVYHLEQGDSVFVAAHTSAGKTVV
AEYAIAMAHRNMTKTIYTSPIKALSNQKFRDFKETFDDVNIGLITGDVQINPDANCLIMT
TEILRSMLYRGADLIRDVEFVIFDEVHYVNDQDRGVVWEEVIIMLPQHVKFILLSATVPN
TYEFANWIGRTKQKNIYVISTPKRPVPLEINIWAKKELIPVINQNSEFLEANFRKHKEIL
NGESAKGAPSKTDNGRGGSTARGGRGGSNTRDGRGGRGNSTRGGANRGGSRGAGAIGSNK
RKFFTQDGPSKKTWPEIVNYLRKRELLPMVVFVFSKKRCEEYADWLEGINFCNNKEKSQI
HMFIEKSITRLKKEDRDLPQILKTRSLLERGIAVHHGGLLPIVKELIEILFSKGFIKVLF
ATETFAMGLNLPTRTVIFSSIRKHDGNGLRELTPGEFTQMAGRAGRRGLDSTGTVIVMAY
NSPLSIATFKEVTMGVPTRLQSQFRLTYNMILNLLRIEALRVEEMIKYSFSENAKETLQP
EHEKQIKVLQEELQTIEYKSCEICDNDIEKFLELMLAYKEATVNLMQEMVKSPSILHILK
EGRLVAFRDPNDCLKLGFVFKVSLKDAVCVIMTFTKPYKLPNGEPNHLIYFPKADGYRRR
NFPKFQKTDFYMEEVPVTAIEVITKRKFAAPLGKVIKKDVAALNEFNAETNNILDGKTLK
EAINIEKQGLKIHQILLDRTNIRDEIFKLKSIKCPNLSQHIVPKFKAHVIKKKIEELYHL
MSDQNLSLLPDYEKRLAVLKDTEFIDQNHNVLLKGRVACEINSGYELVLTELILDNFLGS
FEPEEIVALLSVFVYEGKTREEEPPIVTPRLAKGKQRIEEIYKKMLCVFNTHQIPLTQDE
AEFLDRKRFAMMNVVYEWARGLSFKEIMEMSPEAEGTVVRVITWLDEICREVKTASIIIG

NSTLHMKMSRAQELIKRDIVFAASLYL
>Q03900
MSNSPTVATLSQEYFDPNIPQILPHEKMYKIQVGKSLFKISGASLSSDGPSFFTEYFSKK
RSPSNNDDSNNDTMESNKNEVLFIDRSAEVFEWIYQHLQGYIIEIKDEVQYTMLFADAMY
YNLPRLRSLLKETDYYFTNIGGQSFKIAKNLFRREGDSPNYFEIYAATVYIDVEELIISK
KLLRPPSHSAPYIPRSSEYFKDLLTLLGGASIDLDDNKRNALIKECRYYRLLNLEQRLIK
SHISYNPITRKEEICLLLKDLSKKGITFPASSAFSTSPYFEDDFCSINECDSLSKTREQP
ANKKIKLDMTEKYNDSWNMLCYKRPFLDKHPRELIFQINSTDCTIILNKESQSIHVDITG
ESAYKFEALFGSHLPNTPSGAPKLKNYQYRFPSDSTQTKIETHYLLPACIYLCDLDINGI
KISQVQTLLTDKNKFNDRVIDVSDPLDLRFCSGLKLYLRKSLWKLAVKDGNIMLIAIKAI
AFNGTKEYYKGYEYL
>Q8N1N5
MHEPSLCANVECPPAHTCPCGVPACSCAHVECPPAHTCRCGVPACSHMPMWSARLLTRAH
VECPPAHTRVHVECPPAHVPMWSAHLLTCADVECHLLTHVPMWSARLLTCPCGVPACSHV
PMRSARLLTRAHAECPPAHTCPCGVPACSHVPMRSARLLTRADVECPPAHTCPCGVPACS
HVPTWSARLITRAHVECSPAHTCRCGVPACSHVPMWSVRLLTRADAECPPAHTCRCGVPA
CSHVPMWSARLLTCRCGVPACSHVPMWSARLLTCRCGVPACSHVPMWSARLLTRAHVECP
PAHTCRRGVPACSRAHMECPPAHTCHCGVPACSHTCRCGVPACSHVPMWSARLLTRAHVE
CPPAHTRAHVECPPAHTCPCGVPACSHTCPCGVPACSHKALAWWFCRFPVLPAESDAVTV
HSTHGGFLIRFYVKDPFYISLHLEIT
>P40855
MAAAEEGCSVGAEADRELEELLESALDDFDKAKPSPAPPSTTTAPDASGPQKRSPGDTAK
DALFASQEKFFQELFDSELASQATAEFEKAMKELAEEEPHLVEQFQKLSEAAGRVGSDMT
SQQEFTSCLKETLSGLAKNATDLQNSSMSEEELTKAMEGLGMDEGDGEGNILPIMQSIMQ
NLLSKDVLYPSLKEITEKYPEWLQSHRESLPPEQFEKYQEQHSVMCKICEQFEAETPTDS
ETTQKARFEMVLDLMQQLQDLGHPPKELAGEMPPGLNFDLDALNLSGPPGASGEQCLIM
>P87299
MKKHKSSIKCFKIDQISQQPETDLVFTKRSECKVKDELFSDKENSILCKQLKELDLVVSS
NKEFLNEKTSDQISFLKPRETVVEKKLANGSIWPNETSHLDKSRDLSSHSNGLDALAMTP
YIVKENNKTHLSPRGNYPSEAEKSCDFYGQPLHFYRENTSPCLYGSSLSNVFTEKSSDYR
HGLGSPTSLVSSNTASKLHLGRKDIVKLSELRNCINSSKDSNQIQELENLLRKEKQRNTE
HEKIIRRMKKELKELHSQFNFAKKLFISALSANQDILDKMNDEFN
>O74778
MSMNSSTEISQEPSIMKPVSSISSFVFNAGFLQGTFSDTTLIIKGETYHLHALFLGRSPV
LLQKLIENNPKGDVHYTIEVETEDPYVTKESCLFVLSTLYCDSPRIPAEVNVCSVLAVSD
LLGLDTLAFEASSLIEKSIRPETMESVIRFLDPNFEGLERLKMGMYPRFTSGLFNKAIQV
MYNTLVSNWNTEYARLLCNLPFEVIKDLLESDKLTVGASSMARYKLANEIVKMRASFRKQ
NHIEGKGDESVVLAFDEGSRGIQLVHIAPGAESRRKIWKATSVR
>Q12107
MNQNGDKNEGKLFQLPSLPPWKTPRFNKANFNNFTTPLRKRSTRVINDDSMPITGEVLEE
RTADDLYGINMDVDEVDYLNTLSHIEEEKQYDYSPFCERNTLRESRIDSFLKAERAAHCL
VFHKVGHLDGIDSYRPDIDIMCGEEANKYDSANPEGNGSMLLESVPGCNKEDLERLSRRE
FVTNSKPNMRRLDDIINHETNALKSFWNDSGLVNSLQSHHLHEEYLLLQEELKNVYKIKC
HDRVPIESLRDKCRRHYSNEDSSFL

>O95571
MAEAVLRVARRQLSQRGGSGAPILLRQMFEPVSCTFTYLLGDRESREAVLIDPVLETAPR
DAQLIKELGLRLLYAVNTHCHADHITGSGLLRSLLPGCQSVISRLSGAQADLHIEDGDSI
RFGRFALETRASPGHTPGCVTFVLNDHSMAFTGDALLIRGCGRTDFQQGCAKTLYHSVHE
KIFTLPGDCLIYPAHDYHGFTVSTVEEERTLNPRLTLSCEEFVKIMGNLNLPKPQQIDFA
VPANMRCGVQTPTA
>P32855
MDYLKPAQKGRRRGLSINSLSETQQSAMNSSLDHLQNDLNRINLQWNRILSDNTNPLELA
LAFLDDTSVGLGHRYEEFNQLKSQIGSHLQDVVNEHSQVFNTNVASYGKAVSSIMQAQEQ
TLNLKNCLKEANEKITTDKGSLQELNDNNLKYTKMIDVLVNIEELLQIPEKIEENIRKEN
FHQVQILLERGFILMNNKSLKTVEILKPINQQLELQEHLLFNNLIEEIHDIMYSKSNKTN
FTRVTNNDIFKIISISHNGFTSLENYLYNIVNIDIMEHSKTINKNLEQFIHDQSLNKGNI
MLQENAATQAPLAPSRNQENEGFNRIGFLLKTINNINKLPVAFNIITERAKEEIHNIIVK
STESIRSKHPSLLKMATSLKNDNHFGLPVQDILSIILRECFWEIFLKLLYAIQCHRAIFE
MSNILQPTSSAKPAFKFNKIWGKLLDEIELLLVRYINDPELISSNNGSIKPINGATNNAP
TLPKRKNPKIFSLEYNIEDNSSVKDQAFELKALLKDIFPGFSVSSNMDLDSIYVKDESFE
QDEPLVPPSVFNMKVILDPFLLFTQSTSTIVPSVLTQNTISSLTFFDDYMNKSFLPKIQM
TMDYLFTVEVESNNPYALELSDENHNIFKTALDFQRLFYNLLNVFNTANTFREKISYCIL
DLLNHFYNYYLGLFNSLIGTSDRHLTRKIITAWLQNGILMDQEQKILNGDETLFHEESIE
LFKEIPHFYQAGKGLSKSDLFNNLTLDTILQFSASVLWILNWLPGLKKAINIDEVSQEPM
LDADRLRSSWTFSESMDLNYSNPSSSPNSLGNLKILLDDKASKKFDETIDGFKTLKFKLI
TILRFNIRALCIYDIGSFFQNTKIWNMDVGSIELDQNIASLISELRRTESKLKQQLPEKE
KNSIFIGLDIVNNYALIKGAKSIKVLNHNGIKKMLRNVNVLQHAYRNLSSEPSKINMNVT
MNFYSLCGSSEAELFEYIKDNELPHCSVEDLKTILRLQFSEEMHRQLKRQSTSSTKGSIK
PSNKRYTEALEKLSNLEKEQSKEGARTKIGKLKSKLNAVHTANEK
>Q08171
MEMAKANGWAVVCTSTNTNTVPIYSKYTRCTELRRVDDNDIYKLATILDVNGCWRKLMSI
IPKRLDAQACSAPGALNYQEIAAKVGLKYTAQQISLIDGTAERLTPGQSISQVMIDEWKT
SGKLNERPTVGVLLQLLVHAEIYSAADFVALHFLNEPKPERPTDGPAAHISLDLCSEDLS
EDMDVEDGASYQPNTSALNAAVEQARGTGMNLDYFDKHMVRRDKSVPQQLENGTSSTVPV
PPPRAARSSRLLKATASNVAPTTASNAPSASNTANVPNLSILNASSKKLAASSEQTLQPQ
NIPNLSILNGSSEAVLMATTSTTLDAGKSDNASNGRSSASTSTATIPNVPLITLLIENSS
CEISDASDATQITSKSTATKTVPDMSTASYNNLPAISALNLNIASGAGELDGNGAKARGD
NADNNSSGTNSLSNDDDEQKEDDDDDDDDDVVDVDDEEADVSLPNLSNSDHNDSSLTTVT
CTSGENSFEFTNDSSSASNDDYTNNIPNLSELQQ
>P40084
MATIEDIKETALIPFQKHRQLSMHEAEVITLEIIGLLCDSECKDEKTLKYLGRFLTPDMY
QDLVDERNLNKRCGYPLCGKSPERIRDPFSMNDTTKKFLLENNPYAYLSHYCSKFHFRCS
QFYQVQLSDEALFARTGVHLFEDPEQDKHDIDFKVTLFEELLREKASEEDIKSLISGLKK
LGLNPDSGTTEKDDTELEDDLSKWLAQIKIVENDNPSILGDFTRED
>Q9HD47
MEPTRDCPLFGGAFSAILPMGAIDVSDLRPVPDNQEVFCHPVTDQSLIVELLELQAHVRG
EAAARYHFEDVGGVQGARAVHVESVQPLSLENLALRGRCQEAWVLSGKQQIAKENQQVAK
DVTLHQALLRLPQYQTDLLLTFNQPPPDNRSSLGPENLSPAPWSLGDFEQLVTSLTLHDP

NIFGPQ
>O94679
MSLDLLSRLKKYIHDEENSDLDSIYAECENAGLTAVVNDVIDTLLLGTSSCFDEDCLEKL
FAICSHFADLSSSVRNKVYDLLTSNISSESAILEDMISANATDFTVPQTNLETTGIAFQL
TVNSLSSSNQLSVIRSSTNTVKGRKKNPTTNSNWNGISHVNALLDAIITLFQKKLSRVWT
TSSERDMFLSLFLKPIYTLMESEINIKNASFRSRLFNIIGLAVQFHNHTTAAETNIIQNL
QYFEHLSEYAADLVHIVTVQFNSVTLAEGIIRTLCSLEFNDNDVKGPKQVALFLVRLSSL
IPNLCLKQLTQLVKLLDSESYTLRCAIIEVLANVVIDQIHDEAQNEMSESVPATVQSLMD
LLSERLLDISPYCRTKVLHVFIKIFDLPIKYPRKRQEIAELVIRCLQDRSSHVRRNAIKL
FSKLLTTHPFSVMHNGLLTRNIWEKGLSIIEEQLNSLQPKQQEKVVDSELEVDENLLEDA
TMIQDDESHEGESHLENSLSEYVDSVPAEEIVKVNLTKRFYLEALQYIDIVEAGAKIISQ
LLFAKNKSEVIESMDFFVFCNSFGISSSKLYIKKMIHLIWVKGTSDEGNNIQNHVLSCYK
TLFFEPPPNSGTNEAANYIARNLISLTYDASLAELTSLEQMLCILMKDGYFSHLVITKLW
QVYSYQKKDISRTQRRGSIIVIGMLALGNTDVVMQGLDHLIQIGLGPPGLEDLVLARYTC
IAIKRIGKDASGSSNINFPNSHTLCQKLCMLLLRPSFSEEWFGLEEQAIEAIYAVAKHPD
ELCTNIILLLTKQLFKPSNHENTTSNDDHAMDEDLDDSPEEETLKDEEEIGIRLAHLIFL
VGHVAIKQLVYIEYCEAEFKRRKADAERLAVQNSNNPINGQETSEYDLITGTSEDDFSEA
MTFIRERELLYGENSLLSRFAPLVVELCSNHKSHNNQSLLLAASLTLSKFMCLSNNFCME
HLPLLITILEKCDNPIIRNNLVIGLADLTVCFNHFIDEISEYLYRRLMDEESSVKKTCFM
TLAFLILAGQIKVKGQLGIMARSLEDEDARISDLARMFFTDFAAKDNSVYNNFIDIFSVL
SRSAEEQDEDDAKFKRIIRFLTSFIEKERHTKQLAERLAARLDRCKTQRQWDHVVYALSL
LPHKADNIQKLIDDGYHE
>Q08985
MARLPLKQFLADNPKKVLVLDGGQGTELENRGIKVANPVWSTIPFISESFWSDESSANRK
IVKEMFNDFLNAGAEILMTTTYQTSYKSVSENTPIRTLSEYNNLLNRIVDFSRNCIGEDK
YLIGCIGPWGAHICREFTGDYGAEPENIDFYQYFKPQLENFNKNDKLDLIGFETIPNIHE
LKAILSWDESILSRPFYIGLSVHEHGVLRDGTTMEEIAQVIKDLGDKINPNFSFLGINCV
SFNQSPDILESLHQALPNMALLAYPNSGEVYDTEKKIWLPNSDKLNSWDTVVKQYISSGA
RIIGGCCRTSPKDIQEISAAVKKYT
>Q5BKC6
MEAGCEGSSPQTLGERTMGEEGERVKPFTPEKAKEVIMSLQQPAIFCNMVFDWPSRHWTA
KHLSKVLEGKQIRFRMGLRSTGTVPQFETECSYVDATLEEFLAWNCDQSRISGPFKKYDH
SKFWAYADYKYFVTLFEDKTDVFQEVMWSDFGFPGRNGQESTLWIGSLGAHTPCHLDSYG
CNLVFQVQGRKRWHLFPPEDTPFLYPTRIPYEESSVFSKINVVNPDLKRFPQFQKARRHM
VTLSPGQVLFVPRHWWHYVESLDPVTVSINSWIELEEDHLARVEEAVTRMLVCTLKTAED
PHHPRTWLNPTEVEETSHEVNSCYLNSAVCAFFDHCERAKEVEMQAPRANGEEPGVQEHM
EVEQARDPSSDVGAGKQEAASPFGPDLVPVTPASEERGGALEGDSQECTSRNGEHCAELP
CARRQQASKGARAEAGQSAPRYPVAPSRVFVSTDDLLDCLVNPQVTRMVAQLLIQGKSL
>O94339
MVIFSSYFSKQMNRFSTPFVFFFKKLNNTLKKSLQFIMRFHSYHPMFICLLRDSSRNENR
KKKVSIGLRTIMRAFSFLMQVATCLIRYSLILTCLVAILLSVLWRIQFAALRMHDLIEEE
LKMFVLQHNCTLADLWSGKCPSSEDE
>O19043
MGVYRVCVSTGASIYAGSKNKVELWLVGQHGEVELGSCLRPSRNKEEEFKVNVSKYLGSL

LFVKLRKKHFLKEDAWFCNWISVQALGAAEDKYWFPCYRWVVGDGVQSLPVGTGCTTVGD
PQGLFQKHREQELEERRKLYQWGSWKEGLILNVAGSKLTDLPVDERFLEDKKIDFEASLA
WGLAELALKNSLNILAPWKTLDDFNRIFWCGRSKLARRVRDSWQEDSLFGYQFLNGANPM
LLRRSVQLPARLVFPPGMEELQAQLEKELKAGTLFEADFALLDNIKANVILYCQQYLAAP
LVMLKLQPDGKLMPMVIQLHLPKIGSSPPPLFLPTDPPMVWLLAKCWVRSSDLQVHELNS
HLLRGHLMAEVFTVATMRCLPSIHPVFKLIVPHLRYTLEINVRARNGLVSDFGIFDQIMS
TGGGGHVQLLQQAGAFLTYRSFCPPDDLADRGLLGVESSFYAQDALRLWEIISRYVQGIM
GLYYKTDEAVRDDLELQSWCREITEIGLQGAQKQGFPTSLQSVAQACHFVTMCIFTCTGQ
HSSIHLGQLDWFTWVPNAPCTMRLPPPTTKDATLETVMATLPNLHQSSLQMSIVWQLGRD
QPIMVPLGQHQEEYFSGPEPRAVLEKFREELAIMDKEIEVRNEKLDIPYEYLRPSIVENS
VAI
>Q03375
MSYNGIGLKSAKGSSTSGHVQRSLASNNRRRPQGSQQQRQQRQNAIKKASHDKASRPLAV
QKQIETHMEKREIEVQVSELRDRLEEEETLSEEQIDKKCEALRAKLTNEWQEQQRMSSLY
TPRKARLTEEQHRHE
>Q9XW16
MSGWNAYIDTMTAAAPSIKRCAIVGAADGSVWARTEADNVFKASEEELKTFVALFNDVTQ
VPAKGADIEGVHYVVPRTEESLIFGKKENTGFFAVKTKSAVLIAVYEGPNEVAAQVRKAV
ESMQTYLNNAGY
>Q08641
MGVADLIKKFESISKEEGDATVDTNSSSKPLKSNDETKELHQQESTAVPQEVDVNEEFEN
EPETINSSRTAEKPLETNLPKPETNEEDEEEGSMSENKIYSKGENADINVNDFQEYKEME
NTGAEVLASSVEESDAIQEGVAEETEGIATPKQKENEKNDESEEESANNASEPAEEYSQS
EEDADIEQSNGKETENAENASQQANDGSTSTTTSKNKKKKNKKKNKKKRNGNVNTNANVD
DSTKTGENDDTTGDTTSSTTSAIQEVNDLEVVDDSCLGIDQQHNREHLKALTQDVKEETL
ENIAHEGRGDNTGDQNAVEKSDFEKSDTEGSRIGRDLPFEFGKRNLTEESDVWDHNAWDN
VEWGEEQVQQAEEKIKEQFKHPVPEFDKKLYNENPARYWDIFYKNNKENFFKDRKWLQIE
FPILYASTRKDAEPVTIFEIGCGAGNTFFPILKDNENENLRIIAADFAPRAVELVKNSEQ
FNPKYGHATVWDLANPDGNLPDGVEPHSVDIAVMIFVFSALAPNQWDQAMDNLHKILKPG
GKIIFRDYGAYDLTQVRFKKNRILEENFYVRGDGTRVYFFSEEKLREIFTKKYFLENKIG
TDRRLLVNRKRQLKMYRCWVQAVFDVPQ
>Q07532
MSTISTTTAPEFIRVKRRRDEDSVQALLIDEGKRVKKQKFIFKLSKTVSSESYQSEQESS
TPLLKLAHEDHRHFVLEQRKKSRRDSDDEKSQQRLAAEGSTVDDDGLPPEINQMVNDYLK
LNKGVEKTERKKPSRKYFTGDSAKIASLPSLDYVFDIYHLEKIHDDEVARYNNEKNIGFV
KIIEHIDLALDEESDPNEARSDDEDSNDENYYQNDYPEDEDDDRSILFGSEGEDIAALGE
EIVIGVNKSRFSSWNDDKIQGSNGYHDVEEEYGDLFNRLGGKSDVLKSINSSNFIDLDGQ
EGEIEISDNEDDSDEGDDIEYPRNEFFPTDVDDPLAHHRDRIFHQLQKKINRS
>Q54RP0
MEKKVEYIKENDKIVLMCNYGFRDMTLNLLKCFEKLSIDKSRYILYCMDDKAYQFFAEFK
GIECQRFSRDDIINSSTSSTQLFHDNNTNDNKGIYSENAESYGDIGFRAICNEKPLVVLD
VLKKGYNVLWTDTDIVWKRDPFIHFYQDINQENQFTNDDDIDLYVQQDDDDICAGFYFIR
SNQRTIKFIQDSINFLNPCIDDQIAMRLFLKSQGINIKSKNILLSLSENDKKDKIRYRLL
DKKLFPNGTNYFNLKITQRDNITPFIIHNNCIIGHRSKKDRFIEYGLWYINDDEIDINSN

INNDDENNKEIKLFKNVLKNHTDIITSINSSDDGKLFTTSIDKSIKIWKFENTSGNDTDN
GIFKVLKSKYIHKRGGIWSTFIFSSNNNNNNDYGFENLLTSSHDKTIQYWDNNLQVIQTF
IGHTGIINQLIVIPNSSYFFTCSDDNTIRQFDLNNINFKRVFIGHNGWVSSIAINKNLNT
LYSCSNDGTIRFWDINSGRCLNIIKGNQGGWIRKIIYNDNLNQLISGGNDGTIKIWSCDN
LNNFNDNQYLLKINTNENSSINDLQFDSDTNLIYCAFENGSLKSFKLTSNNNNNNNNNNN
NNNNTINYSLDKVYQGHLNSSINCIHISKSLNLLFSGGFDKQIKSWDL
>Q09793
MFGLNKTPSFGSTGTQNQNTGTSAGTGLFSSNTFGNNTQANTPASTGFGGVTGGAFGQTK
PQTGGSLFGNKPNATSTTPGLNLFGQNPQAAPGGSLFGASTTKPQAPGGLFNQNQTQAQP
AQAAPTGGLFGLSGQNQTQSQTQPAQANTSLFGQSNIGTTGGLFDQNRPNTSTFGQFSTQ
PASAGLFGQSTQPSGSTGFGLSNNTQTTPFFSAAQQQPSTTQLPSNPAINATTRYSSLNA
NTQKFLDDLDKEIFSQIQLAEELQTKLGTVSELVESVPNDVAEVQRRLSSVSTALLIDSD
EIETTKRVVDEDTSNARISSRILDVFKTPGATYPFASNDPLMNYFEQFTENAKKRTDLYA
ATIGELEQHLEQVETTPQNNSPEALLKTIKEEHKLFMALSNRFAQVHDEVKRLQVNTSTS
LPFIS
>Q10951
MTEVECWDDDPDFASDVDNASLFTQSIATTSASTSVDDGFSFDDSISRLNSLSISDVPSA
HSRVEQWNQQVHNLNHELSSNKNRISSNVEGYEDDFSFDEEGEEGNNEFNTLRPNKYQDA
DVDEFNTIRASETASQRPPIPFSSDTLKKTYLSSENDARYPSVSDSPYCESEGFSSFEDD
FEIDPDTDLNSILHRKQNRMDPKASFSSVEQSSLRTPSSAHNDDGFWDDFDIDFNNETES
IFRKKIRSPNTINQKHPYISSTISYQPNVHQDAKYYPLCKDIFPSLANENPHSDNPNLKY
SSKTLSKRDTSSHYPETLKASSKHSSPVKGNSSISSTWTPSNLKIYHSKNSMGLMDLDAL
KTVASNSKYRTKPKNCKTYGDGTELDTLDELPVDYEFELKLRKKQTTKVSGTPKSKHAGS
TQEWHSHTTPRSTSKHENNLNNITNSAKNEHIRSQRQHKTHAAPSKELTKESLSNDQLSV
KEKRRHHKKAPTLIQNLNSPRTPKIVGKMRYNPTKHCWEGNDYAIRDFDTPISPSRPALI
SNISTKKGIQVVGNMVYDPTRLRWIDNSISGQEAEDPFSGFDDLEDTDSTSQYLNENSGS
FNGSINSIINFPDMSEIYDVGPEFEKKQFSEDIQWRKRIDGWFFSFKNDDRSRLWELYNI
LNAEQ
>Q9BY32
MAASLVGKKIVFVTGNAKKLEEVVQILGDKFPCTLVAQKIDLPEYQGEPDEISIQKCQEA
VRQVQGPVLVEDTCLCFNALGGLPGPYIKWFLEKLKPEGLHQLLAGFEDKSAYALCTFAL
STGDPSQPVRLFRGRTSGRIVAPRGCQDFGWDPCFQPDGYEQTYAEMPKAEKNAVSHRFR
ALLELQEYFGSLAA
>Q00597
MAQDSVDLSCDYQFWMQKLSVWDQASTLETQQDTCLHVAQFQEFLRKMYEALKEMDSNTV
IERFPTIGQLLAKACWNPFILAYDESQKILIWCLCCLINKEPQNSGQSKLNSWIQGVLSH
ILSALRFDKEVALFTQGLGYAPIDYYPGLLKNMVLSLASELRENHLNGFNTQRRMAPERV
ASLSRVCVPLITLTDVDPLVEALLICHGREPQEILQPEFFEAVNEAILLKKISLPMSAVV
CLWLRHLPSLEKAMLHLFEKLISSERNCLRRIECFIKDSSLPQAACHPAIFRVVDEMFRC
ALLETDGALEIIATIQVFTQCFVEALEKASKQLRFALKTYFPYTSPSLAMVLLQDPQDIP
RGHWLQTLKHISELLREAVEDQTHGSCGGPFESWFLFIHFGGWAEMVAEQLLMSAAEPPT
ALLWLLAFYYGPRDGRQQRAQTMVQVKAVLGHLLAMSRSSSLSAQDLQTVAGQGTDTDLR
APAQQLIRHLLLNFLLWAPGGHTIAWDVITLMAHTAEITHEIIGFLDQTLYRWNRLGIES
PRSEKLARELLKELRTQV

>O95400
MPKRKVTFQGVGDEEDEDEIIVPKKKLVDPVAGSGGPGSRFKGKHSLDSDEEEDDDDGGS
SKYDILASEDVEGQEAATLPSEGGVRITPFNLQEEMEEGHFDADGNYFLNRDAQIRDSWL
DNIDWVKIRERPPGQRQASDSEEEDSLGQTSMSAQALLEGLLELLLPRETVAGALRRLGA
RGGGKGRKGPGQPSSPQRLDRLSGLADQMVARGNLGVYQETRERLAMRLKGLGCQTLGPH
NPTPPPSLDMFAEELAEEELETPTPTQRGEAESRGDGLVDVMWEYKWENTGDAELYGPFT
SAQMQTWVSEGYFPDGVYCRKLDPPGGQFYNSKRIDFDLYT
>Q9Y2I6
MDEEENHYVSQLREVYSSCDTTGTGFLDRQELTQLCLKLHLEQQLPVLLQTLLGNDHFAR
VNFEEFKEGFVAVLSSNAGVRPSDEDSSSLESAASSAIPPKYVNGSKWYGRRSRPELCDA
ATEARRVPEQQTQASLKSHLWRSASLESVESPKSDEEAESTKEAQNELFEAQGQLQTWDS
EDFGSPQKSCSPSFDTPESQIRGVWEELGVGSSGHLSEQELAVVCQSVGLQGLEKEELED
LFNKLDQDGDGKVSLEEFQLGLFSHEPALLLESSTRVKPSKAWSHYQVPEESGCHTTTTS
SLVSLCSSLRLFSSIDDGSGFAFPDQVLAMWTQEGIQNGREILQSLDFSVDEKVNLLELT
WALDNELMTVDSAVQQAALACYHQELSYQQGQVEQLARERDKARQDLERAEKRNLEFVKE
MDDCHSTLEQLTEKKIKHLEQGYRERLSLLRSEVEAERELFWEQAHRQRAALEWDVGRLQ
AEEAGLREKLTLALKENSRLQKEIVEVVEKLSDSERLALKLQKDLEFVLKDKLEPQSAEL
LAQEERFAAVLKEYELKCRDLQDRNDELQAELEGLWARLPKNRHSPSWSPDGRRRQLPGL
GPAGISFLGNSAPVSIETELMMEQVKEHYQDLRTQLETKVNYYEREIAALKRNFEKERKD
MEQARRREVSVLEGQKADLEELHEKSQEVIWGLQEQLQDTARGPEPEQMGLAPCCTQALC
GLALRHHSHLQQIRREAEAELSGELSGLGALPARRDLTLELEEPPQGPLPRGSQRSEQLE
LERALKLQPCASEKRAQMCVSLALEEEELELARGKRVDGPSLEAEMQALPKDGLVAGSGQ
EGTRGLLPLRPGCGERPLAWLAPGDGRESEEAAGAGPRRRQAQDTEATQSPAPAPAPASH
GPSERWSRMQPCGVDGDIVPKEPEPFGASAAGLEQPGARELPLLGTERDASQTQPRMWEP
PLRPAASCRGQAERLQAIQEERARSWSRGTQEQASEQQARAEGALEPGCHKHSVEVARRG
SLPSHLQLADPQGSWQEQLAAPEEGETKIALEREKDDMETKLLHLEDVVRALEKHVDLRE
NDRLEFHRLSEENTLLKNDLGRVRQELEAAESTHDAQRKEIEVLKKDKEKACSEMEVLNR
QNQNYKDQLSQLNVRVLQLGQEASTHQAQNEEHRVTIQMLTQSLEEVVRSGQQQSDQIQK
LRVELECLNQEHQSLQLPWSELTQTLEESQDQVQGAHLRLRQAQAQHLQEVRLVPQDRVA
ELHRLLSLQGEQARRRLDAQREEHEKQLKATEERVEEAEMILKNMEMLLQEKVDKLKEQF
EKNTKSDLLLKELYVENAHLVRALQATEEKQRGAEKQSRLLEEKVRALNKLVSRIAPAAL
SV
>P78568
MATAQLATFKIPPVSNEPMLSYAPGSPERAGLQAALAEMQSQLPFEVPCIINGQEVRTNN
IQKQPMPHDHARHLCTFHEGSPELVEKATCGALQAKDGWETMPWNDRAAIFLKAADLASG
KYRYKLMAATMLGQGKNTWQAEIDAAAELADFFRFGVSYVEELYAQQPPKNAPGCWNRTE
YRPLEGFVLAVSPFNFTAIGGNLPGSPALVGNVVVWKPAPAATYSNYLVFKILSEAGVPP
GVIQFIPGGAEIVQAAIQSPNFRSLHFTGSTNVFKSLWKDISSNLDKYKVYPRIVGETGG
KNWHVIHKSAEVRNAVLQSVRGAFEYQGQKCSALSRLYVSRSVWENGFKTQYLEEIAKIK
VGPCLDWNNYMGPVIGRRAYDNITGFIKKAKEEGGEVLIGGSGDDSKGFFIQPTVILTKV
PRSTTMVGEIFGPVVTAYVFEDSDYEKTLELIDTTSIYGLTGAIFASERQALLTATNRSR
NAAGNIYYNEKCTGAVVGQQPFGGARGSGTNDKAGSISIFYRFVSARSIKENFVGLEDFH
YPSNLV
>Q99176

MKVKATKLRIKQRRKNKGLNISRLDIIRAEMDVVPSPGLPEKVNEKSKNIPLPEGINLLS
SKEIIDLIQTHRHQLELYVTKFNPLTDFAGKIHAFRDQFKQLEENFEDLHEQKDKVQALL
ENCRILESKYVASWQDYHSEFSKKYGDIALKKKLEQNTKKLDEESSQLETTTRSIDSADD
LDQFIKNYLDIRTQYHLRREKLATWDKQGNLKY
>Q9Y803
MSKKYGGQISFDPPARLEKDQVVAKVVQLKGSALFMVVENNGQELLVEMPPKYRNKIWVR
RNGFVIVDKSEFLEKDNKIDGTILYVVQSPLKNWKKQIYWPKEFADESLNQNDSEESSSS
EEEYDSD
>P32634
MTVLQPPSSVCYPLNLPIVPNPNLDEATRKKLTLECRTGAAVELARSGVFVHGGLTLPLN
LTIINSLQLQKELILYFGKQKDRNADFKTLADWISPEIFFLDLISRTWQRINTTIDTTSE
NELNNGLSFKERLFHSMCFTESNIYIFGGLMVSPHNGYELIATNELWKLDLKTKCWSLIS
ENPQITRRFNHSMHVLNENNENQDTKLIIVGGLDNMDIPVKKIDIFNLRTSLWESESKSD
ENPASKGSSKILVNIDGMPISLSHDSNFSVLIENNQAEIPTLALYYPQREANTSRRGTDD
GSFSTYAHDLDDKSKLPKHHHHHHGDLKYFESDDADENAVKTLMSPIVILPLLGNSQGAR
MTSNPTQNNKENSILQVPFHLQYPSGNYFNYNIVVIGFYPDPQPSNLHCFIYNIASGKWI
RVNIACTECSISMHRFWKLLIWKSHHQALLLGTRTDDFCSPSVQKFDHILSFSLPMLNGY
NKLVNTKHTRTNNGIANSHNLNVNLSLYDHLPYSNSSTIEHTNPYTVTQGYSLDDSGIPR
LTSTATSQFENYSRYITVPLEMESTSSIFPPYAMVLGKDALEIFGKTLSDFEFITADGDS
IGVPVYLLRKRWGRYFDSLLSNGYANTSFNYEFNGDTSNIISFSPHTASKTTKFGNSSQS
SNGSLEKYFSKNGNSKSNSNTSLKKPHSVDFTSSTSSPKQRAISHNKLSPSEPILCADEE
DSRSNTLKQHATGDTGLKETGTSNKRPISTTCSSTGMVFRVPFQDMKNSKLGLSEQSGRS
TRASSVSPPPVYKKSTNDGNDSNCTLSNTPLVYRRASTVGTTTNSSVDDGFSSIRRASHP
LQSYIIAKSSPSSISKASPAEKAFSRRKSSALRFIASPNQSRQTSFASTASTASVVSSTS
GRRRNSNQISHLGSSASLPNSPILPVLNIPLPPQEKIPLEPLPPVPKAPSRRSSSLAEYV
QFGRDSPVASRRSSHSTRKSSSSDARRISNSSLLRNTLDSQLLSNSYGSDIPYEASIQEY
GMNNGRDEEEDGDNQDYGCISPSNIRPIFSTINAININGNFKEGEFFSSKSYINNEKSRR
LSYISNPESVESTNSNNNAIIELEPLLTPRSLYMPWSTASVRAFAEFFYTAQINGKWLLA
PVTLDLLIMAKIYEIPILYELITEVLYKIISKKEEGLSVTCEALLNLFQQKVSRYCNENE
GKIRKQLDSSESYQDTLEIKRSLANIDNGYVDSYLLRNTSMAQSIHYTDDSNGETEIDMH
HTGISSIGSLANRAVPTVFAGGPRDSHNSIGSIAFPSNSGVQNIRRSVSLFSPATKKKSS
LSRETDPLDTSDQFTDDVPDSGPVSRQQNFPRRSSSFTETVPTEPTRYNYQNLDSSKSNR
ASDDKEEQNEQATLQDISNFDKYKVETLQKRNSNDGKDLDRTNDPLKNRGTEIPQNSSNL
ETDPFIRDSFDSDSGSSFRSDSDDLDSQLGILPFTKMNKKLQEQTSQEFDDSIDPLYKIG
SSTPGSSRLHGSFSKYIRPNSQREDGSEYVNISSLENMVSPNALPPVDYVMKSIYRTSVL
VNDSNLMTRTKEAIELSKVLKKLKKKVLQDISQMDDEMRETGKPIFARGSSSPTLSRQHS
DVATPLKQQENTRPALKFASSSPISEGFRKSSIKFSQAPSTQISPRTSVTDFTASQQRRQ
HMNKRFSTQTTHSTSALFMNPAFMPSAVNTGRKESEGHCEDRSATANRTNRKEDATTNDN
DNIAPFPFFGKRR
>Q9P1T7
MRGVRAATAAAVAATAASGLSRREAGGRAGAAAAVVRPPGRKCGRCRRLANFPGRKRRRR
RRKGLGATTGGCGEAVSSLHPAPHSPSSVRPAGRRARRQRRGAGSAERPMSGAGEALAPG
PVGPQRVAEAGGGQLGSTAQGKCDKDNTEKDITQATNSHFTHGEMQDQSIWGNPSDGELI
RTQPQRLPQLQTSAQVPSGEEIGKIKNGHTGLSNGNGIHHGAKHGSADNRKLSAPVSQKM

HRKIQSSLSVNSDISKKSKVNAVFSQKTGSSPEDCCVHCILACLFCEFLTLCNIVLGQAS
CGICTSEACCCCCGDEMGDDCNCPCDMDCGIMDACCESSDCLEICMECCGICFPS
>O96553
MSAQYQRFLKVLEKWPAEKSKVGSGEWTAEPAIKMSGAKIISGTAVAKSIREELRNEVTA
MSKQLADFVPGLRIVQVGGREDSNVYIRMKIKAATEIGIDAAHVQLPRSITEVELLDKIN
DLNEDPRVHGIIVQMPLDCDTPIDSHRITDAVSPEKDVDGLHTVNEGRLAIGDLGGFLPC
TPWGCLELIRRSGVEIAGARAVVLGRSKIVGTPAAELLKWANATVTVCHSKTRNLEEITR
SADILVVGIGVAEMVKGSWIKPGAVVIDCGINVKPDASKASGSKLVGDVDYAEALQVAGH
LTPVPGGVGPMTVAMLMKNTVRSAARFLERLAKSQWALQTLPLKPQRPVPSDIVIARAQK
PKDIAVLAKEIGLEAREVSLYGNKKAKISLSVLERLKDKEVGHYVVVAGMTPTPLGEGKT
TTLMGLVQALGAHKLRNTMAALRQPSQGPTFGIKGGAAGGGYAQVIPMEEFNLHLTGDIH
AVSAANNLLAAQLDTRIFHENTQKDKALYDRLVPAIKGQRKFSPIQLRRLQKLGITKTDP
DTLTADEYGPFARLDIDPDTIMWERVVDINDRYLRTITVGQSPTEKGISRETRFSISVAS
EIMAVLALSRSLEDMKQRLADMVVAFDKRGKPVTADDLGVTGALAVLLKDALEPNLMQSL
EGTPVLVHAGPFANIAHGCNSIIADEVGLKLVGKNGFVCTEAGFGSDIGMEKFCNIKCRT
SGRKPNAMVLVATVRAIKMHGGGAPVTPGAPLNKQYTEENLELVQKGLPNLLQHIENGKA
FGMPVVVSLNAHSADTPAEHELVKKAALEAGAFAAVVSTHWADGGAGAVQLADAVIKACE
QGNQFRLLYDLELPLVDKMNKIATTMYGAGKVVLSPAAEEKVKRLTDAGFGNLPICMSKV
SGSFTGDAKIKGAPKGFTLDVEDVYVSAGAGFVVAMCGEVTKMPGLPTRPAIYDIDLNTE
TGEIEGLF
>P41805
MARRPARCYRYQKNKPYPKSRYNRAVPDSKIRIYDLGKKKATVDEFPLCVHLVSNELEQL
SSEALEAARICANKYMTTVSGRDAFHLRVRVHPFHVLRINKMLSCAGADRLQQGMRGAWG
KPHGLAARVDIGQIIFSVRTKDSNKDVVVEGLRRARYKFPGQQKIILSKKWGFTNLDRPE
YLKKREAGEVKDDGAFVKFLSKKGSLENNIREFPEYFAAQA
>Q9Y7K5
MSNGEFIDQYLLPESGAILQDYLDDYFYTSWYTGNRNLIQLDHYGFPTASNFELNPKSTD
NCGTVDFQGIPWGNKAIGKRKNFRLYRLHTYSLSCNHSDWSPEELSLDTVQVAAEDSFYE
FQRFLKNPNCLRNHHHLCKSVKVVGEHSVIYVSNNSIRMENTLHDNSVQIIDLKNNILQK
KAISCLDTTNAVGCLGTLYGQYCIFNFKNGSFSTRKLSDDSINHIQLFSEKKFILGCNDG
AVYVEDVQRTSPILVYRCSNAVNCVQLFDETPLALVCTDSKDAMLIDIRSNSVSAKLPHS
DSVLTGSWHPNGNILATGGQDTTAKVWDIRALGKSFRNLGSQMSAVTNLCFSPLGESLAV
TEQADFVQLFDTKLFNTSQVIDFFGEIAGTAYSSSGDSLFIGVDDPMLGGILEFRRPLQH
PLSFIF
>Q02046
MSKPGRTILASKVAETFNTEIINNVEEYKKTHNGQGPLLVGFLANNDPAAKMYATWTQKT
SESMGFRYDLRVIEDKDFLEEAIIQANGDDSVNGIMVYFPVFGNAQDQYLQQVVCKEKDV
EGLNHVYYQNLYHNVRYLDKENRLKSILPCTPLAIVKILEFLKIYNNLLPEGNRLYGKKC
IVINRSEIVGRPLAALLANDGATVYSVDVNNIQKFTRGESLKLNKHHVEDLGEYSEDLLK
KCSLDSDVVITGVPSENYKFPTEYIKEGAVCINFACTKNFSDDVKEKASLYVPMTGKVTI
AMLLRNMLRLVRNVELSKEK
>Q9BVW5
MLEPQENGVIDLPDYEHVEDETFPPFPPPASPERQDGEGTEPDEESGNGAPVPVPPKRTV
KRNIPKLDAQRLISERGLPALRHVFDKAKFKGKGHEAEDLKMLIRHMEHWAHRLFPKLQF

EDFIDRVEYLGSKKEVQTCLKRIRLDLPILHEDFVSNNDEVAENNEHDVTSTELDPFLTN
LSESEMFASELSRSLTEEQQQRIERNKQLALERRQAKLLSNSQTLGNDMLMNTPRAHTVE
EVNTDEDQKEESNGLNEDILDNPCNDAIANTLNEEETLLDQSFKNVQQQLDATSRNITEA
R
>Q9VV73
MPNQMRVLDFGDGSVPIQLDYPDSKIFSKCSSYGDRVPGTHWNEITVHHKGRLEYSPNPE
QIILDALYQAIDGADFFPVFYQRGKNMDTMLVRNCKAAIDKLFKQRLSINLKGGASIPIS
IQLGVAQYQRHQITPTFHIARVVTRLMKQLIQRDGVDGLLNLDNFGSHPEFKNLVVSLGN
PSILMNVCQVIHNDDNERFRLNGFILSNNRIRDIRPLTLLANVDYALLDLRGNKIKSAER
LCRALEQFRARELLLENNPIVKISNFPANIKSLESNFELVDGKPFNMLHKIFSPLDVEID
LEVDGARIDTNNMWKLPEFENSQHWHAFMIPDPSHEFNQEVFFDFFFIRLDPTLSNFYPC
YYKYINTEHVFLVRNCFDQIAHLVNNCNLEMTIPTGDRIFRYYLRMNVSTVKQHHVDPEE
CIQKAVSQCYVAQNRMLNLERFHSRECLKDVMVSLSSPKILTYVLSVASRKFMTTCSEIR
LCHNKVLVLDGAHVLGMMGCLRAVDLSHNWVQDLSSIHSLGNLPLKSLVLHGNKLCRNYR
LPSEYVRAVKEVFPQLTTLDGVDLQTNPGQSLQKNFLCDTGAYELVGAFLENYLREFEND
EFRHNLYKYYSENSIFTLTCNYNVVQNHQTPKILQRLSKYNRHARNLRNKDYSKASDGVF
FGCTYIVEILLQLPRVTHDFHSLQTDVMHYNGKGAVIYVAGLLRDEPPSTRNGHGSKTDI
GGVLLGFSRQFVVTFDEANLGLGKRARRLKIANERLHITNPSKTAIRNAFSVNFPDPSER
QAEEDSLDVKDHKLLLFQEVTGLISTWVTSIVEEADWDFERALKLFIQKNADHEIPDLAF
A
>Q9UKK3
MVMGIFANCIFCLKVKYLPQQQKKKLQTDIKENGGKFSFSLNPQCTHIILDNADVLSQYQ
LNSIQKNHVHIANPDFIWKSIREKRLLDVKNYDPYKPLDITPPPDQKASSSEVKTEGLCP
DSATEEEDTVELTEFGMQNVEIPHLPQDFEVAKYNTLEKVGMEGGQEAVVVELQCSRDSR
DCPFLISSHFLLDDGMETRRQFAIKKTSEDASEYFENYIEELKKQGFLLREHFTPEATQL
ASEQLQALLLEEVMNSSTLSQEVSDLVEMIWAEALGHLEHMLLKPVNRISLNDVSKAEGI
LLLVKAALKNGETAEQLQKMMTEFYRLIPHKGTMPKEVNLGLLAKKADLCQLIRDMVNVC
ETNLSKPNPPSLAKYRALRCKIEHVEQNTEEFLRVRKEVLQNHHSKSPVDVLQIFRVGRV
NETTEFLSKLGNVRPLLHGSPVQNIVGILCRGLLLPKVVEDRGVQRTDVGNLGSGIYFSD
SLSTSIKYSHPGETDGTRLLLICDVALGKCMDLHEKDFSLTEAPPGYDSVHGVSQTASVT
TDFEDDEFVVYKTNQVKMKYIIKFSMPGDQIKDFHPSDHTELEEYRPEFSNFSKVEDYQL
PDAKTSSSTKAGLQDASGNLVPLEDVHIKGRIIDTVAQVIVFQTYTNKSHVPIEAKYIFP
LDDKAAVCGFEAFINGKHIVGEIKEKEEAQQEYLEAVTQGHGAYLMSQDAPDVFTVSVGN
LPPKAKVLIKITYITELSILGTVGVFFMPATVAPWQQDKALNENLQDTVEKICIKEIGTK
QSFSLTMSIEMPYVIEFIFSDTHELKQKRTDCKAVISTMEGSSLDSSGFSLHIGLSAAYL
PRMWVEKHPEKESEACMLVFQPDLDVDLPDLASESEVIICLDCSSSMEGVTFLQAKQIAL
HALSLVGEKQKVNIIQFGTGYKELFSYPKHITSNTAAAEFIMSATPTMGNTDFWKTLRYL
SLLYPARGSRNILLVSDGHLQDESLTLQLVKRSRPHTRLFACGIGSTANRHVLRILSQCG
AGVFEYFNAKSKHSWRKQIEDQMTRLCSPSCHSVSVKWQQLNPDAPEALQAPAQVPSLFR
NDRLLVYGFIPHCTQATLCALIQEKEFCTMVSTTELQKTTGTMIHKLAARALIRDYEDGI
LHENETSHEMKKQTLKSLIIKLSKENSLITQFTSFVAVEKRDENESPFPDIPKVSELIAK
EDVDFLPYMSWQGEPQEAVRNQSLLASSEWPELRLSKRKHRKIPFSKRKMELSQPEVSED
FEEDGLGVLPAFTSNLERGGVEKLLDLSWTESCKPTATEPLFKKVSPWETSTSSFFPILA
PAVGSYLTPTTRAHSPASLSFASYRQVASFGSAAPPRQFDASQFSQGPVPGTCADWIPQS

ASCPTGPPQNPPSAPYCGIVFSGSSLSSAQSAPLQHPGGFTTRPSAGTFPELDSPQLHFS
LPTDPDPIRGFGSYHPSAYSPFHFQPSAASLTANLRLPMASALPEALCSQSRTTPVDLCL
LEESVGSLEGSRCPVFAFQSSDTESDELSEVLQDSCFLQIKCDTKDDSIPCFLEVKEEDE
IVCTQHWQDAVPWTELLSLQTEDGFWKLTPELGLILNLNTNGLHSFLKQKGIQSLGVKGR
ECLLDLIATMLVLQFIRTRLEKEGIVFKSLMKMDDPSISRNIPWAFEAIKQASEWVRRTE
GQYPSICPRLELGNDWDSATKQLLGLQPISTVSPLHRVLHYSQG
>Q9USJ7
MIGRRPQGLRAAASLKKQQQLEKQKQEASYELSGNSSPSKENGSENVDNGEMEDETMLVY
TEEDNISQLWGLYEMSREKLENDDIDASVSLVFGTIHEADRILRNTEDISTLPKDFHAAY
SSALLAVSELFEIAQKRLKETNTEESYIDAAIERAQLGLDAPGNESRLFLALARAYLEKV
RVLVWRHDNEESLANIPVTQLVNPYIEKAIQYLRPLAQDSTEYFDALTPDSLRPLYILSS
YLFQFGDQFSEAFLLDVCSIITALWLKSVVDPNTPAYYKLIAQEAVLNNYTTFAEYYMDL
LDNSESNVDDLINKASSWLNNSVDTWNVIYTLDKSPERLLKLADIKMDLAQIVQDEASQD
NYLKEACNAIKEAQGSGVELSPDYVEFVEAYSA
>P28783
MEDKMSQMESSIETIINIFHQYSVRLGHYDTLIQKEFKQLVQKELPNFLKKQKKNEAAIN
EIMEDLDTNVDKQLSFEEFIMLVARLTVASHEEMHNTAPPGQGHRHGPGYGKGGSGSCSG
QGSPDQGSHDLGSHGHGHGHSHGGHGHSHGGHGHSH
>P40160
MKLDTSHMRYLTTDDFRVLQAVEQGSRSHEVVPTPLIHQISGMRSQSGTNRAISDLAKLS
LISKMRNVKYDGYRLTYNGIDYLALKTMLNRDTVYSVGNTIGVGKESDIYKVSDKNGNPR
VMKIHRLGRTSFHSVRNNRDYLKKSNQGANWMHLSRLAANKEYQFMSMLYSKGFKVPEPF
DNSRHIVVMELIEGYPMRRLRKHKNIPKLYSDLMCFIVDLANSGLIHCDFNEFNIMIKDK
LEDENDCGFVVIDFPQCISIQHQDADYYFQRDVDCIRRFFKKKLKYEPKPDSSMLDTEGF
GDGYKYAYPDFKRDVKRTDNLDELVQASGFSKKHPGDRGLETAVESMRNAVYNSDDDMSN
DEAEEENGEGDYSEEDEYYDSELDNESSEDDSEDAQEEENERIIEALSSGVENLKMDKLG
NYILE
>P36040
MSCLVLPLVSVGNIPQLSIDWLLNSQANEWEYLEALDSKYLVEFVGPLDRPEDGSDSLYK
DADMKYSSALEVFYNKKRGLFAIQQRTPLVSVNYLNNFIVEIILPFLSKYNISEICIWDS
LYAMEDENGVIVRPQEVYSLGEFYFDDEAELLSNLHLNDQESMVNNWLHFTPTSFQDKIS
VDQPIFKILFQILNASQRPKALRSIKYCSCLANEGDNSLDSQQFLQWIISQKVIKNAPPI
VKFVRPISWQGAYGMADARDKFVDLYN
>P54637
MISSSMSYRHSTNSVYTLNPHLNIPISTSTTIPPTSFYANNTPEMIQSQSENTNTNNINN
SSSNINNNNNNNTPDSMSMSTSLSSSPSVSFNHLDLNSINNKINNNTTTNNNNNNNNNDD
KFDTNALKLSNTMIIKNNNNNNNNNNNNNNNNNNNNNNNNNNNNNNNNNNNNNNNNNNNN
NNNNNNNSNSNIEINVPSIQFDNEPAMEVDSVAPLNVPSNHTRTTLAMHNTKSLSTSNIG
LLNILPNQQSSSSSSLSSTTTTTTTTSSSLLMPQSLFNNSTYNNHHNNNNSSNAGIVGGL
NGSTSSLPTQAQVQLQQMQQQMQQHQQHQYKKANLSSLSTVVDNNLNNNPMNTSTSSPAQ
PNASPFSFSSSSLFSNSSLSNSGSGSASTTSTSTSSSNSMSSSPPPSLKTSFSQLDEDRE
KMRLEFEMIKKPEMASKKSHKHHQRHYSHNDLDNRKHDEEKFFSALQPNNYGKNRYHDVL
PNESTRVRLTPIESGDGDYINANYINGEVPNSYRYYIACQAPLPSTIKDFWRMVWEERSS
VIVCLTKLEENGKKKADVYYPETSQAQEYGSFWIHLHKKVMFKDIGVSSLHLYKKGEEFP

REVVLLHYTQWPDCGAPPSSSHIRTLSVMVNTFKARGSAKNTNGPVIVHCSAGIGRSGTF
ISININMAKIERFGNDPSQMNISIKDSVLELRRQRRGMVQTLDQYIFIFKVINDVLTDMG
IRSLSSPSKRRSCEMIKSTPMPRLDISIPPPLTFTPKDFQSSISPSTDMIASLSIITQMT
QTLKFPPQQQQDNPFSKSSIKISPSPLNSTNISIPKNQQFQHPFQIQPQLDLNLQQQQQQ
SSQQLNDNPPLNMSSNSIKFPPVTSLSSCHLFEDSKNNDNNNKQQQQQQQQQQKNNQQCS
GFSHFLNNNNNNDNNGSSGGGFNGSFLFNSNNSGSSSTNSECSNNNKNNNNNSNNNNNNN
NNKNSDNNGTKDKDENDSCESPRVTPIKCF
>Q5SW75
MALVTVQRSPTPSTTSSPCASEADSGEEECRSQPRSISESFLTVKGAALFLPRGNGSSTP
RVSHRRNKHAGDLQQHLQAMFILLRPEDNIRLAVRLESTYQNRTRYMVVVSTNGRQDTEE
SIVLGMDFSSNDSSTCTMGLVLPLWSDTLIHLDGDGGFSVSTDNRVHIFKPVSVQAMWSA
LQSLHKACEVARMHNYYPGSLFLTWVSYYESHINSDQSSVNEWNAMQDVQSHRPDSPALF
TDIPTERERTERLIKTKLREIMMQKDLENITSKEIRTELEMQMVCNLREFKEFIDNEMIV
ILGQMDSPTQIFEHVFLGSEWNASNLEDLQNRGVRYILNVTREIDNFFPGVFEYHNIRVY
DEEATDLLAYWNDTYKFISKAKKHGSKCLVHCKMGVSRSASTVIAYAMKEYGWNLDRAYD
YVKERRTVTKPNPSFMRQLEEYQGILLASKQRHNKLWRSHSDSDLSDHHEPICKPGLELN
KKEMTTSADQIAEVKTVENLAAMPTVFMEHVVPQDANQKGLHTKERVICLEFSSQEFRAG
QIEDELNLNDINGCSSGCCLSESKLPLDNCHASKALLQPGQAPDIANKFPDLAVEDLETD
ALKADMNVHLLPMEELTSRLKDLPMSPDLESPSPQASCQAAISDFSTDRIDFFSALEKFV
ELSQETRSRSFSHSRIEELGGGRSEGCRLSVIEVAASEMAADDQRSSSLSNTPHASEESS
VDEDQSKAITELVSPDIIMQSHSENAISVKEIVTEIESISQGVGQVQLKGDILSNPCHTP
KKSTIHELPLERVPAPESKPGHWEQDESFCSVQPELARDSGKCAPEEGCLTTHSSTADLE
EEEPVEGEHDWGPGMHSGAKWCPGSVRRATLEFEERLRQEQENHGTASAGPTLSNRKNSK
NDSSVADLMPKWKSDETTPEHSFFLKEAEPSKGKGKCSGSEAGSLSHCERNPTMPDCELL
EHHSLPAPQDCLGSDSRSKKQEGDLKKQRAVVPNQECDTQAILLPLPKKIEIIEYTPTVT
SLGHTEPGGEATPSKEGEKQGLRKVKMEQSITMFCALDENLNRTLEPSQVSLHPQVLPLP
HSSSECDRPADPNPMLSSPQDKGDCPSTPFKTAAPFVSCSTQGASFSLDYLLPHSVVHLE
GCTEQSSATDNELSPEQASWEDSRGHFLSSGSGMAHTSSPLTNEDLSLINKLGDSVGVLQ
KKLDPSPEACRIPHSSSSENIRDLSHSRGVVKEHAKEIESRVIFQAGFSKTSQMKRSASL
AKLGYLDLCKDYLPDRELVSSESPHLKLLQPFLRTDSGMHALMAHEPSESAGAQQNPQPT
KYSVEQLKTSECIVQSKPVERPSVQYAKEFGYSQQCLLPKARPELTSSEGGLPLLQTQGL
QYTGPSPGLAVAPRQQHGRTHPLRRLKRANDKKRTTNPFYNTM
>Q03036
MREYTSKKELKEEIEKKYEKYDAEFETISESQKDEKVETVDRTPSENLSYQLGWVNLLLE
WEAKEIAGYNVETPAPGYKWNNLGGLYQSFYKKYGIYSIKEQRAKLREAVNEVYKWISTL
SDDELFQAGNRKWATTKAMWPVYKWIHINTVAPFTNFRGKIRKWKRLVPEEQRIKRRKI
>Q8IRY7
MSASAIYRPFPKVKHFCIFPIAMAGSNLLIHLDTIDQNDLIYVERDMNFAQKVGLCFLLY
GDDHSDATYILQKLLAMTRSDFPQSDLLIKFAKSRPETWRRHLVEALCIIGARKVLRRLG
FCWQELRMHYLPHIAGITLHVHPLLKSLYRMCEELSLVQSGRLLLDVREKVESQQAGDPL
RFYDPAYLEIFLLDWLTRRSIKLGDINAAGSDVQLLVGHLKSNGLQAQANLLKDTIISNA
PEPDAAGTAAMAVKQEIESDNQQSYCSTQIDALKLTRENAGIALIINQQKFHRNVSRDNM
KFLSPDPLRRRDGTDVDKERLIEVFSSMGYNVEAYDNVDHMGIIERIRSACDRSLVRDSL
VVFILSHGFEEAVYASNSIAMKITDIEDLLCSYDTLYYKPKLLIIQACQEKLVHKKKPNE

LFRIDVTTVSPDQHIDMLRAMSTVNGYAALRHTQTGSWFIGSLCDAIDRRSASEHIADIL
TIVTNEVSKKRGSNDESMVPNVKSTFRQHVYFPPRL
>P02365
MKLNISYPVNGSQKTFEIDDEHRIRVFFDKRIGQEVDGEAVGDEFKGYVFKISGGNDKQG
FPMKQGVLLPTRIKLLLTKNVSCYRPRRDGERKRKSVRGAIVGPDLAVLALVIVKKGEQE
LEGLTDTTVPKRLGPKRANNIRKFFGLSKEDDVRDFVIRREVTKGEKTYTKAPKIQRLVT
PQRLQRKRHQRALKVRNAQAQREAAAEYAQLLAKRLSERKAEKAEIRKRRASSLKA
>P47017
MSANSKDRNQSNQDAKRQQQNFPKKISEGEADLYLDQYNFTTTAAIVSSVDRKIFVLLRD
GRMLFGVLRTFDQYANLILQDCVERIYFSEENKYAEEDRGIFMIRGENVVMLGEVDIDKE
DQPLEAMERIPFKEAWLTKQKNDEKRFKEETHKGKKMARHGIVYDFHKSDMY
>Q08J23
MGRRSRGRRLQQQQRPEDAEDGAEGGGKRGEAGWEGGYPEIVKENKLFEHYYQELKIVPE
GEWGQFMDALREPLPATLRITGYKSHAKEILHCLKNKYFKELEDLEVDGQKVEVPQPLSW
YPEELAWHTNLSRKILRKSPHLEKFHQFLVSETESGNISRQEAVSMIPPLLLNVRPHHKI
LDMCAAPGSKTTQLIEMLHADMNVPFPEGFVIANDVDNKRCYLLVHQAKRLSSPCIMVVN
HDASSIPRLQIDVDGRKEILFYDRILCDVPCSGDGTMRKNIDVWKKWTTLNSLQLHGLQL
RIATRGAEQLAEGGRMVYSTCSLNPIEDEAVIASLLEKSEGALELADVSNELPGLKWMPG
ITQWKVMTKDGQWFTDWDAVPHSRHTQIRPTMFPPKDPEKLQAMHLERCLRILPHHQNTG
GFFVAVLVKKSSMPWNKRQPKLQGKSAETRESTQLSPADLTEGKPTDPSKLESPSFTGTG
DTEIAHATEDLENNGSKKDGVCGPPPSKKMKLFGFKEDPFVFIPEDDPLFPPIEKFYALD
PSFPRMNLLTRTTEGKKRQLYMVSKELRNVLLNNSEKMKVINTGIKVWCRNNSGEEFDCA
FRLAQEGIYTLYPFINSRIITVSMEDVKILLTQENPFFRKLSSETYSQAKDLAKGSIVLK
YEPDSANPDALQCPIVLCGWRGKASIRTFVPKNERLHYLRMMGLEVLGEKKKEGVILTNE
SAASTGQPDNDVTEGQRAGEPNSPDAEEANSPDVTAGCDPAGVHPPR
>Q9C9C9
MESETLTAKATITTTTLPSHDETKTESTEFEKNQKRYQDLISTFPHEKGWRPKEPLIEYG
GYWWLPSLLEGCIHAQEFFQARPSDFLVCSYPKTGTTWLKALTFAIANRSRFDDSSNPLL
KRNPHEFVPYIEIDFPFFPEVDVLKDKGNTLFSTHIPYELLPDSVVKSGCKMVYIWREPK
DTFISMWTFLHKERTELGPVSNLEESFDMFCRGLSGYGPYLNHILAYWKAYQENPDRILF
LKYETMRADPLPYVKSLAEFMGHGFTAEEEEKGVVEKVVNLCSFETLKNLEANKGEKDRE
DRPGVYANSAYFRKGKVGDWSNYLTPEMAARIDGLMEEKFKGTGLLEHGK
>Q13671
MESPGESGAGSPGAPSPSSFTTGHLAREKPAQDPLYDVPNASGGQAGGPQRPGRVVSLRE
RLLLTRPVWLQLQANAAAALHMLRTEPPGTFLVRKSNTRQCQALCMRLPEASGPSFVSSH
YILESPGGVSLEGSELMFPDLVQLICAYCHTRDILLLPLQLPRAIHHAATHKELEAISHL
GIEFWSSSLNIKAQRGPAGGPVLPQLKARSPQELDQGTGAALCFFNPLFPGDLGPTKREK
FKRSFKVRVSTETSSPLSPPAVPPPPVPVLPGAVPSQTERLPPCQLLRRESSVGYRVPAG
SGPSLPPMPSLQEVDCGSPSSSEEEGVPGSRGSPATSPHLGRRRPLLRSMSAAFCSLLAP
ERQVGRAAAALMQDRHTAAGQLVQDLLTQVRAGPEPQELQGIRQALSRARAMLSAELGPE
KLLSPKRLEHVLEKSLHCSVLKPLRPILAARLRRRLAADGSLGRLAEGLRLARAQGPGAF
GSHLSLPSPVELEQVRQKLLQLLRTYSPSAQVKRLLQACKLLYMALRTQEGEGAGADEFL
PLLSLVLAHCDLPELLLEAEYMSELLEPSLLTGEGGYYLTSLSASLALLSGLGQAHTLPL
SPVQELRRSLSLWEQRRLPATHCFQHLLRVAYQDPSSGCTSKTLAVPPEASIATLNQLCA

TKFRVTQPNTFGLFLYKEQGYHRLPPGALAHRLPTTGYLVYRRAEWPETQGAVTEEEGSG
QSEARSRGEEQGCQGDGDAGVKASPRDIREQSETTAEGGQGQAQEGPAQPGEPEAEGSRA
AEE
>O88737
MGNEASLEGGAGEGPLPPGGSGLGPGPGAGKPPSALAGGGQLPVAGAARAAGPPTPGLGP
VPGPGPGPGPGSVPRRLDPKEPLGSQRTTSPTPKQASATAPGRESPRETRAQGPSGQEAE
SPRRTLQVDSRTQRSGRSPSVSPDRGSTPTSPYSVPQIAPLPSSTLCPICKTSDLTSTPS
QPNFNTCTQCHNKVCNQCGFNPNPHLTQVKEWLCLNCQMQRALGMDMTTAPRSKSQQQLH
SPALSPAHSPAKQPLGKPEQERSPRGPGATQSGPRQAEAARATSVPGPTQATAPPEVGRV
SPQPPLSTKPSTAEPRPPAGEAQGKSATTVPSGLGAGEQTQEGLTGKLFGLGASLLTQAS
TLMSVQPEADTQGQPSPSKGPPKIVFSDASKEAGPRPPGSGPGPGPTPGAKTEPGARTGP
GSGPGALAKTGGTASPKHGRAEHQAASKAAAKPKTMPKERAAACPLCQAELNVGSRGPAN
YNTCTACKLQVCNLCGFNPTPHLVEKTEWLCLNCQTKRLLEGSLGEPAPLPLPTPQQPPA
GVPHRAAGAAPLKQKGPQGLGQPSGSLPAKASPQATKASPQATKASPQATKASPQTTKAS
PQAKPLRATEPSKTSSSAQEKKTATPAKAEPVPKPPPETTVPPGTPKAKSGVKRTDPATP
VVKPVPEAPKGGEAEEPVPKPYSQDLSRSPQSLSDTGYSSDGVSSSQSEITGVVQQEVEQ
LDSAGVTGPRPPSPSELHKVGSSLRPSLEAQAVAPSAEWSKPPRSSSSAVEDQKRRPHSL
SIMPEAFDSDEELGDILEEDDSLAWGRQREQQDTAESSDDFGSQLRHDYVEDSSEGGLSP
LPPQPPARADMTDEEFMRRQILEMSAEEDNLEEDDTAVSGRGLAKHSAQKASARPRPESS
QEPKRRLPHNATTGYEELLSEAGPAEPTDSSGALQGGLRRFKTIELNSTGSYGHELDLGQ
GPDPNLDREPELEMESLTGSPEDRSRGEHSSTLPASTPSYTSGTSPTSLSSLEEDSDSSP
SRRQRLEEAKQQRKARHRSHGPLLPTIEDSSEEEELREEEELLREQEKMREVEQQRIRST
ARKTRRDKEELRAQRRRERSKTPPSNLSPIEDASPTEELRQAAEMEELHRSSCSEYSPSP
SLDSEAETLDGGPTRLYKSGSEYNLPAFMSLYSPTETPSGSSTTPSSGRPLKSAEEAYED
MMRKAEMLQRQQGQVAGARGPHGGPSQPTGPRSQGSFEYQDTQDHDYGGRASQPVAESTP
AGLGAAVYEEILQTSQSIARMRQASSRDLGFTEDKKKEKQFLNAESAYMDPMKQNGGPLT
PGTSPTQLAAPVSFSTSTSSDSSGGRVIPDVRVTQHFAKEPQDPLKLHSSPVSSTLTSKE
VGMTFSQGPGSPATTASPTRGYMTPTSPAGSERSPSTSSTIHSYGQPPTTANYGSQTEEL
PHAPSGPPGSGRAPREKPLSGGDSEVGAPQPSRGYSYFTGSSPPLSPSTPSESPTFSPGK
LGPRATAEFSTQTPSLTLSSDIPRSPGPPSPMVAQGTQTPHRPSTPRLVWQQSSQEAPIM
VITLASDASSQTRMVHASASTSPLCSPTDSQPTSHSYSQTTPPSASQMPSEPAGPPGFPR
APSAGTDGPLALYGWGALPAENISLCRISSVPGTSRVEPGPRPPGTAVVDLRTAVKPTPI
ILTDQGMDLTSLAVEARKYGLALDPVSGRQSTAVQPLVINLNAQEQTHTFLATATTVSIT
MASSVLMAQQKQPVVYGDPFQSRLDFGQGSGSPVCLAQVKQVEQAVQTAPYRGGPRGRPR
EAKFARYNLPNQVTPLARRDILITQMGTAQGVGLKPGPVPEPGAEPHRATPAELRSHAPP
GTRKPHTVVVQMGEGTAGTVTTLLPEEPAGALDLTGMRPESQLACCDMVYKFPFGSSCTG
TFHPAPSAPDKSVTDTALPGQSSGPFYSPRDPEPPEPLTFRTQGVVGPGPHEEQRPYPQG
LPGRLYSSMSDTNLAEAGLNYHAQRLGQLFQGPGRDSAVDLSSLKHSYSLGFADGRYLGQ
GLQYGSFTDLRHPTDLLSHPLPLRRYSSVSNIYSDHRYGPRGDAVGFQEASLAQYSATTA
REISRMCAALNSMDQYGGRHGSGSGGPDLVQYQPQHGPGLSAPQGLAPLRSGLLGNPTYP
EGQPSPGNLAQYGPAASQATAVRQLLPSTATVRAADGMIYSTINTPIAATLPITTQPASV
LRPMVRGGMYRPYVSGGVTAVPLTSLTRVPMIAPRVPLGPAGLYRYPAPRFPIASSVPPA
EGPVYLGKPAATKASGAGGPPRPELPAGVAREEPFSTTAPAVIKEAPVAPAPGPAPAPPP
GQKPAGEAAAGSGSGVLSRPASEKEEASQEDRQRKQQEQLLQLERERVELEKLRQLRLQE

ELERERVELQRHREEEQLLVQRELQELQTIKQHVLQQQQEERQAQFALQREQLAQQRLQL
EQIQQLQQQLQLQLEEQKQRQKAPFPATCEAPSRGPPPAATELAQNGQYWPPLTHAAFIA
VAGTEGPGQPREPVLHRGLPSSASDMSLQTEEQWEAGRSGIKKRHSMPRLRDACEPESGP
DPSTVRRIADSSVQTDDEEGEGRYLVTRRRRTRRSADCSVQTDDEDNADWEQPVRRRRSR
LSRHSDSGSDSKHDATASSSTTAAATARAMSSVGIQTISDCSVQTEPEQLPRVSPAIHIT
AATDPKVEIVRYISAPEKTGRGESLACQTEPDGQAQGVAGPQLIGPTAISPYLPGIQIVT
PGALGRFEKKKPDPLEIGYQAHLPPESLSQLVSRQPPKSPQVLYSPVSPLSPHRLLDTSF
ASSERLNKAHVSPQKQFIADSTLRQQTLPRPMKTLQRSLSDPKPLSPTAEESAKERFSLY
QHQGGLGSQVSVLPPNGLVRKVKRTLPSPPPEEAHLPLAGQVPSQLYAASLLQRGLAGPT
TVPATKASLLRELDRDLRLVEHESTKLRKKQAELDEEEKEIDAKLKYLELGITQRKESLA
KDRGGRDYPPLRGLGEHRDYLSDSELNQLRLQGCTTPAGQYVDYPASAAVPATPSGPTAF
QQPRFPPAAPQYTAGSSGPTQNGFPAHQAPTYTGPSTYPAPTYPPGTGYPAEPGLPSQPA
FHPTGHYAAPTPMPTTQSAPFPVQADSRAAHQKPRQTSLADLEQKVPTNYEVIGSPAVTM
SSAPPETGYSGPAVSGSYEQGKAPEHPRGSDRSSVSQSPAPTYPSDSHYTSLEQNVPRNY
VMIDDISELTKDSTPTASESQRLEPLGPGGVSGRPGKDPGEPAVLEGPTLPCCYGRGEEE
SEEDSYDPRGKSGHHRSMESNGRPSTHYYGDSDYRHGARADKYGPGPMGPKHPSKSLAPA
AISSKRSKHRKQGMEQKISKFSPIEEAKDVESDLASYPPPTVSSSLTSRGRKFQDEITYG
LKKNVYEQQRYYGVSSRDAAEEDERMYGSSSRSRMASAYSGEKLSSHDYSSRGKGYERER
DTAERLQKAGSKPSSLSMAHGRARPPMRSQASEEESPVSPLGRPRPAGGALPPGDTCPQF
CSSHSMPDVQEHVKDGPRAHAYKREEGYMLDDSHCVVSDSEAYHLGQEETDWFDKPRDAR
SDRFRHHGGHTVSSSQKRGPARHSYHDYDEPPEEGLWPHDEGGPGRHTSAKEHRHHSDHG
RHSGRHAGEEPGRRAAKPHARDMGRHEARPHPQASPAPAMQKKGQPGYPSSADYSQSSRA
PSAYHHASESKKGSRQAHTGPSALQPKADTQAQPQMQGRQAAPGPQQSQPPSSRQTPSGT
ASRQPQTQQQQQQQQQQQQGLGQQAPQQAPSQARLQPQSQPTTRGTAPAASQPAGKPQPGP
TTAPGPQPAGPPRAEQASSSKPPAAKAPQQGRAPQAQTTPGPGPAGAKPGARPGGTPGAP
AGQPGAEGESVFSKILPGGAAEQAGKLTEAVSAFGKKFSSFW
>P10155
MEESVNQMQPLNEKQIANSQDGYVWQVTDMNRLHRFLCFGSEGGTYYIKEQKLGLENAEA
LIRLIEDGRGCEVIQEIKSFSQEGRTTKQEPMLFALAICSQCSDISTKQAAFKAVSEVCR
IPTHLFTFIQFKKDLKESMKCGMWGRALRKAIADWYNEKGGMALALAVTKYKQRNGWSHK
DLLRLSHLKPSSEGLAIVTKYITKGWKEVHELYKEKALSVETEKLLKYLEAVEKVKRTRD
ELEVIHLIEEHRLVREHLLTNHLKSKEVWKALLQEMPLTALLRNLGKMTANSVLEPGNSE
VSLVCEKLCNEKLLKKARIHPFHILIALETYKTGHGLRGKLKWRPDEEILKALDAAFYKT
FKTVEPTGKRFLLAVDVSASMNQRVLGSILNASTVAAAMCMVVTRTEKDSYVVAFSDEMV
PCPVTTDMTLQQVLMAMSQIPAGGTDCSLPMIWAQKTNTPADVFIVFTDNETFAGGVHPA
IALREYRKKMDIPAKLIVCGMTSNGFTIADPDDRGMLDMCGFDTGALDVIRNFTLDMI
>Q05881
MSTGSSDRKDDVKLLELLNSIDEQFLVPYKKPEDLRKISSTTKLQGSTPTKELDKLASVL
KAHCTKIGIVCKPGTFDNNHKVVITEIQNFSRPLFYLLSLFPLFYNNKDCPKYFTDQLDE
STLQLLDGLRDFIAELQERLKNDENASLDKERLTSVGKIFNACDSLSNCSKAGPYGILAN
ILKDNVAIMDDTMNEIKEWLEEPDFSANSDDIFLDFEDSESESDSQKEEFDQEKVYENIK
LFFDGFTRKIKLIKLLVSTFRKTLVSKDFTPKRNQAETLDSIHTYLKEIQLLLDEVVSTV
QFEPKNFTNEEVKEEQAALVAVTKKVLIQMSKLYEGDPKRKKWIDTWEIKFNELF
>Q9H160

MLGQQQQQLYSSAALLTGERSRLLTCYVQDYLECVESLPHDMQRNVSVLRELDNKYQETL
KEIDDVYEKYKKEDDLNQKKRLQQLLQRALINSQELGDEKIQIVTQMLELVENRARQMEL
HSQCFQDPAESERASDKAKMDSSQPERSSRRPRRQRTSESRDLCHMANGIEDCDDQPPKE
KKSKSAKKKKRSKAKQEREASPVEFAIDPNEPTYCLCNQVSYGEMIGCDNEQCPIEWFHF
SCVSLTYKPKGKWYCPKCRGDNEKTMDKSTEKTKKDRRSR
>P00488
MSETSRTAFGGRRAVPPNNSNAAEDDLPTVELQGVVPRGVNLQEFLNVTSVHLFKERWDT
NKVDHHTDKYENNKLIVRRGQSFYVQIDFSRPYDPRRDLFRVEYVIGRYPQENKGTYIPV
PIVSELQSGKWGAKIVMREDRSVRLSIQSSPKCIVGKFRMYVAVWTPYGVLRTSRNPETD
TYILFNPWCEDDAVYLDNEKEREEYVLNDIGVIFYGEVNDIKTRSWSYGQFEDGILDTCL
YVMDRAQMDLSGRGNPIKVSRVGSAMVNAKDDEGVLVGSWDNIYAYGVPPSAWTGSVDIL
LEYRSSENPVRYGQCWVFAGVFNTFLRCLGIPARIVTNYFSAHDNDANLQMDIFLEEDGN
VNSKLTKDSVWNYHCWNEAWMTRPDLPVGFGGWQAVDSTPQENSDGMYRCGPASVQAIKH
GHVCFQFDAPFVFAEVNSDLIYITAKKDGTHVVENVDATHIGKLIVTKQIGGDGMMDITD
TYKFQEGQEEERLALETALMYGAKKPLNTEGVMKSRSNVDMDFEVENAVLGKDFKLSITF
RNNSHNRYTITAYLSANITFYTGVPKAEFKKETFDVTLEPLSFKKEAVLIQAGEYMGQLL
EQASLHFFVTARINETRDVLAKQKSTVLTIPEIIIKVRGTQVVGSDMTVTVQFTNPLKET
LRNVWVHLDGPGVTRPMKKMFREIRPNSTVQWEEVCRPWVSGHRKLIASMSSDSLRHVYG
ELDVQIQRRPSM
>P87125
MEDKEGRFRVECIENVHYVTDMFCKYPLKLIAPKTKLDFSILYIMSYGGGLVSGDRVALD
IIVGKNATLCIQSQGNTKLYKQIPGKPATQQKLDVEVGTNALCLLLQDPVQPFGDSNYIQ
TQNFVLEDETSSLALLDWTLHGRSHINEQWSMRSYVSKNCIQMKIPASNQRKTLLRDVLK
IFDEPNLHIGLKAERMHHFECIGNLYLIGPKFLKTKEAVLNQYRNKEKRISKTTDSSQMK
KIIWTACEIRSVTIIKFAAYNTETARNFLLKLFSDYASFLDHETLRAFWY
>O15085
MSVRLPQSIDRLSSLSSLGDSAPERKSPSHHRQPSDASETTGLVQRCVIIQKDQHGFGFT
VSGDRIVLVQSVRPGGAAMKAGVKEGDRIIKVNGTMVTNSSHLEVVKLIKSGAYVALTLL
GSSPSSMGISGLQQDPSPAGAPRITSVIPSPPPPPPLPPPQRITGPKPLQDPEVQKHATQ
ILRNMLRQEEKELQDILPLYGDTSQRPSEGRLSLDSQEGDSGLDSGTERFPSLSESLMNR
NSVLSDPGLDSPRTSPVIMARVAQHHRRQGSDAAVPSTGDQGVDQSPKPLIIGPEEDYDP
GYFNNESDIIFQDLEKLKSRPAHLGVFLRYIFSQADPSPLLFYLCAEVYQQASPKDSRSL
GKDIWNIFLEKNAPLRVKIPEMLQAEIDSRLRNSEDARGVLCEAQEAAMPEIQEQIHDYR
TKRTLGLGSLYGENDLLDLDGDPLRERQVAEKQLAALGDILSKYEEDRSAPMDFALNTYM
SHAGIRLREARPSNTAEKAQSAPDKDKWLPFFPKTKKSSNSKKEKDALEDKKRNPILKYI
GKPKSSSQSTFHIPLSPVEVKPGNVRNIIQHFENNQQYDAPEPGTQRLSTGSFPEDLLES
DSSRSEIRLGRSESLKGREEMKRSRKAENVPRSRSDVDMDAAAEATRLHQSASSSTSSLS
TRSLENPTPPFTPKMGRRSIESPSLGFCTDTLLPHLLEDDLGQLSDLEPEPDAQNWQHTV
GKDVVAGLTQREIDRQEVINELFVTEASHLRTLRVLDLIFYQRMKKENLMPREELARLFP
NLPELIEIHNSWCEAMKKLREEGPIIKEISDLMLARFDGPAREELQQVAAQFCSYQSIAL
ELIKTKQRKESRFQLFMQEAESHPQCRRLQLRDLIISEMQRLTKYPLLLESIIKHTEGGT
SEHEKLCRARDQCREILKYVNEAVKQTENRHRLEGYQKRLDATALERASNPLAAEFKSLD
LTTRKMIHEGPLTWRISKDKTLDLHVLLLEDLLVLLQKQDEKLLLKCHSKTAVGSSDSKQ
TFSPVLKLNAVLIRSVATDKRAFFIICTSKLGPPQIYELVALTSSDKNTWMELLEEAVRN

ATRHPGAAPMPVHPPPPGPREPAQQGPTPSRVELDDSDVFHGEPEPEELPGGTGSQQRVQ
GKHQVLLEDPEQEGSAEEEELGVLPCPSTSLDGENRGIRTRNPIHLAFPGPLFMEGLADS
ALEDVENLRHLILWSLLPGHTMETQAAQEPEDDLTPTPSVISVTSHPWDPGSPGQAPPGG
EGDNTQLAGLEGERPEQEDMGLCSLEHLPPRTRNSGIWESPELDRNLAEDASSTEAAGGY
KVVRKAEVAGSKVVPALPESGQSEPGPPEVEGGTKATGNCFYVSMPSGPPDSSTDHSEAP
MSPPQPDSLPAGQTEPQPQLQGGNDDPRRPSRSPPSLALRDVGMIFHTIEQLTLKLNRLK
DMELAHRELLKSLGGESSGGTTPVGSFHTEAARWTDGSLSPPAKEPLASDSRNSHELGPC
PEDGSDAPLEDSTADAAASPGP
>P46060
MASEDIAKLAETLAKTQVAGGQLSFKGKSLKLNTAEDAKDVIKEIEDFDSLEALRLEGNT
VGVEAARVIAKALEKKSELKRCHWSDMFTGRLRTEIPPALISLGEGLITAGAQLVELDLS
DNAFGPDGVQGFEALLKSSACFTLQELKLNNCGMGIGGGKILAAALTECHRKSSAQGKPL
ALKVFVAGRNRLENDGATALAEAFRVIGTLEEVHMPQNGINHPGITALAQAFAVNPLLRV
INLNDNTFTEKGAVAMAETLKTLRQVEVINFGDCLVRSKGAVAIADAIRGGLPKLKELNL
SFCEIKRDAALAVAEAMADKAELEKLDLNGNTLGEEGCEQLQEVLEGFNMAKVLASLSDD
EDEEEEEEGEEEEEEAEEEEEEDEEEEEEEEEEEEEEPQQRGQGEKSATPSRKILDPNTG
EPAPVLSSPPPADVSTFLAFPSPEKLLRLGPKSSVLIAQQTDTSDPEKVVSAFLKVSSVF
KDEATVRMAVQDAVDALMQKAFNSSSFNSNTFLTRLLVHMGLLKSEDKVKAIANLYGPLM
ALNHMVQQDYFPKALAPLLLAFVTKPNSALESCSFARHSLLQTLYKV
>Q9T041
MSTPTTSGSAAKPTRPARSSSLATRSCSNSGSLTSFQAMVELKQKILTSISKLADRDTYQ
IAVEDLEKTIQSLTPETLPMFLNCLYDSCSDPKPAVKKECLHLLSYVCSLHCDSTAAHLT
KIIAQIVKRLKDSDSGVRDACRDTIGALSGIYLKGKEEGTNTGSASLAVGLFVKPLFEAM
GEQNKVVQSGASMCMARMVESAASPPVTSFQKLCPRICKLLSNSSFLAKASLLPVVSSLS
QVGAIAPQSLESLLESIHDCLGSTDWVTRKAAAETLTALASHSSGLIKEKTDSTITVLET
CRFDKIKPVRESVTEALQLWKKISGKYVDGASDDSKLSASEQLGSEKNGEKRSNLADLMK
KEASDGSTLSPDSASKGKGCFPEKAVGLLKKKAPVLSDKDFNPEFFQRLERRQSVEVVVP
RRCKNNDEEESGLDDLNAMGSSNRLKNTQADDKQVKGRFDGNGSQARTSGDDKAGVVNGK
ETPGHHAPVSNTDNQSEGSFTSNRGNWSAIQRQLLQLERQQTNLMNMLQEFIGGSHDSMV
TLEGRVRGLERIVEDMARDLSISSGRRANLTAGFGKYNSFANYPTGKYNGRAPGERGSQT
DGAMRGRMWNSDMADDWFIPPHAASRNGQAGPRRSPRSEQYENEHMGNGRRGWDNKASGT
IRFGEGPSARSVWQASKDEATLEAIRVAGEDGAVPRPTRVAVAPEAEAMGDDDNEGQERD
PIWVSWSNAMHSLRVGDIDAAYAEVLCAGDQHLVIKLMDKTGPSLDQMSNEIANEALNFI
SQFLLDHSLYDICLSWSQQLLELVLQDGADTFGVPMELKTEILYNLQDACSTMDPPEDWE
GPAPEQLVVQLASVWEIDLQQFDK
>Q9H204
MAAPLGGMFSGQPPGPPQAPPGLPGQASLLQAAPGAPRPSSSTLVDELESSFEACFASLV
SQDYVNGTDQEEIRTGVDQCIQKFLDIARQTECFFLQKRLQLSVQKPEQVIKEDVSELRN
ELQRKDALVQKHLTKLRHWQQVLEDINVQHKKPADIPQGSLAYLEQASANIPAPLKPT
>P54633
MSISLKINIVGANTVKTLRFAPDMCIQECCTHIFEKTNEGGPDHGLYQAHIEGKQSARWL
AMEKTLQFYDINSDQQLDYKKKHRPQKFKLLDGTIKTQLVDESQNVSEIVNSICKKMGIK
NPEEYSLMNSAGAWLNNTQILSEQGISENDITVLMKKFFFNDANIDRNDPVQLHLLFVQC
RDGIIEGKYPTQREESLALSALQCQVQLGDYNPTKHVPGFLTLKDYLPLQWLKSKGVEKD

IFKEHKKLVSMTEVNAKYRYVQLCRSLKTYGMTSFDVKIREYGKKKMVDHILGITREQML
LMLTETKEVIMTHPLKHIKRWAATDKSFTLDFGDHETEYLILQTPNPEQISQLIGGYIEI
IMKARKDSSKVIEKEDTAMGVEEVMAVKKGSVANSSSYMGYGAGGGGANQLQPSQQIPIT
DLKSALRATDLLIGELGGFRSSTGATPQNFTRSFTTLTPQQFKHQLISHTNAMAIAAQGL
FQDMTTPPPTGGIAAFQQAITKRAQIIMAELNTVGTAAKNAGYFPDMASFSDEIIGVATK
LSESMARLLAIGSTIQGTDCDEKSQKAAQTEIFNVQSLVTLMMAACDNEYVTDSSSKLLI
ECAKNVSAAIADMLVVGNSKVEFIDDELLLGQIQNTLKSTSLTSDELLSTTENLASTSCH
PESRKQITNITQSALNQSNALLTAFKSGEIPEQDYNLLNARVSDIIESVNLINYAMDCSE
REYKISITSNGVEVGEGEILAGTNLTEEFATVANDLTNAIMTMRSNLKNPDTVMESYKMV
AGHANRLITCTKAVASRADTQSQQRLFNSTNAVFESVANLSNHCRSYIKNPEQEAHTFQI
VETAGHLQFLTQNMSTDAGKIACITSLRDYSKEMIAQVSSLISTSRTSSQYLPDANGITL
LKGAKDVSDALSKLMVGIKKVVLDPKSEATQMELLTLAQKQSLPPMNLVSTCKRFAPKIS
DPNQKQRLIFSSDAAAQSVQKLMKAGEAYKRICGHIEIEEALEVFDSTIADLETTEIAIA
GGFLDAVSGTTREGAAELLMVAIKDLNKVNNELVTDIRVNPARLGDLVKSATESASSVAI
SAKTLICATTGKQVQKKLMGITKQLMIDMEQLIRASRSVASNPNDAASELLLDAASNDVS
ISTAALVGSTANVDCKELDEASADISNLLSLKMGSLESILSQPTEEFAFYVEEIASSTKA
LNAASQQVVAMARNKNLKGLGASAKITASALSTLVSHAQNAIVLTENEATKNAILASTVA
LGGQIIGLLDFSKARIANYKDPIYDQNLINQAKSVEDHLVKVGRSLGGDGNNTICDEAVD
RIIEATRSLDKTILPDTSGLQTNAHLEMLHQQSLLAITQASKKLGSITSNLVNSKNNSDL
VGSGSTDAAEAIIEMIEAAKHVVHCSISTSSPDILLPAKSILDASQMLTANQADVNHVLS
HARTIAACTQQLLGITRERASQFNEQDEQQVQVRDGIVKSTQQLAHATSSLARAVKSVTS
KEPGAKAMISQSLKDLESAINNLLITSSVPASERGIGIADFNKLMSTCRSVSTASSQLII
SASSCSQKPKDIELSSILSENAVLMTNSLKDIIKVTSSMMPGVNFCEEAIEIAQRAISDL
SSVALSVAVGSFDSSANNKEGLSHVESQERLVDVTKKIGTGINDLLKASRQSPEAIGISA
KALSFIAPSLVNTTKPALATAPDADAQNDLVTESKNVGDSILKLCQASLIASSNPSKETY
QIIVNKCVDASEAMSKLVAQISSGVNLYKELDESLDRIRKSVVQTSAKDAPKDSENRGYQ
EYKEELSNLTKNLALSLKTIVATDGNNLVSISTISKDIANYISDIAHVSSAILATTSDQK
IRDSIITSSRQVIVSTGDIVNHIKVNSTDKANSSQAKVNDSYRATNDNITRFLQSLKQGA
IGEILSDAAIDQIRKVISDLDGYSLFAAAGQLENDQSSQSTMNEVTKQQHLKNLQKDTIT
QAKLLIVSSSQLVGSSRGTQEHLGSATTKVANTVSSLVKTAKDIASVLADTTSQQDILSA
SKALSISSQQMVLATKDAQRFKKDATAFRSLGKSAEAVAEAVGQFLTSVYTAISDAGKGI
KELEKSIVQVANYHEKPDTVLSNKDATAEIFAQSARDLAKSSIEIVTSYTSSQDDLVKSS
QAVVSNVQSFISNSKGVIALLGNGNDDLKSKVLENVKQTTGDMLALLQCVKDQDKNGSTS
IADATRSISDRVHSVVTLSKSLPGGQNIVVEEDNVLEDLEALAEDELSACARSIEEATAK
LIAARPQSKSKNGKLDAEGVAATIVDASSAIAKAVAKLVNSAAVAQSKRREDQIASGSVY
KADPTWSNGLISAAKGVGAATHRLVEAAMKSATGKAEEEELIATARSVAAATALLVSASR
AKSGDDYQSQAAHSHLSTAARQVASATSDLVAAAKAATIFDEQQQEEEQEQFNFTGSKVK
ELEQQMKILKLEKELETARRQMLNSRKQNYNKN
>P06101
MAIDYSKWDKIELSDDSDVEVHPNVDKKSFIKWKQQSIHEQRFKRNQDIKNLETQVDMYS
HLNKRVDRILSNLPESSLTDLPAVTKFLNANFDKMEKSKGENVDPEIATYNEMVEDLFEQ
LAKDLDKEGKDSKSPSLIRDAILKHRAKIDSVTVEAKKKLDELYKEKNAHISSEDIHTGF
DSSFMNKQKGGAKPLEATPSEALSSAAESNILNKLAKSSVPQTFIDFKDDPMKLAKETEE
FGKISINEYSKSQKFLLEHLPIISEQQKDALMMKAFEYQLHGDDKMTLQVIHQSELMAYI

KEIYDMKKIPYLNPMELSNVINMFFEKVIFNKDKPMGKESFLRSVQEKFLHIQKRSKILQ
QEEMDESNAEGVETIQLKSLDDSTELEVNLPDFNSKDPEEMKKVKVFKTLIPEKMQEAIM
TKNLDNINKVFEDIPIEEAEKLLEVFNDIDIIGIKAILENEKDFQSLKDQYEQDHEDATM
ENLSLNDRDGGGDNHEEVKHTADTVD
>P47057
MTDKGKNDLTSKAKDKARGNPEKPPYWFEIIVSDPQKRTGDPGSSSGYVSYQISTKTNNT
SFYDNRGDPESIIVVHRRYSDLLLLHDILLNRFPTCIIPPLPDKKVFQYIAGDRFSQRFT
QKRCHSLQNFLRRVSLHPDLSQSKVFKTFLVSKDWESHRKVLQDSLQPNKDEVTDAFMNA
FKTVHKQNEEFTEIREKSDKLDRTVTKIDKLFHKVVKKNDSMSEDYTKLGSNLQELQELV
TGENEELAAKLKIFNEGVTQLSYGLQDLTKYLDYEYIVDLKDLEHYIDSMRQLIKLKDQK
QIDYEELSDYLTRSIKEKNNLISGYGGSNFFANKLEELAGINQEASRREKINKLEGKITS
LTGELENAKKVADGFEQECLKEIDHFESVKTAEIKKSLGSLADHHIEFYERILEAWEKVD
DSL
>Q12040
MTKEVPYYCDNDDNNIIRLFIIRHGQTEHNVKKILQGHKDTSINPTGEEQATKLGHYLRS
RGIHFDKVVSSDLKRCRQTTALVLKHSKQENVPTSYTSGLRERYMGVIEGMQITEAEKYA
DKHGEGSFRNFGEKSDDFVARLTGCVEEEVAEASNEGVKNLALVSHGGAIRMILQWLKYE
NHQAHKIIVFNTSVTIVDYVKDSKQFIVRRVGNTQHLGDGEFVVSDLRLR
>Q8K558
MDCYLLLLLLLLGLAGQGSADSHPEVLQAPVGSSILVQCHYRLQDVRALKVWCQFLQEGC
HPLVTSAVDRRAPGNGRIFLTDLGGGLLQVEMVTLQEEDTGEYGCVVEGAAGPQTLHRVS
LLVLPPVPGPREGEEAEDEKETYRIGTGSLLEDPSLDPSASAGPHEFRRRENSIPLIWGA
VLLLALVVVAVVIFAVMARKKGNRLVVCGPSQSTGVPGMDPPSAAHRSSDSGLPSDIPHV
RLDSPPSFDSIYTGSSLDPPSSEPPAPPSQPPLPPKVLMSSKSVTYATVVFPGGDKGKIA
SCEPVQDPPNSQTPPSK
>Q8N488
MTMGDKKSPTRPKRQAKPAADEGFWDCSVCTFRNSAEAFKCSICDVRKGTSTRKPRINSQ
LVAQQVAQQYATPPPPKKEKKEKVEKQDKEKPEKDKEISPSVTKKNTNKKTKPKSDILKD
PPSEANSIQSANATTKTSETNHTSRPRLKNVDRSTAQQLAVTVGNVTVIITDFKEKTRSS
STSSSTVTSSAGSEQQNQSSSGSESTDKGSSRSSTPKGDMSAVNDESF
>P28240
MPIPVGNTKNDFAALQAKLDADAAEIEKWWSDSRWSKTKRNYSARDIAVRRGTFPPIEYP
SSVMARKLFKVLEKHHNEGTVSKTFGALDPVQISQMAKYLDTIYISGWQCSSTASTSNEP
GPDLADYPMDTVPNKVEHLFKAQLFHDRKQLEARSKAKSQEELDEMGAPIDYLTPIVADA
DAGHGGLTAVFKLTKMFIERGAAGIHMEDQTSTNKKCGHMAGRCVIPVQEHVNRLVTIRM
CADIMHSDLIVVARTDSEAATLISSTIDTRDHYFIVGATNPNIEPFAEVLNDAIMSGASG
QELADIEQKWCRDAGLKLFHEAVIDEIERSALSNKQELIKKFTSKVGPLTETSHREAKKL
AKEILGHEIFFDWELPRVREGLYRYRGGTQCSIMRARAFAPYADLVWMESNYPDFQQAKE
FAEGVKEKFPDQWLAYNLSPSFNWPKAMSVDEQHTFIQRLGDLGYIWQFITLAGLHTNAL
AVHNFSRDFAKDGMKAYAQNVQQREMDDGVDVLKHQKWSGAEYIDGLLKLAQGGVSATAA
MGTGVTEDQFKENGVKK
>P40492
MAEKSIFNEPDVDFHLKLNQQLFHIPYELLSKRIKHTQAVINKETKSLHEHTAALNQIFE
HNDVEHDELALAKITEMIRKVDHIERFLNTQIKSYCQILNRIKKRLEFFHELKDIKSQNS

GTSHNGNNEGTRTKLIQWYQSYTNILIGDYLTRNNPIKYNSETKDHWNSGVVFLKQSQLD
DLIDYDVLLEANRISTSLLHERNLLPLISWINENKKTLTKKSSILEFQARLQEYIELLKV
DNYTDAIVCFQRFLLPFVKSNFTDLKLASGLLIFIKYCNDQKPTSSTSSGFDTEEIKSQS
LPMKKDRIFQHFFHKSLPRITSKPAVNTTDYDKSSLINLQSGDFERYLNLLDDQRWSVLN
DLFLSDFYSMYGISQNDPLLIYLSLGISSLKTRDCLHPSDDENGNQETETATTAEKEVED
LQLFTLHSLKRKNCPVCSETFKPITQALPFAHHIQSQLFENPILLPNGNVYDSKKLKKLA
KTLKKQNLISLNPGQIMDPVDMKIFCESDSIKMYPT
>Q9Y813
MSTKSKDQHARVSYLYQASQLLFRNVQEPTLSRHYISTAKDVSQKSVMRIHPDIKRTICK
GCNSLLVPGKSCSIRFEEPSRKNPSIDRVLWICKKCAFKKRFSDKSC
>Q9NPP4
MNFIKDNSRALIQRMGMTVIKQITDDLFVWNVLNREEVNIICCEKVEQDAARGIIHMILK
KGSESCNLFLKSLKEWNYPLFQDLNGQSLFHQTSEGDLDDLAQDLKDLYHTPSFLNFYPL
GEDIDIIFNLKSTFTEPVLWRKDQHHHRVEQLTLNGLLQALQSPCIIEGESGKGKSTLLQ
RIAMLWGSGKCKALTKFKFVFFLRLSRAQGGLFETLCDQLLDIPGTIRKQTFMAMLLKLR
QRVLFLLDGYNEFKPQNCPEIEALIKENHRFKNMVIVTTTTECLRHIRQFGALTAEVGDM
TEDSAQALIREVLIKELAEGLLLQIQKSRCLRNLMKTPLFVVITCAIQMGESEFHSHTQT
TLFHTFYDLLIQKNKHKHKGVAASDFIRSLDHCGDLALEGVFSHKFDFELQDVSSVNEDV
LLTTGLLCKYTAQRFKPKYKFFHKSFQEYTAGRRLSSLLTSHEPEEVTKGNGYLQKMVSI
SDITSTYSSLLRYTCGSSVEATRAVMKHLAAVYQHGCLLGLSIAKRPLWRQESLQSVKNT
TEQEILKAININSFVECGIHLYQESTSKSALSQEFEAFFQGKSLYINSGNIPDYLFDFFE
HLPNCASALDFIKLDFYGGAMASWEKAAEDTGGIHMEEAPETYIPSRAVSLFFNWKQEFR
TLEVTLRDFSKLNKQDIRYLGKIFSSATSLRLQIKRCAGVAGSLSLVLSTCKNIYSLMVE
ASPLTIEDERHITSVTNLKTLSIHDLQNQRLPGGLTDSLGNLKNLTKLIMDNIKMNEEDA
IKLAEGLKNLKKMCLFHLTHLSDIGEGMDYIVKSLSSEPCDLEEIQLVSCCLSANAVKIL
AQNLHNLVKLSILDLSENYLEKDGNEALHELIDRMNVLEQLTALMLPWGCDVQGSLSSLL
KHLEEVPQLVKLGLKNWRLTDTEIRILGAFFGKNPLKNFQQLNLAGNRVSSDGWLAFMGV
FENLKQLVFFDFSTKEFLPDPALVRKLSQVLSKLTFLQEARLVGWQFDDDDLSVITGAFK
LVTA
>Q9Y664
MMGEAAVAAGPCPLREDSFTRFSSQSNVYGLAGGAGGRGELLAATLKGKVLGFRYQDLRQ
KIRPVAKELQFNYIPVDAEIVSIDTFNKSPPKRGLVVGITFIKDSGDKGSPFLNIYCDYE
PGSEYNLDSIAQSCLNLELQFTPFQLCHAEVQVGDQLETVFLLSGNDPAIHLYKENEGLH
QFEEQPVENLFPELTNLTSSVLWLDVHNFPGTSRRLSALGCQSGYVRVAHVDQRSREVLQ
MWSVLQDGPISRVIVFSLSAAKETKDRPLQDEYSVLVASMLEPAVVYRDLLNRGLEDQLL
LPGSDQFDSVLCSLVTDVDLDGRPEVLVATYGQELLCYKYRGPESGLPEAQHGFHLLWQR
SFSSPLLAMAHVDLTGDGLQELAVVSLKGVHILQHSLIQASELVLTRLRHQVEQRRRRLQ
GLEDGAGAGPAENAAS
>Q06688
MDFKASGTKFKKTGKKVVPAKAFEEIVQTNRDVLKKSAFLSDLLENLQPHLNNIKKIRCV
AIGNFKEDFPATYQFALLLEITDYIKSEDERDVVVSLYDPIFTKEEIQYLKSLGSKWLIE
EEFSENDAIDYESVLYFLPHAPLDLTENILSSQRPHLWLANNMISHTDRYTKAKLCENYP
NLSKLVHYLQTNAPPEVKKAHDVDGFATFIPKRKRKNRNNSSKLKVTPSDIDYDSIAPKF
KSCQILTDFDEGKYLKEKPWINSFSDLTLHAIEY

>O74434
MLLKVSSCEPLVPIKQWIHTSQLDLSDSSSLVADLLYNIIKFNFSGLEDKRAIFTGYESI
KRYTIGNEMFLQLAKDGFALPVSMSYGLFLEETDTLELRALPRSEGLHRGCEVLVLRLDS
PNELKENIREWSSKFMNSKASQKQQMLSPETMQKVDLAGRASPRYQANDSWISKKRNAPL
SSISQYANMPENYALNTKKSKISNENDTIFKANFQQNKYESLHAKQISASDLSLHQEFVS
ASIVQQPELNSLLTSQSKNSTPSESDSSSSESSSSVSDSSDLSSTSDSSSGDESSDTARQ
SSSDTISSNNVSVSVSLNTANEFSFPQGTFKAENEAVESEVAPSSSTPPTVEEISYLHYD
EPSQYYFSSPSKLSSETTKVHRGCENTEKPSEEGKNFTTPLSDSVESENTWSNTLRSSVE
DDNTGVSDDNKKSKLNAEFDGHVNNISVRPPGSGSSATKARNLRRKKARILKRLMQESND
TFSANESTVVANVPDSYLTKNDEEDSASLASLKVAPKYVSHPVSLSSNSFHTEFPIGSLM
RFTVMDLNPVTCTPEISEKTGRVVDCSEEKVKIQLDLGDRLDLKFDVNGEVIRNRYGTTP
DEELIEGIATYEWSSLSNVHKLELTN
>P14708
MSQQHTLPVTLSPALSQELLKTVPPPVNTQQEQMKQPTPLPPPCQKVPVELPVEVPSKQE
EKHMTAVKGLPEQECEQQQQEPQEQELQQQHWEQHEEHQKAENPEQQLKQEKAQRDQQLN
EQLEEEKKLLDQRLDQELVKRDEQLGMKKEQLLELPEQQEQHLKHLEQQEGQLELPEQQE
GQLKHLEQQEGQLKHLEQQEGQLEVPEEQVGQLKYLEQQEGQLKHLDQQEGQLKHLDQQE
GQLKHLDQQEGQLKHLDQQEGQLKHLDQQEGQLELPEQQEGQLKHLEQQEGQLKHLEHEE
GQLEVPEEQVGQLKYLEQQEGQLKHLDQQEGQLELPEQQEGQLKHLEQQEGQLKHLEHQK
GQLEVPEEQVGQLKYLEQQEGQLKHLDQQEGQLELPEQQEGQLKHLEQQEGQLKHLEHQE
GQLEVPEEQVGQLKYLEQQEGQLKHLDQQEGQLKHLDQQEKQLELPEQQVGQLKHLEQQE
GQLEVPEEQVGQLKYLEQQEGQLKHLDQQEGQLELPEQQEGQLKHLEQQEGQLKHLEHQE
GQLEVPEEQVGQLKYLEQQEGQLKHLDQQEGQLKHLDQQEKQLELPEQQVGQLKHLEQQE
GQLEHLEGQEGQLEHLEHQEGQLGLPEQQVWQLKQLEKQEGQPKNLEEEEGQLKHLVQQE
GQLEQQEGQVEHLEEQVGQLKHLEEQEGQLKYLEQQQGQLEVPEQQVGQPKHLEQEEKQL
ELPEQQEGQLKHLEKQEAQLELPEQQVGQPKHLEQQEKQLEHPEQKDGQLKHLEQQEGQL
KNLEQQKGQLEQPVFAPAPGQVQDIQPALPTKGEVLLPVEQQQQKQEVQWPPKHK
>Q9N5M6
MDQSKMRYTNQFRKTPQKPTSTEVGNHHTPAHSPMAQHETSMWNFNSLNPYFSMLNMNDG
MNYARHQQNHIVTSRPPTPLTDLMSLRSFQSFPNVFMPVSRSRTSSFIQSDTDSSRLEPD
DFSQNVRYCSTEIDRNNSSSKNDHLKYSRPALSRNSRSFTRSNNVLPTWSLDSNGEMRSR
LSLSEVLDSGDLMKFAVDKTGCQFLEKAVKGSLTSYQKFQLFEQVIGRKDDFLKLSTNIF
GNYFVQEIIGMSLTTYDDDNIKRQEKLKNFISSQMTDMCLDKFACRVIQSSLQNMDLSLA
CKLVQALPRDARLIAICVDQNANHVIQKVVAVIPLKNWEFIVDFVATPEHLRQICFDKYG
CRVVQTIIEKLTADSINVDLTSAAQHLRERALQRLMTSVTNRCQELATNEYANYIIQHIV
SNDDLAVYRECIIEKCLMRNLLSLSQEKFASHVVEKAFLHAPMELLAEMMDEIFDGYMPH
PGTGKDALDIMMFHQFGNYVVQCMLTICCDAVSGRRQTKEGSYDHANSFQVWLKKLHSRV
TKERHRLSRFSSGKKMIETLAHLRSTHPIYGLQSSGHESFKTDCFSTASEHDGLELEKNG
IEEGNLRLMRTFSP
>P41000
MTKKNAFKNSEDMKKYHLSEFNSEATSLTRPSPKSLQHVPCKFFRQGTCTSGKNCIFSHD
LELATEKTICKYFQKGNCKFGSKCALEHVLPDGRKVKTRAFAPSTTAMGSSSQNISAAPM
ANIISNNDKILPMTTMASATASEEKNRIKDEALVIKKEESNVAIPSEVTVAANAFSASTE
DVYSIVGDSLSKKASVKDFSDVTGIETIPAYVEATNGSSTVRSPHAKRSLSSISVKSSTS

PFSGSKFLSSSSYPHTPEAHLNSNHISPASFGSGIRTRNIFNPESMSLGLKPPILNRSYS
ASMAPGFSMNTFTATGNLGRPTKSPSVPTSVGSNKSRKFPGINGSTLTATPSSLENLYPL
SSRRSVPNLISSLGTSPSTFSSQFLKSTDRTHSFTSKLQSFNPVGTSLLASSLGTSQEDS
VNYDIPDEFANEEDFIPNSLQELLTPEELERKMSHGDEAVSSSSASRFMSKVSSNLNSGN
PTPYNSYNGTPTSSRFVAFFDRHRQESEKATPPSLNKVSQEPLTATTPKNLGNLTAISET
LENGQTLKSNMASSIEKSKTSTEVVASYDTTLDEETQFQMDEA
>Q96WV5
MEMLTKFESRSSRAKGVAFHPTQPWILTSLHNGRIQLWDYRMGTLLDRFDGHDGPVRGIA
FHPTQPLFVSGGDDYKVNVWNYKSRKLLFSLCGHMDYVRVCTFHHEYPWILSCSDDQTIR
IWNWQSRNCIAILTGHSHYVMCAAFHPSEDLIVSASLDQTVRVWDISGLRMKNAAPVSMS
LEDQLAQAHNSISNDLFGSTDAIVKFVLEGHDRGVNWCAFHPTLPLILSAGDDRLVKLWR
MTASKAWEVDTCRGHFNNVSCCLFHPHQELILSASEDKTIRVWDLNRRTAVQTFRRDNDR
FWFITVHPKLNLFAAAHDSGVMVFKLERERPAHALNINTLLYVNKEKSIVSYDLLRAQST
TVASVKHLGSAWLPPRSLSYNPAEKVALLTSSADNGVYELVNVSSRSNSLPLKDNIKGPG
DDAIFVARNRFAVFSRSDQTIEIKDLSNKVTKTIQLPEKTRDIFFAGMGHVLLSTATQVH
LFDLQQKKIVSSFNANRVKYVVWSNDNSQAALLGKHYVYIVKKNLELITSIHETIRIKSA
VWVENNVLLYATLDHLKYALMSGDTGVIKTLESTLYLVKAKGNMVFALNRAAEPVSFEID
PTEYLFKLALLRKDYEQVLHLIQNSNLVGQAIIAYLQKKGYPEIALQFVEDPSTRFELAL
ECGNLETALELARTIDRPEVWSRLASDAMSYGNHKIAEITFQKLRYFEKLSFLYLITGNA
EKLQKMAIIAEKRNDTLSLFQNSLYLNEVESRINILEQAGMYPIAYLTAKSNGLEEKAQQ
ILSHCNKTEEEIKLPSLGSAFTTPVPVNETYTHNWPLLDTSHSTFEKSLQERMEQLAIER
QEEQESEEEYEEVEQSLMDVVDEMSDLAESVPEEEVDGWEVEDLAPEEAVNDVVDDASAF
VGADEIFLWKRNSPLAADHIAAGDFESAMKILNKQVGAINFSPLKTRFLEIYTASRVYLP
TISGLDPLVSYVRRNAETAERSQALPFITRNLASIKSHELHEAYRLVKANKILEAQICFR
SIIYLALTTVANSEEEADEISALIDECCRYIVALSCELERRRLGEEDTKRALELSYYFAS
ADLQPMHSIIALRLAINASHKLKNYKSASFLGNKLLQLAESGPAAEAANRAITLGDRNPH
DAFEIEYDPHVEMRICPKTLTPVYSGDDFDVCSVCGAVYHKGYVNEVCTVCDVGGIGQKG
TGRRFFA
>P15290
MGYSKTLVAGLFAMLLLAPAVLATDPDPLQDFCVADLDGKAVSVNGHTCKPMSEAGDDFL
FSSKLAKAGNTSTPNGSAVTELDVAEWPGTNTLGVSMNRVDFAPGGTNPPHIHPRATEIG
IVMKGELLVGILGSLDSGNKLYSRVVRAGETFLIPRGLMHFQFNVGKTEASMVVSFNSQN
PGIVFVPLTLFGSNPPIPTPVLTKALRVEARVVELLKSKFAAGF
>Q9VJE5
MSDDTSASGGTSAPFPSPVTADPEPGATASKLPGPIRSNIPTPATSGTGIPQPSKMKAPS
SFGSTGSVSKIGRPCCNHTTPKSGPPPREATSMSRESDDNLSSINSAYTDNSSAVLTANT
EQFIIGQRVWLGGTRPGQIAFIGDTHFAAGEWAGVVLDEPNGKNDGCVSGKRYFQCEPKR
GIFSRLTRLTTYPLAGAQTPTSPLAKSSPDRSRTVSPTASIRSSMLRSPGIGGKNGMAVG
DRVIVSSGFGSRPGILRYLGETQFAPGNWCGVELDEPSGKNDGTVDDIRYFECKPKYGVF
VPIAKVSLSPSSKKTRLSRTGSRESLTSIGTMNSIATTATSRMRMNAQQRKSSTPVKPIL
ATPKSQFSMQDLLREKQQHVEKLMVERDLDREDAQNQALQLQKNINELKARIVELESALD
NERKKTEELQCSIDEAQFCGDELNAQSQVYKEKIHDLESKITKLVSATPSLQSILPPDLP
SDDGALQEEIAKLQEKMTIQQKEVESRIAEQLEEEQRLRENVKYLNEQIATLQSELVSKD
EALEKFSLSECGIENLRRELELLKEENEKQAQEAQAEFTRKLAEKSVEVLRLSSELQNLK

ATSDSLESERVNKTDECEILQTEVRMRDEQIRELNQQLDEVTTQLNVQKADSSALDDMLR
LQKEGTEEKSTLLEKTEKELVQSKEQAAKTLNDKEQLEKQISDLKQLAEQEKLVREMTEN
AINQIQLEKESIEQQLALKQNELEDFQKKQSESEVHLQEIKAQNTQKDFELVESGESLKK
LQQQLEQKTLGHEKLQAALEELKKEKETIIKEKEQELQQLQSKSAESESALKVVQVQLEQ
LQQQAAASGEEGSKTVAKLHDEISQLKSQAEETQSELKSTQSNLEAKSKQLEAANGSLEE
EAKKSGHLLEQITKLKSEVGETQAALSSCHTDVESKTKQLEAANAALEKVNKEYAESRAE
ASDLQDKVKEITDTLHAELQAERSSSSALHTKLSKFSDEIATGHKELTSKADAWSQEMLQ
KEKELQELRQQLQDSQDSQTKLKAEGERKEKSFEESIKNLQEEVTKAKTENLELSTGTQT
TIKDLQERLEITNAELQHKEKMASEDAQKIADLKTLVEAIQVANANISATNAELSTVLEV
LQAEKSETNHIFELFEMEADMNSERLIEKVTGIKEELKETHLQLDERQKKFEELEEKLKQ
AQQSEQKLQQESQTSKEKLTEIQQSLQELQDSVKQKEELVQNLEEKVRESSSIIEAQNTK
LNESNVQLENKTSCLKETQDQLLESQKKEKQLQEEAAKLSGELQQVQEANGDIKDSLVKV
EELVKVLEEKLQAATSQLDAQQATNKELQELLVKSQENEGNLQGESLAVTEKLQQLEQAN
GELKEALCQKENGLKELQGKLDESNTVLESQKKSHNEIQDKLEQAQQKERTLQEETSKLA
EQLSQLKQANEELQKSLQQKQLLLEKGNEFDTQLAEYQKVIDEMDDAASVKSALLEQLQN
RVAELETALRQANDAQKTAYLETKELRRQLESLELEKSREVLSLKAQMNGASSRSGKGDE
VESLDIETSLAKINFLNSIIADMQQKNDALKAKVQTLETLPMDFTKPHAFDALTKRKPAP
RLFCDICDEFDQHDTEDCPIQGSEDQDYSTPSSESNNNEKERKLPAPRKYCDSCEVFGHD
TSECADDETY
>O43087
MDEYIPFDDIVRQYDPDYTGKVSIQAFLEIVDDVDALRLNPEAPLLDNEQRQSAQDFIKD
NSEIVVSTSEIKNLFYELTGLDPDTLPVNKLALRENGVLPRKSVAKPQKISENRIKRKDM
FYQDASYITPRKGSPLSHSTPLSMFRTKNEYGSNKGFSHINKENADDSLIQQLYERIELQ
AAELRSKDEQVKELNARNAKLLEELDSSEEACKSCYTQAKTWEKKFREALRDSKEYAAQL
QTIHEEYEQQQAHIVRMEELIHAVEKERKTETDYMKKESLSEQKERGAFMESNMILEEKV
AHLQLENEQLRLFFKEKAPQPFQNHPPYQNLKITFPSPFFHIPYIPKTETLNDSQFAAGL
SLLASELESQKNLLKKFENLKKKSSKDFLSPSTILSNAFSKVSLPNSLILRVLFFSLLFI
IGIHIFYFLFFHVATIQQWPFLFWLPSTKFDNRWSPT
>Q08601
MKMSLEVYLNYHQRRPTRFTIMYPGSGRYTYNNAGGNNGYQRPMAPPPNQQYGQQYGQQY
EQQYGQQYGQQNDQQFSQQYAPPPGPPPMAYNRPVYPPPQFQQEQAKAQLSNGYNNPNVN
ASNMYGPPQNMSLPPPQTQTIQGTDQPYQYSQCTGRRKALIIGINYIGSKNQLRGCINDA
HNIFNFLTNGYGYSSDDIVILTDDQNDLVRVPTRANMIRAMQWLVKDAQPNDSLFLHYSG
HGGQTEDLDGDEEDGMDDVIYPVDFETQGPIIDDEMHDIMVKPLQQGVRLTALFDSCHSG
TVLDLPYTYSTKGIIKEPNIWKDVGQDGLQAAISYATGNRAALIGSLGSIFKTVKGGMGN
NVDRERVRQIKFSAADVVMLSGSKDNQTSADAVEDGQNTGAMSHAFIKVMTLQPQQSYLS
LLQNMRKELAGKYSQKPQLSSSHPIDVNLQFIM
>O43663
MRRSEVLAEESIVCLQKALNHLREIWELIGIPEDQRLQRTEVVKKHIKELLDMMIAEEES
LKERLIKSISVCQKELNTLCSELHVEPFQEEGETTILQLEKDLRTQVELMRKQKKERKQE
LKLLQEQDQELCEILCMPHYDIDSASVPSLEELNQFRQHVTTLRETKASRREEFVSIKRQ
IILCMEELDHTPDTSFERDVVCEDEDAFCLSLENIATLQKLLRQLEMQKSQNEAVCEGLR
TQIRELWDRLQIPEEEREAVATIMSGSKAKVRKALQLEVDRLEELKMQNMKKVIEAIRVE
LVQYWDQCFYSQEQRQAFAPFCAEDYTESLLQLHDAEIVRLKNYYEVHKELFEGVQKWEE

TWRLFLEFERKASDPNRFTNRGGNLLKEEKQRAKLQKMLPKLEEELKARIELWEQEHSKA
FMVNGQKFMEYVAEQWEMHRLEKERAKQERQLKNKKQTETEMLYGSAPRTPSKRRGLAPN
TPGKARKLNTTTMSNATANSSIRPIFGGTVYHSPVSRLPPSGSKPVAASTCSGKKTPRTG
RHGANKENLELNGSILSGGYPGSAPLQRNFSINSVASTYSEFAKDPSLSDSSTVGLQREL
SKASKSDATSGILNSTNIQS
>Q86AS5
MKFNYPETRRDDSVFDIFKSTEKGSVKVYDPYRHLEDQQSPETKKWVDEENKITRSFLDQ
DNTSEKISNEIMKMLNFERFDWFRRRGSKLFFSRNPNTLNQNIIYLIDIDQISISKDGKS
SAKGFENAIEFLNPNTYSKDGTWSLKSFVISKSGDHVCFSYSKAGSDWEEIAVKKIITTN
ELKTNKDDEEEKEDLKKKNCLHYAVVDLPDSINWCKFTSIKWDENETGFIYNRYPKPEKV
SDDDKGTETDTNLNNKVYYHKLGDANESFDRVVFECPENPQWIFGTEFSHDHSSLFISAF
RDCNVEHNLYVIRNFQEAIANKSAFKVEALIDNFDACYYYITNTKQGEYFFLTNLSAPFN
RLISIQLNDDQPIVPNSKSKLEFKEIIPEKDYVLESVSRSSQEKFYVSYQKHVQDIIEVY
DFNGKYLKDIKLPGPGSASLSATEYHDHIFINFSNLVSPSVTYYMDSKNDELLLFKEPHI
EGFKSSDYECKQVFYESPKDKTKIPMFIAYKKTTDITSGNAPTYMTGYGGFNISYTQSFS
IRNIYFLNKFNGIFVIANIRGGGEYGKAWHEAGSKKNKQNCFDDFIGAAEYLIKENYTNQ
NKLAVRGGSNGGLLMGAISNQRPDLFKCVVADVGVMDMLRFHLHTIGSNWVSDYGRSDNP
DDFDVLIKYSPLNNVPKDSNQYPSIMLCTGDHDDRVIPAHSYKFISELQYQLGKKVDTPL
LIRVDKDSGHGAGKGLSKQNNEIADIFNFFSKVLNVKLNF
>Q91YR1
MSHQTGIQASEDVKEIFARARNGKYRLLKISIENEQLVVGSCSPPSDSWEQDYDSFVLPL
LEDKQPCYVLFRLDSQNAQGYEWIFIAWSPDHSHVRQKMLYAATRATLKKEFGGGHIKDE
VFGTVKEDVSLHGYKKYLLSQSSPAPLTAAEEELRQIKINEVQTDVSVDTKHQTLQGVAF
PISRDAFQALEKLSKKQLNYVQLEIDIKNETIILANTENTELRDLPKRIPKDSARYHFFL
YKHSHEGDYLESVVFIYSMPGYTCSIRERMLYSSCKSPLLEIVERQLQMDVIRKIEIDNG
DELTADFLYDEVHPKQHAHKQSFAKPKGPAGKRGIRRLIRGPAEAEATTD
>P39518
MAAPDYALTDLIESDPRFESLKTRLAGYTKGSDEYIEELYSQLPLTSYPRYKTFLKKQAV
AISNPDNEAGFSSIYRSSLSSENLVSCVDKNLRTAYDHFMFSARRWPQRDCLGSRPIDKA
TGTWEETFRFESYSTVSKRCHNIGSGILSLVNTKRKRPLEANDFVVAILSHNNPEWILTD
LACQAYSLTNTALYETLGPNTSEYILNLTEAPILIFAKSNMYHVLKMVPDMKFVNTLVCM
DELTHDELRMLNESLLPVKCNSLNEKITFFSLEQVEQVGCFNKIPAIPPTPDSLYTISFT
SGTTGLPKGVEMSHRNIASGIAFAFSTFRIPPDKRNQQLYDMCFLPLAHIFERMVIAYDL
AIGFGIGFLHKPDPTVLVEDLKILKPYAVALVPRILTRFEAGIKNALDKSTVQRNVANTI
LDSKSARFTARGGPDKSIMNFLVYHRVLIDKIRDSLGLSNNSFIITGSAPISKDTLLFLR
SALDIGIRQGYGLTETFAGVCLSEPFEKDVGSCGAIGISAECRLKSVPEMGYHADKDLKG
ELQIRGPQVFERYFKNPNETSKAVDQDGWFSTGDVAFIDGKGRISVIDRVKNFFKLAHGE
YIAPEKIENIYLSSCPYITQIFVFGDPLKTFLVGIVGVDVDAAQPILAAKHPEVKTWTKE
VLVENLNRNKKLRKEFLNKINKCTDGLQGFEKLHNIKVGLEPLTLEDDVVTPTFKIKRAK
ASKFFKDTLDQLYAEGSLVKTEKL
>Q921A4
MEKVPGDMEIERRERNEELSEAERKAVQATWARLYANCEDVGVAILVRFFVNFPSAKQYF
SQFKHMEDPLEMERSPQLRKHACRVMGALNTVVENLHDPDKVSSVLALVGKAHALKHKVE
PMYFKILSGVILDVIAEEFANDFPVETQKAWTKLRGLIYSHVTAAYKEVGWVQQVPNTTT

LPATLPSSGP
>O75602
MSQRQVLQVFEQYQKARTQFVQMVAELATRPQNIETLQNAGVMSLLRTLLLDVVPTIQQT
AALALGRLANYNDDLAEAVVKCDILPQLVYSLAEQNRFYKKAAAFVLRAVGKHSPQLAQA
IVDCGALDTLVICLEDFDPGVKEAAAWALRYIARHNAELSQAVVDAGAVPLLVLCIQEPE
IALKRIAASALSDIAKHSPELAQTVVDAGAVAHLAQMILNPDAKLKHQILSALSQVSKHS
VDLAEMVVEAEIFPVVLTCLKDKDEYVKKNASTLIREIAKHTPELSQLVVNAGGVAAVID
CIGSCKGNTRLPGIMMLGYVAAHSENLAMAVIISKGVPQLSVCLSEEPEDHIKAAAAWAL
GQIGRHTPEHARAVAVTNTLPVLLSLYMSTESSEDLQVKSKKAIKNILQKCTYLPALEPF
LYDAPPNILKHVVGQFSKVLPHDSKARRLFVTSGGLKKVQEIKAEPGSLLQEYINSINSC
YPEEIVRYYSPGYSDTLLQRVDSYQPLNN
>Q07653
MNMNESISKDGQGEEEQNNFSFGGKPGSYDSNSDSAQRKKSFSTTKPTEYNLPKEQPEST
SKNLETKAKNILLPWRKKHNKDSETPHEDTEADANRRANVTSDVNPVSADTKSSSGPNAT
ITTHGYSYVKTTTPAATSEQSKVKTSPPTSHEHSNIKASPTAHRHSKGDAGHPSIATTHN
HSTSKAATSPVTHTHGHSSATTSPVTHTHGHASVKTTSPTNTHEHSKANTGPSATATTHG
HINVKTTHPVSHGHSGSSTGPKSTAAAQDHSSTKTNPSVTHGHTSVKDNSSATKGYSNTD
SNSDRDVIPGSFRGMTGTDVNPVDPSVYTSTGPKSNVSSGMNAVDPSVYTDTSSKSADRR
KYSGNTATGPPQDTIKEIAQNVKMDESEQTGLKNDQVSGSDAIQQQTMEPEPKAAVGTSG
FVSQQPSYHDSNKNIQHPEKNKVDNKNISERAAEKFNIERDDILESADDYQQKNIKSKTD
SNWGPIEYSSSAGKNKNLQDVVIPSSMKEKFDSGTSGSQNMPKAGTELGHMKYNDNGRDN
LQYVAGSQAGSQNTNNNIDMSPRHEAEWSGLSNDATTRNNVVSPAMKDEDMNEDSTKPHQ
YGLDYLDDVEDYHENDIDDYSNAKKNDLYSKKAYQGKPSDYNYEQREKIPGTFEPDTLSK
SVQKQDEDPLSPRQTTNRAGMETARDESLGNYEYSNTSGNKKLSDLSKNKSGPTPTRSNF
IDQIEPRRAKTTQDIASDAKDFTNNPETGTTGNVDTTGRMGAKSKTFSSNPFDDSKNTDT
HLENANVAAFDNSRSGDTTYSKSGDAETAAYDNIKNADPTYAKSQDITGMTHDQEPSSEQ
KASYGSGGNSQNQEYSSDDNIDVNKNAKVLEEDAPGYKREVDLKNKRRTDLGGADASNAY
AAEVGNFPSLIDPHVPTYGFKDTNTSSSQKPSEGTYPETTSYSIHNETTSQGRKVSVGSM
GSGKSKHHHNHHRHSRQNSSKGSDYDYNNSTHSAEHTPRHHQYGSDEGEQDYHDDEQGEE
QAGKQSFMGRVRKSISGGTFGFRSEI
>Q91YE9
MSQTSLKHKKKNEPGMRYSKESLDAEKRKDSDKTGARLSTQGSQELPLHNTDSRGYVVRN
QWSRTSRSPSTGAPSVDEPRSRNTAIKVEAPNSSTTSRTSSASPSQHETSPPPQTSEKSS
TQQTPQNRPITQLESQPPTPPETEPNSRRTSAKMYTGSDPWVHRENREPRDLQLRDYAYS
CDSRQGMPKTREYPRTPPTEWKPYAQRRLQYGTSVDMEPEYISDGPQHRQRQQTEEDEVD
EAYWTSVSMLYEKIPSCARPRPPKPKHAITIAVSSRALFNMVDDRKIYEEEGLEKYMEYQ
LTNENVILTPGPAFRFVKALQHVNSRLRDLYPDEQDLFDIVLMTNNHAQVGVRLINSVNH
YGLLIDRFCLTGGKSPIGYLKAYLTNLYLSADSEKVQEAIKEGIASATMYAGAKDMAYCD
TQLRVAFDGDAVLFSDESEHIAKDHGLDKFFQHETLFENKPLAQGPLKSFLEDLGKLQKK
FYAKDERLLCPIRTYLVTARSAASSGARVLKTLRRWGLEIDEALFLAGAPKGPILVKIRP
HIFFDDQMFHIESAQKFGTITAHVPYGIAQKRN
>Q9Y820
MSLNDFLQTLSNAVGEKNSNTLEKCIILDPSEPSFQQLSKTLFLDRKGKNKLNGTIQKEF
GRIRGWKELVTTYFEYVENAASSPDQRKWELLQNLYSNLTTCFSHIDGAWLCTIVKRVSK

LYVKLSLQLDTSTPTLEDSFDGNGFIQRKYVSDASRNVLRTFNSILSDRQQNINPSKKDA
IFCIANLLCLLYFRLKQIRLCQTIQANVISSGADISRATMAELVTFRYYLGRCHLYQRKI
HQAKDHLLFSFLQCPDECYHQKRLSLIYLTTCLLILGKSPTKGLLEKYKLTAAFEPLIKA
LKSGDIKSFRLSLEDNSRRKWFIKRGIYLTLLDRCEIILWRNLFRKVFLFTFEQSQKTPH
VSGSYLLTAARLSTNDNSYDMDDVECICVSLIDQGYIKGYIIHASSTLVLKKDPSFGFSV
IESLMPIARNDHAEKEFFHANA
>Q91783
MSSQNSFMSSKKDDKGKNIQVVVRCRPFNQLERKASSHSVLECESQRKEVCVRTGEVNDK
LGKKTYTFDMVFGPAAKQIDVYRSVVCPILDEVIMGYNCTIFAYGQTGTGKTFTMEGERS
SDEEFTWEQDPLAGIIPRTLHQIFEKLSEIGTEFSVKVSLLEIYNEELFDLLSPSPDVGE
RLQMFDDPRNKRGVIIKGLEEISVHNKDEVYQILERGAAKRKTASTLMNAYSSRSHSVFS
VTIHMKETTIDGEELVKIGKLNLVDLAGSENIGRSGAVDKRAREAGNINQSLLTLGRVIT
ALVERAPHIPYRESKLTRILQDSLGGRTKTSIIATVSPASINLEETMSTLDYASRAKNIM
NKPEVNQKLTKKALIKEYTEEIERLKRELATAREKNGVYLSNENYEQLQGKVLSQEEMIT
EYSEKIAAMEEEIKRIGELFADNKKELEECTTILQCKEKELEATQNNLQESKEQLAQEAF
VVSAMETTEKKLHGTANKLLSTVRETTRDVSGLHEKLDRKRAVEQHNSQVHENFAEQINR
RFSVIQQTVDEYSVKQQGMLDFYTNSIDDLLGASSSALSATATAVAKSFASVQETVSKQV
SHSVEEILKQETLSSQAKDDLQKLMTAHRTGLEQALRTDLLPVVTAVLDLNSHLSHCLQS
FLGVADKIDSHKEDMNSFFTEHSRSLHKLRLDSSSALSSIQSEYESLKEEIATAQSTHSE
GVNNLISSLQNQLNLLAMETRQQFSGFLSKGGKLQESVGCLQQDLDLVSSDAIECISSHH
SKFTEQSQAVTVEIRQLAGSNMSTLEESSKQCEKLTNSINTICQESQQWCESAGQKMDSL
LEEQVCYLHSSKKQIQTLHKDVEDGCGSSVVEITDRVNVQCQAQEKALTSLVEQVKDDKE
MLGEQRLELNEQVQSGLNKVHVYLKEELRNDVPTGTTPQRRDYVYPSLLIKTKPRDVLLE
QFRQQQQEYLESICSVISEAVEPPVEQDSLEDEPPVAVNDSVISEKSCIDLSMTCQEKGG
VPFFQQKKALRKEKENRGNATLLERSKIMDEVEQSLPKSKLPLRMQN
>Q42688
MAAGSVGVFATDEKIGSLLDQSITRHFLSTVTDQQGKICAEYVWIGGSMHDVRSKSRTLS
TIPTKPEDLPHWNYDGSSTGQAPGHDSEVYLIPRSIFKDPFRGGDNILVMCDCYEPPKVN
PDGTLAAPKPIPTNTRFACAEVMEKAKKEEPWFGIEQEYTLLNAITKWPLGWPKGGYPAP
QGPYYCSAGAGVAIGRDVAEVHYRLCLAAGVNISGVNAEVLPSQWEYQVGPCEGITMGDH
MWMSRYIMYRVCEMFNVEVSFDPKPIPGDWNGSGGHTNYSTKATRTAPDGWKVIQEHCAK
LEARHAVHIAAYGEGNERRLTGKHETSSMSDFSWGVANRGCSIRVGRMVPVEKSGYYEDR
RPASNLDAYVVTRLIVETTILL
>O60093
MDFIVVKRSRKKKNGRGKFPKVSNTITQGDTDLWSQDTLQRKLDNSREKLEYSEFLKSVQ
SQLSEFKDPIHKCIILGLGRIHTLTASLQLSLFFEIMKIFNLQPRFCSFYDPAFLKDDVE
FLENKGFCVLPDPPTPSCLKYTLLYMPHCPTSLYETWLAAYVNDDPRHFIMCGNNLQLYV
DNKPSKEIVSTYPNVYKMCTKNYYTRLLFPEFPNVYAFNDLSFHFHDAQSFVEKKQPSKF
VDASSVAEHPS
>P48534
MGKSYPTVSPDYQKAIEKAKRKLRGFIAEKKCAPLILRLAWHSAGTFDSKTKTGGPFGTI
KHQAELAHGANNGLDIAVRLLEPIKEQFPIVSYADFYQLAGVVAVEITGGPEVPFHPGRE
DKPEPPPEGRLPDATKGSDHLRDVFGKAMGLSDQDIVALSGGHTIGAAHKERSGFEGPWT
SNPLIFDNSYFTELLTGEKDGLLQLPSDKALLTDSVFRPLVEKYAADEDVFFADYAEAHL

KLSELGFAEA
>Q3E7A4
MHPQLEAERFHSCLDFINALDKCHQKEYYKRIFGLCNNEKDALNKCLKEASLNNKKRAVI
ESRIKRADVEKRWKKIEEEEYGEDAILKTILDRQYAKKKQESDNDANSK
>Q6IUP1
MASGGHERANEDYRVSGITGCSKTPQPETQDSLQTSSQSSALCTAPVAAANLGPSLRRNV
VSERERRRRISLSCEHLRALLPQFDGRREDMASVLEMSVYFLQLAHSMDPSWEQLSVPQP
PQEMWHMWQGDVLQVTLANQIADSKPDSGIAKPSAVSRVQDPPCFGMLDTDQSQATERES
ELLERPSSCPGHRQSALSFSEPESSSLGPGLPPWIPHSWQPATPEASDIVPGGSHQVASL
AGDPESSGMLAEEANLVLASVPDARYTTGAGSDVVDGAPFLMTTNPDWWLGSVEGRGGPA
LARSSPVDGAEPSFIGDPELCSQELQAGPGELWGLDFGSPGLALKDEADSIFPDFFP
>P04711
MASTKAPGPGEKHHSIDAQLRQLVPGKVSEDDKLIEYDALLVDRFLNILQDLHGPSLREF
VQECYEVSADYEGKGDTTKLGELGAKLTGLAPADAILVASSILHMLNLANLAEEVQIAHR
RRNSKLKKGGFADEGSATTESDIEETLKRLVSEVGKSPEEVFEALKNQTVDLVFTAHPTQ
SARRSLLQKNARIRNCLTQLNAKDITDDDKQELDEALQREIQAAFRTDEIRRAQPTPQAE
MRYGMSYIHETVWKGVPKFLRRVDTALKNIGINERLPYNVSLIRFSSWMGGDRDGNPRVT
PEVTRDVCLLARMMAANLYIDQIEELMFELSMWRCNDELRVRAEELHSSSGSKVTKYYIE
FWKQIPPNEPYRVILGHVRDKLYNTRERARHLLASGVSEISAESSFTSIEEFLEPLELCY
KSLCDCGDKAIADGSLLDLLRQVFTFGLSLVKLDIRQESERHTDVIDAITTHLGIGSYRE
WPEDKRQEWLLSELRGKRPLLPPDLPQTDEIADVIGAFHVLAELPPDSFGPYIISMATAP
SDVLAVELLQRECGVRQPLPVVPLFERLADLQSAPASVERLFSVDWYMDRIKGKQQVMVG
YSDSGKDAGRLSAAWQLYRAQEEMAQVAKRYGVKLTLFHGRGGTVGRGGGPTHLAILSQP
PDTINGSIRVTVQGEVIEFCFGEEHLCFQTLQRFTAATLEHGMHPPVSPKPEWRKLMDEM
AVVATEEYRSVVVKEARFVEYFRSATPETEYGRMNIGSRPAKRRPGGGITTLRAIPWIFS
WTQTRFHLPVWLGVGAAFKFAIDKDVRNFQVLKEMYNEWPFFRVTLDLLEMVFAKGDPGI
AGLYDELLVAEELKPFGKQLRDKYVETQQLLLQIAGHKDILEGDPFLKQGLVLRNPYITT
LNVFQAYTLKRIRDPNFKVTPQPPLSKEFADENKPAGLVKLNPASEYPPGLEDTLILTMK
GIAAGMQNTG
>Q8GZA8
MANNEGEMQCGSMLFKQEELQEMSGVNVGGDYVEVMCGCTSHRYGDAVARLRVFPTGDLE
ITCECTPGCDEDKLTPAAFEKHSGRETARKWKNNVWVIIGGEKVPLSKTVLLKYYNESSK
KCSRSNRSQGAKVCHRDEFVGCNDCGKERRFRLRSRDECRLHHNAMGDPNWKCSDFPYDK
ITCEEEEERGSRKVYRGCTRSPSCKGCTSCVCFGCELCRFSECTCQTCVDFTSNVKA
>Q86WP2
MAQHDFAPAWLNFPTPPSSTKSSLNFEKHSENFAWTENRYDVNRRRHNSSDGFDSAIGRP
NGGNFGRKEKNGWRTHGRNGTENINHRGGYHGGSSRSRSSIFHAGKSQGLHENNIPDNET
GRKEDKRERKQFEAEDFPSLNPEYEREPNHNKSLAAGVWEYPPNPKSRAPRMLVIKKGNT
KDLQLSGFPVVGNLPSQPVKNGTGPSVYKGLVPKPAAPPTKPTQWKSQTKENKVGTSFPH
ESTFGVGNFNAFKSTAKNFSPSTNSVKECNRSNSSSPVDKLNQQPRLTKLTRMRTDKKSE
FLKALKRDRVEEEHEDESRAGSEKDDDSFNLHNSNSTHQERDINRNFDENEIPQENGNAS
VISQQIIRSSTFPQTDVLSSSLEAEHRLLKEMGWQEDSENDETCAPLTEDEMREFQVISE
QLQKNGLRKNGILKNGLICDFKFGPWKNSTFKPTTENDDTETSSSDTSDDDDV
>Q86BY9

MNSQVMYHNVPTPPGNVSLAAAAPDGGLLYAGIRCINYISAPPANGEQPQVVTMSTRINI
LALDVSPMWGLGNGGPTKPFAIVGDDLSVQVWDCALGEAVIGHKAHQHQHEARDVRVVHH
TTNSVLMSYLANGNILSMDASDLVIYCVASNTYCRRSTFISPRNHQLTMVRCSPYNDNLF
AVGTAMGNVLVCDLRKMNIVYKFHGHKAPICGLAWREVPAAEDEKTNNLALSAEEWRSRN
GGQEEKPKTKPPPLTKSKAAESDDPFDIYNFDHLEYEFGAPIAERRRKSSEDCGGEFVGL
EKPAGAAVLDFVEACESVKAELLASRQEDKTQHVEVTLHDCEPTKPTGPLSDASTISNKN
DASDSTEGSLEVIQYSSSSDDAVIVDGEAAKPKREVLHHIYHQAEVHASGTPQTKSEPQS
NLQVVPAISAETISLTSVNSTHLETLLVSIDGDEVMMIWNTNTGAHAGKNYSKSKTAGKL
NNVYWLNNHVIVSLSRHQLFFWSVEFERKMLRYKISKDKSHSCHLQDIVSFACDSSKEMI
WLCRNNRQIGMMNPKTGRMADFYGTVAFGVRAMAECPDDMNKIALGCSDRRVAFFDISKL
TTSCLPIDSVYVSSNVYCLAWSPNCLELAFGTFDGTVGILDVERMKVKTHLRTPHKKEVY
SLVWQDHFIYFIVNRVLGFFDLRKSKIEPTIVNCISRPSYLSIRDSFLFVGTDDGLLQIH
ERDSGMEKSWSPFIRQSALFARYVTDIAWCPLDSNKFAVSGNDRSVYVMEFQPTERNWKT
LHTFTANTEKASITSMRWSHTQKHLLLTFHIEGKVCLWNCNAPEKPPLTITYHCPMWCGM
FLPTNENIIMCSGKALSVELIDIKDALEGDEKSICPKVDALLNVKWASKSLTQPYAPVLT
AAEKKRQRRDQRKAAAKLEVDVANKDQKKIQESVTAVIDNPTNDKCTQETPVEEMLEALS
LDKEQNNRSAKECPKCKEQSPDSFTHSRTCLYLTQKELNKSALEKLAIVLTEDSAKIDKS
VLISKLFSSKVMAKELIATELTNLKHSNTKDIAPLCLAMSTFKLREELEQHIANKTLTER
HVSLAPSVSFTLWQDCCRAYAKQMEEKGYIMHAATYLFSQGMQSEAIKLFLANEYYKEAL
VHARICLPATDPLIKTVINNWLEHLEGTGNFAAAALICVLDNEMLRGYSYLRKYRNCTPE
IADLMDQIKRIGQLGGVLDGCAPNEPIHNGSTAEH
>O94271
MLNGEKSALGEMPSNSNSSSKLNAKSPNFIPSSSNIPRSSAKTKEHSADRKPHRNSEKKT
QGMPRKNQQLASSERKTKNKKRLEKQSSAIADSIGESLDDPQTVYDEHLFDILSARTNKR
GQINLNHLLNFQFTPRTNSNAFSAPPRRSRGYNTYGQGSGHHPMDKSRYVNANYRFVVSP
IGDYQSQKLDPDSPVKWEDVWQVLCSSDFQLAACPFCLEEKPVAARMSRCGHVYCFSCLL
RFVETPTAAEVKAAETSGTKIVKCGHRSCPICWDSIRLRDVHPIRWVEDKEFQKLEEGKS
VCLRLYQRNNGSILAFPRSCRSFALDGSFHSDEIPNFTMSGAAYARIIIGSHDYMVQQHL
LEIEQLQQIAAEDISLYGSADTYYERITQILLDRISSLSESVNEQNIKSLQTDIDNLCLQ
SNSLKQLSEVDDLNDVSGSEIADAYLFYQPFAHSHIYLSPLDIRILKSAFGSYENFPDEL
VPRVERISSGHLVNSELRQRFKYMAHLPEGCEVAFIECDWSKIIPKEVLLTFKSEISKRR
KQRKAQEMREERYRQRAERDTEEQIYSELNMQRPVPKTVVHEDPAEAFPTLGSHHQFEST
DEVNTDESTLSTAKTVWGTRALANIQNDTDDQDDLDGWKVDWDKVAQLSAPSKNSKNKKK
KKLVLLSTGAAHR
>Q55DH8
MNKKLEQLEKFKTNDKNPVYSTTNTGVSLSDDANSLKAGPRGPTLLEDFVLREKITHFDH
ERIPERIVHARGTGAHGYFLSYKDHSKLTKADFLSKQDKKTPVFIRISTVQGPRGSADTV
RDVHGFAVKFYTDEGNYDLVGNNMPVFFIQDASSFPDFVHAVKMEPQNEMPTGGSAHDTF
YDFCGLKPESAHSVLWVMSDRGIPISLRHQQGFGVHSYRFINQEGKSTFVKLHWKPLSGT
CSLLWDEAQKIAGKDCDYHRRRFWEDIESGDFPQWELGAQLLDEDLQKKFDFDILDPTKL
IPEELTPVIPLGRMVIDRNPDNFFAETEQVAFCVSHVVPGIDFSNDPLLQGRIFSYLDTQ
LSRLGGPNFNEIPINRPVCPFANNQRDGIHRMTINKGGASYFPNSIDKGYPLLKEPSANK
GGFRPYPENISGTKSYDRSETFEDHFSQATMFWNSMSQHEKNHIIAAYTFEISKCSRPEV
RTRYVNNILVNIDSVLAEKVAKNLGVKIEPTSTKPIKKIMVKPSPALSQPNLLSGDIVSR

RISVIIEKGVDYDDVINFKDDMEKRGAMVMLVSSTLAQVECSGGEMLSPKGTIIGNPSIF
FDAVYVPKSTEEATKILSDDGNFLHYILEAFKHLKTIAFGGSVSVIKELLRLPQDHGLLL
GNGYKDITEQFFYSLAHHRVWERESKVSKIPA
>Q9BZG8
MRRQVMAVLVVSGAAEQGGRDGPGRGRAPRGRVANQIPPEILKNPQLQAAIRVLPSNYNF
EIPKTIWRIQQAQAKKVALQMPEGLLLFACTIVDILERFTEAEVMVMGDVTYGACCVDDF
TARALGADFLVHYGHSCLIPMDTSAQDFRVLYVFVDIRIDTTHLLDSLRLTFPPATALAL
VSTIQFVSTLQAAAQELKAEYRVSVPQCKPLSPGEILGCTSPRLSKEVEAVVYLGDGRFH
LESVMIANPNVPAYRYDPYSKVLSREHYDHQRMQAARQEAIATARSAKSWGLILGTLGRQ
GSPKILEHLESRLRALGLSFVRLLLSEIFPSKLSLLPEVDVWVQVACPRLSIDWGTAFPK
PLLTPYEAAVALRDISWQQPYPMDFYAGSSLGPWTVNHGQDRRPHAPGRPARGKVQEGSA
RPPSAVACEDCSCRDEKVAPLAP
>Q9Y613
MAGGEDRGDGEPVSVVTVRVQYLEDTDPFACANFPEPRRAPTCSLDGALPLGAQIPAVHR
LLGAPLKLEDCALQVSPSGYYLDTELSLEEQREMLEGFYEEISKGRKPTLILRTQLSVRV
NAILEKLYSSSGPELRRSLFSLKQIFQEDKDLVPEFVHSEGLSCLIRVGAAADHNYQSYI
LRALGQLMLFVDGMLGVVAHSDTIQWLYTLCASLSRLVVKTALKLLLVFVEYSENNAPLF
IRAVNSVASTTGAPPWANLVSILEEKNGADPELLVYTVTLINKTLAALPDQDSFYDVTDA
LEQQGMEALVQRHLGTAGTDVDLRTQLVLYENALKLEDGDIEEAPGAGGRRERRKPSSEE
GKRSRRSLEGGGCPARAPEPGPTGPASPVGPTSSTGPALLTGPASSPVGPPSGLQASVNL
FPTISVAPSADTSSERSIYKARFLENVAAAETEKQVALAQGRAETLAGAMPNEAGGHPDA
RQLWDSPETAPAARTPQSPAPCVLLRAQRSLAPEPKEPLIPASPKAEPIWELPTRAPRLS
IGDLDFSDLGEDEDQDMLNVESVEAGKDIPAPSPPLPLLSGVPPPPPLPPPPPIKGPFPP
PPPLPLAAPLPHSVPDSSALPTKRKTVKLFWRELKLAGGHGVSASRFGPCATLWASLDPV
SVDTARLEHLFESRAKEVLPSKKAGEGRRTMTTVLDPKRSNAINIGLTTLPPVHVIKAAL
LNFDEFAVSKDGIEKLLTMMPTEEERQKIEEAQLANPDIPLGPAENFLMTLASIGGLAAR
LQLWAFKLDYDSMEREIAEPLFDLKVGMEQLVQNATFRCILATLLAVGNFLNGSQSSGFE
LSYLEKVSEVKDTVRRQSLLHHLCSLVLQTRPESSDLYSEIPALTRCAKVDFEQLTENLG
QLERRSRAAEESLRSLAKHELAPALRARLTHFLDQCARRVAMLRIVHRRVCNRFHAFLLY
LGYTPQAAREVRIMQFCHTLREFALEYRTCRERVLQQQQKQATYRERNKTRGRMITETEK
FSGVAGEAPSNPSVPVAVSSGPGRGDADSHASMKSLLTSRPEDTTHNRRSRGMVQSSSPI
MPTVGPSTASPEEPPGSSLPSDTSDEIMDLLVQSVTKSSPRALAARERKRSRGNRKSLRR
TLKSGLGDDLVQALGLSKGPGLEV
>Q9UER7
MATANSIIVLDDDDEDEAAAQPGPSHPLPNAASPGAEAPSSSEPHGARGSSSSGGKKCYK
LENEKLFEEFLELCKMQTADHPEVVPFLYNRQQRAHSLFLASAEFCNILSRVLSRARSRP
AKLYVYINELCTVLKAHSAKKKLNLAPAATTSNEPSGNNPPTHLSLDPTNAENTASQSPR
TRGSRRQIQRLEQLLALYVAEIRRLQEKELDLSELDDPDSAYLQEARLKRKLIRLFGRLC
ELKDCSSLTGRVIEQRIPYRGTRYPEVNRRIERLINKPGPDTFPDYGDVLRAVEKAAARH
SLGLPRQQLQLMAQDAFRDVGIRLQERRHLDLIYNFGCHLTDDYRPGVDPALSDPVLARR
LRENRSLAMSRLDEVISKYAMLQDKSEEGERKKRRARLQGTSSHSADTPEASLDSGEGPS
GMASQGCPSASRAETDDEDDEESDEEEEEEEEEEEEEATDSEEEEDLEQMQEGQEDDEEE
DEEEEAAAGKDGDKSPMSSLQISNEKNLEPGKQISRSSGEQQNKGRIVSPSLLSEEPLAP
SSIDAESNGEQPEELTLEEESPVSQLFELEIEALPLDTPSSVETDISSSRKQSEEPFTTV

LENGAGMVSSTSFNGGVSPHNWGDSGPPCKKSRKEKKQTGSGPLGNSYVERQRSVHEKNG
KKICTLPSPPSPLASLAPVADSSTRVDSPSHGLVTSSLCIPSPARLSQTPHSQPPRPGTC
KTSVATQCDPEEIIVLSDSD
>O13652
MSKNGAPKYVDGGRKIMDEDLRRDLEILRLILLRLSHAADDTSPLDVFNAYESVLRENGL
NPARHRSYIRILYRLISSKKLSWNDEWRNLMRRIGFSQVSSDLNTPLFPLNNTDMENDPF
IVNGDDYRSRSNQQKSSQFASEVAQTIKSQTAMFRRADQYRSQKDKILLANSLDFMLEKY
RYYKKIDAVYLRKADAVYRAFIMLKYFPRWVQKLRNIQSRIYSAGEAYRLILIGQMVNRW
RQKTRERLHQKLRFQKNAFLLDVLLKWSALTVNWNRKHEYMLKRRVFTNWMSKRRRNESL
IQKANVFFQHSVLAHSFKTWKANLSIKNAATLYEKKVVFMSFNRWHANYESHVLDLQKAE
LFSQKAYYSIALHKWKLRIEELSDLMNKADDLYEVNLLQRMLVLWRRKATTYEKIDFWMD
GHDVDIVKSAFFKWKNKFIKVDRNFELADTILQNTFLTKWRLAVRVKVVQEKRNKDRLIV
TFRFWYYLMKAKSFQHKRDHRLLTIAWEAWKDNYQRRIKLQKEAQELQLTRIQKIAKQYL
IKWRISLLDIKELSIRAEKLHTTNEIIGVWNQWVVSYNRKVGMQQNADLFFKRKTLRVVF
EHWRYLRETNMLLRLENKVVDFREVQSEKLVLRCWNAWVARLLSVSQMLDTAELSTQKVV
VRAYEAWSFRYNHQKEIMSQADQFYNERLMRSAMVFWRYQLRAIKFYFNISDWKRREFNR
QTLRNAFDAWHLLMFRVTSLVMQADRFHEQKKNKLLGKYFRKWLQRMQLNQEESSLLEAE
ATIEGNRTSRYMHTFDTTLPMQNDEFTDESVNFENRWKQELKTPISRFSKFLRPIPPNTR

>O74407
MSEMEKQVENLSLEAKNEKPKEVILGEDGKPLSKKALKKLEKEREKEQKRKEREAREAEE
KKKREANEIDYSAGKYGDLPLNRSTARPGRTYTQISDISAKNDGQTVLLRARVYTSRLQG
NKMCFFSLRQKYDTIQALAVVNKDTISKQMVKWCGSISLESIVLVEGIVKKSPEIIKSAT
VQDAEIHISSIYVISPIKKNLPFLVEDAGRSEEQIRESEENAAEGDSKFVRVNLDTRLDN
RVLDLRTPTNQAIFDIQAGICQAFREFLLSNSFNEIHTPKMSGASSEGGSNVFKIQYFKT
DGFLSQSPQLYKQMLIAADRERVFEIGPVFRAEDSNTYRHMTEFTGLDLEMAFNEHYHEV
MEFIEKLFLYIFKTIREKYAKQVAVVRQQYPSEDFILPDADRIRFHFKDAVKLLKEAGYR
KQLVPGQKVPEDEEFHYCEDPEFDDFSTPEERALGQIVREKYHTDFYVIDKYPSSVRPFY
TMPDPEDPRYSNSYDFFMKGQEIMSGAQRIHDPELLVERMKALGVSPDVGLQQYIDAFAI
GCPPHAGGGIGLERVVMFYLNLPNIRLASSFPRDPKRLLP
>P46969
MVKPIIAPSILASDFANLGCECHKVINAGADWLHIDVMDGHFVPNITLGQPIVTSLRRSV
PRPGDASNTEKKPTAFFDCHMMVENPEKWVDDFAKCGADQFTFHYEATQDPLHLVKLIKS
KGIKAACAIKPGTSVDVLFELAPHLDMALVMTVEPGFGGQKFMEDMMPKVETLRAKFPHL
NIQVDGGLGKETIPKAAKAGANVIVAGTSVFTAADPHDVISFMKEEVSKELRSRDLLD
>Q9XXU9
MSSATSGEYWLISVPGEKGANDAWDKLNRSTGNTSTNSKYLIPDLKVGTLDQLVGLSDDL
SKLDTSAEAVIRKLVQYFTEVLEEDKSKIAENLVIGNKDMKTYVTKFQWEGAKYPLKQSL
KVLSEIIGKQISQIDNDLKVKSLTYNNLKNALASMDRKTVGSLLTKDLADLVKADDFVLN
SEYLQTVIVVVPKISVKEWEQKYATLSSMVVPGSSKLLTEEGEHALYTVTLFKKVIDEFK
NTARENKFIVRDFVYDEETLKAGRTERDKLMAEKQRQYAPLIRWLKINFGEIFAAYIHIK
ALRVFVESVLRYGLPVNFQAAVIEPAKGQQKKLRQELHKLYIHLDGSAAGPIDTLEDSPA
LMSLGVNEYYPYVFFKLNIDFLNK
>O94619

MADPGLRSGVGLPSQQGQKHDLQKDQKQPHVNNADRTTQSLLNSYIYDYLIKKDYCEAAR
AFGREAQVQTLVRSQEETNSLAKRHKRMSPVAVKHEGISNNESSDENMNVNNGNLDSFSS
SSAPPPPPILPIDSAGGFLIEWWNVFWDIYNARRGQGSEPAKAYMSHISNLRKKSRLNLQ
EIQKNSLHTGNTSHPYANASFPHDPANAMGQQIDSSQFHQGAGGLNDRNQHLMRQAMLNN
QSRETFPPTAAQLQQLKQLHYRQLQSVQQQQKQHQQKKTPQSGSTPQMQNTTSQPTTHDT
HPPKQQGPISDFRSIPSSPKTEGAPSNAQFRPSLPATPNGSVPQSNPLYDTTGLNGGQYP
VVQNSAQPLLHEINFASNRNPHLKQGGAVPSSTLPQQQKSLDKPKPAQQPSTGQFSGNQM
NQYGFSNSPYSQNMLYNFNGNANPSRLNPALKNYMEELKLLEQQNKKRLLLVSQEKERKG
YTSASPDRPLSQTITESSVAKTKSTTPKSTDTPTEATTSPVKVSTKNSNTTENLNGINES
NMPMLQNGLPLRTSGDHPSNYSNLIENSSTSDTNNADNGMDVMGNWQLQQTHSSRPTPNA
SSPLDVRSKQKPSSANSNAPTPAPTVNTTNPESSTNEATSVGPALEPSQGANVHKSDSEL
DNQNQSGKSNPDTSATPSAPTESTTVATKSSDNQLLDVGNSTDIDAALLNDFDFDKFLKD
TSTGDDLWFGLFNLPDNEDSTAA
>Q92556
MPPPADIVKVAIEWPGAYPKLMEIDQKKPLSAIIKEVCDGWSLANHEYFALQHADSSNFY
ITEKNRNEIKNGTILRLTTSPAQNAQQLHERIQSSSMDAKLEALKDLASLSRDVTFAQEF
INLDGISLLTQMVESGTERYQKLQKIMKPCFGDMLSFTLTAFVELMDHGIVSWDTFSVAF
IKKIASFVNKSAIDISILQRSLAILESMVLNSHDLYQKVAQEITIGQLIPHLQGSDQEIQ
TYTIAVINALFLKAPDERRQEMANILAQKQLRSIILTHVIRAQRAINNEMAHQLYVLQVL
TFNLLEDRMMTKMDPQDQAQRDIIFELRRIAFDAESEPNNSSGSMEKRKSMYTRDYKKLG
FINHVNPAMDFTQTPPGMLALDNMLYFAKHHQDAYIRIVLENSSREDKHECPFGRSSIEL
TKMLCEILKVGELPSETCNDFHPMFFTHDRSFEEFFCICIQLLNKTWKEMRATSEDFNKV
MQVVKEQVMRALTTKPSSLDQFKSKLQNLSYTEILKIRQSERMNQEDFQSRPILELKEKI
QPEILELIKQQRLNRLVEGTCFRKLNARRRQDKFWYCRLSPNHKVLHYGDLEESPQGEVP
HDSLQDKLPVADIKAVVTGKDCPHMKEKGALKQNKEVLELAFSILYDSNCQLNFIAPDKH
EYCIWTDGLNALLGKDMMSDLTRNDLDTLLSMEIKLRLLDLENIQIPDAPPPIPKEPSNY
DFVYDCN
>Q9CY02
MAPFQSNKDLISTGIKEFNVLLDQQVFDDPLISEEDMVIVVHDWVNLYTNYYKKLVHGEQ
EEQDRAMTEFQQELSTLGSQFLAKYRTFLKSKEPPSNTLPSS
>P50503
MDPRKVSELRAFVKMCRQDPSVLHTEEMRFLREWVESMGGKVPPATHKAKSEENTKEEKR
DKTTEDNIKTEEPSSEESDLEIDNEGVIEADTDAPQEMGDENAEITEAMMDEANEKKGAA
IDALNDGELQKAIDLFTDAIKLNPRLAILYAKRASVFVKLQKPNAAIRDCDRAIEINPDS
AQPYKWRGKAHRLLGHWEEAARDLALACKLDYDEDASAMLREVQPRAQKIAEHRRKYERK
REEREIKERIERVKKAREEHEKAQREEEARRQSGSQFGSFPGGFPGGMPGNFPGGMPGMG
GAMPGMAGMPGLNEILSDPEVLAAMQDPEVMVAFQDVAQNPSNMSKYQNNPKVMNLISKL
SAKFGGHS
>P51656
MDPTVVLITGCSSGIGMHLAVRLASDRSQSFKVYATLRDLKAQGPLLEAARTQGCPPGSL
EILELDVRDSKSVAAAQACVTEGRVDVLVCNAGRGLFGPLEAHELNAVGAVLDVNVLGTI
RMLQAFLPDMKRRHSGRVLVTASVGGLMGLPFHEVYCASKFALEGLCESLAILLPLFGVH
VSLIECGAVHTAFYEKLVGGPGGALERADAQTRHLFAHYLRGYEQALSEAQDPEEVTELF
LTAMRAPQPALRYFSTNRFLPLARMRTEDPSGSSYVAAMHQEAFSNLQTQENAKAGAQVP

GVSDTASSALICLPECAIPRVASELGWSASDKPGQDNSCYQQKI
>Q03336
MSSIKIECVLRENYRCGESPVWEEASKCLLFVDIPSKTVCRWDSISNRVQRVGVDAPVSS
VALRQSGGYVATIGTKFCALNWEDQSVFILAMVDEDKKNNRFNDGKVDPAGRYFAGTMAE
ETAPAVLERHQGSLYSLFPDHSVKKYFDQVDISNGLDWSLDHKIFYYIDSLSYTVDAFDY
DLPTGQISNRRTVYKMEKDEQIPDGMCIDVEGKLWVACYNGGRVIRLDPETGKRLQTVKL
PVDKTTSCCFGGKDYSEMYVTCARDGMSAEGLLRQPDAGNIFKITGLGVKGIAPYSYAG
>Q12427
MSENQKEVSPPQAISVKSEASSSIFSKPISTSSPAGLAAAQRVTPGKLSTLLLEMGPLAI
RHITQTLCLDIPCFKDLSSSKQRRLIMSAMESGDKEKSVVFEKIGWGQWSAKRVDPANFD
KELEATNFANAKVKDLISQESQRRKSNNSNSNSGGKVEMPMKVEHNITNIDGATTPPTAV
ASTTIPVNIKRSKSPLAAANVVYIDENALASEDEDEEFDEDDHHLHYQNKSRNSSNNFGK
SSNGDPYSFGRRRSQVVFADSTPENIEHEIIAQKIRPLLRNRRRSSIKPHTPFISKLNTH
QDSSYLSPNTTSTTTPSNNNSNSNQAKIDLEKLTATSEPTSRRASRLSVSKESSIRSTLF
PNKNYLIVTTNPNSKATSVSTSPKLEEQMNVSSNPIVLSDKEKHRVASQHLNGESSPQLV
PHSHHQPHSDTDEEDWESIGAASLRNNSLAPNIDSVASSTNGVVSPKPTNPSFTNSQNGD
IEPPLQHDQQKHEQQPRNGEDNSAAFLLMSLKS
>P15941
MTPGTQSPFFLLLLLTVLTVVTGSGHASSTPGGEKETSATQRSSVPSSTEKNAVSMTSSV
LSSHSPGSGSSTTQGQDVTLAPATEPASGSAATWGQDVTSVPVTRPALGSTTPPAHDVTS
APDNKPAPGSTAPPAHGVTSAPDTRPAPGSTAPPAHGVTSAPDTRPAPGSTAPPAHGVTS
APDTRPAPGSTAPPAHGVTSAPDTRPAPGSTAPPAHGVTSAPDTRPAPGSTAPPAHGVTS
APDTRPAPGSTAPPAHGVTSAPDTRPAPGSTAPPAHGVTSAPDTRPAPGSTAPPAHGVTS
APDTRPAPGSTAPPAHGVTSAPDTRPAPGSTAPPAHGVTSAPDTRPAPGSTAPPAHGVTS
APDTRPAPGSTAPPAHGVTSAPDTRPAPGSTAPPAHGVTSAPDTRPAPGSTAPPAHGVTS
APDTRPAPGSTAPPAHGVTSAPDTRPAPGSTAPPAHGVTSAPDTRPAPGSTAPPAHGVTS
APDTRPAPGSTAPPAHGVTSAPDTRPAPGSTAPPAHGVTSAPDTRPAPGSTAPPAHGVTS
APDTRPAPGSTAPPAHGVTSAPDTRPAPGSTAPPAHGVTSAPDTRPAPGSTAPPAHGVTS
APDTRPAPGSTAPPAHGVTSAPDTRPAPGSTAPPAHGVTSAPDTRPAPGSTAPPAHGVTS
APDTRPAPGSTAPPAHGVTSAPDTRPAPGSTAPPAHGVTSAPDTRPAPGSTAPPAHGVTS
APDTRPAPGSTAPPAHGVTSAPDTRPAPGSTAPPAHGVTSAPDTRPAPGSTAPPAHGVTS
APDTRPAPGSTAPPAHGVTSAPDTRPAPGSTAPPAHGVTSAPDTRPAPGSTAPPAHGVTS
APDTRPAPGSTAPPAHGVTSAPDTRPAPGSTAPPAHGVTSAPDTRPAPGSTAPPAHGVTS
APDTRPAPGSTAPPAHGVTSAPDTRPAPGSTAPPAHGVTSAPDNRPALGSTAPPVHNVTS
ASGSASGSASTLVHNGTSARATTTPASKSTPFSIPSHHSDTPTTLASHSTKTDASSTHHS
SVPPLTSSNHSTSPQLSTGVSFFFLSFHISNLQFNSSLEDPSTDYYQELQRDISEMFLQI
YKQGGFLGLSNIKFRPGSVVVQLTLAFREGTINVHDVETQFNQYKTEAASRYNLTISDVS
VSDVPFPFSAQSGAGVPGWGIALLVLVCVLVALAIVYLIALAVCQCRRKNYGQLDIFPAR
DTYHPMSEYPTYHTHGRYVPPSSTDRSPYEKVSAGNGGSSLSYTNPAVAAASANL
>Q9H6Z4
MADLANEEKPAIAPPVFVFQKDKGQKSPAEQKNLSDSGEEPRGEAEAPHHGTGHPESAGE
HALEPPAPAGASASTPPPPAPEAQLPPFPRELAGRSAGGSSPEGGEDSDREDGNYCPPVK
RERTSSLTQFPPSQSEERSSGFRLKPPTLIHGQAPSAGLPSQKPKEQQRSVLRPAVLQAP
QPKALSQTVPSSGTNGVSLPADCTGAVPAASPDTAAWRSPSEAADEVCALEEKEPQKNES

SNASEEEACEKKDPATQQAFVFGQNLRDRVKLINESVDEADMENAGHPSADTPTATNYFL
QYISSSLENSTNSADASSNKFVFGQNMSERVLSPPKLNEVSSDANRENAAAESGSESSSQ
EATPEKESLAESAAAYTKATARKCLLEKVEVITGEEAESNVLQMQCKLFVFDKTSQSWVE
RGRGLLRLNDMASTDDGTLQSRLVMRTQGSLRLILNTKLWAQMQIDKASEKSIRITAMDT
EDQGVKVFLISASSKDTGQLYAALHHRILALRSRVEQEQEAKMPAPEPGAAPSNEEDDSD
DDDVLAPSGATAAGAGDEGDGQTTGST
>P47116
MAGNGKDKEVDKSPSVSTLKLLGKRLFNSSSHTDNSSLLLSAEQLGNGRSLRKRPTSPSI
SGSGSGGNSPSSSAGARQRSASLHRRKNNASVGFSNGSVSSHKSSVALQDLIKHNNNPYL
NSPSDILGTGTGIASTRDRDRAVLDREKEKERARNKERNTHHAGLPQRSNSMASHHFPNE
NIVYNPYGISPNHARPDTAFADTLNTNKENDLSFYMHDGNSKIRMLPLPIANPNDFLPED
MKQYSVHLTDNFVFDTDNKPIGSGGSSEVRKVKSSYRQKDVYALKKLNMIYHESPEKFYK
RCSKEFIIAKHLSHNVHITNTFYLLKVPTTTYTTRGWGFIMELGVKDLFQLMERTGWKNV
PFNEKYCLFKQVAQGIKFCHDNGIAHRDLKPENVLISKEGICKLTDFGISDWYHVIPHDY
TSPVKTCQGMIGSPPYTPPEVMYFDAKKHYPEKFQKPYNPLAMDSYALGIMLITMINNII
PFIDSCNTDARFREFEVSYDNFINHQNPHFRDKGCHKPGPGSEYSLARNFKNTDATRIAW
RLADPNPATRYTMDDLFNDPFFQQIETCVEPNDDDLVRVPELRKSTSTNDFSENSLDAPH
DQEVIHTSNPFLKKETLTSKPRSMLEIAESPSLKQKSKVKDSAKTKTHDVGDEGGNESTK
PKQQDKKENLKKDEVKNGDKDKVIEEATTTNVDSILEKPTPTSTKVEDNLSEDDSTMKEL
KSMLNSTPTTPTHNGPTPLPAKAGTQLDKRMSDLSLKSETPASTKNFSAPNVSSSSNSLR
SLGSPSVSSSKKKKVIHHHLDITNSVTNMSSVSAFISR
>O43572
MRGAGPSPRQSPRTLRPDPGPAMSFFRRKVKGKEQEKTSDVKSIKASISVHSPQKSTKNH
ALLEAAGPSHVAINAISANMDSFSSSRTATLKKQPSHMEAAHFGDLGRSCLDYQTQETKS
SLSKTLEQVLHDTIVLPYFIQFMELRRMEHLVKFWLEAESFHSTTWSRIRAHSLNTVKQS
SLAEPVSPSKKHETTASFLTDSLDKRLEDSGSAQLFMTHSEGIDLNNRTNSTQNHLLLSQ
ECDSAHSLRLEMARAGTHQVSMETQESSSTLTVASRNSPASPLKELSGKLMKSIEQDAVN
TFTKYISPDAAKPIPITEAMRNDIIARICGEDGQVDPNCFVLAQSIVFSAMEQEHFSEFL
RSHHFCKYQIEVLTSGTVYLADILFCESALFYFSEYMEKEDAVNILQFWLAADNFQSQLA
AKKGQYDGQEAQNDAMILYDKYFSLQATHPLGFDDVVRLEIESNICREGGPLPNCFTTPL
RQAWTTMEKVFLPGFLSSNLYYKYLNDLIHSVRGDEFLGGNVSLTAPGSVGPPDESHPGS
SDSSASQSSVKKASIKILKNFDEAIIVDAASLDPESLYQRTYAGKMTFGRVSDLGQFIRE
SEPEPDVRKSKGSMFSQAMKKWVQGNTDEAQEELAWKIAKMIVSDIMQQAQYDQPLEKST
KL
>Q9P7B8
MAEGDFKNDSTGCIGDSVEFTTFKKRTGKSLGNRRKKRGLSGWEPYFQEPELTAEEKLEN
ETLYSRNIPLTVRMERFIQRYRSRRKWSDPTRIRLFTMYLDLGGVSTGQKQFTGGTDINN
DAKAREVSAQTTTDYIEYDILDEYDIDFKWVAGVFLSSHILYNAGLTKEEDLQIACQIVR
NFLLSVLHNNVAPEFEDNIRDACLLAEQAETELISNKRLSTKLPGRLQRALASIHVDNYK
GLWDKSQSDRTSDSSVNFIESNNTKFMPNDELFEPDGISRFSTQQARDYIQRTLGPRYLT
GKVVEQEYLTVKLVSKTLLNFSNQSLCKAVFIVWDPPGSKYSQDTNKERLEVILESDLLN
NTVDGTHLEGSFTFLDNGLTILDTVLAVLPTFYEEVKDE
>P53893
MPRVESETYKRLQNDPSCIRNICIVAHVDHGKTSLSDSLLASNGIISQRLAGKIRFLDAR

PDEQLRGITMESSAISLYFRVLRKQEGSDEPLVSEHLVNLIDSPGHIDFSSEVSAASRLC
DGAVVLVDVVEGVCSQTVTVLRQCWTEKLKPILVLNKIDRLITELQLTPQEAYIHLSKVI
EQVNSVIGSFFANERQLDDLFWREQLEKNENAEYIEKDDSGIYFNPTDNNVIFASAIDGW
GFNIGQLAKFYEQKLGAKRENLQKVLWGDFYMDPKTKKIINNKGLKGRSLKPLFTSLILE
NIWKIYQNIITSRDSEMVEKIAKTLNIKLLARDLRSKDDKQLLRTIMGQWLPVSTAVLLT
VIEKLPSPLESQTDRLNTILVSESDTAAMDPRLLKAMKTCDKEGPVSAYVSKMLSIPREE
LPVESKRIASSDELMERSRKAREEALNAAKHAGMVENMAMMDLNDNSKNTSDLYKRAKDT
VMTPEVGEQTKPKPSRNNDVFCVVSEPSSALDLEFEYEGEDDSDSQDNFGLDFVPTDIDP
NDPLSSMFEYEEEDPLLESIKQISEDVNDEVDDIFDEKEECLVAFARIYSGTLRVGQEIS
VLGPKYDPKCPEEHIETAIITHLYLFMGKELVPLDVCPSGNIVGIRGLAGKVLKSGTLIE
KGVQGVNLAGVNFHFTPIVRVAVEPANPVEMSKLVRGLKLLDQADPCVHTYVENTGEHIL
CTAGELHLERCLKDLTERFAGIEITHSEPAIPYRETFLSASDMNPPQNSQLGRGVHELLL
SQYKITFRTFPLSGKVTDFLSQHQNSIKNILKTSTSSMDPVIESTGSSFLDKKSLLVAFE
EVINQEEKSRELLSGFKVKLAGFGPSRVGCNILLSQDNLLGSLFEGTPAAFEYSDSIKNG
FQLAVSEGPLANEPVQGMCVLVESVHKMSQDEIESIEDPRYQQHIVDLSGRLITSTRDAI
HEAFLDWSPRIMWAIYSCDIQTSVDVLGKVYAVILQRHGKIISEEMKEGTPFFQIEAHVP
VVEAFGLSEDIRKRTSGAAQPQLVFSGFECIDLDPFWVPTTEEELEELGDTADRENIARK
HMNAIRRRKGLFIEEKVVENAEKQRTLKKN
>P15496
MTADNNSMPHGAVSSYAKLVQNQTPEDILEEFPEIIPLQQRPNTRSSETSNDESGETCFS
GHDEEQIKLMNENCIVLDWDDNAIGAGTKKVCHLMENIEKGLLHRAFSVFIFNEQGELLL
QQRATEKITFPDLWTNTCCSHPLCIDDELGLKGKLDDKIKGAITAAVRKLDHELGIPEDE
TKTRGKFHFLNRIHYMAPSNEPWGEHEIDYILFYKINAKENLTVNPNVNEVRDFKWVSPN
DLKTMFADPSYKFTPWFKIICENYLFNWWEQLDDLSEVENDRQIHRML
>Q10180
MKMPNTLHSVSISTDFPSPNVESEAADVNEYSSTSKFETLGNQSSTNLGFSNSLQDKWDC
WKRDLWSIWWSLSRKATRFYRWLSRSLKWRPVTYETVYPQTYETQMSEPREVTTIVKDLN
NGDSFVLNVTEPVDPEFLRANIPPVHRKHLPPRLSLVASPGTIPTRGVVVLEDWINPLLS
ERCKLLLQSELCNQDSYDCPGYICVYRFEQKNNPSVVLGDSTLIQISRVSDLQRHLREYP
KNCAFSRSVLEIFPDPGKTSKPCQVSFKVERLVHKELNEYLSWMQPFTCDSCGSLHENWL
QIDSKQWDQIRGVILRWVEYSRVIYA
>Q8W575
MIGAVNSVEAVITSIQGLSGSPEDLSALHDLLRGAQDSLRAEPGVNFSTLDQLDASKHSL
GYLYFLEVLTCGPVSKEKAAYEIPIIARFINSCDAGQIRLASYKFVSLCKILKDHVIALG
DPLRGVGPLLNAVQKLQVSSKRLTALHPDVLQLCLQAKSYKSGFSILSDDIVEIDQPRDF
FLYSYYGGMICIGLKRFQKALELLYNVVTAPMHQVNAIALEAYKKYILVSLIHNGQFTNT
LPKCASTAAQRSFKNYTGPYIELGNCYNDGKIGELEALVVARNAEFEEDKNLGLVKQAVS
SLYKRNILRLTQKYLTLSLQDIANMVQLGNAKEAEMHVLQMIQDGQIHALINQKDGMVRF
LEDPEQYKSSEMIEIMDSVIQRTIGLSKNLLAMDESLSCDPLYLGKVGRERQRYDFGDDF
DTVPQKFSM
>Q12084
MDRKAVEEKRIVISIGGGHATGVGAIALDLQNTFKSLYNSINIRVINLDNMIEGNIKSYN
NNDYDFDNILNLVYEKHAVTSQNDMIQHDYEDPIDLIIVCGCYALYDKRINEISQLKVFL
DSDADKRLISLIKKKNVGSNEQLAQLITEYMDHLRPEMQQYIEPTRTFADLIIPSTNENL

GRAVLVDGIVKAIEDTKSQIEGNNTNNKIRPRLWDFEAETMDLEKDRYYDLS
>Q99627
MPVAVMAESAFSFKKLLDQCENQELEAPGGIATPPVYGQLLALYLLHNDMNNARYLWKRI
PPAIKSANSELGGIWSVGQRIWQRDFPGIYTTINAHQWSETVQPIMEALRDATRRRAFAL
VSQAYTSIIADDFAAFVGLPVEEAVKGILEQGWQADSTTRMVLPRKPVAGALDVSFNKFI
PLSEPAPVPPIPNEQQLARLTDYVAFLEN
>Q8R512
MSSPLASLSKTRKVPLESEPVNPGRRGIRIYGDEDEVDMVNDGQDSEEKISLPSCYGGIG
RQGLMIHDSELLTSMARKLQELEQQLKARNEEMLSKEQKILALEDLVQTLQQHQSSTTRE
EELETQCIQLQRQVGEMERFLNDYGLQWVGEPMDQENSEGKIISESDERDWMKAKKFWKP
GDSIVPPEVDFDRLLSSLQDLSELVVEGEAQVTPVPGGAQFRTLEPIPLKLYRNGIMMFD
GPFRPFYDPYTQRCLRDILDGFFPSELQRLYPDGVPFKVSDLRNQVYPEDGLGPFPGEGR
VVGRQKIRKVTDRVEETSGSRMTAEKFLNRLPKCVIRQGEVIDIRGPIRDTLQNCCPMPV
RIQEIIVETPALASERQRTQESPNMPVPPLSMLRIKSENGEQAFLLMMRPEDTIGDVRNL
LAQARDMDSAAFEILSTFPPTVYRDDTVTLQAAGLVPNATLLLRTRRVLPANPSFGTDSG
PGSLP
>Q8WN98
MKDPDPSQVYRPDMDPEAAKDKGSFRNYTSGPLLDRVFRTYKLMHTWQTVDFVRKKHAQF
GGFSYKRMTVLEAVDMLDGLVDESDPDVDFPNSFHAFQTAEGIRKAHPDKDWFHLVGLLH
DLGKVLVLAGEPQWAVVGDTFPVGCRPQASVVFCDSTFQDNPDLQDPVYSTELGMYQPHC
GLENALMSWGHDEYMYQMMKFNKFSLPGEAFYIIRFHSFYPWHTGGDYRQLCNEQDLAML
PWVQEFNKFDLYTKGSDMPDVDELRPYYQGLIDKYCPGVLCW
>Q9CW46
MAADVSVTHRPPLSPEAEAEAETPETVDRRAPEQELPPLDPEEIRKRLEHTERQFRNRRK
ILIRGLPGDVTNQEVHDLLSDYELKYCFVDKYKGTAFVTLLNGEQAEAAINTFHQSRLRE
RELSVQLQPTDALLCVANLPPSLTQAQFEELVRPFGSLERCFLVYSERTGHSKGYGFAEY
MKKDSAARAKSDLLGKPLGPRTLYVHWTDAGQLTPALLHSRCLCVDHLPPGFSDVDALRR
ALSVVYTPTFCQLACGQDGQLKGFAVLEYETAEMAEAAQERADGQALGDSHLRVSFCAPG
PPGRSMLAALIAAQATALNRGKGLLPEPNLLQLLNNLGPSASLQLLLNPLLHGGASGKQG
LLGAPPAMPLLSGPALSTALLQLALQSQSQNQSQGQKKPGILGDSPLGTLQAGAQPSNSL
LGELSAGGGLAPELPPRRGKPQPLLPPLLGPSGGDREPMGLGPPATQLTPPPAPVGLRGS
NHRGLPKDSGPLPTPPGVSLLGEPPKDYRIPLNPYLNLHSLLPSSNLAGKETRGWGGSGR
GRRPAEPPLPSPAVPGGGSGSNNGNKAFQMKSRLLSPIASNRLPPEPGLPDSYGFDYPTD
VGPRRLFSHPREPTLGAHGPSRHKMSPPPSSFNEPRSGGGSGGPLSHFYSGSPTSYFTSG
LQAGLKQSHLNKAVGSSPMGSSEGLLGLGPGPNGHSHLLKTPLGGQKRSFSHLLPSPEPS
PEGSYVGQHSQGLGGHYADSYLKRKRIF
>Q9CQS7
MMVTLVTRKDIPPWVKVPEDLKDPEVFQVQSLVLKYLFGPQGSRMSHIEQVSQAMFELKN
LESPEELIEVFIYGSQNNKIRAKWMLQSMAERYHLRQQKGVLKLEESMKTLELGQCIE
>Q9BPZ3
MKDPSRSSTSPSIINEDVIINGHSHEDDNPFAEYMWMENEEEFNRQIEEELWEEEFIERC
FQEMLEEEEEHEWFIPARDLPQTMDQIQDQFNDLVISDGSSLEDLVVKSNLNPNAKEFVP
GVKYGNI
>Q12387

MSDKIQEEILGLVSRSNFKQCYAKLGQLQKQFPNALYFKILETYVKFKQSPGKFDYNKLL
EEPYGLKGTTITGDTRSLEFLHNFFVELGKYDEALHVYERGNFKFPSYELSYHWFMKALE
DSNYNQMSKASLQLAKYSDSGNLPKRAYYFWNAISILAVSRFQENTLSDPKKILLSRLAR
QSLLDLKPFQNVQEIIVYCLVLDELFPQSREISEEIVAITFANFDTSVNLYLKNFILKHT
KLLNSPQKLFEVCSKLIEKGLDDYELITNLIDAAYKLSKSKDEVKQWIDENLGDSRNTRL
ARLKLDIMYTDSVSESSLSYYLSKYHNKPCCSIDLNHYSGHINIDMLKSIMSKYDPEDKD
LIHHCNILELGLIGSDSINNYNKFKGTLEKKSVTDYSSCSTFLLEIVKDKCKKTNPELKD
VLLCITILENYQAKDPHNFDTMCWLIVLYMYLGLVPDAYFHFINLKIKNVQTDSLDYMIF
SRFSTLFPNKQSDFYSKTFHEHNNLYDTSLANLPRYIQVAFERNSYSKILGMLEMRDKLM
KSYTRWTKTLENLQFSRLCNDKRGHLLQKLHEDWRSLEMTQSVSFSDNRDFSILDENFAQ
FLNRGKILEYANLNEESIFLTLIRELIIEALPNGEKTEQISALLKKLPSINLEELLNNNL
TEVESASFLIFFEIYENNGKNLHDLISRLMKVPINAKQNWMVSHTYLTKMATLKTLDSLK
RIKDKEIQKLIKNSLKELRSCCDDVFKGYSKALVQAYEELKKDECGNLLKELDVKAENVK
NIKNSLLGIQKSVRNL
>Q07362
MTTTSTTSVDGRTSSTLKATLSASGPNSNGPTPAVLPQKPKLTGWAQAAAKALPRQQQQQ
QQARKDDSVAVQPANTKTKTIASTAPPANIKGSSTANGSSTNKKFKRANKQPYNREEVRS
YMHKLFQSYTAGEKSHSMKTYKQVLSETASGRVSTATDWGTVSSSKNKNKKYGCLSDIAK
VLRNQ
>O95996
MASSVAPYEQLVRQVEALKAENSHLRQELRDNSSHLSKLETETSGMKEVLKHLQGKLEQE
ARVLVSSGQTEVLEQLKALQMDITSLYNLKFQPPTLGPEPAARTPEGSPVHGSGPSKDSF
GELSRATIRLLEELDRERCFLLNEIEKEEKEKLWYYSQLQGLSKRLDELPHVETQFSMQM
DLIRQQLEFEAQHIRSLMEERFGTSDEMVQRAQIRASRLEQIDKELLEAQDRVQQTEPQA
LLAVKSVPVDEDPETEVPTHPEDGTPQPGNSKVEVVFWLLSMLATRDQEDTARTLLAMSS
SPESCVAMRRSGCLPLLLQILHGTEAAAGGRAGAPGAPGAKDARMRANAALHNIVFSQPD
QGLARKEMRVLHVLEQIRAYCETCWDWLQARDGGPEGGGAGSAPIPIEPQICQATCAVMK
LSFDEEYRRAMNELGGLQAVAELLQVDYEMHKMTRDPLNLALRRYAGMTLTNLTFGDVAN
KATLCARRGCMEAIVAQLASDSEELHQVVSSILRNLSWRADINSKKVLREAGSVTALVQC
VLRATKESTLKSVLSALWNLSAHSTENKAAICQVDGALGFLVSTLTYKCQSNSLAIIESG
GGILRNVSSLVATREDYRQVLRDHNCLQTLLQHLTSHSLTIVSNACGTLWNLSARSARDQ
ELLWDLGAVGMLRNLVHSKHKMIAMGSAAALRNLLAHRPAKHQAAATAVSPGSCVPSLYV
RKQRALEAELDARHLAQALEHLEKQGPPAAEAATKKPLPPLRHLDGLAQDYASDSGCFDD
DDAPSSLAAAAATGEPASPAALSLFLGSPFLQGQALARTPPTRRGGKEAEKDTSGEAAVA
AKAKAKLALAVARIDQLVEDISALHTSSDDSFSLSSGDPGQEAPREGRAQSCSPCRGPEG
GRREAGSRAHPLLRLKAAHASLSNDSLNSGSASDGYCPREHMLPCPLAALASRREDPRCG
QPRPSRLDLDLPGCQAEPPAREATSADARVRTIKLSPTYQHVPLLEGASRAGAEPLAGPG
ISPGARKQAWLPADHLSKVPEKLAAAPLSVASKALQKLAAQEGPLSLSRCSSLSSLSSAG
RPGPSEGGDLDDSDSSLEGLEEAGPSEAELDSTWRAPGATSLPVAIPAPRRNRGRGLGVE
DATPSSSSENYVQETPLVLSRCSSVSSLGSFESPSIASSIPSEPCSGQGSGTISPSELPD
SPGQTMPPSRSKTPPLAPAPQGPPEATQFSLQWESYVKRFLDIADCRERCRLPSELDAGS
VRFTVEKPDENFSCASSLSALALHEHYVQQDVELRLLPSACPERGGGAGGAGLHFAGHRR
REEGPAPTGSRPRGAADQELELLRECLGAAVPARLRKVASALVPGRRALPVPVYMLVPAP
APAQEDDSCTDSAEGTPVNFSSAASLSDETLQGPPRDQPGGPAGRQRPTGRPTSARQAMG

HRHKAGGAGRSAEQSRGAGKNRAGLELPLGRPPSAPADKDGSKPGRTRGDGALQSLCLTT
PTEEAVYCFYGNDSDEEPPAAAPTPTHRRTSAIPRAFTRERPQGRKEAPAPSKAAPAAPP
PARTQPSLIADETPPCYSLSSSASSLSEPEPSEPPAVHPRGREPAVTKDPGPGGGRDSSP
SPRAAEELLQRCISSALPRRRPPVSGLRRRKPRATRLDERPAEGSRERGEEAAGSDRASD
LDSVEWRAIQEGANSIVTWLHQAAAATREASSESDSILSFVSGLSVGSTLQPPKHRKGRQ
AEGEMGSARRPEKRGAASVKTSGSPRSPAGPEKPRGTQKTTPGVPAVLRGRTVIYVPSPA
PRAQPKGTPGPRATPRKVAPPCLAQPAAPAKVPSPGQQRSRSLHRPAKTSELATLSQPPR
SATPPARLAKTPSSSSSQTSPASQPLPRKRPPVTQAAGALPGPGASPVPKTPARTLLAKQ
HKTQRSPVRIPFMQRPARRGPPPLARAVPEPGPRGRAGTEAGPGARGGRLGLVRVASALS
SGSESSDRSGFRRQLTFIKESPGLRRRRSELSSAESAASAPQGASPRRGRPALPAVFLCS
SRCEELRAAPRQGPAPARQRPPAARPSPGERPARRTTSESPSRLPVRAPAARPETVKRYA
SLPHISVARRPDGAVPAAPASADAARRSSDGEPRPLPRVAAPGTTWRRIRDEDVPHILRS
TLPATALPLRGSTPEDAPAGPPPRKTSDAVVQTEEVAAPKTNSSTSPSLETREPPGAPAG
GQLSLLGSDVDGPSLAKAPISAPFVHEGLGVAVGGFPASRHGSPSRSARVPPFNYVPSPM
VVAATTDSAAEKAPATASATLLE
>Q16222
MNINDLKLTLSKAGQEHLLRFWNELEEAQQVELYAELQAMNFEELNFFFQKAIEGFNQSS
HQKNVDARMEPVPREVLGSATRDQDQLQAWESEGLFQISQNKVAVLLLAGGQGTRLGVAY
PKGMYDVGLPSRKTLFQIQAERILKLQQVAEKYYGNKCIIPWYIMTSGRTMESTKEFFTK
HKYFGLKKENVIFFQQGMLPAMSFDGKIILEEKNKVSMAPDGNGGLYRALAAQNIVEDME
QRGIWSIHVYCVDNILVKVADPRFIGFCIQKGADCGAKVVEKTNPTEPVGVVCRVDGVYQ
VVEYSEISLATAQKRSSDGRLLFNAGNIANHFFTVPFLRDVVNVYEPQLQHHVAQKKIPY
VDTQGQLIKPDKPNGIKMEKFVFDIFQFAKKFVVYEVLREDEFSPLKNADSQNGKDNPTT
ARHALMSLHHCWVLNAGGHFIDENGSRLPAIPRSATNGKSETITADVNHNLKDANDVPIQ
CEISPLISYAGEGLESYVADKEFHAPLIIDENGVHELVKNGI
>P53333
MSTATIQDEDIKFQRENWEMIRSHVSPIISNLTMDNLQESHRDLFQVNILIGRNIICKNV
VDFTLNKQNGRLIPALSALIALLNSDIPDIGETLAKELMLMFVQQFNRKDYVSCGNILQC
LSILFLYDVIHEIVILQILLLLLEKNSLRLVIAVMKICGWKLALVSKKTHDMIWEKLRYI
LQTQELSSTLRESLETLFEIRQKDYKSGSQGLFILDPTSYTVHTHSYIVSDEDEANKELG
NFEKCENFNELTMAFDTLRQKLLINNTSDTNEGSNSQLQIYDMTSTNDVEFKKKIYLVLK
SSLSGDEAAHKLLKLKIANNLKKSVVDIIIKSSLQESTFSKFYSILSERMITFHRSWQTA
YNETFEQNYTQDIEDYETDQLRILGKFWGHLISYEFLPMDCLKIIKLTEEESCPQGRIFI
KFLFQELVNELGLDELQLRLNSSKLDGMFPLEGDAEHIRYSINFFTAIGLGLLTEDMRSR
LTIIQEVEDAEEEEKKLREEEELEKLRKKARESQPTQGPKIHESRLFLQKDTRENSRSRS
PFTVETRKRARSRTPPRGSRNHRNRSRTPPARRQRHR
>P61758
MAAVKDSCGKGEMATGNGRRLHLGIPEAVFVEDVDSFMKQPGNETADTVLKKLDEQYQKY
KFMELNLAQKKRRLKGQIPEIKQTLEILKYMQKKKESTNSMETRFLLADNLYCKASVPPT
DKMCLWLGANVMLEYDIDEAQALLEKNLSTATKNLDSLEEDLDFLRDQFTTTEVNMARVY
NWDVKRRNKDDSTKNKA
>P51003
MPFPVTTQGSQQTQPPQKHYGITSPISLAAPKETDCVLTQKLIETLKPFGVFEEEEELQR
RILILGKLNNLVKEWIREISESKNLPQSVIENVGGKIFTFGSYRLGVHTKGADIDALCVA

PRHVDRSDFFTSFYDKLKLQEEVKDLRAVEEAFVPVIKLCFDGIEIDILFARLALQTIPE
DLDLRDDSLLKNLDIRCIRSLNGCRVTDEILHLVPNIDNFRLTLRAIKLWAKRHNIYSNI
LGFLGGVSWAMLVARTCQLYPNAIASTLVHKFFLVFSKWEWPNPVLLKQPEECNLNLPVW
DPRVNPSDRYHLMPIITPAYPQQNSTYNVSVSTRMVMVEEFKQGLAITDEILLSKAEWSK
LFEAPNFFQKYKHYIVLLASAPTEKQRLEWVGLVESKIRILVGSLEKNEFITLAHVNPQS
FPAPKENPDKEEFRTMWVIGLVFKKTENSENLSVDLTYDIQSFTDTVYRQAINSKMFEVD
MKIAAMHVKRKQLHQLLPNHVLQKKKHSTEGVKLTALNDSSLDLSMDSDNSMSVPSPTS
ATKTSPLNSSGSSQGRNSPAPAVTAASVTNIQATEVSVPQVNSSESSGGTSSESIPQTAT
QPAISPPPKPTVSRVVSSTRLVNPPPRSSGNAATSGNAATKIPTPIVGVKRTSSPHKEES
PKKTKTEEDETSEDANCLALSGHDKTEAKEQLDTETSTTQSETIQTAASLLASQKTSSTD
LSDIPALPANPIPVIKNSIKLRLNR
>Q9UDY8
MSLLGDPLQALPPSAAPTGPLLAPPAGATLNRLREPLLRRLSELLDQAPEGRGWRRLAEL
AGSRGRLRLSCLDLEQCSLKVLEPEGSPSLCLLKLMGEKGCTVTELSDFLQAMEHTEVLQ
LLSPPGIKITVNPESKAVLAGQFVKLCCRATGHPFVQYQWFKMNKEIPNGNTSELIFNAV
HVKDAGFYVCRVNNNFTFEFSQWSQLDVCDIPESFQRSVDGVSESKLQICVEPTSQKLMP
GSTLVLQCVAVGSPIPHYQWFKNELPLTHETKKLYMVPYVDLEHQGTYWCHVYNDRDSQD
SKKVEIIIGRTDEAVECTEDELNNLGHPDNKEQTTDQPLAKDKVALLIGNMNYREHPKLK
APLVDVYELTNLLRQLDFKVVSLLDLTEYEMRNAVDEFLLLLDKGVYGLLYYAGHGYENF
GNSFMVPVDAPNPYRSENCLCVQNILKLMQEKETGLNVFLLDMCRKRNDYDDTIPILDAL
KVTANIVFGYATCQGAEAFEIQHSGLANGIFMKFLKDRLLEDKKITVLLDEVAEDMGKCH
LTKGKQALEIRSSLSEKRALTDPIQGTEYSAESLVRNLQWAKAHELPESMCLKFDCGVQI
QLGFAAEFSNVMIIYTSIVYKPPEIIMCDAYVTDFPLDLDIDPKDANKGTPEETGSYLVS
KDLPKHCLYTRLSSLQKLKEHLVFTVCLSYQYSGLEDTVEDKQEVNVGKPLIAKLDMHRG
LGRKTCFQTCLMSNGPYQSSAATSGGAGHYHSLQDPFHGVYHSHPGNPSNVTPADSCHCS
RTPDAFISSFAHHASCHFSRSNVPVETTDEIPFSFSDRLRISEK
>Q04868
MGSVQRQDLVLFSDQSVLPAHFFQDSNSHNLFFITHQSCTQPLWMINALVETHVLGSPSS
LNESSSSMLPSSTRSHAVLASFIHEQNYFTNSLNKLKIPSNNYNVLDFLSDFIVNNIHNK
PRDKILSDVLAKFSAAIQNNPTDTIVIIEQPELLLSLVSGLTCSELNNKFITPLLRQCKV
LIIVSNSDIFNIDEYDASVHSSNLQNFYKSSFIKSMINLNLNPLKTGFAKDVTGSLHVCR
GGAPIATSNTSLHVVENEYLYLNEKESTKLFYR
>P25655
MLSATYRDLNTASNLETSKEKQAAQIVIAQISLLFTTLNNDNFESVEREIRHILDRSSVD
IYIKVWERLLTLSSRDILQAGKFLLQENLLHRLLLEFAKDLPKKSTDLIELLKERTFNNQ
EFQKQTGITLSLFIDLFDKSANKDIIESLDRSSQINDFKTIKMNHTNYLRNFFLQTTPET
LESNLRDLLHSLEGESLNDLLALLLSEILSPGSQNLQNDPTRSWLTPPMVLDATNRGNVI
ARSISSLQANQINWNRVFNLMSTKYFLSAPLMPTTASLSCLFAALHDGPVIDEFFSCDWK
VIFKLDLAIQLHKWSVQNGCFDLLNAEGTRKVSETIPNTKQSLLYLLSIASLNLELFLQR
EELSDGPMLAYFQECFFEDFNYAPEYLILALVKEMKRFVLLIENRTVIDEILITLLIQVH
NKSPSSFKDVISTITDDSKIVDAAKIIINSDDAPIANFLKSLLDTGRLDTVINKLPFNEA
FKILPCARQIGWEGFDTFLKTKVSPSNVDVVLESLEVQTKMTDTNAPFRSLKTFDLFAFH
SLIEVLNKCPLDVLQLQRFESLEFSLLIRFPRLINFGFGHDEAILANGDIAGINNDIEKE
MQNYLQKMYSGELAIKDVIELLRRLRDSDLPRDQEVFTCITHAVIAESTFFQDYPLDALA

TTSVLFGSMILFQLLRGFVLDVAFRIIMRFAKEPPESKMFKFAVQAIYAFRIRLAEYPQY
CKDLLRDVPALKSQAQVYQSIVEAATLANAPKERSRPVQEMIPLKFFAVDEVSCQINQEG
APKDVVEKVLFVLNNVTLANLNNKVDELKKSLTPNYFSWFSTYLVTQRAKTEPNYHDLYS
KVIVAMGSGLLHQFMVNVTLRQLFVLLSTKDEQAIDKKHLKNLASWLGCITLALNKPIKH
KNIAFREMLIEAYKENRLEIVVPFVTKILQRASESKIFKPPNPWTVGILKLLIELNEKAN
WKLSLTFEVEVLLKSFNLTTKSLKPSNFINTPEVIETLSGALGSITLEQQQTEQQRQIIL
MQQHQQQMLIYQQRQQQQQQRQQQQQHHISANTIADQQAAFGGEGSISHDNPFNNLLGST
IFVTHPDLKRVFQMALAKSVREILLEVVEKSSGIAVVTTTKIILKDFATEVDESKLKTAA
IIMVRHLAQSLARATSIEPLKEGIRSTMQSLAPNLMSLSSSPAEELDTAINENIGIALVL
IEKASMDKSTQDLADQLMQAIAIRRYHKERRADQPFITQNTNPYSLSLPEPLGLKNTGVT
PQQFRVYEEFGKNIPNLDVIPFAGLPAHAPPMTQNVGLTQPQQQQAQMPTQILTSEQIRA
QQQQQQLQKSRLNQPSQSAQPPGVNVPNPQGGIAAVQSDLEQNQRVLVHLMDILVSQIKE
NATKNNLAELGDQNQIKTIIFQILTFIAKSAQKDQLALKVSQAVVNSLFATSESPLCREV
LSLLLEKLCSLSLVARKDVVWWLVYALDSRKFNVPVIRSLLEVNLIDATELDNVLVTAMK
NKMENSTEFAMKLIQNTVLSDDPILMRMDFIKTLEHLASSEDENVKKFIKEFEDTKIMPV
RKGTKTTRTEKLYLVFTEWVKLLQRVENNDVITTVFIKQLVEKGVISDTDNLLTFVKSSL
ELSVSSFKESDPTDEVFIAIDALGSLIIKLLILQGFKDDTRRDYINAIFSVIVLVFAKDH
SQEGTTFNERPYFRLFSNILYEWATIRTHNFVRISDSSTRQELIEFDSVFYNTFSGYLHA
LQPFAFPGFSFAWVTLLSHRMLLPIMLRLPNKIGWEKLMLLIIDLFKFLDQYTSKHAVSD
AVSVVYKGTLRVILGISNDMPSFLIENHYELMNNLPPTYFQLKNVILSAIPKNMTVPNPY
DVDLNMEDIPACKELPEVFFDPVIDLHSLKKPVDNYLRIPSNSLLRTILSAIYKDTYDIK
KGVGYDFLSVDSKLIRAIVLHVGIEAGIEYKRTSSNAVFNTKSSYYTLLFNLIQNGSIEM
KYQIILSIVEQLRYPNIHTYWFSFVLMNMFKSDEWNDQKLEVQEIILRNFLKRIIVNKPH
TWGVSVFFTQLINNNDINLLDLPFVQSVPEIKLILQQLVKYSKKYTTSEQDDQSATINRR
QTPLQSNA
>Q9BZJ0
MTATVENLTFQKDTLGNAVDKNTSRLELRSYSLAGRHGSTEPLVLAWSSQFRRLTWGCAL
DALHRSPCVAASQHGVTHLIRSSRTPHSTRCRKEDAQPGHHGNGAASVTAQARGQRSVLQ
VPLPVPRSCLFSESFVVSVSSQSRFLASVPGTGVQRSTAADMAASTAAGKQRIPKVAKVK
NKAPAEVQITAEQLLREAKERELELLPPPPQQKITDEEELNDYKLRKRKTFEDNIRKNRT
VISNWIKYAQWEESLKEIQRARSIYERALDVDYRNITLWLKYAEMEMKNRQVNHARNIWD
RAITTLPRVNQFWYKYTYMEEMLGNVAGARQVFERWMEWQPEEQAWHSYINFELRYKEVD
RARTIYERFVLVHPDVKNWIKYARFEEKHAYFAHARKVYERAVEFFGDEHMDEHLYVAFA
KFEENQKEFERVRVIYKYALDRISKQDAQELFKNYTIFEKKFGDRRGIEDIIVSKRRFQY
EEEVKANPHNYDAWFDYLRLVESDAEAEAVREVYERAIANVPPIQEKRHWKRYIYLWINY
ALYEELEAKDPERTRQVYQASLELIPHKKFTFAKMWILYAQFEIRQKNLSLARRALGTSI
GKCPKNKLFKVYIELELQLREFDRCRKLYEKFLEFGPENCTSWIKFAELETILGDIDRAR
AIYELAISQPRLDMPEVLWKSYIDFEIEQEETERTRNLYRRLLQRTQHVKVWISFAQFEL
SSGKEGSLTKCRQIYEEANKTMRNCEEKEERLMLLESWRSFEEEFGTASDKERVDKLMPE
KVKKRRKVQTDDGSDAGWEEYFDYIFPEDAANQPNLKLLAMAKLWKKQQQEKEDAEHHPD
EDVDESES
>Q9T052
MAYHPVYNETMSMGGGSSNEFGQWLDKQLVPFDTSSGSLRVELLHGNLDIWVKEAKHLPN
MDGFHNTLVGGMFFGLGRRNHKVDGENSSKITSDPYVTVSISGAVIGRTFVISNSENPVW

MQHFDVPVAHSAAKVHFVVKDSDIIGSQIIGAVEIPTEQLCSGNRIEGLFPILNSRGKPC
KQGAVLSLSIQYIPMERMRLYQKGVGFGVECVGVPGTYFPLRKGGRVTLYQDAHVDDGTL
PSVHLDGGIQYRHGKCWEDMADAIRRARRLIYITGWSVFHPVRLVRRNNDPTQGTLGELL
KVKSQEGVRVLVLVWDDPTSRSLLGFSTKGLMNTSDEETRRFFKHSSVQVLLCPRYGGKG
HSFIKKSEVETIYTHHQKTMIVDAEAAQNRRKIVAFVGGLDLCNGRFDTPKHPLFRTLKT
IHKDDFHNPNFVTTADDGPREPWHDLHSKIDGPAAYDVLANFEERWMKASKPRGIGRLRT
SSDDSLLRLDRIPDIMGLSEASSANDNDPESWHVQVFRSIDSSSVKGFPKDPKEATGRNL
LCGKNILIDMSIHAAYVKAIRSAQHFIYIENQYFLGSSFNWDSNKNLGANNLIPMEIALK
IANKIRAREKFAAYIVIPMWPEGAPTSNPIQRILYWQHKTMQMMYQTIYKALVEVGLDGQ
LEPQDFLNFFCLGTREVGTREVPDGTVSVYNSPRKPPQLNAAQVQALKSRRFMIYVHSKG
MVVDDEFVLIGSANINQRSLEGTRDTEIAMGGYQPHHSWAKKGSRPRGQIFGYRMSLWAE
HLGFLEQEFEEPENMECVRRVRQLSELNWRQYAAEEVTEMPGHLLKYPVQVDRTGKVSSL
PGYETFPDLGGKIIGSFLVVEENLTI
>Q9LK23
MGSGQWHMEKRSTLKNDSFVKEYNPVTETGSLSIIVLGASGDLAKKKTFPALFNLFHQGF
LNPDEVHIFGYARSKITDEELRDKIRGYLVDEKNASKKTEALSKFLKLIKYVSGPYDSEE
GFKRLDKAILEHEISKKTAEGSSRRLFYLALPPSVYPPVSKMIKAWCTNKSDLGGWTRIV
VEKPFGKDLESAEQLSSQIGALFEEPQIYRIDHYLGKELVQNMLVLRFANRLFLPLWNRD
NIANVQIVFREDFGTEGRGGYFDEYGIIRDIIQNHLLQVLCLVAMEKPISLKPEHIRDEK
VKVLQSVIPIKDEEVVLGQYEGYRDDPTVPNDSNTPTFATTILRINNERWEGVPFILKAG
KAMSSKKADIRIQFKDVPGDIFKCQNQGRNEFVIRLQPSEAMYMKLTVKQPGLEMQTVQS
ELDLSYKQRYQDVSIPEAYERLILDTIRGDQQHFVRRDELKAAWEIFTPLLHRIDKGEVK
SVPYKQGSRGPAEADQLLKKAGYMQTHGYIWIPPTL
>Q923K9
MESNHKSGDGLSGTQKEAALRALVQRTGYSLVQENGQRKYGGPPPGWDTTPPERGCEIFI
GKLPRDLFEDELIPLCEKIGKIYEMRMMMDFNGNNRGYAFVTFSNKQEAKNAIKQLNNYE
IRNGRLLGVCASVDNCRLFVGGIPKTKKREEILSEMKKVTEGVVDVIVYPSAADKTKNRG
FAFVEYESHRAAAMARRRLLPGRIQLWGHPIAVDWAEPEVEVDEDTMSSVKILYVRNLML
STSEEMIEKEFNSIKPGAVERVKKIRDYAFVHFSNREDAVEAMKALNGKVLDGSPIEVTL
AKPVDKDSYVRYTRGTGGRNTMLQEYTYPLSHVYDPTTTYLGAPVFYTPQAYAAIPSLHF
PATKGHLSNRALIRTPSVREIYMNVPVGAAGVRGLGGRGYLAYTGLGRGYQVKGDKRQDK
LYDLLPGMELTPMNTISLKPQGVKLAPQILEEICQKNNWGQPVYQLHSAIGQDQRQLFLY
KVTIPALASQNPAIHPFTPPKLSAYVDEAKRYAAEHTLQTLGIPTEGGDAGTTAPTATSA
TVFPGYAVPSATAPVSTAQLKQAVTLGQDLAAYTTYEVYPTFAVTTRGDGYGTF
>O74741
MRSVPQLERLIPALYLAHGSPFLMLPQSSDDEVFSNDSKLGGLHYQFLEQLGPFLLEKFR
PKGIIVFSAHYESRGSVEVYSRDDENPLFYDYYGFPDYLYQIKFHSKGSKRIADQIISAL
KEYQIPAKTVSGDRGLDHGVFVPFKIMFPDGLNIPLIEVSMHTLDPMQLYKVGQALQSLR
KEYLIVSGGLNIHTFEDLSAFNEDTAADGYKEFQLDILKAIETDKQNDRLNKLLGLQLHP
YFRKAHPREEHFVPLYVAAGLGSSGKSKVVCDLYGAVSAFFGIDE
>P32316
MTISNLLKQRVRYAPYLKKVKEAHELIPLFKNGQYLGWSGFTGVGTPKAVPEALIDHVEK
NNLQGKLRFNLFVGASAGPEENRWAEHDMIIKRAPHQVGKPIAKAINQGRIEFFDKHLSM
FPQDLTYGFYTRERKDNKILDYTIIEATAIKEDGSIVPGPSVGGSPEFITVSDKVIIEVN

TATPSFEGIHDIDMPVNPPFRKPYPYLKVDDKCGVDSIPVDPEKVVAIVESTMRDQVPPN
TPSDDMSRAIAGHLVEFFRNEVKHGRLPENLLPLQSGIGNIANAVIEGLAGAQFKHLTVW
TEVLQDSFLDLFENGSLDYATATSVRLTEKGFDRAFANWENFKHRLCLRSQVVSNNPEMI
RRLGVIAMNTPVEVDIYAHANSTNVNGSRMLNGLGGSADFLRNAKLSIMHAPSARPTKVD
PTGISTIVPMASHVDQTEHDLDILVTDQGLADLRGLSPKERAREIINKCAHPDYQALLTD
YLDRAEHYAKKHNCLHEPHMLKNAFKFHTNLAEKGTMKVDSWEPVD
>P39549
MNKYLFDHKIWSTPYYFYCEEDCHRLFLSFIEGRTFEKPTSNAEENVQETEAGESFTLNP
GEDFQNCFPRQRIL
>P37840
MDVFMKGLSKAKEGVVAAAEKTKQGVAEAAGKTKEGVLYVGSKTKEGVVHGVATVAEKTK
EQVTNVGGAVVTGVTAVAQKTVEGAGSIAAATGFVKKDQLGKNEEGAPQEGILEDMPVDP
DNEAYEMPSEEGYQDYEPEA
>P07511
MATAVNGAPRDAALWSSHEQMLAQPLKDSDAEVYDIIKKESNRQRVGLELIASENFASRA
VLEALGSCLNNKYSEGYPGQRYYGGTEHIDELETLCQKRALQAYGLDPQCWGVNVQPYSG
SPANFAVYTALVEPHGRIMGLDLPDGGHLTHGFMTDKKKISATSIFFESMAYKVNPDTGY
IDYDRLEENARLFHPKLIIAGTSCYSRNLDYGRLRKIADENGAYLMADMAHISGLVVAGV
VPSPFEHCHVVTTTTHKTLRGCRAGMIFYRRGVRSVDPKTGKEILYNLESLINSAVFPGL
QGGPHNHAIAGVAVALKQAMTPEFKEYQRQVVANCRALSAALVELGYKIVTGGSDNHLIL
VDLRSKGTDGGRAEKVLEACSIACNKNTCPGDKSALRPSGLRLGTPALTSRGLLEKDFQK
VAHFIHRGIELTVQIQDDTGPRATLKEFKEKLAGDEKHQRAVRALRQEVESFAALFPLPG
LPGF
>Q9NRC8
MAAGGLSRSERKAAERVRRLREEQQRERLRQVSRILRKAAAERSAEEGRLLAESADLVTE
LQGRSRRREGLKRRQEEVCDDPEELRGKVRELASAVRNAKYLVVYTGAGISTAASIPDYR
GPNGVWTLLQKGRSVSAADLSEAEPTLTHMSITRLHEQKLVQHVVSQNCDGLHLRSGLPR
TAISELHGNMYIEVCTSCVPNREYVRVFDVTERTALHRHQTGRTCHKCGTQLRDTIVHFG
ERGTLGQPLNWEAATEAASRADTILCLGSSLKVLKKYPRLWCMTKPPSRRPKLYIVNLQW
TPKDDWAALKLHGKCDDVMRLLMAELGLEIPAYSRWQDPIFSLATPLRAGEEGSHSRKSL
CRSREEAPPGDRGAPLSSAPILGGWFGRGCTKRTKRKKVT
>Q13901
MAGEEINEDYPVEIHEYLSAFENSIGAVDEMLKTMMSVSRNELLQKLDPLEQAKVDLVSA
YTLNSMFWVYLATQGVNPKEHPVKQELERIRVYMNRVKEITDKKKAGKLDRGAASRFVKN
ALWEPKSKNASKVANKGKSKS
>P46414
MSNVRVSNGSPSLERMDARQADHPKPSACRNLFGPVNHEELTRDLEKHCRDMEEASQRKW
NFDFQNHKPLEGRYEWQEVERGSLPEFYYRPPRPPKSACKVLAQESQDVSGSRQAVPLIG
SQANSEDRHLVDQMPDSSDNQAGLAEQCPGMRKRPAAEDSSSQNKRANRTEENVSDGSPN
AGTVEQTPKKPGLRRQT
>Q14978
MADAGIRRVVPSDLYPLVLGFLRDNQLSEVANKFAKATGATQQDANASSLLDIYSFWLKS
AKVPERKLQANGPVAKKAKKKASSSDSEDSSEEEEEVQGPPAKKAAVPAKRVGLPPGKAA
AKASESSSSEESSDDDDEEDQKKQPVQKGVKPQAKAAKAPPKKAKSSDSDSDSSSEDEPP

KNQKPKITPVTVKAQTKAPPKPARAAPKIANGKAASSSSSSSSSSSSSDDSEEEKAAATPK
KTVPKKQVVAKAPVKAATTPTRKSSSSEDSSSDEEEEQKKPMKNKPGPYSSVPPPSAPPP
KKSLGTQPPKKAVEKQQPVESSEDSSDESDSSSEEEKKPPTKAVVSKATTKPPPAKKAAE
SSSDSSDSDSSEDDEAPSKPAGTTKNSSNKPAVTTKSPAVKPAAAPKQPVGGGQKLLTRK
ADSSSSEEESSSSEEEKTKKMVATTKPKATAKAALSLPAKQAPQGSRDSSSDSDSSSSEE
EEEKTSKSAVKKKPQKVAGGAAPSKPASAKKGKAESSNSSSSDDSSEEEEEKLKGKGSPR
PQAPKANGTSALTAQNGKAAKNSEEEEEEKKKAAVVVSKSGSLKKRKQNEAAKEAETPQA
KKIKLQTPNTFPKRKKGEKRASSPFRRVREEEIEVDSRVADNSFDAKRGAAGDWGERANQ
VLKFTKGKSFRHEKTKKKRGSYRGGSISVQVNSIKFDSE
>Q8N6F7
MGNSLLRENRRQQNTQEMPWNVRMQSPKQRTSRCWDHHIAEGCFCLPWKKILIFEKRQDS
QNENERMSSTPIQDNVDQTYSEELCYTLINHRVLCTRPSGNSAEEYYENVPCKAERPRES
LGGTETEYSLLHMPSTDPRHARSPEDEYELLMPHRISSHFLQQPRPLMAPSETQFSHL
>O74397
MCGILFALAEDRDNLLESPSFSALRKRIRARGPDFFGKHKLNSGPWNLHFESSVLHLRGP
SDHLTPQPHVDSFGNVLCWNGEIWQINHSDHHKFTLNRNENDGAKLFELLNNNPGDIEKI
LGSIQGPFAFVYYQVRTNTLWWGRDRLGRRSLLYSLRDSNFVLSSVGNSSDFREVEPGFH
SVQVDLIPKINFSKSLMQLEKTNPLPPVDESFLMGSISSDTVDTLHTYLIKALQDRVLTI
PKLCSSNLDCSHYSRVCVLYSGGVDCGVLARLMHDIVPNNESIDLINVAFENPRFTETYR
DRETGLLPDNFDAYDVCPDRQTGLQGWQELISVCPARKWNFVAINVPYTEVCEAQEIVKT
LIYPNDSVMDLSIGLAFYFASQGRGVLLQNVDDIKLKHQLPSYTIKAKVLISGLGADEQL
GGYMRHLRAFERKGMAGLEEELQLDIDRIPHRNLGRDDRVMSNQGKEVRYPFLDERLMAL
FSKMKTTDKMRLDLVGGDKLILRELGRRLGCPLASQEKKRAIQFGSKAAKMTPGTGRTSG
RKKLEVCI
>P00915
MASPDWGYDDKNGPEQWSKLYPIANGNNQSPVDIKTSETKHDTSLKPISVSYNPATAKEI
INVGHSFHVNFEDNDNRSVLKGGPFSDSYRLFQFHFHWGSTNEHGSEHTVDGVKYSAELH
VAHWNSAKYSSLAEAASKADGLAVIGVLMKVGEANPKLQKVLDALQAIKTKGKRAPFTNF
DPSTLLPSSLDFWTYPGSLTHPPLYESVTWIICKESISVSSEQLAQFRSLLSNVEGDNAV
PMQHNNRPTQPLKGRTVRASF
>O74465
MEQVQDEIWKLSTLDAWEMVNKNTEVVFDEIPEPETLSEMKRHPLYSNIFNADNNTTSFT
EQIETSETSKTQDSEGNKVDKNLKENKSIRRKRSIDNDYELSNVKRNDITSGKNREFENE
HHPASDTSSWRELPSIPTLEELTSKSVELPSNNIYGGYKSFEDYLSIHYRLLREDAVSPL
RESVLRYKVNPNYITGSSLAVYDHVRIDGYTISSSVIAAKLSFSVRAKKKIKWATSRRLI
SGSLVLLSNDDFQTFRIGTVCARPLSGLNKHPHEIDVKFEDISISLDPREEYVMIEATSG
YWEAYKHVLRSLQRLSASTFPMKDYLVHCKSNQETAKHIQNNPRIRINSILKNNSQKIVN
ALEPFGPGEYILDSSQLKAYQSMLTKRLSIIQGPPGTGKSFVTLKAIETLLENTHSHVLP
ILVACQTNHAVDQILIRLLHQGASVMRLGSRTKDPEIAAVTIFQKAKHTKHSFKAAYNEI
RHKKQRLIKQITNIMHNFNLEFVTLSYLHSKGIITTSQLESLRNNTEWISSVAENGEKTE
EELISIWLGDAKVELITPSEITDGFEEELQIDPEKLEEIQKEAEDSGALMEEELRGKFIN
LRCKYLFSKLTTLHEKEIDTLLTIPNIWDIPEYSRGIIYCRWLESAYAAAEKELNRLYRF
YLKVDRERIGFSNKRAAILLRGANVIGMTTTGLNKYRDILERINPKICFIEEAADVLEGP
IIPAVFPSLEQLVLIGDHKQLRPGCSTYALRQDPFNLSISMFERLVENDMEYTRLTMQRR

MHPQIRRLVSSVYEDLSDYEITKYWPSIPGMGEIRRFFLTHSRIEDNDGFASKINLFEAQ
MLVQFAVYLINNGVEPQKITCLTFYAAQKDLIERLLSESLNREKHFIKVATVDGYQGEEN
DVVLLSLVRNNDRTEVGFLSSPHRVCVSLSRARRGLFIFGNAQLVAESNPLWWDAINTLM
NDETIQGLGDHLPLFTKDGTIYVNGKKSKQFQFNFLTHSVDPVELLDVNMRLTRK
>Q86UB2
MPNVAETERSNDSGNGEHKSERKSPEENLQGAVKSFCTSASGAPLGPKGDGHYPWSCPVT
HTREKIYAICSDYAFLNQATSIYKTPNPSRSPCLPDSTSLSAGNNSSRYIGIPTSTSEII
YNEENSLENLSNSLGKLPLAWEIDKSEFDGVTTNSKHKSGNAKKQVSKRKTSDKKGRYQK
ECPQHSPLEDIKQRKVLDLRRWYCISRPQYKTSCGISSLISCWNFLYSTMGAGNLPPITQ
EEALHILGFQPPFEDIRFGPFTGNTTLMRWFRQINDHFHVKGCSYVLYKPHGKNKTAGET
ASGALSKLTRGLKDESLAYIYHCQNHYFCPIGFEATPVKANKAFSRGPLSPQEVEYWILI
GESSRKHPAIHCKKWADIVTDLNTQNPEYLDIRHLERGLQYRKTKKVGGNLHCIIAFQRL
NWQRFGLWNFPFGTIRQESQPPTHAQGIAKSESEDNISKKQHGRLGRSFSASFHQDSAWK
KMSSIHERRNSGYQGYSDYDGND
>P34937
MGRKFFVGGNWKCNGTVEQVEAIVQTLNAGQIVSPDVVEVVVSPPYVFLPIVKAKLRPEI
QVAAQNCWVKKGGAFTGEVSAEMLANLGVPWVILGHSERRSLLGESSEFVGEKVAYALAQ
GLKVIACVGETLEQREAGSTMEVVAEQTKAIAGKIKDWSNGVVAYEPVWAIGTGKVATPA
QAQEVHANLRDWLKTNVSPEVAESTRIIYGGSVTGASCKELAAQADVDGFLVGGASLKPE
FIDIINAAAVKSA
>O95359
MGNENSTSDNQRTLSAQTPRSAQPPGNSQNIKRKQQDTPGSPDHRDASSIGSVGLGGFCT
ASESSASLDPCLVSPEVTEPRKDPQGARGPEGSLLPSPPPSQEREHPSSSMPFAECPPEG
CLASPAAAPEDGPQTQSPRREPAPNAPGDIAAAFPAERDSSTPYQEIAAVPSAGRERQPK
EEGQKSSFSFSSGIDQSPGMSPVPLREPMKAPLCGEGDQPGGFESQEKEAAGGFPPAESR
QGVASVQVTPEAPAAAQQGTESSAVLEKSPLKPMAPIPQDPAPRASDRERGQGEAPPQYL
TDDLEFLRACHLPRSNSGAAPEAEVNAASQESCQQPVGAYLPHAELPWGLPSPALVPEAG
GSGKEALDTIDVQGHPQTGMRGTKPNQVVCVAAGGQPEGGLPVSPEPSLLTPTEEAHPAS
SLASFPAAQIPIAVEEPGSSSRESVSKAGMPVSADAAKEVVDAGLVGLERQVSDLGSKGE
HPEGDPGEVPAPSPQERGEHLNTEQSHEVQPGVPPPPLPKEQSHEVQPGAPPPPLPKAPS
ESARGPPGPTDGAKVHEDSTSPAVAKEGSRSPGDSPGGKEEAPEPPDGGDPGNLQGEDSQ
AFSSKRDPEVGKDELSKPSSDAESRDHPSSHSAQPPRKGGAGHTDGPHSQTAEADASGLP
HKLGEEDPVLPPVPDGAGEPTVPEGAIWEGSGLQPKCPDTLQSREGLGRMESFLTLESEK
SDFPPTPVAEVAPKAQEGESTLEIRKMGSCDGEGLLTSPDQPRGPACDASRQEFHAGVPH
PPQGENLAADLGLTALILDQDQQGIPSCPGEGWIRGAASEWPLLSSEKHLQPSQAQPETS
IFDVLKEQAQPPENGKETSPSHPGFKDQGADSSQIHVPVEPQEDNNLPTHGGQEQALGSE
LQSQLPKGTLSDTPTSSPTDMVWESSLTEESELSAPTRQKLPALGEKRPEGACGDGQSSR
VSPPAADVLKDFSLAGNFSRKETCCTGQGPNKSQQALADALEEGSQHEEACQRHPGASEA
ADGCSPLWGLSKREMASGNTGEAPPCQPDSVALLDAVPCLPALAPASPGVTPTQDAPETE
ACDETQEGRQQPVPAPQQKMECWATSDAESPKLLASFPSAGEQGGEAGAAETGGSAGAGD
PGKQQAPEKPGEATLSCGLLQTEHCLTSGEEASTSALRESCQAEHPMASCQDALLPAREL
GGIPRSTMDFSTHQAVPDPKELLLSGPPEVAAPDTPYLHVDSAAQRGAEDSGVKAVSSAD
PRAPGESPCPVGEPPLALENAASLKLFAGSLAPLLQPGAAGGEIPAVQASSGSPKARTTE
GPVDSMPCLDRMPLLAKGKQATGEEKAATAPGAGAKASGEGMAGDAAGETEGSMERMGEP

SQDPKQGTSGGVDTSSEQIATLTGFPDFREHIAKIFEKPVLGALATPGEKAGAGRSAVGK
DLTRPLGPEKLLDGPPGVDVTLLPAPPARLQVEKKQQLAGEAEISHLALQDPASDKLLGP
AGLTWERNLPGAGVGKEMAGVPPTLREDERPEGPGAAWPGLEGQAYSQLERSRQELASGL
PSPAATQELPVERAAAFQVAPHSHGEEAVAQDRIPSGKQHQETSACDSPHGEDGPGDFAH
TGVPGHVPRSTCAPSPQREVLTVPEANSEPWTLDTLGGERRPGVTAGILEMRNALGNQST
PAPPTGEVADTPLEPGKVAGAAGEAEGDITLSTAETQACASGDLPEAGTTRTFSVVAGDL
VLPGSCQDPACSDKAPGMEGTAALHGDSPARPQQAKEQPGPERPIPAGDGKVCVSSPPEP
DETHDPKLQHLAPEELHTDRESPRPGPSMLPSVPKKDAPRVMDKVTSDETRGAEGTESSP
VADDIIQPAAPADLESPTLAASSYHGDVVGQVSTDLIAQSISPAAAHAGLPPSAAEHIVS
PSAPAGDRVEASTPSCPDPAKDLSRSSDSEEAFETPESTTPVKAPPAPPPPPPEVIPEPE
VSTQPPPEEPGCGSETVPVPDGPRSDSVEGSPFRPPSHSFSAVFDEDKPIASSGTYNLDF
DNIELVDTFQTLEPRASDAKNQEGKVNTRRKSTDSVPISKSTLSRSLSLQASDFDGASSS
GNPEAVALAPDAYSTGSSSASSTLKRTKKPRPPSLKKKQTTKKPTETPPVKETQQEPDEE
SLVPSGENLASETKTESAKTEGPSPALLEETPLEPAVGPKAACPLDSESAEGVVPPASGG
GRVQNSPPVGRKTLPLTTAPEAGEVTPSDSGGQEDSPAKGLSVRLEFDYSEDKSSWDNQQ
ENPPPTKKIGKKPVAKMPLRRPKMKKTPEKLDNTPASPPRSPAEPNDIPIAKGTYTFDID
KWDDPNFNPFSSTSKMQESPKLPQQSYNFDPDTCDESVDPFKTSSKTPSSPSKSPASFEI
PASAMEANGVDGDGLNKPAKKKKTPLKTDTFRVKKSPKRSPLSDPPSQDPTPAATPETPP
VISAVVHATDEEKLAVTNQKWTCMTVDLEADKQDYPQPSDLSTFVNETKFSSPTEELDYR
NSYEIEYMEKIGSSLPQDDDAPKKQALYLMFDTSQESPVKSSPVRMSESPTPCSGSSFEE
TEALVNTAAKNQHPVPRGLAPNQESHLQVPEKSSQKELEAMGLGTPSEAIEITAPEGSFA
SADALLSRLAHPVSLCGALDYLEPDLAEKNPPLFAQKLQEELEFAIMRIEALKLARQIAL
ASRSHQDAKREAAHPTDVSISKTALYSRIGTAEVEKPAGLLFQQPDLDSALQIARAEIIT
KEREVSEWKDKYEESRREVMEMRKIVAEYEKTIAQMIEDEQREKSVSHQTVQQLVLEKEQ
ALADLNSVEKSLADLFRRYEKMKEVLEGFRKNEEVLKRCAQEYLSRVKKEEQRYQALKVH
AEEKLDRANAEIAQVRGKAQQEQAAHQASLRKEQLRVDALERTLEQKNKEIEELTKICDE
LIAKMGKS
>Q12754
MDQDKVAFLLELEDKLAKIRSQVNSKLENQKHIAIILTAVEENIAGQATNDVSKNIVNYI
ISFMSLLDQAVDPSTHEIKDIQLASSSTYLLDLIFHYSPKVLLRSKFSEILTKIAPCITA
EKANAPLIRAAIGCLESLLIAQDAQAWNNTYDLNVTPKRGLQGILELSLDVRPKVRKRAL
DAVHAVLLNPPVAPTAEHVAAVFVADFCDKQLAGILNDLSNLSNKQLKAQKTKEDINASV
MRSLRLITSVVSTGQWPSSQIEPLCDVLLGVTKSSEQYLVSASFECFESMFKTMAETTIS
SGLAENKYLRVLDTIFALKPSNVDTLLTKSWIAVVIKGMSTYATHQPLKALRKIPGVFHI
MCTYLASETPEVYQAASQCLISILSESVKDDLLLYTPSVDEKVFKNVDEIISQIAKTFID
FLSIRYSHCSREILKILVAAFNKFRYRSNPHFLKSLKIVDTWRVNEEQFMDLRNEIELVI
GASISAMGPEMILAEAPLNLDNPSSERPGRAWLLPLIRDYTKNANLATFQNELAPYIKSF
QSKFDKVPEESIQLRVFQTIVDQIWSTLPRFCELPMDLRESFTDEFASELSSLLYSEVEL
RTTICHALKVLAESNVSYAEESSSHNVLLLQRFPISEAQKNIEYLSTKSTNLLAVLFNVY
TQTTPNARSYILETIDQYLKITSKEDLEKTFNNVCGLLKNSMNEESSGNVNKEKKKPQLT
ATLLDLIICMITYLPVSSYSALFSMFSLTVNSADALIQKRAYRIITKLSELKSGSTAVAQ
FISDIENVMVDSASSVQTSAKAARLTAIKTIVELLPLDHLDFIVRTVAEVILSTKDVNEK
SRETAFDTLICMGRKMNEPNGIIKLFQIPGYDPTTPDQSSSISEFFKIISAGLIGESQHM
VSSSITGYACLVFEFKNELDSGILMDIYDTIELYLTSNSREIVKSAIGFTKVCVLGLPEE

LMRPKVPELLLKLLRWSHEHTGHFKAKVKHIIERLIRRFGYDYIEANFPEEDRRLLTNIR
KMRNRNKRKDEEVTTGVSDVAATKGSRFMSAFDEAVYGSDEENDNGSDQEENVAGGKMKN
GAKQFIVESGDNPLDLLDSQTLAHISSTRPKKFNKNQNRARFNDDAFNFDSEGKLVVKGQ
PKPSTNVDDPLSAVTSGINAYLEAVKSGPVRGQRNKLKFRKNGKDSDEFGDDDDGEKDSR
LMRGRVNQGNKIGKHNKKGPKFKSRKKL
>Q9Z266
MAAAGSAAVSGAGTPVAGPTGRDLFAEGLLEFLRPAVQQLDSHVHAVRESQVELREQIDN
LATELCRINEDQKVALDLDPYVKKLLNARRRVVLVNNILQNAQERLRRLNHSVAKETARR
RAMLDSGVYPPGSPSK
>Q5T1C6
MLRSCAARLRTLGALCLPPVGRRLPGSEPRPELRSFSSEEVILKDCSVPNPSWNKDLRLL
FDQFMKKCEDGSWKRLPSYKRTPTEWIQDFKTHFLDPKLMKEEQMSQAQLFTRSFDDGLG
FEYVMFYNDIEKRMVCLFQGGPYLEGPPGFIHGGAIATMIDATVGMCAMMAGGIVMTANL
NINYKRPIPLCSVVMINSQLDKVEGRKFFVSCNVQSVDEKTLYSEATSLFIKLNPAKSLT

>Q8NDI1
MASVWKRLQRVGKHASKFQFVASYQELMVECTKKWQPDKLVVVWTRRSRRKSSKAHSWQP
GIKNPYRGVVVWPVPENIEITVTLFKDPHAEEFEDKEWTFVIENESPSGRRKALATSSIN
MKQYASPMPTQTDVKLKFKPLSKKVVSAALQFSLSCIFLREGKATDEDMQSLASLMSMKQ
ADIGNLDDFEEDNEDDDENRVNQEEKAAKITGIVNQLNALSSLDEDQDDCIKQANMRSAK
SASSSEELINKLNFLDEAEKDLATVNSNPFDDPDAAELNPFGDPDSEEPITETASPRKTE
DSFYNNSYNPFKEVQTPQYLNPFDEPEAFVTIKDSPPQSTKRKNIRPVDMSKYLYADSSK
TEEEELDESNPFYEPKSTPPPNNLVNPVQELETERRVKRKAPAPPVLSPKTGVLNENTVS
AGKDLSTSPKPSPIPSPVLGRKPNASQSLLVWCKEVTKNYRGVKITNFTTSWRNGLSFCA
ILHHFRPDLIDYKSLNPQDIKENNKKAYDGFASIGISRLLEPSDMVLLAIPDKLTVMTYL
YQIRAHFSGQELNVVQIEENSSKSTYKVGNYETDTNSSVDQEKFYAELSDLKREPELQQP
ISGAVDFLSQDDSVFVNDSGVGESESEHQTPDDHLSPSTASPYCRRTKSDTEPQKSQQSS
GRTSGSDDPGICSNTDSTQAQVLLGKKRLLKAETLELSDLYVSDKKKDMSPPFICEETDE
QKLQTLDIGSNLEKEKLENSRSLECRSDPESPIKKTSLSPTSKLGYSYSRDLDLAKKKHA
SLRQTESDPDADRTTLNHADHSSKIVQHRLLSRQEELKERARVLLEQARRDAALKAGNKH
NTNTATPFCNRQLSDQQDEERRRQLRERARQLIAEARSGVKMSELPSYGEMAAEKLKERS
KASGDENDNIEIDTNEEIPEGFVVGGGDELTNLENDLDTPEQNSKLVDLKLKKLLEVQPQ
VANSPSSAAQKAVTESSEQDMKSGTEDLRTERLQKTTERFRNPVVFSKDSTVRKTQLQSF
SQYIENRPEMKRQRSIQEDTKKGNEEKAAITETQRKPSEDEVLNKGFKDTSQYVVGELAA
LENEQKQIDTRAALVEKRLRYLMDTGRNTEEEEAMMQEWFMLVNKKNALIRRMNQLSLLE
KEHDLERRYELLNRELRAMLAIEDWQKTEAQKRREQLLLDELVALVNKRDALVRDLDAQE
KQAEEEDEHLERTLEQNKGKMAKKEEKCVLQ
>P21374
MSRNVDKANSVLVRFQEQQAESAGGYKDYSRYQRPRNVSKVKSIKEANEWKRQVSKEIKQ
KSTRIYDPSLNEMQIAELNDELNNLFKEWKRWQWHIDHTLMEKKTKRKRLEDSHVLMNSG
KLINGKRYFGRALELPEVKEWLKQSQRQNDGGSINTKCIPKDRNDFYYHGKVTAALTEFE
ANWTSILKAHYNVPVNEDEEEMSRQTQEIHVPTLADMEHWLVQRRKKKLMDELNL
>O75534
MSFDPNLLHNNGHNGYPNGTSAALRETGVIEKLLTSYGFIQCSERQARLFFHCSQYNGNL

QDLKVGDDVEFEVSSDRRTGKPIAVKLVKIKQEILPEERMNGQVVCAVPHNLESKSPAAP
GQSPTGSVCYERNGEVFYLTYTPEDVEGNVQLETGDKINFVIDNNKHTGAVSARNIMLLK
KKQARCQGVVCAMKEAFGFIERGDVVKEIFFHYSEFKGDLETLQPGDDVEFTIKDRNGKE
VATDVRLLPQGTVIFEDISIEHFEGTVTKVIPKVPSKNQNDPLPGRIKVDFVIPKELPFG
DKDTKSKVTLLEGDHVRFNISTDRRDKLERATNIEVLSNTFQFTNEAREMGVIAAMRDGF
GFIKCVDRDVRMFFHFSEILDGNQLHIADEVEFTVVPDMLSAQRNHAIRIKKLPKGTVSF
HSHSDHRFLGTVEKEATFSNPKTTSPNKGKEKEAEDGIIAYDDCGVKLTIAFQAKDVEGS
TSPQIGDKVEFSISDKQRPGQQVATCVRLLGRNSNSKRLLGYVATLKDNFGFIETANHDK
EIFFHYSEFSGDVDSLELGDMVEYSLSKGKGNKVSAEKVNKTHSVNGITEEADPTIYSGK
VIRPLRSVDPTQTEYQGMIEIVEEGDMKGEVYPFGIVGMANKGDCLQKGESVKFQLCVLG
QNAQTMAYNITPLRRATVECVKDQFGFINYEVGDSKKLFFHVKEVQDGIELQAGDEVEFS
VILNQRTGKCSACNVWRVCEGPKAVAAPRPDRLVNRLKNITLDDASAPRLMVLRQPRGPD
NSMGFGAERKIRQAGVID
>Q06672
MSTQYIDETAFVQAEQGKTNLMFSDEKQQARFELGVSMVIYKWDALDVAVENSWGGPDSA
EKRDWITGIVVDLFKNEKVVDAALIEETLLYAMIDEFETNVEDDSALPIAVEVINIYNDC
FNLNYNKVEKLYLEWQEKQRTKKSKRVVHIEGDDDEDDEDVEDYDDEDEDEEMDEVVPDL
VSSKPEPIVDEDGFELVQPKGRRKH
>P0C2I0
MAHFKEYQVIGRRLPTESVPEPKLFRMRIFASNEVIAKSRYWYFLQKLHKVKKASGEIVS
INQINEAHPTKVKNFGVWVRYDSRSGTHNMYKEIRDVSRVAAVETLYQDMAARHRARFRS
IHILKVAEIEKTADVKRQYVKQFLTKDLKFPLPHRVQKSTKTFSYKRPSTFY
>P20485
MVQESRPGSVRSYSVGYQARSRSSSQRRHSLTRQRSSQRLIRTISIESDVSNITDDDDLR
AVNEGVAGVQLDVSETANKGPRRASATDVTDSLGSTSSEYIEIPFVKETLDASLPSDYLK
QDILNLIQSLKISKWYNNKKIQPVAQDMNLVKISGAMTNAIFKVEYPKLPSLLLRIYGPN
IDNIIDREYELQILARLSLKNIGPSLYGCFVNGRFEQFLENSKTLTKDDIRNWKNSQRIA
RRMKELHVGVPLLSSERKNGSACWQKINQWLRTIEKVDQWVGDPKNIENSLLCENWSKFM
DIVDRYHKWLISQEQGIEQVNKNLIFCHNDAQYGNLLFTAPVMNTPSLYTAPSSTSLTSQ
SSSLFPSSSNVIVDDIINPPKQEQSQDSKLVVIDFEYAGANPAAYDLANHLSEWMYDYNN
AKAPHQCHADRYPDKEQVLNFLYSYVSHLRGGAKEPIDEEVQRLYKSIIQWRPTVQLFWS
LWAILQSGKLEKKEASTAITREEIGPNGKKYIIKTEPESPEEDFVENDDEPEAGVSIDTF
DYMAYGRDKIAVFWGDLIGLGIITEEECKNFSSFKFLDTSYL
>Q42806
MANIDIEGILKQQQPYDGRVPKTKIVCTLGPASRSVEMTEKLLRAGMNVARFNFSHGTHD
YHQETLNNLKTAMHNTGILCAVMLDTKGPEIRTGFLKDGKPIQLKEGQEVTITTDYDIKG
DPEMISMSYKKLPVHLKPGNTILCSDGTITLTVLSCDPDAGTVRCRCENTATLGERKNVN
LPGVVVDLPTLTEKDKEDILGWGVPNKIDMIALSFVRKGSDLVNVRKVLGPHAKNIQLMS
KVENQEGVLNFDEILRETDAFMVARGDLGMEIPVEKIFLAQKMMIYKCNLVGKPVVTATQ
MLESMIKSPRPTRAEATDVANAVLDGTDCVMLSGESAAGAYPELAVKIMARICIEAESSL
DYGAIFKEMIRSTPLPMSPLESLASSAVRTANKAKAKLIVVLTRGGSTAKLVAKYRPAVP
ILSVVVPVLSTDSFDWTCSDETPARHSLIYRGLIPILGEGSAKATDAESTEVILEAALKS
ATERALCKPGDAVVALHRIGAASVIKICIVK
>P47120

MSTNFEKHFQENVDECTLEQLRDILVNKSGKTVLANRFRALFNLKTVAEEFATKPEEAKK
AIEYIAESFVNDKSELLKHEVAYVLGQTKNLDAAPTLRHVMLDQNQEPMVRHEAAEALGA
LGDKDSLDDLNKAAKEDPHVAVRETCELAINRINWTHGGAKDKENLQQSLYSSIDPAPPL
PLEKDATIPELQALLNDPKQPLFQRYRAMFRLRDIGTDEAILALATGFSAESSLFKHEIA
YVFGQIGSPAAVPSLIEVLGRKEEAPMVRHEAAEALGAIASPEVVDVLKSYLNDEVDVVR
ESCIVALDMYDYENSNELEYAPTAN
>Q14442
MEDERSFSDICGGRLALQRRYYSPSCREFCLSCPRLSLRSLTAVTCTVWLAAYGLFTLCE
NSMILSAAIFITLLGLLGYLHFVKIDQETLLIIDSLGIQMTSSYASGKESTTFIEMGKVK
DIVINEAIYMQKVIYYLCILLKDPVEPHGISQVVPVFQSAKPRLDCLIEVYRSCQEILAH
QKATSTSP
>Q921W0
MDDTLFQLKFTAKQLEKLAKKAEKDSKAEQAKVKKALQQKNVECARVYAENAIRKKNEGV
NWLRMASRVDAVASKVQTAVTMKGVTKNMAQVTKALDKALSAMDLQKVSAVMDRFEQQVQ
NLDVHTSVMEDSVSSATTLTTPQEQVDSLIVQIAEENGLEVLDQLSQLPEGASAVGESSV
RSQEDQLSRRLAALRN
>P53996
MSSNECFKCGRSGHWARECPTGGGRGRGMRSRGRGGFTSDRGFQFVSSSLPDICYRCGES
GHLAKDCDLQEDEACYNCGRGGHIAKDCKEPKREREQCCYNCGKPGHLARDCDHADEQKC
YSCGEFGHIQKDCTKVKCYRCGETGHVAINCSKTSEVNCYRCGESGHLARECTIEATA
>Q8K1H1
MLEADLVSKMLRAVLQSHKNGIVLPRLQGEYRSLTGDWIPFKQLGYPTLEAYLRSVPAVV
RIEASRSGEIVCYAVACTETARIAQLVARQRTSKRKIGRQINCQMRVKKAMPFFLEGKPK
ATLRQPGFASDYSISRKPNSALLRDRGSALGVKADVDMPPYPDTPVQRHASMSANSRFSP
KSSLPASFQTHISRACPTEVNDNLNQTVEKPNITPPASYTNKMDEVQNRIKEILDKHNNG
IWISKLPHFYKEFYKEDLNQGVLQQFEHWPHICTVEKPCGGGQDSLLYPARREQPLKSDQ
DPEKELPPPPPAPKQEVPSQGSPAVMPDVKEKVAELLGKYSSGLWASALPKAFEDMYKVK
FPEDALKNLASLSDVCTINYISGNTQKAILYAKLPLPTDKILKDEGQAQGDFDIKSMIEQ
EYLQIEKNMAESADEFLEDITVPPLVIPTEASPSVLVVELSNTNDVVIRYVGKDYSAAQE
LMEDEMKEFYSKNPRVTPIQTVHVGQLLAVNAEEDAWLRAQIISTDENKIKVCYVDYGFC
ENIEKSKAYRLNPRFCSLSFQATKCKLAGLEVLNDDPDLVKAVESLTCGKIFAVEILDKS
DVPLVVLYDTSGEDDININATCLKAICDRSLQVHLQVDAMYTNVKVTNICSDGTLYCQVP
CKGLNKLNDLLHKTEDYFHCKHMTSEYFISLPFCGKICLFHCKGKWLRVEITNVHSSRAL
DVQFLDSGNSTSVKVSELREIPPRFLQEMLAIPPQAIKCCLADLPQSIGMWTPDAVLWLR
DSVLNCSDCSIKVTKMDETKGVAYVYLFTPNNFPDPHRSINRQITNADLWKHQKDVFLSA
VSTAASSPGNRNGGTPAPGSPAESLRKSHPEVIKKSVLDHTSSFSLEELPPPVHLSRSGE
HMDVYVPVACHPGHFVIQPWQEIHKLEVLMEEMILYYSVSEERHIAVERDQVYAAKVENK
WYRVLLKGILTNGLVSVYELDYGKHELVNIRKVQPLVDVFRKLPFQAVTAQLAGVKCSQW
SEEASMVFRNHVEKKALVALVQTVVEHTNPWDRKVVLYLVDTSLPDTDTWIHDFMSQYLL
ELSKVN
>P25892
MAETNPELSDLMAQTNKKIVPKFTEIFPVEDVNYPYSAFIASVRKDVIKHCTDHKGIFQP
VLPPEKKVPELWFYTELKTRTSSITLAIRMDNLYLVGFRTPGGVWWEFGKAGDTHLLGDN
PRWLGFGGRYQDLIGNKGLETVTMGRAEMTRAVNDLAKKKKMATLEEEEVQMQMQMPEAA

ELAAAAAAADPQADTKSKLVKLVVMVCEGLRFNTVSRTVDAGFNSQHGVTLTVTQGKQVQ
KWDRISKAAFEWADHPTAVIPDMQKLGIKDKNEAARIVALVKNQTTAAAAAATAASADND
DDEA
>P78968
MGQGSSKHADSKLDSYPSFSRSDTQGSIKSLKSLKTVLGKGKDSNHDRRTSTDTTHSRHR
YPETPPSLPPPPSPGILATSPAVLQKHQQEDSGNSSQSPTSPHPSNQPAMLSPSTAASQH
HHHHSSSSSYAVSPTSPTSPTSSGPIGSNFDSASEHNGPVYPQDQQGPVIIPNSAISSTD
PDDPETVVSLNVDEMIQRLIHVGYSRKSSKSVCLKNAEITSICMAVREIFLSQPTLLELT
PPVKIVGDVHGQYSDLIRLFEMCGFPPSSNYLFLGDYVDRGKQSLETILLLFLYKIRYPE
NFFLLRGNHECANITRVYGFYDECKRRCNIKIWKTFINTFNCLPIASVVAGKIFCVHGGL
SPSLSHMDDIREIPRPTDVPDYGLLNDLLWSDPADTENDWEDNERGVSFVFNKNVIRQFL
AKHDFDLICRAHMVVEDGYEFFNDRTLCTVFSAPNYCGEFDNWGAVMSVNSELLCSFELI
KPLDQAAIRRELKKSKRSGMAIYQSPPAEQVTQSV
>Q9V3I8
MLAHNLGFHKKRLFSNMKAVLQDRGVIGLSLEECDLERTLLGGQSFRWRSICDGNRTKYG
GVVFNTYWVLQQEESFITYEAYGTSSPLATKDYSSLISDYLRVDFDLKVNQKDWLSKDDN
FVKFLSKPVRLLSQEPFENIFSFLCSQNNNIKRISSMIEWFCATFGTKIGHFNGADAYTF
PTINRFHDIPCEDLNAQLRAAKFGYRAKFIAQTLQEIQKKGGQNWFISLKSMPFEKAREE
LTLLPGIGYKVADCICLMSMGHLESVPVDIHIYRIAQNYYLPHLTGQKNVTKKIYEEVSK
HFQKLHGKYAGWAQAILFSADLSQFQNTSTVACKKKSNKKPKK
>O43236
MDRSLGWQGNSVPEDRTEAGIKRFLEDTTDDGELSKFVKDFSGNASCHPPEAKTWASRPQ
VPEPRPQAPDLYDDDLEFRPPSRPQSSDNQQYFCAPAPLSPSARPRSPWGKLDPYDSSED
DKEYVGFATLPNQVHRKSVKKGFDFTLMVAGESGLGKSTLVNSLFLTDLYRDRKLLGAEE
RIMQTVEITKHAVDIEEKGVRLRLTIVDTPGFGDAVNNTECWKPVAEYIDQQFEQYFRDE
SGLNRKNIQDNRVHCCLYFISPFGHGLRPLDVEFMKALHQRVNIVPILAKADTLTPPEVD
HKKRKIREEIEHFGIKIYQFPDCDSDEDEDFKLQDQALKESIPFAVIGSNTVVEARGRRV
RGRLYPWGIVEVENPGHCDFVKLRTMLVRTHMQDLKDVTRETHYENYRAQCIQSMTRLVV
KERNRNKLTRESGTDFPIPAVPPGTDPETEKLIREKDEELRRMQEMLHKIQKQMKENY
>Q9SZF7
MDTLFRLVSLQQQQQSDSIITNQSSLSRTSTTTTGSPQTAYHYNFPQNDVVEECFNFFMD
EEDLSSSSSHHNHHNHNNPNTYYSPFTTPTQYHPATSSTPSSTAAAAALASPYSSSGHHN
DPSAFSIPQTPPSFDFSANAKWADSVLLEAARAFSDKDTARAQQILWTLNELSSPYGDTE
QKLASYFLQALFNRMTGSGERCYRTMVTAAATEKTCSFESTRKTVLKFQEVSPWATFGHV
AANGAILEAVDGEAKIHIVDISSTFCTQWPTLLEALATRSDDTPHLRLTTVVVANKFVND
QTASHRMMKEIGNRMEKFARLMGVPFKFNIIHHVGDLSEFDLNELDVKPDEVLAINCVGA
MHGIASRGSPRDAVISSFRRLRPRIVTVVEEEADLVGEEEGGFDDEFLRGFGECLRWFRV
CFESWEESFPRTSNERLMLERAAGRAIVDLVACEPSDSTERRETARKWSRRMRNSGFGAV
GYSDEVADDVRALLRRYKEGVWSMVQCPDAAGIFLCWRDQPVVWASAWRPT
>Q969Z4
MKPSLLCRPLSCFLMLLPWPLATLTSTTLWQCPPGEEPDLDPGQGTLCRPCPPGTFSAAW
GSSPCQPHARCSLWRRLEAQVGMATRDTLCGDCWPGWFGPWGVPRVPCQPCSWAPLGTHG
CDEWGRRARRGVEVAAGASSGGETRQPGNGTRAGGPEETAAQYAVIAIVPVFCLMGLLGI
LVCNLLKRKGYHCTAHKEVGPGPGGGGSGINPAYRTEDANEDTIGVLVRLITEKKENAAA

LEELLKEYHSKQLVQTSHRPVSKLPPAPPNVPHICPHRHHLHTVQGLASLSGPCCSRCSQ
KKWPEVLLSPEAVAATTPVPSLLPNPTRVPKAGAKAGRQGEITILSVGRFRVARIPEQRT
SSMVSEVKTITEAGPSWGDLPDSPQPGLPPEQQALLGSGGSRTKWLKPPAENKAEENRYV
VRLSESNLVI
>Q53GS7
MPSEGRCWETLKALRSSDKGRLCYYRDWLLRREDVLEECMSLPKLSSYSGWVVEHVLPHM
QENQPLSETSPSSTSASALDQPSFVPKSPDASSAFSPASPATPNGTKGKDESQHTESMVL
QSSRGIKVEGCVRMYELVHRMKGTEGLRLWQEEQERKVQALSEMASEQLKRFDEWKELKQ
HKEFQDLREVMEKSSREALGHQEKLKAEHRHRAKILNLKLREAEQQRVKQAEQERLRKEE
GQIRLRALYALQEEMLQLSQQLDASEQHKALLKVDLAAFQTRGNQLCSLISGIIRASSES
SYPTAESQAEAERALREMRDLLMNLGQEITRACEDKRRQDEEEAQVKLQEAQMQQGPEAH
KEPPAPSQGPGGKQNEDLQVKVQDITMQWYQQLQDASMQCVLTFEGLTNSKDSQAKKIKM
DLQKAATIPVSQISTIAGSKLKEIFDKIHSLLSGKPVQSGGRSVSVTLNPQGLDFVQYKL
AEKFVKQGEEEVASHHEAAFPIAVVASGIWELHPRVGDLILAHLHKKCPYSVPFYPTFKE
GMALEDYQRMLGYQVKDSKVEQQDNFLKRMSGMIRLYAAIIQLRWPYGNRQEIHPHGLNH
GWRWLAQILNMEPLSDVTATLLFDFLEVCGNALMKQYQVQFWKMLILIKEDYFPRIEAIT
SSGQMGSFIRLKQFLEKCLQHKDIPVPKGFLTSSFWRS
>O35215
MPFVELETNLPASRIPAGLENRLCAATATILDKPEDRVSVTIRPGMTLLMNKSTEPCAHL
LVSSIGVVGTAEQNRTHSASFFKFLTEELSLDQDRIVIRFFPLEAWQIGKKGTVMTFL
>Q9Z2Q1
MKLKEIDRTAMQAWSPAQNHPIYLATGTSAQQLDATFSTNASLEIFELDLSDPSLDMKSC
ATFSSSHRYHKLIWGPHKMDSKGDVSGVLIAGGENGNIILYDPSKIIAGDKEVVIAQKDK
HTGPVRALDVNIFQTNLVASGANESEIYIWDLNNFATPMTPGAKTQPPEDISCIAWNRQV
QHILASASPSGRATVWDLRKNEPIIKVSDHSNRMHCSGLAWHPDVATQMVLASEDDRLPV
VQMWDLRFASSPLRVLENHARGILAIAWSMADPELLLSCGKDAKILCSNPNTGEVLYELP
TNTQWCFDIQWCPRNPAVLSAASFDGRIRVYSIMGGSIDGLRQKQVDKLSSSFGNLDPFG
TGQPLPPLQIPQQTSQHSIVLPLKKPPKWIRRPVGASFSFGGKLVTFENVTGQPQQGAEQ
PRRQPVFISQVVTEKDFLSRSEQLQHVVQSQGFISYCQKKIDASQTDFEKNVWSFLKVNF
EEDSRGKYLELLGYRREDLGEKIALALNRVDGSDVALKDSDRVAQSDGEESPAEEGQLLG
ERIKEEKQECDFLPSAGGGTFNISVSGDIDGLITRALLTGNFESAVDLCLHDNRMADAII
LAIAGGQELLAQTQKKYFAKSQSKITRLITAVVMKNWKEIVESCDLKNWREALAAVLTYA
KPDEFSALCDLLGARLESEGDSLLRTQACLCYICAGNVERLVACWTKAQDGSNPLSLQDL
IEKVVILRKAVQLTQALDTNTVGALLAEKMSQYANLLAAQGSIAAALAFLPDNTNQPDIV
QLRDRLCRAQGRSVPGQESSRSSYEGQPLPKGGPGPLAGHPQVSRVQSQQYYPQVRIAPT
VTTWSDRTPTALPSHPPAACPSDTQGGNPPPPGFIMHGNVVPNSPAPLPTSPGHMHSQPP
PYPQPQPYQPAQQYSLGTGGSAVYRPQQPVAPPASKRYPNAPYVSPVASYSGQPHMYTAQ
PASSPTSSSAPLPPPPSSGASFQHGGPGAPPSSSAYALPPGTTGTPPAASELPASQRTGP
QNGWNDPPALNRVPKKKKLPENFMPPVPITSPIMNPGGDPQPQGLQQQPSASGPRSSHAS
FPQPHLAGGQPFHGIQQPLAQTGMPPSFSKPNTEGAPGAPIGNTIQHVQALPTEKITKKP
IPDEHLILKTTFEDLIQRCLSSATDPQTKRKLDDASKRLECLYDKLRDQTLSPTIISGLH
SIARSIETRNYSEGLTVHTHIVSTSNFSETSAFMPVLKVVLSQASKLGV
>Q96M11
MEELLPDGQIWANMDPEERMLAAATAFTHICAGQGEGDVRREAQSIQYDPYSKASVAPGK

RPALPVQLQYPHVESNVPSETVSEASQRLRKPVMKRKVLRRKPDGEVLVTDESIISESES
GTENDQDLWDLRQRLMNVQFQEDKESSFDVSQKFNLPHEYQGISQDQLICSLQREGMGSP
AYEQDLIVASRPKSFILPKLDQLSRNRGKTDRVARYFEYKRDWDSIRLPGEDHRKELRWG
VREQMLCRAEPQSKPQHIYVPNNYLVPTEKKRSALRWGVRCDLANGVIPRKLPFPLSPS
>Q9UJZ1
MLARAARGTGALLLRGSLLASGRAPRRASSGLPRNTVVLFVPQQEAWVVERMGRFHRILE
PGLNILIPVLDRIRYVQSLKEIVINVPEQSAVTLDNVTLQIDGVLYLRIMDPYKASYGVE
DPEYAVTQLAQTTMRSELGKLSLDKVFRERESLNASIVDAINQAADCWGIRCLRYEIKDI
HVPPRVKESMQMQVEAERRKRATVLESEGTRESAINVAEGKKQAQILASEAEKAEQINQA
AGEASAVLAKAKAKAEAIRILAAALTQHNGDAAASLTVAEQYVSAFSKLAKDSNTILLPS
NPGDVTSMVAQAMGVYGALTKAPVPGTPDSLSSGSSRDVQGTDASLDEELDRVKMS
>P49672
MATACVKSLESVQCGTCEKTIANGTEFYALLFDKHPDLRHYFKGNENLTGADVKKSDHFK
KQGQRLLLACHVLAHLENDPASFKAYAREIVDPHLRMSVHLEPKLWSEFWPIWLDYLSTK
ESVDDATKNAWLALGKKFSDECLDHLKNLGQPH
>O74944
MENAKDFIGETIKENGLLTIEDDGSSSSDEEATLKRRLAGPSVLKSGQENVNQKKINEII
YEASKGSKFFEAEQKRDRELRLRIEKVQVEVEKYQSKLRFDKAFQREWTIRQESVDTTVE
DFRAKRDLTQIIVHVDCDAFYASIEELKNPKLKSLPMAVGKSVLCTANYVARKFGVRSAM
PEFIARKICPDLVVIPLNLSEYAIKSKEIQNVLAQYDSNLCPASIDEFYMNLTSHLRLQE
LAFTVENITMVVEKIRKQVHEETGVTVSCGIAANKLLAKIASNKRKPNNQFFIPFDEIGI
SKFMNDLPVREVSGIGRVLEQQLLGLEIKTCGDIQRNLVILSYIFLPKSFQNLLRCSYGF
GTTILDEYGESKRKTIGSEATFSSNLSSPSIIEYKLRLLVQNVSENLQKRGLVTNSIAIK
YKTSEFQVHTKQKSIGQFIHSESDLLKPALQLLRQSYPMTIRLLGVRATKLVSKSRCLAM
QLKFQSQNTVPCPVCQKNIENELGILNQHVDLCLNVETVKSLINTDHTANPTIKKRKSNT
LDTYFLE
>Q9UUF1
MASIRQAFVKSLRFYDIGYNATDPVFRGIYHEKLKHPDDFDSIISRAKAVGVEKMMITGD
NVENSEEALNLATNYECFTSTVGVHPCQAQCFLRHSEGPEDYLVKLEALANKGKASGKVV
AFGEFGLDYDRLHYAPADVQKMYFEEQLKVAVRVQLPLFLHSRNAENDFFAILEKYLPEL
PKKGVVHSFTGSIDEMRRCIEHGLYVGVNGCSLKTEENLEVVRAIPLEKMLLETDAPWCE
VRPSHAGHQFLKTKLPFDSCKKERFKEGCMIRGRNEPCNTYIVAEIVAALKDISLEELSE
QIWENSINLLSK
>P42826
MLCSVIQRQTREVSNTMSLDSYYLGFDLSTQQLKCLAINQDLKIVHSETVEFEKDLPHYH
TKKGVYIHGDTIECPVAMWLEALDLVLSKYREAKFPLNKVMAVSGSCQQHGSVYWSSQAE
SLLEQLNKKPEKDLLHYVSSVAFARQTAPNWQDHSTAKQCQEFEECIGGPEKMAQLTGSR
AHFRFTGPQILKIAQLEPEAYEKTKTISLVSNFLTSILVGHLVELEEADACGMNLYDIRE
RKFSDELLHLIDSSSKDKTIRQKLMRAPMKNLIAGTICKYFIEKYGFNTNCKVSPMTGDN
LATICSLPLRKNDVLVSLGTSTTVLLVTDKYHPSPNYHLFIHPTLPNHYMGMICYCNGSL
ARERIRDELNKERENNYEKTNDWTLFNQAVLDDSESSENELGVYFPLGEIVPSVKAINKR
VIFNPKTGMIEREVAKFKDKRHDAKNIVESQALSCRVRISPLLSDSNASSQQRLNEDTIV
KFDYDESPLRDYLNKRPERTFFVGGASKNDAIVKKFAQVIGATKGNFRLETPNSCALGGC
YKAMWSLLYDSNKIAVPFDKFLNDNFPWHVMESISDVDNENWDRYNSKIVPLSELEKTLI

>P32264
MKDANESKSYTIVIKLGSSSLVDEKTKEPKLAIMSLIVETVVKLRRMGHKVIIVSSGGIA
VGLRTMRMNKRPKHLAEVQAIAAIGQGRLIGRWDLLFSQFDQRIAQILLTRNDILDWTQY
KNAQNTINELLNMGVIPIVNENDTLSVREIKFGDNDTLSAITSALIHADYLFLLTDVDCL
YTDNPRTNPDAMPILVVPDLSKGLPGVNTAGGSGSDVGTGGMETKLVAADLATNAGVHTL
IMKSDTPANIGRIVEYMQTLELDDENKVKQAYNGDLTDLQKREFEKLKALNVPLHTKFIA
NDNKHHLKNREFWILHGLVSKGAVVIDQGAYAALTRKNKAGLLPAGVIDVQGTFHELECV
DIKVGKKLPDGTLDPDFPLQTVGKARCNYTSSELTKIKGLHSDQIEEELGYNDSEYVAHR
ENLAFPPR
>Q9C115
MVKGSVTNTSSIVKQFEKSSIKHETETIAFAKQFINECIREFQEQKDNVVEEKNTDQNHQ
NQNQEGVIIEIYQELLQKVDLISSELRQNLSSLQLRFQEVERDTGHDLLNVLNSLSQEAR
LAQKVLEEDGSQQGSQLIQGLLTCFQSTGN
>O94584
MVRSRVSPGLKLQSDASNQSNDKENKLLAYVNDNRHFSLIRALHLADLITLMNGFCGVMS
IFSSLRYCLSGQQSAFHLWNAMYFMPFALFFDFLDGKVARWRGKSSLMGQELDSLADLIS
FGVSPAVFAFCCGFQTFLDTVILSLFVLCGLTRLARFNVSVNSIPKDGSGKSQFFEGTPI
PTTLSLVTVCGVCILKGKTHENLPWGEWCGNTPFAFHPLVVLFVLSGIAMTSKKLKVPKS
TFLLHSLKYV
>P61970
MGDKPIWEQIGSSFIQHYYQLFDNDRTQLGAIYIDASCLTWEGQQFQGKAAIVEKLSSLP
FQKIQHSITAQDHQPTPDSCIISMVVGQLKADEDPIMGFHQMFLLKNINDAWVCTNDMFR
LALHNFG
>Q04601
MSSPINDYFIDYNPLFPIFATRIAKGLAIYRVSDHARLAVIPIRNINLVANYDWDTTTGK
FLSIFFKDGTIRIHDIFKDGRLVSFLRIPSTKISKGIWDRIPLRYEPNNRDFACNIIDDL
PKLIRFVKDSKRINIVPYTQPNSLWRGPDEDDLDSNEKLDVHVVFNEGNDKITVFFNGDY
AVFLSVDNIENENSLKSIIKVQDGFYQCFYEDGTVQTLNLGPLLQSKSSVNLLNYIMVIK
ELIGYMLTHLEFINRELATPYLDFVKRLCDEAYGYGKLKSELEALFLLGEISCDLEDWLC
NSVGEKNFKRWKYLGCEAYQKTVQILTLIFVPACERIIIYVEKLRAILQAFSIQNKLSYT
SDLTAVEVLLKSSQKLLTMTLNSIIGLGRDETLFEKFFIWFNDRLHEALDEDYKLKFQFE
DDLYFGYDLLSYFDRILSKKGTEPSSIIDVKLYRDLINSMSDMEKDIAQSNVNSHIQQHI
LVDLKTDVFAQKYPSSQINLLDAIKLPKHNYIVYLIQVTKHNSAQEPFSEENKKKLYIGT
LKDENLGIISKESSVKIPALFKSYRLSSTRFVPNRVHSLLRDIGLSDSNYHSSHVTDYRG
ENYENEEDDGTIAIPAYIRENRENDDFIACTAKVSVDGRSASLVFPKEKQNV
>Q9DFS6
MATAEPETSTNPSNSEEKNEENEQQIVSLEDYIKHPLQNRWALWFFKNDKSKTWQANLRL
ISKFDTVEDFWALYNHIQLSSNLMSGCDYSLFKDGIEPMWEDERNKRGGRWLITLSKQQR
RADLDRFWLETLLCLVGEAFDDHSDDVCGAVVNIRTKGDKIAIWTTDYENKDAIVHIGRV
YKERLGVPPKVIIGYQSHADTATKSGSTTKNKFVV
>P40453
MLDDDKGTAMHPHITPFTPEYSNELLRRVQDLYHEDIKHYYPQLKLEKLLDLLEHTEYLF
ELYLDSIHHDRPNDALTAFIIGCYYVFLIIPQSLQFQTRNKSYSIYTDLKKMYENEMNMT

NVVLMVKKEIGVVLDESVKHGAGIEHRITKKRAFSVPADDLSGQVASLSLDTAAPQDHGL
KGTFTEDDAEQSSPVWTAPNLEPNDQLKLALLPEVIPTPAFREPERKTSVPVRPSVLLED
VPSIYHEDDTSFASLNPPFREITADRSVTHRKDSYHSVYMVDSGNLKEDNDDLFNVENDG
FIQSLDILQKQSIITAPELFSILSNRVEREKVLLIDLRIPQRSAINHIVAPNLVNVDPNL
LWDKQTNTPIYKDDILEHLLKENENFINRNKFDYIVYYTDVKTFMTINFDYAFIFFYLML
TSQKTPLTTVPTTLLGGYEKWKKTLHSYAQEYHISIEDYLYRPYSQKARLQQEQQQQQQQ
PDSQDSFSAKESSTKVPEPPSWKPPDLPIRLRKRPPPPPPVSMPTTPEIPPPLPPKIMVH
SQVSSISRKPPIPAKQHVKKEQLNSNEIIQRKRQHQHQHYDQQILQPQRAYNIPTIERSP
NVYVSLSITGLRNLGNTCYINSMIQCLFAAKTFRTLFISSKYKSYLQPIRSNGSHYSPKL
SNSLSMLFNKMYLNGGCSVVPTGFLKVINQLRPDLKIPDDQQDTQEFLMILLDRLHDELS
DQQHVANDYPNLLLYNADALKVSNNEYKHWFDKNVIGNGISPIDDIFQGQMENSLQCKRC
GYTTFNYSTFYVLSLAIPRRSMKLSKLGRSTEKRVKLEDCINMFTSDEVLSGENAWDCPR
CGPTASVSTSVSALENEPSIVKSKKKKSRFFTLHTGTKRRHLDFFGDGITEGHNSNNNNT
TIFERERSRSPFRMLGGSGKRSSSSTPFSTGGNDSNNSSDYKNKKLTTVKTINFVTLPKI
LVIHLSRFYYDLTKKNNTVVTYPLILNIILKNNDTMKYKLFGVVNHTGTLISGHYTSLVN
KDLEHNVNIGRSKWYYFDDEVVKADRKHGSDKNLKISSSDVYVLFYERVYD
>Q6I9Y2
MGAVTDDEVIRKRLLIDGDGAGDDRRINLLVKSFIKWCNSGSQEEGYSQYQRMLSTLSQC
EFSMGKTLLVYDMNLREMENYEKIYKEIECSIAGAHEKIAECKKQILQAKRIRKNRQEYD
ALAKVIQHHPDRHETLKELEALGKELEHLSHIKESVEDKLELRRKQFHVLLSTIHELQQT
LENDEKLSEVEEAQEASMESDPKP
>O60057
MLAQNSREVLDKDLESHIPGAYSPAPPSSSLPTQNESNLQQSEKPLKHFENKKFKGEDVF
AHSISKPFDGQNDEEKPSLYSHLTNELYETHDSSDYFGTYMEREESGHDEEEADKDASFT
GYYNSRNNDEEGSELADSQMLPMTESFADIEDIHHHQHEDEFSSSNKDKGFTYEKPVTEL
PPKRPPYHYESSSTSISFVNGGPNFRKVKSGLLKPDADAASNLSTTSLVNQEYVMKQVTP
NEYSMEYLRESFPQTPMGTEASGYSFDRTPHKSDIQASDRLNALNEKLLQGIQQSHQPYH
YTNVKDSQGSHQTKVTIEENMEAPSHQAANRTTLTDSPLGIVEDETTILTDLEPSGPGLQ
KRASETSMSTNVSLPYYQNGRRVRNILTPYPTVSLASTMTSETEDVSEERI
>O74738
MEKLLHEALQNGCKLHKSVEFIQSRDDNACFGSYIAVAQNDIAPDQLLISCPFEYAITYN
KAKEELKKLNPNFESCNPHITLCTFLALESLKGIQSKWYGYIEYLPKTFNTPLYFNENDN
AFLISTNAYSAAQERLHIWKHEYQEALSLHPSPTERFTFDLYIWSATVFSSRCFSSNLIY
KDSESTPILLPLIDSLNHKPKQPILWNSDFQDEKSVQLISQELVAKGNQLFNNYGPKGNE
ELLMGYGFCLPDNPFDTVTLKVAIHPDLPHKDQKAAILENDCQFQLSNLVFFLPKSPDKE
IFQKILQCLAVVTASSLELRKLTAHLLTGDLASYVPSLRGQIKSLEVLLMYIDSRADLLL
KSNPQVSPTSERQVWAKIYRDSQINILQDSITYVKNYMEESLQKTYKPLPNLLQYLILNS
ISIFLLQHPLFAPLSHAIESLYGSTDAEALVATDEQDILMILICVYCLSISEKLPFSISM
LVEGYPAVANPEGVEVFEILDEMFFQQFTNVFGESKHFNKENVSWALQLVNDESLDFSGF
TFIIAHN
>Q9W799
MTSIIKLTTLVGAQEESAVCYLLQVDEFRFLLDCGWDENFSMDIIDSVKKYVHQVDAVLL
SHPDPLHLGALPYAVGKLGLNCAIYATIPVYKMGQMFMYDLYQSRHNTEDFSLFSLDDVD
CAFDKIQQLKYNQIVHLKGKGHGLSITPLPAGHMIGGTIWKIVKDGEEEIVYAVDFNHKR

EIHLNGCSLEMINRPSLLITDSFNATYVQPRRKQRDEQLLTNVLETLRGDGNVLIAVDTA
GRVLELAQLLDQIWRTKDAGLGVYSLALLNNVSYNVVEFSKSQVEWMSDKLMRCFEDKRN
NPFQFRHLTLCHGYSDLARVPSPKVVLASQPDLECGFSRELFIQWCQDPKNSVILTYRTT
PGTLARFLIDHPSERIIDIELRKRVKLEGKELEEYVEKEKLKKEAAKKLEQSKEADLDSS
DDSDVEEDIDQITSHKAKHDLMMKNEGSRKGSFFKQAKKSYPMFPAPEDRIKWDEYGEII
KPEDFLVPELQVTEDEKTKLESGLTNGDEPMDQDLSDVPTKCVSTTESMEIKARVTYIDY
EGRSDGDSIKKIINQMKPRQLIIVHGPPDATQDLAEACRAFGGKDIKVYTPKLHETVDAT
SETHIYQVRLKDSLVSSLKFCKAKDTELAWIDGVLDMRVSKVDTGVILEERELKDEGEDM
EMQVDTQVMDASTIAQQKVIKSLFGDDDKEFSEESEIIPTLEPLPSNEVPGHQSVFMNEP
RLSDFKQVLLREGIHAEFVGGVLVCNNMVAVRRTETGRIGLEGCLCEDFFKIRELLYEQY
AIV
>Q96Q89
MESNFNQEGVPRPSYVFSADPIARPSEINFDGIKLDLSHEFSLVAPNTEANSFESKDYLQ
VCLRIRPFTQSEKELESEGCVHILDSQTVVLKEPQCILGRLSEKSSGQMAQKFSFSKVFG
PATTQKEFFQGCIMQPVKDLLKGQSRLIFTYGLTNSGKTYTFQGTEENIGILPRTLNVLF
DSLQERLYTKMNLKPHRSREYLRLSSEQEKEEIASKSALLRQIKEVTVHNDSDDTLYGSL
TNSLNISEFEESIKDYEQANLNMANSIKFSVWVSFFEIYNEYIYDLFVPVSSKFQKRKML
RLSQDVKGYSFIKDLQWIQVSDSKEAYRLLKLGIKHQSVAFTKLNNASSRSHSIFTVKIL
QIEDSEMSRVIRVSELSLCDLAGSERTMKTQNEGERLRETGNINTSLLTLGKCINVLKNS
EKSKFQQHVPFRESKLTHYFQSFFNGKGKICMIVNISQCYLAYDETLNVLKFSAIAQKVC
VPDTLNSSQEKLFGPVKSSQDVSLDSNSNSKILNVKRATISWENSLEDLMEDEDLVEELE
NAEETQNVETKLLDEDLDKTLEENKAFISHEEKRKLLDLIEDLKKKLINEKKEKLTLEFK
IREEVTQEFTQYWAQREADFKETLLQEREILEENAERRLAIFKDLVGKCDTREEAAKDIC
ATKVETEETHNYVGFEDIIDSLQDNVADIKKQAEIAHLYIASLPDPQEATACLELKFNQI
KAELAKTKGELIKTKEELKKRENESDSLIQELETSNKKIITQNQRIKELINIIDQKEDTI
NEFQNLKSHMENTFKCNDKADTSSLIINNKLICNETVEVPKDSKSKICSERKRVNENELQ
QDEPPAKKGSIHVSSAITEDQKKSEEVRPNIAEIEDIRVLQENNEGLRAFLLTIENELKN
EKEEKAELNKQIVHFQQELSLSEKKNLTLSKEVQQIQSNYDIAIAELHVQKSKNQEQEEK
IMKLSNEIETATRSITNNVSQIKLMHTKIDELRTLDSVSQISNIDLLNLRDLSNGSEEDN
LPNTQLDLLGNDYLVSKQVKEYRIQEPNRENSFHSSIEAIWEECKEIVKASSKKSHQIEE
LEQQIEKLQAEVKGYKDENNRLKEKEHKNQDDLLKEKETLIQQLKEELQEKNVTLDVQIQ
HVVEGKRALSELTQGVTCYKAKIKELETILETQKVERSHSAKLEQDILEKESIILKLERN
LKEFQEHLQDSVKNTKDLNVKELKLKEEITQLTNNLQDMKHLLQLKEEEEETNRQETEKL
KEELSASSARTQNLKADLQRKEEDYADLKEKLTDAKKQIKQVQKEVSVMRDEDKLLRIKI
NELEKKKNQCSQELDMKQRTIQQLKEQLNNQKVEEAIQQYERACKDLNVKEKIIEDMRMT
LEEQEQTQVEQDQVLEAKLEEVERLATELEKWKEKCNDLETKNNQRSNKEHENNTDVLGK
LTNLQDELQESEQKYNADRKKWLEEKMMLITQAKEAENIRNKEMKKYAEDRERFFKQQNE
MEILTAQLTEKDSDLQKWREERDQLVAALEIQLKALISSNVQKDNEIEQLKRIISETSKI
ETQIMDIKPKRISSADPDKLQTEPLSTSFEISRNKIEDGSVVLDSCEVSTENDQSTRFPK
PELEIQFTPLQPNKMAVKHPGCTTPVTVKIPKARKRKSNEMEEDLVKCENKKNATPRTNL
KFPISDDRNSSVKKEQKVAIRPSSKKTYSLRSQASIIGVNLATKKKEGTLQKFGDFLQHS
PSILQSKAKKIIETMSSSKLSNVEASKENVSQPKRAKRKLYTSEISSPIDISGQVILMDQ
KMKESDHQIIKRRLRTKTAK
>Q01780

MAPPSTREPRVLSATSATKSDGEMVLPGFPDADSFVKFALGSVVAVTKASGGLPQFGDEY
DFYRSFPGFQAFCETQGDRLLQCMSRVMQYHGCRSNIKDRSKVTELEDKFDLLVDANDVI
LERVGILLDEASGVNKNQQPVLPAGLQVPKTVVSSWNRKAAEYGKKAKSETFRLLHAKNI
IRPQLKFREKIDNSNTPFLPKIFIKPNAQKPLPQALSKERRERPQDRPEDLDVPPALADF
IHQQRTQQVEQDMFAHPYQYELNHFTPADAVLQKPQPQLYRPIEETPCHFISSLDELVEL
NEKLLNCQEFAVDLEHHSYRSFLGLTCLMQISTRTEDFIIDTLELRSDMYILNESLTDPA
IVKVFHGADSDIEWLQKDFGLYVVNMFDTHQAARLLNLGRHSLDHLLKLYCNVDSNKQYQ
LADWRIRPLPEEMLSYARDDTHYLLYIYDKMRLEMWERGNGQPVQLQVVWQRSRDICLKK
FIKPIFTDESYLELYRKQKKHLNTQQLTAFQLLFAWRDKTARREDESYGYVLPNHMMLKI
AEELPKEPQGIIACCNPVPPLVRQQINEMHLLIQQAREMPLLKSEVAAGVKKSGPLPSAE
RLENVLFGPHDCSHAPPDGYPIIPTSGSVPVQKQASLFPDEKEDNLLGTTCLIATAVITL
FNEPSAEDSKKGPLTVAQKKAQNIMESFENPFRMFLPSLGHRAPVSQAAKFDPSTKIYEI
SNRWKLAQVQVQKDSKEAVKKKAAEQTAAREQAKEACKAAAEQAISVRQQVVLENAAKKR
ERATSDPRTTEQKQEKKRLKISKKPKDPEPPEKEFTPYDYSQSDFKAFAGNSKSKVSSQF
DPNKQTPSGKKCIAAKKIKQSVGNKSMSFPTGKSDRGFRYNWPQR
>O14980
MPAIMTMLADHAARQLLDFSQKLDINLLDNVVNCLYHGEGAQQRMAQEVLTHLKEHPDAW
TRVDTILEFSQNMNTKYYGLQILENVIKTRWKILPRNQCEGIKKYVVGLIIKTSSDPTCV
EKEKVYIGKLNMILVQILKQEWPKHWPTFISDIVGASRTSESLCQNNMVILKLLSEEVFD
FSSGQITQVKSKHLKDSMCNEFSQIFQLCQFVMENSQNAPLVHATLETLLRFLNWIPLGY
IFETKLISTLIYKFLNVPMFRNVSLKCLTEIAGVSVSQYEEQFVTLFTLTMMQLKQMLPL
NTNIRLAYSNGKDDEQNFIQNLSLFLCTFLKEHDQLIEKRLNLRETLMEALHYMLLVSEV
EETEIFKICLEYWNHLAAELYRESPFSTSASPLLSGSQHFDVPPRRQLYLPMLFKVRLLM
VSRMAKPEEVLVVENDQGEVVREFMKDTDSINLYKNMRETLVYLTHLDYVDTERIMTEKL
HNQVNGTEWSWKNLNTLCWAIGSISGAMHEEDEKRFLVTVIKDLLGLCEQKRGKDNKAII
ASNIMYIVGQYPRFLRAHWKFLKTVVNKLFEFMHETHDGVQDMACDTFIKIAQKCRRHFV
QVQVGEVMPFIDEILNNINTIICDLQPQQVHTFYEAVGYMIGAQTDQTVQEHLIEKYMLL
PNQVWDSIIQQATKNVDILKDPETVKQLGSILKTNVRACKAVGHPFVIQLGRIYLDMLNV
YKCLSENISAAIQANGEMVTKQPLIRSMRTVKRETLKLISGWVSRSNDPQMVAENFVPPL
LDAVLIDYQRNVPAAREPEVLSTMAIIVNKLGGHITAEIPQIFDAVFECTLNMINKDFEE
YPEHRTNFFLLLQAVNSHCFPAFLAIPPTQFKLVLDSIIWAFKHTMRNVADTGLQILFTL
LQNVAQEEAAAQSFYQTYFCDILQHIFSVVTDTSHTAGLTMHASILAYMFNLVEEGKIST
SLNPGNPVNNQIFLQEYVANLLKSAFPHLQDAQVKLFVTGLFSLNQDIPAFKEHLRDFLV
QIKEFAGEDTSDLFLEEREIALRQADEEKHKRQMSVPGIFNPHEIPEEMCD
>Q9Y265
MKIEEVKSTTKTQRIASHSHVKGLGLDESGLAKQAASGLVGQENAREACGVIVELIKSKK
MAGRAVLLAGPPGTGKTALALAIAQELGSKVPFCPMVGSEVYSTEIKKTEVLMENFRRAI
GLRIKETKEVYEGEVTELTPCETENPMGGYGKTISHVIIGLKTAKGTKQLKLDPSIFESL
QKERVEAGDVIYIEANSGAVKRQGRCDTYATEFDLEAEEYVPLPKGDVHKKKEIIQDVTL
HDLDVANARPQGGQDILSMMGQLMKPKKTEITDKLRGEINKVVNKYIDQGIAELVPGVLF
VDEVHMLDIECFTYLHRALESSIAPIVIFASNRGNCVIRGTEDITSPHGIPLDLLDRVMI
IRTMLYTPQEMKQIIKIRAQTEGINISEEALNHLGEIGTKTTLRYSVQLLTPANLLAKIN
GKDSIEKEHVEEISELFYDAKSSAKILADQQDKYMK
>Q8R0L1

MATALSPPRGPKLKGAPPSHYYESFLEKKGPCDQDYRKFWAGLQGLAICFYNSNRDLQPL
EKLDLRLFSKLRDEALLGSSRDTAYHFSLVLRDQEVKFKVESLESCEMWKGFILTVVELR
VPSNLTLLPGHLYMMAEVLTKEEVRRAAEVPWCFLQVSRLEAQLLLERYPECGNLLLRPG
GDGKDSVSVTTRQILNGSPVVKHYKVKRAGSKYVIDVEDPFSCPSLEAVVNYFVTHTKRA
LVPFLLDEDYEKVLGFVDSDRENGESAWAVPSFRASGPALPANVLKPLPPVPVSVSSQED
KLPQLPPLPQLPDTDENYVTPIEDSPAAEYMNQDVSLSSQAVPLKPKKPARLPAKPPKPS
VVPKPDLKAITSVWTRKLGGSSSQASSLVTRLGDITAELEEKLQKRRALEH
>Q12778
MAEAPQVVEIDPDFEPLPRPRSCTWPLPRPEFSQSNSATSSPAPSGSAAANPDAAAGLPS
ASAAAVSADFMSNLSLLEESEDFPQAPGSVAAAVAAAAAAAATGGLCGDFQGPEAGCLHP
APPQPPPPGPLSQHPPVPPAAAGPLAGQPRKSSSSRRNAWGNLSYADLITKAIESSAEKR
LTLSQIYEWMVKSVPYFKDKGDSNSSAGWKNSIRHNLSLHSKFIRVQNEGTGKSSWWMLN
PEGGKSGKSPRRRAASMDNNSKFAKSRSRAAKKKASLQSGQEGAGDSPGSQFSKWPASPG
SHSNDDFDNWSTFRPRTSSNASTISGRLSPIMTEQDDLGEGDVHSMVYPPSAAKMASTLP
SLSEISNPENMENLLDNLNLLSSPTSLTVSTQSSPGTMMQQTPCYSFAPPNTSLNSPSPN
YQKYTYGQSSMSPLPQMPIQTLQDNKSSYGGMSQYNCAPGLLKELLTSDSPPHNDIMTPV
DPGVAQPNSRVLGQNVMMGPNSVMSTYGSQASHNKMMNPSSHTHPGHAQQTSAVNGRPLP
HTVSTMPHTSGMNRLTQVKTPVQVPLPHPMQMSALGGYSSVSSCNGYGRMGLLHQEKLPS
DLDGMFIERLDCDMESIIRNDLMDGDTLDFNFDNVLPNQSFPHSVKTTTHSWVSG
>O94612
MADSLVHSLASSSQLEAFDSFEYAEELCTLGSEWIQEAGVLLNLTQNCVIVCLILFRRYC
TLYPPRVPDLDAIVMACVSIGSKTTETPASVQDICNVVVYLKERFKDTNFEARGFIAHDL
YSEEMYSSRNRLSNMELEVLRALNFDTHIVIPHKLAIHYLQTLQLIDNKKLLQITWNFLN
DASRTRLCVLYPPFSLACGCIAMAARVIGMKLPKDWYRVFDTTKEEIDSLTSILENFYKT
SAIAHKTLYLIFTEQVSA
>O73932
MNKLYIGNLSENVSPTDLESLFKESKIPFTGQFLVKSGYAFVDCPDETWAMKAIDTLSGK
VELHGKVIEVEHSVPKRQRSRKLQIRNIPPHLQWEVLDSLLAQYGTVENCEQVNTESETA
VVNVTYANKEHARQGLEKLNGYQLENYSLKVTYIPDEMATPQAPSQQLQQQPQQQHPQGR
RGFGQRGPARQGSPGAAARPKPQTEVPLRMLVPTQFVGAIIGKEGATIRNITKQTQSKID
IHRKENAGAAEKPITIHSTPEGCSAACKIIMEIMQKEAQDTKFTEEIPLKILAHNNFVGR
LIGKEGRNLKKIEQDTDTKITISPLQDLTLYNPERTITVKGSIEPCAKAEEEIMKKIRES
YENDIAAMNLQAHLIPGLNLNALGLFPSSSSGMPPPSVGVPSPTSSTSYPPFGQQPESET
VHLFIPALAVGAIIGKQGQHIKQLSRFAGASIKIAPAEGPDAKLRMVIITGPPEAQFKAQ
GRIYGKLKEENFFGPKEEVKLETHIKVPSYAAGRVIGKGGKTVNELQNLTSAEVVVPRDQ
TPDENDEVVVKITGHFYASQLAQRKIQEILAQVRRQQQQQQKTVQSGQPQPRRK
>P14713
MVSGVGGSGGGRGGGRGGEEEPSSSHTPNNRRGGEQAQSSGTKSLRPRSNTESMSKAIQQ
YTVDARLHAVFEQSGESGKSFDYSQSLKTTTYGSSVPEQQITAYLSRIQRGGYIQPFGCM
IAVDESSFRIIGYSENAREMLGIMPQSVPTLEKPEILAMGTDVRSLFTSSSSILLERAFV
AREITLLNPVWIHSKNTGKPFYAILHRIDVGVVIDLEPARTEDPALSIAGAVQSQKLAVR
AISQLQALPGGDIKLLCDTVVESVRDLTGYDRVMVYKFHEDEHGEVVAESKRDDLEPYIG
LHYPATDIPQASRFLFKQNRVRMIVDCNATPVLVVQDDRLTQSMCLVGSTLRAPHGCHSQ
YMANMGSIASLAMAVIINGNEDDGSNVASGRSSMRLWGLVVCHHTSSRCIPFPLRYACEF

LMQAFGLQLNMELQLALQMSEKRVLRTQTLLCDMLLRDSPAGIVTQSPSIMDLVKCDGAA
FLYHGKYYPLGVAPSEVQIKDVVEWLLANHADSTGLSTDSLGDAGYPGAAALGDAVCGMA
VAYITKRDFLFWFRSHTAKEIKWGGAKHHPEDKDDGQRMHPRSSFQAFLEVVKSRSQPWE
TAEMDAIHSLQLILRDSFKESEAAMNSKVVDGVVQPCRDMAGEQGIDELGAVAREMVRLI
ETATVPIFAVDAGGCINGWNAKIAELTGLSVEEAMGKSLVSDLIYKENEATVNKLLSRAL
RGDEEKNVEVKLKTFSPELQGKAVFVVVNACSSKDYLNNIVGVCFVGQDVTSQKIVMDKF
INIQGDYKAIVHSPNPLIPPIFAADENTCCLEWNMAMEKLTGWSRSEVIGKMIVGEVFGS
CCMLKGPDALTKFMIVLHNAIGGQDTDKFPFPFFDRNGKFVQALLTANKRVSLEGKVIGA
FCFLQIPSPELQQALAVQRRQDTECFTKAKELAYICQVIKNPLSGMRFANSLLEATDLNE
DQKQLLETSVSCEKQISRIVGDMDLESIEDGSFVLKREEFFLGSVINAIVSQAMFLLRDR
GLQLIRDIPEEIKSIEVFGDQIRIQQLLAEFLLSIIRYAPSQEWVEIHLSQLSKQMADGF
AAIRTEFRMACPGEGLPPELVRDMFHSSRWTSPEGLGLSVCRKILKLMNGEVQYIRESER
SYFLIILELPVPRKRPLSTASGSGDMMLMMPY
>P53615
MSATESSSIFTLSHNSNLQDILAANAKWASQMNNIQPTLFPDHNAKGQSPHTLFIGCSDS
RYNENCLGVLPGEVFTWKNVANICHSEDLTLKATLEFAIICLKVNKVIICGHTDCGGIKT
CLTNQREALPKVNCSHLYKYLDDIDTMYHEESQNLIHLKTQREKSHYLSHCNVKRQFNRI
IENPTVQTAVQNGELQVYGLLYNVEDGLLQTVSTYTKVTPK
>A6X982
MRYPNLLFLALPISEHYIEESLKYFKLTSNDPMILSGFRGPRVSHLTIGMIPVKNDEDVL
KCMDFLYNKEDEIRKSYGEKKITIDLKGTSFFGKSPQEAKVLYATPVDKHNGNDLQAFCE
WLKVIFTEHNLFTKDARPLTLHCTLLNSRYIKYQGRRIRFFNSEPFMEKYGQFLWAHNIE
LDKLSIMKTGAVGEPGNMYYEELASIPLLVND
>Q06504
MTTIQPFEPVDLFKTNNVNLDILTENFPLEFYFEYMIIWPDLFFKSSEMTVDPTFKHNIS
GYMMAKTEGKTTEWHTHITAVTVAPRFRRISLASKLCNTLETMTDVMPHEVNFIDLFVKC
NNQLAIKLYEKLGYSVYRRVVGYYNSAEDGYPDTLKKVDDNKDAFDMRKAMARDRNRSVR
PDGRSHKCYPHDVRF
>P83939
MAVAPPSYCFVAFPPRAKDGLVVFGKNSARPRDEVQEVVYFSAADHEPESKVECTYISIN
QVPRTHAIVISRPAWLWGAEMGANEHGVCIANEAINAREPAAETEALLGMDLVRLGLERG
ATAKEALDVIVALLEEHGQGGNYYEDANSCHSFQSAFLIVDREEAWVLETVGKYWAAEKI
TEGVKCICNQLSLTTKIDAEHPELRSYAQSQGWWMGEDEFNFSEVFSPADDHLTCCSGRD
TLEKQEESITVQTMIDVLRDKASGVCVDSESFLTTASVVSVLPQNGSSPCIHYFTGTPDP
SRSIFKPFIFVDDVKLVPKAQSPCFGDDDPAKKEPRFQEKPDRRHELYKAHEWARAVLES
DEEQGQKLRKTMLELEKQGLEAMEEILTSSDPLDPTEVGDLFYDCVDTEIKFFK
>Q9NZJ0
MLFNSVLRQPQLGVLRNGWSSQYPLQSLLTGYQCSGNDEHTSYGETGVPVPPFGCTFSSA
PNMEHVLAVANEEGFVRLYNTESQSFRKKCFKEWMAHWNAVFDLAWVPGELKLVTAAGDQ
TAKFWDVKAGELIGTCKGHQCSLKSVAFSKFEKAVFCTGGRDGNIMVWDTRCNKKDGFYR
QVNQISGAHNTSDKQTPSKPKKKQNSKGLAPSVDFQQSVTVVLFQDENTLVSAGAVDGII
KVWDLRKNYTAYRQEPIASKSFLYPGSSTRKLGYSSLILDSTGSTLFANCTDDNIYMFNM
TGLKTSPVAIFNGHQNSTFYVKSSLSPDDQFLVSGSSDEAAYIWKVSTPWQPPTVLLGHS
QEVTSVCWCPSDFTKIATCSDDNTLKIWRLNRGLEEKPGGDKLSTVGWASQKKKESRPGL

VTVTSSQSTPAKAPRAKCNPSNSSPSSAACAPSCAGDLPLPSNTPTFSIKTSPAKARSPI
NRRGSVSSVSPKPPSSFKMSIRNWVTRTPSSSPPITPPASETKIMSPRKALIPVSQKSSQ
AEACSESRNRVKRRLDSSCLESVKQKCVKSCNCVTELDGQVENLHLDLCCLAGNQEDLSK
DSLGPTKSSKIEGAGTSISEPPSPISPYASESCGTLPLPLRPCGEGSEMVGKENSSPENK
NWLLAMAAKRKAENPSPRSPSSQTPNSRRQSGKTLPSPVTITPSSMRKICTYFHRKSQED
FCGPEHSTEL
>Q9BZZ5
MPTVEELYRNYGILADATEQVGQHKDAYQVILDGVKGGTKEKRLAAQFIPKFFKHFPELA
DSAINAQLDLCEDEDVSIRRQAIKELPQFATGENLPRVADILTQLLQTDDSAEFNLVNNA
LLSIFKMDAKGTLGGLFSQILQGEDIVRERAIKFLSTKLKTLPDEVLTKEVEELILTESK
KVLEDVTGEEFVLFMKILSGLKSLQTVSGRQQLVELVAEQADLEQTFNPSDPDCVDRLLQ
CTRQAVPLFSKNVHSTRFVTYFCEQVLPNLGTLTTPVEGLDIQLEVLKLLAEMSSFCGDM
EKLETNLRKLFDKLLEYMPLPPEEAENGENAGNEEPKLQFSYVECLLYSFHQLGRKLPDF
LTAKLNAEKLKDFKIRLQYFARGLQVYIRQLRLALQGKTGEALKTEENKIKVVALKITNN
INVLIKDLFHIPPSYKSTVTLSWKPVQKVEIGQKRASEDTTSGSPPKKSSAGPKRDARQI
YNPPSGKYSSNLGNFNYGERFRLGTRNMRD
>Q9Y4R8
MEPAPSEVRLAVREAIHALSSSEDGGHIFCTLESLKRYLGEMEPPALPREKEEFASAHFS
PVLRCLASRLSPAWLELLPHGRLEELWASFFLEGPADQAFLVLMETIEGAAGPSFRLMKM
ARLLARFLREGRLAVLMEAQCRQQTQPGFILLRETLLGKVVALPDHLGNRLQQENLAEFF
PQNYFRLLGEEVVRVLQAVVDSLQGGLDSSVSFVSQVLGKACVHGRQQEILGVLVPRLAA
LTQGSYLHQRVCWRLVEQVPDRAMEAVLTGLVEAALGPEVLSRLLGNLVVKNKKAQFVMT
QKLLFLQSRLTTPMLQSLLGHLAMDSQRRPLLLQVLKELLETWGSSSAIRHTPLPQQRHV
SKAVLICLAQLGEPELRDSRDELLASMMAGVKCRLDSSLPPVRRLGMIVAEVVSARIHPE
GPPLKFQYEEDELSLELLALASPQPAGDGASEAGTSLVPATAEPPAETPAEIVDGGVPQA
QLAGSDSDLDSDDEFVPYDMSGDRELKSSKAPAYVRDCVEALTTSEDIERWEAALRALEG
LVYRSPTATREVSVELAKVLLHLEEKTCVVGFAGLRQRALVAVTVTDPAPVADYLTSQFY
ALNYSLRQRMDILDVLTLAAQELSRPGCLGRTPQPGSPSPNTPCLPEAAVSQPGSAVASD
WRVVVEERIRSKTQRLSKGGPRQGPAGSPSRFNSVAGHFFFPLLQRFDRPLVTFDLLGED
QLVLGRLAHTLGALMCLAVNTTVAVAMGKALLEFVWALRFHIDAYVRQGLLSAVSSVLLS
LPAARLLEDLMDELLEARSWLADVAEKDPDEDCRTLALRALLLLQRLKNRLLPPASP
>Q9UUA1
MRKWEIANEVCVDEKQDRDAEEIEDEGKNIILGIVSQLRPNMDLSKVTFPTFVLEPKSML
ERITNFMSHPDLLLNVQKTADPEMRFLDVVRFYMSGWHIRPRGVKKPLNPILGETFTGFW
KFPPSNSKDPNLQKLHGIAVYAAEQVCHHPPISAYFYLCPEYKVRIDGVVKPRSRFLGNS
AASIMEGIASIKLQDLDEEYLITQPNVYARGILFGKMRLELGDHVTVRCPKTDLQADIEF
KVKGFISGTYNSIAGKVKRVSTGEVLYKISGKWDSEMSVESVKTGETRPLLDVSKHDVYL
VSARPLEEQGERESQRLWYKTCQAVIARDQTTATETKSAIEDRQREEAKQREIENVQWKP
KYFKMIAPEEYILNFDIPNGKSDLEVLCALDEFIPIFSEVDRIAFHKFLSENTKPEDSSI
HKHSRDASGDSTKGFAEHMPAPARRRRPQSTASFVTYRSDNGSVDEYHDAQLPDPNTLSK
LHEEQDPAL
>Q9P2Y5
MSASASVGGPVPQPPPGPAAALPPGSAARALHVELPSQQRRLRHLRNIAARNIVNRNGHQ
LLDTYFTLHLCSTEKIYKEFYRSEVIKNSLNPTWRSLDFGIMPDRLDTSVSCFVVKIWGG

KENIYQLLIEWKVCLDGLKYLGQQIHARNQNEIIFGLNDGYYGAPFEHKGYSNAQKTILL
QVDQNCVRNSYDVFSLLRLHRAQCAIKQTQVTVQKIGKEIEEKLRLTSTSNELKKKSECL
QLKILVLQNELERQKKALGREVALLHKQQIALQDKGSAFSAEHLKLQLQKESLNELRKEC
TAKRELFLKTNAQLTIRCRQLLSELSYIYPIDLNEHKDYFVCGVKLPNSEDFQAKDDGSI
AVALGYTAHLVSMISFFLQVPLRYPIIHKGSRSTIKDNINDKLTEKEREFPLYPKGGEKL
QFDYGVYLLNKNIAQLRYQHGLGTPDLRQTLPNLKNFMEHGLMVRCDRHHTSSAIPVPKR
QSSIFGGADVGFSGGIPSPDKGHRKRASSENERLQYKTPPPSYNSALAQPVTTVPSMGET
ERKITSLSSSLDTSLDFSKENKKKGEDLVGSLNGGHANVHPSQEQGEALSGHRATVNGTL
LPSEQAGSASVQLPGEFHPVSEAELCCTVEQAEEIIGLEATGFASGDQLEAFNCIPVDSA
VAVECDEQVLGEFEEFSRRIYALNENVSSFRRPRRSSDK
>Q9D0P7
MEDLKEDIKFIVDETLDFGGLSPSDSHEEEDITVLVSPEKPLRRGLAHRSNPNEVAPALQ
GVRFSLGPLSPEKLEEILDEANRLAAQLEECALKDRERAGTGPGRPSPRGKPSPRRETFV
LKDSPVRDLLPTVSSWSTPPPSSLAGLRSSDKKGSARAVRVASGKKPSSIKKESPTCNLF
PASKSPGRSPLAQPILPPRRKTGFGARTTASPPIPVRPVPQSSASNSQCSSRLQGAAVKS
SSRLPVPSAIPKPATRVPLIGRSLPPGKGALAPDSLSTQKGHPSAIGHRASVSQKTNLPT
TSAARGRTTSAARGRAQPLRKAAVPGPTR
>Q9R0C0
MSVPEPPPPDGVLTGPSDSLEAGEPTPGLSDTSPDEGLIEDFPVDDRAVEHLVGGLLSHY
LPDLQRSKRALQELTQNQVVLLDTLEQEISKFKECHSMLDINALFTEAKHYHAKLVTIRK
EMLLLHEKTSKLKKRALKLQQKRQREELEREQQREKEFEREKQLTAKPAKRT
>P78545
MAATCEISNIFSNYFSAMYSSEDSTLASVPPAATFGADDLVLTLSNPQMSLEGTEKASWL
GEQPQFWSKTQVLDWISYQVEKNKYDASAIDFSRCDMDGATLCNCALEELRLVFGPLGDQ
LHAQLRDLTSSSSDELSWIIELLEKDGMAFQEALDPGPFDQGSPFAQELLDDGQQASPYH
PGSCGAGAPSPGSSDVSTAGTGASRSSHSSDSGGSDVDLDPTDGKLFPSDGFRDCKKGDP
KHGKRKRGRPRKLSKEYWDCLEGKKSKHAPRGTHLWEFIRDILIHPELNEGLMKWENRHE
GVFKFLRSEAVAQLWGQKKKNSNMTYEKLSRAMRYYYKREILERVDGRRLVYKFGKNSSG
WKEEEVLQSRN
>Q8K400
MRKFNIRKVLDGLTAGSSSASQQQQQQQHPPGNREPEIQETLQSEHFQLCKTVRHGFPYQ
PSALAFDPVQKILAVGTQTGALRLFGRPGAECYCQHDSGAAVIQLQFLINEGALVSALAD
DTLHLWNLRQKRPAVLHSLKFCRERVTFCHLPFQSKWLYVGTERGNIHIVNVESFTLSGY
VIMWNKAIELSSKSHPGPVVHISDNPMDEGKLPIGFESGTVVLWDLKSKKADYRYTYDEA
IHSVAWHHEGKQFICSHSDGTLTIWNVRSPAKPVQTITPHGKQLKDGKKPEPCKPILKVE
FKTTRSGEPFIILSGGLSYDTVGRRPCLTVMHGKSTAVLEMDYSIVDFLTLCETPYPNDF
QEPYAVVVLLEKDLVLIDLAQNGYPIFENPYPLSIHESPVTCCEYFADCPVDLIPALYSV
GARQKRQGYSKKEWPINGGNWGLGAQSYPEIIITGHADGSVKFWDASAITLQVLYKLKTS
KVFEKSRNKDDRQNTDIVDEDPYAIQIISWCPESRMLCIAGVSAHVIIYRFSKQEVLTEV
IPMLEVRLLYEINDVDTPEGEQPPPLSTPVGSSNPQPIPPQSHPSTSSSSSDGLRDNVPC
LKVKNSPLKQSPGYQTELVIQLVWVGGEPPQQITSLALNSSYGLVVFGNCNGIAMVDYLQ
KAVLLNLSTIELYGSNDPYRREPRSPRKSRQPSGAGLCDITEGTVVPEDRCKSPTSGSSS
PHNSDDEQKVNNFIEKVKTQSRKFSKMVANDLAKMSRKLSLPTDLKPDLDVKDNSFSRSR
SSSVTSIDKESRETISALHFCETLTRKADSSPSPCLWVGTTVGTAFVITLNLPPGPEQRL

LQPVIVSPSGTILRLKGAILRMAFLDATGCLMSPAYEPWKEHNVAEEKDEKEKLKKRRPV
SVSPSSSQEISENQYAVICSEKQAKVMSLPTQSCAYKQNITETSFVLRGDIVALSNSVCL
ACFCANGHIMTFSLPSLRPLLDVYYLPLTNMRIARTFCFANNGQALYLVSPTEIQRLTYS
QETCENLQEMLGELFTPVETPEAPNRGFFKGLFGGGAQSLDREELFGESSSGKASRSLAQ
HIPGPGGIEGVKGAASGVVGELARARLALDERGQKLSDLEERTAAMMSSADSFSKHAHEM
MLKYKDKKWYQF
>Q96PC2
MVVQNSADAGDMRAGVQLEPFLHQVGGHMSVMKYDEHTVCKPLVSREQRFYESLPLAMKR
FTPQYKGTVTVHLWKDSTGHLSLVANPVKESQEPFKVSTESAAVAIWQTLQQTTGSNGSD
CTLAQWPHAQLARSPKESPAKALLRSEPHLNTPAFSLVEDTNGNQVERKSFNPWGLQCHQ
AHLTRLCSEYPENKRHRFLLLENVVSQYTHPCVLDLKMGTRQHGDDASEEKKARHMRKCA
QSTSACLGVRICGMQVYQTDKKYFLCKDKYYGRKLSVEGFRQALYQFLHNGSHLRRELLE
PILHQLRALLSVIRSQSSYRFYSSSLLVIYDGQEPPERAPGSPHPHEAPQAAHGSSPGGL
TKVDIRMIDFAHTTYKGYWNEHTTYDGPDPGYIFGLENLIRILQDIQEGE
>Q9H8N7
MASVLSRRLGKRSLLGARVLGPSASEGPSAAPPSEPLLEGAAPQPFTTSDDTPCQEQPKE
VLKAPSTSGLQQVAFQPGQKVYVVWYGGQECTGLVEQHSWMEGQVTVWLLEQKLQVCCRVE
EVWLAELQGPCPQAPPLEPGAQALAYRPVSRNIDVPKRKSDAVEMDEMMAAMVLTSLSCS
PVVQSPPGTEANFSASRAACDPWKESGDISDSGSSTTSGHWSGSSGVSTPSPPHPQASPK
YLGDAFGSPQTDHGFETDPDPFLLDEPAPRKRKNSVKVMYKCLWPNCGKVLRSIVGIKRH
VKALHLGDTVDSDQFKREEDFYYTEVQLKEESAAAAAAAAAGTPVPGTPTSEPAPTPSMT
GLPLSALPPPLHKAQSSGPEHPGPESSLPSGALSKSAPGSFWHIQADHAYQALPSFQIPV
SPHIYTSVSWAAAPSAACSLSPVRSRSLSFSEPQQPAPAMKSHLIVTSPPRAQSGARKAR
GEAKKCRKVYGIEHRDQWCTACRWKKACQRFLD
>P20052
MESTSGERSENIHEDQGIPKVILPADFNKCSRTDLVVLISRMLVSLIAINENSATKKSDD
QITLTRYHSKIPPNISIFNYFIRLTKFSSLEHCVLMTSLYYIDLLQTVYPDFTLNSLTAH
RFLLTATTVATKGLCDSFSTNAHYAKVGGVRCHELNILENDFLKRVNYRIIPRDHNITLC
SIEQKQKKFVIDKNALGSLDLDSYSYVNRPKSGYNVLDKYYRRIVQLVGSFNASPDKSRK
VDYVLPPNIDIVSESGSQTTQLKGSSSPNSHSSQKRYSEAKDAHIYNKRSKPD
>P04183
MSCINLPTVLPGSPSKTRGQIQVILGPMFSGKSTELMRRVRRFQIAQYKCLVIKYAKDTR
YSSSFCTHDRNTMEALPACLLRDVAQEALGVAVIGIDEGQFFPDIVEFCEAMANAGKTVI
VAALDGTFQRKPFGAILNLVPLAESVVKLTAVCMECFREAAYTKRLGTEKEVEVIGGADK
YHSVCRLCYFKKASGQPAGPDNKENCPVPGKPGEAVAARKLFAPQQILQCSPAN
>Q49MI3
MPWRRRRNRVSALEGGREEEAPPEAAAVPPALLTSPQQTEAAAERILLRGIFEIGRDSCD
VVLSERALRWRPIQPERPAGDSKYDLLCKEEFIELKDIFSVKLKRRCSVKQQRSGTLLGI
TLFICLKKEQNKLKNSTLDLINLSEDHCDIWFRQFKKILAGFPNRPKSLKILLNPQSHKK
EATQVYYEKVEPLLKLAGIKTDVTIMEYEGHALSLLKECELQGFDGGHRKPLFAIHWSVQ
RLFTGMQTLEPSVVCVGGDGSASEVAHALLLRAQKNAGMETDRILTPVRAQLPLGLIPAG
STNVLAHSLHGVPHVITATLHIIMGHVQLVDVCTFSTAGKLLRFGFSAMFGFGGRTLALA
EKYRWMSPNQRRDFAVVKALAKLKAEDCEISFLPFNSSDDVQERRAQGSPKSDCNDQWQM
IQGQFLNVSIMAIPCLCSVAPRGLAPNTRLNNGSMALIIARNTSRPEFIKHLKRYASVKN

QFNFPFVETYTVEEVKVHPRNNTGGYNPEEEEDETASENCFPWNVDGDLMEVASEVHIRL
HPRLISLYGGSMEEMIPK
>O94347
MTSGSPKITNLLTRVKRIIPPLLDTFHKGQAGRVGVFGGCQHYTGAPYYSSMSSMLFGSD
QSHIFCEKEAANVIKSYSPDLIVHPFLREKDKAGPEDSVDKCFELIKPMMGRLHAIVIGP
GLGRDEWMQEIMAKVIEYARKNDMPMVIDADGLWLIQQRPELVSGYHNVILTPNVIEFKR
LCDKLDIKSDGPDACNQLAGKLNLLIIQKGQSDIISDGATAYACSVPGGLKRCGGQGDIL
TGILATFLAWRHAYLSKEWDTEGNMDAKECLFLAAFGASACTRWCSRLAFKECGRATQST
DLVRHVGKAYNALMEDEIPSVEEKIKD
>Q8C0J2
MSSGLRAADFPRWKRHIAEELRRRDRLQRQAFEEIILQYTKLLEKSDLHSVLTQKLQAEK
HDMPNRHEISPGHDGAWNDSQLQEMAQLRIKHQEELTELHKKRGELAQLVIDLNNQMQQK
DKEIQMNEAKISEYLQTISDLETNCLDLRTKLQDLEVANQTLKDEYDALQITFTALEEKL
RKTTEENQELVTRWMAEKAQEANRLNAENEKDSRRRQARLQKELAEAAKEPLPVEQDDDI
EVIVDETSDHTEETSPVRAVSRAATKRLSQPAGGLLDSITNIFGRRSVSSIPVPQDIMDT
HPASGKDVRVPTTASYVFDAHDGEVNAVQFSPGSRLLATGGMDRRVKLWEAFGDKCEFKG
SLSGSNAGITSIEFDSAGAYLLAASNDFASRIWTVDDYRLRHTLTGHSGKVLSAKFLLDN
ARIVSGSHDRTLKLWDLRSKVCIKTVFAGSSCNDIVCTEQCVMSGHFDKKIRFWDIRSES
VVREMELLGKITALDLNPERTELLSCSRDDLLKVIDLRTNAVKQTFSAPGFKCGSDWTRV
VFSPDGSYVAAGSAEGSLYVWSVLTGKVEKVLSKQHSSSINAVAWAPSGLHVVSVDKGSR
AVLWAQP
>O94527
MSGISLSLRQLDYRDWFQRKISRDIYGNSTWLTGIDLQKELTGHTGCVNTLDWSADGEFL
LSGSDDTRLIVWDVFNEYKPRHLISTGHVQNIFSAKFVPYSNNRQILSASGDKLIKLFDL
DSSKEGGMDHGMETQTRCWSCALDSVKNIVPCDNGHTFLVCSEDGTARQYDIREPHVCNQ
DLDCPSILVNYNPYRINLYTITMSPSNPYYFAIGGTHPYAFLYDRRMVKKSFRDDWTMNT
SPEKDCRCVRKFSPDGSCNSQGILDRYITCCQFSAANPNELLVSWNSDYVYLFHVHEDKS
YTPTFNKIEDSNKKPSKPSLLQTQPLKRKNYSPWYKNNFGASTPASRVSRNPYTAAQPRK
HTFYQMYENIEKFFTTENGGLYESIVSGRLSHFSRSIQYVKDAIYFLENYNYIPDSNGLN
HSIRVSALRYWRACVSILALMDDTVSLEPNTIIQAGWGWLYDFMNWVTRYLLGISDHWAL
QMSPPTNVARQNFVLCDPDEPSRVLFSNPSEMIRAFARIDTNDLSSVRRFFWIHKVLRGC
LLLISSDIYWEQFQPWDSSTSDVTSSQRLDDENGFLTLLEPPVNYENEVESSSGENIVSM
YTGHSDLNDDDDDYQDEESYSYASDDDDESDEDSDEGPTLLSLRMKKRKAVEPNVPVNTH
VKSYYGHCNVESIKNVNFYGQNDEYVMSGSDDGRFFIWDKLNASILAIIHGDSEAVNVIE
GHPRCPTLAVSGIDSTVKIFNTENTPPSGCSRNHTSNSYKIIATNEMNRQQGSRDSYITS
RMLSHLAYRAHLDDGFGHEVLDTDACSIM
>O60158
MTENEKSRSLPAERNPLYKDDTLDHTPLIPKCRAQVIEFPDGPATFVRLKCTNPESKVPH
FLMRMAKDSSISATSMFRSAFPKATQEEEDLEMRWIRDNLNPIEDKRVAGLWVPPADALA
LAKDYSMTPFINALLEASSTPSTYATPSRPTAQKSETSEGEPESSTSATTTSVARRTRQR
LAEHLENSKKTILQHDNKEEDKEIHSEENETKDEIKSEKKEPEIKKQEGGSSTEKVGQPS
SSDDKAKGSTSKDQPSEEEEKTSDIQDRKIKTPIKPSLLGKIRSSVNKGMTDVASQVNRG
MTDVASQVNKGVNGVASQVNKGMNGVANQVNKGVTGVASQVRKPVGKLEKKFENLEKSIG
DTLKSSIRSSPKSKKRSREDFEENEDYNAMVPVKRSRITKLESEVYYEKRKVRALGGIAI

GLGVGAILPFLF
>Q9HAP2
MAADVFMCSPRRPRSRGRQVLLKPQVSEDDDDSDTDEPSPPPASGAATPARAHASAAPPP
PRAGPGREEPPRRQQIIHSGHFMVSSPHREHPPKKGYDFDTVNKQTCQTYSFGKTSSCHL
SIDASLTKLFECMTLAYSGKLVSPKWKNFKGLKLQWRDKIRLNNAIWRAWYMQYLEKRKN
PVCHFVTPLDGSVDVDEHRRPEAITTEGKYWKSRIEIVIREYHKWRTYFKKRLQQHKDED
LSSLVQDDDMLYWHKHGDGWKTPVPMEEDPLLDTDMLMSEFSDTLFSTLSSHQPVAWPNP
REIAHLGNADMIQPGLIPLQPNLDFMDTFEPFQDLFSSSRSIFGSMLPASASAPVPDPNN
PPAQESILPTTALPTVSLPDSLIAPPTAPSLAHMDEQGCEHTSRTEDPFIQPTDFGPSEP
PLSVPQPFLPVFTMPLLSPSPAPPPISPVLPLVPPPATALNPPAPPTFHQPQKFAGVNKA
PSVITHTASATLTHDAPATTFSQSQGLVITTHHPAPSAAPCGLALSPVTRPPQPRLTFVH
PKPVSLTGGRPKQPHKIVPAPKPEPVSLVLKNARIAPAAFSGQPQAVIMTSGPLKREGML
ASTVSQSNVVIAPAAIARAPGVPEFHSSILVTDLGHGTSSPPAPVSRLFPSTAQDPLGKG
EQVPLHGGSPQVTVTGPSRDCPNSGQASPCASEQSPSPQSPQNNCSGKSDPKNVAALKNR
QMKHISAEQKRRFNIKMCFDMLNSLISNNSKLTSHAITLQKTVEYITKLQQERGQMQEEA
RRLREEIEELNATIISCQQLLPATGVPVTRRQFDHMKDMFDEYVKTRTLQNWKFWIFSII
IKPLFESFKGMVSTSSLEELHRTALSWLDQHCSLPILRPMVLSTLRQLSTSTSILTDPAQ
LPEQASKAVTRIGKRLGES
>Q05809
MLLFSSFFSHNKCPKQKKGPQPLKVVKGTSKSCAKQGKIKGKEKLWQAKTGRLVMSETGE
TSEYYKQALEEYKEVQEDEDPDVWDTRISKTGCYVENLALQLCHAETGDWRQCFNEMALF
RKCWEKNGNRERVSTVDVDGTTSKDSEKKK
>O14772
MAAARDPPEVSLREATQRKLRRFSELRGKLVARGEFWDIVAITAADEKQELAYNQQLSEK
LKRKELPLGVQYHVFVDPAGAKIGNGGSTLCALQCLEKLYGDKWNSFTILLIHSGGYSQR
LPNASALGKIFTALPLGNPIYQMLELKLAMYIDFPLNMNPGILVTCADDIELYSIGEFEF
IRFDKPGFTALAHPSSLTIGTTHGVFVLDPFDDLKHRDLEYRSCHRFLHKPSIEKMYQFN
AVCRPGNFCQQDFAGGDIADLKLDSDYVYTDSLFYMDHKSAKMLLAFYEKIGTLSCEIDA
YGDFLQALGPGATVEYTRNTSNVIKEESELVEMRQRIFHLLKGTSLNVVVLNNSKFYHIG
TTEEYLFYFTSDNSLKSELGLQSITFSIFPDIPECSGKTSCIIQSILDSRCSVAPGSVVE
YSRLGPDVSVGENCIISGSYILTKAALPAHSFVCSLSLKMNRCLKYATMAFGVQDNLKKS
VKTLSDIKLLQFFGVCFLSCLDVWNLKVTEELFSGNKTCLSLWTARIFPVCSSLSDSVIT
SLKMLNAVKNKSAFSLNSYKLLSIEEMLIYKDVEDMITYREQIFLEISLKSSLM
>P40026
MGSVNSSPNEEFETVPDSQISGFDSPLIPTSVGSYFRDDDDDEKVHPNFISDPENDSLNS
DEEFSSLENSDLNLSGAKAESGDDFDPILKRTIISKRKAPSNNEDEEIVKTPRKLVNYVP
LKIFNLGDSFDDTITTTVAKLQDLKKEILDSPRSNKSIVITSNTVAKSELQKSIKFSGSI
PEIYLDVVTKETISDKYKDWHFISKNCHYEQLMDLEMKDTAYSFLFGSSRSQGKVPEFVH
LKCPSITNLLVLFGVNQEKCNSLKINYEKKENSRYDNLCTIFPVNKMLKFLMYFYSDDDN
DDVREFFLKAFICLILDRKVFNAMESDHRLCFKVLELFNEAHFINSYFEIVDKNDFFLHY
RLLQIFPHLQSALLRRRFSEKQGRTETIQQNIIKEFNEFFDCKNYKNLLYFILTMYGSKF
IPFGPKCQVTEYFKDCILDISNETTNDVEISILKGILNLFSKIR
>P39683
MSEPVIKSLLDTDMYKITMHAAVFTNFPDVTVTYKYTNRSSQLTFNKEAINWLKEQFSYL

GNLRFTEEEIEYLKQEIPYLPSAYIKYISSSNYKLHPEEQISFTSEEIEGKPTHYKLKIL
VSGSWKDTILYEIPLLSLISEAYFKFVDIDWDYENQLEQAEKKAETLFDNGIRFSEFGTR
RRRSLKAQDLIMQGIMKAVNGNPDRNKSLLLGTSNILFAKKYGVKPIGTVAHEWVMGVAS
ISEDYLHANKNAMDCWINTFGAKNAGLALTDTFGTDDFLKSFRPPYSDAYVGVRQDSGDP
VEYTKKISHHYHDVLKLPKFSKIICYSDSLNVEKAITYSHAAKENGMLATFGIGTNFTND
FRKKSEPQVKSEPLNIVIKLLEVNGNHAIKISDNLGKNMGDPATVKRVKEELGYTERSWS
GDNEAHRWT
>P53886
MTSKPSTRNDYLPRETHNGEYTGDSPEWQLQINITNKIGGINGDIWLSRDGRSVKWCIED
QCLRQFTYNQKIIKAGYIDFEKTPDCFVVVLSDIAHVYMLKNGGSTTVCFPFQIGNAFWY
ANGVILERETSASFMDGGYDLKPIEFDLKHKYITLTDPMAPFGLISITNTFKGGINSASG
NKTDILQDFQLVLFPSDKDKCIAVFLDRNSKVLRFYYSRILSSDQSRKGELTISSTKKTG
LDAAGNSQKSGGISKDLRKFSHLTRRSTSINSNSHDFNAAERVLSGNVGNASGRTDIFAL
PSSCSRRSLSATLDRMGNNIAPTNRAAPSGFFDSSSANTATHSNITPVSQPMQQQQQEYL
NQTATSSKDIVLTEISSLKLPDDIIFTSRRLSSILSKLKFLSLRFERREGLLIFHEPTHF
CKIWLIDLLPDVLDSIPFKIYGNSPQNMIRLENLKLKEPSRIQAMYIHELLESCLILVSE
GQNKEEYKACLYDPFVKITSPSKNISEELTKQNSLPSLQKLFPYPETSFTKLCFEAVKYI
TSPAFNISFIFLWQSAYSILLSRANDDVVGGLKMEHDAFSLVLSLLILPIPSSSAQEYQE
YKEIYERDLFQHLKQDSEITSSVLPRIVIGLHLIREEYSLNVLCRNEHALLGQFLRFATA
AMGWPDLWQSYYVPKMDSESKTFLHPREQNSTFFHPLDEPPSITKSLYSITENSSIPLCP
FISFSRLVATDTQVELRITPRSFKILGLYELVHSPNFLPDYVLGILSSFKVDKDELQTYP
LGILVPLQNILKILEDKLSEVRDNLELLDRADLQRCSAIINSIRSDSKEVLKRGQRDSYM
LCKVPLAKNRSSLSKKPSDIYSILSEIVKSASQVPLDGSAMRMSNIQDDEDIDEGRSLKL
NAGLIFSEDKRFTHVVSLLAYYRPTKTQFFTTKTEYAQILAQKKYFAKIMALRTCTNGVG
WGAVAYATEKPISTQKWVIQPLNLISVFPDDTKITVKAPEDIAHDIVEWGQFHAGVSSGL
RISKKATGITGSWIAFNKPKELDAYHGGFLLGLGLNGHLKNLEEWHIYNYLSPRNTHISI
GLLLGMSSSMKGSMDSKLIKVISVHLVAFLPSGSSDLNIDLKLQTAGIIGMGMLYLNSRH
KRMSDSIFAQLVSLLNVNDEMVADEEYRLAAGISLGLINLGAGQTKLRKWDSSLLGLGDD
LPEDVYDSSDVEQNVMYEDLTTKLLEIVTSTYDVENDWIPENSQIGAVIAIMFLFLKSNN
FGISNMLKVDLKEILKANINTRPELLMYREWASNMILWEFIGDDLSFIMKDVDIGVKFSE
LNTDLLPIYYTMAGRILAMGIRFASTGNLKIRNILLSLVDKFLPLYQYPGKQNLDFRLTI
SVINVLTNVIVVSLSMVMCASGDLEVLRRVKYLHEVASGPYSDLFQEIPSSKSDVSGVTQ
VTSNTNTPGNSDRERVDETAASLDDERSSNGSDISDPTAYLEDKKDIDDHYGKFISTNLA
LGFLFLGSGQYALNTSTLESIAFLSMSVLPTYTTPHPLQELKHFWSIAVEPRCLVIKDIS
TGDAVNNVPIELVVEEDVEKEEVIREISTPCLLPDFSKIKSIRVKMHGYFPLEVNFTKDY
SASDFFSGGTIIYIQRKSESVFENKASFRNVEDIHVALKRKAAESKNYSRLNLKNEQGNT
TSSQLVESLGIQDLTMVELDTLLSAGNNTALTDSESYNLGLLCSDKNSGDILDCQLELWY
KSFGPHDE
>Q68CZ1
MSGPTDETAGDLPVKDTGLNLFGMGGLQETSTTRTMKSRQAVSRVSREELEDRFLRLHDE
NILLKQHARKQEDKIKRMATKLIRLVNDKKRYERVGGGPKRLGRDVEMEEMIEQLQEKVH
ELEKQNETLKNRLISAKQQLQTQGYRQTPYNNVQSRINTGRRKANENAGLQECPRKGIKF
QDADVAETPHPMFTKYGNSLLEEARGEIRNLENVIQSQRGQIEELEHLAEILKTQLRRKE
NEIELSLLQLREQQATDQRSNIRDNVEMIKLHKQLVEKSNALSAMEGKFIQLQEKQRTLR

ISHDALMANGDELNMQLKEQRLKCCSLEKQLHSMKFSERRIEELQDRINDLEKERELLKE
NYDKLYDSAFSAAHEEQWKLKEQQLKVQIAQLETALKSDLTDKTEILDRLKTERDQNEKL
VQENRELQLQYLEQKQQLDELKKRIKLYNQENDINADELSEALLLIKAQKEQKNGDLSFL
VKVDSEINKDLERSMRELQATHAETVQELEKTRNMLIMQHKINKDYQMEVEAVTRKMENL
QQDYELKVEQYVHLLDIRAARIHKLEAQLKDIAYDTKQYKFKPEIMPDDSVDEFGETIHL
ERGENLFEIHINKVTFSSEVLQASGDKEPVTFCTYAFYDFELQTTPVVRGLHPEYNFTSQ
YLVHVNDLFLQYIQKNTITLEVHQAYSTEYETIAACQLKFHEILEKSGRIFCTASLIGTK
GDIPNFGTVEYWFRLRVPMDQAIRLYRERAKALGYITSNFKGPEHMQSLSQQAPKTAQLS
STDSTDGNLNELHITIRCCNHLQSRASHLQPHPYVVYKFFDFADHDTAIIPSSNDPQFDD
HMYFPVPMNMDLDRYLKSESLSFYVFDDSDTQENIYIGKVNVPLISLAHDRCISGIFELT
DHQKHPAGTIHVILKWKFAYLPPSGSITTEDLGNFIRSEEPEVVQRLPPASSVSTLVLAP
RPKPRQRLTPVDKKVSFVDIMPHQSDETSPPLEDRKEISPEVEHIPEIEINMLTVPHVPK
VSQEGSVDEVKENTEKMQQGKDDVSLLSEGQLAEQSLASSEDETEITEDLEPEVEEDMSA
SDSDDCIIPGPISKNIKQSLALSPGLGCSSAISAHCNFRLPGSSDFPASASQVDGITGAC
HHSQPSEKIRIEIIALSLNDSQVTMDDTIQRLFVECRFYSLPAEETPVSLPKPKSGQWVY
YNYSNVIYVDKENNKAKRDILKAILQKQEMPNRSLRFTVVSDPPEDEQDLECEDIGVAHV
DLADMFQEGRDLIEQNIDVFDARADGEGIGKLRVTVEALHALQSVYKQYRDDLEA
>Q02887
MKVLQFNQDATCCVVAASSHQISIFNCDPFGKCFEIDTKNSKKKTSNNNGSASNSESRNN
EESILITNGSRDRTDAEEEEDNEDNALVTGNILKEGEFVIEMLFSTSLIAIADRGQGLNK
GKKLKIVNTKRKCTICEIVFPHEIVDVVMNRKRMCVLLESDQIFIYDISCMKPLETIDLW
EDHYKRSQANSFSNASNTGTLEGDSANLNRVATNLLANATQKSVNGSNPSVRTRRNSLRS
KIRPRMVLSNDDRSILCFTAYSSPKKNKPNSEALYDVVIYDTLNVTPVNYLNSVHKGNVA
CLAVSHDGKLLATASDKGTIIRVFHTGVDSDYMSSRSLFKEFRRGTRLCNLYQLAFDKSM
TMIGCVGDTDTIHLFKLDDASNSLPGDNSSNGHWNEEEYILASNSNPSMGTPKEIPLSKP
RIANYFSKKIKSSIPNQNLSRNFAYITVNESNRSCLGFPDEFPNQVYIASDDGTFSIYSI
PSKPGECVLTKNNKFT
>P21262
MAQYQTWEEFSRAAEKLYLADPMKARVVLKYRHSDGSLCIKVTDDLVCLVYRTDQAQDVK
KIEKFHSQLMRLMVAKESRSVAMETD
>P70347
MSLKRHSLRRNACHLETRAGIPTILYSDATGQRGMDKNIGEQLNRAYEAFRQACMDRDSA
VRELQQKQTENYEQRIREQQEQLSFQQNLIDRLKSQLLLVDSSRDNSYGYVPLLEDSDRR
KNNLTLDEPHDKVKLGTLRDKQSKVRRQEVSSGKESAKGLNIPLHHERDNIEKTFWDLKE
EFHRICLLAKAQKDHLSKLNIPDIATDTQCSVPIQCTDKTEKQEALFKPQAKDDINRGMS
CVTAVTPRGLGRDEEDTSFESLSKFNVKFPPMDNDSIFLHSTPEAPSILAPATPETVCQD
RFNMEVRDNPGNFVKTEETLFEIQGIDPITSAIQNLKTTDKTNPSNLRATCLPAGDHNVF
YVNTFPLQDPPDAPFPSLDSPGKAVRGPQQPFWKPFLNQDTDLVVPSDSDSELLKPLVCE
FCQELFPPSITSRGDFLRHLNTHFNGET
>Q80Z29
MNAAAEAEFNILLATDSYKVTHYKQYPPNTSKVYSYFECREKKTENSKVRKVKYEETVFY
GLQYILNKYLKGKVVTKEKIQEAKEVYREHFQDDVFNERGWNYILEKYDGHLPIEVKAVP
EGSVIPRGNVLFTVENTDPECYWLTNWIETILVQSWYPITVATNSREQKKILAKYLLETS
GNLDGLEYKLHDFGYRGVSSQETAGIGASAHLVNFKGTDTVAGIALIKKYYGTKDPVPGY

SVPAAEHSTITAWGKDHEKDAFEHIVTQFSSVPVSVVSDSYDIYNACEKIWGEDLRHLIV
SRSTEAPLIIRPDSGNPLDTVLKVLDILGKKFPVSENSKGYKLLPPYLRVIQGDGVDINT
LQEIVEGMKQKKWSIENVSFGSGGALLQKLTRDLLNCSFKCSYVVTNGLGVNVFKDPVAD
PNKRSKKGRLSLHRTPAGTFVTLEEGKGDLEEYGHDLLHTVFKNGKVTKSYSFDEVRKNA
QLNMEQDVAPH
>Q9QXA1
MVSKPRTEWSTVLSHLVLAGVSLHAAVSSVQCTNGHLMCAGCFIHLLADARLKEEQATCP
NCRCEISKSLCCRNLAVEKAVSELPSECGFCLRQFPRSLLERHQKEECQDRVTQCKYKRI
GCPWHGPFHELTVHEAACAHPTKTGNELMEILDEMDQSHRKEMQLYNSIFSLLSFEKIGY
TEVQFRPYRTDDFITRLYYETPRFTVLNQTWVLKARVNDSERNPNLSCKRTLSFQLLLKS
KVTAPLECSFLLLKGPYDDVRISPVIYHFVFTNESNETDYVPLPIIDSVECNKLLAAKNI
NLRLFLFQIQK
>Q54QT7
MLTNNEIYQEVQKFAKGYDLNQDGVLTSQEIYYSLLKKMNGNSYEASKATGVLCSTIDIN
KDGKFSYHEIAKYCADNAKKLIEQNADIAALADVEAFLLRFDKDKDRKLNKTEFVEYFKG
GTDTPYSDRDYVLKIIDLDKDGCVSANELQEWFKKKRIDYASRPHC
>P34036
MEDQQINVDSPTSGTNTPPVVTPTTSVISEQTLEETVKYLCKICPTLLDGDQSVFQNNLS
NQIPPENMNKLKKFISDSKIPVLLIQKTNPTINSSNQTSTTTSSSSSSDDNTLTSSQQQS
KESFNFEIEVKFGGENKSTLAIVKRIPESIVEYSSNKSIASQLQVLNLGDGSPMDTLHNY
IHNSVAPFVRSYILSASKDDATTPSGGSADKSITSQNLDKEMKQSIGAVNQKIAELEISL
YNCKQQVQIPEVTLAINPEIKSISKRLKETTGRTIKPDDLGDKASSPEFLNLLQAGTTTW
AKNIQNVTKHNLIENLPSDVSTSQEINFWIELETSLQNIDQQLKSPEVEVTLATLRQAKR
FIASAPFETDTIGVRKAMDKVQSYKTLFKDFPITPLLTATDLDSISSSVAAIFSHLKKTK
NPYYPIPRYLSFLEAIGRDMCNKVYQILRQKNLMNIDYNDFEHLNRSVRALFTLWDDQFG
AFRDILRDLAKKRGNERIPLIVNIDNRIQLVIIKIGKFRKQHEDLKNVVSNVLPGSQLGG
GVVQTNTSNPTSPQKQEINAIEEINQAYLEFKEIDVLQLSKEGEEIWDAVVKRYNSRTDR
VETYITVKLRDRLATAKNANEMFRVYQKFKDLLKRPKIRGATHEYESQLIERVKEDIRVL
HDKFKMQYNNSEAYYMSQLRDLPPVSGAIIWARQIERQLDTYMKRVANVLGDSWESDAEG
QKLKSESDQFRHKLNTDHIFSKWADETEKRSFDISGRILTIVKRGNKLALDINFDSHIIM
LFKEVRNLQWLGFRVPLKISFISQGAKQVYPFAVSLKETLRTYAQTSGKVTPEFSTLVAS
YKRDVQANITEGFRLKWETIPKVDPYVRKLSTSINNFRDKVDDLIVKYSEIKKQLDGLKS
CPFKSESFNEIIANIQKVVDELNLANYSNLPQWVTQLDAQVESMLIERLIDAINSWVQLI
EGKEDQKDSQSTSGSSNKGGKLNRMNYSIRNKSDEENSSDLTQPQQSQQQQQTISIKPKL
EKTIHEIVIRNQILSLSPPLEVARVNWIDQLHSWLNICCDLPRIQSSRYDESAMVHRGGV
DSKKQSTFRDMLPKLPQGSLESAYSAITNKLEQVQQYVSIWLQYQSLWDMDSSFVYSKLG
DDLNKWQLLLNQIKKSRSTFDNSSTEKQFGPLTIDYTQVQASVNNKYDYWHKDILGHFGS
KLAEKMNQFYETISSSRQELEKLSVETVSTEEAVHFIIQIQDMKKKLSSWEADLRYYRTG
QDLLQRQRFSFPNDWLDCERVEGEWSAFNEILNRKNATISEAIPQLQAKILQESKSINDR
IKDFIDEWTANKPLQGSIKHSTALETLKIFEGRLIRLREESDRLSKAKQALDLTDTTGSS
SSDQDRLVPVEEEIQDLKAVWVELSNTWQEIDSLKETAWSAIIPRKVRKSLEDTLQKLKN
LPNRIRQYSAFDHAQNLIKIYLKGNAIITDLHSEAIKDRHWKILKKRLNTNWIITELTLG
SIWDSDLARNENIYREVITAAQGEIALEEFLKGVREFWTTLELDLVNYQRKCKLVRGWDD
LFNKLAEHLNSISAMKMSPYYKVFEEEANHWDDRLNKVRSLLDVWIDVQRRWVYLEGIFS

GSGDINQLLPAESTRFKSINSEFIAILKKVSGAPLILEVLAIERIQQTMERLSDLLGKVQ
KALGEYLERQRSAFARFYFVGDEDLLEIIGNSKDIIKIQKHFRKMFAGLANLTLDDEKTT
IIGMSSAEGETVTFKKPISIANGPKIHEWLTMVESEMKSTLATLLSESLQHFNQVDVNDH
SKYSEWVDNYPTQLVLLTSQIVWSTQVDQALGGGTLQQSKIQEQLQSIEQTTQMILNNLA
DSVLQDLSAQKRKKFEHLITELVHQRDVVRQLQKCKNLTGNKDFDWLYHMRYYYDATQEN
VLHKLVIHMANATFYYGFEYLGIGERLVQTPLTDRCYLTLTQALESRMGGNPFGPAGTGK
TETVKALGSQLGRFVLVFCCDEGFDLQAMSRIFVGLCQCGAWGCFDEFNRLEERILSAVS
QQIQTIQVALKENSKEVELLGGKNISLHQDMGIFVTMNPGYAGRSNLPDNLKKLFRSMAM
IKPDREMIAQVMLYSQGFKTAEVLAGKIVPLFKLCQEQLSAQSHYDFGLRALKSVLVSAG
GIKRKCQPPQLPPITDAESKTKADQIYCQYEIGVLLNSINDTMIPKLVADDIPLIQSLLL
DVFPGSQLQPIQMDQLRKKIQEIAKQRHLVTKQEWVEKILQLHQILNINHGVMMVGPSGG
GKTTSWEVYLEAIEQVDNIKSEAHVMDPKAITKDQLFGSLDLTTREWTDGLFTATLRRII
DNVRGESTKRHWIIFDGDVDPEWVENLNSLLDDNKLLTLPNGERLALPNNVRVMFEVQDL
KYATLATISRCGMVWFSEEILTTQMIFQNYLDTLSNEPFDPQEKEQQKRNENAQLQQQQQ
TTITSPILTSPPTTSSSSRSTTSTTSMIPAGLKVQKECAAIISQYFEPGGLVHKVLEDAG
QRPHIMDFTRLRVLNSFFSLMNRSIVNVIEYNQLHSDFPMSPENQSNYITNRLLYSLMWG
LGGSMGLVERENFSKFIQTIAITPVPANTIPLLDYSVSIDDANWSLWKNKVPSVEVETHK
VASPDVVIPTVDTTRHVDVLHAWLSEHRPLILCGPPGSGKTMTLTSTLRAFPDFEVVSLN
FSSATTPELLLKTFDHHCEYKRTPSGETVLRPTQLGKWLVVFCDEINLPSTDKYGTQRVI
TFIRQMVEKGGFWRTSDHTWIKLDKIQFVGACNPPTDAGRVQLTHRFLRHAPILLVDFPS
TSSLTQIYGTFNRALMKLLPNLRSFADNLTDAMVEFYSESQKRFTPDIQAHYIYSPRELS
RWDRALLEAIQTMDGCTLEGLVRLWAHEALRLFQDRLVETEEKEWTDKKIDEVALKHFPS
VNLDALKRPILYSNWLTKDYQPVNRSDLREYVKARLKVFYEEELDVPLVLFNEVLDHILR
IDRVFRQPQGHALLIGVSGGGKSVLSRFVAWMNGLSIYTIKVNNNYKSSDFDDDLRMLLK
RAGCKEEKICFIFDESNVLESSFLERMNTLLAGGEVPGLFEGEEFTALMHACKETAQRNG
LILDSEEELYKYFTSQVRRNLHVVFTMNPASPDFHNRSATSPALFNRCVLDWFGEWSPEA
LFQVGSEFTRNLDLENPQYIAPPVFIQEAEIMGNNLMAIPPSHRDAVVSSLVYIHQTIGE
ANIRLLKRQGRQNYVTPRHYLDFINQVVLLINEKRDQLEEEQLHLNIGLKKLRDTEAQVK
DLQVSLAQKNRELDVKNEQANQKLKQMVQDQQAAEIKQKDARELQVQLDVRNKEIAVQKV
KAYADLEKAEPAIIEAQEAVSTIKKKHLDEIKSLPKPPTPVKLAMEAVCLMLGGKKLEWA
DIRKKIMEPNFITSIINYDTKKMMTPKIREAITKGYLEDPGFDYETVNRASKACGPLVKW
ATAQTYYSEILDRIKPLREEVEQLENAANELKLKQDEIVATITALEKSIATYKEEYATLI
RETEQIKTESSKVKNKVDRSIALLDNLNSERGRWEQQSENFNTQMSTVVGDVVLASAFLA
YIGFFDQNFRTDLMRKWMIRLDSVGIKFKSDLSVPSFLSKPEERLNWHANSLPSDELCIE
NAIMLKRFNRYPLVIDPSGQAMEFLMNQYADKKITKTSFLDSSFMKNLESALRFGCPLLV
QDVENIDPVLNPVLNKEIRKKGGRILIRLGDQDVDFSPSFMIFLFTRDPTAHFTPDLCSR
VTFVNFTVTPSSLQSQCLHEALKTERPDTHKKRSDLLKIQGEFQVKLRILEKSLLNALSQ
ASGNILDDDSVISTLETLKKETTEIALKVEETETVMQEISEVSALYNPMALSCSRVYFAM
EELSQFHLYQFSLRAFLDIFYNLLNNNPNLVDKKDPNERLVYLSKDIFSMTFNRVTRTLL
NDDKLTFALQLTIISVKGTSNEIEESEWDFLLKGGDNLTSIKETIPQLDSLLSTTQQKWL
ICLRQQVPSFSKLVDHIQQNSSDWKQFFGKDQVGEPIIPESWIVAQAQLSNQQSTIVSNF
RKILLMKAFHSDRVLQYSHSFVCSVFGEDFLNTQELDMANIVEKEVKSSSPLLLCSVPGY
DASSKVDDLALQLHKQYKSFAIGSPEGFELAEKSIYAAAKSGTWVLLKNIHLAPQWLVQL
EKKLHSLSPHPSFRLFMTSEIHPALPANLLRMSNVFSYENPPGVKANLLHTFIGIPATRM

DKQPAERSRIYFLLAWFHAIIQERLRYIPLGWTKFFEFNDADLRGALDSIDYWVDLYSKG
RSNIDPDKIPWIAVRTILGSTIYGGRIDNEFDMRLLYSFLEQLFTPSAFNPDFPLVPSIG
LSVPEGTTRAHFMKWIEALPEISTPIWLGLPENAESLLLSNKARKMINDLQKMQSSEEDG
EDDQVSGSSKKESSSSSSEDKGKAKLRATITEWTKLLPKPLKQLKRTTQNIKDPLFRCFE
REISTGGKLVKKITNDLANLLELISGNIKSTNYLRSLTTSISKGIVPKEWKWYSVPETIS
LSVWISDFSKRMQQLSEISESSDYSSIQVWLGGLLNPEAYITATRQSASQLNGWSLENLR
LHASSLGKISSEGGASFNVKGMALEGAVWNNDQLTPTDILSTPISIATLTWKDKDDPIFN
NSSSKLSVPVYLNETRSELLFSIDLPYDQSTSKQNWYQRSVSISSWKSDI

>Q6J9G1

MGEKGHLSRVLLECSLSDKLCVVREKQYEVIIVPALLVGGFLILLAIILWLFIRGQRSQR
QSPGPRGTASVPASRGRSQEAAGHGEKVLLPLKETSVEGFLRAATPRLAKLQVPREQLLE
VLEQIHSGSCGTLYHATMTTKDHPKPKSVVLKALEDPVGLQEVQDFIGRIQFYQYLGKHK
NLVQLEGCCTERLPLYMMLEDVVPGDLLSFLWTCRRDVMTMDGLLYDLTEKQIYHIGKQI
LLALEFLQEKHLFHGDVAARNILIQSDLTPKLCHLGLAYEVHAHGAISSARSSTIPLKWL
APERLLLRPASIRGDIWSFGILLYEMVTLGAPPYPEVPPTSILQYLQRKKIMKRPSSCSH
AMYNIMKCCWRWSEDSRPLLGQLLQRLEAASRSADDKAVLQVPELVVPELYADVAGIRAE
SISYSFSVL

>Q09739

MPPKGLSLAEKRRRLEAIFHDSKDFFQLKEVEKLGSKKQIVLQTVKDVLQSLVDDNIVKT
EKIGTSNYYWSFPSDAKRSRESVLGSLQAQLDDLKQKSKTLDENISFEKSKRDNEGTEND
ANQYTLELLHAKESELKLLKTQLSNLNHCNPETFELKNENTKKYMEAANLWTDQIHTLIA
FCRDMGADTNQIREYCSIPEDLDDLQLPIL

>Q07799

MKHNRQMPDESLENIKVLLNPKLGKPVKSLTSAQSKACYHTLISNKNLNKTSDEYEKLLA
NYILLCDEKYLCKTVIPDSRFWAILCDNCQKLRSETLVANLIRIFNVALKCQDSNKNEVI
VSICHISRENSQLIGILLQLLSQRPIHIPLFTDTILCITLFLKCSLTLCETSLSHAVEFV
PRILILLFQYNFPASMSELLYIEDLQPLILEEFVPLKQRLINFLSSVSIDDYSCSLKADL
LTAIKDNSVFQKGLEMEMGDLPSINLLNAYDTFTFLNSPNGSFKRLYTEQLLFGENDFPL
YEAIFKLSDQFRRLFNLSGKKENQYSDSERDLKLQIATAVLNRQTCFYKTLELFLRFWIE
SLAKSQSDLVSLLNLAIITLKYVCLSSSDLEAAIQTKSLLKTQVVALDSMRYKFARTLQL
DSIKKEQYRTWSSSIASFDTMLSGQVRDYVRHQRLLQLQKGTWVYAENPLNPEAGTPKVY
FLIVSDNHANLLAREFETQTNDLPYLFDNKILTSPGSEALANGRTKVVVLKHITSFKSIE
LTTPSRRTSSNVYIKLDEANVYTGVELKDRNDRTVLKFYLDTEEGRYIWLDGLKLISPFQ
HEDISEDTKEQIDTLFDLRKNVQMINLNVRQDIIVPPPEPSDEDEDEEFYNLETLKKVTQ
NFYFD

>Q12221

MDNKRLYNGNLSNIPEVIDPGITIPIYEEDIRNDTRMNTNARSVRVSDKRGRSSSTSPQK
IGSYRTRAGRFSDTLTNLLPSISAKLHHSKKSTPVVVVPPTSSTPDSLNSTTYAPRVSSD
SFTVATPLSLQSTTTRTRTRNNTVSSQITASSSLTTDVGNATSANIWSANAESNTSSSPL
FDYPLATSYFEPLTRFKSTDNYTLPQTAQLNSFLEKNGNPNIWSSAGNSNTDHLNTPIVN
RQRSQSQSTTNRVYTDAPYYQQPAQNYQVQVPPRVPKSTSISPVILDDVDPASINWITAN
QKVPLVNQISALLPTNTISISNVFPLQPTQQHQQNAVNLTSTSLATLCSQYGKVLSARTL
RGLNMALVEFSTVESAICALEALQGKELSKVGAPSTVSFARVLPMYEQPLNVNGFNNTPK
QPLLQEQLNHGVLNYQLQQSLQQPELQQQPTSFNQPNLTYCNPTQNLSHLQLSSNENEPY

PFPLPPPSLSDSKKDILHTISSFKLEYDHLELNHLLQNALKNKGVSDTNYFGPLPEHNSK
VPKRKDTFDAPKLRELRKQFDSNSLSTIEMEQLAIVMLDQLPELSSDYLGNTVIQKLFEN
SSNIIRDIMLRKCNKYLTSMGVHKNGTWVCQKIIKMANTPRQINLVTSGVSDYCTPLFND
QFGNYVIQGILKFGFPWNSFIFESVLSHFWTIVQNRYGSRAVRACLEADSIITQCQLLTI
TSLIIVLSPYLATDTNGTLLITWLLDTCTLPNKNLILCDKLVNKNLVKLCCHKLGSLTVL
KILNLRGGEEEALSKNKIIHAIFDGPISSDSILFQILDEGNYGPTFIYKVLTSRILDNSV
RDEAITKIRQLILNSNINLQSRQLLEEVGLSSAGISPKQSSKNHRKQHPQGFHSPGRARG
VSVSSVRSSNSRHNSVIQMNNAGPTPALNFNPAPMSEINSYFNNQQVVYSGNQNQNQNGN
SNGLDELNSQFDSFRIANGTNLSLPIVNLPNVSNNNNNYNNSGYSSQMNPLSRSVSHNNN
NNTNNYNNNDNDNNNNNNNNNNNNNNNNNNNNNNSNNSNNNNNNDTSLYRYRSYGY
>Q58A45
MDGGALTDTSLTDSYFSTSFIGVNGFGSPVETKYPLMQRMTNSSSSPSLLNDSAKPYSAH
DPLTSPASSLFNDFGALNISQRRKPRKYRLGMLEERLVPMGSKARKAKNPIGCLADRCKS
GVPINMVWWNRVTENNLQTPNPTASEFIPKGGSTSRLSNVSQSNMSAFSQVFSHPSMGSP
ATAGLAPGMSLSAGSSPLHSPKITPHTSPAPRRRSHTPNPASYMVPSSASTSVNNPVSQT
PSSGQVIQKETVGGTTYFYTDTTPAPLTGMVFPNYHIYPPTAPHVAYMQPKANAPSFFMA
DELRQELINRHLITMAQIDQADMPAVPTEVDSYHSLFPLEPLPPPNRIQKSSNFGYITSC
YKAVNSKDDLPYCLRRIHGFRLVNTKCMVLVDMWKKIQHSNIVTLREVFTTKAFAEPSLV
FAYDFHAGGETMMSRHFNDPNADAYFTKRKWGQHEGPLPRQHAGLLPESLIWAYIVQLSS
ALRTIHTAGLACRVMDPTKILITGKTRLRVNCVGVFDVLTFDNSQNNNPLALMAQYQQAD
LISLGKVVLALACNSLAGIQRENLQKAMELVTINYSSDLKNLILYLLTDQNRMRSVNDIM
PMIGARFYTQLDAAQMRNDVIEEDLAKEVQNGRLFRLLAKLGTINERPEFQKDPTWSETG
DRYLLKLFRDHLFHQVTEAGAPWIDLSHIISCLNKLDAGVPEKISLISRDEKSVLVVTYS
DLKRCFENTFQELIAAANGQL
>Q3E731
MSGNPGSSLSALRPTPPERGSFPLDHDGECTKYMQEYLKCMQLVQNENAMNCRLLAKDYL
RCRMDHQLMDYDEWSHLGLPEDAPGNNGKTIKDATDNK
>P05743
MAKQSLDVSSDRRKARKAYFTAPSSQRRVLLSAPLSKELRAQYGIKALPIRRDDEVLVVR
GSKKGQEGKISSVYRLKFAVQVDKVTKEKVNGASVPINLHPSKLVITKLHLDKDRKALIQ
RKGGKLE
>Q9BXJ9
MPAVSLPPKENALFKRILRCYEHKQYRNGLKFCKQILSNPKFAEHGETLAMKGLTLNCLG
KKEEAYELVRRGLRNDLKSHVCWHVYGLLQRSDKKYDEAIKCYRNALKWDKDNLQILRDL
SLLQIQMRDLEGYRETRYQLLQLRPAQRASWIGYAIAYHLLEDYEMAAKILEEFRKTQQT
SPDKVDYEYSELLLYQNQVLREAGLYREALEHLCTYEKQICDKLAVEETKGELLLQLCRL
EDAADVYRGLQERNPENWAYYKGLEKALKPANMLERLKIYEEAWTKYPRGLVPRRLPLNF
LSGEKFKECLDKFLRMNFSKGCPPVFNTLRSLYKDKEKVAIIEELVVGYETSLKSCRLFN
PNDDGKEEPPTTLLWVQYYLAQHYDKIGQPSIALEYINTAIESTPTLIELFLVKAKIYKH
AGNIKEAARWMDEAQALDTADRFINSKCAKYMLKANLIKEAEEMCSKFTREGTSAVENLN
EMQCMWFQTECAQAYKAMNKFGEALKKCHEIERHFIEITDDQFDFHTYCMRKITLRSYVD
LLKLEDVLRQHPFYFKAARIAIEIYLKLHDNPLTDENKEHEADTANMSDKELKKLRNKQR
RAQKKAQIEEEKKNAEKEKQQRNQKKKKDDDDEEIGGPKEELIPEKLAKVETPLEEAIKF
LTPLKNLVKNKIETHLFAFEIYFRKEKFLLMLQSVKRAFAIDSSHPWLHECMIRLFNTAV

CESKDLSDTVRTVLKQEMNRLFGATNPKNFNETFLKRNSDSLPHRLSAAKMVYYLDPSSQ
KRAIELATTLDESLTNRNLQTCMEVLEALYDGSLGDCKEAAEIYRANCHKLFPYALAFMP
PGYEEDMKITVNGDSSAEAEELANEI
>P14306
MNQAIDFAQASIDSYKKHGILEDVIHDTSFQPSGILAVEYSSSAPVAMGNTLPTEKARSK
PQFQFTFNKQMQKSVPQANAYVPQDDDLFTLVMTDPDAPSKTDHKWSEFCHLVECDLKLL
NEATHETSGATEFFASEFNTKGSNTLIEYMGPAPPKGSGPHRYVFLLYKQPKGVDSSKFS
KIKDRPNWGYGTPATGVGKWAKENNLQLVASNFFYAETK
>Q9C0V7
MAFNKQAESKKPNFLVIVADDLGWSDVSPFGSEIHTPNIERLAKEGVRLTNFHTASACSP
TRSMLLSGTDNHIAGLGQMAETVRRFSKVWGGKPGYEGYLNDRVAALPEILQEAGYYTTM
SGKWHLGLTPDRYPSKRGFKESFALLPGGGNHFAYEPGTRENPAVPFLPPLYTHNHDPVD
HKSLKNFYSSNYFAEKLIDQLKNREKSQSFFAYLPFTAPHWPLQSPKEYINKYRGRYSEG
PDVLRKNRLQAQKDLGLIPENVIPAPVDGMGTKSWDELTTEEKEFSARTMEVYAAMVELL
DLNIGRVIDYLKTIGELDNTFVIFMSDNGAEGSVLEAIPVLSTKPPVKYFDNSLENLGNY
NSFIWYGPRWAQAATAPSRLSKGFITEGGIRCPAIIRYPPLIKPDIISDEFVTVMDILPT
ILELAEVPHPGHKFQGRDVVIPRGKPWIDHFVHGKPVHKDTDFSGWELFGQRAIRKGNYK
AIYVPKEGIKTEWELYDLSQDKGELENLAKVHPDILNELIEYWLVYEAETGVVTAPDDFV
AENVGLFKPAKHNL
>Q9UUH7
MTTLESLDMPEMTEVEDDTVDIHIDNSKVALLFSKSKVFVHPTSKMKDNISGYLSLSKSK
ALGNSSVAGSDILLSWVPDSFLKNRPRDLSVFQNAETLSNGSIREWVEIPQHLDYSFSVR
LCSIYSIIFRPPRYGWNYGSIVINLRDSGESLPPLFFHDDECISTIEYGKQITRDRFDPF
DESGNMFWGGTHLLMQLKKYASLEQSSHESQLYLVNPSPEDTVAFQSVELQKVISNNRLN
SSSTPPTPRSSSSIFNPFRRALHDLSFTVLERFSRVTNYGKSEVDRLMEHKVTKSILPHL
PRELQVLLESKRVQKLTEEYDPARMFLARWAEGIVEQSESNNSQPVNNAGVWTDAQREED
SSLGPFELVYIEERVKRDDPLSVEQWNSMFNAHGKLQVDVHRVLGIIFHGGIQPSLRKEV
WPFLLSVYPWDSTSEERRVIYLSLQEEYCTLKRKWYEDIHKQFNDRWFIEQRNRIEKDVH
RTDRQHEYFQIEDLPHPDPQSTFTGTNMNMEMMKDILLTYNEYDTELGYVQGMSDLLAPI
YVTFNDNALTFWGMVGLMKRLHFNFLRDQSGMHRQLDTLRLLIEFMDPELFAHLEKTDSS
NLFCFFRMLLIYFKREFDWEVLLKLWDVLFTNYLSYDYHIFVAYAIAERHREVLLNQTSA
FDEVLKYFNELSGKLALEPTLICAEQCFYQFKNKLALIDRKQMEETNSDEDGSKETDLPT
IAPYLRNLLKTSQPQYTPSGKQE
>P61960
MSKVSFKITLTSDPRLPYKVLSVPESTPFTAVLKFAAEEFKVPAATSAIITNDGIGINPA
QTAGNVFLKHGSELRIIPRDRVGSC
>O13674
MAIMHKIGCAGSEVETDRVLEKDQANNGHFVDSDCCFQETGSTGTANSICTLNDSEDDNS
SLNSVDNNECTNLDCGGKKHKNLSPNPSFHVNINAAEFIPKSHNGYAPKSMNPPPEQLDS
PCYPHFDQDSSSVIYPSPPSTYYPNMYVSANTFIPMPYAHYTDNMYHAKPVNRPNFLPRE
GTPPPFAQQPAEQFSPFNSEYESLLDFRALILGNLPVDYKISELLSLIHSGPLEKIQSNP
GKRRIIITFLDSADAFFFYERYHPRGFIFHGRPLKLTFARPSPLPESIQNFCSNTAASRN
VFIGNLPSSYHEKEIEEAFGKFGKIEHIKILSKKNIAFVHFLNIRDAIKVVRTLSCDPDY
HSWRIFFGSDRCANHPPSFDERSCFTSKQNPDTTSDRCRQQESKDNGNRTVFLGNLHTKT

KGHEICDLIRHGGLQDFHYIPEKHICFVTFITYSAANAFHDYLAEEEVLLHGRRIKVGWG
KESGPLPRVLEDSILMGASRNVYFSHISDSLTTEELELILRQYGEIESIKYLKNRSSGFV
AYTNISNAMKAVNGLPIHPLFKKSKIRYAPDRCEQELQKSKSNSPIQQNVPLQQFITPPF
SYPVAYCPAIPPGSDPILNFGIQYQPFIPFTAVPSFPFNCNVPNYPQTSDHEND
>P22224
MDQEGQPLLSKDFQQVLLATASGNNSSWTERAVLNNESTDAVKHEPALGQNDVFDLDPLS
FDKWVPFLRRALDKNQLDPVIDELENSIEDNFQGLELQLLQDSQMNDKLETSIDEIANIQ
GMVQDTLSSEISKFQIRLSESANELIVKKQMYVNNKKISLKISEATILITKVVRILELSS
KCQELITERKFFKVLQNLDSLEKLYLQEFKNYNFQFLIEIYNSIPFLQKVTKDECINLIR
NSLNLNLGKNLIKVGQEFVAIYENELLPQWLETRSKMKLTNFKFNSPIEISMRDESFLAK
LNLGEFFQLDDFHDSIMIFQNLNELSVLSGEFNKEYELRKTKLMYPLIWKKNKTAAYQMD
SLLRGTGTTPGSTAHDVSTDDPFTQSLSLHFLQDYFLKILGFLLYDINLNKATEFILVDN
NYNSTNEFWDGLMDRLSPYLSYFIDEKLKTEEDMIKLKDFLCIYVAILENFKLNIEPLYK
ILVSIFEKFCSVSLRAFDDEFQILLNDDDFMPLSINDKTLYEKVLKICWMKEGEHLSLPD
PTNGEPFAVTLPFSPLYPMTCTLAKKTYSKITAFLSIFYRHELHTLNNILVKTMDDIFND
IVNKKIRSKLESTSREEIAQILVNLDYFIIAAKEFSNFMTRENILQNPDMEIRLSSIKYL
AESRKLAETKLIELIDSKISDILETIEIDWQITEVRQDPDISIIDLAQFLEMMFASTLQN
LPYSVQTLLIFREFDSLTRQFMGLLLHDTPSTITHESIMNFEVDVNYLESIIPRIFPSTP
GTIDSNGYQSPMTPSTPTFPNANGVDAPTLFENNIKSLEATFMELKQCIELLKTQGKDYN
EPEIRLRKYSRIRQEDAALLLSKIQHFVSSVEGANGDDTSVMDSSSIFNSESASVIDSNT
SRIAKFFNRR
>O74414
MLILGLTGSIATGKSTVSREFQEKYHIKIIDADVLARKVVEPNTPCLIKIQKEFGNEVLH
EDGTLNRAKLGQAVFQDAGKRSLLNSIIHPAVRLEMLKELLRCYVRGYSIVILDVPLLFE
AKMQFICWKTICVSCDKSIQKQRLLARNPELTAEDAENRVQAQMPLELKCQLADIVIENN
SDLETLYENIHNVLPLITPSYFFTLLCLILPPLQITLQVIAFVSQKKKVSEFRKHI
>P33402
MSRRKISSESFSSLGSDYLETSPEEEGECPLSRLCWNGSRSPPGPLEPSPAAAAAAAAPA
PTPAASAAAAAATAGARRVQRRRRVNLDSLGESISRLTAPSPQTIQQTLKRTLQYYEHQV
IGYRDAEKNFHNISNRCSYADHSNKEEIEDVSGILQCTANILGLKFEEIQKRFGEEFFNI
CFHENERVLRAVGGTLQDFFNGFDALLEHIRTSFGKQATLESPSFLCKELPEGTLMLHYF
HPHHIVGFAMLGMIKAAGKKIYRLDVEVEQVANEKLCSDVSNPGNCSCLTFLIKECENTN
IMKNLPQGTSQVPADLRISINTFCRAFPFHLMFDPSMSVLQLGEGLRKQLRCDTHKVLKF
EDCFEIVSPKVNATFERVLLRLSTPFVIRTKPEASGSENKDKVMEVKGQMIHVPESNSIL
FLGSPCVDKLDELMGRGLHLSDIPIHDATRDVILVGEQAKAQDGLKKRMDKLKATLERTH
QALEEEKKKTVDLLYSIFPGDVAQQLWQGQQVQARKFDDVTMLFSDIVGFTAICAQCTPM
QVISMLNELYTRFDHQCGFLDIYKVETIGDAYCVAAGLHRKSLCHAKPIALMALKMMELS
EEVLTPDGRPIQMRIGIHSGSVLAGVVGVRMPRYCLFGNNVTLASKFESGSHPRRINVSP
TTYQLLKREESFTFIPRSREELPDNFPKEIPGICYFLEVRTGPKPPKPSLSSSRIKKVSY
NIGTMFLRETSL
>P07170
MSSSESIRMVLIGPPGAGKGTQAPNLQERFHAAHLATGDMLRSQIAKGTQLGLEAKKIMD
QGGLVSDDIMVNMIKDELTNNPACKNGFILDGFPRTIPQAEKLDQMLKEQGTPLEKAIEL
KVDDELLVARITGRLIHPASGRSYHKIFNPPKEDMKDDVTGEALVQRSDDNADALKKRLA

AYHAQTEPIVDFYKKTGIWAGVDASQPPATVWADILNKLGKD
>Q12508
MSELLDSFETEFAKFYTDSNLEETNLQKCLDHTHEFKSQLKKLKAHLNKHIQESKPEVYN
KLSDKEKQKFKRKRELIIEKLSKSQRQWDHSVKKQIKYVSQQSNRFNKSTLNKLKEFDID
SVYVNKLPKETMENVNEAIGYHILRYSIDNMPLGNKNEAFQYLKDVYGITNKESTEFIEM
GQIVHDLKKGDTESCLKWCSNEMESLSSNHTALSSLKFDLYTLSAMQIVKHGNPVELYYQ
ITQNAPLDCFRHREKELMQNVVPLLTKSLIGQPIEDIDSKVNKELKECTSLFIKEYCAAK
HIFFDSPLFLIVLSGLISFQFFIKYKTIRELAHVDWTTKDELPFDVKLPDFLTHFHPIFI
CPVLKEETTTENPPYSLACHHIISKKALDRLSKNGTITFKCPYCPVNTSMSSTKKVRFVM
L
>Q9URX8
MDINDPSFSSTENGIYELLSRGYAAIEAATERDLKLPELGDIIGQVHAPEYISQVLSGWK
PFYLRSVVNIPDRIFEQYNRTECFTQMGLFAEIQRAWITVDNRLFLWDYLSGQNFQAYED
LSHTIVNVKLVRPKANVFVSEIQHLLVIATSQEMLLLGVTIDEKTGELSFFSTGIQISVQ
GINVNCIVSSEDGRIFFSGNKDPNLYEFSYQLEEGWFSRRCSKINITGSVFDNFIPSFFS
FGTHGDGIKQIAVDDSRSLLYVLRETSSVSCYELTKNGVNRCVFYSFSSMISQAQMLNAT
SPLLDPRTTQIVSIVPIPAYESQQIYCVAITSTGCRFYMRGGRGPISHYAPSNSTLSSTP
PSTLQLTFVRFPPPMQVENYASSRNYPANPFFLQNQSTSQQQPERSSAVKTTPMKCSSLS
NIYTSDLFFAISSSNTNEGDVVCCTAPEVGRIANAWQSGTQPSLIESSMYVPIKGFVQDI
KCIQNSRERNELVSQFNTPPPTFAILTNTGVYVVVHRRPIDVLASAIRMGPSLSSGIDGQ
VQLFFESVGRAEGCATCLGIVSGCLDQGDFSHAAANFSGSTTKLAQADLLDIVKKYYIEF
GGKAFIDQSRYNNQYDSSSLEFVRLSGCHDGLASSISRIIRNVWKNHVIIAKKMQNKRIH
YAPAFNATEILKIQSGLLYLSTFLENNKSFIEGLNSPNTLIGSSNVADEIAVQAEHRALS
ALLLVLQQIVEGISFLLFLNDTGVSDFHEIVSSTSIDIQKSCSNMTFGEFFTSKRGREVT
KELVNSLVNRHLQSGGNIDMVSQLLRKKCGSFCSADDVLIFKAVESLKKAKDTVDIEERQ
SLIELSYTLFKKAAHVFTPEDLRLAVEEYKSLNAYTTAVNLALHVASARDDRNQALSYLV
DGMPENDPRREPFESRTKCYSYIFEILDSLESQMSNDSSAIKVDVYDTIQRSKDELFHYC
FYDWYSFKGLTDRLIEIDSPYIQSYLERNSTKDMKIADLLWQYYAKREQYYQASIVLYDL
ATTHLAFSLEQRIEYLTRAKGFGSCHVPNSLRHKMNKVMLSVLEQLDVASIQDDVLIAIR
GDMRIPTSKREELSKQLDGEIIPLSDLFNNYADPLGYGEICLSIFQCADYRGINEILNCW
ESIIKTTHENAIISPVGSSPVEAVSSTLKNLTLRFSQSENVFPIEQIIDITERYAFDQQG
EAVATGWVIDTFLGAGVSHELIFIVLNQLYDRREKPWQGKDRLFFLIKEVTHLLKLWHEV
SVRAGVAQTSKPSFDAPLVLEAIEKYKNALGAPDTATKSCKENLISLDSEIRQTY
>O74815
MSEKKHSCEEKSLQLYPLSNKIRHVNPNISALPSETDISESLTNDLTKLEISNYAPFNRD
LETFSDLIFGLTNIQKKSLYSLQLGRSRGYALYSDENEKYLWSINTKITSTEQREVLLVK
SNNKKFDTSIVLKHTHLSSKSQNEKKARVKVFRQEIWALKTLSHPCVVQLLNYYVSSAEL
ILVENYCMGGDLYHYTKKHHSDFSLEFVGRIFSELVHTVAYLHSKCLIHRDLKLENILLT
QPYNVIKTIDNWKNYPNALIQISDFELSIFVDSKNHLVQSSCGSQEYAPPEVYMGIAHDG
FRADAWSLGIVLFALLEGRLPFDSYPTLDPENVRIKRYVQRLVRCDYTWHLCKSPFKRST
GNTNDNDDPSWRFRLFVKKLLKNRDQRSTPTELLKDFNKHGNFTLPLLENVTI
>Q9P775
MSIVSSVAFVPRGYASEFPKSYDIDEIEYERINQLSKLKLEDARADLQSSMEDSNKANGN
GEETTDGAEGVLQTSEEVDDELKVYNLDTYDDDEEEAETEGPAAMFSNIRGLAYHENGEK

DPYITIDPQEQDDLEREEMQILPTDSLLLAARTEDNLSHVEVYVYEPTEENLYVHHDFLL
PTFPLCLEWLDYKVGTSDNAPGNYVAVGTFDPEIEIWDLDIIDAVYPAAVLGAGASQVNK
KKKKSKKINDSYHTDAVLALSSNRNAHNLLVSGSADTTLKLWDLSTCNCVKSFTYHSDKV
SCLDWYSKAPSVLLSGSYDKTAKIADLRLEEAPSSFQVTSDVENVAWDQHSENNFFIGTD
NGIVYYCDARNLSKSVWQLQAHDGPISCLSVNPSVPSFVATGSTDRVVKLWNTSDSSPKM
VVSRDLDVGRVFTCSFTTDESTAFHLAASGSKGVVRVWDTATNPGVRKAFESRVTEEVTK
KERIVQLEDRGAGEDSSDDDDYEDIEDDDDQDAEMS
>Q8K1N4
MSRKQSQKDSSGFIFDLQSNTVLAQGGTFENMKEKINAVRAIVPNKSNNEIILVLQHFDN
CVDKTVQAFMEGSASEVLKEWIVTGKKKNKKKKSKPKPASEASGSAPDSSKSAPIQEEQP
ASSEKGSINGYHVNGAINDAESVDSLSEGLETLSIDARELEDPEFAAAETLDRTGSVLEN
GVSDFEPKSLTAHSISNVQQSRNAAKSLSRTTPGAQVSNLGMENVPLSSTNKKLGSNIEK
SVKDLQRCTVSLARYRVVVKEEMDASIKKMKQAFAELQSCLMDREVALLAEMDKVKAEAM
EILLSRQKKAELLKKMTDVAVRMSEEQLVELRADIKHFVSERKYDEDLGRVARFTCDVET
LKQSIDSFGQVSHPKNSYSTRSRCSLVAPVSLSGPSDGSAASSSPDASVPSLPGANKRNC
APREASAAMTNSSDRPCQAHREVFPGNRRGGQGYRAQSQKTADPSNPGRHDSVGRYRNSS
WYSSGPRYQGVPPQAPGNAGERSRPYSAGTNGTGAISEPSPPKPSFKKGLPQRKPRASQA
EAANS
>Q9NAD6
MMGSSSQELQTALTDVSKTCHHLWEENKDLQGRFVNELGELQRLQMVIAQLEQQQRLENV
FTVKQQMTELQKRAATLYEHLTQKRNDIVIKLNDGTNFATMLQTQLIGEKLFSWKNAQKL
AQIGMPFDNREQLLDEIQIEFEFLADQNWQLNMFSCWMLDLLRRAPQLNDGLAQATIGKL
TAITEQLNKLLFMLVSQSFIVSVQPEPVLKTQHKFVTEVRLLIGDKLGIRQHLVNTNVSV
KIIAEDEAKQLSVDYDAHKEIRNNKTVGTISNDFEKLTMNERGHLAAKFNNSKLTRIAHR
KPPPKGASDLKCAASMQAATDQKYALLFFITPFQMGNLSKEEQFDVWTLSLPIMVTVHGS
QDCDAQVAILWHRAFASISRNPNTTDVTAVTWDNLAIMLRNKFSLFTGARRPLSDSDLAY
LSEKMLMPNVADQKPITFHRFAKQAMRDDLPFSFWEWFFSIMQLIKQKLLKFWDEGWCIG
FISKNDASQSMMMCQHSSFLLRFSDSQTGAVSIGFVCEEADGQKIPFHLAPFTIKDLDQL
SLASRIASCPQLKDIRYMYPAIDKEEMLRFFESEERHRVGGGDSPTGYIQSEIVMVAKTN
GNFRRMSNAPSMFGADSPSPLSVQSKLDWSPGEVHQNHMMEMSDELGQILTVSDMSGDVE
TLLGPAFKNNITNYNPHDGNHQHNLHFVDMSQQGMMQQHHNQFYPS
>Q13352
MPVKRSLKLDGLLEENSFDPSKITRKKSVITYSPTTGTCQMSLFASPTSSEEQKHRNGLS
NEKRKKLNHPSLTESKESTTKDNDEFMMLLSKVEKLSEEIMEIMQNLSSIQALEGSRELE
NLIGISCASHFLKREMQKTKELMTKVNKQKLFEKSTGLPHKASRHLDSYEFLKAILN
>P23731
SLKQSQESSEYEIAYRSTIQPRTAVRTQSRQSGAYSTGAVSGGGGRVLKMVTEMGSASIG
GISPALSANAAKSFLEATDKEKKEMQGLNDRLGNYIDRVKKLEEQNRKLVADLDELRGRW
GKDTSEIKIQYSDSLRDARKEIDDGARRKAEIDVKVARLRDDLAELRNRYEDVQHRRESD
REKINQWQHAIEDAQSELEMLRARWRQLTEEEKRLNGDNARIWEELQKARNDLDEETLGR
IDFQNQVQTLMEELEFLRRVHEQEVKELQALLAQAPADTREFFKNELALAIRDIKDEYDY
IAKQGKQDMESWYKLKVSEVQGSANRANMESSYQREEVKRMRDNIGDLRGKLGDLEAKNA
LLEKEVQNLNYQLNDDQRQYEAALNDRDATLRRMREECQTLVAELQALLDTKQMLDAEIA
IYRKMLEGEESRVGLRQMVEQVVKTHSLQQQEDTDSTRNVRGEVSTKTTFQRSAKGNVTI

SECDPNGKFITLENTHRSKDENLGEHRLKRKLDNRREIVYTIPPNTVLKAGRTMKIYARD
QGGIHNPPDTLVFDGENTWGIGANVVTSLINKDGDERATHTQKTIQTGQ
>Q9P7W8
MSQVIQGAVEEPVYSNKSLTNENDSFLSLIESFSQERGVPIDINPKIGRKPILLYELYKK
VIKRGGYDAVSATEDGWTNIAEEFNQSDPARSAGILQNVYFKYLISWEIHDHWHLLLPPP
STLELSENRQNVLERVKVFNSCSPPKPTNPITIIDNDKTHSFKKFYKSLVKEDEENGIEK
KINEAMNSSASQPLPNSTNFASTTFPSVPFHPLPVDSGLQKYIDRNSNLTPPALGAGVPG
PPLLVRVALALKSQQDKEVDWAIGHLIKISFERHQEFKLERLPSLAECLVYSLGFQVTKA
KQVSDLTDISLCMDRAIGIALVLRNSVLSVENAKHVAQTKLVISVLEASIRCAKTFNNLE
FLHYCLDISEMISSYLHVEDEKNVFYLALCEFLNSSDYSILIATLRTLARLALNDRNNRL
LQDLKSNVIANIIALVETDNEEIVAAALDFLYQYTTYRTNTSNLLASQEAWMLVDQLIRL
MMYQAQERFMTVTIENSESNPAVKHEATSSISLPMEELQQLVAMREPERSVKWMRCCFEP
SSDDYVLQTDIWQLYCSDMERAQGPNVMGISPADFIKVSSHAFYNARAMTVSTPQLPVEY
VINGIKRRKFPTSVNGMRYQPCRWCLDSGKECGELLLGTPLLHSHLQEMHIFPQILETGK
CRWSDCKYEIQRLTPASELSHYQLLSHIVTHLHDDSLETLVEGRKLSPSREFRIPLLLTA
VDDQGDATGIALTATLVLRNLVRSKQGKMLFSAIESRVLEVSSLNPAVAPYVSEMLLGQI

>P37108
MVLLESEQFLTELTRLFQKCRTSGSVYITLKKYDGRTKPIPKKGTVEGFEPADNKCLLRA
TDGKKKISTVVSSKEVNKFQMAYSNLLRANMDGLKKRDKKNKTKKTKAAAAAAAAAPAAA
ATAPTTAATTAATAAQ
>Q9DB96
MAAPEVLESDVSSSITLLKNLQEQVMAVTAQIQALTTKVRAGTYSTEKGLSFLEVKDQLL
LMYLMDLSHLILDKASGASLQGHPAVLRLVEIRTVLEKLRPLDQKLKYQIDKLVKTAVTG
SLSENDPLRFKPHPSNMVSKLSSEDEEESEAEEGQSEASGKKSAKGSAKKYVPPRLVPVH
YDETEAEREQKRLEKAKRRALSSSVIRELKEQYSDAPEEIRDARHPHVTRQSQEDQHRVN
YEESMMVRLSVSKREKGLRRRASAMSSQLHSLTHFSDISALTGGTAHLDEDQNPVKKRKK
LPKKGRKKKGFRRRW
>Q06408
MAPVTIEKFVNQEERHLVSNRSATIPFGEYIFKRLLSIDTKSVFGVPGDFNLSLLEYLYS
PSVESAGLRWVGTCNELNAAYAADGYSRYSNKIGCLITTYGVGELSALNGIAGSFAENVK
VLHIVGVAKSIDSRSSNFSDRNLHHLVPQLHDSNFKGPNHKVYHDMVKDRVACSVAYLED
IETACDQVDNVIRDIYKYSKPGYIFVPADFADMSVTCDNLVNVPRISQQDCIVYPSENQL
SDIINKITSWIYSSKTPAILGDVLTDRYGVSNFLNKLICKTGIWNFSTVMGKSVIDESNP
TYMGQYNGKEGLKQVYEHFELCDLVLHFGVDINEINNGHYTFTYKPNAKIIQFHPNYIRL
VDTRQGNEQMFKGINFAPILKELYKRIDVSKLSLQYDSNVTQYTNETMRLEDPTNGQSSI
ITQVHLQKTMPKFLNPGDVVVCETGSFQFSVRDFAFPSQLKYISQGFFLSIGMALPAALG
VGIAMQDHSNAHINGGNVKEDYKPRLILFEGDGAAQMTIQELSTILKCNIPLEVIIWNNN
GYTIERAIMGPTRSYNDVMSWKWTKLFEAFGDFDGKYTNSTLIQCPSKLALKLEELKNSN
KRSGIELLEVKLGELDFPEQLKCMVEAAALKRNKK
>Q08962
MRQLTEEETKVVFEKLAGYIGRNISFLVDNKELPHVFRLQKDRVYYVPDHVAKLATSVAR
PNLMSLGICLGKFTKTGKFRLHITSLTVLAKHAKYKIWIKPNGEMPFLYGNHVLKAHVGK
MSDDIPEHAGVIVFAMNDVPLGFGVSAKSTSESRNMQPTGIVAFRQADIGEYLRDEDTLF

T
>Q9FVX0
MSSSSRNLSQENPIPRPNLAKTRTSLRDVGNRRAPLGDITNQKNGSRNPSPSSTLVNCSN
KIGQSKKAPKPALSRNWNLGILDSGLPPKPNAKSNIIVPYEDTELLQSDDSLLCSSPALS
LDASPTQSDPSISTHDSLTNHVVDYMVESTTDDGNDDDDDEIVNIDSDLMDPQLCASFAC
DIYEHLRVSEVNKRPALDYMERTQSSINASMRSILIDWLVEVAEEYRLSPETLYLAVNYV
DRYLTGNAINKQNLQLLGVTCMMIAAKYEEVCVPQVEDFCYITDNTYLRNELLEMESSVL
NYLKFELTTPTAKCFLRRFLRAAQGRKEVPSLLSECLACYLTELSLLDYAMLRYAPSLVA
ASAVFLAQYTLHPSRKPWNATLEHYTSYRAKHMEACVKNLLQLCNEKLSSDVVAIRKKYS
QHKYKFAAKKLCPTSLPQELFL
>Q12129
MTQYNFIIDASAFEKGLGNIKRWCSDCTEAVTLNFYIPTFTLNELDFLQQRRKSFAARES
LKFIDRLDDSKFANLKVFIEFPEVLDIILWSDVMEHNDSSGKINIAKLPKRLKNLLKSCI
YKCYLEGNEGLHWFLISEDPQIREMAMQCNIPSCSIVDVDSILSKDMNDKSFRESEKFNN
MMLKNGTKEESENGREIIRTNFNKTVYASRGTGELWSP
>P20075
MASRKDERAAKEERAQAAAELAAKELRDVNQDRERGIKVVEHKEEVSGGPGVIGSILKSV
QGTLGQAKEVVVGKAHDTAEVSRENTDYAYDKGREGGDVAAQKAEEAKEKAKMAKDTTMG
KAGEYKDYTAQKAEEAKEKAAQKAEETKEKAGEYKNYTAQKAGEAKDTTLGKAGEYKDYA
AQKAAEAKDTTAQKAAEAKEKTGEYKDYAAQKAAEAKVLAAQKAAEAKDTTGKDGEYKDY
AAQKAAEAKDATMQKTGEYKDYAAQKTAETKDATMEKAKEYKEYAAQKAAEAKDATMQKT
GEYKDYSAQKAAETKDATMEKTKEYKDYTAQKAAETKDATMEKAKEAKDTTVQKTGEYKD
YAAEKAKEGKDVTVEKAKEGKDTTVGKMTELKDSAADAARKAMDMFLGKKEEVKGKAGET
AEAAKEKYEDTEFAARKKMEELKLQEEGVKDEAKQRAEADRETAGDRGSAAKGTIFGAMG
SVKDAIVGKLTMPSDVVKDKQQQEAVIKVDETRPGAVAEALKAADQMHGQAFNDVGKMGD
EEVIVERKETRQGKM
>Q1K9C2
MSVKASINNSQEAVSRIAFRCSDALYVVHPNNSILNGALTESLKDLKKFETLNVSGKVPH
VLPLKSHADPFAHIADAILAEEEVASTQPKQITSVVASADALFFATPHLYKLAHEPLVAH
VAIESTEAFDFASVRDTGFVILFSGNRPGDSSEAALEDTLETASLAHRLALKLNTGVLHF
YSPVYDTTAALENIETLPSKEDAQHARVAHIPIEEKQEDSEKEGNIKEAFVPPKFDQPER
DAATSEYLESLSIKPFEYSGSDDATDVLLVFGSAASELAKAAVTSSVAVAIVRVLRPWLP
SKLQEVLPTSTKRLTVLEPITSLPRKWDPLYLDVLSSFVASGSSIELFAVRYGLSSSEQA
TEIIKAVRDNLSGALKPSLVCDFTDGVSQVFVPTPPSIEEAYHKLLHRVFKSRLNIVNDP
ASSATKQNIPSRLIISPQFALGSVLEYENQRRAFCDEVATLLKEKNSSVSSESLEVLSNW
IVSVDNLESPVDPELVISELKKDSSAPIKSLLDRSEFFTNVSHWIIGSDAWAYDLGNSAL
HQVLCLEKNVNLLIVDTQPYSTREAVRSSSRKKDIGLYAMNFGNAYVASTALYSSYTQLI
SALLEADKFKGPSVVLAYLPYHSADDDAITVLQETKKAVDIGYWPLYRWTPALEDGEYSD
FKLDSERIRRELKTFLERDNYLTQLTLRVPSLARTLTQSFGAEVRHQQNVDSRNALNKLI
EGLSGPPLTILFASDGGTAENVAKRLQNRASARGSKCKIMAMDDFPIEELGNEKNVVVLV
STAGQGEFPQNGREFWEAIKGADLNLSELKYGVFGFGDYEYWPRKEDKIYYNRPGKQLDA
RFVELGAAPLVTLGLGNDQDPDGWETAYNLWEPELWKALGLDNVEIDIDEPKPITNEDIK
QASNFLRGTIFEGLADESTGALAESDCQLTKFHGIYMQDDRDIRDERKKQGLEPAYGFMI
RARMPAGVCTPEQWIAMDDISTKWGNHTLKITTRQTFQWHGVLKKNLRNTIRNIVKVFLT

TLGACGDVARNVTCSSTPNNETIHDQLFAVSKQISNELLPTTSSYHEIWIEDPETVEKRK
VAGEAVQDVEPLYGPTYLPRKFKVGVAAPPYNDVDVYTNDVALIAIIENDKVLGFNVGIG
GGMGTTHNNKKTYPRLATVVGYVLTDKIMEVVKAILIVQRDNGDRENRKHARLKYTVDTL
GVSTFVEKVEEVLGYKFEEARDHPQFIKNHDDFEGWHKTEKNKYWRSIFVENGRIENNGI
LQFKTGLRELAERLYTEKSEAEFRLTANQHVILFNVAENELGWINEHMAKYKLDNNAFSG
LRLSSAACVALPTCGLAMAESERYLPKLITKVEEIVYEAGLQKDSIVMRMTGCPNGCSRP
WVAEIACVGKAPNTYNLMLGGGFYGQRLNKLYRSSVQEKEILNLLRPLIKRYALEREDGE
HFGDWVIRAGIITAVENGGANGAVHEGVSPEAF
>P40459
MKIFHTVEEVVQWRTQELRETRFRETIGFVPTMGCLHSGHASLISQSVKENTYTVVSIFV
NPSQFAPTEDLDNYPRTLPDDIKLLESLKVDVLFAPNAHVMYPQGIPLDIEEQKGPFVSV
LGLSEKLEGKTRPNFFRGVATVVTKLFNIVMADVAYFGQKDIQQFIVLQCMVDELFVNTR
LQMMPIVRNNNGLALSSRNKYLCPESLKISENLYRGLKAAENAIRRLAPGGRLSRSEIID
TVTQIWAPYVDSHDFKIDYVSLADFKTLDELSDVENTSEQQPIVISCAVYVTDREKPDTV
VRLIDNIVI
>Q9P7X5
MLANALRLVSNSKKIKYDYDIQKENSVQVGPWTVYSASKKGTNEEVSVFTFDKKNLSTLL
KRGSIDSNLKTNYVLELLRKDVSSLSRLRHPSLLQVVEPLEESKSSMSFVTRRIQSMLQD
FIKSSNGGFSNYGSSANGKSSGNALEEVEIQKGLLQIIDGLVFLHGSAKVIHYNIRPSSV
VVDAKGDWKLCGFSFSQSVESARYEFNDYDFGIPSSLQQSMDFLAPEYITHEIAGPESDV
FSFGCLIYSIFNKNQSIINANNHLLSYEKEITSLNSPTFIESKNLPSENLKSLLKETLAV
DPKQRASMFELERSPYFTGSAIAALRFLESFPEKLPSEKVSFMESLSKNLTTFPYRIQSQ
KILPTLLDHLNDQKLVPSLLPCIFEISKGLDSSIFSSKVFTAIFPIISAANSYPERVPLC
IFQYMDCLKSKLPSGEFLSKIVPFIYGCFENSSLNVQTTSIQILGTLLDIIDVTTVKSSI
CPKLYHSFSVTNQLDVKVAILDTFNVFINQKFLDSFAIVDKLLPVLEKVKTREPTVVMGM
VTVYISAGAIIPEETVHEQVIPRLWILSVSPSLSLEQYNKCMREIRSLSDAVQKSHAKKL
QSKPSSVVPNRITTDPFSSQTKEATSKPSSISPNKATTNIFTSQASLSSQGVARETSSAS
SYRSYSQRASTPAVTAKSSFHYATPTSGLSNFNSVTPSSSASLYPPLIPSEARTPSVQPA
NRRVTTPVVNQNTVTSDSSNDLGGWKSLL
>Q03562
MNCEVKHALHCAVLVAWIVCFAYFCGVFTEPVEGSVPESPVASYGLIWTVCLYLLRFTAL
LVLPQCLCNLGGLMMFNAFREKVQLKAAPLLSPFVCFRVVTKGNFPLLVKENIDTNMKTC
FEAGMENFIFEVVTDKAINLPPNPRVREVVVPTVYKTKSGAKFKARALQYCLEDDVNILQ
PTDWIVHLDEETLLTTNAICGILNFCEDGKHQFGQGVITYANGDIVNWLTTLSDSFRVAD
DMGKLRFQFKLFHKPLFGWKGSYVVTQVEAERDVSYDHGMEGSIAEDCFFSMVAMKHGYS
FDFIEGEMHEKSPFTMWDFLQQRKRWLQGILLTVHSSKIAVVHKALLALSLYAWATMPLT
SLQVFLCPLFPLPRCLPFDFLLSFVGALNLYMYIFGVVKSFSHKYRNSLLRLAMYLAGAL
MTIPFNILIENAAVLVGMFGRKDQFYIVNKDIQTV
>Q08726
MPFAQIVIGPPGSGKSTYCNGCSQFFNAIGRHSQVVNMDPANDALPYPCAVDIRDFITLE
EIMQEQQLGPNGGLMYAVESLDNSIDLFILQIKSLVEEEKAYLVFDCPGQVELFTHHSSL
FNIFKKMEKELDIRFCVVNLIDCFYMTSPSQYISILLLALRSMLMMDLPHINVFSKIDML
KSYGELPFRLDYYTEVQDLDYLEPYIEKEGSSVLGKKYSKLTETIKELVSDFNLVSFEVL
SVDDKESMINLQGVIDKANGYIFGASEVGGDTVWAEASREGALIANYDIQDRWIDNKEKY

DKEEEEKRTALLKEQELQNKAVDVNEEDEWENALKEWEEKQGMDFVR
>Q9C0X3
MASCCIFCAQFVLNRNLLEANKYKLSEITFKVYISIRRLLIVVITLYRRIVECCASLCSL
IFSKYYLHYKTFRILTIIQSWSDKVSVLPNRLYCLPFCNAFIEFVKKLTET
>O14016
MTDSQTETHLSLILSDTAFPLSSFSYSYGLESYLSHQQVRDVNAFFNFLPLSLNSVLHTN
LPTVKAAWESPQQYSEIEDFFESTQTCTIAQKVSTMQGKSLLNIWTKSLSFFVTSTDVFK
YLDEYERRVRSKKALGHFPVVWGVVCRALGLSLERTCYLFLLGHAKSICSAAVRLDVLTS
FQYVSTLAHPQTESLLRDSSQLALNMQLEDTAQSWYTLDLWQGRHSLLYSRIFNS
>Q8TEU7
MNSPVDPGARQALRKKPPERTPEDLNTIYSYLHGMEILSNLREHQLRLMSARARYERYSG
NQVLFCSETIARCWYILLSGSVLVKGSMVLPPCSFGKQFGGKRGCDCLVLEPSEMIVVEN
AKDNEDSILQREIPARQSRRRFRKINYKGERQTITDDVEVNSYLSLPADLTKMHLTENPH
PQVTHVSSSQSGCSIASDSGSSSLSDIYQATESEVGDVDLTRLPEGPVDSEDDEEEDEEI
DRTDPLQGRDLVRECLEKEPADKTDDDIEQLLEFMHQLPAFANMTMSVRRELCSVMIFEV
VEQAGAIILEDGQELDSWYVILNGTVEISHPDGKVENLFMGNSFGITPTLDKQYMHGIVR
TKVDDCQFVCIAQQDYWRILNHVEKNTHKVEEEGEIVMVHEHRELDRSGTRKGHIVIKAT
PERLIMHLIEEHSIVDPTYIEDFLLTYRTFLESPLDVGIKLLEWFKIDSLRDKVTRIVLL
WVNNHFNDFEGDPAMTRFLEEFEKNLEDTKMNGHLRLLNIACAAKAKWRQVVLQKASRES
PLQFSLNGGSEKGFGIFVEGVEPGSEAADSGLKRGDQIMEVNGQNFENITFMKAVEILRN
NTHLALTVKTNIFVFKELPFRTEQEKSGVPHIPKIAKKKSNRHSIQHVPGDIEQTSQEKG
SKKVKANTASGGRNKIRKILDKTRFSILPPKLFSDGGLSQSQDDSIVGTRHCRHSLAIMP
IPGTLSSSSPDLLQPTTSMLDFSNPSDIPDQVIRVFKVDQQSCYIIISKDTTAKEVVFHA
VHEFGLTGASDTYSLCEVSVTPEGVIKQRRLPDQFSKLADRIQLNGRYYLKNNMETETLC
SDEDAQELVKESQLSMLQLSTIEVATQLSMRDFDLFRNIEPTEYIDDLFKLNSKTGNTHL
KRFEDIVNQETFWVASEILTEANQLKRMKIIKHFIKIALHCRECKNFNSMFAIISGLNLA
SVARLRGTWEKLPSKYEKHLQDLQDIFDPSRNMAKYRNILSSQSMQPPIIPLFPVVKKDM
TFLHEGNDSKVDGLVNFEKLRMISKEIRQVVRMTSANMDPAMMFRQRSLSQGSTNSNMLD
VQGGAHKKRARRSSLLNAKKLYEDAQMARKVKQYLSSLDVETDEEKFQMMSLQWEPAYGT
LTKNLSEKRSAKSSEMSPVPMRSAGQTTKAHLHQPHRVSQVLQVPAVNLHPIRKKGQTKD
PALNTSLPQKVLGTTEEISGKKHTEDTISVASSLHSSPPASPQGSPHKGYTLIPSAKSDN
LSDSSHSEISSRSSIVSNCSVDSMSAALQDERCSSQALAVPESTGALEKTEHASGIGDHS
QHGPGWTLLKPSLIKCLAVSSSVSNEEISQEHIIIEAADSGRGSWTSCSSSSHDNFQSLP
NPKSWDFLNSYRHTHLDDPIAEVEPTDSEPYSCSKSCSRTCGQCKGSLERKSWTSSSSLS
DTYEPNYGTVKRRVLESTPAESSEGLDPKDATDPVYKTVTSSTEKGLIVYCVTSPKKDDR
YREPPPTPPGYLGISLADLKEGPHTHLKPPDYSVAVQRSKMMHNSLSRLPPASLSSNLVA
CVPSKIVTQPQRHNLQPFHPKLGDVTDADSEADENEQVSAV
>Q02197
MEVDSILGSLSITDDFDQLVDVTSLFDELCSKLKPEAIVKDPRFDLFEGTHSLEVNNSKL
DSSLIELTAEEIEFDVNVAYDPPLASVAAIADRLLRCVISWLNDYQTLPTTVLSCRYTES
LLSSLVKGTTAGSSWCTGNILYDKVLGSCILGVCYLTKFVQKLLSAGIVFEEEDLNFNNM
GFNTFDNLPGQDVVINSLTESLQILEAYSDDSLHLTMLKHILKIIICLVHLEDHLTDYST
KTSHLDELIENANSVNGIFPQLQLSPPKGAFSTYIQKHRSNQFPPRKITKLPTDYSGFIT
LANDVKTILLVDKAESALETYQFAKFFNKLEQRHVIARILFPLFFIRDDRTVLGKFSYTQ

FYLLHVKEFSAQTPSEFESSIGNELIQESSNMLLEWYQNCSQNTCRYRQGFNRQLILWDS
LQAQFESVNSQVYCSWTYFMKLSSMIEFSLKGFDLDIYKPFEAYSMFWYVYYLSHHLETF
LKDSQNDIESNINAIHSMNKKLKKLKAGEKKDQLRLKYRFAMDNEMEQLQATKQFLNYLL
KEINITKSLCLIEVFQFAILKSFGLIDNKNSTPSKFSNERLIHNLRFKPFNSIGVPELPE
YEVFQQTLKDFVIEEKGAAFDIKLERATNFIETEVRNVVSSIDEIMQGIKGGDNNGVLVT
GTRLVQELSLEYYCKLKHTSKALSVNSKVIVNTLKKNIKNKDSHEYKVELVHTTEGWNYF
PIQTLRIKQDRYK
>Q9UT76
MSTINVEHTYPAVSSLIADLKSRKVQGPFAVAVETALVMRQVISQTRWSTVDQLIDTVRA
VGSTLVKAQPTEFSCGNIIRRILRLIREEYQELLKTADENEKLIVSSSNSSSPSQKRDIP
SNEKLVQSHEPVSVQMYSSMLNLLGRPTLESPTHSKTVGDSRVTGGMDMRAVIISGIQDV
IDELDKINTDIEVQSMDHLHSNEIILTQGCSKTVEAFLRFAAKKRKFSVIVAEGFPNNQK
GSHAMAKRLAQAGIDTTVISDATIFAIMSRVNKVILGTHAILGNGGLVTYSGAQLVAQAA
RHHATPVVVCSGIYKLSPVYPYDLESIIQLSSPDKIMSFNEGDLISRAEILNPYYDYIPP
DLVDLFITNLGGYPPSYLYRIMNDTYDASDTIL
>Q08634
MDEKVELILVPCHSIWKSSSHPSDNSVNLGQLPEYWHLAPFQYEGNDHLAFIKHGLTAIK
LLLQRFDTATVIFSGSQTKKEAGAISEAQSYYFLFEKLIRYVMSNDNIDVPNFDNELRLL
LKEVKNLLSSQNVNVDELFYGGSITTEEFSLDSFDNLIYSIYRFEEVNKKFPQKITIIGF
AFKMPRFISCHAKAIDYPQSNITYIGIDPKPANYNQTQLSKYYDDLVQMEDKNALSLFSS
DWYATKDRLLTKKRSRNPFNRTAPYAQNIFCKENGKRIEGIEDDEEYFETKIKCKMPWSS
PRQ
>Q9P7L2
MATIYACRGFHRVPVKLSPSSTLQEVILSSYKQLGFSDWHNLELLHGDKKVDTSLLLRLS
GIINGAKLIVKESATNQSGKSSSSISPQSKKIKVALQLPGAARIIDEASSETSIKQLLER
HSLLTKVSHVLINGRNFKSEEFDNPLLLYGIREGSILIRLFPIKAQQSIVSEQAPVSQTF
NGDVKEKKNADLMDIESENKKDDIVESFPKYPVDAHKLLEPLPTPIPSLPSTPSSYQNLP
SQSLTGESLPTVSNQEKDEGVIEKVAVNNTPSVSSKSPFPKKKSFSSMLAQVKKEKAENN
GSDGYDLQPTKSQLELYQSILRKRANQVSSTSLTKSSSPKPLPSSAIVKFDFGNGKSIVH
EFSKDDNIETLRAFVASHLSPEESTSFQLTFSNYEALPTTGLIVEHIGRAVVRVHTISDP
VYAQR
>P05153
MAPELKTEVNIISKVIQGDLESLPPQVREFIESNAKLCQPESIHICDGSEEENKKILDIM
VEQGMIKKLSKYENCWLALTNPRDVARIESKTVIITQEQRDTIPIPKTGSSQLGRWMSEE
DFEKAFNTRFPGCMQGRTMYVIPFSMGPIGSPLAKIGIELTDSPYVVASMRMMTRMGTAA
LKALGNGEFVKCLHSVGCPLPLKEPLINNWPCNPELTLIAHLPDRREIISFGSGYGGNSL
LGKKCFALRIASRIAKEEGWLAEHMLILGITNPEGEKKYFAAAFPSACGKTNLAMMNPSR
PGWKIECVGDDIAWMKFDELGNLRAINPENGFFGVAPGTSIKTNPNAIKTIFKNTIFTNV
AETSDGGVYWEGIDEPLPPGVTLTSWKNKDWTPDNGEPCAHPNSRFCTPASQCPIMDPAW
ESPEGVPIEGIIFGGRRPAGVPLVYEAFNWQHGVFIGAAMRSEATAAAEHKGKIIMHDPF
AMRPFFGYNFGKYLAHWLSMAHRPAAKLPRIFHVNWFRKDSQGKFLWPGYGENSRVLEWM
FNRIQGKASAKSTAIGYIPADTALNLKGLEDINLTELFNISKEFWEKEVEEIKQYFEGQV
NADLPYEIERELLALEMRIKQL
>Q96PI1

MSSQQQQRQQQQCPPQRAQQQQVKQPCQPPPVKCQETCAPKTKDPCAPQVKKQCPPKGTI
IPAQQKCPSAQQASKSKQK
>P38217
MASTWKPAEDYVLQLATLLQNCMSPNPEIRNNAMEAMENFQLQPEFLNYLCYILIEGESD
DVLKQHYSLQDLQNNRATAGMLLKNSMLGGNNLIKSNSHDLGYVKSNIIHGLYNSNNNLV
SNVTGIVITTLFSTYYRQHRDDPTGLQMLYQLLELTSNGNEPSIKALSKIMEDSAQFFQL
EWSGNTKPMEALLDSFFRFISNPNFSPVIRSESVKCINTVIPLQTQSFIVRLDKFLEIIF
QLAQNDENDLVRAQICISFSFLLEFRPDKLVSHLDGIVQFMLHLITTVNEEKVAIEACEF
LHAFATSPNIPEHILQPYVKDIVPILLSKMVYNEESIVLLEASNDDDAFLEDKDEDIKPI
APRIVKKKEAGNGEDADDNEDDDDDDDDEDGDVDTQWNLRKCSAATLDVMTNILPHQVMD
IAFPFLREHLGSDRWFIREATILALGAMAEGGMKYFNDGLPALIPFLVEQLNDKWAPVRK
MTCWTLSRFSPWILQDHTEFLIPVLEPIINTLMDKKKDVQEAAISSVAVFIENADSELVE
TLFYSQLLTSFDKCLKYYKKKNLIILYDAIGRFAEKCALDETAMQIILPPLIEKWALLSD
SDKELWPLLECLSCVASSLGERFMPMAPEVYNRAFRILCHCVELEAKSHQDPTIVVPEKD
FIITSLDLIDGLVQGLGAHSQDLLFPQGTKDLTILKIMLECLQDPVHEVRQSCFALLGDI
VYFFNSELVIGNLEDFLKLIGTEIMHNDDSDGTPAVINAIWALGLISERIDLNTYIIDMS
RIILDLFTTNTQIVDSSVMENLSVTIGKMGLTHPEVFSSGAFANDSNWNKWCLSVNALDD
VEEKSSAYMGFLKIINLTSTEVTMSNDTIHKIVTGLSSNVEANVFAQEIYTFLMNHSAQI
SAINFTPDEISFLQQFTS
>Q8TCU4
MEPEDLPWPGELEEEEEEEEEEEEEEEEAAAAAAANVDDVVVVEEVEEEAGRELDSDSHY
GPQHLESIDDEEDEEAKAWLQAHPGRILPPLSPPQHRYSEGERTSLEKIVPLTCHVWQQI
VYQGNSRTQISDTNVVCLETTAQRGSGDDQKTESWHCLPQEMDSSQTLDTSQTRFNVRTE
DTEVTDFPSLEEGILTQSENQVKEPNRDLFCSPLLVIQDSFASPDLPLLTCLTQDQEFAP
DSLFHQSELSFAPLRGIPDKSEDTEWSSRPSEVSEALFQATAEVASDLASSRFSVSQHPL
IGSTAVGSQCPFLPSEQGNNEETISSVDELKIPKDCDRYDDLCSYMSWKTRKDTQWPENN
LADKDQVSVATSFDITDENIATKRSDHFDAARSYGQYWTQEDSSKQAETYLTKGLQGKVE
SDVITLDGLNENAVVCSERVAELQRKPTRESEYHSSDLRMLRMSPDTVPKAPKHLKAGDT
SKGGIAKVTQSNLKSGITTTPVDSDIGSHLSLSLEDLSQLAVSSLETTTGQHTDTLNQKT
LADTHLTEETLKVTAIPEPADQKTATPTVLSSSHSHRGKPSIFYQQGLPDSHLTEEALKV
SAAPGLADQTTGMSTLTSTSYSHREKPGTFYQQELPESNLTEEPLEVSAAPGPVEQKTGI
PTVSSTSHSHVEDLLFFYRQTLPDGHLTDQALKVSAVSGPADQKTGTATVLSTPHSHREK
PGIFYQQEFADSHQTEETLTKVSATPGPADQKTEIPAVQSSSYSQREKPSILYPQDLADS
HLPEEGLKVSAVAGPADQKTGLPTVPSSAYSHREKLLVFYQQALLDSHLPEEALKVSAVS
GPADGKTGTPAVTSTSSASSSLGEKPSAFYQQTLPNSHLTEEALKVSIVPGPGDQKTGIP
SAPSSFYSHREKPIIFSQQTLPDFLFPEEALKVSAVSVLAAQKTGTPTVSSNSHSHSEKS
SVFYQQELPDSDLPRESLKMSAIPGLTDQKTVPTPTVPSGSFSHREKPSIFYQQEWPDSY
ATEKALKVSTGPGPADQKTEIPAVQSSSYPQREKPSVLYPQVLSDSHLPEESLKVSAFPG
PADQMTDTPAVPSTFYSQREKPGIFYQQTLPESHLPKEALKISVAPGLADQKTGTPTVTS
TSYSQHREKPSIFHQQALPGTHIPEEAQKVSAVTGPGNQKTWIPRVLSTFYSQREKPGIF
YQQTLPGSHIPEEAQKVSPVLGPADQKTGTPTPTSASYSHTEKPGIFYQQVLPDNHPTEE
ALKISVASEPVDQTTGTPAVTSTSYSQYREKPSIFYQQSLPSSHLTEEAKNVSAVPGPAD
QKTVIPILPSTFYSHTEKPGVFYQQVLPHSHPTEEALKISVASEPVDQTTGTPTVTSTSY
SQHTEKPSIFYQQSLPGSHLTEEAKNVSAVPGPGDRKTGIPTLPSTFYSHTEKPGSFYQQ

VLPHSHLPEEALEVSVAPGPVDQTIGTPTVTSPSSSFGEKPIVIYKQAFPEGHLPEESLK
VSVAPGPVGQTTGAPTITSPSYSQHRAKSGSFYQLALLGSQIPEEALRVSSAPGPADQTT
GIPTITSTSYSFGEKPIVNYKQAFPDGHLPEEALKVSIVSGPTEKKTDIPAGPLGSSALG
EKPITFYRQALLDSPLNKEVVKVSAAPGPADQKTETLPVHSTSYSNRGKPVIFYQQTLSD
SHLPEEALKVPPVPGPDAQKTETPSVSSSLYSYREKPIVFYQQALPDSELTQEALKVSAV
PQPADQKTGLSTVTSSFYSHTEKPNISYQQELPDSHLTEEALKVSNVPGPADQKTGVSTV
TSTSYSHREKPIVSYQRELPHFTEAGLKILRVPGPADQKTGINILPSNSYPQREHSVISY
EQELPDLTEVTLKAIGVPGPADQKTGIQIASSSSYSNREKASIFHQQELPDVTEEALNVF
VVPGQGDRKTEIPTVPLSYYSRREKPSVISQQELPDSHLTEEALKVSPVSIPAEQKTGIP
IGLSSSYSHSHKEKLKISTVHIPDDQKTEFPAATLSSYSQIEKPKISTVIGPNDQKTPSQ
TAFHSSYSQTVKPNILFQQQLPDRDQSKGILKISAVPELTDVNTGKPVSLSSSYFHREKS
NIFSPQELPGSHVTEDVLKVSTIPGPAGQKTVLPTALPSSFSHREKPDIFYQKDLPDRHL
TEDALKISSALGQADQITGLQTVPSGTYSHGENHKLVSEHVQRLIDNLNSSDSSVSSNNV
LLNSQADDRVVINKPESAGFRDVGSEEIQDAENSAKTLKEIRTLLMEAENMALKRCNFPA
PLARFRDISDISFIQSKKVVCFKEPSSTGVSNGDLLHRQPFTEESPSSRCIQKDIGTQTN
LKCRRGIENWEFISSTTVRSPLQEAESKVSMALEETLRQYQAAKSVMRSEPEGCSGTIGN
KIIIPMMTVIKSDSSSDASDGNGSCSWDSNLPESLESVSDVLLNFFPYVSPKTSITDSRE
EEGVSESEDGGGSSVDSLAAHVKNLLQCESSLNHAKEILRNAEEEESRVRAHAWNMKFNL
AHDCGYSISELNEDDRRKVEEIKAELFGHGRTTDLSKGLQSPRGMGCKPEAVCSHIIIES
HEKGCFRTLTSEHPQLDRHPCAFRSAGPSEMTRGRQNPSSCRAKHVNLSASLDQNNSHFK
VWNSLQLKSHSPFQNFIPDEFKISKGLRMPFDEKMDPWLSELVEPAFVPPKEVDFHSSSQ
MPSPEPMKKFTTSITFSSHRHSKCISNSSVVKVGVTEGSQCTGASVGVFNSHFTEEQNPP
RDLKQKTSSPSSFKMHSNSQDKEVTILAEGRRQSQKLPVDFERSFQEEKPLERSDFTGSH
SEPSTRANCSNFKEIQISDNHTLISMGRPSSTLGVNRSSSRLGVKEKNVTITPDLPSCIF
LEQRELFEQSKAPRADDHVRKHHSPSPQHQDYVAPDLPSCIFLEQRELFEQCKAPYVDHQ
MRENHSPLPQGQDSIASDLPSPISLEQCQSKAPGVDDQMNKHHFPLPQGQDCVVEKNNQH
KPKSHISNINVEAKFNTVVSQSAPNHCTLAASASTPPSNRKALSCVHITLCPKTSSKLDS
GTLDERFHSLDAASKARMNSEFNFDLHTVSSRSLEPTSKLLTSKPVAQDQESLGFLGPKS
SLDFQVVQPSLPDSNTITQDLKTIPSQNSQIVTSRQIQVNISDFEGHSNPEGTPVFADRL
PEKMKTPLSAFSEKLSSDAVTQITTESPEKTLFSSEIFINAEDRGHEIIEPGNQKLRKAP
VKFASSSSVQQVTFSRGTDGQPLLLPYKPSGSTKMYYVPQLRQIPPSPDSKSDTTVESSH
SGSNDAIAPDFPAQVLGTRDDDLSATVNIKHKEGIYSKRVVTKASLPVGEKPLQNENADA
SVQVLITGDENLSDKKQQEIHSTRAVTEAAQAKEKESLQKDTADSSAAAAAEHSAQVGDP
EMKNLPDTKAITQKEEIHRKKTVPEEAWPNNKESLQINIEESECHSEFENTTRSVFRSAK
FYIHHPVHLPSDQDICHESLGKSVFMRHSWKDFFQHHPDKHREHMCLPLPYQNMDKTKTD
YTRIKSLSINVNLGNKEVMDTTKSQVRDYPKHNGQISDPQRDQKVTPEQTTQHTVSLNEL
WNKYRERQRQQRQPELGDRKELSLVDRLDRLAKILQNPITHSLQVSESTHDDSRGERSVK
EWSGRQQQRNKLQKKKRFKSLEKSHKNTGELKKSKVLSHHRAGRSNQIKIEQIKFDKYIL
SKQPGFNYISNTSSDCRPSEESELLTDTTTNILSGTTSTVESDILTQTDREVALHERSSS
VSTIDTARLIQAFGHERVCLSPRRIKLYSSITNQQRRYLEKRSKHSKKVLNTGHPLVTSE
HTRRRHIQVANHVISSDSISSSASSFLSSNSTFCNKQNVHMLNKGIQAGNLEIVNGAKKH
TRDVGITFPTPSSSEAKLEENSDVTSWSEEKREEKMLFTGYPEDRKLKKNKKNSHEGVSW
FVPVENVESRSKKENVPNTCGPGISWFEPITKTRPWREPLREQNCQGQHLDGRGYLAGPG
REAGRDLLKPFVRATLQESLQFHRPDFISRSGERIKRLKLIVQERKLQSMLQTERDALFN

IDRERQGHQNRMCPLPKRVFLAIQKNKPISKKEMIQRSKRIYEQLPEVQKKREEEKRKSE
YKSYRLRAQLYKKRVTNQLLGRKVPWD
>P02212
PSVQGAAAQLTADVKKDLRDSWKVIGSDKKGNGVALMTTLFADNQETIGYFKRLGNVSQG
MANDKLRGHSITLMYALQNFIDQLDNTDDLVCVVEKFAVNHITRKISAAEFGKINGPIKK
VLASKNFGDKYANAWAKLVAVVQAAL
>P38788
MSSPVIGITFGNTSSSIAYINPKNDVDVIANPDGERAIPSALSYVGEDEYHGGQALQQLI
RNPKNTIINFRDFIGLPFDKCDVSKCANGAPAVEVDGKVGFVISRGEGKEEKLTVDEVVS
RHLNRLKLAAEDYIGSAVKEAVLTVPTNFSEEQKTALKASAAKIGLQIVQFINEPSAALL
AHAEQFPFEKDVNVVVADFGGIRSDAAVIAVRNGIFTILATAHDLSLGGDNLDTELVEYF
ASEFQKKYQANPRKNARSLAKLKANSSITKKTLSNATSATISIDSLADGFDYHASINRMR
YELVANKVFAQFSSFVDSVIAKAELDPLDIDAVLLTGGVSFTPKLTTNLEYTLPESVEIL
GPQNKNASNNPNELAASGAALQARLISDYDADELAEALQPVIVNTPHLKKPIGLIGAKGE
FHPVLLAETSFPVQKKLTLKQAKGDFLIGVYEGDHHIEEKTLEPIPKEENAEEDDESEWS
DDEPEVVREKLYTLGTKLMELGIKNANGVEIIFNINKDGALRVTARDLKTGNAVKGEL
>Q02908
MARHGKGPKTNKKKLAPEKERFIQCCADITLELTDSLTSGTTREINLNGLITKYSKKYKL
KQQPRLTDIINSIPDQYKKYLLPKLKAKPVRTASGIAVVAVMCKPHRCPHIAYTGNICVY
CPGGPDSDFEYSTQSYTGYEPTSMRAIRARYDPYEQARGRVEQLKQLGHSIDKVEYVLMG
GTFMSLPKEYREDFIVKLHNALSGFNGNDIDEAILYSQQSLTKCVGITIETRPDYCTQTH
LDDMLKYGCTRLEIGVQSLYEDVARDTNRGHTVRSVCETFAVSKDAGYKVVSHMMPDLPN
VGMERDIEQFKEYFENPDFRTDGLKIYPTLVIRGTGLYELWKTGRYKSYSANALVDLVAR
ILALVPPWTRIYRVQRDIPMPLVTSGVDNGNLRELALARMKDLGTTCRDVRTREVGIQEV
HHKVQPDQVELIRRDYYANGGWETFLSYEDPKKDILIGLLRLRKASKKYTYRKEFTSQRT
SIVRELHVYGSVVPLHSRDPRKFQHQGFGTLLMEEAERIAKEEHGSEKISVISGVGVRNY
YGKLGYELDGPYMSKRI
>Q9C826
MSTNTESSSYSSLPSQRLLGKVALITGGATGIGESIVRLFHKHGAKVCIVDLQDDLGGEV
CKSLLRGESKETAFFIHGDVRVEDDISNAVDFAVKNFGTLDILINNAGLCGAPCPDIRNY
SLSEFEMTFDVNVKGAFLSMKHAARVMIPEKKGSIVSLCSVGGVVGGVGPHSYVGSKHAV
LGLTRSVAAELGQHGIRVNCVSPYAVATKLALAHLPEEERTEDAFVGFRNFAAANANLKG
VELTVDDVANAVLFLASDDSRYISGDNLMIDGGFTCTNHSFKVFR
>O04847
MPKSWPIVISSHSFCFLPNSEQERKMKDLNFHAATLSEEESLRELKAFDETKAGVKGIVD
TGITKIPRIFIDQPKNLDRISVCRGKSDIKIPVINLNGLSSNSEIRREIVEKIGEASEKY
GFFQIVNHGIPQDVMDKMVDGVRKFHEQDDQIKRQYYSRDRFNKNFLYSSNYVLIPGIAC
NWRDTMECIMNSNQPDPQEFPDVCRDILMKYSNYVRNLGLILFELLSEALGLKPNHLEEM
DCAEGLILLGHYYPACPQPELTFGTSKHSDSGFLTILMQDQIGGLQILLENQWIDVPFIP
GALVINIADLLQLITNDKFKSVEHRVLANKVGPRISVAVAFGIKTQTQEGVSPRLYGPIK
ELISEENPPIYKEVTVKDFITIRFAKRFDDSSSLSPFRLNN
>P38164
MGLIKKVTHWSYDNLIDYLSVNPTRDEVTHYKVDPENESDESIIKLHTVKDFGSITCLDY
SESEIGMIGVGEKNGYLRIFNISGQNSSSPASHAPVGLNANNETSMTNASGGKAAQAENI

VGSVSNLKDTQGYPVSETNYDIRVRAKKQRCINSLGINTNGLIAMGLDRNKHDSSLQIWD
MNYHDDSHETINPMFSYCTNESIVSLKFLNDTSVLAASTKFLKEIDVRSPNPIYQHPTRL
TYDIKLNPFNDWQFSTYGDDGTLAIWDRRKLSDQASLGDLNVASPLLTFEKLVGSGAASR
KYMNSCFRWSCVRNNEFATLHRGDTIKRWRLGYYCDSNRDIAADDDNEMNIENLFVSSVH
DTNTMYDRVATFDYIPRSNNGTSLICMRQSGTIYRMPISEVCSKAILNNRNSLLLSNFEN
TEIDEIRVNNEHEKSNLENVKTILKNLSFEDLDVSEDYFPSGHDEPNNEIEYSELSEEEN
EGSNDVLDSKRGFELFWKPEKLLEKDISVIMRTRASLGYGLDPMNTVEMIDSSKNLQNNA
YIRNTWRWIAIAKASVDDGTMVSGDLDLGYEGVIGIWNGINGISNQDRYRQETILSDKQL
NKEMEKIIKLRRKNRDRNSPIANAAGSPKYVQRRLCLIISGWDLSRSDYEDKYNIIMKNG
HYEKAAAWAVFFGDIPKAVEILGSAKKERLRLIATAIAGYLAYKDLPGNNAWRQQCRKMS
SELDDPYLRVIFAFIADNDWWDILYEPAISLRERLGVALRFLNDTDLTTFLDRTSSTVIE
NGELEGLILTGITPNGIDLLQSYVNKTSDVQSAALISIFGSPRYFRDQRVDEWIQTYRDM
LKSWELFSMRARFDVLRSKLSRTKTGVLTADIKPRQIYIQCQNCKQNINTPRTSSPSSAV
STSAGNYKNGEAYRRNNADYKKFNTGSSEAQAADEKPRHKYCCPHCGSSFPRCAICLMPL
GTSNLPFVINGTQSRDPMQTEDSQDGANRELVSRKLKLNEWFSFCLSCNHGMHAGHAEEW
FDRHNVCPTPGCTCQCNK
>Q9C0C2
MKVSTLRESSAMASPLPREMEEELVPTGSEPGDTRAKPPVKPKPRALPAKPALPAKPSLL
VPVGPRPPRGPLAELPSARKMNMLAGPQPYGGSKRPLPFAPRPAVEASTGGEATQETGKE
EAGKEEPPPLTPPARCAAPGGVRKAPAPFRPASERFAATTVEEILAKMEQPRKEVLASPD
RLWGSRLTFNHDGSSRYGPRTYGTTTAPRDEDGSTLFRGWSQEGPVKSPAECREEHSKTP
EERSLPSDLAFNGDLAKAASSELPADISKPWIPSSPAPSSENGGPASPGLPAEASGSGPG
SPHLHPPDKSSPCHSQLLEAQSPEASQASPCPAVTPSAPSAALPDEGSRHTPSPGLPAEG
APEAPRPSSPPPEVLEPHSLDQPPATSPRPLIEVGELLDLTRTFPSGGEEEAKGDAHLRP
TSLVQRRFSEGVLQSPSQDQEKLGGSLAALPQGQGSQLALDRPFGAESNWSLSQSFEWTF
PTRPSGLGVWRLDSPPPSPITEASEAAEAAEAGNLAVSSREEGVSQQGQGAGSAPSGSGS
SWVQGDDPSMSLTQKGDGESQPQFPAVPLEPLPTTEGTPGLPLQQAEERYESQEPLAGQE
SPLPLATREAALPILEPVLGQEQPAAPDQPCVLFADAPEPGQALPVEEEAVTLARAETTQ
ARTEAQDLCRASPEPPGPESSSRWLDDLLASPPPSGGGARRGAGAELKDTQSPSTCSEGL
LGWSQKDLQSEFGITGDPQPSSFSPSSWCQGASQDYGLGGASPRGDPGLGERDWTSKYGQ
GAGEGSTREWASRCGIGQEEMEASSSQDQSKVSAPGVLTAQDRVVGKPAQLGTQRSQEAD
VQDWEFRKRDSQGTYSSRDAELQDQEFGKRDSLGTYSSRDVSLGDWEFGKRDSLGAYASQ
DANEQGQDLGKRDHHGRYSSQDADEQDWEFQKRDVSLGTYGSRAAEPQEQEFGKSAWIRD
YSSGGSSRTLDAQDRSFGTRPLSSGFSPEEAQQQDEEFEKKIPSVEDSLGEGSRDAGRPG
ERGSGGLFSPSTAHVPDGALGQRDQSSWQNSDASQEVGGHQERQQAGAQGPGSADLEDGE
MGKRGWVGEFSLSVGPQREAAFSPGQQDWSRDFCIEASERSYQFGIIGNDRVSGAGFSPS
SKMEGGHFVPPGKTTAGSVDWTDQLGLRNLEVSSCVGSGGSSEARESAVGQMGWSGGLSL
RDMNLTGCLESGGSEEPGGIGVGEKDWTSDVNVKSKDLAEVGEGGGHSQARESGVGQTDW
SGVEAGEFLKSRERGVGQADWTPDLGLRNMAPGAVCSPGESKELGVGQMDWGNNLGLRDL
EVTCDPDSGGSQGLRGCGVGQMDWTQDLAPQNVELFGAPSEAREHGVGGVSQCPEPGLRH
NGSLSPGLEARDPLEARELGVGETSGPETQGEDYSSSSLEPHPADPGMETGEALSFGASP
GRCPARPPPSGSQGLLEEMLAASSSKAVARRESAASGLGGLLEEEGAGAGAAQEEVLEPG
RDSPPSWRPQPDGEASQTEDVDGTWGSSAARWSDQGPAQTSRRPSQGPPARSPSQDFSFI
EDTEILDSAMYRSRANLGRKRGHRAPVIRPGGTLGLSEAADSDAHLFQDSTEPRASRVPS

SDEEVVEEPQSRRTRMSLGTKGLKVNLFPGLSPSALKAKLRPRNRSAEEGELAESKSSQK
ESAVQRSKSCKVPGLGKPLTLPPKPEKSSGSEGSSPNWLQALKLKKKKV
>P13864
MPARTAPARVPALASPAGSLPDHVRRRLKDLERDGLTEKECVREKLNLLHEFLQTEIKSQ
LCDLETKLHKEELSEEGYLAKVKSLLNKDLSLENGTHTLTQKANGCPANGSRPTWRAEMA
DSNRSPRSRPKPRGPRRSKSDSDTLSVETSPSSVATRRTTRQTTITAHFTKGPTKRKPKE
ESEEGNSAESAAEERDQDKKRRVVDTESGAAAAVEKLEEVTAGTQLGPEEPCEQEDDNRS
LRRHTRELSLRRKSKEDPDREARPETHLDEDEDGKKDKRSSRPRSQPRDPAAKRRPKEAE
PEQVAPETPEDRDEDEREEKRRKTTRKKLESHTVPVQSRSERKAAQSKSVIPKINSPKCP
ECGQHLDDPNLKYQQHPEDAVDEPQMLTSEKLSIYDSTSTWFDTYEDSPMHRFTSFSVYC
SRGHLCPVDTGLIEKNVELYFSGCAKAIHDENPSMEGGINGKNLGPINQWWLSGFDGGEK
VLIGFSTAFAEYILMEPSKEYEPIFGLMQEKIYISKIVVEFLQNNPDAVYEDLINKIETT
VPPSTINVNRFTEDSLLRHAQFVVSQVESYDEAKDDDETPIFLSPCMRALIHLAGVSLGQ
RRATRRVMGATKEKDKAPTKATTTKLVYQIFDTFFSEQIEKYDKEDKENAMKRRRCGVCE
VCQQPECGKCKACKDMVKFGGTGRSKQACLKRRCPNLAVKEADDDEEADDDVSEMPSPKK
LHQGKKKKQNKDRISWLGQPMKIEENRTYYQKVSIDEEMLEVGDCVSVIPDDSSKPLYLA
RVTALWEDKNGQMMFHAHWFCAGTDTVLGATSDPLELFLVGECENMQLSYIHSKVKVIYK
APSENWAMEGGTDPETTLPGAEDGKTYFFQLWYNQEYARFESPPKTQPTEDNKHKFCLSC
IRLAELRQKEMPKVLEQIEEVDGRVYCSSITKNGVVYRLGDSVYLPPEAFTFNIKVASPV
KRPKKDPVNETLYPEHYRKYSDYIKGSNLDAPEPYRIGRIKEIHCGKKKGKVNEADIKLR
LYKFYRPENTHRSYNGSYHTDINMLYWSDEEAVVNFSDVQGRCTVEYGEDLLESIQDYSQ
GGPDRFYFLEAYNSKTKNFEDPPNHARSPGNKGKGKGKGKGKGKHQVSEPKEPEAAIKLP
KLRTLDVFSGCGGLSEGFHQAGISETLWAIEMWDPAAQAFRLNNPGTTVFTEDCNVLLKL
VMAGEVTNSLGQRLPQKGDVEMLCGGPPCQGFSGMNRFNSRTYSKFKNSLVVSFLSYCDY
YRPRFFLLENVRNFVSYRRSMVLKLTLRCLVRMGYQCTFGVLQAGQYGVAQTRRRAIILA
AAPGEKLPLFPEPLHVFAPRACQLSVVVDDKKFVSNITRLSSGPFRTITVRDTMSDLPEI
QNGASNSEIPYNGEPLSWFQRQLRGSHYQPILRDHICKDMSPLVAARMRHIPLFPGSDWR
DLPNIQVRLGDGVIAHKLQYTFHDVKNGYSSTGALRGVCSCAEGKACDPESRQFSTLIPW
CLPHTGNRHNHWAGLYGRLEWDGFFSTTVTNPEPMGKQGRVLHPEQHRVVSVRECARSQG
FPDSYRFFGNILDRHRQVGNAVPPPLAKAIGLEIKLCLLSSARESASAAVKAKEEAATKD

>Q9N2M8
MAPRRNSNISSSGNSTQQQHQQQLQQQHQTQQHQLLQFYAENVNSSGVLMAPMQGTGGVG
GVGGGGMVHCCLPSGDCRKLDTLIHLNELSLADCIRVLCNNENCSAGQYMHRECFEWWEA
SVLQALKANGRARSWSERQRLQHLWTKKGYELVQKACSCKCGRGQLRKDLDWVPPSSQGV
IYLNGSGNRPTLANGSLSEDDDKKKAKKKRNRNNNNNNGGGGGGGAGVNGNTKTPLSNNN
GNSYAGLTPNPNVGVIGSGLPHNNGNTASNGSSGNNGSSGVLQTSALATFSNILNTNNVL
GLDLRARAGSLSSSGAGSGSTSPSDSQSSGEISVSPVQQLLPQQQQQQQSLLIQPLGPA
FGSNLLQNGLGLSKNLLIAPQQQQQQQLQQQQQQQNNLPALANISNFKPLASYEQQQLVQ
QQKNKEVELYSDRVRSTSGCNGIFSRRLDFSSFNLLPKTRLNSYQVKIEDEGNHGNDETR
LFILSSLAQSQMSRVACILCEEPLLVFDRYPLVDGSFFLSPKQHSSGCIEVKYEGRTLYL
TCVCMSCLDGTSSSRAINCRFCKEPWDGSSLVLGTMYAYDIFAAMPCCAERFKCNNCFKM
LMHPQQRLSFYSDYSHGVTCPYCNTQDTHFVKPLTFCYAKTTATRLPTLAXHEAPLESPV
GTGATTTQVPNAQGSPTASGCSSNTIASKQPPKQPLYNAAIDYSAPLQQQINPDLIGAHP

HAQQHQQQVRQQQQQQQQQQPQQQQQQQQQQTQQQQSQQQQQQQQQQHQPVVSTFQRGNF
PQQSHKTMNMMAMADVMSKYQQQQHQQQQQQRQQQHNLQPQQQHATQKGLEGLLLTNTNT
SNNNSSSSSSSSSISNLIISHNSASNTGNKMQPTWGQSDAISALWGCSSSSSGCSSGSGSQ
PSLSPTASSNGNDGSKMMLWAAQSSPQNAATLRDGFKELQRQQPPQQQVPQQQPHAASPT
ASLTSSSSSSNGWSASHYGKPNIWELPGSTGQRTPASSHDIFTDLLCNLSISQDNSSQQA
SSKADASDVSSNSSSSAGELLEAANIWRFPEYASSQLYMEEPTGGAAACMQLFNDYLNMN

>O14348
MNDGFADDELSLPKATVQKMVSDILPVDLTFTKEARDLLIECCVEFIHLVSSEANEICEK
EAKKTIAAEHIIKALENLEFKEYIAEALEVAAEHKEQQKNREKKSSKFEQSGVSRDELLR
QQEELLSRARERFKNQNIAHDNHTTTAIPVPTASETETKEN
>Q99288
MCEENVHVSEDVAGSHGSFTNARPRLIVLIRHGESESNKNKEVNGYIPNHLISLTKTGQI
QARQAGIDLLRVLNVDDHNLVEDLAKKYIKDESSRRTLPLKDYTRLSREKDTNIVFYTSP
YRRARETLKGILDVIDEYNELNSGVRICEDMRYDPHGKQKHAFWPRGLNNTGGVYENNED
NICEGKPGKCYLQYRVKDEPRIREQDFGNFQKINSMQDVMKKRSTYGHFFFRFPHGESAA
DVYDRVASFQETLFRHFHDRQERRPRDVVVLVTHGIYSRVFLMKWFRWTYEEFESFTNVP
NGSVMVMELDESINRYVLRTVLPKWTDCEGDLTT
>Q9CXW3
MASVLEELQKDLEEVKVLLEKSTRKRLRDTLTSEKSKIETELKNKMQQKSQKKPELDNEK
PAAVVAPLTTGYTVKISNYGWDQSDKFVKIYITLTGVHQVPTENVQVHFTERSFDLLVKN
LNGKNYSMIVNNLLKPISVESSSKKVKTDTVIILCRKKAENTRWDYLTQVEKECKEKEKP
SYDTEADPSEGLMNVLKKIYEDGDDDMKRTINKAWVESREKQAREDTEF
>Q12265
MSMSNIVVFGGDSHPELVTKICENLDIHPSKVELGKFSNGETNIALRESVREKDVYIIQS
GCGQVNDTFMQLLILISACKSASASRVTAVMPYLCYSRQPDIPYTAKGAPIISKPKENYT
FESHPGTPVSSSLMTQRPGAESSLKSLDSAIRSTINLENPQPIRTPNSSATANNNFDIKK
TLSFSRIPMIPGGKLQNTSNSTDAGELFNAQNAGYKLWVVQAGTLIAHLLSAAGADHVIT
MDLHDPQFPGFFDIPVDNLYCKPIAQNYIQHRIPDYQDAVIVSPDAGGAKRATAIADALE
LSFALIHKERRSQLLKGPPDATLTSGGSLPVSPRPLVTTLVSSQNTTSSGATGVAALEMK
KTTSTSSTSSQSSNSSKFVQTTMLVGDVRNKVCIIVDDLVDTSYTITRAAKLLKDQGSTK
VYALITHGVFSGDALERIGQSSIDKLIISNTVPQDRTLQYLGKDRVDVIDVSCIIGEAIR
RIHNGESISMLFEHGW
>Q9BUB5
MVSSQKLEKPIEMGSSEPLPIADGDRRRKKKRRGRATDSLPGKFEDMYKLTSELLGEGAY
AKVQGAVSLQNGKEYAVKIIEKQAGHSRSRVFREVETLYQCQGNKNILELIEFFEDDTRF
YLVFEKLQGGSILAHIQKQKHFNEREASRVVRDVAAALDFLHTKDKVSLCHLGWSAMAPS
GLTAAPTSLGSSDPPTSASQVAGTTGIAHRDLKPENILCESPEKVSPVKICDFDLGSGMK
LNNSCTPITTPELTTPCGSAEYMAPEVVEVFTDQATFYDKRCDLWSLGVVLYIMLSGYPP
FVGHCGADCGWDRGEVCRVCQNKLFESIQEGKYEFPDKDWAHISSEAKDLISKLLVRDAK
QRLSAAQVLQHPWVQGQAPEKGLPTPQVLQRNSSTMDLTLFAAEAIALNRQLSQHEENEL
AEEPEALADGLCSMKLSPPCKSRLARRALAQAGRGEDRSPPTAL
>Q9SWR3
MAAVTGLYGSIDEFLNHCSQSGDSAYSALRSLLERLEKPDTRTEARIFLAHLQKKLDNDG

ASQRCLETYHFQIQDIYLDRNEGTGYQNRKKFTMMVIPSIFMPEDWSFTFYEGINRHPDS
IFKDKTVAELGCGNGWISIAIAEKWLPLKVYGLDINPRAVKISWINLYLNAFDEDGQPVY
DSESKTLLDRVEFYESDLLSYCRDNHIELERIVGCIPQILNPNPDAMSKLVTENASEEFL
HSLSNYCALQGFVEDQFGLGLIARAVEEGIDVIKPMGIMIFNMGGRPGQGVCKRLFERRG
LSVNKLWQTKILQASDTDISALVEIEKNNPHRFEFFMGLVGDRPICARTAWAFGKACGRI
SHALSVYSCQLRHPNEVKKIFEFLKNGFHDISNSLDLSFEDDSVADEKIPFLAYLAGVLK
DGSRFPYEPPTGNKRFRDLIASFMKTYHHVPLSTDNVAIFPSRATAIENSLRLFTPRLAI
VEEHLTCNLPRQWLTSLEIEQTRDSKTPIDGITVIEAPRQSDLMIELIKKLKPQVVVTGI
AQFEAVTSSAFEHLLRVTREIGSRLFIDISDQFELSSLPSSIGVLKYLARTPLPSHAAII
CGLLRNRVYTDLEVAFVISEEQTIFDALTRTVELLQGNTALISQYYYGCLFHELLSFQIP
DRRQTAEREAENVEASDIDMIGFSSSAISVLSQSELSVRVTEKSSLLHMDVDQIFLPTPT
PVKAAIFESFARQNVTETECDVTPILRQFILNTWNFSVEHSAEFIYADFPLALFNKLVLC
CIEEGGSLCMPAGSNGNYAAAAKFLNANIMSIPTEAEVGFKLTAKQLSSVLETVHKPWVY
ISGPTINPTGLLYSNEEMKSLLTVCARYGARTIIDTSFSGIKFNSQDWDGWNLDASLAGL
TGNPSFSVCLLGGLFFKIPTGGLSYGFLVLKSGFLADSFRSSFSGLNKPHNTVRYTAKKL
LELGEQKGNLTGAAQGQEKLLATRLKRLKETLENCGWEVIEARGGVSVIAKPSAYLGKNI
KLEKDGSTWVTKLDGTNIREAMLRATGLCINGPSWTGIPDYCRFTFALEDGDFDRALDCI
VKFNQLVK
>Q13162
MEALPLLAATTPDHGRHRRLLLLPLLLFLLPAGAVQGWETEERPRTREEECHFYAGGQVY
PGEASRVSVADHSLHLSKAKISKPAPYWEGTAVIDGEFKELKLTDYRGKYLVFFFYPLDF
TFVCPTEIIAFGDRLEEFRSINTEVVACSVDSQFTHLAWINTPRRQGGLGPIRIPLLSDL
THQISKDYGVYLEDSGHTLRGLFIIDDKGILRQITLNDLPVGRSVDETLRLVQAFQYTDK
HGEVCPAGWKPGSETIIPDPAGKLKYFDKLN
>Q9NY74
MSRRRKHDDSPSPKKTPHKTVAAEECGSVVEPGRRRLRSARGSWPCGAREGPPGPVRQRE
QPPTAALCSKSNPEERYETPKRALKMDSLSSSFSSPNDPDGQNDIFWDQNSPLTKQLGKG
RKKQIYTTDSDEISHIVNRIAPQDEKPTTNSMLDMWIGETAIPCTPSVAKGKSRAKISCT
KLKTQSQEEELMKLAKQFDKNMEELDVIQEQNKRNYDFTQMISETEILSNYKDNIQMWSL
HNIVPEIDNATKKPIKGNTKISVANNQNSSQKPFDQIAEAAFNAIFDGSTQKCSGQLSQE
LPEAFWSTSNTTFVKTNALKEEKIITNETLVIEKLSNKTPRSLSSQVDTPIMTKSCVTSC
TKEPETSNKYIDAFTTSDFEDDWENLLGSEPFAMQNIDMPELFPSKTAHVTDQKEICTFN
SKTVKNTSRANTSPDARLGDSKVLQDLSSKTYDRELIDAEYRFSPNSNKSNKLSTGNKMK
FENSSNKIVIQDEIQNCIVTSNLTKIKEDILTNSTEASERKSALNTRYSNEQKNKCILNQ
SIKAPVNTDLFGSANLGSKTSVSNPNQTSASKVGSFFDDWNDPSFANEIIKACHQLDNTW
EADDVDDDLLYQACDDIERLTQQQDIRKDSKTSESICEINNNSEHGAKLTQQQDIRKDSK
TSESICEINNNSEHGAKNMFAISKQGSNLVQSKHLNPGSISVQTSLTNSSQIDKPMKMEK
GEMYGNSPRFLGATNLTMYSKISNCQINNLHVSYTNTDVPIQVNSSKLVLPGSSSLNVTS
DHMNTEITTYKKKLSTNQPCHKTVTDEAQSNLNTTVGFSKFTFTRMKNSQILSQFNQNCI
TGSMSDTKITQGVEKKKGVNPLLEEAVGQQSLVKLSESLKQSSKEEEEKNRKCSPEEIQR
KRQEALVRRMAKARASSVNAAPTSFL
>P26786
MSAPQAKILSQAPTELELQVAQAFVELENSSPELKAELRPLQFKSIREIDVAGGKKALAI
FVPVPSLAGFHKVQTKLTRELEKKFQDRHVIFLAERRILPKPSRTSRQVQKRPRSRTLTA

VHDKILEDLVFPTEIVGKRVRYLVGGNKIQKVLLDSKDVQQIDYKLESFQAVYNKLTGKQ
IVFEIPSETH
>Q8N3C0
MALPRLTGALRSFSNVTKQDNYNEEVADLKIKRSKLHEQVLDLGLTWKKIIKFLNEKLEK
SKMQSINEDLKDILHAAKQIVGTDNGREAIESGAAFLFMTFHLKDSVGHKETKAIKQMFG
PFPSSSATAACNATNRIISHFSQDDLTALVQMTEKEHGDRVFFGKNLAFSFDMHDLDHFD
ELPINGETQKTISLDYKKFLNEHLQEACTPELKPVEKTNGSFLWCEVEKYLNSTLKEMTE
VPRVEDLCCTLYDMLASIKSGDELQDELFELLGPEGLELIEKLLQNRITIVDRFLNSSND
HRFQALQDNCKKILGENAKPNYGCQVTIQSEQEKQLMKQYRREEKRIARREKKAGEDLEV
SEGLMCFDPKELRIQREQALLNARSVPILSRQRDADVEKIHYPHVYDSQAEAMKTSAFIA
GAKMILPEGIQRENNKLYEEVRIPYSEPMPLSFEEKPVYIQDLDEIGQLAFKGMKRLNRI
QSIVFETAYNTNENMLICAPTGAGKTNIAMLTVLHEIRQHFQQGVIKKNEFKIVYVAPMK
ALAAEMTDYFSRRLEPLGIIVKELTGDMQLSKSEILRTQMLVTTPEKWDVVTRKSVGDVA
LSQIVRLLILDEVHLLHEDRGPVLESIVARTLRQVESTQSMIRILGLSATLPNYLDVATF
LHVNPYIGLFFFDGRFRPVPLGQTFLGIKCANKMQQLNNMDEVCYENVLKQVKAGHQVMV
FVHARNATVRTAMSLIERAKNCGHIPFFFPTQGHDYVLAEKQVQRSRNKQVRELFPDGFS
IHHAGMLRQDRNLVENLFSNGHIKVLVCTATLAWGVNLPAHAVIIKGTQIYAAKRGSFVD
LGILDVMQIFGRAGRPQFDKFGEGIIITTHDKLSHYLTLLTQRNPIESQFLESLADNLNA
EIALGTVTNVEEAVKWISYTYLYVRMRANPLAYGISHKAYQIDPTLRKHREQLVIEVGRK
LDKAQMIRFEERTGYFSSTDLGRTASHYYIKYNTIETFNELFDAHKTEGDIFAIVSKAEE
FDQIKVREEEIEELDTLLSNFCELSTPGGVENSYGKINILLQTYISRGEMDSFSLISDSA
YVAQNAARIVRALFEIALRKRWPTMTYRLLNLSKVIDKRLWGWASPLRQFSILPPHILTR
LEEKKLTVDKLKDMRKDEIGHILHHVNIGLKVKQCVHQIPSVMMEASIQPITRTVLRVTL
SIYADFTWNDQVHGTVGEPWWIWVEDPTNDHIYHSEYFLALKKQVISKEAQLLVFTIPIF
EPLPSQYYIRAVSDRWLGAEAVCIINFQHLILPERHPPHTELLDLQPLPITALGCKAYEA
LYNFSHFNPVQTQIFHTLYHTDCNVLLGAPTGSGKTVAAELAIFRVFNKYPTSKAVYIAP
LKALVRERMDDWKVRIEEKLGKKVIELTGDVTPDMKSIAKADLIVTTPEKWDGVSRSWQN
RNYVQQVTILIIDEIHLLGEERGPVLEVIVSRTNFISSHTEKPVRIVGLSTALANARDLA
DWLNIKQMGLFNFRPSVRPVPLEVHIQGFPGQHYCPRMASMNKPAFQAIRSHSPAKPVLI
FVSSRRQTRLTALELIAFLATEEDPKQWLNMDEREMENIIATVRDSNLKLTLAFGIGMHH
AGLHERDRKTVEELFVNCKVQVLIATSTLAWGVNFPAHLVIIKGTEYYDGKTRRYVDFPI
TDVLQMMGRAGRPQFDDQGKAVILVHDIKKDFYKKFLYEPFPVESSLLGVLSDHLNAEIA
GGTITSKQDALDYITWTYFFRRLIMNPSYYNLGDVSHDSVNKFLSHLIEKSLIELELSYC
IEIGEDNRSIEPLTYGRIASYYYLKHQTVKMFKDRLKPECSTEELLSILSDAEEYTDLPV
RHNEDHMNSELAKCLPIESNPHSFDSPHTKAHLLLQAHLSRAMLPCPDYDTDTKTVLDQA
LRVCQAMLDVAANQGWLVTVLNITNLIQMVIQGRWLKDSSLLTLPNIENHHLHLFKKWKP
IMKGPHARGRTSIESLPELIHACGGKDHVFSSMVESELHAAKTKQAWNFLSHLPVINVGI
SVKGSWDDLVEGHNELSVSTLTADKRDDNKWIKLHADQEYVLQVSLQRVHFGFHKGKPES
CAVTPRFPKSKDEGWFLILGEVDKRELIALKRVGYIRNHHVASLSFYTPEIPGRYIYTLY
FMSDCYLGLDQQYDIYLNVTQASLSAQVNTKVSDSLTDLALK
>P30999
MDDSEVESTASILASVKEQEAQFEKLTRALEEERRHVSAQLERVRVSPQDANSLMANGTL
TRRHQNGRFVGDADLERQKFSDLKLNGPQDHNHLLYSTIPRMQEPGQIVETYTEEDPEGA
MSVVSVETTDDGTTRRTETTVKKVVKTMTTRTVQPVPMGPDGLPVDASAVSNNYIQTLGR

DFRKNGNGGPGPYVGQAGTATLPRNFHYPPDGYGRHYEDGYPGGSDNYGSLSRVTRIEER
YRPSMEGYRAPSRQDVYGPQPQVRVGGSSVDLHRFHPEPYGLEDDQRSMGYDDLDYGMMS
DYGTARRTGTPSDPRRRLRSYEDMIGEEVPPDQYYWAPLAQHERGSLASLDSLRKGMPPP
SNWRQPELPEVIAMLGFRLDAVKSNAAAYLQHLCYRNDKVKTDVRKLKGIPILVGLLDHP
KKEVHLGACGALKNISFGRDQDNKIAIKNCDGVPALVRLLRKARDMDLTEVITGTLWNLS
SHDSIKMEIVDHALHALTDEVIIPHSGWEREPNEDCKPRHIEWESVLTNTAGCLRNVSSE
RSEARRKLRECDGLVDALIFIVQAEIGQKDSDSKLVENCVCLLRNLSYQVHREIPQAERY
QEALPTVANSTGPHAASCFGAKKGKDEWFSRGKKPTEDPANDTVDFPKRTSPARGYELLF
QPEVVRIYISLLKESKTPAILEASAGAIQNLCAGRWTYGRYIRSALRQEKALSAIAELLT
SEHERVVKAASGALRNLAVDARNKELIGKHAIPNLVKNLPGGQQNSSWNFSEDTVVSILN
TINEVIAENLEAAKKLRETQGIEKLVLINKSGNRSEKEVRAAALVLQTIWGYKELRKPLE
KEGWKKSDFQVNLNNASRSQSSHSYDDSTLPLIDRNQKSDKKPDREEIPMSNMGSNTKSL
DNNYSTLNERGDHNRTLDRSGDLGDMEPLKGAPLMQKI
>O74977
MGDISKHNIFSLPEDILAKLELREEYSVEEKTDSANLSNQGDIVDSTQKNISSCVNCQID
NLHTLDERKSHIKSDWHRFNTKRKITKLPPVSQDEFESIIEDLPESLSGSESETNSESEE
DNFQIEKAFKQSLNIGKVDSADENNNQRTNSPLTWFQLSNASAEVPTYIGVYKHMFSGNN
HITKDLLVQQQNHNRQKPLQLAMFMVGGGHFAAMIASNEFNPRDPHVPKVLAQKTIHRYT
TRRKQGGSQGAADNTKGNIHSAGSGLRRYNEQALIKDIQQVFKDWGKLLETCDLIFVRAI
GSSNRSIFFSQPGALISPKDPKLRVFPFTTKRATHSELLRCYKELVTPKISHVDSISIKA
QEEERKRQAEIEKEIRQSRLQEEERKKKKLAKYTEVIISNLKASNIEAFLEYLRSNDLSI
NFQFYPKNVHLHTSTPLHYAVTQKNAKLVAKLLRNGADPAMLNGNGKTPFEISTGNKEVK
DEFLIARHELGESFFDWEAAKVGAPQSREQIQKQRQKAKTKLENQRRDKERQEELRRKEA
MQKIEEQSKRDYDKLHGEGHSLGINNVRKVDELQSLSPEMRMRIEREKRAAAAMKRMQTK

>O95273
MASATAPAAAVPTLASPLEQLRHLAEELRLLLPRVRVGEAQETTEEFNREMFWRRLNEAA
VTVSREATTLTIVFSQLPLPSPQETQKFCEQVHAAIKAFIAVYYLLPKDQGITLRKLVRG
ATLDIVDGMAQLMEVLSVTPTQSPENNDLISYNSVWVACQQMPQIPRDNKAAALLMLTKN
VDFVKDAHEEMEQAVEECDPYSGLLNDTEENNSDNHNHEDDVLGFPSNQDLYWSEDDQEL
IIPCLALVRASKACLKKIRMLVAENGKKDQVAQLDDIVDISDEISPSVDDLALSIYPPMC
HLTVRINSAKLVSVLKKALEITKASHVTPQPEDSWIPLLINAIDHCMNRIKELTQSELEL

>P56537
MAVRASFENNCEIGCFAKLTNTYCLVAIGGSENFYSVFEGELSDTIPVVHASIAGCRIIG
RMCVGNRHGLLVPNNTTDQELQHIRNSLPDTVQIRRVEERLSALGNVTTCNDYVALVHPD
LDRETEEILADVLKVEVFRQTVADQVLVGSYCVFSNQGGLVHPKTSIEDQDELSSLLQVP
LVAGTVNRGSEVIAAGMVVNDWCAFCGLDTTSTELSVVESVFKLNEAQPSTIATSMRDSL
IDSLT
>P40031
MGKNHFLKDFSALPEDVLIENERGITLLGYPLFSPKILLPHVDPPQFQRLNTENGSLIAL
SKNTISNFIELYPIDLSTERTAGSSSSQMTKWFVLMDYKEKYDIDDQGWCYSWNFNNSRW
KSKNGLVRRRVWVRLPTTSHGLD
>Q9P6Q1

MSTIHSCLDLIRSQIQQSANGRNVLLVAVSKFHPVETLMEAYNAGQRHFGENYMQEFLKK
VELMPDDVQWHFIGSLQSSKCKKIASVKNLYSIETIDTEKKARLVNSAREALQLPLNVYI
QVNTSGEENKGGVTPSKVLELCKQVQDMKYLRLKGLMTIGSISNSQLSDHNPDFQVLSDL
RESLQNELGIPLQLSMGMSSDYLLAIKYGSDSVRVGSSIFGSRPTEKPSDVHISASK
>P38170
MTTQLRYENNDDDERVEYNLFTNRSTMMANFEEWIKMATDNKINSRNSWNFALIDYFYDL
DVLKDGENNINFQKASATLDGCIKIYSSRVDSVTTETGKLLSGLAQRKTNGASNGDDSNG
GNGEGLGGDSDEANIEIDPLTGMPISNDPDVNNTRRRVYNRVLETTLVEFETIKMKELDQ
ELIIDPLFKKALVDFDEGGAKSLLLNTLNIDNTARVIFDASIKDTQNVGQGKLQRKEEEL
IERDSLVDDENEPSQSLISTRNDSTVNDSVISAPSMEDEILSLGMDFIKFDQIAVCEISG
SIEQLRNVVEDINQAKDFIENVNNRFDNFLTEEELQAAVPDNAEDDSDGFDMGMQQELCY
PDENHDNTSHDEQDDDNVNSTTGSIFEKDLMAYFDENLNRNWRGREHWKVRNFKKANLVN
KESDLLEETRTTIGDTTDKNTTDDKSMDTKKKHKQKKVLEIDFFKTDDSFEDKVFASKGR
TKIDMPIKNRKNDTHYLLPDDFHFSTDRITRLFIKPAQKMSLFSHRKHTRGDVSSGLFEK
STVSANHSNNDIPTIADEHFWADNYERKEQEEKEKEQSKEVGDVVGGALDNPFEDDMDGV
DFNQAFEGTDDNEEASVKLDLQDDEDHKFPIRENKVTYSRVSKKVDVRRLKKNVWRSINN
LIQEHDSRKNREQSSNDSETHTEDESTKELKFSDIIQGISKMYSDDTLKDISTSFCFICL
LHLANEHGLQITHTENYNDLIVNYEDLATTQAAS
>Q12202
MEENQLTCLIVGETPAAQFLGWRLSLSNSFIILVSQYVSSDELVAWKSTKLGANFYTPNI
LTKDIKELHHKLKHNGANTYSIDIVLVSAISLQSFETTCRLLSDYTNDNTTVLISTDFGC
ELEPIAISYFGGKCKCVISISCEVECRQLSLGSYALVNDDQCVITLGLTYCDANFEASPT
ILENTKAASLELQGIKGSNVRRFLLGLTVAKWMKSKLILDPKQMALKMWELLIPKISLNI
LSIIYEQFDYEKMLENKSTEIIFKDLVKELLGICFAQCGSKIARFLLVKSQGEEEINFGK
IVEYCKGKKLQLINSTANEHPEYLSLPFEPYCFYHRFEYPAQILLHQPIILAKKYGVSCS
NLNFLYGFYTRLLTLSGLSINGGRCEHALSMLDSRIGGGINVASGINSGQDFTDGDNEDQ
DKGKNRVDKNVKEGSFISLTQRFTMTSPLGVNDPALPADLEKLYLGAEYISNCDANTSGG
QKRVKSPTKADTGYDDKHLPDDTIHTFEDEYLADEDDFSCLGIDKRSSTKPTKPLEKFGV
VAVPHFIRRFSIKRSSKDKSNDMKRPYTTSSLELQLRSNHFMFAKEYQDLHRQLYYEVKP
RTQSELDARRRNYSELESQMWKIKHRFNIHRGALPRPRTNPYELLLDHIDVLNRGNTGDI
LRFTTSRYGGVDTYDSILRDQSTIMELLDKRCAYSPPVLFDNEAKERDHDRDCYQDYNHD
HRSH
>O74363
MSNFDDLQILDQLQTSARFRGDTRIRRFKGSNNSKSPFGLNPSSRISQWPRITFIRAKDN
YNPAEELCQLIDEQNAHNEAKHQSKEIMLPKETNVKSCEDLSKTGEIKALDIVLFKAITS
KGDTLKVKKIPEYAEAFDTTKNVEVSSFPEKSMNENVLIQHSLNLAPSDKEISSTEESEQ
LCYKEQESEKELYSKDNDDSLDILNVPNLVNDDIANNNAAPPPPLAQAQEQLSTENEDEF
DIDDTTDKMTTFTMNKFADLSVLEEDDDDEDEDEELEGEKEEEEEEKEKPEISNYDETEG
KVNFSLEDSESLEFDEGSDDYDYAKLTTELVGNTDSLAEDEDDILEEDEDEDEEELLAIF
DASDDSEIEAYLSIGDTLQNDFTIYDYDSEDEEYNNPSTQKTEKVSQSKKGAKVKEEKGL
KKDRKLPKKMRKAQKKLERKAGKAVAARMGDAFSNSLDDESMNLYAELQEQWLKDKSKKA
RRRAEREKLRSEGLLGKKSKKKLLRESQKPSSSDSDNASLTRIDKIFINDVYQRMQQFKH
SAIEEISLPPCRKYVRRLVHALANDLNLKSRSYGSGNKRYTMLSKTHKFDASSVDLVSLT
RIMERLQTRVEYQSFSKSGRKSRMTVSSVRSSKATRVYDGQIVGEDAPEISKENPGRRLL

EKLGWYAGKGLGHPENEGSKDSLRAIVKVSRSGLG
>P49957
MEINQAAEKEQEYVHKVYNEIAPHFSQTRYKPWPIVTQFLKTRPMGSIGIDVGCGNGKYL
GVNPDIYIIGSDRSDGLIECARGINPSYNLLVADGLNLPHKNETFDFAISIAVVHHWSTR
ERRVEVIRHVLSKLRQGGQALIYCWALEQGSSRRGYHEGMEQDVFVPWVLPKSKSKPKTK
STPPAKVKTRPKPNLMNIPPKERSEYLQRWKEEQQRSKSLDDNDEKQQQDQEQEREEVKY
RYYHLYREGELAEDCRQAGAAVHSEGFERDNWWVVAQKR
>P53258
MSFFDSLRQKAPFLDKLADSFTPTLTRDEKFRLKYKLPANENILEDTNAEVSFATSIKDG
KGHSDRVNNKGRKTAYVYSGRLFLTPHFLVFRDAFDHSSCVLILNISTIKRVERSPSESY
EFALLVTLYTGAKVLIQFIGIRYRSEQFCDKLKLNLKENIPNAKTLPAFLETSYSEFLIA
KNILGKKDITVPRAGLGQHFKYPGNPTMVKEKAKLRLWFDYFRENGRNLAVVQTPMFRKL
IRIGVPNRMRGEIWELCSGAMYMRYANSGEYERILNENAGKTSQAIDEIEKDLKRSLPEY
SAYQTEEGIQRLRNVLTAYSWKNPDVGYCQAMNIVVAGFLIFMSEEQAFWCLCNLCDIYV
PGYYSKTMYGTLLDQRVFESFVEDRMPVLWEYILQHDIQLSVVSLPWFLSLFFTSMPLEY
AVRIMDIFFMNGSITLFQVALAVLKINADDILQADDDGMFIAIIKHYFQTLGQSAHPDSS
DIKYRQITKFQELLVTAFKEFSVISEEMAMHARHKYEKGIFQNIETFMKRTQLRHMPKTF
NLSSDDLSNIYDMFYQSIETYKISMGTGSSNMGFEVFIQFLSKFCDSCRPCEKDKDPAFR
KQKRNFLQRLFDNWDSAHIGELTLNDVVTGLDKLVTVDLLQAINYFFSLYDTDGDGELHR
EEVLQLSEGLLLLTEPWKSGRYVDLLTKKRIEDDIAENIIKESGGEIATMNQIELPTGVT
IDEEKYKVEQAERYLKAASNFLQRSFEYAKAVDLAEEVNLIDLSDDEGEEKRTVKQKQLE
SIKANAALDPTHPKVIDLPTFRMIILADETYELFFSNTLRSSVHVDEHVNIDNKNKVLRS
MFDGILADGKRVAEQVRRRVDSVATRSSIASVESTPTAAASSITTKEEKYDDLDDFTSEH
QPENEELLQSSWFEIDDANETSTKAIQERSFEPLSANSSEEKSNLIEFEA
>Q62768
MSLLCVGVKKAKFDGAQEKFNTYVTLKVQNVKSTTIAVRGSQPSWEQDFMFEINRLDLGL
TVEVWNKGLIWDTMVGTVWIPLRTIRQSNEEGPGEWLTLDSQAIMADSEICGTKDPTFHR
ILLDAHFELPLDIPEEEARYWAKKLEQLNAMRDQDEYSFQDQQDKPLPVPSSQCCNWNYF
GWGEQNDDPDSAVDDRDSDYRSETSNSIPPPYYTTSQPNASVHQYSVRPPPLGSRESYSD
SMHSYEEFSEPRALSPTGSSRYASSGELSQGSSQLSEDFDPDEHSLQGSELDDERDRDSY
HSCHSSVSYHKDSPRWDQDEEDLEDLEDLEDEELPEEEELEEEELEEEEELEEEELELEE
EEEVPDDLASYTQQEDTTVAEPKEFKRISFPTAAPQKEDKVSAVPIEAPDVSKGIPKAAT
PEEKAAAECAQEAEPPKSEESFRSREAEEGQEGQDAMSRAKANWLRAFNKVRMQLQEARG
EGEMSKSLWFKGGPGGGLIIIDSMPDIRKRKPIPLVSDLAMSLVQSRKAGITSALASSTL
NNEELKNHVYKKTLQALIYPISCTTPHNFEVWTATTPTYCYECEGLLWGIARQGMRCTEC
GVKCHEKCQDLLNADCLQRAAEKSSKHGAEDRTQNIIMVLKDRMKIRERNKPEIFELIQE
VFAVTKSAHTQQMKAVKQSVLDGTSKWSAKISITVVCAQGLQAKDKTGSSDPYVTVQVGK
TKKRTKTIYGNLNPVWEENFHFECHNSSDRIKVRVLDEDDDIKSRVKQRFKRESDDFLGQ
TIIEVRTLSGEMDVWYNLDKRTDKSAVSGAIRLHISVEIKGEEKVAPYHVQYTCLHENLF
HFVTDVQNNGVVKIPDAKGDDAWKVYYDETAQEIVDEFAMRYGVESIYQAMTHFACLSSK
YMCPGVPAVMSTLLANINAYYAHTTASTNVSASDRFAASNFGKERFVKLLDQLHNSLRID
LSMYRNNFPASSPERLQDLKSTVDLLTSITFFRMKVQELQSPPRASQVVKDCVKACLNST
YEYIFNNCHELYGREYQTDPAKKGEVPPEEQGPSIKNLDFWSKLITLIVSIIEEDKNSYT
PCLNQFPQELNVGKISAEVMWSLFAQDMKYAMEEHDKHRLCKSADYMNLHFKVKWLYNEY

VAELPTFKDRVPEYPAWFEPFVIQWLDENEEVSRDFLHGALERDKKDGFQQTSEHALFSC
SVVDVFSQLNQSFEIIKKLECPDPQIVGHYMRRFAKTISNVLLQYADIVSKDFASYCSKE
KEKVPCILMNNTQQLRVQLEKMFEAMGGKELDAEASGTLKELQVKLNNVLDELSHVFATS
FQPHIEECVRQMGDILSQVKGTGNVPASACSSVAQDADNVLQPIMDLLDSNLTLFAKICE
KTVLKRVLKELWKLVMNTMERTIVLPPLTDQTMIGTLLRKHGKGLEKGRVKLPSHSDGTQ
MIFNAAKELGQLSKLKDHMVREEAKSLTPKQCAVVELALDTIKQYFHAGGVGLKKTFLEK
SPDLQSLRYALSLYTQATDLLIKTFVQTQSAQVHGGKGTRFTLSEDVCPEMGSGVEDPVG
EVSVHVELFTHPGTGEQKVTVKVVAANDLKWQTSGIFRPFIEVNIVGPQLSDKKRKFATK
SKNNSWAPKYNESFQFSLSADAGPECYELQVCVKDYCFAREDRTVELAVLQLRELAQRGS
AACWLPLGRRIHMDDTGLTVLRILSQRSNDEVAKEFVKLKSDTRSAEEGGAAPAP
>Q9VIP8
MFPQRSSELYRTPVTHQPPPALELAGNLEYPNLNIADLNARLENLSPRLHDCWDSIAIND
HNHLALATNRREGRQWWGMLFGYGRDQMHHMSVDSANFKLQAEHTVNIVRYAEDDFLLVA
LGDTRLQAWSTYSKVRDSQSPYCLFLVGESSAHPTPISQLSVFKADPRTAVSGSADSTLN
VWDLSGADMVSTYRSRSSHTDKLTGLATPAASVDKFVTCDRGGCARLWDVRAAAPSSTCL
YADASHVLSFTSAAWAAASELQGDNHIYLGDYDGKVHTLDIRVPRKLAETREYFDKGHVA
QLLINGPHLAAMSNLPASVKVANVQAGHEFIYTHQDTHSRLTDAVWTDDSTLITIGHGRK
MVTHAIK
>P18510
MEICRGLRSHLITLLLFLFHSETICRPSGRKSSKMQAFRIWDVNQKTFYLRNNQLVAGYL
QGPNVNLEEKIDVVPIEPHALFLGIHGGKMCLSCVKSGDETRLQLEAVNITDLSENRKQD
KRFAFIRSDSGPTTSFESAACPGWFLCTAMEADQPVSLTNMPDEGVMVTKFYFQEDE
>P10664
MSRPQVTVHSLTGEATANALPLPAVFSAPIRPDIVHTVFTSVNKNKRQAYAVSEKAGHQT
SAESWGTGRAVARIPRVGGGGTGRSGQGAFGNMCRGGRMFAPTKTWRKWNVKVNHNEKRY
ATASAIAATAVASLVLARGHRVEKIPEIPLVVSTDLESIQKTKEAVAALKAVGAHSDLLK
VLKSKKLRAGKGKYRNRRWTQRRGPLVVYAEDNGIVKALRNVPGVETANVASLNLLQLAP
GAHLGRFVIWTEAAFTKLDQVWGSETVASSKVGYTLPSHIISTSDVTRIINSSEIQSAIR
PAGQATQKRTHVLKKNPLKNKQVLLRLNPYAKVFAAEKLGSKKAEKTGTKPAAVFTETLK
HD
>P40307
MEYLIGIQGPDYVLVASDRVAASNIVQMKDDHDKMFKMSEKILLLCVGEAGDTVQFAEYI
QKNVQLYKMRNGYELSPTAAANFTRRNLADCLRSRTPYHVNLLLAGYDEHEGPALYYMDY
LAALAKAPFAAHGYGAFLTLSILDRYYTPTISRERAVELLRKCLEELQKRFILNLPTFSV
RVIDKDGIHNLENITFTKRSS
>P56508
MHARDWFLVFIAIFIPPLAVWLKRGFFTKDLLINFLLFLLGFFPGLIHALYVISCHPYEE
NEARYSHLSSSDDNYGSLA
>O01422
MGDEYMDDDEDYGFEYEDDSGSEPDVDMENQYYTAKGLRSDGKLDEAIKSFEKVLELEGE
KGEWGFKALKQMIKITFGQNRLEKMLEYYRQLLTYIKSAVTKNYSEKSINAILDYISTSR
QMDLLQHFYETTLDALKDAKNERLWFKTNTKLGKLFFDLHEFTKLEKIVKQLKVSCKNEQ
GEEDQRKGTQLLEIYALEIQMYTEQKNNKALKWVYELATQAIHTKSAIPHPLILGTIREC
GGKMHLRDGRFLDAHTDFFEAFKNYDESGSPRRTTCLKYLVLANMLIKSDINPFDSQEAK

PFKNEPEIVAMTQMVQAYQDNDIQAFEQIMAAHQDSIMADPFIREHTEELMNNIRTQVLL
RLIRPYTNVRISYLSQKLKVSQKEVIHLLVDAILDDGLEAKINEESGMIEMPKNKKKMMV
TSLVVPNAGDQGTTKSDSKPGTSSEPSTTTSVTSSILQGPPATSSCHQELSMDGLRVWAE
RIDSIQSNIGTRIKF
>Q8C8H8
MELKKDSNAVAIDMLLIVHSEKRRAAQATHLDPQANPGALLQNRGGFQGVRNGIRKWQEL
EENSFQGNLPEKQCLQQPQVITSYDNQGTQLTVEIHPQDAMPQLLKKFSLAKRLQGDKNG
NMRPRQPGGKDAHAYPWDRSSLKSMPLDLRLFEKLDASASQVTVKSGLNELVSDLLQEAH
SDLERVRAIWIWICHHIEYDVEAAQEKDRQAFKPTDILRTQKTNCDGYAGLFERMCRVAG
VQCVTVPGYSKGFGYQTGQSFSGEFDHAWNAVYLEGRWHLVDSTWGSGLVDTTTSKFTFL
YNEFYFLTHPALFIEDHFPDNKNWQLLKPPQSLRQFENSMYHKSEFYNKGMLSAHPETSM
IRTVNGKATITIESRAPTLFMFMLNGKQEHGLLSLRKNGMKLEVYPPTMGTHKLQIFAKG
NSEIYSSVLEYTLKCNYVDFSVQLPSELHQPVGPSWFSEQMGITKPSHSDPIIHTSDGRC
AISFSVEEGVSVLASLHGDDGPITEETQRRYIFQLNRGKRTELKVQLPHAGKFALKIFVK
KRQEQGNFIFVFNYLLCCANTKVNWPMFPESFGNWGQDNELLEPLSGVLPANRNVAFKLK
LHGIAKALVKGQDTWPLTLNPEGYWEGSCNTAGCQEVYVMVLENANHNFYSYILKYKVND
Q
>Q12523
MDPFHNGNKRSSISFGSSQRQPYNKNNYLSGTNGPSSAAQDQGRGPSPFGMSGNTTNGGN
SKRNSGCDLSATYYASRSPMYSPLDFSPPVFSPNHSQLQQARGYAANIPVVSNLMNPSMA
SVCEYQSHYPLFGLDWSADDYVCLGSYKEDSRNKLQVLHSNDLLSWESVVDADVVYPVSK
IQWVPSQLYPRKLATCSDSLRIWSVSPEERQFQEQINLSLCKYNRQHPANPAAADDMKVI
GTFPPITSFDWNTVDTNLIISSSIDTTCIVWDLQSSHYVKTQLIAHDSEVFDVRFLTKST
QLFASCGGDGSVRVFDLRSLAHSTIIYEPPSSSVSGATAGTITPSSKGSDALLRLEPSPY
DPNVLATFAADSNKIIILDMRNPESPILNLQGHGSSVNGIKWHPTKRNVLLSCGDDCQVL
YWDLNSSFMEINAAGSKSPSIHGTSLEDPDGDTEMTDGGAGSGLNEDPLSLNNNSKQVCK
TLETPNMMYANKTQEINNIAWRPQRGDWFGCVSGKKFQNVRVL
>P30622
MSMLKPSGLKAPTKILKPGSTALKTPTAVVAPVEKTISSEKASSTPSSETQEEFVDDFRV
GERVWVNGNKPGFIQFLGETQFAPGQWAGIVLDEPIGKNDGSVAGVRYFQCEPLKGIFTR
PSKLTRKVQAEDEANGLQTTPASRATSPLCTSTASMVSSSPSTPSNIPQKPSQPAAKEPS
ATPPISNLTKTASESISNLSEAGSIKKGERELKIGDRVLVGGTKAGVVRFLGETDFAKGE
WCGVELDEPLGKNDGAVAGTRYFQCQPKYGLFAPVHKVTKIGFPSTTPAKAKANAVRRVM
ATTSASLKRSPSASSLSSMSSVASSVSSRPSRTGLLTETSSRYARKISGTTALQEALKEK
QQHIEQLLAERDLERAEVAKATSHVGEIEQELALARDGHDQHVLELEAKMDQLRTMVEAA
DREKVELLNQLEEEKRKVEDLQFRVEEESITKGDLETQTKLEHARIKELEQSLLFEKTKA
DKLQRELEDTRVATVSEKSRIMELEKDLALRVQEVAELRRRLESNKPAGDVDMSLSLLQE
ISSLQEKLEVTRTDHQREITSLKEHFGAREETHQKEIKALYTATEKLSKENESLKSKLEH
ANKENSDVIALWKSKLETAIASHQQAMEELKVSFSKGLGTETAEFAELKTQIEKMRLDYQ
HEIENLQNQQDSERAAHAKEMEALRAKLMKVIKEKENSLEAIRSKLDKAEDQHLVEMEDT
LNKLQEAEIKVKELEVLQAKCNEQTKVIDNFTSQLKATEEKLLDLDALRKASSEGKSEMK
KLRQQLEAAEKQIKHLEIEKNAESSKASSITRELQGRELKLTNLQENLSEVSQVKETLEK
ELQILKEKFAEASEEAVSVQRSMQETVNKLHQKEEQFNMLSSDLEKLRENLADMEAKFRE
KDEREEQLIKAKEKLENDIAEIMKMSGDNSSQLTKMNDELRLKERDVEELQLKLTKANEN

ASFLQKSIEDMTVKAEQSQQEAAKKHEEEKKELERKLSDLEKKMETSHNQCQELKARYER
ATSETKTKHEEILQNLQKTLLDTEDKLKGAREENSGLLQELEELRKQADKAKAAQTAEDA
MQIMEQMTKEKTETLASLEDTKQTNAKLQNELDTLKENNLKNVEELNKSKELLTVENQKM
EEFRKEIETLKQAAAQKSQQLSALQEENVKLAEELGRSRDEVTSHQKLEEERSVLNNQLL
EMKKRESKFIKDADEEKASLQKSISITSALLTEKDAELEKLRNEVTVLRGENASAKSLHS
VVQTLESDKVKLELKVKNLELQLKENKRQLSSSSGNTDTQADEDERAQESQIDFLNSVIV
DLQRKNQDLKMKVEMMSEAALNGNGDDLNNYDSDDQEKQSKKKPRLFCDICDCFDLHDTE
DCPTQAQMSEDPPHSTHHGSRGEERPYCEICEMFGHWATNCNDDETF
>Q07825
MTSKPSTSDGRAHSISHVPGTHMRGTSASHSPRPFRPCADCTCSPGLLSRQGRRASLFLR
QLENSRRSSSMLLNELKGAGGGSSAGNGSVYSCDSLCAVNREVNTTDRLLKLRQEMKKHD
LCCYIVPSCDEHQSEYVSLRDQRRAFISGFSGSAGVACITRDLLNFNDDHPDGKSILSTD
GRYFNQARQELDYNWTLLRQNEDPITWQEWCVREALEMAKGLGNKEGMVLKIGIDPKLIT
FNDYVSFRKMIDTKYDAKGKVELVPVEENLVDSIWPDFETLPERPCNDLLLLKYEFHGEE
FKDKKEKLLKKLNDKASSATTGRNTFIVVALDEICWLLNLRGSDIDYNPVFFSYVAINED
ETILFTNNPFNDDISEYFKINGIEVRPYEQIWEHLTKITSQASSAEHEFLIPDSASWQMV
RCLNTSTNANGAIAKKMTAQNFAIIHSPIDVLKSIKNDIEIKNAHKAQVKDAVCLVQYFA
WLEQQLVGREALIDEYRAAEKLTEIRKTQRNFMGNSFETISSTGSNAAIIHYSPPVENSS
MIDPTKIYLCDSGSQFLEGTTDITRTIHLTKPTKEEMDNYTLVLKGGLALERLIFPENTP
GFNIDAIARQFLWSRGLDYKHGTGHGIGSFLNVHEGPMGVGFRPHLMNFPLRAGNIISNE
PGYYKDGEYGIRIESDMLIKKATEKGNFLKFENMTVVPYCRKLINTKLLNEEEKTQINEY
HARVWRTIVHFLQPQSISYKWLKRETSPL
>Q06706
MVEHDKSGSKRQELRSNMRNLITLNKGKFKPTASTAEGDEDDLSFTLLDSVFDTLSDSIT
CVLGSTDIGAIEVQQFMKDGSRNVLASFNIQTFDDKLLSFVHFADINQLVFVFEQGDIIT
ATYDPVSLDPAETLIEIMGTIDNGIAAAQWSYDEETLAMVTKDRNVVVLSKLFEPISEYH
LEVDDLKISKHVTVGWGKKETQFRGKGARAMEREALASLKASGLVGNQLRDPTMPYMVDT
GDVTALDSHEITISWRGDCDYFAVSSVEEVPDEDDETKSIKRRAFRVFSREGQLDSASEP
VTGMEHQLSWKPQGSLIASIQRKTDLGEEDSVDVIFFERNGLRHGEFDTRLPLDEKVESV
CWNSNSEALAVVLANRIQLWTSKNYHWYLKQELYASDISYVKWHPEKDFTLMFSDAGFIN
IVDFAYKMAQGPTLEPFDNGTSLVVDGRTVNITPLALANVPPPMYYRDFETPGNVLDVAC
SFSNEIYAAINKDVLIFAAVPSIEEMKKGKHPSIVCEFPKSEFTSEVDSLRQVAFINDSI
VGVLLDTDNLSRIALLDIQDITQPTLITIVEVYDKIVLLRSDFDYNHLVYETRDGTVCQL
DAEGQLMEITKFPQLVRDFRVKRVHNTSAEDDDNWSAESSELVAFGITNNGKLFANQVLL
ASAVTSLEITDSFLLFTTAQHNLQFVHLNSTDFKPLPLVEEGVEDERVRAIERGSILVSV
IPSKSSVVLQATRGNLETIYPRIMVLAEVRKNIMAKRYKEAFIVCRTHRINLDILHDYAP
ELFIENLEVFINQIGRVDYLNLFISCLSEDDVTKTKYKETLYSGISKSFGMEPAPLTEMQ
IYMKKKMFDPKTSKVNKICDAVLNVLLSNPEYKKKYLQTIITAYASQNPQNLSAALKLIS
ELENSEEKDSCVTYLCFLQDVNVVYKSALSLYDVSLALLVAQKSQMDPREYLPFLQELQD
NEPLRRKFLIDDYLGNYEKALEHLSEIDKDGNVSEEVIDYVESHDLYKHGLALYRYDSEK
QNVIYNIYAKHLSSNQMYTDAAVAYEMLGKLKEAMGAYQSAKRWREAMSIAVQKFPEEVE
SVAEELISSLTFEHRYVDAADIQLEYLDNVKEAVALYCKAYRYDIASLVAIKAKKDELLE
EVVDPGLGEGFGIIAELLADCKGQINSQLRRLRELRAKKEENPYAFYGQETEQADDVSVA
PSETSTQESFFTRYTGKTGGTAKTGASRRTAKNKRREERKRARGKKGTIYEEEYLVQSVG

RLIERLNQTKPDAVRVVEGLCRRNMREQAHQIQKNFVEVLDLLKANVKEIYSISEKDRER
VNENGEVYYIPEIPVPEIHDFPKSHIVDF
>Q9NXV2
MAENHCELLSPARGGIGAGLGGGLCRRCSAGLGALAQRPGSVSKWVRLNVGGTYFLTTRQ
TLCRDPKSFLYRLCQADPDLDSDKDETGAYLIDRDPTYFGPVLNYLRHGKLVINKDLAEE
GVLEEAEFYNITSLIKLVKDKIRERDSKTSQVPVKHVYRVLQCQEEELTQMVSTMSDGWK
FEQLVSIGSSYNYGNEDQAEFLCVVSKELHNTPYGTASEPSEKAKILQERGSRM
>O94334
MRPTHWSSNQCEVDFTAEALTTLSLSNRNHEEDPIQPVLKNSNLFLLNRDIWSLIINYLD
AFDILRLMHSSRQFYYWLRKSAVDECCFNNNFLNLQPYQRTVPVASDLEWATEVDLYGNP
PILKLQLRDSFVWSMLAKFQGLQTIALDGTGVTISSVTNILLNIPTVKTLSIRWCVGVCS
LSLIEFLQNSKSRTFSLENLYVLGVKGLELLKPVLLDGSEDDTLTSNWHSRVILFQNALD
ALPTTHGNPVECDISRCPLNACKIAGQETELADLFSLQKVPACIYCLREWKKPICRYCID
LRSCLVCDSFVCPSCISLDFDLQIQAFARQHRVISTLGVVYPEREDSCFHKIKAAQWHQI
SPRSLLFQFQEQNHIHHKKIRRKLLAAGWKWPQSQI
>P38340
MDVPADSHIKYEDAIDYWTDVDATVDGVLGGYGEGTVVPTMDVLGSNNFLRKLKSRMLPQ
ENNVKYAVDIGAGIGRVSKTMLHKHAAKIDLVEPVKPFIEQMHVELAELKDKGQIGQIYE
VGMQDWTPDAGKYWLIWCQWCVGHLPDAELVAFLKRCIVGLQPNGTIVVKENNTPTDTDD
FDETDSSVTRSDAKFRQIFEEAGLKLIASERQRGLPRELYPVRMYALKPMPN
>P16861
MQSQDSCYGVAFRSIITNDEALFKKTIHFYHTLGFATVKDFNKFKHGENSLLSSGTSQDS
LREVWLESFKLSEVDASGFRIPQQEATNKAQSQGALLKIRLVMSAPIDETFDTNETATIT
YFSTDLNKIVEKFPKQAEKLSDTLVFLKDPMGNNITFSGLANATDSAPTSKDAFLEATSE
DEIISRASSDASDLLRQTLGSSQKKKKIAVMTSGGDSPGMNAAVRAVVRTGIHFGCDVFA
VYEGYEGLLRGGKYLKKMAWEDVRGWLSEGGTLIGTARSMEFRKREGRRQAAGNLISQGI
DALVVCGGDGSLTGADLFRHEWPSLVDELVAEGRFTKEEVAPYKNLSIVGLVGSIDNDMS
GTDSTIGAYSALERICEMVDYIDATAKSHSRAFVVEVMGRHCGWLALMAGIATGADYIFI
PERAVPHGKWQDELKEVCQRHRSKGRRNNTIIVAEGALDDQLNPVTANDVKDALIELGLD
TKVTILGHVQRGGTAVAHDRWLATLQGVDAVKAVLEFTPETPSPLIGILENKIIRMPLVE
SVKLTKSVATAIENKDFDKAISLRDTEFIELYENFLSTTVKDDGSELLPVSDRLNIGIVH
VGAPSAALNAATRAATLYCLSHGHKPYAIMNGFSGLIQTGEVKELSWIDVENWHNLGGSE
IGTNRSVASEDLGTIAYYFQKNKLDGLIILGGFEGFRSLKQLRDGRTQHPIFNIPMCLIP
ATVSNNVPGTEYSLGVDTCLNALVNYTDDIKQSASATRRRVFVCEVQGGHSGYIASFTGL
ITGAVSVYTPEKKIDLASIREDITLLKENFRHDKGENRNGKLLVRNEQASSVYSTQLLAD
IISEASKGKFGVRTAIPGHVQQGGVPSSKDRVTASRFAVKCIKFIEQWNKKNEASPNTDA
KVLRFKFDTHGEKVPTVEHEDDSAAVICVNGSHVSFKPIANLWENETNVELRKGFEVHWA
EYNKIGDILSGRLKLRAEVAALAAENK
>Q9SBK6
MEVMRILHMNKGNGETSYAKNSIVQSNIISLGRRVMDEALKKLMIRNSEILSFGIADLGC
SSGPNSLLSISNIVETIQNLCHDLDRPVPELSLSLNDLPSNDFNYIFASLPEFYDRVKKR
DNNYESLGFEHGSGGPCFVSAVPGSFYGRLFPRRSLHFVHSSSSLHWLSQVPCGEVNKKD
GVVITADLDNRGKIYLSKTSPKSAHKVYALQFQTDFSVFLRSRSEELVPGGRMVLSFLGR
SSPDPTTEESCYQWELLAQALMSLAKEGIIEEENIDAFNAPYYAASPEELKMAIEKEGSF

SIDRLEISPVDWEGGSISDDSYDIVRFKPEALASGRRVAKTIRAVVEPMLEPTFGQKVMD
ELFERYAKLVGEYVYVSSPRYTIVIVSLLRMG
>P08018
MEDKFANLSLHEKTGKSSIQLNEQTGSDNGSAVKRTSSTSSHYNNINADLHARVKAFQEQ
RALKRSASVGSNQSEQDKGSSQSPKHIQQIVNKPLPPLPVAGSSKVSQRMSSQVVQASSK
STLKNVLDNQETQNITDVNINIDTTKITATTIGVNTGLPATDITPSVSNTASATHKAQLL
NPNRRAPRRPLSTQHPTRPNVAPHKAPAIINTPKQSLSARRAVKLPPGGMSLKMPTKTAQ
QPQQFAPSPSNKKHIETLSNSKVVEGKRSNPGSLINGVQSTSTSSSTEGPHDTVGTTPRT
GNSNNSSNSGSSGGGGLFANFSKYVDIKSGSLNFAGKLSLSSKGIDFSNGSSSRITLDEL
EFLDELGHGNYGNVSKVLHKPTNVIMATKEVRLELDEAKFRQILMELEVLHKCNSPYIVD
FYGAFFIEGAVYMCMEYMDGGSLDKIYDESSEIGGIDEPQLAFIANAVIHGLKELKEQHN
IIHRDVKPTNILCSANQGTVKLCDFGVSGNLVASLAKTNIGCQSYMAPERIKSLNPDRAT
YTVQSDIWSLGLSILEMALGRYPYPPETYDNIFSQLSAIVDGPPPRLPSDKFSSDAQDFV
SLCLQKIPERRPTYAALTEHPWLVKYRNQDVHMSEYITERLERRNKILRERGENGLSKNV
PALHMGGL
>P41819
MGKAAKKKYSGATSSKQVSAEKHLSSVFKFNTDLGQHILKNPLVAQGIVDKAQIRPSDVV
LEVGPGTGNLTVRILEQAKNVVAVEMDPRMAAELTKRVRGTPVEKKLEIMLGDFMKTELP
YFDICISNTPYQISSPLVFKLINQPRPPRVSILMFQREFALRLLARPGDSLYCRLSANVQ
MWANVTHIMKVGKNNFRPPPQVESSVVRLEIKNPRPQVDYNEWDGLLRIVFVRKNRTISA
GFKSTTVMDILEKNYKTFLAMNNEMVDDTKGSMHDVVKEKIDTVLKETDLGDKRAGKCDQ
NDFLRLLYAFHQVGIHFS
>O14468
MSTEKLEASEEPQAPLANTSETNSIKGDTENIVTVFDLANEIEKSLKDVQRQMKENDDEF
SRSIQAIEDKLNKMSR
>O88492
MSASGDGTRVPPKSKGKTLSSFFGSLPGFSSARNLVSHTHSSTSTKDLQTATDPSGTPAP
SSKVSTNSQMAGDAAGLLQPSEQTAGDKDMGSFSVTSSEDAFSGVFGIMDAAKGMVQGGL
GATQSALVGTKEAVSGGVMGAVGVAKGLVKGGLDTSKNVLTNTKDTVTTGVMGAANMAKG
TVQTGLDTTKSVVMGTKDTVATGLAGAVNVAKGTIQGGLDTTKSVVMGTKDTVTTGLTGA
ANVAKGVVQGGLDTTKSVVMGTKDTVTTGLTGAMNVAKGTAQMGIDTSKTVLTGTKDTVC
AGATGAINVAKGAAQGGLDTTKSVLIGTKDTVTTGLTGAVNVAKGAVQGGLDTTKSVVMG
TKDTVTTGLTGAMNVAKGTAQMGLGTSKTVLTGTKDTVCAGLTGAINVAKGAAQGGLDTT
KSVLMGTKDTVTTGLTGAVNVAKGTIQGGLDTTKSVVMGTKDTVTTGLTGAVNVAKGTIQ
GGLDTTKSVVMGTKDTVTTGLTGAVNVAKGAAQGGLDTTKSVVMGTKDTVTTGLTGAMNV
AKGTAQMGLGTSKTVLTGTKDTVCAGLTGAINVAKGAAQGGLDTTKSVLMGTKDTVTTGL
TGAVNVAKGTIQGGLDTTKSVVMGTKDTVTTGLTGAVNVAKGAVQGGLDTTKSVVMGTKD
TVTTGLTGALNVAKGTAQMGIDTSKTVLIGTKDTVCAGATGAINMAKGAAQGGLDTTKSV
LMGTKDTVTTGLTGAINVAKGSAQGGLDTTKSVLIGTKDTVTTGLTGALNVAKGTVQTGL
DTSQRVLTGTKDNVYAGVTGAVNVAKGTIQGGLDTTKSVVMGTKDTVTTGLTGAVNVAKG
AVQGGLDTTKSVVMGTKDTVTTGLTGAMNVAKGTAQMGIDTSKTVLTGTKDTVCAGLTGA
INVAKGATQGGLDTTKSVLMGTKDTVTTGLTGAINVAKGAAQGGLDTTKSVLLGTKDTVT
TGLTGAANVAKETVQMGLDTSKNILMDTKDSICAGATGAITVVKGAAQGGLDTSNAALTG
TMDTAKGTVQTSLDTSKHMLIGMKDTVCAGVTSAMNMAKGIHKNTDTTRDTQSSVLAHSG

NVATNAIHTGVHTVPSSLSGSHSIICHEPSIYRATNHGVGHAILTSTESLCCETSSFSDK
YGLGHVTEPRADTKTLVSGMASSACAATRSVEECGQLAATGFAALPDELKGLGDIFQPMT
TEEQAQLAVSESGPRVLSADRGSYYIRLGDLAPSFRQRAFEHALSHIQHNQFQARAAVAQ
LQEAFQMTDMTMEAACGKLCSDQSLNTMVEAVGSHEMRASVAQDRLCTLAHQLHAAYSSL
VTSLQGLPEVQQQAGQARHSLCKLYGLVSSEAGSELQTEQLAQSSAGVVEAWQGLEVLLE
KLQQNPPLSWLVGPFTSMPCGQL
>Q94AA4
MSTVESSKPKIINGSCGYVLEDVPHLSDYLPGLPTYPNPLQDNPAYSVVKQYFVDADDSV
PQKIVVHKDGPRGIHFRRAGPRQKVYFESDEVHACIVTCGGLCPGLNTVIREIVSSLSYM
YGVKRILGIDGGYRGFYAKNTVSLDSKVVNDIHKRGGTILGTSRGGHDTTKIVDSIQDRG
INQVYIIGGDGTQRGASVIFEEIRRRGLKVAVIGIPKTIDNDIPVIDKSFGFDTAVEEAQ
RAINAAHVEAESIENGIGVVKLMGRYSGFIAMYATLASRDVDCCLIPESPFYLEGEGGLF
EYIEKRLKESGHMVLVIAEGAGQDLMSKSMESMTLKDASGNKLLKDVGLWLSQSIKDHFN
QKKMVMNLKYIDPTYMIRAVPSNASDNVYCTLLAQSAVHGAMAGYTGYISGLVNGRQTYI
PFYRITEKQNHVVITDRMWARLLSSTNQPSFLGPKDVFDNKEKPMSALLDDGNCNGVVDV
PPVTKEITK
>Q05043
MKDLNPEMGKFATTKGPPQDNRGMVDIATLPNFPANRSGTPREEMYLAPNKMETPRILNM
NMVPDYLQKENFSPDFSSATVSAKSSPVNVTHDESLPLGTIESNSTKDSKYAVQRQQQQV
VDFIENNMQLLSSETLNFRSDIMKTLELPIPKRRDIKGNHLSKLLFAKSPLTINTYCQFY
DRRTKRICNQEMIWKDKNSREKHGSRKYQRHLSKVHDVQLTPNNFTEFFDHNSPLFQECY
DYQSRLMRDLLVEPDAKFKEKKKKKKGDVNGNHPETGSSLINHQVQQQNVRELQSKIAMN
DLIEILIDLNIPFSVLDYQPMRNWLIKYSIISTDTLPDEVYFKTDPGVNELEHNSSNLNN
SNSGTPHNHNQNQHTN
>Q38SD2
MSQRPPSMYWCVGPEESAVCPERAMETLNGAGDTGGKPSTRGGDPAARSRRTEGIRAAYR
RGDRGGARDLLEEACDQCASQLEKGQLLSIPAAYGDLEMVRYLLSKRLVELPTEPTDDNP
AVVAAYFGHTAVVQNTLPTEPTDDNPAVVAAYFGHTAVVQELLESLPGPCSPQRLLNWML
ALACQRGHLGVVKLLVLTHGADPESYAVRKNEFPVIVRLPLYAAIKSGNEDIAIFLLRHG
AYFCSYILLDSPDPSKHLLRKYFIEASPLPSSYPGKTALRVKWSHLRLPWVDLDWLIDIS
CQITELDLSANCLATLPSVIPWGLINLRKLNLSDNHLGELPGVQSSDEIICSRLLEIDIS
SNKLSHLPPGFLHLSKLQKLTASKNCLEKLFEEENATNWIGLRKLQELDISDNKLTELPA
LFLHSFKSLNSLNVSRNNLKVFPDPWACPLKCCKASRNALECLPDKMAVFWKNHLKDVDF
SENALKEVPLGLFQLDALMFLRLQGNQLAALPPQEKWTCRQLKTLDLSRNQLGKNEDGLK
TKRIAFFTTRGRQRSGTEAASVLEFPAFLSESLEVLCLNDNHLDTVPPSVCLLKSLSELY
LGNNPGLRELPPELGQLGNLWQLDTEDLTISNVPAEIQKEGPKAMLSYLRAQLRKAEKCK
LMKMIIVGPPRQGKSTLLEILQTGRAPQVVHGEATIRTTKWELQRPAGSRAKVESVEFNV
WDIGGPASMATVNQCFFTDKALYVVVWNLALGEEAVANLQFWLLNIEAKAPNAVVLVVGT
HLDLIEAKFRVERIATLRAYVLALCRSPSGSRATGFPDITFKHLHEISCKSLEGQEGLRQ
LIFHVTCSMKDVGSTIGCQRLAGRLIPRSYLSLQEAVLAEQQRRSRDDDVQYLTDRQLEQ
LVEQTPDNDIKDYEDLQSAISFLIETGTLLHFPDTSHGLRNLYFLDPIWLSECLQRIFNI
KGSRSVAKNGVIRAEDLRMLLVGTGFTQQTEEQYFQFLAKFEIALPVANDSYLLPHLLPS
KPGLDTHGMRHPTANTIQRVFKMSFVPVGFWQRFIARMLISLAEMDLQLFENKKNTKSRN
RKVTIYSFTGNQRNRCSTFRVKRNQTIYWQEGLLVTFDGGYLSVESSDVNWKKKKSGGMK

IVCQSEVRDFSAMAFITDHVNSLIDQWFPALTATESDGTPLMEQYVPCPVCETAWAQHTD
PSEKSEDVQYFDMEDCVLTAIERDFISCPRHPDLPVPLQELVPELFMTDFPARLFLENSK
LEHSEDEGSVLGQGGSGTVIYRARYQGQPVAVKRFHIKKFKNFANVPADTMLRHLRATDA
MKNFSEFRQEASMLHALQHPCIVALIGISIHPLCFALELAPLSSLNTVLSENARDSSFIP
LGHMLTQKIAYQIASGLAYLHKKNIIFCDLKSDNILVWSLDVKEHINIKLSDYGISRQSF
HEGALGVEGTPGYQAPEIRPRIVYDEKVDMFSYGMVLYELLSGQRPALGHHQLQIAKKLS
KGIRPVLGQPEEVQFRRLQALMMECWDTKPEKRPLALSVVSQMKDPTFATFMYELCCGKQ
TAFFSSQGQEYTVVFWDGKEESRNYTVVNTEKGLMEVQRMCCPGMKVSCQLQVQRSLWTA
TEDQKIYIYTLKGMCPLNTPQQALDTPAVVTCFLAVPVIKKNSYLVLAGLADGLVAVFPV
VRGTPKDSCSYLCSHTANRSKFSIADEDARQNPYPVKAMEVVNSGSEVWYSNGPGLLVID
CASLEICRRLEPYMAPSMVTSVVCSSEGRGEEVVWCLDDKANSLVMYHSTTYQLCARYFC
GVPSPLRDMFPVRPLDTEPPAASHTANPKVPEGDSIADVSIMYSEELGTQILIHQESLTD
YCSMSSYSSSPPRQAARSPSSLPSSPASSSSVPFSTDCEDSDMLHTPGAASDRSEHDLTP
MDGETFSQHLQAVKILAVRDLIWVPRRGGDVIVIGLEKDSGAQRGRVIAVLKARELTPHG
VLVDAAVVAKDTVVCTFENENTEWCLAVWRGWGAREFDIFYQSYEELGRLEACTRKRR
>O95171
MSNVTLRKMSPTGNEMKSTTQGTTRKQQDFHEVNKRRTFLQDNSWIKKRPEEEKDENYGR
VVLNRHNSHDALDRKVNERDVPKATISRYSSDDTLDRISDRNDAAKTYKANTLDNQLTNR
SMSMFRSLEVTKLQPGGSLNANTSNTIASTSATTPVKKKRQSWFPPPPPGYNASSSTGTR
RREPGVHPPIPPKPSSPVSSPNQLRQDNRQIHPPKPGVYTETNRSAERNISEELDNLIKM
NKSLNRNQGLDSLFRANPKVEEREKRAKSLESLIYMSTRTDKDGKGIQSLGSPIKVNQRT
DKNEKGRQNLESVAKVDARTNKTSRRSEDLDNATEVNPKGHENTTGKKDLDGLIKVDPET
NKNITRGQSLDNLIKVTPEVKRSNQGSKDLNNFIKVYPGTEKSTEGGQSLDSLIKVTPER
NRTNQGNQDLENLIKVIPSANKSSEQGLDEHINVSPKAVKNTDGKQDLDKLIKVNPEIFT
NNQRNQDLANLIKVNPAVIRNNQSQDLDNLIKVKPSALRNTNRDQNLENLIEVNSHVSEN
KNGSSNTGAKQAGPQDTVVYTRTYVENSKSPKDGYQENISGKYIQTVYSTSDRSVIERDM
CTYCRKPLGVETKMILDELQICCHSTCFKCEICKQPLENLQAGDSIWIYRQTIHCEPCYS
KIMAKWIP
>O13963
MDKKVYQCTVSAPVNIAVIKYWGKRDVALNLPTNSSISVTLSQDDLRTVTTASCSEKFEN
DTLWLNGNAEEIFANKRLRVCVEELRKARLDLEEENDDLDKIGALKLHVVSENNFPTAAG
LASSAAGYAAFCEAIARLYDLPWTPTQLSRIARQGSGSACRSLFGGYVAWEMGELHSGAD
SVAVQVEPVENWPEIRVAVLVASAAKKGVSSTAGMQATVASSTLFQHRIQNIVPQRIQEM
KTAIRERDFETFAKLTMTDSNQFHACCLDTFPPIFYLNDTSRAVIRVVENINATAGKTIA
AYTFDAGPNAVIYFLEENSEIVLNTLYAVTKNAEGWSKQYGSSPVTVDSAAANIVSSGIS
RVILTRVGNGPRVLTIDESLIDASGNPKFIGSH
>Q12182
MTILEELNDSSIPQRLDNHIFFGSVHSLTHTDFLVENNIRFFINVDLSTELISHIYHEVR
SKFAHEIVIVNIDNDSQIPIESDLVRSFHWHNTSLLQQLIHHLDFLSGINNHGEPLTPPP
ESHYRNAYVQFDHPSDSVSILDKLLYGNKSEYSRTNIFQVTNEAKFQVFNDLITIFKYSI
AQGGNTNSNILVLSENGSTDENLISLLMSTVLKENPTFNVYQALQFVKSIAVIPDTVRDE
KILWVTGFINYQELIKKNEMYWGLGSQKGRKLTSFASPISKVERKQRRRDDQNIMRSKLP
QQRQNPFCSTERPKRARCD
>P30792

MGSSGFSWTLPDHPKLPKGKSVAVVVLDGWGEANPDQYNCIHVAQTPVMDSLKNGAPEKW
RLVKAHGTAVGLPSDDDMGNSEVGHNALGAGRIFAQGAKLVDQALASGKIYDGDGFNYIK
ESFESGTLHLIGLLSDGGVHSRLDQLQLLLKGVSERGAKKIRVHILTDGRDVLDGSSIGF
VETLENDLLELRAKGVDAQIASGGGRMYVTMDRYENDWDVVKRGWDAQVLGEAPYKFKSA
LEAVKTLRAQPKANDQYLPPFVIVDDSGNAVGPVLDGDAVVTINFRADRMVMLAKALEYA
DFDNFDRVRVPKIRYAGMLQYDGELKLPSRYLVSPPEIDRTSGEYLVKNGIRTFACSETV
KFGHVTFFWNGNRSGYFDATKEEYVEVPSDSGITFNVAPNMKALEIAEKARDALLSGKFD
QVRVNLPNGDMVGHTGDIEATVVACKAADEAVKIILDAVEQVGGIYLVTADHGNAEDMVK
RNKSGKPLLDKNDRIQILTSHTLQPVPVAIGGPGLHPGVKFRNDIQTPGLANVAATVMNL
HGFEAPADYEQTLIEVADN
>Q9UTK5
MSSGGLEDDIQLVHEFLDVSFEDIKPLVSVNGFAVFISAIKTKVKDINALKDQLVLQEVN
HEHKENVLTKKINFLEQQLQSSNNQAEESRNLISVLRNENESLKTNLENQNKRFDALTTE
NQSLRRANSELQEQSKIASEQLSIAKDQIEALQNENSHLGEQVQSAHQALSDIEERKKQH
MFASSSSRVKEEILVQEKSALVSDLASLQSDHSKVCEKLEVSSRQVQDLEKKLAGLAQQN
TELNEKIQLFEQKRSNYSSDGNISKILETDPTSIKELEEEVETQKRLTALWESKSSELQS
EVAALQEKLTSQQSLYNNVTEELNNNKQQLLISENSLRELQEKYDSVVSELQVVKENKNT
SVSAGVGLFSPLAQKLSAVQNPEFSFTKVYSDNMKLQQKVSSLKLQLDRLTNKFSSFCEQ
VKQRIPVVKQQRSEIVRNNIYMNFLSESLETSNNNLTKVQAELLSTKMRQEACYLQLTAS
RTQCSDLSREVICLMAELDHLNETKSRNVPATVQVALDEYAQNPSTASETLVNKELANFS
SIKEAVSKTLELREKVRALECDVEIQKQTVQYQISNAVKENSNTLSEQIKNLESELNSSK
IKNESLLNERNLLKEMLATSRSSILSHNSSAGNIDDKMKSIDESTRELEKNYEVYRNEMT
AIQESLSKRNQDLLSEMEAIRKELENSKYQQQLSTDRLTNANNDVEAFKKEAKELRSINQ
NLQDIISRQDQRASKFAEELLHVNSLAERLKGELNASKGEKDLRKRTQERLISENDKLLA
ERERLMSLVSDLQTFLNQQQLSDAARKVKFESEKESLSLSLQKLKESNEKMSNDLHSLQK
SLEKSGIEYSSRIKTLMLEKQSLSEDNRKLLDNQQMMEIKLQELNGVIELEKQRFSTLEA
KFTQQKNTSYSEREALLESSLSDLQSKHTSLESQYNYSLRNIEQLQAASKLAEEMVERVK
TEYDEYRLQTSESLEKNHLKITSLEQRIVILQDEIASSSLRCENITKDSETRVALLLEEN
KHLNNELSSHRNAEKQHLEKENDYKQQLLLVTEDLRKTREDYEKELLRHADARSTLQKLR
EDYTKALEQVEDLNKEIALKAGINESQPFPISEKEDPLRQEVYVLKKQNAMLLTQLQSSN
LNFAEITSPSPDLDSVMKLGLSDLQNHVKRISKEMEIISCQRQLLFLENKKLKRTVESSN
RVIADLQRGITEKDVSSTSESVGERSNYLNMVALLNESNKSLRENLERNEEVITELREKI
ETLKTDLANFRLNKEQLESQLQTEKAAVKKLENSNEEYKRHNQEILLSLNSSTSTSSDAS
RLKNELVSKENLIEELNQEIGHLKSELETVKSKSEDLENERAQNQSKIEQLELKNTKLAA
AWRTKYEQVVNKSLEKHNQIRQQLSQKTSELEAKVAECHQLNEQLNKPSATPTATTQSEP
STVSLEEFNSTKEELSSTQRKLSEIMDILNTTKEELEKVRQNSNKSEGTSKDTEIPNEEE
MERKKVMQQEVLRLRSRIAKELQKNELLRKQNQVLQDQVKALQETVVSSEEAESASVHAD
TKDLENLKKTEEMLSVTFQVIFNESISDFSTSTADFTTFVQKEWEKRREILQKDVEEQVA
QSHQKQLDNIRKELEMRNKLKLSMLEKNLARVRAELEQSKKKDSPAILSLEASKNTDSNK
SNSEVPAAQVKEKKLIAKTHSVDTNSPPKRSSSDAGMDVSNDVKKAK
>P33447
MSSWDVSMSNHAGLVFNPIRTVSDNAKPSPSPKPIIKLSVGDPTLDKNLLTSAAQIKKLK
EAIDSQECNGYFPTVGSPEAREAVATWWRNSFVHKEELKSTIVKDNVVLCSGGSHGILMA
ITAICDAGDYALVPQPGFPHYETVCKAYGIGMHFYNCRPENDWEADLDEIRRLKDDKTKL

LIVTNPSNPCGSNFSRKHVEDIVRLAEELRLPLFSDEIYAGMVFKGKDPNATFTSVADFE
TTVPRVILGGTAKNLVVPGWRLGWLLYVDPHGNGPSFLEGLKRVGMLVCGPCTVVQAALG
EALLNTPQEHLDQIVAKIEESAMYLYNHIGECIGLAPTMPRGAMYLMSRIDLEKYRDIKT
DVEFFEKLLEEENVQVLPGTIFHAPGFTRLTTTRPVEVYREAVERIKAFCQRHAAV
>Q86AV6
MSSNSQEDNNKTTEKKNTLTVIDNRTGKSYEIPINHETVKSIDFRAIKEQSIDFGTMIYD
PGYYNTAVCKSQITYIDGDRGILEYRGYPIEQLAEKSSFLEVSYLLIYGDLPSKEQSNLW
NTKIMNHTFIHENLISMMKSFRYDAHPMGMLISSLSAMSTFYPEANPALAGVDIYKNKQL
MNKQIFRILGKLPTIAACAYRHRIGRPYNDPSNTLSYTENFLYMLDRLSESNYKPHPVLT
RALDKLFIIHADHELNCSTATMRQIASTLVDPYTACAGSAGALYGPLHGGANEAVLRMLE
AIGTIENIPKFIEQVKQKKQRLMGFGHRVYKSYDPRAKILKTVTMEIFALLGKNPLMQIA
TELERLALSDSYFIERQLYPNVDFYSGIIYKSMGFPTDMFPVLFSIPRAAGWLAHWVEEL
ADPELRIFRPRQIYMGRRNMNYVPMDARQVQQHNSGEKLSSFSSGFDRRRDVSEELFNFE
DGAIPKTATGSKSQLSASIEQSFGEKISPQSH
>P17883
MSDIKQLLKEAKQELTNRDYEETIEISEKVLKLDPDNYFAHIFLGKALSSLPASNNVSSN
RNLERATNHYVSAAKLVPDNLLAWKGLFLLFRTTEVVPDILSYDEYFDLCGQYADALLKQ
EQSQVELINDIKLLKKTHPDCQKAFYQHLKPGSLMAETIGRHLSTPQDALLNLIKILSNI
ETTEIGKTLSQNRLKLKASDPDYQIKLNSFSWEIIKNSEIDQLYNQLVNILADDQKRSEI
ENQWLEYRIKVLKSMPLDVKKDFFTKVKEMVEDMVLVNHQSLLAWQKYFEWTDYEDLDNM
DAPLIIKYFKKFPKDPLAMILYSWLSSKLSKYDIKSLESANKPPEGHKKTEKETDIKDVD
ETNEDEVKDRVEDEVKDRVEDEVKDQDEEAKEDEEEDLDDIEIGLLEEEVVTVLTENIVK
CKNNILAHRILCQYYLLTKEYEAALPYIKNGISLIAYNIKDLGVHLPLTKREFSLDLATV
YTYVDAPKDHNAALKLYDNILSGDFSNIQAKMGKGIIFIERKNWKDAMTLLTQVHEQSPN
NLEVLSELSWSKAHMGYMDEALAGLDTVIKGIKGMDLRSIDFRALNLWRQAKVYIMKHAS
INDAKQENVKCAFKLLIQSIKILDTFAPGFSTLGDIYCHYYKDHLRAFKCYFKAFDLDAG
DYTAAKYITETYASKPNWQAASSIASRLIKGEKAKAELRSNNWPFRVVGIAHLEKQEESD
SIEWFQSALRVDPNDVESWVGLGQAYHACGRIEASIKVFDKAIQLRPSHTFAQYFKAISL
CDVGEYLESLDILEKVCQEAATEESFQIGLVEVLMRCSLDLYSQGFLLKSVSIAKDTIER
IKIIISELKCENQQVWIYLSQVLRLFIWIESKVDTLPVESLVSIFENSQFSGSEEIDSVD
NIKIDTLLDSTTDDNVSIACKFLILASKYSVSDQKFTDIAGTVRASYWYNIGISELTAFI
TLKEPQYRDAAIFAFKKSIQLQSNTSETWIGLGIATMDINFRVSQHCFIKATALEPKATN
TWFNLAMLGLKKKDTEFAQQVLNKLQSLAPQDSSPWLGMALILEEQGDIIGSSKLFAHSF
ILSNGRSKAAQFMYAKNVLENHINNGDDERDIETVEKLTTASIALEQFFKKSPDSQFALQ
CALLTLERLHHYENANELANRLIGILEKKFEKTQDERELFNFAIIKGQFARIHLGLGNFE
LSIENADLSQGIISESSDEKSMKTKISNHICLGLSYFFLNDFDQTLNQFQELLSISKDSK
HLVVLIAKVLYDVGESDTKEIALQELTEYIATSGADLLVTLTIAAMSILDDKREDLSIIL
EELKALPLSKQIIDKHKDAPYLIEEITKRLYRNDTGKQVWQRSAYFFPNNLKVWERLDKN
IQRRIASNGQNKVTAEEMSKLYCESKNLRSIQRGMFLCPWNVTAVKALNECF
>Q03071
MSQDFVTLVSKDDKEYEISRSAAMISPTLKAMIEGPFRESKGRIELKQFDSHILEKAVEY
LNYNLKYSGVSEDDDEIPEFEIPTEMSLELLLAADYLSI
>O94245
MSISNQSGLDILREVDDEGQENSFLRKTTRKIQQFFKSDPGYQSRKAEYNSPAKATGDVN

INVTSQDWLSKLDRNLSIENLTGKSLGIVVVSGRNKGLIKNPFLGGLVDIVMRRQDKLYL
KSVEQVSSEYSPMQEQVYEFKVDMPLVQKRLGAFLKKNRVDGLSLSMHFSTGNYSLPVLI
RHVLLYDYYTDDMKDSLWRAMIIYVSRQVTSNKYTKFHLWCVQKRIGNIRMYLVRPGDVY
SFQGQTSWAAICNKSYMCNIQLEQSPEVSVKSSLPKYSNEPIFQSKTISQEEVGWLLFML
SIGNHARAFPIQNYLANTVMETVLPVELRCPFLSRSVDCEIFQKNTKRRVMEKWRQEFKK
DLNVCEELEFKNVKKSGYMQPLFDLTMPLGCFDSLETKEMFYLRHNIAGFQADLTPYPMR
I
>Q12468
MNNNDRKSIKTAGGSMSLSNKTVKELRQLLKERYTVEDELTESIALSSMRFKPSQEPEFH
ALSQSSLLKTKLKQQSSTDIPSYTHVLISKLSCEKITHYAVRGGNIEIMGILMGFTLKDN
IVVMDCFNLPVVGTETRVNAQLESYEYMVQYIDEMYNHNDGGDGRDYKGAKLNVVGWFHS
HPGYDCWLSNIDIQTQDLNQRFQDPYVAIVVDPLKSLEDKILRMGAFRTIESKSDDNSAT
SYYELETIIFDSELNRALFETKLNLHCVIEDDESEQISLNRLIDSMKQYSYLMDSKNVRT
RIKLATTSERVSNENKKNIDYQNRSTRSQFCLNTQRGDSTETSSFGSMFSGDNTSDVDME
DRNLTEFDSTDTSLCINGEPSIHVNRVERSSRSTDNFHNSKKRMNSNQERCHDEGNDMLQ
RNVLETDYARAKNRILASKIKQYERLRFYKDTFTL
>Q07589
MSRILVIGAGGVGVITALSLWLKKESDVSLVVRSDYDRVLKHGYTIESCDYGRLEGWRPH
HIYSSVEDAASAADNQGYNYIVVTTKNIIDGPVNSRVSNIIRPVLEKNKELHGPQLTTHI
LLVQNGIDIEKEIWAEFPREQYRYTVLSGIQLIGSTKIGSGHISQVGQDHLSCGAFDPQD
AAAIQAANDFVRMYSNEGQNFVEFDPRVRYSRWKKLLYNAAINTSTALVGLDVPRCLEFG
VNKKSTEIEVFHPAMREIIAIAASEGIIIEEEFITMFTEITRKKVFKPSMCVDCEKGQLM
ELEVILGNPIRIAKRNGVATPTLSILYNLLVLVQAKLKERKGLLKFDEKTATLVDE
>Q9QZK7
MESVEPPVKDGILYQQHVKFGKKCWRKVWALLYAGGPSGVARLESWDVRDGGLGPAGDRS
TGPSRRGERRVIRLADCVSVLPADGESCPRDTGAFLITTTERSHLLAAQHRQSWVDPICQ
LAFPGTGECSSGSGQAESPKRGFVPMEENSIYSSWQEVTEFPVIVQRTEATSRCQLKGPY
LLVLGQDDIQLRETSKPQACFSWPYRFLRKYGSDKGVFSFEAGRRCDSGEGLFAFSSPRA
PDICGVVAAAIARQRERLPELAMSPPCPLPRALSLPSLEPPGELREVAPGFELPTPRKLP
LTDPGPQSLPLLLSPTQEGPASGLYASVCKQTSKHTGTAEHLYENVCMLEASPGLTNGGP
EAQEGPPGGRSPLGSPIYHNTEDLSWPGSAQDSNLEAQYRRLLELELDEAGSAGRSGAQA
GIKAKLVTLLTRERKKGPAPCDRP
>Q6KCA4
MSLTEDNNNTTITIAKGENKEIILHGNPTTGYSWVVDSCEGLSNTVEYVADQHAPGICGC
GGKYHIKITGTQTGEGKIVLVYRRPWAPNANDRTFTLKVNVQ
>P34234
MMKKKPKCQIARTKPSVENVIPYNQFKKTQPRFNGNFSALNNEEYIILFGGGRDLILGSL
TPCSSSHLSNQANPQDTSEYGTDLFILNSCIIIWFNGLGYGLEIPYSSVLYHASRRLPDG
REGLQLEILLTLERDEVLDMLYQSLAPQACEFDGEEAHAFTVRSVELTIRPKYSIYDRHY
NNEIETLFTFENFGVNRGDDLVNNCNEALAVCMDLHGEDVQDQDQEQYQDPSMAFEGAQD
LNATYSGLGDTLHGPPVYQNDGLADDLDGDLVMDNVVSRGGPEASMSMEFYANQNLAGRK
NSRDNE
>P54787
MTDDEKREILKEFDPFSQLEQANGNPDKDVKFKKDDPNRAAAEETNRDISAQDKGDEEPF

YDFQIFIKQLQTPGADPLVKYTKSFLRNFLAQRLLWTVSEEIKLISDFKTFIYDKFTLYE
PFRSLDNSKMRNAKEGMEKLIMGKLYSRCFSPSLYEILQKPLDDEHMKDLTNDDTLLEKI
RHYRFISPIMLDIPDTMPNARLNKFVHLASKELGKINRFKSPRDKMVCVLNASKVIFGLL
KHTKLEQNGADSFIPVLIYCILKGQVRYLVSNVNYIERFRSPDFIRGEEEYYLSSLQAAL
NFIMSLTERSLTIDDHEDFEEAYQRNLKQLAEEKEEEEKKKQLEVPDELQPNGTLLKPLD
EVTNIVISKFNELFSPIGEPTQEEALKSEQSNKEEDVSSLIKKIEENERKDTLNTLQNMF
PDMDPSLIEDVCIAKKSRIGPCVDALLSLSE
>Q12417
MDGNDHKVENLGDVDKFYSRIRWNNQFSYMATLPPHLQSEMEGQKSLLMRYDTYRKESSS
FSGEGKKVTLQHVPTDFSEASQAVISKKDHDTHASAFVNKIFQPEVAEELIVNRYEKLLS
QRPEWHAPWKLSRVINGHLGWVRCVAIDPVDNEWFITGSNDTTMKVWDLATGKLKTTLAG
HVMTVRDVAVSDRHPYLFSVSEDKTVKCWDLEKNQIIRDYYGHLSGVRTVSIHPTLDLIA
TAGRDSVIKLWDMRTRIPVITLVGHKGPINQVQCTPVDPQVVSSSTDATVRLWDVVAGKT
MKVLTHHKRSVRATALHPKEFSVASACTDDIRSWGLAEGSLLTNFESEKTGIINTLSINQ
DDVLFAGGDNGVLSFYDYKSGHKYQSLATREMVGSLEGERSVLCSTFDKTGLRLITGEAD
KSIKIWKQDETATKESEPGLAWNPNLSAKRF
>Q9BU02
MAQGLIEVERKFLPGPGTEERLQELGGTLEYRVTFRDTYYDTPELSLMQADHWLRRREDS
GWELKCPGAAGVLGPHTEYKELTAEPTIVAQLCKVLRADGLGAGDVAAVLGPLGLQEVAS
FVTKRSAWKLVLLGADEEEPQLRVDLDTADFGYAVGEVEALVHEEAEVPTALEKIHRLSS
MLGVPAQETAPAKLIVYLQRFRPQDYQRLLEVNSSRERPQETEDPDHCLG
>Q9Y3C4
MQLTHQLDLFPECRVTLLLFKDVKNAGDLRRKAMEGTIDGSLINPTVIVDPFQILVAANK
AVHLYKLGKMKTRTLSTEIIFNLSPNNNISEALKKFGISANDTSILIVYIEEGEKQINQE
YLISQVEGHQVSLKNLPEIMNITEVKKIYKLSSQEESIGTLLDAIICRMSTKDVL
>O94717
MGKVSANNQSGFFKFYIDTALLLNAEFISSFTNCINSEYNGKVKISYSSSSQQFKVIVYE
EHKREAFSLFEEIVQKLKRESTVYKKPRLDIHPFGQLHYDFTTLLNFQRYLDLGYGIEDV
KIKENEDIVKSKVGKKPTSSDFYIPCLIKPKIITPFEWEGNFKQVIYSPTVYNGSFKRLF
YLPDNNDLLKEFAHATNTKYKASLKDNKIVVLANNDQDINAMLEKMKHIENLVSQKVYEY
PEETLVYAGKIAPCQLKFLKLKFENEILYTAMHTTSIFKSVFNILGSFYTMRLLRNKHDG
DSYELVPVFAKNPSKTPPCNDNFKIFSDDIKLIGCKIFAGVSLNFSSPKPAHRFYELNKT
SSNLSIPVLQKPSNFHSSSTELSDNSIHQGRRAVDPVVNQNNPSNFEEMIMNKLNKLPTI
DKQILGTSSLTHFQDKTTAIEHSINKSNSKQPPRFKFQLPPRPTSNTLPLEPEEELVTRY
SVSSDGNTVDEAITKQSQTFQLVNSNEFNEVNANDVHKSLRQNCAKLDFDDSKSKNLLSV
ECLELDKGSDCSTPKSGSLTPSIDMKFLRLQDEKMDDLGDNYYTILMSSNPVSSYGVGSL
YLFQPKIVCSEKYINHEEIDNMNLKSLHRWLSRSLHVLQSFSGEIELNLEFGVILYPNIS
SDVSACSHGFMNIYKDLNLPRSYFADCITKSVSNIDSLLNTPVKILFGRTEYTYPLIEHE
VLDSKNYFVFKGSLLFTDDKRNKTEDSTVFFSMICSSKLDQFAFYKDSKQSSTCTINFPL
ALWDSRLRIETKVPLNDAILTEFSKSIRFRNVNKDLLLVFGNMEDRVIIHSVTRINENSV
PFNKNLLPHSLEFILKCSKVKSYDISPSSINSKEAFVCLDRSKESKPIESCFSLSIQSVY
MQSQFKYNNSIRAGETAPWKLANKQFIGGAITENVSDLYTAAIIMVNQLGGIHPTI
>Q04839
MPLESIWADAPDEEPIKKQKPSHKRSNNNKKNNNSRWSNESSSNNKKKDSVNKVKNNKGN

HESKTKNKIKETLPREKKPPHSQGKISPVSESLAINPFSQKATEISPPPVSPSKMKTTKT
QSKQDTASKMKLLKKKIEEQREILQKTHHKNQQQQVLMDFLNDEGSSNWVDDDEEELILQ
RLKTSLKI
>Q92597
MSREMQDVDLAEVKPLVEKGETITGLLQEFDVQEQDIETLHGSVHVTLCGTPKGNRPVIL
TYHDIGMNHKTCYNPLFNYEDMQEITQHFAVCHVDAPGQQDGAASFPAGYMYPSMDQLAE
MLPGVLQQFGLKSIIGMGTGAGAYILTRFALNNPEMVEGLVLINVNPCAEGWMDWAASKI
SGWTQALPDMVVSHLFGKEEMQSNVEVVHTYRQHIVNDMNPGNLHLFINAYNSRRDLEIE
RPMPGTHTVTLQCPALLVVGDSSPAVDAVVECNSKLDPTKTTLLKMADCGGLPQISQPAK
LAEAFKYFVQGMGYMPSASMTRLMRSRTASGSSVTSLDGTRSRSHTSEGTRSRSHTSEGT
RSRSHTSEGAHLDITPNSGAAGNSAGPKSMEVSC
>Q9GZY1
MRAFLRNQKYEDMHNIIHILQIRKLRHRLSNFPRLPGILAPETVLLPFCYKVFRKKEKVK
RSQKATEFIDYSIEQSHHAILTPLQTHLTMKGSSMKCSSLSSEAILFTLTLQLTQTLGLE
CCLLYLSKTIHPQII
>P38828
MSVSLEQTLGFRIKVTNVLDVVTEGRLYSFNSSNNTLTIQTTKKNQSPQNFKVIKCTFIK
HLEVIGDKPSFNSFKKQQIKPSYVNVERVEKLLKESVIASKKKELLRGKGVSAEGQFIFD
QIFKTIGDTKWVAKDIIILDDVKVQPPYKVEDIKVLHEGSNQSITLIQRIVERSWEQLEQ
DDGRKGG
>Q84JU6
MDTREINGFASAARSISLPTQPNYSSKPVQEALKHLASINLRELCNEAKVERCRATRDLA
SCGRFVNYVLNPCGHASLCTECCQRCDVCPICRSTLPKFGDRLRLRLYYECVEAGLISRT
HEEASQDSDEDEHQLAADVHRLYSLFDVAMNNNLISVVCHYITNVCMDETAVSSDPVIAF
LLDEVVVKDWVKRTFRSTLAELQEIYNLETKEMQAWLDKLLRCSKQVAGICSVLEVMESA
FKGSVSPQLQDVQTLRENIGKTKQHLDIMVWCIRHGFLDDVRSRYSNFTSWNALVGERKS
NAVKRAWPDAVDQSSDCSVQSASLFIEDALENLEREPEYSQEIGADLEVGRLQKDKRSFL
RSKIEGTSGSYPFENLRTAADMLFLHGGSDLVVAKQAIFLYYLFDRHWTTPEKYWKHTID
DFAATFGITRHSLLESFVFYLLDDHSEEALQEACRILPEICGPETYPKVAQVLLERDNPE
TALMVLRWSGRDGVSELVSIGEAVTALRVRVECGLLSEAFTYQRTLCLKVKENNLKNGAV
KHASDDLDIWSWTEWMEILVNEFCCLSIRRNLVDRIIELPWNPDEEKYLHRCLLDSATDD
PSSAVGSLLVVFYLQRYRYIQAYQVDLRLQKIEEAFVSDNQIGEEVMFRMRSQSHWRKEL
VDRAIDILPVIQQQQVRSGQFSEMEDASEGAKKSDLPDAPDMITSSVPFATTNSVFLQSA
NNARAREPVANNGSPFQPGHMIGNASHDLSHGRLFTNANRGQKSEVRSVTKNLKFGEMST
PFKDLNRARGNSQLQGKRTEESSPEVNVDRYIENNMSSPYLRRITANNPVTVKSSSNHLN
GSSQKPESTFFGTRMQPDKDNFVDLDDPMDMSSSLKDNNNNVLATESRNNSGGLRWRSDE
TSDDEDELTSFGSMPVKGRRRRRFAAR
>Q9UTI7
MSQPLHARFATRAVKNPMILEKERQLTDSKYHILVAATGSVAAIKLTLIVKSLLTYKGVD
VQVVLTDPARNFVEKEDLTALGVNVYNNADDWKNWDGLECPITHIELRRWAHLLLIAPLS
ANTMAKMANGLCDNLLTSLIRAWAPLKPILLAPAMNTLMWTNPITQEHLSAISRIYKNSE
FIMPIEKVLACGDIGMGGMAEWRNIVGRVADKLQLEQKSVLPNAVKNIDGQDDDSSEQTA
AFEEYDDDDDDDVDDNEQSNSMIETSANADITPKASLLPSTTESSISKDHETSQAPLGSE
SVDTQASENVTTKPEPPVPFTSSEYRNTEEEQYLNLIRYILENGQSRPDRTGTGTRSVFA

PPQLRFSLRNNTLPLLTTKRVFLRGVLEELLWFIHGDTNANHLSEKGIHIWDGNGSREFL
DSRGLTDRKVGDLGPIYGFQWRHFGAQYVDCDTDYTNKGVDQLAQVISTLKLNPYDRRII
LSAWNPLAIPEMALPPCHIFCQFYVSEPCKPGGKPQLSSMMYQRSADMGLGVPFNIASYS
LLTHMIAHMCGYEAAEFVHVMGDCHIYNDHLEALQTQLERVPKAFPKLFFKRDAKDIGSI
DSFSVDDFAVEGYNPYGPIKMKMSV
>Q9Y801
MMKLFCLNIFRFLYTTSFISAVLSFNQRPINMQEVREPRLFELLAETRDCNIFSELIMQF
PDYVHLFQQKDQHITVLVPNDAAFQQCKVKPWAIEYKNGVAVKSTDIVHITEQQAQDNIL
KFVERHIITSSPIEWNNEYKTIDGTDVHVIKKEEEIYINESIHVLCRKKASNGEMWVLNA
TLSTPSIRE
>P53687
MTSVSPSPPASRSGSMCSDLPSSLQTEKLAHIIGLDADDEVLRRVTKQLSRSRRIACLTG
AGISCNAGIPDFRSSDGLYDLVKKDCSQYWSIKSGREMFDISLFRDDFKISIFAKFMERL
YSNVQLAKPTKTHKFIAHLKDRNKLLRCYTQNIDGLEESIGLTLSNRKLPLTSFSSHWKN
LDVVQLHGDLKTLSCTKCFQTFPWSRYWSRCLRRGELPLCPDCEALINKRLNEGKRTLGS
NVGILRPNIVLYGENHPSCEIITQGLNLDIIKGNPDFLIIMGTSLKVDGVKQLVKKLSKK
IHDRGGLIILVNKTPIGESSWHGIIDYQIHSDCDNWVTFLESQIPDFFKTQDQIKKLRQL
KREASDLRKQMKAQKDSIGTPPTTPLRTAQGIDIQGNNELNTKIKSLNTVKRKILSPENS
SEEDEEENLDTRKRAKIRPTFGDNQAS
>P49775
MNKPIYFSKFLVTEQVFYKSKYTYALVNLKPIVPGHVLIVPLRTTVLNLSDLTMPESQDY
FKTLQLIHRFIKWQYKADSINVAIQDGPEAGQSVPHLHTHIIPRYKINNVGDLIYDKLDH
WDGNGTLTDWQGRRDEYLGVGGRQARKNNSTSATVDGDELSQGPNVLKPDSQRKVRALTE
MKKEAEDLQARLEEFVSSDPGLTQWL
>Q7Z4T9
MSHAVTIEEPQAQPQVSQTRYRERSRAGSHISSNRAYDFLYDPLFIVSSEKDHTQANIQA
TLIRSRLRKVPRFKTMFSNLIHYPRYSLYWSKSDPVPPFISREWKGHKEKHREALRQLTT
TDASFQMPKEVYEDPEVTGKNRYKYFERPFLPFFQQMPFNVVYAVSKAEPYTFPPTSTKH
LSIPSKSTVGTQTDYRDADVQTDPYSPEYVVCQDSIPELLTLATLTWGRGLPAGQAEVEM
IERAREKRAWEASLPALSDTSQFEKRRKMMNEMERKEWAFREQEIEKLQEIRLEVLKELL
RKREENQNEVNMKHLNARWSKLQEGKEAKMAVIYLQKLLRGRVIQNMMFEGKEKRLELIQ
ELRTCHALQEDEKLVKKAEKQVTLALQRQRNLHEHKVSLVENHLAGLEGRALADMFDFLS
KELVRLQEERRIHAFVMLAERQRRVREAEESGRRQVEKQRLREEDEIFKEVVKVHHSTIS
SYLEDIILNTEANTAEEQARAEIEKMAEKINDIAYEMESRRTYLQSEEIVAELVYSFLIP
EVQKYFVKEKVRNAQRKHILAAHQIIHSYTESMVQKKLTEGEQDEASNAAMLLEKETQNE
NNS
>Q09855
MNQDDNGKNVVSKVSDLTSCSDFSTSSPVPCLNPLSHENNRIDLIRDLLASLSKEGVVAV
YNHVRSLLFTDFTEVFPEEVSLRVFSYLDQLDLCKCKLMSKRWKRLLEDPGIWKALYMQK
GWFVNENVLNEFEAWRRTHKFPQPRFENFLKQQNIIGPYGTMFLPQQFIFDSNGRPLLNW
SYLYKEHAHLDSNWRHGRFLVSTFNNPSIRFPADQDFRATLDSVYCVQYDDEIMVSGSKD
RTVSVWDVNSRFILYKLYGHSGSVLCLDFCRRRNLLVSGSSDSTIIIWDWQNRRPLKVYF
GHTDNVLGVVVSENYIISSSRDHTARVWRLDATSPAEACMHVLRGHLASVNSVQYSSKTG
LIVTASSDRTLRTWDITTGHCIRIIHAHQRGIACAQYNGKFIVSGSSDLTIRIFEASSGK

LLRMLQGHEDLIRTVRFNDEKIVSGGYDGTVRIWNFNTGEQHCVLHNSRNSRVFGLQFDH
RRIIACTHSSEILVWNFDDGLDCTFF
>P38339
MEETAKKPASATVSAKSSHDGGTDDLAHLFSTPEIKKVLNSDVAINALLSRLKQSLLTCE
EFMKFIRKKYAFEEEHVQELSKQYKHFFNIQGSTNSSLKKMIHEVLGFDGKMAQVKQSYI
TALQKMYSEISSLLLTMTKLRKSVKENSKRLEKDVSDAIHSAEKAQSRYNSLCQDWDKLR
MTDPTKTKLTLRGSKTTKEQEEELLRKIDNADLEYKQKVDHSNSLRNTFITKERPRIVQE
LKDLILEIDTAMTIQLQKYTIWTENLVLNTGVTISPLDSTKSMKSFAGSVSNERDLYSFL
NKYNQTGKHSLLINKNLIPVSYKKHPSMNHGQKNKSPPKFAVDPSRNSIPKRMISTHNES
PFLSSSSNTAAVPNANLNSATPSLNTNKQLPPTMASSISSTSNAAGAMSPSSSIVTSDTT
SSITKTLDPGNNSPQIPEELINSLDSDRPISHIQTNNNMPPGVQKNFKTFGVPLESLIEF
EQDMVPAIVRQCIYVIDKFGLDQEGIYRKSANVLDVSKLKEEIDKDPANISMILPSKPHS
DSDIYLVGSLLKTFFASLPDSVLPKALSSEIKVCLQIEDPTTRKNFMHGLIYNLPDAQYW
TLRALVFHLKRVLAHEAQNRMNLRALCIIWGPTIAPANPDDANDVNFQIMAMEVLLEVSD
QAFEPE
>P97443
MENVEVFTSEGKGRGLKATKEFWAADVIFAERAYSAVVFDSLINFVCHTCFKRQEKLHRC
GQCKFAHYCDRTCQKDAWLNHKNECAAIKKYGKVPNENIRLAARIMWRVEREGTGLTEGC
LVSVDDLQNHVEHFGEEEQKELRVDVDTFLQYWPPQSQQFSMQYISHIFGVINCNGFTLS
DQRGLQAVGVGIFPNLGLVNHDCWPNCTVIFNNGNHEAVKSMFHTQMRIELRALGKISEG
EELTVSYIDFLHLSEERRRQLKKQYYFDCSCEHCQKGLKDDLFLAAKEDPKPSQEVVKEM
IQFSKDTLEKIDKARSEGLYHEVVKLCRECLEKQEPVFADTNLYVLRLLSIASEVLSYLQ
AYEEASHYARRMVDGYMKLYHHNNAQLGMAVMRAGLTNWHAGHIEVGHGMICKAYAILLV
THGPSHPITKDLEAMRMQTEMELRMFRQNEFMYHKMREAALNNQPMQVMAEPSNEPAPAL
FHKKQ
>Q9P7W4
MKSEPTSLDFTSSNLRRMNRDHSSNNTNRTVLNLPKEILIIIFSFLDPRSLLSAQCTCKY
WKKLLSDDLSWRTAFFHHFAGDQSQIFSPLGNGTWRQEYLLRSTITRAYEKGKGQTVQYD
CRVGQLTNLYYDFSSGRLYSGNWLTGTISVSDPTTGKVERSLLHASTDGSFTHGLSTMTL
GKQIFGFGFMDGRVGVILMSRQAETPRKFRYCLDSHADSVTCIDALTGDLPPTGEIGMVT
GSDDGSVHCWDVKTGVSLQSFQFRSSQILSLCFRPKYKMLLVDTFNYELNSYQLYLIPGY
ARSRKNEQPILLSSRKCVLTDEEEPPCLMTADCCAGVAFLSRGAPKNCICRVSFKEFLEK
NDNVGVQTSSIPLNGKPTSISLDTNDRVLSKSTPGRGARLLAVGDENGLVYVVNTRTEDP
NKAILRTITAYSNFPITDIYLNEVAMVVGSASGYCGVYDTVTGNFLKKIASARNAARREP
INCILLDSNPLSLKGVITMSKHVKSWSYTIPKPFVNKRSKVLPLRPSVTHDNLSKSSDYS
KNEVEREIMLGLDQIAQERREKMEARQKFEQHFGEGLVGLSEEEIIAYVTMLSQEEEAKR
MVQLSMDVDKIEEDFKENDEQATSSLNALSSNHEPPQEQANVAELNEQEQIELAMRLSLM
EM
>O02732
MAFAEHSPLTPHRRDLCSRSIWLARKIRSDLTALMEAYVKHQGLNENINLDSVDGVPMAS
TDRWSELTEAERLQENLRAYRTFHVMLARLLEDQREHFTPAEDDFHQAIHTIVLQVAAFA
YQLEELMVLLEHKVPPSEADGTPLSVGGGGLFEKKLWGLKVLQELSQWTVRSIRDLRVIS
SHQAGVPAHGSHHVAKDKKM
>O94498

MSKIILKTSRFLLKSPVEEDDFDQITKIKSDPLVSNTQLYGKVESRELCRFMFQHYITDA
RITKTRRKRWVFGIYPLEVSSTFPSICYIGNVGLSKKNVKSDTANLFFEIGPLYWGMGIA
TECVGRVIEFGSENNIQNFIIDPIIGNEASKKVALKLGFEDSGTFVTAYNGLQQHIYKLS
KQIRTG
>P53294
MSTIKVIEVYTQNGLRKVRPYYNRRSAFVKGRWLGKLLIDVLVSEFKLRPRAYYLDQIRK
GTYRLIRDGVPLVPDHLMTTIIKNHDVLETTTHKHEPPVKQWCSQEVEAEDLPGRIAGFN
IVFEDESILVIDKPSGIPVHPTGQFYQNTITELLKLHGVDALPCYRLDKITSGLLILAKN
SQSAGEIQKSIRSRDMIKIYLARVKGRFPHSELILDNENAAETTFEDTSKVTVEMTPIYS
IDPKRQFPVGLSTSKDAITKFYPIRYFSHADETVVACKPITGRTHQIRIHLARLGHPIVN
DSVYCSHITKYPERLKFITQFPRWENQQDLDAEELKVRFQKFVDETKNNCRTMETFCPEC
HTVDLKDPVLSDLELWLHAWKYEEINGKFKFKTDLPKWAQLDNS
>O35826
MEKNGNNRKLRVCVATCNRADYSKLAPIMFGIKTEPAFFELDVVVLGSHLIDDYGNTYRM
IEQDDFDINTRLHTIVRGEDEAAMVESVGLALVKLPDVLNRLKPDIMIVHGDRFDALALA
TSAALMNIRILHIEGGEVSGTIDDSIRHAITKLAHYHVCCTRSAEQHLISMCEDHDRILL
AGCPSYDKLLSAKNKDYMSIIRMWLGDDVKCKDYIVALQHPVTTDIKHSIKMFELTLDAL
ISFNKRTLVLFPNIDAGSKEMVRVMRKKGIEHHPNFRAVKHVPFDQFIQLVAHAGCMIGN
SSCGVREVGAFGTPVINLGTRQIGRETGENVLHVRDADTQDKILQALHLQFGKQYPCSKI
YGDGNAVPRILKFLKSIDLQEPLQKKFCFPPVKENISQDIDHILETLSALAVDLGGTNLR
VAIVSMKGEIVKKYTQFNPKTYEERISLILQMCVEAAAEAVKLNCRILGVGISTGGRVNP
QEGVVLHSTKLIQEWNSVDLRTPLSDTLHLPVWVDNDGNCAAMAERKFGQGKGQENFVTL
ITGTGIGGGIIHQHELIHGSSFCAAELGHLVVSLDGPDCSCGSHGCIEAYASGMALQREA
KKLHDEDLLLVEGMSVPKDEAVGALHLIQAAKLGNVKAQSILRTAGTALGLGVVNILHTM
NPSLVILSGVLASHYIHIVRDVIRQQALSSVQDVDVVVSDLVDPALLGAASMVLDYTTRR
IH
>P98081
MVKSLVAKLSTASSNLSLASTFGGGSGAAEETNYAKHRNDPGRFFGDGVQFKAKLIGILE
VGEARGDRMCQEALQDLKMAIRAAGEHKQRITIHVTIDGLRLRDEKTGDSLYHHPVHKIS
FIAQDMTDSRAFGYIFGSPDSGHRFFGIKTDKAASQVVLAMRDLFQVVFELKKKEIEMAR
QQIQGKSLHDHSSQLASLSSLKSSGLGGMGLGHSDLASGGISSGHALTLLGSSLSTTNGT
SRLGVSLDVAKASGSAAKEVSPESVADLVDLEQELTSLQRGISQMERITPNEPTTSSTGG
AGHPSLAKSASEDDPFGDSFIYVPSYSILPPPPDSGRNRHKPPNKTPDAVTSLDAMLSPP
PGTSSSHGSASAGLQAADNDDDNWLQELDQQNDVFDTSKVVSSSGLGSVLAMAPLASSES
TATPTQQLTEVAAGSGPLADLDIGLSTALGNEEQTSTILSLDAFTDLDPLGTGRTRPYVD
KKYFFQELKNPPKKLLKELSSGSQAGLGLGLSLGQLDGLFPEDSTTISTTTTATNITAG
NPQQNSANTLTSTASTAASLGQLLSTVALNPDPLPAPISIPTSISHSITPSAELKLLLGH
VTNPPNPTGHYYTTEPPTLNSLENPHPPADPVLLPRDTDPFSPTRKKSDPDPFQESDLFA
KLDAFEFEAPPAVPAPSIPNLATETKANVFNGPLQVQLPPEKELQLQQPPSTVRNRPTAS
VSALPSGGALDVISSISNKKMPHLFGQARSFGKSGSDIGSSVNMRRLQESDSLSETEAAP
EPPPRPDSTPYSEPPPLPPKKQFSDLVIRPSPANTTQPPTSGRYEYLNSNVTARRTASSV
DAPPIPLPSRRVGRSDGCFPGPGRPRKPGHTEDDYLAPLGAPPPLLPPPSQGSSARARPQ
RQASLGRPQDIYENKAEILQAQAQAQAQAPEVAPSSNTLAPDITLTQLLTLGMDDLAIKL
NVPASKLSTMTLVQLTAYLSEYLSSEKSQVHSQERRSSPANTAPAPASTAAVFKVNFDQQ

TSFVAKFDDTFGEDEPVMPSGSSDSTFVANFANFNDAPTPVPTVSPVVATVPSADRYAVF
REIIDQELQQQQQETDLMGDLTPPPVDETQAKEISEGLEVNNVGAELPIDALDVKPAPKI
DTKITEVVAQAKDRYAALRDIILVENLFDKPAIATDTQPEKEKDLLQDFPEFSDEFNEDH
DLRQIMDHQNVQTHARDRHGLVDSRGFPTEPSSSALTVGDYDEDEDADAGGESSLDSNEK
DAEPVSGQDQYEKLSTSTQQLDAAAPALEDVQQLQQSLPPKQDQKFLSILTAPGGGTKD
DIEIDELMHRAISNLSLDSRDRVSPATSSAAPSRGAPGLHTPSQFNDVSTSPIPLQKPGM
GPSPVPSQLSAVSQLIDTATKQMMGDKDREKQSWATFDSPKAKGKARLTLPPPPPPASNT
SQPDTVESPCSSDPRDDGWSKQQRRWAKKERQQTSSSSRDLSPWDDETPEYLKRRQLAAA
QMAHPHQPPMQAPPQHTDRHGYYMRHARRMNSCDEDYDYDGEFVARRDQPQHQQQQRKFK
HGLSRSRDNFELESPSWYHHPAHHTWSPQEIEQVRVRSFDRTAYERSSYGPPPPIYDKRG
QLRGKYRGDHRDRERERDRDREYRDYARPSYDFDYENVYEERGGRSPLAYKPGRGGGDYL
YDRERDRDRERDRKSFDRESLESYESATRRRRSFGSGNDVYGSLDSRDDYRGDRERDRER
DREQMKTRSLRKPTTTSGKLRISGDIDYEQDSEQDFQQRSGVRSLQRPNQLGGDVVLPSN
AVVGPQRLRKSSGSSPWDGEEPALPGQKSWKRPASAAETERRLAESRRAVALGQTPSDGE
KERRFRKKTRARSAKDLATVGAPSASTSAPSRSSYGRGIRDNYDYICPGQRNDDDDDDE
DYVDDEPPTDEDKFERLNRRRHEMHQRMLESERRQMERHQPPSLAKLPGQNRTRGVVANS
DYGFVDSYEQTPTPTPRSNASSTGPGGLMMSGGESSAGVTSSKFNFDDGFESDFNQSSPP
PAPAGTASSCNSTPAGPVSANANNGGSKSLFRFSNDFSDREKREQFEMDTPPTSTPPITQ
KLRFDDNVKVSQFDDAAFEDDFAKASFDFEKEQAGSATAGAGGSGAMSRKQNMRTSKLQQ
RQELIKKSESVNIFAKKQEDPFEDDEFFKSPDQEQAMDQHNDDTEGGKFQWSEDANFAKF
DENM
>Q14106
MQLEIKVALNFIISYLYNKLPRRRADLFGEELERLLKKKYEGHWYPEKPLKGSGFRCVHI
GEMVDPVVELAAKRSGLAVEDVRANVPEELSVWIDPFEVSYQIGEKGAVKVLYLDDSEGC
GAPELDKEIKSSFNPDAQVFVPIGSQDSSLSNSPSPSFGQSPSPTFIPRSAQPITFTTAS
FAATKFGSTKMKKGGGAASGGGVASSGAGGQQPPQQPRMARSPTNSLLKHKSLSLSMHSL
NFITANPAPQSQLSPNAKEFVYNGGGSPSLFFDAADGQGSGTPGPFGGSGAGTCNSSSFD
MAQVFGGGANSLFLEKTPFVEGLSYNLNTMQYPSQQFQPVVLAN
>Q9H910
MFQVPDSEGGRAGSRAMKPPGGESSNLFGSPEEATPSSRPNRMASNIFGPTEEPQNIPKR
TNPPGGKGSGIFDESTPVQTRQHLNPPGGKTSDIFGSPVTATSRLAHPNKPKDHVFLCEG
EEPKSDLKAARSIPAGAEPGEKGSARKAGPAKEQEPMPTVDSHEPRLGPRPRSHNKVLNP
PGGKSSISFY
>Q9P7N0
MEVYLSEFINWKLYEFKKINELGTSTFRYYFKGKIVNLKNKRVSSNEVCYMFKLMDDTGI
VELNYSLSKYSPKFEMLRQLQLGISVFVYSDVVCRVSGIRAWKLQIHSKNLESKIEATIS
EPEQIPCPGFGSDNIVPLSLVNDFKDGAFVNVLVAIQWKEEFEIFNNETKKSLPKQTIHV
HDISGSAMIVLIGESQVKSALSWNNETILLIHNAIAYRTNTGMLELSIKDAIIQTLQSFD
GKVLKLLNFSIKRKELIKINAYRLSTLEALEKIASQSGEAFGQVGVLIMQSKIKDLTKEG
GLVYGNDLTGLRFADLISIIVDETGSVKNPKFSQKLLEEITNIAPTELSVLNEEQAMHLE
NVFLYRHYRVCVGFLNKQIYVFSSDEPLKSYI
>O74848
MICKNTFVDYPTTQVRKERVHTYRTLTSATPSLEFFSNENTNRLSEVQCKLTHFLSSSEN
SSSVRNTRTHKFKQLLHYIFFSNRSSSVLGKQNVHNSQTLGSVSFCEKHAIDINYSENVN

KQLNFESHLDDQTCSKRFYFNEQSGSDRYSLKCDSDTDSSTYFGDSASETCSSASNSLYK
QTDLTSLCMFNQNKIQTDWSSIDPMDNDKIVCADFEKNFNILKELLNNTKDNGIDQQIVH
KEHISHLEKIWKNINEESSEDPSLLLLRLEFLLTDLQTAIRKYSDTVSTNSVNEAFFDKI
KIELQAFGVLV
>P34222
MITSFLMEKMTVSSNYTIALWATFTAISFAVGYQLGTSNASSTKKSSATLLRSKEMKEGK
LHNDTDEEESESEDESDEDEDIESTSLNDIPGEVRMALVIRQDLGMTKGKIAAQCCHAAL
SCFRHIATNPARASYNPIMTQRWLNAGQAKITLKCPDKFTMDELYAKAISLGVNAAVIHD
AGRTQIAAGSATVLGLGPAPKAVLDQITGDLKLY
>P38980
MSGGLDCLALKEDDVQKFLASGAHIGSSNLDYQMTQYVYKRKPDGTYIINLRRTWEKLLM
AARIIVAIENPADVCVISSRPYGQRAVLKFGAHTGATPIAGRYTPGTFTNQIQAAFREPR
ILIVTDPRSDHQPVTEASYVNIPVIALCNTDSPLRYVDIAIPCNNKSIHSIGLMWWMLSR
EVLRLRGAISRDVTWEIMCDLYFFRDPEEAEKEEQEARDRATAVKEEPAQPYAEQWSDPV
AVPPAGQPVAEVTDWAADSVKAPVAGIGTYGGPSEDWAVADAPAAPAAPTPAAPSQEFTA
TDDWGGAASNDWAISS
>O94324
MTNPEQLKRILSHEVLLKIEMSKNGIVEYANPAFFELIGYEGDLFQCSFYEYLQSDDEHL
MKKATDNLFRKSISVAHVFAFLRCNPIRSPNYYIQQAKAPFEGKTYIRMLFRGILVDSLY
GKDTRVLWAGKYLYPQRSVINEMDFILFNTLGIGAFILHDHLLGIIKYNYIHVPPPSGKL
CSLCEDNLPEWYFEVHSDFCLVWNDLVRRVFAVQQLINCKKLEIEDIVNKLPTGSNHMVE
ETFLSLPVITVFNGKKNRKQRFRIRSWRSSLNFLVKELDKSIKNFAYLEHRTFLTISNSA
AKDMKREIYEKSLVNWEYDFLVPSKIQDYFYDVHSLLLKNLSSKIKLCNHILMYQATFNE
VKNFLQTYSLNMLSIEMENIEGSLYFGNAQLSNLICVNQYLSEQRPVFFNRLLALGNVEN
NNSIYDDIQKRTERISTIKRHKKYFEIGERLTEKDLIVSKTFKTTRIDYFKAVKGSIEDL
DVRPLKNRQKFVNKFYASIVHFLTESMQFPSHNDRRFGDNTPHSLDEFILLKEINRGAYG
RVYLAKKRSSGKYFALKMIPKSSLDSLKKIKGLLLEKRNMHIQRYGPNTVKLYYAFDSGD
YLCLVMDYFNGGDCETLIQKLGPLPEQWVCQYAAELLNAIELLHQDGIIHHDIKPANMLV
DETGHIRLTDFGLSENVEEKKEVYKLTKRMSFEQKHGNLYEQLQPKKFEFVRYVRNYRGN
IDELEKAESPQQNSDYANDSVQHLLDFDINNMDETAIHMLMNQLEKKENRTFIKKDISGT
PNYMAPEILMGVDTQMGDIWAMGCVIFEMLTGTRPFEANTVKAIWARIERNDIGWTKRVK
ESCSKEAVDLITKLMDPDCNKRLGSNGYQEIKKHPFFRTIKWDNLNSGPGPFVPQTENVE
DLTYFEKNISGSDNINKNNCQTSATLILNGIFAFHPPPKATPADSGTETSNSAAFSASEE
ETTNLTDQKRKDLFSLITKAFKGIDLKALNYNNKATLLRMYDEVDFPKNQQRNKEKFRIQ
KRPNKKYRYHLF
>Q14151
MAETLPGSGDSGPGTASLGPGVAETGTRRLSELRVIDLRAELKKRNLDTGGNKSVLMERL
KKAVKEEGQDPDEIGIELEATSKKSAKRCVKGLKMEEEGTEDNGLEDDSRDGQEDMEASL
ENLQNMGMMDMSVLDETEVANSSAPDFGEDGTDGLLDSFCDSKEYVAAQLRQLPAQPPEH
AVDGEGFKNTLETSSLNFKVTPDIEESLLEPENEKILDILGETCKSEPVKEESSELEQPF
AQDTSSVGPDRKLAEEEDLFDSAHPEEGDLDLASESTAHAQSSKADSLLAVVKREPAEQP
GDGERTDCEPVGLEPAVEQSSAASELAEASSEELAEAPTEAPSPEARDSKEDGRKFDFDA
CNEVPPAPKESSTSEGADQKMSSFKEEKDIKPIIKDEKGRVGSGSGRNLWVSGLSSTTRA
TDLKNLFSKYGKVVGAKVVTNARSPGARCYGFVTMSTSDEATKCISHLHRTELHGRMISV

EKAKNEPAGKKLSDRKECEVKKEKLSSVDRHHSVEIKIEKTVIKKEEKIEKKEEKKPEDI
KKEEKDQDELKPGPTNRSRVTKSGSRGMERTVVMDKSKGEPVISVKTTSRSKERSSKSQD
RKSESKEKRDILSFDKIKEQRERERQRQREREIRETERRREREQREREQRLEAFHERKEK
ARLQRERLQLECQRQRLERERMERERLERERMRVERERRKEQERIHREREELRRQQEQLR
YEQERRPGRRPYDLDRRDDAYWPEGKRVAMEDRYRADFPRPDHRFHDFDHRDRGQYQDHA
IDRREGSRPMMGDHRDGQHYGDDRHGHGGPPERHGRDSRDGWGGYGSDKRLSEGRGLPPP
PRGGRDWGEHNQRLEEHQARAWQGAMDAGAASREHARWQGGERGLSGPSGPGHMASRGGV
AGRGGFAQGGHSQGHVVPGGGLEGGGVASQDRGSRVPHPHPHPPPYPHFTRRY
>Q99PF5
MSDYSTGGPPPGPPPPAGGGGGAAGAGGGPPPGPPGAGDRGGGGPGGGGPGGGGASGGPS
QPPGGGGPGIRKDAFADAVQRARQIAAKIGGDAATTVNNNTPDFGFGGQKRQLEDGDQPD
SKKLASQGDSIGSQLGPIHPPPRTSMTEEYRVPDGMVGLIIGRGGEQINKIQQDSGCKVQ
ISPDSGGLPERSVSLTGAPESVQKAKMMLDDIVSRGRGGPPGQFHDNANGGQNGTVQEIM
IPAGKAGLVIGKGGETIKQLQERAGVKMILIQDGSQNTNVDKPLRIIGDPYKVQQACEMV
MDILRERDQGGFGDRNEYGSRVGGGIDVPVPRHSVGVVIGRSGEMIKKIQNDAGVRIQFK
QDDGTGPEKIAHIMGPPDRCEHAARIINDLLQSLRSGPPGPPGAPGMPPGGRGRGRGQGN
WGPPGGEMTFSIPTHKCGLVIGRGGENVKAINQQTGAFVEISRQLPPNGDPNFKLFVIRG
SPQQIDHAKQLIEEKIEGPLCPVGPGPGGPGPAGPMGPFHPGPFNQGPPGAPPHAGGPPP
HQYPPQGWGNTYPEWQPPAPHDPNKAAAAATDPNAAWAAYYSHYYQQPPGPVPGPAPAPA
APPAQGEPPQPPPTGQSDYTKAWEEYYKKIGQQPQQPGAPPQQDYTKAWEEYYKKQAQVA
TGGGPGAPPGSQPDYSAAWAEYYRQQAAYYGQTPGPGGPQPPSTQQGQQQATEANGYELH
L
>Q08977
MFASAGQQHPQIVPKEEESILNYLLEVRSSLAKLKQNRTQYLNSKDVQTTYQHVLTKVRE
LDDIRKNSHETPAKSAATLIHSTELHNRVDSVLDDVFQLLSLCFLTVGLKNSAPATYASL
STVESLLEHLNESNVFTHHDLSPIKERLEEISKIVEQKNSSPAYDEDGNDDRLREIDNER
KKNKIEEDLLLRAKLKHCKDEYDILEGKLEEIDPSLSTVMEKLFRIRRGLLSLVASAKKT
MSKSDINTNSLLQEQNDLQTNNESLTDDKHLVSQEYVHEKLSVLKNELSELESNRDDSGK
FKSLESHQVAEKGQSVLNGLLDDCHDLVNDLSHQKNGGLTLDPYLQPIYEQLIDIKTTLE
NLMITRRWTLRETDLFSYQKKLNEIDNKRINGKFPTKSQDSKGQSILLYLLRRCYAIIYK
LLESSEPVSEALQPIHNQLSTVRRCLLELKRMGGVNNERELYPYQMKLASLDNLRTEGIF
YDSDGNIPEGQGILNALLAECFDILHELKVEAEEKAQNSTSSDGSDDDDNGESGIDSNSN
DSEPESEYQQE
>O88370
MASSSVPPATAPAAAGGPGPGFGFASKTKKKHFVQQKVKVFRAADPLVGVFLWGVAHSIN
ELSQVPPPVMLLPDDFKASSKIKVNNHLFHRENLPSHFKFKEYCPQVFRNLRDRFAIDDH
DYLVSLTRSPPSETEGSDGRFLISYDRTLVIKEVSSEDIADMHSNLSNYHQYIVKCHGNT
LLPQFLGMYRVSVENEDSYMLVMRNMFSHRLPVHRKYDLKGSLVSREASDKEKVKELPTL
KDMDFLNKNQKVYIGEEEKKVFLEKLKRDVEFLVQLKIMDYSLLLGIHDIIRGSEPEEEG
PVREEESEWDGDCNLTGPPALVGSYGTSPEGIGGYIHSHRPLGPGEFESFIDVYAIRSAE
GAPEGGVFHGLIDILTQYDAKKKAAHAAKTVKHGAGAEISTVHPEQYAKRFLDFISNIFA

>Q9QYP6
MDTLVEDDICILNHEKAHRREAVTPLSAYPGDESVASHFALVTAYEDIKKRLKDSEKENS

FLKKRIRALEERLVGARADEETSSVGREQVNKAYHAYREVCIDRDNLKNQLEKINKDNSE
SLKMLNEQLQSKEVELLQLRTEVETQQVMRNLNPPSSSWEVEKLSCDLKIHGLEQELGLL
RKECSDLRTELQKARQTGPPQEDILQGRDVIRPSLSREEHVPHQGLHHSDNMQHAYWELK
REMSNLHLVTQVQAELLRKLKTSAAVKKACTPVGCVEDLGRDSTKLHLTNFTATYKRHPS
LSPNGKAPCYAPSSPLPGDRKVFSDKAVLQSWTDNERLVPNDGADFPEHSSYGRNSLEDN
SWVFPSPPKSSETAFGENKSKILPLSNLPPLHYLDQQNQNCLYKS
>O74349
MKKKSTDLYGRKNPGMQSMSGSFSSLQIALDESSSNFSTIYEPPDLSGLDAEMIVIVKNL
QKRDIVTKCRALQDLIQWNDPSQFDNEQFLNALAVLFPRLSIEVERHVRLKIFVFMSVLS
SALQKKLAPWLKFYITPWVMGFFDSDRAVSVSAKDSFKNLLSEEKWPHVWLKFGSTIAPI
VTDVFLHEDKESLTDLRFFSNEEAESKVTRVKSSCLLTLSFLFKQTADLENLETDKKIDK
QTFKSSLYENLSTLFKSDEFWSLVSSPQDGVTVSLSDLLLIILKYDKPFVTQYKEKYFKR
VSRMISRLTSLTCVPMLRLLSNMISNFPNEVHQFANDSKRPLSKLFSNLITKRISLPNSG
FYTSLLNLFKSIGAMQLVPSIESVDELCDAFLETANQEQRFLSTEVYDCLLNFLSFVYTD
SSDPQIKDHVRDRLRTIFTRYFKGEFVLRCSTSDFDHCLQSVFDKNSDFASLWNEVLFGF
FNDESMDIETIPFDSLSRNLSLTVQTVLYLKNRNFQTGNEVMSILGPCLSFLMKLSTHKN
ERIACLSASQLITVCHIFSDTTLIKPVKELFQKYLVNDLPSSILKLGPQSPAFTLLRDIL
LLLKDYADLSEPWENVANQFTVSFDELENIRVLNSLPSLFADGKLRGKISLVKTLVEYYD
TAVFAIMQNPGNDWDMIRACIGSKEILVPEETIKNILFTTLEYFLTNDWVDNHLIILCAS
LHSLKAHLPTIFDENKSLYSLLPVILFSKPEDDGHVAAHFESIFSLLKEKALEDSGFSLK
LCSEVQEWSLNKVLNGSINEKLAADKCVLVFNSFGKLPSNFFTFPASFWDAKISSCIPFF
SNKLFIDQDFILGFLDLVASEPINVDMTDVGTQFVHIFHASLYTLYYVETTGCDGDALFN
LTFAYLLIRIYLQNGIQSIIDVPIRDAGIFVESFECYFKGEVVKFMRDKDALTVLNELLI
DDIDGKMPVLFKRFKNLSSAENTTSFSIFAAQGLTDFLIVVSNLLEMDEKHVDVVLGKLG
LSSSKSPIFVSSILEGLKPLEVDSEIIQRIRFKAVNDLTGKLHSANEVSKSLLILNAATT
QQITEKPLLPITRCRLFLENITNWCSESGIKSLELLPVCCFLRFMYYFLPTVFSLSGSYW
NSIFDYIKYAMKMSVVDAPIVKSFELFALRLYNALSKNYEMNSDIKDCIVESNESMNYLL
LKRFLFTHESTLRNSVTARMCNQYLVKLLENCPGKVVRSFQYQEFFPSLCNSNDLQMESV
CMKFLREKLSHELKELTVYYMVESDYEPDVSLCPELLSLAIDFPGDPFVMVSKMEKYEHA
LRVYLLVWDLIFYHFEETTYNIKLSIINQLHAMDLLRPLLNTLVEILNLSYDRPINVDKY
PKIDYNLMDYSSATDRIRCLAIHIYYQCLRHLSSSVRSYWSEVKNRAFTSTVESFTGYNV
SPLLISASLDDVERSIESEDFQSVGDVNVKVNRNTREISFIYNVDEHKLEMAIKIPSVYP
LQNVQVEGIERVGVNERQWRSWILASQSILSSQNGSITDALLVLKKNISMHFEGVEECAI
CYSVLSVERTLPNKRCGTCRHKFHASCLYKWFKSSNSSRCPLCRSSFTFV
>Q8TF40
MAPTLFQKLFSKRTGLGAPGRDARDPDCGFSWPLPEFDPSQIRLIVYQDCERRGRNVLFD
SSVKRRNEDISVSKLCSDAQVKVFGKCCQLKPGGDSSSSLDSSVTSSSDIKDQCLKYQGS
RCSSDANMLGEMMFGSVAMSYKGSTLKIHQIRSPPQLMLSKVFTARTGSSICGSLNTLQD
SLEFINQDNNTLKADNNTVINGLLGNIGLSQFCSPRRAFSEQGPLRLIRSASFFAVHSNP
MDMPGRELNEDRDSGIARSASLSSLLITPFPSPNSSLTRSCASSYQRRWRRSQTTSLENG
VFPRWSIEESFNLSDESCGPNPGIVRKKKIAIGVIFSLSKDEDENNKFNEFFFSHFPLFE
SHMNKLKSAIEQAMKMSRRSADASQRSLAYNRIVDALNEFRTTICNLYTMPRIGEPVWLT
MMSGTPEKNHLCYRFMKEFTFLMENASKNQFLPALITAVLTNHLAWVPTVMPNGQPPIKI
FLEKHSSQSVDMLAKTHPYNPLWAQLGDLYGAIGSPVRLARTVVVGKRQDMVQRLLYFLT

YFIRCSELQETHLLENGEDEAIVMPGTVITTTLEKGEIEESEYVLVTMHRNKSSLLFKES
EEIRTPNCNCKYCSHPLLGQNVENISQQEREDIQNSSKELLGISDECQMISPSDCQEENA
VDVKQYRDKLRTCFDAKLETVVCTGSVPVDKCALSESGLESTEETWQSEKLLDSDSHTGK
AMRSTGMVVEKKPPDKIVPASFSCEAAQTKVTFLIGDSMSPDSDTELRSQAVVDQITRHH
TKPLKEERGAIDQHQETKQTTKDQSGESDTQNMVSEEPCELPCWNHSDPESMSLFDEYFN
DDSIETRTIDDVPFKTSTDSKDHCCMLEFSKILCTKNNKQNNEFCKCIETVPQDSCKTCF
PQQDQRDTLSILVPHGDKESSDKKIAVGTEWDIPRNESSDSALGDSESEDTGHDMTRQVS
SYYGGEQEDWAEEDEIPFPGSKLIEVSAVQPNIANFGRSLLGGYCSSYVPDFVLQGIGSD
ERFRQCLMSDLSHAVQHPVLDEPIAEAVCIIADMDKWTVQVASSQRRVTDNKLGKEVLVS
SLVSNLLHSTLQLYKHNLSPNFCVMHLEDRLQELYFKSKMLSEYLRGQMRVHVKELGVVL
GIESSDLPLLAAVASTHSPYVAQILL
>P42798
MVRISVLNDALKSMYNAEKRGKRQVMIRPSSKVIIKFLIVMQKHGYIGEFEYVDDHRSGK
IVVELNGRLNKCGVISPRFDVGVKEIEGWTARLLPSRQFGYIVLTTSAGIMDHEEARRKN
VGGKVLGFFY
>O14467
MSDWDTNTIIGSRARAGGSGPRANVARSQGQINAARRQGLVVSVDKKYGSTNTRGDNEGQ
RLTKVDRETDIVKPKKLDPNVGRAISRARTDKKMSQKDLATKINEKPTVVNDYEAARAIP
NQQVLSKLERALGVKLRGNNIGSPLGAPKKK
>Q923S8
MAECRASGGGSGGDSLDKSITLPPDEIFRNLENAKRFAIDIGGSLTKLAYYSTVQHKVAK
VRSFDHPGKDAEQDHEPPYEISVQEEITARLHFIKFENTYMEACLDFIRDHLVNTETKVI
QATGGGAYKFKDLIEEKLRLKVDKEDVMTCLIKGCNFVLKNIPHEAFMYQKDSDPEFRFQ
TNHPNIFPYLLVNIGSGVSIVKVETEDRFEWIGGSSIGGGTFWGLGALLTKTKKFDELLQ
LASRGRHANVDMLVQDIYGGAHQTLGLSGNLIASSFGKSATADREFSKEDMAKSLLHMIS
NDIGQLACLYAKLHGLDRVYFGGFFIRGHPVTMRTITYSINFFSKGEVQALFLRHEGYLG
AIGAFLKGAEQDNPNQYSWGENYAASSGLMSTAPELCPTQRARSGTFDLLEMDRLERPLV
NLPLLLDPSSYVPDTVDLTDDALARQYWLTCFEEALDGVVKRAVASQPESVDAAERAEKF
RQKYWGKLQTLRHQPFAYGTLTVRSLLDTREHCLNEFNFPDPYSKVKQKENGLALKCFQS
VTRSLDSLGWEERQLALVKGLLAGNVFDWGAKAVSDVLESDPQFGFEEAKRKLQERPWLV
DSYTKWLQRLKGPPHKCALIFADNSGIDIILGVFPFVRELLCRGIEVILACNSGPALNDV
TYSESLIVAERIAAMDPIICTALREDRLLLVQTGSSPPCLDLSRLDKGLAVLVRERGADL
VVIEGMGRAVHTNYHALLRCESLKLAVVKNAWLAERLGGQLFSVIFKYEVPAE
>P87112
MKSQEQQTPETREKSSSTSESGHPISLDSKTPPPNDSKIESIVKAQILFLLSTLREDQYD
TKLEQIRQLINKNAPRVYHHFLRRLIQGNSYRIFGTGKSSDGLATYKLLLDELKSLTKSW
VMAKRFSDAISGSDSEVFEDFDIEAFLDHFQFSPLERTNLLIGLTTSVKPTLAKKASALL
KNTFQSLLNQLSDPTQQQSIDKNDLDLLREGLFTTDYDRIPLIKVPKFDAIIEKKLANSR
PLTALFGPMKASKATLKSMKEAILKKYPPGTATETDVAYLINSLVAIHDYGAWDTVLMGK
AIVSSFENLNWEAMLSMFDNPKFMITGTPSLVLFFTIFTNAYKLRQTNFTLDFLWVLWRN
PLPQLSIISHIILSPTSMFDIREFNVNPVVSVDSLKDYSEELVNYAKIYEKSNLNCIELV
QILLRLLSEVVTYETSLFLNFLDEKVSAELLLLGTLQLPLAWNPVQESLAFQWCSDFFSN
FKEHKFVFVRIERTNPQFLFAFLRSLWLKDSSSVNQVVEFIIENDFTSHIGNIQPNRFAL
EIAALAAARKALSFQDWLDKKMLEFEDADGLNVFLVEVLDFLMSRAALQKNESEQKEESV

VLSIDTVNVLLTTLMDNVSISEEVSEHIKDVQILCLQVYPRLFSLGHDRDSIVIATNTTN
SFSPDVESEVESYFQALYERRISIGKIVLTLQNFKKSENPRDLDLFACLQHSLFDEYRFF
PDYPLEALALTAVLFGSLIQFELLSFVPLGVALRYVYQALLMPTDSKMFRFGMQALVQFQ
EKLPKYINYCNLILGIPSLQLVRPDIYDSIREMIASNENTEVEKDKLDSFSALANPASPK
APEYTFVSIHVPPLNVPNAEGEIEESVCDNILFAINNLSQLNFNEKLKDVKDNLTPAYLP
WFSHYIVTQRVSREANFLSLYGKFLEELKSSDLYKIVFRDTLQAILDIMNNNAETLSPSE
KTNLKNLGSWLGSITLLRNKPITTLQVSFKDLLLEGIDSGRIDRVLPFVCKVLEKASSSV
IFKPPNPWLMGILKLLVELYQFADFRLNLKFEIELLLNNLNVKMDNIEPSEMYRNHLVQK
ADLEKELPEDVLNAEFPDGTDVITQYIVAASSQITVTDAVAQVFGKPKPAIKNITQLIIQ
QSVLEIISAVVRRSVGIAAITTKSLLQKDFAAESNPSRLLLAARQMAKTLAGNLAMVTCR
EPLQMLMINNFRTIALQDVENVHAAVAQAIDELVSQNLFVASSVIESVASETAIAEIDAE
IEHMIVERVRHRKTTPNLPFVDPAGAANLHLNLPSVLKLSSELTPQQFQLYENFDRLSLS
TIMSNNSFTSLNGLRTDSADSTDALNSNLNNTVENEANQTALNYARNLLIIIGQLFQLAA
QMPYTSMQEVPADHELHELVNQFLTGVASINHPIADHVFLLCAQECCRILLTDSKSPFIL
EVFSAILEYICQASTKTAINVSLYWNFSNDLEKLNLPIILSLIRFGILTSGEVDYHVARG
VRSEQGSGPVTDFAIELLRTAVGGENPMALPGNFVNSITSLYEISESFSGETKQAYEQLV
ETMNKSVSPASEILSDLDNKQQLNDQIIIVFVSWVHLLRNSATNDETKAAFVYQLHKQGI
LSEPELCIQFFRCNLEAVLVAFLEAASVNAPDYFNVDAYASLLVNVVKYTEGSTEGTVSS
KSVLFRKIIALIIGVFAELHNSMAEFVHQKTFFRLFSSILSELDDAKDVLESCFVDIYSV
ILECFLAIQPRSFPAFTFAWLSLISHSYLLPKVLLVNNDKINDLFSEILMSFLKFLDLKD
DIDKAQFKLLYNGFLRIILVLLHDFPGFLATHCYQLIPYIPLECVQLRNMVLSAFPSDLH
LPDPFAQGLKVGRLPEVTRAPLISNSVSASLEKFASALDLEACFSSSKPAEVAKLLLEVY
SSQDTPTKLNEWANYFMLCLIRHATRDSPAKQAPQFQSKSPECVIISTMNRSCDSKCRYF
LLTAIANQLRYPSSHTYYASCCFLYLFKSSSNNPQELLIKEQMTTVLLERIICNRPHPWG
LLITFTELLKNEDYNFWKHPYIKRNDEICRLFDSLHEHVMAPSSANNVSTEEATELMGQV

>P19752
MALFPRGFYGSYGSDPSFTNLFRLLDDFDTYTREVQGSAPETGSRRHTQPTRTFSPKFDV
RETEQTYELHGELPGIDRDNVQIEFTDPQTIVIRGRVERNYTAGTPPAQVAGVLTEKGEP
HSPAAHHATVEDDVDEDNRSVATTATGANNQNNQQVAQRASAPTTEEKPKAPAEKYWVSE
RSIGEFSRTFNFPGRVDQNAVSASLNNGILTITVPKAKKHETIRIAIN
>Q9H832
MAESPTEEAATAGAGAAGPGASSVAGVVGVSGSGGGFGPPFLPDVWAAAAAAGGAGGPGS
GLAPLPGLPPSAAAHGAALLSHWDPTLSSDWDGERTAPQCLLRIKRDIMSIYKEPPPGMF
VVPDTVDMTKIHALITGPFDTPYEGGFFLFVFRCPPDYPIHPPRVKLMTTGNNTVRFNPN
FYRNGKVCLSILGTWTGPAWSPAQSISSVLISIQSLMTENPYHNEPGFEQERHPGDSKNY
NECIRHETIRVAVCDMMEGKCPCPEPLRGVMEKSFLEYYDFYEVACKDRLHLQGQTMQDP
FGEKRGHFDYQSLLMRLGLIRQKVLERLHNENAEMDSDSSSSGTETDLHGSLRV
>P06537
MDSKESLAPPGRDEVPSSLLGRGRGSVMDLYKTLRGGATVKVSASSPSVAAASQADSKQQ
RILLDFSKGSASNAQQQQQQQQPQPDLSKAVSLSMGLYMGETETKVMGNDLGYPQQGQLG
LSSGETDFRLLEESIANLNRSTSRPENPKSSTPAAGCATPTEKEFPQTHSDPSSEQQNRK
SQPGTNGGSVKLYTTDQSTFDILQDLEFSAGSPGKETNESPWRSDLLIDENLLSPLAGED
DPFLLEGDVNEDCKPLILPDTKPKIQDTGDTILSSPSSVALPQVKTEKDDFIELCTPGVI

KQEKLGPVYCQASFSGTNIIGNKMSAISVHGVSTSGGQMYHYDMNTASLSQQQDQKPVFN
VIPPIPVGSENWNRCQGSGEDNLTSLGAMNFAGRSVFSNGYSSPGMRPDVSSPPSSSSTA
TGPPPKLCLVCSDEASVCHYGVLTCGSCKVFFKRAVEGQHNYLCAGRNDCIIDKIRRKNC
PACRYRKCLQAGMNLEARKTKKKIKGIQQATAGVSQDTSENANKTIVPAALPQLTPTLVS
LLEVIEPEVLYAGYDSSVPDSAWRIMTTLNMLGGRQVIAAVKWAKAIPGFRNLHLDDQMT
LLQYSWMFLMAFALGWRSYRQASGNLLCFAPDLIINEQRMTLPCMYDQCKHMLFISTELQ
RLQVSYEEYLCMKTLLLLSSVPKEGLKSQELFDEIRMTYIKELGKAIVKREGNSSQNWQR
FYQLTKLLDSMHDVVENLLSYCFQTFLDKSMSIEFPEMLAEIITNQIPKYSNGNIKKLLF
HQK
>Q95ZG4
MNSTFVQIPKLNINKQVKKKTDSITANTLLPKLMNDTSIQQPNQQQQQQQPQQPQQVTNG
ITILQNYQPQQPQPATTTTQQLNSANNTPTLVSTKKSVIGINEIPEHLLIRDIIYVFQGI
DGTYIKYNKQSDSFKIDENTSNTLVNGEPAYISKPKRDLVYRLCEFGWLFKKVRLFITNN
DFKKTGLTNQSFCSAINDELIELYRIIAILETQVYKKFDMVNYGGGGGGSGGSGSGSGLE
SPSSVSSGGTTTSTEIPFIDGDSLTLIRLFVWIQSPLKRLKVLGTCVDSITVDMKGGEIL
SKIDTLSKHGDQDIRILIHNIMFKICQPLFSMIRLWMFKGEINDPYQEFFIRQYESVQLE
KTWKEKFAIVARLLPSFISLPLSKRILIIGKSINYMKQFCNNFKEDKNDRYYYYNQEDDD
DDDEDHDDNDDDDENENQGEDDEIIERKLLIKESKIIKEKTKELNYINKEVLQEIIELVS
RQSSERLLKIVLNRFKFMNHVKALKKYLLLGQGDFIQYLMDLIGEDLLKPTSQIQRHKLV
GWMDTAIRNSNAQFEEQDIVNRLDIALLPERPGNIGWDIFSLDYHVDTPLNTILSPNDIL
RYKKIFHFMWGIKRVEYSLASIWRKIRSSTSLSILSPIGGDIHKSHLIMNEMVHFISNFQ
YYLMFEVLECSWKNLEKFIDQEATDLDQLIEAHHQYLQDICNKMFLSNSDSCYECFKKLL
SIIIKFTLLQTKLINLSIAIQNEKNFNETHQAQVNKEFNSFRNHLNNLYQEYTTSFYKFQ
SEILKVKVNQDLNPISLQYMLDFNEYYEEKKDN
>Q96KG9
MWFFARDPVRDFPFELIPEPPEGGLPGPWALHRGRKKATGSPVSIFVYDVKPGAEEQTQV
AKAAFKRFKTLRHPNILAYIDGLETEKCLHVVTEAVTPLGIYLKARVEAGGLKELEISWG
LHQIVKALSFLVNDCSLIHNNVCMAAVFVDRAGEWKLGGLDYMYSAQGNGGGPPRKGIPE
LEQYDPPELADSSGRVVREKWSADMWRLGCLIWEVFNGPLPRAAALRNPGKIPKTLVPHY
CELVGANPKVRPNPARFLQNCRAPGGFMSNRFVETNLFLEEIQIKEPAEKQKFFQELSKS
LDAFPEDFCRHKVLPQLLTAFEFGNAGAVVLTPLFKVGKFLSAEEYQQKIIPVVVKMFSS
TDRAMRIRLLQQMEQFIQYLDEPTVNTQIFPHVVHGFLDTNPAIREQTVKSMLLLAPKLN
EANLNVELMKHFARLQAKDEQGPIRCNTTVCLGKIGSYLSASTRHRVLTSAFSRATRDPF
APSRVAGVLGFAATHNLYSMNDCAQKILPVLCGLTVDPEKSVRDQAFKAIRSFLSKLESV
SEDPTQLEEVEKDVHAASSPGMGGAAASWAGWAVTGVSSLTSKLIRSHPTTAPTETNIPQ
RPTPEGVPAPAPTPVPATPTTSGHWETQEEDKDTAEDSSTADRWDDEDWGSLEQEAESVL
AQQDDWSTGGQVSRASQVSNSDHKSSKSPESDWSSWEAEGSWEQGWQEPSSQEPPPDGTR
LASEYNWGGPESSDKGDPFATLSARPSTQPRPDSWGEDNWEGLETDSRQVKAELARKKRE
ERRREMEAKRAERKVAKGPMKLGARKLD
>P32893
MECFVPLRCDLDGSNIEQLRQSHLSRKFIIFDEQLNLWLWFQGNSQENKRFVLQNMIILI
NEAQVTRTSTIDDYFTQVENNENLWRLKNDCCSKILFKSNVVMNNGYNNQIKFVFEYKSV
DANFNNQDSLQDPQAKYTLDKYSSEEILPSFEPVYSWSSAATKSSKNTNNHLEKNNRATH
RVSSKNSEVHEADVSRNPNTFTLKLQYPIFSLLNMRLRNISLKSEHCILSSLDFQTSKAS

EQLTKKFIYPQEHNSFLKLNFQEISYKLIDGTSQIELDPICPLKVPLTAFSYDSISATFK
LVLLPKSTQPHRVKITLAYELELHPNLKLPVRTSWETEVTLKRSMPISSTSSQYSSNNNN
TNHSASFNGAANNVNSGGLANLRLGGVSSSRFSLGAASTTSLVNSKLSNVKFKFINSNIK
VIKGEKFTMRLQIINSSSSPLDLVVYYNNTINPIPSANNVRNSNGINNCGMNNGTIPNSP
LTLENQYQLHNKYRKIAEGIILLSNDYKIPVVPPRETYFADLRFIGIMSGYYGTLSGLKV
LDLNTNELIEVGNGASVLIQ
>Q99JL1
MESSVDEEALHQLYLWVDNIPLSRPKRNLSRDFSDGVLVAELIKFYFPKMVEMHNYVPAN
SLQQKLSNWGHLNRKVLNKLNFSVPDDVMRKIAQCSPGVVELVLIPLRQRLEERQRRQKL
GVGSLQELAPQDSSGYMDMGLPQKVRGEGAPALGEQLREGRPLASRPPGYNQALQGDPSF
VLQIAEKEQELLASQETVQVLQMKVKRLEHLLQLKNVRIDDLSRRLQQAERKQR
>Q76I90
MEALTFPRYSPDDIVAYLRSHVLVGAEARNLTKADLFATLKPEVLHMIFIRILQKVYGIR
LEHFYMMPVNVDIVYPQIFEGFLPVCNLYIHMERFLPVCRVNDFQMSDVINPKAKRTARF
LSGILNFVHFRECRREAYLELQLNYKSAMEKHQQLETANQELEMKLEKLNTVPVEQQAEF
KQLSDDIQELEQLLSHDYRRKTAALQEVISQKKSDITERTRKLNELKVTMATLKEEQEQL
KSKIVESPEELKNYKELMKETVKKLKKSKQEVIEKYEGYRDLVEVLPSCQLEVQLYQKKM
ERQAANVERLASVLSEVRNLEDQLESAQIELKKGKTDEMSLKRLVTAKHERLSTAEIRIK
KKREDVEQYKHTVFEYFNRVQEKRGVVYDKVTAIQKEIQQMRFKIQQLNESAEEEEMKAK
EIYQNLKAGLEKRHDSLIKTAKNYAASREDKIAELQKGLLRVQSPGSSS
>P53303
MSEQKEDLFKPVGEAAAEVEDESIAEQNKANDGVKLTGAQDAMGHPVQEIESLCMNCGKN
GTTRLLLTSIPYFREIIIMSFDCPHCGFKNCEIQPASQIQEKGSRYVLKVECREDFNRQV
IKSETATCKFVELDIEIPAKRGQLTTVEGLLSEMIDDLSQDQEMRKSIDEALYKKIDDFI
QKVKSYINCEPNTIPITFILDDPAGNSWIEYKPGEPQHKWSHTQYVRTDEQNVQVGIITR
DQLEQRRQEQLKQLANRERNPSESVKVGSANPQFLSDATDIENFNNEVQTFRASCPSCTQ
ECETHMKPVNIPHFKEVIIMSTVCDHCGYKSNEVKTGGAIPDKGRRITLYCDDAADLSRD
ILKSETCSMVIPELHLDIQEGTLGGRFTTLEGLLRQVYEELESRIFTQTSDSMDEATKAR
WVEFFAKLKEAIAGKVKFTVIMEDPLAGSYIQNVYAPDPDPNMTIEDYERTKEQNEDLGL
SDIKVE
>O75665
MMAQSNMFTVADVLSQDELRKKLYQTFKDRGILDTLKTQLRNQLIHELMHPVLSGELQPR
SISVEGSSLLIGASNSLVADHLQRCGYEYSLSVFFPESGLAKEKVFTMQDLLQLIKINPT
SSLYKSLVSGSDKENQKGFLMHFLKELAEYHQAKESCNMETQTSSTFNRDSLAEKLQLID
DQFADAYPQRIKFESLEIKLNEYKREIEEQLRAEMCQKLKFFKDTEIAKIKMEAKKKYEK
ELTMFQNDFEKACQAKSEALVLREKSTLERIHKHQEIETKEIYAQRQLLLKDMDLLRGRE
AELKQRVEAFELNQKLQEEKHKSITEALRRQEQNIKSFEETYDRKLKNELLKYQLELKDD
YIIRTNRLIEDERKNKEKAVHLQEELIAINSKKEELNQSVNRVKELELELESVKAQSLAI
TKQNHMLNEKVKEMSDYSLLKEEKLELLAQNKLLKQQLEESRNENLRLLNRLAQPAPELA
VFQKELRKAEKAIVVEHEEFESCRQALHKQLQDEIEHSAQLKAQILGYKASVKSLTTQVA
DLKLQLKQTQTALENEVYCNPKQSVIDRSVNGLINGNVVPCNGEISGDFLNNPFKQENVL
ARMVASRITNYPTAWVEGSSPDSDLEFVANTKARVKELQQEAERLEKAFRSYHRRVIKNS
AKSPLAAKSPPSLHLLEAFKNITSSSPERHIFGEDRVVSEQPQVGTLEERNDVVEALTGS
AASRLRGGTSSRRLSSTPLPKAKRSLESEMYLEGLGRSHIASPSPCPDRMPLPSPTESRH

SLSIPPVSSPPEQKVGLYRRQTELQDKSEFSDVDKLAFKDNEEFESSFESAGNMPRQLEM
GGLSPAGDMSHVDAAAAAVPLSYQHPSVDQKQIEEQKEEEKIREQQVKERRQREERRQSN
LQEVLERERRELEKLYQERKMIEESLKIKIKKELEMENELEMSNQEIKDKSAHSENPLEK
YMKIIQQEQDQESADKSSKKMVQEGSLVDTLQSSDKVESLTGFSHEELDDSW
>P07884
MLKGPLKGCLNMSKKVIVIAGTTGVGKSQLSIQLAQKFNGEVINSDSMQVYKDIPIITNK
HPLQEREGIPHHVMNHVDWSEEYYSHRFETECMNAIEDIHRRGKIPIVVGGTHYYLQTLF
NKRVDTKSSERKLTRKQLDILESTDPDVIYNTLVKCDPDIATKYHPNDYRRVQRMLEIYY
KTGKKPSETFNEQKITLKFDTLFLWLYSKPEPLFQRLDDRVDDMLERGALQEIKQLYEYY
SQNKFTPEQCENGVWQVIGFKEFLPWLTGKTDDNTVKLEDCIERMKTRTRQYAKRQVKWI
KKMLIPDIKGDIYLLDATDLSQWDTNASQRAIAISNDFISNRPIKQERAPKALEELLSKG
ETTMKKLDDWTHYTCNVCRNADGKNVVAIGEKYWKIHLGSRRHKSNLKRNTRQADFEKWK
INKKETVE
>O74423
MAFSALAYNLLAKNFSKGYTDTQIKVRNATTNDSWGPSGTAMAEIAELTYDQNEMLEVMD
IIDRRLNDKGKNWRHVFKSLSLLEYCLHNGSENVVRWAKDNIYIITTLREFVYVDDNGHD
QGQNVRTKAKEITSLLEDEHALKEARGDSRERDRDRDRTRSSRFDDDDDDRAPYEESRLS
RAPSRASRYDDDDRDHRSRRRSRSRRPGRSRSRRRSRRPSPSAEHNSAEENDPELQRVIE
ESKRQAEEDAKRRNMANDSEAELQKAIQLSKEEDEARQRHQREREQQEQAFMGNQQNAYQ
PVDFFGNPVQPQPTGFLQQQPTGFIRPQNTGFVQPQYTGFVQPQHTGFVQPQATGFMQPQ
RTGFVQPQATGFVQPQATGFVQPQATGFMQPQRTGFVQPQATGFMQPQRTGFVQPQATGF
MQPQRTGFVQPQATGFIQPQRTGFVQPQQNGFFNPQPTGYMQPQRTGMMQPQRTGFSQPF
ESNNPFPVMQPQRTGFGQTPNAPMMAPNHTGYVHPQPTGLQRQTTGYTGNNNPYSRPLQS
QSTGILQQQQQQSAPRLEPTKTGSNNPFAQFSNLPSQSTAPATKPMKPVRTGDDRFSNIA
QAISTGNPMGTDSFGNIGLTRVPTQHTGSKFTNSAGQTIQAQATGNTHNPFQSQQATGYY
KQPMQQQQNMQQPYYNQQNYNYQNQQPMQGMQQQSMQPQVGSLIDL
>Q80Z36
MASKRKSTTPCMIPVKTMVLPGASTEAQPVEPLPEGPQQDLPSEAPEASSEAAPNPSSTD
GSALANGHRGTLDGYVYSCKECDFRSQDVTHFVGHMTSEHTDFNKDPHFVCTGCSFLAKT
PEGLSLHNAKCHSGEASFLWNVTKPDNHVVVEQSVPENASSSVLAGESTEGTEIIITKTP
IMKIMKGKAEAKKIHMLKENAPTQPGGEALPKPLAGETEGKEGDHTFINGATPVSQASAN
STKPPHTANGPLIGTVPVLPAGIAQFLSLQQPTVHPQHHPHQPLPTSKALPKVMIPLSSI
PTYNAAMDSNSFLKNSFHKFPYPTKAELCYLTVVTKYPEEQLKIWFTAQRLKQGISWSPE
EIEDARKKMFNTVIQSVPQPTITVLNTPLVASAGNVQHLIQAALPGHAVGQPEGTAGGLL
VTQPLMANGLQASSSSLPLTTASVPKPTAAPINTVCSNTTSAVKVVNAAQSLLTACPSIT
SQAFLDANIYKNKKSHEQLSALKGSFCRNQFPGQSEVEHLTKVTGLSTREVRKWFSDRRY
HCRNLKGTRAMVPGEHGSVLIDSVPEVPFPLSSKVPEVPCVPTATSLVSHPATKRQSWHQ
TPDFTPTKYKERAPEQLRVLESSFAQNPLPPEEELDRLRSETKMTRREIDGWFSERRKRV
NAEETKKADGHAPQEEAEGAEEEGRDEELASELRAPGENGSSEMFLSHTLAERKVSPIKI
NLKNLRVTEASGKSELPGMGMGVCEPEEDGLNKAVEQPPSRVSYKKTAQQRHLLRQLFVQ
TQWPSNQDYDSIMAQTGLPRPEVVRWFGDSRYALKNGQLKWYEDYKRGNFPPGLLVIAPG
NRELLQDYYMTHKMLCEEDLQTLCEKTQMSAQQVKQWFAEKMGEETRAVADTSSEDQGPG
HGEPVAVDKVLGDACAALSENSEAWEPSAPEAGSEPFDTSSPQSGRQLETD
>Q96Q15

MSRRAPGSRLSSGGTNYSRSWNDWQPRTDSASADPGNLKYSSSRDRGGSSSYGLQPSNSA
VVSRQRHDDTRVHADIQNDEKGGYSVNGGSGENTYGRKSLGQELRVNNVTSPEFTSVQHG
SRALATKDMRKSQERSMSYSDESRLSNLLRRITREDDRDRRLATVKQLKEFIQQPENKLV
LVKQLDNILAAVHDVLNESSKLLQELRQEGACCLGLLCASLSYEAEKIFKWIFSKFSSSA
KDEVKLLYLCATYKALETVGEKKAFSSVMQLVMTSLQSILENVDTPELLCKCVKCILLVA
RCYPHIFSTNFRDTVDILVGWHIDHTQKPSLTQQVSGWLQSLEPFWVADLAFSTTLLGQF
LEDMEAYAEDLSHVASGESVDEDVPPPSVSLPKLAALLRVFSTVVRSIGERFSPIRGPPI
TEAYVTDVLYRVMRCVTAANQVFFSEAVLTAANECVGVLLGSLDPSMTIHCDMVITYGLD
QLENCQTCGTDYIISVLNLLTLIVEQINTKLPSSFVEKLFIPSSKLLFLRYHKEKEVVAV
AHAVYQAVLSLKNIPVLETAYKLILGEMTCALNNLLHSLQLPEACSEIKHEAFKNHVFNV
DNAKFVVKFDLSALTTIGNAKNSLIGMWALSPTVFALLSKNLMIVHSDLAVHFPAIQYAV
LYTLYSHCTRHDHFISSSLSSASPSLFDGAVISTVTTATKKHFSIILNLLGILLKKDNLN
QDTRKLLMTWALEAAVLMRKSETYAPLFSLPSFHKFCKGLLANTLVEDVNICLQACSSLH
ALSSSLPDDLLQRCVDVCRVQLVHSGTRIRQAFGKLLKSIPLDVVLSNNNHTEIQEISLA
LRSHMSKAPSNTFHPQDFSDVISFILYGNSHRTGKDNWLERLFYSCQRLDKRDQSTIPRN
LLKTDAVLWQWAIWEAAQFTVLSKLRTPLGRAQDTFQTIEGIIRSLAAHTLNPDQDVSQW
TTADNDEGHGNNQLRLVLLLQYLENLEKLMYNAYEGCANALTSPPKVIRTFFYTNRQTCQ
DWLTRIRLSIMRVGLLAGQPAVTVRHGFDLLTEMKTTSLSQGNELEVTIMMVVEALCELH
CPEAIQGIAVWSSSIVGKNLLWINSVAQQAEGRFEKASVEYQEHLCAMTGVDCCISSFDK
SVLTLANAGRNSASPKHSLNGESRKTVLSKPTDSSPEVINYLGNKACEFYISIADWAAVQ
EWQNAIHDLKKSTSSTSLNLKADFNYIKSLSSFESGKFVECTEQLELLPGENINLLAGGS
KEKIDMKKLLPNMLSPDPRELQKSIEVQLLRSSVCLATALNPIEQDQKWQSITENVVKYL
KQTSRIAIGPLRLSTLTVSQSLPVLSTLQLYCSSALENTVSNRLSTEDCLIPLFSEALRS
CKQHDVRPWMQALRYTMYQNQLLEKIKEQTVPIRSHLMELGLTAAKFARKRGNVSLATRL
LAQCSEVQLGKTTTAQDLVQHFKKLSTQGQVDEKWGPELDIEKTKLLYTAGQSTHAMEML
SSCAISFCKSVKAEYAVAKSILTLAKWIQAEWKEISGQLKQVYRAQHQQNFTGLSTLSKN
ILTLIELPSVNTMEEEYPRIESESTVHIGVGEPDFILGQLYHLSSVQAPEVAKSWAALAS
WAYRWGRKVVDNASQGEGVRLLPREKSEVQNLLPDTITEEEKERIYGILGQAVCRPAGIQ
DEDITLQITESEDNEEDDMVDVIWRQLISSCPWLSELDESATEGVIKVWRKVVDRIFSLY
KLSCSAYFTFLKLNAGQIPLDEDDPRLHLSHRVEQSTDDMIVMATLRLLRLLVKHAGELR
QYLEHGLETTPTAPWRGIIPQLFSRLNHPEVYVRQSICNLLCRVAQDSPHLILYPAIVGT
ISLSSESQASGNKFSTAIPTLLGNIQGEELLVSECEGGSPPASQDSNKDEPKSGLNEDQA
MMQDCYSKIVDKLSSANPTMVLQVQMLVAELRRVTVLWDELWLGVLLQQHMYVLRRIQQL
EDEVKRVQNNNTLRKEEKIAIMREKHTALMKPIVFALEHVRSITAAPAETPHEKWFQDNY
GDAIENALEKLKTPLNPAKPGSSWIPFKEIMLSLQQRAQKRASYILRLEEISPWLAAMTN
TEIALPGEVSARDTVTIHSVGGTITILPTKTKPKKLLFLGSDGKSYPYLFKGLEDLHLDE
RIMQFLSIVNTMFATINRQETPRFHARHYSVTPLGTRSGLIQWVDGATPLFGLYKRWQQR
EAALQAQKAQDSYQTPQNPGIVPRPSELYYSKIGPALKTVGLSLDVSRRDWPLHVMKAVL
EELMEATPPNLLAKELWSSCTTPDEWWRVTQSYARSTAVMSMVGYIIGLGDRHLDNVLID
MTTGEVVHIDYNVCFEKGKSLRVPEKVPFRMTQNIETALGVTGVEGVFRLSCEQVLHIMR
RGRETLLTLLEAFVYDPLVDWTAGGEAGFAGAVYGGGGQQAESKQSKREMEREITRSLFS
SRVAEIKVNWFKNRDEMLVVLPKLDGSLDEYLSLQEQLTDVEKLQGKLLEEIEFLEGAEG
VDHPSHTLQHRYSEHTQLQTQQRAVQEAIQVKLNEFEQWITHYQAAFNNLEATQLASLLQ
EISTQMDLGPPSYVPATAFLQNAGQAHLISQCEQLEGEVGALLQQRRSVLRGCLEQLHHY

ATVALQYPKAIFQKHRIEQWKTWMEELICNTTVERCQELYRKYEMQYAPQPPPTVCQFIT
ATEMTLQRYAADINSRLIRQVERLKQEAVTVPVCEDQLKEIERCIKVFLHENGEEGSLSL
ASVIISALCTLTRRNLMMEGAASSAGEQLVDLTSRDGAWFLEELCSMSGNVTCLVQLLKQ
CHLVPQDLDIPNPMEASETVHLANGVYTSLQELNSNFRQIIFPEALRCLMKGEYTLESML
HELDGLIEQTTDGVPLQTLVESLQAYLRNAAMGLEEETHAHYIDVARLLHAQYGELIQPR
NGSVDETPKMSAGQMLLVAFDGMFAQVETAFSLLVEKLNKMEIPIAWRKIDIIREARSTQ
VNFFDDDNHRQVLEEIFFLKRLQTIKEFFRLCGTFSKTLSGSSSLEDQNTVNGPVQIVNV
KTLFRNSCFSEDQMAKPIKAFTADFVRQLLIGLPNQALGLTLCSFISALGVDIIAQVEAK
DFGAESKVSVDDLCKKAVEHNIQIGKFSQLVMNRATVLASSYDTAWKKHDLVRRLETSIS
SCKTSLQRVQLHIAMFQWQHEDLLINRPQAMSVTPPPRSAILTSMKKKLHTLSQIETSIA
TVQEKLAALESSIEQRLKWAGGANPALAPVLQDFEATIAERRNLVLKESQRASQVTFLCS
NIIHFESLRTRTAEALNLDAALFELIKRCQQMCSFASQFNSSVSELELRLLQRVDTGLEH
PIGSSEWLLSAHKQLTQDMSTQRAIQTEKEQQIETVCETIQNLVDNIKTVLTGHNRQLGD
VKHLLKAMAKDEEAALADGEDVPYENSVRQFLGEYKSWQDNIQTVLFTLVQAMGQVRSQE
HVEMLQEITPTLKELKTQSQSIYNNLVSFASPLVTDATNECSSPTSSATYQPSFAAAVRS
NTGQKTQPDVMSQNARKLIQKNLATSADTPPSTVPGTGKSVACSPKKAVRDPKTGKAVQE
RNSYAVSVWKRVKAKLEGRDVDPNRRMSVAEQVDYVIKEATNLDNLAQLYEGWTAWV
>P42286
MSTDIATQLKGSRSDVEKVRKTVEAKFRELSGDGLPLRYEVNVLRHICLALKDNLHQNSD
LYCDIMGIMLPRVVPCEEKPSLWEAHLSSLRYIHHGLFHQRSIEACQKLYNLIRQQPCRL
QEESDYKIYLDIHLTHFNGFHVLLQKQKLPLEATSQLCYALESLGDLFAAMTQRQISLCA
TLLVQLNESLFGKRSRSFFKSLSFLPSESLAKMFNALLMLLASSTSSNLANLFPECLSLT
LALVQIDMFSPQSNQQMSLQLLRMSKELFRQESNLCYALQLMYYYIKLIFVREPTGDFKR
TYIDLSSKFQHFFEHKVASHAKEQWLADFLVAIQLLQVLIHQSNSKLQSPFQIFWQQFDG
ESSPEIYTAHFQLLQTCASLAVNITRSPLGCSCSHEACKSVRRHCILAYGLCALDAYINW
KPAAEQRANVSPHKPLLGVVKYSMDVAKTMKCLGPTSVEIIKLVRQLTYVADQVTCPEQM
SVLLPLLEPLQKLRPLVADQDMSSLLRRLFKASSHCGDSNIACRIQASYLASITNPARLR
SQVCLYYHNLGKKGTEIKRCVYEWHESTPLPFPLTPDQKKQLYDTDFFALLHYLRSPSTA
HMESLIRCRTSDYHLVLLARQMRKDDSISKKCIEVHDKLRQQRSLSRMDNLCLGHASVGL
LLDALEAQKTKVSTKEITENMFEELLLSKNLWQMNIQREQRLVNYASEAISAFSNFFDRA
DQEPLSANETSIDWEALIDDAIATANALSSMGYQSEEDDAWLLLLRMGRLLEDRFTYLRA
LNHFLSQNEVSSRLNLKLGEEVEVAEELLDDLWPQLKNGKFFKRQQTTVMLCFCHLASYY
ARMECYSHAQLLLLHVEQLREEFPERQGKSDIVLLTLQTVRFRIGYQQRKPTNCRLPTPL
RQLDILLDNVRSFCNLSSLDGGSLQLLLSTLVRESTECSANRLSERLSFSNIALHLVLQS
GLALRAIEVFLAWLWTNLQMESFDKAQSKLRLIEHCLGIKQLNPTSRPEKEAIKDVAISD
LASNMHLLQLVEPIRKQQLLNMASPNLLKMRPHSPNPQLDLDRYITLDVAPANLRENSQL
QCLYFVTGCLHARLRFLQRNSEQLEEFYGRAHNWMQEKPPMSSALYPMLHAQQLYHLNYL
RFARKHVEAISTAQLGLKMRSRAVDINFEYNFLAQLKTAQLELKPVGQDKPQVKILRRAL
VFNHSPEDKKRTATGSVSAVKNTASKVKQSAKKAPRFRIYEELELRPPSATSCSSSGGSG
TENTPPSDHVDLNACQAIEISDDDDSPLVSTKKTQPKSREKAKPKATSKACKVLTLDNSL
EIVETPTITTSTRSTRARLRQPVETPKTATLSSKRTRRQVLEAQAPETESISTRTRHRH
>P07866
MEIFSQKDYYPTPSSNVISYESDCVSKPVNSADLPALIVHLSSPLEGVDYNASADFFLIY
RNFITPQDLHDLLIYRFRWCIREITTNAAKAKRRRIGEVALVRTFVLLRHSILNYFVQDF

LPNITLRLRLIEFLNDKHIEQYPKIISSCIINLKKNWVHCSKLVWENIELNEPDKLDFDA
WLHYSLKDFTQLESLHKRGSRLSIYARQSFASPDFRNQSVLSLYKTSDVFRLPEKLQSSN
SSKNQRSPSMLLFPDNTSNVYSKHRIAKEPSVDNESEDMSDSKQKISHLSKVTLVSTLMK
GVDYPSSYAVDKIMPPTPAKKVEFILNSLYIPEDLNEQSGTLQGTSTTSSLDNNSNSNSR
SNTSSMSVLHRSAIGLLAKWMKNHNRHDSSNDKKFMSAIKPANQKPEMDAFVKYVVSISS
LNRKSSKEEEEEFLNSDSSKFDILSARTIDEVESLLHLQNQLIEKVQTHSNNNRGPTVNV
DCERREHIHDIKILQQNSFKPSNDNFSAMDNLDLYQTVSSIAQSVISLTNTLNKQLQNNE
SNMQPSPSYDALQRRKVKSLTTAYYNKMHGSYSAESMRLFDKDNSSSRTDENGPQRLLFH
ETDKTNSEAITNMTPRRKNHSQSQKSMTSSPLKNVLPDLKESSPLNDSREDTESITYSYD
SELSSSSPPRDTVTKKSRKVRNIVNNTDSPTLKTKTGFLNLREFTFEDTKSLDEKKSTID
GLEKNYDNKENQESEYESTKKLDNSLDASSEANNYDITTRKKHSSCNHKIKQAVVRPASG
RISISRVQSIAITPTKELSIVDPEQNKSNSVIEEISEIEPLNLEYNKKSALYSDTSSTVI
SISTSKLFESAQNSPLKQTQNPQREFPNGTSVSETNRIRLSIAPTIESVVSDLNSITTGS
TVETFETSRDLPVPHQRIINLREEYQRGNQDIISNTSSLHELKTIDLSDSNNDLESPSTH
AKNNKYFFSPDDGSIDVASPMKNVEELKSKFLKNESETNSNISGSVLTMDDIDINDTSSA
RNTRRANSESAFTGSLNKKNLNEIANMLDDSINDDPITVALMKLEGTYEKIPEKPENTKS
SDAIGIKTSKLADEVEMLNLNNLPSFQNSPAEKRKSLLIERRRQTIMNIPFTPDQSEKEG
FTSSSPEKIDVSANVDVAVQAAQIQELIGQYRIHDSRLMISNNESHVPFILMYDSLSVAQ
QMTLIEKEILGEIDWKDLLDLKMKHEGPQVISWLQLLVRNETLSGIDLAISRFNLTVDWI
ISEILLTKSSKMKRNVIQRFIHVADHCRTFQNFNTLMEIILALSSSVVQKFTDAWRLIEP
GDLLTWEELKKIPSLDRNYSTIRNLLNSVNPLVGCVPFIVVYLSDLSANAEKKDWILEDK
VVNYNKFDTNVQIVKNFIQRVQWSKFYTFKVNHELLSKCVYISTLTQEEINELST
>O42227
MALSQGTKKKVCYYYDGDVGNYYYGQGHPMKPHRIRMTHNLLLNYGLYRKMEIYRPHKAS
AEEMTKYHSDDYIKFLRSIRPDNMSEYSKQMQRFNVGEDCPVFDGLFEFCQLSTGGSVAS
AVKLNKQQTDISVNWSGGLHHAKKSEASGFCYVNDIVLAILELLKYHQRVVYIDIDIHHG
DGVEEAFYTTDRVMSVSFHKYGEYFPGTGDLRDIGAGKGKYYAVNYPLRDGIDDESYEAI
FKPVMTKVMEMFQPSAVVLQCGADSLSGDRLGCFNLTIKGHAKCVEFIKTFNLPMLMLGG
GGYTIRNVARCWTYETAVALDSEIPNELPYNDYFEYFGPDFKLHISPSNMTNQNTNEYLE
KIKQRLFENLRMLPHAPGVQMQAIPEDSVHDDSGEEDEEDPDKRISIRSSDKRIACDEEF
SDSEDEGEGGRKNVANFKKVKRVKTEEEKEGEDKKDVKEEEKAKDEKTDSKRVKEETKSV

>Q9Z206
MEPEPAAQKQPRPRRRSRRVSMLSEEPAAGLPADTPGPAANERCSLRRGSSFTFLTPGPH
WDFTLKRKRREKDDDAVSLSSLDLKEPSNKRVRPLARVTSLANLISPVRNGAVRRFGQTI
QSFTLRGDHRSPASAQKSFSRSTVPTPTKRRSSALWSEMLDINMKESLTTREIKRQEAIY
ELSRGEQDLIEDLKLARKAYHDPMLKLSIMSEEELTHIFGDLDAYIPLHEDLLARIGEAT
KPDGTVEQIGHILVNWLPGLNAYRGYCSNQLAAKALLDQKKQDPRVQDFLQRCLESPFSR
KLDLWSFLDIPRSRLVKYPLLLKEILRHTPKDHRDVQLLEEAILIIQGVLSDINLKKGES
ECQYYINKLEYLDEKQKDPRIEASKVLLCHGELKNKSGHKLYIFLFQDILVLTRPVTRNE
RHLYQVYRQPIPVQELVLEDLQDGDVRMGGSFRGAFGNSDKAKNIFRVRFQDPSPGHSHT
LQANDVFHKQQWFNCIRAAIAPFQRAASPLELQGLPDLHEECEENNPSAGNLRAQRRSCV
VPGVMQIDEESALDCGSSVQTVEDTRNMKAQRPQPGLRRARDKAQSGGKKKETLV
>Q2EMV9

MAASGSFPLLVEGSWGPDPPKNLINKLQVYFQSRKKSGGGECEVVPEPGNPARFRVLFSP
EDVRQNVLERGNHELVWQEKGTFKLTVLMPTDPEEASASKKSRKESPEEESKTKEDAVKQ
GDLDITHSPSSGSEKTEDVPKECENISSMVAFENLPEKVSEMVLTILVENISGLPSDDFK
VEVNRDFAVAVVTFQKPIDIKKFIVDCISHRSNQQLQLAPRLLETTNVVRVENLPPGVDE
YQLQLFFENPFNGGGRVARVECFPEESSALVEFCDSKVLDTVMAKTHSYNKMPLSVFPYY
PSLGTALYGEEKPLIKLPASFQESLDLPLWKFFQKNNHLIEEINNEMRCCHCELTWSEIN
GKLTIRPAATLVNHRLSIKTWQRDASAVLSGIKSKYGVELFEVCSPVWDIIKHELESGDD
RVLVEFEKESLNIAGKSEDVQGMSQKIRELIESTTEKLRREEQSLKEKVAISPGKHYLLH
HSGFLKDLSKGFPEMEISYDATAQFLYLKGFRADVYKVKCDIQEKVFSMAQKDVQVSSEV
FEFLQQVDSQRLSKSLFEAQNILAIYELKGTALFLVGSSFKDLAEAETKMLSALSHKQIE
VEDKEVLISNGWKKKVHPLQKRHSSCATIIVQNELTSETPAKVIVTGCVKEVNEIHRQLF
EYLENNMKVERALKIKPSLIVDYLRTDKRLLSKIKKAHVYVHFKPKDYPNSILLTGCKSK
VLECMNLVKEIQDSVCVQRFQTDKAGVRHFFKDKESYYKTEIGRQFGCVIELEEDREEKG
EEEDGEEEEGEEEGESSINEQKCHLQRDIAPGVKLFVLEEDLSRFPVDVVVNAANENLKH
ISGLAQALSKAAGPELQTECDQIVKEGGVVLPGNAVISKAGKLPCHHVIHAVGPRWKGDK
VLECVSLLKKVVRQSLSLAEEHRCRSIAMPAVSAGIFDFPLELCVANIVSAIKEHFQHKR
DTHTLKKIYLVGLPAKVARAFAEAVKTTYKDSLSHTAFPSSLKALVPLGKTPQKQGSLLV
SPEGLRIRLVEEGVQNATTHAIVNSISPDLKLNKGPLSQAFLEKAGPKLQEELTRSGQGV
SVDVGTILQTSGCNLNSRHVFHVVPPPWKSNNSAWSLKIMKNIIRDCLKTTENLSLQSIA
FPAIGTGNLRFPKPEFAKLIISEVLKFSSRNQLKTLQEVQFLLHPKDHENIQAFSDEFDK
RNNGDPSDKNPKAEDTQGIYGSLSSPTLGMHEMNIGPILFQVATGNIIKEVADVIVNSTT
LTFDLKSGVSKAILEGAGQNVEQECSLLAKQSNHGYIVTGGGLLQCKNIIHVVGGNDVKK
SVSCVLEECEQRNYSSICLPAIGTGNAQQDPNVVAKAIIDAIEEFVQKKSVQAVKRVKVV
IFQPHILQFFYDNMKEREGSPAPPKQSPAKQSVMSKIASFLGFPKQASPKKNTLVLEKKI
EHTVFQVCGSGVDSVNKTISWLKELITKEQLSYTNDDECVSDFDMEEYEKLNEIQKELNI
TIEMNQKKTSIQVSGISRDVIKARDEIEGMIKSIRLAKEKESQADYISTYVEWQYIDKNI
TQCFDKMTNMKLEVAWKAKKKDTVVQIHNQDFTVDLSTNTATAPQGQTFTVQRLVKAEAE
IPANWSDMKQDKLLLVNLQTSDPEYNMVASAFRQTCSNFFIEKIERIQNPALWRRYQAYK
KSMDEKNGNVRNEKHLFHGTEASSLPQLNSNGFNRSYAGKNATAYGKGTYFAVKASYSAC
DTYSRPDTNGRKYMYYVRVLTGNYTNGNASLIVPPSRDPQNAADLYDTVTDNDKNPSIFV
VFYDNQTYPEYLITFRQ
>Q07381
MAGHSHRSSLKNGHKSYKSKHASKGALKRLYKGKVEKEPVGTGKPDKQVSKLQRKNKAKQ
LRAQRILDSIENRKLFEGKNGAAKIITIVPLVNDLDPLDILYKLLKCADDEGIMVQEVDS
KRIFNVHIKKFKSNLKIIIPDMTNFLNILDCAKVADFVVFGLSGVQEVDEEFGEQIIRAL
ELQGIASYIGVISNLSAVHEKEKFQLDVKQSLESYFKHFFPSEERVYNLEKNSDALNVLR
TLCQRLPRSINWRDNRGYVVADFVDFVETSPDSGDLVIEGTVRGIGFNANRLVHIPDFGD
FQLNKIEKISESSQKRKIIKEKATDSLSLELDLQTVFESNMNRDTLDEYAPEGTEDWSDY
DEDFEYDGLTTARYDDHGFLPGREQTSKKAAVPKGTSDYQAKWYLDDVIDANEEEEAEQT
NGKDETMMEIDDEMMVEQDNEEVAGDEEYDIEDNEGFEELSPEEEERQLREFRDMEKEDR
EFPDEIELEPSESAIERLKRYRGLKNLYNCDWQVDEKDPSSPAEWKRLLRIGNYKNTKNR
IIKETKNEAQAIAGDRIRMFIRFPKFLLEKIQDPKQLLFAVYGLLLHEHKNAVVNFSLQR
WEQYDKPVPSQEPIVVQYGVRRYTIQPLFSQGSNSPNNVHKYERFLHPDTVSVATCIAPV
DFTQSPAIFFKPSPTDAKNIELIGHGTFLNADHSRILAKRAILTGHPFRFHKTVVTVRYM

FFRPEDVEWFKSIPLFTKSGRSGFIKESLGTHGYFKATFDGKLSAQDVVAMSLYKRMWPM
PSLPWNGM
>Q6GYP7
MFSKKPHGDVKKSTQKVLDTKKDALTRLKHLRIVIENAESIDLKQFFDQHFSHIYYVFFE
NFVTIEASLKQKGHKSQREELDAILFIFEKILQLLPERIHQRWQFHSIGLILKKLLHTGN
SLKIRREGVRLFLLWLQALQDNCSKEQLWMFSCLIPGFSAPQSEYGPRTLDNLINPPLSL
QETQVTIEEVTPLVPPQSGDKGQEDLTSYFLEALLKYIVIQVKSLEWKNKENQERGFSFL
FSHFKKFYLPYIFPNICKENSLYHPVLDIPQIRPKPHYVMIKKDAETNETIYCTKEPFIQ
ARVIVIRWLVSFWLEPKPHSGPNIPGMEGEVLPKNIQRAAASLVSREESKNDTVDKVDKS
AEPEQSHSNTSTLTEREPSSSSLCSIDEEHLTDIEIVRRVFSSKRSNVNFVTEIFRQAFL
LPICEAAAMRKVVKVYQEWIQQEEKPLFMQEPEDTAITCSDIPCSETVADHDSAIEDGEK
REEENGTSTSEHVRNSSWTKNGSYQEAFHVCEEATEQNIQAGTQAVLQVFIINSSNIFLL
EPANEIKNLLDEHTDMCKRILNIYRYMVVQVSMDKKTWEQMLLVLLRVTESVLKMSSQAF
LQFQGKKSMTLAGRLAGPLFQTLIVAWIKANLNVYISRELWDDLLSVLSSLTYWEELATE
WSLTMETLTKVLARNLYSLDLSDLPLDKLSEQKQKKHKGKGVGHEFQKVSVDKSFSRGWS
RDQPGQAPMRQRSATTTGSPGTEKARSIVRQKTVDIDDAQILPRSTRVRHFSQSEDTGNE
VFGALHEEQPLPRSSSTSDILEPFTVERAKVNKEDTSPKLPPLNSETGGNSANVPDLMDE
FIAERLRSGNASTMTRRGSSPGSLEIPKDLPDILNKQNQMRPVDDPGVPSEWTSPASAGS
SDLMSSDSHSDSFSAFQCEGRKFDNFGFGTDIGIPSSADVDLGSGHHQSTEEQEVASLTT
LHLDSETSSLNQQAFSAEVATVTGSESASPVHSALGSRSQTPSPSTLSRAHIEQKDLQLD
EKLHHSVLQTPDDLEISEFPSECCSVMAGGTLTGWHADVATVMWRRMLGILGDVNAIMDP
EIHAQVFDYLCELWQNLAKIRDNLGISADNLTSPSPPVLIPPLRILTPWLFKATMLTDKY
KQGKLHAYKLICNTMKRRQDVSPNRDFLTHFYNIMHCGLLHIDQDIVNTIIKHCSPQFFS
LGLPGATMLIMDFIIAAGRVASSAFLNAPRVEAQVLLGSLVCFPNLYCELPALHPNIPDI
AVSQFTDVKELIIKTVLSSARDEPSGPARCVALCSLGIWICEELVHESHHPQIKEALNVI
CVSLKFTNKTVAHVACNMLHMLVHYVPRLQIHQPQSPLKIIQILIATITHLLPSTEASSY
EMDKRLVVSLLLCLLDWIMALPLKTLLQPVHATGAENDKTEKSVLNCIYKVLHGCVYGAQ
SFSNPKYFPISLSDLASVDYDPFMHLESLKEPEPLHSPDSERSSKLQPVTEVKTQMQQGL
ISIAARTVITHLVNHLGHYPMSGGPAMLTSQVCENHDNHYSESTELSPELFESPNIQFFV
LNNTTLVSCIQIRSEESMPGGGLAAGLVSANSNVRIIVRDLSGKYSWDSAILYGPPIVSG
LPEPTSFILSMSDQEKPEEPPTSNECLEDIAVKDGLSLQLRRFRETVPTWSTIREEEDVL
DELLQYLGTTSPECLQRTGISLNVPAPQPLCISEKQENDVINAILKQYTEEKEFVEKHFN
DLNMKASEQDEPTPQKPQSAFYYCRLLLSILGMNSWDKRRSFHLLKKNEKLLRELRNLDS
RQCRETHKIAVFYVAEGQEDKYSILTNIGGSQAYEDFVAGLGWEVNLTNHCGFMGGLQKN
RSTGLTTPYFATSTVEVIFHVSTRMPSDSDDSLTKKLRHLGNDEVHIVWSEHTRDYRRGI
IPTEFGDVLIVIYPMKNHMFSIQIMKKPEVPFFGPLFDGAIVNGKVLPIMVRSTAINASR
ALKSLIPLYQNFYEERARYLQTIVQHHLEPTTFEDFAAQVFSPAPYHHFPADADH
>O95848
MERIEGASVGRCAASPYLRPLTLHYRQNGAQKSWDFMKTHDSVTVLLFNSSRRSLVLVKQ
FRPAVYAGEVERRFPGSLAAVDQDGPRELQPALPGSAGVTVELCAGLVDQPGLSLEEVAC
KEAWEECGYHLAPSDLRRVATYWSGVGLTGSRQTMFYTEVTDAQRSGPGGGLVEEGELIE
VVHLPLEGAQAFADDPDIPKTLGVIFGVSWFLSQVAPNLDLQ
>Q9UTD5
MSFKSQLLILAQKLSKGGISCELIEFDECILKLEWHTELLDKNDSLLYEQEEDDILSLMN

PMITMHAWIRDSPSFEVPQFFFQPYANGSDPLTKMEQIFELLEGSSQNLAYDALAIGDCP
GTVGIAWYIHPCRTRDYFEMLQIDKEDPKYLSLWLLYIHQVLSPLTQPIIKAVDDAEKS
>Q15555
MPGPTQTLSPNGENNNDIIQDNNGTIIPFRKHTVRGERSYSWGMAVNVYSTSITQETMSR
HDIIAWVNDIVSLNYTKVEQLCSGAAYCQFMDMLFPGCISLKKVKFQAKLEHEYIHNFKL
LQASFKRMNVDKVIPVEKLVKGRFQDNLDFIQWFKKFYDANYDGKEYDPVEARQGQDAIP
PPDPGEQIFNLPKKSHHANSPTAGAAKSSPAAKPGSTPSRPSSAKRASSSGSASKSDKDL
ETQVIQLNEQVHSLKLALEGVEKERDFYFGKLREIELLCQEHGQENDDLVQRLMDILYAS
EEHEGHTEEPEAEEQAHEQQPPQQEEY
>Q9ULZ0
MRASPCISQPAASWHPRPSALRPTAGSGPDTRTPGTVEDGSAPCPAFRSPAVSPCGEEPC
CFQISPAEETLELGRLVSPGNCDTLSPRAAGFYACHVRSLIPCRSTKGRWPLTASAAGLS
RHAQCGPSLGLG
>Q99216
MVAPTALKKATVTPVSGQDGGSSRIIGINNTESIDEDDDDDVLLDDSDNNTAKEEVEGEE
GSRKTHESKTVVVDDQGKPRFTSASKTQGNKIKFESRKIMVPPHRMTPLRNSWTKIYPPL
VEHLKLQVRMNLKTKSVELRTNPKFTTDPGALQKGADFIKAFTLGFDLDDSIALLRLDDL
YIETFEVKDVKTLTGDHLSRAIGRIAGKDGKTKFAIENATRTRIVLADSKIHILGGFTHI
RMARESVVSLILGSPPGKVYGNLRTVASRLKERY
>Q10740
MFLLPFVIRHSSSIYLPTLRFRGLLTVISRNIHISTPHKMLPLSIEQRRPSRSPEYDQST
LSNYKDFAVLHTDLNLSVSFEKSAISGSVTFQLKKLHEGKNKSDELHLDTSYLDVQEVHI
DGSKADFQIEQRKEPLGSRLVINNASCNDNFTLNIQFRTTDKCTALQWLNSKQTKGGKPY
VFSQLEAIHARSLFPCFDTPSVKSTFTASIESPLPVVFSGIRIEDTSKDTNIYRFEQKVP
IPAYLIGIASGDLSSAPIGPRSTVYTEPFRLKDCQWEFENDVEKFIQTAEKIIFEYEWGT
YDILVNVDSYPYGGMESPNMTFATPTLIAHDRSNIDVIAHELAHSWSGNLVTNCSWNHFW
LNEGWTVYLERRIIGAIHGEPTRHFSALIGWSDLQNSIDSMKDPERFSTLVQNLNDNTDP
DDAFSTVPYEKGFNLLFHLETILGGKAEFDPFIRHYFKKFAKKSLDTFQFLDTLYEFYPE
KKEILDSVDWETWLYKPGMPPRPHFITALADNVYQLADKWVEMAQHLKTTEDFRSEFNAI
DIKDFNSNQLVLFLETLTQNGHSNKKPKDFDWAKFPVASRALLDIYQDNIVKSQNAEVVF
KMFKFQIFAKLQEEYKHLADWLGTVGRMKFVRPGYRLLNSVDRRLALATFDKFKDTYHPI
CKALVKQDLGL
>Q96NE9
MNKLNFHNNRVMQDRRSVCIFLPNDESLNIIINVKILCHQLLVQVCDLLRLKDCHLFGLS
VIQNNEHVYMELSQKLYKYCPKEWKKEASKVRQYEVTWGIDQFGPPMIIHFRVQYYVENG
RLISDRAARYYYYWHLRKQVLHSQCVLREEAYFLLAAFALQADLGNFKRNKHYGKYFEPE
AYFPSWVVSKRGKDYILKHIPNMHKDQFALTASEAHLKYIKEAVRLDDVAVHYYRLYKDK
REIEASLTLGLTMRGIQIFQNLDEEKQLLYDFPWTNVGKLVFVGKKFEILPDGLPSARKL
IYYTGCPMRSRHLLQLLSNSHRLYMNLQPVLRHIRKLEENEEKKQYRESYISDNLDLDMD
QLEKRSRASGSSAGSMKHKRLSRHSTASHSSSHTSGIEADTKPRDTGPEDSYSSSAIHRK
LKTCSSMTSHGSSHTSGVESGGKDRLEEDLQDDEIEMLVDDPRDLEQMNEESLEVSPDMC
IYITEDMLMSRKLNGHSGLIVKEIGSSTSSSSETVVKLRGQSTDSLPQTICRKPKTSTDR
HSLSLDDIRLYQKDFLRIAGLCQDTAQSYTFGCGHELDEEGLYCNSCLAQQCINIQDAFP
VKRTSKYFSLDLTHDEVPEFVV

>O59779
MTATIIAAITITTIVIITPMEEITTMTIPMEEITTMTIPMEEITTMTIPMEEITTMTTPM
EEITTITTPMEETTTITPMVETTTILPMAAMTTPMVETTTIPTVETTTTPMVETTTITPM
VETTTITPMVEAMITLMEETMTTPMEETTTILPMAAMTTPMEETTTTTPMVETTTIPTVE
TMTTPMVEAMTILPMAAMTTPMEETTTTPMEETTTTPMVETMITPMVEAMTTPTEVVGRS
MVSTIRTTTPMEAMITPTVETTTLPTAAMTTPVEETTTTPMVETMITPTVVTTTTPMVET
MITPTVVTTTPMVILVMAVVMETTINIVGNNDSIPSQNFKSGKTFVSNANSSNSNDGSSS
KSLDVGSFVNAFKQLNVNDNSSNNNSSGNTDSSTIGYAAVFAAAGKFFSQHSCDLASGNK
SAQEGQNQFLSMVESEAKNLMNKTNYSANQSQNGNSQNSGNPVSQAIDLAKSLFQNRNLL
TKLAQSGVLTNAAQGSSGGLSAAAVSGLIGSFMGSSSNNSNSSNNNSNTSNNNSNTSNNE
SMVSKLSSLSGMASSFLGSSGNNNNNNNSNSGNYNNNNSGNNNQQHQQSSSSLQGLASSF
LNSSSGNSNKQNYNNNNNQNYGNNNNQNYNNNNNSSQGGNSQGGSGLLGQAVNMFLKS
>Q9HDZ0
MSGSCQFLSRETLSSSLGWIPLINALREIFTENVVCPTRLHYPIDEDNPSSTANNILLIM
PCWIPGKFLGVKQVNVFPENTKHGLPSLSSHYLLSDATTGCHLAQLDGNELTSRRTAAAS
ALASSYLSKEDSTSLLIIGSGKVAEKLIHAHCSVRPIRSVRIWNHRFESAKSLASRASKE
LPSIQIDAVPIQNLENAVKCSDIISSATLSKKPIIVGSWINPGTHIDLVGGFTPHMHEAD
SKCIQISNVFVDTRKGALHEAGDLLTPIKEGLFSPNEVIADLFDLCNNKHSGRSQLKNPA
EAITLFKSVGDSREDLAAASLAYKVHNKSS
>P13676
MERQVLLSEPQEAAALYRGLSRQPSLSAACLGPEVTTQYGGLYRTVHTEWTQRDLERMEN
IRFCRQYLVFHDGDSVVFAGPAGNSVETRGELLSRESPSGTMKAVLRKAGGTVSGEEKQF
LEVWEKNRKLKSFNLSALEKHGPVYEDDCFGCLSWSHSETHLLYVAEKKRPKAESFFQTK
ALDISASDDEMARPKKPDQAIKGDQFVFYEDWGETMVSKSIPVLCVLDIDSGNISVLEGV
PENVSPGQAFWAPGDTGVVFVGWWHEPFRLGIRYCTNRRSALYYVDLSGGKCELLSDGSL
AICSPRLSPDQCRIVYLQYPCLAPHHQCSQLCLYDWYTKVTSVVVDIVPRQLGESFSGIY
CSLLPLGCWSADSQRVVFDSAQRSRQDLFAVDTQTGSITSLTAAGSAGSWKLLTIDKDLM
VAQFSTPSLPPSLKVGFLPPPGKEQSVSWVSLEEAEPIPGIHWGVRVLHPPPDQENVQYA
DLDFEAILLQPSNPPDKTQVPMVVMPHGGPHSSFVTAWMLFPAMLCKMGFAVLLVNYRGS
TGFGQDSILSLPGNVGHQDVKDVQFAVEQVLQEEHFDARRVALMGGSHGGFLSCHLIGQY
PETYSACIARNPVINIASMMGSTDIPDWCMVETGFPYSNSCLPDLNVWEEMLDKSPIKYI
PQVKTPVLLMLGQEDRRVPFKQGMEYYRALKARNVPVRLLLYPKSNHALSEVEAESDSFM
NAVLWLHTHLGS
>Q5TCZ1
MLAYCVQDATVVDVEKRRNPSKHYVYIINVTWSDSTSQTIYRRYSKFFDLQMQLLDKFPI
EGGQKDPKQRIIPFLPGKILFRRSHIRDVAVKRLKPIDEYCRALVRLPPHISQCDEVFRF
FEARPEDVNPPKEDYGSSKRKSVWLSSWAESPKKDVTGADATAEPMILEQYVVVSNYKKQ
ENSELSLQAGEVVDVIEKNESGWWFVSTSEEQGWVPATYLEAQNGTRDDSDINTSKTGEV
SKRRKAHLRRLDRRWTLGGMVNRQHSREEKYVTVQPYTSQSKDEIGFEKGVTVEVIRKNL
EGWWYIRYLGKEGWAPASYLKKAKDDLPTRKKNLAGPVEIIGNIMEISNLLNKKASGDKE
TPPAEGEGHEAPIAKKEISLPILCNASNGSAVGVPDRTVSRLAQGSPAVARIAPQRAQIS
SPNLRTRPPPRRESSLGFQLPKPPEPPSVEVEYYTIAEFQSCISDGISFRGGQKAEVIDK
NSGGWWYVQIGEKEGWAPASYIDKRKKPNLSRRTSTLTRPKVPPPAPPSKPKEAEEGPTG
ASESQDSPRKLKYEEPEYDIPAFGFDSEPELSEEPVEDRASGERRPAQPHRPSPASSLQR

ARFKVGESSEDVALEEETIYENEGFRPYAEDTLSARGSSGDSDSPGSSSLSLTRKNSPKS
GSPKSSSLLKLKAEKNAQAEMGKNHSSASFSSSITINTTCCSSSSSSSSSLSKTSGDLKP
RSASDAGIRGTPKVRAKKDADANAGLTSCPRAKPSVRPKPFLNRAESQSQEKMDISTLRR
QLRPTGQLRGGLKGSKSEDSELPPQTASEAPSEGSRRSSSDLITLPATTPPCPTKKEWEG
PATSYMTCSAYQKVQDSEISFPAGVEVQVLEKQESGWWYVRFGELEGWAPSHYLVLDENE
QPDPSGKELDTVPAKGRQNEGKSDSLEKIERRVQALNTVNQSKKATPPIPSKPPGGFGKT
SGTPAVKMRNGVRQVAVRPQSVFVSPPPKDNNLSCALRRNESLTATDGLRGVRRNSSFST
ARSAAAEAKGRLAERAASQGSDSPLLPAQRNSIPVSPVRPKPIEKSQFIHNNLKDVYVSI
ADYEGDEETAGFQEGVSMEVLERNPNGWWYCQILDGVKPFKGWVPSNYLEKKN
>O94435
MTNERHDELACLGIFPLKNPKQLDGVVTPILQLRVKFLAPTDRWWEGLVINKDLSKEESE
ELWKFLQSFDPRSAKFVGRGTPEDHVISYLFEAGKYEFEVFFYQKDHSLKLMGLYDKTQK
QLLRRDSSGDLTSTDKERDVSPVSHSEKPYWDRYDLDQPSNQDVEESRNLVQEPKHRSKK
YDRLGLDELVMEQTASLWKICSRNGMSVDEFLRFIRMGLESNFQHSNQVIPTHF
>P31416
MAYLGKVYKFDREENFDGFLKSIGLSEEQVQKYLQYKPSSQLVKEGDKYKYISVSSDGTK
ETVFESGVETDDVVQGGLPIKTTYTVDGNTVTQVVNSAQGSATFKREYNGDELKVTITSS
EWDGVAYRYYKA
>P38972
MTDYILPGPKALSQFRVDNLIKDINSYTNSTSVINELRSCYIHYVNGIAQNLSEQDTKLL
EVLLTYDSALDIANDPLARQLNDAVANNLPSSALGEDTYLIRVVPRSGTISPWSSKATNI
AHVCGLQDKVQRIERGLALLIKTVPGFPLLENLNDISLKCVYDRMTQQLYLTEPPNTMSI
FTHEEPKPLVHVPLTPKDTKQSPKDILSKANTELGLALDSGEMEYLIHAFVETMKRDPTD
VELFMFAQVNSEHCRHKIFNADWTIDGIKQQFTLFQMIRNTHKLNPEYTISAYSDNAAVL
DSENDAFFFAPNSTTKEWTSTKERIPLLIKVETHNHPTAVSPFPGAATGSGGEIRDEGAT
GRGSKTKCGLSGFSVSDLLIPGNEQPWELNIGKPYHIASALDIMIEAPLGSAAFNNEFGR
PCINGYFRTLTTKVLNHQGKEEIRGFHKPIMIAGGFGTVRPQFALKNTPITPGSCLIVLG
GQSMLIGLGGGAASSVASGEGSADLDFASVQRGNPEMERRCQQVIDACVALGNNNPIQSI
HDVGAGGLSNALPELVHDNDLGAKFDIRKVLSLEPGMSPMEIWCNESQERYVLGVSPQDL
SIFEEICKRERAPFAVVGHATAEQKLIVEDPLLKTTPIDLEMPILFGKPPKMSRETITEA
LNLPEANLSEIPSLQDAIQRVLNLPSVGSKSFLITIGDRSVTGLIDRDQFVGPWQVPVAD
VGVTGTSLGETIISTGEAMAMGEKPVNALISASASAKLSVAESLLNIFAADVKSLNHIKL
SANWMSPASHQGEGSKLYEAVQALGLDLCPALGVAIPVGKDSMSMKMKWDDKEVTAPLSL
NITAFAPVFNTSKTWTPLLNRNTDDSVLVLVDLSAKQETKSLGASALLQVYNQVGNKSPT
VYDNAILKGFLESLIQLHQQKEDIVLAYHDRSDGGLLITLLEMAFASRCGLEINIDGGDL
ESQLTNLFNEELGAVFQISAKNLSKFEKILNENGVAKEYISIVGKPSFQSQEIKIINSTT
NDVIYANSRSELEQTWSKTSYEMQKLRDNPKTAEEEFASITDDRDPGLQYALTYNPADDM
KIGLELSSQRPKVAILREQGVNGQMEMAWCFQQAGFNSVDVTMTDLLEGRFHLDDFIGLA
ACGGFSYGDVLGAGAGWAKSVLYHEGVRSQFSKFFNERQDTFAFGACNGCQFLSRLKDII
PGCENWPSFERNVSEQYEARVCMVQISQEKDNSSEESVFLNGMAGSKLPIAVAHGEGKAT
FSKSAEQLEKFEKDGLCCIRYVDNYGNVTERFPFNPNGSTNGIAGIKSPNGRVLAMMPHP
ERVCRLEANSWYPEGKYEEWGGYGPWIRLFRSARRWVG
>Q9ZWR8
MEQIQMVKVLEKCQVTPPSDTTDVELSLPVTFFDIPWLHLNKMQSLLFYDFPYPRTHFLD

TVIPNLKASLSLTLKHYVPLSGNLLMPIKSGEMPKFQYSRDEGDSITLIVAESDQDFDYL
KGHQLVDSNDLHGLFYVMPRVIRTMQDYKVIPLVAVQVTVFPNRGIAVALTAHHSIADAK
SFVMFINAWAYINKFGKDADLLSANLLPSFDRSIIKDLYGLEETFWNEMQDVLEMFSRFG
SKPPRFNKVRATYVLSLAEIQKLKNKVLNLRGSEPTIRVTTFTMTCGYVWTCMVKSKDDV
VSEESSNDENELEYFSFTADCRGLLTPPCPPNYFGNCLASCVAKATHKELVGDKGLLVAV
AAIGEAIEKRLHNEKGVLADAKTWLSESNGIPSKRFLGITGSPKFDSYGVDFGWGKPAKF
DITSVDYAELIYVIQSRDFEKGVEIGVSLPKIHMDAFAKIFEEGFCSLS
>O74325
MTPVSSDYRPNHIFLPKFRPDKITSECNTPQQQNVIAEGLYNVSEPFPIRNTDEERYLDT
KEIHHTWDNTIKNVVRSIVSIKGSALRSFDTESAGSFCATGFVVNKTLGLILSNRHVVSP
GPISARASFINYEEIDIYPIYRDPVHDFGFFRYDPSSIRFHDVTEISLSPESAKVGIDIR
IIGNDAGEKLSILSSTLARLDRPAPNYGIDNYNDFNTFYYQAASGTSGGSSGSPVLDISG
AAVALNSGGSNSSASSFYLPLDRVVRALRCIENNTPITRGTLLTEFLHWSYDELSRIGLP
REFEYDCRTRVPSSTGLLVVSRVLRNSEVSKALEPGDILIAFKTDSHKSTYIVDFVSLFE
VLDEMVGKTIELHVYRPKRGFLTFQLTVQDLHNVTPSRFLEVGGAVLHDLSYQLARSYQF
SLNSGTYVASSGMLNWSSGTRDFLVTRLANKPTPTLDAFIDVLVQLTDNARVPMHFRVLG
KYEEEFTIVTVDRHFFLASIFSRNDEKGTWDRQSLPPPQPSISRRPSVIPRPQEGSKSME
AIQNALVLVHCRMPYSINGFSSTKLYSGTGVIVSVVPPLIVVDRSVIPVDICDIRLTFQS
MSAMGHLTFLDNRIAVVSCDYLPSNSVQLNFVADFLRTGDECTLAALDEDLQLLTKKTTV
RSVSVVETERSSPPRFRYVNCEVISLMDSLASTGGLVFREVGDDREIVALWISVVHQDVG
GKDYTTKYGLSMSYILPVLERLKLPPSARAQHVPTTAGVEWSHITLAGASTLGLSQTRSS
EFYMKSRENGTIPRPLYVISHLRPLLHKTSLGVGDILLEVNGKMITRLSDLHEFETESEI
KAVILRDGIEMEITIPLYPEYPTFSSRAICWMGAIIHPTHSSVFEQVEPDVDLPGPEGVY
VGSILYGSPALNMLRAAHWIVAVDGHDINTFDDFYHMLLEKPTDTFVQVKQMNRRGATSI
VSVRPDPLFWPTCIIERDSNGRWCTKHLQRKTKEVCS
>P47008
MKFDNDSEKQVFDKLKKAIPGIIKEKCAGYDELYGYKLNPEGLTQEEVDKYYDEKIADRL
TYKLCKAYQFEYSTIVQNLIDILNWRREFNPLSCAYKEVHNTELQNVGILTFDANGDANK
KAVTWNLYGQLVKKKELFQNVDKFVRYRIGLMEKGLSLLDFTSSDNNYMTQVHDYKGVSV
WRMDSDIKNCSKTVIGIFQKYYPELLYAKYFVNVPTVFGWVYDLIKKFVDETTRKKFVVL
TDGSKLGQYLKDCPYEGYGGKDKKNNLTKQNVTNVHPTEYGLYILQKQIIEDVE
>Q92871
MAVTAQAARRKERVLCLFDVDGTLTPARQKIDPEVAAFLQKLRSRVQIGVVGGSDYCKIA
EQLGDGDEVIEKFDYVFAENGTVQYKHGRLLSKQTIQNHLGEELLQDLINFCLSYMALLR
LPKKRGTFIEFRNGMLNISPIGRSCTLEERIEFSELDKKEKIREKFVEALKTEFAGKGLR
FSRGGMISFDVFPEGWDKRYCLDSLDQDSFDTIHFFGNETSPGGNDFEIFADPRTVGHSV
VSPQDTVQRCREIFFPETAHEA
>P37879
MATLQECKVKVDGEPKLSKNELKRRLKAEKKLAEKEAKQKELIEKQLSQAAAAAATNHTA
DNGVGVEEETLDPNQYYKIRSQAIHQLKVSGEDPYPHKFHVDISLTQFIQEYSQLQPGDH
LTDITLKVAGRIHAKRASGGKLIFYDLRGEGVKLQVMANSRNYKSEEEFVRINNKLRRGD
IIGVQGNPGKTKKGELSIIPYEITLLSPCLHMLPHLHFGLKDKETRYRQRYLDLILNDFV
RQKFIVRAKIITYIRSFLDELGFLEIETPMMNIIPGGAVAKPFITYHNELDMNLYMRIAP
ELYHKMLVVGGIDRVYEIGRQFRNEGIDLTHNPEFTTCEFYMAYADYHDLMEITEKMLSG

MVKSITGSYKITYHPDGPEGEAYEIDFTPPFRRISMVEELEKALGVKLPETSLFETEETR
KILDDICVAKAVECPPPRTTARLLDKLVGEFLEVTCINPTFICDHPQIMSPLAKWHRSKE
GLTERFELFVMKKEICNAYTELNDPMRQRQLFEEQAKAKAAGDDEAMFIDENFCTALEYG
LPPTAGWGMGIDRVTMFLTDSNNIKEVLLFPAMKPEDKKETAAATETLESTTAGPSV
>Q9USZ0
MLVEGDKGVLEPVNFVGPVTSLALIHNDKFLCCAQGPCLRIYDVSESRHESDLIKSILLP
FHNRIHGMLPCKQGLFVWGGTYFAVVDVETSQVYYDRISDWIFNAAELGEENKFCVVTSK
NQVHLLSLSKGSWEVTNTISCEKTPLLFCATVQISGNEIYIASASAFQQIYVWKFNYNNP
PSCVSIDTYLVGHEGACFDLKFSSDGRYLCSVSEDRTLRLWSIESSPFLIATGFGHSARV
WRCVFLPNKEIATVSEDLTLRLWTWNDKTLTNTYTYTGHRGKHIWSLVVSSANPIIYTGG
NDGSVRSWDYKTRIQESCISVSSLFTSKTLLASFCFVKCQKLLCLAKNGEMSIIDNKGKK
AIPALTTTSDMSIIRSWKNSFFVAAASNKGTIYVFSYQNTNLLKAIDLHRGKIQQLLTYQ
VKERYFLLSKSFVGKNQFRVYLQEFDFEADKVNLISTIELELPATFDPTSLTVDEEYSNF
LIGSRTGSLALYNIGESNYLSVWRRIHEFDAISSIHIKSSKRDYLLIQTVGRDGYVNLFS
IPRVEKSAVPQELCSKKCCDGILSGGLCKKLKNGKENQWVWGFHASNFFLRDETNETNVF
FIDCGGSHRPWAFSMEMDIQSFASYRANQLYIYSTALNLLQRNSVLQNGLHGREIRAMDF
NPSGDLLLSGSEDTKVTLLEFQSNGNIIPLNSVKFHNSGIQSLTWFSDDILFSTGGLEEL
NVYRLIQKPEAYLKRIVHEKLCKPNKSNKTYDGDLRITDISVVKATHLGERVLWINTVQS
DSTIKAFTYNVDTKQLNCIKSWKYKTVCLVFAEAVIFGSHLLLVVASTDGNVAIWNTFWR
DPQIEPLIIWIKTVHQFCVKSLNISRDQDVLTISTGGDDGAISRSILLLEQISENSVVVN
SLHNYFFAEAHASSVTGVLSITKELLLSVSIDQRILLWKVEGDSLKPILERYTHVADVGG
MIRNNDFLIIYGIGCEVFRLGSLR
>Q06325
MTCLILWYLWLISTFQLEFATASTANTTTTAKSGTSSSTEEPFPVLAVGKDGRGNYYVNS
TFGTPGQRQRLLVDIIQPYINLVSGTSESHNEYSGVYHKHPSYLMNDSTSSVPVSPGQIY
EISFIDGRAVNCTLVTDDMNFTNVSSENSSTALITDLMVTRDNVQFNSGSLSISNVSFFD
IQSSNFKTSGLLGLSGKVTNPGNAIDSSQYTEQSYFLSLLKDADIIESSSYSLWLAGDTS
TYKTYRDPISNCGKLLLGGVDPSLFTGTLGKFDLIPYVDPVSNAVSVGYPIVPLGPIYIV
SNSGQSLNMTSKDFLSPALLDSTSSVSYLPTSTIIQIAVQIAATYVESLDRWLVQCSIAD
MGVSLGFRLRELTIEIPLRDLLSSTYDTSTNSSMFFSSGQEACFLTLYANTNTGVNILGE
AFIKNIYMAMDLEDNTIAIAQAKKVEDDAVTEETNETTASTIIKKIKSGYIPYAKVMNSS
NTRNLTLYPSYRSGYMFTVPGQLTAAYSNGVITGAGRSFYDTSRASTSARPSSTQFDSFS
VSASEEWSNSTNRTSSASGAGVRLSSPYTFNKDPAGHVTRIASLLLLSIFSILIVL
>O74798
MRGELNIPALSRFGKSISASLHHQNGSSRDDNDSNPYENRSSNSGLNRRNSVFGLPSSGL
SSRLSKPSLSSINNSNNSSSNTGGNVLPNPALTPVRNMSNKPPLTWSPSSANLFGTSKVT
SIIGNNVSDVVPPVIKKALASSHGGGMSLSIVLLEPVLYLAGFDLNECQIENPALLRGAL
VLRVAKPANIRGISLSFTGRSRTEWPEGIPVKGHDTYEDKVIISHNWKFYEPTMKDADAP
QHGADVARLVGEQLPLPSSAAASLRGYSVFAPGEYTYNFDLAIPNCFPESVEAKMGWVRY
FLEATVERFGTFKSNLNGRTPVQLVRTPSPASLSSSELINISRDWDERLHYELQVSGKSF
RLGEVVPITFRFLLLDKVRLYKLSISVVESSEYWCRSRKFHRVDPKRRVLLAERSAKHQN
TDNLFETPDEGDGLSSAVFNFNVALPTCLVKERDRLTFDTTYKYIKVRHRLKALLVLSIE
NTENPEKRKYFEINIETPVRILSCRCVKDSTLLPPYESSSQGDNQVLLPCPCRLATTHVE
PTEVTAFTTQSVLASSAPSAGRPAAAQISRPAQLFRIPSTNPPPFDGDVCPPACNTPPPN

YDELFDVLSSISIQDCETDRANDDTILNNRVRRSGTIREEAPHRSLSRTVSRSFEIPR
>P40995
MAYFQDRKTSSRSLPSYINHSTQNLVGPRKDETNLSEYMKLRLLQSPSQVIYNLENTVSL
YRRCLNLRKRLMDISELAAFFDSIHREALNSSFKILEFKDIEFDDPVTEIWLFCRLGYPL
CALFNCLPVKQKLEVNSSVSLENTNVCKASLYRFMLMCKNELGLTDAALFSISEIYKPST
APLVRALQTIELLLKKYEVSNTTKSSSTPSPSTDDNVPTGTLNSLIASGRRVTAELYETE
LKYIQDLEYLSNYMVILQQKQILSQDTILSIFTNLNEILDFQRRFLVGLEMNLSLPVEEQ
RLGALFIALEEGFSVYQVFCTNFPNAQQLIIDNQNQLLKVANLLEPSYELPALLIKPIQR
ICKYPLLLNQLLKGTPSGYQYEEELKQGMACVVRVANQVNETRRIHENRNAIIELEQRVI
DWKGYSLQYFGQLLVWDVVNVCKADIEREYHVYLFEKILLCCKEMSTLKRQARSISMNKK
TKRLDSLQLKGRILTSNITTVVPNHHMGSYAIQIFWRGDPQHESFILKLRNEESHKLWMS
VLNRLLWKNEHGSPKDIRSAASTPANPVYNRSSSQTSKGYNSSDYDLLRTHSLDENVNSP
TSISSPSSKSSPFTKTTSKDTKSATTTDERPSDFIRLNSEESVGTSSLRTSQTTSTIVSN
DSSSTASIPSQISRISQVNSLLNDYNYNRQSHITRVYSGTDDGSSVSIFEDTSSSTKQKI
FDQPTTNDCDVMRPRQYSYSAGMKSDGSLLPSTKHTSLSSSSTSTSLSVRNTTNVKIRLR
LHEVSLVLVVAHDITFDELLAKVEHKIKLCGILKQAVPFRVRLKYVDEDGDFITITSDED
VLMAFETCTFELMDPVHNKGMDTVSLHVVVYF
>Q8K3Y6
MADPGVCCFITKILCAHGGRMTLEELLGEIRLPEAQLYELLETAGPDRFVLLETGGQAGI
TRSVVATTRARVCRRKYCQRPCDSLHLCKLNLLGRCHYAQSQRNLCKYSHDVLSEQNFQI
LKNHELSGLNQEELACLLVQSDPFFLPEICKSYKGEGRKQTCGQPQPCERLHICEHFTRG
NCSYLNCLRSHNLMDRKVLTIMREHGLSPDVVQNIQDICNNKHARRNPPGTRAAHPHRRG
GAHRDRSKSRDRFLHNSLEFLSPVVSPLGSGPPSPDVTSCKDSLEDVSVDVTQKFKYLGT
HDRAQLSPVSSKAAGVQGPSQMRASQEFSEDGNLDDIFSRNRSDSSSSRASAAKVAQRNE
AVAMKMGMEVKGKKEAPDIDRVPFLNSYIDGVTMEKASVSGIPGKKFTANDLENLLLLND
TWKNVAKPQDLQTTGRITDSGQDKAFLQNKYGGNPVWASASTHNAPNGSSQIMDETPNVS
KSSTSGFAIKPAIAGGKEAVYSGVQSPRSQVLAVPGEATTPVQSNRLPQSPLSSSSHRAA
ASGSPGKNSTHTSVSPAIESSRMTSDPDEYLLRYILNPLFRMDNHGPKEICQDHLYKGCQ
QSHCDRSHFHLPYRWQMFVYTTWRDFQDMESIEQAYCDPHVELILIENHQINFQKMTCDS
YPIRRLSTPSYEEKPLSAVFATKWIWYWKNEFNEYIQYGNESPGHTSSDINSAYLESFFQ
SCPRGVLPFQAGSQKYELSFQGMIQTNIASKTQRHVVRRPVFVSSNDVEQKRRGPE
>Q11002
MDDLRGFLRQAGQEFLNAAGEAAMGAAKDVVGSVINEIFIKKEADTKRVLPSIKNMRVLG
EKSSSLGPYSEVQDYETILNSCLASGSLFEDPLFPASNESLQFSRRPDRHIEWLRPHEIA
ENPQFFVEGYSRFDVQQGELGDCWLLAATANLTQESNLFFRVIPAEQSFEENYAGIFHFR
FWQYGKWVDVIIDDRLPTYNGELMYMHSTEKNEFWSALLEKAYAKLHGSYEALKGGSTCE
AMEDFTGGVSEWYDLKEAPGNLFTILQKAAERNSMMGCSIEPDPNVTEAETPQGLIRGHA
YSITKVCLIDIVTPNRQGKIPMIRMRNPWGNEAEWNGPWSDSSPEWRYIPEEQKAEIGLT
FDRDGEFWMSFQDFLNHFDRVEICNLSPDSLTEDQQNSGKRKWEMSMYEGEWTPGVTAGG
CRNFLDTFWHNPQYIITLVDPDEEDEEGQCTVIVALMQKNRRSKRNMGMECLTIGFAIYS
LNDRELENRPQGLNFFRYKSSVGRSPHFINTREVCARFKLPPGHYLIVPSTFDPNEEGEF
IIRVFSETQNNMEENDDHVGYGGKADTITPGFPTPKPIDPQKEGLRRLFDSIAGKDMEVD
WMELKRILDHSMRDDLPKPVVFNRFSNNMAFETQAAGPGDDGAGACGLLSLICGPFLKGT
PFEEQLGMNDQSNKRLIGDNPADGGPVTANAIVDETHGFSKDVCRSMVAMLDADKSGKLG

FEEFETLLSEIAKWKAIFKVYDVENTGRVSGFQLREALNSAGYHLNNRVLNVLGHRYGSR
DGKIAFDDFIMCAVKIKTYIDIFKERDTEKNETATFTLEEWIERTIYS
>P21269
MLRSTISLLMNSAAQKTMTNSNFVLNAPKITLTKVEQNICNLLNDYTDLYNQKYHNKPEP
LTLRITGGWVRDKLLGQGSHDLDIAINVMSGEQFATGLNEYLQQHYAKYGAKPHNIHKID
KNPEKSKHLETATTKLFGVEVDFVNLRSEKYTELSRIPKVCFGTPEEDALRRDATLNALF
YNIHKGEVEDFTKRGLQDLKDGVLRTPLPAKQTFLDDPLRVLRLIRFASRFNFTIDPEVM
AEMGDPQINVAFNSKISRERVGVEMEKILVGPTPLLALQLIQRAHLENVIFFWHNDSSVV
KFNEENCQDMDKINHVYNDNILNSHLKSFIELYPMFLEKLPILREKIGRSPGFQQNFILS
AILSPMANLQIIGNPKKKINNLVSVTESIVKEGLKLSKNDAAVIAKTVDSICSYEEILAK
FADRSQLKKSEIGIFLRNFNGEWETAHFASLSDAFLKIPKLETKKIELLFQNYNEFYSYI
FDNNLNNCHELKPIVDGKQMAKLLQMKPGPWLGKINNEAIRWQFDNPTGTDQELITHLKA
ILPKYL
>P22213
MAVEEIASRKDISLRDMQISAILKMLFLNKDLNNNDNITTITDDIFNQQEIIWKVLILDI
KSTATISSVLRVNDLLKAGITVHSLIKQDRSPLPDVPAIYFVSPTKENIDIIVNDLKSDK
YSEFYINFTSSLPRNLLEDLAQQVSITGKSDKIKQVYDQYLDFIVTEPELFSLEISNAYL
TLNDPKTTEEEITGLCANIADGLFNTVLTINSIPIIRAAKGGPAEIIAEKLGTKLRDFVI
NTNSSSTSTLQGNDSLERGVLIILDRNIDFASMFSHSWIYQCMVFDIFKLSRNTVTIPLE
SKENGTDNTTAKPLATKKYDIEPNDFFWMENSHLPFPEAAENVEAALNTYKEEAAEITRK
TGVTNISDLDPNSNNDTVQIQEVVKKLPELTAKKNTIDTHMNIFAALLSQLESKSLDTFF
EVEQDPGSTKTRSRFLDILKDGKTNNLEDKLRSFIVLYLTSTTGLPKDFVQNVENYFKEN
DYDINALKYVYKLREFMQLSNMSLQNKSLEDGSDSAFKPSNLTLSGIYGLTEGKLQGGVG
SLISGIKKLLPEKKTIPITNVVDAIMDPLNSSQKNLETTDSYLYIDPKITRGSHTRKPKR
QSYNKSLVFVVGGGNYLEYQNLQEWAHSQLHNPKKVMYGSTAITTPAEFLNEISRLGASN
SSNNDA
>P22515
MSSNNSGLSAAGEIDESLYSRQLYVLGKEAMLKMQTSNVLILGLKGLGVEIAKNVVLAGV
KSMTVFDPEPVQLADLSTQFFLTEKDIGQKRGDVTRAKLAELNAYVPVNVLDSLDDVTQL
SQFQVVVATDTVSLEDKVKINEFCHSSGIRFISSETRGLFGNTFVDLGDEFTVLDPTGEE
PRTGMVSDIEPDGTVTMLDDNRHGLEDGNFVRFSEVEGLDKLNDGTLFKVEVLGPFAFRI
GSVKEYGEYKKGGIFTEVKVPRKISFKSLKQQLSNPEFVFSDFAKFDRAAQLHLGFQALH
QFAVRHNGELPRTMNDEDANELIKLVTDLSVQQPEVLGEGVDVNEDLIKELSYQARGDIP
GVVAFFGGLVAQEVLKACSGKFTPLKQFMYFDSLESLPDPKNFPRNEKTTQPVNSRYDNQ
IAVFGLDFQKKIANSKVFLVGSGAIGCEMLKNWALLGLGSGSDGYIVVTDNDSIEKSNLN
RQFLFRPKDVGKNKSEVAAEAVCAMNPDLKGKINAKIDKVGPETEEIFNDSFWESLDFVT
NALDNVDARTYVDRRCVFYRKPLLESGTLGTKGNTQVIIPRLTESYSSSRDPPEKSIPLC
TLRSFPNKIDHTIAWAKSLFQGYFTDSAENVNMYLTQPNFVEQTLKQSGDVKGVLESISD
SLSSKPHNFEDCIKWARLEFEKKFNHDIKQLLFNFPKDAKTSNGEPFWSGAKRAPTPLEF
DIYNNDHFHFVVAGASLRAYNYGIKSDDSNSKPNVDEYKSVIDHMIIPEFTPNANLKIQV
NDDDPDPNANAANGSDEIDQLVSSLPDPSTLAGFKLEPVDFEKDDDTNHHIEFITACSNC
RAQNYFIETADRQKTKFIAGRIIPAIATTTSLVTGLVNLELYKLIDNKTDIEQYKNGFVN
LALPFFGFSEPIASPKGEYNNKKYDKIWDRFDIKGDIKLSDLIEHFEKDEGLEITMLSYG
VSLLYASFFPPKKLKERLNLPITQLVKLVTKKDIPAHVSTMILEICADDKEGEDVEVPFI

TIHL
>Q9UT09
MSPVFLPSGETYDQEHLDDNRVLGYNPLVPAALVQQEIPVSETSRKVITDSRKEIQAILN
KQDDRIIVVVGPCSIHDPKLAMDYAKLLKPKADELQDALCVVMRCYLEKPRTTIGWKGLV
NDPNLDGSFAINKGIRMARQMYCDVTNFGIPLASEMLDNISPQFFADLLSFGAIGARTTE
SQLHRELASALSFPVGFKNGTDGTVGVAIDAIGATAHPHTMLGVTKQGLAAITMTRGNKD
TFIILRGGKKGPNYDAEHVAAVRKDLEKANLPPRIMIDCSHGNSSKNHLNQPKVSKSIAE
QIRNGDSSIVGVMIESHINEGRQDAPIRPGVKDTLKYGVSITDACVSWEQTAPMLDDLAE
AVRARRQNQKSN
>Q3UWR2
MSDLRRKGWWNVPDYFHSPLVFDMEEDKEDYIFGPHDEYLHTLEVHSNTLIQLERWFTPT
GQTRVTVVGPLKARLWVMDMIRKVGSKNNLDQIKGKMMLLQIRDHPLRDRDLELHPESGS
SLWITTMNDTTFVEVPHFLRFPLTVAWLFCGFVRILGIHNFADLHW
>O15360
MSDSWVPNSASGQDPGGRRRAWAELLAGRVKREKYNPERAQKLKESAVRLLRSHQDLNAL
LLEVEGPLCKKLSLSKVIDCDSSEAYANHSSSFIGSALQDQASRLGVPVGILSAGMVASS
VGQICTAPAETSHPVLLTVEQRKKLSSLLEFAQYLLAHSMFSRLSFCQELWKIQSSLLLE
AVWHLHVQGIVSLQELLESHPDMHAVGSWLFRNLCCLCEQMEASCQHADVARAMLSDFVQ
MFVLRGFQKNSDLRRTVEPEKMPQVTVDVLQRMLIFALDALAAGVQEESSTHKIVRCWFG
VFSGHTLGSVISTDPLKRFFSHTLTQILTHSPVLKASDAVQMQREWSFARTHPLLTSLYR
RLFVMLSAEELVGHLQEVLETQEVHWQRVLSFVSALVVCFPEAQQLLEDWVARLMAQAFE
SCQLDSMVTAFLVVRQAALEGPSAFLSYADWFKASFGSTRGYHGCSKKALVFLFTFLSEL
VPFESPRYLQVHILHPPLVPGKYRSLLTDYISLAKTRLADLKVSIENMGLYEDLSSAGDI
TEPHSQALQDVEKAIMVFEHTGNIPVTVMEASIFRRPYYVSHFLPALLTPRVLPKVPDSR
VAFIESLKRADKIPPSLYSTYCQACSAAEEKPEDAALGVRAEPNSAEEPLGQLTAALGEL
RASMTDPSQRDVISAQVAVISERLRAVLGHNEDDSSVEISKIQLSINTPRLEPREHMAVD
LLLTSFCQNLMAASSVAPPERQGPWAALFVRTMCGRVLPAVLTRLCQLLRHQGPSLSAPH
VLGLAALAVHLGESRSALPEVDVGPPAPGAGLPVPALFDSLLTCRTRDSLFFCLKFCTAA
ISYSLCKFSSQSRDTLCSCLSPGLIKKFQFLMFRLFSEARQPLSEEDVASLSWRPLHLPS
ADWQRAALSLWTHRTFREVLKEEDVHLTYQDWLHLELEIQPEADALSDTERQDFHQWAIH
EHFLPESSASGGCDGDLQAACTILVNALMDFHQSSRSYDHSENSDLVFGGRTGNEDIISR
LQEMVADLELQQDLIVPLGHTPSQEHFLFEIFRRRLQALTSGWSVAASLQRQRELLMYKR
ILLRLPSSVLCGSSFQAEQPITARCEQFFHLVNSEMRNFCSHGGALTQDITAHFFRGLLN
ACLRSRDPSLMVDFILAKCQTKCPLILTSALVWWPSLEPVLLCRWRRHCQSPLPRELQKL
QEGRQFASDFLSPEAASPAPNPDWLSAAALHFAIQQVREENIRKQLKKLDCEREELLVFL
FFFSLMGLLSSHLTSNSTTDLPKAFHVCAAILECLEKRKISWLALFQLTESDLRLGRLLL
RVAPDQHTRLLPFAFYSLLSYFHEDAAIREEAFLHVAVDMYLKLVQLFVAGDTSTVSPPA
GRSLELKGQGNPVELITKARLFLLQLIPRCPKKSFSHVAELLADRGDCDPEVSAALQSRQ
QAAPDADLSQEPHLF
>P87064
MVPSFVVYSSWVNGRQRYIRQAFASILFYIIRDTTLSFPSHTTMSTKPETDLQTVLDSEI
KEWHFHIYFHQNNAAEHQAALELRDAVLRLRQDGAFVAVPLFRVNMDPMGPHPVGSYEIW
VPSETFASVFSYLCMNRGRLSILVHPLTREELRDHEIRNAWIGPSFPLNLANLPIKSDEI
PLQYPSLKLGYSSTAHKMSLEERRKLGDDIEAVLRGEKEAARAPHRDA

>Q9P802
MSQTETSHSGYIDFKKESQPSVFQCKKCFQIVGDSNAWVISHREYLSFTLSDAVENSVRV
EDTFKRSDDGLCVYSELSCTRCNEVIGKVYNSTPIYLDDIRDMYTFSMDKLQAYQLGNKT
VNPEGLTRYQVDLEMREDIIKACTVILYIVEKNKL
>Q8R1H0
MSAQTASGPTEDQVEILEYNFNKVNKHPDPTTLCLIAAEAGLTEEQTQKWFKQRLAEWRR
SEGLPSECRSVTD
>P02407
MGRVRTKTVKRASKALIERYYPKLTLDFQTNKRLCDEIATIQSKRLRNKIAGYTTHLMKR
IQKGPVRGISFKLQEEERERKDQYVPEVSALDLSRSNGVLNVDNQTSDLVKSLGLKLPLS
VINVSAQRDRRYRKRV
>Q9USV2
MKPFSPSQTNFEKEQLCFGVYQLKDCYQQVIEALSLGIRVIDSAITYRNEKECEQAIQDF
CHQNVNIKREDITLITKIPDSLQGFERTWKAVEQSLRRTGRPKLDVVLIHSPKWPVRRIE
SWRALLQHQKEGRINKIGVSNYNIHHLEEIISLGLPLPAINQVEFSAFNNRPTFLSYCFN
HGILVQAFSPLTRGYRLSDIRLLDLSLKYNKTPANILLRYCLQKGVSPIFKASSFVHIHE
NVKAEQFMLDPSDMDVMDTWDEEFVSKPTWNPIILP
>Q03780
MFDGFSNNKGKRRSFFRFGSESKNNDSEKSVRKPSVPSTIKKSTNIARTSTAETSAPDIP
PRSPNRNAHSRSHSIQAPLQKETLKNTNPFLNAEDTLGDSLELTQSKEASGNDHKGIEYL
QENNIIGQRTNPFTTSANSNAHFSKIKRSRPPPPPMDMKSITTSISNNTTKEEIESNNDS
ERDSIAISSTHNQHRRQRSEAEKLVDDIENYINEHKVSSGSSLSLDTSENSDTKASQDKL
PVDVMEAPILRNVSAESSLSYVKPLIVDNEEVNKASNGNLVQSDHLKEFSSNLDDGDDKF
SFSTSASGKSTKSLQQVSKDESSGFKAAHFDFAYKSNEHLGSDGSIASARKPLRITNEID
SGSSNEDDDDGLQEKGFVDSESKAFINYASDQGSSIKNDVSTQEPELPSHRRIFRVVNED
RPSFYLNSVNDTGSLTDKHSFDTASSGEYDAKSNFSSQSGLSISKGSKSTVLAALDSNGN
TKSSNKTSELNSLNSISESLVPAAHSFNEHTVTIPATVDLPNPVHDAPSERSVKCSPLTS
VVSNKSEKSVPLVSSYVEELRLKYYKTSNFLQAPPNLPVALKQKNNLIQPKNIKVKLRTS
SKQIGIKHGKVKQKLLALETRNEESDGTATGLKNKINVDHTKEFHKLLGKENETGSISKK
EGTDAEQAEDYLKDIPGDEAYNSDDIMAPLREKRGQNGSVDSVSRSNTVVSYYTRSQNRM
RSGTLDNDYVNRQKLPTHISLQDYRDANARSNISRQDSVSTTNSDVVDLSYSLGHGLRVA
NPDSDPE
>Q99K82
MQSCESSGDSADDPLSRGLRRRGQPRVVVIGAGLAGLAAARALLEQGFTDVTVLEASSHI
GGRVQSVRLGDTTFELGATWIHGSHGNPIYQLAEANGLLEETTDGERSVGRISLYSKNGV
ACYLTNRGCRIPKDVVEEFSDLYNEVYNMTQEFFRHGKPVNAESQNSVGVFTREKVRNRI
RDDPDDTEATKRLKLAMIQQYLKVESCESSSHSIDEVSLSAFGEWTEIPGAHHIIPSGFM
RVVELLAEGIPPHVIQLGKPVRCIHWDQASAHPRGPEIEPRGEGDHNHDTGEGGQSGENP
QQGRWDEDEPWPVVVECEDCEVIPADHVIVTVSLGVLKRQYTSFFRPCLPTEKVAAIHRL
GIGTTDKIFLEFEEPFWGPECNSLQFVWEDEAESCTLTYPPELWYRKICGFDVLYPPERY
GHVLSGWICGEEALVMERCDDEAVAEICTEMLRQFTGNPNIPKPRRILRSAWGSNPYFRG
SYSYTQVGSSGADVEKLAKPLPYTESSKTAPMQVLFSGEATHRKYYSTTHGALLSGQREA
ARLIEMYRDLFQQGP
>Q9H503

MDNMSPRLRAFLSEPIGEKDVCWVDGISHELAINLVTKGINKAYILLGQFLLMHKNEAEF
QRWLICCFGATECEAQQTSHCLKEWCACFL
>O94720
MNYNDTTEFCFPFGQPSFPTSAMTEGSFDGQFSEFDPTFTSPADTALSDIANLPIDLHKD
ALFANAPGKGDVDFDSVSMLQLLSDYPLAFNSTENNQKLQTNPSARWSLLDSMDFDNQRQ
CSDLESAQLGDSGLLKSTILSNSHIDIAALSSSKTSEPTPPFSYVQTPCIPTPSSALIDT
PFPGALDSEFGFDESQAPLFPASDGDCQRAFASISYPTNYGCKLSNLGFMSPQSPVKREL
NDSTSPSKLSESSSSLTGSSSALLSQSEFLGSVPSLSDSIATVDPFFSFESFETDEKARS
LLMDASLKLPQFSTPNLSSNSSSLSLKSTLAEGMKGSTPLAAVKTEKASKAARVMKQKKH
REHVCFNCGVTETPLWRRTSDKLNFLCNACGLYNKQYGVMRPLSPRNKGSSKALENLVCA
NCSSTKTSLWRKDRHGQTVCNACGLYARLHGHNRPIGLKKNKITRRRRGKGPGGEDGMSD
EVKSEFPVLSKSVTMAEILSSKGLESPQLTNSVSVSKMPNTDADVSLEHAKISFDSLDNS
VIVKKEEEIENKFSVSC
>Q32MZ4
MTSPAAAQSREIDCLSPEAQKLAEARLAAKRAARAEAREIRMKELERQQKEEDSERYSRR
SRRNTSASDEDERMSVGSRGSLRVEERPEKDFTEKGSRNMPGLSAATLASLGGTSSRRGS
GDTSISIDTEASIREIKELNELKDQIQDVEGKYMQGLKEMKDSLAEVEEKYKKAMVSNAQ
LDNEKTNFMYQVDTLKDMLLELEEQLAESRRQYEEKNKEFEREKHAHSILQFQFAEVKEA
LKQREEMLEKHGIILNSEIATNGETSDTLNNVGYQGPTKMTKEELNALKSTGDGTLGRAS
EVEVKNEIVANVGKREILHNTEKEQHTEDTVKDCVDIEVFPAGENTEDQKSSEDTAPFLG
TLAGATYEEQVQSQILESSSLPENTVQVESNEVMGAPDDRTRTPLEPSNCWSDLDGGNHT
ENVGEAAVTQVEEQAGTVASCPLGHSDDTVYHDDKCMVEVPQELETSTGHSLEKEFTNQE
AAEPKEVPAHSTEVGRDHNEEEGEETGLRDEKPIKTEVPGSPAGTEGNCQEATGPSTVDT
QNEPLDMKEPDEEKSDQQGEALDSSQKKTKNKKKKNKKKKSPVPVETLKDVKKELTYQNT
DLSEIKEEEQVKSTDRKSAVEAQNEVTENPKQKIAAESSENVDCPENPKIKLDGKLDQEG
DDVQTAAEEVLADGDTLDFEDDTVQSSGPRAGGEELDEGVAKDNAKIDGATQSSPAEPKS
EDADRCTLPEHESPSQDISDACEAESTERCEMSEHPSQTVRKALDSNSLENDDLSAPGRE
PGHFNPESREDTRGGNEKGKSKEDCTMS
>Q969Q6
MDWKEVLRRRLATPNTCPNKKKSEQELKDEEMDLFTKYYSEWKGGRKNTNEFYKTIPRFY
YRLPAEDEVLLQKLREESRAVFLQRKSRELLDNEELQNLWFLLDKHQTPPMIGEEAMINY
ENFLKVGEKAGAKCKQFFTAKVFAKLLHTDSYGRISIMQFFNYVMRKVWLHQTRIGLSLY
DVAGQGYLRESDLENYILELIPTLPQLDGLEKSFYSFYVCTAVRKFFFFLDPLRTGKIKI
QDILACSFLDDLLELRDEELSKESQETNWFSAPSALRVYGQYLNLDKDHNGMLSKEELSR
YGTATMTNVFLDRVFQECLTYDGEMDYKTYLDFVLALENRKEPAALQYIFKLLDIENKGY
LNVFSLNYFFRAIQELMKIHGQDPVSFQDVKDEIFDMVKPKDPLKISLQDLINSNQGDTV
TTILIDLNGFWTYENREALVANDSENSADLDDT
>P18120
MEPETKKTKTDSKKIVLLGGDFCGPEVIAEAVKVLKSVAEASGTEFVFEDRLIGGAAIEK
EGEPITDATLDICRKADSIMLGAVGGAANTVWTTPDGRTDVRPEQGLLKLRKDLNLYANL
RPCQLLSPKLADLSPIRNVEGTDFIIVRELVGGIYFGERKEDDGSGVASDTETYSVPEVE
RIARMAAFLALQHNPPLPVWSLDKANVLASSRLWRKTVTRVLKDEFPQLELNHQLIDSAA
MILIKQPSKMNGIIITTNMFGDIISDEASVIPGSLGLLPSASLASLPDTNEAFGLYEPCH
GSAPDLGKQKVNPIATILSAAMMLKFSLNMKPAGDAVEAAVKESVEAGITTADIGGSSST

SEVGDLLPTRSRSCSRRSKSFLRRIDGRSKLTRLRVGLPAGSASV
>O13715
MSSQQMQLTISGFENRIKILEDLMNEFDSNLYIRLKRECDFKLPKEYDVSIHALTILMKR
HLSNMEPIDCYQDLDTGTLQTMLIFCCTLRMIEHYEKSLSNINFFSLSNDLKRLEEYNTS
CSVLLSLYQRQLKKISTTIESYYLADTLRHNQNILNLCERIYNIQKLLNKREKASVLRH
>O14492
MNGAGPGPAAAAPVPVPVPVPDWRQFCELHAQAAAVDFAHKFCRFLRDNPAYDTPDAGAS
FSRHFAANFLDVFGEEVRRVLVAGPTTRGAAVSAEAMEPELADTSALKAASYGHSRSSED
VSTHAATKARVRKGFSLRNMSLCVVDGVRDMWHRRASPEPDAAAAPRTAEPRDKWTRRLR
LSRTLAAKVELVDIQREGALRFMVADDAAAGSGGSAQWQKCRLLLRRAVAEERFRLEFFV
PPKASRPKVSIPLSAIIEVRTTMPLEMPEKDNTFVLKVENGAEYILETIDSLQKHSWVAD
IQGCVDPGDSEEDTELSCTRGGCLASRVASCSCELLTDAVDLPRPPETTAVGAVVTAPHS
RGRDAVRESLIHVPLETFLQTLESPGGSGSDSNNTGEQGAETDPEAEPELELSDYPWFHG
TLSRVKAAQLVLAGGPRNHGLFVIRQSETRPGEYVLTFNFQGKAKHLRLSLNGHGQCHVQ
HLWFQSVLDMLRHFHTHPIPLESGGSADITLRSYVRAQDPPPEPGPTPPAAPASPACWSD
SPGQHYFSSLAAAACPPASPSDAAGASSSSASSSSAASGPAPPRPVEGQLSARSRSNSAE
RLLEAVAATAAEEPPEAAPGRARAVENQYSFY
>P15007
MSWTASVFLRTSTTSMKFLRLRWPLPRIPQNKSANVAPRFRSKSAVSQLSSGFKFVQIRK
STCDSNEMTIKAIKARQIYDSRGNPTVEVDLTTELGLFRAAVPSGASTGVHEALELRDND
KANYHGKSVLKAVGHVNDTLGPELIKANLDVVDQASIDNFMIKLDGTENKSKFGANAILG
VSLAVAKAGAAKKGVPLYKHIADLAGNKEIILPVPAFNVINGGSHAGNKLAMQEFMILPT
GATSFTEAMKMGSEVYHHLKNVIKAKFGLDATAVGDEGGFAPNIQSNKEALNLISDAIAK
AGYTGKIEIGMDVAASEFYKDGQYDLDFKNEKSDKSQWLPADKLANLYQEFIKDFPIVSI
EDPFDQDHWEAWSNLTGCTDIQIVGDDLTVTNPKRIATAVEKKACNCLLLKVNQIGTVTE
SIAAHLLAKKNGWGTMVSHRSGETEDSFIGDLVVGLSTGQIKTGAPCRSERLAKYNQILR
IEEEIGAGVKFAGKSFRKPQ
>P47093
MSATLKDYLNKRVVIIKVDGECLIASLNGFDKNTNLFITNVFNRISKEFICKAQLLRGSE
IALVGLIDAENDDSLAPIDEKKVPMLKDTKNKIENEHVIWEKVYESKTK
>Q9JJY4
MAAAAFEVPAALTTSESTMAAERAAAPVQAVEPTPASPWTQRTAHDIGGPRTRTGDVVLA
EPADFESLLLSRPVLEGLRAAGFERPSPVQLKAIPLGRCGLDLIVQAKSGTGKTCVFSTI
ALDSLILENYSTQILILAPTREIAVQIHSVITAIGIKMEGLECHVFIGGTPLSQDKTRLK
KCHIAVGSPGRIKQLIELDYLNPGSIRLFILDEADKLLEEGSFQEHINWIYSSLPASKQM
LAVSATYPEVLANALTRYMRDPTFVRLNPSDPSLIGLKQYYQVVNSYPLAHKIFEEKTQH
LQELFSKVPFNQALVFSNLHSRAQHLADILSSKGFPTECISGNMNQNQRLDAMAKLKQFH
CRVLISTDLTSRGIDAEKVNLVVNLDVPLDWETYMHRIGRAGRFGTLGLTVTYCCRGEEE
NMMMKIAQKCNINLLPLPDPIPPGLMEECLNWDVEVKAAMHTYSSPTVATQSPKKQVQKL
ERAFQSQRTPGNQTPSPRNTSASALSARPKHSKPKLPVKSHSECGVLEKAAPPQESGCPA
QLEEQVKNSVQTSVEDSSSNSQHQAKDSSPGSLPKIPCLSSFKVHQPSTLTFAELVDDYE
HYIKEGLEKPVEIIRHYTGPEAQTGNPQNGFVRNRVSEDRAQMLVSSSQSGDSESDSDSC
SSRTSSQSKGNKSYLEGSSDTQLKDTECTPVGGPLSLEQVQNGNDTPTQVEYQEAPETQV
KARHKEGANQRSKQSRRNPARRSSYRVQSEPQEESWYDCHRETTASFSDTYQDYEEYWRA

YYRAWQEYYAAASHSYYWNAQRHPSWMAAYHMNTVYLQEMMRGNQ
>P38777
MTVQIPKLLFLHGFLQNGKVFSEKSSGIRKLLKKANVQCDYIDAPVLLEKKDLPFEMDDE
KWQATLDADVNRAWFYHSEISHELDISEGLKSVVDHIKANGPYDGIVGFSQGAALSSIIT
NKISELVPDHPQFKVSVVISGYSFTEPDPEHPGELRITEKFRDSFAVKPDMKTKMIFIYG
ASDQAVPSVRSKYLYDIYLKAQNGNKEKVLAYEHPGGHMVPNKKDIIRPIVEQITSSLQE
ASE
>Q07085
MGGFLSHLTPEQNVEALKASCGPVRGNIYKHDDVIVDGYLGIPYAKPPVGELRFKKPVTV
DVWTEIKDCYKYGPACVQTGGFEQIAGPRTPTPEEAGCLTLNVFTPRNASSEFKNGRPVM
VYIHGGGYELCASSDFCAYSLSGTLPLKDVVVVSINYRLGVFGFLTTGDNVCPGNFGLWD
QTLALKWVQKHISSFGGDPNCVTVFGQSAGGASTDLLSLSPHSRDLFQRFIPISGTAHCD
FAIRASENQAKIFREFAEFHGFSGRDSSALFKWYQEQSPETLSNVKGYKKSISGFLTFIP
NLDGDFFPKPLDELRKEAPKKQMMTGVTEYEGLMLASMNPAFSPADVGLTLMPQGIYGKD
VVSNPDEIQKIFYEKYVEGVDKSDELAMRKKLCEALGDEFFNVGVIQAAKNAAKHGNEVY
FYTFEYVNPDSFGMWDGMMPFKAAVHCTELRYLLGEGVYSKFEPTEEDRKVMETTTTLFS
NFAKYGNPNGKGATAEIWEKYSLNRPERHYRISYPKCEMRDVYHEGRIQFLEKIDGDSDK
YQNWSMERRSRQRFNFF
>O42980
MSSSPTVLELFDVCFKQEDVDSLKKPNWFTDVSIDYVDELIEHLWFPSYPNQANEILLLR
PSLVFLLAEAAISPEELKVALPKKLMNCKYLFMPINDLDKHAAGSGGSHWSLMVASIPDG
QCYYYDSLSNGKTKDCRSALARVSDLFKKKFTIECMPVQQQRNGYDCGAHVCAFTLELVR
RLLHSPMPTSSMWNLSTFQPDVTAIREQLSRCLDHIINSLGTRVSGDFDEDFPTGTVFFD
LESHLPLLDVALPVLPKSSDSSETSHESSNSNLKKSSESGSTNHHNNHESDKDLHHEGHH
HHHHHHHHHHSHDDDPSSPAEKKQNHVPSPSEKIQDHVPSPSEKKQDRVPSPSNNKEDHL
PLLSDEKLDKSAIDKIEPTPLPSVHMNSHIAKGELPKFHNSTDNPFLTPPEELVSGDFPF

>Q8IWL8
MSEGGGQVSCIFAAPTRLCRWPALIECGVNLTQPLCEWMIQVARDRTLSLAWEVASLLTL
SSSEVGLEGVGTIWPSSYSSEESSRNGAEQGRQLSIEGPFQGQNCPSHPAAALPLPMRGE
SQATSCQV
>O95149
MEELSQALASSFSVSQDLNSTAAPHPRLSQYKSKYSSLEQSERRRRLLELQKSKRLDYVN
HARRLAEDDWTGMESEEENKKDDEEMDIDTVKKLPKHYANQLMLSEWLIDVPSDLGQEWI
VVVCPVGKRALIVASRGSTSAYTKSGYCVNRFSSLLPGGNRRNSTAKDYTILDCIYNEVN
QTYYVLDVMCWRGHPFYDCQTDFRFYWMHSKLPEEEGLGEKTKLNPFKFVGLKNFPCTPE
SLCDVLSMDFPFEVDGLLFYHKQTHYSPGSTPLVGWLRPYMVSDVLGVAVPAGPLTTKPD
YAGHQLQQIMEHKKSQKEGMKEKLTHKASENGHYELEHLSTPKLKGSSHSPDHPGCLMEN

>O75936
MACTIQKAEALDGAHLMQILWYDEEESLYPAVWLRDNCPCSDCYLDSAKARKLLVEALDV
NIGIKGLIFDRKKVYITWPDEHYSEFQADWLKKRCFSKQARAKLQRELFFPECQYWGSEL
QLPTLDFEDVLRYDEHAYKWLSTLKKVGIVRLTGASDKPGEVSKLGKRMGFLYLTFYGHT
WQVQDKIDANNVAYTTGKLSFHTDYPALHHPPGVQLLHCIKQTVTGGDSEIVDGFNVCQK

LKKNNPQAFQILSSTFVDFTDIGVDYCDFSVQSKHKIIELDDKGQVVRINFNNATRDTIF
DVPVERVQPFYAALKEFVDLMNSKESKFTFKMNPGDVITFDNWRLLHGRRSYEAGTEISR
HLEGAYADWDVVMSRLRILRQRVENGN
>Q04007
MDTVGTDAAAASINERRFAQSTSPKVSVKSQDSLFLITYSNMQQTVVQASLADRYPSLKK
LNILLYIDIPTIDYYNDEMTHNKLSRLNKRFKLHRLRNSIAQSFSNTSTAEDNDKFWEEL
KSLISSRSTPENKFDLNVLVSSSGSLRYVETIRFLVEKLFNSFKDLYVQKKLNLCFQINV
SPTSLKWFSTFLNAELLNLKIINWQNIGSFTKTIQNSKSLPFKEYYTKLNEKFTGSNQSN
GSMQDQTVLDSIVIVTNSTGVKALLTLLSDHPLTSLISQESIKALHEYSDAVNEDKGDDQ
SNTSLKRNSSSLLNFQNSVLTSNKDKSVRIRSLSINRKSNRAHMFKTNESITTIPSTSIN
NLIGQESNLRKQPSGTALHLQSHLHPHSRSQSYSSSNMSRSPSPFPYGKTPSNDELVYDE
LNNQINEVQDRAKNEEIVLYNNNNYDDYTKERGEQEQDRTSYADEYGFNYDDEEGGNEDN
YDDDEDDDDDDDDDDESDDEGLSFYAPSILSRSGSSTDVLSSGIDSMAKNSKETRGRFRS
LSLMDPALQKPFNQKFPNSQQPDSAGASSPKRSTSSNHFTNVYVHDGDFDGTDTINNKKN
LSSATLIKRKSLMNRNLAPSISNGLIPPEFISRISTPSTSASSSNSSLNDMSTVSNAFSK
LLNDTSKKQKFLNSPIPQHTQQASPLLMRNNSNSNLLFEKNLINKSFEELRRQPSVNLFS
TLMNGNMEKNGLALNFKSRTPTDALMANSIKNSNNSSHRLLNLEEEDQIMSGSLPKERED
DNDSTNSTIVPNHPDNDNYNDNDNDNNTGINSNNFNLNLYDDNDSAGFTDVTTEGVKYSN
SNSTVTKPVYKKAVTLDLYGEDDMDNMGGWVLGGNAR
>O13516
MPRAPRTYSKTYSTPKRPYESSRLDAELKLAGEFGLKNKKEIYRISFQLSKIRRAARDLL
TRDEKDPKRLFEGNALIRRLVRVGVLSEDKKKLDYVLALKVEDFLERRLQTQVYKLGLAK
SVHHARVLITQRHIAVGKQIVNIPSFMVRLDSEKHIDFAPTSPFGGARPGRVARRNAARK
AEASGEAADEADEADEE
>Q07834
MDNFKIYSTVITTAFLQVPHLYTTNRLWKPIEAPFLVEFLQKRISSKELKNTKAICHIDP
SWVNLNASFIRDDMISIKATTDDMDLDAICRISLPLPMNTNDLTAELEKMKRILLDLSEK
FNLELIITKEPAYFTPEQTGESKELCIYVHALGFRSNLMECEPQLLAFVDLIKKNGMTLP
PQHYIIEPMELNSYSVLPLYMGVDMENFKHISRAFKTSIYAPSLITLSRDLKANPQIFFS
GAVHSLSLLARKTLRESISVNSKSFFYRRLTNITPGKLLFIRKYYQQKVNQLILKYQSLI
RVTNEYIEFQSISTNLLEMVIKNFTIQVLHEIVEVQISLNENCAMSPELIIDSFFGHTGN
QIVVITPKEDSFNQLIVVGNQSSTDEASDTSILHYLSDFIMGSNQVINPNLRQIKAIFEI
HPDFEDFISGKKNGKLTRIMELSACLIQLEMEEEDDNLYLNLVSDSFPDFKESFKNVINE
FPAEESFFIPEVCHRPIIGTGGSLIQATMRKHNVFIQFSNSFNLPQNKISMIRYDNVIIR
CPRKNKANICLAKNDLKQIVQEYDSLQSKTLIRFSSGQYRHILHVNGQKNIIGQIEKNEN
VYIMIPLKEPLDGTSQLSIQGNDENASRAANELVNSAFGYEYEFKIDQEIDPNKEYEFYN
LIVVPFLQIMNIIVTFEKDLITFTFEKDTNENTLTKAIELLSNYLETQKTKIIFKKIIKK
FVLGSASSKSNTSNSNTNGNFRSMNNAKSRTTIDNTSQSGASPQRHKMPVITTVGGAQAI
KGYIPNTYYNGYGYGYGYTYEYDYNYANSNKAQTNNRHKYQNGRK
>Q6R556
MDNAHRQTQAHLDAACFWQIWQRFDKDEKGYIKETELDAFFDDLLAKFGIEDTLMEENVQ
KMKEQLMVGHDISKEGRILMKELASMFLSEDENFLLFFRLETPLDNSVEFMQIWRKYDAD
SSGFISAAELSNFLRDLFLHHKKVISEAELEEYTSTMEKIFDRNKDGRLDLNDLARILAL
QENFLLQFKMDASSTEERKRDFEKIFAHYDVSKTGALEGPEVDGFVKDMMELVQPSISGV

DLDKFREILLRHCDVNKDGKIQKSELALCLGLKINP
>Q9US52
MFQGKCAFFDETVPSALIALWQLHNGEAKFLAEGYENFDYAFSMHLRKHVRLPNYKSVQC
RSPWWIAEQVAVSRSKSYVSEPVIHLNVMEPLYVDHRIRSVYLQPLPSTLSFLNGELP
>Q9USG8
MSTHSGDSTKQQHYRSSDPYSGRRPIPTIPKFFRDRKQRAEKKEEQQREQTENEKLFDPI
TQRDVEINDVHFDYAKTYDDPSFTVPNQSIQGSSLPSEKPYLSSNQPTNVYKQHQDDLAP
PEADNQITRDVPISDEKTNILFFPSPSIDLSYVSKEVKQKTGQYSLFAYIFSLVISWFFT
HSIIISAVLPLAISSCMYLWMQNIYAVAKDAEWGAEQKRGEYARLNLIPESAEWMNHLLE
KVWPLINPEMFSSVADQIEDVMQASIPSFVENVRVASLDQGSHPVRVVSIRSLPSGEASE
SFSEKQASEAEHKDEPEQQRKQFYNFELCLAYHAKPVEDATSTSARASNLHLRIVFYPGI
KGTVGFPLPIWVEIKGFVARIRFRCELMPEVPFLKNVTFSLMGLPELNVSAVPVAEGGVN
IFGLPLISKFVNDAISAAANEYVSPKSMTIDLSKTLLGDDIKKEVNALGVIFVHINRAED
LSKQDVNGLSDAYITVGFHKFGKPLYCTRVVKQDLNPIWNEYAFIPVFPDQVKAGEKISI
ELWDSDRFSPDDVVGRTKIGLHLLIQDSGKMHERCDTLTGISEDTSLPGRVFYEIGYFPR
AEFKPSLKTSGHDITIPRSMRDDPAFQNPHGSLDNKEEEAAVTTAPDEEYPSGILSFTVH
QAVNLQMNHPTGTFGNVSGNYNTSPAQSVGDVTAEEGSELPSSYVCVDLDDTLVYKTRTK
VFTSNPIYNAGSEKFVKDWRNAMLCFTVRDFKLREHDSILGVVNIPLATTLTTSSQLTKW
YPIQGGIGFGSVRISILFRSMKLKIPRNLLGWDIGTLEFMDRQIVAEGTGSVSDVSFSSI
RVNIAGVKITAKSSTSNSSSTAEYHVRSRHAVIPVNNRYRSAVVFEFRKQLQRKHNVFAM
VWLVDLEDNVEQNIRVPIFTSSKPAHVLQNMIDFDHPDKESEFKIIGYLSTRICFHRGLD
DSHEQLVDNDDEAAIFETYRCLKSMGLRRGYVKDMKNPLADQRASLDESRETTTASSKFE
SDDSVDTEDEETTTDRTPIECTQTVSMVDPNVNGDIQGRNSLGTMNSNERNLEQEFISLG
YASKNRPKAHAQEGTNQPGASENVEPVLADDSDAVTIHSNISSDDQKRKLVNADDRELEK
RLHRGPYNSKIVRTGEWVKDGAKMGWRNLRRKFALNGRQPDVETEISK
>Q7T019
MPFYDNVYYPYPSDPPGTHSSAGIPSLLSSPQSQPSSGSQSRPAPSTMSGPLTSSGASTS
IPPPFKFRSRRENVDWRRINAVDVDRVACEMDFQALQEHINAVTFCSVEGERCHRCQSPV
DPALIKLFRLAQLTVEYLLHSQDCLSISLQAAEERLLAEAREREQICVQLQKKTQDAKAL
KEELKQRKKIIASQQAMFSAGISANYHKCQHCEKAFMNASFLQSHMQRRHPSEFDMKLMT
DNQKKIQTVKLQDEINKLQEQLTLVTSQMETQQKDYTAKQEKELIQRQEEFKRQLEIWKE
EEKMRMNSKIDEVKQACQRDMDSINQRNRNLETELLKLQQKNIQESMQSVQTQPNASTSN
EHWQEVVKLQQKLHKQEVKWTGKMQKMKEDHDREKSLLQEELCKVSSAVSEGMEESRRQV
QELSHRLQEQQQIITSQNKQMKQISSKPPTITVQREGVSTPSPETKAKVVVSEQSNSVHK
LDPIVELSEEDKDSSSISESPTENRSWQKEVQELLKNPGLRRDMRLAAQHNLDDRLQSLG
IKGVSGLSKNLYKSSMTQIISDRRKKLEEDPVYRRALKEISHKLEQRVKERNTEQPVKSK
LHEQVVQSRPRSSSFPSTVTRVMSGPASKQQRTPQPVPRSRTNVPHKTSTPLQHRRTPPF
SSDEDSSEEEEEEEEEEEESSDEESPQMQKKTVLVNSSTAKAQNTAKTQSTAQSVRSAVAL
TSAEPTNVTTLSDSDWTDGSEMEEINLSQLHKHTDQNGNLKNVTHSNVKALGKSLEKQLA
ARGPKKPAGGVNTFLEKPTDVRNTRQNAKKELKYSDDDDDDDDDWDISSLEDVPAVAKPT
QCPVPVRKSLDKSQDTSTSVWGSSTGKGHKPGLTDAGTASTLKSSLVTVSDWDDSDEI
>O35543
MPNYKLLYFNMRGRAEIIRYIFAYLDIKYEDHRIEQADWPKIKPTLPFGKIPVLEVEGLT
LHQSLAIARYLTKNTDLAGKTELEQCQVDAVVDTLDDFMSLFPWAEENQDLKERTFNDLL

TRQAPHLLKDLDTYLGDKEWFIGNYVTWADFYWDICSTTLLVLKPDLLGIYPRLVSLRNK
VQAIPAISAWILKRPQTKL
>Q9UTB0
MHTYCNTETVQLYRFIAMDQWSDNKDAIGMLSESLKEKVVTNEIAPALKEVKNHKYNFET
EILNTGNVWNNDSKNGESGSAFGLDKIFQSSDSGNIAEGLELDLGNIMLETHNLESPAWK
HADYDGVAASVNDNNNDWKFFVFDEREIDSMLQLFVRDFRADKPALRLAPSKLFYLASRF
AFFYMPKEKELGTVLLNAFLCEVNQVTQQHPNDMVLCVQWLANVSLLLFYLKKDNKLDDL
TVDIQNRCSELMNSLYITICQDAMRRMNENLEEGMVKYTGIQGLEDILRSRSWNLLRRRP
TNDASTSPRSTPSASPRSITKIIASTLHLLEVFYIHPLIRAQCIEQLFSWLGARLFNIVI
SNKKYLSRAAAMETRFNISSLEEWSQTNSPKLQKPFDYPDEDLKVDLISKLLSLVQLLQW
LQCLYRLSEDEDPRALQETLESLDALNPRQIYTAAKLYRPDITETKVSKTFLKKLDAFHE
EKLREKINSSDKGDVSYEFELLKDETVFSPLKLPTKAQLINAYSYIVSGNGFNEDRKIVF
QPHVSNILIDKLEENGLSMERAELPTSVFEDELQRREWRPDQEVEELLST
>Q8IR79
MHHQQRLRANGGRGGTGLGAGSGPVSGGHSPLCAHCRGQLLPHPEEPIVMALGQQWHCDC
FRCSVCEGHLHNWYFEREGLLYCREDYYGRFGDACQQCMAVITGPVMVAGEHKFHPECFC
CTACGSFIGEGESYALVERSKLYCGQCYGKRSCQPADAKARITTAGKPMHSIRLVEIPKD
ATPGLRVDGVALDDGCPTVRITDLFCNFFLWLTSMAEQCCGAPYRIDVNLTNLHIGDRIL
EVNGTPVSDSSVEQIDKLIRSNEKMLQLTVEHDPVQVCRSCSQADIQRAMSASTLILPLS
TSASSVEVGRERLYKTPGEQGTKARKLRQATNASTTIPPAAGATAMTQLKEKERCSSLSK
LLDEQHQAQQHSAHPQLYDLSRTQSCRVVQKPQRIFRATDLVIGEKLGEGFFGKVFKVTH
RQSGEVMVLKELHRADEEAQRNFIKEVAVLRLLDHRHVLKFIGVLYKDKKLHMVTEYVAG
GCLKELIHDPAQVLPWPQRVRLARDIACGMSYLHSMNIIHRDLNSMNCLVREDRSVIVAD
FGLARSVDAPRLPSGNMTPGGYGSGANSDAPMSPSGTLRRSKSRQRRQRYTVVGNPYWMA
PEMMKGLKYDEKVDVFSFGIMLCEIIGRVEADPDFMPRNSDFSLNQQEFREKFCAQCPEP
FVKVAFVCCDLNPDMRPCFETLHVWLQRLADDLAADRVPPERLLHEIETFQEWYASSEDA
LSPTSQRSLNNLDELVKSAVDSEISPVEKEKENMVIKPQDIPKSPHLGKDFSPSGERLRD
SMRARRRQRFLGAQEERRNLTPDTESKERALKKALKKCRPFGERGYLVDLRAGAELQLED
VRDLNTYSDVDSSCDTSLNYHDVNNLPAAQEDENTVKPGKEELLEESTNKPSNQESQHHR
LAIDDMRTRLNQCRSKFEHLEEASRRNFNQSQHSMKNFFKTPPVALKMFQRLEHEAAALN
GGNNCPPPPPRTQRINQTPIFGRKNPPVAIVGQKLQHAESLEDLASSGVAKQLATPAPKR
SKATATTKGGQSSNPPLFLPPSLNISVALNSNGNVTTTTNTNSSCPPSASDWLPKKHKLT
LPLPSAQQQRTSSNHRLPMCNNKGKTLKPLPSRTGSQGIPASNCVSPTRSSRPGSPTKHL
AQRHTAATAQRLTNAAATHQQQHQQQSSKTTRLNILSPEKVHRLGARLTDQKQKMREEAA
ATASSVGGAGCAAGTAAGSLNGHRTIGSSGTPNSAVGERRRRAAPSPPVRTHFNTRC
>P38986
MKSDSVEITTICPDVENSQFVVQSNCPETIPEILKSQNAAVNGSGIACQQRSLPRIKILG
TGGTIASKAIDSSQTAGYHVDLTIQDLLDAIPDISKVCDIEYEQLCNVDSKDINEDILYK
IYKGVSESLQAFDGIVITHGTDTLSETAFFIESTIDAGDVPIVFVGSMRPSTSVSADGPM
NLYQAICIASNPKSRGRGVLVSLNDQISSGYYITKTNANSLDSFNVRQGYLGNFVNNEIH
YYYPPVKPQGCHKFKLRVDGKHFKLPEVCILYAHQAFPPAIVNLVADKYDGIVLATMGAG
SLPEEVNETCMKLSLPIVYSKRSMDGMVPIANVPKKGSKEDNLIASGYLSPEKSRILLQL
CLAGNYTLEEIKHVFTGVYGG
>P38314

MASTSNTFPPSQSNSSNNLPTSRHASIVEMLSTPPLLPHVQVNDTDDKEQPEESTPPTAT
AAAPGPGCAATPAPLRDEKPQFKLSAVPMTQTPSQCLSCVHAQKWQHIPLSQLIEQNKLI
FVPGSISVEEAFNTLIKYHLNSIPVESFPGDMNCFTFDYNDLNSYLLLVLNKITVSNKQL
TADCQNGKPVPVGEMVKLTPKNPFYKLPENESLSTVMGILGSGVHRVAITNEEMTKVKGI
LSQRRLIKYLWDNARSFTSLEPLLNSSLQDLHIGVLNIQSKPTSRQSRVISIQGEEPLIM
GLYKMHVERISSIAVIDKQGNLLGNISVTDVKHVTRTSQYPLLHKTCRHFISVILNSRGL
ETGKDSFPIFHVYPSSSLARTLAKLVATKSHRLWIVQPPESSTSASSTNLTAANTAANAV
SATAQSSANGATPMSKSSSSTSLNSHSPLMTAMEDPPSPRSSAIAIPPPSPASSTNTPNL
FEKEYRTGKLIGVVSLTDIINLLARKQTGNKEVDPQSARRQRGSIAM
>Q21166
MTAVSANGKTTEKHENGAHTNGTTNGTTNGSMNGNEISHVQKLQPVYYKPPQNLETFELS
LRNHFEEKTNKKFADYREFHRFTCDNYGIFWEDLLKLSDVKLHQNYNQVIDHNLKINERP
RWFNGATLNYTENVIERGTATDIAVLNASIEETVTEYTYDNLRKDVYRIATSLRNYGIGP
GDTVCGFVPNTYDTLVAVFATAAVGAAWCSASVDFGPAGVLDRFRQVHPKVLFTVNHVTY
[truncated: 502,299 more chars]
